# Functional dissection of the *ash2 *and *ash1 *transcriptomes provides insights into the transcriptional basis of wing phenotypes and reveals conserved protein interactions

**DOI:** 10.1186/gb-2007-8-4-r67

**Published:** 2007-04-28

**Authors:** Sergi Beltran, Mireia Angulo, Miguel Pignatelli, Florenci Serras, Montserrat Corominas

**Affiliations:** 1 Departament de Genètica, Universitat de Barcelona, Diagonal 645, 08028 Barcelona, Spain

## Abstract

**Background:**

The trithorax group (trxG) genes *absent*, *small or homeotic discs 1 *(*ash1*) and *2 *(*ash2*) were isolated in a screen for mutants with abnormal imaginal discs. Mutations in either gene cause homeotic transformations but Hox genes are not their only targets. Although analysis of double mutants revealed that *ash2 *and *ash1 *mutations enhance each other's phenotypes, suggesting they are functionally related, it was shown that these proteins are subunits of distinct complexes.

**Results:**

The analysis of wing imaginal disc transcriptomes from *ash2 *and *ash1 *mutants showed that they are highly similar. Functional annotation of regulated genes using Gene Ontology allowed identification of severely affected groups of genes that could be correlated to the wing phenotypes observed. Comparison of the differentially expressed genes with those from other genome-wide analyses revealed similarities between ASH2 and Sin3A, suggesting a putative functional relationship. Coimmunoprecipitation studies and immunolocalization on polytene chromosomes demonstrated that ASH2 and Sin3A interact with HCF (host-cell factor). The results of nucleosome western blots and clonal analysis indicated that ASH2 is necessary for trimethylation of the Lys4 on histone 3 (H3K4).

**Conclusion:**

The similarity between the transcriptomes of *ash2 *and *ash1 *mutants supports a model in which the two genes act together to maintain stable states of transcription. Like in humans, both ASH2 and Sin3A bind HCF. Finally, the reduction of H3K4 trimethylation in *ash2 *mutants is the first evidence in *Drosophila *regarding the molecular function of this trxG gene.

## Abstract 2

Analysis of the gene expression profiles of wing imaginal discs from *ash2 *and *ash1 *mutants shows that they are highly similar, supporting a model in which they act together to maintain stable states of transcription.

## Background

During early development, transcription factors and signalling molecules initiate a cascade of developmental decisions that culminates in lineage restriction, cell determination and cell differentiation. However, commitment to a particular cell fate in the early embryo must be maintained throughout development, even after the factors that specified the cell fate are no longer present. The trithorax group (trxG) and Polycomb group (PcG) proteins are positive and negative regulators, respectively, that are involved in maintaining heritable patterns of transcription during development and differentiation (recently reviewed in [1-3]). Although the way in which trxG and PcG proteins recognize their target genes is not fully understood, Polycomb and trithorax response elements (generally termed PREs) are known to play an important role in this process, since they represent the DNA sequences upon which trxG and PcG complexes are assembled (for a review, see [3]). Genetic studies in *Drosophila *have shown that mutations in trxG and PcG genes result in flies with homeotic transformations due to inappropriate expression of Hox genes [4-6]. However, Hox genes are not the only targets [7-9]. The *absent*, *small or homeotic discs *2 (*ash2*) gene is a member of the trxG that was discovered, together with *ash1*, in a screen for late larval and early pupal lethal mutations that generated abnormal imaginal discs [10]. Mutations in *ash2 *and *ash1 *cause the homeotic transformations expected for this group and, in the case of *ash2*, a variety of pattern-formation defects in the wings [7,10-13]. Moreover, since analysis of double mutants reveals that *ash2 *and *ash1 *mutations enhance each other's phenotypes, it is likely that the genes are functionally related [13].

Using the fly wing as a model system, several approaches have been used to gain insight into the role of ASH2. Loss-of-function mutations in *ash2 *result in extra vein tissue and we have demonstrated through genetic analysis, clonal analysis and expression analysis of candidate genes that *ash2 *is involved in the maintenance of intervein cell identity [11,12]. Further insights have been gained using microarrays covering one-third of the *Drosophila *genome to analyze gene expression in whole larvae, an approach that identified several genes involved in the cell cycle, cell proliferation and cell adhesion, which are regulated by *ash2 *in larval tissues [7].

PcG and trxG proteins are assembled into dynamic multimeric complexes, several of which have been purified from organisms ranging from yeast to humans [14,15]. Although no biochemical studies in *Drosophila *have fully described the complexes in which ASH1 and ASH2 are involved, it has been shown that they are subunits of distinct complexes [16] and, in addition, it has been reported that ASH2 binds SKTL, a putative nuclear phosphatidylinositol 4-phosphate 5-kinase [17].

In *Saccharomyces cerevisiae*, two proteins (Bre2 and Spp1) have been proposed to associate to give a full ASH2 analogue in the complex termed Set1C [18,19] or COMPASS [20], which can methylate the Lys4 on histone 3 (H3K4) [18,19]. In mammals, ASH2 has been found in the COMPASS counterpart [21], in ASCOM [22], in two Menin-containing complexes [23,24] and in a histone methyltransferase complex bound by host cell factor 1 (HCF-1) [25]. It has recently been shown that mammalian ASH2 plays a role in the trimethylation of H3K4, a modification associated with transcriptionally active genes [26]. The presence of ASH2 in such a wide variety of complexes is an indication that it must be involved in the regulation of many different processes. ASH1 contains a SET domain and is an H3K4, H3K9 and H4K20 methyltransferase [27,28] required to prevent transcriptional repression by members of the PcG in *Drosophila *[29].

Systematic examination of gene expression patterns using whole-genome techniques, principally microarrays, allows the identification of most or all genes engaged in specific developmental processes [30,31]. However, simply listing genes associated with a certain tumor or developmental stage is insufficient to identify the biological processes in which these genes are involved, and comparative approaches are probably necessary to obtain a broader, yet more informative, picture of the phenomena under investigation. The work presented here is the first attempt to compare three alleles (*ash2*^112411^, *ash2*^*I*1 ^and *ash1*^22^) from two trxG proteins (ASH2 and ASH1) by analysis of their transcriptome using tools based on Gene Ontology (GO) [32,33] and comparisons with published genome-wide data arising from microarray analysis or *in silico *predictions. Using this approach, we have been able to finely dissect the biological processes altered by mutations in *ash2 *and *ash1 *and to assess the similarity between them. We show that, despite the different nature of the *ash2*^112411 ^and *ash2*^*I*1 ^alleles, their transcriptomes are very similar. Furthermore, we present data indicating that ASH1 and ASH2 act upon similar biological processes and genes, supporting the observation that *ash2 *and *ash1 *are functionally related. Moreover, since the number of regulated genes common to both *ash2 *and *Sin3A *mutants is higher than expected, we performed immunoprecipitation experiments that show that, as already reported in humans, Sin3A and ASH2 can both bind HCF [25]. Finally, we have detected a severe reduction of H3K4 trimethylation in *ash2 *mutants, which suggests that ASH2 might play a role in methyltransferase activity while associating with complexes similar to those found in yeast and humans.

## Results and discussion

### Whole genome analysis of *ash1 *and *ash2 *mutant wing imaginal disc

**Figure 1:**
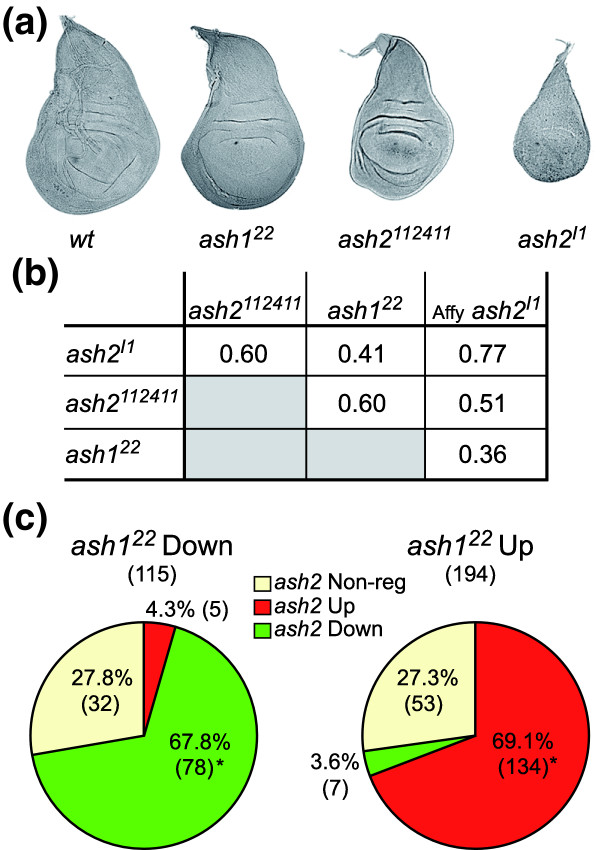
Similarity of *ash2 *and *ash1 *mutant transcriptomes. **(a) **Wing imaginal discs of *wt *and *ash1*^22^, *ash2*^112411 ^and *ash2*^*I*1 ^homozygotes. **(b) **Pearson's correlation coefficients between the normalized mean log_2 _ratios of the different experimental conditions. **(c) **Comparison of gene expression changes in *ash1*^22 ^and *ash2 *mutants. The circles represent the genes that were downregulated (left) or upregulated (right) with a greater than 1.5-fold change in *ash1*^22 ^mutants. The percentage of these genes that were also downregulated (green) or upregulated (red) in one or both of the *ash2 *alleles is shown. The absolute number of genes is shown in brackets. *Statistically significant overlap: *p *= 4.76 × 10^-56 ^(left) and *p *= 2.40 × 10^-104 ^(right).

To characterize the molecular signature of *ash1 *and *ash2 *mutant wing discs, we compared whole genome expression profiles in wing imaginal discs from bromophenol blue-staged third instar larvae for the alleles *ash2*^*I*1^, *ash2*^112411 ^and *ash1*^22 ^with those from an isogenic line used as a common reference. The *ash2*^*I*1 ^allele is lethal in late larval or early pupal stages whereas *ash2*^112411 ^is a weaker allele that, although lethal at the pharate stage, presents a low percentage of adults [11,12]. The null *ash1*^22 ^allele is a late larval/early pupal lethal [34]. The wing disc phenotype associated with *ash2*^*I*1 ^discs involves abnormal shape and severe reduction in size, while *ash2*^112411 ^and *ash1*^22 ^discs are less affected (Figure 1a).

We performed a series of two-color microarray experiments with these alleles and obtained, for each of them, lists of genes showing greater than 1.5- or 2.0-fold changes in expression (Additional data files 2-4). The number of genes that were misregulated in each experimental condition is summarized in Additional data file 1 and all data are deposited in the Gene Expression Omnibus (GEO) database [35,36] with accession number GSE4923. In accordance with the phenotypes of these alleles, there were many less misregulated genes in *ash1 *than in *ash2 *mutants.

To estimate the reproducibility of the results across platforms, two Affymetrix GeneChips were hybridized in a different laboratory with independently extracted *ash2*^*I*1 ^RNA and reference RNA, respectively. We calculated Pearson's correlation coefficients for all the microarray results, including Affymetrix (Figure 1b). The highest similarity observed was between the two-color and the Affymetrix microarrays hybridized with *ash2*^*I*1 ^and reference RNA, highlighting the quality of the data obtained.

### Functional dissection of the *ash1 *and *ash2 *transcriptomes

**Figure 2:**
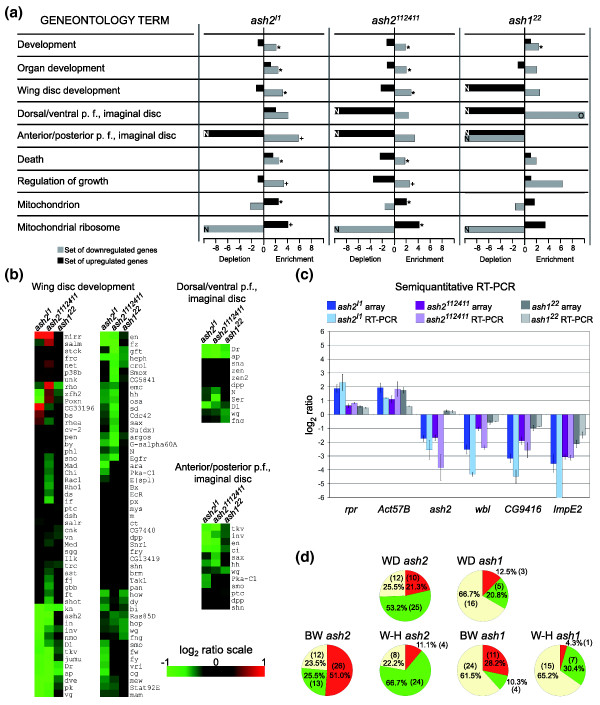
Whole genome expression analysis of *ash2 *and *ash1 *mutants. **(a) **GO annotation of genes that were upregulated (black bar) or downregulated (grey bar) in each allele (see text for details). The length of the bar indicates the fold change. N indicates that no genes were found with a given term in this set. O indicates over 10 times. An asterisk indicates FDR adjusted *p *value < 0.01 and a plus sign indicates <0.05 (FDR adjusted *p *values for each GO annotation are detailed in Additional data file 21); p.f., pattern formation. **(b) **Expression maps including all the genes belonging to GO terms 'Wing disc development', 'Anterior/posterior pattern formation, imaginal disc' and 'Dorsal/ventral pattern formation, imaginal disc'. All the genes from the array having these GO terms are represented irrespective of log_2_ratios or *p *values. Red indicates upregulation and green downregulation (see scale bar). **(c) **Semi-quantitative RT-PCR and microarray results for six genes. The height of the bar is proportional to the log_2 _ratio observed for each gene in the microarray or RT-PCR experiments in all three alleles. The expression level of all genes was statistically significant across microarray replicate experiments in all three mutant alleles (*p *< 0.05) except for *ash2 *in the *ash1*^22 ^allele. Error bars represent standard error in microarray or semiquantitative RT-PCR analyses, respectively. **(d) **Comparison of genes that were upregulated or downregulated in *ash2 *and *ash1 *mutants with the genes preferentially expressed in the wing disc (WD) according to Klebes *et al*. [37] and in the body wall (BW) and wing hinge (W-H) according to Butler *et al*. [38]. The full circle encompasses the total number of genes with valid log_2 _ratios in one or both of the *ash2 *alleles or in the *ash1 *allele for each comparison. The number of genes that were downregulated (green), upregulated (red) or showed no change in expression (yellow) over 1.5 times in each comparison is shown in brackets.

Comparison of genes showing significant levels of misexpression between *ash2 *and *ash1 *alleles revealed an extremely high and significant overlap between genes regulated in the same direction (Figure 1c) and the correlation coefficients were consistent with the wing disc phenotypes and lethality phases observed for the different alleles. Since we also intended to assess the similarity between the three alleles in more general terms, automatic functional annotation of all results was performed based on GO (Additional data files 5-16 and 22 and 23). Although this type of annotation is very useful to obtain a description of the transcriptome, it can be misleading if the absolute number of genes that fall into each class is used as the only factor for selection of relevant classes. Consequently, we identified which GO terms were significantly over-represented in each mutant allele under study. If *ash2 *and *ash1 *did not have specific functions, we would expect misregulated genes to fall into various GO categories and that the proportion of the genes in each category would be the same as the proportion of all *Drosophila *genes found in that category. However, as shown in the examples of GO terms given to misregulated genes in Figure 2a, more genes than expected by chance were involved in certain processes or functions. The bars (grey for downregulated and black for upregulated genes) indicate whether there are more (positive values) or less (negative values) genes with a given GO term than expected with a random distribution. For instance, out of the 526 underexpressed genes identified in *ash2*^*I*1 ^mutants, 440 were annotated in the GO database. If these genes were randomly distributed, we would expect 6 of them to have the GO term 'wing disc development', since only 1.3% of all *Drosophila *genes with GO annotations have that term. However, 18 of the 440 genes held that descriptor (4.1%), representing a 3-fold enrichment in relation to the number expected by chance. This change corresponds to the size of the grey bar (downregulated genes) in the row 'wing disc development' for the *ash2*^*I*1 ^allele (see also Additional data file 5).

While the two *ash2 *alleles affected the same processes almost identically, the *ash1 *mutant displayed some slight differences (Figure 2a,b). Even though the significantly misexpressed genes were not identical between the three alleles, the expression of most genes tended to change in the same direction. The great similarity observed between the transcriptomes correlates with the similarities between the mutant phenotypes, indicating that the data obtained is reliable and that the functional relationship between *ash2 *and *ash1 *found by genetic interactions [13] is maintained at the transcriptional level.

**Figure 3:**
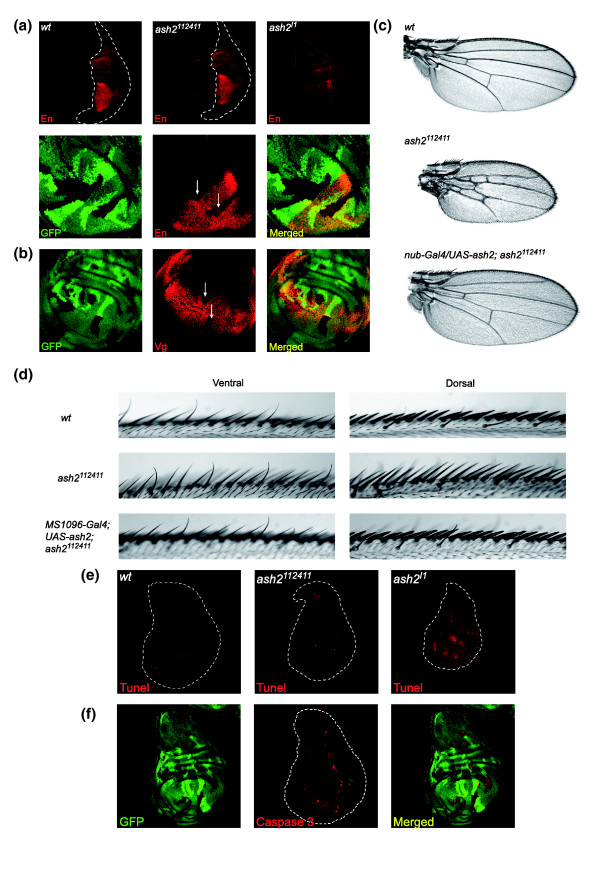
Gene expression and phenotypes of *ash2 *mutants. **(a) **Engrailed (En) expression (dotted line) in *wt *and *ash2 *mutant wing discs (top panels). Reduction of En levels (white arrows) in *ash2*^*I*1 ^homozygous cells (bottom panels). **(b) **Reduction of Vg levels (white arrows) in *ash2*^*I*1 ^homozygous cells. **(c) **Wing phenotype of *ash2*^112411 ^homozygotes (central picture) is rescued to *wt *(top picture) by expression of *ash2 *in *ash2*^112411 ^homozygotes (bottom picture). **(d) **Dorsal bristles of the wing margin in *ash2*^112411 ^homozygotes are partially ventralized (compare central panels with upper panels). Dorsal bristles from *ash2*^112411 ^homozygotes are *wt *if *ash2 *is ectopically expressed with *MS-1096-Gal4 *driver (bottom panels). **(e) **Increased cell death in *ash2*^*I*1 ^homozygous wing discs in comparison to *wt *and *ash2*^112411 ^homozygous wing discs as seen by TUNEL staining (red). **(f) **Apoptosis in *ash2*^*I*1 ^homozygous cells. Apoptotic cells are identified by staining with anti-cleaved Caspase 3. In (a,b,f), *wt *cells are bright green, heterozygous cells are light green and *ash2*^*I*1 ^homozygous cells are black. In (e,f), dotted lines outline the discs. In all wing discs, anterior is left and dorsal up.

We found more underexpressed and fewer overexpressed genes with the terms 'development' and/or 'organ development' than expected by chance, supporting the view that trxG proteins are usually involved in maintaining the activated state of genes involved in such processes. For some of those genes (*ash2*, *rpr*, *Act57B*, *wbl *and *ImpE2*) we confirmed their change of expression by performing semi-quantitative RT-PCR (Figure 2c). For others, like *en *or *vg*, we went one step further and analyzed their protein levels by immunohistochemistry and clonal analysis. In those genes, a correlation was observed between RNA expression and protein levels (Figure 3a,b). Some of the genes identified in this analysis may help to understand the mutant phenotypes observed, thereby establishing a link between the transcriptome and the phenotype.

#### Wing development

As expected based on the function of trxG genes, we found more downregulated genes belonging to the GO class 'wing disc development' than predicted by chance (Figure 2a,b). To identify other misregulated genes that might be involved in wing disc development despite not being annotated as such by GO, we compared our results with available microarray data listing genes predominantly expressed in the whole wing disc in comparison to leg disc [37] and in different parts (wing hinge or body wall) of the wing disc [38].

According to the functional GO annotation of the 67 wing disc-specific genes [37], the terms 'development', 'wing disc development' and 'transcription factor activity' rank amongst the top 10 statistically significant categories (Additional data file 17). Amongst these 67 genes, more were downregulated than upregulated in *ash2 *and *ash1 *mutants (Figure 2d; Additional data file 18). These results support the GO classification of *ash2 *and *ash1 *transcriptomes and shows that the partial picture it provides is sufficient to classify results in a manner that enables successful comparison of transcriptomes beyond the gene to gene level. Regarding wing disc parts [38], there were more upregulated than downregulated genes that were related to the body wall in both *ash2 *and *ash1 *mutants. In contrast, the number of downregulated wing-hinge genes was much greater than the number of upregulated genes (Figure 2d; Additional data file 18). These results suggest that ASH2 and ASH1 may play a more essential role in the wing hinge than in the body wall; this possibility is supported by the wing phenotypes (Figure 3c) [11,12] and the finding that more cell death is detected in the wing pouch in *ash2*^*I*1 ^(Figure 3e).

#### Pattern formation

Going downwards in the hierarchy of GO classification (Figure 2a), it is striking to find that genes that are downregulated in *ash2 *and/or *ash1 *mutants are enriched with 'dorsal/ventral pattern formation, imaginal disc' or 'anterior/posterior pattern formation, imaginal disc' annotations. With these annotations the differences are obvious and radical: no genes holding these descriptors are overexpressed in either *ash2 *or *ash1 *alleles. Again, all three alleles displayed similar patterns of misregulated genes (Figure 2b).

In the case of 'anterior/posterior pattern formation, imaginal disc', it is worth mentioning *engrailed *(*en*) and *invected *(*inv*), which are expressed in the posterior compartment of wing imaginal discs and are required to maintain its identity [39]. Although it was reported that En levels did not seem to be reduced in whole discs from other *ash2 *alleles [6], we show here that En/Inv levels are reduced in *ash2*^*I*1 ^clones and that their pattern of expression is severely altered in whole *ash2*^*I*1 ^discs (Figure 3a). An example of a downregulated gene in *ash2 *from the 'dorsal/ventral pattern formation, imaginal disc' class is *Drop *(*Dr*; Figure 2b). Absence of this gene leads, among other consequences, to a phenotype in which the dorsal wing margin bristles resemble the ventral ones [40]. A similar phenotype is observed in *ash2*^112411 ^adult wings (Figure 3d). The fact that *en *and *Dr *have putative PREs nearby [41] turns them into good candidates to be primary targets of PcG and trxG proteins. Furthermore, the rescue of wing pattern in general and the ventral wing margin bristles in particular upon overexpression of *ash2 *in an *ash2*^112411 ^mutant background (Figures 3c,d) highlights the importance of *ash2 *in the positive regulation of genes involved in wing morphogenesis.

#### Proliferation and apoptosis

In a previous report, we showed that the expression level of genes involved in cell growth was altered in *ash2*^*I*1 ^mutants [7]. We show here that this also holds true for *ash1 *mutants, since there are more downregulated and less upregulated genes annotated as 'regulation of growth' than expected by chance (Figure 2a). One of these downregulated genes in *ash2 *mutants is *vestigial *(*vg*); vg is essential for wing development and no wings are formed in mutant flies due to extensive cell death [42,43]. The reduced Vg levels in cells lacking *ash2 *(Figure 3b) could explain the smaller size of *ash2 *mutant wings since Vg has been linked to cell survival and cell proliferation [44]. The gradient of downregulation observed for the *vg *transcript in *ash2*^*I*1^, *ash2*^112411^, and *ash1*^22 ^mutants (Figure 2b) matches well with the size differences observed in their mutant wing imaginal discs (Figure 1a). Ectopic expression of ASH2 in *ash2*^*I*1 ^and *ash2*^112411 ^mutants increases the size of wing imaginal discs (data not shown) and wings (Figure 3c), demonstrating that the reduction in size is due to the absence of ASH2 or reduced levels of the protein. Although the detection of a PRE sequence near *vg *supports ASH2 acting directly upon this gene, it cannot be ruled out that the combined weak downregulation observed in some of its regulatory proteins may also play a role. Other genes from the same class affected in *ash2 *mutants include Cyclin A (CycA), which has reduced protein levels in homozygous *ash2*^*I*1 ^cells [7], and the growth promoter Cdk4 [45], a Cyclin D-interacting protein that displays the greatest reduction in expression amongst the 'regulation of growth' genes in *ash2*^*I*1 ^wing discs.

The smaller size of wing discs and adult wings can be explained by a lack of growth and/or an enhancement of cell death. The levels of the pro-apoptotic gene *reaper *(*rpr*) were increased in *ash2 *and *ash1 *mutants (Figure 2c), suggesting that cell death plays a role in the reduction of wing disc size. TUNEL assays on third instar larvae wing imaginal discs of *ash2*^*I*1 ^and *ash2*^112411 ^showed a striking pattern in the former, where although dying cells could be found scattered around the whole disc, there was a high concentration of them in the prospective intervein regions of the wing pouch (Figure 3e). This resembles the pattern observed upon DNA damage-induced cell death through irradiation [46] and suggests that genes involved in wing patterning might somehow influence the onset of cell death. Moreover, cleaved Caspase 3 was predominantly found in the wing pouch of *ash2*^*I*1 ^wing imaginal discs (data not shown) and was highly restricted to *ash2*^*I*1 ^homozygous cells, as revealed by clonal analysis (Figure 3f). Taken together, these results indicate that ASH2 is essential for cell survival and to avoid apoptosis in some specific areas of the wing disc. In normal conditions, ASH2 may maintain activated states of transcription of specific intervein region genes that could themselves be anti-apoptotic or activators of other factors with this propriety.

In conclusion, the remarkable similarities between the transcriptomes of *ash2 *and *ash1 *seen by gene to gene comparisons, GO annotations and comparisons with other genome-wide data strongly suggest that these two genes are closely related. However, differences between them are also observed. For example, the fact that they cannot substitute each other and that they do not display exactly the same phenotypes indicates that they may play singular and independent roles.

### *ash2 *mutants are enriched in mitochondrial and/or ribosomal genes similarly to Sin3A-deficient cells

To gain insight into the specific functions of *ash2 *and *ash1*, we compared their expression profiles with those of other proteins that also act at the chromatin level. In a recent study, Affymetrix GeneChips were used to establish a relationship between *dmyc *and *Polycomb *(*Pc*) in *Drosophila *embryos [47]. Although PcG and trxG genes act upon some identical target genes in an antagonistic manner, we did not detect a significant overlap between the *ash2 *or *ash1 *and *Pc *sets of misregulated genes. We also compared our results with those obtained in larval null mutants of the *Nurf301 *subunit of the nucleosome remodeling factor complex NURF [48], and cells with reduced levels of Sin3A, a component of a multimeric histone deacetylase complex that includes Rpd3 [49]. While comparison of *ash2 *and *ash1 *transcriptomes with those from *Nurf301 *mutants did not reveal any significant overlap, intriguing coincidences were found when comparing *ash2*- and *Sin3A*-regulated genes.

**Figure 4:**
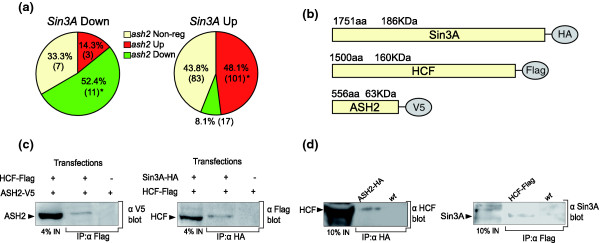
Functional and physical interaction between ASH2 and Sin3A mediated by HCF. **(a) **ASH2 and Sin3A mutants share a large number of commonly misregulated genes. Each full circle encompasses the total number of genes that are downregulated (left) or upregulated (right) in *Sin3A*-deficient cells (according to Pile *et al*. [43]) and have valid log_2 _ratios in one or both of the *ash2 *alleles. The number of genes that are downregulated (green), upregulated (red) or do not display altered expression (yellow) over 1.5 times in one or both of the *ash2 *alleles is shown in brackets. An asterisk indicates statistically significant overlap: *p *= 1.06 × 10^-4 ^(left), *p *= 5.88 × 10^-32 ^(right). **(b) **Diagram showing Sin3A-HA, HCF-Flag and ASH2-V5 fusion proteins. The number of amino acids and the predicted molecular weight without counting tags are indicated above each construct. **(c) **HCF interacts with ASH2 and Sin3A in S2 cells. Anti-Flag immunoprecipitations were performed using cells expressing HCF-Flag and ASH2-V5, or ASH2-V5 alone as a negative control, and immunoblotted with anti-V5 (left). Anti-HA immunoprecipitations were performed with S2 cells transfected with Sin3-HA and HCF-Flag, or HCF-Flag alone as a negative control, and immunoblotted with anti-Flag (right). Input lane shows 4% of the total extract volume used for co-immunoprecipitations. **(d) **HCF interacts with ASH2 and Sin3A in embryos. Co-immunoprecipitation experiments were performed in transgenic embryos overexpressing ASH2-HA (left) or HCF-Flag (right) with *daughterless-*gal4. *wt *embryos were used as a negative control. Input lane shows 10% of the total extract volume used for co-immunoprecipitations.

The genes upregulated in S2 and Kc *Drosophila *cell lines with a Sin3A deficiency generated by RNA interference are enriched with GO annotations related to the mitochondrion [49] (Additional data file 19), a trend also observed in genes upregulated in *ash2 *mutants (Figure 2a; Additional data files 6 and 10). The upregulation of genes related to mitochondria and/or ribosomes is not a general effect, since this is not observed in either the Pc and Nurf301 sets of misregulated genes [47,48] or in other mutants analyzed with our microarrays (data not shown). In addition, a gene to gene comparison between the misregulated genes for *Sin3A *and those for *ash2 *indicated that many of them tend to change in the same direction (Figure 4a; Additional data file 20). One possibility could be that *Sin3A *or another subunit of the deacetylase complex is a target of ASH2. However, from the two core members of the complex (Sin3A and Rpd3), only a slight reduction in the levels of *Sin3A *is observed in the *ash2*^112411 ^mutant allele. Moreover, RT-PCR analysis in both *ash2 *mutants confirms that there are no major differences in *Sin3A *expression levels (data not shown), suggesting that *Sin3A *and *Rpd3 *are unlikely to be ASH2 target genes. Taking into account the important differences between these arrays (wing disc versus tissue culture cells and two-color microarray platform versus Affymetrix GeneChips), the large number of commonly regulated genes between the two analyses suggests a putative functional relationship between ASH2 and Sin3A.

### ASH2 and Sin3A interact with HCF and colocalize on polytene chromosomes

In recent years, several studies in mammals have reported the presence of ASH2 in multimeric complexes homologous to the yeast Set1 complex that can methylate H3K4 [21-26,50]. Strikingly, one variant of this activation-related complex is tethered together by HCF-1 with the Sin3/histone deacetylase (HDAC) complex. The latter is associated with repression activities and includes, amongst others, Sin3, RbAp48 and the Rpd3 homologues HDAC-1 and HDAC-2 [25].

**Figure 5:**
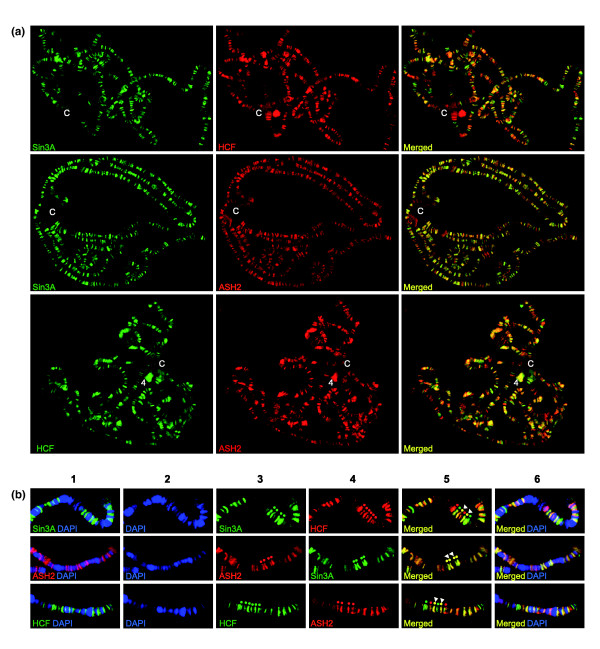
Immunodetection of ASH2, Sin3A and HCF on polytene chromosomes. **(a) **Images of all the chromosomes showing partial colocalization of Sin3A and HCF (top panel), Sin3A and ASH2 (central panel), and HCF and ASH2 (bottom panel). Note that only HCF is found in the chromocenter (C) and that no Sin3A is detected in chromosome 4 (4). **(b) **Details of the 2L chromosome arm. Sin3A, ASH2 and HCF staining does not overlap with DAPI (columns 1-3). Sin3A and HCF (top panel), ASH2 and Sin3A (central panel), and HCF and ASH2 (bottom panel) colocalize at many sites (columns 3-5). Green and red dots in columns 3-4 indicate strongly stained sites. Green and red dots in column 5 indicate a weak colocalization (more green staining or more red staining) and yellow dots indicate bands with strong colocalization. Arrowheads indicate sites of triple colocalization between ASH2, HCF and Sin3A. Antibodies used were anti-Sin3A, anti-HCF and anti-HA (for ASH2-HA detection).

To assess a putative physical interaction between ASH2 and Sin3A in *Drosophila*, we performed co-immunoprecipitation experiments in S2 cells and embryos and immunocytochemical detection of ASH2, Sin3A and HCF on salivary gland polytene chromosomes. As expected for proteins that regulate transcription, all three were located in the nucleus (Additional data file 24) and co-immunoprecipitation experiments demonstrated that HCF binds both Sin3A and ASH2 (Figure 4b-d). The interaction between HCF and ASH2 is easily detected but the interaction between HCF and Sin3A is only visible by a faint band in the embryo extract. Moreover, the HCF-ASH2 interaction seems to be more stable than that of HCF-Sin3A, since the former resists more stringent conditions in terms of lysis buffer composition (see Materials and methods). Although we were not able to co-immunoprecipitate Sin3A and ASH2, we cannot rule out the possibility that they are tethered together by HCF, as occurs in their human counterparts [25]. Although this putative association of ASH2 and Sin3A through HCF might only happen in certain cell contexts, as previously suggested in humans [25], it is supported by the results of immunohistochemistry on polytene chromosomes showing that these proteins colocalize at several loci (Figure 5a,b). ASH2 binding patterns are highly coincident with those of Sin3A and HCF and all of them are non-overlapping with DAPI counterstaining, with the exception of HCF at the chromocenter. Comparison of bands bound by Sin3A, HCF and ASH2 revealed that some sites are shared between all three (Figure 5b), suggesting the existence of a complex containing these proteins. Although it might seem surprising that proteins with opposite transcriptional outcomes might be found in the same complex, this is not an exception. For example, the trxG human Brahma nucleosome remodelling complex (hBrm) contains Sin3, HDAC-1, HDAC-2 and RpAb48, all of them proteins from the human Sin3 histone deacetylase complex [51]. Furthermore, these four proteins and hBrm are also found in the ALL-1 (acute lymphoblastic leukemia - 1) complex, which exhibits chromatin remodeling and histone acetylation, deacetylation and methylation activities [52].

### ASH2 is required for trimethylation of H3K4

**Figure 6:**
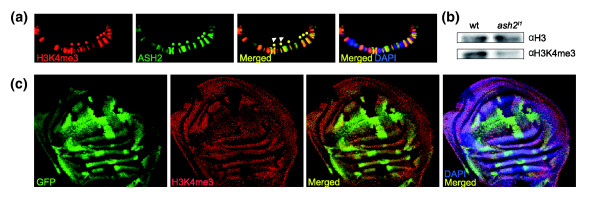
H3K4 trimethylation is severely reduced in *ash2*^*I*1^mutants. **(a) **Colocalization of trimethylated H3K4 (red) with ASH2 (green) in the polytene chromosome 2L arm. **(b) **Detection by western blot of H3K4 trimethylation on *wt *and *ash2*^*I*1 ^histone extracts. H3 was used as a loading control. **(c) **Detection of H3K4 trimethylation (red) in *wt *(bright green), heterozygous (green) and *ash2*^*I*1 ^homozygous (black) wing disc cells. Note that in the latter cells H3K4 trimethylation is severely reduced. Antibodies used were anti-HA (for ASH2-HA detection), anti-trimethylated H3K4 and anti-H3. Nuclei are labelled with DAPI (blue).

Consistent with the H3K4 methyltransferase activity reported for the Set1/ASH2 complex in humans [25], we found that ASH2 colocalizes on polytene chromosomes with trimethylated H3K4 (Figure 6a). To gain insight into the molecular function of ASH2, we compared the levels of H3K4 trimethylation in histones purified from *wt *and *ash2*^*I*1 ^mutant flies, respectively, by western blot (Figure 6b). The lack of ASH2 results in a dramatic reduction of H3K4 trimethylation, indicating that ASH2 is involved in this modification. Analysis of H3K4 trimethylation *in vivo *confirmed the results, since this epigenetic mark was below detectable levels in *ash2*^*I*1 ^mutant clones (Figure 6c). The results presented here are the first obtained in *Drosophila *that show that ASH2 plays a role in the chromatin modification machinery. Although the nature of this regulation remains unclear, the fact that ASH2 does not have any known domain with histone methyltransferase activity suggests that it could be acting in a complex as a structural platform to facilitate the interaction between nucleosomes and proteins with enzymatic activity. This hypothesis is supported by the presence in ASH2 of a PHD domain, which has the ability to bind methylated lysines [53]. However, other proteins might act to recognize and present H3 in a similar way to human WDR5 [54,55].

## Conclusion

The evidence presented here suggests that, like in humans, *Drosophila *might harbor an ASH2 containing complex that could be tethered together with Sin3A by HCF. Although generally associated with opposite transcriptional activities, it has been proposed that these proteins may cooperate in transcriptional regulation [25]. The Sin3A complex is generally involved in transcriptional silencing, but in at least one case it is necessary for the activation of certain genes [56]. Rpd3 is also bound to active promoters in *Drosophila *[57], and in *S*. *cerevisiae *it is preferentially associated with promoters that direct high transcriptional activity [58]. Likewise, it is feasible that canonical positive regulators such as ASH2 may also play a role as negative regulators in certain contexts, and finding so many upregulated genes related to mitochondria in *ash2 *mutants makes this idea attractive. An appealing hypothesis to explain the existence of positive and negative regulators of chromatin remodeling in the same complex is that they could be necessary to maintain an equilibrium between the different histone modifications and to rapidly switch between activated or repressed states of transcription. Alternatively, the activities of these complexes are perhaps sequential, since some deacetylation seems to be required to enable histone methylation [59]. Another explanation for the coexistence of different modifying activities is that they could be targeted to transcription factors, as occurs with the *Drosophila *T-cell factor, which can be repressed by acetylation driven by dCBP, a protein that, paradoxically, acts as a co-activator of other transcription factors [60]. For example, dCBP interacts and colocalizes with ASH1 on polytene chromosomes [61] and is also found with Trithorax (Trx) in the activating TAC1 acetylation complex [62]. Those observations have further implications since *ash1 *and *trx*, together with *ash2*, were found to be functionally related [63] and several other lines of evidence point in the same direction. ASH1 and Trx co-immunoprecipitate in embryonic extracts and colocalize on polytene chromosomes [64], and accumulation of Trx is reduced on *ash1 *mutant polytene chromosomes [65]. ASH1 is an H3K4, H3K9 and H4K20 histone methyltransferase [27,28] and H3K4 dimethylation is severely reduced in *ash1*-mutant polytene chromosomes but only mildly in *ash2*-mutant chromosomes [28].

Byrd and Shearn [28] proposed a model to explain the coordinated action of the ASH1, Trx and ASH2 proteins. In that model, ASH1 and Trx would be required to acetylate and mono- and/or dimethylate histones, and a putative Set1-ASH2 complex would be involved in subsequent H3K4 methylations. This hypothesis is strongly supported by the demonstration that the yeast and human ASH2 homologues are essential for H3K4 trimethylation but not for mono- or dimethylation [26,66,67]. In addition, ASH2 accumulation is reduced on *ash1*-mutant polytene chromosomes but in the reverse situation ASH1 accumulation is not reduced on *ash2*-mutant chromosomes [17], indicating that ASH2 needs ASH1 or its activity to be recruited. Our results also support this model. Firstly, the expression profiles from *ash2 *and *ash1 *mutant alleles reveal that they are highly coincident at both the gene to gene and functional levels, indicating a functional relationship between them. Secondly, the results from co-immunoprecipitation assays, immunolocalization on polytene chromosomes and the reduction of H3K4 trimethylation in *ash2*^*I*1 ^mutant cells suggest the existence of a Set1-ASH2 complex in *Drosophila *involved in H3K4 trimethylation, filling a gap in the proposed model. Finally, since HCF interacts with both ASH2 and Sin3A, it is tempting to speculate that, in *Drosophila*, these proteins could also be subunits of multimeric complexes similar to those found in mammals. However, the fact that the genes in common between *ash2 *and *ash1 *mutants differ from those that are common between *ash2 *mutants and *Sin3A*-defficient cells suggests that different processes such as pattern formation, development or general metabolism may be regulated by ASH2 upon its association with distinct complexes.

## Materials and methods

### *Drosophila *strains

All *Drosophila *strains and crosses were kept on standard media with 0.025% bromophenol blue at 25°C. The reference line used in all cases was the *w*^1118^;+;+ isogenic line from the DrosDel Collection [68]. To reduce the differences in the biological background between the alleles under study and the reference strain, we transferred chromosomes X, Y and 2 from the isogenic line to the TM6c-balanced *ash2*^*I*1^, *ash2*^112411 ^and *ash1*^22 ^alleles. The latter was provided by A Shearn and was studied in homozygosis by combining it with *ash1*^22^F2A, a kind gift from J Muller [29].

To generate UAS-*ash2 *transgenic flies, full-length *ash2-RC *and three copies of the HA (hemagglutinin) epitope were cloned between the *Eco*RI and *Not*I sites of the pUAST plasmid [69]. To generate UAS-*HCF *transgenic flies, full-length *HCF-RC *amplified from the pACXT-T7-*HCF*-Flag (kindly provided by A Wilson [70]) was introduced between the *Not*I and *Kpn*I sites of the pUAST plasmid [69]. *MS1096*-Gal4 and *nub*-Gal4 drivers were used for rescue experiments, *sgs3*-Gal4 and *Act5C*-Gal4 for polytene chromosome immunolocalization and *da*-Gal4 for co-immunoprecipitation experiments in embryos.

### Two-color microarray production, sample preparation and hybridization

Microarrays were printed on Corning UltraGAPS slides (Corning, NY, USA) at the Plataforma de Transcriptòmica (SCT-PCB, Universitat de Barcelona, Spain) using the *Drosophila *Genome Oligo Set version 1.1 (Operon Biotechnologies Inc., Huntsville, AL, USA), a collection of 14,593 probes representing 13,577 *Drosophila *genes with Flybase ID. The 70mer *Arabidopsis *sequences from TIGR [71] and spots with no material or with buffer were also printed to be used as *spike-in *and negative controls, respectively. The microarray annotation is deposited in the Gene Expression Omnibus (GEO) database [35,36] with number GPL3797.

Wandering blue-gut staged Tb+ early third instar larvae were selected in all cases to extract total RNA from wing discs using the RNeasy Protect Mini Kit (Qiagen Inc., Valencia, CA, USA). At least two independent total RNA extractions were carried out from wing imaginal discs for *w*^1118^;+;*ash2*^*I*1^, *w*^1118^;+;*ash2*^112411^, *w*^1118^;+;*ash1*^22^/*ash1*^22^F2A and *w*^1118^;+;+ strains. Quality was assessed in all samples using the Eukaryote Total RNA Nano Assay on a 2100 Bioanalyzer (Agilent Technologies Inc., Santa Clara, CA, USA). Total RNA from *w*^1118^;+;+ wing imaginal discs was pooled and used as a common reference against *ash2*^*I*1^, *ash2*^112411 ^and *ash1*^22^/*ash1*^22^F2A. Four microarrays were hybridized for each experiment in biological replicate pairs, such that the total RNA from the sample used as a starting material came from different extractions. Both arrays from each pair were hybridized with the same amplified RNA from sample and common reference (obtained using the Amino-Allyl Messageamp II aRNA Amplification Kit from Ambion Inc., Austin, TX, USA) but with dyes Cy3 and Cy5 (GE Healthcare UK Ltd, Buckinghamshire, UK) swapped to take dye-bias into account.

### Two-color microarray analysis and data mining

GenePix Results (GPR) data files were obtained for each microarray with an Axon 4000B scanner and GenePix Pro 6 (Molecular Devices Corp., Sunnyvale, CA, USA). All GPR files were analyzed with the Limma package from BioConductor [72,73] using the same criteria. The spots not fulfilling the quality thresholds (based on spot size, foreground versus background signals, saturation, coincidence between differently calculated ratio measures and R^2 ^of regression ratio) were eliminated from the analysis, and the data were background corrected with the *normexp *method and normalized using OLIN [74]. Between-array normalization was carried out independently for each set of four arrays using the *mad *method from OLIN, and a linear model was fitted and corrected with False Discovery Rate (FDR) [72]. We obtained lists of genes that were differentially expressed (had an FDR-corrected *p *value of less than 0.05 and at least two spots from the four replicate arrays that passed the quality filters) over 1.5- or 2.0-fold in the mutants compared to the reference strain.

A script was written to collect all GO terms for each gene on the array and to assess whether they are enriched with regulated genes by using a hypergeometric distribution. *P *values of each term are adjusted with the FDR procedure [75] to correct for multiple testing. The output from that script can be used as input for another one that draws pie charts (available at [76]). Calculations of the statistical significance of the number of overlapping genes between different sets were also corrected for multiple testing in a similar way.

### Affymetrix array analysis

Two Affymetrix GeneChip *Drosophila *Genome 2.0 arrays (Affymetrix Inc.) were hybridized at the Unitat de Genòmica (IDIBAPS, Barcelona, Spain) with amplified RNA from wing imaginal discs of either *ash2*^*I*1 ^or the common reference. Analysis was performed with the Affymetrix GeneChip Operating Software (GCOS) and only spots called 'Present' (detectable expression) were taken into account for calculation of Pearson's correlation coefficients. The number of spots involved in each pairwise comparison in Figure 1b (from left to right and top to bottom) were 7,817, 7,170, 6,147, 9,051 and 6,436.

### Semi-quantitative RT-PCR

Reverse transcription reactions with independently extracted RNA from all mutant alleles and the reference were used to synthesize cDNA with Superscript III (Invitrogen Corp., Carlsbad, CA, USA) according to the manufacturer's instructions. One microliter of each RT reaction was used in each PCR reaction, in which the test and the reference gene (*mRpL9*) were amplified together. Samples from 22, 26, 30 and 34 cycles were run on 2% agarose gels to estimate saturating conditions. Samples with two discrete non-saturated bands were quantified using a DNA 1000 Assay in a 2100 Bioanalyzer (Agilent Technologies Inc., Santa Clara, CA, USA). Expression values of test genes were normalized using the reference gene. Four measures were taken for each gene to evaluate standard error.

### Clonal analysis, immunohistochemistry and TUNEL assay

Clones were generated at 52 ± 6 hours after egg lay by FLP (Flipase)-mediated mitotic recombination [77] with a 30 minute heat shock at 37°C. Immunohistochemistry was performed according to standard protocols with the primary antibodies mouse 4D9 anti-En/Inv (Developmental Studies Hybridoma Bank, University of Iowa, IA, USA), rabbit anti-Vg (kindly provided by S Carroll), rabbit anti-cleaved Caspase 3 (Cell Signalling Technology Inc., Danvers, MA, USA) and rabbit anti-trimethyl H3K4 (Abcam Inc., Cambridge, MA, USA) used at concentrations of 1:10, 1:20,1:100 and 1:200, respectively. Rhodamine Red-conjugated secondary antibodies were from Jackson ImmunoResearch Laboratories (West Grove, PA, USA). Dying cells were detected by TUNEL assay, labeling DNA 3'-OH ends with Chromatide BODIPY Texas Red-14-dUTP (Invitrogen Corp.) for 1.5 hours at 37°C with terminal deoxynucleotidyl transferase (Roche Diagnostics, Indianapolis, IN, USA). In all cases, the discs were mounted in Slowfade Light Antifade (Invitrogen Corp.) and images collected using a Leica TCS 4D confocal laser scanning microscope.

### Constructs, cellular localization, co-immunoprecipitation assays and western blot

S2 cells were maintained at 25°C in *Drosophila *Schneider's medium containing 10% fetal bovine serum and 1% penicillin/streptomycin (Invitrogen Corp.). Cells were transfected with Effectene (Qiagen Inc.), according to the manufacturer's protocol and collected 48 hours later. Three different constructs were used containing full-length coding regions of either *ash2*, *Sin3A *or *HCF*. pAc5.1-*ash2*-V5-His A was produced by inserting *ash2*, obtained from the Berkeley *Drosophila *Genome Project clone LD31680, into pAc5.1-V5-His A (Invitrogen Corp.). pAc5.1-*Sin3A*-HA-His B was produced by inserting *Sin3A*, obtained from the p3NB-*Sin3A *plasmid provided by D Pauli [78], into a pAc5.1-V5-His B in which V5 was substituted for HA.

For immunofluorescence analysis, transfected S2 cells were plated onto coverslips previously coated with 0.5 mg/ml concanavalin A for 1 hour and fixed with 4% paraformaldehyde. The antibodies used were as follows: rabbit anti-V5 (1:1,000; Sigma-Aldrich Corp., St Louis, MO, USA), mouse anti-HA (1:200; Roche Diagnostics), rabbit anti-Flag (1:100; Sigma-Aldrich Corp.), donkey anti-rabbit FITC (1:400; Jackson ImmunoResearch Laboratories), and goat anti-mouse FITC (1:400; Jackson ImmunoResearch Laboratories). Rhodamine phalloidin (Invitrogen Corp.) was used at a dilution of 1:40. Cell nuclei were stained with DAPI. Cells were mounted in Mowiol-Dabco and visualized under a Leica spectral microscope.

For co-immunoprecipitation assays, cells were washed twice with cold phosphate-buffered saline and lysed and disrupted by freezing and thawing cycles. For anti-Flag immunoprecipitation, the lysis buffer was 50 mM Tris HCl, pH 7.4, 150 mM NaCl, 1 mM EDTA, 1% Triton X-100 + complete protease inhibitors (Roche Diagnostics). The whole-cell extract was pre-cleared for 1 hour at 4°C with protein A-Sepharose and incubated with 40 μl of equilibrated EZview Red anti-Flag M2 AffinityGel beads (Sigma-Aldrich Corp.) for at least 3 hours at 4°C. The immunoprecipitates were washed three times for 15 minutes in lysis buffer (in the second wash the concentration of NaCl was increased to 300 mM) and eluted by boiling in loading buffer containing SDS. For anti-HA immunoprecipitations, we used the protocol described above with the following modifications: cells were lysed and washed in 20 mM HEPES, pH 7.9, 20% glycerol, 100 mM NaCl, 0.2 mM EDTA, 0.1% NP-40 + complete protease inhibitors and cell extracts were incubated without pre-clearing with 75 μl of anti-HA affinity matrix (Roche Diagnostics). For co-immunoprecipitation experiments from embryo extracts, 0-16 h embryos were dechorionated and rinsed extensively, incubated with 50 mM Tris pH 8, 200 mM NaCl, 5 mM EDTA, 0.5% NP40 + complete protease inhibitors, homogenized and centrifuged. The supernatant was collected, sonicated and mixed with 40 μl of EZview Red anti-Flag M2 AffinityGel beads or anti-HA affinity matrix to perform the co-immunoprecipitation experiments as described above. In all cases, samples were run on 7.5-10% SDS-PAGE gels and transferred to PVDF membranes. The following antibodies from Sigma-Aldrich Corp. were used for western blots: rabbit anti-V5 (1:2,000), rabbit anti-Flag (1:400), sheep anti-mouse peroxidase (1:3,000) and goat anti-rabbit peroxidase (1:3,000). The mouse anti-HA (1:1,000) was from Roche Diagnostics and rabbit anti-Sin3A (1:500) and rabbit anti-HCF (1:5,000) were kindly provided by D Wassarman and J Workman, respectively. Proteins were detected with the EZL system (Biological Industries Ltd., Kibbutz Beit Haemek, Israel).

To compare the levels of trimethylated H3K4 between *wt *and *ash2*^*I*1 ^larvae, histones were obtained by acidic extraction, dialyzed and separated on 15% SDS-PAGE gels. Immunoblots were performed with anti-H3 (1:2,000) and anti-trimethyl H3K4 (1:5,000) antibodies (Abcam Inc.).

### Staining of polytene chromosomes

Salivary glands of *wt *and UAS-*ash2 *transgenic flies were dissected in Gohen buffer, fixed for 2 minutes and transferred to a solution with acetic acid and formaldehyde for 3 minutes before squashing to spread the polytene chromosomes. Staining was performed by O/N incubation at 4°C with the following antibodies: mouse anti-HA (1:200), rabbit anti-Sin3A (1:100), rat anti-HCF (1:50) and rabbit anti-trimethyl H3K4 (1:200). FITC- or rhodamine red-conjugated secondary antibodies (1:200) were obtained from Jackson ImmunoResearch Laboratories and preparations were mounted with Mowiol-DAPI to stain the DNA. Chromosomes were visualized under a Leica microscope.

## Additional data files

The following additional data are available with the online version of this paper. Additional data file 1 is a table listing the number of unique genes up- and downregulated over 1.5-fold (log_2 _ratio = 0.58) or 2.0-fold (log_2 _ratio = 1) in each mutant allele. Additional data files 2, 3 and 4 are tables listing the genes downregulated and upregulated over 1.5- and 2.0-fold in *ash2*^*I*1^, *ash2*^112411 ^and *ash1*^22^, respectively. Additional data files 5, 9 and 13 are tables containing the GO annotations of the genes downregulated over 1.5-fold in *ash2*^*I*1^, *ash2*^112411 ^and *ash1*^22^, respectively. Additional data files 6, 10 and 14 are tables containing the GO annotation of the genes upregulated over 1.5-fold in *ash2*^*I*1^, *ash2*^112411 ^and *ash1*^22^, respectively. Additional data files 7, 11 and 15 are tables containing the GO annotation of the genes downregulated over 2.0-fold in *ash2*^*I*1^, *ash2*^112411 ^and *ash1*^22^, respectively. Additional data files 8, 12 and 16 are tables containing the GO annotations of the genes upregulated over 2.0-fold in *ash2*^*I*1^, *ash2*^112411 ^and *ash1*^22^, respectively. Additional data file 17 is a table containing the GO annotations of wing disc genes identified by Klebes *et al*. [37]. Additional data file 18 is a table listing the genes misregulated in *ash2 *and *ash1 *mutants preferentially expressed in the wing disc and in the prospective wing-hinge or body wall region. Additional data file 19 is a table containing the GO annotations of the genes upregulated in *Sin3A *deficient cells according to Pile *et al*. [49]. Additional data file 20 is a table listing the genes misregulated in *Sin3A *deficient cells that are also misexpressed in *ash2 *and/or *ash1 *mutants. Additional data file 21 is a table containing the FDR adjusted *p *values for the GO terms displayed in Figure 2a. Additional data file 22 is a figure containing pie charts showing the distribution of regulated genes in Molecular function GO classes. Additional data file 23 is a figure containing pie charts showing the distribution of regulated genes in Biological process GO classes. Additional data file 24 is a figure showing that ASH2, HCF and Sin3A are found in the nucleus.

## Supplementary Material

Additional data file 1 Unique genes up- and downregulated over 1.5-fold (log_2 _ratio = 0.58) or 2.0-fold (log_2 _ratio = 1) in each mutant allele Click here for file

Additional data file 2 Genes downregulated and upregulated over 1.5- and 2.0-fold in *ash2*^*I*1^ Click here for file

Additional data file 3 Genes downregulated and upregulated over 1.5- and 2.0-fold in *ash2*^112411^ Click here for file

Additional data file 4 Genes downregulated and upregulated over 1.5- and 2.0-fold in *ash1*^22^ Click here for file

Additional data file 5 GO annotations of the genes downregulated over 1.5-fold in *ash2*^*I*1^ Click here for file

Additional data file 6 GO annotations of the genes upregulated over 1.5-fold in *ash2*^*I*1^ Click here for file

Additional data file 7 GO annotations of the genes downregulated over 2.0-fold in *ash2*^*I*1^ Click here for file

Additional data file 8 GO annotations of the genes upregulated over 2.0-fold in *ash2*^*I*1^ Click here for file

Additional data file 9 GO annotations of the genes downregulated over 1.5-fold in *ash2*^112411^ Click here for file

Additional data file 10 GO annotations of the genes upregulated over 1.5-fold in *ash2*^112411^ Click here for file

Additional data file 11 GO annotations of the genes downregulated over 2.0-fold in *ash2*^112411^ Click here for file

Additional data file 12 GO annotations of the genes upregulated over 2.0-fold in *ash2*^112411^ Click here for file

Additional data file 13 GO annotations of the genes downregulated over 1.5-fold in *ash1*^22^ Click here for file

Additional data file 14 GO annotations of the genes upregulated over 1.5-fold in *ash1*^22^ Click here for file

Additional data file 15 GO annotations of the genes downregulated over 2.0-fold in *ash1*^22^ Click here for file

Additional data file 16 GO annotations of the genes upregulated over 2.0-fold in *ash1*^22^ Click here for file

Additional data file 17 GO annotations of wing disc genes identified by Klebes *et al*. [37] Click here for file

Additional data file 18 Genes misregulated in *ash2 *and *ash1 *mutants preferentially expressed in the wing disc and in the prospective wing-hinge or body wall region Click here for file

Additional data file 19 GO annotations of the genes upregulated in *Sin3A *deficient cells according to Pile *et al*. [49] Click here for file

Additional data file 20 Genes misregulated in *Sin3A *deficient cells that are also misexpressed in *ash2 *and/or *ash1 *mutants Click here for file

Additional data file 21 FDR adjusted *p *values for the GO terms displayed in Figure 2a Click here for file

Additional data file 22 Distribution of regulated genes in Molecular function GO classes Click here for file

Additional data file 23 Distribution of regulated genes in Biological process GO classes Click here for file

Additional data file 24 ASH2, HCF and Sin3A are found in the nucleus Click here for file
