# Supplementary material for: Functional dissection of the ash2 and ash1 transcriptomes provides insights into the transcriptional basis of wing phenotypes and reveals conserved protein interactions
Source: Genome Biol. 2007 Apr 28;8(4):R67. doi: 10.1186/gb-2007-8-4-r67 (PMC1896016; doi:10.1186/gb-2007-8-4-r67)
Supplement: Additional data file 1 — Unique genes up- and downregulated over 1.5-fold (log2 ratio = 0.58) or 2.0-fold (log2 ratio = 1) in each mutant allele [file gb-2007-8-4-r67-S1.pdf]

**Number of Unique Genes**	***ash2*^{I1}**	***ash2*^{112411}**	***ash1*^{22}**
$\log_2$ratio > 1	**179**	**272**	**59**
$\log_2$ratio <-1	**178**	**174**	**22**
$\log_2$ratio > 0.58	**426**	**782**	**194**
$\log_2$ratio <-0.58	**526**	**939**	**115**
