# Supplementary material for: Functional dissection of the ash2 and ash1 transcriptomes provides insights into the transcriptional basis of wing phenotypes and reveals conserved protein interactions
Source: Genome Biol. 2007 Apr 28;8(4):R67. doi: 10.1186/gb-2007-8-4-r67 (PMC1896016; doi:10.1186/gb-2007-8-4-r67)
Supplement: Additional data file 5 — GO annotations of the genes downregulated over 1.5-fold in ash2I1 [file gb-2007-8-4-r67-S5.html]

  

---

  

|  |  |
| --- | --- |
| Go Statistics | Reg File:**ash2I1\_D1.5x.txt.fbgns** (526 genes -- 86 skipped)  Ref File: **ref.fbgns** (13577 genes -- 4663 skipped)  Database: **go\_200507-termdb.rdf-xml** |

---

  

Fields Description

| Pos | Go Term | Ontology | Levels | Observed | Expected | Possibles | p-value(Adj) | Go term description | Genes with the GO term |
| --- | --- | --- | --- | --- | --- | --- | --- | --- | --- |
| 1 | GO:0007275 | P | 2, | 149 | 73.300 (x 2.033) | 1485 (0.100) | 2.51e-16 | development | 18w Abi Amph Ance Arf79F CG1632 CG17084 CG30011 CG3770 CG40410 CG5096 CG6965 CG7860 CG9211 Cad96Ca Cdk4 Cip4 CkIIalpha CkIIbeta Crc CrebA CtBP CycT Dl Doc1 Doc2 Doc3 Dp Dr Eip71CD Eip74EF Eip75B Eph Fas3 Fs(2)Ket Galpha49B HLHm7 Hrb27C Hsp26 Hsp27 Ice ImpE2 ImpE3 InR LanB2 Optix Pi3K21B Poxn Pten Ptp99A S Sb Sema-1a Sema-1b Sema-2a Sry-alpha TER94 Tl Trl Wnt2 a6 ana ap ara arr ash2 asp betaTub56D blot bnb br btsz bun cdc2 cg ci cib cic ck cyc dap dve eIF-4E ed edl emp en esn fax fra fwd glec glu grh grn hbs heph hth in inv inx2 jumu kis klu knk l(2)01424 lid lola malpha mbt mod(mdg4) mth ninA nmo nonA-l numb ogre opa otk pbl pdm2 ph-p pim pk pll polo pon rasp rost sc serpin-27A sgl shanti shf smi35A spen sqd stai stan th tkv toe tok trh trn tws usp vg wbl |
| 2 | GO:0048731 | P | 3, | 72 | 29.320 (x 2.456) | 594 (0.121) | 2.8e-10 | system development | CG7860 CG9211 Cad96Ca Cdk4 CkIIbeta Crc CrebA Dl Dr Eip71CD Eip74EF Eip75B Eph Fas3 Galpha49B HLHm7 InR Poxn Pten Ptp99A S Sema-1a Sema-1b Sema-2a Tl Wnt2 ana ap ara bnb br bun cdc2 cib cyc dap eIF-4E ed emp en fax fra glec glu grh hbs hth inv jumu l(2)01424 lola mbt ninA numb ogre otk pbl pdm2 pon sc sgl shanti shf smi35A spen stai stan tkv toe trh trn usp |
| 3 | GO:0048513 | P | 3, | 78 | 33.072 (x 2.359) | 670 (0.116) | 3.21e-10 | organ development | Amph CG30011 CG40410 CG7860 CkIIalpha CkIIbeta CrebA Dl Doc1 Dr Eip71CD Eip74EF Eip75B Galpha49B HLHm7 Ice ImpE2 ImpE3 InR LanB2 Optix Poxn Pten S Sb Sema-2a TER94 Tl Wnt2 ap ara arr ash2 betaTub56D br bun cg ci cib ck dap dve eIF-4E ed edl emp en grh grn hbs heph hth in inv inx2 jumu klu l(2)01424 malpha mbt nmo nonA-l numb opa pbl pim pk pll rost sc sgl stan th tkv trh tws usp vg |
| 4 | GO:0009653 | P | 3, | 75 | 31.689 (x 2.367) | 642 (0.117) | 4.21e-10 | morphogenesis | 18w Abi Amph Ance Arf79F CG3770 Cdk4 CycT Dl Doc1 Dp Dr Eph Fas3 Fs(2)Ket Galpha49B Ice ImpE2 ImpE3 InR LanB2 Pi3K21B Poxn Pten Ptp99A S Sb Sema-1a Sema-1b Sema-2a TER94 ap ara arr ash2 blot br btsz bun cg cib ck dve eIF-4E edl fax fra grh grn heph hth in inx2 jumu klu lola mbt ninA nmo numb otk pbl ph-p pim pk sc sgl spen sqd stan th tkv trh usp vg |
| 5 | GO:0007399 | P | 4, | 57 | 23.101 (x 2.467) | 468 (0.122) | 5.44e-08 | nervous system development | CG9211 Cad96Ca CkIIbeta Crc Dl Dr Eph Fas3 Galpha49B HLHm7 InR Poxn Ptp99A S Sema-1a Sema-1b Sema-2a Tl ana ap ara bnb br bun cdc2 cib cyc ed en fax fra glec glu grh hbs hth inv jumu lola mbt ninA numb ogre otk pbl pdm2 pon sc shf smi35A spen stai stan tkv toe trh usp |
| 6 | GO:0050789 | P | 2, | 126 | 74.288 (x 1.696) | 1505 (0.084) | 7.35e-08 | regulation of biological process | Aac11 Abi Amph B52 BEST:GH02921 CG11079 CG14217 CG15835 CG30011 CG3136 CG33525 CG3424 CG40160 CG40410 CG4914 CG6654 CG7379 CG8092 CG8165 CG9211 CREG Caf1 Cdk4 Cks CrebA CtBP CycB3 CycT D19A Dl Doc1 Doc2 Doc3 Dp Dr Dref Dsp1 Eb1 Eip74EF Eip75B GATAd HLHm7 HmgD HmgZ Hrb27C Hus1-like Ice InR Jafrac2 Nek2 Optix Pi3K21B Poxn Pten Rab8 RacGAP50C Rbf2 Rm62 Rpn9 S TER94 Thd1 Tl Trl ald alphaTub84B ana ap ara ash2 br btsz bun cdc2 cg charybde ci cic cyc dap dve eIF-4E ed edl en for fwd fzy grh grn hth inv jumu kis klu l(2)01424 lid lola mbt mdy mod(mdg4) nmo numb opa pAbp pbl pdm2 ph-p pll sc scylla serpin-27A shanti smi35A spen sqd stan th tkv toe trh tws usp woc zf30C zwilch |
| 7 | GO:0050791 | P | 3, | 113 | 66.094 (x 1.710) | 1339 (0.084) | 5.04e-07 | regulation of physiological process | Aac11 Abi Amph B52 BEST:GH02921 CG11079 CG14217 CG15835 CG30011 CG3136 CG33525 CG40160 CG40410 CG4914 CG6654 CG7379 CG8092 CG8165 CREG Caf1 Cdk4 Cks CrebA CtBP CycB3 CycT D19A Doc1 Doc2 Doc3 Dp Dr Dref Dsp1 Eb1 Eip74EF Eip75B GATAd HLHm7 HmgD HmgZ Hrb27C Hus1-like Ice InR Jafrac2 Nek2 Optix Pi3K21B Poxn Pten Rab8 Rbf2 Rm62 Rpn9 TER94 Thd1 Tl Trl ald alphaTub84B ana ap ara ash2 br bun cdc2 cg ci cic cyc dap dve eIF-4E edl en for fwd fzy grh grn hth inv jumu kis klu l(2)01424 lid lola mdy mod(mdg4) nmo opa pAbp pdm2 ph-p pll sc serpin-27A shanti smi35A spen sqd th tkv toe trh tws usp woc zf30C zwilch |
| 8 | GO:0050794 | P | 3, | 115 | 67.969 (x 1.692) | 1377 (0.084) | 5.61e-07 | regulation of cellular process | Aac11 Abi B52 BEST:GH02921 CG11079 CG14217 CG15835 CG30011 CG3136 CG33525 CG40160 CG40410 CG4914 CG6654 CG7379 CG8092 CG8165 CG9211 CREG Caf1 Cdk4 Cks CrebA CtBP CycB3 CycT D19A Dl Doc1 Doc2 Doc3 Dp Dr Dref Dsp1 Eb1 Eip74EF Eip75B GATAd HLHm7 HmgD HmgZ Hus1-like Ice InR Jafrac2 Nek2 Optix Pi3K21B Poxn Pten Rab8 RacGAP50C Rbf2 Rm62 Rpn9 S Thd1 Tl Trl ald alphaTub84B ana ap ara ash2 br bun cdc2 cg ci cic cyc dap dve eIF-4E ed edl en fwd fzy grh grn hth inv jumu kis klu l(2)01424 lid lola mbt mdy mod(mdg4) nmo numb opa pAbp pdm2 ph-p sc shanti smi35A spen sqd stan th tkv toe trh tws usp woc zf30C zwilch |
| 9 | GO:0051244 | P | 4, | 106 | 64.021 (x 1.656) | 1297 (0.082) | 8.84e-06 | regulation of cellular physiological process | Aac11 Abi B52 BEST:GH02921 CG11079 CG14217 CG15835 CG30011 CG3136 CG33525 CG40160 CG40410 CG4914 CG6654 CG7379 CG8092 CG8165 CREG Caf1 Cdk4 Cks CrebA CtBP CycB3 CycT D19A Doc1 Doc2 Doc3 Dp Dr Dref Dsp1 Eb1 Eip74EF Eip75B GATAd HLHm7 HmgD HmgZ Hus1-like Ice InR Jafrac2 Nek2 Optix Pi3K21B Poxn Pten Rab8 Rbf2 Rm62 Rpn9 Thd1 Trl ald alphaTub84B ana ap ara ash2 br bun cdc2 cg ci cic cyc dap dve eIF-4E edl en fwd fzy grh grn hth inv jumu kis klu l(2)01424 lid lola mdy mod(mdg4) nmo opa pAbp pdm2 ph-p sc shanti smi35A spen sqd th tkv toe trh tws usp woc zf30C zwilch |
| 10 | GO:0007155 | P | 3, | 38 | 14.956 (x 2.541) | 303 (0.125) | 1.93e-05 | cell adhesion | 18w Arf79F CG12199 CG16974 CG17419 CG33171 CG4054 CG5096 CG9211 Cad74A Cad87A Cad96Ca Dl Fas3 LanB2 PFE Pten Rapgap1 Sema-1b Sema-2a Tl Tsp26A Tsp39D Tsp66E Tsp96F ed emp fra glec hbs kal-1 ninA otk p120ctn pbl shf stan trn |
| 11 | GO:0007154 | P | 3, | 117 | 74.880 (x 1.563) | 1517 (0.077) | 2.95e-05 | cell communication | 18w Abi Amph Arf79F CG11033 CG11438 CG12199 CG14217 CG15835 CG16974 CG17064 CG17084 CG17493 CG17919 CG2108 CG30440 CG32158 CG33171 CG40410 CG4054 CG5096 CG5522 CG6954 CG6965 CG9066 CG9211 CaMKI Cad74A Cad87A Cad96Ca Cdk4 Cip4 CkIIalpha CkIIbeta Dl ETH Eip75B Eph Galpha49B InR LanB2 M6 Mpk2 Nek2 Nep2 PFE Pi3K21B Pli Pten Rab8 RacGAP50C Rapgap1 Rhp S Sema-1a Sema-1b Sema-2a Snap Tl Tsp26A Tsp39D Tsp66E Tsp96F Wnt2 arr br bun ced-6 ci cyc ed edl fax for fra fwd hbs heph inx2 inx3 kal-1 klu knk lola malpha mav mbt mod(mdg4) mth nmo numb ogre olf413 ome otk p120ctn pAbp para pbl pll polo rasp scf serpin-27A sgl shf spen stai stan tkv trh trn tws usp vimar wbl wgn |
| 12 | GO:0009887 | P | 4, | 39 | 16.585 (x 2.352) | 336 (0.116) | 8.47e-05 | organ morphogenesis | Amph Dl Doc1 Dr Ice ImpE2 ImpE3 LanB2 Poxn Pten S Sb TER94 ap ara arr ash2 br bun cg dve grn heph hth in inx2 jumu klu mbt nmo numb pbl pim pk sgl stan tkv trh vg |
| 13 | GO:0048699 | P | 6, | 26 | 8.885 (x 2.926) | 180 (0.144) | 0.000109 | generation of neurons | Dr Eph Fas3 Galpha49B InR Ptp99A Sema-1a Sema-1b Sema-2a ana ap cdc2 ed fax fra jumu lola ninA numb otk pbl pon sc spen stan usp |
| 14 | GO:0007444 | P | 4, | 36 | 14.907 (x 2.415) | 302 (0.119) | 0.000116 | imaginal disc development | Amph CG40410 Dl Dr ImpE2 ImpE3 InR Poxn S Sb TER94 ap ara arr ash2 br bun cg ci ck dve en heph hth in inv jumu klu mbt nmo pk stan th tkv tws vg |
| 15 | GO:0030154 | P | 3, | 51 | 25.174 (x 2.026) | 510 (0.100) | 0.000116 | cell differentiation | Amph Ance Dl Dr Eph Fas3 Galpha49B Hrb27C InR Pten Ptp99A S Sema-1a Sema-1b Sema-2a TER94 Tl ap ara asp br bun cdc2 edl fax fra grh hbs heph hth in jumu lola malpha mbt ninA numb otk pbl pk pon rost sc spen sqd stan th tkv trh usp wbl |
| 16 | GO:0002165 | P | 4, | 39 | 17.029 (x 2.290) | 345 (0.113) | 0.000118 | larval or pupal development (sensu Insecta) | Amph Ance CG7860 CrebA Dl Dr Eip71CD Eip74EF Eip75B ImpE2 ImpE3 Poxn S Sb TER94 ap ara arr ash2 br bun cg cib dve eIF-4E emp heph hth in jumu klu l(2)01424 mbt nmo pk stan tkv usp vg |
| 17 | GO:0022008 | P | 5, | 27 | 9.527 (x 2.834) | 193 (0.140) | 0.000119 | neurogenesis | Dr Eph Fas3 Galpha49B InR Ptp99A Sema-1a Sema-1b Sema-2a ana ap bnb cdc2 ed fax fra jumu lola ninA numb otk pbl pon sc spen stan usp |
| 18 | GO:0003704 | F | 4, | 16 | 3.899 (x 4.103) | 79 (0.203) | 0.000139 | specific RNA polymerase II transcription factor activity | Dp Eip74EF Eip75B HLHm7 Trl ap ara br ci cyc en grh lola opa pdm2 sc |
| 19 | GO:0007165 | P | 4, | 100 | 63.724 (x 1.569) | 1291 (0.077) | 0.000157 | signal transduction | 18w Abi Arf79F CG11438 CG14217 CG15835 CG17084 CG17493 CG17919 CG2108 CG30440 CG32158 CG33171 CG40410 CG5522 CG6954 CG6965 CG9066 CG9211 CaMKI Cad74A Cad87A Cad96Ca Cdk4 Cip4 CkIIalpha CkIIbeta Dl ETH Eip75B Eph Galpha49B InR LanB2 Mpk2 Nek2 Nep2 PFE Pi3K21B Pli Pten Rab8 RacGAP50C Rapgap1 Rhp S Sema-1a Sema-1b Sema-2a Tl Tsp26A Tsp39D Tsp66E Tsp96F Wnt2 arr bun ced-6 ci cyc ed edl for fra fwd hbs heph inx2 inx3 klu knk malpha mav mbt mth nmo numb ogre ome otk p120ctn pbl pll polo rasp scf serpin-27A sgl shf spen stai stan tkv trh trn tws usp vimar wbl wgn |
| 20 | GO:0000902 | P | 4, 5, | 38 | 16.684 (x 2.278) | 338 (0.112) | 0.00016 | cellular morphogenesis | Abi Amph Arf79F CG3770 Cdk4 CycT Dl Eph Fas3 Fs(2)Ket Galpha49B InR Pi3K21B Pten Ptp99A S Sema-1a Sema-1b Sema-2a TER94 ap ara btsz eIF-4E edl fax fra grh in lola mbt ninA nmo otk pbl pk spen stan |
| 21 | GO:0009791 | P | 3, | 39 | 17.572 (x 2.219) | 356 (0.110) | 0.000201 | post-embryonic development | Amph Ance CG7860 CrebA Dl Dr Eip71CD Eip74EF Eip75B ImpE2 ImpE3 Poxn S Sb TER94 ap ara arr ash2 br bun cg cib dve eIF-4E emp heph hth in jumu klu l(2)01424 mbt nmo pk stan tkv usp vg |
| 22 | GO:0009888 | P | 3, | 42 | 19.646 (x 2.138) | 398 (0.106) | 0.000204 | tissue development | CG30011 CG9211 Cad96Ca Cip4 Dl Doc1 Doc2 Doc3 Dr Eip74EF Galpha49B HLHm7 LanB2 Poxn Pten S Sema-1a Sema-1b Sema-2a ap ara cyc en fra grn hbs in inv ninA nonA-l numb pbl pdm2 pk rost sgl shf smi35A spen stan toe trh |
| 23 | GO:0016265 | P | 3, | 31 | 12.389 (x 2.502) | 251 (0.124) | 0.000206 | death | Aac11 CG14217 CG6680 CG7379 CG7860 Dl Dp Eip71CD Eip74EF Eip75B Ice Jafrac2 Pten Rep4 ap br bun ced-6 eIF-4E emp klu l(2)01424 mdy mod(mdg4) nmo pcs smi35A th trn usp wgn |
| 24 | GO:0019222 | P | 4, | 83 | 50.545 (x 1.642) | 1024 (0.081) | 0.000212 | regulation of metabolism | B52 BEST:GH02921 CG11079 CG15835 CG30011 CG3136 CG33525 CG40160 CG40410 CG4914 CG6654 CG7379 CG8092 CG8165 CREG Caf1 CrebA CtBP CycT D19A Doc1 Doc2 Doc3 Dp Dr Dref Dsp1 Eip74EF Eip75B GATAd HLHm7 HmgD HmgZ Optix Poxn Rab8 Rbf2 Rm62 Thd1 Tl Trl ap ara ash2 br bun cdc2 cg ci cic cyc dap dve edl en fwd fzy grh grn hth inv jumu kis klu l(2)01424 lid lola mod(mdg4) opa pAbp pdm2 ph-p pll sc serpin-27A shanti spen sqd toe trh usp woc zf30C |
| 25 | GO:0016337 | P | 4, | 20 | 6.121 (x 3.268) | 124 (0.161) | 0.000213 | cell-cell adhesion | CG9211 Cad74A Cad87A Cad96Ca Dl Fas3 LanB2 Sema-1b Sema-2a Tsp26A Tsp39D Tsp66E Tsp96F ed glec hbs otk shf stan trn |
| 26 | GO:0003677 | F | 4, | 70 | 40.574 (x 1.725) | 822 (0.085) | 0.000254 | DNA binding | BEST:GH02921 BEST:LD29214 BcDNA:GM10765 CG11033 CG30011 CG3136 CG33525 CG4914 CG6812 CG7379 CG8092 CG9342 CrebA Doc1 Doc2 Doc3 Dp Dp1 Dr Dref Dsp1 Eip74EF Eip75B GATAd HLHm7 His4r HmgD HmgZ Hrb27C Mcm7 Optix Pep Poxn Ptp99A Rab8 Rbf2 Thd1 Top2 Trl ap ara ash2 br btsz bun cg ci cic cyc dve edl en glu grh grn hth inv jumu kis lid opa pdm2 ph-p sc scf toe trh usp woc zf30C |
| 27 | GO:0012501 | P | 5, | 30 | 12.241 (x 2.451) | 248 (0.121) | 0.000361 | programmed cell death | Aac11 CG14217 CG6680 CG7379 CG7860 Dl Dp Eip71CD Eip74EF Eip75B Ice Jafrac2 Pten Rep4 br bun ced-6 eIF-4E emp klu l(2)01424 mdy mod(mdg4) nmo pcs smi35A th trn usp wgn |
| 28 | GO:0008219 | P | 4, | 30 | 12.340 (x 2.431) | 250 (0.120) | 0.000396 | cell death | Aac11 CG14217 CG6680 CG7379 CG7860 Dl Dp Eip71CD Eip74EF Eip75B Ice Jafrac2 Pten Rep4 br bun ced-6 eIF-4E emp klu l(2)01424 mdy mod(mdg4) nmo pcs smi35A th trn usp wgn |
| 29 | GO:0006355 | P | 8, | 67 | 38.847 (x 1.725) | 787 (0.085) | 0.000403 | regulation of transcription, DNA-dependent | BEST:GH02921 CG15835 CG30011 CG3136 CG33525 CG4914 CG6654 CG7379 CG8092 CG8165 Caf1 CrebA CtBP CycT D19A Doc1 Doc2 Doc3 Dp Dr Dref Dsp1 Eip74EF Eip75B GATAd HLHm7 HmgD HmgZ Optix Poxn Rab8 Rbf2 Thd1 Trl ap ara ash2 br bun cdc2 cg ci cic cyc dve edl en grh grn hth inv jumu kis klu lid lola mod(mdg4) opa pdm2 ph-p sc shanti toe trh usp woc zf30C |
| 30 | GO:0007552 | P | 4, | 30 | 12.488 (x 2.402) | 253 (0.119) | 0.000457 | metamorphosis | Amph Ance Dl Dr ImpE2 ImpE3 Poxn S Sb TER94 ap ara arr ash2 br bun cg dve heph hth in jumu klu mbt nmo pk stan tkv usp vg |
| 31 | GO:0019219 | P | 6, | 73 | 43.832 (x 1.665) | 888 (0.082) | 0.000458 | regulation of nucleobase, nucleoside, nucleotide and nucleic acid metabolism | B52 BEST:GH02921 CG11079 CG15835 CG30011 CG3136 CG33525 CG4914 CG6654 CG7379 CG8092 CG8165 CREG Caf1 CrebA CtBP CycT D19A Doc1 Doc2 Doc3 Dp Dr Dref Dsp1 Eip74EF Eip75B GATAd HLHm7 HmgD HmgZ Optix Poxn Rab8 Rbf2 Rm62 Thd1 Trl ap ara ash2 br bun cdc2 cg ci cic cyc dve edl en grh grn hth inv jumu kis klu lid lola mod(mdg4) opa pdm2 ph-p sc shanti spen sqd toe trh usp woc zf30C |
| 32 | GO:0005515 | F | 3, | 94 | 60.911 (x 1.543) | 1234 (0.076) | 0.000468 | protein binding | Abi Amph B52 CG10359 CG11275 CG15835 CG1632 CG17493 CG17952 CG3136 CG31453 CG32137 CG5319 CG6891 CG6966 CG7668 CG9057 CG9906 CG9924 CREG CaMKI Caf1 Cdk4 Cip4 Cks Crc CrebA CtBP CycB3 Dl DnaJ-1 Dp Dsp1 ETH Eb1 Fs(2)Ket Hsp23 InR Incenp Pi3K21B Pten Rep4 Rhp Rm62 Sema-1a Sema-1b Sema-2a Set Sh Sry-alpha Tl Trl Tsp26A Tsp39D Tsp66E Tsp96F Wnt2 alphaTub84B arr asp betaTub56D bip1 br bun cdc2 ced-6 cib cic ck cyc d edl in loj lola mav mbt mod(mdg4) numb otk pAbp pcs pk pll scf shf spen stai stan tkv trh usp vimar wgn |
| 33 | GO:0048518 | P | 3, | 27 | 10.761 (x 2.509) | 218 (0.124) | 0.000528 | positive regulation of biological process | Abi CG14217 CG7379 CG9211 Cks Dp Eip75B InR Jafrac2 Pi3K21B Trl ara ash2 br btsz ci cyc eIF-4E klu lola mbt mod(mdg4) pAbp smi35A stan tkv usp |
| 34 | GO:0045449 | P | 7, | 69 | 41.019 (x 1.682) | 831 (0.083) | 0.000542 | regulation of transcription | BEST:GH02921 CG15835 CG30011 CG3136 CG33525 CG4914 CG6654 CG7379 CG8092 CG8165 CREG Caf1 CrebA CtBP CycT D19A Doc1 Doc2 Doc3 Dp Dr Dref Dsp1 Eip74EF Eip75B GATAd HLHm7 HmgD HmgZ Optix Poxn Rab8 Rbf2 Thd1 Trl ap ara ash2 br bun cdc2 cg ci cic cyc dve edl en grh grn hth inv jumu kis klu lid lola mod(mdg4) opa pdm2 ph-p sc shanti spen toe trh usp woc zf30C |
| 35 | GO:0007423 | P | 4, | 28 | 11.353 (x 2.466) | 230 (0.122) | 0.000546 | sensory organ development | Amph CkIIalpha Dl HLHm7 Ice Optix Poxn S TER94 ara arr br bun dap ed edl hth jumu klu malpha mbt nmo numb pk sc stan tkv tws |
| 36 | GO:0000904 | P | 5, 6, | 23 | 8.342 (x 2.757) | 169 (0.136) | 0.000552 | cellular morphogenesis during differentiation | Amph Dl Eph Fas3 Galpha49B InR Ptp99A S Sema-1a Sema-1b Sema-2a TER94 ap edl fax fra lola mbt ninA otk pbl spen stan |
| 37 | GO:0005623 | C | 2, | 271 | 227.848 (x 1.189) | 4616 (0.059) | 0.000763 | cell | 18w Act42A Amph Ance B52 BEST:CK02656 BEST:GH02921 BEST:LD04971 BEST:LD29214 BG:DS00004.11 BcDNA:GH12558 BcDNA:GM10765 BcDNA:LD41548 CBP CG10657 CG11079 CG11550 CG11739 CG12048 CG14076 CG14214 CG14439 CG15088 CG15435 CG1607 CG1632 CG17084 CG17493 CG17838 CG17952 CG1907 CG1911 CG1924 CG2118 CG2200 CG30011 CG31121 CG3136 CG31738 CG32158 CG3305 CG33113 CG33171 CG33525 CG3424 CG3823 CG3921 CG40410 CG4496 CG4586 CG4914 CG6654 CG6776 CG6782 CG6812 CG6891 CG6930 CG6965 CG8092 CG8679 CG9057 CG9211 CG9342 CG9894 CREG CaMKI Cad74A Cad87A Cad96Ca Caf1 CkIIalpha CkIIbeta Crc CrebA CtBP CycB3 CycT Cyp310a1 D19A Dl DnaJ-1 Doc1 Doc2 Doc3 Dp Dp1 Dr Dref Dsp1 EG:171D11.1 EG:BACN33B1.2 ERp60 Eb1 Eip74EF Eip75B Eno Eph Fas3 Fs(2)Ket GATAd GalNAc-T1 Galpha49B Gapdh1 Gapdh2 Gfat1 HLHm7 His4r HmgD HmgZ Hrb27C Ice Idh ImpE2 ImpE3 InR Incenp Ir Klp10A M6 Mcm7 Mgstl Mpk2 Nek2 Nep2 Nrv1 Optix Pdi Pep Pepck Pgi Pi3K21B Poxn Ppt1 Prosalpha6 Ptp99A Rbf2 Rep4 Rhp Rm62 RnrS RpS12 Rpn9 S Sb Sema-1a Sema-1b Sema-2a Set Sh Snap Sry-alpha TER94 Tapdelta Thd1 Tl Top2 Trl Tsp26A Tsp39D Tsp66E Tsp96F Uch Ucp4B Ugt Vha100-2 alpha-Man-IIb alphaTub84B ap ara arr ash2 asp betaTub56D bip1 blot br btsz bun cdc2 cg ci cib cic ck crp cyc d dap dve eIF-4E east ed edl emp en esn for fra fzy glec glu grh grn gukh hbs heph hth in inv inx2 inx3 jumu kis klu l(1)G0320 l(2)01424 l(2)01810 lid lig loj lola mbt mdy mnd mod(mdg4) mth ninA nonA-l numb ogre ome opa otk p120ctn pAbp pain para pbl pcs pdm2 pgant5 ph-p pk pll polo pon prominin-like rasp rost rpk sc shanti shu smi35A spen sqd stai stan sut1 th tkv toe trh trn tws usp vg wbl wgn woc zf30C |
| 38 | GO:0044464 | C | 2, 3, | 271 | 227.848 (x 1.189) | 4616 (0.059) | 0.000783 | cell part | 18w Act42A Amph Ance B52 BEST:CK02656 BEST:GH02921 BEST:LD04971 BEST:LD29214 BG:DS00004.11 BcDNA:GH12558 BcDNA:GM10765 BcDNA:LD41548 CBP CG10657 CG11079 CG11550 CG11739 CG12048 CG14076 CG14214 CG14439 CG15088 CG15435 CG1607 CG1632 CG17084 CG17493 CG17838 CG17952 CG1907 CG1911 CG1924 CG2118 CG2200 CG30011 CG31121 CG3136 CG31738 CG32158 CG3305 CG33113 CG33171 CG33525 CG3424 CG3823 CG3921 CG40410 CG4496 CG4586 CG4914 CG6654 CG6776 CG6782 CG6812 CG6891 CG6930 CG6965 CG8092 CG8679 CG9057 CG9211 CG9342 CG9894 CREG CaMKI Cad74A Cad87A Cad96Ca Caf1 CkIIalpha CkIIbeta Crc CrebA CtBP CycB3 CycT Cyp310a1 D19A Dl DnaJ-1 Doc1 Doc2 Doc3 Dp Dp1 Dr Dref Dsp1 EG:171D11.1 EG:BACN33B1.2 ERp60 Eb1 Eip74EF Eip75B Eno Eph Fas3 Fs(2)Ket GATAd GalNAc-T1 Galpha49B Gapdh1 Gapdh2 Gfat1 HLHm7 His4r HmgD HmgZ Hrb27C Ice Idh ImpE2 ImpE3 InR Incenp Ir Klp10A M6 Mcm7 Mgstl Mpk2 Nek2 Nep2 Nrv1 Optix Pdi Pep Pepck Pgi Pi3K21B Poxn Ppt1 Prosalpha6 Ptp99A Rbf2 Rep4 Rhp Rm62 RnrS RpS12 Rpn9 S Sb Sema-1a Sema-1b Sema-2a Set Sh Snap Sry-alpha TER94 Tapdelta Thd1 Tl Top2 Trl Tsp26A Tsp39D Tsp66E Tsp96F Uch Ucp4B Ugt Vha100-2 alpha-Man-IIb alphaTub84B ap ara arr ash2 asp betaTub56D bip1 blot br btsz bun cdc2 cg ci cib cic ck crp cyc d dap dve eIF-4E east ed edl emp en esn for fra fzy glec glu grh grn gukh hbs heph hth in inv inx2 inx3 jumu kis klu l(1)G0320 l(2)01424 l(2)01810 lid lig loj lola mbt mdy mnd mod(mdg4) mth ninA nonA-l numb ogre ome opa otk p120ctn pAbp pain para pbl pcs pdm2 pgant5 ph-p pk pll polo pon prominin-like rasp rost rpk sc shanti shu smi35A spen sqd stai stan sut1 th tkv toe trh trn tws usp vg wbl wgn woc zf30C |
| 39 | GO:0035220 | P | 5, | 18 | 5.726 (x 3.144) | 116 (0.155) | 0.000798 | wing disc development | Dl Dr Poxn ap ara ash2 cg ci dve en heph in inv jumu nmo pk tkv vg |
| 40 | GO:0046698 | P | 5, | 29 | 12.389 (x 2.341) | 251 (0.116) | 0.000862 | metamorphosis (sensu Insecta) | Amph Ance Dl Dr ImpE2 ImpE3 Poxn S Sb TER94 ap ara arr ash2 br bun cg dve heph hth in jumu klu mbt nmo pk stan tkv vg |
| 41 | GO:0007560 | P | 5, 6, | 28 | 11.847 (x 2.364) | 240 (0.117) | 0.000984 | imaginal disc morphogenesis | Amph Dl Dr ImpE2 ImpE3 Poxn S Sb TER94 ap ara arr ash2 br bun cg dve heph hth in jumu klu mbt nmo pk stan tkv vg |
| 42 | GO:0000278 | P | 5, | 30 | 13.229 (x 2.268) | 268 (0.112) | 0.00109 | mitotic cell cycle | CG17493 CG1911 CG32137 CG40410 CG4454 Cdk4 Cks CycB3 Eb1 Klp10A Nek2 Rpn9 TER94 Top2 Trl ald alphaTub84B betaTub56D cdc2 ci dap eIF-4E east fzy glu pim polo sc tws zwilch |
| 43 | GO:0031323 | P | 5, | 77 | 48.670 (x 1.582) | 986 (0.078) | 0.00115 | regulation of cellular metabolism | B52 BEST:GH02921 CG11079 CG15835 CG30011 CG3136 CG33525 CG40160 CG4914 CG6654 CG7379 CG8092 CG8165 CREG Caf1 CrebA CtBP CycT D19A Doc1 Doc2 Doc3 Dp Dr Dref Dsp1 Eip74EF Eip75B GATAd HLHm7 HmgD HmgZ Optix Poxn Rab8 Rbf2 Rm62 Thd1 Trl ap ara ash2 br bun cdc2 cg ci cic cyc dap dve edl en grh grn hth inv jumu kis klu l(2)01424 lid lola mod(mdg4) opa pAbp pdm2 ph-p sc shanti spen sqd toe trh usp woc zf30C |
| 44 | GO:0009987 | P | 2, | 366 | 331.012 (x 1.106) | 6706 (0.055) | 0.00124 | cellular process | 18w Aac11 Abi Act42A Amph Ance Arf79F Argk B52 BEST:CK02656 BEST:GH02921 BEST:LD04971 BEST:LD22483 BG:DS00004.11 BcDNA:GH02901 BcDNA:GH02976 BcDNA:GH12558 BcDNA:GM10765 BcDNA:LD41548 Best2 CG10657 CG11033 CG11079 CG11142 CG11438 CG11550 CG11739 CG11824 CG12048 CG12199 CG14214 CG14217 CG14439 CG15088 CG15835 CG1607 CG1630 CG1632 CG16974 CG17052 CG17064 CG17084 CG17323 CG17419 CG17493 CG17919 CG18853 CG1893 CG1907 CG1911 CG1924 CG1998 CG2108 CG2118 CG2200 CG2852 CG30011 CG30440 CG31121 CG3136 CG31453 CG31472 CG31738 CG31915 CG32137 CG32158 CG32499 CG32632 CG33113 CG33116 CG33138 CG33171 CG3328 CG33525 CG3424 CG3590 CG3770 CG3823 CG40160 CG40410 CG4054 CG4454 CG4502 CG4586 CG4914 CG5096 CG5319 CG5390 CG5466 CG5522 CG5794 CG5873 CG6287 CG6391 CG6654 CG6680 CG6767 CG6776 CG6812 CG6904 CG6946 CG6954 CG6965 CG6966 CG7379 CG7860 CG8092 CG8165 CG8963 CG9027 CG9057 CG9066 CG9135 CG9211 CG9307 CG9342 CG9906 CREG CaMKI Cad74A Cad87A Cad96Ca Caf1 Cdk4 Cip4 CkIIalpha CkIIbeta Cks Crc CrebA CtBP CycB3 CycT Cyp310a1 D19A Dl DnaJ-1 Doc1 Doc2 Doc3 Dp Dp1 Dr Dref Dsp1 EG:171D11.1 ERp60 ESTS:39C10S ETH Eb1 Eip55E Eip71CD Eip74EF Eip75B Eno Eph Fas3 Fkbp13 Fs(2)Ket GATAd GalNAc-T1 Galpha49B Gapdh1 Gapdh2 Gfat1 GlyP GstS1 HLHm7 Hex-A His4r HmgD HmgZ Hrb27C Hsp23 Hsp26 Hsp27 Hsp67Ba Hus1-like Ice Idh InR Incenp Ir Jafrac2 Klp10A LanB2 Lsp2 M6 Mcm7 Mgstl Mpk2 Nek2 Nep2 Nrv1 Obp99a Optix Oscillin PFE Pdi Pepck Pgi Pi3K21B Pli Poxn Ppt1 Prosalpha6 Pten Ptp99A Rab8 RacGAP50C Rapgap1 Rbf2 Rep4 Rhp Rm62 RnrS RpS12 Rpn9 S Sb Sema-1a Sema-1b Sema-2a Set Sh Snap Spn1 Spn5 Sry-alpha TER94 Thd1 Tl Top2 Tpi Trl Ts Tsp26A Tsp39D Tsp66E Tsp96F Uch Ucp4B Ugt Vha100-2 Wnt2 ald alphaTub84B ana ap ara arr ash2 asp betaTub56D blot br btsz bun cdc2 ced-6 cg ci cib cic ck cyc d dUTPase dap dve eIF-4E east ed edl emp en esn fax for fra fwd fzy glec glu grh grn hbs heph hth in inv inx2 inx3 jumu kal-1 kis klu knk l(1)G0320 l(2)01424 l(2)01810 lid loj lola malpha mav mbt mdy mnd mod(mdg4) mth ninA nmo nonA-l numb ogre olf413 ome opa otk p120ctn pAbp pain para pbl pcs pdm2 pgant5 ph-p pim pk pll polo pon prominin-like rasp rost rpk sc scf serpin-27A sgl shanti shf shu smi35A spen sqd stai stan sut1 th tkv toe tok trh trn tws usp vg vimar wbl wgn woc zf30C zwilch |
| 45 | GO:0051726 | P | 5, | 24 | 9.823 (x 2.443) | 199 (0.121) | 0.00198 | regulation of cell cycle | Abi CG40410 CG7379 Cdk4 Cks CycB3 Dp Dref Eb1 Hus1-like Nek2 Pten Rbf2 Rpn9 ald alphaTub84B cdc2 ci dap fzy jumu sc tws zwilch |
| 46 | GO:0000074 | P | 6, | 24 | 9.823 (x 2.443) | 199 (0.121) | 0.00203 | regulation of progression through cell cycle | Abi CG40410 CG7379 Cdk4 Cks CycB3 Dp Dref Eb1 Hus1-like Nek2 Pten Rbf2 Rpn9 ald alphaTub84B cdc2 ci dap fzy jumu sc tws zwilch |
| 47 | GO:0016043 | P | 4, | 99 | 68.068 (x 1.454) | 1379 (0.072) | 0.00206 | cell organization and biogenesis | Abi Act42A Amph Arf79F CG14214 CG1893 CG1907 CG1911 CG1924 CG2108 CG2852 CG32137 CG33113 CG33171 CG3770 CG9057 CG9906 Caf1 Cdk4 Cip4 Crc CycB3 CycT Dl Dp1 Dref Dsp1 Eb1 Eph Fas3 Fs(2)Ket Galpha49B GstS1 His4r HmgD HmgZ InR Incenp Klp10A M6 Pi3K21B Pten Ptp99A Rab8 Rhp S Sb Sema-1a Sema-1b Sema-2a Set Snap Sry-alpha TER94 Trl Ucp4B alphaTub84B ap ara arr ash2 asp betaTub56D btsz cib ck d eIF-4E edl esn fax for fra fwd glu grh in jumu kis l(1)G0320 loj lola mbt mod(mdg4) ninA nmo otk pbl ph-p pk pll polo prominin-like spen sqd stai stan vg wbl |
| 48 | GO:0007088 | P | 7, 8, | 9 | 1.777 (x 5.065) | 36 (0.250) | 0.00207 | regulation of mitosis | CG40410 Cks Nek2 Rpn9 ald alphaTub84B fzy tws zwilch |
| 49 | GO:0016477 | P | 5, 6, | 22 | 8.638 (x 2.547) | 175 (0.126) | 0.0021 | cell migration | Fas3 Galpha49B InR Ptp99A Sema-1a Sema-1b Sema-2a ap fra lola mod(mdg4) ninA opa otk pbl sgl spen sqd stai th trn usp |
| 50 | GO:0048522 | P | 4, | 23 | 9.329 (x 2.465) | 189 (0.122) | 0.00233 | positive regulation of cellular process | CG14217 CG7379 CG9211 Cks Dp Eip75B InR Jafrac2 Trl ara ash2 br ci cyc eIF-4E klu lola mod(mdg4) pAbp smi35A stan tkv usp |
| 51 | GO:0006351 | P | 7, | 70 | 44.227 (x 1.583) | 896 (0.078) | 0.00241 | transcription, DNA-dependent | BEST:GH02921 CG15835 CG30011 CG3136 CG31453 CG33525 CG4914 CG5319 CG6654 CG7379 CG8092 CG8165 Caf1 CrebA CtBP CycT D19A Doc1 Doc2 Doc3 Dp Dr Dref Dsp1 Eip74EF Eip75B GATAd HLHm7 HmgD HmgZ Optix Poxn Rab8 Rbf2 Thd1 Top2 Trl ap ara ash2 br bun cdc2 cg ci cic cyc dve edl en grh grn hth inv jumu kis klu lid lola mod(mdg4) opa pdm2 ph-p sc shanti toe trh usp woc zf30C |
| 52 | GO:0006357 | P | 9, | 48 | 27.000 (x 1.778) | 547 (0.088) | 0.00247 | regulation of transcription from RNA polymerase II promoter | CG15835 CG30011 CG6654 CG8165 Caf1 CrebA CtBP CycT D19A Doc1 Doc2 Doc3 Dp Dr Dref Dsp1 Eip74EF Eip75B HLHm7 HmgZ Optix Poxn Rbf2 Trl ap ara br bun cdc2 cg ci cyc en grh grn hth inv kis klu lola mod(mdg4) opa pdm2 sc toe trh usp zf30C |
| 53 | GO:0048468 | P | 4, | 34 | 16.783 (x 2.026) | 340 (0.100) | 0.0025 | cell development | Amph Ance Dl Eph Fas3 Galpha49B Hrb27C InR Ptp99A S Sema-1a Sema-1b Sema-2a TER94 ap asp edl fax fra hbs heph lola mbt ninA otk pbl rost spen sqd stan th tkv usp wbl |
| 54 | GO:0007049 | P | 4, | 43 | 23.249 (x 1.850) | 471 (0.091) | 0.0025 | cell cycle | Abi CG17493 CG1911 CG32137 CG40410 CG4454 CG7379 Cdk4 Cip4 Cks CycB3 CycT Dp Dref Eb1 Hus1-like Klp10A Nek2 Pten Rbf2 Rpn9 TER94 Top2 Trl ald alphaTub84B asp betaTub56D cdc2 ci dap eIF-4E east fwd fzy glu jumu pbl pim polo sc tws zwilch |
| 55 | GO:0007389 | P | 3, | 28 | 12.636 (x 2.216) | 256 (0.109) | 0.00252 | pattern specification | Cdk4 CrebA Dl Dp Dr Hrb27C TER94 Tl ap ci edl en hth inv kis knk opa pll rasp serpin-27A sgl spen sqd stan tkv tok tws wbl |
| 56 | GO:0051242 | P | 5, | 21 | 8.243 (x 2.548) | 167 (0.126) | 0.00272 | positive regulation of cellular physiological process | CG14217 CG7379 Cks Dp Eip75B InR Jafrac2 Trl ara ash2 br ci cyc eIF-4E klu lola mod(mdg4) pAbp smi35A tkv usp |
| 57 | GO:0048812 | P | 7, 8, 10, | 17 | 5.973 (x 2.846) | 121 (0.140) | 0.00312 | neurite morphogenesis | Eph Fas3 Galpha49B InR Ptp99A Sema-1a Sema-1b Sema-2a ap fax fra lola ninA otk pbl spen stan |
| 58 | GO:0007409 | P | 8, 9, 11, | 17 | 5.973 (x 2.846) | 121 (0.140) | 0.00317 | axonogenesis | Eph Fas3 Galpha49B InR Ptp99A Sema-1a Sema-1b Sema-2a ap fax fra lola ninA otk pbl spen stan |
| 59 | GO:0043119 | P | 4, | 21 | 8.342 (x 2.517) | 169 (0.124) | 0.00318 | positive regulation of physiological process | CG14217 CG7379 Cks Dp Eip75B InR Jafrac2 Trl ara ash2 br ci cyc eIF-4E klu lola mod(mdg4) pAbp smi35A tkv usp |
| 60 | GO:0048667 | P | 6, 7, 9, | 17 | 5.973 (x 2.846) | 121 (0.140) | 0.00323 | neuron morphogenesis during differentiation | Eph Fas3 Galpha49B InR Ptp99A Sema-1a Sema-1b Sema-2a ap fax fra lola ninA otk pbl spen stan |
| 61 | GO:0048519 | P | 3, | 31 | 15.055 (x 2.059) | 305 (0.102) | 0.00347 | negative regulation of biological process | Aac11 CG11079 CG33525 CG40410 CREG Caf1 CtBP Dl Pten RacGAP50C Rbf2 ana bun charybde ci cic cyc dap ed edl en fwd nmo numb ph-p scylla serpin-27A sqd stan th usp |
| 62 | GO:0006350 | P | 6, | 72 | 46.646 (x 1.544) | 945 (0.076) | 0.00348 | transcription | BEST:GH02921 CG15835 CG30011 CG3136 CG31453 CG33525 CG4914 CG5319 CG6654 CG7379 CG8092 CG8165 CREG Caf1 CrebA CtBP CycT D19A Doc1 Doc2 Doc3 Dp Dr Dref Dsp1 Eip74EF Eip75B GATAd HLHm7 HmgD HmgZ Optix Poxn Rab8 Rbf2 Thd1 Top2 Trl ap ara ash2 br bun cdc2 cg ci cic cyc dve edl en grh grn hth inv jumu kis klu lid lola mod(mdg4) opa pdm2 ph-p sc shanti spen toe trh usp woc zf30C |
| 63 | GO:0007398 | P | 4, | 26 | 11.698 (x 2.223) | 237 (0.110) | 0.00362 | ectoderm development | CG9211 Cad96Ca Dl Dr HLHm7 Poxn Sema-1a Sema-1b Sema-2a ap ara cyc en fra hbs in inv numb pdm2 pk shf smi35A spen stan toe trh |
| 64 | GO:0005783 | C | 5, 6, 7, 8, | 16 | 5.578 (x 2.869) | 113 (0.142) | 0.00421 | endoplasmic reticulum | BG:DS00004.11 CBP CG14214 CG1924 CG33113 Crc ERp60 Pdi S Snap TER94 Tapdelta Ugt l(1)G0320 shanti wbl |
| 65 | GO:0005634 | C | 5, 6, 7, 8, | 105 | 75.225 (x 1.396) | 1524 (0.069) | 0.00422 | nucleus | B52 BEST:GH02921 BEST:LD29214 BcDNA:GM10765 CG15435 CG17838 CG17952 CG1911 CG30011 CG3136 CG33525 CG40410 CG4496 CG4914 CG6654 CG6930 CG8092 CG8679 CG9342 CG9894 CREG Caf1 CkIIalpha CkIIbeta CrebA CtBP CycB3 CycT D19A Doc1 Doc2 Doc3 Dp Dp1 Dr Dref Dsp1 Eip74EF Eip75B Fs(2)Ket GATAd HLHm7 HmgD HmgZ Hrb27C Mcm7 Mpk2 Optix Pep Poxn Ptp99A Rbf2 Rm62 Set Thd1 Top2 Trl Ugt ap ara ash2 bip1 br btsz bun cdc2 cg ci cic crp cyc dap dve east edl en esn glu grh grn heph hth inv jumu kis klu lid lola mod(mdg4) nonA-l numb opa pbl pdm2 ph-p pk sc spen sqd toe trh usp vg woc zf30C |
| 66 | GO:0007067 | P | 7, | 25 | 11.155 (x 2.241) | 226 (0.111) | 0.00422 | mitosis | CG17493 CG1911 CG32137 CG40410 CG4454 Cdk4 Cks CycB3 Klp10A Nek2 Rpn9 TER94 Top2 ald alphaTub84B betaTub56D cdc2 eIF-4E east fzy glu pim polo tws zwilch |
| 67 | GO:0000087 | P | 6, | 25 | 11.205 (x 2.231) | 227 (0.110) | 0.00427 | M phase of mitotic cell cycle | CG17493 CG1911 CG32137 CG40410 CG4454 Cdk4 Cks CycB3 Klp10A Nek2 Rpn9 TER94 Top2 ald alphaTub84B betaTub56D cdc2 eIF-4E east fzy glu pim polo tws zwilch |
| 68 | GO:0005886 | C | 4, 5, | 46 | 26.359 (x 1.745) | 534 (0.086) | 0.0043 | plasma membrane | 18w Amph CG15088 Cad74A Cad87A Cad96Ca Dl Eph Fas3 Galpha49B ImpE3 InR M6 Nrv1 Ptp99A S Sb Sema-1a Sema-1b Sh Sry-alpha Tl arr blot ed emp for fra hbs in inx2 inx3 mbt mod(mdg4) ogre otk p120ctn para pll prominin-like rasp rost stan tkv trn wgn |
| 69 | GO:0030528 | F | 2, | 63 | 39.735 (x 1.585) | 805 (0.078) | 0.00432 | transcription regulator activity | BEST:GH02921 BEST:LD29214 CG11033 CG15835 CG30011 CG31453 CG33525 CG4914 CG5319 CG6654 CG8165 CREG CrebA CtBP CycT D19A Doc1 Doc2 Doc3 Dp Dr Dref Dsp1 Eip74EF Eip75B GATAd HLHm7 HmgZ Optix Poxn Rbf2 Trl ap ara ash2 br bun cg ci cic crp cyc dve en grh grn hth inv jumu klu lid lola mod(mdg4) nonA-l opa pdm2 sc spen toe trh usp woc zf30C |
| 70 | GO:0007476 | P | 6, 7, 8, | 15 | 5.084 (x 2.950) | 103 (0.146) | 0.00459 | wing morphogenesis | Dl Dr Poxn ap ara ash2 cg dve heph in jumu nmo pk tkv vg |
| 71 | GO:0030234 | F | 2, | 34 | 17.622 (x 1.929) | 357 (0.095) | 0.00499 | enzyme regulator activity | Abi BEST:GH02921 CG11079 CG17919 CG30440 CG31915 CG32354 CG40160 CG5392 CG5522 CG6680 CG9135 CkIIbeta Cks CycB3 CycT Pi3K21B Pli Pten RacGAP50C Rapgap1 Rhp Rpn9 Set Spn1 Spn43Aa Spn5 dap for pbl pcs serpin-27A tws vimar |
| 72 | GO:0005622 | C | 3, 4, | 199 | 162.643 (x 1.224) | 3295 (0.060) | 0.00501 | intracellular | 18w Act42A Amph B52 BEST:GH02921 BEST:LD29214 BG:DS00004.11 BcDNA:GH12558 BcDNA:GM10765 BcDNA:LD41548 CBP CG10657 CG11079 CG11550 CG14214 CG15435 CG17493 CG17838 CG17952 CG1907 CG1911 CG1924 CG2118 CG2200 CG30011 CG3136 CG31738 CG33113 CG33171 CG33525 CG3823 CG40410 CG4496 CG4586 CG4914 CG6654 CG6776 CG6782 CG6812 CG6891 CG6930 CG8092 CG8679 CG9057 CG9342 CG9894 CREG CaMKI Caf1 CkIIalpha CkIIbeta Crc CrebA CtBP CycB3 CycT D19A DnaJ-1 Doc1 Doc2 Doc3 Dp Dp1 Dr Dref Dsp1 EG:171D11.1 EG:BACN33B1.2 ERp60 Eb1 Eip74EF Eip75B Eno Fs(2)Ket GATAd GalNAc-T1 Gapdh1 Gapdh2 Gfat1 HLHm7 His4r HmgD HmgZ Hrb27C Ice Idh ImpE2 Incenp Klp10A Mcm7 Mgstl Mpk2 Nek2 Optix Pdi Pep Pepck Pgi Pi3K21B Poxn Ppt1 Prosalpha6 Ptp99A Rbf2 Rep4 Rhp Rm62 RnrS RpS12 Rpn9 S Set Snap Sry-alpha TER94 Tapdelta Thd1 Top2 Trl Uch Ucp4B Ugt Vha100-2 alpha-Man-IIb alphaTub84B ap ara ash2 asp betaTub56D bip1 br btsz bun cdc2 cg ci cib cic ck crp cyc d dap dve eIF-4E east edl emp en esn for fzy glu grh grn heph hth inv jumu kis klu l(1)G0320 l(2)01424 lid lig lola mdy mod(mdg4) nonA-l numb opa p120ctn pAbp pbl pcs pdm2 pgant5 ph-p pk pll polo pon sc shanti shu smi35A spen sqd stai th toe trh tws usp vg wbl woc zf30C |
| 73 | GO:0006366 | P | 8, | 55 | 33.664 (x 1.634) | 682 (0.081) | 0.00506 | transcription from RNA polymerase II promoter | CG15835 CG30011 CG31453 CG5319 CG6654 CG8165 Caf1 CrebA CtBP CycT D19A Doc1 Doc2 Doc3 Dp Dr Dref Dsp1 Eip74EF Eip75B HLHm7 HmgZ Optix Poxn Rbf2 Top2 Trl ap ara ash2 br bun cdc2 cg ci cyc en grh grn hth inv jumu kis klu lola mod(mdg4) opa pdm2 ph-p sc toe trh usp woc zf30C |
| 74 | GO:0007411 | P | 6, 7, 9, 10, 12, | 13 | 4.048 (x 3.212) | 82 (0.159) | 0.00508 | axon guidance | Fas3 Galpha49B InR Ptp99A Sema-1a Sema-1b Sema-2a ap fra lola ninA otk spen |
| 75 | GO:0007472 | P | 6, 7, | 15 | 5.183 (x 2.894) | 105 (0.143) | 0.00534 | wing disc morphogenesis | Dl Dr Poxn ap ara ash2 cg dve heph in jumu nmo pk tkv vg |
| 76 | GO:0003700 | F | 3, 5, | 36 | 19.201 (x 1.875) | 389 (0.093) | 0.0054 | transcription factor activity | BEST:GH02921 CG30011 CG4914 CrebA Doc1 Doc2 Doc3 Dp Dr Dref Eip74EF Eip75B GATAd HLHm7 Optix Poxn ap ara br bun cg ci cic cyc dve en grn hth inv jumu opa pdm2 toe trh usp zf30C |
| 77 | GO:0048737 | P | 4, | 17 | 6.417 (x 2.649) | 130 (0.131) | 0.00566 | appendage development (sensu Endopterygota) | Dl Dr Poxn ap ara ash2 cg ck dve heph in jumu nmo pk th tkv vg |
| 78 | GO:0048666 | P | 5, 8, | 18 | 7.009 (x 2.568) | 142 (0.127) | 0.00567 | neuron development | Eph Fas3 Galpha49B InR Ptp99A Sema-1a Sema-1b Sema-2a ap fax fra lola ninA otk pbl spen stan usp |
| 79 | GO:0007447 | P | 4, 5, | 9 | 2.123 (x 4.240) | 43 (0.209) | 0.00569 | imaginal disc pattern formation | Dl Dr ap ci en hth inv tkv tws |
| 80 | GO:0035114 | P | 5, | 17 | 6.417 (x 2.649) | 130 (0.131) | 0.00573 | appendage morphogenesis (sensu Endopterygota) | Dl Dr Poxn ap ara ash2 cg ck dve heph in jumu nmo pk th tkv vg |
| 81 | GO:0031175 | P | 6, 9, | 18 | 7.009 (x 2.568) | 142 (0.127) | 0.00574 | neurite development | Eph Fas3 Galpha49B InR Ptp99A Sema-1a Sema-1b Sema-2a ap fax fra lola ninA otk pbl spen stan usp |
| 82 | GO:0007166 | P | 5, | 53 | 32.529 (x 1.629) | 659 (0.080) | 0.00648 | cell surface receptor linked signal transduction | 18w Arf79F CG11438 CG17084 CG30440 CG32158 CG5522 CG6965 CG9211 Cad96Ca CkIIalpha Dl ETH Eph Galpha49B InR PFE Pi3K21B Pli Pten RacGAP50C S Sema-1b Sema-2a Tl Wnt2 arr bun ced-6 ci ed edl heph knk malpha mav mth nmo numb ome otk pbl pll rasp serpin-27A sgl shf spen stan tkv tws wbl wgn |
| 83 | GO:0003702 | F | 3, | 27 | 13.130 (x 2.056) | 266 (0.102) | 0.00684 | RNA polymerase II transcription factor activity | BEST:LD29214 CrebA Dp Eip74EF Eip75B GATAd HLHm7 Optix Trl ap ara br bun ci cic crp cyc en grh grn inv lola mod(mdg4) opa pdm2 sc trh |
| 84 | GO:0009790 | P | 3, | 33 | 17.326 (x 1.905) | 351 (0.094) | 0.00686 | embryonic development | 18w CG40410 Cdk4 CtBP Doc1 InR LanB2 S Sema-1b Tl Trl a6 ci cic edl en inx2 jumu kis knk opa pbl pim pll polo rasp sgl spen stan tkv trh tws wbl |
| 85 | GO:0048523 | P | 4, | 28 | 13.821 (x 2.026) | 280 (0.100) | 0.00686 | negative regulation of cellular process | Aac11 CG11079 CG33525 CG40410 CREG Caf1 CtBP Dl Pten RacGAP50C Rbf2 ana bun ci cic cyc dap ed edl en fwd nmo numb ph-p sqd stan th usp |
| 86 | GO:0007431 | P | 5, | 15 | 5.380 (x 2.788) | 109 (0.138) | 0.00693 | salivary gland development | CG7860 CrebA Eip71CD Eip74EF Eip75B Sema-2a br bun eIF-4E emp hth l(2)01424 tkv trh usp |
| 87 | GO:0035272 | P | 4, | 15 | 5.380 (x 2.788) | 109 (0.138) | 0.00701 | exocrine system development | CG7860 CrebA Eip71CD Eip74EF Eip75B Sema-2a br bun eIF-4E emp hth l(2)01424 tkv trh usp |
| 88 | GO:0005694 | C | 5, 6, 7, 8, | 21 | 9.082 (x 2.312) | 184 (0.114) | 0.00705 | chromosome | CG1911 Caf1 CycT Dp Dp1 Dsp1 His4r HmgD Incenp Pep Rbf2 Top2 Trl fzy glu jumu kis mod(mdg4) polo sqd usp |
| 89 | GO:0035107 | P | 4, | 17 | 6.565 (x 2.590) | 133 (0.128) | 0.00711 | appendage morphogenesis | Dl Dr Poxn ap ara ash2 cg ck dve heph in jumu nmo pk th tkv vg |
| 90 | GO:0048736 | P | 3, | 17 | 6.565 (x 2.590) | 133 (0.128) | 0.0072 | appendage development | Dl Dr Poxn ap ara ash2 cg ck dve heph in jumu nmo pk th tkv vg |
| 91 | GO:0016055 | P | 6, | 10 | 2.715 (x 3.683) | 55 (0.182) | 0.00732 | Wnt receptor signaling pathway | CkIIalpha RacGAP50C Wnt2 arr nmo sgl shf spen stan tws |
| 92 | GO:0044427 | C | 4, 5, 6, 7, 8, 9, | 18 | 7.305 (x 2.464) | 148 (0.122) | 0.00818 | chromosomal part | CG1911 Caf1 CycT Dp Dp1 Dsp1 His4r HmgD Pep Rbf2 Trl fzy glu jumu kis mod(mdg4) polo sqd |
| 93 | GO:0030182 | P | 4, 7, | 18 | 7.355 (x 2.447) | 149 (0.121) | 0.0087 | neuron differentiation | Eph Fas3 Galpha49B InR Ptp99A Sema-1a Sema-1b Sema-2a ap fax fra lola ninA otk pbl spen stan usp |
| 94 | GO:0007059 | P | 4, | 16 | 6.121 (x 2.614) | 124 (0.129) | 0.00876 | chromosome segregation | CG17493 CG1911 CG40410 Dp1 Eb1 Incenp Klp10A ald alphaTub84B betaTub56D eIF-4E east glu pim polo tws |
| 95 | GO:0014016 | P | 4, 7, | 7 | 1.431 (x 4.890) | 29 (0.241) | 0.00923 | neuroblast differentiation | Dr cdc2 jumu numb pon sc spen |
| 96 | GO:0007400 | P | 6, 9, | 7 | 1.431 (x 4.890) | 29 (0.241) | 0.00933 | neuroblast fate determination | Dr cdc2 jumu numb pon sc spen |
| 97 | GO:0006325 | P | 8, | 17 | 6.812 (x 2.496) | 138 (0.123) | 0.00939 | establishment and/or maintenance of chromatin architecture | CG1911 Caf1 Dsp1 His4r HmgD HmgZ Incenp Set Trl ash2 eIF-4E glu jumu kis lola mod(mdg4) ph-p |
| 98 | GO:0014017 | P | 5, 8, | 7 | 1.431 (x 4.890) | 29 (0.241) | 0.00943 | neuroblast fate commitment | Dr cdc2 jumu numb pon sc spen |
| 99 | GO:0006323 | P | 7, | 17 | 6.812 (x 2.496) | 138 (0.123) | 0.00949 | DNA packaging | CG1911 Caf1 Dsp1 His4r HmgD HmgZ Incenp Set Trl ash2 eIF-4E glu jumu kis lola mod(mdg4) ph-p |
| 100 | GO:0044424 | C | 3, 4, 5, | 191 | 157.460 (x 1.213) | 3190 (0.060) | 0.00956 | intracellular part | 18w Act42A Amph B52 BEST:GH02921 BEST:LD29214 BG:DS00004.11 BcDNA:GH12558 BcDNA:GM10765 BcDNA:LD41548 CBP CG11079 CG14214 CG15435 CG17493 CG17838 CG17952 CG1907 CG1911 CG1924 CG2118 CG2200 CG30011 CG3136 CG31738 CG33113 CG33171 CG33525 CG40410 CG4496 CG4586 CG4914 CG6654 CG6776 CG6782 CG6930 CG8092 CG8679 CG9057 CG9342 CG9894 CREG CaMKI Caf1 CkIIalpha CkIIbeta Crc CrebA CtBP CycB3 CycT D19A DnaJ-1 Doc1 Doc2 Doc3 Dp Dp1 Dr Dref Dsp1 EG:171D11.1 EG:BACN33B1.2 ERp60 Eb1 Eip74EF Eip75B Eno Fs(2)Ket GATAd GalNAc-T1 Gapdh1 Gapdh2 Gfat1 HLHm7 His4r HmgD HmgZ Hrb27C Ice Idh ImpE2 Incenp Klp10A Mcm7 Mgstl Mpk2 Nek2 Optix Pdi Pep Pepck Pgi Pi3K21B Poxn Ppt1 Prosalpha6 Ptp99A Rbf2 Rm62 RnrS RpS12 Rpn9 S Set Snap Sry-alpha TER94 Tapdelta Thd1 Top2 Trl Ucp4B Ugt Vha100-2 alpha-Man-IIb alphaTub84B ap ara ash2 asp betaTub56D bip1 br btsz bun cdc2 cg ci cib cic ck crp cyc d dap dve eIF-4E east edl emp en esn for fzy glu grh grn heph hth inv jumu kis klu l(1)G0320 l(2)01424 lid lig lola mdy mod(mdg4) nonA-l numb opa p120ctn pAbp pbl pcs pdm2 pgant5 ph-p pk pll polo pon sc shanti shu smi35A spen sqd stai th toe trh tws usp vg wbl woc zf30C |
| 101 | GO:0009262 | P | 7, | 3 | 0.197 (x 15.194) | 4 (0.750) | 0.00981 | deoxyribonucleotide metabolism | RnrS Ts dUTPase |
| 102 | GO:0019955 | F | 4, | 3 | 0.197 (x 15.194) | 4 (0.750) | 0.00991 | cytokine binding | Tl tkv wgn |
| 103 | GO:0051301 | P | 4, | 17 | 6.861 (x 2.478) | 139 (0.122) | 0.00993 | cell division | Act42A CG4454 Caf1 CycB3 Dl RacGAP50C Snap Sry-alpha Tl asp cdc2 fwd numb pbl polo pon tkv |
| 104 | GO:0016051 | P | 6, 7, | 9 | 2.419 (x 3.721) | 49 (0.184) | 0.0118 | carbohydrate biosynthesis | CG31915 CG6904 GalNAc-T1 Gfat1 Pepck Pgi Tpi pgant5 sgl |
| 105 | GO:0001654 | P | 5, | 20 | 8.934 (x 2.239) | 181 (0.110) | 0.0122 | eye development | Amph CkIIalpha Dl HLHm7 Ice Optix S TER94 ara arr br bun hth jumu klu mbt nmo pk stan tkv |
| 106 | GO:0016271 | P | 4, | 11 | 3.554 (x 3.095) | 72 (0.153) | 0.0147 | tissue death | CG7860 Eip71CD Eip74EF Eip75B ap br bun eIF-4E emp l(2)01424 usp |
| 107 | GO:0043067 | P | 5, 6, | 15 | 5.874 (x 2.554) | 119 (0.126) | 0.0148 | regulation of programmed cell death | Aac11 CG14217 CG7379 Dp Eip75B Ice Jafrac2 br klu mdy mod(mdg4) nmo smi35A th usp |
| 108 | GO:0040008 | P | 3, | 10 | 3.011 (x 3.321) | 61 (0.164) | 0.0149 | regulation of growth | CG3424 Cdk4 InR Pi3K21B Pten btsz charybde eIF-4E scylla tkv |
| 109 | GO:0007559 | P | 5, | 11 | 3.554 (x 3.095) | 72 (0.153) | 0.0149 | histolysis | CG7860 Eip71CD Eip74EF Eip75B ap br bun eIF-4E emp l(2)01424 usp |
| 110 | GO:0007167 | P | 6, | 19 | 8.490 (x 2.238) | 172 (0.110) | 0.016 | enzyme linked receptor protein signaling pathway | CG5522 Cad96Ca Eph InR PFE Pi3K21B Pten S bun ced-6 ed edl knk mav otk pll sgl spen tkv |
| 111 | GO:0035214 | P | 5, | 18 | 7.898 (x 2.279) | 160 (0.113) | 0.0169 | eye-antennal disc development | Amph Dl Poxn S TER94 ara arr br bun ck hth klu mbt nmo pk stan th tkv |
| 112 | GO:0007456 | P | 6, | 19 | 8.539 (x 2.225) | 173 (0.110) | 0.017 | eye development (sensu Endopterygota) | Amph CkIIalpha Dl HLHm7 Optix S TER94 ara arr br bun hth jumu klu mbt nmo pk stan tkv |
| 113 | GO:0051674 | P | 4, | 25 | 12.686 (x 1.971) | 257 (0.097) | 0.017 | localization of cell | Abi Fas3 Galpha49B InR Ptp99A Sema-1a Sema-1b Sema-2a alphaTub84B ap betaTub56D fra lola mod(mdg4) ninA opa otk pbl sgl spen sqd stai th trn usp |
| 114 | GO:0006928 | P | 4, 5, | 25 | 12.686 (x 1.971) | 257 (0.097) | 0.0172 | cell motility | Abi Fas3 Galpha49B InR Ptp99A Sema-1a Sema-1b Sema-2a alphaTub84B ap betaTub56D fra lola mod(mdg4) ninA opa otk pbl sgl spen sqd stai th trn usp |
| 115 | GO:0009953 | P | 4, | 11 | 3.653 (x 3.011) | 74 (0.149) | 0.0175 | dorsal/ventral pattern formation | CrebA Dl Dp Dr Tl ap pll serpin-27A sqd tok wbl |
| 116 | GO:0000279 | P | 5, | 27 | 14.166 (x 1.906) | 287 (0.094) | 0.0176 | M phase | CG17493 CG1911 CG32137 CG40410 CG4454 Cdk4 Cks CycB3 Klp10A Nek2 Rpn9 TER94 Top2 ald alphaTub84B betaTub56D cdc2 eIF-4E east fwd fzy glu pbl pim polo tws zwilch |
| 117 | GO:0045165 | P | 4, | 20 | 9.329 (x 2.144) | 189 (0.106) | 0.019 | cell fate commitment | Dl Dr Pten S ap ara br bun cdc2 grh hth jumu malpha numb pon sc spen stan tkv trh |
| 118 | GO:0006950 | P | 3, | 30 | 16.486 (x 1.820) | 334 (0.090) | 0.0193 | response to stress | 18w BcDNA:GM10765 CG17084 CG18853 CG40410 CG6965 Caf1 CycT DnaJ-1 Eip71CD GstS1 Hsp23 Hsp26 Hsp27 Hsp67Ba Hus1-like Mpk2 Pten Thd1 Tl Tsp96F ald br emp mth ninA pain pll serpin-27A tws |
| 119 | GO:0040011 | P | 3, | 25 | 12.883 (x 1.941) | 261 (0.096) | 0.0202 | locomotion | Abi Fas3 Galpha49B InR Ptp99A Sema-1a Sema-1b Sema-2a alphaTub84B ap betaTub56D fra lola mod(mdg4) ninA opa otk pbl sgl spen sqd stai th trn usp |
| 120 | GO:0019887 | F | 4, | 7 | 1.728 (x 4.052) | 35 (0.200) | 0.0249 | protein kinase regulator activity | CkIIbeta Cks CycB3 CycT dap for pcs |
| 121 | GO:0005700 | C | 6, 7, 8, 9, | 8 | 2.221 (x 3.602) | 45 (0.178) | 0.0255 | polytene chromosome | Caf1 CycT Dp1 Pep jumu mod(mdg4) sqd usp |
| 122 | GO:0005488 | F | 2, | 235 | 204.057 (x 1.152) | 4134 (0.057) | 0.0256 | binding | Abi Amph Ance Arf79F B52 BEST:CK02656 BEST:GH02921 BEST:LD29214 BcDNA:GH02976 BcDNA:GM10765 BcDNA:LD21403 BcDNA:LD41548 CBP CG10359 CG10657 CG11033 CG11142 CG11151 CG11275 CG13848 CG14217 CG15435 CG15835 CG1632 CG17052 CG17419 CG17493 CG17838 CG17919 CG17952 CG18853 CG1907 CG1911 CG1924 CG2108 CG2118 CG30011 CG31121 CG3136 CG31453 CG32137 CG32499 CG33113 CG33525 CG3800 CG3823 CG3921 CG40410 CG4496 CG4914 CG5319 CG6049 CG6654 CG6812 CG6891 CG6930 CG6946 CG6966 CG7379 CG7668 CG8092 CG8963 CG9027 CG9057 CG9134 CG9307 CG9342 CG9906 CG9924 CREG CaMKI Cad74A Cad87A Cad96Ca Caf1 Cdk4 Cip4 CkIIalpha Cks Crc CrebA CtBP CycB3 D19A Dl DnaJ-1 Doc1 Doc2 Doc3 Dp Dp1 Dr Dref Dsp1 ETH Eb1 Eip74EF Eip75B Eph Fkbp13 Fs(2)Ket GATAd GNBP3 Galpha49B Gfat1 HLHm7 Hex-A His4r HmgD HmgZ Hrb27C Hsp23 InR Incenp Klp10A LanB2 Mcm7 Mpk2 Nek2 Nep2 Obp99a Optix Pep Pepck Pi3K21B Poxn Pten Ptp99A Rab8 RacGAP50C Rbf2 Rep4 RhoGAP71E Rhp Rm62 Sema-1a Sema-1b Sema-2a Set Sh Sry-alpha TER94 Tapdelta Thd1 Tl Top2 Trl Tsp26A Tsp39D Tsp66E Tsp96F Ucp4B Vha100-2 Wnt2 a6 ald alphaTub84B ap ara arr ash2 asp betaTub56D bip1 blot br btsz bun cdc2 ced-6 cg ci cib cic ck cyc d dve eIF-4E edl en esn for glec glu grh grn heph hth in inv jumu kis klu l(1)G0320 l(2)01424 lid loj lola mav mbt mdy mod(mdg4) nmo nonA-l numb olf413 ome opa otk pAbp pcs pdm2 ph-p pk pll polo sc scf shf smi35A spen sqd stai stan th tkv toe tok trh usp vimar wgn woc zf30C |
| 123 | GO:0040007 | P | 2, | 12 | 4.442 (x 2.701) | 90 (0.133) | 0.026 | growth | CG3424 Cdk4 InR Pi3K21B Pten btsz charybde eIF-4E mav ninA scylla tkv |
| 124 | GO:0009628 | P | 3, | 32 | 18.362 (x 1.743) | 372 (0.086) | 0.0262 | response to abiotic stimulus | Aldh-III BcDNA:GH04753 CG11711 CG17323 CG40410 CG6776 CG8588 Crc CycT DnaJ-1 Eip75B Fas3 Galpha49B GstS1 Hsp23 Hsp26 Hsp27 Hsp67Ba InR Mgstl Mpk2 Obp99a Sema-2a Sh br cyc mth ogre pain para smi35A usp |
| 125 | GO:0019207 | F | 3, | 10 | 3.307 (x 3.024) | 67 (0.149) | 0.0266 | kinase regulator activity | CG17919 CkIIbeta Cks CycB3 CycT Pi3K21B Pli dap for pcs |
| 126 | GO:0050793 | P | 3, | 11 | 3.899 (x 2.821) | 79 (0.139) | 0.0279 | regulation of development | Dl Dr Eip74EF Tl ana br bun ed mbt pbl usp |
| 127 | GO:0000003 | P | 2, | 40 | 24.730 (x 1.617) | 501 (0.080) | 0.0284 | reproduction | Ance CG11151 Caf1 Dl Dp Eip74EF Eip75B Fas3 Fs(2)Ket Hrb27C InR Poxn S Set Sh TER94 asp bun cdc2 ci dap esn fwd heph lig loj mdy mod(mdg4) opa para ph-p pk polo shu sqd stai th tkv usp wbl |
| 128 | GO:0043118 | P | 4, | 23 | 11.896 (x 1.933) | 241 (0.095) | 0.0307 | negative regulation of physiological process | Aac11 CG11079 CG33525 CG40410 CREG Caf1 CtBP Pten Rbf2 ana ci cic cyc dap edl en fwd nmo ph-p serpin-27A sqd th usp |
| 129 | GO:0007001 | P | 7, | 17 | 7.750 (x 2.194) | 157 (0.108) | 0.0314 | chromosome organization and biogenesis (sensu Eukaryota) | CG1911 Caf1 Dsp1 His4r HmgD HmgZ Incenp Set Trl ash2 eIF-4E glu jumu kis lola mod(mdg4) ph-p |
| 130 | GO:0009408 | P | 4, 5, | 8 | 2.320 (x 3.448) | 47 (0.170) | 0.0318 | response to heat | CycT DnaJ-1 Hsp23 Hsp26 Hsp27 Hsp67Ba mth pain |
| 131 | GO:0048732 | P | 4, | 15 | 6.466 (x 2.320) | 131 (0.115) | 0.0324 | gland development | CG7860 CrebA Eip71CD Eip74EF Eip75B Sema-2a br bun eIF-4E emp hth l(2)01424 tkv trh usp |
| 132 | GO:0051276 | P | 6, | 18 | 8.490 (x 2.120) | 172 (0.105) | 0.0335 | chromosome organization and biogenesis | CG1911 Caf1 Dp1 Dsp1 His4r HmgD HmgZ Incenp Set Trl ash2 eIF-4E glu jumu kis lola mod(mdg4) ph-p |
| 133 | GO:0016861 | F | 5, | 3 | 0.296 (x 10.130) | 6 (0.500) | 0.0344 | intramolecular oxidoreductase activity, interconverting aldoses and ketoses | Oscillin Pgi Tpi |
| 134 | GO:0048100 | P | 6, 7, | 4 | 0.592 (x 6.753) | 12 (0.333) | 0.0345 | wing disc anterior/posterior pattern formation | ci en inv tkv |
| 135 | GO:0006094 | P | 8, 9, 10, | 3 | 0.296 (x 10.130) | 6 (0.500) | 0.0347 | gluconeogenesis | Pepck Pgi Tpi |
| 136 | GO:0051243 | P | 5, | 22 | 11.452 (x 1.921) | 232 (0.095) | 0.035 | negative regulation of cellular physiological process | Aac11 CG11079 CG33525 CG40410 CREG Caf1 CtBP Pten Rbf2 ana ci cic cyc dap edl en fwd nmo ph-p sqd th usp |
| 137 | GO:0006066 | P | 5, | 17 | 7.947 (x 2.139) | 161 (0.106) | 0.0351 | alcohol metabolism | BEST:LD22483 CG1630 CG1998 Eno Gapdh1 Gapdh2 Gfat1 Hex-A Oscillin Pepck Pgi Tpi arr knk mdy olf413 woc |
| 138 | GO:0030215 | F | 4, 5, | 2 | 0.099 (x 20.259) | 2 (1.000) | 0.0352 | semaphorin receptor binding | Sema-1a otk |
| 139 | GO:0035221 | P | 5, 6, | 2 | 0.099 (x 20.259) | 2 (1.000) | 0.0355 | genital disc pattern formation | ci en |
| 140 | GO:0035224 | P | 6, 7, | 2 | 0.099 (x 20.259) | 2 (1.000) | 0.0357 | genital disc anterior/posterior pattern formation | ci en |
| 141 | GO:0007156 | P | 5, | 6 | 1.431 (x 4.192) | 29 (0.207) | 0.0358 | homophilic cell adhesion | Cad74A Cad87A Cad96Ca Fas3 ed stan |
| 142 | GO:0048749 | P | 7, | 15 | 6.565 (x 2.285) | 133 (0.113) | 0.0358 | compound eye development (sensu Endopterygota) | Amph Dl S TER94 ara arr br bun hth klu mbt nmo pk stan tkv |
| 143 | GO:0006641 | P | 7, 8, 9, | 2 | 0.099 (x 20.259) | 2 (1.000) | 0.036 | triacylglycerol metabolism | CG9342 mdy |
| 144 | GO:0002009 | P | 4, | 15 | 6.565 (x 2.285) | 133 (0.113) | 0.0361 | morphogenesis of an epithelium | Dl Dp InR Sb ara blot in inx2 jumu nmo ph-p pk sqd stan tkv |
| 145 | GO:0035007 | P | 5, 6, 8, | 2 | 0.099 (x 20.259) | 2 (1.000) | 0.0362 | regulation of melanization defense response | Tl serpin-27A |
| 146 | GO:0001745 | P | 7, 8, | 15 | 6.565 (x 2.285) | 133 (0.113) | 0.0363 | compound eye morphogenesis (sensu Endopterygota) | Amph Dl S TER94 ara arr br bun hth klu mbt nmo pk stan tkv |
| 147 | GO:0008475 | F | 6, | 2 | 0.099 (x 20.259) | 2 (1.000) | 0.0365 | procollagen-lysine 5-dioxygenase activity | CG31915 CG6199 |
| 148 | GO:0035222 | P | 5, 6, | 5 | 0.987 (x 5.065) | 20 (0.250) | 0.0367 | wing disc pattern formation | ap ci en inv tkv |
| 149 | GO:0007289 | P | 6, 9, | 2 | 0.099 (x 20.259) | 2 (1.000) | 0.0367 | spermatid nuclear differentiation | Ance th |
| 150 | GO:0048102 | P | 6, | 10 | 3.505 (x 2.853) | 71 (0.141) | 0.0369 | autophagic cell death | CG7860 Eip71CD Eip74EF Eip75B br bun eIF-4E emp l(2)01424 usp |
| 151 | GO:0031347 | P | 5, | 2 | 0.099 (x 20.259) | 2 (1.000) | 0.037 | regulation of defense response | Tl serpin-27A |
| 152 | GO:0035071 | P | 7, | 10 | 3.505 (x 2.853) | 71 (0.141) | 0.0371 | salivary gland cell autophagic cell death | CG7860 Eip71CD Eip74EF Eip75B br bun eIF-4E emp l(2)01424 usp |
| 153 | GO:0035070 | P | 6, | 10 | 3.505 (x 2.853) | 71 (0.141) | 0.0374 | salivary gland histolysis | CG7860 Eip71CD Eip74EF Eip75B br bun eIF-4E emp l(2)01424 usp |
| 154 | GO:0016616 | F | 5, | 11 | 4.146 (x 2.653) | 84 (0.131) | 0.0374 | oxidoreductase activity, acting on the CH-OH group of donors, NAD or NADP as acceptor | BEST:LD22483 BcDNA:GH12558 CG11151 CG2767 CG31937 CG6287 CtBP Idh LanB2 Pgi sgl |
| 155 | GO:0000910 | P | 5, | 11 | 4.146 (x 2.653) | 84 (0.131) | 0.0377 | cytokinesis | Act42A CG4454 Caf1 CycB3 RacGAP50C Snap Sry-alpha Tl fwd pbl polo |
| 156 | GO:0048592 | P | 5, 6, | 16 | 7.355 (x 2.175) | 149 (0.107) | 0.038 | eye morphogenesis | Amph Dl Ice S TER94 ara arr br bun hth klu mbt nmo pk stan tkv |
| 157 | GO:0050896 | P | 2, | 78 | 57.702 (x 1.352) | 1169 (0.067) | 0.0391 | response to stimulus | 18w Aldh-III BEST:GH02921 BcDNA:GH04753 BcDNA:GM10765 CG10359 CG11711 CG11824 CG12199 CG17084 CG17323 CG18853 CG2852 CG30011 CG40410 CG5873 CG6776 CG6965 CG7668 CG8588 CG9027 Caf1 CkIIalpha CkIIbeta Crc CycT DnaJ-1 Eip71CD Eip75B Fas3 GNBP3 Galpha49B GstS1 Hsp23 Hsp26 Hsp27 Hsp67Ba Hus1-like InR Jafrac2 Mgstl Mpk2 Obp99a PFE Pli Poxn Pten Sb Sema-2a Sh Thd1 Tl Tsp66E Tsp96F ald br ced-6 ck cyc d emp for fra lig loj mth ninA numb ogre otk pain para pll serpin-27A smi35A trn tws usp |
| 158 | GO:0001709 | P | 5, | 13 | 5.430 (x 2.394) | 110 (0.118) | 0.0396 | cell fate determination | Dr Pten S bun cdc2 grh jumu numb pon sc spen tkv trh |
| 159 | GO:0004672 | F | 6, | 26 | 14.561 (x 1.786) | 295 (0.088) | 0.0398 | protein kinase activity | CG14217 CG40410 CaMKI Cad96Ca Cdk4 CkIIalpha CkIIbeta Cks CycT Eph InR Mpk2 Nek2 PFE ald asp cdc2 for mbt nmo otk pll polo shf smi35A tkv |
| 160 | GO:0016773 | F | 5, | 30 | 17.622 (x 1.702) | 357 (0.084) | 0.0399 | phosphotransferase activity, alcohol group as acceptor | CG14217 CG1630 CG40410 CaMKI Cad96Ca Cdk4 CkIIalpha CkIIbeta Cks CycT Eph Hex-A InR Mpk2 Nek2 PFE Pi3K21B ald asp cdc2 for fwd mbt nmo otk pll polo shf smi35A tkv |
| 161 | GO:0006915 | P | 6, | 18 | 8.786 (x 2.049) | 178 (0.101) | 0.0401 | apoptosis | Aac11 CG14217 CG6680 CG7379 Dp Ice Jafrac2 Pten Rep4 ced-6 emp mdy mod(mdg4) nmo smi35A th trn wgn |
| 162 | GO:0016339 | P | 5, | 5 | 1.037 (x 4.824) | 21 (0.238) | 0.0405 | calcium-dependent cell-cell adhesion | Cad74A Cad87A Cad96Ca Fas3 stan |
| 163 | GO:0007169 | P | 7, | 14 | 6.121 (x 2.287) | 124 (0.113) | 0.0415 | transmembrane receptor protein tyrosine kinase signaling pathway | CG5522 Cad96Ca Eph InR Pi3K21B Pten S ced-6 ed edl knk otk sgl spen |
| 164 | GO:0009892 | P | 5, | 17 | 8.144 (x 2.087) | 165 (0.103) | 0.0419 | negative regulation of metabolism | CG11079 CG33525 CREG Caf1 CtBP Rbf2 ci cic cyc dap edl en fwd ph-p serpin-27A sqd usp |
| 165 | GO:0008283 | P | 4, | 25 | 13.920 (x 1.796) | 282 (0.089) | 0.0422 | cell proliferation | CG6654 CG6966 Cad96Ca Cdk4 CkIIalpha D19A Dl Eip75B HLHm7 InR Pi3K21B Poxn Pten RacGAP50C Tl ana cg klu mbt nmo opa pll shf smi35A toe |
| 166 | GO:0009607 | P | 3, | 40 | 25.766 (x 1.552) | 522 (0.077) | 0.0455 | response to biotic stimulus | 18w Aldh-III BEST:GH02921 BcDNA:GH04753 CG10359 CG11824 CG12199 CG17323 CG2852 CG30011 CG5873 CG6776 CG7668 CG9027 DnaJ-1 Eip71CD GNBP3 GstS1 Hsp23 Hsp26 Hsp27 Hsp67Ba Jafrac2 Mgstl Mpk2 PFE Pli Sb Tl Tsp66E Tsp96F br ced-6 emp fra otk pll serpin-27A trn tws |
| 167 | GO:0043068 | P | 6, 7, | 10 | 3.702 (x 2.701) | 75 (0.133) | 0.0466 | positive regulation of programmed cell death | CG14217 CG7379 Dp Eip75B Jafrac2 br klu mod(mdg4) smi35A usp |
| 168 | GO:0016199 | P | 6, 8, 9, 11, 12, 14, | 3 | 0.346 (x 8.682) | 7 (0.429) | 0.0469 | axon midline choice point recognition | Galpha49B fra lola |
| 169 | GO:0006468 | P | 8, | 25 | 14.068 (x 1.777) | 285 (0.088) | 0.0473 | protein amino acid phosphorylation | CG14217 CG40410 CaMKI Cad96Ca Cdk4 CkIIalpha CkIIbeta Cks Eph InR Incenp Mpk2 Nek2 PFE Pi3K21B ald cdc2 for mbt nmo otk pll polo smi35A tkv |
| 170 | GO:0008037 | P | 3, | 5 | 1.086 (x 4.604) | 22 (0.227) | 0.0474 | cell recognition | Fas3 Galpha49B Sema-2a fra lola |
| 171 | GO:0045927 | P | 4, | 5 | 1.086 (x 4.604) | 22 (0.227) | 0.0477 | positive regulation of growth | InR Pi3K21B btsz eIF-4E tkv |
| 172 | GO:0050875 | P | 3, | 332 | 306.825 (x 1.082) | 6216 (0.053) | 0.0479 | cellular physiological process | Aac11 Abi Act42A Amph Ance Arf79F Argk B52 BEST:CK02656 BEST:GH02921 BEST:LD04971 BEST:LD22483 BG:DS00004.11 BcDNA:GH02901 BcDNA:GH02976 BcDNA:GH12558 BcDNA:GM10765 BcDNA:LD41548 Best2 CG10657 CG11033 CG11079 CG11142 CG11438 CG11550 CG11739 CG11824 CG12048 CG12199 CG14214 CG14217 CG14439 CG15088 CG15835 CG1607 CG1630 CG1632 CG17052 CG17323 CG17419 CG17493 CG18853 CG1893 CG1907 CG1911 CG1924 CG1998 CG2108 CG2118 CG2200 CG2852 CG30011 CG31121 CG3136 CG31453 CG31472 CG31738 CG31915 CG32137 CG32158 CG32499 CG32632 CG33113 CG33116 CG33138 CG33171 CG3328 CG33525 CG3424 CG3590 CG3770 CG3823 CG40160 CG40410 CG4454 CG4502 CG4586 CG4914 CG5319 CG5390 CG5466 CG5794 CG5873 CG6287 CG6391 CG6654 CG6680 CG6767 CG6776 CG6812 CG6904 CG6946 CG6966 CG7379 CG7860 CG8092 CG8165 CG8963 CG9027 CG9057 CG9135 CG9307 CG9342 CG9906 CREG CaMKI Cad96Ca Caf1 Cdk4 Cip4 CkIIalpha CkIIbeta Cks Crc CrebA CtBP CycB3 CycT Cyp310a1 D19A Dl DnaJ-1 Doc1 Doc2 Doc3 Dp Dp1 Dr Dref Dsp1 EG:171D11.1 ERp60 ESTS:39C10S Eb1 Eip55E Eip71CD Eip74EF Eip75B Eno Eph Fas3 Fkbp13 Fs(2)Ket GATAd GalNAc-T1 Galpha49B Gapdh1 Gapdh2 Gfat1 GlyP GstS1 HLHm7 Hex-A His4r HmgD HmgZ Hrb27C Hsp23 Hsp26 Hsp27 Hsp67Ba Hus1-like Ice Idh InR Incenp Ir Jafrac2 Klp10A LanB2 Lsp2 M6 Mcm7 Mgstl Mpk2 Nek2 Nep2 Nrv1 Obp99a Optix Oscillin PFE Pdi Pepck Pgi Pi3K21B Poxn Ppt1 Prosalpha6 Pten Ptp99A Rab8 RacGAP50C Rbf2 Rep4 Rhp Rm62 RnrS RpS12 Rpn9 S Sb Sema-1a Sema-1b Sema-2a Set Sh Snap Spn1 Spn5 Sry-alpha TER94 Thd1 Tl Top2 Tpi Trl Ts Uch Ucp4B Ugt Vha100-2 ald alphaTub84B ana ap ara arr ash2 asp betaTub56D blot br btsz bun cdc2 ced-6 cg ci cib cic ck cyc d dUTPase dap dve eIF-4E east edl emp en esn fax for fra fwd fzy glu grh grn heph hth in inv jumu kis klu knk l(1)G0320 l(2)01424 l(2)01810 lid loj lola mav mbt mdy mnd mod(mdg4) mth ninA nmo nonA-l numb ogre olf413 ome opa otk pAbp pain para pbl pcs pdm2 pgant5 ph-p pim pk pll polo pon prominin-like rasp rpk sc serpin-27A sgl shanti shf shu smi35A spen sqd stai stan sut1 th tkv toe tok trh trn tws usp vg wbl wgn woc zf30C zwilch |
| 173 | GO:0008038 | P | 4, | 5 | 1.086 (x 4.604) | 22 (0.227) | 0.048 | neuron recognition | Fas3 Galpha49B Sema-2a fra lola |
| 174 | GO:0005791 | C | 6, 7, 8, 9, | 4 | 0.691 (x 5.788) | 14 (0.286) | 0.0487 | rough endoplasmic reticulum | CG14214 Tapdelta Ugt l(1)G0320 |
| 175 | GO:0004674 | F | 7, | 20 | 10.464 (x 1.911) | 212 (0.094) | 0.049 | protein serine/threonine kinase activity | CG14217 CG40410 CaMKI Cdk4 CkIIalpha CkIIbeta Cks CycT Mpk2 Nek2 PFE ald cdc2 for mbt nmo pll polo smi35A tkv |
| 176 | GO:0045793 | P | 6, 7, | 4 | 0.691 (x 5.788) | 14 (0.286) | 0.049 | positive regulation of cell size | InR Pi3K21B btsz eIF-4E |
| 177 | GO:0048748 | P | 6, 7, | 15 | 6.960 (x 2.155) | 141 (0.106) | 0.0491 | eye morphogenesis (sensu Endopterygota) | Amph Dl S TER94 ara arr br bun hth klu mbt nmo pk stan tkv |
| 178 | GO:0007448 | P | 5, 6, | 4 | 0.691 (x 5.788) | 14 (0.286) | 0.0493 | anterior/posterior pattern formation, imaginal disc | ci en inv tkv |
| 179 | GO:0007582 | P | 2, | 360 | 337.231 (x 1.068) | 6832 (0.053) | 0.0515 | physiological process | 18w Aac11 Abi Act42A Aldh-III Amph Ance Arf79F Argk B52 BEST:CK02656 BEST:GH02921 BEST:LD04971 BEST:LD22483 BG:DS00004.11 BcDNA:GH02901 BcDNA:GH02976 BcDNA:GH12558 BcDNA:GM10765 BcDNA:LD41548 Best2 CG10657 CG11033 CG11079 CG11142 CG11438 CG11550 CG11739 CG11824 CG12048 CG12199 CG14214 CG14217 CG14439 CG1471 CG15088 CG15835 CG1607 CG1630 CG1632 CG16974 CG17052 CG17084 CG17323 CG17419 CG17493 CG18853 CG1893 CG1907 CG1911 CG1924 CG1998 CG2108 CG2118 CG2200 CG2852 CG30011 CG31121 CG3136 CG31453 CG31472 CG31738 CG31915 CG31937 CG32137 CG32158 CG32499 CG32632 CG33113 CG33116 CG33138 CG33171 CG3328 CG33525 CG3424 CG3590 CG3770 CG3823 CG3842 CG40160 CG40410 CG4054 CG4454 CG4502 CG4586 CG4914 CG5096 CG5319 CG5390 CG5466 CG5731 CG5794 CG5873 CG6199 CG6287 CG6391 CG6654 CG6680 CG6767 CG6776 CG6812 CG6904 CG6946 CG6965 CG6966 CG7379 CG7675 CG7860 CG8092 CG8165 CG8963 CG9027 CG9057 CG9066 CG9135 CG9307 CG9342 CG9906 CREG CaMKI Cad96Ca Caf1 Cdk4 Cip4 CkIIalpha CkIIbeta Cks Crc CrebA CtBP CycB3 CycT Cyp310a1 D19A Dl DnaJ-1 Doc1 Doc2 Doc3 Dp Dp1 Dr Dref Dsp1 EG:118B3.2 EG:171D11.1 EG:BACN33B1.2 ERp60 ESTS:39C10S ETH Eb1 Eip55E Eip71CD Eip74EF Eip75B Eno Eph Fas3 Fkbp13 Fs(2)Ket GATAd GNBP3 GalNAc-T1 Galpha49B Gapdh1 Gapdh2 Gfat1 GlyP GstS1 HLHm7 Hex-A Hexo1 His4r HmgD HmgZ Hrb27C Hsp23 Hsp26 Hsp27 Hsp67Ba Hus1-like Ice Idh InR Incenp Ir Jafrac2 Klp10A LanB2 Lsp2 M6 Mcm7 Mgstl Mpk2 Nek2 Nep2 Nrv1 Obp99a Optix Oscillin PFE Pdi Pepck Pgi Pi3K21B Pli Poxn Ppt1 Prosalpha6 Pten Ptp99A Rab8 RacGAP50C Rbf2 Reg-5 Rep4 RhoGAP71E Rhp Rm62 RnrS RpS12 Rpn9 S Sb Sema-1a Sema-1b Sema-2a Set Sh Snap Spn1 Spn5 Sry-alpha TER94 Tapdelta Thd1 Tl Top2 Tpi Trl Ts Tsp96F Uch Ucp4B Ugt Vha100-2 ald alpha-Man-IIb alphaTub84B ana ap ara arr ash2 asp betaTub56D blot br btsz bun cdc2 ced-6 cg ci cib cic ck cyc d dUTPase dap dve eIF-4E east edl emp en esn fax fbp for fra fwd fzy glu grh grn gukh heph hth in inv jumu kal-1 kis klu knk l(1)G0320 l(2)01424 l(2)01810 lid loj lola mav mbt mdy mnd mod(mdg4) mth ninA nmo nonA-l numb ogre olf413 ome opa otk pAbp pain para pbl pcs pdm2 pgant5 ph-p pim pk pll polo pon prominin-like rasp rpk sc serpin-27A sgl shanti shf shu smi35A spen sqd stai stan sut1 th tkv toe tok trh trn tws usp vg wbl wgn woc zf30C zwilch |
| 180 | GO:0044264 | P | 6, 7, | 10 | 3.801 (x 2.631) | 77 (0.130) | 0.0528 | cellular polysaccharide metabolism | BcDNA:GH02976 CG11142 CG17052 CG31915 CG32499 CG33138 CG6904 CG9307 GlyP sgl |
| 181 | GO:0006952 | P | 4, | 39 | 25.371 (x 1.537) | 514 (0.076) | 0.0552 | defense response | 18w Aldh-III BEST:GH02921 BcDNA:GH04753 CG10359 CG11824 CG12199 CG17323 CG2852 CG30011 CG5873 CG6776 CG7668 CG9027 DnaJ-1 Eip71CD GNBP3 GstS1 Hsp23 Hsp26 Hsp27 Hsp67Ba Jafrac2 Mgstl Mpk2 PFE Pli Sb Tl Tsp66E Tsp96F ced-6 emp fra otk pll serpin-27A trn tws |
| 182 | GO:0044262 | P | 6, | 24 | 13.574 (x 1.768) | 275 (0.087) | 0.0556 | cellular carbohydrate metabolism | BEST:LD22483 BcDNA:GH02976 CG11142 CG1630 CG17052 CG31915 CG32499 CG33138 CG6904 CG9307 Eno GalNAc-T1 Gapdh1 Gapdh2 Gfat1 GlyP Hex-A Idh Oscillin Pepck Pgi Tpi pgant5 sgl |
| 183 | GO:0009266 | P | 4, | 8 | 2.665 (x 3.001) | 54 (0.148) | 0.0556 | response to temperature stimulus | CycT DnaJ-1 Hsp23 Hsp26 Hsp27 Hsp67Ba mth pain |
| 184 | GO:0043231 | C | 4, 5, 6, 7, | 138 | 113.875 (x 1.212) | 2307 (0.060) | 0.056 | intracellular membrane-bound organelle | B52 BEST:GH02921 BEST:LD29214 BG:DS00004.11 BcDNA:GH12558 BcDNA:GM10765 CBP CG11079 CG14214 CG15435 CG17838 CG17952 CG1907 CG1911 CG1924 CG2118 CG30011 CG3136 CG33113 CG33525 CG40410 CG4496 CG4586 CG4914 CG6654 CG6782 CG6930 CG8092 CG8679 CG9342 CG9894 CREG Caf1 CkIIalpha CkIIbeta Crc CrebA CtBP CycB3 CycT D19A Doc1 Doc2 Doc3 Dp Dp1 Dr Dref Dsp1 EG:171D11.1 EG:BACN33B1.2 ERp60 Eip74EF Eip75B Fs(2)Ket GATAd GalNAc-T1 HLHm7 HmgD HmgZ Hrb27C Idh Mcm7 Mgstl Mpk2 Optix Pdi Pep Pepck Poxn Ppt1 Ptp99A Rbf2 Rm62 S Set Snap TER94 Tapdelta Thd1 Top2 Trl Ucp4B Ugt Vha100-2 alpha-Man-IIb ap ara ash2 bip1 br btsz bun cdc2 cg ci cic crp cyc dap dve east edl emp en esn glu grh grn heph hth inv jumu kis klu l(1)G0320 lid lola mod(mdg4) nonA-l numb opa pbl pdm2 pgant5 ph-p pk sc shanti spen sqd toe trh usp vg wbl woc zf30C |
| 185 | GO:0016301 | F | 5, | 32 | 19.794 (x 1.617) | 401 (0.080) | 0.0561 | kinase activity | Argk CG14217 CG1630 CG40410 CG6767 CaMKI Cad96Ca Cdk4 CkIIalpha CkIIbeta Cks CycT Eph Hex-A InR Mpk2 Nek2 PFE Pi3K21B ald asp cdc2 for fwd mbt nmo otk pll polo shf smi35A tkv |
| 186 | GO:0043227 | C | 3, | 138 | 113.974 (x 1.211) | 2309 (0.060) | 0.0572 | membrane-bound organelle | B52 BEST:GH02921 BEST:LD29214 BG:DS00004.11 BcDNA:GH12558 BcDNA:GM10765 CBP CG11079 CG14214 CG15435 CG17838 CG17952 CG1907 CG1911 CG1924 CG2118 CG30011 CG3136 CG33113 CG33525 CG40410 CG4496 CG4586 CG4914 CG6654 CG6782 CG6930 CG8092 CG8679 CG9342 CG9894 CREG Caf1 CkIIalpha CkIIbeta Crc CrebA CtBP CycB3 CycT D19A Doc1 Doc2 Doc3 Dp Dp1 Dr Dref Dsp1 EG:171D11.1 EG:BACN33B1.2 ERp60 Eip74EF Eip75B Fs(2)Ket GATAd GalNAc-T1 HLHm7 HmgD HmgZ Hrb27C Idh Mcm7 Mgstl Mpk2 Optix Pdi Pep Pepck Poxn Ppt1 Ptp99A Rbf2 Rm62 S Set Snap TER94 Tapdelta Thd1 Top2 Trl Ucp4B Ugt Vha100-2 alpha-Man-IIb ap ara ash2 bip1 br btsz bun cdc2 cg ci cic crp cyc dap dve east edl emp en esn glu grh grn heph hth inv jumu kis klu l(1)G0320 lid lola mod(mdg4) nonA-l numb opa pbl pdm2 pgant5 ph-p pk sc shanti spen sqd toe trh usp vg wbl woc zf30C |
| 187 | GO:0000793 | C | 6, 7, 8, 9, | 4 | 0.740 (x 5.402) | 15 (0.267) | 0.0598 | condensed chromosome | CG1911 Incenp glu polo |
| 188 | GO:0007093 | P | 8, 9, | 4 | 0.740 (x 5.402) | 15 (0.267) | 0.0602 | mitotic checkpoint | CG40410 ald alphaTub84B zwilch |
| 189 | GO:0016614 | F | 4, | 13 | 5.825 (x 2.232) | 118 (0.110) | 0.0612 | oxidoreductase activity, acting on CH-OH group of donors | BEST:LD22483 BcDNA:GH12558 CG11151 CG2767 CG31937 CG3842 CG6287 CG7675 CtBP Idh LanB2 Pgi sgl |
| 190 | GO:0005243 | F | 5, | 3 | 0.395 (x 7.597) | 8 (0.375) | 0.0616 | gap-junction forming channel activity | inx2 inx3 ogre |
| 191 | GO:0000271 | P | 7, 8, | 3 | 0.395 (x 7.597) | 8 (0.375) | 0.0619 | polysaccharide biosynthesis | CG31915 CG6904 sgl |
| 192 | GO:0006996 | P | 5, | 51 | 35.638 (x 1.431) | 722 (0.071) | 0.062 | organelle organization and biogenesis | Abi Act42A CG1911 CG33171 CG9057 Caf1 Cdk4 Cip4 CycB3 CycT Dp1 Dref Dsp1 Eb1 Fs(2)Ket Galpha49B GstS1 His4r HmgD HmgZ Incenp Klp10A M6 Pten Rhp Sb Set Sry-alpha TER94 Trl alphaTub84B ash2 asp betaTub56D cib eIF-4E esn for fra fwd glu jumu kis lola mbt mod(mdg4) pbl ph-p polo stai vg |
| 193 | GO:0043284 | P | 6, 7, | 3 | 0.395 (x 7.597) | 8 (0.375) | 0.0622 | biopolymer biosynthesis | CG31915 CG6904 sgl |
| 194 | GO:0005921 | C | 7, 8, 9, | 3 | 0.395 (x 7.597) | 8 (0.375) | 0.0626 | gap junction | inx2 inx3 ogre |
| 195 | GO:0046879 | P | 5, 6, | 3 | 0.395 (x 7.597) | 8 (0.375) | 0.0629 | hormone secretion | CG15835 CG9066 stai |
| 196 | GO:0007455 | P | 6, 7, | 15 | 7.207 (x 2.081) | 146 (0.103) | 0.063 | eye-antennal disc morphogenesis | Amph Dl S TER94 ara arr br bun hth klu mbt nmo pk stan tkv |
| 197 | GO:0008361 | P | 5, 6, | 6 | 1.678 (x 3.575) | 34 (0.176) | 0.0639 | regulation of cell size | Cdk4 InR Pi3K21B Pten btsz eIF-4E |
| 198 | GO:0019953 | P | 3, | 35 | 22.508 (x 1.555) | 456 (0.077) | 0.0655 | sexual reproduction | Ance CG11151 Caf1 Dl Dp Eip74EF Eip75B Fas3 Fs(2)Ket Hrb27C InR S Set TER94 asp bun cdc2 ci dap esn fwd heph mdy mod(mdg4) opa ph-p pk polo shu sqd stai th tkv usp wbl |
| 199 | GO:0007610 | P | 3, | 18 | 9.428 (x 1.909) | 191 (0.094) | 0.0678 | behavior | CG11711 CG8588 CkIIalpha CkIIbeta Crc Fas3 Obp99a Poxn Sema-2a Sh cyc for lig loj numb ogre para smi35A |
| 200 | GO:0005976 | P | 6, | 16 | 7.996 (x 2.001) | 162 (0.099) | 0.0679 | polysaccharide metabolism | BcDNA:GH02976 CG11142 CG17052 CG17323 CG31915 CG32499 CG33138 CG5731 CG6904 CG9307 GNBP3 GalNAc-T1 GlyP Hexo1 pgant5 sgl |
| 201 | GO:0004702 | F | 4, 8, | 17 | 8.737 (x 1.946) | 177 (0.096) | 0.0696 | receptor signaling protein serine/threonine kinase activity | CG14217 CG40410 CaMKI Cdk4 CkIIalpha CkIIbeta Mpk2 Nek2 PFE cdc2 for mbt nmo pll polo smi35A tkv |
| 202 | GO:0045934 | P | 7, | 13 | 5.973 (x 2.177) | 121 (0.107) | 0.0707 | negative regulation of nucleobase, nucleoside, nucleotide and nucleic acid metabolism | CG11079 CG33525 CREG Caf1 CtBP Rbf2 ci cic cyc edl en ph-p usp |
| 203 | GO:0016620 | F | 5, | 4 | 0.790 (x 5.065) | 16 (0.250) | 0.0707 | oxidoreductase activity, acting on the aldehyde or oxo group of donors, NAD or NADP as acceptor | Aldh-III EG:171D11.1 Gapdh1 Gapdh2 |
| 204 | GO:0007367 | P | 6, 7, | 4 | 0.790 (x 5.065) | 16 (0.250) | 0.071 | segment polarity determination | en rasp sgl stan |
| 205 | GO:0043566 | F | 5, | 5 | 1.234 (x 4.052) | 25 (0.200) | 0.0711 | structure-specific DNA binding | Dp1 Dsp1 Hrb27C Pep Thd1 |
| 206 | GO:0003680 | F | 6, | 2 | 0.148 (x 13.506) | 3 (0.667) | 0.0717 | AT DNA binding | HmgD dve |
| 207 | GO:0008340 | P | 4, | 7 | 2.271 (x 3.083) | 46 (0.152) | 0.072 | determination of adult life span | CG17084 CG6965 Hsp26 Hsp27 InR fwd mth |
| 208 | GO:0007386 | P | 4, | 2 | 0.148 (x 13.506) | 3 (0.667) | 0.072 | compartment specification | en inv |
| 209 | GO:0007568 | P | 3, | 7 | 2.271 (x 3.083) | 46 (0.152) | 0.0723 | aging | CG17084 CG6965 Hsp26 Hsp27 InR fwd mth |
| 210 | GO:0035006 | P | 5, 7, | 2 | 0.148 (x 13.506) | 3 (0.667) | 0.0723 | melanization defense response | Tl serpin-27A |
| 211 | GO:0009219 | P | 8, | 2 | 0.148 (x 13.506) | 3 (0.667) | 0.0727 | pyrimidine deoxyribonucleotide metabolism | Ts dUTPase |
| 212 | GO:0005371 | F | 6, | 2 | 0.148 (x 13.506) | 3 (0.667) | 0.073 | tricarboxylate carrier activity | CG11739 CG6782 |
| 213 | GO:0004682 | F | 9, | 2 | 0.148 (x 13.506) | 3 (0.667) | 0.0734 | protein kinase CK2 activity | CkIIalpha CkIIbeta |
| 214 | GO:0007417 | P | 5, | 13 | 6.022 (x 2.159) | 122 (0.107) | 0.0735 | central nervous system development | CkIIbeta Crc Dl Dr br cib en grh hth mbt numb sc stan |
| 215 | GO:0007424 | P | 4, | 12 | 5.380 (x 2.230) | 109 (0.110) | 0.0736 | tracheal system development (sensu Insecta) | Cdk4 Dl Pten S Wnt2 dap grh sgl shanti tkv trh trn |
| 216 | GO:0044238 | P | 4, | 251 | 225.578 (x 1.113) | 4570 (0.055) | 0.0739 | primary metabolism | Ance Arf79F B52 BEST:CK02656 BEST:GH02921 BEST:LD04971 BEST:LD22483 BG:DS00004.11 BcDNA:GH02901 BcDNA:GH02976 BcDNA:GH12558 BcDNA:GM10765 BcDNA:LD41548 CG10657 CG11033 CG11079 CG11142 CG11438 CG11824 CG14217 CG1471 CG15088 CG15835 CG1607 CG1630 CG1632 CG17052 CG17323 CG18853 CG1893 CG1911 CG1924 CG1998 CG2118 CG2200 CG2852 CG30011 CG3136 CG31453 CG31738 CG31915 CG32158 CG32499 CG32632 CG33116 CG33138 CG33171 CG3328 CG33525 CG3590 CG40160 CG40410 CG4502 CG4586 CG4914 CG5319 CG5390 CG5731 CG5794 CG6199 CG6287 CG6391 CG6654 CG6680 CG6767 CG6904 CG6946 CG7379 CG7860 CG8092 CG8165 CG8963 CG9057 CG9135 CG9307 CG9342 CG9906 CREG CaMKI Cad96Ca Caf1 Cdk4 CkIIalpha CkIIbeta Cks Crc CrebA CtBP CycT Cyp310a1 D19A DnaJ-1 Doc1 Doc2 Doc3 Dp Dr Dref Dsp1 EG:171D11.1 ERp60 ESTS:39C10S Eb1 Eip55E Eip71CD Eip74EF Eip75B Eno Eph Fkbp13 GATAd GNBP3 GalNAc-T1 Galpha49B Gapdh1 Gapdh2 Gfat1 GlyP HLHm7 Hex-A Hexo1 His4r HmgD HmgZ Hrb27C Hsp23 Hsp26 Hsp27 Hsp67Ba Hus1-like Ice Idh InR Incenp Lsp2 Mcm7 Mgstl Mpk2 Nek2 Nep2 Optix Oscillin PFE Pdi Pepck Pgi Pi3K21B Poxn Ppt1 Prosalpha6 Pten Ptp99A Rab8 Rbf2 Rep4 Rm62 RnrS RpS12 Rpn9 Sb Set Spn1 Spn5 TER94 Thd1 Top2 Tpi Trl Ts Uch Ugt ald alpha-Man-IIb alphaTub84B ap ara arr ash2 betaTub56D blot br bun cdc2 cg ci cic cyc dUTPase dap dve eIF-4E east edl en fbp for fra fwd fzy glu grh grn heph hth inv jumu kis klu knk l(1)G0320 l(2)01424 l(2)01810 lid lola mbt mdy mnd mod(mdg4) nmo nonA-l olf413 ome opa otk pAbp pbl pdm2 pgant5 ph-p pll polo rasp sc sgl shanti shf shu smi35A spen sqd sut1 th tkv toe tok trh tws usp wbl woc zf30C |
| 217 | GO:0043226 | C | 2, | 156 | 132.484 (x 1.178) | 2684 (0.058) | 0.0761 | organelle | Act42A B52 BEST:GH02921 BEST:LD29214 BG:DS00004.11 BcDNA:GH12558 BcDNA:GM10765 CBP CG11079 CG14214 CG15435 CG17493 CG17838 CG17952 CG1907 CG1911 CG1924 CG2118 CG30011 CG3136 CG31738 CG33113 CG33525 CG40410 CG4496 CG4586 CG4914 CG6654 CG6782 CG6930 CG8092 CG8679 CG9342 CG9894 CREG Caf1 CkIIalpha CkIIbeta Crc CrebA CtBP CycB3 CycT D19A Doc1 Doc2 Doc3 Dp Dp1 Dr Dref Dsp1 EG:171D11.1 EG:BACN33B1.2 ERp60 Eb1 Eip74EF Eip75B Fs(2)Ket GATAd GalNAc-T1 HLHm7 His4r HmgD HmgZ Hrb27C Idh Incenp Klp10A Mcm7 Mgstl Mpk2 Nek2 Optix Pdi Pep Pepck Poxn Ppt1 Ptp99A Rbf2 Rm62 RpS12 S Set Snap TER94 Tapdelta Thd1 Top2 Trl Ucp4B Ugt Vha100-2 alpha-Man-IIb alphaTub84B ap ara ash2 asp betaTub56D bip1 br btsz bun cdc2 cg ci cic ck crp cyc d dap dve east edl emp en esn fzy glu grh grn heph hth inv jumu kis klu l(1)G0320 lid lola mod(mdg4) nonA-l numb opa p120ctn pbl pdm2 pgant5 ph-p pk polo sc shanti spen sqd stai toe trh usp vg wbl woc zf30C |
| 218 | GO:0043229 | C | 3, 4, 5, 6, | 156 | 132.484 (x 1.178) | 2684 (0.058) | 0.0764 | intracellular organelle | Act42A B52 BEST:GH02921 BEST:LD29214 BG:DS00004.11 BcDNA:GH12558 BcDNA:GM10765 CBP CG11079 CG14214 CG15435 CG17493 CG17838 CG17952 CG1907 CG1911 CG1924 CG2118 CG30011 CG3136 CG31738 CG33113 CG33525 CG40410 CG4496 CG4586 CG4914 CG6654 CG6782 CG6930 CG8092 CG8679 CG9342 CG9894 CREG Caf1 CkIIalpha CkIIbeta Crc CrebA CtBP CycB3 CycT D19A Doc1 Doc2 Doc3 Dp Dp1 Dr Dref Dsp1 EG:171D11.1 EG:BACN33B1.2 ERp60 Eb1 Eip74EF Eip75B Fs(2)Ket GATAd GalNAc-T1 HLHm7 His4r HmgD HmgZ Hrb27C Idh Incenp Klp10A Mcm7 Mgstl Mpk2 Nek2 Optix Pdi Pep Pepck Poxn Ppt1 Ptp99A Rbf2 Rm62 RpS12 S Set Snap TER94 Tapdelta Thd1 Top2 Trl Ucp4B Ugt Vha100-2 alpha-Man-IIb alphaTub84B ap ara ash2 asp betaTub56D bip1 br btsz bun cdc2 cg ci cic ck crp cyc d dap dve east edl emp en esn fzy glu grh grn heph hth inv jumu kis klu l(1)G0320 lid lola mod(mdg4) nonA-l numb opa p120ctn pbl pdm2 pgant5 ph-p pk polo sc shanti spen sqd stai toe trh usp vg wbl woc zf30C |
| 219 | GO:0007276 | P | 4, | 34 | 22.064 (x 1.541) | 447 (0.076) | 0.0766 | gametogenesis | Ance CG11151 Caf1 Dl Dp Eip74EF Eip75B Fas3 Fs(2)Ket Hrb27C InR S Set TER94 asp bun cdc2 ci dap esn fwd heph mdy mod(mdg4) opa ph-p pk shu sqd stai th tkv usp wbl |
| 220 | GO:0005703 | C | 5, 6, 7, 8, 9, 10, | 3 | 0.444 (x 6.753) | 9 (0.333) | 0.0772 | polytene chromosome puff | CycT Pep sqd |
| 221 | GO:0006073 | P | 7, 8, | 3 | 0.444 (x 6.753) | 9 (0.333) | 0.0776 | glucan metabolism | CG33138 CG6904 GlyP |
| 222 | GO:0035072 | P | 7, 8, 9, 10, 11, | 3 | 0.444 (x 6.753) | 9 (0.333) | 0.0779 | ecdysone-mediated induction of salivary gland cell autophagic cell death | Eip75B br usp |
| 223 | GO:0005977 | P | 8, 9, | 3 | 0.444 (x 6.753) | 9 (0.333) | 0.0783 | glycogen metabolism | CG33138 CG6904 GlyP |
| 224 | GO:0016860 | F | 4, | 5 | 1.283 (x 3.896) | 26 (0.192) | 0.0784 | intramolecular oxidoreductase activity | ERp60 Oscillin Pdi Pgi Tpi |
| 225 | GO:0035161 | P | 5, 6, | 3 | 0.444 (x 6.753) | 9 (0.333) | 0.0786 | imaginal disc lineage restriction | Dl ap en |
| 226 | GO:0051093 | P | 4, | 5 | 1.283 (x 3.896) | 26 (0.192) | 0.0787 | negative regulation of development | Dl ana bun ed usp |
| 227 | GO:0016481 | P | 8, | 12 | 5.479 (x 2.190) | 111 (0.108) | 0.0807 | negative regulation of transcription | CG33525 CREG Caf1 CtBP Rbf2 ci cic cyc edl en ph-p usp |
| 228 | GO:0006006 | P | 8, 9, | 8 | 2.962 (x 2.701) | 60 (0.133) | 0.0845 | glucose metabolism | BEST:LD22483 Eno Gapdh1 Gapdh2 Hex-A Pepck Pgi Tpi |
| 229 | GO:0035282 | P | 3, | 13 | 6.219 (x 2.090) | 126 (0.103) | 0.0869 | segmentation | Cdk4 Dl ap bun ci en kis knk opa rasp sgl spen stan |
| 230 | GO:0007365 | P | 5, 6, | 5 | 1.333 (x 3.752) | 27 (0.185) | 0.0884 | periodic partitioning | en opa rasp sgl stan |
| 231 | GO:0007419 | P | 4, 6, | 5 | 1.333 (x 3.752) | 27 (0.185) | 0.0888 | ventral cord development | Dl Dr grh numb sc |
| 232 | GO:0031324 | P | 6, | 15 | 7.651 (x 1.961) | 155 (0.097) | 0.089 | negative regulation of cellular metabolism | CG11079 CG33525 CREG Caf1 CtBP Rbf2 ci cic cyc dap edl en ph-p sqd usp |
| 233 | GO:0019199 | F | 5, 7, | 7 | 2.419 (x 2.894) | 49 (0.143) | 0.0904 | transmembrane receptor protein kinase activity | Cad96Ca Eph InR PFE otk pll tkv |
| 234 | GO:0004857 | F | 3, | 13 | 6.269 (x 2.074) | 127 (0.102) | 0.0906 | enzyme inhibitor activity | CG11079 CG17919 CG32354 CG5392 CG6680 Pten Set Spn1 Spn43Aa Spn5 dap pcs serpin-27A |
| 235 | GO:0005996 | P | 6, 7, | 11 | 4.936 (x 2.228) | 100 (0.110) | 0.0928 | monosaccharide metabolism | BEST:LD22483 CG1630 Eno Gapdh1 Gapdh2 Gfat1 Hex-A Oscillin Pepck Pgi Tpi |
| 236 | GO:0006333 | P | 9, | 11 | 4.936 (x 2.228) | 100 (0.110) | 0.0932 | chromatin assembly or disassembly | CG1911 Caf1 Dsp1 His4r HmgZ Set glu kis lola mod(mdg4) ph-p |
| 237 | GO:0007292 | P | 5, | 25 | 15.302 (x 1.634) | 310 (0.081) | 0.0964 | female gamete generation | CG11151 Caf1 Dl Dp Eip74EF Eip75B Fas3 Fs(2)Ket Hrb27C InR S TER94 asp bun ci dap mdy mod(mdg4) ph-p shu sqd th tkv usp wbl |
| 238 | GO:0031577 | P | 8, | 3 | 0.494 (x 6.078) | 10 (0.300) | 0.0981 | spindle checkpoint | ald alphaTub84B zwilch |
| 239 | GO:0046165 | P | 6, | 3 | 0.494 (x 6.078) | 10 (0.300) | 0.0985 | alcohol biosynthesis | Pepck Pgi Tpi |
| 240 | GO:0035078 | P | 6, 7, 8, 9, 10, | 3 | 0.494 (x 6.078) | 10 (0.300) | 0.0989 | induction of programmed cell death by ecdysone | Eip75B br usp |
| 241 | GO:0004871 | F | 2, | 68 | 51.977 (x 1.308) | 1053 (0.065) | 0.0992 | signal transducer activity | 18w Abi CG10359 CG14076 CG14217 CG1632 CG17084 CG17419 CG17952 CG33113 CG3921 CG40410 CG5096 CG6965 CG6966 CG7668 CG9066 CG9211 CaMKI Cad96Ca Cdk4 CkIIalpha CkIIbeta Dl ETH Eb1 Eip75B Eph GNBP3 Galpha49B InR Mpk2 Nek2 PFE Ptp99A RacGAP50C Sema-1a Sema-1b Sema-2a Tl Tsp26A Tsp39D Tsp66E Tsp96F Wnt2 arr cdc2 ced-6 cyc emp for fra mav mbt mth nmo numb otk pbl pll polo shf smi35A stan tkv trh usp wgn |
| 242 | GO:0006564 | P | 9, 10, | 3 | 0.494 (x 6.078) | 10 (0.300) | 0.0993 | L-serine biosynthesis | CG6287 CtBP ESTS:39C10S |
| 243 | GO:0007094 | P | 9, 10, | 3 | 0.494 (x 6.078) | 10 (0.300) | 0.0997 | mitotic spindle checkpoint | ald alphaTub84B zwilch |
| 244 | GO:0046364 | P | 7, 8, | 3 | 0.494 (x 6.078) | 10 (0.300) | 0.1 | monosaccharide biosynthesis | Pepck Pgi Tpi |
| 245 | GO:0006096 | P | 8, 10, 11, | 6 | 1.925 (x 3.117) | 39 (0.154) | 0.1 | glycolysis | Eno Gapdh1 Gapdh2 Hex-A Pgi Tpi |
| 246 | GO:0019319 | P | 8, 9, | 3 | 0.494 (x 6.078) | 10 (0.300) | 0.101 | hexose biosynthesis | Pepck Pgi Tpi |
| 247 | GO:0009880 | P | 4, | 13 | 6.368 (x 2.042) | 129 (0.101) | 0.101 | embryonic pattern specification | Cdk4 Tl ci edl en kis knk opa pll rasp sgl spen stan |
| 248 | GO:0046365 | P | 7, 8, | 7 | 2.517 (x 2.781) | 51 (0.137) | 0.104 | monosaccharide catabolism | BEST:LD22483 Eno Gapdh1 Gapdh2 Hex-A Pgi Tpi |
| 249 | GO:0006007 | P | 9, 10, | 7 | 2.517 (x 2.781) | 51 (0.137) | 0.104 | glucose catabolism | BEST:LD22483 Eno Gapdh1 Gapdh2 Hex-A Pgi Tpi |
| 250 | GO:0007422 | P | 5, | 9 | 3.751 (x 2.399) | 76 (0.118) | 0.105 | peripheral nervous system development | Crc Dl S bun glu hth numb pbl sc |
| 251 | GO:0019320 | P | 8, 9, | 7 | 2.517 (x 2.781) | 51 (0.137) | 0.105 | hexose catabolism | BEST:LD22483 Eno Gapdh1 Gapdh2 Hex-A Pgi Tpi |
| 252 | GO:0043283 | P | 5, | 102 | 83.123 (x 1.227) | 1684 (0.061) | 0.105 | biopolymer metabolism | Arf79F B52 BG:DS00004.11 BcDNA:GH02976 BcDNA:GM10765 CG11142 CG14217 CG17052 CG17323 CG18853 CG1911 CG31915 CG32499 CG32632 CG33138 CG33171 CG40410 CG4502 CG5731 CG5794 CG6904 CG6946 CG7860 CG9307 CaMKI Cad96Ca Caf1 Cdk4 CkIIalpha CkIIbeta Cks Dp Dref Dsp1 ERp60 Eip71CD Eph GNBP3 GalNAc-T1 Galpha49B GlyP Hexo1 His4r HmgD HmgZ Hrb27C Hus1-like InR Incenp Mcm7 Mgstl Mpk2 Nek2 PFE Pdi Pi3K21B Ppt1 Prosalpha6 Pten Ptp99A Rep4 Rm62 RnrS Set Thd1 Top2 Trl Ts Uch Ugt ald ash2 blot cdc2 dap eIF-4E for fzy glu heph jumu kis lola mbt mdy mod(mdg4) nmo nonA-l otk pAbp pgant5 ph-p pll polo rasp sgl smi35A sqd th tkv tws wbl |
| 253 | GO:0046164 | P | 6, | 7 | 2.517 (x 2.781) | 51 (0.137) | 0.105 | alcohol catabolism | BEST:LD22483 Eno Gapdh1 Gapdh2 Hex-A Pgi Tpi |
| 254 | GO:0004616 | F | 6, | 2 | 0.197 (x 10.130) | 4 (0.500) | 0.106 | phosphogluconate dehydrogenase (decarboxylating) activity | BEST:LD22483 Pgi |
| 255 | GO:0008615 | P | 9, | 2 | 0.197 (x 10.130) | 4 (0.500) | 0.106 | pyridoxine biosynthesis | CG31472 ESTS:39C10S |
| 256 | GO:0009570 | C | 6, 7, 8, 9, 10, 11, | 2 | 0.197 (x 10.130) | 4 (0.500) | 0.106 | chloroplast stroma | EG:BACN33B1.2 emp |
| 257 | GO:0044434 | C | 5, 6, 7, 8, 9, 10, | 2 | 0.197 (x 10.130) | 4 (0.500) | 0.107 | chloroplast part | EG:BACN33B1.2 emp |
| 258 | GO:0006457 | P | 7, | 13 | 6.466 (x 2.010) | 131 (0.099) | 0.107 | protein folding | CG1924 CG2852 CG9906 Crc DnaJ-1 ERp60 Fkbp13 Hsp23 Hsp26 Hsp27 Hsp67Ba Pdi shu |
| 259 | GO:0008614 | P | 8, | 2 | 0.197 (x 10.130) | 4 (0.500) | 0.107 | pyridoxine metabolism | CG31472 ESTS:39C10S |
| 260 | GO:0000075 | P | 7, | 4 | 0.938 (x 4.265) | 19 (0.211) | 0.107 | cell cycle checkpoint | CG40410 ald alphaTub84B zwilch |
| 261 | GO:0016853 | F | 3, | 10 | 4.492 (x 2.226) | 91 (0.110) | 0.107 | isomerase activity | CG2852 ERp60 Fkbp13 Oscillin Pdi Pgi Top2 Tpi scf shu |
| 262 | GO:0015977 | P | 5, | 2 | 0.197 (x 10.130) | 4 (0.500) | 0.108 | carbon utilization by fixation of carbon dioxide | EG:BACN33B1.2 emp |
| 263 | GO:0044435 | C | 4, 5, 6, 7, 8, 9, | 2 | 0.197 (x 10.130) | 4 (0.500) | 0.108 | plastid part | EG:BACN33B1.2 emp |
| 264 | GO:0007051 | P | 9, | 5 | 1.431 (x 3.493) | 29 (0.172) | 0.108 | spindle organization and biogenesis | CycB3 Eb1 fwd pbl polo |
| 265 | GO:0048492 | C | 3, 5, 6, 7, 8, | 2 | 0.197 (x 10.130) | 4 (0.500) | 0.108 | ribulose bisphosphate carboxylase complex | EG:BACN33B1.2 emp |
| 266 | GO:0042816 | P | 7, | 2 | 0.197 (x 10.130) | 4 (0.500) | 0.109 | vitamin B6 metabolism | CG31472 ESTS:39C10S |
| 267 | GO:0009507 | C | 6, 7, 8, 9, | 2 | 0.197 (x 10.130) | 4 (0.500) | 0.109 | chloroplast | EG:BACN33B1.2 emp |
| 268 | GO:0009573 | C | 4, 6, 7, 8, 9, 10, 11, 12, | 2 | 0.197 (x 10.130) | 4 (0.500) | 0.109 | ribulose bisphosphate carboxylase complex (sensu Magnoliophyta) | EG:BACN33B1.2 emp |
| 269 | GO:0000301 | P | 7, 8, 9, 10, | 2 | 0.197 (x 10.130) | 4 (0.500) | 0.11 | retrograde transport, vesicle recycling within Golgi | S wbl |
| 270 | GO:0045477 | P | 7, 8, 10, | 2 | 0.197 (x 10.130) | 4 (0.500) | 0.11 | regulation of nurse cell apoptosis | Dp mdy |
| 271 | GO:0000042 | P | 8, 9, 10, 11, | 2 | 0.197 (x 10.130) | 4 (0.500) | 0.111 | protein targeting to Golgi | S wbl |
| 272 | GO:0007561 | P | 6, 7, | 2 | 0.197 (x 10.130) | 4 (0.500) | 0.111 | imaginal disc eversion | ImpE2 ImpE3 |
| 273 | GO:0000785 | C | 5, 6, 7, 8, 9, 10, | 9 | 3.801 (x 2.368) | 77 (0.117) | 0.111 | chromatin | Dp1 Dsp1 His4r HmgD Trl glu kis mod(mdg4) sqd |
| 274 | GO:0008063 | P | 6, | 5 | 1.481 (x 3.377) | 30 (0.167) | 0.111 | Toll signaling pathway | Pli Tl pll serpin-27A wbl |
| 275 | GO:0006563 | P | 8, 9, | 3 | 0.543 (x 5.525) | 11 (0.273) | 0.111 | L-serine metabolism | CG6287 CtBP ESTS:39C10S |
| 276 | GO:0005701 | C | 5, 6, 7, 8, 9, 10, | 2 | 0.197 (x 10.130) | 4 (0.500) | 0.112 | polytene chromosome chromocenter | Dp1 jumu |
| 277 | GO:0051225 | P | 7, 10, | 3 | 0.543 (x 5.525) | 11 (0.273) | 0.112 | spindle assembly | Eb1 pbl polo |
| 278 | GO:0035189 | C | 4, 7, 8, 9, 10, 11, 12, 13, 14, | 2 | 0.197 (x 10.130) | 4 (0.500) | 0.112 | Rb-E2F complex | Dp Rbf2 |
| 279 | GO:0042461 | P | 5, 6, 7, | 7 | 2.567 (x 2.727) | 52 (0.135) | 0.112 | photoreceptor cell development | Amph Dl S TER94 edl mbt stan |
| 280 | GO:0035081 | P | 8, 9, | 3 | 0.543 (x 5.525) | 11 (0.273) | 0.112 | induction of programmed cell death by hormones | Eip75B br usp |
| 281 | GO:0016984 | F | 6, | 2 | 0.197 (x 10.130) | 4 (0.500) | 0.112 | ribulose-bisphosphate carboxylase activity | EG:BACN33B1.2 emp |
| 282 | GO:0015980 | P | 6, | 12 | 5.825 (x 2.060) | 118 (0.102) | 0.112 | energy derivation by oxidation of organic compounds | BEST:LD22483 CG33138 CG6904 Eno Gapdh1 Gapdh2 GlyP Hex-A Idh Pepck Pgi Tpi |
| 283 | GO:0040034 | P | 4, | 3 | 0.543 (x 5.525) | 11 (0.273) | 0.113 | regulation of development, heterochronic | Eip74EF br usp |
| 284 | GO:0051321 | P | 5, | 10 | 4.541 (x 2.202) | 92 (0.109) | 0.113 | meiotic cell cycle | CG40410 Top2 ald asp cdc2 eIF-4E east fwd pbl polo |
| 285 | GO:0009536 | C | 5, 6, 7, 8, | 2 | 0.197 (x 10.130) | 4 (0.500) | 0.113 | plastid | EG:BACN33B1.2 emp |
| 286 | GO:0001558 | P | 4, 5, 7, 8, | 4 | 0.987 (x 4.052) | 20 (0.200) | 0.113 | regulation of cell growth | Cdk4 InR Pten eIF-4E |
| 287 | GO:0007111 | P | 6, 7, | 2 | 0.197 (x 10.130) | 4 (0.500) | 0.113 | cytokinesis after meiosis II | fwd pbl |
| 288 | GO:0003697 | F | 6, | 4 | 0.987 (x 4.052) | 20 (0.200) | 0.113 | single-stranded DNA binding | Dp1 Dsp1 Hrb27C Pep |
| 289 | GO:0005057 | F | 3, | 20 | 11.847 (x 1.688) | 240 (0.083) | 0.113 | receptor signaling protein activity | CG14217 CG17419 CG33113 CG40410 CaMKI Cdk4 CkIIalpha CkIIbeta Mpk2 Nek2 PFE cdc2 for mbt nmo pll polo smi35A stan tkv |
| 290 | GO:0042819 | P | 8, | 2 | 0.197 (x 10.130) | 4 (0.500) | 0.114 | vitamin B6 biosynthesis | CG31472 ESTS:39C10S |
| 291 | GO:0009532 | C | 5, 6, 7, 8, 9, 10, | 2 | 0.197 (x 10.130) | 4 (0.500) | 0.114 | plastid stroma | EG:BACN33B1.2 emp |
| 292 | GO:0048477 | P | 6, | 23 | 14.265 (x 1.612) | 289 (0.080) | 0.119 | oogenesis | Caf1 Dl Dp Eip74EF Eip75B Fas3 Fs(2)Ket Hrb27C S TER94 asp bun ci dap mdy mod(mdg4) ph-p shu sqd th tkv usp wbl |
| 293 | GO:0006259 | P | 6, | 28 | 18.313 (x 1.529) | 371 (0.075) | 0.12 | DNA metabolism | BcDNA:GM10765 CG18853 CG1911 Caf1 Dp Dref Dsp1 His4r HmgD HmgZ Hus1-like Incenp Mcm7 Rep4 RnrS Set Thd1 Top2 Trl Ts ash2 eIF-4E glu jumu kis lola mod(mdg4) ph-p |
| 294 | GO:0045595 | P | 4, | 6 | 2.073 (x 2.894) | 42 (0.143) | 0.12 | regulation of cell differentiation | Dl Dr Tl bun mbt usp |
| 295 | GO:0019318 | P | 7, 8, | 9 | 3.949 (x 2.279) | 80 (0.113) | 0.122 | hexose metabolism | BEST:LD22483 CG1630 Eno Gapdh1 Gapdh2 Hex-A Pepck Pgi Tpi |
| 296 | GO:0007224 | P | 6, | 5 | 1.530 (x 3.268) | 31 (0.161) | 0.124 | smoothened signaling pathway | CG9211 ci rasp sgl shf |
| 297 | GO:0007469 | P | 6, | 4 | 1.037 (x 3.859) | 21 (0.190) | 0.131 | antennal development | Dl Poxn ck th |
| 298 | GO:0001736 | P | 5, 6, | 6 | 2.123 (x 2.827) | 43 (0.140) | 0.131 | establishment of planar polarity | Dl ara in nmo pk stan |
| 299 | GO:0007164 | P | 4, | 6 | 2.123 (x 2.827) | 43 (0.140) | 0.132 | establishment of tissue polarity | Dl ara in nmo pk stan |
| 300 | GO:0031523 | C | 3, 5, 6, 7, 8, 9, 10, | 3 | 0.592 (x 5.065) | 12 (0.250) | 0.133 | Myb complex | Caf1 Dp Rbf2 |
| 301 | GO:0016198 | P | 5, 7, 8, 10, 11, 13, | 3 | 0.592 (x 5.065) | 12 (0.250) | 0.133 | axon choice point recognition | Galpha49B fra lola |
| 302 | GO:0000212 | P | 6, 10, | 3 | 0.592 (x 5.065) | 12 (0.250) | 0.134 | meiotic spindle organization and biogenesis | fwd pbl polo |
| 303 | GO:0030867 | C | 5, 6, 7, 8, 9, 10, 11, | 3 | 0.592 (x 5.065) | 12 (0.250) | 0.134 | rough endoplasmic reticulum membrane | CG14214 Tapdelta l(1)G0320 |
| 304 | GO:0030707 | P | 8, | 12 | 6.121 (x 1.961) | 124 (0.097) | 0.134 | ovarian follicle cell development (sensu Insecta) | Caf1 Dl Dp Fas3 Fs(2)Ket bun ci ph-p sqd th tkv usp |
| 305 | GO:0000070 | P | 6, 8, | 5 | 1.580 (x 3.165) | 32 (0.156) | 0.134 | mitotic sister chromatid segregation | CG1911 Klp10A eIF-4E glu pim |
| 306 | GO:0009993 | P | 7, | 22 | 13.673 (x 1.609) | 277 (0.079) | 0.134 | oogenesis (sensu Insecta) | Caf1 Dl Dp Eip74EF Eip75B Fas3 Fs(2)Ket Hrb27C S TER94 asp bun ci dap mdy mod(mdg4) ph-p shu sqd th tkv usp |
| 307 | GO:0009991 | P | 4, | 3 | 0.592 (x 5.065) | 12 (0.250) | 0.135 | response to extracellular stimulus | InR Pten mth |
| 308 | GO:0000819 | P | 5, | 5 | 1.580 (x 3.165) | 32 (0.156) | 0.135 | sister chromatid segregation | CG1911 Klp10A eIF-4E glu pim |
| 309 | GO:0045786 | P | 6, 7, | 3 | 0.592 (x 5.065) | 12 (0.250) | 0.135 | negative regulation of progression through cell cycle | CG40410 Pten dap |
| 310 | GO:0030178 | P | 6, 7, 8, | 3 | 0.592 (x 5.065) | 12 (0.250) | 0.136 | negative regulation of Wnt receptor signaling pathway | RacGAP50C nmo stan |
| 311 | GO:0031667 | P | 5, | 3 | 0.592 (x 5.065) | 12 (0.250) | 0.136 | response to nutrient levels | InR Pten mth |
| 312 | GO:0042051 | P | 7, 8, 9, 10, | 6 | 2.172 (x 2.763) | 44 (0.136) | 0.14 | eye photoreceptor development (sensu Endopterygota) | Amph Dl S TER94 mbt stan |
| 313 | GO:0003682 | F | 3, | 9 | 4.097 (x 2.197) | 83 (0.108) | 0.144 | chromatin binding | Caf1 Dsp1 HmgZ Mcm7 glu jumu kis mod(mdg4) ph-p |
| 314 | GO:0030332 | F | 4, | 2 | 0.247 (x 8.104) | 5 (0.400) | 0.145 | cyclin binding | Cdk4 Set |
| 315 | GO:0007096 | P | 8, 9, | 2 | 0.247 (x 8.104) | 5 (0.400) | 0.145 | regulation of exit from mitosis | Rpn9 fzy |
| 316 | GO:0042594 | P | 4, 6, | 2 | 0.247 (x 8.104) | 5 (0.400) | 0.145 | response to starvation | Pten mth |
| 317 | GO:0042462 | P | 6, 7, 8, | 6 | 2.221 (x 2.701) | 45 (0.133) | 0.146 | eye photoreceptor cell development | Amph Dl S TER94 mbt stan |
| 318 | GO:0007451 | P | 6, 7, | 2 | 0.247 (x 8.104) | 5 (0.400) | 0.146 | dorsal/ventral lineage restriction, imaginal disc | Dl ap |
| 319 | GO:0046530 | P | 4, | 10 | 4.837 (x 2.067) | 98 (0.102) | 0.146 | photoreceptor cell differentiation | Amph Dl S TER94 br bun edl hth mbt stan |
| 320 | GO:0015976 | P | 4, | 2 | 0.247 (x 8.104) | 5 (0.400) | 0.146 | carbon utilization | EG:BACN33B1.2 emp |
| 321 | GO:0006638 | P | 6, 7, | 2 | 0.247 (x 8.104) | 5 (0.400) | 0.147 | neutral lipid metabolism | CG9342 mdy |
| 322 | GO:0050768 | P | 5, 8, | 2 | 0.247 (x 8.104) | 5 (0.400) | 0.147 | negative regulation of neurogenesis | ana ed |
| 323 | GO:0012502 | P | 7, 8, | 8 | 3.455 (x 2.315) | 70 (0.114) | 0.147 | induction of programmed cell death | CG14217 CG7379 Eip75B Jafrac2 br mod(mdg4) smi35A usp |
| 324 | GO:0009950 | P | 5, | 7 | 2.814 (x 2.488) | 57 (0.123) | 0.148 | dorsal/ventral axis specification | Dp Tl pll serpin-27A sqd tok wbl |
| 325 | GO:0004860 | F | 5, | 2 | 0.247 (x 8.104) | 5 (0.400) | 0.148 | protein kinase inhibitor activity | dap pcs |
| 326 | GO:0045810 | P | 7, 8, 9, | 2 | 0.247 (x 8.104) | 5 (0.400) | 0.148 | negative regulation of frizzled signaling pathway | nmo stan |
| 327 | GO:0007110 | P | 6, 7, | 2 | 0.247 (x 8.104) | 5 (0.400) | 0.149 | cytokinesis after meiosis I | fwd pbl |
| 328 | GO:0008039 | P | 5, | 2 | 0.247 (x 8.104) | 5 (0.400) | 0.149 | synaptic target recognition | Fas3 Sema-2a |
| 329 | GO:0042067 | P | 6, 7, 8, 9, | 5 | 1.629 (x 3.070) | 33 (0.152) | 0.149 | establishment of ommatidial polarity (sensu Endopterygota) | Dl ara nmo pk stan |
| 330 | GO:0006639 | P | 6, 7, 8, | 2 | 0.247 (x 8.104) | 5 (0.400) | 0.15 | acylglycerol metabolism | CG9342 mdy |
| 331 | GO:0007050 | P | 7, 8, | 2 | 0.247 (x 8.104) | 5 (0.400) | 0.15 | cell cycle arrest | CG40410 dap |
| 332 | GO:0045180 | C | 4, 5, 6, 7, 8, 9, 10, | 2 | 0.247 (x 8.104) | 5 (0.400) | 0.15 | basal cortex | numb pon |
| 333 | GO:0035186 | P | 5, 7, | 2 | 0.247 (x 8.104) | 5 (0.400) | 0.151 | syncytial blastoderm mitotic cell cycle | CG40410 Trl |
| 334 | GO:0008152 | P | 3, | 270 | 249.616 (x 1.082) | 5057 (0.053) | 0.159 | metabolism | Abi Aldh-III Ance Arf79F Argk B52 BEST:CK02656 BEST:GH02921 BEST:LD04971 BEST:LD22483 BG:DS00004.11 BcDNA:GH02901 BcDNA:GH02976 BcDNA:GH12558 BcDNA:GM10765 BcDNA:LD41548 CG10657 CG11033 CG11079 CG11142 CG11438 CG11824 CG12199 CG14217 CG1471 CG15088 CG15835 CG1607 CG1630 CG1632 CG17052 CG17323 CG18853 CG1893 CG1911 CG1924 CG1998 CG2118 CG2200 CG2852 CG30011 CG3136 CG31453 CG31472 CG31738 CG31915 CG31937 CG32158 CG32499 CG32632 CG33116 CG33138 CG33171 CG3328 CG33525 CG3590 CG3842 CG40160 CG40410 CG4502 CG4586 CG4914 CG5319 CG5390 CG5731 CG5794 CG5873 CG6199 CG6287 CG6391 CG6654 CG6680 CG6767 CG6776 CG6904 CG6946 CG7379 CG7675 CG7860 CG8092 CG8165 CG8963 CG9027 CG9057 CG9135 CG9307 CG9342 CG9906 CREG CaMKI Cad96Ca Caf1 Cdk4 CkIIalpha CkIIbeta Cks Crc CrebA CtBP CycT Cyp310a1 D19A DnaJ-1 Doc1 Doc2 Doc3 Dp Dr Dref Dsp1 EG:171D11.1 EG:BACN33B1.2 ERp60 ESTS:39C10S Eb1 Eip55E Eip71CD Eip74EF Eip75B Eno Eph Fkbp13 GATAd GNBP3 GalNAc-T1 Galpha49B Gapdh1 Gapdh2 Gfat1 GlyP GstS1 HLHm7 Hex-A Hexo1 His4r HmgD HmgZ Hrb27C Hsp23 Hsp26 Hsp27 Hsp67Ba Hus1-like Ice Idh InR Incenp Jafrac2 Lsp2 Mcm7 Mgstl Mpk2 Nek2 Nep2 Optix Oscillin PFE Pdi Pepck Pgi Pi3K21B Poxn Ppt1 Prosalpha6 Pten Ptp99A Rab8 Rbf2 Rep4 RhoGAP71E Rm62 RnrS RpS12 Rpn9 Sb Set Spn1 Spn5 TER94 Thd1 Tl Top2 Tpi Trl Ts Uch Ugt ald alpha-Man-IIb alphaTub84B ap ara arr ash2 betaTub56D blot br bun cdc2 cg ci cic cyc dUTPase dap dve eIF-4E east edl emp en fbp for fra fwd fzy glu grh grn heph hth inv jumu kis klu knk l(1)G0320 l(2)01424 l(2)01810 lid lola mbt mdy mnd mod(mdg4) mth nmo nonA-l olf413 ome opa otk pAbp pbl pdm2 pgant5 ph-p pll polo rasp sc serpin-27A sgl shanti shf shu smi35A spen sqd sut1 th tkv toe tok trh tws usp wbl woc zf30C |
| 335 | GO:0006767 | P | 6, | 4 | 1.135 (x 3.523) | 23 (0.174) | 0.16 | water-soluble vitamin metabolism | BEST:LD22483 CG31472 ESTS:39C10S Tpi |
| 336 | GO:0044237 | P | 4, | 249 | 228.490 (x 1.090) | 4629 (0.054) | 0.161 | cellular metabolism | Ance Arf79F Argk B52 BEST:GH02921 BEST:LD04971 BEST:LD22483 BG:DS00004.11 BcDNA:GH02901 BcDNA:GH02976 BcDNA:GH12558 BcDNA:GM10765 BcDNA:LD41548 CG10657 CG11033 CG11079 CG11142 CG11438 CG11824 CG12199 CG14217 CG15088 CG15835 CG1607 CG1630 CG1632 CG17052 CG17323 CG18853 CG1911 CG1924 CG1998 CG2118 CG2200 CG2852 CG30011 CG3136 CG31453 CG31472 CG31738 CG31915 CG32158 CG32499 CG32632 CG33116 CG33138 CG33171 CG3328 CG33525 CG3590 CG40160 CG40410 CG4502 CG4586 CG4914 CG5319 CG5390 CG5794 CG5873 CG6287 CG6391 CG6654 CG6680 CG6767 CG6776 CG6904 CG6946 CG7379 CG7860 CG8092 CG8165 CG8963 CG9027 CG9135 CG9307 CG9342 CG9906 CREG CaMKI Cad96Ca Caf1 Cdk4 CkIIalpha CkIIbeta Cks Crc CrebA CtBP CycT Cyp310a1 D19A DnaJ-1 Doc1 Doc2 Doc3 Dp Dr Dref Dsp1 EG:171D11.1 ERp60 ESTS:39C10S Eip55E Eip71CD Eip74EF Eip75B Eno Eph Fkbp13 GATAd GalNAc-T1 Galpha49B Gapdh1 Gapdh2 Gfat1 GlyP GstS1 HLHm7 Hex-A His4r HmgD HmgZ Hrb27C Hsp23 Hsp26 Hsp27 Hsp67Ba Hus1-like Ice Idh InR Incenp Jafrac2 Lsp2 Mcm7 Mgstl Mpk2 Nek2 Nep2 Optix Oscillin PFE Pdi Pepck Pgi Pi3K21B Poxn Ppt1 Prosalpha6 Pten Ptp99A Rab8 Rbf2 Rep4 Rm62 RnrS RpS12 Rpn9 Sb Set Spn1 Spn5 TER94 Thd1 Tl Top2 Tpi Trl Ts Uch Ugt ald alphaTub84B ap ara arr ash2 betaTub56D blot br bun cdc2 cg ci cic cyc dUTPase dap dve eIF-4E east edl en for fra fwd fzy glu grh grn heph hth inv jumu kis klu knk l(1)G0320 l(2)01424 l(2)01810 lid lola mbt mdy mnd mod(mdg4) mth nmo nonA-l olf413 ome opa otk pAbp pdm2 pgant5 ph-p pll polo rasp sc serpin-27A sgl shanti shf shu smi35A spen sqd th tkv toe tok trh tws usp wbl woc zf30C |
| 337 | GO:0009057 | P | 5, | 16 | 9.379 (x 1.706) | 190 (0.084) | 0.165 | macromolecule catabolism | BEST:LD22483 BcDNA:GM10765 CG5794 CG7860 Eno Gapdh1 Gapdh2 Hex-A Pgi Prosalpha6 Rep4 Tpi Uch fzy sqd th |
| 338 | GO:0006139 | P | 5, | 104 | 87.418 (x 1.190) | 1771 (0.059) | 0.168 | nucleobase, nucleoside, nucleotide and nucleic acid metabolism | B52 BEST:GH02921 BEST:LD04971 BEST:LD22483 BcDNA:GM10765 CG11079 CG15835 CG18853 CG1911 CG30011 CG3136 CG31453 CG32158 CG33525 CG3590 CG4914 CG5319 CG6654 CG6767 CG6946 CG7379 CG8092 CG8165 CREG Caf1 CrebA CtBP CycT D19A Doc1 Doc2 Doc3 Dp Dr Dref Dsp1 EG:171D11.1 Eip74EF Eip75B GATAd HLHm7 His4r HmgD HmgZ Hrb27C Hus1-like Incenp Mcm7 Optix Poxn Rab8 Rbf2 Rep4 Rm62 RnrS Set Thd1 Top2 Tpi Trl Ts ap ara ash2 blot br bun cdc2 cg ci cic cyc dUTPase dve eIF-4E edl en glu grh grn heph hth inv jumu kis klu lid lola mdy mod(mdg4) nonA-l opa pAbp pdm2 ph-p sc shanti spen sqd toe trh usp woc zf30C |
| 339 | GO:0001751 | P | 6, 7, 8, 9, | 8 | 3.603 (x 2.220) | 73 (0.110) | 0.172 | eye photoreceptor cell differentiation (sensu Endopterygota) | Amph Dl S TER94 bun hth mbt stan |
| 340 | GO:0045893 | P | 9, | 6 | 2.320 (x 2.586) | 47 (0.128) | 0.173 | positive regulation of transcription, DNA-dependent | Trl ara ash2 ci cyc lola |
| 341 | GO:0000226 | P | 8, | 9 | 4.294 (x 2.096) | 87 (0.103) | 0.174 | microtubule cytoskeleton organization and biogenesis | CycB3 Eb1 TER94 alphaTub84B asp betaTub56D fwd pbl polo |
| 342 | GO:0040014 | P | 4, | 3 | 0.691 (x 4.341) | 14 (0.214) | 0.178 | regulation of body size | InR Pten btsz |
| 343 | GO:0035264 | P | 3, | 3 | 0.691 (x 4.341) | 14 (0.214) | 0.179 | body growth | InR Pten btsz |
| 344 | GO:0009070 | P | 8, 9, | 3 | 0.691 (x 4.341) | 14 (0.214) | 0.179 | serine family amino acid biosynthesis | CG6287 CtBP ESTS:39C10S |
| 345 | GO:0016903 | F | 4, | 4 | 1.185 (x 3.377) | 24 (0.167) | 0.179 | oxidoreductase activity, acting on the aldehyde or oxo group of donors | Aldh-III EG:171D11.1 Gapdh1 Gapdh2 |
| 346 | GO:0006112 | P | 7, | 3 | 0.691 (x 4.341) | 14 (0.214) | 0.18 | energy reserve metabolism | CG33138 CG6904 GlyP |
| 347 | GO:0030261 | P | 7, | 4 | 1.185 (x 3.377) | 24 (0.167) | 0.18 | chromosome condensation | CG1911 Dp1 eIF-4E glu |
| 348 | GO:0008286 | P | 8, | 3 | 0.691 (x 4.341) | 14 (0.214) | 0.18 | insulin receptor signaling pathway | InR Pi3K21B Pten |
| 349 | GO:0019842 | F | 3, | 4 | 1.185 (x 3.377) | 24 (0.167) | 0.18 | vitamin binding | CG10657 CG13848 CG2118 CG3823 |
| 350 | GO:0007474 | P | 7, 8, 9, | 3 | 0.691 (x 4.341) | 14 (0.214) | 0.181 | wing vein specification | Dl ara nmo |
| 351 | GO:0009893 | P | 5, | 8 | 3.653 (x 2.190) | 74 (0.108) | 0.181 | positive regulation of metabolism | Trl ara ash2 ci cyc lola pAbp usp |
| 352 | GO:0045034 | P | 6, 7, 10, | 3 | 0.691 (x 4.341) | 14 (0.214) | 0.181 | neuroblast division | cdc2 numb pon |
| 353 | GO:0031325 | P | 6, | 8 | 3.653 (x 2.190) | 74 (0.108) | 0.181 | positive regulation of cellular metabolism | Trl ara ash2 ci cyc lola pAbp usp |
| 354 | GO:0009966 | P | 4, 5, | 10 | 5.084 (x 1.967) | 103 (0.097) | 0.187 | regulation of signal transduction | CG9211 Pten RacGAP50C S ed edl klu nmo numb stan |
| 355 | GO:0045476 | P | 7, 9, | 2 | 0.296 (x 6.753) | 6 (0.333) | 0.188 | nurse cell apoptosis | Dp mdy |
| 356 | GO:0000796 | C | 3, 5, 6, 7, 8, 9, 10, | 2 | 0.296 (x 6.753) | 6 (0.333) | 0.189 | condensin complex | CG1911 glu |
| 357 | GO:0046486 | P | 6, 7, | 2 | 0.296 (x 6.753) | 6 (0.333) | 0.189 | glycerolipid metabolism | CG9342 mdy |
| 358 | GO:0016321 | P | 6, 9, | 4 | 1.234 (x 3.241) | 25 (0.160) | 0.19 | female meiosis chromosome segregation | CG40410 ald eIF-4E east |
| 359 | GO:0009968 | P | 5, 6, | 6 | 2.419 (x 2.481) | 49 (0.122) | 0.19 | negative regulation of signal transduction | Pten RacGAP50C ed nmo numb stan |
| 360 | GO:0004500 | F | 6, | 2 | 0.296 (x 6.753) | 6 (0.333) | 0.19 | dopamine beta-monooxygenase activity | knk olf413 |
| 361 | GO:0007420 | P | 4, 6, | 6 | 2.419 (x 2.481) | 49 (0.122) | 0.19 | brain development | CkIIbeta Dr cib hth mbt stan |
| 362 | GO:0005956 | C | 3, 4, 5, 6, | 2 | 0.296 (x 6.753) | 6 (0.333) | 0.19 | protein kinase CK2 complex | CkIIalpha CkIIbeta |
| 363 | GO:0008594 | P | 6, 7, 8, | 6 | 2.419 (x 2.481) | 49 (0.122) | 0.191 | photoreceptor cell morphogenesis (sensu Endopterygota) | Amph Dl S TER94 mbt stan |
| 364 | GO:0005788 | C | 4, 5, 6, 7, 8, 9, 10, | 2 | 0.296 (x 6.753) | 6 (0.333) | 0.191 | endoplasmic reticulum lumen | Crc Pdi |
| 365 | GO:0017048 | F | 7, | 2 | 0.296 (x 6.753) | 6 (0.333) | 0.191 | Rho GTPase binding | Cip4 Rhp |
| 366 | GO:0018990 | P | 7, | 2 | 0.296 (x 6.753) | 6 (0.333) | 0.192 | ecdysis (sensu Insecta) | ETH Eip75B |
| 367 | GO:0045178 | C | 3, 4, | 2 | 0.296 (x 6.753) | 6 (0.333) | 0.192 | basal part of cell | numb pon |
| 368 | GO:0001754 | P | 5, 6, 7, | 8 | 3.751 (x 2.133) | 76 (0.105) | 0.192 | eye photoreceptor cell differentiation | Amph Dl S TER94 bun hth mbt stan |
| 369 | GO:0007352 | P | 6, | 2 | 0.296 (x 6.753) | 6 (0.333) | 0.193 | zygotic determination of dorsal/ventral axis | Tl pll |
| 370 | GO:0015286 | F | 6, | 2 | 0.296 (x 6.753) | 6 (0.333) | 0.193 | innexin channel activity | inx2 inx3 |
| 371 | GO:0008590 | P | 6, 7, 8, | 2 | 0.296 (x 6.753) | 6 (0.333) | 0.194 | regulation of frizzled signaling pathway | nmo stan |
| 372 | GO:0042394 | P | 6, | 2 | 0.296 (x 6.753) | 6 (0.333) | 0.195 | ecdysis (sensu Protostomia and Nematoda) | ETH Eip75B |
| 373 | GO:0006662 | P | 5, | 2 | 0.296 (x 6.753) | 6 (0.333) | 0.195 | glycerol ether metabolism | CG9342 mdy |
| 374 | GO:0031887 | P | 8, 9, 10, | 1 | 0.049 (x 20.259) | 1 (1.000) | 0.195 | lipid particle transport along microtubule | CG9057 |
| 375 | GO:0030036 | P | 8, | 9 | 4.788 (x 1.880) | 97 (0.093) | 0.195 | actin cytoskeleton organization and biogenesis | Abi Cip4 CycT Fs(2)Ket Pten Sb Sry-alpha asp pbl |
| 376 | GO:0009162 | P | 8, | 1 | 0.049 (x 20.259) | 1 (1.000) | 0.195 | deoxyribonucleoside monophosphate metabolism | Ts |
| 377 | GO:0030029 | P | 7, | 9 | 4.788 (x 1.880) | 97 (0.093) | 0.196 | actin filament-based process | Abi Cip4 CycT Fs(2)Ket Pten Sb Sry-alpha asp pbl |
| 378 | GO:0030496 | C | 3, 4, | 2 | 0.296 (x 6.753) | 6 (0.333) | 0.196 | midbody | Incenp Nek2 |
| 379 | GO:0017145 | P | 5, | 5 | 1.826 (x 2.738) | 37 (0.135) | 0.196 | stem cell division | asp cdc2 numb pon tkv |
| 380 | GO:0006970 | P | 4, | 1 | 0.049 (x 20.259) | 1 (1.000) | 0.196 | response to osmotic stress | Mpk2 |
| 381 | GO:0009605 | P | 3, | 10 | 5.183 (x 1.929) | 105 (0.095) | 0.196 | response to external stimulus | 18w Galpha49B InR Pten emp mth ninA ogre pain serpin-27A |
| 382 | GO:0035035 | F | 5, | 1 | 0.049 (x 20.259) | 1 (1.000) | 0.196 | histone acetyltransferase binding | Caf1 |
| 383 | GO:0045132 | P | 5, 8, | 5 | 1.826 (x 2.738) | 37 (0.135) | 0.196 | meiotic chromosome segregation | CG40410 ald eIF-4E east polo |
| 384 | GO:0008486 | F | 7, | 1 | 0.049 (x 20.259) | 1 (1.000) | 0.197 | diphosphoinositol-polyphosphate diphosphatase activity | CG6391 |
| 385 | GO:0048511 | P | 3, | 5 | 1.826 (x 2.738) | 37 (0.135) | 0.197 | rhythmic process | CkIIalpha CkIIbeta Reg-5 cyc numb |
| 386 | GO:0005899 | C | 4, 5, 6, 7, 8, 9, | 1 | 0.049 (x 20.259) | 1 (1.000) | 0.197 | insulin receptor complex | InR |
| 387 | GO:0006231 | P | 10, 11, | 1 | 0.049 (x 20.259) | 1 (1.000) | 0.197 | dTMP biosynthesis | Ts |
| 388 | GO:0004693 | F | 8, | 3 | 0.740 (x 4.052) | 15 (0.200) | 0.198 | cyclin-dependent protein kinase activity | Cdk4 Cks cdc2 |
| 389 | GO:0007481 | P | 6, 7, | 1 | 0.049 (x 20.259) | 1 (1.000) | 0.198 | haltere disc morphogenesis | ap |
| 390 | GO:0016508 | F | 6, | 1 | 0.049 (x 20.259) | 1 (1.000) | 0.198 | long-chain-enoyl-CoA hydratase activity | BcDNA:GH12558 |
| 391 | GO:0051327 | P | 6, | 9 | 4.492 (x 2.004) | 91 (0.099) | 0.198 | M phase of meiotic cell cycle | CG40410 Top2 ald cdc2 eIF-4E east fwd pbl polo |
| 392 | GO:0007562 | P | 3, | 3 | 0.740 (x 4.052) | 15 (0.200) | 0.198 | eclosion | CkIIbeta br cyc |
| 393 | GO:0017165 | F | 7, | 1 | 0.049 (x 20.259) | 1 (1.000) | 0.198 | dipeptidase E activity | CG2200 |
| 394 | GO:0007450 | P | 5, 6, | 3 | 0.740 (x 4.052) | 15 (0.200) | 0.199 | dorsal/ventral pattern formation, imaginal disc | Dl Dr ap |
| 395 | GO:0046073 | P | 10, | 1 | 0.049 (x 20.259) | 1 (1.000) | 0.199 | dTMP metabolism | Ts |
| 396 | GO:0018345 | P | 9, 10, | 1 | 0.049 (x 20.259) | 1 (1.000) | 0.199 | protein palmitoylation | rasp |
| 397 | GO:0006090 | P | 7, | 3 | 0.740 (x 4.052) | 15 (0.200) | 0.199 | pyruvate metabolism | Pepck Pgi Tpi |
| 398 | GO:0007058 | P | 9, 10, 13, | 1 | 0.049 (x 20.259) | 1 (1.000) | 0.199 | female meiosis II spindle assembly (sensu Metazoa) | polo |
| 399 | GO:0035075 | P | 5, 6, 7, | 3 | 0.740 (x 4.052) | 15 (0.200) | 0.2 | response to ecdysone | Eip75B br usp |
| 400 | GO:0000089 | P | 7, 8, | 1 | 0.049 (x 20.259) | 1 (1.000) | 0.2 | mitotic metaphase | east |
| 401 | GO:0048545 | P | 5, 6, | 3 | 0.740 (x 4.052) | 15 (0.200) | 0.2 | response to steroid hormone stimulus | Eip75B br usp |
| 402 | GO:0035014 | F | 4, | 1 | 0.049 (x 20.259) | 1 (1.000) | 0.2 | phosphoinositide 3-kinase regulator activity | Pi3K21B |
| 403 | GO:0007498 | P | 4, | 16 | 9.773 (x 1.637) | 198 (0.081) | 0.2 | mesoderm development | CG30011 Cip4 Dl Doc1 Doc2 Doc3 Eip74EF Galpha49B Poxn S grn nonA-l pbl rost sgl spen |
| 404 | GO:0005003 | F | 7, 9, | 1 | 0.049 (x 20.259) | 1 (1.000) | 0.201 | ephrin receptor activity | Eph |
| 405 | GO:0050821 | P | 8, | 1 | 0.049 (x 20.259) | 1 (1.000) | 0.201 | protein stabilization | shf |
| 406 | GO:0008456 | F | 7, | 1 | 0.049 (x 20.259) | 1 (1.000) | 0.201 | alpha-N-acetylgalactosaminidase activity | CG5731 |
| 407 | GO:0042221 | P | 4, | 20 | 12.982 (x 1.541) | 263 (0.076) | 0.202 | response to chemical stimulus | Aldh-III BcDNA:GH04753 CG11711 CG17323 CG6776 CG8588 Crc Eip75B Fas3 GstS1 InR Mgstl Obp99a Sh br cyc mth para smi35A usp |
| 408 | GO:0006513 | P | 10, | 1 | 0.049 (x 20.259) | 1 (1.000) | 0.202 | protein monoubiquitination | th |
| 409 | GO:0042577 | F | 7, | 1 | 0.049 (x 20.259) | 1 (1.000) | 0.202 | lipid phosphatase activity | Pten |
| 410 | GO:0006335 | P | 8, 12, | 1 | 0.049 (x 20.259) | 1 (1.000) | 0.202 | DNA replication-dependent nucleosome assembly | Caf1 |
| 411 | GO:0017049 | F | 8, | 1 | 0.049 (x 20.259) | 1 (1.000) | 0.203 | GTP-Rho binding | Rhp |
| 412 | GO:0000705 | P | 9, | 1 | 0.049 (x 20.259) | 1 (1.000) | 0.203 | achiasmate meiosis I | east |
| 413 | GO:0035312 | F | 8, 9, | 1 | 0.049 (x 20.259) | 1 (1.000) | 0.204 | 5'-3' exodeoxyribonuclease activity | BcDNA:GM10765 |
| 414 | GO:0050431 | F | 5, | 1 | 0.049 (x 20.259) | 1 (1.000) | 0.204 | transforming growth factor beta binding | tkv |
| 415 | GO:0006097 | P | 7, 8, | 1 | 0.049 (x 20.259) | 1 (1.000) | 0.204 | glyoxylate cycle | Idh |
| 416 | GO:0009176 | P | 9, | 1 | 0.049 (x 20.259) | 1 (1.000) | 0.205 | pyrimidine deoxyribonucleoside monophosphate metabolism | Ts |
| 417 | GO:0017129 | F | 4, | 1 | 0.049 (x 20.259) | 1 (1.000) | 0.205 | triglyceride binding | CG9342 |
| 418 | GO:0016978 | F | 6, | 1 | 0.049 (x 20.259) | 1 (1.000) | 0.206 | lipoate-protein ligase B activity | CG6767 |
| 419 | GO:0004714 | F | 6, 8, | 4 | 1.431 (x 2.794) | 29 (0.138) | 0.206 | transmembrane receptor protein tyrosine kinase activity | Cad96Ca Eph InR otk |
| 420 | GO:0007406 | P | 6, 7, 8, 9, | 1 | 0.049 (x 20.259) | 1 (1.000) | 0.206 | negative regulation of neuroblast proliferation | ana |
| 421 | GO:0007010 | P | 6, | 31 | 22.212 (x 1.396) | 450 (0.069) | 0.206 | cytoskeleton organization and biogenesis | Abi Act42A CG33171 CG9057 Cip4 CycB3 CycT Eb1 Fs(2)Ket Galpha49B GstS1 Klp10A M6 Pten Rhp Sb Sry-alpha TER94 alphaTub84B asp betaTub56D cib esn for fra fwd mbt pbl polo stai vg |
| 422 | GO:0005975 | P | 5, | 33 | 23.940 (x 1.378) | 485 (0.068) | 0.206 | carbohydrate metabolism | BEST:LD22483 BcDNA:GH02976 CG11142 CG1630 CG17052 CG17323 CG31915 CG32499 CG33138 CG5731 CG6287 CG6904 CG9307 Eno GNBP3 GalNAc-T1 Gapdh1 Gapdh2 Gfat1 GlyP Hex-A Hexo1 Idh Oscillin Pepck Pgi Tpi alpha-Man-IIb fbp l(2)01810 pgant5 sgl sut1 |
| 423 | GO:0004537 | F | 7, | 1 | 0.049 (x 20.259) | 1 (1.000) | 0.206 | caspase-activated deoxyribonuclease activity | Rep4 |
| 424 | GO:0007622 | P | 4, | 4 | 1.283 (x 3.117) | 26 (0.154) | 0.207 | rhythmic behavior | CkIIalpha CkIIbeta cyc numb |
| 425 | GO:0005031 | F | 6, | 1 | 0.049 (x 20.259) | 1 (1.000) | 0.207 | tumor necrosis factor receptor activity | wgn |
| 426 | GO:0016201 | P | 6, | 1 | 0.049 (x 20.259) | 1 (1.000) | 0.207 | synaptic target inhibition | Sema-2a |
| 427 | GO:0042083 | F | 6, | 1 | 0.049 (x 20.259) | 1 (1.000) | 0.208 | 5,10-methylenetetrahydrofolate-dependent methyltransferase activity | Ts |
| 428 | GO:0004028 | F | 6, | 1 | 0.049 (x 20.259) | 1 (1.000) | 0.208 | 3-chloroallyl aldehyde dehydrogenase activity | Aldh-III |
| 429 | GO:0045941 | P | 8, | 7 | 3.159 (x 2.216) | 64 (0.109) | 0.208 | positive regulation of transcription | Trl ara ash2 ci cyc lola usp |
| 430 | GO:0008476 | F | 6, | 1 | 0.049 (x 20.259) | 1 (1.000) | 0.208 | protein-tyrosine sulfotransferase activity | CG32632 |
| 431 | GO:0030292 | F | 6, | 1 | 0.049 (x 20.259) | 1 (1.000) | 0.209 | protein tyrosine kinase inhibitor activity | pcs |
| 432 | GO:0005626 | C | 4, 5, | 1 | 0.049 (x 20.259) | 1 (1.000) | 0.209 | insoluble fraction | Top2 |
| 433 | GO:0004799 | F | 7, | 1 | 0.049 (x 20.259) | 1 (1.000) | 0.21 | thymidylate synthase activity | Ts |
| 434 | GO:0008184 | F | 7, | 1 | 0.049 (x 20.259) | 1 (1.000) | 0.21 | glycogen phosphorylase activity | GlyP |
| 435 | GO:0004684 | F | 9, | 1 | 0.049 (x 20.259) | 1 (1.000) | 0.21 | calmodulin-dependent protein kinase I activity | CaMKI |
| 436 | GO:0017160 | F | 7, | 1 | 0.049 (x 20.259) | 1 (1.000) | 0.211 | Ral GTPase binding | vimar |
| 437 | GO:0007267 | P | 4, | 30 | 22.114 (x 1.357) | 448 (0.067) | 0.211 | cell-cell signaling | Amph Arf79F CG11033 CG12199 CG15835 CG16974 CG17064 CG4054 CG5096 CG9066 CaMKI Dl Eip75B M6 PFE Snap br fax for kal-1 lola mod(mdg4) mth olf413 otk pAbp para shf stai trn |
| 438 | GO:0042500 | F | 7, | 1 | 0.049 (x 20.259) | 1 (1.000) | 0.211 | aspartic endopeptidase activity, intramembrane cleaving | shanti |
| 439 | GO:0016245 | P | 9, 10, | 1 | 0.049 (x 20.259) | 1 (1.000) | 0.212 | hyperphosphorylation of RNA polymerase II | cdc2 |
| 440 | GO:0004373 | F | 7, 8, | 1 | 0.049 (x 20.259) | 1 (1.000) | 0.212 | glycogen (starch) synthase activity | CG6904 |
| 441 | GO:0004861 | F | 6, | 1 | 0.049 (x 20.259) | 1 (1.000) | 0.212 | cyclin-dependent protein kinase inhibitor activity | dap |
| 442 | GO:0005874 | C | 5, 6, 7, 8, 9, 10, | 5 | 2.073 (x 2.412) | 42 (0.119) | 0.213 | microtubule | Eb1 Klp10A alphaTub84B betaTub56D vg |
| 443 | GO:0047429 | F | 7, | 1 | 0.049 (x 20.259) | 1 (1.000) | 0.213 | nucleoside-triphosphate diphosphatase activity | dUTPase |
| 444 | GO:0031647 | P | 7, | 1 | 0.049 (x 20.259) | 1 (1.000) | 0.213 | regulation of protein stability | shf |
| 445 | GO:0051179 | P | 3, | 101 | 87.418 (x 1.155) | 1771 (0.057) | 0.214 | localization | Abi Amph Arf79F BEST:CK02656 BEST:LD04971 Best2 CG10657 CG11550 CG11739 CG12048 CG14214 CG14439 CG15088 CG15835 CG1607 CG17419 CG1893 CG1907 CG1924 CG2108 CG2852 CG31121 CG32137 CG33113 CG33171 CG3424 CG3823 CG6812 CG9057 CG9066 CG9342 CG9906 Crc Eb1 Fas3 Fs(2)Ket Galpha49B Hrb27C InR Incenp Ir Klp10A LanB2 Lsp2 Nrv1 Obp99a Ptp99A Rab8 S Sema-1a Sema-1b Sema-2a Sh Snap TER94 Tapdelta Ucp4B Vha100-2 alpha-Man-IIb alphaTub84B ap arr asp betaTub56D blot btsz cic ck d fra gukh jumu l(1)G0320 l(2)01810 loj lola mnd mod(mdg4) mth ninA opa otk pain para pbl pk pll polo pon prominin-like rpk sgl spen sqd stai sut1 th trn usp vg wbl |
| 446 | GO:0008348 | P | 7, 8, | 1 | 0.049 (x 20.259) | 1 (1.000) | 0.214 | negative regulation of antimicrobial humoral response | Mpk2 |
| 447 | GO:0051235 | P | 4, | 3 | 0.888 (x 3.377) | 18 (0.167) | 0.214 | maintenance of localization | CG9057 Tapdelta pk |
| 448 | GO:0004867 | F | 6, | 7 | 3.455 (x 2.026) | 70 (0.100) | 0.214 | serine-type endopeptidase inhibitor activity | CG5392 CG6680 Pten Spn1 Spn43Aa Spn5 serpin-27A |
| 449 | GO:0009116 | P | 6, | 3 | 0.888 (x 3.377) | 18 (0.167) | 0.214 | nucleoside metabolism | CG11079 CG6767 dUTPase |
| 450 | GO:0035011 | P | 6, 7, 8, | 1 | 0.049 (x 20.259) | 1 (1.000) | 0.214 | melanotic encapsulation of foreign target | serpin-27A |
| 451 | GO:0007222 | P | 7, | 3 | 0.888 (x 3.377) | 18 (0.167) | 0.215 | frizzled signaling pathway | CkIIalpha nmo stan |
| 452 | GO:0007425 | P | 5, 6, | 2 | 0.395 (x 5.065) | 8 (0.250) | 0.215 | tracheal epithelial cell fate determination (sensu Insecta) | Pten trh |
| 453 | GO:0004062 | F | 6, | 1 | 0.049 (x 20.259) | 1 (1.000) | 0.215 | aryl sulfotransferase activity | CG16733 |
| 454 | GO:0009069 | P | 7, 8, | 3 | 0.888 (x 3.377) | 18 (0.167) | 0.215 | serine family amino acid metabolism | CG6287 CtBP ESTS:39C10S |
| 455 | GO:0035004 | F | 7, | 2 | 0.395 (x 5.065) | 8 (0.250) | 0.215 | phosphoinositide 3-kinase activity | Pi3K21B fwd |
| 456 | GO:0000015 | C | 3, 6, 7, 8, 9, 10, | 1 | 0.049 (x 20.259) | 1 (1.000) | 0.215 | phosphopyruvate hydratase complex | Eno |
| 457 | GO:0042659 | P | 5, 6, | 3 | 0.888 (x 3.377) | 18 (0.167) | 0.215 | regulation of cell fate specification | Dl Dr bun |
| 458 | GO:0004365 | F | 7, | 2 | 0.395 (x 5.065) | 8 (0.250) | 0.215 | glyceraldehyde-3-phosphate dehydrogenase (phosphorylating) activity | Gapdh1 Gapdh2 |
| 459 | GO:0007147 | P | 9, | 1 | 0.049 (x 20.259) | 1 (1.000) | 0.215 | female meiosis II | polo |
| 460 | GO:0004707 | F | 5, 9, | 2 | 0.395 (x 5.065) | 8 (0.250) | 0.216 | MAP kinase activity | Mpk2 nmo |
| 461 | GO:0019432 | P | 8, 9, 10, | 1 | 0.049 (x 20.259) | 1 (1.000) | 0.216 | triacylglycerol biosynthesis | mdy |
| 462 | GO:0016604 | C | 6, 7, 8, 9, 10, 11, 12, 13, | 2 | 0.395 (x 5.065) | 8 (0.250) | 0.216 | nuclear body | B52 sqd |
| 463 | GO:0007225 | P | 7, 9, | 1 | 0.049 (x 20.259) | 1 (1.000) | 0.216 | patched ligand processing | rasp |
| 464 | GO:0008943 | F | 6, | 2 | 0.395 (x 5.065) | 8 (0.250) | 0.216 | glyceraldehyde-3-phosphate dehydrogenase activity | Gapdh1 Gapdh2 |
| 465 | GO:0045315 | P | 8, 9, 10, 11, 12, | 1 | 0.049 (x 20.259) | 1 (1.000) | 0.217 | positive regulation of eye photoreceptor development (sensu Endopterygota) | mbt |
| 466 | GO:0005784 | C | 3, 4, 5, 6, 7, 8, 9, 10, 11, 12, | 2 | 0.395 (x 5.065) | 8 (0.250) | 0.217 | translocon complex | CG14214 Tapdelta |
| 467 | GO:0000700 | F | 7, | 1 | 0.049 (x 20.259) | 1 (1.000) | 0.217 | mismatch base pair DNA N-glycosylase activity | Thd1 |
| 468 | GO:0006582 | P | 6, | 2 | 0.395 (x 5.065) | 8 (0.250) | 0.217 | melanin metabolism | Tl serpin-27A |
| 469 | GO:0051226 | P | 7, 8, 11, | 2 | 0.395 (x 5.065) | 8 (0.250) | 0.218 | meiotic spindle assembly | pbl polo |
| 470 | GO:0000738 | P | 7, 8, | 1 | 0.049 (x 20.259) | 1 (1.000) | 0.218 | DNA catabolism, exonucleolytic | BcDNA:GM10765 |
| 471 | GO:0009886 | P | 4, | 2 | 0.395 (x 5.065) | 8 (0.250) | 0.218 | post-embryonic morphogenesis | br cib |
| 472 | GO:0019911 | F | 3, | 1 | 0.049 (x 20.259) | 1 (1.000) | 0.218 | structural constituent of myelin sheath | M6 |
| 473 | GO:0051056 | P | 5, 6, 7, | 2 | 0.395 (x 5.065) | 8 (0.250) | 0.218 | regulation of small GTPase mediated signal transduction | edl klu |
| 474 | GO:0008106 | F | 7, | 1 | 0.049 (x 20.259) | 1 (1.000) | 0.219 | alcohol dehydrogenase (NADP+) activity | CG2767 |
| 475 | GO:0045978 | P | 8, | 1 | 0.049 (x 20.259) | 1 (1.000) | 0.219 | negative regulation of nucleoside metabolism | CG11079 |
| 476 | GO:0051656 | P | 5, | 4 | 1.481 (x 2.701) | 30 (0.133) | 0.219 | establishment of organelle localization | Eb1 Incenp asp polo |
| 477 | GO:0004866 | F | 5, | 8 | 4.196 (x 1.907) | 85 (0.094) | 0.219 | endopeptidase inhibitor activity | CG32354 CG5392 CG6680 Pten Spn1 Spn43Aa Spn5 serpin-27A |
| 478 | GO:0003844 | F | 6, | 1 | 0.049 (x 20.259) | 1 (1.000) | 0.219 | 1,4-alpha-glucan branching enzyme activity | CG33138 |
| 479 | GO:0016485 | P | 8, | 4 | 1.481 (x 2.701) | 30 (0.133) | 0.22 | protein processing | BG:DS00004.11 InR rasp wbl |
| 480 | GO:0042499 | F | 8, | 1 | 0.049 (x 20.259) | 1 (1.000) | 0.22 | signal peptide peptidase activity | shanti |
| 481 | GO:0016049 | P | 3, 4, 6, 7, | 4 | 1.481 (x 2.701) | 30 (0.133) | 0.22 | cell growth | Cdk4 InR Pten eIF-4E |
| 482 | GO:0015272 | F | 8, 9, 10, | 1 | 0.049 (x 20.259) | 1 (1.000) | 0.22 | ATP-activated inward rectifier potassium channel activity | Ir |
| 483 | GO:0008717 | F | 6, | 1 | 0.049 (x 20.259) | 1 (1.000) | 0.221 | D-alanyl-D-alanine endopeptidase activity | shanti |
| 484 | GO:0051707 | P | 4, | 11 | 6.466 (x 1.701) | 131 (0.084) | 0.221 | response to other organism | 18w CG10359 CG7668 GNBP3 Mpk2 Tl Tsp96F br emp pll serpin-27A |
| 485 | GO:0046839 | P | 7, 8, | 1 | 0.049 (x 20.259) | 1 (1.000) | 0.221 | phospholipid dephosphorylation | Pten |
| 486 | GO:0008022 | F | 4, | 1 | 0.049 (x 20.259) | 1 (1.000) | 0.222 | protein C-terminus binding | CtBP |
| 487 | GO:0007181 | P | 9, | 1 | 0.049 (x 20.259) | 1 (1.000) | 0.222 | transforming growth factor beta receptor complex assembly | tkv |
| 488 | GO:0004144 | F | 9, | 1 | 0.049 (x 20.259) | 1 (1.000) | 0.223 | diacylglycerol O-acyltransferase activity | mdy |
| 489 | GO:0004450 | F | 7, | 1 | 0.049 (x 20.259) | 1 (1.000) | 0.223 | isocitrate dehydrogenase (NADP+) activity | Idh |
| 490 | GO:0045935 | P | 7, | 7 | 3.208 (x 2.182) | 65 (0.108) | 0.223 | positive regulation of nucleobase, nucleoside, nucleotide and nucleic acid metabolism | Trl ara ash2 ci cyc lola usp |
| 491 | GO:0009103 | P | 6, 7, 8, 9, | 1 | 0.049 (x 20.259) | 1 (1.000) | 0.224 | lipopolysaccharide biosynthesis | CG31915 |
| 492 | GO:0009725 | P | 4, 5, | 3 | 0.790 (x 3.799) | 16 (0.188) | 0.224 | response to hormone stimulus | Eip75B br usp |
| 493 | GO:0018009 | P | 10, 12, 13, | 1 | 0.049 (x 20.259) | 1 (1.000) | 0.224 | N-terminal peptidyl-L-cysteine N-palmitoylation | rasp |
| 494 | GO:0008263 | F | 8, | 1 | 0.049 (x 20.259) | 1 (1.000) | 0.224 | pyrimidine-specific mismatch base pair DNA N-glycosylase activity | Thd1 |
| 495 | GO:0016538 | F | 5, | 3 | 0.790 (x 3.799) | 16 (0.188) | 0.225 | cyclin-dependent protein kinase regulator activity | Cks CycB3 CycT |
| 496 | GO:0044432 | C | 4, 5, 6, 7, 8, 9, | 6 | 2.567 (x 2.338) | 52 (0.115) | 0.225 | endoplasmic reticulum part | BG:DS00004.11 CG14214 Crc Pdi Tapdelta l(1)G0320 |
| 497 | GO:0045811 | P | 7, 8, 9, | 1 | 0.049 (x 20.259) | 1 (1.000) | 0.225 | positive regulation of frizzled signaling pathway | stan |
| 498 | GO:0030111 | P | 5, 6, 7, | 3 | 0.790 (x 3.799) | 16 (0.188) | 0.225 | regulation of Wnt receptor signaling pathway | RacGAP50C nmo stan |
| 499 | GO:0007163 | P | 5, 6, | 7 | 3.505 (x 1.997) | 71 (0.099) | 0.225 | establishment and/or maintenance of cell polarity | CG3770 Dl ara in nmo pk stan |
| 500 | GO:0001738 | P | 5, | 6 | 2.567 (x 2.338) | 52 (0.115) | 0.225 | morphogenesis of a polarized epithelium | Dl ara in nmo pk stan |
| 501 | GO:0045842 | P | 8, 9, 10, | 1 | 0.049 (x 20.259) | 1 (1.000) | 0.225 | positive regulation of mitotic metaphase/anaphase transition | Cks |
| 502 | GO:0035193 | P | 5, 6, | 2 | 0.346 (x 5.788) | 7 (0.286) | 0.226 | central nervous system remodeling (sensu Insecta) | br cib |
| 503 | GO:0042058 | P | 5, 6, 9, | 3 | 0.790 (x 3.799) | 16 (0.188) | 0.226 | regulation of epidermal growth factor receptor signaling pathway | S ed edl |
| 504 | GO:0006500 | P | 11, 12, | 1 | 0.049 (x 20.259) | 1 (1.000) | 0.226 | N-terminal protein palmitoylation | rasp |
| 505 | GO:0016607 | C | 7, 8, 9, 10, 11, 12, 13, 14, | 2 | 0.346 (x 5.788) | 7 (0.286) | 0.226 | nuclear speck | B52 sqd |
| 506 | GO:0046666 | P | 6, 7, | 3 | 0.790 (x 3.799) | 16 (0.188) | 0.226 | retinal cell programmed cell death | Dl Ice klu |
| 507 | GO:0008653 | P | 6, 7, 8, | 1 | 0.049 (x 20.259) | 1 (1.000) | 0.226 | lipopolysaccharide metabolism | CG31915 |
| 508 | GO:0007009 | P | 6, | 2 | 0.346 (x 5.788) | 7 (0.286) | 0.227 | plasma membrane organization and biogenesis | CG1893 grh |
| 509 | GO:0042803 | F | 5, | 3 | 0.790 (x 3.799) | 16 (0.188) | 0.227 | protein homodimerization activity | CG3136 CrebA bun |
| 510 | GO:0031348 | P | 6, | 1 | 0.049 (x 20.259) | 1 (1.000) | 0.227 | negative regulation of defense response | serpin-27A |
| 511 | GO:0019838 | F | 4, | 2 | 0.346 (x 5.788) | 7 (0.286) | 0.227 | growth factor binding | InR tkv |
| 512 | GO:0005009 | F | 7, 9, | 1 | 0.049 (x 20.259) | 1 (1.000) | 0.227 | insulin receptor activity | InR |
| 513 | GO:0042802 | F | 4, | 3 | 0.790 (x 3.799) | 16 (0.188) | 0.227 | identical protein binding | CG3136 CrebA bun |
| 514 | GO:0004634 | F | 6, | 1 | 0.049 (x 20.259) | 1 (1.000) | 0.228 | phosphopyruvate hydratase activity | Eno |
| 515 | GO:0042981 | P | 6, 7, | 10 | 5.380 (x 1.859) | 109 (0.092) | 0.228 | regulation of apoptosis | Aac11 CG14217 CG7379 Dp Jafrac2 mdy mod(mdg4) nmo smi35A th |
| 516 | GO:0030258 | P | 6, 7, | 2 | 0.346 (x 5.788) | 7 (0.286) | 0.228 | lipid modification | Pi3K21B Pten |
| 517 | GO:0007143 | P | 8, | 5 | 1.925 (x 2.597) | 39 (0.128) | 0.228 | female meiosis | CG40410 ald eIF-4E east polo |
| 518 | GO:0016564 | F | 3, | 6 | 2.814 (x 2.133) | 57 (0.105) | 0.228 | transcriptional repressor activity | CG33525 CREG CtBP Dsp1 ci en |
| 519 | GO:0004018 | F | 6, | 1 | 0.049 (x 20.259) | 1 (1.000) | 0.228 | adenylosuccinate lyase activity | CG3590 |
| 520 | GO:0009081 | P | 7, 8, | 2 | 0.346 (x 5.788) | 7 (0.286) | 0.228 | branched chain family amino acid metabolism | CG2118 EG:171D11.1 |
| 521 | GO:0005815 | C | 5, 6, 7, 8, | 5 | 1.925 (x 2.597) | 39 (0.128) | 0.228 | microtubule organizing center | Nek2 asp fzy p120ctn polo |
| 522 | GO:0005509 | F | 5, | 17 | 10.761 (x 1.580) | 218 (0.078) | 0.229 | calcium ion binding | CBP CG17493 CG1924 CG33113 CG9906 Cad74A Cad87A Cad96Ca Crc Dl Fkbp13 l(1)G0320 lola ome scf stan tok |
| 523 | GO:0018318 | P | 10, 11, | 1 | 0.049 (x 20.259) | 1 (1.000) | 0.229 | protein amino acid palmitoylation | rasp |
| 524 | GO:0007631 | P | 4, | 2 | 0.346 (x 5.788) | 7 (0.286) | 0.229 | feeding behavior | Sema-2a for |
| 525 | GO:0030414 | F | 4, | 8 | 4.245 (x 1.885) | 86 (0.093) | 0.229 | protease inhibitor activity | CG32354 CG5392 CG6680 Pten Spn1 Spn43Aa Spn5 serpin-27A |
| 526 | GO:0008113 | F | 6, | 1 | 0.049 (x 20.259) | 1 (1.000) | 0.229 | protein-methionine-S-oxide reductase activity | Eip71CD |
| 527 | GO:0008187 | F | 5, | 2 | 0.346 (x 5.788) | 7 (0.286) | 0.23 | poly-pyrimidine tract binding | heph nonA-l |
| 528 | GO:0048151 | P | 8, | 1 | 0.049 (x 20.259) | 1 (1.000) | 0.23 | hyperphosphorylation | cdc2 |
| 529 | GO:0030530 | C | 4, 5, 6, 7, 8, 9, 10, | 2 | 0.346 (x 5.788) | 7 (0.286) | 0.23 | heterogeneous nuclear ribonucleoprotein complex | CG17838 Pep |
| 530 | GO:0003979 | F | 6, | 1 | 0.049 (x 20.259) | 1 (1.000) | 0.23 | UDP-glucose 6-dehydrogenase activity | sgl |
| 531 | GO:0046578 | P | 6, 7, 8, | 2 | 0.346 (x 5.788) | 7 (0.286) | 0.231 | regulation of Ras protein signal transduction | edl klu |
| 532 | GO:0007029 | P | 6, | 1 | 0.049 (x 20.259) | 1 (1.000) | 0.231 | endoplasmic reticulum organization and biogenesis | TER94 |
| 533 | GO:0015929 | F | 6, | 2 | 0.346 (x 5.788) | 7 (0.286) | 0.231 | hexosaminidase activity | CG5731 Hexo1 |
| 534 | GO:0042479 | P | 8, 9, 10, | 1 | 0.049 (x 20.259) | 1 (1.000) | 0.231 | positive regulation of eye photoreceptor cell development | mbt |
| 535 | GO:0030054 | C | 5, 6, 7, | 7 | 3.258 (x 2.149) | 66 (0.106) | 0.231 | cell junction | Fas3 InR inx2 inx3 mbt ogre p120ctn |
| 536 | GO:0004648 | F | 6, | 1 | 0.049 (x 20.259) | 1 (1.000) | 0.232 | phosphoserine transaminase activity | ESTS:39C10S |
| 537 | GO:0016320 | P | 7, | 1 | 0.049 (x 20.259) | 1 (1.000) | 0.232 | endoplasmic reticulum membrane fusion | TER94 |
| 538 | GO:0046856 | P | 8, 9, | 1 | 0.049 (x 20.259) | 1 (1.000) | 0.233 | phosphoinositide dephosphorylation | Pten |
| 539 | GO:0004347 | F | 6, | 1 | 0.049 (x 20.259) | 1 (1.000) | 0.233 | glucose-6-phosphate isomerase activity | Pgi |
| 540 | GO:0044265 | P | 6, | 14 | 8.490 (x 1.649) | 172 (0.081) | 0.234 | cellular macromolecule catabolism | BEST:LD22483 CG5794 CG7860 Eno Gapdh1 Gapdh2 Hex-A Pgi Prosalpha6 Tpi Uch fzy sqd th |
| 541 | GO:0030726 | P | 6, 7, | 1 | 0.049 (x 20.259) | 1 (1.000) | 0.234 | testicular ring canal formation | fwd |
| 542 | GO:0042048 | P | 5, 6, | 6 | 2.616 (x 2.293) | 53 (0.113) | 0.234 | olfactory behavior | CG11711 CG8588 Crc Fas3 Obp99a smi35A |
| 543 | GO:0009118 | P | 7, | 1 | 0.049 (x 20.259) | 1 (1.000) | 0.234 | regulation of nucleoside metabolism | CG11079 |
| 544 | GO:0008356 | P | 5, | 6 | 2.616 (x 2.293) | 53 (0.113) | 0.235 | asymmetric cell division | Dl asp cdc2 numb pon tkv |
| 545 | GO:0004887 | F | 5, | 1 | 0.049 (x 20.259) | 1 (1.000) | 0.235 | thyroid hormone receptor activity | Eip75B |
| 546 | GO:0005521 | F | 4, | 1 | 0.049 (x 20.259) | 1 (1.000) | 0.235 | lamin binding | CG17952 |
| 547 | GO:0046080 | P | 10, | 1 | 0.049 (x 20.259) | 1 (1.000) | 0.236 | dUTP metabolism | dUTPase |
| 548 | GO:0046487 | P | 6, 7, | 1 | 0.049 (x 20.259) | 1 (1.000) | 0.236 | glyoxylate metabolism | Idh |
| 549 | GO:0005035 | F | 5, | 1 | 0.049 (x 20.259) | 1 (1.000) | 0.237 | death receptor activity | wgn |
| 550 | GO:0018198 | P | 9, | 1 | 0.049 (x 20.259) | 1 (1.000) | 0.237 | peptidyl-cysteine modification | rasp |
| 551 | GO:0044275 | P | 7, | 7 | 3.307 (x 2.117) | 67 (0.104) | 0.238 | cellular carbohydrate catabolism | BEST:LD22483 Eno Gapdh1 Gapdh2 Hex-A Pgi Tpi |
| 552 | GO:0019218 | P | 6, 7, 8, | 1 | 0.049 (x 20.259) | 1 (1.000) | 0.238 | regulation of steroid metabolism | Eip75B |
| 553 | GO:0031109 | P | 9, | 3 | 0.938 (x 3.199) | 19 (0.158) | 0.238 | microtubule polymerization or depolymerization | alphaTub84B betaTub56D fwd |
| 554 | GO:0016052 | P | 6, | 7 | 3.307 (x 2.117) | 67 (0.104) | 0.238 | carbohydrate catabolism | BEST:LD22483 Eno Gapdh1 Gapdh2 Hex-A Pgi Tpi |
| 555 | GO:0042756 | P | 5, | 1 | 0.049 (x 20.259) | 1 (1.000) | 0.238 | drinking behavior | Sema-2a |
| 556 | GO:0009620 | P | 5, | 3 | 0.938 (x 3.199) | 19 (0.158) | 0.239 | response to fungus | GNBP3 Tl pll |
| 557 | GO:0003676 | F | 3, | 99 | 86.035 (x 1.151) | 1743 (0.057) | 0.239 | nucleic acid binding | B52 BEST:GH02921 BEST:LD29214 BcDNA:GM10765 BcDNA:LD21403 CG11033 CG15435 CG17838 CG18853 CG1911 CG30011 CG3136 CG33525 CG3800 CG4496 CG4914 CG6049 CG6654 CG6812 CG6930 CG6946 CG7379 CG8092 CG8963 CG9342 CrebA D19A Doc1 Doc2 Doc3 Dp Dp1 Dr Dref Dsp1 Eip74EF Eip75B GATAd HLHm7 His4r HmgD HmgZ Hrb27C Mcm7 Optix Pep Poxn Ptp99A Rab8 Rbf2 Rep4 Rm62 Thd1 Top2 Trl ap ara ash2 br btsz bun cg ci cic cyc dve eIF-4E edl en esn glu grh grn heph hth inv jumu kis klu l(2)01424 lid lola mdy nonA-l numb opa pAbp pdm2 ph-p pk sc scf spen sqd toe trh usp woc zf30C |
| 558 | GO:0042132 | F | 8, | 1 | 0.049 (x 20.259) | 1 (1.000) | 0.239 | fructose-bisphosphatase activity | fbp |
| 559 | GO:0006800 | P | 5, | 7 | 3.307 (x 2.117) | 67 (0.104) | 0.239 | oxygen and reactive oxygen species metabolism | CG12199 CG5873 CG6776 CG9027 GstS1 Jafrac2 mth |
| 560 | GO:0045596 | P | 5, | 3 | 0.938 (x 3.199) | 19 (0.158) | 0.239 | negative regulation of cell differentiation | Dl bun usp |
| 561 | GO:0005911 | C | 6, 7, 8, | 5 | 2.172 (x 2.302) | 44 (0.114) | 0.239 | intercellular junction | Fas3 InR inx2 inx3 ogre |
| 562 | GO:0007467 | P | 5, | 8 | 4.294 (x 1.863) | 87 (0.092) | 0.239 | photoreceptor cell differentiation (sensu Endopterygota) | Amph Dl S TER94 bun hth mbt stan |
| 563 | GO:0007350 | P | 4, 5, | 9 | 5.035 (x 1.788) | 102 (0.088) | 0.239 | blastoderm segmentation | Cdk4 ci en kis knk opa rasp sgl stan |
| 564 | GO:0004807 | F | 6, | 1 | 0.049 (x 20.259) | 1 (1.000) | 0.24 | triose-phosphate isomerase activity | Tpi |
| 565 | GO:0004342 | F | 4, 6, | 1 | 0.049 (x 20.259) | 1 (1.000) | 0.24 | glucosamine-6-phosphate deaminase activity | Oscillin |
| 566 | GO:0009157 | P | 8, 9, | 1 | 0.049 (x 20.259) | 1 (1.000) | 0.241 | deoxyribonucleoside monophosphate biosynthesis | Ts |
| 567 | GO:0009177 | P | 9, 10, | 1 | 0.049 (x 20.259) | 1 (1.000) | 0.241 | pyrimidine deoxyribonucleoside monophosphate biosynthesis | Ts |
| 568 | GO:0016314 | F | 8, | 1 | 0.049 (x 20.259) | 1 (1.000) | 0.242 | phosphatidylinositol-3,4,5-trisphosphate 3-phosphatase activity | Pten |
| 569 | GO:0035009 | P | 6, 7, 9, | 1 | 0.049 (x 20.259) | 1 (1.000) | 0.242 | negative regulation of melanization defense response | serpin-27A |
| 570 | GO:0007553 | P | 7, 8, 9, | 1 | 0.049 (x 20.259) | 1 (1.000) | 0.243 | regulation of ecdysteroid metabolism | Eip75B |
| 571 | GO:0051295 | P | 6, 7, 8, 10, | 1 | 0.049 (x 20.259) | 1 (1.000) | 0.243 | establishment of meiotic spindle localization | asp |
| 572 | GO:0004343 | F | 9, | 1 | 0.049 (x 20.259) | 1 (1.000) | 0.244 | glucosamine 6-phosphate N-acetyltransferase activity | CG1969 |
| 573 | GO:0046684 | P | 7, | 1 | 0.049 (x 20.259) | 1 (1.000) | 0.244 | response to pyrethroid | para |
| 574 | GO:0035293 | P | 5, 7, 8, 9, 11, | 1 | 0.049 (x 20.259) | 1 (1.000) | 0.245 | larval cuticle pattern formation (sensu Insecta) | CrebA |
| 575 | GO:0007388 | P | 5, | 1 | 0.049 (x 20.259) | 1 (1.000) | 0.246 | posterior compartment specification | en |
| 576 | GO:0051234 | P | 4, | 97 | 84.308 (x 1.151) | 1708 (0.057) | 0.246 | establishment of localization | Abi Amph Arf79F BEST:CK02656 BEST:LD04971 Best2 CG10657 CG11550 CG11739 CG12048 CG14214 CG14439 CG15088 CG15835 CG1607 CG17419 CG1893 CG1907 CG1924 CG2108 CG2852 CG31121 CG32137 CG33113 CG33171 CG3424 CG3823 CG6812 CG9057 CG9066 CG9342 CG9906 Crc Eb1 Fas3 Fs(2)Ket Galpha49B InR Incenp Ir Klp10A LanB2 Lsp2 Nrv1 Obp99a Ptp99A Rab8 S Sema-1a Sema-1b Sema-2a Sh Snap TER94 Ucp4B Vha100-2 alpha-Man-IIb alphaTub84B ap arr asp betaTub56D blot btsz cic ck d fra jumu l(1)G0320 l(2)01810 loj lola mnd mod(mdg4) mth ninA opa otk pain para pbl pk pll polo prominin-like rpk sgl spen sqd stai sut1 th trn usp vg wbl |
| 577 | GO:0043170 | P | 4, | 169 | 153.807 (x 1.099) | 3116 (0.054) | 0.246 | macromolecule metabolism | Ance Arf79F B52 BEST:GH02921 BEST:LD22483 BG:DS00004.11 BcDNA:GH02976 BcDNA:GM10765 BcDNA:LD41548 CG10657 CG11033 CG11142 CG11824 CG14217 CG1630 CG1632 CG17052 CG17323 CG18853 CG1911 CG1924 CG2200 CG2852 CG31738 CG31915 CG32499 CG32632 CG33138 CG33171 CG3328 CG40160 CG40410 CG4502 CG4914 CG5390 CG5731 CG5794 CG6199 CG6287 CG6680 CG6904 CG6946 CG7860 CG8963 CG9135 CG9307 CG9906 CaMKI Cad96Ca Caf1 Cdk4 CkIIalpha CkIIbeta Cks Crc D19A DnaJ-1 Dp Dref Dsp1 ERp60 Eb1 Eip71CD Eno Eph Fkbp13 GNBP3 GalNAc-T1 Galpha49B Gapdh1 Gapdh2 Gfat1 GlyP Hex-A Hexo1 His4r HmgD HmgZ Hrb27C Hsp23 Hsp26 Hsp27 Hsp67Ba Hus1-like Ice Idh InR Incenp Lsp2 Mcm7 Mgstl Mpk2 Nek2 Nep2 Oscillin PFE Pdi Pepck Pgi Pi3K21B Ppt1 Prosalpha6 Pten Ptp99A Rep4 Rm62 RnrS RpS12 Rpn9 Sb Set Spn1 Spn5 TER94 Thd1 Top2 Tpi Trl Ts Uch Ugt ald alpha-Man-IIb alphaTub84B ash2 betaTub56D blot cdc2 dap eIF-4E east fbp for fra fwd fzy glu heph jumu kis l(1)G0320 l(2)01424 l(2)01810 lola mbt mdy mod(mdg4) nmo nonA-l ome otk pAbp pbl pgant5 ph-p pll polo rasp sgl shf shu smi35A sqd sut1 th tkv tok tws wbl |
| 578 | GO:0048057 | P | 8, 9, 10, 11, | 1 | 0.049 (x 20.259) | 1 (1.000) | 0.246 | R3/R4 development (sensu Endopterygota) | stan |
| 579 | GO:0008414 | F | 4, | 1 | 0.049 (x 20.259) | 1 (1.000) | 0.247 | CDP-alcohol phosphotransferase activity | CG33116 |
| 580 | GO:0045840 | P | 7, 8, 9, | 1 | 0.049 (x 20.259) | 1 (1.000) | 0.247 | positive regulation of mitosis | Cks |
| 581 | GO:0016310 | P | 7, | 31 | 22.755 (x 1.362) | 461 (0.067) | 0.248 | phosphorylation | Argk CG14217 CG1630 CG40410 CaMKI Cad96Ca Cdk4 CkIIalpha CkIIbeta Cks Eph InR Incenp Mpk2 Nek2 PFE Pi3K21B ald blot cdc2 edl for fwd jumu mbt nmo otk pll polo smi35A tkv |
| 582 | GO:0004428 | F | 6, | 3 | 0.839 (x 3.575) | 17 (0.176) | 0.248 | inositol or phosphatidylinositol kinase activity | CG1630 Pi3K21B fwd |
| 583 | GO:0003980 | F | 7, 8, | 1 | 0.049 (x 20.259) | 1 (1.000) | 0.248 | UDP-glucose:glycoprotein glucosyltransferase activity | Ugt |
| 584 | GO:0006914 | P | 4, | 3 | 0.839 (x 3.575) | 17 (0.176) | 0.248 | autophagy | Eip74EF Pten br |
| 585 | GO:0004491 | F | 6, | 1 | 0.049 (x 20.259) | 1 (1.000) | 0.248 | methylmalonate-semialdehyde dehydrogenase (acylating) activity | EG:171D11.1 |
| 586 | GO:0016715 | F | 5, | 2 | 0.444 (x 4.502) | 9 (0.222) | 0.249 | oxidoreductase activity, acting on paired donors, with incorporation or reduction of molecular oxygen, reduced ascorbate as one donor, and incorporation of one atom of oxygen | knk olf413 |
| 587 | GO:0007635 | P | 4, 5, | 6 | 2.665 (x 2.251) | 54 (0.111) | 0.249 | chemosensory behavior | CG11711 CG8588 Crc Fas3 Obp99a smi35A |
| 588 | GO:0043120 | F | 5, | 1 | 0.049 (x 20.259) | 1 (1.000) | 0.249 | tumor necrosis factor binding | wgn |
| 589 | GO:0046845 | P | 5, 6, | 2 | 0.444 (x 4.502) | 9 (0.222) | 0.249 | branched duct epithelial cell fate determination (sensu Insecta) | S tkv |
| 590 | GO:0007509 | P | 7, 8, | 2 | 0.444 (x 4.502) | 9 (0.222) | 0.249 | mesoderm migration | pbl sgl |
| 591 | GO:0007112 | P | 6, | 1 | 0.049 (x 20.259) | 1 (1.000) | 0.25 | male meiosis cytokinesis | fwd |
| 592 | GO:0000776 | C | 5, 6, 7, 8, 9, 10, 11, | 2 | 0.444 (x 4.502) | 9 (0.222) | 0.25 | kinetochore | fzy polo |
| 593 | GO:0003785 | F | 6, | 1 | 0.049 (x 20.259) | 1 (1.000) | 0.25 | actin monomer binding | cib |
| 594 | GO:0008069 | P | 6, 7, 9, | 2 | 0.444 (x 4.502) | 9 (0.222) | 0.25 | dorsal/ventral axis determination, follicular epithelium (sensu Insecta) | Dp sqd |
| 595 | GO:0000096 | P | 6, 7, 8, | 2 | 0.444 (x 4.502) | 9 (0.222) | 0.251 | sulfur amino acid metabolism | Eip55E Eip71CD |
| 596 | GO:0004170 | F | 8, | 1 | 0.049 (x 20.259) | 1 (1.000) | 0.251 | dUTP diphosphatase activity | dUTPase |
| 597 | GO:0007464 | P | 7, 8, 9, 10, 11, | 2 | 0.444 (x 4.502) | 9 (0.222) | 0.251 | R3/R4 cell fate commitment | Dl stan |
| 598 | GO:0016684 | F | 4, | 4 | 1.580 (x 2.532) | 32 (0.125) | 0.251 | oxidoreductase activity, acting on peroxide as acceptor | CG12199 CG5873 GstS1 Jafrac2 |
| 599 | GO:0006092 | P | 7, | 9 | 5.133 (x 1.753) | 104 (0.087) | 0.251 | main pathways of carbohydrate metabolism | BEST:LD22483 Eno Gapdh1 Gapdh2 Hex-A Idh Pepck Pgi Tpi |
| 600 | GO:0048056 | P | 7, 8, 9, 10, | 2 | 0.444 (x 4.502) | 9 (0.222) | 0.251 | R3/R4 cell differentiation (sensu Endopterygota) | Dl stan |
| 601 | GO:0051640 | P | 4, | 4 | 1.580 (x 2.532) | 32 (0.125) | 0.252 | organelle localization | Eb1 Incenp asp polo |
| 602 | GO:0005112 | F | 4, 5, | 2 | 0.444 (x 4.502) | 9 (0.222) | 0.252 | Notch binding | Dl numb |
| 603 | GO:0006206 | P | 7, | 4 | 1.580 (x 2.532) | 32 (0.125) | 0.252 | pyrimidine base metabolism | CG6767 EG:171D11.1 RnrS Ts |
| 604 | GO:0045185 | P | 5, | 2 | 0.444 (x 4.502) | 9 (0.222) | 0.252 | maintenance of protein localization | Tapdelta pk |
| 605 | GO:0004601 | F | 3, 5, | 4 | 1.580 (x 2.532) | 32 (0.125) | 0.253 | peroxidase activity | CG12199 CG5873 GstS1 Jafrac2 |
| 606 | GO:0008195 | F | 7, | 2 | 0.444 (x 4.502) | 9 (0.222) | 0.253 | phosphatidate phosphatase activity | CG11438 mod(mdg4) |
| 607 | GO:0007632 | P | 4, 6, | 2 | 0.444 (x 4.502) | 9 (0.222) | 0.253 | visual behavior | Sema-2a ogre |
| 608 | GO:0035251 | F | 6, 7, | 2 | 0.444 (x 4.502) | 9 (0.222) | 0.254 | UDP-glucosyltransferase activity | CG6904 Ugt |
| 609 | GO:0008431 | F | 4, | 2 | 0.444 (x 4.502) | 9 (0.222) | 0.254 | vitamin E binding | CG13848 CG3823 |
| 610 | GO:0007549 | P | 4, | 2 | 0.444 (x 4.502) | 9 (0.222) | 0.254 | dosage compensation | Trl sc |
| 611 | GO:0005048 | F | 4, | 2 | 0.444 (x 4.502) | 9 (0.222) | 0.255 | signal sequence binding | Tapdelta l(1)G0320 |
| 612 | GO:0006966 | P | 7, 8, 9, | 2 | 0.444 (x 4.502) | 9 (0.222) | 0.255 | antifungal humoral response (sensu Protostomia) | Tl pll |
| 613 | GO:0042364 | P | 7, | 2 | 0.444 (x 4.502) | 9 (0.222) | 0.256 | water-soluble vitamin biosynthesis | CG31472 ESTS:39C10S |
| 614 | GO:0004675 | F | 5, 6, 8, 9, | 3 | 0.987 (x 3.039) | 20 (0.150) | 0.258 | transmembrane receptor protein serine/threonine kinase activity | PFE pll tkv |
| 615 | GO:0045926 | P | 4, | 3 | 0.987 (x 3.039) | 20 (0.150) | 0.258 | negative regulation of growth | Pten charybde scylla |
| 616 | GO:0016318 | P | 7, 8, 9, 10, | 3 | 0.987 (x 3.039) | 20 (0.150) | 0.258 | ommatidial rotation | Dl nmo stan |
| 617 | GO:0007015 | P | 9, | 6 | 2.962 (x 2.026) | 60 (0.100) | 0.262 | actin filament organization | CycT Fs(2)Ket Pten Sb Sry-alpha asp |
| 618 | GO:0006260 | P | 7, | 10 | 5.973 (x 1.674) | 121 (0.083) | 0.265 | DNA replication | BcDNA:GM10765 Caf1 Dp Dref Dsp1 Mcm7 RnrS Set Thd1 Top2 |
| 619 | GO:0005813 | C | 5, 6, 7, 8, 9, 10, | 4 | 1.629 (x 2.456) | 33 (0.121) | 0.271 | centrosome | Nek2 fzy p120ctn polo |
| 620 | GO:0016125 | P | 6, 7, 8, | 4 | 1.629 (x 2.456) | 33 (0.121) | 0.271 | sterol metabolism | CG1998 arr mdy woc |
| 621 | GO:0048598 | P | 4, | 9 | 5.232 (x 1.720) | 106 (0.085) | 0.271 | embryonic morphogenesis | 18w InR LanB2 inx2 jumu pbl pim tkv trh |
| 622 | GO:0008354 | P | 5, 6, 7, | 4 | 1.629 (x 2.456) | 33 (0.121) | 0.271 | germ cell migration | mod(mdg4) opa stai th |
| 623 | GO:0043565 | F | 5, | 4 | 1.629 (x 2.456) | 33 (0.121) | 0.272 | sequence-specific DNA binding | Dp1 HmgD Mcm7 dve |
| 624 | GO:0009611 | P | 4, | 4 | 1.629 (x 2.456) | 33 (0.121) | 0.272 | response to wounding | 18w emp ninA serpin-27A |
| 625 | GO:0016772 | F | 4, | 32 | 24.730 (x 1.294) | 501 (0.064) | 0.275 | transferase activity, transferring phosphorus-containing groups | Argk CG14217 CG1630 CG40410 CG6767 CaMKI Cad96Ca Cdk4 CkIIalpha CkIIbeta Cks CycT Eph Hex-A InR Mpk2 Nek2 PFE Pi3K21B ald asp cdc2 for fwd mbt nmo otk pll polo shf smi35A tkv |
| 626 | GO:0048565 | P | 4, | 6 | 3.060 (x 1.961) | 62 (0.097) | 0.282 | gut development | dve inx2 opa pbl pim trh |
| 627 | GO:0016864 | F | 5, | 2 | 0.494 (x 4.052) | 10 (0.200) | 0.282 | intramolecular oxidoreductase activity, transposing S-S bonds | ERp60 Pdi |
| 628 | GO:0008062 | P | 4, 6, | 2 | 0.494 (x 4.052) | 10 (0.200) | 0.283 | eclosion rhythm | CkIIbeta cyc |
| 629 | GO:0045321 | P | 5, 6, | 2 | 0.494 (x 4.052) | 10 (0.200) | 0.283 | leukocyte activation | 18w emp |
| 630 | GO:0006308 | P | 6, 7, | 2 | 0.494 (x 4.052) | 10 (0.200) | 0.284 | DNA catabolism | BcDNA:GM10765 Rep4 |
| 631 | GO:0009263 | P | 7, 8, | 1 | 0.099 (x 10.130) | 2 (0.500) | 0.284 | deoxyribonucleotide biosynthesis | Ts |
| 632 | GO:0008309 | F | 9, | 1 | 0.099 (x 10.130) | 2 (0.500) | 0.284 | double-stranded DNA specific exodeoxyribonuclease activity | BcDNA:GM10765 |
| 633 | GO:0051653 | P | 5, 6, | 2 | 0.494 (x 4.052) | 10 (0.200) | 0.284 | spindle localization | Eb1 asp |
| 634 | GO:0008284 | P | 6, | 1 | 0.099 (x 10.130) | 2 (0.500) | 0.284 | positive regulation of cell proliferation | InR |
| 635 | GO:0007346 | P | 6, 7, | 3 | 1.037 (x 2.894) | 21 (0.143) | 0.284 | regulation of progression through mitotic cell cycle | CG40410 ci sc |
| 636 | GO:0042116 | P | 6, 7, | 2 | 0.494 (x 4.052) | 10 (0.200) | 0.284 | macrophage activation | 18w emp |
| 637 | GO:0005520 | F | 5, | 1 | 0.099 (x 10.130) | 2 (0.500) | 0.285 | insulin-like growth factor binding | InR |
| 638 | GO:0003756 | F | 6, | 2 | 0.494 (x 4.052) | 10 (0.200) | 0.285 | protein disulfide isomerase activity | ERp60 Pdi |
| 639 | GO:0019210 | F | 4, | 3 | 1.037 (x 2.894) | 21 (0.143) | 0.285 | kinase inhibitor activity | CG17919 dap pcs |
| 640 | GO:0009211 | P | 9, | 1 | 0.099 (x 10.130) | 2 (0.500) | 0.285 | pyrimidine deoxyribonucleoside triphosphate metabolism | dUTPase |
| 641 | GO:0006954 | P | 5, 6, | 2 | 0.494 (x 4.052) | 10 (0.200) | 0.285 | inflammatory response | 18w emp |
| 642 | GO:0050954 | P | 4, 6, | 3 | 1.037 (x 2.894) | 21 (0.143) | 0.285 | sensory perception of mechanical stimulus | ck d pain |
| 643 | GO:0051323 | P | 6, | 1 | 0.099 (x 10.130) | 2 (0.500) | 0.286 | metaphase | east |
| 644 | GO:0008362 | P | 9, | 2 | 0.494 (x 4.052) | 10 (0.200) | 0.286 | embryonic cuticle biosynthesis (sensu Insecta) | grh knk |
| 645 | GO:0035157 | P | 7, 8, | 1 | 0.099 (x 10.130) | 2 (0.500) | 0.286 | negative regulation of fusion cell fate specification | Dl |
| 646 | GO:0001737 | P | 6, 7, 8, 9, 10, 11, | 2 | 0.494 (x 4.052) | 10 (0.200) | 0.286 | establishment of wing hair orientation | in pk |
| 647 | GO:0042246 | P | 4, 5, 6, | 1 | 0.099 (x 10.130) | 2 (0.500) | 0.286 | tissue regeneration | ninA |
| 648 | GO:0030693 | F | 7, | 2 | 0.494 (x 4.052) | 10 (0.200) | 0.287 | caspase activity | CG3328 Ice |
| 649 | GO:0006944 | P | 6, | 1 | 0.099 (x 10.130) | 2 (0.500) | 0.287 | membrane fusion | TER94 |
| 650 | GO:0048132 | P | 6, 7, | 1 | 0.099 (x 10.130) | 2 (0.500) | 0.287 | female germ-line stem cell division | asp |
| 651 | GO:0005041 | F | 6, | 2 | 0.494 (x 4.052) | 10 (0.200) | 0.287 | low-density lipoprotein receptor activity | CG1632 arr |
| 652 | GO:0035216 | P | 5, | 1 | 0.099 (x 10.130) | 2 (0.500) | 0.288 | haltere disc development | ap |
| 653 | GO:0030431 | P | 4, | 2 | 0.494 (x 4.052) | 10 (0.200) | 0.288 | sleep | Sh cyc |
| 654 | GO:0009120 | P | 7, | 1 | 0.099 (x 10.130) | 2 (0.500) | 0.288 | deoxyribonucleoside metabolism | dUTPase |
| 655 | GO:0040018 | P | 5, | 2 | 0.494 (x 4.052) | 10 (0.200) | 0.288 | positive regulation of body size | InR btsz |
| 656 | GO:0004613 | F | 7, | 1 | 0.099 (x 10.130) | 2 (0.500) | 0.288 | phosphoenolpyruvate carboxykinase (GTP) activity | Pepck |
| 657 | GO:0030169 | F | 5, | 2 | 0.494 (x 4.052) | 10 (0.200) | 0.289 | low-density lipoprotein binding | CG1632 arr |
| 658 | GO:0016740 | F | 3, | 57 | 47.732 (x 1.194) | 967 (0.059) | 0.289 | transferase activity | Abi Argk BcDNA:GH04753 CG11307 CG14217 CG1630 CG16733 CG17323 CG1969 CG32632 CG33116 CG33138 CG33171 CG40410 CG6767 CG6776 CG6904 CG7379 CaMKI Cad96Ca Caf1 Cdk4 CkIIalpha CkIIbeta Cks CycT ESTS:39C10S Eph GalNAc-T1 Gfat1 GlyP GstS1 Hex-A InR Mgstl Mpk2 Nek2 PFE Pi3K21B Ts Ugt ald asp cdc2 for fwd mbt mdy nmo otk pgant5 pll polo rasp shf smi35A tkv |
| 659 | GO:0016671 | F | 5, | 1 | 0.099 (x 10.130) | 2 (0.500) | 0.289 | oxidoreductase activity, acting on sulfur group of donors, disulfide as acceptor | Eip71CD |
| 660 | GO:0007623 | P | 4, | 4 | 1.777 (x 2.251) | 36 (0.111) | 0.289 | circadian rhythm | CkIIalpha CkIIbeta Reg-5 cyc |
| 661 | GO:0016862 | F | 5, | 2 | 0.494 (x 4.052) | 10 (0.200) | 0.289 | intramolecular oxidoreductase activity, interconverting keto- and enol-groups | ERp60 Pdi |
| 662 | GO:0035044 | P | 6, 9, | 1 | 0.099 (x 10.130) | 2 (0.500) | 0.289 | sperm aster formation | polo |
| 663 | GO:0004364 | F | 5, | 4 | 1.777 (x 2.251) | 36 (0.111) | 0.289 | glutathione transferase activity | BcDNA:GH04753 CG6776 GstS1 Mgstl |
| 664 | GO:0030713 | P | 9, | 2 | 0.494 (x 4.052) | 10 (0.200) | 0.289 | stalk formation (sensu Insecta) | Dl ph-p |
| 665 | GO:0046125 | P | 8, | 1 | 0.099 (x 10.130) | 2 (0.500) | 0.29 | pyrimidine deoxyribonucleoside metabolism | dUTPase |
| 666 | GO:0006766 | P | 5, | 4 | 1.777 (x 2.251) | 36 (0.111) | 0.29 | vitamin metabolism | BEST:LD22483 CG31472 ESTS:39C10S Tpi |
| 667 | GO:0051293 | P | 6, 7, 9, | 2 | 0.494 (x 4.052) | 10 (0.200) | 0.29 | establishment of spindle localization | Eb1 asp |
| 668 | GO:0006081 | P | 5, | 1 | 0.099 (x 10.130) | 2 (0.500) | 0.29 | aldehyde metabolism | Idh |
| 669 | GO:0016209 | F | 2, | 4 | 1.777 (x 2.251) | 36 (0.111) | 0.29 | antioxidant activity | CG12199 CG5873 GstS1 Jafrac2 |
| 670 | GO:0007629 | P | 6, | 2 | 0.543 (x 3.683) | 11 (0.182) | 0.29 | flight behavior | Sema-2a Sh |
| 671 | GO:0008297 | F | 9, | 1 | 0.099 (x 10.130) | 2 (0.500) | 0.29 | single-stranded DNA specific exodeoxyribonuclease activity | BcDNA:GM10765 |
| 672 | GO:0006220 | P | 7, | 2 | 0.494 (x 4.052) | 10 (0.200) | 0.29 | pyrimidine nucleotide metabolism | Ts dUTPase |
| 673 | GO:0008034 | F | 4, | 2 | 0.543 (x 3.683) | 11 (0.182) | 0.291 | lipoprotein binding | CG1632 arr |
| 674 | GO:0017051 | F | 6, | 1 | 0.099 (x 10.130) | 2 (0.500) | 0.291 | retinol dehydratase activity | CG16733 |
| 675 | GO:0003916 | F | 4, 5, | 2 | 0.494 (x 4.052) | 10 (0.200) | 0.291 | DNA topoisomerase activity | Top2 scf |
| 676 | GO:0030228 | F | 5, | 2 | 0.543 (x 3.683) | 11 (0.182) | 0.291 | lipoprotein receptor activity | CG1632 arr |
| 677 | GO:0035030 | C | 4, 7, 8, 9, 10, 11, | 1 | 0.099 (x 10.130) | 2 (0.500) | 0.291 | phosphoinositide 3-kinase complex, class IA | Pi3K21B |
| 678 | GO:0001727 | F | 6, | 2 | 0.543 (x 3.683) | 11 (0.182) | 0.291 | lipid kinase activity | Pi3K21B fwd |
| 679 | GO:0006796 | P | 6, | 37 | 29.814 (x 1.241) | 604 (0.061) | 0.292 | phosphate metabolism | Argk CG11438 CG14217 CG1630 CG40410 CaMKI Cad96Ca Cdk4 CkIIalpha CkIIbeta Cks Eph InR Incenp Mpk2 Nek2 PFE Pi3K21B Pten Ptp99A ald blot cdc2 dap edl for fwd jumu l(2)01810 mbt nmo otk pll polo smi35A tkv tws |
| 680 | GO:0004617 | F | 6, | 1 | 0.099 (x 10.130) | 2 (0.500) | 0.292 | phosphoglycerate dehydrogenase activity | CG6287 |
| 681 | GO:0046668 | P | 6, 7, 8, | 2 | 0.543 (x 3.683) | 11 (0.182) | 0.292 | regulation of retinal programmed cell death | Ice klu |
| 682 | GO:0006793 | P | 5, | 37 | 29.814 (x 1.241) | 604 (0.061) | 0.292 | phosphorus metabolism | Argk CG11438 CG14217 CG1630 CG40410 CaMKI Cad96Ca Cdk4 CkIIalpha CkIIbeta Cks Eph InR Incenp Mpk2 Nek2 PFE Pi3K21B Pten Ptp99A ald blot cdc2 dap edl for fwd jumu l(2)01810 mbt nmo otk pll polo smi35A tkv tws |
| 683 | GO:0006607 | P | 8, 9, 10, 11, | 1 | 0.099 (x 10.130) | 2 (0.500) | 0.292 | NLS-bearing substrate import into nucleus | Fs(2)Ket |
| 684 | GO:0004680 | F | 8, | 2 | 0.543 (x 3.683) | 11 (0.182) | 0.292 | casein kinase activity | CkIIalpha CkIIbeta |
| 685 | GO:0030272 | F | 6, | 1 | 0.099 (x 10.130) | 2 (0.500) | 0.292 | 5-formyltetrahydrofolate cyclo-ligase activity | CG11079 |
| 686 | GO:0007174 | P | 9, | 1 | 0.099 (x 10.130) | 2 (0.500) | 0.293 | epidermal growth factor ligand processing | S |
| 687 | GO:0007202 | P | 8, 9, | 1 | 0.099 (x 10.130) | 2 (0.500) | 0.293 | phospholipase C activation | Galpha49B |
| 688 | GO:0045859 | P | 6, | 3 | 1.135 (x 2.642) | 23 (0.130) | 0.293 | regulation of protein kinase activity | Abi dap mbt |
| 689 | GO:0004749 | F | 6, 7, | 1 | 0.099 (x 10.130) | 2 (0.500) | 0.294 | ribose phosphate diphosphokinase activity | CG6767 |
| 690 | GO:0051338 | P | 4, | 3 | 1.135 (x 2.642) | 23 (0.130) | 0.294 | regulation of transferase activity | Abi dap mbt |
| 691 | GO:0000254 | F | 6, | 1 | 0.099 (x 10.130) | 2 (0.500) | 0.294 | C-4 methylsterol oxidase activity | CG1998 |
| 692 | GO:0043549 | P | 5, | 3 | 1.135 (x 2.642) | 23 (0.130) | 0.294 | regulation of kinase activity | Abi dap mbt |
| 693 | GO:0016322 | P | 7, 10, | 1 | 0.099 (x 10.130) | 2 (0.500) | 0.294 | neuron remodeling | usp |
| 694 | GO:0016319 | P | 5, 7, | 3 | 1.135 (x 2.642) | 23 (0.130) | 0.295 | mushroom body development | CkIIbeta mbt stan |
| 695 | GO:0016509 | F | 6, | 1 | 0.099 (x 10.130) | 2 (0.500) | 0.295 | long-chain-3-hydroxyacyl-CoA dehydrogenase activity | BcDNA:GH12558 |
| 696 | GO:0016333 | P | 5, | 3 | 1.135 (x 2.642) | 23 (0.130) | 0.295 | morphogenesis of follicular epithelium | Dp ph-p sqd |
| 697 | GO:0015397 | F | 6, 7, | 1 | 0.099 (x 10.130) | 2 (0.500) | 0.295 | equilibrative nucleoside transporter, nitrobenzyl-thioinosine-insensitive activity | BEST:LD04971 |
| 698 | GO:0008230 | C | 4, 7, 8, 9, 10, 11, 12, 13, 14, | 1 | 0.099 (x 10.130) | 2 (0.500) | 0.296 | ecdysone receptor holocomplex | usp |
| 699 | GO:0048150 | P | 5, 7, | 1 | 0.099 (x 10.130) | 2 (0.500) | 0.296 | behavioral response to ether | Sh |
| 700 | GO:0017121 | P | 7, | 1 | 0.099 (x 10.130) | 2 (0.500) | 0.297 | phospholipid scrambling | CG1893 |
| 701 | GO:0005500 | F | 4, | 1 | 0.099 (x 10.130) | 2 (0.500) | 0.297 | juvenile hormone binding | usp |
| 702 | GO:0009221 | P | 8, 9, | 1 | 0.099 (x 10.130) | 2 (0.500) | 0.297 | pyrimidine deoxyribonucleotide biosynthesis | Ts |
| 703 | GO:0043147 | P | 7, 9, 10, 11, 12, 14, | 1 | 0.099 (x 10.130) | 2 (0.500) | 0.298 | meiotic spindle stabilization | fwd |
| 704 | GO:0005575 | C | 1, | 299 | 285.403 (x 1.048) | 5782 (0.052) | 0.298 | cellular\_component | 18w Act42A Amph Ance B52 BEST:CK02656 BEST:GH02921 BEST:LD04971 BEST:LD29214 BG:DS00004.11 BcDNA:GH02976 BcDNA:GH12558 BcDNA:GM10765 BcDNA:LD41548 CBP CG10657 CG11079 CG11142 CG11550 CG11711 CG11739 CG12048 CG14076 CG14214 CG14439 CG15088 CG15435 CG1607 CG1632 CG17052 CG17084 CG17493 CG17838 CG17952 CG1907 CG1911 CG1924 CG2118 CG2200 CG30011 CG31121 CG31125 CG31320 CG3136 CG31738 CG31749 CG31997 CG32158 CG32354 CG32415 CG32499 CG32711 CG32827 CG3305 CG33113 CG33171 CG33525 CG3424 CG3823 CG3921 CG40410 CG4496 CG4586 CG4914 CG6654 CG6776 CG6782 CG6812 CG6891 CG6930 CG6965 CG8092 CG8588 CG8679 CG9057 CG9211 CG9307 CG9342 CG9894 CREG CaMKI Cad74A Cad87A Cad96Ca Caf1 CkIIalpha CkIIbeta Crc CrebA CtBP CycB3 CycT Cyp310a1 D19A Dl DnaJ-1 Doc1 Doc2 Doc3 Dp Dp1 Dr Dref Dsp1 EG:171D11.1 EG:BACN33B1.2 ERp60 Eb1 Eip74EF Eip75B Eno Eph Fas3 Fs(2)Ket GATAd GalNAc-T1 Galpha49B Gapdh1 Gapdh2 Gfat1 GstS1 HLHm7 His4r HmgD HmgZ Hrb27C Ice Idh ImpE2 ImpE3 InR Incenp Ir Jafrac2 Klp10A LanB2 Lsp2 M6 Mcm7 Mgstl Mpk2 Nek2 Nep2 Nrv1 Obp99a Optix Pdi Pep Pepck Pgi Pi3K21B Pli Poxn Ppt1 Prosalpha6 Ptp99A Rbf2 Rep4 Rhp Rm62 RnrS RpS12 Rpn9 S Sb Sema-1a Sema-1b Sema-2a Set Sh Snap Sry-alpha TER94 Tapdelta Thd1 Tl Top2 Trl Tsp26A Tsp39D Tsp66E Tsp96F Uch Ucp4B Ugt Vha100-2 Wnt2 a6 alpha-Man-IIb alphaTub84B ana ap ara arr ash2 asp betaTub56D bip1 blot bnb br btsz bun cdc2 cg ci cib cic ck crp cyc d dap dve eIF-4E east ed edl emp en esn for fra fzy glec glu grh grn gukh hbs heph hth in inv inx2 inx3 jumu kal-1 kis klu l(1)G0320 l(2)01424 l(2)01810 l(3)neo38 lid lig loj lola mbt mdy mnd mod(mdg4) mth ninA nonA-l numb ogre ome opa otk p120ctn pAbp pain para pbl pcs pdm2 pgant5 ph-p pk pll polo pon prominin-like rasp rost rpk sc shanti shf shu smi35A spen sqd stai stan sut1 th tkv toe trh trn tws usp vg wbl wgn woc zf30C |
| 705 | GO:0046680 | P | 7, | 1 | 0.099 (x 10.130) | 2 (0.500) | 0.298 | response to DDT | para |
| 706 | GO:0009130 | P | 8, 9, | 1 | 0.099 (x 10.130) | 2 (0.500) | 0.299 | pyrimidine nucleoside monophosphate biosynthesis | Ts |
| 707 | GO:0003904 | F | 6, | 1 | 0.099 (x 10.130) | 2 (0.500) | 0.299 | deoxyribodipyrimidine photo-lyase activity | CG18853 |
| 708 | GO:0045313 | P | 9, 10, 11, 12, | 1 | 0.099 (x 10.130) | 2 (0.500) | 0.299 | rhabdomere membrane biogenesis | Amph |
| 709 | GO:0009129 | P | 8, | 1 | 0.099 (x 10.130) | 2 (0.500) | 0.3 | pyrimidine nucleoside monophosphate metabolism | Ts |
| 710 | GO:0004645 | F | 6, | 1 | 0.099 (x 10.130) | 2 (0.500) | 0.3 | phosphorylase activity | GlyP |
| 711 | GO:0001700 | P | 5, | 10 | 6.368 (x 1.570) | 129 (0.078) | 0.301 | embryonic development (sensu Insecta) | CG40410 CtBP Doc1 InR Sema-1b a6 cic jumu tkv tws |
| 712 | GO:0008311 | F | 9, 10, | 1 | 0.099 (x 10.130) | 2 (0.500) | 0.301 | double-stranded DNA specific 3'-5' exodeoxyribonuclease activity | BcDNA:GM10765 |
| 713 | GO:0031099 | P | 4, | 1 | 0.099 (x 10.130) | 2 (0.500) | 0.301 | regeneration | ninA |
| 714 | GO:0007443 | P | 5, 9, 10, | 3 | 1.086 (x 2.763) | 22 (0.136) | 0.302 | Malpighian tubule morphogenesis | pbl pim trh |
| 715 | GO:0045787 | P | 6, 7, | 1 | 0.099 (x 10.130) | 2 (0.500) | 0.302 | positive regulation of progression through cell cycle | Cks |
| 716 | GO:0031267 | F | 6, | 3 | 1.086 (x 2.763) | 22 (0.136) | 0.302 | small GTPase binding | Cip4 Rhp vimar |
| 717 | GO:0005811 | C | 5, 6, 7, 8, | 1 | 0.099 (x 10.130) | 2 (0.500) | 0.302 | lipid particle | CG9057 |
| 718 | GO:0040003 | P | 8, | 3 | 1.086 (x 2.763) | 22 (0.136) | 0.302 | cuticle biosynthesis (sensu Insecta) | CrebA grh knk |
| 719 | GO:0005845 | C | 3, 4, 5, 6, | 1 | 0.099 (x 10.130) | 2 (0.500) | 0.303 | mRNA cap complex | l(2)01424 |
| 720 | GO:0045892 | P | 9, | 8 | 4.788 (x 1.671) | 97 (0.082) | 0.303 | negative regulation of transcription, DNA-dependent | CG33525 Caf1 CtBP Rbf2 ci cyc ph-p usp |
| 721 | GO:0030246 | F | 3, | 9 | 5.578 (x 1.614) | 113 (0.080) | 0.303 | carbohydrate binding | BcDNA:GH02976 CG11142 CG17052 CG32499 CG3921 CG9134 CG9307 Gfat1 glec |
| 722 | GO:0007178 | P | 7, | 5 | 2.517 (x 1.986) | 51 (0.098) | 0.303 | transmembrane receptor protein serine/threonine kinase signaling pathway | PFE bun mav pll tkv |
| 723 | GO:0048613 | P | 7, 8, | 3 | 1.086 (x 2.763) | 22 (0.136) | 0.303 | embryonic ectodermal gut morphogenesis | pbl pim trh |
| 724 | GO:0004360 | F | 6, | 1 | 0.099 (x 10.130) | 2 (0.500) | 0.303 | glutamine-fructose-6-phosphate transaminase (isomerizing) activity | Gfat1 |
| 725 | GO:0048558 | P | 6, 7, | 3 | 1.086 (x 2.763) | 22 (0.136) | 0.303 | embryonic gut morphogenesis | pbl pim trh |
| 726 | GO:0008310 | F | 9, 10, | 1 | 0.099 (x 10.130) | 2 (0.500) | 0.303 | single-stranded DNA specific 3'-5' exodeoxyribonuclease activity | BcDNA:GM10765 |
| 727 | GO:0046983 | F | 4, | 5 | 2.419 (x 2.067) | 49 (0.102) | 0.303 | protein dimerization activity | CG3136 CrebA bun cyc trh |
| 728 | GO:0004748 | F | 6, | 1 | 0.099 (x 10.130) | 2 (0.500) | 0.304 | ribonucleoside-diphosphate reductase activity | RnrS |
| 729 | GO:0048619 | P | 8, 9, | 3 | 1.086 (x 2.763) | 22 (0.136) | 0.304 | embryonic hindgut morphogenesis | pbl pim trh |
| 730 | GO:0006464 | P | 7, | 52 | 43.289 (x 1.201) | 877 (0.059) | 0.304 | protein modification | Arf79F BG:DS00004.11 CG14217 CG32632 CG33171 CG40410 CG4502 CG5794 CG7860 CaMKI Cad96Ca Caf1 Cdk4 CkIIalpha CkIIbeta Cks ERp60 Eip71CD Eph GalNAc-T1 Galpha49B InR Incenp Mgstl Mpk2 Nek2 PFE Pdi Pi3K21B Ppt1 Prosalpha6 Pten Ptp99A Uch Ugt ald cdc2 dap for fzy mbt nmo otk pgant5 pll polo rasp smi35A th tkv tws wbl |
| 731 | GO:0048256 | F | 8, | 1 | 0.099 (x 10.130) | 2 (0.500) | 0.304 | flap endonuclease activity | BcDNA:GM10765 |
| 732 | GO:0048557 | P | 6, | 3 | 1.086 (x 2.763) | 22 (0.136) | 0.304 | embryonic digestive tract morphogenesis | pbl pim trh |
| 733 | GO:0004485 | F | 6, | 1 | 0.099 (x 10.130) | 2 (0.500) | 0.305 | methylcrotonoyl-CoA carboxylase activity | CG2118 |
| 734 | GO:0048611 | P | 6, | 3 | 1.086 (x 2.763) | 22 (0.136) | 0.305 | embryonic ectodermal gut development | pbl pim trh |
| 735 | GO:0004611 | F | 6, | 1 | 0.099 (x 10.130) | 2 (0.500) | 0.305 | phosphoenolpyruvate carboxykinase activity | Pepck |
| 736 | GO:0048566 | P | 5, | 3 | 1.086 (x 2.763) | 22 (0.136) | 0.305 | embryonic gut development | pbl pim trh |
| 737 | GO:0016020 | C | 3, 4, | 103 | 92.107 (x 1.118) | 1866 (0.055) | 0.306 | membrane | 18w Amph Ance BEST:LD04971 BG:DS00004.11 CG11739 CG12048 CG14076 CG14214 CG14439 CG15088 CG1607 CG1632 CG17084 CG17952 CG1907 CG1924 CG32158 CG3305 CG3424 CG3921 CG6812 CG6965 CG8679 CG9211 Cad74A Cad87A Cad96Ca Cyp310a1 Dl Eph Fas3 Fs(2)Ket Galpha49B ImpE2 ImpE3 InR Ir M6 Mgstl Nep2 Nrv1 Ptp99A S Sb Sema-1a Sema-1b Sema-2a Sh Snap Sry-alpha Tapdelta Tl Tsp26A Tsp39D Tsp66E Tsp96F Ucp4B Ugt Vha100-2 alpha-Man-IIb arr blot btsz ci ed emp for fra glec gukh hbs in inx2 inx3 jumu l(1)G0320 l(2)01810 loj mbt mnd mod(mdg4) mth ninA ogre ome otk p120ctn pain para pbl pk pll prominin-like rasp rost rpk shanti stan sut1 tkv trn wgn |
| 738 | GO:0030730 | P | 5, 7, | 1 | 0.099 (x 10.130) | 2 (0.500) | 0.306 | sequestering of triacylglycerol | CG9057 |
| 739 | GO:0007592 | P | 7, | 3 | 1.086 (x 2.763) | 22 (0.136) | 0.306 | cuticle biosynthesis (sensu Protostomia and Nematoda) | CrebA grh knk |
| 740 | GO:0004123 | F | 5, | 1 | 0.099 (x 10.130) | 2 (0.500) | 0.306 | cystathionine gamma-lyase activity | Eip55E |
| 741 | GO:0046903 | P | 5, | 16 | 11.402 (x 1.403) | 231 (0.069) | 0.306 | secretion | Amph Arf79F CG14214 CG15835 CG1924 CG9066 CG9906 Crc Rab8 S Snap TER94 alpha-Man-IIb mth stai wbl |
| 742 | GO:0016728 | F | 5, | 1 | 0.099 (x 10.130) | 2 (0.500) | 0.306 | oxidoreductase activity, acting on CH2 groups, disulfide as acceptor | RnrS |
| 743 | GO:0008442 | F | 6, | 1 | 0.099 (x 10.130) | 2 (0.500) | 0.307 | 3-hydroxyisobutyrate dehydrogenase activity | BEST:LD22483 |
| 744 | GO:0009186 | P | 8, | 1 | 0.099 (x 10.130) | 2 (0.500) | 0.307 | deoxyribonucleoside diphosphate metabolism | RnrS |
| 745 | GO:0008303 | C | 3, 6, 7, 8, 9, 10, | 1 | 0.099 (x 10.130) | 2 (0.500) | 0.308 | caspase complex | Ice |
| 746 | GO:0009200 | P | 8, | 1 | 0.099 (x 10.130) | 2 (0.500) | 0.308 | deoxyribonucleoside triphosphate metabolism | dUTPase |
| 747 | GO:0008474 | F | 6, | 1 | 0.099 (x 10.130) | 2 (0.500) | 0.309 | palmitoyl-(protein) hydrolase activity | Ppt1 |
| 748 | GO:0004572 | F | 8, | 1 | 0.099 (x 10.130) | 2 (0.500) | 0.309 | mannosyl-oligosaccharide 1,3-1,6-alpha-mannosidase activity | alpha-Man-IIb |
| 749 | GO:0004884 | F | 6, | 1 | 0.099 (x 10.130) | 2 (0.500) | 0.31 | ecdysteroid hormone receptor activity | usp |
| 750 | GO:0043065 | P | 7, 8, | 6 | 3.159 (x 1.899) | 64 (0.094) | 0.31 | positive regulation of apoptosis | CG14217 CG7379 Dp Jafrac2 mod(mdg4) smi35A |
| 751 | GO:0017128 | F | 5, | 1 | 0.099 (x 10.130) | 2 (0.500) | 0.31 | phospholipid scramblase activity | CG1893 |
| 752 | GO:0030097 | P | 5, | 6 | 3.159 (x 1.899) | 64 (0.094) | 0.31 | hemopoiesis | CG30011 Galpha49B Tl grn nonA-l pll |
| 753 | GO:0045850 | P | 8, 9, 11, | 1 | 0.099 (x 10.130) | 2 (0.500) | 0.311 | positive regulation of nurse cell apoptosis | Dp |
| 754 | GO:0016507 | C | 3, 5, 6, 7, 8, 9, 10, 11, 12, | 1 | 0.099 (x 10.130) | 2 (0.500) | 0.311 | fatty acid beta-oxidation multienzyme complex | BcDNA:GH12558 |
| 755 | GO:0000022 | P | 7, 11, | 1 | 0.099 (x 10.130) | 2 (0.500) | 0.312 | mitotic spindle elongation | Eb1 |
| 756 | GO:0042787 | P | 10, 11, 12, | 1 | 0.099 (x 10.130) | 2 (0.500) | 0.312 | protein ubiquitination during ubiquitin-dependent protein catabolism | th |
| 757 | GO:0040015 | P | 5, | 1 | 0.099 (x 10.130) | 2 (0.500) | 0.312 | negative regulation of body size | Pten |
| 758 | GO:0005042 | F | 6, | 1 | 0.099 (x 10.130) | 2 (0.500) | 0.313 | netrin receptor activity | fra |
| 759 | GO:0005971 | C | 3, 5, 6, 7, 8, | 1 | 0.099 (x 10.130) | 2 (0.500) | 0.313 | ribonucleoside-diphosphate reductase complex | RnrS |
| 760 | GO:0005102 | F | 3, 4, | 19 | 13.772 (x 1.380) | 279 (0.068) | 0.314 | receptor binding | CG10359 CG6966 CG7668 Dl ETH Eb1 Sema-1a Sema-1b Sema-2a Tsp26A Tsp39D Tsp66E Tsp96F Wnt2 ced-6 mav numb otk shf |
| 761 | GO:0008255 | F | 6, 7, | 1 | 0.099 (x 10.130) | 2 (0.500) | 0.314 | ecdysis-triggering hormone activity | ETH |
| 762 | GO:0048512 | P | 5, | 3 | 1.185 (x 2.532) | 24 (0.125) | 0.319 | circadian behavior | CkIIalpha CkIIbeta cyc |
| 763 | GO:0016490 | F | 3, | 3 | 1.185 (x 2.532) | 24 (0.125) | 0.319 | structural constituent of peritrophic membrane (sensu Insecta) | BcDNA:GH02976 CG11142 CG17052 |
| 764 | GO:0019899 | F | 4, | 5 | 2.567 (x 1.948) | 52 (0.096) | 0.32 | enzyme binding | Caf1 Cip4 Pi3K21B Rhp vimar |
| 765 | GO:0008587 | P | 7, 8, 9, | 3 | 1.185 (x 2.532) | 24 (0.125) | 0.32 | wing margin morphogenesis | Dl heph vg |
| 766 | GO:0000122 | P | 10, | 5 | 2.567 (x 1.948) | 52 (0.096) | 0.32 | negative regulation of transcription from RNA polymerase II promoter | Caf1 CtBP Rbf2 ci cyc |
| 767 | GO:0007076 | P | 7, 8, 9, | 2 | 0.592 (x 3.377) | 12 (0.167) | 0.322 | mitotic chromosome condensation | eIF-4E glu |
| 768 | GO:0046527 | F | 6, | 2 | 0.592 (x 3.377) | 12 (0.167) | 0.322 | glucosyltransferase activity | CG6904 Ugt |
| 769 | GO:0000915 | P | 7, 11, | 2 | 0.592 (x 3.377) | 12 (0.167) | 0.323 | cytokinesis, contractile ring formation | Sry-alpha pbl |
| 770 | GO:0046667 | P | 7, 8, 9, | 2 | 0.592 (x 3.377) | 12 (0.167) | 0.323 | retinal cell programmed cell death (sensu Endopterygota) | Dl klu |
| 771 | GO:0000912 | P | 6, 10, | 2 | 0.592 (x 3.377) | 12 (0.167) | 0.323 | cytokinesis, formation of actomyosin apparatus | Sry-alpha pbl |
| 772 | GO:0006740 | P | 10, 11, | 2 | 0.592 (x 3.377) | 12 (0.167) | 0.324 | NADPH regeneration | BEST:LD22483 Tpi |
| 773 | GO:0031519 | C | 3, 5, 6, 7, 8, 9, 10, | 2 | 0.592 (x 3.377) | 12 (0.167) | 0.324 | PcG protein complex | Caf1 ph-p |
| 774 | GO:0008045 | P | 7, 8, 10, 11, 13, | 2 | 0.592 (x 3.377) | 12 (0.167) | 0.325 | motor axon guidance | Ptp99A fra |
| 775 | GO:0031032 | P | 9, | 2 | 0.592 (x 3.377) | 12 (0.167) | 0.325 | actomyosin structure organization and biogenesis | Sry-alpha pbl |
| 776 | GO:0045298 | C | 3, 5, 6, 7, 8, 9, 10, 11, | 2 | 0.592 (x 3.377) | 12 (0.167) | 0.326 | tubulin | alphaTub84B betaTub56D |
| 777 | GO:0000082 | P | 7, | 2 | 0.592 (x 3.377) | 12 (0.167) | 0.326 | G1/S transition of mitotic cell cycle | cdc2 dap |
| 778 | GO:0046785 | P | 8, 10, | 2 | 0.592 (x 3.377) | 12 (0.167) | 0.326 | microtubule polymerization | alphaTub84B betaTub56D |
| 779 | GO:0035172 | P | 5, 6, | 2 | 0.592 (x 3.377) | 12 (0.167) | 0.327 | hemocyte proliferation (sensu Arthropoda) | Tl pll |
| 780 | GO:0030307 | P | 5, 6, 7, 8, 9, | 2 | 0.592 (x 3.377) | 12 (0.167) | 0.327 | positive regulation of cell growth | InR eIF-4E |
| 781 | GO:0006739 | P | 9, 10, | 2 | 0.592 (x 3.377) | 12 (0.167) | 0.328 | NADP metabolism | BEST:LD22483 Tpi |
| 782 | GO:0006098 | P | 8, 10, 11, 12, | 2 | 0.592 (x 3.377) | 12 (0.167) | 0.328 | pentose-phosphate shunt | BEST:LD22483 Tpi |
| 783 | GO:0001708 | P | 5, | 5 | 2.616 (x 1.911) | 53 (0.094) | 0.331 | cell fate specification | Dl Dr ara bun malpha |
| 784 | GO:0009613 | P | 4, 5, | 8 | 4.936 (x 1.621) | 100 (0.080) | 0.333 | response to pest, pathogen or parasite | 18w Mpk2 Tl Tsp96F br emp pll serpin-27A |
| 785 | GO:0007265 | P | 7, | 3 | 1.234 (x 2.431) | 25 (0.120) | 0.339 | Ras protein signal transduction | Rapgap1 edl klu |
| 786 | GO:0051020 | F | 5, | 3 | 1.234 (x 2.431) | 25 (0.120) | 0.339 | GTPase binding | Cip4 Rhp vimar |
| 787 | GO:0048562 | P | 5, | 3 | 1.234 (x 2.431) | 25 (0.120) | 0.34 | embryonic organ morphogenesis | pbl pim trh |
| 788 | GO:0005279 | F | 5, 6, | 4 | 1.925 (x 2.078) | 39 (0.103) | 0.34 | amino acid-polyamine transporter activity | CG15088 CG1607 CG3424 mnd |
| 789 | GO:0035265 | P | 3, | 3 | 1.234 (x 2.431) | 25 (0.120) | 0.34 | organ growth | InR Pi3K21B Pten |
| 790 | GO:0007173 | P | 8, | 4 | 1.925 (x 2.078) | 39 (0.103) | 0.341 | epidermal growth factor receptor signaling pathway | S ed edl spen |
| 791 | GO:0050767 | P | 4, 7, | 3 | 1.234 (x 2.431) | 25 (0.120) | 0.341 | regulation of neurogenesis | ana ed pbl |
| 792 | GO:0035218 | P | 5, | 4 | 1.925 (x 2.078) | 39 (0.103) | 0.341 | leg disc development | Dl Poxn ap hth |
| 793 | GO:0046620 | P | 4, | 3 | 1.234 (x 2.431) | 25 (0.120) | 0.341 | regulation of organ size | InR Pi3K21B Pten |
| 794 | GO:0015203 | F | 4, | 4 | 1.925 (x 2.078) | 39 (0.103) | 0.341 | polyamine transporter activity | CG15088 CG1607 CG3424 mnd |
| 795 | GO:0051239 | P | 4, | 3 | 1.234 (x 2.431) | 25 (0.120) | 0.341 | regulation of organismal physiological process | Amph cyc for |
| 796 | GO:0000158 | F | 9, | 1 | 0.148 (x 6.753) | 3 (0.333) | 0.342 | protein phosphatase type 2A activity | tws |
| 797 | GO:0001518 | C | 3, 5, 6, 7, 8, 9, | 1 | 0.148 (x 6.753) | 3 (0.333) | 0.343 | voltage-gated sodium channel complex | para |
| 798 | GO:0005581 | C | 3, 4, 5, | 1 | 0.148 (x 6.753) | 3 (0.333) | 0.343 | collagen | CG33171 |
| 799 | GO:0015280 | F | 6, 7, 8, | 1 | 0.148 (x 6.753) | 3 (0.333) | 0.343 | amiloride-sensitive sodium channel activity | rpk |
| 800 | GO:0008360 | P | 5, 6, | 7 | 4.196 (x 1.668) | 85 (0.082) | 0.343 | regulation of cell shape | Abi Arf79F CycT Fs(2)Ket Pten grh pbl |
| 801 | GO:0005498 | F | 4, | 1 | 0.148 (x 6.753) | 3 (0.333) | 0.344 | sterol carrier activity | CG11151 |
| 802 | GO:0007113 | P | 5, | 1 | 0.148 (x 6.753) | 3 (0.333) | 0.344 | endomitotic cell cycle | Dp |
| 803 | GO:0046942 | P | 6, 7, | 5 | 2.665 (x 1.876) | 54 (0.093) | 0.344 | carboxylic acid transport | CG15088 CG1607 CG1907 CG3424 mnd |
| 804 | GO:0007219 | P | 6, | 4 | 2.024 (x 1.976) | 41 (0.098) | 0.345 | Notch signaling pathway | Dl heph malpha numb |
| 805 | GO:0035156 | P | 6, 7, | 1 | 0.148 (x 6.753) | 3 (0.333) | 0.345 | fusion cell fate specification | Dl |
| 806 | GO:0015849 | P | 5, 6, | 5 | 2.665 (x 1.876) | 54 (0.093) | 0.345 | organic acid transport | CG15088 CG1607 CG1907 CG3424 mnd |
| 807 | GO:0042175 | C | 4, 5, 6, | 4 | 2.024 (x 1.976) | 41 (0.098) | 0.345 | nuclear envelope-endoplasmic reticulum network | BG:DS00004.11 CG14214 Tapdelta l(1)G0320 |
| 808 | GO:0008024 | C | 4, 7, 8, 9, 10, 11, 12, 13, 14, | 1 | 0.148 (x 6.753) | 3 (0.333) | 0.345 | transcription elongation factor complex b | CycT |
| 809 | GO:0043146 | P | 8, 9, 10, 11, 13, | 1 | 0.148 (x 6.753) | 3 (0.333) | 0.345 | spindle stabilization | fwd |
| 810 | GO:0006264 | P | 8, 9, | 1 | 0.148 (x 6.753) | 3 (0.333) | 0.346 | mitochondrial DNA replication | Dref |
| 811 | GO:0030540 | P | 5, | 1 | 0.148 (x 6.753) | 3 (0.333) | 0.346 | female genitalia development | en |
| 812 | GO:0019233 | P | 4, 6, | 1 | 0.148 (x 6.753) | 3 (0.333) | 0.347 | sensory perception of pain | pain |
| 813 | GO:0015743 | P | 8, 9, | 1 | 0.148 (x 6.753) | 3 (0.333) | 0.347 | malate transport | CG1907 |
| 814 | GO:0045314 | P | 7, 8, 9, 10, 11, | 1 | 0.148 (x 6.753) | 3 (0.333) | 0.347 | regulation of eye photoreceptor development (sensu Endopterygota) | mbt |
| 815 | GO:0008409 | F | 7, | 1 | 0.148 (x 6.753) | 3 (0.333) | 0.348 | 5'-3' exonuclease activity | BcDNA:GM10765 |
| 816 | GO:0006040 | P | 6, 7, | 6 | 3.554 (x 1.688) | 72 (0.083) | 0.348 | amino sugar metabolism | BcDNA:GH02976 CG11142 CG17052 CG32499 CG9307 Oscillin |
| 817 | GO:0042551 | P | 6, 9, | 1 | 0.148 (x 6.753) | 3 (0.333) | 0.348 | neuron maturation | usp |
| 818 | GO:0016979 | F | 5, | 1 | 0.148 (x 6.753) | 3 (0.333) | 0.349 | lipoate-protein ligase activity | CG6767 |
| 819 | GO:0016064 | P | 6, 7, | 1 | 0.148 (x 6.753) | 3 (0.333) | 0.349 | immunoglobulin mediated immune response | Tsp96F |
| 820 | GO:0007403 | P | 6, 9, | 1 | 0.148 (x 6.753) | 3 (0.333) | 0.349 | glial cell fate determination | spen |
| 821 | GO:0004824 | F | 7, | 1 | 0.148 (x 6.753) | 3 (0.333) | 0.35 | lysine-tRNA ligase activity | mdy |
| 822 | GO:0004054 | F | 6, 7, | 1 | 0.148 (x 6.753) | 3 (0.333) | 0.35 | arginine kinase activity | Argk |
| 823 | GO:0019915 | P | 6, | 1 | 0.148 (x 6.753) | 3 (0.333) | 0.35 | sequestering of lipid | CG9057 |
| 824 | GO:0016200 | P | 6, | 1 | 0.148 (x 6.753) | 3 (0.333) | 0.351 | synaptic target attraction | Fas3 |
| 825 | GO:0021781 | P | 5, 8, | 1 | 0.148 (x 6.753) | 3 (0.333) | 0.351 | glial cell fate commitment | spen |
| 826 | GO:0000775 | C | 5, 6, 7, 8, 9, 10, | 3 | 1.333 (x 2.251) | 27 (0.111) | 0.352 | chromosome, pericentric region | Trl fzy polo |
| 827 | GO:0009250 | P | 8, 9, | 1 | 0.148 (x 6.753) | 3 (0.333) | 0.352 | glucan biosynthesis | CG6904 |
| 828 | GO:0042335 | P | 6, | 3 | 1.333 (x 2.251) | 27 (0.111) | 0.352 | cuticle biosynthesis | CrebA grh knk |
| 829 | GO:0005025 | F | 7, 8, 10, 11, | 1 | 0.148 (x 6.753) | 3 (0.333) | 0.352 | transforming growth factor beta receptor activity, type I | tkv |
| 830 | GO:0042127 | P | 5, | 3 | 1.333 (x 2.251) | 27 (0.111) | 0.352 | regulation of cell proliferation | InR Pi3K21B ana |
| 831 | GO:0008054 | P | 7, 11, 12, 13, | 1 | 0.148 (x 6.753) | 3 (0.333) | 0.353 | cyclin catabolism | fzy |
| 832 | GO:0044274 | P | 5, | 3 | 1.333 (x 2.251) | 27 (0.111) | 0.353 | organismal biosynthesis | CrebA grh knk |
| 833 | GO:0051231 | P | 10, | 1 | 0.148 (x 6.753) | 3 (0.333) | 0.353 | spindle elongation | Eb1 |
| 834 | GO:0048469 | P | 5, | 3 | 1.333 (x 2.251) | 27 (0.111) | 0.353 | cell maturation | hbs rost usp |
| 835 | GO:0030262 | P | 8, | 1 | 0.148 (x 6.753) | 3 (0.333) | 0.353 | apoptotic nuclear changes | Rep4 |
| 836 | GO:0005789 | C | 4, 5, 6, 7, 8, 9, 10, | 4 | 1.974 (x 2.026) | 40 (0.100) | 0.353 | endoplasmic reticulum membrane | BG:DS00004.11 CG14214 Tapdelta l(1)G0320 |
| 837 | GO:0004872 | F | 3, | 33 | 27.296 (x 1.209) | 553 (0.060) | 0.353 | receptor activity | 18w CG14076 CG1632 CG17084 CG17952 CG3921 CG5096 CG6965 CG9066 CG9211 Cad96Ca Eip75B Eph GNBP3 InR PFE Ptp99A Sema-1a Sema-1b Sema-2a Tl arr emp fra mth otk pbl pll shf stan tkv usp wgn |
| 838 | GO:0015630 | C | 6, 7, 8, 9, | 13 | 9.181 (x 1.416) | 186 (0.070) | 0.354 | microtubule cytoskeleton | CycB3 Eb1 Klp10A Nek2 TER94 alphaTub84B asp betaTub56D fzy p120ctn polo stai vg |
| 839 | GO:0008104 | P | 4, | 34 | 27.938 (x 1.217) | 566 (0.060) | 0.354 | protein localization | Amph Arf79F CG14214 CG1924 CG2108 CG2852 CG32137 CG33113 CG9906 Crc Fs(2)Ket Incenp Klp10A Rab8 S Snap TER94 Tapdelta alpha-Man-IIb alphaTub84B arr betaTub56D ck d gukh jumu l(1)G0320 loj pk pll polo pon prominin-like wbl |
| 840 | GO:0016882 | F | 5, | 1 | 0.148 (x 6.753) | 3 (0.333) | 0.354 | cyclo-ligase activity | CG11079 |
| 841 | GO:0048567 | P | 6, 7, | 4 | 1.974 (x 2.026) | 40 (0.100) | 0.354 | ectodermal gut morphogenesis | inx2 pbl pim trh |
| 842 | GO:0003696 | F | 6, | 1 | 0.148 (x 6.753) | 3 (0.333) | 0.354 | satellite DNA binding | Dp1 |
| 843 | GO:0007439 | P | 5, | 4 | 1.974 (x 2.026) | 40 (0.100) | 0.354 | ectodermal gut development | inx2 pbl pim trh |
| 844 | GO:0004430 | F | 8, | 1 | 0.148 (x 6.753) | 3 (0.333) | 0.355 | 1-phosphatidylinositol 4-kinase activity | fwd |
| 845 | GO:0048547 | P | 5, 6, | 4 | 1.974 (x 2.026) | 40 (0.100) | 0.355 | gut morphogenesis | inx2 pbl pim trh |
| 846 | GO:0045448 | P | 4, 6, | 2 | 0.642 (x 3.117) | 13 (0.154) | 0.355 | mitotic cell cycle, embryonic | CG40410 Trl |
| 847 | GO:0040029 | P | 3, | 6 | 3.455 (x 1.736) | 70 (0.086) | 0.355 | regulation of gene expression, epigenetic | Caf1 Rm62 Trl ash2 ph-p sc |
| 848 | GO:0035062 | C | 8, 9, 10, 11, 12, 13, 14, 15, | 1 | 0.148 (x 6.753) | 3 (0.333) | 0.355 | omega speckle | sqd |
| 849 | GO:0048546 | P | 5, | 4 | 1.974 (x 2.026) | 40 (0.100) | 0.355 | digestive tract morphogenesis | inx2 pbl pim trh |
| 850 | GO:0006891 | P | 6, 7, 8, 9, | 2 | 0.642 (x 3.117) | 13 (0.154) | 0.355 | intra-Golgi vesicle-mediated transport | S wbl |
| 851 | GO:0051227 | P | 7, 8, 11, | 1 | 0.148 (x 6.753) | 3 (0.333) | 0.355 | mitotic spindle assembly | Eb1 |
| 852 | GO:0046552 | P | 5, | 4 | 1.974 (x 2.026) | 40 (0.100) | 0.356 | photoreceptor cell fate commitment | Dl br hth stan |
| 853 | GO:0035285 | P | 4, 5, | 2 | 0.642 (x 3.117) | 13 (0.154) | 0.356 | appendage segmentation | Dl ap |
| 854 | GO:0007313 | P | 7, 9, 10, 12, | 1 | 0.148 (x 6.753) | 3 (0.333) | 0.356 | maternal determination of dorsal/ventral axis, oocyte, soma encoded | wbl |
| 855 | GO:0008203 | P | 7, 8, 9, | 3 | 1.283 (x 2.338) | 26 (0.115) | 0.356 | cholesterol metabolism | CG1998 arr mdy |
| 856 | GO:0007459 | P | 6, | 4 | 1.974 (x 2.026) | 40 (0.100) | 0.356 | photoreceptor fate commitment (sensu Endopterygota) | Dl br hth stan |
| 857 | GO:0009312 | P | 7, 8, | 2 | 0.642 (x 3.117) | 13 (0.154) | 0.356 | oligosaccharide biosynthesis | GalNAc-T1 pgant5 |
| 858 | GO:0006769 | P | 8, 9, | 2 | 0.691 (x 2.894) | 14 (0.143) | 0.356 | nicotinamide metabolism | BEST:LD22483 Tpi |
| 859 | GO:0000014 | F | 8, | 1 | 0.148 (x 6.753) | 3 (0.333) | 0.356 | single-stranded DNA specific endodeoxyribonuclease activity | BcDNA:GM10765 |
| 860 | GO:0005272 | F | 6, 7, | 3 | 1.283 (x 2.338) | 26 (0.115) | 0.356 | sodium channel activity | CG12048 para rpk |
| 861 | GO:0035286 | P | 5, 6, 7, | 2 | 0.642 (x 3.117) | 13 (0.154) | 0.356 | leg segmentation | Dl ap |
| 862 | GO:0006997 | P | 6, | 2 | 0.691 (x 2.894) | 14 (0.143) | 0.357 | nuclear organization and biogenesis | mod(mdg4) polo |
| 863 | GO:0007486 | P | 6, 7, | 1 | 0.148 (x 6.753) | 3 (0.333) | 0.357 | female genitalia development (sensu Endopterygota) | en |
| 864 | GO:0006936 | P | 4, | 7 | 4.245 (x 1.649) | 86 (0.081) | 0.357 | muscle contraction | Amph CG31738 CaMKI EG:118B3.2 GstS1 for para |
| 865 | GO:0019220 | P | 7, | 2 | 0.642 (x 3.117) | 13 (0.154) | 0.357 | regulation of phosphate metabolism | dap edl |
| 866 | GO:0019732 | P | 6, 7, 8, | 2 | 0.691 (x 2.894) | 14 (0.143) | 0.357 | antifungal humoral response | Tl pll |
| 867 | GO:0035017 | P | 4, 7, | 1 | 0.148 (x 6.753) | 3 (0.333) | 0.357 | cuticle pattern formation | CrebA |
| 868 | GO:0007479 | P | 6, 7, | 2 | 0.691 (x 2.894) | 14 (0.143) | 0.357 | leg disc proximal/distal pattern formation | ap hth |
| 869 | GO:0000792 | C | 6, 7, 8, 9, 10, 11, | 2 | 0.642 (x 3.117) | 13 (0.154) | 0.357 | heterochromatin | Dp1 Trl |
| 870 | GO:0001666 | P | 4, | 1 | 0.148 (x 6.753) | 3 (0.333) | 0.357 | response to hypoxia | ald |
| 871 | GO:0008239 | F | 7, | 2 | 0.691 (x 2.894) | 14 (0.143) | 0.358 | dipeptidyl-peptidase activity | CG2200 ome |
| 872 | GO:0051174 | P | 6, | 2 | 0.642 (x 3.117) | 13 (0.154) | 0.358 | regulation of phosphorus metabolism | dap edl |
| 873 | GO:0009374 | F | 4, | 1 | 0.148 (x 6.753) | 3 (0.333) | 0.358 | biotin binding | CG2118 |
| 874 | GO:0035317 | P | 7, 8, 9, 10, | 2 | 0.691 (x 2.894) | 14 (0.143) | 0.358 | wing hair organization and biogenesis | in pk |
| 875 | GO:0007428 | P | 5, 6, | 2 | 0.642 (x 3.117) | 13 (0.154) | 0.358 | primary tracheal branching (sensu Insecta) | grh sgl |
| 876 | GO:0042826 | F | 5, | 1 | 0.148 (x 6.753) | 3 (0.333) | 0.358 | histone deacetylase binding | Caf1 |
| 877 | GO:0035223 | P | 5, 6, | 2 | 0.691 (x 2.894) | 14 (0.143) | 0.359 | leg disc pattern formation | ap hth |
| 878 | GO:0000165 | P | 7, | 8 | 5.084 (x 1.574) | 103 (0.078) | 0.359 | MAPKKK cascade | CG14217 CG30440 CG5522 Mpk2 ced-6 mbt nmo tws |
| 879 | GO:0005721 | C | 6, 7, 8, 9, 10, 11, 12, | 1 | 0.148 (x 6.753) | 3 (0.333) | 0.359 | centric heterochromatin | Trl |
| 880 | GO:0009996 | P | 6, 7, | 2 | 0.642 (x 3.117) | 13 (0.154) | 0.359 | negative regulation of cell fate specification | Dl bun |
| 881 | GO:0042052 | P | 8, 9, 10, 11, | 2 | 0.691 (x 2.894) | 14 (0.143) | 0.359 | rhabdomere development | Amph TER94 |
| 882 | GO:0035010 | P | 5, 6, | 1 | 0.148 (x 6.753) | 3 (0.333) | 0.359 | encapsulation of foreign target | serpin-27A |
| 883 | GO:0006584 | P | 7, 8, | 2 | 0.642 (x 3.117) | 13 (0.154) | 0.359 | catecholamine metabolism | knk olf413 |
| 884 | GO:0018958 | P | 6, | 2 | 0.691 (x 2.894) | 14 (0.143) | 0.359 | phenol metabolism | knk olf413 |
| 885 | GO:0032042 | P | 7, 8, | 1 | 0.148 (x 6.753) | 3 (0.333) | 0.36 | mitochondrial DNA metabolism | Dref |
| 886 | GO:0016799 | F | 5, | 4 | 2.073 (x 1.929) | 42 (0.095) | 0.36 | hydrolase activity, hydrolyzing N-glycosyl compounds | CG9307 Hexo1 Thd1 alpha-Man-IIb |
| 887 | GO:0004653 | F | 7, | 2 | 0.691 (x 2.894) | 14 (0.143) | 0.36 | polypeptide N-acetylgalactosaminyltransferase activity | GalNAc-T1 pgant5 |
| 888 | GO:0008353 | F | 8, | 1 | 0.148 (x 6.753) | 3 (0.333) | 0.36 | RNA polymerase subunit kinase activity | CycT |
| 889 | GO:0030177 | P | 6, 7, 8, | 1 | 0.148 (x 6.753) | 3 (0.333) | 0.36 | positive regulation of Wnt receptor signaling pathway | stan |
| 890 | GO:0004303 | F | 7, | 1 | 0.148 (x 6.753) | 3 (0.333) | 0.361 | estradiol 17-beta-dehydrogenase activity | CG11151 |
| 891 | GO:0001672 | P | 6, 8, 10, | 1 | 0.148 (x 6.753) | 3 (0.333) | 0.361 | regulation of chromatin assembly or disassembly | mod(mdg4) |
| 892 | GO:0006044 | P | 8, 9, | 6 | 3.505 (x 1.712) | 71 (0.085) | 0.361 | N-acetylglucosamine metabolism | BcDNA:GH02976 CG11142 CG17052 CG32499 CG9307 Oscillin |
| 893 | GO:0007126 | P | 7, | 7 | 4.294 (x 1.630) | 87 (0.080) | 0.361 | meiosis | CG40410 Top2 ald cdc2 eIF-4E east polo |
| 894 | GO:0000281 | P | 6, 7, | 1 | 0.148 (x 6.753) | 3 (0.333) | 0.362 | cytokinesis after mitosis | CycB3 |
| 895 | GO:0006041 | P | 7, 8, | 6 | 3.505 (x 1.712) | 71 (0.085) | 0.362 | glucosamine metabolism | BcDNA:GH02976 CG11142 CG17052 CG32499 CG9307 Oscillin |
| 896 | GO:0006430 | P | 9, 10, 11, | 1 | 0.148 (x 6.753) | 3 (0.333) | 0.362 | lysyl-tRNA aminoacylation | mdy |
| 897 | GO:0045472 | P | 6, | 1 | 0.148 (x 6.753) | 3 (0.333) | 0.363 | response to ether | Sh |
| 898 | GO:0035310 | P | 6, 7, | 1 | 0.148 (x 6.753) | 3 (0.333) | 0.363 | notum cell fate specification | ara |
| 899 | GO:0006937 | P | 5, | 1 | 0.148 (x 6.753) | 3 (0.333) | 0.363 | regulation of muscle contraction | Amph |
| 900 | GO:0004733 | F | 6, | 1 | 0.148 (x 6.753) | 3 (0.333) | 0.364 | pyridoxamine-phosphate oxidase activity | CG31472 |
| 901 | GO:0030308 | P | 5, 6, 7, 8, 9, | 1 | 0.148 (x 6.753) | 3 (0.333) | 0.364 | negative regulation of cell growth | Pten |
| 902 | GO:0035046 | P | 6, 8, 9, | 1 | 0.148 (x 6.753) | 3 (0.333) | 0.365 | pronuclear migration | polo |
| 903 | GO:0042734 | C | 3, 4, 5, | 1 | 0.148 (x 6.753) | 3 (0.333) | 0.365 | presynaptic membrane | gukh |
| 904 | GO:0015742 | P | 8, 9, | 1 | 0.148 (x 6.753) | 3 (0.333) | 0.366 | alpha-ketoglutarate transport | CG1907 |
| 905 | GO:0005978 | P | 9, 10, | 1 | 0.148 (x 6.753) | 3 (0.333) | 0.366 | glycogen biosynthesis | CG6904 |
| 906 | GO:0004692 | F | 9, | 1 | 0.148 (x 6.753) | 3 (0.333) | 0.366 | cGMP-dependent protein kinase activity | for |
| 907 | GO:0006621 | P | 6, | 1 | 0.148 (x 6.753) | 3 (0.333) | 0.367 | protein retention in ER | Tapdelta |
| 908 | GO:0019724 | P | 7, 8, | 1 | 0.148 (x 6.753) | 3 (0.333) | 0.367 | B cell mediated immunity | Tsp96F |
| 909 | GO:0051310 | P | 5, 7, 8, | 1 | 0.148 (x 6.753) | 3 (0.333) | 0.368 | metaphase plate congression | Incenp |
| 910 | GO:0010092 | P | 5, | 1 | 0.148 (x 6.753) | 3 (0.333) | 0.368 | specification of organ identity | hth |
| 911 | GO:0015395 | F | 5, 6, | 1 | 0.148 (x 6.753) | 3 (0.333) | 0.369 | nucleoside transporter activity, down a concentration gradient | BEST:LD04971 |
| 912 | GO:0006309 | P | 7, 8, 9, | 1 | 0.148 (x 6.753) | 3 (0.333) | 0.369 | DNA fragmentation during apoptosis | Rep4 |
| 913 | GO:0006573 | P | 8, 9, | 1 | 0.148 (x 6.753) | 3 (0.333) | 0.37 | valine metabolism | EG:171D11.1 |
| 914 | GO:0016842 | F | 5, | 1 | 0.148 (x 6.753) | 3 (0.333) | 0.37 | amidine-lyase activity | CG3590 |
| 915 | GO:0004772 | F | 8, | 1 | 0.148 (x 6.753) | 3 (0.333) | 0.37 | sterol O-acyltransferase activity | mdy |
| 916 | GO:0019098 | P | 3, 4, | 5 | 2.863 (x 1.746) | 58 (0.086) | 0.372 | reproductive behavior | Poxn Sh lig loj para |
| 917 | GO:0005085 | F | 4, | 5 | 2.863 (x 1.746) | 58 (0.086) | 0.372 | guanyl-nucleotide exchange factor activity | CG30440 CG5522 CG9135 pbl vimar |
| 918 | GO:0043412 | P | 6, | 52 | 45.264 (x 1.149) | 917 (0.057) | 0.373 | biopolymer modification | Arf79F BG:DS00004.11 CG14217 CG32632 CG33171 CG40410 CG4502 CG5794 CG7860 CaMKI Cad96Ca Caf1 Cdk4 CkIIalpha CkIIbeta Cks ERp60 Eip71CD Eph GalNAc-T1 Galpha49B InR Incenp Mgstl Mpk2 Nek2 PFE Pdi Pi3K21B Ppt1 Prosalpha6 Pten Ptp99A Uch Ugt ald cdc2 dap for fzy mbt nmo otk pgant5 pll polo rasp smi35A th tkv tws wbl |
| 919 | GO:0003712 | F | 3, 5, | 6 | 3.653 (x 1.643) | 74 (0.081) | 0.373 | transcription cofactor activity | CG15835 CG31453 CtBP Dsp1 cic spen |
| 920 | GO:0005819 | C | 5, 6, 7, 8, 9, 10, | 3 | 1.382 (x 2.171) | 28 (0.107) | 0.373 | spindle | Klp10A fzy polo |
| 921 | GO:0048534 | P | 4, | 6 | 3.653 (x 1.643) | 74 (0.081) | 0.373 | hemopoietic or lymphoid organ development | CG30011 Galpha49B Tl grn nonA-l pll |
| 922 | GO:0035110 | P | 6, | 3 | 1.382 (x 2.171) | 28 (0.107) | 0.373 | leg morphogenesis | Dl Poxn ap |
| 923 | GO:0006814 | P | 8, 9, | 4 | 2.123 (x 1.885) | 43 (0.093) | 0.378 | sodium ion transport | CG12048 Nrv1 para rpk |
| 924 | GO:0048729 | P | 4, | 4 | 2.123 (x 1.885) | 43 (0.093) | 0.378 | tissue morphogenesis | in pbl pk sgl |
| 925 | GO:0007530 | P | 3, | 4 | 2.123 (x 1.885) | 43 (0.093) | 0.379 | sex determination | br lola mod(mdg4) sc |
| 926 | GO:0015631 | F | 5, | 7 | 4.492 (x 1.558) | 91 (0.077) | 0.38 | tubulin binding | CG32137 Eb1 Incenp alphaTub84B asp betaTub56D stai |
| 927 | GO:0009798 | P | 4, | 11 | 7.848 (x 1.402) | 159 (0.069) | 0.38 | axis specification | Dl Dp Hrb27C TER94 Tl knk pll serpin-27A sqd tok wbl |
| 928 | GO:0006887 | P | 6, 7, | 9 | 6.170 (x 1.459) | 125 (0.072) | 0.385 | exocytosis | Amph CG1924 CG9906 Crc Rab8 Snap TER94 mth wbl |
| 929 | GO:0007307 | P | 9, 10, 11, | 2 | 0.740 (x 2.701) | 15 (0.133) | 0.388 | chorion gene amplification | Caf1 Dp |
| 930 | GO:0007435 | P | 6, | 2 | 0.740 (x 2.701) | 15 (0.133) | 0.388 | salivary gland morphogenesis | CrebA trh |
| 931 | GO:0009308 | P | 5, | 23 | 18.560 (x 1.239) | 376 (0.061) | 0.388 | amine metabolism | BcDNA:GH02976 CG11142 CG15088 CG1607 CG17052 CG2118 CG32499 CG40160 CG6287 CG7860 CG9307 CtBP EG:171D11.1 ESTS:39C10S Eip55E Eip71CD Oscillin blot knk mdy mnd olf413 sgl |
| 932 | GO:0019362 | P | 7, 8, | 2 | 0.740 (x 2.701) | 15 (0.133) | 0.388 | pyridine nucleotide metabolism | BEST:LD22483 Tpi |
| 933 | GO:0008301 | F | 5, | 1 | 0.197 (x 5.065) | 4 (0.250) | 0.388 | DNA bending activity | HmgD |
| 934 | GO:0009003 | F | 5, | 1 | 0.197 (x 5.065) | 4 (0.250) | 0.389 | signal peptidase activity | BG:DS00004.11 |
| 935 | GO:0007432 | P | 6, | 2 | 0.740 (x 2.701) | 15 (0.133) | 0.389 | salivary gland determination | hth tkv |
| 936 | GO:0046331 | P | 6, | 1 | 0.197 (x 5.065) | 4 (0.250) | 0.389 | lateral inhibition | Dl |
| 937 | GO:0016806 | F | 6, | 2 | 0.740 (x 2.701) | 15 (0.133) | 0.389 | dipeptidyl-peptidase and tripeptidyl-peptidase activity | CG2200 ome |
| 938 | GO:0004274 | F | 6, 8, | 1 | 0.197 (x 5.065) | 4 (0.250) | 0.39 | dipeptidyl-peptidase IV activity | ome |
| 939 | GO:0016542 | P | 7, 8, | 2 | 0.740 (x 2.701) | 15 (0.133) | 0.39 | male courtship behavior (sensu Insecta) | Poxn para |
| 940 | GO:0046463 | P | 7, 8, 9, | 1 | 0.197 (x 5.065) | 4 (0.250) | 0.39 | acylglycerol biosynthesis | mdy |
| 941 | GO:0009792 | P | 4, | 13 | 9.625 (x 1.351) | 195 (0.067) | 0.39 | embryonic development (sensu Metazoa) | CG40410 CtBP Doc1 InR Sema-1b a6 cic jumu pbl sgl tkv tws wbl |
| 942 | GO:0035215 | P | 5, | 2 | 0.740 (x 2.701) | 15 (0.133) | 0.39 | genital disc development | ci en |
| 943 | GO:0007242 | P | 5, | 31 | 25.865 (x 1.199) | 524 (0.059) | 0.39 | intracellular signaling cascade | CG14217 CG17493 CG2108 CG30440 CG32158 CG40410 CG5522 CaMKI Cdk4 CkIIalpha Eip75B Galpha49B Mpk2 Nek2 Pi3K21B Rab8 RacGAP50C Rapgap1 ced-6 edl for fwd klu mbt nmo numb polo scf stai tws vimar |
| 944 | GO:0005787 | C | 3, 4, 5, 6, 7, 8, 9, 10, 11, | 1 | 0.197 (x 5.065) | 4 (0.250) | 0.39 | signal peptidase complex | BG:DS00004.11 |
| 945 | GO:0007052 | P | 6, 10, | 2 | 0.740 (x 2.701) | 15 (0.133) | 0.391 | mitotic spindle organization and biogenesis | CycB3 Eb1 |
| 946 | GO:0009306 | P | 6, | 1 | 0.197 (x 5.065) | 4 (0.250) | 0.391 | protein secretion | alpha-Man-IIb |
| 947 | GO:0019226 | P | 5, | 22 | 17.918 (x 1.228) | 363 (0.061) | 0.391 | transmission of nerve impulse | Amph Arf79F CG11033 CG12199 CG16974 CG4054 CG5096 CaMKI M6 PFE Snap br fax for kal-1 lola mod(mdg4) mth olf413 pAbp para trn |
| 948 | GO:0009311 | P | 6, | 2 | 0.740 (x 2.701) | 15 (0.133) | 0.391 | oligosaccharide metabolism | GalNAc-T1 pgant5 |
| 949 | GO:0043543 | P | 8, | 2 | 0.790 (x 2.532) | 16 (0.125) | 0.391 | protein amino acid acylation | Caf1 rasp |
| 950 | GO:0006551 | P | 8, 9, | 1 | 0.197 (x 5.065) | 4 (0.250) | 0.391 | leucine metabolism | CG2118 |
| 951 | GO:0006277 | P | 8, | 2 | 0.790 (x 2.532) | 16 (0.125) | 0.391 | DNA amplification | Caf1 Dp |
| 952 | GO:0016589 | C | 5, 8, 9, 10, 11, 12, 13, 14, 15, | 1 | 0.197 (x 5.065) | 4 (0.250) | 0.392 | NURF complex | Caf1 |
| 953 | GO:0035108 | P | 5, | 3 | 1.431 (x 2.096) | 29 (0.103) | 0.392 | limb morphogenesis | Dl Poxn ap |
| 954 | GO:0051347 | P | 5, | 2 | 0.790 (x 2.532) | 16 (0.125) | 0.392 | positive regulation of transferase activity | Abi mbt |
| 955 | GO:0016573 | P | 9, 10, 12, | 1 | 0.197 (x 5.065) | 4 (0.250) | 0.392 | histone acetylation | Caf1 |
| 956 | GO:0007179 | P | 8, | 3 | 1.431 (x 2.096) | 29 (0.103) | 0.392 | transforming growth factor beta receptor signaling pathway | bun mav tkv |
| 957 | GO:0045860 | P | 6, 7, | 2 | 0.790 (x 2.532) | 16 (0.125) | 0.392 | positive regulation of protein kinase activity | Abi mbt |
| 958 | GO:0048099 | P | 6, 7, | 1 | 0.197 (x 5.065) | 4 (0.250) | 0.392 | anterior/posterior lineage restriction, imaginal disc | en |
| 959 | GO:0007520 | P | 7, 8, 10, 11, | 2 | 0.790 (x 2.532) | 16 (0.125) | 0.393 | myoblast fusion | hbs rost |
| 960 | GO:0042157 | P | 7, | 3 | 1.431 (x 2.096) | 29 (0.103) | 0.393 | lipoprotein metabolism | CG33171 Ppt1 rasp |
| 961 | GO:0016421 | F | 5, | 1 | 0.197 (x 5.065) | 4 (0.250) | 0.393 | CoA carboxylase activity | CG2118 |
| 962 | GO:0005887 | C | 6, 7, 8, | 13 | 9.675 (x 1.344) | 196 (0.066) | 0.393 | integral to plasma membrane | CG15088 Cad74A Cad87A Cad96Ca InR Nrv1 Sh Tl blot emp para stan wgn |
| 963 | GO:0006497 | P | 8, 9, | 3 | 1.431 (x 2.096) | 29 (0.103) | 0.393 | protein amino acid lipidation | CG33171 Ppt1 rasp |
| 964 | GO:0004396 | F | 6, 7, | 1 | 0.197 (x 5.065) | 4 (0.250) | 0.393 | hexokinase activity | Hex-A |
| 965 | GO:0007017 | P | 7, | 13 | 9.675 (x 1.344) | 196 (0.066) | 0.393 | microtubule-based process | CG9057 CycB3 Eb1 Klp10A TER94 alphaTub84B asp betaTub56D fwd pbl polo stai vg |
| 966 | GO:0042158 | P | 7, 8, | 3 | 1.431 (x 2.096) | 29 (0.103) | 0.393 | lipoprotein biosynthesis | CG33171 Ppt1 rasp |
| 967 | GO:0017040 | F | 6, | 1 | 0.197 (x 5.065) | 4 (0.250) | 0.393 | ceramidase activity | CG1471 |
| 968 | GO:0005242 | F | 7, 8, 9, | 1 | 0.197 (x 5.065) | 4 (0.250) | 0.394 | inward rectifier potassium channel activity | Ir |
| 969 | GO:0008379 | F | 4, 6, | 1 | 0.197 (x 5.065) | 4 (0.250) | 0.394 | thioredoxin peroxidase activity | Jafrac2 |
| 970 | GO:0005737 | C | 4, 5, 6, | 81 | 73.300 (x 1.105) | 1485 (0.055) | 0.394 | cytoplasm | 18w Amph BG:DS00004.11 BcDNA:GH12558 BcDNA:LD41548 CBP CG11079 CG14214 CG1907 CG1924 CG2118 CG2200 CG31738 CG33113 CG33171 CG4586 CG6776 CG6782 CG9057 CaMKI CkIIalpha CkIIbeta Crc DnaJ-1 EG:171D11.1 EG:BACN33B1.2 ERp60 Eno Fs(2)Ket GalNAc-T1 Gapdh1 Gapdh2 Gfat1 Ice Idh ImpE2 Mgstl Nek2 Pdi Pepck Pgi Pi3K21B Ppt1 RnrS RpS12 S Snap Sry-alpha TER94 Tapdelta Ucp4B Ugt Vha100-2 alpha-Man-IIb asp btsz bun ci cib eIF-4E emp fzy glu l(1)G0320 lig mdy numb p120ctn pAbp pcs pgant5 pk pll polo pon shanti shu smi35A sqd tws wbl |
| 971 | GO:0031010 | C | 4, 7, 8, 9, 10, 11, 12, 13, 14, | 1 | 0.197 (x 5.065) | 4 (0.250) | 0.395 | ISWI complex | Caf1 |
| 972 | GO:0035301 | C | 3, 4, 5, 6, | 1 | 0.197 (x 5.065) | 4 (0.250) | 0.395 | Hedgehog signaling complex | ci |
| 973 | GO:0008078 | P | 6, 7, 8, 9, | 1 | 0.197 (x 5.065) | 4 (0.250) | 0.395 | mesodermal cell migration | pbl |
| 974 | GO:0005785 | C | 4, 5, 6, 7, 8, 9, 10, 11, 12, | 1 | 0.197 (x 5.065) | 4 (0.250) | 0.396 | signal recognition particle receptor complex | l(1)G0320 |
| 975 | GO:0035308 | P | 7, 8, 9, 10, | 1 | 0.197 (x 5.065) | 4 (0.250) | 0.396 | negative regulation of protein amino acid dephosphorylation | dap |
| 976 | GO:0016529 | C | 6, 7, 8, 9, | 1 | 0.197 (x 5.065) | 4 (0.250) | 0.397 | sarcoplasmic reticulum | CBP |
| 977 | GO:0016765 | F | 4, | 5 | 2.962 (x 1.688) | 60 (0.083) | 0.397 | transferase activity, transferring alkyl or aryl (other than methyl) groups | BcDNA:GH04753 CG33171 CG6776 GstS1 Mgstl |
| 978 | GO:0007060 | P | 6, 9, | 1 | 0.197 (x 5.065) | 4 (0.250) | 0.397 | male meiosis chromosome segregation | polo |
| 979 | GO:0046670 | P | 7, 8, 9, | 1 | 0.197 (x 5.065) | 4 (0.250) | 0.397 | positive regulation of retinal programmed cell death | klu |
| 980 | GO:0015014 | P | 8, 9, 10, | 1 | 0.197 (x 5.065) | 4 (0.250) | 0.398 | heparan sulfate proteoglycan biosynthesis, polysaccharide chain biosynthesis | sgl |
| 981 | GO:0046627 | P | 6, 7, 10, | 1 | 0.197 (x 5.065) | 4 (0.250) | 0.398 | negative regulation of insulin receptor signaling pathway | Pten |
| 982 | GO:0042478 | P | 7, 8, 9, | 1 | 0.197 (x 5.065) | 4 (0.250) | 0.399 | regulation of eye photoreceptor cell development | mbt |
| 983 | GO:0003913 | F | 5, | 1 | 0.197 (x 5.065) | 4 (0.250) | 0.399 | DNA photolyase activity | CG18853 |
| 984 | GO:0044459 | C | 4, 5, 6, | 20 | 15.943 (x 1.254) | 323 (0.062) | 0.399 | plasma membrane part | CG15088 Cad74A Cad87A Cad96Ca Fas3 Galpha49B InR Nrv1 Sh Tl blot emp inx2 inx3 mbt ogre p120ctn para stan wgn |
| 985 | GO:0035155 | P | 7, 8, | 1 | 0.197 (x 5.065) | 4 (0.250) | 0.399 | negative regulation of terminal cell fate specification | Dl |
| 986 | GO:0006284 | P | 6, 8, | 1 | 0.197 (x 5.065) | 4 (0.250) | 0.4 | base-excision repair | BcDNA:GM10765 |
| 987 | GO:0007243 | P | 6, | 9 | 6.269 (x 1.436) | 127 (0.071) | 0.4 | protein kinase cascade | CG14217 CG30440 CG5522 Cdk4 Mpk2 ced-6 mbt nmo tws |
| 988 | GO:0015837 | P | 5, 6, | 4 | 2.271 (x 1.762) | 46 (0.087) | 0.4 | amine transport | CG15088 CG1607 CG3424 mnd |
| 989 | GO:0051303 | P | 6, 7, | 1 | 0.197 (x 5.065) | 4 (0.250) | 0.4 | establishment of chromosome localization | Incenp |
| 990 | GO:0043285 | P | 6, | 9 | 6.269 (x 1.436) | 127 (0.071) | 0.4 | biopolymer catabolism | BcDNA:GM10765 CG5794 CG7860 Prosalpha6 Rep4 Uch fzy sqd th |
| 991 | GO:0006917 | P | 8, 9, | 5 | 3.060 (x 1.634) | 62 (0.081) | 0.4 | induction of apoptosis | CG14217 CG7379 Jafrac2 mod(mdg4) smi35A |
| 992 | GO:0006865 | P | 6, 7, 8, | 4 | 2.271 (x 1.762) | 46 (0.087) | 0.4 | amino acid transport | CG15088 CG1607 CG3424 mnd |
| 993 | GO:0006921 | P | 8, | 1 | 0.197 (x 5.065) | 4 (0.250) | 0.401 | disassembly of cell structures during apoptosis | Rep4 |
| 994 | GO:0008150 | P | 1, | 405 | 398.932 (x 1.015) | 8082 (0.050) | 0.401 | biological\_process | 18w Aac11 Abi Act42A Aldh-III Amph Ance Arf79F Argk B52 BEST:CK02656 BEST:GH02921 BEST:LD04971 BEST:LD22483 BG:DS00004.11 BcDNA:GH02901 BcDNA:GH02976 BcDNA:GH04753 BcDNA:GH12558 BcDNA:GM10765 BcDNA:LD41548 Best2 CG10359 CG10657 CG11033 CG11079 CG11142 CG11151 CG11438 CG11550 CG11711 CG11739 CG11824 CG12048 CG12199 CG14214 CG14217 CG14439 CG1471 CG15088 CG15835 CG1607 CG1630 CG1632 CG16974 CG17052 CG17064 CG17084 CG17323 CG17419 CG17493 CG17919 CG18853 CG1893 CG1907 CG1911 CG1924 CG1998 CG2108 CG2118 CG2200 CG2852 CG30011 CG30440 CG31121 CG31125 CG31320 CG3136 CG31453 CG31472 CG31738 CG31749 CG31915 CG31937 CG31997 CG32137 CG32158 CG32415 CG32499 CG32632 CG32711 CG32827 CG33113 CG33116 CG33138 CG33171 CG3328 CG33525 CG3424 CG3590 CG3770 CG3823 CG3842 CG40160 CG40410 CG4054 CG4454 CG4502 CG4586 CG4914 CG5096 CG5319 CG5390 CG5466 CG5522 CG5731 CG5794 CG5873 CG6199 CG6287 CG6391 CG6654 CG6680 CG6767 CG6776 CG6812 CG6904 CG6946 CG6954 CG6965 CG6966 CG7379 CG7668 CG7675 CG7860 CG8092 CG8165 CG8588 CG8963 CG9027 CG9057 CG9066 CG9135 CG9211 CG9307 CG9342 CG9906 CREG CaMKI Cad74A Cad87A Cad96Ca Caf1 Cdk4 Cip4 CkIIalpha CkIIbeta Cks Crc CrebA CtBP CycB3 CycT Cyp310a1 D19A Dl DnaJ-1 Doc1 Doc2 Doc3 Dp Dp1 Dr Dref Dsp1 EG:118B3.2 EG:171D11.1 EG:BACN33B1.2 ERp60 ESTS:39C10S ETH Eb1 Eip55E Eip71CD Eip74EF Eip75B Eno Eph Fas3 Fkbp13 Fs(2)Ket GATAd GNBP3 GalNAc-T1 Galpha49B Gapdh1 Gapdh2 Gfat1 GlyP GstS1 HLHm7 Hex-A Hexo1 His4r HmgD HmgZ Hrb27C Hsp23 Hsp26 Hsp27 Hsp67Ba Hus1-like Ice Idh ImpE2 ImpE3 InR Incenp Ir Jafrac2 Klp10A LanB2 Lsp2 M6 Mcm7 Mgstl Mpk2 Nek2 Nep2 Nrv1 Obp99a Optix Oscillin PFE Pdi Pepck Pgi Pi3K21B Pli Poxn Ppt1 Prosalpha6 Pten Ptp99A Rab8 RacGAP50C Rapgap1 Rbf2 Reg-5 Rep4 RhoGAP71E Rhp Rm62 RnrS RpS12 Rpn9 S Sb Sema-1a Sema-1b Sema-2a Set Sh Snap Spn1 Spn5 Sry-alpha TER94 Tapdelta Thd1 Tl Top2 Tpi Trl Ts Tsp26A Tsp39D Tsp66E Tsp96F Uch Ucp4B Ugt Vha100-2 Wnt2 a6 ald alpha-Man-IIb alphaTub84B ana ap ara arr ash2 asp betaTub56D bip1 blot bnb br btsz bun cdc2 ced-6 cg charybde ci cib cic ck cyc d dUTPase dap dve eIF-4E east ed edl emp en esn fax fbp for fra fwd fzy glec glu grh grn gukh hbs heph hth in inv inx2 inx3 jumu kal-1 kis klu knk l(1)G0320 l(2)01424 l(2)01810 l(3)neo38 lid lig loj lola malpha mav mbt mdy mnd mod(mdg4) mth ninA nmo nonA-l numb ogre olf413 ome opa otk p120ctn pAbp pain para pbl pcs pdm2 pgant5 ph-p pim pk pll polo pon prominin-like rasp rost rpk sc scf scylla serpin-27A sgl shanti shf shu smi35A spen sqd stai stan sut1 th tkv toe tok trh trn tws usp vg vimar wbl wgn woc zf30C zwilch |
| 995 | GO:0030723 | P | 7, 9, | 1 | 0.197 (x 5.065) | 4 (0.250) | 0.401 | ovarian fusome organization and biogenesis | asp |
| 996 | GO:0016584 | P | 11, | 1 | 0.197 (x 5.065) | 4 (0.250) | 0.401 | nucleosome spacing | Caf1 |
| 997 | GO:0046460 | P | 6, 7, 8, | 1 | 0.197 (x 5.065) | 4 (0.250) | 0.402 | neutral lipid biosynthesis | mdy |
| 998 | GO:0005625 | C | 4, 5, | 1 | 0.197 (x 5.065) | 4 (0.250) | 0.402 | soluble fraction | Top2 |
| 999 | GO:0046777 | P | 9, 10, | 1 | 0.197 (x 5.065) | 4 (0.250) | 0.403 | protein amino acid autophosphorylation | InR |
| 1000 | GO:0007056 | P | 8, 9, 12, | 1 | 0.197 (x 5.065) | 4 (0.250) | 0.403 | female meiotic spindle assembly (sensu Metazoa) | polo |
| 1001 | GO:0016725 | F | 4, | 1 | 0.197 (x 5.065) | 4 (0.250) | 0.403 | oxidoreductase activity, acting on CH2 groups | RnrS |
| 1002 | GO:0030695 | F | 3, | 9 | 6.417 (x 1.403) | 130 (0.069) | 0.404 | GTPase regulator activity | Abi CG30440 CG5522 CG9135 RacGAP50C Rapgap1 Rhp pbl vimar |
| 1003 | GO:0008363 | P | 6, 8, 10, | 1 | 0.197 (x 5.065) | 4 (0.250) | 0.404 | larval cuticle biosynthesis (sensu Insecta) | CrebA |
| 1004 | GO:0009952 | P | 4, | 9 | 6.417 (x 1.403) | 130 (0.069) | 0.404 | anterior/posterior pattern formation | Dl Hrb27C TER94 ci en inv knk sqd tkv |
| 1005 | GO:0003918 | F | 5, 6, | 1 | 0.197 (x 5.065) | 4 (0.250) | 0.404 | DNA topoisomerase (ATP-hydrolyzing) activity | Top2 |
| 1006 | GO:0000002 | P | 7, | 1 | 0.197 (x 5.065) | 4 (0.250) | 0.405 | mitochondrial genome maintenance | Dref |
| 1007 | GO:0016528 | C | 5, 6, 7, | 1 | 0.197 (x 5.065) | 4 (0.250) | 0.405 | sarcoplasm | CBP |
| 1008 | GO:0005828 | C | 7, 8, 9, 10, 11, 12, | 1 | 0.197 (x 5.065) | 4 (0.250) | 0.405 | kinetochore microtubule | Klp10A |
| 1009 | GO:0000737 | P | 7, 8, | 1 | 0.197 (x 5.065) | 4 (0.250) | 0.406 | DNA catabolism, endonucleolytic | BcDNA:GM10765 |
| 1010 | GO:0046626 | P | 5, 6, 9, | 1 | 0.197 (x 5.065) | 4 (0.250) | 0.406 | regulation of insulin receptor signaling pathway | Pten |
| 1011 | GO:0019216 | P | 5, 6, | 1 | 0.197 (x 5.065) | 4 (0.250) | 0.407 | regulation of lipid metabolism | Eip75B |
| 1012 | GO:0035305 | P | 8, 9, | 1 | 0.197 (x 5.065) | 4 (0.250) | 0.407 | negative regulation of dephosphorylation | dap |
| 1013 | GO:0050000 | P | 5, 6, | 1 | 0.197 (x 5.065) | 4 (0.250) | 0.408 | chromosome localization | Incenp |
| 1014 | GO:0045880 | P | 6, 7, 8, | 1 | 0.197 (x 5.065) | 4 (0.250) | 0.408 | positive regulation of smoothened signaling pathway | CG9211 |
| 1015 | GO:0000916 | P | 6, | 1 | 0.197 (x 5.065) | 4 (0.250) | 0.408 | cytokinesis, contractile ring contraction | fwd |
| 1016 | GO:0016328 | C | 5, 6, 7, | 1 | 0.197 (x 5.065) | 4 (0.250) | 0.409 | lateral plasma membrane | Fas3 |
| 1017 | GO:0019202 | F | 6, | 1 | 0.197 (x 5.065) | 4 (0.250) | 0.409 | amino acid kinase activity | Argk |
| 1018 | GO:0019898 | C | 4, 5, 6, | 4 | 2.221 (x 1.801) | 45 (0.089) | 0.409 | extrinsic to membrane | Galpha49B ImpE2 ImpE3 Snap |
| 1019 | GO:0006465 | P | 6, 9, | 1 | 0.197 (x 5.065) | 4 (0.250) | 0.41 | signal peptide processing | BG:DS00004.11 |
| 1020 | GO:0005578 | C | 3, 4, | 4 | 2.221 (x 1.801) | 45 (0.089) | 0.41 | extracellular matrix (sensu Metazoa) | CG33171 LanB2 kal-1 shf |
| 1021 | GO:0045168 | P | 5, | 1 | 0.197 (x 5.065) | 4 (0.250) | 0.41 | cell-cell signaling during cell fate commitment | Dl |
| 1022 | GO:0031226 | C | 5, 6, 7, | 13 | 9.773 (x 1.330) | 198 (0.066) | 0.41 | intrinsic to plasma membrane | CG15088 Cad74A Cad87A Cad96Ca InR Nrv1 Sh Tl blot emp para stan wgn |
| 1023 | GO:0016830 | F | 4, | 4 | 2.221 (x 1.801) | 45 (0.089) | 0.41 | carbon-carbon lyase activity | CG18853 EG:BACN33B1.2 Pepck emp |
| 1024 | GO:0006213 | P | 7, | 1 | 0.197 (x 5.065) | 4 (0.250) | 0.41 | pyrimidine nucleoside metabolism | dUTPase |
| 1025 | GO:0031012 | C | 2, | 4 | 2.221 (x 1.801) | 45 (0.089) | 0.411 | extracellular matrix | CG33171 LanB2 kal-1 shf |
| 1026 | GO:0008159 | F | 4, | 1 | 0.197 (x 5.065) | 4 (0.250) | 0.411 | positive transcription elongation factor activity | CycT |
| 1027 | GO:0046835 | P | 6, 8, | 1 | 0.197 (x 5.065) | 4 (0.250) | 0.411 | carbohydrate phosphorylation | CG1630 |
| 1028 | GO:0043161 | P | 10, 11, 12, | 1 | 0.197 (x 5.065) | 4 (0.250) | 0.412 | proteasomal ubiquitin-dependent protein catabolism | fzy |
| 1029 | GO:0007348 | P | 6, 8, 9, | 1 | 0.197 (x 5.065) | 4 (0.250) | 0.412 | regulation of progression through syncytial blastoderm mitotic cell cycle | CG40410 |
| 1030 | GO:0046853 | P | 7, 9, 10, | 1 | 0.197 (x 5.065) | 4 (0.250) | 0.413 | inositol and derivative phosphorylation | CG1630 |
| 1031 | GO:0004563 | F | 7, | 1 | 0.197 (x 5.065) | 4 (0.250) | 0.413 | beta-N-acetylhexosaminidase activity | Hexo1 |
| 1032 | GO:0046672 | P | 8, 9, 10, 11, | 1 | 0.197 (x 5.065) | 4 (0.250) | 0.413 | positive regulation of retinal cell programmed cell death (sensu Endopterygota) | klu |
| 1033 | GO:0030162 | P | 6, 7, 8, | 3 | 1.481 (x 2.026) | 30 (0.100) | 0.414 | regulation of proteolysis | CG40410 fzy pll |
| 1034 | GO:0015367 | F | 6, 8, | 1 | 0.197 (x 5.065) | 4 (0.250) | 0.414 | oxoglutarate:malate antiporter activity | CG1907 |
| 1035 | GO:0007591 | P | 6, | 3 | 1.481 (x 2.026) | 30 (0.100) | 0.414 | molting cycle (sensu Insecta) | CrebA ETH Eip75B |
| 1036 | GO:0005006 | F | 7, 9, | 1 | 0.197 (x 5.065) | 4 (0.250) | 0.414 | epidermal growth factor receptor activity | InR |
| 1037 | GO:0005248 | F | 6, 7, 8, | 1 | 0.197 (x 5.065) | 4 (0.250) | 0.415 | voltage-gated sodium channel activity | para |
| 1038 | GO:0008061 | F | 5, | 5 | 3.110 (x 1.608) | 63 (0.079) | 0.416 | chitin binding | BcDNA:GH02976 CG11142 CG17052 CG32499 CG9307 |
| 1039 | GO:0006030 | P | 7, 8, 9, 10, | 5 | 3.110 (x 1.608) | 63 (0.079) | 0.417 | chitin metabolism | BcDNA:GH02976 CG11142 CG17052 CG32499 CG9307 |
| 1040 | GO:0015171 | F | 4, 5, | 4 | 2.320 (x 1.724) | 47 (0.085) | 0.419 | amino acid transporter activity | CG15088 CG1607 CG3424 mnd |
| 1041 | GO:0007293 | P | 8, | 4 | 2.320 (x 1.724) | 47 (0.085) | 0.42 | egg chamber formation (sensu Insecta) | Dl asp dap ph-p |
| 1042 | GO:0035315 | P | 5, 7, 8, | 2 | 0.839 (x 2.383) | 17 (0.118) | 0.42 | hair cell differentiation | in pk |
| 1043 | GO:0005328 | F | 4, 7, 9, | 2 | 0.839 (x 2.383) | 17 (0.118) | 0.42 | neurotransmitter:sodium symporter activity | CG15088 blot |
| 1044 | GO:0006979 | P | 4, 5, 6, | 2 | 0.839 (x 2.383) | 17 (0.118) | 0.421 | response to oxidative stress | GstS1 mth |
| 1045 | GO:0006839 | P | 6, 7, 8, | 2 | 0.839 (x 2.383) | 17 (0.118) | 0.421 | mitochondrial transport | CG1907 Ucp4B |
| 1046 | GO:0035316 | P | 6, 7, 8, 9, | 2 | 0.839 (x 2.383) | 17 (0.118) | 0.421 | trichome organization and biogenesis (sensu Insecta) | in pk |
| 1047 | GO:0044260 | P | 5, | 114 | 106.224 (x 1.073) | 2152 (0.053) | 0.422 | cellular macromolecule metabolism | Ance Arf79F BEST:GH02921 BG:DS00004.11 BcDNA:GH02976 BcDNA:LD41548 CG10657 CG11033 CG11142 CG11824 CG14217 CG1632 CG17052 CG1924 CG2200 CG2852 CG31738 CG31915 CG32499 CG32632 CG33138 CG33171 CG3328 CG40160 CG40410 CG4502 CG4914 CG5390 CG5794 CG6680 CG6904 CG7860 CG8963 CG9135 CG9307 CG9906 CaMKI Cad96Ca Caf1 Cdk4 CkIIalpha CkIIbeta Cks Crc D19A DnaJ-1 ERp60 Eip71CD Eph Fkbp13 GalNAc-T1 Galpha49B GlyP Hsp23 Hsp26 Hsp27 Hsp67Ba Ice InR Incenp Lsp2 Mgstl Mpk2 Nek2 Nep2 PFE Pdi Pi3K21B Ppt1 Prosalpha6 Pten Ptp99A RpS12 Rpn9 Sb Spn1 Spn5 TER94 Uch Ugt ald alphaTub84B betaTub56D blot cdc2 dap eIF-4E east for fra fwd fzy l(1)G0320 l(2)01424 mbt mdy nmo ome otk pAbp pgant5 pll polo rasp sgl shf shu smi35A sqd th tkv tok tws wbl |
| 1048 | GO:0008146 | F | 5, | 2 | 0.839 (x 2.383) | 17 (0.118) | 0.422 | sulfotransferase activity | CG16733 CG32632 |
| 1049 | GO:0003714 | F | 4, 6, | 2 | 0.839 (x 2.383) | 17 (0.118) | 0.422 | transcription corepressor activity | CtBP Dsp1 |
| 1050 | GO:0009913 | P | 4, 6, 7, | 2 | 0.839 (x 2.383) | 17 (0.118) | 0.423 | epidermal cell differentiation | in pk |
| 1051 | GO:0008586 | P | 7, 8, 9, | 2 | 0.839 (x 2.383) | 17 (0.118) | 0.423 | wing vein morphogenesis | Dl heph |
| 1052 | GO:0001775 | P | 4, | 2 | 0.839 (x 2.383) | 17 (0.118) | 0.423 | cell activation | 18w emp |
| 1053 | GO:0016334 | P | 6, | 2 | 0.839 (x 2.383) | 17 (0.118) | 0.424 | establishment and/or maintenance of polarity of follicular epithelium | Dp sqd |
| 1054 | GO:0048730 | P | 5, 6, | 2 | 0.839 (x 2.383) | 17 (0.118) | 0.424 | epidermis morphogenesis | in pk |
| 1055 | GO:0006471 | P | 8, | 2 | 0.839 (x 2.383) | 17 (0.118) | 0.425 | protein amino acid ADP-ribosylation | Arf79F Galpha49B |
| 1056 | GO:0035309 | P | 6, | 1 | 0.247 (x 4.052) | 5 (0.200) | 0.427 | wing and notum subfield formation | ara |
| 1057 | GO:0050982 | P | 5, 6, | 1 | 0.247 (x 4.052) | 5 (0.200) | 0.427 | detection of mechanical stimulus | pain |
| 1058 | GO:0008605 | F | 5, | 1 | 0.247 (x 4.052) | 5 (0.200) | 0.427 | protein kinase CK2 regulator activity | CkIIbeta |
| 1059 | GO:0016790 | F | 5, | 3 | 1.580 (x 1.899) | 32 (0.094) | 0.428 | thiolester hydrolase activity | CG5794 Ppt1 Uch |
| 1060 | GO:0006697 | P | 8, 9, 10, | 1 | 0.247 (x 4.052) | 5 (0.200) | 0.428 | ecdysone biosynthesis | woc |
| 1061 | GO:0045017 | P | 6, 7, 8, | 1 | 0.247 (x 4.052) | 5 (0.200) | 0.428 | glycerolipid biosynthesis | mdy |
| 1062 | GO:0004181 | F | 7, | 3 | 1.580 (x 1.899) | 32 (0.094) | 0.428 | metallocarboxypeptidase activity | Lsp2 east fra |
| 1063 | GO:0046854 | P | 8, 9, 10, 11, | 1 | 0.247 (x 4.052) | 5 (0.200) | 0.428 | phosphoinositide phosphorylation | Pi3K21B |
| 1064 | GO:0016044 | P | 5, | 3 | 1.580 (x 1.899) | 32 (0.094) | 0.428 | membrane organization and biogenesis | CG1893 TER94 grh |
| 1065 | GO:0007485 | P | 6, 7, | 1 | 0.247 (x 4.052) | 5 (0.200) | 0.429 | male genitalia development (sensu Endopterygota) | en |
| 1066 | GO:0004182 | F | 8, | 3 | 1.580 (x 1.899) | 32 (0.094) | 0.429 | carboxypeptidase A activity | Lsp2 east fra |
| 1067 | GO:0017032 | F | 7, 8, 9, | 1 | 0.247 (x 4.052) | 5 (0.200) | 0.429 | potassium:amino acid symporter activity | CG15088 |
| 1068 | GO:0008368 | F | 4, | 1 | 0.247 (x 4.052) | 5 (0.200) | 0.43 | Gram-negative bacterial binding | GNBP3 |
| 1069 | GO:0051766 | F | 7, | 1 | 0.247 (x 4.052) | 5 (0.200) | 0.43 | inositol trisphosphate kinase activity | CG1630 |
| 1070 | GO:0016705 | F | 4, | 5 | 3.258 (x 1.535) | 66 (0.076) | 0.43 | oxidoreductase activity, acting on paired donors, with incorporation or reduction of molecular oxygen | CG1998 CG31915 CG6199 knk olf413 |
| 1071 | GO:0035277 | P | 4, 5, | 1 | 0.247 (x 4.052) | 5 (0.200) | 0.43 | spiracle morphogenesis | trh |
| 1072 | GO:0006835 | P | 7, 8, | 1 | 0.247 (x 4.052) | 5 (0.200) | 0.431 | dicarboxylic acid transport | CG1907 |
| 1073 | GO:0050974 | P | 5, 6, 7, | 1 | 0.247 (x 4.052) | 5 (0.200) | 0.431 | detection of mechanical stimulus during sensory perception | pain |
| 1074 | GO:0046976 | F | 9, 10, | 1 | 0.247 (x 4.052) | 5 (0.200) | 0.431 | histone lysine N-methyltransferase activity (H3-K27 specific) | Caf1 |
| 1075 | GO:0009719 | P | 3, | 9 | 6.565 (x 1.371) | 133 (0.068) | 0.431 | response to endogenous stimulus | BcDNA:GM10765 CG18853 CG40410 Caf1 Eip75B Hus1-like Thd1 br usp |
| 1076 | GO:0016778 | F | 5, | 1 | 0.247 (x 4.052) | 5 (0.200) | 0.432 | diphosphotransferase activity | CG6767 |
| 1077 | GO:0016192 | P | 5, 6, | 18 | 14.611 (x 1.232) | 296 (0.061) | 0.432 | vesicle-mediated transport | Amph Arf79F CG14214 CG1924 CG2108 CG9906 Crc Rab8 S Snap TER94 arr btsz ck d l(1)G0320 mth wbl |
| 1078 | GO:0043406 | P | 7, 8, | 1 | 0.247 (x 4.052) | 5 (0.200) | 0.432 | positive regulation of MAPK activity | mbt |
| 1079 | GO:0016303 | F | 8, | 1 | 0.247 (x 4.052) | 5 (0.200) | 0.433 | phosphatidylinositol 3-kinase activity | Pi3K21B |
| 1080 | GO:0017026 | F | 7, | 1 | 0.247 (x 4.052) | 5 (0.200) | 0.433 | procollagen C-endopeptidase activity | tok |
| 1081 | GO:0007091 | P | 8, | 1 | 0.247 (x 4.052) | 5 (0.200) | 0.433 | mitotic metaphase/anaphase transition | Cks |
| 1082 | GO:0035098 | C | 4, 6, 7, 8, 9, 10, 11, 12, 13, 14, | 1 | 0.247 (x 4.052) | 5 (0.200) | 0.434 | ESC/E(Z) complex | Caf1 |
| 1083 | GO:0016885 | F | 4, | 1 | 0.247 (x 4.052) | 5 (0.200) | 0.434 | ligase activity, forming carbon-carbon bonds | CG2118 |
| 1084 | GO:0016572 | P | 9, 12, | 1 | 0.247 (x 4.052) | 5 (0.200) | 0.435 | histone phosphorylation | Incenp |
| 1085 | GO:0008367 | F | 3, | 1 | 0.247 (x 4.052) | 5 (0.200) | 0.435 | bacterial binding | GNBP3 |
| 1086 | GO:0005637 | C | 5, 6, 7, 8, 9, 10, 11, 12, 13, | 1 | 0.247 (x 4.052) | 5 (0.200) | 0.435 | nuclear inner membrane | CG17952 |
| 1087 | GO:0016909 | F | 6, 10, | 1 | 0.247 (x 4.052) | 5 (0.200) | 0.436 | SAP kinase activity | Mpk2 |
| 1088 | GO:0007266 | P | 7, | 1 | 0.247 (x 4.052) | 5 (0.200) | 0.436 | Rho protein signal transduction | RacGAP50C |
| 1089 | GO:0006616 | P | 9, 10, 11, 12, 13, | 1 | 0.247 (x 4.052) | 5 (0.200) | 0.436 | SRP-dependent cotranslational protein targeting to membrane, translocation | CG14214 |
| 1090 | GO:0045468 | P | 9, 10, 11, 12, | 1 | 0.247 (x 4.052) | 5 (0.200) | 0.437 | regulation of R8 spacing | Dl |
| 1091 | GO:0008603 | F | 5, | 1 | 0.247 (x 4.052) | 5 (0.200) | 0.437 | cAMP-dependent protein kinase regulator activity | for |
| 1092 | GO:0017124 | F | 5, | 1 | 0.247 (x 4.052) | 5 (0.200) | 0.438 | SH3 domain binding | pcs |
| 1093 | GO:0030536 | P | 5, | 1 | 0.247 (x 4.052) | 5 (0.200) | 0.438 | larval feeding behavior | for |
| 1094 | GO:0007540 | P | 6, | 1 | 0.247 (x 4.052) | 5 (0.200) | 0.438 | sex determination, establishment of X:A ratio | sc |
| 1095 | GO:0000287 | F | 5, | 1 | 0.247 (x 4.052) | 5 (0.200) | 0.439 | magnesium ion binding | RhoGAP71E |
| 1096 | GO:0042325 | P | 8, | 1 | 0.247 (x 4.052) | 5 (0.200) | 0.439 | regulation of phosphorylation | edl |
| 1097 | GO:0008315 | P | 9, | 1 | 0.247 (x 4.052) | 5 (0.200) | 0.44 | meiotic G2/MI transition | cdc2 |
| 1098 | GO:0046504 | P | 6, | 1 | 0.247 (x 4.052) | 5 (0.200) | 0.44 | glycerol ether biosynthesis | mdy |
| 1099 | GO:0042336 | P | 6, 8, | 1 | 0.247 (x 4.052) | 5 (0.200) | 0.44 | cuticle biosynthesis during molting (sensu Protostomia and Nematoda) | CrebA |
| 1100 | GO:0008440 | F | 8, | 1 | 0.247 (x 4.052) | 5 (0.200) | 0.441 | inositol trisphosphate 3-kinase activity | CG1630 |
| 1101 | GO:0000187 | P | 8, 9, | 1 | 0.247 (x 4.052) | 5 (0.200) | 0.441 | activation of MAPK activity | mbt |
| 1102 | GO:0007344 | P | 6, 8, | 1 | 0.247 (x 4.052) | 5 (0.200) | 0.442 | pronuclear fusion | polo |
| 1103 | GO:0035154 | P | 6, 7, | 1 | 0.247 (x 4.052) | 5 (0.200) | 0.442 | terminal cell fate specification | Dl |
| 1104 | GO:0005942 | C | 3, 6, 7, 8, 9, 10, | 1 | 0.247 (x 4.052) | 5 (0.200) | 0.442 | phosphoinositide 3-kinase complex | Pi3K21B |
| 1105 | GO:0017077 | F | 4, | 1 | 0.247 (x 4.052) | 5 (0.200) | 0.443 | oxidative phosphorylation uncoupler activity | Ucp4B |
| 1106 | GO:0007405 | P | 5, 7, | 1 | 0.247 (x 4.052) | 5 (0.200) | 0.443 | neuroblast proliferation | ana |
| 1107 | GO:0035239 | P | 4, | 5 | 3.208 (x 1.558) | 65 (0.077) | 0.444 | tube morphogenesis | grh pbl pim sgl trh |
| 1108 | GO:0000079 | P | 7, | 1 | 0.247 (x 4.052) | 5 (0.200) | 0.444 | regulation of cyclin-dependent protein kinase activity | dap |
| 1109 | GO:0042337 | P | 7, 9, | 1 | 0.247 (x 4.052) | 5 (0.200) | 0.444 | cuticle biosynthesis during molting (sensu Insecta) | CrebA |
| 1110 | GO:0045887 | P | 5, 6, 8, 9, 10, 11, | 1 | 0.247 (x 4.052) | 5 (0.200) | 0.444 | positive regulation of synaptic growth at neuromuscular junction | tkv |
| 1111 | GO:0051603 | P | 8, 9, | 6 | 4.048 (x 1.482) | 82 (0.073) | 0.445 | proteolysis during cellular protein catabolism | CG5794 CG7860 Prosalpha6 Uch fzy th |
| 1112 | GO:0030037 | P | 5, 10, | 1 | 0.247 (x 4.052) | 5 (0.200) | 0.445 | actin filament reorganization during cell cycle | asp |
| 1113 | GO:0044257 | P | 7, 8, | 6 | 4.048 (x 1.482) | 82 (0.073) | 0.445 | cellular protein catabolism | CG5794 CG7860 Prosalpha6 Uch fzy th |
| 1114 | GO:0030071 | P | 8, 9, | 1 | 0.247 (x 4.052) | 5 (0.200) | 0.445 | regulation of mitotic metaphase/anaphase transition | Cks |
| 1115 | GO:0006807 | P | 4, | 23 | 19.300 (x 1.192) | 391 (0.059) | 0.446 | nitrogen compound metabolism | BcDNA:GH02976 CG11142 CG15088 CG1607 CG17052 CG2118 CG32499 CG40160 CG6287 CG7860 CG9307 CtBP EG:171D11.1 ESTS:39C10S Eip55E Eip71CD Oscillin blot knk mdy mnd olf413 sgl |
| 1116 | GO:0000077 | P | 6, 9, | 1 | 0.247 (x 4.052) | 5 (0.200) | 0.446 | DNA damage checkpoint | CG40410 |
| 1117 | GO:0019104 | F | 6, | 1 | 0.247 (x 4.052) | 5 (0.200) | 0.446 | DNA N-glycosylase activity | Thd1 |
| 1118 | GO:0016831 | F | 5, | 3 | 1.678 (x 1.788) | 34 (0.088) | 0.446 | carboxy-lyase activity | EG:BACN33B1.2 Pepck emp |
| 1119 | GO:0030247 | F | 4, | 5 | 3.307 (x 1.512) | 67 (0.075) | 0.446 | polysaccharide binding | BcDNA:GH02976 CG11142 CG17052 CG32499 CG9307 |
| 1120 | GO:0000940 | C | 5, 6, 7, 8, 9, 10, 11, 12, 13, | 1 | 0.247 (x 4.052) | 5 (0.200) | 0.446 | outer kinetochore of condensed chromosome | polo |
| 1121 | GO:0001763 | P | 4, | 3 | 1.678 (x 1.788) | 34 (0.088) | 0.447 | morphogenesis of a branching structure | grh sgl tkv |
| 1122 | GO:0005024 | F | 6, 7, 9, 10, | 1 | 0.247 (x 4.052) | 5 (0.200) | 0.447 | transforming growth factor beta receptor activity | tkv |
| 1123 | GO:0045792 | P | 6, 7, | 1 | 0.247 (x 4.052) | 5 (0.200) | 0.447 | negative regulation of cell size | Pten |
| 1124 | GO:0045463 | P | 8, 9, 10, 11, | 1 | 0.247 (x 4.052) | 5 (0.200) | 0.448 | R8 development | Dl |
| 1125 | GO:0035159 | P | 7, | 1 | 0.247 (x 4.052) | 5 (0.200) | 0.448 | regulation of tracheal tube length | grh |
| 1126 | GO:0007005 | P | 6, | 2 | 0.888 (x 2.251) | 18 (0.111) | 0.448 | mitochondrion organization and biogenesis | Cdk4 Dref |
| 1127 | GO:0008623 | C | 4, 7, 8, 9, 10, 11, 12, 13, 14, | 1 | 0.247 (x 4.052) | 5 (0.200) | 0.449 | chromatin accessibility complex | Top2 |
| 1128 | GO:0050832 | P | 5, 6, | 2 | 0.888 (x 2.251) | 18 (0.111) | 0.449 | defense response to fungus | Tl pll |
| 1129 | GO:0004726 | F | 9, | 1 | 0.247 (x 4.052) | 5 (0.200) | 0.449 | non-membrane spanning protein tyrosine phosphatase activity | Pten |
| 1130 | GO:0008376 | F | 6, | 2 | 0.888 (x 2.251) | 18 (0.111) | 0.449 | acetylgalactosaminyltransferase activity | GalNAc-T1 pgant5 |
| 1131 | GO:0007141 | P | 9, | 1 | 0.247 (x 4.052) | 5 (0.200) | 0.449 | male meiosis I | cdc2 |
| 1132 | GO:0042303 | P | 4, | 3 | 1.629 (x 1.842) | 33 (0.091) | 0.449 | molting cycle | CrebA ETH Eip75B |
| 1133 | GO:0009954 | P | 4, | 2 | 0.888 (x 2.251) | 18 (0.111) | 0.45 | proximal/distal pattern formation | ap hth |
| 1134 | GO:0008101 | P | 9, | 1 | 0.247 (x 4.052) | 5 (0.200) | 0.45 | decapentaplegic receptor signaling pathway | bun |
| 1135 | GO:0018988 | P | 5, | 3 | 1.629 (x 1.842) | 33 (0.091) | 0.45 | molting cycle (sensu Protostomia and Nematoda) | CrebA ETH Eip75B |
| 1136 | GO:0019209 | F | 4, | 2 | 0.888 (x 2.251) | 18 (0.111) | 0.45 | kinase activator activity | CycB3 CycT |
| 1137 | GO:0006516 | P | 8, 9, 10, | 1 | 0.247 (x 4.052) | 5 (0.200) | 0.45 | glycoprotein catabolism | CG7860 |
| 1138 | GO:0042706 | P | 6, 7, 8, | 3 | 1.629 (x 1.842) | 33 (0.091) | 0.45 | eye photoreceptor cell fate commitment | Dl hth stan |
| 1139 | GO:0007449 | P | 5, 6, | 2 | 0.888 (x 2.251) | 18 (0.111) | 0.45 | proximal/distal pattern formation, imaginal disc | ap hth |
| 1140 | GO:0000741 | P | 7, | 1 | 0.247 (x 4.052) | 5 (0.200) | 0.451 | karyogamy | polo |
| 1141 | GO:0007619 | P | 5, 6, | 3 | 1.629 (x 1.842) | 33 (0.091) | 0.451 | courtship behavior | Poxn Sh para |
| 1142 | GO:0045746 | P | 6, 7, 8, | 1 | 0.247 (x 4.052) | 5 (0.200) | 0.451 | negative regulation of Notch signaling pathway | numb |
| 1143 | GO:0007297 | P | 6, 7, 9, | 3 | 1.629 (x 1.842) | 33 (0.091) | 0.451 | follicle cell migration (sensu Insecta) | sqd th usp |
| 1144 | GO:0001752 | P | 7, 8, 9, 10, | 3 | 1.629 (x 1.842) | 33 (0.091) | 0.451 | eye photoreceptor fate commitment (sensu Endopterygota) | Dl hth stan |
| 1145 | GO:0050839 | F | 4, | 2 | 0.938 (x 2.133) | 19 (0.105) | 0.455 | cell adhesion molecule binding | otk stan |
| 1146 | GO:0003707 | F | 5, | 2 | 0.938 (x 2.133) | 19 (0.105) | 0.456 | steroid hormone receptor activity | Eip75B usp |
| 1147 | GO:0007140 | P | 8, | 2 | 0.938 (x 2.133) | 19 (0.105) | 0.456 | male meiosis | cdc2 polo |
| 1148 | GO:0016769 | F | 4, | 2 | 0.938 (x 2.133) | 19 (0.105) | 0.457 | transferase activity, transferring nitrogenous groups | ESTS:39C10S Gfat1 |
| 1149 | GO:0008483 | F | 5, | 2 | 0.938 (x 2.133) | 19 (0.105) | 0.457 | transaminase activity | ESTS:39C10S Gfat1 |
| 1150 | GO:0005667 | C | 3, 6, 7, 8, 9, 10, 11, 12, 13, | 6 | 4.196 (x 1.430) | 85 (0.071) | 0.458 | transcription factor complex | Caf1 Dp Dref Rbf2 toe usp |
| 1151 | GO:0035304 | P | 6, 7, 9, | 1 | 0.296 (x 3.377) | 6 (0.167) | 0.463 | regulation of protein amino acid dephosphorylation | dap |
| 1152 | GO:0009056 | P | 4, | 19 | 16.141 (x 1.177) | 327 (0.058) | 0.463 | catabolism | BEST:LD22483 BcDNA:GM10765 CG5794 CG7860 EG:171D11.1 Eno Gapdh1 Gapdh2 Hex-A Idh Pgi Prosalpha6 Rep4 Tpi Uch br fzy sqd th |
| 1153 | GO:0000280 | P | 4, | 1 | 0.296 (x 3.377) | 6 (0.167) | 0.463 | nuclear division | Trl |
| 1154 | GO:0000779 | C | 6, 7, 8, 9, 10, 11, | 1 | 0.296 (x 3.377) | 6 (0.167) | 0.463 | condensed chromosome, pericentric region | polo |
| 1155 | GO:0007626 | P | 4, | 5 | 3.455 (x 1.447) | 70 (0.071) | 0.464 | locomotory behavior | CkIIalpha Sema-2a Sh cyc for |
| 1156 | GO:0030708 | P | 7, 8, 9, | 1 | 0.296 (x 3.377) | 6 (0.167) | 0.464 | female germ-line cyst encapsulation (sensu Insecta) | ph-p |
| 1157 | GO:0019203 | F | 7, | 1 | 0.296 (x 3.377) | 6 (0.167) | 0.464 | carbohydrate phosphatase activity | fbp |
| 1158 | GO:0003690 | F | 6, | 1 | 0.296 (x 3.377) | 6 (0.167) | 0.465 | double-stranded DNA binding | Thd1 |
| 1159 | GO:0004176 | F | 5, | 1 | 0.296 (x 3.377) | 6 (0.167) | 0.465 | ATP-dependent peptidase activity | TER94 |
| 1160 | GO:0005524 | F | 6, | 37 | 33.170 (x 1.115) | 672 (0.055) | 0.465 | ATP binding | BEST:CK02656 CG14217 CG2118 CG31121 CG31453 CG40410 CaMKI Cad96Ca Cdk4 CkIIalpha Eph Hex-A InR Klp10A Mcm7 Mpk2 Nek2 Rm62 TER94 Top2 ald blot cdc2 ck d for glu jumu kis mbt mdy nmo otk pll polo smi35A tkv |
| 1161 | GO:0008158 | F | 5, | 1 | 0.296 (x 3.377) | 6 (0.167) | 0.465 | hedgehog receptor activity | CG9211 |
| 1162 | GO:0043405 | P | 7, | 1 | 0.296 (x 3.377) | 6 (0.167) | 0.466 | regulation of MAPK activity | mbt |
| 1163 | GO:0016335 | P | 5, 6, 7, | 1 | 0.296 (x 3.377) | 6 (0.167) | 0.466 | morphogenesis of larval imaginal disc epithelium | Sb |
| 1164 | GO:0003997 | F | 6, | 1 | 0.296 (x 3.377) | 6 (0.167) | 0.466 | acyl-CoA oxidase activity | CG4586 |
| 1165 | GO:0009314 | P | 4, | 4 | 2.616 (x 1.529) | 53 (0.075) | 0.467 | response to radiation | CG40410 Galpha49B Sema-2a ogre |
| 1166 | GO:0005856 | C | 5, 6, 7, 8, | 17 | 14.315 (x 1.188) | 290 (0.059) | 0.467 | cytoskeleton | Act42A CG17493 CycB3 Eb1 Klp10A Nek2 TER94 alphaTub84B asp betaTub56D ck d fzy p120ctn polo stai vg |
| 1167 | GO:0009147 | P | 8, | 1 | 0.296 (x 3.377) | 6 (0.167) | 0.467 | pyrimidine nucleoside triphosphate metabolism | dUTPase |
| 1168 | GO:0008329 | F | 4, | 1 | 0.296 (x 3.377) | 6 (0.167) | 0.467 | pattern recognition receptor activity | GNBP3 |
| 1169 | GO:0016829 | F | 3, | 10 | 7.750 (x 1.290) | 157 (0.064) | 0.468 | lyase activity | BcDNA:GH12558 CG16733 CG18853 CG32158 CG3590 EG:BACN33B1.2 Eip55E Eno Pepck emp |
| 1170 | GO:0048568 | P | 4, | 3 | 1.728 (x 1.736) | 35 (0.086) | 0.468 | embryonic organ development | pbl pim trh |
| 1171 | GO:0007461 | P | 9, 10, 11, 12, | 1 | 0.296 (x 3.377) | 6 (0.167) | 0.468 | restriction of R8 fate | Dl |
| 1172 | GO:0016358 | P | 7, 10, | 3 | 1.728 (x 1.736) | 35 (0.086) | 0.468 | dendrite development | fra stan usp |
| 1173 | GO:0031111 | P | 7, 11, | 1 | 0.296 (x 3.377) | 6 (0.167) | 0.468 | negative regulation of microtubule polymerization or depolymerization | fwd |
| 1174 | GO:0008601 | F | 5, | 1 | 0.296 (x 3.377) | 6 (0.167) | 0.468 | protein phosphatase type 2A regulator activity | tws |
| 1175 | GO:0031570 | P | 8, | 1 | 0.296 (x 3.377) | 6 (0.167) | 0.469 | DNA integrity checkpoint | CG40410 |
| 1176 | GO:0007487 | P | 6, | 1 | 0.296 (x 3.377) | 6 (0.167) | 0.469 | analia development (sensu Endopterygota) | en |
| 1177 | GO:0009132 | P | 7, | 1 | 0.296 (x 3.377) | 6 (0.167) | 0.47 | nucleoside diphosphate metabolism | RnrS |
| 1178 | GO:0046834 | P | 7, 8, | 1 | 0.296 (x 3.377) | 6 (0.167) | 0.47 | lipid phosphorylation | Pi3K21B |
| 1179 | GO:0005483 | F | 4, | 1 | 0.296 (x 3.377) | 6 (0.167) | 0.47 | soluble NSF attachment protein activity | Snap |
| 1180 | GO:0016840 | F | 4, | 1 | 0.296 (x 3.377) | 6 (0.167) | 0.471 | carbon-nitrogen lyase activity | CG3590 |
| 1181 | GO:0042181 | P | 6, | 1 | 0.296 (x 3.377) | 6 (0.167) | 0.471 | ketone biosynthesis | woc |
| 1182 | GO:0007414 | P | 9, 10, 12, | 1 | 0.296 (x 3.377) | 6 (0.167) | 0.472 | axonal defasciculation | Ptp99A |
| 1183 | GO:0006422 | P | 9, 10, 11, | 1 | 0.296 (x 3.377) | 6 (0.167) | 0.472 | aspartyl-tRNA aminoacylation | mdy |
| 1184 | GO:0050874 | P | 3, | 49 | 44.425 (x 1.103) | 900 (0.054) | 0.472 | organismal physiological process | 18w Amph Arf79F CG11033 CG12199 CG16974 CG17084 CG31738 CG4054 CG5096 CG6965 CaMKI CrebA EG:118B3.2 ETH Eip75B Galpha49B GstS1 Hsp26 Hsp27 InR M6 Mpk2 Obp99a PFE Pli Sh Snap Tl Tsp96F br ck cyc d emp fax for fwd kal-1 lola mod(mdg4) mth olf413 pAbp pain para pll serpin-27A trn |
| 1185 | GO:0005528 | F | 5, | 1 | 0.296 (x 3.377) | 6 (0.167) | 0.472 | FK506 binding | Fkbp13 |
| 1186 | GO:0004815 | F | 7, | 1 | 0.296 (x 3.377) | 6 (0.167) | 0.473 | aspartate-tRNA ligase activity | mdy |
| 1187 | GO:0051649 | P | 5, 6, | 35 | 31.344 (x 1.117) | 635 (0.055) | 0.473 | establishment of cellular localization | Amph Arf79F CG14214 CG1907 CG1924 CG2108 CG2852 CG32137 CG33113 CG9057 CG9906 Crc Eb1 Fs(2)Ket Incenp Klp10A Rab8 S Snap TER94 Ucp4B alphaTub84B arr asp betaTub56D ck d l(1)G0320 loj pll polo prominin-like sqd vg wbl |
| 1188 | GO:0019198 | F | 5, 8, | 1 | 0.296 (x 3.377) | 6 (0.167) | 0.473 | transmembrane receptor protein phosphatase activity | Ptp99A |
| 1189 | GO:0042026 | P | 8, | 1 | 0.296 (x 3.377) | 6 (0.167) | 0.473 | protein refolding | Hsp27 |
| 1190 | GO:0048148 | P | 5, 7, 8, | 1 | 0.296 (x 3.377) | 6 (0.167) | 0.474 | behavioral response to cocaine | cyc |
| 1191 | GO:0008296 | F | 8, 9, | 1 | 0.296 (x 3.377) | 6 (0.167) | 0.474 | 3'-5'-exodeoxyribonuclease activity | BcDNA:GM10765 |
| 1192 | GO:0007415 | P | 10, 11, 13, | 1 | 0.296 (x 3.377) | 6 (0.167) | 0.475 | defasciculation of motor neuron axon | Ptp99A |
| 1193 | GO:0045456 | P | 7, 8, 9, | 1 | 0.296 (x 3.377) | 6 (0.167) | 0.475 | ecdysteroid biosynthesis | woc |
| 1194 | GO:0005527 | F | 4, | 1 | 0.296 (x 3.377) | 6 (0.167) | 0.475 | macrolide binding | Fkbp13 |
| 1195 | GO:0005616 | C | 3, 4, 5, | 1 | 0.296 (x 3.377) | 6 (0.167) | 0.476 | larval serum protein complex | Lsp2 |
| 1196 | GO:0044248 | P | 5, | 18 | 15.104 (x 1.192) | 306 (0.059) | 0.476 | cellular catabolism | BEST:LD22483 BcDNA:GM10765 CG5794 CG7860 EG:171D11.1 Eno Gapdh1 Gapdh2 Hex-A Idh Pgi Prosalpha6 Rep4 Tpi Uch fzy sqd th |
| 1197 | GO:0007296 | P | 6, | 1 | 0.296 (x 3.377) | 6 (0.167) | 0.476 | vitellogenesis | InR |
| 1198 | GO:0048190 | P | 6, 7, | 1 | 0.296 (x 3.377) | 6 (0.167) | 0.477 | wing disc dorsal/ventral pattern formation | ap |
| 1199 | GO:0009794 | P | 5, 7, 8, | 1 | 0.296 (x 3.377) | 6 (0.167) | 0.477 | regulation of progression through embryonic mitotic cell cycle | CG40410 |
| 1200 | GO:0000777 | C | 6, 7, 8, 9, 10, 11, 12, | 1 | 0.296 (x 3.377) | 6 (0.167) | 0.477 | condensed chromosome kinetochore | polo |
| 1201 | GO:0005275 | F | 3, | 4 | 2.567 (x 1.558) | 52 (0.077) | 0.478 | amine transporter activity | CG15088 CG1607 CG3424 mnd |
| 1202 | GO:0016805 | F | 6, | 1 | 0.296 (x 3.377) | 6 (0.167) | 0.478 | dipeptidase activity | CG2200 |
| 1203 | GO:0051641 | P | 4, 5, | 35 | 31.393 (x 1.115) | 636 (0.055) | 0.478 | cellular localization | Amph Arf79F CG14214 CG1907 CG1924 CG2108 CG2852 CG32137 CG33113 CG9057 CG9906 Crc Eb1 Fs(2)Ket Incenp Klp10A Rab8 S Snap TER94 Ucp4B alphaTub84B arr asp betaTub56D ck d l(1)G0320 loj pll polo prominin-like sqd vg wbl |
| 1204 | GO:0045317 | P | 7, 8, 9, 10, | 1 | 0.296 (x 3.377) | 6 (0.167) | 0.478 | equator specification | ara |
| 1205 | GO:0006955 | P | 4, 5, | 8 | 6.022 (x 1.328) | 122 (0.066) | 0.478 | immune response | 18w Mpk2 Pli Tl Tsp96F emp pll serpin-27A |
| 1206 | GO:0005001 | F | 6, 9, | 1 | 0.296 (x 3.377) | 6 (0.167) | 0.479 | transmembrane receptor protein tyrosine phosphatase activity | Ptp99A |
| 1207 | GO:0004690 | F | 8, | 1 | 0.296 (x 3.377) | 6 (0.167) | 0.479 | cyclic nucleotide-dependent protein kinase activity | for |
| 1208 | GO:0005337 | F | 4, | 1 | 0.296 (x 3.377) | 6 (0.167) | 0.479 | nucleoside transporter activity | BEST:LD04971 |
| 1209 | GO:0007026 | P | 7, 8, 10, 12, | 1 | 0.296 (x 3.377) | 6 (0.167) | 0.48 | negative regulation of microtubule depolymerization | fwd |
| 1210 | GO:0005201 | F | 3, | 1 | 0.296 (x 3.377) | 6 (0.167) | 0.48 | extracellular matrix structural constituent | CG33171 |
| 1211 | GO:0046622 | P | 5, | 1 | 0.296 (x 3.377) | 6 (0.167) | 0.481 | positive regulation of organ size | InR |
| 1212 | GO:0016126 | P | 7, 8, 9, | 1 | 0.296 (x 3.377) | 6 (0.167) | 0.481 | sterol biosynthesis | woc |
| 1213 | GO:0035303 | P | 8, | 1 | 0.296 (x 3.377) | 6 (0.167) | 0.481 | regulation of dephosphorylation | dap |
| 1214 | GO:0005099 | F | 5, | 1 | 0.296 (x 3.377) | 6 (0.167) | 0.482 | Ras GTPase activator activity | Rapgap1 |
| 1215 | GO:0016918 | F | 4, 5, | 1 | 0.296 (x 3.377) | 6 (0.167) | 0.482 | retinal binding | CG10657 |
| 1216 | GO:0007314 | P | 6, 8, 9, 11, | 4 | 2.764 (x 1.447) | 56 (0.071) | 0.482 | oocyte anterior/posterior axis determination | Dl Hrb27C TER94 sqd |
| 1217 | GO:0019094 | P | 6, 10, 12, 13, 15, | 3 | 1.826 (x 1.643) | 37 (0.081) | 0.483 | pole plasm mRNA localization | Hrb27C TER94 sqd |
| 1218 | GO:0016846 | F | 4, | 1 | 0.296 (x 3.377) | 6 (0.167) | 0.483 | carbon-sulfur lyase activity | Eip55E |
| 1219 | GO:0016709 | F | 5, | 1 | 0.346 (x 2.894) | 7 (0.143) | 0.483 | oxidoreductase activity, acting on paired donors, with incorporation or reduction of molecular oxygen, NAD or NADH as one donor, and incorporation of one atom of oxygen | CG1998 |
| 1220 | GO:0045944 | P | 10, | 2 | 0.987 (x 2.026) | 20 (0.100) | 0.483 | positive regulation of transcription from RNA polymerase II promoter | ci cyc |
| 1221 | GO:0046580 | P | 7, 8, 9, | 1 | 0.296 (x 3.377) | 6 (0.167) | 0.483 | negative regulation of Ras protein signal transduction | klu |
| 1222 | GO:0004448 | F | 6, | 1 | 0.346 (x 2.894) | 7 (0.143) | 0.483 | isocitrate dehydrogenase activity | Idh |
| 1223 | GO:0008134 | F | 4, | 6 | 4.393 (x 1.366) | 89 (0.067) | 0.483 | transcription factor binding | CG15835 CG31453 CtBP Dsp1 cic spen |
| 1224 | GO:0009124 | P | 7, 8, | 2 | 1.037 (x 1.929) | 21 (0.095) | 0.483 | nucleoside monophosphate biosynthesis | CG6767 Ts |
| 1225 | GO:0016782 | F | 4, | 2 | 0.987 (x 2.026) | 20 (0.100) | 0.483 | transferase activity, transferring sulfur-containing groups | CG16733 CG32632 |
| 1226 | GO:0045946 | P | 8, 9, 10, | 1 | 0.346 (x 2.894) | 7 (0.143) | 0.483 | positive regulation of translation | pAbp |
| 1227 | GO:0045936 | P | 7, 8, | 1 | 0.296 (x 3.377) | 6 (0.167) | 0.484 | negative regulation of phosphate metabolism | dap |
| 1228 | GO:0009117 | P | 6, | 10 | 7.848 (x 1.274) | 159 (0.063) | 0.484 | nucleotide metabolism | BEST:LD22483 CG32158 CG3590 CG6767 RnrS Tpi Ts blot dUTPase jumu |
| 1229 | GO:0051325 | P | 5, | 2 | 1.037 (x 1.929) | 21 (0.095) | 0.484 | interphase | cdc2 dap |
| 1230 | GO:0015924 | F | 7, | 1 | 0.346 (x 2.894) | 7 (0.143) | 0.484 | mannosyl-oligosaccharide mannosidase activity | alpha-Man-IIb |
| 1231 | GO:0007317 | P | 4, 8, 12, 14, 15, 17, | 2 | 0.987 (x 2.026) | 20 (0.100) | 0.484 | regulation of pole plasm oskar mRNA localization | Hrb27C TER94 |
| 1232 | GO:0007458 | P | 8, 9, | 1 | 0.296 (x 3.377) | 6 (0.167) | 0.484 | progression of morphogenetic furrow (sensu Endopterygota) | br |
| 1233 | GO:0005829 | C | 5, 6, 7, 8, | 11 | 9.082 (x 1.211) | 184 (0.060) | 0.484 | cytosol | CaMKI CkIIalpha CkIIbeta Eno Ice Pi3K21B RpS12 Sry-alpha cib eIF-4E smi35A |
| 1234 | GO:0007480 | P | 7, 8, | 2 | 1.037 (x 1.929) | 21 (0.095) | 0.484 | leg morphogenesis (sensu Endopterygota) | Dl Poxn |
| 1235 | GO:0035102 | C | 4, 6, 7, 8, 9, 10, 11, | 1 | 0.346 (x 2.894) | 7 (0.143) | 0.484 | PRC1 complex | ph-p |
| 1236 | GO:0016798 | F | 4, | 7 | 5.282 (x 1.325) | 107 (0.065) | 0.484 | hydrolase activity, acting on glycosyl bonds | CG33138 CG5731 CG9307 GNBP3 Hexo1 Thd1 alpha-Man-IIb |
| 1237 | GO:0007605 | P | 5, 7, | 2 | 0.987 (x 2.026) | 20 (0.100) | 0.484 | sensory perception of sound | ck d |
| 1238 | GO:0035153 | P | 5, 6, | 1 | 0.296 (x 3.377) | 6 (0.167) | 0.484 | tracheal epithelial cell type specification | Dl |
| 1239 | GO:0006265 | P | 7, | 1 | 0.346 (x 2.894) | 7 (0.143) | 0.485 | DNA topological change | Top2 |
| 1240 | GO:0009123 | P | 7, | 2 | 1.037 (x 1.929) | 21 (0.095) | 0.485 | nucleoside monophosphate metabolism | CG6767 Ts |
| 1241 | GO:0007286 | P | 5, 8, | 3 | 1.777 (x 1.688) | 36 (0.083) | 0.485 | spermatid development | Ance heph th |
| 1242 | GO:0008320 | F | 4, | 2 | 0.987 (x 2.026) | 20 (0.100) | 0.485 | protein carrier activity | Fs(2)Ket loj |
| 1243 | GO:0008046 | F | 5, | 1 | 0.296 (x 3.377) | 6 (0.167) | 0.485 | axon guidance receptor activity | fra |
| 1244 | GO:0042745 | P | 5, 6, | 1 | 0.346 (x 2.894) | 7 (0.143) | 0.485 | circadian sleep/wake cycle | cyc |
| 1245 | GO:0010033 | P | 5, | 2 | 1.037 (x 1.929) | 21 (0.095) | 0.485 | response to organic substance | Sh cyc |
| 1246 | GO:0007442 | P | 7, 8, | 3 | 1.777 (x 1.688) | 36 (0.083) | 0.485 | hindgut morphogenesis | pbl pim trh |
| 1247 | GO:0008049 | P | 6, 7, | 2 | 0.987 (x 2.026) | 20 (0.100) | 0.485 | male courtship behavior | Poxn para |
| 1248 | GO:0005678 | C | 4, 7, 8, 9, 10, 11, 12, 13, 14, | 1 | 0.296 (x 3.377) | 6 (0.167) | 0.485 | chromatin assembly complex | Caf1 |
| 1249 | GO:0000922 | C | 5, 6, 7, 8, 9, 10, 11, | 1 | 0.346 (x 2.894) | 7 (0.143) | 0.485 | spindle pole | polo |
| 1250 | GO:0051329 | P | 6, | 2 | 1.037 (x 1.929) | 21 (0.095) | 0.485 | interphase of mitotic cell cycle | cdc2 dap |
| 1251 | GO:0048515 | P | 4, 7, | 3 | 1.777 (x 1.688) | 36 (0.083) | 0.485 | spermatid differentiation | Ance heph th |
| 1252 | GO:0045475 | P | 5, 6, | 2 | 0.987 (x 2.026) | 20 (0.100) | 0.486 | locomotor rhythm | CkIIalpha cyc |
| 1253 | GO:0007484 | P | 5, 6, | 1 | 0.346 (x 2.894) | 7 (0.143) | 0.486 | genitalia development (sensu Endopterygota) | en |
| 1254 | GO:0009994 | P | 4, 7, | 6 | 4.294 (x 1.397) | 87 (0.069) | 0.486 | oocyte differentiation | Dl Hrb27C TER94 asp sqd wbl |
| 1255 | GO:0009110 | P | 6, | 2 | 1.037 (x 1.929) | 21 (0.095) | 0.486 | vitamin biosynthesis | CG31472 ESTS:39C10S |
| 1256 | GO:0043235 | C | 3, | 3 | 1.777 (x 1.688) | 36 (0.083) | 0.486 | receptor complex | InR l(1)G0320 usp |
| 1257 | GO:0006733 | P | 7, | 2 | 0.987 (x 2.026) | 20 (0.100) | 0.486 | oxidoreduction coenzyme metabolism | BEST:LD22483 Tpi |
| 1258 | GO:0016540 | P | 9, | 1 | 0.346 (x 2.894) | 7 (0.143) | 0.486 | protein autoprocessing | InR |
| 1259 | GO:0003755 | F | 5, | 2 | 1.037 (x 1.929) | 21 (0.095) | 0.486 | peptidyl-prolyl cis-trans isomerase activity | CG2852 Fkbp13 |
| 1260 | GO:0048193 | P | 6, 7, 8, | 3 | 1.777 (x 1.688) | 36 (0.083) | 0.486 | Golgi vesicle transport | S Snap wbl |
| 1261 | GO:0050654 | P | 6, 8, | 1 | 0.346 (x 2.894) | 7 (0.143) | 0.486 | chondroitin sulfate proteoglycan metabolism | sgl |
| 1262 | GO:0005326 | F | 3, | 2 | 0.987 (x 2.026) | 20 (0.100) | 0.486 | neurotransmitter transporter activity | CG15088 blot |
| 1263 | GO:0051258 | P | 7, | 2 | 1.037 (x 1.929) | 21 (0.095) | 0.487 | protein polymerization | alphaTub84B betaTub56D |
| 1264 | GO:0004785 | F | 6, | 1 | 0.346 (x 2.894) | 7 (0.143) | 0.487 | copper, zinc superoxide dismutase activity | CG9027 |
| 1265 | GO:0016411 | F | 8, | 1 | 0.346 (x 2.894) | 7 (0.143) | 0.487 | acylglycerol O-acyltransferase activity | mdy |
| 1266 | GO:0015932 | F | 3, | 1 | 0.346 (x 2.894) | 7 (0.143) | 0.487 | nucleobase, nucleoside, nucleotide and nucleic acid transporter activity | BEST:LD04971 |
| 1267 | GO:0048096 | P | 5, 10, 11, | 1 | 0.346 (x 2.894) | 7 (0.143) | 0.488 | chromatin-mediated maintenance of transcription | ash2 |
| 1268 | GO:0008355 | P | 6, 7, | 3 | 1.925 (x 1.558) | 39 (0.077) | 0.488 | olfactory learning | CG11711 CG8588 Fas3 |
| 1269 | GO:0042749 | P | 5, 6, 7, | 1 | 0.346 (x 2.894) | 7 (0.143) | 0.488 | regulation of circadian sleep/wake cycle | cyc |
| 1270 | GO:0012505 | C | 4, 5, | 9 | 7.108 (x 1.266) | 144 (0.062) | 0.488 | endomembrane system | BG:DS00004.11 CG14214 CG17952 CG8679 Fs(2)Ket Tapdelta Ugt alpha-Man-IIb l(1)G0320 |
| 1271 | GO:0046532 | P | 5, | 1 | 0.346 (x 2.894) | 7 (0.143) | 0.489 | regulation of photoreceptor cell differentiation | mbt |
| 1272 | GO:0042220 | P | 6, 7, | 1 | 0.346 (x 2.894) | 7 (0.143) | 0.489 | response to cocaine | cyc |
| 1273 | GO:0030204 | P | 7, 8, 9, | 1 | 0.346 (x 2.894) | 7 (0.143) | 0.489 | chondroitin sulfate metabolism | sgl |
| 1274 | GO:0006020 | P | 8, 9, | 1 | 0.346 (x 2.894) | 7 (0.143) | 0.49 | myo-inositol metabolism | CG1630 |
| 1275 | GO:0030145 | F | 6, | 1 | 0.346 (x 2.894) | 7 (0.143) | 0.49 | manganese ion binding | BcDNA:LD41548 |
| 1276 | GO:0042766 | P | 10, | 1 | 0.346 (x 2.894) | 7 (0.143) | 0.49 | nucleosome mobilization | Caf1 |
| 1277 | GO:0046669 | P | 7, 8, 9, 10, | 1 | 0.346 (x 2.894) | 7 (0.143) | 0.491 | regulation of retinal cell programmed cell death (sensu Endopterygota) | klu |
| 1278 | GO:0007402 | P | 5, 6, | 1 | 0.346 (x 2.894) | 7 (0.143) | 0.491 | ganglion mother cell fate determination | grh |
| 1279 | GO:0043279 | P | 6, | 1 | 0.346 (x 2.894) | 7 (0.143) | 0.492 | response to alkaloid | cyc |
| 1280 | GO:0050802 | P | 6, 7, | 1 | 0.346 (x 2.894) | 7 (0.143) | 0.492 | circadian sleep/wake cycle, sleep | cyc |
| 1281 | GO:0035288 | P | 6, 7, | 1 | 0.346 (x 2.894) | 7 (0.143) | 0.492 | anterior head segmentation | en |
| 1282 | GO:0045727 | P | 7, 8, 9, | 1 | 0.346 (x 2.894) | 7 (0.143) | 0.493 | positive regulation of protein biosynthesis | pAbp |
| 1283 | GO:0000159 | C | 4, 5, 6, | 1 | 0.346 (x 2.894) | 7 (0.143) | 0.493 | protein phosphatase type 2A complex | tws |
| 1284 | GO:0030163 | P | 6, 7, | 6 | 4.442 (x 1.351) | 90 (0.067) | 0.493 | protein catabolism | CG5794 CG7860 Prosalpha6 Uch fzy th |
| 1285 | GO:0008205 | P | 7, 8, 9, | 1 | 0.346 (x 2.894) | 7 (0.143) | 0.493 | ecdysone metabolism | woc |
| 1286 | GO:0007517 | P | 4, | 7 | 5.331 (x 1.313) | 108 (0.065) | 0.494 | muscle development | Dr Wnt2 ap betaTub56D hbs rost tkv |
| 1287 | GO:0019840 | F | 3, | 1 | 0.346 (x 2.894) | 7 (0.143) | 0.494 | isoprenoid binding | CG10657 |
| 1288 | GO:0007030 | P | 6, | 1 | 0.346 (x 2.894) | 7 (0.143) | 0.494 | Golgi organization and biogenesis | TER94 |
| 1289 | GO:0048284 | P | 6, | 1 | 0.346 (x 2.894) | 7 (0.143) | 0.495 | organelle fusion | polo |
| 1290 | GO:0042393 | F | 4, | 1 | 0.346 (x 2.894) | 7 (0.143) | 0.495 | histone binding | Caf1 |
| 1291 | GO:0004033 | F | 6, | 1 | 0.346 (x 2.894) | 7 (0.143) | 0.495 | aldo-keto reductase activity | CG2767 |
| 1292 | GO:0050650 | P | 7, 8, 9, | 1 | 0.346 (x 2.894) | 7 (0.143) | 0.496 | chondroitin sulfate proteoglycan biosynthesis | sgl |
| 1293 | GO:0045673 | P | 6, | 1 | 0.346 (x 2.894) | 7 (0.143) | 0.496 | regulation of photoreceptor differentiation (sensu Endopterygota) | mbt |
| 1294 | GO:0045815 | P | 4, | 1 | 0.346 (x 2.894) | 7 (0.143) | 0.496 | positive regulation of gene expression, epigenetic | ash2 |
| 1295 | GO:0007304 | P | 8, 9, | 4 | 2.715 (x 1.473) | 55 (0.073) | 0.496 | eggshell formation (sensu Insecta) | Caf1 Dp Fs(2)Ket bun |
| 1296 | GO:0048806 | P | 4, | 1 | 0.346 (x 2.894) | 7 (0.143) | 0.497 | genitalia development | en |
| 1297 | GO:0007617 | P | 4, 5, | 4 | 2.715 (x 1.473) | 55 (0.073) | 0.497 | mating behavior | Poxn Sh lig para |
| 1298 | GO:0016202 | P | 6, | 1 | 0.346 (x 2.894) | 7 (0.143) | 0.497 | regulation of striated muscle development | hbs |
| 1299 | GO:0030703 | P | 7, | 4 | 2.715 (x 1.473) | 55 (0.073) | 0.497 | eggshell formation | Caf1 Dp Fs(2)Ket bun |
| 1300 | GO:0008092 | F | 4, | 14 | 11.748 (x 1.192) | 238 (0.059) | 0.497 | cytoskeletal protein binding | CG32137 CG6891 Eb1 Hsp23 Incenp Pten Sry-alpha alphaTub84B asp betaTub56D cib ck d stai |
| 1301 | GO:0048138 | P | 5, 6, | 1 | 0.346 (x 2.894) | 7 (0.143) | 0.498 | germ-line cyst encapsulation | ph-p |
| 1302 | GO:0051705 | P | 3, | 4 | 2.715 (x 1.473) | 55 (0.073) | 0.498 | behavioral interaction between organisms | Poxn Sh lig para |
| 1303 | GO:0005386 | F | 3, | 25 | 22.360 (x 1.118) | 453 (0.055) | 0.498 | carrier activity | BEST:LD04971 CG10657 CG11739 CG13848 CG14214 CG15088 CG1607 CG1893 CG1907 CG3424 CG3823 CG6782 CG6812 CG9342 Fs(2)Ket LanB2 Nrv1 Ucp4B Vha100-2 blot jumu l(2)01810 loj mnd sut1 |
| 1304 | GO:0006267 | P | 9, | 1 | 0.346 (x 2.894) | 7 (0.143) | 0.498 | pre-replicative complex formation and maintenance | Mcm7 |
| 1305 | GO:0006916 | P | 8, 9, | 3 | 1.876 (x 1.599) | 38 (0.079) | 0.498 | anti-apoptosis | Aac11 nmo th |
| 1306 | GO:0051058 | P | 6, 7, 8, | 1 | 0.346 (x 2.894) | 7 (0.143) | 0.498 | negative regulation of small GTPase mediated signal transduction | klu |
| 1307 | GO:0007316 | P | 5, 9, 11, 12, 14, | 3 | 1.876 (x 1.599) | 38 (0.079) | 0.499 | pole plasm RNA localization | Hrb27C TER94 sqd |
| 1308 | GO:0035097 | C | 3, 6, 7, 8, 9, 10, 11, 12, 13, | 1 | 0.346 (x 2.894) | 7 (0.143) | 0.499 | histone methyltransferase complex | Caf1 |
| 1309 | GO:0004246 | F | 6, 7, | 1 | 0.346 (x 2.894) | 7 (0.143) | 0.499 | peptidyl-dipeptidase A activity | Ance |
| 1310 | GO:0031532 | P | 9, | 1 | 0.346 (x 2.894) | 7 (0.143) | 0.499 | actin cytoskeleton reorganization | asp |
| 1311 | GO:0030206 | P | 8, 9, 10, | 1 | 0.346 (x 2.894) | 7 (0.143) | 0.5 | chondroitin sulfate biosynthesis | sgl |
| 1312 | GO:0016641 | F | 5, | 1 | 0.346 (x 2.894) | 7 (0.143) | 0.5 | oxidoreductase activity, acting on the CH-NH2 group of donors, oxygen as acceptor | CG31472 |
| 1313 | GO:0030865 | P | 7, | 1 | 0.346 (x 2.894) | 7 (0.143) | 0.501 | cortical cytoskeleton organization and biogenesis | Abi |
| 1314 | GO:0004467 | F | 6, | 1 | 0.346 (x 2.894) | 7 (0.143) | 0.501 | long-chain-fatty-acid-CoA ligase activity | BcDNA:GH02901 |
| 1315 | GO:0042752 | P | 4, 5, | 1 | 0.346 (x 2.894) | 7 (0.143) | 0.501 | regulation of circadian rhythm | cyc |
| 1316 | GO:0006511 | P | 9, 10, 11, | 5 | 3.702 (x 1.351) | 75 (0.067) | 0.502 | ubiquitin-dependent protein catabolism | CG5794 Prosalpha6 Uch fzy th |
| 1317 | GO:0030866 | P | 8, 9, | 1 | 0.346 (x 2.894) | 7 (0.143) | 0.502 | cortical actin cytoskeleton organization and biogenesis | Abi |
| 1318 | GO:0048139 | P | 6, 7, | 1 | 0.346 (x 2.894) | 7 (0.143) | 0.502 | female germ-line cyst encapsulation | ph-p |
| 1319 | GO:0031114 | P | 7, 9, 11, | 1 | 0.346 (x 2.894) | 7 (0.143) | 0.503 | regulation of microtubule depolymerization | fwd |
| 1320 | GO:0007620 | P | 6, 7, | 1 | 0.346 (x 2.894) | 7 (0.143) | 0.503 | copulation | lig |
| 1321 | GO:0046974 | F | 9, 10, | 1 | 0.346 (x 2.894) | 7 (0.143) | 0.503 | histone lysine N-methyltransferase activity (H3-K9 specific) | Caf1 |
| 1322 | GO:0008238 | F | 5, | 7 | 5.380 (x 1.301) | 109 (0.064) | 0.504 | exopeptidase activity | Ance BcDNA:LD41548 CG2200 Lsp2 east fra ome |
| 1323 | GO:0010001 | P | 4, 7, | 1 | 0.346 (x 2.894) | 7 (0.143) | 0.504 | glial cell differentiation | spen |
| 1324 | GO:0005501 | F | 4, | 1 | 0.346 (x 2.894) | 7 (0.143) | 0.504 | retinoid binding | CG10657 |
| 1325 | GO:0007019 | P | 8, 10, | 1 | 0.346 (x 2.894) | 7 (0.143) | 0.505 | microtubule depolymerization | fwd |
| 1326 | GO:0005654 | C | 5, 6, 7, 8, 9, 10, 11, | 13 | 11.057 (x 1.176) | 224 (0.058) | 0.505 | nucleoplasm | B52 Caf1 CycT Dp Dref Hrb27C Mcm7 Rbf2 Top2 ap sqd toe usp |
| 1327 | GO:0016859 | F | 4, | 2 | 1.135 (x 1.762) | 23 (0.087) | 0.505 | cis-trans isomerase activity | CG2852 Fkbp13 |
| 1328 | GO:0008241 | F | 6, | 1 | 0.346 (x 2.894) | 7 (0.143) | 0.505 | peptidyl-dipeptidase activity | Ance |
| 1329 | GO:0009967 | P | 5, 6, | 2 | 1.135 (x 1.762) | 23 (0.087) | 0.505 | positive regulation of signal transduction | CG9211 stan |
| 1330 | GO:0031328 | P | 7, | 1 | 0.346 (x 2.894) | 7 (0.143) | 0.505 | positive regulation of cellular biosynthesis | pAbp |
| 1331 | GO:0045445 | P | 5, 7, 8, | 2 | 1.135 (x 1.762) | 23 (0.087) | 0.506 | myoblast differentiation | hbs rost |
| 1332 | GO:0048589 | P | 3, | 3 | 1.974 (x 1.519) | 40 (0.075) | 0.506 | developmental growth | InR ninA tkv |
| 1333 | GO:0005100 | F | 5, | 1 | 0.346 (x 2.894) | 7 (0.143) | 0.506 | Rho GTPase activator activity | RacGAP50C |
| 1334 | GO:0007281 | P | 5, | 7 | 5.528 (x 1.266) | 112 (0.062) | 0.506 | germ cell development | Dl Hrb27C TER94 asp sqd tkv wbl |
| 1335 | GO:0009299 | P | 8, | 2 | 1.135 (x 1.762) | 23 (0.087) | 0.506 | mRNA transcription | Top2 ph-p |
| 1336 | GO:0006268 | P | 9, | 1 | 0.346 (x 2.894) | 7 (0.143) | 0.506 | DNA unwinding during replication | Dsp1 |
| 1337 | GO:0019395 | P | 7, 8, | 2 | 1.135 (x 1.762) | 23 (0.087) | 0.506 | fatty acid oxidation | BcDNA:GH12558 CG4586 |
| 1338 | GO:0006836 | P | 5, 6, | 2 | 1.086 (x 1.842) | 22 (0.091) | 0.506 | neurotransmitter transport | CG15088 blot |
| 1339 | GO:0009891 | P | 6, | 1 | 0.346 (x 2.894) | 7 (0.143) | 0.507 | positive regulation of biosynthesis | pAbp |
| 1340 | GO:0007478 | P | 6, 7, | 2 | 1.135 (x 1.762) | 23 (0.087) | 0.507 | leg disc morphogenesis | Dl Poxn |
| 1341 | GO:0042078 | P | 6, | 2 | 1.086 (x 1.842) | 22 (0.091) | 0.507 | germ-line stem cell division | asp tkv |
| 1342 | GO:0045187 | P | 6, 7, 8, | 1 | 0.346 (x 2.894) | 7 (0.143) | 0.507 | regulation of circadian sleep/wake cycle, sleep | cyc |
| 1343 | GO:0035109 | P | 6, | 2 | 1.135 (x 1.762) | 23 (0.087) | 0.507 | limb morphogenesis (sensu Endopterygota) | Dl Poxn |
| 1344 | GO:0048627 | P | 5, 6, 8, 9, | 2 | 1.086 (x 1.842) | 22 (0.091) | 0.507 | myoblast development | hbs rost |
| 1345 | GO:0016461 | C | 4, 6, 7, 8, 9, 10, 11, | 1 | 0.346 (x 2.894) | 7 (0.143) | 0.507 | unconventional myosin | ck |
| 1346 | GO:0044420 | C | 2, 3, | 2 | 1.086 (x 1.842) | 22 (0.091) | 0.508 | extracellular matrix part | CG33171 LanB2 |
| 1347 | GO:0044430 | C | 4, 5, 6, 7, 8, 9, | 14 | 11.847 (x 1.182) | 240 (0.058) | 0.508 | cytoskeletal part | Act42A Eb1 Klp10A Nek2 alphaTub84B asp betaTub56D ck d fzy p120ctn polo stai vg |
| 1348 | GO:0015645 | F | 5, | 1 | 0.346 (x 2.894) | 7 (0.143) | 0.508 | fatty-acid ligase activity | BcDNA:GH02901 |
| 1349 | GO:0030554 | F | 5, | 37 | 34.009 (x 1.088) | 689 (0.054) | 0.508 | adenyl nucleotide binding | BEST:CK02656 CG14217 CG2118 CG31121 CG31453 CG40410 CaMKI Cad96Ca Cdk4 CkIIalpha Eph Hex-A InR Klp10A Mcm7 Mpk2 Nek2 Rm62 TER94 Top2 ald blot cdc2 ck d for glu jumu kis mbt mdy nmo otk pll polo smi35A tkv |
| 1350 | GO:0008544 | P | 5, | 2 | 1.086 (x 1.842) | 22 (0.091) | 0.508 | epidermis development | in pk |
| 1351 | GO:0007584 | P | 5, 6, | 1 | 0.346 (x 2.894) | 7 (0.143) | 0.508 | response to nutrient | InR |
| 1352 | GO:0043085 | P | 4, | 2 | 1.086 (x 1.842) | 22 (0.091) | 0.508 | positive regulation of enzyme activity | Abi mbt |
| 1353 | GO:0016056 | P | 7, 8, 9, | 1 | 0.346 (x 2.894) | 7 (0.143) | 0.508 | rhodopsin mediated signaling | Galpha49B |
| 1354 | GO:0005044 | F | 5, | 2 | 1.086 (x 1.842) | 22 (0.091) | 0.509 | scavenger receptor activity | CG3921 emp |
| 1355 | GO:0031110 | P | 6, 10, | 1 | 0.346 (x 2.894) | 7 (0.143) | 0.509 | regulation of microtubule polymerization or depolymerization | fwd |
| 1356 | GO:0006635 | P | 8, 9, | 2 | 1.086 (x 1.842) | 22 (0.091) | 0.509 | fatty acid beta-oxidation | BcDNA:GH12558 CG4586 |
| 1357 | GO:0003727 | F | 5, | 2 | 1.086 (x 1.842) | 22 (0.091) | 0.51 | single-stranded RNA binding | Pep pAbp |
| 1358 | GO:0005319 | F | 3, | 2 | 1.086 (x 1.842) | 22 (0.091) | 0.51 | lipid transporter activity | CG1893 CG9342 |
| 1359 | GO:0016491 | F | 3, | 34 | 30.801 (x 1.104) | 624 (0.054) | 0.51 | oxidoreductase activity | Aldh-III BEST:LD22483 BcDNA:GH12558 CG11151 CG12199 CG1998 CG2767 CG30427 CG31472 CG31915 CG31937 CG3842 CG4586 CG5873 CG6199 CG6287 CG7675 CG9027 CtBP Cyp310a1 EG:171D11.1 Eip71CD Gapdh1 Gapdh2 GstS1 Idh Jafrac2 LanB2 Pgi RnrS Ts knk olf413 sgl |
| 1360 | GO:0048628 | P | 6, 7, 9, 10, | 2 | 1.086 (x 1.842) | 22 (0.091) | 0.51 | myoblast maturation | hbs rost |
| 1361 | GO:0008017 | F | 6, | 5 | 3.751 (x 1.333) | 76 (0.066) | 0.512 | microtubule binding | CG32137 Eb1 Incenp asp stai |
| 1362 | GO:0007309 | P | 5, 7, 8, 10, | 5 | 3.751 (x 1.333) | 76 (0.066) | 0.513 | oocyte axis determination | Dl Hrb27C TER94 sqd wbl |
| 1363 | GO:0051704 | P | 2, | 4 | 2.863 (x 1.397) | 58 (0.069) | 0.513 | interaction between organisms | Poxn Sh lig para |
| 1364 | GO:0042063 | P | 6, | 2 | 1.185 (x 1.688) | 24 (0.083) | 0.515 | gliogenesis | bnb spen |
| 1365 | GO:0046982 | F | 5, | 2 | 1.185 (x 1.688) | 24 (0.083) | 0.516 | protein heterodimerization activity | cyc trh |
| 1366 | GO:0007494 | P | 5, | 2 | 1.185 (x 1.688) | 24 (0.083) | 0.516 | midgut development | dve opa |
| 1367 | GO:0004879 | F | 4, | 2 | 1.185 (x 1.688) | 24 (0.083) | 0.516 | ligand-dependent nuclear receptor activity | Eip75B usp |
| 1368 | GO:0051246 | P | 5, 6, | 8 | 6.565 (x 1.219) | 133 (0.060) | 0.517 | regulation of protein metabolism | CG40410 dap fwd fzy l(2)01424 pAbp pll sqd |
| 1369 | GO:0016570 | P | 8, 11, | 2 | 1.185 (x 1.688) | 24 (0.083) | 0.517 | histone modification | Caf1 Incenp |
| 1370 | GO:0006334 | P | 7, 11, | 2 | 1.185 (x 1.688) | 24 (0.083) | 0.517 | nucleosome assembly | Caf1 Set |
| 1371 | GO:0000184 | P | 9, | 1 | 0.395 (x 2.532) | 8 (0.125) | 0.517 | mRNA catabolism, nonsense-mediated decay | sqd |
| 1372 | GO:0016569 | P | 10, | 2 | 1.185 (x 1.688) | 24 (0.083) | 0.518 | covalent chromatin modification | Caf1 Incenp |
| 1373 | GO:0045705 | P | 8, | 1 | 0.395 (x 2.532) | 8 (0.125) | 0.518 | negative regulation of salivary gland determination | tkv |
| 1374 | GO:0006967 | P | 7, 8, 9, | 1 | 0.395 (x 2.532) | 8 (0.125) | 0.518 | positive regulation of antifungal peptide biosynthesis | Tl |
| 1375 | GO:0006801 | P | 6, | 1 | 0.395 (x 2.532) | 8 (0.125) | 0.518 | superoxide metabolism | CG9027 |
| 1376 | GO:0043228 | C | 3, | 38 | 35.095 (x 1.083) | 711 (0.053) | 0.518 | non-membrane-bound organelle | Act42A CG17493 CG1911 CG31738 Caf1 CycB3 CycT Dp Dp1 Dsp1 Eb1 His4r HmgD Incenp Klp10A Nek2 Pep Rbf2 RpS12 TER94 Top2 Trl alphaTub84B asp betaTub56D ck d fzy glu jumu kis mod(mdg4) p120ctn polo sqd stai usp vg |
| 1377 | GO:0004067 | F | 6, | 1 | 0.395 (x 2.532) | 8 (0.125) | 0.519 | asparaginase activity | CG7860 |
| 1378 | GO:0043232 | C | 4, 5, 6, 7, | 38 | 35.095 (x 1.083) | 711 (0.053) | 0.519 | intracellular non-membrane-bound organelle | Act42A CG17493 CG1911 CG31738 Caf1 CycB3 CycT Dp Dp1 Dsp1 Eb1 His4r HmgD Incenp Klp10A Nek2 Pep Rbf2 RpS12 TER94 Top2 Trl alphaTub84B asp betaTub56D ck d fzy glu jumu kis mod(mdg4) p120ctn polo sqd stai usp vg |
| 1379 | GO:0005344 | F | 3, | 1 | 0.395 (x 2.532) | 8 (0.125) | 0.519 | oxygen transporter activity | Lsp2 |
| 1380 | GO:0016721 | F | 4, | 1 | 0.395 (x 2.532) | 8 (0.125) | 0.519 | oxidoreductase activity, acting on superoxide radicals as acceptor | CG9027 |
| 1381 | GO:0005391 | F | 6, 7, 9, 14, | 1 | 0.395 (x 2.532) | 8 (0.125) | 0.52 | sodium:potassium-exchanging ATPase activity | Nrv1 |
| 1382 | GO:0042180 | P | 5, | 1 | 0.395 (x 2.532) | 8 (0.125) | 0.52 | ketone metabolism | woc |
| 1383 | GO:0008429 | F | 5, | 1 | 0.395 (x 2.532) | 8 (0.125) | 0.521 | phosphatidylethanolamine binding | CG17919 |
| 1384 | GO:0008023 | C | 3, 6, 7, 8, 9, 10, 11, 12, 13, | 1 | 0.395 (x 2.532) | 8 (0.125) | 0.521 | transcription elongation factor complex | CycT |
| 1385 | GO:0005890 | C | 3, 5, 6, 7, 8, 9, | 1 | 0.395 (x 2.532) | 8 (0.125) | 0.521 | sodium:potassium-exchanging ATPase complex | Nrv1 |
| 1386 | GO:0042562 | F | 3, | 1 | 0.395 (x 2.532) | 8 (0.125) | 0.522 | hormone binding | usp |
| 1387 | GO:0008047 | F | 3, | 6 | 4.689 (x 1.280) | 95 (0.063) | 0.522 | enzyme activator activity | BEST:GH02921 CG40160 CycB3 CycT RacGAP50C Rapgap1 |
| 1388 | GO:0005089 | F | 5, | 1 | 0.395 (x 2.532) | 8 (0.125) | 0.522 | Rho guanyl-nucleotide exchange factor activity | pbl |
| 1389 | GO:0006518 | P | 5, | 1 | 0.395 (x 2.532) | 8 (0.125) | 0.522 | peptide metabolism | BG:DS00004.11 |
| 1390 | GO:0004896 | F | 5, | 1 | 0.395 (x 2.532) | 8 (0.125) | 0.523 | hematopoietin/interferon-class (D200-domain) cytokine receptor activity | InR |
| 1391 | GO:0004602 | F | 4, 6, | 1 | 0.395 (x 2.532) | 8 (0.125) | 0.523 | glutathione peroxidase activity | GstS1 |
| 1392 | GO:0016895 | F | 8, | 1 | 0.395 (x 2.532) | 8 (0.125) | 0.524 | exodeoxyribonuclease activity, producing 5'-phosphomonoesters | BcDNA:GM10765 |
| 1393 | GO:0006376 | P | 8, 11, 13, | 1 | 0.395 (x 2.532) | 8 (0.125) | 0.524 | mRNA splice site selection | B52 |
| 1394 | GO:0004683 | F | 8, | 1 | 0.395 (x 2.532) | 8 (0.125) | 0.524 | calmodulin regulated protein kinase activity | CaMKI |
| 1395 | GO:0048113 | P | 8, 10, 11, 13, | 3 | 2.024 (x 1.482) | 41 (0.073) | 0.525 | pole plasm assembly (sensu Insecta) | Hrb27C TER94 sqd |
| 1396 | GO:0016350 | P | 9, | 1 | 0.395 (x 2.532) | 8 (0.125) | 0.525 | maintenance of oocyte identity (sensu Insecta) | dap |
| 1397 | GO:0016229 | F | 6, | 1 | 0.395 (x 2.532) | 8 (0.125) | 0.525 | steroid dehydrogenase activity | CG11151 |
| 1398 | GO:0043632 | P | 7, | 5 | 3.801 (x 1.316) | 77 (0.065) | 0.525 | modification-dependent macromolecule catabolism | CG5794 Prosalpha6 Uch fzy th |
| 1399 | GO:0015301 | F | 5, 7, | 1 | 0.395 (x 2.532) | 8 (0.125) | 0.526 | anion:anion antiporter activity | CG1907 |
| 1400 | GO:0009112 | P | 6, | 5 | 3.850 (x 1.299) | 78 (0.064) | 0.526 | nucleobase metabolism | CG3590 CG6767 EG:171D11.1 RnrS Ts |
| 1401 | GO:0035295 | P | 3, | 5 | 3.801 (x 1.316) | 77 (0.065) | 0.526 | tube development | grh pbl pim sgl trh |
| 1402 | GO:0045704 | P | 7, | 1 | 0.395 (x 2.532) | 8 (0.125) | 0.526 | regulation of salivary gland determination | tkv |
| 1403 | GO:0006959 | P | 5, 6, | 5 | 3.850 (x 1.299) | 78 (0.064) | 0.526 | humoral immune response | 18w Mpk2 Tl Tsp96F pll |
| 1404 | GO:0019941 | P | 8, 9, 10, | 5 | 3.801 (x 1.316) | 77 (0.065) | 0.526 | modification-dependent protein catabolism | CG5794 Prosalpha6 Uch fzy th |
| 1405 | GO:0045735 | F | 2, | 1 | 0.395 (x 2.532) | 8 (0.125) | 0.526 | nutrient reservoir activity | Lsp2 |
| 1406 | GO:0048637 | P | 6, | 3 | 2.073 (x 1.447) | 42 (0.071) | 0.526 | skeletal muscle development | hbs rost tkv |
| 1407 | GO:0007611 | P | 4, | 4 | 2.962 (x 1.351) | 60 (0.067) | 0.526 | learning and/or memory | CG11711 CG8588 Fas3 Sh |
| 1408 | GO:0000086 | P | 7, | 1 | 0.395 (x 2.532) | 8 (0.125) | 0.527 | G2/M transition of mitotic cell cycle | cdc2 |
| 1409 | GO:0043066 | P | 7, 8, | 3 | 2.073 (x 1.447) | 42 (0.071) | 0.527 | negative regulation of apoptosis | Aac11 nmo th |
| 1410 | GO:0005952 | C | 3, 4, 5, 6, | 1 | 0.395 (x 2.532) | 8 (0.125) | 0.527 | cAMP-dependent protein kinase complex | for |
| 1411 | GO:0048747 | P | 5, | 3 | 2.073 (x 1.447) | 42 (0.071) | 0.527 | muscle fiber development | hbs rost tkv |
| 1412 | GO:0008541 | C | 3, 4, 5, 6, 7, 8, | 1 | 0.395 (x 2.532) | 8 (0.125) | 0.527 | proteasome regulatory particle, lid subcomplex (sensu Eukaryota) | Rpn9 |
| 1413 | GO:0043069 | P | 6, 7, | 3 | 2.073 (x 1.447) | 42 (0.071) | 0.528 | negative regulation of programmed cell death | Aac11 nmo th |
| 1414 | GO:0045184 | P | 5, | 28 | 25.667 (x 1.091) | 520 (0.054) | 0.528 | establishment of protein localization | Amph Arf79F CG14214 CG1924 CG2108 CG2852 CG32137 CG33113 CG9906 Crc Fs(2)Ket Klp10A Rab8 S Snap TER94 alpha-Man-IIb alphaTub84B arr betaTub56D ck d l(1)G0320 loj pk pll prominin-like wbl |
| 1415 | GO:0004784 | F | 5, | 1 | 0.395 (x 2.532) | 8 (0.125) | 0.528 | superoxide dismutase activity | CG9027 |
| 1416 | GO:0007306 | P | 9, 10, | 3 | 2.073 (x 1.447) | 42 (0.071) | 0.528 | insect chorion formation | Caf1 Dp bun |
| 1417 | GO:0042770 | P | 5, | 1 | 0.395 (x 2.532) | 8 (0.125) | 0.528 | DNA damage response, signal transduction | CG40410 |
| 1418 | GO:0048741 | P | 6, 7, | 3 | 2.073 (x 1.447) | 42 (0.071) | 0.528 | skeletal muscle fiber development | hbs rost tkv |
| 1419 | GO:0043062 | P | 3, | 3 | 2.073 (x 1.447) | 42 (0.071) | 0.529 | extracellular structure organization and biogenesis | Tl fwd tkv |
| 1420 | GO:0006338 | P | 10, | 3 | 2.073 (x 1.447) | 42 (0.071) | 0.529 | chromatin remodeling | Caf1 ash2 ph-p |
| 1421 | GO:0007264 | P | 6, | 7 | 5.726 (x 1.223) | 116 (0.060) | 0.533 | small GTPase mediated signal transduction | CG2108 CG5522 Rab8 RacGAP50C Rapgap1 edl klu |
| 1422 | GO:0016459 | C | 3, 5, 6, 7, 8, 9, 10, | 2 | 1.234 (x 1.621) | 25 (0.080) | 0.536 | myosin | ck d |
| 1423 | GO:0007308 | P | 6, 7, 9, | 5 | 3.899 (x 1.282) | 79 (0.063) | 0.536 | oocyte construction | Dl Hrb27C TER94 sqd wbl |
| 1424 | GO:0005764 | C | 7, 8, 9, 10, | 2 | 1.234 (x 1.621) | 25 (0.080) | 0.537 | lysosome | Ppt1 emp |
| 1425 | GO:0016585 | C | 3, 6, 7, 8, 9, 10, 11, 12, 13, | 2 | 1.234 (x 1.621) | 25 (0.080) | 0.537 | chromatin remodeling complex | Caf1 Top2 |
| 1426 | GO:0000323 | C | 6, 7, 8, 9, | 2 | 1.234 (x 1.621) | 25 (0.080) | 0.537 | lytic vacuole | Ppt1 emp |
| 1427 | GO:0004888 | F | 4, | 23 | 20.978 (x 1.096) | 425 (0.054) | 0.538 | transmembrane receptor activity | 18w CG14076 CG1632 CG17084 CG3921 CG6965 CG9211 Cad96Ca Eph InR PFE Ptp99A Tl arr emp fra mth otk pbl pll stan tkv wgn |
| 1428 | GO:0007507 | P | 5, | 4 | 3.011 (x 1.328) | 61 (0.066) | 0.54 | heart development | Doc1 numb sgl tkv |
| 1429 | GO:0004553 | F | 5, | 6 | 4.837 (x 1.240) | 98 (0.061) | 0.54 | hydrolase activity, hydrolyzing O-glycosyl compounds | CG33138 CG5731 CG9307 GNBP3 Hexo1 alpha-Man-IIb |
| 1430 | GO:0016568 | P | 9, | 4 | 3.011 (x 1.328) | 61 (0.066) | 0.54 | chromatin modification | Caf1 Incenp ash2 ph-p |
| 1431 | GO:0006261 | P | 8, | 4 | 3.011 (x 1.328) | 61 (0.066) | 0.54 | DNA-dependent DNA replication | Dref Dsp1 Mcm7 Thd1 |
| 1432 | GO:0007612 | P | 5, | 3 | 2.123 (x 1.413) | 43 (0.070) | 0.541 | learning | CG11711 CG8588 Fas3 |
| 1433 | GO:0009796 | P | 4, 5, | 1 | 0.444 (x 2.251) | 9 (0.111) | 0.541 | cellularization (sensu Metazoa) | CG40410 |
| 1434 | GO:0004180 | F | 6, | 3 | 2.123 (x 1.413) | 43 (0.070) | 0.542 | carboxypeptidase activity | Lsp2 east fra |
| 1435 | GO:0009288 | C | 5, 6, | 1 | 0.444 (x 2.251) | 9 (0.111) | 0.542 | flagellum (sensu Bacteria) | lola |
| 1436 | GO:0005635 | C | 4, 5, 6, 7, 8, 9, 10, | 4 | 3.060 (x 1.307) | 62 (0.065) | 0.542 | nuclear envelope | CG17952 CG8679 Fs(2)Ket Ugt |
| 1437 | GO:0051248 | P | 6, 7, | 3 | 2.123 (x 1.413) | 43 (0.070) | 0.542 | negative regulation of protein metabolism | dap fwd sqd |
| 1438 | GO:0003730 | F | 6, | 1 | 0.444 (x 2.251) | 9 (0.111) | 0.542 | mRNA 3'-UTR binding | sqd |
| 1439 | GO:0007315 | P | 7, 9, 10, 12, | 3 | 2.123 (x 1.413) | 43 (0.070) | 0.542 | pole plasm assembly | Hrb27C TER94 sqd |
| 1440 | GO:0018346 | P | 10, 11, | 1 | 0.444 (x 2.251) | 9 (0.111) | 0.542 | protein amino acid prenylation | CG33171 |
| 1441 | GO:0005160 | F | 4, 5, | 1 | 0.444 (x 2.251) | 9 (0.111) | 0.543 | transforming growth factor beta receptor binding | mav |
| 1442 | GO:0007618 | P | 5, 6, | 1 | 0.444 (x 2.251) | 9 (0.111) | 0.543 | mating | lig |
| 1443 | GO:0000090 | P | 7, 8, | 1 | 0.444 (x 2.251) | 9 (0.111) | 0.544 | mitotic anaphase | tws |
| 1444 | GO:0051247 | P | 6, 7, | 1 | 0.444 (x 2.251) | 9 (0.111) | 0.544 | positive regulation of protein metabolism | pAbp |
| 1445 | GO:0006298 | P | 6, 8, 10, | 1 | 0.444 (x 2.251) | 9 (0.111) | 0.544 | mismatch repair | Thd1 |
| 1446 | GO:0001539 | P | 5, 6, | 1 | 0.444 (x 2.251) | 9 (0.111) | 0.545 | ciliary or flagellar motility | lola |
| 1447 | GO:0004221 | F | 6, 8, | 2 | 1.283 (x 1.558) | 26 (0.077) | 0.545 | ubiquitin thiolesterase activity | CG5794 Uch |
| 1448 | GO:0030201 | P | 6, 8, | 1 | 0.444 (x 2.251) | 9 (0.111) | 0.545 | heparan sulfate proteoglycan metabolism | sgl |
| 1449 | GO:0051128 | P | 5, | 2 | 1.283 (x 1.558) | 26 (0.077) | 0.545 | regulation of cell organization and biogenesis | fwd mod(mdg4) |
| 1450 | GO:0016458 | P | 6, | 3 | 2.172 (x 1.381) | 44 (0.068) | 0.545 | gene silencing | Caf1 Rm62 ph-p |
| 1451 | GO:0001871 | F | 3, | 5 | 3.949 (x 1.266) | 80 (0.062) | 0.545 | pattern binding | BcDNA:GH02976 CG11142 CG17052 CG32499 CG9307 |
| 1452 | GO:0030720 | P | 9, | 1 | 0.444 (x 2.251) | 9 (0.111) | 0.545 | oocyte localization during oogenesis | Dl |
| 1453 | GO:0048332 | P | 5, | 2 | 1.283 (x 1.558) | 26 (0.077) | 0.546 | mesoderm morphogenesis | pbl sgl |
| 1454 | GO:0050790 | P | 3, | 3 | 2.172 (x 1.381) | 44 (0.068) | 0.546 | regulation of catalytic activity | Abi dap mbt |
| 1455 | GO:0048489 | P | 6, 7, | 5 | 3.949 (x 1.266) | 80 (0.062) | 0.546 | synaptic vesicle transport | Amph Arf79F Snap btsz mth |
| 1456 | GO:0005548 | F | 4, | 1 | 0.444 (x 2.251) | 9 (0.111) | 0.546 | phospholipid transporter activity | CG1893 |
| 1457 | GO:0001707 | P | 6, 7, | 2 | 1.283 (x 1.558) | 26 (0.077) | 0.546 | mesoderm formation | pbl sgl |
| 1458 | GO:0009582 | P | 4, 5, | 3 | 2.172 (x 1.381) | 44 (0.068) | 0.546 | detection of abiotic stimulus | Galpha49B ogre pain |
| 1459 | GO:0005656 | C | 3, 6, 7, 8, 9, 10, 11, 12, 13, | 1 | 0.444 (x 2.251) | 9 (0.111) | 0.546 | pre-replicative complex | Mcm7 |
| 1460 | GO:0048599 | P | 5, 6, 8, | 5 | 3.949 (x 1.266) | 80 (0.062) | 0.546 | oocyte development | Dl Hrb27C TER94 sqd wbl |
| 1461 | GO:0008344 | P | 5, | 2 | 1.283 (x 1.558) | 26 (0.077) | 0.546 | adult locomotory behavior | Sema-2a Sh |
| 1462 | GO:0016027 | C | 3, 6, 7, 8, | 1 | 0.444 (x 2.251) | 9 (0.111) | 0.547 | inaD signaling complex | Galpha49B |
| 1463 | GO:0051129 | P | 6, | 1 | 0.444 (x 2.251) | 9 (0.111) | 0.547 | negative regulation of cell organization and biogenesis | fwd |
| 1464 | GO:0042676 | P | 5, | 1 | 0.444 (x 2.251) | 9 (0.111) | 0.547 | cone cell fate commitment | Dl |
| 1465 | GO:0045045 | P | 5, 6, | 12 | 10.613 (x 1.131) | 215 (0.056) | 0.547 | secretory pathway | Amph Arf79F CG14214 CG1924 CG9906 Crc Rab8 S Snap TER94 mth wbl |
| 1466 | GO:0006402 | P | 8, | 1 | 0.444 (x 2.251) | 9 (0.111) | 0.548 | mRNA catabolism | sqd |
| 1467 | GO:0019212 | F | 4, 5, | 1 | 0.444 (x 2.251) | 9 (0.111) | 0.548 | phosphatase inhibitor activity | Set |
| 1468 | GO:0045478 | P | 6, | 1 | 0.444 (x 2.251) | 9 (0.111) | 0.548 | fusome organization and biogenesis | asp |
| 1469 | GO:0015672 | P | 7, 8, | 10 | 8.638 (x 1.158) | 175 (0.057) | 0.549 | monovalent inorganic cation transport | CG12048 Ir Nrv1 Sh Ucp4B Vha100-2 blot jumu para rpk |
| 1470 | GO:0015012 | P | 7, 8, 9, | 1 | 0.444 (x 2.251) | 9 (0.111) | 0.549 | heparan sulfate proteoglycan biosynthesis | sgl |
| 1471 | GO:0004559 | F | 7, | 1 | 0.444 (x 2.251) | 9 (0.111) | 0.549 | alpha-mannosidase activity | alpha-Man-IIb |
| 1472 | GO:0007466 | P | 6, 9, 10, | 1 | 0.444 (x 2.251) | 9 (0.111) | 0.55 | cone cell fate commitment (sensu Endopterygota) | Dl |
| 1473 | GO:0006519 | P | 5, | 16 | 14.463 (x 1.106) | 293 (0.055) | 0.55 | amino acid and derivative metabolism | CG15088 CG1607 CG2118 CG40160 CG6287 CG7860 CtBP EG:171D11.1 ESTS:39C10S Eip55E Eip71CD blot knk mdy mnd olf413 |
| 1474 | GO:0008439 | F | 4, 7, | 1 | 0.444 (x 2.251) | 9 (0.111) | 0.55 | monophenol monooxygenase activator activity | BEST:GH02921 |
| 1475 | GO:0050906 | P | 4, 5, 6, | 1 | 0.444 (x 2.251) | 9 (0.111) | 0.55 | detection of stimulus during sensory perception | pain |
| 1476 | GO:0018342 | P | 9, 10, | 1 | 0.444 (x 2.251) | 9 (0.111) | 0.551 | protein prenylation | CG33171 |
| 1477 | GO:0045005 | P | 9, | 1 | 0.444 (x 2.251) | 9 (0.111) | 0.551 | maintenance of fidelity during DNA-dependent DNA replication | Thd1 |
| 1478 | GO:0007282 | P | 6, | 1 | 0.444 (x 2.251) | 9 (0.111) | 0.551 | cystoblast division | asp |
| 1479 | GO:0007460 | P | 8, 9, 10, 11, | 1 | 0.444 (x 2.251) | 9 (0.111) | 0.552 | R8 cell fate commitment | Dl |
| 1480 | GO:0006221 | P | 7, 8, | 1 | 0.444 (x 2.251) | 9 (0.111) | 0.552 | pyrimidine nucleotide biosynthesis | Ts |
| 1481 | GO:0040001 | P | 6, 7, 8, 10, | 1 | 0.444 (x 2.251) | 9 (0.111) | 0.553 | establishment of mitotic spindle localization | Eb1 |
| 1482 | GO:0019730 | P | 6, 7, | 4 | 3.110 (x 1.286) | 63 (0.063) | 0.554 | antimicrobial humoral response | 18w Mpk2 Tl pll |
| 1483 | GO:0019752 | P | 6, | 21 | 19.349 (x 1.085) | 392 (0.054) | 0.557 | carboxylic acid metabolism | BcDNA:GH02901 BcDNA:GH12558 CG15088 CG1607 CG2118 CG40160 CG4586 CG6287 CG7860 CtBP EG:171D11.1 ESTS:39C10S Eip55E Eip71CD Idh Pepck Pgi Tpi blot mdy mnd |
| 1484 | GO:0006082 | P | 5, | 21 | 19.349 (x 1.085) | 392 (0.054) | 0.558 | organic acid metabolism | BcDNA:GH02901 BcDNA:GH12558 CG15088 CG1607 CG2118 CG40160 CG4586 CG6287 CG7860 CtBP EG:171D11.1 ESTS:39C10S Eip55E Eip71CD Idh Pepck Pgi Tpi blot mdy mnd |
| 1485 | GO:0015290 | F | 4, | 12 | 10.711 (x 1.120) | 217 (0.055) | 0.562 | electrochemical potential-driven transporter activity | BEST:LD04971 CG11739 CG15088 CG1607 CG1907 CG3424 CG6782 LanB2 blot l(2)01810 mnd sut1 |
| 1486 | GO:0006960 | P | 7, 8, | 3 | 2.221 (x 1.351) | 45 (0.067) | 0.562 | antimicrobial humoral response (sensu Protostomia) | 18w Tl pll |
| 1487 | GO:0015291 | F | 5, | 12 | 10.711 (x 1.120) | 217 (0.055) | 0.562 | porter activity | BEST:LD04971 CG11739 CG15088 CG1607 CG1907 CG3424 CG6782 LanB2 blot l(2)01810 mnd sut1 |
| 1488 | GO:0009581 | P | 4, 5, | 3 | 2.221 (x 1.351) | 45 (0.067) | 0.563 | detection of external stimulus | Galpha49B ogre pain |
| 1489 | GO:0048112 | P | 7, 9, 10, 12, | 3 | 2.221 (x 1.351) | 45 (0.067) | 0.563 | oocyte anterior/posterior axis determination (sensu Insecta) | Hrb27C TER94 sqd |
| 1490 | GO:0031507 | P | 11, | 2 | 1.333 (x 1.501) | 27 (0.074) | 0.564 | heterochromatin formation | Caf1 ph-p |
| 1491 | GO:0017076 | F | 4, | 44 | 41.907 (x 1.050) | 849 (0.052) | 0.564 | purine nucleotide binding | Arf79F BEST:CK02656 CG14217 CG2108 CG2118 CG31121 CG31453 CG40410 CaMKI Cad96Ca Cdk4 CkIIalpha Eph Galpha49B Hex-A InR Klp10A Mcm7 Mpk2 Nek2 Pepck Rab8 Rm62 TER94 Top2 ald alphaTub84B betaTub56D blot cdc2 ck d for glu jumu kis mbt mdy nmo otk pll polo smi35A tkv |
| 1492 | GO:0006342 | P | 5, 7, 10, 12, | 2 | 1.333 (x 1.501) | 27 (0.074) | 0.564 | chromatin silencing | Caf1 ph-p |
| 1493 | GO:0007298 | P | 7, 8, 10, | 2 | 1.333 (x 1.501) | 27 (0.074) | 0.564 | border follicle cell migration (sensu Insecta) | th usp |
| 1494 | GO:0002168 | P | 5, | 2 | 1.333 (x 1.501) | 27 (0.074) | 0.565 | larval development (sensu Insecta) | CrebA Sb |
| 1495 | GO:0045814 | P | 4, | 2 | 1.333 (x 1.501) | 27 (0.074) | 0.565 | negative regulation of gene expression, epigenetic | Caf1 ph-p |
| 1496 | GO:0005507 | F | 6, | 2 | 1.333 (x 1.501) | 27 (0.074) | 0.565 | copper ion binding | Vha100-2 olf413 |
| 1497 | GO:0042692 | P | 4, | 2 | 1.333 (x 1.501) | 27 (0.074) | 0.566 | muscle cell differentiation | hbs rost |
| 1498 | GO:0006023 | P | 7, 8, | 1 | 0.494 (x 2.026) | 10 (0.100) | 0.569 | aminoglycan biosynthesis | sgl |
| 1499 | GO:0030725 | P | 5, | 1 | 0.494 (x 2.026) | 10 (0.100) | 0.57 | ring canal formation | fwd |
| 1500 | GO:0030534 | P | 4, | 3 | 2.271 (x 1.321) | 46 (0.065) | 0.57 | adult behavior | Sema-2a Sh cyc |
| 1501 | GO:0042059 | P | 6, 7, 10, | 1 | 0.494 (x 2.026) | 10 (0.100) | 0.57 | negative regulation of epidermal growth factor receptor signaling pathway | ed |
| 1502 | GO:0046621 | P | 5, | 1 | 0.494 (x 2.026) | 10 (0.100) | 0.571 | negative regulation of organ size | Pten |
| 1503 | GO:0004178 | F | 7, | 1 | 0.494 (x 2.026) | 10 (0.100) | 0.571 | leucyl aminopeptidase activity | BcDNA:LD41548 |
| 1504 | GO:0043063 | P | 4, | 1 | 0.494 (x 2.026) | 10 (0.100) | 0.571 | intercellular bridge organization and biogenesis | fwd |
| 1505 | GO:0008016 | P | 5, | 1 | 0.494 (x 2.026) | 10 (0.100) | 0.572 | regulation of heart contraction | for |
| 1506 | GO:0006024 | P | 8, 9, | 1 | 0.494 (x 2.026) | 10 (0.100) | 0.572 | glycosaminoglycan biosynthesis | sgl |
| 1507 | GO:0045433 | P | 10, 11, | 1 | 0.494 (x 2.026) | 10 (0.100) | 0.572 | male courtship behavior (sensu Insecta), song production | para |
| 1508 | GO:0016775 | F | 5, | 1 | 0.494 (x 2.026) | 10 (0.100) | 0.573 | phosphotransferase activity, nitrogenous group as acceptor | Argk |
| 1509 | GO:0004024 | F | 7, | 1 | 0.494 (x 2.026) | 10 (0.100) | 0.573 | alcohol dehydrogenase activity, zinc-dependent | LanB2 |
| 1510 | GO:0009190 | P | 7, 8, | 1 | 0.494 (x 2.026) | 10 (0.100) | 0.574 | cyclic nucleotide biosynthesis | CG32158 |
| 1511 | GO:0008593 | P | 5, 6, 7, | 1 | 0.494 (x 2.026) | 10 (0.100) | 0.574 | regulation of Notch signaling pathway | numb |
| 1512 | GO:0016545 | P | 9, 10, | 1 | 0.494 (x 2.026) | 10 (0.100) | 0.574 | male courtship behavior (sensu Insecta), wing vibration | para |
| 1513 | GO:0009612 | P | 4, | 1 | 0.494 (x 2.026) | 10 (0.100) | 0.575 | response to mechanical stimulus | pain |
| 1514 | GO:0051017 | P | 10, | 1 | 0.494 (x 2.026) | 10 (0.100) | 0.575 | actin filament bundle formation | Sb |
| 1515 | GO:0007223 | P | 7, | 1 | 0.494 (x 2.026) | 10 (0.100) | 0.576 | frizzled-2 signaling pathway | Wnt2 |
| 1516 | GO:0006022 | P | 6, 7, | 1 | 0.494 (x 2.026) | 10 (0.100) | 0.576 | aminoglycan metabolism | sgl |
| 1517 | GO:0030203 | P | 7, 8, | 1 | 0.494 (x 2.026) | 10 (0.100) | 0.576 | glycosaminoglycan metabolism | sgl |
| 1518 | GO:0008015 | P | 4, | 1 | 0.494 (x 2.026) | 10 (0.100) | 0.577 | circulation | for |
| 1519 | GO:0019783 | F | 6, | 2 | 1.382 (x 1.447) | 28 (0.071) | 0.579 | small conjugating protein-specific protease activity | CG5794 Uch |
| 1520 | GO:0007300 | P | 8, | 2 | 1.382 (x 1.447) | 28 (0.071) | 0.58 | nurse cell to oocyte transport (sensu Insecta) | Dp mdy |
| 1521 | GO:0004843 | F | 7, | 2 | 1.382 (x 1.447) | 28 (0.071) | 0.58 | ubiquitin-specific protease activity | CG5794 Uch |
| 1522 | GO:0001704 | P | 5, 6, | 2 | 1.382 (x 1.447) | 28 (0.071) | 0.58 | formation of primary germ layer | pbl sgl |
| 1523 | GO:0031098 | P | 6, | 3 | 2.320 (x 1.293) | 47 (0.064) | 0.584 | stress-activated protein kinase signaling pathway | CG14217 Mpk2 mbt |
| 1524 | GO:0044267 | P | 6, | 105 | 102.621 (x 1.023) | 2079 (0.051) | 0.584 | cellular protein metabolism | Ance Arf79F BEST:GH02921 BG:DS00004.11 BcDNA:LD41548 CG10657 CG11033 CG11824 CG14217 CG1632 CG1924 CG2200 CG2852 CG31738 CG32632 CG33171 CG3328 CG40160 CG40410 CG4502 CG4914 CG5390 CG5794 CG6680 CG7860 CG8963 CG9135 CG9906 CaMKI Cad96Ca Caf1 Cdk4 CkIIalpha CkIIbeta Cks Crc D19A DnaJ-1 ERp60 Eip71CD Eph Fkbp13 GalNAc-T1 Galpha49B Hsp23 Hsp26 Hsp27 Hsp67Ba Ice InR Incenp Lsp2 Mgstl Mpk2 Nek2 Nep2 PFE Pdi Pi3K21B Ppt1 Prosalpha6 Pten Ptp99A RpS12 Rpn9 Sb Spn1 Spn5 TER94 Uch Ugt ald alphaTub84B betaTub56D blot cdc2 dap eIF-4E east for fra fwd fzy l(1)G0320 l(2)01424 mbt mdy nmo ome otk pAbp pgant5 pll polo rasp sgl shf shu smi35A sqd th tkv tok tws wbl |
| 1525 | GO:0044421 | C | 2, 3, | 5 | 4.196 (x 1.192) | 85 (0.059) | 0.584 | extracellular region part | CG33171 LanB2 Lsp2 kal-1 shf |
| 1526 | GO:0005529 | F | 4, | 3 | 2.320 (x 1.293) | 47 (0.064) | 0.584 | sugar binding | CG3921 CG9134 Gfat1 |
| 1527 | GO:0008610 | P | 5, 6, 7, | 5 | 4.196 (x 1.192) | 85 (0.059) | 0.585 | lipid biosynthesis | CG2118 CG31915 CG33116 mdy woc |
| 1528 | GO:0007254 | P | 7, 8, | 3 | 2.320 (x 1.293) | 47 (0.064) | 0.585 | JNK cascade | CG14217 Mpk2 mbt |
| 1529 | GO:0004713 | F | 7, | 5 | 4.196 (x 1.192) | 85 (0.059) | 0.585 | protein-tyrosine kinase activity | Cad96Ca Eph InR otk shf |
| 1530 | GO:0030030 | P | 5, 6, | 3 | 2.320 (x 1.293) | 47 (0.064) | 0.585 | cell projection organization and biogenesis | Abi in pk |
| 1531 | GO:0015268 | F | 4, | 10 | 8.984 (x 1.113) | 182 (0.055) | 0.585 | alpha-type channel activity | CG12048 CG14076 Ir Sh inx2 inx3 ogre pain para rpk |
| 1532 | GO:0015267 | F | 3, | 10 | 8.984 (x 1.113) | 182 (0.055) | 0.586 | channel or pore class transporter activity | CG12048 CG14076 Ir Sh inx2 inx3 ogre pain para rpk |
| 1533 | GO:0006520 | P | 6, 7, | 14 | 12.883 (x 1.087) | 261 (0.054) | 0.586 | amino acid metabolism | CG15088 CG1607 CG2118 CG40160 CG6287 CG7860 CtBP EG:171D11.1 ESTS:39C10S Eip55E Eip71CD blot mdy mnd |
| 1534 | GO:0019538 | P | 5, | 110 | 107.754 (x 1.021) | 2183 (0.050) | 0.592 | protein metabolism | Ance Arf79F B52 BEST:GH02921 BG:DS00004.11 BcDNA:LD41548 CG10657 CG11033 CG11824 CG14217 CG1632 CG1924 CG2200 CG2852 CG31738 CG32632 CG33171 CG3328 CG40160 CG40410 CG4502 CG4914 CG5390 CG5794 CG6199 CG6680 CG7860 CG8963 CG9135 CG9906 CaMKI Cad96Ca Caf1 Cdk4 CkIIalpha CkIIbeta Cks Crc D19A DnaJ-1 ERp60 Eb1 Eip71CD Eph Fkbp13 GalNAc-T1 Galpha49B Hsp23 Hsp26 Hsp27 Hsp67Ba Ice InR Incenp Lsp2 Mgstl Mpk2 Nek2 Nep2 PFE Pdi Pi3K21B Ppt1 Prosalpha6 Pten Ptp99A RpS12 Rpn9 Sb Set Spn1 Spn5 TER94 Uch Ugt ald alphaTub84B betaTub56D blot cdc2 dap eIF-4E east for fra fwd fzy l(1)G0320 l(2)01424 mbt mdy nmo ome otk pAbp pbl pgant5 pll polo rasp sgl shf shu smi35A sqd th tkv tok tws wbl |
| 1535 | GO:0019904 | F | 4, | 1 | 0.543 (x 1.842) | 11 (0.091) | 0.593 | protein domain specific binding | pcs |
| 1536 | GO:0048065 | P | 8, 9, | 1 | 0.543 (x 1.842) | 11 (0.091) | 0.593 | male courtship behavior (sensu Insecta), wing extension | para |
| 1537 | GO:0005786 | C | 5, 6, 7, 8, 9, | 1 | 0.543 (x 1.842) | 11 (0.091) | 0.594 | signal recognition particle (sensu Eukaryota) | Tapdelta |
| 1538 | GO:0044451 | C | 5, 6, 7, 8, 9, 10, 11, 12, | 11 | 10.020 (x 1.098) | 203 (0.054) | 0.594 | nucleoplasm part | B52 Caf1 CycT Dp Dref Mcm7 Rbf2 Top2 sqd toe usp |
| 1539 | GO:0042674 | P | 5, 8, 9, | 1 | 0.543 (x 1.842) | 11 (0.091) | 0.594 | cone cell differentiation (sensu Endopterygota) | Dl |
| 1540 | GO:0042060 | P | 5, | 1 | 0.543 (x 1.842) | 11 (0.091) | 0.595 | wound healing | ninA |
| 1541 | GO:0006446 | P | 8, 9, 10, | 1 | 0.543 (x 1.842) | 11 (0.091) | 0.595 | regulation of translational initiation | l(2)01424 |
| 1542 | GO:0008345 | P | 5, | 1 | 0.543 (x 1.842) | 11 (0.091) | 0.595 | larval locomotory behavior | for |
| 1543 | GO:0016246 | P | 6, 9, | 1 | 0.543 (x 1.842) | 11 (0.091) | 0.596 | RNA interference | Rm62 |
| 1544 | GO:0000339 | F | 5, | 1 | 0.543 (x 1.842) | 11 (0.091) | 0.596 | RNA cap binding | eIF-4E |
| 1545 | GO:0016634 | F | 5, | 1 | 0.543 (x 1.842) | 11 (0.091) | 0.596 | oxidoreductase activity, acting on the CH-CH group of donors, oxygen as acceptor | CG4586 |
| 1546 | GO:0003774 | F | 2, | 5 | 4.294 (x 1.164) | 87 (0.057) | 0.597 | motor activity | Act42A Klp10A ck d lola |
| 1547 | GO:0030509 | P | 8, | 1 | 0.543 (x 1.842) | 11 (0.091) | 0.597 | BMP signaling pathway | tkv |
| 1548 | GO:0007157 | P | 5, | 1 | 0.543 (x 1.842) | 11 (0.091) | 0.597 | heterophilic cell adhesion | glec |
| 1549 | GO:0007379 | P | 4, 5, | 2 | 1.431 (x 1.397) | 29 (0.069) | 0.597 | segment specification | kis spen |
| 1550 | GO:0042675 | P | 4, | 1 | 0.543 (x 1.842) | 11 (0.091) | 0.598 | cone cell differentiation | Dl |
| 1551 | GO:0005795 | C | 5, 6, 7, 8, 9, 10, | 2 | 1.431 (x 1.397) | 29 (0.069) | 0.598 | Golgi stack | GalNAc-T1 pgant5 |
| 1552 | GO:0030718 | P | 4, | 1 | 0.543 (x 1.842) | 11 (0.091) | 0.598 | germ-line stem cell maintenance | tkv |
| 1553 | GO:0007416 | P | 5, 6, | 2 | 1.431 (x 1.397) | 29 (0.069) | 0.598 | synaptogenesis | Tl tkv |
| 1554 | GO:0007390 | P | 6, | 1 | 0.543 (x 1.842) | 11 (0.091) | 0.598 | germ-band shortening | InR |
| 1555 | GO:0007349 | P | 3, 4, | 2 | 1.431 (x 1.397) | 29 (0.069) | 0.599 | cellularization | CG40410 Sry-alpha |
| 1556 | GO:0016866 | F | 4, | 1 | 0.543 (x 1.842) | 11 (0.091) | 0.599 | intramolecular transferase activity | Tpi |
| 1557 | GO:0030176 | C | 6, 7, 8, 9, 10, 11, 12, | 1 | 0.543 (x 1.842) | 11 (0.091) | 0.599 | integral to endoplasmic reticulum membrane | CG14214 |
| 1558 | GO:0007413 | P | 9, 10, 12, | 1 | 0.543 (x 1.842) | 11 (0.091) | 0.6 | axonal fasciculation | Fas3 |
| 1559 | GO:0009416 | P | 5, | 3 | 2.369 (x 1.266) | 48 (0.062) | 0.6 | response to light stimulus | Galpha49B Sema-2a ogre |
| 1560 | GO:0016667 | F | 4, | 1 | 0.543 (x 1.842) | 11 (0.091) | 0.6 | oxidoreductase activity, acting on sulfur group of donors | Eip71CD |
| 1561 | GO:0048500 | C | 4, 5, 6, 7, 8, | 1 | 0.543 (x 1.842) | 11 (0.091) | 0.6 | signal recognition particle | Tapdelta |
| 1562 | GO:0031227 | C | 5, 6, 7, 8, 9, 10, 11, | 1 | 0.543 (x 1.842) | 11 (0.091) | 0.601 | intrinsic to endoplasmic reticulum membrane | CG14214 |
| 1563 | GO:0035111 | P | 6, 7, 8, | 1 | 0.543 (x 1.842) | 11 (0.091) | 0.601 | leg joint morphogenesis | Dl |
| 1564 | GO:0016511 | F | 7, | 1 | 0.543 (x 1.842) | 11 (0.091) | 0.601 | endothelin-converting enzyme activity | Nep2 |
| 1565 | GO:0016281 | C | 3, 5, 6, 7, 8, | 1 | 0.543 (x 1.842) | 11 (0.091) | 0.602 | eukaryotic translation initiation factor 4F complex | eIF-4E |
| 1566 | GO:0019861 | C | 4, 5, | 1 | 0.543 (x 1.842) | 11 (0.091) | 0.602 | flagellum | lola |
| 1567 | GO:0005876 | C | 6, 7, 8, 9, 10, 11, | 1 | 0.543 (x 1.842) | 11 (0.091) | 0.603 | spindle microtubule | Klp10A |
| 1568 | GO:0000381 | P | 10, 11, 13, | 3 | 2.419 (x 1.240) | 49 (0.061) | 0.606 | regulation of alternative nuclear mRNA splicing, via spliceosome | B52 Rm62 sqd |
| 1569 | GO:0008298 | P | 5, | 3 | 2.419 (x 1.240) | 49 (0.061) | 0.606 | intracellular mRNA localization | Hrb27C TER94 sqd |
| 1570 | GO:0006576 | P | 6, 7, | 2 | 1.481 (x 1.351) | 30 (0.067) | 0.606 | biogenic amine metabolism | knk olf413 |
| 1571 | GO:0005912 | C | 6, 7, 8, | 3 | 2.419 (x 1.240) | 49 (0.061) | 0.606 | adherens junction | Fas3 mbt p120ctn |
| 1572 | GO:0031589 | P | 4, | 2 | 1.481 (x 1.351) | 30 (0.067) | 0.607 | cell-substrate adhesion | CG33171 LanB2 |
| 1573 | GO:0051606 | P | 3, 4, | 3 | 2.419 (x 1.240) | 49 (0.061) | 0.607 | detection of stimulus | Galpha49B ogre pain |
| 1574 | GO:0007160 | P | 5, | 2 | 1.481 (x 1.351) | 30 (0.067) | 0.607 | cell-matrix adhesion | CG33171 LanB2 |
| 1575 | GO:0007519 | P | 5, | 3 | 2.419 (x 1.240) | 49 (0.061) | 0.607 | striated muscle development | hbs rost tkv |
| 1576 | GO:0048646 | P | 4, | 2 | 1.481 (x 1.351) | 30 (0.067) | 0.607 | anatomical structure formation | pbl sgl |
| 1577 | GO:0000380 | P | 10, 12, | 3 | 2.419 (x 1.240) | 49 (0.061) | 0.608 | alternative nuclear mRNA splicing, via spliceosome | B52 Rm62 sqd |
| 1578 | GO:0004725 | F | 8, | 2 | 1.481 (x 1.351) | 30 (0.067) | 0.608 | protein tyrosine phosphatase activity | Pten Ptp99A |
| 1579 | GO:0006886 | P | 6, 7, 8, | 26 | 24.976 (x 1.041) | 506 (0.051) | 0.613 | intracellular protein transport | Amph Arf79F CG14214 CG1924 CG2108 CG2852 CG32137 CG33113 CG9906 Crc Fs(2)Ket Klp10A Rab8 S Snap TER94 alphaTub84B arr betaTub56D ck d l(1)G0320 loj pll prominin-like wbl |
| 1580 | GO:0042742 | P | 5, 6, | 4 | 3.406 (x 1.174) | 69 (0.058) | 0.613 | defense response to bacterium | 18w CG10359 CG7668 Tl |
| 1581 | GO:0042446 | P | 6, | 1 | 0.592 (x 1.688) | 12 (0.083) | 0.621 | hormone biosynthesis | woc |
| 1582 | GO:0005496 | F | 3, | 1 | 0.592 (x 1.688) | 12 (0.083) | 0.621 | steroid binding | CG11151 |
| 1583 | GO:0018991 | P | 4, 5, | 1 | 0.592 (x 1.688) | 12 (0.083) | 0.622 | oviposition | loj |
| 1584 | GO:0051322 | P | 6, | 1 | 0.592 (x 1.688) | 12 (0.083) | 0.622 | anaphase | tws |
| 1585 | GO:0048754 | P | 5, | 2 | 1.530 (x 1.307) | 31 (0.065) | 0.622 | branching morphogenesis of a tube | grh sgl |
| 1586 | GO:0008589 | P | 5, 6, 7, | 1 | 0.592 (x 1.688) | 12 (0.083) | 0.622 | regulation of smoothened signaling pathway | CG9211 |
| 1587 | GO:0007127 | P | 8, | 2 | 1.530 (x 1.307) | 31 (0.065) | 0.622 | meiosis I | cdc2 east |
| 1588 | GO:0007426 | P | 5, | 1 | 0.592 (x 1.688) | 12 (0.083) | 0.623 | tracheal outgrowth (sensu Insecta) | grh |
| 1589 | GO:0019236 | P | 5, | 1 | 0.592 (x 1.688) | 12 (0.083) | 0.623 | response to pheromone | Obp99a |
| 1590 | GO:0008543 | P | 8, | 1 | 0.592 (x 1.688) | 12 (0.083) | 0.624 | fibroblast growth factor receptor signaling pathway | sgl |
| 1591 | GO:0007440 | P | 7, 8, | 1 | 0.592 (x 1.688) | 12 (0.083) | 0.624 | foregut morphogenesis | inx2 |
| 1592 | GO:0006694 | P | 6, 7, 8, | 1 | 0.592 (x 1.688) | 12 (0.083) | 0.624 | steroid biosynthesis | woc |
| 1593 | GO:0009636 | P | 5, | 7 | 6.368 (x 1.099) | 129 (0.054) | 0.624 | response to toxin | Aldh-III BcDNA:GH04753 CG17323 CG6776 GstS1 Mgstl para |
| 1594 | GO:0016082 | P | 8, 9, 10, | 1 | 0.592 (x 1.688) | 12 (0.083) | 0.625 | synaptic vesicle priming | Snap |
| 1595 | GO:0016758 | F | 5, | 7 | 6.368 (x 1.099) | 129 (0.054) | 0.625 | transferase activity, transferring hexosyl groups | CG17323 CG33138 CG6904 GalNAc-T1 GlyP Ugt pgant5 |
| 1596 | GO:0005794 | C | 5, 6, 7, 8, | 5 | 4.442 (x 1.126) | 90 (0.056) | 0.625 | Golgi apparatus | GalNAc-T1 S Snap alpha-Man-IIb pgant5 |
| 1597 | GO:0016638 | F | 4, | 1 | 0.592 (x 1.688) | 12 (0.083) | 0.625 | oxidoreductase activity, acting on the CH-NH2 group of donors | CG31472 |
| 1598 | GO:0008318 | F | 6, | 1 | 0.592 (x 1.688) | 12 (0.083) | 0.626 | protein prenyltransferase activity | CG33171 |
| 1599 | GO:0009165 | P | 6, 7, | 6 | 5.430 (x 1.105) | 110 (0.055) | 0.627 | nucleotide biosynthesis | CG32158 CG3590 CG6767 Ts blot jumu |
| 1600 | GO:0005244 | F | 5, 6, | 3 | 2.517 (x 1.192) | 51 (0.059) | 0.631 | voltage-gated ion channel activity | Ir Sh para |
| 1601 | GO:0031497 | P | 10, | 3 | 2.517 (x 1.192) | 51 (0.059) | 0.631 | chromatin assembly | Caf1 Set ph-p |
| 1602 | GO:0005083 | F | 4, | 5 | 4.492 (x 1.113) | 91 (0.055) | 0.637 | small GTPase regulator activity | Abi RacGAP50C Rapgap1 Rhp pbl |
| 1603 | GO:0007283 | P | 6, | 6 | 5.479 (x 1.095) | 111 (0.054) | 0.637 | spermatogenesis | Ance Set cdc2 fwd heph th |
| 1604 | GO:0048232 | P | 5, | 6 | 5.479 (x 1.095) | 111 (0.054) | 0.637 | male gamete generation | Ance Set cdc2 fwd heph th |
| 1605 | GO:0030198 | P | 4, | 2 | 1.580 (x 1.266) | 32 (0.062) | 0.639 | extracellular matrix organization and biogenesis | Tl tkv |
| 1606 | GO:0045451 | P | 7, 11, 13, 14, 16, | 2 | 1.580 (x 1.266) | 32 (0.062) | 0.639 | pole plasm oskar mRNA localization | Hrb27C TER94 |
| 1607 | GO:0008105 | P | 5, | 2 | 1.580 (x 1.266) | 32 (0.062) | 0.64 | asymmetric protein localization | jumu pon |
| 1608 | GO:0019208 | F | 3, | 2 | 1.580 (x 1.266) | 32 (0.062) | 0.64 | phosphatase regulator activity | Set tws |
| 1609 | GO:0050808 | P | 5, | 2 | 1.580 (x 1.266) | 32 (0.062) | 0.641 | synapse organization and biogenesis | Tl tkv |
| 1610 | GO:0031047 | P | 7, | 1 | 0.642 (x 1.558) | 13 (0.077) | 0.641 | RNA-mediated gene silencing | Rm62 |
| 1611 | GO:0046907 | P | 5, 6, 7, | 31 | 30.406 (x 1.020) | 616 (0.050) | 0.641 | intracellular transport | Amph Arf79F CG14214 CG1907 CG1924 CG2108 CG2852 CG32137 CG33113 CG9057 CG9906 Crc Fs(2)Ket Klp10A Rab8 S Snap TER94 Ucp4B alphaTub84B arr betaTub56D ck d l(1)G0320 loj pll prominin-like sqd vg wbl |
| 1612 | GO:0019888 | F | 4, | 2 | 1.580 (x 1.266) | 32 (0.062) | 0.641 | protein phosphatase regulator activity | Set tws |
| 1613 | GO:0005416 | F | 6, 7, 8, | 1 | 0.642 (x 1.558) | 13 (0.077) | 0.641 | cation:amino acid symporter activity | CG15088 |
| 1614 | GO:0007602 | P | 6, 7, | 2 | 1.580 (x 1.266) | 32 (0.062) | 0.641 | phototransduction | Galpha49B ogre |
| 1615 | GO:0008347 | P | 6, 7, | 1 | 0.642 (x 1.558) | 13 (0.077) | 0.641 | glial cell migration | spen |
| 1616 | GO:0019900 | F | 5, | 1 | 0.642 (x 1.558) | 13 (0.077) | 0.642 | kinase binding | Pi3K21B |
| 1617 | GO:0016279 | F | 7, 8, | 1 | 0.642 (x 1.558) | 13 (0.077) | 0.642 | protein-lysine N-methyltransferase activity | Caf1 |
| 1618 | GO:0003711 | F | 3, | 1 | 0.642 (x 1.558) | 13 (0.077) | 0.643 | transcriptional elongation regulator activity | CycT |
| 1619 | GO:0007338 | P | 5, | 1 | 0.642 (x 1.558) | 13 (0.077) | 0.643 | fertilization (sensu Metazoa) | polo |
| 1620 | GO:0008582 | P | 4, 5, 7, 8, 9, 10, | 1 | 0.642 (x 1.558) | 13 (0.077) | 0.643 | regulation of synaptic growth at neuromuscular junction | tkv |
| 1621 | GO:0008533 | F | 7, | 1 | 0.642 (x 1.558) | 13 (0.077) | 0.644 | astacin activity | tok |
| 1622 | GO:0035290 | P | 5, 6, | 1 | 0.642 (x 1.558) | 13 (0.077) | 0.644 | trunk segmentation | en |
| 1623 | GO:0006473 | P | 9, | 1 | 0.642 (x 1.558) | 13 (0.077) | 0.645 | protein amino acid acetylation | Caf1 |
| 1624 | GO:0046943 | F | 4, | 4 | 3.554 (x 1.126) | 72 (0.056) | 0.645 | carboxylic acid transporter activity | CG15088 CG1607 CG3424 mnd |
| 1625 | GO:0008194 | F | 5, | 5 | 4.541 (x 1.101) | 92 (0.054) | 0.645 | UDP-glycosyltransferase activity | CG17323 CG6904 GalNAc-T1 Ugt pgant5 |
| 1626 | GO:0018024 | F | 8, 9, | 1 | 0.642 (x 1.558) | 13 (0.077) | 0.645 | histone-lysine N-methyltransferase activity | Caf1 |
| 1627 | GO:0008652 | P | 7, 8, | 4 | 3.554 (x 1.126) | 72 (0.056) | 0.645 | amino acid biosynthesis | CG40160 CG6287 CtBP ESTS:39C10S |
| 1628 | GO:0016571 | P | 9, 10, 12, | 1 | 0.642 (x 1.558) | 13 (0.077) | 0.645 | histone methylation | Caf1 |
| 1629 | GO:0045465 | P | 7, 8, 9, 10, | 1 | 0.642 (x 1.558) | 13 (0.077) | 0.646 | R8 cell differentiation | Dl |
| 1630 | GO:0016065 | P | 6, 7, | 3 | 2.616 (x 1.147) | 53 (0.057) | 0.646 | humoral defense mechanism (sensu Protostomia) | 18w Tl pll |
| 1631 | GO:0045169 | C | 5, 6, 7, 8, | 1 | 0.642 (x 1.558) | 13 (0.077) | 0.646 | fusome | TER94 |
| 1632 | GO:0015031 | P | 5, 6, | 26 | 25.519 (x 1.019) | 517 (0.050) | 0.646 | protein transport | Amph Arf79F CG14214 CG1924 CG2108 CG2852 CG32137 CG33113 CG9906 Crc Fs(2)Ket Klp10A Rab8 S Snap TER94 alphaTub84B arr betaTub56D ck d l(1)G0320 loj pll prominin-like wbl |
| 1633 | GO:0048024 | P | 9, 10, 12, | 3 | 2.616 (x 1.147) | 53 (0.057) | 0.646 | regulation of nuclear mRNA splicing, via spliceosome | B52 Rm62 sqd |
| 1634 | GO:0016278 | F | 7, | 1 | 0.642 (x 1.558) | 13 (0.077) | 0.647 | lysine N-methyltransferase activity | Caf1 |
| 1635 | GO:0050684 | P | 8, 9, | 3 | 2.616 (x 1.147) | 53 (0.057) | 0.647 | regulation of mRNA processing | B52 Rm62 sqd |
| 1636 | GO:0016441 | P | 4, 7, | 1 | 0.642 (x 1.558) | 13 (0.077) | 0.647 | posttranscriptional gene silencing | Rm62 |
| 1637 | GO:0048111 | P | 6, 8, 9, 11, | 3 | 2.616 (x 1.147) | 53 (0.057) | 0.647 | oocyte axis determination (sensu Insecta) | Hrb27C TER94 sqd |
| 1638 | GO:0007218 | P | 7, | 2 | 1.629 (x 1.228) | 33 (0.061) | 0.647 | neuropeptide signaling pathway | ETH stan |
| 1639 | GO:0035194 | P | 5, 8, | 1 | 0.642 (x 1.558) | 13 (0.077) | 0.647 | RNA-mediated posttranscriptional gene silencing | Rm62 |
| 1640 | GO:0019200 | F | 6, | 2 | 1.629 (x 1.228) | 33 (0.061) | 0.648 | carbohydrate kinase activity | CG6767 Hex-A |
| 1641 | GO:0007539 | P | 5, | 1 | 0.642 (x 1.558) | 13 (0.077) | 0.648 | primary sex determination, soma | sc |
| 1642 | GO:0005249 | F | 6, 7, 8, | 2 | 1.629 (x 1.228) | 33 (0.061) | 0.648 | voltage-gated potassium channel activity | Ir Sh |
| 1643 | GO:0008103 | P | 6, 8, 9, 10, 11, | 1 | 0.642 (x 1.558) | 13 (0.077) | 0.648 | oocyte microtubule cytoskeleton polarization | TER94 |
| 1644 | GO:0000166 | F | 3, | 44 | 43.339 (x 1.015) | 878 (0.050) | 0.648 | nucleotide binding | Arf79F BEST:CK02656 CG14217 CG2108 CG2118 CG31121 CG31453 CG40410 CaMKI Cad96Ca Cdk4 CkIIalpha Eph Galpha49B Hex-A InR Klp10A Mcm7 Mpk2 Nek2 Pepck Rab8 Rm62 TER94 Top2 ald alphaTub84B betaTub56D blot cdc2 ck d for glu jumu kis mbt mdy nmo otk pll polo smi35A tkv |
| 1645 | GO:0031981 | C | 4, 5, 6, 7, 8, 9, 10, | 14 | 13.574 (x 1.031) | 275 (0.051) | 0.648 | nuclear lumen | B52 Caf1 CycT Dp Dref Hrb27C Mcm7 Rbf2 Top2 ap jumu sqd toe usp |
| 1646 | GO:0019221 | P | 6, | 2 | 1.629 (x 1.228) | 33 (0.061) | 0.648 | cytokine and chemokine mediated signaling pathway | 18w Tl |
| 1647 | GO:0004529 | F | 7, | 1 | 0.642 (x 1.558) | 13 (0.077) | 0.649 | exodeoxyribonuclease activity | BcDNA:GM10765 |
| 1648 | GO:0044448 | C | 5, 6, 7, 8, 9, | 2 | 1.629 (x 1.228) | 33 (0.061) | 0.649 | cell cortex part | numb pon |
| 1649 | GO:0005605 | C | 3, 4, 5, 6, | 1 | 0.642 (x 1.558) | 13 (0.077) | 0.649 | basal lamina | LanB2 |
| 1650 | GO:0044255 | P | 5, 6, | 17 | 16.536 (x 1.028) | 335 (0.051) | 0.649 | cellular lipid metabolism | BcDNA:GH02901 BcDNA:GH12558 CG17323 CG1998 CG2118 CG31915 CG33116 CG4586 CG6391 CG9342 Cyp310a1 Eip75B Pi3K21B Pten arr mdy woc |
| 1651 | GO:0016706 | F | 5, | 2 | 1.629 (x 1.228) | 33 (0.061) | 0.649 | oxidoreductase activity, acting on paired donors, with incorporation or reduction of molecular oxygen, 2-oxoglutarate as one donor, and incorporation of one atom each of oxygen into both donors | CG31915 CG6199 |
| 1652 | GO:0016021 | C | 5, 6, 7, | 47 | 46.596 (x 1.009) | 944 (0.050) | 0.656 | integral to membrane | 18w CG14214 CG14439 CG15088 CG17084 CG1924 CG32158 CG6965 CG9211 Cad74A Cad87A Cad96Ca Dl Fas3 Fs(2)Ket InR Nrv1 S Sema-1a Sh Tapdelta Tl Tsp26A Tsp39D Tsp66E Tsp96F blot ed emp glec inx2 inx3 jumu l(2)01810 mth ninA ogre pain para pbl prominin-like rasp rost shanti stan sut1 wgn |
| 1653 | GO:0009617 | P | 5, | 4 | 3.653 (x 1.095) | 74 (0.054) | 0.657 | response to bacterium | 18w CG10359 CG7668 Tl |
| 1654 | GO:0005342 | F | 3, | 4 | 3.653 (x 1.095) | 74 (0.054) | 0.658 | organic acid transporter activity | CG15088 CG1607 CG3424 mnd |
| 1655 | GO:0051261 | P | 7, | 1 | 0.691 (x 1.447) | 14 (0.071) | 0.658 | protein depolymerization | fwd |
| 1656 | GO:0016197 | P | 6, 7, 8, | 1 | 0.691 (x 1.447) | 14 (0.071) | 0.659 | endosome transport | Arf79F |
| 1657 | GO:0007475 | P | 8, 9, 10, | 1 | 0.691 (x 1.447) | 14 (0.071) | 0.659 | apposition of dorsal and ventral wing surfaces | Dl |
| 1658 | GO:0051252 | P | 7, | 3 | 2.665 (x 1.126) | 54 (0.056) | 0.659 | regulation of RNA metabolism | B52 Rm62 sqd |
| 1659 | GO:0006270 | P | 9, | 1 | 0.691 (x 1.447) | 14 (0.071) | 0.659 | DNA replication initiation | Mcm7 |
| 1660 | GO:0048110 | P | 7, 8, 10, | 3 | 2.665 (x 1.126) | 54 (0.056) | 0.659 | oocyte construction (sensu Insecta) | Hrb27C TER94 sqd |
| 1661 | GO:0017085 | P | 6, | 1 | 0.691 (x 1.447) | 14 (0.071) | 0.66 | response to insecticide | para |
| 1662 | GO:0000502 | C | 3, 4, 5, 6, | 3 | 2.665 (x 1.126) | 54 (0.056) | 0.66 | proteasome complex (sensu Eukaryota) | Prosalpha6 Rpn9 TER94 |
| 1663 | GO:0004556 | F | 7, | 1 | 0.691 (x 1.447) | 14 (0.071) | 0.66 | alpha-amylase activity | CG33138 |
| 1664 | GO:0005773 | C | 5, 6, 7, 8, | 3 | 2.665 (x 1.126) | 54 (0.056) | 0.66 | vacuole | Ppt1 Vha100-2 emp |
| 1665 | GO:0000245 | P | 7, 10, 12, | 1 | 0.691 (x 1.447) | 14 (0.071) | 0.66 | spliceosome assembly | B52 |
| 1666 | GO:0008202 | P | 6, 7, | 7 | 6.713 (x 1.043) | 136 (0.051) | 0.661 | steroid metabolism | CG17323 CG1998 Cyp310a1 Eip75B arr mdy woc |
| 1667 | GO:0030166 | P | 7, 8, | 1 | 0.691 (x 1.447) | 14 (0.071) | 0.661 | proteoglycan biosynthesis | sgl |
| 1668 | GO:0016160 | F | 6, | 1 | 0.691 (x 1.447) | 14 (0.071) | 0.661 | amylase activity | CG33138 |
| 1669 | GO:0005261 | F | 5, 6, | 6 | 5.676 (x 1.057) | 115 (0.052) | 0.661 | cation channel activity | CG12048 Ir Sh pain para rpk |
| 1670 | GO:0016203 | P | 5, | 1 | 0.691 (x 1.447) | 14 (0.071) | 0.662 | muscle attachment | betaTub56D |
| 1671 | GO:0004568 | F | 6, | 1 | 0.691 (x 1.447) | 14 (0.071) | 0.662 | chitinase activity | CG9307 |
| 1672 | GO:0048488 | P | 7, 8, | 2 | 1.678 (x 1.192) | 34 (0.059) | 0.662 | synaptic vesicle endocytosis | Amph Arf79F |
| 1673 | GO:0003688 | F | 6, | 1 | 0.691 (x 1.447) | 14 (0.071) | 0.662 | DNA replication origin binding | Mcm7 |
| 1674 | GO:0006521 | P | 6, 7, 8, | 1 | 0.691 (x 1.447) | 14 (0.071) | 0.663 | regulation of amino acid metabolism | CG40160 |
| 1675 | GO:0009566 | P | 4, | 1 | 0.691 (x 1.447) | 14 (0.071) | 0.663 | fertilization | polo |
| 1676 | GO:0051124 | P | 4, 6, 7, 8, 9, | 1 | 0.691 (x 1.447) | 14 (0.071) | 0.664 | synaptic growth at neuromuscular junction | tkv |
| 1677 | GO:0016360 | P | 6, | 1 | 0.691 (x 1.447) | 14 (0.071) | 0.664 | sensory organ precursor cell fate determination | numb |
| 1678 | GO:0046660 | P | 4, | 1 | 0.691 (x 1.447) | 14 (0.071) | 0.664 | female sex differentiation | en |
| 1679 | GO:0031224 | C | 4, 5, 6, | 47 | 46.744 (x 1.005) | 947 (0.050) | 0.665 | intrinsic to membrane | 18w CG14214 CG14439 CG15088 CG17084 CG1924 CG32158 CG6965 CG9211 Cad74A Cad87A Cad96Ca Dl Fas3 Fs(2)Ket InR Nrv1 S Sema-1a Sh Tapdelta Tl Tsp26A Tsp39D Tsp66E Tsp96F blot ed emp glec inx2 inx3 jumu l(2)01810 mth ninA ogre pain para pbl prominin-like rasp rost shanti stan sut1 wgn |
| 1680 | GO:0006144 | P | 7, | 3 | 2.715 (x 1.105) | 55 (0.055) | 0.666 | purine base metabolism | CG3590 CG6767 RnrS |
| 1681 | GO:0016563 | F | 3, | 3 | 2.715 (x 1.105) | 55 (0.055) | 0.666 | transcriptional activator activity | ci lola sc |
| 1682 | GO:0000267 | C | 3, 4, | 5 | 4.739 (x 1.055) | 96 (0.052) | 0.668 | cell fraction | 18w Cyp310a1 Mgstl Top2 glec |
| 1683 | GO:0006461 | P | 6, | 6 | 5.775 (x 1.039) | 117 (0.051) | 0.673 | protein complex assembly | B52 Caf1 Eb1 Set pbl polo |
| 1684 | GO:0009583 | P | 5, 6, | 2 | 1.728 (x 1.158) | 35 (0.057) | 0.673 | detection of light stimulus | Galpha49B ogre |
| 1685 | GO:0008235 | F | 6, | 4 | 3.751 (x 1.066) | 76 (0.053) | 0.673 | metalloexopeptidase activity | BcDNA:LD41548 Lsp2 east fra |
| 1686 | GO:0004022 | F | 6, | 1 | 0.740 (x 1.351) | 15 (0.067) | 0.68 | alcohol dehydrogenase activity | LanB2 |
| 1687 | GO:0016836 | F | 5, | 3 | 2.764 (x 1.085) | 56 (0.054) | 0.68 | hydro-lyase activity | BcDNA:GH12558 CG16733 Eno |
| 1688 | GO:0006029 | P | 7, | 1 | 0.740 (x 1.351) | 15 (0.067) | 0.68 | proteoglycan metabolism | sgl |
| 1689 | GO:0035151 | P | 6, | 1 | 0.740 (x 1.351) | 15 (0.067) | 0.68 | regulation of tracheal tube size | grh |
| 1690 | GO:0035289 | P | 6, 7, | 1 | 0.740 (x 1.351) | 15 (0.067) | 0.681 | posterior head segmentation | en |
| 1691 | GO:0006888 | P | 6, 7, 8, 9, | 1 | 0.740 (x 1.351) | 15 (0.067) | 0.681 | ER to Golgi vesicle-mediated transport | Snap |
| 1692 | GO:0048015 | P | 7, | 1 | 0.740 (x 1.351) | 15 (0.067) | 0.682 | phosphoinositide-mediated signaling | Galpha49B |
| 1693 | GO:0005355 | F | 7, | 1 | 0.740 (x 1.351) | 15 (0.067) | 0.682 | glucose transporter activity | sut1 |
| 1694 | GO:0007538 | P | 4, | 1 | 0.740 (x 1.351) | 15 (0.067) | 0.682 | primary sex determination | sc |
| 1695 | GO:0006974 | P | 4, | 6 | 5.825 (x 1.030) | 118 (0.051) | 0.683 | response to DNA damage stimulus | BcDNA:GM10765 CG18853 CG40410 Caf1 Hus1-like Thd1 |
| 1696 | GO:0016757 | F | 4, | 8 | 7.848 (x 1.019) | 159 (0.050) | 0.683 | transferase activity, transferring glycosyl groups | CG11307 CG17323 CG33138 CG6904 GalNAc-T1 GlyP Ugt pgant5 |
| 1697 | GO:0000786 | C | 3, 5, 6, 7, 8, 9, 10, 11, | 1 | 0.740 (x 1.351) | 15 (0.067) | 0.683 | nucleosome | His4r |
| 1698 | GO:0015923 | F | 6, | 1 | 0.740 (x 1.351) | 15 (0.067) | 0.683 | mannosidase activity | alpha-Man-IIb |
| 1699 | GO:0007200 | P | 7, 8, | 1 | 0.740 (x 1.351) | 15 (0.067) | 0.684 | G-protein signaling, coupled to IP3 second messenger (phospholipase C activating) | Galpha49B |
| 1700 | GO:0019827 | P | 3, | 1 | 0.740 (x 1.351) | 15 (0.067) | 0.684 | stem cell maintenance | tkv |
| 1701 | GO:0051052 | P | 7, | 1 | 0.740 (x 1.351) | 15 (0.067) | 0.684 | regulation of DNA metabolism | mod(mdg4) |
| 1702 | GO:0006898 | P | 7, 8, | 2 | 1.777 (x 1.126) | 36 (0.056) | 0.685 | receptor mediated endocytosis | Amph arr |
| 1703 | GO:0008285 | P | 6, | 1 | 0.740 (x 1.351) | 15 (0.067) | 0.685 | negative regulation of cell proliferation | ana |
| 1704 | GO:0006963 | P | 7, 8, 9, | 1 | 0.740 (x 1.351) | 15 (0.067) | 0.685 | positive regulation of antibacterial peptide biosynthesis | Tl |
| 1705 | GO:0015992 | P | 6, 7, 8, 9, | 4 | 3.850 (x 1.039) | 78 (0.051) | 0.69 | proton transport | Ucp4B Vha100-2 blot jumu |
| 1706 | GO:0006818 | P | 5, 6, | 4 | 3.850 (x 1.039) | 78 (0.051) | 0.691 | hydrogen transport | Ucp4B Vha100-2 blot jumu |
| 1707 | GO:0006629 | P | 5, | 24 | 24.187 (x 0.992) | 490 (0.049) | 0.697 | lipid metabolism | BEST:CK02656 BcDNA:GH02901 BcDNA:GH12558 CG11438 CG1471 CG17323 CG1893 CG1998 CG2118 CG31915 CG33116 CG4586 CG6391 CG9057 CG9342 Cyp310a1 Eip75B Pi3K21B Pten arr mdy sgl usp woc |
| 1708 | GO:0007492 | P | 4, | 1 | 0.790 (x 1.266) | 16 (0.062) | 0.7 | endoderm development | Poxn |
| 1709 | GO:0006289 | P | 6, 8, | 1 | 0.790 (x 1.266) | 16 (0.062) | 0.7 | nucleotide-excision repair | Thd1 |
| 1710 | GO:0042054 | F | 7, | 1 | 0.790 (x 1.266) | 16 (0.062) | 0.7 | histone methyltransferase activity | Caf1 |
| 1711 | GO:0008334 | P | 8, | 1 | 0.790 (x 1.266) | 16 (0.062) | 0.701 | histone mRNA metabolism | pAbp |
| 1712 | GO:0015931 | P | 5, 6, | 2 | 1.826 (x 1.095) | 37 (0.054) | 0.701 | nucleobase, nucleoside, nucleotide and nucleic acid transport | BEST:LD04971 sqd |
| 1713 | GO:0006869 | P | 5, 6, | 3 | 2.863 (x 1.048) | 58 (0.052) | 0.701 | lipid transport | BEST:CK02656 CG1893 CG9342 |
| 1714 | GO:0030537 | P | 4, | 1 | 0.790 (x 1.266) | 16 (0.062) | 0.701 | larval behavior | for |
| 1715 | GO:0004536 | F | 6, | 2 | 1.826 (x 1.095) | 37 (0.054) | 0.701 | deoxyribonuclease activity | BcDNA:GM10765 Rep4 |
| 1716 | GO:0006813 | P | 8, 9, | 3 | 2.863 (x 1.048) | 58 (0.052) | 0.702 | potassium ion transport | Ir Nrv1 Sh |
| 1717 | GO:0006398 | P | 9, 10, | 1 | 0.790 (x 1.266) | 16 (0.062) | 0.702 | histone mRNA 3'-end processing | pAbp |
| 1718 | GO:0008143 | F | 6, | 1 | 0.790 (x 1.266) | 16 (0.062) | 0.702 | poly(A) binding | pAbp |
| 1719 | GO:0009586 | P | 6, 7, 8, | 1 | 0.790 (x 1.266) | 16 (0.062) | 0.702 | rhodopsin mediated phototransduction | Galpha49B |
| 1720 | GO:0004659 | F | 5, | 1 | 0.790 (x 1.266) | 16 (0.062) | 0.703 | prenyltransferase activity | CG33171 |
| 1721 | GO:0000220 | C | 4, 5, 6, 7, 8, 9, 10, 11, 12, | 1 | 0.790 (x 1.266) | 16 (0.062) | 0.703 | hydrogen-transporting ATPase V0 domain | Vha100-2 |
| 1722 | GO:0004016 | F | 4, 5, | 1 | 0.790 (x 1.266) | 16 (0.062) | 0.704 | adenylate cyclase activity | CG32158 |
| 1723 | GO:0007097 | P | 7, 8, | 1 | 0.790 (x 1.266) | 16 (0.062) | 0.704 | nuclear migration | polo |
| 1724 | GO:0007291 | P | 6, 9, | 1 | 0.790 (x 1.266) | 16 (0.062) | 0.704 | sperm individualization | Ance |
| 1725 | GO:0006614 | P | 8, 9, 10, 11, 12, | 1 | 0.790 (x 1.266) | 16 (0.062) | 0.705 | SRP-dependent cotranslational protein targeting to membrane | CG14214 |
| 1726 | GO:0005918 | C | 7, 8, 9, 10, | 1 | 0.790 (x 1.266) | 16 (0.062) | 0.705 | septate junction | Fas3 |
| 1727 | GO:0000578 | P | 5, | 3 | 2.912 (x 1.030) | 59 (0.051) | 0.708 | embryonic axis specification | Tl knk pll |
| 1728 | GO:0006790 | P | 5, | 3 | 2.912 (x 1.030) | 59 (0.051) | 0.709 | sulfur metabolism | Eip55E Eip71CD sgl |
| 1729 | GO:0019731 | P | 6, 7, 8, | 2 | 1.876 (x 1.066) | 38 (0.053) | 0.712 | antibacterial humoral response | 18w Tl |
| 1730 | GO:0003779 | F | 5, | 6 | 6.071 (x 0.988) | 123 (0.049) | 0.718 | actin binding | CG6891 Hsp23 Pten Sry-alpha cib ck |
| 1731 | GO:0043413 | P | 7, | 4 | 3.998 (x 1.000) | 81 (0.049) | 0.718 | biopolymer glycosylation | CG7860 GalNAc-T1 Ugt pgant5 |
| 1732 | GO:0006486 | P | 8, 9, | 4 | 3.998 (x 1.000) | 81 (0.049) | 0.718 | protein amino acid glycosylation | CG7860 GalNAc-T1 Ugt pgant5 |
| 1733 | GO:0035150 | P | 5, | 1 | 0.839 (x 1.192) | 17 (0.059) | 0.719 | regulation of tube size | grh |
| 1734 | GO:0050770 | P | 5, 8, 9, 10, 12, | 1 | 0.839 (x 1.192) | 17 (0.059) | 0.719 | regulation of axonogenesis | pbl |
| 1735 | GO:0006406 | P | 8, 9, 10, 11, | 1 | 0.839 (x 1.192) | 17 (0.059) | 0.719 | mRNA export from nucleus | sqd |
| 1736 | GO:0016478 | P | 8, 9, 10, | 1 | 0.839 (x 1.192) | 17 (0.059) | 0.72 | negative regulation of translation | sqd |
| 1737 | GO:0045047 | P | 6, 7, 8, 9, 10, | 1 | 0.839 (x 1.192) | 17 (0.059) | 0.72 | protein targeting to ER | CG14214 |
| 1738 | GO:0006725 | P | 5, | 7 | 7.157 (x 0.978) | 145 (0.048) | 0.72 | aromatic compound metabolism | CG3590 CG6767 EG:171D11.1 RnrS Ts knk olf413 |
| 1739 | GO:0044425 | C | 3, 4, 5, | 59 | 60.022 (x 0.983) | 1216 (0.049) | 0.721 | membrane part | 18w BG:DS00004.11 CG14214 CG14439 CG15088 CG17084 CG17952 CG1924 CG32158 CG6965 CG9211 Cad74A Cad87A Cad96Ca Dl Fas3 Fs(2)Ket Galpha49B ImpE2 ImpE3 InR Mgstl Nrv1 S Sema-1a Sh Snap Tapdelta Tl Tsp26A Tsp39D Tsp66E Tsp96F Vha100-2 alpha-Man-IIb blot ed emp glec inx2 inx3 jumu l(1)G0320 l(2)01810 mbt mth ninA ogre p120ctn pain para pbl prominin-like rasp rost shanti stan sut1 wgn |
| 1740 | GO:0046843 | P | 10, 11, | 1 | 0.839 (x 1.192) | 17 (0.059) | 0.721 | dorsal appendage formation | bun |
| 1741 | GO:0045202 | C | 2, | 2 | 1.925 (x 1.039) | 39 (0.051) | 0.721 | synapse | Amph gukh |
| 1742 | GO:0007018 | P | 7, 8, 9, | 5 | 5.084 (x 0.983) | 103 (0.049) | 0.721 | microtubule-based movement | CG9057 Klp10A alphaTub84B betaTub56D vg |
| 1743 | GO:0051028 | P | 7, 8, 9, | 1 | 0.839 (x 1.192) | 17 (0.059) | 0.721 | mRNA transport | sqd |
| 1744 | GO:0018993 | P | 4, | 1 | 0.839 (x 1.192) | 17 (0.059) | 0.721 | somatic sex determination | sc |
| 1745 | GO:0007259 | P | 7, | 1 | 0.839 (x 1.192) | 17 (0.059) | 0.722 | JAK-STAT cascade | Cdk4 |
| 1746 | GO:0016331 | P | 5, | 4 | 4.048 (x 0.988) | 82 (0.049) | 0.722 | morphogenesis of embryonic epithelium | InR inx2 jumu tkv |
| 1747 | GO:0043169 | F | 4, | 43 | 43.783 (x 0.982) | 887 (0.048) | 0.722 | cation binding | Ance BcDNA:LD41548 CBP CG11033 CG15435 CG17419 CG17493 CG1924 CG33113 CG4496 CG6654 CG6930 CG8092 CG9906 Cad74A Cad87A Cad96Ca Crc D19A Dl Fkbp13 LanB2 Nep2 Pep Trl Vha100-2 ap br cg ci esn klu l(1)G0320 lola olf413 ome opa pk scf stan th tok zf30C |
| 1748 | GO:0050830 | P | 6, 7, | 1 | 0.839 (x 1.192) | 17 (0.059) | 0.722 | defense response to Gram-positive bacterium | Tl |
| 1749 | GO:0006613 | P | 7, 8, 9, 10, 11, | 1 | 0.839 (x 1.192) | 17 (0.059) | 0.723 | cotranslational protein targeting to membrane | CG14214 |
| 1750 | GO:0000059 | P | 8, 9, 10, 11, | 1 | 0.839 (x 1.192) | 17 (0.059) | 0.723 | protein import into nucleus, docking | Fs(2)Ket |
| 1751 | GO:0008052 | P | 5, | 1 | 0.839 (x 1.192) | 17 (0.059) | 0.724 | sensory organ determination | numb |
| 1752 | GO:0040023 | P | 6, 7, | 1 | 0.839 (x 1.192) | 17 (0.059) | 0.724 | establishment of nucleus localization | polo |
| 1753 | GO:0044444 | C | 4, 5, 6, 7, | 56 | 57.110 (x 0.981) | 1157 (0.048) | 0.727 | cytoplasmic part | BG:DS00004.11 BcDNA:GH12558 CBP CG11079 CG14214 CG1907 CG1924 CG2118 CG31738 CG33113 CG4586 CG6782 CG9057 CaMKI CkIIalpha CkIIbeta Crc EG:171D11.1 EG:BACN33B1.2 ERp60 Eno GalNAc-T1 Ice Idh Mgstl Nek2 Pdi Pepck Pi3K21B Ppt1 RnrS RpS12 S Snap Sry-alpha TER94 Tapdelta Ucp4B Ugt Vha100-2 alpha-Man-IIb asp btsz cib eIF-4E emp fzy l(1)G0320 numb p120ctn pgant5 polo pon shanti smi35A wbl |
| 1754 | GO:0043167 | F | 3, | 45 | 46.004 (x 0.978) | 932 (0.048) | 0.728 | ion binding | Ance BcDNA:LD41548 CBP CG11033 CG15435 CG17419 CG17493 CG1924 CG33113 CG4496 CG6654 CG6930 CG8092 CG9027 CG9906 Cad74A Cad87A Cad96Ca Crc D19A Dl Fkbp13 LanB2 Nep2 Pep RhoGAP71E Trl Vha100-2 ap br cg ci esn klu l(1)G0320 lola olf413 ome opa pk scf stan th tok zf30C |
| 1755 | GO:0046872 | F | 4, | 45 | 46.004 (x 0.978) | 932 (0.048) | 0.729 | metal ion binding | Ance BcDNA:LD41548 CBP CG11033 CG15435 CG17419 CG17493 CG1924 CG33113 CG4496 CG6654 CG6930 CG8092 CG9027 CG9906 Cad74A Cad87A Cad96Ca Crc D19A Dl Fkbp13 LanB2 Nep2 Pep RhoGAP71E Trl Vha100-2 ap br cg ci esn klu l(1)G0320 lola olf413 ome opa pk scf stan th tok zf30C |
| 1756 | GO:0030705 | P | 6, 7, 8, | 5 | 5.133 (x 0.974) | 104 (0.048) | 0.729 | cytoskeleton-dependent intracellular transport | CG9057 Klp10A alphaTub84B betaTub56D vg |
| 1757 | GO:0009101 | P | 7, 8, | 4 | 4.097 (x 0.976) | 83 (0.048) | 0.732 | glycoprotein biosynthesis | CG7860 GalNAc-T1 Ugt pgant5 |
| 1758 | GO:0030384 | P | 9, 10, | 1 | 0.888 (x 1.126) | 18 (0.056) | 0.734 | phosphoinositide metabolism | Pi3K21B |
| 1759 | GO:0030001 | P | 7, 8, | 7 | 7.256 (x 0.965) | 147 (0.048) | 0.734 | metal ion transport | CG12048 Ir Nrv1 Sh pain para rpk |
| 1760 | GO:0051647 | P | 5, 6, | 1 | 0.888 (x 1.126) | 18 (0.056) | 0.734 | nucleus localization | polo |
| 1761 | GO:0005351 | F | 5, 6, | 2 | 1.974 (x 1.013) | 40 (0.050) | 0.735 | sugar porter activity | LanB2 sut1 |
| 1762 | GO:0015149 | F | 6, | 1 | 0.888 (x 1.126) | 18 (0.056) | 0.735 | hexose transporter activity | sut1 |
| 1763 | GO:0006368 | P | 9, | 1 | 0.888 (x 1.126) | 18 (0.056) | 0.735 | RNA elongation from RNA polymerase II promoter | CycT |
| 1764 | GO:0005741 | C | 5, 6, 7, 8, 9, 10, 11, 12, | 1 | 0.888 (x 1.126) | 18 (0.056) | 0.736 | mitochondrial outer membrane | Mgstl |
| 1765 | GO:0042493 | P | 5, | 1 | 0.888 (x 1.126) | 18 (0.056) | 0.736 | response to drug | cyc |
| 1766 | GO:0016835 | F | 4, | 3 | 3.060 (x 0.980) | 62 (0.048) | 0.736 | carbon-oxygen lyase activity | BcDNA:GH12558 CG16733 Eno |
| 1767 | GO:0045610 | P | 5, 7, | 1 | 0.888 (x 1.126) | 18 (0.056) | 0.737 | regulation of hemocyte differentiation | Tl |
| 1768 | GO:0044271 | P | 5, 6, | 4 | 4.146 (x 0.965) | 84 (0.048) | 0.737 | nitrogen compound biosynthesis | CG40160 CG6287 CtBP ESTS:39C10S |
| 1769 | GO:0051082 | F | 4, | 3 | 3.060 (x 0.980) | 62 (0.048) | 0.737 | unfolded protein binding | CG9906 Crc DnaJ-1 |
| 1770 | GO:0035287 | P | 5, 6, | 1 | 0.888 (x 1.126) | 18 (0.056) | 0.737 | head segmentation | en |
| 1771 | GO:0009309 | P | 6, 7, | 4 | 4.146 (x 0.965) | 84 (0.048) | 0.737 | amine biosynthesis | CG40160 CG6287 CtBP ESTS:39C10S |
| 1772 | GO:0015370 | F | 6, 8, | 3 | 3.060 (x 0.980) | 62 (0.048) | 0.737 | solute:sodium symporter activity | CG15088 blot l(2)01810 |
| 1773 | GO:0005316 | F | 7, 8, 9, | 1 | 0.888 (x 1.126) | 18 (0.056) | 0.737 | high affinity inorganic phosphate:sodium symporter activity | l(2)01810 |
| 1774 | GO:0005884 | C | 5, 6, 7, 8, 9, 10, | 1 | 0.888 (x 1.126) | 18 (0.056) | 0.738 | actin filament | Act42A |
| 1775 | GO:0015294 | F | 5, 7, | 3 | 3.110 (x 0.965) | 63 (0.048) | 0.744 | solute:cation symporter activity | CG15088 blot l(2)01810 |
| 1776 | GO:0006470 | P | 8, | 4 | 4.196 (x 0.953) | 85 (0.047) | 0.747 | protein amino acid dephosphorylation | Pten Ptp99A dap tws |
| 1777 | GO:0031968 | C | 4, 5, 6, 7, 8, 9, | 1 | 0.938 (x 1.066) | 19 (0.053) | 0.752 | organelle outer membrane | Mgstl |
| 1778 | GO:0019722 | P | 7, | 3 | 3.159 (x 0.950) | 64 (0.047) | 0.753 | calcium-mediated signaling | CG17493 CaMKI scf |
| 1779 | GO:0006612 | P | 8, 9, 10, | 1 | 0.938 (x 1.066) | 19 (0.053) | 0.753 | protein targeting to membrane | CG14214 |
| 1780 | GO:0031301 | C | 5, 6, 7, 8, 9, 10, | 1 | 0.938 (x 1.066) | 19 (0.053) | 0.753 | integral to organelle membrane | CG14214 |
| 1781 | GO:0009100 | P | 7, | 4 | 4.245 (x 0.942) | 86 (0.047) | 0.754 | glycoprotein metabolism | CG7860 GalNAc-T1 Ugt pgant5 |
| 1782 | GO:0006650 | P | 8, 9, | 1 | 0.938 (x 1.066) | 19 (0.053) | 0.754 | glycerophospholipid metabolism | Pi3K21B |
| 1783 | GO:0004437 | F | 7, | 1 | 0.938 (x 1.066) | 19 (0.053) | 0.754 | inositol or phosphatidylinositol phosphatase activity | Pten |
| 1784 | GO:0044272 | P | 6, | 1 | 0.938 (x 1.066) | 19 (0.053) | 0.755 | sulfur compound biosynthesis | sgl |
| 1785 | GO:0043190 | C | 3, 4, | 2 | 2.073 (x 0.965) | 42 (0.048) | 0.755 | ATP-binding cassette (ABC) transporter complex | BEST:CK02656 CG31121 |
| 1786 | GO:0031300 | C | 4, 5, 6, 7, 8, 9, | 1 | 0.938 (x 1.066) | 19 (0.053) | 0.755 | intrinsic to organelle membrane | CG14214 |
| 1787 | GO:0019867 | C | 4, 5, 6, | 1 | 0.938 (x 1.066) | 19 (0.053) | 0.755 | outer membrane | Mgstl |
| 1788 | GO:0016311 | P | 7, | 5 | 5.331 (x 0.938) | 108 (0.046) | 0.756 | dephosphorylation | CG11438 Pten Ptp99A dap tws |
| 1789 | GO:0004190 | F | 6, | 1 | 0.938 (x 1.066) | 19 (0.053) | 0.756 | aspartic-type endopeptidase activity | shanti |
| 1790 | GO:0030706 | P | 5, 8, 9, | 1 | 0.938 (x 1.066) | 19 (0.053) | 0.756 | oocyte differentiation (sensu Insecta) | asp |
| 1791 | GO:0003924 | F | 8, | 6 | 6.417 (x 0.935) | 130 (0.046) | 0.759 | GTPase activity | Arf79F CG2108 Galpha49B Rab8 alphaTub84B betaTub56D |
| 1792 | GO:0044431 | C | 4, 5, 6, 7, 8, 9, | 3 | 3.208 (x 0.935) | 65 (0.046) | 0.763 | Golgi apparatus part | GalNAc-T1 alpha-Man-IIb pgant5 |
| 1793 | GO:0009156 | P | 8, 9, | 1 | 0.987 (x 1.013) | 20 (0.050) | 0.767 | ribonucleoside monophosphate biosynthesis | CG6767 |
| 1794 | GO:0009161 | P | 8, | 1 | 0.987 (x 1.013) | 20 (0.050) | 0.767 | ribonucleoside monophosphate metabolism | CG6767 |
| 1795 | GO:0019932 | P | 6, | 4 | 4.344 (x 0.921) | 88 (0.045) | 0.767 | second-messenger-mediated signaling | CG17493 CaMKI Galpha49B scf |
| 1796 | GO:0008276 | F | 6, | 1 | 0.987 (x 1.013) | 20 (0.050) | 0.768 | protein methyltransferase activity | Caf1 |
| 1797 | GO:0006281 | P | 5, 7, | 5 | 5.430 (x 0.921) | 110 (0.045) | 0.768 | DNA repair | BcDNA:GM10765 CG18853 Caf1 Hus1-like Thd1 |
| 1798 | GO:0008289 | F | 3, | 3 | 3.258 (x 0.921) | 66 (0.045) | 0.768 | lipid binding | CG17919 CG9342 RacGAP50C |
| 1799 | GO:0008374 | F | 7, | 1 | 0.987 (x 1.013) | 20 (0.050) | 0.768 | O-acyltransferase activity | mdy |
| 1800 | GO:0051169 | P | 6, 7, 8, | 3 | 3.258 (x 0.921) | 66 (0.045) | 0.768 | nuclear transport | Fs(2)Ket pll sqd |
| 1801 | GO:0015145 | F | 5, | 1 | 0.987 (x 1.013) | 20 (0.050) | 0.769 | monosaccharide transporter activity | sut1 |
| 1802 | GO:0006817 | P | 8, 9, | 2 | 2.123 (x 0.942) | 43 (0.047) | 0.769 | phosphate transport | CG33171 l(2)01810 |
| 1803 | GO:0005604 | C | 3, 4, 5, | 1 | 0.987 (x 1.013) | 20 (0.050) | 0.769 | basement membrane | LanB2 |
| 1804 | GO:0005576 | C | 2, | 18 | 19.053 (x 0.945) | 386 (0.047) | 0.769 | extracellular region | Ance BcDNA:GH02976 CG11142 CG17052 CG32354 CG32499 CG33171 CG9307 ImpE2 Jafrac2 LanB2 Lsp2 Obp99a Sema-2a Wnt2 ana kal-1 shf |
| 1805 | GO:0016028 | C | 5, 6, 7, | 1 | 0.987 (x 1.013) | 20 (0.050) | 0.769 | rhabdomere | Galpha49B |
| 1806 | GO:0007528 | P | 7, 8, | 1 | 0.987 (x 1.013) | 20 (0.050) | 0.77 | neuromuscular junction development | tkv |
| 1807 | GO:0008632 | P | 7, | 1 | 0.987 (x 1.013) | 20 (0.050) | 0.77 | apoptotic program | Rep4 |
| 1808 | GO:0000160 | P | 5, | 1 | 0.987 (x 1.013) | 20 (0.050) | 0.771 | two-component signal transduction system (phosphorelay) | Rab8 |
| 1809 | GO:0008144 | F | 3, | 1 | 0.987 (x 1.013) | 20 (0.050) | 0.771 | drug binding | Fkbp13 |
| 1810 | GO:0016877 | F | 4, | 1 | 0.987 (x 1.013) | 20 (0.050) | 0.772 | ligase activity, forming carbon-sulfur bonds | BcDNA:GH02901 |
| 1811 | GO:0044428 | C | 4, 5, 6, 7, 8, 9, | 24 | 25.421 (x 0.944) | 515 (0.047) | 0.777 | nuclear part | B52 CG17838 CG17952 CG8679 Caf1 CycT Dp Dref Fs(2)Ket HmgD Hrb27C Mcm7 Pep Rbf2 Top2 Ugt ap heph jumu nonA-l ph-p sqd toe usp |
| 1812 | GO:0007362 | P | 8, 9, | 1 | 1.037 (x 0.965) | 21 (0.048) | 0.781 | terminal region determination | knk |
| 1813 | GO:0030952 | P | 7, | 1 | 1.037 (x 0.965) | 21 (0.048) | 0.782 | establishment and/or maintenance of cytoskeleton polarity | TER94 |
| 1814 | GO:0035152 | P | 5, | 1 | 1.037 (x 0.965) | 21 (0.048) | 0.782 | regulation of tracheal tube architecture | grh |
| 1815 | GO:0006405 | P | 7, 8, 9, 10, | 1 | 1.037 (x 0.965) | 21 (0.048) | 0.782 | RNA export from nucleus | sqd |
| 1816 | GO:0005436 | F | 6, 7, 8, | 1 | 1.037 (x 0.965) | 21 (0.048) | 0.783 | sodium:phosphate symporter activity | l(2)01810 |
| 1817 | GO:0030951 | P | 8, 9, | 1 | 1.037 (x 0.965) | 21 (0.048) | 0.783 | establishment and/or maintenance of microtubule cytoskeleton polarity | TER94 |
| 1818 | GO:0008138 | F | 8, | 1 | 1.037 (x 0.965) | 21 (0.048) | 0.784 | protein tyrosine/serine/threonine phosphatase activity | Pten |
| 1819 | GO:0015300 | F | 7, | 1 | 1.037 (x 0.965) | 21 (0.048) | 0.784 | solute:solute antiporter activity | CG1907 |
| 1820 | GO:0005096 | F | 4, | 2 | 2.221 (x 0.900) | 45 (0.044) | 0.784 | GTPase activator activity | RacGAP50C Rapgap1 |
| 1821 | GO:0017148 | P | 7, 8, 9, | 1 | 1.037 (x 0.965) | 21 (0.048) | 0.785 | negative regulation of protein biosynthesis | sqd |
| 1822 | GO:0002164 | P | 4, | 2 | 2.221 (x 0.900) | 45 (0.044) | 0.785 | larval development | CrebA Sb |
| 1823 | GO:0005834 | C | 3, 6, 7, 8, | 1 | 1.037 (x 0.965) | 21 (0.048) | 0.785 | heterotrimeric G-protein complex | Galpha49B |
| 1824 | GO:0005938 | C | 5, 6, 7, 8, | 2 | 2.221 (x 0.900) | 45 (0.044) | 0.785 | cell cortex | numb pon |
| 1825 | GO:0016325 | P | 7, 8, 9, 10, | 1 | 1.037 (x 0.965) | 21 (0.048) | 0.785 | oocyte microtubule cytoskeleton organization | TER94 |
| 1826 | GO:0016579 | P | 9, | 1 | 1.037 (x 0.965) | 21 (0.048) | 0.786 | protein deubiquitination | Uch |
| 1827 | GO:0009260 | P | 7, 8, | 4 | 4.492 (x 0.891) | 91 (0.044) | 0.79 | ribonucleotide biosynthesis | CG3590 CG6767 blot jumu |
| 1828 | GO:0009259 | P | 7, | 4 | 4.541 (x 0.881) | 92 (0.043) | 0.795 | ribonucleotide metabolism | CG3590 CG6767 blot jumu |
| 1829 | GO:0009890 | P | 6, | 1 | 1.086 (x 0.921) | 22 (0.045) | 0.795 | negative regulation of biosynthesis | sqd |
| 1830 | GO:0009401 | P | 6, 7, | 1 | 1.086 (x 0.921) | 22 (0.045) | 0.795 | phosphoenolpyruvate-dependent sugar phosphotransferase system | LanB2 |
| 1831 | GO:0006354 | P | 8, | 1 | 1.086 (x 0.921) | 22 (0.045) | 0.796 | RNA elongation | CycT |
| 1832 | GO:0009059 | P | 5, 6, | 24 | 25.766 (x 0.931) | 522 (0.046) | 0.796 | macromolecule biosynthesis | CG31738 CG31915 CG33171 CG6904 CG7860 CG8963 GalNAc-T1 Gfat1 Pepck Pgi Ppt1 RpS12 Tpi Ugt blot eIF-4E l(1)G0320 l(2)01424 mdy pAbp pgant5 rasp sgl sqd |
| 1833 | GO:0000790 | C | 6, 7, 8, 9, 10, 11, 12, | 1 | 1.086 (x 0.921) | 22 (0.045) | 0.796 | nuclear chromatin | HmgD |
| 1834 | GO:0007446 | P | 4, 5, | 1 | 1.086 (x 0.921) | 22 (0.045) | 0.797 | imaginal disc growth | InR |
| 1835 | GO:0016079 | P | 7, 8, 9, | 2 | 2.271 (x 0.881) | 46 (0.043) | 0.797 | synaptic vesicle exocytosis | Snap mth |
| 1836 | GO:0005615 | C | 3, 4, | 1 | 1.086 (x 0.921) | 22 (0.045) | 0.797 | extracellular space | Lsp2 |
| 1837 | GO:0042440 | P | 5, | 2 | 2.271 (x 0.881) | 46 (0.043) | 0.798 | pigment metabolism | Tl serpin-27A |
| 1838 | GO:0008076 | C | 3, 5, 6, 7, 8, 9, | 1 | 1.086 (x 0.921) | 22 (0.045) | 0.798 | voltage-gated potassium channel complex | Sh |
| 1839 | GO:0031327 | P | 7, | 1 | 1.086 (x 0.921) | 22 (0.045) | 0.798 | negative regulation of cellular biosynthesis | sqd |
| 1840 | GO:0007427 | P | 5, 6, 7, | 1 | 1.086 (x 0.921) | 22 (0.045) | 0.798 | tracheal epithelial cell migration (sensu Insecta) | sgl |
| 1841 | GO:0044453 | C | 4, 5, 6, 7, 8, 9, 10, 11, 12, | 2 | 2.320 (x 0.862) | 47 (0.043) | 0.802 | nuclear membrane part | CG17952 Fs(2)Ket |
| 1842 | GO:0031965 | C | 5, 6, 7, 8, 9, 10, 11, | 2 | 2.320 (x 0.862) | 47 (0.043) | 0.803 | nuclear membrane | CG17952 Fs(2)Ket |
| 1843 | GO:0051119 | F | 4, | 2 | 2.320 (x 0.862) | 47 (0.043) | 0.803 | sugar transporter activity | LanB2 sut1 |
| 1844 | GO:0005525 | F | 6, | 7 | 7.947 (x 0.881) | 161 (0.043) | 0.803 | GTP binding | Arf79F CG2108 Galpha49B Pepck Rab8 alphaTub84B betaTub56D |
| 1845 | GO:0008234 | F | 5, | 4 | 4.591 (x 0.871) | 93 (0.043) | 0.803 | cysteine-type peptidase activity | CG3328 CG5794 Ice Uch |
| 1846 | GO:0008213 | P | 8, | 1 | 1.135 (x 0.881) | 23 (0.043) | 0.804 | protein amino acid alkylation | Caf1 |
| 1847 | GO:0016811 | F | 5, | 2 | 2.320 (x 0.862) | 47 (0.043) | 0.804 | hydrolase activity, acting on carbon-nitrogen (but not peptide) bonds, in linear amides | CG1471 CG7860 |
| 1848 | GO:0005624 | C | 4, 5, | 4 | 4.591 (x 0.871) | 93 (0.043) | 0.804 | membrane fraction | 18w Cyp310a1 Mgstl glec |
| 1849 | GO:0006479 | P | 8, 9, | 1 | 1.135 (x 0.881) | 23 (0.043) | 0.804 | protein amino acid methylation | Caf1 |
| 1850 | GO:0006606 | P | 7, 8, 9, 10, | 2 | 2.320 (x 0.862) | 47 (0.043) | 0.804 | protein import into nucleus | Fs(2)Ket pll |
| 1851 | GO:0006508 | P | 7, | 35 | 37.415 (x 0.935) | 758 (0.046) | 0.804 | proteolysis | Ance BEST:GH02921 BG:DS00004.11 BcDNA:LD41548 CG11033 CG11824 CG1632 CG2200 CG3328 CG40160 CG40410 CG4914 CG5390 CG5794 CG6680 CG7860 CG9135 D19A Ice Lsp2 Nep2 Prosalpha6 Rpn9 Sb Spn1 Spn5 TER94 Uch east fra fzy ome pll th tok |
| 1852 | GO:0000139 | C | 4, 5, 6, 7, 8, 9, 10, | 1 | 1.135 (x 0.881) | 23 (0.043) | 0.805 | Golgi membrane | alpha-Man-IIb |
| 1853 | GO:0016407 | F | 7, | 2 | 2.320 (x 0.862) | 47 (0.043) | 0.805 | acetyltransferase activity | CG1969 CG7379 |
| 1854 | GO:0019239 | F | 3, | 1 | 1.135 (x 0.881) | 23 (0.043) | 0.805 | deaminase activity | Oscillin |
| 1855 | GO:0045087 | P | 5, 6, | 1 | 1.135 (x 0.881) | 23 (0.043) | 0.805 | innate immune response | Tl |
| 1856 | GO:0006961 | P | 7, 8, 9, | 1 | 1.135 (x 0.881) | 23 (0.043) | 0.806 | antibacterial humoral response (sensu Protostomia) | 18w |
| 1857 | GO:0005838 | C | 3, 4, 5, 6, 7, | 1 | 1.135 (x 0.881) | 23 (0.043) | 0.806 | proteasome regulatory particle (sensu Eukaryota) | Rpn9 |
| 1858 | GO:0042445 | P | 5, | 1 | 1.135 (x 0.881) | 23 (0.043) | 0.807 | hormone metabolism | woc |
| 1859 | GO:0006401 | P | 7, | 1 | 1.135 (x 0.881) | 23 (0.043) | 0.807 | RNA catabolism | sqd |
| 1860 | GO:0004245 | F | 7, | 1 | 1.135 (x 0.881) | 23 (0.043) | 0.808 | neprilysin activity | Nep2 |
| 1861 | GO:0009141 | P | 7, | 3 | 3.505 (x 0.856) | 71 (0.042) | 0.808 | nucleoside triphosphate metabolism | blot dUTPase jumu |
| 1862 | GO:0051170 | P | 7, 8, 9, | 2 | 2.369 (x 0.844) | 48 (0.042) | 0.808 | nuclear import | Fs(2)Ket pll |
| 1863 | GO:0006913 | P | 6, 7, 8, | 3 | 3.505 (x 0.856) | 71 (0.042) | 0.808 | nucleocytoplasmic transport | Fs(2)Ket pll sqd |
| 1864 | GO:0005516 | F | 4, | 3 | 3.505 (x 0.856) | 71 (0.042) | 0.809 | calmodulin binding | CG17493 CaMKI scf |
| 1865 | GO:0003729 | F | 5, | 14 | 15.450 (x 0.906) | 313 (0.045) | 0.809 | mRNA binding | B52 CG17838 CG6049 CG6946 CG8963 Hrb27C Rm62 Top2 heph l(2)01424 nonA-l pAbp spen sqd |
| 1866 | GO:0019001 | F | 5, | 7 | 7.996 (x 0.875) | 162 (0.043) | 0.809 | guanyl nucleotide binding | Arf79F CG2108 Galpha49B Pepck Rab8 alphaTub84B betaTub56D |
| 1867 | GO:0042625 | F | 4, 5, 7, 12, | 4 | 4.689 (x 0.853) | 95 (0.042) | 0.811 | ATPase activity, coupled to transmembrane movement of ions | Nrv1 Vha100-2 blot jumu |
| 1868 | GO:0006445 | P | 7, 8, 9, | 3 | 3.554 (x 0.844) | 72 (0.042) | 0.811 | regulation of translation | l(2)01424 pAbp sqd |
| 1869 | GO:0008237 | F | 5, | 8 | 9.132 (x 0.876) | 185 (0.043) | 0.814 | metallopeptidase activity | Ance BcDNA:LD41548 D19A Lsp2 Nep2 east fra tok |
| 1870 | GO:0006575 | P | 6, | 2 | 2.419 (x 0.827) | 49 (0.041) | 0.815 | amino acid derivative metabolism | knk olf413 |
| 1871 | GO:0015450 | F | 4, 6, | 1 | 1.185 (x 0.844) | 24 (0.042) | 0.815 | protein translocase activity | CG14214 |
| 1872 | GO:0009063 | P | 7, 8, | 2 | 2.419 (x 0.827) | 49 (0.041) | 0.815 | amino acid catabolism | CG7860 EG:171D11.1 |
| 1873 | GO:0050658 | P | 6, 7, 8, | 1 | 1.185 (x 0.844) | 24 (0.042) | 0.816 | RNA transport | sqd |
| 1874 | GO:0050877 | P | 4, | 28 | 30.406 (x 0.921) | 616 (0.045) | 0.816 | neurophysiological process | Amph Arf79F CG11033 CG12199 CG16974 CG4054 CG5096 CaMKI Galpha49B M6 Obp99a PFE Sh Snap br ck d fax for kal-1 lola mod(mdg4) mth olf413 pAbp pain para trn |
| 1875 | GO:0005216 | F | 4, 5, | 7 | 8.095 (x 0.865) | 164 (0.043) | 0.816 | ion channel activity | CG12048 CG14076 Ir Sh pain para rpk |
| 1876 | GO:0015293 | F | 6, | 3 | 3.603 (x 0.833) | 73 (0.041) | 0.816 | symporter activity | CG15088 blot l(2)01810 |
| 1877 | GO:0016323 | C | 5, 6, 7, | 1 | 1.185 (x 0.844) | 24 (0.042) | 0.816 | basolateral plasma membrane | Fas3 |
| 1878 | GO:0016462 | F | 6, | 24 | 26.210 (x 0.916) | 531 (0.045) | 0.816 | pyrophosphatase activity | Arf79F BEST:CK02656 CG2108 CG31121 CG31453 CG6391 Galpha49B Mcm7 Nrv1 Rab8 RhoGAP71E Rm62 TER94 Top2 Vha100-2 alphaTub84B betaTub56D blot ck d dUTPase glu jumu kis |
| 1879 | GO:0009948 | P | 5, | 5 | 5.874 (x 0.851) | 119 (0.042) | 0.816 | anterior/posterior axis specification | Dl Hrb27C TER94 knk sqd |
| 1880 | GO:0051236 | P | 5, | 1 | 1.185 (x 0.844) | 24 (0.042) | 0.816 | establishment of RNA localization | sqd |
| 1881 | GO:0050657 | P | 6, 7, | 1 | 1.185 (x 0.844) | 24 (0.042) | 0.817 | nucleic acid transport | sqd |
| 1882 | GO:0031202 | F | 5, | 1 | 1.185 (x 0.844) | 24 (0.042) | 0.817 | RNA splicing factor activity, transesterification mechanism | B52 |
| 1883 | GO:0008407 | P | 4, 5, | 1 | 1.185 (x 0.844) | 24 (0.042) | 0.818 | bristle morphogenesis | sc |
| 1884 | GO:0005913 | C | 7, 8, 9, | 1 | 1.185 (x 0.844) | 24 (0.042) | 0.818 | cell-cell adherens junction | Fas3 |
| 1885 | GO:0015399 | F | 4, | 5 | 5.923 (x 0.844) | 120 (0.042) | 0.822 | primary active transporter activity | CG14214 Nrv1 Vha100-2 blot jumu |
| 1886 | GO:0015405 | F | 5, | 5 | 5.923 (x 0.844) | 120 (0.042) | 0.822 | P-P-bond-hydrolysis-driven transporter activity | CG14214 Nrv1 Vha100-2 blot jumu |
| 1887 | GO:0007601 | P | 5, 7, | 3 | 3.653 (x 0.821) | 74 (0.041) | 0.823 | visual perception | Galpha49B ck d |
| 1888 | GO:0015629 | C | 6, 7, 8, 9, | 3 | 3.653 (x 0.821) | 74 (0.041) | 0.823 | actin cytoskeleton | Act42A ck d |
| 1889 | GO:0050953 | P | 4, 6, | 3 | 3.653 (x 0.821) | 74 (0.041) | 0.824 | sensory perception of light stimulus | Galpha49B ck d |
| 1890 | GO:0005262 | F | 6, 7, | 1 | 1.234 (x 0.810) | 25 (0.040) | 0.825 | calcium channel activity | pain |
| 1891 | GO:0005839 | C | 3, 4, 5, 6, 7, | 1 | 1.234 (x 0.810) | 25 (0.040) | 0.825 | proteasome core complex (sensu Eukaryota) | Prosalpha6 |
| 1892 | GO:0016796 | F | 7, | 1 | 1.234 (x 0.810) | 25 (0.040) | 0.826 | exonuclease activity, active with either ribo- or deoxyribonucleic acids and producing 5'-phosphomonoesters | BcDNA:GM10765 |
| 1893 | GO:0004970 | F | 6, | 1 | 1.234 (x 0.810) | 25 (0.040) | 0.826 | ionotropic glutamate receptor activity | CG14076 |
| 1894 | GO:0008565 | F | 3, | 3 | 3.702 (x 0.810) | 75 (0.040) | 0.83 | protein transporter activity | CG14214 Fs(2)Ket loj |
| 1895 | GO:0006417 | P | 6, 7, 8, | 3 | 3.702 (x 0.810) | 75 (0.040) | 0.83 | regulation of protein biosynthesis | l(2)01424 pAbp sqd |
| 1896 | GO:0008654 | P | 7, 8, 9, | 1 | 1.283 (x 0.779) | 26 (0.038) | 0.836 | phospholipid biosynthesis | CG33116 |
| 1897 | GO:0007310 | P | 6, 8, 9, 11, | 1 | 1.283 (x 0.779) | 26 (0.038) | 0.836 | oocyte dorsal/ventral axis determination | wbl |
| 1898 | GO:0008287 | C | 3, 4, | 1 | 1.283 (x 0.779) | 26 (0.038) | 0.837 | protein serine/threonine phosphatase complex | tws |
| 1899 | GO:0019992 | F | 4, | 1 | 1.283 (x 0.779) | 26 (0.038) | 0.837 | diacylglycerol binding | RacGAP50C |
| 1900 | GO:0044270 | P | 5, 6, | 2 | 2.567 (x 0.779) | 52 (0.038) | 0.837 | nitrogen compound catabolism | CG7860 EG:171D11.1 |
| 1901 | GO:0004383 | F | 4, 5, | 1 | 1.283 (x 0.779) | 26 (0.038) | 0.838 | guanylate cyclase activity | CG32158 |
| 1902 | GO:0009310 | P | 6, 7, | 2 | 2.567 (x 0.779) | 52 (0.038) | 0.838 | amine catabolism | CG7860 EG:171D11.1 |
| 1903 | GO:0004520 | F | 7, | 1 | 1.283 (x 0.779) | 26 (0.038) | 0.838 | endodeoxyribonuclease activity | BcDNA:GM10765 |
| 1904 | GO:0005267 | F | 6, 7, | 2 | 2.567 (x 0.779) | 52 (0.038) | 0.838 | potassium channel activity | Ir Sh |
| 1905 | GO:0015114 | F | 6, | 1 | 1.283 (x 0.779) | 26 (0.038) | 0.838 | phosphate transporter activity | l(2)01810 |
| 1906 | GO:0007391 | P | 6, | 3 | 3.751 (x 0.800) | 76 (0.039) | 0.839 | dorsal closure | InR jumu tkv |
| 1907 | GO:0003684 | F | 5, | 1 | 1.283 (x 0.779) | 26 (0.038) | 0.839 | damaged DNA binding | BcDNA:GM10765 |
| 1908 | GO:0006644 | P | 7, 8, | 3 | 3.751 (x 0.800) | 76 (0.039) | 0.839 | phospholipid metabolism | CG33116 CG6391 Pi3K21B |
| 1909 | GO:0003723 | F | 4, | 16 | 18.214 (x 0.878) | 369 (0.043) | 0.844 | RNA binding | B52 CG17838 CG6049 CG6946 CG8963 Hrb27C Pep Rm62 Top2 eIF-4E heph l(2)01424 nonA-l pAbp spen sqd |
| 1910 | GO:0051168 | P | 7, 8, 9, | 1 | 1.333 (x 0.750) | 27 (0.037) | 0.847 | nuclear export | sqd |
| 1911 | GO:0005215 | F | 2, | 44 | 47.781 (x 0.921) | 968 (0.045) | 0.847 | transporter activity | BEST:CK02656 BEST:LD04971 CG10657 CG11550 CG11739 CG12048 CG13848 CG14076 CG14214 CG14439 CG15088 CG1607 CG1893 CG1907 CG31121 CG3424 CG3823 CG6782 CG6812 CG9342 Fs(2)Ket Ir LanB2 Lsp2 Nrv1 Sh Snap Ucp4B Vha100-2 blot btsz cic ck inx2 inx3 jumu l(2)01810 loj mnd ogre pain para rpk sut1 |
| 1912 | GO:0007354 | P | 7, 8, | 1 | 1.333 (x 0.750) | 27 (0.037) | 0.847 | zygotic determination of anterior/posterior axis, embryo | knk |
| 1913 | GO:0005543 | F | 4, | 1 | 1.333 (x 0.750) | 27 (0.037) | 0.848 | phospholipid binding | CG17919 |
| 1914 | GO:0044456 | C | 2, 3, | 1 | 1.333 (x 0.750) | 27 (0.037) | 0.848 | synapse part | gukh |
| 1915 | GO:0005234 | F | 8, 9, | 1 | 1.333 (x 0.750) | 27 (0.037) | 0.849 | glutamate-gated ion channel activity | CG14076 |
| 1916 | GO:0000398 | P | 9, 11, | 6 | 7.355 (x 0.816) | 149 (0.040) | 0.852 | nuclear mRNA splicing, via spliceosome | B52 CG6946 Rm62 heph nonA-l sqd |
| 1917 | GO:0000377 | P | 10, | 6 | 7.355 (x 0.816) | 149 (0.040) | 0.852 | RNA splicing, via transesterification reactions with bulged adenosine as nucleophile | B52 CG6946 Rm62 heph nonA-l sqd |
| 1918 | GO:0000375 | P | 9, | 6 | 7.355 (x 0.816) | 149 (0.040) | 0.853 | RNA splicing, via transesterification reactions | B52 CG6946 Rm62 heph nonA-l sqd |
| 1919 | GO:0015698 | P | 7, 8, | 2 | 2.665 (x 0.750) | 54 (0.037) | 0.854 | inorganic anion transport | CG33171 l(2)01810 |
| 1920 | GO:0009889 | P | 5, | 3 | 3.899 (x 0.769) | 79 (0.038) | 0.855 | regulation of biosynthesis | l(2)01424 pAbp sqd |
| 1921 | GO:0031326 | P | 6, | 3 | 3.899 (x 0.769) | 79 (0.038) | 0.855 | regulation of cellular biosynthesis | l(2)01424 pAbp sqd |
| 1922 | GO:0006631 | P | 6, 7, | 4 | 5.084 (x 0.787) | 103 (0.039) | 0.855 | fatty acid metabolism | BcDNA:GH02901 BcDNA:GH12558 CG2118 CG4586 |
| 1923 | GO:0008080 | F | 8, | 1 | 1.382 (x 0.724) | 28 (0.036) | 0.856 | N-acetyltransferase activity | CG1969 |
| 1924 | GO:0004091 | F | 6, | 1 | 1.382 (x 0.724) | 28 (0.036) | 0.857 | carboxylesterase activity | CG4382 |
| 1925 | GO:0008408 | F | 7, | 1 | 1.382 (x 0.724) | 28 (0.036) | 0.857 | 3'-5' exonuclease activity | BcDNA:GM10765 |
| 1926 | GO:0016817 | F | 4, | 24 | 27.050 (x 0.887) | 548 (0.044) | 0.859 | hydrolase activity, acting on acid anhydrides | Arf79F BEST:CK02656 CG2108 CG31121 CG31453 CG6391 Galpha49B Mcm7 Nrv1 Rab8 RhoGAP71E Rm62 TER94 Top2 Vha100-2 alphaTub84B betaTub56D blot ck d dUTPase glu jumu kis |
| 1927 | GO:0016818 | F | 5, | 24 | 27.050 (x 0.887) | 548 (0.044) | 0.859 | hydrolase activity, acting on acid anhydrides, in phosphorus-containing anhydrides | Arf79F BEST:CK02656 CG2108 CG31121 CG31453 CG6391 Galpha49B Mcm7 Nrv1 Rab8 RhoGAP71E Rm62 TER94 Top2 Vha100-2 alphaTub84B betaTub56D blot ck d dUTPase glu jumu kis |
| 1928 | GO:0017038 | P | 6, 7, | 2 | 2.715 (x 0.737) | 55 (0.036) | 0.859 | protein import | Fs(2)Ket pll |
| 1929 | GO:0043492 | F | 3, 10, | 6 | 7.453 (x 0.805) | 151 (0.040) | 0.859 | ATPase activity, coupled to movement of substances | BEST:CK02656 CG31121 Nrv1 Vha100-2 blot jumu |
| 1930 | GO:0042626 | F | 4, 6, 11, | 6 | 7.453 (x 0.805) | 151 (0.040) | 0.86 | ATPase activity, coupled to transmembrane movement of substances | BEST:CK02656 CG31121 Nrv1 Vha100-2 blot jumu |
| 1931 | GO:0004197 | F | 6, | 3 | 3.998 (x 0.750) | 81 (0.037) | 0.862 | cysteine-type endopeptidase activity | CG3328 CG5794 Ice |
| 1932 | GO:0031123 | P | 8, | 1 | 1.431 (x 0.699) | 29 (0.034) | 0.863 | RNA 3'-end processing | pAbp |
| 1933 | GO:0031124 | P | 9, | 1 | 1.431 (x 0.699) | 29 (0.034) | 0.863 | mRNA 3'-end processing | pAbp |
| 1934 | GO:0003743 | F | 4, 5, | 2 | 2.764 (x 0.724) | 56 (0.036) | 0.863 | translation initiation factor activity | eIF-4E l(2)01424 |
| 1935 | GO:0009975 | F | 3, | 1 | 1.431 (x 0.699) | 29 (0.034) | 0.863 | cyclase activity | CG32158 |
| 1936 | GO:0045055 | P | 6, 7, | 4 | 5.183 (x 0.772) | 105 (0.038) | 0.864 | regulated secretory pathway | Amph Arf79F Snap mth |
| 1937 | GO:0016471 | C | 3, 4, 5, 6, 7, 8, 9, 10, 11, | 1 | 1.431 (x 0.699) | 29 (0.034) | 0.864 | hydrogen-translocating V-type ATPase complex | Vha100-2 |
| 1938 | GO:0007269 | P | 6, 7, 8, | 4 | 5.183 (x 0.772) | 105 (0.038) | 0.864 | neurotransmitter secretion | Amph Arf79F Snap mth |
| 1939 | GO:0005478 | F | 3, | 1 | 1.431 (x 0.699) | 29 (0.034) | 0.864 | intracellular transporter activity | Snap |
| 1940 | GO:0006397 | P | 8, | 8 | 9.773 (x 0.819) | 198 (0.040) | 0.865 | mRNA processing | B52 CG6946 Hrb27C Rm62 heph nonA-l pAbp sqd |
| 1941 | GO:0016820 | F | 5, | 6 | 7.503 (x 0.800) | 152 (0.039) | 0.865 | hydrolase activity, acting on acid anhydrides, catalyzing transmembrane movement of substances | BEST:CK02656 CG31121 Nrv1 Vha100-2 blot jumu |
| 1942 | GO:0006633 | P | 6, 7, 8, | 1 | 1.431 (x 0.699) | 29 (0.034) | 0.865 | fatty acid biosynthesis | CG2118 |
| 1943 | GO:0006816 | P | 8, 9, | 1 | 1.431 (x 0.699) | 29 (0.034) | 0.865 | calcium ion transport | pain |
| 1944 | GO:0044446 | C | 3, 4, 5, 6, 7, | 65 | 70.289 (x 0.925) | 1424 (0.046) | 0.87 | intracellular organelle part | Act42A B52 BG:DS00004.11 BcDNA:GH12558 CG14214 CG17838 CG17952 CG1907 CG1911 CG2118 CG6782 CG8679 Caf1 Crc CycT Dp Dp1 Dref Dsp1 EG:BACN33B1.2 Eb1 Fs(2)Ket GalNAc-T1 His4r HmgD Hrb27C Klp10A Mcm7 Mgstl Nek2 Pdi Pep Rbf2 RpS12 Tapdelta Top2 Trl Ucp4B Ugt Vha100-2 alpha-Man-IIb alphaTub84B ap asp betaTub56D ck d emp fzy glu heph jumu kis l(1)G0320 mod(mdg4) nonA-l p120ctn pgant5 ph-p polo sqd stai toe usp vg |
| 1945 | GO:0044422 | C | 2, 3, | 65 | 70.289 (x 0.925) | 1424 (0.046) | 0.871 | organelle part | Act42A B52 BG:DS00004.11 BcDNA:GH12558 CG14214 CG17838 CG17952 CG1907 CG1911 CG2118 CG6782 CG8679 Caf1 Crc CycT Dp Dp1 Dref Dsp1 EG:BACN33B1.2 Eb1 Fs(2)Ket GalNAc-T1 His4r HmgD Hrb27C Klp10A Mcm7 Mgstl Nek2 Pdi Pep Rbf2 RpS12 Tapdelta Top2 Trl Ucp4B Ugt Vha100-2 alpha-Man-IIb alphaTub84B ap asp betaTub56D ck d emp fzy glu heph jumu kis l(1)G0320 mod(mdg4) nonA-l p120ctn pgant5 ph-p polo sqd stai toe usp vg |
| 1946 | GO:0042386 | P | 4, 6, | 1 | 1.481 (x 0.675) | 30 (0.033) | 0.871 | hemocyte differentiation (sensu Arthropoda) | Tl |
| 1947 | GO:0016627 | F | 4, | 1 | 1.481 (x 0.675) | 30 (0.033) | 0.871 | oxidoreductase activity, acting on the CH-CH group of donors | CG4586 |
| 1948 | GO:0005871 | C | 4, 6, 7, 8, 9, 10, 11, | 1 | 1.481 (x 0.675) | 30 (0.033) | 0.872 | kinesin complex | Klp10A |
| 1949 | GO:0019201 | F | 7, | 1 | 1.481 (x 0.675) | 30 (0.033) | 0.872 | nucleotide kinase activity | CG6767 |
| 1950 | GO:0016849 | F | 4, | 1 | 1.481 (x 0.675) | 30 (0.033) | 0.873 | phosphorus-oxygen lyase activity | CG32158 |
| 1951 | GO:0009058 | P | 4, | 40 | 44.326 (x 0.902) | 898 (0.045) | 0.873 | biosynthesis | CG2118 CG31472 CG31738 CG31915 CG32158 CG33116 CG33171 CG3590 CG40160 CG6287 CG6767 CG6904 CG7860 CG8963 CrebA CtBP ESTS:39C10S GalNAc-T1 Gfat1 Pepck Pgi Ppt1 RpS12 Tpi Ts Ugt blot eIF-4E grh jumu knk l(1)G0320 l(2)01424 mdy pAbp pgant5 rasp sgl sqd woc |
| 1952 | GO:0008380 | P | 8, | 6 | 7.651 (x 0.784) | 155 (0.039) | 0.873 | RNA splicing | B52 CG6946 Rm62 heph nonA-l sqd |
| 1953 | GO:0016876 | F | 5, | 2 | 2.863 (x 0.699) | 58 (0.034) | 0.876 | ligase activity, forming aminoacyl-tRNA and related compounds | blot mdy |
| 1954 | GO:0004812 | F | 6, | 2 | 2.863 (x 0.699) | 58 (0.034) | 0.876 | aminoacyl-tRNA ligase activity | blot mdy |
| 1955 | GO:0046467 | P | 6, 7, 8, | 1 | 1.530 (x 0.654) | 31 (0.032) | 0.877 | membrane lipid biosynthesis | CG33116 |
| 1956 | GO:0003824 | F | 2, | 179 | 186.632 (x 0.959) | 3781 (0.047) | 0.877 | catalytic activity | Abi Aldh-III Ance Arf79F Argk BEST:CK02656 BEST:GH02921 BEST:LD22483 BG:DS00004.11 BcDNA:GH02901 BcDNA:GH04753 BcDNA:GH12558 BcDNA:GM10765 BcDNA:LD41548 CG11079 CG11151 CG11307 CG11438 CG11824 CG12199 CG14217 CG1471 CG1630 CG1632 CG16733 CG17323 CG18853 CG1969 CG1998 CG2108 CG2118 CG2200 CG2767 CG2852 CG30427 CG31121 CG31453 CG31472 CG31915 CG31937 CG32158 CG32499 CG32632 CG33116 CG33138 CG33171 CG3328 CG3590 CG3842 CG40160 CG40410 CG4382 CG4502 CG4586 CG4914 CG5390 CG5731 CG5794 CG5873 CG6199 CG6287 CG6391 CG6767 CG6776 CG6904 CG7379 CG7675 CG7860 CG9027 CG9307 CaMKI Cad96Ca Caf1 Cdk4 CkIIalpha CkIIbeta Cks CtBP CycT Cyp310a1 D19A EG:171D11.1 EG:BACN33B1.2 ERp60 ESTS:39C10S Eip55E Eip71CD Eno Eph Fkbp13 GNBP3 GalNAc-T1 Galpha49B Gapdh1 Gapdh2 Gfat1 GlyP GstS1 Hex-A Hexo1 Ice Idh InR Jafrac2 LanB2 Lsp2 Mcm7 Mgstl Mpk2 Nek2 Nep2 Nrv1 Oscillin PFE Pdi Pepck Pgi Pi3K21B Ppt1 Prosalpha6 Pten Ptp99A Rab8 Rep4 RhoGAP71E Rm62 RnrS Rpn9 Sb TER94 Thd1 Top2 Tpi Ts Uch Ugt Vha100-2 ald alpha-Man-IIb alphaTub84B asp betaTub56D blot br cdc2 ck d dUTPase east emp fbp for fra fwd glu jumu kis knk mbt mdy mod(mdg4) nmo olf413 ome otk pgant5 pll polo rasp scf sgl shanti shf shu smi35A th tkv tok tws |
| 1957 | GO:0007369 | P | 5, | 2 | 2.863 (x 0.699) | 58 (0.034) | 0.877 | gastrulation | pbl sgl |
| 1958 | GO:0042995 | C | 3, 4, | 1 | 1.530 (x 0.654) | 31 (0.032) | 0.877 | cell projection | lola |
| 1959 | GO:0004497 | F | 4, | 4 | 5.380 (x 0.743) | 109 (0.037) | 0.877 | monooxygenase activity | CG1998 Cyp310a1 knk olf413 |
| 1960 | GO:0016875 | F | 4, | 2 | 2.863 (x 0.699) | 58 (0.034) | 0.877 | ligase activity, forming carbon-oxygen bonds | blot mdy |
| 1961 | GO:0016410 | F | 7, | 1 | 1.530 (x 0.654) | 31 (0.032) | 0.878 | N-acyltransferase activity | CG1969 |
| 1962 | GO:0046394 | P | 7, | 1 | 1.530 (x 0.654) | 31 (0.032) | 0.878 | carboxylic acid biosynthesis | CG2118 |
| 1963 | GO:0015926 | F | 6, | 1 | 1.530 (x 0.654) | 31 (0.032) | 0.878 | glucosidase activity | GNBP3 |
| 1964 | GO:0006812 | P | 6, 7, | 16 | 18.905 (x 0.846) | 383 (0.042) | 0.878 | cation transport | CG11739 CG12048 CG15088 CG1907 CG6812 Ir Nrv1 Sh Ucp4B Vha100-2 blot jumu l(2)01810 pain para rpk |
| 1965 | GO:0015297 | F | 6, | 1 | 1.530 (x 0.654) | 31 (0.032) | 0.879 | antiporter activity | CG1907 |
| 1966 | GO:0042277 | F | 3, | 2 | 2.912 (x 0.687) | 59 (0.034) | 0.879 | peptide binding | Tapdelta l(1)G0320 |
| 1967 | GO:0016053 | P | 6, | 1 | 1.530 (x 0.654) | 31 (0.032) | 0.879 | organic acid biosynthesis | CG2118 |
| 1968 | GO:0006413 | P | 8, 9, | 2 | 2.912 (x 0.687) | 59 (0.034) | 0.88 | translational initiation | eIF-4E l(2)01424 |
| 1969 | GO:0016810 | F | 4, | 3 | 4.196 (x 0.715) | 85 (0.035) | 0.881 | hydrolase activity, acting on carbon-nitrogen (but not peptide) bonds | CG1471 CG32499 CG7860 |
| 1970 | GO:0006810 | P | 4, 5, | 67 | 72.906 (x 0.919) | 1477 (0.045) | 0.882 | transport | Amph Arf79F BEST:CK02656 BEST:LD04971 Best2 CG10657 CG11550 CG11739 CG12048 CG14214 CG14439 CG15088 CG1607 CG17419 CG1893 CG1907 CG1924 CG2108 CG2852 CG31121 CG32137 CG33113 CG33171 CG3424 CG3823 CG6812 CG9057 CG9342 CG9906 Crc Fs(2)Ket Ir Klp10A LanB2 Lsp2 Nrv1 Obp99a Rab8 S Sh Snap TER94 Ucp4B Vha100-2 alphaTub84B arr betaTub56D blot btsz cic ck d jumu l(1)G0320 l(2)01810 loj mnd mth pain para pll prominin-like rpk sqd sut1 vg wbl |
| 1971 | GO:0006418 | P | 8, 9, 10, | 2 | 2.962 (x 0.675) | 60 (0.033) | 0.882 | tRNA aminoacylation for protein translation | blot mdy |
| 1972 | GO:0043039 | P | 8, 9, | 2 | 2.962 (x 0.675) | 60 (0.033) | 0.883 | tRNA aminoacylation | blot mdy |
| 1973 | GO:0031974 | C | 2, | 18 | 21.274 (x 0.846) | 431 (0.042) | 0.883 | membrane-enclosed lumen | B52 BcDNA:GH12558 CG2118 Caf1 Crc CycT Dp Dref Hrb27C Mcm7 Pdi Rbf2 Top2 ap jumu sqd toe usp |
| 1974 | GO:0050909 | P | 5, 7, | 2 | 2.962 (x 0.675) | 60 (0.033) | 0.883 | sensory perception of taste | Galpha49B Sh |
| 1975 | GO:0030031 | P | 6, 7, | 1 | 1.580 (x 0.633) | 32 (0.031) | 0.884 | cell projection biogenesis | Abi |
| 1976 | GO:0043233 | C | 3, 4, | 18 | 21.274 (x 0.846) | 431 (0.042) | 0.884 | organelle lumen | B52 BcDNA:GH12558 CG2118 Caf1 Crc CycT Dp Dref Hrb27C Mcm7 Pdi Rbf2 Top2 ap jumu sqd toe usp |
| 1977 | GO:0005774 | C | 5, 6, 7, 8, 9, 10, | 1 | 1.580 (x 0.633) | 32 (0.031) | 0.884 | vacuolar membrane | Vha100-2 |
| 1978 | GO:0044437 | C | 4, 5, 6, 7, 8, 9, | 1 | 1.580 (x 0.633) | 32 (0.031) | 0.884 | vacuolar part | Vha100-2 |
| 1979 | GO:0019205 | F | 6, | 1 | 1.580 (x 0.633) | 32 (0.031) | 0.885 | nucleobase, nucleoside, nucleotide kinase activity | CG6767 |
| 1980 | GO:0043296 | C | 6, 7, 8, 9, | 1 | 1.580 (x 0.633) | 32 (0.031) | 0.885 | apical junction complex | Fas3 |
| 1981 | GO:0004840 | F | 4, | 1 | 1.629 (x 0.614) | 33 (0.030) | 0.889 | ubiquitin conjugating enzyme activity | CG4502 |
| 1982 | GO:0016071 | P | 7, | 8 | 10.267 (x 0.779) | 208 (0.038) | 0.889 | mRNA metabolism | B52 CG6946 Hrb27C Rm62 heph nonA-l pAbp sqd |
| 1983 | GO:0043038 | P | 7, 8, | 2 | 3.011 (x 0.664) | 61 (0.033) | 0.89 | amino acid activation | blot mdy |
| 1984 | GO:0006643 | P | 6, 7, | 3 | 4.294 (x 0.699) | 87 (0.034) | 0.89 | membrane lipid metabolism | CG33116 CG6391 Pi3K21B |
| 1985 | GO:0008293 | P | 8, | 1 | 1.629 (x 0.614) | 33 (0.030) | 0.89 | torso signaling pathway | knk |
| 1986 | GO:0008083 | F | 4, 5, | 1 | 1.629 (x 0.614) | 33 (0.030) | 0.89 | growth factor activity | mav |
| 1987 | GO:0006605 | P | 7, 8, 9, | 9 | 11.402 (x 0.789) | 231 (0.039) | 0.89 | protein targeting | CG14214 CG2852 CG32137 Fs(2)Ket Klp10A S l(1)G0320 pll wbl |
| 1988 | GO:0008639 | F | 3, | 1 | 1.678 (x 0.596) | 34 (0.029) | 0.895 | small protein conjugating enzyme activity | CG4502 |
| 1989 | GO:0004721 | F | 7, | 3 | 4.393 (x 0.683) | 89 (0.034) | 0.896 | phosphoprotein phosphatase activity | Pten Ptp99A tws |
| 1990 | GO:0015020 | F | 6, | 1 | 1.678 (x 0.596) | 34 (0.029) | 0.896 | glucuronosyltransferase activity | CG17323 |
| 1991 | GO:0009152 | P | 8, 9, | 3 | 4.393 (x 0.683) | 89 (0.034) | 0.896 | purine ribonucleotide biosynthesis | CG3590 blot jumu |
| 1992 | GO:0008170 | F | 6, | 1 | 1.678 (x 0.596) | 34 (0.029) | 0.896 | N-methyltransferase activity | Caf1 |
| 1993 | GO:0009187 | P | 7, | 1 | 1.678 (x 0.596) | 34 (0.029) | 0.897 | cyclic nucleotide metabolism | CG32158 |
| 1994 | GO:0046933 | F | 7, | 2 | 3.110 (x 0.643) | 63 (0.032) | 0.897 | hydrogen-transporting ATP synthase activity, rotational mechanism | blot jumu |
| 1995 | GO:0005184 | F | 5, 6, | 1 | 1.678 (x 0.596) | 34 (0.029) | 0.897 | neuropeptide hormone activity | ETH |
| 1996 | GO:0044249 | P | 5, | 36 | 40.969 (x 0.879) | 830 (0.043) | 0.897 | cellular biosynthesis | CG2118 CG31472 CG31738 CG31915 CG32158 CG33171 CG3590 CG40160 CG6287 CG6767 CG6904 CG7860 CG8963 CtBP ESTS:39C10S GalNAc-T1 Gfat1 Pepck Pgi Ppt1 RpS12 Tpi Ts Ugt blot eIF-4E jumu l(1)G0320 l(2)01424 mdy pAbp pgant5 rasp sgl sqd woc |
| 1997 | GO:0046961 | F | 6, 7, 9, 14, | 2 | 3.110 (x 0.643) | 63 (0.032) | 0.898 | hydrogen-transporting ATPase activity, rotational mechanism | blot jumu |
| 1998 | GO:0042579 | C | 5, 6, 7, 8, | 1 | 1.678 (x 0.596) | 34 (0.029) | 0.898 | microbody | CG4586 |
| 1999 | GO:0005777 | C | 6, 7, 8, 9, | 1 | 1.678 (x 0.596) | 34 (0.029) | 0.898 | peroxisome | CG4586 |
| 2000 | GO:0008415 | F | 6, | 4 | 5.676 (x 0.705) | 115 (0.035) | 0.898 | acyltransferase activity | CG1969 CG7379 mdy rasp |
| 2001 | GO:0009150 | P | 8, | 3 | 4.442 (x 0.675) | 90 (0.033) | 0.899 | purine ribonucleotide metabolism | CG3590 blot jumu |
| 2002 | GO:0046483 | P | 5, | 5 | 6.910 (x 0.724) | 140 (0.036) | 0.899 | heterocycle metabolism | CG3590 CG6767 EG:171D11.1 RnrS Ts |
| 2003 | GO:0006811 | P | 5, 6, | 19 | 22.805 (x 0.833) | 462 (0.041) | 0.899 | ion transport | Best2 CG11739 CG12048 CG15088 CG17419 CG1907 CG33171 CG6812 Ir Nrv1 Sh Ucp4B Vha100-2 blot jumu l(2)01810 pain para rpk |
| 2004 | GO:0015986 | P | 7, 8, 9, 10, 11, 12, | 2 | 3.159 (x 0.633) | 64 (0.031) | 0.9 | ATP synthesis coupled proton transport | blot jumu |
| 2005 | GO:0006753 | P | 8, | 2 | 3.159 (x 0.633) | 64 (0.031) | 0.9 | nucleoside phosphate metabolism | blot jumu |
| 2006 | GO:0006754 | P | 7, 8, 9, 10, 11, | 2 | 3.159 (x 0.633) | 64 (0.031) | 0.9 | ATP biosynthesis | blot jumu |
| 2007 | GO:0015985 | P | 7, 8, 9, 10, | 2 | 3.159 (x 0.633) | 64 (0.031) | 0.901 | energy coupled proton transport, down electrochemical gradient | blot jumu |
| 2008 | GO:0018193 | P | 8, | 1 | 1.728 (x 0.579) | 35 (0.029) | 0.901 | peptidyl-amino acid modification | rasp |
| 2009 | GO:0005875 | C | 3, 5, 6, 7, 8, 9, 10, | 4 | 5.726 (x 0.699) | 116 (0.034) | 0.901 | microtubule associated complex | Eb1 Klp10A asp stai |
| 2010 | GO:0006897 | P | 6, 7, | 4 | 5.726 (x 0.699) | 116 (0.034) | 0.901 | endocytosis | Amph Arf79F Rab8 arr |
| 2011 | GO:0006164 | P | 7, 8, | 3 | 4.492 (x 0.668) | 91 (0.033) | 0.902 | purine nucleotide biosynthesis | CG3590 blot jumu |
| 2012 | GO:0001505 | P | 7, | 4 | 5.775 (x 0.693) | 117 (0.034) | 0.904 | regulation of neurotransmitter levels | Amph Arf79F Snap mth |
| 2013 | GO:0046034 | P | 6, 10, | 2 | 3.208 (x 0.623) | 65 (0.031) | 0.905 | ATP metabolism | blot jumu |
| 2014 | GO:0005681 | C | 4, 5, 6, 7, 8, 9, 10, | 3 | 4.541 (x 0.661) | 92 (0.033) | 0.905 | spliceosome complex | B52 heph nonA-l |
| 2015 | GO:0019897 | C | 5, 6, 7, | 1 | 1.777 (x 0.563) | 36 (0.028) | 0.906 | extrinsic to plasma membrane | Galpha49B |
| 2016 | GO:0006163 | P | 7, | 3 | 4.541 (x 0.661) | 92 (0.033) | 0.906 | purine nucleotide metabolism | CG3590 blot jumu |
| 2017 | GO:0044445 | C | 5, 6, 7, 8, 9, | 4 | 5.825 (x 0.687) | 118 (0.034) | 0.907 | cytosolic part | Eno Ice Pi3K21B RpS12 |
| 2018 | GO:0006403 | P | 4, | 3 | 4.591 (x 0.654) | 93 (0.032) | 0.91 | RNA localization | Hrb27C TER94 sqd |
| 2019 | GO:0008270 | F | 6, | 25 | 29.715 (x 0.841) | 602 (0.042) | 0.911 | zinc ion binding | Ance CG11033 CG15435 CG17419 CG4496 CG6654 CG6930 CG8092 D19A LanB2 Nep2 Pep Trl ap br cg ci esn klu lola opa pk th tok zf30C |
| 2020 | GO:0016327 | C | 5, 6, 7, | 1 | 1.826 (x 0.548) | 37 (0.027) | 0.911 | apicolateral plasma membrane | Fas3 |
| 2021 | GO:0015276 | F | 5, 6, | 2 | 3.307 (x 0.605) | 67 (0.030) | 0.915 | ligand-gated ion channel activity | CG14076 rpk |
| 2022 | GO:0009206 | P | 9, 10, | 2 | 3.357 (x 0.596) | 68 (0.029) | 0.917 | purine ribonucleoside triphosphate biosynthesis | blot jumu |
| 2023 | GO:0016469 | C | 3, 6, 7, 8, | 2 | 3.357 (x 0.596) | 68 (0.029) | 0.918 | proton-transporting two-sector ATPase complex | blot jumu |
| 2024 | GO:0009145 | P | 8, 9, | 2 | 3.357 (x 0.596) | 68 (0.029) | 0.918 | purine nucleoside triphosphate biosynthesis | blot jumu |
| 2025 | GO:0016747 | F | 5, | 4 | 5.973 (x 0.670) | 121 (0.033) | 0.918 | transferase activity, transferring groups other than amino-acyl groups | CG1969 CG7379 mdy rasp |
| 2026 | GO:0008094 | F | 10, | 1 | 1.876 (x 0.533) | 38 (0.026) | 0.918 | DNA-dependent ATPase activity | Mcm7 |
| 2027 | GO:0046914 | F | 5, | 28 | 33.368 (x 0.839) | 676 (0.041) | 0.919 | transition metal ion binding | Ance BcDNA:LD41548 CG11033 CG15435 CG17419 CG4496 CG6654 CG6930 CG8092 D19A LanB2 Nep2 Pep Trl Vha100-2 ap br cg ci esn klu lola olf413 opa pk th tok zf30C |
| 2028 | GO:0009201 | P | 8, 9, | 2 | 3.357 (x 0.596) | 68 (0.029) | 0.919 | ribonucleoside triphosphate biosynthesis | blot jumu |
| 2029 | GO:0008066 | F | 5, | 1 | 1.876 (x 0.533) | 38 (0.026) | 0.919 | glutamate receptor activity | CG14076 |
| 2030 | GO:0016283 | C | 3, 5, 6, 7, 8, | 1 | 1.925 (x 0.519) | 39 (0.026) | 0.919 | eukaryotic 48S initiation complex | RpS12 |
| 2031 | GO:0019748 | P | 4, | 2 | 3.357 (x 0.596) | 68 (0.029) | 0.919 | secondary metabolism | Tl serpin-27A |
| 2032 | GO:0015296 | F | 5, 7, | 1 | 1.925 (x 0.519) | 39 (0.026) | 0.919 | anion:cation symporter activity | l(2)01810 |
| 2033 | GO:0005843 | C | 4, 5, 6, 7, 8, 9, 10, 11, | 1 | 1.925 (x 0.519) | 39 (0.026) | 0.92 | cytosolic small ribosomal subunit (sensu Eukaryota) | RpS12 |
| 2034 | GO:0008236 | F | 5, | 11 | 14.463 (x 0.761) | 293 (0.038) | 0.92 | serine-type peptidase activity | BEST:GH02921 BG:DS00004.11 CG11824 CG1632 CG2200 CG40160 CG4914 CG5390 Sb TER94 ome |
| 2035 | GO:0004722 | F | 8, | 1 | 1.925 (x 0.519) | 39 (0.026) | 0.92 | protein serine/threonine phosphatase activity | tws |
| 2036 | GO:0009142 | P | 7, 8, | 2 | 3.406 (x 0.587) | 69 (0.029) | 0.921 | nucleoside triphosphate biosynthesis | blot jumu |
| 2037 | GO:0004004 | F | 5, 11, | 1 | 1.974 (x 0.506) | 40 (0.025) | 0.921 | ATP-dependent RNA helicase activity | Rm62 |
| 2038 | GO:0019829 | F | 5, 6, 8, 13, | 2 | 3.406 (x 0.587) | 69 (0.029) | 0.921 | cation-transporting ATPase activity | blot jumu |
| 2039 | GO:0043414 | P | 7, | 1 | 1.974 (x 0.506) | 40 (0.025) | 0.921 | biopolymer methylation | Caf1 |
| 2040 | GO:0009199 | P | 8, | 2 | 3.406 (x 0.587) | 69 (0.029) | 0.921 | ribonucleoside triphosphate metabolism | blot jumu |
| 2041 | GO:0004177 | F | 6, | 1 | 1.974 (x 0.506) | 40 (0.025) | 0.922 | aminopeptidase activity | BcDNA:LD41548 |
| 2042 | GO:0009205 | P | 9, | 2 | 3.406 (x 0.587) | 69 (0.029) | 0.922 | purine ribonucleoside triphosphate metabolism | blot jumu |
| 2043 | GO:0005200 | F | 3, | 11 | 14.413 (x 0.763) | 292 (0.038) | 0.922 | structural constituent of cytoskeleton | Act42A CG31738 CG32137 EG:118B3.2 Klp10A alphaTub84B betaTub56D ck d esn pk |
| 2044 | GO:0015674 | P | 7, 8, | 1 | 1.974 (x 0.506) | 40 (0.025) | 0.922 | di-, tri-valent inorganic cation transport | pain |
| 2045 | GO:0009144 | P | 8, | 2 | 3.406 (x 0.587) | 69 (0.029) | 0.922 | purine nucleoside triphosphate metabolism | blot jumu |
| 2046 | GO:0008010 | F | 5, | 1 | 1.974 (x 0.506) | 40 (0.025) | 0.922 | structural constituent of larval cuticle (sensu Insecta) | CG8502 |
| 2047 | GO:0005643 | C | 3, 5, 6, 7, 8, 9, 10, 11, 12, 13, | 1 | 1.974 (x 0.506) | 40 (0.025) | 0.923 | nuclear pore | Fs(2)Ket |
| 2048 | GO:0046930 | C | 6, 7, 8, | 1 | 1.974 (x 0.506) | 40 (0.025) | 0.923 | pore complex | Fs(2)Ket |
| 2049 | GO:0017111 | F | 7, | 21 | 25.816 (x 0.813) | 523 (0.040) | 0.923 | nucleoside-triphosphatase activity | Arf79F BEST:CK02656 CG2108 CG31121 CG31453 Galpha49B Mcm7 Nrv1 Rab8 Rm62 TER94 Top2 Vha100-2 alphaTub84B betaTub56D blot ck d glu jumu kis |
| 2050 | GO:0008186 | F | 10, | 1 | 1.974 (x 0.506) | 40 (0.025) | 0.924 | RNA-dependent ATPase activity | Rm62 |
| 2051 | GO:0004222 | F | 6, | 2 | 3.505 (x 0.571) | 71 (0.028) | 0.924 | metalloendopeptidase activity | Nep2 tok |
| 2052 | GO:0015103 | F | 5, | 1 | 1.974 (x 0.506) | 40 (0.025) | 0.924 | inorganic anion transporter activity | l(2)01810 |
| 2053 | GO:0042302 | F | 3, | 3 | 4.887 (x 0.614) | 99 (0.030) | 0.925 | structural constituent of cuticle | CG15757 CG8502 CG8634 |
| 2054 | GO:0004527 | F | 6, | 1 | 2.024 (x 0.494) | 41 (0.024) | 0.926 | exonuclease activity | BcDNA:GM10765 |
| 2055 | GO:0007268 | P | 6, | 8 | 11.155 (x 0.717) | 226 (0.035) | 0.927 | synaptic transmission | Amph Arf79F CaMKI Snap mth olf413 pAbp para |
| 2056 | GO:0006752 | P | 7, | 2 | 3.554 (x 0.563) | 72 (0.028) | 0.927 | group transfer coenzyme metabolism | blot jumu |
| 2057 | GO:0015662 | F | 5, 6, 8, 13, | 2 | 3.554 (x 0.563) | 72 (0.028) | 0.928 | ATPase activity, coupled to transmembrane movement of ions, phosphorylative mechanism | Nrv1 Vha100-2 |
| 2058 | GO:0016746 | F | 4, | 4 | 6.269 (x 0.638) | 127 (0.031) | 0.929 | transferase activity, transferring acyl groups | CG1969 CG7379 mdy rasp |
| 2059 | GO:0016251 | F | 4, | 3 | 4.985 (x 0.602) | 101 (0.030) | 0.931 | general RNA polymerase II transcription factor activity | BEST:LD29214 GATAd grn |
| 2060 | GO:0016887 | F | 8, | 14 | 18.313 (x 0.764) | 371 (0.038) | 0.933 | ATPase activity | BEST:CK02656 CG31121 Mcm7 Nrv1 Rm62 TER94 Top2 Vha100-2 blot ck d glu jumu kis |
| 2061 | GO:0017157 | P | 6, 7, 8, | 1 | 2.123 (x 0.471) | 43 (0.023) | 0.935 | regulation of exocytosis | Rab8 |
| 2062 | GO:0044454 | C | 5, 6, 7, 8, 9, 10, 11, | 1 | 2.123 (x 0.471) | 43 (0.023) | 0.936 | nuclear chromosome part | HmgD |
| 2063 | GO:0015078 | F | 6, | 3 | 5.084 (x 0.590) | 103 (0.029) | 0.938 | hydrogen ion transporter activity | Vha100-2 blot jumu |
| 2064 | GO:0016791 | F | 6, | 6 | 8.984 (x 0.668) | 182 (0.033) | 0.939 | phosphoric monoester hydrolase activity | CG11438 Pten Ptp99A fbp mod(mdg4) tws |
| 2065 | GO:0007186 | P | 6, | 11 | 15.055 (x 0.731) | 305 (0.036) | 0.942 | G-protein coupled receptor protein signaling pathway | CG11438 CG17084 CG30440 CG32158 CG5522 CG6965 ETH Galpha49B mth pbl stan |
| 2066 | GO:0008233 | F | 4, | 26 | 32.134 (x 0.809) | 651 (0.040) | 0.943 | peptidase activity | Ance BEST:GH02921 BG:DS00004.11 BcDNA:LD41548 CG11824 CG1632 CG2200 CG3328 CG40160 CG4914 CG5390 CG5794 D19A Ice Lsp2 Nep2 Prosalpha6 Rpn9 Sb TER94 Uch east fra ome shanti tok |
| 2067 | GO:0015077 | F | 5, | 3 | 5.183 (x 0.579) | 105 (0.029) | 0.944 | monovalent inorganic cation transporter activity | Vha100-2 blot jumu |
| 2068 | GO:0005231 | F | 7, 8, | 1 | 2.221 (x 0.450) | 45 (0.022) | 0.944 | excitatory extracellular ligand-gated ion channel activity | CG14076 |
| 2069 | GO:0000228 | C | 5, 6, 7, 8, 9, 10, | 1 | 2.271 (x 0.440) | 46 (0.022) | 0.949 | nuclear chromosome | HmgD |
| 2070 | GO:0043037 | P | 7, 8, | 6 | 9.181 (x 0.654) | 186 (0.032) | 0.949 | translation | blot eIF-4E l(2)01424 mdy pAbp sqd |
| 2071 | GO:0009060 | P | 8, | 1 | 2.320 (x 0.431) | 47 (0.021) | 0.952 | aerobic respiration | Idh |
| 2072 | GO:0006099 | P | 8, 9, | 1 | 2.320 (x 0.431) | 47 (0.021) | 0.952 | tricarboxylic acid cycle | Idh |
| 2073 | GO:0046356 | P | 8, | 1 | 2.320 (x 0.431) | 47 (0.021) | 0.953 | acetyl-CoA catabolism | Idh |
| 2074 | GO:0045333 | P | 7, | 1 | 2.320 (x 0.431) | 47 (0.021) | 0.953 | cellular respiration | Idh |
| 2075 | GO:0009109 | P | 7, | 1 | 2.369 (x 0.422) | 48 (0.021) | 0.956 | coenzyme catabolism | Idh |
| 2076 | GO:0006084 | P | 7, | 1 | 2.369 (x 0.422) | 48 (0.021) | 0.956 | acetyl-CoA metabolism | Idh |
| 2077 | GO:0015144 | F | 3, | 2 | 3.998 (x 0.500) | 81 (0.025) | 0.957 | carbohydrate transporter activity | LanB2 sut1 |
| 2078 | GO:0008643 | P | 5, 6, | 2 | 3.998 (x 0.500) | 81 (0.025) | 0.957 | carbohydrate transport | LanB2 sut1 |
| 2079 | GO:0042598 | C | 5, 6, | 2 | 4.097 (x 0.488) | 83 (0.024) | 0.958 | vesicular fraction | Cyp310a1 Mgstl |
| 2080 | GO:0008553 | F | 6, 7, 9, 14, | 1 | 2.419 (x 0.413) | 49 (0.020) | 0.958 | hydrogen-exporting ATPase activity, phosphorylative mechanism | Vha100-2 |
| 2081 | GO:0006091 | P | 5, | 19 | 24.927 (x 0.762) | 505 (0.038) | 0.958 | generation of precursor metabolites and energy | BEST:LD22483 CG33138 CG40160 CG6904 Cyp310a1 ERp60 Eno Gapdh1 Gapdh2 GlyP Hex-A Idh Pdi Pepck Pgi Tpi blot jumu sgl |
| 2082 | GO:0008026 | F | 4, 10, | 2 | 4.097 (x 0.488) | 83 (0.024) | 0.958 | ATP-dependent helicase activity | Rm62 kis |
| 2083 | GO:0008135 | F | 3, 4, | 2 | 4.048 (x 0.494) | 82 (0.024) | 0.958 | translation factor activity, nucleic acid binding | eIF-4E l(2)01424 |
| 2084 | GO:0004519 | F | 6, | 1 | 2.419 (x 0.413) | 49 (0.020) | 0.958 | endonuclease activity | BcDNA:GM10765 |
| 2085 | GO:0007548 | P | 3, | 1 | 2.468 (x 0.405) | 50 (0.020) | 0.958 | sex differentiation | en |
| 2086 | GO:0005792 | C | 6, 7, | 2 | 4.097 (x 0.488) | 83 (0.024) | 0.958 | microsome | Cyp310a1 Mgstl |
| 2087 | GO:0051187 | P | 6, | 1 | 2.419 (x 0.413) | 49 (0.020) | 0.959 | cofactor catabolism | Idh |
| 2088 | GO:0003777 | F | 3, | 1 | 2.468 (x 0.405) | 50 (0.020) | 0.959 | microtubule motor activity | Klp10A |
| 2089 | GO:0008595 | P | 6, 7, | 1 | 2.468 (x 0.405) | 50 (0.020) | 0.959 | determination of anterior/posterior axis, embryo | knk |
| 2090 | GO:0004386 | F | 3, | 3 | 5.528 (x 0.543) | 112 (0.027) | 0.96 | helicase activity | Mcm7 Rm62 kis |
| 2091 | GO:0008021 | C | 8, 9, 10, 11, 12, | 1 | 2.517 (x 0.397) | 51 (0.020) | 0.96 | synaptic vesicle | btsz |
| 2092 | GO:0007351 | P | 5, 6, | 1 | 2.468 (x 0.405) | 50 (0.020) | 0.96 | regional subdivision | knk |
| 2093 | GO:0003678 | F | 4, | 1 | 2.517 (x 0.397) | 51 (0.020) | 0.96 | DNA helicase activity | Mcm7 |
| 2094 | GO:0005230 | F | 6, 7, | 1 | 2.468 (x 0.405) | 50 (0.020) | 0.96 | extracellular ligand-gated ion channel activity | CG14076 |
| 2095 | GO:0008168 | F | 5, | 2 | 4.146 (x 0.482) | 84 (0.024) | 0.96 | methyltransferase activity | Caf1 Ts |
| 2096 | GO:0045182 | F | 2, | 2 | 4.196 (x 0.477) | 85 (0.024) | 0.962 | translation regulator activity | eIF-4E l(2)01424 |
| 2097 | GO:0016741 | F | 4, | 2 | 4.196 (x 0.477) | 85 (0.024) | 0.962 | transferase activity, transferring one-carbon groups | Caf1 Ts |
| 2098 | GO:0006352 | P | 8, | 2 | 4.245 (x 0.471) | 86 (0.023) | 0.964 | transcription initiation | ara cdc2 |
| 2099 | GO:0005179 | F | 4, 5, | 1 | 2.616 (x 0.382) | 53 (0.019) | 0.965 | hormone activity | ETH |
| 2100 | GO:0042578 | F | 5, | 6 | 9.872 (x 0.608) | 200 (0.030) | 0.968 | phosphoric ester hydrolase activity | CG11438 Pten Ptp99A fbp mod(mdg4) tws |
| 2101 | GO:0005730 | C | 5, 6, 7, 8, 9, 10, 11, | 1 | 2.665 (x 0.375) | 54 (0.019) | 0.968 | nucleolus | jumu |
| 2102 | GO:0006399 | P | 7, | 2 | 4.344 (x 0.460) | 88 (0.023) | 0.968 | tRNA metabolism | blot mdy |
| 2103 | GO:0006396 | P | 7, | 8 | 12.587 (x 0.636) | 255 (0.031) | 0.972 | RNA processing | B52 CG6946 Hrb27C Rm62 heph nonA-l pAbp sqd |
| 2104 | GO:0042623 | F | 9, | 11 | 16.289 (x 0.675) | 330 (0.033) | 0.972 | ATPase activity, coupled | BEST:CK02656 CG31121 Mcm7 Nrv1 Rm62 Vha100-2 blot ck d jumu kis |
| 2105 | GO:0004518 | F | 5, | 2 | 4.442 (x 0.450) | 90 (0.022) | 0.972 | nuclease activity | BcDNA:GM10765 Rep4 |
| 2106 | GO:0030136 | C | 7, 8, 9, 10, 11, | 1 | 2.764 (x 0.362) | 56 (0.018) | 0.972 | clathrin-coated vesicle | btsz |
| 2107 | GO:0004252 | F | 6, | 8 | 12.686 (x 0.631) | 257 (0.031) | 0.974 | serine-type endopeptidase activity | BEST:GH02921 CG11824 CG1632 CG40160 CG4914 CG5390 Sb TER94 |
| 2108 | GO:0009108 | P | 7, | 2 | 4.640 (x 0.431) | 94 (0.021) | 0.977 | coenzyme biosynthesis | blot jumu |
| 2109 | GO:0008509 | F | 4, | 2 | 4.640 (x 0.431) | 94 (0.021) | 0.978 | anion transporter activity | CG1907 l(2)01810 |
| 2110 | GO:0003724 | F | 4, | 1 | 2.912 (x 0.343) | 59 (0.017) | 0.978 | RNA helicase activity | Rm62 |
| 2111 | GO:0004263 | F | 7, | 6 | 10.267 (x 0.584) | 208 (0.029) | 0.978 | chymotrypsin activity | BEST:GH02921 CG11824 CG40160 CG4914 CG5390 Sb |
| 2112 | GO:0051049 | P | 5, 6, | 1 | 2.912 (x 0.343) | 59 (0.017) | 0.978 | regulation of transport | Rab8 |
| 2113 | GO:0016282 | C | 3, 5, 6, 7, 8, | 1 | 2.962 (x 0.338) | 60 (0.017) | 0.979 | eukaryotic 43S preinitiation complex | RpS12 |
| 2114 | GO:0004295 | F | 7, | 7 | 11.600 (x 0.603) | 235 (0.030) | 0.979 | trypsin activity | BEST:GH02921 CG11824 CG1632 CG40160 CG4914 CG5390 Sb |
| 2115 | GO:0008757 | F | 6, | 1 | 2.912 (x 0.343) | 59 (0.017) | 0.979 | S-adenosylmethionine-dependent methyltransferase activity | Caf1 |
| 2116 | GO:0051189 | P | 5, 7, | 1 | 2.962 (x 0.338) | 60 (0.017) | 0.979 | prosthetic group metabolism | CG10657 |
| 2117 | GO:0006732 | P | 6, | 7 | 11.797 (x 0.593) | 239 (0.029) | 0.981 | coenzyme metabolism | BEST:LD22483 CG10657 CG31472 Idh Tpi blot jumu |
| 2118 | GO:0031975 | C | 2, | 8 | 13.179 (x 0.607) | 267 (0.030) | 0.981 | envelope | CG17952 CG1907 CG6782 CG8679 Fs(2)Ket Mgstl Ucp4B Ugt |
| 2119 | GO:0030529 | C | 3, 4, 5, 6, | 10 | 15.697 (x 0.637) | 318 (0.031) | 0.982 | ribonucleoprotein complex | B52 CG17838 CG31738 Hrb27C Pep RpS12 Tapdelta heph nonA-l sqd |
| 2120 | GO:0031967 | C | 3, 4, 5, 6, 7, 8, | 8 | 13.179 (x 0.607) | 267 (0.030) | 0.982 | organelle envelope | CG17952 CG1907 CG6782 CG8679 Fs(2)Ket Mgstl Ucp4B Ugt |
| 2121 | GO:0006412 | P | 6, 7, | 17 | 24.384 (x 0.697) | 494 (0.034) | 0.984 | protein biosynthesis | CG31738 CG33171 CG7860 CG8963 GalNAc-T1 Ppt1 RpS12 Ugt blot eIF-4E l(1)G0320 l(2)01424 mdy pAbp pgant5 rasp sqd |
| 2122 | GO:0006820 | P | 6, 7, | 2 | 4.936 (x 0.405) | 100 (0.020) | 0.984 | anion transport | CG33171 l(2)01810 |
| 2123 | GO:0004175 | F | 5, | 16 | 23.249 (x 0.688) | 471 (0.034) | 0.984 | endopeptidase activity | BEST:GH02921 CG11824 CG1632 CG3328 CG40160 CG4914 CG5390 CG5794 Ice Nep2 Prosalpha6 Rpn9 Sb TER94 shanti tok |
| 2124 | GO:0003674 | F | 1, | 400 | 409.002 (x 0.978) | 8286 (0.048) | 0.984 | molecular\_function | 18w Abi Act42A Aldh-III Amph Ance Arf79F Argk B52 BEST:CK02656 BEST:GH02921 BEST:LD04971 BEST:LD22483 BEST:LD29214 BG:DS00004.11 BcDNA:GH02901 BcDNA:GH02976 BcDNA:GH04753 BcDNA:GH12558 BcDNA:GM10765 BcDNA:LD21403 BcDNA:LD41548 CBP CG10359 CG10657 CG11033 CG11079 CG11142 CG11151 CG11275 CG11307 CG11438 CG11550 CG11711 CG11739 CG11824 CG12048 CG12199 CG13848 CG14076 CG14214 CG14217 CG14439 CG1471 CG15088 CG15435 CG15757 CG15835 CG1607 CG1630 CG1632 CG16733 CG17052 CG17084 CG17323 CG17419 CG17493 CG17838 CG17919 CG17952 CG18853 CG1893 CG1907 CG1911 CG1924 CG1969 CG1998 CG2108 CG2118 CG2200 CG2767 CG2852 CG30011 CG30427 CG30440 CG31121 CG31125 CG31320 CG3136 CG31453 CG31472 CG31738 CG31749 CG31915 CG31937 CG31997 CG32137 CG32158 CG32354 CG32415 CG32499 CG32632 CG32711 CG32827 CG33113 CG33116 CG33138 CG33171 CG3328 CG33525 CG3424 CG3590 CG3800 CG3823 CG3842 CG3921 CG40160 CG40410 CG4382 CG4496 CG4502 CG4586 CG4914 CG5096 CG5319 CG5390 CG5392 CG5522 CG5731 CG5794 CG5873 CG6049 CG6199 CG6287 CG6391 CG6654 CG6680 CG6767 CG6776 CG6782 CG6812 CG6891 CG6904 CG6930 CG6946 CG6965 CG6966 CG7379 CG7668 CG7675 CG7860 CG8092 CG8165 CG8502 CG8588 CG8634 CG8963 CG9027 CG9057 CG9066 CG9134 CG9135 CG9211 CG9307 CG9342 CG9906 CG9924 CREG CaMKI Cad74A Cad87A Cad96Ca Caf1 Cdk4 Cip4 CkIIalpha CkIIbeta Cks Crc CrebA CtBP CycB3 CycT Cyp310a1 D19A Dl DnaJ-1 Doc1 Doc2 Doc3 Dp Dp1 Dr Dref Dsp1 EG:118B3.2 EG:171D11.1 EG:BACN33B1.2 ERp60 ESTS:39C10S ETH Eb1 Eip55E Eip71CD Eip74EF Eip75B Eno Eph Fkbp13 Fs(2)Ket GATAd GNBP3 GalNAc-T1 Galpha49B Gapdh1 Gapdh2 Gfat1 GlyP GstS1 HLHm7 Hex-A Hexo1 His4r HmgD HmgZ Hrb27C Hsp23 Ice Idh InR Incenp Ir Jafrac2 Klp10A LanB2 Lsp2 M6 Mcm7 Mgstl Mpk2 Nek2 Nep2 Nrv1 Obp99a Optix Oscillin PFE Pdi Pep Pepck Pgi Pi3K21B Pli Poxn Ppt1 Prosalpha6 Pten Ptp99A Rab8 RacGAP50C Rapgap1 Rbf2 Rep4 RhoGAP71E Rhp Rm62 RnrS RpS12 Rpn9 Sb Sema-1a Sema-1b Sema-2a Set Sh Snap Spn1 Spn43Aa Spn5 Sry-alpha TER94 Tapdelta Thd1 Tl Top2 Tpi Trl Ts Tsp26A Tsp39D Tsp66E Tsp96F Uch Ucp4B Ugt Vha100-2 Wnt2 a6 ald alpha-Man-IIb alphaTub84B ap ara arr ash2 asp betaTub56D bip1 blot bnb br btsz bun cdc2 ced-6 cg ci cib cic ck crp cyc d dUTPase dap dve eIF-4E east edl emp en esn fbp for fra fwd glec glu grh grn heph hth in inv inx2 inx3 jumu kis klu knk l(1)G0320 l(2)01424 l(2)01810 l(3)neo38 lid loj lola mav mbt mdy mnd mod(mdg4) mth nmo nonA-l numb ogre olf413 ome opa otk pAbp pain para pbl pcs pdm2 pgant5 ph-p pk pll polo rasp rpk sc scf serpin-27A sgl shanti shf shu smi35A spen sqd stai stan sut1 th tkv toe tok trh trn tws usp vg vimar wgn woc zf30C |
| 2125 | GO:0008324 | F | 4, | 13 | 19.695 (x 0.660) | 399 (0.033) | 0.984 | cation transporter activity | CG12048 CG15088 CG6812 Ir Nrv1 Sh Vha100-2 blot jumu l(2)01810 pain para rpk |
| 2126 | GO:0043234 | C | 2, | 64 | 77.940 (x 0.821) | 1579 (0.041) | 0.99 | protein complex | B52 BEST:CK02656 BG:DS00004.11 BcDNA:GH12558 CG14214 CG17838 CG1911 CG31121 CG31738 Caf1 CkIIalpha CkIIbeta CycT Dp Dref EG:BACN33B1.2 Eb1 Eno Fs(2)Ket Galpha49B His4r Hrb27C Ice InR Klp10A Lsp2 Mcm7 Nrv1 Pep Pi3K21B Prosalpha6 Rbf2 RnrS RpS12 Rpn9 Sh TER94 Tapdelta Top2 Vha100-2 alphaTub84B asp betaTub56D blot ci ck d eIF-4E emp for glu heph jumu l(1)G0320 l(2)01424 nonA-l para ph-p sqd stai th toe tws usp |
| 2127 | GO:0030135 | C | 6, 7, 8, 9, 10, | 1 | 3.357 (x 0.298) | 68 (0.015) | 0.99 | coated vesicle | btsz |
| 2128 | GO:0051188 | P | 6, | 2 | 5.183 (x 0.386) | 105 (0.019) | 0.99 | cofactor biosynthesis | blot jumu |
| 2129 | GO:0051186 | P | 5, | 7 | 12.488 (x 0.561) | 253 (0.028) | 0.991 | cofactor metabolism | BEST:LD22483 CG10657 CG31472 Idh Tpi blot jumu |
| 2130 | GO:0015935 | C | 3, 4, 5, 6, 7, 8, 9, | 1 | 3.406 (x 0.294) | 69 (0.014) | 0.991 | small ribosomal subunit | RpS12 |
| 2131 | GO:0031090 | C | 4, 5, 6, 7, 8, | 11 | 17.819 (x 0.617) | 361 (0.030) | 0.992 | organelle membrane | BG:DS00004.11 CG14214 CG17952 CG1907 Fs(2)Ket Mgstl Tapdelta Ucp4B Vha100-2 alpha-Man-IIb l(1)G0320 |
| 2132 | GO:0016070 | P | 6, | 11 | 18.115 (x 0.607) | 367 (0.030) | 0.996 | RNA metabolism | B52 CG6946 Hrb27C Rm62 blot eIF-4E heph mdy nonA-l pAbp sqd |
| 2133 | GO:0006512 | P | 8, | 6 | 11.550 (x 0.519) | 234 (0.026) | 0.996 | ubiquitin cycle | CG4502 CG5794 Prosalpha6 Uch fzy th |
| 2134 | GO:0005549 | F | 3, | 2 | 5.676 (x 0.352) | 115 (0.017) | 0.998 | odorant binding | Obp99a a6 |
| 2135 | GO:0000004 | P | 2, | 9 | 34.602 (x 0.260) | 701 (0.013) | 1 | biological process unknown | CG31125 CG31320 CG31749 CG31997 CG32415 CG32711 CG32827 bip1 l(3)neo38 |
| 2136 | GO:0016788 | F | 4, | 14 | 22.459 (x 0.623) | 455 (0.031) | 1 | hydrolase activity, acting on ester bonds | BcDNA:GM10765 CG11438 CG1471 CG4382 CG5794 Ppt1 Pten Ptp99A Rep4 Uch br fbp mod(mdg4) tws |
| 2137 | GO:0005554 | F | 2, | 11 | 37.119 (x 0.296) | 752 (0.015) | 1 | molecular function unknown | CG11711 CG31125 CG31320 CG31749 CG31997 CG32415 CG32711 CG32827 CG8588 bnb l(3)neo38 |
| 2138 | GO:0005198 | F | 2, | 25 | 36.428 (x 0.686) | 738 (0.034) | 1 | structural molecule activity | Act42A BcDNA:GH02976 CG11142 CG15757 CG17052 CG31738 CG32137 CG33171 CG8502 CG8634 EG:118B3.2 Klp10A LanB2 M6 RpS12 alphaTub84B betaTub56D ck d esn lola pk stan trn vg |
| 2139 | GO:0015075 | F | 3, | 15 | 23.940 (x 0.627) | 485 (0.031) | 1 | ion transporter activity | CG12048 CG14076 CG15088 CG1907 CG6812 Ir Nrv1 Sh Vha100-2 blot jumu l(2)01810 pain para rpk |
| 2140 | GO:0031982 | C | 3, | 1 | 4.245 (x 0.236) | 86 (0.012) | 1 | vesicle | btsz |
| 2141 | GO:0006367 | P | 9, | 1 | 4.146 (x 0.241) | 84 (0.012) | 1 | transcription initiation from RNA polymerase II promoter | cdc2 |
| 2142 | GO:0016789 | F | 5, | 2 | 5.923 (x 0.338) | 120 (0.017) | 1 | carboxylic ester hydrolase activity | CG1471 CG4382 |
| 2143 | GO:0008372 | C | 2, | 14 | 40.179 (x 0.348) | 814 (0.017) | 1 | cellular component unknown | CG11711 CG31125 CG31320 CG31749 CG31997 CG32415 CG32711 CG32827 CG8588 GstS1 Pli a6 bnb l(3)neo38 |
| 2144 | GO:0031410 | C | 4, 5, 6, 7, 8, | 1 | 4.097 (x 0.244) | 83 (0.012) | 1 | cytoplasmic vesicle | btsz |
| 2145 | GO:0006858 | P | 5, 6, | 2 | 6.071 (x 0.329) | 123 (0.016) | 1 | extracellular transport | CG15088 l(2)01810 |
| 2146 | GO:0031988 | C | 4, | 1 | 4.245 (x 0.236) | 86 (0.012) | 1 | membrane-bound vesicle | btsz |
| 2147 | GO:0005214 | F | 4, | 1 | 3.998 (x 0.250) | 81 (0.012) | 1 | structural constituent of cuticle (sensu Insecta) | CG8502 |
| 2148 | GO:0001584 | F | 6, | 1 | 10.514 (x 0.095) | 213 (0.005) | 1 | rhodopsin-like receptor activity | pbl |
| 2149 | GO:0016023 | C | 5, 6, 7, 8, 9, | 1 | 4.097 (x 0.244) | 83 (0.012) | 1 | cytoplasmic membrane-bound vesicle | btsz |
| 2150 | GO:0016298 | F | 6, | 1 | 4.344 (x 0.230) | 88 (0.011) | 1 | lipase activity | CG1471 |
| 2151 | GO:0007606 | P | 4, 6, | 3 | 8.194 (x 0.366) | 166 (0.018) | 1 | sensory perception of chemical stimulus | Galpha49B Obp99a Sh |
| 2152 | GO:0006118 | P | 6, | 5 | 16.437 (x 0.304) | 333 (0.015) | 1 | electron transport | CG40160 Cyp310a1 ERp60 Pdi sgl |
| 2153 | GO:0005830 | C | 5, 6, 7, 8, 9, 10, | 1 | 4.442 (x 0.225) | 90 (0.011) | 1 | cytosolic ribosome (sensu Eukaryota) | RpS12 |
| 2154 | GO:0016881 | F | 5, | 1 | 7.799 (x 0.128) | 158 (0.006) | 1 | acid-amino acid ligase activity | th |
| 2155 | GO:0007600 | P | 3, 5, | 6 | 12.735 (x 0.471) | 258 (0.023) | 1 | sensory perception | Galpha49B Obp99a Sh ck d pain |
| 2156 | GO:0044429 | C | 4, 5, 6, 7, 8, 9, | 6 | 17.029 (x 0.352) | 345 (0.017) | 1 | mitochondrial part | BcDNA:GH12558 CG1907 CG2118 CG6782 Mgstl Ucp4B |
| 2157 | GO:0016787 | F | 3, | 70 | 88.652 (x 0.790) | 1796 (0.039) | 1 | hydrolase activity | Ance Arf79F BEST:CK02656 BEST:GH02921 BG:DS00004.11 BcDNA:GM10765 BcDNA:LD41548 CG11438 CG11824 CG1471 CG1632 CG2108 CG2200 CG31121 CG31453 CG32499 CG33138 CG3328 CG40160 CG4382 CG4914 CG5390 CG5731 CG5794 CG6391 CG7860 CG9307 D19A GNBP3 Galpha49B Hexo1 Ice Lsp2 Mcm7 Nep2 Nrv1 Ppt1 Prosalpha6 Pten Ptp99A Rab8 Rep4 RhoGAP71E Rm62 Rpn9 Sb TER94 Thd1 Top2 Uch Vha100-2 alpha-Man-IIb alphaTub84B betaTub56D blot br ck d dUTPase east fbp fra glu jumu kis mod(mdg4) ome shanti tok tws |
| 2158 | GO:0005739 | C | 5, 6, 7, 8, | 10 | 22.805 (x 0.439) | 462 (0.022) | 1 | mitochondrion | BcDNA:GH12558 CG11079 CG1907 CG2118 CG6782 EG:171D11.1 Idh Mgstl Pepck Ucp4B |
| 2159 | GO:0005740 | C | 4, 5, 6, 7, 8, 9, 10, | 4 | 10.119 (x 0.395) | 205 (0.020) | 1 | mitochondrial envelope | CG1907 CG6782 Mgstl Ucp4B |
| 2160 | GO:0019866 | C | 4, 5, 6, 7, 8, 9, | 3 | 8.589 (x 0.349) | 174 (0.017) | 1 | organelle inner membrane | CG17952 CG1907 Ucp4B |
| 2161 | GO:0005840 | C | 4, 5, 6, 7, 8, | 2 | 9.329 (x 0.214) | 189 (0.011) | 1 | ribosome | CG31738 RpS12 |
| 2162 | GO:0016879 | F | 4, | 3 | 8.984 (x 0.334) | 182 (0.016) | 1 | ligase activity, forming carbon-nitrogen bonds | CG11079 CG6767 th |
| 2163 | GO:0004930 | F | 5, | 5 | 12.686 (x 0.394) | 257 (0.019) | 1 | G-protein coupled receptor activity | CG17084 CG6965 mth pbl stan |
| 2164 | GO:0016567 | P | 9, | 1 | 5.578 (x 0.179) | 113 (0.009) | 1 | protein ubiquitination | th |
| 2165 | GO:0006119 | P | 6, 8, | 2 | 6.960 (x 0.287) | 141 (0.014) | 1 | oxidative phosphorylation | blot jumu |
| 2166 | GO:0003735 | F | 3, | 2 | 9.280 (x 0.216) | 188 (0.011) | 1 | structural constituent of ribosome | CG31738 RpS12 |
| 2167 | GO:0005759 | C | 5, 6, 7, 8, 9, 10, 11, | 2 | 7.157 (x 0.279) | 145 (0.014) | 1 | mitochondrial matrix | BcDNA:GH12558 CG2118 |
| 2168 | GO:0016874 | F | 3, | 7 | 15.647 (x 0.447) | 317 (0.022) | 1 | ligase activity | BcDNA:GH02901 CG11079 CG2118 CG6767 blot mdy th |
| 2169 | GO:0031966 | C | 5, 6, 7, 8, 9, 10, 11, | 3 | 9.230 (x 0.325) | 187 (0.016) | 1 | mitochondrial membrane | CG1907 Mgstl Ucp4B |
| 2170 | GO:0004842 | F | 7, | 1 | 6.861 (x 0.146) | 139 (0.007) | 1 | ubiquitin-protein ligase activity | th |
| 2171 | GO:0000151 | C | 3, 4, 5, 6, | 1 | 6.219 (x 0.161) | 126 (0.008) | 1 | ubiquitin ligase complex | th |
| 2172 | GO:0031980 | C | 4, 5, 6, 7, 8, 9, 10, | 2 | 7.157 (x 0.279) | 145 (0.014) | 1 | mitochondrial lumen | BcDNA:GH12558 CG2118 |
| 2173 | GO:0005743 | C | 5, 6, 7, 8, 9, 10, 11, 12, | 2 | 8.342 (x 0.240) | 169 (0.012) | 1 | mitochondrial inner membrane | CG1907 Ucp4B |
| 2174 | GO:0019787 | F | 6, | 1 | 6.861 (x 0.146) | 139 (0.007) | 1 | small conjugating protein ligase activity | th |

  

---

Regulated Genes that don't have GO terms
  

BG:DS00180.3 BG:DS07721.3 BcDNA:GH11415 BcDNA:LD24702 CG10424 CG10522 CG10555 CG11509 CG11577 CG11882 CG12004 CG12452 CG12546 CG12708 CG13200 CG13373 CG13676 CG13698 CG13827 CG14132 CG14301 CG1499 CG14997 CG15415 CG15628 CG15905 CG17032 CG17163 CG17383 CG18212 CG18349 CG1962 CG2016 CG2083 CG2467 CG2791 CG30183 CG30423 CG32373 CG32541 CG33129 CG3570 CG3625 CG3678 CG3880 CG40294 CG40354 CG4089 CG4098 CG4332 CG4877 CG5126 CG5175 CG6040 CG6169 CG6234 CG6398 CG6448 CG6617 CG6900 CG6959 CG6983 CG7047 CG7110 CG7702 CG7802 CG7872 CG8031 CG8420 CG8580 CG8600 CG9257 CG9266 CG9416 CG9427 CG9526 CG9628 CG9723 EG:152A3.3 EG:25E8.4 EG:80H7.11 Fcp3C l(2)05714 l(2)k09913 miple yuri
