# Supplementary material for: Functional dissection of the ash2 and ash1 transcriptomes provides insights into the transcriptional basis of wing phenotypes and reveals conserved protein interactions
Source: Genome Biol. 2007 Apr 28;8(4):R67. doi: 10.1186/gb-2007-8-4-r67 (PMC1896016; doi:10.1186/gb-2007-8-4-r67)
Supplement: Additional data file 6 — GO annotations of the genes upregulated over 1.5-fold in ash2I1 [file gb-2007-8-4-r67-S6.html]

  

---

  

|  |  |
| --- | --- |
| Go Statistics | Reg File: **ash2I1\_U1.5x.txt.fbgns** (426 genes -- 125 skipped)  Ref File: **ref.fbgns** (13577 genes -- 4663 skipped)  Database: **go\_200507-termdb.rdf-xml** |

---

  

Fields Description

| Pos | Go Term | Ontology | Levels | Observed | Expected | Possibles | p-value(Adj) | Go term description | Genes with the GO term |
| --- | --- | --- | --- | --- | --- | --- | --- | --- | --- |
| 1 | GO:0005739 | C | 5, 6, 7, 8, | 38 | 15.600 (x 2.436) | 462 (0.082) | 0.000416 | mitochondrion | Aats-leu Aldh Bmcp CG10214 CG12264 CG12954 CG3192 CG32649 CG33002 CG3803 CG4769 CG4866 CG5037 CG5548 CG5703 CG6512 CG7791 CG7842 CG7955 CG8004 CG8199 CG8226 CG8798 Gdh JhI-1 Las Pdsw Tim10 Tim9a bcn92 mRpL22 mRpL22-24 mRpS14 mRpS18a mRpS21 mRpS33 rpr tko |
| 2 | GO:0016765 | F | 4, | 12 | 2.026 (x 5.923) | 60 (0.200) | 0.000455 | transferase activity, transferring alkyl or aryl (other than methyl) groups | CG5037 CG8327 GstD3 GstD5 GstD6 GstD9 GstE1 GstE3 GstE5 GstE6 GstE7 Rep |
| 3 | GO:0042221 | P | 4, | 25 | 8.881 (x 2.815) | 263 (0.095) | 0.00105 | response to chemical stimulus | BEST:CK01227 BG:DS01219.1 CG11897 CG31793 CG7627 CG7955 GstD3 GstD6 GstD9 GstE1 GstE3 GstE5 GstE6 GstE7 Hmgcr Jheh3 Ugt86Da Ugt86Di W cher ftz-f1 puc rpr wun wun2 |
| 4 | GO:0004364 | F | 5, | 9 | 1.216 (x 7.404) | 36 (0.250) | 0.00112 | glutathione transferase activity | GstD3 GstD5 GstD6 GstD9 GstE1 GstE3 GstE5 GstE6 GstE7 |
| 5 | GO:0044429 | C | 4, 5, 6, 7, 8, 9, | 29 | 11.650 (x 2.489) | 345 (0.084) | 0.00158 | mitochondrial part | Aats-leu Aldh Bmcp CG12954 CG3192 CG33002 CG3803 CG4769 CG4866 CG5037 CG5548 CG5703 CG7955 CG8004 CG8199 CG8226 CG8798 Gdh Pdsw Tim10 Tim9a bcn92 mRpL22 mRpL22-24 mRpS14 mRpS18a mRpS21 mRpS33 tko |
| 6 | GO:0044444 | C | 4, 5, 6, 7, | 66 | 39.069 (x 1.689) | 1157 (0.057) | 0.00171 | cytoplasmic part | Aats-leu Aldh Bmcp CG10214 CG10306 CG12264 CG12954 CG3192 CG32549 CG32649 CG33002 CG33158 CG3803 CG4769 CG4866 CG4917 CG5037 CG5317 CG5338 CG5548 CG5703 CG6512 CG6764 CG7014 CG7791 CG7842 CG7955 CG8004 CG8199 CG8226 CG8630 CG8798 Gclc Gdh HBS1 Hmgcr Jafrac1 JhI-1 Las Mhc Mlc1 Mlc2 Pdsw REG Rep RpL17A RpS4 Sulf1 Tim10 Tim9a bcn92 desat1 dia eIF6 l(3)01239 mRpL22 mRpL22-24 mRpS14 mRpS18a mRpS21 mRpS33 na rpr tko unc-13 wupA |
| 7 | GO:0009636 | P | 5, | 16 | 4.356 (x 3.673) | 129 (0.124) | 0.00171 | response to toxin | BEST:CK01227 CG11897 CG31793 CG7627 CG7955 GstD3 GstD6 GstD9 GstE1 GstE3 GstE5 GstE6 GstE7 Jheh3 Ugt86Da Ugt86Di |
| 8 | GO:0009628 | P | 3, | 30 | 12.561 (x 2.388) | 372 (0.081) | 0.00176 | response to abiotic stimulus | Arr2 BEST:CK01227 BG:DS01219.1 CG11897 CG31793 CG7627 CG7955 GstD3 GstD6 GstD9 GstE1 GstE3 GstE5 GstE6 GstE7 Hmgcr Jheh3 TotA Ugt86Da Ugt86Di W cher ftz-f1 ppk puc rpr sda tko wun wun2 |
| 9 | GO:0000723 | P | 9, | 4 | 0.203 (x 19.743) | 6 (0.667) | 0.00286 | telomere maintenance | Irbp Ku80 mre11 rad50 |
| 10 | GO:0032200 | P | 8, | 4 | 0.203 (x 19.743) | 6 (0.667) | 0.00317 | telomere organization and biogenesis | Irbp Ku80 mre11 rad50 |
| 11 | GO:0000146 | F | 3, | 3 | 0.101 (x 29.615) | 3 (1.000) | 0.00501 | microfilament motor activity | Mhc Mlc1 Mlc2 |
| 12 | GO:0005859 | C | 5, 6, 7, 8, 9, 10, 11, 12, | 3 | 0.101 (x 29.615) | 3 (1.000) | 0.00546 | muscle myosin | Mhc Mlc1 Mlc2 |
| 13 | GO:0003735 | F | 3, | 18 | 6.348 (x 2.835) | 188 (0.096) | 0.00763 | structural constituent of ribosome | CG12954 CG33002 CG4364 CG4866 CG5317 CG5338 CG6764 CG7014 RpL17A RpS4 mRpL22 mRpL22-24 mRpS14 mRpS18a mRpS21 mRpS33 na tko |
| 14 | GO:0005737 | C | 4, 5, 6, | 76 | 50.144 (x 1.516) | 1485 (0.051) | 0.00838 | cytoplasm | Aats-leu Aldh Bmcp CG10214 CG10306 CG10861 CG12264 CG12954 CG3192 CG32549 CG32649 CG33002 CG33158 CG3803 CG4769 CG4866 CG4917 CG5037 CG5317 CG5338 CG5548 CG5703 CG6330 CG6512 CG6764 CG7014 CG7791 CG7842 CG7955 CG8004 CG8199 CG8226 CG8630 CG8798 CG9358 Gclc Gdh Gs2 HBS1 Hmgcr Jafrac1 JhI-1 La Las Mhc Mlc1 Mlc2 Mlp60A Nxt1 Pdsw Ptp61F REG Rep RpL17A RpS4 Sulf1 Tim10 Tim9a bcn92 desat1 dia eIF6 ftz-f1 huntingtin l(3)01239 mRpL22 mRpL22-24 mRpS14 mRpS18a mRpS21 mRpS33 na rpr tko unc-13 wupA |
| 15 | GO:0031980 | C | 4, 5, 6, 7, 8, 9, 10, | 15 | 4.896 (x 3.064) | 145 (0.103) | 0.0107 | mitochondrial lumen | Aats-leu Aldh CG12954 CG33002 CG4866 CG8199 CG8798 Gdh mRpL22 mRpL22-24 mRpS14 mRpS18a mRpS21 mRpS33 tko |
| 16 | GO:0005759 | C | 5, 6, 7, 8, 9, 10, 11, | 15 | 4.896 (x 3.064) | 145 (0.103) | 0.0114 | mitochondrial matrix | Aats-leu Aldh CG12954 CG33002 CG4866 CG8199 CG8798 Gdh mRpL22 mRpL22-24 mRpS14 mRpS18a mRpS21 mRpS33 tko |
| 17 | GO:0050896 | P | 2, | 62 | 39.474 (x 1.571) | 1169 (0.053) | 0.0135 | response to stimulus | Arr2 BEST:CK01227 BG:DS01219.1 CG10460 CG11897 CG15629 CG17227 CG18522 CG31793 CG40006 CG4917 CG5397 CG7627 CG7955 CG9273 CG9358 CG9460 DDB1 Gdh Gr39a GstD3 GstD6 GstD9 GstE1 GstE3 GstE5 GstE6 GstE7 Hmgcr Irbp Jafrac1 Jheh3 Ku80 Obp83g TepIV Thor TotA Traf1 Tsf1 Tsf3 Ugt86Da Ugt86Di W agt cher eiger fru ftz-f1 hay ken mre11 mus205 na os ppk puc rad50 rpr sda tko wun wun2 |
| 18 | GO:0016460 | C | 4, 6, 7, 8, 9, 10, 11, | 3 | 0.135 (x 22.211) | 4 (0.750) | 0.0138 | myosin II | Mhc Mlc1 Mlc2 |
| 19 | GO:0005761 | C | 5, 6, 7, 8, 9, 10, 11, 12, | 10 | 2.499 (x 4.002) | 74 (0.135) | 0.0138 | mitochondrial ribosome | CG12954 CG33002 CG4866 mRpL22 mRpL22-24 mRpS14 mRpS18a mRpS21 mRpS33 tko |
| 20 | GO:0000313 | C | 5, 6, 7, 8, 9, | 10 | 2.499 (x 4.002) | 74 (0.135) | 0.0146 | organellar ribosome | CG12954 CG33002 CG4866 mRpL22 mRpL22-24 mRpS14 mRpS18a mRpS21 mRpS33 tko |
| 21 | GO:0005840 | C | 4, 5, 6, 7, 8, | 17 | 6.382 (x 2.664) | 189 (0.090) | 0.0162 | ribosome | CG12954 CG33002 CG4866 CG5317 CG5338 CG6764 CG7014 RpL17A RpS4 mRpL22 mRpL22-24 mRpS14 mRpS18a mRpS21 mRpS33 na tko |
| 22 | GO:0044249 | P | 5, | 47 | 28.027 (x 1.677) | 830 (0.057) | 0.0186 | cellular biosynthesis | Aats-leu Act57B BcDNA:LD32788 CG10306 CG12264 CG12954 CG13645 CG2246 CG2453 CG31373 CG33002 CG33158 CG4866 CG5037 CG5177 CG5317 CG5338 CG6764 CG6950 CG7014 CG7842 CG8199 CG8327 CG8630 Dgp-1 Gclc Gs2 HBS1 Imp Las Mocs1 Rep RpL17A RpS4 Thor apt desat1 eIF6 mRpL22 mRpL22-24 mRpS14 mRpS18a mRpS21 mRpS33 na rpr tko |
| 23 | GO:0044449 | C | 5, 6, 7, 8, 9, | 4 | 0.371 (x 10.769) | 11 (0.364) | 0.0239 | contractile fiber part | Mhc Mlc1 Mlc2 wupA |
| 24 | GO:0009058 | P | 4, | 49 | 30.323 (x 1.616) | 898 (0.055) | 0.0296 | biosynthesis | Aats-leu Act57B BcDNA:LD32788 CG10306 CG12264 CG12954 CG13645 CG2246 CG2453 CG31373 CG33002 CG33158 CG4866 CG5037 CG5177 CG5317 CG5338 CG6764 CG6950 CG7014 CG7842 CG8199 CG8327 CG8630 Dgp-1 Gclc Gs2 HBS1 Hmgcr Imp Las Lcp65Ag1 Mocs1 Rep RpL17A RpS4 Thor apt desat1 eIF6 mRpL22 mRpL22-24 mRpS14 mRpS18a mRpS21 mRpS33 na rpr tko |
| 25 | GO:0009719 | P | 3, | 13 | 4.491 (x 2.895) | 133 (0.098) | 0.0339 | response to endogenous stimulus | CG17227 CG9273 DDB1 Irbp Ku80 W agt ftz-f1 hay mre11 mus205 rad50 rpr |
| 26 | GO:0009059 | P | 5, 6, | 32 | 17.626 (x 1.815) | 522 (0.061) | 0.0413 | macromolecule biosynthesis | Aats-leu Act57B CG10306 CG12954 CG31373 CG33002 CG33158 CG4866 CG5177 CG5317 CG5338 CG6764 CG7014 Dgp-1 Gclc HBS1 Imp Rep RpL17A RpS4 Thor apt eIF6 mRpL22 mRpL22-24 mRpS14 mRpS18a mRpS21 mRpS33 na rpr tko |
| 27 | GO:0043292 | C | 5, 6, 7, 8, | 4 | 0.439 (x 9.112) | 13 (0.308) | 0.0418 | contractile fiber | Mhc Mlc1 Mlc2 wupA |
| 28 | GO:0030239 | P | 7, 8, 10, 11, | 3 | 0.203 (x 14.807) | 6 (0.500) | 0.0428 | myofibril assembly | CG6803 Mhc wupA |
| 29 | GO:0016226 | P | 7, | 2 | 0.068 (x 29.615) | 2 (1.000) | 0.0527 | iron-sulfur cluster assembly | CG12264 CG7955 |
| 30 | GO:0031163 | P | 6, | 2 | 0.068 (x 29.615) | 2 (1.000) | 0.0543 | metallo-sulfur cluster assembly | CG12264 CG7955 |
| 31 | GO:0006935 | P | 5, 6, | 3 | 0.236 (x 12.692) | 7 (0.429) | 0.0543 | chemotaxis | Hmgcr wun wun2 |
| 32 | GO:0006412 | P | 6, 7, | 30 | 16.681 (x 1.798) | 494 (0.061) | 0.0547 | protein biosynthesis | Aats-leu CG10306 CG12954 CG31373 CG33002 CG33158 CG4866 CG5317 CG5338 CG6764 CG7014 Dgp-1 Gclc HBS1 Imp Rep RpL17A RpS4 Thor apt eIF6 mRpL22 mRpL22-24 mRpS14 mRpS18a mRpS21 mRpS33 na rpr tko |
| 33 | GO:0035233 | P | 6, 7, 8, | 2 | 0.068 (x 29.615) | 2 (1.000) | 0.056 | germ cell repulsion | wun wun2 |
| 34 | GO:0035096 | P | 6, 7, | 2 | 0.068 (x 29.615) | 2 (1.000) | 0.0578 | larval midgut cell programmed cell death | W rpr |
| 35 | GO:0019740 | P | 5, | 2 | 0.068 (x 29.615) | 2 (1.000) | 0.0597 | nitrogen utilization | CG12264 CG9836 |
| 36 | GO:0030529 | C | 3, 4, 5, 6, | 22 | 10.738 (x 2.049) | 318 (0.069) | 0.06 | ribonucleoprotein complex | CG12954 CG31922 CG33002 CG4866 CG5205 CG5317 CG5338 CG6610 CG6764 CG6876 CG7014 La RpL17A RpS4 mRpL22 mRpL22-24 mRpS14 mRpS18a mRpS21 mRpS33 na tko |
| 37 | GO:0006800 | P | 5, | 8 | 2.262 (x 3.536) | 67 (0.119) | 0.0757 | oxygen and reactive oxygen species metabolism | CG18522 GstE1 GstE3 GstE5 GstE6 GstE7 Jafrac1 puc |
| 38 | GO:0009055 | F | 4, | 7 | 1.790 (x 3.911) | 53 (0.132) | 0.0766 | electron carrier activity | CG3192 CG4769 CG5548 CG5703 CG5946 Fdxh Pdsw |
| 39 | GO:0002168 | P | 5, | 5 | 0.912 (x 5.484) | 27 (0.185) | 0.0771 | larval development (sensu Insecta) | Lcp65Ag1 Mmp1 Traf1 rad50 sas |
| 40 | GO:0006974 | P | 4, | 11 | 3.985 (x 2.761) | 118 (0.093) | 0.0822 | response to DNA damage stimulus | CG17227 CG9273 DDB1 Irbp Ku80 agt hay mre11 mus205 rad50 rpr |
| 41 | GO:0015935 | C | 3, 4, 5, 6, 7, 8, 9, | 8 | 2.330 (x 3.434) | 69 (0.116) | 0.0826 | small ribosomal subunit | CG5338 CG7014 RpS4 mRpS14 mRpS18a mRpS21 mRpS33 tko |
| 42 | GO:0004357 | F | 6, | 2 | 0.101 (x 19.743) | 3 (0.667) | 0.0955 | glutamate-cysteine ligase activity | CG4917 Gclc |
| 43 | GO:0000726 | P | 6, 8, | 2 | 0.101 (x 19.743) | 3 (0.667) | 0.0973 | non-recombinational repair | Irbp Ku80 |
| 44 | GO:0044446 | C | 3, 4, 5, 6, 7, | 66 | 48.084 (x 1.373) | 1424 (0.046) | 0.0989 | intracellular organelle part | Aats-leu Act57B Aldh Bmcp CG12954 CG31611 CG3192 CG31922 CG33002 CG3371 CG3803 CG4769 CG4866 CG5037 CG5205 CG5317 CG5338 CG5548 CG5703 CG6610 CG6764 CG6876 CG7014 CG7339 CG7955 CG8004 CG8199 CG8226 CG8798 CG9273 EG:52C10.1 GV1 Gdh Hmgcr Ku80 LamC Mhc Mlc1 Mlc2 Nxt1 Pcaf Pdsw Rep RpA-70 RpL17A RpS4 Ssb-c31a Ssrp Sulf1 Taf12L Tim10 Tim9a bcn92 cul-2 dia hay mRpL22 mRpL22-24 mRpS14 mRpS18a mRpS21 mRpS33 mus205 rad50 tko wupA |
| 45 | GO:0005662 | C | 3, 5, 6, 7, 8, 9, 10, 11, 12, 13, 14, | 2 | 0.101 (x 19.743) | 3 (0.667) | 0.0991 | DNA replication factor A complex | CG9273 RpA-70 |
| 46 | GO:0044422 | C | 2, 3, | 66 | 48.084 (x 1.373) | 1424 (0.046) | 0.101 | organelle part | Aats-leu Act57B Aldh Bmcp CG12954 CG31611 CG3192 CG31922 CG33002 CG3371 CG3803 CG4769 CG4866 CG5037 CG5205 CG5317 CG5338 CG5548 CG5703 CG6610 CG6764 CG6876 CG7014 CG7339 CG7955 CG8004 CG8199 CG8226 CG8798 CG9273 EG:52C10.1 GV1 Gdh Hmgcr Ku80 LamC Mhc Mlc1 Mlc2 Nxt1 Pcaf Pdsw Rep RpA-70 RpL17A RpS4 Ssb-c31a Ssrp Sulf1 Taf12L Tim10 Tim9a bcn92 cul-2 dia hay mRpL22 mRpL22-24 mRpS14 mRpS18a mRpS21 mRpS33 mus205 rad50 tko wupA |
| 47 | GO:0006303 | P | 7, 9, | 2 | 0.101 (x 19.743) | 3 (0.667) | 0.101 | double-strand break repair via nonhomologous end joining | Irbp Ku80 |
| 48 | GO:0000314 | C | 4, 5, 6, 7, 8, 9, 10, | 5 | 1.013 (x 4.936) | 30 (0.167) | 0.101 | organellar small ribosomal subunit | mRpS14 mRpS18a mRpS21 mRpS33 tko |
| 49 | GO:0006302 | P | 6, 8, | 3 | 0.338 (x 8.884) | 10 (0.300) | 0.102 | double-strand break repair | Irbp Ku80 mre11 |
| 50 | GO:0017109 | C | 3, 5, 6, 7, 8, | 2 | 0.101 (x 19.743) | 3 (0.667) | 0.103 | glutamate-cysteine ligase complex | CG4917 Gclc |
| 51 | GO:0004869 | F | 6, | 3 | 0.304 (x 9.872) | 9 (0.333) | 0.103 | cysteine protease inhibitor activity | CG10460 CG8066 Cys |
| 52 | GO:0005763 | C | 5, 6, 7, 8, 9, 10, 11, 12, 13, | 5 | 1.013 (x 4.936) | 30 (0.167) | 0.104 | mitochondrial small ribosomal subunit | mRpS14 mRpS18a mRpS21 mRpS33 tko |
| 53 | GO:0006281 | P | 5, 7, | 10 | 3.714 (x 2.692) | 110 (0.091) | 0.104 | DNA repair | CG17227 CG9273 DDB1 Irbp Ku80 agt hay mre11 mus205 rad50 |
| 54 | GO:0003697 | F | 6, | 4 | 0.675 (x 5.923) | 20 (0.200) | 0.104 | single-stranded DNA binding | CG9273 RpA-70 Ssb-c31a Ssrp |
| 55 | GO:0042330 | P | 4, 5, | 3 | 0.338 (x 8.884) | 10 (0.300) | 0.104 | taxis | Hmgcr wun wun2 |
| 56 | GO:0016651 | F | 4, | 6 | 1.452 (x 4.132) | 43 (0.140) | 0.105 | oxidoreductase activity, acting on NADH or NADPH | CG3192 CG5548 CG5703 CG5946 Pdsw bcn92 |
| 57 | GO:0009399 | P | 5, | 2 | 0.101 (x 19.743) | 3 (0.667) | 0.105 | nitrogen fixation | CG11897 Gs2 |
| 58 | GO:0009607 | P | 3, | 30 | 17.626 (x 1.702) | 522 (0.057) | 0.106 | response to biotic stimulus | BEST:CK01227 CG11897 CG18522 CG31793 CG40006 CG5397 CG7627 CG7955 CG9358 CG9460 GstD3 GstD6 GstD9 GstE1 GstE3 GstE5 GstE6 GstE7 Jafrac1 Jheh3 TepIV Thor TotA Traf1 Tsf1 Tsf3 Ugt86Da Ugt86Di eiger os |
| 59 | GO:0035069 | P | 6, | 2 | 0.101 (x 19.743) | 3 (0.667) | 0.107 | larval midgut histolysis | W rpr |
| 60 | GO:0005506 | F | 6, | 6 | 1.452 (x 4.132) | 43 (0.140) | 0.107 | iron ion binding | CG8630 Las Mocs1 Tsf1 Tsf3 desat1 |
| 61 | GO:0035234 | P | 6, 7, 8, | 2 | 0.101 (x 19.743) | 3 (0.667) | 0.109 | germ cell programmed cell death | wun wun2 |
| 62 | GO:0004386 | F | 3, | 10 | 3.782 (x 2.644) | 112 (0.089) | 0.114 | helicase activity | CG10445 CG1582 CG31755 CG5205 CG5800 CG6204 Dbp80 Irbp Ku80 hay |
| 63 | GO:0008083 | F | 4, 5, | 5 | 1.114 (x 4.487) | 33 (0.152) | 0.116 | growth factor activity | CG2989 Chit Idgf2 Idgf3 sog |
| 64 | GO:0006626 | P | 8, 9, 10, | 5 | 1.148 (x 4.355) | 34 (0.147) | 0.13 | protein targeting to mitochondrion | CG7791 CG8004 CG8226 Tim10 Tim9a |
| 65 | GO:0031974 | C | 2, | 25 | 14.554 (x 1.718) | 431 (0.058) | 0.136 | membrane-enclosed lumen | Aats-leu Aldh CG12954 CG33002 CG4866 CG6610 CG7339 CG8199 CG8798 EG:52C10.1 Gdh Nxt1 Pcaf Ssb-c31a Ssrp Taf12L hay mRpL22 mRpL22-24 mRpS14 mRpS18a mRpS21 mRpS33 mus205 tko |
| 66 | GO:0043233 | C | 3, 4, | 25 | 14.554 (x 1.718) | 431 (0.058) | 0.138 | organelle lumen | Aats-leu Aldh CG12954 CG33002 CG4866 CG6610 CG7339 CG8199 CG8798 EG:52C10.1 Gdh Nxt1 Pcaf Ssb-c31a Ssrp Taf12L hay mRpL22 mRpL22-24 mRpS14 mRpS18a mRpS21 mRpS33 mus205 tko |
| 67 | GO:0003715 | F | 3, | 2 | 0.135 (x 14.807) | 4 (0.500) | 0.149 | transcription termination factor activity | CG10445 La |
| 68 | GO:0006538 | P | 9, 10, | 2 | 0.135 (x 14.807) | 4 (0.500) | 0.151 | glutamate catabolism | Gdh Gs2 |
| 69 | GO:0008026 | F | 4, 10, | 8 | 2.803 (x 2.854) | 83 (0.096) | 0.152 | ATP-dependent helicase activity | CG1582 CG31755 CG5205 CG5800 Dbp80 Irbp Ku80 hay |
| 70 | GO:0006826 | P | 9, 10, | 2 | 0.135 (x 14.807) | 4 (0.500) | 0.153 | iron ion transport | Tsf1 Tsf3 |
| 71 | GO:0003678 | F | 4, | 6 | 1.722 (x 3.484) | 51 (0.118) | 0.157 | DNA helicase activity | CG10445 CG1582 CG6204 Irbp Ku80 hay |
| 72 | GO:0006952 | P | 4, | 28 | 17.356 (x 1.613) | 514 (0.054) | 0.179 | defense response | BEST:CK01227 CG11897 CG18522 CG31793 CG40006 CG5397 CG7627 CG7955 CG9460 GstD3 GstD6 GstD9 GstE1 GstE3 GstE5 GstE6 GstE7 Jafrac1 Jheh3 TepIV Thor TotA Traf1 Tsf1 Ugt86Da Ugt86Di eiger os |
| 73 | GO:0004529 | F | 7, | 3 | 0.439 (x 6.834) | 13 (0.231) | 0.183 | exodeoxyribonuclease activity | CG10214 mre11 rad50 |
| 74 | GO:0031966 | C | 5, 6, 7, 8, 9, 10, 11, | 13 | 6.314 (x 2.059) | 187 (0.070) | 0.185 | mitochondrial membrane | Bmcp CG3192 CG3803 CG4769 CG5548 CG5703 CG7955 CG8004 CG8226 Pdsw Tim10 Tim9a bcn92 |
| 75 | GO:0004857 | F | 3, | 10 | 4.288 (x 2.332) | 127 (0.079) | 0.187 | enzyme inhibitor activity | CG10460 CG17124 CG40006 CG7054 CG7722 CG8066 CG9460 Cys TepIV puc |
| 76 | GO:0042721 | C | 3, 5, 6, 7, 8, 9, 10, 11, 12, 13, | 2 | 0.169 (x 11.846) | 5 (0.400) | 0.188 | mitochondrial inner membrane protein insertion complex | Tim10 Tim9a |
| 77 | GO:0031975 | C | 2, | 17 | 9.016 (x 1.886) | 267 (0.064) | 0.188 | envelope | Bmcp CG3192 CG3803 CG4769 CG5037 CG5548 CG5703 CG7955 CG8004 CG8226 GV1 LamC Nxt1 Pdsw Tim10 Tim9a bcn92 |
| 78 | GO:0006388 | P | 9, 10, | 2 | 0.169 (x 11.846) | 5 (0.400) | 0.19 | tRNA splicing | CG7163 JhI-1 |
| 79 | GO:0048102 | P | 6, | 7 | 2.397 (x 2.920) | 71 (0.099) | 0.19 | autophagic cell death | CG10861 EP2237 Mmp1 Traf1 W ftz-f1 rpr |
| 80 | GO:0031967 | C | 3, 4, 5, 6, 7, 8, | 17 | 9.016 (x 1.886) | 267 (0.064) | 0.191 | organelle envelope | Bmcp CG3192 CG3803 CG4769 CG5037 CG5548 CG5703 CG7955 CG8004 CG8226 GV1 LamC Nxt1 Pdsw Tim10 Tim9a bcn92 |
| 81 | GO:0043566 | F | 5, | 4 | 0.844 (x 4.738) | 25 (0.160) | 0.191 | structure-specific DNA binding | CG9273 RpA-70 Ssb-c31a Ssrp |
| 82 | GO:0000394 | P | 9, | 2 | 0.169 (x 11.846) | 5 (0.400) | 0.192 | RNA splicing, via endonucleolytic cleavage and ligation | CG7163 JhI-1 |
| 83 | GO:0035070 | P | 6, | 7 | 2.397 (x 2.920) | 71 (0.099) | 0.193 | salivary gland histolysis | CG10861 EP2237 Mmp1 Traf1 W ftz-f1 rpr |
| 84 | GO:0005381 | F | 6, | 2 | 0.169 (x 11.846) | 5 (0.400) | 0.195 | iron ion transporter activity | Tsf1 Tsf3 |
| 85 | GO:0035071 | P | 7, | 7 | 2.397 (x 2.920) | 71 (0.099) | 0.195 | salivary gland cell autophagic cell death | CG10861 EP2237 Mmp1 Traf1 W ftz-f1 rpr |
| 86 | GO:0005740 | C | 4, 5, 6, 7, 8, 9, 10, | 14 | 6.922 (x 2.022) | 205 (0.068) | 0.196 | mitochondrial envelope | Bmcp CG3192 CG3803 CG4769 CG5037 CG5548 CG5703 CG7955 CG8004 CG8226 Pdsw Tim10 Tim9a bcn92 |
| 87 | GO:0007638 | P | 4, 5, | 2 | 0.169 (x 11.846) | 5 (0.400) | 0.197 | mechanosensory behavior | sda tko |
| 88 | GO:0004222 | F | 6, | 7 | 2.397 (x 2.920) | 71 (0.099) | 0.198 | metalloendopeptidase activity | CG14869 CG4096 CG4933 CG6512 CG7791 Ide Mmp1 |
| 89 | GO:0008084 | F | 5, 6, | 2 | 0.169 (x 11.846) | 5 (0.400) | 0.199 | imaginal disc growth factor activity | Idgf2 Idgf3 |
| 90 | GO:0016271 | P | 4, | 7 | 2.431 (x 2.879) | 72 (0.097) | 0.2 | tissue death | CG10861 EP2237 Mmp1 Traf1 W ftz-f1 rpr |
| 91 | GO:0006310 | P | 7, | 5 | 1.384 (x 3.612) | 41 (0.122) | 0.201 | DNA recombination | CG17227 CG9273 Irbp Ku80 rad50 |
| 92 | GO:0007559 | P | 5, | 7 | 2.431 (x 2.879) | 72 (0.097) | 0.202 | histolysis | CG10861 EP2237 Mmp1 Traf1 W ftz-f1 rpr |
| 93 | GO:0035075 | P | 5, 6, 7, | 3 | 0.507 (x 5.923) | 15 (0.200) | 0.213 | response to ecdysone | W ftz-f1 rpr |
| 94 | GO:0043190 | C | 3, 4, | 5 | 1.418 (x 3.526) | 42 (0.119) | 0.213 | ATP-binding cassette (ABC) transporter complex | BEST:CK01227 CG1718 CG31793 CG7627 CG7955 |
| 95 | GO:0007610 | P | 3, | 13 | 6.450 (x 2.016) | 191 (0.068) | 0.214 | behavior | BG:DS01219.1 CG10460 Gdh Hmgcr cher fru ken na ppk sda tko wun wun2 |
| 96 | GO:0048545 | P | 5, 6, | 3 | 0.507 (x 5.923) | 15 (0.200) | 0.215 | response to steroid hormone stimulus | W ftz-f1 rpr |
| 97 | GO:0050136 | F | 6, | 4 | 0.945 (x 4.231) | 28 (0.143) | 0.219 | NADH dehydrogenase (quinone) activity | CG3192 CG5548 CG5703 Pdsw |
| 98 | GO:0016655 | F | 5, | 4 | 0.945 (x 4.231) | 28 (0.143) | 0.221 | oxidoreductase activity, acting on NADH or NADPH, quinone or similar compound as acceptor | CG3192 CG5548 CG5703 Pdsw |
| 99 | GO:0003724 | F | 4, | 6 | 1.992 (x 3.012) | 59 (0.102) | 0.223 | RNA helicase activity | CG1582 CG31755 CG5205 CG5800 CG6204 Dbp80 |
| 100 | GO:0008137 | F | 6, 7, | 4 | 0.945 (x 4.231) | 28 (0.143) | 0.224 | NADH dehydrogenase (ubiquinone) activity | CG3192 CG5548 CG5703 Pdsw |
| 101 | GO:0007007 | P | 7, 8, | 2 | 0.203 (x 9.872) | 6 (0.333) | 0.227 | inner mitochondrial membrane organization and biogenesis | Tim10 Tim9a |
| 102 | GO:0004032 | F | 7, | 2 | 0.203 (x 9.872) | 6 (0.333) | 0.229 | aldehyde reductase activity | BcDNA:GH10614 CG10638 |
| 103 | GO:0019866 | C | 4, 5, 6, 7, 8, 9, | 12 | 5.875 (x 2.042) | 174 (0.069) | 0.23 | organelle inner membrane | Bmcp CG3192 CG3803 CG4769 CG5548 CG5703 CG7955 LamC Pdsw Tim10 Tim9a bcn92 |
| 104 | GO:0006611 | P | 8, 9, 10, | 2 | 0.203 (x 9.872) | 6 (0.333) | 0.232 | protein export from nucleus | Nmd3 Nxt1 |
| 105 | GO:0045039 | P | 7, 8, 9, 10, 11, | 2 | 0.203 (x 9.872) | 6 (0.333) | 0.234 | protein import into mitochondrial inner membrane | Tim10 Tim9a |
| 106 | GO:0006289 | P | 6, 8, | 3 | 0.540 (x 5.553) | 16 (0.188) | 0.236 | nucleotide-excision repair | DDB1 hay mre11 |
| 107 | GO:0016846 | F | 4, | 2 | 0.203 (x 9.872) | 6 (0.333) | 0.236 | carbon-sulfur lyase activity | CG12264 CG6950 |
| 108 | GO:0009725 | P | 4, 5, | 3 | 0.540 (x 5.553) | 16 (0.188) | 0.238 | response to hormone stimulus | W ftz-f1 rpr |
| 109 | GO:0042775 | P | 8, 10, | 6 | 2.060 (x 2.913) | 61 (0.098) | 0.239 | ATP synthesis coupled electron transport (sensu Eukaryota) | CG3192 CG32649 CG4769 CG5548 CG5703 Pdsw |
| 110 | GO:0002164 | P | 4, | 5 | 1.520 (x 3.291) | 45 (0.111) | 0.246 | larval development | Lcp65Ag1 Mmp1 Traf1 rad50 sas |
| 111 | GO:0003824 | F | 2, | 146 | 127.673 (x 1.144) | 3781 (0.039) | 0.247 | catalytic activity | Aats-leu Act57B Aldh Arf84F Ate1 BEST:CK01227 BG:DS01068.5 BcDNA:GH10614 BcDNA:LD23830 BcDNA:LD32788 CAH1 CAH2 CG10214 CG10445 CG10527 CG10638 CG10877 CG1134 CG11837 CG11858 CG11897 CG12224 CG12264 CG1299 CG13645 CG14869 CG14935 CG15629 CG1582 CG15879 CG17108 CG1718 CG17227 CG17843 CG18522 CG18547 CG1882 CG2064 CG2065 CG2246 CG2277 CG2453 CG2885 CG2989 CG3008 CG31063 CG31373 CG3168 CG31713 CG31755 CG31793 CG3192 CG32549 CG32649 CG4096 CG4267 CG4769 CG4917 CG4933 CG5037 CG5044 CG5162 CG5177 CG5205 CG5317 CG5397 CG5548 CG5703 CG5800 CG5946 CG6204 CG6330 CG6357 CG6512 CG6950 CG7163 CG7339 CG7627 CG7791 CG7828 CG7842 CG7955 CG7997 CG8199 CG8327 CG8630 CG8756 CG8798 CG9358 CG9372 CG9790 Chit DDB1 Dbp80 Dgp-1 Fdxh Gclc Gdh Gs2 GstD3 GstD5 GstD6 GstD9 GstE1 GstE3 GstE5 GstE6 GstE7 Hmgcr Ide Idgf2 Idgf3 ImpL3 Irbp Jafrac1 JhI-1 Jheh3 Ku80 Las Mhc Mlc1 Mlc2 Mmp1 Mocs1 Pcaf Pdsw Ptp61F R Rep Sulf1 Ugt86Da Ugt86Di agt alpha-Est10 bcn92 desat1 hay huntingtin isopeptidase-T-3 mre11 mus205 puc rad50 sda wun wun2 |
| 112 | GO:0003674 | F | 1, | 289 | 279.794 (x 1.033) | 8286 (0.035) | 0.247 | molecular\_function | Aats-leu Act57B Aldh Aly Arf84F Arr2 Ate1 BEST:CK01227 BG:DS01068.5 BG:DS01219.1 BG:DS02740.9 BG:DS03431.1 BcDNA:GH10614 BcDNA:LD23830 BcDNA:LD32788 Bmcp CAH1 CAH2 CG10126 CG10214 CG10306 CG10444 CG10445 CG10460 CG10527 CG10638 CG10861 CG10877 CG1134 CG11837 CG11858 CG11897 CG11907 CG12224 CG12264 CG12876 CG12954 CG1299 CG13586 CG13645 CG13692 CG14869 CG14935 CG15629 CG1582 CG15879 CG17108 CG17124 CG1718 CG17227 CG17843 CG17904 CG18522 CG18547 CG1882 CG2064 CG2065 CG2246 CG2277 CG2453 CG2885 CG2989 CG3008 CG30152 CG30343 CG3036 CG31063 CG31279 CG31373 CG3153 CG31601 CG31611 CG31666 CG3168 CG31713 CG31755 CG31793 CG3192 CG31922 CG32017 CG32021 CG32207 CG3244 CG32442 CG32448 CG32549 CG32582 CG32625 CG32649 CG32756 CG33002 CG33158 CG3371 CG3860 CG40006 CG4096 CG4115 CG4267 CG4364 CG4511 CG4769 CG4858 CG4866 CG4917 CG4933 CG5037 CG5044 CG5162 CG5177 CG5205 CG5317 CG5338 CG5397 CG5535 CG5548 CG5558 CG5703 CG5800 CG5946 CG6204 CG6272 CG6305 CG6330 CG6357 CG6512 CG6574 CG6739 CG6764 CG6876 CG6950 CG7014 CG7054 CG7163 CG7194 CG7339 CG7447 CG7515 CG7571 CG7627 CG7722 CG7791 CG7828 CG7842 CG7955 CG7997 CG8066 CG8199 CG8226 CG8327 CG8630 CG8756 CG8798 CG9077 CG9273 CG9358 CG9372 CG9460 CG9790 CG9862 Chit Cp36 Cys DDB1 Dbp80 Dgp-1 EG:52C10.1 EP2237 Ets21C Fdxh GABA-B-R2 GV1 Gasp Gclc Gdh Gr39a Gs2 GstD3 GstD5 GstD6 GstD9 GstE1 GstE3 GstE5 GstE6 GstE7 HBS1 HLHmgamma Hmgcr Ide Idgf2 Idgf3 Imp ImpL3 Irbp Jafrac1 JhI-1 JhI-26 Jheh3 Ku80 La LamC Las Lcp65Ag1 Mhc Mlc1 Mlc2 Mlp60A Mmp1 Mocs1 NP15.6 Nmd3 Nxt1 Obp83g PQBP-1 Pcaf Pdsw Ptp61F R REG Rep RpA-70 RpL17A RpS4 SP1173 Ssb-c31a Ssrp Sulf1 TMS1 Taf12L TepIV Thor Tim10 Tim9a TpnC41C Traf1 Tsf1 Tsf3 Tsp42El Ugt86Da Ugt86Di ab agt alpha-Est10 apt bcn92 cher desat1 dia dlp eIF6 eiger fau fru ftz-f1 glob1 hay huntingtin isopeptidase-T-3 ken l(3)01239 lama loco mRpL22 mRpL22-24 mRpS14 mRpS18a mRpS21 mRpS33 melt mirr mre11 mus205 na os osp ppk puc rad50 sas sda sog tko toy trol unc-13 wun wun2 wupA yellow-b |
| 113 | GO:0042773 | P | 7, 9, | 6 | 2.094 (x 2.866) | 62 (0.097) | 0.248 | ATP synthesis coupled electron transport | CG3192 CG32649 CG4769 CG5548 CG5703 Pdsw |
| 114 | GO:0007280 | P | 6, 7, 8, | 3 | 0.574 (x 5.226) | 17 (0.176) | 0.252 | pole cell migration | Hmgcr wun wun2 |
| 115 | GO:0005747 | C | 4, 5, 6, 7, 8, 9, 10, 11, 12, 13, 14, | 4 | 1.249 (x 3.202) | 37 (0.108) | 0.254 | respiratory chain complex I (sensu Eukaryota) | CG3192 CG5548 CG5703 Pdsw |
| 116 | GO:0045271 | C | 3, 4, 5, 6, | 4 | 1.249 (x 3.202) | 37 (0.108) | 0.255 | respiratory chain complex I | CG3192 CG5548 CG5703 Pdsw |
| 117 | GO:0000217 | F | 6, | 1 | 0.034 (x 29.615) | 1 (1.000) | 0.256 | DNA secondary structure binding | Ssrp |
| 118 | GO:0015931 | P | 5, 6, | 4 | 1.249 (x 3.202) | 37 (0.108) | 0.256 | nucleobase, nucleoside, nucleotide and nucleic acid transport | Aly CG11907 La Nxt1 |
| 119 | GO:0030017 | C | 6, 7, 8, 9, 10, | 2 | 0.304 (x 6.581) | 9 (0.222) | 0.256 | sarcomere | Mhc wupA |
| 120 | GO:0016598 | P | 8, 10, 11, 12, | 1 | 0.034 (x 29.615) | 1 (1.000) | 0.257 | protein arginylation | Ate1 |
| 121 | GO:0035072 | P | 7, 8, 9, 10, 11, | 2 | 0.304 (x 6.581) | 9 (0.222) | 0.258 | ecdysone-mediated induction of salivary gland cell autophagic cell death | W rpr |
| 122 | GO:0008846 | F | 7, | 1 | 0.034 (x 29.615) | 1 (1.000) | 0.258 | endopeptidase La activity | CG8798 |
| 123 | GO:0007618 | P | 5, 6, | 2 | 0.304 (x 6.581) | 9 (0.222) | 0.259 | mating | fru ken |
| 124 | GO:0031327 | P | 7, | 3 | 0.743 (x 4.038) | 22 (0.136) | 0.259 | negative regulation of cellular biosynthesis | Thor apt rpr |
| 125 | GO:0000045 | P | 6, | 1 | 0.034 (x 29.615) | 1 (1.000) | 0.26 | autophagic vacuole formation | CG10861 |
| 126 | GO:0008514 | F | 5, | 2 | 0.304 (x 6.581) | 9 (0.222) | 0.26 | organic anion transporter activity | BEST:CK01227 CG7571 |
| 127 | GO:0009890 | P | 6, | 3 | 0.743 (x 4.038) | 22 (0.136) | 0.26 | negative regulation of biosynthesis | Thor apt rpr |
| 128 | GO:0006409 | P | 8, 9, 10, 11, | 1 | 0.034 (x 29.615) | 1 (1.000) | 0.261 | tRNA export from nucleus | Nxt1 |
| 129 | GO:0008195 | F | 7, | 2 | 0.304 (x 6.581) | 9 (0.222) | 0.261 | phosphatidate phosphatase activity | wun wun2 |
| 130 | GO:0048627 | P | 5, 6, 8, 9, | 3 | 0.743 (x 4.038) | 22 (0.136) | 0.262 | myoblast development | CG6803 Mhc wupA |
| 131 | GO:0016732 | F | 5, | 1 | 0.034 (x 29.615) | 1 (1.000) | 0.262 | oxidoreductase activity, acting on iron-sulfur proteins as donors, dinitrogen as acceptor | CG11897 |
| 132 | GO:0030016 | C | 6, 7, 8, 9, | 2 | 0.304 (x 6.581) | 9 (0.222) | 0.262 | myofibril | Mhc wupA |
| 133 | GO:0048628 | P | 6, 7, 9, 10, | 3 | 0.743 (x 4.038) | 22 (0.136) | 0.263 | myoblast maturation | CG6803 Mhc wupA |
| 134 | GO:0008538 | F | 4, | 1 | 0.034 (x 29.615) | 1 (1.000) | 0.263 | proteasome activator activity | REG |
| 135 | GO:0043228 | C | 3, | 33 | 24.008 (x 1.375) | 711 (0.046) | 0.263 | non-membrane-bound organelle | Act57B CG12954 CG31611 CG33002 CG3371 CG4866 CG5317 CG5338 CG6610 CG6764 CG7014 CG9273 EG:52C10.1 LamC Mhc Mlc1 Mlc2 Pcaf Rep RpA-70 RpL17A RpS4 Ssrp dia mRpL22 mRpL22-24 mRpS14 mRpS18a mRpS21 mRpS33 na tko wupA |
| 136 | GO:0006536 | P | 8, 9, | 2 | 0.304 (x 6.581) | 9 (0.222) | 0.264 | glutamate metabolism | Gdh Gs2 |
| 137 | GO:0043232 | C | 4, 5, 6, 7, | 33 | 24.008 (x 1.375) | 711 (0.046) | 0.265 | intracellular non-membrane-bound organelle | Act57B CG12954 CG31611 CG33002 CG3371 CG4866 CG5317 CG5338 CG6610 CG6764 CG7014 CG9273 EG:52C10.1 LamC Mhc Mlc1 Mlc2 Pcaf Rep RpA-70 RpL17A RpS4 Ssrp dia mRpL22 mRpL22-24 mRpS14 mRpS18a mRpS21 mRpS33 na tko wupA |
| 138 | GO:0003894 | F | 7, | 1 | 0.034 (x 29.615) | 1 (1.000) | 0.265 | zeta DNA polymerase activity | mus205 |
| 139 | GO:0006307 | P | 6, 8, | 1 | 0.034 (x 29.615) | 1 (1.000) | 0.266 | DNA dealkylation | agt |
| 140 | GO:0004081 | F | 9, | 1 | 0.034 (x 29.615) | 1 (1.000) | 0.267 | bis(5'-nucleosyl)-tetraphosphatase (asymmetrical) activity | CG31713 |
| 141 | GO:0015629 | C | 6, 7, 8, 9, | 6 | 2.499 (x 2.401) | 74 (0.081) | 0.268 | actin cytoskeleton | Act57B Mhc Mlc1 Mlc2 dia wupA |
| 142 | GO:0005198 | F | 2, | 34 | 24.920 (x 1.364) | 738 (0.046) | 0.268 | structural molecule activity | Act57B BG:DS02740.9 CG12954 CG33002 CG4364 CG4866 CG5162 CG5317 CG5338 CG6305 CG6764 CG7014 CG9077 Cp36 Gasp LamC Lcp65Ag1 Mhc Mmp1 RpL17A RpS4 cher dia dlp mRpL22 mRpL22-24 mRpS14 mRpS18a mRpS21 mRpS33 na tko trol wupA |
| 143 | GO:0003860 | F | 7, | 1 | 0.034 (x 29.615) | 1 (1.000) | 0.269 | 3-hydroxyisobutyryl-CoA hydrolase activity | CG5044 |
| 144 | GO:0035001 | P | 4, 5, | 1 | 0.034 (x 29.615) | 1 (1.000) | 0.27 | dorsal trunk growth | Mmp1 |
| 145 | GO:0016163 | F | 6, | 1 | 0.034 (x 29.615) | 1 (1.000) | 0.272 | nitrogenase activity | CG11897 |
| 146 | GO:0015671 | P | 6, 7, | 1 | 0.034 (x 29.615) | 1 (1.000) | 0.273 | oxygen transport | glob1 |
| 147 | GO:0006522 | P | 8, 9, | 1 | 0.034 (x 29.615) | 1 (1.000) | 0.274 | alanine metabolism | CG12264 |
| 148 | GO:0009078 | P | 7, 8, | 1 | 0.034 (x 29.615) | 1 (1.000) | 0.276 | pyruvate family amino acid metabolism | CG12264 |
| 149 | GO:0018741 | F | 6, | 1 | 0.034 (x 29.615) | 1 (1.000) | 0.277 | alkyl sulfatase activity | Sulf1 |
| 150 | GO:0008152 | P | 3, | 186 | 170.760 (x 1.089) | 5057 (0.037) | 0.278 | metabolism | Aats-leu Act57B Aldh Aly Arf84F Ate1 BG:DS03431.1 BcDNA:LD32788 CAH1 CAH2 CG10214 CG10306 CG10444 CG10445 CG10638 CG10877 CG1134 CG11837 CG11897 CG11907 CG12264 CG12954 CG1299 CG13645 CG13692 CG14869 CG14935 CG15629 CG17227 CG17843 CG18522 CG1882 CG2064 CG2065 CG2246 CG2277 CG2453 CG2885 CG2989 CG3008 CG3036 CG31373 CG31611 CG3168 CG3192 CG32549 CG32649 CG33002 CG33158 CG3803 CG3860 CG4096 CG4267 CG4364 CG4511 CG4769 CG4866 CG4933 CG5037 CG5044 CG5162 CG5177 CG5205 CG5317 CG5338 CG5535 CG5548 CG5558 CG5703 CG5800 CG5946 CG6204 CG6272 CG6330 CG6512 CG6574 CG6610 CG6764 CG6803 CG6876 CG6950 CG7014 CG7163 CG7339 CG7722 CG7791 CG7828 CG7842 CG7955 CG7997 CG8199 CG8327 CG8630 CG8756 CG8798 CG9273 CG9358 CG9372 CG9460 CG9836 CG9862 Chit DDB1 Dbp80 Dgp-1 EP2237 Ets21C Fdxh Gasp Gclc Gdh Gs2 GstE1 GstE3 GstE5 GstE6 GstE7 HBS1 HLHmgamma Hmgcr Ide Idgf2 Idgf3 Imp ImpL3 Irbp Jafrac1 JhI-1 Jheh3 Ku80 La Las Lcp65Ag1 Mlc1 Mmp1 Mocs1 Nmd3 Pcaf Pdsw Ptp61F REG Rep RpA-70 RpL17A RpS4 Ssb-c31a Ssrp Sulf1 TMS1 Taf12L Thor Ugt86Da Ugt86Di W ab agt apt bcn92 cul-2 desat1 dia eIF6 fru ftz-f1 hay huntingtin l(3)01239 mRpL22 mRpL22-24 mRpS14 mRpS18a mRpS21 mRpS33 mirr mre11 mus205 na puc rad50 rpr sda tko toy trol wun wun2 |
| 151 | GO:0046916 | P | 8, | 2 | 0.236 (x 8.461) | 7 (0.286) | 0.278 | transition metal ion homeostasis | Tsf1 Tsf3 |
| 152 | GO:0006636 | P | 7, 8, | 1 | 0.034 (x 29.615) | 1 (1.000) | 0.279 | fatty acid desaturation | CG5946 |
| 153 | GO:0008169 | F | 6, | 1 | 0.034 (x 29.615) | 1 (1.000) | 0.28 | C-methyltransferase activity | CG2453 |
| 154 | GO:0006879 | P | 8, 9, | 2 | 0.236 (x 8.461) | 7 (0.286) | 0.28 | iron ion homeostasis | Tsf1 Tsf3 |
| 155 | GO:0006417 | P | 6, 7, 8, | 6 | 2.533 (x 2.369) | 75 (0.080) | 0.281 | regulation of protein biosynthesis | CG5317 HBS1 Imp Thor apt rpr |
| 156 | GO:0005829 | C | 5, 6, 7, 8, | 12 | 6.213 (x 1.931) | 184 (0.065) | 0.281 | cytosol | CG32549 CG5317 CG5338 CG6764 CG7014 HBS1 Jafrac1 REG RpL17A RpS4 eIF6 l(3)01239 |
| 157 | GO:0042254 | P | 6, | 4 | 1.081 (x 3.702) | 32 (0.125) | 0.281 | ribosome biogenesis and assembly | CG32409 CG6764 EG:52C10.1 Nmd3 |
| 158 | GO:0030870 | C | 3, 5, 6, 7, 8, 9, 10, | 1 | 0.034 (x 29.615) | 1 (1.000) | 0.282 | Mre11 complex | rad50 |
| 159 | GO:0045445 | P | 5, 7, 8, | 3 | 0.777 (x 3.863) | 23 (0.130) | 0.282 | myoblast differentiation | CG6803 Mhc wupA |
| 160 | GO:0008199 | F | 7, | 2 | 0.236 (x 8.461) | 7 (0.286) | 0.282 | ferric iron binding | Tsf1 Tsf3 |
| 161 | GO:0008495 | F | 7, | 1 | 0.034 (x 29.615) | 1 (1.000) | 0.283 | protoheme IX farnesyltransferase activity | CG5037 |
| 162 | GO:0009612 | P | 4, | 2 | 0.338 (x 5.923) | 10 (0.200) | 0.284 | response to mechanical stimulus | sda tko |
| 163 | GO:0004128 | F | 5, 6, | 1 | 0.034 (x 29.615) | 1 (1.000) | 0.285 | cytochrome-b5 reductase activity | CG5946 |
| 164 | GO:0009065 | P | 8, 9, | 2 | 0.236 (x 8.461) | 7 (0.286) | 0.285 | glutamine family amino acid catabolism | Gdh Gs2 |
| 165 | GO:0048609 | P | 4, | 2 | 0.338 (x 5.923) | 10 (0.200) | 0.286 | reproductive organismal physiological process | Gdh ken |
| 166 | GO:0048033 | P | 7, 8, | 1 | 0.034 (x 29.615) | 1 (1.000) | 0.286 | heme o metabolism | CG5037 |
| 167 | GO:0043596 | C | 6, 7, 8, 9, 10, 11, 12, | 2 | 0.338 (x 5.923) | 10 (0.200) | 0.287 | replication fork (sensu Eukaryota) | CG9273 RpA-70 |
| 168 | GO:0015934 | C | 3, 4, 5, 6, 7, 8, 9, | 7 | 3.242 (x 2.159) | 96 (0.073) | 0.287 | large ribosomal subunit | CG12954 CG33002 CG5317 CG6764 RpL17A mRpL22 mRpL22-24 |
| 169 | GO:0007620 | P | 6, 7, | 2 | 0.236 (x 8.461) | 7 (0.286) | 0.287 | copulation | fru ken |
| 170 | GO:0004677 | F | 8, | 1 | 0.034 (x 29.615) | 1 (1.000) | 0.288 | DNA-dependent protein kinase activity | Irbp |
| 171 | GO:0030894 | C | 3, 5, 6, 7, 8, 9, 10, 11, | 2 | 0.338 (x 5.923) | 10 (0.200) | 0.288 | replisome | CG9273 RpA-70 |
| 172 | GO:0007320 | P | 5, 6, | 2 | 0.338 (x 5.923) | 10 (0.200) | 0.289 | insemination | Gdh ken |
| 173 | GO:0006542 | P | 9, 10, | 1 | 0.034 (x 29.615) | 1 (1.000) | 0.289 | glutamine biosynthesis | Gs2 |
| 174 | GO:0007006 | P | 6, 7, | 2 | 0.236 (x 8.461) | 7 (0.286) | 0.29 | mitochondrial membrane organization and biogenesis | Tim10 Tim9a |
| 175 | GO:0043601 | C | 4, 6, 7, 8, 9, 10, 11, 12, 13, | 2 | 0.338 (x 5.923) | 10 (0.200) | 0.29 | replisome (sensu Eukaryota) | CG9273 RpA-70 |
| 176 | GO:0018348 | P | 11, 12, | 1 | 0.034 (x 29.615) | 1 (1.000) | 0.291 | protein amino acid geranylgeranylation | Rep |
| 177 | GO:0035078 | P | 6, 7, 8, 9, 10, | 2 | 0.338 (x 5.923) | 10 (0.200) | 0.292 | induction of programmed cell death by ecdysone | W rpr |
| 178 | GO:0004033 | F | 6, | 2 | 0.236 (x 8.461) | 7 (0.286) | 0.292 | aldo-keto reductase activity | BcDNA:GH10614 CG10638 |
| 179 | GO:0042828 |  | 5, 6, | 1 | 0.034 (x 29.615) | 1 (1.000) | 0.292 |  | eiger |
| 180 | GO:0050876 | P | 3, | 2 | 0.338 (x 5.923) | 10 (0.200) | 0.293 | reproductive physiological process | Gdh ken |
| 181 | GO:0051031 | P | 7, 8, 9, | 1 | 0.034 (x 29.615) | 1 (1.000) | 0.294 | tRNA transport | Nxt1 |
| 182 | GO:0006941 | P | 5, | 1 | 0.034 (x 29.615) | 1 (1.000) | 0.296 | striated muscle contraction | Mhc |
| 183 | GO:0030580 | F | 7, | 1 | 0.034 (x 29.615) | 1 (1.000) | 0.297 | quinone cofactor methyltransferase activity | CG2453 |
| 184 | GO:0008267 | F | 4, | 1 | 0.034 (x 29.615) | 1 (1.000) | 0.299 | poly-glutamine tract binding | PQBP-1 |
| 185 | GO:0016887 | F | 8, | 19 | 12.528 (x 1.517) | 371 (0.051) | 0.3 | ATPase activity | BEST:CK01227 CG11897 CG1582 CG1718 CG31755 CG31793 CG5205 CG5800 CG6512 CG7627 CG7955 Dbp80 Irbp Ku80 Mhc Mlc1 Mlc2 hay rad50 |
| 186 | GO:0016903 | F | 4, | 3 | 0.810 (x 3.702) | 24 (0.125) | 0.3 | oxidoreductase activity, acting on the aldehyde or oxo group of donors | Aldh CG8199 DDB1 |
| 187 | GO:0004243 | F | 7, | 1 | 0.034 (x 29.615) | 1 (1.000) | 0.301 | mitochondrial intermediate peptidase activity | CG7791 |
| 188 | GO:0044445 | C | 5, 6, 7, 8, 9, | 8 | 3.985 (x 2.008) | 118 (0.068) | 0.301 | cytosolic part | CG5317 CG5338 CG6764 CG7014 REG RpL17A RpS4 l(3)01239 |
| 189 | GO:0015450 | F | 4, 6, | 3 | 0.810 (x 3.702) | 24 (0.125) | 0.301 | protein translocase activity | CG8226 Tim10 Tim9a |
| 190 | GO:0000055 | P | 9, 10, 11, 12, | 1 | 0.034 (x 29.615) | 1 (1.000) | 0.302 | ribosomal large subunit export from nucleus | Nmd3 |
| 191 | GO:0008796 | F | 8, | 1 | 0.034 (x 29.615) | 1 (1.000) | 0.304 | bis(5'-nucleosyl)-tetraphosphatase activity | CG31713 |
| 192 | GO:0008425 | F | 8, | 1 | 0.034 (x 29.615) | 1 (1.000) | 0.306 | 2-polyprenyl-6-methoxy-1,4-benzoquinone methyltransferase activity | CG2453 |
| 193 | GO:0005958 | C | 4, | 1 | 0.034 (x 29.615) | 1 (1.000) | 0.308 | DNA-dependent protein kinase complex | Ku80 |
| 194 | GO:0035182 | C | 3, 4, 5, 6, | 1 | 0.034 (x 29.615) | 1 (1.000) | 0.309 | ring canal outer rim | cher |
| 195 | GO:0045497 | P | 7, | 1 | 0.068 (x 14.807) | 2 (0.500) | 0.31 | female analia development (sensu Endopterygota) | ken |
| 196 | GO:0004866 | F | 5, | 7 | 2.870 (x 2.439) | 85 (0.082) | 0.31 | endopeptidase inhibitor activity | CG10460 CG40006 CG7722 CG8066 CG9460 Cys TepIV |
| 197 | GO:0008627 | P | 10, 11, | 1 | 0.068 (x 14.807) | 2 (0.500) | 0.311 | induction of apoptosis by ionic changes | rpr |
| 198 | GO:0009079 | P | 8, 9, | 1 | 0.034 (x 29.615) | 1 (1.000) | 0.311 | pyruvate family amino acid biosynthesis | CG12264 |
| 199 | GO:0008463 | F | 6, | 1 | 0.068 (x 14.807) | 2 (0.500) | 0.311 | formylmethionine deformylase activity | CG31373 |
| 200 | GO:0008450 | F | 7, | 1 | 0.068 (x 14.807) | 2 (0.500) | 0.312 | O-sialoglycoprotein endopeptidase activity | CG4933 |
| 201 | GO:0006429 | P | 9, 10, 11, | 1 | 0.034 (x 29.615) | 1 (1.000) | 0.313 | leucyl-tRNA aminoacylation | Aats-leu |
| 202 | GO:0003717 | F | 4, | 1 | 0.068 (x 14.807) | 2 (0.500) | 0.313 | RNA polymerase II transcription termination factor activity | CG10445 |
| 203 | GO:0045111 | C | 6, 7, 8, 9, | 1 | 0.068 (x 14.807) | 2 (0.500) | 0.314 | intermediate filament cytoskeleton | LamC |
| 204 | GO:0004527 | F | 6, | 4 | 1.384 (x 2.889) | 41 (0.098) | 0.315 | exonuclease activity | CG10214 mre11 mus205 rad50 |
| 205 | GO:0008537 | C | 3, 6, 7, 8, 9, 10, | 1 | 0.034 (x 29.615) | 1 (1.000) | 0.315 | proteasome activator complex | REG |
| 206 | GO:0005882 | C | 5, 6, 7, 8, 9, 10, | 1 | 0.068 (x 14.807) | 2 (0.500) | 0.315 | intermediate filament | LamC |
| 207 | GO:0004459 | F | 6, | 1 | 0.068 (x 14.807) | 2 (0.500) | 0.316 | L-lactate dehydrogenase activity | ImpL3 |
| 208 | GO:0045947 | P | 9, 10, 11, | 1 | 0.034 (x 29.615) | 1 (1.000) | 0.317 | negative regulation of translational initiation | Thor |
| 209 | GO:0017086 | C | 3, 5, 6, 7, 8, 9, 10, 11, 12, | 1 | 0.068 (x 14.807) | 2 (0.500) | 0.317 | 3-methyl-2-oxobutanoate dehydrogenase (lipoamide) complex | CG8199 |
| 210 | GO:0005861 | C | 3, 5, 6, 7, 8, 9, 10, 11, 12, | 1 | 0.068 (x 14.807) | 2 (0.500) | 0.318 | troponin complex | wupA |
| 211 | GO:0008190 | F | 5, | 1 | 0.034 (x 29.615) | 1 (1.000) | 0.319 | eukaryotic initiation factor 4E binding | Thor |
| 212 | GO:0048471 | C | 5, 6, 7, 8, | 1 | 0.068 (x 14.807) | 2 (0.500) | 0.319 | perinuclear region | Rep |
| 213 | GO:0015886 | P | 6, 7, | 1 | 0.068 (x 14.807) | 2 (0.500) | 0.32 | heme transport | CG7955 |
| 214 | GO:0004231 | F | 7, | 1 | 0.034 (x 29.615) | 1 (1.000) | 0.321 | insulysin activity | Ide |
| 215 | GO:0030241 | P | 8, 9, 11, 12, | 1 | 0.068 (x 14.807) | 2 (0.500) | 0.321 | muscle thick filament assembly | CG6803 |
| 216 | GO:0004311 | F | 6, | 1 | 0.068 (x 14.807) | 2 (0.500) | 0.322 | farnesyltranstransferase activity | CG5037 |
| 217 | GO:0016282 | C | 3, 5, 6, 7, 8, | 5 | 2.026 (x 2.468) | 60 (0.083) | 0.322 | eukaryotic 43S preinitiation complex | CG10306 CG33158 CG5338 CG7014 RpS4 |
| 218 | GO:0005744 | C | 3, 5, 6, 7, 8, 9, 10, 11, 12, 13, | 2 | 0.439 (x 4.556) | 13 (0.154) | 0.322 | mitochondrial inner membrane presequence translocase complex | Tim10 Tim9a |
| 219 | GO:0004019 | F | 5, | 1 | 0.034 (x 29.615) | 1 (1.000) | 0.323 | adenylosuccinate synthase activity | BcDNA:LD32788 |
| 220 | GO:0004823 | F | 7, | 1 | 0.068 (x 14.807) | 2 (0.500) | 0.323 | leucine-tRNA ligase activity | Aats-leu |
| 221 | GO:0009889 | P | 5, | 6 | 2.668 (x 2.249) | 79 (0.076) | 0.323 | regulation of biosynthesis | CG5317 HBS1 Imp Thor apt rpr |
| 222 | GO:0051189 | P | 5, 7, | 5 | 2.026 (x 2.468) | 60 (0.083) | 0.323 | prosthetic group metabolism | CG10444 CG13645 CG32649 CG6574 Las |
| 223 | GO:0016798 | F | 4, | 7 | 3.613 (x 1.937) | 107 (0.065) | 0.323 | hydrolase activity, acting on glycosyl bonds | CG14935 CG17843 CG2989 CG7997 Chit Idgf2 Idgf3 |
| 224 | GO:0007277 | P | 5, | 2 | 0.371 (x 5.384) | 11 (0.182) | 0.324 | pole cell development | dia wun2 |
| 225 | GO:0016030 | F | 5, | 1 | 0.068 (x 14.807) | 2 (0.500) | 0.324 | metarhodopsin binding | Arr2 |
| 226 | GO:0001700 | P | 5, | 8 | 4.356 (x 1.837) | 129 (0.062) | 0.324 | embryonic development (sensu Insecta) | EP2237 R Traf1 W mirr os puc rpr |
| 227 | GO:0006936 | P | 4, | 6 | 2.904 (x 2.066) | 86 (0.070) | 0.324 | muscle contraction | Mhc Mlc1 Mlc2 TpnC41C cher wupA |
| 228 | GO:0030511 | P | 6, 7, 10, | 1 | 0.034 (x 29.615) | 1 (1.000) | 0.324 | positive regulation of transforming growth factor beta receptor signaling pathway | sog |
| 229 | GO:0031326 | P | 6, | 6 | 2.668 (x 2.249) | 79 (0.076) | 0.325 | regulation of cellular biosynthesis | CG5317 HBS1 Imp Thor apt rpr |
| 230 | GO:0016459 | C | 3, 5, 6, 7, 8, 9, 10, | 3 | 0.844 (x 3.554) | 25 (0.120) | 0.325 | myosin | Mhc Mlc1 Mlc2 |
| 231 | GO:0000041 | P | 8, 9, | 2 | 0.371 (x 5.384) | 11 (0.182) | 0.325 | transition metal ion transport | Tsf1 Tsf3 |
| 232 | GO:0004314 | F | 8, 9, | 1 | 0.068 (x 14.807) | 2 (0.500) | 0.325 | [acyl-carrier protein] S-malonyltransferase activity | CG7842 |
| 233 | GO:0030414 | F | 4, | 7 | 2.904 (x 2.410) | 86 (0.081) | 0.326 | protease inhibitor activity | CG10460 CG40006 CG7722 CG8066 CG9460 Cys TepIV |
| 234 | GO:0016652 | F | 5, | 1 | 0.068 (x 14.807) | 2 (0.500) | 0.326 | oxidoreductase activity, acting on NADH or NADPH, NAD or NADP as acceptor | CG5946 |
| 235 | GO:0035081 | P | 8, 9, | 2 | 0.371 (x 5.384) | 11 (0.182) | 0.326 | induction of programmed cell death by hormones | W rpr |
| 236 | GO:0016543 | P | 8, 9, | 1 | 0.034 (x 29.615) | 1 (1.000) | 0.326 | male courtship behavior (sensu Insecta), orientation | fru |
| 237 | GO:0016799 | F | 5, | 4 | 1.418 (x 2.820) | 42 (0.095) | 0.327 | hydrolase activity, hydrolyzing N-glycosyl compounds | CG2989 Chit Idgf2 Idgf3 |
| 238 | GO:0006855 | P | 6, 7, | 1 | 0.068 (x 14.807) | 2 (0.500) | 0.327 | multidrug transport | BEST:CK01227 |
| 239 | GO:0016236 | P | 5, | 1 | 0.068 (x 14.807) | 2 (0.500) | 0.328 | macroautophagy | CG10861 |
| 240 | GO:0004420 | F | 6, | 1 | 0.034 (x 29.615) | 1 (1.000) | 0.328 | hydroxymethylglutaryl-CoA reductase (NADPH) activity | Hmgcr |
| 241 | GO:0019008 | C | 4, | 1 | 0.068 (x 14.807) | 2 (0.500) | 0.329 | molybdopterin synthase complex | Mocs1 |
| 242 | GO:0004353 | F | 6, | 1 | 0.068 (x 14.807) | 2 (0.500) | 0.33 | glutamate dehydrogenase [NAD(P)+] activity | Gdh |
| 243 | GO:0015669 | P | 5, 6, | 1 | 0.034 (x 29.615) | 1 (1.000) | 0.331 | gas transport | glob1 |
| 244 | GO:0005523 | F | 5, | 1 | 0.068 (x 14.807) | 2 (0.500) | 0.331 | tropomyosin binding | wupA |
| 245 | GO:0006081 | P | 5, | 1 | 0.068 (x 14.807) | 2 (0.500) | 0.332 | aldehyde metabolism | CG10638 |
| 246 | GO:0009618 |  | 6, 7, | 1 | 0.034 (x 29.615) | 1 (1.000) | 0.333 |  | eiger |
| 247 | GO:0045153 | F | 5, | 1 | 0.068 (x 14.807) | 2 (0.500) | 0.333 | electron transporter, transferring electrons within CoQH2-cytochrome c reductase complex activity | CG4769 |
| 248 | GO:0003989 | F | 6, | 1 | 0.068 (x 14.807) | 2 (0.500) | 0.334 | acetyl-CoA carboxylase activity | CG17108 |
| 249 | GO:0007254 | P | 7, 8, | 4 | 1.587 (x 2.520) | 47 (0.085) | 0.334 | JNK cascade | EP2237 Traf1 eiger puc |
| 250 | GO:0017117 | C | 3, 5, 6, 7, 8, 9, 10, | 1 | 0.034 (x 29.615) | 1 (1.000) | 0.335 | single-stranded DNA-dependent ATP-dependent DNA helicase complex | Ku80 |
| 251 | GO:0031098 | P | 6, | 4 | 1.587 (x 2.520) | 47 (0.085) | 0.335 | stress-activated protein kinase signaling pathway | EP2237 Traf1 eiger puc |
| 252 | GO:0009107 | P | 8, 9, | 1 | 0.068 (x 14.807) | 2 (0.500) | 0.335 | lipoate biosynthesis | Las |
| 253 | GO:0006633 | P | 6, 7, 8, | 3 | 0.979 (x 3.064) | 29 (0.103) | 0.336 | fatty acid biosynthesis | CG7842 CG8630 desat1 |
| 254 | GO:0005164 | F | 5, 6, | 1 | 0.068 (x 14.807) | 2 (0.500) | 0.336 | tumor necrosis factor receptor binding | eiger |
| 255 | GO:0016491 | F | 3, | 28 | 21.071 (x 1.329) | 624 (0.045) | 0.337 | oxidoreductase activity | Aldh BcDNA:GH10614 CG10638 CG11897 CG12224 CG15629 CG17843 CG18522 CG18547 CG2064 CG2065 CG3192 CG4769 CG5044 CG5548 CG5703 CG5946 CG8199 CG8630 DDB1 Fdxh Gdh Hmgcr ImpL3 Jafrac1 Pdsw bcn92 desat1 |
| 256 | GO:0018344 | P | 10, 11, | 1 | 0.034 (x 29.615) | 1 (1.000) | 0.337 | protein geranylgeranylation | Rep |
| 257 | GO:0008097 | F | 5, | 1 | 0.068 (x 14.807) | 2 (0.500) | 0.338 | 5S rRNA binding | La |
| 258 | GO:0005743 | C | 5, 6, 7, 8, 9, 10, 11, 12, | 11 | 5.707 (x 1.928) | 169 (0.065) | 0.338 | mitochondrial inner membrane | Bmcp CG3192 CG3803 CG4769 CG5548 CG5703 CG7955 Pdsw Tim10 Tim9a bcn92 |
| 259 | GO:0005638 | C | 5, 6, 7, 8, 9, 10, 11, 12, 13, 14, | 1 | 0.068 (x 14.807) | 2 (0.500) | 0.339 | lamin filament | LamC |
| 260 | GO:0008946 | F | 7, 9, | 1 | 0.034 (x 29.615) | 1 (1.000) | 0.339 | oligonucleotidase activity | CG10214 |
| 261 | GO:0048732 | P | 4, | 8 | 4.423 (x 1.809) | 131 (0.061) | 0.34 | gland development | CG10861 EP2237 Mmp1 Traf1 W ftz-f1 rpr sog |
| 262 | GO:0004123 | F | 5, | 1 | 0.068 (x 14.807) | 2 (0.500) | 0.34 | cystathionine gamma-lyase activity | CG12264 |
| 263 | GO:0008559 | F | 4, 5, 7, 12, | 2 | 0.270 (x 7.404) | 8 (0.250) | 0.34 | xenobiotic-transporting ATPase activity | CG11897 CG7627 |
| 264 | GO:0042623 | F | 9, | 17 | 11.143 (x 1.526) | 330 (0.052) | 0.34 | ATPase activity, coupled | BEST:CK01227 CG11897 CG1582 CG1718 CG31755 CG31793 CG5205 CG5800 CG7627 CG7955 Dbp80 Irbp Ku80 Mhc Mlc1 Mlc2 hay |
| 265 | GO:0042981 | P | 6, 7, | 7 | 3.681 (x 1.902) | 109 (0.064) | 0.341 | regulation of apoptosis | CG12876 CG7188 Mmp1 Traf1 W eiger rpr |
| 266 | GO:0019674 | P | 9, 10, | 1 | 0.068 (x 14.807) | 2 (0.500) | 0.341 | NAD metabolism | CG13645 |
| 267 | GO:0017140 | F | 3, | 1 | 0.034 (x 29.615) | 1 (1.000) | 0.341 | lipoic acid synthase activity | Las |
| 268 | GO:0007431 | P | 5, | 7 | 3.681 (x 1.902) | 109 (0.064) | 0.341 | salivary gland development | CG10861 EP2237 Mmp1 Traf1 W ftz-f1 rpr |
| 269 | GO:0003863 | F | 6, | 1 | 0.068 (x 14.807) | 2 (0.500) | 0.342 | 3-methyl-2-oxobutanoate dehydrogenase (2-methylpropanoyl-transferring) activity | CG8199 |
| 270 | GO:0004768 | F | 6, | 2 | 0.270 (x 7.404) | 8 (0.250) | 0.342 | stearoyl-CoA 9-desaturase activity | CG8630 desat1 |
| 271 | GO:0035272 | P | 4, | 7 | 3.681 (x 1.902) | 109 (0.064) | 0.342 | exocrine system development | CG10861 EP2237 Mmp1 Traf1 W ftz-f1 rpr |
| 272 | GO:0044237 | P | 4, | 173 | 156.308 (x 1.107) | 4629 (0.037) | 0.343 | cellular metabolism | Aats-leu Act57B Aldh Aly Arf84F Ate1 BG:DS03431.1 BcDNA:LD32788 CAH1 CAH2 CG10214 CG10306 CG10444 CG10445 CG10638 CG10877 CG1134 CG11837 CG11907 CG12264 CG12954 CG1299 CG13645 CG13692 CG14869 CG17227 CG17843 CG18522 CG1882 CG2246 CG2277 CG2453 CG2885 CG2989 CG3008 CG3036 CG31373 CG31611 CG3168 CG3192 CG32549 CG32649 CG33002 CG33158 CG3803 CG3860 CG4096 CG4364 CG4511 CG4769 CG4866 CG4933 CG5037 CG5044 CG5162 CG5177 CG5205 CG5317 CG5338 CG5535 CG5548 CG5558 CG5703 CG5800 CG5946 CG6204 CG6272 CG6330 CG6512 CG6574 CG6610 CG6764 CG6876 CG6950 CG7014 CG7163 CG7339 CG7722 CG7791 CG7828 CG7842 CG7955 CG8199 CG8327 CG8630 CG8756 CG8798 CG9273 CG9358 CG9372 CG9460 CG9862 Chit DDB1 Dbp80 Dgp-1 EP2237 Ets21C Fdxh Gasp Gclc Gdh Gs2 GstE1 GstE3 GstE5 GstE6 GstE7 HBS1 HLHmgamma Hmgcr Ide Imp ImpL3 Irbp Jafrac1 JhI-1 Jheh3 Ku80 La Las Mlc1 Mmp1 Mocs1 Nmd3 Pcaf Pdsw Ptp61F REG Rep RpA-70 RpL17A RpS4 Ssb-c31a Ssrp Sulf1 TMS1 Taf12L Thor Ugt86Da Ugt86Di W ab agt apt bcn92 cul-2 desat1 eIF6 fru ftz-f1 hay huntingtin l(3)01239 mRpL22 mRpL22-24 mRpS14 mRpS18a mRpS21 mRpS33 mirr mre11 mus205 na puc rad50 rpr sda tko toy trol wun wun2 |
| 273 | GO:0002046 | F | 4, | 1 | 0.068 (x 14.807) | 2 (0.500) | 0.343 | opsin binding | Arr2 |
| 274 | GO:0006523 | P | 9, 10, | 1 | 0.034 (x 29.615) | 1 (1.000) | 0.343 | alanine biosynthesis | CG12264 |
| 275 | GO:0009435 | P | 8, 9, 10, 11, | 1 | 0.068 (x 14.807) | 2 (0.500) | 0.344 | NAD biosynthesis | CG13645 |
| 276 | GO:0012501 | P | 5, | 13 | 8.374 (x 1.552) | 248 (0.052) | 0.345 | programmed cell death | CG10861 CG12876 CG40006 CG7188 EP2237 Mmp1 Traf1 W eiger ftz-f1 rpr wun wun2 |
| 277 | GO:0042910 | F | 3, | 2 | 0.270 (x 7.404) | 8 (0.250) | 0.345 | xenobiotic transporter activity | CG11897 CG7627 |
| 278 | GO:0006408 | P | 8, 9, 10, 11, | 1 | 0.068 (x 14.807) | 2 (0.500) | 0.345 | snRNA export from nucleus | Nxt1 |
| 279 | GO:0003718 | F | 4, | 1 | 0.034 (x 29.615) | 1 (1.000) | 0.346 | RNA polymerase III transcription termination factor activity | La |
| 280 | GO:0017111 | F | 7, | 24 | 17.660 (x 1.359) | 523 (0.046) | 0.346 | nucleoside-triphosphatase activity | Arf84F BEST:CK01227 BcDNA:LD23830 CG11897 CG1582 CG1718 CG2885 CG31755 CG31793 CG5205 CG5800 CG6512 CG7627 CG7955 CG8798 Dbp80 Irbp Ku80 Mhc Mlc1 Mlc2 R hay rad50 |
| 281 | GO:0048047 | P | 5, 6, | 1 | 0.068 (x 14.807) | 2 (0.500) | 0.346 | mating behavior, sex discrimination | fru |
| 282 | GO:0008203 | P | 7, 8, 9, | 3 | 0.878 (x 3.417) | 26 (0.115) | 0.347 | cholesterol metabolism | CG3860 CG5946 Hmgcr |
| 283 | GO:0004766 | F | 5, | 1 | 0.068 (x 14.807) | 2 (0.500) | 0.348 | spermidine synthase activity | CG8327 |
| 284 | GO:0006858 | P | 5, 6, | 8 | 4.153 (x 1.926) | 123 (0.065) | 0.348 | extracellular transport | BEST:CK01227 BG:DS03431.1 CG10444 CG11897 CG3036 CG31793 CG7627 CG7955 |
| 285 | GO:0008098 | F | 6, | 1 | 0.034 (x 29.615) | 1 (1.000) | 0.348 | 5S rRNA primary transcript binding | La |
| 286 | GO:0016215 | F | 5, | 2 | 0.270 (x 7.404) | 8 (0.250) | 0.348 | CoA desaturase activity | CG8630 desat1 |
| 287 | GO:0007396 | P | 7, | 1 | 0.068 (x 14.807) | 2 (0.500) | 0.349 | suture of dorsal opening | puc |
| 288 | GO:0006120 | P | 9, 11, | 4 | 1.182 (x 3.385) | 35 (0.114) | 0.35 | mitochondrial electron transport, NADH to ubiquinone | CG3192 CG5548 CG5703 Pdsw |
| 289 | GO:0004749 | F | 6, 7, | 1 | 0.068 (x 14.807) | 2 (0.500) | 0.35 | ribose phosphate diphosphokinase activity | CG2246 |
| 290 | GO:0016992 | F | 6, | 1 | 0.034 (x 29.615) | 1 (1.000) | 0.35 | lipoate synthase activity | Las |
| 291 | GO:0016462 | F | 6, | 25 | 17.930 (x 1.394) | 531 (0.047) | 0.35 | pyrophosphatase activity | Arf84F BEST:CK01227 BcDNA:LD23830 CG11897 CG1582 CG1718 CG2885 CG31713 CG31755 CG31793 CG5205 CG5800 CG6512 CG7627 CG7955 CG8798 Dbp80 Irbp Ku80 Mhc Mlc1 Mlc2 R hay rad50 |
| 292 | GO:0035101 | C | 4, 7, 8, 9, 10, 11, 12, 13, 14, | 1 | 0.068 (x 14.807) | 2 (0.500) | 0.351 | FACT complex | Ssrp |
| 293 | GO:0008033 | P | 8, | 2 | 0.473 (x 4.231) | 14 (0.143) | 0.351 | tRNA processing | CG7163 JhI-1 |
| 294 | GO:0032155 | C | 3, 4, 5, | 1 | 0.068 (x 14.807) | 2 (0.500) | 0.352 | cell division site part | dia |
| 295 | GO:0005084 | F | 5, | 1 | 0.034 (x 29.615) | 1 (1.000) | 0.352 | Rab escort protein activity | Rep |
| 296 | GO:0006720 | P | 6, 7, | 2 | 0.473 (x 4.231) | 14 (0.143) | 0.352 | isoprenoid metabolism | Hmgcr Jheh3 |
| 297 | GO:0006917 | P | 8, 9, | 5 | 2.094 (x 2.388) | 62 (0.081) | 0.353 | induction of apoptosis | CG12876 Traf1 W eiger rpr |
| 298 | GO:0004568 | F | 6, | 2 | 0.473 (x 4.231) | 14 (0.143) | 0.353 | chitinase activity | CG2989 Chit |
| 299 | GO:0016847 | F | 5, | 1 | 0.068 (x 14.807) | 2 (0.500) | 0.354 | 1-aminocyclopropane-1-carboxylate synthase activity | CG6950 |
| 300 | GO:0016420 | F | 7, | 1 | 0.068 (x 14.807) | 2 (0.500) | 0.355 | malonyltransferase activity | CG7842 |
| 301 | GO:0003908 | F | 7, | 1 | 0.034 (x 29.615) | 1 (1.000) | 0.355 | methylated-DNA-[protein]-cysteine S-methyltransferase activity | agt |
| 302 | GO:0005826 | C | 4, 5, 6, 7, 8, 9, 10, | 1 | 0.068 (x 14.807) | 2 (0.500) | 0.356 | contractile ring | dia |
| 303 | GO:0008219 | P | 4, | 13 | 8.442 (x 1.540) | 250 (0.052) | 0.357 | cell death | CG10861 CG12876 CG40006 CG7188 EP2237 Mmp1 Traf1 W eiger ftz-f1 rpr wun wun2 |
| 304 | GO:0051030 | P | 7, 8, 9, | 1 | 0.068 (x 14.807) | 2 (0.500) | 0.357 | snRNA transport | Nxt1 |
| 305 | GO:0016212 | F | 6, | 1 | 0.034 (x 29.615) | 1 (1.000) | 0.357 | kynurenine-oxoglutarate transaminase activity | CG6950 |
| 306 | GO:0016817 | F | 4, | 27 | 18.504 (x 1.459) | 548 (0.049) | 0.357 | hydrolase activity, acting on acid anhydrides | Arf84F BEST:CK01227 BcDNA:LD23830 CG11897 CG1582 CG1718 CG2885 CG31063 CG31713 CG31755 CG31793 CG5205 CG5800 CG6512 CG7627 CG7955 CG8798 Dbp80 Dgp-1 Irbp Ku80 Mhc Mlc1 Mlc2 R hay rad50 |
| 307 | GO:0051246 | P | 5, 6, | 8 | 4.491 (x 1.781) | 133 (0.060) | 0.358 | regulation of protein metabolism | Ate1 CG5317 HBS1 Imp Thor W apt rpr |
| 308 | GO:0045496 | P | 7, | 1 | 0.068 (x 14.807) | 2 (0.500) | 0.358 | male analia development (sensu Endopterygota) | ken |
| 309 | GO:0004057 | F | 6, | 1 | 0.034 (x 29.615) | 1 (1.000) | 0.36 | arginyltransferase activity | Ate1 |
| 310 | GO:0016419 | F | 8, | 1 | 0.068 (x 14.807) | 2 (0.500) | 0.36 | S-malonyltransferase activity | CG7842 |
| 311 | GO:0016818 | F | 5, | 27 | 18.504 (x 1.459) | 548 (0.049) | 0.36 | hydrolase activity, acting on acid anhydrides, in phosphorus-containing anhydrides | Arf84F BEST:CK01227 BcDNA:LD23830 CG11897 CG1582 CG1718 CG2885 CG31063 CG31713 CG31755 CG31793 CG5205 CG5800 CG6512 CG7627 CG7955 CG8798 Dbp80 Dgp-1 Irbp Ku80 Mhc Mlc1 Mlc2 R hay rad50 |
| 312 | GO:0045214 | P | 8, 9, 11, 12, | 1 | 0.068 (x 14.807) | 2 (0.500) | 0.361 | sarcomere organization | wupA |
| 313 | GO:0006122 | P | 9, 11, | 2 | 0.405 (x 4.936) | 12 (0.167) | 0.361 | mitochondrial electron transport, ubiquinol to cytochrome c | CG32649 CG4769 |
| 314 | GO:0006631 | P | 6, 7, | 7 | 3.478 (x 2.013) | 103 (0.068) | 0.362 | fatty acid metabolism | CG10877 CG5044 CG5946 CG7842 CG8199 CG8630 desat1 |
| 315 | GO:0031369 | F | 4, | 1 | 0.034 (x 29.615) | 1 (1.000) | 0.362 | translation initiation factor binding | Thor |
| 316 | GO:0004457 | F | 5, | 1 | 0.068 (x 14.807) | 2 (0.500) | 0.362 | lactate dehydrogenase activity | ImpL3 |
| 317 | GO:0005496 | F | 3, | 2 | 0.405 (x 4.936) | 12 (0.167) | 0.362 | steroid binding | CG3860 ftz-f1 |
| 318 | GO:0015397 | F | 6, 7, | 1 | 0.068 (x 14.807) | 2 (0.500) | 0.363 | equilibrative nucleoside transporter, nitrobenzyl-thioinosine-insensitive activity | CG11907 |
| 319 | GO:0005746 | C | 5, 6, 7, 8, 9, 10, 11, 12, 13, | 5 | 2.330 (x 2.146) | 69 (0.072) | 0.364 | mitochondrial electron transport chain | CG3192 CG4769 CG5548 CG5703 Pdsw |
| 320 | GO:0007519 | P | 5, | 4 | 1.655 (x 2.418) | 49 (0.082) | 0.364 | striated muscle development | CG6803 Mhc Mlp60A wupA |
| 321 | GO:0016265 | P | 3, | 13 | 8.476 (x 1.534) | 251 (0.052) | 0.364 | death | CG10861 CG12876 CG40006 CG7188 EP2237 Mmp1 Traf1 W eiger ftz-f1 rpr wun wun2 |
| 322 | GO:0015239 | F | 4, | 2 | 0.405 (x 4.936) | 12 (0.167) | 0.364 | multidrug transporter activity | CG11897 CG7627 |
| 323 | GO:0006950 | P | 3, | 17 | 11.278 (x 1.507) | 334 (0.051) | 0.364 | response to stress | CG17227 CG9273 DDB1 GstE1 Irbp Ku80 TepIV Thor TotA agt eiger hay mre11 mus205 puc rad50 rpr |
| 324 | GO:0005830 | C | 5, 6, 7, 8, 9, 10, | 6 | 3.039 (x 1.974) | 90 (0.067) | 0.364 | cytosolic ribosome (sensu Eukaryota) | CG5317 CG5338 CG6764 CG7014 RpL17A RpS4 |
| 325 | GO:0048034 | P | 8, 9, | 1 | 0.034 (x 29.615) | 1 (1.000) | 0.364 | heme o biosynthesis | CG5037 |
| 326 | GO:0004551 | F | 7, | 1 | 0.068 (x 14.807) | 2 (0.500) | 0.365 | nucleotide diphosphatase activity | CG31713 |
| 327 | GO:0042692 | P | 4, | 3 | 0.912 (x 3.291) | 27 (0.111) | 0.365 | muscle cell differentiation | CG6803 Mhc wupA |
| 328 | GO:0005652 | C | 5, 6, 7, 8, 9, 10, 11, | 1 | 0.101 (x 9.872) | 3 (0.333) | 0.366 | nuclear lamina | LamC |
| 329 | GO:0032153 | C | 3, 4, | 1 | 0.068 (x 14.807) | 2 (0.500) | 0.366 | cell division site | dia |
| 330 | GO:0043231 | C | 4, 5, 6, 7, | 90 | 77.901 (x 1.155) | 2307 (0.039) | 0.366 | intracellular membrane-bound organelle | Aats-leu Aldh BcDNA:LD23830 Bmcp CG10214 CG10306 CG10445 CG12264 CG12954 CG31611 CG31666 CG3192 CG31922 CG32409 CG32649 CG33002 CG3371 CG3803 CG4769 CG4866 CG5037 CG5205 CG5548 CG5703 CG6272 CG6512 CG6610 CG6876 CG7163 CG7339 CG7791 CG7842 CG7955 CG8004 CG8199 CG8226 CG8630 CG8798 CG9273 DDB1 EG:52C10.1 EP2237 Ets21C GV1 Gdh HLHmgamma Hmgcr Irbp JhI-1 Ku80 La LamC Las Mlp60A Nxt1 Pcaf Pdsw Ptp61F REG Rep RpA-70 Ssb-c31a Ssrp Sulf1 Taf12L Tim10 Tim9a ab apt bcn92 cul-2 desat1 fru ftz-f1 hay ken mRpL22 mRpL22-24 mRpS14 mRpS18a mRpS21 mRpS33 mirr mre11 mus205 rad50 rpr tko toy unc-13 |
| 331 | GO:0051168 | P | 7, 8, 9, | 3 | 0.912 (x 3.291) | 27 (0.111) | 0.366 | nuclear export | La Nmd3 Nxt1 |
| 332 | GO:0046351 | P | 7, 8, | 1 | 0.101 (x 9.872) | 3 (0.333) | 0.367 | disaccharide biosynthesis | CG5177 |
| 333 | GO:0004557 | F | 7, | 1 | 0.034 (x 29.615) | 1 (1.000) | 0.367 | alpha-galactosidase activity | CG7997 |
| 334 | GO:0031033 | P | 9, | 1 | 0.068 (x 14.807) | 2 (0.500) | 0.367 | myosin filament assembly or disassembly | CG6803 |
| 335 | GO:0007486 | P | 6, 7, | 1 | 0.101 (x 9.872) | 3 (0.333) | 0.367 | female genitalia development (sensu Endopterygota) | ken |
| 336 | GO:0048469 | P | 5, | 3 | 0.912 (x 3.291) | 27 (0.111) | 0.368 | cell maturation | CG6803 Mhc wupA |
| 337 | GO:0016787 | F | 3, | 70 | 60.646 (x 1.154) | 1796 (0.039) | 0.368 | hydrolase activity | Arf84F BEST:CK01227 BG:DS01068.5 BcDNA:LD23830 CG10214 CG11897 CG1299 CG14869 CG14935 CG1582 CG15879 CG1718 CG17843 CG1882 CG2277 CG2885 CG2989 CG31063 CG31373 CG31713 CG31755 CG31793 CG32549 CG4096 CG4267 CG4933 CG5044 CG5162 CG5177 CG5205 CG5317 CG5397 CG5800 CG6357 CG6512 CG7627 CG7791 CG7955 CG7997 CG8756 CG8798 CG9372 Chit Dbp80 Dgp-1 Ide Idgf2 Idgf3 Irbp JhI-1 Jheh3 Ku80 Mhc Mlc1 Mlc2 Mmp1 Ptp61F R Sulf1 alpha-Est10 hay huntingtin isopeptidase-T-3 mre11 mus205 puc rad50 sda wun wun2 |
| 338 | GO:0016125 | P | 6, 7, 8, | 3 | 1.114 (x 2.692) | 33 (0.091) | 0.368 | sterol metabolism | CG3860 CG5946 Hmgcr |
| 339 | GO:0004659 | F | 5, | 2 | 0.540 (x 3.702) | 16 (0.125) | 0.368 | prenyltransferase activity | CG5037 Rep |
| 340 | GO:0004029 | F | 6, | 1 | 0.101 (x 9.872) | 3 (0.333) | 0.368 | aldehyde dehydrogenase (NAD) activity | Aldh |
| 341 | GO:0031034 | P | 7, 10, | 1 | 0.068 (x 14.807) | 2 (0.500) | 0.368 | myosin filament assembly | CG6803 |
| 342 | GO:0009792 | P | 4, | 12 | 6.585 (x 1.822) | 195 (0.062) | 0.369 | embryonic development (sensu Metazoa) | CG9358 EP2237 Ptp61F R Traf1 W dia mirr os puc rpr sog |
| 343 | GO:0008354 | P | 5, 6, 7, | 3 | 1.114 (x 2.692) | 33 (0.091) | 0.369 | germ cell migration | Hmgcr wun wun2 |
| 344 | GO:0004089 | F | 6, | 2 | 0.540 (x 3.702) | 16 (0.125) | 0.369 | carbonate dehydratase activity | CAH1 CAH2 |
| 345 | GO:0004104 | F | 6, | 1 | 0.101 (x 9.872) | 3 (0.333) | 0.369 | cholinesterase activity | alpha-Est10 |
| 346 | GO:0000054 | P | 8, 9, 10, 11, | 1 | 0.034 (x 29.615) | 1 (1.000) | 0.37 | ribosome export from nucleus | Nmd3 |
| 347 | GO:0044455 | C | 4, 5, 6, 7, 8, 9, 10, 11, 12, | 8 | 3.714 (x 2.154) | 110 (0.073) | 0.37 | mitochondrial membrane part | CG3192 CG4769 CG5548 CG5703 CG8226 Pdsw Tim10 Tim9a |
| 348 | GO:0009106 | P | 7, 8, | 1 | 0.068 (x 14.807) | 2 (0.500) | 0.37 | lipoate metabolism | Las |
| 349 | GO:0016620 | F | 5, | 2 | 0.540 (x 3.702) | 16 (0.125) | 0.37 | oxidoreductase activity, acting on the aldehyde or oxo group of donors, NAD or NADP as acceptor | Aldh DDB1 |
| 350 | GO:0045213 | P | 7, 8, | 1 | 0.101 (x 9.872) | 3 (0.333) | 0.37 | neurotransmitter receptor metabolism | Gs2 |
| 351 | GO:0005578 | C | 3, 4, | 4 | 1.520 (x 2.632) | 45 (0.089) | 0.37 | extracellular matrix (sensu Metazoa) | Mmp1 dlp os trol |
| 352 | GO:0035183 | C | 3, 4, 5, 6, | 1 | 0.101 (x 9.872) | 3 (0.333) | 0.371 | ring canal inner rim | cher |
| 353 | GO:0009105 | P | 7, 8, | 1 | 0.068 (x 14.807) | 2 (0.500) | 0.371 | lipoic acid biosynthesis | Las |
| 354 | GO:0043227 | C | 3, | 90 | 77.968 (x 1.154) | 2309 (0.039) | 0.371 | membrane-bound organelle | Aats-leu Aldh BcDNA:LD23830 Bmcp CG10214 CG10306 CG10445 CG12264 CG12954 CG31611 CG31666 CG3192 CG31922 CG32409 CG32649 CG33002 CG3371 CG3803 CG4769 CG4866 CG5037 CG5205 CG5548 CG5703 CG6272 CG6512 CG6610 CG6876 CG7163 CG7339 CG7791 CG7842 CG7955 CG8004 CG8199 CG8226 CG8630 CG8798 CG9273 DDB1 EG:52C10.1 EP2237 Ets21C GV1 Gdh HLHmgamma Hmgcr Irbp JhI-1 Ku80 La LamC Las Mlp60A Nxt1 Pcaf Pdsw Ptp61F REG Rep RpA-70 Ssb-c31a Ssrp Sulf1 Taf12L Tim10 Tim9a ab apt bcn92 cul-2 desat1 fru ftz-f1 hay ken mRpL22 mRpL22-24 mRpS14 mRpS18a mRpS21 mRpS33 mirr mre11 mus205 rad50 rpr tko toy unc-13 |
| 355 | GO:0016053 | P | 6, | 3 | 1.047 (x 2.866) | 31 (0.097) | 0.372 | organic acid biosynthesis | CG7842 CG8630 desat1 |
| 356 | GO:0031012 | C | 2, | 4 | 1.520 (x 2.632) | 45 (0.089) | 0.372 | extracellular matrix | Mmp1 dlp os trol |
| 357 | GO:0004003 | F | 5, 11, | 3 | 0.709 (x 4.231) | 21 (0.143) | 0.372 | ATP-dependent DNA helicase activity | Irbp Ku80 hay |
| 358 | GO:0005863 | C | 6, 7, 8, 9, 10, 11, 12, | 1 | 0.101 (x 9.872) | 3 (0.333) | 0.372 | striated muscle thick filament | Mhc |
| 359 | GO:0008579 | F | 9, | 1 | 0.034 (x 29.615) | 1 (1.000) | 0.372 | JUN kinase phosphatase activity | puc |
| 360 | GO:0019551 | P | 7, 8, 9, 10, 11, | 1 | 0.068 (x 14.807) | 2 (0.500) | 0.372 | glutamate catabolism to 2-oxoglutarate | Gdh |
| 361 | GO:0046394 | P | 7, | 3 | 1.047 (x 2.866) | 31 (0.097) | 0.373 | carboxylic acid biosynthesis | CG7842 CG8630 desat1 |
| 362 | GO:0004805 | F | 8, | 1 | 0.101 (x 9.872) | 3 (0.333) | 0.373 | trehalose-phosphatase activity | CG5177 |
| 363 | GO:0005762 | C | 5, 6, 7, 8, 9, 10, 11, 12, 13, | 4 | 1.520 (x 2.632) | 45 (0.089) | 0.373 | mitochondrial large ribosomal subunit | CG12954 CG33002 mRpL22 mRpL22-24 |
| 364 | GO:0004301 | F | 7, | 1 | 0.101 (x 9.872) | 3 (0.333) | 0.374 | epoxide hydrolase activity | Jheh3 |
| 365 | GO:0042586 | F | 6, | 1 | 0.068 (x 14.807) | 2 (0.500) | 0.374 | peptide deformylase activity | CG31373 |
| 366 | GO:0000315 | C | 4, 5, 6, 7, 8, 9, 10, | 4 | 1.520 (x 2.632) | 45 (0.089) | 0.374 | organellar large ribosomal subunit | CG12954 CG33002 mRpL22 mRpL22-24 |
| 367 | GO:0017148 | P | 7, 8, 9, | 3 | 0.709 (x 4.231) | 21 (0.143) | 0.375 | negative regulation of protein biosynthesis | Thor apt rpr |
| 368 | GO:0043112 | P | 7, | 1 | 0.101 (x 9.872) | 3 (0.333) | 0.375 | receptor metabolism | Gs2 |
| 369 | GO:0040040 | P | 4, 5, | 1 | 0.034 (x 29.615) | 1 (1.000) | 0.375 | thermosensory behavior | ppk |
| 370 | GO:0003954 | F | 5, | 4 | 1.216 (x 3.291) | 36 (0.111) | 0.375 | NADH dehydrogenase activity | CG3192 CG5548 CG5703 Pdsw |
| 371 | GO:0007084 | P | 6, 8, 9, | 1 | 0.068 (x 14.807) | 2 (0.500) | 0.375 | mitotic nuclear envelope reassembly | LamC |
| 372 | GO:0008449 | F | 6, | 1 | 0.101 (x 9.872) | 3 (0.333) | 0.376 | N-acetylglucosamine-6-sulfatase activity | Sulf1 |
| 373 | GO:0051188 | P | 6, | 7 | 3.546 (x 1.974) | 105 (0.067) | 0.376 | cofactor biosynthesis | CG13645 CG2453 CG5037 CG8199 Gclc Las Mocs1 |
| 374 | GO:0043226 | C | 2, | 103 | 90.631 (x 1.136) | 2684 (0.038) | 0.376 | organelle | Aats-leu Act57B Aldh BcDNA:LD23830 Bmcp CG10214 CG10306 CG10445 CG12264 CG12954 CG31611 CG31666 CG3192 CG31922 CG32409 CG32649 CG33002 CG3371 CG3803 CG4769 CG4866 CG5037 CG5205 CG5317 CG5338 CG5548 CG5703 CG6272 CG6512 CG6610 CG6764 CG6876 CG7014 CG7163 CG7339 CG7791 CG7842 CG7955 CG8004 CG8199 CG8226 CG8630 CG8798 CG9273 DDB1 EG:52C10.1 EP2237 Ets21C GV1 Gdh HLHmgamma Hmgcr Irbp JhI-1 Ku80 La LamC Las Mhc Mlc1 Mlc2 Mlp60A Nxt1 Pcaf Pdsw Ptp61F REG Rep RpA-70 RpL17A RpS4 Ssb-c31a Ssrp Sulf1 Taf12L Tim10 Tim9a ab apt bcn92 cul-2 desat1 dia fru ftz-f1 hay ken mRpL22 mRpL22-24 mRpS14 mRpS18a mRpS21 mRpS33 mirr mre11 mus205 na rad50 rpr tko toy unc-13 wupA |
| 375 | GO:0012502 | P | 7, 8, | 5 | 2.364 (x 2.115) | 70 (0.071) | 0.376 | induction of programmed cell death | CG12876 Traf1 W eiger rpr |
| 376 | GO:0006103 | P | 6, 7, 9, | 1 | 0.068 (x 14.807) | 2 (0.500) | 0.376 | 2-oxoglutarate metabolism | Gdh |
| 377 | GO:0008302 | P | 7, 10, | 1 | 0.101 (x 9.872) | 3 (0.333) | 0.376 | ring canal formation, actin assembly | cher |
| 378 | GO:0007548 | P | 3, | 4 | 1.688 (x 2.369) | 50 (0.080) | 0.377 | sex differentiation | CG7194 Hmgcr fru ken |
| 379 | GO:0009605 | P | 3, | 7 | 3.546 (x 1.974) | 105 (0.067) | 0.377 | response to external stimulus | Arr2 Hmgcr puc sda tko wun wun2 |
| 380 | GO:0043229 | C | 3, 4, 5, 6, | 103 | 90.631 (x 1.136) | 2684 (0.038) | 0.377 | intracellular organelle | Aats-leu Act57B Aldh BcDNA:LD23830 Bmcp CG10214 CG10306 CG10445 CG12264 CG12954 CG31611 CG31666 CG3192 CG31922 CG32409 CG32649 CG33002 CG3371 CG3803 CG4769 CG4866 CG5037 CG5205 CG5317 CG5338 CG5548 CG5703 CG6272 CG6512 CG6610 CG6764 CG6876 CG7014 CG7163 CG7339 CG7791 CG7842 CG7955 CG8004 CG8199 CG8226 CG8630 CG8798 CG9273 DDB1 EG:52C10.1 EP2237 Ets21C GV1 Gdh HLHmgamma Hmgcr Irbp JhI-1 Ku80 La LamC Las Mhc Mlc1 Mlc2 Mlp60A Nxt1 Pcaf Pdsw Ptp61F REG Rep RpA-70 RpL17A RpS4 Ssb-c31a Ssrp Sulf1 Taf12L Tim10 Tim9a ab apt bcn92 cul-2 desat1 dia fru ftz-f1 hay ken mRpL22 mRpL22-24 mRpS14 mRpS18a mRpS21 mRpS33 mirr mre11 mus205 na rad50 rpr tko toy unc-13 wupA |
| 381 | GO:0007626 | P | 4, | 5 | 2.364 (x 2.115) | 70 (0.071) | 0.377 | locomotory behavior | Hmgcr na ppk wun wun2 |
| 382 | GO:0016060 | P | 8, 9, 10, | 1 | 0.101 (x 9.872) | 3 (0.333) | 0.377 | metarhodopsin inactivation | Arr2 |
| 383 | GO:0016035 | C | 4, 5, 6, 7, 8, 9, 10, 11, 12, 13, | 1 | 0.034 (x 29.615) | 1 (1.000) | 0.377 | zeta DNA polymerase complex | mus205 |
| 384 | GO:0035232 | P | 6, 7, 8, | 1 | 0.068 (x 14.807) | 2 (0.500) | 0.378 | germ cell attraction | Hmgcr |
| 385 | GO:0001672 | P | 6, 8, 10, | 1 | 0.101 (x 9.872) | 3 (0.333) | 0.378 | regulation of chromatin assembly or disassembly | Ssrp |
| 386 | GO:0043065 | P | 7, 8, | 5 | 2.161 (x 2.314) | 64 (0.078) | 0.378 | positive regulation of apoptosis | CG12876 Traf1 W eiger rpr |
| 387 | GO:0004356 | F | 7, | 1 | 0.101 (x 9.872) | 3 (0.333) | 0.379 | glutamate-ammonia ligase activity | Gs2 |
| 388 | GO:0019010 | F | 7, | 1 | 0.034 (x 29.615) | 1 (1.000) | 0.38 | farnesoic acid O-methyltransferase activity | CG10527 |
| 389 | GO:0005992 | P | 8, 9, | 1 | 0.101 (x 9.872) | 3 (0.333) | 0.38 | trehalose biosynthesis | CG5177 |
| 390 | GO:0017116 | F | 6, 12, | 1 | 0.101 (x 9.872) | 3 (0.333) | 0.381 | single-stranded DNA-dependent ATP-dependent DNA helicase activity | Ku80 |
| 391 | GO:0016639 | F | 5, | 1 | 0.101 (x 9.872) | 3 (0.333) | 0.382 | oxidoreductase activity, acting on the CH-NH2 group of donors, NAD or NADP as acceptor | Gdh |
| 392 | GO:0016433 | F | 8, | 1 | 0.101 (x 9.872) | 3 (0.333) | 0.383 | rRNA (adenine) methyltransferase activity | CG11837 |
| 393 | GO:0009617 | P | 5, | 5 | 2.499 (x 2.001) | 74 (0.068) | 0.383 | response to bacterium | CG9358 TepIV Thor Tsf3 eiger |
| 394 | GO:0005125 | F | 4, 5, | 2 | 0.507 (x 3.949) | 15 (0.133) | 0.384 | cytokine activity | eiger os |
| 395 | GO:0019363 | P | 7, 8, 9, | 1 | 0.101 (x 9.872) | 3 (0.333) | 0.384 | pyridine nucleotide biosynthesis | CG13645 |
| 396 | GO:0043142 | F | 11, | 1 | 0.101 (x 9.872) | 3 (0.333) | 0.385 | single-stranded DNA-dependent ATPase activity | Ku80 |
| 397 | GO:0008624 | P | 9, 10, | 1 | 0.101 (x 9.872) | 3 (0.333) | 0.386 | induction of apoptosis by extracellular signals | rpr |
| 398 | GO:0030540 | P | 5, | 1 | 0.101 (x 9.872) | 3 (0.333) | 0.387 | female genitalia development | ken |
| 399 | GO:0015020 | F | 6, | 3 | 1.148 (x 2.613) | 34 (0.088) | 0.388 | glucuronosyltransferase activity | Act57B Ugt86Da Ugt86Di |
| 400 | GO:0017017 | F | 9, | 1 | 0.101 (x 9.872) | 3 (0.333) | 0.388 | MAP kinase phosphatase activity | puc |
| 401 | GO:0043234 | C | 2, | 62 | 53.318 (x 1.163) | 1579 (0.039) | 0.388 | protein complex | BEST:CK01227 CG10306 CG1134 CG12954 CG1718 CG31611 CG31793 CG3192 CG31922 CG33002 CG33158 CG4769 CG4866 CG4917 CG5205 CG5317 CG5338 CG5548 CG5703 CG6610 CG6764 CG6876 CG7014 CG7339 CG7627 CG7955 CG8199 CG8226 CG9273 Gclc Ku80 La Mhc Mlc1 Mlc2 Mocs1 Pcaf Pdsw REG Rep RpA-70 RpL17A RpS4 Ssb-c31a Ssrp Taf12L Tim10 Tim9a cul-2 hay l(3)01239 mRpL22 mRpL22-24 mRpS14 mRpS18a mRpS21 mRpS33 mus205 na rad50 tko wupA |
| 402 | GO:0016289 | F | 6, | 1 | 0.101 (x 9.872) | 3 (0.333) | 0.389 | CoA hydrolase activity | CG5044 |
| 403 | GO:0005542 | F | 4, | 1 | 0.101 (x 9.872) | 3 (0.333) | 0.39 | folic acid binding | CG6574 |
| 404 | GO:0008171 | F | 6, | 1 | 0.101 (x 9.872) | 3 (0.333) | 0.391 | O-methyltransferase activity | CG10527 |
| 405 | GO:0000273 | P | 6, 7, | 1 | 0.101 (x 9.872) | 3 (0.333) | 0.392 | lipoic acid metabolism | Las |
| 406 | GO:0043067 | P | 5, 6, | 7 | 4.018 (x 1.742) | 119 (0.059) | 0.392 | regulation of programmed cell death | CG12876 CG7188 Mmp1 Traf1 W eiger rpr |
| 407 | GO:0004850 | F | 6, | 1 | 0.101 (x 9.872) | 3 (0.333) | 0.393 | uridine phosphorylase activity | CG6330 |
| 408 | GO:0046949 | P | 8, 9, | 1 | 0.101 (x 9.872) | 3 (0.333) | 0.394 | acyl-CoA biosynthesis | CG8199 |
| 409 | GO:0016979 | F | 5, | 1 | 0.101 (x 9.872) | 3 (0.333) | 0.395 | lipoate-protein ligase activity | Las |
| 410 | GO:0016044 | P | 5, | 3 | 1.081 (x 2.776) | 32 (0.094) | 0.395 | membrane organization and biogenesis | LamC Tim10 Tim9a |
| 411 | GO:0007028 | P | 5, | 4 | 1.823 (x 2.194) | 54 (0.074) | 0.395 | cytoplasm organization and biogenesis | CG32409 CG6764 EG:52C10.1 Nmd3 |
| 412 | GO:0043068 | P | 6, 7, | 5 | 2.533 (x 1.974) | 75 (0.067) | 0.395 | positive regulation of programmed cell death | CG12876 Traf1 W eiger rpr |
| 413 | GO:0016211 | F | 6, | 1 | 0.101 (x 9.872) | 3 (0.333) | 0.396 | ammonia ligase activity | Gs2 |
| 414 | GO:0009894 | P | 5, | 1 | 0.101 (x 9.872) | 3 (0.333) | 0.397 | regulation of catabolism | Ate1 |
| 415 | GO:0008172 | F | 6, | 1 | 0.101 (x 9.872) | 3 (0.333) | 0.398 | S-methyltransferase activity | agt |
| 416 | GO:0015347 | F | 6, | 1 | 0.101 (x 9.872) | 3 (0.333) | 0.399 | sodium-independent organic anion transporter activity | CG7571 |
| 417 | GO:0003746 | F | 4, 5, | 2 | 0.574 (x 3.484) | 17 (0.118) | 0.399 | translation elongation factor activity | CG33158 HBS1 |
| 418 | GO:0016478 | P | 8, 9, 10, | 2 | 0.574 (x 3.484) | 17 (0.118) | 0.4 | negative regulation of translation | Thor apt |
| 419 | GO:0008518 | F | 6, | 1 | 0.101 (x 9.872) | 3 (0.333) | 0.4 | reduced folate carrier activity | CG6574 |
| 420 | GO:0006471 | P | 8, | 2 | 0.574 (x 3.484) | 17 (0.118) | 0.401 | protein amino acid ADP-ribosylation | Arf84F CG13692 |
| 421 | GO:0006541 | P | 8, 9, | 1 | 0.101 (x 9.872) | 3 (0.333) | 0.401 | glutamine metabolism | Gs2 |
| 422 | GO:0006979 | P | 4, 5, 6, | 2 | 0.574 (x 3.484) | 17 (0.118) | 0.402 | response to oxidative stress | GstE1 puc |
| 423 | GO:0042176 | P | 6, 7, 8, | 1 | 0.101 (x 9.872) | 3 (0.333) | 0.402 | regulation of protein catabolism | Ate1 |
| 424 | GO:0016880 | F | 5, | 1 | 0.101 (x 9.872) | 3 (0.333) | 0.403 | acid-ammonia (or amide) ligase activity | Gs2 |
| 425 | GO:0008096 | F | 8, | 1 | 0.101 (x 9.872) | 3 (0.333) | 0.404 | juvenile hormone epoxide hydrolase activity | Jheh3 |
| 426 | GO:0005525 | F | 6, | 9 | 5.437 (x 1.655) | 161 (0.056) | 0.405 | GTP binding | Arf84F BcDNA:LD23830 BcDNA:LD32788 CG13692 CG2885 CG33158 Dgp-1 HBS1 R |
| 427 | GO:0019003 | F | 6, | 1 | 0.101 (x 9.872) | 3 (0.333) | 0.405 | GDP binding | R |
| 428 | GO:0015395 | F | 5, 6, | 1 | 0.101 (x 9.872) | 3 (0.333) | 0.406 | nucleoside transporter activity, down a concentration gradient | CG11907 |
| 429 | GO:0006445 | P | 7, 8, 9, | 5 | 2.431 (x 2.057) | 72 (0.069) | 0.407 | regulation of translation | CG5317 HBS1 Imp Thor apt |
| 430 | GO:0007617 | P | 4, 5, | 4 | 1.857 (x 2.154) | 55 (0.073) | 0.407 | mating behavior | Gdh fru ken tko |
| 431 | GO:0000179 | F | 9, | 1 | 0.101 (x 9.872) | 3 (0.333) | 0.407 | rRNA (adenine-N6,N6-)-dimethyltransferase activity | CG11837 |
| 432 | GO:0006144 | P | 7, | 4 | 1.857 (x 2.154) | 55 (0.073) | 0.408 | purine base metabolism | CG18522 CG2246 CG2277 CG32549 |
| 433 | GO:0005818 | C | 5, 6, 7, 8, 9, 10, 11, | 1 | 0.101 (x 9.872) | 3 (0.333) | 0.408 | aster | Rep |
| 434 | GO:0016563 | F | 3, | 4 | 1.857 (x 2.154) | 55 (0.073) | 0.409 | transcriptional activator activity | Aly EP2237 Ssb-c31a mirr |
| 435 | GO:0031672 | C | 6, 7, 8, 9, 10, 11, | 1 | 0.101 (x 9.872) | 3 (0.333) | 0.409 | A band | Mhc |
| 436 | GO:0043451 | P | 6, | 1 | 0.135 (x 7.404) | 4 (0.250) | 0.41 | alkene catabolism | Jheh3 |
| 437 | GO:0051705 | P | 3, | 4 | 1.857 (x 2.154) | 55 (0.073) | 0.41 | behavioral interaction between organisms | Gdh fru ken tko |
| 438 | GO:0050910 | P | 6, 7, 8, | 1 | 0.135 (x 7.404) | 4 (0.250) | 0.41 | detection of mechanical stimulus during sensory perception of sound | tko |
| 439 | GO:0016730 | F | 4, | 1 | 0.101 (x 9.872) | 3 (0.333) | 0.41 | oxidoreductase activity, acting on iron-sulfur proteins as donors | CG11897 |
| 440 | GO:0046693 | P | 7, 8, | 1 | 0.135 (x 7.404) | 4 (0.250) | 0.411 | sperm storage | Gdh |
| 441 | GO:0008295 | P | 9, 10, | 1 | 0.101 (x 9.872) | 3 (0.333) | 0.412 | spermidine biosynthesis | CG8327 |
| 442 | GO:0015232 | F | 4, | 1 | 0.135 (x 7.404) | 4 (0.250) | 0.412 | heme transporter activity | CG7955 |
| 443 | GO:0015925 | F | 6, | 1 | 0.101 (x 9.872) | 3 (0.333) | 0.413 | galactosidase activity | CG7997 |
| 444 | GO:0004217 | F | 7, | 1 | 0.135 (x 7.404) | 4 (0.250) | 0.413 | cathepsin L activity | CG6357 |
| 445 | GO:0004772 | F | 8, | 1 | 0.101 (x 9.872) | 3 (0.333) | 0.414 | sterol O-acyltransferase activity | CG5397 |
| 446 | GO:0005625 | C | 4, 5, | 1 | 0.135 (x 7.404) | 4 (0.250) | 0.414 | soluble fraction | Arr2 |
| 447 | GO:0019001 | F | 5, | 9 | 5.470 (x 1.645) | 162 (0.056) | 0.414 | guanyl nucleotide binding | Arf84F BcDNA:LD23830 BcDNA:LD32788 CG13692 CG2885 CG33158 Dgp-1 HBS1 R |
| 448 | GO:0009108 | P | 7, | 6 | 3.174 (x 1.890) | 94 (0.064) | 0.415 | coenzyme biosynthesis | CG13645 CG2453 CG8199 Gclc Las Mocs1 |
| 449 | GO:0016107 | P | 7, 9, 10, 11, | 1 | 0.135 (x 7.404) | 4 (0.250) | 0.415 | sesquiterpenoid catabolism | Jheh3 |
| 450 | GO:0008363 | P | 6, 8, 10, | 1 | 0.135 (x 7.404) | 4 (0.250) | 0.416 | larval cuticle biosynthesis (sensu Insecta) | Lcp65Ag1 |
| 451 | GO:0031968 | C | 4, 5, 6, 7, 8, 9, | 2 | 0.642 (x 3.117) | 19 (0.105) | 0.416 | organelle outer membrane | CG8004 CG8226 |
| 452 | GO:0006032 | P | 8, 9, 10, 11, | 1 | 0.135 (x 7.404) | 4 (0.250) | 0.416 | chitin catabolism | Chit |
| 453 | GO:0009064 | P | 7, 8, | 2 | 0.642 (x 3.117) | 19 (0.105) | 0.417 | glutamine family amino acid metabolism | Gdh Gs2 |
| 454 | GO:0051763 | P | 8, | 1 | 0.135 (x 7.404) | 4 (0.250) | 0.417 | sesquiterpene catabolism | Jheh3 |
| 455 | GO:0044272 | P | 6, | 2 | 0.642 (x 3.117) | 19 (0.105) | 0.417 | sulfur compound biosynthesis | Act57B Gclc |
| 456 | GO:0006719 | P | 7, 8, 10, 11, 12, | 1 | 0.135 (x 7.404) | 4 (0.250) | 0.418 | juvenile hormone catabolism | Jheh3 |
| 457 | GO:0005657 | C | 5, 6, 7, 8, 9, 10, | 2 | 0.642 (x 3.117) | 19 (0.105) | 0.418 | replication fork | CG9273 RpA-70 |
| 458 | GO:0019098 | P | 3, 4, | 4 | 1.958 (x 2.042) | 58 (0.069) | 0.418 | reproductive behavior | Gdh fru ken tko |
| 459 | GO:0003918 | F | 5, 6, | 1 | 0.135 (x 7.404) | 4 (0.250) | 0.419 | DNA topoisomerase (ATP-hydrolyzing) activity | CG3168 |
| 460 | GO:0019867 | C | 4, 5, 6, | 2 | 0.642 (x 3.117) | 19 (0.105) | 0.419 | outer membrane | CG8004 CG8226 |
| 461 | GO:0051704 | P | 2, | 4 | 1.958 (x 2.042) | 58 (0.069) | 0.419 | interaction between organisms | Gdh fru ken tko |
| 462 | GO:0030512 | P | 6, 7, 10, | 1 | 0.135 (x 7.404) | 4 (0.250) | 0.42 | negative regulation of transforming growth factor beta receptor signaling pathway | sog |
| 463 | GO:0006510 | P | 8, | 2 | 0.642 (x 3.117) | 19 (0.105) | 0.42 | ATP-dependent proteolysis | CG6512 CG8798 |
| 464 | GO:0000272 | P | 7, | 1 | 0.135 (x 7.404) | 4 (0.250) | 0.421 | polysaccharide catabolism | Chit |
| 465 | GO:0046915 | F | 5, | 2 | 0.642 (x 3.117) | 19 (0.105) | 0.421 | transition metal ion transporter activity | Tsf1 Tsf3 |
| 466 | GO:0016769 | F | 4, | 2 | 0.642 (x 3.117) | 19 (0.105) | 0.422 | transferase activity, transferring nitrogenous groups | CG12264 CG6950 |
| 467 | GO:0046348 | P | 7, 8, | 1 | 0.135 (x 7.404) | 4 (0.250) | 0.422 | amino sugar catabolism | Chit |
| 468 | GO:0008094 | F | 10, | 3 | 1.283 (x 2.338) | 38 (0.079) | 0.422 | DNA-dependent ATPase activity | Irbp Ku80 hay |
| 469 | GO:0008483 | F | 5, | 2 | 0.642 (x 3.117) | 19 (0.105) | 0.422 | transaminase activity | CG12264 CG6950 |
| 470 | GO:0008300 | P | 7, 8, | 1 | 0.135 (x 7.404) | 4 (0.250) | 0.422 | isoprenoid catabolism | Jheh3 |
| 471 | GO:0015238 | F | 3, | 2 | 0.642 (x 3.117) | 19 (0.105) | 0.423 | drug transporter activity | CG11897 CG7627 |
| 472 | GO:0016062 | P | 7, 8, 9, | 1 | 0.135 (x 7.404) | 4 (0.250) | 0.423 | adaptation of rhodopsin mediated signaling | Arr2 |
| 473 | GO:0016097 | P | 8, 9, | 1 | 0.135 (x 7.404) | 4 (0.250) | 0.424 | polyisoprenoid catabolism | Jheh3 |
| 474 | GO:0016421 | F | 5, | 1 | 0.135 (x 7.404) | 4 (0.250) | 0.425 | CoA carboxylase activity | CG17108 |
| 475 | GO:0009986 | C | 3, 4, | 1 | 0.135 (x 7.404) | 4 (0.250) | 0.426 | cell surface | Sulf1 |
| 476 | GO:0016836 | F | 5, | 4 | 1.891 (x 2.115) | 56 (0.071) | 0.426 | hydro-lyase activity | CAH1 CAH2 CG10877 CG5044 |
| 477 | GO:0016746 | F | 4, | 7 | 4.288 (x 1.632) | 127 (0.055) | 0.427 | transferase activity, transferring acyl groups | Ate1 CG10877 CG5037 CG5397 CG7842 Pcaf Rep |
| 478 | GO:0016973 | P | 9, 10, 11, 12, | 1 | 0.135 (x 7.404) | 4 (0.250) | 0.427 | poly(A)+ mRNA export from nucleus | Nxt1 |
| 479 | GO:0008757 | F | 6, | 4 | 1.992 (x 2.008) | 59 (0.068) | 0.427 | S-adenosylmethionine-dependent methyltransferase activity | CG10527 CG11837 CG2453 CG8327 |
| 480 | GO:0005968 | C | 4, | 1 | 0.135 (x 7.404) | 4 (0.250) | 0.428 | Rab-protein geranylgeranyltransferase complex | Rep |
| 481 | GO:0016803 | F | 5, | 1 | 0.135 (x 7.404) | 4 (0.250) | 0.429 | ether hydrolase activity | Jheh3 |
| 482 | GO:0006034 | P | 8, 9, 10, 11, | 1 | 0.135 (x 7.404) | 4 (0.250) | 0.43 | cuticle chitin metabolism | Chit |
| 483 | GO:0008379 | F | 4, 6, | 1 | 0.135 (x 7.404) | 4 (0.250) | 0.431 | thioredoxin peroxidase activity | Jafrac1 |
| 484 | GO:0005741 | C | 5, 6, 7, 8, 9, 10, 11, 12, | 2 | 0.608 (x 3.291) | 18 (0.111) | 0.431 | mitochondrial outer membrane | CG8004 CG8226 |
| 485 | GO:0045880 | P | 6, 7, 8, | 1 | 0.135 (x 7.404) | 4 (0.250) | 0.431 | positive regulation of smoothened signaling pathway | dlp |
| 486 | GO:0007005 | P | 6, | 2 | 0.608 (x 3.291) | 18 (0.111) | 0.432 | mitochondrion organization and biogenesis | Tim10 Tim9a |
| 487 | GO:0044247 | P | 7, 8, | 1 | 0.135 (x 7.404) | 4 (0.250) | 0.432 | cellular polysaccharide catabolism | Chit |
| 488 | GO:0009116 | P | 6, | 2 | 0.608 (x 3.291) | 18 (0.111) | 0.433 | nucleoside metabolism | CG2246 CG6330 |
| 489 | GO:0043021 | F | 3, | 1 | 0.135 (x 7.404) | 4 (0.250) | 0.433 | ribonucleoprotein binding | EG:52C10.1 |
| 490 | GO:0009112 | P | 6, | 5 | 2.634 (x 1.898) | 78 (0.064) | 0.434 | nucleobase metabolism | CG18522 CG2246 CG2277 CG32549 CG6330 |
| 491 | GO:0008216 | P | 8, 9, | 1 | 0.135 (x 7.404) | 4 (0.250) | 0.434 | spermidine metabolism | CG8327 |
| 492 | GO:0008142 | F | 4, | 1 | 0.135 (x 7.404) | 4 (0.250) | 0.435 | oxysterol binding | CG3860 |
| 493 | GO:0006259 | P | 6, | 17 | 12.528 (x 1.357) | 371 (0.046) | 0.436 | DNA metabolism | CG17227 CG31611 CG3168 CG6204 CG9273 DDB1 Irbp Ku80 RpA-70 Ssrp agt fru hay mre11 mus205 rad50 trol |
| 494 | GO:0004463 | F | 6, | 1 | 0.135 (x 7.404) | 4 (0.250) | 0.436 | leukotriene-A4 hydrolase activity | Jheh3 |
| 495 | GO:0007111 | P | 6, 7, | 1 | 0.135 (x 7.404) | 4 (0.250) | 0.437 | cytokinesis after meiosis II | dia |
| 496 | GO:0044424 | C | 3, 4, 5, | 117 | 107.717 (x 1.086) | 3190 (0.037) | 0.437 | intracellular part | Aats-leu Act57B Aldh BcDNA:LD23830 Bmcp CG10214 CG10306 CG10445 CG10861 CG1134 CG12264 CG12954 CG31611 CG31666 CG3192 CG31922 CG32409 CG32549 CG32649 CG33002 CG33158 CG3371 CG3803 CG4769 CG4866 CG4917 CG5037 CG5205 CG5317 CG5338 CG5548 CG5703 CG6272 CG6330 CG6512 CG6610 CG6764 CG6876 CG7014 CG7163 CG7339 CG7791 CG7842 CG7955 CG8004 CG8199 CG8226 CG8630 CG8798 CG9273 CG9358 DDB1 EG:52C10.1 EP2237 Ets21C GV1 Gclc Gdh Gs2 HBS1 HLHmgamma Hmgcr Irbp Jafrac1 JhI-1 Ku80 La LamC Las Mhc Mlc1 Mlc2 Mlp60A Nxt1 Pcaf Pdsw Ptp61F REG Rep RpA-70 RpL17A RpS4 Ssb-c31a Ssrp Sulf1 Taf12L Tim10 Tim9a ab apt bcn92 cul-2 desat1 dia eIF6 fru ftz-f1 hay huntingtin ken l(3)01239 mRpL22 mRpL22-24 mRpS14 mRpS18a mRpS21 mRpS33 mirr mre11 mus205 na rad50 rpr tko toy unc-13 wupA |
| 497 | GO:0006036 | P | 9, 10, 11, 12, | 1 | 0.135 (x 7.404) | 4 (0.250) | 0.438 | cuticle chitin catabolism | Chit |
| 498 | GO:0042575 | C | 3, 4, 5, 6, | 1 | 0.135 (x 7.404) | 4 (0.250) | 0.439 | DNA polymerase complex | mus205 |
| 499 | GO:0019985 | P | 6, 8, | 1 | 0.135 (x 7.404) | 4 (0.250) | 0.44 | bypass DNA synthesis | mus205 |
| 500 | GO:0006046 | P | 9, 10, | 1 | 0.135 (x 7.404) | 4 (0.250) | 0.441 | N-acetylglucosamine catabolism | Chit |
| 501 | GO:0006043 | P | 8, 9, | 1 | 0.135 (x 7.404) | 4 (0.250) | 0.442 | glucosamine catabolism | Chit |
| 502 | GO:0005843 | C | 4, 5, 6, 7, 8, 9, 10, 11, | 3 | 1.317 (x 2.278) | 39 (0.077) | 0.442 | cytosolic small ribosomal subunit (sensu Eukaryota) | CG5338 CG7014 RpS4 |
| 503 | GO:0030707 | P | 8, | 7 | 4.187 (x 1.672) | 124 (0.056) | 0.442 | ovarian follicle cell development (sensu Insecta) | CG9358 Cp36 R cher loco os puc |
| 504 | GO:0008632 | P | 7, | 2 | 0.675 (x 2.961) | 20 (0.100) | 0.442 | apoptotic program | W rpr |
| 505 | GO:0046247 | P | 7, | 1 | 0.135 (x 7.404) | 4 (0.250) | 0.443 | terpene catabolism | Jheh3 |
| 506 | GO:0016283 | C | 3, 5, 6, 7, 8, | 3 | 1.317 (x 2.278) | 39 (0.077) | 0.443 | eukaryotic 48S initiation complex | CG5338 CG7014 RpS4 |
| 507 | GO:0008049 | P | 6, 7, | 2 | 0.675 (x 2.961) | 20 (0.100) | 0.443 | male courtship behavior | fru tko |
| 508 | GO:0007517 | P | 4, | 6 | 3.647 (x 1.645) | 108 (0.056) | 0.444 | muscle development | CG6803 Mhc Mlp60A fru toy wupA |
| 509 | GO:0016115 | P | 6, 8, 9, 10, | 1 | 0.135 (x 7.404) | 4 (0.250) | 0.444 | terpenoid catabolism | Jheh3 |
| 510 | GO:0030005 | P | 7, | 2 | 0.675 (x 2.961) | 20 (0.100) | 0.444 | di-, tri-valent inorganic cation homeostasis | Tsf1 Tsf3 |
| 511 | GO:0007110 | P | 6, 7, | 1 | 0.169 (x 5.923) | 5 (0.200) | 0.444 | cytokinesis after meiosis I | dia |
| 512 | GO:0005622 | C | 3, 4, | 120 | 111.263 (x 1.079) | 3295 (0.036) | 0.444 | intracellular | Aats-leu Act57B Aldh BG:DS02740.9 BcDNA:LD23830 Bmcp CG10214 CG10306 CG10445 CG10861 CG1134 CG12264 CG12954 CG17227 CG31611 CG31666 CG3192 CG31922 CG32409 CG32549 CG32649 CG33002 CG33158 CG3371 CG3803 CG4364 CG4769 CG4866 CG4917 CG5037 CG5205 CG5317 CG5338 CG5548 CG5703 CG6272 CG6330 CG6512 CG6610 CG6764 CG6876 CG7014 CG7163 CG7339 CG7791 CG7842 CG7955 CG8004 CG8199 CG8226 CG8630 CG8798 CG9273 CG9358 DDB1 EG:52C10.1 EP2237 Ets21C GV1 Gclc Gdh Gs2 HBS1 HLHmgamma Hmgcr Irbp Jafrac1 JhI-1 Ku80 La LamC Las Mhc Mlc1 Mlc2 Mlp60A Nxt1 Pcaf Pdsw Ptp61F REG Rep RpA-70 RpL17A RpS4 Ssb-c31a Ssrp Sulf1 Taf12L Tim10 Tim9a ab apt bcn92 cul-2 desat1 dia eIF6 fru ftz-f1 hay huntingtin ken l(3)01239 mRpL22 mRpL22-24 mRpS14 mRpS18a mRpS21 mRpS33 mirr mre11 mus205 na rad50 rpr tko toy unc-13 wupA |
| 513 | GO:0031468 | P | 7, 8, | 1 | 0.135 (x 7.404) | 4 (0.250) | 0.445 | nuclear envelope reassembly | LamC |
| 514 | GO:0035270 | P | 4, | 1 | 0.169 (x 5.923) | 5 (0.200) | 0.445 | endocrine system development | sog |
| 515 | GO:0007605 | P | 5, 7, | 2 | 0.675 (x 2.961) | 20 (0.100) | 0.445 | sensory perception of sound | CG4917 tko |
| 516 | GO:0016741 | F | 4, | 5 | 2.870 (x 1.742) | 85 (0.059) | 0.445 | transferase activity, transferring one-carbon groups | CG10527 CG11837 CG2453 CG8327 agt |
| 517 | GO:0046844 | P | 10, 11, | 1 | 0.135 (x 7.404) | 4 (0.250) | 0.446 | micropyle formation | puc |
| 518 | GO:0016972 | F | 6, | 1 | 0.169 (x 5.923) | 5 (0.200) | 0.446 | thiol oxidase activity | CG17843 |
| 519 | GO:0006730 | P | 5, | 2 | 0.675 (x 2.961) | 20 (0.100) | 0.446 | one-carbon compound metabolism | CAH1 CAH2 |
| 520 | GO:0044421 | C | 2, 3, | 5 | 2.870 (x 1.742) | 85 (0.059) | 0.446 | extracellular region part | Mmp1 cher dlp os trol |
| 521 | GO:0046329 | P | 6, 7, 9, 10, | 1 | 0.169 (x 5.923) | 5 (0.200) | 0.447 | negative regulation of JNK cascade | puc |
| 522 | GO:0006749 | P | 6, 7, | 1 | 0.135 (x 7.404) | 4 (0.250) | 0.447 | glutathione metabolism | Gclc |
| 523 | GO:0006596 | P | 8, 9, | 1 | 0.169 (x 5.923) | 5 (0.200) | 0.447 | polyamine biosynthesis | CG8327 |
| 524 | GO:0004663 | F | 8, | 1 | 0.135 (x 7.404) | 4 (0.250) | 0.448 | Rab-protein geranylgeranyltransferase activity | Rep |
| 525 | GO:0050982 | P | 5, 6, | 1 | 0.169 (x 5.923) | 5 (0.200) | 0.448 | detection of mechanical stimulus | tko |
| 526 | GO:0004536 | F | 6, | 3 | 1.249 (x 2.401) | 37 (0.081) | 0.449 | deoxyribonuclease activity | CG10214 mre11 rad50 |
| 527 | GO:0006750 | P | 7, 8, | 1 | 0.135 (x 7.404) | 4 (0.250) | 0.449 | glutathione biosynthesis | Gclc |
| 528 | GO:0015326 | F | 5, 6, 7, | 1 | 0.169 (x 5.923) | 5 (0.200) | 0.449 | cationic amino acid transporter activity | CG5535 |
| 529 | GO:0007540 | P | 6, | 1 | 0.169 (x 5.923) | 5 (0.200) | 0.45 | sex determination, establishment of X:A ratio | os |
| 530 | GO:0006998 | P | 6, 7, | 1 | 0.169 (x 5.923) | 5 (0.200) | 0.451 | nuclear membrane organization and biogenesis | LamC |
| 531 | GO:0051186 | P | 5, | 12 | 8.543 (x 1.405) | 253 (0.047) | 0.451 | cofactor metabolism | CG10444 CG12264 CG13645 CG2453 CG32649 CG5037 CG6574 CG7955 CG8199 Gclc Las Mocs1 |
| 532 | GO:0035271 | P | 5, | 1 | 0.169 (x 5.923) | 5 (0.200) | 0.451 | ring gland development | sog |
| 533 | GO:0016417 | F | 7, | 1 | 0.169 (x 5.923) | 5 (0.200) | 0.452 | S-acyltransferase activity | CG7842 |
| 534 | GO:0042336 | P | 6, 8, | 1 | 0.169 (x 5.923) | 5 (0.200) | 0.453 | cuticle biosynthesis during molting (sensu Protostomia and Nematoda) | Lcp65Ag1 |
| 535 | GO:0006030 | P | 7, 8, 9, 10, | 4 | 2.127 (x 1.880) | 63 (0.063) | 0.454 | chitin metabolism | CG2989 CG8756 Chit Gasp |
| 536 | GO:0007378 | P | 8, | 1 | 0.169 (x 5.923) | 5 (0.200) | 0.454 | amnioserosa formation | sog |
| 537 | GO:0002009 | P | 4, | 7 | 4.491 (x 1.559) | 133 (0.053) | 0.455 | morphogenesis of an epithelium | EP2237 R Traf1 cher loco mirr puc |
| 538 | GO:0007485 | P | 6, 7, | 1 | 0.169 (x 5.923) | 5 (0.200) | 0.455 | male genitalia development (sensu Endopterygota) | ken |
| 539 | GO:0003910 | F | 6, | 1 | 0.169 (x 5.923) | 5 (0.200) | 0.456 | DNA ligase (ATP) activity | CG17227 |
| 540 | GO:0042337 | P | 7, 9, | 1 | 0.169 (x 5.923) | 5 (0.200) | 0.456 | cuticle biosynthesis during molting (sensu Insecta) | Lcp65Ag1 |
| 541 | GO:0005991 | P | 8, | 1 | 0.169 (x 5.923) | 5 (0.200) | 0.457 | trehalose metabolism | CG5177 |
| 542 | GO:0009008 | F | 6, | 1 | 0.169 (x 5.923) | 5 (0.200) | 0.458 | DNA-methyltransferase activity | agt |
| 543 | GO:0005865 | C | 5, 6, 7, 8, 9, 10, 11, | 1 | 0.169 (x 5.923) | 5 (0.200) | 0.459 | striated muscle thin filament | wupA |
| 544 | GO:0030234 | F | 2, | 16 | 12.055 (x 1.327) | 357 (0.045) | 0.46 | enzyme regulator activity | CG10460 CG13692 CG17124 CG40006 CG7054 CG7515 CG7722 CG8066 CG9460 CG9790 Cys REG Rep TepIV loco puc |
| 545 | GO:0050974 | P | 5, 6, 7, | 1 | 0.169 (x 5.923) | 5 (0.200) | 0.46 | detection of mechanical stimulus during sensory perception | tko |
| 546 | GO:0016778 | F | 5, | 1 | 0.169 (x 5.923) | 5 (0.200) | 0.461 | diphosphotransferase activity | CG2246 |
| 547 | GO:0045454 | P | 5, | 1 | 0.169 (x 5.923) | 5 (0.200) | 0.462 | cell redox homeostasis | Jafrac1 |
| 548 | GO:0042447 | P | 6, | 1 | 0.169 (x 5.923) | 5 (0.200) | 0.462 | hormone catabolism | Jheh3 |
| 549 | GO:0004839 | F | 4, | 1 | 0.169 (x 5.923) | 5 (0.200) | 0.463 | ubiquitin activating enzyme activity | CG7828 |
| 550 | GO:0007319 | P | 9, 10, 11, | 1 | 0.169 (x 5.923) | 5 (0.200) | 0.464 | negative regulation of oskar mRNA translation | apt |
| 551 | GO:0016971 | F | 7, | 1 | 0.169 (x 5.923) | 5 (0.200) | 0.465 | flavin-linked sulfhydryl oxidase activity | CG17843 |
| 552 | GO:0006873 | P | 5, | 2 | 0.743 (x 2.692) | 22 (0.091) | 0.465 | cell ion homeostasis | Tsf1 Tsf3 |
| 553 | GO:0048637 | P | 6, | 3 | 1.418 (x 2.115) | 42 (0.071) | 0.466 | skeletal muscle development | CG6803 Mhc wupA |
| 554 | GO:0045792 | P | 6, 7, | 1 | 0.169 (x 5.923) | 5 (0.200) | 0.466 | negative regulation of cell size | Thor |
| 555 | GO:0003713 | F | 4, 6, | 2 | 0.743 (x 2.692) | 22 (0.091) | 0.466 | transcription coactivator activity | Aly Ssb-c31a |
| 556 | GO:0048747 | P | 5, | 3 | 1.418 (x 2.115) | 42 (0.071) | 0.466 | muscle fiber development | CG6803 Mhc wupA |
| 557 | GO:0008168 | F | 5, | 5 | 2.836 (x 1.763) | 84 (0.060) | 0.467 | methyltransferase activity | CG10527 CG11837 CG2453 CG8327 agt |
| 558 | GO:0030003 | P | 6, | 2 | 0.743 (x 2.692) | 22 (0.091) | 0.467 | cation homeostasis | Tsf1 Tsf3 |
| 559 | GO:0016885 | F | 4, | 1 | 0.169 (x 5.923) | 5 (0.200) | 0.467 | ligase activity, forming carbon-carbon bonds | CG17108 |
| 560 | GO:0048741 | P | 6, 7, | 3 | 1.418 (x 2.115) | 42 (0.071) | 0.467 | skeletal muscle fiber development | CG6803 Mhc wupA |
| 561 | GO:0006875 | P | 7, | 2 | 0.743 (x 2.692) | 22 (0.091) | 0.468 | metal ion homeostasis | Tsf1 Tsf3 |
| 562 | GO:0051181 | P | 5, 6, | 1 | 0.169 (x 5.923) | 5 (0.200) | 0.468 | cofactor transport | CG7955 |
| 563 | GO:0008253 | F | 8, | 1 | 0.169 (x 5.923) | 5 (0.200) | 0.468 | 5'-nucleotidase activity | CG32549 |
| 564 | GO:0005850 | C | 3, 4, 5, 6, 7, 8, 9, | 1 | 0.169 (x 5.923) | 5 (0.200) | 0.469 | eukaryotic translation initiation factor 2 complex | CG33158 |
| 565 | GO:0006807 | P | 4, | 17 | 13.203 (x 1.288) | 391 (0.043) | 0.469 | nitrogen compound metabolism | Aats-leu Act57B BG:DS03431.1 CG11897 CG12264 CG2989 CG5535 CG6950 CG8199 CG8327 CG8756 CG9836 Chit Gasp Gclc Gdh Gs2 |
| 566 | GO:0005637 | C | 5, 6, 7, 8, 9, 10, 11, 12, 13, | 1 | 0.169 (x 5.923) | 5 (0.200) | 0.47 | nuclear inner membrane | LamC |
| 567 | GO:0007362 | P | 8, 9, | 2 | 0.709 (x 2.820) | 21 (0.095) | 0.471 | terminal region determination | CG9358 sog |
| 568 | GO:0008101 | P | 9, | 1 | 0.169 (x 5.923) | 5 (0.200) | 0.471 | decapentaplegic receptor signaling pathway | dlp |
| 569 | GO:0019210 | F | 4, | 2 | 0.709 (x 2.820) | 21 (0.095) | 0.471 | kinase inhibitor activity | CG7054 puc |
| 570 | GO:0006415 | P | 8, 9, | 1 | 0.169 (x 5.923) | 5 (0.200) | 0.472 | translational termination | HBS1 |
| 571 | GO:0006405 | P | 7, 8, 9, 10, | 2 | 0.709 (x 2.820) | 21 (0.095) | 0.472 | RNA export from nucleus | La Nxt1 |
| 572 | GO:0006100 | P | 8, | 2 | 0.709 (x 2.820) | 21 (0.095) | 0.473 | tricarboxylic acid cycle intermediate metabolism | Gdh ImpL3 |
| 573 | GO:0016835 | F | 4, | 4 | 2.094 (x 1.911) | 62 (0.065) | 0.474 | carbon-oxygen lyase activity | CAH1 CAH2 CG10877 CG5044 |
| 574 | GO:0050954 | P | 4, 6, | 2 | 0.709 (x 2.820) | 21 (0.095) | 0.474 | sensory perception of mechanical stimulus | CG4917 tko |
| 575 | GO:0008237 | F | 5, | 9 | 6.247 (x 1.441) | 185 (0.049) | 0.475 | metallopeptidase activity | CG14869 CG4096 CG4933 CG6512 CG7791 Ide Mmp1 huntingtin sda |
| 576 | GO:0019752 | P | 6, | 17 | 13.237 (x 1.284) | 392 (0.043) | 0.475 | carboxylic acid metabolism | Aats-leu Aldh BG:DS03431.1 CG10877 CG12264 CG5044 CG5535 CG5946 CG6950 CG7842 CG8199 CG8630 Gclc Gdh Gs2 Las desat1 |
| 577 | GO:0006082 | P | 5, | 17 | 13.237 (x 1.284) | 392 (0.043) | 0.476 | organic acid metabolism | Aats-leu Aldh BG:DS03431.1 CG10877 CG12264 CG5044 CG5535 CG5946 CG6950 CG7842 CG8199 CG8630 Gclc Gdh Gs2 Las desat1 |
| 578 | GO:0017157 | P | 6, 7, 8, | 3 | 1.452 (x 2.066) | 43 (0.070) | 0.477 | regulation of exocytosis | Arf84F CG2885 R |
| 579 | GO:0001736 | P | 5, 6, | 3 | 1.452 (x 2.066) | 43 (0.070) | 0.478 | establishment of planar polarity | EP2237 mirr puc |
| 580 | GO:0007164 | P | 4, | 3 | 1.452 (x 2.066) | 43 (0.070) | 0.479 | establishment of tissue polarity | EP2237 mirr puc |
| 581 | GO:0005984 | P | 7, | 1 | 0.203 (x 4.936) | 6 (0.167) | 0.479 | disaccharide metabolism | CG5177 |
| 582 | GO:0043037 | P | 7, 8, | 9 | 6.281 (x 1.433) | 186 (0.048) | 0.479 | translation | Aats-leu CG10306 CG33158 CG5317 HBS1 Imp Thor apt eIF6 |
| 583 | GO:0007530 | P | 3, | 3 | 1.452 (x 2.066) | 43 (0.070) | 0.48 | sex determination | ab fru os |
| 584 | GO:0004661 | F | 7, | 1 | 0.203 (x 4.936) | 6 (0.167) | 0.48 | protein geranylgeranyltransferase activity | Rep |
| 585 | GO:0016072 | P | 7, | 3 | 1.452 (x 2.066) | 43 (0.070) | 0.48 | rRNA metabolism | CG11837 CG4364 CG5558 |
| 586 | GO:0019720 | P | 8, | 1 | 0.203 (x 4.936) | 6 (0.167) | 0.481 | Mo-molybdopterin cofactor metabolism | Mocs1 |
| 587 | GO:0051248 | P | 6, 7, | 3 | 1.452 (x 2.066) | 43 (0.070) | 0.481 | negative regulation of protein metabolism | Thor apt rpr |
| 588 | GO:0016670 | F | 5, | 1 | 0.203 (x 4.936) | 6 (0.167) | 0.481 | oxidoreductase activity, acting on sulfur group of donors, oxygen as acceptor | CG17843 |
| 589 | GO:0004518 | F | 5, | 5 | 3.039 (x 1.645) | 90 (0.056) | 0.482 | nuclease activity | CG10214 JhI-1 mre11 mus205 rad50 |
| 590 | GO:0030708 | P | 7, 8, 9, | 1 | 0.203 (x 4.936) | 6 (0.167) | 0.482 | female germ-line cyst encapsulation (sensu Insecta) | cher |
| 591 | GO:0016740 | F | 3, | 38 | 32.653 (x 1.164) | 967 (0.039) | 0.483 | transferase activity | Act57B Ate1 CG10527 CG10877 CG11837 CG12264 CG13645 CG2246 CG2453 CG3008 CG32649 CG5037 CG5397 CG6330 CG6950 CG7163 CG7339 CG7842 CG8327 CG9358 CG9790 GstD3 GstD5 GstD6 GstD9 GstE1 GstE3 GstE5 GstE6 GstE7 Irbp Las Pcaf Rep Ugt86Da Ugt86Di agt mus205 |
| 592 | GO:0019203 | F | 7, | 1 | 0.203 (x 4.936) | 6 (0.167) | 0.483 | carbohydrate phosphatase activity | CG5177 |
| 593 | GO:0004176 | F | 5, | 1 | 0.203 (x 4.936) | 6 (0.167) | 0.484 | ATP-dependent peptidase activity | CG8798 |
| 594 | GO:0006124 | P | 7, | 1 | 0.203 (x 4.936) | 6 (0.167) | 0.484 | ferredoxin metabolism | Fdxh |
| 595 | GO:0048522 | P | 4, | 9 | 6.382 (x 1.410) | 189 (0.048) | 0.485 | positive regulation of cellular process | CG12876 EP2237 Traf1 W dlp eiger mirr rpr sog |
| 596 | GO:0006961 | P | 7, 8, 9, | 2 | 0.777 (x 2.575) | 23 (0.087) | 0.485 | antibacterial humoral response (sensu Protostomia) | TepIV Thor |
| 597 | GO:0048190 | P | 6, 7, | 1 | 0.203 (x 4.936) | 6 (0.167) | 0.485 | wing disc dorsal/ventral pattern formation | dlp |
| 598 | GO:0005976 | P | 6, | 8 | 5.470 (x 1.462) | 162 (0.049) | 0.486 | polysaccharide metabolism | CG2989 CG7997 CG8756 Chit Gasp Sulf1 Ugt86Da Ugt86Di |
| 599 | GO:0006401 | P | 7, | 2 | 0.777 (x 2.575) | 23 (0.087) | 0.486 | RNA catabolism | CG10214 CG6204 |
| 600 | GO:0043545 | P | 7, | 1 | 0.203 (x 4.936) | 6 (0.167) | 0.486 | molybdopterin cofactor metabolism | Mocs1 |
| 601 | GO:0009967 | P | 5, 6, | 2 | 0.777 (x 2.575) | 23 (0.087) | 0.487 | positive regulation of signal transduction | dlp sog |
| 602 | GO:0003909 | F | 5, | 1 | 0.203 (x 4.936) | 6 (0.167) | 0.487 | DNA ligase activity | CG17227 |
| 603 | GO:0016333 | P | 5, | 2 | 0.777 (x 2.575) | 23 (0.087) | 0.488 | morphogenesis of follicular epithelium | cher loco |
| 604 | GO:0006106 | P | 8, 9, | 1 | 0.203 (x 4.936) | 6 (0.167) | 0.488 | fumarate metabolism | Gdh |
| 605 | GO:0017015 | P | 5, 6, 9, | 1 | 0.203 (x 4.936) | 6 (0.167) | 0.489 | regulation of transforming growth factor beta receptor signaling pathway | sog |
| 606 | GO:0008523 | F | 7, 9, | 1 | 0.203 (x 4.936) | 6 (0.167) | 0.489 | sodium-dependent multivitamin transporter activity | CG10444 |
| 607 | GO:0050658 | P | 6, 7, 8, | 2 | 0.810 (x 2.468) | 24 (0.083) | 0.49 | RNA transport | La Nxt1 |
| 608 | GO:0045317 | P | 7, 8, 9, 10, | 1 | 0.203 (x 4.936) | 6 (0.167) | 0.49 | equator specification | mirr |
| 609 | GO:0042063 | P | 6, | 2 | 0.810 (x 2.468) | 24 (0.083) | 0.491 | gliogenesis | BG:DS02740.9 loco |
| 610 | GO:0007487 | P | 6, | 1 | 0.203 (x 4.936) | 6 (0.167) | 0.491 | analia development (sensu Endopterygota) | ken |
| 611 | GO:0010004 | P | 7, | 2 | 0.810 (x 2.468) | 24 (0.083) | 0.491 | gastrulation (sensu Insecta) | Ptp61F sog |
| 612 | GO:0046483 | P | 5, | 7 | 4.727 (x 1.481) | 140 (0.050) | 0.492 | heterocycle metabolism | CG18522 CG2246 CG2277 CG32549 CG5037 CG6330 Las |
| 613 | GO:0006777 | P | 8, 9, | 1 | 0.203 (x 4.936) | 6 (0.167) | 0.492 | Mo-molybdopterin cofactor biosynthesis | Mocs1 |
| 614 | GO:0001703 | P | 6, | 2 | 0.810 (x 2.468) | 24 (0.083) | 0.492 | gastrulation (sensu Protostomia) | Ptp61F sog |
| 615 | GO:0005337 | F | 4, | 1 | 0.203 (x 4.936) | 6 (0.167) | 0.493 | nucleoside transporter activity | CG11907 |
| 616 | GO:0051236 | P | 5, | 2 | 0.810 (x 2.468) | 24 (0.083) | 0.493 | establishment of RNA localization | La Nxt1 |
| 617 | GO:0008415 | F | 6, | 6 | 3.883 (x 1.545) | 115 (0.052) | 0.493 | acyltransferase activity | CG10877 CG5037 CG5397 CG7842 Pcaf Rep |
| 618 | GO:0030510 | P | 5, 6, 9, | 1 | 0.203 (x 4.936) | 6 (0.167) | 0.493 | regulation of BMP signaling pathway | sog |
| 619 | GO:0015082 | F | 5, | 2 | 0.810 (x 2.468) | 24 (0.083) | 0.494 | di-, tri-valent inorganic cation transporter activity | Tsf1 Tsf3 |
| 620 | GO:0006595 | P | 7, 8, | 1 | 0.203 (x 4.936) | 6 (0.167) | 0.494 | polyamine metabolism | CG8327 |
| 621 | GO:0050657 | P | 6, 7, | 2 | 0.810 (x 2.468) | 24 (0.083) | 0.495 | nucleic acid transport | La Nxt1 |
| 622 | GO:0000154 | P | 8, | 1 | 0.203 (x 4.936) | 6 (0.167) | 0.495 | rRNA modification | CG11837 |
| 623 | GO:0017048 | F | 7, | 1 | 0.203 (x 4.936) | 6 (0.167) | 0.496 | Rho GTPase binding | dia |
| 624 | GO:0045178 | C | 3, 4, | 1 | 0.203 (x 4.936) | 6 (0.167) | 0.497 | basal part of cell | BG:DS01219.1 |
| 625 | GO:0016788 | F | 4, | 19 | 15.364 (x 1.237) | 455 (0.042) | 0.5 | hydrolase activity, acting on ester bonds | CG10214 CG2277 CG32549 CG4267 CG5044 CG5162 CG5177 CG5397 JhI-1 Ptp61F Sulf1 alpha-Est10 isopeptidase-T-3 mre11 mus205 puc rad50 wun wun2 |
| 626 | GO:0006119 | P | 6, 8, | 7 | 4.761 (x 1.470) | 141 (0.050) | 0.502 | oxidative phosphorylation | CG3192 CG32649 CG3803 CG4769 CG5548 CG5703 Pdsw |
| 627 | GO:0003676 | F | 3, | 65 | 58.856 (x 1.104) | 1743 (0.037) | 0.505 | nucleic acid binding | Aats-leu Aly CG10214 CG10306 CG10445 CG11837 CG1582 CG17227 CG2885 CG31611 CG31666 CG3168 CG31922 CG33158 CG3371 CG3860 CG4364 CG4866 CG5317 CG5338 CG5558 CG5800 CG6204 CG6272 CG6764 CG6876 CG7014 CG7163 CG7339 CG9273 CG9862 DDB1 Dbp80 EP2237 Ets21C GV1 Gclc HBS1 HLHmgamma Imp Irbp Ku80 La Nmd3 RpA-70 RpL17A RpS4 Ssb-c31a Ssrp ab agt apt eIF6 fru ftz-f1 hay ken mRpS14 mirr mre11 mus205 rad50 tko toy trol |
| 628 | GO:0035193 | P | 5, 6, | 1 | 0.236 (x 4.231) | 7 (0.143) | 0.507 | central nervous system remodeling (sensu Insecta) | rpr |
| 629 | GO:0048806 | P | 4, | 1 | 0.236 (x 4.231) | 7 (0.143) | 0.507 | genitalia development | ken |
| 630 | GO:0006265 | P | 7, | 1 | 0.236 (x 4.231) | 7 (0.143) | 0.508 | DNA topological change | CG3168 |
| 631 | GO:0016202 | P | 6, | 1 | 0.236 (x 4.231) | 7 (0.143) | 0.509 | regulation of striated muscle development | Mlp60A |
| 632 | GO:0048138 | P | 5, 6, | 1 | 0.236 (x 4.231) | 7 (0.143) | 0.51 | germ-line cyst encapsulation | cher |
| 633 | GO:0016755 | F | 5, | 1 | 0.236 (x 4.231) | 7 (0.143) | 0.511 | transferase activity, transferring amino-acyl groups | Ate1 |
| 634 | GO:0000124 | C | 5, 8, 9, 10, 11, 12, 13, 14, 15, | 1 | 0.236 (x 4.231) | 7 (0.143) | 0.511 | SAGA complex | Pcaf |
| 635 | GO:0016624 | F | 5, | 1 | 0.236 (x 4.231) | 7 (0.143) | 0.512 | oxidoreductase activity, acting on the aldehyde or oxo group of donors, disulfide as acceptor | CG8199 |
| 636 | GO:0000922 | C | 5, 6, 7, 8, 9, 10, 11, | 1 | 0.236 (x 4.231) | 7 (0.143) | 0.513 | spindle pole | Rep |
| 637 | GO:0007484 | P | 5, 6, | 1 | 0.236 (x 4.231) | 7 (0.143) | 0.514 | genitalia development (sensu Endopterygota) | ken |
| 638 | GO:0006725 | P | 5, | 7 | 4.896 (x 1.430) | 145 (0.048) | 0.514 | aromatic compound metabolism | CG18522 CG1882 CG2246 CG2277 CG32549 CG6330 Mocs1 |
| 639 | GO:0019992 | F | 4, | 2 | 0.878 (x 2.278) | 26 (0.077) | 0.514 | diacylglycerol binding | CG9358 unc-13 |
| 640 | GO:0050654 | P | 6, 8, | 1 | 0.236 (x 4.231) | 7 (0.143) | 0.514 | chondroitin sulfate proteoglycan metabolism | Act57B |
| 641 | GO:0004520 | F | 7, | 2 | 0.878 (x 2.278) | 26 (0.077) | 0.515 | endodeoxyribonuclease activity | mre11 rad50 |
| 642 | GO:0006044 | P | 8, 9, | 4 | 2.397 (x 1.668) | 71 (0.056) | 0.515 | N-acetylglucosamine metabolism | CG2989 CG8756 Chit Gasp |
| 643 | GO:0016272 | C | 3, 6, 7, 8, 9, 10, | 1 | 0.236 (x 4.231) | 7 (0.143) | 0.515 | prefoldin complex | l(3)01239 |
| 644 | GO:0008406 | P | 5, | 2 | 0.844 (x 2.369) | 25 (0.080) | 0.515 | gonad development | CG7194 Hmgcr |
| 645 | GO:0003684 | F | 5, | 2 | 0.878 (x 2.278) | 26 (0.077) | 0.516 | damaged DNA binding | DDB1 rad50 |
| 646 | GO:0006041 | P | 7, 8, | 4 | 2.397 (x 1.668) | 71 (0.056) | 0.516 | glucosamine metabolism | CG2989 CG8756 Chit Gasp |
| 647 | GO:0015932 | F | 3, | 1 | 0.236 (x 4.231) | 7 (0.143) | 0.516 | nucleobase, nucleoside, nucleotide and nucleic acid transporter activity | CG11907 |
| 648 | GO:0045137 | P | 4, | 2 | 0.844 (x 2.369) | 25 (0.080) | 0.516 | development of primary sexual characteristics | CG7194 Hmgcr |
| 649 | GO:0048731 | P | 3, | 24 | 20.058 (x 1.197) | 594 (0.040) | 0.517 | system development | BG:DS02740.9 CG10861 EP2237 Gs2 HLHmgamma Mmp1 Ptp61F Traf1 Tsp42El W ab apt fru ftz-f1 loco melt mfas mirr puc rpr sog toy trol wupA |
| 650 | GO:0004965 | F | 6, 7, | 1 | 0.236 (x 4.231) | 7 (0.143) | 0.517 | GABA-B receptor activity | GABA-B-R2 |
| 651 | GO:0030206 | P | 8, 9, 10, | 1 | 0.236 (x 4.231) | 7 (0.143) | 0.518 | chondroitin sulfate biosynthesis | Act57B |
| 652 | GO:0042600 | C | 4, 5, | 1 | 0.236 (x 4.231) | 7 (0.143) | 0.518 | chorion | Cp36 |
| 653 | GO:0009790 | P | 3, | 15 | 11.852 (x 1.266) | 351 (0.043) | 0.518 | embryonic development | CG9358 EP2237 Ptp61F R Traf1 W dia dlp ftz-f1 mirr os puc rpr sog wun |
| 654 | GO:0006714 | P | 6, 8, 9, 10, | 1 | 0.236 (x 4.231) | 7 (0.143) | 0.519 | sesquiterpenoid metabolism | Jheh3 |
| 655 | GO:0030204 | P | 7, 8, 9, | 1 | 0.236 (x 4.231) | 7 (0.143) | 0.52 | chondroitin sulfate metabolism | Act57B |
| 656 | GO:0046661 | P | 4, | 1 | 0.236 (x 4.231) | 7 (0.143) | 0.521 | male sex differentiation | fru |
| 657 | GO:0048139 | P | 6, 7, | 1 | 0.236 (x 4.231) | 7 (0.143) | 0.522 | female germ-line cyst encapsulation | cher |
| 658 | GO:0030145 | F | 6, | 1 | 0.236 (x 4.231) | 7 (0.143) | 0.522 | manganese ion binding | mre11 |
| 659 | GO:0048103 | P | 6, | 1 | 0.236 (x 4.231) | 7 (0.143) | 0.523 | somatic stem cell division | os |
| 660 | GO:0007305 | P | 9, 10, | 1 | 0.236 (x 4.231) | 7 (0.143) | 0.524 | vitelline membrane formation (sensu Insecta) | Cp36 |
| 661 | GO:0016801 | F | 4, | 1 | 0.236 (x 4.231) | 7 (0.143) | 0.525 | hydrolase activity, acting on ether bonds | Jheh3 |
| 662 | GO:0010001 | P | 4, 7, | 1 | 0.236 (x 4.231) | 7 (0.143) | 0.526 | glial cell differentiation | loco |
| 663 | GO:0004312 | F | 7, | 1 | 0.236 (x 4.231) | 7 (0.143) | 0.526 | fatty-acid synthase activity | CG7842 |
| 664 | GO:0006260 | P | 7, | 6 | 4.086 (x 1.468) | 121 (0.050) | 0.526 | DNA replication | CG17227 CG6204 CG9273 Irbp RpA-70 mus205 |
| 665 | GO:0006040 | P | 6, 7, | 4 | 2.431 (x 1.645) | 72 (0.056) | 0.527 | amino sugar metabolism | CG2989 CG8756 Chit Gasp |
| 666 | GO:0016747 | F | 5, | 6 | 4.086 (x 1.468) | 121 (0.050) | 0.527 | transferase activity, transferring groups other than amino-acyl groups | CG10877 CG5037 CG5397 CG7842 Pcaf Rep |
| 667 | GO:0008641 | F | 3, | 1 | 0.236 (x 4.231) | 7 (0.143) | 0.527 | small protein activating enzyme activity | CG7828 |
| 668 | GO:0006716 | P | 6, 7, 9, 10, 11, | 1 | 0.236 (x 4.231) | 7 (0.143) | 0.528 | juvenile hormone metabolism | Jheh3 |
| 669 | GO:0004864 | F | 5, 6, | 1 | 0.236 (x 4.231) | 7 (0.143) | 0.529 | protein phosphatase inhibitor activity | CG17124 |
| 670 | GO:0051761 | P | 7, | 1 | 0.236 (x 4.231) | 7 (0.143) | 0.53 | sesquiterpene metabolism | Jheh3 |
| 671 | GO:0008088 | P | 8, 9, 10, | 1 | 0.236 (x 4.231) | 7 (0.143) | 0.531 | axon cargo transport | huntingtin |
| 672 | GO:0016015 | F | 3, | 1 | 0.236 (x 4.231) | 7 (0.143) | 0.531 | morphogen activity | os |
| 673 | GO:0050650 | P | 7, 8, 9, | 1 | 0.236 (x 4.231) | 7 (0.143) | 0.532 | chondroitin sulfate proteoglycan biosynthesis | Act57B |
| 674 | GO:0030704 | P | 8, | 1 | 0.236 (x 4.231) | 7 (0.143) | 0.533 | vitelline membrane formation | Cp36 |
| 675 | GO:0004519 | F | 6, | 3 | 1.655 (x 1.813) | 49 (0.061) | 0.534 | endonuclease activity | JhI-1 mre11 rad50 |
| 676 | GO:0009063 | P | 7, 8, | 3 | 1.655 (x 1.813) | 49 (0.061) | 0.535 | amino acid catabolism | CG8199 Gdh Gs2 |
| 677 | GO:0016895 | F | 8, | 1 | 0.270 (x 3.702) | 8 (0.125) | 0.535 | exodeoxyribonuclease activity, producing 5'-phosphomonoesters | CG10214 |
| 678 | GO:0043449 | P | 5, | 1 | 0.270 (x 3.702) | 8 (0.125) | 0.536 | alkene metabolism | Jheh3 |
| 679 | GO:0007365 | P | 5, 6, | 2 | 0.912 (x 2.194) | 27 (0.074) | 0.536 | periodic partitioning | dlp ftz-f1 |
| 680 | GO:0008649 | F | 7, | 1 | 0.270 (x 3.702) | 8 (0.125) | 0.537 | rRNA methyltransferase activity | CG11837 |
| 681 | GO:0050801 | P | 4, | 2 | 0.912 (x 2.194) | 27 (0.074) | 0.537 | ion homeostasis | Tsf1 Tsf3 |
| 682 | GO:0008535 | P | 7, | 1 | 0.270 (x 3.702) | 8 (0.125) | 0.537 | cytochrome c oxidase complex assembly | CG3803 |
| 683 | GO:0007354 | P | 7, 8, | 2 | 0.912 (x 2.194) | 27 (0.074) | 0.538 | zygotic determination of anterior/posterior axis, embryo | CG9358 sog |
| 684 | GO:0016886 | F | 4, | 1 | 0.270 (x 3.702) | 8 (0.125) | 0.538 | ligase activity, forming phosphoric ester bonds | CG17227 |
| 685 | GO:0005666 | C | 4, 5, 6, 7, 8, 9, 10, 11, 12, 13, | 1 | 0.270 (x 3.702) | 8 (0.125) | 0.539 | DNA-directed RNA polymerase III complex | CG7339 |
| 686 | GO:0042214 | P | 6, | 1 | 0.270 (x 3.702) | 8 (0.125) | 0.54 | terpene metabolism | Jheh3 |
| 687 | GO:0030312 | C | 3, 4, | 1 | 0.270 (x 3.702) | 8 (0.125) | 0.54 | external encapsulating structure | Cp36 |
| 688 | GO:0007300 | P | 8, | 2 | 0.945 (x 2.115) | 28 (0.071) | 0.541 | nurse cell to oocyte transport (sensu Insecta) | cher loco |
| 689 | GO:0005344 | F | 3, | 1 | 0.270 (x 3.702) | 8 (0.125) | 0.541 | oxygen transporter activity | glob1 |
| 690 | GO:0008408 | F | 7, | 2 | 0.945 (x 2.115) | 28 (0.071) | 0.542 | 3'-5' exonuclease activity | CG10214 mus205 |
| 691 | GO:0004365 | F | 7, | 1 | 0.270 (x 3.702) | 8 (0.125) | 0.542 | glyceraldehyde-3-phosphate dehydrogenase (phosphorylating) activity | DDB1 |
| 692 | GO:0015711 | P | 7, 8, | 1 | 0.270 (x 3.702) | 8 (0.125) | 0.543 | organic anion transport | CG7571 |
| 693 | GO:0045735 | F | 2, | 1 | 0.270 (x 3.702) | 8 (0.125) | 0.543 | nutrient reservoir activity | Idgf2 |
| 694 | GO:0051226 | P | 7, 8, 11, | 1 | 0.270 (x 3.702) | 8 (0.125) | 0.544 | meiotic spindle assembly | dia |
| 695 | GO:0009886 | P | 4, | 1 | 0.270 (x 3.702) | 8 (0.125) | 0.545 | post-embryonic morphogenesis | rpr |
| 696 | GO:0007279 | P | 6, | 1 | 0.270 (x 3.702) | 8 (0.125) | 0.546 | pole cell formation | dia |
| 697 | GO:0042180 | P | 5, | 1 | 0.270 (x 3.702) | 8 (0.125) | 0.547 | ketone metabolism | Gdh |
| 698 | GO:0008565 | F | 3, | 4 | 2.533 (x 1.579) | 75 (0.053) | 0.547 | protein transporter activity | CG8226 Nxt1 Tim10 Tim9a |
| 699 | GO:0004553 | F | 5, | 5 | 3.309 (x 1.511) | 98 (0.051) | 0.547 | hydrolase activity, hydrolyzing O-glycosyl compounds | CG14935 CG17843 CG2989 CG7997 Chit |
| 700 | GO:0046692 | P | 6, 7, | 1 | 0.270 (x 3.702) | 8 (0.125) | 0.547 | sperm competition | Gdh |
| 701 | GO:0005842 | C | 4, 5, 6, 7, 8, 9, 10, 11, | 3 | 1.722 (x 1.742) | 51 (0.059) | 0.548 | cytosolic large ribosomal subunit (sensu Eukaryota) | CG5317 CG6764 RpL17A |
| 702 | GO:0008252 | F | 7, | 1 | 0.270 (x 3.702) | 8 (0.125) | 0.548 | nucleotidase activity | CG32549 |
| 703 | GO:0008429 | F | 5, | 1 | 0.270 (x 3.702) | 8 (0.125) | 0.549 | phosphatidylethanolamine binding | CG7054 |
| 704 | GO:0008060 | F | 5, | 1 | 0.270 (x 3.702) | 8 (0.125) | 0.55 | ARF GTPase activator activity | CG13692 |
| 705 | GO:0016096 | P | 7, 8, | 1 | 0.270 (x 3.702) | 8 (0.125) | 0.551 | polyisoprenoid metabolism | Jheh3 |
| 706 | GO:0005742 | C | 3, 5, 6, 7, 8, 9, 10, 11, 12, 13, | 1 | 0.270 (x 3.702) | 8 (0.125) | 0.551 | mitochondrial outer membrane translocase complex | CG8226 |
| 707 | GO:0007377 | P | 8, | 1 | 0.270 (x 3.702) | 8 (0.125) | 0.552 | germ-band extension | Ptp61F |
| 708 | GO:0031090 | C | 4, 5, 6, 7, 8, | 15 | 12.190 (x 1.231) | 361 (0.042) | 0.553 | organelle membrane | Bmcp CG3192 CG3803 CG4769 CG5548 CG5703 CG7955 CG8004 CG8226 Hmgcr LamC Pdsw Tim10 Tim9a bcn92 |
| 709 | GO:0008023 | C | 3, 6, 7, 8, 9, 10, 11, 12, 13, | 1 | 0.270 (x 3.702) | 8 (0.125) | 0.553 | transcription elongation factor complex | Ssrp |
| 710 | GO:0016299 | F | 4, | 1 | 0.270 (x 3.702) | 8 (0.125) | 0.554 | regulator of G-protein signaling activity | loco |
| 711 | GO:0006721 | P | 5, 7, 8, 9, | 1 | 0.270 (x 3.702) | 8 (0.125) | 0.555 | terpenoid metabolism | Jheh3 |
| 712 | GO:0008943 | F | 6, | 1 | 0.270 (x 3.702) | 8 (0.125) | 0.555 | glyceraldehyde-3-phosphate dehydrogenase activity | DDB1 |
| 713 | GO:0006915 | P | 6, | 8 | 6.011 (x 1.331) | 178 (0.045) | 0.561 | apoptosis | CG12876 CG40006 CG7188 Mmp1 Traf1 W eiger rpr |
| 714 | GO:0007179 | P | 8, | 2 | 0.979 (x 2.042) | 29 (0.069) | 0.561 | transforming growth factor beta receptor signaling pathway | dlp sog |
| 715 | GO:0044270 | P | 5, 6, | 3 | 1.756 (x 1.709) | 52 (0.058) | 0.562 | nitrogen compound catabolism | CG8199 Gdh Gs2 |
| 716 | GO:0043170 | P | 4, | 111 | 105.218 (x 1.055) | 3116 (0.036) | 0.562 | macromolecule metabolism | Aats-leu Act57B Arf84F Ate1 CG10214 CG10306 CG10444 CG10445 CG1134 CG11837 CG12954 CG1299 CG13645 CG13692 CG14869 CG14935 CG17227 CG2989 CG3008 CG3036 CG31373 CG31611 CG3168 CG32649 CG33002 CG33158 CG3803 CG4096 CG4364 CG4866 CG4933 CG5177 CG5205 CG5317 CG5338 CG5558 CG6204 CG6512 CG6574 CG6610 CG6764 CG6803 CG6876 CG7014 CG7163 CG7722 CG7791 CG7828 CG7997 CG8756 CG8798 CG9273 CG9358 CG9372 CG9460 Chit DDB1 Dgp-1 Fdxh Gasp Gclc Gdh Gs2 HBS1 Ide Imp ImpL3 Irbp JhI-1 Ku80 La Las Mlc1 Mmp1 Nmd3 Ptp61F REG Rep RpA-70 RpL17A RpS4 Ssrp Sulf1 Thor Ugt86Da Ugt86Di W agt apt cul-2 dia eIF6 fru hay huntingtin l(3)01239 mRpL22 mRpL22-24 mRpS14 mRpS18a mRpS21 mRpS33 mre11 mus205 na puc rad50 rpr sda tko trol |
| 717 | GO:0007391 | P | 6, | 4 | 2.566 (x 1.559) | 76 (0.053) | 0.562 | dorsal closure | EP2237 R Traf1 puc |
| 718 | GO:0005682 | C | 5, 6, 7, 8, 9, 10, 11, 12, | 1 | 0.304 (x 3.291) | 9 (0.111) | 0.562 | snRNP U5 | CG5205 |
| 719 | GO:0005941 | C | 3, | 3 | 1.756 (x 1.709) | 52 (0.058) | 0.562 | unlocalized protein complex | Ku80 Mocs1 Rep |
| 720 | GO:0009796 | P | 4, 5, | 1 | 0.304 (x 3.291) | 9 (0.111) | 0.563 | cellularization (sensu Metazoa) | dia |
| 721 | GO:0001738 | P | 5, | 3 | 1.756 (x 1.709) | 52 (0.058) | 0.563 | morphogenesis of a polarized epithelium | EP2237 mirr puc |
| 722 | GO:0048513 | P | 3, | 26 | 22.624 (x 1.149) | 670 (0.039) | 0.563 | organ development | Act57B CG10861 CG6803 CG7194 EP2237 HLHmgamma Hmgcr Idgf2 Idgf3 Mhc Mlp60A Mmp1 Traf1 W apt dlp fru ftz-f1 ken mirr os puc rpr sog toy wupA |
| 723 | GO:0016070 | P | 6, | 15 | 12.393 (x 1.210) | 367 (0.041) | 0.564 | RNA metabolism | Aats-leu CG10214 CG10445 CG11837 CG4364 CG5205 CG5558 CG6204 CG6610 CG6876 CG7163 JhI-1 La Nmd3 hay |
| 724 | GO:0019212 | F | 4, 5, | 1 | 0.304 (x 3.291) | 9 (0.111) | 0.564 | phosphatase inhibitor activity | CG17124 |
| 725 | GO:0009310 | P | 6, 7, | 3 | 1.756 (x 1.709) | 52 (0.058) | 0.564 | amine catabolism | CG8199 Gdh Gs2 |
| 726 | GO:0007243 | P | 6, | 6 | 4.288 (x 1.399) | 127 (0.047) | 0.564 | protein kinase cascade | CG7515 EP2237 Traf1 eiger os puc |
| 727 | GO:0008069 | P | 6, 7, 9, | 1 | 0.304 (x 3.291) | 9 (0.111) | 0.564 | dorsal/ventral axis determination, follicular epithelium (sensu Insecta) | loco |
| 728 | GO:0008079 | F | 4, 5, | 1 | 0.304 (x 3.291) | 9 (0.111) | 0.565 | translation termination factor activity | HBS1 |
| 729 | GO:0007046 | P | 7, | 2 | 1.013 (x 1.974) | 30 (0.067) | 0.565 | ribosome biogenesis | EG:52C10.1 Nmd3 |
| 730 | GO:0000165 | P | 7, | 5 | 3.478 (x 1.438) | 103 (0.049) | 0.566 | MAPKKK cascade | CG7515 EP2237 Traf1 eiger puc |
| 731 | GO:0009880 | P | 4, | 6 | 4.356 (x 1.377) | 129 (0.047) | 0.566 | embryonic pattern specification | CG9358 dlp ftz-f1 os sog wun |
| 732 | GO:0008277 | P | 5, 6, 7, | 1 | 0.304 (x 3.291) | 9 (0.111) | 0.566 | regulation of G-protein coupled receptor protein signaling pathway | loco |
| 733 | GO:0008380 | P | 8, | 7 | 5.234 (x 1.337) | 155 (0.045) | 0.566 | RNA splicing | CG10445 CG5205 CG6610 CG6876 CG7163 JhI-1 hay |
| 734 | GO:0004725 | F | 8, | 2 | 1.013 (x 1.974) | 30 (0.067) | 0.566 | protein tyrosine phosphatase activity | Ptp61F puc |
| 735 | GO:0046914 | F | 5, | 26 | 22.827 (x 1.139) | 676 (0.038) | 0.566 | transition metal ion binding | CAH1 CAH2 CG1134 CG14869 CG31666 CG31922 CG4096 CG4933 CG7163 CG7791 CG8630 EP2237 Ide Las Mlp60A Mmp1 Mocs1 Traf1 Tsf1 Tsf3 ab desat1 fru ken mre11 sda |
| 736 | GO:0018346 | P | 10, 11, | 1 | 0.304 (x 3.291) | 9 (0.111) | 0.567 | protein amino acid prenylation | Rep |
| 737 | GO:0030162 | P | 6, 7, 8, | 2 | 1.013 (x 1.974) | 30 (0.067) | 0.567 | regulation of proteolysis | W rpr |
| 738 | GO:0008484 | F | 5, | 1 | 0.304 (x 3.291) | 9 (0.111) | 0.567 | sulfuric ester hydrolase activity | Sulf1 |
| 739 | GO:0015012 | P | 7, 8, 9, | 1 | 0.304 (x 3.291) | 9 (0.111) | 0.568 | heparan sulfate proteoglycan biosynthesis | Act57B |
| 740 | GO:0005675 | C | 4, 7, 8, 9, 10, 11, 12, 13, 14, | 1 | 0.304 (x 3.291) | 9 (0.111) | 0.569 | transcription factor TFIIH complex | hay |
| 741 | GO:0007350 | P | 4, 5, | 5 | 3.444 (x 1.452) | 102 (0.049) | 0.569 | blastoderm segmentation | CG9358 dlp ftz-f1 os sog |
| 742 | GO:0003747 | F | 5, 6, | 1 | 0.304 (x 3.291) | 9 (0.111) | 0.57 | translation release factor activity | HBS1 |
| 743 | GO:0044264 | P | 6, 7, | 4 | 2.600 (x 1.538) | 77 (0.052) | 0.57 | cellular polysaccharide metabolism | CG2989 CG8756 Chit Gasp |
| 744 | GO:0050906 | P | 4, 5, 6, | 1 | 0.304 (x 3.291) | 9 (0.111) | 0.57 | detection of stimulus during sensory perception | tko |
| 745 | GO:0018342 | P | 9, 10, | 1 | 0.304 (x 3.291) | 9 (0.111) | 0.571 | protein prenylation | Rep |
| 746 | GO:0030201 | P | 6, 8, | 1 | 0.304 (x 3.291) | 9 (0.111) | 0.572 | heparan sulfate proteoglycan metabolism | Act57B |
| 747 | GO:0005688 | C | 5, 6, 7, 8, 9, 10, 11, 12, | 1 | 0.304 (x 3.291) | 9 (0.111) | 0.573 | snRNP U6 | CG6610 |
| 748 | GO:0005213 | F | 3, | 1 | 0.304 (x 3.291) | 9 (0.111) | 0.574 | structural constituent of chorion (sensu Insecta) | Cp36 |
| 749 | GO:0005730 | C | 5, 6, 7, 8, 9, 10, 11, | 3 | 1.823 (x 1.645) | 54 (0.056) | 0.574 | nucleolus | CG6610 EG:52C10.1 Ssrp |
| 750 | GO:0008299 | P | 6, 7, 8, | 1 | 0.304 (x 3.291) | 9 (0.111) | 0.574 | isoprenoid biosynthesis | Hmgcr |
| 751 | GO:0042364 | P | 7, | 1 | 0.304 (x 3.291) | 9 (0.111) | 0.575 | water-soluble vitamin biosynthesis | CG13645 |
| 752 | GO:0009166 | P | 6, 7, | 1 | 0.304 (x 3.291) | 9 (0.111) | 0.576 | nucleotide catabolism | CG6330 |
| 753 | GO:0016065 | P | 6, 7, | 3 | 1.790 (x 1.676) | 53 (0.057) | 0.576 | humoral defense mechanism (sensu Protostomia) | TepIV Thor TotA |
| 754 | GO:0007301 | P | 6, 9, | 1 | 0.304 (x 3.291) | 9 (0.111) | 0.577 | ovarian ring canal formation | cher |
| 755 | GO:0002165 | P | 4, | 14 | 11.650 (x 1.202) | 345 (0.041) | 0.578 | larval or pupal development (sensu Insecta) | CG10861 CG9358 EP2237 Lcp65Ag1 Mmp1 Traf1 W ftz-f1 mirr puc rad50 rpr sas sog |
| 756 | GO:0007001 | P | 7, | 7 | 5.301 (x 1.320) | 157 (0.045) | 0.583 | chromosome organization and biogenesis (sensu Eukaryota) | CG31611 Irbp Ku80 Ssrp fru mre11 rad50 |
| 757 | GO:0006023 | P | 7, 8, | 1 | 0.338 (x 2.961) | 10 (0.100) | 0.583 | aminoglycan biosynthesis | Act57B |
| 758 | GO:0007398 | P | 4, | 10 | 8.003 (x 1.250) | 237 (0.042) | 0.583 | ectoderm development | BG:DS02740.9 CG5397 HLHmgamma Tsp42El melt mfas mirr puc sog toy |
| 759 | GO:0016829 | F | 3, | 7 | 5.301 (x 1.320) | 157 (0.045) | 0.583 | lyase activity | CAH1 CAH2 CG10877 CG12264 CG5044 CG6950 rad50 |
| 760 | GO:0015926 | F | 6, | 2 | 1.047 (x 1.911) | 31 (0.065) | 0.583 | glucosidase activity | CG14935 CG17843 |
| 761 | GO:0030725 | P | 5, | 1 | 0.338 (x 2.961) | 10 (0.100) | 0.584 | ring canal formation | cher |
| 762 | GO:0007224 | P | 6, | 2 | 1.047 (x 1.911) | 31 (0.065) | 0.584 | smoothened signaling pathway | dlp mirr |
| 763 | GO:0051707 | P | 4, | 6 | 4.423 (x 1.356) | 131 (0.046) | 0.585 | response to other organism | CG9358 TepIV Thor TotA Tsf3 eiger |
| 764 | GO:0006783 | P | 7, 8, | 1 | 0.338 (x 2.961) | 10 (0.100) | 0.585 | heme biosynthesis | CG5037 |
| 765 | GO:0016891 | F | 8, | 1 | 0.338 (x 2.961) | 10 (0.100) | 0.585 | endoribonuclease activity, producing 5'-phosphomonoesters | JhI-1 |
| 766 | GO:0004402 | F | 10, | 1 | 0.338 (x 2.961) | 10 (0.100) | 0.586 | histone acetyltransferase activity | Pcaf |
| 767 | GO:0043063 | P | 4, | 1 | 0.338 (x 2.961) | 10 (0.100) | 0.587 | intercellular bridge organization and biogenesis | cher |
| 768 | GO:0017038 | P | 6, 7, | 3 | 1.857 (x 1.615) | 55 (0.055) | 0.587 | protein import | Nxt1 Tim10 Tim9a |
| 769 | GO:0016482 | P | 6, 7, 8, | 1 | 0.338 (x 2.961) | 10 (0.100) | 0.588 | cytoplasmic transport | loco |
| 770 | GO:0016790 | F | 5, | 2 | 1.081 (x 1.851) | 32 (0.062) | 0.588 | thiolester hydrolase activity | CG5044 isopeptidase-T-3 |
| 771 | GO:0051087 | F | 4, | 1 | 0.338 (x 2.961) | 10 (0.100) | 0.588 | chaperone binding | l(3)01239 |
| 772 | GO:0046873 | F | 4, | 2 | 1.081 (x 1.851) | 32 (0.062) | 0.589 | metal ion transporter activity | Tsf1 Tsf3 |
| 773 | GO:0004468 | F | 9, | 1 | 0.338 (x 2.961) | 10 (0.100) | 0.589 | lysine N-acetyltransferase activity | Pcaf |
| 774 | GO:0006206 | P | 7, | 2 | 1.081 (x 1.851) | 32 (0.062) | 0.589 | pyrimidine base metabolism | CG2246 CG6330 |
| 775 | GO:0007303 | P | 7, 8, 9, | 1 | 0.338 (x 2.961) | 10 (0.100) | 0.59 | cytoplasmic transport, nurse cell to oocyte | loco |
| 776 | GO:0006024 | P | 8, 9, | 1 | 0.338 (x 2.961) | 10 (0.100) | 0.591 | glycosaminoglycan biosynthesis | Act57B |
| 777 | GO:0046011 | P | 8, 9, 10, | 1 | 0.338 (x 2.961) | 10 (0.100) | 0.591 | regulation of oskar mRNA translation | apt |
| 778 | GO:0016059 | P | 7, 8, 9, | 1 | 0.338 (x 2.961) | 10 (0.100) | 0.592 | deactivation of rhodopsin mediated signaling | Arr2 |
| 779 | GO:0044242 | P | 6, 7, | 1 | 0.338 (x 2.961) | 10 (0.100) | 0.593 | cellular lipid catabolism | Jheh3 |
| 780 | GO:0045433 | P | 10, 11, | 1 | 0.338 (x 2.961) | 10 (0.100) | 0.594 | male courtship behavior (sensu Insecta), song production | fru |
| 781 | GO:0006022 | P | 6, 7, | 1 | 0.338 (x 2.961) | 10 (0.100) | 0.595 | aminoglycan metabolism | Act57B |
| 782 | GO:0030203 | P | 7, 8, | 1 | 0.338 (x 2.961) | 10 (0.100) | 0.595 | glycosaminoglycan metabolism | Act57B |
| 783 | GO:0006732 | P | 6, | 10 | 8.070 (x 1.239) | 239 (0.042) | 0.596 | coenzyme metabolism | CG10444 CG13645 CG2453 CG32649 CG5037 CG6574 CG8199 Gclc Las Mocs1 |
| 784 | GO:0030713 | P | 9, | 1 | 0.338 (x 2.961) | 10 (0.100) | 0.596 | stalk formation (sensu Insecta) | R |
| 785 | GO:0044428 | C | 4, 5, 6, 7, 8, 9, | 20 | 17.390 (x 1.150) | 515 (0.039) | 0.596 | nuclear part | CG31922 CG5205 CG6610 CG6876 CG7339 CG9273 EG:52C10.1 GV1 Ku80 LamC Nxt1 Pcaf RpA-70 Ssb-c31a Ssrp Taf12L cul-2 hay mus205 rad50 |
| 786 | GO:0009308 | P | 5, | 15 | 12.696 (x 1.181) | 376 (0.040) | 0.596 | amine metabolism | Aats-leu Act57B BG:DS03431.1 CG12264 CG2989 CG5535 CG6950 CG8199 CG8327 CG8756 Chit Gasp Gclc Gdh Gs2 |
| 787 | GO:0015893 | P | 5, 6, | 1 | 0.338 (x 2.961) | 10 (0.100) | 0.597 | drug transport | BEST:CK01227 |
| 788 | GO:0003916 | F | 4, 5, | 1 | 0.338 (x 2.961) | 10 (0.100) | 0.598 | DNA topoisomerase activity | CG3168 |
| 789 | GO:0016331 | P | 5, | 4 | 2.769 (x 1.445) | 82 (0.049) | 0.598 | morphogenesis of embryonic epithelium | EP2237 R Traf1 puc |
| 790 | GO:0016545 | P | 9, 10, | 1 | 0.338 (x 2.961) | 10 (0.100) | 0.598 | male courtship behavior (sensu Insecta), wing vibration | fru |
| 791 | GO:0008135 | F | 3, 4, | 4 | 2.769 (x 1.445) | 82 (0.049) | 0.598 | translation factor activity, nucleic acid binding | CG10306 CG33158 HBS1 eIF6 |
| 792 | GO:0016616 | F | 5, | 4 | 2.836 (x 1.410) | 84 (0.048) | 0.607 | oxidoreductase activity, acting on the CH-OH group of donors, NAD or NADP as acceptor | BcDNA:GH10614 CG10638 Hmgcr ImpL3 |
| 793 | GO:0044271 | P | 5, 6, | 4 | 2.836 (x 1.410) | 84 (0.048) | 0.607 | nitrogen compound biosynthesis | CG12264 CG6950 CG8327 Gs2 |
| 794 | GO:0009791 | P | 3, | 14 | 12.021 (x 1.165) | 356 (0.039) | 0.608 | post-embryonic development | CG10861 CG9358 EP2237 Lcp65Ag1 Mmp1 Traf1 W ftz-f1 mirr puc rad50 rpr sas sog |
| 795 | GO:0048518 | P | 3, | 9 | 7.361 (x 1.223) | 218 (0.041) | 0.608 | positive regulation of biological process | CG12876 EP2237 Traf1 W dlp eiger mirr rpr sog |
| 796 | GO:0009309 | P | 6, 7, | 4 | 2.836 (x 1.410) | 84 (0.048) | 0.608 | amine biosynthesis | CG12264 CG6950 CG8327 Gs2 |
| 797 | GO:0040034 | P | 4, | 1 | 0.371 (x 2.692) | 11 (0.091) | 0.609 | regulation of development, heterochronic | ftz-f1 |
| 798 | GO:0000910 | P | 5, | 4 | 2.836 (x 1.410) | 84 (0.048) | 0.609 | cytokinesis | Act57B Mhc R dia |
| 799 | GO:0042067 | P | 6, 7, 8, 9, | 2 | 1.114 (x 1.795) | 33 (0.061) | 0.609 | establishment of ommatidial polarity (sensu Endopterygota) | mirr puc |
| 800 | GO:0005750 | C | 4, 5, 6, 7, 8, 9, 10, 11, 12, 13, 14, | 1 | 0.371 (x 2.692) | 11 (0.091) | 0.61 | respiratory chain complex III (sensu Eukaryota) | CG4769 |
| 801 | GO:0007619 | P | 5, 6, | 2 | 1.114 (x 1.795) | 33 (0.061) | 0.61 | courtship behavior | fru tko |
| 802 | GO:0008307 | F | 3, | 1 | 0.371 (x 2.692) | 11 (0.091) | 0.61 | structural constituent of muscle | Mhc |
| 803 | GO:0008293 | P | 8, | 2 | 1.114 (x 1.795) | 33 (0.061) | 0.611 | torso signaling pathway | CG9358 sog |
| 804 | GO:0006458 | P | 8, | 1 | 0.371 (x 2.692) | 11 (0.091) | 0.611 | 'de novo' protein folding | l(3)01239 |
| 805 | GO:0045285 | C | 3, 4, 5, 6, 7, | 1 | 0.371 (x 2.692) | 11 (0.091) | 0.612 | ubiquinol-cytochrome-c reductase complex | CG4769 |
| 806 | GO:0048065 | P | 8, 9, | 1 | 0.371 (x 2.692) | 11 (0.091) | 0.613 | male courtship behavior (sensu Insecta), wing extension | fru |
| 807 | GO:0016667 | F | 4, | 1 | 0.371 (x 2.692) | 11 (0.091) | 0.613 | oxidoreductase activity, acting on sulfur group of donors | CG17843 |
| 808 | GO:0042060 | P | 5, | 1 | 0.371 (x 2.692) | 11 (0.091) | 0.614 | wound healing | puc |
| 809 | GO:0006790 | P | 5, | 3 | 1.992 (x 1.506) | 59 (0.051) | 0.614 | sulfur metabolism | Act57B Gclc Sulf1 |
| 810 | GO:0006446 | P | 8, 9, 10, | 1 | 0.371 (x 2.692) | 11 (0.091) | 0.615 | regulation of translational initiation | Thor |
| 811 | GO:0006413 | P | 8, 9, | 3 | 1.992 (x 1.506) | 59 (0.051) | 0.615 | translational initiation | CG10306 Thor eIF6 |
| 812 | GO:0045182 | F | 2, | 4 | 2.870 (x 1.394) | 85 (0.047) | 0.616 | translation regulator activity | CG10306 CG33158 HBS1 eIF6 |
| 813 | GO:0008345 | P | 5, | 1 | 0.371 (x 2.692) | 11 (0.091) | 0.616 | larval locomotory behavior | ppk |
| 814 | GO:0051049 | P | 5, 6, | 3 | 1.992 (x 1.506) | 59 (0.051) | 0.616 | regulation of transport | Arf84F CG2885 R |
| 815 | GO:0005667 | C | 3, 6, 7, 8, 9, 10, 11, 12, 13, | 4 | 2.870 (x 1.394) | 85 (0.047) | 0.616 | transcription factor complex | Pcaf Ssb-c31a Taf12L hay |
| 816 | GO:0006383 | P | 8, | 1 | 0.371 (x 2.692) | 11 (0.091) | 0.616 | transcription from RNA polymerase III promoter | CG7339 |
| 817 | GO:0008610 | P | 5, 6, 7, | 4 | 2.870 (x 1.394) | 85 (0.047) | 0.617 | lipid biosynthesis | CG7842 CG8630 Hmgcr desat1 |
| 818 | GO:0045275 | C | 3, 4, 5, 6, | 1 | 0.371 (x 2.692) | 11 (0.091) | 0.617 | respiratory chain complex III | CG4769 |
| 819 | GO:0005576 | C | 2, | 15 | 13.034 (x 1.151) | 386 (0.039) | 0.617 | extracellular region | CG13586 CG2989 CG8756 Chit Gasp Idgf2 Idgf3 Lcp65Ag1 Mmp1 Tsf1 Tsf3 cher dlp os trol |
| 820 | GO:0042168 | P | 6, 7, | 1 | 0.371 (x 2.692) | 11 (0.091) | 0.618 | heme metabolism | CG5037 |
| 821 | GO:0050875 | P | 3, | 214 | 209.896 (x 1.020) | 6216 (0.034) | 0.618 | cellular physiological process | Aats-leu Act57B Aldh Aly Arf84F Arr2 Ate1 BEST:CK01227 BG:DS03431.1 BcDNA:LD23830 BcDNA:LD32788 Bmcp CAH1 CAH2 CG10214 CG10306 CG10444 CG10445 CG10638 CG10861 CG10877 CG1134 CG11837 CG11897 CG11907 CG12264 CG12876 CG12954 CG1299 CG13645 CG13692 CG14869 CG17108 CG1718 CG17227 CG17843 CG18522 CG1882 CG2246 CG2277 CG2453 CG2885 CG2989 CG3008 CG3036 CG31373 CG31611 CG3168 CG31793 CG3192 CG32409 CG32549 CG32649 CG33002 CG33158 CG3803 CG3860 CG40006 CG4096 CG4364 CG4511 CG4769 CG4866 CG4933 CG5037 CG5044 CG5162 CG5177 CG5205 CG5317 CG5338 CG5535 CG5548 CG5558 CG5703 CG5800 CG5946 CG6204 CG6272 CG6330 CG6512 CG6574 CG6610 CG6764 CG6803 CG6876 CG6950 CG7014 CG7163 CG7188 CG7339 CG7571 CG7627 CG7722 CG7791 CG7828 CG7842 CG7955 CG8004 CG8199 CG8226 CG8327 CG8630 CG8756 CG8798 CG9273 CG9358 CG9372 CG9460 CG9790 CG9862 Chit DDB1 Dbp80 Dgp-1 EG:52C10.1 EP2237 Ets21C Fdxh Gasp Gclc Gdh Gs2 GstE1 GstE3 GstE5 GstE6 GstE7 HBS1 HLHmgamma Hmgcr Ide Imp ImpL3 Irbp Jafrac1 JhI-1 Jheh3 Ku80 La LamC Las Mhc Mlc1 Mlp60A Mmp1 Mocs1 Nmd3 Nxt1 Obp83g Pcaf Pdsw Ptp61F R REG Rep RpA-70 RpL17A RpS4 Ssb-c31a Ssrp Sulf1 TMS1 Taf12L TepIV Thor Tim10 Tim9a Traf1 Tsf1 Tsf3 Ugt86Da Ugt86Di W ab agt apt bcn92 cher cul-2 desat1 dia eIF6 eiger fru ftz-f1 glob1 hay huntingtin l(3)01239 loco mRpL22 mRpL22-24 mRpS14 mRpS18a mRpS21 mRpS33 mfas mirr mre11 mus205 na os ppk puc rad50 rpr sda tko toy trol unc-13 wun wun2 |
| 822 | GO:0007369 | P | 5, | 3 | 1.958 (x 1.532) | 58 (0.052) | 0.618 | gastrulation | CG9358 Ptp61F sog |
| 823 | GO:0030509 | P | 8, | 1 | 0.371 (x 2.692) | 11 (0.091) | 0.619 | BMP signaling pathway | sog |
| 824 | GO:0051225 | P | 7, 10, | 1 | 0.371 (x 2.692) | 11 (0.091) | 0.619 | spindle assembly | dia |
| 825 | GO:0046668 | P | 6, 7, 8, | 1 | 0.371 (x 2.692) | 11 (0.091) | 0.62 | regulation of retinal programmed cell death | W |
| 826 | GO:0006779 | P | 7, | 1 | 0.371 (x 2.692) | 11 (0.091) | 0.621 | porphyrin biosynthesis | CG5037 |
| 827 | GO:0005245 | F | 6, 7, 8, | 1 | 0.371 (x 2.692) | 11 (0.091) | 0.622 | voltage-gated calcium channel activity | na |
| 828 | GO:0007390 | P | 6, | 1 | 0.371 (x 2.692) | 11 (0.091) | 0.623 | germ-band shortening | EP2237 |
| 829 | GO:0007249 | P | 7, | 1 | 0.371 (x 2.692) | 11 (0.091) | 0.623 | I-kappaB kinase/NF-kappaB cascade | Traf1 |
| 830 | GO:0007440 | P | 7, 8, | 1 | 0.405 (x 2.468) | 12 (0.083) | 0.624 | foregut morphogenesis | os |
| 831 | GO:0003774 | F | 2, | 4 | 2.938 (x 1.362) | 87 (0.046) | 0.624 | motor activity | Act57B Mhc Mlc1 Mlc2 |
| 832 | GO:0000915 | P | 7, 11, | 1 | 0.405 (x 2.468) | 12 (0.083) | 0.624 | cytokinesis, contractile ring formation | dia |
| 833 | GO:0051301 | P | 4, | 6 | 4.694 (x 1.278) | 139 (0.043) | 0.625 | cell division | Act57B Mhc R dia os trol |
| 834 | GO:0046667 | P | 7, 8, 9, | 1 | 0.405 (x 2.468) | 12 (0.083) | 0.625 | retinal cell programmed cell death (sensu Endopterygota) | W |
| 835 | GO:0042559 | P | 7, | 1 | 0.405 (x 2.468) | 12 (0.083) | 0.626 | pteridine and derivative biosynthesis | Mocs1 |
| 836 | GO:0007015 | P | 9, | 3 | 2.026 (x 1.481) | 60 (0.050) | 0.626 | actin filament organization | cher dia puc |
| 837 | GO:0043087 | P | 5, | 1 | 0.405 (x 2.468) | 12 (0.083) | 0.627 | regulation of GTPase activity | Rep |
| 838 | GO:0007611 | P | 4, | 3 | 2.026 (x 1.481) | 60 (0.050) | 0.627 | learning and/or memory | BG:DS01219.1 CG10460 cher |
| 839 | GO:0051184 | F | 3, | 1 | 0.405 (x 2.468) | 12 (0.083) | 0.627 | cofactor transporter activity | CG7955 |
| 840 | GO:0008067 | F | 6, | 1 | 0.405 (x 2.468) | 12 (0.083) | 0.628 | metabotropic glutamate, GABA-B-like receptor activity | GABA-B-R2 |
| 841 | GO:0009084 | P | 8, 9, | 1 | 0.405 (x 2.468) | 12 (0.083) | 0.629 | glutamine family amino acid biosynthesis | Gs2 |
| 842 | GO:0000912 | P | 6, 10, | 1 | 0.405 (x 2.468) | 12 (0.083) | 0.63 | cytokinesis, formation of actomyosin apparatus | dia |
| 843 | GO:0000003 | P | 2, | 19 | 16.917 (x 1.123) | 501 (0.038) | 0.63 | reproduction | CG5162 CG9358 Cp36 Gdh Hmgcr Ptp61F R Taf12L cher dia fru ken l(3)01239 loco os puc tko wun wun2 |
| 844 | GO:0051242 | P | 5, | 7 | 5.639 (x 1.241) | 167 (0.042) | 0.63 | positive regulation of cellular physiological process | CG12876 EP2237 Traf1 W eiger mirr rpr |
| 845 | GO:0016082 | P | 8, 9, 10, | 1 | 0.405 (x 2.468) | 12 (0.083) | 0.63 | synaptic vesicle priming | unc-13 |
| 846 | GO:0030246 | F | 3, | 5 | 3.816 (x 1.310) | 113 (0.044) | 0.631 | carbohydrate binding | CG2989 CG3244 CG4115 CG8756 Gasp |
| 847 | GO:0006118 | P | 6, | 13 | 11.244 (x 1.156) | 333 (0.039) | 0.631 | electron transport | CG17843 CG18522 CG3192 CG32649 CG3803 CG4511 CG4769 CG5548 CG5703 CG5946 Pdsw TMS1 bcn92 |
| 848 | GO:0016638 | F | 4, | 1 | 0.405 (x 2.468) | 12 (0.083) | 0.631 | oxidoreductase activity, acting on the CH-NH2 group of donors | Gdh |
| 849 | GO:0044255 | P | 5, 6, | 13 | 11.312 (x 1.149) | 335 (0.039) | 0.631 | cellular lipid metabolism | CG10877 CG3860 CG5044 CG5162 CG5946 CG7842 CG8199 CG8630 Hmgcr Jheh3 Ugt86Da Ugt86Di desat1 |
| 850 | GO:0005783 | C | 5, 6, 7, 8, | 5 | 3.816 (x 1.310) | 113 (0.044) | 0.632 | endoplasmic reticulum | CG6512 CG8630 Hmgcr Sulf1 desat1 |
| 851 | GO:0008589 | P | 5, 6, 7, | 1 | 0.405 (x 2.468) | 12 (0.083) | 0.632 | regulation of smoothened signaling pathway | dlp |
| 852 | GO:0016198 | P | 5, 7, 8, 10, 11, 13, | 1 | 0.405 (x 2.468) | 12 (0.083) | 0.633 | axon choice point recognition | ab |
| 853 | GO:0000123 | C | 4, 7, 8, 9, 10, 11, 12, 13, 14, | 1 | 0.405 (x 2.468) | 12 (0.083) | 0.633 | histone acetyltransferase complex | Pcaf |
| 854 | GO:0000212 | P | 6, 10, | 1 | 0.405 (x 2.468) | 12 (0.083) | 0.634 | meiotic spindle organization and biogenesis | dia |
| 855 | GO:0008318 | F | 6, | 1 | 0.405 (x 2.468) | 12 (0.083) | 0.635 | protein prenyltransferase activity | Rep |
| 856 | GO:0043119 | P | 4, | 7 | 5.707 (x 1.227) | 169 (0.041) | 0.635 | positive regulation of physiological process | CG12876 EP2237 Traf1 W eiger mirr rpr |
| 857 | GO:0031032 | P | 9, | 1 | 0.405 (x 2.468) | 12 (0.083) | 0.636 | actomyosin structure organization and biogenesis | dia |
| 858 | GO:0006399 | P | 7, | 4 | 2.972 (x 1.346) | 88 (0.045) | 0.636 | tRNA metabolism | Aats-leu CG7163 JhI-1 La |
| 859 | GO:0005635 | C | 4, 5, 6, 7, 8, 9, 10, | 3 | 2.094 (x 1.433) | 62 (0.048) | 0.64 | nuclear envelope | GV1 LamC Nxt1 |
| 860 | GO:0000166 | F | 3, | 32 | 29.648 (x 1.079) | 878 (0.036) | 0.64 | nucleotide binding | Aats-leu Arf84F BEST:CK01227 BcDNA:LD23830 BcDNA:LD32788 CG10445 CG11897 CG13692 CG1582 CG1718 CG17227 CG17904 CG2885 CG3168 CG31793 CG33158 CG4511 CG4858 CG5205 CG5800 CG6512 CG7627 CG7955 CG8798 Dbp80 Dgp-1 HBS1 Mhc R hay mus205 rad50 |
| 861 | GO:0030532 | C | 4, 5, 6, 7, 8, 9, 10, | 3 | 2.094 (x 1.433) | 62 (0.048) | 0.64 | small nuclear ribonucleoprotein complex | CG31922 CG5205 CG6610 |
| 862 | GO:0007582 | P | 2, | 234 | 230.697 (x 1.014) | 6832 (0.034) | 0.644 | physiological process | Aats-leu Act57B Aldh Aly Arf84F Arr2 Ate1 BEST:CK01227 BG:DS01219.1 BG:DS03431.1 BcDNA:LD23830 BcDNA:LD32788 Bmcp CAH1 CAH2 CG10214 CG10306 CG10444 CG10445 CG10638 CG10861 CG10877 CG1134 CG11837 CG11897 CG11907 CG12264 CG12876 CG12954 CG1299 CG13645 CG13692 CG14869 CG14935 CG15629 CG17108 CG1718 CG17227 CG17843 CG18522 CG1882 CG2064 CG2065 CG2246 CG2277 CG2453 CG2885 CG2989 CG3008 CG3036 CG31373 CG31611 CG3168 CG31793 CG3192 CG32409 CG32549 CG32649 CG33002 CG33158 CG3803 CG3860 CG40006 CG4096 CG4267 CG4364 CG4511 CG4769 CG4866 CG4917 CG4933 CG5037 CG5044 CG5162 CG5177 CG5205 CG5317 CG5338 CG5535 CG5548 CG5558 CG5703 CG5800 CG5946 CG6204 CG6272 CG6330 CG6512 CG6574 CG6610 CG6764 CG6803 CG6876 CG6950 CG7014 CG7163 CG7188 CG7339 CG7571 CG7627 CG7722 CG7791 CG7828 CG7842 CG7955 CG7997 CG8004 CG8199 CG8226 CG8327 CG8630 CG8756 CG8798 CG9273 CG9358 CG9372 CG9460 CG9790 CG9836 CG9862 Chit DDB1 Dbp80 Dgp-1 EG:52C10.1 EP2237 Ets21C Fdxh GABA-B-R2 Gasp Gclc Gdh Gr39a Gs2 GstE1 GstE3 GstE5 GstE6 GstE7 HBS1 HLHmgamma Hmgcr Ide Idgf2 Idgf3 Imp ImpL3 Irbp Jafrac1 JhI-1 Jheh3 Ku80 La LamC Las Lcp65Ag1 Mhc Mlc1 Mlc2 Mlp60A Mmp1 Mocs1 Nmd3 Nxt1 Obp83g Pcaf Pdsw Ptp61F R REG Rep RpA-70 RpL17A RpS4 Ssb-c31a Ssrp Sulf1 TMS1 Taf12L TepIV Thor Tim10 Tim9a TotA TpnC41C Traf1 Tsf1 Tsf3 Tsp42El Ugt86Da Ugt86Di W ab agt apt bcn92 cher cul-2 desat1 dia eIF6 eiger fru ftz-f1 glob1 hay huntingtin ken l(3)01239 loco mRpL22 mRpL22-24 mRpS14 mRpS18a mRpS21 mRpS33 mfas mirr mre11 mus205 na os ppk puc rad50 rpr sda tko toy trol unc-13 wun wun2 wupA |
| 863 | GO:0004558 | F | 7, | 1 | 0.439 (x 2.278) | 13 (0.077) | 0.648 | alpha-glucosidase activity | CG14935 |
| 864 | GO:0045448 | P | 4, 6, | 1 | 0.439 (x 2.278) | 13 (0.077) | 0.649 | mitotic cell cycle, embryonic | dia |
| 865 | GO:0006461 | P | 6, | 5 | 3.951 (x 1.266) | 117 (0.043) | 0.649 | protein complex assembly | CG31611 CG3803 CG4364 CG6803 dia |
| 866 | GO:0051276 | P | 6, | 7 | 5.808 (x 1.205) | 172 (0.041) | 0.65 | chromosome organization and biogenesis | CG31611 Irbp Ku80 Ssrp fru mre11 rad50 |
| 867 | GO:0005416 | F | 6, 7, 8, | 1 | 0.439 (x 2.278) | 13 (0.077) | 0.65 | cation:amino acid symporter activity | BG:DS03431.1 |
| 868 | GO:0006778 | P | 6, | 1 | 0.439 (x 2.278) | 13 (0.077) | 0.651 | porphyrin metabolism | CG5037 |
| 869 | GO:0005689 | C | 5, 6, 7, 8, 9, 10, 11, | 1 | 0.439 (x 2.278) | 13 (0.077) | 0.651 | minor (U12-dependent) spliceosome complex | CG5205 |
| 870 | GO:0017145 | P | 5, | 2 | 1.249 (x 1.601) | 37 (0.054) | 0.652 | stem cell division | os trol |
| 871 | GO:0016566 | F | 4, | 1 | 0.439 (x 2.278) | 13 (0.077) | 0.652 | specific transcriptional repressor activity | HLHmgamma |
| 872 | GO:0007428 | P | 5, 6, | 1 | 0.439 (x 2.278) | 13 (0.077) | 0.653 | primary tracheal branching (sensu Insecta) | apt |
| 873 | GO:0016893 | F | 7, | 1 | 0.439 (x 2.278) | 13 (0.077) | 0.654 | endonuclease activity, active with either ribo- or deoxyribonucleic acids and producing 5'-phosphomonoesters | JhI-1 |
| 874 | GO:0008061 | F | 5, | 3 | 2.127 (x 1.410) | 63 (0.048) | 0.654 | chitin binding | CG2989 CG8756 Gasp |
| 875 | GO:0007616 | P | 6, | 1 | 0.439 (x 2.278) | 13 (0.077) | 0.654 | long-term memory | CG10460 |
| 876 | GO:0042558 | P | 6, | 1 | 0.439 (x 2.278) | 13 (0.077) | 0.655 | pteridine and derivative metabolism | Mocs1 |
| 877 | GO:0046328 | P | 5, 6, 8, 9, | 1 | 0.439 (x 2.278) | 13 (0.077) | 0.656 | regulation of JNK cascade | puc |
| 878 | GO:0007539 | P | 5, | 1 | 0.439 (x 2.278) | 13 (0.077) | 0.657 | primary sex determination, soma | os |
| 879 | GO:0016779 | F | 5, | 4 | 3.073 (x 1.302) | 91 (0.044) | 0.661 | nucleotidyltransferase activity | CG13645 CG7163 CG7339 mus205 |
| 880 | GO:0006916 | P | 8, 9, | 2 | 1.283 (x 1.559) | 38 (0.053) | 0.661 | anti-apoptosis | CG7188 Mmp1 |
| 881 | GO:0019731 | P | 6, 7, 8, | 2 | 1.283 (x 1.559) | 38 (0.053) | 0.662 | antibacterial humoral response | TepIV Thor |
| 882 | GO:0004556 | F | 7, | 1 | 0.473 (x 2.115) | 14 (0.071) | 0.67 | alpha-amylase activity | CG14935 |
| 883 | GO:0016197 | P | 6, 7, 8, | 1 | 0.473 (x 2.115) | 14 (0.071) | 0.671 | endosome transport | Arf84F |
| 884 | GO:0006769 | P | 8, 9, | 1 | 0.473 (x 2.115) | 14 (0.071) | 0.672 | nicotinamide metabolism | CG13645 |
| 885 | GO:0006997 | P | 6, | 1 | 0.473 (x 2.115) | 14 (0.071) | 0.673 | nuclear organization and biogenesis | LamC |
| 886 | GO:0030166 | P | 7, 8, | 1 | 0.473 (x 2.115) | 14 (0.071) | 0.673 | proteoglycan biosynthesis | Act57B |
| 887 | GO:0016160 | F | 6, | 1 | 0.473 (x 2.115) | 14 (0.071) | 0.674 | amylase activity | CG14935 |
| 888 | GO:0008234 | F | 5, | 4 | 3.140 (x 1.274) | 93 (0.043) | 0.675 | cysteine-type peptidase activity | CG5317 CG6357 Mlc1 isopeptidase-T-3 |
| 889 | GO:0051169 | P | 6, 7, 8, | 3 | 2.229 (x 1.346) | 66 (0.045) | 0.675 | nuclear transport | La Nmd3 Nxt1 |
| 890 | GO:0019438 | P | 6, | 1 | 0.473 (x 2.115) | 14 (0.071) | 0.675 | aromatic compound biosynthesis | Mocs1 |
| 891 | GO:0019725 | P | 4, | 3 | 2.229 (x 1.346) | 66 (0.045) | 0.676 | cell homeostasis | Jafrac1 Tsf1 Tsf3 |
| 892 | GO:0045034 | P | 6, 7, 10, | 1 | 0.473 (x 2.115) | 14 (0.071) | 0.676 | neuroblast division | trol |
| 893 | GO:0008289 | F | 3, | 3 | 2.229 (x 1.346) | 66 (0.045) | 0.676 | lipid binding | CG7054 CG9358 unc-13 |
| 894 | GO:0006112 | P | 7, | 1 | 0.473 (x 2.115) | 14 (0.071) | 0.676 | energy reserve metabolism | CG5177 |
| 895 | GO:0051336 | P | 4, | 1 | 0.473 (x 2.115) | 14 (0.071) | 0.677 | regulation of hydrolase activity | Rep |
| 896 | GO:0042401 | P | 7, 8, | 1 | 0.473 (x 2.115) | 14 (0.071) | 0.678 | biogenic amine biosynthesis | CG8327 |
| 897 | GO:0000175 | F | 8, 9, | 1 | 0.473 (x 2.115) | 14 (0.071) | 0.679 | 3'-5'-exoribonuclease activity | CG10214 |
| 898 | GO:0005279 | F | 5, 6, | 2 | 1.317 (x 1.519) | 39 (0.051) | 0.679 | amino acid-polyamine transporter activity | BG:DS03431.1 CG5535 |
| 899 | GO:0046660 | P | 4, | 1 | 0.473 (x 2.115) | 14 (0.071) | 0.679 | female sex differentiation | ken |
| 900 | GO:0006096 | P | 8, 10, 11, | 2 | 1.317 (x 1.519) | 39 (0.051) | 0.68 | glycolysis | DDB1 ImpL3 |
| 901 | GO:0008355 | P | 6, 7, | 2 | 1.317 (x 1.519) | 39 (0.051) | 0.681 | olfactory learning | BG:DS01219.1 cher |
| 902 | GO:0015203 | F | 4, | 2 | 1.317 (x 1.519) | 39 (0.051) | 0.681 | polyamine transporter activity | BG:DS03431.1 CG5535 |
| 903 | GO:0004004 | F | 5, 11, | 2 | 1.351 (x 1.481) | 40 (0.050) | 0.685 | ATP-dependent RNA helicase activity | CG31755 Dbp80 |
| 904 | GO:0019207 | F | 3, | 3 | 2.262 (x 1.326) | 67 (0.045) | 0.686 | kinase regulator activity | CG7054 CG9790 puc |
| 905 | GO:0015674 | P | 7, 8, | 2 | 1.351 (x 1.481) | 40 (0.050) | 0.686 | di-, tri-valent inorganic cation transport | Tsf1 Tsf3 |
| 906 | GO:0044275 | P | 7, | 3 | 2.262 (x 1.326) | 67 (0.045) | 0.686 | cellular carbohydrate catabolism | Chit DDB1 ImpL3 |
| 907 | GO:0008186 | F | 10, | 2 | 1.351 (x 1.481) | 40 (0.050) | 0.687 | RNA-dependent ATPase activity | CG31755 Dbp80 |
| 908 | GO:0016052 | P | 6, | 3 | 2.262 (x 1.326) | 67 (0.045) | 0.687 | carbohydrate catabolism | Chit DDB1 ImpL3 |
| 909 | GO:0016542 | P | 7, 8, | 1 | 0.507 (x 1.974) | 15 (0.067) | 0.687 | male courtship behavior (sensu Insecta) | fru |
| 910 | GO:0030247 | F | 4, | 3 | 2.262 (x 1.326) | 67 (0.045) | 0.688 | polysaccharide binding | CG2989 CG8756 Gasp |
| 911 | GO:0006090 | P | 7, | 1 | 0.507 (x 1.974) | 15 (0.067) | 0.688 | pyruvate metabolism | Aldh |
| 912 | GO:0035215 | P | 5, | 1 | 0.507 (x 1.974) | 15 (0.067) | 0.689 | genital disc development | ken |
| 913 | GO:0004693 | F | 8, | 1 | 0.507 (x 1.974) | 15 (0.067) | 0.689 | cyclin-dependent protein kinase activity | CG9790 |
| 914 | GO:0007538 | P | 4, | 1 | 0.507 (x 1.974) | 15 (0.067) | 0.69 | primary sex determination | os |
| 915 | GO:0005684 | C | 5, 6, 7, 8, 9, 10, 11, | 2 | 1.384 (x 1.445) | 41 (0.049) | 0.691 | major (U2-dependent) spliceosome | CG5205 CG6610 |
| 916 | GO:0000786 | C | 3, 5, 6, 7, 8, 9, 10, 11, | 1 | 0.507 (x 1.974) | 15 (0.067) | 0.691 | nucleosome | CG31611 |
| 917 | GO:0006029 | P | 7, | 1 | 0.507 (x 1.974) | 15 (0.067) | 0.692 | proteoglycan metabolism | Act57B |
| 918 | GO:0043648 | P | 7, | 1 | 0.507 (x 1.974) | 15 (0.067) | 0.692 | dicarboxylic acid metabolism | Gdh |
| 919 | GO:0007450 | P | 5, 6, | 1 | 0.507 (x 1.974) | 15 (0.067) | 0.693 | dorsal/ventral pattern formation, imaginal disc | dlp |
| 920 | GO:0005852 | C | 3, 4, 5, 6, 7, 8, 9, | 1 | 0.507 (x 1.974) | 15 (0.067) | 0.694 | eukaryotic translation initiation factor 3 complex | CG10306 |
| 921 | GO:0016820 | F | 5, | 6 | 5.133 (x 1.169) | 152 (0.039) | 0.694 | hydrolase activity, acting on acid anhydrides, catalyzing transmembrane movement of substances | BEST:CK01227 CG11897 CG1718 CG31793 CG7627 CG7955 |
| 922 | GO:0016324 | C | 4, 5, 6, 7, | 1 | 0.507 (x 1.974) | 15 (0.067) | 0.695 | apical plasma membrane | sas |
| 923 | GO:0043492 | F | 3, 10, | 6 | 5.099 (x 1.177) | 151 (0.040) | 0.695 | ATPase activity, coupled to movement of substances | BEST:CK01227 CG11897 CG1718 CG31793 CG7627 CG7955 |
| 924 | GO:0051052 | P | 7, | 1 | 0.507 (x 1.974) | 15 (0.067) | 0.695 | regulation of DNA metabolism | Ssrp |
| 925 | GO:0042626 | F | 4, 6, 11, | 6 | 5.099 (x 1.177) | 151 (0.040) | 0.696 | ATPase activity, coupled to transmembrane movement of substances | BEST:CK01227 CG11897 CG1718 CG31793 CG7627 CG7955 |
| 926 | GO:0019362 | P | 7, 8, | 1 | 0.507 (x 1.974) | 15 (0.067) | 0.696 | pyridine nucleotide metabolism | CG13645 |
| 927 | GO:0007492 | P | 4, | 1 | 0.540 (x 1.851) | 16 (0.062) | 0.701 | endoderm development | toy |
| 928 | GO:0045171 | C | 3, 4, | 1 | 0.540 (x 1.851) | 16 (0.062) | 0.702 | intercellular bridge | cher |
| 929 | GO:0046666 | P | 6, 7, | 1 | 0.540 (x 1.851) | 16 (0.062) | 0.703 | retinal cell programmed cell death | W |
| 930 | GO:0017076 | F | 4, | 30 | 28.668 (x 1.046) | 849 (0.035) | 0.703 | purine nucleotide binding | Aats-leu Arf84F BEST:CK01227 BcDNA:LD23830 BcDNA:LD32788 CG10445 CG11897 CG13692 CG1582 CG1718 CG17227 CG2885 CG3168 CG31793 CG33158 CG4511 CG4858 CG5205 CG5800 CG6512 CG7627 CG7955 CG8798 Dbp80 Dgp-1 HBS1 Mhc R hay rad50 |
| 931 | GO:0007399 | P | 4, | 17 | 15.803 (x 1.076) | 468 (0.036) | 0.703 | nervous system development | BG:DS02740.9 Gs2 HLHmgamma Ptp61F Tsp42El ab apt fru loco melt mfas mirr puc rpr toy trol wupA |
| 932 | GO:0009586 | P | 6, 7, 8, | 1 | 0.540 (x 1.851) | 16 (0.062) | 0.703 | rhodopsin mediated phototransduction | Arr2 |
| 933 | GO:0008334 | P | 8, | 1 | 0.540 (x 1.851) | 16 (0.062) | 0.704 | histone mRNA metabolism | Nmd3 |
| 934 | GO:0016791 | F | 6, | 7 | 6.146 (x 1.139) | 182 (0.038) | 0.705 | phosphoric monoester hydrolase activity | CG2277 CG32549 CG5177 Ptp61F puc wun wun2 |
| 935 | GO:0043066 | P | 7, 8, | 2 | 1.418 (x 1.410) | 42 (0.048) | 0.705 | negative regulation of apoptosis | CG7188 Mmp1 |
| 936 | GO:0042803 | F | 5, | 1 | 0.540 (x 1.851) | 16 (0.062) | 0.705 | protein homodimerization activity | wun |
| 937 | GO:0030036 | P | 8, | 4 | 3.275 (x 1.221) | 97 (0.041) | 0.705 | actin cytoskeleton organization and biogenesis | CG6803 cher dia puc |
| 938 | GO:0043069 | P | 6, 7, | 2 | 1.418 (x 1.410) | 42 (0.048) | 0.706 | negative regulation of programmed cell death | CG7188 Mmp1 |
| 939 | GO:0009451 | P | 7, | 1 | 0.540 (x 1.851) | 16 (0.062) | 0.706 | RNA modification | CG11837 |
| 940 | GO:0030029 | P | 7, | 4 | 3.275 (x 1.221) | 97 (0.041) | 0.706 | actin filament-based process | CG6803 cher dia puc |
| 941 | GO:0007306 | P | 9, 10, | 2 | 1.418 (x 1.410) | 42 (0.048) | 0.706 | insect chorion formation | Cp36 puc |
| 942 | GO:0030537 | P | 4, | 1 | 0.540 (x 1.851) | 16 (0.062) | 0.706 | larval behavior | ppk |
| 943 | GO:0043062 | P | 3, | 2 | 1.418 (x 1.410) | 42 (0.048) | 0.707 | extracellular structure organization and biogenesis | Gs2 cher |
| 944 | GO:0045172 | C | 4, 5, | 1 | 0.540 (x 1.851) | 16 (0.062) | 0.707 | ring canal (sensu Insecta) | cher |
| 945 | GO:0016538 | F | 5, | 1 | 0.540 (x 1.851) | 16 (0.062) | 0.708 | cyclin-dependent protein kinase regulator activity | CG9790 |
| 946 | GO:0042398 | P | 6, 7, | 1 | 0.540 (x 1.851) | 16 (0.062) | 0.709 | amino acid derivative biosynthesis | CG8327 |
| 947 | GO:0044454 | C | 5, 6, 7, 8, 9, 10, 11, | 2 | 1.452 (x 1.377) | 43 (0.047) | 0.709 | nuclear chromosome part | CG9273 RpA-70 |
| 948 | GO:0042802 | F | 4, | 1 | 0.540 (x 1.851) | 16 (0.062) | 0.709 | identical protein binding | wun |
| 949 | GO:0007612 | P | 5, | 2 | 1.452 (x 1.377) | 43 (0.047) | 0.709 | learning | BG:DS01219.1 cher |
| 950 | GO:0000152 | C | 4, 5, 6, 7, 8, 9, 10, | 1 | 0.540 (x 1.851) | 16 (0.062) | 0.71 | nuclear ubiquitin ligase complex | cul-2 |
| 951 | GO:0006398 | P | 9, 10, | 1 | 0.540 (x 1.851) | 16 (0.062) | 0.711 | histone mRNA 3'-end processing | Nmd3 |
| 952 | GO:0035282 | P | 3, | 5 | 4.255 (x 1.175) | 126 (0.040) | 0.711 | segmentation | CG9358 dlp ftz-f1 os sog |
| 953 | GO:0007367 | P | 6, 7, | 1 | 0.540 (x 1.851) | 16 (0.062) | 0.712 | segment polarity determination | dlp |
| 954 | GO:0004867 | F | 6, | 3 | 2.364 (x 1.269) | 70 (0.043) | 0.712 | serine-type endopeptidase inhibitor activity | CG40006 CG7722 CG9460 |
| 955 | GO:0005516 | F | 4, | 3 | 2.397 (x 1.251) | 71 (0.042) | 0.712 | calmodulin binding | Mlc2 TpnC41C unc-13 |
| 956 | GO:0006913 | P | 6, 7, 8, | 3 | 2.397 (x 1.251) | 71 (0.042) | 0.713 | nucleocytoplasmic transport | La Nmd3 Nxt1 |
| 957 | GO:0046843 | P | 10, 11, | 1 | 0.574 (x 1.742) | 17 (0.059) | 0.718 | dorsal appendage formation | puc |
| 958 | GO:0051028 | P | 7, 8, 9, | 1 | 0.574 (x 1.742) | 17 (0.059) | 0.719 | mRNA transport | Nxt1 |
| 959 | GO:0018993 | P | 4, | 1 | 0.574 (x 1.742) | 17 (0.059) | 0.72 | somatic sex determination | os |
| 960 | GO:0007259 | P | 7, | 1 | 0.574 (x 1.742) | 17 (0.059) | 0.721 | JAK-STAT cascade | os |
| 961 | GO:0008173 | F | 6, | 1 | 0.574 (x 1.742) | 17 (0.059) | 0.721 | RNA methyltransferase activity | CG11837 |
| 962 | GO:0009613 | P | 4, 5, | 4 | 3.377 (x 1.185) | 100 (0.040) | 0.722 | response to pest, pathogen or parasite | TepIV Thor TotA eiger |
| 963 | GO:0006406 | P | 8, 9, 10, 11, | 1 | 0.574 (x 1.742) | 17 (0.059) | 0.722 | mRNA export from nucleus | Nxt1 |
| 964 | GO:0006914 | P | 4, | 1 | 0.574 (x 1.742) | 17 (0.059) | 0.723 | autophagy | CG10861 |
| 965 | GO:0008586 | P | 7, 8, 9, | 1 | 0.574 (x 1.742) | 17 (0.059) | 0.724 | wing vein morphogenesis | sog |
| 966 | GO:0008652 | P | 7, 8, | 3 | 2.431 (x 1.234) | 72 (0.042) | 0.724 | amino acid biosynthesis | CG12264 CG6950 Gs2 |
| 967 | GO:0009582 | P | 4, 5, | 2 | 1.486 (x 1.346) | 44 (0.045) | 0.724 | detection of abiotic stimulus | Arr2 tko |
| 968 | GO:0005328 | F | 4, 7, 9, | 1 | 0.574 (x 1.742) | 17 (0.059) | 0.724 | neurotransmitter:sodium symporter activity | BG:DS03431.1 |
| 969 | GO:0004540 | F | 6, | 2 | 1.486 (x 1.346) | 44 (0.045) | 0.725 | ribonuclease activity | CG10214 JhI-1 |
| 970 | GO:0016334 | P | 6, | 1 | 0.574 (x 1.742) | 17 (0.059) | 0.725 | establishment and/or maintenance of polarity of follicular epithelium | loco |
| 971 | GO:0006839 | P | 6, 7, 8, | 1 | 0.574 (x 1.742) | 17 (0.059) | 0.726 | mitochondrial transport | Bmcp |
| 972 | GO:0005096 | F | 4, | 2 | 1.520 (x 1.316) | 45 (0.044) | 0.728 | GTPase activator activity | CG13692 loco |
| 973 | GO:0015293 | F | 6, | 3 | 2.465 (x 1.217) | 73 (0.041) | 0.729 | symporter activity | BG:DS03431.1 CG10444 CG3036 |
| 974 | GO:0009581 | P | 4, 5, | 2 | 1.520 (x 1.316) | 45 (0.044) | 0.729 | detection of external stimulus | Arr2 tko |
| 975 | GO:0031981 | C | 4, 5, 6, 7, 8, 9, 10, | 10 | 9.286 (x 1.077) | 275 (0.036) | 0.73 | nuclear lumen | CG6610 CG7339 EG:52C10.1 Nxt1 Pcaf Ssb-c31a Ssrp Taf12L hay mus205 |
| 976 | GO:0006960 | P | 7, 8, | 2 | 1.520 (x 1.316) | 45 (0.044) | 0.73 | antimicrobial humoral response (sensu Protostomia) | TepIV Thor |
| 977 | GO:0015290 | F | 4, | 8 | 7.327 (x 1.092) | 217 (0.037) | 0.73 | electrochemical potential-driven transporter activity | BG:DS03431.1 CG10444 CG11907 CG3036 CG5535 CG6574 CG7571 TepIV |
| 978 | GO:0015291 | F | 5, | 8 | 7.327 (x 1.092) | 217 (0.037) | 0.731 | porter activity | BG:DS03431.1 CG10444 CG11907 CG3036 CG5535 CG6574 CG7571 TepIV |
| 979 | GO:0005386 | F | 3, | 16 | 15.296 (x 1.046) | 453 (0.035) | 0.733 | carrier activity | BG:DS03431.1 Bmcp CG10444 CG11907 CG3036 CG5535 CG6574 CG7571 CG8226 CG9358 TepIV Tim10 Tim9a Tsf1 Tsf3 glob1 |
| 980 | GO:0000228 | C | 5, 6, 7, 8, 9, 10, | 2 | 1.553 (x 1.288) | 46 (0.043) | 0.734 | nuclear chromosome | CG9273 RpA-70 |
| 981 | GO:0042493 | P | 5, | 1 | 0.608 (x 1.645) | 18 (0.056) | 0.735 | response to drug | BEST:CK01227 |
| 982 | GO:0008340 | P | 4, | 2 | 1.553 (x 1.288) | 46 (0.043) | 0.735 | determination of adult life span | cher puc |
| 983 | GO:0006305 | P | 8, | 1 | 0.608 (x 1.645) | 18 (0.056) | 0.735 | DNA alkylation | trol |
| 984 | GO:0015837 | P | 5, 6, | 2 | 1.553 (x 1.288) | 46 (0.043) | 0.735 | amine transport | BG:DS03431.1 CG5535 |
| 985 | GO:0003712 | F | 3, 5, | 3 | 2.499 (x 1.201) | 74 (0.041) | 0.736 | transcription cofactor activity | Aly Ssb-c31a ftz-f1 |
| 986 | GO:0016896 | F | 8, | 1 | 0.608 (x 1.645) | 18 (0.056) | 0.736 | exoribonuclease activity, producing 5'-phosphomonoesters | CG10214 |
| 987 | GO:0007568 | P | 3, | 2 | 1.553 (x 1.288) | 46 (0.043) | 0.736 | aging | cher puc |
| 988 | GO:0007601 | P | 5, 7, | 3 | 2.499 (x 1.201) | 74 (0.041) | 0.737 | visual perception | Arr2 CG15629 CG4917 |
| 989 | GO:0006368 | P | 9, | 1 | 0.608 (x 1.645) | 18 (0.056) | 0.737 | RNA elongation from RNA polymerase II promoter | CG7339 |
| 990 | GO:0006865 | P | 6, 7, 8, | 2 | 1.553 (x 1.288) | 46 (0.043) | 0.737 | amino acid transport | BG:DS03431.1 CG5535 |
| 991 | GO:0044248 | P | 5, | 11 | 10.333 (x 1.065) | 306 (0.036) | 0.737 | cellular catabolism | Ate1 CG10214 CG6204 CG6330 CG8199 Chit DDB1 Gdh Gs2 ImpL3 Jheh3 |
| 992 | GO:0050953 | P | 4, 6, | 3 | 2.499 (x 1.201) | 74 (0.041) | 0.737 | sensory perception of light stimulus | Arr2 CG15629 CG4917 |
| 993 | GO:0006306 | P | 4, 8, 9, | 1 | 0.608 (x 1.645) | 18 (0.056) | 0.738 | DNA methylation | trol |
| 994 | GO:0009953 | P | 4, | 3 | 2.499 (x 1.201) | 74 (0.041) | 0.738 | dorsal/ventral pattern formation | dlp loco sog |
| 995 | GO:0009966 | P | 4, 5, | 4 | 3.478 (x 1.150) | 103 (0.039) | 0.738 | regulation of signal transduction | dlp loco puc sog |
| 996 | GO:0005884 | C | 5, 6, 7, 8, 9, 10, | 1 | 0.608 (x 1.645) | 18 (0.056) | 0.738 | actin filament | Act57B |
| 997 | GO:0005102 | F | 3, 4, | 10 | 9.421 (x 1.061) | 279 (0.036) | 0.742 | receptor binding | CG13586 CG2989 CG3153 Chit Idgf2 Idgf3 Traf1 eiger os sog |
| 998 | GO:0007422 | P | 5, | 3 | 2.566 (x 1.169) | 76 (0.039) | 0.748 | peripheral nervous system development | melt mirr puc |
| 999 | GO:0005529 | F | 4, | 2 | 1.587 (x 1.260) | 47 (0.043) | 0.749 | sugar binding | CG3244 CG4115 |
| 1000 | GO:0042592 | P | 3, | 3 | 2.566 (x 1.169) | 76 (0.039) | 0.749 | homeostasis | Jafrac1 Tsf1 Tsf3 |
| 1001 | GO:0007269 | P | 6, 7, 8, | 4 | 3.546 (x 1.128) | 105 (0.038) | 0.749 | neurotransmitter secretion | Arf84F CG3168 Rep unc-13 |
| 1002 | GO:0015171 | F | 4, 5, | 2 | 1.587 (x 1.260) | 47 (0.043) | 0.75 | amino acid transporter activity | BG:DS03431.1 CG5535 |
| 1003 | GO:0045055 | P | 6, 7, | 4 | 3.546 (x 1.128) | 105 (0.038) | 0.75 | regulated secretory pathway | Arf84F CG3168 Rep unc-13 |
| 1004 | GO:0006414 | P | 8, 9, | 1 | 0.642 (x 1.559) | 19 (0.053) | 0.75 | translational elongation | CG33158 |
| 1005 | GO:0003707 | F | 5, | 1 | 0.642 (x 1.559) | 19 (0.053) | 0.751 | steroid hormone receptor activity | ftz-f1 |
| 1006 | GO:0016917 | F | 5, | 1 | 0.642 (x 1.559) | 19 (0.053) | 0.751 | GABA receptor activity | GABA-B-R2 |
| 1007 | GO:0007140 | P | 8, | 1 | 0.642 (x 1.559) | 19 (0.053) | 0.752 | male meiosis | Taf12L |
| 1008 | GO:0050874 | P | 3, | 31 | 30.390 (x 1.020) | 900 (0.034) | 0.753 | organismal physiological process | Arf84F Arr2 CG15629 CG3168 CG4917 CG9358 GABA-B-R2 Gdh Gr39a Gs2 Lcp65Ag1 Mhc Mlc1 Mlc2 Obp83g Rep TepIV Thor TotA TpnC41C Tsp42El ab apt cher eiger fru ken puc tko unc-13 wupA |
| 1009 | GO:0016763 | F | 5, | 1 | 0.642 (x 1.559) | 19 (0.053) | 0.753 | transferase activity, transferring pentosyl groups | CG6330 |
| 1010 | GO:0048598 | P | 4, | 4 | 3.579 (x 1.118) | 106 (0.038) | 0.754 | embryonic morphogenesis | EP2237 R Traf1 puc |
| 1011 | GO:0001558 | P | 4, 5, 7, 8, | 1 | 0.675 (x 1.481) | 20 (0.050) | 0.759 | regulation of cell growth | Thor |
| 1012 | GO:0016311 | P | 7, | 4 | 3.647 (x 1.097) | 108 (0.037) | 0.759 | dephosphorylation | Ptp61F puc wun wun2 |
| 1013 | GO:0005654 | C | 5, 6, 7, 8, 9, 10, 11, | 8 | 7.564 (x 1.058) | 224 (0.036) | 0.759 | nucleoplasm | CG7339 Nxt1 Pcaf Ssb-c31a Ssrp Taf12L hay mus205 |
| 1014 | GO:0035222 | P | 5, 6, | 1 | 0.675 (x 1.481) | 20 (0.050) | 0.759 | wing disc pattern formation | dlp |
| 1015 | GO:0008202 | P | 6, 7, | 5 | 4.592 (x 1.089) | 136 (0.037) | 0.76 | steroid metabolism | CG3860 CG5946 Hmgcr Ugt86Da Ugt86Di |
| 1016 | GO:0006637 | P | 7, 8, | 1 | 0.675 (x 1.481) | 20 (0.050) | 0.76 | acyl-CoA metabolism | CG8199 |
| 1017 | GO:0005604 | C | 3, 4, 5, | 1 | 0.675 (x 1.481) | 20 (0.050) | 0.761 | basement membrane | trol |
| 1018 | GO:0016284 | F | 7, | 1 | 0.675 (x 1.481) | 20 (0.050) | 0.762 | alanine aminopeptidase activity | sda |
| 1019 | GO:0016028 | C | 5, 6, 7, | 1 | 0.675 (x 1.481) | 20 (0.050) | 0.762 | rhabdomere | Arr2 |
| 1020 | GO:0000160 | P | 5, | 1 | 0.675 (x 1.481) | 20 (0.050) | 0.763 | two-component signal transduction system (phosphorelay) | CG2885 |
| 1021 | GO:0006733 | P | 7, | 1 | 0.675 (x 1.481) | 20 (0.050) | 0.764 | oxidoreduction coenzyme metabolism | CG13645 |
| 1022 | GO:0008374 | F | 7, | 1 | 0.675 (x 1.481) | 20 (0.050) | 0.765 | O-acyltransferase activity | CG5397 |
| 1023 | GO:0005326 | F | 3, | 1 | 0.675 (x 1.481) | 20 (0.050) | 0.765 | neurotransmitter transporter activity | BG:DS03431.1 |
| 1024 | GO:0005732 | C | 4, 5, 6, 7, 8, 9, 10, 11, 12, 13, | 1 | 0.675 (x 1.481) | 20 (0.050) | 0.766 | small nucleolar ribonucleoprotein complex | CG6610 |
| 1025 | GO:0016318 | P | 7, 8, 9, 10, | 1 | 0.675 (x 1.481) | 20 (0.050) | 0.767 | ommatidial rotation | puc |
| 1026 | GO:0005488 | F | 2, | 140 | 139.593 (x 1.003) | 4134 (0.034) | 0.767 | binding | Aats-leu Aly Arf84F Arr2 BEST:CK01227 BG:DS01219.1 BG:DS02740.9 BcDNA:LD23830 BcDNA:LD32788 Bmcp CAH1 CAH2 CG10126 CG10214 CG10306 CG10445 CG1134 CG11837 CG11897 CG13586 CG13692 CG14869 CG1582 CG1718 CG17227 CG17904 CG2885 CG2989 CG31063 CG3153 CG31611 CG31666 CG3168 CG31793 CG31922 CG3244 CG33158 CG3371 CG3860 CG4096 CG4115 CG4364 CG4511 CG4858 CG4866 CG4933 CG5205 CG5317 CG5338 CG5558 CG5800 CG6204 CG6272 CG6512 CG6574 CG6764 CG6876 CG7014 CG7054 CG7163 CG7339 CG7447 CG7627 CG7791 CG7955 CG8630 CG8756 CG8798 CG9273 CG9358 CG9862 Chit DDB1 Dbp80 Dgp-1 EG:52C10.1 EP2237 Ets21C GV1 Gasp Gclc HBS1 HLHmgamma Ide Idgf2 Idgf3 Imp Irbp Ku80 La Las Mhc Mlc2 Mlp60A Mmp1 Mocs1 Nmd3 Obp83g PQBP-1 R RpA-70 RpL17A RpS4 Ssb-c31a Ssrp Thor TpnC41C Traf1 Tsf1 Tsf3 ab agt apt cher desat1 dia dlp eIF6 eiger fru ftz-f1 hay huntingtin ken l(3)01239 loco mRpS14 mirr mre11 mus205 os rad50 sda sog tko toy trol unc-13 wun wupA |
| 1027 | GO:0000381 | P | 10, 11, 13, | 2 | 1.655 (x 1.209) | 49 (0.041) | 0.767 | regulation of alternative nuclear mRNA splicing, via spliceosome | CG10445 hay |
| 1028 | GO:0004179 | F | 7, 8, | 1 | 0.675 (x 1.481) | 20 (0.050) | 0.768 | membrane alanyl aminopeptidase activity | sda |
| 1029 | GO:0006959 | P | 5, 6, | 3 | 2.634 (x 1.139) | 78 (0.038) | 0.768 | humoral immune response | TepIV Thor TotA |
| 1030 | GO:0016051 | P | 6, 7, | 2 | 1.655 (x 1.209) | 49 (0.041) | 0.768 | carbohydrate biosynthesis | Act57B CG5177 |
| 1031 | GO:0006396 | P | 7, | 9 | 8.611 (x 1.045) | 255 (0.035) | 0.769 | RNA processing | CG10445 CG5205 CG6610 CG6876 CG7163 JhI-1 La Nmd3 hay |
| 1032 | GO:0046983 | F | 4, | 2 | 1.655 (x 1.209) | 49 (0.041) | 0.769 | protein dimerization activity | CG6272 wun |
| 1033 | GO:0009968 | P | 5, 6, | 2 | 1.655 (x 1.209) | 49 (0.041) | 0.77 | negative regulation of signal transduction | puc sog |
| 1034 | GO:0051606 | P | 3, 4, | 2 | 1.655 (x 1.209) | 49 (0.041) | 0.77 | detection of stimulus | Arr2 tko |
| 1035 | GO:0000380 | P | 10, 12, | 2 | 1.655 (x 1.209) | 49 (0.041) | 0.771 | alternative nuclear mRNA splicing, via spliceosome | CG10445 hay |
| 1036 | GO:0008595 | P | 6, 7, | 2 | 1.688 (x 1.185) | 50 (0.040) | 0.771 | determination of anterior/posterior axis, embryo | CG9358 sog |
| 1037 | GO:0007351 | P | 5, 6, | 2 | 1.688 (x 1.185) | 50 (0.040) | 0.772 | regional subdivision | CG9358 sog |
| 1038 | GO:0001871 | F | 3, | 3 | 2.701 (x 1.111) | 80 (0.037) | 0.775 | pattern binding | CG2989 CG8756 Gasp |
| 1039 | GO:0005214 | F | 4, | 3 | 2.735 (x 1.097) | 81 (0.037) | 0.775 | structural constituent of cuticle (sensu Insecta) | CG6305 CG9077 Lcp65Ag1 |
| 1040 | GO:0004521 | F | 7, | 1 | 0.709 (x 1.410) | 21 (0.048) | 0.776 | endoribonuclease activity | JhI-1 |
| 1041 | GO:0004197 | F | 6, | 3 | 2.735 (x 1.097) | 81 (0.037) | 0.776 | cysteine-type endopeptidase activity | CG5317 CG6357 Mlc1 |
| 1042 | GO:0046164 | P | 6, | 2 | 1.722 (x 1.161) | 51 (0.039) | 0.776 | alcohol catabolism | DDB1 ImpL3 |
| 1043 | GO:0007469 | P | 6, | 1 | 0.709 (x 1.410) | 21 (0.048) | 0.776 | antennal development | W |
| 1044 | GO:0008643 | P | 5, 6, | 3 | 2.735 (x 1.097) | 81 (0.037) | 0.776 | carbohydrate transport | CG3036 CG3168 TepIV |
| 1045 | GO:0046365 | P | 7, 8, | 2 | 1.722 (x 1.161) | 51 (0.039) | 0.777 | monosaccharide catabolism | DDB1 ImpL3 |
| 1046 | GO:0009110 | P | 6, | 1 | 0.709 (x 1.410) | 21 (0.048) | 0.777 | vitamin biosynthesis | CG13645 |
| 1047 | GO:0008021 | C | 8, 9, 10, 11, 12, | 2 | 1.722 (x 1.161) | 51 (0.039) | 0.778 | synaptic vesicle | Rep unc-13 |
| 1048 | GO:0042578 | F | 5, | 7 | 6.753 (x 1.037) | 200 (0.035) | 0.778 | phosphoric ester hydrolase activity | CG2277 CG32549 CG5177 Ptp61F puc wun wun2 |
| 1049 | GO:0003755 | F | 5, | 1 | 0.709 (x 1.410) | 21 (0.048) | 0.778 | peptidyl-prolyl cis-trans isomerase activity | CG11858 |
| 1050 | GO:0006007 | P | 9, 10, | 2 | 1.722 (x 1.161) | 51 (0.039) | 0.778 | glucose catabolism | DDB1 ImpL3 |
| 1051 | GO:0005436 | F | 6, 7, 8, | 1 | 0.709 (x 1.410) | 21 (0.048) | 0.779 | sodium:phosphate symporter activity | CG3036 |
| 1052 | GO:0007178 | P | 7, | 2 | 1.722 (x 1.161) | 51 (0.039) | 0.779 | transmembrane receptor protein serine/threonine kinase signaling pathway | dlp sog |
| 1053 | GO:0016042 | P | 5, 6, | 1 | 0.709 (x 1.410) | 21 (0.048) | 0.779 | lipid catabolism | Jheh3 |
| 1054 | GO:0019320 | P | 8, 9, | 2 | 1.722 (x 1.161) | 51 (0.039) | 0.78 | hexose catabolism | DDB1 ImpL3 |
| 1055 | GO:0008138 | F | 8, | 1 | 0.709 (x 1.410) | 21 (0.048) | 0.78 | protein tyrosine/serine/threonine phosphatase activity | puc |
| 1056 | GO:0008544 | P | 5, | 1 | 0.743 (x 1.346) | 22 (0.045) | 0.78 | epidermis development | puc |
| 1057 | GO:0006520 | P | 6, 7, | 9 | 8.813 (x 1.021) | 261 (0.034) | 0.78 | amino acid metabolism | Aats-leu BG:DS03431.1 CG12264 CG5535 CG6950 CG8199 Gclc Gdh Gs2 |
| 1058 | GO:0006836 | P | 5, 6, | 1 | 0.743 (x 1.346) | 22 (0.045) | 0.781 | neurotransmitter transport | BG:DS03431.1 |
| 1059 | GO:0006519 | P | 5, | 10 | 9.894 (x 1.011) | 293 (0.034) | 0.781 | amino acid and derivative metabolism | Aats-leu BG:DS03431.1 CG12264 CG5535 CG6950 CG8199 CG8327 Gclc Gdh Gs2 |
| 1060 | GO:0044451 | C | 5, 6, 7, 8, 9, 10, 11, 12, | 7 | 6.855 (x 1.021) | 203 (0.034) | 0.782 | nucleoplasm part | CG7339 Pcaf Ssb-c31a Ssrp Taf12L hay mus205 |
| 1061 | GO:0042078 | P | 6, | 1 | 0.743 (x 1.346) | 22 (0.045) | 0.782 | germ-line stem cell division | os |
| 1062 | GO:0006635 | P | 8, 9, | 1 | 0.743 (x 1.346) | 22 (0.045) | 0.782 | fatty acid beta-oxidation | CG5044 |
| 1063 | GO:0009401 | P | 6, 7, | 1 | 0.743 (x 1.346) | 22 (0.045) | 0.783 | phosphoenolpyruvate-dependent sugar phosphotransferase system | TepIV |
| 1064 | GO:0043169 | F | 4, | 30 | 29.951 (x 1.002) | 887 (0.034) | 0.784 | cation binding | CAH1 CAH2 CG10126 CG1134 CG14869 CG31666 CG31922 CG4096 CG4933 CG7163 CG7447 CG7791 CG8630 EP2237 Ide Las Mlc2 Mlp60A Mmp1 Mocs1 TpnC41C Traf1 Tsf1 Tsf3 ab desat1 fru ken mre11 sda |
| 1065 | GO:0003727 | F | 5, | 1 | 0.743 (x 1.346) | 22 (0.045) | 0.784 | single-stranded RNA binding | Ssrp |
| 1066 | GO:0044265 | P | 6, | 6 | 5.808 (x 1.033) | 172 (0.035) | 0.784 | cellular macromolecule catabolism | Ate1 CG10214 CG6204 Chit DDB1 ImpL3 |
| 1067 | GO:0006354 | P | 8, | 1 | 0.743 (x 1.346) | 22 (0.045) | 0.785 | RNA elongation | CG7339 |
| 1068 | GO:0031267 | F | 6, | 1 | 0.743 (x 1.346) | 22 (0.045) | 0.785 | small GTPase binding | dia |
| 1069 | GO:0008037 | P | 3, | 1 | 0.743 (x 1.346) | 22 (0.045) | 0.786 | cell recognition | ab |
| 1070 | GO:0005275 | F | 3, | 2 | 1.756 (x 1.139) | 52 (0.038) | 0.787 | amine transporter activity | BG:DS03431.1 CG5535 |
| 1071 | GO:0008038 | P | 4, | 1 | 0.743 (x 1.346) | 22 (0.045) | 0.787 | neuron recognition | ab |
| 1072 | GO:0042048 | P | 5, 6, | 2 | 1.790 (x 1.118) | 53 (0.038) | 0.787 | olfactory behavior | BG:DS01219.1 cher |
| 1073 | GO:0040003 | P | 8, | 1 | 0.743 (x 1.346) | 22 (0.045) | 0.788 | cuticle biosynthesis (sensu Insecta) | Lcp65Ag1 |
| 1074 | GO:0008356 | P | 5, | 2 | 1.790 (x 1.118) | 53 (0.038) | 0.788 | asymmetric cell division | os trol |
| 1075 | GO:0007592 | P | 7, | 1 | 0.743 (x 1.346) | 22 (0.045) | 0.788 | cuticle biosynthesis (sensu Protostomia and Nematoda) | Lcp65Ag1 |
| 1076 | GO:0050684 | P | 8, 9, | 2 | 1.790 (x 1.118) | 53 (0.038) | 0.788 | regulation of mRNA processing | CG10445 hay |
| 1077 | GO:0044420 | C | 2, 3, | 1 | 0.743 (x 1.346) | 22 (0.045) | 0.789 | extracellular matrix part | trol |
| 1078 | GO:0044238 | P | 4, | 154 | 154.316 (x 0.998) | 4570 (0.034) | 0.789 | primary metabolism | Aats-leu Act57B Aly Arf84F Ate1 BG:DS03431.1 BcDNA:LD32788 CG10214 CG10306 CG10444 CG10445 CG10877 CG1134 CG11837 CG11907 CG12264 CG12954 CG1299 CG13645 CG13692 CG14869 CG14935 CG17227 CG18522 CG2246 CG2277 CG2885 CG2989 CG3008 CG3036 CG31373 CG31611 CG3168 CG32549 CG32649 CG33002 CG33158 CG3803 CG3860 CG4096 CG4267 CG4364 CG4866 CG4933 CG5044 CG5162 CG5177 CG5205 CG5317 CG5338 CG5535 CG5558 CG5800 CG5946 CG6204 CG6272 CG6330 CG6512 CG6574 CG6610 CG6764 CG6803 CG6876 CG6950 CG7014 CG7163 CG7339 CG7722 CG7791 CG7828 CG7842 CG7997 CG8199 CG8327 CG8630 CG8756 CG8798 CG9273 CG9358 CG9372 CG9460 CG9862 Chit DDB1 Dbp80 Dgp-1 EP2237 Ets21C Fdxh Gasp Gclc Gdh Gs2 HBS1 HLHmgamma Hmgcr Ide Imp ImpL3 Irbp JhI-1 Jheh3 Ku80 La Las Mlc1 Mmp1 Nmd3 Pcaf Ptp61F REG Rep RpA-70 RpL17A RpS4 Ssb-c31a Ssrp Sulf1 Taf12L Thor Ugt86Da Ugt86Di W ab agt apt cul-2 desat1 dia eIF6 fru ftz-f1 hay huntingtin l(3)01239 mRpL22 mRpL22-24 mRpS14 mRpS18a mRpS21 mRpS33 mirr mre11 mus205 na puc rad50 rpr sda tko toy trol wun wun2 |
| 1079 | GO:0048024 | P | 9, 10, 12, | 2 | 1.790 (x 1.118) | 53 (0.038) | 0.789 | regulation of nuclear mRNA splicing, via spliceosome | CG10445 hay |
| 1080 | GO:0006091 | P | 5, | 17 | 17.052 (x 0.997) | 505 (0.034) | 0.791 | generation of precursor metabolites and energy | CG17843 CG18522 CG3192 CG32649 CG3803 CG4511 CG4769 CG5177 CG5548 CG5703 CG5946 DDB1 Gdh ImpL3 Pdsw TMS1 bcn92 |
| 1081 | GO:0015849 | P | 5, 6, | 2 | 1.823 (x 1.097) | 54 (0.037) | 0.791 | organic acid transport | BG:DS03431.1 CG5535 |
| 1082 | GO:0009056 | P | 4, | 11 | 11.042 (x 0.996) | 327 (0.034) | 0.791 | catabolism | Ate1 CG10214 CG6204 CG6330 CG8199 Chit DDB1 Gdh Gs2 ImpL3 Jheh3 |
| 1083 | GO:0051252 | P | 7, | 2 | 1.823 (x 1.097) | 54 (0.037) | 0.792 | regulation of RNA metabolism | CG10445 hay |
| 1084 | GO:0008258 | P | 6, | 1 | 0.777 (x 1.288) | 23 (0.043) | 0.792 | head involution | W |
| 1085 | GO:0009266 | P | 4, | 2 | 1.823 (x 1.097) | 54 (0.037) | 0.792 | response to temperature stimulus | TotA ppk |
| 1086 | GO:0016859 | F | 4, | 1 | 0.777 (x 1.288) | 23 (0.043) | 0.793 | cis-trans isomerase activity | CG11858 |
| 1087 | GO:0016477 | P | 5, 6, | 6 | 5.909 (x 1.015) | 175 (0.034) | 0.793 | cell migration | Hmgcr Ptp61F ab os wun wun2 |
| 1088 | GO:0007635 | P | 4, 5, | 2 | 1.823 (x 1.097) | 54 (0.037) | 0.793 | chemosensory behavior | BG:DS01219.1 cher |
| 1089 | GO:0006767 | P | 6, | 1 | 0.777 (x 1.288) | 23 (0.043) | 0.793 | water-soluble vitamin metabolism | CG13645 |
| 1090 | GO:0046942 | P | 6, 7, | 2 | 1.823 (x 1.097) | 54 (0.037) | 0.794 | carboxylic acid transport | BG:DS03431.1 CG5535 |
| 1091 | GO:0019395 | P | 7, 8, | 1 | 0.777 (x 1.288) | 23 (0.043) | 0.794 | fatty acid oxidation | CG5044 |
| 1092 | GO:0007276 | P | 4, | 15 | 15.094 (x 0.994) | 447 (0.034) | 0.794 | gametogenesis | CG5162 CG9358 Cp36 Hmgcr Ptp61F R Taf12L cher dia l(3)01239 loco os puc wun wun2 |
| 1093 | GO:0042445 | P | 5, | 1 | 0.777 (x 1.288) | 23 (0.043) | 0.795 | hormone metabolism | Jheh3 |
| 1094 | GO:0006304 | P | 7, | 1 | 0.777 (x 1.288) | 23 (0.043) | 0.795 | DNA modification | trol |
| 1095 | GO:0004532 | F | 7, | 1 | 0.777 (x 1.288) | 23 (0.043) | 0.796 | exoribonuclease activity | CG10214 |
| 1096 | GO:0006897 | P | 6, 7, | 4 | 3.917 (x 1.021) | 116 (0.034) | 0.797 | endocytosis | Arf84F Arr2 CG2885 R |
| 1097 | GO:0007264 | P | 6, | 4 | 3.917 (x 1.021) | 116 (0.034) | 0.797 | small GTPase mediated signal transduction | Arf84F CG2885 CG9358 R |
| 1098 | GO:0031202 | F | 5, | 1 | 0.810 (x 1.234) | 24 (0.042) | 0.8 | RNA splicing factor activity, transesterification mechanism | CG6876 |
| 1099 | GO:0004879 | F | 4, | 1 | 0.810 (x 1.234) | 24 (0.042) | 0.801 | ligand-dependent nuclear receptor activity | ftz-f1 |
| 1100 | GO:0030880 | C | 3, 4, 5, 6, | 1 | 0.810 (x 1.234) | 24 (0.042) | 0.802 | RNA polymerase complex | CG7339 |
| 1101 | GO:0007304 | P | 8, 9, | 2 | 1.857 (x 1.077) | 55 (0.036) | 0.802 | eggshell formation (sensu Insecta) | Cp36 puc |
| 1102 | GO:0006334 | P | 7, 11, | 1 | 0.810 (x 1.234) | 24 (0.042) | 0.802 | nucleosome assembly | CG31611 |
| 1103 | GO:0030703 | P | 7, | 2 | 1.857 (x 1.077) | 55 (0.036) | 0.802 | eggshell formation | Cp36 puc |
| 1104 | GO:0046872 | F | 4, | 31 | 31.471 (x 0.985) | 932 (0.033) | 0.803 | metal ion binding | CAH1 CAH2 CG10126 CG1134 CG14869 CG31063 CG31666 CG31922 CG4096 CG4933 CG7163 CG7447 CG7791 CG8630 EP2237 Ide Las Mlc2 Mlp60A Mmp1 Mocs1 TpnC41C Traf1 Tsf1 Tsf3 ab desat1 fru ken mre11 sda |
| 1105 | GO:0019842 | F | 3, | 1 | 0.810 (x 1.234) | 24 (0.042) | 0.803 | vitamin binding | CG6574 |
| 1106 | GO:0043167 | F | 3, | 31 | 31.471 (x 0.985) | 932 (0.033) | 0.803 | ion binding | CAH1 CAH2 CG10126 CG1134 CG14869 CG31063 CG31666 CG31922 CG4096 CG4933 CG7163 CG7447 CG7791 CG8630 EP2237 Ide Las Mlc2 Mlp60A Mmp1 Mocs1 TpnC41C Traf1 Tsf1 Tsf3 ab desat1 fru ken mre11 sda |
| 1107 | GO:0000398 | P | 9, 11, | 5 | 5.031 (x 0.994) | 149 (0.034) | 0.804 | nuclear mRNA splicing, via spliceosome | CG10445 CG5205 CG6610 CG6876 hay |
| 1108 | GO:0046982 | F | 5, | 1 | 0.810 (x 1.234) | 24 (0.042) | 0.804 | protein heterodimerization activity | CG6272 |
| 1109 | GO:0044430 | C | 4, 5, 6, 7, 8, 9, | 8 | 8.104 (x 0.987) | 240 (0.033) | 0.804 | cytoskeletal part | Act57B LamC Mhc Mlc1 Mlc2 Rep dia wupA |
| 1110 | GO:0001505 | P | 7, | 4 | 3.951 (x 1.012) | 117 (0.034) | 0.804 | regulation of neurotransmitter levels | Arf84F CG3168 Rep unc-13 |
| 1111 | GO:0000377 | P | 10, | 5 | 5.031 (x 0.994) | 149 (0.034) | 0.804 | RNA splicing, via transesterification reactions with bulged adenosine as nucleophile | CG10445 CG5205 CG6610 CG6876 hay |
| 1112 | GO:0016490 | F | 3, | 1 | 0.810 (x 1.234) | 24 (0.042) | 0.804 | structural constituent of peritrophic membrane (sensu Insecta) | Gasp |
| 1113 | GO:0030136 | C | 7, 8, 9, 10, 11, | 2 | 1.891 (x 1.058) | 56 (0.036) | 0.805 | clathrin-coated vesicle | Rep unc-13 |
| 1114 | GO:0000375 | P | 9, | 5 | 5.031 (x 0.994) | 149 (0.034) | 0.805 | RNA splicing, via transesterification reactions | CG10445 CG5205 CG6610 CG6876 hay |
| 1115 | GO:0016614 | F | 4, | 4 | 3.985 (x 1.004) | 118 (0.034) | 0.805 | oxidoreductase activity, acting on CH-OH group of donors | BcDNA:GH10614 CG10638 Hmgcr ImpL3 |
| 1116 | GO:0003743 | F | 4, 5, | 2 | 1.891 (x 1.058) | 56 (0.036) | 0.805 | translation initiation factor activity | CG10306 eIF6 |
| 1117 | GO:0015980 | P | 6, | 4 | 3.985 (x 1.004) | 118 (0.034) | 0.806 | energy derivation by oxidation of organic compounds | CG5177 DDB1 Gdh ImpL3 |
| 1118 | GO:0001654 | P | 5, | 6 | 6.112 (x 0.982) | 181 (0.033) | 0.807 | eye development | EP2237 HLHmgamma W mirr puc toy |
| 1119 | GO:0007265 | P | 7, | 1 | 0.844 (x 1.185) | 25 (0.040) | 0.808 | Ras protein signal transduction | CG9358 |
| 1120 | GO:0005262 | F | 6, 7, | 1 | 0.844 (x 1.185) | 25 (0.040) | 0.809 | calcium channel activity | na |
| 1121 | GO:0009950 | P | 5, | 2 | 1.925 (x 1.039) | 57 (0.035) | 0.809 | dorsal/ventral axis specification | loco sog |
| 1122 | GO:0051020 | F | 5, | 1 | 0.844 (x 1.185) | 25 (0.040) | 0.809 | GTPase binding | dia |
| 1123 | GO:0016796 | F | 7, | 1 | 0.844 (x 1.185) | 25 (0.040) | 0.81 | exonuclease activity, active with either ribo- or deoxyribonucleic acids and producing 5'-phosphomonoesters | CG10214 |
| 1124 | GO:0007613 | P | 5, | 1 | 0.844 (x 1.185) | 25 (0.040) | 0.811 | memory | CG10460 |
| 1125 | GO:0019953 | P | 3, | 15 | 15.398 (x 0.974) | 456 (0.033) | 0.811 | sexual reproduction | CG5162 CG9358 Cp36 Hmgcr Ptp61F R Taf12L cher dia l(3)01239 loco os puc wun wun2 |
| 1126 | GO:0035265 | P | 3, | 1 | 0.844 (x 1.185) | 25 (0.040) | 0.812 | organ growth | W |
| 1127 | GO:0005975 | P | 5, | 16 | 16.377 (x 0.977) | 485 (0.033) | 0.812 | carbohydrate metabolism | Act57B CG14935 CG2989 CG3036 CG3168 CG5177 CG7997 CG8756 Chit DDB1 Gasp Gdh ImpL3 Sulf1 Ugt86Da Ugt86Di |
| 1128 | GO:0046620 | P | 4, | 1 | 0.844 (x 1.185) | 25 (0.040) | 0.812 | regulation of organ size | W |
| 1129 | GO:0016879 | F | 4, | 6 | 6.146 (x 0.976) | 182 (0.033) | 0.813 | ligase activity, forming carbon-nitrogen bonds | BcDNA:LD32788 CG1134 CG4917 Gclc Gs2 Las |
| 1130 | GO:0008134 | F | 4, | 3 | 3.005 (x 0.998) | 89 (0.034) | 0.813 | transcription factor binding | Aly Ssb-c31a ftz-f1 |
| 1131 | GO:0007186 | P | 6, | 10 | 10.299 (x 0.971) | 305 (0.033) | 0.814 | G-protein coupled receptor protein signaling pathway | Arf84F Arr2 CG13586 CG7515 GABA-B-R2 Ide R loco wun wun2 |
| 1132 | GO:0004221 | F | 6, 8, | 1 | 0.878 (x 1.139) | 26 (0.038) | 0.815 | ubiquitin thiolesterase activity | isopeptidase-T-3 |
| 1133 | GO:0051128 | P | 5, | 1 | 0.878 (x 1.139) | 26 (0.038) | 0.816 | regulation of cell organization and biogenesis | Ssrp |
| 1134 | GO:0005575 | C | 1, | 194 | 195.241 (x 0.994) | 5782 (0.034) | 0.816 | cellular\_component | Aats-leu Act57B Aldh Arr2 BEST:CK01227 BG:DS01219.1 BG:DS02740.9 BG:DS03431.1 BcDNA:LD23830 Bmcp CG10214 CG10306 CG10444 CG10445 CG10861 CG1134 CG11837 CG11897 CG11907 CG12264 CG12954 CG13586 CG17108 CG1718 CG17227 CG2989 CG30152 CG30343 CG3036 CG31279 CG31601 CG31611 CG31666 CG3168 CG31793 CG3192 CG31922 CG32017 CG32021 CG32207 CG32409 CG32442 CG32448 CG32549 CG32582 CG32625 CG32649 CG32756 CG33002 CG33158 CG3371 CG3803 CG40006 CG4364 CG4769 CG4866 CG4917 CG5037 CG5205 CG5317 CG5338 CG5535 CG5548 CG5703 CG6272 CG6330 CG6512 CG6574 CG6610 CG6739 CG6764 CG6876 CG7014 CG7163 CG7188 CG7194 CG7339 CG7571 CG7627 CG7791 CG7842 CG7955 CG8004 CG8199 CG8226 CG8327 CG8630 CG8756 CG8798 CG9273 CG9358 Chit Cp36 DDB1 Dgp-1 EG:52C10.1 EP2237 Ets21C GABA-B-R2 GV1 Gasp Gclc Gdh Gr39a Gs2 GstD3 HBS1 HLHmgamma Hmgcr Ide Idgf2 Idgf3 Irbp Jafrac1 JhI-1 JhI-26 Jheh3 Ku80 La LamC Las Lcp65Ag1 Mhc Mlc1 Mlc2 Mlp60A Mmp1 Mocs1 NP15.6 Nxt1 Obp83g Pcaf Pdsw Ptp61F REG Rep RpA-70 RpL17A RpS4 SP1173 Ssb-c31a Ssrp Sulf1 TMS1 Taf12L Tim10 Tim9a Tsf1 Tsf3 Tsp42El ab apt bcn92 cher cul-2 desat1 dia dlp eIF6 eiger fau fru ftz-f1 hay huntingtin ken l(3)01239 lama mRpL22 mRpL22-24 mRpS14 mRpS18a mRpS21 mRpS33 mfas mirr mre11 mus205 na os osp ppk rad50 rpr sas sog tko toy trol unc-13 wun wun2 wupA yellow-b |
| 1135 | GO:0044452 | C | 5, 6, 7, 8, 9, 10, 11, 12, | 1 | 0.878 (x 1.139) | 26 (0.038) | 0.817 | nucleolar part | CG6610 |
| 1136 | GO:0044262 | P | 6, | 9 | 9.286 (x 0.969) | 275 (0.033) | 0.817 | cellular carbohydrate metabolism | Act57B CG2989 CG5177 CG8756 Chit DDB1 Gasp Gdh ImpL3 |
| 1137 | GO:0006629 | P | 5, | 16 | 16.546 (x 0.967) | 490 (0.033) | 0.817 | lipid metabolism | CG10877 CG3860 CG4267 CG5044 CG5162 CG5946 CG7842 CG8199 CG8630 Hmgcr Jheh3 Ugt86Da Ugt86Di desat1 wun wun2 |
| 1138 | GO:0003887 | F | 6, | 1 | 0.878 (x 1.139) | 26 (0.038) | 0.817 | DNA-directed DNA polymerase activity | mus205 |
| 1139 | GO:0007275 | P | 2, | 49 | 50.144 (x 0.977) | 1485 (0.033) | 0.818 | development | Act57B BG:DS02740.9 CG10861 CG5397 CG6739 CG6803 CG7194 CG9358 EP2237 Gs2 HLHmgamma Hmgcr Idgf2 Idgf3 Lcp65Ag1 Mhc Mlc1 Mlp60A Mmp1 Ptp61F R Sulf1 Thor Traf1 Tsp42El W ab apt cher dia dlp fru ftz-f1 ken loco melt mfas mirr os puc rad50 rpr sas sog toy trol wun wun2 wupA |
| 1140 | GO:0006955 | P | 4, 5, | 4 | 4.120 (x 0.971) | 122 (0.033) | 0.818 | immune response | TepIV Thor TotA eiger |
| 1141 | GO:0005272 | F | 6, 7, | 1 | 0.878 (x 1.139) | 26 (0.038) | 0.818 | sodium channel activity | ppk |
| 1142 | GO:0009653 | P | 3, | 21 | 21.678 (x 0.969) | 642 (0.033) | 0.818 | morphogenesis | Act57B CG7194 CG9358 EP2237 Hmgcr Ptp61F R Thor Traf1 W ab apt cher ftz-f1 loco mfas mirr os puc rpr sog |
| 1143 | GO:0003723 | F | 4, | 12 | 12.460 (x 0.963) | 369 (0.033) | 0.818 | RNA binding | Aats-leu Aly CG4364 CG4866 CG6876 CG9862 Imp La Nmd3 RpS4 Ssrp apt |
| 1144 | GO:0009993 | P | 7, | 9 | 9.353 (x 0.962) | 277 (0.032) | 0.818 | oogenesis (sensu Insecta) | CG9358 Cp36 Ptp61F R cher l(3)01239 loco os puc |
| 1145 | GO:0051327 | P | 6, | 3 | 3.073 (x 0.976) | 91 (0.033) | 0.818 | M phase of meiotic cell cycle | Taf12L dia mre11 |
| 1146 | GO:0045177 | C | 3, 4, | 1 | 0.878 (x 1.139) | 26 (0.038) | 0.819 | apical part of cell | sas |
| 1147 | GO:0000578 | P | 5, | 2 | 1.992 (x 1.004) | 59 (0.034) | 0.819 | embryonic axis specification | CG9358 sog |
| 1148 | GO:0005083 | F | 4, | 3 | 3.073 (x 0.976) | 91 (0.033) | 0.819 | small GTPase regulator activity | CG13692 Rep loco |
| 1149 | GO:0015114 | F | 6, | 1 | 0.878 (x 1.139) | 26 (0.038) | 0.819 | phosphate transporter activity | CG3036 |
| 1150 | GO:0008344 | P | 5, | 1 | 0.878 (x 1.139) | 26 (0.038) | 0.82 | adult locomotory behavior | na |
| 1151 | GO:0003677 | F | 4, | 27 | 27.757 (x 0.973) | 822 (0.033) | 0.821 | DNA binding | CG10445 CG2885 CG31611 CG31666 CG3168 CG3371 CG6272 CG9273 DDB1 Ets21C GV1 HLHmgamma Irbp Ku80 RpA-70 Ssb-c31a Ssrp apt fru ftz-f1 hay ken mirr mus205 rad50 toy trol |
| 1152 | GO:0040007 | P | 2, | 3 | 3.039 (x 0.987) | 90 (0.033) | 0.821 | growth | Mmp1 Thor W |
| 1153 | GO:0051321 | P | 5, | 3 | 3.107 (x 0.966) | 92 (0.033) | 0.821 | meiotic cell cycle | Taf12L dia mre11 |
| 1154 | GO:0003779 | F | 5, | 4 | 4.153 (x 0.963) | 123 (0.033) | 0.822 | actin binding | BG:DS02740.9 cher dia wupA |
| 1155 | GO:0006006 | P | 8, 9, | 2 | 2.026 (x 0.987) | 60 (0.033) | 0.822 | glucose metabolism | DDB1 ImpL3 |
| 1156 | GO:0008194 | F | 5, | 3 | 3.107 (x 0.966) | 92 (0.033) | 0.822 | UDP-glycosyltransferase activity | Act57B Ugt86Da Ugt86Di |
| 1157 | GO:0009987 | P | 2, | 225 | 226.442 (x 0.994) | 6706 (0.034) | 0.822 | cellular process | Aats-leu Act57B Aldh Aly Arf84F Arr2 Ate1 BEST:CK01227 BG:DS03431.1 BcDNA:LD23830 BcDNA:LD32788 Bmcp CAH1 CAH2 CG10214 CG10306 CG10444 CG10445 CG10638 CG10861 CG10877 CG1134 CG11837 CG11897 CG11907 CG12264 CG12876 CG12954 CG1299 CG13586 CG13645 CG13692 CG14869 CG17108 CG1718 CG17227 CG17843 CG18522 CG1882 CG2246 CG2277 CG2453 CG2885 CG2989 CG3008 CG3036 CG31373 CG31611 CG3168 CG31793 CG3192 CG32409 CG32549 CG32649 CG33002 CG33158 CG3803 CG3860 CG40006 CG4096 CG4364 CG4511 CG4769 CG4866 CG4933 CG5037 CG5044 CG5162 CG5177 CG5205 CG5317 CG5338 CG5535 CG5548 CG5558 CG5703 CG5800 CG5946 CG6204 CG6272 CG6330 CG6512 CG6574 CG6610 CG6764 CG6803 CG6876 CG6950 CG7014 CG7054 CG7163 CG7188 CG7339 CG7515 CG7571 CG7627 CG7722 CG7791 CG7828 CG7842 CG7955 CG8004 CG8199 CG8226 CG8327 CG8630 CG8756 CG8798 CG9273 CG9358 CG9372 CG9460 CG9790 CG9862 Chit DDB1 Dbp80 Dgp-1 EG:52C10.1 EP2237 Ets21C Fdxh GABA-B-R2 Gasp Gclc Gdh Gs2 GstE1 GstE3 GstE5 GstE6 GstE7 HBS1 HLHmgamma Hmgcr Ide Idgf2 Idgf3 Imp ImpL3 Irbp Jafrac1 JhI-1 Jheh3 Ku80 La LamC Las Mhc Mlc1 Mlp60A Mmp1 Mocs1 Nmd3 Nxt1 Obp83g Pcaf Pdsw Ptp61F R REG Rep RpA-70 RpL17A RpS4 Ssb-c31a Ssrp Sulf1 TMS1 Taf12L TepIV Thor Tim10 Tim9a TpnC41C Traf1 Tsf1 Tsf3 Tsp42El Ugt86Da Ugt86Di W ab agt apt bcn92 cher cul-2 desat1 dia dlp eIF6 eiger fru ftz-f1 glob1 hay huntingtin l(3)01239 loco mRpL22 mRpL22-24 mRpS14 mRpS18a mRpS21 mRpS33 mfas mirr mre11 mus205 na os ppk puc rad50 rpr sda sog tko toy trol unc-13 wun wun2 wupA |
| 1158 | GO:0016337 | P | 4, | 4 | 4.187 (x 0.955) | 124 (0.032) | 0.823 | cell-cell adhesion | CG14869 CG4096 mfas trol |
| 1159 | GO:0005681 | C | 4, 5, 6, 7, 8, 9, 10, | 3 | 3.107 (x 0.966) | 92 (0.033) | 0.823 | spliceosome complex | CG5205 CG6610 CG6876 |
| 1160 | GO:0007292 | P | 5, | 10 | 10.468 (x 0.955) | 310 (0.032) | 0.823 | female gamete generation | CG5162 CG9358 Cp36 Ptp61F R cher l(3)01239 loco os puc |
| 1161 | GO:0005543 | F | 4, | 1 | 0.912 (x 1.097) | 27 (0.037) | 0.823 | phospholipid binding | CG7054 |
| 1162 | GO:0000775 | C | 5, 6, 7, 8, 9, 10, | 1 | 0.912 (x 1.097) | 27 (0.037) | 0.824 | chromosome, pericentric region | CG3371 |
| 1163 | GO:0007298 | P | 7, 8, 10, | 1 | 0.912 (x 1.097) | 27 (0.037) | 0.825 | border follicle cell migration (sensu Insecta) | os |
| 1164 | GO:0044274 | P | 5, | 1 | 0.912 (x 1.097) | 27 (0.037) | 0.826 | organismal biosynthesis | Lcp65Ag1 |
| 1165 | GO:0042335 | P | 6, | 1 | 0.912 (x 1.097) | 27 (0.037) | 0.826 | cuticle biosynthesis | Lcp65Ag1 |
| 1166 | GO:0005669 | C | 4, 7, 8, 9, 10, 11, 12, 13, 14, | 1 | 0.945 (x 1.058) | 28 (0.036) | 0.828 | transcription factor TFIID complex | Taf12L |
| 1167 | GO:0006403 | P | 4, | 3 | 3.140 (x 0.955) | 93 (0.032) | 0.828 | RNA localization | CG9862 La Nxt1 |
| 1168 | GO:0019783 | F | 6, | 1 | 0.945 (x 1.058) | 28 (0.036) | 0.829 | small conjugating protein-specific protease activity | isopeptidase-T-3 |
| 1169 | GO:0006887 | P | 6, 7, | 4 | 4.221 (x 0.948) | 125 (0.032) | 0.829 | exocytosis | Arf84F CG2885 R unc-13 |
| 1170 | GO:0007507 | P | 5, | 2 | 2.060 (x 0.971) | 61 (0.033) | 0.829 | heart development | Act57B apt |
| 1171 | GO:0008080 | F | 8, | 1 | 0.945 (x 1.058) | 28 (0.036) | 0.829 | N-acetyltransferase activity | Pcaf |
| 1172 | GO:0008509 | F | 4, | 3 | 3.174 (x 0.945) | 94 (0.032) | 0.83 | anion transporter activity | BEST:CK01227 CG3036 CG7571 |
| 1173 | GO:0040008 | P | 3, | 2 | 2.060 (x 0.971) | 61 (0.033) | 0.83 | regulation of growth | Thor W |
| 1174 | GO:0004843 | F | 7, | 1 | 0.945 (x 1.058) | 28 (0.036) | 0.83 | ubiquitin-specific protease activity | isopeptidase-T-3 |
| 1175 | GO:0006261 | P | 8, | 2 | 2.060 (x 0.971) | 61 (0.033) | 0.831 | DNA-dependent DNA replication | CG9273 RpA-70 |
| 1176 | GO:0004091 | F | 6, | 1 | 0.945 (x 1.058) | 28 (0.036) | 0.831 | carboxylesterase activity | alpha-Est10 |
| 1177 | GO:0003729 | F | 5, | 10 | 10.569 (x 0.946) | 313 (0.032) | 0.831 | mRNA binding | Aats-leu Aly CG4364 CG4866 CG9862 Imp La Nmd3 RpS4 apt |
| 1178 | GO:0005819 | C | 5, 6, 7, 8, 9, 10, | 1 | 0.945 (x 1.058) | 28 (0.036) | 0.832 | spindle | Rep |
| 1179 | GO:0007051 | P | 9, | 1 | 0.979 (x 1.021) | 29 (0.034) | 0.833 | spindle organization and biogenesis | dia |
| 1180 | GO:0014016 | P | 4, 7, | 1 | 0.979 (x 1.021) | 29 (0.034) | 0.833 | neuroblast differentiation | trol |
| 1181 | GO:0016874 | F | 3, | 10 | 10.704 (x 0.934) | 317 (0.032) | 0.833 | ligase activity | Aats-leu BcDNA:LD32788 CG1134 CG17108 CG17227 CG4917 CG7828 Gclc Gs2 Las |
| 1182 | GO:0019730 | P | 6, 7, | 2 | 2.127 (x 0.940) | 63 (0.032) | 0.834 | antimicrobial humoral response | TepIV Thor |
| 1183 | GO:0007400 | P | 6, 9, | 1 | 0.979 (x 1.021) | 29 (0.034) | 0.834 | neuroblast fate determination | trol |
| 1184 | GO:0015370 | F | 6, 8, | 2 | 2.094 (x 0.955) | 62 (0.032) | 0.834 | solute:sodium symporter activity | BG:DS03431.1 CG10444 |
| 1185 | GO:0015294 | F | 5, 7, | 2 | 2.127 (x 0.940) | 63 (0.032) | 0.834 | solute:cation symporter activity | BG:DS03431.1 CG10444 |
| 1186 | GO:0042157 | P | 7, | 1 | 0.979 (x 1.021) | 29 (0.034) | 0.835 | lipoprotein metabolism | Rep |
| 1187 | GO:0009057 | P | 5, | 6 | 6.416 (x 0.935) | 190 (0.032) | 0.835 | macromolecule catabolism | Ate1 CG10214 CG6204 Chit DDB1 ImpL3 |
| 1188 | GO:0006497 | P | 8, 9, | 1 | 0.979 (x 1.021) | 29 (0.034) | 0.835 | protein amino acid lipidation | Rep |
| 1189 | GO:0043285 | P | 6, | 4 | 4.288 (x 0.933) | 127 (0.031) | 0.836 | biopolymer catabolism | Ate1 CG10214 CG6204 Chit |
| 1190 | GO:0005795 | C | 5, 6, 7, 8, 9, 10, | 1 | 0.979 (x 1.021) | 29 (0.034) | 0.836 | Golgi stack | Sulf1 |
| 1191 | GO:0008047 | F | 3, | 3 | 3.208 (x 0.935) | 95 (0.032) | 0.837 | enzyme activator activity | CG13692 REG loco |
| 1192 | GO:0007416 | P | 5, 6, | 1 | 0.979 (x 1.021) | 29 (0.034) | 0.837 | synaptogenesis | Gs2 |
| 1193 | GO:0003924 | F | 8, | 4 | 4.390 (x 0.911) | 130 (0.031) | 0.837 | GTPase activity | Arf84F BcDNA:LD23830 CG2885 R |
| 1194 | GO:0008063 | P | 6, | 1 | 1.013 (x 0.987) | 30 (0.033) | 0.837 | Toll signaling pathway | Traf1 |
| 1195 | GO:0031123 | P | 8, | 1 | 0.979 (x 1.021) | 29 (0.034) | 0.837 | RNA 3'-end processing | Nmd3 |
| 1196 | GO:0030695 | F | 3, | 4 | 4.390 (x 0.911) | 130 (0.031) | 0.838 | GTPase regulator activity | CG13692 CG7515 Rep loco |
| 1197 | GO:0048477 | P | 6, | 9 | 9.759 (x 0.922) | 289 (0.031) | 0.838 | oogenesis | CG9358 Cp36 Ptp61F R cher l(3)01239 loco os puc |
| 1198 | GO:0005515 | F | 3, | 40 | 41.669 (x 0.960) | 1234 (0.032) | 0.838 | protein binding | Aly Arr2 BG:DS01219.1 BG:DS02740.9 CG13586 CG2989 CG3153 CG31666 CG6272 Chit Gclc Idgf2 Idgf3 Mhc Mlc2 Mlp60A Nmd3 PQBP-1 Ssb-c31a Ssrp Thor TpnC41C Traf1 ab cher dia dlp eiger fru ftz-f1 huntingtin ken l(3)01239 loco mirr os sog unc-13 wun wupA |
| 1199 | GO:0006576 | P | 6, 7, | 1 | 1.013 (x 0.987) | 30 (0.033) | 0.838 | biogenic amine metabolism | CG8327 |
| 1200 | GO:0007268 | P | 6, | 7 | 7.631 (x 0.917) | 226 (0.031) | 0.838 | synaptic transmission | Arf84F CG3168 GABA-B-R2 Gs2 Rep apt unc-13 |
| 1201 | GO:0031124 | P | 9, | 1 | 0.979 (x 1.021) | 29 (0.034) | 0.838 | mRNA 3'-end processing | Nmd3 |
| 1202 | GO:0007591 | P | 6, | 1 | 1.013 (x 0.987) | 30 (0.033) | 0.839 | molting cycle (sensu Insecta) | Lcp65Ag1 |
| 1203 | GO:0006066 | P | 5, | 5 | 5.437 (x 0.920) | 161 (0.031) | 0.839 | alcohol metabolism | CG3860 CG5946 DDB1 Hmgcr ImpL3 |
| 1204 | GO:0022008 | P | 5, | 6 | 6.517 (x 0.921) | 193 (0.031) | 0.839 | neurogenesis | BG:DS02740.9 Ptp61F ab loco mfas trol |
| 1205 | GO:0014017 | P | 5, 8, | 1 | 0.979 (x 1.021) | 29 (0.034) | 0.839 | neuroblast fate commitment | trol |
| 1206 | GO:0007389 | P | 3, | 8 | 8.644 (x 0.925) | 256 (0.031) | 0.839 | pattern specification | CG9358 Sulf1 dlp ftz-f1 loco os sog wun |
| 1207 | GO:0016485 | P | 8, | 1 | 1.013 (x 0.987) | 30 (0.033) | 0.839 | protein processing | CG7791 |
| 1208 | GO:0007349 | P | 3, 4, | 1 | 0.979 (x 1.021) | 29 (0.034) | 0.84 | cellularization | dia |
| 1209 | GO:0031589 | P | 4, | 1 | 1.013 (x 0.987) | 30 (0.033) | 0.84 | cell-substrate adhesion | trol |
| 1210 | GO:0007274 | P | 7, | 1 | 0.979 (x 1.021) | 29 (0.034) | 0.84 | neuromuscular synaptic transmission | apt |
| 1211 | GO:0007160 | P | 5, | 1 | 1.013 (x 0.987) | 30 (0.033) | 0.841 | cell-matrix adhesion | trol |
| 1212 | GO:0045941 | P | 8, | 2 | 2.161 (x 0.925) | 64 (0.031) | 0.841 | positive regulation of transcription | EP2237 mirr |
| 1213 | GO:0042158 | P | 7, 8, | 1 | 0.979 (x 1.021) | 29 (0.034) | 0.841 | lipoprotein biosynthesis | Rep |
| 1214 | GO:0019201 | F | 7, | 1 | 1.013 (x 0.987) | 30 (0.033) | 0.841 | nucleotide kinase activity | CG2246 |
| 1215 | GO:0003899 | F | 6, | 1 | 1.013 (x 0.987) | 30 (0.033) | 0.842 | DNA-directed RNA polymerase activity | CG7339 |
| 1216 | GO:0045935 | P | 7, | 2 | 2.195 (x 0.911) | 65 (0.031) | 0.842 | positive regulation of nucleobase, nucleoside, nucleotide and nucleic acid metabolism | EP2237 mirr |
| 1217 | GO:0016049 | P | 3, 4, 6, 7, | 1 | 1.013 (x 0.987) | 30 (0.033) | 0.843 | cell growth | Thor |
| 1218 | GO:0048754 | P | 5, | 1 | 1.047 (x 0.955) | 31 (0.032) | 0.845 | branching morphogenesis of a tube | apt |
| 1219 | GO:0005623 | C | 2, | 153 | 155.869 (x 0.982) | 4616 (0.033) | 0.845 | cell | Aats-leu Act57B Aldh Arr2 BEST:CK01227 BG:DS01219.1 BG:DS02740.9 BG:DS03431.1 BcDNA:LD23830 Bmcp CG10214 CG10306 CG10444 CG10445 CG10861 CG1134 CG11897 CG11907 CG12264 CG12954 CG17108 CG1718 CG17227 CG3036 CG31611 CG31666 CG3168 CG31793 CG3192 CG31922 CG32409 CG32549 CG32649 CG33002 CG33158 CG3371 CG3803 CG40006 CG4364 CG4769 CG4866 CG4917 CG5037 CG5205 CG5317 CG5338 CG5535 CG5548 CG5703 CG6272 CG6330 CG6512 CG6574 CG6610 CG6739 CG6764 CG6876 CG7014 CG7163 CG7188 CG7339 CG7571 CG7627 CG7791 CG7842 CG7955 CG8004 CG8199 CG8226 CG8630 CG8798 CG9273 CG9358 Cp36 DDB1 EG:52C10.1 EP2237 Ets21C GABA-B-R2 GV1 Gclc Gdh Gr39a Gs2 HBS1 HLHmgamma Hmgcr Ide Irbp Jafrac1 JhI-1 Jheh3 Ku80 La LamC Las Mhc Mlc1 Mlc2 Mlp60A Nxt1 Pcaf Pdsw Ptp61F REG Rep RpA-70 RpL17A RpS4 Ssb-c31a Ssrp Sulf1 TMS1 Taf12L Tim10 Tim9a Tsp42El ab apt bcn92 cul-2 desat1 dia eIF6 eiger fru ftz-f1 hay huntingtin ken l(3)01239 mRpL22 mRpL22-24 mRpS14 mRpS18a mRpS21 mRpS33 mfas mirr mre11 mus205 na ppk rad50 rpr sas sog tko toy unc-13 wun wun2 wupA |
| 1220 | GO:0016410 | F | 7, | 1 | 1.047 (x 0.955) | 31 (0.032) | 0.846 | N-acyltransferase activity | Pcaf |
| 1221 | GO:0044464 | C | 2, 3, | 153 | 155.869 (x 0.982) | 4616 (0.033) | 0.846 | cell part | Aats-leu Act57B Aldh Arr2 BEST:CK01227 BG:DS01219.1 BG:DS02740.9 BG:DS03431.1 BcDNA:LD23830 Bmcp CG10214 CG10306 CG10444 CG10445 CG10861 CG1134 CG11897 CG11907 CG12264 CG12954 CG17108 CG1718 CG17227 CG3036 CG31611 CG31666 CG3168 CG31793 CG3192 CG31922 CG32409 CG32549 CG32649 CG33002 CG33158 CG3371 CG3803 CG40006 CG4364 CG4769 CG4866 CG4917 CG5037 CG5205 CG5317 CG5338 CG5535 CG5548 CG5703 CG6272 CG6330 CG6512 CG6574 CG6610 CG6739 CG6764 CG6876 CG7014 CG7163 CG7188 CG7339 CG7571 CG7627 CG7791 CG7842 CG7955 CG8004 CG8199 CG8226 CG8630 CG8798 CG9273 CG9358 Cp36 DDB1 EG:52C10.1 EP2237 Ets21C GABA-B-R2 GV1 Gclc Gdh Gr39a Gs2 HBS1 HLHmgamma Hmgcr Ide Irbp Jafrac1 JhI-1 Jheh3 Ku80 La LamC Las Mhc Mlc1 Mlc2 Mlp60A Nxt1 Pcaf Pdsw Ptp61F REG Rep RpA-70 RpL17A RpS4 Ssb-c31a Ssrp Sulf1 TMS1 Taf12L Tim10 Tim9a Tsp42El ab apt bcn92 cul-2 desat1 dia eIF6 eiger fru ftz-f1 hay huntingtin ken l(3)01239 mRpL22 mRpL22-24 mRpS14 mRpS18a mRpS21 mRpS33 mfas mirr mre11 mus205 na ppk rad50 rpr sas sog tko toy unc-13 wun wun2 wupA |
| 1222 | GO:0042302 | F | 3, | 3 | 3.343 (x 0.897) | 99 (0.030) | 0.846 | structural constituent of cuticle | CG6305 CG9077 Lcp65Ag1 |
| 1223 | GO:0008270 | F | 6, | 19 | 20.328 (x 0.935) | 602 (0.032) | 0.846 | zinc ion binding | CAH1 CAH2 CG1134 CG14869 CG31666 CG31922 CG4096 CG4933 CG7163 CG7791 EP2237 Ide Mlp60A Mmp1 Traf1 ab fru ken sda |
| 1224 | GO:0007267 | P | 4, | 14 | 15.128 (x 0.925) | 448 (0.031) | 0.847 | cell-cell signaling | Arf84F CG2989 CG3168 Chit GABA-B-R2 Gs2 Idgf2 Idgf3 Rep Tsp42El ab apt fru unc-13 |
| 1225 | GO:0016705 | F | 4, | 2 | 2.229 (x 0.897) | 66 (0.030) | 0.847 | oxidoreductase activity, acting on paired donors, with incorporation or reduction of molecular oxygen | CG8630 desat1 |
| 1226 | GO:0004181 | F | 7, | 1 | 1.081 (x 0.925) | 32 (0.031) | 0.848 | metallocarboxypeptidase activity | huntingtin |
| 1227 | GO:0019208 | F | 3, | 1 | 1.081 (x 0.925) | 32 (0.031) | 0.849 | phosphatase regulator activity | CG17124 |
| 1228 | GO:0006605 | P | 7, 8, 9, | 7 | 7.800 (x 0.897) | 231 (0.030) | 0.849 | protein targeting | CG7791 CG8004 CG8226 Nmd3 Nxt1 Tim10 Tim9a |
| 1229 | GO:0050808 | P | 5, | 1 | 1.081 (x 0.925) | 32 (0.031) | 0.85 | synapse organization and biogenesis | Gs2 |
| 1230 | GO:0019205 | F | 6, | 1 | 1.081 (x 0.925) | 32 (0.031) | 0.85 | nucleobase, nucleoside, nucleotide kinase activity | CG2246 |
| 1231 | GO:0006333 | P | 9, | 3 | 3.377 (x 0.888) | 100 (0.030) | 0.851 | chromatin assembly or disassembly | CG31611 Ssrp fru |
| 1232 | GO:0019888 | F | 4, | 1 | 1.081 (x 0.925) | 32 (0.031) | 0.851 | protein phosphatase regulator activity | CG17124 |
| 1233 | GO:0016684 | F | 4, | 1 | 1.081 (x 0.925) | 32 (0.031) | 0.852 | oxidoreductase activity, acting on peroxide as acceptor | Jafrac1 |
| 1234 | GO:0030198 | P | 4, | 1 | 1.081 (x 0.925) | 32 (0.031) | 0.853 | extracellular matrix organization and biogenesis | Gs2 |
| 1235 | GO:0044260 | P | 5, | 70 | 72.667 (x 0.963) | 2152 (0.033) | 0.853 | cellular macromolecule metabolism | Aats-leu Act57B Arf84F Ate1 CG10306 CG10444 CG1134 CG12954 CG1299 CG13645 CG13692 CG14869 CG2989 CG3008 CG31373 CG32649 CG33002 CG33158 CG4096 CG4866 CG4933 CG5317 CG5338 CG6512 CG6574 CG6764 CG7014 CG7722 CG7791 CG7828 CG8756 CG8798 CG9358 CG9372 CG9460 Chit Dgp-1 Fdxh Gasp Gclc Gs2 HBS1 Ide Imp Las Mlc1 Mmp1 Ptp61F REG Rep RpL17A RpS4 Thor W apt cul-2 eIF6 huntingtin l(3)01239 mRpL22 mRpL22-24 mRpS14 mRpS18a mRpS21 mRpS33 na puc rpr sda tko |
| 1236 | GO:0006397 | P | 8, | 6 | 6.686 (x 0.897) | 198 (0.030) | 0.853 | mRNA processing | CG10445 CG5205 CG6610 CG6876 Nmd3 hay |
| 1237 | GO:0007602 | P | 6, 7, | 1 | 1.081 (x 0.925) | 32 (0.031) | 0.853 | phototransduction | Arr2 |
| 1238 | GO:0004182 | F | 8, | 1 | 1.081 (x 0.925) | 32 (0.031) | 0.854 | carboxypeptidase A activity | huntingtin |
| 1239 | GO:0019538 | P | 5, | 71 | 73.714 (x 0.963) | 2183 (0.033) | 0.854 | protein metabolism | Aats-leu Act57B Arf84F Ate1 CG10306 CG10444 CG1134 CG12954 CG1299 CG13645 CG13692 CG14869 CG3008 CG31373 CG31611 CG32649 CG33002 CG33158 CG3803 CG4096 CG4364 CG4866 CG4933 CG5317 CG5338 CG6512 CG6574 CG6764 CG6803 CG7014 CG7722 CG7791 CG7828 CG8798 CG9358 CG9372 CG9460 Dgp-1 Fdxh Gclc Gs2 HBS1 Ide Imp Las Mlc1 Mmp1 Ptp61F REG Rep RpL17A RpS4 Thor W apt cul-2 dia eIF6 huntingtin l(3)01239 mRpL22 mRpL22-24 mRpS14 mRpS18a mRpS21 mRpS33 na puc rpr sda tko |
| 1240 | GO:0007218 | P | 7, | 1 | 1.114 (x 0.897) | 33 (0.030) | 0.854 | neuropeptide signaling pathway | CG13586 |
| 1241 | GO:0008105 | P | 5, | 1 | 1.081 (x 0.925) | 32 (0.031) | 0.855 | asymmetric protein localization | BG:DS01219.1 |
| 1242 | GO:0019200 | F | 6, | 1 | 1.114 (x 0.897) | 33 (0.030) | 0.855 | carbohydrate kinase activity | CG2246 |
| 1243 | GO:0019748 | P | 4, | 2 | 2.296 (x 0.871) | 68 (0.029) | 0.855 | secondary metabolism | CG5037 Jheh3 |
| 1244 | GO:0004601 | F | 3, 5, | 1 | 1.081 (x 0.925) | 32 (0.031) | 0.855 | peroxidase activity | Jafrac1 |
| 1245 | GO:0007297 | P | 6, 7, 9, | 1 | 1.114 (x 0.897) | 33 (0.030) | 0.856 | follicle cell migration (sensu Insecta) | os |
| 1246 | GO:0030135 | C | 6, 7, 8, 9, 10, | 2 | 2.296 (x 0.871) | 68 (0.029) | 0.856 | coated vesicle | Rep unc-13 |
| 1247 | GO:0042303 | P | 4, | 1 | 1.114 (x 0.897) | 33 (0.030) | 0.856 | molting cycle | Lcp65Ag1 |
| 1248 | GO:0009611 | P | 4, | 1 | 1.114 (x 0.897) | 33 (0.030) | 0.857 | response to wounding | puc |
| 1249 | GO:0018988 | P | 5, | 1 | 1.114 (x 0.897) | 33 (0.030) | 0.858 | molting cycle (sensu Protostomia and Nematoda) | Lcp65Ag1 |
| 1250 | GO:0042742 | P | 5, 6, | 2 | 2.330 (x 0.858) | 69 (0.029) | 0.858 | defense response to bacterium | TepIV Thor |
| 1251 | GO:0019221 | P | 6, | 1 | 1.114 (x 0.897) | 33 (0.030) | 0.859 | cytokine and chemokine mediated signaling pathway | Traf1 |
| 1252 | GO:0044448 | C | 5, 6, 7, 8, 9, | 1 | 1.114 (x 0.897) | 33 (0.030) | 0.859 | cell cortex part | dia |
| 1253 | GO:0005524 | F | 6, | 21 | 22.691 (x 0.925) | 672 (0.031) | 0.86 | ATP binding | Aats-leu BEST:CK01227 CG10445 CG11897 CG1582 CG1718 CG17227 CG3168 CG31793 CG4511 CG4858 CG5205 CG5800 CG6512 CG7627 CG7955 CG8798 Dbp80 Mhc hay rad50 |
| 1254 | GO:0005184 | F | 5, 6, | 1 | 1.148 (x 0.871) | 34 (0.029) | 0.864 | neuropeptide hormone activity | CG13586 |
| 1255 | GO:0006092 | P | 7, | 3 | 3.512 (x 0.854) | 104 (0.029) | 0.864 | main pathways of carbohydrate metabolism | DDB1 Gdh ImpL3 |
| 1256 | GO:0008361 | P | 5, 6, | 1 | 1.148 (x 0.871) | 34 (0.029) | 0.865 | regulation of cell size | Thor |
| 1257 | GO:0048488 | P | 7, 8, | 1 | 1.148 (x 0.871) | 34 (0.029) | 0.865 | synaptic vesicle endocytosis | Arf84F |
| 1258 | GO:0001763 | P | 4, | 1 | 1.148 (x 0.871) | 34 (0.029) | 0.866 | morphogenesis of a branching structure | apt |
| 1259 | GO:0008150 | P | 1, | 271 | 272.906 (x 0.993) | 8082 (0.034) | 0.867 | biological\_process | Aats-leu Act57B Aldh Aly Arf84F Arr2 Ate1 BEST:CK01227 BG:DS01219.1 BG:DS02740.9 BG:DS03431.1 BcDNA:LD23830 BcDNA:LD32788 Bmcp CAH1 CAH2 CG10214 CG10306 CG10444 CG10445 CG10460 CG10638 CG10861 CG10877 CG1134 CG11837 CG11897 CG11907 CG12264 CG12876 CG12954 CG1299 CG13586 CG13645 CG13692 CG14869 CG14935 CG15629 CG17108 CG1718 CG17227 CG17843 CG18522 CG1882 CG2064 CG2065 CG2246 CG2277 CG2453 CG2885 CG2989 CG3008 CG30152 CG30343 CG3036 CG31279 CG31373 CG31601 CG31611 CG3168 CG31793 CG3192 CG31922 CG32017 CG32021 CG32207 CG32409 CG32442 CG32448 CG32549 CG32582 CG32625 CG32649 CG32756 CG33002 CG33158 CG3803 CG3860 CG40006 CG4096 CG4267 CG4364 CG4511 CG4769 CG4866 CG4917 CG4933 CG5037 CG5044 CG5162 CG5177 CG5205 CG5317 CG5338 CG5397 CG5535 CG5548 CG5558 CG5703 CG5800 CG5946 CG6204 CG6272 CG6330 CG6512 CG6574 CG6610 CG6739 CG6764 CG6803 CG6876 CG6950 CG7014 CG7054 CG7163 CG7188 CG7194 CG7339 CG7515 CG7571 CG7627 CG7722 CG7791 CG7828 CG7842 CG7955 CG7997 CG8004 CG8199 CG8226 CG8327 CG8630 CG8756 CG8798 CG9273 CG9358 CG9372 CG9460 CG9790 CG9836 CG9862 Chit Cp36 DDB1 Dbp80 Dgp-1 EG:52C10.1 EP2237 Ets21C Fdxh GABA-B-R2 GV1 Gasp Gclc Gdh Gr39a Gs2 GstD3 GstD6 GstD9 GstE1 GstE3 GstE5 GstE6 GstE7 HBS1 HLHmgamma Hmgcr Ide Idgf2 Idgf3 Imp ImpL3 Irbp Jafrac1 JhI-1 JhI-26 Jheh3 Ku80 La LamC Las Lcp65Ag1 Mhc Mlc1 Mlc2 Mlp60A Mmp1 Mocs1 NP15.6 Nmd3 Nxt1 Obp83g Pcaf Pdsw Ptp61F R REG Rep RpA-70 RpL17A RpS4 SP1173 Ssb-c31a Ssrp Sulf1 TMS1 Taf12L TepIV Thor Tim10 Tim9a TotA TpnC41C Traf1 Tsf1 Tsf3 Tsp42El Ugt86Da Ugt86Di W ab agt apt bcn92 cher cul-2 desat1 dia dlp eIF6 eiger fau fru ftz-f1 glob1 hay huntingtin ken l(3)01239 lama loco mRpL22 mRpL22-24 mRpS14 mRpS18a mRpS21 mRpS33 melt mfas mirr mre11 mus205 na os osp ppk puc rad50 rpr sas sda sog tko toy trol unc-13 wun wun2 wupA yellow-b |
| 1260 | GO:0019887 | F | 4, | 1 | 1.182 (x 0.846) | 35 (0.029) | 0.872 | protein kinase regulator activity | CG9790 |
| 1261 | GO:0007163 | P | 5, 6, | 2 | 2.397 (x 0.834) | 71 (0.028) | 0.872 | establishment and/or maintenance of cell polarity | mirr puc |
| 1262 | GO:0007444 | P | 4, | 9 | 10.198 (x 0.883) | 302 (0.030) | 0.872 | imaginal disc development | Idgf2 Idgf3 W dlp ken mirr puc sog toy |
| 1263 | GO:0009583 | P | 5, 6, | 1 | 1.182 (x 0.846) | 35 (0.029) | 0.872 | detection of light stimulus | Arr2 |
| 1264 | GO:0009888 | P | 3, | 12 | 13.439 (x 0.893) | 398 (0.030) | 0.873 | tissue development | BG:DS02740.9 CG5397 HLHmgamma Mlc1 Mlp60A Tsp42El melt mfas mirr puc sog toy |
| 1265 | GO:0019204 | F | 7, | 1 | 1.182 (x 0.846) | 35 (0.029) | 0.873 | nucleotide phosphatase activity | CG2277 |
| 1266 | GO:0007456 | P | 6, | 5 | 5.842 (x 0.856) | 173 (0.029) | 0.874 | eye development (sensu Endopterygota) | HLHmgamma W mirr puc toy |
| 1267 | GO:0046943 | F | 4, | 2 | 2.431 (x 0.823) | 72 (0.028) | 0.876 | carboxylic acid transporter activity | BG:DS03431.1 CG5535 |
| 1268 | GO:0009887 | P | 4, | 10 | 11.346 (x 0.881) | 336 (0.030) | 0.876 | organ morphogenesis | Act57B CG7194 EP2237 Hmgcr W apt mirr os puc sog |
| 1269 | GO:0007442 | P | 7, 8, | 1 | 1.216 (x 0.823) | 36 (0.028) | 0.878 | hindgut morphogenesis | os |
| 1270 | GO:0007623 | P | 4, | 1 | 1.216 (x 0.823) | 36 (0.028) | 0.879 | circadian rhythm | na |
| 1271 | GO:0016209 | F | 2, | 1 | 1.216 (x 0.823) | 36 (0.028) | 0.879 | antioxidant activity | Jafrac1 |
| 1272 | GO:0019897 | C | 5, 6, 7, | 1 | 1.216 (x 0.823) | 36 (0.028) | 0.88 | extrinsic to plasma membrane | CG9358 |
| 1273 | GO:0006898 | P | 7, 8, | 1 | 1.216 (x 0.823) | 36 (0.028) | 0.881 | receptor mediated endocytosis | R |
| 1274 | GO:0016071 | P | 7, | 6 | 7.024 (x 0.854) | 208 (0.029) | 0.881 | mRNA metabolism | CG10445 CG5205 CG6610 CG6876 Nmd3 hay |
| 1275 | GO:0006766 | P | 5, | 1 | 1.216 (x 0.823) | 36 (0.028) | 0.882 | vitamin metabolism | CG13645 |
| 1276 | GO:0005342 | F | 3, | 2 | 2.499 (x 0.800) | 74 (0.027) | 0.887 | organic acid transporter activity | BG:DS03431.1 CG5535 |
| 1277 | GO:0031325 | P | 6, | 2 | 2.499 (x 0.800) | 74 (0.027) | 0.887 | positive regulation of cellular metabolism | EP2237 mirr |
| 1278 | GO:0048511 | P | 3, | 1 | 1.249 (x 0.800) | 37 (0.027) | 0.887 | rhythmic process | na |
| 1279 | GO:0009893 | P | 5, | 2 | 2.499 (x 0.800) | 74 (0.027) | 0.888 | positive regulation of metabolism | EP2237 mirr |
| 1280 | GO:0012505 | C | 4, 5, | 4 | 4.862 (x 0.823) | 144 (0.028) | 0.888 | endomembrane system | GV1 Hmgcr LamC Nxt1 |
| 1281 | GO:0008372 | C | 2, | 25 | 27.486 (x 0.910) | 814 (0.031) | 0.889 | cellular component unknown | CG11837 CG30152 CG30343 CG31279 CG31601 CG32017 CG32021 CG32207 CG32442 CG32448 CG32582 CG32625 CG32756 CG7194 CG8327 Dgp-1 GstD3 JhI-26 NP15.6 Obp83g SP1173 fau lama osp yellow-b |
| 1282 | GO:0030554 | F | 5, | 21 | 23.266 (x 0.903) | 689 (0.030) | 0.889 | adenyl nucleotide binding | Aats-leu BEST:CK01227 CG10445 CG11897 CG1582 CG1718 CG17227 CG3168 CG31793 CG4511 CG4858 CG5205 CG5800 CG6512 CG7627 CG7955 CG8798 Dbp80 Mhc hay rad50 |
| 1283 | GO:0009165 | P | 6, 7, | 3 | 3.714 (x 0.808) | 110 (0.027) | 0.889 | nucleotide biosynthesis | BcDNA:LD32788 CG13645 CG2246 |
| 1284 | GO:0008066 | F | 5, | 1 | 1.283 (x 0.779) | 38 (0.026) | 0.893 | glutamate receptor activity | GABA-B-R2 |
| 1285 | GO:0007283 | P | 6, | 3 | 3.748 (x 0.800) | 111 (0.027) | 0.893 | spermatogenesis | CG9358 Taf12L dia |
| 1286 | GO:0004175 | F | 5, | 14 | 15.904 (x 0.880) | 471 (0.030) | 0.893 | endopeptidase activity | BG:DS01068.5 CG1299 CG14869 CG4096 CG4933 CG5317 CG6357 CG6512 CG7791 CG8798 CG9372 Ide Mlc1 Mmp1 |
| 1287 | GO:0005634 | C | 5, 6, 7, 8, | 48 | 51.461 (x 0.933) | 1524 (0.031) | 0.894 | nucleus | BcDNA:LD23830 CG10214 CG10306 CG10445 CG31611 CG31666 CG31922 CG32409 CG3371 CG5205 CG6272 CG6610 CG6876 CG7163 CG7339 CG9273 DDB1 EG:52C10.1 EP2237 Ets21C GV1 HLHmgamma Irbp JhI-1 Ku80 La LamC Mlp60A Nxt1 Pcaf Ptp61F REG RpA-70 Ssb-c31a Ssrp Taf12L ab apt cul-2 fru ftz-f1 hay ken mirr mre11 mus205 rad50 toy |
| 1288 | GO:0048232 | P | 5, | 3 | 3.748 (x 0.800) | 111 (0.027) | 0.894 | male gamete generation | CG9358 Taf12L dia |
| 1289 | GO:0008235 | F | 6, | 2 | 2.566 (x 0.779) | 76 (0.026) | 0.896 | metalloexopeptidase activity | huntingtin sda |
| 1290 | GO:0006139 | P | 5, | 56 | 59.802 (x 0.936) | 1771 (0.032) | 0.896 | nucleobase, nucleoside, nucleotide and nucleic acid metabolism | Aats-leu Aly BcDNA:LD32788 CG10214 CG10445 CG11837 CG11907 CG13645 CG17227 CG18522 CG2246 CG2277 CG2885 CG31611 CG3168 CG32549 CG4364 CG5205 CG5558 CG5800 CG6204 CG6272 CG6330 CG6610 CG6876 CG7163 CG7339 CG9273 CG9862 DDB1 Dbp80 EP2237 Ets21C HLHmgamma Irbp JhI-1 Ku80 La Nmd3 Pcaf RpA-70 Ssb-c31a Ssrp Taf12L ab agt apt fru ftz-f1 hay mirr mre11 mus205 rad50 toy trol |
| 1291 | GO:0048523 | P | 4, | 8 | 9.455 (x 0.846) | 280 (0.029) | 0.897 | negative regulation of cellular process | CG7188 HLHmgamma Mmp1 Thor apt puc rpr sog |
| 1292 | GO:0048547 | P | 5, 6, | 1 | 1.351 (x 0.740) | 40 (0.025) | 0.899 | gut morphogenesis | os |
| 1293 | GO:0048546 | P | 5, | 1 | 1.351 (x 0.740) | 40 (0.025) | 0.9 | digestive tract morphogenesis | os |
| 1294 | GO:0045045 | P | 5, 6, | 6 | 7.260 (x 0.826) | 215 (0.028) | 0.9 | secretory pathway | Arf84F CG2885 CG3168 R Rep unc-13 |
| 1295 | GO:0046552 | P | 5, | 1 | 1.351 (x 0.740) | 40 (0.025) | 0.9 | photoreceptor cell fate commitment | R |
| 1296 | GO:0015296 | F | 5, 7, | 1 | 1.317 (x 0.759) | 39 (0.026) | 0.901 | anion:cation symporter activity | CG3036 |
| 1297 | GO:0044267 | P | 6, | 66 | 70.202 (x 0.940) | 2079 (0.032) | 0.901 | cellular protein metabolism | Aats-leu Act57B Arf84F Ate1 CG10306 CG10444 CG1134 CG12954 CG1299 CG13645 CG13692 CG14869 CG3008 CG31373 CG32649 CG33002 CG33158 CG4096 CG4866 CG4933 CG5317 CG5338 CG6512 CG6574 CG6764 CG7014 CG7722 CG7791 CG7828 CG8798 CG9358 CG9372 CG9460 Dgp-1 Fdxh Gclc Gs2 HBS1 Ide Imp Las Mlc1 Mmp1 Ptp61F REG Rep RpL17A RpS4 Thor W apt cul-2 eIF6 huntingtin l(3)01239 mRpL22 mRpL22-24 mRpS14 mRpS18a mRpS21 mRpS33 na puc rpr sda tko |
| 1298 | GO:0000004 | P | 2, | 21 | 23.671 (x 0.887) | 701 (0.030) | 0.901 | biological process unknown | CG30152 CG30343 CG31279 CG31601 CG31922 CG32017 CG32021 CG32207 CG32442 CG32448 CG32582 CG32625 CG32756 GV1 JhI-26 NP15.6 SP1173 fau lama osp yellow-b |
| 1299 | GO:0043414 | P | 7, | 1 | 1.351 (x 0.740) | 40 (0.025) | 0.901 | biopolymer methylation | trol |
| 1300 | GO:0005694 | C | 5, 6, 7, 8, | 5 | 6.213 (x 0.805) | 184 (0.027) | 0.901 | chromosome | CG31611 CG3371 CG9273 Pcaf RpA-70 |
| 1301 | GO:0016567 | P | 9, | 3 | 3.816 (x 0.786) | 113 (0.027) | 0.901 | protein ubiquitination | CG1134 W rpr |
| 1302 | GO:0046148 | P | 6, | 1 | 1.317 (x 0.759) | 39 (0.026) | 0.901 | pigment biosynthesis | CG5037 |
| 1303 | GO:0044427 | C | 4, 5, 6, 7, 8, 9, | 4 | 4.998 (x 0.800) | 148 (0.027) | 0.901 | chromosomal part | CG31611 CG3371 CG9273 RpA-70 |
| 1304 | GO:0048589 | P | 3, | 1 | 1.351 (x 0.740) | 40 (0.025) | 0.902 | developmental growth | Mmp1 |
| 1305 | GO:0003704 | F | 4, | 2 | 2.668 (x 0.750) | 79 (0.025) | 0.902 | specific RNA polymerase II transcription factor activity | ab toy |
| 1306 | GO:0005789 | C | 4, 5, 6, 7, 8, 9, 10, | 1 | 1.351 (x 0.740) | 40 (0.025) | 0.902 | endoplasmic reticulum membrane | Hmgcr |
| 1307 | GO:0004177 | F | 6, | 1 | 1.351 (x 0.740) | 40 (0.025) | 0.903 | aminopeptidase activity | sda |
| 1308 | GO:0005351 | F | 5, 6, | 1 | 1.351 (x 0.740) | 40 (0.025) | 0.904 | sugar porter activity | TepIV |
| 1309 | GO:0007459 | P | 6, | 1 | 1.351 (x 0.740) | 40 (0.025) | 0.904 | photoreceptor fate commitment (sensu Endopterygota) | R |
| 1310 | GO:0035220 | P | 5, | 3 | 3.917 (x 0.766) | 116 (0.026) | 0.904 | wing disc development | dlp mirr sog |
| 1311 | GO:0004620 | F | 7, | 1 | 1.384 (x 0.722) | 41 (0.024) | 0.905 | phospholipase activity | CG4267 |
| 1312 | GO:0048567 | P | 6, 7, | 1 | 1.351 (x 0.740) | 40 (0.025) | 0.905 | ectodermal gut morphogenesis | os |
| 1313 | GO:0019318 | P | 7, 8, | 2 | 2.701 (x 0.740) | 80 (0.025) | 0.905 | hexose metabolism | DDB1 ImpL3 |
| 1314 | GO:0042175 | C | 4, 5, 6, | 1 | 1.384 (x 0.722) | 41 (0.024) | 0.906 | nuclear envelope-endoplasmic reticulum network | Hmgcr |
| 1315 | GO:0007439 | P | 5, | 1 | 1.351 (x 0.740) | 40 (0.025) | 0.906 | ectodermal gut development | os |
| 1316 | GO:0048489 | P | 6, 7, | 2 | 2.701 (x 0.740) | 80 (0.025) | 0.906 | synaptic vesicle transport | Arf84F unc-13 |
| 1317 | GO:0000279 | P | 5, | 8 | 9.691 (x 0.825) | 287 (0.028) | 0.906 | M phase | CG6512 CG9790 CG9862 Mhc R Taf12L dia mre11 |
| 1318 | GO:0007219 | P | 6, | 1 | 1.384 (x 0.722) | 41 (0.024) | 0.906 | Notch signaling pathway | HLHmgamma |
| 1319 | GO:0048592 | P | 5, 6, | 4 | 5.031 (x 0.795) | 149 (0.027) | 0.906 | eye morphogenesis | EP2237 W mirr puc |
| 1320 | GO:0008010 | F | 5, | 1 | 1.351 (x 0.740) | 40 (0.025) | 0.906 | structural constituent of larval cuticle (sensu Insecta) | Lcp65Ag1 |
| 1321 | GO:0015103 | F | 5, | 1 | 1.351 (x 0.740) | 40 (0.025) | 0.907 | inorganic anion transporter activity | CG3036 |
| 1322 | GO:0015144 | F | 3, | 2 | 2.735 (x 0.731) | 81 (0.025) | 0.911 | carbohydrate transporter activity | CG3168 TepIV |
| 1323 | GO:0015399 | F | 4, | 3 | 4.052 (x 0.740) | 120 (0.025) | 0.916 | primary active transporter activity | CG8226 Tim10 Tim9a |
| 1324 | GO:0015405 | F | 5, | 3 | 4.052 (x 0.740) | 120 (0.025) | 0.917 | P-P-bond-hydrolysis-driven transporter activity | CG8226 Tim10 Tim9a |
| 1325 | GO:0005856 | C | 5, 6, 7, 8, | 8 | 9.792 (x 0.817) | 290 (0.028) | 0.917 | cytoskeleton | Act57B LamC Mhc Mlc1 Mlc2 Rep dia wupA |
| 1326 | GO:0007447 | P | 4, 5, | 1 | 1.452 (x 0.689) | 43 (0.023) | 0.917 | imaginal disc pattern formation | dlp |
| 1327 | GO:0007411 | P | 6, 7, 9, 10, 12, | 2 | 2.769 (x 0.722) | 82 (0.024) | 0.917 | axon guidance | Ptp61F ab |
| 1328 | GO:0016789 | F | 5, | 3 | 4.052 (x 0.740) | 120 (0.025) | 0.917 | carboxylic ester hydrolase activity | CG4267 CG5162 alpha-Est10 |
| 1329 | GO:0031324 | P | 6, | 4 | 5.234 (x 0.764) | 155 (0.026) | 0.917 | negative regulation of cellular metabolism | HLHmgamma Thor apt rpr |
| 1330 | GO:0006817 | P | 8, 9, | 1 | 1.452 (x 0.689) | 43 (0.023) | 0.917 | phosphate transport | CG3036 |
| 1331 | GO:0016591 | C | 3, 6, 7, 8, 9, 10, 11, 12, 13, | 2 | 2.803 (x 0.714) | 83 (0.024) | 0.917 | DNA-directed RNA polymerase II, holoenzyme | Taf12L hay |
| 1332 | GO:0007600 | P | 3, 5, | 7 | 8.712 (x 0.803) | 258 (0.027) | 0.918 | sensory perception | Arr2 CG15629 CG4917 CG9358 Gr39a Obp83g tko |
| 1333 | GO:0006367 | P | 9, | 2 | 2.836 (x 0.705) | 84 (0.024) | 0.918 | transcription initiation from RNA polymerase II promoter | Taf12L hay |
| 1334 | GO:0048812 | P | 7, 8, 10, | 3 | 4.086 (x 0.734) | 121 (0.025) | 0.918 | neurite morphogenesis | Ptp61F ab mfas |
| 1335 | GO:0006814 | P | 8, 9, | 1 | 1.452 (x 0.689) | 43 (0.023) | 0.918 | sodium ion transport | ppk |
| 1336 | GO:0016023 | C | 5, 6, 7, 8, 9, | 2 | 2.803 (x 0.714) | 83 (0.024) | 0.918 | cytoplasmic membrane-bound vesicle | Rep unc-13 |
| 1337 | GO:0009408 | P | 4, 5, | 1 | 1.587 (x 0.630) | 47 (0.021) | 0.918 | response to heat | TotA |
| 1338 | GO:0007270 | P | 7, | 1 | 1.553 (x 0.644) | 46 (0.022) | 0.918 | nerve-nerve synaptic transmission | GABA-B-R2 |
| 1339 | GO:0006996 | P | 5, | 21 | 24.380 (x 0.861) | 722 (0.029) | 0.918 | organelle organization and biogenesis | Act57B CG10861 CG31611 CG32409 CG6764 CG6803 EG:52C10.1 Irbp Ku80 LamC Nmd3 Ssrp Tim10 Tim9a cher dia fru huntingtin mre11 puc rad50 |
| 1340 | GO:0048667 | P | 6, 7, 9, | 3 | 4.086 (x 0.734) | 121 (0.025) | 0.919 | neuron morphogenesis during differentiation | Ptp61F ab mfas |
| 1341 | GO:0004180 | F | 6, | 1 | 1.452 (x 0.689) | 43 (0.023) | 0.919 | carboxypeptidase activity | huntingtin |
| 1342 | GO:0005700 | C | 6, 7, 8, 9, | 1 | 1.520 (x 0.658) | 45 (0.022) | 0.919 | polytene chromosome | Pcaf |
| 1343 | GO:0031410 | C | 4, 5, 6, 7, 8, | 2 | 2.803 (x 0.714) | 83 (0.024) | 0.919 | cytoplasmic vesicle | Rep unc-13 |
| 1344 | GO:0007293 | P | 8, | 1 | 1.587 (x 0.630) | 47 (0.021) | 0.919 | egg chamber formation (sensu Insecta) | cher |
| 1345 | GO:0016079 | P | 7, 8, 9, | 1 | 1.553 (x 0.644) | 46 (0.022) | 0.919 | synaptic vesicle exocytosis | unc-13 |
| 1346 | GO:0016298 | F | 6, | 2 | 2.972 (x 0.673) | 88 (0.023) | 0.919 | lipase activity | CG4267 CG5162 |
| 1347 | GO:0007409 | P | 8, 9, 11, | 3 | 4.086 (x 0.734) | 121 (0.025) | 0.919 | axonogenesis | Ptp61F ab mfas |
| 1348 | GO:0019898 | C | 4, 5, 6, | 1 | 1.520 (x 0.658) | 45 (0.022) | 0.92 | extrinsic to membrane | CG9358 |
| 1349 | GO:0050790 | P | 3, | 1 | 1.486 (x 0.673) | 44 (0.023) | 0.92 | regulation of catalytic activity | Rep |
| 1350 | GO:0016811 | F | 5, | 1 | 1.587 (x 0.630) | 47 (0.021) | 0.92 | hydrolase activity, acting on carbon-nitrogen (but not peptide) bonds, in linear amides | CG31373 |
| 1351 | GO:0007067 | P | 7, | 6 | 7.631 (x 0.786) | 226 (0.027) | 0.92 | mitosis | CG6512 CG9790 CG9862 Mhc R dia |
| 1352 | GO:0042440 | P | 5, | 1 | 1.553 (x 0.644) | 46 (0.022) | 0.92 | pigment metabolism | CG5037 |
| 1353 | GO:0031982 | C | 3, | 2 | 2.904 (x 0.689) | 86 (0.023) | 0.92 | vesicle | Rep unc-13 |
| 1354 | GO:0009117 | P | 6, | 4 | 5.369 (x 0.745) | 159 (0.025) | 0.92 | nucleotide metabolism | BcDNA:LD32788 CG13645 CG2246 CG6330 |
| 1355 | GO:0046903 | P | 5, | 6 | 7.800 (x 0.769) | 231 (0.026) | 0.92 | secretion | Arf84F CG2885 CG3168 R Rep unc-13 |
| 1356 | GO:0005938 | C | 5, 6, 7, 8, | 1 | 1.520 (x 0.658) | 45 (0.022) | 0.92 | cell cortex | dia |
| 1357 | GO:0019226 | P | 5, | 10 | 12.257 (x 0.816) | 363 (0.028) | 0.92 | transmission of nerve impulse | Arf84F CG3168 GABA-B-R2 Gs2 Rep Tsp42El ab apt fru unc-13 |
| 1358 | GO:0006606 | P | 7, 8, 9, 10, | 1 | 1.587 (x 0.630) | 47 (0.021) | 0.92 | protein import into nucleus | Nxt1 |
| 1359 | GO:0030534 | P | 4, | 1 | 1.553 (x 0.644) | 46 (0.022) | 0.92 | adult behavior | na |
| 1360 | GO:0000087 | P | 6, | 6 | 7.665 (x 0.783) | 227 (0.026) | 0.92 | M phase of mitotic cell cycle | CG6512 CG9790 CG9862 Mhc R dia |
| 1361 | GO:0006352 | P | 8, | 2 | 2.904 (x 0.689) | 86 (0.023) | 0.921 | transcription initiation | Taf12L hay |
| 1362 | GO:0007126 | P | 7, | 2 | 2.938 (x 0.681) | 87 (0.023) | 0.921 | meiosis | Taf12L mre11 |
| 1363 | GO:0016757 | F | 4, | 4 | 5.369 (x 0.745) | 159 (0.025) | 0.921 | transferase activity, transferring glycosyl groups | Act57B CG6330 Ugt86Da Ugt86Di |
| 1364 | GO:0043283 | P | 5, | 52 | 56.864 (x 0.914) | 1684 (0.031) | 0.921 | biopolymer metabolism | Aats-leu Arf84F Ate1 CG10214 CG10445 CG1134 CG11837 CG13692 CG17227 CG2989 CG3008 CG31373 CG31611 CG3168 CG4364 CG5205 CG5558 CG6204 CG6610 CG6876 CG7163 CG7791 CG7828 CG7997 CG8756 CG9273 CG9358 Chit DDB1 Gasp Irbp JhI-1 Ku80 La Nmd3 Ptp61F Rep RpA-70 Ssrp Sulf1 Ugt86Da Ugt86Di W agt fru hay mre11 mus205 puc rad50 rpr trol |
| 1365 | GO:0005554 | F | 2, | 22 | 25.393 (x 0.866) | 752 (0.029) | 0.921 | molecular function unknown | CG10861 CG30152 CG30343 CG31279 CG31601 CG32017 CG32021 CG32207 CG32442 CG32448 CG32582 CG32625 CG32756 CG7194 JhI-26 NP15.6 SP1173 fau lama melt osp yellow-b |
| 1366 | GO:0007423 | P | 4, | 6 | 7.766 (x 0.773) | 230 (0.026) | 0.921 | sensory organ development | EP2237 HLHmgamma W mirr puc toy |
| 1367 | GO:0016407 | F | 7, | 1 | 1.587 (x 0.630) | 47 (0.021) | 0.921 | acetyltransferase activity | Pcaf |
| 1368 | GO:0008360 | P | 5, 6, | 2 | 2.870 (x 0.697) | 85 (0.024) | 0.921 | regulation of cell shape | R puc |
| 1369 | GO:0031988 | C | 4, | 2 | 2.904 (x 0.689) | 86 (0.023) | 0.921 | membrane-bound vesicle | Rep unc-13 |
| 1370 | GO:0035214 | P | 5, | 4 | 5.403 (x 0.740) | 160 (0.025) | 0.921 | eye-antennal disc development | W mirr puc toy |
| 1371 | GO:0007417 | P | 5, | 3 | 4.120 (x 0.728) | 122 (0.025) | 0.922 | central nervous system development | apt fru rpr |
| 1372 | GO:0044453 | C | 4, 5, 6, 7, 8, 9, 10, 11, 12, | 1 | 1.587 (x 0.630) | 47 (0.021) | 0.922 | nuclear membrane part | LamC |
| 1373 | GO:0016810 | F | 4, | 2 | 2.870 (x 0.697) | 85 (0.024) | 0.922 | hydrolase activity, acting on carbon-nitrogen (but not peptide) bonds | CG31373 CG8756 |
| 1374 | GO:0000278 | P | 5, | 7 | 9.050 (x 0.774) | 268 (0.026) | 0.922 | mitotic cell cycle | CG6512 CG9790 CG9862 LamC Mhc R dia |
| 1375 | GO:0031965 | C | 5, 6, 7, 8, 9, 10, 11, | 1 | 1.587 (x 0.630) | 47 (0.021) | 0.922 | nuclear membrane | LamC |
| 1376 | GO:0006470 | P | 8, | 2 | 2.870 (x 0.697) | 85 (0.024) | 0.922 | protein amino acid dephosphorylation | Ptp61F puc |
| 1377 | GO:0009416 | P | 5, | 1 | 1.621 (x 0.617) | 48 (0.021) | 0.923 | response to light stimulus | Arr2 |
| 1378 | GO:0051119 | F | 4, | 1 | 1.587 (x 0.630) | 47 (0.021) | 0.923 | sugar transporter activity | TepIV |
| 1379 | GO:0051243 | P | 5, | 6 | 7.834 (x 0.766) | 232 (0.026) | 0.923 | negative regulation of cellular physiological process | CG7188 HLHmgamma Mmp1 Thor apt rpr |
| 1380 | GO:0051170 | P | 7, 8, 9, | 1 | 1.621 (x 0.617) | 48 (0.021) | 0.923 | nuclear import | Nxt1 |
| 1381 | GO:0004721 | F | 7, | 2 | 3.005 (x 0.665) | 89 (0.022) | 0.924 | phosphoprotein phosphatase activity | Ptp61F puc |
| 1382 | GO:0009892 | P | 5, | 4 | 5.572 (x 0.718) | 165 (0.024) | 0.928 | negative regulation of metabolism | HLHmgamma Thor apt rpr |
| 1383 | GO:0006575 | P | 6, | 1 | 1.655 (x 0.604) | 49 (0.020) | 0.928 | amino acid derivative metabolism | CG8327 |
| 1384 | GO:0048519 | P | 3, | 8 | 10.299 (x 0.777) | 305 (0.026) | 0.929 | negative regulation of biological process | CG7188 HLHmgamma Mmp1 Thor apt puc rpr sog |
| 1385 | GO:0016758 | F | 5, | 3 | 4.356 (x 0.689) | 129 (0.023) | 0.929 | transferase activity, transferring hexosyl groups | Act57B Ugt86Da Ugt86Di |
| 1386 | GO:0016853 | F | 3, | 2 | 3.073 (x 0.651) | 91 (0.022) | 0.929 | isomerase activity | CG11858 CG3168 |
| 1387 | GO:0048468 | P | 4, | 9 | 11.481 (x 0.784) | 340 (0.026) | 0.93 | cell development | CG6803 Mhc Ptp61F ab dia mfas os wun2 wupA |
| 1388 | GO:0048737 | P | 4, | 3 | 4.390 (x 0.683) | 130 (0.023) | 0.93 | appendage development (sensu Endopterygota) | W mirr sog |
| 1389 | GO:0035114 | P | 5, | 3 | 4.390 (x 0.683) | 130 (0.023) | 0.931 | appendage morphogenesis (sensu Endopterygota) | W mirr sog |
| 1390 | GO:0008092 | F | 4, | 6 | 8.037 (x 0.747) | 238 (0.025) | 0.932 | cytoskeletal protein binding | BG:DS02740.9 Mhc cher dia huntingtin wupA |
| 1391 | GO:0005244 | F | 5, 6, | 1 | 1.722 (x 0.581) | 51 (0.020) | 0.936 | voltage-gated ion channel activity | na |
| 1392 | GO:0031497 | P | 10, | 1 | 1.722 (x 0.581) | 51 (0.020) | 0.937 | chromatin assembly | CG31611 |
| 1393 | GO:0005624 | C | 4, 5, | 2 | 3.140 (x 0.637) | 93 (0.022) | 0.937 | membrane fraction | Arr2 Jheh3 |
| 1394 | GO:0019899 | F | 4, | 1 | 1.756 (x 0.570) | 52 (0.019) | 0.937 | enzyme binding | dia |
| 1395 | GO:0043118 | P | 4, | 6 | 8.138 (x 0.737) | 241 (0.025) | 0.938 | negative regulation of physiological process | CG7188 HLHmgamma Mmp1 Thor apt rpr |
| 1396 | GO:0000122 | P | 10, | 1 | 1.756 (x 0.570) | 52 (0.019) | 0.938 | negative regulation of transcription from RNA polymerase II promoter | HLHmgamma |
| 1397 | GO:0048749 | P | 7, | 3 | 4.491 (x 0.668) | 133 (0.023) | 0.938 | compound eye development (sensu Endopterygota) | W mirr puc |
| 1398 | GO:0044432 | C | 4, 5, 6, 7, 8, 9, | 1 | 1.756 (x 0.570) | 52 (0.019) | 0.939 | endoplasmic reticulum part | Hmgcr |
| 1399 | GO:0035107 | P | 4, | 3 | 4.491 (x 0.668) | 133 (0.023) | 0.939 | appendage morphogenesis | W mirr sog |
| 1400 | GO:0005215 | F | 2, | 28 | 32.687 (x 0.857) | 968 (0.029) | 0.939 | transporter activity | BEST:CK01227 BG:DS03431.1 Bmcp CG10444 CG11897 CG11907 CG14935 CG17108 CG1718 CG3036 CG3168 CG31793 CG5535 CG6574 CG7571 CG7627 CG7955 CG8226 CG9358 Nxt1 TepIV Tim10 Tim9a Tsf1 Tsf3 glob1 na ppk |
| 1401 | GO:0048736 | P | 3, | 3 | 4.491 (x 0.668) | 133 (0.023) | 0.94 | appendage development | W mirr sog |
| 1402 | GO:0001745 | P | 7, 8, | 3 | 4.491 (x 0.668) | 133 (0.023) | 0.94 | compound eye morphogenesis (sensu Endopterygota) | W mirr puc |
| 1403 | GO:0008527 | F | 7, | 1 | 1.790 (x 0.559) | 53 (0.019) | 0.941 | taste receptor activity | Gr39a |
| 1404 | GO:0000267 | C | 3, 4, | 2 | 3.242 (x 0.617) | 96 (0.021) | 0.941 | cell fraction | Arr2 Jheh3 |
| 1405 | GO:0009314 | P | 4, | 1 | 1.790 (x 0.559) | 53 (0.019) | 0.942 | response to radiation | Arr2 |
| 1406 | GO:0050877 | P | 4, | 17 | 20.801 (x 0.817) | 616 (0.028) | 0.942 | neurophysiological process | Arf84F Arr2 CG15629 CG3168 CG4917 CG9358 GABA-B-R2 Gr39a Gs2 Obp83g Rep Tsp42El ab apt fru tko unc-13 |
| 1407 | GO:0005179 | F | 4, 5, | 1 | 1.790 (x 0.559) | 53 (0.019) | 0.942 | hormone activity | CG13586 |
| 1408 | GO:0015698 | P | 7, 8, | 1 | 1.823 (x 0.548) | 54 (0.019) | 0.944 | inorganic anion transport | CG3036 |
| 1409 | GO:0000502 | C | 3, 4, 5, 6, | 1 | 1.823 (x 0.548) | 54 (0.019) | 0.945 | proteasome complex (sensu Eukaryota) | REG |
| 1410 | GO:0006323 | P | 7, | 3 | 4.660 (x 0.644) | 138 (0.022) | 0.949 | DNA packaging | CG31611 Ssrp fru |
| 1411 | GO:0016055 | P | 6, | 1 | 1.857 (x 0.538) | 55 (0.018) | 0.95 | Wnt receptor signaling pathway | dlp |
| 1412 | GO:0006325 | P | 8, | 3 | 4.660 (x 0.644) | 138 (0.022) | 0.95 | establishment and/or maintenance of chromatin architecture | CG31611 Ssrp fru |
| 1413 | GO:0005085 | F | 4, | 1 | 1.958 (x 0.511) | 58 (0.017) | 0.954 | guanyl-nucleotide exchange factor activity | CG7515 |
| 1414 | GO:0046698 | P | 5, | 6 | 8.476 (x 0.708) | 251 (0.024) | 0.954 | metamorphosis (sensu Insecta) | CG9358 W ftz-f1 mirr puc sog |
| 1415 | GO:0016876 | F | 5, | 1 | 1.958 (x 0.511) | 58 (0.017) | 0.955 | ligase activity, forming aminoacyl-tRNA and related compounds | Aats-leu |
| 1416 | GO:0006820 | P | 6, 7, | 2 | 3.377 (x 0.592) | 100 (0.020) | 0.955 | anion transport | CG3036 CG7571 |
| 1417 | GO:0004812 | F | 6, | 1 | 1.958 (x 0.511) | 58 (0.017) | 0.955 | aminoacyl-tRNA ligase activity | Aats-leu |
| 1418 | GO:0005996 | P | 6, 7, | 2 | 3.377 (x 0.592) | 100 (0.020) | 0.956 | monosaccharide metabolism | DDB1 ImpL3 |
| 1419 | GO:0016251 | F | 4, | 2 | 3.410 (x 0.586) | 101 (0.020) | 0.956 | general RNA polymerase II transcription factor activity | Taf12L hay |
| 1420 | GO:0016875 | F | 4, | 1 | 1.958 (x 0.511) | 58 (0.017) | 0.956 | ligase activity, forming carbon-oxygen bonds | Aats-leu |
| 1421 | GO:0016564 | F | 3, | 1 | 1.925 (x 0.520) | 57 (0.018) | 0.956 | transcriptional repressor activity | HLHmgamma |
| 1422 | GO:0048748 | P | 6, 7, | 3 | 4.761 (x 0.630) | 141 (0.021) | 0.956 | eye morphogenesis (sensu Endopterygota) | W mirr puc |
| 1423 | GO:0006508 | P | 7, | 21 | 25.595 (x 0.820) | 758 (0.028) | 0.956 | proteolysis | Ate1 CG1299 CG14869 CG4096 CG4933 CG5317 CG6512 CG7722 CG7791 CG8798 CG9372 CG9460 Ide Mlc1 Mmp1 REG W cul-2 huntingtin rpr sda |
| 1424 | GO:0048666 | P | 5, 8, | 3 | 4.795 (x 0.626) | 142 (0.021) | 0.956 | neuron development | Ptp61F ab mfas |
| 1425 | GO:0007552 | P | 4, | 6 | 8.543 (x 0.702) | 253 (0.024) | 0.956 | metamorphosis | CG9358 W ftz-f1 mirr puc sog |
| 1426 | GO:0006869 | P | 5, 6, | 1 | 1.958 (x 0.511) | 58 (0.017) | 0.957 | lipid transport | CG3860 |
| 1427 | GO:0048699 | P | 6, | 4 | 6.078 (x 0.658) | 180 (0.022) | 0.957 | generation of neurons | Ptp61F ab mfas trol |
| 1428 | GO:0007476 | P | 6, 7, 8, | 2 | 3.478 (x 0.575) | 103 (0.019) | 0.957 | wing morphogenesis | mirr sog |
| 1429 | GO:0031175 | P | 6, 9, | 3 | 4.795 (x 0.626) | 142 (0.021) | 0.957 | neurite development | Ptp61F ab mfas |
| 1430 | GO:0008104 | P | 4, | 15 | 19.112 (x 0.785) | 566 (0.027) | 0.957 | protein localization | Arf84F Arr2 BG:DS01219.1 CG2885 CG7791 CG8004 CG8226 Nmd3 Nxt1 R Rep Tim10 Tim9a cher dia |
| 1431 | GO:0005200 | F | 3, | 7 | 9.860 (x 0.710) | 292 (0.024) | 0.957 | structural constituent of cytoskeleton | Act57B BG:DS02740.9 LamC Mhc cher dia wupA |
| 1432 | GO:0051674 | P | 4, | 6 | 8.678 (x 0.691) | 257 (0.023) | 0.958 | localization of cell | Hmgcr Ptp61F ab os wun wun2 |
| 1433 | GO:0043039 | P | 8, 9, | 1 | 2.026 (x 0.494) | 60 (0.017) | 0.959 | tRNA aminoacylation | Aats-leu |
| 1434 | GO:0006928 | P | 4, 5, | 6 | 8.678 (x 0.691) | 257 (0.023) | 0.959 | cell motility | Hmgcr Ptp61F ab os wun wun2 |
| 1435 | GO:0006418 | P | 8, 9, 10, | 1 | 2.026 (x 0.494) | 60 (0.017) | 0.959 | tRNA aminoacylation for protein translation | Aats-leu |
| 1436 | GO:0007472 | P | 6, 7, | 2 | 3.546 (x 0.564) | 105 (0.019) | 0.959 | wing disc morphogenesis | mirr sog |
| 1437 | GO:0007455 | P | 6, 7, | 3 | 4.930 (x 0.609) | 146 (0.021) | 0.96 | eye-antennal disc morphogenesis | W mirr puc |
| 1438 | GO:0050909 | P | 5, 7, | 1 | 2.026 (x 0.494) | 60 (0.017) | 0.96 | sensory perception of taste | Gr39a |
| 1439 | GO:0043038 | P | 7, 8, | 1 | 2.060 (x 0.485) | 61 (0.016) | 0.961 | amino acid activation | Aats-leu |
| 1440 | GO:0030001 | P | 7, 8, | 3 | 4.964 (x 0.604) | 147 (0.020) | 0.962 | metal ion transport | Tsf1 Tsf3 ppk |
| 1441 | GO:0040011 | P | 3, | 6 | 8.813 (x 0.681) | 261 (0.023) | 0.963 | locomotion | Hmgcr Ptp61F ab os wun wun2 |
| 1442 | GO:0048565 | P | 4, | 1 | 2.094 (x 0.478) | 62 (0.016) | 0.964 | gut development | os |
| 1443 | GO:0030182 | P | 4, 7, | 3 | 5.031 (x 0.596) | 149 (0.020) | 0.966 | neuron differentiation | Ptp61F ab mfas |
| 1444 | GO:0008238 | F | 5, | 2 | 3.681 (x 0.543) | 109 (0.018) | 0.968 | exopeptidase activity | huntingtin sda |
| 1445 | GO:0007424 | P | 4, | 2 | 3.681 (x 0.543) | 109 (0.018) | 0.969 | tracheal system development (sensu Insecta) | Mmp1 apt |
| 1446 | GO:0019722 | P | 7, | 1 | 2.161 (x 0.463) | 64 (0.016) | 0.97 | calcium-mediated signaling | TpnC41C |
| 1447 | GO:0003702 | F | 3, | 6 | 8.982 (x 0.668) | 266 (0.023) | 0.97 | RNA polymerase II transcription factor activity | Taf12L ab apt fru hay toy |
| 1448 | GO:0044431 | C | 4, 5, 6, 7, 8, 9, | 1 | 2.195 (x 0.456) | 65 (0.015) | 0.971 | Golgi apparatus part | Sulf1 |
| 1449 | GO:0007155 | P | 3, | 7 | 10.231 (x 0.684) | 303 (0.023) | 0.971 | cell adhesion | CG14869 CG40006 CG4096 R dlp mfas trol |
| 1450 | GO:0035239 | P | 4, | 1 | 2.195 (x 0.456) | 65 (0.015) | 0.971 | tube morphogenesis | apt |
| 1451 | GO:0046907 | P | 5, 6, 7, | 16 | 20.801 (x 0.769) | 616 (0.026) | 0.972 | intracellular transport | Arf84F Arr2 Bmcp CG2885 CG7791 CG8004 CG8226 La Nmd3 Nxt1 R Rep Tim10 Tim9a huntingtin loco |
| 1452 | GO:0008233 | F | 4, | 17 | 21.982 (x 0.773) | 651 (0.026) | 0.972 | peptidase activity | BG:DS01068.5 CG1299 CG14869 CG4096 CG4933 CG5317 CG6357 CG6512 CG7791 CG8798 CG9372 Ide Mlc1 Mmp1 huntingtin isopeptidase-T-3 sda |
| 1453 | GO:0005261 | F | 5, 6, | 2 | 3.883 (x 0.515) | 115 (0.017) | 0.981 | cation channel activity | na ppk |
| 1454 | GO:0016881 | F | 5, | 3 | 5.335 (x 0.562) | 158 (0.019) | 0.983 | acid-amino acid ligase activity | CG1134 CG4917 Gclc |
| 1455 | GO:0009798 | P | 4, | 3 | 5.369 (x 0.559) | 159 (0.019) | 0.985 | axis specification | CG9358 loco sog |
| 1456 | GO:0040029 | P | 3, | 1 | 2.364 (x 0.423) | 70 (0.014) | 0.986 | regulation of gene expression, epigenetic | trol |
| 1457 | GO:0009948 | P | 5, | 2 | 4.018 (x 0.498) | 119 (0.017) | 0.99 | anterior/posterior axis specification | CG9358 sog |
| 1458 | GO:0051649 | P | 5, 6, | 16 | 21.442 (x 0.746) | 635 (0.025) | 0.99 | establishment of cellular localization | Arf84F Arr2 Bmcp CG2885 CG7791 CG8004 CG8226 La Nmd3 Nxt1 R Rep Tim10 Tim9a huntingtin loco |
| 1459 | GO:0016020 | C | 3, 4, | 54 | 63.009 (x 0.857) | 1866 (0.029) | 0.99 | membrane | Arr2 BEST:CK01227 BG:DS01219.1 BG:DS03431.1 Bmcp CG10444 CG11897 CG11907 CG17108 CG3036 CG3168 CG31793 CG3192 CG3803 CG40006 CG4769 CG5037 CG5535 CG5548 CG5703 CG6512 CG6574 CG6739 CG7188 CG7571 CG7627 CG7955 CG8004 CG8226 CG8630 CG9358 GABA-B-R2 GV1 Gr39a Hmgcr Ide LamC Nxt1 Pdsw Rep TMS1 Tim10 Tim9a Tsp42El bcn92 desat1 eiger mfas na ppk sas sog wun wun2 |
| 1460 | GO:0051179 | P | 3, | 51 | 59.802 (x 0.853) | 1771 (0.029) | 0.99 | localization | Aly Arf84F Arr2 BEST:CK01227 BG:DS01219.1 BG:DS03431.1 Bmcp CG10444 CG11897 CG11907 CG17108 CG1718 CG2885 CG3036 CG3168 CG31793 CG3860 CG5535 CG6574 CG7571 CG7627 CG7791 CG7955 CG8004 CG8226 CG9862 Hmgcr La Nmd3 Nxt1 Obp83g Ptp61F R Rep TepIV Tim10 Tim9a Tsf1 Tsf3 ab cher dia glob1 huntingtin loco na os ppk unc-13 wun wun2 |
| 1461 | GO:0051641 | P | 4, 5, | 16 | 21.476 (x 0.745) | 636 (0.025) | 0.99 | cellular localization | Arf84F Arr2 Bmcp CG2885 CG7791 CG8004 CG8226 La Nmd3 Nxt1 R Rep Tim10 Tim9a huntingtin loco |
| 1462 | GO:0006366 | P | 8, | 17 | 23.029 (x 0.738) | 682 (0.025) | 0.995 | transcription from RNA polymerase II promoter | Aly CG10445 CG7339 EP2237 Ets21C HLHmgamma Pcaf Ssb-c31a Ssrp Taf12L ab apt fru ftz-f1 hay mirr toy |
| 1463 | GO:0000151 | C | 3, 4, 5, 6, | 2 | 4.255 (x 0.470) | 126 (0.016) | 0.995 | ubiquitin ligase complex | CG1134 cul-2 |
| 1464 | GO:0050793 | P | 3, | 1 | 2.668 (x 0.375) | 79 (0.013) | 0.995 | regulation of development | ftz-f1 |
| 1465 | GO:0007167 | P | 6, | 3 | 5.808 (x 0.517) | 172 (0.017) | 0.995 | enzyme linked receptor protein signaling pathway | CG9358 dlp sog |
| 1466 | GO:0016043 | P | 4, | 38 | 46.565 (x 0.816) | 1379 (0.028) | 0.995 | cell organization and biogenesis | Act57B Arf84F Arr2 Bmcp CG10861 CG2885 CG31611 CG32409 CG6764 CG6803 CG7791 CG8004 CG8226 EG:52C10.1 Irbp Ku80 La LamC Nmd3 Nxt1 Ptp61F R Rep Ssrp Thor Tim10 Tim9a ab cher dia fru huntingtin loco mfas mirr mre11 puc rad50 |
| 1467 | GO:0035295 | P | 3, | 1 | 2.600 (x 0.385) | 77 (0.013) | 0.996 | tube development | apt |
| 1468 | GO:0006810 | P | 4, 5, | 41 | 49.874 (x 0.822) | 1477 (0.028) | 0.996 | transport | Aly Arf84F Arr2 BEST:CK01227 BG:DS03431.1 Bmcp CG10444 CG11897 CG11907 CG17108 CG1718 CG2885 CG3036 CG3168 CG31793 CG3860 CG5535 CG6574 CG7571 CG7627 CG7791 CG7955 CG8004 CG8226 La Nmd3 Nxt1 Obp83g R Rep TepIV Tim10 Tim9a Tsf1 Tsf3 glob1 huntingtin loco na ppk unc-13 |
| 1469 | GO:0000902 | P | 4, 5, | 7 | 11.413 (x 0.613) | 338 (0.021) | 0.996 | cellular morphogenesis | Ptp61F R Thor ab mfas mirr puc |
| 1470 | GO:0006357 | P | 9, | 13 | 18.471 (x 0.704) | 547 (0.024) | 0.996 | regulation of transcription from RNA polymerase II promoter | CG10445 EP2237 Ets21C HLHmgamma Pcaf Ssb-c31a Ssrp ab apt fru ftz-f1 mirr toy |
| 1471 | GO:0030154 | P | 3, | 12 | 17.221 (x 0.697) | 510 (0.024) | 0.996 | cell differentiation | CG6803 Mhc Ptp61F R ab dia loco mfas os trol wun2 wupA |
| 1472 | GO:0051603 | P | 8, 9, | 1 | 2.769 (x 0.361) | 82 (0.012) | 0.996 | proteolysis during cellular protein catabolism | Ate1 |
| 1473 | GO:0005792 | C | 6, 7, | 1 | 2.803 (x 0.357) | 83 (0.012) | 0.996 | microsome | Jheh3 |
| 1474 | GO:0043632 | P | 7, | 1 | 2.600 (x 0.385) | 77 (0.013) | 0.996 | modification-dependent macromolecule catabolism | Ate1 |
| 1475 | GO:0045184 | P | 5, | 12 | 17.559 (x 0.683) | 520 (0.023) | 0.996 | establishment of protein localization | Arf84F Arr2 CG2885 CG7791 CG8004 CG8226 Nmd3 Nxt1 R Rep Tim10 Tim9a |
| 1476 | GO:0044257 | P | 7, 8, | 1 | 2.769 (x 0.361) | 82 (0.012) | 0.997 | cellular protein catabolism | Ate1 |
| 1477 | GO:0015031 | P | 5, 6, | 12 | 17.458 (x 0.687) | 517 (0.023) | 0.997 | protein transport | Arf84F Arr2 CG2885 CG7791 CG8004 CG8226 Nmd3 Nxt1 R Rep Tim10 Tim9a |
| 1478 | GO:0042598 | C | 5, 6, | 1 | 2.803 (x 0.357) | 83 (0.012) | 0.997 | vesicular fraction | Jheh3 |
| 1479 | GO:0019941 | P | 8, 9, 10, | 1 | 2.600 (x 0.385) | 77 (0.013) | 0.997 | modification-dependent protein catabolism | Ate1 |
| 1480 | GO:0006511 | P | 9, 10, 11, | 1 | 2.533 (x 0.395) | 75 (0.013) | 0.997 | ubiquitin-dependent protein catabolism | Ate1 |
| 1481 | GO:0007166 | P | 5, | 16 | 22.253 (x 0.719) | 659 (0.024) | 0.997 | cell surface receptor linked signal transduction | Arf84F Arr2 CG13586 CG7515 CG9358 GABA-B-R2 HLHmgamma Ide R Traf1 dlp loco mirr sog wun wun2 |
| 1482 | GO:0000904 | P | 5, 6, | 3 | 5.707 (x 0.526) | 169 (0.018) | 0.997 | cellular morphogenesis during differentiation | Ptp61F ab mfas |
| 1483 | GO:0016192 | P | 5, 6, | 6 | 9.995 (x 0.600) | 296 (0.020) | 0.997 | vesicle-mediated transport | Arf84F Arr2 CG2885 R Rep unc-13 |
| 1484 | GO:0007049 | P | 4, | 11 | 15.904 (x 0.692) | 471 (0.023) | 0.997 | cell cycle | CG6204 CG6512 CG9790 CG9862 LamC Mhc R Taf12L cul-2 dia mre11 |
| 1485 | GO:0007165 | P | 4, | 35 | 43.593 (x 0.803) | 1291 (0.027) | 0.997 | signal transduction | Arf84F Arr2 CG12876 CG13586 CG14869 CG2885 CG2989 CG4096 CG7054 CG7515 CG9358 Chit EP2237 GABA-B-R2 HLHmgamma Ide Idgf2 Idgf3 Ptp61F R TpnC41C Traf1 dlp eiger ftz-f1 loco mfas mirr os puc sog trol unc-13 wun wun2 |
| 1486 | GO:0006886 | P | 6, 7, 8, | 12 | 17.086 (x 0.702) | 506 (0.024) | 0.997 | intracellular protein transport | Arf84F Arr2 CG2885 CG7791 CG8004 CG8226 Nmd3 Nxt1 R Rep Tim10 Tim9a |
| 1487 | GO:0007242 | P | 5, | 12 | 17.694 (x 0.678) | 524 (0.023) | 0.997 | intracellular signaling cascade | Arf84F CG2885 CG7515 CG9358 EP2237 R TpnC41C Traf1 eiger os puc unc-13 |
| 1488 | GO:0005509 | F | 5, | 4 | 7.361 (x 0.543) | 218 (0.018) | 0.998 | calcium ion binding | CG10126 CG7447 Mlc2 TpnC41C |
| 1489 | GO:0000785 | C | 5, 6, 7, 8, 9, 10, | 1 | 2.600 (x 0.385) | 77 (0.013) | 0.998 | chromatin | CG31611 |
| 1490 | GO:0007169 | P | 7, | 2 | 4.187 (x 0.478) | 124 (0.016) | 0.998 | transmembrane receptor protein tyrosine kinase signaling pathway | CG9358 sog |
| 1491 | GO:0008017 | F | 6, | 1 | 2.566 (x 0.390) | 76 (0.013) | 0.998 | microtubule binding | huntingtin |
| 1492 | GO:0009952 | P | 4, | 2 | 4.390 (x 0.456) | 130 (0.015) | 0.998 | anterior/posterior pattern formation | CG9358 sog |
| 1493 | GO:0007154 | P | 3, | 42 | 51.225 (x 0.820) | 1517 (0.028) | 0.998 | cell communication | Arf84F Arr2 CG12876 CG13586 CG14869 CG2885 CG2989 CG3168 CG4096 CG7054 CG7515 CG9358 Chit EP2237 GABA-B-R2 Gs2 HLHmgamma Ide Idgf2 Idgf3 Ptp61F R Rep TpnC41C Traf1 Tsp42El ab apt dlp eiger fru ftz-f1 loco mfas mirr os puc sog trol unc-13 wun wun2 |
| 1494 | GO:0006644 | P | 7, 8, | 1 | 2.566 (x 0.390) | 76 (0.013) | 0.998 | phospholipid metabolism | CG5162 |
| 1495 | GO:0004888 | F | 4, | 4 | 14.351 (x 0.279) | 425 (0.009) | 1 | transmembrane receptor activity | CG6739 GABA-B-R2 Gr39a Ide |
| 1496 | GO:0044425 | C | 3, 4, 5, | 32 | 41.061 (x 0.779) | 1216 (0.026) | 1 | membrane part | Arr2 BEST:CK01227 BG:DS03431.1 CG11897 CG17108 CG3036 CG3168 CG31793 CG3192 CG4769 CG5037 CG5548 CG5703 CG7188 CG7627 CG7955 CG8004 CG8226 CG9358 GABA-B-R2 Gr39a Hmgcr Ide LamC Pdsw Tim10 Tim9a Tsp42El sas sog wun wun2 |
| 1497 | GO:0006464 | P | 7, | 14 | 29.614 (x 0.473) | 877 (0.016) | 1 | protein modification | Arf84F Ate1 CG1134 CG13692 CG3008 CG31373 CG7791 CG7828 CG9358 Ptp61F Rep W puc rpr |
| 1498 | GO:0015631 | F | 5, | 1 | 3.073 (x 0.325) | 91 (0.011) | 1 | tubulin binding | huntingtin |
| 1499 | GO:0006812 | P | 6, 7, | 8 | 12.933 (x 0.619) | 383 (0.021) | 1 | cation transport | BG:DS03431.1 Bmcp CG10444 CG3036 Tsf1 Tsf3 na ppk |
| 1500 | GO:0006468 | P | 8, | 2 | 9.624 (x 0.208) | 285 (0.007) | 1 | protein amino acid phosphorylation | CG3008 CG9358 |
| 1501 | GO:0006796 | P | 6, | 14 | 20.395 (x 0.686) | 604 (0.023) | 1 | phosphate metabolism | CG3008 CG3036 CG3192 CG32649 CG3803 CG4769 CG5548 CG5703 CG9358 Pdsw Ptp61F puc wun wun2 |
| 1502 | GO:0003700 | F | 3, 5, | 8 | 13.135 (x 0.609) | 389 (0.021) | 1 | transcription factor activity | CG31666 Ets21C HLHmgamma fru ftz-f1 ken mirr toy |
| 1503 | GO:0030163 | P | 6, 7, | 1 | 3.039 (x 0.329) | 90 (0.011) | 1 | protein catabolism | Ate1 |
| 1504 | GO:0000226 | P | 8, | 1 | 2.938 (x 0.340) | 87 (0.011) | 1 | microtubule cytoskeleton organization and biogenesis | dia |
| 1505 | GO:0006164 | P | 7, 8, | 1 | 3.073 (x 0.325) | 91 (0.011) | 1 | purine nucleotide biosynthesis | BcDNA:LD32788 |
| 1506 | GO:0043412 | P | 6, | 16 | 30.964 (x 0.517) | 917 (0.017) | 1 | biopolymer modification | Arf84F Ate1 CG1134 CG11837 CG13692 CG3008 CG31373 CG7791 CG7828 CG9358 Ptp61F Rep W puc rpr trol |
| 1507 | GO:0019219 | P | 6, | 16 | 29.985 (x 0.534) | 888 (0.018) | 1 | regulation of nucleobase, nucleoside, nucleotide and nucleic acid metabolism | CG10445 CG2885 CG6272 EP2237 Ets21C HLHmgamma Pcaf Ssb-c31a Ssrp ab apt fru ftz-f1 hay mirr toy |
| 1508 | GO:0006163 | P | 7, | 1 | 3.107 (x 0.322) | 92 (0.011) | 1 | purine nucleotide metabolism | BcDNA:LD32788 |
| 1509 | GO:0006793 | P | 5, | 14 | 20.395 (x 0.686) | 604 (0.023) | 1 | phosphorus metabolism | CG3008 CG3036 CG3192 CG32649 CG3803 CG4769 CG5548 CG5703 CG9358 Pdsw Ptp61F puc wun wun2 |
| 1510 | GO:0015630 | C | 6, 7, 8, 9, | 1 | 6.281 (x 0.159) | 186 (0.005) | 1 | microtubule cytoskeleton | Rep |
| 1511 | GO:0019932 | P | 6, | 1 | 2.972 (x 0.337) | 88 (0.011) | 1 | second-messenger-mediated signaling | TpnC41C |
| 1512 | GO:0005794 | C | 5, 6, 7, 8, | 1 | 3.039 (x 0.329) | 90 (0.011) | 1 | Golgi apparatus | Sulf1 |
| 1513 | GO:0006512 | P | 8, | 4 | 7.902 (x 0.506) | 234 (0.017) | 1 | ubiquitin cycle | Ate1 CG1134 W rpr |
| 1514 | GO:0051234 | P | 4, | 47 | 57.674 (x 0.815) | 1708 (0.028) | 1 | establishment of localization | Aly Arf84F Arr2 BEST:CK01227 BG:DS03431.1 Bmcp CG10444 CG11897 CG11907 CG17108 CG1718 CG2885 CG3036 CG3168 CG31793 CG3860 CG5535 CG6574 CG7571 CG7627 CG7791 CG7955 CG8004 CG8226 Hmgcr La Nmd3 Nxt1 Obp83g Ptp61F R Rep TepIV Tim10 Tim9a Tsf1 Tsf3 ab glob1 huntingtin loco na os ppk unc-13 wun wun2 |
| 1515 | GO:0006643 | P | 6, 7, | 1 | 2.938 (x 0.340) | 87 (0.011) | 1 | membrane lipid metabolism | CG5162 |
| 1516 | GO:0015672 | P | 7, 8, | 1 | 5.909 (x 0.169) | 175 (0.006) | 1 | monovalent inorganic cation transport | ppk |
| 1517 | GO:0045449 | P | 7, | 15 | 28.060 (x 0.535) | 831 (0.018) | 1 | regulation of transcription | CG10445 CG2885 CG6272 EP2237 Ets21C HLHmgamma Pcaf Ssb-c31a Ssrp ab apt fru ftz-f1 mirr toy |
| 1518 | GO:0006350 | P | 6, | 19 | 31.910 (x 0.595) | 945 (0.020) | 1 | transcription | Aly CG10445 CG2885 CG6272 CG7339 EP2237 Ets21C HLHmgamma Pcaf Ssb-c31a Ssrp Taf12L ab apt fru ftz-f1 hay mirr toy |
| 1519 | GO:0004295 | F | 7, | 2 | 7.935 (x 0.252) | 235 (0.009) | 1 | trypsin activity | CG1299 CG9372 |
| 1520 | GO:0004872 | F | 3, | 9 | 18.673 (x 0.482) | 553 (0.016) | 1 | receptor activity | CG40006 CG6739 GABA-B-R2 Gr39a Ide Ptp61F ftz-f1 sas unc-13 |
| 1521 | GO:0007010 | P | 6, | 7 | 15.195 (x 0.461) | 450 (0.016) | 1 | cytoskeleton organization and biogenesis | Act57B CG6803 LamC cher dia huntingtin puc |
| 1522 | GO:0006355 | P | 8, | 15 | 26.575 (x 0.564) | 787 (0.019) | 1 | regulation of transcription, DNA-dependent | CG10445 CG2885 CG6272 EP2237 Ets21C HLHmgamma Pcaf Ssb-c31a Ssrp ab apt fru ftz-f1 mirr toy |
| 1523 | GO:0044459 | C | 4, 5, 6, | 4 | 10.907 (x 0.367) | 323 (0.012) | 1 | plasma membrane part | Arr2 BG:DS03431.1 CG9358 sas |
| 1524 | GO:0001584 | F | 6, | 2 | 7.192 (x 0.278) | 213 (0.009) | 1 | rhodopsin-like receptor activity | Gr39a Ide |
| 1525 | GO:0031323 | P | 5, | 21 | 33.294 (x 0.631) | 986 (0.021) | 1 | regulation of cellular metabolism | CG10445 CG2885 CG5317 CG6272 EP2237 Ets21C HBS1 HLHmgamma Imp Pcaf Ssb-c31a Ssrp Thor ab apt fru ftz-f1 hay mirr rpr toy |
| 1526 | GO:0016773 | F | 5, | 5 | 12.055 (x 0.415) | 357 (0.014) | 1 | phosphotransferase activity, alcohol group as acceptor | CG3008 CG32649 CG9358 CG9790 Irbp |
| 1527 | GO:0016301 | F | 5, | 6 | 13.541 (x 0.443) | 401 (0.015) | 1 | kinase activity | CG2246 CG3008 CG32649 CG9358 CG9790 Irbp |
| 1528 | GO:0046530 | P | 4, | 1 | 3.309 (x 0.302) | 98 (0.010) | 1 | photoreceptor cell differentiation | R |
| 1529 | GO:0045892 | P | 9, | 1 | 3.275 (x 0.305) | 97 (0.010) | 1 | negative regulation of transcription, DNA-dependent | HLHmgamma |
| 1530 | GO:0007560 | P | 5, 6, | 4 | 8.104 (x 0.494) | 240 (0.017) | 1 | imaginal disc morphogenesis | W mirr puc sog |
| 1531 | GO:0031224 | C | 4, 5, 6, | 20 | 31.977 (x 0.625) | 947 (0.021) | 1 | intrinsic to membrane | BEST:CK01227 BG:DS03431.1 CG11897 CG17108 CG3036 CG3168 CG31793 CG5037 CG7188 CG7627 CG7955 GABA-B-R2 Gr39a Hmgcr Ide Tsp42El sas sog wun wun2 |
| 1532 | GO:0008324 | F | 4, | 8 | 13.473 (x 0.594) | 399 (0.020) | 1 | cation transporter activity | BG:DS03431.1 CG10444 CG3036 CG5535 Tsf1 Tsf3 na ppk |
| 1533 | GO:0007498 | P | 4, | 3 | 6.686 (x 0.449) | 198 (0.015) | 1 | mesoderm development | Mlc1 Mlp60A toy |
| 1534 | GO:0030528 | F | 2, | 19 | 27.183 (x 0.699) | 805 (0.024) | 1 | transcription regulator activity | Aly CG10445 CG31666 CG5317 EP2237 Ets21C HLHmgamma La Ssb-c31a Ssrp Taf12L ab apt fru ftz-f1 hay ken mirr toy |
| 1535 | GO:0004263 | F | 7, | 2 | 7.024 (x 0.285) | 208 (0.010) | 1 | chymotrypsin activity | CG1299 CG9372 |
| 1536 | GO:0005057 | F | 3, | 4 | 8.104 (x 0.494) | 240 (0.017) | 1 | receptor signaling protein activity | CG12876 TMS1 Tsp42El loco |
| 1537 | GO:0006351 | P | 7, | 19 | 30.255 (x 0.628) | 896 (0.021) | 1 | transcription, DNA-dependent | Aly CG10445 CG2885 CG6272 CG7339 EP2237 Ets21C HLHmgamma Pcaf Ssb-c31a Ssrp Taf12L ab apt fru ftz-f1 hay mirr toy |
| 1538 | GO:0016021 | C | 5, 6, 7, | 20 | 31.876 (x 0.627) | 944 (0.021) | 1 | integral to membrane | BEST:CK01227 BG:DS03431.1 CG11897 CG17108 CG3036 CG3168 CG31793 CG5037 CG7188 CG7627 CG7955 GABA-B-R2 Gr39a Hmgcr Ide Tsp42El sas sog wun wun2 |
| 1539 | GO:0051726 | P | 5, | 2 | 6.720 (x 0.298) | 199 (0.010) | 1 | regulation of cell cycle | CG9790 cul-2 |
| 1540 | GO:0004930 | F | 5, | 3 | 8.678 (x 0.346) | 257 (0.012) | 1 | G-protein coupled receptor activity | GABA-B-R2 Gr39a Ide |
| 1541 | GO:0000074 | P | 6, | 2 | 6.720 (x 0.298) | 199 (0.010) | 1 | regulation of progression through cell cycle | CG9790 cul-2 |
| 1542 | GO:0015075 | F | 3, | 10 | 16.377 (x 0.611) | 485 (0.021) | 1 | ion transporter activity | BEST:CK01227 BG:DS03431.1 CG10444 CG3036 CG5535 CG7571 Tsf1 Tsf3 na ppk |
| 1543 | GO:0016481 | P | 8, | 1 | 3.748 (x 0.267) | 111 (0.009) | 1 | negative regulation of transcription | HLHmgamma |
| 1544 | GO:0019787 | F | 6, | 1 | 4.694 (x 0.213) | 139 (0.007) | 1 | small conjugating protein ligase activity | CG1134 |
| 1545 | GO:0007281 | P | 5, | 1 | 3.782 (x 0.264) | 112 (0.009) | 1 | germ cell development | os |
| 1546 | GO:0007606 | P | 4, 6, | 2 | 5.605 (x 0.357) | 166 (0.012) | 1 | sensory perception of chemical stimulus | Gr39a Obp83g |
| 1547 | GO:0001709 | P | 5, | 1 | 3.714 (x 0.269) | 110 (0.009) | 1 | cell fate determination | trol |
| 1548 | GO:0004842 | F | 7, | 1 | 4.694 (x 0.213) | 139 (0.007) | 1 | ubiquitin-protein ligase activity | CG1134 |
| 1549 | GO:0007018 | P | 7, 8, 9, | 1 | 3.478 (x 0.288) | 103 (0.010) | 1 | microtubule-based movement | huntingtin |
| 1550 | GO:0016772 | F | 4, | 10 | 16.917 (x 0.591) | 501 (0.020) | 1 | transferase activity, transferring phosphorus-containing groups | CG13645 CG2246 CG3008 CG32649 CG7163 CG7339 CG9358 CG9790 Irbp mus205 |
| 1551 | GO:0031226 | C | 5, 6, 7, | 2 | 6.686 (x 0.299) | 198 (0.010) | 1 | intrinsic to plasma membrane | BG:DS03431.1 sas |
| 1552 | GO:0030705 | P | 6, 7, 8, | 1 | 3.512 (x 0.285) | 104 (0.010) | 1 | cytoskeleton-dependent intracellular transport | huntingtin |
| 1553 | GO:0005887 | C | 6, 7, 8, | 2 | 6.618 (x 0.302) | 196 (0.010) | 1 | integral to plasma membrane | BG:DS03431.1 sas |
| 1554 | GO:0005549 | F | 3, | 1 | 3.883 (x 0.258) | 115 (0.009) | 1 | odorant binding | Obp83g |
| 1555 | GO:0050794 | P | 3, | 35 | 46.497 (x 0.753) | 1377 (0.025) | 1 | regulation of cellular process | Arf84F CG10445 CG12876 CG2885 CG5317 CG6272 CG7188 CG9790 EP2237 Ets21C HBS1 HLHmgamma Imp Mmp1 Pcaf R Ssb-c31a Ssrp Thor Traf1 W ab apt cul-2 dlp eiger fru ftz-f1 hay loco mirr puc rpr sog toy |
| 1556 | GO:0004672 | F | 6, | 5 | 9.961 (x 0.502) | 295 (0.017) | 1 | protein kinase activity | CG3008 CG32649 CG9358 CG9790 Irbp |
| 1557 | GO:0007017 | P | 7, | 2 | 6.618 (x 0.302) | 196 (0.010) | 1 | microtubule-based process | dia huntingtin |
| 1558 | GO:0008236 | F | 5, | 4 | 9.894 (x 0.404) | 293 (0.014) | 1 | serine-type peptidase activity | BG:DS01068.5 CG1299 CG8798 CG9372 |
| 1559 | GO:0006811 | P | 5, 6, | 9 | 15.600 (x 0.577) | 462 (0.019) | 1 | ion transport | BG:DS03431.1 Bmcp CG10444 CG3036 CG7571 Tsf1 Tsf3 na ppk |
| 1560 | GO:0050791 | P | 3, | 32 | 45.214 (x 0.708) | 1339 (0.024) | 1 | regulation of physiological process | Arf84F Ate1 CG10445 CG12876 CG2885 CG5317 CG6272 CG7188 CG9790 EP2237 Ets21C HBS1 HLHmgamma Imp Mmp1 Pcaf R Ssb-c31a Ssrp Thor Traf1 W ab apt cul-2 eiger fru ftz-f1 hay mirr rpr toy |
| 1561 | GO:0016310 | P | 7, | 9 | 15.567 (x 0.578) | 461 (0.020) | 1 | phosphorylation | CG3008 CG3192 CG32649 CG3803 CG4769 CG5548 CG5703 CG9358 Pdsw |
| 1562 | GO:0006457 | P | 7, | 1 | 4.423 (x 0.226) | 131 (0.008) | 1 | protein folding | l(3)01239 |
| 1563 | GO:0019222 | P | 4, | 23 | 34.578 (x 0.665) | 1024 (0.022) | 1 | regulation of metabolism | Ate1 CG10445 CG2885 CG5317 CG6272 EP2237 Ets21C HBS1 HLHmgamma Imp Pcaf Ssb-c31a Ssrp Thor W ab apt fru ftz-f1 hay mirr rpr toy |
| 1564 | GO:0005216 | F | 4, 5, | 2 | 5.538 (x 0.361) | 164 (0.012) | 1 | ion channel activity | na ppk |
| 1565 | GO:0051244 | P | 4, | 31 | 43.796 (x 0.708) | 1297 (0.024) | 1 | regulation of cellular physiological process | Arf84F CG10445 CG12876 CG2885 CG5317 CG6272 CG7188 CG9790 EP2237 Ets21C HBS1 HLHmgamma Imp Mmp1 Pcaf R Ssb-c31a Ssrp Thor Traf1 W ab apt cul-2 eiger fru ftz-f1 hay mirr rpr toy |
| 1566 | GO:0004252 | F | 6, | 4 | 8.678 (x 0.461) | 257 (0.016) | 1 | serine-type endopeptidase activity | BG:DS01068.5 CG1299 CG8798 CG9372 |
| 1567 | GO:0015268 | F | 4, | 2 | 6.146 (x 0.325) | 182 (0.011) | 1 | alpha-type channel activity | na ppk |
| 1568 | GO:0045165 | P | 4, | 2 | 6.382 (x 0.313) | 189 (0.011) | 1 | cell fate commitment | R trol |
| 1569 | GO:0050789 | P | 2, | 38 | 50.819 (x 0.748) | 1505 (0.025) | 1 | regulation of biological process | Arf84F Ate1 CG10445 CG12876 CG2885 CG5317 CG6272 CG7188 CG9790 EP2237 Ets21C HBS1 HLHmgamma Imp Mmp1 Pcaf R Rep Ssb-c31a Ssrp Thor Traf1 W ab apt cul-2 dlp eiger fru ftz-f1 hay loco mirr puc rpr sog toy trol |
| 1570 | GO:0045934 | P | 7, | 1 | 4.086 (x 0.245) | 121 (0.008) | 1 | negative regulation of nucleobase, nucleoside, nucleotide and nucleic acid metabolism | HLHmgamma |
| 1571 | GO:0005886 | C | 4, 5, | 10 | 18.032 (x 0.555) | 534 (0.019) | 1 | plasma membrane | Arr2 BG:DS01219.1 BG:DS03431.1 CG9358 TMS1 mfas sas sog wun wun2 |
| 1572 | GO:0004674 | F | 7, | 3 | 7.159 (x 0.419) | 212 (0.014) | 1 | protein serine/threonine kinase activity | CG9358 CG9790 Irbp |
| 1573 | GO:0008283 | P | 4, | 4 | 9.522 (x 0.420) | 282 (0.014) | 1 | cell proliferation | BcDNA:LD23830 HLHmgamma Mlp60A toy |
| 1574 | GO:0004871 | F | 2, | 24 | 35.557 (x 0.675) | 1053 (0.023) | 1 | signal transducer activity | Arf84F CG12876 CG13586 CG2989 CG3153 CG40006 CG6739 Chit GABA-B-R2 Gr39a Ide Idgf2 Idgf3 Ptp61F TMS1 Traf1 Tsp42El eiger ftz-f1 loco os sas sog unc-13 |
| 1575 | GO:0015267 | F | 3, | 2 | 6.146 (x 0.325) | 182 (0.011) | 1 | channel or pore class transporter activity | na ppk |
| 1576 | GO:0007059 | P | 4, | 1 | 4.187 (x 0.239) | 124 (0.008) | 1 | chromosome segregation | CG9862 |

  

---

Regulated Genes that don't have GO terms
  

BG:DS01368.1 BcDNA:GH03693 BcDNA:GH03753 BcDNA:GH07269 BcDNA:LD21529 BcDNA:RE39515 CG10005 CG10075 CG10189 CG10337 CG10428 CG10463 CG10559 CG10570 CG10675 CG10916 CG11009 CG11086 CG11279 CG1146 CG11847 CG11852 CG11893 CG12505 CG12868 CG13623 CG13822 CG13886 CG13941 CG14121 CG14696 CG14907 CG14984 CG15173 CG15611 CG15675 CG1572 CG15784 CG15863 CG15917 CG17219 CG17327 CG17574 CG17681 CG17734 CG17738 CG18294 CG18358 CG18410 CG18596 CG18600 CG18643 CG1868 CG2471 CG2641 CG2909 CG2948 CG30196 CG30373 CG30493 CG31633 CG31781 CG31955 CG32069 CG32425 CG32500 CG3280 CG33056 CG33107 CG33201 CG3448 CG40115 CG40164 CG40169 CG40188 CG40260 CG40295 CG40329 CG4975 CG5174 CG5325 CG5359 CG5664 CG5792 CG5903 CG5953 CG5961 CG5989 CG6013 CG6043 CG6066 CG6073 CG6171 CG6353 CG6643 CG7168 CG7341 CG7506 CG8034 CG8206 CG8369 CG8486 CG8927 CG9192 CG9231 CG9328 CG9336 CG9338 CG9350 CG9422 CG9646 CG9667 CG9815 CG9945 CG9972 CG9996 Chmp1 EG:131F2.3 GstE8 His2B:CG17949 Ip259 Max-element SP558 l(1)G0136 l(2)35Bg
