# Supplementary material for: Functional dissection of the ash2 and ash1 transcriptomes provides insights into the transcriptional basis of wing phenotypes and reveals conserved protein interactions
Source: Genome Biol. 2007 Apr 28;8(4):R67. doi: 10.1186/gb-2007-8-4-r67 (PMC1896016; doi:10.1186/gb-2007-8-4-r67)
Supplement: Additional data file 7 — GO annotations of the genes downregulated over 2.0-fold in ash2I1 [file gb-2007-8-4-r67-S7.html]

  

---

  

|  |  |
| --- | --- |
| Go Statistics | Reg File: **ash2I1\_D2.0x.txt.fbgns** (178 genes -- 27 skipped)  Ref File: **ref.fbgns** (13577 genes -- 4663 skipped)  Database: **go\_200507-termdb.rdf-xml** |

---

  

Fields Description

| Pos | Go Term | Ontology | Levels | Observed | Expected | Possibles | p-value(Adj) | Go term description | Genes with the GO term |
| --- | --- | --- | --- | --- | --- | --- | --- | --- | --- |
| 1 | GO:0007275 | P | 2, | 56 | 25.155 (x 2.226) | 1485 (0.038) | 1.16e-06 | development | Abi CG3770 CG40410 Cdk4 Doc1 Doc2 Doc3 Eip71CD Eip75B Fas3 Hrb27C Hsp26 Hsp27 ImpE2 ImpE3 Optix Poxn Sb Sema-1b Tl Trl Wnt2 ana arr ash2 br dap dve edl en esn fax fra glec grn in inv klu malpha mth ninA ogre pbl pk rost sc serpin-27A sgl smi35A stai th toe tok trn vg wbl |
| 2 | GO:0048513 | P | 3, | 30 | 11.350 (x 2.643) | 670 (0.045) | 0.000411 | organ development | CG40410 Doc1 Eip71CD Eip75B ImpE2 ImpE3 Optix Poxn Sb Tl Wnt2 arr ash2 br dap dve edl en grn in inv klu malpha pbl pk rost sc sgl th vg |
| 3 | GO:0009653 | P | 3, | 27 | 10.875 (x 2.483) | 642 (0.042) | 0.00355 | morphogenesis | Abi CG3770 Cdk4 Doc1 Fas3 ImpE2 ImpE3 Poxn Sb Sema-1b arr ash2 br dve edl fax fra grn in klu ninA pbl pk sc sgl th vg |
| 4 | GO:0048731 | P | 3, | 25 | 10.062 (x 2.485) | 594 (0.042) | 0.00598 | system development | Cdk4 Eip71CD Eip75B Fas3 Poxn Sema-1b Tl Wnt2 ana br dap en fax fra glec inv ninA ogre pbl sc sgl smi35A stai toe trn |
| 5 | GO:0007444 | P | 4, | 16 | 5.116 (x 3.128) | 302 (0.053) | 0.0124 | imaginal disc development | CG40410 ImpE2 ImpE3 Poxn Sb arr ash2 br dve en in inv klu pk th vg |
| 6 | GO:0009607 | P | 3, | 21 | 8.842 (x 2.375) | 522 (0.040) | 0.0323 | response to biotic stimulus | BEST:GH02921 CG10359 CG17323 CG2852 CG5873 CG7668 CG9027 Eip71CD GNBP3 Hsp23 Hsp26 Hsp27 Hsp67Ba PFE Sb Tl Tsp66E br fra serpin-27A trn |
| 7 | GO:0009887 | P | 4, | 16 | 5.692 (x 2.811) | 336 (0.048) | 0.0362 | organ morphogenesis | Doc1 ImpE2 ImpE3 Poxn Sb arr ash2 br dve grn in klu pbl pk sgl vg |
| 8 | GO:0031347 | P | 5, | 2 | 0.034 (x 59.033) | 2 (1.000) | 0.0396 | regulation of defense response | Tl serpin-27A |
| 9 | GO:0035007 | P | 5, 6, 8, | 2 | 0.034 (x 59.033) | 2 (1.000) | 0.0445 | regulation of melanization defense response | Tl serpin-27A |
| 10 | GO:0006952 | P | 4, | 20 | 8.707 (x 2.297) | 514 (0.039) | 0.0458 | defense response | BEST:GH02921 CG10359 CG17323 CG2852 CG5873 CG7668 CG9027 Eip71CD GNBP3 Hsp23 Hsp26 Hsp27 Hsp67Ba PFE Sb Tl Tsp66E fra serpin-27A trn |
| 11 | GO:0009888 | P | 3, | 17 | 6.742 (x 2.522) | 398 (0.043) | 0.0491 | tissue development | Doc1 Doc2 Doc3 Poxn Sema-1b en fra grn in inv ninA pbl pk rost sgl smi35A toe |
| 12 | GO:0035006 | P | 5, 7, | 2 | 0.051 (x 39.355) | 3 (0.667) | 0.066 | melanization defense response | Tl serpin-27A |
| 13 | GO:0007399 | P | 4, | 18 | 7.928 (x 2.271) | 468 (0.038) | 0.0672 | nervous system development | Fas3 Poxn Sema-1b Tl ana br en fax fra glec inv ninA ogre pbl sc smi35A stai toe |
| 14 | GO:0035220 | P | 5, | 8 | 1.965 (x 4.071) | 116 (0.069) | 0.0681 | wing disc development | Poxn ash2 dve en in inv pk vg |
| 15 | GO:0007386 | P | 4, | 2 | 0.051 (x 39.355) | 3 (0.667) | 0.0704 | compartment specification | en inv |
| 16 | GO:0007560 | P | 5, 6, | 12 | 4.066 (x 2.952) | 240 (0.050) | 0.0726 | imaginal disc morphogenesis | ImpE2 ImpE3 Poxn Sb arr ash2 br dve in klu pk vg |
| 17 | GO:0050791 | P | 3, | 38 | 22.682 (x 1.675) | 1339 (0.028) | 0.0734 | regulation of physiological process | Abi BEST:GH02921 CG40410 CG4914 CREG Caf1 Cdk4 D19A Doc1 Doc2 Doc3 Dsp1 Eip75B HmgZ Hrb27C Optix Poxn Rpn9 Tl Trl ana ash2 br dap dve edl en for grn inv klu mdy sc serpin-27A smi35A th toe zwilch |
| 18 | GO:0009408 | P | 4, 5, | 5 | 0.796 (x 6.280) | 47 (0.106) | 0.0742 | response to heat | Hsp23 Hsp26 Hsp27 Hsp67Ba mth |
| 19 | GO:0007552 | P | 4, | 12 | 4.286 (x 2.800) | 253 (0.047) | 0.0747 | metamorphosis | ImpE2 ImpE3 Poxn Sb arr ash2 br dve in klu pk vg |
| 20 | GO:0046698 | P | 5, | 12 | 4.252 (x 2.822) | 251 (0.048) | 0.0775 | metamorphosis (sensu Insecta) | ImpE2 ImpE3 Poxn Sb arr ash2 br dve in klu pk vg |
| 21 | GO:0007561 | P | 6, 7, | 2 | 0.068 (x 29.517) | 4 (0.500) | 0.095 | imaginal disc eversion | ImpE2 ImpE3 |
| 22 | GO:0050896 | P | 2, | 33 | 19.802 (x 1.666) | 1169 (0.028) | 0.0983 | response to stimulus | BEST:GH02921 CG10359 CG17323 CG2852 CG40410 CG5873 CG7668 CG8588 CG9027 Caf1 Eip71CD Eip75B Fas3 GNBP3 Hsp23 Hsp26 Hsp27 Hsp67Ba Obp99a PFE Poxn Sb Tl Tsp66E br for fra mth ninA ogre serpin-27A smi35A trn |
| 23 | GO:0019955 | F | 4, | 2 | 0.068 (x 29.517) | 4 (0.500) | 0.0995 | cytokine binding | Tl wgn |
| 24 | GO:0007155 | P | 3, | 13 | 5.133 (x 2.533) | 303 (0.043) | 0.101 | cell adhesion | CG33171 CG4054 Fas3 PFE Sema-1b Tl Tsp66E fra glec kal-1 ninA pbl trn |
| 25 | GO:0009266 | P | 4, | 5 | 0.915 (x 5.466) | 54 (0.093) | 0.102 | response to temperature stimulus | Hsp23 Hsp26 Hsp27 Hsp67Ba mth |
| 26 | GO:0002165 | P | 4, | 14 | 5.844 (x 2.396) | 345 (0.041) | 0.105 | larval or pupal development (sensu Insecta) | Eip71CD Eip75B ImpE2 ImpE3 Poxn Sb arr ash2 br dve in klu pk vg |
| 27 | GO:0016477 | P | 5, 6, | 9 | 2.964 (x 3.036) | 175 (0.051) | 0.119 | cell migration | Fas3 Sema-1b fra ninA pbl sgl stai th trn |
| 28 | GO:0009791 | P | 3, | 14 | 6.031 (x 2.322) | 356 (0.039) | 0.121 | post-embryonic development | Eip71CD Eip75B ImpE2 ImpE3 Poxn Sb arr ash2 br dve in klu pk vg |
| 29 | GO:0007050 | P | 7, 8, | 2 | 0.085 (x 23.613) | 5 (0.400) | 0.123 | cell cycle arrest | CG40410 dap |
| 30 | GO:0035186 | P | 5, 7, | 2 | 0.085 (x 23.613) | 5 (0.400) | 0.128 | syncytial blastoderm mitotic cell cycle | CG40410 Trl |
| 31 | GO:0050789 | P | 2, | 39 | 25.494 (x 1.530) | 1505 (0.026) | 0.133 | regulation of biological process | Abi BEST:GH02921 CG40410 CG4914 CREG Caf1 Cdk4 D19A Doc1 Doc2 Doc3 Dsp1 Eip75B HmgZ Hrb27C Optix Poxn Rpn9 Tl Trl ana ash2 br dap dve edl en for grn inv klu mdy pbl sc serpin-27A smi35A th toe zwilch |
| 32 | GO:0009628 | P | 3, | 14 | 6.302 (x 2.222) | 372 (0.038) | 0.153 | response to abiotic stimulus | CG17323 CG40410 CG8588 Eip75B Fas3 Hsp23 Hsp26 Hsp27 Hsp67Ba Obp99a br mth ogre smi35A |
| 33 | GO:0016861 | F | 5, | 2 | 0.102 (x 19.678) | 6 (0.333) | 0.155 | intramolecular oxidoreductase activity, interconverting aldoses and ketoses | Oscillin Tpi |
| 34 | GO:0006094 | P | 8, 9, 10, | 2 | 0.102 (x 19.678) | 6 (0.333) | 0.16 | gluconeogenesis | Pepck Tpi |
| 35 | GO:0031887 | P | 8, 9, 10, | 1 | 0.017 (x 59.033) | 1 (1.000) | 0.173 | lipid particle transport along microtubule | CG9057 |
| 36 | GO:0035035 | F | 5, | 1 | 0.017 (x 59.033) | 1 (1.000) | 0.175 | histone acetyltransferase binding | Caf1 |
| 37 | GO:0004144 | F | 9, | 1 | 0.017 (x 59.033) | 1 (1.000) | 0.176 | diacylglycerol O-acyltransferase activity | mdy |
| 38 | GO:0004450 | F | 7, | 1 | 0.017 (x 59.033) | 1 (1.000) | 0.178 | isocitrate dehydrogenase (NADP+) activity | Idh |
| 39 | GO:0008456 | F | 7, | 1 | 0.017 (x 59.033) | 1 (1.000) | 0.179 | alpha-N-acetylgalactosaminidase activity | CG5731 |
| 40 | GO:0006357 | P | 9, | 18 | 9.266 (x 1.943) | 547 (0.033) | 0.179 | regulation of transcription from RNA polymerase II promoter | Caf1 D19A Doc1 Doc2 Doc3 Dsp1 Eip75B HmgZ Optix Poxn Trl br en grn inv klu sc toe |
| 41 | GO:0006513 | P | 10, | 1 | 0.017 (x 59.033) | 1 (1.000) | 0.181 | protein monoubiquitination | th |
| 42 | GO:0046578 | P | 6, 7, 8, | 2 | 0.119 (x 16.867) | 7 (0.286) | 0.181 | regulation of Ras protein signal transduction | edl klu |
| 43 | GO:0006335 | P | 8, 12, | 1 | 0.017 (x 59.033) | 1 (1.000) | 0.182 | DNA replication-dependent nucleosome assembly | Caf1 |
| 44 | GO:0048729 | P | 4, | 4 | 0.728 (x 5.491) | 43 (0.093) | 0.183 | tissue morphogenesis | in pbl pk sgl |
| 45 | GO:0006582 | P | 6, | 2 | 0.136 (x 14.758) | 8 (0.250) | 0.183 | melanin metabolism | Tl serpin-27A |
| 46 | GO:0050794 | P | 3, | 35 | 23.326 (x 1.500) | 1377 (0.025) | 0.183 | regulation of cellular process | Abi BEST:GH02921 CG40410 CG4914 CREG Caf1 Cdk4 D19A Doc1 Doc2 Doc3 Dsp1 Eip75B HmgZ Optix Poxn Rpn9 Tl Trl ana ash2 br dap dve edl en grn inv klu mdy sc smi35A th toe zwilch |
| 47 | GO:0007476 | P | 6, 7, 8, | 6 | 1.745 (x 3.439) | 103 (0.058) | 0.184 | wing morphogenesis | Poxn ash2 dve in pk vg |
| 48 | GO:0031348 | P | 6, | 1 | 0.017 (x 59.033) | 1 (1.000) | 0.184 | negative regulation of defense response | serpin-27A |
| 49 | GO:0048737 | P | 4, | 7 | 2.202 (x 3.179) | 130 (0.054) | 0.184 | appendage development (sensu Endopterygota) | Poxn ash2 dve in pk th vg |
| 50 | GO:0006355 | P | 8, | 23 | 13.332 (x 1.725) | 787 (0.029) | 0.185 | regulation of transcription, DNA-dependent | BEST:GH02921 CG4914 Caf1 D19A Doc1 Doc2 Doc3 Dsp1 Eip75B HmgZ Optix Poxn Trl ash2 br dve edl en grn inv klu sc toe |
| 51 | GO:0019222 | P | 4, | 28 | 17.346 (x 1.614) | 1024 (0.027) | 0.185 | regulation of metabolism | BEST:GH02921 CG40410 CG4914 CREG Caf1 D19A Doc1 Doc2 Doc3 Dsp1 Eip75B HmgZ Optix Poxn Tl Trl ash2 br dap dve edl en grn inv klu sc serpin-27A toe |
| 52 | GO:0007423 | P | 4, | 10 | 3.896 (x 2.567) | 230 (0.043) | 0.186 | sensory organ development | Optix Poxn arr br dap edl klu malpha pk sc |
| 53 | GO:0006097 | P | 7, 8, | 1 | 0.017 (x 59.033) | 1 (1.000) | 0.186 | glyoxylate cycle | Idh |
| 54 | GO:0051244 | P | 4, | 34 | 21.971 (x 1.548) | 1297 (0.026) | 0.186 | regulation of cellular physiological process | Abi BEST:GH02921 CG40410 CG4914 CREG Caf1 Cdk4 D19A Doc1 Doc2 Doc3 Dsp1 Eip75B HmgZ Optix Poxn Rpn9 Trl ana ash2 br dap dve edl en grn inv klu mdy sc smi35A th toe zwilch |
| 55 | GO:0005975 | P | 5, | 16 | 8.216 (x 1.947) | 485 (0.033) | 0.186 | carbohydrate metabolism | BcDNA:GH02976 CG17052 CG17323 CG5731 CG6287 GNBP3 Gfat1 GlyP Idh Oscillin Pepck Tpi alpha-Man-IIb fbp l(2)01810 sgl |
| 56 | GO:0051056 | P | 5, 6, 7, | 2 | 0.136 (x 14.758) | 8 (0.250) | 0.186 | regulation of small GTPase mediated signal transduction | edl klu |
| 57 | GO:0008113 | F | 6, | 1 | 0.017 (x 59.033) | 1 (1.000) | 0.187 | protein-methionine-S-oxide reductase activity | Eip71CD |
| 58 | GO:0003700 | F | 3, 5, | 14 | 6.590 (x 2.125) | 389 (0.036) | 0.188 | transcription factor activity | BEST:GH02921 CG4914 Doc1 Doc2 Doc3 Eip75B Optix Poxn br dve en grn inv toe |
| 59 | GO:0035114 | P | 5, | 7 | 2.202 (x 3.179) | 130 (0.054) | 0.188 | appendage morphogenesis (sensu Endopterygota) | Poxn ash2 dve in pk th vg |
| 60 | GO:0003979 | F | 6, | 1 | 0.017 (x 59.033) | 1 (1.000) | 0.189 | UDP-glucose 6-dehydrogenase activity | sgl |
| 61 | GO:0019842 | F | 3, | 3 | 0.407 (x 7.379) | 24 (0.125) | 0.189 | vitamin binding | CG10657 CG13848 CG3823 |
| 62 | GO:0030154 | P | 3, | 17 | 8.639 (x 1.968) | 510 (0.033) | 0.19 | cell differentiation | Fas3 Hrb27C Sema-1b Tl br edl fax fra in malpha ninA pbl pk rost sc th wbl |
| 63 | GO:0016978 | F | 6, | 1 | 0.017 (x 59.033) | 1 (1.000) | 0.191 | lipoate-protein ligase B activity | CG6767 |
| 64 | GO:0035107 | P | 4, | 7 | 2.253 (x 3.107) | 133 (0.053) | 0.191 | appendage morphogenesis | Poxn ash2 dve in pk th vg |
| 65 | GO:0045449 | P | 7, | 24 | 14.077 (x 1.705) | 831 (0.029) | 0.191 | regulation of transcription | BEST:GH02921 CG4914 CREG Caf1 D19A Doc1 Doc2 Doc3 Dsp1 Eip75B HmgZ Optix Poxn Trl ash2 br dve edl en grn inv klu sc toe |
| 66 | GO:0045448 | P | 4, 6, | 2 | 0.220 (x 9.082) | 13 (0.154) | 0.192 | mitotic cell cycle, embryonic | CG40410 Trl |
| 67 | GO:0007398 | P | 4, | 9 | 4.015 (x 2.242) | 237 (0.038) | 0.192 | ectoderm development | Poxn Sema-1b en fra in inv pk smi35A toe |
| 68 | GO:0007406 | P | 6, 7, 8, 9, | 1 | 0.017 (x 59.033) | 1 (1.000) | 0.192 | negative regulation of neuroblast proliferation | ana |
| 69 | GO:0007498 | P | 4, | 8 | 3.354 (x 2.385) | 198 (0.040) | 0.193 | mesoderm development | Doc1 Doc2 Doc3 Poxn grn pbl rost sgl |
| 70 | GO:0019220 | P | 7, | 2 | 0.220 (x 9.082) | 13 (0.154) | 0.193 | regulation of phosphate metabolism | dap edl |
| 71 | GO:0005031 | F | 6, | 1 | 0.017 (x 59.033) | 1 (1.000) | 0.194 | tumor necrosis factor receptor activity | wgn |
| 72 | GO:0005976 | P | 6, | 7 | 2.744 (x 2.551) | 162 (0.043) | 0.194 | polysaccharide metabolism | BcDNA:GH02976 CG17052 CG17323 CG5731 GNBP3 GlyP sgl |
| 73 | GO:0007509 | P | 7, 8, | 2 | 0.152 (x 13.118) | 9 (0.222) | 0.194 | mesoderm migration | pbl sgl |
| 74 | GO:0007154 | P | 3, | 36 | 25.697 (x 1.401) | 1517 (0.024) | 0.195 | cell communication | Abi CG17064 CG17919 CG30440 CG33171 CG40410 CG4054 Cdk4 Eip75B Nep2 PFE Sema-1b Snap Tl Tsp66E Wnt2 arr br edl fax for fra kal-1 klu malpha mav mth ogre ome pbl serpin-27A sgl stai trn wbl wgn |
| 75 | GO:0048736 | P | 3, | 7 | 2.253 (x 3.107) | 133 (0.053) | 0.195 | appendage development | Poxn ash2 dve in pk th vg |
| 76 | GO:0051174 | P | 6, | 2 | 0.220 (x 9.082) | 13 (0.154) | 0.195 | regulation of phosphorus metabolism | dap edl |
| 77 | GO:0004648 | F | 6, | 1 | 0.017 (x 59.033) | 1 (1.000) | 0.196 | phosphoserine transaminase activity | ESTS:39C10S |
| 78 | GO:0003677 | F | 4, | 22 | 13.924 (x 1.580) | 822 (0.027) | 0.198 | DNA binding | BEST:GH02921 CG4914 Doc1 Doc2 Doc3 Dsp1 Eip75B His4r HmgZ Hrb27C Optix Poxn Trl ash2 br dve edl en grn inv sc toe |
| 79 | GO:0035072 | P | 7, 8, 9, 10, 11, | 2 | 0.152 (x 13.118) | 9 (0.222) | 0.198 | ecdysone-mediated induction of salivary gland cell autophagic cell death | Eip75B br |
| 80 | GO:0007472 | P | 6, 7, | 6 | 1.779 (x 3.373) | 105 (0.057) | 0.198 | wing disc morphogenesis | Poxn ash2 dve in pk vg |
| 81 | GO:0004887 | F | 5, | 1 | 0.017 (x 59.033) | 1 (1.000) | 0.198 | thyroid hormone receptor activity | Eip75B |
| 82 | GO:0046487 | P | 6, 7, | 1 | 0.017 (x 59.033) | 1 (1.000) | 0.2 | glyoxylate metabolism | Idh |
| 83 | GO:0000096 | P | 6, 7, 8, | 2 | 0.152 (x 13.118) | 9 (0.222) | 0.201 | sulfur amino acid metabolism | Eip55E Eip71CD |
| 84 | GO:0005035 | F | 5, | 1 | 0.017 (x 59.033) | 1 (1.000) | 0.202 | death receptor activity | wgn |
| 85 | GO:0006366 | P | 8, | 19 | 11.553 (x 1.645) | 682 (0.028) | 0.202 | transcription from RNA polymerase II promoter | Caf1 D19A Doc1 Doc2 Doc3 Dsp1 Eip75B HmgZ Optix Poxn Trl ash2 br en grn inv klu sc toe |
| 86 | GO:0043118 | P | 4, | 9 | 4.082 (x 2.205) | 241 (0.037) | 0.202 | negative regulation of physiological process | CG40410 CREG Caf1 ana dap edl en serpin-27A th |
| 87 | GO:0042048 | P | 5, 6, | 4 | 0.898 (x 4.455) | 53 (0.075) | 0.203 | olfactory behavior | CG8588 Fas3 Obp99a smi35A |
| 88 | GO:0019218 | P | 6, 7, 8, | 1 | 0.017 (x 59.033) | 1 (1.000) | 0.203 | regulation of steroid metabolism | Eip75B |
| 89 | GO:0051674 | P | 4, | 10 | 4.353 (x 2.297) | 257 (0.039) | 0.204 | localization of cell | Abi Fas3 Sema-1b fra ninA pbl sgl stai th trn |
| 90 | GO:0007549 | P | 4, | 2 | 0.152 (x 13.118) | 9 (0.222) | 0.204 | dosage compensation | Trl sc |
| 91 | GO:0000902 | P | 4, 5, | 12 | 5.726 (x 2.096) | 338 (0.036) | 0.205 | cellular morphogenesis | Abi CG3770 Cdk4 Fas3 Sema-1b edl fax fra in ninA pbl pk |
| 92 | GO:0042132 | F | 8, | 1 | 0.017 (x 59.033) | 1 (1.000) | 0.205 | fructose-bisphosphatase activity | fbp |
| 93 | GO:0006928 | P | 4, 5, | 10 | 4.353 (x 2.297) | 257 (0.039) | 0.206 | cell motility | Abi Fas3 Sema-1b fra ninA pbl sgl stai th trn |
| 94 | GO:0001737 | P | 6, 7, 8, 9, 10, 11, | 2 | 0.169 (x 11.807) | 10 (0.200) | 0.206 | establishment of wing hair orientation | in pk |
| 95 | GO:0016051 | P | 6, 7, | 4 | 0.830 (x 4.819) | 49 (0.082) | 0.207 | carbohydrate biosynthesis | Gfat1 Pepck Tpi sgl |
| 96 | GO:0004807 | F | 6, | 1 | 0.017 (x 59.033) | 1 (1.000) | 0.207 | triose-phosphate isomerase activity | Tpi |
| 97 | GO:0008431 | F | 4, | 2 | 0.152 (x 13.118) | 9 (0.222) | 0.208 | vitamin E binding | CG13848 CG3823 |
| 98 | GO:0009070 | P | 8, 9, | 2 | 0.237 (x 8.433) | 14 (0.143) | 0.208 | serine family amino acid biosynthesis | CG6287 ESTS:39C10S |
| 99 | GO:0006564 | P | 9, 10, | 2 | 0.169 (x 11.807) | 10 (0.200) | 0.209 | L-serine biosynthesis | CG6287 ESTS:39C10S |
| 100 | GO:0008184 | F | 7, | 1 | 0.017 (x 59.033) | 1 (1.000) | 0.209 | glycogen phosphorylase activity | GlyP |
| 101 | GO:0035317 | P | 7, 8, 9, 10, | 2 | 0.237 (x 8.433) | 14 (0.143) | 0.21 | wing hair organization and biogenesis | in pk |
| 102 | GO:0004342 | F | 4, 6, | 1 | 0.017 (x 59.033) | 1 (1.000) | 0.212 | glucosamine-6-phosphate deaminase activity | Oscillin |
| 103 | GO:0007448 | P | 5, 6, | 2 | 0.237 (x 8.433) | 14 (0.143) | 0.212 | anterior/posterior pattern formation, imaginal disc | en inv |
| 104 | GO:0046165 | P | 6, | 2 | 0.169 (x 11.807) | 10 (0.200) | 0.212 | alcohol biosynthesis | Pepck Tpi |
| 105 | GO:0007088 | P | 7, 8, | 3 | 0.610 (x 4.919) | 36 (0.083) | 0.212 | regulation of mitosis | CG40410 Rpn9 zwilch |
| 106 | GO:0040011 | P | 3, | 10 | 4.421 (x 2.262) | 261 (0.038) | 0.213 | locomotion | Abi Fas3 Sema-1b fra ninA pbl sgl stai th trn |
| 107 | GO:0007635 | P | 4, 5, | 4 | 0.915 (x 4.373) | 54 (0.074) | 0.213 | chemosensory behavior | CG8588 Fas3 Obp99a smi35A |
| 108 | GO:0004861 | F | 6, | 1 | 0.017 (x 59.033) | 1 (1.000) | 0.214 | cyclin-dependent protein kinase inhibitor activity | dap |
| 109 | GO:0051707 | P | 4, | 6 | 2.219 (x 2.704) | 131 (0.046) | 0.215 | response to other organism | CG10359 CG7668 GNBP3 Tl br serpin-27A |
| 110 | GO:0046364 | P | 7, 8, | 2 | 0.169 (x 11.807) | 10 (0.200) | 0.216 | monosaccharide biosynthesis | Pepck Tpi |
| 111 | GO:0035009 | P | 6, 7, 9, | 1 | 0.017 (x 59.033) | 1 (1.000) | 0.216 | negative regulation of melanization defense response | serpin-27A |
| 112 | GO:0006563 | P | 8, 9, | 2 | 0.186 (x 10.733) | 11 (0.182) | 0.216 | L-serine metabolism | CG6287 ESTS:39C10S |
| 113 | GO:0006457 | P | 7, | 6 | 2.219 (x 2.704) | 131 (0.046) | 0.216 | protein folding | CG2852 Fkbp13 Hsp23 Hsp26 Hsp27 Hsp67Ba |
| 114 | GO:0003704 | F | 4, | 5 | 1.338 (x 3.736) | 79 (0.063) | 0.217 | specific RNA polymerase II transcription factor activity | Eip75B Trl br en sc |
| 115 | GO:0007553 | P | 7, 8, 9, | 1 | 0.017 (x 59.033) | 1 (1.000) | 0.218 | regulation of ecdysteroid metabolism | Eip75B |
| 116 | GO:0035081 | P | 8, 9, | 2 | 0.186 (x 10.733) | 11 (0.182) | 0.219 | induction of programmed cell death by hormones | Eip75B br |
| 117 | GO:0019319 | P | 8, 9, | 2 | 0.169 (x 11.807) | 10 (0.200) | 0.219 | hexose biosynthesis | Pepck Tpi |
| 118 | GO:0008063 | P | 6, | 3 | 0.508 (x 5.903) | 30 (0.100) | 0.219 | Toll signaling pathway | Tl serpin-27A wbl |
| 119 | GO:0019219 | P | 6, | 24 | 15.042 (x 1.595) | 888 (0.027) | 0.219 | regulation of nucleobase, nucleoside, nucleotide and nucleic acid metabolism | BEST:GH02921 CG4914 CREG Caf1 D19A Doc1 Doc2 Doc3 Dsp1 Eip75B HmgZ Optix Poxn Trl ash2 br dve edl en grn inv klu sc toe |
| 120 | GO:0006950 | P | 3, | 12 | 5.658 (x 2.121) | 334 (0.036) | 0.22 | response to stress | CG40410 Caf1 Eip71CD Hsp23 Hsp26 Hsp27 Hsp67Ba Tl br mth ninA serpin-27A |
| 121 | GO:0035011 | P | 6, 7, 8, | 1 | 0.017 (x 59.033) | 1 (1.000) | 0.22 | melanotic encapsulation of foreign target | serpin-27A |
| 122 | GO:0000910 | P | 5, | 5 | 1.423 (x 3.514) | 84 (0.060) | 0.221 | cytokinesis | Act42A Caf1 Snap Tl pbl |
| 123 | GO:0000904 | P | 5, 6, | 7 | 2.863 (x 2.445) | 169 (0.041) | 0.221 | cellular morphogenesis during differentiation | Fas3 Sema-1b edl fax fra ninA pbl |
| 124 | GO:0048699 | P | 6, | 8 | 3.049 (x 2.624) | 180 (0.044) | 0.222 | generation of neurons | Fas3 Sema-1b ana fax fra ninA pbl sc |
| 125 | GO:0035078 | P | 6, 7, 8, 9, 10, | 2 | 0.169 (x 11.807) | 10 (0.200) | 0.222 | induction of programmed cell death by ecdysone | Eip75B br |
| 126 | GO:0012501 | P | 5, | 9 | 4.201 (x 2.142) | 248 (0.036) | 0.223 | programmed cell death | Eip71CD Eip75B br klu mdy smi35A th trn wgn |
| 127 | GO:0004062 | F | 6, | 1 | 0.017 (x 59.033) | 1 (1.000) | 0.223 | aryl sulfotransferase activity | CG16733 |
| 128 | GO:0007389 | P | 3, | 10 | 4.337 (x 2.306) | 256 (0.039) | 0.223 | pattern specification | Cdk4 Hrb27C Tl edl en inv serpin-27A sgl tok wbl |
| 129 | GO:0006090 | P | 7, | 2 | 0.254 (x 7.871) | 15 (0.133) | 0.223 | pyruvate metabolism | Pepck Tpi |
| 130 | GO:0008219 | P | 4, | 9 | 4.235 (x 2.125) | 250 (0.036) | 0.223 | cell death | Eip71CD Eip75B br klu mdy smi35A th trn wgn |
| 131 | GO:0031323 | P | 5, | 25 | 16.702 (x 1.497) | 986 (0.025) | 0.223 | regulation of cellular metabolism | BEST:GH02921 CG4914 CREG Caf1 D19A Doc1 Doc2 Doc3 Dsp1 Eip75B HmgZ Optix Poxn Trl ash2 br dap dve edl en grn inv klu sc toe |
| 132 | GO:0035075 | P | 5, 6, 7, | 2 | 0.254 (x 7.871) | 15 (0.133) | 0.225 | response to ecdysone | Eip75B br |
| 133 | GO:0007388 | P | 5, | 1 | 0.017 (x 59.033) | 1 (1.000) | 0.225 | posterior compartment specification | en |
| 134 | GO:0048545 | P | 5, 6, | 2 | 0.254 (x 7.871) | 15 (0.133) | 0.226 | response to steroid hormone stimulus | Eip75B br |
| 135 | GO:0016265 | P | 3, | 9 | 4.252 (x 2.117) | 251 (0.036) | 0.227 | death | Eip71CD Eip75B br klu mdy smi35A th trn wgn |
| 136 | GO:0019432 | P | 8, 9, 10, | 1 | 0.017 (x 59.033) | 1 (1.000) | 0.228 | triacylglycerol biosynthesis | mdy |
| 137 | GO:0007093 | P | 8, 9, | 2 | 0.254 (x 7.871) | 15 (0.133) | 0.228 | mitotic checkpoint | CG40410 zwilch |
| 138 | GO:0004645 | F | 6, | 1 | 0.034 (x 29.517) | 2 (0.500) | 0.228 | phosphorylase activity | GlyP |
| 139 | GO:0031099 | P | 4, | 1 | 0.034 (x 29.517) | 2 (0.500) | 0.229 | regeneration | ninA |
| 140 | GO:0043120 | F | 5, | 1 | 0.017 (x 59.033) | 1 (1.000) | 0.23 | tumor necrosis factor binding | wgn |
| 141 | GO:0042246 | P | 4, 5, 6, | 1 | 0.034 (x 29.517) | 2 (0.500) | 0.231 | tissue regeneration | ninA |
| 142 | GO:0048100 | P | 6, 7, | 2 | 0.203 (x 9.839) | 12 (0.167) | 0.231 | wing disc anterior/posterior pattern formation | en inv |
| 143 | GO:0005811 | C | 5, 6, 7, 8, | 1 | 0.034 (x 29.517) | 2 (0.500) | 0.232 | lipid particle | CG9057 |
| 144 | GO:0009950 | P | 5, | 4 | 0.966 (x 4.143) | 57 (0.070) | 0.232 | dorsal/ventral axis specification | Tl serpin-27A tok wbl |
| 145 | GO:0004360 | F | 6, | 1 | 0.034 (x 29.517) | 2 (0.500) | 0.233 | glutamine-fructose-6-phosphate transaminase (isomerizing) activity | Gfat1 |
| 146 | GO:0006351 | P | 7, | 23 | 15.178 (x 1.515) | 896 (0.026) | 0.233 | transcription, DNA-dependent | BEST:GH02921 CG4914 Caf1 D19A Doc1 Doc2 Doc3 Dsp1 Eip75B HmgZ Optix Poxn Trl ash2 br dve edl en grn inv klu sc toe |
| 147 | GO:0043067 | P | 5, 6, | 6 | 2.016 (x 2.976) | 119 (0.050) | 0.233 | regulation of programmed cell death | Eip75B br klu mdy smi35A th |
| 148 | GO:0045786 | P | 6, 7, | 2 | 0.203 (x 9.839) | 12 (0.167) | 0.234 | negative regulation of progression through cell cycle | CG40410 dap |
| 149 | GO:0006350 | P | 6, | 24 | 16.008 (x 1.499) | 945 (0.025) | 0.234 | transcription | BEST:GH02921 CG4914 CREG Caf1 D19A Doc1 Doc2 Doc3 Dsp1 Eip75B HmgZ Optix Poxn Trl ash2 br dve edl en grn inv klu sc toe |
| 150 | GO:0007289 | P | 6, 9, | 1 | 0.034 (x 29.517) | 2 (0.500) | 0.234 | spermatid nuclear differentiation | th |
| 151 | GO:0022008 | P | 5, | 8 | 3.269 (x 2.447) | 193 (0.041) | 0.235 | neurogenesis | Fas3 Sema-1b ana fax fra ninA pbl sc |
| 152 | GO:0004613 | F | 7, | 1 | 0.034 (x 29.517) | 2 (0.500) | 0.236 | phosphoenolpyruvate carboxykinase (GTP) activity | Pepck |
| 153 | GO:0016671 | F | 5, | 1 | 0.034 (x 29.517) | 2 (0.500) | 0.237 | oxidoreductase activity, acting on sulfur group of donors, disulfide as acceptor | Eip71CD |
| 154 | GO:0048812 | P | 7, 8, 10, | 6 | 2.050 (x 2.927) | 121 (0.050) | 0.237 | neurite morphogenesis | Fas3 Sema-1b fax fra ninA pbl |
| 155 | GO:0035221 | P | 5, 6, | 1 | 0.034 (x 29.517) | 2 (0.500) | 0.238 | genital disc pattern formation | en |
| 156 | GO:0006081 | P | 5, | 1 | 0.034 (x 29.517) | 2 (0.500) | 0.24 | aldehyde metabolism | Idh |
| 157 | GO:0048667 | P | 6, 7, 9, | 6 | 2.050 (x 2.927) | 121 (0.050) | 0.24 | neuron morphogenesis during differentiation | Fas3 Sema-1b fax fra ninA pbl |
| 158 | GO:0006323 | P | 7, | 6 | 2.338 (x 2.567) | 138 (0.043) | 0.24 | DNA packaging | Caf1 Dsp1 His4r HmgZ Trl ash2 |
| 159 | GO:0004611 | F | 6, | 1 | 0.034 (x 29.517) | 2 (0.500) | 0.241 | phosphoenolpyruvate carboxykinase activity | Pepck |
| 160 | GO:0009953 | P | 4, | 4 | 1.254 (x 3.191) | 74 (0.054) | 0.241 | dorsal/ventral pattern formation | Tl serpin-27A tok wbl |
| 161 | GO:0048468 | P | 4, | 11 | 5.759 (x 1.910) | 340 (0.032) | 0.242 | cell development | Fas3 Hrb27C Sema-1b edl fax fra ninA pbl rost th wbl |
| 162 | GO:0006325 | P | 8, | 6 | 2.338 (x 2.567) | 138 (0.043) | 0.242 | establishment and/or maintenance of chromatin architecture | Caf1 Dsp1 His4r HmgZ Trl ash2 |
| 163 | GO:0009725 | P | 4, 5, | 2 | 0.271 (x 7.379) | 16 (0.125) | 0.242 | response to hormone stimulus | Eip75B br |
| 164 | GO:0009790 | P | 3, | 11 | 5.946 (x 1.850) | 351 (0.031) | 0.242 | embryonic development | CG40410 Cdk4 Doc1 Sema-1b Tl Trl edl en pbl sgl wbl |
| 165 | GO:0030730 | P | 5, 7, | 1 | 0.034 (x 29.517) | 2 (0.500) | 0.243 | sequestering of triacylglycerol | CG9057 |
| 166 | GO:0007409 | P | 8, 9, 11, | 6 | 2.050 (x 2.927) | 121 (0.050) | 0.243 | axonogenesis | Fas3 Sema-1b fax fra ninA pbl |
| 167 | GO:0051235 | P | 4, | 2 | 0.305 (x 6.559) | 18 (0.111) | 0.243 | maintenance of localization | CG9057 pk |
| 168 | GO:0030528 | F | 2, | 22 | 13.636 (x 1.613) | 805 (0.027) | 0.243 | transcription regulator activity | BEST:GH02921 CG4914 CREG D19A Doc1 Doc2 Doc3 Dsp1 Eip75B HmgZ Optix Poxn Trl ash2 br dve en grn inv klu sc toe |
| 169 | GO:0016043 | P | 4, | 32 | 23.360 (x 1.370) | 1379 (0.023) | 0.243 | cell organization and biogenesis | Abi Act42A CG2852 CG33113 CG33171 CG3770 CG9057 Caf1 Cdk4 Dsp1 Fas3 His4r HmgZ Sb Sema-1b Snap Trl arr ash2 edl esn fax for fra in l(1)G0320 ninA pbl pk stai vg wbl |
| 170 | GO:0007367 | P | 6, 7, | 2 | 0.271 (x 7.379) | 16 (0.125) | 0.244 | segment polarity determination | en sgl |
| 171 | GO:0009069 | P | 7, 8, | 2 | 0.305 (x 6.559) | 18 (0.111) | 0.244 | serine family amino acid metabolism | CG6287 ESTS:39C10S |
| 172 | GO:0004123 | F | 5, | 1 | 0.034 (x 29.517) | 2 (0.500) | 0.244 | cystathionine gamma-lyase activity | Eip55E |
| 173 | GO:0017051 | F | 6, | 1 | 0.034 (x 29.517) | 2 (0.500) | 0.245 | retinol dehydratase activity | CG16733 |
| 174 | GO:0007424 | P | 4, | 5 | 1.846 (x 2.708) | 109 (0.046) | 0.246 | tracheal system development (sensu Insecta) | Cdk4 Wnt2 dap sgl trn |
| 175 | GO:0004617 | F | 6, | 1 | 0.034 (x 29.517) | 2 (0.500) | 0.247 | phosphoglycerate dehydrogenase activity | CG6287 |
| 176 | GO:0040029 | P | 3, | 4 | 1.186 (x 3.373) | 70 (0.057) | 0.247 | regulation of gene expression, epigenetic | Caf1 Trl ash2 sc |
| 177 | GO:0035224 | P | 6, 7, | 1 | 0.034 (x 29.517) | 2 (0.500) | 0.248 | genital disc anterior/posterior pattern formation | en |
| 178 | GO:0043068 | P | 6, 7, | 4 | 1.270 (x 3.148) | 75 (0.053) | 0.249 | positive regulation of programmed cell death | Eip75B br klu smi35A |
| 179 | GO:0004749 | F | 6, 7, | 1 | 0.034 (x 29.517) | 2 (0.500) | 0.25 | ribose phosphate diphosphokinase activity | CG6767 |
| 180 | GO:0004572 | F | 8, | 1 | 0.034 (x 29.517) | 2 (0.500) | 0.251 | mannosyl-oligosaccharide 1,3-1,6-alpha-mannosidase activity | alpha-Man-IIb |
| 181 | GO:0006641 | P | 7, 8, 9, | 1 | 0.034 (x 29.517) | 2 (0.500) | 0.253 | triacylglycerol metabolism | mdy |
| 182 | GO:0015397 | F | 6, 7, | 1 | 0.034 (x 29.517) | 2 (0.500) | 0.254 | equilibrative nucleoside transporter, nitrobenzyl-thioinosine-insensitive activity | BEST:LD04971 |
| 183 | GO:0042787 | P | 10, 11, 12, | 1 | 0.034 (x 29.517) | 2 (0.500) | 0.256 | protein ubiquitination during ubiquitin-dependent protein catabolism | th |
| 184 | GO:0009620 | P | 5, | 2 | 0.322 (x 6.214) | 19 (0.105) | 0.256 | response to fungus | GNBP3 Tl |
| 185 | GO:0009913 | P | 4, 6, 7, | 2 | 0.288 (x 6.945) | 17 (0.118) | 0.256 | epidermal cell differentiation | in pk |
| 186 | GO:0016769 | F | 4, | 2 | 0.322 (x 6.214) | 19 (0.105) | 0.257 | transferase activity, transferring nitrogenous groups | ESTS:39C10S Gfat1 |
| 187 | GO:0030182 | P | 4, 7, | 6 | 2.524 (x 2.377) | 149 (0.040) | 0.257 | neuron differentiation | Fas3 Sema-1b fax fra ninA pbl |
| 188 | GO:0005042 | F | 6, | 1 | 0.034 (x 29.517) | 2 (0.500) | 0.257 | netrin receptor activity | fra |
| 189 | GO:0048666 | P | 5, 8, | 6 | 2.405 (x 2.494) | 142 (0.042) | 0.258 | neuron development | Fas3 Sema-1b fax fra ninA pbl |
| 190 | GO:0035315 | P | 5, 7, 8, | 2 | 0.288 (x 6.945) | 17 (0.118) | 0.258 | hair cell differentiation | in pk |
| 191 | GO:0000075 | P | 7, | 2 | 0.322 (x 6.214) | 19 (0.105) | 0.259 | cell cycle checkpoint | CG40410 zwilch |
| 192 | GO:0030234 | F | 2, | 11 | 6.047 (x 1.819) | 357 (0.031) | 0.259 | enzyme regulator activity | Abi BEST:GH02921 CG17919 CG30440 Rpn9 Spn1 Spn43Aa dap for pbl serpin-27A |
| 193 | GO:0031175 | P | 6, 9, | 6 | 2.405 (x 2.494) | 142 (0.042) | 0.259 | neurite development | Fas3 Sema-1b fax fra ninA pbl |
| 194 | GO:0048730 | P | 5, 6, | 2 | 0.288 (x 6.945) | 17 (0.118) | 0.259 | epidermis morphogenesis | in pk |
| 195 | GO:0044264 | P | 6, 7, | 4 | 1.304 (x 3.067) | 77 (0.052) | 0.26 | cellular polysaccharide metabolism | BcDNA:GH02976 CG17052 GlyP sgl |
| 196 | GO:0008483 | F | 5, | 2 | 0.322 (x 6.214) | 19 (0.105) | 0.26 | transaminase activity | ESTS:39C10S Gfat1 |
| 197 | GO:0030246 | F | 3, | 5 | 1.914 (x 2.612) | 113 (0.044) | 0.26 | carbohydrate binding | BcDNA:GH02976 CG17052 CG9134 Gfat1 glec |
| 198 | GO:0007165 | P | 4, | 30 | 21.869 (x 1.372) | 1291 (0.023) | 0.261 | signal transduction | Abi CG17919 CG30440 CG33171 CG40410 Cdk4 Eip75B Nep2 PFE Sema-1b Tl Tsp66E Wnt2 arr edl for fra klu malpha mav mth ogre ome pbl serpin-27A sgl stai trn wbl wgn |
| 199 | GO:0019898 | C | 4, 5, 6, | 3 | 0.762 (x 3.936) | 45 (0.067) | 0.261 | extrinsic to membrane | ImpE2 ImpE3 Snap |
| 200 | GO:0035316 | P | 6, 7, 8, 9, | 2 | 0.288 (x 6.945) | 17 (0.118) | 0.261 | trichome organization and biogenesis (sensu Insecta) | in pk |
| 201 | GO:0005783 | C | 5, 6, 7, 8, | 5 | 1.914 (x 2.612) | 113 (0.044) | 0.261 | endoplasmic reticulum | CBP CG33113 Snap l(1)G0320 wbl |
| 202 | GO:0005634 | C | 5, 6, 7, 8, | 35 | 25.816 (x 1.356) | 1524 (0.023) | 0.262 | nucleus | BEST:GH02921 CG17838 CG40410 CG4914 CG6930 CG9894 CREG Caf1 D19A Doc1 Doc2 Doc3 Dsp1 Eip75B HmgZ Hrb27C Optix Poxn Trl ash2 bip1 br dap dve edl en esn grn inv klu pbl pk sc toe vg |
| 203 | GO:0009987 | P | 2, | 123 | 113.597 (x 1.083) | 6706 (0.018) | 0.262 | cellular process | Abi Act42A Argk BEST:GH02921 BEST:LD04971 BcDNA:GH02976 BcDNA:LD41548 CG10657 CG14439 CG1607 CG17052 CG17064 CG17323 CG17919 CG2852 CG30440 CG31121 CG33113 CG33171 CG3770 CG3823 CG40410 CG4054 CG4586 CG4914 CG5390 CG5466 CG5794 CG5873 CG6287 CG6767 CG9027 CG9057 CREG Caf1 Cdk4 Cyp310a1 D19A Doc1 Doc2 Doc3 Dsp1 ESTS:39C10S Eip55E Eip71CD Eip75B Fas3 Fkbp13 Gfat1 GlyP His4r HmgZ Hrb27C Hsp23 Hsp26 Hsp27 Hsp67Ba Idh Lsp2 Nep2 Nrv1 Obp99a Optix Oscillin PFE Pepck Poxn RpS12 Rpn9 Sb Sema-1b Snap Spn1 Tl Tpi Trl Tsp66E Wnt2 ana arr ash2 br dap dve edl en esn fax for fra glec grn in inv kal-1 klu l(1)G0320 l(2)01810 malpha mav mdy mnd mth ninA ogre ome pbl pk rost rpk sc serpin-27A sgl smi35A stai th toe tok trn vg wbl wgn zwilch |
| 204 | GO:0008340 | P | 4, | 3 | 0.779 (x 3.850) | 46 (0.065) | 0.262 | determination of adult life span | Hsp26 Hsp27 mth |
| 205 | GO:0007610 | P | 3, | 7 | 3.235 (x 2.164) | 191 (0.037) | 0.262 | behavior | CG8588 Fas3 Obp99a Poxn for ogre smi35A |
| 206 | GO:0051243 | P | 5, | 8 | 3.930 (x 2.036) | 232 (0.034) | 0.263 | negative regulation of cellular physiological process | CG40410 CREG Caf1 ana dap edl en th |
| 207 | GO:0007568 | P | 3, | 3 | 0.779 (x 3.850) | 46 (0.065) | 0.263 | aging | Hsp26 Hsp27 mth |
| 208 | GO:0044262 | P | 6, | 9 | 4.658 (x 1.932) | 275 (0.033) | 0.264 | cellular carbohydrate metabolism | BcDNA:GH02976 CG17052 Gfat1 GlyP Idh Oscillin Pepck Tpi sgl |
| 209 | GO:0035222 | P | 5, 6, | 2 | 0.339 (x 5.903) | 20 (0.100) | 0.264 | wing disc pattern formation | en inv |
| 210 | GO:0030030 | P | 5, 6, | 3 | 0.796 (x 3.768) | 47 (0.064) | 0.265 | cell projection organization and biogenesis | Abi in pk |
| 211 | GO:0050793 | P | 3, | 4 | 1.338 (x 2.989) | 79 (0.051) | 0.265 | regulation of development | Tl ana br pbl |
| 212 | GO:0003697 | F | 6, | 2 | 0.339 (x 5.903) | 20 (0.100) | 0.265 | single-stranded DNA binding | Dsp1 Hrb27C |
| 213 | GO:0007411 | P | 6, 7, 9, 10, 12, | 4 | 1.389 (x 2.880) | 82 (0.049) | 0.265 | axon guidance | Fas3 Sema-1b fra ninA |
| 214 | GO:0007001 | P | 7, | 6 | 2.660 (x 2.256) | 157 (0.038) | 0.266 | chromosome organization and biogenesis (sensu Eukaryota) | Caf1 Dsp1 His4r HmgZ Trl ash2 |
| 215 | GO:0008283 | P | 4, | 9 | 4.777 (x 1.884) | 282 (0.032) | 0.266 | cell proliferation | Cdk4 D19A Eip75B Poxn Tl ana klu smi35A toe |
| 216 | GO:0003680 | F | 6, | 1 | 0.051 (x 19.678) | 3 (0.333) | 0.267 | AT DNA binding | dve |
| 217 | GO:0007313 | P | 7, 9, 10, 12, | 1 | 0.051 (x 19.678) | 3 (0.333) | 0.268 | maternal determination of dorsal/ventral axis, oocyte, soma encoded | wbl |
| 218 | GO:0007486 | P | 6, 7, | 1 | 0.051 (x 19.678) | 3 (0.333) | 0.269 | female genitalia development (sensu Endopterygota) | en |
| 219 | GO:0042826 | F | 5, | 1 | 0.051 (x 19.678) | 3 (0.333) | 0.27 | histone deacetylase binding | Caf1 |
| 220 | GO:0005721 | C | 6, 7, 8, 9, 10, 11, 12, | 1 | 0.051 (x 19.678) | 3 (0.333) | 0.271 | centric heterochromatin | Trl |
| 221 | GO:0008544 | P | 5, | 2 | 0.373 (x 5.367) | 22 (0.091) | 0.272 | epidermis development | in pk |
| 222 | GO:0035010 | P | 5, 6, | 1 | 0.051 (x 19.678) | 3 (0.333) | 0.273 | encapsulation of foreign target | serpin-27A |
| 223 | GO:0051726 | P | 5, | 7 | 3.371 (x 2.077) | 199 (0.035) | 0.273 | regulation of cell cycle | Abi CG40410 Cdk4 Rpn9 dap sc zwilch |
| 224 | GO:0008037 | P | 3, | 2 | 0.373 (x 5.367) | 22 (0.091) | 0.274 | cell recognition | Fas3 fra |
| 225 | GO:0005581 | C | 3, 4, 5, | 1 | 0.051 (x 19.678) | 3 (0.333) | 0.274 | collagen | CG33171 |
| 226 | GO:0000074 | P | 6, | 7 | 3.371 (x 2.077) | 199 (0.035) | 0.274 | regulation of progression through cell cycle | Abi CG40410 Cdk4 Rpn9 dap sc zwilch |
| 227 | GO:0008038 | P | 4, | 2 | 0.373 (x 5.367) | 22 (0.091) | 0.275 | neuron recognition | Fas3 fra |
| 228 | GO:0015280 | F | 6, 7, 8, | 1 | 0.051 (x 19.678) | 3 (0.333) | 0.275 | amiloride-sensitive sodium channel activity | rpk |
| 229 | GO:0030540 | P | 5, | 1 | 0.051 (x 19.678) | 3 (0.333) | 0.276 | female genitalia development | en |
| 230 | GO:0006430 | P | 9, 10, 11, | 1 | 0.051 (x 19.678) | 3 (0.333) | 0.277 | lysyl-tRNA aminoacylation | mdy |
| 231 | GO:0035214 | P | 5, | 6 | 2.710 (x 2.214) | 160 (0.037) | 0.278 | eye-antennal disc development | Poxn arr br klu pk th |
| 232 | GO:0016979 | F | 5, | 1 | 0.051 (x 19.678) | 3 (0.333) | 0.279 | lipoate-protein ligase activity | CG6767 |
| 233 | GO:0009308 | P | 5, | 11 | 6.369 (x 1.727) | 376 (0.029) | 0.279 | amine metabolism | BcDNA:GH02976 CG1607 CG17052 CG6287 ESTS:39C10S Eip55E Eip71CD Oscillin mdy mnd sgl |
| 234 | GO:0004824 | F | 7, | 1 | 0.051 (x 19.678) | 3 (0.333) | 0.28 | lysine-tRNA ligase activity | mdy |
| 235 | GO:0007346 | P | 6, 7, | 2 | 0.356 (x 5.622) | 21 (0.095) | 0.281 | regulation of progression through mitotic cell cycle | CG40410 sc |
| 236 | GO:0004054 | F | 6, 7, | 1 | 0.051 (x 19.678) | 3 (0.333) | 0.281 | arginine kinase activity | Argk |
| 237 | GO:0019210 | F | 4, | 2 | 0.356 (x 5.622) | 21 (0.095) | 0.282 | kinase inhibitor activity | CG17919 dap |
| 238 | GO:0019915 | P | 6, | 1 | 0.051 (x 19.678) | 3 (0.333) | 0.282 | sequestering of lipid | CG9057 |
| 239 | GO:0007469 | P | 6, | 2 | 0.356 (x 5.622) | 21 (0.095) | 0.283 | antennal development | Poxn th |
| 240 | GO:0016200 | P | 6, | 1 | 0.051 (x 19.678) | 3 (0.333) | 0.284 | synaptic target attraction | Fas3 |
| 241 | GO:0006066 | P | 5, | 6 | 2.727 (x 2.200) | 161 (0.037) | 0.284 | alcohol metabolism | Gfat1 Oscillin Pepck Tpi arr mdy |
| 242 | GO:0007265 | P | 7, | 2 | 0.423 (x 4.723) | 25 (0.080) | 0.284 | Ras protein signal transduction | edl klu |
| 243 | GO:0003755 | F | 5, | 2 | 0.356 (x 5.622) | 21 (0.095) | 0.284 | peptidyl-prolyl cis-trans isomerase activity | CG2852 Fkbp13 |
| 244 | GO:0043566 | F | 5, | 2 | 0.423 (x 4.723) | 25 (0.080) | 0.285 | structure-specific DNA binding | Dsp1 Hrb27C |
| 245 | GO:0004692 | F | 9, | 1 | 0.051 (x 19.678) | 3 (0.333) | 0.285 | cGMP-dependent protein kinase activity | for |
| 246 | GO:0016584 | P | 11, | 1 | 0.068 (x 14.758) | 4 (0.250) | 0.286 | nucleosome spacing | Caf1 |
| 247 | GO:0016859 | F | 4, | 2 | 0.390 (x 5.133) | 23 (0.087) | 0.286 | cis-trans isomerase activity | CG2852 Fkbp13 |
| 248 | GO:0050767 | P | 4, 7, | 2 | 0.423 (x 4.723) | 25 (0.080) | 0.286 | regulation of neurogenesis | ana pbl |
| 249 | GO:0015395 | F | 5, 6, | 1 | 0.051 (x 19.678) | 3 (0.333) | 0.286 | nucleoside transporter activity, down a concentration gradient | BEST:LD04971 |
| 250 | GO:0043119 | P | 4, | 6 | 2.863 (x 2.096) | 169 (0.036) | 0.286 | positive regulation of physiological process | Eip75B Trl ash2 br klu smi35A |
| 251 | GO:0046460 | P | 6, 7, 8, | 1 | 0.068 (x 14.758) | 4 (0.250) | 0.287 | neutral lipid biosynthesis | mdy |
| 252 | GO:0045859 | P | 6, | 2 | 0.390 (x 5.133) | 23 (0.087) | 0.287 | regulation of protein kinase activity | Abi dap |
| 253 | GO:0019752 | P | 6, | 11 | 6.640 (x 1.657) | 392 (0.028) | 0.287 | carboxylic acid metabolism | CG1607 CG4586 CG6287 ESTS:39C10S Eip55E Eip71CD Idh Pepck Tpi mdy mnd |
| 254 | GO:0009880 | P | 4, | 5 | 2.185 (x 2.288) | 129 (0.039) | 0.287 | embryonic pattern specification | Cdk4 Tl edl en sgl |
| 255 | GO:0016853 | F | 3, | 4 | 1.542 (x 2.595) | 91 (0.044) | 0.287 | isomerase activity | CG2852 Fkbp13 Oscillin Tpi |
| 256 | GO:0008615 | P | 9, | 1 | 0.068 (x 14.758) | 4 (0.250) | 0.288 | pyridoxine biosynthesis | ESTS:39C10S |
| 257 | GO:0004772 | F | 8, | 1 | 0.051 (x 19.678) | 3 (0.333) | 0.288 | sterol O-acyltransferase activity | mdy |
| 258 | GO:0006807 | P | 4, | 11 | 6.623 (x 1.661) | 391 (0.028) | 0.288 | nitrogen compound metabolism | BcDNA:GH02976 CG1607 CG17052 CG6287 ESTS:39C10S Eip55E Eip71CD Oscillin mdy mnd sgl |
| 259 | GO:0005886 | C | 4, 5, | 14 | 9.046 (x 1.548) | 534 (0.026) | 0.288 | plasma membrane | Fas3 ImpE3 Nrv1 Sb Sema-1b Tl arr for fra in ogre rost trn wgn |
| 260 | GO:0006767 | P | 6, | 2 | 0.390 (x 5.133) | 23 (0.087) | 0.288 | water-soluble vitamin metabolism | ESTS:39C10S Tpi |
| 261 | GO:0006082 | P | 5, | 11 | 6.640 (x 1.657) | 392 (0.028) | 0.288 | organic acid metabolism | CG1607 CG4586 CG6287 ESTS:39C10S Eip55E Eip71CD Idh Pepck Tpi mdy mnd |
| 262 | GO:0004274 | F | 6, 8, | 1 | 0.068 (x 14.758) | 4 (0.250) | 0.289 | dipeptidyl-peptidase IV activity | ome |
| 263 | GO:0043549 | P | 5, | 2 | 0.390 (x 5.133) | 23 (0.087) | 0.289 | regulation of kinase activity | Abi dap |
| 264 | GO:0046463 | P | 7, 8, 9, | 1 | 0.068 (x 14.758) | 4 (0.250) | 0.29 | acylglycerol biosynthesis | mdy |
| 265 | GO:0051338 | P | 4, | 2 | 0.390 (x 5.133) | 23 (0.087) | 0.29 | regulation of transferase activity | Abi dap |
| 266 | GO:0016528 | C | 5, 6, 7, | 1 | 0.068 (x 14.758) | 4 (0.250) | 0.291 | sarcoplasm | CBP |
| 267 | GO:0009306 | P | 6, | 1 | 0.068 (x 14.758) | 4 (0.250) | 0.292 | protein secretion | alpha-Man-IIb |
| 268 | GO:0016589 | C | 5, 8, 9, 10, 11, 12, 13, 14, 15, | 1 | 0.068 (x 14.758) | 4 (0.250) | 0.293 | NURF complex | Caf1 |
| 269 | GO:0008203 | P | 7, 8, 9, | 2 | 0.440 (x 4.541) | 26 (0.077) | 0.293 | cholesterol metabolism | arr mdy |
| 270 | GO:0016573 | P | 9, 10, 12, | 1 | 0.068 (x 14.758) | 4 (0.250) | 0.294 | histone acetylation | Caf1 |
| 271 | GO:0048332 | P | 5, | 2 | 0.440 (x 4.541) | 26 (0.077) | 0.294 | mesoderm morphogenesis | pbl sgl |
| 272 | GO:0016564 | F | 3, | 3 | 0.966 (x 3.107) | 57 (0.053) | 0.294 | transcriptional repressor activity | CREG Dsp1 en |
| 273 | GO:0048099 | P | 6, 7, | 1 | 0.068 (x 14.758) | 4 (0.250) | 0.295 | anterior/posterior lineage restriction, imaginal disc | en |
| 274 | GO:0016337 | P | 4, | 5 | 2.101 (x 2.380) | 124 (0.040) | 0.295 | cell-cell adhesion | Fas3 Sema-1b Tsp66E glec trn |
| 275 | GO:0016860 | F | 4, | 2 | 0.440 (x 4.541) | 26 (0.077) | 0.295 | intramolecular oxidoreductase activity | Oscillin Tpi |
| 276 | GO:0051276 | P | 6, | 6 | 2.914 (x 2.059) | 172 (0.035) | 0.295 | chromosome organization and biogenesis | Caf1 Dsp1 His4r HmgZ Trl ash2 |
| 277 | GO:0019216 | P | 5, 6, | 1 | 0.068 (x 14.758) | 4 (0.250) | 0.296 | regulation of lipid metabolism | Eip75B |
| 278 | GO:0001707 | P | 6, 7, | 2 | 0.440 (x 4.541) | 26 (0.077) | 0.296 | mesoderm formation | pbl sgl |
| 279 | GO:0035305 | P | 8, 9, | 1 | 0.068 (x 14.758) | 4 (0.250) | 0.297 | negative regulation of dephosphorylation | dap |
| 280 | GO:0008614 | P | 8, | 1 | 0.068 (x 14.758) | 4 (0.250) | 0.298 | pyridoxine metabolism | ESTS:39C10S |
| 281 | GO:0031010 | C | 4, 7, 8, 9, 10, 11, 12, 13, 14, | 1 | 0.068 (x 14.758) | 4 (0.250) | 0.299 | ISWI complex | Caf1 |
| 282 | GO:0016328 | C | 5, 6, 7, | 1 | 0.068 (x 14.758) | 4 (0.250) | 0.3 | lateral plasma membrane | Fas3 |
| 283 | GO:0019202 | F | 6, | 1 | 0.068 (x 14.758) | 4 (0.250) | 0.301 | amino acid kinase activity | Argk |
| 284 | GO:0007111 | P | 6, 7, | 1 | 0.068 (x 14.758) | 4 (0.250) | 0.302 | cytokinesis after meiosis II | pbl |
| 285 | GO:0030332 | F | 4, | 1 | 0.085 (x 11.807) | 5 (0.200) | 0.303 | cyclin binding | Cdk4 |
| 286 | GO:0003702 | F | 3, | 8 | 4.506 (x 1.775) | 266 (0.030) | 0.303 | RNA polymerase II transcription factor activity | Eip75B Optix Trl br en grn inv sc |
| 287 | GO:0048519 | P | 3, | 9 | 5.167 (x 1.742) | 305 (0.030) | 0.303 | negative regulation of biological process | CG40410 CREG Caf1 ana dap edl en serpin-27A th |
| 288 | GO:0008078 | P | 6, 7, 8, 9, | 1 | 0.068 (x 14.758) | 4 (0.250) | 0.303 | mesodermal cell migration | pbl |
| 289 | GO:0008603 | F | 5, | 1 | 0.085 (x 11.807) | 5 (0.200) | 0.304 | cAMP-dependent protein kinase regulator activity | for |
| 290 | GO:0009892 | P | 5, | 6 | 2.795 (x 2.147) | 165 (0.036) | 0.304 | negative regulation of metabolism | CREG Caf1 dap edl en serpin-27A |
| 291 | GO:0007096 | P | 8, 9, | 1 | 0.085 (x 11.807) | 5 (0.200) | 0.304 | regulation of exit from mitosis | Rpn9 |
| 292 | GO:0005785 | C | 4, 5, 6, 7, 8, 9, 10, 11, 12, | 1 | 0.068 (x 14.758) | 4 (0.250) | 0.304 | signal recognition particle receptor complex | l(1)G0320 |
| 293 | GO:0016490 | F | 3, | 2 | 0.407 (x 4.919) | 24 (0.083) | 0.305 | structural constituent of peritrophic membrane (sensu Insecta) | BcDNA:GH02976 CG17052 |
| 294 | GO:0007110 | P | 6, 7, | 1 | 0.085 (x 11.807) | 5 (0.200) | 0.305 | cytokinesis after meiosis I | pbl |
| 295 | GO:0035308 | P | 7, 8, 9, 10, | 1 | 0.068 (x 14.758) | 4 (0.250) | 0.306 | negative regulation of protein amino acid dephosphorylation | dap |
| 296 | GO:0006996 | P | 5, | 18 | 12.230 (x 1.472) | 722 (0.025) | 0.306 | organelle organization and biogenesis | Abi Act42A CG33171 CG9057 Caf1 Cdk4 Dsp1 His4r HmgZ Sb Trl ash2 esn for fra pbl stai vg |
| 297 | GO:0042594 | P | 4, 6, | 1 | 0.085 (x 11.807) | 5 (0.200) | 0.306 | response to starvation | mth |
| 298 | GO:0042816 | P | 7, | 1 | 0.068 (x 14.758) | 4 (0.250) | 0.307 | vitamin B6 metabolism | ESTS:39C10S |
| 299 | GO:0005623 | C | 2, | 88 | 78.193 (x 1.125) | 4616 (0.019) | 0.307 | cell | Act42A BEST:GH02921 BEST:LD04971 BcDNA:LD41548 CBP CG10657 CG14076 CG14439 CG1607 CG17838 CG31121 CG33113 CG33171 CG3823 CG40410 CG4586 CG4914 CG6930 CG9057 CG9894 CREG Caf1 Cyp310a1 D19A Doc1 Doc2 Doc3 Dsp1 Eip75B Fas3 Gfat1 His4r HmgZ Hrb27C Idh ImpE2 ImpE3 Nep2 Nrv1 Optix Pepck Poxn RpS12 Rpn9 Sb Sema-1b Snap Tl Trl Tsp66E alpha-Man-IIb arr ash2 bip1 br dap dve edl en esn for fra glec grn in inv klu l(1)G0320 l(2)01810 mdy mnd mth ninA ogre ome pbl pk rost rpk sc smi35A stai th toe trn vg wbl wgn |
| 300 | GO:0030536 | P | 5, | 1 | 0.085 (x 11.807) | 5 (0.200) | 0.307 | larval feeding behavior | for |
| 301 | GO:0016529 | C | 6, 7, 8, 9, | 1 | 0.068 (x 14.758) | 4 (0.250) | 0.308 | sarcoplasmic reticulum | CBP |
| 302 | GO:0044464 | C | 2, 3, | 88 | 78.193 (x 1.125) | 4616 (0.019) | 0.308 | cell part | Act42A BEST:GH02921 BEST:LD04971 BcDNA:LD41548 CBP CG10657 CG14076 CG14439 CG1607 CG17838 CG31121 CG33113 CG33171 CG3823 CG40410 CG4586 CG4914 CG6930 CG9057 CG9894 CREG Caf1 Cyp310a1 D19A Doc1 Doc2 Doc3 Dsp1 Eip75B Fas3 Gfat1 His4r HmgZ Hrb27C Idh ImpE2 ImpE3 Nep2 Nrv1 Optix Pepck Poxn RpS12 Rpn9 Sb Sema-1b Snap Tl Trl Tsp66E alpha-Man-IIb arr ash2 bip1 br dap dve edl en esn for fra glec grn in inv klu l(1)G0320 l(2)01810 mdy mnd mth ninA ogre ome pbl pk rost rpk sc smi35A stai th toe trn vg wbl wgn |
| 303 | GO:0007540 | P | 6, | 1 | 0.085 (x 11.807) | 5 (0.200) | 0.308 | sex determination, establishment of X:A ratio | sc |
| 304 | GO:0008039 | P | 5, | 1 | 0.085 (x 11.807) | 5 (0.200) | 0.309 | synaptic target recognition | Fas3 |
| 305 | GO:0007365 | P | 5, 6, | 2 | 0.457 (x 4.373) | 27 (0.074) | 0.309 | periodic partitioning | en sgl |
| 306 | GO:0042819 | P | 8, | 1 | 0.068 (x 14.758) | 4 (0.250) | 0.309 | vitamin B6 biosynthesis | ESTS:39C10S |
| 307 | GO:0006639 | P | 6, 7, 8, | 1 | 0.085 (x 11.807) | 5 (0.200) | 0.31 | acylglycerol metabolism | mdy |
| 308 | GO:0046670 | P | 7, 8, 9, | 1 | 0.068 (x 14.758) | 4 (0.250) | 0.31 | positive regulation of retinal programmed cell death | klu |
| 309 | GO:0042325 | P | 8, | 1 | 0.085 (x 11.807) | 5 (0.200) | 0.311 | regulation of phosphorylation | edl |
| 310 | GO:0000301 | P | 7, 8, 9, 10, | 1 | 0.068 (x 14.758) | 4 (0.250) | 0.311 | retrograde transport, vesicle recycling within Golgi | wbl |
| 311 | GO:0004857 | F | 3, | 5 | 2.151 (x 2.324) | 127 (0.039) | 0.311 | enzyme inhibitor activity | CG17919 Spn1 Spn43Aa dap serpin-27A |
| 312 | GO:0046504 | P | 6, | 1 | 0.085 (x 11.807) | 5 (0.200) | 0.312 | glycerol ether biosynthesis | mdy |
| 313 | GO:0051242 | P | 5, | 6 | 2.829 (x 2.121) | 167 (0.036) | 0.312 | positive regulation of cellular physiological process | Eip75B Trl ash2 br klu smi35A |
| 314 | GO:0048518 | P | 3, | 7 | 3.693 (x 1.896) | 218 (0.032) | 0.312 | positive regulation of biological process | Abi Eip75B Trl ash2 br klu smi35A |
| 315 | GO:0015014 | P | 8, 9, 10, | 1 | 0.068 (x 14.758) | 4 (0.250) | 0.313 | heparan sulfate proteoglycan biosynthesis, polysaccharide chain biosynthesis | sgl |
| 316 | GO:0045017 | P | 6, 7, 8, | 1 | 0.085 (x 11.807) | 5 (0.200) | 0.313 | glycerolipid biosynthesis | mdy |
| 317 | GO:0007267 | P | 4, | 12 | 7.589 (x 1.581) | 448 (0.027) | 0.313 | cell-cell signaling | CG17064 CG4054 Eip75B PFE Snap br fax for kal-1 mth stai trn |
| 318 | GO:0045477 | P | 7, 8, 10, | 1 | 0.068 (x 14.758) | 4 (0.250) | 0.314 | regulation of nurse cell apoptosis | mdy |
| 319 | GO:0007485 | P | 6, 7, | 1 | 0.085 (x 11.807) | 5 (0.200) | 0.314 | male genitalia development (sensu Endopterygota) | en |
| 320 | GO:0042221 | P | 4, | 8 | 4.455 (x 1.796) | 263 (0.030) | 0.314 | response to chemical stimulus | CG17323 CG8588 Eip75B Fas3 Obp99a br mth smi35A |
| 321 | GO:0006790 | P | 5, | 3 | 0.999 (x 3.002) | 59 (0.051) | 0.314 | sulfur metabolism | Eip55E Eip71CD sgl |
| 322 | GO:0008368 | F | 4, | 1 | 0.085 (x 11.807) | 5 (0.200) | 0.315 | Gram-negative bacterial binding | GNBP3 |
| 323 | GO:0007348 | P | 6, 8, 9, | 1 | 0.068 (x 14.758) | 4 (0.250) | 0.315 | regulation of progression through syncytial blastoderm mitotic cell cycle | CG40410 |
| 324 | GO:0007405 | P | 5, 7, | 1 | 0.085 (x 11.807) | 5 (0.200) | 0.316 | neuroblast proliferation | ana |
| 325 | GO:0046672 | P | 8, 9, 10, 11, | 1 | 0.068 (x 14.758) | 4 (0.250) | 0.316 | positive regulation of retinal cell programmed cell death (sensu Endopterygota) | klu |
| 326 | GO:0000079 | P | 7, | 1 | 0.085 (x 11.807) | 5 (0.200) | 0.317 | regulation of cyclin-dependent protein kinase activity | dap |
| 327 | GO:0000042 | P | 8, 9, 10, 11, | 1 | 0.068 (x 14.758) | 4 (0.250) | 0.317 | protein targeting to Golgi | wbl |
| 328 | GO:0006638 | P | 6, 7, | 1 | 0.085 (x 11.807) | 5 (0.200) | 0.318 | neutral lipid metabolism | mdy |
| 329 | GO:0016055 | P | 6, | 3 | 0.932 (x 3.220) | 55 (0.055) | 0.318 | Wnt receptor signaling pathway | Wnt2 arr sgl |
| 330 | GO:0000077 | P | 6, 9, | 1 | 0.085 (x 11.807) | 5 (0.200) | 0.319 | DNA damage checkpoint | CG40410 |
| 331 | GO:0051301 | P | 4, | 5 | 2.355 (x 2.123) | 139 (0.036) | 0.319 | cell division | Act42A Caf1 Snap Tl pbl |
| 332 | GO:0046976 | F | 9, 10, | 1 | 0.085 (x 11.807) | 5 (0.200) | 0.32 | histone lysine N-methyltransferase activity (H3-K27 specific) | Caf1 |
| 333 | GO:0016778 | F | 5, | 1 | 0.085 (x 11.807) | 5 (0.200) | 0.321 | diphosphotransferase activity | CG6767 |
| 334 | GO:0050875 | P | 3, | 113 | 105.297 (x 1.073) | 6216 (0.018) | 0.322 | cellular physiological process | Abi Act42A Argk BEST:GH02921 BEST:LD04971 BcDNA:GH02976 BcDNA:LD41548 CG10657 CG14439 CG1607 CG17052 CG17323 CG2852 CG31121 CG33113 CG33171 CG3770 CG3823 CG40410 CG4586 CG4914 CG5390 CG5466 CG5794 CG5873 CG6287 CG6767 CG9027 CG9057 CREG Caf1 Cdk4 Cyp310a1 D19A Doc1 Doc2 Doc3 Dsp1 ESTS:39C10S Eip55E Eip71CD Eip75B Fas3 Fkbp13 Gfat1 GlyP His4r HmgZ Hrb27C Hsp23 Hsp26 Hsp27 Hsp67Ba Idh Lsp2 Nep2 Nrv1 Obp99a Optix Oscillin PFE Pepck Poxn RpS12 Rpn9 Sb Sema-1b Snap Spn1 Tl Tpi Trl ana arr ash2 br dap dve edl en esn fax for fra grn in inv klu l(1)G0320 l(2)01810 mav mdy mnd mth ninA ogre ome pbl pk rpk sc serpin-27A sgl smi35A stai th toe tok trn vg wbl wgn zwilch |
| 335 | GO:0050768 | P | 5, 8, | 1 | 0.085 (x 11.807) | 5 (0.200) | 0.322 | negative regulation of neurogenesis | ana |
| 336 | GO:0045476 | P | 7, 9, | 1 | 0.102 (x 9.839) | 6 (0.167) | 0.322 | nurse cell apoptosis | mdy |
| 337 | GO:0017026 | F | 7, | 1 | 0.085 (x 11.807) | 5 (0.200) | 0.323 | procollagen C-endopeptidase activity | tok |
| 338 | GO:0035304 | P | 6, 7, 9, | 1 | 0.102 (x 9.839) | 6 (0.167) | 0.323 | regulation of protein amino acid dephosphorylation | dap |
| 339 | GO:0008202 | P | 6, 7, | 5 | 2.304 (x 2.170) | 136 (0.037) | 0.323 | steroid metabolism | CG17323 Cyp310a1 Eip75B arr mdy |
| 340 | GO:0035098 | C | 4, 6, 7, 8, 9, 10, 11, 12, 13, 14, | 1 | 0.085 (x 11.807) | 5 (0.200) | 0.324 | ESC/E(Z) complex | Caf1 |
| 341 | GO:0000280 | P | 4, | 1 | 0.102 (x 9.839) | 6 (0.167) | 0.324 | nuclear division | Trl |
| 342 | GO:0001704 | P | 5, 6, | 2 | 0.474 (x 4.217) | 28 (0.071) | 0.324 | formation of primary germ layer | pbl sgl |
| 343 | GO:0007352 | P | 6, | 1 | 0.102 (x 9.839) | 6 (0.167) | 0.325 | zygotic determination of dorsal/ventral axis | Tl |
| 344 | GO:0008367 | F | 3, | 1 | 0.085 (x 11.807) | 5 (0.200) | 0.325 | bacterial binding | GNBP3 |
| 345 | GO:0005527 | F | 4, | 1 | 0.102 (x 9.839) | 6 (0.167) | 0.325 | macrolide binding | Fkbp13 |
| 346 | GO:0004860 | F | 5, | 1 | 0.085 (x 11.807) | 5 (0.200) | 0.326 | protein kinase inhibitor activity | dap |
| 347 | GO:0042394 | P | 6, | 1 | 0.102 (x 9.839) | 6 (0.167) | 0.326 | ecdysis (sensu Protostomia and Nematoda) | Eip75B |
| 348 | GO:0006333 | P | 9, | 4 | 1.694 (x 2.361) | 100 (0.040) | 0.327 | chromatin assembly or disassembly | Caf1 Dsp1 His4r HmgZ |
| 349 | GO:0019203 | F | 7, | 1 | 0.102 (x 9.839) | 6 (0.167) | 0.327 | carbohydrate phosphatase activity | fbp |
| 350 | GO:0005996 | P | 6, 7, | 4 | 1.694 (x 2.361) | 100 (0.040) | 0.328 | monosaccharide metabolism | Gfat1 Oscillin Pepck Tpi |
| 351 | GO:0005616 | C | 3, 4, 5, | 1 | 0.102 (x 9.839) | 6 (0.167) | 0.328 | larval serum protein complex | Lsp2 |
| 352 | GO:0009794 | P | 5, 7, 8, | 1 | 0.102 (x 9.839) | 6 (0.167) | 0.329 | regulation of progression through embryonic mitotic cell cycle | CG40410 |
| 353 | GO:0016335 | P | 5, 6, 7, | 1 | 0.102 (x 9.839) | 6 (0.167) | 0.33 | morphogenesis of larval imaginal disc epithelium | Sb |
| 354 | GO:0006662 | P | 5, | 1 | 0.102 (x 9.839) | 6 (0.167) | 0.331 | glycerol ether metabolism | mdy |
| 355 | GO:0003997 | F | 6, | 1 | 0.102 (x 9.839) | 6 (0.167) | 0.332 | acyl-CoA oxidase activity | CG4586 |
| 356 | GO:0048646 | P | 4, | 2 | 0.508 (x 3.936) | 30 (0.067) | 0.332 | anatomical structure formation | pbl sgl |
| 357 | GO:0007166 | P | 5, | 16 | 11.163 (x 1.433) | 659 (0.024) | 0.332 | cell surface receptor linked signal transduction | CG30440 PFE Sema-1b Tl Wnt2 arr edl malpha mav mth ome pbl serpin-27A sgl wbl wgn |
| 358 | GO:0004181 | F | 7, | 2 | 0.542 (x 3.690) | 32 (0.062) | 0.333 | metallocarboxypeptidase activity | Lsp2 fra |
| 359 | GO:0046486 | P | 6, 7, | 1 | 0.102 (x 9.839) | 6 (0.167) | 0.333 | glycerolipid metabolism | mdy |
| 360 | GO:0004182 | F | 8, | 2 | 0.542 (x 3.690) | 32 (0.062) | 0.333 | carboxypeptidase A activity | Lsp2 fra |
| 361 | GO:0008329 | F | 4, | 1 | 0.102 (x 9.839) | 6 (0.167) | 0.333 | pattern recognition receptor activity | GNBP3 |
| 362 | GO:0048522 | P | 4, | 6 | 3.202 (x 1.874) | 189 (0.032) | 0.334 | positive regulation of cellular process | Eip75B Trl ash2 br klu smi35A |
| 363 | GO:0009605 | P | 3, | 4 | 1.779 (x 2.249) | 105 (0.038) | 0.334 | response to external stimulus | mth ninA ogre serpin-27A |
| 364 | GO:0048523 | P | 4, | 8 | 4.743 (x 1.687) | 280 (0.029) | 0.334 | negative regulation of cellular process | CG40410 CREG Caf1 ana dap edl en th |
| 365 | GO:0004690 | F | 8, | 1 | 0.102 (x 9.839) | 6 (0.167) | 0.334 | cyclic nucleotide-dependent protein kinase activity | for |
| 366 | GO:0045815 | P | 4, | 1 | 0.119 (x 8.433) | 7 (0.143) | 0.335 | positive regulation of gene expression, epigenetic | ash2 |
| 367 | GO:0009792 | P | 4, | 6 | 3.303 (x 1.816) | 195 (0.031) | 0.335 | embryonic development (sensu Metazoa) | CG40410 Doc1 Sema-1b pbl sgl wbl |
| 368 | GO:0004448 | F | 6, | 1 | 0.119 (x 8.433) | 7 (0.143) | 0.335 | isocitrate dehydrogenase activity | Idh |
| 369 | GO:0031570 | P | 8, | 1 | 0.102 (x 9.839) | 6 (0.167) | 0.335 | DNA integrity checkpoint | CG40410 |
| 370 | GO:0008238 | F | 5, | 4 | 1.846 (x 2.166) | 109 (0.037) | 0.336 | exopeptidase activity | BcDNA:LD41548 Lsp2 fra ome |
| 371 | GO:0035193 | P | 5, 6, | 1 | 0.119 (x 8.433) | 7 (0.143) | 0.336 | central nervous system remodeling (sensu Insecta) | br |
| 372 | GO:0007487 | P | 6, | 1 | 0.102 (x 9.839) | 6 (0.167) | 0.336 | analia development (sensu Endopterygota) | en |
| 373 | GO:0048806 | P | 4, | 1 | 0.119 (x 8.433) | 7 (0.143) | 0.337 | genitalia development | en |
| 374 | GO:0005483 | F | 4, | 1 | 0.102 (x 9.839) | 6 (0.167) | 0.337 | soluble NSF attachment protein activity | Snap |
| 375 | GO:0015924 | F | 7, | 1 | 0.119 (x 8.433) | 7 (0.143) | 0.338 | mannosyl-oligosaccharide mannosidase activity | alpha-Man-IIb |
| 376 | GO:0019207 | F | 3, | 3 | 1.135 (x 2.643) | 67 (0.045) | 0.338 | kinase regulator activity | CG17919 dap for |
| 377 | GO:0005337 | F | 4, | 1 | 0.102 (x 9.839) | 6 (0.167) | 0.338 | nucleoside transporter activity | BEST:LD04971 |
| 378 | GO:0051058 | P | 6, 7, 8, | 1 | 0.119 (x 8.433) | 7 (0.143) | 0.339 | negative regulation of small GTPase mediated signal transduction | klu |
| 379 | GO:0004867 | F | 6, | 3 | 1.186 (x 2.530) | 70 (0.043) | 0.339 | serine-type endopeptidase inhibitor activity | Spn1 Spn43Aa serpin-27A |
| 380 | GO:0006800 | P | 5, | 3 | 1.135 (x 2.643) | 67 (0.045) | 0.339 | oxygen and reactive oxygen species metabolism | CG5873 CG9027 mth |
| 381 | GO:0005201 | F | 3, | 1 | 0.102 (x 9.839) | 6 (0.167) | 0.339 | extracellular matrix structural constituent | CG33171 |
| 382 | GO:0007484 | P | 5, 6, | 1 | 0.119 (x 8.433) | 7 (0.143) | 0.339 | genitalia development (sensu Endopterygota) | en |
| 383 | GO:0012502 | P | 7, 8, | 3 | 1.186 (x 2.530) | 70 (0.043) | 0.339 | induction of programmed cell death | Eip75B br smi35A |
| 384 | GO:0006422 | P | 9, 10, 11, | 1 | 0.102 (x 9.839) | 6 (0.167) | 0.34 | aspartyl-tRNA aminoacylation | mdy |
| 385 | GO:0035097 | C | 3, 6, 7, 8, 9, 10, 11, 12, 13, | 1 | 0.119 (x 8.433) | 7 (0.143) | 0.34 | histone methyltransferase complex | Caf1 |
| 386 | GO:0008237 | F | 5, | 6 | 3.134 (x 1.915) | 185 (0.032) | 0.341 | metallopeptidase activity | BcDNA:LD41548 D19A Lsp2 Nep2 fra tok |
| 387 | GO:0035303 | P | 8, | 1 | 0.102 (x 9.839) | 6 (0.167) | 0.341 | regulation of dephosphorylation | dap |
| 388 | GO:0016199 | P | 6, 8, 9, 11, 12, 14, | 1 | 0.119 (x 8.433) | 7 (0.143) | 0.341 | axon midline choice point recognition | fra |
| 389 | GO:0050654 | P | 6, 8, | 1 | 0.119 (x 8.433) | 7 (0.143) | 0.342 | chondroitin sulfate proteoglycan metabolism | sgl |
| 390 | GO:0005528 | F | 5, | 1 | 0.102 (x 9.839) | 6 (0.167) | 0.342 | FK506 binding | Fkbp13 |
| 391 | GO:0004785 | F | 6, | 1 | 0.119 (x 8.433) | 7 (0.143) | 0.343 | copper, zinc superoxide dismutase activity | CG9027 |
| 392 | GO:0016481 | P | 8, | 4 | 1.880 (x 2.127) | 111 (0.036) | 0.343 | negative regulation of transcription | CREG Caf1 edl en |
| 393 | GO:0004815 | F | 7, | 1 | 0.102 (x 9.839) | 6 (0.167) | 0.343 | aspartate-tRNA ligase activity | mdy |
| 394 | GO:0048102 | P | 6, | 3 | 1.203 (x 2.494) | 71 (0.042) | 0.343 | autophagic cell death | Eip71CD Eip75B br |
| 395 | GO:0007631 | P | 4, | 1 | 0.119 (x 8.433) | 7 (0.143) | 0.343 | feeding behavior | for |
| 396 | GO:0007163 | P | 5, 6, | 3 | 1.203 (x 2.494) | 71 (0.042) | 0.344 | establishment and/or maintenance of cell polarity | CG3770 in pk |
| 397 | GO:0016846 | F | 4, | 1 | 0.102 (x 9.839) | 6 (0.167) | 0.344 | carbon-sulfur lyase activity | Eip55E |
| 398 | GO:0009611 | P | 4, | 2 | 0.559 (x 3.578) | 33 (0.061) | 0.344 | response to wounding | ninA serpin-27A |
| 399 | GO:0048477 | P | 6, | 8 | 4.896 (x 1.634) | 289 (0.028) | 0.344 | oogenesis | Caf1 Eip75B Fas3 Hrb27C dap mdy th wbl |
| 400 | GO:0016411 | F | 8, | 1 | 0.119 (x 8.433) | 7 (0.143) | 0.344 | acylglycerol O-acyltransferase activity | mdy |
| 401 | GO:0035070 | P | 6, | 3 | 1.203 (x 2.494) | 71 (0.042) | 0.345 | salivary gland histolysis | Eip71CD Eip75B br |
| 402 | GO:0016125 | P | 6, 7, 8, | 2 | 0.559 (x 3.578) | 33 (0.061) | 0.345 | sterol metabolism | arr mdy |
| 403 | GO:0016918 | F | 4, 5, | 1 | 0.102 (x 9.839) | 6 (0.167) | 0.345 | retinal binding | CG10657 |
| 404 | GO:0015932 | F | 3, | 1 | 0.119 (x 8.433) | 7 (0.143) | 0.345 | nucleobase, nucleoside, nucleotide and nucleic acid transporter activity | BEST:LD04971 |
| 405 | GO:0006044 | P | 8, 9, | 3 | 1.203 (x 2.494) | 71 (0.042) | 0.345 | N-acetylglucosamine metabolism | BcDNA:GH02976 CG17052 Oscillin |
| 406 | GO:0019887 | F | 4, | 2 | 0.593 (x 3.373) | 35 (0.057) | 0.346 | protein kinase regulator activity | dap for |
| 407 | GO:0008354 | P | 5, 6, 7, | 2 | 0.559 (x 3.578) | 33 (0.061) | 0.346 | germ cell migration | stai th |
| 408 | GO:0046580 | P | 7, 8, 9, | 1 | 0.102 (x 9.839) | 6 (0.167) | 0.346 | negative regulation of Ras protein signal transduction | klu |
| 409 | GO:0030206 | P | 8, 9, 10, | 1 | 0.119 (x 8.433) | 7 (0.143) | 0.346 | chondroitin sulfate biosynthesis | sgl |
| 410 | GO:0035071 | P | 7, | 3 | 1.203 (x 2.494) | 71 (0.042) | 0.346 | salivary gland cell autophagic cell death | Eip71CD Eip75B br |
| 411 | GO:0005089 | F | 5, | 1 | 0.136 (x 7.379) | 8 (0.125) | 0.346 | Rho guanyl-nucleotide exchange factor activity | pbl |
| 412 | GO:0045936 | P | 7, 8, | 1 | 0.102 (x 9.839) | 6 (0.167) | 0.347 | negative regulation of phosphate metabolism | dap |
| 413 | GO:0048096 | P | 5, 10, 11, | 1 | 0.119 (x 8.433) | 7 (0.143) | 0.347 | chromatin-mediated maintenance of transcription | ash2 |
| 414 | GO:0006041 | P | 7, 8, | 3 | 1.203 (x 2.494) | 71 (0.042) | 0.347 | glucosamine metabolism | BcDNA:GH02976 CG17052 Oscillin |
| 415 | GO:0000271 | P | 7, 8, | 1 | 0.136 (x 7.379) | 8 (0.125) | 0.347 | polysaccharide biosynthesis | sgl |
| 416 | GO:0030865 | P | 7, | 1 | 0.119 (x 8.433) | 7 (0.143) | 0.348 | cortical cytoskeleton organization and biogenesis | Abi |
| 417 | GO:0043284 | P | 6, 7, | 1 | 0.136 (x 7.379) | 8 (0.125) | 0.348 | biopolymer biosynthesis | sgl |
| 418 | GO:0042026 | P | 8, | 1 | 0.102 (x 9.839) | 6 (0.167) | 0.348 | protein refolding | Hsp27 |
| 419 | GO:0006967 | P | 7, 8, 9, | 1 | 0.136 (x 7.379) | 8 (0.125) | 0.349 | positive regulation of antifungal peptide biosynthesis | Tl |
| 420 | GO:0030204 | P | 7, 8, 9, | 1 | 0.119 (x 8.433) | 7 (0.143) | 0.349 | chondroitin sulfate metabolism | sgl |
| 421 | GO:0007458 | P | 8, 9, | 1 | 0.102 (x 9.839) | 6 (0.167) | 0.349 | progression of morphogenetic furrow (sensu Endopterygota) | br |
| 422 | GO:0006801 | P | 6, | 1 | 0.136 (x 7.379) | 8 (0.125) | 0.349 | superoxide metabolism | CG9027 |
| 423 | GO:0030866 | P | 8, 9, | 1 | 0.119 (x 8.433) | 7 (0.143) | 0.349 | cortical actin cytoskeleton organization and biogenesis | Abi |
| 424 | GO:0018990 | P | 7, | 1 | 0.102 (x 9.839) | 6 (0.167) | 0.35 | ecdysis (sensu Insecta) | Eip75B |
| 425 | GO:0016350 | P | 9, | 1 | 0.136 (x 7.379) | 8 (0.125) | 0.35 | maintenance of oocyte identity (sensu Insecta) | dap |
| 426 | GO:0031324 | P | 6, | 5 | 2.626 (x 1.904) | 155 (0.032) | 0.35 | negative regulation of cellular metabolism | CREG Caf1 dap edl en |
| 427 | GO:0030145 | F | 6, | 1 | 0.119 (x 8.433) | 7 (0.143) | 0.35 | manganese ion binding | BcDNA:LD41548 |
| 428 | GO:0016271 | P | 4, | 3 | 1.220 (x 2.460) | 72 (0.042) | 0.35 | tissue death | Eip71CD Eip75B br |
| 429 | GO:0008046 | F | 5, | 1 | 0.102 (x 9.839) | 6 (0.167) | 0.351 | axon guidance receptor activity | fra |
| 430 | GO:0005344 | F | 3, | 1 | 0.136 (x 7.379) | 8 (0.125) | 0.351 | oxygen transporter activity | Lsp2 |
| 431 | GO:0042766 | P | 10, | 1 | 0.119 (x 8.433) | 7 (0.143) | 0.351 | nucleosome mobilization | Caf1 |
| 432 | GO:0007559 | P | 5, | 3 | 1.220 (x 2.460) | 72 (0.042) | 0.351 | histolysis | Eip71CD Eip75B br |
| 433 | GO:0048193 | P | 6, 7, 8, | 2 | 0.610 (x 3.280) | 36 (0.056) | 0.351 | Golgi vesicle transport | Snap wbl |
| 434 | GO:0016721 | F | 4, | 1 | 0.136 (x 7.379) | 8 (0.125) | 0.352 | oxidoreductase activity, acting on superoxide radicals as acceptor | CG9027 |
| 435 | GO:0005678 | C | 4, 7, 8, 9, 10, 11, 12, 13, 14, | 1 | 0.102 (x 9.839) | 6 (0.167) | 0.352 | chromatin assembly complex | Caf1 |
| 436 | GO:0009617 | P | 5, | 3 | 1.254 (x 2.393) | 74 (0.041) | 0.352 | response to bacterium | CG10359 CG7668 Tl |
| 437 | GO:0006040 | P | 6, 7, | 3 | 1.220 (x 2.460) | 72 (0.042) | 0.352 | amino sugar metabolism | BcDNA:GH02976 CG17052 Oscillin |
| 438 | GO:0046974 | F | 9, 10, | 1 | 0.119 (x 8.433) | 7 (0.143) | 0.352 | histone lysine N-methyltransferase activity (H3-K9 specific) | Caf1 |
| 439 | GO:0006766 | P | 5, | 2 | 0.610 (x 3.280) | 36 (0.056) | 0.352 | vitamin metabolism | ESTS:39C10S Tpi |
| 440 | GO:0045735 | F | 2, | 1 | 0.136 (x 7.379) | 8 (0.125) | 0.352 | nutrient reservoir activity | Lsp2 |
| 441 | GO:0046669 | P | 7, 8, 9, 10, | 1 | 0.119 (x 8.433) | 7 (0.143) | 0.353 | regulation of retinal cell programmed cell death (sensu Endopterygota) | klu |
| 442 | GO:0051226 | P | 7, 8, 11, | 1 | 0.136 (x 7.379) | 8 (0.125) | 0.353 | meiotic spindle assembly | pbl |
| 443 | GO:0005501 | F | 4, | 1 | 0.119 (x 8.433) | 7 (0.143) | 0.354 | retinoid binding | CG10657 |
| 444 | GO:0009886 | P | 4, | 1 | 0.136 (x 7.379) | 8 (0.125) | 0.354 | post-embryonic morphogenesis | br |
| 445 | GO:0005391 | F | 6, 7, 9, 14, | 1 | 0.136 (x 7.379) | 8 (0.125) | 0.355 | sodium:potassium-exchanging ATPase activity | Nrv1 |
| 446 | GO:0030530 | C | 4, 5, 6, 7, 8, 9, 10, | 1 | 0.119 (x 8.433) | 7 (0.143) | 0.355 | heterogeneous nuclear ribonucleoprotein complex | CG17838 |
| 447 | GO:0005243 | F | 5, | 1 | 0.136 (x 7.379) | 8 (0.125) | 0.355 | gap-junction forming channel activity | ogre |
| 448 | GO:0006268 | P | 9, | 1 | 0.119 (x 8.433) | 7 (0.143) | 0.356 | DNA unwinding during replication | Dsp1 |
| 449 | GO:0005952 | C | 3, 4, 5, 6, | 1 | 0.136 (x 7.379) | 8 (0.125) | 0.356 | cAMP-dependent protein kinase complex | for |
| 450 | GO:0035288 | P | 6, 7, | 1 | 0.119 (x 8.433) | 7 (0.143) | 0.357 | anterior head segmentation | en |
| 451 | GO:0008429 | F | 5, | 1 | 0.136 (x 7.379) | 8 (0.125) | 0.357 | phosphatidylethanolamine binding | CG17919 |
| 452 | GO:0042742 | P | 5, 6, | 3 | 1.169 (x 2.567) | 69 (0.043) | 0.357 | defense response to bacterium | CG10359 CG7668 Tl |
| 453 | GO:0019840 | F | 3, | 1 | 0.119 (x 8.433) | 7 (0.143) | 0.357 | isoprenoid binding | CG10657 |
| 454 | GO:0005921 | C | 7, 8, 9, | 1 | 0.136 (x 7.379) | 8 (0.125) | 0.358 | gap junction | ogre |
| 455 | GO:0009798 | P | 4, | 5 | 2.693 (x 1.856) | 159 (0.031) | 0.358 | axis specification | Hrb27C Tl serpin-27A tok wbl |
| 456 | GO:0042393 | F | 4, | 1 | 0.119 (x 8.433) | 7 (0.143) | 0.358 | histone binding | Caf1 |
| 457 | GO:0008541 | C | 3, 4, 5, 6, 7, 8, | 1 | 0.136 (x 7.379) | 8 (0.125) | 0.359 | proteasome regulatory particle, lid subcomplex (sensu Eukaryota) | Rpn9 |
| 458 | GO:0015929 | F | 6, | 1 | 0.119 (x 8.433) | 7 (0.143) | 0.359 | hexosaminidase activity | CG5731 |
| 459 | GO:0004784 | F | 5, | 1 | 0.136 (x 7.379) | 8 (0.125) | 0.359 | superoxide dismutase activity | CG9027 |
| 460 | GO:0050650 | P | 7, 8, 9, | 1 | 0.119 (x 8.433) | 7 (0.143) | 0.36 | chondroitin sulfate proteoglycan biosynthesis | sgl |
| 461 | GO:0046879 | P | 5, 6, | 1 | 0.136 (x 7.379) | 8 (0.125) | 0.36 | hormone secretion | stai |
| 462 | GO:0042770 | P | 5, | 1 | 0.136 (x 7.379) | 8 (0.125) | 0.361 | DNA damage response, signal transduction | CG40410 |
| 463 | GO:0005890 | C | 3, 5, 6, 7, 8, 9, | 1 | 0.136 (x 7.379) | 8 (0.125) | 0.362 | sodium:potassium-exchanging ATPase complex | Nrv1 |
| 464 | GO:0009796 | P | 4, 5, | 1 | 0.152 (x 6.559) | 9 (0.111) | 0.364 | cellularization (sensu Metazoa) | CG40410 |
| 465 | GO:0006073 | P | 7, 8, | 1 | 0.152 (x 6.559) | 9 (0.111) | 0.365 | glucan metabolism | GlyP |
| 466 | GO:0018346 | P | 10, 11, | 1 | 0.152 (x 6.559) | 9 (0.111) | 0.366 | protein amino acid prenylation | CG33171 |
| 467 | GO:0005160 | F | 4, 5, | 1 | 0.152 (x 6.559) | 9 (0.111) | 0.366 | transforming growth factor beta receptor binding | mav |
| 468 | GO:0015012 | P | 7, 8, 9, | 1 | 0.152 (x 6.559) | 9 (0.111) | 0.367 | heparan sulfate proteoglycan biosynthesis | sgl |
| 469 | GO:0007276 | P | 4, | 11 | 7.572 (x 1.453) | 447 (0.025) | 0.368 | gametogenesis | Caf1 Eip75B Fas3 Hrb27C dap esn mdy pk stai th wbl |
| 470 | GO:0045185 | P | 5, | 1 | 0.152 (x 6.559) | 9 (0.111) | 0.368 | maintenance of protein localization | pk |
| 471 | GO:0004559 | F | 7, | 1 | 0.152 (x 6.559) | 9 (0.111) | 0.369 | alpha-mannosidase activity | alpha-Man-IIb |
| 472 | GO:0005977 | P | 8, 9, | 1 | 0.152 (x 6.559) | 9 (0.111) | 0.369 | glycogen metabolism | GlyP |
| 473 | GO:0008439 | F | 4, 7, | 1 | 0.152 (x 6.559) | 9 (0.111) | 0.37 | monophenol monooxygenase activator activity | BEST:GH02921 |
| 474 | GO:0008235 | F | 6, | 3 | 1.287 (x 2.330) | 76 (0.039) | 0.37 | metalloexopeptidase activity | BcDNA:LD41548 Lsp2 fra |
| 475 | GO:0035161 | P | 5, 6, | 1 | 0.152 (x 6.559) | 9 (0.111) | 0.371 | imaginal disc lineage restriction | en |
| 476 | GO:0018342 | P | 9, 10, | 1 | 0.152 (x 6.559) | 9 (0.111) | 0.372 | protein prenylation | CG33171 |
| 477 | GO:0005048 | F | 4, | 1 | 0.152 (x 6.559) | 9 (0.111) | 0.373 | signal sequence binding | l(1)G0320 |
| 478 | GO:0030201 | P | 6, 8, | 1 | 0.152 (x 6.559) | 9 (0.111) | 0.373 | heparan sulfate proteoglycan metabolism | sgl |
| 479 | GO:0006966 | P | 7, 8, 9, | 1 | 0.152 (x 6.559) | 9 (0.111) | 0.374 | antifungal humoral response (sensu Protostomia) | Tl |
| 480 | GO:0016614 | F | 4, | 4 | 1.999 (x 2.001) | 118 (0.034) | 0.374 | oxidoreductase activity, acting on CH-OH group of donors | CG3842 CG6287 Idh sgl |
| 481 | GO:0007010 | P | 6, | 11 | 7.623 (x 1.443) | 450 (0.024) | 0.374 | cytoskeleton organization and biogenesis | Abi Act42A CG33171 CG9057 Sb esn for fra pbl stai vg |
| 482 | GO:0005279 | F | 5, 6, | 2 | 0.661 (x 3.027) | 39 (0.051) | 0.375 | amino acid-polyamine transporter activity | CG1607 mnd |
| 483 | GO:0007632 | P | 4, 6, | 1 | 0.152 (x 6.559) | 9 (0.111) | 0.375 | visual behavior | ogre |
| 484 | GO:0015980 | P | 6, | 4 | 1.999 (x 2.001) | 118 (0.034) | 0.375 | energy derivation by oxidation of organic compounds | GlyP Idh Pepck Tpi |
| 485 | GO:0000785 | C | 5, 6, 7, 8, 9, 10, | 3 | 1.304 (x 2.300) | 77 (0.039) | 0.375 | chromatin | Dsp1 His4r Trl |
| 486 | GO:0000003 | P | 2, | 12 | 8.487 (x 1.414) | 501 (0.024) | 0.375 | reproduction | Caf1 Eip75B Fas3 Hrb27C Poxn dap esn mdy pk stai th wbl |
| 487 | GO:0008355 | P | 6, 7, | 2 | 0.661 (x 3.027) | 39 (0.051) | 0.375 | olfactory learning | CG8588 Fas3 |
| 488 | GO:0042364 | P | 7, | 1 | 0.152 (x 6.559) | 9 (0.111) | 0.376 | water-soluble vitamin biosynthesis | ESTS:39C10S |
| 489 | GO:0015203 | F | 4, | 2 | 0.661 (x 3.027) | 39 (0.051) | 0.376 | polyamine transporter activity | CG1607 mnd |
| 490 | GO:0045934 | P | 7, | 4 | 2.050 (x 1.952) | 121 (0.033) | 0.38 | negative regulation of nucleobase, nucleoside, nucleotide and nucleic acid metabolism | CREG Caf1 edl en |
| 491 | GO:0019953 | P | 3, | 11 | 7.724 (x 1.424) | 456 (0.024) | 0.382 | sexual reproduction | Caf1 Eip75B Fas3 Hrb27C dap esn mdy pk stai th wbl |
| 492 | GO:0006023 | P | 7, 8, | 1 | 0.169 (x 5.903) | 10 (0.100) | 0.387 | aminoglycan biosynthesis | sgl |
| 493 | GO:0051017 | P | 10, | 1 | 0.169 (x 5.903) | 10 (0.100) | 0.388 | actin filament bundle formation | Sb |
| 494 | GO:0031577 | P | 8, | 1 | 0.169 (x 5.903) | 10 (0.100) | 0.389 | spindle checkpoint | zwilch |
| 495 | GO:0005041 | F | 6, | 1 | 0.169 (x 5.903) | 10 (0.100) | 0.389 | low-density lipoprotein receptor activity | arr |
| 496 | GO:0044238 | P | 4, | 84 | 77.414 (x 1.085) | 4570 (0.018) | 0.39 | primary metabolism | BEST:GH02921 BEST:LD04971 BcDNA:GH02976 BcDNA:LD41548 CG10657 CG1607 CG17052 CG17323 CG2852 CG33171 CG40410 CG4586 CG4914 CG5390 CG5731 CG5794 CG6287 CG6767 CG9057 CREG Caf1 Cdk4 Cyp310a1 D19A Doc1 Doc2 Doc3 Dsp1 ESTS:39C10S Eip55E Eip71CD Eip75B Fkbp13 GNBP3 Gfat1 GlyP His4r HmgZ Hrb27C Hsp23 Hsp26 Hsp27 Hsp67Ba Idh Lsp2 Nep2 Optix Oscillin PFE Pepck Poxn RpS12 Rpn9 Sb Spn1 Tpi Trl alpha-Man-IIb arr ash2 br dap dve edl en fbp for fra grn inv klu l(1)G0320 l(2)01810 mdy mnd ome pbl sc sgl smi35A th toe tok wbl |
| 497 | GO:0006338 | P | 10, | 2 | 0.711 (x 2.811) | 42 (0.048) | 0.39 | chromatin remodeling | Caf1 ash2 |
| 498 | GO:0004178 | F | 7, | 1 | 0.169 (x 5.903) | 10 (0.100) | 0.39 | leucyl aminopeptidase activity | BcDNA:LD41548 |
| 499 | GO:0007094 | P | 9, 10, | 1 | 0.169 (x 5.903) | 10 (0.100) | 0.391 | mitotic spindle checkpoint | zwilch |
| 500 | GO:0008016 | P | 5, | 1 | 0.169 (x 5.903) | 10 (0.100) | 0.392 | regulation of heart contraction | for |
| 501 | GO:0006520 | P | 6, 7, | 7 | 4.421 (x 1.583) | 261 (0.027) | 0.392 | amino acid metabolism | CG1607 CG6287 ESTS:39C10S Eip55E Eip71CD mdy mnd |
| 502 | GO:0006024 | P | 8, 9, | 1 | 0.169 (x 5.903) | 10 (0.100) | 0.392 | glycosaminoglycan biosynthesis | sgl |
| 503 | GO:0007223 | P | 7, | 1 | 0.169 (x 5.903) | 10 (0.100) | 0.393 | frizzled-2 signaling pathway | Wnt2 |
| 504 | GO:0006022 | P | 6, 7, | 1 | 0.169 (x 5.903) | 10 (0.100) | 0.394 | aminoglycan metabolism | sgl |
| 505 | GO:0007292 | P | 5, | 8 | 5.251 (x 1.523) | 310 (0.026) | 0.394 | female gamete generation | Caf1 Eip75B Fas3 Hrb27C dap mdy th wbl |
| 506 | GO:0030169 | F | 5, | 1 | 0.169 (x 5.903) | 10 (0.100) | 0.395 | low-density lipoprotein binding | arr |
| 507 | GO:0030203 | P | 7, 8, | 1 | 0.169 (x 5.903) | 10 (0.100) | 0.396 | glycosaminoglycan metabolism | sgl |
| 508 | GO:0016775 | F | 5, | 1 | 0.169 (x 5.903) | 10 (0.100) | 0.396 | phosphotransferase activity, nitrogenous group as acceptor | Argk |
| 509 | GO:0040034 | P | 4, | 1 | 0.186 (x 5.367) | 11 (0.091) | 0.396 | regulation of development, heterochronic | br |
| 510 | GO:0005509 | F | 5, | 6 | 3.693 (x 1.625) | 218 (0.028) | 0.397 | calcium ion binding | CBP CG33113 Fkbp13 l(1)G0320 ome tok |
| 511 | GO:0007447 | P | 4, 5, | 2 | 0.728 (x 2.746) | 43 (0.047) | 0.397 | imaginal disc pattern formation | en inv |
| 512 | GO:0008034 | F | 4, | 1 | 0.186 (x 5.367) | 11 (0.091) | 0.397 | lipoprotein binding | arr |
| 513 | GO:0008015 | P | 4, | 1 | 0.169 (x 5.903) | 10 (0.100) | 0.397 | circulation | for |
| 514 | GO:0006817 | P | 8, 9, | 2 | 0.728 (x 2.746) | 43 (0.047) | 0.398 | phosphate transport | CG33171 l(2)01810 |
| 515 | GO:0007413 | P | 9, 10, 12, | 1 | 0.186 (x 5.367) | 11 (0.091) | 0.398 | axonal fasciculation | Fas3 |
| 516 | GO:0019226 | P | 5, | 9 | 6.149 (x 1.464) | 363 (0.025) | 0.398 | transmission of nerve impulse | CG4054 PFE Snap br fax for kal-1 mth trn |
| 517 | GO:0001736 | P | 5, 6, | 2 | 0.728 (x 2.746) | 43 (0.047) | 0.399 | establishment of planar polarity | in pk |
| 518 | GO:0030228 | F | 5, | 1 | 0.186 (x 5.367) | 11 (0.091) | 0.399 | lipoprotein receptor activity | arr |
| 519 | GO:0003682 | F | 3, | 3 | 1.406 (x 2.134) | 83 (0.036) | 0.399 | chromatin binding | Caf1 Dsp1 HmgZ |
| 520 | GO:0006814 | P | 8, 9, | 2 | 0.728 (x 2.746) | 43 (0.047) | 0.399 | sodium ion transport | Nrv1 rpk |
| 521 | GO:0016667 | F | 4, | 1 | 0.186 (x 5.367) | 11 (0.091) | 0.399 | oxidoreductase activity, acting on sulfur group of donors | Eip71CD |
| 522 | GO:0008152 | P | 3, | 92 | 85.664 (x 1.074) | 5057 (0.018) | 0.4 | metabolism | Abi Argk BEST:GH02921 BEST:LD04971 BcDNA:GH02976 BcDNA:LD41548 CG10657 CG1607 CG17052 CG17323 CG2852 CG33171 CG3842 CG40410 CG4586 CG4914 CG5390 CG5731 CG5794 CG5873 CG6287 CG6767 CG9027 CG9057 CREG Caf1 Cdk4 Cyp310a1 D19A Doc1 Doc2 Doc3 Dsp1 ESTS:39C10S Eip55E Eip71CD Eip75B Fkbp13 GNBP3 Gfat1 GlyP His4r HmgZ Hrb27C Hsp23 Hsp26 Hsp27 Hsp67Ba Idh Lsp2 Nep2 Optix Oscillin PFE Pepck Poxn RpS12 Rpn9 Sb Spn1 Tl Tpi Trl alpha-Man-IIb arr ash2 br dap dve edl en fbp for fra grn inv klu l(1)G0320 l(2)01810 mdy mnd mth ome pbl sc serpin-27A sgl smi35A th toe tok wbl |
| 523 | GO:0007164 | P | 4, | 2 | 0.728 (x 2.746) | 43 (0.047) | 0.4 | establishment of tissue polarity | in pk |
| 524 | GO:0042060 | P | 5, | 1 | 0.186 (x 5.367) | 11 (0.091) | 0.4 | wound healing | ninA |
| 525 | GO:0004180 | F | 6, | 2 | 0.728 (x 2.746) | 43 (0.047) | 0.401 | carboxypeptidase activity | Lsp2 fra |
| 526 | GO:0008345 | P | 5, | 1 | 0.186 (x 5.367) | 11 (0.091) | 0.401 | larval locomotory behavior | for |
| 527 | GO:0004871 | F | 2, | 22 | 17.837 (x 1.233) | 1053 (0.021) | 0.401 | signal transducer activity | Abi CG10359 CG14076 CG33113 CG40410 CG7668 Cdk4 Eip75B GNBP3 PFE Sema-1b Tl Tsp66E Wnt2 arr for fra mav mth pbl smi35A wgn |
| 528 | GO:0007612 | P | 5, | 2 | 0.728 (x 2.746) | 43 (0.047) | 0.402 | learning | CG8588 Fas3 |
| 529 | GO:0016634 | F | 5, | 1 | 0.186 (x 5.367) | 11 (0.091) | 0.402 | oxidoreductase activity, acting on the CH-CH group of donors, oxygen as acceptor | CG4586 |
| 530 | GO:0004866 | F | 5, | 3 | 1.440 (x 2.084) | 85 (0.035) | 0.402 | endopeptidase inhibitor activity | Spn1 Spn43Aa serpin-27A |
| 531 | GO:0007157 | P | 5, | 1 | 0.186 (x 5.367) | 11 (0.091) | 0.402 | heterophilic cell adhesion | glec |
| 532 | GO:0007530 | P | 3, | 2 | 0.728 (x 2.746) | 43 (0.047) | 0.402 | sex determination | br sc |
| 533 | GO:0044421 | C | 2, 3, | 3 | 1.440 (x 2.084) | 85 (0.035) | 0.403 | extracellular region part | CG33171 Lsp2 kal-1 |
| 534 | GO:0016511 | F | 7, | 1 | 0.186 (x 5.367) | 11 (0.091) | 0.403 | endothelin-converting enzyme activity | Nep2 |
| 535 | GO:0035172 | P | 5, 6, | 1 | 0.203 (x 4.919) | 12 (0.083) | 0.404 | hemocyte proliferation (sensu Arthropoda) | Tl |
| 536 | GO:0051225 | P | 7, 10, | 1 | 0.186 (x 5.367) | 11 (0.091) | 0.404 | spindle assembly | pbl |
| 537 | GO:0000915 | P | 7, 11, | 1 | 0.203 (x 4.919) | 12 (0.083) | 0.404 | cytokinesis, contractile ring formation | pbl |
| 538 | GO:0005578 | C | 3, 4, | 2 | 0.762 (x 2.624) | 45 (0.044) | 0.404 | extracellular matrix (sensu Metazoa) | CG33171 kal-1 |
| 539 | GO:0050790 | P | 3, | 2 | 0.745 (x 2.683) | 44 (0.045) | 0.405 | regulation of catalytic activity | Abi dap |
| 540 | GO:0046668 | P | 6, 7, 8, | 1 | 0.186 (x 5.367) | 11 (0.091) | 0.405 | regulation of retinal programmed cell death | klu |
| 541 | GO:0016616 | F | 5, | 3 | 1.423 (x 2.108) | 84 (0.036) | 0.405 | oxidoreductase activity, acting on the CH-OH group of donors, NAD or NADP as acceptor | CG6287 Idh sgl |
| 542 | GO:0046667 | P | 7, 8, 9, | 1 | 0.203 (x 4.919) | 12 (0.083) | 0.405 | retinal cell programmed cell death (sensu Endopterygota) | klu |
| 543 | GO:0007456 | P | 6, | 5 | 2.931 (x 1.706) | 173 (0.029) | 0.405 | eye development (sensu Endopterygota) | Optix arr br klu pk |
| 544 | GO:0031012 | C | 2, | 2 | 0.762 (x 2.624) | 45 (0.044) | 0.405 | extracellular matrix | CG33171 kal-1 |
| 545 | GO:0051179 | P | 3, | 35 | 30.000 (x 1.167) | 1771 (0.020) | 0.405 | localization | Abi BEST:LD04971 CG10657 CG14439 CG1607 CG2852 CG31121 CG33113 CG33171 CG3823 CG9057 Fas3 Hrb27C Lsp2 Nrv1 Obp99a Sema-1b Snap alpha-Man-IIb arr fra l(1)G0320 l(2)01810 mnd mth ninA pbl pk rpk sgl stai th trn vg wbl |
| 546 | GO:0005911 | C | 6, 7, 8, | 2 | 0.745 (x 2.683) | 44 (0.045) | 0.405 | intercellular junction | Fas3 ogre |
| 547 | GO:0016866 | F | 4, | 1 | 0.186 (x 5.367) | 11 (0.091) | 0.405 | intramolecular transferase activity | Tpi |
| 548 | GO:0051234 | P | 4, | 34 | 28.933 (x 1.175) | 1708 (0.020) | 0.406 | establishment of localization | Abi BEST:LD04971 CG10657 CG14439 CG1607 CG2852 CG31121 CG33113 CG33171 CG3823 CG9057 Fas3 Lsp2 Nrv1 Obp99a Sema-1b Snap alpha-Man-IIb arr fra l(1)G0320 l(2)01810 mnd mth ninA pbl pk rpk sgl stai th trn vg wbl |
| 549 | GO:0000278 | P | 5, | 7 | 4.540 (x 1.542) | 268 (0.026) | 0.406 | mitotic cell cycle | CG40410 Cdk4 Rpn9 Trl dap sc zwilch |
| 550 | GO:0000912 | P | 6, 10, | 1 | 0.203 (x 4.919) | 12 (0.083) | 0.406 | cytokinesis, formation of actomyosin apparatus | pbl |
| 551 | GO:0009991 | P | 4, | 1 | 0.203 (x 4.919) | 12 (0.083) | 0.406 | response to extracellular stimulus | mth |
| 552 | GO:0045893 | P | 9, | 2 | 0.796 (x 2.512) | 47 (0.043) | 0.407 | positive regulation of transcription, DNA-dependent | Trl ash2 |
| 553 | GO:0031523 | C | 3, 5, 6, 7, 8, 9, 10, | 1 | 0.203 (x 4.919) | 12 (0.083) | 0.407 | Myb complex | Caf1 |
| 554 | GO:0005529 | F | 4, | 2 | 0.796 (x 2.512) | 47 (0.043) | 0.408 | sugar binding | CG9134 Gfat1 |
| 555 | GO:0030414 | F | 4, | 3 | 1.457 (x 2.059) | 86 (0.035) | 0.408 | protease inhibitor activity | Spn1 Spn43Aa serpin-27A |
| 556 | GO:0006740 | P | 10, 11, | 1 | 0.203 (x 4.919) | 12 (0.083) | 0.408 | NADPH regeneration | Tpi |
| 557 | GO:0009993 | P | 7, | 7 | 4.692 (x 1.492) | 277 (0.025) | 0.408 | oogenesis (sensu Insecta) | Caf1 Eip75B Fas3 Hrb27C dap mdy th |
| 558 | GO:0015171 | F | 4, 5, | 2 | 0.796 (x 2.512) | 47 (0.043) | 0.408 | amino acid transporter activity | CG1607 mnd |
| 559 | GO:0048749 | P | 7, | 4 | 2.253 (x 1.775) | 133 (0.030) | 0.408 | compound eye development (sensu Endopterygota) | arr br klu pk |
| 560 | GO:0031519 | C | 3, 5, 6, 7, 8, 9, 10, | 1 | 0.203 (x 4.919) | 12 (0.083) | 0.409 | PcG protein complex | Caf1 |
| 561 | GO:0009719 | P | 3, | 4 | 2.253 (x 1.775) | 133 (0.030) | 0.409 | response to endogenous stimulus | CG40410 Caf1 Eip75B br |
| 562 | GO:0016082 | P | 8, 9, 10, | 1 | 0.203 (x 4.919) | 12 (0.083) | 0.409 | synaptic vesicle priming | Snap |
| 563 | GO:0001745 | P | 7, 8, | 4 | 2.253 (x 1.775) | 133 (0.030) | 0.41 | compound eye morphogenesis (sensu Endopterygota) | arr br klu pk |
| 564 | GO:0016198 | P | 5, 7, 8, 10, 11, 13, | 1 | 0.203 (x 4.919) | 12 (0.083) | 0.41 | axon choice point recognition | fra |
| 565 | GO:0006739 | P | 9, 10, | 1 | 0.203 (x 4.919) | 12 (0.083) | 0.411 | NADP metabolism | Tpi |
| 566 | GO:0031667 | P | 5, | 1 | 0.203 (x 4.919) | 12 (0.083) | 0.411 | response to nutrient levels | mth |
| 567 | GO:0006098 | P | 8, 10, 11, 12, | 1 | 0.203 (x 4.919) | 12 (0.083) | 0.412 | pentose-phosphate shunt | Tpi |
| 568 | GO:0000212 | P | 6, 10, | 1 | 0.203 (x 4.919) | 12 (0.083) | 0.413 | meiotic spindle organization and biogenesis | pbl |
| 569 | GO:0004702 | F | 4, 8, | 5 | 2.998 (x 1.668) | 177 (0.028) | 0.413 | receptor signaling protein serine/threonine kinase activity | CG40410 Cdk4 PFE for smi35A |
| 570 | GO:0008318 | F | 6, | 1 | 0.203 (x 4.919) | 12 (0.083) | 0.414 | protein prenyltransferase activity | CG33171 |
| 571 | GO:0015837 | P | 5, 6, | 2 | 0.779 (x 2.567) | 46 (0.043) | 0.414 | amine transport | CG1607 mnd |
| 572 | GO:0001654 | P | 5, | 5 | 3.066 (x 1.631) | 181 (0.028) | 0.414 | eye development | Optix arr br klu pk |
| 573 | GO:0019236 | P | 5, | 1 | 0.203 (x 4.919) | 12 (0.083) | 0.414 | response to pheromone | Obp99a |
| 574 | GO:0016079 | P | 7, 8, 9, | 2 | 0.779 (x 2.567) | 46 (0.043) | 0.414 | synaptic vesicle exocytosis | Snap mth |
| 575 | GO:0030867 | C | 5, 6, 7, 8, 9, 10, 11, | 1 | 0.203 (x 4.919) | 12 (0.083) | 0.415 | rough endoplasmic reticulum membrane | l(1)G0320 |
| 576 | GO:0042440 | P | 5, | 2 | 0.779 (x 2.567) | 46 (0.043) | 0.415 | pigment metabolism | Tl serpin-27A |
| 577 | GO:0008045 | P | 7, 8, 10, 11, 13, | 1 | 0.203 (x 4.919) | 12 (0.083) | 0.416 | motor axon guidance | fra |
| 578 | GO:0006915 | P | 6, | 5 | 3.015 (x 1.658) | 178 (0.028) | 0.416 | apoptosis | mdy smi35A th trn wgn |
| 579 | GO:0006865 | P | 6, 7, 8, | 2 | 0.779 (x 2.567) | 46 (0.043) | 0.416 | amino acid transport | CG1607 mnd |
| 580 | GO:0008543 | P | 8, | 1 | 0.203 (x 4.919) | 12 (0.083) | 0.417 | fibroblast growth factor receptor signaling pathway | sgl |
| 581 | GO:0031032 | P | 9, | 1 | 0.203 (x 4.919) | 12 (0.083) | 0.417 | actomyosin structure organization and biogenesis | pbl |
| 582 | GO:0000082 | P | 7, | 1 | 0.203 (x 4.919) | 12 (0.083) | 0.418 | G1/S transition of mitotic cell cycle | dap |
| 583 | GO:0016571 | P | 9, 10, 12, | 1 | 0.220 (x 4.541) | 13 (0.077) | 0.419 | histone methylation | Caf1 |
| 584 | GO:0040007 | P | 2, | 3 | 1.525 (x 1.968) | 90 (0.033) | 0.419 | growth | Cdk4 mav ninA |
| 585 | GO:0006891 | P | 6, 7, 8, 9, | 1 | 0.220 (x 4.541) | 13 (0.077) | 0.42 | intra-Golgi vesicle-mediated transport | wbl |
| 586 | GO:0016278 | F | 7, | 1 | 0.220 (x 4.541) | 13 (0.077) | 0.42 | lysine N-methyltransferase activity | Caf1 |
| 587 | GO:0000792 | C | 6, 7, 8, 9, 10, 11, | 1 | 0.220 (x 4.541) | 13 (0.077) | 0.421 | heterochromatin | Trl |
| 588 | GO:0016279 | F | 7, 8, | 1 | 0.220 (x 4.541) | 13 (0.077) | 0.422 | protein-lysine N-methyltransferase activity | Caf1 |
| 589 | GO:0007428 | P | 5, 6, | 1 | 0.220 (x 4.541) | 13 (0.077) | 0.423 | primary tracheal branching (sensu Insecta) | sgl |
| 590 | GO:0007539 | P | 5, | 1 | 0.220 (x 4.541) | 13 (0.077) | 0.423 | primary sex determination, soma | sc |
| 591 | GO:0044237 | P | 4, | 84 | 78.414 (x 1.071) | 4629 (0.018) | 0.424 | cellular metabolism | Argk BEST:GH02921 BEST:LD04971 BcDNA:GH02976 BcDNA:LD41548 CG10657 CG1607 CG17052 CG17323 CG2852 CG33171 CG40410 CG4586 CG4914 CG5390 CG5794 CG5873 CG6287 CG6767 CG9027 CREG Caf1 Cdk4 Cyp310a1 D19A Doc1 Doc2 Doc3 Dsp1 ESTS:39C10S Eip55E Eip71CD Eip75B Fkbp13 Gfat1 GlyP His4r HmgZ Hrb27C Hsp23 Hsp26 Hsp27 Hsp67Ba Idh Lsp2 Nep2 Optix Oscillin PFE Pepck Poxn RpS12 Rpn9 Sb Spn1 Tl Tpi Trl arr ash2 br dap dve edl en for fra grn inv klu l(1)G0320 l(2)01810 mdy mnd mth ome sc serpin-27A sgl smi35A th toe tok wbl |
| 592 | GO:0008533 | F | 7, | 1 | 0.220 (x 4.541) | 13 (0.077) | 0.424 | astacin activity | tok |
| 593 | GO:0043231 | C | 4, 5, 6, 7, | 44 | 39.080 (x 1.126) | 2307 (0.019) | 0.424 | intracellular membrane-bound organelle | BEST:GH02921 CBP CG17838 CG33113 CG40410 CG4586 CG4914 CG6930 CG9894 CREG Caf1 D19A Doc1 Doc2 Doc3 Dsp1 Eip75B HmgZ Hrb27C Idh Optix Pepck Poxn Snap Trl alpha-Man-IIb ash2 bip1 br dap dve edl en esn grn inv klu l(1)G0320 pbl pk sc toe vg wbl |
| 594 | GO:0035290 | P | 5, 6, | 1 | 0.220 (x 4.541) | 13 (0.077) | 0.425 | trunk segmentation | en |
| 595 | GO:0006473 | P | 9, | 1 | 0.220 (x 4.541) | 13 (0.077) | 0.425 | protein amino acid acetylation | Caf1 |
| 596 | GO:0018024 | F | 8, 9, | 1 | 0.220 (x 4.541) | 13 (0.077) | 0.426 | histone-lysine N-methyltransferase activity | Caf1 |
| 597 | GO:0043227 | C | 3, | 44 | 39.114 (x 1.125) | 2309 (0.019) | 0.427 | membrane-bound organelle | BEST:GH02921 CBP CG17838 CG33113 CG40410 CG4586 CG4914 CG6930 CG9894 CREG Caf1 D19A Doc1 Doc2 Doc3 Dsp1 Eip75B HmgZ Hrb27C Idh Optix Pepck Poxn Snap Trl alpha-Man-IIb ash2 bip1 br dap dve edl en esn grn inv klu l(1)G0320 pbl pk sc toe vg wbl |
| 598 | GO:0043170 | P | 4, | 58 | 52.784 (x 1.099) | 3116 (0.019) | 0.433 | macromolecule metabolism | BEST:GH02921 BcDNA:GH02976 BcDNA:LD41548 CG10657 CG17052 CG17323 CG2852 CG33171 CG40410 CG4914 CG5390 CG5731 CG5794 CG6287 Caf1 Cdk4 D19A Dsp1 Eip71CD Fkbp13 GNBP3 Gfat1 GlyP His4r HmgZ Hrb27C Hsp23 Hsp26 Hsp27 Hsp67Ba Idh Lsp2 Nep2 Oscillin PFE Pepck RpS12 Rpn9 Sb Spn1 Tpi Trl alpha-Man-IIb ash2 dap fbp for fra l(1)G0320 l(2)01810 mdy ome pbl sgl smi35A th tok wbl |
| 599 | GO:0005576 | C | 2, | 9 | 6.539 (x 1.376) | 386 (0.023) | 0.433 | extracellular region | BcDNA:GH02976 CG17052 CG33171 ImpE2 Lsp2 Obp99a Wnt2 ana kal-1 |
| 600 | GO:0005791 | C | 6, 7, 8, 9, | 1 | 0.237 (x 4.217) | 14 (0.071) | 0.438 | rough endoplasmic reticulum | l(1)G0320 |
| 601 | GO:0006769 | P | 8, 9, | 1 | 0.237 (x 4.217) | 14 (0.071) | 0.439 | nicotinamide metabolism | Tpi |
| 602 | GO:0007178 | P | 7, | 2 | 0.864 (x 2.315) | 51 (0.039) | 0.439 | transmembrane receptor protein serine/threonine kinase signaling pathway | PFE mav |
| 603 | GO:0019732 | P | 6, 7, 8, | 1 | 0.237 (x 4.217) | 14 (0.071) | 0.439 | antifungal humoral response | Tl |
| 604 | GO:0030166 | P | 7, 8, | 1 | 0.237 (x 4.217) | 14 (0.071) | 0.44 | proteoglycan biosynthesis | sgl |
| 605 | GO:0008239 | F | 7, | 1 | 0.237 (x 4.217) | 14 (0.071) | 0.441 | dipeptidyl-peptidase activity | ome |
| 606 | GO:0006112 | P | 7, | 1 | 0.237 (x 4.217) | 14 (0.071) | 0.442 | energy reserve metabolism | GlyP |
| 607 | GO:0006508 | P | 7, | 16 | 12.840 (x 1.246) | 758 (0.021) | 0.442 | proteolysis | BEST:GH02921 BcDNA:LD41548 CG40410 CG4914 CG5390 CG5794 D19A Lsp2 Nep2 Rpn9 Sb Spn1 fra ome th tok |
| 608 | GO:0046660 | P | 4, | 1 | 0.237 (x 4.217) | 14 (0.071) | 0.442 | female sex differentiation | en |
| 609 | GO:0048748 | P | 6, 7, | 4 | 2.388 (x 1.675) | 141 (0.028) | 0.444 | eye morphogenesis (sensu Endopterygota) | arr br klu pk |
| 610 | GO:0030036 | P | 8, | 3 | 1.643 (x 1.826) | 97 (0.031) | 0.449 | actin cytoskeleton organization and biogenesis | Abi Sb pbl |
| 611 | GO:0005275 | F | 3, | 2 | 0.881 (x 2.271) | 52 (0.038) | 0.45 | amine transporter activity | CG1607 mnd |
| 612 | GO:0030029 | P | 7, | 3 | 1.643 (x 1.826) | 97 (0.031) | 0.45 | actin filament-based process | Abi Sb pbl |
| 613 | GO:0009314 | P | 4, | 2 | 0.898 (x 2.228) | 53 (0.038) | 0.45 | response to radiation | CG40410 ogre |
| 614 | GO:0001738 | P | 5, | 2 | 0.881 (x 2.271) | 52 (0.038) | 0.45 | morphogenesis of a polarized epithelium | in pk |
| 615 | GO:0016542 | P | 7, 8, | 1 | 0.254 (x 3.936) | 15 (0.067) | 0.451 | male courtship behavior (sensu Insecta) | Poxn |
| 616 | GO:0007307 | P | 9, 10, 11, | 1 | 0.254 (x 3.936) | 15 (0.067) | 0.451 | chorion gene amplification | Caf1 |
| 617 | GO:0035215 | P | 5, | 1 | 0.254 (x 3.936) | 15 (0.067) | 0.452 | genital disc development | en |
| 618 | GO:0006519 | P | 5, | 7 | 4.963 (x 1.410) | 293 (0.024) | 0.452 | amino acid and derivative metabolism | CG1607 CG6287 ESTS:39C10S Eip55E Eip71CD mdy mnd |
| 619 | GO:0005057 | F | 3, | 6 | 4.066 (x 1.476) | 240 (0.025) | 0.453 | receptor signaling protein activity | CG33113 CG40410 Cdk4 PFE for smi35A |
| 620 | GO:0004693 | F | 8, | 1 | 0.254 (x 3.936) | 15 (0.067) | 0.453 | cyclin-dependent protein kinase activity | Cdk4 |
| 621 | GO:0007538 | P | 4, | 1 | 0.254 (x 3.936) | 15 (0.067) | 0.454 | primary sex determination | sc |
| 622 | GO:0000786 | C | 3, 5, 6, 7, 8, 9, 10, 11, | 1 | 0.254 (x 3.936) | 15 (0.067) | 0.454 | nucleosome | His4r |
| 623 | GO:0006029 | P | 7, | 1 | 0.254 (x 3.936) | 15 (0.067) | 0.455 | proteoglycan metabolism | sgl |
| 624 | GO:0015923 | F | 6, | 1 | 0.254 (x 3.936) | 15 (0.067) | 0.456 | mannosidase activity | alpha-Man-IIb |
| 625 | GO:0007562 | P | 3, | 1 | 0.254 (x 3.936) | 15 (0.067) | 0.456 | eclosion | br |
| 626 | GO:0004553 | F | 5, | 3 | 1.660 (x 1.807) | 98 (0.031) | 0.456 | hydrolase activity, hydrolyzing O-glycosyl compounds | CG5731 GNBP3 alpha-Man-IIb |
| 627 | GO:0035289 | P | 6, 7, | 1 | 0.254 (x 3.936) | 15 (0.067) | 0.457 | posterior head segmentation | en |
| 628 | GO:0015698 | P | 7, 8, | 2 | 0.915 (x 2.186) | 54 (0.037) | 0.457 | inorganic anion transport | CG33171 l(2)01810 |
| 629 | GO:0019362 | P | 7, 8, | 1 | 0.254 (x 3.936) | 15 (0.067) | 0.458 | pyridine nucleotide metabolism | Tpi |
| 630 | GO:0015849 | P | 5, 6, | 2 | 0.915 (x 2.186) | 54 (0.037) | 0.458 | organic acid transport | CG1607 mnd |
| 631 | GO:0008285 | P | 6, | 1 | 0.254 (x 3.936) | 15 (0.067) | 0.459 | negative regulation of cell proliferation | ana |
| 632 | GO:0046942 | P | 6, 7, | 2 | 0.915 (x 2.186) | 54 (0.037) | 0.459 | carboxylic acid transport | CG1607 mnd |
| 633 | GO:0006963 | P | 7, 8, 9, | 1 | 0.254 (x 3.936) | 15 (0.067) | 0.459 | positive regulation of antibacterial peptide biosynthesis | Tl |
| 634 | GO:0007492 | P | 4, | 1 | 0.271 (x 3.690) | 16 (0.062) | 0.46 | endoderm development | Poxn |
| 635 | GO:0016806 | F | 6, | 1 | 0.254 (x 3.936) | 15 (0.067) | 0.46 | dipeptidyl-peptidase and tripeptidyl-peptidase activity | ome |
| 636 | GO:0007582 | P | 2, | 120 | 115.732 (x 1.037) | 6832 (0.018) | 0.46 | physiological process | Abi Act42A Argk BEST:GH02921 BEST:LD04971 BcDNA:GH02976 BcDNA:LD41548 CG10657 CG14439 CG1607 CG17052 CG17323 CG2852 CG31121 CG33113 CG33171 CG3770 CG3823 CG3842 CG40410 CG4054 CG4586 CG4914 CG5390 CG5466 CG5731 CG5794 CG5873 CG6287 CG6767 CG9027 CG9057 CREG Caf1 Cdk4 Cyp310a1 D19A Doc1 Doc2 Doc3 Dsp1 ESTS:39C10S Eip55E Eip71CD Eip75B Fas3 Fkbp13 GNBP3 Gfat1 GlyP His4r HmgZ Hrb27C Hsp23 Hsp26 Hsp27 Hsp67Ba Idh Lsp2 Nep2 Nrv1 Obp99a Optix Oscillin PFE Pepck Poxn RpS12 Rpn9 Sb Sema-1b Snap Spn1 Tl Tpi Trl alpha-Man-IIb ana arr ash2 br dap dve edl en esn fax fbp for fra grn in inv kal-1 klu l(1)G0320 l(2)01810 mav mdy mnd mth ninA ogre ome pbl pk rpk sc serpin-27A sgl smi35A stai th toe tok trn vg wbl wgn zwilch |
| 637 | GO:0009613 | P | 4, 5, | 3 | 1.694 (x 1.771) | 100 (0.030) | 0.46 | response to pest, pathogen or parasite | Tl br serpin-27A |
| 638 | GO:0043543 | P | 8, | 1 | 0.271 (x 3.690) | 16 (0.062) | 0.461 | protein amino acid acylation | Caf1 |
| 639 | GO:0006888 | P | 6, 7, 8, 9, | 1 | 0.254 (x 3.936) | 15 (0.067) | 0.461 | ER to Golgi vesicle-mediated transport | Snap |
| 640 | GO:0042302 | F | 3, | 3 | 1.677 (x 1.789) | 99 (0.030) | 0.461 | structural constituent of cuticle | CG15757 CG8502 CG8634 |
| 641 | GO:0007455 | P | 6, 7, | 4 | 2.473 (x 1.617) | 146 (0.027) | 0.461 | eye-antennal disc morphogenesis | arr br klu pk |
| 642 | GO:0006277 | P | 8, | 1 | 0.271 (x 3.690) | 16 (0.062) | 0.461 | DNA amplification | Caf1 |
| 643 | GO:0046666 | P | 6, 7, | 1 | 0.271 (x 3.690) | 16 (0.062) | 0.462 | retinal cell programmed cell death | klu |
| 644 | GO:0042054 | F | 7, | 1 | 0.271 (x 3.690) | 16 (0.062) | 0.463 | histone methyltransferase activity | Caf1 |
| 645 | GO:0004659 | F | 5, | 1 | 0.271 (x 3.690) | 16 (0.062) | 0.463 | prenyltransferase activity | CG33171 |
| 646 | GO:0044427 | C | 4, 5, 6, 7, 8, 9, | 4 | 2.507 (x 1.595) | 148 (0.027) | 0.464 | chromosomal part | Caf1 Dsp1 His4r Trl |
| 647 | GO:0030537 | P | 4, | 1 | 0.271 (x 3.690) | 16 (0.062) | 0.464 | larval behavior | for |
| 648 | GO:0051347 | P | 5, | 1 | 0.271 (x 3.690) | 16 (0.062) | 0.465 | positive regulation of transferase activity | Abi |
| 649 | GO:0045860 | P | 6, 7, | 1 | 0.271 (x 3.690) | 16 (0.062) | 0.466 | positive regulation of protein kinase activity | Abi |
| 650 | GO:0007520 | P | 7, 8, 10, 11, | 1 | 0.271 (x 3.690) | 16 (0.062) | 0.466 | myoblast fusion | rost |
| 651 | GO:0005918 | C | 7, 8, 9, 10, | 1 | 0.271 (x 3.690) | 16 (0.062) | 0.467 | septate junction | Fas3 |
| 652 | GO:0042058 | P | 5, 6, 9, | 1 | 0.271 (x 3.690) | 16 (0.062) | 0.468 | regulation of epidermal growth factor receptor signaling pathway | edl |
| 653 | GO:0048592 | P | 5, 6, | 4 | 2.524 (x 1.585) | 149 (0.027) | 0.47 | eye morphogenesis | arr br klu pk |
| 654 | GO:0007350 | P | 4, 5, | 3 | 1.728 (x 1.736) | 102 (0.029) | 0.476 | blastoderm segmentation | Cdk4 en sgl |
| 655 | GO:0018993 | P | 4, | 1 | 0.288 (x 3.473) | 17 (0.059) | 0.476 | somatic sex determination | sc |
| 656 | GO:0007259 | P | 7, | 1 | 0.288 (x 3.473) | 17 (0.059) | 0.477 | JAK-STAT cascade | Cdk4 |
| 657 | GO:0050770 | P | 5, 8, 9, 10, 12, | 1 | 0.288 (x 3.473) | 17 (0.059) | 0.477 | regulation of axonogenesis | pbl |
| 658 | GO:0050830 | P | 6, 7, | 1 | 0.288 (x 3.473) | 17 (0.059) | 0.478 | defense response to Gram-positive bacterium | Tl |
| 659 | GO:0006914 | P | 4, | 1 | 0.288 (x 3.473) | 17 (0.059) | 0.479 | autophagy | br |
| 660 | GO:0006979 | P | 4, 5, 6, | 1 | 0.288 (x 3.473) | 17 (0.059) | 0.48 | response to oxidative stress | mth |
| 661 | GO:0008146 | F | 5, | 1 | 0.288 (x 3.473) | 17 (0.059) | 0.48 | sulfotransferase activity | CG16733 |
| 662 | GO:0003714 | F | 4, 6, | 1 | 0.288 (x 3.473) | 17 (0.059) | 0.481 | transcription corepressor activity | Dsp1 |
| 663 | GO:0005085 | F | 4, | 2 | 0.982 (x 2.036) | 58 (0.034) | 0.485 | guanyl-nucleotide exchange factor activity | CG30440 pbl |
| 664 | GO:0006092 | P | 7, | 3 | 1.762 (x 1.703) | 104 (0.029) | 0.485 | main pathways of carbohydrate metabolism | Idh Pepck Tpi |
| 665 | GO:0007369 | P | 5, | 2 | 0.982 (x 2.036) | 58 (0.034) | 0.485 | gastrulation | pbl sgl |
| 666 | GO:0007005 | P | 6, | 1 | 0.305 (x 3.280) | 18 (0.056) | 0.491 | mitochondrion organization and biogenesis | Cdk4 |
| 667 | GO:0050832 | P | 5, 6, | 1 | 0.305 (x 3.280) | 18 (0.056) | 0.492 | defense response to fungus | Tl |
| 668 | GO:0045610 | P | 5, 7, | 1 | 0.305 (x 3.280) | 18 (0.056) | 0.492 | regulation of hemocyte differentiation | Tl |
| 669 | GO:0009116 | P | 6, | 1 | 0.305 (x 3.280) | 18 (0.056) | 0.493 | nucleoside metabolism | CG6767 |
| 670 | GO:0005515 | F | 3, | 24 | 20.904 (x 1.148) | 1234 (0.019) | 0.493 | protein binding | Abi CG10359 CG11275 CG7668 CG9057 CREG Caf1 Cdk4 Dsp1 Hsp23 Sema-1b Tl Trl Tsp66E Wnt2 arr bip1 br edl in mav pk stai wgn |
| 671 | GO:0035287 | P | 5, 6, | 1 | 0.305 (x 3.280) | 18 (0.056) | 0.494 | head segmentation | en |
| 672 | GO:0005316 | F | 7, 8, 9, | 1 | 0.305 (x 3.280) | 18 (0.056) | 0.495 | high affinity inorganic phosphate:sodium symporter activity | l(2)01810 |
| 673 | GO:0005622 | C | 3, 4, | 60 | 55.816 (x 1.075) | 3295 (0.018) | 0.495 | intracellular | Act42A BEST:GH02921 BcDNA:LD41548 CBP CG10657 CG17838 CG33113 CG33171 CG3823 CG40410 CG4586 CG4914 CG6930 CG9057 CG9894 CREG Caf1 D19A Doc1 Doc2 Doc3 Dsp1 Eip75B Gfat1 His4r HmgZ Hrb27C Idh ImpE2 Optix Pepck Poxn RpS12 Rpn9 Snap Trl alpha-Man-IIb ash2 bip1 br dap dve edl en esn for grn inv klu l(1)G0320 mdy pbl pk sc smi35A stai th toe vg wbl |
| 674 | GO:0005884 | C | 5, 6, 7, 8, 9, 10, | 1 | 0.305 (x 3.280) | 18 (0.056) | 0.495 | actin filament | Act42A |
| 675 | GO:0006006 | P | 8, 9, | 2 | 1.016 (x 1.968) | 60 (0.033) | 0.499 | glucose metabolism | Pepck Tpi |
| 676 | GO:0007611 | P | 4, | 2 | 1.016 (x 1.968) | 60 (0.033) | 0.5 | learning and/or memory | CG8588 Fas3 |
| 677 | GO:0016798 | F | 4, | 3 | 1.813 (x 1.655) | 107 (0.028) | 0.502 | hydrolase activity, acting on glycosyl bonds | CG5731 GNBP3 alpha-Man-IIb |
| 678 | GO:0044424 | C | 3, 4, 5, | 58 | 54.037 (x 1.073) | 3190 (0.018) | 0.507 | intracellular part | Act42A BEST:GH02921 BcDNA:LD41548 CBP CG17838 CG33113 CG33171 CG40410 CG4586 CG4914 CG6930 CG9057 CG9894 CREG Caf1 D19A Doc1 Doc2 Doc3 Dsp1 Eip75B Gfat1 His4r HmgZ Hrb27C Idh ImpE2 Optix Pepck Poxn RpS12 Rpn9 Snap Trl alpha-Man-IIb ash2 bip1 br dap dve edl en esn for grn inv klu l(1)G0320 mdy pbl pk sc smi35A stai th toe vg wbl |
| 679 | GO:0007507 | P | 5, | 2 | 1.033 (x 1.936) | 61 (0.033) | 0.508 | heart development | Doc1 sgl |
| 680 | GO:0044272 | P | 6, | 1 | 0.322 (x 3.107) | 19 (0.053) | 0.508 | sulfur compound biosynthesis | sgl |
| 681 | GO:0016568 | P | 9, | 2 | 1.033 (x 1.936) | 61 (0.033) | 0.509 | chromatin modification | Caf1 ash2 |
| 682 | GO:0003707 | F | 5, | 1 | 0.322 (x 3.107) | 19 (0.053) | 0.509 | steroid hormone receptor activity | Eip75B |
| 683 | GO:0042981 | P | 6, 7, | 3 | 1.846 (x 1.625) | 109 (0.028) | 0.513 | regulation of apoptosis | mdy smi35A th |
| 684 | GO:0007431 | P | 5, | 3 | 1.846 (x 1.625) | 109 (0.028) | 0.513 | salivary gland development | Eip71CD Eip75B br |
| 685 | GO:0035272 | P | 4, | 3 | 1.846 (x 1.625) | 109 (0.028) | 0.514 | exocrine system development | Eip71CD Eip75B br |
| 686 | GO:0048565 | P | 4, | 2 | 1.050 (x 1.904) | 62 (0.032) | 0.515 | gut development | dve pbl |
| 687 | GO:0005488 | F | 2, | 74 | 70.028 (x 1.057) | 4134 (0.018) | 0.515 | binding | Abi BEST:GH02921 BcDNA:GH02976 BcDNA:LD41548 CBP CG10359 CG10657 CG11275 CG13848 CG17052 CG17838 CG17919 CG31121 CG33113 CG3823 CG40410 CG4914 CG6930 CG7668 CG9027 CG9057 CG9134 CREG Caf1 Cdk4 D19A Doc1 Doc2 Doc3 Dsp1 Eip75B Fkbp13 GNBP3 Gfat1 His4r HmgZ Hrb27C Hsp23 Nep2 Obp99a Optix Pepck Poxn Sema-1b Tl Trl Tsp66E Wnt2 arr ash2 bip1 br dve edl en esn for glec grn in inv klu l(1)G0320 mav mdy ome pk sc smi35A stai th toe tok wgn |
| 688 | GO:0001558 | P | 4, 5, 7, 8, | 1 | 0.339 (x 2.952) | 20 (0.050) | 0.517 | regulation of cell growth | Cdk4 |
| 689 | GO:0004674 | F | 7, | 5 | 3.591 (x 1.392) | 212 (0.024) | 0.517 | protein serine/threonine kinase activity | CG40410 Cdk4 PFE for smi35A |
| 690 | GO:0009156 | P | 8, 9, | 1 | 0.339 (x 2.952) | 20 (0.050) | 0.518 | ribonucleoside monophosphate biosynthesis | CG6767 |
| 691 | GO:0008049 | P | 6, 7, | 1 | 0.339 (x 2.952) | 20 (0.050) | 0.518 | male courtship behavior | Poxn |
| 692 | GO:0009161 | P | 8, | 1 | 0.339 (x 2.952) | 20 (0.050) | 0.519 | ribonucleoside monophosphate metabolism | CG6767 |
| 693 | GO:0006733 | P | 7, | 1 | 0.339 (x 2.952) | 20 (0.050) | 0.52 | oxidoreduction coenzyme metabolism | Tpi |
| 694 | GO:0008276 | F | 6, | 1 | 0.339 (x 2.952) | 20 (0.050) | 0.521 | protein methyltransferase activity | Caf1 |
| 695 | GO:0008374 | F | 7, | 1 | 0.339 (x 2.952) | 20 (0.050) | 0.521 | O-acyltransferase activity | mdy |
| 696 | GO:0004675 | F | 5, 6, 8, 9, | 1 | 0.339 (x 2.952) | 20 (0.050) | 0.522 | transmembrane receptor protein serine/threonine kinase activity | PFE |
| 697 | GO:0008144 | F | 3, | 1 | 0.339 (x 2.952) | 20 (0.050) | 0.523 | drug binding | Fkbp13 |
| 698 | GO:0016782 | F | 4, | 1 | 0.339 (x 2.952) | 20 (0.050) | 0.524 | transferase activity, transferring sulfur-containing groups | CG16733 |
| 699 | GO:0006030 | P | 7, 8, 9, 10, | 2 | 1.067 (x 1.874) | 63 (0.032) | 0.524 | chitin metabolism | BcDNA:GH02976 CG17052 |
| 700 | GO:0007317 | P | 4, 8, 12, 14, 15, 17, | 1 | 0.339 (x 2.952) | 20 (0.050) | 0.524 | regulation of pole plasm oskar mRNA localization | Hrb27C |
| 701 | GO:0045941 | P | 8, | 2 | 1.084 (x 1.845) | 64 (0.031) | 0.525 | positive regulation of transcription | Trl ash2 |
| 702 | GO:0008061 | F | 5, | 2 | 1.067 (x 1.874) | 63 (0.032) | 0.525 | chitin binding | BcDNA:GH02976 CG17052 |
| 703 | GO:0030097 | P | 5, | 2 | 1.084 (x 1.845) | 64 (0.031) | 0.526 | hemopoiesis | Tl grn |
| 704 | GO:0045935 | P | 7, | 2 | 1.101 (x 1.816) | 65 (0.031) | 0.529 | positive regulation of nucleobase, nucleoside, nucleotide and nucleic acid metabolism | Trl ash2 |
| 705 | GO:0035239 | P | 4, | 2 | 1.101 (x 1.816) | 65 (0.031) | 0.529 | tube morphogenesis | pbl sgl |
| 706 | GO:0009123 | P | 7, | 1 | 0.356 (x 2.811) | 21 (0.048) | 0.53 | nucleoside monophosphate metabolism | CG6767 |
| 707 | GO:0051329 | P | 6, | 1 | 0.356 (x 2.811) | 21 (0.048) | 0.531 | interphase of mitotic cell cycle | dap |
| 708 | GO:0009110 | P | 6, | 1 | 0.356 (x 2.811) | 21 (0.048) | 0.532 | vitamin biosynthesis | ESTS:39C10S |
| 709 | GO:0016339 | P | 5, | 1 | 0.356 (x 2.811) | 21 (0.048) | 0.532 | calcium-dependent cell-cell adhesion | Fas3 |
| 710 | GO:0005436 | F | 6, 7, 8, | 1 | 0.356 (x 2.811) | 21 (0.048) | 0.533 | sodium:phosphate symporter activity | l(2)01810 |
| 711 | GO:0009124 | P | 7, 8, | 1 | 0.356 (x 2.811) | 21 (0.048) | 0.534 | nucleoside monophosphate biosynthesis | CG6767 |
| 712 | GO:0051325 | P | 5, | 1 | 0.356 (x 2.811) | 21 (0.048) | 0.535 | interphase | dap |
| 713 | GO:0007480 | P | 7, 8, | 1 | 0.356 (x 2.811) | 21 (0.048) | 0.535 | leg morphogenesis (sensu Endopterygota) | Poxn |
| 714 | GO:0015276 | F | 5, 6, | 2 | 1.135 (x 1.762) | 67 (0.030) | 0.537 | ligand-gated ion channel activity | CG14076 rpk |
| 715 | GO:0048558 | P | 6, 7, | 1 | 0.373 (x 2.683) | 22 (0.045) | 0.537 | embryonic gut morphogenesis | pbl |
| 716 | GO:0030247 | F | 4, | 2 | 1.135 (x 1.762) | 67 (0.030) | 0.538 | polysaccharide binding | BcDNA:GH02976 CG17052 |
| 717 | GO:0048619 | P | 8, 9, | 1 | 0.373 (x 2.683) | 22 (0.045) | 0.538 | embryonic hindgut morphogenesis | pbl |
| 718 | GO:0043085 | P | 4, | 1 | 0.373 (x 2.683) | 22 (0.045) | 0.539 | positive regulation of enzyme activity | Abi |
| 719 | GO:0030054 | C | 5, 6, 7, | 2 | 1.118 (x 1.789) | 66 (0.030) | 0.539 | cell junction | Fas3 ogre |
| 720 | GO:0007443 | P | 5, 9, 10, | 1 | 0.373 (x 2.683) | 22 (0.045) | 0.54 | Malpighian tubule morphogenesis | pbl |
| 721 | GO:0008233 | F | 4, | 13 | 11.028 (x 1.179) | 651 (0.020) | 0.54 | peptidase activity | BEST:GH02921 BcDNA:LD41548 CG4914 CG5390 CG5794 D19A Lsp2 Nep2 Rpn9 Sb fra ome tok |
| 722 | GO:0006635 | P | 8, 9, | 1 | 0.373 (x 2.683) | 22 (0.045) | 0.54 | fatty acid beta-oxidation | CG4586 |
| 723 | GO:0048557 | P | 6, | 1 | 0.373 (x 2.683) | 22 (0.045) | 0.541 | embryonic digestive tract morphogenesis | pbl |
| 724 | GO:0048627 | P | 5, 6, 8, 9, | 1 | 0.373 (x 2.683) | 22 (0.045) | 0.542 | myoblast development | rost |
| 725 | GO:0005615 | C | 3, 4, | 1 | 0.373 (x 2.683) | 22 (0.045) | 0.543 | extracellular space | Lsp2 |
| 726 | GO:0048628 | P | 6, 7, 9, 10, | 1 | 0.373 (x 2.683) | 22 (0.045) | 0.543 | myoblast maturation | rost |
| 727 | GO:0048611 | P | 6, | 1 | 0.373 (x 2.683) | 22 (0.045) | 0.544 | embryonic ectodermal gut development | pbl |
| 728 | GO:0048566 | P | 5, | 1 | 0.373 (x 2.683) | 22 (0.045) | 0.545 | embryonic gut development | pbl |
| 729 | GO:0044420 | C | 2, 3, | 1 | 0.373 (x 2.683) | 22 (0.045) | 0.546 | extracellular matrix part | CG33171 |
| 730 | GO:0048613 | P | 7, 8, | 1 | 0.373 (x 2.683) | 22 (0.045) | 0.546 | embryonic ectodermal gut morphogenesis | pbl |
| 731 | GO:0019239 | F | 3, | 1 | 0.390 (x 2.567) | 23 (0.043) | 0.547 | deaminase activity | Oscillin |
| 732 | GO:0019748 | P | 4, | 2 | 1.152 (x 1.736) | 68 (0.029) | 0.547 | secondary metabolism | Tl serpin-27A |
| 733 | GO:0007427 | P | 5, 6, 7, | 1 | 0.373 (x 2.683) | 22 (0.045) | 0.547 | tracheal epithelial cell migration (sensu Insecta) | sgl |
| 734 | GO:0045087 | P | 5, 6, | 1 | 0.390 (x 2.567) | 23 (0.043) | 0.548 | innate immune response | Tl |
| 735 | GO:0005838 | C | 3, 4, 5, 6, 7, | 1 | 0.390 (x 2.567) | 23 (0.043) | 0.548 | proteasome regulatory particle (sensu Eukaryota) | Rpn9 |
| 736 | GO:0019395 | P | 7, 8, | 1 | 0.390 (x 2.567) | 23 (0.043) | 0.549 | fatty acid oxidation | CG4586 |
| 737 | GO:0008213 | P | 8, | 1 | 0.390 (x 2.567) | 23 (0.043) | 0.55 | protein amino acid alkylation | Caf1 |
| 738 | GO:0006479 | P | 8, 9, | 1 | 0.390 (x 2.567) | 23 (0.043) | 0.55 | protein amino acid methylation | Caf1 |
| 739 | GO:0007478 | P | 6, 7, | 1 | 0.390 (x 2.567) | 23 (0.043) | 0.551 | leg disc morphogenesis | Poxn |
| 740 | GO:0045445 | P | 5, 7, 8, | 1 | 0.390 (x 2.567) | 23 (0.043) | 0.552 | myoblast differentiation | rost |
| 741 | GO:0035109 | P | 6, | 1 | 0.390 (x 2.567) | 23 (0.043) | 0.553 | limb morphogenesis (sensu Endopterygota) | Poxn |
| 742 | GO:0000139 | C | 4, 5, 6, 7, 8, 9, 10, | 1 | 0.390 (x 2.567) | 23 (0.043) | 0.553 | Golgi membrane | alpha-Man-IIb |
| 743 | GO:0004245 | F | 7, | 1 | 0.390 (x 2.567) | 23 (0.043) | 0.554 | neprilysin activity | Nep2 |
| 744 | GO:0007494 | P | 5, | 1 | 0.407 (x 2.460) | 24 (0.042) | 0.557 | midgut development | dve |
| 745 | GO:0004879 | F | 4, | 1 | 0.407 (x 2.460) | 24 (0.042) | 0.558 | ligand-dependent nuclear receptor activity | Eip75B |
| 746 | GO:0016323 | C | 5, 6, 7, | 1 | 0.407 (x 2.460) | 24 (0.042) | 0.559 | basolateral plasma membrane | Fas3 |
| 747 | GO:0007167 | P | 6, | 4 | 2.914 (x 1.373) | 172 (0.023) | 0.559 | enzyme linked receptor protein signaling pathway | PFE edl mav sgl |
| 748 | GO:0016570 | P | 8, 11, | 1 | 0.407 (x 2.460) | 24 (0.042) | 0.559 | histone modification | Caf1 |
| 749 | GO:0008407 | P | 4, 5, | 1 | 0.407 (x 2.460) | 24 (0.042) | 0.56 | bristle morphogenesis | sc |
| 750 | GO:0004222 | F | 6, | 2 | 1.203 (x 1.663) | 71 (0.028) | 0.56 | metalloendopeptidase activity | Nep2 tok |
| 751 | GO:0006334 | P | 7, 11, | 1 | 0.407 (x 2.460) | 24 (0.042) | 0.561 | nucleosome assembly | Caf1 |
| 752 | GO:0004872 | F | 3, | 11 | 9.368 (x 1.174) | 553 (0.020) | 0.561 | receptor activity | CG14076 Eip75B GNBP3 PFE Sema-1b Tl arr fra mth pbl wgn |
| 753 | GO:0005102 | F | 3, 4, | 6 | 4.726 (x 1.270) | 279 (0.022) | 0.561 | receptor binding | CG10359 CG7668 Sema-1b Tsp66E Wnt2 mav |
| 754 | GO:0008587 | P | 7, 8, 9, | 1 | 0.407 (x 2.460) | 24 (0.042) | 0.562 | wing margin morphogenesis | vg |
| 755 | GO:0005913 | C | 7, 8, 9, | 1 | 0.407 (x 2.460) | 24 (0.042) | 0.562 | cell-cell adherens junction | Fas3 |
| 756 | GO:0016569 | P | 10, | 1 | 0.407 (x 2.460) | 24 (0.042) | 0.563 | covalent chromatin modification | Caf1 |
| 757 | GO:0007417 | P | 5, | 3 | 2.067 (x 1.452) | 122 (0.025) | 0.564 | central nervous system development | br en sc |
| 758 | GO:0044260 | P | 5, | 39 | 36.454 (x 1.070) | 2152 (0.018) | 0.564 | cellular macromolecule metabolism | BEST:GH02921 BcDNA:GH02976 BcDNA:LD41548 CG10657 CG17052 CG2852 CG33171 CG40410 CG4914 CG5390 CG5794 Caf1 Cdk4 D19A Eip71CD Fkbp13 GlyP Hsp23 Hsp26 Hsp27 Hsp67Ba Lsp2 Nep2 PFE RpS12 Rpn9 Sb Spn1 dap for fra l(1)G0320 mdy ome sgl smi35A th tok wbl |
| 759 | GO:0016020 | C | 3, 4, | 34 | 31.609 (x 1.076) | 1866 (0.018) | 0.567 | membrane | BEST:LD04971 CG14076 CG14439 CG1607 Cyp310a1 Fas3 ImpE2 ImpE3 Nep2 Nrv1 Sb Sema-1b Snap Tl Tsp66E alpha-Man-IIb arr for fra glec in l(1)G0320 l(2)01810 mnd mth ninA ogre ome pbl pk rost rpk trn wgn |
| 760 | GO:0004970 | F | 6, | 1 | 0.423 (x 2.361) | 25 (0.040) | 0.567 | ionotropic glutamate receptor activity | CG14076 |
| 761 | GO:0046943 | F | 4, | 2 | 1.220 (x 1.640) | 72 (0.028) | 0.568 | carboxylic acid transporter activity | CG1607 mnd |
| 762 | GO:0016321 | P | 6, 9, | 1 | 0.423 (x 2.361) | 25 (0.040) | 0.568 | female meiosis chromosome segregation | CG40410 |
| 763 | GO:0008652 | P | 7, 8, | 2 | 1.220 (x 1.640) | 72 (0.028) | 0.568 | amino acid biosynthesis | CG6287 ESTS:39C10S |
| 764 | GO:0016585 | C | 3, 6, 7, 8, 9, 10, 11, 12, 13, | 1 | 0.423 (x 2.361) | 25 (0.040) | 0.569 | chromatin remodeling complex | Caf1 |
| 765 | GO:0048562 | P | 5, | 1 | 0.423 (x 2.361) | 25 (0.040) | 0.57 | embryonic organ morphogenesis | pbl |
| 766 | GO:0051239 | P | 4, | 1 | 0.423 (x 2.361) | 25 (0.040) | 0.57 | regulation of organismal physiological process | for |
| 767 | GO:0030707 | P | 8, | 3 | 2.101 (x 1.428) | 124 (0.024) | 0.571 | ovarian follicle cell development (sensu Insecta) | Caf1 Fas3 th |
| 768 | GO:0043226 | C | 2, | 48 | 45.466 (x 1.056) | 2684 (0.018) | 0.575 | organelle | Act42A BEST:GH02921 CBP CG17838 CG33113 CG40410 CG4586 CG4914 CG6930 CG9894 CREG Caf1 D19A Doc1 Doc2 Doc3 Dsp1 Eip75B His4r HmgZ Hrb27C Idh Optix Pepck Poxn RpS12 Snap Trl alpha-Man-IIb ash2 bip1 br dap dve edl en esn grn inv klu l(1)G0320 pbl pk sc stai toe vg wbl |
| 769 | GO:0004221 | F | 6, 8, | 1 | 0.440 (x 2.271) | 26 (0.038) | 0.575 | ubiquitin thiolesterase activity | CG5794 |
| 770 | GO:0046903 | P | 5, | 5 | 3.913 (x 1.278) | 231 (0.022) | 0.575 | secretion | Snap alpha-Man-IIb mth stai wbl |
| 771 | GO:0043229 | C | 3, 4, 5, 6, | 48 | 45.466 (x 1.056) | 2684 (0.018) | 0.575 | intracellular organelle | Act42A BEST:GH02921 CBP CG17838 CG33113 CG40410 CG4586 CG4914 CG6930 CG9894 CREG Caf1 D19A Doc1 Doc2 Doc3 Dsp1 Eip75B His4r HmgZ Hrb27C Idh Optix Pepck Poxn RpS12 Snap Trl alpha-Man-IIb ash2 bip1 br dap dve edl en esn grn inv klu l(1)G0320 pbl pk sc stai toe vg wbl |
| 772 | GO:0005272 | F | 6, 7, | 1 | 0.440 (x 2.271) | 26 (0.038) | 0.576 | sodium channel activity | rpk |
| 773 | GO:0006887 | P | 6, 7, | 3 | 2.117 (x 1.417) | 125 (0.024) | 0.576 | exocytosis | Snap mth wbl |
| 774 | GO:0035282 | P | 3, | 3 | 2.134 (x 1.406) | 126 (0.024) | 0.576 | segmentation | Cdk4 en sgl |
| 775 | GO:0051093 | P | 4, | 1 | 0.440 (x 2.271) | 26 (0.038) | 0.576 | negative regulation of development | ana |
| 776 | GO:0005342 | F | 3, | 2 | 1.254 (x 1.595) | 74 (0.027) | 0.577 | organic acid transporter activity | CG1607 mnd |
| 777 | GO:0007310 | P | 6, 8, 9, 11, | 1 | 0.440 (x 2.271) | 26 (0.038) | 0.577 | oocyte dorsal/ventral axis determination | wbl |
| 778 | GO:0031325 | P | 6, | 2 | 1.254 (x 1.595) | 74 (0.027) | 0.577 | positive regulation of cellular metabolism | Trl ash2 |
| 779 | GO:0015114 | F | 6, | 1 | 0.440 (x 2.271) | 26 (0.038) | 0.578 | phosphate transporter activity | l(2)01810 |
| 780 | GO:0009893 | P | 5, | 2 | 1.254 (x 1.595) | 74 (0.027) | 0.578 | positive regulation of metabolism | Trl ash2 |
| 781 | GO:0048534 | P | 4, | 2 | 1.254 (x 1.595) | 74 (0.027) | 0.579 | hemopoietic or lymphoid organ development | Tl grn |
| 782 | GO:0007422 | P | 5, | 2 | 1.287 (x 1.554) | 76 (0.026) | 0.581 | peripheral nervous system development | pbl sc |
| 783 | GO:0006511 | P | 9, 10, 11, | 2 | 1.270 (x 1.574) | 75 (0.027) | 0.581 | ubiquitin-dependent protein catabolism | CG5794 th |
| 784 | GO:0007309 | P | 5, 7, 8, 10, | 2 | 1.287 (x 1.554) | 76 (0.026) | 0.581 | oocyte axis determination | Hrb27C wbl |
| 785 | GO:0005543 | F | 4, | 1 | 0.457 (x 2.186) | 27 (0.037) | 0.582 | phospholipid binding | CG17919 |
| 786 | GO:0042127 | P | 5, | 1 | 0.457 (x 2.186) | 27 (0.037) | 0.583 | regulation of cell proliferation | ana |
| 787 | GO:0031507 | P | 11, | 1 | 0.457 (x 2.186) | 27 (0.037) | 0.583 | heterochromatin formation | Caf1 |
| 788 | GO:0000775 | C | 5, 6, 7, 8, 9, 10, | 1 | 0.457 (x 2.186) | 27 (0.037) | 0.584 | chromosome, pericentric region | Trl |
| 789 | GO:0007419 | P | 4, 6, | 1 | 0.457 (x 2.186) | 27 (0.037) | 0.585 | ventral cord development | sc |
| 790 | GO:0042692 | P | 4, | 1 | 0.457 (x 2.186) | 27 (0.037) | 0.586 | muscle cell differentiation | rost |
| 791 | GO:0001700 | P | 5, | 3 | 2.185 (x 1.373) | 129 (0.023) | 0.586 | embryonic development (sensu Insecta) | CG40410 Doc1 Sema-1b |
| 792 | GO:0006342 | P | 5, 7, 10, 12, | 1 | 0.457 (x 2.186) | 27 (0.037) | 0.586 | chromatin silencing | Caf1 |
| 793 | GO:0007298 | P | 7, 8, 10, | 1 | 0.457 (x 2.186) | 27 (0.037) | 0.587 | border follicle cell migration (sensu Insecta) | th |
| 794 | GO:0035295 | P | 3, | 2 | 1.304 (x 1.533) | 77 (0.026) | 0.587 | tube development | pbl sgl |
| 795 | GO:0048469 | P | 5, | 1 | 0.457 (x 2.186) | 27 (0.037) | 0.588 | cell maturation | rost |
| 796 | GO:0043632 | P | 7, | 2 | 1.304 (x 1.533) | 77 (0.026) | 0.588 | modification-dependent macromolecule catabolism | CG5794 th |
| 797 | GO:0019783 | F | 6, | 1 | 0.474 (x 2.108) | 28 (0.036) | 0.588 | small conjugating protein-specific protease activity | CG5794 |
| 798 | GO:0019941 | P | 8, 9, 10, | 2 | 1.304 (x 1.533) | 77 (0.026) | 0.589 | modification-dependent protein catabolism | CG5794 th |
| 799 | GO:0002168 | P | 5, | 1 | 0.457 (x 2.186) | 27 (0.037) | 0.589 | larval development (sensu Insecta) | Sb |
| 800 | GO:0007300 | P | 8, | 1 | 0.474 (x 2.108) | 28 (0.036) | 0.589 | nurse cell to oocyte transport (sensu Insecta) | mdy |
| 801 | GO:0005234 | F | 8, 9, | 1 | 0.457 (x 2.186) | 27 (0.037) | 0.589 | glutamate-gated ion channel activity | CG14076 |
| 802 | GO:0030695 | F | 3, | 3 | 2.202 (x 1.362) | 130 (0.023) | 0.59 | GTPase regulator activity | Abi CG30440 pbl |
| 803 | GO:0035110 | P | 6, | 1 | 0.474 (x 2.108) | 28 (0.036) | 0.59 | leg morphogenesis | Poxn |
| 804 | GO:0005694 | C | 5, 6, 7, 8, | 4 | 3.117 (x 1.283) | 184 (0.022) | 0.59 | chromosome | Caf1 Dsp1 His4r Trl |
| 805 | GO:0045814 | P | 4, | 1 | 0.457 (x 2.186) | 27 (0.037) | 0.59 | negative regulation of gene expression, epigenetic | Caf1 |
| 806 | GO:0009952 | P | 4, | 3 | 2.202 (x 1.362) | 130 (0.023) | 0.59 | anterior/posterior pattern formation | Hrb27C en inv |
| 807 | GO:0004843 | F | 7, | 1 | 0.474 (x 2.108) | 28 (0.036) | 0.591 | ubiquitin-specific protease activity | CG5794 |
| 808 | GO:0004091 | F | 6, | 1 | 0.474 (x 2.108) | 28 (0.036) | 0.591 | carboxylesterase activity | CG4382 |
| 809 | GO:0048732 | P | 4, | 3 | 2.219 (x 1.352) | 131 (0.023) | 0.592 | gland development | Eip71CD Eip75B br |
| 810 | GO:0007051 | P | 9, | 1 | 0.491 (x 2.036) | 29 (0.034) | 0.594 | spindle organization and biogenesis | pbl |
| 811 | GO:0007156 | P | 5, | 1 | 0.491 (x 2.036) | 29 (0.034) | 0.594 | homophilic cell adhesion | Fas3 |
| 812 | GO:0001871 | F | 3, | 2 | 1.355 (x 1.476) | 80 (0.025) | 0.595 | pattern binding | BcDNA:GH02976 CG17052 |
| 813 | GO:0002009 | P | 4, | 3 | 2.253 (x 1.332) | 133 (0.023) | 0.595 | morphogenesis of an epithelium | Sb in pk |
| 814 | GO:0014016 | P | 4, 7, | 1 | 0.491 (x 2.036) | 29 (0.034) | 0.595 | neuroblast differentiation | sc |
| 815 | GO:0048599 | P | 5, 6, 8, | 2 | 1.355 (x 1.476) | 80 (0.025) | 0.595 | oocyte development | Hrb27C wbl |
| 816 | GO:0035108 | P | 5, | 1 | 0.491 (x 2.036) | 29 (0.034) | 0.596 | limb morphogenesis | Poxn |
| 817 | GO:0019318 | P | 7, 8, | 2 | 1.355 (x 1.476) | 80 (0.025) | 0.596 | hexose metabolism | Pepck Tpi |
| 818 | GO:0007179 | P | 8, | 1 | 0.491 (x 2.036) | 29 (0.034) | 0.597 | transforming growth factor beta receptor signaling pathway | mav |
| 819 | GO:0048489 | P | 6, 7, | 2 | 1.355 (x 1.476) | 80 (0.025) | 0.597 | synaptic vesicle transport | Snap mth |
| 820 | GO:0007400 | P | 6, 9, | 1 | 0.491 (x 2.036) | 29 (0.034) | 0.597 | neuroblast fate determination | sc |
| 821 | GO:0042157 | P | 7, | 1 | 0.491 (x 2.036) | 29 (0.034) | 0.598 | lipoprotein metabolism | CG33171 |
| 822 | GO:0007308 | P | 6, 7, 9, | 2 | 1.338 (x 1.495) | 79 (0.025) | 0.599 | oocyte construction | Hrb27C wbl |
| 823 | GO:0005478 | F | 3, | 1 | 0.491 (x 2.036) | 29 (0.034) | 0.599 | intracellular transporter activity | Snap |
| 824 | GO:0007591 | P | 6, | 1 | 0.508 (x 1.968) | 30 (0.033) | 0.599 | molting cycle (sensu Insecta) | Eip75B |
| 825 | GO:0006497 | P | 8, 9, | 1 | 0.491 (x 2.036) | 29 (0.034) | 0.6 | protein amino acid lipidation | CG33171 |
| 826 | GO:0016485 | P | 8, | 1 | 0.508 (x 1.968) | 30 (0.033) | 0.6 | protein processing | wbl |
| 827 | GO:0007416 | P | 5, 6, | 1 | 0.491 (x 2.036) | 29 (0.034) | 0.6 | synaptogenesis | Tl |
| 828 | GO:0031589 | P | 4, | 1 | 0.508 (x 1.968) | 30 (0.033) | 0.601 | cell-substrate adhesion | CG33171 |
| 829 | GO:0014017 | P | 5, 8, | 1 | 0.491 (x 2.036) | 29 (0.034) | 0.601 | neuroblast fate commitment | sc |
| 830 | GO:0007160 | P | 5, | 1 | 0.508 (x 1.968) | 30 (0.033) | 0.601 | cell-matrix adhesion | CG33171 |
| 831 | GO:0007349 | P | 3, 4, | 1 | 0.491 (x 2.036) | 29 (0.034) | 0.602 | cellularization | CG40410 |
| 832 | GO:0042386 | P | 4, 6, | 1 | 0.508 (x 1.968) | 30 (0.033) | 0.602 | hemocyte differentiation (sensu Arthropoda) | Tl |
| 833 | GO:0042158 | P | 7, 8, | 1 | 0.491 (x 2.036) | 29 (0.034) | 0.602 | lipoprotein biosynthesis | CG33171 |
| 834 | GO:0007049 | P | 4, | 9 | 7.979 (x 1.128) | 471 (0.019) | 0.603 | cell cycle | Abi CG40410 Cdk4 Rpn9 Trl dap pbl sc zwilch |
| 835 | GO:0016627 | F | 4, | 1 | 0.508 (x 1.968) | 30 (0.033) | 0.603 | oxidoreductase activity, acting on the CH-CH group of donors | CG4586 |
| 836 | GO:0019201 | F | 7, | 1 | 0.508 (x 1.968) | 30 (0.033) | 0.604 | nucleotide kinase activity | CG6767 |
| 837 | GO:0051603 | P | 8, 9, | 2 | 1.389 (x 1.440) | 82 (0.024) | 0.604 | proteolysis during cellular protein catabolism | CG5794 th |
| 838 | GO:0016049 | P | 3, 4, 6, 7, | 1 | 0.508 (x 1.968) | 30 (0.033) | 0.604 | cell growth | Cdk4 |
| 839 | GO:0044257 | P | 7, 8, | 2 | 1.389 (x 1.440) | 82 (0.024) | 0.605 | cellular protein catabolism | CG5794 th |
| 840 | GO:0030162 | P | 6, 7, 8, | 1 | 0.508 (x 1.968) | 30 (0.033) | 0.605 | regulation of proteolysis | CG40410 |
| 841 | GO:0015926 | F | 6, | 1 | 0.525 (x 1.904) | 31 (0.032) | 0.609 | glucosidase activity | GNBP3 |
| 842 | GO:0048754 | P | 5, | 1 | 0.525 (x 1.904) | 31 (0.032) | 0.609 | branching morphogenesis of a tube | sgl |
| 843 | GO:0007224 | P | 6, | 1 | 0.525 (x 1.904) | 31 (0.032) | 0.61 | smoothened signaling pathway | sgl |
| 844 | GO:0046872 | F | 4, | 17 | 15.788 (x 1.077) | 932 (0.018) | 0.61 | metal ion binding | BcDNA:LD41548 CBP CG33113 CG6930 CG9027 D19A Fkbp13 Nep2 Trl br esn klu l(1)G0320 ome pk th tok |
| 845 | GO:0043167 | F | 3, | 17 | 15.788 (x 1.077) | 932 (0.018) | 0.611 | ion binding | BcDNA:LD41548 CBP CG33113 CG6930 CG9027 D19A Fkbp13 Nep2 Trl br esn klu l(1)G0320 ome pk th tok |
| 846 | GO:0016790 | F | 5, | 1 | 0.542 (x 1.845) | 32 (0.031) | 0.614 | thiolester hydrolase activity | CG5794 |
| 847 | GO:0030031 | P | 6, 7, | 1 | 0.542 (x 1.845) | 32 (0.031) | 0.615 | cell projection biogenesis | Abi |
| 848 | GO:0050808 | P | 5, | 1 | 0.542 (x 1.845) | 32 (0.031) | 0.615 | synapse organization and biogenesis | Tl |
| 849 | GO:0008360 | P | 5, 6, | 2 | 1.440 (x 1.389) | 85 (0.024) | 0.616 | regulation of cell shape | Abi pbl |
| 850 | GO:0019205 | F | 6, | 1 | 0.542 (x 1.845) | 32 (0.031) | 0.616 | nucleobase, nucleoside, nucleotide kinase activity | CG6767 |
| 851 | GO:0005667 | C | 3, 6, 7, 8, 9, 10, 11, 12, 13, | 2 | 1.440 (x 1.389) | 85 (0.024) | 0.616 | transcription factor complex | Caf1 toe |
| 852 | GO:0044271 | P | 5, 6, | 2 | 1.423 (x 1.406) | 84 (0.024) | 0.616 | nitrogen compound biosynthesis | CG6287 ESTS:39C10S |
| 853 | GO:0016684 | F | 4, | 1 | 0.542 (x 1.845) | 32 (0.031) | 0.617 | oxidoreductase activity, acting on peroxide as acceptor | CG5873 |
| 854 | GO:0009309 | P | 6, 7, | 2 | 1.423 (x 1.406) | 84 (0.024) | 0.617 | amine biosynthesis | CG6287 ESTS:39C10S |
| 855 | GO:0007017 | P | 7, | 4 | 3.320 (x 1.205) | 196 (0.020) | 0.617 | microtubule-based process | CG9057 pbl stai vg |
| 856 | GO:0030198 | P | 4, | 1 | 0.542 (x 1.845) | 32 (0.031) | 0.617 | extracellular matrix organization and biogenesis | Tl |
| 857 | GO:0007602 | P | 6, 7, | 1 | 0.542 (x 1.845) | 32 (0.031) | 0.618 | phototransduction | ogre |
| 858 | GO:0045451 | P | 7, 11, 13, 14, 16, | 1 | 0.542 (x 1.845) | 32 (0.031) | 0.619 | pole plasm oskar mRNA localization | Hrb27C |
| 859 | GO:0004888 | F | 4, | 8 | 7.199 (x 1.111) | 425 (0.019) | 0.619 | transmembrane receptor activity | CG14076 PFE Tl arr fra mth pbl wgn |
| 860 | GO:0008083 | F | 4, 5, | 1 | 0.559 (x 1.789) | 33 (0.030) | 0.62 | growth factor activity | mav |
| 861 | GO:0006206 | P | 7, | 1 | 0.542 (x 1.845) | 32 (0.031) | 0.62 | pyrimidine base metabolism | CG6767 |
| 862 | GO:0042067 | P | 6, 7, 8, 9, | 1 | 0.559 (x 1.789) | 33 (0.030) | 0.62 | establishment of ommatidial polarity (sensu Endopterygota) | pk |
| 863 | GO:0004601 | F | 3, 5, | 1 | 0.542 (x 1.845) | 32 (0.031) | 0.62 | peroxidase activity | CG5873 |
| 864 | GO:0019200 | F | 6, | 1 | 0.559 (x 1.789) | 33 (0.030) | 0.621 | carbohydrate kinase activity | CG6767 |
| 865 | GO:0043296 | C | 6, 7, 8, 9, | 1 | 0.542 (x 1.845) | 32 (0.031) | 0.621 | apical junction complex | Fas3 |
| 866 | GO:0043565 | F | 5, | 1 | 0.559 (x 1.789) | 33 (0.030) | 0.622 | sequence-specific DNA binding | dve |
| 867 | GO:0043169 | F | 4, | 16 | 15.025 (x 1.065) | 887 (0.018) | 0.622 | cation binding | BcDNA:LD41548 CBP CG33113 CG6930 D19A Fkbp13 Nep2 Trl br esn klu l(1)G0320 ome pk th tok |
| 868 | GO:0007619 | P | 5, 6, | 1 | 0.559 (x 1.789) | 33 (0.030) | 0.622 | courtship behavior | Poxn |
| 869 | GO:0009994 | P | 4, 7, | 2 | 1.474 (x 1.357) | 87 (0.023) | 0.623 | oocyte differentiation | Hrb27C wbl |
| 870 | GO:0007297 | P | 6, 7, 9, | 1 | 0.559 (x 1.789) | 33 (0.030) | 0.623 | follicle cell migration (sensu Insecta) | th |
| 871 | GO:0042303 | P | 4, | 1 | 0.559 (x 1.789) | 33 (0.030) | 0.624 | molting cycle | Eip75B |
| 872 | GO:0018988 | P | 5, | 1 | 0.559 (x 1.789) | 33 (0.030) | 0.625 | molting cycle (sensu Protostomia and Nematoda) | Eip75B |
| 873 | GO:0019221 | P | 6, | 1 | 0.559 (x 1.789) | 33 (0.030) | 0.625 | cytokine and chemokine mediated signaling pathway | Tl |
| 874 | GO:0008170 | F | 6, | 1 | 0.576 (x 1.736) | 34 (0.029) | 0.626 | N-methyltransferase activity | Caf1 |
| 875 | GO:0042579 | C | 5, 6, 7, 8, | 1 | 0.576 (x 1.736) | 34 (0.029) | 0.627 | microbody | CG4586 |
| 876 | GO:0008361 | P | 5, 6, | 1 | 0.576 (x 1.736) | 34 (0.029) | 0.628 | regulation of cell size | Cdk4 |
| 877 | GO:0016831 | F | 5, | 1 | 0.576 (x 1.736) | 34 (0.029) | 0.628 | carboxy-lyase activity | Pepck |
| 878 | GO:0015020 | F | 6, | 1 | 0.576 (x 1.736) | 34 (0.029) | 0.629 | glucuronosyltransferase activity | CG17323 |
| 879 | GO:0005777 | C | 6, 7, 8, 9, | 1 | 0.576 (x 1.736) | 34 (0.029) | 0.63 | peroxisome | CG4586 |
| 880 | GO:0001763 | P | 4, | 1 | 0.576 (x 1.736) | 34 (0.029) | 0.631 | morphogenesis of a branching structure | sgl |
| 881 | GO:0009583 | P | 5, 6, | 1 | 0.593 (x 1.687) | 35 (0.029) | 0.638 | detection of light stimulus | ogre |
| 882 | GO:0016358 | P | 7, 10, | 1 | 0.593 (x 1.687) | 35 (0.029) | 0.638 | dendrite development | fra |
| 883 | GO:0030163 | P | 6, 7, | 2 | 1.525 (x 1.312) | 90 (0.022) | 0.639 | protein catabolism | CG5794 th |
| 884 | GO:0048568 | P | 4, | 1 | 0.593 (x 1.687) | 35 (0.029) | 0.639 | embryonic organ development | pbl |
| 885 | GO:0005794 | C | 5, 6, 7, 8, | 2 | 1.525 (x 1.312) | 90 (0.022) | 0.64 | Golgi apparatus | Snap alpha-Man-IIb |
| 886 | GO:0007286 | P | 5, 8, | 1 | 0.610 (x 1.640) | 36 (0.028) | 0.643 | spermatid development | th |
| 887 | GO:0007442 | P | 7, 8, | 1 | 0.610 (x 1.640) | 36 (0.028) | 0.644 | hindgut morphogenesis | pbl |
| 888 | GO:0048515 | P | 4, 7, | 1 | 0.610 (x 1.640) | 36 (0.028) | 0.645 | spermatid differentiation | th |
| 889 | GO:0051327 | P | 6, | 2 | 1.542 (x 1.297) | 91 (0.022) | 0.645 | M phase of meiotic cell cycle | CG40410 pbl |
| 890 | GO:0016209 | F | 2, | 1 | 0.610 (x 1.640) | 36 (0.028) | 0.646 | antioxidant activity | CG5873 |
| 891 | GO:0005083 | F | 4, | 2 | 1.542 (x 1.297) | 91 (0.022) | 0.646 | small GTPase regulator activity | Abi pbl |
| 892 | GO:0043235 | C | 3, | 1 | 0.610 (x 1.640) | 36 (0.028) | 0.646 | receptor complex | l(1)G0320 |
| 893 | GO:0006898 | P | 7, 8, | 1 | 0.610 (x 1.640) | 36 (0.028) | 0.647 | receptor mediated endocytosis | arr |
| 894 | GO:0051321 | P | 5, | 2 | 1.558 (x 1.283) | 92 (0.022) | 0.648 | meiotic cell cycle | CG40410 pbl |
| 895 | GO:0005624 | C | 4, 5, | 2 | 1.575 (x 1.270) | 93 (0.022) | 0.652 | membrane fraction | Cyp310a1 glec |
| 896 | GO:0045132 | P | 5, 8, | 1 | 0.627 (x 1.595) | 37 (0.027) | 0.653 | meiotic chromosome segregation | CG40410 |
| 897 | GO:0004263 | F | 7, | 4 | 3.523 (x 1.135) | 208 (0.019) | 0.653 | chymotrypsin activity | BEST:GH02921 CG4914 CG5390 Sb |
| 898 | GO:0015931 | P | 5, 6, | 1 | 0.627 (x 1.595) | 37 (0.027) | 0.653 | nucleobase, nucleoside, nucleotide and nucleic acid transport | BEST:LD04971 |
| 899 | GO:0044267 | P | 6, | 36 | 35.218 (x 1.022) | 2079 (0.017) | 0.654 | cellular protein metabolism | BEST:GH02921 BcDNA:LD41548 CG10657 CG2852 CG33171 CG40410 CG4914 CG5390 CG5794 Caf1 Cdk4 D19A Eip71CD Fkbp13 Hsp23 Hsp26 Hsp27 Hsp67Ba Lsp2 Nep2 PFE RpS12 Rpn9 Sb Spn1 dap for fra l(1)G0320 mdy ome sgl smi35A th tok wbl |
| 900 | GO:0019094 | P | 6, 10, 12, 13, 15, | 1 | 0.627 (x 1.595) | 37 (0.027) | 0.654 | pole plasm mRNA localization | Hrb27C |
| 901 | GO:0016327 | C | 5, 6, 7, | 1 | 0.627 (x 1.595) | 37 (0.027) | 0.655 | apicolateral plasma membrane | Fas3 |
| 902 | GO:0007316 | P | 5, 9, 11, 12, 14, | 1 | 0.644 (x 1.554) | 38 (0.026) | 0.66 | pole plasm RNA localization | Hrb27C |
| 903 | GO:0006916 | P | 8, 9, | 1 | 0.644 (x 1.554) | 38 (0.026) | 0.661 | anti-apoptosis | th |
| 904 | GO:0008066 | F | 5, | 1 | 0.644 (x 1.554) | 38 (0.026) | 0.662 | glutamate receptor activity | CG14076 |
| 905 | GO:0019731 | P | 6, 7, 8, | 1 | 0.644 (x 1.554) | 38 (0.026) | 0.662 | antibacterial humoral response | Tl |
| 906 | GO:0015296 | F | 5, 7, | 1 | 0.661 (x 1.514) | 39 (0.026) | 0.666 | anion:cation symporter activity | l(2)01810 |
| 907 | GO:0007143 | P | 8, | 1 | 0.661 (x 1.514) | 39 (0.026) | 0.667 | female meiosis | CG40410 |
| 908 | GO:0007173 | P | 8, | 1 | 0.661 (x 1.514) | 39 (0.026) | 0.668 | epidermal growth factor receptor signaling pathway | edl |
| 909 | GO:0005843 | C | 4, 5, 6, 7, 8, 9, 10, 11, | 1 | 0.661 (x 1.514) | 39 (0.026) | 0.669 | cytosolic small ribosomal subunit (sensu Eukaryota) | RpS12 |
| 910 | GO:0006096 | P | 8, 10, 11, | 1 | 0.661 (x 1.514) | 39 (0.026) | 0.669 | glycolysis | Tpi |
| 911 | GO:0048547 | P | 5, 6, | 1 | 0.678 (x 1.476) | 40 (0.025) | 0.67 | gut morphogenesis | pbl |
| 912 | GO:0016283 | C | 3, 5, 6, 7, 8, | 1 | 0.661 (x 1.514) | 39 (0.026) | 0.67 | eukaryotic 48S initiation complex | RpS12 |
| 913 | GO:0048546 | P | 5, | 1 | 0.678 (x 1.476) | 40 (0.025) | 0.67 | digestive tract morphogenesis | pbl |
| 914 | GO:0000267 | C | 3, 4, | 2 | 1.626 (x 1.230) | 96 (0.021) | 0.67 | cell fraction | Cyp310a1 glec |
| 915 | GO:0046530 | P | 4, | 2 | 1.660 (x 1.205) | 98 (0.020) | 0.671 | photoreceptor cell differentiation | br edl |
| 916 | GO:0035218 | P | 5, | 1 | 0.661 (x 1.514) | 39 (0.026) | 0.671 | leg disc development | Poxn |
| 917 | GO:0046552 | P | 5, | 1 | 0.678 (x 1.476) | 40 (0.025) | 0.671 | photoreceptor cell fate commitment | br |
| 918 | GO:0043414 | P | 7, | 1 | 0.678 (x 1.476) | 40 (0.025) | 0.672 | biopolymer methylation | Caf1 |
| 919 | GO:0048589 | P | 3, | 1 | 0.678 (x 1.476) | 40 (0.025) | 0.672 | developmental growth | ninA |
| 920 | GO:0016829 | F | 3, | 3 | 2.660 (x 1.128) | 157 (0.019) | 0.673 | lyase activity | CG16733 Eip55E Pepck |
| 921 | GO:0005789 | C | 4, 5, 6, 7, 8, 9, 10, | 1 | 0.678 (x 1.476) | 40 (0.025) | 0.673 | endoplasmic reticulum membrane | l(1)G0320 |
| 922 | GO:0004177 | F | 6, | 1 | 0.678 (x 1.476) | 40 (0.025) | 0.674 | aminopeptidase activity | BcDNA:LD41548 |
| 923 | GO:0042175 | C | 4, 5, 6, | 1 | 0.695 (x 1.440) | 41 (0.024) | 0.675 | nuclear envelope-endoplasmic reticulum network | l(1)G0320 |
| 924 | GO:0007459 | P | 6, | 1 | 0.678 (x 1.476) | 40 (0.025) | 0.675 | photoreceptor fate commitment (sensu Endopterygota) | br |
| 925 | GO:0007219 | P | 6, | 1 | 0.695 (x 1.440) | 41 (0.024) | 0.675 | Notch signaling pathway | malpha |
| 926 | GO:0048567 | P | 6, 7, | 1 | 0.678 (x 1.476) | 40 (0.025) | 0.675 | ectodermal gut morphogenesis | pbl |
| 927 | GO:0048113 | P | 8, 10, 11, 13, | 1 | 0.695 (x 1.440) | 41 (0.024) | 0.676 | pole plasm assembly (sensu Insecta) | Hrb27C |
| 928 | GO:0005386 | F | 3, | 8 | 7.674 (x 1.043) | 453 (0.018) | 0.676 | carrier activity | BEST:LD04971 CG10657 CG13848 CG1607 CG3823 Nrv1 l(2)01810 mnd |
| 929 | GO:0007439 | P | 5, | 1 | 0.678 (x 1.476) | 40 (0.025) | 0.676 | ectodermal gut development | pbl |
| 930 | GO:0044255 | P | 5, 6, | 6 | 5.675 (x 1.057) | 335 (0.018) | 0.677 | cellular lipid metabolism | CG17323 CG4586 Cyp310a1 Eip75B arr mdy |
| 931 | GO:0008010 | F | 5, | 1 | 0.678 (x 1.476) | 40 (0.025) | 0.677 | structural constituent of larval cuticle (sensu Insecta) | CG8502 |
| 932 | GO:0043190 | C | 3, 4, | 1 | 0.711 (x 1.406) | 42 (0.024) | 0.677 | ATP-binding cassette (ABC) transporter complex | CG31121 |
| 933 | GO:0015290 | F | 4, | 4 | 3.676 (x 1.088) | 217 (0.018) | 0.677 | electrochemical potential-driven transporter activity | BEST:LD04971 CG1607 l(2)01810 mnd |
| 934 | GO:0015103 | F | 5, | 1 | 0.678 (x 1.476) | 40 (0.025) | 0.678 | inorganic anion transporter activity | l(2)01810 |
| 935 | GO:0048637 | P | 6, | 1 | 0.711 (x 1.406) | 42 (0.024) | 0.678 | skeletal muscle development | rost |
| 936 | GO:0015291 | F | 5, | 4 | 3.676 (x 1.088) | 217 (0.018) | 0.678 | porter activity | BEST:LD04971 CG1607 l(2)01810 mnd |
| 937 | GO:0006820 | P | 6, 7, | 2 | 1.694 (x 1.181) | 100 (0.020) | 0.678 | anion transport | CG33171 l(2)01810 |
| 938 | GO:0043066 | P | 7, 8, | 1 | 0.711 (x 1.406) | 42 (0.024) | 0.679 | negative regulation of apoptosis | th |
| 939 | GO:0045595 | P | 4, | 1 | 0.711 (x 1.406) | 42 (0.024) | 0.679 | regulation of cell differentiation | Tl |
| 940 | GO:0016799 | F | 5, | 1 | 0.711 (x 1.406) | 42 (0.024) | 0.68 | hydrolase activity, hydrolyzing N-glycosyl compounds | alpha-Man-IIb |
| 941 | GO:0048747 | P | 5, | 1 | 0.711 (x 1.406) | 42 (0.024) | 0.681 | muscle fiber development | rost |
| 942 | GO:0043069 | P | 6, 7, | 1 | 0.711 (x 1.406) | 42 (0.024) | 0.681 | negative regulation of programmed cell death | th |
| 943 | GO:0007306 | P | 9, 10, | 1 | 0.711 (x 1.406) | 42 (0.024) | 0.682 | insect chorion formation | Caf1 |
| 944 | GO:0048741 | P | 6, 7, | 1 | 0.711 (x 1.406) | 42 (0.024) | 0.683 | skeletal muscle fiber development | rost |
| 945 | GO:0005874 | C | 5, 6, 7, 8, 9, 10, | 1 | 0.711 (x 1.406) | 42 (0.024) | 0.684 | microtubule | vg |
| 946 | GO:0043062 | P | 3, | 1 | 0.711 (x 1.406) | 42 (0.024) | 0.684 | extracellular structure organization and biogenesis | Tl |
| 947 | GO:0051248 | P | 6, 7, | 1 | 0.728 (x 1.373) | 43 (0.023) | 0.687 | negative regulation of protein metabolism | dap |
| 948 | GO:0007315 | P | 7, 9, 10, 12, | 1 | 0.728 (x 1.373) | 43 (0.023) | 0.687 | pole plasm assembly | Hrb27C |
| 949 | GO:0007018 | P | 7, 8, 9, | 2 | 1.745 (x 1.146) | 103 (0.019) | 0.688 | microtubule-based movement | CG9057 vg |
| 950 | GO:0016301 | F | 5, | 7 | 6.793 (x 1.031) | 401 (0.017) | 0.688 | kinase activity | Argk CG40410 CG6767 Cdk4 PFE for smi35A |
| 951 | GO:0009966 | P | 4, 5, | 2 | 1.745 (x 1.146) | 103 (0.019) | 0.689 | regulation of signal transduction | edl klu |
| 952 | GO:0016458 | P | 6, | 1 | 0.745 (x 1.342) | 44 (0.023) | 0.693 | gene silencing | Caf1 |
| 953 | GO:0016830 | F | 4, | 1 | 0.762 (x 1.312) | 45 (0.022) | 0.693 | carbon-carbon lyase activity | Pepck |
| 954 | GO:0019538 | P | 5, | 37 | 36.979 (x 1.001) | 2183 (0.017) | 0.693 | protein metabolism | BEST:GH02921 BcDNA:LD41548 CG10657 CG2852 CG33171 CG40410 CG4914 CG5390 CG5794 Caf1 Cdk4 D19A Eip71CD Fkbp13 Hsp23 Hsp26 Hsp27 Hsp67Ba Lsp2 Nep2 PFE RpS12 Rpn9 Sb Spn1 dap for fra l(1)G0320 mdy ome pbl sgl smi35A th tok wbl |
| 955 | GO:0009582 | P | 4, 5, | 1 | 0.745 (x 1.342) | 44 (0.023) | 0.694 | detection of abiotic stimulus | ogre |
| 956 | GO:0030705 | P | 6, 7, 8, | 2 | 1.762 (x 1.135) | 104 (0.019) | 0.694 | cytoskeleton-dependent intracellular transport | CG9057 vg |
| 957 | GO:0005231 | F | 7, 8, | 1 | 0.762 (x 1.312) | 45 (0.022) | 0.694 | excitatory extracellular ligand-gated ion channel activity | CG14076 |
| 958 | GO:0000279 | P | 5, | 5 | 4.862 (x 1.028) | 287 (0.017) | 0.694 | M phase | CG40410 Cdk4 Rpn9 pbl zwilch |
| 959 | GO:0000087 | P | 6, | 4 | 3.845 (x 1.040) | 227 (0.018) | 0.695 | M phase of mitotic cell cycle | CG40410 Cdk4 Rpn9 zwilch |
| 960 | GO:0005700 | C | 6, 7, 8, 9, | 1 | 0.762 (x 1.312) | 45 (0.022) | 0.695 | polytene chromosome | Caf1 |
| 961 | GO:0007269 | P | 6, 7, 8, | 2 | 1.779 (x 1.124) | 105 (0.019) | 0.695 | neurotransmitter secretion | Snap mth |
| 962 | GO:0009581 | P | 4, 5, | 1 | 0.762 (x 1.312) | 45 (0.022) | 0.695 | detection of external stimulus | ogre |
| 963 | GO:0045055 | P | 6, 7, | 2 | 1.779 (x 1.124) | 105 (0.019) | 0.696 | regulated secretory pathway | Snap mth |
| 964 | GO:0006468 | P | 8, | 5 | 4.828 (x 1.036) | 285 (0.018) | 0.696 | protein amino acid phosphorylation | CG40410 Cdk4 PFE for smi35A |
| 965 | GO:0006139 | P | 5, | 30 | 30.000 (x 1.000) | 1771 (0.017) | 0.696 | nucleobase, nucleoside, nucleotide and nucleic acid metabolism | BEST:GH02921 BEST:LD04971 CG4914 CG6767 CREG Caf1 D19A Doc1 Doc2 Doc3 Dsp1 Eip75B His4r HmgZ Hrb27C Optix Poxn Tpi Trl ash2 br dve edl en grn inv klu mdy sc toe |
| 966 | GO:0005575 | C | 1, | 98 | 97.945 (x 1.001) | 5782 (0.017) | 0.696 | cellular\_component | Act42A BEST:GH02921 BEST:LD04971 BcDNA:GH02976 BcDNA:LD41548 CBP CG10657 CG14076 CG14439 CG1607 CG17052 CG17838 CG31121 CG31125 CG31997 CG33113 CG33171 CG3823 CG40410 CG4586 CG4914 CG6930 CG8588 CG9057 CG9894 CREG Caf1 Cyp310a1 D19A Doc1 Doc2 Doc3 Dsp1 Eip75B Fas3 Gfat1 His4r HmgZ Hrb27C Idh ImpE2 ImpE3 Lsp2 Nep2 Nrv1 Obp99a Optix Pepck Poxn RpS12 Rpn9 Sb Sema-1b Snap Tl Trl Tsp66E Wnt2 alpha-Man-IIb ana arr ash2 bip1 br dap dve edl en esn for fra glec grn in inv kal-1 klu l(1)G0320 l(2)01810 mdy mnd mth ninA ogre ome pbl pk rost rpk sc smi35A stai th toe trn vg wbl wgn |
| 967 | GO:0006960 | P | 7, 8, | 1 | 0.762 (x 1.312) | 45 (0.022) | 0.696 | antimicrobial humoral response (sensu Protostomia) | Tl |
| 968 | GO:0007067 | P | 7, | 4 | 3.828 (x 1.045) | 226 (0.018) | 0.696 | mitosis | CG40410 Cdk4 Rpn9 zwilch |
| 969 | GO:0002164 | P | 4, | 1 | 0.762 (x 1.312) | 45 (0.022) | 0.697 | larval development | Sb |
| 970 | GO:0048112 | P | 7, 9, 10, 12, | 1 | 0.762 (x 1.312) | 45 (0.022) | 0.698 | oocyte anterior/posterior axis determination (sensu Insecta) | Hrb27C |
| 971 | GO:0007517 | P | 4, | 2 | 1.829 (x 1.093) | 108 (0.019) | 0.706 | muscle development | Wnt2 rost |
| 972 | GO:0004175 | F | 5, | 8 | 7.979 (x 1.003) | 471 (0.017) | 0.706 | endopeptidase activity | BEST:GH02921 CG4914 CG5390 CG5794 Nep2 Rpn9 Sb tok |
| 973 | GO:0007293 | P | 8, | 1 | 0.796 (x 1.256) | 47 (0.021) | 0.707 | egg chamber formation (sensu Insecta) | dap |
| 974 | GO:0009060 | P | 8, | 1 | 0.796 (x 1.256) | 47 (0.021) | 0.708 | aerobic respiration | Idh |
| 975 | GO:0006099 | P | 8, 9, | 1 | 0.796 (x 1.256) | 47 (0.021) | 0.708 | tricarboxylic acid cycle | Idh |
| 976 | GO:0046356 | P | 8, | 1 | 0.796 (x 1.256) | 47 (0.021) | 0.709 | acetyl-CoA catabolism | Idh |
| 977 | GO:0045333 | P | 7, | 1 | 0.796 (x 1.256) | 47 (0.021) | 0.71 | cellular respiration | Idh |
| 978 | GO:0008236 | F | 5, | 5 | 4.963 (x 1.007) | 293 (0.017) | 0.712 | serine-type peptidase activity | BEST:GH02921 CG4914 CG5390 Sb ome |
| 979 | GO:0044265 | P | 6, | 3 | 2.914 (x 1.030) | 172 (0.017) | 0.714 | cellular macromolecule catabolism | CG5794 Tpi th |
| 980 | GO:0009109 | P | 7, | 1 | 0.813 (x 1.230) | 48 (0.021) | 0.714 | coenzyme catabolism | Idh |
| 981 | GO:0009416 | P | 5, | 1 | 0.813 (x 1.230) | 48 (0.021) | 0.714 | response to light stimulus | ogre |
| 982 | GO:0006084 | P | 7, | 1 | 0.813 (x 1.230) | 48 (0.021) | 0.715 | acetyl-CoA metabolism | Idh |
| 983 | GO:0008298 | P | 5, | 1 | 0.830 (x 1.205) | 49 (0.020) | 0.715 | intracellular mRNA localization | Hrb27C |
| 984 | GO:0004672 | F | 6, | 5 | 4.997 (x 1.001) | 295 (0.017) | 0.716 | protein kinase activity | CG40410 Cdk4 PFE for smi35A |
| 985 | GO:0005912 | C | 6, 7, 8, | 1 | 0.830 (x 1.205) | 49 (0.020) | 0.716 | adherens junction | Fas3 |
| 986 | GO:0007281 | P | 5, | 2 | 1.897 (x 1.054) | 112 (0.018) | 0.716 | germ cell development | Hrb27C wbl |
| 987 | GO:0051606 | P | 3, 4, | 1 | 0.830 (x 1.205) | 49 (0.020) | 0.717 | detection of stimulus | ogre |
| 988 | GO:0007519 | P | 5, | 1 | 0.830 (x 1.205) | 49 (0.020) | 0.717 | striated muscle development | rost |
| 989 | GO:0019199 | F | 5, 7, | 1 | 0.830 (x 1.205) | 49 (0.020) | 0.718 | transmembrane receptor protein kinase activity | PFE |
| 990 | GO:0050874 | P | 3, | 15 | 15.246 (x 0.984) | 900 (0.017) | 0.718 | organismal physiological process | CG4054 Eip75B Hsp26 Hsp27 Obp99a PFE Snap Tl br fax for kal-1 mth serpin-27A trn |
| 991 | GO:0051187 | P | 6, | 1 | 0.830 (x 1.205) | 49 (0.020) | 0.719 | cofactor catabolism | Idh |
| 992 | GO:0004295 | F | 7, | 4 | 3.981 (x 1.005) | 235 (0.017) | 0.719 | trypsin activity | BEST:GH02921 CG4914 CG5390 Sb |
| 993 | GO:0016192 | P | 5, 6, | 5 | 5.014 (x 0.997) | 296 (0.017) | 0.719 | vesicle-mediated transport | Snap arr l(1)G0320 mth wbl |
| 994 | GO:0008270 | F | 6, | 10 | 10.198 (x 0.981) | 602 (0.017) | 0.72 | zinc ion binding | CG6930 D19A Nep2 Trl br esn klu pk th tok |
| 995 | GO:0007548 | P | 3, | 1 | 0.847 (x 1.181) | 50 (0.020) | 0.722 | sex differentiation | en |
| 996 | GO:0003676 | F | 3, | 29 | 29.526 (x 0.982) | 1743 (0.017) | 0.722 | nucleic acid binding | BEST:GH02921 CG17838 CG4914 CG6930 D19A Doc1 Doc2 Doc3 Dsp1 Eip75B His4r HmgZ Hrb27C Optix Poxn Trl ash2 br dve edl en esn grn inv klu mdy pk sc toe |
| 997 | GO:0005230 | F | 6, 7, | 1 | 0.847 (x 1.181) | 50 (0.020) | 0.722 | extracellular ligand-gated ion channel activity | CG14076 |
| 998 | GO:0016740 | F | 3, | 16 | 16.381 (x 0.977) | 967 (0.017) | 0.725 | transferase activity | Abi Argk CG16733 CG17323 CG33171 CG40410 CG6767 Caf1 Cdk4 ESTS:39C10S Gfat1 GlyP PFE for mdy smi35A |
| 999 | GO:0046164 | P | 6, | 1 | 0.864 (x 1.158) | 51 (0.020) | 0.726 | alcohol catabolism | Tpi |
| 1000 | GO:0046365 | P | 7, 8, | 1 | 0.864 (x 1.158) | 51 (0.020) | 0.726 | monosaccharide catabolism | Tpi |
| 1001 | GO:0031497 | P | 10, | 1 | 0.864 (x 1.158) | 51 (0.020) | 0.727 | chromatin assembly | Caf1 |
| 1002 | GO:0006007 | P | 9, 10, | 1 | 0.864 (x 1.158) | 51 (0.020) | 0.728 | glucose catabolism | Tpi |
| 1003 | GO:0019320 | P | 8, 9, | 1 | 0.864 (x 1.158) | 51 (0.020) | 0.728 | hexose catabolism | Tpi |
| 1004 | GO:0019899 | F | 4, | 1 | 0.881 (x 1.135) | 52 (0.019) | 0.731 | enzyme binding | Caf1 |
| 1005 | GO:0000122 | P | 10, | 1 | 0.881 (x 1.135) | 52 (0.019) | 0.731 | negative regulation of transcription from RNA polymerase II promoter | Caf1 |
| 1006 | GO:0007264 | P | 6, | 2 | 1.965 (x 1.018) | 116 (0.017) | 0.732 | small GTPase mediated signal transduction | edl klu |
| 1007 | GO:0044432 | C | 4, 5, 6, 7, 8, 9, | 1 | 0.881 (x 1.135) | 52 (0.019) | 0.732 | endoplasmic reticulum part | l(1)G0320 |
| 1008 | GO:0042461 | P | 5, 6, 7, | 1 | 0.881 (x 1.135) | 52 (0.019) | 0.733 | photoreceptor cell development | edl |
| 1009 | GO:0006461 | P | 6, | 2 | 1.982 (x 1.009) | 117 (0.017) | 0.733 | protein complex assembly | Caf1 pbl |
| 1010 | GO:0001505 | P | 7, | 2 | 1.982 (x 1.009) | 117 (0.017) | 0.734 | regulation of neurotransmitter levels | Snap mth |
| 1011 | GO:0006629 | P | 5, | 8 | 8.300 (x 0.964) | 490 (0.016) | 0.734 | lipid metabolism | CG17323 CG4586 CG9057 Cyp310a1 Eip75B arr mdy sgl |
| 1012 | GO:0016065 | P | 6, 7, | 1 | 0.898 (x 1.114) | 53 (0.019) | 0.735 | humoral defense mechanism (sensu Protostomia) | Tl |
| 1013 | GO:0006974 | P | 4, | 2 | 1.999 (x 1.001) | 118 (0.017) | 0.736 | response to DNA damage stimulus | CG40410 Caf1 |
| 1014 | GO:0001708 | P | 5, | 1 | 0.898 (x 1.114) | 53 (0.019) | 0.736 | cell fate specification | malpha |
| 1015 | GO:0015268 | F | 4, | 3 | 3.083 (x 0.973) | 182 (0.016) | 0.736 | alpha-type channel activity | CG14076 ogre rpk |
| 1016 | GO:0048111 | P | 6, 8, 9, 11, | 1 | 0.898 (x 1.114) | 53 (0.019) | 0.736 | oocyte axis determination (sensu Insecta) | Hrb27C |
| 1017 | GO:0046914 | F | 5, | 11 | 11.451 (x 0.961) | 676 (0.016) | 0.737 | transition metal ion binding | BcDNA:LD41548 CG6930 D19A Nep2 Trl br esn klu pk th tok |
| 1018 | GO:0015267 | F | 3, | 3 | 3.083 (x 0.973) | 182 (0.016) | 0.737 | channel or pore class transporter activity | CG14076 ogre rpk |
| 1019 | GO:0000502 | C | 3, 4, 5, 6, | 1 | 0.915 (x 1.093) | 54 (0.019) | 0.738 | proteasome complex (sensu Eukaryota) | Rpn9 |
| 1020 | GO:0005198 | F | 2, | 12 | 12.501 (x 0.960) | 738 (0.016) | 0.738 | structural molecule activity | Act42A BcDNA:GH02976 CG15757 CG17052 CG33171 CG8502 CG8634 RpS12 esn pk trn vg |
| 1021 | GO:0006259 | P | 6, | 6 | 6.285 (x 0.955) | 371 (0.016) | 0.738 | DNA metabolism | Caf1 Dsp1 His4r HmgZ Trl ash2 |
| 1022 | GO:0048110 | P | 7, 8, 10, | 1 | 0.915 (x 1.093) | 54 (0.019) | 0.738 | oocyte construction (sensu Insecta) | Hrb27C |
| 1023 | GO:0050877 | P | 4, | 10 | 10.435 (x 0.958) | 616 (0.016) | 0.739 | neurophysiological process | CG4054 Obp99a PFE Snap br fax for kal-1 mth trn |
| 1024 | GO:0007304 | P | 8, 9, | 1 | 0.932 (x 1.073) | 55 (0.018) | 0.741 | eggshell formation (sensu Insecta) | Caf1 |
| 1025 | GO:0006260 | P | 7, | 2 | 2.050 (x 0.976) | 121 (0.017) | 0.742 | DNA replication | Caf1 Dsp1 |
| 1026 | GO:0007617 | P | 4, 5, | 1 | 0.932 (x 1.073) | 55 (0.018) | 0.742 | mating behavior | Poxn |
| 1027 | GO:0006144 | P | 7, | 1 | 0.932 (x 1.073) | 55 (0.018) | 0.742 | purine base metabolism | CG6767 |
| 1028 | GO:0030703 | P | 7, | 1 | 0.932 (x 1.073) | 55 (0.018) | 0.743 | eggshell formation | Caf1 |
| 1029 | GO:0016563 | F | 3, | 1 | 0.932 (x 1.073) | 55 (0.018) | 0.744 | transcriptional activator activity | sc |
| 1030 | GO:0051705 | P | 3, | 1 | 0.932 (x 1.073) | 55 (0.018) | 0.744 | behavioral interaction between organisms | Poxn |
| 1031 | GO:0007314 | P | 6, 8, 9, 11, | 1 | 0.949 (x 1.054) | 56 (0.018) | 0.746 | oocyte anterior/posterior axis determination | Hrb27C |
| 1032 | GO:0006955 | P | 4, 5, | 2 | 2.067 (x 0.968) | 122 (0.016) | 0.746 | immune response | Tl serpin-27A |
| 1033 | GO:0016836 | F | 5, | 1 | 0.949 (x 1.054) | 56 (0.018) | 0.747 | hydro-lyase activity | CG16733 |
| 1034 | GO:0016491 | F | 3, | 10 | 10.570 (x 0.946) | 624 (0.016) | 0.748 | oxidoreductase activity | CG30427 CG3842 CG4586 CG5873 CG6287 CG9027 Cyp310a1 Eip71CD Idh sgl |
| 1035 | GO:0045165 | P | 4, | 3 | 3.202 (x 0.937) | 189 (0.016) | 0.754 | cell fate commitment | br malpha sc |
| 1036 | GO:0007169 | P | 7, | 2 | 2.101 (x 0.952) | 124 (0.016) | 0.754 | transmembrane receptor protein tyrosine kinase signaling pathway | edl sgl |
| 1037 | GO:0019098 | P | 3, 4, | 1 | 0.982 (x 1.018) | 58 (0.017) | 0.754 | reproductive behavior | Poxn |
| 1038 | GO:0051704 | P | 2, | 1 | 0.982 (x 1.018) | 58 (0.017) | 0.755 | interaction between organisms | Poxn |
| 1039 | GO:0016876 | F | 5, | 1 | 0.982 (x 1.018) | 58 (0.017) | 0.756 | ligase activity, forming aminoacyl-tRNA and related compounds | mdy |
| 1040 | GO:0004812 | F | 6, | 1 | 0.982 (x 1.018) | 58 (0.017) | 0.757 | aminoacyl-tRNA ligase activity | mdy |
| 1041 | GO:0016875 | F | 4, | 1 | 0.982 (x 1.018) | 58 (0.017) | 0.757 | ligase activity, forming carbon-oxygen bonds | mdy |
| 1042 | GO:0009057 | P | 5, | 3 | 3.219 (x 0.932) | 190 (0.016) | 0.757 | macromolecule catabolism | CG5794 Tpi th |
| 1043 | GO:0006813 | P | 8, 9, | 1 | 0.982 (x 1.018) | 58 (0.017) | 0.758 | potassium ion transport | Nrv1 |
| 1044 | GO:0043285 | P | 6, | 2 | 2.151 (x 0.930) | 127 (0.016) | 0.76 | biopolymer catabolism | CG5794 th |
| 1045 | GO:0008757 | F | 6, | 1 | 0.999 (x 1.001) | 59 (0.017) | 0.76 | S-adenosylmethionine-dependent methyltransferase activity | Caf1 |
| 1046 | GO:0007243 | P | 6, | 2 | 2.151 (x 0.930) | 127 (0.016) | 0.76 | protein kinase cascade | CG30440 Cdk4 |
| 1047 | GO:0042277 | F | 3, | 1 | 0.999 (x 1.001) | 59 (0.017) | 0.76 | peptide binding | l(1)G0320 |
| 1048 | GO:0016282 | C | 3, 5, 6, 7, 8, | 1 | 1.016 (x 0.984) | 60 (0.017) | 0.761 | eukaryotic 43S preinitiation complex | RpS12 |
| 1049 | GO:0004252 | F | 6, | 4 | 4.353 (x 0.919) | 257 (0.016) | 0.761 | serine-type endopeptidase activity | BEST:GH02921 CG4914 CG5390 Sb |
| 1050 | GO:0000578 | P | 5, | 1 | 0.999 (x 1.001) | 59 (0.017) | 0.761 | embryonic axis specification | Tl |
| 1051 | GO:0051189 | P | 5, 7, | 1 | 1.016 (x 0.984) | 60 (0.017) | 0.761 | prosthetic group metabolism | CG10657 |
| 1052 | GO:0044459 | C | 4, 5, 6, | 5 | 5.472 (x 0.914) | 323 (0.015) | 0.762 | plasma membrane part | Fas3 Nrv1 Tl ogre wgn |
| 1053 | GO:0007015 | P | 9, | 1 | 1.016 (x 0.984) | 60 (0.017) | 0.762 | actin filament organization | Sb |
| 1054 | GO:0016765 | F | 4, | 1 | 1.016 (x 0.984) | 60 (0.017) | 0.763 | transferase activity, transferring alkyl or aryl (other than methyl) groups | CG33171 |
| 1055 | GO:0043039 | P | 8, 9, | 1 | 1.016 (x 0.984) | 60 (0.017) | 0.764 | tRNA aminoacylation | mdy |
| 1056 | GO:0016758 | F | 5, | 2 | 2.185 (x 0.915) | 129 (0.016) | 0.764 | transferase activity, transferring hexosyl groups | CG17323 GlyP |
| 1057 | GO:0043038 | P | 7, 8, | 1 | 1.033 (x 0.968) | 61 (0.016) | 0.764 | amino acid activation | mdy |
| 1058 | GO:0006418 | P | 8, 9, 10, | 1 | 1.016 (x 0.984) | 60 (0.017) | 0.764 | tRNA aminoacylation for protein translation | mdy |
| 1059 | GO:0005887 | C | 6, 7, 8, | 3 | 3.320 (x 0.904) | 196 (0.015) | 0.765 | integral to plasma membrane | Nrv1 Tl wgn |
| 1060 | GO:0040008 | P | 3, | 1 | 1.033 (x 0.968) | 61 (0.016) | 0.765 | regulation of growth | Cdk4 |
| 1061 | GO:0006261 | P | 8, | 1 | 1.033 (x 0.968) | 61 (0.016) | 0.766 | DNA-dependent DNA replication | Dsp1 |
| 1062 | GO:0009056 | P | 4, | 5 | 5.539 (x 0.903) | 327 (0.015) | 0.768 | catabolism | CG5794 Idh Tpi br th |
| 1063 | GO:0016835 | F | 4, | 1 | 1.050 (x 0.952) | 62 (0.016) | 0.768 | carbon-oxygen lyase activity | CG16733 |
| 1064 | GO:0015370 | F | 6, 8, | 1 | 1.050 (x 0.952) | 62 (0.016) | 0.769 | solute:sodium symporter activity | l(2)01810 |
| 1065 | GO:0031226 | C | 5, 6, 7, | 3 | 3.354 (x 0.894) | 198 (0.015) | 0.769 | intrinsic to plasma membrane | Nrv1 Tl wgn |
| 1066 | GO:0006917 | P | 8, 9, | 1 | 1.050 (x 0.952) | 62 (0.016) | 0.77 | induction of apoptosis | smi35A |
| 1067 | GO:0019730 | P | 6, 7, | 1 | 1.067 (x 0.937) | 63 (0.016) | 0.772 | antimicrobial humoral response | Tl |
| 1068 | GO:0015294 | F | 5, 7, | 1 | 1.067 (x 0.937) | 63 (0.016) | 0.773 | solute:cation symporter activity | l(2)01810 |
| 1069 | GO:0045184 | P | 5, | 8 | 8.809 (x 0.908) | 520 (0.015) | 0.773 | establishment of protein localization | CG2852 CG33113 Snap alpha-Man-IIb arr l(1)G0320 pk wbl |
| 1070 | GO:0051246 | P | 5, 6, | 2 | 2.253 (x 0.888) | 133 (0.015) | 0.774 | regulation of protein metabolism | CG40410 dap |
| 1071 | GO:0043065 | P | 7, 8, | 1 | 1.084 (x 0.922) | 64 (0.016) | 0.776 | positive regulation of apoptosis | smi35A |
| 1072 | GO:0009059 | P | 5, 6, | 8 | 8.842 (x 0.905) | 522 (0.015) | 0.776 | macromolecule biosynthesis | CG33171 Gfat1 Pepck RpS12 Tpi l(1)G0320 mdy sgl |
| 1073 | GO:0008150 | P | 1, | 136 | 136.906 (x 0.993) | 8082 (0.017) | 0.776 | biological\_process | Abi Act42A Argk BEST:GH02921 BEST:LD04971 BcDNA:GH02976 BcDNA:LD41548 CG10359 CG10657 CG14439 CG1607 CG17052 CG17064 CG17323 CG17919 CG2852 CG30440 CG31121 CG31125 CG31997 CG33113 CG33171 CG3770 CG3823 CG3842 CG40410 CG4054 CG4586 CG4914 CG5390 CG5466 CG5731 CG5794 CG5873 CG6287 CG6767 CG7668 CG8588 CG9027 CG9057 CREG Caf1 Cdk4 Cyp310a1 D19A Doc1 Doc2 Doc3 Dsp1 ESTS:39C10S Eip55E Eip71CD Eip75B Fas3 Fkbp13 GNBP3 Gfat1 GlyP His4r HmgZ Hrb27C Hsp23 Hsp26 Hsp27 Hsp67Ba Idh ImpE2 ImpE3 Lsp2 Nep2 Nrv1 Obp99a Optix Oscillin PFE Pepck Poxn RpS12 Rpn9 Sb Sema-1b Snap Spn1 Tl Tpi Trl Tsp66E Wnt2 alpha-Man-IIb ana arr ash2 bip1 br dap dve edl en esn fax fbp for fra glec grn in inv kal-1 klu l(1)G0320 l(2)01810 malpha mav mdy mnd mth ninA ogre ome pbl pk rost rpk sc serpin-27A sgl smi35A stai th toe tok trn vg wbl wgn zwilch |
| 1074 | GO:0007242 | P | 5, | 8 | 8.876 (x 0.901) | 524 (0.015) | 0.779 | intracellular signaling cascade | CG30440 CG40410 Cdk4 Eip75B edl for klu stai |
| 1075 | GO:0016310 | P | 7, | 7 | 7.809 (x 0.896) | 461 (0.015) | 0.779 | phosphorylation | Argk CG40410 Cdk4 PFE edl for smi35A |
| 1076 | GO:0044431 | C | 4, 5, 6, 7, 8, 9, | 1 | 1.101 (x 0.908) | 65 (0.015) | 0.78 | Golgi apparatus part | alpha-Man-IIb |
| 1077 | GO:0008289 | F | 3, | 1 | 1.118 (x 0.894) | 66 (0.015) | 0.786 | lipid binding | CG17919 |
| 1078 | GO:0044275 | P | 7, | 1 | 1.135 (x 0.881) | 67 (0.015) | 0.79 | cellular carbohydrate catabolism | Tpi |
| 1079 | GO:0016052 | P | 6, | 1 | 1.135 (x 0.881) | 67 (0.015) | 0.791 | carbohydrate catabolism | Tpi |
| 1080 | GO:0005215 | F | 2, | 15 | 16.398 (x 0.915) | 968 (0.015) | 0.791 | transporter activity | BEST:LD04971 CG10657 CG13848 CG14076 CG14439 CG1607 CG31121 CG3823 Lsp2 Nrv1 Snap l(2)01810 mnd ogre rpk |
| 1081 | GO:0015935 | C | 3, 4, 5, 6, 7, 8, 9, | 1 | 1.169 (x 0.856) | 69 (0.014) | 0.802 | small ribosomal subunit | RpS12 |
| 1082 | GO:0007626 | P | 4, | 1 | 1.186 (x 0.843) | 70 (0.014) | 0.807 | locomotory behavior | for |
| 1083 | GO:0006796 | P | 6, | 9 | 10.232 (x 0.880) | 604 (0.015) | 0.81 | phosphate metabolism | Argk CG40410 Cdk4 PFE dap edl for l(2)01810 smi35A |
| 1084 | GO:0006793 | P | 5, | 9 | 10.232 (x 0.880) | 604 (0.015) | 0.811 | phosphorus metabolism | Argk CG40410 Cdk4 PFE dap edl for l(2)01810 smi35A |
| 1085 | GO:0012505 | C | 4, 5, | 2 | 2.439 (x 0.820) | 144 (0.014) | 0.812 | endomembrane system | alpha-Man-IIb l(1)G0320 |
| 1086 | GO:0015662 | F | 5, 6, 8, 13, | 1 | 1.220 (x 0.820) | 72 (0.014) | 0.816 | ATPase activity, coupled to transmembrane movement of ions, phosphorylative mechanism | Nrv1 |
| 1087 | GO:0045045 | P | 5, 6, | 3 | 3.642 (x 0.824) | 215 (0.014) | 0.816 | secretory pathway | Snap mth wbl |
| 1088 | GO:0015293 | F | 6, | 1 | 1.237 (x 0.809) | 73 (0.014) | 0.82 | symporter activity | l(2)01810 |
| 1089 | GO:0030001 | P | 7, 8, | 2 | 2.490 (x 0.803) | 147 (0.014) | 0.821 | metal ion transport | Nrv1 rpk |
| 1090 | GO:0003712 | F | 3, 5, | 1 | 1.254 (x 0.798) | 74 (0.014) | 0.823 | transcription cofactor activity | Dsp1 |
| 1091 | GO:0015629 | C | 6, 7, 8, 9, | 1 | 1.254 (x 0.798) | 74 (0.014) | 0.824 | actin cytoskeleton | Act42A |
| 1092 | GO:0043492 | F | 3, 10, | 2 | 2.558 (x 0.782) | 151 (0.013) | 0.832 | ATPase activity, coupled to movement of substances | CG31121 Nrv1 |
| 1093 | GO:0042626 | F | 4, 6, 11, | 2 | 2.558 (x 0.782) | 151 (0.013) | 0.833 | ATPase activity, coupled to transmembrane movement of substances | CG31121 Nrv1 |
| 1094 | GO:0016773 | F | 5, | 5 | 6.047 (x 0.827) | 357 (0.014) | 0.833 | phosphotransferase activity, alcohol group as acceptor | CG40410 Cdk4 PFE for smi35A |
| 1095 | GO:0008017 | F | 6, | 1 | 1.287 (x 0.777) | 76 (0.013) | 0.833 | microtubule binding | stai |
| 1096 | GO:0016820 | F | 5, | 2 | 2.575 (x 0.777) | 152 (0.013) | 0.835 | hydrolase activity, acting on acid anhydrides, catalyzing transmembrane movement of substances | CG31121 Nrv1 |
| 1097 | GO:0043283 | P | 5, | 26 | 28.526 (x 0.911) | 1684 (0.015) | 0.835 | biopolymer metabolism | BcDNA:GH02976 CG17052 CG17323 CG33171 CG40410 CG5731 CG5794 Caf1 Cdk4 Dsp1 Eip71CD GNBP3 GlyP His4r HmgZ Hrb27C PFE Trl ash2 dap for mdy sgl smi35A th wbl |
| 1098 | GO:0005654 | C | 5, 6, 7, 8, 9, 10, 11, | 3 | 3.794 (x 0.791) | 224 (0.013) | 0.838 | nucleoplasm | Caf1 Hrb27C toe |
| 1099 | GO:0006959 | P | 5, 6, | 1 | 1.321 (x 0.757) | 78 (0.013) | 0.838 | humoral immune response | Tl |
| 1100 | GO:0009112 | P | 6, | 1 | 1.321 (x 0.757) | 78 (0.013) | 0.838 | nucleobase metabolism | CG6767 |
| 1101 | GO:0008104 | P | 4, | 8 | 9.588 (x 0.834) | 566 (0.014) | 0.851 | protein localization | CG2852 CG33113 Snap alpha-Man-IIb arr l(1)G0320 pk wbl |
| 1102 | GO:0005214 | F | 4, | 1 | 1.372 (x 0.729) | 81 (0.012) | 0.851 | structural constituent of cuticle (sensu Insecta) | CG8502 |
| 1103 | GO:0016772 | F | 4, | 7 | 8.487 (x 0.825) | 501 (0.014) | 0.851 | transferase activity, transferring phosphorus-containing groups | Argk CG40410 CG6767 Cdk4 PFE for smi35A |
| 1104 | GO:0004197 | F | 6, | 1 | 1.372 (x 0.729) | 81 (0.012) | 0.852 | cysteine-type endopeptidase activity | CG5794 |
| 1105 | GO:0009117 | P | 6, | 2 | 2.693 (x 0.743) | 159 (0.013) | 0.852 | nucleotide metabolism | CG6767 Tpi |
| 1106 | GO:0016757 | F | 4, | 2 | 2.693 (x 0.743) | 159 (0.013) | 0.853 | transferase activity, transferring glycosyl groups | CG17323 GlyP |
| 1107 | GO:0006605 | P | 7, 8, 9, | 3 | 3.913 (x 0.767) | 231 (0.013) | 0.854 | protein targeting | CG2852 l(1)G0320 wbl |
| 1108 | GO:0005792 | C | 6, 7, | 1 | 1.406 (x 0.711) | 83 (0.012) | 0.855 | microsome | Cyp310a1 |
| 1109 | GO:0042598 | C | 5, 6, | 1 | 1.406 (x 0.711) | 83 (0.012) | 0.856 | vesicular fraction | Cyp310a1 |
| 1110 | GO:0008168 | F | 5, | 1 | 1.423 (x 0.703) | 84 (0.012) | 0.859 | methyltransferase activity | Caf1 |
| 1111 | GO:0006470 | P | 8, | 1 | 1.440 (x 0.695) | 85 (0.012) | 0.86 | protein amino acid dephosphorylation | dap |
| 1112 | GO:0016741 | F | 4, | 1 | 1.440 (x 0.695) | 85 (0.012) | 0.861 | transferase activity, transferring one-carbon groups | Caf1 |
| 1113 | GO:0008610 | P | 5, 6, 7, | 1 | 1.440 (x 0.695) | 85 (0.012) | 0.861 | lipid biosynthesis | mdy |
| 1114 | GO:0044248 | P | 5, | 4 | 5.184 (x 0.772) | 306 (0.013) | 0.862 | cellular catabolism | CG5794 Idh Tpi th |
| 1115 | GO:0044425 | C | 3, 4, 5, | 18 | 20.599 (x 0.874) | 1216 (0.015) | 0.862 | membrane part | CG14439 Fas3 ImpE2 ImpE3 Nrv1 Snap Tl Tsp66E alpha-Man-IIb glec l(1)G0320 l(2)01810 mth ninA ogre pbl rost wgn |
| 1116 | GO:0005216 | F | 4, 5, | 2 | 2.778 (x 0.720) | 164 (0.012) | 0.862 | ion channel activity | CG14076 rpk |
| 1117 | GO:0006936 | P | 4, | 1 | 1.457 (x 0.686) | 86 (0.012) | 0.863 | muscle contraction | for |
| 1118 | GO:0007126 | P | 7, | 1 | 1.474 (x 0.679) | 87 (0.011) | 0.864 | meiosis | CG40410 |
| 1119 | GO:0003774 | F | 2, | 1 | 1.474 (x 0.679) | 87 (0.011) | 0.865 | motor activity | Act42A |
| 1120 | GO:0000226 | P | 8, | 1 | 1.474 (x 0.679) | 87 (0.011) | 0.866 | microtubule cytoskeleton organization and biogenesis | pbl |
| 1121 | GO:0044430 | C | 4, 5, 6, 7, 8, 9, | 3 | 4.066 (x 0.738) | 240 (0.013) | 0.866 | cytoskeletal part | Act42A stai vg |
| 1122 | GO:0006399 | P | 7, | 1 | 1.491 (x 0.671) | 88 (0.011) | 0.866 | tRNA metabolism | mdy |
| 1123 | GO:0006810 | P | 4, 5, | 22 | 25.020 (x 0.879) | 1477 (0.015) | 0.866 | transport | BEST:LD04971 CG10657 CG14439 CG1607 CG2852 CG31121 CG33113 CG33171 CG3823 CG9057 Lsp2 Nrv1 Obp99a Snap arr l(1)G0320 l(2)01810 mnd mth rpk vg wbl |
| 1124 | GO:0006732 | P | 6, | 3 | 4.049 (x 0.741) | 239 (0.013) | 0.866 | coenzyme metabolism | CG10657 Idh Tpi |
| 1125 | GO:0008134 | F | 4, | 1 | 1.508 (x 0.663) | 89 (0.011) | 0.869 | transcription factor binding | Dsp1 |
| 1126 | GO:0005830 | C | 5, 6, 7, 8, 9, 10, | 1 | 1.525 (x 0.656) | 90 (0.011) | 0.873 | cytosolic ribosome (sensu Eukaryota) | RpS12 |
| 1127 | GO:0009260 | P | 7, 8, | 1 | 1.542 (x 0.649) | 91 (0.011) | 0.875 | ribonucleotide biosynthesis | CG6767 |
| 1128 | GO:0015631 | F | 5, | 1 | 1.542 (x 0.649) | 91 (0.011) | 0.876 | tubulin binding | stai |
| 1129 | GO:0008194 | F | 5, | 1 | 1.558 (x 0.642) | 92 (0.011) | 0.878 | UDP-glycosyltransferase activity | CG17323 |
| 1130 | GO:0009259 | P | 7, | 1 | 1.558 (x 0.642) | 92 (0.011) | 0.878 | ribonucleotide metabolism | CG6767 |
| 1131 | GO:0008234 | F | 5, | 1 | 1.575 (x 0.635) | 93 (0.011) | 0.88 | cysteine-type peptidase activity | CG5794 |
| 1132 | GO:0006403 | P | 4, | 1 | 1.575 (x 0.635) | 93 (0.011) | 0.881 | RNA localization | Hrb27C |
| 1133 | GO:0008509 | F | 4, | 1 | 1.592 (x 0.628) | 94 (0.011) | 0.882 | anion transporter activity | l(2)01810 |
| 1134 | GO:0015672 | P | 7, 8, | 2 | 2.964 (x 0.675) | 175 (0.011) | 0.883 | monovalent inorganic cation transport | Nrv1 rpk |
| 1135 | GO:0042625 | F | 4, 5, 7, 12, | 1 | 1.609 (x 0.621) | 95 (0.011) | 0.884 | ATPase activity, coupled to transmembrane movement of ions | Nrv1 |
| 1136 | GO:0008047 | F | 3, | 1 | 1.609 (x 0.621) | 95 (0.011) | 0.885 | enzyme activator activity | BEST:GH02921 |
| 1137 | GO:0051186 | P | 5, | 3 | 4.286 (x 0.700) | 253 (0.012) | 0.887 | cofactor metabolism | CG10657 Idh Tpi |
| 1138 | GO:0045892 | P | 9, | 1 | 1.643 (x 0.609) | 97 (0.010) | 0.89 | negative regulation of transcription, DNA-dependent | Caf1 |
| 1139 | GO:0016879 | F | 4, | 2 | 3.083 (x 0.649) | 182 (0.011) | 0.897 | ligase activity, forming carbon-nitrogen bonds | CG6767 th |
| 1140 | GO:0006464 | P | 7, | 12 | 14.856 (x 0.808) | 877 (0.014) | 0.9 | protein modification | CG33171 CG40410 CG5794 Caf1 Cdk4 Eip71CD PFE dap for smi35A th wbl |
| 1141 | GO:0016251 | F | 4, | 1 | 1.711 (x 0.584) | 101 (0.010) | 0.9 | general RNA polymerase II transcription factor activity | grn |
| 1142 | GO:0016021 | C | 5, 6, 7, | 13 | 15.991 (x 0.813) | 944 (0.014) | 0.9 | integral to membrane | CG14439 Fas3 Nrv1 Tl Tsp66E glec l(2)01810 mth ninA ogre pbl rost wgn |
| 1143 | GO:0005829 | C | 5, 6, 7, 8, | 2 | 3.117 (x 0.642) | 184 (0.011) | 0.901 | cytosol | RpS12 smi35A |
| 1144 | GO:0031224 | C | 4, 5, 6, | 13 | 16.042 (x 0.810) | 947 (0.014) | 0.902 | intrinsic to membrane | CG14439 Fas3 Nrv1 Tl Tsp66E glec l(2)01810 mth ninA ogre pbl rost wgn |
| 1145 | GO:0000165 | P | 7, | 1 | 1.745 (x 0.573) | 103 (0.010) | 0.903 | MAPKKK cascade | CG30440 |
| 1146 | GO:0046907 | P | 5, 6, 7, | 8 | 10.435 (x 0.767) | 616 (0.013) | 0.903 | intracellular transport | CG2852 CG33113 CG9057 Snap arr l(1)G0320 vg wbl |
| 1147 | GO:0015630 | C | 6, 7, 8, 9, | 2 | 3.151 (x 0.635) | 186 (0.011) | 0.903 | microtubule cytoskeleton | stai vg |
| 1148 | GO:0006631 | P | 6, 7, | 1 | 1.745 (x 0.573) | 103 (0.010) | 0.903 | fatty acid metabolism | CG4586 |
| 1149 | GO:0048598 | P | 4, | 1 | 1.796 (x 0.557) | 106 (0.009) | 0.911 | embryonic morphogenesis | pbl |
| 1150 | GO:0016311 | P | 7, | 1 | 1.829 (x 0.547) | 108 (0.009) | 0.916 | dephosphorylation | dap |
| 1151 | GO:0044249 | P | 5, | 11 | 14.060 (x 0.782) | 830 (0.013) | 0.916 | cellular biosynthesis | CG33171 CG6287 CG6767 ESTS:39C10S Gfat1 Pepck RpS12 Tpi l(1)G0320 mdy sgl |
| 1152 | GO:0004497 | F | 4, | 1 | 1.846 (x 0.542) | 109 (0.009) | 0.918 | monooxygenase activity | Cyp310a1 |
| 1153 | GO:0001709 | P | 5, | 1 | 1.863 (x 0.537) | 110 (0.009) | 0.918 | cell fate determination | sc |
| 1154 | GO:0031981 | C | 4, 5, 6, 7, 8, 9, 10, | 3 | 4.658 (x 0.644) | 275 (0.011) | 0.918 | nuclear lumen | Caf1 Hrb27C toe |
| 1155 | GO:0051649 | P | 5, 6, | 8 | 10.757 (x 0.744) | 635 (0.013) | 0.918 | establishment of cellular localization | CG2852 CG33113 CG9057 Snap arr l(1)G0320 vg wbl |
| 1156 | GO:0007283 | P | 6, | 1 | 1.880 (x 0.532) | 111 (0.009) | 0.919 | spermatogenesis | th |
| 1157 | GO:0051641 | P | 4, 5, | 8 | 10.774 (x 0.743) | 636 (0.013) | 0.919 | cellular localization | CG2852 CG33113 CG9057 Snap arr l(1)G0320 vg wbl |
| 1158 | GO:0009165 | P | 6, 7, | 1 | 1.863 (x 0.537) | 110 (0.009) | 0.919 | nucleotide biosynthesis | CG6767 |
| 1159 | GO:0048232 | P | 5, | 1 | 1.880 (x 0.532) | 111 (0.009) | 0.919 | male gamete generation | th |
| 1160 | GO:0006281 | P | 5, 7, | 1 | 1.863 (x 0.537) | 110 (0.009) | 0.92 | DNA repair | Caf1 |
| 1161 | GO:0016567 | P | 9, | 1 | 1.914 (x 0.522) | 113 (0.009) | 0.922 | protein ubiquitination | th |
| 1162 | GO:0006091 | P | 5, | 6 | 8.555 (x 0.701) | 505 (0.012) | 0.923 | generation of precursor metabolites and energy | Cyp310a1 GlyP Idh Pepck Tpi sgl |
| 1163 | GO:0006886 | P | 6, 7, 8, | 6 | 8.571 (x 0.700) | 506 (0.012) | 0.924 | intracellular protein transport | CG2852 CG33113 Snap arr l(1)G0320 wbl |
| 1164 | GO:0043412 | P | 6, | 12 | 15.534 (x 0.773) | 917 (0.013) | 0.924 | biopolymer modification | CG33171 CG40410 CG5794 Caf1 Cdk4 Eip71CD PFE dap for smi35A th wbl |
| 1165 | GO:0005549 | F | 3, | 1 | 1.948 (x 0.513) | 115 (0.009) | 0.925 | odorant binding | Obp99a |
| 1166 | GO:0005875 | C | 3, 5, 6, 7, 8, 9, 10, | 1 | 1.965 (x 0.509) | 116 (0.009) | 0.925 | microtubule associated complex | stai |
| 1167 | GO:0044451 | C | 5, 6, 7, 8, 9, 10, 11, 12, | 2 | 3.439 (x 0.582) | 203 (0.010) | 0.925 | nucleoplasm part | Caf1 toe |
| 1168 | GO:0008415 | F | 6, | 1 | 1.948 (x 0.513) | 115 (0.009) | 0.925 | acyltransferase activity | mdy |
| 1169 | GO:0006897 | P | 6, 7, | 1 | 1.965 (x 0.509) | 116 (0.009) | 0.925 | endocytosis | arr |
| 1170 | GO:0005261 | F | 5, 6, | 1 | 1.948 (x 0.513) | 115 (0.009) | 0.926 | cation channel activity | rpk |
| 1171 | GO:0044445 | C | 5, 6, 7, 8, 9, | 1 | 1.999 (x 0.500) | 118 (0.008) | 0.926 | cytosolic part | RpS12 |
| 1172 | GO:0015399 | F | 4, | 1 | 2.033 (x 0.492) | 120 (0.008) | 0.928 | primary active transporter activity | Nrv1 |
| 1173 | GO:0009948 | P | 5, | 1 | 2.016 (x 0.496) | 119 (0.008) | 0.928 | anterior/posterior axis specification | Hrb27C |
| 1174 | GO:0005856 | C | 5, 6, 7, 8, | 3 | 4.912 (x 0.611) | 290 (0.010) | 0.928 | cytoskeleton | Act42A stai vg |
| 1175 | GO:0016747 | F | 5, | 1 | 2.050 (x 0.488) | 121 (0.008) | 0.929 | transferase activity, transferring groups other than amino-acyl groups | mdy |
| 1176 | GO:0015405 | F | 5, | 1 | 2.033 (x 0.492) | 120 (0.008) | 0.929 | P-P-bond-hydrolysis-driven transporter activity | Nrv1 |
| 1177 | GO:0016789 | F | 5, | 1 | 2.033 (x 0.492) | 120 (0.008) | 0.93 | carboxylic ester hydrolase activity | CG4382 |
| 1178 | GO:0005200 | F | 3, | 3 | 4.946 (x 0.607) | 292 (0.010) | 0.93 | structural constituent of cytoskeleton | Act42A esn pk |
| 1179 | GO:0003779 | F | 5, | 1 | 2.084 (x 0.480) | 123 (0.008) | 0.93 | actin binding | Hsp23 |
| 1180 | GO:0015031 | P | 5, 6, | 6 | 8.758 (x 0.685) | 517 (0.012) | 0.931 | protein transport | CG2852 CG33113 Snap arr l(1)G0320 wbl |
| 1181 | GO:0006858 | P | 5, 6, | 1 | 2.084 (x 0.480) | 123 (0.008) | 0.931 | extracellular transport | l(2)01810 |
| 1182 | GO:0007059 | P | 4, | 1 | 2.101 (x 0.476) | 124 (0.008) | 0.932 | chromosome segregation | CG40410 |
| 1183 | GO:0000151 | C | 3, 4, 5, 6, | 1 | 2.134 (x 0.469) | 126 (0.008) | 0.935 | ubiquitin ligase complex | th |
| 1184 | GO:0016746 | F | 4, | 1 | 2.151 (x 0.465) | 127 (0.008) | 0.936 | transferase activity, transferring acyl groups | mdy |
| 1185 | GO:0009636 | P | 5, | 1 | 2.185 (x 0.458) | 129 (0.008) | 0.94 | response to toxin | CG17323 |
| 1186 | GO:0007186 | P | 6, | 3 | 5.167 (x 0.581) | 305 (0.010) | 0.942 | G-protein coupled receptor protein signaling pathway | CG30440 mth pbl |
| 1187 | GO:0007268 | P | 6, | 2 | 3.828 (x 0.522) | 226 (0.009) | 0.947 | synaptic transmission | Snap mth |
| 1188 | GO:0009058 | P | 4, | 11 | 15.212 (x 0.723) | 898 (0.012) | 0.952 | biosynthesis | CG33171 CG6287 CG6767 ESTS:39C10S Gfat1 Pepck RpS12 Tpi l(1)G0320 mdy sgl |
| 1189 | GO:0006512 | P | 8, | 2 | 3.964 (x 0.505) | 234 (0.009) | 0.952 | ubiquitin cycle | CG5794 th |
| 1190 | GO:0030529 | C | 3, 4, 5, 6, | 3 | 5.387 (x 0.557) | 318 (0.009) | 0.953 | ribonucleoprotein complex | CG17838 Hrb27C RpS12 |
| 1191 | GO:0016874 | F | 3, | 3 | 5.370 (x 0.559) | 317 (0.009) | 0.953 | ligase activity | CG6767 mdy th |
| 1192 | GO:0019787 | F | 6, | 1 | 2.355 (x 0.425) | 139 (0.007) | 0.954 | small conjugating protein ligase activity | th |
| 1193 | GO:0046483 | P | 5, | 1 | 2.372 (x 0.422) | 140 (0.007) | 0.954 | heterocycle metabolism | CG6767 |
| 1194 | GO:0004842 | F | 7, | 1 | 2.355 (x 0.425) | 139 (0.007) | 0.955 | ubiquitin-protein ligase activity | th |
| 1195 | GO:0008092 | F | 4, | 2 | 4.032 (x 0.496) | 238 (0.008) | 0.957 | cytoskeletal protein binding | Hsp23 stai |
| 1196 | GO:0006725 | P | 5, | 1 | 2.456 (x 0.407) | 145 (0.007) | 0.958 | aromatic compound metabolism | CG6767 |
| 1197 | GO:0043228 | C | 3, | 8 | 12.044 (x 0.664) | 711 (0.011) | 0.962 | non-membrane-bound organelle | Act42A Caf1 Dsp1 His4r RpS12 Trl stai vg |
| 1198 | GO:0043232 | C | 4, 5, 6, 7, | 8 | 12.044 (x 0.664) | 711 (0.011) | 0.963 | intracellular non-membrane-bound organelle | Act42A Caf1 Dsp1 His4r RpS12 Trl stai vg |
| 1199 | GO:0016881 | F | 5, | 1 | 2.676 (x 0.374) | 158 (0.006) | 0.973 | acid-amino acid ligase activity | th |
| 1200 | GO:0004930 | F | 5, | 2 | 4.353 (x 0.459) | 257 (0.008) | 0.974 | G-protein coupled receptor activity | mth pbl |
| 1201 | GO:0005525 | F | 6, | 1 | 2.727 (x 0.367) | 161 (0.006) | 0.975 | GTP binding | Pepck |
| 1202 | GO:0019001 | F | 5, | 1 | 2.744 (x 0.364) | 162 (0.006) | 0.975 | guanyl nucleotide binding | Pepck |
| 1203 | GO:0007606 | P | 4, 6, | 1 | 2.812 (x 0.356) | 166 (0.006) | 0.979 | sensory perception of chemical stimulus | Obp99a |
| 1204 | GO:0016788 | F | 4, | 4 | 7.708 (x 0.519) | 455 (0.009) | 0.99 | hydrolase activity, acting on ester bonds | CG4382 CG5794 br fbp |
| 1205 | GO:0005840 | C | 4, 5, 6, 7, 8, | 1 | 3.202 (x 0.312) | 189 (0.005) | 0.991 | ribosome | RpS12 |
| 1206 | GO:0003735 | F | 3, | 1 | 3.185 (x 0.314) | 188 (0.005) | 0.991 | structural constituent of ribosome | RpS12 |
| 1207 | GO:0006811 | P | 5, 6, | 4 | 7.826 (x 0.511) | 462 (0.009) | 0.992 | ion transport | CG33171 Nrv1 l(2)01810 rpk |
| 1208 | GO:0016791 | F | 6, | 1 | 3.083 (x 0.324) | 182 (0.005) | 0.992 | phosphoric monoester hydrolase activity | fbp |
| 1209 | GO:0003824 | F | 2, | 54 | 64.049 (x 0.843) | 3781 (0.014) | 0.992 | catalytic activity | Abi Argk BEST:GH02921 BcDNA:LD41548 CG16733 CG17323 CG2852 CG30427 CG31121 CG33171 CG3842 CG40410 CG4382 CG4586 CG4914 CG5390 CG5731 CG5794 CG5873 CG6287 CG6767 CG9027 Caf1 Cdk4 Cyp310a1 D19A ESTS:39C10S Eip55E Eip71CD Fkbp13 GNBP3 Gfat1 GlyP Idh Lsp2 Nep2 Nrv1 Oscillin PFE Pepck Rpn9 Sb Tpi alpha-Man-IIb br fbp for fra mdy ome sgl smi35A th tok |
| 1210 | GO:0043037 | P | 7, 8, | 1 | 3.151 (x 0.317) | 186 (0.005) | 0.992 | translation | mdy |
| 1211 | GO:0005737 | C | 4, 5, 6, | 18 | 25.155 (x 0.716) | 1485 (0.012) | 0.992 | cytoplasm | BcDNA:LD41548 CBP CG33113 CG33171 CG4586 CG9057 Gfat1 Idh ImpE2 Pepck RpS12 Snap alpha-Man-IIb l(1)G0320 mdy pk smi35A wbl |
| 1212 | GO:0006812 | P | 6, 7, | 3 | 6.488 (x 0.462) | 383 (0.008) | 0.993 | cation transport | Nrv1 l(2)01810 rpk |
| 1213 | GO:0042578 | F | 5, | 1 | 3.388 (x 0.295) | 200 (0.005) | 0.995 | phosphoric ester hydrolase activity | fbp |
| 1214 | GO:0008324 | F | 4, | 3 | 6.759 (x 0.444) | 399 (0.008) | 0.995 | cation transporter activity | Nrv1 l(2)01810 rpk |
| 1215 | GO:0015075 | F | 3, | 4 | 8.216 (x 0.487) | 485 (0.008) | 0.996 | ion transporter activity | CG14076 Nrv1 l(2)01810 rpk |
| 1216 | GO:0006397 | P | 8, | 1 | 3.354 (x 0.298) | 198 (0.005) | 0.996 | mRNA processing | Hrb27C |
| 1217 | GO:0003729 | F | 5, | 2 | 5.302 (x 0.377) | 313 (0.006) | 0.996 | mRNA binding | CG17838 Hrb27C |
| 1218 | GO:0016071 | P | 7, | 1 | 3.523 (x 0.284) | 208 (0.005) | 0.996 | mRNA metabolism | Hrb27C |
| 1219 | GO:0006412 | P | 6, 7, | 4 | 8.368 (x 0.478) | 494 (0.008) | 0.997 | protein biosynthesis | CG33171 RpS12 l(1)G0320 mdy |
| 1220 | GO:0030554 | F | 5, | 6 | 11.671 (x 0.514) | 689 (0.009) | 0.997 | adenyl nucleotide binding | CG31121 CG40410 Cdk4 for mdy smi35A |
| 1221 | GO:0005524 | F | 6, | 6 | 11.383 (x 0.527) | 672 (0.009) | 0.998 | ATP binding | CG31121 CG40410 Cdk4 for mdy smi35A |
| 1222 | GO:0031974 | C | 2, | 3 | 7.301 (x 0.411) | 431 (0.007) | 0.998 | membrane-enclosed lumen | Caf1 Hrb27C toe |
| 1223 | GO:0001584 | F | 6, | 1 | 3.608 (x 0.277) | 213 (0.005) | 0.998 | rhodopsin-like receptor activity | pbl |
| 1224 | GO:0044444 | C | 4, 5, 6, 7, | 12 | 19.599 (x 0.612) | 1157 (0.010) | 0.998 | cytoplasmic part | CBP CG33113 CG4586 CG9057 Idh Pepck RpS12 Snap alpha-Man-IIb l(1)G0320 smi35A wbl |
| 1225 | GO:0016787 | F | 3, | 21 | 30.424 (x 0.690) | 1796 (0.012) | 0.998 | hydrolase activity | BEST:GH02921 BcDNA:LD41548 CG31121 CG4382 CG4914 CG5390 CG5731 CG5794 D19A GNBP3 Lsp2 Nep2 Nrv1 Rpn9 Sb alpha-Man-IIb br fbp fra ome tok |
| 1226 | GO:0043233 | C | 3, 4, | 3 | 7.301 (x 0.411) | 431 (0.007) | 0.999 | organelle lumen | Caf1 Hrb27C toe |
| 1227 | GO:0044428 | C | 4, 5, 6, 7, 8, 9, | 4 | 8.724 (x 0.459) | 515 (0.008) | 0.999 | nuclear part | CG17838 Caf1 Hrb27C toe |
| 1228 | GO:0006118 | P | 6, | 2 | 5.641 (x 0.355) | 333 (0.006) | 0.999 | electron transport | Cyp310a1 sgl |
| 1229 | GO:0042623 | F | 9, | 2 | 5.590 (x 0.358) | 330 (0.006) | 1 | ATPase activity, coupled | CG31121 Nrv1 |
| 1230 | GO:0007600 | P | 3, 5, | 1 | 4.370 (x 0.229) | 258 (0.004) | 1 | sensory perception | Obp99a |
| 1231 | GO:0008372 | C | 2, | 3 | 13.789 (x 0.218) | 814 (0.004) | 1 | cellular component unknown | CG31125 CG31997 CG8588 |
| 1232 | GO:0016887 | F | 8, | 2 | 6.285 (x 0.318) | 371 (0.005) | 1 | ATPase activity | CG31121 Nrv1 |
| 1233 | GO:0005554 | F | 2, | 3 | 12.739 (x 0.236) | 752 (0.004) | 1 | molecular function unknown | CG31125 CG31997 CG8588 |
| 1234 | GO:0006396 | P | 7, | 1 | 4.320 (x 0.232) | 255 (0.004) | 1 | RNA processing | Hrb27C |
| 1235 | GO:0000004 | P | 2, | 3 | 11.875 (x 0.253) | 701 (0.004) | 1 | biological process unknown | CG31125 CG31997 bip1 |
| 1236 | GO:0003723 | F | 4, | 2 | 6.251 (x 0.320) | 369 (0.005) | 1 | RNA binding | CG17838 Hrb27C |
| 1237 | GO:0031090 | C | 4, 5, 6, 7, 8, | 2 | 6.115 (x 0.327) | 361 (0.006) | 1 | organelle membrane | alpha-Man-IIb l(1)G0320 |
| 1238 | GO:0016817 | F | 4, | 2 | 9.283 (x 0.215) | 548 (0.004) | 1 | hydrolase activity, acting on acid anhydrides | CG31121 Nrv1 |
| 1239 | GO:0016070 | P | 6, | 2 | 6.217 (x 0.322) | 367 (0.005) | 1 | RNA metabolism | Hrb27C mdy |
| 1240 | GO:0017076 | F | 4, | 7 | 14.382 (x 0.487) | 849 (0.008) | 1 | purine nucleotide binding | CG31121 CG40410 Cdk4 Pepck for mdy smi35A |
| 1241 | GO:0016818 | F | 5, | 2 | 9.283 (x 0.215) | 548 (0.004) | 1 | hydrolase activity, acting on acid anhydrides, in phosphorus-containing anhydrides | CG31121 Nrv1 |
| 1242 | GO:0016462 | F | 6, | 2 | 8.995 (x 0.222) | 531 (0.004) | 1 | pyrophosphatase activity | CG31121 Nrv1 |
| 1243 | GO:0017111 | F | 7, | 2 | 8.859 (x 0.226) | 523 (0.004) | 1 | nucleoside-triphosphatase activity | CG31121 Nrv1 |
| 1244 | GO:0044446 | C | 3, 4, 5, 6, 7, | 13 | 24.122 (x 0.539) | 1424 (0.009) | 1 | intracellular organelle part | Act42A CG17838 Caf1 Dsp1 His4r Hrb27C RpS12 Trl alpha-Man-IIb l(1)G0320 stai toe vg |
| 1245 | GO:0000166 | F | 3, | 7 | 14.873 (x 0.471) | 878 (0.008) | 1 | nucleotide binding | CG31121 CG40410 Cdk4 Pepck for mdy smi35A |
| 1246 | GO:0003674 | F | 1, | 132 | 140.362 (x 0.940) | 8286 (0.016) | 1 | molecular\_function | Abi Act42A Argk BEST:GH02921 BEST:LD04971 BcDNA:GH02976 BcDNA:LD41548 CBP CG10359 CG10657 CG11275 CG13848 CG14076 CG14439 CG15757 CG1607 CG16733 CG17052 CG17323 CG17838 CG17919 CG2852 CG30427 CG30440 CG31121 CG31125 CG31997 CG33113 CG33171 CG3823 CG3842 CG40410 CG4382 CG4586 CG4914 CG5390 CG5731 CG5794 CG5873 CG6287 CG6767 CG6930 CG7668 CG8502 CG8588 CG8634 CG9027 CG9057 CG9134 CREG Caf1 Cdk4 Cyp310a1 D19A Doc1 Doc2 Doc3 Dsp1 ESTS:39C10S Eip55E Eip71CD Eip75B Fkbp13 GNBP3 Gfat1 GlyP His4r HmgZ Hrb27C Hsp23 Idh Lsp2 Nep2 Nrv1 Obp99a Optix Oscillin PFE Pepck Poxn RpS12 Rpn9 Sb Sema-1b Snap Spn1 Spn43Aa Tl Tpi Trl Tsp66E Wnt2 alpha-Man-IIb arr ash2 bip1 br dap dve edl en esn fbp for fra glec grn in inv klu l(1)G0320 l(2)01810 mav mdy mnd mth ogre ome pbl pk rpk sc serpin-27A sgl smi35A stai th toe tok trn vg wgn |
| 1247 | GO:0043234 | C | 2, | 14 | 26.748 (x 0.523) | 1579 (0.009) | 1 | protein complex | CG17838 CG31121 Caf1 His4r Hrb27C Lsp2 Nrv1 RpS12 Rpn9 for l(1)G0320 stai th toe |
| 1248 | GO:0044422 | C | 2, 3, | 13 | 24.122 (x 0.539) | 1424 (0.009) | 1 | organelle part | Act42A CG17838 Caf1 Dsp1 His4r Hrb27C RpS12 Trl alpha-Man-IIb l(1)G0320 stai toe vg |
| 1249 | GO:0005739 | C | 5, 6, 7, 8, | 2 | 7.826 (x 0.256) | 462 (0.004) | 1 | mitochondrion | Idh Pepck |

  

---

Regulated Genes that don't have GO terms
  

BG:DS07721.3 BcDNA:GH11415 CG11509 CG13676 CG13698 CG14132 CG15628 CG15905 CG17032 CG18212 CG18349 CG2016 CG2083 CG2791 CG30423 CG32373 CG32541 CG3880 CG40294 CG5175 CG6234 CG7047 CG7802 CG9266 CG9416 CG9628 miple
