# Supplementary material for: Functional dissection of the ash2 and ash1 transcriptomes provides insights into the transcriptional basis of wing phenotypes and reveals conserved protein interactions
Source: Genome Biol. 2007 Apr 28;8(4):R67. doi: 10.1186/gb-2007-8-4-r67 (PMC1896016; doi:10.1186/gb-2007-8-4-r67)
Supplement: Additional data file 8 — GO annotations of the genes upregulated over 2.0-fold in ash2I1 [file gb-2007-8-4-r67-S8.html]

  

---

  

|  |  |
| --- | --- |
| Go Statistics | Reg File: **ash2I1\_U2.0x.txt.fbgns** (179 genes -- 53 skipped)  Ref File: **ref.fbgns** (13577 genes -- 4663 skipped)  Database: **go\_200507-termdb.rdf-xml** |

---

  

Fields Description

| Pos | Go Term | Ontology | Levels | Observed | Expected | Possibles | p-value(Adj) | Go term description | Genes with the GO term |
| --- | --- | --- | --- | --- | --- | --- | --- | --- | --- |
| 1 | GO:0004364 | F | 5, | 8 | 0.509 (x 15.721) | 36 (0.222) | 2.71e-05 | glutathione transferase activity | GstD3 GstD6 GstD9 GstE1 GstE3 GstE5 GstE6 GstE7 |
| 2 | GO:0042221 | P | 4, | 17 | 3.718 (x 4.573) | 263 (0.065) | 7.34e-05 | response to chemical stimulus | BEST:CK01227 BG:DS01219.1 GstD3 GstD6 GstD9 GstE1 GstE3 GstE5 GstE6 GstE7 Jheh3 Ugt86Da Ugt86Di cher rpr wun wun2 |
| 3 | GO:0009636 | P | 5, | 12 | 1.823 (x 6.581) | 129 (0.093) | 7.9e-05 | response to toxin | BEST:CK01227 GstD3 GstD6 GstD9 GstE1 GstE3 GstE5 GstE6 GstE7 Jheh3 Ugt86Da Ugt86Di |
| 4 | GO:0009628 | P | 3, | 19 | 5.258 (x 3.613) | 372 (0.051) | 0.00025 | response to abiotic stimulus | BEST:CK01227 BG:DS01219.1 GstD3 GstD6 GstD9 GstE1 GstE3 GstE5 GstE6 GstE7 Jheh3 TotA Ugt86Da Ugt86Di cher rpr sda wun wun2 |
| 5 | GO:0016765 | F | 4, | 8 | 0.848 (x 9.433) | 60 (0.133) | 0.000345 | transferase activity, transferring alkyl or aryl (other than methyl) groups | GstD3 GstD6 GstD9 GstE1 GstE3 GstE5 GstE6 GstE7 |
| 6 | GO:0000146 | F | 3, | 3 | 0.042 (x 70.746) | 3 (1.000) | 0.000385 | microfilament motor activity | Mhc Mlc1 Mlc2 |
| 7 | GO:0005859 | C | 5, 6, 7, 8, 9, 10, 11, 12, | 3 | 0.042 (x 70.746) | 3 (1.000) | 0.00045 | muscle myosin | Mhc Mlc1 Mlc2 |
| 8 | GO:0009607 | P | 3, | 21 | 7.379 (x 2.846) | 522 (0.040) | 0.00115 | response to biotic stimulus | BEST:CK01227 CG18522 CG5397 CG9460 GstD3 GstD6 GstD9 GstE1 GstE3 GstE5 GstE6 GstE7 Jheh3 TepIV Thor TotA Tsf1 Tsf3 Ugt86Da Ugt86Di os |
| 9 | GO:0044449 | C | 5, 6, 7, 8, 9, | 4 | 0.155 (x 25.726) | 11 (0.364) | 0.00126 | contractile fiber part | Mhc Mlc1 Mlc2 wupA |
| 10 | GO:0016460 | C | 4, 6, 7, 8, 9, 10, 11, | 3 | 0.057 (x 53.060) | 4 (0.750) | 0.00133 | myosin II | Mhc Mlc1 Mlc2 |
| 11 | GO:0050896 | P | 2, | 34 | 16.524 (x 2.058) | 1169 (0.029) | 0.00196 | response to stimulus | BEST:CK01227 BG:DS01219.1 CG18522 CG4917 CG5397 CG9460 GstD3 GstD6 GstD9 GstE1 GstE3 GstE5 GstE6 GstE7 Irbp Jheh3 Ku80 TepIV Thor TotA Tsf1 Tsf3 Ugt86Da Ugt86Di agt cher hay na os rad50 rpr sda wun wun2 |
| 12 | GO:0043292 | C | 5, 6, 7, 8, | 4 | 0.184 (x 21.768) | 13 (0.308) | 0.00201 | contractile fiber | Mhc Mlc1 Mlc2 wupA |
| 13 | GO:0006952 | P | 4, | 20 | 7.265 (x 2.753) | 514 (0.039) | 0.00237 | defense response | BEST:CK01227 CG18522 CG5397 CG9460 GstD3 GstD6 GstD9 GstE1 GstE3 GstE5 GstE6 GstE7 Jheh3 TepIV Thor TotA Tsf1 Ugt86Da Ugt86Di os |
| 14 | GO:0030239 | P | 7, 8, 10, 11, | 3 | 0.085 (x 35.373) | 6 (0.500) | 0.00327 | myofibril assembly | CG6803 Mhc wupA |
| 15 | GO:0000723 | P | 9, | 3 | 0.085 (x 35.373) | 6 (0.500) | 0.00349 | telomere maintenance | Irbp Ku80 rad50 |
| 16 | GO:0032200 | P | 8, | 3 | 0.085 (x 35.373) | 6 (0.500) | 0.00374 | telomere organization and biogenesis | Irbp Ku80 rad50 |
| 17 | GO:0019740 | P | 5, | 2 | 0.028 (x 70.746) | 2 (1.000) | 0.0108 | nitrogen utilization | CG12264 CG9836 |
| 18 | GO:0035233 | P | 6, 7, 8, | 2 | 0.028 (x 70.746) | 2 (1.000) | 0.0114 | germ cell repulsion | wun wun2 |
| 19 | GO:0006800 | P | 5, | 6 | 0.947 (x 6.335) | 67 (0.090) | 0.018 | oxygen and reactive oxygen species metabolism | CG18522 GstE1 GstE3 GstE5 GstE6 GstE7 |
| 20 | GO:0000726 | P | 6, 8, | 2 | 0.042 (x 47.164) | 3 (0.667) | 0.0262 | non-recombinational repair | Irbp Ku80 |
| 21 | GO:0035234 | P | 6, 7, 8, | 2 | 0.042 (x 47.164) | 3 (0.667) | 0.0274 | germ cell programmed cell death | wun wun2 |
| 22 | GO:0006303 | P | 7, 9, | 2 | 0.042 (x 47.164) | 3 (0.667) | 0.0288 | double-strand break repair via nonhomologous end joining | Irbp Ku80 |
| 23 | GO:0006826 | P | 9, 10, | 2 | 0.057 (x 35.373) | 4 (0.500) | 0.0476 | iron ion transport | Tsf1 Tsf3 |
| 24 | GO:0008083 | F | 4, 5, | 4 | 0.466 (x 8.575) | 33 (0.121) | 0.0482 | growth factor activity | CG2989 Chit Idgf2 sog |
| 25 | GO:0005381 | F | 6, | 2 | 0.071 (x 28.298) | 5 (0.400) | 0.0754 | iron ion transporter activity | Tsf1 Tsf3 |
| 26 | GO:0046916 | P | 8, | 2 | 0.099 (x 20.213) | 7 (0.286) | 0.102 | transition metal ion homeostasis | Tsf1 Tsf3 |
| 27 | GO:0006879 | P | 8, 9, | 2 | 0.099 (x 20.213) | 7 (0.286) | 0.105 | iron ion homeostasis | Tsf1 Tsf3 |
| 28 | GO:0048627 | P | 5, 6, 8, 9, | 3 | 0.311 (x 9.647) | 22 (0.136) | 0.107 | myoblast development | CG6803 Mhc wupA |
| 29 | GO:0005506 | F | 6, | 4 | 0.608 (x 6.581) | 43 (0.093) | 0.107 | iron ion binding | Las Mocs1 Tsf1 Tsf3 |
| 30 | GO:0008199 | F | 7, | 2 | 0.099 (x 20.213) | 7 (0.286) | 0.108 | ferric iron binding | Tsf1 Tsf3 |
| 31 | GO:0031327 | P | 7, | 3 | 0.311 (x 9.647) | 22 (0.136) | 0.11 | negative regulation of cellular biosynthesis | Thor apt rpr |
| 32 | GO:0004003 | F | 5, 11, | 3 | 0.297 (x 10.107) | 21 (0.143) | 0.11 | ATP-dependent DNA helicase activity | Irbp Ku80 hay |
| 33 | GO:0006935 | P | 5, 6, | 2 | 0.099 (x 20.213) | 7 (0.286) | 0.111 | chemotaxis | wun wun2 |
| 34 | GO:0009890 | P | 6, | 3 | 0.311 (x 9.647) | 22 (0.136) | 0.114 | negative regulation of biosynthesis | Thor apt rpr |
| 35 | GO:0015629 | C | 6, 7, 8, 9, | 5 | 1.046 (x 4.780) | 74 (0.068) | 0.114 | actin cytoskeleton | Act57B Mhc Mlc1 Mlc2 wupA |
| 36 | GO:0045445 | P | 5, 7, 8, | 3 | 0.325 (x 9.228) | 23 (0.130) | 0.114 | myoblast differentiation | CG6803 Mhc wupA |
| 37 | GO:0017148 | P | 7, 8, 9, | 3 | 0.297 (x 10.107) | 21 (0.143) | 0.115 | negative regulation of protein biosynthesis | Thor apt rpr |
| 38 | GO:0048628 | P | 6, 7, 9, 10, | 3 | 0.311 (x 9.647) | 22 (0.136) | 0.118 | myoblast maturation | CG6803 Mhc wupA |
| 39 | GO:0016459 | C | 3, 5, 6, 7, 8, 9, 10, | 3 | 0.353 (x 8.490) | 25 (0.120) | 0.124 | myosin | Mhc Mlc1 Mlc2 |
| 40 | GO:0007519 | P | 5, | 4 | 0.693 (x 5.775) | 49 (0.082) | 0.124 | striated muscle development | CG6803 Mhc Mlp60A wupA |
| 41 | GO:0008195 | F | 7, | 2 | 0.127 (x 15.721) | 9 (0.222) | 0.139 | phosphatidate phosphatase activity | wun wun2 |
| 42 | GO:0030016 | C | 6, 7, 8, 9, | 2 | 0.127 (x 15.721) | 9 (0.222) | 0.142 | myofibril | Mhc wupA |
| 43 | GO:0006974 | P | 4, | 6 | 1.668 (x 3.597) | 118 (0.051) | 0.143 | response to DNA damage stimulus | Irbp Ku80 agt hay rad50 rpr |
| 44 | GO:0048469 | P | 5, | 3 | 0.382 (x 7.861) | 27 (0.111) | 0.143 | cell maturation | CG6803 Mhc wupA |
| 45 | GO:0030017 | C | 6, 7, 8, 9, 10, | 2 | 0.127 (x 15.721) | 9 (0.222) | 0.145 | sarcomere | Mhc wupA |
| 46 | GO:0042692 | P | 4, | 3 | 0.382 (x 7.861) | 27 (0.111) | 0.147 | muscle cell differentiation | CG6803 Mhc wupA |
| 47 | GO:0006936 | P | 4, | 5 | 1.216 (x 4.113) | 86 (0.058) | 0.149 | muscle contraction | Mhc Mlc1 Mlc2 cher wupA |
| 48 | GO:0008026 | F | 4, 10, | 5 | 1.173 (x 4.262) | 83 (0.060) | 0.15 | ATP-dependent helicase activity | CG31755 CG5205 Irbp Ku80 hay |
| 49 | GO:0006302 | P | 6, 8, | 2 | 0.141 (x 14.149) | 10 (0.200) | 0.162 | double-strand break repair | Irbp Ku80 |
| 50 | GO:0042330 | P | 4, 5, | 2 | 0.141 (x 14.149) | 10 (0.200) | 0.165 | taxis | wun wun2 |
| 51 | GO:0035182 | C | 3, 4, 5, 6, | 1 | 0.014 (x 70.746) | 1 (1.000) | 0.187 | ring canal outer rim | cher |
| 52 | GO:0009079 | P | 8, 9, | 1 | 0.014 (x 70.746) | 1 (1.000) | 0.189 | pyruvate family amino acid biosynthesis | CG12264 |
| 53 | GO:0045947 | P | 9, 10, 11, | 1 | 0.014 (x 70.746) | 1 (1.000) | 0.192 | negative regulation of translational initiation | Thor |
| 54 | GO:0000041 | P | 8, 9, | 2 | 0.155 (x 12.863) | 11 (0.182) | 0.192 | transition metal ion transport | Tsf1 Tsf3 |
| 55 | GO:0008190 | F | 5, | 1 | 0.014 (x 70.746) | 1 (1.000) | 0.195 | eukaryotic initiation factor 4E binding | Thor |
| 56 | GO:0030511 | P | 6, 7, 10, | 1 | 0.014 (x 70.746) | 1 (1.000) | 0.197 | positive regulation of transforming growth factor beta receptor signaling pathway | sog |
| 57 | GO:0006307 | P | 6, 8, | 1 | 0.014 (x 70.746) | 1 (1.000) | 0.2 | DNA dealkylation | agt |
| 58 | GO:0015669 | P | 5, 6, | 1 | 0.014 (x 70.746) | 1 (1.000) | 0.203 | gas transport | glob1 |
| 59 | GO:0016887 | F | 8, | 11 | 5.244 (x 2.098) | 371 (0.030) | 0.205 | ATPase activity | BEST:CK01227 CG31755 CG5205 CG6512 Irbp Ku80 Mhc Mlc1 Mlc2 hay rad50 |
| 60 | GO:0008094 | F | 10, | 3 | 0.537 (x 5.585) | 38 (0.079) | 0.206 | DNA-dependent ATPase activity | Irbp Ku80 hay |
| 61 | GO:0017117 | C | 3, 5, 6, 7, 8, 9, 10, | 1 | 0.014 (x 70.746) | 1 (1.000) | 0.206 | single-stranded DNA-dependent ATP-dependent DNA helicase complex | Ku80 |
| 62 | GO:0004568 | F | 6, | 2 | 0.198 (x 10.107) | 14 (0.143) | 0.208 | chitinase activity | CG2989 Chit |
| 63 | GO:0017140 | F | 3, | 1 | 0.014 (x 70.746) | 1 (1.000) | 0.209 | lipoic acid synthase activity | Las |
| 64 | GO:0009719 | P | 3, | 6 | 1.880 (x 3.192) | 133 (0.045) | 0.212 | response to endogenous stimulus | Irbp Ku80 agt hay rad50 rpr |
| 65 | GO:0015671 | P | 6, 7, | 1 | 0.014 (x 70.746) | 1 (1.000) | 0.213 | oxygen transport | glob1 |
| 66 | GO:0006522 | P | 8, 9, | 1 | 0.014 (x 70.746) | 1 (1.000) | 0.216 | alanine metabolism | CG12264 |
| 67 | GO:0009078 | P | 7, 8, | 1 | 0.014 (x 70.746) | 1 (1.000) | 0.219 | pyruvate family amino acid metabolism | CG12264 |
| 68 | GO:0015020 | F | 6, | 3 | 0.481 (x 6.242) | 34 (0.088) | 0.221 | glucuronosyltransferase activity | Act57B Ugt86Da Ugt86Di |
| 69 | GO:0030870 | C | 3, 5, 6, 7, 8, 9, 10, | 1 | 0.014 (x 70.746) | 1 (1.000) | 0.223 | Mre11 complex | rad50 |
| 70 | GO:0008627 | P | 10, 11, | 1 | 0.028 (x 35.373) | 2 (0.500) | 0.225 | induction of apoptosis by ionic changes | rpr |
| 71 | GO:0006523 | P | 9, 10, | 1 | 0.014 (x 70.746) | 1 (1.000) | 0.227 | alanine biosynthesis | CG12264 |
| 72 | GO:0016226 | P | 7, | 1 | 0.028 (x 35.373) | 2 (0.500) | 0.227 | iron-sulfur cluster assembly | CG12264 |
| 73 | GO:0008463 | F | 6, | 1 | 0.028 (x 35.373) | 2 (0.500) | 0.229 | formylmethionine deformylase activity | CG31373 |
| 74 | GO:0007517 | P | 4, | 5 | 1.527 (x 3.275) | 108 (0.046) | 0.229 | muscle development | CG6803 Mhc Mlp60A toy wupA |
| 75 | GO:0046915 | F | 5, | 2 | 0.269 (x 7.447) | 19 (0.105) | 0.23 | transition metal ion transporter activity | Tsf1 Tsf3 |
| 76 | GO:0016992 | F | 6, | 1 | 0.014 (x 70.746) | 1 (1.000) | 0.23 | lipoate synthase activity | Las |
| 77 | GO:0045111 | C | 6, 7, 8, 9, | 1 | 0.028 (x 35.373) | 2 (0.500) | 0.231 | intermediate filament cytoskeleton | LamC |
| 78 | GO:0005882 | C | 5, 6, 7, 8, 9, 10, | 1 | 0.028 (x 35.373) | 2 (0.500) | 0.233 | intermediate filament | LamC |
| 79 | GO:0003908 | F | 7, | 1 | 0.014 (x 70.746) | 1 (1.000) | 0.234 | methylated-DNA-[protein]-cysteine S-methyltransferase activity | agt |
| 80 | GO:0004459 | F | 6, | 1 | 0.028 (x 35.373) | 2 (0.500) | 0.235 | L-lactate dehydrogenase activity | ImpL3 |
| 81 | GO:0016420 | F | 7, | 1 | 0.028 (x 35.373) | 2 (0.500) | 0.237 | malonyltransferase activity | CG7842 |
| 82 | GO:0004677 | F | 8, | 1 | 0.014 (x 70.746) | 1 (1.000) | 0.238 | DNA-dependent protein kinase activity | Irbp |
| 83 | GO:0035096 | P | 6, 7, | 1 | 0.028 (x 35.373) | 2 (0.500) | 0.239 | larval midgut cell programmed cell death | rpr |
| 84 | GO:0006310 | P | 7, | 3 | 0.580 (x 5.177) | 41 (0.073) | 0.24 | DNA recombination | Irbp Ku80 rad50 |
| 85 | GO:0005861 | C | 3, 5, 6, 7, 8, 9, 10, 11, 12, | 1 | 0.028 (x 35.373) | 2 (0.500) | 0.241 | troponin complex | wupA |
| 86 | GO:0006950 | P | 3, | 10 | 4.721 (x 2.118) | 334 (0.030) | 0.241 | response to stress | GstE1 Irbp Ku80 TepIV Thor TotA agt hay rad50 rpr |
| 87 | GO:0048637 | P | 6, | 3 | 0.594 (x 5.053) | 42 (0.071) | 0.241 | skeletal muscle development | CG6803 Mhc wupA |
| 88 | GO:0031369 | F | 4, | 1 | 0.014 (x 70.746) | 1 (1.000) | 0.243 | translation initiation factor binding | Thor |
| 89 | GO:0031163 | P | 6, | 1 | 0.028 (x 35.373) | 2 (0.500) | 0.243 | metallo-sulfur cluster assembly | CG12264 |
| 90 | GO:0006281 | P | 5, 7, | 5 | 1.555 (x 3.216) | 110 (0.045) | 0.243 | DNA repair | Irbp Ku80 agt hay rad50 |
| 91 | GO:0048741 | P | 6, 7, | 3 | 0.594 (x 5.053) | 42 (0.071) | 0.244 | skeletal muscle fiber development | CG6803 Mhc wupA |
| 92 | GO:0030241 | P | 8, 9, 11, 12, | 1 | 0.028 (x 35.373) | 2 (0.500) | 0.245 | muscle thick filament assembly | CG6803 |
| 93 | GO:0006941 | P | 5, | 1 | 0.014 (x 70.746) | 1 (1.000) | 0.247 | striated muscle contraction | Mhc |
| 94 | GO:0016799 | F | 5, | 3 | 0.594 (x 5.053) | 42 (0.071) | 0.247 | hydrolase activity, hydrolyzing N-glycosyl compounds | CG2989 Chit Idgf2 |
| 95 | GO:0016419 | F | 8, | 1 | 0.028 (x 35.373) | 2 (0.500) | 0.247 | S-malonyltransferase activity | CG7842 |
| 96 | GO:0051248 | P | 6, 7, | 3 | 0.608 (x 4.936) | 43 (0.070) | 0.248 | negative regulation of protein metabolism | Thor apt rpr |
| 97 | GO:0016818 | F | 5, | 14 | 7.746 (x 1.807) | 548 (0.026) | 0.248 | hydrolase activity, acting on acid anhydrides, in phosphorus-containing anhydrides | BEST:CK01227 CG2885 CG31063 CG31755 CG5205 CG6512 Dgp-1 Irbp Ku80 Mhc Mlc1 Mlc2 hay rad50 |
| 98 | GO:0045214 | P | 8, 9, 11, 12, | 1 | 0.028 (x 35.373) | 2 (0.500) | 0.25 | sarcomere organization | wupA |
| 99 | GO:0048747 | P | 5, | 3 | 0.594 (x 5.053) | 42 (0.071) | 0.25 | muscle fiber development | CG6803 Mhc wupA |
| 100 | GO:0004386 | F | 3, | 5 | 1.583 (x 3.158) | 112 (0.045) | 0.251 | helicase activity | CG31755 CG5205 Irbp Ku80 hay |
| 101 | GO:0016817 | F | 4, | 14 | 7.746 (x 1.807) | 548 (0.026) | 0.251 | hydrolase activity, acting on acid anhydrides | BEST:CK01227 CG2885 CG31063 CG31755 CG5205 CG6512 Dgp-1 Irbp Ku80 Mhc Mlc1 Mlc2 hay rad50 |
| 102 | GO:0005958 | C | 4, | 1 | 0.014 (x 70.746) | 1 (1.000) | 0.251 | DNA-dependent protein kinase complex | Ku80 |
| 103 | GO:0030005 | P | 7, | 2 | 0.283 (x 7.075) | 20 (0.100) | 0.252 | di-, tri-valent inorganic cation homeostasis | Tsf1 Tsf3 |
| 104 | GO:0004457 | F | 5, | 1 | 0.028 (x 35.373) | 2 (0.500) | 0.252 | lactate dehydrogenase activity | ImpL3 |
| 105 | GO:0007280 | P | 6, 7, 8, | 2 | 0.240 (x 8.323) | 17 (0.118) | 0.252 | pole cell migration | wun wun2 |
| 106 | GO:0015397 | F | 6, 7, | 1 | 0.028 (x 35.373) | 2 (0.500) | 0.254 | equilibrative nucleoside transporter, nitrobenzyl-thioinosine-insensitive activity | CG11907 |
| 107 | GO:0016478 | P | 8, 9, 10, | 2 | 0.240 (x 8.323) | 17 (0.118) | 0.255 | negative regulation of translation | Thor apt |
| 108 | GO:0019010 | F | 7, | 1 | 0.014 (x 70.746) | 1 (1.000) | 0.256 | farnesoic acid O-methyltransferase activity | CG10527 |
| 109 | GO:0004314 | F | 8, 9, | 1 | 0.028 (x 35.373) | 2 (0.500) | 0.257 | [acyl-carrier protein] S-malonyltransferase activity | CG7842 |
| 110 | GO:0005542 | F | 4, | 1 | 0.042 (x 23.582) | 3 (0.333) | 0.257 | folic acid binding | CG6574 |
| 111 | GO:0009112 | P | 6, | 4 | 1.103 (x 3.628) | 78 (0.051) | 0.259 | nucleobase metabolism | CG18522 CG2277 CG32549 CG6330 |
| 112 | GO:0008171 | F | 6, | 1 | 0.042 (x 23.582) | 3 (0.333) | 0.259 | O-methyltransferase activity | CG10527 |
| 113 | GO:0006855 | P | 6, 7, | 1 | 0.028 (x 35.373) | 2 (0.500) | 0.259 | multidrug transport | BEST:CK01227 |
| 114 | GO:0006144 | P | 7, | 3 | 0.777 (x 3.859) | 55 (0.055) | 0.26 | purine base metabolism | CG18522 CG2277 CG32549 |
| 115 | GO:0005652 | C | 5, 6, 7, 8, 9, 10, 11, | 1 | 0.042 (x 23.582) | 3 (0.333) | 0.26 | nuclear lamina | LamC |
| 116 | GO:0031033 | P | 9, | 1 | 0.028 (x 35.373) | 2 (0.500) | 0.261 | myosin filament assembly or disassembly | CG6803 |
| 117 | GO:0000273 | P | 6, 7, | 1 | 0.042 (x 23.582) | 3 (0.333) | 0.262 | lipoic acid metabolism | Las |
| 118 | GO:0004357 | F | 6, | 1 | 0.042 (x 23.582) | 3 (0.333) | 0.264 | glutamate-cysteine ligase activity | CG4917 |
| 119 | GO:0031034 | P | 7, 10, | 1 | 0.028 (x 35.373) | 2 (0.500) | 0.264 | myosin filament assembly | CG6803 |
| 120 | GO:0004857 | F | 3, | 5 | 1.795 (x 2.785) | 127 (0.039) | 0.265 | enzyme inhibitor activity | CG17124 CG7054 CG7722 CG9460 TepIV |
| 121 | GO:0046351 | P | 7, 8, | 1 | 0.042 (x 23.582) | 3 (0.333) | 0.266 | disaccharide biosynthesis | CG5177 |
| 122 | GO:0019008 | C | 4, | 1 | 0.028 (x 35.373) | 2 (0.500) | 0.267 | molybdopterin synthase complex | Mocs1 |
| 123 | GO:0042623 | F | 9, | 9 | 4.665 (x 1.929) | 330 (0.027) | 0.267 | ATPase activity, coupled | BEST:CK01227 CG31755 CG5205 Irbp Ku80 Mhc Mlc1 Mlc2 hay |
| 124 | GO:0004850 | F | 6, | 1 | 0.042 (x 23.582) | 3 (0.333) | 0.267 | uridine phosphorylase activity | CG6330 |
| 125 | GO:0003774 | F | 2, | 4 | 1.230 (x 3.253) | 87 (0.046) | 0.268 | motor activity | Act57B Mhc Mlc1 Mlc2 |
| 126 | GO:0004029 | F | 6, | 1 | 0.042 (x 23.582) | 3 (0.333) | 0.269 | aldehyde dehydrogenase (NAD) activity | Aldh |
| 127 | GO:0005523 | F | 5, | 1 | 0.028 (x 35.373) | 2 (0.500) | 0.269 | tropomyosin binding | wupA |
| 128 | GO:0015082 | F | 5, | 2 | 0.339 (x 5.896) | 24 (0.083) | 0.27 | di-, tri-valent inorganic cation transporter activity | Tsf1 Tsf3 |
| 129 | GO:0003678 | F | 4, | 3 | 0.721 (x 4.162) | 51 (0.059) | 0.27 | DNA helicase activity | Irbp Ku80 hay |
| 130 | GO:0016979 | F | 5, | 1 | 0.042 (x 23.582) | 3 (0.333) | 0.271 | lipoate-protein ligase activity | Las |
| 131 | GO:0009106 | P | 7, 8, | 1 | 0.028 (x 35.373) | 2 (0.500) | 0.272 | lipoate metabolism | Las |
| 132 | GO:0017109 | C | 3, 5, 6, 7, 8, | 1 | 0.042 (x 23.582) | 3 (0.333) | 0.273 | glutamate-cysteine ligase complex | CG4917 |
| 133 | GO:0008172 | F | 6, | 1 | 0.042 (x 23.582) | 3 (0.333) | 0.274 | S-methyltransferase activity | agt |
| 134 | GO:0006081 | P | 5, | 1 | 0.028 (x 35.373) | 2 (0.500) | 0.275 | aldehyde metabolism | CG10638 |
| 135 | GO:0035183 | C | 3, 4, 5, 6, | 1 | 0.042 (x 23.582) | 3 (0.333) | 0.276 | ring canal inner rim | cher |
| 136 | GO:0009105 | P | 7, 8, | 1 | 0.028 (x 35.373) | 2 (0.500) | 0.277 | lipoic acid biosynthesis | Las |
| 137 | GO:0008518 | F | 6, | 1 | 0.042 (x 23.582) | 3 (0.333) | 0.278 | reduced folate carrier activity | CG6574 |
| 138 | GO:0043451 | P | 6, | 1 | 0.057 (x 17.687) | 4 (0.250) | 0.279 | alkene catabolism | Jheh3 |
| 139 | GO:0005863 | C | 6, 7, 8, 9, 10, 11, 12, | 1 | 0.042 (x 23.582) | 3 (0.333) | 0.28 | striated muscle thick filament | Mhc |
| 140 | GO:0042586 | F | 6, | 1 | 0.028 (x 35.373) | 2 (0.500) | 0.28 | peptide deformylase activity | CG31373 |
| 141 | GO:0004217 | F | 7, | 1 | 0.057 (x 17.687) | 4 (0.250) | 0.281 | cathepsin L activity | CG6357 |
| 142 | GO:0004463 | F | 6, | 1 | 0.057 (x 17.687) | 4 (0.250) | 0.282 | leukotriene-A4 hydrolase activity | Jheh3 |
| 143 | GO:0004805 | F | 8, | 1 | 0.042 (x 23.582) | 3 (0.333) | 0.282 | trehalose-phosphatase activity | CG5177 |
| 144 | GO:0003989 | F | 6, | 1 | 0.028 (x 35.373) | 2 (0.500) | 0.283 | acetyl-CoA carboxylase activity | CG17108 |
| 145 | GO:0016107 | P | 7, 9, 10, 11, | 1 | 0.057 (x 17.687) | 4 (0.250) | 0.283 | sesquiterpenoid catabolism | Jheh3 |
| 146 | GO:0004301 | F | 7, | 1 | 0.042 (x 23.582) | 3 (0.333) | 0.284 | epoxide hydrolase activity | Jheh3 |
| 147 | GO:0016787 | F | 3, | 34 | 25.387 (x 1.339) | 1796 (0.019) | 0.285 | hydrolase activity | BEST:CK01227 BG:DS01068.5 CG1299 CG14935 CG2277 CG2885 CG2989 CG31063 CG31373 CG31755 CG32549 CG4267 CG5162 CG5177 CG5205 CG5397 CG6357 CG6512 CG9372 Chit Dgp-1 Idgf2 Irbp Jheh3 Ku80 Mhc Mlc1 Mlc2 hay huntingtin rad50 sda wun wun2 |
| 148 | GO:0008363 | P | 6, 8, 10, | 1 | 0.057 (x 17.687) | 4 (0.250) | 0.285 | larval cuticle biosynthesis (sensu Insecta) | Lcp65Ag1 |
| 149 | GO:0008096 | F | 8, | 1 | 0.042 (x 23.582) | 3 (0.333) | 0.286 | juvenile hormone epoxide hydrolase activity | Jheh3 |
| 150 | GO:0009107 | P | 8, 9, | 1 | 0.028 (x 35.373) | 2 (0.500) | 0.286 | lipoate biosynthesis | Las |
| 151 | GO:0006032 | P | 8, 9, 10, 11, | 1 | 0.057 (x 17.687) | 4 (0.250) | 0.286 | chitin catabolism | Chit |
| 152 | GO:0006873 | P | 5, | 2 | 0.311 (x 6.431) | 22 (0.091) | 0.286 | cell ion homeostasis | Tsf1 Tsf3 |
| 153 | GO:0016065 | P | 6, 7, | 3 | 0.749 (x 4.004) | 53 (0.057) | 0.287 | humoral defense mechanism (sensu Protostomia) | TepIV Thor TotA |
| 154 | GO:0008302 | P | 7, 10, | 1 | 0.042 (x 23.582) | 3 (0.333) | 0.288 | ring canal formation, actin assembly | cher |
| 155 | GO:0051763 | P | 8, | 1 | 0.057 (x 17.687) | 4 (0.250) | 0.288 | sesquiterpene catabolism | Jheh3 |
| 156 | GO:0046483 | P | 5, | 5 | 1.979 (x 2.527) | 140 (0.036) | 0.289 | heterocycle metabolism | CG18522 CG2277 CG32549 CG6330 Las |
| 157 | GO:0030003 | P | 6, | 2 | 0.311 (x 6.431) | 22 (0.091) | 0.289 | cation homeostasis | Tsf1 Tsf3 |
| 158 | GO:0007084 | P | 6, 8, 9, | 1 | 0.028 (x 35.373) | 2 (0.500) | 0.289 | mitotic nuclear envelope reassembly | LamC |
| 159 | GO:0006719 | P | 7, 8, 10, 11, 12, | 1 | 0.057 (x 17.687) | 4 (0.250) | 0.29 | juvenile hormone catabolism | Jheh3 |
| 160 | GO:0015395 | F | 5, 6, | 1 | 0.042 (x 23.582) | 3 (0.333) | 0.29 | nucleoside transporter activity, down a concentration gradient | CG11907 |
| 161 | GO:0006875 | P | 7, | 2 | 0.311 (x 6.431) | 22 (0.091) | 0.291 | metal ion homeostasis | Tsf1 Tsf3 |
| 162 | GO:0006036 | P | 9, 10, 11, 12, | 1 | 0.057 (x 17.687) | 4 (0.250) | 0.291 | cuticle chitin catabolism | Chit |
| 163 | GO:0005638 | C | 5, 6, 7, 8, 9, 10, 11, 12, 13, 14, | 1 | 0.028 (x 35.373) | 2 (0.500) | 0.292 | lamin filament | LamC |
| 164 | GO:0035069 | P | 6, | 1 | 0.042 (x 23.582) | 3 (0.333) | 0.292 | larval midgut histolysis | rpr |
| 165 | GO:0030512 | P | 6, 7, 10, | 1 | 0.057 (x 17.687) | 4 (0.250) | 0.293 | negative regulation of transforming growth factor beta receptor signaling pathway | sog |
| 166 | GO:0031672 | C | 6, 7, 8, 9, 10, 11, | 1 | 0.042 (x 23.582) | 3 (0.333) | 0.294 | A band | Mhc |
| 167 | GO:0000272 | P | 7, | 1 | 0.057 (x 17.687) | 4 (0.250) | 0.294 | polysaccharide catabolism | Chit |
| 168 | GO:0004123 | F | 5, | 1 | 0.028 (x 35.373) | 2 (0.500) | 0.295 | cystathionine gamma-lyase activity | CG12264 |
| 169 | GO:0046348 | P | 7, 8, | 1 | 0.057 (x 17.687) | 4 (0.250) | 0.296 | amino sugar catabolism | Chit |
| 170 | GO:0005992 | P | 8, 9, | 1 | 0.042 (x 23.582) | 3 (0.333) | 0.296 | trehalose biosynthesis | CG5177 |
| 171 | GO:0008300 | P | 7, 8, | 1 | 0.057 (x 17.687) | 4 (0.250) | 0.297 | isoprenoid catabolism | Jheh3 |
| 172 | GO:0006030 | P | 7, 8, 9, 10, | 3 | 0.891 (x 3.369) | 63 (0.048) | 0.298 | chitin metabolism | CG2989 Chit Gasp |
| 173 | GO:0017116 | F | 6, 12, | 1 | 0.042 (x 23.582) | 3 (0.333) | 0.298 | single-stranded DNA-dependent ATP-dependent DNA helicase activity | Ku80 |
| 174 | GO:0006046 | P | 9, 10, | 1 | 0.057 (x 17.687) | 4 (0.250) | 0.299 | N-acetylglucosamine catabolism | Chit |
| 175 | GO:0043142 | F | 11, | 1 | 0.042 (x 23.582) | 3 (0.333) | 0.301 | single-stranded DNA-dependent ATPase activity | Ku80 |
| 176 | GO:0006043 | P | 8, 9, | 1 | 0.057 (x 17.687) | 4 (0.250) | 0.301 | glucosamine catabolism | Chit |
| 177 | GO:0003824 | F | 2, | 63 | 53.445 (x 1.179) | 3781 (0.017) | 0.302 | catalytic activity | Act57B Aldh BEST:CK01227 BG:DS01068.5 CAH2 CG10527 CG10638 CG12224 CG12264 CG1299 CG14935 CG17108 CG18522 CG18547 CG2065 CG2277 CG2885 CG2989 CG3008 CG31063 CG31373 CG31755 CG32549 CG4267 CG4917 CG5162 CG5177 CG5205 CG5397 CG6330 CG6357 CG6512 CG7842 CG9372 Chit Dgp-1 GstD3 GstD6 GstD9 GstE1 GstE3 GstE5 GstE6 GstE7 Idgf2 ImpL3 Irbp Jheh3 Ku80 Las Mhc Mlc1 Mlc2 Mocs1 Ugt86Da Ugt86Di agt hay huntingtin rad50 sda wun wun2 |
| 178 | GO:0016097 | P | 8, 9, | 1 | 0.057 (x 17.687) | 4 (0.250) | 0.302 | polyisoprenoid catabolism | Jheh3 |
| 179 | GO:0008624 | P | 9, 10, | 1 | 0.042 (x 23.582) | 3 (0.333) | 0.303 | induction of apoptosis by extracellular signals | rpr |
| 180 | GO:0006961 | P | 7, 8, 9, | 2 | 0.325 (x 6.152) | 23 (0.087) | 0.304 | antibacterial humoral response (sensu Protostomia) | TepIV Thor |
| 181 | GO:0046247 | P | 7, | 1 | 0.057 (x 17.687) | 4 (0.250) | 0.304 | terpene catabolism | Jheh3 |
| 182 | GO:0004772 | F | 8, | 1 | 0.042 (x 23.582) | 3 (0.333) | 0.305 | sterol O-acyltransferase activity | CG5397 |
| 183 | GO:0016421 | F | 5, | 1 | 0.057 (x 17.687) | 4 (0.250) | 0.306 | CoA carboxylase activity | CG17108 |
| 184 | GO:0016740 | F | 3, | 20 | 13.669 (x 1.463) | 967 (0.021) | 0.307 | transferase activity | Act57B CG10527 CG12264 CG3008 CG5397 CG6330 CG7842 GstD3 GstD6 GstD9 GstE1 GstE3 GstE5 GstE6 GstE7 Irbp Las Ugt86Da Ugt86Di agt |
| 185 | GO:0016115 | P | 6, 8, 9, 10, | 1 | 0.057 (x 17.687) | 4 (0.250) | 0.308 | terpenoid catabolism | Jheh3 |
| 186 | GO:0016282 | C | 3, 5, 6, 7, 8, | 3 | 0.848 (x 3.537) | 60 (0.050) | 0.309 | eukaryotic 43S preinitiation complex | CG33158 CG7014 RpS4 |
| 187 | GO:0031468 | P | 7, 8, | 1 | 0.057 (x 17.687) | 4 (0.250) | 0.309 | nuclear envelope reassembly | LamC |
| 188 | GO:0042337 | P | 7, 9, | 1 | 0.071 (x 14.149) | 5 (0.200) | 0.31 | cuticle biosynthesis during molting (sensu Insecta) | Lcp65Ag1 |
| 189 | GO:0051189 | P | 5, 7, | 3 | 0.848 (x 3.537) | 60 (0.050) | 0.311 | prosthetic group metabolism | CG10444 CG6574 Las |
| 190 | GO:0007610 | P | 3, | 6 | 2.700 (x 2.222) | 191 (0.031) | 0.311 | behavior | BG:DS01219.1 cher na sda wun wun2 |
| 191 | GO:0005991 | P | 8, | 1 | 0.071 (x 14.149) | 5 (0.200) | 0.311 | trehalose metabolism | CG5177 |
| 192 | GO:0016803 | F | 5, | 1 | 0.057 (x 17.687) | 4 (0.250) | 0.311 | ether hydrolase activity | Jheh3 |
| 193 | GO:0009008 | F | 6, | 1 | 0.071 (x 14.149) | 5 (0.200) | 0.313 | DNA-methyltransferase activity | agt |
| 194 | GO:0006034 | P | 8, 9, 10, 11, | 1 | 0.057 (x 17.687) | 4 (0.250) | 0.313 | cuticle chitin metabolism | Chit |
| 195 | GO:0035270 | P | 4, | 1 | 0.071 (x 14.149) | 5 (0.200) | 0.314 | endocrine system development | sog |
| 196 | GO:0044247 | P | 7, 8, | 1 | 0.057 (x 17.687) | 4 (0.250) | 0.315 | cellular polysaccharide catabolism | Chit |
| 197 | GO:0006725 | P | 5, | 5 | 2.050 (x 2.440) | 145 (0.034) | 0.315 | aromatic compound metabolism | CG18522 CG2277 CG32549 CG6330 Mocs1 |
| 198 | GO:0005865 | C | 5, 6, 7, 8, 9, 10, 11, | 1 | 0.071 (x 14.149) | 5 (0.200) | 0.316 | striated muscle thin filament | wupA |
| 199 | GO:0002168 | P | 5, | 2 | 0.382 (x 5.240) | 27 (0.074) | 0.316 | larval development (sensu Insecta) | Lcp65Ag1 rad50 |
| 200 | GO:0042447 | P | 6, | 1 | 0.071 (x 14.149) | 5 (0.200) | 0.317 | hormone catabolism | Jheh3 |
| 201 | GO:0050801 | P | 4, | 2 | 0.382 (x 5.240) | 27 (0.074) | 0.318 | ion homeostasis | Tsf1 Tsf3 |
| 202 | GO:0007540 | P | 6, | 1 | 0.071 (x 14.149) | 5 (0.200) | 0.319 | sex determination, establishment of X:A ratio | os |
| 203 | GO:0007319 | P | 9, 10, 11, | 1 | 0.071 (x 14.149) | 5 (0.200) | 0.32 | negative regulation of oskar mRNA translation | apt |
| 204 | GO:0006998 | P | 6, 7, | 1 | 0.071 (x 14.149) | 5 (0.200) | 0.322 | nuclear membrane organization and biogenesis | LamC |
| 205 | GO:0016462 | F | 6, | 12 | 7.506 (x 1.599) | 531 (0.023) | 0.322 | pyrophosphatase activity | BEST:CK01227 CG2885 CG31755 CG5205 CG6512 Irbp Ku80 Mhc Mlc1 Mlc2 hay rad50 |
| 206 | GO:0016798 | F | 4, | 4 | 1.512 (x 2.645) | 107 (0.037) | 0.323 | hydrolase activity, acting on glycosyl bonds | CG14935 CG2989 Chit Idgf2 |
| 207 | GO:0035271 | P | 5, | 1 | 0.071 (x 14.149) | 5 (0.200) | 0.323 | ring gland development | sog |
| 208 | GO:0045792 | P | 6, 7, | 1 | 0.071 (x 14.149) | 5 (0.200) | 0.325 | negative regulation of cell size | Thor |
| 209 | GO:0017111 | F | 7, | 12 | 7.393 (x 1.623) | 523 (0.023) | 0.326 | nucleoside-triphosphatase activity | BEST:CK01227 CG2885 CG31755 CG5205 CG6512 Irbp Ku80 Mhc Mlc1 Mlc2 hay rad50 |
| 210 | GO:0016417 | F | 7, | 1 | 0.071 (x 14.149) | 5 (0.200) | 0.326 | S-acyltransferase activity | CG7842 |
| 211 | GO:0016885 | F | 4, | 1 | 0.071 (x 14.149) | 5 (0.200) | 0.328 | ligase activity, forming carbon-carbon bonds | CG17108 |
| 212 | GO:0042336 | P | 6, 8, | 1 | 0.071 (x 14.149) | 5 (0.200) | 0.329 | cuticle biosynthesis during molting (sensu Protostomia and Nematoda) | Lcp65Ag1 |
| 213 | GO:0007378 | P | 8, | 1 | 0.071 (x 14.149) | 5 (0.200) | 0.331 | amnioserosa formation | sog |
| 214 | GO:0007638 | P | 4, 5, | 1 | 0.071 (x 14.149) | 5 (0.200) | 0.333 | mechanosensory behavior | sda |
| 215 | GO:0046873 | F | 4, | 2 | 0.452 (x 4.422) | 32 (0.062) | 0.334 | metal ion transporter activity | Tsf1 Tsf3 |
| 216 | GO:0008253 | F | 8, | 1 | 0.071 (x 14.149) | 5 (0.200) | 0.334 | 5'-nucleotidase activity | CG32549 |
| 217 | GO:0005850 | C | 3, 4, 5, 6, 7, 8, 9, | 1 | 0.071 (x 14.149) | 5 (0.200) | 0.336 | eukaryotic translation initiation factor 2 complex | CG33158 |
| 218 | GO:0004032 | F | 7, | 1 | 0.085 (x 11.791) | 6 (0.167) | 0.337 | aldehyde reductase activity | CG10638 |
| 219 | GO:0005637 | C | 5, 6, 7, 8, 9, 10, 11, 12, 13, | 1 | 0.071 (x 14.149) | 5 (0.200) | 0.338 | nuclear inner membrane | LamC |
| 220 | GO:0006777 | P | 8, 9, | 1 | 0.085 (x 11.791) | 6 (0.167) | 0.338 | Mo-molybdopterin cofactor biosynthesis | Mocs1 |
| 221 | GO:0007626 | P | 4, | 3 | 0.989 (x 3.032) | 70 (0.043) | 0.339 | locomotory behavior | na wun wun2 |
| 222 | GO:0005337 | F | 4, | 1 | 0.085 (x 11.791) | 6 (0.167) | 0.339 | nucleoside transporter activity | CG11907 |
| 223 | GO:0008084 | F | 5, 6, | 1 | 0.071 (x 14.149) | 5 (0.200) | 0.339 | imaginal disc growth factor activity | Idgf2 |
| 224 | GO:0030510 | P | 5, 6, 9, | 1 | 0.085 (x 11.791) | 6 (0.167) | 0.341 | regulation of BMP signaling pathway | sog |
| 225 | GO:0005984 | P | 7, | 1 | 0.085 (x 11.791) | 6 (0.167) | 0.342 | disaccharide metabolism | CG5177 |
| 226 | GO:0019720 | P | 8, | 1 | 0.085 (x 11.791) | 6 (0.167) | 0.344 | Mo-molybdopterin cofactor metabolism | Mocs1 |
| 227 | GO:0015293 | F | 6, | 3 | 1.032 (x 2.907) | 73 (0.041) | 0.344 | symporter activity | BG:DS03431.1 CG10444 CG3036 |
| 228 | GO:0030708 | P | 7, 8, 9, | 1 | 0.085 (x 11.791) | 6 (0.167) | 0.345 | female germ-line cyst encapsulation (sensu Insecta) | cher |
| 229 | GO:0006044 | P | 8, 9, | 3 | 1.004 (x 2.989) | 71 (0.042) | 0.346 | N-acetylglucosamine metabolism | CG2989 Chit Gasp |
| 230 | GO:0005976 | P | 6, | 5 | 2.290 (x 2.184) | 162 (0.031) | 0.346 | polysaccharide metabolism | CG2989 Chit Gasp Ugt86Da Ugt86Di |
| 231 | GO:0019203 | F | 7, | 1 | 0.085 (x 11.791) | 6 (0.167) | 0.347 | carbohydrate phosphatase activity | CG5177 |
| 232 | GO:0006041 | P | 7, 8, | 3 | 1.004 (x 2.989) | 71 (0.042) | 0.348 | glucosamine metabolism | CG2989 Chit Gasp |
| 233 | GO:0016846 | F | 4, | 1 | 0.085 (x 11.791) | 6 (0.167) | 0.348 | carbon-sulfur lyase activity | CG12264 |
| 234 | GO:0008354 | P | 5, 6, 7, | 2 | 0.466 (x 4.288) | 33 (0.061) | 0.349 | germ cell migration | wun wun2 |
| 235 | GO:0043545 | P | 7, | 1 | 0.085 (x 11.791) | 6 (0.167) | 0.35 | molybdopterin cofactor metabolism | Mocs1 |
| 236 | GO:0044264 | P | 6, 7, | 3 | 1.088 (x 2.756) | 77 (0.039) | 0.35 | cellular polysaccharide metabolism | CG2989 Chit Gasp |
| 237 | GO:0048139 | P | 6, 7, | 1 | 0.099 (x 10.107) | 7 (0.143) | 0.35 | female germ-line cyst encapsulation | cher |
| 238 | GO:0005576 | C | 2, | 9 | 5.456 (x 1.650) | 386 (0.023) | 0.351 | extracellular region | CG2989 Chit Gasp Idgf2 Lcp65Ag1 Tsf1 Tsf3 cher os |
| 239 | GO:0015291 | F | 5, | 6 | 3.067 (x 1.956) | 217 (0.028) | 0.351 | porter activity | BG:DS03431.1 CG10444 CG11907 CG3036 CG6574 TepIV |
| 240 | GO:0017015 | P | 5, 6, 9, | 1 | 0.085 (x 11.791) | 6 (0.167) | 0.351 | regulation of transforming growth factor beta receptor signaling pathway | sog |
| 241 | GO:0006858 | P | 5, 6, | 4 | 1.739 (x 2.301) | 123 (0.033) | 0.351 | extracellular transport | BEST:CK01227 BG:DS03431.1 CG10444 CG3036 |
| 242 | GO:0035193 | P | 5, 6, | 1 | 0.099 (x 10.107) | 7 (0.143) | 0.351 | central nervous system remodeling (sensu Insecta) | rpr |
| 243 | GO:0015290 | F | 4, | 6 | 3.067 (x 1.956) | 217 (0.028) | 0.353 | electrochemical potential-driven transporter activity | BG:DS03431.1 CG10444 CG11907 CG3036 CG6574 TepIV |
| 244 | GO:0048103 | P | 6, | 1 | 0.099 (x 10.107) | 7 (0.143) | 0.353 | somatic stem cell division | os |
| 245 | GO:0008523 | F | 7, 9, | 1 | 0.085 (x 11.791) | 6 (0.167) | 0.353 | sodium-dependent multivitamin transporter activity | CG10444 |
| 246 | GO:0009617 | P | 5, | 3 | 1.046 (x 2.868) | 74 (0.041) | 0.354 | response to bacterium | TepIV Thor Tsf3 |
| 247 | GO:0007305 | P | 9, 10, | 1 | 0.099 (x 10.107) | 7 (0.143) | 0.354 | vitelline membrane formation (sensu Insecta) | Cp36 |
| 248 | GO:0006040 | P | 6, 7, | 3 | 1.018 (x 2.948) | 72 (0.042) | 0.354 | amino sugar metabolism | CG2989 Chit Gasp |
| 249 | GO:0045178 | C | 3, 4, | 1 | 0.085 (x 11.791) | 6 (0.167) | 0.354 | basal part of cell | BG:DS01219.1 |
| 250 | GO:0016801 | F | 4, | 1 | 0.099 (x 10.107) | 7 (0.143) | 0.356 | hydrolase activity, acting on ether bonds | Jheh3 |
| 251 | GO:0006959 | P | 5, 6, | 3 | 1.103 (x 2.721) | 78 (0.038) | 0.356 | humoral immune response | TepIV Thor TotA |
| 252 | GO:0016202 | P | 6, | 1 | 0.099 (x 10.107) | 7 (0.143) | 0.357 | regulation of striated muscle development | Mlp60A |
| 253 | GO:0048138 | P | 5, 6, | 1 | 0.099 (x 10.107) | 7 (0.143) | 0.358 | germ-line cyst encapsulation | cher |
| 254 | GO:0004312 | F | 7, | 1 | 0.099 (x 10.107) | 7 (0.143) | 0.36 | fatty-acid synthase activity | CG7842 |
| 255 | GO:0006417 | P | 6, 7, 8, | 3 | 1.060 (x 2.830) | 75 (0.040) | 0.361 | regulation of protein biosynthesis | Thor apt rpr |
| 256 | GO:0050654 | P | 6, 8, | 1 | 0.099 (x 10.107) | 7 (0.143) | 0.361 | chondroitin sulfate proteoglycan metabolism | Act57B |
| 257 | GO:0006716 | P | 6, 7, 9, 10, 11, | 1 | 0.099 (x 10.107) | 7 (0.143) | 0.362 | juvenile hormone metabolism | Jheh3 |
| 258 | GO:0009889 | P | 5, | 3 | 1.117 (x 2.687) | 79 (0.038) | 0.363 | regulation of biosynthesis | Thor apt rpr |
| 259 | GO:0019731 | P | 6, 7, 8, | 2 | 0.537 (x 3.723) | 38 (0.053) | 0.363 | antibacterial humoral response | TepIV Thor |
| 260 | GO:0015932 | F | 3, | 1 | 0.099 (x 10.107) | 7 (0.143) | 0.364 | nucleobase, nucleoside, nucleotide and nucleic acid transporter activity | CG11907 |
| 261 | GO:0031326 | P | 6, | 3 | 1.117 (x 2.687) | 79 (0.038) | 0.364 | regulation of cellular biosynthesis | Thor apt rpr |
| 262 | GO:0004864 | F | 5, 6, | 1 | 0.099 (x 10.107) | 7 (0.143) | 0.365 | protein phosphatase inhibitor activity | CG17124 |
| 263 | GO:0030206 | P | 8, 9, 10, | 1 | 0.099 (x 10.107) | 7 (0.143) | 0.367 | chondroitin sulfate biosynthesis | Act57B |
| 264 | GO:0042600 | C | 4, 5, | 1 | 0.099 (x 10.107) | 7 (0.143) | 0.368 | chorion | Cp36 |
| 265 | GO:0045735 | F | 2, | 1 | 0.113 (x 8.843) | 8 (0.125) | 0.37 | nutrient reservoir activity | Idgf2 |
| 266 | GO:0051761 | P | 7, | 1 | 0.099 (x 10.107) | 7 (0.143) | 0.37 | sesquiterpene metabolism | Jheh3 |
| 267 | GO:0009886 | P | 4, | 1 | 0.113 (x 8.843) | 8 (0.125) | 0.371 | post-embryonic morphogenesis | rpr |
| 268 | GO:0008088 | P | 8, 9, 10, | 1 | 0.099 (x 10.107) | 7 (0.143) | 0.371 | axon cargo transport | huntingtin |
| 269 | GO:0008252 | F | 7, | 1 | 0.113 (x 8.843) | 8 (0.125) | 0.372 | nucleotidase activity | CG32549 |
| 270 | GO:0016283 | C | 3, 5, 6, 7, 8, | 2 | 0.551 (x 3.628) | 39 (0.051) | 0.373 | eukaryotic 48S initiation complex | CG7014 RpS4 |
| 271 | GO:0016015 | F | 3, | 1 | 0.099 (x 10.107) | 7 (0.143) | 0.373 | morphogen activity | os |
| 272 | GO:0043449 | P | 5, | 1 | 0.113 (x 8.843) | 8 (0.125) | 0.373 | alkene metabolism | Jheh3 |
| 273 | GO:0015674 | P | 7, 8, | 2 | 0.565 (x 3.537) | 40 (0.050) | 0.374 | di-, tri-valent inorganic cation transport | Tsf1 Tsf3 |
| 274 | GO:0008355 | P | 6, 7, | 2 | 0.551 (x 3.628) | 39 (0.051) | 0.374 | olfactory learning | BG:DS01219.1 cher |
| 275 | GO:0006714 | P | 6, 8, 9, 10, | 1 | 0.099 (x 10.107) | 7 (0.143) | 0.374 | sesquiterpenoid metabolism | Jheh3 |
| 276 | GO:0008429 | F | 5, | 1 | 0.113 (x 8.843) | 8 (0.125) | 0.375 | phosphatidylethanolamine binding | CG7054 |
| 277 | GO:0005843 | C | 4, 5, 6, 7, 8, 9, 10, 11, | 2 | 0.551 (x 3.628) | 39 (0.051) | 0.375 | cytosolic small ribosomal subunit (sensu Eukaryota) | CG7014 RpS4 |
| 278 | GO:0050650 | P | 7, 8, 9, | 1 | 0.099 (x 10.107) | 7 (0.143) | 0.376 | chondroitin sulfate proteoglycan biosynthesis | Act57B |
| 279 | GO:0016096 | P | 7, 8, | 1 | 0.113 (x 8.843) | 8 (0.125) | 0.376 | polyisoprenoid metabolism | Jheh3 |
| 280 | GO:0004033 | F | 6, | 1 | 0.099 (x 10.107) | 7 (0.143) | 0.377 | aldo-keto reductase activity | CG10638 |
| 281 | GO:0042214 | P | 6, | 1 | 0.113 (x 8.843) | 8 (0.125) | 0.377 | terpene metabolism | Jheh3 |
| 282 | GO:0030204 | P | 7, 8, 9, | 1 | 0.099 (x 10.107) | 7 (0.143) | 0.379 | chondroitin sulfate metabolism | Act57B |
| 283 | GO:0030312 | C | 3, 4, | 1 | 0.113 (x 8.843) | 8 (0.125) | 0.379 | external encapsulating structure | Cp36 |
| 284 | GO:0005344 | F | 3, | 1 | 0.113 (x 8.843) | 8 (0.125) | 0.38 | oxygen transporter activity | glob1 |
| 285 | GO:0030704 | P | 8, | 1 | 0.099 (x 10.107) | 7 (0.143) | 0.38 | vitelline membrane formation | Cp36 |
| 286 | GO:0006721 | P | 5, 7, 8, 9, | 1 | 0.113 (x 8.843) | 8 (0.125) | 0.382 | terpenoid metabolism | Jheh3 |
| 287 | GO:0051707 | P | 4, | 4 | 1.852 (x 2.160) | 131 (0.031) | 0.39 | response to other organism | TepIV Thor TotA Tsf3 |
| 288 | GO:0016791 | F | 6, | 5 | 2.573 (x 1.944) | 182 (0.027) | 0.391 | phosphoric monoester hydrolase activity | CG2277 CG32549 CG5177 wun wun2 |
| 289 | GO:0005682 | C | 5, 6, 7, 8, 9, 10, 11, 12, | 1 | 0.127 (x 7.861) | 9 (0.111) | 0.392 | snRNP U5 | CG5205 |
| 290 | GO:0008514 | F | 5, | 1 | 0.127 (x 7.861) | 9 (0.111) | 0.394 | organic anion transporter activity | BEST:CK01227 |
| 291 | GO:0019212 | F | 4, 5, | 1 | 0.127 (x 7.861) | 9 (0.111) | 0.395 | phosphatase inhibitor activity | CG17124 |
| 292 | GO:0030414 | F | 4, | 3 | 1.216 (x 2.468) | 86 (0.035) | 0.396 | protease inhibitor activity | CG7722 CG9460 TepIV |
| 293 | GO:0030201 | P | 6, 8, | 1 | 0.127 (x 7.861) | 9 (0.111) | 0.396 | heparan sulfate proteoglycan metabolism | Act57B |
| 294 | GO:0005213 | F | 3, | 1 | 0.127 (x 7.861) | 9 (0.111) | 0.397 | structural constituent of chorion (sensu Insecta) | Cp36 |
| 295 | GO:0035072 | P | 7, 8, 9, 10, 11, | 1 | 0.127 (x 7.861) | 9 (0.111) | 0.399 | ecdysone-mediated induction of salivary gland cell autophagic cell death | rpr |
| 296 | GO:0007612 | P | 5, | 2 | 0.608 (x 3.291) | 43 (0.047) | 0.399 | learning | BG:DS01219.1 cher |
| 297 | GO:0015012 | P | 7, 8, 9, | 1 | 0.127 (x 7.861) | 9 (0.111) | 0.4 | heparan sulfate proteoglycan biosynthesis | Act57B |
| 298 | GO:0044430 | C | 4, 5, 6, 7, 8, 9, | 6 | 3.392 (x 1.769) | 240 (0.025) | 0.401 | cytoskeletal part | Act57B LamC Mhc Mlc1 Mlc2 wupA |
| 299 | GO:0005675 | C | 4, 7, 8, 9, 10, 11, 12, 13, 14, | 1 | 0.127 (x 7.861) | 9 (0.111) | 0.402 | transcription factor TFIIH complex | hay |
| 300 | GO:0004866 | F | 5, | 3 | 1.201 (x 2.497) | 85 (0.035) | 0.402 | endopeptidase inhibitor activity | CG7722 CG9460 TepIV |
| 301 | GO:0009059 | P | 5, 6, | 11 | 7.379 (x 1.491) | 522 (0.021) | 0.403 | macromolecule biosynthesis | Act57B CG31373 CG33158 CG5177 CG7014 Dgp-1 RpS4 Thor apt na rpr |
| 302 | GO:0009166 | P | 6, 7, | 1 | 0.127 (x 7.861) | 9 (0.111) | 0.403 | nucleotide catabolism | CG6330 |
| 303 | GO:0007301 | P | 6, 9, | 1 | 0.127 (x 7.861) | 9 (0.111) | 0.404 | ovarian ring canal formation | cher |
| 304 | GO:0006023 | P | 7, 8, | 1 | 0.141 (x 7.075) | 10 (0.100) | 0.411 | aminoglycan biosynthesis | Act57B |
| 305 | GO:0030725 | P | 5, | 1 | 0.141 (x 7.075) | 10 (0.100) | 0.412 | ring canal formation | cher |
| 306 | GO:0006024 | P | 8, 9, | 1 | 0.141 (x 7.075) | 10 (0.100) | 0.413 | glycosaminoglycan biosynthesis | Act57B |
| 307 | GO:0009612 | P | 4, | 1 | 0.141 (x 7.075) | 10 (0.100) | 0.415 | response to mechanical stimulus | sda |
| 308 | GO:0046011 | P | 8, 9, 10, | 1 | 0.141 (x 7.075) | 10 (0.100) | 0.416 | regulation of oskar mRNA translation | apt |
| 309 | GO:0044242 | P | 6, 7, | 1 | 0.141 (x 7.075) | 10 (0.100) | 0.417 | cellular lipid catabolism | Jheh3 |
| 310 | GO:0006022 | P | 6, 7, | 1 | 0.141 (x 7.075) | 10 (0.100) | 0.419 | aminoglycan metabolism | Act57B |
| 311 | GO:0030203 | P | 7, 8, | 1 | 0.141 (x 7.075) | 10 (0.100) | 0.42 | glycosaminoglycan metabolism | Act57B |
| 312 | GO:0015893 | P | 5, 6, | 1 | 0.141 (x 7.075) | 10 (0.100) | 0.422 | drug transport | BEST:CK01227 |
| 313 | GO:0035078 | P | 6, 7, 8, 9, 10, | 1 | 0.141 (x 7.075) | 10 (0.100) | 0.423 | induction of programmed cell death by ecdysone | rpr |
| 314 | GO:0043063 | P | 4, | 1 | 0.141 (x 7.075) | 10 (0.100) | 0.424 | intercellular bridge organization and biogenesis | cher |
| 315 | GO:0006960 | P | 7, 8, | 2 | 0.636 (x 3.144) | 45 (0.044) | 0.426 | antimicrobial humoral response (sensu Protostomia) | TepIV Thor |
| 316 | GO:0002164 | P | 4, | 2 | 0.636 (x 3.144) | 45 (0.044) | 0.427 | larval development | Lcp65Ag1 rad50 |
| 317 | GO:0008194 | F | 5, | 3 | 1.300 (x 2.307) | 92 (0.033) | 0.435 | UDP-glycosyltransferase activity | Act57B Ugt86Da Ugt86Di |
| 318 | GO:0030509 | P | 8, | 1 | 0.155 (x 6.431) | 11 (0.091) | 0.439 | BMP signaling pathway | sog |
| 319 | GO:0007277 | P | 5, | 1 | 0.155 (x 6.431) | 11 (0.091) | 0.44 | pole cell development | wun2 |
| 320 | GO:0008307 | F | 3, | 1 | 0.155 (x 6.431) | 11 (0.091) | 0.442 | structural constituent of muscle | Mhc |
| 321 | GO:0006446 | P | 8, 9, 10, | 1 | 0.155 (x 6.431) | 11 (0.091) | 0.443 | regulation of translational initiation | Thor |
| 322 | GO:0035081 | P | 8, 9, | 1 | 0.155 (x 6.431) | 11 (0.091) | 0.445 | induction of programmed cell death by hormones | rpr |
| 323 | GO:0005975 | P | 5, | 10 | 6.856 (x 1.459) | 485 (0.021) | 0.446 | carbohydrate metabolism | Act57B CG14935 CG2989 CG3036 CG5177 Chit Gasp ImpL3 Ugt86Da Ugt86Di |
| 324 | GO:0005245 | F | 6, 7, 8, | 1 | 0.155 (x 6.431) | 11 (0.091) | 0.446 | voltage-gated calcium channel activity | na |
| 325 | GO:0016051 | P | 6, 7, | 2 | 0.693 (x 2.888) | 49 (0.041) | 0.456 | carbohydrate biosynthesis | Act57B CG5177 |
| 326 | GO:0042578 | F | 5, | 5 | 2.827 (x 1.769) | 200 (0.025) | 0.458 | phosphoric ester hydrolase activity | CG2277 CG32549 CG5177 wun wun2 |
| 327 | GO:0046983 | F | 4, | 2 | 0.693 (x 2.888) | 49 (0.041) | 0.458 | protein dimerization activity | CG6272 wun |
| 328 | GO:0016082 | P | 8, 9, 10, | 1 | 0.170 (x 5.896) | 12 (0.083) | 0.466 | synaptic vesicle priming | unc-13 |
| 329 | GO:0007440 | P | 7, 8, | 1 | 0.170 (x 5.896) | 12 (0.083) | 0.467 | foregut morphogenesis | os |
| 330 | GO:0042559 | P | 7, | 1 | 0.170 (x 5.896) | 12 (0.083) | 0.469 | pteridine and derivative biosynthesis | Mocs1 |
| 331 | GO:0004553 | F | 5, | 3 | 1.385 (x 2.166) | 98 (0.031) | 0.476 | hydrolase activity, hydrolyzing O-glycosyl compounds | CG14935 CG2989 Chit |
| 332 | GO:0004558 | F | 7, | 1 | 0.184 (x 5.442) | 13 (0.077) | 0.486 | alpha-glucosidase activity | CG14935 |
| 333 | GO:0007428 | P | 5, 6, | 1 | 0.184 (x 5.442) | 13 (0.077) | 0.488 | primary tracheal branching (sensu Insecta) | apt |
| 334 | GO:0042558 | P | 6, | 1 | 0.184 (x 5.442) | 13 (0.077) | 0.489 | pteridine and derivative metabolism | Mocs1 |
| 335 | GO:0005416 | F | 6, 7, 8, | 1 | 0.184 (x 5.442) | 13 (0.077) | 0.491 | cation:amino acid symporter activity | BG:DS03431.1 |
| 336 | GO:0005689 | C | 5, 6, 7, 8, 9, 10, 11, | 1 | 0.184 (x 5.442) | 13 (0.077) | 0.492 | minor (U12-dependent) spliceosome complex | CG5205 |
| 337 | GO:0005941 | C | 3, | 2 | 0.735 (x 2.721) | 52 (0.038) | 0.492 | unlocalized protein complex | Ku80 Mocs1 |
| 338 | GO:0009613 | P | 4, 5, | 3 | 1.414 (x 2.122) | 100 (0.030) | 0.493 | response to pest, pathogen or parasite | TepIV Thor TotA |
| 339 | GO:0007539 | P | 5, | 1 | 0.184 (x 5.442) | 13 (0.077) | 0.494 | primary sex determination, soma | os |
| 340 | GO:0042048 | P | 5, 6, | 2 | 0.749 (x 2.670) | 53 (0.038) | 0.494 | olfactory behavior | BG:DS01219.1 cher |
| 341 | GO:0004529 | F | 7, | 1 | 0.184 (x 5.442) | 13 (0.077) | 0.495 | exodeoxyribonuclease activity | rad50 |
| 342 | GO:0006112 | P | 7, | 1 | 0.198 (x 5.053) | 14 (0.071) | 0.507 | energy reserve metabolism | CG5177 |
| 343 | GO:0007635 | P | 4, 5, | 2 | 0.763 (x 2.620) | 54 (0.037) | 0.507 | chemosensory behavior | BG:DS01219.1 cher |
| 344 | GO:0004556 | F | 7, | 1 | 0.198 (x 5.053) | 14 (0.071) | 0.508 | alpha-amylase activity | CG14935 |
| 345 | GO:0007001 | P | 7, | 4 | 2.219 (x 1.802) | 157 (0.025) | 0.508 | chromosome organization and biogenesis (sensu Eukaryota) | CG31611 Irbp Ku80 rad50 |
| 346 | GO:0006997 | P | 6, | 1 | 0.198 (x 5.053) | 14 (0.071) | 0.51 | nuclear organization and biogenesis | LamC |
| 347 | GO:0030166 | P | 7, 8, | 1 | 0.198 (x 5.053) | 14 (0.071) | 0.511 | proteoglycan biosynthesis | Act57B |
| 348 | GO:0016160 | F | 6, | 1 | 0.198 (x 5.053) | 14 (0.071) | 0.513 | amylase activity | CG14935 |
| 349 | GO:0019438 | P | 6, | 1 | 0.198 (x 5.053) | 14 (0.071) | 0.514 | aromatic compound biosynthesis | Mocs1 |
| 350 | GO:0006720 | P | 6, 7, | 1 | 0.198 (x 5.053) | 14 (0.071) | 0.516 | isoprenoid metabolism | Jheh3 |
| 351 | GO:0009605 | P | 3, | 3 | 1.484 (x 2.021) | 105 (0.029) | 0.517 | response to external stimulus | sda wun wun2 |
| 352 | GO:0016757 | F | 4, | 4 | 2.247 (x 1.780) | 159 (0.025) | 0.521 | transferase activity, transferring glycosyl groups | Act57B CG6330 Ugt86Da Ugt86Di |
| 353 | GO:0000786 | C | 3, 5, 6, 7, 8, 9, 10, 11, | 1 | 0.212 (x 4.716) | 15 (0.067) | 0.523 | nucleosome | CG31611 |
| 354 | GO:0044262 | P | 6, | 6 | 3.887 (x 1.544) | 275 (0.022) | 0.523 | cellular carbohydrate metabolism | Act57B CG2989 CG5177 Chit Gasp ImpL3 |
| 355 | GO:0016788 | F | 4, | 9 | 6.431 (x 1.399) | 455 (0.020) | 0.523 | hydrolase activity, acting on ester bonds | CG2277 CG32549 CG4267 CG5162 CG5177 CG5397 rad50 wun wun2 |
| 356 | GO:0044249 | P | 5, | 15 | 11.732 (x 1.279) | 830 (0.018) | 0.523 | cellular biosynthesis | Act57B CG12264 CG31373 CG33158 CG5177 CG7014 CG7842 Dgp-1 Las Mocs1 RpS4 Thor apt na rpr |
| 357 | GO:0006029 | P | 7, | 1 | 0.212 (x 4.716) | 15 (0.067) | 0.524 | proteoglycan metabolism | Act57B |
| 358 | GO:0035075 | P | 5, 6, 7, | 1 | 0.212 (x 4.716) | 15 (0.067) | 0.526 | response to ecdysone | rpr |
| 359 | GO:0006090 | P | 7, | 1 | 0.212 (x 4.716) | 15 (0.067) | 0.527 | pyruvate metabolism | Aldh |
| 360 | GO:0005386 | F | 3, | 9 | 6.403 (x 1.406) | 453 (0.020) | 0.529 | carrier activity | BG:DS03431.1 CG10444 CG11907 CG3036 CG6574 TepIV Tsf1 Tsf3 glob1 |
| 361 | GO:0005125 | F | 4, 5, | 1 | 0.212 (x 4.716) | 15 (0.067) | 0.529 | cytokine activity | os |
| 362 | GO:0048545 | P | 5, 6, | 1 | 0.212 (x 4.716) | 15 (0.067) | 0.53 | response to steroid hormone stimulus | rpr |
| 363 | GO:0007492 | P | 4, | 1 | 0.226 (x 4.422) | 16 (0.062) | 0.53 | endoderm development | toy |
| 364 | GO:0007538 | P | 4, | 1 | 0.212 (x 4.716) | 15 (0.067) | 0.532 | primary sex determination | os |
| 365 | GO:0045171 | C | 3, 4, | 1 | 0.226 (x 4.422) | 16 (0.062) | 0.532 | intercellular bridge | cher |
| 366 | GO:0009058 | P | 4, | 16 | 12.693 (x 1.261) | 898 (0.018) | 0.532 | biosynthesis | Act57B CG12264 CG31373 CG33158 CG5177 CG7014 CG7842 Dgp-1 Las Lcp65Ag1 Mocs1 RpS4 Thor apt na rpr |
| 367 | GO:0045172 | C | 4, 5, | 1 | 0.226 (x 4.422) | 16 (0.062) | 0.533 | ring canal (sensu Insecta) | cher |
| 368 | GO:0042802 | F | 4, | 1 | 0.226 (x 4.422) | 16 (0.062) | 0.534 | identical protein binding | wun |
| 369 | GO:0006289 | P | 6, 8, | 1 | 0.226 (x 4.422) | 16 (0.062) | 0.536 | nucleotide-excision repair | hay |
| 370 | GO:0000152 | C | 4, 5, 6, 7, 8, 9, 10, | 1 | 0.226 (x 4.422) | 16 (0.062) | 0.537 | nuclear ubiquitin ligase complex | cul-2 |
| 371 | GO:0042803 | F | 5, | 1 | 0.226 (x 4.422) | 16 (0.062) | 0.539 | protein homodimerization activity | wun |
| 372 | GO:0007611 | P | 4, | 2 | 0.848 (x 2.358) | 60 (0.033) | 0.54 | learning and/or memory | BG:DS01219.1 cher |
| 373 | GO:0004089 | F | 6, | 1 | 0.226 (x 4.422) | 16 (0.062) | 0.54 | carbonate dehydratase activity | CAH2 |
| 374 | GO:0016620 | F | 5, | 1 | 0.226 (x 4.422) | 16 (0.062) | 0.542 | oxidoreductase activity, acting on the aldehyde or oxo group of donors, NAD or NADP as acceptor | Aldh |
| 375 | GO:0003724 | F | 4, | 2 | 0.834 (x 2.398) | 59 (0.034) | 0.542 | RNA helicase activity | CG31755 CG5205 |
| 376 | GO:0005102 | F | 3, 4, | 6 | 3.944 (x 1.521) | 279 (0.022) | 0.542 | receptor binding | CG2989 CG3153 Chit Idgf2 os sog |
| 377 | GO:0009725 | P | 4, 5, | 1 | 0.226 (x 4.422) | 16 (0.062) | 0.543 | response to hormone stimulus | rpr |
| 378 | GO:0003746 | F | 4, 5, | 1 | 0.240 (x 4.162) | 17 (0.059) | 0.546 | translation elongation factor activity | CG33158 |
| 379 | GO:0018993 | P | 4, | 1 | 0.240 (x 4.162) | 17 (0.059) | 0.548 | somatic sex determination | os |
| 380 | GO:0007259 | P | 7, | 1 | 0.240 (x 4.162) | 17 (0.059) | 0.549 | JAK-STAT cascade | os |
| 381 | GO:0006979 | P | 4, 5, 6, | 1 | 0.240 (x 4.162) | 17 (0.059) | 0.551 | response to oxidative stress | GstE1 |
| 382 | GO:0007507 | P | 5, | 2 | 0.862 (x 2.320) | 61 (0.033) | 0.552 | heart development | Act57B apt |
| 383 | GO:0008586 | P | 7, 8, 9, | 1 | 0.240 (x 4.162) | 17 (0.059) | 0.552 | wing vein morphogenesis | sog |
| 384 | GO:0015370 | F | 6, 8, | 2 | 0.876 (x 2.282) | 62 (0.032) | 0.553 | solute:sodium symporter activity | BG:DS03431.1 CG10444 |
| 385 | GO:0030246 | F | 3, | 3 | 1.597 (x 1.878) | 113 (0.027) | 0.553 | carbohydrate binding | CG2989 CG3244 Gasp |
| 386 | GO:0005328 | F | 4, 7, 9, | 1 | 0.240 (x 4.162) | 17 (0.059) | 0.554 | neurotransmitter:sodium symporter activity | BG:DS03431.1 |
| 387 | GO:0009116 | P | 6, | 1 | 0.254 (x 3.930) | 18 (0.056) | 0.562 | nucleoside metabolism | CG6330 |
| 388 | GO:0008061 | F | 5, | 2 | 0.891 (x 2.246) | 63 (0.032) | 0.562 | chitin binding | CG2989 Gasp |
| 389 | GO:0005856 | C | 5, 6, 7, 8, | 6 | 4.099 (x 1.464) | 290 (0.021) | 0.562 | cytoskeleton | Act57B LamC Mhc Mlc1 Mlc2 wupA |
| 390 | GO:0005741 | C | 5, 6, 7, 8, 9, 10, 11, 12, | 1 | 0.254 (x 3.930) | 18 (0.056) | 0.563 | mitochondrial outer membrane | CG8004 |
| 391 | GO:0019730 | P | 6, 7, | 2 | 0.891 (x 2.246) | 63 (0.032) | 0.564 | antimicrobial humoral response | TepIV Thor |
| 392 | GO:0042493 | P | 5, | 1 | 0.254 (x 3.930) | 18 (0.056) | 0.564 | response to drug | BEST:CK01227 |
| 393 | GO:0015294 | F | 5, 7, | 2 | 0.891 (x 2.246) | 63 (0.032) | 0.565 | solute:cation symporter activity | BG:DS03431.1 CG10444 |
| 394 | GO:0005884 | C | 5, 6, 7, 8, 9, 10, | 1 | 0.254 (x 3.930) | 18 (0.056) | 0.566 | actin filament | Act57B |
| 395 | GO:0051276 | P | 6, | 4 | 2.431 (x 1.645) | 172 (0.023) | 0.566 | chromosome organization and biogenesis | CG31611 Irbp Ku80 rad50 |
| 396 | GO:0031968 | C | 4, 5, 6, 7, 8, 9, | 1 | 0.269 (x 3.723) | 19 (0.053) | 0.574 | organelle outer membrane | CG8004 |
| 397 | GO:0006510 | P | 8, | 1 | 0.269 (x 3.723) | 19 (0.053) | 0.576 | ATP-dependent proteolysis | CG6512 |
| 398 | GO:0019725 | P | 4, | 2 | 0.933 (x 2.144) | 66 (0.030) | 0.576 | cell homeostasis | Tsf1 Tsf3 |
| 399 | GO:0006955 | P | 4, 5, | 3 | 1.724 (x 1.740) | 122 (0.025) | 0.577 | immune response | TepIV Thor TotA |
| 400 | GO:0044272 | P | 6, | 1 | 0.269 (x 3.723) | 19 (0.053) | 0.577 | sulfur compound biosynthesis | Act57B |
| 401 | GO:0001558 | P | 4, 5, 7, 8, | 1 | 0.283 (x 3.537) | 20 (0.050) | 0.577 | regulation of cell growth | Thor |
| 402 | GO:0008289 | F | 3, | 2 | 0.933 (x 2.144) | 66 (0.030) | 0.578 | lipid binding | CG7054 unc-13 |
| 403 | GO:0016769 | F | 4, | 1 | 0.269 (x 3.723) | 19 (0.053) | 0.579 | transferase activity, transferring nitrogenous groups | CG12264 |
| 404 | GO:0000160 | P | 5, | 1 | 0.283 (x 3.537) | 20 (0.050) | 0.579 | two-component signal transduction system (phosphorelay) | CG2885 |
| 405 | GO:0016763 | F | 5, | 1 | 0.269 (x 3.723) | 19 (0.053) | 0.58 | transferase activity, transferring pentosyl groups | CG6330 |
| 406 | GO:0008374 | F | 7, | 1 | 0.283 (x 3.537) | 20 (0.050) | 0.58 | O-acyltransferase activity | CG5397 |
| 407 | GO:0019867 | C | 4, 5, 6, | 1 | 0.269 (x 3.723) | 19 (0.053) | 0.581 | outer membrane | CG8004 |
| 408 | GO:0016284 | F | 7, | 1 | 0.283 (x 3.537) | 20 (0.050) | 0.582 | alanine aminopeptidase activity | sda |
| 409 | GO:0016052 | P | 6, | 2 | 0.947 (x 2.112) | 67 (0.030) | 0.582 | carbohydrate catabolism | Chit ImpL3 |
| 410 | GO:0008483 | F | 5, | 1 | 0.269 (x 3.723) | 19 (0.053) | 0.583 | transaminase activity | CG12264 |
| 411 | GO:0005326 | F | 3, | 1 | 0.283 (x 3.537) | 20 (0.050) | 0.583 | neurotransmitter transporter activity | BG:DS03431.1 |
| 412 | GO:0005198 | F | 2, | 13 | 10.432 (x 1.246) | 738 (0.018) | 0.583 | structural molecule activity | Act57B CG5162 CG6305 CG7014 Cp36 Gasp LamC Lcp65Ag1 Mhc RpS4 cher na wupA |
| 413 | GO:0044275 | P | 7, | 2 | 0.947 (x 2.112) | 67 (0.030) | 0.583 | cellular carbohydrate catabolism | Chit ImpL3 |
| 414 | GO:0006414 | P | 8, 9, | 1 | 0.269 (x 3.723) | 19 (0.053) | 0.584 | translational elongation | CG33158 |
| 415 | GO:0008632 | P | 7, | 1 | 0.283 (x 3.537) | 20 (0.050) | 0.584 | apoptotic program | rpr |
| 416 | GO:0030247 | F | 4, | 2 | 0.947 (x 2.112) | 67 (0.030) | 0.585 | polysaccharide binding | CG2989 Gasp |
| 417 | GO:0007398 | P | 4, | 5 | 3.350 (x 1.493) | 237 (0.021) | 0.585 | ectoderm development | CG5397 Tsp42El mfas sog toy |
| 418 | GO:0048513 | P | 3, | 12 | 9.470 (x 1.267) | 670 (0.018) | 0.586 | organ development | Act57B CG6803 CG7194 Idgf2 Mhc Mlp60A apt os rpr sog toy wupA |
| 419 | GO:0004179 | F | 7, 8, | 1 | 0.283 (x 3.537) | 20 (0.050) | 0.586 | membrane alanyl aminopeptidase activity | sda |
| 420 | GO:0007605 | P | 5, 7, | 1 | 0.283 (x 3.537) | 20 (0.050) | 0.587 | sensory perception of sound | CG4917 |
| 421 | GO:0007362 | P | 8, 9, | 1 | 0.297 (x 3.369) | 21 (0.048) | 0.588 | terminal region determination | sog |
| 422 | GO:0006730 | P | 5, | 1 | 0.283 (x 3.537) | 20 (0.050) | 0.589 | one-carbon compound metabolism | CAH2 |
| 423 | GO:0015935 | C | 3, 4, 5, 6, 7, 8, 9, | 2 | 0.975 (x 2.051) | 69 (0.029) | 0.59 | small ribosomal subunit | CG7014 RpS4 |
| 424 | GO:0019210 | F | 4, | 1 | 0.297 (x 3.369) | 21 (0.048) | 0.59 | kinase inhibitor activity | CG7054 |
| 425 | GO:0030707 | P | 8, | 3 | 1.753 (x 1.712) | 124 (0.024) | 0.59 | ovarian follicle cell development (sensu Insecta) | Cp36 cher os |
| 426 | GO:0004867 | F | 6, | 2 | 0.989 (x 2.021) | 70 (0.029) | 0.591 | serine-type endopeptidase inhibitor activity | CG7722 CG9460 |
| 427 | GO:0005436 | F | 6, 7, 8, | 1 | 0.297 (x 3.369) | 21 (0.048) | 0.591 | sodium:phosphate symporter activity | CG3036 |
| 428 | GO:0042742 | P | 5, 6, | 2 | 0.975 (x 2.051) | 69 (0.029) | 0.591 | defense response to bacterium | TepIV Thor |
| 429 | GO:0006100 | P | 8, | 1 | 0.297 (x 3.369) | 21 (0.048) | 0.592 | tricarboxylic acid cycle intermediate metabolism | ImpL3 |
| 430 | GO:0016042 | P | 5, 6, | 1 | 0.297 (x 3.369) | 21 (0.048) | 0.594 | lipid catabolism | Jheh3 |
| 431 | GO:0050954 | P | 4, 6, | 1 | 0.297 (x 3.369) | 21 (0.048) | 0.595 | sensory perception of mechanical stimulus | CG4917 |
| 432 | GO:0006412 | P | 6, 7, | 9 | 6.983 (x 1.289) | 494 (0.018) | 0.597 | protein biosynthesis | CG31373 CG33158 CG7014 Dgp-1 RpS4 Thor apt na rpr |
| 433 | GO:0005516 | F | 4, | 2 | 1.004 (x 1.993) | 71 (0.028) | 0.6 | calmodulin binding | Mlc2 unc-13 |
| 434 | GO:0006836 | P | 5, 6, | 1 | 0.311 (x 3.216) | 22 (0.045) | 0.601 | neurotransmitter transport | BG:DS03431.1 |
| 435 | GO:0042078 | P | 6, | 1 | 0.311 (x 3.216) | 22 (0.045) | 0.602 | germ-line stem cell division | os |
| 436 | GO:0006445 | P | 7, 8, 9, | 2 | 1.018 (x 1.965) | 72 (0.028) | 0.603 | regulation of translation | Thor apt |
| 437 | GO:0009401 | P | 6, 7, | 1 | 0.311 (x 3.216) | 22 (0.045) | 0.604 | phosphoenolpyruvate-dependent sugar phosphotransferase system | TepIV |
| 438 | GO:0040003 | P | 8, | 1 | 0.311 (x 3.216) | 22 (0.045) | 0.605 | cuticle biosynthesis (sensu Insecta) | Lcp65Ag1 |
| 439 | GO:0007592 | P | 7, | 1 | 0.311 (x 3.216) | 22 (0.045) | 0.607 | cuticle biosynthesis (sensu Protostomia and Nematoda) | Lcp65Ag1 |
| 440 | GO:0009880 | P | 4, | 3 | 1.823 (x 1.645) | 129 (0.023) | 0.61 | embryonic pattern specification | os sog wun |
| 441 | GO:0016758 | F | 5, | 3 | 1.823 (x 1.645) | 129 (0.023) | 0.611 | transferase activity, transferring hexosyl groups | Act57B Ugt86Da Ugt86Di |
| 442 | GO:0009967 | P | 5, 6, | 1 | 0.325 (x 3.076) | 23 (0.043) | 0.616 | positive regulation of signal transduction | sog |
| 443 | GO:0016333 | P | 5, | 1 | 0.325 (x 3.076) | 23 (0.043) | 0.617 | morphogenesis of follicular epithelium | cher |
| 444 | GO:0042445 | P | 5, | 1 | 0.325 (x 3.076) | 23 (0.043) | 0.618 | hormone metabolism | Jheh3 |
| 445 | GO:0051246 | P | 5, 6, | 3 | 1.880 (x 1.596) | 133 (0.023) | 0.625 | regulation of protein metabolism | Thor apt rpr |
| 446 | GO:0001703 | P | 6, | 1 | 0.339 (x 2.948) | 24 (0.042) | 0.626 | gastrulation (sensu Protostomia) | sog |
| 447 | GO:0008235 | F | 6, | 2 | 1.074 (x 1.862) | 76 (0.026) | 0.626 | metalloexopeptidase activity | huntingtin sda |
| 448 | GO:0016903 | F | 4, | 1 | 0.339 (x 2.948) | 24 (0.042) | 0.627 | oxidoreductase activity, acting on the aldehyde or oxo group of donors | Aldh |
| 449 | GO:0042592 | P | 3, | 2 | 1.074 (x 1.862) | 76 (0.026) | 0.627 | homeostasis | Tsf1 Tsf3 |
| 450 | GO:0031202 | F | 5, | 1 | 0.339 (x 2.948) | 24 (0.042) | 0.628 | RNA splicing factor activity, transesterification mechanism | CG6876 |
| 451 | GO:0006334 | P | 7, 11, | 1 | 0.339 (x 2.948) | 24 (0.042) | 0.63 | nucleosome assembly | CG31611 |
| 452 | GO:0019842 | F | 3, | 1 | 0.339 (x 2.948) | 24 (0.042) | 0.631 | vitamin binding | CG6574 |
| 453 | GO:0051186 | P | 5, | 5 | 3.576 (x 1.398) | 253 (0.020) | 0.631 | cofactor metabolism | CG10444 CG12264 CG6574 Las Mocs1 |
| 454 | GO:0010004 | P | 7, | 1 | 0.339 (x 2.948) | 24 (0.042) | 0.632 | gastrulation (sensu Insecta) | sog |
| 455 | GO:0046982 | F | 5, | 1 | 0.339 (x 2.948) | 24 (0.042) | 0.634 | protein heterodimerization activity | CG6272 |
| 456 | GO:0016490 | F | 3, | 1 | 0.339 (x 2.948) | 24 (0.042) | 0.635 | structural constituent of peritrophic membrane (sensu Insecta) | Gasp |
| 457 | GO:0045137 | P | 4, | 1 | 0.353 (x 2.830) | 25 (0.040) | 0.639 | development of primary sexual characteristics | CG7194 |
| 458 | GO:0008406 | P | 5, | 1 | 0.353 (x 2.830) | 25 (0.040) | 0.64 | gonad development | CG7194 |
| 459 | GO:0005262 | F | 6, 7, | 1 | 0.353 (x 2.830) | 25 (0.040) | 0.642 | calcium channel activity | na |
| 460 | GO:0019992 | F | 4, | 1 | 0.368 (x 2.721) | 26 (0.038) | 0.653 | diacylglycerol binding | unc-13 |
| 461 | GO:0015114 | F | 6, | 1 | 0.368 (x 2.721) | 26 (0.038) | 0.654 | phosphate transporter activity | CG3036 |
| 462 | GO:0003684 | F | 5, | 1 | 0.368 (x 2.721) | 26 (0.038) | 0.656 | damaged DNA binding | rad50 |
| 463 | GO:0004520 | F | 7, | 1 | 0.368 (x 2.721) | 26 (0.038) | 0.657 | endodeoxyribonuclease activity | rad50 |
| 464 | GO:0001871 | F | 3, | 2 | 1.131 (x 1.769) | 80 (0.025) | 0.658 | pattern binding | CG2989 Gasp |
| 465 | GO:0005543 | F | 4, | 1 | 0.382 (x 2.620) | 27 (0.037) | 0.658 | phospholipid binding | CG7054 |
| 466 | GO:0051301 | P | 4, | 3 | 1.965 (x 1.527) | 139 (0.022) | 0.658 | cell division | Act57B Mhc os |
| 467 | GO:0008344 | P | 5, | 1 | 0.368 (x 2.721) | 26 (0.038) | 0.658 | adult locomotory behavior | na |
| 468 | GO:0007298 | P | 7, 8, 10, | 1 | 0.382 (x 2.620) | 27 (0.037) | 0.659 | border follicle cell migration (sensu Insecta) | os |
| 469 | GO:0044274 | P | 5, | 1 | 0.382 (x 2.620) | 27 (0.037) | 0.661 | organismal biosynthesis | Lcp65Ag1 |
| 470 | GO:0006807 | P | 4, | 7 | 5.527 (x 1.267) | 391 (0.018) | 0.661 | nitrogen compound metabolism | Act57B BG:DS03431.1 CG12264 CG2989 CG9836 Chit Gasp |
| 471 | GO:0005214 | F | 4, | 2 | 1.145 (x 1.747) | 81 (0.025) | 0.662 | structural constituent of cuticle (sensu Insecta) | CG6305 Lcp65Ag1 |
| 472 | GO:0007354 | P | 7, 8, | 1 | 0.382 (x 2.620) | 27 (0.037) | 0.662 | zygotic determination of anterior/posterior axis, embryo | sog |
| 473 | GO:0008643 | P | 5, 6, | 2 | 1.145 (x 1.747) | 81 (0.025) | 0.663 | carbohydrate transport | CG3036 TepIV |
| 474 | GO:0042335 | P | 6, | 1 | 0.382 (x 2.620) | 27 (0.037) | 0.663 | cuticle biosynthesis | Lcp65Ag1 |
| 475 | GO:0004197 | F | 6, | 2 | 1.145 (x 1.747) | 81 (0.025) | 0.664 | cysteine-type endopeptidase activity | CG6357 Mlc1 |
| 476 | GO:0007300 | P | 8, | 1 | 0.396 (x 2.527) | 28 (0.036) | 0.675 | nurse cell to oocyte transport (sensu Insecta) | cher |
| 477 | GO:0003674 | F | 1, | 119 | 117.123 (x 1.016) | 8286 (0.014) | 0.675 | molecular\_function | Act57B Aldh BEST:CK01227 BG:DS01068.5 BG:DS01219.1 BG:DS03431.1 CAH2 CG10126 CG10444 CG10527 CG10638 CG11907 CG12224 CG12264 CG1299 CG14935 CG17108 CG17124 CG17904 CG18522 CG18547 CG2065 CG2277 CG2885 CG2989 CG3008 CG3036 CG31063 CG31279 CG31373 CG3153 CG31601 CG31611 CG31666 CG31755 CG32017 CG32021 CG32207 CG3244 CG32448 CG32549 CG32625 CG33158 CG4267 CG4511 CG4858 CG4917 CG5162 CG5177 CG5205 CG5397 CG6272 CG6305 CG6330 CG6357 CG6512 CG6574 CG6876 CG7014 CG7054 CG7194 CG7447 CG7722 CG7842 CG9372 CG9460 Chit Cp36 Dgp-1 Ets21C Gasp GstD3 GstD6 GstD9 GstE1 GstE3 GstE5 GstE6 GstE7 Idgf2 ImpL3 Irbp JhI-26 Jheh3 Ku80 LamC Las Lcp65Ag1 Mhc Mlc1 Mlc2 Mlp60A Mocs1 RpS4 TepIV Thor Tsf1 Tsf3 Tsp42El Ugt86Da Ugt86Di agt apt cher fau glob1 hay huntingtin na os rad50 sda sog toy unc-13 wun wun2 wupA yellow-b |
| 478 | GO:0000910 | P | 5, | 2 | 1.187 (x 1.684) | 84 (0.024) | 0.678 | cytokinesis | Act57B Mhc |
| 479 | GO:0009888 | P | 3, | 7 | 5.626 (x 1.244) | 398 (0.018) | 0.679 | tissue development | CG5397 Mlc1 Mlp60A Tsp42El mfas sog toy |
| 480 | GO:0016616 | F | 5, | 2 | 1.187 (x 1.684) | 84 (0.024) | 0.68 | oxidoreductase activity, acting on the CH-OH group of donors, NAD or NADP as acceptor | CG10638 ImpL3 |
| 481 | GO:0008168 | F | 5, | 2 | 1.187 (x 1.684) | 84 (0.024) | 0.681 | methyltransferase activity | CG10527 agt |
| 482 | GO:0016741 | F | 4, | 2 | 1.201 (x 1.665) | 85 (0.024) | 0.682 | transferase activity, transferring one-carbon groups | CG10527 agt |
| 483 | GO:0044421 | C | 2, 3, | 2 | 1.201 (x 1.665) | 85 (0.024) | 0.683 | extracellular region part | cher os |
| 484 | GO:0006633 | P | 6, 7, 8, | 1 | 0.410 (x 2.440) | 29 (0.034) | 0.684 | fatty acid biosynthesis | CG7842 |
| 485 | GO:0007179 | P | 8, | 1 | 0.410 (x 2.440) | 29 (0.034) | 0.686 | transforming growth factor beta receptor signaling pathway | sog |
| 486 | GO:0007274 | P | 7, | 1 | 0.410 (x 2.440) | 29 (0.034) | 0.687 | neuromuscular synaptic transmission | apt |
| 487 | GO:0007591 | P | 6, | 1 | 0.424 (x 2.358) | 30 (0.033) | 0.696 | molting cycle (sensu Insecta) | Lcp65Ag1 |
| 488 | GO:0048468 | P | 4, | 6 | 4.806 (x 1.248) | 340 (0.018) | 0.697 | cell development | CG6803 Mhc mfas os wun2 wupA |
| 489 | GO:0016049 | P | 3, 4, 6, 7, | 1 | 0.424 (x 2.358) | 30 (0.033) | 0.698 | cell growth | Thor |
| 490 | GO:0030162 | P | 6, 7, 8, | 1 | 0.424 (x 2.358) | 30 (0.033) | 0.699 | regulation of proteolysis | rpr |
| 491 | GO:0000377 | P | 10, | 3 | 2.106 (x 1.424) | 149 (0.020) | 0.699 | RNA splicing, via transesterification reactions with bulged adenosine as nucleophile | CG5205 CG6876 hay |
| 492 | GO:0000398 | P | 9, 11, | 3 | 2.106 (x 1.424) | 149 (0.020) | 0.701 | nuclear mRNA splicing, via spliceosome | CG5205 CG6876 hay |
| 493 | GO:0016298 | F | 6, | 2 | 1.244 (x 1.608) | 88 (0.023) | 0.701 | lipase activity | CG4267 CG5162 |
| 494 | GO:0015926 | F | 6, | 1 | 0.438 (x 2.282) | 31 (0.032) | 0.702 | glucosidase activity | CG14935 |
| 495 | GO:0000375 | P | 9, | 3 | 2.106 (x 1.424) | 149 (0.020) | 0.702 | RNA splicing, via transesterification reactions | CG5205 CG6876 hay |
| 496 | GO:0048754 | P | 5, | 1 | 0.438 (x 2.282) | 31 (0.032) | 0.703 | branching morphogenesis of a tube | apt |
| 497 | GO:0016053 | P | 6, | 1 | 0.438 (x 2.282) | 31 (0.032) | 0.704 | organic acid biosynthesis | CG7842 |
| 498 | GO:0046394 | P | 7, | 1 | 0.438 (x 2.282) | 31 (0.032) | 0.706 | carboxylic acid biosynthesis | CG7842 |
| 499 | GO:0019888 | F | 4, | 1 | 0.452 (x 2.211) | 32 (0.031) | 0.707 | protein phosphatase regulator activity | CG17124 |
| 500 | GO:0004181 | F | 7, | 1 | 0.452 (x 2.211) | 32 (0.031) | 0.708 | metallocarboxypeptidase activity | huntingtin |
| 501 | GO:0019208 | F | 3, | 1 | 0.452 (x 2.211) | 32 (0.031) | 0.71 | phosphatase regulator activity | CG17124 |
| 502 | GO:0044444 | C | 4, 5, 6, 7, | 18 | 16.354 (x 1.101) | 1157 (0.016) | 0.71 | cytoplasmic part | Aldh CG12264 CG32549 CG33158 CG4917 CG6512 CG7014 CG7842 CG8004 Las Mhc Mlc1 Mlc2 RpS4 na rpr unc-13 wupA |
| 503 | GO:0006206 | P | 7, | 1 | 0.452 (x 2.211) | 32 (0.031) | 0.711 | pyrimidine base metabolism | CG6330 |
| 504 | GO:0016044 | P | 5, | 1 | 0.452 (x 2.211) | 32 (0.031) | 0.712 | membrane organization and biogenesis | LamC |
| 505 | GO:0005830 | C | 5, 6, 7, 8, 9, 10, | 2 | 1.272 (x 1.572) | 90 (0.022) | 0.713 | cytosolic ribosome (sensu Eukaryota) | CG7014 RpS4 |
| 506 | GO:0008380 | P | 8, | 3 | 2.191 (x 1.369) | 155 (0.019) | 0.713 | RNA splicing | CG5205 CG6876 hay |
| 507 | GO:0004182 | F | 8, | 1 | 0.452 (x 2.211) | 32 (0.031) | 0.714 | carboxypeptidase A activity | huntingtin |
| 508 | GO:0008372 | C | 2, | 13 | 11.506 (x 1.130) | 814 (0.016) | 0.714 | cellular component unknown | CG31279 CG31601 CG32017 CG32021 CG32207 CG32448 CG32625 CG7194 Dgp-1 GstD3 JhI-26 fau yellow-b |
| 509 | GO:0031324 | P | 6, | 3 | 2.191 (x 1.369) | 155 (0.019) | 0.715 | negative regulation of cellular metabolism | Thor apt rpr |
| 510 | GO:0008105 | P | 5, | 1 | 0.452 (x 2.211) | 32 (0.031) | 0.715 | asymmetric protein localization | BG:DS01219.1 |
| 511 | GO:0042303 | P | 4, | 1 | 0.466 (x 2.144) | 33 (0.030) | 0.716 | molting cycle | Lcp65Ag1 |
| 512 | GO:0008293 | P | 8, | 1 | 0.466 (x 2.144) | 33 (0.030) | 0.717 | torso signaling pathway | sog |
| 513 | GO:0007297 | P | 6, 7, 9, | 1 | 0.466 (x 2.144) | 33 (0.030) | 0.718 | follicle cell migration (sensu Insecta) | os |
| 514 | GO:0008234 | F | 5, | 2 | 1.315 (x 1.521) | 93 (0.022) | 0.72 | cysteine-type peptidase activity | CG6357 Mlc1 |
| 515 | GO:0005681 | C | 4, 5, 6, 7, 8, 9, 10, | 2 | 1.300 (x 1.538) | 92 (0.022) | 0.72 | spliceosome complex | CG5205 CG6876 |
| 516 | GO:0018988 | P | 5, | 1 | 0.466 (x 2.144) | 33 (0.030) | 0.72 | molting cycle (sensu Protostomia and Nematoda) | Lcp65Ag1 |
| 517 | GO:0009108 | P | 7, | 2 | 1.329 (x 1.505) | 94 (0.021) | 0.721 | coenzyme biosynthesis | Las Mocs1 |
| 518 | GO:0008509 | F | 4, | 2 | 1.329 (x 1.505) | 94 (0.021) | 0.722 | anion transporter activity | BEST:CK01227 CG3036 |
| 519 | GO:0008361 | P | 5, 6, | 1 | 0.481 (x 2.081) | 34 (0.029) | 0.723 | regulation of cell size | Thor |
| 520 | GO:0006626 | P | 8, 9, 10, | 1 | 0.481 (x 2.081) | 34 (0.029) | 0.724 | protein targeting to mitochondrion | CG8004 |
| 521 | GO:0016829 | F | 3, | 3 | 2.219 (x 1.352) | 157 (0.019) | 0.725 | lyase activity | CAH2 CG12264 rad50 |
| 522 | GO:0001763 | P | 4, | 1 | 0.481 (x 2.081) | 34 (0.029) | 0.725 | morphogenesis of a branching structure | apt |
| 523 | GO:0006629 | P | 5, | 8 | 6.926 (x 1.155) | 490 (0.016) | 0.729 | lipid metabolism | CG4267 CG5162 CG7842 Jheh3 Ugt86Da Ugt86Di wun wun2 |
| 524 | GO:0019204 | F | 7, | 1 | 0.495 (x 2.021) | 35 (0.029) | 0.733 | nucleotide phosphatase activity | CG2277 |
| 525 | GO:0005200 | F | 3, | 5 | 4.127 (x 1.211) | 292 (0.017) | 0.739 | structural constituent of cytoskeleton | Act57B LamC Mhc cher wupA |
| 526 | GO:0030036 | P | 8, | 2 | 1.371 (x 1.459) | 97 (0.021) | 0.74 | actin cytoskeleton organization and biogenesis | CG6803 cher |
| 527 | GO:0007442 | P | 7, 8, | 1 | 0.509 (x 1.965) | 36 (0.028) | 0.741 | hindgut morphogenesis | os |
| 528 | GO:0005525 | F | 6, | 3 | 2.276 (x 1.318) | 161 (0.019) | 0.741 | GTP binding | CG2885 CG33158 Dgp-1 |
| 529 | GO:0019001 | F | 5, | 3 | 2.290 (x 1.310) | 162 (0.019) | 0.741 | guanyl nucleotide binding | CG2885 CG33158 Dgp-1 |
| 530 | GO:0030029 | P | 7, | 2 | 1.371 (x 1.459) | 97 (0.021) | 0.742 | actin filament-based process | CG6803 cher |
| 531 | GO:0007623 | P | 4, | 1 | 0.509 (x 1.965) | 36 (0.028) | 0.742 | circadian rhythm | na |
| 532 | GO:0004536 | F | 6, | 1 | 0.523 (x 1.912) | 37 (0.027) | 0.748 | deoxyribonuclease activity | rad50 |
| 533 | GO:0015931 | P | 5, 6, | 1 | 0.523 (x 1.912) | 37 (0.027) | 0.75 | nucleobase, nucleoside, nucleotide and nucleic acid transport | CG11907 |
| 534 | GO:0017145 | P | 5, | 1 | 0.523 (x 1.912) | 37 (0.027) | 0.751 | stem cell division | os |
| 535 | GO:0048511 | P | 3, | 1 | 0.523 (x 1.912) | 37 (0.027) | 0.752 | rhythmic process | na |
| 536 | GO:0042302 | F | 3, | 2 | 1.399 (x 1.429) | 99 (0.020) | 0.753 | structural constituent of cuticle | CG6305 Lcp65Ag1 |
| 537 | GO:0009892 | P | 5, | 3 | 2.332 (x 1.286) | 165 (0.018) | 0.754 | negative regulation of metabolism | Thor apt rpr |
| 538 | GO:0008092 | F | 4, | 4 | 3.364 (x 1.189) | 238 (0.017) | 0.769 | cytoskeletal protein binding | Mhc cher huntingtin wupA |
| 539 | GO:0048547 | P | 5, 6, | 1 | 0.565 (x 1.769) | 40 (0.025) | 0.769 | gut morphogenesis | os |
| 540 | GO:0015296 | F | 5, 7, | 1 | 0.551 (x 1.814) | 39 (0.026) | 0.77 | anion:cation symporter activity | CG3036 |
| 541 | GO:0048546 | P | 5, | 1 | 0.565 (x 1.769) | 40 (0.025) | 0.77 | digestive tract morphogenesis | os |
| 542 | GO:0006259 | P | 6, | 6 | 5.244 (x 1.144) | 371 (0.016) | 0.771 | DNA metabolism | CG31611 Irbp Ku80 agt hay rad50 |
| 543 | GO:0006096 | P | 8, 10, 11, | 1 | 0.551 (x 1.814) | 39 (0.026) | 0.771 | glycolysis | ImpL3 |
| 544 | GO:0007350 | P | 4, 5, | 2 | 1.442 (x 1.387) | 102 (0.020) | 0.772 | blastoderm segmentation | os sog |
| 545 | GO:0048567 | P | 6, 7, | 1 | 0.565 (x 1.769) | 40 (0.025) | 0.772 | ectodermal gut morphogenesis | os |
| 546 | GO:0005684 | C | 5, 6, 7, 8, 9, 10, 11, | 1 | 0.580 (x 1.726) | 41 (0.024) | 0.772 | major (U2-dependent) spliceosome | CG5205 |
| 547 | GO:0051188 | P | 6, | 2 | 1.484 (x 1.348) | 105 (0.019) | 0.772 | cofactor biosynthesis | Las Mocs1 |
| 548 | GO:0006732 | P | 6, | 4 | 3.378 (x 1.184) | 239 (0.017) | 0.773 | coenzyme metabolism | CG10444 CG6574 Las Mocs1 |
| 549 | GO:0005279 | F | 5, 6, | 1 | 0.551 (x 1.814) | 39 (0.026) | 0.773 | amino acid-polyamine transporter activity | BG:DS03431.1 |
| 550 | GO:0007275 | P | 2, | 22 | 20.991 (x 1.048) | 1485 (0.015) | 0.773 | development | Act57B CG5397 CG6803 CG7194 Idgf2 Lcp65Ag1 Mhc Mlc1 Mlp60A Thor Tsp42El apt cher mfas os rad50 rpr sog toy wun wun2 wupA |
| 551 | GO:0007439 | P | 5, | 1 | 0.565 (x 1.769) | 40 (0.025) | 0.773 | ectodermal gut development | os |
| 552 | GO:0004620 | F | 7, | 1 | 0.580 (x 1.726) | 41 (0.024) | 0.773 | phospholipase activity | CG4267 |
| 553 | GO:0009308 | P | 5, | 6 | 5.315 (x 1.129) | 376 (0.016) | 0.774 | amine metabolism | Act57B BG:DS03431.1 CG12264 CG2989 Chit Gasp |
| 554 | GO:0015203 | F | 4, | 1 | 0.551 (x 1.814) | 39 (0.026) | 0.774 | polyamine transporter activity | BG:DS03431.1 |
| 555 | GO:0004004 | F | 5, 11, | 1 | 0.565 (x 1.769) | 40 (0.025) | 0.775 | ATP-dependent RNA helicase activity | CG31755 |
| 556 | GO:0004527 | F | 6, | 1 | 0.580 (x 1.726) | 41 (0.024) | 0.775 | exonuclease activity | rad50 |
| 557 | GO:0008010 | F | 5, | 1 | 0.565 (x 1.769) | 40 (0.025) | 0.776 | structural constituent of larval cuticle (sensu Insecta) | Lcp65Ag1 |
| 558 | GO:0008186 | F | 10, | 1 | 0.565 (x 1.769) | 40 (0.025) | 0.778 | RNA-dependent ATPase activity | CG31755 |
| 559 | GO:0004177 | F | 6, | 1 | 0.565 (x 1.769) | 40 (0.025) | 0.779 | aminopeptidase activity | sda |
| 560 | GO:0015103 | F | 5, | 1 | 0.565 (x 1.769) | 40 (0.025) | 0.78 | inorganic anion transporter activity | CG3036 |
| 561 | GO:0016477 | P | 5, 6, | 3 | 2.474 (x 1.213) | 175 (0.017) | 0.781 | cell migration | os wun wun2 |
| 562 | GO:0043190 | C | 3, 4, | 1 | 0.594 (x 1.684) | 42 (0.024) | 0.782 | ATP-binding cassette (ABC) transporter complex | BEST:CK01227 |
| 563 | GO:0005351 | F | 5, 6, | 1 | 0.565 (x 1.769) | 40 (0.025) | 0.782 | sugar porter activity | TepIV |
| 564 | GO:0043062 | P | 3, | 1 | 0.594 (x 1.684) | 42 (0.024) | 0.783 | extracellular structure organization and biogenesis | cher |
| 565 | GO:0016311 | P | 7, | 2 | 1.527 (x 1.310) | 108 (0.019) | 0.783 | dephosphorylation | wun wun2 |
| 566 | GO:0006817 | P | 8, 9, | 1 | 0.608 (x 1.645) | 43 (0.023) | 0.784 | phosphate transport | CG3036 |
| 567 | GO:0007306 | P | 9, 10, | 1 | 0.594 (x 1.684) | 42 (0.024) | 0.784 | insect chorion formation | Cp36 |
| 568 | GO:0017157 | P | 6, 7, 8, | 1 | 0.608 (x 1.645) | 43 (0.023) | 0.785 | regulation of exocytosis | CG2885 |
| 569 | GO:0004180 | F | 6, | 1 | 0.608 (x 1.645) | 43 (0.023) | 0.787 | carboxypeptidase activity | huntingtin |
| 570 | GO:0007530 | P | 3, | 1 | 0.608 (x 1.645) | 43 (0.023) | 0.788 | sex determination | os |
| 571 | GO:0006812 | P | 6, 7, | 6 | 5.414 (x 1.108) | 383 (0.016) | 0.789 | cation transport | BG:DS03431.1 CG10444 CG3036 Tsf1 Tsf3 na |
| 572 | GO:0008238 | F | 5, | 2 | 1.541 (x 1.298) | 109 (0.018) | 0.79 | exopeptidase activity | huntingtin sda |
| 573 | GO:0030529 | C | 3, 4, 5, 6, | 5 | 4.495 (x 1.112) | 318 (0.016) | 0.801 | ribonucleoprotein complex | CG5205 CG6876 CG7014 RpS4 na |
| 574 | GO:0009408 | P | 4, 5, | 1 | 0.664 (x 1.505) | 47 (0.021) | 0.805 | response to heat | TotA |
| 575 | GO:0031012 | C | 2, | 1 | 0.636 (x 1.572) | 45 (0.022) | 0.806 | extracellular matrix | os |
| 576 | GO:0007293 | P | 8, | 1 | 0.664 (x 1.505) | 47 (0.021) | 0.806 | egg chamber formation (sensu Insecta) | cher |
| 577 | GO:0016811 | F | 5, | 1 | 0.664 (x 1.505) | 47 (0.021) | 0.807 | hydrolase activity, acting on carbon-nitrogen (but not peptide) bonds, in linear amides | CG31373 |
| 578 | GO:0005578 | C | 3, 4, | 1 | 0.636 (x 1.572) | 45 (0.022) | 0.807 | extracellular matrix (sensu Metazoa) | os |
| 579 | GO:0043037 | P | 7, 8, | 3 | 2.629 (x 1.141) | 186 (0.016) | 0.808 | translation | CG33158 Thor apt |
| 580 | GO:0005515 | F | 3, | 18 | 17.443 (x 1.032) | 1234 (0.015) | 0.808 | protein binding | BG:DS01219.1 CG2989 CG3153 CG31666 CG6272 Chit Idgf2 Mhc Mlc2 Mlp60A Thor cher huntingtin os sog unc-13 wun wupA |
| 581 | GO:0005529 | F | 4, | 1 | 0.664 (x 1.505) | 47 (0.021) | 0.809 | sugar binding | CG3244 |
| 582 | GO:0016079 | P | 7, 8, 9, | 1 | 0.650 (x 1.538) | 46 (0.022) | 0.809 | synaptic vesicle exocytosis | unc-13 |
| 583 | GO:0000381 | P | 10, 11, 13, | 1 | 0.693 (x 1.444) | 49 (0.020) | 0.809 | regulation of alternative nuclear mRNA splicing, via spliceosome | hay |
| 584 | GO:0044453 | C | 4, 5, 6, 7, 8, 9, 10, 11, 12, | 1 | 0.664 (x 1.505) | 47 (0.021) | 0.81 | nuclear membrane part | LamC |
| 585 | GO:0006865 | P | 6, 7, 8, | 1 | 0.650 (x 1.538) | 46 (0.022) | 0.81 | amino acid transport | BG:DS03431.1 |
| 586 | GO:0000380 | P | 10, 12, | 1 | 0.693 (x 1.444) | 49 (0.020) | 0.811 | alternative nuclear mRNA splicing, via spliceosome | hay |
| 587 | GO:0009887 | P | 4, | 5 | 4.749 (x 1.053) | 336 (0.015) | 0.811 | organ morphogenesis | Act57B CG7194 apt os sog |
| 588 | GO:0007417 | P | 5, | 2 | 1.724 (x 1.160) | 122 (0.016) | 0.811 | central nervous system development | apt rpr |
| 589 | GO:0005829 | C | 5, 6, 7, 8, | 3 | 2.601 (x 1.153) | 184 (0.016) | 0.811 | cytosol | CG32549 CG7014 RpS4 |
| 590 | GO:0008152 | P | 3, | 72 | 71.481 (x 1.007) | 5057 (0.014) | 0.811 | metabolism | Act57B Aldh BG:DS03431.1 CAH2 CG10444 CG10638 CG11907 CG12264 CG1299 CG14935 CG18522 CG2065 CG2277 CG2885 CG2989 CG3008 CG3036 CG31373 CG31611 CG32549 CG33158 CG4267 CG4511 CG5162 CG5177 CG5205 CG6272 CG6330 CG6512 CG6574 CG6803 CG6876 CG7014 CG7722 CG7842 CG9372 CG9460 CG9836 Chit Dgp-1 Ets21C Gasp GstE1 GstE3 GstE5 GstE6 GstE7 Idgf2 ImpL3 Irbp Jheh3 Ku80 Las Lcp65Ag1 Mlc1 Mocs1 RpS4 Thor Ugt86Da Ugt86Di agt apt cul-2 hay huntingtin na rad50 rpr sda toy wun wun2 |
| 591 | GO:0015171 | F | 4, 5, | 1 | 0.664 (x 1.505) | 47 (0.021) | 0.811 | amino acid transporter activity | BG:DS03431.1 |
| 592 | GO:0030534 | P | 4, | 1 | 0.650 (x 1.538) | 46 (0.022) | 0.812 | adult behavior | na |
| 593 | GO:0016614 | F | 4, | 2 | 1.668 (x 1.199) | 118 (0.017) | 0.812 | oxidoreductase activity, acting on CH-OH group of donors | CG10638 ImpL3 |
| 594 | GO:0008021 | C | 8, 9, 10, 11, 12, | 1 | 0.721 (x 1.387) | 51 (0.020) | 0.812 | synaptic vesicle | unc-13 |
| 595 | GO:0004519 | F | 6, | 1 | 0.693 (x 1.444) | 49 (0.020) | 0.812 | endonuclease activity | rad50 |
| 596 | GO:0006461 | P | 6, | 2 | 1.654 (x 1.209) | 117 (0.017) | 0.812 | protein complex assembly | CG31611 CG6803 |
| 597 | GO:0008415 | F | 6, | 2 | 1.626 (x 1.230) | 115 (0.017) | 0.812 | acyltransferase activity | CG5397 CG7842 |
| 598 | GO:0031965 | C | 5, 6, 7, 8, 9, 10, 11, | 1 | 0.664 (x 1.505) | 47 (0.021) | 0.813 | nuclear membrane | LamC |
| 599 | GO:0044445 | C | 5, 6, 7, 8, 9, | 2 | 1.668 (x 1.199) | 118 (0.017) | 0.813 | cytosolic part | CG7014 RpS4 |
| 600 | GO:0008340 | P | 4, | 1 | 0.650 (x 1.538) | 46 (0.022) | 0.813 | determination of adult life span | cher |
| 601 | GO:0046164 | P | 6, | 1 | 0.721 (x 1.387) | 51 (0.020) | 0.813 | alcohol catabolism | ImpL3 |
| 602 | GO:0005840 | C | 4, 5, 6, 7, 8, | 3 | 2.672 (x 1.123) | 189 (0.016) | 0.813 | ribosome | CG7014 RpS4 na |
| 603 | GO:0009968 | P | 5, 6, | 1 | 0.693 (x 1.444) | 49 (0.020) | 0.813 | negative regulation of signal transduction | sog |
| 604 | GO:0051119 | F | 4, | 1 | 0.664 (x 1.505) | 47 (0.021) | 0.814 | sugar transporter activity | TepIV |
| 605 | GO:0015980 | P | 6, | 2 | 1.668 (x 1.199) | 118 (0.017) | 0.814 | energy derivation by oxidation of organic compounds | CG5177 ImpL3 |
| 606 | GO:0008237 | F | 5, | 3 | 2.615 (x 1.147) | 185 (0.016) | 0.814 | metallopeptidase activity | CG6512 huntingtin sda |
| 607 | GO:0015837 | P | 5, 6, | 1 | 0.650 (x 1.538) | 46 (0.022) | 0.814 | amine transport | BG:DS03431.1 |
| 608 | GO:0005244 | F | 5, 6, | 1 | 0.721 (x 1.387) | 51 (0.020) | 0.814 | voltage-gated ion channel activity | na |
| 609 | GO:0005554 | F | 2, | 11 | 10.630 (x 1.035) | 752 (0.015) | 0.815 | molecular function unknown | CG31279 CG31601 CG32017 CG32021 CG32207 CG32448 CG32625 CG7194 JhI-26 fau yellow-b |
| 610 | GO:0050874 | P | 3, | 13 | 12.722 (x 1.022) | 900 (0.014) | 0.815 | organismal physiological process | CG4917 Lcp65Ag1 Mhc Mlc1 Mlc2 TepIV Thor TotA Tsp42El apt cher unc-13 wupA |
| 611 | GO:0003779 | F | 5, | 2 | 1.739 (x 1.150) | 123 (0.016) | 0.815 | actin binding | cher wupA |
| 612 | GO:0008595 | P | 6, 7, | 1 | 0.707 (x 1.415) | 50 (0.020) | 0.815 | determination of anterior/posterior axis, embryo | sog |
| 613 | GO:0003735 | F | 3, | 3 | 2.657 (x 1.129) | 188 (0.016) | 0.816 | structural constituent of ribosome | CG7014 RpS4 na |
| 614 | GO:0046365 | P | 7, 8, | 1 | 0.721 (x 1.387) | 51 (0.020) | 0.816 | monosaccharide catabolism | ImpL3 |
| 615 | GO:0007568 | P | 3, | 1 | 0.650 (x 1.538) | 46 (0.022) | 0.816 | aging | cher |
| 616 | GO:0008324 | F | 4, | 6 | 5.640 (x 1.064) | 399 (0.015) | 0.816 | cation transporter activity | BG:DS03431.1 CG10444 CG3036 Tsf1 Tsf3 na |
| 617 | GO:0005275 | F | 3, | 1 | 0.735 (x 1.361) | 52 (0.019) | 0.816 | amine transporter activity | BG:DS03431.1 |
| 618 | GO:0009792 | P | 4, | 3 | 2.756 (x 1.088) | 195 (0.015) | 0.816 | embryonic development (sensu Metazoa) | os rpr sog |
| 619 | GO:0007548 | P | 3, | 1 | 0.707 (x 1.415) | 50 (0.020) | 0.817 | sex differentiation | CG7194 |
| 620 | GO:0016789 | F | 5, | 2 | 1.696 (x 1.179) | 120 (0.017) | 0.817 | carboxylic ester hydrolase activity | CG4267 CG5162 |
| 621 | GO:0005739 | C | 5, 6, 7, 8, | 7 | 6.530 (x 1.072) | 462 (0.015) | 0.817 | mitochondrion | Aldh CG12264 CG6512 CG7842 CG8004 Las rpr |
| 622 | GO:0006007 | P | 9, 10, | 1 | 0.721 (x 1.387) | 51 (0.020) | 0.817 | glucose catabolism | ImpL3 |
| 623 | GO:0016747 | F | 5, | 2 | 1.710 (x 1.169) | 121 (0.017) | 0.817 | transferase activity, transferring groups other than amino-acyl groups | CG5397 CG7842 |
| 624 | GO:0015698 | P | 7, 8, | 1 | 0.763 (x 1.310) | 54 (0.019) | 0.817 | inorganic anion transport | CG3036 |
| 625 | GO:0007351 | P | 5, 6, | 1 | 0.707 (x 1.415) | 50 (0.020) | 0.818 | regional subdivision | sog |
| 626 | GO:0007178 | P | 7, | 1 | 0.721 (x 1.387) | 51 (0.020) | 0.818 | transmembrane receptor protein serine/threonine kinase signaling pathway | sog |
| 627 | GO:0016746 | F | 4, | 2 | 1.795 (x 1.114) | 127 (0.016) | 0.819 | transferase activity, transferring acyl groups | CG5397 CG7842 |
| 628 | GO:0015849 | P | 5, 6, | 1 | 0.763 (x 1.310) | 54 (0.019) | 0.819 | organic acid transport | BG:DS03431.1 |
| 629 | GO:0044255 | P | 5, 6, | 5 | 4.735 (x 1.056) | 335 (0.015) | 0.82 | cellular lipid metabolism | CG5162 CG7842 Jheh3 Ugt86Da Ugt86Di |
| 630 | GO:0019320 | P | 8, 9, | 1 | 0.721 (x 1.387) | 51 (0.020) | 0.82 | hexose catabolism | ImpL3 |
| 631 | GO:0051252 | P | 7, | 1 | 0.763 (x 1.310) | 54 (0.019) | 0.82 | regulation of RNA metabolism | hay |
| 632 | GO:0031497 | P | 10, | 1 | 0.721 (x 1.387) | 51 (0.020) | 0.821 | chromatin assembly | CG31611 |
| 633 | GO:0046942 | P | 6, 7, | 1 | 0.763 (x 1.310) | 54 (0.019) | 0.821 | carboxylic acid transport | BG:DS03431.1 |
| 634 | GO:0050684 | P | 8, 9, | 1 | 0.749 (x 1.335) | 53 (0.019) | 0.821 | regulation of mRNA processing | hay |
| 635 | GO:0007498 | P | 4, | 3 | 2.799 (x 1.072) | 198 (0.015) | 0.822 | mesoderm development | Mlc1 Mlp60A toy |
| 636 | GO:0035282 | P | 3, | 2 | 1.781 (x 1.123) | 126 (0.016) | 0.822 | segmentation | os sog |
| 637 | GO:0009266 | P | 4, | 1 | 0.763 (x 1.310) | 54 (0.019) | 0.823 | response to temperature stimulus | TotA |
| 638 | GO:0008356 | P | 5, | 1 | 0.749 (x 1.335) | 53 (0.019) | 0.823 | asymmetric cell division | os |
| 639 | GO:0005737 | C | 4, 5, 6, | 21 | 20.991 (x 1.000) | 1485 (0.014) | 0.823 | cytoplasm | Aldh CG12264 CG32549 CG33158 CG4917 CG6330 CG6512 CG7014 CG7842 CG8004 Las Mhc Mlc1 Mlc2 Mlp60A RpS4 huntingtin na rpr unc-13 wupA |
| 640 | GO:0000004 | P | 2, | 10 | 9.909 (x 1.009) | 701 (0.014) | 0.823 | biological process unknown | CG31279 CG31601 CG32017 CG32021 CG32207 CG32448 CG32625 JhI-26 fau yellow-b |
| 641 | GO:0006397 | P | 8, | 3 | 2.799 (x 1.072) | 198 (0.015) | 0.823 | mRNA processing | CG5205 CG6876 hay |
| 642 | GO:0015075 | F | 3, | 7 | 6.856 (x 1.021) | 485 (0.014) | 0.824 | ion transporter activity | BEST:CK01227 BG:DS03431.1 CG10444 CG3036 Tsf1 Tsf3 na |
| 643 | GO:0007304 | P | 8, 9, | 1 | 0.777 (x 1.286) | 55 (0.018) | 0.824 | eggshell formation (sensu Insecta) | Cp36 |
| 644 | GO:0048024 | P | 9, 10, 12, | 1 | 0.749 (x 1.335) | 53 (0.019) | 0.824 | regulation of nuclear mRNA splicing, via spliceosome | hay |
| 645 | GO:0006887 | P | 6, 7, | 2 | 1.767 (x 1.132) | 125 (0.016) | 0.825 | exocytosis | CG2885 unc-13 |
| 646 | GO:0030703 | P | 7, | 1 | 0.777 (x 1.286) | 55 (0.018) | 0.825 | eggshell formation | Cp36 |
| 647 | GO:0001700 | P | 5, | 2 | 1.823 (x 1.097) | 129 (0.016) | 0.828 | embryonic development (sensu Insecta) | os rpr |
| 648 | GO:0030136 | C | 7, 8, 9, 10, 11, | 1 | 0.792 (x 1.263) | 56 (0.018) | 0.83 | clathrin-coated vesicle | unc-13 |
| 649 | GO:0016836 | F | 5, | 1 | 0.792 (x 1.263) | 56 (0.018) | 0.831 | hydro-lyase activity | CAH2 |
| 650 | GO:0043228 | C | 3, | 10 | 10.050 (x 0.995) | 711 (0.014) | 0.833 | non-membrane-bound organelle | Act57B CG31611 CG7014 LamC Mhc Mlc1 Mlc2 RpS4 na wupA |
| 651 | GO:0048732 | P | 4, | 2 | 1.852 (x 1.080) | 131 (0.015) | 0.834 | gland development | rpr sog |
| 652 | GO:0009950 | P | 5, | 1 | 0.806 (x 1.241) | 57 (0.018) | 0.834 | dorsal/ventral axis specification | sog |
| 653 | GO:0043232 | C | 4, 5, 6, 7, | 10 | 10.050 (x 0.995) | 711 (0.014) | 0.835 | intracellular non-membrane-bound organelle | Act57B CG31611 CG7014 LamC Mhc Mlc1 Mlc2 RpS4 na wupA |
| 654 | GO:0009653 | P | 3, | 9 | 9.075 (x 0.992) | 642 (0.014) | 0.838 | morphogenesis | Act57B CG7194 Thor apt cher mfas os rpr sog |
| 655 | GO:0007369 | P | 5, | 1 | 0.820 (x 1.220) | 58 (0.017) | 0.84 | gastrulation | sog |
| 656 | GO:0008757 | F | 6, | 1 | 0.834 (x 1.199) | 59 (0.017) | 0.84 | S-adenosylmethionine-dependent methyltransferase activity | CG10527 |
| 657 | GO:0048523 | P | 4, | 4 | 3.958 (x 1.011) | 280 (0.014) | 0.84 | negative regulation of cellular process | Thor apt rpr sog |
| 658 | GO:0006790 | P | 5, | 1 | 0.834 (x 1.199) | 59 (0.017) | 0.841 | sulfur metabolism | Act57B |
| 659 | GO:0006413 | P | 8, 9, | 1 | 0.834 (x 1.199) | 59 (0.017) | 0.843 | translational initiation | Thor |
| 660 | GO:0016071 | P | 7, | 3 | 2.940 (x 1.020) | 208 (0.014) | 0.843 | mRNA metabolism | CG5205 CG6876 hay |
| 661 | GO:0030234 | F | 2, | 5 | 5.046 (x 0.991) | 357 (0.014) | 0.843 | enzyme regulator activity | CG17124 CG7054 CG7722 CG9460 TepIV |
| 662 | GO:0044237 | P | 4, | 65 | 65.431 (x 0.993) | 4629 (0.014) | 0.844 | cellular metabolism | Act57B Aldh BG:DS03431.1 CAH2 CG10444 CG10638 CG11907 CG12264 CG1299 CG18522 CG2277 CG2885 CG2989 CG3008 CG3036 CG31373 CG31611 CG32549 CG33158 CG4511 CG5162 CG5177 CG5205 CG6272 CG6330 CG6512 CG6574 CG6876 CG7014 CG7722 CG7842 CG9372 CG9460 Chit Dgp-1 Ets21C Gasp GstE1 GstE3 GstE5 GstE6 GstE7 ImpL3 Irbp Jheh3 Ku80 Las Mlc1 Mocs1 RpS4 Thor Ugt86Da Ugt86Di agt apt cul-2 hay huntingtin na rad50 rpr sda toy wun wun2 |
| 663 | GO:0000578 | P | 5, | 1 | 0.834 (x 1.199) | 59 (0.017) | 0.844 | embryonic axis specification | sog |
| 664 | GO:0051049 | P | 5, 6, | 1 | 0.834 (x 1.199) | 59 (0.017) | 0.845 | regulation of transport | CG2885 |
| 665 | GO:0008202 | P | 6, 7, | 2 | 1.922 (x 1.040) | 136 (0.015) | 0.845 | steroid metabolism | Ugt86Da Ugt86Di |
| 666 | GO:0007015 | P | 9, | 1 | 0.848 (x 1.179) | 60 (0.017) | 0.845 | actin filament organization | cher |
| 667 | GO:0006006 | P | 8, 9, | 1 | 0.848 (x 1.179) | 60 (0.017) | 0.846 | glucose metabolism | ImpL3 |
| 668 | GO:0040008 | P | 3, | 1 | 0.862 (x 1.160) | 61 (0.016) | 0.852 | regulation of growth | Thor |
| 669 | GO:0005635 | C | 4, 5, 6, 7, 8, 9, 10, | 1 | 0.876 (x 1.141) | 62 (0.016) | 0.854 | nuclear envelope | LamC |
| 670 | GO:0048565 | P | 4, | 1 | 0.876 (x 1.141) | 62 (0.016) | 0.855 | gut development | os |
| 671 | GO:0006917 | P | 8, 9, | 1 | 0.876 (x 1.141) | 62 (0.016) | 0.856 | induction of apoptosis | rpr |
| 672 | GO:0016835 | F | 4, | 1 | 0.876 (x 1.141) | 62 (0.016) | 0.858 | carbon-oxygen lyase activity | CAH2 |
| 673 | GO:0030532 | C | 4, 5, 6, 7, 8, 9, 10, | 1 | 0.876 (x 1.141) | 62 (0.016) | 0.859 | small nuclear ribonucleoprotein complex | CG5205 |
| 674 | GO:0000166 | F | 3, | 12 | 12.411 (x 0.967) | 878 (0.014) | 0.861 | nucleotide binding | BEST:CK01227 CG17904 CG2885 CG33158 CG4511 CG4858 CG5205 CG6512 Dgp-1 Mhc hay rad50 |
| 675 | GO:0005509 | F | 5, | 3 | 3.081 (x 0.974) | 218 (0.014) | 0.866 | calcium ion binding | CG10126 CG7447 Mlc2 |
| 676 | GO:0044428 | C | 4, 5, 6, 7, 8, 9, | 7 | 7.280 (x 0.962) | 515 (0.014) | 0.866 | nuclear part | CG5205 CG6876 Ku80 LamC cul-2 hay rad50 |
| 677 | GO:0043065 | P | 7, 8, | 1 | 0.905 (x 1.105) | 64 (0.016) | 0.867 | positive regulation of apoptosis | rpr |
| 678 | GO:0035239 | P | 4, | 1 | 0.919 (x 1.088) | 65 (0.015) | 0.873 | tube morphogenesis | apt |
| 679 | GO:0007276 | P | 4, | 6 | 6.318 (x 0.950) | 447 (0.013) | 0.879 | gametogenesis | CG5162 Cp36 cher os wun wun2 |
| 680 | GO:0007267 | P | 4, | 6 | 6.333 (x 0.947) | 448 (0.013) | 0.881 | cell-cell signaling | CG2989 Chit Idgf2 Tsp42El apt unc-13 |
| 681 | GO:0019207 | F | 3, | 1 | 0.947 (x 1.056) | 67 (0.015) | 0.885 | kinase regulator activity | CG7054 |
| 682 | GO:0003677 | F | 4, | 11 | 11.619 (x 0.947) | 822 (0.013) | 0.886 | DNA binding | CG2885 CG31611 CG31666 CG6272 Ets21C Irbp Ku80 apt hay rad50 toy |
| 683 | GO:0030135 | C | 6, 7, 8, 9, 10, | 1 | 0.961 (x 1.040) | 68 (0.015) | 0.887 | coated vesicle | unc-13 |
| 684 | GO:0030001 | P | 7, 8, | 2 | 2.078 (x 0.963) | 147 (0.014) | 0.888 | metal ion transport | Tsf1 Tsf3 |
| 685 | GO:0019748 | P | 4, | 1 | 0.961 (x 1.040) | 68 (0.015) | 0.889 | secondary metabolism | Jheh3 |
| 686 | GO:0005215 | F | 2, | 13 | 13.683 (x 0.950) | 968 (0.013) | 0.889 | transporter activity | BEST:CK01227 BG:DS03431.1 CG10444 CG11907 CG14935 CG17108 CG3036 CG6574 TepIV Tsf1 Tsf3 glob1 na |
| 687 | GO:0007292 | P | 5, | 4 | 4.382 (x 0.913) | 310 (0.013) | 0.896 | female gamete generation | CG5162 Cp36 cher os |
| 688 | GO:0048519 | P | 3, | 4 | 4.311 (x 0.928) | 305 (0.013) | 0.896 | negative regulation of biological process | Thor apt rpr sog |
| 689 | GO:0006811 | P | 5, 6, | 6 | 6.530 (x 0.919) | 462 (0.013) | 0.897 | ion transport | BG:DS03431.1 CG10444 CG3036 Tsf1 Tsf3 na |
| 690 | GO:0019953 | P | 3, | 6 | 6.446 (x 0.931) | 456 (0.013) | 0.897 | sexual reproduction | CG5162 Cp36 cher os wun wun2 |
| 691 | GO:0044248 | P | 5, | 4 | 4.325 (x 0.925) | 306 (0.013) | 0.897 | cellular catabolism | CG6330 Chit ImpL3 Jheh3 |
| 692 | GO:0012502 | P | 7, 8, | 1 | 0.989 (x 1.011) | 70 (0.014) | 0.897 | induction of programmed cell death | rpr |
| 693 | GO:0006508 | P | 7, | 10 | 10.714 (x 0.933) | 758 (0.013) | 0.898 | proteolysis | CG1299 CG6512 CG7722 CG9372 CG9460 Mlc1 cul-2 huntingtin rpr sda |
| 694 | GO:0016271 | P | 4, | 1 | 1.018 (x 0.983) | 72 (0.014) | 0.898 | tissue death | rpr |
| 695 | GO:0048102 | P | 6, | 1 | 1.004 (x 0.996) | 71 (0.014) | 0.898 | autophagic cell death | rpr |
| 696 | GO:0007559 | P | 5, | 1 | 1.018 (x 0.983) | 72 (0.014) | 0.899 | histolysis | rpr |
| 697 | GO:0035071 | P | 7, | 1 | 1.004 (x 0.996) | 71 (0.014) | 0.9 | salivary gland cell autophagic cell death | rpr |
| 698 | GO:0008652 | P | 7, 8, | 1 | 1.018 (x 0.983) | 72 (0.014) | 0.9 | amino acid biosynthesis | CG12264 |
| 699 | GO:0004222 | F | 6, | 1 | 1.004 (x 0.996) | 71 (0.014) | 0.901 | metalloendopeptidase activity | CG6512 |
| 700 | GO:0051243 | P | 5, | 3 | 3.279 (x 0.915) | 232 (0.013) | 0.902 | negative regulation of cellular physiological process | Thor apt rpr |
| 701 | GO:0046943 | F | 4, | 1 | 1.018 (x 0.983) | 72 (0.014) | 0.902 | carboxylic acid transporter activity | BG:DS03431.1 |
| 702 | GO:0035070 | P | 6, | 1 | 1.004 (x 0.996) | 71 (0.014) | 0.902 | salivary gland histolysis | rpr |
| 703 | GO:0005342 | F | 3, | 1 | 1.046 (x 0.956) | 74 (0.014) | 0.904 | organic acid transporter activity | BG:DS03431.1 |
| 704 | GO:0019752 | P | 6, | 5 | 5.541 (x 0.902) | 392 (0.013) | 0.905 | carboxylic acid metabolism | Aldh BG:DS03431.1 CG12264 CG7842 Las |
| 705 | GO:0009953 | P | 4, | 1 | 1.046 (x 0.956) | 74 (0.014) | 0.906 | dorsal/ventral pattern formation | sog |
| 706 | GO:0006082 | P | 5, | 5 | 5.541 (x 0.902) | 392 (0.013) | 0.906 | organic acid metabolism | Aldh BG:DS03431.1 CG12264 CG7842 Las |
| 707 | GO:0043068 | P | 6, 7, | 1 | 1.060 (x 0.943) | 75 (0.013) | 0.906 | positive regulation of programmed cell death | rpr |
| 708 | GO:0007399 | P | 4, | 6 | 6.615 (x 0.907) | 468 (0.013) | 0.906 | nervous system development | Tsp42El apt mfas rpr toy wupA |
| 709 | GO:0007601 | P | 5, 7, | 1 | 1.046 (x 0.956) | 74 (0.014) | 0.907 | visual perception | CG4917 |
| 710 | GO:0050953 | P | 4, 6, | 1 | 1.046 (x 0.956) | 74 (0.014) | 0.908 | sensory perception of light stimulus | CG4917 |
| 711 | GO:0008017 | F | 6, | 1 | 1.074 (x 0.931) | 76 (0.013) | 0.909 | microtubule binding | huntingtin |
| 712 | GO:0004175 | F | 5, | 6 | 6.658 (x 0.901) | 471 (0.013) | 0.91 | endopeptidase activity | BG:DS01068.5 CG1299 CG6357 CG6512 CG9372 Mlc1 |
| 713 | GO:0006644 | P | 7, 8, | 1 | 1.074 (x 0.931) | 76 (0.013) | 0.91 | phospholipid metabolism | CG5162 |
| 714 | GO:0035295 | P | 3, | 1 | 1.088 (x 0.919) | 77 (0.013) | 0.91 | tube development | apt |
| 715 | GO:0017076 | F | 4, | 11 | 12.001 (x 0.917) | 849 (0.013) | 0.911 | purine nucleotide binding | BEST:CK01227 CG2885 CG33158 CG4511 CG4858 CG5205 CG6512 Dgp-1 Mhc hay rad50 |
| 716 | GO:0000785 | C | 5, 6, 7, 8, 9, 10, | 1 | 1.088 (x 0.919) | 77 (0.013) | 0.912 | chromatin | CG31611 |
| 717 | GO:0043118 | P | 4, | 3 | 3.407 (x 0.881) | 241 (0.012) | 0.913 | negative regulation of physiological process | Thor apt rpr |
| 718 | GO:0046872 | F | 4, | 12 | 13.174 (x 0.911) | 932 (0.013) | 0.921 | metal ion binding | CAH2 CG10126 CG31063 CG31666 CG7447 Las Mlc2 Mlp60A Mocs1 Tsf1 Tsf3 sda |
| 719 | GO:0043167 | F | 3, | 12 | 13.174 (x 0.911) | 932 (0.013) | 0.922 | ion binding | CAH2 CG10126 CG31063 CG31666 CG7447 Las Mlc2 Mlp60A Mocs1 Tsf1 Tsf3 sda |
| 720 | GO:0003704 | F | 4, | 1 | 1.117 (x 0.896) | 79 (0.013) | 0.922 | specific RNA polymerase II transcription factor activity | toy |
| 721 | GO:0019318 | P | 7, 8, | 1 | 1.131 (x 0.884) | 80 (0.013) | 0.923 | hexose metabolism | ImpL3 |
| 722 | GO:0048489 | P | 6, 7, | 1 | 1.131 (x 0.884) | 80 (0.013) | 0.924 | synaptic vesicle transport | unc-13 |
| 723 | GO:0006928 | P | 4, 5, | 3 | 3.633 (x 0.826) | 257 (0.012) | 0.925 | cell motility | os wun wun2 |
| 724 | GO:0008233 | F | 4, | 8 | 9.202 (x 0.869) | 651 (0.012) | 0.925 | peptidase activity | BG:DS01068.5 CG1299 CG6357 CG6512 CG9372 Mlc1 huntingtin sda |
| 725 | GO:0012501 | P | 5, | 3 | 3.505 (x 0.856) | 248 (0.012) | 0.925 | programmed cell death | rpr wun wun2 |
| 726 | GO:0006643 | P | 6, 7, | 1 | 1.230 (x 0.813) | 87 (0.011) | 0.926 | membrane lipid metabolism | CG5162 |
| 727 | GO:0004252 | F | 6, | 3 | 3.633 (x 0.826) | 257 (0.012) | 0.926 | serine-type endopeptidase activity | BG:DS01068.5 CG1299 CG9372 |
| 728 | GO:0009056 | P | 4, | 4 | 4.622 (x 0.865) | 327 (0.012) | 0.926 | catabolism | CG6330 Chit ImpL3 Jheh3 |
| 729 | GO:0031982 | C | 3, | 1 | 1.216 (x 0.823) | 86 (0.012) | 0.927 | vesicle | unc-13 |
| 730 | GO:0015144 | F | 3, | 1 | 1.145 (x 0.873) | 81 (0.012) | 0.927 | carbohydrate transporter activity | TepIV |
| 731 | GO:0006396 | P | 7, | 3 | 3.604 (x 0.832) | 255 (0.012) | 0.927 | RNA processing | CG5205 CG6876 hay |
| 732 | GO:0005792 | C | 6, 7, | 1 | 1.173 (x 0.852) | 83 (0.012) | 0.927 | microsome | Jheh3 |
| 733 | GO:0051674 | P | 4, | 3 | 3.633 (x 0.826) | 257 (0.012) | 0.927 | localization of cell | os wun wun2 |
| 734 | GO:0016810 | F | 4, | 1 | 1.201 (x 0.832) | 85 (0.012) | 0.927 | hydrolase activity, acting on carbon-nitrogen (but not peptide) bonds | CG31373 |
| 735 | GO:0006352 | P | 8, | 1 | 1.216 (x 0.823) | 86 (0.012) | 0.928 | transcription initiation | hay |
| 736 | GO:0016591 | C | 3, 6, 7, 8, 9, 10, 11, 12, 13, | 1 | 1.173 (x 0.852) | 83 (0.012) | 0.928 | DNA-directed RNA polymerase II, holoenzyme | hay |
| 737 | GO:0045182 | F | 2, | 1 | 1.201 (x 0.832) | 85 (0.012) | 0.929 | translation regulator activity | CG33158 |
| 738 | GO:0007389 | P | 3, | 3 | 3.619 (x 0.829) | 256 (0.012) | 0.929 | pattern specification | os sog wun |
| 739 | GO:0031988 | C | 4, | 1 | 1.216 (x 0.823) | 86 (0.012) | 0.929 | membrane-bound vesicle | unc-13 |
| 740 | GO:0006367 | P | 9, | 1 | 1.187 (x 0.842) | 84 (0.012) | 0.929 | transcription initiation from RNA polymerase II promoter | hay |
| 741 | GO:0016023 | C | 5, 6, 7, 8, 9, | 1 | 1.173 (x 0.852) | 83 (0.012) | 0.93 | cytoplasmic membrane-bound vesicle | unc-13 |
| 742 | GO:0008610 | P | 5, 6, 7, | 1 | 1.201 (x 0.832) | 85 (0.012) | 0.93 | lipid biosynthesis | CG7842 |
| 743 | GO:0008219 | P | 4, | 3 | 3.534 (x 0.849) | 250 (0.012) | 0.93 | cell death | rpr wun wun2 |
| 744 | GO:0008135 | F | 3, 4, | 1 | 1.159 (x 0.863) | 82 (0.012) | 0.93 | translation factor activity, nucleic acid binding | CG33158 |
| 745 | GO:0044271 | P | 5, 6, | 1 | 1.187 (x 0.842) | 84 (0.012) | 0.93 | nitrogen compound biosynthesis | CG12264 |
| 746 | GO:0042598 | C | 5, 6, | 1 | 1.173 (x 0.852) | 83 (0.012) | 0.931 | vesicular fraction | Jheh3 |
| 747 | GO:0006996 | P | 5, | 9 | 10.206 (x 0.882) | 722 (0.012) | 0.931 | organelle organization and biogenesis | Act57B CG31611 CG6803 Irbp Ku80 LamC cher huntingtin rad50 |
| 748 | GO:0005667 | C | 3, 6, 7, 8, 9, 10, 11, 12, 13, | 1 | 1.201 (x 0.832) | 85 (0.012) | 0.931 | transcription factor complex | hay |
| 749 | GO:0009309 | P | 6, 7, | 1 | 1.187 (x 0.842) | 84 (0.012) | 0.932 | amine biosynthesis | CG12264 |
| 750 | GO:0044265 | P | 6, | 2 | 2.431 (x 0.823) | 172 (0.012) | 0.932 | cellular macromolecule catabolism | Chit ImpL3 |
| 751 | GO:0016265 | P | 3, | 3 | 3.548 (x 0.846) | 251 (0.012) | 0.932 | death | rpr wun wun2 |
| 752 | GO:0031410 | C | 4, 5, 6, 7, 8, | 1 | 1.173 (x 0.852) | 83 (0.012) | 0.932 | cytoplasmic vesicle | unc-13 |
| 753 | GO:0040011 | P | 3, | 3 | 3.689 (x 0.813) | 261 (0.011) | 0.933 | locomotion | os wun wun2 |
| 754 | GO:0000003 | P | 2, | 6 | 7.082 (x 0.847) | 501 (0.012) | 0.934 | reproduction | CG5162 Cp36 cher os wun wun2 |
| 755 | GO:0043169 | F | 4, | 11 | 12.538 (x 0.877) | 887 (0.012) | 0.934 | cation binding | CAH2 CG10126 CG31666 CG7447 Las Mlc2 Mlp60A Mocs1 Tsf1 Tsf3 sda |
| 756 | GO:0002165 | P | 4, | 4 | 4.877 (x 0.820) | 345 (0.012) | 0.935 | larval or pupal development (sensu Insecta) | Lcp65Ag1 rad50 rpr sog |
| 757 | GO:0004518 | F | 5, | 1 | 1.272 (x 0.786) | 90 (0.011) | 0.935 | nuclease activity | rad50 |
| 758 | GO:0040007 | P | 2, | 1 | 1.272 (x 0.786) | 90 (0.011) | 0.937 | growth | Thor |
| 759 | GO:0015631 | F | 5, | 1 | 1.286 (x 0.777) | 91 (0.011) | 0.938 | tubulin binding | huntingtin |
| 760 | GO:0003702 | F | 3, | 3 | 3.760 (x 0.798) | 266 (0.011) | 0.941 | RNA polymerase II transcription factor activity | apt hay toy |
| 761 | GO:0016879 | F | 4, | 2 | 2.573 (x 0.777) | 182 (0.011) | 0.942 | ligase activity, forming carbon-nitrogen bonds | CG4917 Las |
| 762 | GO:0005624 | C | 4, 5, | 1 | 1.315 (x 0.761) | 93 (0.011) | 0.942 | membrane fraction | Jheh3 |
| 763 | GO:0009790 | P | 3, | 4 | 4.961 (x 0.806) | 351 (0.011) | 0.942 | embryonic development | os rpr sog wun |
| 764 | GO:0000278 | P | 5, | 3 | 3.788 (x 0.792) | 268 (0.011) | 0.943 | mitotic cell cycle | CG6512 LamC Mhc |
| 765 | GO:0030154 | P | 3, | 6 | 7.209 (x 0.832) | 510 (0.012) | 0.943 | cell differentiation | CG6803 Mhc mfas os wun2 wupA |
| 766 | GO:0048731 | P | 3, | 7 | 8.396 (x 0.834) | 594 (0.012) | 0.948 | system development | Tsp42El apt mfas rpr sog toy wupA |
| 767 | GO:0005524 | F | 6, | 8 | 9.499 (x 0.842) | 672 (0.012) | 0.949 | ATP binding | BEST:CK01227 CG4511 CG4858 CG5205 CG6512 Mhc hay rad50 |
| 768 | GO:0009791 | P | 3, | 4 | 5.032 (x 0.795) | 356 (0.011) | 0.949 | post-embryonic development | Lcp65Ag1 rad50 rpr sog |
| 769 | GO:0043170 | P | 4, | 41 | 44.045 (x 0.931) | 3116 (0.013) | 0.95 | macromolecule metabolism | Act57B CG10444 CG1299 CG14935 CG2989 CG3008 CG3036 CG31373 CG31611 CG33158 CG5177 CG5205 CG6512 CG6574 CG6803 CG6876 CG7014 CG7722 CG9372 CG9460 Chit Dgp-1 Gasp ImpL3 Irbp Ku80 Las Mlc1 RpS4 Thor Ugt86Da Ugt86Di agt apt cul-2 hay huntingtin na rad50 rpr sda |
| 770 | GO:0000267 | C | 3, 4, | 1 | 1.357 (x 0.737) | 96 (0.010) | 0.95 | cell fraction | Jheh3 |
| 771 | GO:0046914 | F | 5, | 8 | 9.555 (x 0.837) | 676 (0.012) | 0.95 | transition metal ion binding | CAH2 CG31666 Las Mlp60A Mocs1 Tsf1 Tsf3 sda |
| 772 | GO:0048522 | P | 4, | 2 | 2.672 (x 0.749) | 189 (0.011) | 0.952 | positive regulation of cellular process | rpr sog |
| 773 | GO:0009057 | P | 5, | 2 | 2.686 (x 0.745) | 190 (0.011) | 0.954 | macromolecule catabolism | Chit ImpL3 |
| 774 | GO:0009993 | P | 7, | 3 | 3.915 (x 0.766) | 277 (0.011) | 0.955 | oogenesis (sensu Insecta) | Cp36 cher os |
| 775 | GO:0006333 | P | 9, | 1 | 1.414 (x 0.707) | 100 (0.010) | 0.958 | chromatin assembly or disassembly | CG31611 |
| 776 | GO:0006820 | P | 6, 7, | 1 | 1.414 (x 0.707) | 100 (0.010) | 0.959 | anion transport | CG3036 |
| 777 | GO:0005996 | P | 6, 7, | 1 | 1.414 (x 0.707) | 100 (0.010) | 0.96 | monosaccharide metabolism | ImpL3 |
| 778 | GO:0016251 | F | 4, | 1 | 1.428 (x 0.700) | 101 (0.010) | 0.961 | general RNA polymerase II transcription factor activity | hay |
| 779 | GO:0006631 | P | 6, 7, | 1 | 1.456 (x 0.687) | 103 (0.010) | 0.962 | fatty acid metabolism | CG7842 |
| 780 | GO:0030705 | P | 6, 7, 8, | 1 | 1.470 (x 0.680) | 104 (0.010) | 0.962 | cytoskeleton-dependent intracellular transport | huntingtin |
| 781 | GO:0045055 | P | 6, 7, | 1 | 1.484 (x 0.674) | 105 (0.010) | 0.963 | regulated secretory pathway | unc-13 |
| 782 | GO:0007476 | P | 6, 7, 8, | 1 | 1.456 (x 0.687) | 103 (0.010) | 0.963 | wing morphogenesis | sog |
| 783 | GO:0006092 | P | 7, | 1 | 1.470 (x 0.680) | 104 (0.010) | 0.964 | main pathways of carbohydrate metabolism | ImpL3 |
| 784 | GO:0007269 | P | 6, 7, 8, | 1 | 1.484 (x 0.674) | 105 (0.010) | 0.964 | neurotransmitter secretion | unc-13 |
| 785 | GO:0030554 | F | 5, | 8 | 9.739 (x 0.821) | 689 (0.012) | 0.964 | adenyl nucleotide binding | BEST:CK01227 CG4511 CG4858 CG5205 CG6512 Mhc hay rad50 |
| 786 | GO:0007018 | P | 7, 8, 9, | 1 | 1.456 (x 0.687) | 103 (0.010) | 0.964 | microtubule-based movement | huntingtin |
| 787 | GO:0005886 | C | 4, 5, | 6 | 7.548 (x 0.795) | 534 (0.011) | 0.965 | plasma membrane | BG:DS01219.1 BG:DS03431.1 mfas sog wun wun2 |
| 788 | GO:0007010 | P | 6, | 5 | 6.361 (x 0.786) | 450 (0.011) | 0.965 | cytoskeleton organization and biogenesis | Act57B CG6803 LamC cher huntingtin |
| 789 | GO:0007472 | P | 6, 7, | 1 | 1.484 (x 0.674) | 105 (0.010) | 0.965 | wing disc morphogenesis | sog |
| 790 | GO:0048477 | P | 6, | 3 | 4.085 (x 0.734) | 289 (0.010) | 0.966 | oogenesis | Cp36 cher os |
| 791 | GO:0009966 | P | 4, 5, | 1 | 1.456 (x 0.687) | 103 (0.010) | 0.966 | regulation of signal transduction | sog |
| 792 | GO:0035272 | P | 4, | 1 | 1.541 (x 0.649) | 109 (0.009) | 0.97 | exocrine system development | rpr |
| 793 | GO:0007424 | P | 4, | 1 | 1.541 (x 0.649) | 109 (0.009) | 0.971 | tracheal system development (sensu Insecta) | apt |
| 794 | GO:0042981 | P | 6, 7, | 1 | 1.541 (x 0.649) | 109 (0.009) | 0.972 | regulation of apoptosis | rpr |
| 795 | GO:0008236 | F | 5, | 3 | 4.142 (x 0.724) | 293 (0.010) | 0.973 | serine-type peptidase activity | BG:DS01068.5 CG1299 CG9372 |
| 796 | GO:0016491 | F | 3, | 7 | 8.820 (x 0.794) | 624 (0.011) | 0.973 | oxidoreductase activity | Aldh CG10638 CG12224 CG18522 CG18547 CG2065 ImpL3 |
| 797 | GO:0007431 | P | 5, | 1 | 1.541 (x 0.649) | 109 (0.009) | 0.973 | salivary gland development | rpr |
| 798 | GO:0004263 | F | 7, | 2 | 2.940 (x 0.680) | 208 (0.010) | 0.977 | chymotrypsin activity | CG1299 CG9372 |
| 799 | GO:0007281 | P | 5, | 1 | 1.583 (x 0.632) | 112 (0.009) | 0.978 | germ cell development | os |
| 800 | GO:0005783 | C | 5, 6, 7, 8, | 1 | 1.597 (x 0.626) | 113 (0.009) | 0.979 | endoplasmic reticulum | CG6512 |
| 801 | GO:0016567 | P | 9, | 1 | 1.597 (x 0.626) | 113 (0.009) | 0.98 | protein ubiquitination | rpr |
| 802 | GO:0001505 | P | 7, | 1 | 1.654 (x 0.605) | 117 (0.009) | 0.982 | regulation of neurotransmitter levels | unc-13 |
| 803 | GO:0045045 | P | 5, 6, | 2 | 3.039 (x 0.658) | 215 (0.009) | 0.982 | secretory pathway | CG2885 unc-13 |
| 804 | GO:0044446 | C | 3, 4, 5, 6, 7, | 17 | 20.128 (x 0.845) | 1424 (0.012) | 0.982 | intracellular organelle part | Act57B Aldh CG31611 CG5205 CG6876 CG7014 CG8004 Ku80 LamC Mhc Mlc1 Mlc2 RpS4 cul-2 hay rad50 wupA |
| 805 | GO:0007444 | P | 4, | 3 | 4.269 (x 0.703) | 302 (0.010) | 0.983 | imaginal disc development | Idgf2 sog toy |
| 806 | GO:0007264 | P | 6, | 1 | 1.640 (x 0.610) | 116 (0.009) | 0.983 | small GTPase mediated signal transduction | CG2885 |
| 807 | GO:0044422 | C | 2, 3, | 17 | 20.128 (x 0.845) | 1424 (0.012) | 0.983 | organelle part | Act57B Aldh CG31611 CG5205 CG6876 CG7014 CG8004 Ku80 LamC Mhc Mlc1 Mlc2 RpS4 cul-2 hay rad50 wupA |
| 808 | GO:0005261 | F | 5, 6, | 1 | 1.626 (x 0.615) | 115 (0.009) | 0.984 | cation channel activity | na |
| 809 | GO:0035220 | P | 5, | 1 | 1.640 (x 0.610) | 116 (0.009) | 0.984 | wing disc development | sog |
| 810 | GO:0048518 | P | 3, | 2 | 3.081 (x 0.649) | 218 (0.009) | 0.984 | positive regulation of biological process | rpr sog |
| 811 | GO:0043067 | P | 5, 6, | 1 | 1.682 (x 0.595) | 119 (0.008) | 0.986 | regulation of programmed cell death | rpr |
| 812 | GO:0006897 | P | 6, 7, | 1 | 1.640 (x 0.610) | 116 (0.009) | 0.986 | endocytosis | CG2885 |
| 813 | GO:0006260 | P | 7, | 1 | 1.710 (x 0.585) | 121 (0.008) | 0.986 | DNA replication | Irbp |
| 814 | GO:0009948 | P | 5, | 1 | 1.682 (x 0.595) | 119 (0.008) | 0.987 | anterior/posterior axis specification | sog |
| 815 | GO:0048667 | P | 6, 7, 9, | 1 | 1.710 (x 0.585) | 121 (0.008) | 0.987 | neuron morphogenesis during differentiation | mfas |
| 816 | GO:0048812 | P | 7, 8, 10, | 1 | 1.710 (x 0.585) | 121 (0.008) | 0.988 | neurite morphogenesis | mfas |
| 817 | GO:0007409 | P | 8, 9, 11, | 1 | 1.710 (x 0.585) | 121 (0.008) | 0.989 | axonogenesis | mfas |
| 818 | GO:0016874 | F | 3, | 3 | 4.481 (x 0.670) | 317 (0.009) | 0.991 | ligase activity | CG17108 CG4917 Las |
| 819 | GO:0000151 | C | 3, 4, 5, 6, | 1 | 1.781 (x 0.561) | 126 (0.008) | 0.992 | ubiquitin ligase complex | cul-2 |
| 820 | GO:0007268 | P | 6, | 2 | 3.195 (x 0.626) | 226 (0.009) | 0.992 | synaptic transmission | apt unc-13 |
| 821 | GO:0016337 | P | 4, | 1 | 1.753 (x 0.571) | 124 (0.008) | 0.992 | cell-cell adhesion | mfas |
| 822 | GO:0043285 | P | 6, | 1 | 1.795 (x 0.557) | 127 (0.008) | 0.992 | biopolymer catabolism | Chit |
| 823 | GO:0000087 | P | 6, | 2 | 3.209 (x 0.623) | 227 (0.009) | 0.993 | M phase of mitotic cell cycle | CG6512 Mhc |
| 824 | GO:0007067 | P | 7, | 2 | 3.195 (x 0.626) | 226 (0.009) | 0.993 | mitosis | CG6512 Mhc |
| 825 | GO:0007169 | P | 7, | 1 | 1.753 (x 0.571) | 124 (0.008) | 0.993 | transmembrane receptor protein tyrosine kinase signaling pathway | sog |
| 826 | GO:0007243 | P | 6, | 1 | 1.795 (x 0.557) | 127 (0.008) | 0.994 | protein kinase cascade | os |
| 827 | GO:0009952 | P | 4, | 1 | 1.838 (x 0.544) | 130 (0.008) | 0.994 | anterior/posterior pattern formation | sog |
| 828 | GO:0051179 | P | 3, | 21 | 25.033 (x 0.839) | 1771 (0.012) | 0.995 | localization | BEST:CK01227 BG:DS01219.1 BG:DS03431.1 CG10444 CG11907 CG17108 CG2885 CG3036 CG6574 CG8004 TepIV Tsf1 Tsf3 cher glob1 huntingtin na os unc-13 wun wun2 |
| 829 | GO:0002009 | P | 4, | 1 | 1.880 (x 0.532) | 133 (0.008) | 0.995 | morphogenesis of an epithelium | cher |
| 830 | GO:0035114 | P | 5, | 1 | 1.838 (x 0.544) | 130 (0.008) | 0.996 | appendage morphogenesis (sensu Endopterygota) | sog |
| 831 | GO:0035107 | P | 4, | 1 | 1.880 (x 0.532) | 133 (0.008) | 0.996 | appendage morphogenesis | sog |
| 832 | GO:0004295 | F | 7, | 2 | 3.322 (x 0.602) | 235 (0.009) | 0.996 | trypsin activity | CG1299 CG9372 |
| 833 | GO:0046903 | P | 5, | 2 | 3.265 (x 0.613) | 231 (0.009) | 0.997 | secretion | CG2885 unc-13 |
| 834 | GO:0003924 | F | 8, | 1 | 1.838 (x 0.544) | 130 (0.008) | 0.997 | GTPase activity | CG2885 |
| 835 | GO:0044260 | P | 5, | 26 | 30.419 (x 0.855) | 2152 (0.012) | 0.997 | cellular macromolecule metabolism | Act57B CG10444 CG1299 CG2989 CG3008 CG31373 CG33158 CG6512 CG6574 CG7014 CG7722 CG9372 CG9460 Chit Dgp-1 Gasp Las Mlc1 RpS4 Thor apt cul-2 huntingtin na rpr sda |
| 836 | GO:0048736 | P | 3, | 1 | 1.880 (x 0.532) | 133 (0.008) | 0.997 | appendage development | sog |
| 837 | GO:0048737 | P | 4, | 1 | 1.838 (x 0.544) | 130 (0.008) | 0.998 | appendage development (sensu Endopterygota) | sog |
| 838 | GO:0043412 | P | 6, | 3 | 12.962 (x 0.231) | 917 (0.003) | 1 | biopolymer modification | CG3008 CG31373 rpr |
| 839 | GO:0006464 | P | 7, | 3 | 12.396 (x 0.242) | 877 (0.003) | 1 | protein modification | CG3008 CG31373 rpr |
| 840 | GO:0004872 | F | 3, | 1 | 7.817 (x 0.128) | 553 (0.002) | 1 | receptor activity | unc-13 |
| 841 | GO:0007582 | P | 2, | 92 | 96.571 (x 0.953) | 6832 (0.013) | 1 | physiological process | Act57B Aldh BEST:CK01227 BG:DS01219.1 BG:DS03431.1 CAH2 CG10444 CG10638 CG11907 CG12264 CG1299 CG14935 CG17108 CG18522 CG2065 CG2277 CG2885 CG2989 CG3008 CG3036 CG31373 CG31611 CG32549 CG33158 CG4267 CG4511 CG4917 CG5162 CG5177 CG5205 CG6272 CG6330 CG6512 CG6574 CG6803 CG6876 CG7014 CG7722 CG7842 CG8004 CG9372 CG9460 CG9836 Chit Dgp-1 Ets21C Gasp GstE1 GstE3 GstE5 GstE6 GstE7 Idgf2 ImpL3 Irbp Jheh3 Ku80 LamC Las Lcp65Ag1 Mhc Mlc1 Mlc2 Mlp60A Mocs1 RpS4 TepIV Thor TotA Tsf1 Tsf3 Tsp42El Ugt86Da Ugt86Di agt apt cher cul-2 glob1 hay huntingtin mfas na os rad50 rpr sda toy unc-13 wun wun2 wupA |
| 842 | GO:0050789 | P | 2, | 10 | 21.273 (x 0.470) | 1505 (0.007) | 1 | regulation of biological process | CG2885 CG6272 Ets21C Thor apt cul-2 hay rpr sog toy |
| 843 | GO:0051244 | P | 4, | 9 | 18.333 (x 0.491) | 1297 (0.007) | 1 | regulation of cellular physiological process | CG2885 CG6272 Ets21C Thor apt cul-2 hay rpr toy |
| 844 | GO:0016310 | P | 7, | 1 | 6.516 (x 0.153) | 461 (0.002) | 1 | phosphorylation | CG3008 |
| 845 | GO:0050791 | P | 3, | 9 | 18.927 (x 0.476) | 1339 (0.007) | 1 | regulation of physiological process | CG2885 CG6272 Ets21C Thor apt cul-2 hay rpr toy |
| 846 | GO:0007166 | P | 5, | 3 | 9.315 (x 0.322) | 659 (0.005) | 1 | cell surface receptor linked signal transduction | sog wun wun2 |
| 847 | GO:0006323 | P | 7, | 1 | 1.951 (x 0.513) | 138 (0.007) | 1 | DNA packaging | CG31611 |
| 848 | GO:0050794 | P | 3, | 10 | 19.464 (x 0.514) | 1377 (0.007) | 1 | regulation of cellular process | CG2885 CG6272 Ets21C Thor apt cul-2 hay rpr sog toy |
| 849 | GO:0006325 | P | 8, | 1 | 1.951 (x 0.513) | 138 (0.007) | 1 | establishment and/or maintenance of chromatin architecture | CG31611 |
| 850 | GO:0044464 | C | 2, 3, | 51 | 65.247 (x 0.782) | 4616 (0.011) | 1 | cell part | Act57B Aldh BEST:CK01227 BG:DS01219.1 BG:DS03431.1 CG10444 CG11907 CG12264 CG17108 CG3036 CG31611 CG31666 CG32549 CG33158 CG4917 CG5205 CG6272 CG6330 CG6512 CG6574 CG6876 CG7014 CG7842 CG8004 Cp36 Ets21C Irbp Jheh3 Ku80 LamC Las Mhc Mlc1 Mlc2 Mlp60A RpS4 Tsp42El apt cul-2 hay huntingtin mfas na rad50 rpr sog toy unc-13 wun wun2 wupA |
| 851 | GO:0005623 | C | 2, | 51 | 65.247 (x 0.782) | 4616 (0.011) | 1 | cell | Act57B Aldh BEST:CK01227 BG:DS01219.1 BG:DS03431.1 CG10444 CG11907 CG12264 CG17108 CG3036 CG31611 CG31666 CG32549 CG33158 CG4917 CG5205 CG6272 CG6330 CG6512 CG6574 CG6876 CG7014 CG7842 CG8004 Cp36 Ets21C Irbp Jheh3 Ku80 LamC Las Mhc Mlc1 Mlc2 Mlp60A RpS4 Tsp42El apt cul-2 hay huntingtin mfas na rad50 rpr sog toy unc-13 wun wun2 wupA |
| 852 | GO:0045184 | P | 5, | 2 | 7.350 (x 0.272) | 520 (0.004) | 1 | establishment of protein localization | CG2885 CG8004 |
| 853 | GO:0015031 | P | 5, 6, | 2 | 7.308 (x 0.274) | 517 (0.004) | 1 | protein transport | CG2885 CG8004 |
| 854 | GO:0051641 | P | 4, 5, | 3 | 8.990 (x 0.334) | 636 (0.005) | 1 | cellular localization | CG2885 CG8004 huntingtin |
| 855 | GO:0051649 | P | 5, 6, | 3 | 8.976 (x 0.334) | 635 (0.005) | 1 | establishment of cellular localization | CG2885 CG8004 huntingtin |
| 856 | GO:0048666 | P | 5, 8, | 1 | 2.007 (x 0.498) | 142 (0.007) | 1 | neuron development | mfas |
| 857 | GO:0006886 | P | 6, 7, 8, | 2 | 7.152 (x 0.280) | 506 (0.004) | 1 | intracellular protein transport | CG2885 CG8004 |
| 858 | GO:0016772 | F | 4, | 2 | 7.082 (x 0.282) | 501 (0.004) | 1 | transferase activity, transferring phosphorus-containing groups | CG3008 Irbp |
| 859 | GO:0031175 | P | 6, 9, | 1 | 2.007 (x 0.498) | 142 (0.007) | 1 | neurite development | mfas |
| 860 | GO:0006350 | P | 6, | 6 | 13.358 (x 0.449) | 945 (0.006) | 1 | transcription | CG2885 CG6272 Ets21C apt hay toy |
| 861 | GO:0005759 | C | 5, 6, 7, 8, 9, 10, 11, | 1 | 2.050 (x 0.488) | 145 (0.007) | 1 | mitochondrial matrix | Aldh |
| 862 | GO:0030528 | F | 2, | 5 | 11.379 (x 0.439) | 805 (0.006) | 1 | transcription regulator activity | CG31666 Ets21C apt hay toy |
| 863 | GO:0012505 | C | 4, 5, | 1 | 2.035 (x 0.491) | 144 (0.007) | 1 | endomembrane system | LamC |
| 864 | GO:0046907 | P | 5, 6, 7, | 3 | 8.707 (x 0.345) | 616 (0.005) | 1 | intracellular transport | CG2885 CG8004 huntingtin |
| 865 | GO:0044459 | C | 4, 5, 6, | 1 | 4.566 (x 0.219) | 323 (0.003) | 1 | plasma membrane part | BG:DS03431.1 |
| 866 | GO:0043234 | C | 2, | 18 | 22.319 (x 0.806) | 1579 (0.011) | 1 | protein complex | BEST:CK01227 CG31611 CG33158 CG4917 CG5205 CG6876 CG7014 Ku80 Mhc Mlc1 Mlc2 Mocs1 RpS4 cul-2 hay na rad50 wupA |
| 867 | GO:0031980 | C | 4, 5, 6, 7, 8, 9, 10, | 1 | 2.050 (x 0.488) | 145 (0.007) | 1 | mitochondrial lumen | Aldh |
| 868 | GO:0006351 | P | 7, | 6 | 12.665 (x 0.474) | 896 (0.007) | 1 | transcription, DNA-dependent | CG2885 CG6272 Ets21C apt hay toy |
| 869 | GO:0045449 | P | 7, | 5 | 11.746 (x 0.426) | 831 (0.006) | 1 | regulation of transcription | CG2885 CG6272 Ets21C apt toy |
| 870 | GO:0019219 | P | 6, | 6 | 12.552 (x 0.478) | 888 (0.007) | 1 | regulation of nucleobase, nucleoside, nucleotide and nucleic acid metabolism | CG2885 CG6272 Ets21C apt hay toy |
| 871 | GO:0006355 | P | 8, | 5 | 11.124 (x 0.449) | 787 (0.006) | 1 | regulation of transcription, DNA-dependent | CG2885 CG6272 Ets21C apt toy |
| 872 | GO:0006366 | P | 8, | 4 | 9.640 (x 0.415) | 682 (0.006) | 1 | transcription from RNA polymerase II promoter | Ets21C apt hay toy |
| 873 | GO:0016020 | C | 3, 4, | 17 | 26.376 (x 0.645) | 1866 (0.009) | 1 | membrane | BEST:CK01227 BG:DS01219.1 BG:DS03431.1 CG10444 CG11907 CG17108 CG3036 CG6512 CG6574 CG8004 LamC Tsp42El mfas na sog wun wun2 |
| 874 | GO:0007154 | P | 3, | 13 | 21.443 (x 0.606) | 1517 (0.009) | 1 | cell communication | CG2885 CG2989 CG7054 Chit Idgf2 Tsp42El apt mfas os sog unc-13 wun wun2 |
| 875 | GO:0004871 | F | 2, | 8 | 14.884 (x 0.537) | 1053 (0.008) | 1 | signal transducer activity | CG2989 CG3153 Chit Idgf2 Tsp42El os sog unc-13 |
| 876 | GO:0007155 | P | 3, | 1 | 4.283 (x 0.233) | 303 (0.003) | 1 | cell adhesion | mfas |
| 877 | GO:0044427 | C | 4, 5, 6, 7, 8, 9, | 1 | 2.092 (x 0.478) | 148 (0.007) | 1 | chromosomal part | CG31611 |
| 878 | GO:0031974 | C | 2, | 2 | 6.092 (x 0.328) | 431 (0.005) | 1 | membrane-enclosed lumen | Aldh hay |
| 879 | GO:0030182 | P | 4, 7, | 1 | 2.106 (x 0.475) | 149 (0.007) | 1 | neuron differentiation | mfas |
| 880 | GO:0043233 | C | 3, 4, | 2 | 6.092 (x 0.328) | 431 (0.005) | 1 | organelle lumen | Aldh hay |
| 881 | GO:0003676 | F | 3, | 16 | 24.637 (x 0.649) | 1743 (0.009) | 1 | nucleic acid binding | CG2885 CG31611 CG31666 CG33158 CG6272 CG6876 CG7014 Ets21C Irbp Ku80 RpS4 agt apt hay rad50 toy |
| 882 | GO:0043492 | F | 3, 10, | 1 | 2.134 (x 0.469) | 151 (0.007) | 1 | ATPase activity, coupled to movement of substances | BEST:CK01227 |
| 883 | GO:0006357 | P | 9, | 3 | 7.732 (x 0.388) | 547 (0.005) | 1 | regulation of transcription from RNA polymerase II promoter | Ets21C apt toy |
| 884 | GO:0043227 | C | 3, | 23 | 32.638 (x 0.705) | 2309 (0.010) | 1 | membrane-bound organelle | Aldh CG12264 CG31611 CG31666 CG5205 CG6272 CG6512 CG6876 CG7842 CG8004 Ets21C Irbp Ku80 LamC Las Mlp60A apt cul-2 hay rad50 rpr toy unc-13 |
| 885 | GO:0035214 | P | 5, | 1 | 2.262 (x 0.442) | 160 (0.006) | 1 | eye-antennal disc development | toy |
| 886 | GO:0006520 | P | 6, 7, | 2 | 3.689 (x 0.542) | 261 (0.008) | 1 | amino acid metabolism | BG:DS03431.1 CG12264 |
| 887 | GO:0016820 | F | 5, | 1 | 2.149 (x 0.465) | 152 (0.007) | 1 | hydrolase activity, acting on acid anhydrides, catalyzing transmembrane movement of substances | BEST:CK01227 |
| 888 | GO:0006066 | P | 5, | 1 | 2.276 (x 0.439) | 161 (0.006) | 1 | alcohol metabolism | ImpL3 |
| 889 | GO:0003723 | F | 4, | 3 | 5.216 (x 0.575) | 369 (0.008) | 1 | RNA binding | CG6876 RpS4 apt |
| 890 | GO:0042626 | F | 4, 6, 11, | 1 | 2.134 (x 0.469) | 151 (0.007) | 1 | ATPase activity, coupled to transmembrane movement of substances | BEST:CK01227 |
| 891 | GO:0006468 | P | 8, | 1 | 4.028 (x 0.248) | 285 (0.004) | 1 | protein amino acid phosphorylation | CG3008 |
| 892 | GO:0044238 | P | 4, | 58 | 64.597 (x 0.898) | 4570 (0.013) | 1 | primary metabolism | Act57B BG:DS03431.1 CG10444 CG11907 CG12264 CG1299 CG14935 CG18522 CG2277 CG2885 CG2989 CG3008 CG3036 CG31373 CG31611 CG32549 CG33158 CG4267 CG5162 CG5177 CG5205 CG6272 CG6330 CG6512 CG6574 CG6803 CG6876 CG7014 CG7722 CG7842 CG9372 CG9460 Chit Dgp-1 Ets21C Gasp ImpL3 Irbp Jheh3 Ku80 Las Mlc1 RpS4 Thor Ugt86Da Ugt86Di agt apt cul-2 hay huntingtin na rad50 rpr sda toy wun wun2 |
| 893 | GO:0006091 | P | 5, | 4 | 7.138 (x 0.560) | 505 (0.008) | 1 | generation of precursor metabolites and energy | CG18522 CG4511 CG5177 ImpL3 |
| 894 | GO:0007186 | P | 6, | 2 | 4.311 (x 0.464) | 305 (0.007) | 1 | G-protein coupled receptor protein signaling pathway | wun wun2 |
| 895 | GO:0043231 | C | 4, 5, 6, 7, | 23 | 32.610 (x 0.705) | 2307 (0.010) | 1 | intracellular membrane-bound organelle | Aldh CG12264 CG31611 CG31666 CG5205 CG6272 CG6512 CG6876 CG7842 CG8004 Ets21C Irbp Ku80 LamC Las Mlp60A apt cul-2 hay rad50 rpr toy unc-13 |
| 896 | GO:0043226 | C | 2, | 31 | 37.939 (x 0.817) | 2684 (0.012) | 1 | organelle | Act57B Aldh CG12264 CG31611 CG31666 CG5205 CG6272 CG6512 CG6876 CG7014 CG7842 CG8004 Ets21C Irbp Ku80 LamC Las Mhc Mlc1 Mlc2 Mlp60A RpS4 apt cul-2 hay na rad50 rpr toy unc-13 wupA |
| 897 | GO:0009117 | P | 6, | 1 | 2.247 (x 0.445) | 159 (0.006) | 1 | nucleotide metabolism | CG6330 |
| 898 | GO:0006810 | P | 4, 5, | 16 | 20.877 (x 0.766) | 1477 (0.011) | 1 | transport | BEST:CK01227 BG:DS03431.1 CG10444 CG11907 CG17108 CG2885 CG3036 CG6574 CG8004 TepIV Tsf1 Tsf3 glob1 huntingtin na unc-13 |
| 899 | GO:0019222 | P | 4, | 8 | 14.474 (x 0.553) | 1024 (0.008) | 1 | regulation of metabolism | CG2885 CG6272 Ets21C Thor apt hay rpr toy |
| 900 | GO:0051234 | P | 4, | 19 | 24.143 (x 0.787) | 1708 (0.011) | 1 | establishment of localization | BEST:CK01227 BG:DS03431.1 CG10444 CG11907 CG17108 CG2885 CG3036 CG6574 CG8004 TepIV Tsf1 Tsf3 glob1 huntingtin na os unc-13 wun wun2 |
| 901 | GO:0031966 | C | 5, 6, 7, 8, 9, 10, 11, | 1 | 2.643 (x 0.378) | 187 (0.005) | 1 | mitochondrial membrane | CG8004 |
| 902 | GO:0015268 | F | 4, | 1 | 2.573 (x 0.389) | 182 (0.005) | 1 | alpha-type channel activity | na |
| 903 | GO:0044425 | C | 3, 4, 5, | 10 | 17.188 (x 0.582) | 1216 (0.008) | 1 | membrane part | BEST:CK01227 BG:DS03431.1 CG17108 CG3036 CG8004 LamC Tsp42El sog wun wun2 |
| 904 | GO:0008283 | P | 4, | 2 | 3.986 (x 0.502) | 282 (0.007) | 1 | cell proliferation | Mlp60A toy |
| 905 | GO:0044267 | P | 6, | 23 | 29.387 (x 0.783) | 2079 (0.011) | 1 | cellular protein metabolism | Act57B CG10444 CG1299 CG3008 CG31373 CG33158 CG6512 CG6574 CG7014 CG7722 CG9372 CG9460 Dgp-1 Las Mlc1 RpS4 Thor apt cul-2 huntingtin na rpr sda |
| 906 | GO:0005216 | F | 4, 5, | 1 | 2.318 (x 0.431) | 164 (0.006) | 1 | ion channel activity | na |
| 907 | GO:0007049 | P | 4, | 4 | 6.658 (x 0.601) | 471 (0.008) | 1 | cell cycle | CG6512 LamC Mhc cul-2 |
| 908 | GO:0016070 | P | 6, | 3 | 5.188 (x 0.578) | 367 (0.008) | 1 | RNA metabolism | CG5205 CG6876 hay |
| 909 | GO:0043229 | C | 3, 4, 5, 6, | 31 | 37.939 (x 0.817) | 2684 (0.012) | 1 | intracellular organelle | Act57B Aldh CG12264 CG31611 CG31666 CG5205 CG6272 CG6512 CG6876 CG7014 CG7842 CG8004 Ets21C Irbp Ku80 LamC Las Mhc Mlc1 Mlc2 Mlp60A RpS4 apt cul-2 hay na rad50 rpr toy unc-13 wupA |
| 910 | GO:0005622 | C | 3, 4, | 36 | 46.575 (x 0.773) | 3295 (0.011) | 1 | intracellular | Act57B Aldh CG12264 CG31611 CG31666 CG32549 CG33158 CG4917 CG5205 CG6272 CG6330 CG6512 CG6876 CG7014 CG7842 CG8004 Ets21C Irbp Ku80 LamC Las Mhc Mlc1 Mlc2 Mlp60A RpS4 apt cul-2 hay huntingtin na rad50 rpr toy unc-13 wupA |
| 911 | GO:0051726 | P | 5, | 1 | 2.813 (x 0.356) | 199 (0.005) | 1 | regulation of cell cycle | cul-2 |
| 912 | GO:0009798 | P | 4, | 1 | 2.247 (x 0.445) | 159 (0.006) | 1 | axis specification | sog |
| 913 | GO:0019866 | C | 4, 5, 6, 7, 8, 9, | 1 | 2.460 (x 0.407) | 174 (0.006) | 1 | organelle inner membrane | LamC |
| 914 | GO:0043119 | P | 4, | 1 | 2.389 (x 0.419) | 169 (0.006) | 1 | positive regulation of physiological process | rpr |
| 915 | GO:0019538 | P | 5, | 25 | 30.857 (x 0.810) | 2183 (0.011) | 1 | protein metabolism | Act57B CG10444 CG1299 CG3008 CG31373 CG31611 CG33158 CG6512 CG6574 CG6803 CG7014 CG7722 CG9372 CG9460 Dgp-1 Las Mlc1 RpS4 Thor apt cul-2 huntingtin na rpr sda |
| 916 | GO:0007017 | P | 7, | 1 | 2.770 (x 0.361) | 196 (0.005) | 1 | microtubule-based process | huntingtin |
| 917 | GO:0016881 | F | 5, | 1 | 2.233 (x 0.448) | 158 (0.006) | 1 | acid-amino acid ligase activity | CG4917 |
| 918 | GO:0001654 | P | 5, | 1 | 2.558 (x 0.391) | 181 (0.006) | 1 | eye development | toy |
| 919 | GO:0005694 | C | 5, 6, 7, 8, | 1 | 2.601 (x 0.384) | 184 (0.005) | 1 | chromosome | CG31611 |
| 920 | GO:0015267 | F | 3, | 1 | 2.573 (x 0.389) | 182 (0.005) | 1 | channel or pore class transporter activity | na |
| 921 | GO:0051242 | P | 5, | 1 | 2.361 (x 0.424) | 167 (0.006) | 1 | positive regulation of cellular physiological process | rpr |
| 922 | GO:0031975 | C | 2, | 2 | 3.774 (x 0.530) | 267 (0.007) | 1 | envelope | CG8004 LamC |
| 923 | GO:0009987 | P | 2, | 85 | 94.790 (x 0.897) | 6706 (0.013) | 1 | cellular process | Act57B Aldh BEST:CK01227 BG:DS03431.1 CAH2 CG10444 CG10638 CG11907 CG12264 CG1299 CG17108 CG18522 CG2277 CG2885 CG2989 CG3008 CG3036 CG31373 CG31611 CG32549 CG33158 CG4511 CG5162 CG5177 CG5205 CG6272 CG6330 CG6512 CG6574 CG6803 CG6876 CG7014 CG7054 CG7722 CG7842 CG8004 CG9372 CG9460 Chit Dgp-1 Ets21C Gasp GstE1 GstE3 GstE5 GstE6 GstE7 Idgf2 ImpL3 Irbp Jheh3 Ku80 LamC Las Mhc Mlc1 Mlp60A Mocs1 RpS4 TepIV Thor Tsf1 Tsf3 Tsp42El Ugt86Da Ugt86Di agt apt cher cul-2 glob1 hay huntingtin mfas na os rad50 rpr sda sog toy unc-13 wun wun2 wupA |
| 924 | GO:0019226 | P | 5, | 3 | 5.131 (x 0.585) | 363 (0.008) | 1 | transmission of nerve impulse | Tsp42El apt unc-13 |
| 925 | GO:0003700 | F | 3, 5, | 3 | 5.499 (x 0.546) | 389 (0.008) | 1 | transcription factor activity | CG31666 Ets21C toy |
| 926 | GO:0000074 | P | 6, | 1 | 2.813 (x 0.356) | 199 (0.005) | 1 | regulation of progression through cell cycle | cul-2 |
| 927 | GO:0022008 | P | 5, | 1 | 2.728 (x 0.367) | 193 (0.005) | 1 | neurogenesis | mfas |
| 928 | GO:0031226 | C | 5, 6, 7, | 1 | 2.799 (x 0.357) | 198 (0.005) | 1 | intrinsic to plasma membrane | BG:DS03431.1 |
| 929 | GO:0000904 | P | 5, 6, | 1 | 2.389 (x 0.419) | 169 (0.006) | 1 | cellular morphogenesis during differentiation | mfas |
| 930 | GO:0005488 | F | 2, | 52 | 58.434 (x 0.890) | 4134 (0.013) | 1 | binding | BEST:CK01227 BG:DS01219.1 CAH2 CG10126 CG17904 CG2885 CG2989 CG31063 CG3153 CG31611 CG31666 CG3244 CG33158 CG4511 CG4858 CG5205 CG6272 CG6512 CG6574 CG6876 CG7014 CG7054 CG7447 Chit Dgp-1 Ets21C Gasp Idgf2 Irbp Ku80 Las Mhc Mlc2 Mlp60A Mocs1 RpS4 Thor Tsf1 Tsf3 agt apt cher hay huntingtin os rad50 sda sog toy unc-13 wun wupA |
| 931 | GO:0043283 | P | 5, | 16 | 23.803 (x 0.672) | 1684 (0.010) | 1 | biopolymer metabolism | CG2989 CG3008 CG31373 CG31611 CG5205 CG6876 Chit Gasp Irbp Ku80 Ugt86Da Ugt86Di agt hay rad50 rpr |
| 932 | GO:0016192 | P | 5, 6, | 2 | 4.184 (x 0.478) | 296 (0.007) | 1 | vesicle-mediated transport | CG2885 unc-13 |
| 933 | GO:0005740 | C | 4, 5, 6, 7, 8, 9, 10, | 1 | 2.898 (x 0.345) | 205 (0.005) | 1 | mitochondrial envelope | CG8004 |
| 934 | GO:0005887 | C | 6, 7, 8, | 1 | 2.770 (x 0.361) | 196 (0.005) | 1 | integral to plasma membrane | BG:DS03431.1 |
| 935 | GO:0003729 | F | 5, | 2 | 4.424 (x 0.452) | 313 (0.006) | 1 | mRNA binding | RpS4 apt |
| 936 | GO:0000279 | P | 5, | 2 | 4.057 (x 0.493) | 287 (0.007) | 1 | M phase | CG6512 Mhc |
| 937 | GO:0050875 | P | 3, | 80 | 87.864 (x 0.911) | 6216 (0.013) | 1 | cellular physiological process | Act57B Aldh BEST:CK01227 BG:DS03431.1 CAH2 CG10444 CG10638 CG11907 CG12264 CG1299 CG17108 CG18522 CG2277 CG2885 CG2989 CG3008 CG3036 CG31373 CG31611 CG32549 CG33158 CG4511 CG5162 CG5177 CG5205 CG6272 CG6330 CG6512 CG6574 CG6803 CG6876 CG7014 CG7722 CG7842 CG8004 CG9372 CG9460 Chit Dgp-1 Ets21C Gasp GstE1 GstE3 GstE5 GstE6 GstE7 ImpL3 Irbp Jheh3 Ku80 LamC Las Mhc Mlc1 Mlp60A Mocs1 RpS4 TepIV Thor Tsf1 Tsf3 Ugt86Da Ugt86Di agt apt cher cul-2 glob1 hay huntingtin mfas na os rad50 rpr sda toy unc-13 wun wun2 |
| 938 | GO:0004672 | F | 6, | 2 | 4.170 (x 0.480) | 295 (0.007) | 1 | protein kinase activity | CG3008 Irbp |
| 939 | GO:0007165 | P | 4, | 11 | 18.248 (x 0.603) | 1291 (0.009) | 1 | signal transduction | CG2885 CG2989 CG7054 Chit Idgf2 mfas os sog unc-13 wun wun2 |
| 940 | GO:0031967 | C | 3, 4, 5, 6, 7, 8, | 2 | 3.774 (x 0.530) | 267 (0.007) | 1 | organelle envelope | CG8004 LamC |
| 941 | GO:0007167 | P | 6, | 1 | 2.431 (x 0.411) | 172 (0.006) | 1 | enzyme linked receptor protein signaling pathway | sog |
| 942 | GO:0005575 | C | 1, | 74 | 81.729 (x 0.905) | 5782 (0.013) | 1 | cellular\_component | Act57B Aldh BEST:CK01227 BG:DS01219.1 BG:DS03431.1 CG10444 CG11907 CG12264 CG17108 CG2989 CG3036 CG31279 CG31601 CG31611 CG31666 CG32017 CG32021 CG32207 CG32448 CG32549 CG32625 CG33158 CG4917 CG5205 CG6272 CG6330 CG6512 CG6574 CG6876 CG7014 CG7194 CG7842 CG8004 Chit Cp36 Dgp-1 Ets21C Gasp GstD3 Idgf2 Irbp JhI-26 Jheh3 Ku80 LamC Las Lcp65Ag1 Mhc Mlc1 Mlc2 Mlp60A Mocs1 RpS4 Tsf1 Tsf3 Tsp42El apt cher cul-2 fau hay huntingtin mfas na os rad50 rpr sog toy unc-13 wun wun2 wupA yellow-b |
| 943 | GO:0006519 | P | 5, | 2 | 4.142 (x 0.483) | 293 (0.007) | 1 | amino acid and derivative metabolism | BG:DS03431.1 CG12264 |
| 944 | GO:0007456 | P | 6, | 1 | 2.445 (x 0.409) | 173 (0.006) | 1 | eye development (sensu Endopterygota) | toy |
| 945 | GO:0044451 | C | 5, 6, 7, 8, 9, 10, 11, 12, | 1 | 2.869 (x 0.349) | 203 (0.005) | 1 | nucleoplasm part | hay |
| 946 | GO:0031090 | C | 4, 5, 6, 7, 8, | 2 | 5.103 (x 0.392) | 361 (0.006) | 1 | organelle membrane | CG8004 LamC |
| 947 | GO:0007600 | P | 3, 5, | 1 | 3.647 (x 0.274) | 258 (0.004) | 1 | sensory perception | CG4917 |
| 948 | GO:0007560 | P | 5, 6, | 1 | 3.392 (x 0.295) | 240 (0.004) | 1 | imaginal disc morphogenesis | sog |
| 949 | GO:0050877 | P | 4, | 4 | 8.707 (x 0.459) | 616 (0.006) | 1 | neurophysiological process | CG4917 Tsp42El apt unc-13 |
| 950 | GO:0007242 | P | 5, | 3 | 7.407 (x 0.405) | 524 (0.006) | 1 | intracellular signaling cascade | CG2885 os unc-13 |
| 951 | GO:0006796 | P | 6, | 4 | 8.538 (x 0.469) | 604 (0.007) | 1 | phosphate metabolism | CG3008 CG3036 wun wun2 |
| 952 | GO:0048699 | P | 6, | 1 | 2.544 (x 0.393) | 180 (0.006) | 1 | generation of neurons | mfas |
| 953 | GO:0006512 | P | 8, | 1 | 3.308 (x 0.302) | 234 (0.004) | 1 | ubiquitin cycle | rpr |
| 954 | GO:0016301 | F | 5, | 2 | 5.668 (x 0.353) | 401 (0.005) | 1 | kinase activity | CG3008 Irbp |
| 955 | GO:0008150 | P | 1, | 110 | 114.240 (x 0.963) | 8082 (0.014) | 1 | biological\_process | Act57B Aldh BEST:CK01227 BG:DS01219.1 BG:DS03431.1 CAH2 CG10444 CG10638 CG11907 CG12264 CG1299 CG14935 CG17108 CG18522 CG2065 CG2277 CG2885 CG2989 CG3008 CG3036 CG31279 CG31373 CG31601 CG31611 CG32017 CG32021 CG32207 CG32448 CG32549 CG32625 CG33158 CG4267 CG4511 CG4917 CG5162 CG5177 CG5205 CG5397 CG6272 CG6330 CG6512 CG6574 CG6803 CG6876 CG7014 CG7054 CG7194 CG7722 CG7842 CG8004 CG9372 CG9460 CG9836 Chit Cp36 Dgp-1 Ets21C Gasp GstD3 GstD6 GstD9 GstE1 GstE3 GstE5 GstE6 GstE7 Idgf2 ImpL3 Irbp JhI-26 Jheh3 Ku80 LamC Las Lcp65Ag1 Mhc Mlc1 Mlc2 Mlp60A Mocs1 RpS4 TepIV Thor TotA Tsf1 Tsf3 Tsp42El Ugt86Da Ugt86Di agt apt cher cul-2 fau glob1 hay huntingtin mfas na os rad50 rpr sda sog toy unc-13 wun wun2 wupA yellow-b |
| 956 | GO:0044424 | C | 3, 4, 5, | 36 | 45.091 (x 0.798) | 3190 (0.011) | 1 | intracellular part | Act57B Aldh CG12264 CG31611 CG31666 CG32549 CG33158 CG4917 CG5205 CG6272 CG6330 CG6512 CG6876 CG7014 CG7842 CG8004 Ets21C Irbp Ku80 LamC Las Mhc Mlc1 Mlc2 Mlp60A RpS4 apt cul-2 hay huntingtin na rad50 rpr toy unc-13 wupA |
| 957 | GO:0031981 | C | 4, 5, 6, 7, 8, 9, 10, | 1 | 3.887 (x 0.257) | 275 (0.004) | 1 | nuclear lumen | hay |
| 958 | GO:0005057 | F | 3, | 1 | 3.392 (x 0.295) | 240 (0.004) | 1 | receptor signaling protein activity | Tsp42El |
| 959 | GO:0006793 | P | 5, | 4 | 8.538 (x 0.469) | 604 (0.007) | 1 | phosphorus metabolism | CG3008 CG3036 wun wun2 |
| 960 | GO:0006915 | P | 6, | 1 | 2.516 (x 0.397) | 178 (0.006) | 1 | apoptosis | rpr |
| 961 | GO:0031224 | C | 4, 5, 6, | 8 | 13.386 (x 0.598) | 947 (0.008) | 1 | intrinsic to membrane | BEST:CK01227 BG:DS03431.1 CG17108 CG3036 Tsp42El sog wun wun2 |
| 962 | GO:0031323 | P | 5, | 8 | 13.937 (x 0.574) | 986 (0.008) | 1 | regulation of cellular metabolism | CG2885 CG6272 Ets21C Thor apt hay rpr toy |
| 963 | GO:0004674 | F | 7, | 1 | 2.997 (x 0.334) | 212 (0.005) | 1 | protein serine/threonine kinase activity | Irbp |
| 964 | GO:0016773 | F | 5, | 2 | 5.046 (x 0.396) | 357 (0.006) | 1 | phosphotransferase activity, alcohol group as acceptor | CG3008 Irbp |
| 965 | GO:0006118 | P | 6, | 2 | 4.707 (x 0.425) | 333 (0.006) | 1 | electron transport | CG18522 CG4511 |
| 966 | GO:0008270 | F | 6, | 4 | 8.509 (x 0.470) | 602 (0.007) | 1 | zinc ion binding | CAH2 CG31666 Mlp60A sda |
| 967 | GO:0016043 | P | 4, | 13 | 19.492 (x 0.667) | 1379 (0.009) | 1 | cell organization and biogenesis | Act57B CG2885 CG31611 CG6803 CG8004 Irbp Ku80 LamC Thor cher huntingtin mfas rad50 |
| 968 | GO:0007552 | P | 4, | 1 | 3.576 (x 0.280) | 253 (0.004) | 1 | metamorphosis | sog |
| 969 | GO:0000902 | P | 4, 5, | 2 | 4.778 (x 0.419) | 338 (0.006) | 1 | cellular morphogenesis | Thor mfas |
| 970 | GO:0046698 | P | 5, | 1 | 3.548 (x 0.282) | 251 (0.004) | 1 | metamorphosis (sensu Insecta) | sog |
| 971 | GO:0006605 | P | 7, 8, 9, | 1 | 3.265 (x 0.306) | 231 (0.004) | 1 | protein targeting | CG8004 |
| 972 | GO:0005654 | C | 5, 6, 7, 8, 9, 10, 11, | 1 | 3.166 (x 0.316) | 224 (0.004) | 1 | nucleoplasm | hay |
| 973 | GO:0006139 | P | 5, | 18 | 25.033 (x 0.719) | 1771 (0.010) | 1 | nucleobase, nucleoside, nucleotide and nucleic acid metabolism | CG11907 CG18522 CG2277 CG2885 CG31611 CG32549 CG5205 CG6272 CG6330 CG6876 Ets21C Irbp Ku80 agt apt hay rad50 toy |
| 974 | GO:0016021 | C | 5, 6, 7, | 8 | 13.344 (x 0.600) | 944 (0.008) | 1 | integral to membrane | BEST:CK01227 BG:DS03431.1 CG17108 CG3036 Tsp42El sog wun wun2 |
| 975 | GO:0044429 | C | 4, 5, 6, 7, 8, 9, | 2 | 4.877 (x 0.410) | 345 (0.006) | 1 | mitochondrial part | Aldh CG8004 |
| 976 | GO:0007423 | P | 4, | 1 | 3.251 (x 0.308) | 230 (0.004) | 1 | sensory organ development | toy |
| 977 | GO:0005634 | C | 5, 6, 7, 8, | 15 | 21.542 (x 0.696) | 1524 (0.010) | 1 | nucleus | CG31611 CG31666 CG5205 CG6272 CG6876 Ets21C Irbp Ku80 LamC Mlp60A apt cul-2 hay rad50 toy |
| 978 | GO:0008104 | P | 4, | 4 | 8.000 (x 0.500) | 566 (0.007) | 1 | protein localization | BG:DS01219.1 CG2885 CG8004 cher |

  

---

Regulated Genes that don't have GO terms
  

CG10075 CG10189 CG10337 CG10559 CG10675 CG10916 CG11086 CG1146 CG11852 CG11893 CG12505 CG12868 CG13822 CG13886 CG14907 CG15611 CG15675 CG1572 CG15784 CG17681 CG18294 CG18358 CG18410 CG18596 CG18643 CG2909 CG30196 CG31633 CG31781 CG31955 CG32425 CG3280 CG3448 CG40115 CG40164 CG40169 CG40188 CG40260 CG40295 CG5174 CG5953 CG6353 CG7506 CG8369 CG8486 CG9192 CG9336 CG9338 CG9350 CG9667 CG9815 Max-element SP558
