# Supplementary material for: Functional dissection of the ash2 and ash1 transcriptomes provides insights into the transcriptional basis of wing phenotypes and reveals conserved protein interactions
Source: Genome Biol. 2007 Apr 28;8(4):R67. doi: 10.1186/gb-2007-8-4-r67 (PMC1896016; doi:10.1186/gb-2007-8-4-r67)
Supplement: Additional data file 9 — GO annotations of the genes downregulated over 1.5-fold in ash2112411 [file gb-2007-8-4-r67-S9.html]

  

---

  

|  |  |
| --- | --- |
| Go Statistics | Reg File: **ash2112411\_D1.5x.txt.fbgns** (939 genes -- 192 skipped)  Ref File: **ref.fbgns** (13577 genes -- 4663 skipped)  Database: **go\_200507-termdb.rdf-xml** |

---

  

Fields Description

| Pos | Go Term | Ontology | Levels | Observed | Expected | Possibles | p-value(Adj) | Go term description | Genes with the GO term |
| --- | --- | --- | --- | --- | --- | --- | --- | --- | --- |
| 1 | GO:0007275 | P | 2, | 233 | 124.444 (x 1.872) | 1485 (0.157) | 1.57e-21 | development | 14-3-3epsilon 14-3-3zeta 18w Abi Act5C Akap200 Akt1 Ald Amph Arf79F BicD Btk29A CG12896 CG1900 CG30011 CG3227 CG40410 CG5830 CG5841 CG6896 CG7161 CG8104 CG8924 CaMKII Cat Cdc42 Cdk4 Chc Cip4 CkIIalpha CkIIbeta Cont CtBP CycA CycT Cyp1 Dlc90F Dll Dp Dr EG:BACH59J11.2 Eip63E Eip75B Es2 Fas2 Fas3 Fs(2)Ket G-salpha60A Galpha49B Gap1 Gef26 Got2 Hem Hrb27C Hsp26 Hsp27 ImpE2 ImpE3 Indy Lac Lam LanA LanB2 Mbs Moe Msp-300 Mtl NetA Nop60B Optix PR2 Paip2 Pen Pp1-87B Ptp99A Rop S Sb Scm Sdc SelD Sema-1b Sema-2a Ser Sin3A Smox Sox14 Src64B Su(dx) Tm1 Trxr-1 Vang Vap-33-1 Vha44 Wnt2 X11L abs alpha-Cat alpha-Spec ana aop ap argos arm ash2 asp barr baz bel betaTub56D bib bl bnb br brk brm btsz capu cdc2 chb ci cib cic ck cora crb crc crol csul csw cta da dac dome dve ebi ed edl egl en ena esn exd fat2 fax fdl flfl fra fwd fz gft gish glec gol grn guf gus hdc heph hh hts in inx2 jar jing jumu kay kis klar klu kn knk l(1)G0148 l(2)01424 l(2)gl larp lgs lid lola lqf lwr mask mbt mod msi msn mth ninA nonA-l ogre otk ovo par-6 pav pbl ph-p pk plexA pll pnut polo pon rg rin sc sca scrib sd sdk serpin-27A sgl shg sima smi35A spag sqd ss stai stan stc sty th tkv toe tok trio trn tub tud vav vg wbl zip |
| 2 | GO:0009653 | P | 3, | 133 | 53.800 (x 2.472) | 642 (0.207) | 2.49e-21 | morphogenesis | 18w Abi Akt1 Amph Arf79F Btk29A CG1900 CG5841 CG6896 CG7161 CaMKII Cdc42 Cdk4 Cont CycT Dll Dp Dr Eip63E Fas2 Fas3 Fs(2)Ket G-salpha60A Galpha49B Gef26 Hem ImpE2 ImpE3 Lac Lam LanA LanB2 Mbs Moe Mtl NetA Paip2 Ptp99A S Sb Scm Sdc Sema-1b Sema-2a Ser Smox Src64B Su(dx) Vang X11L abs alpha-Cat alpha-Spec aop ap argos arm ash2 barr baz bl br brm btsz chb cib ck cora crb crc crol csw cta da dac dome dve ebi edl ena fat2 fax flfl fra fz gft gol grn hdc heph hh in inx2 jar jumu kay klar klu kn l(2)gl lola lqf mbt msn ninA otk ovo par-6 pbl ph-p pk plexA pnut rg rin sc sca scrib sd sdk sgl shg spag sqd ss stan sty th tkv trio vav vg zip |
| 3 | GO:0016043 | P | 4, | 217 | 115.561 (x 1.878) | 1379 (0.157) | 3.3e-20 | cell organization and biogenesis | 14-3-3epsilon 14-3-3zeta AP-1gamma ATPsyn-beta Abi Act42A Act5C Akt1 Amph Arf79F BcDNA:LD09009 BicD CG10542 CG10695 CG11207 CG11305 CG11856 CG1418 CG1472 CG17143 CG17184 CG1893 CG1900 CG1907 CG1911 CG2108 CG2158 CG2852 CG2980 CG31617 CG32137 CG32164 CG3249 CG32672 CG33113 CG33214 CG5064 CG5434 CG5728 CG6838 CG7161 CG8104 CG8155 CG8243 CG9057 CG9906 CaMKII Caf1 Cap-G Cdc42 Cdk4 Cen190 Chc Cip4 Cont CycT Cyp1 Dlc90F Dref Dsp1 E(z) EG:34F3.8 Eb1 Fas2 Fas3 Fs(2)Ket Galpha49B Gef26 Hem HmgD HmgZ Hsp60 Iswi KP78b Karybeta3 Klp10A Lac Lam LanA MICAL-like Map60 Mapmodulin Mbs Mcm5 Mi-2 Moe Msp-300 Mtl Myb NetA Nlp Nop60B Ote Paip2 Pak3 Pcl Pen Pi3K68D Pp1-87B Psc Ptp99A Rab10 Ranbp16 RhoBTB Rhp S SMC1 Sb Sdc Sema-1b Sema-2a Set Snap Spt6 Src64B Su(var)3-9 Su(z)12 Ucp4B Vang Vap-33-1 Vha55 X11L abs alpha-Adaptin alpha-Cat alpha-Spec alphaTub84B aop ap argos arm ash2 asp barr baz betaTub56D bl brm btsz capu cav chb cib ck cora crb cta d dac dalao dpa dre4 edl egl ena esn fax fra fwd fz garz gp210 hh hts in jar jumu katanin-60 kay kel kis klar krz l(1)dd4 l(2)gl larp lola lqf lwr mask mbt mor msk msn mus209 ninA nop5 nudC otk ovo par-6 pav pbl pfk ph-p pk plexA pll pnut polo prominin-like rg rin sca scrib sec23 shg slmb sqd stai stan syndapin trio vav vg wbl zip |
| 4 | GO:0030154 | P | 3, | 102 | 42.738 (x 2.387) | 510 (0.200) | 9.39e-15 | cell differentiation | 14-3-3epsilon 14-3-3zeta Act5C Amph BicD Cdc42 Chc Dlc90F Dr Fas2 Fas3 Galpha49B Gef26 Hem Hrb27C Lac LanA Mbs Moe NetA Nop60B Pen Ptp99A Rop S Sdc Sema-1b Sema-2a Ser Smox Tm1 alpha-Spec aop ap argos arm asp baz bel bl br capu cdc2 chb crb csul csw da dac dome ebi edl egl ena exd fax fra fz guf gus hdc heph hh hts in jar jumu kay klar kn l(2)gl lola lqf mask mbt mod msi msn ninA otk ovo par-6 pbl pk plexA pnut pon rg rin sc sca sdk shg sqd stan sty th tkv trio tud wbl zip |
| 5 | GO:0005488 | F | 2, | 456 | 346.432 (x 1.316) | 4134 (0.110) | 1.72e-14 | binding | 14-3-3epsilon 14-3-3zeta ATPsyn-beta ATbp Aats-ala Aats-glupro Abi Ack Akap200 Akt1 Amph Arf79F B52 BEAF-32 BEST:CK01140 Bap170 BcDNA:GH02976 BcDNA:GH03163 BcDNA:GM10765 BcDNA:LD09009 BcDNA:LD23371 BcDNA:LD23876 BcDNA:LD41548 BicD Brf Btk29A CBP CG10103 CG10211 CG10354 CG10423 CG10542 CG10602 CG10657 CG10990 CG11069 CG11123 CG11142 CG11207 CG11228 CG11275 CG11305 CG11856 CG12130 CG12190 CG12299 CG12391 CG13350 CG1354 CG13848 CG13895 CG1418 CG14217 CG14231 CG1472 CG15141 CG1529 CG15835 CG1600 CG1647 CG1677 CG17036 CG17271 CG17309 CG17361 CG17419 CG17493 CG17514 CG17765 CG17838 CG17919 CG17952 CG1815 CG1826 CG1869 CG1900 CG1907 CG1911 CG2108 CG2118 CG2316 CG2947 CG30011 CG31169 CG31258 CG31301 CG31453 CG31617 CG31716 CG31739 CG32099 CG32137 CG3249 CG32640 CG32672 CG33113 CG33123 CG33214 CG33525 CG3823 CG3847 CG40410 CG4509 CG4612 CG4749 CG4914 CG5064 CG5319 CG5434 CG5728 CG5789 CG5841 CG6049 CG6227 CG6418 CG6791 CG6812 CG6835 CG6854 CG6930 CG6946 CG7008 CG7102 CG7154 CG7441 CG7668 CG7878 CG7922 CG7987 CG8089 CG8092 CG8443 CG8478 CG8789 CG8863 CG8902 CG8924 CG8963 CG9025 CG9027 CG9057 CG9104 CG9342 CG9373 CG9425 CG9503 CG9598 CG9684 CG9809 CG9906 CG9924 CREG CaMKII Cad87A Caf1 CalpB Cat Cbp80 CdGAPr Cdc42 Cdk4 Cen190 Cip4 CkIIalpha CkIalpha Cks Cont CtBP D19A D19B DNApol-delta DNApol-epsilon Dhh1 Dll DnaJ-1 Dp Dr Dref Dsp1 E(z) EG:115C2.6 EG:196F3.2 EG:86E4.2 Eb1 Eip63E Eip75B Elf Fkbp13 Fs(2)Ket G-salpha60A GATAd GNBP3 Galpha49B Gef26 Gfat1 Gprk1 Hcf Hem Hlc HmgD HmgZ Hmr Hmu Hrb27C Hrb87F Hsc70-3 Hsp23 Hsp60 Iswi KP78b Karybeta3 Klp10A Lam LanA LanB2 M(2)21AB MICAL-like Map60 Mapmodulin Mbs Mcm5 Mcm6 Mcm7 Mcr Mdr49 Mekk1 Mi-2 Moe Mpcp Msp-300 Mtl Myb NUCB1 Nek2 Nep2 Nlp Nop60B Optix Os-C Ote PR2 Paip2 Pak3 Pcl Pen Pep Pepck Pi3K68D Psa Psc Ptp99A Rab10 RacGAP50C Ranbp16 Rbf2 Rbp2 RhoBTB RhoGAP19D RhoGAP1A Rhp Rm62 Rop RpII140 RpL13 RpL15 RpL22 RpL23a RpL3 RpS6 Rrp1 SMC1 Scm Sdc SelD Sema-1b Sema-2a Ser Set Sh3beta Sin3A Sip1 Slip1 Sox14 Spt6 Src64B Su(var)3-9 Su(z)12 TBPH Taf2 Thd1 Tis11 Tm1 Tom34 Top2 Top3beta Trap1 Trxr-1 Tsp66E Uba2 Ucp4B Vha44 Vha55 Wnt2 Wsck X11L XNP abs ald alpha-Cat alpha-Spec alphaTub84B aop ap argos arm ash2 asp baz bel betaTub56D bib bl br brk brm bsf btsz capu cav cdc2 chb ci cib cic ck cora crb crc crol csul csw cta d d4 da dalao dnk dome dpa dre4 dve eIF-4B eIF3-S9 eIF5 ebi edl egl en ena endos esn exd fat2 fz gfzf gish glec gol grn heph hh hts in jar jim jing jumu katanin-60 kay kel kis klar klu kn l(1)G0148 l(1)dd4 l(2)01424 l(2)gl lds lgs lid lmg lola lwr mask mav mbt mdy me31B mod mor msi msk msn mus209 nonA-l nop5 olf413 ome otk ovo pAbp par-6 pav pfk ph-p pk pll pnut polo qkr54B rg rin sc sca scf scrib sd sec23 shg sima smi35A spag sqd ss stai stan stc sty su(f) th tkv toe tok tos tub tud vav viaf1 vig vimar wgn woc yps zf30C zip |
| 6 | GO:0005622 | C | 3, 4, | 379 | 276.124 (x 1.373) | 3295 (0.115) | 3.49e-13 | intracellular | 14-3-3epsilon 14-3-3zeta 18w AP-1gamma ATPsyn-beta ATbp Aats-glupro Ack Acon Acox57D-p Act42A Act5C Amph ApepP Arc92 B52 BEAF-32 BG:DS00004.11 Bap170 BcDNA:GH04962 BcDNA:GH12558 BcDNA:GM10765 BcDNA:LD23876 BcDNA:LD41548 BicD Brf Btk29A CBP CG10211 CG10354 CG10423 CG10473 CG10542 CG10657 CG10695 CG11188 CG11207 CG11305 CG11856 CG12252 CG12299 CG12391 CG13895 CG1472 CG15093 CG1529 CG1647 CG1677 CG17361 CG1746 CG17493 CG17838 CG17952 CG1815 CG1907 CG1911 CG2097 CG2118 CG2158 CG30011 CG31258 CG31301 CG31363 CG31617 CG31716 CG31739 CG32164 CG33113 CG33123 CG33525 CG3605 CG3823 CG3847 CG40410 CG4238 CG4914 CG5028 CG5064 CG5434 CG5728 CG5789 CG5841 CG6064 CG6227 CG6394 CG6543 CG6638 CG6673 CG6791 CG6812 CG6930 CG7008 CG7145 CG7154 CG7263 CG7987 CG8089 CG8092 CG8426 CG8443 CG8478 CG8924 CG9057 CG9104 CG9149 CG9153 CG9342 CG9425 CG9894 CREG CaMKII Caf1 CalpB Cap-G Cat Cbp80 Cen190 Chc CkIIalpha CkIIbeta CkIalpha CtBP CycA CycT Cyp1 D12 D19A D19B DNApol-delta DNApol-epsilon Dlc90F Dll DnaJ-1 Dp Dr Dref Dsp1 E(z) EG:115C2.6 ERp60 Eb1 Eip75B Elf Eno Es2 Fas2 Fs(2)Ket Fur1 GATAd Gapdh1 Gapdh2 Gdh Gfat1 Got2 Hcf Hem HmgD HmgZ Hrb27C Hrb87F Hsc70-3 Hsp60 Idh ImpE2 Iswi Karybeta3 Klp10A Lam Map60 Mapmodulin Mcm5 Mcm6 Mcm7 Mi-2 Moe Mpcp Msp-300 Myb NUCB1 Nek2 Nlp Nop60B Optix Ote Pcl Pen Pep Pepck Pgi Pi3K68D Pp1-87B Psc Ptp99A Ranbp16 Rbf2 Rbp2 Rhp Rm62 RnrL Rop RpII140 RpL13 RpL15 RpL22 RpL23a RpL3 RpS12 RpS6 Rrp1 S SMC1 Scm Set Sin3A Smox Snap Sox14 Spt6 Ste12DOR Ste:CG33236 Su(dx) Su(var)3-9 Su(z)12 Taf2 Thd1 Thiolase Tis11 Tm1 Tom34 Top2 TppII Trxr-1 Uba2 UbcD6 Uch Ucp4B Vap-33-1 Vha44 Vha55 XNP abs ade3 alpha-Adaptin alpha-Cat alpha-Spec alphaTub84B aop ap arm ash2 asp barr baz betaTub56D bl br brk brm bsf btsz capu cav cdc2 chb ci cib cic ck cora crc crol crp csul csw d d4 da dac dalao dnk dpa dre4 dve eIF-4B eIF3-S9 eIF5 east ebi edl egl en esn exd fz fzy garz gft gfzf gol grn hdc heph hh hts jar jim jing jumu katanin-60 kay kel kis klar klu kn l(1)G0334 l(1)dd4 l(2)01424 l(2)dtl l(2)gl larp lds lgs lid lig lin19 lmg lola lwr mdy mod mor msk mus209 nonA-l nop5 nudC ovo p120ctn pAbp par-6 pav pbl pfk ph-p pk pll pnut polo pon qkr54B rg rin sc scrib sd sec23 sima simj slmb smi35A sqd ss stai stc sty su(f) th toe tub tud vg viaf1 vig wbl woc yps zf30C zip |
| 7 | GO:0005515 | F | 3, | 181 | 103.410 (x 1.750) | 1234 (0.147) | 4.3e-13 | protein binding | 14-3-3epsilon 14-3-3zeta Aats-glupro Abi Ack Akap200 Amph B52 BEAF-32 Bap170 BcDNA:GH03163 BicD Brf CG11207 CG11228 CG11275 CG11856 CG12190 CG1418 CG1472 CG15835 CG17493 CG17765 CG17952 CG1815 CG1826 CG2947 CG31453 CG32137 CG3249 CG32640 CG32672 CG33214 CG5319 CG5841 CG7008 CG7102 CG7668 CG8863 CG8924 CG9025 CG9057 CG9598 CG9906 CG9924 CREG CaMKII Caf1 CalpB CdGAPr Cdk4 Cen190 Cip4 Cks CtBP DnaJ-1 Dp Dsp1 E(z) EG:196F3.2 Eb1 Eip63E Fs(2)Ket Gef26 Hcf Hem Hsp23 Hsp60 Karybeta3 Lam LanA MICAL-like Map60 Mapmodulin Mbs Mcr Moe Msp-300 Nlp Ote Paip2 Pcl Pen Ranbp16 RhoBTB RhoGAP19D RhoGAP1A Rhp Rm62 Rop Sdc Sema-1b Sema-2a Ser Set Sin3A Sip1 Slip1 Su(z)12 Tm1 Trap1 Tsp66E Uba2 Wnt2 X11L alpha-Cat alpha-Spec alphaTub84B aop argos arm asp baz betaTub56D bl br brm capu cdc2 chb cib cic ck cora crb crc csul csw d dalao dome dre4 ebi edl ena endos fat2 fz hh hts in jar katanin-60 kay kel klar l(1)dd4 l(2)gl lgs lola lwr mask mav mbt mod mor msk otk pAbp par-6 pfk pk pll pnut rg rin sca scf scrib sec23 shg spag stai stan sty tkv tub viaf1 vimar wgn zip |
| 8 | GO:0050789 | P | 2, | 209 | 126.120 (x 1.657) | 1505 (0.139) | 4.72e-13 | regulation of biological process | 14-3-3epsilon 14-3-3zeta ATbp Aac11 Abi Akap200 Akt1 Amph B52 BEAF-32 Bap170 BcDNA:LD23876 Brf CG10473 CG10990 CG11207 CG11228 CG11305 CG12190 CG12299 CG14217 CG15141 CG15835 CG17309 CG17514 CG17765 CG1815 CG1900 CG30011 CG30372 CG31258 CG31716 CG33525 CG40160 CG40410 CG4914 CG5434 CG5728 CG5841 CG6227 CG6838 CG7263 CG7987 CG8092 CG8165 CG8243 CG8400 CG8426 CG8443 CG8924 CREG CaMKII Caf1 Cdc42 Cdk4 CkIalpha Cks CtBP CycA CycT D12 D19A D19B Dll Dp Dr Dref Dsp1 E(z) Eb1 Eip63E Eip75B Elf Fas2 G-salpha60A GATAd Gap1 Gprk1 HmgD HmgZ Hrb27C Hrb87F Hus1-like Iswi LanA Mbs Mi-2 Myb Nek2 Optix Paip2 Pcl Psc Rab10 RacGAP50C Rbf2 Rm62 Rop RpS6 S Scm Ser Sin3A Smox Sox14 Src64B Ste:CG33236 Su(var)3-9 Su(z)12 TBPH Taf2 Thd1 Uba2 UbcD6 Vha44 XNP ald alphaTub84B ana aop ap argos arm ash2 bl br brk brm btsz cdc2 charybde ci cic crc crol d4 da dalao dre4 dve eIF-4B eIF3-S9 eIF5 ebi ed edl en ena exd fat2 fwd fzy gft gol grn hdc hh jim jing jumu kay kis klu kn l(2)01424 l(2)gl lds lgs lid lin19 lola lqf mbt mdy mor msi msn mus209 ovo pAbp pbl ph-p pll rin sc scrib scylla sd sdk serpin-27A sima simj slmb smi35A sqd ss stan stc sty th tkv toe trio vig woc yps zf30C zip zwilch |
| 9 | GO:0044424 | C | 3, 4, 5, | 366 | 267.324 (x 1.369) | 3190 (0.115) | 2.28e-12 | intracellular part | 14-3-3epsilon 14-3-3zeta 18w AP-1gamma ATPsyn-beta ATbp Aats-glupro Ack Acon Acox57D-p Act42A Act5C Amph ApepP Arc92 B52 BEAF-32 BG:DS00004.11 Bap170 BcDNA:GH04962 BcDNA:GH12558 BcDNA:GM10765 BcDNA:LD23876 BcDNA:LD41548 BicD Brf Btk29A CBP CG10211 CG10354 CG10423 CG10473 CG10542 CG10695 CG11188 CG11207 CG11305 CG11856 CG12299 CG12391 CG13895 CG1472 CG15093 CG1529 CG1647 CG1677 CG17361 CG1746 CG17493 CG17838 CG17952 CG1815 CG1907 CG1911 CG2097 CG2118 CG2158 CG30011 CG31258 CG31363 CG31617 CG31716 CG31739 CG32164 CG33113 CG33123 CG33525 CG3605 CG3847 CG40410 CG4914 CG5028 CG5064 CG5434 CG5728 CG5789 CG5841 CG6064 CG6227 CG6394 CG6543 CG6638 CG6673 CG6791 CG6930 CG7008 CG7145 CG7154 CG7263 CG7987 CG8089 CG8092 CG8426 CG8443 CG8478 CG8924 CG9057 CG9104 CG9149 CG9342 CG9425 CG9894 CREG CaMKII Caf1 CalpB Cap-G Cat Cbp80 Cen190 Chc CkIIalpha CkIIbeta CkIalpha CtBP CycA CycT Cyp1 D12 D19A D19B DNApol-delta DNApol-epsilon Dlc90F Dll DnaJ-1 Dp Dr Dref Dsp1 E(z) EG:115C2.6 ERp60 Eb1 Eip75B Elf Eno Es2 Fas2 Fs(2)Ket Fur1 GATAd Gapdh1 Gapdh2 Gdh Gfat1 Got2 Hcf Hem HmgD HmgZ Hrb27C Hrb87F Hsc70-3 Hsp60 Idh ImpE2 Iswi Karybeta3 Klp10A Lam Map60 Mapmodulin Mcm5 Mcm6 Mcm7 Mi-2 Moe Mpcp Msp-300 Myb NUCB1 Nek2 Nlp Nop60B Optix Ote Pcl Pen Pep Pepck Pgi Pi3K68D Pp1-87B Psc Ptp99A Ranbp16 Rbf2 Rbp2 Rm62 RnrL Rop RpII140 RpL13 RpL15 RpL22 RpL23a RpL3 RpS12 RpS6 Rrp1 S SMC1 Scm Set Sin3A Snap Sox14 Spt6 Ste12DOR Ste:CG33236 Su(var)3-9 Su(z)12 Taf2 Thd1 Thiolase Tis11 Tm1 Tom34 Top2 TppII Trxr-1 Uba2 UbcD6 Ucp4B Vap-33-1 Vha44 Vha55 XNP abs ade3 alpha-Adaptin alpha-Cat alpha-Spec alphaTub84B aop ap arm ash2 asp barr baz betaTub56D bl br brk brm bsf btsz capu cav cdc2 chb ci cib cic ck cora crc crol crp csul csw d d4 da dac dalao dnk dpa dre4 dve eIF-4B eIF3-S9 eIF5 east ebi edl en esn exd fz fzy garz gft gfzf gol grn hdc heph hh hts jar jim jing jumu katanin-60 kay kel kis klar klu kn l(1)G0334 l(1)dd4 l(2)01424 l(2)dtl l(2)gl larp lds lgs lid lig lin19 lmg lola lwr mdy mod mor msk mus209 nonA-l nop5 nudC ovo p120ctn pAbp par-6 pav pbl pfk ph-p pk pll pnut polo pon qkr54B rg rin sc scrib sd sec23 sima simj slmb smi35A sqd ss stai stc su(f) th toe tub tud vg viaf1 vig wbl woc yps zf30C zip |
| 10 | GO:0000902 | P | 4, 5, | 73 | 28.325 (x 2.577) | 338 (0.216) | 3.22e-12 | cellular morphogenesis | Abi Akt1 Amph Arf79F CG1900 CG7161 CaMKII Cdc42 Cdk4 Cont CycT Fas2 Fas3 Fs(2)Ket Galpha49B Gef26 Hem Lac LanA Mbs Moe NetA Paip2 Ptp99A S Sdc Sema-1b Sema-2a Vang X11L abs alpha-Cat alpha-Spec aop ap argos arm baz btsz chb cora crb cta dac edl ena fax fra fz hh in kay klar l(2)gl lola mbt msn ninA otk ovo par-6 pbl pk plexA pnut rin sca scrib shg stan trio vav zip |
| 11 | GO:0048731 | P | 3, | 106 | 49.778 (x 2.129) | 594 (0.178) | 3.34e-12 | system development | Akap200 Akt1 CG8104 Cdc42 Cdk4 CkIIbeta Cont CycA Cyp1 Dll Dr EG:BACH59J11.2 Eip75B Es2 Fas2 Fas3 Galpha49B Gef26 Got2 Hem Lac Lam LanA NetA PR2 Pp1-87B Ptp99A S Sdc Sema-1b Sema-2a Ser Sin3A Smox Sox14 Src64B Tm1 Wnt2 abs ana ap argos arm barr baz bib bnb br brk cdc2 chb cib cora crb csw da dac dome ed en ena exd fat2 fax fdl fra gish glec hdc hh jar jing jumu kay klar l(1)G0148 l(2)01424 l(2)gl larp lola mbt msi ninA ogre otk par-6 pav pbl plexA pon sc sca sd sgl shg sima smi35A stai stan stc sty tkv toe trio trn zip |
| 12 | GO:0007444 | P | 4, | 68 | 25.308 (x 2.687) | 302 (0.225) | 3.47e-12 | imaginal disc development | 14-3-3epsilon Amph Btk29A CG40410 CG5841 CG6896 Cdc42 Dll Dr Fas2 G-salpha60A ImpE2 ImpE3 Mbs Moe S Sb SelD Ser Smox Su(dx) Vang aop ap argos arm ash2 bl br brm ci ck crb crol csw da dac dve ebi en exd fat2 fz gft heph hh in jumu kay klar klu kn l(2)gl mbt pk pnut rg rin sca scrib sd sdk spag ss stan th tkv vg |
| 13 | GO:0048468 | P | 4, | 73 | 28.492 (x 2.562) | 340 (0.215) | 4.11e-12 | cell development | 14-3-3epsilon 14-3-3zeta Act5C Amph BicD Cdc42 Chc Dlc90F Fas2 Fas3 Galpha49B Gef26 Hem Hrb27C Lac LanA Mbs Moe NetA Nop60B Pen Ptp99A Rop S Sdc Sema-1b Sema-2a Smox Tm1 alpha-Spec aop ap argos arm asp baz bel capu chb crb csul dac edl egl ena fax fra gus heph hh hts jar klar lola mbt mod msn ninA otk par-6 pbl plexA pnut sca shg sqd stan th tkv trio tud wbl zip |
| 14 | GO:0002009 | P | 4, | 41 | 11.146 (x 3.679) | 133 (0.308) | 1.05e-11 | morphogenesis of an epithelium | Btk29A Cdc42 Dp Eip63E Mbs Moe Mtl Sb Scm Src64B Vang aop argos arm barr baz cora crb csw dome ena flfl fz in inx2 jar jumu kay l(2)gl msn par-6 ph-p pk rin sca scrib shg sqd stan tkv zip |
| 15 | GO:0048477 | P | 6, | 65 | 24.218 (x 2.684) | 289 (0.225) | 1.07e-11 | oogenesis | 14-3-3epsilon 14-3-3zeta BicD Btk29A Caf1 Cdc42 Dp Eip75B Fas3 Fs(2)Ket Hem Hrb27C Mbs Mcm6 Moe Myb Pen Pp1-87B Rop S Scm Src64B Su(var)3-9 Tm1 alpha-Cat alpha-Spec argos arm asp baz bib brm capu chb ci csul csw da dome egl endos gus hh hts jar jing kay kel mdy mor msn mus209 nudC ovo par-6 ph-p shg sqd sty th tkv tud wbl yps zip |
| 16 | GO:0048513 | P | 3, | 113 | 56.147 (x 2.013) | 670 (0.169) | 1.45e-11 | organ development | 14-3-3epsilon Akap200 Amph Btk29A CG30011 CG3227 CG40410 CG5841 CG6896 CaMKII Cdc42 CkIIalpha CkIIbeta Cyp1 Dll Dr Eip75B Fas2 G-salpha60A Galpha49B Hem ImpE2 ImpE3 LanA LanB2 Mbs Moe Optix PR2 Pen S Sb SelD Sema-2a Ser Smox Sox14 Src64B Su(dx) Vang Vap-33-1 Wnt2 aop ap argos arm ash2 barr betaTub56D bl br brk brm ci cib ck cora crb crol csw da dac dome dve ebi ed edl en exd fat2 fdl fz gft grn heph hh in inx2 jumu kay klar klu kn l(1)G0148 l(2)01424 l(2)gl larp lqf lwr mbt msn nonA-l pbl pk pll pnut rg rin sc sca scrib sd sdk sgl shg spag ss stan th tkv tub vg zip |
| 17 | GO:0050794 | P | 3, | 189 | 115.394 (x 1.638) | 1377 (0.137) | 2.98e-11 | regulation of cellular process | 14-3-3epsilon 14-3-3zeta ATbp Aac11 Abi Akap200 Akt1 B52 BEAF-32 Bap170 BcDNA:LD23876 Brf CG10473 CG10990 CG11207 CG11228 CG11305 CG12190 CG12299 CG14217 CG15141 CG15835 CG17514 CG17765 CG1815 CG1900 CG30011 CG31258 CG31716 CG33525 CG40160 CG40410 CG4914 CG5434 CG5728 CG5841 CG6227 CG7263 CG7987 CG8092 CG8165 CG8400 CG8426 CG8443 CG8924 CREG CaMKII Caf1 Cdk4 CkIalpha Cks CtBP CycA CycT D12 D19A D19B Dll Dp Dr Dref Dsp1 E(z) Eb1 Eip63E Eip75B Elf Fas2 GATAd Gap1 Gprk1 HmgD HmgZ Hrb87F Hus1-like Iswi LanA Mbs Mi-2 Myb Nek2 Optix Paip2 Pcl Psc Rab10 RacGAP50C Rbf2 Rm62 RpS6 S Scm Ser Sin3A Smox Sox14 Src64B Su(var)3-9 Su(z)12 TBPH Taf2 Thd1 Uba2 UbcD6 Vha44 XNP ald alphaTub84B ana aop ap argos arm ash2 bl br brk brm cdc2 ci cic crc crol d4 da dalao dre4 dve eIF-4B eIF3-S9 eIF5 ebi ed edl en ena exd fwd fzy gft gol grn hdc hh jim jing jumu kay kis klu kn l(2)01424 l(2)gl lds lgs lid lin19 lola lqf mbt mdy mor msi mus209 ovo pAbp ph-p rin sc scrib sd sdk sima simj slmb smi35A sqd ss stan stc sty th tkv toe woc yps zf30C zip zwilch |
| 18 | GO:0009993 | P | 7, | 62 | 23.213 (x 2.671) | 277 (0.224) | 4.44e-11 | oogenesis (sensu Insecta) | 14-3-3epsilon 14-3-3zeta BicD Btk29A Caf1 Cdc42 Dp Eip75B Fas3 Fs(2)Ket Hrb27C Mbs Mcm6 Moe Myb Pen Pp1-87B Rop S Scm Src64B Su(var)3-9 Tm1 alpha-Cat alpha-Spec argos arm asp baz bib brm capu chb ci csw da dome egl endos gus hh hts jar jing kay kel mdy mor msn mus209 nudC ovo par-6 ph-p shg sqd sty th tkv tud yps zip |
| 19 | GO:0005634 | C | 5, 6, 7, 8, | 203 | 127.712 (x 1.590) | 1524 (0.133) | 5.1e-11 | nucleus | 14-3-3epsilon 14-3-3zeta ATbp Arc92 B52 BEAF-32 Bap170 BcDNA:GM10765 BcDNA:LD23876 Brf CG10211 CG10354 CG10473 CG11188 CG11305 CG11856 CG12299 CG12391 CG13895 CG1529 CG1647 CG1677 CG17361 CG17838 CG17952 CG1911 CG2097 CG2158 CG30011 CG31258 CG31617 CG32164 CG33525 CG3605 CG3847 CG40410 CG4914 CG5728 CG5789 CG6064 CG6227 CG6791 CG6930 CG7154 CG7263 CG7987 CG8089 CG8092 CG8426 CG8478 CG8924 CG9104 CG9342 CG9425 CG9894 CREG Caf1 Cbp80 Cen190 CkIIalpha CkIIbeta CkIalpha CtBP CycA CycT Cyp1 D12 D19A D19B DNApol-delta DNApol-epsilon Dll Dp Dr Dref Dsp1 E(z) EG:115C2.6 Eip75B Es2 Fs(2)Ket GATAd Hcf HmgD HmgZ Hrb27C Hrb87F Iswi Karybeta3 Lam Mcm5 Mcm6 Mcm7 Mi-2 Myb Nlp Nop60B Optix Ote Pcl Pen Pep Psc Ptp99A Ranbp16 Rbf2 Rm62 RpII140 Rrp1 SMC1 Scm Set Sin3A Sox14 Spt6 Ste12DOR Ste:CG33236 Su(var)3-9 Su(z)12 Taf2 Thd1 Tis11 Top2 Uba2 UbcD6 XNP abs aop ap ash2 barr bl br brk brm btsz capu cav cdc2 chb ci cic crc crol crp d4 da dac dalao dpa dre4 dve east ebi edl en esn exd gft gol grn heph hh jim jing jumu kay kis klar klu kn lds lgs lid lin19 lmg lola lwr mod mor msk mus209 nonA-l ovo pav pbl pfk ph-p pk rin sc sd sima simj sqd ss stc su(f) toe vg woc yps zf30C |
| 20 | GO:0007276 | P | 4, | 84 | 37.459 (x 2.242) | 447 (0.188) | 7.29e-11 | gametogenesis | 14-3-3epsilon 14-3-3zeta Act5C BicD Btk29A Caf1 Cdc42 Chc Dlc90F Dp Eip75B Fas3 Fpps Fs(2)Ket Hem Hrb27C Mbs Mcm6 Moe Myb Nop60B Pen Pp1-87B Rop S Scm Set Src64B Ste12DOR Ste:CG33236 Su(var)3-9 Tm1 alpha-Cat alpha-Spec argos arm asp baz bel bib brm capu cdc2 chb ci csul csw da dome egl endos esn fwd gish gus heph hh hts jar jing kay kel mdy mod mor msn mus209 nudC ovo par-6 ph-p pk qkr54B scrib shg sqd stai sty th tkv tud wbl yps zip |
| 21 | GO:0019953 | P | 3, | 85 | 38.213 (x 2.224) | 456 (0.186) | 7.94e-11 | sexual reproduction | 14-3-3epsilon 14-3-3zeta Act5C BicD Btk29A Caf1 Cdc42 Chc Dlc90F Dp Eip75B Fas3 Fpps Fs(2)Ket Hem Hrb27C Mbs Mcm6 Moe Myb Nop60B Pen Pp1-87B Rop S Scm Set Src64B Ste12DOR Ste:CG33236 Su(var)3-9 Tm1 alpha-Cat alpha-Spec argos arm asp baz bel bib brm capu cdc2 chb ci csul csw da dome egl endos esn fwd gish gus heph hh hts jar jing kay kel mdy mod mor msn mus209 nudC ovo par-6 ph-p pk polo qkr54B scrib shg sqd stai sty th tkv tud wbl yps zip |
| 22 | GO:0007399 | P | 4, | 86 | 39.219 (x 2.193) | 468 (0.184) | 1.19e-10 | nervous system development | CG8104 Cdc42 CkIIbeta Cont CycA Dll Dr EG:BACH59J11.2 Es2 Fas2 Fas3 Galpha49B Gef26 Got2 Hem Lac LanA NetA Pp1-87B Ptp99A S Sdc Sema-1b Sema-2a Ser Sin3A Smox Sox14 Src64B Tm1 abs ana ap argos arm barr baz bib bnb br brk cdc2 chb cib crb csw da dac ed en ena exd fax fdl fra gish glec hh jar jing jumu kay l(2)gl lola mbt msi ninA ogre otk par-6 pav pbl plexA pon sc sca sd shg sima smi35A stai stan stc tkv toe trio |
| 23 | GO:0009987 | P | 2, | 638 | 561.968 (x 1.135) | 6706 (0.095) | 1.24e-10 | cellular process | 14-3-3epsilon 14-3-3zeta 18w AP-1gamma ATPsyn-beta ATbp Aac11 Aats-ala Aats-glupro Abi Ack Acon Acox57D-p Act42A Act5C Akap200 Akt1 Ald Amph ApepP Arc92 Arf79F B52 BEAF-32 BEST:CK01140 BEST:LD22483 BG:DS00004.11 BG:DS07473.1 Bap170 BcDNA:GH02901 BcDNA:GH02976 BcDNA:GH12558 BcDNA:GM10765 BcDNA:LD09009 BcDNA:LD22910 BcDNA:LD23371 BcDNA:LD23876 BcDNA:LD41548 BicD Brf BthD Btk29A CG10211 CG10354 CG10423 CG10473 CG10542 CG10602 CG10657 CG10695 CG10960 CG10990 CG11055 CG11069 CG11123 CG11142 CG11207 CG11228 CG11305 CG11451 CG11836 CG11856 CG12006 CG12048 CG12130 CG12190 CG12199 CG12252 CG12299 CG12896 CG13852 CG1418 CG14217 CG14222 CG14231 CG1440 CG14439 CG14670 CG1472 CG14882 CG15093 CG1514 CG15141 CG1544 CG1550 CG15835 CG17036 CG17064 CG17143 CG17184 CG17309 CG17419 CG1746 CG17493 CG17514 CG17598 CG17765 CG17919 CG18030 CG1815 CG1869 CG1893 CG1900 CG1906 CG1907 CG1911 CG2097 CG2108 CG2118 CG2158 CG2316 CG2852 CG2947 CG2980 CG30011 CG30025 CG30372 CG30440 CG31169 CG31251 CG31258 CG31453 CG31472 CG31559 CG31617 CG31640 CG31716 CG31739 CG32099 CG32137 CG32158 CG32164 CG32447 CG3249 CG32632 CG32640 CG32672 CG33113 CG33116 CG33123 CG33138 CG33145 CG33214 CG3328 CG33525 CG3590 CG3605 CG3823 CG40160 CG40410 CG4238 CG4454 CG4509 CG4612 CG4670 CG4749 CG4914 CG5028 CG5064 CG5191 CG5319 CG5390 CG5434 CG5466 CG5505 CG5522 CG5687 CG5728 CG5789 CG5841 CG5873 CG6227 CG6287 CG6391 CG6394 CG6418 CG6543 CG6638 CG6673 CG6680 CG6767 CG6812 CG6835 CG6838 CG6854 CG6904 CG6946 CG6954 CG7008 CG7145 CG7161 CG7263 CG7288 CG7441 CG7461 CG7720 CG7878 CG7922 CG7987 CG8036 CG8092 CG8104 CG8155 CG8165 CG8188 CG8243 CG8400 CG8426 CG8443 CG8494 CG8789 CG8863 CG8924 CG8963 CG9027 CG9057 CG9135 CG9153 CG9246 CG9342 CG9425 CG9471 CG9503 CG9809 CG9906 CREG CaMKII Cad87A Caf1 CalpB Cap-G Cat Cbp80 CdGAPr Cdc42 Cdk4 Cen190 Chc Cip4 CkIIalpha CkIIbeta CkIalpha Cks Cont CtBP CycA CycG CycT Cyp1 Cyp6v1 D12 D19A D19B DNApol-delta DNApol-epsilon Dhh1 Dlc90F Dll DnaJ-1 Dp Dr Dref Dsp1 E(z) EG:196F3.2 EG:34F3.8 EG:86E4.2 EG:BACH59J11.2 ERp60 ESTS:39C10S Eb1 Eip55E Eip63E Eip75B Elf Eno Fas2 Fas3 Fkbp13 Fpps Fs(2)Ket Fur1 G-salpha60A GATAd Galpha49B Gap1 Gapdh1 Gapdh2 Gdh Gef26 Gfat1 GlyP Got2 Gprk1 Hcf Hem Hlc HmgD HmgZ Hrb27C Hrb87F Hsc70-3 Hsp23 Hsp26 Hsp27 Hsp60 Hsp67Bc Hus1-like Idh ImpL2 Indy Iswi Jheh1 Jheh2 KP78b Karybeta3 Klp10A Lac Lam LanA LanB2 M(2)21AB MICAL-like Map60 Mapmodulin Mbs Mcm5 Mcm6 Mcm7 Mcr Mdr49 Mekk1 Mi-2 Moe Mpcp Msp-300 Mtl Myb NTPase Nek2 Nep2 NetA Nlp Nop60B Nrv1 Optix Ote PFE PR2 Paf-AHalpha Paip2 Pak3 Pcl Pen Pepck Pgi Pi3K68D Pld Pli Pp1-87B Pp2C1 Psa Psc Ptp99A Rab10 RacGAP50C Ranbp16 Rbf2 Rbp2 Rgl RhoBTB RhoGAP19D RhoGAP1A RhoGEF3 Rhp Rm62 RnrL Rop RpII140 RpL13 RpL15 RpL22 RpL23a RpL3 RpS12 RpS6 Rrp1 S SMC1 Sb Scm Sdc SelD Sema-1b Sema-2a Ser Set Sh3beta Sin3A Sip1 Smox Snap Sox14 Spn5 Spt6 Src64B Su(dx) Su(var)3-9 Su(z)12 TBPH Taf2 Thd1 Thiolase Tis11 Tm1 Tom34 Top2 Top3beta TppII Tpr2 Trap1 Trxr-1 Tsp66E Uba1 Uba2 UbcD2 UbcD6 Uch Ucp4B Vang Vap-33-1 Vha44 Vha55 Wnt2 Wsck X11L XNP abs ade2 ade3 ald alpha-Adaptin alpha-Cat alpha-Spec alphaTub84B ana aop ap argos arm ash2 asp barr baz bel betaTub56D bib bl br brk brm btsz capu cav cdc2 chb ci cib cic ck cora crb crc crol csul csw cta d d4 da dac dalao deltaTry dnk dome dpa dre4 dve eIF-4B eIF3-S9 eIF5 east ebi ed edl egl en ena endos esn exd fat2 fax flfl fra fwd fz fzy garz gft gish glec gol gp210 grn guf gus hdc heph hh hts in inx2 inx3 jar jim jing jumu katanin-60 kay kel kis klar klu kn knk krz l(1)G0148 l(1)G0334 l(1)dd4 l(2)01424 l(2)08717 l(2)dtl l(2)gl larp lds lgs lid lin19 lmg lola lqf lwr mask mav mbt mdy me31B mod mor msi msk msn mth mus209 ninA nonA-l nop5 nudC ogre olf413 ome otk ovo p120ctn pAbp pain par-6 pav pbl pfk ph-p pk plexA pll pnut polo pon prominin-like qkr54B r-l rg rin rpk sc sca scf scrib sd sdk sec23 serpin-27A sgl shg sima simj slmb smi35A spag sqd ss stai stan stc sty su(f) syndapin th tkv toe tok tos trio trn tub tud vav vg vig vimar wbl wds wgn woc yps zf30C zip zwilch |
| 24 | GO:0007292 | P | 5, | 65 | 25.978 (x 2.502) | 310 (0.210) | 2.08e-10 | female gamete generation | 14-3-3epsilon 14-3-3zeta BicD Btk29A Caf1 Cdc42 Dp Eip75B Fas3 Fs(2)Ket Hem Hrb27C Mbs Mcm6 Moe Myb Pen Pp1-87B Rop S Scm Src64B Su(var)3-9 Tm1 alpha-Cat alpha-Spec argos arm asp baz bib brm capu chb ci csul csw da dome egl endos gus hh hts jar jing kay kel mdy mor msn mus209 nudC ovo par-6 ph-p shg sqd sty th tkv tud wbl yps zip |
| 25 | GO:0006996 | P | 5, | 115 | 60.504 (x 1.901) | 722 (0.159) | 2.98e-10 | organelle organization and biogenesis | 14-3-3epsilon 14-3-3zeta ATPsyn-beta Abi Act42A Act5C BcDNA:LD09009 BicD CG10542 CG10695 CG11207 CG11305 CG17184 CG1911 CG31617 CG32672 CG5728 CG8104 CG8243 CG9057 Caf1 Cap-G Cdc42 Cdk4 Cen190 Cip4 CycT Dlc90F Dref Dsp1 E(z) Eb1 Fs(2)Ket Galpha49B Hem HmgD HmgZ Iswi KP78b Klp10A Lam MICAL-like Map60 Mapmodulin Mcm5 Mi-2 Moe Msp-300 Mtl Myb Nlp Nop60B Ote Pak3 Pcl Pp1-87B Psc RhoBTB Rhp SMC1 Sb Set Spt6 Src64B Su(var)3-9 Su(z)12 Vha55 alpha-Cat alpha-Spec alphaTub84B arm ash2 asp barr baz betaTub56D brm cav chb cib cora cta dalao dpa dre4 egl ena esn fra fwd hts jar jumu katanin-60 kis klar l(1)dd4 larp lola mask mbt mor mus209 nop5 pav pbl pfk ph-p pnut polo sec23 stai trio vav vg |
| 26 | GO:0050875 | P | 3, | 601 | 520.906 (x 1.154) | 6216 (0.097) | 3.28e-10 | cellular physiological process | 14-3-3epsilon 14-3-3zeta AP-1gamma ATPsyn-beta ATbp Aac11 Aats-ala Aats-glupro Abi Ack Acon Acox57D-p Act42A Act5C Akap200 Akt1 Ald Amph ApepP Arc92 Arf79F B52 BEAF-32 BEST:CK01140 BEST:LD22483 BG:DS00004.11 BG:DS07473.1 Bap170 BcDNA:GH02901 BcDNA:GH02976 BcDNA:GH12558 BcDNA:GM10765 BcDNA:LD09009 BcDNA:LD22910 BcDNA:LD23371 BcDNA:LD23876 BcDNA:LD41548 BicD Brf BthD Btk29A CG10211 CG10354 CG10423 CG10473 CG10542 CG10602 CG10657 CG10695 CG10960 CG10990 CG11055 CG11069 CG11123 CG11142 CG11207 CG11228 CG11305 CG11451 CG11836 CG11856 CG12006 CG12048 CG12130 CG12190 CG12199 CG12252 CG12299 CG12896 CG13852 CG1418 CG14217 CG14222 CG14231 CG1440 CG14439 CG14670 CG1472 CG14882 CG15093 CG15141 CG1544 CG1550 CG15835 CG17036 CG17143 CG17184 CG17309 CG17419 CG1746 CG17493 CG17514 CG17598 CG17765 CG18030 CG1815 CG1869 CG1893 CG1900 CG1906 CG1907 CG1911 CG2097 CG2108 CG2118 CG2158 CG2316 CG2852 CG2947 CG2980 CG30011 CG30025 CG31169 CG31251 CG31258 CG31453 CG31472 CG31559 CG31617 CG31716 CG31739 CG32099 CG32137 CG32158 CG32164 CG3249 CG32632 CG32640 CG32672 CG33113 CG33116 CG33123 CG33138 CG33145 CG33214 CG3328 CG33525 CG3590 CG3605 CG3823 CG40160 CG40410 CG4238 CG4454 CG4509 CG4612 CG4670 CG4749 CG4914 CG5028 CG5064 CG5191 CG5319 CG5390 CG5434 CG5466 CG5505 CG5687 CG5728 CG5789 CG5841 CG5873 CG6227 CG6287 CG6391 CG6394 CG6418 CG6543 CG6638 CG6673 CG6680 CG6767 CG6812 CG6835 CG6838 CG6854 CG6904 CG6946 CG7008 CG7145 CG7161 CG7263 CG7288 CG7441 CG7461 CG7720 CG7878 CG7922 CG7987 CG8036 CG8092 CG8104 CG8155 CG8165 CG8188 CG8243 CG8400 CG8426 CG8443 CG8494 CG8789 CG8863 CG8924 CG8963 CG9027 CG9057 CG9135 CG9153 CG9246 CG9342 CG9425 CG9471 CG9503 CG9809 CG9906 CREG CaMKII Caf1 CalpB Cap-G Cat Cbp80 Cdc42 Cdk4 Cen190 Chc Cip4 CkIIalpha CkIIbeta CkIalpha Cks Cont CtBP CycA CycG CycT Cyp1 Cyp6v1 D12 D19A D19B DNApol-delta DNApol-epsilon Dhh1 Dlc90F Dll DnaJ-1 Dp Dr Dref Dsp1 E(z) EG:196F3.2 EG:34F3.8 EG:86E4.2 ERp60 ESTS:39C10S Eb1 Eip55E Eip63E Eip75B Elf Eno Fas2 Fas3 Fkbp13 Fpps Fs(2)Ket Fur1 GATAd Galpha49B Gap1 Gapdh1 Gapdh2 Gdh Gef26 Gfat1 GlyP Got2 Gprk1 Hcf Hem Hlc HmgD HmgZ Hrb27C Hrb87F Hsc70-3 Hsp23 Hsp26 Hsp27 Hsp60 Hsp67Bc Hus1-like Idh Indy Iswi Jheh1 Jheh2 KP78b Karybeta3 Klp10A Lac Lam LanA LanB2 M(2)21AB MICAL-like Map60 Mapmodulin Mbs Mcm5 Mcm6 Mcm7 Mcr Mdr49 Mekk1 Mi-2 Moe Mpcp Msp-300 Mtl Myb NTPase Nek2 Nep2 NetA Nlp Nop60B Nrv1 Optix Ote PFE PR2 Paf-AHalpha Paip2 Pak3 Pcl Pen Pepck Pgi Pi3K68D Pld Pp1-87B Pp2C1 Psa Psc Ptp99A Rab10 RacGAP50C Ranbp16 Rbf2 Rbp2 RhoBTB Rhp Rm62 RnrL Rop RpII140 RpL13 RpL15 RpL22 RpL23a RpL3 RpS12 RpS6 Rrp1 S SMC1 Sb Scm Sdc SelD Sema-1b Sema-2a Ser Set Sh3beta Sin3A Sip1 Smox Snap Sox14 Spn5 Spt6 Src64B Su(dx) Su(var)3-9 Su(z)12 TBPH Taf2 Thd1 Thiolase Tis11 Tom34 Top2 Top3beta TppII Tpr2 Trap1 Trxr-1 Uba1 Uba2 UbcD2 UbcD6 Uch Ucp4B Vang Vap-33-1 Vha44 Vha55 Wsck X11L XNP abs ade2 ade3 ald alpha-Adaptin alpha-Cat alpha-Spec alphaTub84B ana aop ap argos arm ash2 asp barr baz bel betaTub56D bib bl br brk brm btsz capu cav cdc2 chb ci cib cic ck cora crb crc crol csul csw cta d d4 da dac dalao deltaTry dnk dome dpa dre4 dve eIF-4B eIF3-S9 eIF5 east ebi edl egl en ena endos esn exd fat2 fax fra fwd fz fzy garz gft gish gol gp210 grn heph hh hts in jar jim jing jumu katanin-60 kay kel kis klar klu kn knk krz l(1)G0148 l(1)G0334 l(1)dd4 l(2)01424 l(2)08717 l(2)dtl l(2)gl larp lds lid lin19 lmg lola lqf lwr mask mav mbt mdy me31B mod mor msi msk msn mth mus209 ninA nonA-l nop5 nudC ogre olf413 ome otk ovo pAbp pain par-6 pav pbl pfk ph-p pk plexA pll pnut polo pon prominin-like qkr54B r-l rg rin rpk sc sca scrib sd sec23 serpin-27A sgl shg sima simj slmb smi35A spag sqd ss stai stan stc su(f) syndapin th tkv toe tok tos trio trn tub tud vav vg vig wbl wds wgn woc yps zf30C zip zwilch |
| 27 | GO:0009887 | P | 4, | 68 | 28.157 (x 2.415) | 336 (0.202) | 3.29e-10 | organ morphogenesis | Amph CG5841 CG6896 Cdc42 Dll Dr Fas2 G-salpha60A ImpE2 ImpE3 LanA LanB2 Mbs Moe S Sb Smox Su(dx) Vang aop ap argos arm ash2 barr br brm crb crol csw da dac dome dve ebi fat2 fz gft grn heph hh in inx2 jumu kay klar klu kn l(2)gl lqf mbt msn pbl pk pnut rg rin sca scrib sd sdk sgl shg spag stan tkv vg zip |
| 28 | GO:0000003 | P | 2, | 88 | 41.984 (x 2.096) | 501 (0.176) | 7.01e-10 | reproduction | 14-3-3epsilon 14-3-3zeta Act5C BicD Btk29A CaMKII Caf1 Cdc42 Chc Dlc90F Dp Eip75B Fas3 Fpps Fs(2)Ket Gdh Hem Hrb27C Mbs Mcm6 Moe Myb Nop60B Pen Pp1-87B Rop S Scm Set Src64B Ste12DOR Ste:CG33236 Su(var)3-9 Tm1 alpha-Cat alpha-Spec argos arm asp baz bel bib brm capu cdc2 chb ci csul csw da dome egl endos esn fwd gish gus heph hh hts jar jing kay kel lig mdy mod mor msn mus209 nudC ovo par-6 ph-p pk polo qkr54B scrib shg sqd stai sty th tkv tud wbl yps zip |
| 29 | GO:0046698 | P | 5, | 55 | 21.034 (x 2.615) | 251 (0.219) | 1.58e-09 | metamorphosis (sensu Insecta) | Amph CG5841 CG6896 Cdc42 Dr Eip63E G-salpha60A ImpE2 ImpE3 Mbs Moe S Sb Smox Su(dx) Vang aop ap argos arm ash2 br brm crb crc crol csw da dac dve ebi fz gft heph hh in jumu kay klar klu kn l(2)gl mbt pk pnut rg rin sca scrib sd sdk spag stan tkv vg |
| 30 | GO:0007552 | P | 4, | 55 | 21.202 (x 2.594) | 253 (0.217) | 2.12e-09 | metamorphosis | Amph CG5841 CG6896 Cdc42 Dr Eip63E G-salpha60A ImpE2 ImpE3 Mbs Moe S Sb Smox Su(dx) Vang aop ap argos arm ash2 br brm crb crc crol csw da dac dve ebi fz gft heph hh in jumu kay klar klu kn l(2)gl mbt pk pnut rg rin sca scrib sd sdk spag stan tkv vg |
| 31 | GO:0007560 | P | 5, 6, | 53 | 20.112 (x 2.635) | 240 (0.221) | 2.57e-09 | imaginal disc morphogenesis | Amph CG5841 CG6896 Cdc42 Dr G-salpha60A ImpE2 ImpE3 Mbs Moe S Sb Smox Su(dx) Vang aop ap argos arm ash2 br brm crb crol csw da dac dve ebi fz gft heph hh in jumu kay klar klu kn l(2)gl mbt pk pnut rg rin sca scrib sd sdk spag stan tkv vg |
| 32 | GO:0002165 | P | 4, | 67 | 28.911 (x 2.317) | 345 (0.194) | 2.74e-09 | larval or pupal development (sensu Insecta) | Akap200 Amph CG5841 CG6896 Cdc42 Cyp1 Dr Eip63E Eip75B G-salpha60A ImpE2 ImpE3 Mbs Moe PR2 S Sb Ser Smox Su(dx) Vang Vha44 alpha-Spec aop ap argos arm ash2 br brm cib crb crc crol csw da dac dve ebi fz gft heph hh in jumu kay klar klu kn l(1)G0148 l(2)01424 l(2)gl larp mbt pk pnut rg rin sca scrib sd sdk spag stan tkv vg zip |
| 33 | GO:0050791 | P | 3, | 177 | 112.209 (x 1.577) | 1339 (0.132) | 3.47e-09 | regulation of physiological process | 14-3-3epsilon 14-3-3zeta ATbp Aac11 Abi Akt1 Amph B52 BEAF-32 Bap170 BcDNA:LD23876 Brf CG10473 CG10990 CG11207 CG11228 CG11305 CG12190 CG12299 CG14217 CG15141 CG15835 CG17514 CG17765 CG1815 CG1900 CG30011 CG31258 CG31716 CG33525 CG40160 CG40410 CG4914 CG5434 CG5728 CG5841 CG6227 CG7263 CG7987 CG8092 CG8165 CG8400 CG8426 CG8443 CG8924 CREG CaMKII Caf1 Cdk4 CkIalpha Cks CtBP CycA CycT D12 D19A D19B Dll Dp Dr Dref Dsp1 E(z) Eb1 Eip63E Eip75B Elf Fas2 GATAd HmgD HmgZ Hrb27C Hrb87F Hus1-like Iswi LanA Mi-2 Myb Nek2 Optix Paip2 Pcl Psc Rab10 Rbf2 Rm62 Rop RpS6 Scm Sin3A Smox Sox14 Src64B Su(var)3-9 Su(z)12 TBPH Taf2 Thd1 Uba2 UbcD6 Vha44 XNP ald alphaTub84B ana aop ap ash2 bl br brk brm cdc2 ci cic crc crol d4 da dalao dre4 dve eIF-4B eIF3-S9 eIF5 ebi edl en ena exd fwd fzy gft gol grn hh jim jing jumu kay kis klu kn l(2)01424 l(2)gl lds lid lin19 lola mdy mor msi mus209 ovo pAbp ph-p pll rin sc scrib sd serpin-27A sima simj slmb smi35A sqd ss stc th tkv toe woc yps zf30C zip zwilch |
| 34 | GO:0051244 | P | 4, | 171 | 108.690 (x 1.573) | 1297 (0.132) | 1.02e-08 | regulation of cellular physiological process | 14-3-3epsilon 14-3-3zeta ATbp Aac11 Abi Akt1 B52 BEAF-32 Bap170 BcDNA:LD23876 Brf CG10473 CG10990 CG11207 CG11228 CG11305 CG12190 CG12299 CG14217 CG15141 CG15835 CG17514 CG17765 CG1815 CG1900 CG30011 CG31258 CG31716 CG33525 CG40160 CG40410 CG4914 CG5434 CG5728 CG5841 CG6227 CG7263 CG7987 CG8092 CG8165 CG8400 CG8426 CG8443 CG8924 CREG CaMKII Caf1 Cdk4 Cks CtBP CycA CycT D12 D19A D19B Dll Dp Dr Dref Dsp1 E(z) Eb1 Eip63E Eip75B Elf Fas2 GATAd HmgD HmgZ Hrb87F Hus1-like Iswi LanA Mi-2 Myb Nek2 Optix Paip2 Pcl Psc Rab10 Rbf2 Rm62 RpS6 Scm Sin3A Smox Sox14 Src64B Su(var)3-9 Su(z)12 TBPH Taf2 Thd1 Uba2 UbcD6 Vha44 XNP ald alphaTub84B ana aop ap ash2 bl br brk brm cdc2 ci cic crc crol d4 da dalao dre4 dve eIF-4B eIF3-S9 eIF5 ebi edl en ena exd fwd fzy gft gol grn hh jim jing jumu kay kis klu kn l(2)01424 l(2)gl lds lid lin19 lola mdy mor msi mus209 ovo pAbp ph-p rin sc scrib sd sima simj slmb smi35A sqd ss stc th tkv toe woc yps zf30C zip zwilch |
| 35 | GO:0009791 | P | 3, | 67 | 29.833 (x 2.246) | 356 (0.188) | 1.06e-08 | post-embryonic development | Akap200 Amph CG5841 CG6896 Cdc42 Cyp1 Dr Eip63E Eip75B G-salpha60A ImpE2 ImpE3 Mbs Moe PR2 S Sb Ser Smox Su(dx) Vang Vha44 alpha-Spec aop ap argos arm ash2 br brm cib crb crc crol csw da dac dve ebi fz gft heph hh in jumu kay klar klu kn l(1)G0148 l(2)01424 l(2)gl larp mbt pk pnut rg rin sca scrib sd sdk spag stan tkv vg zip |
| 36 | GO:0003676 | F | 3, | 213 | 146.065 (x 1.458) | 1743 (0.122) | 3.26e-08 | nucleic acid binding | ATbp Aats-ala Aats-glupro B52 BEAF-32 Bap170 BcDNA:GM10765 BcDNA:LD23876 CG10103 CG10211 CG10354 CG10423 CG10990 CG11123 CG11305 CG12299 CG12391 CG13350 CG13895 CG15141 CG1529 CG1647 CG1677 CG17361 CG17514 CG17838 CG1815 CG1911 CG30011 CG31169 CG31258 CG31301 CG31617 CG31716 CG31739 CG3249 CG33123 CG33525 CG3847 CG4612 CG4749 CG4914 CG5064 CG5434 CG5728 CG5789 CG6049 CG6227 CG6418 CG6791 CG6812 CG6854 CG6930 CG6946 CG7008 CG7154 CG7878 CG7922 CG7987 CG8089 CG8092 CG8443 CG8478 CG8924 CG8963 CG9104 CG9342 CG9373 CG9425 CG9684 CG9809 Cbp80 Cen190 D19A D19B DNApol-delta DNApol-epsilon Dhh1 Dll Dp Dr Dref Dsp1 E(z) EG:115C2.6 Eip75B Elf GATAd Hcf Hlc HmgD HmgZ Hmr Hmu Hrb27C Hrb87F Iswi LanA Mcm5 Mcm6 Mcm7 Mi-2 Msp-300 Myb NUCB1 Nlp Nop60B Optix Pcl Pep Psc Ptp99A Rab10 Rbf2 Rbp2 Rm62 RpII140 RpL13 RpL15 RpL22 RpL23a RpL3 RpS6 Rrp1 SMC1 Sin3A Sox14 Spt6 Su(var)3-9 Su(z)12 TBPH Taf2 Thd1 Tis11 Top2 Top3beta XNP abs aop ap ash2 bel bl br brk brm bsf btsz capu cav ci cic crc crol d4 da dalao dpa dre4 dve eIF-4B eIF3-S9 eIF5 ebi edl egl en esn exd gfzf grn heph jim jing jumu kay kis klu kn l(2)01424 lds lid lola mask mdy me31B mod msi mus209 nonA-l nop5 ovo pAbp pfk ph-p pk qkr54B rin sc scf sd sima sqd ss stc su(f) toe tos tud vig woc yps zf30C |
| 37 | GO:0000904 | P | 5, 6, | 40 | 14.162 (x 2.824) | 169 (0.237) | 7.17e-08 | cellular morphogenesis during differentiation | Amph Cdc42 Fas2 Fas3 Galpha49B Gef26 Hem Lac LanA Mbs Moe NetA Ptp99A S Sdc Sema-1b Sema-2a aop ap argos chb crb dac edl ena fax fra klar lola mbt msn ninA otk pbl plexA pnut sca shg stan trio |
| 38 | GO:0030707 | P | 8, | 33 | 10.391 (x 3.176) | 124 (0.266) | 8.69e-08 | ovarian follicle cell development (sensu Insecta) | Caf1 Cdc42 Dp Fas3 Fs(2)Ket Mcm6 Myb Scm alpha-Spec argos arm baz bib capu ci csw da dome gus hh jar jing kay kel mus209 par-6 ph-p shg sqd sty th tkv zip |
| 39 | GO:0007293 | P | 8, | 19 | 3.939 (x 4.824) | 47 (0.404) | 1.46e-07 | egg chamber formation (sensu Insecta) | 14-3-3epsilon 14-3-3zeta BicD Btk29A Pen Scm Src64B alpha-Cat alpha-Spec arm asp baz chb egl hts kel par-6 ph-p shg |
| 40 | GO:0019222 | P | 4, | 138 | 85.812 (x 1.608) | 1024 (0.135) | 2.09e-07 | regulation of metabolism | ATbp B52 BEAF-32 Bap170 BcDNA:LD23876 Brf CG11207 CG11228 CG11305 CG12190 CG12299 CG15141 CG15835 CG17514 CG1815 CG30011 CG31258 CG31716 CG33525 CG40160 CG40410 CG4914 CG5434 CG5728 CG6227 CG7987 CG8092 CG8165 CG8426 CG8443 CG8924 CREG Caf1 CkIalpha CtBP CycT D12 D19A D19B Dll Dp Dr Dref Dsp1 E(z) Eip75B Elf GATAd HmgD HmgZ Hrb87F Iswi Mi-2 Myb Optix Paip2 Pcl Psc Rab10 Rbf2 Rm62 RpS6 Scm Sin3A Smox Sox14 Src64B Su(var)3-9 Su(z)12 TBPH Taf2 Thd1 Uba2 Vha44 XNP aop ap ash2 bl br brk brm cdc2 ci cic crc crol d4 da dalao dre4 dve eIF-4B eIF3-S9 eIF5 ebi edl en ena exd fwd fzy gft gol grn hh jim jing jumu kay kis klu kn l(2)01424 lds lid lola mor msi mus209 ovo pAbp ph-p pll rin sc sd serpin-27A sima simj slmb sqd ss stc toe woc yps zf30C |
| 41 | GO:0022008 | P | 5, | 42 | 16.174 (x 2.597) | 193 (0.218) | 3.72e-07 | neurogenesis | Cdc42 Dr Fas2 Fas3 Galpha49B Gef26 Hem Lac LanA NetA Ptp99A Sdc Sema-1b Sema-2a Smox Tm1 ana ap argos arm baz bnb cdc2 chb ed ena fax fra gish hh jar jumu lola ninA otk pbl plexA pon sc shg stan trio |
| 42 | GO:0043226 | C | 2, | 295 | 224.921 (x 1.312) | 2684 (0.110) | 4.42e-07 | organelle | 14-3-3epsilon 14-3-3zeta AP-1gamma ATPsyn-beta ATbp Acon Acox57D-p Act42A Act5C Arc92 B52 BEAF-32 BG:DS00004.11 Bap170 BcDNA:GH04962 BcDNA:GH12558 BcDNA:GM10765 BcDNA:LD23876 BicD Brf CBP CG10211 CG10354 CG10423 CG10473 CG10695 CG11188 CG11207 CG11305 CG11856 CG12299 CG12391 CG13895 CG1472 CG15093 CG1529 CG1647 CG1677 CG17361 CG1746 CG17493 CG17838 CG17952 CG1907 CG1911 CG2097 CG2118 CG2158 CG30011 CG31258 CG31363 CG31617 CG32164 CG33113 CG33525 CG3605 CG3847 CG40410 CG4914 CG5028 CG5728 CG5789 CG6064 CG6227 CG6394 CG6543 CG6638 CG6791 CG6930 CG7145 CG7154 CG7263 CG7987 CG8089 CG8092 CG8426 CG8478 CG8924 CG9104 CG9342 CG9425 CG9894 CREG Caf1 Cap-G Cat Cbp80 Cen190 Chc CkIIalpha CkIIbeta CkIalpha CtBP CycA CycT Cyp1 D12 D19A D19B DNApol-delta DNApol-epsilon Dlc90F Dll Dp Dr Dref Dsp1 E(z) EG:115C2.6 ERp60 Eb1 Eip75B Es2 Fs(2)Ket Fur1 GATAd Gdh Got2 Hcf HmgD HmgZ Hrb27C Hrb87F Hsc70-3 Hsp60 Idh Iswi Karybeta3 Klp10A Lam Map60 Mapmodulin Mcm5 Mcm6 Mcm7 Mi-2 Moe Mpcp Msp-300 Myb NUCB1 Nek2 Nlp Nop60B Optix Ote Pcl Pen Pep Pepck Psc Ptp99A Ranbp16 Rbf2 Rm62 Rop RpII140 RpL13 RpL15 RpL22 RpL23a RpL3 RpS12 RpS6 Rrp1 S SMC1 Scm Set Sin3A Snap Sox14 Spt6 Ste12DOR Ste:CG33236 Su(var)3-9 Su(z)12 Taf2 Thd1 Thiolase Tis11 Tm1 Tom34 Top2 Trxr-1 Uba2 UbcD6 Ucp4B Vap-33-1 Vha44 Vha55 XNP abs alpha-Adaptin alpha-Cat alpha-Spec alphaTub84B aop ap ash2 asp barr betaTub56D bl br brk brm btsz capu cav cdc2 chb ci cic ck cora crc crol crp d d4 da dac dalao dnk dpa dre4 dve east ebi edl en esn exd fzy garz gft gol grn heph hh hts jar jim jing jumu katanin-60 kay kis klar klu kn l(1)dd4 l(2)gl lds lgs lid lin19 lmg lola lwr mod mor msk mus209 nonA-l nop5 ovo p120ctn pav pbl pfk ph-p pk pnut polo rin sc sd sec23 sima simj sqd ss stai stc su(f) toe tud vg wbl woc yps zf30C zip |
| 43 | GO:0007163 | P | 5, 6, | 23 | 5.950 (x 3.866) | 71 (0.324) | 4.47e-07 | establishment and/or maintenance of cell polarity | Cont Moe Vang X11L abs alpha-Cat argos arm baz cora crb fz in kay l(2)gl par-6 pk rin sca scrib shg stan zip |
| 44 | GO:0043229 | C | 3, 4, 5, 6, | 295 | 224.921 (x 1.312) | 2684 (0.110) | 4.52e-07 | intracellular organelle | 14-3-3epsilon 14-3-3zeta AP-1gamma ATPsyn-beta ATbp Acon Acox57D-p Act42A Act5C Arc92 B52 BEAF-32 BG:DS00004.11 Bap170 BcDNA:GH04962 BcDNA:GH12558 BcDNA:GM10765 BcDNA:LD23876 BicD Brf CBP CG10211 CG10354 CG10423 CG10473 CG10695 CG11188 CG11207 CG11305 CG11856 CG12299 CG12391 CG13895 CG1472 CG15093 CG1529 CG1647 CG1677 CG17361 CG1746 CG17493 CG17838 CG17952 CG1907 CG1911 CG2097 CG2118 CG2158 CG30011 CG31258 CG31363 CG31617 CG32164 CG33113 CG33525 CG3605 CG3847 CG40410 CG4914 CG5028 CG5728 CG5789 CG6064 CG6227 CG6394 CG6543 CG6638 CG6791 CG6930 CG7145 CG7154 CG7263 CG7987 CG8089 CG8092 CG8426 CG8478 CG8924 CG9104 CG9342 CG9425 CG9894 CREG Caf1 Cap-G Cat Cbp80 Cen190 Chc CkIIalpha CkIIbeta CkIalpha CtBP CycA CycT Cyp1 D12 D19A D19B DNApol-delta DNApol-epsilon Dlc90F Dll Dp Dr Dref Dsp1 E(z) EG:115C2.6 ERp60 Eb1 Eip75B Es2 Fs(2)Ket Fur1 GATAd Gdh Got2 Hcf HmgD HmgZ Hrb27C Hrb87F Hsc70-3 Hsp60 Idh Iswi Karybeta3 Klp10A Lam Map60 Mapmodulin Mcm5 Mcm6 Mcm7 Mi-2 Moe Mpcp Msp-300 Myb NUCB1 Nek2 Nlp Nop60B Optix Ote Pcl Pen Pep Pepck Psc Ptp99A Ranbp16 Rbf2 Rm62 Rop RpII140 RpL13 RpL15 RpL22 RpL23a RpL3 RpS12 RpS6 Rrp1 S SMC1 Scm Set Sin3A Snap Sox14 Spt6 Ste12DOR Ste:CG33236 Su(var)3-9 Su(z)12 Taf2 Thd1 Thiolase Tis11 Tm1 Tom34 Top2 Trxr-1 Uba2 UbcD6 Ucp4B Vap-33-1 Vha44 Vha55 XNP abs alpha-Adaptin alpha-Cat alpha-Spec alphaTub84B aop ap ash2 asp barr betaTub56D bl br brk brm btsz capu cav cdc2 chb ci cic ck cora crc crol crp d d4 da dac dalao dnk dpa dre4 dve east ebi edl en esn exd fzy garz gft gol grn heph hh hts jar jim jing jumu katanin-60 kay kis klar klu kn l(1)dd4 l(2)gl lds lgs lid lin19 lmg lola lwr mod mor msk mus209 nonA-l nop5 ovo p120ctn pav pbl pfk ph-p pk pnut polo rin sc sd sec23 sima simj sqd ss stai stc su(f) toe tud vg wbl woc yps zf30C zip |
| 45 | GO:0007010 | P | 6, | 74 | 37.710 (x 1.962) | 450 (0.164) | 4.93e-07 | cytoskeleton organization and biogenesis | 14-3-3epsilon 14-3-3zeta ATPsyn-beta Abi Act42A Act5C BicD CG10542 CG10695 CG11207 CG17184 CG32672 CG8104 CG8243 CG9057 Cdc42 Cen190 Cip4 CycT Dlc90F Eb1 Fs(2)Ket Galpha49B Hem KP78b Klp10A Lam MICAL-like Map60 Mapmodulin Moe Msp-300 Mtl Myb Pak3 RhoBTB Rhp Sb Src64B Vha55 alpha-Cat alpha-Spec alphaTub84B arm asp baz betaTub56D chb cib cora cta dpa egl ena esn fra fwd hts jar katanin-60 klar l(1)dd4 larp mask mbt mus209 pav pbl pnut polo stai trio vav vg |
| 46 | GO:0016477 | P | 5, 6, | 39 | 14.665 (x 2.659) | 175 (0.223) | 5.83e-07 | cell migration | Cdc42 Fas3 Fpps Galpha49B LanA NetA Ptp99A Sdc Sema-1b Sema-2a ap argos baz chb csw dome ena fra gish hh jar jing kay kel lola ninA otk par-6 pbl plexA scrib sgl shg sqd stai th trio trn zip |
| 47 | GO:0007049 | P | 4, | 76 | 39.470 (x 1.926) | 471 (0.161) | 6.85e-07 | cell cycle | 14-3-3epsilon 14-3-3zeta Abi BicD CG11207 CG13852 CG17493 CG1911 CG31251 CG32137 CG40410 CG4454 CG5841 CG9425 Cap-G Cdk4 Cip4 Cks CycA CycT Dlc90F Dp Dref Eb1 Eip63E Gap1 Gprk1 Hus1-like Klp10A Lam LanA Msp-300 Myb Nek2 Nop60B Pp1-87B Rbf2 SMC1 Top2 UbcD6 ald alphaTub84B asp barr baz betaTub56D cdc2 chb ci csul da dpa east ebi fwd fzy gft hh jar jumu l(1)dd4 l(2)gl larp lin19 lmg lwr mus209 pav pbl pnut polo sc sca scrib zip zwilch |
| 48 | GO:0048699 | P | 6, | 39 | 15.084 (x 2.585) | 180 (0.217) | 1.28e-06 | generation of neurons | Cdc42 Dr Fas2 Fas3 Galpha49B Gef26 Hem Lac LanA NetA Ptp99A Sdc Sema-1b Sema-2a Smox Tm1 ana ap argos arm baz cdc2 chb ed ena fax fra jar jumu lola ninA otk pbl plexA pon sc shg stan trio |
| 49 | GO:0005623 | C | 2, | 458 | 386.824 (x 1.184) | 4616 (0.099) | 1.55e-06 | cell | 14-3-3epsilon 14-3-3zeta 18w AP-1gamma ATPsyn-beta ATbp Aats-glupro Ack Acon Acox57D-p Act42A Act5C Akt1 Amph ApepP Arc92 B52 BEAF-32 BEST:CK01140 BG:DS00004.11 BG:DS07473.1 Bap170 BcDNA:GH04962 BcDNA:GH12558 BcDNA:GM10765 BcDNA:LD23876 BcDNA:LD41548 BicD Brf Btk29A CBP CG10211 CG10354 CG10423 CG10473 CG10542 CG10657 CG10695 CG10960 CG11069 CG11188 CG11207 CG11305 CG11856 CG12048 CG12130 CG12252 CG12299 CG12391 CG13895 CG14439 CG1472 CG15093 CG1529 CG1647 CG1677 CG17036 CG17361 CG1746 CG17493 CG17598 CG17838 CG17952 CG1815 CG1906 CG1907 CG1911 CG2097 CG2118 CG2158 CG2316 CG30011 CG31258 CG31301 CG31363 CG31617 CG31716 CG31739 CG32158 CG32164 CG32447 CG3305 CG33113 CG33123 CG33145 CG33214 CG33525 CG3605 CG3823 CG3847 CG40410 CG4238 CG4509 CG4914 CG5028 CG5064 CG5434 CG5687 CG5728 CG5789 CG5841 CG6064 CG6227 CG6394 CG6543 CG6638 CG6673 CG6791 CG6812 CG6930 CG7008 CG7145 CG7154 CG7263 CG7720 CG7987 CG8089 CG8092 CG8426 CG8443 CG8478 CG8924 CG9057 CG9104 CG9149 CG9153 CG9342 CG9425 CG9894 CREG CaMKII Cad87A Caf1 CalpB Cap-G Cat Cbp80 Cen190 Chc CkIIalpha CkIIbeta CkIalpha Cont CtBP CycA CycT Cyp1 Cyp6v1 D12 D19A D19B DNApol-delta DNApol-epsilon Dlc90F Dll DnaJ-1 Dp Dr Dref Dsp1 E(z) EG:115C2.6 EG:196F3.2 EG:86E4.2 ERp60 Eb1 Eip75B Elf Eno Es2 Fas2 Fas3 Fs(2)Ket Fur1 G-salpha60A GATAd Galpha49B Gap1 Gapdh1 Gapdh2 Gdh Gfat1 Got2 Hcf Hem HmgD HmgZ Hmu Hrb27C Hrb87F Hsc70-3 Hsp60 Idh ImpE2 ImpE3 Indy Iswi Jheh2 Karybeta3 Klp10A Lac Lam LanA Map60 Mapmodulin Mbs Mcm5 Mcm6 Mcm7 Mdr49 Mi-2 Moe Mpcp Msp-300 Myb NUCB1 Nek2 Nep2 Nlp Nop60B Nrv1 Optix Ote PR2 Pcl Pen Pep Pepck Pgi Pi3K68D Pp1-87B Pp2C1 Psc Ptp99A Ranbp16 Rbf2 Rbp2 Rhp Rm62 RnrL Rop RpII140 RpL13 RpL15 RpL22 RpL23a RpL3 RpS12 RpS6 Rrp1 S SMC1 Sb Scm Sdc Sema-1b Sema-2a Ser Set Sin3A Smox Snap Sox14 Spt6 Ste12DOR Ste:CG33236 Su(dx) Su(var)3-9 Su(z)12 Taf2 Thd1 Thiolase Tis11 Tm1 Tom34 Top2 TppII Trxr-1 Tsp66E Uba2 UbcD6 Uch Ucp4B Vang Vap-33-1 Vha44 Vha55 Wsck XNP abs ade3 alpha-Adaptin alpha-Cat alpha-Spec alphaTub84B aop ap arm ash2 asp barr baz betaTub56D bib bl br brk brm bsf btsz capu cav cdc2 chb ci cib cic ck cora crb crc crol crp csul csw cta d d4 da dac dalao dnk dome dpa dre4 dve eIF-4B eIF3-S9 eIF5 east ebi ed edl egl en esn exd fat2 fra fz fzy garz gft gfzf glec gol gp210 grn hdc heph hh hts in inx2 inx3 jar jim jing jumu katanin-60 kay kel kis klar klu kn l(1)G0334 l(1)dd4 l(2)01424 l(2)08717 l(2)dtl l(2)gl larp lds lgs lid lig lin19 lmg lola lwr mbt mdy mod mor msk mth mus209 ninA nonA-l nop5 nudC ogre ome otk ovo p120ctn pAbp pain par-6 pav pbl pfk ph-p pk plexA pll pnut polo pon prominin-like qkr54B rg rin rpk sc scrib sd sdk sec23 shg sima simj slmb smi35A sqd ss stai stan stc sty su(f) th tkv toe trio trn tub tud vg viaf1 vig wbl wgn woc yps zf30C zip |
| 50 | GO:0044464 | C | 2, 3, | 458 | 386.824 (x 1.184) | 4616 (0.099) | 1.58e-06 | cell part | 14-3-3epsilon 14-3-3zeta 18w AP-1gamma ATPsyn-beta ATbp Aats-glupro Ack Acon Acox57D-p Act42A Act5C Akt1 Amph ApepP Arc92 B52 BEAF-32 BEST:CK01140 BG:DS00004.11 BG:DS07473.1 Bap170 BcDNA:GH04962 BcDNA:GH12558 BcDNA:GM10765 BcDNA:LD23876 BcDNA:LD41548 BicD Brf Btk29A CBP CG10211 CG10354 CG10423 CG10473 CG10542 CG10657 CG10695 CG10960 CG11069 CG11188 CG11207 CG11305 CG11856 CG12048 CG12130 CG12252 CG12299 CG12391 CG13895 CG14439 CG1472 CG15093 CG1529 CG1647 CG1677 CG17036 CG17361 CG1746 CG17493 CG17598 CG17838 CG17952 CG1815 CG1906 CG1907 CG1911 CG2097 CG2118 CG2158 CG2316 CG30011 CG31258 CG31301 CG31363 CG31617 CG31716 CG31739 CG32158 CG32164 CG32447 CG3305 CG33113 CG33123 CG33145 CG33214 CG33525 CG3605 CG3823 CG3847 CG40410 CG4238 CG4509 CG4914 CG5028 CG5064 CG5434 CG5687 CG5728 CG5789 CG5841 CG6064 CG6227 CG6394 CG6543 CG6638 CG6673 CG6791 CG6812 CG6930 CG7008 CG7145 CG7154 CG7263 CG7720 CG7987 CG8089 CG8092 CG8426 CG8443 CG8478 CG8924 CG9057 CG9104 CG9149 CG9153 CG9342 CG9425 CG9894 CREG CaMKII Cad87A Caf1 CalpB Cap-G Cat Cbp80 Cen190 Chc CkIIalpha CkIIbeta CkIalpha Cont CtBP CycA CycT Cyp1 Cyp6v1 D12 D19A D19B DNApol-delta DNApol-epsilon Dlc90F Dll DnaJ-1 Dp Dr Dref Dsp1 E(z) EG:115C2.6 EG:196F3.2 EG:86E4.2 ERp60 Eb1 Eip75B Elf Eno Es2 Fas2 Fas3 Fs(2)Ket Fur1 G-salpha60A GATAd Galpha49B Gap1 Gapdh1 Gapdh2 Gdh Gfat1 Got2 Hcf Hem HmgD HmgZ Hmu Hrb27C Hrb87F Hsc70-3 Hsp60 Idh ImpE2 ImpE3 Indy Iswi Jheh2 Karybeta3 Klp10A Lac Lam LanA Map60 Mapmodulin Mbs Mcm5 Mcm6 Mcm7 Mdr49 Mi-2 Moe Mpcp Msp-300 Myb NUCB1 Nek2 Nep2 Nlp Nop60B Nrv1 Optix Ote PR2 Pcl Pen Pep Pepck Pgi Pi3K68D Pp1-87B Pp2C1 Psc Ptp99A Ranbp16 Rbf2 Rbp2 Rhp Rm62 RnrL Rop RpII140 RpL13 RpL15 RpL22 RpL23a RpL3 RpS12 RpS6 Rrp1 S SMC1 Sb Scm Sdc Sema-1b Sema-2a Ser Set Sin3A Smox Snap Sox14 Spt6 Ste12DOR Ste:CG33236 Su(dx) Su(var)3-9 Su(z)12 Taf2 Thd1 Thiolase Tis11 Tm1 Tom34 Top2 TppII Trxr-1 Tsp66E Uba2 UbcD6 Uch Ucp4B Vang Vap-33-1 Vha44 Vha55 Wsck XNP abs ade3 alpha-Adaptin alpha-Cat alpha-Spec alphaTub84B aop ap arm ash2 asp barr baz betaTub56D bib bl br brk brm bsf btsz capu cav cdc2 chb ci cib cic ck cora crb crc crol crp csul csw cta d d4 da dac dalao dnk dome dpa dre4 dve eIF-4B eIF3-S9 eIF5 east ebi ed edl egl en esn exd fat2 fra fz fzy garz gft gfzf glec gol gp210 grn hdc heph hh hts in inx2 inx3 jar jim jing jumu katanin-60 kay kel kis klar klu kn l(1)G0334 l(1)dd4 l(2)01424 l(2)08717 l(2)dtl l(2)gl larp lds lgs lid lig lin19 lmg lola lwr mbt mdy mod mor msk mth mus209 ninA nonA-l nop5 nudC ogre ome otk ovo p120ctn pAbp pain par-6 pav pbl pfk ph-p pk plexA pll pnut polo pon prominin-like qkr54B rg rin rpk sc scrib sd sdk sec23 shg sima simj slmb smi35A sqd ss stai stan stc sty su(f) th tkv toe trio trn tub tud vg viaf1 vig wbl wgn woc yps zf30C zip |
| 51 | GO:0035107 | P | 4, | 32 | 11.146 (x 2.871) | 133 (0.241) | 1.63e-06 | appendage morphogenesis | CG5841 CG6896 Cdc42 Dll Dr G-salpha60A Mbs Ser Smox Su(dx) ap argos ash2 bl brm ck crol dac dve fz gft heph hh in jumu kn pk sd ss th tkv vg |
| 52 | GO:0048736 | P | 3, | 32 | 11.146 (x 2.871) | 133 (0.241) | 1.67e-06 | appendage development | CG5841 CG6896 Cdc42 Dll Dr G-salpha60A Mbs Ser Smox Su(dx) ap argos ash2 bl brm ck crol dac dve fz gft heph hh in jumu kn pk sd ss th tkv vg |
| 53 | GO:0005911 | C | 6, 7, 8, | 17 | 3.687 (x 4.611) | 44 (0.386) | 1.72e-06 | intercellular junction | CG2097 Cont Fas3 Lac Moe alpha-Cat arm baz cora crb inx2 inx3 l(2)gl ogre par-6 scrib shg |
| 54 | GO:0000278 | P | 5, | 50 | 22.459 (x 2.226) | 268 (0.187) | 2.26e-06 | mitotic cell cycle | 14-3-3epsilon 14-3-3zeta BicD CG11207 CG13852 CG17493 CG1911 CG31251 CG32137 CG40410 CG4454 Cap-G Cdk4 Cks CycA Eb1 Eip63E Gap1 Klp10A Lam Msp-300 Myb Nek2 Nop60B Pp1-87B SMC1 Top2 ald alphaTub84B barr baz betaTub56D cdc2 chb ci csul dpa east fzy hh jar larp lmg mus209 pav pnut polo sc zip zwilch |
| 55 | GO:0040011 | P | 3, | 49 | 21.872 (x 2.240) | 261 (0.188) | 2.48e-06 | locomotion | ATPsyn-beta Abi CG17184 CG8104 Cdc42 Fas3 Fpps Galpha49B LanA NetA Pp1-87B Ptp99A Sdc Sema-1b Sema-2a Vha55 alpha-Cat alphaTub84B ap argos baz betaTub56D chb csw cta dome ena fra gish hh jar jing kay kel lola ninA otk par-6 pbl plexA scrib sgl shg sqd stai th trio trn zip |
| 56 | GO:0009790 | P | 3, | 60 | 29.414 (x 2.040) | 351 (0.171) | 2.73e-06 | embryonic development | 14-3-3zeta 18w Btk29A CG40410 Cdc42 Cdk4 CtBP CycA Eip63E Gap1 LanA LanB2 Mbs Mtl S Sema-1b Src64B aop arm barr baz ci cic cora crb csw cta dome edl en ena flfl gol hh inx2 jar jumu kay kis kn knk l(2)gl lgs lqf lwr msn par-6 pbl pll polo scrib sgl shg stan sty tkv tub tud wbl zip |
| 57 | GO:0017076 | F | 4, | 115 | 71.147 (x 1.616) | 849 (0.135) | 3.03e-06 | purine nucleotide binding | ATPsyn-beta Aats-ala Aats-glupro Ack Akt1 Arf79F BEST:CK01140 BcDNA:LD09009 BcDNA:LD23371 Btk29A CG11069 CG11228 CG1354 CG14217 CG17309 CG1900 CG2108 CG2118 CG2316 CG31453 CG31739 CG33123 CG40410 CG5789 CG6227 CG6418 CG6835 CG7441 CG7878 CG7922 CG8789 CG8902 CG9425 CG9503 CaMKII Cdc42 Cdk4 CkIIalpha CkIalpha Dhh1 Eip63E Elf G-salpha60A Galpha49B Gprk1 Hlc Hsc70-3 Hsp60 Iswi KP78b Klp10A M(2)21AB Mcm5 Mcm6 Mcm7 Mdr49 Mekk1 Mi-2 Mtl Nek2 PR2 Pak3 Pepck Rab10 RhoBTB Rm62 SMC1 SelD Sin3A Src64B Su(var)3-9 Tom34 Top2 Top3beta Trap1 Vha44 Vha55 Wsck X11L XNP abs ald alphaTub84B bel betaTub56D brm cdc2 chb ck cta d dnk dome dpa ebi fat2 gish jar jumu katanin-60 kis l(1)G0148 lds mbt mdy me31B msn otk pav pll pnut polo smi35A tkv zip |
| 58 | GO:0003677 | F | 4, | 112 | 68.884 (x 1.626) | 822 (0.136) | 3.32e-06 | DNA binding | BEAF-32 Bap170 BcDNA:GM10765 BcDNA:LD23876 CG10211 CG13350 CG13895 CG15141 CG1815 CG30011 CG31169 CG31258 CG31617 CG33525 CG4914 CG5789 CG6812 CG6854 CG7154 CG8092 CG8924 CG9104 CG9342 DNApol-delta DNApol-epsilon Dll Dp Dr Dref Dsp1 E(z) Eip75B GATAd Hcf HmgD HmgZ Hmr Hrb27C Iswi Mcm5 Mcm6 Mcm7 Mi-2 Msp-300 Myb NUCB1 Nop60B Optix Pcl Pep Psc Ptp99A Rab10 Rbf2 RpII140 SMC1 Sin3A Sox14 Su(z)12 Taf2 Thd1 Tis11 Top2 Top3beta XNP aop ap ash2 br brk brm btsz capu cav ci cic crc d4 da dalao dpa dre4 dve ebi edl en exd grn jim jing jumu kay kis kn lds lid mod mus209 ovo pfk ph-p sc scf sd sima ss stc toe tos woc yps zf30C |
| 59 | GO:0007456 | P | 6, | 37 | 14.498 (x 2.552) | 173 (0.214) | 3.44e-06 | eye development (sensu Endopterygota) | Amph CG5841 CkIIalpha Fas2 Mbs Moe Optix S Ser Vang aop argos arm br crb csw da dac ebi fz gft hh jumu kay klar klu mbt msn pk pnut rg rin sca sdk shg stan tkv |
| 60 | GO:0051674 | P | 4, | 48 | 21.537 (x 2.229) | 257 (0.187) | 3.63e-06 | localization of cell | ATPsyn-beta Abi CG17184 CG8104 Cdc42 Fas3 Fpps Galpha49B LanA NetA Ptp99A Sdc Sema-1b Sema-2a Vha55 alpha-Cat alphaTub84B ap argos baz betaTub56D chb csw cta dome ena fra gish hh jar jing kay kel lola ninA otk par-6 pbl plexA scrib sgl shg sqd stai th trio trn zip |
| 61 | GO:0006928 | P | 4, 5, | 48 | 21.537 (x 2.229) | 257 (0.187) | 3.69e-06 | cell motility | ATPsyn-beta Abi CG17184 CG8104 Cdc42 Fas3 Fpps Galpha49B LanA NetA Ptp99A Sdc Sema-1b Sema-2a Vha55 alpha-Cat alphaTub84B ap argos baz betaTub56D chb csw cta dome ena fra gish hh jar jing kay kel lola ninA otk par-6 pbl plexA scrib sgl shg sqd stai th trio trn zip |
| 62 | GO:0043296 | C | 6, 7, 8, 9, | 14 | 2.682 (x 5.221) | 32 (0.438) | 3.78e-06 | apical junction complex | CG2097 Cont Fas3 Lac Moe alpha-Cat arm baz cora crb l(2)gl par-6 scrib shg |
| 63 | GO:0016327 | C | 5, 6, 7, | 15 | 3.101 (x 4.838) | 37 (0.405) | 4.4e-06 | apicolateral plasma membrane | CG2097 Cont Fas3 Lac Moe alpha-Cat arm baz cora crb dome l(2)gl par-6 scrib shg |
| 64 | GO:0048598 | P | 4, | 27 | 8.883 (x 3.040) | 106 (0.255) | 4.55e-06 | embryonic morphogenesis | 18w Btk29A Cdc42 LanA LanB2 Mbs Mtl Src64B aop arm barr baz cora crb ena flfl inx2 jar jumu kay l(2)gl msn par-6 pbl scrib tkv zip |
| 65 | GO:0000166 | F | 3, | 117 | 73.577 (x 1.590) | 878 (0.133) | 4.97e-06 | nucleotide binding | ATPsyn-beta Aats-ala Aats-glupro Ack Akt1 Arf79F BEST:CK01140 BcDNA:LD09009 BcDNA:LD23371 Btk29A CG11069 CG11228 CG1354 CG14217 CG17309 CG1900 CG2108 CG2118 CG2316 CG31453 CG31739 CG33123 CG40410 CG5789 CG6227 CG6418 CG6835 CG7441 CG7878 CG7922 CG8789 CG8902 CG9425 CG9503 CaMKII Cdc42 Cdk4 CkIIalpha CkIalpha DNApol-delta DNApol-epsilon Dhh1 Eip63E Elf G-salpha60A Galpha49B Gprk1 Hlc Hsc70-3 Hsp60 Iswi KP78b Klp10A M(2)21AB Mcm5 Mcm6 Mcm7 Mdr49 Mekk1 Mi-2 Mtl Nek2 PR2 Pak3 Pepck Rab10 RhoBTB Rm62 SMC1 SelD Sin3A Src64B Su(var)3-9 Tom34 Top2 Top3beta Trap1 Vha44 Vha55 Wsck X11L XNP abs ald alphaTub84B bel betaTub56D brm cdc2 chb ck cta d dnk dome dpa ebi fat2 gish jar jumu katanin-60 kis l(1)G0148 lds mbt mdy me31B msn otk pav pll pnut polo smi35A tkv zip |
| 66 | GO:0048748 | P | 6, 7, | 32 | 11.816 (x 2.708) | 141 (0.227) | 5.6e-06 | eye morphogenesis (sensu Endopterygota) | Amph Fas2 Mbs Moe S Vang aop argos arm br crb csw da dac ebi fz gft hh kay klar klu mbt msn pk pnut rg rin sca sdk shg stan tkv |
| 67 | GO:0007423 | P | 4, | 44 | 19.274 (x 2.283) | 230 (0.191) | 5.69e-06 | sensory organ development | Amph CG3227 CG5841 CkIIalpha Fas2 Hem Mbs Moe Optix S Ser Vang aop argos arm br crb csw da dac ebi ed edl fz gft hh jumu kay klar klu l(2)gl mbt msn pk pnut rg rin sc sca sd sdk shg stan tkv |
| 68 | GO:0048737 | P | 4, | 30 | 10.894 (x 2.754) | 130 (0.231) | 8.83e-06 | appendage development (sensu Endopterygota) | CG5841 CG6896 Cdc42 Dll Dr G-salpha60A Mbs Smox Su(dx) ap argos ash2 brm ck crol dac dve fz gft heph hh in jumu kn pk sd ss th tkv vg |
| 69 | GO:0048518 | P | 3, | 42 | 18.269 (x 2.299) | 218 (0.193) | 8.85e-06 | positive regulation of biological process | Abi Akt1 Bap170 CG10473 CG10990 CG11228 CG14217 CG17765 CG5841 CG7263 CG8400 Cks Dp Eip75B G-salpha60A Iswi aop arm ash2 bl br brm btsz ci da dalao gft hh klu l(2)gl lgs lola mbt mor msn pAbp scrib slmb smi35A stan tkv zip |
| 70 | GO:0030054 | C | 5, 6, 7, | 20 | 5.531 (x 3.616) | 66 (0.303) | 8.9e-06 | cell junction | CG2097 Cont Fas3 Lac Moe Sdc alpha-Cat arm baz cora crb inx2 inx3 l(2)gl mbt ogre p120ctn par-6 scrib shg |
| 71 | GO:0035114 | P | 5, | 30 | 10.894 (x 2.754) | 130 (0.231) | 8.95e-06 | appendage morphogenesis (sensu Endopterygota) | CG5841 CG6896 Cdc42 Dll Dr G-salpha60A Mbs Smox Su(dx) ap argos ash2 brm ck crol dac dve fz gft heph hh in jumu kn pk sd ss th tkv vg |
| 72 | GO:0001654 | P | 5, | 37 | 15.168 (x 2.439) | 181 (0.204) | 9.53e-06 | eye development | Amph CG5841 CkIIalpha Fas2 Mbs Moe Optix S Ser Vang aop argos arm br crb csw da dac ebi fz gft hh jumu kay klar klu mbt msn pk pnut rg rin sca sdk shg stan tkv |
| 73 | GO:0007154 | P | 3, | 179 | 127.126 (x 1.408) | 1517 (0.118) | 9.71e-06 | cell communication | 14-3-3epsilon 14-3-3zeta 18w AP-1gamma Abi Ack Akap200 Akt1 Amph Arf79F Btk29A CG10602 CG11228 CG11451 CG12199 CG14217 CG1514 CG15835 CG17064 CG17309 CG17493 CG17765 CG17919 CG1815 CG1900 CG2108 CG30372 CG30440 CG31640 CG32158 CG32447 CG3249 CG40410 CG4509 CG5522 CG5841 CG6954 CG8243 CG8400 CG8789 CG8924 CaMKII Cad87A CdGAPr Cdc42 Cdk4 Chc Cip4 CkIIalpha CkIIbeta CkIalpha Eip75B Fas2 G-salpha60A Galpha49B Gap1 Gef26 Got2 Gprk1 KP78b Lac LanA LanB2 Mcr Mekk1 Mtl Nek2 Nep2 NetA PFE PR2 Pak3 Pi3K68D Pld Pli Rab10 RacGAP50C Rgl RhoBTB RhoGAP19D RhoGAP1A RhoGEF3 Rhp Rop S Sema-1b Sema-2a Ser Smox Snap Spt6 Src64B Su(dx) Tis11 Tsp66E Vap-33-1 Wnt2 X11L alpha-Adaptin alpha-Spec aop argos arm bib bl br brk capu ci crb csul csw cta dome ebi ed edl endos fat2 fax flfl fra fwd fz gish gus heph hh inx2 inx3 kay klu knk krz l(2)gl lgs lola lqf mask mav mbt msi msk msn mth ogre olf413 ome otk p120ctn pAbp pav pbl plexA pll polo rin sca scf scrib sec23 serpin-27A sgl shg sima slmb ss stai stan sty syndapin tkv trio trn tub vav vimar wbl wgn |
| 74 | GO:0043227 | C | 3, | 253 | 193.496 (x 1.308) | 2309 (0.110) | 9.77e-06 | membrane-bound organelle | 14-3-3epsilon 14-3-3zeta AP-1gamma ATPsyn-beta ATbp Acon Acox57D-p Arc92 B52 BEAF-32 BG:DS00004.11 Bap170 BcDNA:GH04962 BcDNA:GH12558 BcDNA:GM10765 BcDNA:LD23876 Brf CBP CG10211 CG10354 CG10473 CG11188 CG11305 CG11856 CG12299 CG12391 CG13895 CG1472 CG15093 CG1529 CG1647 CG1677 CG17361 CG1746 CG17838 CG17952 CG1907 CG1911 CG2097 CG2118 CG2158 CG30011 CG31258 CG31617 CG32164 CG33113 CG33525 CG3605 CG3847 CG40410 CG4914 CG5028 CG5728 CG5789 CG6064 CG6227 CG6394 CG6543 CG6638 CG6791 CG6930 CG7145 CG7154 CG7263 CG7987 CG8089 CG8092 CG8426 CG8478 CG8924 CG9104 CG9342 CG9425 CG9894 CREG Caf1 Cat Cbp80 Cen190 Chc CkIIalpha CkIIbeta CkIalpha CtBP CycA CycT Cyp1 D12 D19A D19B DNApol-delta DNApol-epsilon Dll Dp Dr Dref Dsp1 E(z) EG:115C2.6 ERp60 Eip75B Es2 Fs(2)Ket Fur1 GATAd Gdh Got2 Hcf HmgD HmgZ Hrb27C Hrb87F Hsc70-3 Hsp60 Idh Iswi Karybeta3 Lam Mcm5 Mcm6 Mcm7 Mi-2 Mpcp Myb NUCB1 Nlp Nop60B Optix Ote Pcl Pen Pep Pepck Psc Ptp99A Ranbp16 Rbf2 Rm62 Rop RpII140 Rrp1 S SMC1 Scm Set Sin3A Snap Sox14 Spt6 Ste12DOR Ste:CG33236 Su(var)3-9 Su(z)12 Taf2 Thd1 Thiolase Tis11 Tom34 Top2 Trxr-1 Uba2 UbcD6 Ucp4B Vap-33-1 Vha44 Vha55 XNP abs alpha-Adaptin alpha-Spec aop ap ash2 barr bl br brk brm btsz capu cav cdc2 chb ci cic crc crol crp d4 da dac dalao dnk dpa dre4 dve east ebi edl en esn exd garz gft gol grn heph hh jar jim jing jumu kay kis klar klu kn l(2)gl lds lgs lid lin19 lmg lola lwr mod mor msk mus209 nonA-l ovo pav pbl pfk ph-p pk rin sc sd sec23 sima simj sqd ss stc su(f) toe tud vg wbl woc yps zf30C |
| 75 | GO:0005524 | F | 6, | 94 | 56.314 (x 1.669) | 672 (0.140) | 9.92e-06 | ATP binding | ATPsyn-beta Aats-ala Aats-glupro Ack Akt1 BEST:CK01140 BcDNA:LD09009 BcDNA:LD23371 Btk29A CG11069 CG11228 CG14217 CG17309 CG2118 CG2316 CG31453 CG31739 CG33123 CG40410 CG5789 CG6227 CG6418 CG6835 CG7441 CG7878 CG7922 CG8789 CG9425 CaMKII Cdk4 CkIIalpha CkIalpha Dhh1 Eip63E Gprk1 Hlc Hsc70-3 Hsp60 Iswi KP78b Klp10A M(2)21AB Mcm5 Mcm6 Mcm7 Mdr49 Mekk1 Mi-2 Nek2 PR2 Pak3 Rm62 SMC1 SelD Sin3A Src64B Tom34 Top2 Top3beta Trap1 Vha44 Vha55 Wsck X11L XNP abs ald bel brm cdc2 ck d dnk dome dpa fat2 gish jar jumu katanin-60 kis l(1)G0148 lds mbt mdy me31B msn otk pav pll polo smi35A tkv zip |
| 76 | GO:0007165 | P | 4, | 157 | 108.187 (x 1.451) | 1291 (0.122) | 1e-05 | signal transduction | 14-3-3epsilon 14-3-3zeta 18w Abi Ack Akap200 Akt1 Arf79F Btk29A CG10602 CG11228 CG11451 CG14217 CG1514 CG15835 CG17309 CG17493 CG17765 CG17919 CG1815 CG1900 CG2108 CG30372 CG30440 CG31640 CG32158 CG32447 CG3249 CG40410 CG4509 CG5522 CG5841 CG6954 CG8243 CG8400 CG8789 CaMKII Cad87A CdGAPr Cdc42 Cdk4 Cip4 CkIIalpha CkIIbeta CkIalpha Eip75B Fas2 G-salpha60A Galpha49B Gap1 Gef26 Gprk1 KP78b Lac LanA LanB2 Mcr Mekk1 Mtl Nek2 Nep2 NetA PFE PR2 Pak3 Pi3K68D Pld Pli Rab10 RacGAP50C Rgl RhoBTB RhoGAP19D RhoGAP1A RhoGEF3 Rhp S Sema-1b Sema-2a Ser Smox Spt6 Src64B Su(dx) Tis11 Tsp66E Wnt2 aop argos arm bib bl brk capu ci crb csul csw cta dome ebi ed edl fat2 flfl fra fwd fz gish gus heph hh inx2 inx3 kay klu knk krz l(2)gl lgs lqf mask mav mbt msk msn mth ogre ome otk p120ctn pav pbl plexA pll polo rin scf sec23 serpin-27A sgl shg sima slmb ss stai stan sty syndapin tkv trio trn tub vav vimar wbl wgn |
| 77 | GO:0043231 | C | 4, 5, 6, 7, | 252 | 193.328 (x 1.303) | 2307 (0.109) | 1.32e-05 | intracellular membrane-bound organelle | 14-3-3epsilon 14-3-3zeta AP-1gamma ATPsyn-beta ATbp Acon Acox57D-p Arc92 B52 BEAF-32 BG:DS00004.11 Bap170 BcDNA:GH04962 BcDNA:GH12558 BcDNA:GM10765 BcDNA:LD23876 Brf CBP CG10211 CG10354 CG10473 CG11188 CG11305 CG11856 CG12299 CG12391 CG13895 CG1472 CG15093 CG1529 CG1647 CG1677 CG17361 CG1746 CG17838 CG17952 CG1907 CG1911 CG2097 CG2118 CG2158 CG30011 CG31258 CG31617 CG32164 CG33113 CG33525 CG3605 CG3847 CG40410 CG4914 CG5028 CG5728 CG5789 CG6064 CG6227 CG6394 CG6543 CG6638 CG6791 CG6930 CG7145 CG7154 CG7263 CG7987 CG8089 CG8092 CG8426 CG8478 CG8924 CG9104 CG9342 CG9425 CG9894 CREG Caf1 Cat Cbp80 Cen190 Chc CkIIalpha CkIIbeta CkIalpha CtBP CycA CycT Cyp1 D12 D19A D19B DNApol-delta DNApol-epsilon Dll Dp Dr Dref Dsp1 E(z) EG:115C2.6 ERp60 Eip75B Es2 Fs(2)Ket Fur1 GATAd Gdh Got2 Hcf HmgD HmgZ Hrb27C Hrb87F Hsc70-3 Hsp60 Idh Iswi Karybeta3 Lam Mcm5 Mcm6 Mcm7 Mi-2 Mpcp Myb NUCB1 Nlp Nop60B Optix Ote Pcl Pen Pep Pepck Psc Ptp99A Ranbp16 Rbf2 Rm62 RpII140 Rrp1 S SMC1 Scm Set Sin3A Snap Sox14 Spt6 Ste12DOR Ste:CG33236 Su(var)3-9 Su(z)12 Taf2 Thd1 Thiolase Tis11 Tom34 Top2 Trxr-1 Uba2 UbcD6 Ucp4B Vap-33-1 Vha44 Vha55 XNP abs alpha-Adaptin alpha-Spec aop ap ash2 barr bl br brk brm btsz capu cav cdc2 chb ci cic crc crol crp d4 da dac dalao dnk dpa dre4 dve east ebi edl en esn exd garz gft gol grn heph hh jar jim jing jumu kay kis klar klu kn l(2)gl lds lgs lid lin19 lmg lola lwr mod mor msk mus209 nonA-l ovo pav pbl pfk ph-p pk rin sc sd sec23 sima simj sqd ss stc su(f) toe tud vg wbl woc yps zf30C |
| 78 | GO:0031323 | P | 5, | 126 | 82.628 (x 1.525) | 986 (0.128) | 1.37e-05 | regulation of cellular metabolism | ATbp B52 BEAF-32 Bap170 BcDNA:LD23876 Brf CG11228 CG11305 CG12190 CG12299 CG15141 CG15835 CG17514 CG1815 CG30011 CG31258 CG31716 CG33525 CG40160 CG4914 CG5434 CG5728 CG6227 CG7987 CG8092 CG8165 CG8426 CG8443 CG8924 CREG Caf1 CtBP CycT D12 D19A D19B Dll Dp Dr Dref Dsp1 E(z) Eip75B Elf GATAd HmgD HmgZ Hrb87F Iswi Mi-2 Myb Optix Paip2 Pcl Psc Rab10 Rbf2 Rm62 RpS6 Scm Sin3A Smox Sox14 Su(var)3-9 Su(z)12 TBPH Taf2 Thd1 Uba2 Vha44 XNP aop ap ash2 bl br brk brm cdc2 ci cic crc crol d4 da dalao dre4 dve eIF-4B eIF3-S9 eIF5 edl en exd gol grn hh jim jing jumu kay kis klu kn l(2)01424 lds lid lola mor msi mus209 ovo pAbp ph-p rin sc sd sima simj sqd ss stc toe woc yps zf30C |
| 79 | GO:0030554 | F | 5, | 95 | 57.739 (x 1.645) | 689 (0.138) | 1.61e-05 | adenyl nucleotide binding | ATPsyn-beta Aats-ala Aats-glupro Ack Akt1 BEST:CK01140 BcDNA:LD09009 BcDNA:LD23371 Btk29A CG11069 CG11228 CG14217 CG17309 CG2118 CG2316 CG31453 CG31739 CG33123 CG40410 CG5789 CG6227 CG6418 CG6835 CG7441 CG7878 CG7922 CG8789 CG9425 CG9503 CaMKII Cdk4 CkIIalpha CkIalpha Dhh1 Eip63E Gprk1 Hlc Hsc70-3 Hsp60 Iswi KP78b Klp10A M(2)21AB Mcm5 Mcm6 Mcm7 Mdr49 Mekk1 Mi-2 Nek2 PR2 Pak3 Rm62 SMC1 SelD Sin3A Src64B Tom34 Top2 Top3beta Trap1 Vha44 Vha55 Wsck X11L XNP abs ald bel brm cdc2 ck d dnk dome dpa fat2 gish jar jumu katanin-60 kis l(1)G0148 lds mbt mdy me31B msn otk pav pll polo smi35A tkv zip |
| 80 | GO:0048519 | P | 3, | 52 | 25.559 (x 2.034) | 305 (0.170) | 1.66e-05 | negative regulation of biological process | Aac11 Akt1 CG11207 CG11228 CG11305 CG17309 CG31716 CG33525 CG40410 CREG Caf1 CkIalpha CtBP CycA E(z) Myb Paip2 Pcl Psc RacGAP50C Rbf2 Sin3A Su(var)3-9 Su(z)12 ana aop argos brk charybde ci cic ed edl en fat2 fwd hdc hh l(2)gl lqf msi ph-p scrib scylla sdk serpin-27A simj slmb sqd stan sty th |
| 81 | GO:0048592 | P | 5, 6, | 32 | 12.486 (x 2.563) | 149 (0.215) | 1.74e-05 | eye morphogenesis | Amph Fas2 Mbs Moe S Vang aop argos arm br crb csw da dac ebi fz gft hh kay klar klu mbt msn pk pnut rg rin sca sdk shg stan tkv |
| 82 | GO:0007242 | P | 5, | 77 | 43.912 (x 1.754) | 524 (0.147) | 1.75e-05 | intracellular signaling cascade | 14-3-3epsilon 14-3-3zeta Ack Akap200 Btk29A CG11228 CG14217 CG1514 CG17309 CG17493 CG17765 CG1900 CG2108 CG30372 CG30440 CG32158 CG40410 CG5522 CG8400 CG8789 CaMKII CdGAPr Cdc42 Cdk4 CkIIalpha CkIalpha Eip75B G-salpha60A Galpha49B Gap1 Gef26 Gprk1 KP78b Mekk1 Mtl Nek2 PR2 Pak3 Pi3K68D Pld Rab10 RacGAP50C Rgl RhoBTB RhoGAP19D RhoGAP1A Smox Spt6 Src64B Tis11 aop arm capu csul csw dome edl flfl fwd gish gus hh kay klu lqf mbt msn polo rin scf slmb stai sty syndapin trio vav vimar |
| 83 | GO:0051276 | P | 6, | 35 | 14.414 (x 2.428) | 172 (0.203) | 1.96e-05 | chromosome organization and biogenesis | BcDNA:LD09009 CG11305 CG1911 CG31617 Caf1 Cap-G Dsp1 E(z) HmgD HmgZ Iswi Mcm5 Mi-2 Nlp Pcl Pp1-87B Psc SMC1 Set Spt6 Su(var)3-9 Su(z)12 ash2 barr brm cav dalao dre4 jumu kis larp lola mor pfk ph-p |
| 84 | GO:0016331 | P | 5, | 22 | 6.872 (x 3.202) | 82 (0.268) | 2e-05 | morphogenesis of embryonic epithelium | Btk29A Cdc42 Mbs Mtl Src64B aop arm baz cora crb ena flfl inx2 jar jumu kay l(2)gl msn par-6 scrib tkv zip |
| 85 | GO:0030695 | F | 3, | 29 | 10.894 (x 2.662) | 130 (0.223) | 2.44e-05 | GTPase regulator activity | Abi CG11856 CG17184 CG30372 CG30440 CG5522 CG6838 CG8155 CG8243 CG9135 CG9153 CdGAPr Gap1 Gef26 RacGAP50C Rgl RhoGAP19D RhoGAP1A RhoGEF3 Rhp eIF5 garz msk msn pbl sec23 trio vav vimar |
| 86 | GO:0035220 | P | 5, | 27 | 9.721 (x 2.778) | 116 (0.233) | 2.44e-05 | wing disc development | CG5841 CG6896 Cdc42 Dr G-salpha60A Mbs Smox Su(dx) ap argos ash2 brm ci crol dve en fz gft heph hh in jumu kn pk sd tkv vg |
| 87 | GO:0007476 | P | 6, 7, 8, | 25 | 8.631 (x 2.896) | 103 (0.243) | 2.66e-05 | wing morphogenesis | CG5841 CG6896 Cdc42 Dr G-salpha60A Mbs Smox Su(dx) ap argos ash2 brm crol dve fz gft heph hh in jumu kn pk sd tkv vg |
| 88 | GO:0035214 | P | 5, | 33 | 13.408 (x 2.461) | 160 (0.206) | 2.82e-05 | eye-antennal disc development | Amph Dll Mbs Moe S Vang aop argos arm br ck crb csw da dac ebi fz gft hh kay klar klu mbt pk pnut rg rin sca sdk ss stan th tkv |
| 89 | GO:0006259 | P | 6, | 59 | 31.090 (x 1.898) | 371 (0.159) | 2.85e-05 | DNA metabolism | BcDNA:GM10765 BcDNA:LD09009 CG10354 CG11305 CG1911 CG31617 CG5841 CG7263 CG7922 CG9425 Caf1 CkIalpha CycG DNApol-delta DNApol-epsilon Dp Dref Dsp1 E(z) HmgD HmgZ Hus1-like Iswi Mcm5 Mcm6 Mcm7 Mi-2 Myb Nlp Pcl Psc RnrL Rrp1 SMC1 Set Spt6 Su(var)3-9 Su(z)12 Taf2 Thd1 Tom34 Top2 Top3beta UbcD6 XNP ash2 brm dalao dpa dre4 jumu kis lds lola mor mus209 pfk ph-p tos |
| 90 | GO:0051301 | P | 4, | 30 | 11.648 (x 2.575) | 139 (0.216) | 3.18e-05 | cell division | Act42A Act5C CG11207 CG13852 CG4454 Caf1 Cap-G Msp-300 Myb RacGAP50C Rop Snap alpha-Adaptin arm asp baz cdc2 chb fwd hh hts jar pav pbl pnut polo pon shg tkv zip |
| 91 | GO:0046530 | P | 4, | 24 | 8.212 (x 2.922) | 98 (0.245) | 3.54e-05 | photoreceptor cell differentiation | 14-3-3zeta Amph Mbs Moe S aop br crb csw da dac edl fz hh kay klar mask mbt msn pnut rin sca sdk stan |
| 92 | GO:0048749 | P | 7, | 29 | 11.146 (x 2.602) | 133 (0.218) | 3.61e-05 | compound eye development (sensu Endopterygota) | Amph Mbs Moe S Vang aop argos arm br crb csw da dac ebi fz gft hh kay klar klu mbt pk pnut rg rin sca sdk stan tkv |
| 93 | GO:0007389 | P | 3, | 45 | 21.453 (x 2.098) | 256 (0.176) | 3.61e-05 | pattern specification | 14-3-3epsilon 14-3-3zeta BicD Cdk4 Dll Dp Dr Gap1 Hrb27C Moe Rop Ser Tm1 ap baz capu ci csul csw dac dome edl egl en exd gus hh kis kn knk lgs lwr par-6 pll serpin-27A sgl shg sqd stan sty tkv tok tub tud wbl |
| 94 | GO:0007281 | P | 5, | 26 | 9.386 (x 2.770) | 112 (0.232) | 3.61e-05 | germ cell development | 14-3-3epsilon 14-3-3zeta BicD Hem Hrb27C Moe Nop60B Rop Tm1 alpha-Spec arm asp baz capu chb csul egl gus hh hts par-6 shg sqd tkv tud wbl |
| 95 | GO:0007472 | P | 6, 7, | 25 | 8.799 (x 2.841) | 105 (0.238) | 3.62e-05 | wing disc morphogenesis | CG5841 CG6896 Cdc42 Dr G-salpha60A Mbs Smox Su(dx) ap argos ash2 brm crol dve fz gft heph hh in jumu kn pk sd tkv vg |
| 96 | GO:0005913 | C | 7, 8, 9, | 11 | 2.011 (x 5.469) | 24 (0.458) | 3.63e-05 | cell-cell adherens junction | Cont Fas3 Lac alpha-Cat arm baz cora crb l(2)gl scrib shg |
| 97 | GO:0001745 | P | 7, 8, | 29 | 11.146 (x 2.602) | 133 (0.218) | 3.64e-05 | compound eye morphogenesis (sensu Endopterygota) | Amph Mbs Moe S Vang aop argos arm br crb csw da dac ebi fz gft hh kay klar klu mbt pk pnut rg rin sca sdk stan tkv |
| 98 | GO:0044427 | C | 4, 5, 6, 7, 8, 9, | 31 | 12.403 (x 2.499) | 148 (0.209) | 3.83e-05 | chromosomal part | BEAF-32 CG13895 CG1911 CG31617 Caf1 Cap-G CycT DNApol-epsilon Dp Dsp1 E(z) HmgD Hrb87F Mi-2 Myb Pep Psc Rbf2 SMC1 Sin3A Su(var)3-9 Su(z)12 barr bl cav chb fzy jumu kis polo sqd |
| 99 | GO:0006139 | P | 5, | 199 | 148.411 (x 1.341) | 1771 (0.112) | 4.72e-05 | nucleobase, nucleoside, nucleotide and nucleic acid metabolism | ATPsyn-beta ATbp Aats-ala Aats-glupro Arc92 B52 BEAF-32 BEST:LD22483 Bap170 BcDNA:GM10765 BcDNA:LD09009 BcDNA:LD23876 Brf CG10354 CG11123 CG11228 CG11305 CG12190 CG12252 CG12299 CG15093 CG15141 CG15835 CG1746 CG1815 CG1911 CG2097 CG30011 CG31258 CG31453 CG31617 CG31716 CG31739 CG32158 CG33123 CG33525 CG3590 CG3605 CG4612 CG4749 CG4914 CG5191 CG5319 CG5728 CG5841 CG6227 CG6418 CG6767 CG6854 CG6946 CG7008 CG7263 CG7441 CG7878 CG7922 CG7987 CG8036 CG8092 CG8165 CG8426 CG8924 CG9425 CREG Caf1 Cbp80 CkIalpha CtBP CycG CycT D12 D19A D19B DNApol-delta DNApol-epsilon Dhh1 Dll Dp Dr Dref Dsp1 E(z) Eip75B GATAd Hcf Hlc HmgD HmgZ Hrb27C Hrb87F Hus1-like Iswi Mcm5 Mcm6 Mcm7 Mi-2 Myb NTPase Nlp Nop60B Optix Pcl Psc Rab10 Rbf2 Rm62 RnrL RpII140 Rrp1 SMC1 Scm Set Sin3A Smox Sox14 Spt6 Su(var)3-9 Su(z)12 TBPH Taf2 Thd1 Tis11 Tom34 Top2 Top3beta Uba2 UbcD6 Vha44 Vha55 X11L XNP abs ade2 ade3 aop ap ash2 bel bl br brk brm cdc2 ci cic crc crol d4 da dac dalao dnk dome dpa dre4 dve edl en exd gol grn heph hh jim jing jumu kay kis klu kn lds lid lola mdy me31B mor msi mus209 nonA-l nop5 ovo pAbp pfk ph-p qkr54B r-l sc sd sima simj sqd ss stc su(f) toe tos vig woc yps zf30C |
| 100 | GO:0009994 | P | 4, 7, | 22 | 7.291 (x 3.018) | 87 (0.253) | 4.88e-05 | oocyte differentiation | 14-3-3epsilon 14-3-3zeta BicD Hem Hrb27C Moe Rop Tm1 alpha-Spec asp baz capu chb csul egl gus hts par-6 shg sqd tud wbl |
| 101 | GO:0048812 | P | 7, 8, 10, | 27 | 10.140 (x 2.663) | 121 (0.223) | 4.91e-05 | neurite morphogenesis | Cdc42 Fas2 Fas3 Galpha49B Gef26 Hem Lac LanA NetA Ptp99A Sdc Sema-1b Sema-2a ap argos chb ena fax fra lola ninA otk pbl plexA shg stan trio |
| 102 | GO:0007409 | P | 8, 9, 11, | 27 | 10.140 (x 2.663) | 121 (0.223) | 4.96e-05 | axonogenesis | Cdc42 Fas2 Fas3 Galpha49B Gef26 Hem Lac LanA NetA Ptp99A Sdc Sema-1b Sema-2a ap argos chb ena fax fra lola ninA otk pbl plexA shg stan trio |
| 103 | GO:0048667 | P | 6, 7, 9, | 27 | 10.140 (x 2.663) | 121 (0.223) | 5.01e-05 | neuron morphogenesis during differentiation | Cdc42 Fas2 Fas3 Galpha49B Gef26 Hem Lac LanA NetA Ptp99A Sdc Sema-1b Sema-2a ap argos chb ena fax fra lola ninA otk pbl plexA shg stan trio |
| 104 | GO:0048522 | P | 4, | 36 | 15.838 (x 2.273) | 189 (0.190) | 5.67e-05 | positive regulation of cellular process | Akt1 Bap170 CG10473 CG10990 CG11228 CG14217 CG17765 CG5841 CG7263 CG8400 Cks Dp Eip75B Iswi aop arm ash2 bl br brm ci da dalao gft hh klu l(2)gl lgs lola mor pAbp slmb smi35A stan tkv zip |
| 105 | GO:0046578 | P | 6, 7, 8, | 6 | 0.587 (x 10.228) | 7 (0.857) | 5.85e-05 | regulation of Ras protein signal transduction | Akap200 Gap1 aop edl klu sty |
| 106 | GO:0051656 | P | 5, | 12 | 2.514 (x 4.773) | 30 (0.400) | 6.05e-05 | establishment of organelle localization | BicD Eb1 Lam asp baz chb egl jar klar msn nudC polo |
| 107 | GO:0043283 | P | 5, | 190 | 141.120 (x 1.346) | 1684 (0.113) | 6.32e-05 | biopolymer metabolism | Aats-ala Aats-glupro Ack Akt1 Arf79F B52 BG:DS00004.11 BcDNA:GH02976 BcDNA:GH04962 BcDNA:GM10765 BcDNA:LD09009 BcDNA:LD22910 BcDNA:LD23371 Btk29A CG10354 CG10542 CG11123 CG11142 CG11228 CG11305 CG12006 CG12130 CG14217 CG14222 CG14670 CG15141 CG1550 CG17309 CG17598 CG1815 CG1869 CG1906 CG1911 CG2097 CG31617 CG31716 CG31739 CG32099 CG32632 CG33123 CG33138 CG33145 CG3605 CG40410 CG4238 CG4612 CG4670 CG4749 CG5191 CG5505 CG5728 CG5841 CG6227 CG6394 CG6904 CG6946 CG7263 CG7288 CG7441 CG7922 CG8188 CG8494 CG8789 CG9153 CG9425 CaMKII Caf1 CalpB Cbp80 Cdk4 CkIIalpha CkIIbeta CkIalpha Cks CycG DNApol-delta DNApol-epsilon Dp Dref Dsp1 E(z) EG:86E4.2 ERp60 Eip63E GNBP3 Galpha49B GlyP Gprk1 Hexo1 HmgD HmgZ Hrb27C Hrb87F Hus1-like Iswi KP78b Mcm5 Mcm6 Mcm7 Mekk1 Mi-2 Myb Nek2 Nlp Nop60B PFE PR2 Pak3 Pcl Pi3K68D Pp1-87B Pp2C1 Psc Ptp99A Rm62 RnrL Rrp1 SMC1 Set Spt6 Src64B Su(dx) Su(var)3-9 Su(z)12 TBPH Taf2 Thd1 Tis11 Tom34 Top2 Top3beta Uba1 Uba2 UbcD2 UbcD6 Uch Wsck X11L XNP ald ash2 bl brm cdc2 csw d4 dalao dome dpa dre4 fat2 fdl fzy gish gol heph hh jumu kis l(1)G0148 lds lmg lola lwr mbt mdy mor msi msn mus209 nonA-l nop5 otk pAbp pfk ph-p plexA pll polo sgl slmb smi35A sqd stc su(f) syndapin th tkv tos wbl |
| 108 | GO:0048523 | P | 4, | 47 | 23.464 (x 2.003) | 280 (0.168) | 6.91e-05 | negative regulation of cellular process | Aac11 Akt1 CG11207 CG11228 CG11305 CG31716 CG33525 CG40410 CREG Caf1 CkIalpha CtBP CycA E(z) Myb Paip2 Pcl Psc RacGAP50C Rbf2 Sin3A Su(var)3-9 Su(z)12 ana aop argos brk ci cic ed edl en fwd hdc hh l(2)gl lqf msi ph-p scrib sdk simj slmb sqd stan sty th |
| 109 | GO:0006325 | P | 8, | 29 | 11.565 (x 2.508) | 138 (0.210) | 6.96e-05 | establishment and/or maintenance of chromatin architecture | BcDNA:LD09009 CG11305 CG1911 CG31617 Caf1 Dsp1 E(z) HmgD HmgZ Iswi Mi-2 Nlp Pcl Psc SMC1 Set Spt6 Su(var)3-9 Su(z)12 ash2 brm dalao dre4 jumu kis lola mor pfk ph-p |
| 110 | GO:0006323 | P | 7, | 29 | 11.565 (x 2.508) | 138 (0.210) | 7.02e-05 | DNA packaging | BcDNA:LD09009 CG11305 CG1911 CG31617 Caf1 Dsp1 E(z) HmgD HmgZ Iswi Mi-2 Nlp Pcl Psc SMC1 Set Spt6 Su(var)3-9 Su(z)12 ash2 brm dalao dre4 jumu kis lola mor pfk ph-p |
| 111 | GO:0001738 | P | 5, | 16 | 4.358 (x 3.672) | 52 (0.308) | 7.38e-05 | morphogenesis of a polarized epithelium | Vang argos baz crb fz in kay l(2)gl msn par-6 pk rin sca scrib stan zip |
| 112 | GO:0005694 | C | 5, 6, 7, 8, | 35 | 15.419 (x 2.270) | 184 (0.190) | 7.52e-05 | chromosome | 14-3-3epsilon BEAF-32 CG13895 CG1911 CG31617 Caf1 Cap-G CycT DNApol-epsilon Dp Dsp1 E(z) HmgD Hrb87F Mi-2 Myb Pep Psc Rbf2 SMC1 Sin3A Spt6 Su(var)3-9 Su(z)12 Top2 barr bl cav chb fzy jumu kis pfk polo sqd |
| 113 | GO:0003682 | F | 3, | 21 | 6.955 (x 3.019) | 83 (0.253) | 7.55e-05 | chromatin binding | BEAF-32 BicD CG11305 CG7154 Caf1 Dsp1 HmgZ Mcm5 Mcm6 Mcm7 Mi-2 SMC1 Scm Sin3A Spt6 Su(var)3-9 dalao jumu kis mor ph-p |
| 114 | GO:0043119 | P | 4, | 33 | 14.162 (x 2.330) | 169 (0.195) | 7.81e-05 | positive regulation of physiological process | Akt1 Bap170 CG10473 CG10990 CG11228 CG14217 CG17765 CG5841 CG7263 CG8400 Cks Dp Eip75B Iswi aop ash2 bl br brm ci da dalao gft klu l(2)gl lola mor pAbp scrib slmb smi35A tkv zip |
| 115 | GO:0051649 | P | 5, 6, | 86 | 53.213 (x 1.616) | 635 (0.135) | 8.66e-05 | establishment of cellular localization | AP-1gamma Akt1 Amph Arf79F BicD CG10695 CG11856 CG1418 CG1472 CG17143 CG1900 CG1907 CG2108 CG2158 CG2852 CG2980 CG32137 CG32164 CG3249 CG33113 CG33214 CG5064 CG5434 CG6838 CG8155 CG9057 CG9906 Chc Cyp1 Dlc90F EG:34F3.8 Eb1 Fs(2)Ket Hsp60 Karybeta3 Klp10A Lam Pen Pi3K68D Rab10 Ranbp16 S Snap Ucp4B Vap-33-1 X11L alpha-Adaptin alphaTub84B asp baz betaTub56D bl chb ck cora d egl garz gp210 hh jar katanin-60 kel klar krz l(2)gl lqf lwr mask msk msn nudC par-6 pav pll polo prominin-like rg rin scrib sec23 slmb sqd syndapin vg wbl |
| 116 | GO:0051641 | P | 4, 5, | 86 | 53.297 (x 1.614) | 636 (0.135) | 9.16e-05 | cellular localization | AP-1gamma Akt1 Amph Arf79F BicD CG10695 CG11856 CG1418 CG1472 CG17143 CG1900 CG1907 CG2108 CG2158 CG2852 CG2980 CG32137 CG32164 CG3249 CG33113 CG33214 CG5064 CG5434 CG6838 CG8155 CG9057 CG9906 Chc Cyp1 Dlc90F EG:34F3.8 Eb1 Fs(2)Ket Hsp60 Karybeta3 Klp10A Lam Pen Pi3K68D Rab10 Ranbp16 S Snap Ucp4B Vap-33-1 X11L alpha-Adaptin alphaTub84B asp baz betaTub56D bl chb ck cora d egl garz gp210 hh jar katanin-60 kel klar krz l(2)gl lqf lwr mask msk msn nudC par-6 pav pll polo prominin-like rg rin scrib sec23 slmb sqd syndapin vg wbl |
| 117 | GO:0007067 | P | 7, | 40 | 18.939 (x 2.112) | 226 (0.177) | 9.48e-05 | mitosis | 14-3-3epsilon BicD CG11207 CG13852 CG17493 CG1911 CG31251 CG32137 CG40410 CG4454 Cap-G Cdk4 Cks CycA Eip63E Gap1 Klp10A Msp-300 Myb Nek2 Nop60B Pp1-87B SMC1 Top2 ald alphaTub84B barr betaTub56D cdc2 chb csul east fzy larp lmg pav pnut polo zip zwilch |
| 118 | GO:0000087 | P | 6, | 40 | 19.023 (x 2.103) | 227 (0.176) | 0.000105 | M phase of mitotic cell cycle | 14-3-3epsilon BicD CG11207 CG13852 CG17493 CG1911 CG31251 CG32137 CG40410 CG4454 Cap-G Cdk4 Cks CycA Eip63E Gap1 Klp10A Msp-300 Myb Nek2 Nop60B Pp1-87B SMC1 Top2 ald alphaTub84B barr betaTub56D cdc2 chb csul east fzy larp lmg pav pnut polo zip zwilch |
| 119 | GO:0048666 | P | 5, 8, | 29 | 11.900 (x 2.437) | 142 (0.204) | 0.000116 | neuron development | Cdc42 Fas2 Fas3 Galpha49B Gef26 Hem Lac LanA NetA Ptp99A Sdc Sema-1b Sema-2a Smox Tm1 ap argos chb ena fax fra lola ninA otk pbl plexA shg stan trio |
| 120 | GO:0031175 | P | 6, 9, | 29 | 11.900 (x 2.437) | 142 (0.204) | 0.000117 | neurite development | Cdc42 Fas2 Fas3 Galpha49B Gef26 Hem Lac LanA NetA Ptp99A Sdc Sema-1b Sema-2a Smox Tm1 ap argos chb ena fax fra lola ninA otk pbl plexA shg stan trio |
| 121 | GO:0051640 | P | 4, | 12 | 2.682 (x 4.475) | 32 (0.375) | 0.000118 | organelle localization | BicD Eb1 Lam asp baz chb egl jar klar msn nudC polo |
| 122 | GO:0008104 | P | 4, | 78 | 47.431 (x 1.644) | 566 (0.138) | 0.000126 | protein localization | AP-1gamma Akap200 Akt1 Amph Arf79F CG11856 CG1418 CG1472 CG1900 CG2108 CG2158 CG2852 CG32137 CG32164 CG3249 CG33113 CG33214 CG5064 CG5434 CG5841 CG6838 CG8155 CG9906 Chc Cyp1 Dlc90F EG:34F3.8 Fs(2)Ket Hsp60 Karybeta3 Klp10A Moe Pen Pi3K68D Rab10 Ranbp16 RhoBTB S Snap Vap-33-1 X11L alpha-Adaptin alphaTub84B arm baz betaTub56D bl ck cora crb d fz garz gp210 hh jar jumu katanin-60 krz l(2)gl lqf lwr mask msk par-6 pav pk pll polo pon prominin-like rg rin scrib sec23 slmb syndapin wbl |
| 123 | GO:0045165 | P | 4, | 35 | 15.838 (x 2.210) | 189 (0.185) | 0.000129 | cell fate commitment | BicD Dr S alpha-Spec aop ap argos arm baz bl br cdc2 csw da dome ebi egl fz hdc hh hts jar jumu kay l(2)gl lqf msi pon rin sc sca stan sty tkv tud |
| 124 | GO:0030234 | F | 2, | 55 | 29.917 (x 1.838) | 357 (0.154) | 0.00013 | enzyme regulator activity | 14-3-3epsilon 14-3-3zeta Abi CG11856 CG13852 CG17184 CG17514 CG17919 CG30372 CG30440 CG40160 CG5522 CG6680 CG6838 CG6896 CG7219 CG8155 CG8243 CG9135 CG9153 CdGAPr CkIIbeta Cks CycA CycG CycT Cyp1 Gap1 Gef26 Mapmodulin Mcr Pli RacGAP50C Rgl RhoGAP19D RhoGAP1A RhoGEF3 Rhp Set Spn43Aa Spn5 Ste12DOR Ste:CG33236 eIF5 garz guf msk msn pbl plexA sec23 serpin-27A trio vav vimar |
| 125 | GO:0019219 | P | 6, | 111 | 74.415 (x 1.492) | 888 (0.125) | 0.000139 | regulation of nucleobase, nucleoside, nucleotide and nucleic acid metabolism | ATbp B52 BEAF-32 Bap170 BcDNA:LD23876 Brf CG11228 CG11305 CG12190 CG12299 CG15141 CG15835 CG1815 CG30011 CG31258 CG31716 CG33525 CG4914 CG5728 CG6227 CG7987 CG8092 CG8165 CG8426 CG8924 CREG Caf1 CtBP CycT D12 D19A D19B Dll Dp Dr Dref Dsp1 E(z) Eip75B GATAd HmgD HmgZ Hrb87F Iswi Mi-2 Myb Optix Pcl Psc Rab10 Rbf2 Rm62 Scm Sin3A Smox Sox14 Su(var)3-9 Su(z)12 TBPH Taf2 Thd1 Uba2 XNP aop ap ash2 bl br brk brm cdc2 ci cic crc crol d4 da dalao dre4 dve edl en exd gol grn hh jim jing jumu kay kis klu kn lds lid lola mor mus209 ovo ph-p sc sd sima simj sqd ss stc toe woc yps zf30C |
| 126 | GO:0005912 | C | 6, 7, 8, | 15 | 4.106 (x 3.653) | 49 (0.306) | 0.000141 | adherens junction | Cont Fas3 Lac Moe Sdc alpha-Cat arm baz cora crb l(2)gl mbt p120ctn scrib shg |
| 127 | GO:0048599 | P | 5, 6, 8, | 20 | 6.704 (x 2.983) | 80 (0.250) | 0.000141 | oocyte development | 14-3-3epsilon 14-3-3zeta BicD Hem Hrb27C Moe Rop Tm1 alpha-Spec baz capu chb csul egl gus par-6 shg sqd tud wbl |
| 128 | GO:0001700 | P | 5, | 27 | 10.810 (x 2.498) | 129 (0.209) | 0.000142 | embryonic development (sensu Insecta) | Btk29A CG40410 Cdc42 CtBP Eip63E Mbs Mtl Sema-1b Src64B aop arm barr cic cora crb ena flfl jar jumu kay kn l(2)gl msn scrib shg tkv zip |
| 129 | GO:0007467 | P | 5, | 21 | 7.291 (x 2.880) | 87 (0.241) | 0.000147 | photoreceptor cell differentiation (sensu Endopterygota) | 14-3-3zeta Amph Mbs Moe S aop crb csw da dac fz hh kay klar mask mbt msn pnut sca sdk stan |
| 130 | GO:0051242 | P | 5, | 32 | 13.995 (x 2.287) | 167 (0.192) | 0.000147 | positive regulation of cellular physiological process | Akt1 Bap170 CG10473 CG10990 CG11228 CG14217 CG17765 CG5841 CG7263 CG8400 Cks Dp Eip75B Iswi aop ash2 bl br brm ci da dalao gft klu l(2)gl lola mor pAbp slmb smi35A tkv zip |
| 131 | GO:0051056 | P | 5, 6, 7, | 6 | 0.670 (x 8.950) | 8 (0.750) | 0.000174 | regulation of small GTPase mediated signal transduction | Akap200 Gap1 aop edl klu sty |
| 132 | GO:0007455 | P | 6, 7, | 29 | 12.235 (x 2.370) | 146 (0.199) | 0.000187 | eye-antennal disc morphogenesis | Amph Mbs Moe S Vang aop argos arm br crb csw da dac ebi fz gft hh kay klar klu mbt pk pnut rg rin sca sdk stan tkv |
| 133 | GO:0016337 | P | 4, | 26 | 10.391 (x 2.502) | 124 (0.210) | 0.000196 | cell-cell adhesion | CG4509 Cad87A Cat Cont Fas2 Fas3 Lac LanA LanB2 NetA Sema-1b Sema-2a Ser Tsp66E alpha-Spec arm baz crb ed fat2 fz glec otk shg stan trn |
| 134 | GO:0007411 | P | 6, 7, 9, 10, 12, | 20 | 6.872 (x 2.911) | 82 (0.244) | 0.000199 | axon guidance | Cdc42 Fas3 Galpha49B LanA NetA Ptp99A Sdc Sema-1b Sema-2a ap argos chb ena fra lola ninA otk plexA shg trio |
| 135 | GO:0007391 | P | 6, | 19 | 6.369 (x 2.983) | 76 (0.250) | 0.000224 | dorsal closure | Btk29A Cdc42 Mbs Mtl Src64B aop arm cora crb ena flfl jar jumu kay l(2)gl msn scrib tkv zip |
| 136 | GO:0051246 | P | 5, 6, | 27 | 11.146 (x 2.423) | 133 (0.203) | 0.000243 | regulation of protein metabolism | CG11207 CG17514 CG40410 CG5434 CG8443 CkIalpha Elf Paip2 RpS6 Src64B bl eIF-4B eIF3-S9 eIF5 ebi ena fwd fzy gft hh l(2)01424 msi pAbp pll rin slmb sqd |
| 137 | GO:0000279 | P | 5, | 46 | 24.051 (x 1.913) | 287 (0.160) | 0.000246 | M phase | 14-3-3epsilon BicD CG11207 CG13852 CG17493 CG1911 CG31251 CG32137 CG40410 CG4454 Cap-G Cdk4 Cks CycA Eip63E Gap1 Klp10A LanA Msp-300 Myb Nek2 Nop60B Pp1-87B SMC1 Top2 ald alphaTub84B barr betaTub56D cdc2 chb csul east fwd fzy l(1)dd4 larp lmg lwr pav pbl pnut polo sca zip zwilch |
| 138 | GO:0030182 | P | 4, 7, | 29 | 12.486 (x 2.323) | 149 (0.195) | 0.00027 | neuron differentiation | Cdc42 Fas2 Fas3 Galpha49B Gef26 Hem Lac LanA NetA Ptp99A Sdc Sema-1b Sema-2a Smox Tm1 ap argos chb ena fax fra lola ninA otk pbl plexA shg stan trio |
| 139 | GO:0007001 | P | 7, | 30 | 13.157 (x 2.280) | 157 (0.191) | 0.000279 | chromosome organization and biogenesis (sensu Eukaryota) | BcDNA:LD09009 CG11305 CG1911 CG31617 Caf1 Dsp1 E(z) HmgD HmgZ Iswi Mi-2 Nlp Pcl Psc SMC1 Set Spt6 Su(var)3-9 Su(z)12 ash2 brm cav dalao dre4 jumu kis lola mor pfk ph-p |
| 140 | GO:0044428 | C | 4, 5, 6, 7, 8, 9, | 71 | 43.157 (x 1.645) | 515 (0.138) | 0.000293 | nuclear part | Arc92 B52 BEAF-32 Bap170 Brf CG11305 CG11856 CG17838 CG17952 CG2097 CG2158 CG32164 CG3605 CG5728 CG6227 Caf1 Cbp80 CycA CycT Cyp1 DNApol-epsilon Dp Dref E(z) Fs(2)Ket HmgD Hrb27C Hrb87F Iswi Karybeta3 Lam Mcm5 Mcm6 Mcm7 Mi-2 Nlp Nop60B Ote Pen Pep Psc Ranbp16 Rbf2 RpII140 Scm Sin3A Spt6 Su(z)12 Taf2 Top2 ap bl brm cav dac dalao dre4 gft heph jumu klar lds lin19 lmg mor msk nonA-l ph-p sqd su(f) toe |
| 141 | GO:0005737 | C | 4, 5, 6, | 167 | 124.444 (x 1.342) | 1485 (0.112) | 0.0003 | cytoplasm | 14-3-3epsilon 18w AP-1gamma ATPsyn-beta Aats-glupro Ack Acon Acox57D-p Amph ApepP BG:DS00004.11 BcDNA:GH04962 BcDNA:GH12558 BcDNA:LD41548 Btk29A CBP CG10423 CG11207 CG1472 CG15093 CG1746 CG1907 CG2118 CG31739 CG32164 CG33113 CG33123 CG5028 CG5064 CG5434 CG5841 CG6064 CG6394 CG6543 CG6638 CG6673 CG7145 CG7263 CG8443 CG9057 CG9149 CalpB Cat Cbp80 Cen190 Chc CkIIalpha CkIIbeta CkIalpha CycA Cyp1 DnaJ-1 ERp60 Elf Eno Fas2 Fs(2)Ket Fur1 Gapdh1 Gapdh2 Gdh Gfat1 Got2 Hem Hsc70-3 Hsp60 Idh ImpE2 Karybeta3 Map60 Moe Mpcp NUCB1 Nek2 Pen Pepck Pgi Pi3K68D Pp1-87B Ranbp16 Rbp2 RnrL Rop RpL13 RpL15 RpL22 RpL23a RpL3 RpS12 RpS6 S Snap Ste12DOR Ste:CG33236 Su(var)3-9 Thiolase Tm1 Tom34 TppII Trxr-1 Uba2 Ucp4B Vap-33-1 Vha44 Vha55 ade3 alpha-Adaptin alpha-Cat alpha-Spec arm asp baz bl bsf btsz chb ci cib csul csw dnk eIF-4B eIF3-S9 eIF5 exd fz fzy garz gfzf hdc hh hts jar katanin-60 kay kel l(1)G0334 l(1)dd4 l(2)dtl l(2)gl larp lig mdy msk nudC p120ctn pAbp par-6 pav pk pll pnut polo pon qkr54B rg rin scrib sec23 smi35A sqd stc tub tud viaf1 wbl zip |
| 142 | GO:0045171 | C | 3, 4, | 8 | 1.341 (x 5.967) | 16 (0.500) | 0.000319 | intercellular bridge | 14-3-3epsilon 14-3-3zeta Btk29A Src64B chb hts kel pnut |
| 143 | GO:0045172 | C | 4, 5, | 8 | 1.341 (x 5.967) | 16 (0.500) | 0.000321 | ring canal (sensu Insecta) | 14-3-3epsilon 14-3-3zeta Btk29A Src64B chb hts kel pnut |
| 144 | GO:0007308 | P | 6, 7, 9, | 19 | 6.620 (x 2.870) | 79 (0.241) | 0.000381 | oocyte construction | 14-3-3epsilon 14-3-3zeta BicD Hrb27C Moe Rop Tm1 alpha-Spec baz capu chb csul egl gus par-6 shg sqd tud wbl |
| 145 | GO:0045449 | P | 7, | 103 | 69.638 (x 1.479) | 831 (0.124) | 0.000391 | regulation of transcription | ATbp BEAF-32 Bap170 BcDNA:LD23876 Brf CG11228 CG11305 CG12190 CG12299 CG15141 CG15835 CG1815 CG30011 CG31258 CG31716 CG33525 CG4914 CG7987 CG8092 CG8165 CG8426 CG8924 CREG Caf1 CtBP CycT D12 D19A D19B Dll Dp Dr Dref Dsp1 E(z) Eip75B GATAd HmgD HmgZ Iswi Mi-2 Myb Optix Pcl Psc Rab10 Rbf2 Scm Sin3A Smox Sox14 Su(var)3-9 Su(z)12 TBPH Taf2 Thd1 Uba2 XNP aop ap ash2 br brk brm cdc2 ci cic crc crol d4 da dalao dre4 dve edl en exd gol grn hh jim jing jumu kay kis klu kn lds lid lola mor ovo ph-p sc sd sima simj ss stc toe woc yps zf30C |
| 146 | GO:0046580 | P | 7, 8, 9, | 5 | 0.503 (x 9.944) | 6 (0.833) | 0.000434 | negative regulation of Ras protein signal transduction | Akap200 Gap1 aop klu sty |
| 147 | GO:0006338 | P | 10, | 13 | 3.520 (x 3.694) | 42 (0.310) | 0.000442 | chromatin remodeling | CG11305 Caf1 E(z) Iswi Psc Su(var)3-9 Su(z)12 ash2 brm dalao mor pfk ph-p |
| 148 | GO:0006350 | P | 6, | 114 | 79.192 (x 1.440) | 945 (0.121) | 0.000452 | transcription | ATbp Arc92 BEAF-32 Bap170 BcDNA:LD23876 Brf CG11228 CG11305 CG12190 CG12252 CG12299 CG15141 CG15835 CG1815 CG30011 CG31258 CG31453 CG31716 CG33525 CG4914 CG5319 CG5841 CG7008 CG7987 CG8092 CG8165 CG8426 CG8924 CREG Caf1 CtBP CycT D12 D19A D19B Dll Dp Dr Dref Dsp1 E(z) Eip75B GATAd HmgD HmgZ Iswi Mi-2 Myb Optix Pcl Psc Rab10 Rbf2 RpII140 Scm Sin3A Smox Sox14 Spt6 Su(var)3-9 Su(z)12 TBPH Taf2 Thd1 Top2 Uba2 XNP aop ap ash2 bl br brk brm cdc2 ci cic crc crol d4 da dac dalao dre4 dve edl en exd gol grn hh jim jing jumu kay kis klu kn lds lid lola mor ovo ph-p sc sd sima simj ss stc toe woc yps zf30C |
| 149 | GO:0000226 | P | 8, | 20 | 7.291 (x 2.743) | 87 (0.230) | 0.000458 | microtubule cytoskeleton organization and biogenesis | 14-3-3epsilon 14-3-3zeta BicD CG11207 Eb1 Myb alphaTub84B asp baz betaTub56D chb dpa egl fwd jar l(1)dd4 larp mus209 pbl polo |
| 150 | GO:0006355 | P | 8, | 98 | 65.951 (x 1.486) | 787 (0.125) | 0.000509 | regulation of transcription, DNA-dependent | ATbp BEAF-32 Bap170 BcDNA:LD23876 Brf CG11228 CG11305 CG12190 CG12299 CG15141 CG15835 CG1815 CG30011 CG31258 CG31716 CG33525 CG4914 CG7987 CG8092 CG8165 CG8426 CG8924 Caf1 CtBP CycT D12 D19A D19B Dll Dp Dr Dref Dsp1 E(z) Eip75B GATAd HmgD HmgZ Iswi Mi-2 Myb Optix Pcl Psc Rab10 Rbf2 Sin3A Smox Sox14 Su(var)3-9 Su(z)12 TBPH Taf2 Thd1 XNP aop ap ash2 br brk brm cdc2 ci cic crc crol d4 da dalao dre4 dve edl en exd gol grn hh jim jumu kay kis klu kn lds lid lola mor ph-p sc sd sima simj ss stc toe woc yps zf30C |
| 151 | GO:0007059 | P | 4, | 25 | 10.391 (x 2.406) | 124 (0.202) | 0.000517 | chromosome segregation | 14-3-3zeta CG17493 CG1911 CG40410 Cap-G CycA Eb1 Gap1 Klp10A LanA Msp-300 Nop60B Pp1-87B SMC1 ald alphaTub84B barr betaTub56D chb east larp lwr pav polo sca |
| 152 | GO:0009792 | P | 4, | 34 | 16.341 (x 2.081) | 195 (0.174) | 0.000532 | embryonic development (sensu Metazoa) | Btk29A CG40410 Cdc42 CtBP Eip63E Mbs Mtl Sema-1b Src64B aop arm barr baz cic cora crb cta ena flfl gol jar jumu kay kn l(2)gl lqf msn pbl scrib sgl shg tkv wbl zip |
| 153 | GO:0007582 | P | 2, | 616 | 572.527 (x 1.076) | 6832 (0.090) | 0.000598 | physiological process | 14-3-3epsilon 14-3-3zeta 18w AP-1gamma ATPsyn-beta ATbp Aac11 Aats-ala Aats-glupro Abi Ack Acon Acox57D-p Act42A Act5C Akap200 Akt1 Ald Amph ApepP Arc92 Arf79F B52 BEAF-32 BEST:CK01140 BEST:LD22483 BG:DS00004.11 BG:DS07473.1 Bap170 BcDNA:GH02901 BcDNA:GH02976 BcDNA:GH04962 BcDNA:GH12558 BcDNA:GM10765 BcDNA:LD09009 BcDNA:LD22910 BcDNA:LD23371 BcDNA:LD23876 BcDNA:LD41548 BicD Brf BthD Btk29A CG10211 CG10354 CG10423 CG10473 CG10542 CG10602 CG10657 CG10695 CG10960 CG10990 CG11055 CG11069 CG11123 CG11142 CG11207 CG11228 CG1129 CG11305 CG11451 CG11836 CG11856 CG12006 CG12048 CG12130 CG12190 CG12199 CG12252 CG12299 CG12896 CG13852 CG1418 CG14217 CG14222 CG14231 CG1440 CG14411 CG14439 CG14670 CG1472 CG14882 CG15093 CG15141 CG1544 CG1550 CG15835 CG17036 CG17121 CG17143 CG17184 CG17309 CG17419 CG1746 CG17493 CG17514 CG17598 CG17765 CG18030 CG1815 CG1869 CG1893 CG1900 CG1906 CG1907 CG1911 CG2097 CG2108 CG2118 CG2158 CG2316 CG2852 CG2947 CG2980 CG30011 CG30025 CG31169 CG31251 CG31258 CG31453 CG31472 CG31559 CG31617 CG31716 CG31739 CG32099 CG32137 CG32158 CG32164 CG3249 CG32632 CG32640 CG32672 CG33113 CG33116 CG33123 CG33138 CG33145 CG33214 CG3328 CG33525 CG3590 CG3605 CG3823 CG40160 CG40410 CG4238 CG4454 CG4509 CG4612 CG4670 CG4749 CG4914 CG5028 CG5064 CG5191 CG5319 CG5390 CG5434 CG5466 CG5505 CG5687 CG5728 CG5789 CG5841 CG5873 CG6227 CG6287 CG6391 CG6394 CG6418 CG6543 CG6638 CG6673 CG6680 CG6767 CG6812 CG6835 CG6838 CG6854 CG6904 CG6946 CG7008 CG7145 CG7161 CG7263 CG7288 CG7441 CG7461 CG7675 CG7720 CG7878 CG7922 CG7987 CG8036 CG8092 CG8104 CG8155 CG8165 CG8188 CG8243 CG8400 CG8426 CG8443 CG8494 CG8789 CG8863 CG8902 CG8924 CG8963 CG9027 CG9057 CG9135 CG9153 CG9246 CG9342 CG9425 CG9471 CG9503 CG9809 CG9906 CREG CaMKII Caf1 CalpB Cap-G Cat Cbp80 Cdc42 Cdk4 Cen190 Chc Cip4 CkIIalpha CkIIbeta CkIalpha Cks Cont CtBP CycA CycG CycT Cyp1 Cyp6v1 D12 D19A D19B DNApol-delta DNApol-epsilon Dhh1 Dlc90F Dll DnaJ-1 Dp Dr Dref Dsp1 E(z) EG:196F3.2 EG:34F3.8 EG:86E4.2 ERp60 ESTS:39C10S Eb1 Eip55E Eip63E Eip75B Elf Eno Fas2 Fas3 Fkbp13 Fpps Fs(2)Ket Fur1 G-salpha60A GATAd GNBP3 Galpha49B Gap1 Gapdh1 Gapdh2 Gdh Gef26 Gfat1 GlyP Got2 Gprk1 Hcf Hem Hexo1 Hlc HmgD HmgZ Hmu Hrb27C Hrb87F Hsc70-3 Hsp23 Hsp26 Hsp27 Hsp60 Hsp67Bc Hus1-like Idh Indy Iswi Jheh1 Jheh2 KP78b Karybeta3 Klp10A Lac Lam LanA LanB2 M(2)21AB MICAL-like Map60 Mapmodulin Mbs Mcm5 Mcm6 Mcm7 Mcr Mdr49 Mekk1 Mi-2 Moe Mpcp Msp-300 Mtl Myb NTPase Nek2 Nep2 NetA Nlp Nop60B Nrv1 Optix Ote PFE PR2 Paf-AHalpha Paip2 Pak3 Pcl Pen Pepck Pgi Pi3K68D Pld Pli Pp1-87B Pp2C1 Psa Psc Ptp99A Rab10 RacGAP50C Ranbp16 Rbf2 Rbp2 RhoBTB Rhp Rm62 RnrL Rop RpII140 RpL13 RpL15 RpL22 RpL23a RpL3 RpS12 RpS6 Rrp1 S SMC1 Sb Scm Sdc SelD Sema-1b Sema-2a Ser Set Sh3beta Sin3A Sip1 Smox Snap Sox14 Spn5 Spt6 Src64B Su(dx) Su(var)3-9 Su(z)12 TBPH Taf2 Thd1 Thiolase Tis11 Tm1 Tom34 Top2 Top3beta TppII Tpr2 Trap1 Trxr-1 Uba1 Uba2 UbcD2 UbcD6 Uch Ucp4B Vang Vap-33-1 Vha44 Vha55 Wsck X11L XNP abs ade2 ade3 ald alpha-Adaptin alpha-Cat alpha-Spec alphaTub84B ana aop ap argos arm ash2 asp barr baz bel betaTub56D bib bl br brk brm btsz capu cav cdc2 chb ci cib cic ck cora crb crc crol csul csw cta d d4 da dac dalao deltaTry dnk dome dpa dre4 dve eIF-4B eIF3-S9 eIF5 east ebi edl egl en ena endos esn exd fat2 fax fbp fdl fra fwd fz fzy garz gft gish gol gp210 grn heph hh hts in jar jim jing jumu katanin-60 kay kel kis klar klu kn knk krz l(1)G0148 l(1)G0334 l(1)dd4 l(2)01424 l(2)08717 l(2)dtl l(2)gl larp lds lid lin19 lmg lola lqf lwr mask mav mbt mdy me31B mod mor msi msk msn mth mus209 ninA nonA-l nop5 nudC ogre olf413 ome otk ovo pAbp pain par-6 pav pbl pfk ph-p pk plexA pll pnut polo pon prominin-like qkr54B r-l rg rin rpk sc sca scrib sd sec23 serpin-27A sgl shg sima simj slmb smi35A spag sqd ss stai stan stc su(f) syndapin th tkv toe tok tos trio trn tub tud vav vg vig wbl wds wgn woc yps zf30C zip zwilch |
| 154 | GO:0006351 | P | 7, | 108 | 75.085 (x 1.438) | 896 (0.121) | 0.000761 | transcription, DNA-dependent | ATbp Arc92 BEAF-32 Bap170 BcDNA:LD23876 Brf CG11228 CG11305 CG12190 CG12252 CG12299 CG15141 CG15835 CG1815 CG30011 CG31258 CG31453 CG31716 CG33525 CG4914 CG5319 CG7008 CG7987 CG8092 CG8165 CG8426 CG8924 Caf1 CtBP CycT D12 D19A D19B Dll Dp Dr Dref Dsp1 E(z) Eip75B GATAd HmgD HmgZ Iswi Mi-2 Myb Optix Pcl Psc Rab10 Rbf2 RpII140 Sin3A Smox Sox14 Spt6 Su(var)3-9 Su(z)12 TBPH Taf2 Thd1 Top2 XNP aop ap ash2 bl br brk brm cdc2 ci cic crc crol d4 da dalao dre4 dve edl en exd gol grn hh jim jumu kay kis klu kn lds lid lola mor ovo ph-p sc sd sima simj ss stc toe woc yps zf30C |
| 155 | GO:0007297 | P | 6, 7, 9, | 11 | 2.765 (x 3.978) | 33 (0.333) | 0.000827 | follicle cell migration (sensu Insecta) | baz dome jar jing kay kel par-6 shg sqd th zip |
| 156 | GO:0045197 | P | 7, 8, | 8 | 1.508 (x 5.304) | 18 (0.444) | 0.000854 | establishment and/or maintenance of epithelial cell polarity | Cont Moe baz crb fz l(2)gl par-6 scrib |
| 157 | GO:0000910 | P | 5, | 19 | 7.039 (x 2.699) | 84 (0.226) | 0.000865 | cytokinesis | Act42A Act5C CG11207 CG13852 CG4454 Caf1 Cap-G Msp-300 Myb RacGAP50C Rop Snap baz fwd pav pbl pnut polo zip |
| 158 | GO:0005083 | F | 4, | 20 | 7.626 (x 2.623) | 91 (0.220) | 0.000867 | small GTPase regulator activity | Abi CG11856 CG17184 CG30372 CG6838 CG8155 CG8243 Gap1 RacGAP50C Rgl RhoGAP19D RhoGEF3 Rhp eIF5 msk msn pbl sec23 trio vav |
| 159 | GO:0007155 | P | 3, | 46 | 25.392 (x 1.812) | 303 (0.152) | 0.000869 | cell adhesion | 18w Arf79F CG12199 CG17419 CG31640 CG33214 CG4509 CG8243 Cad87A Cat Cont EG:BACH59J11.2 Fas2 Fas3 ImpL2 Lac LanA LanB2 NetA PFE Sema-1b Sema-2a Ser Tsp66E alpha-Cat alpha-Spec arm baz crb crol ed fat2 fra fz glec ninA otk p120ctn par-6 pbl scrib sdk shg stan trn vav |
| 160 | GO:0030725 | P | 5, | 6 | 0.838 (x 7.160) | 10 (0.600) | 0.000911 | ring canal formation | Btk29A Pen Src64B fwd hts kel |
| 161 | GO:0045216 | P | 6, | 9 | 1.927 (x 4.669) | 23 (0.391) | 0.000916 | intercellular junction assembly and maintenance | Cont arm baz cora crb jar l(2)gl par-6 scrib |
| 162 | GO:0043063 | P | 4, | 6 | 0.838 (x 7.160) | 10 (0.600) | 0.000917 | intercellular bridge organization and biogenesis | Btk29A Pen Src64B fwd hts kel |
| 163 | GO:0043228 | C | 3, | 89 | 59.582 (x 1.494) | 711 (0.125) | 0.00092 | non-membrane-bound organelle | 14-3-3epsilon Act42A Act5C BEAF-32 BicD CG10423 CG10695 CG11207 CG13895 CG17493 CG1911 CG31363 CG31617 CG5728 Caf1 Cap-G Cen190 CycA CycT DNApol-epsilon Dlc90F Dp Dsp1 E(z) Eb1 HmgD Hrb87F Klp10A Lam Map60 Mapmodulin Mi-2 Moe Msp-300 Myb Nek2 Nlp Nop60B Pen Pep Psc Rbf2 RpL13 RpL15 RpL22 RpL23a RpL3 RpS12 RpS6 SMC1 Sin3A Spt6 Su(var)3-9 Su(z)12 Tm1 Top2 alpha-Cat alpha-Spec alphaTub84B asp barr betaTub56D bl cav chb ck cora d fzy hts jar jumu katanin-60 kis klar l(1)dd4 l(2)gl nop5 p120ctn pav pfk pnut polo sqd stai stc tud vg zip |
| 164 | GO:0016333 | P | 5, | 9 | 1.927 (x 4.669) | 23 (0.391) | 0.000922 | morphogenesis of follicular epithelium | Dp Scm crb csw l(2)gl ph-p scrib shg sqd |
| 165 | GO:0030010 | P | 6, 7, | 6 | 0.838 (x 7.160) | 10 (0.600) | 0.000922 | establishment of cell polarity | abs baz crb fz l(2)gl zip |
| 166 | GO:0043232 | C | 4, 5, 6, 7, | 89 | 59.582 (x 1.494) | 711 (0.125) | 0.000926 | intracellular non-membrane-bound organelle | 14-3-3epsilon Act42A Act5C BEAF-32 BicD CG10423 CG10695 CG11207 CG13895 CG17493 CG1911 CG31363 CG31617 CG5728 Caf1 Cap-G Cen190 CycA CycT DNApol-epsilon Dlc90F Dp Dsp1 E(z) Eb1 HmgD Hrb87F Klp10A Lam Map60 Mapmodulin Mi-2 Moe Msp-300 Myb Nek2 Nlp Nop60B Pen Pep Psc Rbf2 RpL13 RpL15 RpL22 RpL23a RpL3 RpS12 RpS6 SMC1 Sin3A Spt6 Su(var)3-9 Su(z)12 Tm1 Top2 alpha-Cat alpha-Spec alphaTub84B asp barr betaTub56D bl cav chb ck cora d fzy hts jar jumu katanin-60 kis klar l(1)dd4 l(2)gl nop5 p120ctn pav pfk pnut polo sqd stai stc tud vg zip |
| 167 | GO:0043170 | P | 4, | 310 | 261.123 (x 1.187) | 3116 (0.099) | 0.00103 | macromolecule metabolism | AP-1gamma Aats-ala Aats-glupro Ack Acon Akt1 Ald ApepP Arf79F B52 BEST:LD22483 BG:DS00004.11 BcDNA:GH02976 BcDNA:GH04962 BcDNA:GM10765 BcDNA:LD09009 BcDNA:LD22910 BcDNA:LD23371 BcDNA:LD41548 Btk29A CG10354 CG10423 CG10542 CG10602 CG10657 CG10960 CG10990 CG11123 CG11142 CG11207 CG11228 CG1129 CG11305 CG11836 CG12006 CG12130 CG14217 CG14222 CG14231 CG1440 CG14411 CG14670 CG15093 CG15141 CG1544 CG1550 CG17036 CG17309 CG17514 CG17598 CG18030 CG1815 CG1869 CG1906 CG1911 CG2097 CG2852 CG2947 CG30025 CG31169 CG31617 CG31716 CG31739 CG32099 CG32632 CG32640 CG33123 CG33138 CG33145 CG3328 CG3605 CG40160 CG40410 CG4238 CG4612 CG4670 CG4749 CG4914 CG5028 CG5191 CG5390 CG5434 CG5505 CG5687 CG5728 CG5841 CG6227 CG6287 CG6394 CG6680 CG6835 CG6904 CG6946 CG7263 CG7288 CG7441 CG7720 CG7922 CG8036 CG8188 CG8443 CG8494 CG8789 CG8863 CG8963 CG9135 CG9153 CG9425 CG9906 CaMKII Caf1 CalpB Cbp80 Cdc42 Cdk4 CkIIalpha CkIIbeta CkIalpha Cks Cont CycG Cyp1 D19A D19B DNApol-delta DNApol-epsilon DnaJ-1 Dp Dref Dsp1 E(z) EG:86E4.2 ERp60 Eb1 Eip63E Elf Eno Fkbp13 Fur1 GNBP3 Galpha49B Gapdh1 Gapdh2 Gdh Gfat1 GlyP Got2 Gprk1 Hexo1 HmgD HmgZ Hmu Hrb27C Hrb87F Hsc70-3 Hsp23 Hsp26 Hsp27 Hsp60 Hsp67Bc Hus1-like Idh Iswi KP78b LanA Mcm5 Mcm6 Mcm7 Mcr Mekk1 Mi-2 Myb Nek2 Nep2 Nlp Nop60B PFE PR2 Paip2 Pak3 Pcl Pepck Pgi Pi3K68D Pp1-87B Pp2C1 Psa Psc Ptp99A Rbp2 Rm62 RnrL RpL13 RpL15 RpL22 RpL23a RpL3 RpS12 RpS6 Rrp1 SMC1 Sb Set Spn5 Spt6 Src64B Su(dx) Su(var)3-9 Su(z)12 TBPH Taf2 Thd1 Tis11 Tom34 Top2 Top3beta TppII Tpr2 Trap1 Uba1 Uba2 UbcD2 UbcD6 Uch Wsck X11L XNP ald alpha-Adaptin alphaTub84B arm ash2 baz betaTub56D bl brm cdc2 cora crb csw d4 da dalao deltaTry dome dpa dre4 eIF-4B eIF3-S9 eIF5 east ebi ena fat2 fbp fdl fra fwd fzy gft gish gol heph hh hts jumu kis l(1)G0148 l(1)G0334 l(2)01424 l(2)08717 l(2)gl larp lds lin19 lmg lola lwr mbt mdy mor msi msn mus209 nonA-l nop5 ome otk pAbp par-6 pbl pfk ph-p plexA pll polo rin scrib sgl slmb smi35A sqd stc su(f) syndapin th tkv tok tos wbl |
| 168 | GO:0006333 | P | 9, | 21 | 8.380 (x 2.506) | 100 (0.210) | 0.00107 | chromatin assembly or disassembly | CG11305 CG1911 CG31617 Caf1 Dsp1 E(z) HmgZ Iswi Mi-2 Nlp Psc SMC1 Set Spt6 Su(var)3-9 Su(z)12 dalao dre4 kis lola ph-p |
| 169 | GO:0042461 | P | 5, 6, 7, | 14 | 4.358 (x 3.213) | 52 (0.269) | 0.00107 | photoreceptor cell development | Amph Mbs Moe S aop crb dac edl klar mbt msn pnut sca stan |
| 170 | GO:0030528 | F | 2, | 98 | 67.460 (x 1.453) | 805 (0.122) | 0.0011 | transcription regulator activity | 14-3-3epsilon 14-3-3zeta ATbp Arc92 Bap170 BcDNA:LD23876 Brf CG11305 CG12190 CG12299 CG15835 CG30011 CG31453 CG31716 CG33525 CG4914 CG5319 CG6854 CG7008 CG7987 CG8165 CG8426 CG8924 CG9104 CREG CtBP CycT D19A D19B Dll Dp Dr Dref Dsp1 E(z) Eip75B GATAd Hcf HmgZ Hmr Iswi Mi-2 Myb Optix Pcl Psc Rbf2 Scm Sin3A Smox Sox14 Spt6 TBPH Taf2 XNP aop ap ash2 br brk brm ci cic crc crol crp d4 da dac dalao dre4 dve en exd gol grn jim jing jumu kay klu kn lds lid lola mor nonA-l ovo sc sd sima simj ss stc toe woc yps zf30C |
| 171 | GO:0045184 | P | 5, | 69 | 43.576 (x 1.583) | 520 (0.133) | 0.00111 | establishment of protein localization | AP-1gamma Akt1 Amph Arf79F CG11856 CG1418 CG1472 CG1900 CG2108 CG2158 CG2852 CG32137 CG32164 CG3249 CG33113 CG33214 CG5064 CG5434 CG6838 CG8155 CG9906 Chc Cyp1 Dlc90F EG:34F3.8 Fs(2)Ket Hsp60 Karybeta3 Klp10A Pen Pi3K68D Rab10 Ranbp16 RhoBTB S Snap Vap-33-1 X11L alpha-Adaptin alphaTub84B baz betaTub56D bl ck cora d garz gp210 hh jar katanin-60 krz l(2)gl lqf lwr mask msk par-6 pav pk pll prominin-like rg rin scrib sec23 slmb syndapin wbl |
| 172 | GO:0050793 | P | 3, | 18 | 6.620 (x 2.719) | 79 (0.228) | 0.00113 | regulation of development | Cdc42 Dr LanA Mbs Ser ana aop argos br ed hdc kn lqf mbt pbl sdk sty trio |
| 173 | GO:0051058 | P | 6, 7, 8, | 5 | 0.587 (x 8.524) | 7 (0.714) | 0.00118 | negative regulation of small GTPase mediated signal transduction | Akap200 Gap1 aop klu sty |
| 174 | GO:0007156 | P | 5, | 10 | 2.430 (x 4.115) | 29 (0.345) | 0.00118 | homophilic cell adhesion | CG4509 Cad87A Fas2 Fas3 Lac ed fat2 fz shg stan |
| 175 | GO:0007051 | P | 9, | 10 | 2.430 (x 4.115) | 29 (0.345) | 0.00119 | spindle organization and biogenesis | CG11207 Eb1 Myb chb dpa fwd larp mus209 pbl polo |
| 176 | GO:0008092 | F | 4, | 38 | 19.945 (x 1.905) | 238 (0.160) | 0.00119 | cytoskeletal protein binding | Aats-glupro CG1826 CG32137 CG32672 Cen190 Eb1 Hsp23 MICAL-like Map60 Mapmodulin Mbs Moe Msp-300 Sdc Tm1 alpha-Cat alpha-Spec alphaTub84B arm asp betaTub56D capu chb cib ck cora crb d ena hts jar katanin-60 kel l(1)dd4 l(2)gl pnut stai zip |
| 177 | GO:0035088 | P | 6, 7, | 8 | 1.592 (x 5.024) | 19 (0.421) | 0.0012 | establishment and/or maintenance of apical/basal cell polarity | Cont Moe baz crb fz l(2)gl par-6 scrib |
| 178 | GO:0007264 | P | 6, | 23 | 9.721 (x 2.366) | 116 (0.198) | 0.0012 | small GTPase mediated signal transduction | 14-3-3epsilon 14-3-3zeta Akap200 CG1900 CG2108 CG30372 CG5522 Cdc42 Gap1 Gef26 Mtl Rab10 RacGAP50C Rgl RhoBTB aop edl flfl klu rin sty trio vav |
| 179 | GO:0001751 | P | 6, 7, 8, 9, | 17 | 6.117 (x 2.779) | 73 (0.233) | 0.00129 | eye photoreceptor cell differentiation (sensu Endopterygota) | Amph Mbs Moe S aop crb csw da dac fz hh kay klar mbt pnut sca stan |
| 180 | GO:0006886 | P | 6, 7, 8, | 67 | 42.403 (x 1.580) | 506 (0.132) | 0.00143 | intracellular protein transport | AP-1gamma Akt1 Amph Arf79F CG11856 CG1418 CG1472 CG1900 CG2108 CG2158 CG2852 CG32137 CG32164 CG3249 CG33113 CG33214 CG5064 CG5434 CG6838 CG8155 CG9906 Chc Cyp1 Dlc90F EG:34F3.8 Fs(2)Ket Hsp60 Karybeta3 Klp10A Pen Pi3K68D Rab10 Ranbp16 S Snap Vap-33-1 X11L alpha-Adaptin alphaTub84B baz betaTub56D bl ck cora d garz gp210 hh jar katanin-60 krz l(2)gl lqf lwr mask msk par-6 pav pll prominin-like rg rin scrib sec23 slmb syndapin wbl |
| 181 | GO:0009892 | P | 5, | 29 | 13.827 (x 2.097) | 165 (0.176) | 0.00146 | negative regulation of metabolism | CG11207 CG11228 CG11305 CG31716 CG33525 CREG Caf1 CtBP E(z) Paip2 Pcl Psc Rbf2 Sin3A Su(var)3-9 Su(z)12 aop brk ci cic edl en fwd hh msi ph-p serpin-27A simj sqd |
| 182 | GO:0051243 | P | 5, | 37 | 19.442 (x 1.903) | 232 (0.159) | 0.00147 | negative regulation of cellular physiological process | Aac11 Akt1 CG11207 CG11228 CG11305 CG31716 CG33525 CG40410 CREG Caf1 CtBP CycA E(z) Myb Paip2 Pcl Psc Rbf2 Sin3A Su(var)3-9 Su(z)12 ana aop brk ci cic edl en fwd hh l(2)gl msi ph-p scrib simj sqd th |
| 183 | GO:0043118 | P | 4, | 38 | 20.196 (x 1.882) | 241 (0.158) | 0.0015 | negative regulation of physiological process | Aac11 Akt1 CG11207 CG11228 CG11305 CG31716 CG33525 CG40410 CREG Caf1 CtBP CycA E(z) Myb Paip2 Pcl Psc Rbf2 Sin3A Su(var)3-9 Su(z)12 ana aop brk ci cic edl en fwd hh l(2)gl msi ph-p scrib serpin-27A simj sqd th |
| 184 | GO:0035215 | P | 5, | 7 | 1.257 (x 5.569) | 15 (0.467) | 0.00151 | genital disc development | Btk29A Dll Fas2 ci dac en hh |
| 185 | GO:0015031 | P | 5, 6, | 68 | 43.325 (x 1.570) | 517 (0.132) | 0.00151 | protein transport | AP-1gamma Akt1 Amph Arf79F CG11856 CG1418 CG1472 CG1900 CG2108 CG2158 CG2852 CG32137 CG32164 CG3249 CG33113 CG33214 CG5064 CG5434 CG6838 CG8155 CG9906 Chc Cyp1 Dlc90F EG:34F3.8 Fs(2)Ket Hsp60 Karybeta3 Klp10A Pen Pi3K68D Rab10 Ranbp16 RhoBTB S Snap Vap-33-1 X11L alpha-Adaptin alphaTub84B baz betaTub56D bl ck cora d garz gp210 hh jar katanin-60 krz l(2)gl lqf lwr mask msk par-6 pav pll prominin-like rg rin scrib sec23 slmb syndapin wbl |
| 186 | GO:0005654 | C | 5, 6, 7, 8, 9, 10, 11, | 36 | 18.771 (x 1.918) | 224 (0.161) | 0.00151 | nucleoplasm | Arc92 B52 Bap170 Brf CG11305 CG2097 Caf1 CycT Cyp1 Dp Dref E(z) Hrb27C Hrb87F Iswi Mcm5 Mcm6 Mcm7 Mi-2 Rbf2 RpII140 Sin3A Su(z)12 Taf2 Top2 ap bl brm dac dalao dre4 lds mor sqd su(f) toe |
| 187 | GO:0009966 | P | 4, 5, | 21 | 8.631 (x 2.433) | 103 (0.204) | 0.00152 | regulation of signal transduction | Akap200 CG5841 CkIalpha Gap1 Gprk1 RacGAP50C S aop argos arm brk ebi ed edl hh klu l(2)gl lgs slmb stan sty |
| 188 | GO:0045186 | P | 8, 9, | 6 | 0.922 (x 6.509) | 11 (0.545) | 0.0016 | zonula adherens assembly | arm baz crb l(2)gl par-6 scrib |
| 189 | GO:0003729 | F | 5, | 46 | 26.230 (x 1.754) | 313 (0.147) | 0.00161 | mRNA binding | Aats-ala Aats-glupro B52 CG11123 CG17838 CG31716 CG31739 CG33123 CG4612 CG5064 CG5728 CG6049 CG6227 CG6946 CG8963 CG9373 CG9809 Hmu Hrb27C Hrb87F LanA Nop60B Rbp2 Rm62 RpL23a Spt6 TBPH Tis11 Top2 bl bsf eIF-4B eIF3-S9 heph l(2)01424 mod msi nonA-l nop5 pAbp qkr54B rin sqd su(f) vig yps |
| 190 | GO:0016481 | P | 8, | 22 | 9.302 (x 2.365) | 111 (0.198) | 0.00161 | negative regulation of transcription | CG11228 CG11305 CG31716 CG33525 CREG Caf1 CtBP E(z) Pcl Psc Rbf2 Sin3A Su(var)3-9 Su(z)12 aop brk ci cic edl en ph-p simj |
| 191 | GO:0016568 | P | 9, | 15 | 5.112 (x 2.934) | 61 (0.246) | 0.00162 | chromatin modification | BcDNA:LD09009 CG11305 Caf1 E(z) Iswi Pcl Psc Su(var)3-9 Su(z)12 ash2 brm dalao mor pfk ph-p |
| 192 | GO:0007265 | P | 7, | 9 | 2.095 (x 4.296) | 25 (0.360) | 0.00168 | Ras protein signal transduction | 14-3-3epsilon 14-3-3zeta Akap200 Gap1 aop edl klu rin sty |
| 193 | GO:0043068 | P | 6, 7, | 17 | 6.285 (x 2.705) | 75 (0.227) | 0.0017 | positive regulation of programmed cell death | CG10473 CG10990 CG11228 CG14217 CG17765 CG7263 CG8400 Dp Eip75B aop bl br gft klu l(2)gl smi35A zip |
| 194 | GO:0043297 | P | 8, | 8 | 1.676 (x 4.773) | 20 (0.400) | 0.00171 | apical junction assembly | Cont arm baz cora crb l(2)gl par-6 scrib |
| 195 | GO:0009798 | P | 4, | 28 | 13.324 (x 2.101) | 159 (0.176) | 0.00173 | axis specification | 14-3-3epsilon 14-3-3zeta BicD Dp Gap1 Hrb27C Moe Rop Tm1 baz capu csul csw egl gus hh knk lwr par-6 pll serpin-27A shg sqd sty tok tub tud wbl |
| 196 | GO:0006950 | P | 3, | 48 | 27.989 (x 1.715) | 334 (0.144) | 0.00194 | response to stress | 14-3-3epsilon 18w BcDNA:GM10765 BthD CG32640 CG33123 CG40410 CG6227 CG8863 Caf1 Cat Cdc42 CkIalpha CycG CycT DNApol-delta DNApol-epsilon DnaJ-1 Hsc70-3 Hsp23 Hsp26 Hsp27 Hsp60 Hsp67Bc Hus1-like Mekk1 Rrp1 Thd1 Tom34 Trap1 UbcD6 XNP ald br crol da kay kn lds mth mus209 ninA pain pll scrib serpin-27A tos tub |
| 197 | GO:0008594 | P | 6, 7, 8, | 13 | 4.106 (x 3.166) | 49 (0.265) | 0.00196 | photoreceptor cell morphogenesis (sensu Endopterygota) | Amph Mbs Moe S aop crb dac klar mbt msn pnut sca stan |
| 198 | GO:0001754 | P | 5, 6, 7, | 17 | 6.369 (x 2.669) | 76 (0.224) | 0.00197 | eye photoreceptor cell differentiation | Amph Mbs Moe S aop crb csw da dac fz hh kay klar mbt pnut sca stan |
| 199 | GO:0007309 | P | 5, 7, 8, 10, | 17 | 6.369 (x 2.669) | 76 (0.224) | 0.00198 | oocyte axis determination | 14-3-3epsilon 14-3-3zeta BicD Hrb27C Moe Rop Tm1 baz capu csul egl gus par-6 shg sqd tud wbl |
| 200 | GO:0005886 | C | 4, 5, | 69 | 44.750 (x 1.542) | 534 (0.129) | 0.00215 | plasma membrane | 18w Akt1 Amph BEST:CK01140 Btk29A CG17036 CG2097 CG4509 Cad87A Cat Chc Cont EG:196F3.2 Fas2 Fas3 Fur1 G-salpha60A Galpha49B Gap1 Hem ImpE3 Indy Lac Mdr49 Moe Msp-300 Nrv1 Ptp99A S Sb Sdc Sema-1b Ser Wsck alpha-Adaptin alpha-Cat alpha-Spec arm baz bib cora crb cta dome ed fat2 fra fz in inx2 inx3 l(2)gl mbt ogre otk p120ctn par-6 pll pnut prominin-like scrib sdk shg stan sty tkv trio trn wgn |
| 201 | GO:0006366 | P | 8, | 84 | 57.152 (x 1.470) | 682 (0.123) | 0.00219 | transcription from RNA polymerase II promoter | ATbp Arc92 BEAF-32 BcDNA:LD23876 Brf CG11228 CG12190 CG12252 CG12299 CG15835 CG30011 CG31453 CG31716 CG5319 CG7008 CG7987 CG8165 CG8426 CG8924 Caf1 CtBP CycT D19A D19B Dll Dp Dr Dref Dsp1 E(z) Eip75B HmgZ Iswi Mi-2 Myb Optix Pcl Psc Rbf2 RpII140 Sin3A Smox Sox14 Spt6 TBPH Taf2 Top2 XNP aop ap ash2 bl br brk brm cdc2 ci crol d4 da dre4 en gol grn hh jim jumu kis klu kn lds lola mor ovo ph-p sc sd sima ss stc toe woc yps zf30C |
| 202 | GO:0007097 | P | 7, 8, | 7 | 1.341 (x 5.221) | 16 (0.438) | 0.00225 | nuclear migration | BicD Lam egl klar msn nudC polo |
| 203 | GO:0005918 | C | 7, 8, 9, 10, | 7 | 1.341 (x 5.221) | 16 (0.438) | 0.00226 | septate junction | Cont Fas3 Lac cora crb l(2)gl scrib |
| 204 | GO:0007043 | P | 7, | 8 | 1.760 (x 4.546) | 21 (0.381) | 0.00242 | intercellular junction assembly | Cont arm baz cora crb l(2)gl par-6 scrib |
| 205 | GO:0007017 | P | 7, | 32 | 16.425 (x 1.948) | 196 (0.163) | 0.00244 | microtubule-based process | 14-3-3epsilon 14-3-3zeta BicD CG10695 CG11207 CG9057 Cen190 Dlc90F Eb1 Klp10A Map60 Mapmodulin Myb alphaTub84B asp baz betaTub56D chb dpa egl fwd jar katanin-60 klar l(1)dd4 larp mus209 pav pbl polo stai vg |
| 206 | GO:0016895 | F | 8, | 5 | 0.670 (x 7.458) | 8 (0.625) | 0.00248 | exodeoxyribonuclease activity, producing 5'-phosphomonoesters | BcDNA:GM10765 DNApol-delta DNApol-epsilon Rrp1 tos |
| 207 | GO:0046907 | P | 5, 6, 7, | 77 | 51.621 (x 1.492) | 616 (0.125) | 0.00251 | intracellular transport | AP-1gamma Akt1 Amph Arf79F CG10695 CG11856 CG1418 CG1472 CG17143 CG1900 CG1907 CG2108 CG2158 CG2852 CG2980 CG32137 CG32164 CG3249 CG33113 CG33214 CG5064 CG5434 CG6838 CG8155 CG9057 CG9906 Chc Cyp1 Dlc90F EG:34F3.8 Fs(2)Ket Hsp60 Karybeta3 Klp10A Pen Pi3K68D Rab10 Ranbp16 S Snap Ucp4B Vap-33-1 X11L alpha-Adaptin alphaTub84B baz betaTub56D bl ck cora d garz gp210 hh jar katanin-60 kel klar krz l(2)gl lqf lwr mask msk par-6 pav pll prominin-like rg rin scrib sec23 slmb sqd syndapin vg wbl |
| 208 | GO:0008105 | P | 5, | 10 | 2.682 (x 3.729) | 32 (0.312) | 0.00252 | asymmetric protein localization | Moe baz fz jar jumu l(2)gl mask par-6 pon scrib |
| 209 | GO:0042051 | P | 7, 8, 9, 10, | 12 | 3.687 (x 3.254) | 44 (0.273) | 0.00252 | eye photoreceptor development (sensu Endopterygota) | Amph Mbs Moe S aop crb dac klar mbt pnut sca stan |
| 210 | GO:0008360 | P | 5, 6, | 18 | 7.123 (x 2.527) | 85 (0.212) | 0.00252 | regulation of cell shape | Abi Akt1 Arf79F CG1900 Cdc42 CycT Fas2 Fs(2)Ket Hem alpha-Spec arm cta ena l(2)gl msn par-6 pbl vav |
| 211 | GO:0031519 | C | 3, 5, 6, 7, 8, 9, 10, | 6 | 1.006 (x 5.967) | 12 (0.500) | 0.00266 | PcG protein complex | Caf1 E(z) Psc Scm Su(z)12 ph-p |
| 212 | GO:0003723 | F | 4, | 51 | 30.922 (x 1.649) | 369 (0.138) | 0.003 | RNA binding | Aats-ala Aats-glupro B52 CG11123 CG17838 CG31716 CG31739 CG33123 CG4612 CG5064 CG5434 CG5728 CG6049 CG6227 CG6946 CG8963 CG9373 CG9809 Cbp80 Hmu Hrb27C Hrb87F LanA Msp-300 Nop60B Pep Rbp2 Rm62 RpL23a Spt6 Su(var)3-9 TBPH Tis11 Top2 bl bsf eIF-4B eIF3-S9 heph l(2)01424 mod msi nonA-l nop5 pAbp qkr54B rin sqd su(f) vig yps |
| 213 | GO:0005085 | F | 4, | 14 | 4.860 (x 2.880) | 58 (0.241) | 0.00302 | guanyl-nucleotide exchange factor activity | CG30440 CG5522 CG9135 CG9153 CdGAPr Gef26 Rgl RhoGAP1A RhoGEF3 garz pbl trio vav vimar |
| 214 | GO:0042462 | P | 6, 7, 8, | 12 | 3.771 (x 3.182) | 45 (0.267) | 0.00306 | eye photoreceptor cell development | Amph Mbs Moe S aop crb dac klar mbt pnut sca stan |
| 215 | GO:0007424 | P | 4, | 21 | 9.134 (x 2.299) | 109 (0.193) | 0.00307 | tracheal system development (sensu Insecta) | Akt1 Cdk4 Fas2 Lac Lam S Wnt2 arm barr cora csw dome fat2 hdc hh jing sgl shg sty tkv trn |
| 216 | GO:0006461 | P | 6, | 22 | 9.805 (x 2.244) | 117 (0.188) | 0.0031 | protein complex assembly | AP-1gamma B52 CG31617 Caf1 Cont Eb1 Hsc70-3 Iswi Set Tpr2 alpha-Adaptin arm baz cora crb hts l(2)gl larp par-6 pbl polo scrib |
| 217 | GO:0044238 | P | 4, | 429 | 382.969 (x 1.120) | 4570 (0.094) | 0.00311 | primary metabolism | 14-3-3zeta AP-1gamma ATPsyn-beta ATbp Aats-ala Aats-glupro Ack Acon Acox57D-p Akt1 Ald ApepP Arc92 Arf79F B52 BEAF-32 BEST:LD22483 BG:DS00004.11 Bap170 BcDNA:GH02901 BcDNA:GH02976 BcDNA:GH04962 BcDNA:GH12558 BcDNA:GM10765 BcDNA:LD09009 BcDNA:LD22910 BcDNA:LD23371 BcDNA:LD23876 BcDNA:LD41548 Brf Btk29A CG10354 CG10423 CG10542 CG10602 CG10657 CG10960 CG10990 CG11055 CG11123 CG11142 CG11207 CG11228 CG1129 CG11305 CG11836 CG12006 CG12130 CG12190 CG12252 CG12299 CG14217 CG14222 CG14231 CG1440 CG14411 CG14670 CG15093 CG15141 CG1544 CG1550 CG15835 CG17036 CG17309 CG1746 CG17514 CG17598 CG18030 CG1815 CG1869 CG1893 CG1906 CG1911 CG2097 CG2118 CG2316 CG2852 CG2947 CG30011 CG30025 CG31169 CG31258 CG31453 CG31617 CG31716 CG31739 CG32099 CG32158 CG32632 CG32640 CG33116 CG33123 CG33138 CG33145 CG3328 CG33525 CG3590 CG3605 CG40160 CG40410 CG4238 CG4612 CG4670 CG4749 CG4914 CG5028 CG5191 CG5319 CG5390 CG5434 CG5505 CG5687 CG5728 CG5841 CG6227 CG6287 CG6391 CG6394 CG6418 CG6543 CG6638 CG6680 CG6767 CG6835 CG6854 CG6904 CG6946 CG7008 CG7145 CG7263 CG7288 CG7441 CG7461 CG7720 CG7878 CG7922 CG7987 CG8036 CG8092 CG8165 CG8188 CG8426 CG8443 CG8494 CG8789 CG8863 CG8924 CG8963 CG9057 CG9135 CG9153 CG9342 CG9425 CG9906 CREG CaMKII Caf1 CalpB Cbp80 Cdc42 Cdk4 CkIIalpha CkIIbeta CkIalpha Cks Cont CtBP CycG CycT Cyp1 D12 D19A D19B DNApol-delta DNApol-epsilon Dhh1 Dll DnaJ-1 Dp Dr Dref Dsp1 E(z) EG:86E4.2 ERp60 ESTS:39C10S Eb1 Eip55E Eip63E Eip75B Elf Eno Fkbp13 Fpps Fur1 GATAd GNBP3 Galpha49B Gapdh1 Gapdh2 Gdh Gfat1 GlyP Got2 Gprk1 Hcf Hexo1 Hlc HmgD HmgZ Hmu Hrb27C Hrb87F Hsc70-3 Hsp23 Hsp26 Hsp27 Hsp60 Hsp67Bc Hus1-like Idh Iswi Jheh1 Jheh2 KP78b LanA M(2)21AB Mcm5 Mcm6 Mcm7 Mcr Mekk1 Mi-2 Myb NTPase Nek2 Nep2 Nlp Nop60B Optix PFE PR2 Paf-AHalpha Paip2 Pak3 Pcl Pepck Pgi Pi3K68D Pld Pp1-87B Pp2C1 Psa Psc Ptp99A Rab10 Rbf2 Rbp2 Rm62 RnrL RpII140 RpL13 RpL15 RpL22 RpL23a RpL3 RpS12 RpS6 Rrp1 SMC1 Sb Scm SelD Set Sin3A Smox Sox14 Spn5 Spt6 Src64B Su(dx) Su(var)3-9 Su(z)12 TBPH Taf2 Thd1 Thiolase Tis11 Tom34 Top2 Top3beta TppII Tpr2 Trap1 Uba1 Uba2 UbcD2 UbcD6 Uch Vha44 Vha55 Wsck X11L XNP abs ade2 ade3 ald alpha-Adaptin alphaTub84B aop ap arm ash2 baz bel betaTub56D bl br brk brm cdc2 ci cic cora crb crc crol csw d4 da dac dalao deltaTry dnk dome dpa dre4 dve eIF-4B eIF3-S9 eIF5 east ebi edl en ena exd fat2 fbp fdl fra fwd fzy gft gish gol grn heph hh hts jim jing jumu kay kis klu kn knk l(1)G0148 l(1)G0334 l(1)dd4 l(2)01424 l(2)08717 l(2)gl larp lds lid lin19 lmg lola lwr mbt mdy me31B mor msi msn mus209 nonA-l nop5 olf413 ome otk ovo pAbp par-6 pbl pfk ph-p plexA pll polo qkr54B r-l rin sc scrib sd sgl sima simj slmb smi35A sqd ss stc su(f) syndapin th tkv toe tok tos vig wbl woc yps zf30C |
| 218 | GO:0009888 | P | 3, | 54 | 33.353 (x 1.619) | 398 (0.136) | 0.00312 | tissue development | Ald CG12896 CG30011 CG5830 CG8104 Cdc42 Cip4 Cont Dll Dr EG:BACH59J11.2 Fas2 Galpha49B LanA LanB2 Mbs Msp-300 NetA S Sema-1b Sema-2a Ser Sox14 aop ap arm bib crb crol csw dac en exd fra fz gol grn hh in msi ninA nonA-l ovo pbl pk plexA sca sgl shg sima smi35A stan sty toe |
| 219 | GO:0005815 | C | 5, 6, 7, 8, | 11 | 3.268 (x 3.366) | 39 (0.282) | 0.00318 | microtubule organizing center | Cen190 Map60 Nek2 asp chb fzy katanin-60 l(1)dd4 p120ctn pav polo |
| 220 | GO:0040023 | P | 6, 7, | 7 | 1.425 (x 4.914) | 17 (0.412) | 0.00326 | establishment of nucleus localization | BicD Lam egl klar msn nudC polo |
| 221 | GO:0009401 | P | 6, 7, | 8 | 1.844 (x 4.339) | 22 (0.364) | 0.00327 | phosphoenolpyruvate-dependent sugar phosphotransferase system | CG11451 CG9246 CG9809 LanB2 PR2 Psa alpha-Cat l(2)dtl |
| 222 | GO:0019899 | F | 4, | 13 | 4.358 (x 2.983) | 52 (0.250) | 0.00332 | enzyme binding | Akap200 CG11856 CG1418 CG1815 CG3249 Caf1 Cip4 Ranbp16 Rhp baz msk rg vimar |
| 223 | GO:0051169 | P | 6, 7, 8, | 15 | 5.531 (x 2.712) | 66 (0.227) | 0.00356 | nuclear transport | Akt1 CG17143 CG2980 CG32164 Fs(2)Ket Karybeta3 Pen Ranbp16 hh lwr msk pll rin slmb sqd |
| 224 | GO:0005856 | C | 5, 6, 7, 8, | 42 | 24.302 (x 1.728) | 290 (0.145) | 0.00372 | cytoskeleton | Act42A Act5C BicD CG10695 CG11207 CG17493 CG31363 Cen190 Dlc90F Eb1 Klp10A Lam Map60 Mapmodulin Moe Msp-300 Nek2 Pen Tm1 alpha-Cat alpha-Spec alphaTub84B asp betaTub56D chb ck cora d fzy jar katanin-60 klar l(1)dd4 l(2)gl nop5 p120ctn pav pnut polo stai vg zip |
| 225 | GO:0043067 | P | 5, 6, | 22 | 9.972 (x 2.206) | 119 (0.185) | 0.00386 | regulation of programmed cell death | Aac11 Akt1 CG10473 CG10990 CG11228 CG14217 CG17765 CG7263 CG8400 Dp Eip75B Myb aop bl br gft klu l(2)gl mdy smi35A th zip |
| 226 | GO:0042048 | P | 5, 6, | 13 | 4.441 (x 2.927) | 53 (0.245) | 0.00398 | olfactory behavior | 14-3-3zeta BEST:GH15083 CG11711 CG8588 Fas2 Fas3 Pp1-87B Src64B gp210 scrib slmb smi21F smi35A |
| 227 | GO:0008283 | P | 4, | 41 | 23.632 (x 1.735) | 282 (0.145) | 0.00399 | cell proliferation | 14-3-3zeta CG11228 CG12299 CG5841 CG7987 Cdk4 CkIIalpha D19A D19B Eip75B Hcf Myb Pak3 Pen RacGAP50C SelD Ser Su(z)12 ana bl brm crb crol dac eIF-4B hh jim klu l(2)gl lwr mask mbt mod pll qkr54B scrib smi35A spag toe tub wds |
| 228 | GO:0008134 | F | 4, | 18 | 7.458 (x 2.413) | 89 (0.202) | 0.00421 | transcription factor binding | Bap170 Brf CG12190 CG15835 CG31453 CG7008 CtBP Dsp1 E(z) Hcf Sin3A bl brm cic dalao dre4 kay mor |
| 229 | GO:0044451 | C | 5, 6, 7, 8, 9, 10, 11, 12, | 32 | 17.012 (x 1.881) | 203 (0.158) | 0.00421 | nucleoplasm part | Arc92 B52 Bap170 Brf CG11305 CG2097 Caf1 CycT Cyp1 Dp Dref E(z) Hrb87F Iswi Mcm5 Mcm6 Mcm7 Mi-2 Rbf2 RpII140 Sin3A Su(z)12 Taf2 Top2 bl brm dalao dre4 mor sqd su(f) toe |
| 230 | GO:0004386 | F | 3, | 21 | 9.386 (x 2.237) | 112 (0.188) | 0.00423 | helicase activity | CG6227 CG6418 CG7878 CG7922 CG9425 Dhh1 Hlc Iswi Mcm5 Mcm6 Mcm7 Mi-2 Rm62 XNP abs bel brm dpa kis lds me31B |
| 231 | GO:0051179 | P | 3, | 185 | 148.411 (x 1.247) | 1771 (0.104) | 0.00437 | localization | AP-1gamma ATPsyn-beta Abi Akap200 Akt1 Amph Arf79F BEST:CK01140 BG:DS07473.1 BicD CG10211 CG10657 CG10695 CG10960 CG11069 CG11451 CG11856 CG12048 CG1418 CG14439 CG1472 CG15835 CG17036 CG17143 CG17184 CG17419 CG1746 CG1893 CG1900 CG1907 CG2108 CG2158 CG2316 CG2852 CG2980 CG32137 CG32164 CG3249 CG33113 CG33214 CG3823 CG4509 CG5064 CG5434 CG5687 CG5789 CG5841 CG6812 CG6838 CG7720 CG8104 CG8155 CG9057 CG9246 CG9342 CG9809 CG9906 Cdc42 Chc Cyp1 DNApol-delta Dlc90F EG:196F3.2 EG:34F3.8 Eb1 Fas3 Fpps Fs(2)Ket Galpha49B Hrb27C Hrb87F Hsp60 Indy Karybeta3 Klp10A Lam LanA LanB2 Mdr49 Moe Mpcp NetA Nrv1 PR2 Pen Pi3K68D Psa Ptp99A Rab10 Ranbp16 RhoBTB Rop S Sdc Sema-1b Sema-2a Sin3A Sip1 Snap Tm1 Ucp4B Vap-33-1 Vha44 Vha55 X11L alpha-Adaptin alpha-Cat alphaTub84B ap argos arm asp baz betaTub56D bib bl btsz capu chb cic ck cora crb csw cta d dome egl ena fra fz garz gish gp210 hh jar jing jumu katanin-60 kay kel klar krz l(2)08717 l(2)dtl l(2)gl lola lqf lwr mask msk msn mth ninA nudC otk pain par-6 pav pbl pk plexA pll pnut polo pon prominin-like rg rin rpk scrib sec23 sgl shg slmb sqd stai syndapin th trio trn tud vg wbl zip |
| 232 | GO:0006606 | P | 7, 8, 9, 10, | 12 | 3.939 (x 3.047) | 47 (0.255) | 0.00439 | protein import into nucleus | Akt1 CG32164 Fs(2)Ket Karybeta3 Pen Ranbp16 hh lwr msk pll rin slmb |
| 233 | GO:0030030 | P | 5, 6, | 12 | 3.939 (x 3.047) | 47 (0.255) | 0.00441 | cell projection organization and biogenesis | Abi CG7161 CaMKII Cdc42 Hem Mbs fz hh in ovo pk shg |
| 234 | GO:0031981 | C | 4, 5, 6, 7, 8, 9, 10, | 40 | 23.045 (x 1.736) | 275 (0.145) | 0.0045 | nuclear lumen | Arc92 B52 Bap170 Brf CG11305 CG2097 CG5728 Caf1 CycT Cyp1 Dp Dref E(z) Hrb27C Hrb87F Iswi Mcm5 Mcm6 Mcm7 Mi-2 Nlp Nop60B Rbf2 RpII140 Sin3A Su(z)12 Taf2 Top2 ap bl brm dac dalao dre4 jumu lds mor sqd su(f) toe |
| 235 | GO:0035003 | C | 7, 8, 9, 10, | 5 | 0.754 (x 6.629) | 9 (0.556) | 0.00454 | subapical complex | Moe baz crb par-6 scrib |
| 236 | GO:0007301 | P | 6, 9, | 5 | 0.754 (x 6.629) | 9 (0.556) | 0.00456 | ovarian ring canal formation | Btk29A Pen Src64B hts kel |
| 237 | GO:0006260 | P | 7, | 22 | 10.140 (x 2.170) | 121 (0.182) | 0.0046 | DNA replication | BcDNA:GM10765 CG5841 CG9425 Caf1 DNApol-delta DNApol-epsilon Dp Dref Dsp1 Mcm5 Mcm6 Mcm7 Myb RnrL Set Thd1 Tom34 Top2 Top3beta dpa dre4 mus209 |
| 238 | GO:0006605 | P | 7, 8, 9, | 35 | 19.358 (x 1.808) | 231 (0.152) | 0.0046 | protein targeting | Akt1 CG11856 CG2158 CG2852 CG32137 CG32164 CG3249 CG5064 CG5434 Cyp1 Fs(2)Ket Hsp60 Karybeta3 Klp10A Pen Pi3K68D Ranbp16 S X11L baz bl cora gp210 hh lwr mask msk par-6 pav pll rg rin scrib slmb wbl |
| 239 | GO:0009266 | P | 4, | 13 | 4.525 (x 2.873) | 54 (0.241) | 0.0046 | response to temperature stimulus | CG32640 CG6227 CycT DnaJ-1 Hsc70-3 Hsp23 Hsp26 Hsp27 Hsp60 Hsp67Bc crol mth pain |
| 240 | GO:0045934 | P | 7, | 22 | 10.140 (x 2.170) | 121 (0.182) | 0.00462 | negative regulation of nucleobase, nucleoside, nucleotide and nucleic acid metabolism | CG11228 CG11305 CG31716 CG33525 CREG Caf1 CtBP E(z) Pcl Psc Rbf2 Sin3A Su(var)3-9 Su(z)12 aop brk ci cic edl en ph-p simj |
| 241 | GO:0040007 | P | 2, | 18 | 7.542 (x 2.387) | 90 (0.200) | 0.00462 | growth | Akt1 CG11228 CG17309 Cdk4 Fas2 Hem Paip2 bl brk btsz charybde fat2 hh mav ninA scylla spag tkv |
| 242 | GO:0051647 | P | 5, 6, | 7 | 1.508 (x 4.641) | 18 (0.389) | 0.00462 | nucleus localization | BicD Lam egl klar msn nudC polo |
| 243 | GO:0007635 | P | 4, 5, | 13 | 4.525 (x 2.873) | 54 (0.241) | 0.00462 | chemosensory behavior | 14-3-3zeta BEST:GH15083 CG11711 CG8588 Fas2 Fas3 Pp1-87B Src64B gp210 scrib slmb smi21F smi35A |
| 244 | GO:0019887 | F | 4, | 10 | 2.933 (x 3.409) | 35 (0.286) | 0.00485 | protein kinase regulator activity | 14-3-3epsilon 14-3-3zeta CkIIbeta Cks CycA CycG CycT Cyp1 Ste12DOR Ste:CG33236 |
| 245 | GO:0004672 | F | 6, | 42 | 24.721 (x 1.699) | 295 (0.142) | 0.00491 | protein kinase activity | Ack Akt1 BcDNA:LD09009 BcDNA:LD23371 Btk29A CG11228 CG14217 CG17309 CG31640 CG40410 CG8789 CaMKII Cdk4 CkIIalpha CkIIbeta CkIalpha Cks CycT Eip63E Gprk1 KP78b Mekk1 Nek2 PFE PR2 Pak3 Src64B Wsck ald asp cdc2 fat2 gish l(1)G0148 mbt msn otk plexA pll polo smi35A tkv |
| 246 | GO:0051170 | P | 7, 8, 9, | 12 | 4.022 (x 2.983) | 48 (0.250) | 0.00513 | nuclear import | Akt1 CG32164 Fs(2)Ket Karybeta3 Pen Ranbp16 hh lwr msk pll rin slmb |
| 247 | GO:0031324 | P | 6, | 26 | 12.989 (x 2.002) | 155 (0.168) | 0.0052 | negative regulation of cellular metabolism | CG11228 CG11305 CG31716 CG33525 CREG Caf1 CtBP E(z) Paip2 Pcl Psc Rbf2 Sin3A Su(var)3-9 Su(z)12 aop brk ci cic edl en hh msi ph-p simj sqd |
| 248 | GO:0016265 | P | 3, | 37 | 21.034 (x 1.759) | 251 (0.147) | 0.00537 | death | Aac11 Akap200 Akt1 CG10473 CG10990 CG11228 CG14217 CG17765 CG6680 CG7263 CG8400 Cyp1 Dp Eip75B Hem Myb PR2 abs aop ap bl br gft kel klu l(1)G0148 l(2)01424 l(2)gl larp mdy pnut qkr54B smi35A th trn wgn zip |
| 249 | GO:0045595 | P | 4, | 11 | 3.520 (x 3.125) | 42 (0.262) | 0.00564 | regulation of cell differentiation | Dr Mbs Ser aop argos hdc kn lqf mbt sdk sty |
| 250 | GO:0009628 | P | 3, | 50 | 31.174 (x 1.604) | 372 (0.134) | 0.00565 | response to abiotic stimulus | 14-3-3epsilon 14-3-3zeta BEST:GH15083 BthD CG11711 CG32640 CG33123 CG40410 CG5789 CG6227 CG6673 CG8588 Cat CycT DnaJ-1 Eip75B Fas2 Fas3 G-salpha60A Galpha49B Hsc70-3 Hsp23 Hsp26 Hsp27 Hsp60 Hsp67Bc Jheh1 Jheh2 Mdr49 Pp1-87B Rop Sema-2a Src64B br crol da endos gfzf gp210 hh l(2)gl mth ogre pain scrib slmb smi21F smi35A ss zip |
| 251 | GO:0043062 | P | 3, | 11 | 3.520 (x 3.125) | 42 (0.262) | 0.00566 | extracellular structure organization and biogenesis | Btk29A Fas2 Got2 Pen Src64B baz fwd hts kel par-6 tkv |
| 252 | GO:0007088 | P | 7, 8, | 10 | 3.017 (x 3.315) | 36 (0.278) | 0.00602 | regulation of mitosis | 14-3-3epsilon CG40410 Cks CycA Myb Nek2 ald alphaTub84B fzy zwilch |
| 253 | GO:0007420 | P | 4, 6, | 12 | 4.106 (x 2.922) | 49 (0.245) | 0.00611 | brain development | CkIIbeta Dr Fas2 Smox Src64B cib dac exd fdl mbt shg stan |
| 254 | GO:0012502 | P | 7, 8, | 15 | 5.866 (x 2.557) | 70 (0.214) | 0.00613 | induction of programmed cell death | CG10473 CG10990 CG11228 CG14217 CG17765 CG7263 CG8400 Eip75B aop bl br gft l(2)gl smi35A zip |
| 255 | GO:0045314 | P | 7, 8, 9, 10, 11, | 3 | 0.251 (x 11.933) | 3 (1.000) | 0.00634 | regulation of eye photoreceptor development (sensu Endopterygota) | Mbs aop mbt |
| 256 | GO:0005914 | C | 8, 9, 10, | 3 | 0.251 (x 11.933) | 3 (1.000) | 0.00636 | spot adherens junction | alpha-Cat arm shg |
| 257 | GO:0035062 | C | 8, 9, 10, 11, 12, 13, 14, 15, | 3 | 0.251 (x 11.933) | 3 (1.000) | 0.00639 | omega speckle | Hrb87F bl sqd |
| 258 | GO:0016335 | P | 5, 6, 7, | 4 | 0.503 (x 7.955) | 6 (0.667) | 0.00673 | morphogenesis of larval imaginal disc epithelium | Moe Sb l(2)gl scrib |
| 259 | GO:0001736 | P | 5, 6, | 11 | 3.603 (x 3.053) | 43 (0.256) | 0.00675 | establishment of planar polarity | Vang argos fz in kay msn pk rin sca stan zip |
| 260 | GO:0005956 | C | 3, 4, 5, 6, | 4 | 0.503 (x 7.955) | 6 (0.667) | 0.00675 | protein kinase CK2 complex | CkIIalpha CkIIbeta Ste12DOR Ste:CG33236 |
| 261 | GO:0007164 | P | 4, | 11 | 3.603 (x 3.053) | 43 (0.256) | 0.00677 | establishment of tissue polarity | Vang argos fz in kay msn pk rin sca stan zip |
| 262 | GO:0008296 | F | 8, 9, | 4 | 0.503 (x 7.955) | 6 (0.667) | 0.00678 | 3'-5'-exodeoxyribonuclease activity | BcDNA:GM10765 DNApol-delta DNApol-epsilon Rrp1 |
| 263 | GO:0007612 | P | 5, | 11 | 3.603 (x 3.053) | 43 (0.256) | 0.0068 | learning | 14-3-3epsilon 14-3-3zeta BEST:GH15083 CG11711 CG8588 Fas2 Fas3 Pp1-87B Src64B gp210 slmb |
| 264 | GO:0045196 | P | 6, 7, | 4 | 0.503 (x 7.955) | 6 (0.667) | 0.00681 | establishment and/or maintenance of neuroblast polarity | baz l(2)gl par-6 zip |
| 265 | GO:0006913 | P | 6, 7, 8, | 15 | 5.950 (x 2.521) | 71 (0.211) | 0.00689 | nucleocytoplasmic transport | Akt1 CG17143 CG2980 CG32164 Fs(2)Ket Karybeta3 Pen Ranbp16 hh lwr msk pll rin slmb sqd |
| 266 | GO:0016585 | C | 3, 6, 7, 8, 9, 10, 11, 12, 13, | 8 | 2.095 (x 3.819) | 25 (0.320) | 0.00726 | chromatin remodeling complex | Bap170 CG11305 Caf1 Iswi Top2 brm dalao mor |
| 267 | GO:0016321 | P | 6, 9, | 8 | 2.095 (x 3.819) | 25 (0.320) | 0.00729 | female meiosis chromosome segregation | CG40410 Gap1 LanA ald east lwr pav sca |
| 268 | GO:0009889 | P | 5, | 16 | 6.620 (x 2.417) | 79 (0.203) | 0.00732 | regulation of biosynthesis | CG17514 CG5434 CG8443 Elf Paip2 RpS6 Vha44 bl eIF-4B eIF3-S9 eIF5 l(2)01424 msi pAbp rin sqd |
| 269 | GO:0031326 | P | 6, | 16 | 6.620 (x 2.417) | 79 (0.203) | 0.00735 | regulation of cellular biosynthesis | CG17514 CG5434 CG8443 Elf Paip2 RpS6 Vha44 bl eIF-4B eIF3-S9 eIF5 l(2)01424 msi pAbp rin sqd |
| 270 | GO:0019901 | F | 6, | 5 | 0.838 (x 5.967) | 10 (0.500) | 0.00737 | protein kinase binding | Akap200 CG1815 CG3249 baz rg |
| 271 | GO:0051653 | P | 5, 6, | 5 | 0.838 (x 5.967) | 10 (0.500) | 0.0074 | spindle localization | Eb1 asp baz chb jar |
| 272 | GO:0051293 | P | 6, 7, 9, | 5 | 0.838 (x 5.967) | 10 (0.500) | 0.00742 | establishment of spindle localization | Eb1 asp baz chb jar |
| 273 | GO:0016332 | P | 6, | 5 | 0.838 (x 5.967) | 10 (0.500) | 0.00745 | establishment and/or maintenance of polarity of embryonic epithelium | baz crb l(2)gl par-6 scrib |
| 274 | GO:0004674 | F | 7, | 32 | 17.766 (x 1.801) | 212 (0.151) | 0.00761 | protein serine/threonine kinase activity | Akt1 BcDNA:LD09009 BcDNA:LD23371 CG11228 CG14217 CG17309 CG40410 CG8789 CaMKII Cdk4 CkIIalpha CkIIbeta CkIalpha Cks CycT Eip63E Gprk1 KP78b Mekk1 Nek2 PFE Pak3 ald cdc2 gish l(1)G0148 mbt msn pll polo smi35A tkv |
| 275 | GO:0012501 | P | 5, | 36 | 20.783 (x 1.732) | 248 (0.145) | 0.00768 | programmed cell death | Aac11 Akap200 Akt1 CG10473 CG10990 CG11228 CG14217 CG17765 CG6680 CG7263 CG8400 Cyp1 Dp Eip75B Hem Myb PR2 abs aop bl br gft kel klu l(1)G0148 l(2)01424 l(2)gl larp mdy pnut qkr54B smi35A th trn wgn zip |
| 276 | GO:0006445 | P | 7, 8, 9, | 15 | 6.034 (x 2.486) | 72 (0.208) | 0.00772 | regulation of translation | CG17514 CG5434 CG8443 Elf Paip2 RpS6 bl eIF-4B eIF3-S9 eIF5 l(2)01424 msi pAbp rin sqd |
| 277 | GO:0006468 | P | 8, | 40 | 23.883 (x 1.675) | 285 (0.140) | 0.00787 | protein amino acid phosphorylation | Ack Akt1 BcDNA:LD09009 BcDNA:LD23371 Btk29A CG11228 CG14217 CG17309 CG40410 CG8789 CaMKII Cdk4 CkIIalpha CkIIbeta CkIalpha Cks Eip63E Gprk1 KP78b Mekk1 Nek2 PFE PR2 Pak3 Src64B Wsck ald cdc2 fat2 gish l(1)G0148 mbt msn otk plexA pll polo smi35A syndapin tkv |
| 278 | GO:0007243 | P | 6, | 22 | 10.643 (x 2.067) | 127 (0.173) | 0.00792 | protein kinase cascade | Btk29A CG14217 CG30440 CG5522 CG8789 Cdc42 Cdk4 Gap1 Gef26 Gprk1 Mekk1 Pak3 Src64B aop arm csul dome kay mbt msn slmb sty |
| 279 | GO:0000165 | P | 7, | 19 | 8.631 (x 2.201) | 103 (0.184) | 0.00805 | MAPKKK cascade | Btk29A CG14217 CG30440 CG5522 CG8789 Cdc42 Gap1 Gef26 Gprk1 Mekk1 Pak3 Src64B aop arm csul kay mbt msn sty |
| 280 | GO:0005057 | F | 3, | 35 | 20.112 (x 1.740) | 240 (0.146) | 0.00817 | receptor signaling protein activity | Akt1 CG11228 CG14217 CG17419 CG31640 CG33113 CG40410 CG5841 CG8789 CG9025 CaMKII Cdk4 CkIIalpha CkIIbeta CkIalpha Eip63E Gprk1 KP78b Mekk1 Nek2 PFE Pak3 Smox argos cdc2 csw gish l(1)G0148 mbt msn pll polo smi35A stan tkv |
| 281 | GO:0007052 | P | 6, 10, | 6 | 1.257 (x 4.773) | 15 (0.400) | 0.00868 | mitotic spindle organization and biogenesis | CG11207 Eb1 chb dpa larp mus209 |
| 282 | GO:0008219 | P | 4, | 36 | 20.950 (x 1.718) | 250 (0.144) | 0.0087 | cell death | Aac11 Akap200 Akt1 CG10473 CG10990 CG11228 CG14217 CG17765 CG6680 CG7263 CG8400 Cyp1 Dp Eip75B Hem Myb PR2 abs aop bl br gft kel klu l(1)G0148 l(2)01424 l(2)gl larp mdy pnut qkr54B smi35A th trn wgn zip |
| 283 | GO:0043412 | P | 6, | 103 | 76.845 (x 1.340) | 917 (0.112) | 0.00904 | biopolymer modification | Ack Akt1 Arf79F BG:DS00004.11 BcDNA:LD09009 BcDNA:LD22910 BcDNA:LD23371 Btk29A CG10542 CG11228 CG12006 CG12130 CG14217 CG14222 CG14670 CG15141 CG1550 CG17309 CG17598 CG1815 CG1906 CG31716 CG32099 CG32632 CG33145 CG40410 CG4238 CG4670 CG5505 CG5841 CG6394 CG7288 CG7922 CG8188 CG8494 CG8789 CG9153 CG9425 CaMKII Caf1 CalpB Cdk4 CkIIalpha CkIIbeta CkIalpha Cks E(z) EG:86E4.2 ERp60 Eip63E Galpha49B Gprk1 KP78b Mekk1 Mi-2 Nek2 Nop60B PFE PR2 Pak3 Pi3K68D Pp1-87B Pp2C1 Psc Ptp99A Src64B Su(dx) Su(var)3-9 Su(z)12 Taf2 Top3beta Uba1 Uba2 UbcD2 UbcD6 Uch Wsck ald cdc2 csw d4 dome fat2 fzy gish gol hh l(1)G0148 lmg lwr mbt msn otk plexA pll polo slmb smi35A stc syndapin th tkv wbl |
| 284 | GO:0051093 | P | 4, | 8 | 2.179 (x 3.672) | 26 (0.308) | 0.00915 | negative regulation of development | ana aop argos ed hdc lqf sdk sty |
| 285 | GO:0005938 | C | 5, 6, 7, 8, | 11 | 3.771 (x 2.917) | 45 (0.244) | 0.00931 | cell cortex | CG5841 alpha-Spec baz fz kel l(2)gl msk par-6 pnut pon zip |
| 286 | GO:0030036 | P | 8, | 18 | 8.129 (x 2.214) | 97 (0.186) | 0.0099 | actin cytoskeleton organization and biogenesis | Abi Cdc42 Cip4 CycT Fs(2)Ket Hem Moe Pak3 Sb Src64B asp ena hts pav pbl pnut trio vav |
| 287 | GO:0016874 | F | 3, | 43 | 26.565 (x 1.619) | 317 (0.136) | 0.00992 | ligase activity | Aats-ala Aats-glupro BcDNA:GH02901 CG10542 CG14670 CG15141 CG1550 CG1815 CG2118 CG31716 CG31739 CG33123 CG4238 CG5191 CG5841 CG6767 CG6835 CG6854 CG7441 CG8188 CG9153 CG9425 Mi-2 Psc Su(dx) Tom34 Top3beta Uba1 Uba2 UbcD2 UbcD6 X11L ade2 ade3 d4 gft gol lmg lwr mdy slmb stc th |
| 288 | GO:0030029 | P | 7, | 18 | 8.129 (x 2.214) | 97 (0.186) | 0.00993 | actin filament-based process | Abi Cdc42 Cip4 CycT Fs(2)Ket Hem Moe Pak3 Sb Src64B asp ena hts pav pbl pnut trio vav |
| 289 | GO:0003712 | F | 3, 5, | 15 | 6.201 (x 2.419) | 74 (0.203) | 0.00995 | transcription cofactor activity | Bap170 CG12190 CG15835 CG31453 CG7008 CtBP Dsp1 E(z) Hcf Sin3A brm cic dalao dre4 mor |
| 290 | GO:0044446 | C | 3, 4, 5, 6, 7, | 150 | 119.332 (x 1.257) | 1424 (0.105) | 0.0102 | intracellular organelle part | AP-1gamma ATPsyn-beta Act42A Act5C Arc92 B52 BEAF-32 BG:DS00004.11 Bap170 BcDNA:GH04962 BcDNA:GH12558 Brf CG10423 CG10695 CG11207 CG11305 CG11856 CG13895 CG1472 CG1746 CG17838 CG17952 CG1907 CG1911 CG2097 CG2118 CG2158 CG31617 CG32164 CG3605 CG5028 CG5728 CG6227 CG6394 CG6543 CG6638 CG7145 CG7263 Caf1 Cap-G Cbp80 Cen190 Chc CycA CycT Cyp1 DNApol-epsilon Dlc90F Dp Dref Dsp1 E(z) Eb1 Fs(2)Ket Fur1 Gdh Got2 HmgD Hrb27C Hrb87F Hsp60 Iswi Karybeta3 Klp10A Lam Map60 Mapmodulin Mcm5 Mcm6 Mcm7 Mi-2 Mpcp Myb Nek2 Nlp Nop60B Ote Pen Pep Psc Ranbp16 Rbf2 Rop RpII140 RpL13 RpL15 RpL22 RpL23a RpL3 RpS12 RpS6 SMC1 Scm Sin3A Spt6 Su(var)3-9 Su(z)12 Taf2 Thiolase Tm1 Tom34 Top2 Ucp4B Vha44 Vha55 alpha-Adaptin alpha-Spec alphaTub84B ap asp barr betaTub56D bl brm cav chb ck d dac dalao dre4 fzy garz gft heph jar jumu katanin-60 kis klar l(1)dd4 lds lin19 lmg mor msk nonA-l nop5 p120ctn pav ph-p pnut polo sec23 sqd stai su(f) toe vg zip |
| 291 | GO:0044422 | C | 2, 3, | 150 | 119.332 (x 1.257) | 1424 (0.105) | 0.0103 | organelle part | AP-1gamma ATPsyn-beta Act42A Act5C Arc92 B52 BEAF-32 BG:DS00004.11 Bap170 BcDNA:GH04962 BcDNA:GH12558 Brf CG10423 CG10695 CG11207 CG11305 CG11856 CG13895 CG1472 CG1746 CG17838 CG17952 CG1907 CG1911 CG2097 CG2118 CG2158 CG31617 CG32164 CG3605 CG5028 CG5728 CG6227 CG6394 CG6543 CG6638 CG7145 CG7263 Caf1 Cap-G Cbp80 Cen190 Chc CycA CycT Cyp1 DNApol-epsilon Dlc90F Dp Dref Dsp1 E(z) Eb1 Fs(2)Ket Fur1 Gdh Got2 HmgD Hrb27C Hrb87F Hsp60 Iswi Karybeta3 Klp10A Lam Map60 Mapmodulin Mcm5 Mcm6 Mcm7 Mi-2 Mpcp Myb Nek2 Nlp Nop60B Ote Pen Pep Psc Ranbp16 Rbf2 Rop RpII140 RpL13 RpL15 RpL22 RpL23a RpL3 RpS12 RpS6 SMC1 Scm Sin3A Spt6 Su(var)3-9 Su(z)12 Taf2 Thiolase Tm1 Tom34 Top2 Ucp4B Vha44 Vha55 alpha-Adaptin alpha-Spec alphaTub84B ap asp barr betaTub56D bl brm cav chb ck d dac dalao dre4 fzy garz gft heph jar jumu katanin-60 kis klar l(1)dd4 lds lin19 lmg mor msk nonA-l nop5 p120ctn pav ph-p pnut polo sec23 sqd stai su(f) toe vg zip |
| 292 | GO:0008355 | P | 6, 7, | 10 | 3.268 (x 3.060) | 39 (0.256) | 0.0103 | olfactory learning | 14-3-3zeta BEST:GH15083 CG11711 CG8588 Fas2 Fas3 Pp1-87B Src64B gp210 slmb |
| 293 | GO:0051726 | P | 5, | 30 | 16.676 (x 1.799) | 199 (0.151) | 0.0104 | regulation of cell cycle | 14-3-3epsilon 14-3-3zeta Abi CG40410 Cdk4 Cks CycA Dp Dref Eb1 Eip63E Hus1-like Myb Nek2 Rbf2 UbcD6 ald alphaTub84B cdc2 ci ebi fzy gft hh jumu l(2)gl lin19 sc scrib zwilch |
| 294 | GO:0019207 | F | 3, | 14 | 5.615 (x 2.493) | 67 (0.209) | 0.0105 | kinase regulator activity | 14-3-3epsilon 14-3-3zeta CG13852 CG17514 CG17919 CkIIbeta Cks CycA CycG CycT Cyp1 Pli Ste12DOR Ste:CG33236 |
| 295 | GO:0000074 | P | 6, | 30 | 16.676 (x 1.799) | 199 (0.151) | 0.0105 | regulation of progression through cell cycle | 14-3-3epsilon 14-3-3zeta Abi CG40410 Cdk4 Cks CycA Dp Dref Eb1 Eip63E Hus1-like Myb Nek2 Rbf2 UbcD6 ald alphaTub84B cdc2 ci ebi fzy gft hh jumu l(2)gl lin19 sc scrib zwilch |
| 296 | GO:0007417 | P | 5, | 21 | 10.224 (x 2.054) | 122 (0.172) | 0.0106 | central nervous system development | CkIIbeta Dr Fas2 Hem LanA Smox Src64B br cib csw dac en exd fdl jing kay mbt sc shg stan trio |
| 297 | GO:0007611 | P | 4, | 13 | 5.028 (x 2.585) | 60 (0.217) | 0.0108 | learning and/or memory | 14-3-3epsilon 14-3-3zeta BEST:GH15083 CG11711 CG8588 CaMKII Fas2 Fas3 G-salpha60A Pp1-87B Src64B gp210 slmb |
| 298 | GO:0016339 | P | 5, | 7 | 1.760 (x 3.978) | 21 (0.333) | 0.0108 | calcium-dependent cell-cell adhesion | CG4509 Cad87A Cat Fas3 fat2 shg stan |
| 299 | GO:0008356 | P | 5, | 12 | 4.441 (x 2.702) | 53 (0.226) | 0.011 | asymmetric cell division | alpha-Adaptin arm asp baz cdc2 chb hh hts jar pon shg tkv |
| 300 | GO:0005813 | C | 5, 6, 7, 8, 9, 10, | 9 | 2.765 (x 3.254) | 33 (0.273) | 0.011 | centrosome | Cen190 Map60 Nek2 chb fzy katanin-60 p120ctn pav polo |
| 301 | GO:0006417 | P | 6, 7, 8, | 15 | 6.285 (x 2.387) | 75 (0.200) | 0.011 | regulation of protein biosynthesis | CG17514 CG5434 CG8443 Elf Paip2 RpS6 bl eIF-4B eIF3-S9 eIF5 l(2)01424 msi pAbp rin sqd |
| 302 | GO:0043565 | F | 5, | 9 | 2.765 (x 3.254) | 33 (0.273) | 0.011 | sequence-specific DNA binding | HmgD Mcm5 Mcm6 Mcm7 Msp-300 Nop60B cav dpa dve |
| 303 | GO:0042058 | P | 5, 6, 9, | 6 | 1.341 (x 4.475) | 16 (0.375) | 0.012 | regulation of epidermal growth factor receptor signaling pathway | S argos ebi ed edl sty |
| 304 | GO:0009408 | P | 4, 5, | 11 | 3.939 (x 2.793) | 47 (0.234) | 0.012 | response to heat | CG32640 CycT DnaJ-1 Hsc70-3 Hsp23 Hsp26 Hsp27 Hsp60 Hsp67Bc mth pain |
| 305 | GO:0031098 | P | 6, | 11 | 3.939 (x 2.793) | 47 (0.234) | 0.0121 | stress-activated protein kinase signaling pathway | Btk29A CG14217 CG8789 Cdc42 Mekk1 Src64B aop arm kay mbt msn |
| 306 | GO:0044453 | C | 4, 5, 6, 7, 8, 9, 10, 11, 12, | 11 | 3.939 (x 2.793) | 47 (0.234) | 0.0121 | nuclear membrane part | CG11856 CG17952 CG2158 CG32164 Fs(2)Ket Karybeta3 Lam Ote Pen Ranbp16 msk |
| 307 | GO:0051234 | P | 4, | 175 | 143.132 (x 1.223) | 1708 (0.102) | 0.0121 | establishment of localization | AP-1gamma ATPsyn-beta Abi Akt1 Amph Arf79F BEST:CK01140 BG:DS07473.1 BicD CG10211 CG10657 CG10695 CG10960 CG11069 CG11451 CG11856 CG12048 CG1418 CG14439 CG1472 CG15835 CG17036 CG17143 CG17184 CG17419 CG1746 CG1893 CG1900 CG1907 CG2108 CG2158 CG2316 CG2852 CG2980 CG32137 CG32164 CG3249 CG33113 CG33214 CG3823 CG4509 CG5064 CG5434 CG5687 CG5789 CG5841 CG6812 CG6838 CG7720 CG8104 CG8155 CG9057 CG9246 CG9342 CG9809 CG9906 Cdc42 Chc Cyp1 DNApol-delta Dlc90F EG:196F3.2 EG:34F3.8 Eb1 Fas3 Fpps Fs(2)Ket Galpha49B Hsp60 Indy Karybeta3 Klp10A Lam LanA LanB2 Mdr49 Mpcp NetA Nrv1 PR2 Pen Pi3K68D Psa Ptp99A Rab10 Ranbp16 RhoBTB Rop S Sdc Sema-1b Sema-2a Sin3A Sip1 Snap Ucp4B Vap-33-1 Vha44 Vha55 X11L alpha-Adaptin alpha-Cat alphaTub84B ap argos asp baz betaTub56D bib bl btsz chb cic ck cora csw cta d dome egl ena fra garz gish gp210 hh jar jing jumu katanin-60 kay kel klar krz l(2)08717 l(2)dtl l(2)gl lola lqf lwr mask msk msn mth ninA nudC otk pain par-6 pav pbl pk plexA pll pnut polo prominin-like rg rin rpk scrib sec23 sgl shg slmb sqd stai syndapin th trio trn tud vg wbl zip |
| 308 | GO:0031965 | C | 5, 6, 7, 8, 9, 10, 11, | 11 | 3.939 (x 2.793) | 47 (0.234) | 0.0122 | nuclear membrane | CG11856 CG17952 CG2158 CG32164 Fs(2)Ket Karybeta3 Lam Ote Pen Ranbp16 msk |
| 309 | GO:0007254 | P | 7, 8, | 11 | 3.939 (x 2.793) | 47 (0.234) | 0.0122 | JNK cascade | Btk29A CG14217 CG8789 Cdc42 Mekk1 Src64B aop arm kay mbt msn |
| 310 | GO:0004004 | F | 5, 11, | 10 | 3.352 (x 2.983) | 40 (0.250) | 0.0122 | ATP-dependent RNA helicase activity | CG6227 CG6418 CG7878 CG7922 Dhh1 Hlc Rm62 abs bel me31B |
| 311 | GO:0007028 | P | 5, | 12 | 4.525 (x 2.652) | 54 (0.222) | 0.0122 | cytoplasm organization and biogenesis | CG5728 Cont Nop60B arm baz cora crb jar l(2)gl nop5 par-6 scrib |
| 312 | GO:0016616 | F | 5, | 16 | 7.039 (x 2.273) | 84 (0.190) | 0.0122 | oxidoreductase activity, acting on the CH-OH group of donors, NAD or NADP as acceptor | BEST:LD22483 BcDNA:GH12558 CG15093 CG1600 CG17121 CG2767 CG31169 CG5028 CG6287 CtBP Es2 Idh LanB2 Pgi Thiolase sgl |
| 313 | GO:0007484 | P | 5, 6, | 4 | 0.587 (x 6.819) | 7 (0.571) | 0.0122 | genitalia development (sensu Endopterygota) | Btk29A Dll Fas2 en |
| 314 | GO:0008186 | F | 10, | 10 | 3.352 (x 2.983) | 40 (0.250) | 0.0123 | RNA-dependent ATPase activity | CG6227 CG6418 CG7878 CG7922 Dhh1 Hlc Rm62 abs bel me31B |
| 315 | GO:0048110 | P | 7, 8, 10, | 12 | 4.525 (x 2.652) | 54 (0.222) | 0.0123 | oocyte construction (sensu Insecta) | 14-3-3epsilon 14-3-3zeta BicD Hrb27C Moe Rop Tm1 capu chb egl sqd tud |
| 316 | GO:0016607 | C | 7, 8, 9, 10, 11, 12, 13, 14, | 4 | 0.587 (x 6.819) | 7 (0.571) | 0.0123 | nuclear speck | B52 Hrb87F bl sqd |
| 317 | GO:0006357 | P | 9, | 66 | 45.839 (x 1.440) | 547 (0.121) | 0.0123 | regulation of transcription from RNA polymerase II promoter | ATbp BEAF-32 BcDNA:LD23876 CG11228 CG12190 CG12299 CG15835 CG30011 CG31716 CG7987 CG8165 CG8426 CG8924 Caf1 CtBP CycT D19A D19B Dll Dp Dr Dref Dsp1 E(z) Eip75B HmgZ Iswi Mi-2 Myb Optix Pcl Psc Rbf2 Sin3A Smox Sox14 TBPH Taf2 XNP aop ap br brk brm cdc2 ci crol d4 da dre4 en grn hh jim kis klu lds lola mor sc sd sima stc toe yps zf30C |
| 318 | GO:0046532 | P | 5, | 4 | 0.587 (x 6.819) | 7 (0.571) | 0.0123 | regulation of photoreceptor cell differentiation | Mbs aop mbt sdk |
| 319 | GO:0040008 | P | 3, | 13 | 5.112 (x 2.543) | 61 (0.213) | 0.0123 | regulation of growth | Akt1 CG11228 CG17309 Cdk4 Fas2 Paip2 brk btsz charybde fat2 hh scylla tkv |
| 320 | GO:0030658 | C | 6, 7, 8, 9, 10, 11, | 4 | 0.587 (x 6.819) | 7 (0.571) | 0.0124 | transport vesicle membrane | AP-1gamma CG1472 alpha-Adaptin sec23 |
| 321 | GO:0045673 | P | 6, | 4 | 0.587 (x 6.819) | 7 (0.571) | 0.0124 | regulation of photoreceptor differentiation (sensu Endopterygota) | Mbs aop mbt sdk |
| 322 | GO:0048806 | P | 4, | 4 | 0.587 (x 6.819) | 7 (0.571) | 0.0124 | genitalia development | Btk29A Dll Fas2 en |
| 323 | GO:0048130 | P | 8, 9, 10, 11, | 4 | 0.587 (x 6.819) | 7 (0.571) | 0.0125 | oocyte microtubule cytoskeleton organization (sensu Insecta) | 14-3-3epsilon 14-3-3zeta BicD chb |
| 324 | GO:0046974 | F | 9, 10, | 4 | 0.587 (x 6.819) | 7 (0.571) | 0.0125 | histone lysine N-methyltransferase activity (H3-K9 specific) | Caf1 E(z) Su(var)3-9 Su(z)12 |
| 325 | GO:0005829 | C | 5, 6, 7, 8, | 28 | 15.419 (x 1.816) | 184 (0.152) | 0.0125 | cytosol | ApepP CG10423 CG9149 CkIIalpha CkIIbeta Cyp1 Elf Eno Karybeta3 Pi3K68D Rbp2 Rop RpL13 RpL15 RpL22 RpL23a RpL3 RpS12 RpS6 Su(var)3-9 cib eIF-4B eIF3-S9 eIF5 l(2)gl larp rin smi35A |
| 326 | GO:0030660 | C | 5, 6, 7, 8, 9, 10, 11, | 4 | 0.587 (x 6.819) | 7 (0.571) | 0.0126 | Golgi-associated vesicle membrane | AP-1gamma CG1472 alpha-Adaptin sec23 |
| 327 | GO:0031267 | F | 6, | 7 | 1.844 (x 3.797) | 22 (0.318) | 0.0133 | small GTPase binding | CG11856 CG1418 Cip4 Ranbp16 Rhp msk vimar |
| 328 | GO:0008544 | P | 5, | 7 | 1.844 (x 3.797) | 22 (0.318) | 0.0133 | epidermis development | Cdc42 Mbs fz hh in ovo pk |
| 329 | GO:0007431 | P | 5, | 19 | 9.134 (x 2.080) | 109 (0.174) | 0.0137 | salivary gland development | Akap200 Cyp1 Eip75B PR2 Sema-2a Ser br brk cora crb exd fat2 klar l(1)G0148 l(2)01424 l(2)gl larp tkv zip |
| 330 | GO:0035272 | P | 4, | 19 | 9.134 (x 2.080) | 109 (0.174) | 0.0138 | exocrine system development | Akap200 Cyp1 Eip75B PR2 Sema-2a Ser br brk cora crb exd fat2 klar l(1)G0148 l(2)01424 l(2)gl larp tkv zip |
| 331 | GO:0016563 | F | 3, | 12 | 4.609 (x 2.604) | 55 (0.218) | 0.0139 | transcriptional activator activity | Bap170 CG7008 Hcf Myb brm ci da dalao lola mor sc sima |
| 332 | GO:0017038 | P | 6, 7, | 12 | 4.609 (x 2.604) | 55 (0.218) | 0.0139 | protein import | Akt1 CG32164 Fs(2)Ket Karybeta3 Pen Ranbp16 hh lwr msk pll rin slmb |
| 333 | GO:0016462 | F | 6, | 64 | 44.498 (x 1.438) | 531 (0.121) | 0.0142 | pyrophosphatase activity | ATPsyn-beta Arf79F BEST:CK01140 CG11069 CG1746 CG1900 CG2108 CG2316 CG31453 CG5789 CG6210 CG6227 CG6391 CG6418 CG7878 CG7922 CG8902 CG9425 Cdc42 Dhh1 Dlc90F Elf G-salpha60A Galpha49B Hlc Hsc70-3 Hsp60 Iswi Mcm5 Mcm6 Mcm7 Mdr49 Mi-2 Mtl NTPase Nlp Nrv1 Rab10 RhoBTB Rm62 SMC1 Sin3A Top2 Vha44 Vha55 abs alphaTub84B bel betaTub56D brm ck cta d dome dpa jar jumu katanin-60 kis klar lds me31B pnut zip |
| 334 | GO:0001709 | P | 5, | 19 | 9.218 (x 2.061) | 110 (0.173) | 0.0152 | cell fate determination | BicD Dr S alpha-Spec aop arm baz cdc2 dome egl fz hts jar jumu msi pon sc tkv tud |
| 335 | GO:0035315 | P | 5, 7, 8, | 6 | 1.425 (x 4.212) | 17 (0.353) | 0.0153 | hair cell differentiation | Cdc42 Mbs fz in ovo pk |
| 336 | GO:0035316 | P | 6, 7, 8, 9, | 6 | 1.425 (x 4.212) | 17 (0.353) | 0.0154 | trichome organization and biogenesis (sensu Insecta) | Cdc42 Mbs fz in ovo pk |
| 337 | GO:0009913 | P | 4, 6, 7, | 6 | 1.425 (x 4.212) | 17 (0.353) | 0.0154 | epidermal cell differentiation | Cdc42 Mbs fz in ovo pk |
| 338 | GO:0016334 | P | 6, | 6 | 1.425 (x 4.212) | 17 (0.353) | 0.0155 | establishment and/or maintenance of polarity of follicular epithelium | Dp crb csw l(2)gl scrib sqd |
| 339 | GO:0017111 | F | 7, | 63 | 43.828 (x 1.437) | 523 (0.120) | 0.0155 | nucleoside-triphosphatase activity | ATPsyn-beta Arf79F BEST:CK01140 CG11069 CG1746 CG1900 CG2108 CG2316 CG31453 CG5789 CG6210 CG6227 CG6418 CG7878 CG7922 CG8902 CG9425 Cdc42 Dhh1 Dlc90F Elf G-salpha60A Galpha49B Hlc Hsc70-3 Hsp60 Iswi Mcm5 Mcm6 Mcm7 Mdr49 Mi-2 Mtl NTPase Nlp Nrv1 Rab10 RhoBTB Rm62 SMC1 Sin3A Top2 Vha44 Vha55 abs alphaTub84B bel betaTub56D brm ck cta d dome dpa jar jumu katanin-60 kis klar lds me31B pnut zip |
| 340 | GO:0048730 | P | 5, 6, | 6 | 1.425 (x 4.212) | 17 (0.353) | 0.0155 | epidermis morphogenesis | Cdc42 Mbs fz in ovo pk |
| 341 | GO:0014016 | P | 4, 7, | 8 | 2.430 (x 3.292) | 29 (0.276) | 0.0166 | neuroblast differentiation | Dr arm baz cdc2 jar jumu pon sc |
| 342 | GO:0007400 | P | 6, 9, | 8 | 2.430 (x 3.292) | 29 (0.276) | 0.0167 | neuroblast fate determination | Dr arm baz cdc2 jar jumu pon sc |
| 343 | GO:0003704 | F | 4, | 15 | 6.620 (x 2.266) | 79 (0.190) | 0.0167 | specific RNA polymerase II transcription factor activity | Dll Dp Eip75B Scm ap br ci da en exd kay kn lola sc sd |
| 344 | GO:0014017 | P | 5, 8, | 8 | 2.430 (x 3.292) | 29 (0.276) | 0.0167 | neuroblast fate commitment | Dr arm baz cdc2 jar jumu pon sc |
| 345 | GO:0016319 | P | 5, 7, | 7 | 1.927 (x 3.632) | 23 (0.304) | 0.0169 | mushroom body development | CkIIbeta Fas2 Smox Src64B dac mbt stan |
| 346 | GO:0004616 | F | 6, | 3 | 0.335 (x 8.950) | 4 (0.750) | 0.0171 | phosphogluconate dehydrogenase (decarboxylating) activity | BEST:LD22483 CG15093 Pgi |
| 347 | GO:0016336 | P | 6, 7, 8, | 3 | 0.335 (x 8.950) | 4 (0.750) | 0.0171 | establishment and/or maintenance of polarity of larval imaginal disc epithelium | Moe l(2)gl scrib |
| 348 | GO:0016803 | F | 5, | 3 | 0.335 (x 8.950) | 4 (0.750) | 0.0171 | ether hydrolase activity | CG10602 Jheh1 Jheh2 |
| 349 | GO:0004463 | F | 6, | 3 | 0.335 (x 8.950) | 4 (0.750) | 0.0172 | leukotriene-A4 hydrolase activity | CG10602 Jheh1 Jheh2 |
| 350 | GO:0006464 | P | 7, | 97 | 73.493 (x 1.320) | 877 (0.111) | 0.0172 | protein modification | Ack Akt1 Arf79F BG:DS00004.11 BcDNA:LD09009 BcDNA:LD22910 BcDNA:LD23371 Btk29A CG10542 CG11228 CG12006 CG12130 CG14217 CG14222 CG14670 CG15141 CG1550 CG17309 CG17598 CG1815 CG1906 CG31716 CG32099 CG32632 CG33145 CG40410 CG4238 CG4670 CG5505 CG5841 CG6394 CG7288 CG8188 CG8494 CG8789 CG9153 CaMKII Caf1 CalpB Cdk4 CkIIalpha CkIIbeta CkIalpha Cks E(z) EG:86E4.2 ERp60 Eip63E Galpha49B Gprk1 KP78b Mekk1 Mi-2 Nek2 PFE PR2 Pak3 Pp1-87B Pp2C1 Psc Ptp99A Src64B Su(dx) Su(var)3-9 Su(z)12 Uba1 Uba2 UbcD2 UbcD6 Uch Wsck ald cdc2 csw d4 dome fat2 fzy gish gol hh l(1)G0148 lmg lwr mbt msn otk plexA pll polo slmb smi35A stc syndapin th tkv wbl |
| 351 | GO:0007167 | P | 6, | 26 | 14.414 (x 1.804) | 172 (0.151) | 0.0172 | enzyme linked receptor protein signaling pathway | Akt1 CG5522 Cdc42 Gap1 PFE Rgl S Smox argos brk csw ebi ed edl knk l(2)gl mask mav msk otk plexA pll sgl slmb sty tkv |
| 352 | GO:0030097 | P | 5, | 13 | 5.363 (x 2.424) | 64 (0.203) | 0.0172 | hemopoiesis | CG30011 Galpha49B Ser Sox14 aop brm exd grn kn lwr nonA-l pll tub |
| 353 | GO:0051018 | F | 7, | 3 | 0.335 (x 8.950) | 4 (0.750) | 0.0172 | protein kinase A binding | Akap200 CG3249 rg |
| 354 | GO:0042478 | P | 7, 8, 9, | 3 | 0.335 (x 8.950) | 4 (0.750) | 0.0173 | regulation of eye photoreceptor cell development | Mbs aop mbt |
| 355 | GO:0019538 | P | 5, | 216 | 182.937 (x 1.181) | 2183 (0.099) | 0.0173 | protein metabolism | AP-1gamma Aats-ala Aats-glupro Ack Akt1 ApepP Arf79F B52 BG:DS00004.11 BcDNA:LD09009 BcDNA:LD22910 BcDNA:LD23371 BcDNA:LD41548 Btk29A CG10423 CG10542 CG10602 CG10657 CG10990 CG11207 CG11228 CG11836 CG12006 CG12130 CG14217 CG14222 CG14231 CG1440 CG14411 CG14670 CG15141 CG1550 CG17036 CG17309 CG17514 CG17598 CG18030 CG1815 CG1906 CG2852 CG2947 CG30025 CG31617 CG31716 CG31739 CG32099 CG32632 CG32640 CG33123 CG33145 CG3328 CG40160 CG40410 CG4238 CG4670 CG4914 CG5191 CG5390 CG5434 CG5505 CG5687 CG5841 CG6394 CG6680 CG6835 CG7263 CG7288 CG7441 CG7720 CG8188 CG8443 CG8494 CG8789 CG8863 CG8963 CG9135 CG9153 CG9425 CG9906 CaMKII Caf1 CalpB Cbp80 Cdc42 Cdk4 CkIIalpha CkIIbeta CkIalpha Cks Cont Cyp1 D19A D19B DnaJ-1 E(z) EG:86E4.2 ERp60 Eb1 Eip63E Elf Fkbp13 Fur1 Galpha49B Got2 Gprk1 Hsc70-3 Hsp23 Hsp26 Hsp27 Hsp60 Hsp67Bc Iswi KP78b LanA Mcr Mekk1 Mi-2 Nek2 Nep2 PFE PR2 Paip2 Pak3 Pp1-87B Pp2C1 Psa Psc Ptp99A Rbp2 RpL13 RpL15 RpL22 RpL23a RpL3 RpS12 RpS6 Sb Set Spn5 Src64B Su(dx) Su(var)3-9 Su(z)12 Top3beta TppII Tpr2 Trap1 Uba1 Uba2 UbcD2 UbcD6 Uch Wsck X11L ald alpha-Adaptin alphaTub84B arm baz betaTub56D bl cdc2 cora crb csw d4 da deltaTry dome dre4 eIF-4B eIF3-S9 eIF5 east ebi ena fat2 fra fwd fzy gft gish gol hh hts l(1)G0148 l(2)01424 l(2)gl larp lin19 lmg lwr mbt mdy msi msn ome otk pAbp par-6 pbl plexA pll polo rin scrib sgl slmb smi35A sqd stc syndapin th tkv tok wbl |
| 356 | GO:0030723 | P | 7, 9, | 3 | 0.335 (x 8.950) | 4 (0.750) | 0.0173 | ovarian fusome organization and biogenesis | asp chb hts |
| 357 | GO:0016584 | P | 11, | 3 | 0.335 (x 8.950) | 4 (0.750) | 0.0174 | nucleosome spacing | Caf1 Iswi Nlp |
| 358 | GO:0000916 | P | 6, | 3 | 0.335 (x 8.950) | 4 (0.750) | 0.0174 | cytokinesis, contractile ring contraction | CG11207 fwd pav |
| 359 | GO:0019955 | F | 4, | 3 | 0.335 (x 8.950) | 4 (0.750) | 0.0175 | cytokine binding | dome tkv wgn |
| 360 | GO:0016564 | F | 3, | 12 | 4.777 (x 2.512) | 57 (0.211) | 0.0177 | transcriptional repressor activity | CG31716 CG33525 CREG CtBP Dsp1 Mi-2 Sin3A aop brk ci en simj |
| 361 | GO:0007286 | P | 5, 8, | 9 | 3.017 (x 2.983) | 36 (0.250) | 0.0178 | spermatid development | Act5C Chc Dlc90F Pen bel heph jar mod th |
| 362 | GO:0016817 | F | 4, | 65 | 45.923 (x 1.415) | 548 (0.119) | 0.0178 | hydrolase activity, acting on acid anhydrides | ATPsyn-beta Arf79F BEST:CK01140 CG11069 CG1354 CG1746 CG1900 CG2108 CG2316 CG31453 CG5789 CG6210 CG6227 CG6391 CG6418 CG7878 CG7922 CG8902 CG9425 Cdc42 Dhh1 Dlc90F Elf G-salpha60A Galpha49B Hlc Hsc70-3 Hsp60 Iswi Mcm5 Mcm6 Mcm7 Mdr49 Mi-2 Mtl NTPase Nlp Nrv1 Rab10 RhoBTB Rm62 SMC1 Sin3A Top2 Vha44 Vha55 abs alphaTub84B bel betaTub56D brm ck cta d dome dpa jar jumu katanin-60 kis klar lds me31B pnut zip |
| 363 | GO:0048515 | P | 4, 7, | 9 | 3.017 (x 2.983) | 36 (0.250) | 0.0178 | spermatid differentiation | Act5C Chc Dlc90F Pen bel heph jar mod th |
| 364 | GO:0016818 | F | 5, | 65 | 45.923 (x 1.415) | 548 (0.119) | 0.0178 | hydrolase activity, acting on acid anhydrides, in phosphorus-containing anhydrides | ATPsyn-beta Arf79F BEST:CK01140 CG11069 CG1354 CG1746 CG1900 CG2108 CG2316 CG31453 CG5789 CG6210 CG6227 CG6391 CG6418 CG7878 CG7922 CG8902 CG9425 Cdc42 Dhh1 Dlc90F Elf G-salpha60A Galpha49B Hlc Hsc70-3 Hsp60 Iswi Mcm5 Mcm6 Mcm7 Mdr49 Mi-2 Mtl NTPase Nlp Nrv1 Rab10 RhoBTB Rm62 SMC1 Sin3A Top2 Vha44 Vha55 abs alphaTub84B bel betaTub56D brm ck cta d dome dpa jar jumu katanin-60 kis klar lds me31B pnut zip |
| 365 | GO:0007447 | P | 4, 5, | 10 | 3.603 (x 2.775) | 43 (0.233) | 0.0184 | imaginal disc pattern formation | Dll Dr Ser ap ci dac en exd hh tkv |
| 366 | GO:0035239 | P | 4, | 13 | 5.447 (x 2.387) | 65 (0.200) | 0.0189 | tube morphogenesis | Fas2 Lac Lam arm barr cora csw hdc pbl sgl shg sty zip |
| 367 | GO:0016604 | C | 6, 7, 8, 9, 10, 11, 12, 13, | 4 | 0.670 (x 5.967) | 8 (0.500) | 0.0195 | nuclear body | B52 Hrb87F bl sqd |
| 368 | GO:0035060 | C | 4, 7, 8, 9, 10, 11, 12, 13, 14, | 4 | 0.670 (x 5.967) | 8 (0.500) | 0.0196 | brahma complex | Bap170 brm dalao mor |
| 369 | GO:0030162 | P | 6, 7, 8, | 8 | 2.514 (x 3.182) | 30 (0.267) | 0.0196 | regulation of proteolysis | CG40410 CkIalpha ebi fzy gft hh pll slmb |
| 370 | GO:0016350 | P | 9, | 4 | 0.670 (x 5.967) | 8 (0.500) | 0.0196 | maintenance of oocyte identity (sensu Insecta) | 14-3-3epsilon 14-3-3zeta baz par-6 |
| 371 | GO:0003702 | F | 3, | 36 | 22.291 (x 1.615) | 266 (0.135) | 0.02 | RNA polymerase II transcription factor activity | Arc92 Brf Dll Dp Eip75B GATAd Iswi Optix Scm Sin3A Spt6 Taf2 XNP ap br brk brm ci cic crol crp da dac dalao en exd grn kay kn lola mor ovo sc sd sima stc |
| 372 | GO:0048732 | P | 4, | 21 | 10.978 (x 1.913) | 131 (0.160) | 0.0209 | gland development | Akap200 Cyp1 Eip75B PR2 Pen Sema-2a Ser br brk cora crb exd fat2 klar kn l(1)G0148 l(2)01424 l(2)gl larp tkv zip |
| 373 | GO:0017145 | P | 5, | 9 | 3.101 (x 2.903) | 37 (0.243) | 0.021 | stem cell division | arm asp baz cdc2 hh jar pon shg tkv |
| 374 | GO:0031497 | P | 10, | 11 | 4.274 (x 2.574) | 51 (0.216) | 0.021 | chromatin assembly | CG11305 CG31617 Caf1 E(z) Iswi Nlp Psc Set Su(var)3-9 Su(z)12 ph-p |
| 375 | GO:0045132 | P | 5, 8, | 9 | 3.101 (x 2.903) | 37 (0.243) | 0.0211 | meiotic chromosome segregation | CG40410 Gap1 LanA ald east lwr pav polo sca |
| 376 | GO:0016458 | P | 6, | 10 | 3.687 (x 2.712) | 44 (0.227) | 0.0214 | gene silencing | CG11305 Caf1 E(z) Psc Rm62 Scm Su(var)3-9 Su(z)12 ph-p vig |
| 377 | GO:0009953 | P | 4, | 14 | 6.201 (x 2.258) | 74 (0.189) | 0.0218 | dorsal/ventral pattern formation | BicD Dp Dr Ser ap csw egl lwr pll serpin-27A sqd tok tub wbl |
| 378 | GO:0019900 | F | 5, | 5 | 1.089 (x 4.590) | 13 (0.385) | 0.0218 | kinase binding | Akap200 CG1815 CG3249 baz rg |
| 379 | GO:0048534 | P | 4, | 14 | 6.201 (x 2.258) | 74 (0.189) | 0.0218 | hemopoietic or lymphoid organ development | CG30011 Galpha49B Pen Ser Sox14 aop brm exd grn kn lwr nonA-l pll tub |
| 380 | GO:0004529 | F | 7, | 5 | 1.089 (x 4.590) | 13 (0.385) | 0.0219 | exodeoxyribonuclease activity | BcDNA:GM10765 DNApol-delta DNApol-epsilon Rrp1 tos |
| 381 | GO:0044430 | C | 4, 5, 6, 7, 8, 9, | 33 | 20.112 (x 1.641) | 240 (0.138) | 0.0223 | cytoskeletal part | Act42A Act5C CG10695 CG11207 Cen190 Dlc90F Eb1 Klp10A Lam Map60 Mapmodulin Nek2 Tm1 alpha-Spec alphaTub84B asp betaTub56D chb ck d fzy jar katanin-60 klar l(1)dd4 nop5 p120ctn pav pnut polo stai vg zip |
| 382 | GO:0006800 | P | 5, | 13 | 5.615 (x 2.315) | 67 (0.194) | 0.0239 | oxygen and reactive oxygen species metabolism | BthD CG10211 CG12199 CG12896 CG33123 CG5873 CG6673 CG7263 CG9027 Cat Sh3beta da mth |
| 383 | GO:0004702 | F | 4, 8, | 26 | 14.833 (x 1.753) | 177 (0.147) | 0.0241 | receptor signaling protein serine/threonine kinase activity | Akt1 CG11228 CG14217 CG40410 CG8789 CaMKII Cdk4 CkIIalpha CkIIbeta CkIalpha Eip63E Gprk1 KP78b Mekk1 Nek2 PFE Pak3 cdc2 gish l(1)G0148 mbt msn pll polo smi35A tkv |
| 384 | GO:0043037 | P | 7, 8, | 27 | 15.587 (x 1.732) | 186 (0.145) | 0.0241 | translation | Aats-ala Aats-glupro CG17514 CG31739 CG33123 CG5191 CG5434 CG7441 CG8443 CG9425 Elf Paip2 Rbp2 RpS6 Su(var)3-9 Top3beta X11L bl eIF-4B eIF3-S9 eIF5 l(2)01424 mdy msi pAbp rin sqd |
| 385 | GO:0009719 | P | 3, | 21 | 11.146 (x 1.884) | 133 (0.158) | 0.0243 | response to endogenous stimulus | 14-3-3epsilon BcDNA:GM10765 CG40410 Caf1 CkIalpha CycG DNApol-delta DNApol-epsilon Eip75B Hus1-like Rrp1 Thd1 Tom34 UbcD6 XNP br l(2)gl lds mus209 tos zip |
| 386 | GO:0015631 | F | 5, | 16 | 7.626 (x 2.098) | 91 (0.176) | 0.0243 | tubulin binding | CG32137 CG32672 Cen190 Eb1 Map60 Mapmodulin alpha-Spec alphaTub84B asp betaTub56D chb jar katanin-60 l(1)dd4 pnut stai |
| 387 | GO:0005700 | C | 6, 7, 8, 9, | 10 | 3.771 (x 2.652) | 45 (0.222) | 0.0247 | polytene chromosome | Caf1 CycT Hrb87F Mi-2 Pep Spt6 Su(z)12 jumu pfk sqd |
| 388 | GO:0016773 | F | 5, | 45 | 29.917 (x 1.504) | 357 (0.126) | 0.0249 | phosphotransferase activity, alcohol group as acceptor | Ack Akt1 BcDNA:LD09009 BcDNA:LD23371 Btk29A CG11228 CG14217 CG17309 CG31640 CG40410 CG8789 CaMKII Cdk4 CkIIalpha CkIIbeta CkIalpha Cks CycT Eip63E Gprk1 KP78b Mekk1 Nek2 PFE PR2 Pak3 Pi3K68D Src64B Wsck ald asp cdc2 dnk fat2 fwd gish l(1)G0148 mbt msn otk plexA pll polo smi35A tkv |
| 389 | GO:0030706 | P | 5, 8, 9, | 6 | 1.592 (x 3.768) | 19 (0.316) | 0.0253 | oocyte differentiation (sensu Insecta) | BicD alpha-Spec asp baz egl hts |
| 390 | GO:0051020 | F | 5, | 7 | 2.095 (x 3.341) | 25 (0.280) | 0.0253 | GTPase binding | CG11856 CG1418 Cip4 Ranbp16 Rhp msk vimar |
| 391 | GO:0045596 | P | 5, | 6 | 1.592 (x 3.768) | 19 (0.316) | 0.0254 | negative regulation of cell differentiation | aop argos hdc lqf sdk sty |
| 392 | GO:0008017 | F | 6, | 14 | 6.369 (x 2.198) | 76 (0.184) | 0.0272 | microtubule binding | CG32137 CG32672 Cen190 Eb1 Map60 Mapmodulin alpha-Spec asp chb jar katanin-60 l(1)dd4 pnut stai |
| 393 | GO:0048111 | P | 6, 8, 9, 11, | 11 | 4.441 (x 2.477) | 53 (0.208) | 0.0274 | oocyte axis determination (sensu Insecta) | 14-3-3epsilon 14-3-3zeta BicD Hrb27C Moe Rop Tm1 capu egl sqd tud |
| 394 | GO:0044237 | P | 4, | 423 | 387.914 (x 1.090) | 4629 (0.091) | 0.0282 | cellular metabolism | 14-3-3zeta ATPsyn-beta ATbp Aats-ala Aats-glupro Ack Acon Acox57D-p Akt1 Ald ApepP Arc92 Arf79F B52 BEAF-32 BEST:LD22483 BG:DS00004.11 Bap170 BcDNA:GH02901 BcDNA:GH02976 BcDNA:GH12558 BcDNA:GM10765 BcDNA:LD09009 BcDNA:LD22910 BcDNA:LD23371 BcDNA:LD23876 BcDNA:LD41548 Brf BthD Btk29A CG10211 CG10354 CG10423 CG10542 CG10602 CG10657 CG10990 CG11055 CG11123 CG11142 CG11207 CG11228 CG11305 CG11836 CG12006 CG12130 CG12190 CG12199 CG12252 CG12299 CG12896 CG14217 CG14222 CG14231 CG1440 CG14670 CG14882 CG15093 CG15141 CG1544 CG1550 CG15835 CG17036 CG17309 CG1746 CG17514 CG17598 CG18030 CG1815 CG1869 CG1906 CG1911 CG2097 CG2118 CG2852 CG2947 CG30011 CG30025 CG31169 CG31258 CG31453 CG31472 CG31559 CG31617 CG31716 CG31739 CG32099 CG32158 CG32632 CG32640 CG33116 CG33123 CG33138 CG33145 CG3328 CG33525 CG3590 CG3605 CG40160 CG40410 CG4238 CG4612 CG4670 CG4749 CG4914 CG5028 CG5191 CG5319 CG5390 CG5434 CG5505 CG5687 CG5728 CG5841 CG5873 CG6227 CG6287 CG6391 CG6394 CG6418 CG6543 CG6638 CG6673 CG6680 CG6767 CG6835 CG6854 CG6904 CG6946 CG7008 CG7145 CG7263 CG7288 CG7441 CG7461 CG7720 CG7878 CG7922 CG7987 CG8036 CG8092 CG8165 CG8188 CG8426 CG8443 CG8494 CG8789 CG8863 CG8924 CG8963 CG9027 CG9135 CG9153 CG9342 CG9425 CG9471 CG9503 CG9906 CREG CaMKII Caf1 CalpB Cat Cbp80 Cdc42 Cdk4 CkIIalpha CkIIbeta CkIalpha Cks CtBP CycG CycT Cyp1 Cyp6v1 D12 D19A D19B DNApol-delta DNApol-epsilon Dhh1 Dll DnaJ-1 Dp Dr Dref Dsp1 E(z) EG:86E4.2 ERp60 ESTS:39C10S Eip55E Eip63E Eip75B Elf Eno Fkbp13 Fpps Fur1 GATAd Galpha49B Gapdh1 Gapdh2 Gdh Gfat1 GlyP Got2 Gprk1 Hcf Hlc HmgD HmgZ Hrb27C Hrb87F Hsc70-3 Hsp23 Hsp26 Hsp27 Hsp60 Hsp67Bc Hus1-like Idh Iswi Jheh1 Jheh2 KP78b LanA M(2)21AB Mcm5 Mcm6 Mcm7 Mcr Mekk1 Mi-2 Mpcp Myb NTPase Nek2 Nep2 Nlp Nop60B Optix PFE PR2 Paf-AHalpha Paip2 Pak3 Pcl Pepck Pgi Pi3K68D Pld Pp1-87B Pp2C1 Psa Psc Ptp99A Rab10 Rbf2 Rbp2 Rm62 RnrL RpII140 RpL13 RpL15 RpL22 RpL23a RpL3 RpS12 RpS6 Rrp1 SMC1 Sb Scm SelD Set Sh3beta Sin3A Smox Sox14 Spn5 Spt6 Src64B Su(dx) Su(var)3-9 Su(z)12 TBPH Taf2 Thd1 Thiolase Tis11 Tom34 Top2 Top3beta TppII Tpr2 Trap1 Trxr-1 Uba1 Uba2 UbcD2 UbcD6 Uch Vha44 Vha55 Wsck X11L XNP abs ade2 ade3 ald alphaTub84B aop ap ash2 bel betaTub56D bl br brk brm cdc2 ci cic crc crol csw d4 da dac dalao deltaTry dnk dome dpa dre4 dve eIF-4B eIF3-S9 eIF5 east ebi edl en ena exd fat2 fra fwd fzy gft gish gol grn heph hh jim jing jumu kay kis klu kn knk l(1)G0148 l(1)G0334 l(1)dd4 l(2)01424 l(2)08717 lds lid lin19 lmg lola lwr mbt mdy me31B mor msi msn mth mus209 nonA-l nop5 olf413 ome otk ovo pAbp pfk ph-p plexA pll polo qkr54B r-l rg rin sc sd serpin-27A sgl sima simj slmb smi35A sqd ss stc su(f) syndapin th tkv toe tok tos vig wbl woc yps zf30C |
| 395 | GO:0000070 | P | 6, 8, | 8 | 2.682 (x 2.983) | 32 (0.250) | 0.0282 | mitotic sister chromatid segregation | CG1911 Cap-G CycA Klp10A Pp1-87B barr chb larp |
| 396 | GO:0000819 | P | 5, | 8 | 2.682 (x 2.983) | 32 (0.250) | 0.0283 | sister chromatid segregation | CG1911 Cap-G CycA Klp10A Pp1-87B barr chb larp |
| 397 | GO:0007143 | P | 8, | 9 | 3.268 (x 2.754) | 39 (0.231) | 0.029 | female meiosis | CG40410 Gap1 LanA ald east lwr pav polo sca |
| 398 | GO:0006261 | P | 8, | 12 | 5.112 (x 2.347) | 61 (0.197) | 0.029 | DNA-dependent DNA replication | DNApol-delta DNApol-epsilon Dref Dsp1 Mcm5 Mcm6 Mcm7 Thd1 Top3beta dpa dre4 mus209 |
| 399 | GO:0005703 | C | 5, 6, 7, 8, 9, 10, | 4 | 0.754 (x 5.304) | 9 (0.444) | 0.0298 | polytene chromosome puff | CycT Hrb87F Pep sqd |
| 400 | GO:0035072 | P | 7, 8, 9, 10, 11, | 4 | 0.754 (x 5.304) | 9 (0.444) | 0.0299 | ecdysone-mediated induction of salivary gland cell autophagic cell death | Eip75B br l(2)gl zip |
| 401 | GO:0004713 | F | 7, | 15 | 7.123 (x 2.106) | 85 (0.176) | 0.0299 | protein-tyrosine kinase activity | Ack Akt1 Btk29A CG17309 CG31640 CG8789 Eip63E KP78b PR2 Src64B Wsck fat2 msn otk plexA |
| 402 | GO:0007632 | P | 4, 6, | 4 | 0.754 (x 5.304) | 9 (0.444) | 0.0299 | visual behavior | G-salpha60A Pp1-87B Sema-2a ogre |
| 403 | GO:0035317 | P | 7, 8, 9, 10, | 5 | 1.173 (x 4.262) | 14 (0.357) | 0.0299 | wing hair organization and biogenesis | Cdc42 Mbs fz in pk |
| 404 | GO:0000785 | C | 5, 6, 7, 8, 9, 10, | 14 | 6.453 (x 2.170) | 77 (0.182) | 0.03 | chromatin | BEAF-32 CG31617 Dsp1 E(z) HmgD Hrb87F Mi-2 Psc Sin3A Su(var)3-9 Su(z)12 bl kis sqd |
| 405 | GO:0045478 | P | 6, | 4 | 0.754 (x 5.304) | 9 (0.444) | 0.03 | fusome organization and biogenesis | alpha-Spec asp chb hts |
| 406 | GO:0035295 | P | 3, | 14 | 6.453 (x 2.170) | 77 (0.182) | 0.0301 | tube development | Fas2 Lac Lam arm barr cora csw hdc lqf pbl sgl shg sty zip |
| 407 | GO:0035161 | P | 5, 6, | 4 | 0.754 (x 5.304) | 9 (0.444) | 0.0301 | imaginal disc lineage restriction | Ser ap en hh |
| 408 | GO:0040001 | P | 6, 7, 8, 10, | 4 | 0.754 (x 5.304) | 9 (0.444) | 0.0302 | establishment of mitotic spindle localization | Eb1 baz chb jar |
| 409 | GO:0045177 | C | 3, 4, | 7 | 2.179 (x 3.213) | 26 (0.269) | 0.0306 | apical part of cell | CG5841 baz crb dome par-6 pnut zip |
| 410 | GO:0007398 | P | 4, | 32 | 19.861 (x 1.611) | 237 (0.135) | 0.0315 | ectoderm development | CG8104 Cdc42 Cont Dll Dr EG:BACH59J11.2 Fas2 Mbs NetA Sema-1b Sema-2a Ser Sox14 ap arm bib crb dac en fra fz hh in msi ovo pk plexA sca sima smi35A stan toe |
| 411 | GO:0007528 | P | 7, 8, | 6 | 1.676 (x 3.580) | 20 (0.300) | 0.032 | neuromuscular junction development | CaMKII Fas2 G-salpha60A Hem Vap-33-1 tkv |
| 412 | GO:0005635 | C | 4, 5, 6, 7, 8, 9, 10, | 12 | 5.196 (x 2.310) | 62 (0.194) | 0.0321 | nuclear envelope | CG11856 CG17952 CG2158 CG32164 Fs(2)Ket Karybeta3 Lam Ote Pen Ranbp16 klar msk |
| 413 | GO:0016879 | F | 4, | 26 | 15.252 (x 1.705) | 182 (0.143) | 0.0327 | ligase activity, forming carbon-nitrogen bonds | CG10542 CG14670 CG15141 CG1550 CG1815 CG31716 CG4238 CG5191 CG5841 CG6767 CG6835 CG6854 CG9153 Mi-2 Psc Su(dx) Uba1 ade2 ade3 d4 gft gol lmg slmb stc th |
| 414 | GO:0040029 | P | 3, | 13 | 5.866 (x 2.216) | 70 (0.186) | 0.0327 | regulation of gene expression, epigenetic | CG11305 Caf1 E(z) Psc Rm62 Su(var)3-9 Su(z)12 Taf2 ash2 brm ph-p sc vig |
| 415 | GO:0042067 | P | 6, 7, 8, 9, | 8 | 2.765 (x 2.893) | 33 (0.242) | 0.0329 | establishment of ommatidial polarity (sensu Endopterygota) | Vang argos fz kay pk rin sca stan |
| 416 | GO:0046552 | P | 5, | 9 | 3.352 (x 2.685) | 40 (0.225) | 0.033 | photoreceptor cell fate commitment | br csw da fz hh kay rin sca stan |
| 417 | GO:0005351 | F | 5, 6, | 9 | 3.352 (x 2.685) | 40 (0.225) | 0.0331 | sugar porter activity | CG10960 CG11451 CG9246 CG9809 LanB2 PR2 Psa alpha-Cat l(2)dtl |
| 418 | GO:0007459 | P | 6, | 9 | 3.352 (x 2.685) | 40 (0.225) | 0.0332 | photoreceptor fate commitment (sensu Endopterygota) | br csw da fz hh kay rin sca stan |
| 419 | GO:0030332 | F | 4, | 3 | 0.419 (x 7.160) | 5 (0.600) | 0.0337 | cyclin binding | Cdk4 Eip63E Set |
| 420 | GO:0008605 | F | 5, | 3 | 0.419 (x 7.160) | 5 (0.600) | 0.0338 | protein kinase CK2 regulator activity | CkIIbeta Ste12DOR Ste:CG33236 |
| 421 | GO:0007485 | P | 6, 7, | 3 | 0.419 (x 7.160) | 5 (0.600) | 0.0338 | male genitalia development (sensu Endopterygota) | Btk29A Fas2 en |
| 422 | GO:0046976 | F | 9, 10, | 3 | 0.419 (x 7.160) | 5 (0.600) | 0.0339 | histone lysine N-methyltransferase activity (H3-K27 specific) | Caf1 E(z) Su(z)12 |
| 423 | GO:0035098 | C | 4, 6, 7, 8, 9, 10, 11, 12, 13, 14, | 3 | 0.419 (x 7.160) | 5 (0.600) | 0.034 | ESC/E(Z) complex | Caf1 E(z) Su(z)12 |
| 424 | GO:0005637 | C | 5, 6, 7, 8, 9, 10, 11, 12, 13, | 3 | 0.419 (x 7.160) | 5 (0.600) | 0.0341 | nuclear inner membrane | CG17952 Lam Ote |
| 425 | GO:0007266 | P | 7, | 3 | 0.419 (x 7.160) | 5 (0.600) | 0.0342 | Rho protein signal transduction | RacGAP50C trio vav |
| 426 | GO:0031507 | P | 11, | 7 | 2.263 (x 3.094) | 27 (0.259) | 0.0365 | heterochromatin formation | CG11305 Caf1 E(z) Psc Su(var)3-9 Su(z)12 ph-p |
| 427 | GO:0006342 | P | 5, 7, 10, 12, | 7 | 2.263 (x 3.094) | 27 (0.259) | 0.0366 | chromatin silencing | CG11305 Caf1 E(z) Psc Su(var)3-9 Su(z)12 ph-p |
| 428 | GO:0007298 | P | 7, 8, 10, | 7 | 2.263 (x 3.094) | 27 (0.259) | 0.0367 | border follicle cell migration (sensu Insecta) | baz dome jar jing par-6 shg th |
| 429 | GO:0002168 | P | 5, | 7 | 2.263 (x 3.094) | 27 (0.259) | 0.0368 | larval development (sensu Insecta) | Eip63E Moe Sb Ser alpha-Spec l(2)gl scrib |
| 430 | GO:0045814 | P | 4, | 7 | 2.263 (x 3.094) | 27 (0.259) | 0.0369 | negative regulation of gene expression, epigenetic | CG11305 Caf1 E(z) Psc Su(var)3-9 Su(z)12 ph-p |
| 431 | GO:0007314 | P | 6, 8, 9, 11, | 11 | 4.693 (x 2.344) | 56 (0.196) | 0.0383 | oocyte anterior/posterior axis determination | BicD Hrb27C Moe Rop Tm1 capu csul gus shg sqd tud |
| 432 | GO:0007307 | P | 9, 10, 11, | 5 | 1.257 (x 3.978) | 15 (0.333) | 0.0384 | chorion gene amplification | Caf1 Dp Mcm6 Myb mus209 |
| 433 | GO:0004715 | F | 8, | 5 | 1.257 (x 3.978) | 15 (0.333) | 0.0385 | non-membrane spanning protein tyrosine kinase activity | Ack Btk29A CG17309 PR2 Src64B |
| 434 | GO:0001763 | P | 4, | 8 | 2.849 (x 2.808) | 34 (0.235) | 0.0385 | morphogenesis of a branching structure | Lam arm csw hdc sgl shg sty tkv |
| 435 | GO:0016324 | C | 4, 5, 6, 7, | 5 | 1.257 (x 3.978) | 15 (0.333) | 0.0386 | apical plasma membrane | baz crb dome par-6 pnut |
| 436 | GO:0007093 | P | 8, 9, | 5 | 1.257 (x 3.978) | 15 (0.333) | 0.0387 | mitotic checkpoint | 14-3-3epsilon CG40410 ald alphaTub84B zwilch |
| 437 | GO:0048489 | P | 6, 7, | 14 | 6.704 (x 2.088) | 80 (0.175) | 0.0392 | synaptic vesicle transport | AP-1gamma Amph Arf79F Chc Rop Snap Vap-33-1 X11L alpha-Adaptin btsz l(2)gl lqf mth syndapin |
| 438 | GO:0043065 | P | 7, 8, | 12 | 5.363 (x 2.237) | 64 (0.188) | 0.0394 | positive regulation of apoptosis | CG10473 CG10990 CG11228 CG14217 CG17765 CG7263 CG8400 Dp aop bl gft smi35A |
| 439 | GO:0050896 | P | 2, | 121 | 97.963 (x 1.235) | 1169 (0.104) | 0.0399 | response to stimulus | 14-3-3epsilon 14-3-3zeta 18w Ack BEST:GH15083 BcDNA:GM10765 BthD Btk29A CG10211 CG10602 CG11652 CG11711 CG11836 CG12199 CG12896 CG17121 CG2852 CG30011 CG32640 CG33123 CG40410 CG5789 CG5873 CG6227 CG6673 CG7263 CG7668 CG8588 CG8863 CG8902 CG9027 CaMKII Caf1 Cat Cdc42 CkIIalpha CkIIbeta CkIalpha CycG CycT Cyp1 DNApol-delta DNApol-epsilon DnaJ-1 EG:BACH59J11.2 Eip75B Fas2 Fas3 G-salpha60A GNBP3 Galpha49B Gdh Hsc70-3 Hsp23 Hsp26 Hsp27 Hsp60 Hsp67Bc Hus1-like Jheh1 Jheh2 Mcr Mdr49 Mekk1 PFE PR2 Pi3K68D Pli Pp1-87B Rop RpS6 Rrp1 Sb Sema-2a Src64B Thd1 Tom34 Trap1 Tsp66E UbcD6 XNP ald br ck crb crol d da dome ebi endos fra gfzf gp210 hh jar kay kn krz l(2)gl lds lig lqf mth mus209 ninA ogre otk pain pll sca scrib serpin-27A slmb smi21F smi35A ss tos trn tub zip |
| 440 | GO:0045892 | P | 9, | 16 | 8.129 (x 1.968) | 97 (0.165) | 0.0406 | negative regulation of transcription, DNA-dependent | CG11228 CG11305 CG33525 Caf1 CtBP E(z) Pcl Psc Rbf2 Sin3A Su(var)3-9 Su(z)12 brk ci ph-p simj |
| 441 | GO:0015630 | C | 6, 7, 8, 9, | 26 | 15.587 (x 1.668) | 186 (0.140) | 0.0409 | microtubule cytoskeleton | BicD CG10695 CG11207 CG31363 Cen190 Dlc90F Eb1 Klp10A Map60 Mapmodulin Nek2 alphaTub84B asp betaTub56D chb fzy jar katanin-60 klar l(1)dd4 nop5 p120ctn pav polo stai vg |
| 442 | GO:0006272 | P | 10, | 2 | 0.168 (x 11.933) | 2 (1.000) | 0.0414 | leading strand elongation | DNApol-delta mus209 |
| 443 | GO:0008309 | F | 9, | 2 | 0.168 (x 11.933) | 2 (1.000) | 0.0415 | double-stranded DNA specific exodeoxyribonuclease activity | BcDNA:GM10765 Rrp1 |
| 444 | GO:0035019 | P | 4, | 2 | 0.168 (x 11.933) | 2 (1.000) | 0.0416 | somatic stem cell maintenance | arm shg |
| 445 | GO:0042623 | F | 9, | 41 | 27.654 (x 1.483) | 330 (0.124) | 0.0416 | ATPase activity, coupled | ATPsyn-beta BEST:CK01140 CG11069 CG1746 CG2316 CG5789 CG6210 CG6227 CG6418 CG7878 CG7922 Dhh1 Dlc90F Hlc Hsp60 Iswi Mcm5 Mcm6 Mcm7 Mdr49 Mi-2 Nlp Nrv1 Rm62 Sin3A Vha44 Vha55 abs bel brm ck d dome dpa jar jumu kis klar lds me31B zip |
| 446 | GO:0035221 | P | 5, 6, | 2 | 0.168 (x 11.933) | 2 (1.000) | 0.0417 | genital disc pattern formation | ci en |
| 447 | GO:0006915 | P | 6, | 25 | 14.917 (x 1.676) | 178 (0.140) | 0.0417 | apoptosis | Aac11 Akt1 CG10473 CG10990 CG11228 CG14217 CG17765 CG6680 CG7263 CG8400 Dp Hem Myb abs aop bl gft kel mdy pnut qkr54B smi35A th trn wgn |
| 448 | GO:0008426 | F | 6, | 2 | 0.168 (x 11.933) | 2 (1.000) | 0.0418 | protein kinase C inhibitor activity | 14-3-3epsilon 14-3-3zeta |
| 449 | GO:0008297 | F | 9, | 2 | 0.168 (x 11.933) | 2 (1.000) | 0.0419 | single-stranded DNA specific exodeoxyribonuclease activity | BcDNA:GM10765 DNApol-delta |
| 450 | GO:0035224 | P | 6, 7, | 2 | 0.168 (x 11.933) | 2 (1.000) | 0.042 | genital disc anterior/posterior pattern formation | ci en |
| 451 | GO:0006607 | P | 8, 9, 10, 11, | 2 | 0.168 (x 11.933) | 2 (1.000) | 0.0421 | NLS-bearing substrate import into nucleus | Fs(2)Ket Pen |
| 452 | GO:0006641 | P | 7, 8, 9, | 2 | 0.168 (x 11.933) | 2 (1.000) | 0.0422 | triacylglycerol metabolism | CG9342 mdy |
| 453 | GO:0045200 | P | 7, 8, | 2 | 0.168 (x 11.933) | 2 (1.000) | 0.0423 | establishment of neuroblast polarity | l(2)gl zip |
| 454 | GO:0016509 | F | 6, | 2 | 0.168 (x 11.933) | 2 (1.000) | 0.0423 | long-chain-3-hydroxyacyl-CoA dehydrogenase activity | BcDNA:GH12558 Thiolase |
| 455 | GO:0045217 | P | 7, | 2 | 0.168 (x 11.933) | 2 (1.000) | 0.0424 | intercellular junction maintenance | crb jar |
| 456 | GO:0051050 | P | 6, 7, | 2 | 0.168 (x 11.933) | 2 (1.000) | 0.0425 | positive regulation of transport | CG5841 slmb |
| 457 | GO:0045313 | P | 9, 10, 11, 12, | 2 | 0.168 (x 11.933) | 2 (1.000) | 0.0426 | rhabdomere membrane biogenesis | Amph Moe |
| 458 | GO:0008311 | F | 9, 10, | 2 | 0.168 (x 11.933) | 2 (1.000) | 0.0427 | double-stranded DNA specific 3'-5' exodeoxyribonuclease activity | BcDNA:GM10765 Rrp1 |
| 459 | GO:0035078 | P | 6, 7, 8, 9, 10, | 4 | 0.838 (x 4.773) | 10 (0.400) | 0.0427 | induction of programmed cell death by ecdysone | Eip75B br l(2)gl zip |
| 460 | GO:0048129 | P | 7, 9, 10, 11, 12, | 2 | 0.168 (x 11.933) | 2 (1.000) | 0.0428 | oocyte microtubule cytoskeleton polarization (sensu Insecta) | 14-3-3epsilon 14-3-3zeta |
| 461 | GO:0016342 | C | 3, 6, 7, 8, | 2 | 0.168 (x 11.933) | 2 (1.000) | 0.0429 | catenin complex | alpha-Cat arm |
| 462 | GO:0008310 | F | 9, 10, | 2 | 0.168 (x 11.933) | 2 (1.000) | 0.043 | single-stranded DNA specific 3'-5' exodeoxyribonuclease activity | BcDNA:GM10765 DNApol-delta |
| 463 | GO:0004863 | F | 7, | 2 | 0.168 (x 11.933) | 2 (1.000) | 0.0431 | diacylglycerol-activated phospholipid-dependent protein kinase C inhibitor activity | 14-3-3epsilon 14-3-3zeta |
| 464 | GO:0008442 | F | 6, | 2 | 0.168 (x 11.933) | 2 (1.000) | 0.0432 | 3-hydroxyisobutyrate dehydrogenase activity | BEST:LD22483 CG15093 |
| 465 | GO:0009950 | P | 5, | 11 | 4.777 (x 2.303) | 57 (0.193) | 0.0432 | dorsal/ventral axis specification | BicD Dp csw egl lwr pll serpin-27A sqd tok tub wbl |
| 466 | GO:0016507 | C | 3, 5, 6, 7, 8, 9, 10, 11, 12, | 2 | 0.168 (x 11.933) | 2 (1.000) | 0.0433 | fatty acid beta-oxidation multienzyme complex | BcDNA:GH12558 Thiolase |
| 467 | GO:0000022 | P | 7, 11, | 2 | 0.168 (x 11.933) | 2 (1.000) | 0.0434 | mitotic spindle elongation | Eb1 chb |
| 468 | GO:0007300 | P | 8, | 7 | 2.346 (x 2.983) | 28 (0.250) | 0.0434 | nurse cell to oocyte transport (sensu Insecta) | Btk29A Dp Pen Src64B hts kel mdy |
| 469 | GO:0031974 | C | 2, | 51 | 36.118 (x 1.412) | 431 (0.118) | 0.0435 | membrane-enclosed lumen | Arc92 B52 Bap170 BcDNA:GH12558 Brf CG11305 CG2097 CG2118 CG5028 CG5728 CG6543 CG6638 CG7145 CG7263 Caf1 CycT Cyp1 Dp Dref E(z) Gdh Got2 Hrb27C Hrb87F Hsp60 Iswi Mcm5 Mcm6 Mcm7 Mi-2 Nlp Nop60B Rbf2 RpII140 Sin3A Su(z)12 Taf2 Thiolase Top2 ap bl brm dac dalao dre4 jumu lds mor sqd su(f) toe |
| 470 | GO:0005819 | C | 5, 6, 7, 8, 9, 10, | 7 | 2.346 (x 2.983) | 28 (0.250) | 0.0435 | spindle | CG11207 Klp10A chb fzy l(1)dd4 pav polo |
| 471 | GO:0043233 | C | 3, 4, | 51 | 36.118 (x 1.412) | 431 (0.118) | 0.0436 | organelle lumen | Arc92 B52 Bap170 BcDNA:GH12558 Brf CG11305 CG2097 CG2118 CG5028 CG5728 CG6543 CG6638 CG7145 CG7263 Caf1 CycT Cyp1 Dp Dref E(z) Gdh Got2 Hrb27C Hrb87F Hsp60 Iswi Mcm5 Mcm6 Mcm7 Mi-2 Nlp Nop60B Rbf2 RpII140 Sin3A Su(z)12 Taf2 Thiolase Top2 ap bl brm dac dalao dre4 jumu lds mor sqd su(f) toe |
| 472 | GO:0044459 | C | 4, 5, 6, | 40 | 27.068 (x 1.478) | 323 (0.124) | 0.0451 | plasma membrane part | CG2097 CG4509 Cad87A Chc Cont EG:196F3.2 Fas3 G-salpha60A Galpha49B Lac Mdr49 Moe Msp-300 Nrv1 Sdc Ser alpha-Adaptin alpha-Cat arm baz bib cora crb cta dome fat2 fz inx2 inx3 l(2)gl mbt ogre p120ctn par-6 pnut scrib sdk shg stan wgn |
| 473 | GO:0008037 | P | 3, | 6 | 1.844 (x 3.254) | 22 (0.273) | 0.0461 | cell recognition | Fas2 Fas3 Galpha49B Sema-2a fra lola |
| 474 | GO:0003713 | F | 4, 6, | 6 | 1.844 (x 3.254) | 22 (0.273) | 0.0462 | transcription coactivator activity | Bap170 CG7008 Hcf brm dalao mor |
| 475 | GO:0008038 | P | 4, | 6 | 1.844 (x 3.254) | 22 (0.273) | 0.0463 | neuron recognition | Fas2 Fas3 Galpha49B Sema-2a fra lola |
| 476 | GO:0006277 | P | 8, | 5 | 1.341 (x 3.729) | 16 (0.312) | 0.0474 | DNA amplification | Caf1 Dp Mcm6 Myb mus209 |
| 477 | GO:0048729 | P | 4, | 9 | 3.603 (x 2.498) | 43 (0.209) | 0.0474 | tissue morphogenesis | Cdc42 Mbs fz gol in ovo pbl pk sgl |
| 478 | GO:0016538 | F | 5, | 5 | 1.341 (x 3.729) | 16 (0.312) | 0.0475 | cyclin-dependent protein kinase regulator activity | Cks CycA CycG CycT Cyp1 |
| 479 | GO:0007367 | P | 6, 7, | 5 | 1.341 (x 3.729) | 16 (0.312) | 0.0476 | segment polarity determination | en hh lgs sgl stan |
| 480 | GO:0016209 | F | 2, | 8 | 3.017 (x 2.652) | 36 (0.222) | 0.0495 | antioxidant activity | CG10211 CG12199 CG12896 CG33123 CG5873 Cat Trxr-1 da |
| 481 | GO:0008026 | F | 4, 10, | 14 | 6.955 (x 2.013) | 83 (0.169) | 0.0496 | ATP-dependent helicase activity | CG6227 CG6418 CG7878 CG7922 Dhh1 Hlc Iswi Mi-2 Rm62 abs bel kis lds me31B |
| 482 | GO:0003678 | F | 4, | 10 | 4.274 (x 2.340) | 51 (0.196) | 0.0504 | DNA helicase activity | CG9425 Iswi Mcm5 Mcm6 Mcm7 Mi-2 XNP brm dpa lds |
| 483 | GO:0042981 | P | 6, 7, | 17 | 9.134 (x 1.861) | 109 (0.156) | 0.0512 | regulation of apoptosis | Aac11 Akt1 CG10473 CG10990 CG11228 CG14217 CG17765 CG7263 CG8400 Dp Myb aop bl gft mdy smi35A th |
| 484 | GO:0015980 | P | 6, | 18 | 9.888 (x 1.820) | 118 (0.153) | 0.052 | energy derivation by oxidation of organic compounds | Acon Ald BEST:LD22483 CG15093 CG1544 CG33138 CG5028 CG6904 CG8036 Eno Gapdh1 Gapdh2 Gdh GlyP Idh Pepck Pgi l(1)G0334 |
| 485 | GO:0016614 | F | 4, | 18 | 9.888 (x 1.820) | 118 (0.153) | 0.0521 | oxidoreductase activity, acting on CH-OH group of donors | BEST:LD22483 BcDNA:GH12558 CG15093 CG1600 CG17121 CG2767 CG31169 CG5028 CG6287 CG7675 CG9503 CtBP Es2 Idh LanB2 Pgi Thiolase sgl |
| 486 | GO:0051321 | P | 5, | 15 | 7.710 (x 1.946) | 92 (0.163) | 0.0522 | meiotic cell cycle | CG40410 Gap1 LanA Top2 ald asp cdc2 east fwd l(1)dd4 lwr pav pbl polo sca |
| 487 | GO:0030708 | P | 7, 8, 9, | 3 | 0.503 (x 5.967) | 6 (0.500) | 0.0546 | female germ-line cyst encapsulation (sensu Insecta) | Scm ph-p shg |
| 488 | GO:0000796 | C | 3, 5, 6, 7, 8, 9, 10, | 3 | 0.503 (x 5.967) | 6 (0.500) | 0.0547 | condensin complex | CG1911 Cap-G barr |
| 489 | GO:0007487 | P | 6, | 3 | 0.503 (x 5.967) | 6 (0.500) | 0.0548 | analia development (sensu Endopterygota) | Dll en hh |
| 490 | GO:0045175 | P | 6, | 3 | 0.503 (x 5.967) | 6 (0.500) | 0.0549 | basal protein localization | baz jar l(2)gl |
| 491 | GO:0007352 | P | 6, | 3 | 0.503 (x 5.967) | 6 (0.500) | 0.055 | zygotic determination of dorsal/ventral axis | lwr pll tub |
| 492 | GO:0045170 | C | 5, 6, 7, 8, | 3 | 0.503 (x 5.967) | 6 (0.500) | 0.0551 | spectrosome | CycA alpha-Spec hts |
| 493 | GO:0019904 | F | 4, | 4 | 0.922 (x 4.339) | 11 (0.364) | 0.056 | protein domain specific binding | 14-3-3epsilon 14-3-3zeta Ack rin |
| 494 | GO:0051225 | P | 7, 10, | 4 | 0.922 (x 4.339) | 11 (0.364) | 0.0561 | spindle assembly | Eb1 larp pbl polo |
| 495 | GO:0035081 | P | 8, 9, | 4 | 0.922 (x 4.339) | 11 (0.364) | 0.0563 | induction of programmed cell death by hormones | Eip75B br l(2)gl zip |
| 496 | GO:0004680 | F | 8, | 4 | 0.922 (x 4.339) | 11 (0.364) | 0.0564 | casein kinase activity | CkIIalpha CkIIbeta CkIalpha gish |
| 497 | GO:0004536 | F | 6, | 8 | 3.101 (x 2.580) | 37 (0.216) | 0.0568 | deoxyribonuclease activity | BcDNA:GM10765 CG7922 CG9425 DNApol-delta DNApol-epsilon Rrp1 Top3beta tos |
| 498 | GO:0006979 | P | 4, 5, 6, | 5 | 1.425 (x 3.510) | 17 (0.294) | 0.0599 | response to oxidative stress | BthD CG33123 Cat da mth |
| 499 | GO:0000059 | P | 8, 9, 10, 11, | 5 | 1.425 (x 3.510) | 17 (0.294) | 0.06 | protein import into nucleus, docking | CG32164 Fs(2)Ket Pen Ranbp16 msk |
| 500 | GO:0005096 | F | 4, | 9 | 3.771 (x 2.387) | 45 (0.200) | 0.0614 | GTPase activator activity | CG30372 CG6838 CG8243 CdGAPr Gap1 RacGAP50C RhoGAP1A sec23 vav |
| 501 | GO:0019898 | C | 4, 5, 6, | 9 | 3.771 (x 2.387) | 45 (0.200) | 0.0615 | extrinsic to membrane | G-salpha60A Galpha49B ImpE2 ImpE3 Lac Snap alpha-Cat arm cta |
| 502 | GO:0016887 | F | 8, | 44 | 31.090 (x 1.415) | 371 (0.119) | 0.0625 | ATPase activity | ATPsyn-beta BEST:CK01140 CG11069 CG1746 CG2316 CG5789 CG6210 CG6227 CG6418 CG7878 CG7922 Dhh1 Dlc90F Hlc Hsc70-3 Hsp60 Iswi Mcm5 Mcm6 Mcm7 Mdr49 Mi-2 Nlp Nrv1 Rm62 SMC1 Sin3A Top2 Vha44 Vha55 abs bel brm ck d dome dpa jar jumu kis klar lds me31B zip |
| 503 | GO:0009952 | P | 4, | 19 | 10.894 (x 1.744) | 130 (0.146) | 0.0647 | anterior/posterior pattern formation | BicD Gap1 Hrb27C Moe Rop Tm1 capu ci csul csw en gus hh knk shg sqd sty tkv tud |
| 504 | GO:0008094 | F | 10, | 8 | 3.184 (x 2.512) | 38 (0.211) | 0.0659 | DNA-dependent ATPase activity | Iswi Mcm5 Mcm6 Mcm7 Mi-2 brm dpa lds |
| 505 | GO:0030261 | P | 7, | 6 | 2.011 (x 2.983) | 24 (0.250) | 0.0677 | chromosome condensation | CG1911 Cap-G Mcm5 Pp1-87B barr larp |
| 506 | GO:0048754 | P | 5, | 7 | 2.598 (x 2.695) | 31 (0.226) | 0.0686 | branching morphogenesis of a tube | Lam arm csw hdc sgl shg sty |
| 507 | GO:0006917 | P | 8, 9, | 11 | 5.196 (x 2.117) | 62 (0.177) | 0.0712 | induction of apoptosis | CG10473 CG10990 CG11228 CG14217 CG17765 CG7263 CG8400 aop bl gft smi35A |
| 508 | GO:0008152 | P | 3, | 453 | 423.780 (x 1.069) | 5057 (0.090) | 0.0718 | metabolism | 14-3-3zeta AP-1gamma ATPsyn-beta ATbp Aats-ala Aats-glupro Abi Ack Acon Acox57D-p Akt1 Ald ApepP Arc92 Arf79F B52 BEAF-32 BEST:CK01140 BEST:LD22483 BG:DS00004.11 Bap170 BcDNA:GH02901 BcDNA:GH02976 BcDNA:GH04962 BcDNA:GH12558 BcDNA:GM10765 BcDNA:LD09009 BcDNA:LD22910 BcDNA:LD23371 BcDNA:LD23876 BcDNA:LD41548 Brf BthD Btk29A CG10211 CG10354 CG10423 CG10542 CG10602 CG10657 CG10960 CG10990 CG11055 CG11123 CG11142 CG11207 CG11228 CG1129 CG11305 CG11836 CG12006 CG12130 CG12190 CG12199 CG12252 CG12299 CG12896 CG14217 CG14222 CG14231 CG1440 CG14411 CG14670 CG14882 CG15093 CG15141 CG1544 CG1550 CG15835 CG17036 CG17121 CG17309 CG1746 CG17514 CG17598 CG18030 CG1815 CG1869 CG1893 CG1906 CG1911 CG2097 CG2118 CG2316 CG2852 CG2947 CG30011 CG30025 CG31169 CG31258 CG31453 CG31472 CG31559 CG31617 CG31716 CG31739 CG32099 CG32158 CG32632 CG32640 CG33116 CG33123 CG33138 CG33145 CG3328 CG33525 CG3590 CG3605 CG40160 CG40410 CG4238 CG4612 CG4670 CG4749 CG4914 CG5028 CG5191 CG5319 CG5390 CG5434 CG5505 CG5687 CG5728 CG5841 CG5873 CG6227 CG6287 CG6391 CG6394 CG6418 CG6543 CG6638 CG6673 CG6680 CG6767 CG6835 CG6854 CG6904 CG6946 CG7008 CG7145 CG7263 CG7288 CG7441 CG7461 CG7675 CG7720 CG7878 CG7922 CG7987 CG8036 CG8092 CG8165 CG8188 CG8426 CG8443 CG8494 CG8789 CG8863 CG8924 CG8963 CG9027 CG9057 CG9135 CG9153 CG9342 CG9425 CG9471 CG9503 CG9906 CREG CaMKII Caf1 CalpB Cat Cbp80 Cdc42 Cdk4 CkIIalpha CkIIbeta CkIalpha Cks Cont CtBP CycG CycT Cyp1 Cyp6v1 D12 D19A D19B DNApol-delta DNApol-epsilon Dhh1 Dll DnaJ-1 Dp Dr Dref Dsp1 E(z) EG:86E4.2 ERp60 ESTS:39C10S Eb1 Eip55E Eip63E Eip75B Elf Eno Fkbp13 Fpps Fur1 GATAd GNBP3 Galpha49B Gapdh1 Gapdh2 Gdh Gfat1 GlyP Got2 Gprk1 Hcf Hexo1 Hlc HmgD HmgZ Hmu Hrb27C Hrb87F Hsc70-3 Hsp23 Hsp26 Hsp27 Hsp60 Hsp67Bc Hus1-like Idh Iswi Jheh1 Jheh2 KP78b LanA M(2)21AB Mcm5 Mcm6 Mcm7 Mcr Mekk1 Mi-2 Mpcp Myb NTPase Nek2 Nep2 Nlp Nop60B Optix PFE PR2 Paf-AHalpha Paip2 Pak3 Pcl Pepck Pgi Pi3K68D Pld Pp1-87B Pp2C1 Psa Psc Ptp99A Rab10 Rbf2 Rbp2 Rm62 RnrL RpII140 RpL13 RpL15 RpL22 RpL23a RpL3 RpS12 RpS6 Rrp1 SMC1 Sb Scm SelD Set Sh3beta Sin3A Smox Sox14 Spn5 Spt6 Src64B Su(dx) Su(var)3-9 Su(z)12 TBPH Taf2 Thd1 Thiolase Tis11 Tom34 Top2 Top3beta TppII Tpr2 Trap1 Trxr-1 Uba1 Uba2 UbcD2 UbcD6 Uch Vha44 Vha55 Wsck X11L XNP abs ade2 ade3 ald alpha-Adaptin alphaTub84B aop ap arm ash2 baz bel betaTub56D bl br brk brm cdc2 ci cic cora crb crc crol csw d4 da dac dalao deltaTry dnk dome dpa dre4 dve eIF-4B eIF3-S9 eIF5 east ebi edl en ena exd fat2 fbp fdl fra fwd fzy gft gish gol grn heph hh hts jim jing jumu kay kis klu kn knk l(1)G0148 l(1)G0334 l(1)dd4 l(2)01424 l(2)08717 l(2)gl larp lds lid lin19 lmg lola lwr mbt mdy me31B mor msi msn mth mus209 nonA-l nop5 olf413 ome otk ovo pAbp par-6 pbl pfk ph-p plexA pll polo qkr54B r-l rg rin sc scrib sd serpin-27A sgl sima simj slmb smi35A sqd ss stc su(f) syndapin th tkv toe tok tos vig wbl woc yps zf30C |
| 509 | GO:0045179 | C | 4, 5, 6, 7, 8, 9, 10, | 4 | 1.006 (x 3.978) | 12 (0.333) | 0.0749 | apical cortex | CG5841 baz par-6 zip |
| 510 | GO:0003779 | F | 5, | 18 | 10.307 (x 1.746) | 123 (0.146) | 0.075 | actin binding | Aats-glupro CG1826 Hsp23 MICAL-like Moe Msp-300 Tm1 alpha-Cat alpha-Spec capu cib ck cora ena hts jar kel pnut |
| 511 | GO:0031523 | C | 3, 5, 6, 7, 8, 9, 10, | 4 | 1.006 (x 3.978) | 12 (0.333) | 0.0751 | Myb complex | Caf1 Dp Myb Rbf2 |
| 512 | GO:0007294 | P | 6, 7, 9, 10, | 5 | 1.508 (x 3.315) | 18 (0.278) | 0.0751 | oocyte fate determination (sensu Insecta) | BicD alpha-Spec baz egl hts |
| 513 | GO:0035172 | P | 5, 6, | 4 | 1.006 (x 3.978) | 12 (0.333) | 0.0752 | hemocyte proliferation (sensu Arthropoda) | brm lwr pll tub |
| 514 | GO:0007222 | P | 7, | 5 | 1.508 (x 3.315) | 18 (0.278) | 0.0753 | frizzled signaling pathway | CkIIalpha fz lgs slmb stan |
| 515 | GO:0007173 | P | 8, | 8 | 3.268 (x 2.448) | 39 (0.205) | 0.0753 | epidermal growth factor receptor signaling pathway | S argos csw ebi ed edl msk sty |
| 516 | GO:0048100 | P | 6, 7, | 4 | 1.006 (x 3.978) | 12 (0.333) | 0.0754 | wing disc anterior/posterior pattern formation | ci en hh tkv |
| 517 | GO:0009954 | P | 4, | 5 | 1.508 (x 3.315) | 18 (0.278) | 0.0754 | proximal/distal pattern formation | Dll ap dac exd hh |
| 518 | GO:0016082 | P | 8, 9, 10, | 4 | 1.006 (x 3.978) | 12 (0.333) | 0.0755 | synaptic vesicle priming | Rop Snap Vap-33-1 l(2)gl |
| 519 | GO:0042659 | P | 5, 6, | 5 | 1.508 (x 3.315) | 18 (0.278) | 0.0756 | regulation of cell fate specification | Dr argos hdc lqf sty |
| 520 | GO:0007449 | P | 5, 6, | 5 | 1.508 (x 3.315) | 18 (0.278) | 0.0757 | proximal/distal pattern formation, imaginal disc | Dll ap dac exd hh |
| 521 | GO:0051119 | F | 4, | 9 | 3.939 (x 2.285) | 47 (0.191) | 0.0781 | sugar transporter activity | CG10960 CG11451 CG9246 CG9809 LanB2 PR2 Psa alpha-Cat l(2)dtl |
| 522 | GO:0016044 | P | 5, | 7 | 2.682 (x 2.610) | 32 (0.219) | 0.0785 | membrane organization and biogenesis | CG1893 Lam Ote alpha-Spec crb jar klar |
| 523 | GO:0030031 | P | 6, 7, | 7 | 2.682 (x 2.610) | 32 (0.219) | 0.0787 | cell projection biogenesis | Abi CG7161 CaMKII Cdc42 Hem hh shg |
| 524 | GO:0016055 | P | 6, | 10 | 4.609 (x 2.170) | 55 (0.182) | 0.0788 | Wnt receptor signaling pathway | CkIIalpha CkIalpha RacGAP50C Wnt2 arm fz lgs sgl slmb stan |
| 525 | GO:0016684 | F | 4, | 7 | 2.682 (x 2.610) | 32 (0.219) | 0.0788 | oxidoreductase activity, acting on peroxide as acceptor | CG10211 CG12199 CG12896 CG33123 CG5873 Cat da |
| 526 | GO:0004601 | F | 3, 5, | 7 | 2.682 (x 2.610) | 32 (0.219) | 0.079 | peroxidase activity | CG10211 CG12199 CG12896 CG33123 CG5873 Cat da |
| 527 | GO:0016796 | F | 7, | 6 | 2.095 (x 2.864) | 25 (0.240) | 0.0794 | exonuclease activity, active with either ribo- or deoxyribonucleic acids and producing 5'-phosphomonoesters | BcDNA:GM10765 CG10354 DNApol-delta DNApol-epsilon Rrp1 tos |
| 528 | GO:0043566 | F | 5, | 6 | 2.095 (x 2.864) | 25 (0.240) | 0.0795 | structure-specific DNA binding | BEAF-32 Dsp1 Hrb27C Pep Thd1 stc |
| 529 | GO:0035102 | C | 4, 6, 7, 8, 9, 10, 11, | 3 | 0.587 (x 5.114) | 7 (0.429) | 0.0803 | PRC1 complex | Psc Scm ph-p |
| 530 | GO:0000922 | C | 5, 6, 7, 8, 9, 10, 11, | 3 | 0.587 (x 5.114) | 7 (0.429) | 0.0804 | spindle pole | chb l(1)dd4 polo |
| 531 | GO:0016199 | P | 6, 8, 9, 11, 12, 14, | 3 | 0.587 (x 5.114) | 7 (0.429) | 0.0806 | axon midline choice point recognition | Galpha49B fra lola |
| 532 | GO:0006714 | P | 6, 8, 9, 10, | 3 | 0.587 (x 5.114) | 7 (0.429) | 0.0807 | sesquiterpenoid metabolism | Jheh1 Jheh2 Vha44 |
| 533 | GO:0005915 | C | 7, 8, 9, 10, | 3 | 0.587 (x 5.114) | 7 (0.429) | 0.0809 | zonula adherens | alpha-Cat arm shg |
| 534 | GO:0035089 | P | 7, 8, | 3 | 0.587 (x 5.114) | 7 (0.429) | 0.081 | establishment of apical/basal cell polarity | baz crb fz |
| 535 | GO:0042766 | P | 10, | 3 | 0.587 (x 5.114) | 7 (0.429) | 0.0812 | nucleosome mobilization | Caf1 Iswi Mi-2 |
| 536 | GO:0016801 | F | 4, | 3 | 0.587 (x 5.114) | 7 (0.429) | 0.0813 | hydrolase activity, acting on ether bonds | CG10602 Jheh1 Jheh2 |
| 537 | GO:0035288 | P | 6, 7, | 3 | 0.587 (x 5.114) | 7 (0.429) | 0.0815 | anterior head segmentation | en hh kn |
| 538 | GO:0009081 | P | 7, 8, | 3 | 0.587 (x 5.114) | 7 (0.429) | 0.0816 | branched chain family amino acid metabolism | CG15093 CG2118 CG6638 |
| 539 | GO:0005643 | C | 3, 5, 6, 7, 8, 9, 10, 11, 12, 13, | 8 | 3.352 (x 2.387) | 40 (0.200) | 0.0817 | nuclear pore | CG11856 CG2158 CG32164 Fs(2)Ket Karybeta3 Pen Ranbp16 msk |
| 540 | GO:0006716 | P | 6, 7, 9, 10, 11, | 3 | 0.587 (x 5.114) | 7 (0.429) | 0.0818 | juvenile hormone metabolism | Jheh1 Jheh2 Vha44 |
| 541 | GO:0046930 | C | 6, 7, 8, | 8 | 3.352 (x 2.387) | 40 (0.200) | 0.0819 | pore complex | CG11856 CG2158 CG32164 Fs(2)Ket Karybeta3 Pen Ranbp16 msk |
| 542 | GO:0048138 | P | 5, 6, | 3 | 0.587 (x 5.114) | 7 (0.429) | 0.0819 | germ-line cyst encapsulation | Scm ph-p shg |
| 543 | GO:0006267 | P | 9, | 3 | 0.587 (x 5.114) | 7 (0.429) | 0.0821 | pre-replicative complex formation and maintenance | Mcm5 Mcm6 Mcm7 |
| 544 | GO:0035097 | C | 3, 6, 7, 8, 9, 10, 11, 12, 13, | 3 | 0.587 (x 5.114) | 7 (0.429) | 0.0822 | histone methyltransferase complex | Caf1 E(z) Su(z)12 |
| 545 | GO:0030865 | P | 7, | 3 | 0.587 (x 5.114) | 7 (0.429) | 0.0824 | cortical cytoskeleton organization and biogenesis | Abi Cdc42 Hem |
| 546 | GO:0030866 | P | 8, 9, | 3 | 0.587 (x 5.114) | 7 (0.429) | 0.0825 | cortical actin cytoskeleton organization and biogenesis | Abi Cdc42 Hem |
| 547 | GO:0048139 | P | 6, 7, | 3 | 0.587 (x 5.114) | 7 (0.429) | 0.0827 | female germ-line cyst encapsulation | Scm ph-p shg |
| 548 | GO:0030530 | C | 4, 5, 6, 7, 8, 9, 10, | 3 | 0.587 (x 5.114) | 7 (0.429) | 0.0829 | heterogeneous nuclear ribonucleoprotein complex | CG17838 Hrb87F Pep |
| 549 | GO:0006268 | P | 9, | 3 | 0.587 (x 5.114) | 7 (0.429) | 0.083 | DNA unwinding during replication | Dsp1 Top3beta dre4 |
| 550 | GO:0016461 | C | 4, 6, 7, 8, 9, 10, 11, | 3 | 0.587 (x 5.114) | 7 (0.429) | 0.0832 | unconventional myosin | ck jar zip |
| 551 | GO:0051761 | P | 7, | 3 | 0.587 (x 5.114) | 7 (0.429) | 0.0833 | sesquiterpene metabolism | Jheh1 Jheh2 Vha44 |
| 552 | GO:0012505 | C | 4, 5, | 20 | 12.067 (x 1.657) | 144 (0.139) | 0.0845 | endomembrane system | AP-1gamma BG:DS00004.11 CG11856 CG1472 CG17952 CG2158 CG32164 Chc Fs(2)Ket Fur1 Karybeta3 Lam Ote Pen Ranbp16 Rop alpha-Adaptin klar msk sec23 |
| 553 | GO:0016301 | F | 5, | 46 | 33.604 (x 1.369) | 401 (0.115) | 0.0858 | kinase activity | Ack Akt1 BcDNA:LD09009 BcDNA:LD23371 Btk29A CG11228 CG14217 CG17309 CG31640 CG40410 CG6767 CG8789 CaMKII Cdk4 CkIIalpha CkIIbeta CkIalpha Cks CycT Eip63E Gprk1 KP78b Mekk1 Nek2 PFE PR2 Pak3 Pi3K68D Src64B Wsck ald asp cdc2 dnk fat2 fwd gish l(1)G0148 mbt msn otk plexA pll polo smi35A tkv |
| 554 | GO:0050839 | F | 4, | 5 | 1.592 (x 3.140) | 19 (0.263) | 0.0876 | cell adhesion molecule binding | alpha-Cat fat2 otk shg stan |
| 555 | GO:0042706 | P | 6, 7, 8, | 7 | 2.765 (x 2.531) | 33 (0.212) | 0.0876 | eye photoreceptor cell fate commitment | csw da fz hh kay sca stan |
| 556 | GO:0000075 | P | 7, | 5 | 1.592 (x 3.140) | 19 (0.263) | 0.0877 | cell cycle checkpoint | 14-3-3epsilon CG40410 ald alphaTub84B zwilch |
| 557 | GO:0001752 | P | 7, 8, 9, 10, | 7 | 2.765 (x 2.531) | 33 (0.212) | 0.0878 | eye photoreceptor fate commitment (sensu Endopterygota) | csw da fz hh kay sca stan |
| 558 | GO:0044448 | C | 5, 6, 7, 8, 9, | 7 | 2.765 (x 2.531) | 33 (0.212) | 0.088 | cell cortex part | CG5841 alpha-Spec baz par-6 pnut pon zip |
| 559 | GO:0051128 | P | 5, | 6 | 2.179 (x 2.754) | 26 (0.231) | 0.0907 | regulation of cell organization and biogenesis | CG11207 CaMKII Src64B dre4 ena fwd |
| 560 | GO:0003680 | F | 6, | 2 | 0.251 (x 7.955) | 3 (0.667) | 0.0915 | AT DNA binding | HmgD dve |
| 561 | GO:0042309 | P | 5, | 2 | 0.251 (x 7.955) | 3 (0.667) | 0.0917 | homoiothermy | CG6227 crol |
| 562 | GO:0035183 | C | 3, 4, 5, 6, | 2 | 0.251 (x 7.955) | 3 (0.667) | 0.0918 | ring canal inner rim | hts kel |
| 563 | GO:0005080 | F | 7, | 2 | 0.251 (x 7.955) | 3 (0.667) | 0.092 | protein kinase C binding | CG1815 baz |
| 564 | GO:0050825 | F | 4, | 2 | 0.251 (x 7.955) | 3 (0.667) | 0.0921 | ice binding | CG6227 crol |
| 565 | GO:0008024 | C | 4, 7, 8, 9, 10, 11, 12, 13, 14, | 2 | 0.251 (x 7.955) | 3 (0.667) | 0.0923 | transcription elongation factor complex b | CycT Cyp1 |
| 566 | GO:0043146 | P | 8, 9, 10, 11, 13, | 2 | 0.251 (x 7.955) | 3 (0.667) | 0.0924 | spindle stabilization | CG11207 fwd |
| 567 | GO:0051327 | P | 6, | 14 | 7.626 (x 1.836) | 91 (0.154) | 0.0925 | M phase of meiotic cell cycle | CG40410 Gap1 LanA Top2 ald cdc2 east fwd l(1)dd4 lwr pav pbl polo sca |
| 568 | GO:0004301 | F | 7, | 2 | 0.251 (x 7.955) | 3 (0.667) | 0.0926 | epoxide hydrolase activity | Jheh1 Jheh2 |
| 569 | GO:0016279 | F | 7, 8, | 4 | 1.089 (x 3.672) | 13 (0.308) | 0.0926 | protein-lysine N-methyltransferase activity | Caf1 E(z) Su(var)3-9 Su(z)12 |
| 570 | GO:0042221 | P | 4, | 32 | 22.040 (x 1.452) | 263 (0.122) | 0.0926 | response to chemical stimulus | 14-3-3zeta BEST:GH15083 BthD CG11711 CG33123 CG5789 CG6673 CG8588 Cat Eip75B Fas2 Fas3 G-salpha60A Jheh1 Jheh2 Mdr49 Pp1-87B Src64B br da endos gfzf gp210 hh l(2)gl mth scrib slmb smi21F smi35A ss zip |
| 571 | GO:0008150 | P | 1, | 693 | 677.278 (x 1.023) | 8082 (0.086) | 0.0927 | biological\_process | 14-3-3epsilon 14-3-3zeta 18w AP-1gamma ATPsyn-beta ATbp Aac11 Aats-ala Aats-glupro Abi Ack Acon Acox57D-p Act42A Act5C Akap200 Akt1 Ald Amph ApepP Arc92 Arf79F Atu B52 BEAF-32 BEST:CK01140 BEST:GH15083 BEST:LD22483 BG:DS00004.11 BG:DS07473.1 Bap170 BcDNA:GH02901 BcDNA:GH02976 BcDNA:GH04962 BcDNA:GH12558 BcDNA:GM10765 BcDNA:LD09009 BcDNA:LD22910 BcDNA:LD23371 BcDNA:LD23876 BcDNA:LD41548 BicD Brf BthD Btk29A CG10211 CG10354 CG10423 CG10473 CG10542 CG10602 CG10657 CG10695 CG10960 CG10990 CG11055 CG11069 CG11123 CG11142 CG11188 CG11207 CG11228 CG1129 CG11305 CG11451 CG11652 CG11711 CG11836 CG11856 CG12006 CG12048 CG12130 CG12190 CG12199 CG12252 CG12299 CG12896 CG13852 CG1418 CG14217 CG14222 CG14231 CG1440 CG14411 CG14439 CG14670 CG1472 CG14882 CG15093 CG1514 CG15141 CG1544 CG1550 CG15835 CG17036 CG17064 CG17121 CG17143 CG17184 CG17309 CG17419 CG1746 CG17493 CG17514 CG17598 CG17765 CG17919 CG18030 CG1815 CG1869 CG1893 CG1900 CG1906 CG1907 CG1911 CG2097 CG2108 CG2118 CG2158 CG2316 CG2852 CG2947 CG2980 CG30011 CG30025 CG30372 CG30440 CG31125 CG31169 CG31195 CG31251 CG31258 CG31363 CG31453 CG31472 CG31559 CG31617 CG31640 CG31658 CG31710 CG31716 CG31739 CG31749 CG31997 CG32099 CG32108 CG32137 CG32158 CG32163 CG32164 CG32243 CG32251 CG32267 CG3227 CG32415 CG32447 CG3249 CG32534 CG32629 CG32632 CG32640 CG32663 CG32672 CG32783 CG32797 CG33113 CG33116 CG33123 CG33138 CG33145 CG33214 CG3328 CG33455 CG33525 CG3590 CG3605 CG3823 CG40160 CG40410 CG4238 CG4454 CG4509 CG4612 CG4670 CG4749 CG4914 CG5028 CG5064 CG5191 CG5319 CG5390 CG5434 CG5466 CG5505 CG5522 CG5687 CG5728 CG5789 CG5830 CG5841 CG5873 CG6210 CG6227 CG6287 CG6391 CG6394 CG6418 CG6543 CG6638 CG6673 CG6680 CG6767 CG6812 CG6835 CG6838 CG6854 CG6896 CG6904 CG6946 CG6954 CG7008 CG7145 CG7161 CG7263 CG7288 CG7441 CG7461 CG7668 CG7675 CG7720 CG7878 CG7922 CG7987 CG8036 CG8092 CG8104 CG8155 CG8165 CG8188 CG8243 CG8400 CG8426 CG8443 CG8494 CG8588 CG8789 CG8863 CG8902 CG8924 CG8963 CG9027 CG9057 CG9135 CG9153 CG9246 CG9342 CG9425 CG9471 CG9503 CG9809 CG9906 CREG CaMKII Cad87A Caf1 CalpB Cap-G Cat Cbp80 CdGAPr Cdc42 Cdk4 Cen190 Chc Cip4 CkIIalpha CkIIbeta CkIalpha Cks Cont CtBP CycA CycG CycT Cyp1 Cyp6v1 D12 D19A D19B DNApol-delta DNApol-epsilon Dhh1 Dlc90F Dll DnaJ-1 Dp Dr Dref Dsp1 E(z) EG:196F3.2 EG:34F3.8 EG:86E4.2 EG:BACH59J11.2 ERp60 ESTS:39C10S Eb1 Eip55E Eip63E Eip75B Elf Eno Es2 Fas2 Fas3 Fkbp13 Fpps Fs(2)Ket Fur1 G-salpha60A GATAd GNBP3 Galpha49B Gap1 Gapdh1 Gapdh2 Gdh Gef26 Gfat1 GlyP Got2 Gprk1 Hcf Hem Hexo1 Hlc HmgD HmgZ Hmu Hrb27C Hrb87F Hsc70-3 Hsp23 Hsp26 Hsp27 Hsp60 Hsp67Bc Hus1-like Idh ImpE2 ImpE3 ImpL2 Indy Iswi Jheh1 Jheh2 KP78b Karybeta3 Klp10A Lac Lam LanA LanB2 M(2)21AB MESR6 MICAL-like Map60 Mapmodulin Mbs Mcm5 Mcm6 Mcm7 Mcr Mdr49 Mekk1 Mi-2 Moe Mpcp Msp-300 Mtl Myb NTPase Nek2 Nep2 NetA Nlp Nop60B Nrv1 Optix Os-C Ote PFE PR2 Paf-AHalpha Paip2 Pak3 Pcl Pen Pepck Pgi Pi3K68D Pld Pli Pp1-87B Pp2C1 Psa Psc Ptp99A Rab10 RacGAP50C Ranbp16 Rbf2 Rbp2 Rgl RhoBTB RhoGAP19D RhoGAP1A RhoGEF3 Rhp Rm62 RnrL Rop RpII140 RpL13 RpL15 RpL22 RpL23a RpL3 RpS12 RpS6 Rrp1 S SMC1 Sb Scm Sdc SelD Sema-1b Sema-2a Ser Set Sh3beta Sin3A Sip1 Slip1 Smox Snap Sox14 Spn5 Spt6 Src64B Ste12DOR Ste:CG33236 Su(dx) Su(var)3-9 Su(z)12 TBPH Taf2 Thd1 Thiolase Tis11 Tm1 Tom34 Top2 Top3beta TppII Tpr2 Trap1 Trxr-1 Tsp66E Uba1 Uba2 UbcD2 UbcD6 Uch Ucp4B Vang Vap-33-1 Vha44 Vha55 Wnt2 Wsck X11L XNP abs ade2 ade3 ald alpha-Adaptin alpha-Cat alpha-Spec alphaTub84B ana aop ap argos arm ash2 asp barr baz bel betaTub56D bib bl bnb br brk brm btsz capu cav cdc2 charybde chb ci cib cic ck cora crb crc crol csul csw cta d d4 da dac dalao deltaTry dnk dome dpa dre4 dve eIF-4B eIF3-S9 eIF5 east ebi ed edl egl en ena endos esn exd fat2 fax fbp fdl flfl fra fwd fz fzy garz gft gfzf gish glec gol gp210 grn guf gus hdc heph hh hts in inx2 inx3 jar jim jing jumu katanin-60 kay kel kis klar klu kn knk krz l(1)G0148 l(1)G0334 l(1)dd4 l(2)01424 l(2)08717 l(2)dtl l(2)gl larp lds lgs lid lig lin19 lmg lola lqf lwr mask mav mbt mdy me31B mod mor msi msk msn mth mus209 ninA nonA-l nop5 nudC ogre olf413 ome otk ovo p120ctn pAbp pain par-6 pav pbl pfk ph-p pk plexA pll pnut polo pon prominin-like qkr54B r-l rg rin rpk sc sca scf scrib scylla sd sdk sec23 serpin-27A sgl shg sima simj slmb smi21F smi35A spag sqd ss stai stan stc sty su(f) syndapin th tkv toe tok tos trio trn tub tud vav vg vig vimar wbl wds wgn woc yps zf30C zip zwilch |
| 572 | GO:0015666 | F | 8, | 2 | 0.251 (x 7.955) | 3 (0.667) | 0.0927 | restriction endodeoxyribonuclease activity | CG7922 CG9425 |
| 573 | GO:0030133 | C | 6, 7, 8, 9, 10, | 4 | 1.089 (x 3.672) | 13 (0.308) | 0.0928 | transport vesicle | AP-1gamma CG1472 alpha-Adaptin sec23 |
| 574 | GO:0009968 | P | 5, 6, | 9 | 4.106 (x 2.192) | 49 (0.184) | 0.0929 | negative regulation of signal transduction | CkIalpha RacGAP50C argos brk ed l(2)gl slmb stan sty |
| 575 | GO:0008302 | P | 7, 10, | 2 | 0.251 (x 7.955) | 3 (0.667) | 0.0929 | ring canal formation, actin assembly | Src64B hts |
| 576 | GO:0018024 | F | 8, 9, | 4 | 1.089 (x 3.672) | 13 (0.308) | 0.093 | histone-lysine N-methyltransferase activity | Caf1 E(z) Su(var)3-9 Su(z)12 |
| 577 | GO:0050826 | P | 5, 6, | 2 | 0.251 (x 7.955) | 3 (0.667) | 0.093 | response to freezing | CG6227 crol |
| 578 | GO:0016571 | P | 9, 10, 12, | 4 | 1.089 (x 3.672) | 13 (0.308) | 0.0931 | histone methylation | Caf1 E(z) Su(var)3-9 Su(z)12 |
| 579 | GO:0030127 | C | 5, 6, 7, 8, 9, 10, 11, 12, 13, | 2 | 0.251 (x 7.955) | 3 (0.667) | 0.0932 | COPII vesicle coat | CG1472 sec23 |
| 580 | GO:0045169 | C | 5, 6, 7, 8, | 4 | 1.089 (x 3.672) | 13 (0.308) | 0.0933 | fusome | CycA alpha-Spec chb hts |
| 581 | GO:0008409 | F | 7, | 2 | 0.251 (x 7.955) | 3 (0.667) | 0.0934 | 5'-3' exonuclease activity | BcDNA:GM10765 CG10354 |
| 582 | GO:0006974 | P | 4, | 17 | 9.888 (x 1.719) | 118 (0.144) | 0.0934 | response to DNA damage stimulus | 14-3-3epsilon BcDNA:GM10765 CG40410 Caf1 CkIalpha CycG DNApol-delta DNApol-epsilon Hus1-like Rrp1 Thd1 Tom34 UbcD6 XNP lds mus209 tos |
| 583 | GO:0016278 | F | 7, | 4 | 1.089 (x 3.672) | 13 (0.308) | 0.0935 | lysine N-methyltransferase activity | Caf1 E(z) Su(var)3-9 Su(z)12 |
| 584 | GO:0050824 | F | 3, | 2 | 0.251 (x 7.955) | 3 (0.667) | 0.0935 | water binding | CG6227 crol |
| 585 | GO:0009996 | P | 6, 7, | 4 | 1.089 (x 3.672) | 13 (0.308) | 0.0936 | negative regulation of cell fate specification | argos hdc lqf sty |
| 586 | GO:0004824 | F | 7, | 2 | 0.251 (x 7.955) | 3 (0.667) | 0.0937 | lysine-tRNA ligase activity | CG31739 mdy |
| 587 | GO:0005200 | F | 3, | 35 | 24.470 (x 1.430) | 292 (0.120) | 0.0938 | structural constituent of cytoskeleton | Act42A Act5C CG10542 CG1826 CG31251 CG32137 CG32672 CG6896 Dlc90F Klp10A Lam MICAL-like Mbs Moe alpha-Cat alpha-Spec alphaTub84B arm betaTub56D capu ck cora d ena esn hts jar l(1)dd4 l(2)gl mask pav pk pnut syndapin zip |
| 588 | GO:0007386 | P | 4, | 2 | 0.251 (x 7.955) | 3 (0.667) | 0.0938 | compartment specification | en hh |
| 589 | GO:0051231 | P | 10, | 2 | 0.251 (x 7.955) | 3 (0.667) | 0.094 | spindle elongation | Eb1 chb |
| 590 | GO:0008096 | F | 8, | 2 | 0.251 (x 7.955) | 3 (0.667) | 0.0942 | juvenile hormone epoxide hydrolase activity | Jheh1 Jheh2 |
| 591 | GO:0048488 | P | 7, 8, | 7 | 2.849 (x 2.457) | 34 (0.206) | 0.0942 | synaptic vesicle endocytosis | AP-1gamma Amph Arf79F Chc alpha-Adaptin lqf syndapin |
| 592 | GO:0046533 | P | 6, | 2 | 0.251 (x 7.955) | 3 (0.667) | 0.0943 | negative regulation of photoreceptor cell differentiation | aop sdk |
| 593 | GO:0017018 | F | 10, | 2 | 0.251 (x 7.955) | 3 (0.667) | 0.0945 | myosin phosphatase activity | Mbs Pp1-87B |
| 594 | GO:0030134 | C | 6, 7, 8, 9, 10, 11, | 2 | 0.251 (x 7.955) | 3 (0.667) | 0.0946 | ER to Golgi transport vesicle | CG1472 sec23 |
| 595 | GO:0051227 | P | 7, 8, 11, | 2 | 0.251 (x 7.955) | 3 (0.667) | 0.0948 | mitotic spindle assembly | Eb1 larp |
| 596 | GO:0001659 | P | 4, | 2 | 0.251 (x 7.955) | 3 (0.667) | 0.095 | thermoregulation | CG6227 crol |
| 597 | GO:0016888 | F | 8, | 2 | 0.251 (x 7.955) | 3 (0.667) | 0.0951 | endodeoxyribonuclease activity, producing 5'-phosphomonoesters | CG7922 CG9425 |
| 598 | GO:0045674 | P | 7, | 2 | 0.251 (x 7.955) | 3 (0.667) | 0.0953 | negative regulation of photoreceptor differentiation (sensu Endopterygota) | aop sdk |
| 599 | GO:0030177 | P | 6, 7, 8, | 2 | 0.251 (x 7.955) | 3 (0.667) | 0.0955 | positive regulation of Wnt receptor signaling pathway | lgs stan |
| 600 | GO:0012507 | C | 6, 7, 8, 9, 10, 11, 12, | 2 | 0.251 (x 7.955) | 3 (0.667) | 0.0956 | ER to Golgi transport vesicle membrane | CG1472 sec23 |
| 601 | GO:0015668 | F | 9, | 2 | 0.251 (x 7.955) | 3 (0.667) | 0.0958 | Type III site-specific deoxyribonuclease activity | CG7922 CG9425 |
| 602 | GO:0006430 | P | 9, 10, 11, | 2 | 0.251 (x 7.955) | 3 (0.667) | 0.096 | lysyl-tRNA aminoacylation | CG31739 mdy |
| 603 | GO:0030132 | C | 5, 6, 7, 8, 9, 10, | 2 | 0.251 (x 7.955) | 3 (0.667) | 0.0961 | clathrin coat of coated pit | Chc alpha-Adaptin |
| 604 | GO:0030139 | C | 6, 7, 8, 9, 10, | 2 | 0.251 (x 7.955) | 3 (0.667) | 0.0963 | endocytic vesicle | alpha-Adaptin jar |
| 605 | GO:0004682 | F | 9, | 2 | 0.251 (x 7.955) | 3 (0.667) | 0.0965 | protein kinase CK2 activity | CkIIalpha CkIIbeta |
| 606 | GO:0009307 | P | 8, | 2 | 0.251 (x 7.955) | 3 (0.667) | 0.0966 | DNA restriction-modification system | CG7922 CG9425 |
| 607 | GO:0009880 | P | 4, | 18 | 10.810 (x 1.665) | 129 (0.140) | 0.0988 | embryonic pattern specification | Cdk4 Gap1 ci csw dome edl en hh kis kn knk lgs lwr pll sgl stan sty tub |
| 608 | GO:0035222 | P | 5, 6, | 5 | 1.676 (x 2.983) | 20 (0.250) | 0.099 | wing disc pattern formation | ap ci en hh tkv |
| 609 | GO:0030716 | P | 6, | 5 | 1.676 (x 2.983) | 20 (0.250) | 0.0991 | oocyte fate determination | BicD alpha-Spec baz egl hts |
| 610 | GO:0008276 | F | 6, | 5 | 1.676 (x 2.983) | 20 (0.250) | 0.0993 | protein methyltransferase activity | Caf1 E(z) Su(var)3-9 Su(z)12 csul |
| 611 | GO:0045926 | P | 4, | 5 | 1.676 (x 2.983) | 20 (0.250) | 0.0995 | negative regulation of growth | CG11228 CG17309 charybde fat2 scylla |
| 612 | GO:0016772 | F | 4, | 55 | 41.984 (x 1.310) | 501 (0.110) | 0.0995 | transferase activity, transferring phosphorus-containing groups | Ack Akt1 BcDNA:LD09009 BcDNA:LD23371 Btk29A CG11228 CG1129 CG14217 CG17309 CG31640 CG32099 CG40410 CG5841 CG6767 CG8789 CaMKII Cdk4 CkIIalpha CkIIbeta CkIalpha Cks CycT DNApol-delta DNApol-epsilon Eip63E Gprk1 KP78b M(2)21AB Mekk1 Nek2 PFE PR2 Pak3 Pi3K68D RpII140 Src64B Wsck ald alpha-Cat asp cdc2 dnk fat2 fwd gish l(1)G0148 mbt msn mus209 otk plexA pll polo smi35A tkv |
| 613 | GO:0016318 | P | 7, 8, 9, 10, | 5 | 1.676 (x 2.983) | 20 (0.250) | 0.0996 | ommatidial rotation | argos fz rin sca stan |
| 614 | GO:0000775 | C | 5, 6, 7, 8, 9, 10, | 6 | 2.263 (x 2.652) | 27 (0.222) | 0.0997 | chromosome, pericentric region | CG13895 Su(var)3-9 cav chb fzy polo |
| 615 | GO:0007283 | P | 6, | 16 | 9.302 (x 1.720) | 111 (0.144) | 0.1 | spermatogenesis | Act5C Chc Dlc90F Pen Set Ste12DOR Ste:CG33236 bel cdc2 fwd gish heph jar mod qkr54B th |
| 616 | GO:0048232 | P | 5, | 16 | 9.302 (x 1.720) | 111 (0.144) | 0.1 | male gamete generation | Act5C Chc Dlc90F Pen Set Ste12DOR Ste:CG33236 bel cdc2 fwd gish heph jar mod qkr54B th |
| 617 | GO:0016310 | P | 7, | 51 | 38.632 (x 1.320) | 461 (0.111) | 0.105 | phosphorylation | ATPsyn-beta Ack Akt1 BcDNA:LD09009 BcDNA:LD23371 Btk29A CG11228 CG14217 CG17309 CG1746 CG40410 CG8789 CaMKII Cdk4 CkIIalpha CkIIbeta CkIalpha Cks Eip63E Gprk1 KP78b Mekk1 Nek2 PFE PR2 Pak3 Pi3K68D Sin3A Src64B Vha44 Vha55 Wsck ald cdc2 dnk dome edl fat2 fwd gish jumu l(1)G0148 mbt msn otk plexA pll polo smi35A syndapin tkv |
| 618 | GO:0043449 | P | 5, | 3 | 0.670 (x 4.475) | 8 (0.375) | 0.105 | alkene metabolism | Jheh1 Jheh2 Vha44 |
| 619 | GO:0008536 | F | 7, | 3 | 0.670 (x 4.475) | 8 (0.375) | 0.106 | Ran GTPase binding | CG11856 Ranbp16 msk |
| 620 | GO:0044421 | C | 2, 3, | 13 | 7.123 (x 1.825) | 85 (0.153) | 0.106 | extracellular region part | 14-3-3epsilon 14-3-3zeta Btk29A LanA LanB2 NetA Src64B chb hts kel l(2)gl pnut sca |
| 621 | GO:0005243 | F | 5, | 3 | 0.670 (x 4.475) | 8 (0.375) | 0.106 | gap-junction forming channel activity | inx2 inx3 ogre |
| 622 | GO:0003724 | F | 4, | 10 | 4.944 (x 2.023) | 59 (0.169) | 0.106 | RNA helicase activity | CG6227 CG6418 CG7878 CG7922 Dhh1 Hlc Rm62 abs bel me31B |
| 623 | GO:0016096 | P | 7, 8, | 3 | 0.670 (x 4.475) | 8 (0.375) | 0.106 | polyisoprenoid metabolism | Jheh1 Jheh2 Vha44 |
| 624 | GO:0008023 | C | 3, 6, 7, 8, 9, 10, 11, 12, 13, | 3 | 0.670 (x 4.475) | 8 (0.375) | 0.106 | transcription elongation factor complex | CycT Cyp1 dre4 |
| 625 | GO:0005089 | F | 5, | 3 | 0.670 (x 4.475) | 8 (0.375) | 0.106 | Rho guanyl-nucleotide exchange factor activity | RhoGEF3 pbl trio |
| 626 | GO:0042214 | P | 6, | 3 | 0.670 (x 4.475) | 8 (0.375) | 0.106 | terpene metabolism | Jheh1 Jheh2 Vha44 |
| 627 | GO:0008060 | F | 5, | 3 | 0.670 (x 4.475) | 8 (0.375) | 0.107 | ARF GTPase activator activity | CG30372 CG6838 CG8243 |
| 628 | GO:0005921 | C | 7, 8, 9, | 3 | 0.670 (x 4.475) | 8 (0.375) | 0.107 | gap junction | inx2 inx3 ogre |
| 629 | GO:0006721 | P | 5, 7, 8, 9, | 3 | 0.670 (x 4.475) | 8 (0.375) | 0.107 | terpenoid metabolism | Jheh1 Jheh2 Vha44 |
| 630 | GO:0007178 | P | 7, | 9 | 4.274 (x 2.106) | 51 (0.176) | 0.107 | transmembrane receptor protein serine/threonine kinase signaling pathway | Cdc42 PFE Smox brk l(2)gl mav pll slmb tkv |
| 631 | GO:0006720 | P | 6, 7, | 4 | 1.173 (x 3.409) | 14 (0.286) | 0.108 | isoprenoid metabolism | Fpps Jheh1 Jheh2 Vha44 |
| 632 | GO:0007315 | P | 7, 9, 10, 12, | 8 | 3.603 (x 2.220) | 43 (0.186) | 0.108 | pole plasm assembly | Hrb27C Moe Rop Tm1 capu csul sqd tud |
| 633 | GO:0044260 | P | 5, | 203 | 180.339 (x 1.126) | 2152 (0.094) | 0.108 | cellular macromolecule metabolism | Aats-ala Aats-glupro Ack Akt1 ApepP Arf79F BG:DS00004.11 BcDNA:GH02976 BcDNA:LD09009 BcDNA:LD22910 BcDNA:LD23371 BcDNA:LD41548 Btk29A CG10423 CG10542 CG10602 CG10657 CG10990 CG11142 CG11207 CG11228 CG11836 CG12006 CG12130 CG14217 CG14222 CG14231 CG1440 CG14670 CG15141 CG1550 CG17036 CG17309 CG17514 CG17598 CG18030 CG1815 CG1869 CG1906 CG2852 CG2947 CG30025 CG31716 CG31739 CG32099 CG32632 CG32640 CG33123 CG33138 CG33145 CG3328 CG40160 CG40410 CG4238 CG4670 CG4914 CG5191 CG5390 CG5434 CG5505 CG5687 CG5841 CG6394 CG6680 CG6835 CG6904 CG7263 CG7288 CG7441 CG7720 CG8188 CG8443 CG8494 CG8789 CG8863 CG8963 CG9135 CG9153 CG9425 CG9906 CaMKII Caf1 CalpB Cbp80 Cdc42 Cdk4 CkIIalpha CkIIbeta CkIalpha Cks Cyp1 D19A D19B DnaJ-1 E(z) EG:86E4.2 ERp60 Eip63E Elf Fkbp13 Fur1 Galpha49B GlyP Got2 Gprk1 Hsc70-3 Hsp23 Hsp26 Hsp27 Hsp60 Hsp67Bc KP78b LanA Mcr Mekk1 Mi-2 Nek2 Nep2 PFE PR2 Paip2 Pak3 Pp1-87B Pp2C1 Psa Psc Ptp99A Rbp2 RpL13 RpL15 RpL22 RpL23a RpL3 RpS12 RpS6 Sb Spn5 Src64B Su(dx) Su(var)3-9 Su(z)12 Top3beta TppII Tpr2 Trap1 Uba1 Uba2 UbcD2 UbcD6 Uch Wsck X11L ald alphaTub84B betaTub56D bl cdc2 csw d4 da deltaTry dome dre4 eIF-4B eIF3-S9 eIF5 east ebi ena fat2 fra fwd fzy gft gish gol hh l(1)G0148 l(2)01424 lin19 lmg lwr mbt mdy msi msn ome otk pAbp plexA pll polo rin sgl slmb smi35A sqd stc syndapin th tkv tok wbl |
| 634 | GO:0009070 | P | 8, 9, | 4 | 1.173 (x 3.409) | 14 (0.286) | 0.108 | serine family amino acid biosynthesis | CG6287 CtBP ESTS:39C10S SelD |
| 635 | GO:0007479 | P | 6, 7, | 4 | 1.173 (x 3.409) | 14 (0.286) | 0.108 | leg disc proximal/distal pattern formation | Dll ap dac exd |
| 636 | GO:0006270 | P | 9, | 4 | 1.173 (x 3.409) | 14 (0.286) | 0.108 | DNA replication initiation | Mcm5 Mcm6 Mcm7 dpa |
| 637 | GO:0035223 | P | 5, 6, | 4 | 1.173 (x 3.409) | 14 (0.286) | 0.108 | leg disc pattern formation | Dll ap dac exd |
| 638 | GO:0003688 | F | 6, | 4 | 1.173 (x 3.409) | 14 (0.286) | 0.109 | DNA replication origin binding | Mcm5 Mcm6 Mcm7 dpa |
| 639 | GO:0045034 | P | 6, 7, 10, | 4 | 1.173 (x 3.409) | 14 (0.286) | 0.109 | neuroblast division | baz cdc2 jar pon |
| 640 | GO:0007448 | P | 5, 6, | 4 | 1.173 (x 3.409) | 14 (0.286) | 0.109 | anterior/posterior pattern formation, imaginal disc | ci en hh tkv |
| 641 | GO:0006418 | P | 8, 9, 10, | 10 | 5.028 (x 1.989) | 60 (0.167) | 0.113 | tRNA aminoacylation for protein translation | Aats-ala Aats-glupro CG31739 CG33123 CG5191 CG7441 CG9425 Top3beta X11L mdy |
| 642 | GO:0006092 | P | 7, | 15 | 8.715 (x 1.721) | 104 (0.144) | 0.113 | main pathways of carbohydrate metabolism | Acon Ald BEST:LD22483 CG15093 CG1544 CG5028 CG8036 Eno Gapdh1 Gapdh2 Gdh Idh Pepck Pgi l(1)G0334 |
| 643 | GO:0007015 | P | 9, | 10 | 5.028 (x 1.989) | 60 (0.167) | 0.113 | actin filament organization | Cdc42 CycT Fs(2)Ket Pak3 Sb Src64B asp ena hts vav |
| 644 | GO:0043039 | P | 8, 9, | 10 | 5.028 (x 1.989) | 60 (0.167) | 0.113 | tRNA aminoacylation | Aats-ala Aats-glupro CG31739 CG33123 CG5191 CG7441 CG9425 Top3beta X11L mdy |
| 645 | GO:0007346 | P | 6, 7, | 5 | 1.760 (x 2.841) | 21 (0.238) | 0.114 | regulation of progression through mitotic cell cycle | CG40410 CycA ci hh sc |
| 646 | GO:0030952 | P | 7, | 5 | 1.760 (x 2.841) | 21 (0.238) | 0.114 | establishment and/or maintenance of cytoskeleton polarity | 14-3-3epsilon 14-3-3zeta BicD chb egl |
| 647 | GO:0030951 | P | 8, 9, | 5 | 1.760 (x 2.841) | 21 (0.238) | 0.115 | establishment and/or maintenance of microtubule cytoskeleton polarity | 14-3-3epsilon 14-3-3zeta BicD chb egl |
| 648 | GO:0050954 | P | 4, 6, | 5 | 1.760 (x 2.841) | 21 (0.238) | 0.115 | sensory perception of mechanical stimulus | ck d ebi jar pain |
| 649 | GO:0016325 | P | 7, 8, 9, 10, | 5 | 1.760 (x 2.841) | 21 (0.238) | 0.115 | oocyte microtubule cytoskeleton organization | 14-3-3epsilon 14-3-3zeta BicD chb egl |
| 650 | GO:0007469 | P | 6, | 5 | 1.760 (x 2.841) | 21 (0.238) | 0.115 | antennal development | Dll ck dac ss th |
| 651 | GO:0016579 | P | 9, | 5 | 1.760 (x 2.841) | 21 (0.238) | 0.115 | protein deubiquitination | BcDNA:LD22910 CG5505 CG7288 CG8494 Uch |
| 652 | GO:0050790 | P | 3, | 8 | 3.687 (x 2.170) | 44 (0.182) | 0.119 | regulation of catalytic activity | Abi CG30372 CG6838 CG8243 G-salpha60A Ste:CG33236 mbt msn |
| 653 | GO:0044267 | P | 6, | 196 | 174.222 (x 1.125) | 2079 (0.094) | 0.12 | cellular protein metabolism | Aats-ala Aats-glupro Ack Akt1 ApepP Arf79F BG:DS00004.11 BcDNA:LD09009 BcDNA:LD22910 BcDNA:LD23371 BcDNA:LD41548 Btk29A CG10423 CG10542 CG10602 CG10657 CG10990 CG11207 CG11228 CG11836 CG12006 CG12130 CG14217 CG14222 CG14231 CG1440 CG14670 CG15141 CG1550 CG17036 CG17309 CG17514 CG17598 CG18030 CG1815 CG1906 CG2852 CG2947 CG30025 CG31716 CG31739 CG32099 CG32632 CG32640 CG33123 CG33145 CG3328 CG40160 CG40410 CG4238 CG4670 CG4914 CG5191 CG5390 CG5434 CG5505 CG5687 CG5841 CG6394 CG6680 CG6835 CG7263 CG7288 CG7441 CG7720 CG8188 CG8443 CG8494 CG8789 CG8863 CG8963 CG9135 CG9153 CG9425 CG9906 CaMKII Caf1 CalpB Cbp80 Cdc42 Cdk4 CkIIalpha CkIIbeta CkIalpha Cks Cyp1 D19A D19B DnaJ-1 E(z) EG:86E4.2 ERp60 Eip63E Elf Fkbp13 Fur1 Galpha49B Got2 Gprk1 Hsc70-3 Hsp23 Hsp26 Hsp27 Hsp60 Hsp67Bc KP78b LanA Mcr Mekk1 Mi-2 Nek2 Nep2 PFE PR2 Paip2 Pak3 Pp1-87B Pp2C1 Psa Psc Ptp99A Rbp2 RpL13 RpL15 RpL22 RpL23a RpL3 RpS12 RpS6 Sb Spn5 Src64B Su(dx) Su(var)3-9 Su(z)12 Top3beta TppII Tpr2 Trap1 Uba1 Uba2 UbcD2 UbcD6 Uch Wsck X11L ald alphaTub84B betaTub56D bl cdc2 csw d4 da deltaTry dome dre4 eIF-4B eIF3-S9 eIF5 east ebi fat2 fra fwd fzy gft gish gol hh l(1)G0148 l(2)01424 lin19 lmg lwr mbt mdy msi msn ome otk pAbp plexA pll polo rin sgl slmb smi35A sqd stc syndapin th tkv tok wbl |
| 654 | GO:0043234 | C | 2, | 152 | 132.321 (x 1.149) | 1579 (0.096) | 0.123 | protein complex | ATPsyn-beta Aats-glupro Arc92 B52 BG:DS00004.11 Bap170 BcDNA:GH04962 BcDNA:GH12558 Brf CG10423 CG10542 CG10695 CG11069 CG11305 CG11856 CG1746 CG17598 CG17838 CG1815 CG1906 CG1911 CG2097 CG2158 CG2316 CG31169 CG31617 CG31716 CG32164 CG3605 CG5064 CG5434 CG5789 CG5841 CG6227 CG7008 CG8443 CaMKII Caf1 Cap-G Cbp80 Cen190 CkIIalpha CkIIbeta CycA CycT Cyp1 DNApol-epsilon Dlc90F Dp Dref E(z) EG:196F3.2 Eb1 Eno Fs(2)Ket G-salpha60A Galpha49B Hrb27C Hrb87F Iswi Karybeta3 Klp10A LanA Map60 Mapmodulin Mbs Mcm5 Mcm6 Mcm7 Mi-2 Msp-300 Myb Nrv1 Pen Pep Pi3K68D Pp1-87B Pp2C1 Psc Ranbp16 Rbf2 RnrL RpII140 RpL13 RpL15 RpL22 RpL23a RpL3 RpS12 RpS6 SMC1 Scm Sin3A Spt6 Ste12DOR Ste:CG33236 Su(var)3-9 Su(z)12 Taf2 Thiolase Tom34 Top2 Uba2 Vha44 Vha55 alpha-Adaptin alpha-Cat alphaTub84B arm asp barr betaTub56D bl brm chb ci ck cta d d4 dalao dome dre4 eIF3-S9 gft gol heph jar jumu katanin-60 klar l(1)G0334 l(1)dd4 l(2)01424 lin19 lmg mor msk nonA-l nop5 pav ph-p sca slmb sqd stai stc su(f) th toe vig zip |
| 655 | GO:0007166 | P | 5, | 69 | 55.225 (x 1.249) | 659 (0.105) | 0.123 | cell surface receptor linked signal transduction | 18w Akt1 Arf79F CG10602 CG30440 CG32158 CG32447 CG5522 CG5841 CG8243 Cdc42 CkIIalpha CkIalpha G-salpha60A Galpha49B Gap1 Gprk1 Mtl PFE Pli RacGAP50C Rgl RhoBTB S Sema-1b Sema-2a Ser Smox Su(dx) Wnt2 argos arm bib brk ci crb csw cta ebi ed edl fz heph hh knk krz l(2)gl lgs mask mav msk mth ome otk pav pbl plexA pll sec23 serpin-27A sgl slmb stan sty tkv trio tub wbl wgn |
| 656 | GO:0007610 | P | 3, | 24 | 16.006 (x 1.499) | 191 (0.126) | 0.123 | behavior | 14-3-3epsilon 14-3-3zeta BEST:GH15083 Btk29A CG11711 CG8588 CaMKII CkIIalpha CkIIbeta Fas2 Fas3 G-salpha60A Gdh Pp1-87B Sema-2a Src64B gp210 hh lig ogre scrib slmb smi21F smi35A |
| 657 | GO:0043038 | P | 7, 8, | 10 | 5.112 (x 1.956) | 61 (0.164) | 0.123 | amino acid activation | Aats-ala Aats-glupro CG31739 CG33123 CG5191 CG7441 CG9425 Top3beta X11L mdy |
| 658 | GO:0007169 | P | 7, | 17 | 10.391 (x 1.636) | 124 (0.137) | 0.123 | transmembrane receptor protein tyrosine kinase signaling pathway | Akt1 CG5522 Gap1 Rgl S argos csw ebi ed edl knk mask msk otk plexA sgl sty |
| 659 | GO:0007179 | P | 8, | 6 | 2.430 (x 2.469) | 29 (0.207) | 0.128 | transforming growth factor beta receptor signaling pathway | Cdc42 brk l(2)gl mav slmb tkv |
| 660 | GO:0004693 | F | 8, | 4 | 1.257 (x 3.182) | 15 (0.267) | 0.132 | cyclin-dependent protein kinase activity | Cdk4 Cks Eip63E cdc2 |
| 661 | GO:0006888 | P | 6, 7, 8, 9, | 4 | 1.257 (x 3.182) | 15 (0.267) | 0.132 | ER to Golgi vesicle-mediated transport | CG1472 Snap garz sec23 |
| 662 | GO:0035075 | P | 5, 6, 7, | 4 | 1.257 (x 3.182) | 15 (0.267) | 0.132 | response to ecdysone | Eip75B br l(2)gl zip |
| 663 | GO:0002164 | P | 4, | 8 | 3.771 (x 2.121) | 45 (0.178) | 0.132 | larval development | Eip63E Moe Sb Ser alpha-Spec kn l(2)gl scrib |
| 664 | GO:0048545 | P | 5, 6, | 4 | 1.257 (x 3.182) | 15 (0.267) | 0.132 | response to steroid hormone stimulus | Eip75B br l(2)gl zip |
| 665 | GO:0048112 | P | 7, 9, 10, 12, | 8 | 3.771 (x 2.121) | 45 (0.178) | 0.132 | oocyte anterior/posterior axis determination (sensu Insecta) | BicD Hrb27C Moe Rop Tm1 capu sqd tud |
| 666 | GO:0000793 | C | 6, 7, 8, 9, | 4 | 1.257 (x 3.182) | 15 (0.267) | 0.133 | condensed chromosome | CG1911 Cap-G barr polo |
| 667 | GO:0019827 | P | 3, | 4 | 1.257 (x 3.182) | 15 (0.267) | 0.133 | stem cell maintenance | arm chb shg tkv |
| 668 | GO:0005875 | C | 3, 5, 6, 7, 8, 9, 10, | 16 | 9.721 (x 1.646) | 116 (0.138) | 0.133 | microtubule associated complex | CG10695 Cen190 Dlc90F Eb1 Klp10A Map60 Mapmodulin asp chb jar katanin-60 klar l(1)dd4 nop5 pav stai |
| 669 | GO:0005798 | C | 5, 6, 7, 8, 9, 10, | 5 | 1.844 (x 2.712) | 22 (0.227) | 0.134 | Golgi-associated vesicle | AP-1gamma CG1472 alpha-Adaptin garz sec23 |
| 670 | GO:0042078 | P | 6, | 5 | 1.844 (x 2.712) | 22 (0.227) | 0.134 | germ-line stem cell division | arm asp hh shg tkv |
| 671 | GO:0000790 | C | 6, 7, 8, 9, 10, 11, 12, | 5 | 1.844 (x 2.712) | 22 (0.227) | 0.134 | nuclear chromatin | BEAF-32 E(z) HmgD Psc Su(z)12 |
| 672 | GO:0016715 | F | 5, | 3 | 0.754 (x 3.978) | 9 (0.333) | 0.136 | oxidoreductase activity, acting on paired donors, with incorporation or reduction of molecular oxygen, reduced ascorbate as one donor, and incorporation of one atom of oxygen | CG12130 knk olf413 |
| 673 | GO:0008069 | P | 6, 7, 9, | 3 | 0.754 (x 3.978) | 9 (0.333) | 0.136 | dorsal/ventral axis determination, follicular epithelium (sensu Insecta) | Dp csw sqd |
| 674 | GO:0006073 | P | 7, 8, | 3 | 0.754 (x 3.978) | 9 (0.333) | 0.136 | glucan metabolism | CG33138 CG6904 GlyP |
| 675 | GO:0000096 | P | 6, 7, 8, | 3 | 0.754 (x 3.978) | 9 (0.333) | 0.137 | sulfur amino acid metabolism | Eip55E M(2)21AB SelD |
| 676 | GO:0007464 | P | 7, 8, 9, 10, 11, | 3 | 0.754 (x 3.978) | 9 (0.333) | 0.137 | R3/R4 cell fate commitment | fz kay stan |
| 677 | GO:0048056 | P | 7, 8, 9, 10, | 3 | 0.754 (x 3.978) | 9 (0.333) | 0.137 | R3/R4 cell differentiation (sensu Endopterygota) | fz kay stan |
| 678 | GO:0005977 | P | 8, 9, | 3 | 0.754 (x 3.978) | 9 (0.333) | 0.137 | glycogen metabolism | CG33138 CG6904 GlyP |
| 679 | GO:0030720 | P | 9, | 3 | 0.754 (x 3.978) | 9 (0.333) | 0.137 | oocyte localization during oogenesis | alpha-Cat arm shg |
| 680 | GO:0005656 | C | 3, 6, 7, 8, 9, 10, 11, 12, 13, | 3 | 0.754 (x 3.978) | 9 (0.333) | 0.138 | pre-replicative complex | Mcm5 Mcm6 Mcm7 |
| 681 | GO:0007430 | P | 5, 6, | 3 | 0.754 (x 3.978) | 9 (0.333) | 0.138 | terminal branching of trachea, cytoplasmic projection extension (sensu Insecta) | Lam hdc sty |
| 682 | GO:0007282 | P | 6, | 3 | 0.754 (x 3.978) | 9 (0.333) | 0.138 | cystoblast division | asp chb hts |
| 683 | GO:0007460 | P | 8, 9, 10, 11, | 3 | 0.754 (x 3.978) | 9 (0.333) | 0.138 | R8 cell fate commitment | da hh sca |
| 684 | GO:0035282 | P | 3, | 17 | 10.559 (x 1.610) | 126 (0.135) | 0.138 | segmentation | Cdk4 Gap1 Ser Su(dx) ap ci csw dome en hh kis kn knk lgs sgl stan sty |
| 685 | GO:0006221 | P | 7, 8, | 3 | 0.754 (x 3.978) | 9 (0.333) | 0.138 | pyrimidine nucleotide biosynthesis | CG6854 dnk r-l |
| 686 | GO:0006512 | P | 8, | 28 | 19.609 (x 1.428) | 234 (0.120) | 0.139 | ubiquitin cycle | BcDNA:LD22910 CG10542 CG15141 CG1815 CG31716 CG4238 CG5505 CG5841 CG7288 CG8188 CG8494 CG9153 Mi-2 Psc Su(dx) Uba1 Uba2 UbcD2 UbcD6 Uch d4 fzy gol lmg lwr slmb stc th |
| 687 | GO:0016107 | P | 7, 9, 10, 11, | 2 | 0.335 (x 5.967) | 4 (0.500) | 0.142 | sesquiterpenoid catabolism | Jheh1 Jheh2 |
| 688 | GO:0008615 | P | 9, | 2 | 0.335 (x 5.967) | 4 (0.500) | 0.142 | pyridoxine biosynthesis | CG31472 ESTS:39C10S |
| 689 | GO:0007304 | P | 8, 9, | 9 | 4.609 (x 1.953) | 55 (0.164) | 0.143 | eggshell formation (sensu Insecta) | Caf1 Cdc42 Dp Fs(2)Ket Mcm6 Myb capu gus mus209 |
| 690 | GO:0051763 | P | 8, | 2 | 0.335 (x 5.967) | 4 (0.500) | 0.143 | sesquiterpene catabolism | Jheh1 Jheh2 |
| 691 | GO:0030703 | P | 7, | 9 | 4.609 (x 1.953) | 55 (0.164) | 0.143 | eggshell formation | Caf1 Cdc42 Dp Fs(2)Ket Mcm6 Myb capu gus mus209 |
| 692 | GO:0016097 | P | 8, 9, | 2 | 0.335 (x 5.967) | 4 (0.500) | 0.143 | polyisoprenoid catabolism | Jheh1 Jheh2 |
| 693 | GO:0012506 | C | 4, 5, 6, 7, 8, 9, | 6 | 2.514 (x 2.387) | 30 (0.200) | 0.143 | vesicle membrane | AP-1gamma CG1472 Chc Rop alpha-Adaptin sec23 |
| 694 | GO:0008340 | P | 4, | 8 | 3.855 (x 2.075) | 46 (0.174) | 0.143 | determination of adult life span | Btk29A Cat Hsp26 Hsp27 Indy Trxr-1 fwd mth |
| 695 | GO:0006551 | P | 8, 9, | 2 | 0.335 (x 5.967) | 4 (0.500) | 0.143 | leucine metabolism | CG2118 CG6638 |
| 696 | GO:0007568 | P | 3, | 8 | 3.855 (x 2.075) | 46 (0.174) | 0.143 | aging | Btk29A Cat Hsp26 Hsp27 Indy Trxr-1 fwd mth |
| 697 | GO:0016589 | C | 5, 8, 9, 10, 11, 12, 13, 14, 15, | 2 | 0.335 (x 5.967) | 4 (0.500) | 0.143 | NURF complex | Caf1 Iswi |
| 698 | GO:0048099 | P | 6, 7, | 2 | 0.335 (x 5.967) | 4 (0.500) | 0.143 | anterior/posterior lineage restriction, imaginal disc | en hh |
| 699 | GO:0009409 | P | 4, 5, | 2 | 0.335 (x 5.967) | 4 (0.500) | 0.144 | response to cold | CG6227 crol |
| 700 | GO:0030721 | P | 6, | 2 | 0.335 (x 5.967) | 4 (0.500) | 0.144 | spectrosome organization and biogenesis | alpha-Spec hts |
| 701 | GO:0008614 | P | 8, | 2 | 0.335 (x 5.967) | 4 (0.500) | 0.144 | pyridoxine metabolism | CG31472 ESTS:39C10S |
| 702 | GO:0031010 | C | 4, 7, 8, 9, 10, 11, 12, 13, 14, | 2 | 0.335 (x 5.967) | 4 (0.500) | 0.144 | ISWI complex | Caf1 Iswi |
| 703 | GO:0042816 | P | 7, | 2 | 0.335 (x 5.967) | 4 (0.500) | 0.144 | vitamin B6 metabolism | CG31472 ESTS:39C10S |
| 704 | GO:0045464 | P | 6, 9, 10, 11, 12, | 2 | 0.335 (x 5.967) | 4 (0.500) | 0.145 | R8 cell fate specification | da hh |
| 705 | GO:0009967 | P | 5, 6, | 5 | 1.927 (x 2.594) | 23 (0.217) | 0.145 | positive regulation of signal transduction | CG5841 arm hh lgs stan |
| 706 | GO:0000097 | P | 7, 8, 9, | 2 | 0.335 (x 5.967) | 4 (0.500) | 0.145 | sulfur amino acid biosynthesis | M(2)21AB SelD |
| 707 | GO:0005575 | C | 1, | 507 | 484.536 (x 1.046) | 5782 (0.088) | 0.145 | cellular\_component | 14-3-3epsilon 14-3-3zeta 18w AP-1gamma ATPsyn-beta ATbp Aats-glupro Ack Acon Acox57D-p Act42A Act5C Akt1 Amph ApepP Arc92 Atu B52 BEAF-32 BEST:CK01140 BEST:GH15083 BG:DS00004.11 BG:DS07473.1 Bap170 BcDNA:GH02976 BcDNA:GH04962 BcDNA:GH12558 BcDNA:GM10765 BcDNA:LD23876 BcDNA:LD41548 BicD Brf BthD Btk29A CBP CG10211 CG10354 CG10423 CG10473 CG10542 CG10657 CG10695 CG10960 CG11069 CG11142 CG11188 CG11207 CG11228 CG11305 CG11711 CG11856 CG12048 CG12130 CG12252 CG12299 CG12391 CG13895 CG14439 CG1472 CG15093 CG1529 CG1647 CG1677 CG17036 CG17361 CG1746 CG17493 CG17598 CG17838 CG17952 CG1815 CG1869 CG1906 CG1907 CG1911 CG2097 CG2118 CG2158 CG2316 CG30011 CG30025 CG31125 CG31169 CG31195 CG31258 CG31301 CG31363 CG31617 CG31658 CG31710 CG31716 CG31739 CG31749 CG31997 CG32108 CG32158 CG32163 CG32164 CG32243 CG32251 CG32267 CG32415 CG32447 CG32534 CG32629 CG32663 CG32783 CG32797 CG3305 CG33113 CG33123 CG33145 CG33214 CG33455 CG33525 CG3605 CG3823 CG3847 CG40410 CG4238 CG4509 CG4914 CG5028 CG5064 CG5434 CG5687 CG5728 CG5789 CG5841 CG6064 CG6210 CG6227 CG6394 CG6543 CG6638 CG6673 CG6791 CG6812 CG6930 CG7008 CG7145 CG7154 CG7263 CG7720 CG7987 CG8089 CG8092 CG8426 CG8443 CG8478 CG8588 CG8924 CG9057 CG9104 CG9149 CG9153 CG9246 CG9342 CG9425 CG9894 CREG CaMKII Cad87A Caf1 CalpB Cap-G Cat Cbp80 Cen190 Chc CkIIalpha CkIIbeta CkIalpha Cont CtBP CycA CycT Cyp1 Cyp6v1 D12 D19A D19B DNApol-delta DNApol-epsilon Dlc90F Dll DnaJ-1 Dp Dr Dref Dsp1 E(z) EG:115C2.6 EG:196F3.2 EG:86E4.2 ERp60 Eb1 Eip75B Elf Eno Es2 Fas2 Fas3 Fs(2)Ket Fur1 G-salpha60A GATAd Galpha49B Gap1 Gapdh1 Gapdh2 Gdh Gfat1 Got2 Hcf Hem HmgD HmgZ Hmu Hrb27C Hrb87F Hsc70-3 Hsp60 Idh ImpE2 ImpE3 ImpL2 Indy Iswi Jheh2 Karybeta3 Klp10A Lac Lam LanA LanB2 MESR6 Map60 Mapmodulin Mbs Mcm5 Mcm6 Mcm7 Mdr49 Mi-2 Moe Mpcp Msp-300 Myb NUCB1 Nek2 Nep2 NetA Nlp Nop60B Nrv1 Optix Os-C Ote PR2 Paip2 Pcl Pen Pep Pepck Pgi Pi3K68D Pli Pp1-87B Pp2C1 Psc Ptp99A Ranbp16 Rbf2 Rbp2 Rhp Rm62 RnrL Rop RpII140 RpL13 RpL15 RpL22 RpL23a RpL3 RpS12 RpS6 Rrp1 S SMC1 Sb Scm Sdc Sema-1b Sema-2a Ser Set Sh3beta Sin3A Slip1 Smox Snap Sox14 Spt6 Src64B Ste12DOR Ste:CG33236 Su(dx) Su(var)3-9 Su(z)12 Taf2 Thd1 Thiolase Tis11 Tm1 Tom34 Top2 TppII Trxr-1 Tsp66E Uba2 UbcD6 Uch Ucp4B Vang Vap-33-1 Vha44 Vha55 Wnt2 Wsck XNP abs ade3 alpha-Adaptin alpha-Cat alpha-Spec alphaTub84B ana aop ap argos arm ash2 asp barr baz betaTub56D bib bl bnb br brk brm bsf btsz capu cav cdc2 chb ci cib cic ck cora crb crc crol crp csul csw cta d d4 da dac dalao deltaTry dnk dome dpa dre4 dve eIF-4B eIF3-S9 eIF5 east ebi ed edl egl en esn exd fat2 fdl fra fz fzy garz gft gfzf glec gol gp210 grn hdc heph hh hts in inx2 inx3 jar jim jing jumu katanin-60 kay kel kis klar klu kn l(1)G0334 l(1)dd4 l(2)01424 l(2)08717 l(2)dtl l(2)gl larp lds lgs lid lig lin19 lmg lola lwr mbt mdy mod mor msk mth mus209 ninA nonA-l nop5 nudC ogre ome otk ovo p120ctn pAbp pain par-6 pav pbl pfk ph-p pk plexA pll pnut polo pon prominin-like qkr54B rg rin rpk sc sca scrib sd sdk sec23 shg sima simj slmb smi35A sqd ss stai stan stc sty su(f) th tkv toe trio trn tub tud vav vg viaf1 vig wbl wgn woc yps zf30C zip |
| 708 | GO:0006304 | P | 7, | 5 | 1.927 (x 2.594) | 23 (0.217) | 0.145 | DNA modification | CG7922 CG9425 Su(var)3-9 Taf2 Top3beta |
| 709 | GO:0000301 | P | 7, 8, 9, 10, | 2 | 0.335 (x 5.967) | 4 (0.500) | 0.145 | retrograde transport, vesicle recycling within Golgi | S wbl |
| 710 | GO:0000139 | C | 4, 5, 6, 7, 8, 9, 10, | 5 | 1.927 (x 2.594) | 23 (0.217) | 0.145 | Golgi membrane | AP-1gamma CG1472 Fur1 alpha-Adaptin sec23 |
| 711 | GO:0045477 | P | 7, 8, 10, | 2 | 0.335 (x 5.967) | 4 (0.500) | 0.145 | regulation of nurse cell apoptosis | Dp mdy |
| 712 | GO:0008258 | P | 6, | 5 | 1.927 (x 2.594) | 23 (0.217) | 0.145 | head involution | Btk29A barr kay shg zip |
| 713 | GO:0016192 | P | 5, 6, | 34 | 24.805 (x 1.371) | 296 (0.115) | 0.145 | vesicle-mediated transport | AP-1gamma Amph Arf79F CG1418 CG1472 CG1900 CG2108 CG5841 CG6838 CG9906 Chc Dlc90F EG:34F3.8 Pi3K68D Rab10 Rop S Snap Vap-33-1 X11L alpha-Adaptin btsz ck d garz jar krz l(2)gl lqf mth pnut sec23 syndapin wbl |
| 714 | GO:0030130 | C | 5, 6, 7, 8, 9, 10, 11, 12, 13, 14, | 2 | 0.335 (x 5.967) | 4 (0.500) | 0.145 | clathrin coat of trans-Golgi network vesicle | AP-1gamma alpha-Adaptin |
| 715 | GO:0006767 | P | 6, | 5 | 1.927 (x 2.594) | 23 (0.217) | 0.145 | water-soluble vitamin metabolism | BEST:LD22483 CG15093 CG31472 CG8036 ESTS:39C10S |
| 716 | GO:0016271 | P | 4, | 11 | 6.034 (x 1.823) | 72 (0.153) | 0.146 | tissue death | Akap200 Cyp1 Eip75B PR2 ap br l(1)G0148 l(2)01424 l(2)gl larp zip |
| 717 | GO:0012510 | C | 6, 7, 8, 9, 10, 11, 12, 13, | 2 | 0.335 (x 5.967) | 4 (0.500) | 0.146 | trans-Golgi network transport vesicle membrane | AP-1gamma alpha-Adaptin |
| 718 | GO:0042445 | P | 5, | 5 | 1.927 (x 2.594) | 23 (0.217) | 0.146 | hormone metabolism | 14-3-3zeta Jheh1 Jheh2 Vha44 woc |
| 719 | GO:0007559 | P | 5, | 11 | 6.034 (x 1.823) | 72 (0.153) | 0.146 | histolysis | Akap200 Cyp1 Eip75B PR2 ap br l(1)G0148 l(2)01424 l(2)gl larp zip |
| 720 | GO:0035155 | P | 7, 8, | 2 | 0.335 (x 5.967) | 4 (0.500) | 0.146 | negative regulation of terminal cell fate specification | hdc sty |
| 721 | GO:0046247 | P | 7, | 2 | 0.335 (x 5.967) | 4 (0.500) | 0.146 | terpene catabolism | Jheh1 Jheh2 |
| 722 | GO:0031468 | P | 7, 8, | 2 | 0.335 (x 5.967) | 4 (0.500) | 0.146 | nuclear envelope reassembly | Lam Ote |
| 723 | GO:0018108 | P | 9, 10, | 2 | 0.335 (x 5.967) | 4 (0.500) | 0.146 | peptidyl-tyrosine phosphorylation | Ack Src64B |
| 724 | GO:0000042 | P | 8, 9, 10, 11, | 2 | 0.335 (x 5.967) | 4 (0.500) | 0.147 | protein targeting to Golgi | S wbl |
| 725 | GO:0043451 | P | 6, | 2 | 0.335 (x 5.967) | 4 (0.500) | 0.147 | alkene catabolism | Jheh1 Jheh2 |
| 726 | GO:0042054 | F | 7, | 4 | 1.341 (x 2.983) | 16 (0.250) | 0.147 | histone methyltransferase activity | Caf1 E(z) Su(var)3-9 Su(z)12 |
| 727 | GO:0007561 | P | 6, 7, | 2 | 0.335 (x 5.967) | 4 (0.500) | 0.147 | imaginal disc eversion | ImpE2 ImpE3 |
| 728 | GO:0009725 | P | 4, 5, | 4 | 1.341 (x 2.983) | 16 (0.250) | 0.147 | response to hormone stimulus | Eip75B br l(2)gl zip |
| 729 | GO:0048135 | P | 6, 7, | 2 | 0.335 (x 5.967) | 4 (0.500) | 0.147 | female germ-line cyst formation | alpha-Spec hts |
| 730 | GO:0009948 | P | 5, | 16 | 9.972 (x 1.604) | 119 (0.134) | 0.147 | anterior/posterior axis specification | BicD Gap1 Hrb27C Moe Rop Tm1 capu csul csw gus hh knk shg sqd sty tud |
| 731 | GO:0030111 | P | 5, 6, 7, | 4 | 1.341 (x 2.983) | 16 (0.250) | 0.147 | regulation of Wnt receptor signaling pathway | RacGAP50C lgs slmb stan |
| 732 | GO:0008135 | F | 3, 4, | 12 | 6.872 (x 1.746) | 82 (0.146) | 0.147 | translation factor activity, nucleic acid binding | CG10990 CG17514 CG5434 CG8443 Elf Rbp2 Su(var)3-9 eIF-4B eIF3-S9 eIF5 l(2)01424 msi |
| 733 | GO:0006719 | P | 7, 8, 10, 11, 12, | 2 | 0.335 (x 5.967) | 4 (0.500) | 0.147 | juvenile hormone catabolism | Jheh1 Jheh2 |
| 734 | GO:0030512 | P | 6, 7, 10, | 2 | 0.335 (x 5.967) | 4 (0.500) | 0.148 | negative regulation of transforming growth factor beta receptor signaling pathway | brk slmb |
| 735 | GO:0008300 | P | 7, 8, | 2 | 0.335 (x 5.967) | 4 (0.500) | 0.148 | isoprenoid catabolism | Jheh1 Jheh2 |
| 736 | GO:0000737 | P | 7, 8, | 2 | 0.335 (x 5.967) | 4 (0.500) | 0.148 | DNA catabolism, endonucleolytic | BcDNA:GM10765 Top3beta |
| 737 | GO:0005905 | C | 5, 6, 7, | 2 | 0.335 (x 5.967) | 4 (0.500) | 0.148 | coated pit | Chc alpha-Adaptin |
| 738 | GO:0019216 | P | 5, 6, | 2 | 0.335 (x 5.967) | 4 (0.500) | 0.149 | regulation of lipid metabolism | Eip75B Vha44 |
| 739 | GO:0035189 | C | 4, 7, 8, 9, 10, 11, 12, 13, 14, | 2 | 0.335 (x 5.967) | 4 (0.500) | 0.149 | Rb-E2F complex | Dp Rbf2 |
| 740 | GO:0046529 | P | 7, 8, | 2 | 0.335 (x 5.967) | 4 (0.500) | 0.149 | imaginal disc fusion, thorax closure | kay spag |
| 741 | GO:0016328 | C | 5, 6, 7, | 2 | 0.335 (x 5.967) | 4 (0.500) | 0.149 | lateral plasma membrane | Fas3 scrib |
| 742 | GO:0007111 | P | 6, 7, | 2 | 0.335 (x 5.967) | 4 (0.500) | 0.149 | cytokinesis after meiosis II | fwd pbl |
| 743 | GO:0005848 | C | 4, 7, 8, 9, 10, 11, 12, 13, 14, | 2 | 0.335 (x 5.967) | 4 (0.500) | 0.15 | mRNA cleavage stimulating factor complex | CG2097 su(f) |
| 744 | GO:0042819 | P | 8, | 2 | 0.335 (x 5.967) | 4 (0.500) | 0.15 | vitamin B6 biosynthesis | CG31472 ESTS:39C10S |
| 745 | GO:0046528 | P | 6, 7, | 2 | 0.335 (x 5.967) | 4 (0.500) | 0.15 | imaginal disc fusion | kay spag |
| 746 | GO:0016115 | P | 6, 8, 9, 10, | 2 | 0.335 (x 5.967) | 4 (0.500) | 0.15 | terpenoid catabolism | Jheh1 Jheh2 |
| 747 | GO:0009165 | P | 6, 7, | 15 | 9.218 (x 1.627) | 110 (0.136) | 0.15 | nucleotide biosynthesis | ATPsyn-beta CG1746 CG32158 CG3590 CG6767 CG6854 Sin3A Vha44 Vha55 ade2 ade3 dnk dome jumu r-l |
| 748 | GO:0004563 | F | 7, | 2 | 0.335 (x 5.967) | 4 (0.500) | 0.15 | beta-N-acetylhexosaminidase activity | Hexo1 fdl |
| 749 | GO:0006281 | P | 5, 7, | 15 | 9.218 (x 1.627) | 110 (0.136) | 0.151 | DNA repair | BcDNA:GM10765 Caf1 CkIalpha CycG DNApol-delta DNApol-epsilon Hus1-like Rrp1 Thd1 Tom34 UbcD6 XNP lds mus209 tos |
| 750 | GO:0016881 | F | 5, | 20 | 13.241 (x 1.511) | 158 (0.127) | 0.151 | acid-amino acid ligase activity | CG10542 CG15141 CG1550 CG1815 CG31716 CG4238 CG5841 CG6835 CG9153 Mi-2 Psc Su(dx) Uba1 d4 gft gol lmg slmb stc th |
| 751 | GO:0007224 | P | 6, | 6 | 2.598 (x 2.310) | 31 (0.194) | 0.151 | smoothened signaling pathway | CkIalpha ci hh pav sgl slmb |
| 752 | GO:0004722 | F | 8, | 7 | 3.268 (x 2.142) | 39 (0.179) | 0.151 | protein serine/threonine phosphatase activity | CG12252 CG17598 CG1906 Mbs Pp1-87B Pp2C1 spag |
| 753 | GO:0004842 | F | 7, | 18 | 11.648 (x 1.545) | 139 (0.129) | 0.154 | ubiquitin-protein ligase activity | CG10542 CG15141 CG1815 CG31716 CG4238 CG5841 CG9153 Mi-2 Psc Su(dx) Uba1 d4 gft gol lmg slmb stc th |
| 754 | GO:0005102 | F | 3, 4, | 32 | 23.380 (x 1.369) | 279 (0.115) | 0.154 | receptor binding | BcDNA:GH03163 CG11207 CG1815 CG3249 CG33214 CG5841 CG7668 CG9025 CdGAPr Eb1 Hem LanA Mcr RhoGAP1A Sema-1b Sema-2a Ser Tsp66E Wnt2 argos arm crb csul endos hh mask mav otk rin sca scrib sty |
| 755 | GO:0005509 | F | 5, | 26 | 18.269 (x 1.423) | 218 (0.119) | 0.154 | calcium ion binding | BEST:CK01140 CBP CG17271 CG17493 CG17765 CG33113 CG4509 CG9906 Cad87A CalpB DNApol-delta EG:86E4.2 Fkbp13 NUCB1 PR2 Ser alpha-Spec crb eIF5 fat2 lola ome scf shg stan tok |
| 756 | GO:0019787 | F | 6, | 18 | 11.648 (x 1.545) | 139 (0.129) | 0.154 | small conjugating protein ligase activity | CG10542 CG15141 CG1815 CG31716 CG4238 CG5841 CG9153 Mi-2 Psc Su(dx) Uba1 d4 gft gol lmg slmb stc th |
| 757 | GO:0015629 | C | 6, 7, 8, 9, | 11 | 6.201 (x 1.774) | 74 (0.149) | 0.158 | actin cytoskeleton | Act42A Act5C Msp-300 Pen Tm1 alpha-Cat alpha-Spec ck d jar zip |
| 758 | GO:0031577 | P | 8, | 3 | 0.838 (x 3.580) | 10 (0.300) | 0.163 | spindle checkpoint | ald alphaTub84B zwilch |
| 759 | GO:0042059 | P | 6, 7, 10, | 3 | 0.838 (x 3.580) | 10 (0.300) | 0.163 | negative regulation of epidermal growth factor receptor signaling pathway | argos ed sty |
| 760 | GO:0016323 | C | 5, 6, 7, | 5 | 2.011 (x 2.486) | 24 (0.208) | 0.163 | basolateral plasma membrane | Fas3 Sdc alpha-Cat l(2)gl scrib |
| 761 | GO:0001746 | P | 7, 8, | 3 | 0.838 (x 3.580) | 10 (0.300) | 0.163 | Bolwig's organ morphogenesis | Fas2 hh shg |
| 762 | GO:0030659 | C | 5, 6, 7, 8, 9, 10, | 5 | 2.011 (x 2.486) | 24 (0.208) | 0.163 | cytoplasmic vesicle membrane | AP-1gamma CG1472 Chc alpha-Adaptin sec23 |
| 763 | GO:0006308 | P | 6, 7, | 3 | 0.838 (x 3.580) | 10 (0.300) | 0.163 | DNA catabolism | BcDNA:GM10765 CG7263 Top3beta |
| 764 | GO:0030662 | C | 6, 7, 8, 9, 10, 11, | 5 | 2.011 (x 2.486) | 24 (0.208) | 0.163 | coated vesicle membrane | AP-1gamma CG1472 Chc alpha-Adaptin sec23 |
| 765 | GO:0008362 | P | 9, | 3 | 0.838 (x 3.580) | 10 (0.300) | 0.163 | embryonic cuticle biosynthesis (sensu Insecta) | Akt1 cora knk |
| 766 | GO:0030120 | C | 5, 6, 7, 8, 9, 10, 11, 12, | 5 | 2.011 (x 2.486) | 24 (0.208) | 0.163 | vesicle coat | AP-1gamma CG1472 Chc alpha-Adaptin sec23 |
| 767 | GO:0007350 | P | 4, 5, | 14 | 8.548 (x 1.638) | 102 (0.137) | 0.164 | blastoderm segmentation | Cdk4 Gap1 ci csw dome en hh kis kn knk lgs sgl stan sty |
| 768 | GO:0001737 | P | 6, 7, 8, 9, 10, 11, | 3 | 0.838 (x 3.580) | 10 (0.300) | 0.164 | establishment of wing hair orientation | fz in pk |
| 769 | GO:0016570 | P | 8, 11, | 5 | 2.011 (x 2.486) | 24 (0.208) | 0.164 | histone modification | BcDNA:LD09009 Caf1 E(z) Su(var)3-9 Su(z)12 |
| 770 | GO:0006564 | P | 9, 10, | 3 | 0.838 (x 3.580) | 10 (0.300) | 0.164 | L-serine biosynthesis | CG6287 CtBP ESTS:39C10S |
| 771 | GO:0019842 | F | 3, | 5 | 2.011 (x 2.486) | 24 (0.208) | 0.164 | vitamin binding | CG10657 CG13848 CG17036 CG2118 CG3823 |
| 772 | GO:0048589 | P | 3, | 7 | 3.352 (x 2.088) | 40 (0.175) | 0.164 | developmental growth | Fas2 bl fat2 hh ninA spag tkv |
| 773 | GO:0007094 | P | 9, 10, | 3 | 0.838 (x 3.580) | 10 (0.300) | 0.164 | mitotic spindle checkpoint | ald alphaTub84B zwilch |
| 774 | GO:0016569 | P | 10, | 5 | 2.011 (x 2.486) | 24 (0.208) | 0.164 | covalent chromatin modification | BcDNA:LD09009 Caf1 E(z) Su(var)3-9 Su(z)12 |
| 775 | GO:0048567 | P | 6, 7, | 7 | 3.352 (x 2.088) | 40 (0.175) | 0.164 | ectodermal gut morphogenesis | barr dome fat2 hh inx2 pbl zip |
| 776 | GO:0030713 | P | 9, | 3 | 0.838 (x 3.580) | 10 (0.300) | 0.164 | stalk formation (sensu Insecta) | Scm hh ph-p |
| 777 | GO:0007439 | P | 5, | 7 | 3.352 (x 2.088) | 40 (0.175) | 0.164 | ectodermal gut development | barr dome fat2 hh inx2 pbl zip |
| 778 | GO:0006220 | P | 7, | 3 | 0.838 (x 3.580) | 10 (0.300) | 0.165 | pyrimidine nucleotide metabolism | CG6854 dnk r-l |
| 779 | GO:0006457 | P | 7, | 17 | 10.978 (x 1.549) | 131 (0.130) | 0.165 | protein folding | CG2852 CG2947 CG32640 CG8863 CG9906 Cyp1 DnaJ-1 ERp60 Fkbp13 Hsc70-3 Hsp23 Hsp26 Hsp27 Hsp60 Hsp67Bc Tpr2 Trap1 |
| 780 | GO:0048547 | P | 5, 6, | 7 | 3.352 (x 2.088) | 40 (0.175) | 0.165 | gut morphogenesis | barr dome fat2 hh inx2 pbl zip |
| 781 | GO:0003916 | F | 4, 5, | 3 | 0.838 (x 3.580) | 10 (0.300) | 0.165 | DNA topoisomerase activity | Top2 Top3beta scf |
| 782 | GO:0048546 | P | 5, | 7 | 3.352 (x 2.088) | 40 (0.175) | 0.165 | digestive tract morphogenesis | barr dome fat2 hh inx2 pbl zip |
| 783 | GO:0046903 | P | 5, | 27 | 19.358 (x 1.395) | 231 (0.117) | 0.169 | secretion | AP-1gamma Amph Arf79F CG1472 CG15835 CG1900 CG5064 CG5434 CG9906 Chc EG:34F3.8 Rab10 Rop S Snap Vap-33-1 X11L alpha-Adaptin garz l(2)gl lqf mth pnut sec23 stai syndapin wbl |
| 784 | GO:0005525 | F | 6, | 20 | 13.492 (x 1.482) | 161 (0.124) | 0.17 | GTP binding | Arf79F CG1354 CG1900 CG2108 CG8902 Cdc42 Elf G-salpha60A Galpha49B Mtl Pepck Rab10 RhoBTB Su(var)3-9 alphaTub84B betaTub56D chb cta ebi pnut |
| 785 | GO:0008298 | P | 5, | 8 | 4.106 (x 1.948) | 49 (0.163) | 0.174 | intracellular mRNA localization | BicD Hrb27C Rop Tm1 capu egl sqd tud |
| 786 | GO:0045182 | F | 2, | 12 | 7.123 (x 1.685) | 85 (0.141) | 0.177 | translation regulator activity | CG10990 CG17514 CG5434 CG8443 Elf Rbp2 Su(var)3-9 eIF-4B eIF3-S9 eIF5 l(2)01424 msi |
| 787 | GO:0016876 | F | 5, | 9 | 4.860 (x 1.852) | 58 (0.155) | 0.178 | ligase activity, forming aminoacyl-tRNA and related compounds | Aats-ala Aats-glupro CG31739 CG33123 CG7441 CG9425 Top3beta X11L mdy |
| 788 | GO:0019001 | F | 5, | 20 | 13.576 (x 1.473) | 162 (0.123) | 0.178 | guanyl nucleotide binding | Arf79F CG1354 CG1900 CG2108 CG8902 Cdc42 Elf G-salpha60A Galpha49B Mtl Pepck Rab10 RhoBTB Su(var)3-9 alphaTub84B betaTub56D chb cta ebi pnut |
| 789 | GO:0004812 | F | 6, | 9 | 4.860 (x 1.852) | 58 (0.155) | 0.179 | aminoacyl-tRNA ligase activity | Aats-ala Aats-glupro CG31739 CG33123 CG7441 CG9425 Top3beta X11L mdy |
| 790 | GO:0016875 | F | 4, | 9 | 4.860 (x 1.852) | 58 (0.155) | 0.179 | ligase activity, forming carbon-oxygen bonds | Aats-ala Aats-glupro CG31739 CG33123 CG7441 CG9425 Top3beta X11L mdy |
| 791 | GO:0007219 | P | 6, | 7 | 3.436 (x 2.037) | 41 (0.171) | 0.182 | Notch signaling pathway | CG5841 Ser Su(dx) bib ebi heph l(2)gl |
| 792 | GO:0048113 | P | 8, 10, 11, 13, | 7 | 3.436 (x 2.037) | 41 (0.171) | 0.182 | pole plasm assembly (sensu Insecta) | Hrb27C Moe Rop Tm1 capu sqd tud |
| 793 | GO:0004527 | F | 6, | 7 | 3.436 (x 2.037) | 41 (0.171) | 0.182 | exonuclease activity | BcDNA:GM10765 CG10354 DNApol-delta DNApol-epsilon Rrp1 egl tos |
| 794 | GO:0008047 | F | 3, | 13 | 7.961 (x 1.633) | 95 (0.137) | 0.185 | enzyme activator activity | 14-3-3zeta CG30372 CG40160 CG6838 CG8243 CdGAPr CycA CycT Gap1 RacGAP50C RhoGAP1A sec23 vav |
| 795 | GO:0050767 | P | 4, 7, | 5 | 2.095 (x 2.387) | 25 (0.200) | 0.186 | regulation of neurogenesis | Cdc42 ana ed pbl trio |
| 796 | GO:0008354 | P | 5, 6, 7, | 6 | 2.765 (x 2.170) | 33 (0.182) | 0.186 | germ cell migration | Fpps hh scrib shg stai th |
| 797 | GO:0009611 | P | 4, | 6 | 2.765 (x 2.170) | 33 (0.182) | 0.187 | response to wounding | 18w Cdc42 kay kn ninA serpin-27A |
| 798 | GO:0031982 | C | 3, | 12 | 7.207 (x 1.665) | 86 (0.140) | 0.188 | vesicle | AP-1gamma CG1472 Chc Rop Vap-33-1 alpha-Adaptin btsz garz hh jar l(2)gl sec23 |
| 799 | GO:0031988 | C | 4, | 12 | 7.207 (x 1.665) | 86 (0.140) | 0.188 | membrane-bound vesicle | AP-1gamma CG1472 Chc Rop Vap-33-1 alpha-Adaptin btsz garz hh jar l(2)gl sec23 |
| 800 | GO:0007548 | P | 3, | 8 | 4.190 (x 1.909) | 50 (0.160) | 0.189 | sex differentiation | Btk29A Dll Fas2 dac en hh scrib shg |
| 801 | GO:0007451 | P | 6, 7, | 2 | 0.419 (x 4.773) | 5 (0.400) | 0.197 | dorsal/ventral lineage restriction, imaginal disc | Ser ap |
| 802 | GO:0017022 | F | 5, | 2 | 0.419 (x 4.773) | 5 (0.400) | 0.197 | myosin binding | Mbs l(2)gl |
| 803 | GO:0018212 | P | 9, | 2 | 0.419 (x 4.773) | 5 (0.400) | 0.197 | peptidyl-tyrosine modification | Ack Src64B |
| 804 | GO:0006638 | P | 6, 7, | 2 | 0.419 (x 4.773) | 5 (0.400) | 0.197 | neutral lipid metabolism | CG9342 mdy |
| 805 | GO:0050768 | P | 5, 8, | 2 | 0.419 (x 4.773) | 5 (0.400) | 0.198 | negative regulation of neurogenesis | ana ed |
| 806 | GO:0009112 | P | 6, | 11 | 6.536 (x 1.683) | 78 (0.141) | 0.198 | nucleobase metabolism | ATPsyn-beta CG1746 CG3590 CG6767 CG6854 RnrL Vha55 ade2 ade3 dnk r-l |
| 807 | GO:0007126 | P | 7, | 12 | 7.291 (x 1.646) | 87 (0.138) | 0.198 | meiosis | CG40410 Gap1 LanA Top2 ald cdc2 east l(1)dd4 lwr pav polo sca |
| 808 | GO:0042447 | P | 6, | 2 | 0.419 (x 4.773) | 5 (0.400) | 0.198 | hormone catabolism | Jheh1 Jheh2 |
| 809 | GO:0042674 | P | 5, 8, 9, | 3 | 0.922 (x 3.254) | 11 (0.273) | 0.198 | cone cell differentiation (sensu Endopterygota) | ebi rg sdk |
| 810 | GO:0008312 | F | 5, | 2 | 0.419 (x 4.773) | 5 (0.400) | 0.198 | 7S RNA binding | CG5064 CG5434 |
| 811 | GO:0042060 | P | 5, | 3 | 0.922 (x 3.254) | 11 (0.273) | 0.198 | wound healing | Cdc42 kay ninA |
| 812 | GO:0004839 | F | 4, | 2 | 0.419 (x 4.773) | 5 (0.400) | 0.198 | ubiquitin activating enzyme activity | Uba1 Uba2 |
| 813 | GO:0030855 | P | 4, 5, | 3 | 0.922 (x 3.254) | 11 (0.273) | 0.198 | epithelial cell differentiation | crb fz zip |
| 814 | GO:0046365 | P | 7, 8, | 8 | 4.274 (x 1.872) | 51 (0.157) | 0.198 | monosaccharide catabolism | Ald BEST:LD22483 CG15093 CG8036 Eno Gapdh1 Gapdh2 Pgi |
| 815 | GO:0007091 | P | 8, | 2 | 0.419 (x 4.773) | 5 (0.400) | 0.199 | mitotic metaphase/anaphase transition | Cks CycA |
| 816 | GO:0006563 | P | 8, 9, | 3 | 0.922 (x 3.254) | 11 (0.273) | 0.199 | L-serine metabolism | CG6287 CtBP ESTS:39C10S |
| 817 | GO:0006007 | P | 9, 10, | 8 | 4.274 (x 1.872) | 51 (0.157) | 0.199 | glucose catabolism | Ald BEST:LD22483 CG15093 CG8036 Eno Gapdh1 Gapdh2 Pgi |
| 818 | GO:0004860 | F | 5, | 2 | 0.419 (x 4.773) | 5 (0.400) | 0.199 | protein kinase inhibitor activity | 14-3-3epsilon 14-3-3zeta |
| 819 | GO:0031461 | C | 4, 5, 6, 7, | 3 | 0.922 (x 3.254) | 11 (0.273) | 0.199 | cullin-RING ubiquitin ligase complex | gft lin19 slmb |
| 820 | GO:0019320 | P | 8, 9, | 8 | 4.274 (x 1.872) | 51 (0.157) | 0.199 | hexose catabolism | Ald BEST:LD22483 CG15093 CG8036 Eno Gapdh1 Gapdh2 Pgi |
| 821 | GO:0007110 | P | 6, 7, | 2 | 0.419 (x 4.773) | 5 (0.400) | 0.199 | cytokinesis after meiosis I | fwd pbl |
| 822 | GO:0019005 | C | 5, 6, 7, 8, | 3 | 0.922 (x 3.254) | 11 (0.273) | 0.199 | SCF ubiquitin ligase complex | gft lin19 slmb |
| 823 | GO:0046164 | P | 6, | 8 | 4.274 (x 1.872) | 51 (0.157) | 0.199 | alcohol catabolism | Ald BEST:LD22483 CG15093 CG8036 Eno Gapdh1 Gapdh2 Pgi |
| 824 | GO:0006998 | P | 6, 7, | 2 | 0.419 (x 4.773) | 5 (0.400) | 0.199 | nuclear membrane organization and biogenesis | Lam Ote |
| 825 | GO:0042675 | P | 4, | 3 | 0.922 (x 3.254) | 11 (0.273) | 0.199 | cone cell differentiation | ebi rg sdk |
| 826 | GO:0008039 | P | 5, | 2 | 0.419 (x 4.773) | 5 (0.400) | 0.2 | synaptic target recognition | Fas3 Sema-2a |
| 827 | GO:0035147 | P | 5, 7, | 3 | 0.922 (x 3.254) | 11 (0.273) | 0.2 | tracheal branch fusion | arm hdc shg |
| 828 | GO:0006639 | P | 6, 7, 8, | 2 | 0.419 (x 4.773) | 5 (0.400) | 0.2 | acylglycerol metabolism | CG9342 mdy |
| 829 | GO:0035146 | P | 6, | 3 | 0.922 (x 3.254) | 11 (0.273) | 0.2 | tube fusion | arm hdc shg |
| 830 | GO:0035154 | P | 6, 7, | 2 | 0.419 (x 4.773) | 5 (0.400) | 0.2 | terminal cell fate specification | hdc sty |
| 831 | GO:0048637 | P | 6, | 7 | 3.520 (x 1.989) | 42 (0.167) | 0.2 | skeletal muscle development | CaMKII Fas2 G-salpha60A Hem Vap-33-1 tkv zip |
| 832 | GO:0019991 | P | 8, 9, | 3 | 0.922 (x 3.254) | 11 (0.273) | 0.2 | septate junction assembly | Cont cora l(2)gl |
| 833 | GO:0045045 | P | 5, 6, | 25 | 18.017 (x 1.388) | 215 (0.116) | 0.2 | secretory pathway | AP-1gamma Amph Arf79F CG1472 CG1900 CG5064 CG5434 CG9906 Chc EG:34F3.8 Rab10 Rop S Snap Vap-33-1 X11L alpha-Adaptin garz l(2)gl lqf mth pnut sec23 syndapin wbl |
| 834 | GO:0030071 | P | 8, 9, | 2 | 0.419 (x 4.773) | 5 (0.400) | 0.2 | regulation of mitotic metaphase/anaphase transition | Cks CycA |
| 835 | GO:0048747 | P | 5, | 7 | 3.520 (x 1.989) | 42 (0.167) | 0.2 | muscle fiber development | CaMKII Fas2 G-salpha60A Hem Vap-33-1 tkv zip |
| 836 | GO:0009069 | P | 7, 8, | 4 | 1.508 (x 2.652) | 18 (0.222) | 0.2 | serine family amino acid metabolism | CG6287 CtBP ESTS:39C10S SelD |
| 837 | GO:0006006 | P | 8, 9, | 9 | 5.028 (x 1.790) | 60 (0.150) | 0.2 | glucose metabolism | Ald BEST:LD22483 CG15093 CG8036 Eno Gapdh1 Gapdh2 Pepck Pgi |
| 838 | GO:0000077 | P | 6, 9, | 2 | 0.419 (x 4.773) | 5 (0.400) | 0.2 | DNA damage checkpoint | 14-3-3epsilon CG40410 |
| 839 | GO:0007306 | P | 9, 10, | 7 | 3.520 (x 1.989) | 42 (0.167) | 0.201 | insect chorion formation | Caf1 Cdc42 Dp Mcm6 Myb gus mus209 |
| 840 | GO:0008623 | C | 4, 7, 8, 9, 10, 11, 12, 13, 14, | 2 | 0.419 (x 4.773) | 5 (0.400) | 0.201 | chromatin accessibility complex | Iswi Top2 |
| 841 | GO:0048741 | P | 6, 7, | 7 | 3.520 (x 1.989) | 42 (0.167) | 0.201 | skeletal muscle fiber development | CaMKII Fas2 G-salpha60A Hem Vap-33-1 tkv zip |
| 842 | GO:0048475 | C | 4, 5, | 5 | 2.179 (x 2.295) | 26 (0.192) | 0.202 | coated membrane | AP-1gamma CG1472 Chc alpha-Adaptin sec23 |
| 843 | GO:0008287 | C | 3, 4, | 5 | 2.179 (x 2.295) | 26 (0.192) | 0.202 | protein serine/threonine phosphatase complex | CG17598 CG1906 Mbs Pp1-87B Pp2C1 |
| 844 | GO:0004221 | F | 6, 8, | 5 | 2.179 (x 2.295) | 26 (0.192) | 0.202 | ubiquitin thiolesterase activity | BcDNA:LD22910 CG5505 CG7288 CG8494 Uch |
| 845 | GO:0004520 | F | 7, | 5 | 2.179 (x 2.295) | 26 (0.192) | 0.202 | endodeoxyribonuclease activity | BcDNA:GM10765 CG7922 CG9425 Rrp1 Top3beta |
| 846 | GO:0030117 | C | 4, 5, 6, 7, 8, | 5 | 2.179 (x 2.295) | 26 (0.192) | 0.203 | membrane coat | AP-1gamma CG1472 Chc alpha-Adaptin sec23 |
| 847 | GO:0045476 | P | 7, 9, | 2 | 0.503 (x 3.978) | 6 (0.333) | 0.205 | nurse cell apoptosis | Dp mdy |
| 848 | GO:0031332 | C | 3, 4, 5, 6, | 2 | 0.503 (x 3.978) | 6 (0.333) | 0.205 | RNAi effector complex | CG7008 vig |
| 849 | GO:0051299 | P | 6, 8, 9, | 2 | 0.503 (x 3.978) | 6 (0.333) | 0.205 | centrosome separation | chb larp |
| 850 | GO:0046486 | P | 6, 7, | 2 | 0.503 (x 3.978) | 6 (0.333) | 0.205 | glycerolipid metabolism | CG9342 mdy |
| 851 | GO:0017015 | P | 5, 6, 9, | 2 | 0.503 (x 3.978) | 6 (0.333) | 0.205 | regulation of transforming growth factor beta receptor signaling pathway | brk slmb |
| 852 | GO:0031111 | P | 7, 11, | 2 | 0.503 (x 3.978) | 6 (0.333) | 0.206 | negative regulation of microtubule polymerization or depolymerization | CG11207 fwd |
| 853 | GO:0004500 | F | 6, | 2 | 0.503 (x 3.978) | 6 (0.333) | 0.206 | dopamine beta-monooxygenase activity | knk olf413 |
| 854 | GO:0045941 | P | 8, | 9 | 5.363 (x 1.678) | 64 (0.141) | 0.206 | positive regulation of transcription | Bap170 Iswi ash2 brm ci da dalao lola mor |
| 855 | GO:0031570 | P | 8, | 2 | 0.503 (x 3.978) | 6 (0.333) | 0.206 | DNA integrity checkpoint | 14-3-3epsilon CG40410 |
| 856 | GO:0000307 | C | 3, 4, 5, 6, | 2 | 0.503 (x 3.978) | 6 (0.333) | 0.206 | cyclin-dependent protein kinase holoenzyme complex | CycA Cyp1 |
| 857 | GO:0016840 | F | 4, | 2 | 0.503 (x 3.978) | 6 (0.333) | 0.206 | carbon-nitrogen lyase activity | CG3590 Hmu |
| 858 | GO:0006422 | P | 9, 10, 11, | 2 | 0.503 (x 3.978) | 6 (0.333) | 0.206 | aspartyl-tRNA aminoacylation | CG31739 mdy |
| 859 | GO:0004815 | F | 7, | 2 | 0.503 (x 3.978) | 6 (0.333) | 0.207 | aspartate-tRNA ligase activity | CG31739 mdy |
| 860 | GO:0051091 | P | 9, | 2 | 0.503 (x 3.978) | 6 (0.333) | 0.207 | positive regulation of transcription factor activity | Uba2 hh |
| 861 | GO:0017048 | F | 7, | 2 | 0.503 (x 3.978) | 6 (0.333) | 0.207 | Rho GTPase binding | Cip4 Rhp |
| 862 | GO:0031410 | C | 4, 5, 6, 7, 8, | 11 | 6.955 (x 1.581) | 83 (0.133) | 0.207 | cytoplasmic vesicle | AP-1gamma CG1472 Chc Vap-33-1 alpha-Adaptin btsz garz hh jar l(2)gl sec23 |
| 863 | GO:0042026 | P | 8, | 2 | 0.503 (x 3.978) | 6 (0.333) | 0.207 | protein refolding | Hsp27 Hsp60 |
| 864 | GO:0016023 | C | 5, 6, 7, 8, 9, | 11 | 6.955 (x 1.581) | 83 (0.133) | 0.207 | cytoplasmic membrane-bound vesicle | AP-1gamma CG1472 Chc Vap-33-1 alpha-Adaptin btsz garz hh jar l(2)gl sec23 |
| 865 | GO:0008013 | F | 4, | 2 | 0.503 (x 3.978) | 6 (0.333) | 0.207 | beta-catenin binding | lgs shg |
| 866 | GO:0016442 | C | 4, 5, 6, 7, | 2 | 0.503 (x 3.978) | 6 (0.333) | 0.208 | RNA-induced silencing complex | CG7008 vig |
| 867 | GO:0015286 | F | 6, | 2 | 0.503 (x 3.978) | 6 (0.333) | 0.208 | innexin channel activity | inx2 inx3 |
| 868 | GO:0008590 | P | 6, 7, 8, | 2 | 0.503 (x 3.978) | 6 (0.333) | 0.208 | regulation of frizzled signaling pathway | slmb stan |
| 869 | GO:0045198 | P | 6, 7, 8, 9, | 2 | 0.503 (x 3.978) | 6 (0.333) | 0.208 | establishment of epithelial cell polarity | crb fz |
| 870 | GO:0051090 | P | 8, | 2 | 0.503 (x 3.978) | 6 (0.333) | 0.208 | regulation of transcription factor activity | Uba2 hh |
| 871 | GO:0044444 | C | 4, 5, 6, 7, | 111 | 96.957 (x 1.145) | 1157 (0.096) | 0.208 | cytoplasmic part | AP-1gamma ATPsyn-beta Acon Acox57D-p ApepP BG:DS00004.11 BcDNA:GH04962 BcDNA:GH12558 CBP CG10423 CG1472 CG15093 CG1746 CG1907 CG2118 CG33113 CG5028 CG5064 CG5434 CG5841 CG6394 CG6543 CG6638 CG7145 CG7263 CG8443 CG9057 CG9149 Cat Cbp80 Cen190 Chc CkIIalpha CkIIbeta CycA Cyp1 ERp60 Elf Eno Fur1 Gdh Got2 Hsc70-3 Hsp60 Idh Karybeta3 Map60 Mpcp NUCB1 Nek2 Pepck Pi3K68D Pp1-87B Rbp2 RnrL Rop RpL13 RpL15 RpL22 RpL23a RpL3 RpS12 RpS6 S Snap Su(var)3-9 Thiolase Tm1 Tom34 Trxr-1 Ucp4B Vap-33-1 Vha44 Vha55 alpha-Adaptin alpha-Spec asp baz btsz chb cib dnk eIF-4B eIF3-S9 eIF5 fz fzy garz hh hts jar katanin-60 kel l(1)G0334 l(1)dd4 l(2)gl larp msk p120ctn par-6 pav pnut polo pon rin sec23 smi35A stc tud wbl zip |
| 872 | GO:0009794 | P | 5, 7, 8, | 2 | 0.503 (x 3.978) | 6 (0.333) | 0.208 | regulation of progression through embryonic mitotic cell cycle | CG40410 CycA |
| 873 | GO:0006662 | P | 5, | 2 | 0.503 (x 3.978) | 6 (0.333) | 0.209 | glycerol ether metabolism | CG9342 mdy |
| 874 | GO:0030859 | P | 5, 6, | 2 | 0.503 (x 3.978) | 6 (0.333) | 0.209 | polarized epithelial cell differentiation | crb fz |
| 875 | GO:0006952 | P | 4, | 52 | 43.074 (x 1.207) | 514 (0.101) | 0.209 | defense response | 18w Ack CG10211 CG10602 CG11652 CG11836 CG12199 CG12896 CG2852 CG30011 CG5789 CG5873 CG6673 CG7263 CG7668 CG8863 CG8902 CG9027 Cat Cyp1 DnaJ-1 EG:BACH59J11.2 GNBP3 Hsc70-3 Hsp23 Hsp26 Hsp27 Hsp67Bc Jheh1 Jheh2 Mcr Mdr49 PFE PR2 Pi3K68D Pli RpS6 Sb Trap1 Tsp66E dome fra gfzf kn otk pll sca scrib serpin-27A ss trn tub |
| 876 | GO:0006094 | P | 8, 9, 10, | 2 | 0.503 (x 3.978) | 6 (0.333) | 0.209 | gluconeogenesis | Pepck Pgi |
| 877 | GO:0006144 | P | 7, | 8 | 4.609 (x 1.736) | 55 (0.145) | 0.209 | purine base metabolism | ATPsyn-beta CG1746 CG3590 CG6767 RnrL Vha55 ade2 ade3 |
| 878 | GO:0007026 | P | 7, 8, 10, 12, | 2 | 0.503 (x 3.978) | 6 (0.333) | 0.209 | negative regulation of microtubule depolymerization | CG11207 fwd |
| 879 | GO:0042810 | P | 5, | 2 | 0.503 (x 3.978) | 6 (0.333) | 0.209 | pheromone metabolism | E(z) ovo |
| 880 | GO:0007312 | P | 8, 9, 11, 12, 14, | 2 | 0.503 (x 3.978) | 6 (0.333) | 0.21 | oocyte nucleus migration during oocyte axis determination | BicD egl |
| 881 | GO:0000228 | C | 5, 6, 7, 8, 9, 10, | 7 | 3.855 (x 1.816) | 46 (0.152) | 0.21 | nuclear chromosome | BEAF-32 DNApol-epsilon E(z) HmgD Psc Su(z)12 cav |
| 882 | GO:0019908 | C | 4, 5, 6, 7, 8, 9, 10, | 2 | 0.503 (x 3.978) | 6 (0.333) | 0.21 | nuclear cyclin-dependent protein kinase holoenzyme complex | CycA Cyp1 |
| 883 | GO:0030496 | C | 3, 4, | 2 | 0.503 (x 3.978) | 6 (0.333) | 0.21 | midbody | Nek2 pav |
| 884 | GO:0030140 | C | 6, 7, 8, 9, 10, 11, 12, | 2 | 0.503 (x 3.978) | 6 (0.333) | 0.21 | trans-Golgi network transport vesicle | AP-1gamma alpha-Adaptin |
| 885 | GO:0048128 | P | 9, 10, 12, 13, 15, | 2 | 0.503 (x 3.978) | 6 (0.333) | 0.21 | oocyte nucleus migration during oocyte axis determination (sensu Insecta) | BicD egl |
| 886 | GO:0007458 | P | 8, 9, | 2 | 0.503 (x 3.978) | 6 (0.333) | 0.211 | progression of morphogenetic furrow (sensu Endopterygota) | br hh |
| 887 | GO:0044454 | C | 5, 6, 7, 8, 9, 10, 11, | 7 | 3.603 (x 1.943) | 43 (0.163) | 0.211 | nuclear chromosome part | BEAF-32 DNApol-epsilon E(z) HmgD Psc Su(z)12 cav |
| 888 | GO:0035153 | P | 5, 6, | 2 | 0.503 (x 3.978) | 6 (0.333) | 0.211 | tracheal epithelial cell type specification | hdc sty |
| 889 | GO:0007530 | P | 3, | 7 | 3.603 (x 1.943) | 43 (0.163) | 0.211 | sex determination | CG8924 Sox14 br da lola ovo sc |
| 890 | GO:0008046 | F | 5, | 2 | 0.503 (x 3.978) | 6 (0.333) | 0.211 | axon guidance receptor activity | fra plexA |
| 891 | GO:0005678 | C | 4, 7, 8, 9, 10, 11, 12, 13, 14, | 2 | 0.503 (x 3.978) | 6 (0.333) | 0.211 | chromatin assembly complex | Caf1 Iswi |
| 892 | GO:0045334 | C | 7, 8, 9, 10, 11, 12, | 1 | 0.084 (x 11.933) | 1 (1.000) | 0.211 | clathrin-coated endocytic vesicle | alpha-Adaptin |
| 893 | GO:0006403 | P | 4, | 12 | 7.793 (x 1.540) | 93 (0.129) | 0.211 | RNA localization | BicD CG17143 CG2980 Dlc90F Hrb27C Hrb87F Rop Tm1 capu egl sqd tud |
| 894 | GO:0004077 | F | 6, | 1 | 0.084 (x 11.933) | 1 (1.000) | 0.211 | biotin-[acetyl-CoA-carboxylase] ligase activity | CG14670 |
| 895 | GO:0030545 | F | 4, | 1 | 0.084 (x 11.933) | 1 (1.000) | 0.212 | receptor regulator activity | argos |
| 896 | GO:0031887 | P | 8, 9, 10, | 1 | 0.084 (x 11.933) | 1 (1.000) | 0.212 | lipid particle transport along microtubule | CG9057 |
| 897 | GO:0005614 | C | 3, 4, | 1 | 0.084 (x 11.933) | 1 (1.000) | 0.212 | interstitial matrix | l(2)gl |
| 898 | GO:0015249 | F | 5, | 1 | 0.084 (x 11.933) | 1 (1.000) | 0.212 | nonselective channel activity | bib |
| 899 | GO:0019949 | F | 4, | 1 | 0.084 (x 11.933) | 1 (1.000) | 0.212 | SUMO conjugating enzyme activity | lwr |
| 900 | GO:0035035 | F | 5, | 1 | 0.084 (x 11.933) | 1 (1.000) | 0.213 | histone acetyltransferase binding | Caf1 |
| 901 | GO:0008486 | F | 7, | 1 | 0.084 (x 11.933) | 1 (1.000) | 0.213 | diphosphoinositol-polyphosphate diphosphatase activity | CG6391 |
| 902 | GO:0030128 | C | 7, 8, 9, 10, 11, 12, 13, 14, | 1 | 0.084 (x 11.933) | 1 (1.000) | 0.213 | clathrin coat of endocytic vesicle | alpha-Adaptin |
| 903 | GO:0003883 | F | 5, | 1 | 0.084 (x 11.933) | 1 (1.000) | 0.213 | CTP synthase activity | CG6854 |
| 904 | GO:0017023 | C | 4, 5, | 1 | 0.084 (x 11.933) | 1 (1.000) | 0.213 | myosin phosphatase complex | Mbs |
| 905 | GO:0007481 | P | 6, 7, | 1 | 0.084 (x 11.933) | 1 (1.000) | 0.213 | haltere disc morphogenesis | ap |
| 906 | GO:0045448 | P | 4, 6, | 3 | 1.089 (x 2.754) | 13 (0.231) | 0.214 | mitotic cell cycle, embryonic | 14-3-3zeta CG40410 CycA |
| 907 | GO:0035074 | P | 6, | 1 | 0.084 (x 11.933) | 1 (1.000) | 0.214 | pupation | crc |
| 908 | GO:0006891 | P | 6, 7, 8, 9, | 3 | 1.089 (x 2.754) | 13 (0.231) | 0.214 | intra-Golgi vesicle-mediated transport | S garz wbl |
| 909 | GO:0009893 | P | 5, | 10 | 6.201 (x 1.613) | 74 (0.135) | 0.214 | positive regulation of metabolism | Bap170 Iswi ash2 brm ci da dalao lola mor pAbp |
| 910 | GO:0016508 | F | 6, | 1 | 0.084 (x 11.933) | 1 (1.000) | 0.214 | long-chain-enoyl-CoA hydratase activity | BcDNA:GH12558 |
| 911 | GO:0016183 | P | 8, 9, 10, | 3 | 1.089 (x 2.754) | 13 (0.231) | 0.214 | synaptic vesicle coating | AP-1gamma Chc alpha-Adaptin |
| 912 | GO:0031325 | P | 6, | 10 | 6.201 (x 1.613) | 74 (0.135) | 0.214 | positive regulation of cellular metabolism | Bap170 Iswi ash2 brm ci da dalao lola mor pAbp |
| 913 | GO:0035285 | P | 4, 5, | 3 | 1.089 (x 2.754) | 13 (0.231) | 0.214 | appendage segmentation | Ser Su(dx) ap |
| 914 | GO:0004522 | F | 9, | 1 | 0.084 (x 11.933) | 1 (1.000) | 0.214 | pancreatic ribonuclease activity | CG10103 |
| 915 | GO:0051297 | P | 7, | 3 | 1.089 (x 2.754) | 13 (0.231) | 0.214 | centrosome organization and biogenesis | Myb chb larp |
| 916 | GO:0007058 | P | 9, 10, 13, | 1 | 0.084 (x 11.933) | 1 (1.000) | 0.214 | female meiosis II spindle assembly (sensu Metazoa) | polo |
| 917 | GO:0035286 | P | 5, 6, 7, | 3 | 1.089 (x 2.754) | 13 (0.231) | 0.214 | leg segmentation | Ser Su(dx) ap |
| 918 | GO:0000089 | P | 7, 8, | 1 | 0.084 (x 11.933) | 1 (1.000) | 0.214 | mitotic metaphase | east |
| 919 | GO:0045465 | P | 7, 8, 9, 10, | 3 | 1.089 (x 2.754) | 13 (0.231) | 0.215 | R8 cell differentiation | da hh sca |
| 920 | GO:0008588 | P | 8, 11, 12, 13, 14, 15, | 1 | 0.084 (x 11.933) | 1 (1.000) | 0.215 | release of cytoplasmic sequestered NF-kappaB | slmb |
| 921 | GO:0007098 | P | 5, 8, | 3 | 1.089 (x 2.754) | 13 (0.231) | 0.215 | centrosome cycle | Myb chb larp |
| 922 | GO:0043295 | F | 4, | 1 | 0.084 (x 11.933) | 1 (1.000) | 0.215 | glutathione binding | gfzf |
| 923 | GO:0006901 | P | 7, 8, | 3 | 1.089 (x 2.754) | 13 (0.231) | 0.215 | vesicle coating | AP-1gamma Chc alpha-Adaptin |
| 924 | GO:0006369 | P | 9, | 1 | 0.084 (x 11.933) | 1 (1.000) | 0.215 | transcription termination from RNA polymerase II promoter | lds |
| 925 | GO:0051223 | P | 6, 7, | 3 | 1.089 (x 2.754) | 13 (0.231) | 0.215 | regulation of protein transport | Akt1 hh slmb |
| 926 | GO:0007190 | P | 7, 10, 11, 12, | 1 | 0.084 (x 11.933) | 1 (1.000) | 0.215 | adenylate cyclase activation | G-salpha60A |
| 927 | GO:0008103 | P | 6, 8, 9, 10, 11, | 3 | 1.089 (x 2.754) | 13 (0.231) | 0.215 | oocyte microtubule cytoskeleton polarization | 14-3-3epsilon 14-3-3zeta BicD |
| 928 | GO:0015988 | P | 7, 8, 9, 10, | 1 | 0.084 (x 11.933) | 1 (1.000) | 0.215 | energy coupled proton transport, against electrochemical gradient | Vha55 |
| 929 | GO:0030586 | F | 5, 6, | 1 | 0.084 (x 11.933) | 1 (1.000) | 0.216 | [methionine synthase] reductase activity | CG14882 |
| 930 | GO:0042542 | P | 6, 7, 8, | 1 | 0.084 (x 11.933) | 1 (1.000) | 0.216 | response to hydrogen peroxide | Cat |
| 931 | GO:0015038 | F | 6, | 1 | 0.084 (x 11.933) | 1 (1.000) | 0.216 | glutathione disulfide oxidoreductase activity | Trxr-1 |
| 932 | GO:0035108 | P | 5, | 5 | 2.430 (x 2.057) | 29 (0.172) | 0.216 | limb morphogenesis | Dll Ser Su(dx) ap dac |
| 933 | GO:0045218 | P | 8, | 1 | 0.084 (x 11.933) | 1 (1.000) | 0.216 | zonula adherens maintenance | crb |
| 934 | GO:0007416 | P | 5, 6, | 5 | 2.430 (x 2.057) | 29 (0.172) | 0.216 | synaptogenesis | Fas2 Got2 baz par-6 tkv |
| 935 | GO:0045841 | P | 8, 9, 10, | 1 | 0.084 (x 11.933) | 1 (1.000) | 0.216 | negative regulation of mitotic metaphase/anaphase transition | CycA |
| 936 | GO:0007349 | P | 3, 4, | 5 | 2.430 (x 2.057) | 29 (0.172) | 0.217 | cellularization | Btk29A CG40410 Cdc42 Src64B pnut |
| 937 | GO:0045294 | F | 4, | 1 | 0.084 (x 11.933) | 1 (1.000) | 0.217 | alpha-catenin binding | arm |
| 938 | GO:0006513 | P | 10, | 1 | 0.084 (x 11.933) | 1 (1.000) | 0.217 | protein monoubiquitination | th |
| 939 | GO:0051222 | P | 7, 8, | 1 | 0.084 (x 11.933) | 1 (1.000) | 0.217 | positive regulation of protein transport | slmb |
| 940 | GO:0044271 | P | 5, 6, | 11 | 7.039 (x 1.563) | 84 (0.131) | 0.217 | nitrogen compound biosynthesis | 14-3-3zeta Acon CG40160 CG5028 CG6287 CG7145 CtBP ESTS:39C10S Got2 M(2)21AB SelD |
| 941 | GO:0004362 | F | 3, 5, 6, 7, | 1 | 0.084 (x 11.933) | 1 (1.000) | 0.217 | glutathione-disulfide reductase activity | Trxr-1 |
| 942 | GO:0009309 | P | 6, 7, | 11 | 7.039 (x 1.563) | 84 (0.131) | 0.217 | amine biosynthesis | 14-3-3zeta Acon CG40160 CG5028 CG6287 CG7145 CtBP ESTS:39C10S Got2 M(2)21AB SelD |
| 943 | GO:0045935 | P | 7, | 9 | 5.447 (x 1.652) | 65 (0.138) | 0.217 | positive regulation of nucleobase, nucleoside, nucleotide and nucleic acid metabolism | Bap170 Iswi ash2 brm ci da dalao lola mor |
| 944 | GO:0008240 | F | 6, | 1 | 0.084 (x 11.933) | 1 (1.000) | 0.217 | tripeptidyl-peptidase activity | TppII |
| 945 | GO:0006335 | P | 8, 12, | 1 | 0.084 (x 11.933) | 1 (1.000) | 0.218 | DNA replication-dependent nucleosome assembly | Caf1 |
| 946 | GO:0018215 | P | 8, | 1 | 0.084 (x 11.933) | 1 (1.000) | 0.218 | protein amino acid phosphopantetheinylation | CG32099 |
| 947 | GO:0009173 | P | 9, | 1 | 0.084 (x 11.933) | 1 (1.000) | 0.218 | pyrimidine ribonucleoside monophosphate metabolism | dnk |
| 948 | GO:0017049 | F | 8, | 1 | 0.084 (x 11.933) | 1 (1.000) | 0.218 | GTP-Rho binding | Rhp |
| 949 | GO:0000705 | P | 9, | 1 | 0.084 (x 11.933) | 1 (1.000) | 0.219 | achiasmate meiosis I | east |
| 950 | GO:0000185 | P | 8, 9, | 1 | 0.084 (x 11.933) | 1 (1.000) | 0.219 | activation of MAPKKK activity | msn |
| 951 | GO:0004721 | F | 7, | 12 | 7.458 (x 1.609) | 89 (0.135) | 0.219 | phosphoprotein phosphatase activity | CG12252 CG14411 CG17598 CG1906 CG6896 Mbs Pp1-87B Pp2C1 Ptp99A csw dome spag |
| 952 | GO:0035312 | F | 8, 9, | 1 | 0.084 (x 11.933) | 1 (1.000) | 0.219 | 5'-3' exodeoxyribonuclease activity | BcDNA:GM10765 |
| 953 | GO:0050431 | F | 5, | 1 | 0.084 (x 11.933) | 1 (1.000) | 0.219 | transforming growth factor beta binding | tkv |
| 954 | GO:0017098 | F | 4, 5, | 1 | 0.084 (x 11.933) | 1 (1.000) | 0.219 | sulfonylurea receptor binding | endos |
| 955 | GO:0006097 | P | 7, 8, | 1 | 0.084 (x 11.933) | 1 (1.000) | 0.22 | glyoxylate cycle | Idh |
| 956 | GO:0018107 | P | 9, 10, | 1 | 0.084 (x 11.933) | 1 (1.000) | 0.22 | peptidyl-threonine phosphorylation | BcDNA:LD09009 |
| 957 | GO:0008542 | P | 5, 6, 7, | 1 | 0.084 (x 11.933) | 1 (1.000) | 0.22 | visual learning | Pp1-87B |
| 958 | GO:0031072 | F | 4, | 1 | 0.084 (x 11.933) | 1 (1.000) | 0.22 | heat shock protein binding | CG2947 |
| 959 | GO:0017129 | F | 4, | 1 | 0.084 (x 11.933) | 1 (1.000) | 0.22 | triglyceride binding | CG9342 |
| 960 | GO:0007498 | P | 4, | 23 | 16.593 (x 1.386) | 198 (0.116) | 0.22 | mesoderm development | Ald CG12896 CG30011 CG5830 Cip4 Galpha49B LanA Msp-300 S Sox14 aop bib crol csw exd gol grn hh nonA-l pbl sgl shg sty |
| 961 | GO:0016978 | F | 6, | 1 | 0.084 (x 11.933) | 1 (1.000) | 0.221 | lipoate-protein ligase B activity | CG6767 |
| 962 | GO:0004642 | F | 6, | 1 | 0.084 (x 11.933) | 1 (1.000) | 0.221 | phosphoribosylformylglycinamidine synthase activity | ade2 |
| 963 | GO:0007406 | P | 6, 7, 8, 9, | 1 | 0.084 (x 11.933) | 1 (1.000) | 0.221 | negative regulation of neuroblast proliferation | ana |
| 964 | GO:0006980 | P | 5, 6, 7, | 1 | 0.084 (x 11.933) | 1 (1.000) | 0.221 | redox signal response | BthD |
| 965 | GO:0031344 | P | 6, 7, | 1 | 0.084 (x 11.933) | 1 (1.000) | 0.221 | regulation of cell projection organization and biogenesis | CaMKII |
| 966 | GO:0005031 | F | 6, | 1 | 0.084 (x 11.933) | 1 (1.000) | 0.222 | tumor necrosis factor receptor activity | wgn |
| 967 | GO:0004080 | F | 6, | 1 | 0.084 (x 11.933) | 1 (1.000) | 0.222 | biotin-[propionyl-CoA-carboxylase (ATP-hydrolyzing)] ligase activity | CG14670 |
| 968 | GO:0004074 | F | 6, | 1 | 0.084 (x 11.933) | 1 (1.000) | 0.222 | biliverdin reductase activity | CG9471 |
| 969 | GO:0016358 | P | 7, 10, | 6 | 2.933 (x 2.046) | 35 (0.171) | 0.222 | dendrite development | Cdc42 Smox Tm1 ena fra stan |
| 970 | GO:0016201 | P | 6, | 1 | 0.084 (x 11.933) | 1 (1.000) | 0.222 | synaptic target inhibition | Sema-2a |
| 971 | GO:0009124 | P | 7, 8, | 4 | 1.760 (x 2.273) | 21 (0.190) | 0.222 | nucleoside monophosphate biosynthesis | CG6767 ade2 ade3 dnk |
| 972 | GO:0005704 | C | 5, 6, 7, 8, 9, 10, | 1 | 0.084 (x 11.933) | 1 (1.000) | 0.222 | polytene chromosome band | Su(z)12 |
| 973 | GO:0009123 | P | 7, | 4 | 1.760 (x 2.273) | 21 (0.190) | 0.223 | nucleoside monophosphate metabolism | CG6767 ade2 ade3 dnk |
| 974 | GO:0008476 | F | 6, | 1 | 0.084 (x 11.933) | 1 (1.000) | 0.223 | protein-tyrosine sulfotransferase activity | CG32632 |
| 975 | GO:0042979 | F | 3, | 1 | 0.084 (x 11.933) | 1 (1.000) | 0.223 | ornithine decarboxylase regulator activity | guf |
| 976 | GO:0030666 | C | 6, 7, 8, 9, 10, 11, | 1 | 0.084 (x 11.933) | 1 (1.000) | 0.223 | endocytic vesicle membrane | alpha-Adaptin |
| 977 | GO:0046500 | P | 6, 7, | 1 | 0.084 (x 11.933) | 1 (1.000) | 0.223 | S-adenosylmethionine metabolism | M(2)21AB |
| 978 | GO:0040037 | P | 6, 7, 10, | 1 | 0.084 (x 11.933) | 1 (1.000) | 0.224 | negative regulation of fibroblast growth factor receptor signaling pathway | sty |
| 979 | GO:0008349 | F | 5, 9, | 1 | 0.084 (x 11.933) | 1 (1.000) | 0.224 | MAP kinase kinase kinase kinase activity | msn |
| 980 | GO:0035184 | F | 8, | 1 | 0.084 (x 11.933) | 1 (1.000) | 0.224 | histone threonine kinase activity | BcDNA:LD09009 |
| 981 | GO:0031109 | P | 9, | 4 | 1.592 (x 2.512) | 19 (0.211) | 0.224 | microtubule polymerization or depolymerization | CG11207 alphaTub84B betaTub56D fwd |
| 982 | GO:0005626 | C | 4, 5, | 1 | 0.084 (x 11.933) | 1 (1.000) | 0.224 | insoluble fraction | Top2 |
| 983 | GO:0009174 | P | 9, 10, | 1 | 0.084 (x 11.933) | 1 (1.000) | 0.224 | pyrimidine ribonucleoside monophosphate biosynthesis | dnk |
| 984 | GO:0044272 | P | 6, | 4 | 1.592 (x 2.512) | 19 (0.211) | 0.224 | sulfur compound biosynthesis | CG6835 M(2)21AB SelD sgl |
| 985 | GO:0008184 | F | 7, | 1 | 0.084 (x 11.933) | 1 (1.000) | 0.225 | glycogen phosphorylase activity | GlyP |
| 986 | GO:0017160 | F | 7, | 1 | 0.084 (x 11.933) | 1 (1.000) | 0.225 | Ral GTPase binding | vimar |
| 987 | GO:0004294 | F | 7, | 1 | 0.084 (x 11.933) | 1 (1.000) | 0.225 | tripeptidyl-peptidase II activity | TppII |
| 988 | GO:0045893 | P | 9, | 7 | 3.939 (x 1.777) | 47 (0.149) | 0.225 | positive regulation of transcription, DNA-dependent | Bap170 ash2 brm ci dalao lola mor |
| 989 | GO:0030192 | F | 3, | 1 | 0.084 (x 11.933) | 1 (1.000) | 0.225 | Hsp70/Hsc70 protein regulator activity | CG2947 |
| 990 | GO:0046602 | P | 7, 8, 10, 11, | 1 | 0.084 (x 11.933) | 1 (1.000) | 0.225 | regulation of mitotic centrosome separation | chb |
| 991 | GO:0030188 | F | 2, | 1 | 0.084 (x 11.933) | 1 (1.000) | 0.226 | chaperone regulator activity | CG2947 |
| 992 | GO:0016245 | P | 9, 10, | 1 | 0.084 (x 11.933) | 1 (1.000) | 0.226 | hyperphosphorylation of RNA polymerase II | cdc2 |
| 993 | GO:0008420 | F | 9, | 1 | 0.084 (x 11.933) | 1 (1.000) | 0.226 | CTD phosphatase activity | CG12252 |
| 994 | GO:0004373 | F | 7, 8, | 1 | 0.084 (x 11.933) | 1 (1.000) | 0.226 | glycogen (starch) synthase activity | CG6904 |
| 995 | GO:0042346 | P | 10, 11, 12, 13, 14, | 1 | 0.084 (x 11.933) | 1 (1.000) | 0.227 | positive regulation of NF-kappaB import into nucleus | slmb |
| 996 | GO:0042087 | P | 7, 8, | 1 | 0.084 (x 11.933) | 1 (1.000) | 0.227 | cell-mediated immune response | kn |
| 997 | GO:0017099 | F | 6, | 1 | 0.084 (x 11.933) | 1 (1.000) | 0.227 | very-long-chain-acyl-CoA dehydrogenase activity | CG7461 |
| 998 | GO:0045337 | P | 7, 8, 9, | 1 | 0.084 (x 11.933) | 1 (1.000) | 0.227 | farnesyl diphosphate biosynthesis | Fpps |
| 999 | GO:0008321 | F | 5, | 1 | 0.084 (x 11.933) | 1 (1.000) | 0.227 | Ral guanyl-nucleotide exchange factor activity | Rgl |
| 1000 | GO:0035011 | P | 6, 7, 8, | 1 | 0.084 (x 11.933) | 1 (1.000) | 0.228 | melanotic encapsulation of foreign target | serpin-27A |
| 1001 | GO:0004062 | F | 6, | 1 | 0.084 (x 11.933) | 1 (1.000) | 0.228 | aryl sulfotransferase activity | CG16733 |
| 1002 | GO:0007365 | P | 5, 6, | 5 | 2.263 (x 2.210) | 27 (0.185) | 0.228 | periodic partitioning | en hh lgs sgl stan |
| 1003 | GO:0000015 | C | 3, 6, 7, 8, 9, 10, | 1 | 0.084 (x 11.933) | 1 (1.000) | 0.228 | phosphopyruvate hydratase complex | Eno |
| 1004 | GO:0004382 | F | 8, | 1 | 0.084 (x 11.933) | 1 (1.000) | 0.228 | guanosine-diphosphatase activity | NTPase |
| 1005 | GO:0042127 | P | 5, | 5 | 2.263 (x 2.210) | 27 (0.185) | 0.228 | regulation of cell proliferation | CG11228 ana hh l(2)gl scrib |
| 1006 | GO:0048102 | P | 6, | 10 | 5.950 (x 1.681) | 71 (0.141) | 0.229 | autophagic cell death | Akap200 Cyp1 Eip75B PR2 br l(1)G0148 l(2)01424 l(2)gl larp zip |
| 1007 | GO:0007418 | P | 6, | 1 | 0.084 (x 11.933) | 1 (1.000) | 0.229 | ventral midline development | csw |
| 1008 | GO:0044433 | C | 4, 5, 6, 7, 8, 9, | 5 | 2.263 (x 2.210) | 27 (0.185) | 0.229 | cytoplasmic vesicle part | AP-1gamma CG1472 Chc alpha-Adaptin sec23 |
| 1009 | GO:0007147 | P | 9, | 1 | 0.084 (x 11.933) | 1 (1.000) | 0.229 | female meiosis II | polo |
| 1010 | GO:0035071 | P | 7, | 10 | 5.950 (x 1.681) | 71 (0.141) | 0.229 | salivary gland cell autophagic cell death | Akap200 Cyp1 Eip75B PR2 br l(1)G0148 l(2)01424 l(2)gl larp zip |
| 1011 | GO:0006552 | P | 9, 10, | 1 | 0.084 (x 11.933) | 1 (1.000) | 0.229 | leucine catabolism | CG6638 |
| 1012 | GO:0035070 | P | 6, | 10 | 5.950 (x 1.681) | 71 (0.141) | 0.229 | salivary gland histolysis | Akap200 Cyp1 Eip75B PR2 br l(1)G0148 l(2)01424 l(2)gl larp zip |
| 1013 | GO:0016233 | P | 10, | 1 | 0.084 (x 11.933) | 1 (1.000) | 0.229 | telomere capping | cav |
| 1014 | GO:0004641 | F | 6, | 1 | 0.084 (x 11.933) | 1 (1.000) | 0.23 | phosphoribosylformylglycinamidine cyclo-ligase activity | ade3 |
| 1015 | GO:0019432 | P | 8, 9, 10, | 1 | 0.084 (x 11.933) | 1 (1.000) | 0.23 | triacylglycerol biosynthesis | mdy |
| 1016 | GO:0007076 | P | 7, 8, 9, | 3 | 1.006 (x 2.983) | 12 (0.250) | 0.23 | mitotic chromosome condensation | Cap-G barr larp |
| 1017 | GO:0004907 | F | 6, | 1 | 0.084 (x 11.933) | 1 (1.000) | 0.23 | interleukin receptor activity | dome |
| 1018 | GO:0003700 | F | 3, 5, | 41 | 32.598 (x 1.258) | 389 (0.105) | 0.23 | transcription factor activity | BcDNA:LD23876 CG30011 CG4914 CG6854 CG9104 Dll Dp Dr Dref Eip75B GATAd Hcf Hmr Myb Optix Pcl Sin3A Sox14 aop ap br brk ci cic crc d4 da dve en exd grn jim jing jumu kay sd sima ss stc toe zf30C |
| 1019 | GO:0018987 | P | 4, | 1 | 0.084 (x 11.933) | 1 (1.000) | 0.23 | osmoregulation | endos |
| 1020 | GO:0042446 | P | 6, | 3 | 1.006 (x 2.983) | 12 (0.250) | 0.23 | hormone biosynthesis | 14-3-3zeta Vha44 woc |
| 1021 | GO:0015141 | F | 6, | 1 | 0.084 (x 11.933) | 1 (1.000) | 0.23 | succinate transporter activity | Indy |
| 1022 | GO:0000915 | P | 7, 11, | 3 | 1.006 (x 2.983) | 12 (0.250) | 0.23 | cytokinesis, contractile ring formation | pav pbl pnut |
| 1023 | GO:0046605 | P | 6, 9, | 1 | 0.084 (x 11.933) | 1 (1.000) | 0.231 | regulation of centrosome cycle | chb |
| 1024 | GO:0000912 | P | 6, 10, | 3 | 1.006 (x 2.983) | 12 (0.250) | 0.231 | cytokinesis, formation of actomyosin apparatus | pav pbl pnut |
| 1025 | GO:0030046 | P | 11, | 1 | 0.084 (x 11.933) | 1 (1.000) | 0.231 | parallel actin filament bundle formation | Src64B |
| 1026 | GO:0006740 | P | 10, 11, | 3 | 1.006 (x 2.983) | 12 (0.250) | 0.231 | NADPH regeneration | BEST:LD22483 CG15093 CG8036 |
| 1027 | GO:0017155 | F | 8, 9, 10, | 1 | 0.084 (x 11.933) | 1 (1.000) | 0.231 | sodium:hydrogen antiporter regulator activity | Sip1 |
| 1028 | GO:0008589 | P | 5, 6, 7, | 3 | 1.006 (x 2.983) | 12 (0.250) | 0.231 | regulation of smoothened signaling pathway | CkIalpha hh slmb |
| 1029 | GO:0045315 | P | 8, 9, 10, 11, 12, | 1 | 0.084 (x 11.933) | 1 (1.000) | 0.231 | positive regulation of eye photoreceptor development (sensu Endopterygota) | mbt |
| 1030 | GO:0004518 | F | 5, | 12 | 7.542 (x 1.591) | 90 (0.133) | 0.231 | nuclease activity | BcDNA:GM10765 CG10103 CG10354 CG7008 CG7922 CG9425 DNApol-delta DNApol-epsilon Rrp1 Top3beta egl tos |
| 1031 | GO:0016198 | P | 5, 7, 8, 10, 11, 13, | 3 | 1.006 (x 2.983) | 12 (0.250) | 0.232 | axon choice point recognition | Galpha49B fra lola |
| 1032 | GO:0042480 | P | 8, 9, 10, | 1 | 0.084 (x 11.933) | 1 (1.000) | 0.232 | negative regulation of eye photoreceptor cell development | aop |
| 1033 | GO:0000212 | P | 6, 10, | 3 | 1.006 (x 2.983) | 12 (0.250) | 0.232 | meiotic spindle organization and biogenesis | fwd pbl polo |
| 1034 | GO:0000700 | F | 7, | 1 | 0.084 (x 11.933) | 1 (1.000) | 0.232 | mismatch base pair DNA N-glycosylase activity | Thd1 |
| 1035 | GO:0048565 | P | 4, | 9 | 5.196 (x 1.732) | 62 (0.145) | 0.232 | gut development | barr dome dve fat2 hh inx2 kay pbl zip |
| 1036 | GO:0006556 | P | 6, 7, 8, 9, 10, | 1 | 0.084 (x 11.933) | 1 (1.000) | 0.232 | S-adenosylmethionine biosynthesis | M(2)21AB |
| 1037 | GO:0008045 | P | 7, 8, 10, 11, 13, | 3 | 1.006 (x 2.983) | 12 (0.250) | 0.232 | motor axon guidance | Ptp99A fra plexA |
| 1038 | GO:0000738 | P | 7, 8, | 1 | 0.084 (x 11.933) | 1 (1.000) | 0.232 | DNA catabolism, exonucleolytic | BcDNA:GM10765 |
| 1039 | GO:0008543 | P | 8, | 3 | 1.006 (x 2.983) | 12 (0.250) | 0.232 | fibroblast growth factor receptor signaling pathway | csw sgl sty |
| 1040 | GO:0008106 | F | 7, | 1 | 0.084 (x 11.933) | 1 (1.000) | 0.232 | alcohol dehydrogenase (NADP+) activity | CG2767 |
| 1041 | GO:0031032 | P | 9, | 3 | 1.006 (x 2.983) | 12 (0.250) | 0.233 | actomyosin structure organization and biogenesis | pav pbl pnut |
| 1042 | GO:0009314 | P | 4, | 8 | 4.441 (x 1.801) | 53 (0.151) | 0.233 | response to radiation | 14-3-3epsilon CG40410 G-salpha60A Galpha49B Pp1-87B Rop Sema-2a ogre |
| 1043 | GO:0042993 | P | 9, 10, 11, 12, 13, | 1 | 0.084 (x 11.933) | 1 (1.000) | 0.233 | positive regulation of transcription factor import into nucleus | slmb |
| 1044 | GO:0007440 | P | 7, 8, | 3 | 1.006 (x 2.983) | 12 (0.250) | 0.233 | foregut morphogenesis | fat2 hh inx2 |
| 1045 | GO:0003844 | F | 6, | 1 | 0.084 (x 11.933) | 1 (1.000) | 0.233 | 1,4-alpha-glucan branching enzyme activity | CG33138 |
| 1046 | GO:0043087 | P | 5, | 3 | 1.006 (x 2.983) | 12 (0.250) | 0.233 | regulation of GTPase activity | CG30372 CG6838 CG8243 |
| 1047 | GO:0003959 | F | 5, | 1 | 0.084 (x 11.933) | 1 (1.000) | 0.233 | NADPH dehydrogenase activity | CG9471 |
| 1048 | GO:0009058 | P | 4, | 86 | 75.253 (x 1.143) | 898 (0.096) | 0.233 | biosynthesis | 14-3-3zeta ATPsyn-beta Aats-ala Aats-glupro Acon Akt1 CG10423 CG10990 CG1129 CG12006 CG1746 CG17514 CG2118 CG31472 CG31739 CG32099 CG32158 CG33116 CG33123 CG33145 CG3590 CG40160 CG5028 CG5191 CG5434 CG6287 CG6394 CG6767 CG6835 CG6854 CG6904 CG7145 CG7441 CG8443 CG8963 CG9425 Cbp80 CtBP EG:86E4.2 ESTS:39C10S Elf Fpps Gfat1 Got2 Hmu M(2)21AB Paip2 Pepck Pgi Rbp2 RpL13 RpL15 RpL22 RpL23a RpL3 RpS12 RpS6 SelD Sin3A Su(var)3-9 Top3beta Vha44 Vha55 X11L ade2 ade3 bl cora dnk dome eIF-4B eIF3-S9 eIF5 jumu knk l(2)01424 mdy msi ovo pAbp r-l rin sgl sqd stc woc |
| 1049 | GO:0006739 | P | 9, 10, | 3 | 1.006 (x 2.983) | 12 (0.250) | 0.233 | NADP metabolism | BEST:LD22483 CG15093 CG8036 |
| 1050 | GO:0043148 | P | 7, 9, 10, 11, 12, 14, | 1 | 0.084 (x 11.933) | 1 (1.000) | 0.233 | mitotic spindle stabilization | CG11207 |
| 1051 | GO:0016723 | F | 5, | 1 | 0.084 (x 11.933) | 1 (1.000) | 0.234 | oxidoreductase activity, oxidizing metal ions, NAD or NADP as acceptor | CG14882 |
| 1052 | GO:0030178 | P | 6, 7, 8, | 3 | 1.006 (x 2.983) | 12 (0.250) | 0.234 | negative regulation of Wnt receptor signaling pathway | RacGAP50C slmb stan |
| 1053 | GO:0004588 | F | 6, | 1 | 0.084 (x 11.933) | 1 (1.000) | 0.234 | orotate phosphoribosyltransferase activity | r-l |
| 1054 | GO:0006098 | P | 8, 10, 11, 12, | 3 | 1.006 (x 2.983) | 12 (0.250) | 0.234 | pentose-phosphate shunt | BEST:LD22483 CG15093 CG8036 |
| 1055 | GO:0004590 | F | 6, | 1 | 0.084 (x 11.933) | 1 (1.000) | 0.234 | orotidine-5'-phosphate decarboxylase activity | r-l |
| 1056 | GO:0042306 | P | 7, 8, 9, 10, 11, | 3 | 1.006 (x 2.983) | 12 (0.250) | 0.234 | regulation of protein import into nucleus | Akt1 hh slmb |
| 1057 | GO:0008022 | F | 4, | 1 | 0.084 (x 11.933) | 1 (1.000) | 0.234 | protein C-terminus binding | CtBP |
| 1058 | GO:0007181 | P | 9, | 1 | 0.084 (x 11.933) | 1 (1.000) | 0.235 | transforming growth factor beta receptor complex assembly | tkv |
| 1059 | GO:0016539 | P | 10, | 1 | 0.084 (x 11.933) | 1 (1.000) | 0.235 | intein-mediated protein splicing | hh |
| 1060 | GO:0009057 | P | 5, | 22 | 15.922 (x 1.382) | 190 (0.116) | 0.235 | macromolecule catabolism | Ald BEST:LD22483 BcDNA:GM10765 BcDNA:LD22910 CG15093 CG5505 CG7263 CG7288 CG8036 CG8494 CkIalpha Eno Gapdh1 Gapdh2 Pgi Tis11 Top3beta Uba2 Uch fzy sqd th |
| 1061 | GO:0000302 | P | 5, 6, 7, | 1 | 0.084 (x 11.933) | 1 (1.000) | 0.235 | response to reactive oxygen species | Cat |
| 1062 | GO:0016285 | F | 7, 8, | 1 | 0.084 (x 11.933) | 1 (1.000) | 0.235 | cytosol alanyl aminopeptidase activity | Psa |
| 1063 | GO:0045055 | P | 6, 7, | 13 | 8.799 (x 1.477) | 105 (0.124) | 0.235 | regulated secretory pathway | AP-1gamma Amph Arf79F Chc Rop Snap Vap-33-1 X11L alpha-Adaptin l(2)gl lqf mth syndapin |
| 1064 | GO:0035318 | P | 8, 9, 10, 11, | 1 | 0.084 (x 11.933) | 1 (1.000) | 0.236 | wing hair outgrowth | Cdc42 |
| 1065 | GO:0006887 | P | 6, 7, | 15 | 10.475 (x 1.432) | 125 (0.120) | 0.236 | exocytosis | Amph CG1900 CG9906 EG:34F3.8 Rab10 Rop Snap Vap-33-1 X11L garz l(2)gl mth pnut sec23 wbl |
| 1066 | GO:0007269 | P | 6, 7, 8, | 13 | 8.799 (x 1.477) | 105 (0.124) | 0.236 | neurotransmitter secretion | AP-1gamma Amph Arf79F Chc Rop Snap Vap-33-1 X11L alpha-Adaptin l(2)gl lqf mth syndapin |
| 1067 | GO:0017078 | F | 4, 6, | 1 | 0.084 (x 11.933) | 1 (1.000) | 0.236 | Hsc70 protein regulator activity | CG2947 |
| 1068 | GO:0009605 | P | 3, | 13 | 8.799 (x 1.477) | 105 (0.124) | 0.236 | response to external stimulus | 14-3-3epsilon 18w Cdc42 Galpha49B endos hh kay kn mth ninA ogre pain serpin-27A |
| 1069 | GO:0004144 | F | 9, | 1 | 0.084 (x 11.933) | 1 (1.000) | 0.236 | diacylglycerol O-acyltransferase activity | mdy |
| 1070 | GO:0004450 | F | 7, | 1 | 0.084 (x 11.933) | 1 (1.000) | 0.236 | isocitrate dehydrogenase (NADP+) activity | Idh |
| 1071 | GO:0005827 | C | 6, 7, 8, 9, 10, 11, 12, | 1 | 0.084 (x 11.933) | 1 (1.000) | 0.236 | polar microtubule | chb |
| 1072 | GO:0001555 | P | 4, 5, 7, 8, 10, | 1 | 0.084 (x 11.933) | 1 (1.000) | 0.237 | oocyte growth | Hem |
| 1073 | GO:0035291 | P | 6, 7, 8, | 1 | 0.084 (x 11.933) | 1 (1.000) | 0.237 | specification of segmental identity, intercalary segment | kn |
| 1074 | GO:0008897 | F | 6, | 1 | 0.084 (x 11.933) | 1 (1.000) | 0.237 | phosphopantetheinyltransferase activity | CG32099 |
| 1075 | GO:0008263 | F | 8, | 1 | 0.084 (x 11.933) | 1 (1.000) | 0.237 | pyrimidine-specific mismatch base pair DNA N-glycosylase activity | Thd1 |
| 1076 | GO:0019965 | F | 5, | 1 | 0.084 (x 11.933) | 1 (1.000) | 0.238 | interleukin binding | dome |
| 1077 | GO:0045811 | P | 7, 8, 9, | 1 | 0.084 (x 11.933) | 1 (1.000) | 0.238 | positive regulation of frizzled signaling pathway | stan |
| 1078 | GO:0042307 | P | 8, 9, 10, 11, 12, | 1 | 0.084 (x 11.933) | 1 (1.000) | 0.238 | positive regulation of protein import into nucleus | slmb |
| 1079 | GO:0030129 | C | 6, 7, 8, 9, 10, 11, 12, 13, 14, | 1 | 0.084 (x 11.933) | 1 (1.000) | 0.238 | clathrin coat of synaptic vesicle | Chc |
| 1080 | GO:0045842 | P | 8, 9, 10, | 1 | 0.084 (x 11.933) | 1 (1.000) | 0.239 | positive regulation of mitotic metaphase/anaphase transition | Cks |
| 1081 | GO:0042656 | F | 6, 10, | 1 | 0.084 (x 11.933) | 1 (1.000) | 0.239 | JUN kinase kinase kinase kinase activity | msn |
| 1082 | GO:0046824 | P | 7, 8, 9, 10, | 1 | 0.084 (x 11.933) | 1 (1.000) | 0.239 | positive regulation of nucleocytoplasmic transport | slmb |
| 1083 | GO:0030547 | F | 5, | 1 | 0.084 (x 11.933) | 1 (1.000) | 0.239 | receptor inhibitor activity | argos |
| 1084 | GO:0008652 | P | 7, 8, | 10 | 6.034 (x 1.657) | 72 (0.139) | 0.24 | amino acid biosynthesis | Acon CG40160 CG5028 CG6287 CG7145 CtBP ESTS:39C10S Got2 M(2)21AB SelD |
| 1085 | GO:0007442 | P | 7, 8, | 6 | 3.017 (x 1.989) | 36 (0.167) | 0.24 | hindgut morphogenesis | barr dome fat2 hh pbl zip |
| 1086 | GO:0030908 | P | 9, | 1 | 0.084 (x 11.933) | 1 (1.000) | 0.24 | protein splicing | hh |
| 1087 | GO:0007422 | P | 5, | 10 | 6.369 (x 1.570) | 76 (0.132) | 0.24 | peripheral nervous system development | CycA LanA S Sin3A barr da pav pbl sc trio |
| 1088 | GO:0008063 | P | 6, | 5 | 2.514 (x 1.989) | 30 (0.167) | 0.24 | Toll signaling pathway | Pli pll serpin-27A tub wbl |
| 1089 | GO:0042348 | P | 9, 10, 11, 12, | 1 | 0.084 (x 11.933) | 1 (1.000) | 0.24 | NF-kappaB import into nucleus | slmb |
| 1090 | GO:0048193 | P | 6, 7, 8, | 6 | 3.017 (x 1.989) | 36 (0.167) | 0.24 | Golgi vesicle transport | CG1472 S Snap garz sec23 wbl |
| 1091 | GO:0031348 | P | 6, | 1 | 0.084 (x 11.933) | 1 (1.000) | 0.24 | negative regulation of defense response | serpin-27A |
| 1092 | GO:0009260 | P | 7, 8, | 12 | 7.626 (x 1.574) | 91 (0.132) | 0.24 | ribonucleotide biosynthesis | ATPsyn-beta CG1746 CG3590 CG6767 Sin3A Vha44 Vha55 ade2 ade3 dnk dome jumu |
| 1093 | GO:0004634 | F | 6, | 1 | 0.084 (x 11.933) | 1 (1.000) | 0.24 | phosphopyruvate hydratase activity | Eno |
| 1094 | GO:0004644 | F | 8, | 1 | 0.084 (x 11.933) | 1 (1.000) | 0.241 | phosphoribosylglycinamide formyltransferase activity | ade3 |
| 1095 | GO:0003847 | F | 6, | 1 | 0.084 (x 11.933) | 1 (1.000) | 0.241 | 1-alkyl-2-acetylglycerophosphocholine esterase activity | Paf-AHalpha |
| 1096 | GO:0019093 | P | 5, | 1 | 0.084 (x 11.933) | 1 (1.000) | 0.241 | mitochondrial RNA localization | tud |
| 1097 | GO:0004018 | F | 6, | 1 | 0.084 (x 11.933) | 1 (1.000) | 0.241 | adenylosuccinate lyase activity | CG3590 |
| 1098 | GO:0018271 | F | 5, | 1 | 0.084 (x 11.933) | 1 (1.000) | 0.242 | biotin-protein ligase activity | CG14670 |
| 1099 | GO:0016220 | F | 6, | 1 | 0.084 (x 11.933) | 1 (1.000) | 0.242 | RAL GDP-dissociation stimulator activity | Rgl |
| 1100 | GO:0048151 | P | 8, | 1 | 0.084 (x 11.933) | 1 (1.000) | 0.242 | hyperphosphorylation | cdc2 |
| 1101 | GO:0003979 | F | 6, | 1 | 0.084 (x 11.933) | 1 (1.000) | 0.242 | UDP-glucose 6-dehydrogenase activity | sgl |
| 1102 | GO:0017020 | F | 5, | 1 | 0.084 (x 11.933) | 1 (1.000) | 0.243 | myosin phosphatase regulator activity | CG6896 |
| 1103 | GO:0004478 | F | 5, | 1 | 0.084 (x 11.933) | 1 (1.000) | 0.243 | methionine adenosyltransferase activity | M(2)21AB |
| 1104 | GO:0007436 | P | 6, 7, | 1 | 0.084 (x 11.933) | 1 (1.000) | 0.243 | larval salivary gland morphogenesis | Ser |
| 1105 | GO:0006230 | P | 10, 11, | 1 | 0.084 (x 11.933) | 1 (1.000) | 0.243 | TMP biosynthesis | dnk |
| 1106 | GO:0045338 | P | 7, 8, | 1 | 0.084 (x 11.933) | 1 (1.000) | 0.244 | farnesyl diphosphate metabolism | Fpps |
| 1107 | GO:0042479 | P | 8, 9, 10, | 1 | 0.084 (x 11.933) | 1 (1.000) | 0.244 | positive regulation of eye photoreceptor cell development | mbt |
| 1108 | GO:0016483 | F | 4, | 1 | 0.084 (x 11.933) | 1 (1.000) | 0.244 | tryptophan hydroxylase activator activity | 14-3-3zeta |
| 1109 | GO:0004648 | F | 6, | 1 | 0.084 (x 11.933) | 1 (1.000) | 0.244 | phosphoserine transaminase activity | ESTS:39C10S |
| 1110 | GO:0016066 |  | 6, 7, | 1 | 0.084 (x 11.933) | 1 (1.000) | 0.245 |  | kn |
| 1111 | GO:0046044 | P | 10, | 1 | 0.084 (x 11.933) | 1 (1.000) | 0.245 | TMP metabolism | dnk |
| 1112 | GO:0035218 | P | 5, | 6 | 3.268 (x 1.836) | 39 (0.154) | 0.245 | leg disc development | Dll Su(dx) ap dac exd hh |
| 1113 | GO:0030122 | C | 5, 6, 7, 8, 9, 10, 11, 12, 13, 14, 15, | 1 | 0.084 (x 11.933) | 1 (1.000) | 0.245 | AP-2 adaptor complex | alpha-Adaptin |
| 1114 | GO:0008852 | F | 9, | 1 | 0.084 (x 11.933) | 1 (1.000) | 0.245 | exodeoxyribonuclease I activity | tos |
| 1115 | GO:0019136 | F | 8, | 1 | 0.084 (x 11.933) | 1 (1.000) | 0.246 | deoxynucleoside kinase activity | dnk |
| 1116 | GO:0004347 | F | 6, | 1 | 0.084 (x 11.933) | 1 (1.000) | 0.246 | glucose-6-phosphate isomerase activity | Pgi |
| 1117 | GO:0019186 | F | 8, | 1 | 0.084 (x 11.933) | 1 (1.000) | 0.246 | acyl-CoA N-acyltransferase activity | CG14222 |
| 1118 | GO:0004630 | F | 7, 8, | 1 | 0.084 (x 11.933) | 1 (1.000) | 0.247 | phospholipase D activity | Pld |
| 1119 | GO:0030726 | P | 6, 7, | 1 | 0.084 (x 11.933) | 1 (1.000) | 0.247 | testicular ring canal formation | fwd |
| 1120 | GO:0030018 | C | 6, 7, 8, 9, 10, 11, | 1 | 0.084 (x 11.933) | 1 (1.000) | 0.247 | Z disc | zip |
| 1121 | GO:0004290 | F | 8, | 1 | 0.084 (x 11.933) | 1 (1.000) | 0.247 | kexin activity | Fur1 |
| 1122 | GO:0004887 | F | 5, | 1 | 0.084 (x 11.933) | 1 (1.000) | 0.248 | thyroid hormone receptor activity | Eip75B |
| 1123 | GO:0005521 | F | 4, | 1 | 0.084 (x 11.933) | 1 (1.000) | 0.248 | lamin binding | CG17952 |
| 1124 | GO:0046487 | P | 6, 7, | 1 | 0.084 (x 11.933) | 1 (1.000) | 0.248 | glyoxylate metabolism | Idh |
| 1125 | GO:0005035 | F | 5, | 1 | 0.084 (x 11.933) | 1 (1.000) | 0.248 | death receptor activity | wgn |
| 1126 | GO:0018210 | P | 9, | 1 | 0.084 (x 11.933) | 1 (1.000) | 0.249 | peptidyl-threonine modification | BcDNA:LD09009 |
| 1127 | GO:0019218 | P | 6, 7, 8, | 1 | 0.084 (x 11.933) | 1 (1.000) | 0.249 | regulation of steroid metabolism | Eip75B |
| 1128 | GO:0042756 | P | 5, | 1 | 0.084 (x 11.933) | 1 (1.000) | 0.249 | drinking behavior | Sema-2a |
| 1129 | GO:0042132 | F | 8, | 1 | 0.084 (x 11.933) | 1 (1.000) | 0.249 | fructose-bisphosphatase activity | fbp |
| 1130 | GO:0009607 | P | 3, | 53 | 43.744 (x 1.212) | 522 (0.102) | 0.249 | response to biotic stimulus | 18w Ack CG10211 CG10602 CG11652 CG11836 CG12199 CG12896 CG2852 CG30011 CG5789 CG5873 CG6673 CG7263 CG7668 CG8863 CG8902 CG9027 Cat Cyp1 DnaJ-1 EG:BACH59J11.2 GNBP3 Hsc70-3 Hsp23 Hsp26 Hsp27 Hsp67Bc Jheh1 Jheh2 Mcr Mdr49 PFE PR2 Pi3K68D Pli RpS6 Sb Trap1 Tsp66E br dome fra gfzf kn otk pll sca scrib serpin-27A ss trn tub |
| 1131 | GO:0019783 | F | 6, | 5 | 2.346 (x 2.131) | 28 (0.179) | 0.25 | small conjugating protein-specific protease activity | BcDNA:LD22910 CG5505 CG7288 CG8494 Uch |
| 1132 | GO:0015281 | F | 6, 7, | 1 | 0.084 (x 11.933) | 1 (1.000) | 0.25 | nonselective cation channel activity | bib |
| 1133 | GO:0006769 | P | 8, 9, | 3 | 1.173 (x 2.557) | 14 (0.214) | 0.25 | nicotinamide metabolism | BEST:LD22483 CG15093 CG8036 |
| 1134 | GO:0007446 | P | 4, 5, | 4 | 1.844 (x 2.170) | 22 (0.182) | 0.25 | imaginal disc growth | bl fat2 hh spag |
| 1135 | GO:0004843 | F | 7, | 5 | 2.346 (x 2.131) | 28 (0.179) | 0.25 | ubiquitin-specific protease activity | BcDNA:LD22910 CG5505 CG7288 CG8494 Uch |
| 1136 | GO:0006997 | P | 6, | 3 | 1.173 (x 2.557) | 14 (0.214) | 0.25 | nuclear organization and biogenesis | Lam Ote polo |
| 1137 | GO:0048019 | F | 4, 5, 6, | 1 | 0.084 (x 11.933) | 1 (1.000) | 0.25 | receptor antagonist activity | argos |
| 1138 | GO:0043085 | P | 4, | 4 | 1.844 (x 2.170) | 22 (0.182) | 0.25 | positive regulation of enzyme activity | Abi G-salpha60A mbt msn |
| 1139 | GO:0008408 | F | 7, | 5 | 2.346 (x 2.131) | 28 (0.179) | 0.25 | 3'-5' exonuclease activity | BcDNA:GM10765 DNApol-delta DNApol-epsilon Rrp1 egl |
| 1140 | GO:0003697 | F | 6, | 4 | 1.676 (x 2.387) | 20 (0.200) | 0.25 | single-stranded DNA binding | Dsp1 Hrb27C Pep stc |
| 1141 | GO:0006112 | P | 7, | 3 | 1.173 (x 2.557) | 14 (0.214) | 0.25 | energy reserve metabolism | CG33138 CG6904 GlyP |
| 1142 | GO:0006635 | P | 8, 9, | 4 | 1.844 (x 2.170) | 22 (0.182) | 0.25 | fatty acid beta-oxidation | Acox57D-p BcDNA:GH12558 CG6543 Thiolase |
| 1143 | GO:0008073 | F | 4, | 1 | 0.084 (x 11.933) | 1 (1.000) | 0.25 | ornithine decarboxylase inhibitor activity | guf |
| 1144 | GO:0051336 | P | 4, | 3 | 1.173 (x 2.557) | 14 (0.214) | 0.25 | regulation of hydrolase activity | CG30372 CG6838 CG8243 |
| 1145 | GO:0035110 | P | 6, | 5 | 2.346 (x 2.131) | 28 (0.179) | 0.25 | leg morphogenesis | Dll Ser Su(dx) ap dac |
| 1146 | GO:0009156 | P | 8, 9, | 4 | 1.676 (x 2.387) | 20 (0.200) | 0.25 | ribonucleoside monophosphate biosynthesis | CG6767 ade2 ade3 dnk |
| 1147 | GO:0003727 | F | 5, | 4 | 1.844 (x 2.170) | 22 (0.182) | 0.25 | single-stranded RNA binding | CG4612 Pep pAbp su(f) |
| 1148 | GO:0019090 | P | 6, 7, 8, 9, | 1 | 0.084 (x 11.933) | 1 (1.000) | 0.251 | mitochondrial rRNA export from mitochondrion | tud |
| 1149 | GO:0030665 | C | 7, 8, 9, 10, 11, 12, | 3 | 1.173 (x 2.557) | 14 (0.214) | 0.251 | clathrin coated vesicle membrane | AP-1gamma Chc alpha-Adaptin |
| 1150 | GO:0009161 | P | 8, | 4 | 1.676 (x 2.387) | 20 (0.200) | 0.251 | ribonucleoside monophosphate metabolism | CG6767 ade2 ade3 dnk |
| 1151 | GO:0008418 | F | 7, | 1 | 0.084 (x 11.933) | 1 (1.000) | 0.251 | protein N-terminal asparagine amidohydrolase activity | SP2637 |
| 1152 | GO:0046822 | P | 6, 7, 8, 9, | 3 | 1.173 (x 2.557) | 14 (0.214) | 0.251 | regulation of nucleocytoplasmic transport | Akt1 hh slmb |
| 1153 | GO:0007605 | P | 5, 7, | 4 | 1.676 (x 2.387) | 20 (0.200) | 0.251 | sensory perception of sound | ck d ebi jar |
| 1154 | GO:0030125 | C | 6, 7, 8, 9, 10, 11, 12, 13, | 3 | 1.173 (x 2.557) | 14 (0.214) | 0.251 | clathrin vesicle coat | AP-1gamma Chc alpha-Adaptin |
| 1155 | GO:0015037 | F | 5, | 1 | 0.084 (x 11.933) | 1 (1.000) | 0.251 | peptide disulfide oxidoreductase activity | Trxr-1 |
| 1156 | GO:0042052 | P | 8, 9, 10, 11, | 3 | 1.173 (x 2.557) | 14 (0.214) | 0.251 | rhabdomere development | Amph Moe crb |
| 1157 | GO:0008320 | F | 4, | 4 | 1.676 (x 2.387) | 20 (0.200) | 0.251 | protein carrier activity | Fs(2)Ket Karybeta3 Pen msk |
| 1158 | GO:0005577 | C | 3, 4, 5, | 1 | 0.084 (x 11.933) | 1 (1.000) | 0.251 | fibrinogen complex | sca |
| 1159 | GO:0007474 | P | 7, 8, 9, | 3 | 1.173 (x 2.557) | 14 (0.214) | 0.251 | wing vein specification | brm hh kn |
| 1160 | GO:0035009 | P | 6, 7, 9, | 1 | 0.084 (x 11.933) | 1 (1.000) | 0.252 | negative regulation of melanization defense response | serpin-27A |
| 1161 | GO:0031023 | P | 6, | 3 | 1.173 (x 2.557) | 14 (0.214) | 0.252 | microtubule organizing center organization and biogenesis | Myb chb larp |
| 1162 | GO:0004637 | F | 5, | 1 | 0.084 (x 11.933) | 1 (1.000) | 0.252 | phosphoribosylamine-glycine ligase activity | ade3 |
| 1163 | GO:0007553 | P | 7, 8, 9, | 1 | 0.084 (x 11.933) | 1 (1.000) | 0.252 | regulation of ecdysteroid metabolism | Eip75B |
| 1164 | GO:0045176 | P | 6, | 2 | 0.587 (x 3.409) | 7 (0.286) | 0.252 | apical protein localization | baz par-6 |
| 1165 | GO:0004448 | F | 6, | 2 | 0.587 (x 3.409) | 7 (0.286) | 0.252 | isocitrate dehydrogenase activity | CG5028 Idh |
| 1166 | GO:0051295 | P | 6, 7, 8, 10, | 1 | 0.084 (x 11.933) | 1 (1.000) | 0.252 | establishment of meiotic spindle localization | asp |
| 1167 | GO:0035193 | P | 5, 6, | 2 | 0.587 (x 3.409) | 7 (0.286) | 0.253 | central nervous system remodeling (sensu Insecta) | br cib |
| 1168 | GO:0004797 | F | 9, | 1 | 0.084 (x 11.933) | 1 (1.000) | 0.253 | thymidine kinase activity | dnk |
| 1169 | GO:0006265 | P | 7, | 2 | 0.587 (x 3.409) | 7 (0.286) | 0.253 | DNA topological change | Top2 Top3beta |
| 1170 | GO:0042345 | P | 9, 10, 11, 12, 13, | 1 | 0.084 (x 11.933) | 1 (1.000) | 0.253 | regulation of NF-kappaB import into nucleus | slmb |
| 1171 | GO:0016540 | P | 9, | 2 | 0.587 (x 3.409) | 7 (0.286) | 0.253 | protein autoprocessing | CalpB hh |
| 1172 | GO:0009259 | P | 7, | 12 | 7.710 (x 1.556) | 92 (0.130) | 0.253 | ribonucleotide metabolism | ATPsyn-beta CG1746 CG3590 CG6767 Sin3A Vha44 Vha55 ade2 ade3 dnk dome jumu |
| 1173 | GO:0004785 | F | 6, | 2 | 0.587 (x 3.409) | 7 (0.286) | 0.253 | copper, zinc superoxide dismutase activity | CG9027 Sh3beta |
| 1174 | GO:0050931 | P | 4, | 1 | 0.084 (x 11.933) | 1 (1.000) | 0.253 | pigment cell differentiation | sdk |
| 1175 | GO:0006189 | P | 11, 12, | 2 | 0.587 (x 3.409) | 7 (0.286) | 0.253 | 'de novo' IMP biosynthesis | ade2 ade3 |
| 1176 | GO:0007388 | P | 5, | 1 | 0.084 (x 11.933) | 1 (1.000) | 0.254 | posterior compartment specification | en |
| 1177 | GO:0048096 | P | 5, 10, 11, | 2 | 0.587 (x 3.409) | 7 (0.286) | 0.254 | chromatin-mediated maintenance of transcription | ash2 brm |
| 1178 | GO:0048057 | P | 8, 9, 10, 11, | 1 | 0.084 (x 11.933) | 1 (1.000) | 0.254 | R3/R4 development (sensu Endopterygota) | stan |
| 1179 | GO:0007009 | P | 6, | 2 | 0.587 (x 3.409) | 7 (0.286) | 0.254 | plasma membrane organization and biogenesis | CG1893 alpha-Spec |
| 1180 | GO:0019838 | F | 4, | 2 | 0.587 (x 3.409) | 7 (0.286) | 0.254 | growth factor binding | CG33214 tkv |
| 1181 | GO:0051489 | P | 7, 8, 9, 10, | 1 | 0.084 (x 11.933) | 1 (1.000) | 0.254 | regulation of filopodium formation | CaMKII |
| 1182 | GO:0000381 | P | 10, 11, 13, | 7 | 4.106 (x 1.705) | 49 (0.143) | 0.254 | regulation of alternative nuclear mRNA splicing, via spliceosome | B52 CG5728 CG6227 Hrb87F Rm62 bl sqd |
| 1183 | GO:0046040 | P | 10, | 2 | 0.587 (x 3.409) | 7 (0.286) | 0.254 | IMP metabolism | ade2 ade3 |
| 1184 | GO:0008494 | F | 4, 5, | 1 | 0.084 (x 11.933) | 1 (1.000) | 0.254 | translation activator activity | CG17514 |
| 1185 | GO:0007519 | P | 5, | 7 | 4.106 (x 1.705) | 49 (0.143) | 0.254 | striated muscle development | CaMKII Fas2 G-salpha60A Hem Vap-33-1 tkv zip |
| 1186 | GO:0000118 | C | 4, 7, 8, 9, 10, 11, 12, 13, 14, | 2 | 0.587 (x 3.409) | 7 (0.286) | 0.254 | histone deacetylase complex | Mi-2 Sin3A |
| 1187 | GO:0000380 | P | 10, 12, | 7 | 4.106 (x 1.705) | 49 (0.143) | 0.255 | alternative nuclear mRNA splicing, via spliceosome | B52 CG5728 CG6227 Hrb87F Rm62 bl sqd |
| 1188 | GO:0048601 | P | 6, 7, 9, | 1 | 0.084 (x 11.933) | 1 (1.000) | 0.255 | oocyte morphogenesis | Hem |
| 1189 | GO:0048103 | P | 6, | 2 | 0.587 (x 3.409) | 7 (0.286) | 0.255 | somatic stem cell division | hh shg |
| 1190 | GO:0042393 | F | 4, | 2 | 0.587 (x 3.409) | 7 (0.286) | 0.255 | histone binding | Caf1 Nlp |
| 1191 | GO:0030544 | F | 5, | 1 | 0.084 (x 11.933) | 1 (1.000) | 0.255 | Hsp70 protein binding | CG2947 |
| 1192 | GO:0048126 | P | 8, 9, 11, 12, 14, | 2 | 0.587 (x 3.409) | 7 (0.286) | 0.255 | establishment of oocyte nucleus localization during oocyte axis determination (sensu Insecta) | BicD egl |
| 1193 | GO:0016219 | F | 5, | 1 | 0.084 (x 11.933) | 1 (1.000) | 0.255 | GDP-dissociation stimulator activity | Rgl |
| 1194 | GO:0045815 | P | 4, | 2 | 0.587 (x 3.409) | 7 (0.286) | 0.255 | positive regulation of gene expression, epigenetic | ash2 brm |
| 1195 | GO:0008414 | F | 4, | 1 | 0.084 (x 11.933) | 1 (1.000) | 0.255 | CDP-alcohol phosphotransferase activity | CG33116 |
| 1196 | GO:0016624 | F | 5, | 2 | 0.587 (x 3.409) | 7 (0.286) | 0.256 | oxidoreductase activity, acting on the aldehyde or oxo group of donors, disulfide as acceptor | CG1544 l(1)G0334 |
| 1197 | GO:0016884 | F | 5, | 2 | 0.587 (x 3.409) | 7 (0.286) | 0.256 | carbon-nitrogen ligase activity, with glutamine as amido-N-donor | CG5191 ade2 |
| 1198 | GO:0051567 | P | 10, 11, 13, | 1 | 0.084 (x 11.933) | 1 (1.000) | 0.256 | histone H3-K9 methylation | Su(var)3-9 |
| 1199 | GO:0005154 | F | 4, 5, | 2 | 0.587 (x 3.409) | 7 (0.286) | 0.256 | epidermal growth factor receptor binding | Ser argos |
| 1200 | GO:0009056 | P | 4, | 34 | 27.403 (x 1.241) | 327 (0.104) | 0.256 | catabolism | Acon Ald BEST:LD22483 BcDNA:GM10765 BcDNA:LD22910 CG15093 CG1544 CG5028 CG5505 CG6638 CG7145 CG7263 CG7288 CG8036 CG8494 CkIalpha DNApol-delta Eno Gapdh1 Gapdh2 Gdh Idh Jheh1 Jheh2 Pgi Tis11 Top3beta Uba2 Uch br fzy l(1)G0334 sqd th |
| 1201 | GO:0045840 | P | 7, 8, 9, | 1 | 0.084 (x 11.933) | 1 (1.000) | 0.256 | positive regulation of mitosis | Cks |
| 1202 | GO:0046112 | P | 6, 7, | 2 | 0.587 (x 3.409) | 7 (0.286) | 0.256 | nucleobase biosynthesis | ade3 r-l |
| 1203 | GO:0043120 | F | 5, | 1 | 0.084 (x 11.933) | 1 (1.000) | 0.256 | tumor necrosis factor binding | wgn |
| 1204 | GO:0008187 | F | 5, | 2 | 0.587 (x 3.409) | 7 (0.286) | 0.256 | poly-pyrimidine tract binding | heph nonA-l |
| 1205 | GO:0040036 | P | 5, 6, 9, | 1 | 0.084 (x 11.933) | 1 (1.000) | 0.257 | regulation of fibroblast growth factor receptor signaling pathway | sty |
| 1206 | GO:0031114 | P | 7, 9, 11, | 2 | 0.587 (x 3.409) | 7 (0.286) | 0.257 | regulation of microtubule depolymerization | CG11207 fwd |
| 1207 | GO:0007620 | P | 6, 7, | 2 | 0.587 (x 3.409) | 7 (0.286) | 0.257 | copulation | Btk29A lig |
| 1208 | GO:0045159 | F | 6, | 1 | 0.084 (x 11.933) | 1 (1.000) | 0.257 | myosin II binding | l(2)gl |
| 1209 | GO:0051662 | P | 8, 10, 11, 13, | 2 | 0.587 (x 3.409) | 7 (0.286) | 0.257 | oocyte nucleus localization during oocyte axis determination (sensu Insecta) | BicD egl |
| 1210 | GO:0007112 | P | 6, | 1 | 0.084 (x 11.933) | 1 (1.000) | 0.257 | male meiosis cytokinesis | fwd |
| 1211 | GO:0006271 | P | 9, | 2 | 0.587 (x 3.409) | 7 (0.286) | 0.257 | DNA strand elongation | DNApol-delta mus209 |
| 1212 | GO:0003785 | F | 6, | 1 | 0.084 (x 11.933) | 1 (1.000) | 0.257 | actin monomer binding | cib |
| 1213 | GO:0007019 | P | 8, 10, | 2 | 0.587 (x 3.409) | 7 (0.286) | 0.257 | microtubule depolymerization | CG11207 fwd |
| 1214 | GO:0005100 | F | 5, | 2 | 0.587 (x 3.409) | 7 (0.286) | 0.258 | Rho GTPase activator activity | RacGAP50C vav |
| 1215 | GO:0045316 | P | 8, 9, 10, 11, 12, | 1 | 0.084 (x 11.933) | 1 (1.000) | 0.258 | negative regulation of eye photoreceptor development (sensu Endopterygota) | aop |
| 1216 | GO:0008641 | F | 3, | 2 | 0.587 (x 3.409) | 7 (0.286) | 0.258 | small protein activating enzyme activity | Uba1 Uba2 |
| 1217 | GO:0001505 | P | 7, | 14 | 9.805 (x 1.428) | 117 (0.120) | 0.258 | regulation of neurotransmitter levels | 14-3-3zeta AP-1gamma Amph Arf79F Chc Rop Snap Vap-33-1 X11L alpha-Adaptin l(2)gl lqf mth syndapin |
| 1218 | GO:0006588 | P | 9, 10, 11, 12, | 1 | 0.084 (x 11.933) | 1 (1.000) | 0.258 | tryptophan hydroxylase activation | 14-3-3zeta |
| 1219 | GO:0031110 | P | 6, 10, | 2 | 0.587 (x 3.409) | 7 (0.286) | 0.258 | regulation of microtubule polymerization or depolymerization | CG11207 fwd |
| 1220 | GO:0008470 | F | 6, | 1 | 0.084 (x 11.933) | 1 (1.000) | 0.258 | isovaleryl-CoA dehydrogenase activity | CG6638 |
| 1221 | GO:0030722 | P | 7, 8, 10, 11, 13, | 2 | 0.587 (x 3.409) | 7 (0.286) | 0.258 | establishment of oocyte nucleus localization during oocyte axis determination | BicD egl |
| 1222 | GO:0015929 | F | 6, | 2 | 0.587 (x 3.409) | 7 (0.286) | 0.259 | hexosaminidase activity | Hexo1 fdl |
| 1223 | GO:0045807 | P | 7, 8, 9, | 1 | 0.084 (x 11.933) | 1 (1.000) | 0.259 | positive regulation of endocytosis | CG5841 |
| 1224 | GO:0006188 | P | 10, 11, | 2 | 0.587 (x 3.409) | 7 (0.286) | 0.259 | IMP biosynthesis | ade2 ade3 |
| 1225 | GO:0030669 | C | 7, 8, 9, 10, 11, 12, 13, | 1 | 0.084 (x 11.933) | 1 (1.000) | 0.259 | clathrin-coated endocytic vesicle membrane | alpha-Adaptin |
| 1226 | GO:0051775 | P | 5, | 1 | 0.084 (x 11.933) | 1 (1.000) | 0.259 | response to redox state | BthD |
| 1227 | GO:0006796 | P | 6, | 59 | 50.616 (x 1.166) | 604 (0.098) | 0.263 | phosphate metabolism | ATPsyn-beta Ack Akt1 BcDNA:LD09009 BcDNA:LD23371 Btk29A CG11228 CG14217 CG17309 CG1746 CG17598 CG1906 CG40410 CG8789 CaMKII Cdk4 CkIIalpha CkIIbeta CkIalpha Cks Eip63E Gprk1 KP78b Mekk1 Mpcp Nek2 PFE PR2 Pak3 Pi3K68D Pp1-87B Pp2C1 Ptp99A Sin3A Src64B Vha44 Vha55 Wsck ald cdc2 csw dnk dome edl fat2 fwd gish jumu l(1)G0148 l(2)08717 mbt msn otk plexA pll polo smi35A syndapin tkv |
| 1228 | GO:0009117 | P | 6, | 18 | 13.324 (x 1.351) | 159 (0.113) | 0.264 | nucleotide metabolism | ATPsyn-beta BEST:LD22483 CG15093 CG1746 CG32158 CG3590 CG6767 CG6854 CG8036 Sin3A Vha44 Vha55 ade2 ade3 dnk dome jumu r-l |
| 1229 | GO:0006793 | P | 5, | 59 | 50.616 (x 1.166) | 604 (0.098) | 0.264 | phosphorus metabolism | ATPsyn-beta Ack Akt1 BcDNA:LD09009 BcDNA:LD23371 Btk29A CG11228 CG14217 CG17309 CG1746 CG17598 CG1906 CG40410 CG8789 CaMKII Cdk4 CkIIalpha CkIIbeta CkIalpha Cks Eip63E Gprk1 KP78b Mekk1 Mpcp Nek2 PFE PR2 Pak3 Pi3K68D Pp1-87B Pp2C1 Ptp99A Sin3A Src64B Vha44 Vha55 Wsck ald cdc2 csw dnk dome edl fat2 fwd gish jumu l(1)G0148 l(2)08717 mbt msn otk plexA pll polo smi35A syndapin tkv |
| 1230 | GO:0000578 | P | 5, | 8 | 4.944 (x 1.618) | 59 (0.136) | 0.265 | embryonic axis specification | Gap1 csw hh knk lwr pll sty tub |
| 1231 | GO:0045859 | P | 6, | 4 | 1.927 (x 2.075) | 23 (0.174) | 0.273 | regulation of protein kinase activity | Abi Ste:CG33236 mbt msn |
| 1232 | GO:0008213 | P | 8, | 4 | 1.927 (x 2.075) | 23 (0.174) | 0.273 | protein amino acid alkylation | Caf1 E(z) Su(var)3-9 Su(z)12 |
| 1233 | GO:0006479 | P | 8, 9, | 4 | 1.927 (x 2.075) | 23 (0.174) | 0.273 | protein amino acid methylation | Caf1 E(z) Su(var)3-9 Su(z)12 |
| 1234 | GO:0051338 | P | 4, | 4 | 1.927 (x 2.075) | 23 (0.174) | 0.273 | regulation of transferase activity | Abi Ste:CG33236 mbt msn |
| 1235 | GO:0019395 | P | 7, 8, | 4 | 1.927 (x 2.075) | 23 (0.174) | 0.274 | fatty acid oxidation | Acox57D-p BcDNA:GH12558 CG6543 Thiolase |
| 1236 | GO:0043549 | P | 5, | 4 | 1.927 (x 2.075) | 23 (0.174) | 0.274 | regulation of kinase activity | Abi Ste:CG33236 mbt msn |
| 1237 | GO:0007435 | P | 6, | 3 | 1.257 (x 2.387) | 15 (0.200) | 0.279 | salivary gland morphogenesis | Ser cora zip |
| 1238 | GO:0035151 | P | 6, | 3 | 1.257 (x 2.387) | 15 (0.200) | 0.279 | regulation of tracheal tube size | Fas2 Lac cora |
| 1239 | GO:0007450 | P | 5, 6, | 3 | 1.257 (x 2.387) | 15 (0.200) | 0.279 | dorsal/ventral pattern formation, imaginal disc | Dr Ser ap |
| 1240 | GO:0035289 | P | 6, 7, | 3 | 1.257 (x 2.387) | 15 (0.200) | 0.279 | posterior head segmentation | en hh kn |
| 1241 | GO:0019362 | P | 7, 8, | 3 | 1.257 (x 2.387) | 15 (0.200) | 0.279 | pyridine nucleotide metabolism | BEST:LD22483 CG15093 CG8036 |
| 1242 | GO:0007432 | P | 6, | 3 | 1.257 (x 2.387) | 15 (0.200) | 0.28 | salivary gland determination | brk exd tkv |
| 1243 | GO:0006090 | P | 7, | 3 | 1.257 (x 2.387) | 15 (0.200) | 0.28 | pyruvate metabolism | Pepck Pgi l(1)G0334 |
| 1244 | GO:0016185 | P | 7, 8, 9, | 3 | 1.257 (x 2.387) | 15 (0.200) | 0.28 | synaptic vesicle budding | AP-1gamma Chc alpha-Adaptin |
| 1245 | GO:0008285 | P | 6, | 3 | 1.257 (x 2.387) | 15 (0.200) | 0.28 | negative regulation of cell proliferation | ana l(2)gl scrib |
| 1246 | GO:0016790 | F | 5, | 5 | 2.682 (x 1.865) | 32 (0.156) | 0.281 | thiolester hydrolase activity | BcDNA:LD22910 CG5505 CG7288 CG8494 Uch |
| 1247 | GO:0030198 | P | 4, | 5 | 2.682 (x 1.865) | 32 (0.156) | 0.281 | extracellular matrix organization and biogenesis | Fas2 Got2 baz par-6 tkv |
| 1248 | GO:0050808 | P | 5, | 5 | 2.682 (x 1.865) | 32 (0.156) | 0.281 | synapse organization and biogenesis | Fas2 Got2 baz par-6 tkv |
| 1249 | GO:0006206 | P | 7, | 5 | 2.682 (x 1.865) | 32 (0.156) | 0.281 | pyrimidine base metabolism | CG6767 CG6854 RnrL dnk r-l |
| 1250 | GO:0003924 | F | 8, | 15 | 10.894 (x 1.377) | 130 (0.115) | 0.282 | GTPase activity | Arf79F CG1900 CG2108 CG8902 Cdc42 Elf G-salpha60A Galpha49B Mtl Rab10 RhoBTB alphaTub84B betaTub56D cta pnut |
| 1251 | GO:0005794 | C | 5, 6, 7, 8, | 11 | 7.542 (x 1.458) | 90 (0.122) | 0.29 | Golgi apparatus | AP-1gamma CG1472 CG6394 Fur1 NUCB1 S Snap alpha-Adaptin alpha-Spec garz sec23 |
| 1252 | GO:0007507 | P | 5, | 8 | 5.112 (x 1.565) | 61 (0.131) | 0.3 | heart development | LanA arm fz hh lqf sgl shg tkv |
| 1253 | GO:0005874 | C | 5, 6, 7, 8, 9, 10, | 6 | 3.520 (x 1.705) | 42 (0.143) | 0.301 | microtubule | Eb1 Klp10A alphaTub84B betaTub56D chb vg |
| 1254 | GO:0050658 | P | 6, 7, 8, | 4 | 2.011 (x 1.989) | 24 (0.167) | 0.301 | RNA transport | CG17143 CG2980 sqd tud |
| 1255 | GO:0051236 | P | 5, | 4 | 2.011 (x 1.989) | 24 (0.167) | 0.301 | establishment of RNA localization | CG17143 CG2980 sqd tud |
| 1256 | GO:0050657 | P | 6, 7, | 4 | 2.011 (x 1.989) | 24 (0.167) | 0.302 | nucleic acid transport | CG17143 CG2980 sqd tud |
| 1257 | GO:0016903 | F | 4, | 4 | 2.011 (x 1.989) | 24 (0.167) | 0.302 | oxidoreductase activity, acting on the aldehyde or oxo group of donors | CG1544 Gapdh1 Gapdh2 l(1)G0334 |
| 1258 | GO:0006334 | P | 7, 11, | 4 | 2.011 (x 1.989) | 24 (0.167) | 0.302 | nucleosome assembly | CG31617 Caf1 Iswi Set |
| 1259 | GO:0008587 | P | 7, 8, 9, | 4 | 2.011 (x 1.989) | 24 (0.167) | 0.302 | wing margin morphogenesis | CG5841 Su(dx) heph vg |
| 1260 | GO:0051663 | P | 6, 7, 9, 10, 12, | 2 | 0.670 (x 2.983) | 8 (0.250) | 0.304 | oocyte nucleus localization during oocyte axis determination | BicD egl |
| 1261 | GO:0045879 | P | 6, 7, 8, | 2 | 0.670 (x 2.983) | 8 (0.250) | 0.305 | negative regulation of smoothened signaling pathway | CkIalpha slmb |
| 1262 | GO:0035004 | F | 7, | 2 | 0.670 (x 2.983) | 8 (0.250) | 0.305 | phosphoinositide 3-kinase activity | Pi3K68D fwd |
| 1263 | GO:0006801 | P | 6, | 2 | 0.670 (x 2.983) | 8 (0.250) | 0.305 | superoxide metabolism | CG9027 Sh3beta |
| 1264 | GO:0016721 | F | 4, | 2 | 0.670 (x 2.983) | 8 (0.250) | 0.305 | oxidoreductase activity, acting on superoxide radicals as acceptor | CG9027 Sh3beta |
| 1265 | GO:0004365 | F | 7, | 2 | 0.670 (x 2.983) | 8 (0.250) | 0.305 | glyceraldehyde-3-phosphate dehydrogenase (phosphorylating) activity | Gapdh1 Gapdh2 |
| 1266 | GO:0004681 | F | 9, | 2 | 0.670 (x 2.983) | 8 (0.250) | 0.306 | casein kinase I activity | CkIalpha gish |
| 1267 | GO:0042180 | P | 5, | 2 | 0.670 (x 2.983) | 8 (0.250) | 0.306 | ketone metabolism | Gdh woc |
| 1268 | GO:0000163 | F | 9, | 2 | 0.670 (x 2.983) | 8 (0.250) | 0.306 | protein phosphatase type 1 activity | Mbs Pp1-87B |
| 1269 | GO:0008943 | F | 6, | 2 | 0.670 (x 2.983) | 8 (0.250) | 0.306 | glyceraldehyde-3-phosphate dehydrogenase activity | Gapdh1 Gapdh2 |
| 1270 | GO:0006518 | P | 5, | 2 | 0.670 (x 2.983) | 8 (0.250) | 0.307 | peptide metabolism | BG:DS00004.11 CG12130 |
| 1271 | GO:0000271 | P | 7, 8, | 2 | 0.670 (x 2.983) | 8 (0.250) | 0.307 | polysaccharide biosynthesis | CG6904 sgl |
| 1272 | GO:0043284 | P | 6, 7, | 2 | 0.670 (x 2.983) | 8 (0.250) | 0.307 | biopolymer biosynthesis | CG6904 sgl |
| 1273 | GO:0051226 | P | 7, 8, 11, | 2 | 0.670 (x 2.983) | 8 (0.250) | 0.307 | meiotic spindle assembly | pbl polo |
| 1274 | GO:0009886 | P | 4, | 2 | 0.670 (x 2.983) | 8 (0.250) | 0.308 | post-embryonic morphogenesis | br cib |
| 1275 | GO:0007506 | P | 5, 6, | 2 | 0.670 (x 2.983) | 8 (0.250) | 0.308 | gonadal mesoderm development | hh shg |
| 1276 | GO:0000086 | P | 7, | 2 | 0.670 (x 2.983) | 8 (0.250) | 0.308 | G2/M transition of mitotic cell cycle | CycA cdc2 |
| 1277 | GO:0004784 | F | 5, | 2 | 0.670 (x 2.983) | 8 (0.250) | 0.308 | superoxide dismutase activity | CG9027 Sh3beta |
| 1278 | GO:0046879 | P | 5, 6, | 2 | 0.670 (x 2.983) | 8 (0.250) | 0.309 | hormone secretion | CG15835 stai |
| 1279 | GO:0005606 | C | 4, 5, 6, 7, 8, | 1 | 0.168 (x 5.967) | 2 (0.500) | 0.309 | laminin-1 | LanA |
| 1280 | GO:0051092 | P | 10, | 1 | 0.168 (x 5.967) | 2 (0.500) | 0.309 | activation of NF-kappaB transcription factor | Uba2 |
| 1281 | GO:0042770 | P | 5, | 2 | 0.670 (x 2.983) | 8 (0.250) | 0.309 | DNA damage response, signal transduction | 14-3-3epsilon CG40410 |
| 1282 | GO:0035212 | P | 4, | 1 | 0.168 (x 5.967) | 2 (0.500) | 0.309 | cell competition (sensu Metazoa) | brk |
| 1283 | GO:0004591 | F | 6, | 1 | 0.168 (x 5.967) | 2 (0.500) | 0.309 | oxoglutarate dehydrogenase (succinyl-transferring) activity | CG1544 |
| 1284 | GO:0008450 | F | 7, | 1 | 0.168 (x 5.967) | 2 (0.500) | 0.309 | O-sialoglycoprotein endopeptidase activity | CG14231 |
| 1285 | GO:0035160 | P | 6, | 1 | 0.168 (x 5.967) | 2 (0.500) | 0.31 | maintenance of tracheal epithelial integrity | Lac |
| 1286 | GO:0004534 | F | 8, 9, | 1 | 0.168 (x 5.967) | 2 (0.500) | 0.31 | 5'-3' exoribonuclease activity | CG10354 |
| 1287 | GO:0045111 | C | 6, 7, 8, 9, | 1 | 0.168 (x 5.967) | 2 (0.500) | 0.31 | intermediate filament cytoskeleton | Lam |
| 1288 | GO:0005882 | C | 5, 6, 7, 8, 9, 10, | 1 | 0.168 (x 5.967) | 2 (0.500) | 0.31 | intermediate filament | Lam |
| 1289 | GO:0016843 | F | 5, | 1 | 0.168 (x 5.967) | 2 (0.500) | 0.311 | amine-lyase activity | Hmu |
| 1290 | GO:0006433 | P | 9, 10, 11, | 1 | 0.168 (x 5.967) | 2 (0.500) | 0.311 | prolyl-tRNA aminoacylation | Aats-glupro |
| 1291 | GO:0030215 | F | 4, 5, | 1 | 0.168 (x 5.967) | 2 (0.500) | 0.311 | semaphorin receptor binding | otk |
| 1292 | GO:0051323 | P | 6, | 1 | 0.168 (x 5.967) | 2 (0.500) | 0.311 | metaphase | east |
| 1293 | GO:0042246 | P | 4, 5, 6, | 1 | 0.168 (x 5.967) | 2 (0.500) | 0.311 | tissue regeneration | ninA |
| 1294 | GO:0005049 | F | 5, | 1 | 0.168 (x 5.967) | 2 (0.500) | 0.312 | nuclear export signal receptor activity | Ranbp16 |
| 1295 | GO:0004823 | F | 7, | 1 | 0.168 (x 5.967) | 2 (0.500) | 0.312 | leucine-tRNA ligase activity | CG33123 |
| 1296 | GO:0017134 | F | 5, | 1 | 0.168 (x 5.967) | 2 (0.500) | 0.312 | fibroblast growth factor binding | CG33214 |
| 1297 | GO:0007557 | P | 6, 7, 8, 9, 10, 11, 12, 13, | 1 | 0.168 (x 5.967) | 2 (0.500) | 0.312 | regulation of juvenile hormone biosynthesis | Vha44 |
| 1298 | GO:0031281 | P | 5, | 1 | 0.168 (x 5.967) | 2 (0.500) | 0.313 | positive regulation of cyclase activity | G-salpha60A |
| 1299 | GO:0048132 | P | 6, 7, | 1 | 0.168 (x 5.967) | 2 (0.500) | 0.313 | female germ-line stem cell division | asp |
| 1300 | GO:0035216 | P | 5, | 1 | 0.168 (x 5.967) | 2 (0.500) | 0.313 | haltere disc development | ap |
| 1301 | GO:0008334 | P | 8, | 3 | 1.341 (x 2.237) | 16 (0.188) | 0.313 | histone mRNA metabolism | CG4612 pAbp su(f) |
| 1302 | GO:0051252 | P | 7, | 7 | 4.525 (x 1.547) | 54 (0.130) | 0.313 | regulation of RNA metabolism | B52 CG5728 CG6227 Hrb87F Rm62 bl sqd |
| 1303 | GO:0000126 | C | 4, 7, 8, 9, 10, 11, 12, 13, 14, | 1 | 0.168 (x 5.967) | 2 (0.500) | 0.313 | transcription factor TFIIIB complex | Brf |
| 1304 | GO:0030118 | C | 5, 6, 7, 8, 9, | 3 | 1.341 (x 2.237) | 16 (0.188) | 0.313 | clathrin coat | AP-1gamma Chc alpha-Adaptin |
| 1305 | GO:0006587 | P | 8, 9, 10, 11, | 1 | 0.168 (x 5.967) | 2 (0.500) | 0.313 | serotonin biosynthesis from tryptophan | 14-3-3zeta |
| 1306 | GO:0051347 | P | 5, | 3 | 1.341 (x 2.237) | 16 (0.188) | 0.314 | positive regulation of transferase activity | Abi mbt msn |
| 1307 | GO:0004830 | F | 7, | 1 | 0.168 (x 5.967) | 2 (0.500) | 0.314 | tryptophan-tRNA ligase activity | CG7441 |
| 1308 | GO:0045860 | P | 6, 7, | 3 | 1.341 (x 2.237) | 16 (0.188) | 0.314 | positive regulation of protein kinase activity | Abi mbt msn |
| 1309 | GO:0051270 | P | 5, 6, | 1 | 0.168 (x 5.967) | 2 (0.500) | 0.314 | regulation of cell motility | LanA |
| 1310 | GO:0006398 | P | 9, 10, | 3 | 1.341 (x 2.237) | 16 (0.188) | 0.314 | histone mRNA 3'-end processing | CG4612 pAbp su(f) |
| 1311 | GO:0035173 | F | 7, | 1 | 0.168 (x 5.967) | 2 (0.500) | 0.314 | histone kinase activity | BcDNA:LD09009 |
| 1312 | GO:0004613 | F | 7, | 1 | 0.168 (x 5.967) | 2 (0.500) | 0.314 | phosphoenolpyruvate carboxykinase (GTP) activity | Pepck |
| 1313 | GO:0008143 | F | 6, | 3 | 1.341 (x 2.237) | 16 (0.188) | 0.314 | poly(A) binding | CG4612 pAbp su(f) |
| 1314 | GO:0030702 | P | 6, 8, 11, 13, | 1 | 0.168 (x 5.967) | 2 (0.500) | 0.314 | chromatin silencing at centromere | Su(var)3-9 |
| 1315 | GO:0007291 | P | 6, 9, | 3 | 1.341 (x 2.237) | 16 (0.188) | 0.315 | sperm individualization | Act5C Chc Pen |
| 1316 | GO:0031490 | F | 4, 6, | 1 | 0.168 (x 5.967) | 2 (0.500) | 0.315 | chromatin DNA binding | BEAF-32 |
| 1317 | GO:0000152 | C | 4, 5, 6, 7, 8, 9, 10, | 3 | 1.341 (x 2.237) | 16 (0.188) | 0.315 | nuclear ubiquitin ligase complex | gft lin19 lmg |
| 1318 | GO:0035044 | P | 6, 9, | 1 | 0.168 (x 5.967) | 2 (0.500) | 0.315 | sperm aster formation | polo |
| 1319 | GO:0035209 | P | 5, | 1 | 0.168 (x 5.967) | 2 (0.500) | 0.315 | pupal development (sensu Insecta) | crc |
| 1320 | GO:0006436 | P | 9, 10, 11, | 1 | 0.168 (x 5.967) | 2 (0.500) | 0.315 | tryptophanyl-tRNA aminoacylation | CG7441 |
| 1321 | GO:0019752 | P | 6, | 39 | 32.850 (x 1.187) | 392 (0.099) | 0.316 | carboxylic acid metabolism | 14-3-3zeta Aats-ala Aats-glupro Acon Acox57D-p BcDNA:GH02901 BcDNA:GH12558 CG11055 CG15093 CG2118 CG31739 CG32099 CG33123 CG40160 CG5028 CG5191 CG6287 CG6543 CG6638 CG7145 CG7441 CG7461 CG9425 CtBP ESTS:39C10S Eip55E Gdh Got2 Idh M(2)21AB Pepck Pgi SelD Thiolase Top3beta X11L l(1)G0334 l(1)dd4 mdy |
| 1322 | GO:0006081 | P | 5, | 1 | 0.168 (x 5.967) | 2 (0.500) | 0.316 | aldehyde metabolism | Idh |
| 1323 | GO:0006082 | P | 5, | 39 | 32.850 (x 1.187) | 392 (0.099) | 0.316 | organic acid metabolism | 14-3-3zeta Aats-ala Aats-glupro Acon Acox57D-p BcDNA:GH02901 BcDNA:GH12558 CG11055 CG15093 CG2118 CG31739 CG32099 CG33123 CG40160 CG5028 CG5191 CG6287 CG6543 CG6638 CG7145 CG7441 CG7461 CG9425 CtBP ESTS:39C10S Eip55E Gdh Got2 Idh M(2)21AB Pepck Pgi SelD Thiolase Top3beta X11L l(1)G0334 l(1)dd4 mdy |
| 1324 | GO:0035231 | P | 7, 8, | 1 | 0.168 (x 5.967) | 2 (0.500) | 0.316 | cytoneme biogenesis | hh |
| 1325 | GO:0045761 | P | 5, 9, 10, | 1 | 0.168 (x 5.967) | 2 (0.500) | 0.316 | regulation of adenylate cyclase activity | G-salpha60A |
| 1326 | GO:0046939 | P | 7, 8, | 1 | 0.168 (x 5.967) | 2 (0.500) | 0.316 | nucleotide phosphorylation | dnk |
| 1327 | GO:0017051 | F | 6, | 1 | 0.168 (x 5.967) | 2 (0.500) | 0.317 | retinol dehydratase activity | CG16733 |
| 1328 | GO:0007483 | P | 6, 7, | 1 | 0.168 (x 5.967) | 2 (0.500) | 0.317 | genital disc morphogenesis | dac |
| 1329 | GO:0035150 | P | 5, | 3 | 1.425 (x 2.106) | 17 (0.176) | 0.317 | regulation of tube size | Fas2 Lac cora |
| 1330 | GO:0051248 | P | 6, 7, | 6 | 3.603 (x 1.665) | 43 (0.140) | 0.317 | negative regulation of protein metabolism | CG11207 Paip2 fwd hh msi sqd |
| 1331 | GO:0006520 | P | 6, 7, | 27 | 21.872 (x 1.234) | 261 (0.103) | 0.317 | amino acid metabolism | 14-3-3zeta Aats-ala Aats-glupro Acon CG15093 CG2118 CG31739 CG33123 CG40160 CG5028 CG5191 CG6287 CG6638 CG7145 CG7441 CG9425 CtBP ESTS:39C10S Eip55E Gdh Got2 M(2)21AB SelD Top3beta X11L l(1)dd4 mdy |
| 1332 | GO:0050770 | P | 5, 8, 9, 10, 12, | 3 | 1.425 (x 2.106) | 17 (0.176) | 0.317 | regulation of axonogenesis | Cdc42 pbl trio |
| 1333 | GO:0030507 | F | 5, | 1 | 0.168 (x 5.967) | 2 (0.500) | 0.317 | spectrin binding | crb |
| 1334 | GO:0006406 | P | 8, 9, 10, 11, | 3 | 1.425 (x 2.106) | 17 (0.176) | 0.317 | mRNA export from nucleus | CG17143 CG2980 sqd |
| 1335 | GO:0019948 | F | 4, | 1 | 0.168 (x 5.967) | 2 (0.500) | 0.317 | SUMO activating enzyme activity | Uba2 |
| 1336 | GO:0016478 | P | 8, 9, 10, | 3 | 1.425 (x 2.106) | 17 (0.176) | 0.317 | negative regulation of translation | Paip2 msi sqd |
| 1337 | GO:0004617 | F | 6, | 1 | 0.168 (x 5.967) | 2 (0.500) | 0.317 | phosphoglycerate dehydrogenase activity | CG6287 |
| 1338 | GO:0006900 | P | 6, 7, | 3 | 1.425 (x 2.106) | 17 (0.176) | 0.318 | vesicle budding | AP-1gamma Chc alpha-Adaptin |
| 1339 | GO:0004161 | F | 5, | 1 | 0.168 (x 5.967) | 2 (0.500) | 0.318 | dimethylallyltranstransferase activity | Fpps |
| 1340 | GO:0051028 | P | 7, 8, 9, | 3 | 1.425 (x 2.106) | 17 (0.176) | 0.318 | mRNA transport | CG17143 CG2980 sqd |
| 1341 | GO:0006531 | P | 8, 9, | 1 | 0.168 (x 5.967) | 2 (0.500) | 0.318 | aspartate metabolism | Got2 |
| 1342 | GO:0006471 | P | 8, | 3 | 1.425 (x 2.106) | 17 (0.176) | 0.318 | protein amino acid ADP-ribosylation | Arf79F CG5841 Galpha49B |
| 1343 | GO:0016590 | C | 5, 8, 9, 10, 11, 12, 13, 14, 15, | 1 | 0.168 (x 5.967) | 2 (0.500) | 0.318 | ACF complex | Iswi |
| 1344 | GO:0007016 | P | 7, | 3 | 1.425 (x 2.106) | 17 (0.176) | 0.318 | cytoskeletal anchoring | alpha-Cat arm mask |
| 1345 | GO:0007396 | P | 7, | 1 | 0.168 (x 5.967) | 2 (0.500) | 0.318 | suture of dorsal opening | Cdc42 |
| 1346 | GO:0044249 | P | 5, | 78 | 69.555 (x 1.121) | 830 (0.094) | 0.319 | cellular biosynthesis | 14-3-3zeta ATPsyn-beta Aats-ala Aats-glupro Acon CG10423 CG10990 CG12006 CG1746 CG17514 CG2118 CG31472 CG31739 CG32099 CG32158 CG33123 CG33145 CG3590 CG40160 CG5028 CG5191 CG5434 CG6287 CG6394 CG6767 CG6835 CG6854 CG6904 CG7145 CG7441 CG8443 CG8963 CG9425 Cbp80 CtBP EG:86E4.2 ESTS:39C10S Elf Gfat1 Got2 M(2)21AB Paip2 Pepck Pgi Rbp2 RpL13 RpL15 RpL22 RpL23a RpL3 RpS12 RpS6 SelD Sin3A Su(var)3-9 Top3beta Vha44 Vha55 X11L ade2 ade3 bl dnk dome eIF-4B eIF3-S9 eIF5 jumu l(2)01424 mdy msi pAbp r-l rin sgl sqd stc woc |
| 1347 | GO:0007174 | P | 9, | 1 | 0.168 (x 5.967) | 2 (0.500) | 0.319 | epidermal growth factor ligand processing | S |
| 1348 | GO:0007202 | P | 8, 9, | 1 | 0.168 (x 5.967) | 2 (0.500) | 0.319 | phospholipase C activation | Galpha49B |
| 1349 | GO:0004749 | F | 6, 7, | 1 | 0.168 (x 5.967) | 2 (0.500) | 0.319 | ribose phosphate diphosphokinase activity | CG6767 |
| 1350 | GO:0035101 | C | 4, 7, 8, 9, 10, 11, 12, 13, 14, | 1 | 0.168 (x 5.967) | 2 (0.500) | 0.319 | FACT complex | dre4 |
| 1351 | GO:0031508 | P | 12, | 1 | 0.168 (x 5.967) | 2 (0.500) | 0.319 | centric heterochromatin formation | Su(var)3-9 |
| 1352 | GO:0004827 | F | 7, | 1 | 0.168 (x 5.967) | 2 (0.500) | 0.32 | proline-tRNA ligase activity | Aats-glupro |
| 1353 | GO:0007267 | P | 4, | 44 | 37.543 (x 1.172) | 448 (0.098) | 0.32 | cell-cell signaling | 14-3-3zeta AP-1gamma Amph Arf79F CG12199 CG15835 CG17064 CG8924 CaMKII Chc Eip75B Fas2 G-salpha60A Got2 Mcr NetA PFE Rop Ser Smox Snap Vap-33-1 X11L alpha-Adaptin alpha-Spec br fax fz hh l(2)gl lola lqf msi mth olf413 otk pAbp sca scrib ss stai syndapin trio trn |
| 1354 | GO:0003985 | F | 9, | 1 | 0.168 (x 5.967) | 2 (0.500) | 0.32 | acetyl-CoA C-acetyltransferase activity | CG9149 |
| 1355 | GO:0004337 | F | 6, | 1 | 0.168 (x 5.967) | 2 (0.500) | 0.32 | geranyltranstransferase activity | Fpps |
| 1356 | GO:0005862 | C | 5, 6, 7, 8, 9, 10, 11, 12, | 1 | 0.168 (x 5.967) | 2 (0.500) | 0.32 | muscle thin filament tropomyosin | Tm1 |
| 1357 | GO:0015459 | F | 5, | 1 | 0.168 (x 5.967) | 2 (0.500) | 0.321 | potassium channel regulator activity | Slip1 |
| 1358 | GO:0045167 | P | 5, 6, | 2 | 0.754 (x 2.652) | 9 (0.222) | 0.321 | asymmetric protein localization during cell fate commitment | jar l(2)gl |
| 1359 | GO:0017110 | F | 7, | 1 | 0.168 (x 5.967) | 2 (0.500) | 0.321 | nucleoside-diphosphatase activity | NTPase |
| 1360 | GO:0008507 | F | 7, 8, 9, 10, | 2 | 0.754 (x 2.652) | 9 (0.222) | 0.321 | sodium:iodide symporter activity | CG5687 CG7720 |
| 1361 | GO:0005113 | F | 4, 5, | 1 | 0.168 (x 5.967) | 2 (0.500) | 0.321 | patched binding | hh |
| 1362 | GO:0046845 | P | 5, 6, | 2 | 0.754 (x 2.652) | 9 (0.222) | 0.321 | branched duct epithelial cell fate determination (sensu Insecta) | S tkv |
| 1363 | GO:0051339 | P | 4, | 1 | 0.168 (x 5.967) | 2 (0.500) | 0.321 | regulation of lyase activity | G-salpha60A |
| 1364 | GO:0007509 | P | 7, 8, | 2 | 0.754 (x 2.652) | 9 (0.222) | 0.321 | mesoderm migration | pbl sgl |
| 1365 | GO:0005846 | C | 3, 5, 6, 7, 8, | 1 | 0.168 (x 5.967) | 2 (0.500) | 0.322 | snRNA cap binding complex | Cbp80 |
| 1366 | GO:0000776 | C | 5, 6, 7, 8, 9, 10, 11, | 2 | 0.754 (x 2.652) | 9 (0.222) | 0.322 | kinetochore | fzy polo |
| 1367 | GO:0017121 | P | 7, | 1 | 0.168 (x 5.967) | 2 (0.500) | 0.322 | phospholipid scrambling | CG1893 |
| 1368 | GO:0003730 | F | 6, | 2 | 0.754 (x 2.652) | 9 (0.222) | 0.322 | mRNA 3'-UTR binding | bsf sqd |
| 1369 | GO:0031510 | C | 4, | 1 | 0.168 (x 5.967) | 2 (0.500) | 0.322 | SUMO activating enzyme complex | Uba2 |
| 1370 | GO:0030017 | C | 6, 7, 8, 9, 10, | 2 | 0.754 (x 2.652) | 9 (0.222) | 0.322 | sarcomere | Tm1 zip |
| 1371 | GO:0043147 | P | 7, 9, 10, 11, 12, 14, | 1 | 0.168 (x 5.967) | 2 (0.500) | 0.322 | meiotic spindle stabilization | fwd |
| 1372 | GO:0007618 | P | 5, 6, | 2 | 0.754 (x 2.652) | 9 (0.222) | 0.322 | mating | Btk29A lig |
| 1373 | GO:0000151 | C | 3, 4, 5, 6, | 14 | 10.559 (x 1.326) | 126 (0.111) | 0.322 | ubiquitin ligase complex | CG10542 CG1815 CG31716 CG5841 Mi-2 Psc d4 gft gol lin19 lmg slmb stc th |
| 1374 | GO:0006165 | P | 8, 9, | 1 | 0.168 (x 5.967) | 2 (0.500) | 0.322 | nucleoside diphosphate phosphorylation | dnk |
| 1375 | GO:0015373 | F | 6, 7, 8, 9, | 2 | 0.754 (x 2.652) | 9 (0.222) | 0.322 | monovalent anion:sodium symporter activity | CG5687 CG7720 |
| 1376 | GO:0016654 | F | 5, | 1 | 0.168 (x 5.967) | 2 (0.500) | 0.323 | oxidoreductase activity, acting on NADH or NADPH, disulfide as acceptor | Trxr-1 |
| 1377 | GO:0006298 | P | 6, 8, 10, | 2 | 0.754 (x 2.652) | 9 (0.222) | 0.323 | mismatch repair | Thd1 mus209 |
| 1378 | GO:0030016 | C | 6, 7, 8, 9, | 2 | 0.754 (x 2.652) | 9 (0.222) | 0.323 | myofibril | Tm1 zip |
| 1379 | GO:0035007 | P | 5, 6, 8, | 1 | 0.168 (x 5.967) | 2 (0.500) | 0.323 | regulation of melanization defense response | serpin-27A |
| 1380 | GO:0004289 | F | 7, | 2 | 0.754 (x 2.652) | 9 (0.222) | 0.323 | subtilase activity | Fur1 TppII |
| 1381 | GO:0019551 | P | 7, 8, 9, 10, 11, | 1 | 0.168 (x 5.967) | 2 (0.500) | 0.323 | glutamate catabolism to 2-oxoglutarate | Gdh |
| 1382 | GO:0008431 | F | 4, | 2 | 0.754 (x 2.652) | 9 (0.222) | 0.323 | vitamin E binding | CG13848 CG3823 |
| 1383 | GO:0045762 | P | 6, 10, 11, | 1 | 0.168 (x 5.967) | 2 (0.500) | 0.323 | positive regulation of adenylate cyclase activity | G-salpha60A |
| 1384 | GO:0051129 | P | 6, | 2 | 0.754 (x 2.652) | 9 (0.222) | 0.324 | negative regulation of cell organization and biogenesis | CG11207 fwd |
| 1385 | GO:0008905 | F | 6, | 1 | 0.168 (x 5.967) | 2 (0.500) | 0.324 | mannose-phosphate guanylyltransferase activity | CG1129 |
| 1386 | GO:0003995 | F | 5, | 2 | 0.754 (x 2.652) | 9 (0.222) | 0.324 | acyl-CoA dehydrogenase activity | CG6638 CG7461 |
| 1387 | GO:0035232 | P | 6, 7, 8, | 1 | 0.168 (x 5.967) | 2 (0.500) | 0.324 | germ cell attraction | hh |
| 1388 | GO:0005667 | C | 3, 6, 7, 8, 9, 10, 11, 12, 13, | 10 | 7.123 (x 1.404) | 85 (0.118) | 0.324 | transcription factor complex | Brf Caf1 Dp Dref Iswi Mi-2 Rbf2 Sin3A Taf2 toe |
| 1389 | GO:0019212 | F | 4, 5, | 2 | 0.754 (x 2.652) | 9 (0.222) | 0.324 | phosphatase inhibitor activity | Mapmodulin Set |
| 1390 | GO:0045823 | P | 6, | 1 | 0.168 (x 5.967) | 2 (0.500) | 0.324 | positive regulation of heart contraction | scrib |
| 1391 | GO:0016408 | F | 7, | 2 | 0.754 (x 2.652) | 9 (0.222) | 0.324 | C-acyltransferase activity | CG9149 Thiolase |
| 1392 | GO:0017154 | F | 5, | 1 | 0.168 (x 5.967) | 2 (0.500) | 0.324 | semaphorin receptor activity | plexA |
| 1393 | GO:0045005 | P | 9, | 2 | 0.754 (x 2.652) | 9 (0.222) | 0.324 | maintenance of fidelity during DNA-dependent DNA replication | Thd1 mus209 |
| 1394 | GO:0017132 | F | 5, | 1 | 0.168 (x 5.967) | 2 (0.500) | 0.325 | cyclic nucleotide-dependent guanyl-nucleotide exchange factor activity | Gef26 |
| 1395 | GO:0006966 | P | 7, 8, 9, | 2 | 0.754 (x 2.652) | 9 (0.222) | 0.325 | antifungal humoral response (sensu Protostomia) | pll tub |
| 1396 | GO:0008361 | P | 5, 6, | 5 | 2.849 (x 1.755) | 34 (0.147) | 0.325 | regulation of cell size | Akt1 Cdk4 Hem Paip2 btsz |
| 1397 | GO:0009130 | P | 8, 9, | 1 | 0.168 (x 5.967) | 2 (0.500) | 0.325 | pyrimidine nucleoside monophosphate biosynthesis | dnk |
| 1398 | GO:0008299 | P | 6, 7, 8, | 2 | 0.754 (x 2.652) | 9 (0.222) | 0.325 | isoprenoid biosynthesis | Fpps Vha44 |
| 1399 | GO:0008170 | F | 6, | 5 | 2.849 (x 1.755) | 34 (0.147) | 0.325 | N-methyltransferase activity | Caf1 E(z) Su(var)3-9 Su(z)12 Taf2 |
| 1400 | GO:0016459 | C | 3, 5, 6, 7, 8, 9, 10, | 4 | 2.095 (x 1.909) | 25 (0.160) | 0.325 | myosin | ck d jar zip |
| 1401 | GO:0003717 | F | 4, | 1 | 0.168 (x 5.967) | 2 (0.500) | 0.325 | RNA polymerase II transcription termination factor activity | lds |
| 1402 | GO:0006536 | P | 8, 9, | 2 | 0.754 (x 2.652) | 9 (0.222) | 0.325 | glutamate metabolism | Gdh Got2 |
| 1403 | GO:0048562 | P | 5, | 4 | 2.095 (x 1.909) | 25 (0.160) | 0.325 | embryonic organ morphogenesis | barr msn pbl zip |
| 1404 | GO:0009129 | P | 8, | 1 | 0.168 (x 5.967) | 2 (0.500) | 0.325 | pyrimidine nucleoside monophosphate metabolism | dnk |
| 1405 | GO:0042364 | P | 7, | 2 | 0.754 (x 2.652) | 9 (0.222) | 0.325 | water-soluble vitamin biosynthesis | CG31472 ESTS:39C10S |
| 1406 | GO:0048024 | P | 9, 10, 12, | 7 | 4.441 (x 1.576) | 53 (0.132) | 0.325 | regulation of nuclear mRNA splicing, via spliceosome | B52 CG5728 CG6227 Hrb87F Rm62 bl sqd |
| 1407 | GO:0004645 | F | 6, | 1 | 0.168 (x 5.967) | 2 (0.500) | 0.326 | phosphorylase activity | GlyP |
| 1408 | GO:0035265 | P | 3, | 4 | 2.095 (x 1.909) | 25 (0.160) | 0.326 | organ growth | Akt1 CG11228 fat2 hh |
| 1409 | GO:0001708 | P | 5, | 7 | 4.441 (x 1.576) | 53 (0.132) | 0.326 | cell fate specification | Dr argos da hdc hh lqf sty |
| 1410 | GO:0031509 | P | 12, | 1 | 0.168 (x 5.967) | 2 (0.500) | 0.326 | telomeric heterochromatin formation | Su(var)3-9 |
| 1411 | GO:0046620 | P | 4, | 4 | 2.095 (x 1.909) | 25 (0.160) | 0.326 | regulation of organ size | Akt1 CG11228 fat2 hh |
| 1412 | GO:0050684 | P | 8, 9, | 7 | 4.441 (x 1.576) | 53 (0.132) | 0.326 | regulation of mRNA processing | B52 CG5728 CG6227 Hrb87F Rm62 bl sqd |
| 1413 | GO:0004069 | F | 6, | 1 | 0.168 (x 5.967) | 2 (0.500) | 0.326 | aspartate transaminase activity | Got2 |
| 1414 | GO:0031099 | P | 4, | 1 | 0.168 (x 5.967) | 2 (0.500) | 0.326 | regeneration | ninA |
| 1415 | GO:0004363 | F | 6, | 1 | 0.168 (x 5.967) | 2 (0.500) | 0.326 | glutathione synthase activity | CG6835 |
| 1416 | GO:0004802 | F | 5, | 1 | 0.168 (x 5.967) | 2 (0.500) | 0.327 | transketolase activity | CG8036 |
| 1417 | GO:0045787 | P | 6, 7, | 1 | 0.168 (x 5.967) | 2 (0.500) | 0.327 | positive regulation of progression through cell cycle | Cks |
| 1418 | GO:0005811 | C | 5, 6, 7, 8, | 1 | 0.168 (x 5.967) | 2 (0.500) | 0.327 | lipid particle | CG9057 |
| 1419 | GO:0005845 | C | 3, 4, 5, 6, | 1 | 0.168 (x 5.967) | 2 (0.500) | 0.327 | mRNA cap complex | l(2)01424 |
| 1420 | GO:0016925 | P | 8, | 1 | 0.168 (x 5.967) | 2 (0.500) | 0.328 | protein sumoylation | Uba2 |
| 1421 | GO:0006855 | P | 6, 7, | 1 | 0.168 (x 5.967) | 2 (0.500) | 0.328 | multidrug transport | Mdr49 |
| 1422 | GO:0044248 | P | 5, | 31 | 25.643 (x 1.209) | 306 (0.101) | 0.328 | cellular catabolism | Acon Ald BEST:LD22483 BcDNA:GM10765 BcDNA:LD22910 CG15093 CG1544 CG5028 CG5505 CG6638 CG7145 CG7263 CG7288 CG8036 CG8494 Eno Gapdh1 Gapdh2 Gdh Idh Jheh1 Jheh2 Pgi Tis11 Top3beta Uba2 Uch fzy l(1)G0334 sqd th |
| 1423 | GO:0005954 | C | 3, 4, 5, 6, | 1 | 0.168 (x 5.967) | 2 (0.500) | 0.328 | calcium- and calmodulin-dependent protein kinase complex | CaMKII |
| 1424 | GO:0004096 | F | 4, 6, | 1 | 0.168 (x 5.967) | 2 (0.500) | 0.328 | catalase activity | Cat |
| 1425 | GO:0004360 | F | 6, | 1 | 0.168 (x 5.967) | 2 (0.500) | 0.329 | glutamine-fructose-6-phosphate transaminase (isomerizing) activity | Gfat1 |
| 1426 | GO:0007289 | P | 6, 9, | 1 | 0.168 (x 5.967) | 2 (0.500) | 0.329 | spermatid nuclear differentiation | th |
| 1427 | GO:0035320 | P | 7, 8, 9, 10, 11, 12, | 1 | 0.168 (x 5.967) | 2 (0.500) | 0.329 | wing hair site selection | fz |
| 1428 | GO:0046885 | P | 7, | 1 | 0.168 (x 5.967) | 2 (0.500) | 0.329 | regulation of hormone biosynthesis | Vha44 |
| 1429 | GO:0003891 | F | 7, | 1 | 0.168 (x 5.967) | 2 (0.500) | 0.33 | delta DNA polymerase activity | DNApol-delta |
| 1430 | GO:0004353 | F | 6, | 1 | 0.168 (x 5.967) | 2 (0.500) | 0.33 | glutamate dehydrogenase [NAD(P)+] activity | Gdh |
| 1431 | GO:0016260 | P | 8, 9, 10, | 1 | 0.168 (x 5.967) | 2 (0.500) | 0.33 | selenocysteine biosynthesis | SelD |
| 1432 | GO:0051240 | P | 5, | 1 | 0.168 (x 5.967) | 2 (0.500) | 0.33 | positive regulation of organismal physiological process | scrib |
| 1433 | GO:0016844 | F | 6, | 1 | 0.168 (x 5.967) | 2 (0.500) | 0.331 | strictosidine synthase activity | Hmu |
| 1434 | GO:0043285 | P | 6, | 14 | 10.643 (x 1.315) | 127 (0.110) | 0.331 | biopolymer catabolism | BcDNA:GM10765 BcDNA:LD22910 CG5505 CG7263 CG7288 CG8494 CkIalpha Tis11 Top3beta Uba2 Uch fzy sqd th |
| 1435 | GO:0045296 | F | 5, | 1 | 0.168 (x 5.967) | 2 (0.500) | 0.331 | cadherin binding | alpha-Cat |
| 1436 | GO:0035203 | P | 6, 7, 8, 9, 10, | 1 | 0.168 (x 5.967) | 2 (0.500) | 0.331 | regulation of lamellocyte differentiation | kn |
| 1437 | GO:0004748 | F | 6, | 1 | 0.168 (x 5.967) | 2 (0.500) | 0.331 | ribonucleoside-diphosphate reductase activity | RnrL |
| 1438 | GO:0007189 | P | 9, 10, | 1 | 0.168 (x 5.967) | 2 (0.500) | 0.332 | G-protein signaling, adenylate cyclase activating pathway | G-salpha60A |
| 1439 | GO:0005638 | C | 5, 6, 7, 8, 9, 10, 11, 12, 13, 14, | 1 | 0.168 (x 5.967) | 2 (0.500) | 0.332 | lamin filament | Lam |
| 1440 | GO:0048256 | F | 8, | 1 | 0.168 (x 5.967) | 2 (0.500) | 0.332 | flap endonuclease activity | BcDNA:GM10765 |
| 1441 | GO:0000784 | C | 6, 7, 8, 9, 10, 11, 12, | 1 | 0.168 (x 5.967) | 2 (0.500) | 0.332 | nuclear chromosome, telomeric region | cav |
| 1442 | GO:0004485 | F | 6, | 1 | 0.168 (x 5.967) | 2 (0.500) | 0.333 | methylcrotonoyl-CoA carboxylase activity | CG2118 |
| 1443 | GO:0004611 | F | 6, | 1 | 0.168 (x 5.967) | 2 (0.500) | 0.333 | phosphoenolpyruvate carboxykinase activity | Pepck |
| 1444 | GO:0030730 | P | 5, 7, | 1 | 0.168 (x 5.967) | 2 (0.500) | 0.333 | sequestering of triacylglycerol | CG9057 |
| 1445 | GO:0004123 | F | 5, | 1 | 0.168 (x 5.967) | 2 (0.500) | 0.333 | cystathionine gamma-lyase activity | Eip55E |
| 1446 | GO:0051349 | P | 5, | 1 | 0.168 (x 5.967) | 2 (0.500) | 0.334 | positive regulation of lyase activity | G-salpha60A |
| 1447 | GO:0007556 | P | 5, 7, 8, 9, 10, 11, 12, | 1 | 0.168 (x 5.967) | 2 (0.500) | 0.334 | regulation of juvenile hormone metabolism | Vha44 |
| 1448 | GO:0031279 | P | 4, | 1 | 0.168 (x 5.967) | 2 (0.500) | 0.334 | regulation of cyclase activity | G-salpha60A |
| 1449 | GO:0016728 | F | 5, | 1 | 0.168 (x 5.967) | 2 (0.500) | 0.334 | oxidoreductase activity, acting on CH2 groups, disulfide as acceptor | RnrL |
| 1450 | GO:0035005 | F | 8, | 1 | 0.168 (x 5.967) | 2 (0.500) | 0.335 | phosphatidylinositol-4-phosphate 3-kinase activity | Pi3K68D |
| 1451 | GO:0004791 | F | 3, 5, 6, | 1 | 0.168 (x 5.967) | 2 (0.500) | 0.335 | thioredoxin-disulfide reductase activity | Trxr-1 |
| 1452 | GO:0031055 | P | 11, | 1 | 0.168 (x 5.967) | 2 (0.500) | 0.335 | chromatin remodeling at centromere | Su(var)3-9 |
| 1453 | GO:0006125 | P | 7, | 1 | 0.168 (x 5.967) | 2 (0.500) | 0.335 | thioredoxin pathway | Trxr-1 |
| 1454 | GO:0004332 | F | 6, | 1 | 0.168 (x 5.967) | 2 (0.500) | 0.336 | fructose-bisphosphate aldolase activity | Ald |
| 1455 | GO:0030155 | P | 4, | 1 | 0.168 (x 5.967) | 2 (0.500) | 0.336 | regulation of cell adhesion | LanA |
| 1456 | GO:0045214 | P | 8, 9, 11, 12, | 1 | 0.168 (x 5.967) | 2 (0.500) | 0.336 | sarcomere organization | zip |
| 1457 | GO:0031347 | P | 5, | 1 | 0.168 (x 5.967) | 2 (0.500) | 0.336 | regulation of defense response | serpin-27A |
| 1458 | GO:0006348 | P | 6, 8, 11, 13, | 1 | 0.168 (x 5.967) | 2 (0.500) | 0.337 | chromatin silencing at telomere | Su(var)3-9 |
| 1459 | GO:0017128 | F | 5, | 1 | 0.168 (x 5.967) | 2 (0.500) | 0.337 | phospholipid scramblase activity | CG1893 |
| 1460 | GO:0006766 | P | 5, | 5 | 3.017 (x 1.657) | 36 (0.139) | 0.337 | vitamin metabolism | BEST:LD22483 CG15093 CG31472 CG8036 ESTS:39C10S |
| 1461 | GO:0000791 | C | 6, 7, 8, 9, 10, 11, | 1 | 0.168 (x 5.967) | 2 (0.500) | 0.337 | euchromatin | Sin3A |
| 1462 | GO:0019897 | C | 5, 6, 7, | 5 | 3.017 (x 1.657) | 36 (0.139) | 0.337 | extrinsic to plasma membrane | G-salpha60A Galpha49B alpha-Cat arm cta |
| 1463 | GO:0004475 | F | 7, | 1 | 0.168 (x 5.967) | 2 (0.500) | 0.337 | mannose-1-phosphate guanylyltransferase activity | CG1129 |
| 1464 | GO:0030727 | P | 7, 8, 9, | 1 | 0.168 (x 5.967) | 2 (0.500) | 0.338 | female germ-line cyst formation (sensu Insecta) | alpha-Spec |
| 1465 | GO:0030100 | P | 6, 7, 8, | 1 | 0.168 (x 5.967) | 2 (0.500) | 0.338 | regulation of endocytosis | CG5841 |
| 1466 | GO:0016259 | P | 7, 8, 9, | 1 | 0.168 (x 5.967) | 2 (0.500) | 0.338 | selenocysteine metabolism | SelD |
| 1467 | GO:0009083 | P | 8, 9, | 1 | 0.168 (x 5.967) | 2 (0.500) | 0.338 | branched chain family amino acid catabolism | CG6638 |
| 1468 | GO:0045850 | P | 8, 9, 11, | 1 | 0.168 (x 5.967) | 2 (0.500) | 0.339 | positive regulation of nurse cell apoptosis | Dp |
| 1469 | GO:0043256 | C | 3, 4, 5, 6, 7, | 1 | 0.168 (x 5.967) | 2 (0.500) | 0.339 | laminin complex | LanA |
| 1470 | GO:0043035 | F | 5, 7, | 1 | 0.168 (x 5.967) | 2 (0.500) | 0.339 | chromatin insulator sequence binding | BEAF-32 |
| 1471 | GO:0019950 | P | 9, 10, 11, | 1 | 0.168 (x 5.967) | 2 (0.500) | 0.339 | SMT3-dependent protein catabolism | Uba2 |
| 1472 | GO:0019747 | P | 6, 7, 8, | 1 | 0.168 (x 5.967) | 2 (0.500) | 0.34 | regulation of isoprenoid metabolism | Vha44 |
| 1473 | GO:0042787 | P | 10, 11, 12, | 1 | 0.168 (x 5.967) | 2 (0.500) | 0.34 | protein ubiquitination during ubiquitin-dependent protein catabolism | th |
| 1474 | GO:0030334 | P | 6, 7, | 1 | 0.168 (x 5.967) | 2 (0.500) | 0.34 | regulation of cell migration | LanA |
| 1475 | GO:0051233 | C | 5, 6, 7, 8, 9, 10, 11, | 1 | 0.168 (x 5.967) | 2 (0.500) | 0.34 | spindle midzone | CG11207 |
| 1476 | GO:0006353 | P | 8, | 1 | 0.168 (x 5.967) | 2 (0.500) | 0.341 | transcription termination | lds |
| 1477 | GO:0007084 | P | 6, 8, 9, | 1 | 0.168 (x 5.967) | 2 (0.500) | 0.341 | mitotic nuclear envelope reassembly | Lam |
| 1478 | GO:0006103 | P | 6, 7, 9, | 1 | 0.168 (x 5.967) | 2 (0.500) | 0.341 | 2-oxoglutarate metabolism | Gdh |
| 1479 | GO:0005042 | F | 6, | 1 | 0.168 (x 5.967) | 2 (0.500) | 0.341 | netrin receptor activity | fra |
| 1480 | GO:0005971 | C | 3, 5, 6, 7, 8, | 1 | 0.168 (x 5.967) | 2 (0.500) | 0.342 | ribonucleoside-diphosphate reductase complex | RnrL |
| 1481 | GO:0035211 | P | 5, | 1 | 0.168 (x 5.967) | 2 (0.500) | 0.342 | spermathecum morphogenesis | dac |
| 1482 | GO:0016079 | P | 7, 8, 9, | 6 | 3.855 (x 1.556) | 46 (0.130) | 0.345 | synaptic vesicle exocytosis | Rop Snap Vap-33-1 X11L l(2)gl mth |
| 1483 | GO:0051168 | P | 7, 8, 9, | 4 | 2.263 (x 1.768) | 27 (0.148) | 0.348 | nuclear export | CG17143 CG2980 Ranbp16 sqd |
| 1484 | GO:0003743 | F | 4, 5, | 7 | 4.693 (x 1.492) | 56 (0.125) | 0.348 | translation initiation factor activity | CG8443 Rbp2 Su(var)3-9 eIF-4B eIF3-S9 eIF5 l(2)01424 |
| 1485 | GO:0042335 | P | 6, | 4 | 2.263 (x 1.768) | 27 (0.148) | 0.348 | cuticle biosynthesis | Akt1 cora knk ovo |
| 1486 | GO:0044274 | P | 5, | 4 | 2.263 (x 1.768) | 27 (0.148) | 0.348 | organismal biosynthesis | Akt1 cora knk ovo |
| 1487 | GO:0045610 | P | 5, 7, | 3 | 1.508 (x 1.989) | 18 (0.167) | 0.35 | regulation of hemocyte differentiation | Ser aop kn |
| 1488 | GO:0035287 | P | 5, 6, | 3 | 1.508 (x 1.989) | 18 (0.167) | 0.35 | head segmentation | en hh kn |
| 1489 | GO:0003774 | F | 2, | 10 | 7.291 (x 1.372) | 87 (0.115) | 0.356 | motor activity | Act42A Act5C CG10695 Klp10A ck d jar lola pav zip |
| 1490 | GO:0044265 | P | 6, | 18 | 14.414 (x 1.249) | 172 (0.105) | 0.359 | cellular macromolecule catabolism | Ald BEST:LD22483 BcDNA:LD22910 CG15093 CG5505 CG7288 CG8036 CG8494 Eno Gapdh1 Gapdh2 Pgi Tis11 Uba2 Uch fzy sqd th |
| 1491 | GO:0019094 | P | 6, 10, 12, 13, 15, | 5 | 3.101 (x 1.613) | 37 (0.135) | 0.362 | pole plasm mRNA localization | Hrb27C Rop Tm1 capu sqd |
| 1492 | GO:0044275 | P | 7, | 8 | 5.615 (x 1.425) | 67 (0.119) | 0.365 | cellular carbohydrate catabolism | Ald BEST:LD22483 CG15093 CG8036 Eno Gapdh1 Gapdh2 Pgi |
| 1493 | GO:0006519 | P | 5, | 29 | 24.554 (x 1.181) | 293 (0.099) | 0.365 | amino acid and derivative metabolism | 14-3-3zeta Aats-ala Aats-glupro Acon CG15093 CG2118 CG31739 CG33123 CG40160 CG5028 CG5191 CG6287 CG6638 CG7145 CG7441 CG9425 CtBP ESTS:39C10S Eip55E Gdh Got2 M(2)21AB SelD Top3beta X11L knk l(1)dd4 mdy olf413 |
| 1494 | GO:0016052 | P | 6, | 8 | 5.615 (x 1.425) | 67 (0.119) | 0.365 | carbohydrate catabolism | Ald BEST:LD22483 CG15093 CG8036 Eno Gapdh1 Gapdh2 Pgi |
| 1495 | GO:0006399 | P | 7, | 10 | 7.374 (x 1.356) | 88 (0.114) | 0.372 | tRNA metabolism | Aats-ala Aats-glupro CG31739 CG33123 CG5191 CG7441 CG9425 Top3beta X11L mdy |
| 1496 | GO:0046621 | P | 5, | 2 | 0.838 (x 2.387) | 10 (0.200) | 0.372 | negative regulation of organ size | CG11228 fat2 |
| 1497 | GO:0016482 | P | 6, 7, 8, | 2 | 0.838 (x 2.387) | 10 (0.200) | 0.372 | cytoplasmic transport | Pen kel |
| 1498 | GO:0046165 | P | 6, | 2 | 0.838 (x 2.387) | 10 (0.200) | 0.373 | alcohol biosynthesis | Pepck Pgi |
| 1499 | GO:0044242 | P | 6, 7, | 2 | 0.838 (x 2.387) | 10 (0.200) | 0.373 | cellular lipid catabolism | Jheh1 Jheh2 |
| 1500 | GO:0004024 | F | 7, | 2 | 0.838 (x 2.387) | 10 (0.200) | 0.373 | alcohol dehydrogenase activity, zinc-dependent | CG1600 LanB2 |
| 1501 | GO:0006732 | P | 6, | 24 | 20.028 (x 1.198) | 239 (0.100) | 0.373 | coenzyme metabolism | ATPsyn-beta Acon BEST:LD22483 CG10657 CG15093 CG1544 CG17036 CG1746 CG31472 CG5028 CG5687 CG6638 CG6835 CG7461 CG7720 CG8036 CG9471 Idh Sin3A Vha44 Vha55 dome jumu l(1)G0334 |
| 1502 | GO:0008593 | P | 5, 6, 7, | 2 | 0.838 (x 2.387) | 10 (0.200) | 0.373 | regulation of Notch signaling pathway | CG5841 l(2)gl |
| 1503 | GO:0051017 | P | 10, | 2 | 0.838 (x 2.387) | 10 (0.200) | 0.374 | actin filament bundle formation | Sb Src64B |
| 1504 | GO:0040018 | P | 5, | 2 | 0.838 (x 2.387) | 10 (0.200) | 0.374 | positive regulation of body size | Akt1 btsz |
| 1505 | GO:0007303 | P | 7, 8, 9, | 2 | 0.838 (x 2.387) | 10 (0.200) | 0.374 | cytoplasmic transport, nurse cell to oocyte | Pen kel |
| 1506 | GO:0035168 | P | 5, 6, 7, 8, | 2 | 0.838 (x 2.387) | 10 (0.200) | 0.374 | lymph gland hemocyte differentiation (sensu Arthropoda) | Ser kn |
| 1507 | GO:0046364 | P | 7, 8, | 2 | 0.838 (x 2.387) | 10 (0.200) | 0.375 | monosaccharide biosynthesis | Pepck Pgi |
| 1508 | GO:0019319 | P | 8, 9, | 2 | 0.838 (x 2.387) | 10 (0.200) | 0.375 | hexose biosynthesis | Pepck Pgi |
| 1509 | GO:0030135 | C | 6, 7, 8, 9, 10, | 8 | 5.698 (x 1.404) | 68 (0.118) | 0.382 | coated vesicle | AP-1gamma CG1472 Chc Vap-33-1 alpha-Adaptin btsz l(2)gl sec23 |
| 1510 | GO:0007316 | P | 5, 9, 11, 12, 14, | 5 | 3.184 (x 1.570) | 38 (0.132) | 0.384 | pole plasm RNA localization | Hrb27C Rop Tm1 capu sqd |
| 1511 | GO:0006612 | P | 8, 9, 10, | 3 | 1.592 (x 1.884) | 19 (0.158) | 0.384 | protein targeting to membrane | CG5064 CG5434 cora |
| 1512 | GO:0009064 | P | 7, 8, | 3 | 1.592 (x 1.884) | 19 (0.158) | 0.385 | glutamine family amino acid metabolism | CG7145 Gdh Got2 |
| 1513 | GO:0009152 | P | 8, 9, | 10 | 7.458 (x 1.341) | 89 (0.112) | 0.385 | purine ribonucleotide biosynthesis | ATPsyn-beta CG1746 CG3590 Sin3A Vha44 Vha55 ade2 ade3 dome jumu |
| 1514 | GO:0009620 | P | 5, | 3 | 1.592 (x 1.884) | 19 (0.158) | 0.385 | response to fungus | GNBP3 pll tub |
| 1515 | GO:0016769 | F | 4, | 3 | 1.592 (x 1.884) | 19 (0.158) | 0.385 | transferase activity, transferring nitrogenous groups | ESTS:39C10S Gfat1 Got2 |
| 1516 | GO:0043167 | F | 3, | 85 | 78.102 (x 1.088) | 932 (0.091) | 0.385 | ion binding | ATbp BEST:CK01140 Bap170 BcDNA:LD41548 CBP CG10542 CG10602 CG12130 CG12299 CG12391 CG14231 CG1529 CG1600 CG1647 CG17271 CG17361 CG17419 CG17493 CG17765 CG1815 CG31716 CG32099 CG33113 CG3847 CG4509 CG5841 CG6791 CG6930 CG7987 CG8089 CG8092 CG8478 CG9027 CG9425 CG9906 Cad87A CalpB Cen190 D19A D19B DNApol-delta EG:115C2.6 EG:86E4.2 Fkbp13 LanB2 MICAL-like Mi-2 NUCB1 Nep2 PR2 Pep Psa Psc Ser Sh3beta Su(var)3-9 Su(z)12 Trxr-1 alpha-Spec ap br ci crb crol d4 eIF5 esn fat2 gol jim jing klu lmg lola olf413 ome ovo pk scf shg stan stc th tok zf30C |
| 1517 | GO:0008483 | F | 5, | 3 | 1.592 (x 1.884) | 19 (0.158) | 0.385 | transaminase activity | ESTS:39C10S Gfat1 Got2 |
| 1518 | GO:0046872 | F | 4, | 85 | 78.102 (x 1.088) | 932 (0.091) | 0.386 | metal ion binding | ATbp BEST:CK01140 Bap170 BcDNA:LD41548 CBP CG10542 CG10602 CG12130 CG12299 CG12391 CG14231 CG1529 CG1600 CG1647 CG17271 CG17361 CG17419 CG17493 CG17765 CG1815 CG31716 CG32099 CG33113 CG3847 CG4509 CG5841 CG6791 CG6930 CG7987 CG8089 CG8092 CG8478 CG9027 CG9425 CG9906 Cad87A CalpB Cen190 D19A D19B DNApol-delta EG:115C2.6 EG:86E4.2 Fkbp13 LanB2 MICAL-like Mi-2 NUCB1 Nep2 PR2 Pep Psa Psc Ser Sh3beta Su(var)3-9 Su(z)12 Trxr-1 alpha-Spec ap br ci crb crol d4 eIF5 esn fat2 gol jim jing klu lmg lola olf413 ome ovo pk scf shg stan stc th tok zf30C |
| 1519 | GO:0009416 | P | 5, | 6 | 4.022 (x 1.492) | 48 (0.125) | 0.386 | response to light stimulus | G-salpha60A Galpha49B Pp1-87B Rop Sema-2a ogre |
| 1520 | GO:0016601 | P | 7, | 1 | 0.251 (x 3.978) | 3 (0.333) | 0.39 | Rac protein signal transduction | flfl |
| 1521 | GO:0001522 | P | 8, | 1 | 0.251 (x 3.978) | 3 (0.333) | 0.391 | pseudouridine synthesis | Nop60B |
| 1522 | GO:0005786 | C | 5, 6, 7, 8, 9, | 2 | 0.922 (x 2.170) | 11 (0.182) | 0.391 | signal recognition particle (sensu Eukaryota) | CG5064 CG5434 |
| 1523 | GO:0043169 | F | 4, | 81 | 74.331 (x 1.090) | 887 (0.091) | 0.391 | cation binding | ATbp BEST:CK01140 Bap170 BcDNA:LD41548 CBP CG10542 CG10602 CG12130 CG12299 CG12391 CG14231 CG1529 CG1600 CG1647 CG17271 CG17361 CG17419 CG17493 CG17765 CG1815 CG31716 CG33113 CG3847 CG4509 CG5841 CG6791 CG6930 CG7987 CG8089 CG8092 CG8478 CG9425 CG9906 Cad87A CalpB Cen190 D19A D19B DNApol-delta EG:115C2.6 EG:86E4.2 Fkbp13 LanB2 MICAL-like Mi-2 NUCB1 Nep2 PR2 Pep Psa Psc Ser Su(var)3-9 Su(z)12 alpha-Spec ap br ci crb crol d4 eIF5 esn fat2 gol jim jing klu lmg lola olf413 ome ovo pk scf shg stan stc th tok zf30C |
| 1524 | GO:0048098 | P | 7, | 1 | 0.251 (x 3.978) | 3 (0.333) | 0.391 | antennal joint development | dac |
| 1525 | GO:0001727 | F | 6, | 2 | 0.922 (x 2.170) | 11 (0.182) | 0.391 | lipid kinase activity | Pi3K68D fwd |
| 1526 | GO:0009113 | P | 7, 8, | 1 | 0.251 (x 3.978) | 3 (0.333) | 0.391 | purine base biosynthesis | ade3 |
| 1527 | GO:0006446 | P | 8, 9, 10, | 2 | 0.922 (x 2.170) | 11 (0.182) | 0.391 | regulation of translational initiation | eIF5 l(2)01424 |
| 1528 | GO:0009241 | P | 7, 8, 9, | 1 | 0.251 (x 3.978) | 3 (0.333) | 0.391 | polyisoprenoid biosynthesis | Vha44 |
| 1529 | GO:0016246 | P | 6, 9, | 2 | 0.922 (x 2.170) | 11 (0.182) | 0.391 | RNA interference | Rm62 vig |
| 1530 | GO:0005539 | F | 5, | 1 | 0.251 (x 3.978) | 3 (0.333) | 0.392 | glycosaminoglycan binding | CG1869 |
| 1531 | GO:0044449 | C | 5, 6, 7, 8, 9, | 2 | 0.922 (x 2.170) | 11 (0.182) | 0.392 | contractile fiber part | Tm1 zip |
| 1532 | GO:0001558 | P | 4, 5, 7, 8, | 3 | 1.676 (x 1.790) | 20 (0.150) | 0.392 | regulation of cell growth | Akt1 Cdk4 Paip2 |
| 1533 | GO:0003842 | F | 6, | 1 | 0.251 (x 3.978) | 3 (0.333) | 0.392 | 1-pyrroline-5-carboxylate dehydrogenase activity | CG7145 |
| 1534 | GO:0035166 | P | 4, 5, 6, | 2 | 0.922 (x 2.170) | 11 (0.182) | 0.392 | post-embryonic hemopoiesis | Ser kn |
| 1535 | GO:0004675 | F | 5, 6, 8, 9, | 3 | 1.676 (x 1.790) | 20 (0.150) | 0.392 | transmembrane receptor protein serine/threonine kinase activity | PFE pll tkv |
| 1536 | GO:0006725 | P | 5, | 15 | 12.151 (x 1.234) | 145 (0.103) | 0.392 | aromatic compound metabolism | 14-3-3zeta ATPsyn-beta CG1746 CG3590 CG6767 CG6854 M(2)21AB RnrL Vha55 ade2 ade3 dnk knk olf413 r-l |
| 1537 | GO:0007395 | P | 6, 7, | 1 | 0.251 (x 3.978) | 3 (0.333) | 0.392 | dorsal closure, spreading of leading edge cells | zip |
| 1538 | GO:0030509 | P | 8, | 2 | 0.922 (x 2.170) | 11 (0.182) | 0.392 | BMP signaling pathway | brk tkv |
| 1539 | GO:0006364 | P | 8, | 3 | 1.676 (x 1.790) | 20 (0.150) | 0.392 | rRNA processing | CG5728 Nop60B nop5 |
| 1540 | GO:0007299 | P | 5, 9, | 1 | 0.251 (x 3.978) | 3 (0.333) | 0.392 | follicle cell adhesion (sensu Insecta) | baz |
| 1541 | GO:0007413 | P | 9, 10, 12, | 2 | 0.922 (x 2.170) | 11 (0.182) | 0.392 | axonal fasciculation | Fas2 Fas3 |
| 1542 | GO:0006733 | P | 7, | 3 | 1.676 (x 1.790) | 20 (0.150) | 0.392 | oxidoreduction coenzyme metabolism | BEST:LD22483 CG15093 CG8036 |
| 1543 | GO:0006273 | P | 10, | 1 | 0.251 (x 3.978) | 3 (0.333) | 0.392 | lagging strand elongation | DNApol-delta |
| 1544 | GO:0016667 | F | 4, | 2 | 0.922 (x 2.170) | 11 (0.182) | 0.393 | oxidoreductase activity, acting on sulfur group of donors | CG4670 Trxr-1 |
| 1545 | GO:0015280 | F | 6, 7, 8, | 1 | 0.251 (x 3.978) | 3 (0.333) | 0.393 | amiloride-sensitive sodium channel activity | rpk |
| 1546 | GO:0048500 | C | 4, 5, 6, 7, 8, | 2 | 0.922 (x 2.170) | 11 (0.182) | 0.393 | signal recognition particle | CG5064 CG5434 |
| 1547 | GO:0007113 | P | 5, | 1 | 0.251 (x 3.978) | 3 (0.333) | 0.393 | endomitotic cell cycle | Dp |
| 1548 | GO:0035111 | P | 6, 7, 8, | 2 | 0.922 (x 2.170) | 11 (0.182) | 0.393 | leg joint morphogenesis | Ser Su(dx) |
| 1549 | GO:0042427 | P | 7, 8, 9, 10, | 1 | 0.251 (x 3.978) | 3 (0.333) | 0.393 | serotonin biosynthesis | 14-3-3zeta |
| 1550 | GO:0006903 | P | 6, 7, | 2 | 0.922 (x 2.170) | 11 (0.182) | 0.393 | vesicle targeting | X11L pnut |
| 1551 | GO:0015144 | F | 3, | 9 | 6.788 (x 1.326) | 81 (0.111) | 0.393 | carbohydrate transporter activity | CG10960 CG11451 CG9246 CG9809 LanB2 PR2 Psa alpha-Cat l(2)dtl |
| 1552 | GO:0006264 | P | 8, 9, | 1 | 0.251 (x 3.978) | 3 (0.333) | 0.393 | mitochondrial DNA replication | Dref |
| 1553 | GO:0048569 | P | 4, | 2 | 0.922 (x 2.170) | 11 (0.182) | 0.393 | post-embryonic organ development | Ser kn |
| 1554 | GO:0004197 | F | 6, | 9 | 6.788 (x 1.326) | 81 (0.111) | 0.394 | cysteine-type endopeptidase activity | BcDNA:LD22910 CG1440 CG3328 CG5505 CG7288 CG8494 CalpB da hh |
| 1555 | GO:0046664 | P | 7, | 1 | 0.251 (x 3.978) | 3 (0.333) | 0.394 | dorsal closure, amnioserosa morphology change | crb |
| 1556 | GO:0035167 | P | 5, 6, 7, | 2 | 0.922 (x 2.170) | 11 (0.182) | 0.394 | lymph gland hemopoiesis (sensu Arthropoda) | Ser kn |
| 1557 | GO:0008643 | P | 5, 6, | 9 | 6.788 (x 1.326) | 81 (0.111) | 0.394 | carbohydrate transport | CG10960 CG11451 CG9246 CG9809 LanB2 PR2 Psa alpha-Cat l(2)dtl |
| 1558 | GO:0007216 | P | 7, | 1 | 0.251 (x 3.978) | 3 (0.333) | 0.394 | metabotropic glutamate receptor signaling pathway | CG32447 |
| 1559 | GO:0005876 | C | 6, 7, 8, 9, 10, 11, | 2 | 0.922 (x 2.170) | 11 (0.182) | 0.394 | spindle microtubule | Klp10A chb |
| 1560 | GO:0030424 | C | 5, 6, | 1 | 0.251 (x 3.978) | 3 (0.333) | 0.394 | axon | Fas2 |
| 1561 | GO:0030540 | P | 5, | 1 | 0.251 (x 3.978) | 3 (0.333) | 0.394 | female genitalia development | en |
| 1562 | GO:0030863 | C | 6, 7, 8, 9, 10, | 1 | 0.251 (x 3.978) | 3 (0.333) | 0.395 | cortical cytoskeleton | alpha-Spec |
| 1563 | GO:0019233 | P | 4, 6, | 1 | 0.251 (x 3.978) | 3 (0.333) | 0.395 | sensory perception of pain | pain |
| 1564 | GO:0045995 | P | 4, | 1 | 0.251 (x 3.978) | 3 (0.333) | 0.395 | regulation of embryonic development | LanA |
| 1565 | GO:0008451 | F | 7, | 1 | 0.251 (x 3.978) | 3 (0.333) | 0.395 | X-Pro aminopeptidase activity | ApepP |
| 1566 | GO:0003917 | F | 5, 6, | 1 | 0.251 (x 3.978) | 3 (0.333) | 0.396 | DNA topoisomerase type I activity | Top3beta |
| 1567 | GO:0015743 | P | 8, 9, | 1 | 0.251 (x 3.978) | 3 (0.333) | 0.396 | malate transport | CG1907 |
| 1568 | GO:0003893 | F | 7, | 1 | 0.251 (x 3.978) | 3 (0.333) | 0.396 | epsilon DNA polymerase activity | DNApol-epsilon |
| 1569 | GO:0046890 | P | 6, 7, 8, | 1 | 0.251 (x 3.978) | 3 (0.333) | 0.396 | regulation of lipid biosynthesis | Vha44 |
| 1570 | GO:0019905 | F | 5, | 1 | 0.251 (x 3.978) | 3 (0.333) | 0.397 | syntaxin binding | Rop |
| 1571 | GO:0016979 | F | 5, | 1 | 0.251 (x 3.978) | 3 (0.333) | 0.397 | lipoate-protein ligase activity | CG6767 |
| 1572 | GO:0048049 | P | 7, 8, | 1 | 0.251 (x 3.978) | 3 (0.333) | 0.397 | embryonic eye morphogenesis (sensu Endopterygota) | msn |
| 1573 | GO:0019915 | P | 6, | 1 | 0.251 (x 3.978) | 3 (0.333) | 0.397 | sequestering of lipid | CG9057 |
| 1574 | GO:0016200 | P | 6, | 1 | 0.251 (x 3.978) | 3 (0.333) | 0.398 | synaptic target attraction | Fas3 |
| 1575 | GO:0008518 | F | 6, | 1 | 0.251 (x 3.978) | 3 (0.333) | 0.398 | reduced folate carrier activity | CG17036 |
| 1576 | GO:0009250 | P | 8, 9, | 1 | 0.251 (x 3.978) | 3 (0.333) | 0.398 | glucan biosynthesis | CG6904 |
| 1577 | GO:0005025 | F | 7, 8, 10, 11, | 1 | 0.251 (x 3.978) | 3 (0.333) | 0.398 | transforming growth factor beta receptor activity, type I | tkv |
| 1578 | GO:0016892 | F | 8, | 1 | 0.251 (x 3.978) | 3 (0.333) | 0.399 | endoribonuclease activity, producing 3'-phosphomonoesters | CG10103 |
| 1579 | GO:0016744 | F | 4, | 1 | 0.251 (x 3.978) | 3 (0.333) | 0.399 | transferase activity, transferring aldehyde or ketonic groups | CG8036 |
| 1580 | GO:0008054 | P | 7, 11, 12, 13, | 1 | 0.251 (x 3.978) | 3 (0.333) | 0.399 | cyclin catabolism | fzy |
| 1581 | GO:0042169 | F | 5, | 1 | 0.251 (x 3.978) | 3 (0.333) | 0.399 | SH2 domain binding | Ack |
| 1582 | GO:0030262 | P | 8, | 1 | 0.251 (x 3.978) | 3 (0.333) | 0.4 | apoptotic nuclear changes | CG7263 |
| 1583 | GO:0016485 | P | 8, | 4 | 2.514 (x 1.591) | 30 (0.133) | 0.4 | protein processing | BG:DS00004.11 CalpB hh wbl |
| 1584 | GO:0005923 | C | 7, 8, 9, 10, | 1 | 0.251 (x 3.978) | 3 (0.333) | 0.4 | tight junction | CG2097 |
| 1585 | GO:0016627 | F | 4, | 4 | 2.514 (x 1.591) | 30 (0.133) | 0.4 | oxidoreductase activity, acting on the CH-CH group of donors | Acox57D-p CG6638 CG7461 CG9471 |
| 1586 | GO:0017101 | C | 3, 4, 5, 6, | 1 | 0.251 (x 3.978) | 3 (0.333) | 0.4 | aminoacyl-tRNA synthetase multienzyme complex | Aats-glupro |
| 1587 | GO:0016049 | P | 3, 4, 6, 7, | 4 | 2.514 (x 1.591) | 30 (0.133) | 0.4 | cell growth | Akt1 Cdk4 Hem Paip2 |
| 1588 | GO:0006163 | P | 7, | 10 | 7.710 (x 1.297) | 92 (0.109) | 0.4 | purine nucleotide metabolism | ATPsyn-beta CG1746 CG3590 Sin3A Vha44 Vha55 ade2 ade3 dome jumu |
| 1589 | GO:0016882 | F | 5, | 1 | 0.251 (x 3.978) | 3 (0.333) | 0.4 | cyclo-ligase activity | ade3 |
| 1590 | GO:0003988 | F | 8, | 1 | 0.251 (x 3.978) | 3 (0.333) | 0.401 | acetyl-CoA C-acyltransferase activity | Thiolase |
| 1591 | GO:0043414 | P | 7, | 5 | 3.352 (x 1.492) | 40 (0.125) | 0.401 | biopolymer methylation | Caf1 E(z) Su(var)3-9 Su(z)12 Taf2 |
| 1592 | GO:0003696 | F | 6, | 1 | 0.251 (x 3.978) | 3 (0.333) | 0.401 | satellite DNA binding | cav |
| 1593 | GO:0008201 | F | 6, | 1 | 0.251 (x 3.978) | 3 (0.333) | 0.401 | heparin binding | CG1869 |
| 1594 | GO:0004430 | F | 8, | 1 | 0.251 (x 3.978) | 3 (0.333) | 0.401 | 1-phosphatidylinositol 4-kinase activity | fwd |
| 1595 | GO:0009150 | P | 8, | 10 | 7.542 (x 1.326) | 90 (0.111) | 0.402 | purine ribonucleotide metabolism | ATPsyn-beta CG1746 CG3590 Sin3A Vha44 Vha55 ade2 ade3 dome jumu |
| 1596 | GO:0004738 | F | 5, | 1 | 0.251 (x 3.978) | 3 (0.333) | 0.402 | pyruvate dehydrogenase activity | l(1)G0334 |
| 1597 | GO:0005652 | C | 5, 6, 7, 8, 9, 10, 11, | 1 | 0.251 (x 3.978) | 3 (0.333) | 0.402 | nuclear lamina | Lam |
| 1598 | GO:0035006 | P | 5, 7, | 1 | 0.251 (x 3.978) | 3 (0.333) | 0.402 | melanization defense response | serpin-27A |
| 1599 | GO:0007313 | P | 7, 9, 10, 12, | 1 | 0.251 (x 3.978) | 3 (0.333) | 0.402 | maternal determination of dorsal/ventral axis, oocyte, soma encoded | wbl |
| 1600 | GO:0004276 | F | 7, | 1 | 0.251 (x 3.978) | 3 (0.333) | 0.403 | furin activity | Fur1 |
| 1601 | GO:0000014 | F | 8, | 1 | 0.251 (x 3.978) | 3 (0.333) | 0.403 | single-stranded DNA specific endodeoxyribonuclease activity | BcDNA:GM10765 |
| 1602 | GO:0007486 | P | 6, 7, | 1 | 0.251 (x 3.978) | 3 (0.333) | 0.403 | female genitalia development (sensu Endopterygota) | en |
| 1603 | GO:0001666 | P | 4, | 1 | 0.251 (x 3.978) | 3 (0.333) | 0.403 | response to hypoxia | ald |
| 1604 | GO:0008965 | F | 6, | 1 | 0.251 (x 3.978) | 3 (0.333) | 0.404 | phosphoenolpyruvate-protein phosphotransferase activity | alpha-Cat |
| 1605 | GO:0009374 | F | 4, | 1 | 0.251 (x 3.978) | 3 (0.333) | 0.404 | biotin binding | CG2118 |
| 1606 | GO:0006424 | P | 9, 10, 11, | 1 | 0.251 (x 3.978) | 3 (0.333) | 0.404 | glutamyl-tRNA aminoacylation | Aats-glupro |
| 1607 | GO:0042826 | F | 5, | 1 | 0.251 (x 3.978) | 3 (0.333) | 0.404 | histone deacetylase binding | Caf1 |
| 1608 | GO:0046246 | P | 7, | 1 | 0.251 (x 3.978) | 3 (0.333) | 0.405 | terpene biosynthesis | Vha44 |
| 1609 | GO:0035010 | P | 5, 6, | 1 | 0.251 (x 3.978) | 3 (0.333) | 0.405 | encapsulation of foreign target | serpin-27A |
| 1610 | GO:0032042 | P | 7, 8, | 1 | 0.251 (x 3.978) | 3 (0.333) | 0.405 | mitochondrial DNA metabolism | Dref |
| 1611 | GO:0045213 | P | 7, 8, | 1 | 0.251 (x 3.978) | 3 (0.333) | 0.405 | neurotransmitter receptor metabolism | Got2 |
| 1612 | GO:0006413 | P | 8, 9, | 7 | 4.944 (x 1.416) | 59 (0.119) | 0.405 | translational initiation | CG8443 Rbp2 Su(var)3-9 eIF-4B eIF3-S9 eIF5 l(2)01424 |
| 1613 | GO:0016114 | P | 6, 8, 9, 10, | 1 | 0.251 (x 3.978) | 3 (0.333) | 0.406 | terpenoid biosynthesis | Vha44 |
| 1614 | GO:0003709 | F | 3, | 1 | 0.251 (x 3.978) | 3 (0.333) | 0.406 | RNA polymerase III transcription factor activity | Brf |
| 1615 | GO:0000900 | F | 4, 5, | 1 | 0.251 (x 3.978) | 3 (0.333) | 0.406 | translation repressor activity, nucleic acid binding | msi |
| 1616 | GO:0017068 | F | 6, | 1 | 0.251 (x 3.978) | 3 (0.333) | 0.406 | glutamyl-tRNA(Gln) amidotransferase activity | CG5191 |
| 1617 | GO:0048048 | P | 6, 7, | 1 | 0.251 (x 3.978) | 3 (0.333) | 0.407 | embryonic eye morphogenesis | msn |
| 1618 | GO:0004504 | F | 6, | 1 | 0.251 (x 3.978) | 3 (0.333) | 0.407 | peptidylglycine monooxygenase activity | CG12130 |
| 1619 | GO:0016581 | C | 5, 8, 9, 10, 11, 12, 13, 14, 15, | 1 | 0.251 (x 3.978) | 3 (0.333) | 0.407 | NuRD complex | Mi-2 |
| 1620 | GO:0051762 | P | 8, | 1 | 0.251 (x 3.978) | 3 (0.333) | 0.407 | sesquiterpene biosynthesis | Vha44 |
| 1621 | GO:0005919 | C | 8, 9, 10, 11, | 1 | 0.251 (x 3.978) | 3 (0.333) | 0.408 | pleated septate junction | Cont |
| 1622 | GO:0008353 | F | 8, | 1 | 0.251 (x 3.978) | 3 (0.333) | 0.408 | RNA polymerase subunit kinase activity | CycT |
| 1623 | GO:0043112 | P | 7, | 1 | 0.251 (x 3.978) | 3 (0.333) | 0.408 | receptor metabolism | Got2 |
| 1624 | GO:0008622 | C | 4, 5, 6, 7, 8, 9, 10, 11, 12, 13, 14, | 1 | 0.251 (x 3.978) | 3 (0.333) | 0.408 | epsilon DNA polymerase complex | DNApol-epsilon |
| 1625 | GO:0016251 | F | 4, | 11 | 8.464 (x 1.300) | 101 (0.109) | 0.409 | general RNA polymerase II transcription factor activity | Arc92 Brf GATAd Iswi Spt6 Taf2 XNP brm dalao grn mor |
| 1626 | GO:0004367 | F | 6, | 1 | 0.251 (x 3.978) | 3 (0.333) | 0.409 | glycerol-3-phosphate dehydrogenase (NAD+) activity | CG31169 |
| 1627 | GO:0001672 | P | 6, 8, 10, | 1 | 0.251 (x 3.978) | 3 (0.333) | 0.409 | regulation of chromatin assembly or disassembly | dre4 |
| 1628 | GO:0016051 | P | 6, 7, | 6 | 4.106 (x 1.461) | 49 (0.122) | 0.409 | carbohydrate biosynthesis | CG6394 CG6904 Gfat1 Pepck Pgi sgl |
| 1629 | GO:0016639 | F | 5, | 1 | 0.251 (x 3.978) | 3 (0.333) | 0.409 | oxidoreductase activity, acting on the CH-NH2 group of donors, NAD or NADP as acceptor | Gdh |
| 1630 | GO:0004519 | F | 6, | 6 | 4.106 (x 1.461) | 49 (0.122) | 0.409 | endonuclease activity | BcDNA:GM10765 CG10103 CG7922 CG9425 Rrp1 Top3beta |
| 1631 | GO:0004300 | F | 6, | 1 | 0.251 (x 3.978) | 3 (0.333) | 0.41 | enoyl-CoA hydratase activity | CG6543 |
| 1632 | GO:0006096 | P | 8, 10, 11, | 5 | 3.268 (x 1.530) | 39 (0.128) | 0.41 | glycolysis | Ald Eno Gapdh1 Gapdh2 Pgi |
| 1633 | GO:0019318 | P | 7, 8, | 9 | 6.704 (x 1.342) | 80 (0.113) | 0.41 | hexose metabolism | Ald BEST:LD22483 CG15093 CG8036 Eno Gapdh1 Gapdh2 Pepck Pgi |
| 1634 | GO:0016401 | F | 7, | 1 | 0.251 (x 3.978) | 3 (0.333) | 0.41 | palmitoyl-CoA oxidase activity | Acox57D-p |
| 1635 | GO:0005542 | F | 4, | 1 | 0.251 (x 3.978) | 3 (0.333) | 0.41 | folic acid binding | CG17036 |
| 1636 | GO:0016106 | P | 7, 9, 10, 11, | 1 | 0.251 (x 3.978) | 3 (0.333) | 0.41 | sesquiterpenoid biosynthesis | Vha44 |
| 1637 | GO:0001748 | P | 8, 9, | 1 | 0.251 (x 3.978) | 3 (0.333) | 0.411 | optic placode development (sensu Endopterygota) | shg |
| 1638 | GO:0043450 | P | 6, | 1 | 0.251 (x 3.978) | 3 (0.333) | 0.411 | alkene biosynthesis | Vha44 |
| 1639 | GO:0006718 | P | 7, 8, 10, 11, 12, | 1 | 0.251 (x 3.978) | 3 (0.333) | 0.411 | juvenile hormone biosynthesis | Vha44 |
| 1640 | GO:0006419 | P | 9, 10, 11, | 1 | 0.251 (x 3.978) | 3 (0.333) | 0.411 | alanyl-tRNA aminoacylation | Aats-ala |
| 1641 | GO:0006937 | P | 5, | 1 | 0.251 (x 3.978) | 3 (0.333) | 0.412 | regulation of muscle contraction | Amph |
| 1642 | GO:0000109 | C | 3, 5, 6, 7, 8, 9, 10, | 1 | 0.251 (x 3.978) | 3 (0.333) | 0.412 | nucleotide-excision repair complex | DNApol-epsilon |
| 1643 | GO:0004733 | F | 6, | 1 | 0.251 (x 3.978) | 3 (0.333) | 0.412 | pyridoxamine-phosphate oxidase activity | CG31472 |
| 1644 | GO:0046219 | P | 7, 8, 9, | 1 | 0.251 (x 3.978) | 3 (0.333) | 0.412 | indolalkylamine biosynthesis | 14-3-3zeta |
| 1645 | GO:0004739 | F | 6, | 1 | 0.251 (x 3.978) | 3 (0.333) | 0.413 | pyruvate dehydrogenase (acetyl-transferring) activity | l(1)G0334 |
| 1646 | GO:0016740 | F | 3, | 87 | 81.035 (x 1.074) | 967 (0.090) | 0.413 | transferase activity | Abi Ack Akt1 BcDNA:LD09009 BcDNA:LD23371 Btk29A CG11228 CG1129 CG11307 CG12006 CG14217 CG14222 CG16733 CG17309 CG31640 CG32099 CG32632 CG33116 CG33138 CG33145 CG40410 CG5505 CG5841 CG6227 CG6394 CG6673 CG6767 CG6904 CG8036 CG8789 CG9149 CaMKII Caf1 Cdk4 CkIIalpha CkIIbeta CkIalpha Cks CycT DNApol-delta DNApol-epsilon E(z) ESTS:39C10S Eip63E Fpps Gfat1 GlyP Got2 Gprk1 KP78b M(2)21AB Mekk1 Nek2 PFE PR2 Pak3 Pi3K68D RpII140 Src64B Su(var)3-9 Su(z)12 Taf2 Thiolase Wsck ade3 ald alpha-Cat asp cdc2 csul dnk fat2 fwd gfzf gish l(1)G0148 mbt mdy msn mus209 otk plexA pll polo r-l smi35A tkv |
| 1647 | GO:0046958 | P | 6, | 1 | 0.251 (x 3.978) | 3 (0.333) | 0.413 | nonassociative learning | 14-3-3epsilon |
| 1648 | GO:0035046 | P | 6, 8, 9, | 1 | 0.251 (x 3.978) | 3 (0.333) | 0.413 | pronuclear migration | polo |
| 1649 | GO:0045839 | P | 7, 8, 9, | 1 | 0.251 (x 3.978) | 3 (0.333) | 0.414 | negative regulation of mitosis | CycA |
| 1650 | GO:0042734 | C | 3, 4, 5, | 1 | 0.251 (x 3.978) | 3 (0.333) | 0.414 | presynaptic membrane | Fas2 |
| 1651 | GO:0016668 | F | 5, | 1 | 0.251 (x 3.978) | 3 (0.333) | 0.414 | oxidoreductase activity, acting on sulfur group of donors, NAD or NADP as acceptor | Trxr-1 |
| 1652 | GO:0008091 | C | 5, 6, 7, 8, 9, 10, 11, 12, | 1 | 0.251 (x 3.978) | 3 (0.333) | 0.414 | spectrin | alpha-Spec |
| 1653 | GO:0004728 | F | 4, 9, | 1 | 0.251 (x 3.978) | 3 (0.333) | 0.415 | receptor signaling protein tyrosine phosphatase activity | csw |
| 1654 | GO:0015742 | P | 8, 9, | 1 | 0.251 (x 3.978) | 3 (0.333) | 0.415 | alpha-ketoglutarate transport | CG1907 |
| 1655 | GO:0005978 | P | 9, 10, | 1 | 0.251 (x 3.978) | 3 (0.333) | 0.415 | glycogen biosynthesis | CG6904 |
| 1656 | GO:0016453 | F | 8, | 1 | 0.251 (x 3.978) | 3 (0.333) | 0.415 | C-acetyltransferase activity | CG9149 |
| 1657 | GO:0016722 | F | 4, | 1 | 0.251 (x 3.978) | 3 (0.333) | 0.416 | oxidoreductase activity, oxidizing metal ions | CG14882 |
| 1658 | GO:0030864 | C | 5, 6, 7, 8, 9, 10, 11, | 1 | 0.251 (x 3.978) | 3 (0.333) | 0.416 | cortical actin cytoskeleton | alpha-Spec |
| 1659 | GO:0008234 | F | 5, | 10 | 7.793 (x 1.283) | 93 (0.108) | 0.416 | cysteine-type peptidase activity | BcDNA:LD22910 CG1440 CG3328 CG5505 CG7288 CG8494 CalpB Uch da hh |
| 1660 | GO:0004757 | F | 6, | 1 | 0.251 (x 3.978) | 3 (0.333) | 0.416 | sepiapterin reductase activity | Es2 |
| 1661 | GO:0016580 | C | 5, 8, 9, 10, 11, 12, 13, 14, 15, | 1 | 0.251 (x 3.978) | 3 (0.333) | 0.416 | Sin3 complex | Sin3A |
| 1662 | GO:0004813 | F | 7, | 1 | 0.251 (x 3.978) | 3 (0.333) | 0.417 | alanine-tRNA ligase activity | Aats-ala |
| 1663 | GO:0010092 | P | 5, | 1 | 0.251 (x 3.978) | 3 (0.333) | 0.417 | specification of organ identity | Dll |
| 1664 | GO:0035170 | P | 6, 7, 8, 9, | 1 | 0.251 (x 3.978) | 3 (0.333) | 0.417 | lymph gland crystal cell differentiation | Ser |
| 1665 | GO:0006573 | P | 8, 9, | 1 | 0.251 (x 3.978) | 3 (0.333) | 0.418 | valine metabolism | CG15093 |
| 1666 | GO:0006309 | P | 7, 8, 9, | 1 | 0.251 (x 3.978) | 3 (0.333) | 0.418 | DNA fragmentation during apoptosis | CG7263 |
| 1667 | GO:0006164 | P | 7, 8, | 10 | 7.626 (x 1.311) | 91 (0.110) | 0.418 | purine nucleotide biosynthesis | ATPsyn-beta CG1746 CG3590 Sin3A Vha44 Vha55 ade2 ade3 dome jumu |
| 1668 | GO:0017137 | F | 7, | 1 | 0.251 (x 3.978) | 3 (0.333) | 0.418 | Rab GTPase binding | CG1418 |
| 1669 | GO:0016628 | F | 5, | 1 | 0.251 (x 3.978) | 3 (0.333) | 0.418 | oxidoreductase activity, acting on the CH-CH group of donors, NAD or NADP as acceptor | CG9471 |
| 1670 | GO:0016842 | F | 5, | 1 | 0.251 (x 3.978) | 3 (0.333) | 0.419 | amidine-lyase activity | CG3590 |
| 1671 | GO:0007614 | P | 6, | 1 | 0.251 (x 3.978) | 3 (0.333) | 0.419 | short-term memory | Fas2 |
| 1672 | GO:0004772 | F | 8, | 1 | 0.251 (x 3.978) | 3 (0.333) | 0.419 | sterol O-acyltransferase activity | mdy |
| 1673 | GO:0040012 | P | 4, | 1 | 0.251 (x 3.978) | 3 (0.333) | 0.419 | regulation of locomotion | LanA |
| 1674 | GO:0008021 | C | 8, 9, 10, 11, 12, | 6 | 4.274 (x 1.404) | 51 (0.118) | 0.42 | synaptic vesicle | AP-1gamma Chc Vap-33-1 alpha-Adaptin btsz l(2)gl |
| 1675 | GO:0019210 | F | 4, | 3 | 1.760 (x 1.705) | 21 (0.143) | 0.421 | kinase inhibitor activity | 14-3-3epsilon 14-3-3zeta CG17919 |
| 1676 | GO:0035152 | P | 5, | 3 | 1.760 (x 1.705) | 21 (0.143) | 0.422 | regulation of tracheal tube architecture | Fas2 Lac cora |
| 1677 | GO:0006405 | P | 7, 8, 9, 10, | 3 | 1.760 (x 1.705) | 21 (0.143) | 0.422 | RNA export from nucleus | CG17143 CG2980 sqd |
| 1678 | GO:0016042 | P | 5, 6, | 3 | 1.760 (x 1.705) | 21 (0.143) | 0.422 | lipid catabolism | DNApol-delta Jheh1 Jheh2 |
| 1679 | GO:0017148 | P | 7, 8, 9, | 3 | 1.760 (x 1.705) | 21 (0.143) | 0.422 | negative regulation of protein biosynthesis | Paip2 msi sqd |
| 1680 | GO:0005834 | C | 3, 6, 7, 8, | 3 | 1.760 (x 1.705) | 21 (0.143) | 0.423 | heterotrimeric G-protein complex | G-salpha60A Galpha49B cta |
| 1681 | GO:0008154 | P | 6, 9, | 3 | 1.760 (x 1.705) | 21 (0.143) | 0.423 | actin polymerization and/or depolymerization | Cdc42 Src64B ena |
| 1682 | GO:0003755 | F | 5, | 3 | 1.760 (x 1.705) | 21 (0.143) | 0.423 | peptidyl-prolyl cis-trans isomerase activity | CG2852 Cyp1 Fkbp13 |
| 1683 | GO:0048542 | P | 5, | 3 | 1.760 (x 1.705) | 21 (0.143) | 0.423 | lymph gland development (sensu Arthropoda) | Pen Ser kn |
| 1684 | GO:0051258 | P | 7, | 3 | 1.760 (x 1.705) | 21 (0.143) | 0.424 | protein polymerization | Cdc42 alphaTub84B betaTub56D |
| 1685 | GO:0007268 | P | 6, | 22 | 18.939 (x 1.162) | 226 (0.097) | 0.429 | synaptic transmission | 14-3-3zeta AP-1gamma Amph Arf79F CaMKII Chc Fas2 G-salpha60A Got2 Rop Snap Vap-33-1 X11L alpha-Adaptin alpha-Spec l(2)gl lqf mth olf413 pAbp scrib syndapin |
| 1686 | GO:0016835 | F | 4, | 7 | 5.196 (x 1.347) | 62 (0.113) | 0.43 | carbon-oxygen lyase activity | Acon BcDNA:GH12558 CG16733 CG6543 Eno Nop60B Rrp1 |
| 1687 | GO:0019237 | F | 6, | 2 | 1.006 (x 1.989) | 12 (0.167) | 0.435 | centromeric DNA binding | Msp-300 Nop60B |
| 1688 | GO:0009059 | P | 5, 6, | 48 | 43.744 (x 1.097) | 522 (0.092) | 0.435 | macromolecule biosynthesis | Aats-ala Aats-glupro CG10423 CG10990 CG12006 CG17514 CG31739 CG33123 CG33145 CG5191 CG5434 CG6394 CG6835 CG6904 CG7441 CG8443 CG8963 CG9425 Cbp80 EG:86E4.2 Elf Gfat1 Paip2 Pepck Pgi Rbp2 RpL13 RpL15 RpL22 RpL23a RpL3 RpS12 RpS6 Su(var)3-9 Top3beta X11L bl eIF-4B eIF3-S9 eIF5 l(2)01424 mdy msi pAbp rin sgl sqd stc |
| 1689 | GO:0015239 | F | 4, | 2 | 1.006 (x 1.989) | 12 (0.167) | 0.435 | multidrug transporter activity | CG5789 Mdr49 |
| 1690 | GO:0051322 | P | 6, | 2 | 1.006 (x 1.989) | 12 (0.167) | 0.436 | anaphase | l(1)dd4 lmg |
| 1691 | GO:0006897 | P | 6, 7, | 12 | 9.721 (x 1.234) | 116 (0.103) | 0.436 | endocytosis | AP-1gamma Amph Arf79F CG1900 CG5841 Chc Pi3K68D Rab10 alpha-Adaptin krz lqf syndapin |
| 1692 | GO:0019213 | F | 4, | 2 | 1.006 (x 1.989) | 12 (0.167) | 0.436 | deacetylase activity | CG11305 Sin3A |
| 1693 | GO:0045298 | C | 3, 5, 6, 7, 8, 9, 10, 11, | 2 | 1.006 (x 1.989) | 12 (0.167) | 0.436 | tubulin | alphaTub84B betaTub56D |
| 1694 | GO:0046785 | P | 8, 10, | 2 | 1.006 (x 1.989) | 12 (0.167) | 0.436 | microtubule polymerization | alphaTub84B betaTub56D |
| 1695 | GO:0004857 | F | 3, | 13 | 10.643 (x 1.221) | 127 (0.102) | 0.437 | enzyme inhibitor activity | 14-3-3epsilon 14-3-3zeta CG17919 CG6680 CG7219 Mapmodulin Mcr Set Spn43Aa Spn5 guf plexA serpin-27A |
| 1696 | GO:0009084 | P | 8, 9, | 2 | 1.006 (x 1.989) | 12 (0.167) | 0.437 | glutamine family amino acid biosynthesis | CG7145 Got2 |
| 1697 | GO:0009991 | P | 4, | 2 | 1.006 (x 1.989) | 12 (0.167) | 0.437 | response to extracellular stimulus | endos mth |
| 1698 | GO:0000122 | P | 10, | 6 | 4.358 (x 1.377) | 52 (0.115) | 0.437 | negative regulation of transcription from RNA polymerase II promoter | CG11228 Caf1 CtBP Rbf2 brk ci |
| 1699 | GO:0045786 | P | 6, 7, | 2 | 1.006 (x 1.989) | 12 (0.167) | 0.437 | negative regulation of progression through cell cycle | CG40410 CycA |
| 1700 | GO:0016638 | F | 4, | 2 | 1.006 (x 1.989) | 12 (0.167) | 0.437 | oxidoreductase activity, acting on the CH-NH2 group of donors | CG31472 Gdh |
| 1701 | GO:0031667 | P | 5, | 2 | 1.006 (x 1.989) | 12 (0.167) | 0.438 | response to nutrient levels | endos mth |
| 1702 | GO:0006066 | P | 5, | 16 | 13.492 (x 1.186) | 161 (0.099) | 0.446 | alcohol metabolism | Ald BEST:LD22483 CG15093 CG31169 CG8036 Eno Fpps Gapdh1 Gapdh2 Gfat1 Pepck Pgi knk mdy olf413 woc |
| 1703 | GO:0006810 | P | 4, 5, | 130 | 123.774 (x 1.050) | 1477 (0.088) | 0.45 | transport | AP-1gamma ATPsyn-beta Akt1 Amph Arf79F BEST:CK01140 BG:DS07473.1 CG10211 CG10657 CG10695 CG10960 CG11069 CG11451 CG11856 CG12048 CG1418 CG14439 CG1472 CG17036 CG17143 CG17419 CG1746 CG1893 CG1900 CG1907 CG2108 CG2158 CG2316 CG2852 CG2980 CG32137 CG32164 CG3249 CG33113 CG33214 CG3823 CG4509 CG5064 CG5434 CG5687 CG5789 CG5841 CG6812 CG6838 CG7720 CG8155 CG9057 CG9246 CG9342 CG9809 CG9906 Chc Cyp1 DNApol-delta Dlc90F EG:196F3.2 EG:34F3.8 Fs(2)Ket Hsp60 Indy Karybeta3 Klp10A LanB2 Mdr49 Mpcp Nrv1 PR2 Pen Pi3K68D Psa Rab10 Ranbp16 RhoBTB Rop S Sin3A Sip1 Snap Ucp4B Vap-33-1 Vha44 Vha55 X11L alpha-Adaptin alpha-Cat alphaTub84B baz betaTub56D bib bl btsz cic ck cora d dome garz gp210 hh jar jumu katanin-60 kel klar krz l(2)08717 l(2)dtl l(2)gl lqf lwr mask msk mth pain par-6 pav pll pnut prominin-like rg rin rpk scrib sec23 slmb sqd syndapin tud vg wbl |
| 1704 | GO:0007443 | P | 5, 9, 10, | 3 | 1.844 (x 1.627) | 22 (0.136) | 0.451 | Malpighian tubule morphogenesis | barr pbl zip |
| 1705 | GO:0009890 | P | 6, | 3 | 1.844 (x 1.627) | 22 (0.136) | 0.451 | negative regulation of biosynthesis | Paip2 msi sqd |
| 1706 | GO:0045927 | P | 4, | 3 | 1.844 (x 1.627) | 22 (0.136) | 0.451 | positive regulation of growth | Akt1 btsz tkv |
| 1707 | GO:0031047 | P | 7, | 2 | 1.089 (x 1.836) | 13 (0.154) | 0.451 | RNA-mediated gene silencing | Rm62 vig |
| 1708 | GO:0040003 | P | 8, | 3 | 1.844 (x 1.627) | 22 (0.136) | 0.451 | cuticle biosynthesis (sensu Insecta) | Akt1 cora knk |
| 1709 | GO:0043292 | C | 5, 6, 7, 8, | 2 | 1.089 (x 1.836) | 13 (0.154) | 0.451 | contractile fiber | Tm1 zip |
| 1710 | GO:0048613 | P | 7, 8, | 3 | 1.844 (x 1.627) | 22 (0.136) | 0.452 | embryonic ectodermal gut morphogenesis | barr pbl zip |
| 1711 | GO:0008347 | P | 6, 7, | 2 | 1.089 (x 1.836) | 13 (0.154) | 0.452 | glial cell migration | gish hh |
| 1712 | GO:0048558 | P | 6, 7, | 3 | 1.844 (x 1.627) | 22 (0.136) | 0.452 | embryonic gut morphogenesis | barr pbl zip |
| 1713 | GO:0003711 | F | 3, | 2 | 1.089 (x 1.836) | 13 (0.154) | 0.452 | transcriptional elongation regulator activity | CycT Spt6 |
| 1714 | GO:0048619 | P | 8, 9, | 3 | 1.844 (x 1.627) | 22 (0.136) | 0.452 | embryonic hindgut morphogenesis | barr pbl zip |
| 1715 | GO:0005849 | C | 3, 6, 7, 8, 9, 10, 11, 12, 13, | 2 | 1.089 (x 1.836) | 13 (0.154) | 0.452 | mRNA cleavage factor complex | CG2097 su(f) |
| 1716 | GO:0008301 | F | 5, | 1 | 0.335 (x 2.983) | 4 (0.250) | 0.452 | DNA bending activity | HmgD |
| 1717 | GO:0031327 | P | 7, | 3 | 1.844 (x 1.627) | 22 (0.136) | 0.452 | negative regulation of cellular biosynthesis | Paip2 msi sqd |
| 1718 | GO:0016893 | F | 7, | 2 | 1.089 (x 1.836) | 13 (0.154) | 0.452 | endonuclease activity, active with either ribo- or deoxyribonucleic acids and producing 5'-phosphomonoesters | CG7922 CG9425 |
| 1719 | GO:0004818 | F | 7, | 1 | 0.335 (x 2.983) | 4 (0.250) | 0.452 | glutamate-tRNA ligase activity | Aats-glupro |
| 1720 | GO:0048557 | P | 6, | 3 | 1.844 (x 1.627) | 22 (0.136) | 0.453 | embryonic digestive tract morphogenesis | barr pbl zip |
| 1721 | GO:0008582 | P | 4, 5, 7, 8, 9, 10, | 2 | 1.089 (x 1.836) | 13 (0.154) | 0.453 | regulation of synaptic growth at neuromuscular junction | Fas2 tkv |
| 1722 | GO:0009003 | F | 5, | 1 | 0.335 (x 2.983) | 4 (0.250) | 0.453 | signal peptidase activity | BG:DS00004.11 |
| 1723 | GO:0030832 | P | 9, | 2 | 1.089 (x 1.836) | 13 (0.154) | 0.453 | regulation of actin filament length | Src64B ena |
| 1724 | GO:0048611 | P | 6, | 3 | 1.844 (x 1.627) | 22 (0.136) | 0.453 | embryonic ectodermal gut development | barr pbl zip |
| 1725 | GO:0008352 | C | 4, 6, 7, 8, 9, 10, 11, | 1 | 0.335 (x 2.983) | 4 (0.250) | 0.453 | katanin | katanin-60 |
| 1726 | GO:0035290 | P | 5, 6, | 2 | 1.089 (x 1.836) | 13 (0.154) | 0.453 | trunk segmentation | en hh |
| 1727 | GO:0046331 | P | 6, | 1 | 0.335 (x 2.983) | 4 (0.250) | 0.453 | lateral inhibition | sca |
| 1728 | GO:0048566 | P | 5, | 3 | 1.844 (x 1.627) | 22 (0.136) | 0.453 | embryonic gut development | barr pbl zip |
| 1729 | GO:0007601 | P | 5, 7, | 8 | 6.201 (x 1.290) | 74 (0.108) | 0.453 | visual perception | CG17121 Galpha49B ck crb d ebi krz lqf |
| 1730 | GO:0006473 | P | 9, | 2 | 1.089 (x 1.836) | 13 (0.154) | 0.453 | protein amino acid acetylation | CG14222 Caf1 |
| 1731 | GO:0008275 | C | 4, 7, 8, 9, 10, 11, | 1 | 0.335 (x 2.983) | 4 (0.250) | 0.453 | gamma-tubulin small complex | l(1)dd4 |
| 1732 | GO:0007592 | P | 7, | 3 | 1.844 (x 1.627) | 22 (0.136) | 0.454 | cuticle biosynthesis (sensu Protostomia and Nematoda) | Akt1 cora knk |
| 1733 | GO:0050953 | P | 4, 6, | 8 | 6.201 (x 1.290) | 74 (0.108) | 0.454 | sensory perception of light stimulus | CG17121 Galpha49B ck crb d ebi krz lqf |
| 1734 | GO:0045451 | P | 7, 11, 13, 14, 16, | 4 | 2.682 (x 1.492) | 32 (0.125) | 0.454 | pole plasm oskar mRNA localization | Hrb27C Rop Tm1 capu |
| 1735 | GO:0048134 | P | 6, | 2 | 1.089 (x 1.836) | 13 (0.154) | 0.454 | germ-line cyst formation | alpha-Spec hts |
| 1736 | GO:0004274 | F | 6, 8, | 1 | 0.335 (x 2.983) | 4 (0.250) | 0.454 | dipeptidyl-peptidase IV activity | ome |
| 1737 | GO:0007427 | P | 5, 6, 7, | 3 | 1.844 (x 1.627) | 22 (0.136) | 0.454 | tracheal epithelial cell migration (sensu Insecta) | csw hh sgl |
| 1738 | GO:0000792 | C | 6, 7, 8, 9, 10, 11, | 2 | 1.089 (x 1.836) | 13 (0.154) | 0.454 | heterochromatin | Psc Su(var)3-9 |
| 1739 | GO:0007215 | P | 6, | 1 | 0.335 (x 2.983) | 4 (0.250) | 0.454 | glutamate signaling pathway | CG32447 |
| 1740 | GO:0008064 | P | 6, 7, 10, | 2 | 1.089 (x 1.836) | 13 (0.154) | 0.454 | regulation of actin polymerization and/or depolymerization | Src64B ena |
| 1741 | GO:0042991 | P | 8, 9, 10, 11, | 1 | 0.335 (x 2.983) | 4 (0.250) | 0.454 | transcription factor import into nucleus | slmb |
| 1742 | GO:0016441 | P | 4, 7, | 2 | 1.089 (x 1.836) | 13 (0.154) | 0.454 | posttranscriptional gene silencing | Rm62 vig |
| 1743 | GO:0004703 | F | 8, | 1 | 0.335 (x 2.983) | 4 (0.250) | 0.454 | G-protein coupled receptor kinase activity | Gprk1 |
| 1744 | GO:0007428 | P | 5, 6, | 2 | 1.089 (x 1.836) | 13 (0.154) | 0.455 | primary tracheal branching (sensu Insecta) | csw sgl |
| 1745 | GO:0003715 | F | 3, | 1 | 0.335 (x 2.983) | 4 (0.250) | 0.455 | transcription termination factor activity | lds |
| 1746 | GO:0048123 | P | 7, 9, 10, 12, | 2 | 1.089 (x 1.836) | 13 (0.154) | 0.455 | oocyte dorsal/ventral axis determination (sensu Insecta) | BicD egl |
| 1747 | GO:0046463 | P | 7, 8, 9, | 1 | 0.335 (x 2.983) | 4 (0.250) | 0.455 | acylglycerol biosynthesis | mdy |
| 1748 | GO:0006397 | P | 8, | 19 | 16.593 (x 1.145) | 198 (0.096) | 0.455 | mRNA processing | B52 CG2097 CG3605 CG4612 CG5728 CG6227 CG6946 Cbp80 Hrb27C Hrb87F Rm62 TBPH bl heph msi nonA-l pAbp sqd su(f) |
| 1749 | GO:0035194 | P | 5, 8, | 2 | 1.089 (x 1.836) | 13 (0.154) | 0.455 | RNA-mediated posttranscriptional gene silencing | Rm62 vig |
| 1750 | GO:0046034 | P | 6, 10, | 7 | 5.447 (x 1.285) | 65 (0.108) | 0.455 | ATP metabolism | ATPsyn-beta CG1746 Sin3A Vha44 Vha55 dome jumu |
| 1751 | GO:0009997 | P | 7, 8, 9, 10, 11, 12, | 1 | 0.335 (x 2.983) | 4 (0.250) | 0.455 | negative regulation of cardioblast cell fate specification | lqf |
| 1752 | GO:0004840 | F | 4, | 4 | 2.765 (x 1.446) | 33 (0.121) | 0.455 | ubiquitin conjugating enzyme activity | CG8188 UbcD2 UbcD6 lwr |
| 1753 | GO:0007539 | P | 5, | 2 | 1.089 (x 1.836) | 13 (0.154) | 0.455 | primary sex determination, soma | da sc |
| 1754 | GO:0044431 | C | 4, 5, 6, 7, 8, 9, | 7 | 5.447 (x 1.285) | 65 (0.108) | 0.455 | Golgi apparatus part | AP-1gamma CG1472 CG6394 Fur1 alpha-Adaptin garz sec23 |
| 1755 | GO:0042813 | F | 5, | 1 | 0.335 (x 2.983) | 4 (0.250) | 0.456 | Wnt receptor activity | fz |
| 1756 | GO:0008293 | P | 8, | 4 | 2.765 (x 1.446) | 33 (0.121) | 0.456 | torso signaling pathway | Gap1 csw knk sty |
| 1757 | GO:0006584 | P | 7, 8, | 2 | 1.089 (x 1.836) | 13 (0.154) | 0.456 | catecholamine metabolism | knk olf413 |
| 1758 | GO:0005787 | C | 3, 4, 5, 6, 7, 8, 9, 10, 11, | 1 | 0.335 (x 2.983) | 4 (0.250) | 0.456 | signal peptidase complex | BG:DS00004.11 |
| 1759 | GO:0005605 | C | 3, 4, 5, 6, | 2 | 1.089 (x 1.836) | 13 (0.154) | 0.456 | basal lamina | LanA LanB2 |
| 1760 | GO:0000781 | C | 5, 6, 7, 8, 9, 10, | 1 | 0.335 (x 2.983) | 4 (0.250) | 0.456 | chromosome, telomeric region | cav |
| 1761 | GO:0008335 | P | 9, | 1 | 0.335 (x 2.983) | 4 (0.250) | 0.456 | ovarian ring canal stabilization | Src64B |
| 1762 | GO:0016859 | F | 4, | 3 | 1.927 (x 1.556) | 23 (0.130) | 0.456 | cis-trans isomerase activity | CG2852 Cyp1 Fkbp13 |
| 1763 | GO:0016573 | P | 9, 10, 12, | 1 | 0.335 (x 2.983) | 4 (0.250) | 0.457 | histone acetylation | Caf1 |
| 1764 | GO:0009299 | P | 8, | 3 | 1.927 (x 1.556) | 23 (0.130) | 0.457 | mRNA transcription | RpII140 Top2 ph-p |
| 1765 | GO:0016421 | F | 5, | 1 | 0.335 (x 2.983) | 4 (0.250) | 0.457 | CoA carboxylase activity | CG2118 |
| 1766 | GO:0007228 | P | 8, 10, | 1 | 0.335 (x 2.983) | 4 (0.250) | 0.457 | activation of hh target transcription factor | hh |
| 1767 | GO:0045502 | F | 4, | 1 | 0.335 (x 2.983) | 4 (0.250) | 0.457 | dynein binding | BicD |
| 1768 | GO:0035301 | C | 3, 4, 5, 6, | 1 | 0.335 (x 2.983) | 4 (0.250) | 0.458 | Hedgehog signaling complex | ci |
| 1769 | GO:0006538 | P | 9, 10, | 1 | 0.335 (x 2.983) | 4 (0.250) | 0.458 | glutamate catabolism | Gdh |
| 1770 | GO:0008078 | P | 6, 7, 8, 9, | 1 | 0.335 (x 2.983) | 4 (0.250) | 0.458 | mesodermal cell migration | pbl |
| 1771 | GO:0016529 | C | 6, 7, 8, 9, | 1 | 0.335 (x 2.983) | 4 (0.250) | 0.458 | sarcoplasmic reticulum | CBP |
| 1772 | GO:0035073 | P | 6, | 1 | 0.335 (x 2.983) | 4 (0.250) | 0.459 | pupariation | crc |
| 1773 | GO:0007060 | P | 6, 9, | 1 | 0.335 (x 2.983) | 4 (0.250) | 0.459 | male meiosis chromosome segregation | polo |
| 1774 | GO:0046670 | P | 7, 8, 9, | 1 | 0.335 (x 2.983) | 4 (0.250) | 0.459 | positive regulation of retinal programmed cell death | klu |
| 1775 | GO:0004685 | F | 9, | 1 | 0.335 (x 2.983) | 4 (0.250) | 0.459 | calcium- and calmodulin-dependent protein kinase activity | CaMKII |
| 1776 | GO:0019099 | P | 5, | 1 | 0.335 (x 2.983) | 4 (0.250) | 0.46 | female germ-line sex determination | ovo |
| 1777 | GO:0015014 | P | 8, 9, 10, | 1 | 0.335 (x 2.983) | 4 (0.250) | 0.46 | heparan sulfate proteoglycan biosynthesis, polysaccharide chain biosynthesis | sgl |
| 1778 | GO:0045571 | P | 6, 7, | 1 | 0.335 (x 2.983) | 4 (0.250) | 0.46 | negative regulation of imaginal disc growth | fat2 |
| 1779 | GO:0007429 | P | 5, 6, | 1 | 0.335 (x 2.983) | 4 (0.250) | 0.46 | secondary tracheal branching (sensu Insecta) | sty |
| 1780 | GO:0020037 | F | 4, | 1 | 0.335 (x 2.983) | 4 (0.250) | 0.461 | heme binding | Cat |
| 1781 | GO:0006284 | P | 6, 8, | 1 | 0.335 (x 2.983) | 4 (0.250) | 0.461 | base-excision repair | BcDNA:GM10765 |
| 1782 | GO:0006921 | P | 8, | 1 | 0.335 (x 2.983) | 4 (0.250) | 0.461 | disassembly of cell structures during apoptosis | CG7263 |
| 1783 | GO:0006749 | P | 6, 7, | 1 | 0.335 (x 2.983) | 4 (0.250) | 0.461 | glutathione metabolism | CG6835 |
| 1784 | GO:0006207 | P | 8, 9, | 1 | 0.335 (x 2.983) | 4 (0.250) | 0.462 | 'de novo' pyrimidine base biosynthesis | r-l |
| 1785 | GO:0007278 | P | 6, | 1 | 0.335 (x 2.983) | 4 (0.250) | 0.462 | pole cell fate determination | tud |
| 1786 | GO:0004198 | F | 7, | 1 | 0.335 (x 2.983) | 4 (0.250) | 0.462 | calpain activity | CalpB |
| 1787 | GO:0042990 | P | 8, 9, 10, 11, 12, | 1 | 0.335 (x 2.983) | 4 (0.250) | 0.462 | regulation of transcription factor import into nucleus | slmb |
| 1788 | GO:0046693 | P | 7, 8, | 1 | 0.335 (x 2.983) | 4 (0.250) | 0.463 | sperm storage | Gdh |
| 1789 | GO:0046460 | P | 6, 7, 8, | 1 | 0.335 (x 2.983) | 4 (0.250) | 0.463 | neutral lipid biosynthesis | mdy |
| 1790 | GO:0005625 | C | 4, 5, | 1 | 0.335 (x 2.983) | 4 (0.250) | 0.463 | soluble fraction | Top2 |
| 1791 | GO:0007056 | P | 8, 9, 12, | 1 | 0.335 (x 2.983) | 4 (0.250) | 0.463 | female meiotic spindle assembly (sensu Metazoa) | polo |
| 1792 | GO:0015986 | P | 7, 8, 9, 10, 11, 12, | 7 | 5.363 (x 1.305) | 64 (0.109) | 0.464 | ATP synthesis coupled proton transport | ATPsyn-beta CG1746 Sin3A Vha44 Vha55 dome jumu |
| 1793 | GO:0016725 | F | 4, | 1 | 0.335 (x 2.983) | 4 (0.250) | 0.464 | oxidoreductase activity, acting on CH2 groups | RnrL |
| 1794 | GO:0006753 | P | 8, | 7 | 5.363 (x 1.305) | 64 (0.109) | 0.464 | nucleoside phosphate metabolism | ATPsyn-beta CG1746 Sin3A Vha44 Vha55 dome jumu |
| 1795 | GO:0004716 | F | 4, 8, | 1 | 0.335 (x 2.983) | 4 (0.250) | 0.464 | receptor signaling protein tyrosine kinase activity | CG31640 |
| 1796 | GO:0006754 | P | 7, 8, 9, 10, 11, | 7 | 5.363 (x 1.305) | 64 (0.109) | 0.464 | ATP biosynthesis | ATPsyn-beta CG1746 Sin3A Vha44 Vha55 dome jumu |
| 1797 | GO:0042689 | P | 6, 8, | 1 | 0.335 (x 2.983) | 4 (0.250) | 0.464 | regulation of crystal cell differentiation | Ser |
| 1798 | GO:0015985 | P | 7, 8, 9, 10, | 7 | 5.363 (x 1.305) | 64 (0.109) | 0.464 | energy coupled proton transport, down electrochemical gradient | ATPsyn-beta CG1746 Sin3A Vha44 Vha55 dome jumu |
| 1799 | GO:0005758 | C | 5, 6, 7, 8, 9, 10, 11, | 1 | 0.335 (x 2.983) | 4 (0.250) | 0.465 | mitochondrial intermembrane space | CG7263 |
| 1800 | GO:0005701 | C | 5, 6, 7, 8, 9, 10, | 1 | 0.335 (x 2.983) | 4 (0.250) | 0.465 | polytene chromosome chromocenter | jumu |
| 1801 | GO:0042428 | P | 6, 8, 9, | 1 | 0.335 (x 2.983) | 4 (0.250) | 0.465 | serotonin metabolism | 14-3-3zeta |
| 1802 | GO:0003918 | F | 5, 6, | 1 | 0.335 (x 2.983) | 4 (0.250) | 0.465 | DNA topoisomerase (ATP-hydrolyzing) activity | Top2 |
| 1803 | GO:0000002 | P | 7, | 1 | 0.335 (x 2.983) | 4 (0.250) | 0.466 | mitochondrial genome maintenance | Dref |
| 1804 | GO:0031228 | C | 5, 6, 7, 8, 9, 10, 11, | 1 | 0.335 (x 2.983) | 4 (0.250) | 0.466 | intrinsic to Golgi membrane | Fur1 |
| 1805 | GO:0016528 | C | 5, 6, 7, | 1 | 0.335 (x 2.983) | 4 (0.250) | 0.466 | sarcoplasm | CBP |
| 1806 | GO:0005828 | C | 7, 8, 9, 10, 11, 12, | 1 | 0.335 (x 2.983) | 4 (0.250) | 0.466 | kinetochore microtubule | Klp10A |
| 1807 | GO:0006561 | P | 9, 10, | 1 | 0.335 (x 2.983) | 4 (0.250) | 0.467 | proline biosynthesis | CG7145 |
| 1808 | GO:0016742 | F | 7, | 1 | 0.335 (x 2.983) | 4 (0.250) | 0.467 | hydroxymethyl-, formyl- and related transferase activity | ade3 |
| 1809 | GO:0009986 | C | 3, 4, | 1 | 0.335 (x 2.983) | 4 (0.250) | 0.467 | cell surface | Hmu |
| 1810 | GO:0005072 | F | 5, | 1 | 0.335 (x 2.983) | 4 (0.250) | 0.467 | transforming growth factor beta receptor, cytoplasmic mediator activity | Smox |
| 1811 | GO:0035210 | P | 5, | 1 | 0.335 (x 2.983) | 4 (0.250) | 0.468 | prepupal development (sensu Insecta) | crc |
| 1812 | GO:0045880 | P | 6, 7, 8, | 1 | 0.335 (x 2.983) | 4 (0.250) | 0.468 | positive regulation of smoothened signaling pathway | hh |
| 1813 | GO:0030173 | C | 6, 7, 8, 9, 10, 11, 12, | 1 | 0.335 (x 2.983) | 4 (0.250) | 0.468 | integral to Golgi membrane | Fur1 |
| 1814 | GO:0051013 | P | 8, | 1 | 0.335 (x 2.983) | 4 (0.250) | 0.469 | microtubule severing | katanin-60 |
| 1815 | GO:0051186 | P | 5, | 24 | 21.202 (x 1.132) | 253 (0.095) | 0.469 | cofactor metabolism | ATPsyn-beta Acon BEST:LD22483 CG10657 CG15093 CG1544 CG17036 CG1746 CG31472 CG5028 CG5687 CG6638 CG6835 CG7461 CG7720 CG8036 CG9471 Idh Sin3A Vha44 Vha55 dome jumu l(1)G0334 |
| 1816 | GO:0046906 | F | 3, | 1 | 0.335 (x 2.983) | 4 (0.250) | 0.469 | tetrapyrrole binding | Cat |
| 1817 | GO:0003994 | F | 6, | 1 | 0.335 (x 2.983) | 4 (0.250) | 0.469 | aconitate hydratase activity | Acon |
| 1818 | GO:0035158 | P | 7, | 1 | 0.335 (x 2.983) | 4 (0.250) | 0.469 | regulation of tracheal tube diameter | Fas2 |
| 1819 | GO:0007100 | P | 7, 9, 10, | 1 | 0.335 (x 2.983) | 4 (0.250) | 0.47 | mitotic centrosome separation | chb |
| 1820 | GO:0016071 | P | 7, | 20 | 17.431 (x 1.147) | 208 (0.096) | 0.47 | mRNA metabolism | B52 CG11123 CG2097 CG3605 CG4612 CG5728 CG6227 CG6946 Cbp80 Hrb27C Hrb87F Rm62 TBPH bl heph msi nonA-l pAbp sqd su(f) |
| 1821 | GO:0030588 | P | 5, | 1 | 0.335 (x 2.983) | 4 (0.250) | 0.47 | pseudocleavage | jar |
| 1822 | GO:0030589 | P | 6, | 1 | 0.335 (x 2.983) | 4 (0.250) | 0.47 | pseudocleavage (sensu Insecta) | jar |
| 1823 | GO:0006465 | P | 6, 9, | 1 | 0.335 (x 2.983) | 4 (0.250) | 0.47 | signal peptide processing | BG:DS00004.11 |
| 1824 | GO:0045254 | C | 3, 5, 6, 7, 8, | 1 | 0.335 (x 2.983) | 4 (0.250) | 0.471 | pyruvate dehydrogenase complex | l(1)G0334 |
| 1825 | GO:0016070 | P | 6, | 34 | 30.755 (x 1.106) | 367 (0.093) | 0.471 | RNA metabolism | Aats-ala Aats-glupro B52 CG11123 CG2097 CG31739 CG33123 CG3605 CG4612 CG4749 CG5191 CG5728 CG6227 CG6946 CG7441 CG9425 Cbp80 Hrb27C Hrb87F Nop60B Rm62 TBPH Tis11 Top3beta X11L bl heph mdy msi nonA-l nop5 pAbp sqd su(f) |
| 1826 | GO:0035263 | P | 4, 6, | 1 | 0.335 (x 2.983) | 4 (0.250) | 0.471 | genital disc sexually dimorphic development | dac |
| 1827 | GO:0030371 | F | 3, | 1 | 0.335 (x 2.983) | 4 (0.250) | 0.471 | translation repressor activity | msi |
| 1828 | GO:0045168 | P | 5, | 1 | 0.335 (x 2.983) | 4 (0.250) | 0.471 | cell-cell signaling during cell fate commitment | sca |
| 1829 | GO:0042575 | C | 3, 4, 5, 6, | 1 | 0.335 (x 2.983) | 4 (0.250) | 0.472 | DNA polymerase complex | DNApol-epsilon |
| 1830 | GO:0030337 | F | 7, | 1 | 0.335 (x 2.983) | 4 (0.250) | 0.472 | DNA polymerase processivity factor activity | mus209 |
| 1831 | GO:0008159 | F | 4, | 1 | 0.335 (x 2.983) | 4 (0.250) | 0.472 | positive transcription elongation factor activity | CycT |
| 1832 | GO:0043161 | P | 10, 11, 12, | 1 | 0.335 (x 2.983) | 4 (0.250) | 0.473 | proteasomal ubiquitin-dependent protein catabolism | fzy |
| 1833 | GO:0007348 | P | 6, 8, 9, | 1 | 0.335 (x 2.983) | 4 (0.250) | 0.473 | regulation of progression through syncytial blastoderm mitotic cell cycle | CG40410 |
| 1834 | GO:0007517 | P | 4, | 11 | 9.050 (x 1.215) | 108 (0.102) | 0.473 | muscle development | CaMKII Dr Fas2 G-salpha60A Hem Vap-33-1 Wnt2 ap betaTub56D tkv zip |
| 1835 | GO:0046672 | P | 8, 9, 10, 11, | 1 | 0.335 (x 2.983) | 4 (0.250) | 0.473 | positive regulation of retinal cell programmed cell death (sensu Endopterygota) | klu |
| 1836 | GO:0030672 | C | 5, 6, 7, 8, 9, 10, 11, 12, 13, | 1 | 0.335 (x 2.983) | 4 (0.250) | 0.473 | synaptic vesicle membrane | Chc |
| 1837 | GO:0019856 | P | 7, 8, | 1 | 0.335 (x 2.983) | 4 (0.250) | 0.474 | pyrimidine base biosynthesis | r-l |
| 1838 | GO:0046330 | P | 6, 7, 9, 10, | 1 | 0.335 (x 2.983) | 4 (0.250) | 0.474 | positive regulation of JNK cascade | arm |
| 1839 | GO:0007227 | P | 7, | 1 | 0.335 (x 2.983) | 4 (0.250) | 0.474 | signal transduction downstream of smoothened | hh |
| 1840 | GO:0015367 | F | 6, 8, | 1 | 0.335 (x 2.983) | 4 (0.250) | 0.474 | oxoglutarate:malate antiporter activity | CG1907 |
| 1841 | GO:0006750 | P | 7, 8, | 1 | 0.335 (x 2.983) | 4 (0.250) | 0.475 | glutathione biosynthesis | CG6835 |
| 1842 | GO:0043632 | P | 7, | 8 | 6.453 (x 1.240) | 77 (0.104) | 0.476 | modification-dependent macromolecule catabolism | BcDNA:LD22910 CG5505 CG7288 CG8494 Uba2 Uch fzy th |
| 1843 | GO:0008270 | F | 6, | 54 | 50.448 (x 1.070) | 602 (0.090) | 0.476 | zinc ion binding | ATbp Bap170 CG10542 CG10602 CG12299 CG12391 CG14231 CG1529 CG1600 CG1647 CG17361 CG17419 CG1815 CG31716 CG3847 CG5841 CG6791 CG6930 CG7987 CG8089 CG8092 CG8478 CG9425 Cen190 D19A D19B EG:115C2.6 LanB2 MICAL-like Mi-2 Nep2 Pep Psa Psc Su(var)3-9 Su(z)12 ap br ci crol d4 esn gol jim jing klu lmg lola ovo pk stc th tok zf30C |
| 1844 | GO:0019941 | P | 8, 9, 10, | 8 | 6.453 (x 1.240) | 77 (0.104) | 0.477 | modification-dependent protein catabolism | BcDNA:LD22910 CG5505 CG7288 CG8494 Uba2 Uch fzy th |
| 1845 | GO:0008639 | F | 3, | 4 | 2.849 (x 1.404) | 34 (0.118) | 0.478 | small protein conjugating enzyme activity | CG8188 UbcD2 UbcD6 lwr |
| 1846 | GO:0042063 | P | 6, | 3 | 2.011 (x 1.492) | 24 (0.125) | 0.49 | gliogenesis | bnb gish hh |
| 1847 | GO:0010004 | P | 7, | 3 | 2.011 (x 1.492) | 24 (0.125) | 0.49 | gastrulation (sensu Insecta) | baz cta shg |
| 1848 | GO:0048512 | P | 5, | 3 | 2.011 (x 1.492) | 24 (0.125) | 0.49 | circadian behavior | CkIIalpha CkIIbeta slmb |
| 1849 | GO:0001703 | P | 6, | 3 | 2.011 (x 1.492) | 24 (0.125) | 0.491 | gastrulation (sensu Protostomia) | baz cta shg |
| 1850 | GO:0008407 | P | 4, 5, | 3 | 2.011 (x 1.492) | 24 (0.125) | 0.491 | bristle morphogenesis | Hem sc sca |
| 1851 | GO:0030136 | C | 7, 8, 9, 10, 11, | 6 | 4.693 (x 1.279) | 56 (0.107) | 0.491 | clathrin-coated vesicle | AP-1gamma Chc Vap-33-1 alpha-Adaptin btsz l(2)gl |
| 1852 | GO:0005996 | P | 6, 7, | 10 | 8.380 (x 1.193) | 100 (0.100) | 0.491 | monosaccharide metabolism | Ald BEST:LD22483 CG15093 CG8036 Eno Gapdh1 Gapdh2 Gfat1 Pepck Pgi |
| 1853 | GO:0016836 | F | 5, | 6 | 4.693 (x 1.279) | 56 (0.107) | 0.491 | hydro-lyase activity | Acon BcDNA:GH12558 CG16733 CG6543 Eno Nop60B |
| 1854 | GO:0051261 | P | 7, | 2 | 1.173 (x 1.705) | 14 (0.143) | 0.492 | protein depolymerization | CG11207 fwd |
| 1855 | GO:0019732 | P | 6, 7, 8, | 2 | 1.173 (x 1.705) | 14 (0.143) | 0.492 | antifungal humoral response | pll tub |
| 1856 | GO:0040014 | P | 4, | 2 | 1.173 (x 1.705) | 14 (0.143) | 0.492 | regulation of body size | Akt1 btsz |
| 1857 | GO:0006379 | P | 9, | 2 | 1.173 (x 1.705) | 14 (0.143) | 0.492 | mRNA cleavage | CG2097 su(f) |
| 1858 | GO:0045793 | P | 6, 7, | 2 | 1.173 (x 1.705) | 14 (0.143) | 0.493 | positive regulation of cell size | Akt1 btsz |
| 1859 | GO:0015992 | P | 6, 7, 8, 9, | 8 | 6.536 (x 1.224) | 78 (0.103) | 0.493 | proton transport | ATPsyn-beta CG1746 Sin3A Ucp4B Vha44 Vha55 dome jumu |
| 1860 | GO:0035264 | P | 3, | 2 | 1.173 (x 1.705) | 14 (0.143) | 0.493 | body growth | Akt1 btsz |
| 1861 | GO:0006818 | P | 5, 6, | 8 | 6.536 (x 1.224) | 78 (0.103) | 0.493 | hydrogen transport | ATPsyn-beta CG1746 Sin3A Ucp4B Vha44 Vha55 dome jumu |
| 1862 | GO:0007062 | P | 5, | 2 | 1.173 (x 1.705) | 14 (0.143) | 0.493 | sister chromatid cohesion | Gap1 SMC1 |
| 1863 | GO:0050803 | P | 7, | 2 | 1.173 (x 1.705) | 14 (0.143) | 0.493 | regulation of synapse structure and function | Fas2 scrib |
| 1864 | GO:0018958 | P | 6, | 2 | 1.173 (x 1.705) | 14 (0.143) | 0.494 | phenol metabolism | knk olf413 |
| 1865 | GO:0051124 | P | 4, 6, 7, 8, 9, | 2 | 1.173 (x 1.705) | 14 (0.143) | 0.494 | synaptic growth at neuromuscular junction | Fas2 tkv |
| 1866 | GO:0000221 | C | 4, 5, 6, 7, 8, 9, 10, 11, 12, | 2 | 1.173 (x 1.705) | 14 (0.143) | 0.494 | hydrogen-transporting ATPase V1 domain | Vha44 Vha55 |
| 1867 | GO:0048568 | P | 4, | 4 | 2.933 (x 1.364) | 35 (0.114) | 0.501 | embryonic organ development | barr msn pbl zip |
| 1868 | GO:0007096 | P | 8, 9, | 1 | 0.419 (x 2.387) | 5 (0.200) | 0.501 | regulation of exit from mitosis | fzy |
| 1869 | GO:0006537 | P | 9, 10, | 1 | 0.419 (x 2.387) | 5 (0.200) | 0.501 | glutamate biosynthesis | Got2 |
| 1870 | GO:0005677 | C | 4, 7, 8, 9, 10, 11, 12, 13, 14, | 1 | 0.419 (x 2.387) | 5 (0.200) | 0.502 | chromatin silencing complex | CG11305 |
| 1871 | GO:0042685 | P | 6, 7, 8, 9, 10, | 1 | 0.419 (x 2.387) | 5 (0.200) | 0.502 | cardioblast cell fate specification | lqf |
| 1872 | GO:0006359 | P | 9, | 1 | 0.419 (x 2.387) | 5 (0.200) | 0.502 | regulation of transcription from RNA polymerase III promoter | Brf |
| 1873 | GO:0017177 | C | 3, 5, 6, 7, 8, 9, 10, | 1 | 0.419 (x 2.387) | 5 (0.200) | 0.503 | alpha-glucosidase II complex | BcDNA:GH04962 |
| 1874 | GO:0016894 | F | 7, | 1 | 0.419 (x 2.387) | 5 (0.200) | 0.503 | endonuclease activity, active with either ribo- or deoxyribonucleic acids and producing 3'-phosphomonoesters | CG10103 |
| 1875 | GO:0042594 | P | 4, 6, | 1 | 0.419 (x 2.387) | 5 (0.200) | 0.503 | response to starvation | mth |
| 1876 | GO:0009142 | P | 7, 8, | 7 | 5.782 (x 1.211) | 69 (0.101) | 0.503 | nucleoside triphosphate biosynthesis | ATPsyn-beta CG1746 Sin3A Vha44 Vha55 dome jumu |
| 1877 | GO:0050982 | P | 5, 6, | 1 | 0.419 (x 2.387) | 5 (0.200) | 0.503 | detection of mechanical stimulus | pain |
| 1878 | GO:0019829 | F | 5, 6, 8, 13, | 7 | 5.782 (x 1.211) | 69 (0.101) | 0.503 | cation-transporting ATPase activity | ATPsyn-beta BEST:CK01140 CG1746 Sin3A Vha55 dome jumu |
| 1879 | GO:0003906 | F | 5, | 1 | 0.419 (x 2.387) | 5 (0.200) | 0.504 | DNA-(apurinic or apyrimidinic site) lyase activity | Rrp1 |
| 1880 | GO:0009199 | P | 8, | 7 | 5.782 (x 1.211) | 69 (0.101) | 0.504 | ribonucleoside triphosphate metabolism | ATPsyn-beta CG1746 Sin3A Vha44 Vha55 dome jumu |
| 1881 | GO:0009218 | P | 8, | 1 | 0.419 (x 2.387) | 5 (0.200) | 0.504 | pyrimidine ribonucleotide metabolism | dnk |
| 1882 | GO:0006898 | P | 7, 8, | 4 | 3.017 (x 1.326) | 36 (0.111) | 0.504 | receptor mediated endocytosis | Amph Chc lqf syndapin |
| 1883 | GO:0009205 | P | 9, | 7 | 5.782 (x 1.211) | 69 (0.101) | 0.504 | purine ribonucleoside triphosphate metabolism | ATPsyn-beta CG1746 Sin3A Vha44 Vha55 dome jumu |
| 1884 | GO:0009060 | P | 8, | 5 | 3.939 (x 1.269) | 47 (0.106) | 0.504 | aerobic respiration | Acon CG1544 CG5028 Idh l(1)G0334 |
| 1885 | GO:0007473 | P | 6, 7, | 1 | 0.419 (x 2.387) | 5 (0.200) | 0.504 | wing disc proximal/distal pattern formation | hh |
| 1886 | GO:0009144 | P | 8, | 7 | 5.782 (x 1.211) | 69 (0.101) | 0.504 | purine nucleoside triphosphate metabolism | ATPsyn-beta CG1746 Sin3A Vha44 Vha55 dome jumu |
| 1887 | GO:0006099 | P | 8, 9, | 5 | 3.939 (x 1.269) | 47 (0.106) | 0.504 | tricarboxylic acid cycle | Acon CG1544 CG5028 Idh l(1)G0334 |
| 1888 | GO:0030119 | C | 3, 4, 5, 6, 7, 8, 9, | 1 | 0.419 (x 2.387) | 5 (0.200) | 0.504 | membrane coat adaptor complex | alpha-Adaptin |
| 1889 | GO:0046356 | P | 8, | 5 | 3.939 (x 1.269) | 47 (0.106) | 0.504 | acetyl-CoA catabolism | Acon CG1544 CG5028 Idh l(1)G0334 |
| 1890 | GO:0006697 | P | 8, 9, 10, | 1 | 0.419 (x 2.387) | 5 (0.200) | 0.505 | ecdysone biosynthesis | woc |
| 1891 | GO:0007369 | P | 5, | 6 | 4.860 (x 1.234) | 58 (0.103) | 0.505 | gastrulation | baz cta gol pbl sgl shg |
| 1892 | GO:0045333 | P | 7, | 5 | 3.939 (x 1.269) | 47 (0.106) | 0.505 | cellular respiration | Acon CG1544 CG5028 Idh l(1)G0334 |
| 1893 | GO:0015385 | F | 7, 8, 9, | 1 | 0.419 (x 2.387) | 5 (0.200) | 0.505 | sodium:hydrogen antiporter activity | Sip1 |
| 1894 | GO:0009220 | P | 8, 9, | 1 | 0.419 (x 2.387) | 5 (0.200) | 0.505 | pyrimidine ribonucleotide biosynthesis | dnk |
| 1895 | GO:0045017 | P | 6, 7, 8, | 1 | 0.419 (x 2.387) | 5 (0.200) | 0.505 | glycerolipid biosynthesis | mdy |
| 1896 | GO:0046854 | P | 8, 9, 10, 11, | 1 | 0.419 (x 2.387) | 5 (0.200) | 0.506 | phosphoinositide phosphorylation | Pi3K68D |
| 1897 | GO:0008368 | F | 4, | 1 | 0.419 (x 2.387) | 5 (0.200) | 0.506 | Gram-negative bacterial binding | GNBP3 |
| 1898 | GO:0006835 | P | 7, 8, | 1 | 0.419 (x 2.387) | 5 (0.200) | 0.506 | dicarboxylic acid transport | CG1907 |
| 1899 | GO:0030163 | P | 6, 7, | 9 | 7.542 (x 1.193) | 90 (0.100) | 0.506 | protein catabolism | BcDNA:LD22910 CG5505 CG7288 CG8494 CkIalpha Uba2 Uch fzy th |
| 1900 | GO:0030131 | C | 4, 5, 6, 7, 8, 9, 10, | 1 | 0.419 (x 2.387) | 5 (0.200) | 0.506 | clathrin adaptor complex | alpha-Adaptin |
| 1901 | GO:0005865 | C | 5, 6, 7, 8, 9, 10, 11, | 1 | 0.419 (x 2.387) | 5 (0.200) | 0.507 | striated muscle thin filament | Tm1 |
| 1902 | GO:0050974 | P | 5, 6, 7, | 1 | 0.419 (x 2.387) | 5 (0.200) | 0.507 | detection of mechanical stimulus during sensory perception | pain |
| 1903 | GO:0030055 | C | 6, 7, 8, | 2 | 1.257 (x 1.591) | 15 (0.133) | 0.507 | cell-matrix junction | Sdc alpha-Cat |
| 1904 | GO:0016778 | F | 5, | 1 | 0.419 (x 2.387) | 5 (0.200) | 0.507 | diphosphotransferase activity | CG6767 |
| 1905 | GO:0004022 | F | 6, | 2 | 1.257 (x 1.591) | 15 (0.133) | 0.507 | alcohol dehydrogenase activity | CG1600 LanB2 |
| 1906 | GO:0042686 | P | 6, 7, 8, 9, 10, 11, | 1 | 0.419 (x 2.387) | 5 (0.200) | 0.507 | regulation of cardioblast cell fate specification | lqf |
| 1907 | GO:0007562 | P | 3, | 2 | 1.257 (x 1.591) | 15 (0.133) | 0.508 | eclosion | CkIIbeta br |
| 1908 | GO:0004709 | F | 5, 9, | 1 | 0.419 (x 2.387) | 5 (0.200) | 0.508 | MAP kinase kinase kinase activity | Mekk1 |
| 1909 | GO:0005852 | C | 3, 4, 5, 6, 7, 8, 9, | 2 | 1.257 (x 1.591) | 15 (0.133) | 0.508 | eukaryotic translation initiation factor 3 complex | CG8443 eIF3-S9 |
| 1910 | GO:0004571 | F | 8, | 1 | 0.419 (x 2.387) | 5 (0.200) | 0.508 | mannosyl-oligosaccharide 1,2-alpha-mannosidase activity | EG:86E4.2 |
| 1911 | GO:0007538 | P | 4, | 2 | 1.257 (x 1.591) | 15 (0.133) | 0.508 | primary sex determination | da sc |
| 1912 | GO:0006631 | P | 6, 7, | 10 | 8.631 (x 1.159) | 103 (0.097) | 0.508 | fatty acid metabolism | Acox57D-p BcDNA:GH02901 BcDNA:GH12558 CG11055 CG2118 CG32099 CG6543 CG6638 CG7461 Thiolase |
| 1913 | GO:0043406 | P | 7, 8, | 1 | 0.419 (x 2.387) | 5 (0.200) | 0.508 | positive regulation of MAPK activity | mbt |
| 1914 | GO:0051052 | P | 7, | 2 | 1.257 (x 1.591) | 15 (0.133) | 0.508 | regulation of DNA metabolism | dre4 mus209 |
| 1915 | GO:0009206 | P | 9, 10, | 7 | 5.698 (x 1.228) | 68 (0.103) | 0.508 | purine ribonucleoside triphosphate biosynthesis | ATPsyn-beta CG1746 Sin3A Vha44 Vha55 dome jumu |
| 1916 | GO:0016303 | F | 8, | 1 | 0.419 (x 2.387) | 5 (0.200) | 0.508 | phosphatidylinositol 3-kinase activity | Pi3K68D |
| 1917 | GO:0016469 | C | 3, 6, 7, 8, | 7 | 5.698 (x 1.228) | 68 (0.103) | 0.509 | proton-transporting two-sector ATPase complex | ATPsyn-beta CG1746 Sin3A Vha44 Vha55 dome jumu |
| 1918 | GO:0016971 | F | 7, | 1 | 0.419 (x 2.387) | 5 (0.200) | 0.509 | flavin-linked sulfhydryl oxidase activity | CG4670 |
| 1919 | GO:0009145 | P | 8, 9, | 7 | 5.698 (x 1.228) | 68 (0.103) | 0.509 | purine nucleoside triphosphate biosynthesis | ATPsyn-beta CG1746 Sin3A Vha44 Vha55 dome jumu |
| 1920 | GO:0017026 | F | 7, | 1 | 0.419 (x 2.387) | 5 (0.200) | 0.509 | procollagen C-endopeptidase activity | tok |
| 1921 | GO:0009201 | P | 8, 9, | 7 | 5.698 (x 1.228) | 68 (0.103) | 0.509 | ribonucleoside triphosphate biosynthesis | ATPsyn-beta CG1746 Sin3A Vha44 Vha55 dome jumu |
| 1922 | GO:0035026 | P | 5, 6, | 1 | 0.419 (x 2.387) | 5 (0.200) | 0.509 | leading edge cell differentiation | zip |
| 1923 | GO:0019748 | P | 4, | 7 | 5.698 (x 1.228) | 68 (0.103) | 0.509 | secondary metabolism | E(z) Jheh1 Jheh2 Vha44 ade3 ovo serpin-27A |
| 1924 | GO:0016885 | F | 4, | 1 | 0.419 (x 2.387) | 5 (0.200) | 0.51 | ligase activity, forming carbon-carbon bonds | CG2118 |
| 1925 | GO:0016572 | P | 9, 12, | 1 | 0.419 (x 2.387) | 5 (0.200) | 0.51 | histone phosphorylation | BcDNA:LD09009 |
| 1926 | GO:0008367 | F | 3, | 1 | 0.419 (x 2.387) | 5 (0.200) | 0.51 | bacterial binding | GNBP3 |
| 1927 | GO:0045810 | P | 7, 8, 9, | 1 | 0.419 (x 2.387) | 5 (0.200) | 0.51 | negative regulation of frizzled signaling pathway | stan |
| 1928 | GO:0007020 | P | 9, | 1 | 0.419 (x 2.387) | 5 (0.200) | 0.511 | microtubule nucleation | l(1)dd4 |
| 1929 | GO:0000049 | F | 5, | 1 | 0.419 (x 2.387) | 5 (0.200) | 0.511 | tRNA binding | Su(var)3-9 |
| 1930 | GO:0016307 | F | 7, | 1 | 0.419 (x 2.387) | 5 (0.200) | 0.511 | phosphatidylinositol phosphate kinase activity | Pi3K68D |
| 1931 | GO:0045468 | P | 9, 10, 11, 12, | 1 | 0.419 (x 2.387) | 5 (0.200) | 0.511 | regulation of R8 spacing | sca |
| 1932 | GO:0004372 | F | 6, | 1 | 0.419 (x 2.387) | 5 (0.200) | 0.512 | glycine hydroxymethyltransferase activity | ade3 |
| 1933 | GO:0017124 | F | 5, | 1 | 0.419 (x 2.387) | 5 (0.200) | 0.512 | SH3 domain binding | rin |
| 1934 | GO:0016972 | F | 6, | 1 | 0.419 (x 2.387) | 5 (0.200) | 0.512 | thiol oxidase activity | CG4670 |
| 1935 | GO:0007374 | P | 8, | 1 | 0.419 (x 2.387) | 5 (0.200) | 0.512 | posterior midgut invagination | cta |
| 1936 | GO:0004871 | F | 2, | 92 | 88.242 (x 1.043) | 1053 (0.087) | 0.513 | signal transducer activity | 18w Abi Akt1 BcDNA:GH03163 CG11207 CG11228 CG14217 CG1514 CG17419 CG17952 CG1815 CG31640 CG32447 CG3249 CG33113 CG33214 CG40410 CG5841 CG7668 CG8789 CG9025 CaMKII CdGAPr Cdk4 CkIIalpha CkIIbeta CkIalpha EG:BACH59J11.2 Eb1 Eip63E Eip75B G-salpha60A GNBP3 Galpha49B Gprk1 Hem Hmu KP78b LanA Mcr Mekk1 Nek2 PFE Pak3 Ptp99A RacGAP50C Ranbp16 RhoGAP1A RhoGEF3 Sdc Sema-1b Sema-2a Ser Smox Tsp66E Wnt2 argos arm cdc2 crb csul csw cta dome endos fat2 fra fz gish hh l(1)G0148 mask mav mbt msn mth otk pbl plexA pll polo rin sca scrib shg sima smi35A ss stan sty tkv wgn |
| 1937 | GO:0007540 | P | 6, | 1 | 0.419 (x 2.387) | 5 (0.200) | 0.513 | sex determination, establishment of X:A ratio | sc |
| 1938 | GO:0000287 | F | 5, | 1 | 0.419 (x 2.387) | 5 (0.200) | 0.513 | magnesium ion binding | CG32099 |
| 1939 | GO:0042325 | P | 8, | 1 | 0.419 (x 2.387) | 5 (0.200) | 0.513 | regulation of phosphorylation | edl |
| 1940 | GO:0008315 | P | 9, | 1 | 0.419 (x 2.387) | 5 (0.200) | 0.514 | meiotic G2/MI transition | cdc2 |
| 1941 | GO:0046504 | P | 6, | 1 | 0.419 (x 2.387) | 5 (0.200) | 0.514 | glycerol ether biosynthesis | mdy |
| 1942 | GO:0005451 | F | 6, 7, 8, | 1 | 0.419 (x 2.387) | 5 (0.200) | 0.514 | monovalent cation:proton antiporter activity | Sip1 |
| 1943 | GO:0000149 | F | 4, | 1 | 0.419 (x 2.387) | 5 (0.200) | 0.514 | SNARE binding | Rop |
| 1944 | GO:0016567 | P | 9, | 11 | 9.469 (x 1.162) | 113 (0.097) | 0.515 | protein ubiquitination | CG10542 CG1815 CG31716 CG5841 Mi-2 Psc d4 gol lmg stc th |
| 1945 | GO:0032156 | C | 6, 7, 8, 9, | 1 | 0.419 (x 2.387) | 5 (0.200) | 0.515 | septin cytoskeleton | pnut |
| 1946 | GO:0000187 | P | 8, 9, | 1 | 0.419 (x 2.387) | 5 (0.200) | 0.515 | activation of MAPK activity | mbt |
| 1947 | GO:0007344 | P | 6, 8, | 1 | 0.419 (x 2.387) | 5 (0.200) | 0.515 | pronuclear fusion | polo |
| 1948 | GO:0005942 | C | 3, 6, 7, 8, 9, 10, | 1 | 0.419 (x 2.387) | 5 (0.200) | 0.515 | phosphoinositide 3-kinase complex | Pi3K68D |
| 1949 | GO:0017077 | F | 4, | 1 | 0.419 (x 2.387) | 5 (0.200) | 0.516 | oxidative phosphorylation uncoupler activity | Ucp4B |
| 1950 | GO:0007541 | P | 6, | 1 | 0.419 (x 2.387) | 5 (0.200) | 0.516 | sex determination, primary response to X:A ratio | da |
[truncated: 125,234 more chars]
