# Supplementary material for: Functional dissection of the ash2 and ash1 transcriptomes provides insights into the transcriptional basis of wing phenotypes and reveals conserved protein interactions
Source: Genome Biol. 2007 Apr 28;8(4):R67. doi: 10.1186/gb-2007-8-4-r67 (PMC1896016; doi:10.1186/gb-2007-8-4-r67)
Supplement: Additional data file 10 — GO annotations of the genes upregulated over 1.5-fold in ash2112411 [file gb-2007-8-4-r67-S10.html]

  

---

  

|  |  |
| --- | --- |
| Go Statistics | Reg File: **ash2112411\_U1.5x.txt.fbgns** (782 genes -- 263 skipped)  Ref File: **ref.fbgns** (13577 genes -- 4663 skipped)  Database: **go\_200507-termdb.rdf-xml** |

---

  

Fields Description

| Pos | Go Term | Ontology | Levels | Observed | Expected | Possibles | p-value(Adj) | Go term description | Genes with the GO term |
| --- | --- | --- | --- | --- | --- | --- | --- | --- | --- |
| 1 | GO:0005840 | C | 4, 5, 6, 7, 8, | 46 | 11.004 (x 4.180) | 189 (0.243) | 2.23e-14 | ribosome | CG12775 CG18767 CG1883 CG2998 CG33002 CG3843 CG4046 CG4866 CG5338 CG6764 CG7014 CG8415 CG8857 Cyp12e1 Dhc98D Pof Rlc1 RpL11 RpL17A RpL27A RpL38 RpL46 RpL8 RpL9 RpP1 RpS17 RpS18 RpS4 RpS9 mRpL11 mRpL14 mRpL2 mRpL21 mRpL22 mRpL22-24 mRpL33 mRpL54 mRpS14 mRpS21 mRpS24 mRpS26 mRpS32 na oho23B sop tko |
| 2 | GO:0003735 | F | 3, | 46 | 10.946 (x 4.202) | 188 (0.245) | 3.56e-14 | structural constituent of ribosome | CG12775 CG18767 CG1883 CG2998 CG33002 CG3843 CG4046 CG4866 CG5338 CG6764 CG7014 CG8415 CG8857 Cyp12e1 Dhc98D Pof Rlc1 RpL11 RpL17A RpL27A RpL38 RpL46 RpL8 RpL9 RpP1 RpS17 RpS18 RpS4 RpS9 mRpL11 mRpL14 mRpL2 mRpL21 mRpL22 mRpL22-24 mRpL33 mRpL54 mRpS14 mRpS21 mRpS24 mRpS26 mRpS32 na oho23B sop tko |
| 3 | GO:0030529 | C | 3, 4, 5, 6, | 59 | 18.515 (x 3.187) | 318 (0.186) | 3.62e-13 | ribonucleoprotein complex | CG10418 CG1249 CG12775 CG13277 CG17266 CG17768 CG18767 CG1883 CG2021 CG2998 CG31184 CG31922 CG31950 CG33002 CG3843 CG4046 CG4279 CG4866 CG5338 CG6610 CG6764 CG7014 CG8415 CG8857 Cyp12e1 DebB Dhc98D Pof Rlc1 RpL11 RpL17A RpL27A RpL38 RpL46 RpL8 RpL9 RpP1 RpS17 RpS18 RpS4 RpS9 SmB mRpL11 mRpL14 mRpL2 mRpL21 mRpL22 mRpL22-24 mRpL33 mRpL54 mRpS14 mRpS21 mRpS24 mRpS26 mRpS32 na oho23B sop tko |
| 4 | GO:0005830 | C | 5, 6, 7, 8, 9, 10, | 24 | 5.240 (x 4.580) | 90 (0.267) | 7.46e-08 | cytosolic ribosome (sensu Eukaryota) | CG12775 CG1883 CG2998 CG3843 CG4046 CG5338 CG6764 CG7014 CG8415 CG8857 RpL11 RpL17A RpL27A RpL38 RpL46 RpL8 RpL9 RpP1 RpS17 RpS18 RpS4 RpS9 oho23B sop |
| 5 | GO:0044445 | C | 5, 6, 7, 8, 9, | 27 | 6.870 (x 3.930) | 118 (0.229) | 1.88e-07 | cytosolic part | CG12775 CG1883 CG2998 CG3843 CG4046 CG5338 CG6764 CG7014 CG7770 CG8415 CG8857 REG RpL11 RpL17A RpL27A RpL38 RpL46 RpL8 RpL9 RpP1 RpS17 RpS18 RpS4 RpS9 l(3)01239 oho23B sop |
| 6 | GO:0005732 | C | 4, 5, 6, 7, 8, 9, 10, 11, 12, 13, | 11 | 1.164 (x 9.446) | 20 (0.550) | 7.5e-07 | small nucleolar ribonucleoprotein complex | CG10418 CG1249 CG13277 CG17768 CG2021 CG31184 CG31950 CG4279 CG6610 DebB SmB |
| 7 | GO:0015934 | C | 3, 4, 5, 6, 7, 8, 9, | 23 | 5.589 (x 4.115) | 96 (0.240) | 1.02e-06 | large ribosomal subunit | CG12775 CG18767 CG33002 CG3843 CG6764 Rlc1 RpL11 RpL17A RpL27A RpL38 RpL46 RpL8 RpL9 RpP1 mRpL11 mRpL14 mRpL2 mRpL21 mRpL22 mRpL22-24 mRpL33 mRpL54 mRpS32 |
| 8 | GO:0005198 | F | 2, | 82 | 42.969 (x 1.908) | 738 (0.111) | 1.03e-06 | structural molecule activity | Act57B Arp11 BG:DS00180.7 BG:DS02740.9 CG12775 CG13889 CG18767 CG1883 CG2555 CG2998 CG31551 CG31876 CG33002 CG3843 CG4046 CG4052 CG4673 CG4866 CG5162 CG5338 CG6124 CG6764 CG6947 CG7014 CG7298 CG7941 CG8415 CG8511 CG8515 CG8857 CG8918 CLIP-190 Con Cp36 Cyp12e1 Dhc98D Edg91 Femcoat Gasp Lcp2 Lcp65Ae Mp20 Myo28B1 Peritrophin-A Pof Rlc1 RpL11 RpL17A RpL27A RpL38 RpL46 RpL8 RpL9 RpP1 RpS17 RpS18 RpS4 RpS9 Sgs4 Vm34Ca chp mRpL11 mRpL14 mRpL2 mRpL21 mRpL22 mRpL22-24 mRpL33 mRpL54 mRpS14 mRpS21 mRpS24 mRpS26 mRpS32 mira na oho23B robl sop tko trol zormin |
| 9 | GO:0015935 | C | 3, 4, 5, 6, 7, 8, 9, | 19 | 4.017 (x 4.729) | 69 (0.275) | 1.54e-06 | small ribosomal subunit | CG1883 CG2998 CG4046 CG5338 CG7014 CG8415 CG8857 RpS17 RpS18 RpS4 RpS9 mRpS14 mRpS21 mRpS24 mRpS26 mRpS32 oho23B sop tko |
| 10 | GO:0044429 | C | 4, 5, 6, 7, 8, 9, | 48 | 20.087 (x 2.390) | 345 (0.139) | 2.11e-06 | mitochondrial part | CG10320 CG11015 CG1140 CG11455 CG12400 CG14482 CG14508 CG17280 CG18767 CG2789 CG31477 CG32174 CG32230 CG33002 CG33066 CG4169 CG4769 CG4866 CG5037 CG5548 CG7181 CG7211 CG7834 CG8004 CoVa EG:152A3.7 Pdsw Rlc1 Tim17b2 Tim9a l(2)06225 mRpL11 mRpL14 mRpL2 mRpL21 mRpL22 mRpL22-24 mRpL33 mRpL54 mRpS14 mRpS21 mRpS24 mRpS26 mRpS32 mtacp1 porin sun tko |
| 11 | GO:0044455 | C | 4, 5, 6, 7, 8, 9, 10, 11, 12, | 23 | 6.405 (x 3.591) | 110 (0.209) | 1.02e-05 | mitochondrial membrane part | CG10320 CG11015 CG11455 CG12400 CG14482 CG14508 CG17280 CG31477 CG32230 CG33066 CG4169 CG4769 CG5548 CG7181 CG7211 CoVa EG:152A3.7 Pdsw Tim17b2 Tim9a l(2)06225 mtacp1 sun |
| 12 | GO:0044452 | C | 5, 6, 7, 8, 9, 10, 11, 12, | 11 | 1.514 (x 7.266) | 26 (0.423) | 1.25e-05 | nucleolar part | CG10418 CG1249 CG13277 CG17768 CG2021 CG31184 CG31950 CG4279 CG6610 DebB SmB |
| 13 | GO:0005843 | C | 4, 5, 6, 7, 8, 9, 10, 11, | 13 | 2.271 (x 5.725) | 39 (0.333) | 1.76e-05 | cytosolic small ribosomal subunit (sensu Eukaryota) | CG1883 CG2998 CG4046 CG5338 CG7014 CG8415 CG8857 RpS17 RpS18 RpS4 RpS9 oho23B sop |
| 14 | GO:0016283 | C | 3, 5, 6, 7, 8, | 13 | 2.271 (x 5.725) | 39 (0.333) | 1.87e-05 | eukaryotic 48S initiation complex | CG1883 CG2998 CG4046 CG5338 CG7014 CG8415 CG8857 RpS17 RpS18 RpS4 RpS9 oho23B sop |
| 15 | GO:0005761 | C | 5, 6, 7, 8, 9, 10, 11, 12, | 18 | 4.309 (x 4.178) | 74 (0.243) | 1.99e-05 | mitochondrial ribosome | CG18767 CG33002 CG4866 Rlc1 mRpL11 mRpL14 mRpL2 mRpL21 mRpL22 mRpL22-24 mRpL33 mRpL54 mRpS14 mRpS21 mRpS24 mRpS26 mRpS32 tko |
| 16 | GO:0000313 | C | 5, 6, 7, 8, 9, | 18 | 4.309 (x 4.178) | 74 (0.243) | 2.15e-05 | organellar ribosome | CG18767 CG33002 CG4866 Rlc1 mRpL11 mRpL14 mRpL2 mRpL21 mRpL22 mRpL22-24 mRpL33 mRpL54 mRpS14 mRpS21 mRpS24 mRpS26 mRpS32 tko |
| 17 | GO:0005739 | C | 5, 6, 7, 8, | 55 | 26.899 (x 2.045) | 462 (0.119) | 2.45e-05 | mitochondrion | CG10320 CG11015 CG1140 CG11455 CG12400 CG14482 CG14508 CG17280 CG18767 CG2789 CG31477 CG32174 CG32230 CG33002 CG33066 CG4095 CG4169 CG4592 CG4769 CG4866 CG5037 CG5548 CG7181 CG7211 CG7834 CG8004 CG8993 CoVa Cyp12e1 Cyp49a1 EG:152A3.7 Las Pdsw Rlc1 Tim17b2 Tim9a l(2)06225 mRpL11 mRpL14 mRpL2 mRpL21 mRpL22 mRpL22-24 mRpL33 mRpL54 mRpS14 mRpS21 mRpS24 mRpS26 mRpS32 mtacp1 porin rpr sun tko |
| 18 | GO:0042775 | P | 8, 10, | 16 | 3.552 (x 4.505) | 61 (0.262) | 2.5e-05 | ATP synthesis coupled electron transport (sensu Eukaryota) | CG10320 CG11015 CG11455 CG12400 CG14482 CG14508 CG17280 CG32230 CG4169 CG4769 CG5548 CG7181 CoVa EG:152A3.7 Pdsw mtacp1 |
| 19 | GO:0042773 | P | 7, 9, | 16 | 3.610 (x 4.432) | 62 (0.258) | 3.02e-05 | ATP synthesis coupled electron transport | CG10320 CG11015 CG11455 CG12400 CG14482 CG14508 CG17280 CG32230 CG4169 CG4769 CG5548 CG7181 CoVa EG:152A3.7 Pdsw mtacp1 |
| 20 | GO:0044444 | C | 4, 5, 6, 7, | 107 | 67.364 (x 1.588) | 1157 (0.092) | 3.35e-05 | cytoplasmic part | CG10166 CG10320 CG11015 CG1140 CG11455 CG11909 CG12400 CG12775 CG14482 CG14508 CG14691 CG17280 CG18767 CG1883 CG2789 CG2998 CG31272 CG31477 CG32174 CG32230 CG32549 CG33002 CG33066 CG3529 CG3843 CG3931 CG4046 CG4095 CG4169 CG4592 CG4769 CG4866 CG5037 CG5189 CG5338 CG5548 CG6764 CG7014 CG7181 CG7211 CG7770 CG7834 CG8004 CG8415 CG8857 CG8993 CLIP-190 CoVa Cyp12e1 Cyp49a1 Dhc98D EG:152A3.7 Las Mlc2 Mp20 Pdsw Pof REG Rab3 Rlc1 RpL11 RpL17A RpL27A RpL38 RpL46 RpL8 RpL9 RpP1 RpS17 RpS18 RpS4 RpS9 Sras SytIV Syx16 Tim17b2 Tim9a Tm1 Vha36 insc l(2)06225 l(3)01239 mRpL11 mRpL14 mRpL2 mRpL21 mRpL22 mRpL22-24 mRpL33 mRpL54 mRpS14 mRpS21 mRpS24 mRpS26 mRpS32 mira mtacp1 na oho23B pip porin rho rpr sop sun tko unc-13 |
| 21 | GO:0005746 | C | 5, 6, 7, 8, 9, 10, 11, 12, 13, | 16 | 4.017 (x 3.983) | 69 (0.232) | 0.000131 | mitochondrial electron transport chain | CG10320 CG11015 CG11455 CG12400 CG14482 CG14508 CG17280 CG32230 CG4169 CG4769 CG5548 CG7181 CoVa EG:152A3.7 Pdsw mtacp1 |
| 22 | GO:0043228 | C | 3, | 72 | 41.397 (x 1.739) | 711 (0.101) | 0.000139 | non-membrane-bound organelle | Act57B Arp11 CG10418 CG1249 CG12775 CG13277 CG15220 CG17768 CG18767 CG1883 CG2021 CG2998 CG31184 CG31611 CG31950 CG33002 CG3843 CG4046 CG4279 CG4866 CG5338 CG6610 CG6764 CG7014 CG8415 CG8857 CLIP-190 Cyp12e1 DebB Dhc98D Eip93F HP1c Mlc2 Myo28B1 Orc6 Pof Rlc1 RpL11 RpL17A RpL27A RpL38 RpL46 RpL8 RpL9 RpP1 RpS17 RpS18 RpS4 RpS9 Rpb4 SmB Tm1 dynactin-subunit-p25 mRpL11 mRpL14 mRpL2 mRpL21 mRpL22 mRpL22-24 mRpL33 mRpL54 mRpS14 mRpS21 mRpS24 mRpS26 mRpS32 na oho23B pip robl sop tko |
| 23 | GO:0043232 | C | 4, 5, 6, 7, | 72 | 41.397 (x 1.739) | 711 (0.101) | 0.000145 | intracellular non-membrane-bound organelle | Act57B Arp11 CG10418 CG1249 CG12775 CG13277 CG15220 CG17768 CG18767 CG1883 CG2021 CG2998 CG31184 CG31611 CG31950 CG33002 CG3843 CG4046 CG4279 CG4866 CG5338 CG6610 CG6764 CG7014 CG8415 CG8857 CLIP-190 Cyp12e1 DebB Dhc98D Eip93F HP1c Mlc2 Myo28B1 Orc6 Pof Rlc1 RpL11 RpL17A RpL27A RpL38 RpL46 RpL8 RpL9 RpP1 RpS17 RpS18 RpS4 RpS9 Rpb4 SmB Tm1 dynactin-subunit-p25 mRpL11 mRpL14 mRpL2 mRpL21 mRpL22 mRpL22-24 mRpL33 mRpL54 mRpS14 mRpS21 mRpS24 mRpS26 mRpS32 na oho23B pip robl sop tko |
| 24 | GO:0005829 | C | 5, 6, 7, 8, | 28 | 10.713 (x 2.614) | 184 (0.152) | 0.000188 | cytosol | CG12775 CG1883 CG2998 CG32549 CG3843 CG4046 CG5338 CG6764 CG7014 CG7770 CG8415 CG8857 REG RpL11 RpL17A RpL27A RpL38 RpL46 RpL8 RpL9 RpP1 RpS17 RpS18 RpS4 RpS9 l(3)01239 oho23B sop |
| 25 | GO:0044446 | C | 3, 4, 5, 6, 7, | 121 | 82.910 (x 1.459) | 1424 (0.085) | 0.000333 | intracellular organelle part | Act57B Arp11 Bro CG10320 CG10418 CG11015 CG1140 CG11455 CG11909 CG12400 CG1249 CG12775 CG13277 CG14482 CG14508 CG15220 CG15398 CG17266 CG17280 CG17768 CG18767 CG1883 CG2021 CG2789 CG2998 CG31184 CG31272 CG31477 CG31611 CG31922 CG31950 CG32174 CG32230 CG33002 CG33066 CG3529 CG3843 CG3931 CG4046 CG4169 CG4279 CG4673 CG4769 CG4866 CG5037 CG5338 CG5548 CG6610 CG6764 CG7014 CG7181 CG7211 CG7339 CG7834 CG8004 CG8219 CG8415 CG8857 CLIP-190 CoVa DebB Dhc98D EG:152A3.7 HP1c Mlc2 Myo28B1 Nxt1 Orc6 Pdsw Rlc1 RpII18 RpL11 RpL17A RpL27A RpL38 RpL46 RpL8 RpL9 RpP1 RpS17 RpS18 RpS4 RpS9 Rpb10 Rpb4 SmB Sras Ssb-c31a Syx16 Taf10b Taf11 TfIIEalpha Tfb2 Tim17b2 Tim9a Tm1 Trap36 Vha36 dynactin-subunit-p25 hay l(2)06225 mRpL11 mRpL14 mRpL2 mRpL21 mRpL22 mRpL22-24 mRpL33 mRpL54 mRpS14 mRpS21 mRpS24 mRpS26 mRpS32 mtacp1 oho23B porin robl sop sun tko |
| 26 | GO:0044422 | C | 2, 3, | 121 | 82.910 (x 1.459) | 1424 (0.085) | 0.000347 | organelle part | Act57B Arp11 Bro CG10320 CG10418 CG11015 CG1140 CG11455 CG11909 CG12400 CG1249 CG12775 CG13277 CG14482 CG14508 CG15220 CG15398 CG17266 CG17280 CG17768 CG18767 CG1883 CG2021 CG2789 CG2998 CG31184 CG31272 CG31477 CG31611 CG31922 CG31950 CG32174 CG32230 CG33002 CG33066 CG3529 CG3843 CG3931 CG4046 CG4169 CG4279 CG4673 CG4769 CG4866 CG5037 CG5338 CG5548 CG6610 CG6764 CG7014 CG7181 CG7211 CG7339 CG7834 CG8004 CG8219 CG8415 CG8857 CLIP-190 CoVa DebB Dhc98D EG:152A3.7 HP1c Mlc2 Myo28B1 Nxt1 Orc6 Pdsw Rlc1 RpII18 RpL11 RpL17A RpL27A RpL38 RpL46 RpL8 RpL9 RpP1 RpS17 RpS18 RpS4 RpS9 Rpb10 Rpb4 SmB Sras Ssb-c31a Syx16 Taf10b Taf11 TfIIEalpha Tfb2 Tim17b2 Tim9a Tm1 Trap36 Vha36 dynactin-subunit-p25 hay l(2)06225 mRpL11 mRpL14 mRpL2 mRpL21 mRpL22 mRpL22-24 mRpL33 mRpL54 mRpS14 mRpS21 mRpS24 mRpS26 mRpS32 mtacp1 oho23B porin robl sop sun tko |
| 27 | GO:0005762 | C | 5, 6, 7, 8, 9, 10, 11, 12, 13, | 12 | 2.620 (x 4.580) | 45 (0.267) | 0.000432 | mitochondrial large ribosomal subunit | CG18767 CG33002 Rlc1 mRpL11 mRpL14 mRpL2 mRpL21 mRpL22 mRpL22-24 mRpL33 mRpL54 mRpS32 |
| 28 | GO:0000315 | C | 4, 5, 6, 7, 8, 9, 10, | 12 | 2.620 (x 4.580) | 45 (0.267) | 0.000448 | organellar large ribosomal subunit | CG18767 CG33002 Rlc1 mRpL11 mRpL14 mRpL2 mRpL21 mRpL22 mRpL22-24 mRpL33 mRpL54 mRpS32 |
| 29 | GO:0005737 | C | 4, 5, 6, | 124 | 86.461 (x 1.434) | 1485 (0.084) | 0.000531 | cytoplasm | Aats-his Abl CG10166 CG10268 CG10320 CG10861 CG11015 CG1140 CG11455 CG11909 CG12400 CG12775 CG14482 CG14508 CG14691 CG17224 CG17280 CG18767 CG1883 CG2789 CG2846 CG2998 CG31272 CG31477 CG32174 CG32230 CG32549 CG33002 CG33066 CG3529 CG3843 CG3931 CG4046 CG4095 CG4169 CG4592 CG4769 CG4866 CG5037 CG5189 CG5338 CG5548 CG6764 CG7014 CG7181 CG7211 CG7770 CG7834 CG8004 CG8219 CG8415 CG8857 CG8993 CLIP-190 CoVa Cpn Cyp12e1 Cyp49a1 Dab Dhc98D Doa EG:152A3.7 EG:BACR7A4.8 Femcoat Gs2 Las Mlc2 Mp20 Nxt1 Pdsw Pof REG Rab3 Rlc1 RpL11 RpL17A RpL27A RpL38 RpL46 RpL8 RpL9 RpP1 RpS17 RpS18 RpS4 RpS9 Sod Sras SytIV Syx16 Tim17b2 Tim9a Tm1 Vha36 insc l(2)06225 l(3)01239 mRpL11 mRpL14 mRpL2 mRpL21 mRpL22 mRpL22-24 mRpL33 mRpL54 mRpS14 mRpS21 mRpS24 mRpS26 mRpS32 mira mtacp1 na oho23B pip porin pum rho rpr shu sop sun tko unc-13 |
| 30 | GO:0006412 | P | 6, 7, | 53 | 28.762 (x 1.843) | 494 (0.107) | 0.000558 | protein biosynthesis | Aats-his CG10092 CG10166 CG12775 CG18767 CG1883 CG2998 CG33002 CG3843 CG40068 CG4046 CG4866 CG5338 CG6764 CG7014 CG8415 CG8857 Cyp12e1 Dhc98D Pof RpL11 RpL17A RpL27A RpL38 RpL46 RpL8 RpL9 RpP1 RpS17 RpS18 RpS4 RpS9 apt betaggt-II gatA mRpL11 mRpL14 mRpL2 mRpL21 mRpL22 mRpL22-24 mRpL33 mRpS14 mRpS21 mRpS24 mRpS26 mRpS32 na oho23B pum rpr sop tko |
| 31 | GO:0009059 | P | 5, 6, | 55 | 30.392 (x 1.810) | 522 (0.105) | 0.000619 | macromolecule biosynthesis | Aats-his Act57B CG10092 CG10166 CG12775 CG18767 CG1883 CG2998 CG33002 CG3843 CG40068 CG4046 CG4866 CG5177 CG5338 CG6764 CG7014 CG8415 CG8857 Cyp12e1 Dhc98D Pof RpL11 RpL17A RpL27A RpL38 RpL46 RpL8 RpL9 RpP1 RpS17 RpS18 RpS4 RpS9 apt betaggt-II gatA mRpL11 mRpL14 mRpL2 mRpL21 mRpL22 mRpL22-24 mRpL33 mRpS14 mRpS21 mRpS24 mRpS26 mRpS32 na oho23B pum rpr sop tko |
| 32 | GO:0005740 | C | 4, 5, 6, 7, 8, 9, 10, | 28 | 11.936 (x 2.346) | 205 (0.137) | 0.00112 | mitochondrial envelope | CG10320 CG11015 CG11455 CG12400 CG14482 CG14508 CG17280 CG2789 CG31477 CG32174 CG32230 CG33066 CG4169 CG4769 CG5037 CG5548 CG7181 CG7211 CG8004 CoVa EG:152A3.7 Pdsw Tim17b2 Tim9a l(2)06225 mtacp1 porin sun |
| 33 | GO:0006119 | P | 6, 8, | 22 | 8.209 (x 2.680) | 141 (0.156) | 0.00113 | oxidative phosphorylation | CG10320 CG11015 CG11455 CG12400 CG14482 CG14508 CG17280 CG31477 CG32230 CG4169 CG4769 CG5548 CG7181 CG7211 CG7834 CoVa EG:152A3.7 Pdsw Vha36 l(2)06225 mtacp1 sun |
| 34 | GO:0031966 | C | 5, 6, 7, 8, 9, 10, 11, | 26 | 10.888 (x 2.388) | 187 (0.139) | 0.00157 | mitochondrial membrane | CG10320 CG11015 CG11455 CG12400 CG14482 CG14508 CG17280 CG31477 CG32174 CG32230 CG33066 CG4169 CG4769 CG5548 CG7181 CG7211 CG8004 CoVa EG:152A3.7 Pdsw Tim17b2 Tim9a l(2)06225 mtacp1 porin sun |
| 35 | GO:0016282 | C | 3, 5, 6, 7, 8, | 13 | 3.493 (x 3.721) | 60 (0.217) | 0.00165 | eukaryotic 43S preinitiation complex | CG1883 CG2998 CG4046 CG5338 CG7014 CG8415 CG8857 RpS17 RpS18 RpS4 RpS9 oho23B sop |
| 36 | GO:0044249 | P | 5, | 76 | 48.325 (x 1.573) | 830 (0.092) | 0.00179 | cellular biosynthesis | Aats-his Ac78C Act57B CG10092 CG10166 CG12775 CG14721 CG18767 CG1883 CG1885 CG2846 CG2998 CG31477 CG32174 CG32626 CG33002 CG3843 CG40068 CG4046 CG4866 CG5037 CG5177 CG5338 CG6764 CG7014 CG7211 CG8415 CG8857 CG9804 Cyp12e1 Dhc98D Dhfr Gs2 Las Pof RpL11 RpL17A RpL27A RpL38 RpL46 RpL8 RpL9 RpP1 RpS17 RpS18 RpS4 RpS9 SamDC Tbh Vha36 apt betaggt-II gatA l(2)06225 l(3)02640 mRpL11 mRpL14 mRpL2 mRpL21 mRpL22 mRpL22-24 mRpL33 mRpS14 mRpS21 mRpS24 mRpS26 mRpS32 mtacp1 na nmdyn-D6 oho23B pum rpr sop sun tko |
| 37 | GO:0005743 | C | 5, 6, 7, 8, 9, 10, 11, 12, | 24 | 9.840 (x 2.439) | 169 (0.142) | 0.00205 | mitochondrial inner membrane | CG10320 CG11015 CG11455 CG12400 CG14482 CG14508 CG17280 CG31477 CG32174 CG32230 CG33066 CG4169 CG4769 CG5548 CG7181 CG7211 CoVa EG:152A3.7 Pdsw Tim17b2 Tim9a l(2)06225 mtacp1 sun |
| 38 | GO:0043234 | C | 2, | 126 | 91.934 (x 1.371) | 1579 (0.080) | 0.00305 | protein complex | BG:DS02740.5 Bro CG10320 CG10418 CG11015 CG11360 CG11455 CG11597 CG11909 CG12400 CG1249 CG12775 CG13277 CG14482 CG14508 CG15220 CG15398 CG17266 CG17280 CG17768 CG18749 CG18767 CG1883 CG2021 CG2998 CG31184 CG31477 CG31611 CG31922 CG31950 CG3215 CG32230 CG33002 CG33066 CG3397 CG3843 CG3931 CG4046 CG4095 CG4169 CG4279 CG4673 CG4769 CG4866 CG5338 CG5382 CG5548 CG6610 CG6763 CG6764 CG7014 CG7181 CG7211 CG7339 CG7770 CG7834 CG8219 CG8415 CG8857 CLIP-190 CoVa Cyp12e1 DebB Dhc98D EG:152A3.7 Iap2 InR Mlc2 Myo28B1 Orc6 Pdsw Pof Prosbeta5 REG Rlc1 RpII18 RpL11 RpL17A RpL27A RpL38 RpL46 RpL8 RpL9 RpP1 RpS17 RpS18 RpS4 RpS9 Rpb10 Rpb4 SmB Ssb-c31a Taf10b Taf11 TfIIEalpha Tfb2 Tim17b2 Tim9a Trap36 Vha36 dynactin-subunit-p25 hay l(2)03659 l(2)06225 l(3)01239 mRpL11 mRpL14 mRpL2 mRpL21 mRpL22 mRpL22-24 mRpL33 mRpL54 mRpS14 mRpS21 mRpS24 mRpS26 mRpS32 mtacp1 na oho23B robl skpA sop sun tko |
| 39 | GO:0005688 | C | 5, 6, 7, 8, 9, 10, 11, 12, | 5 | 0.524 (x 9.542) | 9 (0.556) | 0.00313 | snRNP U6 | CG10418 CG13277 CG2021 CG31184 CG6610 |
| 40 | GO:0019866 | C | 4, 5, 6, 7, 8, 9, | 24 | 10.131 (x 2.369) | 174 (0.138) | 0.00314 | organelle inner membrane | CG10320 CG11015 CG11455 CG12400 CG14482 CG14508 CG17280 CG31477 CG32174 CG32230 CG33066 CG4169 CG4769 CG5548 CG7181 CG7211 CoVa EG:152A3.7 Pdsw Tim17b2 Tim9a l(2)06225 mtacp1 sun |
| 41 | GO:0031974 | C | 2, | 45 | 25.094 (x 1.793) | 431 (0.104) | 0.00376 | membrane-enclosed lumen | Bro CG10418 CG1140 CG1249 CG13277 CG15398 CG17768 CG18767 CG2021 CG31184 CG31950 CG33002 CG4279 CG4866 CG6610 CG7339 CG7834 DebB Nxt1 Rlc1 RpII18 Rpb10 Rpb4 SmB Ssb-c31a Taf10b Taf11 TfIIEalpha Tfb2 Trap36 hay mRpL11 mRpL14 mRpL2 mRpL21 mRpL22 mRpL22-24 mRpL33 mRpL54 mRpS14 mRpS21 mRpS24 mRpS26 mRpS32 tko |
| 42 | GO:0043233 | C | 3, 4, | 45 | 25.094 (x 1.793) | 431 (0.104) | 0.00386 | organelle lumen | Bro CG10418 CG1140 CG1249 CG13277 CG15398 CG17768 CG18767 CG2021 CG31184 CG31950 CG33002 CG4279 CG4866 CG6610 CG7339 CG7834 DebB Nxt1 Rlc1 RpII18 Rpb10 Rpb4 SmB Ssb-c31a Taf10b Taf11 TfIIEalpha Tfb2 Trap36 hay mRpL11 mRpL14 mRpL2 mRpL21 mRpL22 mRpL22-24 mRpL33 mRpL54 mRpS14 mRpS21 mRpS24 mRpS26 mRpS32 tko |
| 43 | GO:0009058 | P | 4, | 79 | 52.284 (x 1.511) | 898 (0.088) | 0.00412 | biosynthesis | Aats-his Ac78C Act57B CG10092 CG10166 CG10268 CG12775 CG14721 CG18767 CG1883 CG1885 CG2846 CG2998 CG31477 CG32174 CG32626 CG33002 CG3843 CG40068 CG4046 CG4866 CG5037 CG5177 CG5338 CG6764 CG7014 CG7211 CG8415 CG8857 CG9804 Cyp12e1 Dhc98D Dhfr Doa Gs2 Las Pof RpL11 RpL17A RpL27A RpL38 RpL46 RpL8 RpL9 RpP1 RpS17 RpS18 RpS4 RpS9 SamDC Tbh Vha36 apt betaggt-II fu12 gatA l(2)06225 l(3)02640 mRpL11 mRpL14 mRpL2 mRpL21 mRpL22 mRpL22-24 mRpL33 mRpS14 mRpS21 mRpS24 mRpS26 mRpS32 mtacp1 na nmdyn-D6 oho23B pum rpr sop sun tko |
| 44 | GO:0005842 | C | 4, 5, 6, 7, 8, 9, 10, 11, | 11 | 2.969 (x 3.704) | 51 (0.216) | 0.00556 | cytosolic large ribosomal subunit (sensu Eukaryota) | CG12775 CG3843 CG6764 RpL11 RpL17A RpL27A RpL38 RpL46 RpL8 RpL9 RpP1 |
| 45 | GO:0016765 | F | 4, | 12 | 3.493 (x 3.435) | 60 (0.200) | 0.00587 | transferase activity, transferring alkyl or aryl (other than methyl) groups | CG17639 CG33177 CG5037 CG5224 GstD6 GstD9 GstE1 GstE5 GstE6 GstE7 betaggt-II l(3)02640 |
| 46 | GO:0009636 | P | 5, | 19 | 7.511 (x 2.530) | 129 (0.147) | 0.00634 | response to toxin | CG11897 CG11898 CG17639 CG18869 CG30022 CG30438 CG31146 CG33177 CG5224 CG6214 GstD6 GstD9 GstE1 GstE5 GstE6 GstE7 Ugt86De Ugt86Di l(2)03659 |
| 47 | GO:0004364 | F | 5, | 9 | 2.096 (x 4.294) | 36 (0.250) | 0.00643 | glutathione transferase activity | CG17639 CG33177 CG5224 GstD6 GstD9 GstE1 GstE5 GstE6 GstE7 |
| 48 | GO:0031975 | C | 2, | 31 | 15.546 (x 1.994) | 267 (0.116) | 0.00648 | envelope | CG10320 CG11015 CG11455 CG12400 CG14482 CG14508 CG17280 CG2789 CG31477 CG32174 CG32230 CG33066 CG4169 CG4673 CG4769 CG5037 CG5548 CG7181 CG7211 CG8004 CG8219 CoVa EG:152A3.7 Nxt1 Pdsw Tim17b2 Tim9a l(2)06225 mtacp1 porin sun |
| 49 | GO:0031967 | C | 3, 4, 5, 6, 7, 8, | 31 | 15.546 (x 1.994) | 267 (0.116) | 0.00661 | organelle envelope | CG10320 CG11015 CG11455 CG12400 CG14482 CG14508 CG17280 CG2789 CG31477 CG32174 CG32230 CG33066 CG4169 CG4673 CG4769 CG5037 CG5548 CG7181 CG7211 CG8004 CG8219 CoVa EG:152A3.7 Nxt1 Pdsw Tim17b2 Tim9a l(2)06225 mtacp1 porin sun |
| 50 | GO:0009055 | F | 4, | 11 | 3.086 (x 3.565) | 53 (0.208) | 0.00704 | electron carrier activity | CG10320 CG11455 CG12400 CG14508 CG32230 CG4769 CG5548 CG7834 EG:152A3.7 Pdsw mtacp1 |
| 51 | GO:0030532 | C | 4, 5, 6, 7, 8, 9, 10, | 12 | 3.610 (x 3.324) | 62 (0.194) | 0.00718 | small nuclear ribonucleoprotein complex | CG10418 CG1249 CG13277 CG17266 CG2021 CG31184 CG31922 CG31950 CG4279 CG6610 DebB SmB |
| 52 | GO:0005730 | C | 5, 6, 7, 8, 9, 10, 11, | 11 | 3.144 (x 3.499) | 54 (0.204) | 0.00807 | nucleolus | CG10418 CG1249 CG13277 CG17768 CG2021 CG31184 CG31950 CG4279 CG6610 DebB SmB |
| 53 | GO:0031980 | C | 4, 5, 6, 7, 8, 9, 10, | 20 | 8.442 (x 2.369) | 145 (0.138) | 0.00907 | mitochondrial lumen | CG1140 CG18767 CG33002 CG4866 CG7834 Rlc1 mRpL11 mRpL14 mRpL2 mRpL21 mRpL22 mRpL22-24 mRpL33 mRpL54 mRpS14 mRpS21 mRpS24 mRpS26 mRpS32 tko |
| 54 | GO:0005759 | C | 5, 6, 7, 8, 9, 10, 11, | 20 | 8.442 (x 2.369) | 145 (0.138) | 0.00924 | mitochondrial matrix | CG1140 CG18767 CG33002 CG4866 CG7834 Rlc1 mRpL11 mRpL14 mRpL2 mRpL21 mRpL22 mRpL22-24 mRpL33 mRpL54 mRpS14 mRpS21 mRpS24 mRpS26 mRpS32 tko |
| 55 | GO:0042221 | P | 4, | 30 | 15.313 (x 1.959) | 263 (0.114) | 0.00999 | response to chemical stimulus | BG:DS01219.1 CG11897 CG11898 CG13889 CG17639 CG18869 CG30022 CG30438 CG31146 CG33177 CG5224 CG6214 CG9381 Eip93F GstD6 GstD9 GstE1 GstE5 GstE6 GstE7 InR Kr-h1 Obp56a Tbh Ugt86De Ugt86Di l(2)03659 rho rpr sra |
| 56 | GO:0006120 | P | 9, 11, | 8 | 2.038 (x 3.926) | 35 (0.229) | 0.0239 | mitochondrial electron transport, NADH to ubiquinone | CG10320 CG11455 CG12400 CG32230 CG5548 EG:152A3.7 Pdsw mtacp1 |
| 57 | GO:0003954 | F | 5, | 8 | 2.096 (x 3.817) | 36 (0.222) | 0.0287 | NADH dehydrogenase activity | CG10320 CG11455 CG12400 CG32230 CG5548 EG:152A3.7 Pdsw mtacp1 |
| 58 | GO:0005747 | C | 4, 5, 6, 7, 8, 9, 10, 11, 12, 13, 14, | 8 | 2.154 (x 3.714) | 37 (0.216) | 0.0336 | respiratory chain complex I (sensu Eukaryota) | CG10320 CG11455 CG12400 CG32230 CG5548 EG:152A3.7 Pdsw mtacp1 |
| 59 | GO:0045271 | C | 3, 4, 5, 6, | 8 | 2.154 (x 3.714) | 37 (0.216) | 0.0342 | respiratory chain complex I | CG10320 CG11455 CG12400 CG32230 CG5548 EG:152A3.7 Pdsw mtacp1 |
| 60 | GO:0006952 | P | 4, | 46 | 29.927 (x 1.537) | 514 (0.089) | 0.0662 | defense response | AnnX Bc BcDNA:GH08420 CG10433 CG11897 CG11898 CG1504 CG17266 CG17639 CG18249 CG18522 CG18869 CG30022 CG30438 CG31146 CG3212 CG33177 CG3355 CG5001 CG5224 CG5397 CG6214 CG6426 CG6435 CG8193 CG9095 Def GstD6 GstD9 GstE1 GstE5 GstE6 GstE7 PGRP-SA Prx6005 Sod Tehao Toll-7 Tsf1 Ugt86De Ugt86Di chp cyp33 hig l(2)03659 upd3 |
| 61 | GO:0005750 | C | 4, 5, 6, 7, 8, 9, 10, 11, 12, 13, 14, | 4 | 0.640 (x 6.246) | 11 (0.364) | 0.0789 | respiratory chain complex III (sensu Eukaryota) | CG14482 CG14508 CG4169 CG4769 |
| 62 | GO:0045285 | C | 3, 4, 5, 6, 7, | 4 | 0.640 (x 6.246) | 11 (0.364) | 0.0802 | ubiquinol-cytochrome-c reductase complex | CG14482 CG14508 CG4169 CG4769 |
| 63 | GO:0045275 | C | 3, 4, 5, 6, | 4 | 0.640 (x 6.246) | 11 (0.364) | 0.0815 | respiratory chain complex III | CG14482 CG14508 CG4169 CG4769 |
| 64 | GO:0009607 | P | 3, | 46 | 30.392 (x 1.514) | 522 (0.088) | 0.084 | response to biotic stimulus | AnnX Bc BcDNA:GH08420 CG10433 CG11897 CG11898 CG1504 CG17266 CG17639 CG18249 CG18522 CG18869 CG30022 CG30438 CG31146 CG3212 CG33177 CG3355 CG5001 CG5224 CG5397 CG6214 CG6426 CG6435 CG8193 CG9095 Def GstD6 GstD9 GstE1 GstE5 GstE6 GstE7 PGRP-SA Prx6005 Sod Tehao Toll-7 Tsf1 Ugt86De Ugt86Di chp cyp33 hig l(2)03659 upd3 |
| 65 | GO:0016651 | F | 4, | 8 | 2.504 (x 3.195) | 43 (0.186) | 0.0843 | oxidoreductase activity, acting on NADH or NADPH | CG10320 CG11455 CG12400 CG32230 CG5548 EG:152A3.7 Pdsw mtacp1 |
| 66 | GO:0046483 | P | 5, | 17 | 8.151 (x 2.086) | 140 (0.121) | 0.086 | heterocycle metabolism | Ahcy13 CG17224 CG18522 CG18749 CG1885 CG2277 CG32549 CG32626 CG5037 CG8360 CG9326 CG9804 Dhfr Las Vha36 l(3)02640 nmdyn-D6 |
| 67 | GO:0045153 | F | 5, | 2 | 0.116 (x 17.175) | 2 (1.000) | 0.0878 | electron transporter, transferring electrons within CoQH2-cytochrome c reductase complex activity | CG14508 CG4769 |
| 68 | GO:0009628 | P | 3, | 35 | 21.659 (x 1.616) | 372 (0.094) | 0.0886 | response to abiotic stimulus | Arr2 BG:DS01219.1 CG11897 CG11898 CG13889 CG17639 CG18869 CG30022 CG30438 CG31146 CG33177 CG5001 CG5224 CG6214 CG9381 Eip93F GstD6 GstD9 GstE1 GstE5 GstE6 GstE7 Hsp22 InR Kr-h1 Obp56a Tbh Ugt86De Ugt86Di l(2)03659 rho rpr sda sra tko |
| 69 | GO:0009107 | P | 8, 9, | 2 | 0.116 (x 17.175) | 2 (1.000) | 0.0891 | lipoate biosynthesis | CG9804 Las |
| 70 | GO:0009106 | P | 7, 8, | 2 | 0.116 (x 17.175) | 2 (1.000) | 0.0904 | lipoate metabolism | CG9804 Las |
| 71 | GO:0009105 | P | 7, 8, | 2 | 0.116 (x 17.175) | 2 (1.000) | 0.0917 | lipoic acid biosynthesis | CG9804 Las |
| 72 | GO:0006122 | P | 9, 11, | 4 | 0.699 (x 5.725) | 12 (0.333) | 0.0975 | mitochondrial electron transport, ubiquinol to cytochrome c | CG14482 CG14508 CG4169 CG4769 |
| 73 | GO:0006123 | P | 9, 11, | 4 | 0.699 (x 5.725) | 12 (0.333) | 0.0989 | mitochondrial electron transport, cytochrome c to oxygen | CG11015 CG17280 CG7181 CoVa |
| 74 | GO:0009112 | P | 6, | 11 | 4.541 (x 2.422) | 78 (0.141) | 0.13 | nucleobase metabolism | Ahcy13 CG17224 CG18522 CG2277 CG32549 CG32626 CG8360 CG9326 Dhfr Vha36 nmdyn-D6 |
| 75 | GO:0000314 | C | 4, 5, 6, 7, 8, 9, 10, | 6 | 1.747 (x 3.435) | 30 (0.200) | 0.164 | organellar small ribosomal subunit | mRpS14 mRpS21 mRpS24 mRpS26 mRpS32 tko |
| 76 | GO:0005763 | C | 5, 6, 7, 8, 9, 10, 11, 12, 13, | 6 | 1.747 (x 3.435) | 30 (0.200) | 0.167 | mitochondrial small ribosomal subunit | mRpS14 mRpS21 mRpS24 mRpS26 mRpS32 tko |
| 77 | GO:0005751 | C | 4, 5, 6, 7, 8, 9, 10, 11, 12, 13, 14, | 4 | 0.815 (x 4.907) | 14 (0.286) | 0.168 | respiratory chain complex IV (sensu Eukaryota) | CG11015 CG17280 CG7181 CoVa |
| 78 | GO:0045277 | C | 3, 4, 5, 6, | 4 | 0.815 (x 4.907) | 14 (0.286) | 0.17 | respiratory chain complex IV | CG11015 CG17280 CG7181 CoVa |
| 79 | GO:0008559 | F | 4, 5, 7, 12, | 3 | 0.466 (x 6.441) | 8 (0.375) | 0.201 | xenobiotic-transporting ATPase activity | CG11897 CG11898 CG6214 |
| 80 | GO:0005344 | F | 3, | 3 | 0.466 (x 6.441) | 8 (0.375) | 0.203 | oxygen transporter activity | Bc CG8193 glob1 |
| 81 | GO:0008624 | P | 9, 10, | 2 | 0.175 (x 11.450) | 3 (0.667) | 0.204 | induction of apoptosis by extracellular signals | Eip93F rpr |
| 82 | GO:0042910 | F | 3, | 3 | 0.466 (x 6.441) | 8 (0.375) | 0.206 | xenobiotic transporter activity | CG11897 CG11898 CG6214 |
| 83 | GO:0000273 | P | 6, 7, | 2 | 0.175 (x 11.450) | 3 (0.667) | 0.207 | lipoic acid metabolism | CG9804 Las |
| 84 | GO:0016716 | F | 5, | 2 | 0.175 (x 11.450) | 3 (0.667) | 0.209 | oxidoreductase activity, acting on paired donors, with incorporation or reduction of molecular oxygen, another compound as one donor, and incorporation of one atom of oxygen | Bc CG8193 |
| 85 | GO:0016979 | F | 5, | 2 | 0.175 (x 11.450) | 3 (0.667) | 0.212 | lipoate-protein ligase activity | CG9804 Las |
| 86 | GO:0009399 | P | 5, | 2 | 0.175 (x 11.450) | 3 (0.667) | 0.214 | nitrogen fixation | CG11897 Gs2 |
| 87 | GO:0004503 | F | 6, | 2 | 0.175 (x 11.450) | 3 (0.667) | 0.217 | monophenol monooxygenase activity | Bc CG8193 |
| 88 | GO:0035069 | P | 6, | 2 | 0.175 (x 11.450) | 3 (0.667) | 0.219 | larval midgut histolysis | Eip93F rpr |
| 89 | GO:0031090 | C | 4, 5, 6, 7, 8, | 32 | 21.019 (x 1.522) | 361 (0.089) | 0.231 | organelle membrane | CG10320 CG11015 CG11455 CG12400 CG14482 CG14508 CG17280 CG31272 CG31477 CG32174 CG32230 CG33066 CG4169 CG4673 CG4769 CG5548 CG7181 CG7211 CG8004 CG8219 CoVa EG:152A3.7 Pdsw Sras Syx16 Tim17b2 Tim9a Vha36 l(2)06225 mtacp1 porin sun |
| 90 | GO:0016675 | F | 4, | 4 | 0.932 (x 4.294) | 16 (0.250) | 0.234 | oxidoreductase activity, acting on heme group of donors | CG11015 CG17280 CG7181 CoVa |
| 91 | GO:0016676 | F | 5, | 4 | 0.932 (x 4.294) | 16 (0.250) | 0.236 | oxidoreductase activity, acting on heme group of donors, oxygen as acceptor | CG11015 CG17280 CG7181 CoVa |
| 92 | GO:0015002 | F | 4, | 4 | 0.932 (x 4.294) | 16 (0.250) | 0.239 | heme-copper terminal oxidase activity | CG11015 CG17280 CG7181 CoVa |
| 93 | GO:0004129 | F | 5, 6, 7, | 4 | 0.932 (x 4.294) | 16 (0.250) | 0.242 | cytochrome-c oxidase activity | CG11015 CG17280 CG7181 CoVa |
| 94 | GO:0006144 | P | 7, | 8 | 3.202 (x 2.498) | 55 (0.145) | 0.266 | purine base metabolism | Ahcy13 CG18522 CG2277 CG32549 CG32626 CG9326 Vha36 nmdyn-D6 |
| 95 | GO:0006800 | P | 5, | 9 | 3.901 (x 2.307) | 67 (0.134) | 0.291 | oxygen and reactive oxygen species metabolism | CG13889 CG18522 CG30022 GstE1 GstE5 GstE6 GstE7 Prx6005 Sod |
| 96 | GO:0031981 | C | 4, 5, 6, 7, 8, 9, 10, | 25 | 16.011 (x 1.561) | 275 (0.091) | 0.337 | nuclear lumen | Bro CG10418 CG1249 CG13277 CG15398 CG17768 CG2021 CG31184 CG31950 CG4279 CG6610 CG7339 DebB Nxt1 RpII18 Rpb10 Rpb4 SmB Ssb-c31a Taf10b Taf11 TfIIEalpha Tfb2 Trap36 hay |
| 97 | GO:0006965 | P | 7, 8, 9, 10, | 2 | 0.233 (x 8.588) | 4 (0.500) | 0.353 | positive regulation of biosynthesis of antibacterial peptides active against anti-Gram-positive bacteria | Def PGRP-SA |
| 98 | GO:0051188 | P | 6, | 12 | 6.113 (x 1.963) | 105 (0.114) | 0.354 | cofactor biosynthesis | CG14721 CG1885 CG31477 CG32174 CG5037 CG7211 CG9804 Las Vha36 l(2)06225 l(3)02640 sun |
| 99 | GO:0050136 | F | 6, | 5 | 1.630 (x 3.067) | 28 (0.179) | 0.386 | NADH dehydrogenase (quinone) activity | CG12400 CG5548 EG:152A3.7 Pdsw mtacp1 |
| 100 | GO:0016655 | F | 5, | 5 | 1.630 (x 3.067) | 28 (0.179) | 0.39 | oxidoreductase activity, acting on NADH or NADPH, quinone or similar compound as acceptor | CG12400 CG5548 EG:152A3.7 Pdsw mtacp1 |
| 101 | GO:0016591 | C | 3, 6, 7, 8, 9, 10, 11, 12, 13, | 10 | 4.833 (x 2.069) | 83 (0.120) | 0.393 | DNA-directed RNA polymerase II, holoenzyme | CG15398 RpII18 Rpb10 Rpb4 Taf10b Taf11 TfIIEalpha Tfb2 Trap36 hay |
| 102 | GO:0008137 | F | 6, 7, | 5 | 1.630 (x 3.067) | 28 (0.179) | 0.394 | NADH dehydrogenase (ubiquinone) activity | CG12400 CG5548 EG:152A3.7 Pdsw mtacp1 |
| 103 | GO:0006779 | P | 7, | 3 | 0.640 (x 4.684) | 11 (0.273) | 0.4 | porphyrin biosynthesis | CG1885 CG5037 l(3)02640 |
| 104 | GO:0005665 | C | 4, 5, 6, 7, 8, 9, 10, 11, 12, 13, 14, | 3 | 0.640 (x 4.684) | 11 (0.273) | 0.404 | DNA-directed RNA polymerase II, core complex | RpII18 Rpb10 Rpb4 |
| 105 | GO:0005544 | F | 5, | 3 | 0.640 (x 4.684) | 11 (0.273) | 0.408 | calcium-dependent phospholipid binding | AnnX CG5559 SytIV |
| 106 | GO:0005753 | C | 4, 5, 6, 7, 8, 9, 10, 11, 12, 13, | 4 | 1.164 (x 3.435) | 20 (0.200) | 0.446 | proton-transporting ATP synthase complex (sensu Eukaryota) | CG31477 CG7211 l(2)06225 sun |
| 107 | GO:0045255 | C | 4, 7, 8, 9, | 4 | 1.164 (x 3.435) | 20 (0.200) | 0.45 | hydrogen-translocating F-type ATPase complex | CG31477 CG7211 l(2)06225 sun |
| 108 | GO:0045259 | C | 3, 4, 5, 6, | 4 | 1.164 (x 3.435) | 20 (0.200) | 0.454 | proton-transporting ATP synthase complex | CG31477 CG7211 l(2)06225 sun |
| 109 | GO:0008011 | F | 5, | 2 | 0.408 (x 4.907) | 7 (0.286) | 0.479 | structural constituent of pupal cuticle (sensu Insecta) | CG31876 Edg91 |
| 110 | GO:0016272 | C | 3, 6, 7, 8, 9, 10, | 2 | 0.408 (x 4.907) | 7 (0.286) | 0.481 | prefoldin complex | CG7770 l(3)01239 |
| 111 | GO:0004965 | F | 6, 7, | 2 | 0.408 (x 4.907) | 7 (0.286) | 0.483 | GABA-B receptor activity | EG:30B8.6 GABA-B-R2 |
| 112 | GO:0001742 | P | 4, | 2 | 0.408 (x 4.907) | 7 (0.286) | 0.485 | oenocyte differentiation | rho salm |
| 113 | GO:0042721 | C | 3, 5, 6, 7, 8, 9, 10, 11, 12, 13, | 2 | 0.291 (x 6.870) | 5 (0.400) | 0.486 | mitochondrial inner membrane protein insertion complex | CG33066 Tim9a |
| 114 | GO:0007305 | P | 9, 10, | 2 | 0.408 (x 4.907) | 7 (0.286) | 0.488 | vitelline membrane formation (sensu Insecta) | Cp36 Vm34Ca |
| 115 | GO:0007006 | P | 6, 7, | 2 | 0.408 (x 4.907) | 7 (0.286) | 0.49 | mitochondrial membrane organization and biogenesis | CG33066 Tim9a |
| 116 | GO:0045454 | P | 5, | 2 | 0.291 (x 6.870) | 5 (0.400) | 0.491 | cell redox homeostasis | Prx6005 SelG |
| 117 | GO:0030704 | P | 8, | 2 | 0.408 (x 4.907) | 7 (0.286) | 0.492 | vitelline membrane formation | Cp36 Vm34Ca |
| 118 | GO:0015239 | F | 4, | 3 | 0.699 (x 4.294) | 12 (0.250) | 0.492 | multidrug transporter activity | CG11897 CG11898 CG6214 |
| 119 | GO:0006091 | P | 5, | 38 | 29.403 (x 1.292) | 505 (0.075) | 0.493 | generation of precursor metabolites and energy | CG10320 CG11015 CG11455 CG12400 CG14482 CG14508 CG17280 CG18011 CG18522 CG2964 CG30499 CG31477 CG32230 CG4095 CG4169 CG4511 CG4769 CG5103 CG5177 CG5548 CG7181 CG7211 CG7834 CG8993 CoVa Cyp12e1 Cyp28d2 Cyp311a1 Cyp49a1 EG:152A3.7 ImpL3 Or59a Pdsw Pglym78 Vha36 l(2)06225 mtacp1 sun |
| 120 | GO:0007611 | P | 4, | 7 | 3.493 (x 2.004) | 60 (0.117) | 0.494 | learning and/or memory | BG:DS01219.1 CG10460 CG9381 Tbh pum rho sra |
| 121 | GO:0004631 | F | 6, | 1 | 0.058 (x 17.175) | 1 (1.000) | 0.494 | phosphomevalonate kinase activity | CG10268 |
| 122 | GO:0007638 | P | 4, 5, | 2 | 0.291 (x 6.870) | 5 (0.400) | 0.495 | mechanosensory behavior | sda tko |
| 123 | GO:0000045 | P | 6, | 1 | 0.058 (x 17.175) | 1 (1.000) | 0.497 | autophagic vacuole formation | CG10861 |
| 124 | GO:0043487 | P | 8, | 1 | 0.058 (x 17.175) | 1 (1.000) | 0.499 | regulation of RNA stability | pum |
| 125 | GO:0008253 | F | 8, | 2 | 0.291 (x 6.870) | 5 (0.400) | 0.499 | 5'-nucleotidase activity | CG32549 CG4827 |
| 126 | GO:0046950 | P | 5, | 1 | 0.058 (x 17.175) | 1 (1.000) | 0.501 | ketone body metabolism | CG1140 |
| 127 | GO:0006118 | P | 6, | 28 | 19.388 (x 1.444) | 333 (0.084) | 0.503 | electron transport | CG10320 CG11015 CG11455 CG12400 CG14482 CG14508 CG17280 CG18011 CG18522 CG32230 CG4169 CG4511 CG4769 CG5548 CG7181 CG7211 CG7834 CG8993 CoVa Cyp12e1 Cyp28d2 Cyp311a1 Cyp49a1 EG:152A3.7 Or59a Pdsw l(2)06225 mtacp1 |
| 128 | GO:0006409 | P | 8, 9, 10, 11, | 1 | 0.058 (x 17.175) | 1 (1.000) | 0.504 | tRNA export from nucleus | Nxt1 |
| 129 | GO:0008084 | F | 5, 6, | 2 | 0.291 (x 6.870) | 5 (0.400) | 0.504 | imaginal disc growth factor activity | Idgf1 Idgf2 |
| 130 | GO:0007492 | P | 4, | 3 | 0.932 (x 3.220) | 16 (0.188) | 0.504 | endoderm development | btl sisA toy |
| 131 | GO:0016732 | F | 5, | 1 | 0.058 (x 17.175) | 1 (1.000) | 0.506 | oxidoreductase activity, acting on iron-sulfur proteins as donors, dinitrogen as acceptor | CG11897 |
| 132 | GO:0009725 | P | 4, 5, | 3 | 0.932 (x 3.220) | 16 (0.188) | 0.507 | response to hormone stimulus | Eip93F Kr-h1 rpr |
| 133 | GO:0008538 | F | 4, | 1 | 0.058 (x 17.175) | 1 (1.000) | 0.509 | proteasome activator activity | REG |
| 134 | GO:0051186 | P | 5, | 21 | 14.730 (x 1.426) | 253 (0.083) | 0.511 | cofactor metabolism | CG10237 CG14721 CG1885 CG2789 CG30499 CG31477 CG32174 CG4095 CG5037 CG5103 CG6574 CG6723 CG7211 CG8498 CG9804 Dhfr Las Vha36 l(2)06225 l(3)02640 sun |
| 135 | GO:0042724 | P | 8, | 1 | 0.058 (x 17.175) | 1 (1.000) | 0.511 | thiamin and derivative biosynthesis | CG14721 |
| 136 | GO:0004793 | F | 6, | 1 | 0.058 (x 17.175) | 1 (1.000) | 0.513 | threonine aldolase activity | CG10184 |
| 137 | GO:0006307 | P | 6, 8, | 1 | 0.058 (x 17.175) | 1 (1.000) | 0.516 | DNA dealkylation | agt |
| 138 | GO:0008503 | F | 5, | 1 | 0.058 (x 17.175) | 1 (1.000) | 0.518 | benzodiazepine receptor activity | CG2789 |
| 139 | GO:0050896 | P | 2, | 80 | 68.063 (x 1.175) | 1169 (0.068) | 0.52 | response to stimulus | AnnX Arr2 BG:DS01219.1 Bc BcDNA:GH08420 CG10433 CG10460 CG11897 CG11898 CG13889 CG1504 CG17266 CG17639 CG18249 CG18522 CG18869 CG30022 CG30438 CG31146 CG3212 CG33177 CG3355 CG5001 CG5224 CG5397 CG6214 CG6426 CG6435 CG8193 CG9095 CG9381 CycG Def Doa Eip93F Gr61a Gr98c GstD6 GstD9 GstE1 GstE5 GstE6 GstE7 Hsp22 InR Kr-h1 Myo28B1 Obp56a Or46a Or59a Or85a Or94a Or98b PGRP-SA Prx6005 Rh7 Sod Tbh Tehao Tfb2 Toll-7 Tsf1 TyrR Ugt86De Ugt86Di agt chp cyp33 hay hep hig l(2)03659 na pum rho rpr sda sra tko upd3 |
| 140 | GO:0046952 | P | 6, | 1 | 0.058 (x 17.175) | 1 (1.000) | 0.521 | ketone body catabolism | CG1140 |
| 141 | GO:0004808 | F | 7, 8, | 1 | 0.058 (x 17.175) | 1 (1.000) | 0.523 | tRNA (5-methylaminomethyl-2-thiouridylate)-methyltransferase activity | EG:BACR7A4.8 |
| 142 | GO:0005899 | C | 4, 5, 6, 7, 8, 9, | 1 | 0.058 (x 17.175) | 1 (1.000) | 0.526 | insulin receptor complex | InR |
| 143 | GO:0004821 | F | 7, | 1 | 0.058 (x 17.175) | 1 (1.000) | 0.529 | histidine-tRNA ligase activity | Aats-his |
| 144 | GO:0042357 | P | 7, 8, | 1 | 0.058 (x 17.175) | 1 (1.000) | 0.531 | thiamin diphosphate metabolism | CG14721 |
| 145 | GO:0016163 | F | 6, | 1 | 0.058 (x 17.175) | 1 (1.000) | 0.534 | nitrogenase activity | CG11897 |
| 146 | GO:0015671 | P | 6, 7, | 1 | 0.058 (x 17.175) | 1 (1.000) | 0.537 | oxygen transport | glob1 |
| 147 | GO:0042723 | P | 7, | 1 | 0.058 (x 17.175) | 1 (1.000) | 0.539 | thiamin and derivative metabolism | CG14721 |
| 148 | GO:0004418 | F | 5, | 1 | 0.058 (x 17.175) | 1 (1.000) | 0.542 | hydroxymethylbilane synthase activity | l(3)02640 |
| 149 | GO:0007185 | P | 7, | 1 | 0.058 (x 17.175) | 1 (1.000) | 0.545 | transmembrane receptor protein tyrosine phosphatase signaling pathway | Gp150 |
| 150 | GO:0008495 | F | 7, | 1 | 0.058 (x 17.175) | 1 (1.000) | 0.547 | protoheme IX farnesyltransferase activity | CG5037 |
| 151 | GO:0030503 | P | 5, 6, | 1 | 0.058 (x 17.175) | 1 (1.000) | 0.55 | regulation of cell redox homeostasis | SelG |
| 152 | GO:0048033 | P | 7, 8, | 1 | 0.058 (x 17.175) | 1 (1.000) | 0.553 | heme o metabolism | CG5037 |
| 153 | GO:0006404 | P | 7, 8, 9, 10, | 1 | 0.058 (x 17.175) | 1 (1.000) | 0.556 | RNA import into nucleus | CG10320 |
| 154 | GO:0007497 | P | 6, | 1 | 0.058 (x 17.175) | 1 (1.000) | 0.559 | posterior midgut development | sisA |
| 155 | GO:0043226 | C | 2, | 175 | 156.271 (x 1.120) | 2684 (0.065) | 0.56 | organelle | Act57B Arp11 BEST:LD29214 Bro CG10166 CG10320 CG10418 CG10669 CG11015 CG11360 CG1140 CG11455 CG11909 CG12361 CG12400 CG1249 CG12605 CG12775 CG13277 CG14482 CG14508 CG14691 CG15220 CG15398 CG17266 CG17280 CG17385 CG17768 CG18011 CG18013 CG18619 CG18767 CG1883 CG2021 CG2789 CG2998 CG31184 CG31272 CG31477 CG31611 CG31922 CG31950 CG32105 CG32174 CG32230 CG32409 CG33002 CG33066 CG3529 CG3843 CG3931 CG4046 CG4095 CG4169 CG4279 CG4592 CG4673 CG4769 CG4866 CG5037 CG5189 CG5338 CG5548 CG6272 CG6610 CG6764 CG7014 CG7181 CG7211 CG7339 CG7834 CG7911 CG8004 CG8219 CG8415 CG8506 CG8857 CG8993 CG9650 CLIP-190 CoVa Cyp12e1 Cyp49a1 DebB Dhc98D Doa EG:152A3.7 EG:BACR7A4.18 Eip93F HP1c Hr38 Hr4 Kr-h1 Las Mlc2 Myo28B1 Nipped-B Nxt1 Orc6 PNUTS Pdsw Pof REG Rab3 Rlc1 RpII18 RpL11 RpL17A RpL27A RpL38 RpL46 RpL8 RpL9 RpP1 RpS17 RpS18 RpS4 RpS9 Rpb10 Rpb4 SmB Sras Ssb-c31a SytIV Syx16 Taf10b Taf11 TfIIEalpha Tfb2 Tim17b2 Tim9a Tm1 Trap36 Vha36 apt bbx cyp33 dmrt93B dynactin-subunit-p25 e(y)2 gt hay l(1)10Bb l(2)06225 l(2)k10201 mRpL11 mRpL14 mRpL2 mRpL21 mRpL22 mRpL22-24 mRpL33 mRpL54 mRpS14 mRpS21 mRpS24 mRpS26 mRpS32 mirr mtacp1 na oho23B pip porin rho robl rpr salm sisA sop sun tko toy unc-13 vvl |
| 156 | GO:0043292 | C | 5, 6, 7, 8, | 3 | 0.757 (x 3.964) | 13 (0.231) | 0.561 | contractile fiber | Mlc2 Mp20 Tm1 |
| 157 | GO:0006597 | P | 9, 10, | 1 | 0.058 (x 17.175) | 1 (1.000) | 0.562 | spermine biosynthesis | SamDC |
| 158 | GO:0015078 | F | 6, | 11 | 5.997 (x 1.834) | 103 (0.107) | 0.564 | hydrogen ion transporter activity | CG11015 CG14482 CG17280 CG31477 CG4169 CG7181 CG7211 CoVa Vha36 l(2)06225 sun |
| 159 | GO:0005009 | F | 7, 9, | 1 | 0.058 (x 17.175) | 1 (1.000) | 0.565 | insulin receptor activity | InR |
| 160 | GO:0043229 | C | 3, 4, 5, 6, | 175 | 156.271 (x 1.120) | 2684 (0.065) | 0.565 | intracellular organelle | Act57B Arp11 BEST:LD29214 Bro CG10166 CG10320 CG10418 CG10669 CG11015 CG11360 CG1140 CG11455 CG11909 CG12361 CG12400 CG1249 CG12605 CG12775 CG13277 CG14482 CG14508 CG14691 CG15220 CG15398 CG17266 CG17280 CG17385 CG17768 CG18011 CG18013 CG18619 CG18767 CG1883 CG2021 CG2789 CG2998 CG31184 CG31272 CG31477 CG31611 CG31922 CG31950 CG32105 CG32174 CG32230 CG32409 CG33002 CG33066 CG3529 CG3843 CG3931 CG4046 CG4095 CG4169 CG4279 CG4592 CG4673 CG4769 CG4866 CG5037 CG5189 CG5338 CG5548 CG6272 CG6610 CG6764 CG7014 CG7181 CG7211 CG7339 CG7834 CG7911 CG8004 CG8219 CG8415 CG8506 CG8857 CG8993 CG9650 CLIP-190 CoVa Cyp12e1 Cyp49a1 DebB Dhc98D Doa EG:152A3.7 EG:BACR7A4.18 Eip93F HP1c Hr38 Hr4 Kr-h1 Las Mlc2 Myo28B1 Nipped-B Nxt1 Orc6 PNUTS Pdsw Pof REG Rab3 Rlc1 RpII18 RpL11 RpL17A RpL27A RpL38 RpL46 RpL8 RpL9 RpP1 RpS17 RpS18 RpS4 RpS9 Rpb10 Rpb4 SmB Sras Ssb-c31a SytIV Syx16 Taf10b Taf11 TfIIEalpha Tfb2 Tim17b2 Tim9a Tm1 Trap36 Vha36 apt bbx cyp33 dmrt93B dynactin-subunit-p25 e(y)2 gt hay l(1)10Bb l(2)06225 l(2)k10201 mRpL11 mRpL14 mRpL2 mRpL21 mRpL22 mRpL22-24 mRpL33 mRpL54 mRpS14 mRpS21 mRpS24 mRpS26 mRpS32 mirr mtacp1 na oho23B pip porin rho robl rpr salm sisA sop sun tko toy unc-13 vvl |
| 161 | GO:0006778 | P | 6, | 3 | 0.757 (x 3.964) | 13 (0.231) | 0.566 | porphyrin metabolism | CG1885 CG5037 l(3)02640 |
| 162 | GO:0006542 | P | 9, 10, | 1 | 0.058 (x 17.175) | 1 (1.000) | 0.568 | glutamine biosynthesis | Gs2 |
| 163 | GO:0001540 | F | 4, | 1 | 0.058 (x 17.175) | 1 (1.000) | 0.571 | beta-amyloid binding | CG32677 |
| 164 | GO:0005744 | C | 3, 5, 6, 7, 8, 9, 10, 11, 12, 13, | 3 | 0.757 (x 3.964) | 13 (0.231) | 0.571 | mitochondrial inner membrane presequence translocase complex | CG33066 Tim17b2 Tim9a |
| 165 | GO:0051031 | P | 7, 8, 9, | 1 | 0.058 (x 17.175) | 1 (1.000) | 0.574 | tRNA transport | Nxt1 |
| 166 | GO:0007616 | P | 6, | 3 | 0.757 (x 3.964) | 13 (0.231) | 0.576 | long-term memory | CG10460 pum sra |
| 167 | GO:0009229 | P | 8, 9, | 1 | 0.058 (x 17.175) | 1 (1.000) | 0.577 | thiamin diphosphate biosynthesis | CG14721 |
| 168 | GO:0008267 | F | 4, | 1 | 0.058 (x 17.175) | 1 (1.000) | 0.58 | poly-glutamine tract binding | PQBP-1 |
| 169 | GO:0006589 | P | 7, 8, 9, 10, | 1 | 0.058 (x 17.175) | 1 (1.000) | 0.583 | octopamine biosynthesis | Tbh |
| 170 | GO:0006772 | P | 8, | 1 | 0.058 (x 17.175) | 1 (1.000) | 0.586 | thiamin metabolism | CG14721 |
| 171 | GO:0017070 | F | 6, | 1 | 0.058 (x 17.175) | 1 (1.000) | 0.59 | U6 snRNA binding | CG17768 |
| 172 | GO:0004712 | F | 7, | 2 | 0.466 (x 4.294) | 8 (0.250) | 0.591 | protein threonine/tyrosine kinase activity | Doa hep |
| 173 | GO:0008319 | F | 7, | 1 | 0.058 (x 17.175) | 1 (1.000) | 0.593 | prenyl protein specific endopeptidase activity | Sras |
| 174 | GO:0008252 | F | 7, | 2 | 0.466 (x 4.294) | 8 (0.250) | 0.593 | nucleotidase activity | CG32549 CG4827 |
| 175 | GO:0005243 | F | 5, | 2 | 0.466 (x 4.294) | 8 (0.250) | 0.596 | gap-junction forming channel activity | inx7 zpg |
| 176 | GO:0004845 | F | 6, | 1 | 0.058 (x 17.175) | 1 (1.000) | 0.596 | uracil phosphoribosyltransferase activity | CG5537 |
| 177 | GO:0005921 | C | 7, 8, 9, | 2 | 0.466 (x 4.294) | 8 (0.250) | 0.598 | gap junction | inx7 zpg |
| 178 | GO:0004836 | F | 4, | 1 | 0.058 (x 17.175) | 1 (1.000) | 0.599 | tyramine-beta hydroxylase activity | Tbh |
| 179 | GO:0015286 | F | 6, | 2 | 0.349 (x 5.725) | 6 (0.333) | 0.601 | innexin channel activity | inx7 zpg |
| 180 | GO:0045931 | P | 7, 8, | 1 | 0.058 (x 17.175) | 1 (1.000) | 0.603 | positive regulation of progression through mitotic cell cycle | skpA |
| 181 | GO:0007438 | P | 5, | 2 | 0.349 (x 5.725) | 6 (0.333) | 0.606 | oenocyte development | rho salm |
| 182 | GO:0008537 | C | 3, 6, 7, 8, 9, 10, | 1 | 0.058 (x 17.175) | 1 (1.000) | 0.606 | proteasome activator complex | REG |
| 183 | GO:0045034 | P | 6, 7, 10, | 3 | 0.815 (x 3.680) | 14 (0.214) | 0.608 | neuroblast division | insc mira trol |
| 184 | GO:0019239 | F | 3, | 4 | 1.339 (x 2.987) | 23 (0.174) | 0.609 | deaminase activity | CG32626 CG8360 Rpb4 l(3)02640 |
| 185 | GO:0008628 | P | 9, 10, 11, | 1 | 0.058 (x 17.175) | 1 (1.000) | 0.61 | induction of apoptosis by hormones | Eip93F |
| 186 | GO:0007007 | P | 7, 8, | 2 | 0.349 (x 5.725) | 6 (0.333) | 0.611 | inner mitochondrial membrane organization and biogenesis | CG33066 Tim9a |
| 187 | GO:0005681 | C | 4, 5, 6, 7, 8, 9, 10, | 10 | 5.357 (x 1.867) | 92 (0.109) | 0.611 | spliceosome complex | CG10418 CG1249 CG13277 CG17266 CG2021 CG31184 CG4279 CG6610 DebB SmB |
| 188 | GO:0006545 | P | 9, 10, | 1 | 0.058 (x 17.175) | 1 (1.000) | 0.613 | glycine biosynthesis | Dhfr |
| 189 | GO:0006767 | P | 6, | 4 | 1.339 (x 2.987) | 23 (0.174) | 0.614 | water-soluble vitamin metabolism | CG14721 CG2846 CG30499 CG5103 |
| 190 | GO:0005214 | F | 4, | 9 | 4.716 (x 1.908) | 81 (0.111) | 0.615 | structural constituent of cuticle (sensu Insecta) | CG2555 CG31876 CG4052 CG7941 CG8511 CG8515 Edg91 Lcp2 Lcp65Ae |
| 191 | GO:0030035 | P | 7, 8, | 2 | 0.349 (x 5.725) | 6 (0.333) | 0.615 | microspike biogenesis | btl hep |
| 192 | GO:0015077 | F | 5, | 11 | 6.113 (x 1.799) | 105 (0.105) | 0.616 | monovalent inorganic cation transporter activity | CG11015 CG14482 CG17280 CG31477 CG4169 CG7181 CG7211 CoVa Vha36 l(2)06225 sun |
| 193 | GO:0008215 | P | 8, 9, | 1 | 0.058 (x 17.175) | 1 (1.000) | 0.617 | spermine metabolism | SamDC |
| 194 | GO:0045039 | P | 7, 8, 9, 10, 11, | 2 | 0.349 (x 5.725) | 6 (0.333) | 0.62 | protein import into mitochondrial inner membrane | CG33066 Tim9a |
| 195 | GO:0008531 | F | 6, | 1 | 0.058 (x 17.175) | 1 (1.000) | 0.62 | riboflavin kinase activity | CG2846 |
| 196 | GO:0006725 | P | 5, | 13 | 8.442 (x 1.540) | 145 (0.090) | 0.621 | aromatic compound metabolism | Ahcy13 CG10184 CG17224 CG18522 CG2277 CG32549 CG32626 CG8360 CG9326 Dhfr Tbh Vha36 nmdyn-D6 |
| 197 | GO:0009117 | P | 6, | 14 | 9.257 (x 1.512) | 159 (0.088) | 0.623 | nucleotide metabolism | Ac78C CG10738 CG17224 CG30499 CG31477 CG32626 CG4827 CG5103 CG7211 Dhfr Vha36 l(2)06225 nmdyn-D6 sun |
| 198 | GO:0004630 | F | 7, 8, | 1 | 0.058 (x 17.175) | 1 (1.000) | 0.624 | phospholipase D activity | Pld |
| 199 | GO:0046847 | P | 8, 9, | 2 | 0.349 (x 5.725) | 6 (0.333) | 0.625 | filopodium formation | btl hep |
| 200 | GO:0008010 | F | 5, | 5 | 2.329 (x 2.147) | 40 (0.125) | 0.627 | structural constituent of larval cuticle (sensu Insecta) | CG2555 CG4052 CG7941 Lcp2 Lcp65Ae |
| 201 | GO:0015669 | P | 5, 6, | 1 | 0.058 (x 17.175) | 1 (1.000) | 0.628 | gas transport | glob1 |
| 202 | GO:0045178 | C | 3, 4, | 2 | 0.349 (x 5.725) | 6 (0.333) | 0.63 | basal part of cell | BG:DS01219.1 mira |
| 203 | GO:0004582 | F | 7, | 1 | 0.058 (x 17.175) | 1 (1.000) | 0.631 | dolichyl-phosphate beta-D-mannosyltransferase activity | CG10166 |
| 204 | GO:0043488 | P | 8, 9, | 1 | 0.058 (x 17.175) | 1 (1.000) | 0.635 | regulation of mRNA stability | pum |
| 205 | GO:0044424 | C | 3, 4, 5, | 201 | 185.731 (x 1.082) | 3190 (0.063) | 0.637 | intracellular part | Aats-his Abl Act57B Arp11 BEST:LD29214 BG:DS02740.5 Bro CG10166 CG10268 CG10320 CG10418 CG10669 CG10861 CG11015 CG11360 CG1140 CG11455 CG11597 CG11909 CG12361 CG12400 CG1249 CG12605 CG12775 CG13277 CG14482 CG14508 CG14691 CG15220 CG15398 CG17224 CG17266 CG17280 CG17385 CG17768 CG18011 CG18013 CG18619 CG18767 CG1883 CG2021 CG2789 CG2846 CG2998 CG31184 CG31272 CG31477 CG31611 CG31922 CG31950 CG32105 CG32174 CG32230 CG32409 CG32549 CG33002 CG33066 CG3529 CG3843 CG3931 CG4046 CG4095 CG4169 CG4279 CG4592 CG4673 CG4769 CG4866 CG5037 CG5189 CG5338 CG5382 CG5548 CG6272 CG6610 CG6764 CG7014 CG7181 CG7211 CG7339 CG7770 CG7834 CG7911 CG8004 CG8219 CG8415 CG8506 CG8857 CG8993 CG9650 CLIP-190 CoVa Cpn Cyp12e1 Cyp49a1 Dab DebB Dhc98D Doa EG:152A3.7 EG:BACR7A4.18 EG:BACR7A4.8 Eip93F Femcoat Gs2 HP1c Hr38 Hr4 Iap2 Kr-h1 Las Mlc2 Mp20 Myo28B1 Nipped-B Nxt1 Orc6 PNUTS Pdsw Pof Prosbeta5 REG Rab3 Rlc1 RpII18 RpL11 RpL17A RpL27A RpL38 RpL46 RpL8 RpL9 RpP1 RpS17 RpS18 RpS4 RpS9 Rpb10 Rpb4 SmB Sod Sras Ssb-c31a SytIV Syx16 Taf10b Taf11 TfIIEalpha Tfb2 Tim17b2 Tim9a Tm1 Trap36 Vha36 apt bbx cyp33 dmrt93B dynactin-subunit-p25 e(y)2 gt hay insc l(1)10Bb l(2)06225 l(2)k10201 l(3)01239 mRpL11 mRpL14 mRpL2 mRpL21 mRpL22 mRpL22-24 mRpL33 mRpL54 mRpS14 mRpS21 mRpS24 mRpS26 mRpS32 mira mirr mtacp1 na oho23B pip porin pum rho robl rpr salm shu sisA skpA sop sun tko toy unc-13 vvl |
| 206 | GO:0019430 | P | 7, | 1 | 0.116 (x 8.588) | 2 (0.500) | 0.637 | removal of superoxide radicals | Sod |
| 207 | GO:0030880 | C | 3, 4, 5, 6, | 4 | 1.397 (x 2.863) | 24 (0.167) | 0.638 | RNA polymerase complex | CG7339 RpII18 Rpb10 Rpb4 |
| 208 | GO:0008093 | F | 5, | 1 | 0.058 (x 17.175) | 1 (1.000) | 0.639 | cytoskeletal adaptor activity | insc |
| 209 | GO:0008627 | P | 10, 11, | 1 | 0.116 (x 8.588) | 2 (0.500) | 0.639 | induction of apoptosis by ionic changes | rpr |
| 210 | GO:0008545 | F | 9, | 1 | 0.116 (x 8.588) | 2 (0.500) | 0.641 | JUN kinase kinase activity | hep |
| 211 | GO:0046982 | F | 5, | 4 | 1.397 (x 2.863) | 24 (0.167) | 0.642 | protein heterodimerization activity | Bro CG6272 gt sisA |
| 212 | GO:0006397 | P | 8, | 16 | 11.528 (x 1.388) | 198 (0.081) | 0.643 | mRNA processing | CG10418 CG10466 CG11360 CG1249 CG13277 CG17266 CG17768 CG2021 CG31184 CG3931 CG4279 CG6610 DebB Doa SmB hay |
| 213 | GO:0017091 | F | 5, | 1 | 0.058 (x 17.175) | 1 (1.000) | 0.643 | AU-specific RNA binding | CG8778 |
| 214 | GO:0008284 | P | 6, | 1 | 0.116 (x 8.588) | 2 (0.500) | 0.643 | positive regulation of cell proliferation | InR |
| 215 | GO:0005520 | F | 5, | 1 | 0.116 (x 8.588) | 2 (0.500) | 0.645 | insulin-like growth factor binding | InR |
| 216 | GO:0006427 | P | 9, 10, 11, | 1 | 0.058 (x 17.175) | 1 (1.000) | 0.646 | histidyl-tRNA aminoacylation | Aats-his |
| 217 | GO:0004459 | F | 6, | 1 | 0.116 (x 8.588) | 2 (0.500) | 0.647 | L-lactate dehydrogenase activity | ImpL3 |
| 218 | GO:0016490 | F | 3, | 4 | 1.397 (x 2.863) | 24 (0.167) | 0.647 | structural constituent of peritrophic membrane (sensu Insecta) | CG6947 CG7298 Gasp Peritrophin-A |
| 219 | GO:0004802 | F | 5, | 1 | 0.116 (x 8.588) | 2 (0.500) | 0.649 | transketolase activity | CG5103 |
| 220 | GO:0004014 | F | 6, | 1 | 0.058 (x 17.175) | 1 (1.000) | 0.65 | adenosylmethionine decarboxylase activity | SamDC |
| 221 | GO:0046087 | P | 9, | 1 | 0.116 (x 8.588) | 2 (0.500) | 0.651 | cytidine metabolism | CG8360 |
| 222 | GO:0004788 | F | 6, | 1 | 0.116 (x 8.588) | 2 (0.500) | 0.653 | thiamin diphosphokinase activity | CG14721 |
| 223 | GO:0017140 | F | 3, | 1 | 0.058 (x 17.175) | 1 (1.000) | 0.654 | lipoic acid synthase activity | Las |
| 224 | GO:0045787 | P | 6, 7, | 1 | 0.116 (x 8.588) | 2 (0.500) | 0.655 | positive regulation of progression through cell cycle | skpA |
| 225 | GO:0030241 | P | 8, 9, 11, 12, | 1 | 0.116 (x 8.588) | 2 (0.500) | 0.657 | muscle thick filament assembly | CG6803 |
| 226 | GO:0008380 | P | 8, | 13 | 9.025 (x 1.441) | 155 (0.084) | 0.658 | RNA splicing | CG10418 CG11360 CG1249 CG13277 CG17266 CG2021 CG31184 CG4279 CG6610 DebB Doa SmB hay |
| 227 | GO:0004750 | F | 6, | 1 | 0.058 (x 17.175) | 1 (1.000) | 0.658 | ribulose-phosphate 3-epimerase activity | CG30499 |
| 228 | GO:0004311 | F | 6, | 1 | 0.116 (x 8.588) | 2 (0.500) | 0.659 | farnesyltranstransferase activity | CG5037 |
| 229 | GO:0005622 | C | 3, 4, | 205 | 191.845 (x 1.069) | 3295 (0.062) | 0.66 | intracellular | Aats-his Abl Act57B Arp11 BEST:LD29214 BG:DS02740.5 BG:DS02740.9 Bro CG10166 CG10237 CG10268 CG10320 CG10418 CG10669 CG10861 CG11015 CG11360 CG1140 CG11455 CG11597 CG11909 CG12361 CG12400 CG1249 CG12605 CG12775 CG13277 CG14482 CG14508 CG14691 CG15220 CG15398 CG17224 CG17266 CG17280 CG17385 CG17768 CG18011 CG18013 CG18619 CG18767 CG1883 CG2021 CG2789 CG2846 CG2998 CG31184 CG31272 CG31477 CG31611 CG31922 CG31950 CG32105 CG32174 CG32230 CG32409 CG32549 CG33002 CG33066 CG3529 CG3843 CG3931 CG4046 CG4095 CG4169 CG4279 CG4592 CG4673 CG4769 CG4866 CG5037 CG5189 CG5338 CG5382 CG5548 CG6272 CG6610 CG6764 CG6891 CG7014 CG7181 CG7211 CG7339 CG7770 CG7834 CG7911 CG8004 CG8152 CG8219 CG8415 CG8506 CG8857 CG8993 CG9650 CLIP-190 CoVa Cpn Cyp12e1 Cyp49a1 Dab DebB Dhc98D Doa EG:152A3.7 EG:BACR7A4.18 EG:BACR7A4.8 Eip93F Femcoat Gs2 HP1c Hr38 Hr4 Iap2 Kr-h1 Las Mlc2 Mp20 Myo28B1 Nipped-B Nxt1 Orc6 PNUTS Pdsw Pof Prosbeta5 REG Rab3 Rlc1 RpII18 RpL11 RpL17A RpL27A RpL38 RpL46 RpL8 RpL9 RpP1 RpS17 RpS18 RpS4 RpS9 Rpb10 Rpb4 SmB Sod Sras Ssb-c31a SytIV Syx16 Taf10b Taf11 TfIIEalpha Tfb2 Tim17b2 Tim9a Tm1 Trap36 Vha36 apt bbx cyp33 dmrt93B dynactin-subunit-p25 e(y)2 gt hay insc l(1)10Bb l(2)06225 l(2)k10201 l(3)01239 mRpL11 mRpL14 mRpL2 mRpL21 mRpL22 mRpL22-24 mRpL33 mRpL54 mRpS14 mRpS21 mRpS24 mRpS26 mRpS32 mira mirr mtacp1 na oho23B pip porin pum rho robl rpr salm shu sisA skpA sop sun tko toy unc-13 vvl |
| 230 | GO:0016030 | F | 5, | 1 | 0.116 (x 8.588) | 2 (0.500) | 0.662 | metarhodopsin binding | Arr2 |
| 231 | GO:0000288 | P | 9, 10, | 1 | 0.058 (x 17.175) | 1 (1.000) | 0.662 | mRNA catabolism, deadenylylation-dependent decay | pum |
| 232 | GO:0030522 | P | 6, | 1 | 0.116 (x 8.588) | 2 (0.500) | 0.664 | intracellular receptor-mediated signaling pathway | Hr4 |
| 233 | GO:0003674 | F | 1, | 492 | 482.436 (x 1.020) | 8286 (0.059) | 0.666 | molecular\_function | Aats-his Abl Ac78C Act57B Ahcy13 Amyrel AnnX Arf84F Arp11 Arr2 BEST:LD29214 BG:DS00180.7 BG:DS01068.5 BG:DS01219.1 BG:DS02740.5 BG:DS02740.9 Bc BcDNA:GH08420 BcDNA:GH08902 BcDNA:GH11110 Bro CAH2 CG10092 CG10104 CG10126 CG10166 CG10184 CG10237 CG10268 CG10320 CG10425 CG10460 CG10466 CG10638 CG10669 CG10674 CG10738 CG10804 CG10861 CG10950 CG10962 CG11015 CG11251 CG11313 CG11318 CG11360 CG1140 CG11455 CG11597 CG11722 CG11897 CG11898 CG11909 CG12022 CG1213 CG12133 CG12175 CG12361 CG12400 CG1249 CG12605 CG12775 CG1299 CG1304 CG13318 CG1342 CG13691 CG13889 CG14076 CG14482 CG14508 CG14691 CG14701 CG14721 CG14825 CG14935 CG15012 CG1504 CG15220 CG15361 CG15398 CG15408 CG15820 CG16712 CG16817 CG17224 CG17266 CG17280 CG17385 CG1756 CG17639 CG17768 CG17821 CG17904 CG18011 CG18013 CG18155 CG18223 CG18249 CG18522 CG18530 CG18619 CG18749 CG18767 CG1883 CG1885 CG18869 CG1939 CG2056 CG2069 CG2185 CG2277 CG2555 CG2789 CG2846 CG2964 CG2998 CG30022 CG30105 CG30126 CG30154 CG30283 CG30334 CG30343 CG3036 CG30438 CG30476 CG30499 CG31146 CG31184 CG31272 CG31477 CG3153 CG31551 CG31601 CG31611 CG31704 CG31715 CG31876 CG31922 CG31957 CG32023 CG32105 CG3212 CG3215 CG32160 CG32174 CG32175 CG32202 CG32207 CG32230 CG32442 CG32448 CG32479 CG32549 CG32582 CG32625 CG32626 CG32627 CG32637 CG32677 CG32692 CG32710 CG32856 CG33002 CG33066 CG33096 CG33128 CG33177 CG33322 CG33543 CG3355 CG3397 CG3529 CG3843 CG3887 CG3931 CG40045 CG40068 CG4046 CG4052 CG4071 CG4095 CG4101 CG4115 CG4169 CG4187 CG4288 CG4386 CG4408 CG4511 CG4592 CG4673 CG4769 CG4805 CG4827 CG4858 CG4866 CG5001 CG5037 CG5103 CG5122 CG5162 CG5177 CG5224 CG5338 CG5382 CG5397 CG5535 CG5537 CG5548 CG5559 CG5639 CG6124 CG6214 CG6272 CG6296 CG6357 CG6426 CG6432 CG6461 CG6574 CG6723 CG6763 CG6764 CG6891 CG6921 CG6947 CG7014 CG7084 CG7181 CG7194 CG7211 CG7298 CG7322 CG7333 CG7339 CG7646 CG7770 CG7777 CG7834 CG7911 CG7941 CG7949 CG8152 CG8193 CG8219 CG8271 CG8360 CG8397 CG8415 CG8498 CG8506 CG8511 CG8515 CG8520 CG8550 CG8756 CG8778 CG8857 CG8916 CG8918 CG8925 CG8979 CG8993 CG9095 CG9164 CG9267 CG9326 CG9372 CG9381 CG9413 CG9602 CG9629 CG9650 CG9790 CG9804 CG9862 CLIP-190 Cad99C Chit CoVa Con Cp36 Cpn CycG Cyp12e1 Cyp28d2 Cyp311a1 Cyp49a1 Dab DebB Dgkepsilon Dhc98D Dhfr Doa EG:152A3.7 EG:30B8.6 EG:52C10.2 EG:63B12.12 EG:80H7.10 EG:9D2.4 EG:BACH7M4.1 EG:BACR7A4.14 EG:BACR7A4.17 EG:BACR7A4.18 EG:BACR7A4.8 Edg91 Eip93F Femcoat GABA-B-R2 Gasp Gp150 Gr61a Gr98c Gs2 GstD6 GstD9 GstE1 GstE5 GstE6 GstE7 HP1c Hmgs Hr38 Hr4 Iap2 Idgf1 Idgf2 ImpL3 InR JhI-26 Karl Kr-h1 Las Lcp2 Lcp65Ae Lip1 Mlc2 Mp20 MtnA Myo28B1 NP15.6 Nipped-B Nxt1 Obp56a Obp58b Or46a Or59a Or85a Or94a Or98b Orc6 PGRP-SA PNUTS PQBP-1 Pdsw Peritrophin-A Pglym78 Pld Pof Prosbeta5 Prx6005 REG RN-tre Rab3 Rep2 Rh7 Rlc1 RpII18 RpL11 RpL17A RpL27A RpL38 RpL46 RpL8 RpL9 RpP1 RpS17 RpS18 RpS4 RpS9 Rpb10 Rpb4 SIP1 SamDC SelG Ser7 Sgs4 SmB Sod Spn6 Sras Ssb-c31a SytIV Syx16 Syx8 Taf10b Taf11 Takl2 Takr99D Tbh Tehao TfIIEalpha Tfb2 Tig Tim17b2 Tim9a Timp Tm1 Toll-7 TpnC41C Trap36 Tsf1 Tsp42El TyrR Ugt86De Ugt86Di Vha36 Vm34Ca agt alpha-Est8 apt bbx betaggt-II btl chp cyp33 dmrt93B e(y)2 fau fu12 gatA glob1 gt hay hep insc inx7 jdp ksr l(1)10Bb l(2)03659 l(2)06225 l(2)k10201 l(3)01239 l(3)02640 lectin-28C mRpL11 mRpL14 mRpL2 mRpL21 mRpL22 mRpL22-24 mRpL33 mRpL54 mRpS14 mRpS21 mRpS24 mRpS26 mRpS32 mira mirr mtacp1 na nmdyn-D6 oho23B pip porin pum ran-like retinin rho robl salm sda shu sisA skpA sop sra sun tko toy trol tsl unc-13 upd3 veli vvl zormin zpg |
| 234 | GO:0030518 | P | 7, | 1 | 0.116 (x 8.588) | 2 (0.500) | 0.666 | steroid hormone receptor signaling pathway | Hr4 |
| 235 | GO:0017148 | P | 7, 8, 9, | 3 | 1.223 (x 2.454) | 21 (0.143) | 0.666 | negative regulation of protein biosynthesis | apt pum rpr |
| 236 | GO:0000036 | F | 4, | 1 | 0.058 (x 17.175) | 1 (1.000) | 0.666 | acyl carrier activity | mtacp1 |
| 237 | GO:0005673 | C | 4, 7, 8, 9, 10, 11, 12, 13, 14, | 1 | 0.116 (x 8.588) | 2 (0.500) | 0.668 | transcription factor TFIIE complex | TfIIEalpha |
| 238 | GO:0016776 | F | 5, | 3 | 1.223 (x 2.454) | 21 (0.143) | 0.668 | phosphotransferase activity, phosphate group as acceptor | CG10268 CG9326 nmdyn-D6 |
| 239 | GO:0046333 | P | 6, 7, 8, 9, | 1 | 0.116 (x 8.588) | 2 (0.500) | 0.67 | octopamine metabolism | Tbh |
| 240 | GO:0015949 | P | 6, | 1 | 0.058 (x 17.175) | 1 (1.000) | 0.671 | nucleobase, nucleoside and nucleotide interconversion | Dhfr |
| 241 | GO:0000398 | P | 9, 11, | 13 | 8.675 (x 1.499) | 149 (0.087) | 0.671 | nuclear mRNA splicing, via spliceosome | CG10418 CG11360 CG1249 CG13277 CG17266 CG2021 CG31184 CG4279 CG6610 DebB Doa SmB hay |
| 242 | GO:0003899 | F | 6, | 4 | 1.747 (x 2.290) | 30 (0.133) | 0.672 | DNA-directed RNA polymerase activity | CG7339 RpII18 Rpb10 Rpb4 |
| 243 | GO:0016236 | P | 5, | 1 | 0.116 (x 8.588) | 2 (0.500) | 0.672 | macroautophagy | CG10861 |
| 244 | GO:0008289 | F | 3, | 7 | 3.843 (x 1.822) | 66 (0.106) | 0.672 | lipid binding | AnnX CG5559 CG8498 Dgkepsilon SytIV ksr unc-13 |
| 245 | GO:0005684 | C | 5, 6, 7, 8, 9, 10, 11, | 5 | 2.387 (x 2.095) | 41 (0.122) | 0.674 | major (U2-dependent) spliceosome | CG10418 CG13277 CG2021 CG31184 CG6610 |
| 246 | GO:0044237 | P | 4, | 285 | 269.514 (x 1.057) | 4629 (0.062) | 0.674 | cellular metabolism | Aats-his Abl Ac78C Act57B Ahcy13 Arf84F BG:DS02740.5 Bc BcDNA:GH08420 BcDNA:GH08902 Bro CAH2 CG10092 CG10104 CG10166 CG10184 CG10237 CG10268 CG10320 CG10418 CG10466 CG10638 CG10669 CG10738 CG11015 CG11251 CG11313 CG11360 CG1140 CG11455 CG11597 CG11909 CG12133 CG12175 CG12361 CG12400 CG1249 CG12605 CG12775 CG1299 CG1304 CG13277 CG13318 CG13889 CG14482 CG14508 CG14721 CG14894 CG15220 CG15398 CG17224 CG17266 CG17280 CG17385 CG17768 CG17821 CG18011 CG18013 CG18223 CG18522 CG18619 CG18749 CG18767 CG1883 CG1885 CG18869 CG2021 CG2056 CG2277 CG2789 CG2846 CG2964 CG2998 CG30022 CG30283 CG3036 CG30438 CG30499 CG31184 CG31477 CG31611 CG31704 CG32105 CG3215 CG32174 CG32230 CG32479 CG32549 CG32626 CG32627 CG33002 CG33128 CG33177 CG3355 CG3843 CG3931 CG40045 CG40068 CG4046 CG4095 CG4169 CG4279 CG4288 CG4386 CG4408 CG4511 CG4769 CG4827 CG4866 CG5001 CG5037 CG5103 CG5122 CG5162 CG5177 CG5338 CG5382 CG5535 CG5537 CG5548 CG6214 CG6272 CG6432 CG6461 CG6574 CG6610 CG6723 CG6763 CG6764 CG6921 CG6947 CG7014 CG7181 CG7211 CG7298 CG7339 CG7770 CG7834 CG8360 CG8415 CG8498 CG8550 CG8756 CG8778 CG8857 CG8918 CG8993 CG9267 CG9326 CG9372 CG9413 CG9602 CG9650 CG9804 CG9862 Chit CoVa CycG Cyp12e1 Cyp28d2 Cyp311a1 Cyp49a1 Dab DebB Dgkepsilon Dhc98D Dhfr Doa EG:152A3.7 EG:9D2.4 EG:BACR7A4.18 EG:BACR7A4.8 Eip93F Gasp Gs2 GstE1 GstE5 GstE6 GstE7 HP1c Hmgs Hr38 Hr4 Hsp22 Iap2 ImpL3 InR Kr-h1 Las Nipped-B Obp58b Or59a Orc6 PGRP-SA PNUTS Pdsw Peritrophin-A Pglym78 Pld Pof Prosbeta5 Prx6005 REG RN-tre RpII18 RpL11 RpL17A RpL27A RpL38 RpL46 RpL8 RpL9 RpP1 RpS17 RpS18 RpS4 RpS9 Rpb10 Rpb4 SamDC Ser7 SmB Sod Spn6 Sras Ssb-c31a Taf10b Taf11 Takl2 Tbh TfIIEalpha Tfb2 Timp Trap36 Ugt86De Ugt86Di Vha36 agt apt bbx betaggt-II btl cyp33 dmrt93B e(y)2 fu12 gatA gt hay hep jdp ksr l(2)06225 l(3)01239 l(3)02640 mRpL11 mRpL14 mRpL2 mRpL21 mRpL22 mRpL22-24 mRpL33 mRpS14 mRpS21 mRpS24 mRpS26 mRpS32 mirr mtacp1 na nmdyn-D6 oho23B pip pum rpr salm sda shu skpA sop sun tko toy trol vvl |
| 247 | GO:0016917 | F | 5, | 3 | 1.106 (x 2.712) | 19 (0.158) | 0.674 | GABA receptor activity | CG8916 EG:30B8.6 GABA-B-R2 |
| 248 | GO:0042302 | F | 3, | 9 | 5.764 (x 1.561) | 99 (0.091) | 0.674 | structural constituent of cuticle | CG2555 CG31876 CG4052 CG7941 CG8511 CG8515 Edg91 Lcp2 Lcp65Ae |
| 249 | GO:0000377 | P | 10, | 13 | 8.675 (x 1.499) | 149 (0.087) | 0.674 | RNA splicing, via transesterification reactions with bulged adenosine as nucleophile | CG10418 CG11360 CG1249 CG13277 CG17266 CG2021 CG31184 CG4279 CG6610 DebB Doa SmB hay |
| 250 | GO:0016513 | C | 4, 7, 8, 9, 10, 11, 12, 13, 14, | 1 | 0.116 (x 8.588) | 2 (0.500) | 0.674 | core-binding factor complex | Bro |
| 251 | GO:0006695 | P | 8, 9, 10, | 1 | 0.058 (x 17.175) | 1 (1.000) | 0.675 | cholesterol biosynthesis | CG10268 |
| 252 | GO:0015238 | F | 3, | 3 | 1.106 (x 2.712) | 19 (0.158) | 0.676 | drug transporter activity | CG11897 CG11898 CG6214 |
| 253 | GO:0008226 | F | 8, | 1 | 0.116 (x 8.588) | 2 (0.500) | 0.677 | tyramine receptor activity | TyrR |
| 254 | GO:0000375 | P | 9, | 13 | 8.675 (x 1.499) | 149 (0.087) | 0.677 | RNA splicing, via transesterification reactions | CG10418 CG11360 CG1249 CG13277 CG17266 CG2021 CG31184 CG4279 CG6610 DebB Doa SmB hay |
| 255 | GO:0009108 | P | 7, | 9 | 5.473 (x 1.644) | 94 (0.096) | 0.677 | coenzyme biosynthesis | CG14721 CG31477 CG32174 CG7211 CG9804 Las Vha36 l(2)06225 sun |
| 256 | GO:0019221 | P | 6, | 4 | 1.921 (x 2.082) | 33 (0.121) | 0.678 | cytokine and chemokine mediated signaling pathway | CG1504 Tehao Toll-7 chp |
| 257 | GO:0044275 | P | 7, | 7 | 3.901 (x 1.794) | 67 (0.104) | 0.679 | cellular carbohydrate catabolism | CG2964 CG30499 CG5103 Chit ImpL3 PGRP-SA Pglym78 |
| 258 | GO:0004852 | F | 6, | 1 | 0.116 (x 8.588) | 2 (0.500) | 0.679 | uroporphyrinogen-III synthase activity | CG1885 |
| 259 | GO:0045167 | P | 5, 6, | 2 | 0.524 (x 3.817) | 9 (0.222) | 0.679 | asymmetric protein localization during cell fate commitment | insc mira |
| 260 | GO:0007496 | P | 6, | 1 | 0.058 (x 17.175) | 1 (1.000) | 0.679 | anterior midgut development | sisA |
| 261 | GO:0006081 | P | 5, | 1 | 0.116 (x 8.588) | 2 (0.500) | 0.681 | aldehyde metabolism | CG10638 |
| 262 | GO:0016052 | P | 6, | 7 | 3.901 (x 1.794) | 67 (0.104) | 0.681 | carbohydrate catabolism | CG2964 CG30499 CG5103 Chit ImpL3 PGRP-SA Pglym78 |
| 263 | GO:0046845 | P | 5, 6, | 2 | 0.524 (x 3.817) | 9 (0.222) | 0.682 | branched duct epithelial cell fate determination (sensu Insecta) | rho salm |
| 264 | GO:0009231 | P | 8, 9, | 1 | 0.116 (x 8.588) | 2 (0.500) | 0.683 | riboflavin biosynthesis | CG2846 |
| 265 | GO:0016992 | F | 6, | 1 | 0.058 (x 17.175) | 1 (1.000) | 0.683 | lipoate synthase activity | Las |
| 266 | GO:0035072 | P | 7, 8, 9, 10, 11, | 2 | 0.524 (x 3.817) | 9 (0.222) | 0.684 | ecdysone-mediated induction of salivary gland cell autophagic cell death | Eip93F rpr |
| 267 | GO:0007608 | P | 5, 7, | 7 | 4.192 (x 1.670) | 72 (0.097) | 0.685 | sensory perception of smell | Obp56a Or46a Or59a Or85a Or94a Or98b TyrR |
| 268 | GO:0042727 | P | 8, | 1 | 0.116 (x 8.588) | 2 (0.500) | 0.685 | riboflavin and derivative biosynthesis | CG2846 |
| 269 | GO:0016310 | P | 7, | 34 | 26.841 (x 1.267) | 461 (0.074) | 0.685 | phosphorylation | Abl CG10320 CG10738 CG11015 CG11455 CG12400 CG14482 CG14508 CG17280 CG2056 CG31477 CG32230 CG4169 CG4769 CG5548 CG6214 CG7181 CG7211 CG7834 CG9267 CoVa Dgkepsilon Doa EG:152A3.7 InR Pdsw Takl2 Vha36 btl hep ksr l(2)06225 mtacp1 sun |
| 270 | GO:0005675 | C | 4, 7, 8, 9, 10, 11, 12, 13, 14, | 2 | 0.524 (x 3.817) | 9 (0.222) | 0.687 | transcription factor TFIIH complex | Tfb2 hay |
| 271 | GO:0008410 | F | 5, | 1 | 0.116 (x 8.588) | 2 (0.500) | 0.688 | CoA-transferase activity | CG1140 |
| 272 | GO:0004146 | F | 6, | 1 | 0.058 (x 17.175) | 1 (1.000) | 0.688 | dihydrofolate reductase activity | Dhfr |
| 273 | GO:0045261 | C | 3, 4, 5, 6, 7, | 2 | 0.524 (x 3.817) | 9 (0.222) | 0.69 | proton-transporting ATP synthase complex, catalytic core F(1) | CG31477 sun |
| 274 | GO:0017133 | C | 4, 5, 6, 7, 8, 9, 10, 11, 12, | 1 | 0.116 (x 8.588) | 2 (0.500) | 0.69 | electron transfer flavoprotein complex (sensu Eukaryota) | CG7834 |
| 275 | GO:0016071 | P | 7, | 17 | 12.110 (x 1.404) | 208 (0.082) | 0.691 | mRNA metabolism | CG10418 CG10466 CG11360 CG1249 CG13277 CG17266 CG17768 CG2021 CG31184 CG3931 CG4279 CG6610 DebB Doa SmB hay pum |
| 276 | GO:0035075 | P | 5, 6, 7, | 3 | 0.873 (x 3.435) | 15 (0.200) | 0.692 | response to ecdysone | Eip93F Kr-h1 rpr |
| 277 | GO:0002046 | F | 4, | 1 | 0.116 (x 8.588) | 2 (0.500) | 0.692 | opsin binding | Arr2 |
| 278 | GO:0003908 | F | 7, | 1 | 0.058 (x 17.175) | 1 (1.000) | 0.692 | methylated-DNA-[protein]-cysteine S-methyltransferase activity | agt |
| 279 | GO:0000275 | C | 4, 5, 6, 7, 8, 9, 10, 11, 12, 13, 14, | 2 | 0.524 (x 3.817) | 9 (0.222) | 0.693 | proton-transporting ATP synthase complex, catalytic core F(1) (sensu Eukaryota) | CG31477 sun |
| 280 | GO:0006408 | P | 8, 9, 10, 11, | 1 | 0.116 (x 8.588) | 2 (0.500) | 0.695 | snRNA export from nucleus | Nxt1 |
| 281 | GO:0005213 | F | 3, | 2 | 0.524 (x 3.817) | 9 (0.222) | 0.695 | structural constituent of chorion (sensu Insecta) | Cp36 Femcoat |
| 282 | GO:0048545 | P | 5, 6, | 3 | 0.873 (x 3.435) | 15 (0.200) | 0.696 | response to steroid hormone stimulus | Eip93F Kr-h1 rpr |
| 283 | GO:0008340 | P | 4, | 5 | 2.678 (x 1.867) | 46 (0.109) | 0.697 | determination of adult life span | Hsp22 InR SelG Sod hep |
| 284 | GO:0004574 | F | 7, | 1 | 0.058 (x 17.175) | 1 (1.000) | 0.697 | oligo-1,6-glucosidase activity | Amyrel |
| 285 | GO:0007174 | P | 9, | 1 | 0.116 (x 8.588) | 2 (0.500) | 0.697 | epidermal growth factor ligand processing | rho |
| 286 | GO:0008121 | F | 6, 7, | 2 | 0.524 (x 3.817) | 9 (0.222) | 0.698 | ubiquinol-cytochrome-c reductase activity | CG14482 CG4169 |
| 287 | GO:0007568 | P | 3, | 5 | 2.678 (x 1.867) | 46 (0.109) | 0.699 | aging | Hsp22 InR SelG Sod hep |
| 288 | GO:0004275 | F | 7, | 1 | 0.116 (x 8.588) | 2 (0.500) | 0.699 | enteropeptidase activity | CG3355 |
| 289 | GO:0016681 | F | 5, | 2 | 0.524 (x 3.817) | 9 (0.222) | 0.701 | oxidoreductase activity, acting on diphenols and related substances as donors, cytochrome as acceptor | CG14482 CG4169 |
| 290 | GO:0030849 | C | 6, 7, 8, 9, | 1 | 0.058 (x 17.175) | 1 (1.000) | 0.701 | autosome | Pof |
| 291 | GO:0005122 | F | 4, 5, | 1 | 0.116 (x 8.588) | 2 (0.500) | 0.702 | torso binding | tsl |
| 292 | GO:0005858 | C | 5, 6, 7, 8, 9, 10, 11, 12, | 2 | 0.640 (x 3.123) | 11 (0.182) | 0.702 | axonemal dynein complex | Dhc98D robl |
| 293 | GO:0006626 | P | 8, 9, 10, | 4 | 1.980 (x 2.021) | 34 (0.118) | 0.704 | protein targeting to mitochondrion | CG33066 CG8004 Tim17b2 Tim9a |
| 294 | GO:0005671 | C | 5, 8, 9, 10, 11, 12, 13, 14, 15, | 1 | 0.116 (x 8.588) | 2 (0.500) | 0.704 | Ada2/Gcn5/Ada3 transcription activator complex | Rpb4 |
| 295 | GO:0009166 | P | 6, 7, | 2 | 0.524 (x 3.817) | 9 (0.222) | 0.704 | nucleotide catabolism | CG17224 CG4827 |
| 296 | GO:0007267 | P | 4, | 32 | 26.084 (x 1.227) | 448 (0.071) | 0.704 | cell-cell signaling | Arf84F CG14691 CG1504 CG1756 CG18249 CG31146 CG31272 CG5559 CG5819 CG8916 Chit GABA-B-R2 Gp150 Gs2 Idgf1 Idgf2 Or46a Or59a Or85a Or94a Rab3 SytIV Syx16 Syx8 Takr99D Tbh Tsp42El TyrR apt pum unc-13 veli |
| 297 | GO:0006458 | P | 8, | 2 | 0.640 (x 3.123) | 11 (0.182) | 0.704 | 'de novo' protein folding | CG7770 l(3)01239 |
| 298 | GO:0007613 | P | 5, | 4 | 1.456 (x 2.748) | 25 (0.160) | 0.705 | memory | CG10460 Tbh pum sra |
| 299 | GO:0031327 | P | 7, | 3 | 1.281 (x 2.342) | 22 (0.136) | 0.705 | negative regulation of cellular biosynthesis | apt pum rpr |
| 300 | GO:0015955 | P | 8, | 1 | 0.058 (x 17.175) | 1 (1.000) | 0.706 | pyrimidine deoxyribonucleotide interconversion | Dhfr |
| 301 | GO:0005930 | C | 4, 5, 6, 7, 8, | 2 | 0.640 (x 3.123) | 11 (0.182) | 0.706 | axoneme | Dhc98D robl |
| 302 | GO:0035096 | P | 6, 7, | 1 | 0.116 (x 8.588) | 2 (0.500) | 0.706 | larval midgut cell programmed cell death | rpr |
| 303 | GO:0009890 | P | 6, | 3 | 1.281 (x 2.342) | 22 (0.136) | 0.708 | negative regulation of biosynthesis | apt pum rpr |
| 304 | GO:0042060 | P | 5, | 2 | 0.640 (x 3.123) | 11 (0.182) | 0.708 | wound healing | Bc hep |
| 305 | GO:0004662 | F | 8, | 1 | 0.116 (x 8.588) | 2 (0.500) | 0.709 | CAAX-protein geranylgeranyltransferase activity | betaggt-II |
| 306 | GO:0044447 | C | 4, 5, 6, 7, 8, 9, | 2 | 0.640 (x 3.123) | 11 (0.182) | 0.71 | axoneme part | Dhc98D robl |
| 307 | GO:0008539 | F | 4, | 1 | 0.058 (x 17.175) | 1 (1.000) | 0.711 | proteasome inhibitor activity | CG8979 |
| 308 | GO:0051030 | P | 7, 8, 9, | 1 | 0.116 (x 8.588) | 2 (0.500) | 0.711 | snRNA transport | Nxt1 |
| 309 | GO:0008152 | P | 3, | 307 | 294.434 (x 1.043) | 5057 (0.061) | 0.712 | metabolism | Aats-his Abl Ac78C Act57B Ahcy13 Amyrel AnnX Arf84F BG:DS02740.5 Bc BcDNA:GH08420 BcDNA:GH08902 Bro CAH2 CG10092 CG10104 CG10166 CG10184 CG10237 CG10268 CG10320 CG10418 CG10425 CG10466 CG10638 CG10669 CG10738 CG10962 CG11015 CG11251 CG11313 CG11360 CG1140 CG11455 CG11597 CG11897 CG11909 CG1213 CG12133 CG12175 CG12361 CG12400 CG1249 CG12605 CG12775 CG1299 CG1304 CG13277 CG13318 CG13889 CG14482 CG14508 CG14721 CG14894 CG14935 CG15220 CG15398 CG15408 CG17224 CG17266 CG17280 CG17385 CG17768 CG17821 CG18011 CG18013 CG18155 CG18223 CG18522 CG18530 CG18619 CG18749 CG18767 CG1883 CG1885 CG18869 CG2021 CG2056 CG2277 CG2789 CG2846 CG2964 CG2998 CG30022 CG30283 CG3036 CG30438 CG30499 CG31184 CG31272 CG31477 CG31611 CG31704 CG32105 CG3215 CG32174 CG32230 CG32479 CG32549 CG32626 CG32627 CG33002 CG33128 CG33177 CG3355 CG3843 CG3931 CG40045 CG40068 CG4046 CG4095 CG4169 CG4279 CG4288 CG4386 CG4408 CG4511 CG4592 CG4769 CG4827 CG4866 CG5001 CG5037 CG5103 CG5122 CG5162 CG5177 CG5338 CG5382 CG5535 CG5537 CG5548 CG6214 CG6272 CG6296 CG6432 CG6461 CG6574 CG6610 CG6723 CG6763 CG6764 CG6803 CG6921 CG6947 CG7014 CG7181 CG7211 CG7298 CG7322 CG7339 CG7770 CG7834 CG8193 CG8360 CG8415 CG8498 CG8550 CG8756 CG8778 CG8857 CG8918 CG8993 CG9267 CG9326 CG9372 CG9413 CG9602 CG9629 CG9650 CG9804 CG9836 CG9862 Chit CoVa CycG Cyp12e1 Cyp28d2 Cyp311a1 Cyp49a1 Dab DebB Dgkepsilon Dhc98D Dhfr Doa EG:152A3.7 EG:9D2.4 EG:BACR7A4.14 EG:BACR7A4.18 EG:BACR7A4.8 Eip93F Gasp Gs2 GstE1 GstE5 GstE6 GstE7 HP1c Hmgs Hr38 Hr4 Hsp22 Iap2 Idgf1 Idgf2 ImpL3 InR Kr-h1 Las Lip1 Nipped-B Obp58b Or59a Orc6 PGRP-SA PNUTS Pdsw Peritrophin-A Pglym78 Pld Pof Prosbeta5 Prx6005 REG RN-tre RpII18 RpL11 RpL17A RpL27A RpL38 RpL46 RpL8 RpL9 RpP1 RpS17 RpS18 RpS4 RpS9 Rpb10 Rpb4 SamDC Ser7 SmB Sod Spn6 Sras Ssb-c31a Taf10b Taf11 Takl2 Tbh TfIIEalpha Tfb2 Timp Trap36 Ugt86De Ugt86Di Vha36 agt apt bbx betaggt-II btl cyp33 dmrt93B e(y)2 fu12 gatA gt hay hep jdp ksr l(2)06225 l(3)01239 l(3)02640 mRpL11 mRpL14 mRpL2 mRpL21 mRpL22 mRpL22-24 mRpL33 mRpS14 mRpS21 mRpS24 mRpS26 mRpS32 mirr mtacp1 na nmdyn-D6 oho23B pip pum rpr salm sda shu skpA sop sun tko toy trol vvl |
| 310 | GO:0044449 | C | 5, 6, 7, 8, 9, | 2 | 0.640 (x 3.123) | 11 (0.182) | 0.713 | contractile fiber part | Mlc2 Tm1 |
| 311 | GO:0005007 | F | 7, 9, | 1 | 0.175 (x 5.725) | 3 (0.333) | 0.713 | fibroblast growth factor receptor activity | btl |
| 312 | GO:0035202 | P | 5, | 1 | 0.116 (x 8.588) | 2 (0.500) | 0.714 | tracheal sac formation (sensu Insecta) | rho |
| 313 | GO:0035006 | P | 5, 7, | 1 | 0.175 (x 5.725) | 3 (0.333) | 0.714 | melanization defense response | Bc |
| 314 | GO:0017145 | P | 5, | 4 | 2.154 (x 1.857) | 37 (0.108) | 0.715 | stem cell division | insc mira pum trol |
| 315 | GO:0042168 | P | 6, 7, | 2 | 0.640 (x 3.123) | 11 (0.182) | 0.715 | heme metabolism | CG1885 CG5037 |
| 316 | GO:0044425 | C | 3, 4, 5, | 81 | 70.799 (x 1.144) | 1216 (0.067) | 0.715 | membrane part | Abl Ac78C Arr2 CG10320 CG10804 CG11015 CG11318 CG11455 CG11897 CG11898 CG1213 CG12400 CG14482 CG14508 CG14691 CG15408 CG17280 CG17821 CG2789 CG3036 CG31272 CG31477 CG3212 CG32230 CG33066 CG3397 CG4101 CG4169 CG4187 CG4288 CG4673 CG4769 CG5037 CG5548 CG6214 CG6763 CG6921 CG7181 CG7188 CG7211 CG7333 CG8004 CG8219 CG8271 CG8916 CG8925 Cad99C CoVa EG:152A3.7 EG:80H7.10 GABA-B-R2 Gr61a Gr98c InR Or46a Or59a Or85a Or94a Or98b PGRP-SA Pdsw Rh7 Sras Syx16 Takr99D Tehao Tim17b2 Tim9a Toll-7 Tsp42El TyrR Vha36 inx7 l(2)03659 l(2)06225 mtacp1 porin rho sun veli zpg |
| 317 | GO:0006858 | P | 5, 6, | 11 | 7.161 (x 1.536) | 123 (0.089) | 0.715 | extracellular transport | CG10804 CG11897 CG11898 CG3036 CG4288 CG6214 CG6723 CG7084 CG7333 CG8925 l(2)03659 |
| 318 | GO:0007268 | P | 6, | 17 | 13.158 (x 1.292) | 226 (0.075) | 0.715 | synaptic transmission | Arf84F CG14691 CG31146 CG31272 CG5559 CG8916 GABA-B-R2 Gs2 Rab3 SytIV Syx16 Syx8 Tbh TyrR apt pum unc-13 |
| 319 | GO:0048034 | P | 8, 9, | 1 | 0.058 (x 17.175) | 1 (1.000) | 0.715 | heme o biosynthesis | CG5037 |
| 320 | GO:0009259 | P | 7, | 8 | 5.357 (x 1.494) | 92 (0.087) | 0.716 | ribonucleotide metabolism | CG31477 CG32626 CG7211 Dhfr Vha36 l(2)06225 nmdyn-D6 sun |
| 321 | GO:0046351 | P | 7, 8, | 1 | 0.175 (x 5.725) | 3 (0.333) | 0.716 | disaccharide biosynthesis | CG5177 |
| 322 | GO:0005862 | C | 5, 6, 7, 8, 9, 10, 11, 12, | 1 | 0.116 (x 8.588) | 2 (0.500) | 0.716 | muscle thin filament tropomyosin | Tm1 |
| 323 | GO:0035081 | P | 8, 9, | 2 | 0.640 (x 3.123) | 11 (0.182) | 0.717 | induction of programmed cell death by hormones | Eip93F rpr |
| 324 | GO:0006732 | P | 6, | 19 | 13.915 (x 1.365) | 239 (0.079) | 0.717 | coenzyme metabolism | CG10237 CG14721 CG2789 CG30499 CG31477 CG32174 CG4095 CG5037 CG5103 CG6574 CG6723 CG7211 CG8498 CG9804 Dhfr Las Vha36 l(2)06225 sun |
| 325 | GO:0006163 | P | 7, | 8 | 5.357 (x 1.494) | 92 (0.087) | 0.717 | purine nucleotide metabolism | CG31477 CG32626 CG7211 Dhfr Vha36 l(2)06225 nmdyn-D6 sun |
| 326 | GO:0007313 | P | 7, 9, 10, 12, | 1 | 0.175 (x 5.725) | 3 (0.333) | 0.718 | maternal determination of dorsal/ventral axis, oocyte, soma encoded | pip |
| 327 | GO:0004457 | F | 5, | 1 | 0.116 (x 8.588) | 2 (0.500) | 0.719 | lactate dehydrogenase activity | ImpL3 |
| 328 | GO:0004092 | F | 9, | 1 | 0.175 (x 5.725) | 3 (0.333) | 0.719 | carnitine O-acetyltransferase activity | CG5122 |
| 329 | GO:0005927 | C | 8, 9, 10, | 1 | 0.058 (x 17.175) | 1 (1.000) | 0.72 | muscle tendon junction | Abl |
| 330 | GO:0019740 | P | 5, | 1 | 0.116 (x 8.588) | 2 (0.500) | 0.721 | nitrogen utilization | CG9836 |
| 331 | GO:0016802 | F | 5, | 1 | 0.175 (x 5.725) | 3 (0.333) | 0.721 | trialkylsulfonium hydrolase activity | Ahcy13 |
| 332 | GO:0048098 | P | 7, | 1 | 0.175 (x 5.725) | 3 (0.333) | 0.723 | antennal joint development | salm |
| 333 | GO:0006771 | P | 8, | 1 | 0.116 (x 8.588) | 2 (0.500) | 0.724 | riboflavin metabolism | CG2846 |
| 334 | GO:0016491 | F | 3, | 43 | 36.331 (x 1.184) | 624 (0.069) | 0.724 | oxidoreductase activity | Bc CG10320 CG10425 CG10638 CG10962 CG11015 CG11455 CG11897 CG12400 CG13889 CG14482 CG14508 CG17280 CG18522 CG18749 CG3215 CG32230 CG3397 CG4169 CG4769 CG5548 CG7181 CG7322 CG7834 CG8193 CG8778 CG8993 CG9629 CoVa Cyp12e1 Cyp28d2 Cyp311a1 Cyp49a1 Dhfr EG:152A3.7 EG:BACR7A4.14 ImpL3 Or59a Pdsw Prx6005 Sod Tbh mtacp1 |
| 335 | GO:0050819 | P | 5, | 1 | 0.175 (x 5.725) | 3 (0.333) | 0.725 | negative regulation of coagulation | AnnX |
| 336 | GO:0015953 | P | 7, | 1 | 0.058 (x 17.175) | 1 (1.000) | 0.725 | pyrimidine nucleotide interconversion | Dhfr |
| 337 | GO:0035314 | P | 6, | 1 | 0.116 (x 8.588) | 2 (0.500) | 0.726 | scab formation | Bc |
| 338 | GO:0000158 | F | 9, | 1 | 0.175 (x 5.725) | 3 (0.333) | 0.726 | protein phosphatase type 2A activity | CG11597 |
| 339 | GO:0000146 | F | 3, | 1 | 0.175 (x 5.725) | 3 (0.333) | 0.728 | microfilament motor activity | Mlc2 |
| 340 | GO:0004394 | F | 6, | 1 | 0.116 (x 8.588) | 2 (0.500) | 0.729 | heparan sulfate 2-O-sulfotransferase activity | pip |
| 341 | GO:0045011 | P | 11, | 1 | 0.175 (x 5.725) | 3 (0.333) | 0.73 | actin cable formation | hep |
| 342 | GO:0046164 | P | 6, | 5 | 2.969 (x 1.684) | 51 (0.098) | 0.73 | alcohol catabolism | CG2964 CG30499 CG5103 ImpL3 Pglym78 |
| 343 | GO:0008260 | F | 6, | 1 | 0.058 (x 17.175) | 1 (1.000) | 0.73 | 3-oxoacid CoA-transferase activity | CG1140 |
| 344 | GO:0031033 | P | 9, | 1 | 0.116 (x 8.588) | 2 (0.500) | 0.731 | myosin filament assembly or disassembly | CG6803 |
| 345 | GO:0046365 | P | 7, 8, | 5 | 2.969 (x 1.684) | 51 (0.098) | 0.731 | monosaccharide catabolism | CG2964 CG30499 CG5103 ImpL3 Pglym78 |
| 346 | GO:0004029 | F | 6, | 1 | 0.175 (x 5.725) | 3 (0.333) | 0.731 | aldehyde dehydrogenase (NAD) activity | CG9629 |
| 347 | GO:0008021 | C | 8, 9, 10, 11, 12, | 5 | 2.969 (x 1.684) | 51 (0.098) | 0.733 | synaptic vesicle | CG14691 CG31272 Rab3 SytIV unc-13 |
| 348 | GO:0007395 | P | 6, 7, | 1 | 0.175 (x 5.725) | 3 (0.333) | 0.733 | dorsal closure, spreading of leading edge cells | hep |
| 349 | GO:0031034 | P | 7, 10, | 1 | 0.116 (x 8.588) | 2 (0.500) | 0.734 | myosin filament assembly | CG6803 |
| 350 | GO:0004558 | F | 7, | 2 | 0.757 (x 2.642) | 13 (0.154) | 0.734 | alpha-glucosidase activity | CG11909 CG14935 |
| 351 | GO:0006007 | P | 9, 10, | 5 | 2.969 (x 1.684) | 51 (0.098) | 0.735 | glucose catabolism | CG2964 CG30499 CG5103 ImpL3 Pglym78 |
| 352 | GO:0004421 | F | 6, | 1 | 0.058 (x 17.175) | 1 (1.000) | 0.735 | hydroxymethylglutaryl-CoA synthase activity | Hmgs |
| 353 | GO:0030032 | P | 7, 8, | 1 | 0.175 (x 5.725) | 3 (0.333) | 0.735 | lamellipodium biogenesis | hep |
| 354 | GO:0016679 | F | 4, | 2 | 0.757 (x 2.642) | 13 (0.154) | 0.736 | oxidoreductase activity, acting on diphenols and related substances as donors | CG14482 CG4169 |
| 355 | GO:0016006 | C | 6, 7, 8, 9, | 1 | 0.116 (x 8.588) | 2 (0.500) | 0.736 | Nebenkern | porin |
| 356 | GO:0019320 | P | 8, 9, | 5 | 2.969 (x 1.684) | 51 (0.098) | 0.736 | hexose catabolism | CG2964 CG30499 CG5103 ImpL3 Pglym78 |
| 357 | GO:0005353 | F | 7, | 1 | 0.175 (x 5.725) | 3 (0.333) | 0.737 | fructose transporter activity | CG15408 |
| 358 | GO:0006733 | P | 7, | 3 | 1.164 (x 2.576) | 20 (0.150) | 0.737 | oxidoreduction coenzyme metabolism | CG30499 CG32174 CG5103 |
| 359 | GO:0007428 | P | 5, 6, | 2 | 0.757 (x 2.642) | 13 (0.154) | 0.738 | primary tracheal branching (sensu Insecta) | apt btl |
| 360 | GO:0045213 | P | 7, 8, | 1 | 0.175 (x 5.725) | 3 (0.333) | 0.739 | neurotransmitter receptor metabolism | Gs2 |
| 361 | GO:0006911 | P | 8, 9, | 1 | 0.116 (x 8.588) | 2 (0.500) | 0.739 | phagocytosis, engulfment | Eip93F |
| 362 | GO:0004194 | F | 7, | 2 | 0.757 (x 2.642) | 13 (0.154) | 0.74 | pepsin A activity | CG10104 CG33128 |
| 363 | GO:0003876 | F | 4, 6, | 1 | 0.058 (x 17.175) | 1 (1.000) | 0.74 | AMP deaminase activity | CG32626 |
| 364 | GO:0008374 | F | 7, | 3 | 1.164 (x 2.576) | 20 (0.150) | 0.74 | O-acyltransferase activity | CG5122 CG5397 fu12 |
| 365 | GO:0000900 | F | 4, 5, | 1 | 0.175 (x 5.725) | 3 (0.333) | 0.74 | translation repressor activity, nucleic acid binding | pum |
| 366 | GO:0046131 | P | 8, | 1 | 0.116 (x 8.588) | 2 (0.500) | 0.742 | pyrimidine ribonucleoside metabolism | CG8360 |
| 367 | GO:0017068 | F | 6, | 1 | 0.175 (x 5.725) | 3 (0.333) | 0.742 | glutamyl-tRNA(Gln) amidotransferase activity | gatA |
| 368 | GO:0016782 | F | 4, | 3 | 1.164 (x 2.576) | 20 (0.150) | 0.743 | transferase activity, transferring sulfur-containing groups | CG1140 CG9164 pip |
| 369 | GO:0017056 | F | 3, | 1 | 0.175 (x 5.725) | 3 (0.333) | 0.744 | structural constituent of nuclear pore | CG4673 |
| 370 | GO:0042726 | P | 7, | 1 | 0.116 (x 8.588) | 2 (0.500) | 0.744 | riboflavin and derivative metabolism | CG2846 |
| 371 | GO:0008191 | F | 6, | 1 | 0.058 (x 17.175) | 1 (1.000) | 0.745 | metalloendopeptidase inhibitor activity | Timp |
| 372 | GO:0045742 | P | 6, 7, 10, | 1 | 0.175 (x 5.725) | 3 (0.333) | 0.746 | positive regulation of epidermal growth factor receptor signaling pathway | rho |
| 373 | GO:0045251 | C | 3, 5, 6, 7, 8, | 1 | 0.116 (x 8.588) | 2 (0.500) | 0.747 | electron transfer flavoprotein complex | CG7834 |
| 374 | GO:0017069 | F | 5, | 1 | 0.175 (x 5.725) | 3 (0.333) | 0.748 | snRNA binding | CG17768 |
| 375 | GO:0003779 | F | 5, | 10 | 7.161 (x 1.396) | 123 (0.081) | 0.749 | actin binding | AnnX Arp11 BG:DS02740.9 CG6891 CG8397 CLIP-190 Mp20 Myo28B1 Tm1 mira |
| 376 | GO:0005667 | C | 3, 6, 7, 8, 9, 10, 11, 12, 13, | 9 | 4.949 (x 1.819) | 85 (0.106) | 0.75 | transcription factor complex | Bro CG15398 Rpb4 Ssb-c31a Taf10b Taf11 TfIIEalpha Tfb2 hay |
| 377 | GO:0007211 | P | 7, | 1 | 0.116 (x 8.588) | 2 (0.500) | 0.75 | octopamine/tyramine signaling pathway | TyrR |
| 378 | GO:0004805 | F | 8, | 1 | 0.175 (x 5.725) | 3 (0.333) | 0.75 | trehalose-phosphatase activity | CG5177 |
| 379 | GO:0001505 | P | 7, | 10 | 6.812 (x 1.468) | 117 (0.085) | 0.75 | regulation of neurotransmitter levels | Arf84F CG14691 CG31272 CG5559 Rab3 SytIV Syx16 Syx8 Tbh unc-13 |
| 380 | GO:0030031 | P | 6, 7, | 4 | 1.863 (x 2.147) | 32 (0.125) | 0.751 | cell projection biogenesis | CG14825 CG2069 btl hep |
| 381 | GO:0009612 | P | 4, | 2 | 0.582 (x 3.435) | 10 (0.200) | 0.751 | response to mechanical stimulus | sda tko |
| 382 | GO:0043112 | P | 7, | 1 | 0.175 (x 5.725) | 3 (0.333) | 0.751 | receptor metabolism | Gs2 |
| 383 | GO:0016358 | P | 7, 10, | 4 | 2.038 (x 1.963) | 35 (0.114) | 0.752 | dendrite development | Tm1 pum robl vvl |
| 384 | GO:0050910 | P | 6, 7, 8, | 1 | 0.233 (x 4.294) | 4 (0.250) | 0.752 | detection of mechanical stimulus during sensory perception of sound | tko |
| 385 | GO:0004367 | F | 6, | 1 | 0.175 (x 5.725) | 3 (0.333) | 0.753 | glycerol-3-phosphate dehydrogenase (NAD+) activity | CG3215 |
| 386 | GO:0042375 | P | 6, | 1 | 0.233 (x 4.294) | 4 (0.250) | 0.753 | quinone cofactor metabolism | CG32174 |
| 387 | GO:0006206 | P | 7, | 4 | 1.863 (x 2.147) | 32 (0.125) | 0.754 | pyrimidine base metabolism | CG17224 CG8360 Dhfr nmdyn-D6 |
| 388 | GO:0006783 | P | 7, 8, | 2 | 0.582 (x 3.435) | 10 (0.200) | 0.754 | heme biosynthesis | CG1885 CG5037 |
| 389 | GO:0007064 | P | 6, 7, 9, | 1 | 0.233 (x 4.294) | 4 (0.250) | 0.755 | mitotic sister chromatid cohesion | Nipped-B |
| 390 | GO:0016060 | P | 8, 9, 10, | 1 | 0.175 (x 5.725) | 3 (0.333) | 0.755 | metarhodopsin inactivation | Arr2 |
| 391 | GO:0017038 | P | 6, 7, | 5 | 3.202 (x 1.561) | 55 (0.091) | 0.756 | protein import | CG10950 CG33066 CG8219 Nxt1 Tim9a |
| 392 | GO:0004217 | F | 7, | 1 | 0.233 (x 4.294) | 4 (0.250) | 0.756 | cathepsin L activity | CG6357 |
| 393 | GO:0008565 | F | 3, | 7 | 4.367 (x 1.603) | 75 (0.093) | 0.756 | protein transporter activity | CG10950 CG33066 CG3529 CG8219 Nxt1 Tim17b2 Tim9a |
| 394 | GO:0008105 | P | 5, | 4 | 1.863 (x 2.147) | 32 (0.125) | 0.756 | asymmetric protein localization | BG:DS01219.1 CG9326 insc mira |
| 395 | GO:0006367 | P | 9, | 7 | 4.891 (x 1.431) | 84 (0.083) | 0.757 | transcription initiation from RNA polymerase II promoter | CG15398 Taf10b Taf11 TfIIEalpha Tfb2 Trap36 hay |
| 396 | GO:0019226 | P | 5, | 25 | 21.135 (x 1.183) | 363 (0.069) | 0.757 | transmission of nerve impulse | Arf84F CG14691 CG1504 CG1756 CG18249 CG31146 CG31272 CG5559 CG5819 CG8916 GABA-B-R2 Gp150 Gs2 Rab3 SytIV Syx16 Syx8 Takr99D Tbh Tsp42El TyrR apt pum unc-13 veli |
| 397 | GO:0051087 | F | 4, | 2 | 0.582 (x 3.435) | 10 (0.200) | 0.757 | chaperone binding | CG7770 l(3)01239 |
| 398 | GO:0009152 | P | 8, 9, | 8 | 5.182 (x 1.544) | 89 (0.090) | 0.757 | purine ribonucleotide biosynthesis | CG31477 CG32626 CG7211 Dhfr Vha36 l(2)06225 nmdyn-D6 sun |
| 399 | GO:0005662 | C | 3, 5, 6, 7, 8, 9, 10, 11, 12, 13, 14, | 1 | 0.175 (x 5.725) | 3 (0.333) | 0.757 | DNA replication factor A complex | CG15220 |
| 400 | GO:0019362 | P | 7, 8, | 2 | 0.873 (x 2.290) | 15 (0.133) | 0.757 | pyridine nucleotide metabolism | CG30499 CG5103 |
| 401 | GO:0005625 | C | 4, 5, | 1 | 0.233 (x 4.294) | 4 (0.250) | 0.758 | soluble fraction | Arr2 |
| 402 | GO:0005911 | C | 6, 7, 8, | 5 | 2.562 (x 1.952) | 44 (0.114) | 0.758 | intercellular junction | Abl InR inx7 veli zpg |
| 403 | GO:0004356 | F | 7, | 1 | 0.175 (x 5.725) | 3 (0.333) | 0.759 | glutamate-ammonia ligase activity | Gs2 |
| 404 | GO:0006963 | P | 7, 8, 9, | 2 | 0.873 (x 2.290) | 15 (0.133) | 0.759 | positive regulation of antibacterial peptide biosynthesis | Def PGRP-SA |
| 405 | GO:0046777 | P | 9, 10, | 1 | 0.233 (x 4.294) | 4 (0.250) | 0.759 | protein amino acid autophosphorylation | InR |
| 406 | GO:0016857 | F | 5, | 2 | 0.582 (x 3.435) | 10 (0.200) | 0.76 | racemase and epimerase activity, acting on carbohydrates and derivatives | BcDNA:GH08902 CG30499 |
| 407 | GO:0003840 | F | 6, | 1 | 0.233 (x 4.294) | 4 (0.250) | 0.761 | gamma-glutamyltransferase activity | CG6461 |
| 408 | GO:0005992 | P | 8, 9, | 1 | 0.175 (x 5.725) | 3 (0.333) | 0.761 | trehalose biosynthesis | CG5177 |
| 409 | GO:0006032 | P | 8, 9, 10, 11, | 1 | 0.233 (x 4.294) | 4 (0.250) | 0.762 | chitin catabolism | Chit |
| 410 | GO:0035078 | P | 6, 7, 8, 9, 10, | 2 | 0.582 (x 3.435) | 10 (0.200) | 0.762 | induction of programmed cell death by ecdysone | Eip93F rpr |
| 411 | GO:0004300 | F | 6, | 1 | 0.175 (x 5.725) | 3 (0.333) | 0.763 | enoyl-CoA hydratase activity | CG8778 |
| 412 | GO:0045426 | P | 7, 8, | 1 | 0.233 (x 4.294) | 4 (0.250) | 0.764 | quinone cofactor biosynthesis | CG32174 |
| 413 | GO:0004814 | F | 7, | 1 | 0.175 (x 5.725) | 3 (0.333) | 0.765 | arginine-tRNA ligase activity | CG10092 |
| 414 | GO:0000272 | P | 7, | 1 | 0.233 (x 4.294) | 4 (0.250) | 0.765 | polysaccharide catabolism | Chit |
| 415 | GO:0030728 | P | 6, | 1 | 0.233 (x 4.294) | 4 (0.250) | 0.767 | ovulation | Tbh |
| 416 | GO:0005542 | F | 4, | 1 | 0.175 (x 5.725) | 3 (0.333) | 0.767 | folic acid binding | CG6574 |
| 417 | GO:0046348 | P | 7, 8, | 1 | 0.233 (x 4.294) | 4 (0.250) | 0.768 | amino sugar catabolism | Chit |
| 418 | GO:0003987 | F | 6, | 1 | 0.175 (x 5.725) | 3 (0.333) | 0.769 | acetate-CoA ligase activity | CG6432 |
| 419 | GO:0008592 | P | 5, 6, 7, | 1 | 0.233 (x 4.294) | 4 (0.250) | 0.77 | regulation of Toll signaling pathway | PGRP-SA |
| 420 | GO:0006420 | P | 9, 10, 11, | 1 | 0.175 (x 5.725) | 3 (0.333) | 0.77 | arginyl-tRNA aminoacylation | CG10092 |
| 421 | GO:0005669 | C | 4, 7, 8, 9, 10, 11, 12, 13, 14, | 3 | 1.630 (x 1.840) | 28 (0.107) | 0.771 | transcription factor TFIID complex | CG15398 Taf10b Taf11 |
| 422 | GO:0005252 | F | 7, 8, 9, | 1 | 0.233 (x 4.294) | 4 (0.250) | 0.771 | open rectifier potassium channel activity | CG1756 |
| 423 | GO:0004850 | F | 6, | 1 | 0.175 (x 5.725) | 3 (0.333) | 0.772 | uridine phosphorylase activity | CG17224 |
| 424 | GO:0004550 | F | 6, 7, | 1 | 0.233 (x 4.294) | 4 (0.250) | 0.773 | nucleoside diphosphate kinase activity | nmdyn-D6 |
| 425 | GO:0016062 | P | 7, 8, 9, | 1 | 0.233 (x 4.294) | 4 (0.250) | 0.774 | adaptation of rhodopsin mediated signaling | Arr2 |
| 426 | GO:0016211 | F | 6, | 1 | 0.175 (x 5.725) | 3 (0.333) | 0.774 | ammonia ligase activity | Gs2 |
| 427 | GO:0008067 | F | 6, | 2 | 0.699 (x 2.863) | 12 (0.167) | 0.776 | metabotropic glutamate, GABA-B-like receptor activity | EG:30B8.6 GABA-B-R2 |
| 428 | GO:0004708 | F | 8, | 1 | 0.233 (x 4.294) | 4 (0.250) | 0.776 | MAP kinase kinase activity | hep |
| 429 | GO:0009150 | P | 8, | 8 | 5.240 (x 1.527) | 90 (0.089) | 0.776 | purine ribonucleotide metabolism | CG31477 CG32626 CG7211 Dhfr Vha36 l(2)06225 nmdyn-D6 sun |
| 430 | GO:0005859 | C | 5, 6, 7, 8, 9, 10, 11, 12, | 1 | 0.175 (x 5.725) | 3 (0.333) | 0.776 | muscle myosin | Mlc2 |
| 431 | GO:0016460 | C | 4, 6, 7, 8, 9, 10, 11, | 1 | 0.233 (x 4.294) | 4 (0.250) | 0.777 | myosin II | Mlc2 |
| 432 | GO:0005486 | F | 5, | 2 | 0.699 (x 2.863) | 12 (0.167) | 0.778 | t-SNARE activity | Syx16 Syx8 |
| 433 | GO:0008172 | F | 6, | 1 | 0.175 (x 5.725) | 3 (0.333) | 0.778 | S-methyltransferase activity | agt |
| 434 | GO:0016973 | P | 9, 10, 11, 12, | 1 | 0.233 (x 4.294) | 4 (0.250) | 0.779 | poly(A)+ mRNA export from nucleus | Nxt1 |
| 435 | GO:0006740 | P | 10, 11, | 2 | 0.699 (x 2.863) | 12 (0.167) | 0.78 | NADPH regeneration | CG30499 CG5103 |
| 436 | GO:0006034 | P | 8, 9, 10, 11, | 1 | 0.233 (x 4.294) | 4 (0.250) | 0.78 | cuticle chitin metabolism | Chit |
| 437 | GO:0006744 | P | 8, 9, | 1 | 0.175 (x 5.725) | 3 (0.333) | 0.78 | ubiquinone biosynthesis | CG32174 |
| 438 | GO:0030136 | C | 7, 8, 9, 10, 11, | 5 | 3.260 (x 1.534) | 56 (0.089) | 0.782 | clathrin-coated vesicle | CG14691 CG31272 Rab3 SytIV unc-13 |
| 439 | GO:0042043 | F | 4, | 1 | 0.233 (x 4.294) | 4 (0.250) | 0.782 | neurexin binding | CG31146 |
| 440 | GO:0008415 | F | 6, | 9 | 6.696 (x 1.344) | 115 (0.078) | 0.782 | acyltransferase activity | CG17821 CG5037 CG5122 CG5397 CG6461 CG6921 Rpb4 betaggt-II fu12 |
| 441 | GO:0042384 | P | 7, 8, | 2 | 0.699 (x 2.863) | 12 (0.167) | 0.782 | cilium biogenesis | CG14825 CG2069 |
| 442 | GO:0004866 | F | 5, | 7 | 4.949 (x 1.414) | 85 (0.082) | 0.782 | endopeptidase inhibitor activity | CG10460 CG1342 CG16712 CG31704 CG5639 Spn6 Timp |
| 443 | GO:0004013 | F | 6, | 1 | 0.175 (x 5.725) | 3 (0.333) | 0.782 | adenosylhomocysteinase activity | Ahcy13 |
| 444 | GO:0046983 | F | 4, | 5 | 2.853 (x 1.753) | 49 (0.102) | 0.782 | protein dimerization activity | Bro CG18619 CG6272 gt sisA |
| 445 | GO:0006766 | P | 5, | 4 | 2.096 (x 1.908) | 36 (0.111) | 0.783 | vitamin metabolism | CG14721 CG2846 CG30499 CG5103 |
| 446 | GO:0016836 | F | 5, | 5 | 3.260 (x 1.534) | 56 (0.089) | 0.783 | hydro-lyase activity | CAH2 CG1885 CG4095 CG4592 CG8778 |
| 447 | GO:0004333 | F | 6, | 1 | 0.233 (x 4.294) | 4 (0.250) | 0.783 | fumarate hydratase activity | CG4095 |
| 448 | GO:0006739 | P | 9, 10, | 2 | 0.699 (x 2.863) | 12 (0.167) | 0.784 | NADP metabolism | CG30499 CG5103 |
| 449 | GO:0016200 | P | 6, | 1 | 0.175 (x 5.725) | 3 (0.333) | 0.784 | synaptic target attraction | Con |
| 450 | GO:0008553 | F | 6, 7, 9, 14, | 5 | 2.853 (x 1.753) | 49 (0.102) | 0.785 | hydrogen-exporting ATPase activity, phosphorylative mechanism | CG31477 CG7211 Vha36 l(2)06225 sun |
| 451 | GO:0009208 | P | 9, | 1 | 0.233 (x 4.294) | 4 (0.250) | 0.785 | pyrimidine ribonucleoside triphosphate metabolism | nmdyn-D6 |
| 452 | GO:0008518 | F | 6, | 1 | 0.175 (x 5.725) | 3 (0.333) | 0.786 | reduced folate carrier activity | CG6574 |
| 453 | GO:0046529 | P | 7, 8, | 1 | 0.233 (x 4.294) | 4 (0.250) | 0.786 | imaginal disc fusion, thorax closure | hep |
| 454 | GO:0006098 | P | 8, 10, 11, 12, | 2 | 0.699 (x 2.863) | 12 (0.167) | 0.786 | pentose-phosphate shunt | CG30499 CG5103 |
| 455 | GO:0044247 | P | 7, 8, | 1 | 0.233 (x 4.294) | 4 (0.250) | 0.788 | cellular polysaccharide catabolism | Chit |
| 456 | GO:0051298 | P | 6, 8, 9, | 1 | 0.175 (x 5.725) | 3 (0.333) | 0.788 | centrosome duplication | skpA |
| 457 | GO:0008216 | P | 8, 9, | 1 | 0.233 (x 4.294) | 4 (0.250) | 0.79 | spermidine metabolism | SamDC |
| 458 | GO:0007269 | P | 6, 7, 8, | 9 | 6.113 (x 1.472) | 105 (0.086) | 0.79 | neurotransmitter secretion | Arf84F CG14691 CG31272 CG5559 Rab3 SytIV Syx16 Syx8 unc-13 |
| 459 | GO:0004126 | F | 4, 6, | 1 | 0.175 (x 5.725) | 3 (0.333) | 0.791 | cytidine deaminase activity | CG8360 |
| 460 | GO:0009260 | P | 7, 8, | 8 | 5.298 (x 1.510) | 91 (0.088) | 0.791 | ribonucleotide biosynthesis | CG31477 CG32626 CG7211 Dhfr Vha36 l(2)06225 nmdyn-D6 sun |
| 461 | GO:0019992 | F | 4, | 3 | 1.514 (x 1.982) | 26 (0.115) | 0.791 | diacylglycerol binding | Dgkepsilon ksr unc-13 |
| 462 | GO:0009209 | P | 9, 10, | 1 | 0.233 (x 4.294) | 4 (0.250) | 0.791 | pyrimidine ribonucleoside triphosphate biosynthesis | nmdyn-D6 |
| 463 | GO:0008355 | P | 6, 7, | 4 | 2.271 (x 1.762) | 39 (0.103) | 0.792 | olfactory learning | BG:DS01219.1 CG9381 rho sra |
| 464 | GO:0009165 | P | 6, 7, | 9 | 6.405 (x 1.405) | 110 (0.082) | 0.792 | nucleotide biosynthesis | Ac78C CG31477 CG32626 CG7211 Dhfr Vha36 l(2)06225 nmdyn-D6 sun |
| 465 | GO:0045055 | P | 6, 7, | 9 | 6.113 (x 1.472) | 105 (0.086) | 0.792 | regulated secretory pathway | Arf84F CG14691 CG31272 CG5559 Rab3 SytIV Syx16 Syx8 unc-13 |
| 466 | GO:0006605 | P | 7, 8, 9, | 17 | 13.450 (x 1.264) | 231 (0.074) | 0.792 | protein targeting | CG10950 CG17266 CG32677 CG33066 CG4187 CG4673 CG8004 CG8219 CG9326 CLIP-190 Nxt1 PNUTS Syx16 Tim17b2 Tim9a cyp33 ran-like |
| 467 | GO:0050818 | P | 4, | 1 | 0.175 (x 5.725) | 3 (0.333) | 0.793 | regulation of coagulation | AnnX |
| 468 | GO:0004556 | F | 7, | 2 | 0.815 (x 2.454) | 14 (0.143) | 0.793 | alpha-amylase activity | Amyrel CG14935 |
| 469 | GO:0046912 | F | 5, | 1 | 0.233 (x 4.294) | 4 (0.250) | 0.793 | transferase activity, transferring acyl groups, acyl groups converted into alkyl on transfer | Hmgs |
| 470 | GO:0016853 | F | 3, | 8 | 5.298 (x 1.510) | 91 (0.088) | 0.793 | isomerase activity | BcDNA:GH08902 CG11251 CG17266 CG30499 CG4592 Pglym78 cyp33 shu |
| 471 | GO:0006538 | P | 9, 10, | 1 | 0.233 (x 4.294) | 4 (0.250) | 0.794 | glutamate catabolism | Gs2 |
| 472 | GO:0006769 | P | 8, 9, | 2 | 0.815 (x 2.454) | 14 (0.143) | 0.794 | nicotinamide metabolism | CG30499 CG5103 |
| 473 | GO:0016744 | F | 4, | 1 | 0.175 (x 5.725) | 3 (0.333) | 0.795 | transferase activity, transferring aldehyde or ketonic groups | CG5103 |
| 474 | GO:0009141 | P | 7, | 6 | 4.134 (x 1.451) | 71 (0.085) | 0.795 | nucleoside triphosphate metabolism | CG31477 CG7211 Vha36 l(2)06225 nmdyn-D6 sun |
| 475 | GO:0006164 | P | 7, 8, | 8 | 5.298 (x 1.510) | 91 (0.088) | 0.795 | purine nucleotide biosynthesis | CG31477 CG32626 CG7211 Dhfr Vha36 l(2)06225 nmdyn-D6 sun |
| 476 | GO:0006030 | P | 7, 8, 9, 10, | 6 | 3.668 (x 1.636) | 63 (0.095) | 0.796 | chitin metabolism | CG6947 CG7298 CG8756 Chit Gasp Peritrophin-A |
| 477 | GO:0046036 | P | 10, | 1 | 0.233 (x 4.294) | 4 (0.250) | 0.796 | CTP metabolism | nmdyn-D6 |
| 478 | GO:0031202 | F | 5, | 3 | 1.397 (x 2.147) | 24 (0.125) | 0.796 | RNA splicing factor activity, transesterification mechanism | CG1249 DebB SmB |
| 479 | GO:0016160 | F | 6, | 2 | 0.815 (x 2.454) | 14 (0.143) | 0.796 | amylase activity | Amyrel CG14935 |
| 480 | GO:0006044 | P | 8, 9, | 6 | 4.134 (x 1.451) | 71 (0.085) | 0.796 | N-acetylglucosamine metabolism | CG6947 CG7298 CG8756 Chit Gasp Peritrophin-A |
| 481 | GO:0044260 | P | 5, | 134 | 125.296 (x 1.069) | 2152 (0.062) | 0.797 | cellular macromolecule metabolism | Aats-his Abl Act57B Arf84F BG:DS02740.5 BcDNA:GH08420 CG10092 CG10104 CG10166 CG10237 CG10466 CG10738 CG11313 CG11360 CG11597 CG12133 CG12775 CG1299 CG1304 CG13318 CG14894 CG17266 CG18223 CG18749 CG18767 CG1883 CG1885 CG2056 CG2789 CG2998 CG30283 CG31704 CG32479 CG32627 CG33002 CG33128 CG33177 CG3355 CG3843 CG40045 CG40068 CG4046 CG4386 CG4408 CG4866 CG5001 CG5338 CG5382 CG6214 CG6461 CG6574 CG6723 CG6763 CG6764 CG6947 CG7014 CG7298 CG7770 CG8415 CG8550 CG8756 CG8857 CG8918 CG9267 CG9372 CG9602 CG9804 Chit Cyp12e1 Dab Dhc98D Doa EG:9D2.4 Gasp Gs2 Hsp22 Iap2 InR Las Obp58b PNUTS Peritrophin-A Pof Prosbeta5 REG RN-tre RpL11 RpL17A RpL27A RpL38 RpL46 RpL8 RpL9 RpP1 RpS17 RpS18 RpS4 RpS9 Ser7 Spn6 Sras Takl2 Timp apt betaggt-II btl cyp33 gatA hep jdp ksr l(3)01239 mRpL11 mRpL14 mRpL2 mRpL21 mRpL22 mRpL22-24 mRpL33 mRpS14 mRpS21 mRpS24 mRpS26 mRpS32 na oho23B pip pum rpr sda shu skpA sop tko |
| 482 | GO:0006541 | P | 8, 9, | 1 | 0.175 (x 5.725) | 3 (0.333) | 0.797 | glutamine metabolism | Gs2 |
| 483 | GO:0006183 | P | 10, 11, | 1 | 0.233 (x 4.294) | 4 (0.250) | 0.798 | GTP biosynthesis | nmdyn-D6 |
| 484 | GO:0006041 | P | 7, 8, | 6 | 4.134 (x 1.451) | 71 (0.085) | 0.798 | glucosamine metabolism | CG6947 CG7298 CG8756 Chit Gasp Peritrophin-A |
| 485 | GO:0016854 | F | 4, | 2 | 0.815 (x 2.454) | 14 (0.143) | 0.798 | racemase and epimerase activity | BcDNA:GH08902 CG30499 |
| 486 | GO:0015450 | F | 4, 6, | 3 | 1.397 (x 2.147) | 24 (0.125) | 0.798 | protein translocase activity | CG33066 Tim17b2 Tim9a |
| 487 | GO:0016880 | F | 5, | 1 | 0.175 (x 5.725) | 3 (0.333) | 0.799 | acid-ammonia (or amide) ligase activity | Gs2 |
| 488 | GO:0030371 | F | 3, | 1 | 0.233 (x 4.294) | 4 (0.250) | 0.799 | translation repressor activity | pum |
| 489 | GO:0042401 | P | 7, 8, | 2 | 0.815 (x 2.454) | 14 (0.143) | 0.8 | biogenic amine biosynthesis | SamDC Tbh |
| 490 | GO:0030414 | F | 4, | 7 | 5.007 (x 1.398) | 86 (0.081) | 0.8 | protease inhibitor activity | CG10460 CG1342 CG16712 CG31704 CG5639 Spn6 Timp |
| 491 | GO:0016251 | F | 4, | 8 | 5.881 (x 1.360) | 101 (0.079) | 0.801 | general RNA polymerase II transcription factor activity | BEST:LD29214 Rpb4 Taf10b Taf11 TfIIEalpha Tfb2 Trap36 hay |
| 492 | GO:0006036 | P | 9, 10, 11, 12, | 1 | 0.233 (x 4.294) | 4 (0.250) | 0.801 | cuticle chitin catabolism | Chit |
| 493 | GO:0042169 | F | 5, | 1 | 0.175 (x 5.725) | 3 (0.333) | 0.801 | SH2 domain binding | Dab |
| 494 | GO:0044463 | C | 3, 4, 5, | 2 | 0.815 (x 2.454) | 14 (0.143) | 0.802 | cell projection part | Dhc98D robl |
| 495 | GO:0042048 | P | 5, 6, | 5 | 3.086 (x 1.620) | 53 (0.094) | 0.802 | olfactory behavior | BG:DS01219.1 CG9381 Obp56a rho sra |
| 496 | GO:0006352 | P | 8, | 7 | 5.007 (x 1.398) | 86 (0.081) | 0.802 | transcription initiation | CG15398 Taf10b Taf11 TfIIEalpha Tfb2 Trap36 hay |
| 497 | GO:0006241 | P | 10, 11, | 1 | 0.233 (x 4.294) | 4 (0.250) | 0.803 | CTP biosynthesis | nmdyn-D6 |
| 498 | GO:0004619 | F | 6, | 1 | 0.175 (x 5.725) | 3 (0.333) | 0.803 | phosphoglycerate mutase activity | Pglym78 |
| 499 | GO:0004659 | F | 5, | 2 | 0.932 (x 2.147) | 16 (0.125) | 0.804 | prenyltransferase activity | CG5037 betaggt-II |
| 500 | GO:0008308 | F | 6, 7, | 1 | 0.233 (x 4.294) | 4 (0.250) | 0.804 | voltage-gated ion-selective channel activity | porin |
| 501 | GO:0007156 | P | 5, | 3 | 1.688 (x 1.777) | 29 (0.103) | 0.804 | homophilic cell adhesion | Cad99C Con chp |
| 502 | GO:0016019 | F | 5, | 1 | 0.175 (x 5.725) | 3 (0.333) | 0.805 | peptidoglycan receptor activity | PGRP-SA |
| 503 | GO:0016538 | F | 5, | 2 | 0.932 (x 2.147) | 16 (0.125) | 0.805 | cyclin-dependent protein kinase regulator activity | CG9790 CycG |
| 504 | GO:0007176 | P | 5, 6, 9, | 1 | 0.233 (x 4.294) | 4 (0.250) | 0.806 | regulation of epidermal growth factor receptor activity | rho |
| 505 | GO:0014016 | P | 4, 7, | 3 | 1.688 (x 1.777) | 29 (0.103) | 0.806 | neuroblast differentiation | insc mira trol |
| 506 | GO:0042398 | P | 6, 7, | 2 | 0.932 (x 2.147) | 16 (0.125) | 0.807 | amino acid derivative biosynthesis | SamDC Tbh |
| 507 | GO:0007612 | P | 5, | 4 | 2.504 (x 1.598) | 43 (0.093) | 0.807 | learning | BG:DS01219.1 CG9381 rho sra |
| 508 | GO:0007400 | P | 6, 9, | 3 | 1.688 (x 1.777) | 29 (0.103) | 0.807 | neuroblast fate determination | insc mira trol |
| 509 | GO:0016730 | F | 4, | 1 | 0.175 (x 5.725) | 3 (0.333) | 0.807 | oxidoreductase activity, acting on iron-sulfur proteins as donors | CG11897 |
| 510 | GO:0006213 | P | 7, | 1 | 0.233 (x 4.294) | 4 (0.250) | 0.807 | pyrimidine nucleoside metabolism | CG8360 |
| 511 | GO:0043231 | C | 4, 5, 6, 7, | 143 | 134.321 (x 1.065) | 2307 (0.062) | 0.808 | intracellular membrane-bound organelle | BEST:LD29214 Bro CG10166 CG10320 CG10418 CG10669 CG11015 CG11360 CG1140 CG11455 CG11909 CG12361 CG12400 CG1249 CG12605 CG13277 CG14482 CG14508 CG14691 CG15220 CG15398 CG17266 CG17280 CG17385 CG17768 CG18011 CG18013 CG18619 CG18767 CG2021 CG2789 CG31184 CG31272 CG31477 CG31611 CG31922 CG31950 CG32105 CG32174 CG32230 CG32409 CG33002 CG33066 CG3529 CG3931 CG4095 CG4169 CG4279 CG4592 CG4673 CG4769 CG4866 CG5037 CG5189 CG5548 CG6272 CG6610 CG7181 CG7211 CG7339 CG7834 CG7911 CG8004 CG8219 CG8506 CG8993 CG9650 CLIP-190 CoVa Cyp12e1 Cyp49a1 DebB Dhc98D Doa EG:152A3.7 EG:BACR7A4.18 Eip93F HP1c Hr38 Hr4 Kr-h1 Las Nipped-B Nxt1 Orc6 PNUTS Pdsw REG Rab3 Rlc1 RpII18 Rpb10 Rpb4 SmB Sras Ssb-c31a SytIV Syx16 Taf10b Taf11 TfIIEalpha Tfb2 Tim17b2 Tim9a Trap36 Vha36 apt bbx cyp33 dmrt93B e(y)2 gt hay l(1)10Bb l(2)06225 l(2)k10201 mRpL11 mRpL14 mRpL2 mRpL21 mRpL22 mRpL22-24 mRpL33 mRpL54 mRpS14 mRpS21 mRpS24 mRpS26 mRpS32 mirr mtacp1 pip porin rho robl rpr salm sisA sun tko toy unc-13 vvl |
| 512 | GO:0006040 | P | 6, 7, | 6 | 4.192 (x 1.431) | 72 (0.083) | 0.809 | amino sugar metabolism | CG6947 CG7298 CG8756 Chit Gasp Peritrophin-A |
| 513 | GO:0014017 | P | 5, 8, | 3 | 1.688 (x 1.777) | 29 (0.103) | 0.809 | neuroblast fate commitment | insc mira trol |
| 514 | GO:0006046 | P | 9, 10, | 1 | 0.233 (x 4.294) | 4 (0.250) | 0.809 | N-acetylglucosamine catabolism | Chit |
| 515 | GO:0008295 | P | 9, 10, | 1 | 0.175 (x 5.725) | 3 (0.333) | 0.81 | spermidine biosynthesis | SamDC |
| 516 | GO:0006752 | P | 7, | 6 | 4.192 (x 1.431) | 72 (0.083) | 0.81 | group transfer coenzyme metabolism | CG31477 CG7211 Dhfr Vha36 l(2)06225 sun |
| 517 | GO:0004989 | F | 8, | 1 | 0.233 (x 4.294) | 4 (0.250) | 0.811 | octopamine receptor activity | TyrR |
| 518 | GO:0004772 | F | 8, | 1 | 0.175 (x 5.725) | 3 (0.333) | 0.812 | sterol O-acyltransferase activity | CG5397 |
| 519 | GO:0006043 | P | 8, 9, | 1 | 0.233 (x 4.294) | 4 (0.250) | 0.812 | glucosamine catabolism | Chit |
| 520 | GO:0008175 | F | 7, | 1 | 0.233 (x 4.294) | 4 (0.250) | 0.814 | tRNA methyltransferase activity | EG:BACR7A4.8 |
| 521 | GO:0009617 | P | 5, | 6 | 4.309 (x 1.393) | 74 (0.081) | 0.814 | response to bacterium | CG6426 CG6435 Def PGRP-SA Tehao Toll-7 |
| 522 | GO:0015629 | C | 6, 7, 8, 9, | 6 | 4.309 (x 1.393) | 74 (0.081) | 0.816 | actin cytoskeleton | Act57B Arp11 Mlc2 Myo28B1 Tm1 dynactin-subunit-p25 |
| 523 | GO:0050830 | P | 6, 7, | 2 | 0.990 (x 2.021) | 17 (0.118) | 0.816 | defense response to Gram-positive bacterium | Def PGRP-SA |
| 524 | GO:0046039 | P | 10, | 1 | 0.233 (x 4.294) | 4 (0.250) | 0.816 | GTP metabolism | nmdyn-D6 |
| 525 | GO:0044267 | P | 6, | 128 | 121.046 (x 1.057) | 2079 (0.062) | 0.816 | cellular protein metabolism | Aats-his Abl Act57B Arf84F BG:DS02740.5 BcDNA:GH08420 CG10092 CG10104 CG10166 CG10237 CG10466 CG10738 CG11313 CG11360 CG11597 CG12133 CG12775 CG1299 CG1304 CG13318 CG14894 CG17266 CG18223 CG18749 CG18767 CG1883 CG1885 CG2056 CG2789 CG2998 CG30283 CG31704 CG32479 CG32627 CG33002 CG33128 CG33177 CG3355 CG3843 CG40045 CG40068 CG4046 CG4386 CG4408 CG4866 CG5001 CG5338 CG5382 CG6214 CG6461 CG6574 CG6723 CG6763 CG6764 CG7014 CG7770 CG8415 CG8550 CG8857 CG8918 CG9267 CG9372 CG9602 CG9804 Cyp12e1 Dab Dhc98D Doa EG:9D2.4 Gs2 Hsp22 Iap2 InR Las Obp58b PNUTS Pof Prosbeta5 REG RN-tre RpL11 RpL17A RpL27A RpL38 RpL46 RpL8 RpL9 RpP1 RpS17 RpS18 RpS4 RpS9 Ser7 Spn6 Sras Takl2 Timp apt betaggt-II btl cyp33 gatA hep jdp ksr l(3)01239 mRpL11 mRpL14 mRpL2 mRpL21 mRpL22 mRpL22-24 mRpL33 mRpS14 mRpS21 mRpS24 mRpS26 mRpS32 na oho23B pip pum rpr sda shu skpA sop tko |
| 526 | GO:0006914 | P | 4, | 2 | 0.990 (x 2.021) | 17 (0.118) | 0.817 | autophagy | CG10861 Eip93F |
| 527 | GO:0007429 | P | 5, 6, | 1 | 0.233 (x 4.294) | 4 (0.250) | 0.818 | secondary tracheal branching (sensu Insecta) | btl |
| 528 | GO:0009206 | P | 9, 10, | 6 | 3.959 (x 1.515) | 68 (0.088) | 0.818 | purine ribonucleoside triphosphate biosynthesis | CG31477 CG7211 Vha36 l(2)06225 nmdyn-D6 sun |
| 529 | GO:0008586 | P | 7, 8, 9, | 2 | 0.990 (x 2.021) | 17 (0.118) | 0.818 | wing vein morphogenesis | rho salm |
| 530 | GO:0016746 | F | 4, | 10 | 7.394 (x 1.352) | 127 (0.079) | 0.819 | transferase activity, transferring acyl groups | CG17821 CG5037 CG5122 CG5397 CG6461 CG6921 Hmgs Rpb4 betaggt-II fu12 |
| 531 | GO:0051252 | P | 7, | 5 | 3.144 (x 1.590) | 54 (0.093) | 0.819 | regulation of RNA metabolism | CG10418 CG11360 Doa hay pum |
| 532 | GO:0016829 | F | 3, | 12 | 9.141 (x 1.313) | 157 (0.076) | 0.819 | lyase activity | Ac78C CAH2 CG10184 CG10738 CG11251 CG1885 CG30022 CG4095 CG4592 CG8778 Hmgs SamDC |
| 533 | GO:0008316 | F | 3, | 1 | 0.233 (x 4.294) | 4 (0.250) | 0.819 | structural constituent of vitelline membrane (sensu Insecta) | Vm34Ca |
| 534 | GO:0043227 | C | 3, | 143 | 134.437 (x 1.064) | 2309 (0.062) | 0.82 | membrane-bound organelle | BEST:LD29214 Bro CG10166 CG10320 CG10418 CG10669 CG11015 CG11360 CG1140 CG11455 CG11909 CG12361 CG12400 CG1249 CG12605 CG13277 CG14482 CG14508 CG14691 CG15220 CG15398 CG17266 CG17280 CG17385 CG17768 CG18011 CG18013 CG18619 CG18767 CG2021 CG2789 CG31184 CG31272 CG31477 CG31611 CG31922 CG31950 CG32105 CG32174 CG32230 CG32409 CG33002 CG33066 CG3529 CG3931 CG4095 CG4169 CG4279 CG4592 CG4673 CG4769 CG4866 CG5037 CG5189 CG5548 CG6272 CG6610 CG7181 CG7211 CG7339 CG7834 CG7911 CG8004 CG8219 CG8506 CG8993 CG9650 CLIP-190 CoVa Cyp12e1 Cyp49a1 DebB Dhc98D Doa EG:152A3.7 EG:BACR7A4.18 Eip93F HP1c Hr38 Hr4 Kr-h1 Las Nipped-B Nxt1 Orc6 PNUTS Pdsw REG Rab3 Rlc1 RpII18 Rpb10 Rpb4 SmB Sras Ssb-c31a SytIV Syx16 Taf10b Taf11 TfIIEalpha Tfb2 Tim17b2 Tim9a Trap36 Vha36 apt bbx cyp33 dmrt93B e(y)2 gt hay l(1)10Bb l(2)06225 l(2)k10201 mRpL11 mRpL14 mRpL2 mRpL21 mRpL22 mRpL22-24 mRpL33 mRpL54 mRpS14 mRpS21 mRpS24 mRpS26 mRpS32 mirr mtacp1 pip porin rho robl rpr salm sisA sun tko toy unc-13 vvl |
| 535 | GO:0016478 | P | 8, 9, 10, | 2 | 0.990 (x 2.021) | 17 (0.118) | 0.82 | negative regulation of translation | apt pum |
| 536 | GO:0009145 | P | 8, 9, | 6 | 3.959 (x 1.515) | 68 (0.088) | 0.82 | purine nucleoside triphosphate biosynthesis | CG31477 CG7211 Vha36 l(2)06225 nmdyn-D6 sun |
| 537 | GO:0007635 | P | 4, 5, | 5 | 3.144 (x 1.590) | 54 (0.093) | 0.821 | chemosensory behavior | BG:DS01219.1 CG9381 Obp56a rho sra |
| 538 | GO:0017124 | F | 5, | 1 | 0.291 (x 3.435) | 5 (0.200) | 0.821 | SH3 domain binding | Dab |
| 539 | GO:0017090 | C | 3, 6, 7, 8, | 1 | 0.233 (x 4.294) | 4 (0.250) | 0.821 | meprin A complex | CG6763 |
| 540 | GO:0005543 | F | 4, | 3 | 1.572 (x 1.908) | 27 (0.111) | 0.821 | phospholipid binding | AnnX CG5559 SytIV |
| 541 | GO:0006979 | P | 4, 5, 6, | 2 | 0.990 (x 2.021) | 17 (0.118) | 0.821 | response to oxidative stress | CG13889 GstE1 |
| 542 | GO:0009201 | P | 8, 9, | 6 | 3.959 (x 1.515) | 68 (0.088) | 0.822 | ribonucleoside triphosphate biosynthesis | CG31477 CG7211 Vha36 l(2)06225 nmdyn-D6 sun |
| 543 | GO:0035270 | P | 4, | 1 | 0.291 (x 3.435) | 5 (0.200) | 0.822 | endocrine system development | gt |
| 544 | GO:0046528 | P | 6, 7, | 1 | 0.233 (x 4.294) | 4 (0.250) | 0.823 | imaginal disc fusion | hep |
| 545 | GO:0008146 | F | 5, | 2 | 0.990 (x 2.021) | 17 (0.118) | 0.823 | sulfotransferase activity | CG9164 pip |
| 546 | GO:0017177 | C | 3, 5, 6, 7, 8, 9, 10, | 1 | 0.291 (x 3.435) | 5 (0.200) | 0.824 | alpha-glucosidase II complex | CG11909 |
| 547 | GO:0004563 | F | 7, | 1 | 0.233 (x 4.294) | 4 (0.250) | 0.824 | beta-N-acetylhexosaminidase activity | CG15012 |
| 548 | GO:0016045 | P | 5, 6, | 1 | 0.291 (x 3.435) | 5 (0.200) | 0.825 | detection of bacterium | PGRP-SA |
| 549 | GO:0046051 | P | 10, | 1 | 0.233 (x 4.294) | 4 (0.250) | 0.826 | UTP metabolism | nmdyn-D6 |
| 550 | GO:0006596 | P | 8, 9, | 1 | 0.291 (x 3.435) | 5 (0.200) | 0.826 | polyamine biosynthesis | SamDC |
| 551 | GO:0030672 | C | 5, 6, 7, 8, 9, 10, 11, 12, 13, | 1 | 0.233 (x 4.294) | 4 (0.250) | 0.828 | synaptic vesicle membrane | CG31272 |
| 552 | GO:0050982 | P | 5, 6, | 1 | 0.291 (x 3.435) | 5 (0.200) | 0.828 | detection of mechanical stimulus | tko |
| 553 | GO:0042462 | P | 6, 7, 8, | 4 | 2.620 (x 1.527) | 45 (0.089) | 0.828 | eye photoreceptor cell development | Cpn Dab Doa chp |
| 554 | GO:0015326 | F | 5, 6, 7, | 1 | 0.291 (x 3.435) | 5 (0.200) | 0.829 | cationic amino acid transporter activity | CG5535 |
| 555 | GO:0006228 | P | 10, 11, | 1 | 0.233 (x 4.294) | 4 (0.250) | 0.83 | UTP biosynthesis | nmdyn-D6 |
| 556 | GO:0009218 | P | 8, | 1 | 0.291 (x 3.435) | 5 (0.200) | 0.831 | pyrimidine ribonucleotide metabolism | nmdyn-D6 |
| 557 | GO:0050817 | P | 3, | 1 | 0.233 (x 4.294) | 4 (0.250) | 0.831 | coagulation | AnnX |
| 558 | GO:0009605 | P | 3, | 8 | 6.113 (x 1.309) | 105 (0.076) | 0.832 | response to external stimulus | Arr2 Bc InR PGRP-SA Toll-7 hep sda tko |
| 559 | GO:0009595 | P | 4, 5, | 1 | 0.291 (x 3.435) | 5 (0.200) | 0.832 | detection of biotic stimulus | PGRP-SA |
| 560 | GO:0046844 | P | 10, 11, | 1 | 0.233 (x 4.294) | 4 (0.250) | 0.833 | micropyle formation | hep |
| 561 | GO:0007540 | P | 6, | 1 | 0.291 (x 3.435) | 5 (0.200) | 0.834 | sex determination, establishment of X:A ratio | sisA |
| 562 | GO:0004238 | F | 7, | 1 | 0.233 (x 4.294) | 4 (0.250) | 0.835 | meprin A activity | CG6763 |
| 563 | GO:0005623 | C | 2, | 276 | 268.757 (x 1.027) | 4616 (0.060) | 0.835 | cell | Aats-his Abl Ac78C Act57B Arp11 Arr2 BEST:LD29214 BG:DS01219.1 BG:DS02740.5 BG:DS02740.9 Bro CG10166 CG10237 CG10268 CG10320 CG10418 CG10669 CG10738 CG10804 CG10861 CG11015 CG11318 CG11360 CG1140 CG11455 CG11597 CG11897 CG11898 CG11909 CG1213 CG12361 CG12400 CG1249 CG12605 CG12775 CG12918 CG13277 CG14076 CG14482 CG14508 CG14691 CG15220 CG15398 CG15408 CG17224 CG17262 CG17266 CG17280 CG17385 CG1756 CG17768 CG17821 CG18011 CG18013 CG18619 CG18767 CG1883 CG2021 CG2789 CG2846 CG2998 CG3036 CG31184 CG31272 CG31477 CG31611 CG31922 CG31950 CG32105 CG3212 CG32174 CG32230 CG32409 CG32549 CG33002 CG33066 CG33543 CG3397 CG3529 CG3843 CG3931 CG4046 CG4095 CG4101 CG4169 CG4187 CG4279 CG4288 CG4592 CG4673 CG4769 CG4805 CG4866 CG5037 CG5189 CG5338 CG5382 CG5535 CG5548 CG6214 CG6272 CG6574 CG6610 CG6723 CG6763 CG6764 CG6891 CG6921 CG7014 CG7181 CG7188 CG7211 CG7333 CG7339 CG7770 CG7777 CG7834 CG7911 CG8004 CG8152 CG8219 CG8271 CG8415 CG8506 CG8550 CG8857 CG8916 CG8925 CG8993 CG9326 CG9413 CG9650 CLIP-190 Cad99C CoVa Con Cp36 Cpn Cyp12e1 Cyp28d2 Cyp311a1 Cyp49a1 Dab DebB Dhc98D Doa EG:152A3.7 EG:80H7.10 EG:9D2.4 EG:BACR7A4.18 EG:BACR7A4.8 Eip93F Femcoat GABA-B-R2 Gp150 Gr61a Gr98c Gs2 HP1c Hr38 Hr4 Iap2 InR Kr-h1 Las Mlc2 Mp20 Myo28B1 Nipped-B Nxt1 Or46a Or59a Or85a Or94a Or98b Orc6 PGRP-SA PNUTS Pdsw Pof Prosbeta5 REG Rab3 Rh7 Rlc1 RpII18 RpL11 RpL17A RpL27A RpL38 RpL46 RpL8 RpL9 RpP1 RpS17 RpS18 RpS4 RpS9 Rpb10 Rpb4 SmB Sod Sras Ssb-c31a SytIV Syx16 Syx8 Taf10b Taf11 Takr99D Tehao TfIIEalpha Tfb2 Tim17b2 Tim9a Tm1 Toll-7 Trap36 Tsp42El TyrR Vha36 apt bbx btl chp cyp33 dmrt93B dynactin-subunit-p25 e(y)2 fu12 gt hay hig insc inx7 l(1)10Bb l(2)03659 l(2)06225 l(2)k10201 l(3)01239 mRpL11 mRpL14 mRpL2 mRpL21 mRpL22 mRpL22-24 mRpL33 mRpL54 mRpS14 mRpS21 mRpS24 mRpS26 mRpS32 mira mirr mtacp1 na oho23B pip porin pum rho robl rpr salm shu sisA skpA sop sun tko toy unc-13 veli vvl zpg |
| 564 | GO:0008039 | P | 5, | 1 | 0.291 (x 3.435) | 5 (0.200) | 0.835 | synaptic target recognition | Con |
| 565 | GO:0005215 | F | 2, | 61 | 56.360 (x 1.082) | 968 (0.063) | 0.835 | transporter activity | Bc CG10237 CG10804 CG10950 CG11015 CG11897 CG11898 CG1213 CG14076 CG14482 CG14691 CG14935 CG15408 CG17280 CG1756 CG2789 CG3036 CG31272 CG31477 CG33066 CG3529 CG4071 CG4169 CG4288 CG4805 CG5535 CG6214 CG6574 CG6723 CG7084 CG7181 CG7211 CG7333 CG7777 CG8193 CG8219 CG8271 CG8498 CG8916 CG8925 CG9413 CoVa EG:9D2.4 Nxt1 SytIV Syx16 Syx8 Tim17b2 Tim9a Tsf1 Vha36 bbx glob1 inx7 l(2)03659 l(2)06225 mtacp1 na porin sun zpg |
| 566 | GO:0009205 | P | 9, | 6 | 4.017 (x 1.494) | 69 (0.087) | 0.836 | purine ribonucleoside triphosphate metabolism | CG31477 CG7211 Vha36 l(2)06225 nmdyn-D6 sun |
| 567 | GO:0015926 | F | 6, | 3 | 1.805 (x 1.662) | 31 (0.097) | 0.836 | glucosidase activity | Amyrel CG11909 CG14935 |
| 568 | GO:0051189 | P | 5, 7, | 5 | 3.493 (x 1.431) | 60 (0.083) | 0.836 | prosthetic group metabolism | CG10237 CG2789 CG6574 CG6723 Las |
| 569 | GO:0044464 | C | 2, 3, | 276 | 268.757 (x 1.027) | 4616 (0.060) | 0.836 | cell part | Aats-his Abl Ac78C Act57B Arp11 Arr2 BEST:LD29214 BG:DS01219.1 BG:DS02740.5 BG:DS02740.9 Bro CG10166 CG10237 CG10268 CG10320 CG10418 CG10669 CG10738 CG10804 CG10861 CG11015 CG11318 CG11360 CG1140 CG11455 CG11597 CG11897 CG11898 CG11909 CG1213 CG12361 CG12400 CG1249 CG12605 CG12775 CG12918 CG13277 CG14076 CG14482 CG14508 CG14691 CG15220 CG15398 CG15408 CG17224 CG17262 CG17266 CG17280 CG17385 CG1756 CG17768 CG17821 CG18011 CG18013 CG18619 CG18767 CG1883 CG2021 CG2789 CG2846 CG2998 CG3036 CG31184 CG31272 CG31477 CG31611 CG31922 CG31950 CG32105 CG3212 CG32174 CG32230 CG32409 CG32549 CG33002 CG33066 CG33543 CG3397 CG3529 CG3843 CG3931 CG4046 CG4095 CG4101 CG4169 CG4187 CG4279 CG4288 CG4592 CG4673 CG4769 CG4805 CG4866 CG5037 CG5189 CG5338 CG5382 CG5535 CG5548 CG6214 CG6272 CG6574 CG6610 CG6723 CG6763 CG6764 CG6891 CG6921 CG7014 CG7181 CG7188 CG7211 CG7333 CG7339 CG7770 CG7777 CG7834 CG7911 CG8004 CG8152 CG8219 CG8271 CG8415 CG8506 CG8550 CG8857 CG8916 CG8925 CG8993 CG9326 CG9413 CG9650 CLIP-190 Cad99C CoVa Con Cp36 Cpn Cyp12e1 Cyp28d2 Cyp311a1 Cyp49a1 Dab DebB Dhc98D Doa EG:152A3.7 EG:80H7.10 EG:9D2.4 EG:BACR7A4.18 EG:BACR7A4.8 Eip93F Femcoat GABA-B-R2 Gp150 Gr61a Gr98c Gs2 HP1c Hr38 Hr4 Iap2 InR Kr-h1 Las Mlc2 Mp20 Myo28B1 Nipped-B Nxt1 Or46a Or59a Or85a Or94a Or98b Orc6 PGRP-SA PNUTS Pdsw Pof Prosbeta5 REG Rab3 Rh7 Rlc1 RpII18 RpL11 RpL17A RpL27A RpL38 RpL46 RpL8 RpL9 RpP1 RpS17 RpS18 RpS4 RpS9 Rpb10 Rpb4 SmB Sod Sras Ssb-c31a SytIV Syx16 Syx8 Taf10b Taf11 Takr99D Tehao TfIIEalpha Tfb2 Tim17b2 Tim9a Tm1 Toll-7 Trap36 Tsp42El TyrR Vha36 apt bbx btl chp cyp33 dmrt93B dynactin-subunit-p25 e(y)2 fu12 gt hay hig insc inx7 l(1)10Bb l(2)03659 l(2)06225 l(2)k10201 l(3)01239 mRpL11 mRpL14 mRpL2 mRpL21 mRpL22 mRpL22-24 mRpL33 mRpL54 mRpS14 mRpS21 mRpS24 mRpS26 mRpS32 mira mirr mtacp1 na oho23B pip porin pum rho robl rpr salm shu sisA skpA sop sun tko toy unc-13 veli vvl zpg |
| 570 | GO:0035271 | P | 5, | 1 | 0.291 (x 3.435) | 5 (0.200) | 0.837 | ring gland development | gt |
| 571 | GO:0006826 | P | 9, 10, | 1 | 0.233 (x 4.294) | 4 (0.250) | 0.837 | iron ion transport | Tsf1 |
| 572 | GO:0016747 | F | 5, | 9 | 7.045 (x 1.278) | 121 (0.074) | 0.837 | transferase activity, transferring groups other than amino-acyl groups | CG17821 CG5037 CG5122 CG5397 CG6461 CG6921 Rpb4 betaggt-II fu12 |
| 573 | GO:0009142 | P | 7, 8, | 6 | 4.017 (x 1.494) | 69 (0.087) | 0.837 | nucleoside triphosphate biosynthesis | CG31477 CG7211 Vha36 l(2)06225 nmdyn-D6 sun |
| 574 | GO:0006006 | P | 8, 9, | 5 | 3.493 (x 1.431) | 60 (0.083) | 0.838 | glucose metabolism | CG2964 CG30499 CG5103 ImpL3 Pglym78 |
| 575 | GO:0009220 | P | 8, 9, | 1 | 0.291 (x 3.435) | 5 (0.200) | 0.838 | pyrimidine ribonucleotide biosynthesis | nmdyn-D6 |
| 576 | GO:0005006 | F | 7, 9, | 1 | 0.233 (x 4.294) | 4 (0.250) | 0.838 | epidermal growth factor receptor activity | InR |
| 577 | GO:0009144 | P | 8, | 6 | 4.017 (x 1.494) | 69 (0.087) | 0.839 | purine nucleoside triphosphate metabolism | CG31477 CG7211 Vha36 l(2)06225 nmdyn-D6 sun |
| 578 | GO:0004165 | F | 6, | 1 | 0.291 (x 3.435) | 5 (0.200) | 0.84 | dodecenoyl-CoA delta-isomerase activity | CG4592 |
| 579 | GO:0008063 | P | 6, | 3 | 1.747 (x 1.718) | 30 (0.100) | 0.84 | Toll signaling pathway | PGRP-SA Tehao pip |
| 580 | GO:0035277 | P | 4, 5, | 1 | 0.291 (x 3.435) | 5 (0.200) | 0.841 | spiracle morphogenesis | salm |
| 581 | GO:0009199 | P | 8, | 6 | 4.017 (x 1.494) | 69 (0.087) | 0.841 | ribonucleoside triphosphate metabolism | CG31477 CG7211 Vha36 l(2)06225 nmdyn-D6 sun |
| 582 | GO:0031589 | P | 4, | 3 | 1.747 (x 1.718) | 30 (0.100) | 0.841 | cell-substrate adhesion | BG:DS00180.7 CG6124 trol |
| 583 | GO:0030246 | F | 3, | 9 | 6.579 (x 1.368) | 113 (0.080) | 0.842 | carbohydrate binding | BG:DS02740.5 CG4115 CG6947 CG7298 CG8756 CG9095 Gasp Peritrophin-A lectin-28C |
| 584 | GO:0005991 | P | 8, | 1 | 0.291 (x 3.435) | 5 (0.200) | 0.842 | trehalose metabolism | CG5177 |
| 585 | GO:0007160 | P | 5, | 3 | 1.747 (x 1.718) | 30 (0.100) | 0.843 | cell-matrix adhesion | BG:DS00180.7 CG6124 trol |
| 586 | GO:0042742 | P | 5, 6, | 6 | 4.017 (x 1.494) | 69 (0.087) | 0.843 | defense response to bacterium | CG6426 CG6435 Def PGRP-SA Tehao Toll-7 |
| 587 | GO:0009008 | F | 6, | 1 | 0.291 (x 3.435) | 5 (0.200) | 0.844 | DNA-methyltransferase activity | agt |
| 588 | GO:0019201 | F | 7, | 3 | 1.747 (x 1.718) | 30 (0.100) | 0.844 | nucleotide kinase activity | CG5537 CG9326 nmdyn-D6 |
| 589 | GO:0042051 | P | 7, 8, 9, 10, | 4 | 2.562 (x 1.561) | 44 (0.091) | 0.845 | eye photoreceptor development (sensu Endopterygota) | Cpn Dab Doa chp |
| 590 | GO:0005865 | C | 5, 6, 7, 8, 9, 10, 11, | 1 | 0.291 (x 3.435) | 5 (0.200) | 0.845 | striated muscle thin filament | Tm1 |
| 591 | GO:0031968 | C | 4, 5, 6, 7, 8, 9, | 2 | 1.106 (x 1.808) | 19 (0.105) | 0.846 | organelle outer membrane | CG8004 porin |
| 592 | GO:0050974 | P | 5, 6, 7, | 1 | 0.291 (x 3.435) | 5 (0.200) | 0.847 | detection of mechanical stimulus during sensory perception | tko |
| 593 | GO:0051170 | P | 7, 8, 9, | 4 | 2.795 (x 1.431) | 48 (0.083) | 0.847 | nuclear import | CG10320 CG10950 CG8219 Nxt1 |
| 594 | GO:0019867 | C | 4, 5, 6, | 2 | 1.106 (x 1.808) | 19 (0.105) | 0.847 | outer membrane | CG8004 porin |
| 595 | GO:0005976 | P | 6, | 11 | 9.432 (x 1.166) | 162 (0.068) | 0.848 | polysaccharide metabolism | CG11909 CG18869 CG30438 CG6947 CG7298 CG8756 Chit Gasp Peritrophin-A Ugt86De Ugt86Di |
| 596 | GO:0045180 | C | 4, 5, 6, 7, 8, 9, 10, | 1 | 0.291 (x 3.435) | 5 (0.200) | 0.848 | basal cortex | mira |
| 597 | GO:0045176 | P | 6, | 1 | 0.408 (x 2.454) | 7 (0.143) | 0.848 | apical protein localization | insc |
| 598 | GO:0003707 | F | 5, | 2 | 1.106 (x 1.808) | 19 (0.105) | 0.849 | steroid hormone receptor activity | Hr38 Hr4 |
| 599 | GO:0035193 | P | 5, 6, | 1 | 0.408 (x 2.454) | 7 (0.143) | 0.85 | central nervous system remodeling (sensu Insecta) | rpr |
| 600 | GO:0004190 | F | 6, | 2 | 1.106 (x 1.808) | 19 (0.105) | 0.85 | aspartic-type endopeptidase activity | CG10104 CG33128 |
| 601 | GO:0016413 | F | 8, | 1 | 0.291 (x 3.435) | 5 (0.200) | 0.85 | O-acetyltransferase activity | CG5122 |
| 602 | GO:0007362 | P | 8, 9, | 2 | 1.223 (x 1.636) | 21 (0.095) | 0.851 | terminal region determination | gt tsl |
| 603 | GO:0035304 | P | 6, 7, 9, | 1 | 0.349 (x 2.863) | 6 (0.167) | 0.851 | regulation of protein amino acid dephosphorylation | PNUTS |
| 604 | GO:0045946 | P | 8, 9, 10, | 1 | 0.408 (x 2.454) | 7 (0.143) | 0.851 | positive regulation of translation | pum |
| 605 | GO:0016763 | F | 5, | 2 | 1.106 (x 1.808) | 19 (0.105) | 0.851 | transferase activity, transferring pentosyl groups | CG17224 CG5537 |
| 606 | GO:0016778 | F | 5, | 1 | 0.291 (x 3.435) | 5 (0.200) | 0.851 | diphosphotransferase activity | CG14721 |
| 607 | GO:0005741 | C | 5, 6, 7, 8, 9, 10, 11, 12, | 2 | 1.048 (x 1.908) | 18 (0.111) | 0.852 | mitochondrial outer membrane | CG8004 porin |
| 608 | GO:0016081 | P | 8, 9, 10, | 2 | 1.223 (x 1.636) | 21 (0.095) | 0.852 | synaptic vesicle docking during exocytosis | Syx16 Syx8 |
| 609 | GO:0016755 | F | 5, | 1 | 0.408 (x 2.454) | 7 (0.143) | 0.852 | transferase activity, transferring amino-acyl groups | CG6461 |
| 610 | GO:0005984 | P | 7, | 1 | 0.349 (x 2.863) | 6 (0.167) | 0.852 | disaccharide metabolism | CG5177 |
| 611 | GO:0008061 | F | 5, | 5 | 3.668 (x 1.363) | 63 (0.079) | 0.852 | chitin binding | CG6947 CG7298 CG8756 Gasp Peritrophin-A |
| 612 | GO:0009123 | P | 7, | 2 | 1.223 (x 1.636) | 21 (0.095) | 0.853 | nucleoside monophosphate metabolism | CG32626 Dhfr |
| 613 | GO:0007319 | P | 9, 10, 11, | 1 | 0.291 (x 3.435) | 5 (0.200) | 0.853 | negative regulation of oskar mRNA translation | apt |
| 614 | GO:0009127 | P | 8, 9, | 2 | 1.048 (x 1.908) | 18 (0.111) | 0.853 | purine nucleoside monophosphate biosynthesis | CG32626 Dhfr |
| 615 | GO:0016884 | F | 5, | 1 | 0.408 (x 2.454) | 7 (0.143) | 0.853 | carbon-nitrogen ligase activity, with glutamine as amido-N-donor | gatA |
| 616 | GO:0004661 | F | 7, | 1 | 0.349 (x 2.863) | 6 (0.167) | 0.853 | protein geranylgeranyltransferase activity | betaggt-II |
| 617 | GO:0046961 | F | 6, 7, 9, 14, | 5 | 3.668 (x 1.363) | 63 (0.079) | 0.854 | hydrogen-transporting ATPase activity, rotational mechanism | CG31477 CG7211 Vha36 l(2)06225 sun |
| 618 | GO:0003755 | F | 5, | 2 | 1.223 (x 1.636) | 21 (0.095) | 0.854 | peptidyl-prolyl cis-trans isomerase activity | CG17266 cyp33 |
| 619 | GO:0046916 | P | 8, | 1 | 0.408 (x 2.454) | 7 (0.143) | 0.854 | transition metal ion homeostasis | Tsf1 |
| 620 | GO:0007005 | P | 6, | 2 | 1.048 (x 1.908) | 18 (0.111) | 0.854 | mitochondrion organization and biogenesis | CG33066 Tim9a |
| 621 | GO:0016209 | F | 2, | 3 | 2.096 (x 1.431) | 36 (0.083) | 0.854 | antioxidant activity | CG13889 Prx6005 Sod |
| 622 | GO:0005381 | F | 6, | 1 | 0.291 (x 3.435) | 5 (0.200) | 0.855 | iron ion transporter activity | Tsf1 |
| 623 | GO:0045494 | P | 6, 8, | 1 | 0.349 (x 2.863) | 6 (0.167) | 0.855 | photoreceptor maintenance | Doa |
| 624 | GO:0046933 | F | 7, | 5 | 3.668 (x 1.363) | 63 (0.079) | 0.855 | hydrogen-transporting ATP synthase activity, rotational mechanism | CG31477 CG7211 Vha36 l(2)06225 sun |
| 625 | GO:0005436 | F | 6, 7, 8, | 2 | 1.223 (x 1.636) | 21 (0.095) | 0.855 | sodium:phosphate symporter activity | CG3036 CG4288 |
| 626 | GO:0009331 | C | 4, | 1 | 0.408 (x 2.454) | 7 (0.143) | 0.855 | glycerol-3-phosphate dehydrogenase complex | CG3215 |
| 627 | GO:0030247 | F | 4, | 5 | 3.901 (x 1.282) | 67 (0.075) | 0.856 | polysaccharide binding | CG6947 CG7298 CG8756 Gasp Peritrophin-A |
| 628 | GO:0015149 | F | 6, | 2 | 1.048 (x 1.908) | 18 (0.111) | 0.856 | hexose transporter activity | CG1213 CG15408 |
| 629 | GO:0019203 | F | 7, | 1 | 0.349 (x 2.863) | 6 (0.167) | 0.856 | carbohydrate phosphatase activity | CG5177 |
| 630 | GO:0016079 | P | 7, 8, 9, | 4 | 2.678 (x 1.494) | 46 (0.087) | 0.856 | synaptic vesicle exocytosis | SytIV Syx16 Syx8 unc-13 |
| 631 | GO:0007217 | P | 7, | 1 | 0.291 (x 3.435) | 5 (0.200) | 0.856 | tachykinin signaling pathway | Takr99D |
| 632 | GO:0006100 | P | 8, | 2 | 1.223 (x 1.636) | 21 (0.095) | 0.856 | tricarboxylic acid cycle intermediate metabolism | CG4095 ImpL3 |
| 633 | GO:0006879 | P | 8, 9, | 1 | 0.408 (x 2.454) | 7 (0.143) | 0.857 | iron ion homeostasis | Tsf1 |
| 634 | GO:0009167 | P | 9, | 2 | 1.048 (x 1.908) | 18 (0.111) | 0.857 | purine ribonucleoside monophosphate metabolism | CG32626 Dhfr |
| 635 | GO:0015355 | F | 6, | 1 | 0.349 (x 2.863) | 6 (0.167) | 0.857 | monocarboxylate porter activity | CG8271 |
| 636 | GO:0009124 | P | 7, 8, | 2 | 1.223 (x 1.636) | 21 (0.095) | 0.857 | nucleoside monophosphate biosynthesis | CG32626 Dhfr |
| 637 | GO:0016832 | F | 5, | 1 | 0.291 (x 3.435) | 5 (0.200) | 0.858 | aldehyde-lyase activity | CG10184 |
| 638 | GO:0016540 | P | 9, | 1 | 0.408 (x 2.454) | 7 (0.143) | 0.858 | protein autoprocessing | InR |
| 639 | GO:0019205 | F | 6, | 3 | 1.863 (x 1.610) | 32 (0.094) | 0.858 | nucleobase, nucleoside, nucleotide kinase activity | CG5537 CG9326 nmdyn-D6 |
| 640 | GO:0050954 | P | 4, 6, | 2 | 1.223 (x 1.636) | 21 (0.095) | 0.859 | sensory perception of mechanical stimulus | Myo28B1 tko |
| 641 | GO:0007296 | P | 6, | 1 | 0.349 (x 2.863) | 6 (0.167) | 0.859 | vitellogenesis | InR |
| 642 | GO:0009168 | P | 9, 10, | 2 | 1.048 (x 1.908) | 18 (0.111) | 0.859 | purine ribonucleoside monophosphate biosynthesis | CG32626 Dhfr |
| 643 | GO:0050654 | P | 6, 8, | 1 | 0.408 (x 2.454) | 7 (0.143) | 0.859 | chondroitin sulfate proteoglycan metabolism | Act57B |
| 644 | GO:0006743 | P | 8, | 1 | 0.291 (x 3.435) | 5 (0.200) | 0.859 | ubiquinone metabolism | CG32174 |
| 645 | GO:0030239 | P | 7, 8, 10, 11, | 1 | 0.349 (x 2.863) | 6 (0.167) | 0.86 | myofibril assembly | CG6803 |
| 646 | GO:0042023 | P | 9, | 1 | 0.408 (x 2.454) | 7 (0.143) | 0.86 | DNA endoreduplication | skpA |
| 647 | GO:0006368 | P | 9, | 2 | 1.048 (x 1.908) | 18 (0.111) | 0.86 | RNA elongation from RNA polymerase II promoter | CG7339 Tfb2 |
| 648 | GO:0006457 | P | 7, | 9 | 7.627 (x 1.180) | 131 (0.069) | 0.861 | protein folding | CG14894 CG17266 CG5001 CG7770 Hsp22 cyp33 jdp l(3)01239 shu |
| 649 | GO:0008199 | F | 7, | 1 | 0.408 (x 2.454) | 7 (0.143) | 0.861 | ferric iron binding | Tsf1 |
| 650 | GO:0016406 | F | 8, | 1 | 0.349 (x 2.863) | 6 (0.167) | 0.861 | carnitine O-acyltransferase activity | CG5122 |
| 651 | GO:0009116 | P | 6, | 2 | 1.048 (x 1.908) | 18 (0.111) | 0.861 | nucleoside metabolism | CG17224 CG8360 |
| 652 | GO:0004785 | F | 6, | 1 | 0.408 (x 2.454) | 7 (0.143) | 0.862 | copper, zinc superoxide dismutase activity | Sod |
| 653 | GO:0009147 | P | 8, | 1 | 0.349 (x 2.863) | 6 (0.167) | 0.863 | pyrimidine nucleoside triphosphate metabolism | nmdyn-D6 |
| 654 | GO:0004984 | F | 7, | 5 | 3.552 (x 1.408) | 61 (0.082) | 0.863 | olfactory receptor activity | Or46a Or59a Or85a Or94a Or98b |
| 655 | GO:0009126 | P | 8, | 2 | 1.048 (x 1.908) | 18 (0.111) | 0.863 | purine nucleoside monophosphate metabolism | CG32626 Dhfr |
| 656 | GO:0016411 | F | 8, | 1 | 0.408 (x 2.454) | 7 (0.143) | 0.864 | acylglycerol O-acyltransferase activity | fu12 |
| 657 | GO:0004192 | F | 7, | 1 | 0.349 (x 2.863) | 6 (0.167) | 0.864 | cathepsin D activity | CG10104 |
| 658 | GO:0005884 | C | 5, 6, 7, 8, 9, 10, | 2 | 1.048 (x 1.908) | 18 (0.111) | 0.864 | actin filament | Act57B Arp11 |
| 659 | GO:0006189 | P | 11, 12, | 1 | 0.408 (x 2.454) | 7 (0.143) | 0.865 | 'de novo' IMP biosynthesis | Dhfr |
| 660 | GO:0003841 | F | 9, | 1 | 0.349 (x 2.863) | 6 (0.167) | 0.865 | 1-acylglycerol-3-phosphate O-acyltransferase activity | fu12 |
| 661 | GO:0030206 | P | 8, 9, 10, | 1 | 0.408 (x 2.454) | 7 (0.143) | 0.866 | chondroitin sulfate biosynthesis | Act57B |
| 662 | GO:0006106 | P | 8, 9, | 1 | 0.349 (x 2.863) | 6 (0.167) | 0.867 | fumarate metabolism | CG4095 |
| 663 | GO:0042600 | C | 4, 5, | 1 | 0.408 (x 2.454) | 7 (0.143) | 0.867 | chorion | Cp36 |
| 664 | GO:0050877 | P | 4, | 39 | 35.865 (x 1.087) | 616 (0.063) | 0.867 | neurophysiological process | Arf84F Arr2 CG14691 CG1504 CG1756 CG18249 CG31146 CG31272 CG5559 CG5819 CG8916 Doa GABA-B-R2 Gp150 Gr61a Gr98c Gs2 Myo28B1 Obp56a Or46a Or59a Or85a Or94a Or98b Rab3 Rh7 SytIV Syx16 Syx8 Takr99D Tbh Tsp42El TyrR apt chp pum tko unc-13 veli |
| 665 | GO:0008329 | F | 4, | 1 | 0.349 (x 2.863) | 6 (0.167) | 0.868 | pattern recognition receptor activity | PGRP-SA |
| 666 | GO:0045263 | C | 3, 4, 5, 6, 7, | 1 | 0.408 (x 2.454) | 7 (0.143) | 0.868 | proton-transporting ATP synthase complex, coupling factor F(o) | l(2)06225 |
| 667 | GO:0045317 | P | 7, 8, 9, 10, | 1 | 0.349 (x 2.863) | 6 (0.167) | 0.869 | equator specification | mirr |
| 668 | GO:0006760 | P | 6, 7, 8, | 1 | 0.408 (x 2.454) | 7 (0.143) | 0.87 | folic acid and derivative metabolism | Dhfr |
| 669 | GO:0006396 | P | 7, | 17 | 14.847 (x 1.145) | 255 (0.067) | 0.87 | RNA processing | CG10418 CG10466 CG11360 CG1249 CG13277 CG17266 CG17768 CG2021 CG31184 CG3931 CG4279 CG6610 DebB Doa EG:BACR7A4.8 SmB hay |
| 670 | GO:0019538 | P | 5, | 131 | 127.101 (x 1.031) | 2183 (0.060) | 0.87 | protein metabolism | Aats-his Abl Act57B Arf84F BG:DS02740.5 BcDNA:GH08420 CG10092 CG10104 CG10166 CG10237 CG10466 CG10738 CG11313 CG11360 CG11597 CG12133 CG12775 CG1299 CG1304 CG13318 CG14894 CG17266 CG18223 CG18749 CG18767 CG1883 CG1885 CG2056 CG2789 CG2998 CG30283 CG31611 CG31704 CG32479 CG32627 CG33002 CG33128 CG33177 CG3355 CG3843 CG40045 CG40068 CG4046 CG4386 CG4408 CG4866 CG5001 CG5338 CG5382 CG6214 CG6461 CG6574 CG6723 CG6763 CG6764 CG6803 CG7014 CG7770 CG8415 CG8550 CG8857 CG8918 CG9267 CG9372 CG9602 CG9804 Cyp12e1 Dab Dhc98D Doa EG:9D2.4 EG:BACR7A4.18 Gs2 Hsp22 Iap2 InR Las Obp58b PNUTS Pof Prosbeta5 REG RN-tre RpL11 RpL17A RpL27A RpL38 RpL46 RpL8 RpL9 RpP1 RpS17 RpS18 RpS4 RpS9 Ser7 Spn6 Sras Takl2 Timp apt betaggt-II btl cyp33 gatA hep jdp ksr l(3)01239 mRpL11 mRpL14 mRpL2 mRpL21 mRpL22 mRpL22-24 mRpL33 mRpS14 mRpS21 mRpS24 mRpS26 mRpS32 na oho23B pip pum rpr sda shu skpA sop tko |
| 671 | GO:0000381 | P | 10, 11, 13, | 4 | 2.853 (x 1.402) | 49 (0.082) | 0.87 | regulation of alternative nuclear mRNA splicing, via spliceosome | CG10418 CG11360 Doa hay |
| 672 | GO:0006754 | P | 7, 8, 9, 10, 11, | 5 | 3.726 (x 1.342) | 64 (0.078) | 0.87 | ATP biosynthesis | CG31477 CG7211 Vha36 l(2)06225 sun |
| 673 | GO:0004500 | F | 6, | 1 | 0.349 (x 2.863) | 6 (0.167) | 0.871 | dopamine beta-monooxygenase activity | Tbh |
| 674 | GO:0004467 | F | 6, | 1 | 0.408 (x 2.454) | 7 (0.143) | 0.871 | long-chain-fatty-acid-CoA ligase activity | CG18155 |
| 675 | GO:0015020 | F | 6, | 3 | 1.980 (x 1.515) | 34 (0.088) | 0.871 | glucuronosyltransferase activity | Act57B Ugt86De Ugt86Di |
| 676 | GO:0008594 | P | 6, 7, 8, | 4 | 2.853 (x 1.402) | 49 (0.082) | 0.871 | photoreceptor cell morphogenesis (sensu Endopterygota) | Cpn Dab Doa chp |
| 677 | GO:0015986 | P | 7, 8, 9, 10, 11, 12, | 5 | 3.726 (x 1.342) | 64 (0.078) | 0.872 | ATP synthesis coupled proton transport | CG31477 CG7211 Vha36 l(2)06225 sun |
| 678 | GO:0042461 | P | 5, 6, 7, | 4 | 3.028 (x 1.321) | 52 (0.077) | 0.872 | photoreceptor cell development | Cpn Dab Doa chp |
| 679 | GO:0000276 | C | 4, 5, 6, 7, 8, 9, 10, 11, 12, 13, 14, | 1 | 0.408 (x 2.454) | 7 (0.143) | 0.872 | proton-transporting ATP synthase complex, coupling factor F(o) (sensu Eukaryota) | l(2)06225 |
| 680 | GO:0030381 | P | 4, 9, 10, | 1 | 0.349 (x 2.863) | 6 (0.167) | 0.872 | eggshell pattern formation (sensu Insecta) | hep |
| 681 | GO:0001763 | P | 4, | 3 | 1.980 (x 1.515) | 34 (0.088) | 0.872 | morphogenesis of a branching structure | apt btl rho |
| 682 | GO:0000380 | P | 10, 12, | 4 | 2.853 (x 1.402) | 49 (0.082) | 0.873 | alternative nuclear mRNA splicing, via spliceosome | CG10418 CG11360 Doa hay |
| 683 | GO:0015985 | P | 7, 8, 9, 10, | 5 | 3.726 (x 1.342) | 64 (0.078) | 0.873 | energy coupled proton transport, down electrochemical gradient | CG31477 CG7211 Vha36 l(2)06225 sun |
| 684 | GO:0019838 | F | 4, | 1 | 0.408 (x 2.454) | 7 (0.143) | 0.873 | growth factor binding | InR |
| 685 | GO:0004032 | F | 7, | 1 | 0.349 (x 2.863) | 6 (0.167) | 0.873 | aldehyde reductase activity | CG10638 |
| 686 | GO:0008320 | F | 4, | 2 | 1.164 (x 1.718) | 20 (0.100) | 0.874 | protein carrier activity | CG10950 CG8219 |
| 687 | GO:0006753 | P | 8, | 5 | 3.726 (x 1.342) | 64 (0.078) | 0.874 | nucleoside phosphate metabolism | CG31477 CG7211 Vha36 l(2)06225 sun |
| 688 | GO:0046040 | P | 10, | 1 | 0.408 (x 2.454) | 7 (0.143) | 0.874 | IMP metabolism | Dhfr |
| 689 | GO:0006611 | P | 8, 9, 10, | 1 | 0.349 (x 2.863) | 6 (0.167) | 0.875 | protein export from nucleus | Nxt1 |
| 690 | GO:0030030 | P | 5, 6, | 4 | 2.736 (x 1.462) | 47 (0.085) | 0.875 | cell projection organization and biogenesis | CG14825 CG2069 btl hep |
| 691 | GO:0044264 | P | 6, 7, | 6 | 4.483 (x 1.338) | 77 (0.078) | 0.875 | cellular polysaccharide metabolism | CG6947 CG7298 CG8756 Chit Gasp Peritrophin-A |
| 692 | GO:0009156 | P | 8, 9, | 2 | 1.164 (x 1.718) | 20 (0.100) | 0.875 | ribonucleoside monophosphate biosynthesis | CG32626 Dhfr |
| 693 | GO:0030204 | P | 7, 8, 9, | 1 | 0.408 (x 2.454) | 7 (0.143) | 0.876 | chondroitin sulfate metabolism | Act57B |
| 694 | GO:0016835 | F | 4, | 5 | 3.610 (x 1.385) | 62 (0.081) | 0.876 | carbon-oxygen lyase activity | CAH2 CG1885 CG4095 CG4592 CG8778 |
| 695 | GO:0046622 | P | 5, | 1 | 0.349 (x 2.863) | 6 (0.167) | 0.876 | positive regulation of organ size | InR |
| 696 | GO:0005529 | F | 4, | 4 | 2.736 (x 1.462) | 47 (0.085) | 0.876 | sugar binding | BG:DS02740.5 CG4115 CG9095 lectin-28C |
| 697 | GO:0046034 | P | 6, 10, | 5 | 3.784 (x 1.321) | 65 (0.077) | 0.876 | ATP metabolism | CG31477 CG7211 Vha36 l(2)06225 sun |
| 698 | GO:0016814 | F | 5, | 2 | 1.164 (x 1.718) | 20 (0.100) | 0.877 | hydrolase activity, acting on carbon-nitrogen (but not peptide) bonds, in cyclic amidines | CG32626 CG8360 |
| 699 | GO:0009065 | P | 8, 9, | 1 | 0.408 (x 2.454) | 7 (0.143) | 0.877 | glutamine family amino acid catabolism | Gs2 |
| 700 | GO:0001871 | F | 3, | 6 | 4.658 (x 1.288) | 80 (0.075) | 0.877 | pattern binding | CG6947 CG7298 CG8756 Gasp PGRP-SA Peritrophin-A |
| 701 | GO:0045175 | P | 6, | 1 | 0.349 (x 2.863) | 6 (0.167) | 0.878 | basal protein localization | insc |
| 702 | GO:0009161 | P | 8, | 2 | 1.164 (x 1.718) | 20 (0.100) | 0.878 | ribonucleoside monophosphate metabolism | CG32626 Dhfr |
| 703 | GO:0000808 | C | 3, 5, 6, 7, 8, 9, 10, | 1 | 0.408 (x 2.454) | 7 (0.143) | 0.878 | origin recognition complex | Orc6 |
| 704 | GO:0019318 | P | 7, 8, | 6 | 4.658 (x 1.288) | 80 (0.075) | 0.878 | hexose metabolism | BcDNA:GH08902 CG2964 CG30499 CG5103 ImpL3 Pglym78 |
| 705 | GO:0016469 | C | 3, 6, 7, 8, | 5 | 3.959 (x 1.263) | 68 (0.074) | 0.879 | proton-transporting two-sector ATPase complex | CG31477 CG7211 Vha36 l(2)06225 sun |
| 706 | GO:0016500 | F | 7, | 1 | 0.349 (x 2.863) | 6 (0.167) | 0.879 | protein-hormone receptor activity | CG4187 |
| 707 | GO:0008150 | P | 1, | 474 | 470.558 (x 1.007) | 8082 (0.059) | 0.879 | biological\_process | Aats-his Abl Ac78C Act57B Ahcy13 Amyrel AnnX Arf84F Arp11 Arr2 BG:DS00180.7 BG:DS01219.1 BG:DS02740.5 BG:DS02740.9 Bc BcDNA:GH08420 BcDNA:GH08902 Bro CAH2 CG10092 CG10104 CG10166 CG10184 CG10237 CG10268 CG10320 CG10418 CG10425 CG10433 CG10460 CG10466 CG10638 CG10669 CG10674 CG10738 CG10804 CG10861 CG10950 CG10962 CG11015 CG11251 CG11313 CG11318 CG11360 CG1140 CG11455 CG11597 CG11722 CG11897 CG11898 CG11909 CG12022 CG1213 CG12133 CG12175 CG12361 CG12400 CG1249 CG12605 CG12775 CG1299 CG1304 CG13277 CG13318 CG13691 CG13889 CG14482 CG14508 CG14691 CG14701 CG14721 CG14825 CG14894 CG14935 CG1504 CG15220 CG15361 CG15398 CG15408 CG16817 CG17224 CG17262 CG17266 CG17280 CG17385 CG1756 CG17639 CG17768 CG17821 CG1796 CG18011 CG18013 CG18155 CG18223 CG18249 CG18522 CG18530 CG18619 CG18749 CG18767 CG1883 CG1885 CG18869 CG1942 CG2021 CG2056 CG2069 CG2185 CG2277 CG2750 CG2789 CG2846 CG2964 CG2998 CG30022 CG30105 CG30126 CG30154 CG30283 CG30334 CG30343 CG3036 CG30438 CG30476 CG30499 CG31146 CG31184 CG31272 CG31477 CG31551 CG31601 CG31611 CG31704 CG31715 CG31922 CG31957 CG32023 CG32105 CG3212 CG3215 CG32160 CG32174 CG32175 CG32202 CG32207 CG32230 CG32409 CG32442 CG32448 CG32479 CG32549 CG32582 CG32625 CG32626 CG32627 CG32637 CG32677 CG32692 CG32710 CG32856 CG33002 CG33066 CG33128 CG33177 CG33322 CG33543 CG3355 CG3397 CG3529 CG3843 CG3887 CG3931 CG40045 CG40068 CG4046 CG4071 CG4095 CG4101 CG4169 CG4187 CG4279 CG4288 CG4386 CG4408 CG4511 CG4592 CG4673 CG4769 CG4805 CG4827 CG4866 CG5001 CG5037 CG5103 CG5122 CG5162 CG5177 CG5224 CG5338 CG5382 CG5397 CG5535 CG5537 CG5548 CG5559 CG5819 CG6124 CG6214 CG6272 CG6296 CG6426 CG6432 CG6435 CG6461 CG6574 CG6610 CG6723 CG6763 CG6764 CG6803 CG6921 CG6947 CG7014 CG7084 CG7181 CG7188 CG7194 CG7211 CG7298 CG7322 CG7333 CG7339 CG7646 CG7770 CG7777 CG7834 CG7949 CG8004 CG8152 CG8193 CG8219 CG8271 CG8360 CG8415 CG8498 CG8550 CG8756 CG8778 CG8857 CG8916 CG8918 CG8925 CG8993 CG9095 CG9267 CG9326 CG9372 CG9381 CG9413 CG9602 CG9629 CG9650 CG9790 CG9804 CG9836 CG9862 CLIP-190 Cad99C Chit CoVa Con Cp36 Cpn CycG Cyp12e1 Cyp28d2 Cyp311a1 Cyp49a1 Dab DebB Def Dgkepsilon Dhc98D Dhfr Doa EG:152A3.7 EG:52C10.2 EG:63B12.12 EG:80H7.10 EG:9D2.4 EG:BACR7A4.14 EG:BACR7A4.18 EG:BACR7A4.8 Eip93F Femcoat GABA-B-R2 Gasp Gp150 Gr61a Gr98c Gs2 GstD6 GstD9 GstE1 GstE5 GstE6 GstE7 HP1c Hmgs Hr38 Hr4 Hsp22 Iap2 Idgf1 Idgf2 ImpL3 InR JhI-26 Karl Kr-h1 Las Lip1 Mlc2 Mp20 Myo28B1 NP15.6 Nipped-B Nxt1 Obp56a Obp58b Or46a Or59a Or85a Or94a Or98b Orc6 PGRP-SA PNUTS Pdsw Peritrophin-A Pglym78 Pld Pof Prosbeta5 Prx6005 REG RN-tre Rab3 Rep2 Rh7 RpII18 RpL11 RpL17A RpL27A RpL38 RpL46 RpL8 RpL9 RpP1 RpS17 RpS18 RpS4 RpS9 Rpb10 Rpb4 SIP1 SamDC SelG Ser7 Sgs4 SmB Sod Spn6 Sras Ssb-c31a SytIV Syx16 Syx8 Taf10b Taf11 Takl2 Takr99D Tbh Tehao TfIIEalpha Tfb2 Tig Tim17b2 Tim9a Timp Tm1 Toll-7 TpnC41C Trap36 Tsf1 Tsp42El TyrR Ugt86De Ugt86Di Vha36 Vm34Ca agt apt bbx betaggt-II btl chp cyp33 dmrt93B dynactin-subunit-p25 e(y)2 fau fu12 gatA glob1 gt hay hep hig insc inx7 jdp ksr l(2)03659 l(2)06225 l(2)k10201 l(3)01239 l(3)02640 lectin-28C mRpL11 mRpL14 mRpL2 mRpL21 mRpL22 mRpL22-24 mRpL33 mRpS14 mRpS21 mRpS24 mRpS26 mRpS32 mira mirr mtacp1 na nmdyn-D6 oho23B pip porin pum ran-like regucalcin retinin rho robl rpr salm sda shu sisA skpA sop sra sun tko toy trol tsl unc-13 upd3 veli vvl zormin zpg |
| 708 | GO:0015145 | F | 5, | 2 | 1.164 (x 1.718) | 20 (0.100) | 0.879 | monosaccharide transporter activity | CG1213 CG15408 |
| 709 | GO:0016801 | F | 4, | 1 | 0.408 (x 2.454) | 7 (0.143) | 0.879 | hydrolase activity, acting on ether bonds | Ahcy13 |
| 710 | GO:0005554 | F | 2, | 47 | 43.784 (x 1.073) | 752 (0.062) | 0.879 | molecular function unknown | CG10674 CG10861 CG11722 CG12022 CG13691 CG14701 CG14825 CG16817 CG2069 CG30105 CG30126 CG30154 CG30334 CG30343 CG30476 CG31601 CG31715 CG32023 CG32160 CG32174 CG32175 CG32202 CG32207 CG32442 CG32448 CG32582 CG32625 CG32637 CG32692 CG32710 CG32856 CG33322 CG33543 CG3887 CG4101 CG7194 CG7949 CG9381 EG:63B12.12 JhI-26 Karl NP15.6 Rep2 SIP1 SelG fau retinin |
| 711 | GO:0030135 | C | 6, 7, 8, 9, 10, | 5 | 3.959 (x 1.263) | 68 (0.074) | 0.88 | coated vesicle | CG14691 CG31272 Rab3 SytIV unc-13 |
| 712 | GO:0006796 | P | 6, | 38 | 35.167 (x 1.081) | 604 (0.063) | 0.88 | phosphate metabolism | Abl CG10320 CG10738 CG11015 CG11455 CG11597 CG12400 CG14482 CG14508 CG17280 CG2056 CG3036 CG31477 CG32230 CG4169 CG4288 CG4769 CG5548 CG6214 CG7181 CG7211 CG7834 CG9267 CoVa Dgkepsilon Doa EG:152A3.7 InR PNUTS Pdsw Takl2 Vha36 btl hep ksr l(2)06225 mtacp1 sun |
| 713 | GO:0004743 | F | 6, | 1 | 0.349 (x 2.863) | 6 (0.167) | 0.88 | pyruvate kinase activity | CG2964 |
| 714 | GO:0016877 | F | 4, | 2 | 1.164 (x 1.718) | 20 (0.100) | 0.88 | ligase activity, forming carbon-sulfur bonds | CG18155 CG6432 |
| 715 | GO:0005501 | F | 4, | 1 | 0.408 (x 2.454) | 7 (0.143) | 0.88 | retinoid binding | CG10237 |
| 716 | GO:0005996 | P | 6, 7, | 7 | 5.822 (x 1.202) | 100 (0.070) | 0.88 | monosaccharide metabolism | BcDNA:GH08902 CG11909 CG2964 CG30499 CG5103 ImpL3 Pglym78 |
| 717 | GO:0006793 | P | 5, | 38 | 35.167 (x 1.081) | 604 (0.063) | 0.881 | phosphorus metabolism | Abl CG10320 CG10738 CG11015 CG11455 CG11597 CG12400 CG14482 CG14508 CG17280 CG2056 CG3036 CG31477 CG32230 CG4169 CG4288 CG4769 CG5548 CG6214 CG7181 CG7211 CG7834 CG9267 CoVa Dgkepsilon Doa EG:152A3.7 InR PNUTS Pdsw Takl2 Vha36 btl hep ksr l(2)06225 mtacp1 sun |
| 718 | GO:0007402 | P | 5, 6, | 1 | 0.408 (x 2.454) | 7 (0.143) | 0.882 | ganglion mother cell fate determination | mira |
| 719 | GO:0016126 | P | 7, 8, 9, | 1 | 0.349 (x 2.863) | 6 (0.167) | 0.882 | sterol biosynthesis | CG10268 |
| 720 | GO:0007605 | P | 5, 7, | 2 | 1.164 (x 1.718) | 20 (0.100) | 0.882 | sensory perception of sound | Myo28B1 tko |
| 721 | GO:0004197 | F | 6, | 6 | 4.716 (x 1.272) | 81 (0.074) | 0.882 | cysteine-type endopeptidase activity | CG1885 CG32479 CG6357 Dab Obp58b Ser7 |
| 722 | GO:0008595 | P | 6, 7, | 4 | 2.911 (x 1.374) | 50 (0.080) | 0.882 | determination of anterior/posterior axis, embryo | gt ksr pum tsl |
| 723 | GO:0006271 | P | 9, | 1 | 0.408 (x 2.454) | 7 (0.143) | 0.883 | DNA strand elongation | CG18013 |
| 724 | GO:0005484 | F | 4, | 2 | 1.164 (x 1.718) | 20 (0.100) | 0.883 | SNAP receptor activity | Syx16 Syx8 |
| 725 | GO:0046008 | P | 5, 6, 7, | 1 | 0.349 (x 2.863) | 6 (0.167) | 0.883 | regulation of female receptivity, post-mating | sra |
| 726 | GO:0007351 | P | 5, 6, | 4 | 2.911 (x 1.374) | 50 (0.080) | 0.883 | regional subdivision | gt ksr pum tsl |
| 727 | GO:0007205 | P | 8, 9, | 1 | 0.466 (x 2.147) | 8 (0.125) | 0.884 | protein kinase C activation | Dgkepsilon |
| 728 | GO:0022008 | P | 5, | 13 | 11.237 (x 1.157) | 193 (0.067) | 0.884 | neurogenesis | Abl BG:DS02740.9 Con InR Tig Tm1 btl insc mira pum robl trol vvl |
| 729 | GO:0031328 | P | 7, | 1 | 0.408 (x 2.454) | 7 (0.143) | 0.884 | positive regulation of cellular biosynthesis | pum |
| 730 | GO:0006730 | P | 5, | 2 | 1.164 (x 1.718) | 20 (0.100) | 0.884 | one-carbon compound metabolism | Ahcy13 CAH2 |
| 731 | GO:0006595 | P | 7, 8, | 1 | 0.349 (x 2.863) | 6 (0.167) | 0.884 | polyamine metabolism | SamDC |
| 732 | GO:0004806 | F | 7, | 3 | 2.154 (x 1.393) | 37 (0.081) | 0.885 | triacylglycerol lipase activity | CG18530 CG6296 Lip1 |
| 733 | GO:0004263 | F | 7, | 14 | 12.110 (x 1.156) | 208 (0.067) | 0.885 | chymotrypsin activity | BcDNA:GH08420 CG11313 CG12133 CG1299 CG1304 CG13318 CG18223 CG2056 CG30283 CG3355 CG4386 CG9372 EG:9D2.4 Ser7 |
| 734 | GO:0042136 | P | 6, 9, | 1 | 0.466 (x 2.147) | 8 (0.125) | 0.885 | neurotransmitter biosynthesis | Tbh |
| 735 | GO:0042049 | P | 5, 8, 9, | 1 | 0.408 (x 2.454) | 7 (0.143) | 0.885 | cell acyl-CoA homeostasis | CG8498 |
| 736 | GO:0008066 | F | 5, | 3 | 2.212 (x 1.356) | 38 (0.079) | 0.885 | glutamate receptor activity | CG14076 EG:30B8.6 GABA-B-R2 |
| 737 | GO:0035303 | P | 8, | 1 | 0.349 (x 2.863) | 6 (0.167) | 0.886 | regulation of dephosphorylation | PNUTS |
| 738 | GO:0004896 | F | 5, | 1 | 0.466 (x 2.147) | 8 (0.125) | 0.886 | hematopoietin/interferon-class (D200-domain) cytokine receptor activity | InR |
| 739 | GO:0008234 | F | 5, | 7 | 5.415 (x 1.293) | 93 (0.075) | 0.886 | cysteine-type peptidase activity | CG1885 CG32479 CG6357 Dab Obp58b RN-tre Ser7 |
| 740 | GO:0045727 | P | 7, 8, 9, | 1 | 0.408 (x 2.454) | 7 (0.143) | 0.887 | positive regulation of protein biosynthesis | pum |
| 741 | GO:0000177 | C | 4, 5, 6, 7, 8, | 1 | 0.466 (x 2.147) | 8 (0.125) | 0.887 | cytoplasmic exosome (RNase complex) | CG3931 |
| 742 | GO:0015226 | F | 4, | 1 | 0.349 (x 2.863) | 6 (0.167) | 0.887 | carnitine transporter activity | CG8925 |
| 743 | GO:0005664 | C | 4, 6, 7, 8, 9, 10, 11, 12, | 1 | 0.408 (x 2.454) | 7 (0.143) | 0.888 | nuclear origin of replication recognition complex | Orc6 |
| 744 | GO:0006403 | P | 4, | 7 | 5.415 (x 1.293) | 93 (0.075) | 0.888 | RNA localization | CG10320 CG9862 Nxt1 Tm1 insc mira ran-like |
| 745 | GO:0007425 | P | 5, 6, | 1 | 0.466 (x 2.147) | 8 (0.125) | 0.888 | tracheal epithelial cell fate determination (sensu Insecta) | vvl |
| 746 | GO:0006066 | P | 5, | 11 | 9.374 (x 1.173) | 161 (0.068) | 0.888 | alcohol metabolism | BcDNA:GH08902 CG10268 CG11909 CG2964 CG30499 CG3215 CG5103 Hmgs ImpL3 Pglym78 Tbh |
| 747 | GO:0004034 | F | 6, | 1 | 0.349 (x 2.863) | 6 (0.167) | 0.889 | aldose 1-epimerase activity | BcDNA:GH08902 |
| 748 | GO:0003713 | F | 4, 6, | 2 | 1.281 (x 1.561) | 22 (0.091) | 0.889 | transcription coactivator activity | Bro Ssb-c31a |
| 749 | GO:0000159 | C | 4, 5, 6, | 1 | 0.408 (x 2.454) | 7 (0.143) | 0.889 | protein phosphatase type 2A complex | CG11597 |
| 750 | GO:0006582 | P | 6, | 1 | 0.466 (x 2.147) | 8 (0.125) | 0.889 | melanin metabolism | Bc |
| 751 | GO:0050874 | P | 3, | 55 | 52.401 (x 1.050) | 900 (0.061) | 0.89 | organismal physiological process | Arf84F Arr2 CG14691 CG1504 CG1756 CG18249 CG31146 CG31272 CG5559 CG5819 CG8916 Def Doa GABA-B-R2 Gp150 Gr61a Gr98c Gs2 Hsp22 InR Mlc2 Mp20 Myo28B1 Obp56a Or46a Or59a Or85a Or94a Or98b PGRP-SA Rab3 Rh7 SelG Sgs4 Sod SytIV Syx16 Syx8 Takr99D Tbh Tehao Tm1 Toll-7 TpnC41C Tsp42El TyrR apt chp hep pum tko unc-13 upd3 veli zormin |
| 752 | GO:0006354 | P | 8, | 2 | 1.281 (x 1.561) | 22 (0.091) | 0.89 | RNA elongation | CG7339 Tfb2 |
| 753 | GO:0016918 | F | 4, 5, | 1 | 0.349 (x 2.863) | 6 (0.167) | 0.89 | retinal binding | CG10237 |
| 754 | GO:0009891 | P | 6, | 1 | 0.408 (x 2.454) | 7 (0.143) | 0.89 | positive regulation of biosynthesis | pum |
| 755 | GO:0004602 | F | 4, 6, | 1 | 0.466 (x 2.147) | 8 (0.125) | 0.891 | glutathione peroxidase activity | Prx6005 |
| 756 | GO:0006936 | P | 4, | 6 | 5.007 (x 1.198) | 86 (0.070) | 0.891 | muscle contraction | Mlc2 Mp20 Tm1 TpnC41C TyrR zormin |
| 757 | GO:0007427 | P | 5, 6, 7, | 2 | 1.281 (x 1.561) | 22 (0.091) | 0.891 | tracheal epithelial cell migration (sensu Insecta) | btl salm |
| 758 | GO:0042026 | P | 8, | 1 | 0.349 (x 2.863) | 6 (0.167) | 0.892 | protein refolding | Hsp22 |
| 759 | GO:0019840 | F | 3, | 1 | 0.408 (x 2.454) | 7 (0.143) | 0.892 | isoprenoid binding | CG10237 |
| 760 | GO:0008356 | P | 5, | 4 | 3.086 (x 1.296) | 53 (0.075) | 0.892 | asymmetric cell division | insc mira pum trol |
| 761 | GO:0008020 | F | 5, 7, | 1 | 0.466 (x 2.147) | 8 (0.125) | 0.892 | G-protein coupled photoreceptor activity | Rh7 |
| 762 | GO:0004867 | F | 6, | 5 | 4.076 (x 1.227) | 70 (0.071) | 0.892 | serine-type endopeptidase inhibitor activity | CG1342 CG16712 CG31704 CG5639 Spn6 |
| 763 | GO:0050684 | P | 8, 9, | 4 | 3.086 (x 1.296) | 53 (0.075) | 0.893 | regulation of mRNA processing | CG10418 CG11360 Doa hay |
| 764 | GO:0051183 | F | 3, | 1 | 0.408 (x 2.454) | 7 (0.143) | 0.893 | vitamin transporter activity | CG8925 |
| 765 | GO:0004385 | F | 6, 8, | 1 | 0.466 (x 2.147) | 8 (0.125) | 0.893 | guanylate kinase activity | CG9326 |
| 766 | GO:0006544 | P | 8, 9, | 1 | 0.349 (x 2.863) | 6 (0.167) | 0.893 | glycine metabolism | Dhfr |
| 767 | GO:0048024 | P | 9, 10, 12, | 4 | 3.086 (x 1.296) | 53 (0.075) | 0.894 | regulation of nuclear mRNA splicing, via spliceosome | CG10418 CG11360 Doa hay |
| 768 | GO:0006967 | P | 7, 8, 9, | 1 | 0.466 (x 2.147) | 8 (0.125) | 0.894 | positive regulation of antifungal peptide biosynthesis | Tehao |
| 769 | GO:0008083 | F | 4, 5, | 3 | 1.921 (x 1.561) | 33 (0.091) | 0.894 | growth factor activity | Chit Idgf1 Idgf2 |
| 770 | GO:0015645 | F | 5, | 1 | 0.408 (x 2.454) | 7 (0.143) | 0.894 | fatty-acid ligase activity | CG18155 |
| 771 | GO:0004040 | F | 6, | 1 | 0.349 (x 2.863) | 6 (0.167) | 0.894 | amidase activity | gatA |
| 772 | GO:0019725 | P | 4, | 5 | 3.843 (x 1.301) | 66 (0.076) | 0.895 | cell homeostasis | CG7777 CG8498 Prx6005 SelG Tsf1 |
| 773 | GO:0000062 | F | 5, | 1 | 0.466 (x 2.147) | 8 (0.125) | 0.895 | acyl-CoA binding | CG8498 |
| 774 | GO:0007584 | P | 5, 6, | 1 | 0.408 (x 2.454) | 7 (0.143) | 0.895 | response to nutrient | InR |
| 775 | GO:0019829 | F | 5, 6, 8, 13, | 5 | 4.017 (x 1.245) | 69 (0.072) | 0.895 | cation-transporting ATPase activity | CG31477 CG7211 Vha36 l(2)06225 sun |
| 776 | GO:0009611 | P | 4, | 3 | 1.921 (x 1.561) | 33 (0.091) | 0.895 | response to wounding | Bc Toll-7 hep |
| 777 | GO:0004995 | F | 6, 7, 9, | 1 | 0.349 (x 2.863) | 6 (0.167) | 0.896 | tachykinin receptor activity | Takr99D |
| 778 | GO:0030054 | C | 5, 6, 7, | 5 | 3.843 (x 1.301) | 66 (0.076) | 0.896 | cell junction | Abl InR inx7 veli zpg |
| 779 | GO:0005666 | C | 4, 5, 6, 7, 8, 9, 10, 11, 12, 13, | 1 | 0.466 (x 2.147) | 8 (0.125) | 0.896 | DNA-directed RNA polymerase III complex | CG7339 |
| 780 | GO:0042393 | F | 4, | 1 | 0.408 (x 2.454) | 7 (0.143) | 0.897 | histone binding | EG:BACR7A4.18 |
| 781 | GO:0008293 | P | 8, | 3 | 1.921 (x 1.561) | 33 (0.091) | 0.897 | torso signaling pathway | gt ksr tsl |
| 782 | GO:0044428 | C | 4, 5, 6, 7, 8, 9, | 32 | 29.985 (x 1.067) | 515 (0.062) | 0.897 | nuclear part | Bro CG10418 CG1249 CG13277 CG15220 CG15398 CG17266 CG17768 CG2021 CG31184 CG31922 CG31950 CG3931 CG4279 CG4673 CG6610 CG7339 CG8219 DebB Nxt1 Orc6 RpII18 Rpb10 Rpb4 SmB Ssb-c31a Taf10b Taf11 TfIIEalpha Tfb2 Trap36 hay |
| 783 | GO:0006801 | P | 6, | 1 | 0.466 (x 2.147) | 8 (0.125) | 0.897 | superoxide metabolism | Sod |
| 784 | GO:0015929 | F | 6, | 1 | 0.408 (x 2.454) | 7 (0.143) | 0.898 | hexosaminidase activity | CG15012 |
| 785 | GO:0006402 | P | 8, | 1 | 0.524 (x 1.908) | 9 (0.111) | 0.899 | mRNA catabolism | pum |
| 786 | GO:0005372 | F | 3, | 1 | 0.466 (x 2.147) | 8 (0.125) | 0.899 | water transporter activity | CG7777 |
| 787 | GO:0009119 | P | 7, | 1 | 0.408 (x 2.454) | 7 (0.143) | 0.899 | ribonucleoside metabolism | CG8360 |
| 788 | GO:0008507 | F | 7, 8, 9, 10, | 1 | 0.524 (x 1.908) | 9 (0.111) | 0.9 | sodium:iodide symporter activity | CG6723 |
| 789 | GO:0016868 | F | 5, | 1 | 0.466 (x 2.147) | 8 (0.125) | 0.9 | intramolecular transferase activity, phosphotransferases | Pglym78 |
| 790 | GO:0009299 | P | 8, | 2 | 1.339 (x 1.494) | 23 (0.087) | 0.9 | mRNA transcription | RpII18 Rpb4 |
| 791 | GO:0050650 | P | 7, 8, 9, | 1 | 0.408 (x 2.454) | 7 (0.143) | 0.9 | chondroitin sulfate proteoglycan biosynthesis | Act57B |
| 792 | GO:0016715 | F | 5, | 1 | 0.524 (x 1.908) | 9 (0.111) | 0.901 | oxidoreductase activity, acting on paired donors, with incorporation or reduction of molecular oxygen, reduced ascorbate as one donor, and incorporation of one atom of oxygen | Tbh |
| 793 | GO:0030312 | C | 3, 4, | 1 | 0.466 (x 2.147) | 8 (0.125) | 0.901 | external encapsulating structure | Cp36 |
| 794 | GO:0016859 | F | 4, | 2 | 1.339 (x 1.494) | 23 (0.087) | 0.901 | cis-trans isomerase activity | CG17266 cyp33 |
| 795 | GO:0003730 | F | 6, | 1 | 0.524 (x 1.908) | 9 (0.111) | 0.902 | mRNA 3'-UTR binding | pum |
| 796 | GO:0004033 | F | 6, | 1 | 0.408 (x 2.454) | 7 (0.143) | 0.902 | aldo-keto reductase activity | CG10638 |
| 797 | GO:0016721 | F | 4, | 1 | 0.466 (x 2.147) | 8 (0.125) | 0.902 | oxidoreductase activity, acting on superoxide radicals as acceptor | Sod |
| 798 | GO:0007525 | P | 5, | 1 | 0.524 (x 1.908) | 9 (0.111) | 0.903 | somatic muscle development | insc |
| 799 | GO:0006188 | P | 10, 11, | 1 | 0.408 (x 2.454) | 7 (0.143) | 0.903 | IMP biosynthesis | Dhfr |
| 800 | GO:0004143 | F | 6, | 1 | 0.466 (x 2.147) | 8 (0.125) | 0.903 | diacylglycerol kinase activity | Dgkepsilon |
| 801 | GO:0030017 | C | 6, 7, 8, 9, 10, | 1 | 0.524 (x 1.908) | 9 (0.111) | 0.904 | sarcomere | Tm1 |
| 802 | GO:0045735 | F | 2, | 1 | 0.466 (x 2.147) | 8 (0.125) | 0.904 | nutrient reservoir activity | Idgf2 |
| 803 | GO:0006631 | P | 6, 7, | 7 | 5.997 (x 1.167) | 103 (0.068) | 0.904 | fatty acid metabolism | CG1140 CG17821 CG6432 CG6921 CG8498 CG8778 mtacp1 |
| 804 | GO:0008277 | P | 5, 6, 7, | 1 | 0.524 (x 1.908) | 9 (0.111) | 0.905 | regulation of G-protein coupled receptor protein signaling pathway | EG:52C10.2 |
| 805 | GO:0009886 | P | 4, | 1 | 0.466 (x 2.147) | 8 (0.125) | 0.906 | post-embryonic morphogenesis | rpr |
| 806 | GO:0030717 | P | 8, | 1 | 0.524 (x 1.908) | 9 (0.111) | 0.906 | karyosome formation | Doa |
| 807 | GO:0001751 | P | 6, 7, 8, 9, | 5 | 4.250 (x 1.176) | 73 (0.068) | 0.906 | eye photoreceptor cell differentiation (sensu Endopterygota) | Cpn Dab Doa chp salm |
| 808 | GO:0007304 | P | 8, 9, | 4 | 3.202 (x 1.249) | 55 (0.073) | 0.907 | eggshell formation (sensu Insecta) | Cp36 Femcoat Vm34Ca hep |
| 809 | GO:0009881 | F | 4, | 1 | 0.466 (x 2.147) | 8 (0.125) | 0.907 | photoreceptor activity | Rh7 |
| 810 | GO:0015012 | P | 7, 8, 9, | 1 | 0.524 (x 1.908) | 9 (0.111) | 0.907 | heparan sulfate proteoglycan biosynthesis | Act57B |
| 811 | GO:0005575 | C | 1, | 340 | 336.646 (x 1.010) | 5782 (0.059) | 0.907 | cellular\_component | Aats-his Abl Ac78C Act57B Arp11 Arr2 BEST:LD29214 BG:DS00180.7 BG:DS01219.1 BG:DS02740.5 BG:DS02740.9 Bro CG10166 CG10237 CG10268 CG10320 CG10418 CG10669 CG10674 CG10738 CG10804 CG10861 CG11015 CG11318 CG11360 CG1140 CG11455 CG11597 CG11722 CG11897 CG11898 CG11909 CG12022 CG1213 CG12361 CG12400 CG1249 CG12605 CG12775 CG12918 CG13277 CG13691 CG14076 CG14482 CG14508 CG14691 CG14701 CG14825 CG15220 CG15361 CG15398 CG15408 CG16817 CG17224 CG17262 CG17266 CG17280 CG17385 CG1756 CG17768 CG17821 CG18011 CG18013 CG18619 CG18749 CG18767 CG1883 CG2021 CG2069 CG2789 CG2846 CG2998 CG30105 CG30126 CG30154 CG30334 CG30343 CG3036 CG30476 CG31184 CG31272 CG31477 CG31601 CG31611 CG31715 CG31922 CG31950 CG31957 CG32023 CG32105 CG3212 CG3215 CG32160 CG32174 CG32175 CG32202 CG32207 CG32230 CG32409 CG32442 CG32448 CG32549 CG32582 CG32625 CG32637 CG32692 CG32710 CG32856 CG33002 CG33066 CG33322 CG33543 CG3397 CG3529 CG3843 CG3887 CG3931 CG4046 CG4095 CG4101 CG4169 CG4187 CG4279 CG4288 CG4592 CG4673 CG4769 CG4805 CG4866 CG5037 CG5189 CG5338 CG5382 CG5535 CG5548 CG6214 CG6272 CG6574 CG6610 CG6723 CG6763 CG6764 CG6891 CG6921 CG6947 CG7014 CG7181 CG7188 CG7194 CG7211 CG7298 CG7333 CG7339 CG7770 CG7777 CG7834 CG7911 CG7949 CG8004 CG8152 CG8219 CG8271 CG8415 CG8506 CG8550 CG8756 CG8857 CG8916 CG8925 CG8993 CG9326 CG9381 CG9413 CG9650 CLIP-190 Cad99C Chit CoVa Con Cp36 Cpn Cyp12e1 Cyp28d2 Cyp311a1 Cyp49a1 Dab DebB Dhc98D Doa EG:152A3.7 EG:63B12.12 EG:80H7.10 EG:9D2.4 EG:BACR7A4.18 EG:BACR7A4.8 Eip93F Femcoat GABA-B-R2 Gasp Gp150 Gr61a Gr98c Gs2 HP1c Hr38 Hr4 Iap2 Idgf1 Idgf2 InR JhI-26 Karl Kr-h1 Las Mlc2 Mp20 Myo28B1 NP15.6 Nipped-B Nxt1 Obp56a Or46a Or59a Or85a Or94a Or98b Orc6 PGRP-SA PNUTS Pdsw Peritrophin-A Pof Prosbeta5 REG Rab3 Rep2 Rh7 Rlc1 RpII18 RpL11 RpL17A RpL27A RpL38 RpL46 RpL8 RpL9 RpP1 RpS17 RpS18 RpS4 RpS9 Rpb10 Rpb4 SIP1 SelG Sgs4 SmB Sod Sras Ssb-c31a SytIV Syx16 Syx8 Taf10b Taf11 Takr99D Tehao TfIIEalpha Tfb2 Tig Tim17b2 Tim9a Timp Tm1 Toll-7 Trap36 Tsf1 Tsp42El TyrR Vha36 apt bbx btl chp cyp33 dmrt93B dynactin-subunit-p25 e(y)2 fau fu12 gt hay hig insc inx7 l(1)10Bb l(2)03659 l(2)06225 l(2)k10201 l(3)01239 mRpL11 mRpL14 mRpL2 mRpL21 mRpL22 mRpL22-24 mRpL33 mRpL54 mRpS14 mRpS21 mRpS24 mRpS26 mRpS32 mira mirr mtacp1 na oho23B pip porin pum retinin rho robl rpr salm shu sisA skpA sop sun tko toy trol tsl unc-13 upd3 veli vvl zpg |
| 812 | GO:0030703 | P | 7, | 4 | 3.202 (x 1.249) | 55 (0.073) | 0.908 | eggshell formation | Cp36 Femcoat Vm34Ca hep |
| 813 | GO:0008202 | P | 6, 7, | 9 | 7.918 (x 1.137) | 136 (0.066) | 0.908 | steroid metabolism | CG10268 CG18869 CG30438 Cyp28d2 Cyp311a1 Cyp49a1 Hmgs Ugt86De Ugt86Di |
| 814 | GO:0004784 | F | 5, | 1 | 0.466 (x 2.147) | 8 (0.125) | 0.908 | superoxide dismutase activity | Sod |
| 815 | GO:0005869 | C | 4, 6, 7, 8, 9, 10, 11, | 1 | 0.524 (x 1.908) | 9 (0.111) | 0.908 | dynactin complex | dynactin-subunit-p25 |
| 816 | GO:0016563 | F | 3, | 4 | 3.202 (x 1.249) | 55 (0.073) | 0.909 | transcriptional activator activity | Bro Nipped-B Ssb-c31a mirr |
| 817 | GO:0016299 | F | 4, | 1 | 0.466 (x 2.147) | 8 (0.125) | 0.909 | regulator of G-protein signaling activity | EG:52C10.2 |
| 818 | GO:0015373 | F | 6, 7, 8, 9, | 1 | 0.524 (x 1.908) | 9 (0.111) | 0.909 | monovalent anion:sodium symporter activity | CG6723 |
| 819 | GO:0015399 | F | 4, | 8 | 6.987 (x 1.145) | 120 (0.067) | 0.909 | primary active transporter activity | CG31477 CG33066 CG7211 Tim17b2 Tim9a Vha36 l(2)06225 sun |
| 820 | GO:0005386 | F | 3, | 28 | 26.375 (x 1.062) | 453 (0.062) | 0.91 | carrier activity | CG10237 CG10804 CG10950 CG1213 CG3036 CG31477 CG33066 CG4071 CG4288 CG5535 CG6574 CG6723 CG7084 CG7211 CG7333 CG7777 CG8219 CG8271 CG8498 CG9413 Tim17b2 Tim9a Tsf1 Vha36 glob1 l(2)06225 mtacp1 sun |
| 821 | GO:0051247 | P | 6, 7, | 1 | 0.524 (x 1.908) | 9 (0.111) | 0.91 | positive regulation of protein metabolism | pum |
| 822 | GO:0015405 | F | 5, | 8 | 6.987 (x 1.145) | 120 (0.067) | 0.91 | P-P-bond-hydrolysis-driven transporter activity | CG31477 CG33066 CG7211 Tim17b2 Tim9a Vha36 l(2)06225 sun |
| 823 | GO:0015296 | F | 5, 7, | 3 | 2.271 (x 1.321) | 39 (0.077) | 0.911 | anion:cation symporter activity | CG3036 CG4288 CG6723 |
| 824 | GO:0008439 | F | 4, 7, | 1 | 0.524 (x 1.908) | 9 (0.111) | 0.911 | monophenol monooxygenase activator activity | CG11313 |
| 825 | GO:0044451 | C | 5, 6, 7, 8, 9, 10, 11, 12, | 13 | 11.819 (x 1.100) | 203 (0.064) | 0.912 | nucleoplasm part | Bro CG15398 CG7339 RpII18 Rpb10 Rpb4 Ssb-c31a Taf10b Taf11 TfIIEalpha Tfb2 Trap36 hay |
| 826 | GO:0006096 | P | 8, 10, 11, | 3 | 2.271 (x 1.321) | 39 (0.077) | 0.912 | glycolysis | CG2964 ImpL3 Pglym78 |
| 827 | GO:0006012 | P | 8, 9, | 1 | 0.524 (x 1.908) | 9 (0.111) | 0.912 | galactose metabolism | BcDNA:GH08902 |
| 828 | GO:0005516 | F | 4, | 5 | 4.134 (x 1.210) | 71 (0.070) | 0.913 | calmodulin binding | CG2185 CG7646 Mlc2 TpnC41C unc-13 |
| 829 | GO:0050906 | P | 4, 5, 6, | 1 | 0.524 (x 1.908) | 9 (0.111) | 0.913 | detection of stimulus during sensory perception | tko |
| 830 | GO:0007430 | P | 5, 6, | 1 | 0.524 (x 1.908) | 9 (0.111) | 0.915 | terminal branching of trachea, cytoplasmic projection extension (sensu Insecta) | btl |
| 831 | GO:0016021 | C | 5, 6, 7, | 57 | 54.963 (x 1.037) | 944 (0.060) | 0.915 | integral to membrane | Ac78C CG10804 CG11318 CG11897 CG11898 CG1213 CG14691 CG15408 CG17821 CG2789 CG3036 CG31272 CG31477 CG3212 CG3397 CG4101 CG4187 CG4288 CG4673 CG5037 CG6214 CG6763 CG6921 CG7188 CG7211 CG7333 CG8219 CG8271 CG8916 CG8925 Cad99C EG:80H7.10 GABA-B-R2 Gr61a Gr98c InR Or46a Or59a Or85a Or94a Or98b PGRP-SA Rh7 Sras Syx16 Takr99D Tehao Toll-7 Tsp42El TyrR Vha36 inx7 l(2)03659 l(2)06225 rho sun zpg |
| 832 | GO:0045239 | C | 3, 5, 6, 7, 8, | 1 | 0.524 (x 1.908) | 9 (0.111) | 0.916 | tricarboxylic acid cycle enzyme complex | CG4095 |
| 833 | GO:0030201 | P | 6, 8, | 1 | 0.524 (x 1.908) | 9 (0.111) | 0.917 | heparan sulfate proteoglycan metabolism | Act57B |
| 834 | GO:0035003 | C | 7, 8, 9, 10, | 1 | 0.524 (x 1.908) | 9 (0.111) | 0.918 | subapical complex | veli |
| 835 | GO:0005794 | C | 5, 6, 7, 8, | 6 | 5.240 (x 1.145) | 90 (0.067) | 0.918 | Golgi apparatus | CG3529 CG5189 CLIP-190 Syx16 pip rho |
| 836 | GO:0030016 | C | 6, 7, 8, 9, | 1 | 0.524 (x 1.908) | 9 (0.111) | 0.919 | myofibril | Tm1 |
| 837 | GO:0016298 | F | 6, | 6 | 5.124 (x 1.171) | 88 (0.068) | 0.919 | lipase activity | CG18530 CG31272 CG5162 CG6296 Lip1 Pld |
| 838 | GO:0000578 | P | 5, | 4 | 3.435 (x 1.164) | 59 (0.068) | 0.919 | embryonic axis specification | gt ksr pum tsl |
| 839 | GO:0008203 | P | 7, 8, 9, | 2 | 1.514 (x 1.321) | 26 (0.077) | 0.92 | cholesterol metabolism | CG10268 Hmgs |
| 840 | GO:0006536 | P | 8, 9, | 1 | 0.524 (x 1.908) | 9 (0.111) | 0.92 | glutamate metabolism | Gs2 |
| 841 | GO:0050658 | P | 6, 7, 8, | 2 | 1.397 (x 1.431) | 24 (0.083) | 0.92 | RNA transport | CG10320 Nxt1 |
| 842 | GO:0004383 | F | 4, 5, | 2 | 1.514 (x 1.321) | 26 (0.077) | 0.921 | guanylate cyclase activity | Ac78C CG10738 |
| 843 | GO:0045924 | P | 4, 5, 6, | 1 | 0.524 (x 1.908) | 9 (0.111) | 0.921 | regulation of female receptivity | sra |
| 844 | GO:0042063 | P | 6, | 2 | 1.397 (x 1.431) | 24 (0.083) | 0.922 | gliogenesis | BG:DS02740.9 btl |
| 845 | GO:0045177 | C | 3, 4, | 2 | 1.514 (x 1.321) | 26 (0.077) | 0.922 | apical part of cell | insc mira |
| 846 | GO:0005504 | F | 4, | 1 | 0.582 (x 1.718) | 10 (0.100) | 0.922 | fatty acid binding | CG8498 |
| 847 | GO:0006072 | P | 8, | 1 | 0.524 (x 1.908) | 9 (0.111) | 0.922 | glycerol-3-phosphate metabolism | CG3215 |
| 848 | GO:0004879 | F | 4, | 2 | 1.397 (x 1.431) | 24 (0.083) | 0.923 | ligand-dependent nuclear receptor activity | Hr38 Hr4 |
| 849 | GO:0015114 | F | 6, | 2 | 1.514 (x 1.321) | 26 (0.077) | 0.923 | phosphate transporter activity | CG3036 CG4288 |
| 850 | GO:0006023 | P | 7, 8, | 1 | 0.582 (x 1.718) | 10 (0.100) | 0.923 | aminoglycan biosynthesis | Act57B |
| 851 | GO:0006221 | P | 7, 8, | 1 | 0.524 (x 1.908) | 9 (0.111) | 0.923 | pyrimidine nucleotide biosynthesis | nmdyn-D6 |
| 852 | GO:0016459 | C | 3, 5, 6, 7, 8, 9, 10, | 2 | 1.456 (x 1.374) | 25 (0.080) | 0.924 | myosin | Mlc2 Myo28B1 |
| 853 | GO:0006334 | P | 7, 11, | 2 | 1.397 (x 1.431) | 24 (0.083) | 0.924 | nucleosome assembly | CG31611 EG:BACR7A4.18 |
| 854 | GO:0007601 | P | 5, 7, | 5 | 4.309 (x 1.160) | 74 (0.068) | 0.924 | visual perception | Arr2 Doa Myo28B1 Rh7 chp |
| 855 | GO:0006954 | P | 5, 6, | 1 | 0.582 (x 1.718) | 10 (0.100) | 0.924 | inflammatory response | Toll-7 |
| 856 | GO:0016020 | C | 3, 4, | 111 | 108.644 (x 1.022) | 1866 (0.059) | 0.924 | membrane | Abl Ac78C Arr2 BG:DS01219.1 CG10320 CG10738 CG10804 CG11015 CG11318 CG11455 CG11897 CG11898 CG1213 CG12400 CG12918 CG14076 CG14482 CG14508 CG14691 CG15408 CG17262 CG17280 CG1756 CG17821 CG2789 CG3036 CG31272 CG31477 CG3212 CG32174 CG32230 CG33066 CG33543 CG3397 CG4101 CG4169 CG4187 CG4288 CG4673 CG4769 CG4805 CG5037 CG5535 CG5548 CG6214 CG6574 CG6723 CG6763 CG6921 CG7181 CG7188 CG7211 CG7333 CG7777 CG8004 CG8219 CG8271 CG8550 CG8916 CG8925 CG9326 CG9413 Cad99C CoVa Con Cyp28d2 Cyp311a1 Cyp49a1 EG:152A3.7 EG:80H7.10 EG:9D2.4 GABA-B-R2 Gp150 Gr61a Gr98c InR Nxt1 Or46a Or59a Or85a Or94a Or98b PGRP-SA Pdsw Rh7 Sras SytIV Syx16 Syx8 Takr99D Tehao Tim17b2 Tim9a Toll-7 Tsp42El TyrR Vha36 btl chp fu12 hig inx7 l(2)03659 l(2)06225 mtacp1 na porin rho sun veli zpg |
| 857 | GO:0042364 | P | 7, | 1 | 0.524 (x 1.908) | 9 (0.111) | 0.924 | water-soluble vitamin biosynthesis | CG2846 |
| 858 | GO:0006629 | P | 5, | 30 | 28.529 (x 1.052) | 490 (0.061) | 0.925 | lipid metabolism | AnnX CG10268 CG1140 CG17821 CG18530 CG18869 CG2789 CG30438 CG31272 CG3215 CG4592 CG5162 CG6296 CG6432 CG6461 CG6921 CG7322 CG8498 CG8778 Cyp28d2 Cyp311a1 Cyp49a1 Dgkepsilon Hmgs Lip1 Pld Ugt86De Ugt86Di fu12 mtacp1 |
| 859 | GO:0031224 | C | 4, 5, 6, | 57 | 55.137 (x 1.034) | 947 (0.060) | 0.925 | intrinsic to membrane | Ac78C CG10804 CG11318 CG11897 CG11898 CG1213 CG14691 CG15408 CG17821 CG2789 CG3036 CG31272 CG31477 CG3212 CG3397 CG4101 CG4187 CG4288 CG4673 CG5037 CG6214 CG6763 CG6921 CG7188 CG7211 CG7333 CG8219 CG8271 CG8916 CG8925 Cad99C EG:80H7.10 GABA-B-R2 Gr61a Gr98c InR Or46a Or59a Or85a Or94a Or98b PGRP-SA Rh7 Sras Syx16 Takr99D Tehao Toll-7 Tsp42El TyrR Vha36 inx7 l(2)03659 l(2)06225 rho sun zpg |
| 860 | GO:0045211 | C | 3, 4, 5, | 2 | 1.456 (x 1.374) | 25 (0.080) | 0.925 | postsynaptic membrane | CG8916 veli |
| 861 | GO:0051236 | P | 5, | 2 | 1.397 (x 1.431) | 24 (0.083) | 0.925 | establishment of RNA localization | CG10320 Nxt1 |
| 862 | GO:0050953 | P | 4, 6, | 5 | 4.309 (x 1.160) | 74 (0.068) | 0.925 | sensory perception of light stimulus | Arr2 Doa Myo28B1 Rh7 chp |
| 863 | GO:0008362 | P | 9, | 1 | 0.582 (x 1.718) | 10 (0.100) | 0.925 | embryonic cuticle biosynthesis (sensu Insecta) | Doa |
| 864 | GO:0001754 | P | 5, 6, 7, | 5 | 4.425 (x 1.130) | 76 (0.066) | 0.925 | eye photoreceptor cell differentiation | Cpn Dab Doa chp salm |
| 865 | GO:0016798 | F | 4, | 7 | 6.230 (x 1.124) | 107 (0.065) | 0.925 | hydrolase activity, acting on glycosyl bonds | Amyrel CG11909 CG14935 CG15012 Chit Idgf1 Idgf2 |
| 866 | GO:0045500 | P | 8, 9, 10, 11, 12, | 1 | 0.524 (x 1.908) | 9 (0.111) | 0.926 | sevenless signaling pathway | Dab |
| 867 | GO:0015662 | F | 5, 6, 8, 13, | 5 | 4.192 (x 1.193) | 72 (0.069) | 0.926 | ATPase activity, coupled to transmembrane movement of ions, phosphorylative mechanism | CG31477 CG7211 Vha36 l(2)06225 sun |
| 868 | GO:0019842 | F | 3, | 2 | 1.397 (x 1.431) | 24 (0.083) | 0.926 | vitamin binding | CG10237 CG6574 |
| 869 | GO:0005654 | C | 5, 6, 7, 8, 9, 10, 11, | 14 | 13.042 (x 1.073) | 224 (0.062) | 0.926 | nucleoplasm | Bro CG15398 CG7339 Nxt1 RpII18 Rpb10 Rpb4 Ssb-c31a Taf10b Taf11 TfIIEalpha Tfb2 Trap36 hay |
| 870 | GO:0004835 | F | 6, | 1 | 0.582 (x 1.718) | 10 (0.100) | 0.926 | tubulin-tyrosine ligase activity | CG8918 |
| 871 | GO:0042592 | P | 3, | 5 | 4.425 (x 1.130) | 76 (0.066) | 0.926 | homeostasis | CG7777 CG8498 Prx6005 SelG Tsf1 |
| 872 | GO:0008431 | F | 4, | 1 | 0.524 (x 1.908) | 9 (0.111) | 0.927 | vitamin E binding | CG10237 |
| 873 | GO:0044262 | P | 6, | 17 | 16.011 (x 1.062) | 275 (0.062) | 0.927 | cellular carbohydrate metabolism | Act57B BcDNA:GH08902 CG11909 CG2964 CG30499 CG4095 CG5103 CG5177 CG6947 CG7298 CG8756 Chit Gasp ImpL3 PGRP-SA Peritrophin-A Pglym78 |
| 874 | GO:0050657 | P | 6, 7, | 2 | 1.397 (x 1.431) | 24 (0.083) | 0.927 | nucleic acid transport | CG10320 Nxt1 |
| 875 | GO:0051017 | P | 10, | 1 | 0.582 (x 1.718) | 10 (0.100) | 0.927 | actin filament bundle formation | hep |
| 876 | GO:0004869 | F | 6, | 1 | 0.524 (x 1.908) | 9 (0.111) | 0.928 | cysteine protease inhibitor activity | CG10460 |
| 877 | GO:0030693 | F | 7, | 1 | 0.582 (x 1.718) | 10 (0.100) | 0.928 | caspase activity | CG1885 |
| 878 | GO:0008513 | F | 6, | 2 | 1.397 (x 1.431) | 24 (0.083) | 0.928 | organic cation porter activity | CG7084 CG7333 |
| 879 | GO:0040001 | P | 6, 7, 8, 10, | 1 | 0.524 (x 1.908) | 9 (0.111) | 0.929 | establishment of mitotic spindle localization | insc |
| 880 | GO:0004402 | F | 10, | 1 | 0.582 (x 1.718) | 10 (0.100) | 0.929 | histone acetyltransferase activity | Rpb4 |
| 881 | GO:0040018 | P | 5, | 1 | 0.582 (x 1.718) | 10 (0.100) | 0.93 | positive regulation of body size | InR |
| 882 | GO:0048149 | P | 5, 7, | 1 | 0.582 (x 1.718) | 10 (0.100) | 0.931 | behavioral response to ethanol | Tbh |
| 883 | GO:0005506 | F | 6, | 3 | 2.504 (x 1.198) | 43 (0.070) | 0.932 | iron ion binding | CG18011 Las Tsf1 |
| 884 | GO:0006071 | P | 7, | 1 | 0.582 (x 1.718) | 10 (0.100) | 0.932 | glycerol metabolism | CG3215 |
| 885 | GO:0004857 | F | 3, | 8 | 7.394 (x 1.082) | 127 (0.063) | 0.932 | enzyme inhibitor activity | CG10460 CG1342 CG16712 CG31704 CG5639 CG8979 Spn6 Timp |
| 886 | GO:0001709 | P | 5, | 7 | 6.405 (x 1.093) | 110 (0.064) | 0.933 | cell fate determination | insc mira pum rho salm trol vvl |
| 887 | GO:0051248 | P | 6, 7, | 3 | 2.504 (x 1.198) | 43 (0.070) | 0.933 | negative regulation of protein metabolism | apt pum rpr |
| 888 | GO:0035058 | P | 8, 9, | 1 | 0.582 (x 1.718) | 10 (0.100) | 0.933 | sensory cilium biogenesis | CG2069 |
| 889 | GO:0000030 | F | 6, | 1 | 0.582 (x 1.718) | 10 (0.100) | 0.935 | mannosyltransferase activity | CG10166 |
| 890 | GO:0004468 | F | 9, | 1 | 0.582 (x 1.718) | 10 (0.100) | 0.936 | lysine N-acetyltransferase activity | Rpb4 |
| 891 | GO:0019751 | P | 6, | 1 | 0.582 (x 1.718) | 10 (0.100) | 0.937 | polyol metabolism | CG3215 |
| 892 | GO:0006024 | P | 8, 9, | 1 | 0.582 (x 1.718) | 10 (0.100) | 0.938 | glycosaminoglycan biosynthesis | Act57B |
| 893 | GO:0016863 | F | 5, | 1 | 0.582 (x 1.718) | 10 (0.100) | 0.939 | intramolecular oxidoreductase activity, transposing C=C bonds | CG4592 |
| 894 | GO:0046011 | P | 8, 9, 10, | 1 | 0.582 (x 1.718) | 10 (0.100) | 0.94 | regulation of oskar mRNA translation | apt |
| 895 | GO:0045321 | P | 5, 6, | 1 | 0.582 (x 1.718) | 10 (0.100) | 0.941 | leukocyte activation | Toll-7 |
| 896 | GO:0016059 | P | 7, 8, 9, | 1 | 0.582 (x 1.718) | 10 (0.100) | 0.942 | deactivation of rhodopsin mediated signaling | Arr2 |
| 897 | GO:0043596 | C | 6, 7, 8, 9, 10, 11, 12, | 1 | 0.582 (x 1.718) | 10 (0.100) | 0.943 | replication fork (sensu Eukaryota) | CG15220 |
| 898 | GO:0006022 | P | 6, 7, | 1 | 0.582 (x 1.718) | 10 (0.100) | 0.944 | aminoglycan metabolism | Act57B |
| 899 | GO:0006887 | P | 6, 7, | 8 | 7.278 (x 1.099) | 125 (0.064) | 0.945 | exocytosis | AnnX Arf84F CG2185 Rab3 SytIV Syx16 Syx8 unc-13 |
| 900 | GO:0030203 | P | 7, 8, | 1 | 0.582 (x 1.718) | 10 (0.100) | 0.945 | glycosaminoglycan metabolism | Act57B |
| 901 | GO:0005975 | P | 5, | 29 | 28.238 (x 1.027) | 485 (0.060) | 0.945 | carbohydrate metabolism | Act57B Amyrel BcDNA:GH08902 CG10166 CG11909 CG1213 CG14935 CG15408 CG18869 CG2964 CG3036 CG30438 CG30499 CG3215 CG4095 CG4288 CG5103 CG5177 CG6947 CG7298 CG8756 Chit Gasp ImpL3 PGRP-SA Peritrophin-A Pglym78 Ugt86De Ugt86Di |
| 902 | GO:0008970 | F | 8, | 1 | 0.582 (x 1.718) | 10 (0.100) | 0.946 | phospholipase A1 activity | CG6296 |
| 903 | GO:0016799 | F | 5, | 3 | 2.445 (x 1.227) | 42 (0.071) | 0.947 | hydrolase activity, hydrolyzing N-glycosyl compounds | Chit Idgf1 Idgf2 |
| 904 | GO:0051653 | P | 5, 6, | 1 | 0.582 (x 1.718) | 10 (0.100) | 0.947 | spindle localization | insc |
| 905 | GO:0007306 | P | 9, 10, | 3 | 2.445 (x 1.227) | 42 (0.071) | 0.948 | insect chorion formation | Cp36 Femcoat hep |
| 906 | GO:0009190 | P | 7, 8, | 1 | 0.582 (x 1.718) | 10 (0.100) | 0.948 | cyclic nucleotide biosynthesis | Ac78C |
| 907 | GO:0044456 | C | 2, 3, | 2 | 1.572 (x 1.272) | 27 (0.074) | 0.949 | synapse part | CG8916 veli |
| 908 | GO:0051293 | P | 6, 7, 9, | 1 | 0.582 (x 1.718) | 10 (0.100) | 0.949 | establishment of spindle localization | insc |
| 909 | GO:0007354 | P | 7, 8, | 2 | 1.572 (x 1.272) | 27 (0.074) | 0.95 | zygotic determination of anterior/posterior axis, embryo | gt tsl |
| 910 | GO:0042116 | P | 6, 7, | 1 | 0.582 (x 1.718) | 10 (0.100) | 0.951 | macrophage activation | Toll-7 |
| 911 | GO:0030894 | C | 3, 5, 6, 7, 8, 9, 10, 11, | 1 | 0.582 (x 1.718) | 10 (0.100) | 0.952 | replisome | CG15220 |
| 912 | GO:0030176 | C | 6, 7, 8, 9, 10, 11, 12, | 1 | 0.640 (x 1.561) | 11 (0.091) | 0.953 | integral to endoplasmic reticulum membrane | Sras |
| 913 | GO:0043601 | C | 4, 6, 7, 8, 9, 10, 11, 12, 13, | 1 | 0.582 (x 1.718) | 10 (0.100) | 0.953 | replisome (sensu Eukaryota) | CG15220 |
| 914 | GO:0030508 | F | 5, | 1 | 0.640 (x 1.561) | 11 (0.091) | 0.954 | thiol-disulfide exchange intermediate activity | CG8993 |
| 915 | GO:0006220 | P | 7, | 1 | 0.582 (x 1.718) | 10 (0.100) | 0.954 | pyrimidine nucleotide metabolism | nmdyn-D6 |
| 916 | GO:0004497 | F | 4, | 7 | 6.346 (x 1.103) | 109 (0.064) | 0.954 | monooxygenase activity | Bc CG8193 Cyp12e1 Cyp28d2 Cyp311a1 Cyp49a1 Tbh |
| 917 | GO:0019904 | F | 4, | 1 | 0.640 (x 1.561) | 11 (0.091) | 0.955 | protein domain specific binding | Dab |
| 918 | GO:0007431 | P | 5, | 7 | 6.346 (x 1.103) | 109 (0.064) | 0.956 | salivary gland development | CG10861 Doa Eip93F btl gt rho rpr |
| 919 | GO:0007413 | P | 9, 10, 12, | 1 | 0.640 (x 1.561) | 11 (0.091) | 0.956 | axonal fasciculation | Con |
| 920 | GO:0035272 | P | 4, | 7 | 6.346 (x 1.103) | 109 (0.064) | 0.957 | exocrine system development | CG10861 Doa Eip93F btl gt rho rpr |
| 921 | GO:0031227 | C | 5, 6, 7, 8, 9, 10, 11, | 1 | 0.640 (x 1.561) | 11 (0.091) | 0.957 | intrinsic to endoplasmic reticulum membrane | Sras |
| 922 | GO:0003723 | F | 4, | 22 | 21.484 (x 1.024) | 369 (0.060) | 0.957 | RNA binding | Aats-his BcDNA:GH11110 CG10092 CG10466 CG1249 CG17768 CG31184 CG3931 CG4866 CG8778 CG9862 DebB Pof RpL8 RpS18 RpS4 RpS9 SmB apt cyp33 mRpL21 pum |
| 923 | GO:0006383 | P | 8, | 1 | 0.640 (x 1.561) | 11 (0.091) | 0.958 | transcription from RNA polymerase III promoter | CG7339 |
| 924 | GO:0000041 | P | 8, 9, | 1 | 0.640 (x 1.561) | 11 (0.091) | 0.959 | transition metal ion transport | Tsf1 |
| 925 | GO:0031461 | C | 4, 5, 6, 7, | 1 | 0.640 (x 1.561) | 11 (0.091) | 0.96 | cullin-RING ubiquitin ligase complex | skpA |
| 926 | GO:0007594 | P | 7, | 1 | 0.640 (x 1.561) | 11 (0.091) | 0.961 | puparial adhesion | Sgs4 |
| 927 | GO:0015992 | P | 6, 7, 8, 9, | 5 | 4.541 (x 1.101) | 78 (0.064) | 0.961 | proton transport | CG31477 CG7211 Vha36 l(2)06225 sun |
| 928 | GO:0016830 | F | 4, | 3 | 2.620 (x 1.145) | 45 (0.067) | 0.962 | carbon-carbon lyase activity | CG10184 CG11251 SamDC |
| 929 | GO:0019005 | C | 5, 6, 7, 8, | 1 | 0.640 (x 1.561) | 11 (0.091) | 0.962 | SCF ubiquitin ligase complex | skpA |
| 930 | GO:0006818 | P | 5, 6, | 5 | 4.541 (x 1.101) | 78 (0.064) | 0.962 | hydrogen transport | CG31477 CG7211 Vha36 l(2)06225 sun |
| 931 | GO:0009581 | P | 4, 5, | 3 | 2.620 (x 1.145) | 45 (0.067) | 0.963 | detection of external stimulus | Arr2 PGRP-SA tko |
| 932 | GO:0032147 | P | 7, 8, | 1 | 0.640 (x 1.561) | 11 (0.091) | 0.963 | activation of protein kinase activity | Dgkepsilon |
| 933 | GO:0048699 | P | 6, | 11 | 10.480 (x 1.050) | 180 (0.061) | 0.963 | generation of neurons | Abl Con InR Tig Tm1 insc mira pum robl trol vvl |
| 934 | GO:0006694 | P | 6, 7, 8, | 1 | 0.699 (x 1.431) | 12 (0.083) | 0.964 | steroid biosynthesis | CG10268 |
| 935 | GO:0005578 | C | 3, 4, | 3 | 2.620 (x 1.145) | 45 (0.067) | 0.964 | extracellular matrix (sensu Metazoa) | Tig Timp trol |
| 936 | GO:0005245 | F | 6, 7, 8, | 1 | 0.640 (x 1.561) | 11 (0.091) | 0.964 | voltage-gated calcium channel activity | na |
| 937 | GO:0019237 | F | 6, | 1 | 0.699 (x 1.431) | 12 (0.083) | 0.965 | centromeric DNA binding | skpA |
| 938 | GO:0031012 | C | 2, | 3 | 2.620 (x 1.145) | 45 (0.067) | 0.965 | extracellular matrix | Tig Timp trol |
| 939 | GO:0007390 | P | 6, | 1 | 0.640 (x 1.561) | 11 (0.091) | 0.965 | germ-band shortening | InR |
| 940 | GO:0030307 | P | 5, 6, 7, 8, 9, | 1 | 0.699 (x 1.431) | 12 (0.083) | 0.965 | positive regulation of cell growth | InR |
| 941 | GO:0005938 | C | 5, 6, 7, 8, | 3 | 2.620 (x 1.145) | 45 (0.067) | 0.966 | cell cortex | CLIP-190 insc mira |
| 942 | GO:0045466 | P | 7, 8, 9, 10, | 2 | 1.630 (x 1.227) | 28 (0.071) | 0.966 | R7 cell differentiation | Dab salm |
| 943 | GO:0050795 | P | 3, 4, | 1 | 0.640 (x 1.561) | 11 (0.091) | 0.966 | regulation of behavior | sra |
| 944 | GO:0042708 | F | 6, | 1 | 0.699 (x 1.431) | 12 (0.083) | 0.966 | elastase activity | BcDNA:GH08420 |
| 945 | GO:0016866 | F | 4, | 1 | 0.640 (x 1.561) | 11 (0.091) | 0.967 | intramolecular transferase activity | Pglym78 |
| 946 | GO:0000176 | C | 4, 5, 6, 7, 8, 9, 10, | 1 | 0.699 (x 1.431) | 12 (0.083) | 0.967 | nuclear exosome (RNase complex) | CG3931 |
| 947 | GO:0019722 | P | 7, | 4 | 3.726 (x 1.073) | 64 (0.062) | 0.968 | calcium-mediated signaling | CG7646 TpnC41C regucalcin sra |
| 948 | GO:0005876 | C | 6, 7, 8, 9, 10, 11, | 1 | 0.640 (x 1.561) | 11 (0.091) | 0.968 | spindle microtubule | CLIP-190 |
| 949 | GO:0009253 | P | 8, | 1 | 0.699 (x 1.431) | 12 (0.083) | 0.968 | peptidoglycan catabolism | PGRP-SA |
| 950 | GO:0051184 | F | 3, | 1 | 0.699 (x 1.431) | 12 (0.083) | 0.969 | cofactor transporter activity | CG8925 |
| 951 | GO:0009084 | P | 8, 9, | 1 | 0.699 (x 1.431) | 12 (0.083) | 0.97 | glutamine family amino acid biosynthesis | Gs2 |
| 952 | GO:0048732 | P | 4, | 8 | 7.627 (x 1.049) | 131 (0.061) | 0.971 | gland development | CG10861 Doa Eip93F btl gt oho23B rho rpr |
| 953 | GO:0045179 | C | 4, 5, 6, 7, 8, 9, 10, | 1 | 0.699 (x 1.431) | 12 (0.083) | 0.971 | apical cortex | insc |
| 954 | GO:0051707 | P | 4, | 8 | 7.627 (x 1.049) | 131 (0.061) | 0.972 | response to other organism | Bc CG6426 CG6435 Def PGRP-SA Tehao Toll-7 upd3 |
| 955 | GO:0009991 | P | 4, | 1 | 0.699 (x 1.431) | 12 (0.083) | 0.972 | response to extracellular stimulus | InR |
| 956 | GO:0045786 | P | 6, 7, | 1 | 0.699 (x 1.431) | 12 (0.083) | 0.973 | negative regulation of progression through cell cycle | pum |
| 957 | GO:0006606 | P | 7, 8, 9, 10, | 3 | 2.736 (x 1.096) | 47 (0.064) | 0.974 | protein import into nucleus | CG10950 CG8219 Nxt1 |
| 958 | GO:0043492 | F | 3, 10, | 9 | 8.792 (x 1.024) | 151 (0.060) | 0.974 | ATPase activity, coupled to movement of substances | CG11897 CG11898 CG31477 CG6214 CG7211 Vha36 l(2)03659 l(2)06225 sun |
| 959 | GO:0016082 | P | 8, 9, 10, | 1 | 0.699 (x 1.431) | 12 (0.083) | 0.974 | synaptic vesicle priming | unc-13 |
| 960 | GO:0042626 | F | 4, 6, 11, | 9 | 8.792 (x 1.024) | 151 (0.060) | 0.975 | ATPase activity, coupled to transmembrane movement of substances | CG11897 CG11898 CG31477 CG6214 CG7211 Vha36 l(2)03659 l(2)06225 sun |
| 961 | GO:0000123 | C | 4, 7, 8, 9, 10, 11, 12, 13, 14, | 1 | 0.699 (x 1.431) | 12 (0.083) | 0.975 | histone acetyltransferase complex | Rpb4 |
| 962 | GO:0004890 | F | 6, | 1 | 0.699 (x 1.431) | 12 (0.083) | 0.976 | GABA-A receptor activity | CG8916 |
| 963 | GO:0000178 | C | 3, 4, 5, 6, | 1 | 0.757 (x 1.321) | 13 (0.077) | 0.977 | exosome (RNase complex) | CG3931 |
| 964 | GO:0007426 | P | 5, | 1 | 0.699 (x 1.431) | 12 (0.083) | 0.977 | tracheal outgrowth (sensu Insecta) | btl |
| 965 | GO:0007263 | P | 6, | 1 | 0.757 (x 1.321) | 13 (0.077) | 0.978 | nitric oxide mediated signal transduction | CG10738 |
| 966 | GO:0031667 | P | 5, | 1 | 0.699 (x 1.431) | 12 (0.083) | 0.978 | response to nutrient levels | InR |
| 967 | GO:0048489 | P | 6, 7, | 5 | 4.658 (x 1.073) | 80 (0.062) | 0.979 | synaptic vesicle transport | Arf84F SytIV Syx16 Syx8 unc-13 |
| 968 | GO:0045465 | P | 7, 8, 9, 10, | 1 | 0.757 (x 1.321) | 13 (0.077) | 0.979 | R8 cell differentiation | salm |
| 969 | GO:0008318 | F | 6, | 1 | 0.699 (x 1.431) | 12 (0.083) | 0.979 | protein prenyltransferase activity | betaggt-II |
| 970 | GO:0045169 | C | 5, 6, 7, 8, | 1 | 0.757 (x 1.321) | 13 (0.077) | 0.98 | fusome | CLIP-190 |
| 971 | GO:0000270 | P | 7, | 1 | 0.699 (x 1.431) | 12 (0.083) | 0.981 | peptidoglycan metabolism | PGRP-SA |
| 972 | GO:0007098 | P | 5, 8, | 1 | 0.757 (x 1.321) | 13 (0.077) | 0.981 | centrosome cycle | skpA |
| 973 | GO:0008745 | F | 6, | 1 | 0.699 (x 1.431) | 12 (0.083) | 0.982 | N-acetylmuramoyl-L-alanine amidase activity | PGRP-SA |
| 974 | GO:0019730 | P | 6, 7, | 4 | 3.668 (x 1.090) | 63 (0.063) | 0.982 | antimicrobial humoral response | Def PGRP-SA Tehao upd3 |
| 975 | GO:0006891 | P | 6, 7, 8, 9, | 1 | 0.757 (x 1.321) | 13 (0.077) | 0.982 | intra-Golgi vesicle-mediated transport | CG3529 |
| 976 | GO:0006904 | P | 7, 8, | 2 | 1.688 (x 1.185) | 29 (0.069) | 0.982 | vesicle docking during exocytosis | Syx16 Syx8 |
| 977 | GO:0004295 | F | 7, | 14 | 13.682 (x 1.023) | 235 (0.060) | 0.982 | trypsin activity | BcDNA:GH08420 CG11313 CG12133 CG1299 CG1304 CG13318 CG18223 CG2056 CG30283 CG3355 CG4386 CG9372 EG:9D2.4 Ser7 |
| 978 | GO:0019236 | P | 5, | 1 | 0.699 (x 1.431) | 12 (0.083) | 0.983 | response to pheromone | Obp56a |
| 979 | GO:0019220 | P | 7, | 1 | 0.757 (x 1.321) | 13 (0.077) | 0.983 | regulation of phosphate metabolism | PNUTS |
| 980 | GO:0048754 | P | 5, | 2 | 1.805 (x 1.108) | 31 (0.065) | 0.983 | branching morphogenesis of a tube | apt btl |
| 981 | GO:0005478 | F | 3, | 2 | 1.688 (x 1.185) | 29 (0.069) | 0.983 | intracellular transporter activity | Syx16 Syx8 |
| 982 | GO:0007270 | P | 7, | 3 | 2.678 (x 1.120) | 46 (0.065) | 0.984 | nerve-nerve synaptic transmission | CG8916 GABA-B-R2 TyrR |
| 983 | GO:0008045 | P | 7, 8, 10, 11, 13, | 1 | 0.699 (x 1.431) | 12 (0.083) | 0.984 | motor axon guidance | vvl |
| 984 | GO:0008347 | P | 6, 7, | 1 | 0.757 (x 1.321) | 13 (0.077) | 0.984 | glial cell migration | btl |
| 985 | GO:0006807 | P | 4, | 23 | 22.765 (x 1.010) | 391 (0.059) | 0.984 | nitrogen compound metabolism | Aats-his Act57B CG10092 CG10184 CG11251 CG11897 CG18749 CG5122 CG5535 CG6461 CG6947 CG7298 CG8756 CG9413 CG9836 Chit Dhfr Gasp Gs2 Peritrophin-A SamDC Tbh gatA |
| 986 | GO:0042995 | C | 3, 4, | 2 | 1.805 (x 1.108) | 31 (0.065) | 0.984 | cell projection | Dhc98D robl |
| 987 | GO:0004714 | F | 6, 8, | 2 | 1.688 (x 1.185) | 29 (0.069) | 0.984 | transmembrane receptor protein tyrosine kinase activity | InR btl |
| 988 | GO:0016820 | F | 5, | 9 | 8.850 (x 1.017) | 152 (0.059) | 0.984 | hydrolase activity, acting on acid anhydrides, catalyzing transmembrane movement of substances | CG11897 CG11898 CG31477 CG6214 CG7211 Vha36 l(2)03659 l(2)06225 sun |
| 989 | GO:0006576 | P | 6, 7, | 2 | 1.747 (x 1.145) | 30 (0.067) | 0.984 | biogenic amine metabolism | SamDC Tbh |
| 990 | GO:0008543 | P | 8, | 1 | 0.699 (x 1.431) | 12 (0.083) | 0.985 | fibroblast growth factor receptor signaling pathway | btl |
| 991 | GO:0042440 | P | 5, | 3 | 2.678 (x 1.120) | 46 (0.065) | 0.985 | pigment metabolism | Bc CG1885 CG5037 |
| 992 | GO:0051174 | P | 6, | 1 | 0.757 (x 1.321) | 13 (0.077) | 0.985 | regulation of phosphorus metabolism | PNUTS |
| 993 | GO:0016197 | P | 6, 7, 8, | 1 | 0.815 (x 1.227) | 14 (0.071) | 0.985 | endosome transport | Arf84F |
| 994 | GO:0006092 | P | 7, | 6 | 6.055 (x 0.991) | 104 (0.058) | 0.985 | main pathways of carbohydrate metabolism | CG2964 CG30499 CG4095 CG5103 ImpL3 Pglym78 |
| 995 | GO:0009975 | F | 3, | 2 | 1.688 (x 1.185) | 29 (0.069) | 0.985 | cyclase activity | Ac78C CG10738 |
| 996 | GO:0006575 | P | 6, | 3 | 2.853 (x 1.052) | 49 (0.061) | 0.985 | amino acid derivative metabolism | CG18749 SamDC Tbh |
| 997 | GO:0005549 | F | 3, | 7 | 6.696 (x 1.045) | 115 (0.061) | 0.985 | odorant binding | Obp56a Obp58b Or46a Or59a Or85a Or94a Or98b |
| 998 | GO:0000004 | P | 2, | 41 | 40.814 (x 1.005) | 701 (0.058) | 0.985 | biological process unknown | CG10674 CG11722 CG12022 CG13691 CG14701 CG16817 CG30105 CG30126 CG30154 CG30334 CG30343 CG30476 CG31601 CG31715 CG31922 CG31957 CG32023 CG32160 CG32175 CG32202 CG32207 CG32442 CG32448 CG32582 CG32625 CG32637 CG32692 CG32710 CG32856 CG33322 CG3887 CG4101 CG7949 CG8152 EG:63B12.12 JhI-26 Karl NP15.6 Rep2 fau retinin |
| 999 | GO:0016485 | P | 8, | 2 | 1.747 (x 1.145) | 30 (0.067) | 0.985 | protein processing | InR pip |
| 1000 | GO:0045471 | P | 6, | 1 | 0.699 (x 1.431) | 12 (0.083) | 0.986 | response to ethanol | Tbh |
| 1001 | GO:0035223 | P | 5, 6, | 1 | 0.815 (x 1.227) | 14 (0.071) | 0.986 | leg disc pattern formation | rho |
| 1002 | GO:0008064 | P | 6, 7, 10, | 1 | 0.757 (x 1.321) | 13 (0.077) | 0.986 | regulation of actin polymerization and/or depolymerization | Abl |
| 1003 | GO:0009613 | P | 4, 5, | 6 | 5.822 (x 1.031) | 100 (0.060) | 0.986 | response to pest, pathogen or parasite | Bc Def PGRP-SA Tehao Toll-7 upd3 |
| 1004 | GO:0015101 | F | 5, | 2 | 1.688 (x 1.185) | 29 (0.069) | 0.986 | organic cation transporter activity | CG7084 CG7333 |
| 1005 | GO:0051606 | P | 3, 4, | 3 | 2.853 (x 1.052) | 49 (0.061) | 0.986 | detection of stimulus | Arr2 PGRP-SA tko |
| 1006 | GO:0016849 | F | 4, | 2 | 1.747 (x 1.145) | 30 (0.067) | 0.986 | phosphorus-oxygen lyase activity | Ac78C CG10738 |
| 1007 | GO:0019732 | P | 6, 7, 8, | 1 | 0.815 (x 1.227) | 14 (0.071) | 0.987 | antifungal humoral response | Tehao |
| 1008 | GO:0016878 | F | 5, | 1 | 0.757 (x 1.321) | 13 (0.077) | 0.987 | acid-thiol ligase activity | CG6432 |
| 1009 | GO:0043170 | P | 4, | 180 | 181.423 (x 0.992) | 3116 (0.058) | 0.987 | macromolecule metabolism | Aats-his Abl Act57B Amyrel Arf84F BG:DS02740.5 BcDNA:GH08420 BcDNA:GH08902 CG10092 CG10104 CG10166 CG10237 CG10418 CG10466 CG10738 CG11313 CG11360 CG11597 CG11909 CG1213 CG12133 CG1249 CG12775 CG1299 CG1304 CG13277 CG13318 CG14894 CG14935 CG15220 CG15408 CG17266 CG17768 CG18013 CG18223 CG18749 CG18767 CG1883 CG1885 CG18869 CG2021 CG2056 CG2789 CG2964 CG2998 CG30283 CG3036 CG30438 CG30499 CG31184 CG31611 CG31704 CG3215 CG32479 CG32627 CG33002 CG33128 CG33177 CG3355 CG3843 CG3931 CG40045 CG40068 CG4046 CG4095 CG4279 CG4288 CG4386 CG4408 CG4866 CG5001 CG5103 CG5177 CG5338 CG5382 CG6214 CG6461 CG6574 CG6610 CG6723 CG6763 CG6764 CG6803 CG6947 CG7014 CG7298 CG7770 CG8415 CG8550 CG8756 CG8857 CG8918 CG9267 CG9372 CG9602 CG9804 Chit CycG Cyp12e1 Dab DebB Dhc98D Dhfr Doa EG:9D2.4 EG:BACR7A4.18 EG:BACR7A4.8 Gasp Gs2 HP1c Hsp22 Iap2 ImpL3 InR Las Obp58b Orc6 PGRP-SA PNUTS Peritrophin-A Pglym78 Pof Prosbeta5 REG RN-tre RpL11 RpL17A RpL27A RpL38 RpL46 RpL8 RpL9 RpP1 RpS17 RpS18 RpS4 RpS9 Ser7 SmB Spn6 Sras Takl2 Tfb2 Timp Ugt86De Ugt86Di agt apt betaggt-II btl cyp33 gatA hay hep jdp ksr l(3)01239 mRpL11 mRpL14 mRpL2 mRpL21 mRpL22 mRpL22-24 mRpL33 mRpS14 mRpS21 mRpS24 mRpS26 mRpS32 na oho23B pip pum rpr sda shu skpA sop tko trol |
| 1010 | GO:0044265 | P | 6, | 10 | 10.014 (x 0.999) | 172 (0.058) | 0.987 | cellular macromolecule catabolism | CG2964 CG30499 CG32479 CG5103 Chit ImpL3 PGRP-SA Pglym78 Prosbeta5 pum |
| 1011 | GO:0007417 | P | 5, | 7 | 7.103 (x 0.985) | 122 (0.057) | 0.987 | central nervous system development | Abl Doa apt btl rho robl rpr |
| 1012 | GO:0048278 | P | 6, 7, | 2 | 1.688 (x 1.185) | 29 (0.069) | 0.987 | vesicle docking | Syx16 Syx8 |
| 1013 | GO:0009063 | P | 7, 8, | 3 | 2.853 (x 1.052) | 49 (0.061) | 0.987 | amino acid catabolism | CG10184 CG11251 Gs2 |
| 1014 | GO:0040014 | P | 4, | 1 | 0.815 (x 1.227) | 14 (0.071) | 0.987 | regulation of body size | InR |
| 1015 | GO:0016566 | F | 4, | 1 | 0.757 (x 1.321) | 13 (0.077) | 0.988 | specific transcriptional repressor activity | gt |
| 1016 | GO:0007274 | P | 7, | 2 | 1.688 (x 1.185) | 29 (0.069) | 0.988 | neuromuscular synaptic transmission | CG31146 apt |
| 1017 | GO:0008033 | P | 8, | 1 | 0.815 (x 1.227) | 14 (0.071) | 0.988 | tRNA processing | EG:BACR7A4.8 |
| 1018 | GO:0003711 | F | 3, | 1 | 0.757 (x 1.321) | 13 (0.077) | 0.989 | transcriptional elongation regulator activity | CG18013 |
| 1019 | GO:0008324 | F | 4, | 23 | 23.231 (x 0.990) | 399 (0.058) | 0.989 | cation transporter activity | CG10804 CG11015 CG14482 CG17280 CG1756 CG3036 CG31477 CG4169 CG4288 CG4805 CG5535 CG6723 CG7084 CG7181 CG7211 CG7333 CG8925 CoVa Tsf1 Vha36 l(2)06225 na sun |
| 1020 | GO:0008286 | P | 8, | 1 | 0.815 (x 1.227) | 14 (0.071) | 0.989 | insulin receptor signaling pathway | InR |
| 1021 | GO:0016646 | F | 5, | 1 | 0.757 (x 1.321) | 13 (0.077) | 0.99 | oxidoreductase activity, acting on the CH-NH group of donors, NAD or NADP as acceptor | Dhfr |
| 1022 | GO:0030166 | P | 7, 8, | 1 | 0.815 (x 1.227) | 14 (0.071) | 0.99 | proteoglycan biosynthesis | Act57B |
| 1023 | GO:0030832 | P | 9, | 1 | 0.757 (x 1.321) | 13 (0.077) | 0.991 | regulation of actin filament length | Abl |
| 1024 | GO:0016789 | F | 5, | 7 | 6.987 (x 1.002) | 120 (0.058) | 0.991 | carboxylic ester hydrolase activity | CG18530 CG31272 CG5162 CG6296 Lip1 Pld alpha-Est8 |
| 1025 | GO:0045793 | P | 6, 7, | 1 | 0.815 (x 1.227) | 14 (0.071) | 0.991 | positive regulation of cell size | InR |
| 1026 | GO:0016405 | F | 5, | 1 | 0.757 (x 1.321) | 13 (0.077) | 0.992 | CoA-ligase activity | CG6432 |
| 1027 | GO:0004252 | F | 6, | 15 | 14.963 (x 1.002) | 257 (0.058) | 0.992 | serine-type endopeptidase activity | BG:DS01068.5 BcDNA:GH08420 CG11313 CG12133 CG1299 CG1304 CG13318 CG18223 CG2056 CG30283 CG3355 CG4386 CG9372 EG:9D2.4 Ser7 |
| 1028 | GO:0015980 | P | 6, | 7 | 6.870 (x 1.019) | 118 (0.059) | 0.992 | energy derivation by oxidation of organic compounds | CG2964 CG30499 CG4095 CG5103 CG5177 ImpL3 Pglym78 |
| 1029 | GO:0035264 | P | 3, | 1 | 0.815 (x 1.227) | 14 (0.071) | 0.992 | body growth | InR |
| 1030 | GO:0042834 | F | 4, | 1 | 0.757 (x 1.321) | 13 (0.077) | 0.993 | peptidoglycan binding | PGRP-SA |
| 1031 | GO:0016023 | C | 5, 6, 7, 8, 9, | 5 | 4.833 (x 1.035) | 83 (0.060) | 0.993 | cytoplasmic membrane-bound vesicle | CG14691 CG31272 Rab3 SytIV unc-13 |
| 1032 | GO:0042052 | P | 8, 9, 10, 11, | 1 | 0.815 (x 1.227) | 14 (0.071) | 0.993 | rhabdomere development | Cpn |
| 1033 | GO:0007539 | P | 5, | 1 | 0.757 (x 1.321) | 13 (0.077) | 0.994 | primary sex determination, soma | sisA |
| 1034 | GO:0031410 | C | 4, 5, 6, 7, 8, | 5 | 4.833 (x 1.035) | 83 (0.060) | 0.994 | cytoplasmic vesicle | CG14691 CG31272 Rab3 SytIV unc-13 |
| 1035 | GO:0048015 | P | 7, | 1 | 0.873 (x 1.145) | 15 (0.067) | 0.994 | phosphoinositide-mediated signaling | Dgkepsilon |
| 1036 | GO:0007062 | P | 5, | 1 | 0.815 (x 1.227) | 14 (0.071) | 0.994 | sister chromatid cohesion | Nipped-B |
| 1037 | GO:0007467 | P | 5, | 5 | 5.065 (x 0.987) | 87 (0.057) | 0.994 | photoreceptor cell differentiation (sensu Endopterygota) | Cpn Dab Doa chp salm |
| 1038 | GO:0051297 | P | 7, | 1 | 0.757 (x 1.321) | 13 (0.077) | 0.994 | centrosome organization and biogenesis | skpA |
| 1039 | GO:0016810 | F | 4, | 5 | 4.949 (x 1.010) | 85 (0.059) | 0.995 | hydrolase activity, acting on carbon-nitrogen (but not peptide) bonds | CG32626 CG8360 CG8756 PGRP-SA gatA |
| 1040 | GO:0005355 | F | 7, | 1 | 0.873 (x 1.145) | 15 (0.067) | 0.995 | glucose transporter activity | CG1213 |
| 1041 | GO:0005244 | F | 5, 6, | 3 | 2.969 (x 1.010) | 51 (0.059) | 0.995 | voltage-gated ion channel activity | CG1756 na porin |
| 1042 | GO:0004568 | F | 6, | 1 | 0.815 (x 1.227) | 14 (0.071) | 0.995 | chitinase activity | Chit |
| 1043 | GO:0008533 | F | 7, | 1 | 0.757 (x 1.321) | 13 (0.077) | 0.995 | astacin activity | CG6763 |
| 1044 | GO:0005125 | F | 4, 5, | 1 | 0.873 (x 1.145) | 15 (0.067) | 0.996 | cytokine activity | upd3 |
| 1045 | GO:0007155 | P | 3, | 17 | 17.642 (x 0.964) | 303 (0.056) | 0.996 | cell adhesion | BG:DS00180.7 CG1504 CG18249 CG33543 CG5819 CG6124 Cad99C Con Gp150 Mp20 RN-tre Tehao Toll-7 chp hig trol veli |
| 1046 | GO:0031497 | P | 10, | 3 | 2.969 (x 1.010) | 51 (0.059) | 0.996 | chromatin assembly | CG31611 EG:BACR7A4.18 Orc6 |
| 1047 | GO:0007474 | P | 7, 8, 9, | 1 | 0.815 (x 1.227) | 14 (0.071) | 0.996 | wing vein specification | rho |
| 1048 | GO:0007218 | P | 7, | 2 | 1.921 (x 1.041) | 33 (0.061) | 0.996 | neuropeptide signaling pathway | CG11318 CG15361 |
| 1049 | GO:0048102 | P | 6, | 4 | 4.134 (x 0.968) | 71 (0.056) | 0.996 | autophagic cell death | CG10861 Doa Eip93F rpr |
| 1050 | GO:0030055 | C | 6, 7, 8, | 1 | 0.873 (x 1.145) | 15 (0.067) | 0.996 | cell-matrix junction | Abl |
| 1051 | GO:0005534 | F | 6, | 1 | 0.757 (x 1.321) | 13 (0.077) | 0.996 | galactose binding | lectin-28C |
| 1052 | GO:0008639 | F | 3, | 2 | 1.980 (x 1.010) | 34 (0.059) | 0.997 | small protein conjugating enzyme activity | CG40045 CG9602 |
| 1053 | GO:0005924 | C | 7, 8, 9, | 1 | 0.815 (x 1.227) | 14 (0.071) | 0.997 | cell-substrate adherens junction | Abl |
| 1054 | GO:0004840 | F | 4, | 2 | 1.921 (x 1.041) | 33 (0.061) | 0.997 | ubiquitin conjugating enzyme activity | CG40045 CG9602 |
| 1055 | GO:0035070 | P | 6, | 4 | 4.134 (x 0.968) | 71 (0.056) | 0.997 | salivary gland histolysis | CG10861 Doa Eip93F rpr |
| 1056 | GO:0035215 | P | 5, | 1 | 0.873 (x 1.145) | 15 (0.067) | 0.997 | genital disc development | btl |
| 1057 | GO:0042133 | P | 5, 8, | 1 | 0.757 (x 1.321) | 13 (0.077) | 0.997 | neurotransmitter metabolism | Tbh |
| 1058 | GO:0009187 | P | 7, | 2 | 1.980 (x 1.010) | 34 (0.059) | 0.997 | cyclic nucleotide metabolism | Ac78C CG10738 |
| 1059 | GO:0009070 | P | 8, 9, | 1 | 0.815 (x 1.227) | 14 (0.071) | 0.998 | serine family amino acid biosynthesis | Dhfr |
| 1060 | GO:0016125 | P | 6, 7, 8, | 2 | 1.921 (x 1.041) | 33 (0.061) | 0.998 | sterol metabolism | CG10268 Hmgs |
| 1061 | GO:0035071 | P | 7, | 4 | 4.134 (x 0.968) | 71 (0.056) | 0.998 | salivary gland cell autophagic cell death | CG10861 Doa Eip93F rpr |
| 1062 | GO:0007435 | P | 6, | 1 | 0.873 (x 1.145) | 15 (0.067) | 0.998 | salivary gland morphogenesis | btl |
| 1063 | GO:0044255 | P | 5, 6, | 19 | 19.505 (x 0.974) | 335 (0.057) | 0.998 | cellular lipid metabolism | CG10268 CG1140 CG17821 CG18869 CG30438 CG5162 CG6432 CG6921 CG8498 CG8778 Cyp28d2 Cyp311a1 Cyp49a1 Hmgs Pld Ugt86De Ugt86Di fu12 mtacp1 |
| 1064 | GO:0016831 | F | 5, | 2 | 1.980 (x 1.010) | 34 (0.059) | 0.998 | carboxy-lyase activity | CG11251 SamDC |
| 1065 | GO:0007600 | P | 3, 5, | 15 | 15.022 (x 0.999) | 258 (0.058) | 0.998 | sensory perception | Arr2 Doa Gr61a Gr98c Myo28B1 Obp56a Or46a Or59a Or85a Or94a Or98b Rh7 TyrR chp tko |
| 1066 | GO:0006584 | P | 7, 8, | 1 | 0.757 (x 1.321) | 13 (0.077) | 0.998 | catecholamine metabolism | Tbh |
| 1067 | GO:0007479 | P | 6, 7, | 1 | 0.815 (x 1.227) | 14 (0.071) | 0.999 | leg disc proximal/distal pattern formation | rho |
| 1068 | GO:0016740 | F | 3, | 56 | 56.302 (x 0.995) | 967 (0.058) | 0.999 | transferase activity | Abl Act57B CG10166 CG10268 CG10738 CG1140 CG14721 CG17224 CG17639 CG17821 CG18869 CG2056 CG2846 CG2964 CG30438 CG33177 CG5037 CG5103 CG5122 CG5224 CG5397 CG5537 CG6214 CG6461 CG6921 CG7339 CG9164 CG9326 CG9790 Dgkepsilon Doa EG:BACR7A4.8 GstD6 GstD9 GstE1 GstE5 GstE6 GstE7 Hmgs InR Las RpII18 Rpb10 Rpb4 Takl2 Ugt86De Ugt86Di agt betaggt-II btl fu12 hep ksr l(3)02640 nmdyn-D6 pip |
| 1069 | GO:0006913 | P | 6, 7, 8, | 4 | 4.134 (x 0.968) | 71 (0.056) | 0.999 | nucleocytoplasmic transport | CG10320 CG10950 CG8219 Nxt1 |
| 1070 | GO:0016705 | F | 4, | 4 | 3.843 (x 1.041) | 66 (0.061) | 0.999 | oxidoreductase activity, acting on paired donors, with incorporation or reduction of molecular oxygen | Bc CG18749 CG8193 Tbh |
| 1071 | GO:0044448 | C | 5, 6, 7, 8, 9, | 2 | 1.921 (x 1.041) | 33 (0.061) | 0.999 | cell cortex part | insc mira |
| 1072 | GO:0004693 | F | 8, | 1 | 0.873 (x 1.145) | 15 (0.067) | 0.999 | cyclin-dependent protein kinase activity | CG9790 |
| 1073 | GO:0007582 | P | 2, | 396 | 397.780 (x 0.996) | 6832 (0.058) | 1 | physiological process | Aats-his Abl Ac78C Act57B Ahcy13 Amyrel AnnX Arf84F Arp11 Arr2 BG:DS01219.1 BG:DS02740.5 Bc BcDNA:GH08420 BcDNA:GH08902 Bro CAH2 CG10092 CG10104 CG10166 CG10184 CG10237 CG10268 CG10320 CG10418 CG10425 CG10466 CG10638 CG10669 CG10738 CG10804 CG10861 CG10950 CG10962 CG11015 CG11251 CG11313 CG11360 CG1140 CG11455 CG11597 CG11897 CG11898 CG11909 CG1213 CG12133 CG12175 CG12361 CG12400 CG1249 CG12605 CG12775 CG1299 CG1304 CG13277 CG13318 CG13889 CG14482 CG14508 CG14691 CG14721 CG14825 CG14894 CG14935 CG1504 CG15220 CG15398 CG15408 CG17224 CG17266 CG17280 CG17385 CG1756 CG17768 CG17821 CG18011 CG18013 CG18155 CG18223 CG18249 CG18522 CG18530 CG18619 CG18749 CG18767 CG1883 CG1885 CG18869 CG2021 CG2056 CG2069 CG2185 CG2277 CG2750 CG2789 CG2846 CG2964 CG2998 CG30022 CG30283 CG3036 CG30438 CG30499 CG31146 CG31184 CG31272 CG31477 CG31551 CG31611 CG31704 CG32105 CG3215 CG32174 CG32230 CG32409 CG32479 CG32549 CG32626 CG32627 CG32677 CG33002 CG33066 CG33128 CG33177 CG3355 CG3397 CG3529 CG3843 CG3931 CG40045 CG40068 CG4046 CG4071 CG4095 CG4169 CG4187 CG4279 CG4288 CG4386 CG4408 CG4511 CG4592 CG4673 CG4769 CG4805 CG4827 CG4866 CG5001 CG5037 CG5103 CG5122 CG5162 CG5177 CG5338 CG5382 CG5535 CG5537 CG5548 CG5559 CG5819 CG6214 CG6272 CG6296 CG6432 CG6461 CG6574 CG6610 CG6723 CG6763 CG6764 CG6803 CG6921 CG6947 CG7014 CG7084 CG7181 CG7188 CG7211 CG7298 CG7322 CG7333 CG7339 CG7770 CG7777 CG7834 CG8004 CG8193 CG8219 CG8271 CG8360 CG8415 CG8498 CG8550 CG8756 CG8778 CG8857 CG8916 CG8918 CG8925 CG8993 CG9267 CG9326 CG9372 CG9413 CG9602 CG9629 CG9650 CG9790 CG9804 CG9836 CG9862 CLIP-190 Chit CoVa Con Cpn CycG Cyp12e1 Cyp28d2 Cyp311a1 Cyp49a1 Dab DebB Def Dgkepsilon Dhc98D Dhfr Doa EG:152A3.7 EG:9D2.4 EG:BACR7A4.14 EG:BACR7A4.18 EG:BACR7A4.8 Eip93F GABA-B-R2 Gasp Gp150 Gr61a Gr98c Gs2 GstE1 GstE5 GstE6 GstE7 HP1c Hmgs Hr38 Hr4 Hsp22 Iap2 Idgf1 Idgf2 ImpL3 InR Kr-h1 Las Lip1 Mlc2 Mp20 Myo28B1 Nipped-B Nxt1 Obp56a Obp58b Or46a Or59a Or85a Or94a Or98b Orc6 PGRP-SA PNUTS Pdsw Peritrophin-A Pglym78 Pld Pof Prosbeta5 Prx6005 REG RN-tre Rab3 Rh7 RpII18 RpL11 RpL17A RpL27A RpL38 RpL46 RpL8 RpL9 RpP1 RpS17 RpS18 RpS4 RpS9 Rpb10 Rpb4 SIP1 SamDC SelG Ser7 Sgs4 SmB Sod Spn6 Sras Ssb-c31a SytIV Syx16 Syx8 Taf10b Taf11 Takl2 Takr99D Tbh Tehao TfIIEalpha Tfb2 Tig Tim17b2 Tim9a Timp Tm1 Toll-7 TpnC41C Trap36 Tsf1 Tsp42El TyrR Ugt86De Ugt86Di Vha36 agt apt bbx betaggt-II btl chp cyp33 dmrt93B dynactin-subunit-p25 e(y)2 fu12 gatA glob1 gt hay hep insc jdp ksr l(2)03659 l(2)06225 l(3)01239 l(3)02640 mRpL11 mRpL14 mRpL2 mRpL21 mRpL22 mRpL22-24 mRpL33 mRpS14 mRpS21 mRpS24 mRpS26 mRpS32 mira mirr mtacp1 na nmdyn-D6 oho23B pip porin pum ran-like rho robl rpr salm sda shu skpA sop sra sun tko toy trol unc-13 upd3 veli vvl zormin |
| 1074 | GO:0009987 | P | 2, | 389 | 390.444 (x 0.996) | 6706 (0.058) | 1 | cellular process | Aats-his Abl Ac78C Act57B Ahcy13 AnnX Arf84F Arp11 Arr2 BG:DS00180.7 BG:DS02740.5 Bc BcDNA:GH08420 BcDNA:GH08902 Bro CAH2 CG10092 CG10104 CG10166 CG10184 CG10237 CG10268 CG10320 CG10418 CG10466 CG10638 CG10669 CG10738 CG10804 CG10861 CG10950 CG11015 CG11251 CG11313 CG11318 CG11360 CG1140 CG11455 CG11597 CG11897 CG11898 CG11909 CG1213 CG12133 CG12175 CG12361 CG12400 CG1249 CG12605 CG12775 CG1299 CG1304 CG13277 CG13318 CG13889 CG14482 CG14508 CG14691 CG14721 CG14825 CG14894 CG1504 CG15220 CG15361 CG15398 CG15408 CG17224 CG17262 CG17266 CG17280 CG17385 CG1756 CG17768 CG17821 CG1796 CG18011 CG18013 CG18223 CG18249 CG18522 CG18619 CG18749 CG18767 CG1883 CG1885 CG18869 CG2021 CG2056 CG2069 CG2185 CG2277 CG2750 CG2789 CG2846 CG2964 CG2998 CG30022 CG30283 CG3036 CG30438 CG30499 CG31146 CG31184 CG31272 CG31477 CG31551 CG31611 CG31704 CG32105 CG3215 CG32174 CG32230 CG32409 CG32479 CG32549 CG32626 CG32627 CG32677 CG33002 CG33066 CG33128 CG33177 CG33543 CG3355 CG3397 CG3529 CG3843 CG3931 CG40045 CG40068 CG4046 CG4071 CG4095 CG4169 CG4187 CG4279 CG4288 CG4386 CG4408 CG4511 CG4673 CG4769 CG4805 CG4827 CG4866 CG5001 CG5037 CG5103 CG5122 CG5162 CG5177 CG5338 CG5382 CG5535 CG5537 CG5548 CG5559 CG5819 CG6124 CG6214 CG6272 CG6432 CG6461 CG6574 CG6610 CG6723 CG6763 CG6764 CG6803 CG6921 CG6947 CG7014 CG7084 CG7181 CG7188 CG7211 CG7298 CG7333 CG7339 CG7646 CG7770 CG7777 CG7834 CG8004 CG8193 CG8219 CG8271 CG8360 CG8415 CG8498 CG8550 CG8756 CG8778 CG8857 CG8916 CG8918 CG8925 CG8993 CG9267 CG9326 CG9372 CG9413 CG9602 CG9650 CG9790 CG9804 CG9862 CLIP-190 Cad99C Chit CoVa Con Cpn CycG Cyp12e1 Cyp28d2 Cyp311a1 Cyp49a1 Dab DebB Dgkepsilon Dhc98D Dhfr Doa EG:152A3.7 EG:52C10.2 EG:9D2.4 EG:BACR7A4.18 EG:BACR7A4.8 Eip93F GABA-B-R2 Gasp Gp150 Gs2 GstE1 GstE5 GstE6 GstE7 HP1c Hmgs Hr38 Hr4 Hsp22 Iap2 Idgf1 Idgf2 ImpL3 InR Kr-h1 Las Mp20 Myo28B1 Nipped-B Nxt1 Obp58b Or46a Or59a Or85a Or94a Orc6 PGRP-SA PNUTS Pdsw Peritrophin-A Pglym78 Pld Pof Prosbeta5 Prx6005 REG RN-tre Rab3 Rh7 RpII18 RpL11 RpL17A RpL27A RpL38 RpL46 RpL8 RpL9 RpP1 RpS17 RpS18 RpS4 RpS9 Rpb10 Rpb4 SIP1 SamDC SelG Ser7 SmB Sod Spn6 Sras Ssb-c31a SytIV Syx16 Syx8 Taf10b Taf11 Takl2 Takr99D Tbh Tehao TfIIEalpha Tfb2 Tig Tim17b2 Tim9a Timp Tm1 Toll-7 TpnC41C Trap36 Tsf1 Tsp42El TyrR Ugt86De Ugt86Di Vha36 agt apt bbx betaggt-II btl chp cyp33 dmrt93B dynactin-subunit-p25 e(y)2 fu12 gatA glob1 gt hay hep hig insc inx7 jdp ksr l(2)03659 l(2)06225 l(3)01239 l(3)02640 mRpL11 mRpL14 mRpL2 mRpL21 mRpL22 mRpL22-24 mRpL33 mRpS14 mRpS21 mRpS24 mRpS26 mRpS32 mira mirr mtacp1 na nmdyn-D6 oho23B pip porin pum ran-like regucalcin rho robl rpr salm sda shu skpA sop sra sun tko toy trol tsl unc-13 upd3 veli vvl zpg |
| 1075 | GO:0006112 | P | 7, | 1 | 0.815 (x 1.227) | 14 (0.071) | 1 | energy reserve metabolism | CG5177 |
| 1076 | GO:0004181 | F | 7, | 2 | 1.863 (x 1.073) | 32 (0.062) | 1 | metallocarboxypeptidase activity | CG32627 CG4408 |
| 1077 | GO:0007610 | P | 3, | 11 | 11.121 (x 0.989) | 191 (0.058) | 1 | behavior | BG:DS01219.1 CG10460 CG9381 Obp56a Tbh na pum rho sda sra tko |
| 1078 | GO:0050789 | P | 2, | 56 | 87.626 (x 0.639) | 1505 (0.037) | 1 | regulation of biological process | Abl AnnX Arf84F Bro CG10418 CG10669 CG11360 CG12175 CG12361 CG12605 CG15398 CG17385 CG18619 CG2750 CG32105 CG40068 CG6272 CG7188 CG9650 CG9790 Dgkepsilon Doa EG:52C10.2 Eip93F HP1c Hr38 Hr4 Iap2 InR Kr-h1 Nipped-B Orc6 PGRP-SA PNUTS Rab3 Rpb4 SelG Ssb-c31a Taf11 Tfb2 apt bbx dmrt93B gt hay hep mirr pum rho rpr salm skpA sra toy trol vvl |
| 1079 | GO:0051169 | P | 6, 7, 8, | 4 | 3.843 (x 1.041) | 66 (0.061) | 1 | nuclear transport | CG10320 CG10950 CG8219 Nxt1 |
| 1080 | GO:0007538 | P | 4, | 1 | 0.873 (x 1.145) | 15 (0.067) | 1 | primary sex determination | sisA |
| 1081 | GO:0007049 | P | 4, | 11 | 27.423 (x 0.401) | 471 (0.023) | 1 | cell cycle | Abl CG2750 CG9790 CG9862 Nipped-B insc pip pum ran-like skpA sra |
| 1082 | GO:0045045 | P | 5, 6, | 12 | 12.518 (x 0.959) | 215 (0.056) | 1 | secretory pathway | AnnX Arf84F CG14691 CG2185 CG31272 CG3529 CG5559 Rab3 SytIV Syx16 Syx8 unc-13 |
| 1083 | GO:0018958 | P | 6, | 1 | 0.815 (x 1.227) | 14 (0.071) | 1 | phenol metabolism | Tbh |
| 1084 | GO:0042254 | P | 6, | 2 | 1.863 (x 1.073) | 32 (0.062) | 1 | ribosome biogenesis and assembly | CG32409 CG6764 |
| 1085 | GO:0018208 | P | 9, | 1 | 0.873 (x 1.145) | 15 (0.067) | 1 | peptidyl-proline modification | CG18749 |
| 1086 | GO:0050791 | P | 3, | 50 | 77.961 (x 0.641) | 1339 (0.037) | 1 | regulation of physiological process | Abl AnnX Arf84F Bro CG10418 CG10669 CG11360 CG12175 CG12361 CG12605 CG15398 CG17385 CG18619 CG2750 CG32105 CG40068 CG6272 CG7188 CG9650 CG9790 Doa Eip93F HP1c Hr38 Hr4 Iap2 InR Kr-h1 Nipped-B Orc6 PNUTS Rab3 Rpb4 SelG Ssb-c31a Taf11 Tfb2 apt bbx dmrt93B gt hay hep mirr pum rpr salm skpA toy vvl |
| 1087 | GO:0050794 | P | 3, | 52 | 80.173 (x 0.649) | 1377 (0.038) | 1 | regulation of cellular process | Abl Arf84F Bro CG10418 CG10669 CG11360 CG12175 CG12361 CG12605 CG15398 CG17385 CG18619 CG2750 CG32105 CG40068 CG6272 CG7188 CG9650 CG9790 Doa EG:52C10.2 Eip93F HP1c Hr38 Hr4 Iap2 InR Kr-h1 Nipped-B Orc6 PGRP-SA PNUTS Rab3 Rpb4 SelG Ssb-c31a Taf11 Tfb2 apt bbx dmrt93B gt hay hep mirr pum rho rpr salm skpA toy vvl |
| 1088 | GO:0000175 | F | 8, 9, | 1 | 0.815 (x 1.227) | 14 (0.071) | 1 | 3'-5'-exoribonuclease activity | CG3931 |
| 1089 | GO:0016684 | F | 4, | 2 | 1.863 (x 1.073) | 32 (0.062) | 1 | oxidoreductase activity, acting on peroxide as acceptor | CG13889 Prx6005 |
| 1090 | GO:0000786 | C | 3, 5, 6, 7, 8, 9, 10, 11, | 1 | 0.873 (x 1.145) | 15 (0.067) | 1 | nucleosome | CG31611 |
| 1091 | GO:0000166 | F | 3, | 29 | 51.120 (x 0.567) | 878 (0.033) | 1 | nucleotide binding | Aats-his Abl Arf84F CG10092 CG10738 CG11897 CG11898 CG14721 CG17904 CG1939 CG2056 CG32627 CG4511 CG4858 CG6214 Doa EG:BACR7A4.17 Gp150 InR Myo28B1 Rab3 Takl2 btl hay hep ksr l(2)03659 nmdyn-D6 ran-like |
| 1092 | GO:0051244 | P | 4, | 49 | 75.515 (x 0.649) | 1297 (0.038) | 1 | regulation of cellular physiological process | Abl Arf84F Bro CG10418 CG10669 CG11360 CG12175 CG12361 CG12605 CG15398 CG17385 CG18619 CG2750 CG32105 CG40068 CG6272 CG7188 CG9650 CG9790 Doa Eip93F HP1c Hr38 Hr4 Iap2 InR Kr-h1 Nipped-B Orc6 PNUTS Rab3 Rpb4 SelG Ssb-c31a Taf11 Tfb2 apt bbx dmrt93B gt hay hep mirr pum rpr salm skpA toy vvl |
| 1093 | GO:0031023 | P | 6, | 1 | 0.815 (x 1.227) | 14 (0.071) | 1 | microtubule organizing center organization and biogenesis | skpA |
| 1094 | GO:0016044 | P | 5, | 2 | 1.863 (x 1.073) | 32 (0.062) | 1 | membrane organization and biogenesis | CG33066 Tim9a |
| 1095 | GO:0031982 | C | 3, | 5 | 5.007 (x 0.999) | 86 (0.058) | 1 | vesicle | CG14691 CG31272 Rab3 SytIV unc-13 |
| 1096 | GO:0006029 | P | 7, | 1 | 0.873 (x 1.145) | 15 (0.067) | 1 | proteoglycan metabolism | Act57B |
| 1097 | GO:0017076 | F | 4, | 28 | 49.431 (x 0.566) | 849 (0.033) | 1 | purine nucleotide binding | Aats-his Abl Arf84F CG10092 CG10738 CG11897 CG11898 CG14721 CG1939 CG2056 CG32627 CG4511 CG4858 CG6214 Doa EG:BACR7A4.17 Gp150 InR Myo28B1 Rab3 Takl2 btl hay hep ksr l(2)03659 nmdyn-D6 ran-like |
| 1098 | GO:0000221 | C | 4, 5, 6, 7, 8, 9, 10, 11, 12, | 1 | 0.815 (x 1.227) | 14 (0.071) | 1 | hydrogen-transporting ATPase V1 domain | Vha36 |
| 1099 | GO:0006996 | P | 5, | 23 | 42.037 (x 0.547) | 722 (0.032) | 1 | organelle organization and biogenesis | Abl Act57B Arp11 CG10861 CG31551 CG31611 CG32409 CG33066 CG6764 CG6803 CLIP-190 Dhc98D Doa EG:BACR7A4.18 HP1c Orc6 Tim9a dynactin-subunit-p25 hep insc mira robl skpA |
| 1100 | GO:0004182 | F | 8, | 2 | 1.863 (x 1.073) | 32 (0.062) | 1 | carboxypeptidase A activity | CG32627 CG4408 |
| 1101 | GO:0031988 | C | 4, | 5 | 5.007 (x 0.999) | 86 (0.058) | 1 | membrane-bound vesicle | CG14691 CG31272 Rab3 SytIV unc-13 |
| 1102 | GO:0019511 | P | 10, | 1 | 0.873 (x 1.145) | 15 (0.067) | 1 | peptidyl-proline hydroxylation | CG18749 |
| 1103 | GO:0016070 | P | 6, | 21 | 21.368 (x 0.983) | 367 (0.057) | 1 | RNA metabolism | Aats-his CG10092 CG10418 CG10466 CG11360 CG1249 CG13277 CG17266 CG17768 CG2021 CG31184 CG3931 CG4279 CG6610 DebB Doa EG:BACR7A4.8 SmB gatA hay pum |
| 1104 | GO:0043412 | P | 6, | 32 | 53.391 (x 0.599) | 917 (0.035) | 1 | biopolymer modification | Abl Arf84F BG:DS02740.5 CG10166 CG10738 CG11360 CG11597 CG18749 CG2056 CG32479 CG33177 CG40045 CG5382 CG6214 CG6461 CG8918 CG9267 CG9602 CG9804 Doa Iap2 InR PNUTS Prosbeta5 Takl2 betaggt-II btl hep ksr pip rpr trol |
| 1105 | GO:0030695 | F | 3, | 1 | 7.569 (x 0.132) | 130 (0.008) | 1 | GTPase regulator activity | EG:52C10.2 |
| 1106 | GO:0006270 | P | 9, | 1 | 0.815 (x 1.227) | 14 (0.071) | 1 | DNA replication initiation | Orc6 |
| 1107 | GO:0004601 | F | 3, 5, | 2 | 1.863 (x 1.073) | 32 (0.062) | 1 | peroxidase activity | CG13889 Prx6005 |
| 1108 | GO:0007200 | P | 7, 8, | 1 | 0.873 (x 1.145) | 15 (0.067) | 1 | G-protein signaling, coupled to IP3 second messenger (phospholipase C activating) | Dgkepsilon |
| 1109 | GO:0007010 | P | 6, | 12 | 26.200 (x 0.458) | 450 (0.027) | 1 | cytoskeleton organization and biogenesis | Abl Act57B Arp11 CG31551 CG6803 CLIP-190 Dhc98D dynactin-subunit-p25 hep insc mira robl |
| 1110 | GO:0007517 | P | 4, | 6 | 6.288 (x 0.954) | 108 (0.056) | 1 | muscle development | CG6803 Mp20 btl dmrt93B insc toy |
| 1111 | GO:0007548 | P | 3, | 3 | 2.911 (x 1.031) | 50 (0.060) | 1 | sex differentiation | CG7194 Doa dmrt93B |
| 1112 | GO:0004386 | F | 3, | 1 | 6.521 (x 0.153) | 112 (0.009) | 1 | helicase activity | hay |
| 1113 | GO:0006464 | P | 7, | 31 | 51.062 (x 0.607) | 877 (0.035) | 1 | protein modification | Abl Arf84F BG:DS02740.5 CG10166 CG10738 CG11360 CG11597 CG18749 CG2056 CG32479 CG33177 CG40045 CG5382 CG6214 CG6461 CG8918 CG9267 CG9602 CG9804 Doa Iap2 InR PNUTS Prosbeta5 Takl2 betaggt-II btl hep ksr pip rpr |
| 1114 | GO:0019932 | P | 6, | 5 | 5.124 (x 0.976) | 88 (0.057) | 1 | second-messenger-mediated signaling | CG7646 Dgkepsilon TpnC41C regucalcin sra |
| 1115 | GO:0043648 | P | 7, | 1 | 0.873 (x 1.145) | 15 (0.067) | 1 | dicarboxylic acid metabolism | CG4095 |
| 1116 | GO:0044270 | P | 5, 6, | 3 | 3.028 (x 0.991) | 52 (0.058) | 1 | nitrogen compound catabolism | CG10184 CG11251 Gs2 |
| 1117 | GO:0043283 | P | 5, | 73 | 98.048 (x 0.745) | 1684 (0.043) | 1 | biopolymer metabolism | Aats-his Abl Arf84F BG:DS02740.5 CG10092 CG10166 CG10418 CG10466 CG10738 CG11360 CG11597 CG11909 CG1249 CG13277 CG15220 CG17266 CG17768 CG18013 CG18749 CG18869 CG2021 CG2056 CG30438 CG31184 CG31611 CG32479 CG33177 CG3931 CG40045 CG4279 CG5382 CG6214 CG6461 CG6610 CG6947 CG7298 CG8756 CG8918 CG9267 CG9602 CG9804 Chit CycG DebB Dhfr Doa EG:BACR7A4.18 EG:BACR7A4.8 Gasp HP1c Iap2 InR Orc6 PNUTS Peritrophin-A Prosbeta5 SmB Takl2 Tfb2 Ugt86De Ugt86Di agt betaggt-II btl gatA hay hep ksr pip pum rpr skpA trol |
| 1118 | GO:0007283 | P | 6, | 1 | 6.463 (x 0.155) | 111 (0.009) | 1 | spermatogenesis | lectin-28C |
| 1119 | GO:0018401 | P | 7, 8, 11, | 1 | 0.873 (x 1.145) | 15 (0.067) | 1 | peptidyl-proline hydroxylation to 4-hydroxy-L-proline | CG18749 |
| 1120 | GO:0009310 | P | 6, 7, | 3 | 3.028 (x 0.991) | 52 (0.058) | 1 | amine catabolism | CG10184 CG11251 Gs2 |
| 1121 | GO:0007169 | P | 7, | 7 | 7.220 (x 0.970) | 124 (0.056) | 1 | transmembrane receptor protein tyrosine kinase signaling pathway | Dab InR btl gt ksr rho tsl |
| 1122 | GO:0048232 | P | 5, | 1 | 6.463 (x 0.155) | 111 (0.009) | 1 | male gamete generation | lectin-28C |
| 1123 | GO:0048666 | P | 5, 8, | 8 | 8.268 (x 0.968) | 142 (0.056) | 1 | neuron development | Abl Con InR Tig Tm1 pum robl vvl |
| 1124 | GO:0019471 | P | 6, 7, | 1 | 0.873 (x 1.145) | 15 (0.067) | 1 | 4-hydroxyproline metabolism | CG18749 |
| 1125 | GO:0000278 | P | 5, | 6 | 15.604 (x 0.385) | 268 (0.022) | 1 | mitotic cell cycle | CG9790 CG9862 Nipped-B insc pum skpA |
| 1126 | GO:0030554 | F | 5, | 24 | 40.116 (x 0.598) | 689 (0.035) | 1 | adenyl nucleotide binding | Aats-his Abl CG10092 CG10738 CG11897 CG11898 CG14721 CG1939 CG2056 CG4511 CG4858 CG6214 Doa EG:BACR7A4.17 Gp150 InR Myo28B1 Takl2 btl hay hep ksr l(2)03659 nmdyn-D6 |
| 1127 | GO:0031175 | P | 6, 9, | 8 | 8.268 (x 0.968) | 142 (0.056) | 1 | neurite development | Abl Con InR Tig Tm1 pum robl vvl |
| 1128 | GO:0035289 | P | 6, 7, | 1 | 0.873 (x 1.145) | 15 (0.067) | 1 | posterior head segmentation | gt |
| 1129 | GO:0019222 | P | 4, | 40 | 59.620 (x 0.671) | 1024 (0.039) | 1 | regulation of metabolism | Abl Bro CG10418 CG10669 CG11360 CG12175 CG12361 CG12605 CG15398 CG17385 CG18619 CG32105 CG40068 CG6272 CG9650 Doa Eip93F HP1c Hr38 Hr4 Kr-h1 Nipped-B Orc6 PNUTS Rpb4 Ssb-c31a Taf11 Tfb2 apt bbx dmrt93B gt hay hep mirr pum rpr salm toy vvl |
| 1130 | GO:0016645 | F | 4, | 1 | 0.932 (x 1.073) | 16 (0.062) | 1 | oxidoreductase activity, acting on the CH-NH group of donors | Dhfr |
| 1131 | GO:0004702 | F | 4, 8, | 3 | 10.305 (x 0.291) | 177 (0.017) | 1 | receptor signaling protein serine/threonine kinase activity | Doa hep ksr |
| 1132 | GO:0000279 | P | 5, | 7 | 16.710 (x 0.419) | 287 (0.024) | 1 | M phase | CG9790 CG9862 Nipped-B pip pum skpA sra |
| 1133 | GO:0007432 | P | 6, | 1 | 0.873 (x 1.145) | 15 (0.067) | 1 | salivary gland determination | rho |
| 1134 | GO:0003677 | F | 4, | 31 | 47.859 (x 0.648) | 822 (0.038) | 1 | DNA binding | BEST:LD29214 CG12361 CG15220 CG15398 CG18619 CG31611 CG32105 CG6272 Eip93F Hr38 Hr4 Kr-h1 Orc6 RpII18 Rpb4 Ssb-c31a Taf10b Taf11 Tfb2 apt bbx dmrt93B e(y)2 gt hay mirr sisA skpA toy trol vvl |
| 1135 | GO:0006289 | P | 6, 8, | 1 | 0.932 (x 1.073) | 16 (0.062) | 1 | nucleotide-excision repair | hay |
| 1136 | GO:0031323 | P | 5, | 39 | 57.408 (x 0.679) | 986 (0.040) | 1 | regulation of cellular metabolism | Bro CG10418 CG10669 CG11360 CG12175 CG12361 CG12605 CG15398 CG17385 CG18619 CG32105 CG40068 CG6272 CG9650 Doa Eip93F HP1c Hr38 Hr4 Kr-h1 Nipped-B Orc6 PNUTS Rpb4 Ssb-c31a Taf11 Tfb2 apt bbx dmrt93B gt hay hep mirr pum rpr salm toy vvl |
| 1137 | GO:0004715 | F | 8, | 1 | 0.873 (x 1.145) | 15 (0.067) | 1 | non-membrane spanning protein tyrosine kinase activity | Abl |
| 1138 | GO:0016271 | P | 4, | 4 | 4.192 (x 0.954) | 72 (0.056) | 1 | tissue death | CG10861 Doa Eip93F rpr |
| 1139 | GO:0005524 | F | 6, | 24 | 39.126 (x 0.613) | 672 (0.036) | 1 | ATP binding | Aats-his Abl CG10092 CG10738 CG11897 CG11898 CG14721 CG1939 CG2056 CG4511 CG4858 CG6214 Doa EG:BACR7A4.17 Gp150 InR Myo28B1 Takl2 btl hay hep ksr l(2)03659 nmdyn-D6 |
| 1140 | GO:0009586 | P | 6, 7, 8, | 1 | 0.932 (x 1.073) | 16 (0.062) | 1 | rhodopsin mediated phototransduction | Arr2 |
| 1141 | GO:0000087 | P | 6, | 5 | 13.217 (x 0.378) | 227 (0.022) | 1 | M phase of mitotic cell cycle | CG9790 CG9862 Nipped-B pum skpA |
| 1142 | GO:0007559 | P | 5, | 4 | 4.192 (x 0.954) | 72 (0.056) | 1 | histolysis | CG10861 Doa Eip93F rpr |
| 1143 | GO:0007067 | P | 7, | 5 | 13.158 (x 0.380) | 226 (0.022) | 1 | mitosis | CG9790 CG9862 Nipped-B pum skpA |
| 1144 | GO:0042803 | F | 5, | 1 | 0.932 (x 1.073) | 16 (0.062) | 1 | protein homodimerization activity | gt |
| 1145 | GO:0016787 | F | 3, | 81 | 104.569 (x 0.775) | 1796 (0.045) | 1 | hydrolase activity | Ahcy13 Amyrel Arf84F BG:DS01068.5 BcDNA:GH08420 CG10104 CG10466 CG11313 CG11597 CG11897 CG11898 CG11909 CG12133 CG1299 CG1304 CG13318 CG14935 CG15012 CG15820 CG18223 CG18530 CG1885 CG2056 CG2277 CG30022 CG30283 CG31272 CG31477 CG32479 CG32549 CG32626 CG32627 CG33128 CG3355 CG3931 CG4386 CG4408 CG4827 CG5162 CG5177 CG5397 CG6214 CG6296 CG6357 CG6461 CG6763 CG7211 CG8360 CG8520 CG8550 CG8756 CG9267 CG9372 Chit Dab Dhc98D EG:9D2.4 Idgf1 Idgf2 Lip1 Mlc2 Myo28B1 Obp58b PGRP-SA Pld Prosbeta5 RN-tre Rab3 Ser7 Sras Vha36 alpha-Est8 gatA hay l(2)03659 l(2)06225 ran-like rho robl sda sun |
| 1146 | GO:0009308 | P | 5, | 21 | 21.892 (x 0.959) | 376 (0.056) | 1 | amine metabolism | Aats-his Act57B CG10092 CG10184 CG11251 CG18749 CG5122 CG5535 CG6461 CG6947 CG7298 CG8756 CG9413 Chit Dhfr Gasp Gs2 Peritrophin-A SamDC Tbh gatA |
| 1147 | GO:0016265 | P | 3, | 6 | 14.614 (x 0.411) | 251 (0.024) | 1 | death | CG10861 CG7188 Doa Eip93F Iap2 rpr |
| 1148 | GO:0019887 | F | 4, | 2 | 2.038 (x 0.981) | 35 (0.057) | 1 | protein kinase regulator activity | CG9790 CycG |
| 1149 | GO:0004089 | F | 6, | 1 | 0.932 (x 1.073) | 16 (0.062) | 1 | carbonate dehydratase activity | CAH2 |
| 1150 | GO:0008219 | P | 4, | 6 | 14.556 (x 0.412) | 250 (0.024) | 1 | cell death | CG10861 CG7188 Doa Eip93F Iap2 rpr |
| 1151 | GO:0045449 | P | 7, | 32 | 48.383 (x 0.661) | 831 (0.039) | 1 | regulation of transcription | Bro CG10669 CG12175 CG12361 CG12605 CG15398 CG17385 CG18619 CG32105 CG6272 CG9650 Eip93F HP1c Hr38 Hr4 Kr-h1 Nipped-B Orc6 Rpb4 Ssb-c31a Taf11 Tfb2 apt bbx dmrt93B gt hep mirr pum salm toy vvl |
| 1152 | GO:0016620 | F | 5, | 1 | 0.932 (x 1.073) | 16 (0.062) | 1 | oxidoreductase activity, acting on the aldehyde or oxo group of donors, NAD or NADP as acceptor | CG9629 |
| 1153 | GO:0046872 | F | 4, | 37 | 54.264 (x 0.682) | 932 (0.040) | 1 | metal ion binding | AnnX BG:DS02740.5 CAH2 CG10126 CG10466 CG10669 CG11360 CG12605 CG17385 CG18011 CG2185 CG31922 CG32105 CG5382 CG6426 CG6763 CG7646 CG8360 CG8506 CG8550 CG9650 Cad99C Cpn Iap2 Kr-h1 Las Mlc2 Mp20 MtnA Rpb4 Sod Tbh TpnC41C Tsf1 l(2)k10201 salm sda |
| 1154 | GO:0016879 | F | 4, | 10 | 10.597 (x 0.944) | 182 (0.055) | 1 | ligase activity, forming carbon-nitrogen bonds | BG:DS02740.5 CG11360 CG5382 CG8918 CG9602 CG9804 Gs2 Iap2 Las gatA |
| 1155 | GO:0043167 | F | 3, | 37 | 54.264 (x 0.682) | 932 (0.040) | 1 | ion binding | AnnX BG:DS02740.5 CAH2 CG10126 CG10466 CG10669 CG11360 CG12605 CG17385 CG18011 CG2185 CG31922 CG32105 CG5382 CG6426 CG6763 CG7646 CG8360 CG8506 CG8550 CG9650 Cad99C Cpn Iap2 Kr-h1 Las Mlc2 Mp20 MtnA Rpb4 Sod Tbh TpnC41C Tsf1 l(2)k10201 salm sda |
| 1156 | GO:0004016 | F | 4, 5, | 1 | 0.932 (x 1.073) | 16 (0.062) | 1 | adenylate cyclase activity | Ac78C |
| 1157 | GO:0012501 | P | 5, | 6 | 14.439 (x 0.416) | 248 (0.024) | 1 | programmed cell death | CG10861 CG7188 Doa Eip93F Iap2 rpr |
| 1158 | GO:0015293 | F | 6, | 4 | 4.250 (x 0.941) | 73 (0.055) | 1 | symporter activity | CG10804 CG3036 CG4288 CG6723 |
| 1159 | GO:0043169 | F | 4, | 35 | 51.644 (x 0.678) | 887 (0.039) | 1 | cation binding | AnnX BG:DS02740.5 CAH2 CG10126 CG10466 CG10669 CG11360 CG12605 CG17385 CG18011 CG2185 CG31922 CG32105 CG5382 CG6426 CG6763 CG7646 CG8360 CG8506 CG8550 CG9650 Cad99C Cpn Iap2 Kr-h1 Las Mlc2 Mp20 Rpb4 Tbh TpnC41C Tsf1 l(2)k10201 salm sda |
| 1160 | GO:0042802 | F | 4, | 1 | 0.932 (x 1.073) | 16 (0.062) | 1 | identical protein binding | gt |
| 1161 | GO:0019001 | F | 5, | 3 | 9.432 (x 0.318) | 162 (0.019) | 1 | guanyl nucleotide binding | Arf84F Rab3 ran-like |
| 1162 | GO:0016817 | F | 4, | 19 | 31.906 (x 0.595) | 548 (0.035) | 1 | hydrolase activity, acting on acid anhydrides | Arf84F CG11897 CG11898 CG31477 CG32627 CG6214 CG7211 CG8520 Dhc98D Mlc2 Myo28B1 Rab3 Vha36 hay l(2)03659 l(2)06225 ran-like robl sun |
| 1163 | GO:0051347 | P | 5, | 1 | 0.932 (x 1.073) | 16 (0.062) | 1 | positive regulation of transferase activity | Dgkepsilon |
| 1164 | GO:0016818 | F | 5, | 19 | 31.906 (x 0.595) | 548 (0.035) | 1 | hydrolase activity, acting on acid anhydrides, in phosphorus-containing anhydrides | Arf84F CG11897 CG11898 CG31477 CG32627 CG6214 CG7211 CG8520 Dhc98D Mlc2 Myo28B1 Rab3 Vha36 hay l(2)03659 l(2)06225 ran-like robl sun |
| 1165 | GO:0045860 | P | 6, 7, | 1 | 0.932 (x 1.073) | 16 (0.062) | 1 | positive regulation of protein kinase activity | Dgkepsilon |
| 1166 | GO:0005525 | F | 6, | 3 | 9.374 (x 0.320) | 161 (0.019) | 1 | GTP binding | Arf84F Rab3 ran-like |
| 1167 | GO:0044248 | P | 5, | 17 | 17.816 (x 0.954) | 306 (0.056) | 1 | cellular catabolism | CG10184 CG11251 CG1140 CG17224 CG2964 CG30499 CG32479 CG4095 CG4827 CG5103 Chit Gs2 ImpL3 PGRP-SA Pglym78 Prosbeta5 pum |
| 1168 | GO:0046914 | F | 5, | 25 | 39.359 (x 0.635) | 676 (0.037) | 1 | transition metal ion binding | BG:DS02740.5 CAH2 CG10466 CG10669 CG11360 CG12605 CG17385 CG18011 CG31922 CG32105 CG5382 CG6763 CG8360 CG8506 CG8550 CG9650 Iap2 Kr-h1 Las Rpb4 Tbh Tsf1 l(2)k10201 salm sda |
| 1169 | GO:0005488 | F | 2, | 212 | 240.694 (x 0.881) | 4134 (0.051) | 1 | binding | Aats-his Abl AnnX Arf84F Arp11 Arr2 BEST:LD29214 BG:DS00180.7 BG:DS01219.1 BG:DS02740.5 BG:DS02740.9 BcDNA:GH11110 Bro CAH2 CG10092 CG10126 CG10237 CG10466 CG10669 CG10738 CG10950 CG11360 CG11897 CG11898 CG12361 CG1249 CG12605 CG12775 CG14721 CG15220 CG15361 CG15398 CG17385 CG17768 CG17904 CG18011 CG18619 CG1883 CG1939 CG2056 CG2185 CG2789 CG2998 CG31146 CG31184 CG3153 CG31611 CG31922 CG31957 CG32105 CG32627 CG32677 CG3529 CG3843 CG3931 CG40068 CG4046 CG4071 CG4115 CG4187 CG4511 CG4858 CG4866 CG5001 CG5338 CG5382 CG5559 CG6124 CG6214 CG6272 CG6426 CG6574 CG6763 CG6764 CG6891 CG6947 CG7014 CG7298 CG7339 CG7646 CG7770 CG7777 CG7911 CG8152 CG8219 CG8360 CG8397 CG8415 CG8498 CG8506 CG8550 CG8756 CG8778 CG8857 CG9095 CG9326 CG9650 CG9862 CLIP-190 Cad99C Chit Cpn Dab DebB Dgkepsilon Doa EG:BACH7M4.1 EG:BACR7A4.17 EG:BACR7A4.18 EG:BACR7A4.8 Eip93F Gasp Gp150 HP1c Hr38 Hr4 Iap2 Idgf1 Idgf2 InR Kr-h1 Las Mlc2 Mp20 MtnA Myo28B1 Obp56a Obp58b Or46a Or59a Or85a Or94a Or98b Orc6 PGRP-SA PNUTS PQBP-1 Peritrophin-A Pof Rab3 RpII18 RpL11 RpL17A RpL27A RpL38 RpL46 RpL8 RpL9 RpP1 RpS17 RpS18 RpS4 RpS9 Rpb10 Rpb4 SmB Sod Ssb-c31a SytIV Taf10b Taf11 Takl2 Takr99D Tbh Tfb2 Tig Tm1 TpnC41C Tsf1 agt apt bbx btl cyp33 dmrt93B e(y)2 gt hay hep insc jdp ksr l(2)03659 l(2)k10201 l(3)01239 lectin-28C mRpL11 mRpL2 mRpL21 mRpS14 mira mirr mtacp1 nmdyn-D6 oho23B pum ran-like rho salm sda sisA skpA sop sra tko toy trol tsl unc-13 upd3 veli vvl |
| 1170 | GO:0042058 | P | 5, 6, 9, | 1 | 0.932 (x 1.073) | 16 (0.062) | 1 | regulation of epidermal growth factor receptor signaling pathway | rho |
| 1171 | GO:0005083 | F | 4, | 1 | 5.298 (x 0.189) | 91 (0.011) | 1 | small GTPase regulator activity | EG:52C10.2 |
| 1172 | GO:0015631 | F | 5, | 1 | 5.298 (x 0.189) | 91 (0.011) | 1 | tubulin binding | CLIP-190 |
| 1173 | GO:0015630 | C | 6, 7, 8, 9, | 4 | 10.829 (x 0.369) | 186 (0.022) | 1 | microtubule cytoskeleton | CLIP-190 Dhc98D dynactin-subunit-p25 robl |
| 1174 | GO:0004518 | F | 5, | 1 | 5.240 (x 0.191) | 90 (0.011) | 1 | nuclease activity | CG3931 |
| 1175 | GO:0004674 | F | 7, | 5 | 12.343 (x 0.405) | 212 (0.024) | 1 | protein serine/threonine kinase activity | CG9790 Doa Takl2 hep ksr |
| 1176 | GO:0008270 | F | 6, | 22 | 35.050 (x 0.628) | 602 (0.037) | 1 | zinc ion binding | BG:DS02740.5 CAH2 CG10466 CG10669 CG11360 CG12605 CG17385 CG18011 CG31922 CG32105 CG5382 CG6763 CG8360 CG8506 CG8550 CG9650 Iap2 Kr-h1 Rpb4 l(2)k10201 salm sda |
| 1177 | GO:0006355 | P | 8, | 31 | 45.822 (x 0.677) | 787 (0.039) | 1 | regulation of transcription, DNA-dependent | Bro CG10669 CG12175 CG12361 CG12605 CG15398 CG17385 CG18619 CG32105 CG6272 CG9650 Eip93F HP1c Hr38 Hr4 Kr-h1 Nipped-B Orc6 Rpb4 Ssb-c31a Taf11 Tfb2 apt bbx dmrt93B gt hep mirr pum toy vvl |
| 1178 | GO:0000226 | P | 8, | 1 | 5.065 (x 0.197) | 87 (0.011) | 1 | microtubule cytoskeleton organization and biogenesis | insc |
| 1179 | GO:0019219 | P | 6, | 36 | 51.702 (x 0.696) | 888 (0.041) | 1 | regulation of nucleobase, nucleoside, nucleotide and nucleic acid metabolism | Bro CG10418 CG10669 CG11360 CG12175 CG12361 CG12605 CG15398 CG17385 CG18619 CG32105 CG6272 CG9650 Doa Eip93F HP1c Hr38 Hr4 Kr-h1 Nipped-B Orc6 Rpb4 Ssb-c31a Taf11 Tfb2 apt bbx dmrt93B gt hay hep mirr pum salm toy vvl |
| 1180 | GO:0009100 | P | 7, | 1 | 5.007 (x 0.200) | 86 (0.012) | 1 | glycoprotein metabolism | CG10166 |
| 1181 | GO:0008283 | P | 4, | 8 | 16.419 (x 0.487) | 282 (0.028) | 1 | cell proliferation | CG10669 CG12605 CG17385 CG2750 InR Kr-h1 btl toy |
| 1182 | GO:0016462 | F | 6, | 19 | 30.916 (x 0.615) | 531 (0.036) | 1 | pyrophosphatase activity | Arf84F CG11897 CG11898 CG31477 CG32627 CG6214 CG7211 CG8520 Dhc98D Mlc2 Myo28B1 Rab3 Vha36 hay l(2)03659 l(2)06225 ran-like robl sun |
| 1183 | GO:0048519 | P | 3, | 9 | 17.758 (x 0.507) | 305 (0.030) | 1 | negative regulation of biological process | AnnX CG7188 Iap2 Orc6 apt gt pum rpr salm |
| 1184 | GO:0006915 | P | 6, | 4 | 10.364 (x 0.386) | 178 (0.022) | 1 | apoptosis | CG7188 Eip93F Iap2 rpr |
| 1185 | GO:0046843 | P | 10, 11, | 1 | 0.990 (x 1.010) | 17 (0.059) | 1 | dorsal appendage formation | hep |
| 1186 | GO:0048523 | P | 4, | 8 | 16.302 (x 0.491) | 280 (0.029) | 1 | negative regulation of cellular process | CG7188 Iap2 Orc6 apt gt pum rpr salm |
| 1187 | GO:0015931 | P | 5, 6, | 2 | 2.154 (x 0.928) | 37 (0.054) | 1 | nucleobase, nucleoside, nucleotide and nucleic acid transport | CG10320 Nxt1 |
| 1188 | GO:0007275 | P | 2, | 67 | 86.461 (x 0.775) | 1485 (0.045) | 1 | development | Abl Act57B BG:DS00180.7 BG:DS02740.9 Bro CG10861 CG12361 CG14825 CG1942 CG2069 CG31146 CG5397 CG6803 CG7194 CG9650 CLIP-190 Con Cpn Dab Doa EG:80H7.10 Eip93F Gp150 Gs2 Hmgs Hr38 Hr4 Hsp22 Idgf1 Idgf2 InR Kr-h1 Mp20 SIP1 SelG Sod Tehao Tig Tm1 Tsp42El apt btl chp dmrt93B gt hep insc ksr l(2)03659 l(2)k10201 mira mirr oho23B pip pum regucalcin rho robl rpr salm sisA toy trol tsl veli vvl zpg |
| 1189 | GO:0006417 | P | 6, 7, 8, | 4 | 4.367 (x 0.916) | 75 (0.053) | 1 | regulation of protein biosynthesis | CG40068 apt pum rpr |
| 1190 | GO:0051028 | P | 7, 8, 9, | 1 | 0.990 (x 1.010) | 17 (0.059) | 1 | mRNA transport | Nxt1 |
| 1191 | GO:0009101 | P | 7, 8, | 1 | 4.833 (x 0.207) | 83 (0.012) | 1 | glycoprotein biosynthesis | CG10166 |
| 1192 | GO:0006886 | P | 6, 7, 8, | 28 | 29.461 (x 0.950) | 506 (0.055) | 1 | intracellular protein transport | AnnX Arf84F Arr2 CG10950 CG17266 CG2185 CG32677 CG33066 CG3529 CG4071 CG4187 CG4673 CG5559 CG8004 CG8219 CG9326 CLIP-190 Myo28B1 Nxt1 PNUTS Rab3 SytIV Syx16 Syx8 Tim17b2 Tim9a cyp33 ran-like |
| 1193 | GO:0004175 | F | 5, | 26 | 27.423 (x 0.948) | 471 (0.055) | 1 | endopeptidase activity | BG:DS01068.5 BcDNA:GH08420 CG10104 CG11313 CG12133 CG1299 CG1304 CG13318 CG18223 CG1885 CG2056 CG30283 CG32479 CG33128 CG3355 CG4386 CG6357 CG6763 CG8550 CG9372 Dab EG:9D2.4 Obp58b Prosbeta5 Ser7 Sras |
| 1194 | GO:0004930 | F | 5, | 14 | 14.963 (x 0.936) | 257 (0.054) | 1 | G-protein coupled receptor activity | CG11318 CG4187 EG:30B8.6 GABA-B-R2 Gr61a Gr98c Or46a Or59a Or85a Or94a Or98b Rh7 Takr99D TyrR |
| 1195 | GO:0008026 | F | 4, 10, | 1 | 4.833 (x 0.207) | 83 (0.012) | 1 | ATP-dependent helicase activity | hay |
| 1196 | GO:0018993 | P | 4, | 1 | 0.990 (x 1.010) | 17 (0.059) | 1 | somatic sex determination | sisA |
| 1197 | GO:0030182 | P | 4, 7, | 8 | 8.675 (x 0.922) | 149 (0.054) | 1 | neuron differentiation | Abl Con InR Tig Tm1 pum robl vvl |
| 1198 | GO:0008236 | F | 5, | 16 | 17.059 (x 0.938) | 293 (0.055) | 1 | serine-type peptidase activity | BG:DS01068.5 BcDNA:GH08420 CG11313 CG12133 CG1299 CG1304 CG13318 CG18223 CG2056 CG30283 CG3355 CG4386 CG9372 EG:9D2.4 Ser7 rho |
| 1199 | GO:0003682 | F | 3, | 1 | 4.833 (x 0.207) | 83 (0.012) | 1 | chromatin binding | HP1c |
| 1200 | GO:0007398 | P | 4, | 13 | 13.799 (x 0.942) | 237 (0.055) | 1 | ectoderm development | BG:DS02740.9 CG12361 CG31146 CG5397 Dab Doa Hr38 Tsp42El btl mirr rho toy vvl |
| 1201 | GO:0006357 | P | 9, | 20 | 31.848 (x 0.628) | 547 (0.037) | 1 | regulation of transcription from RNA polymerase II promoter | Bro CG10669 CG12175 CG12361 CG12605 CG17385 CG32105 CG9650 HP1c Hr38 Hr4 Kr-h1 Rpb4 Ssb-c31a apt gt hep mirr toy vvl |
| 1202 | GO:0017111 | F | 7, | 19 | 30.451 (x 0.624) | 523 (0.036) | 1 | nucleoside-triphosphatase activity | Arf84F CG11897 CG11898 CG31477 CG32627 CG6214 CG7211 CG8520 Dhc98D Mlc2 Myo28B1 Rab3 Vha36 hay l(2)03659 l(2)06225 ran-like robl sun |
| 1203 | GO:0007259 | P | 7, | 1 | 0.990 (x 1.010) | 17 (0.059) | 1 | JAK-STAT cascade | upd3 |
| 1204 | GO:0000003 | P | 2, | 18 | 29.170 (x 0.617) | 501 (0.036) | 1 | reproduction | CG5162 Cp36 Doa Femcoat InR Tbh Tm1 Vm34Ca hep l(3)01239 lectin-28C pip pum rho shu sra tko zpg |
| 1205 | GO:0031325 | P | 6, | 4 | 4.309 (x 0.928) | 74 (0.054) | 1 | positive regulation of cellular metabolism | Bro Eip93F mirr pum |
| 1206 | GO:0006909 | P | 7, 8, | 1 | 0.990 (x 1.010) | 17 (0.059) | 1 | phagocytosis | Eip93F |
| 1207 | GO:0043413 | P | 7, | 1 | 4.716 (x 0.212) | 81 (0.012) | 1 | biopolymer glycosylation | CG10166 |
| 1208 | GO:0001700 | P | 5, | 7 | 7.511 (x 0.932) | 129 (0.054) | 1 | embryonic development (sensu Insecta) | Abl Hmgs InR hep mirr pum rpr |
| 1209 | GO:0006486 | P | 8, 9, | 1 | 4.716 (x 0.212) | 81 (0.012) | 1 | protein amino acid glycosylation | CG10166 |
| 1210 | GO:0019953 | P | 3, | 16 | 26.550 (x 0.603) | 456 (0.035) | 1 | sexual reproduction | CG5162 Cp36 Doa Femcoat InR Tbh Tm1 Vm34Ca hep l(3)01239 lectin-28C pip pum rho shu zpg |
| 1211 | GO:0009893 | P | 5, | 4 | 4.309 (x 0.928) | 74 (0.054) | 1 | positive regulation of metabolism | Bro Eip93F mirr pum |
| 1212 | GO:0008173 | F | 6, | 1 | 0.990 (x 1.010) | 17 (0.059) | 1 | RNA methyltransferase activity | EG:BACR7A4.8 |
| 1213 | GO:0044427 | C | 4, 5, 6, 7, 8, 9, | 4 | 8.617 (x 0.464) | 148 (0.027) | 1 | chromosomal part | CG15220 CG31611 HP1c Orc6 |
| 1214 | GO:0016568 | P | 9, | 1 | 3.552 (x 0.282) | 61 (0.016) | 1 | chromatin modification | Orc6 |
| 1215 | GO:0030001 | P | 7, 8, | 4 | 8.559 (x 0.467) | 147 (0.027) | 1 | metal ion transport | CG1756 CG3397 CG4805 Tsf1 |
| 1216 | GO:0051726 | P | 5, | 5 | 11.586 (x 0.432) | 199 (0.025) | 1 | regulation of cell cycle | Abl CG2750 CG9790 pum skpA |
| 1217 | GO:0008047 | F | 3, | 2 | 5.531 (x 0.362) | 95 (0.021) | 1 | enzyme activator activity | CG11313 REG |
| 1218 | GO:0048565 | P | 4, | 1 | 3.610 (x 0.277) | 62 (0.016) | 1 | gut development | sisA |
| 1219 | GO:0009790 | P | 3, | 13 | 20.436 (x 0.636) | 351 (0.037) | 1 | embryonic development | Abl Doa Hmgs InR SelG gt hep ksr mirr pum rpr salm tsl |
| 1220 | GO:0007059 | P | 4, | 3 | 7.220 (x 0.416) | 124 (0.024) | 1 | chromosome segregation | CG9862 Nipped-B skpA |
| 1221 | GO:0048534 | P | 4, | 4 | 4.309 (x 0.928) | 74 (0.054) | 1 | hemopoietic or lymphoid organ development | CG12361 CG9650 hep oho23B |
| 1222 | GO:0000059 | P | 8, 9, 10, 11, | 1 | 0.990 (x 1.010) | 17 (0.059) | 1 | protein import into nucleus, docking | CG8219 |
| 1223 | GO:0040008 | P | 3, | 1 | 3.552 (x 0.282) | 61 (0.016) | 1 | regulation of growth | InR |
| 1224 | GO:0000074 | P | 6, | 5 | 11.586 (x 0.432) | 199 (0.025) | 1 | regulation of progression through cell cycle | Abl CG2750 CG9790 pum skpA |
| 1225 | GO:0009791 | P | 3, | 13 | 20.727 (x 0.627) | 356 (0.037) | 1 | post-embryonic development | CG10861 Cpn Dab Doa Eip93F Hr4 Kr-h1 chp hep mirr rho rpr salm |
| 1226 | GO:0007015 | P | 9, | 1 | 3.493 (x 0.286) | 60 (0.017) | 1 | actin filament organization | hep |
| 1227 | GO:0051321 | P | 5, | 2 | 5.357 (x 0.373) | 92 (0.022) | 1 | meiotic cell cycle | pip sra |
| 1228 | GO:0016788 | F | 4, | 16 | 26.491 (x 0.604) | 455 (0.035) | 1 | hydrolase activity, acting on ester bonds | CG11597 CG18530 CG2277 CG31272 CG32479 CG32549 CG3931 CG4827 CG5162 CG5177 CG5397 CG6296 CG9267 Lip1 Pld alpha-Est8 |
| 1229 | GO:0008017 | F | 6, | 1 | 4.425 (x 0.226) | 76 (0.013) | 1 | microtubule binding | CLIP-190 |
| 1230 | GO:0008757 | F | 6, | 1 | 3.435 (x 0.291) | 59 (0.017) | 1 | S-adenosylmethionine-dependent methyltransferase activity | EG:BACR7A4.8 |
| 1231 | GO:0007560 | P | 5, 6, | 8 | 13.974 (x 0.573) | 240 (0.033) | 1 | imaginal disc morphogenesis | Cpn Dab Doa chp hep mirr rho salm |
| 1232 | GO:0050793 | P | 3, | 1 | 4.600 (x 0.217) | 79 (0.013) | 1 | regulation of development | hep |
| 1233 | GO:0009653 | P | 3, | 25 | 37.379 (x 0.669) | 642 (0.039) | 1 | morphogenesis | Abl Act57B CG14825 CG2069 CG7194 Con Cpn Dab Doa Hr4 InR Kr-h1 Mp20 SIP1 Tig apt btl chp hep mirr rho rpr salm veli vvl |
| 1234 | GO:0005515 | F | 3, | 58 | 71.847 (x 0.807) | 1234 (0.047) | 1 | protein binding | Abl AnnX Arp11 Arr2 BG:DS00180.7 BG:DS01219.1 BG:DS02740.9 Bro CG10950 CG15361 CG18619 CG2185 CG31146 CG3153 CG32677 CG3529 CG5001 CG6124 CG6272 CG6891 CG7646 CG7770 CG8219 CG8397 CG9326 CLIP-190 Chit Dab EG:BACH7M4.1 EG:BACR7A4.18 Idgf1 Idgf2 InR Mlc2 Mp20 Myo28B1 PQBP-1 RpL11 Ssb-c31a Taf10b Tm1 TpnC41C gt hep insc jdp l(3)01239 mira mirr pum rho sisA sra tsl unc-13 upd3 veli vvl |
| 1235 | GO:0006406 | P | 8, 9, 10, 11, | 1 | 0.990 (x 1.010) | 17 (0.059) | 1 | mRNA export from nucleus | Nxt1 |
| 1236 | GO:0008134 | F | 4, | 2 | 5.182 (x 0.386) | 89 (0.022) | 1 | transcription factor binding | Bro Ssb-c31a |
| 1237 | GO:0006350 | P | 6, | 41 | 55.021 (x 0.745) | 945 (0.043) | 1 | transcription | Bro CG10669 CG12175 CG12361 CG12605 CG15398 CG17385 CG18619 CG32105 CG5382 CG6272 CG7339 CG9650 Eip93F HP1c Hr38 Hr4 Kr-h1 Nipped-B Orc6 RpII18 Rpb10 Rpb4 Ssb-c31a Taf10b Taf11 TfIIEalpha Tfb2 Trap36 apt bbx dmrt93B e(y)2 gt hay hep mirr pum salm toy vvl |
| 1238 | GO:0007276 | P | 4, | 16 | 26.026 (x 0.615) | 447 (0.036) | 1 | gametogenesis | CG5162 Cp36 Doa Femcoat InR Tbh Tm1 Vm34Ca hep l(3)01239 lectin-28C pip pum rho shu zpg |
| 1239 | GO:0051327 | P | 6, | 2 | 5.298 (x 0.377) | 91 (0.022) | 1 | M phase of meiotic cell cycle | pip sra |
| 1240 | GO:0007017 | P | 7, | 5 | 11.412 (x 0.438) | 196 (0.026) | 1 | microtubule-based process | CLIP-190 Dhc98D dynactin-subunit-p25 insc robl |
| 1241 | GO:0007242 | P | 5, | 20 | 30.509 (x 0.656) | 524 (0.038) | 1 | intracellular signaling cascade | Abl Ac78C Arf84F CG10738 CG17262 CG2185 CG7646 Dgkepsilon Hr38 Hr4 Pld Rab3 TpnC41C hep ksr ran-like regucalcin sra unc-13 upd3 |
| 1242 | GO:0006413 | P | 8, 9, | 1 | 3.435 (x 0.291) | 59 (0.017) | 1 | translational initiation | RpS18 |
| 1243 | GO:0035107 | P | 4, | 3 | 7.744 (x 0.387) | 133 (0.023) | 1 | appendage morphogenesis | mirr rho salm |
| 1244 | GO:0040007 | P | 2, | 2 | 5.240 (x 0.382) | 90 (0.022) | 1 | growth | Hr4 InR |
| 1245 | GO:0009887 | P | 4, | 11 | 19.563 (x 0.562) | 336 (0.033) | 1 | organ morphogenesis | Act57B CG7194 Cpn Dab Doa apt chp hep mirr rho salm |
| 1246 | GO:0004721 | F | 7, | 2 | 5.182 (x 0.386) | 89 (0.022) | 1 | phosphoprotein phosphatase activity | CG11597 CG9267 |
| 1247 | GO:0002165 | P | 4, | 13 | 20.087 (x 0.647) | 345 (0.038) | 1 | larval or pupal development (sensu Insecta) | CG10861 Cpn Dab Doa Eip93F Hr4 Kr-h1 chp hep mirr rho rpr salm |
| 1248 | GO:0016311 | P | 7, | 2 | 6.288 (x 0.318) | 108 (0.019) | 1 | dephosphorylation | CG11597 PNUTS |
| 1249 | GO:0016222 | C | 4, | 1 | 0.990 (x 1.010) | 17 (0.059) | 1 | procollagen-proline, 2-oxoglutarate-4-dioxygenase complex | CG18749 |
| 1250 | GO:0048736 | P | 3, | 3 | 7.744 (x 0.387) | 133 (0.023) | 1 | appendage development | mirr rho salm |
| 1251 | GO:0030163 | P | 6, 7, | 2 | 5.240 (x 0.382) | 90 (0.022) | 1 | protein catabolism | CG32479 Prosbeta5 |
| 1252 | GO:0005856 | C | 5, 6, 7, 8, | 9 | 16.885 (x 0.533) | 290 (0.031) | 1 | cytoskeleton | Act57B Arp11 CLIP-190 Dhc98D Mlc2 Myo28B1 Tm1 dynactin-subunit-p25 robl |
| 1253 | GO:0035220 | P | 5, | 3 | 6.754 (x 0.444) | 116 (0.026) | 1 | wing disc development | mirr rho salm |
| 1254 | GO:0005057 | F | 3, | 7 | 13.974 (x 0.501) | 240 (0.029) | 1 | receptor signaling protein activity | Doa EG:52C10.2 EG:80H7.10 Tsp42El hep ksr rho |
| 1255 | GO:0006259 | P | 6, | 13 | 21.601 (x 0.602) | 371 (0.035) | 1 | DNA metabolism | CG15220 CG18013 CG31611 CycG Dhfr EG:BACR7A4.18 HP1c Orc6 Tfb2 agt hay skpA trol |
| 1256 | GO:0007243 | P | 6, | 3 | 7.394 (x 0.406) | 127 (0.024) | 1 | protein kinase cascade | hep ksr upd3 |
| 1257 | GO:0007626 | P | 4, | 1 | 4.076 (x 0.245) | 70 (0.014) | 1 | locomotory behavior | na |
| 1258 | GO:0016043 | P | 4, | 64 | 80.290 (x 0.797) | 1379 (0.046) | 1 | cell organization and biogenesis | Abl Act57B AnnX Arf84F Arp11 Arr2 CG10320 CG10861 CG10950 CG14825 CG17266 CG2069 CG2185 CG31551 CG31611 CG32409 CG32677 CG33066 CG3529 CG4071 CG4187 CG4673 CG5559 CG6764 CG6803 CG8004 CG8219 CG9326 CLIP-190 Con Cpn Dab Dhc98D Doa EG:BACR7A4.18 HP1c InR Mp20 Myo28B1 Nxt1 Orc6 PNUTS Rab3 SIP1 SytIV Syx16 Syx8 Tig Tim17b2 Tim9a btl chp cyp33 dynactin-subunit-p25 hep insc mira mirr porin ran-like robl skpA veli vvl |
| 1259 | GO:0051234 | P | 4, | 84 | 99.445 (x 0.845) | 1708 (0.049) | 1 | establishment of localization | Abl AnnX Arf84F Arr2 Bc CG10237 CG10320 CG10804 CG10950 CG11897 CG11898 CG1213 CG14691 CG15408 CG17266 CG1756 CG2185 CG2789 CG3036 CG31272 CG31477 CG32677 CG33066 CG3397 CG3529 CG4071 CG4187 CG4288 CG4673 CG4805 CG5535 CG5559 CG6214 CG6574 CG6723 CG7084 CG7211 CG7333 CG7777 CG8004 CG8193 CG8219 CG8271 CG8498 CG8916 CG8925 CG9326 CG9413 CLIP-190 Dhc98D EG:9D2.4 Eip93F InR Myo28B1 Nxt1 Obp58b PNUTS Rab3 SytIV Syx16 Syx8 Tig Tim17b2 Tim9a Tsf1 Vha36 bbx btl cyp33 dynactin-subunit-p25 glob1 hep insc l(2)03659 l(2)06225 na porin pum ran-like robl salm sun unc-13 vvl |
| 1260 | GO:0048737 | P | 4, | 3 | 7.569 (x 0.396) | 130 (0.023) | 1 | appendage development (sensu Endopterygota) | mirr rho salm |
| 1261 | GO:0005328 | F | 4, 7, 9, | 1 | 0.990 (x 1.010) | 17 (0.059) | 1 | neurotransmitter:sodium symporter activity | CG10804 |
| 1262 | GO:0016564 | F | 3, | 1 | 3.319 (x 0.301) | 57 (0.018) | 1 | transcriptional repressor activity | gt |
| 1263 | GO:0007126 | P | 7, | 2 | 5.065 (x 0.395) | 87 (0.023) | 1 | meiosis | pip sra |
| 1264 | GO:0000165 | P | 7, | 2 | 5.997 (x 0.334) | 103 (0.019) | 1 | MAPKKK cascade | hep ksr |
| 1265 | GO:0030154 | P | 3, | 21 | 29.694 (x 0.707) | 510 (0.041) | 1 | cell differentiation | Abl CG6803 Con Cpn Dab Doa InR Tig Tm1 chp hep insc mira pip pum rho robl salm trol vvl zpg |
| 1266 | GO:0030528 | F | 2, | 36 | 46.870 (x 0.768) | 805 (0.045) | 1 | transcription regulator activity | BEST:LD29214 Bro CG10669 CG12175 CG12361 CG12605 CG15398 CG17385 CG18013 CG32105 CG5382 CG9650 Eip93F Hr38 Hr4 Kr-h1 Nipped-B Rpb4 Ssb-c31a Taf10b Taf11 TfIIEalpha Tfb2 Trap36 apt bbx dmrt93B e(y)2 gt hay l(1)10Bb mirr salm sisA toy vvl |
| 1267 | GO:0006351 | P | 7, | 39 | 52.168 (x 0.748) | 896 (0.044) | 1 | transcription, DNA-dependent | Bro CG10669 CG12175 CG12361 CG12605 CG15398 CG17385 CG18619 CG32105 CG5382 CG6272 CG7339 CG9650 Eip93F HP1c Hr38 Hr4 Kr-h1 Nipped-B Orc6 RpII18 Rpb10 Rpb4 Ssb-c31a Taf10b Taf11 TfIIEalpha Tfb2 Trap36 apt bbx dmrt93B gt hay hep mirr pum toy vvl |
| 1268 | GO:0035114 | P | 5, | 3 | 7.569 (x 0.396) | 130 (0.023) | 1 | appendage morphogenesis (sensu Endopterygota) | mirr rho salm |
| 1269 | GO:0019798 | F | 7, | 1 | 1.048 (x 0.954) | 18 (0.056) | 1 | procollagen-proline dioxygenase activity | CG18749 |
| 1270 | GO:0005634 | C | 5, 6, 7, 8, | 72 | 88.732 (x 0.811) | 1524 (0.047) | 1 | nucleus | BEST:LD29214 Bro CG10418 CG10669 CG11360 CG12361 CG1249 CG12605 CG13277 CG15220 CG15398 CG17266 CG17385 CG17768 CG18011 CG18013 CG18619 CG2021 CG31184 CG31611 CG31922 CG31950 CG32105 CG32409 CG3931 CG4279 CG4673 CG6272 CG6610 CG7339 CG7911 CG8219 CG8506 CG9650 DebB Doa EG:BACR7A4.18 Eip93F HP1c Hr38 Hr4 Kr-h1 Nipped-B Nxt1 Orc6 PNUTS REG RpII18 Rpb10 Rpb4 SmB Ssb-c31a Taf10b Taf11 TfIIEalpha Tfb2 Trap36 apt bbx cyp33 dmrt93B e(y)2 gt hay l(1)10Bb l(2)k10201 mirr pip salm sisA toy vvl |
| 1271 | GO:0007606 | P | 4, 6, | 9 | 9.665 (x 0.931) | 166 (0.054) | 1 | sensory perception of chemical stimulus | Gr61a Gr98c Obp56a Or46a Or59a Or85a Or94a Or98b TyrR |
| 1272 | GO:0009994 | P | 4, 7, | 2 | 5.065 (x 0.395) | 87 (0.023) | 1 | oocyte differentiation | Tm1 pip |
| 1273 | GO:0007314 | P | 6, 8, 9, 11, | 1 | 3.260 (x 0.307) | 56 (0.018) | 1 | oocyte anterior/posterior axis determination | Tm1 |
| 1274 | GO:0005261 | F | 5, 6, | 3 | 6.696 (x 0.448) | 115 (0.026) | 1 | cation channel activity | CG1756 CG4805 na |
| 1275 | GO:0001775 | P | 4, | 1 | 0.990 (x 1.010) | 17 (0.059) | 1 | cell activation | Toll-7 |
| 1276 | GO:0051243 | P | 5, | 8 | 13.508 (x 0.592) | 232 (0.034) | 1 | negative regulation of cellular physiological process | CG7188 Iap2 Orc6 apt gt pum rpr salm |
| 1277 | GO:0005783 | C | 5, 6, 7, 8, | 3 | 6.579 (x 0.456) | 113 (0.027) | 1 | endoplasmic reticulum | CG10166 CG11909 Sras |
| 1278 | GO:0006323 | P | 7, | 4 | 8.035 (x 0.498) | 138 (0.029) | 1 | DNA packaging | CG31611 EG:BACR7A4.18 HP1c Orc6 |
| 1279 | GO:0004656 | F | 8, | 1 | 1.048 (x 0.954) | 18 (0.056) | 1 | procollagen-proline 4-dioxygenase activity | CG18749 |
| 1280 | GO:0003743 | F | 4, 5, | 1 | 3.260 (x 0.307) | 56 (0.018) | 1 | translation initiation factor activity | CG40068 |
| 1281 | GO:0006471 | P | 8, | 1 | 0.990 (x 1.010) | 17 (0.059) | 1 | protein amino acid ADP-ribosylation | Arf84F |
| 1282 | GO:0006325 | P | 8, | 4 | 8.035 (x 0.498) | 138 (0.029) | 1 | establishment and/or maintenance of chromatin architecture | CG31611 EG:BACR7A4.18 HP1c Orc6 |
| 1283 | GO:0051179 | P | 3, | 88 | 103.113 (x 0.853) | 1771 (0.050) | 1 | localization | Abl AnnX Arf84F Arr2 BG:DS01219.1 Bc CG10237 CG10320 CG10804 CG10950 CG11897 CG11898 CG1213 CG14691 CG15408 CG17266 CG1756 CG2185 CG2789 CG3036 CG31272 CG31477 CG32677 CG33066 CG3397 CG3529 CG4071 CG4187 CG4288 CG4673 CG4805 CG5535 CG5559 CG6214 CG6574 CG6723 CG7084 CG7211 CG7333 CG7777 CG8004 CG8193 CG8219 CG8271 CG8498 CG8916 CG8925 CG9326 CG9413 CG9862 CLIP-190 Dhc98D EG:9D2.4 Eip93F InR Myo28B1 Nxt1 Obp58b PNUTS Rab3 SytIV Syx16 Syx8 Tig Tim17b2 Tim9a Tm1 Tsf1 Vha36 bbx btl cyp33 dynactin-subunit-p25 glob1 hep insc l(2)03659 l(2)06225 mira na porin pum ran-like robl salm sun unc-13 vvl |
| 1284 | GO:0006305 | P | 8, | 1 | 1.048 (x 0.954) | 18 (0.056) | 1 | DNA alkylation | trol |
| 1285 | GO:0045182 | F | 2, | 2 | 4.949 (x 0.404) | 85 (0.024) | 1 | translation regulator activity | CG40068 pum |
| 1286 | GO:0007165 | P | 4, | 62 | 75.166 (x 0.825) | 1291 (0.048) | 1 | signal transduction | Abl Ac78C Arf84F Arr2 BG:DS00180.7 CG10738 CG11318 CG1504 CG15361 CG17262 CG1796 CG18249 CG2185 CG3529 CG4187 CG6124 CG7646 CG8916 CG9326 Cad99C Chit Con Dab Dgkepsilon EG:52C10.2 GABA-B-R2 Gp150 Hr38 Hr4 Idgf1 Idgf2 InR Or46a Or59a Or85a Or94a PGRP-SA Pld Rab3 Rh7 Takr99D Tehao Toll-7 TpnC41C TyrR btl chp gt hep inx7 ksr mirr pip ran-like regucalcin rho sra trol tsl unc-13 upd3 zpg |
| 1287 | GO:0007280 | P | 6, 7, 8, | 1 | 0.990 (x 1.010) | 17 (0.059) | 1 | pole cell migration | pum |
| 1288 | GO:0006470 | P | 8, | 2 | 4.949 (x 0.404) | 85 (0.024) | 1 | protein amino acid dephosphorylation | CG11597 PNUTS |
| 1289 | GO:0009719 | P | 3, | 7 | 7.744 (x 0.904) | 133 (0.053) | 1 | response to endogenous stimulus | CycG Eip93F Kr-h1 Tfb2 agt hay rpr |
| 1290 | GO:0050832 | P | 5, 6, | 1 | 1.048 (x 0.954) | 18 (0.056) | 1 | defense response to fungus | Tehao |
| 1291 | GO:0016741 | F | 4, | 2 | 4.949 (x 0.404) | 85 (0.024) | 1 | transferase activity, transferring one-carbon groups | EG:BACR7A4.8 agt |
| 1292 | GO:0008028 | F | 5, | 1 | 0.990 (x 1.010) | 17 (0.059) | 1 | monocarboxylic acid transporter activity | CG8271 |
| 1293 | GO:0004672 | F | 6, | 11 | 17.176 (x 0.640) | 295 (0.037) | 1 | protein kinase activity | Abl CG10738 CG2056 CG6214 CG9790 Doa InR Takl2 btl hep ksr |
| 1294 | GO:0006811 | P | 5, 6, | 19 | 26.899 (x 0.706) | 462 (0.041) | 1 | ion transport | CG1756 CG3036 CG31477 CG3397 CG4288 CG4805 CG6723 CG7084 CG7211 CG7333 CG8271 CG8916 CG8925 Tsf1 Vha36 l(2)06225 na porin sun |
| 1295 | GO:0048110 | P | 7, 8, 10, | 1 | 3.144 (x 0.318) | 54 (0.019) | 1 | oocyte construction (sensu Insecta) | Tm1 |
| 1296 | GO:0009954 | P | 4, | 1 | 1.048 (x 0.954) | 18 (0.056) | 1 | proximal/distal pattern formation | rho |
| 1297 | GO:0000910 | P | 5, | 2 | 4.891 (x 0.409) | 84 (0.024) | 1 | cytokinesis | Act57B rho |
| 1298 | GO:0045297 | P | 5, 6, | 1 | 0.990 (x 1.010) | 17 (0.059) | 1 | post-mating behavior | sra |
| 1299 | GO:0005773 | C | 5, 6, 7, 8, | 1 | 3.144 (x 0.318) | 54 (0.019) | 1 | vacuole | Vha36 |
| 1300 | GO:0009069 | P | 7, 8, | 1 | 1.048 (x 0.954) | 18 (0.056) | 1 | serine family amino acid metabolism | Dhfr |
| 1301 | GO:0016065 | P | 6, 7, | 1 | 3.086 (x 0.324) | 53 (0.019) | 1 | humoral defense mechanism (sensu Protostomia) | Def |
| 1302 | GO:0008168 | F | 5, | 2 | 4.891 (x 0.409) | 84 (0.024) | 1 | methyltransferase activity | EG:BACR7A4.8 agt |
| 1303 | GO:0030234 | F | 2, | 14 | 20.786 (x 0.674) | 357 (0.039) | 1 | enzyme regulator activity | CG10460 CG11313 CG1342 CG16712 CG31704 CG5639 CG8979 CG9790 CycG EG:52C10.2 PNUTS REG Spn6 Timp |
| 1304 | GO:0006839 | P | 6, 7, 8, | 1 | 0.990 (x 1.010) | 17 (0.059) | 1 | mitochondrial transport | porin |
| 1305 | GO:0009314 | P | 4, | 1 | 3.086 (x 0.324) | 53 (0.019) | 1 | response to radiation | Arr2 |
| 1306 | GO:0016773 | F | 5, | 14 | 20.786 (x 0.674) | 357 (0.039) | 1 | phosphotransferase activity, alcohol group as acceptor | Abl CG10738 CG2056 CG2846 CG2964 CG6214 CG9790 Dgkepsilon Doa InR Takl2 btl hep ksr |
| 1307 | GO:0016896 | F | 8, | 1 | 1.048 (x 0.954) | 18 (0.056) | 1 | exoribonuclease activity, producing 5'-phosphomonoesters | CG3931 |
| 1308 | GO:0005179 | F | 4, 5, | 1 | 3.086 (x 0.324) | 53 (0.019) | 1 | hormone activity | CG15361 |
| 1309 | GO:0003729 | F | 5, | 17 | 18.224 (x 0.933) | 313 (0.054) | 1 | mRNA binding | Aats-his BcDNA:GH11110 CG10092 CG10466 CG31184 CG3931 CG4866 CG9862 Pof RpL8 RpS18 RpS4 RpS9 apt cyp33 mRpL21 pum |
| 1310 | GO:0007422 | P | 5, | 4 | 4.425 (x 0.904) | 76 (0.053) | 1 | peripheral nervous system development | insc mirr rho vvl |
| 1311 | GO:0045610 | P | 5, 7, | 1 | 1.048 (x 0.954) | 18 (0.056) | 1 | regulation of hemocyte differentiation | hep |
| 1312 | GO:0003824 | F | 2, | 202 | 220.141 (x 0.918) | 3781 (0.053) | 1 | catalytic activity | Aats-his Abl Ac78C Act57B Ahcy13 Amyrel Arf84F BG:DS01068.5 BG:DS02740.5 Bc BcDNA:GH08420 BcDNA:GH08902 CAH2 CG10092 CG10104 CG10166 CG10184 CG10268 CG10320 CG10425 CG10466 CG10638 CG10738 CG10962 CG11015 CG11251 CG11313 CG11360 CG1140 CG11455 CG11597 CG11897 CG11898 CG11909 CG12133 CG12400 CG1299 CG1304 CG13318 CG13889 CG14482 CG14508 CG14721 CG14935 CG15012 CG15820 CG17224 CG17266 CG17280 CG17639 CG17821 CG18155 CG18223 CG18522 CG18530 CG18749 CG1885 CG18869 CG2056 CG2277 CG2846 CG2964 CG30022 CG30283 CG30438 CG30499 CG31272 CG31477 CG3215 CG32230 CG32479 CG32549 CG32626 CG32627 CG33096 CG33128 CG33177 CG3355 CG3397 CG3931 CG40045 CG4095 CG4169 CG4386 CG4408 CG4592 CG4769 CG4827 CG5037 CG5103 CG5122 CG5162 CG5177 CG5224 CG5382 CG5397 CG5537 CG5548 CG6214 CG6296 CG6357 CG6432 CG6461 CG6763 CG6921 CG7181 CG7211 CG7322 CG7339 CG7834 CG8193 CG8360 CG8520 CG8550 CG8756 CG8778 CG8918 CG8993 CG9164 CG9267 CG9326 CG9372 CG9602 CG9629 CG9790 CG9804 Chit CoVa Cyp12e1 Cyp28d2 Cyp311a1 Cyp49a1 Dab Dgkepsilon Dhc98D Dhfr Doa EG:152A3.7 EG:9D2.4 EG:BACR7A4.14 EG:BACR7A4.8 Gs2 GstD6 GstD9 GstE1 GstE5 GstE6 GstE7 Hmgs Iap2 Idgf1 Idgf2 ImpL3 InR Las Lip1 Mlc2 Myo28B1 Obp58b Or59a PGRP-SA Pdsw Pglym78 Pld Prosbeta5 Prx6005 RN-tre Rab3 RpII18 Rpb10 Rpb4 SamDC Ser7 Sod Sras Takl2 Tbh Ugt86De Ugt86Di Vha36 agt alpha-Est8 betaggt-II btl cyp33 fu12 gatA hay hep ksr l(2)03659 l(2)06225 l(3)02640 mtacp1 nmdyn-D6 pip ran-like rho robl sda shu sun |
| 1313 | GO:0042923 | F | 4, | 2 | 2.562 (x 0.781) | 44 (0.045) | 1 | neuropeptide binding | CG4187 Takr99D |
| 1314 | GO:0048111 | P | 6, 8, 9, 11, | 1 | 3.086 (x 0.324) | 53 (0.019) | 1 | oocyte axis determination (sensu Insecta) | Tm1 |
| 1315 | GO:0008238 | F | 5, | 3 | 6.346 (x 0.473) | 109 (0.028) | 1 | exopeptidase activity | CG32627 CG4408 sda |
| 1316 | GO:0005792 | C | 6, 7, | 2 | 4.833 (x 0.414) | 83 (0.024) | 1 | microsome | Cyp28d2 Cyp311a1 |
| 1317 | GO:0008135 | F | 3, 4, | 2 | 4.774 (x 0.419) | 82 (0.024) | 1 | translation factor activity, nucleic acid binding | CG40068 pum |
| 1318 | GO:0048029 | F | 5, | 1 | 1.048 (x 0.954) | 18 (0.056) | 1 | monosaccharide binding | lectin-28C |
| 1319 | GO:0050790 | P | 3, | 2 | 2.562 (x 0.781) | 44 (0.045) | 1 | regulation of catalytic activity | Dgkepsilon PNUTS |
| 1320 | GO:0042598 | C | 5, 6, | 2 | 4.833 (x 0.414) | 83 (0.024) | 1 | vesicular fraction | Cyp28d2 Cyp311a1 |
| 1321 | GO:0051603 | P | 8, 9, | 2 | 4.774 (x 0.419) | 82 (0.024) | 1 | proteolysis during cellular protein catabolism | CG32479 Prosbeta5 |
| 1322 | GO:0008544 | P | 5, | 1 | 1.281 (x 0.781) | 22 (0.045) | 1 | epidermis development | Hr38 |
| 1323 | GO:0042578 | F | 5, | 7 | 11.645 (x 0.601) | 200 (0.035) | 1 | phosphoric ester hydrolase activity | CG11597 CG2277 CG32549 CG4827 CG5177 CG9267 Pld |
| 1324 | GO:0048599 | P | 5, 6, 8, | 2 | 4.658 (x 0.429) | 80 (0.025) | 1 | oocyte development | Tm1 pip |
| 1325 | GO:0016887 | F | 8, | 15 | 21.601 (x 0.694) | 371 (0.040) | 1 | ATPase activity | CG11897 CG11898 CG31477 CG6214 CG7211 CG8520 Dhc98D Mlc2 Myo28B1 Vha36 hay l(2)03659 l(2)06225 robl sun |
| 1326 | GO:0008188 | F | 5, 6, 8, | 2 | 2.562 (x 0.781) | 44 (0.045) | 1 | neuropeptide receptor activity | CG4187 Takr99D |
| 1327 | GO:0044257 | P | 7, 8, | 2 | 4.774 (x 0.419) | 82 (0.024) | 1 | cellular protein catabolism | CG32479 Prosbeta5 |
| 1328 | GO:0006306 | P | 4, 8, 9, | 1 | 1.048 (x 0.954) | 18 (0.056) | 1 | DNA methylation | trol |
| 1329 | GO:0048037 | F | 3, | 2 | 2.212 (x 0.904) | 38 (0.053) | 1 | cofactor binding | CG8498 mtacp1 |
| 1330 | GO:0003924 | F | 8, | 4 | 7.569 (x 0.528) | 130 (0.031) | 1 | GTPase activity | Arf84F CG32627 Rab3 ran-like |
| 1331 | GO:0042625 | F | 4, 5, 7, 12, | 5 | 5.531 (x 0.904) | 95 (0.053) | 1 | ATPase activity, coupled to transmembrane movement of ions | CG31477 CG7211 Vha36 l(2)06225 sun |
| 1332 | GO:0007308 | P | 6, 7, 9, | 2 | 4.600 (x 0.435) | 79 (0.025) | 1 | oocyte construction | Tm1 pip |
| 1333 | GO:0048598 | P | 4, | 3 | 6.172 (x 0.486) | 106 (0.028) | 1 | embryonic morphogenesis | Abl InR hep |
| 1334 | GO:0007472 | P | 6, 7, | 3 | 6.113 (x 0.491) | 105 (0.029) | 1 | wing disc morphogenesis | mirr rho salm |
| 1335 | GO:0007498 | P | 4, | 7 | 11.528 (x 0.607) | 198 (0.035) | 1 | mesoderm development | BG:DS00180.7 CG12361 CG1942 CG9650 btl dmrt93B toy |
| 1336 | GO:0043085 | P | 4, | 1 | 1.281 (x 0.781) | 22 (0.045) | 1 | positive regulation of enzyme activity | Dgkepsilon |
| 1337 | GO:0000122 | P | 10, | 1 | 3.028 (x 0.330) | 52 (0.019) | 1 | negative regulation of transcription from RNA polymerase II promoter | gt |
| 1338 | GO:0003777 | F | 3, | 1 | 2.911 (x 0.344) | 50 (0.020) | 1 | microtubule motor activity | Dhc98D |
| 1339 | GO:0003678 | F | 4, | 1 | 2.969 (x 0.337) | 51 (0.020) | 1 | DNA helicase activity | hay |
| 1340 | GO:0009582 | P | 4, 5, | 2 | 2.562 (x 0.781) | 44 (0.045) | 1 | detection of abiotic stimulus | Arr2 tko |
| 1341 | GO:0004713 | F | 7, | 4 | 4.949 (x 0.808) | 85 (0.047) | 1 | protein-tyrosine kinase activity | Abl InR btl ksr |
| 1342 | GO:0048477 | P | 6, | 11 | 16.826 (x 0.654) | 289 (0.038) | 1 | oogenesis | Cp36 Doa Femcoat Tm1 Vm34Ca hep l(3)01239 pip pum rho shu |
| 1343 | GO:0006468 | P | 8, | 11 | 16.594 (x 0.663) | 285 (0.039) | 1 | protein amino acid phosphorylation | Abl CG10738 CG2056 CG6214 CG9267 Doa InR Takl2 btl hep ksr |
| 1344 | GO:0005912 | C | 6, 7, 8, | 1 | 2.853 (x 0.351) | 49 (0.020) | 1 | adherens junction | Abl |
| 1345 | GO:0001654 | P | 5, | 9 | 10.538 (x 0.854) | 181 (0.050) | 1 | eye development | Bro Cpn Dab Doa Gp150 chp mirr salm toy |
| 1346 | GO:0035287 | P | 5, 6, | 1 | 1.048 (x 0.954) | 18 (0.056) | 1 | head segmentation | gt |
| 1347 | GO:0006916 | P | 8, 9, | 2 | 2.212 (x 0.904) | 38 (0.053) | 1 | anti-apoptosis | CG7188 Iap2 |
| 1348 | GO:0007001 | P | 7, | 5 | 9.141 (x 0.547) | 157 (0.032) | 1 | chromosome organization and biogenesis (sensu Eukaryota) | CG31611 Doa EG:BACR7A4.18 HP1c Orc6 |
| 1349 | GO:0005267 | F | 6, 7, | 1 | 3.028 (x 0.330) | 52 (0.019) | 1 | potassium channel activity | CG1756 |
| 1350 | GO:0006836 | P | 5, 6, | 1 | 1.281 (x 0.781) | 22 (0.045) | 1 | neurotransmitter transport | CG10804 |
| 1351 | GO:0045935 | P | 7, | 3 | 3.784 (x 0.793) | 65 (0.046) | 1 | positive regulation of nucleobase, nucleoside, nucleotide and nucleic acid metabolism | Bro Eip93F mirr |
| 1352 | GO:0002009 | P | 4, | 4 | 7.744 (x 0.517) | 133 (0.030) | 1 | morphogenesis of an epithelium | Abl InR hep mirr |
| 1353 | GO:0007519 | P | 5, | 1 | 2.853 (x 0.351) | 49 (0.020) | 1 | striated muscle development | CG6803 |
| 1354 | GO:0043118 | P | 4, | 9 | 14.032 (x 0.641) | 241 (0.037) | 1 | negative regulation of physiological process | AnnX CG7188 Iap2 Orc6 apt gt pum rpr salm |
| 1355 | GO:0040011 | P | 3, | 10 | 15.196 (x 0.658) | 261 (0.038) | 1 | locomotion | Abl Dhc98D InR Tig btl hep pum robl salm vvl |
| 1356 | GO:0044430 | C | 4, 5, 6, 7, 8, 9, | 9 | 13.974 (x 0.644) | 240 (0.037) | 1 | cytoskeletal part | Act57B Arp11 CLIP-190 Dhc98D Mlc2 Myo28B1 Tm1 dynactin-subunit-p25 robl |
| 1357 | GO:0006366 | P | 8, | 31 | 39.708 (x 0.781) | 682 (0.045) | 1 | transcription from RNA polymerase II promoter | Bro CG10669 CG12175 CG12361 CG12605 CG15398 CG17385 CG32105 CG5382 CG7339 CG9650 HP1c Hr38 Hr4 Kr-h1 RpII18 Rpb10 Rpb4 Ssb-c31a Taf10b Taf11 TfIIEalpha Tfb2 Trap36 apt gt hay hep mirr toy vvl |
| 1358 | GO:0005316 | F | 7, 8, 9, | 1 | 1.048 (x 0.954) | 18 (0.056) | 1 | high affinity inorganic phosphate:sodium symporter activity | CG4288 |
| 1359 | GO:0019731 | P | 6, 7, 8, | 2 | 2.212 (x 0.904) | 38 (0.053) | 1 | antibacterial humoral response | Def PGRP-SA |
| 1360 | GO:0000904 | P | 5, 6, | 9 | 9.840 (x 0.915) | 169 (0.053) | 1 | cellular morphogenesis during differentiation | Abl Con Cpn Dab Doa InR Tig chp vvl |
| 1361 | GO:0008081 | F | 6, | 1 | 1.281 (x 0.781) | 22 (0.045) | 1 | phosphoric diester hydrolase activity | Pld |
| 1362 | GO:0051187 | P | 6, | 1 | 2.853 (x 0.351) | 49 (0.020) | 1 | cofactor catabolism | CG4095 |
| 1363 | GO:0016192 | P | 5, 6, | 15 | 17.234 (x 0.870) | 296 (0.051) | 1 | vesicle-mediated transport | AnnX Arf84F Arr2 CG2185 CG3529 CG5559 CLIP-190 Eip93F Myo28B1 Rab3 SytIV Syx16 Syx8 ran-like unc-13 |
| 1364 | GO:0030705 | P | 6, 7, 8, | 3 | 6.055 (x 0.495) | 104 (0.029) | 1 | cytoskeleton-dependent intracellular transport | Dhc98D dynactin-subunit-p25 robl |
| 1365 | GO:0009109 | P | 7, | 1 | 2.795 (x 0.358) | 48 (0.021) | 1 | coenzyme catabolism | CG4095 |
| 1366 | GO:0008258 | P | 6, | 1 | 1.339 (x 0.747) | 23 (0.043) | 1 | head involution | pum |
| 1367 | GO:0008104 | P | 4, | 31 | 32.954 (x 0.941) | 566 (0.055) | 1 | protein localization | AnnX Arf84F Arr2 BG:DS01219.1 CG10950 CG17266 CG2185 CG32677 CG33066 CG3529 CG4071 CG4187 CG4673 CG5559 CG8004 CG8219 CG9326 CLIP-190 Myo28B1 Nxt1 PNUTS Rab3 SytIV Syx16 Syx8 Tim17b2 Tim9a cyp33 insc mira ran-like |
| 1368 | GO:0007449 | P | 5, 6, | 1 | 1.048 (x 0.954) | 18 (0.056) | 1 | proximal/distal pattern formation, imaginal disc | rho |
| 1369 | GO:0035282 | P | 3, | 6 | 7.336 (x 0.818) | 126 (0.048) | 1 | segmentation | Doa gt ksr pum salm tsl |
| 1370 | GO:0006873 | P | 5, | 1 | 1.281 (x 0.781) | 22 (0.045) | 1 | cell ion homeostasis | Tsf1 |
| 1371 | GO:0009416 | P | 5, | 1 | 2.795 (x 0.358) | 48 (0.021) | 1 | response to light stimulus | Arr2 |
| 1372 | GO:0005576 | C | 2, | 20 | 22.474 (x 0.890) | 386 (0.052) | 1 | extracellular region | BG:DS00180.7 CG15361 CG6947 CG7298 CG8756 Chit Gasp Idgf1 Idgf2 Obp56a PGRP-SA Peritrophin-A Sgs4 Tig Timp Tsf1 hig trol tsl upd3 |
| 1373 | GO:0045087 | P | 5, 6, | 1 | 1.339 (x 0.747) | 23 (0.043) | 1 | innate immune response | PGRP-SA |
| 1374 | GO:0006084 | P | 7, | 1 | 2.795 (x 0.358) | 48 (0.021) | 1 | acetyl-CoA metabolism | CG4095 |
| 1375 | GO:0007465 | P | 8, 9, 10, 11, | 1 | 1.048 (x 0.954) | 18 (0.056) | 1 | R7 cell fate commitment | Dab |
| 1376 | GO:0042078 | P | 6, | 1 | 1.281 (x 0.781) | 22 (0.045) | 1 | germ-line stem cell division | pum |
| 1377 | GO:0009948 | P | 5, | 6 | 6.929 (x 0.866) | 119 (0.050) | 1 | anterior/posterior axis specification | Tm1 gt ksr pum regucalcin tsl |
| 1378 | GO:0007476 | P | 6, 7, 8, | 3 | 5.997 (x 0.500) | 103 (0.029) | 1 | wing morphogenesis | mirr rho salm |
| 1379 | GO:0006961 | P | 7, 8, 9, | 1 | 1.339 (x 0.747) | 23 (0.043) | 1 | antibacterial humoral response (sensu Protostomia) | Def |
| 1380 | GO:0005279 | F | 5, 6, | 2 | 2.271 (x 0.881) | 39 (0.051) | 1 | amino acid-polyamine transporter activity | CG5535 CG9413 |
| 1381 | GO:0001558 | P | 4, 5, 7, 8, | 1 | 1.164 (x 0.859) | 20 (0.050) | 1 | regulation of cell growth | InR |
| 1382 | GO:0005044 | F | 5, | 1 | 1.281 (x 0.781) | 22 (0.045) | 1 | scavenger receptor activity | CG3212 |
| 1383 | GO:0015144 | F | 3, | 4 | 4.716 (x 0.848) | 81 (0.049) | 1 | carbohydrate transporter activity | CG1213 CG15408 CG7084 CG7333 |
| 1384 | GO:0007018 | P | 7, 8, 9, | 3 | 5.997 (x 0.500) | 103 (0.029) | 1 | microtubule-based movement | Dhc98D dynactin-subunit-p25 robl |
| 1385 | GO:0015075 | F | 3, | 26 | 28.238 (x 0.921) | 485 (0.054) | 1 | ion transporter activity | CG10804 CG11015 CG14076 CG14482 CG17280 CG1756 CG3036 CG31477 CG4169 CG4288 CG4805 CG5535 CG6723 CG7084 CG7181 CG7211 CG7333 CG8916 CG8925 CoVa Tsf1 Vha36 l(2)06225 na porin sun |
| 1386 | GO:0045859 | P | 6, | 1 | 1.339 (x 0.747) | 23 (0.043) | 1 | regulation of protein kinase activity | Dgkepsilon |
| 1387 | GO:0046903 | P | 5, | 12 | 13.450 (x 0.892) | 231 (0.052) | 1 | secretion | AnnX Arf84F CG14691 CG2185 CG31272 CG3529 CG5559 Rab3 SytIV Syx16 Syx8 unc-13 |
| 1388 | GO:0042067 | P | 6, 7, 8, 9, | 1 | 1.921 (x 0.520) | 33 (0.030) | 1 | establishment of ommatidial polarity (sensu Endopterygota) | mirr |
| 1389 | GO:0045202 | C | 2, | 2 | 2.271 (x 0.881) | 39 (0.051) | 1 | synapse | CG8916 veli |
| 1390 | GO:0045944 | P | 10, | 1 | 1.164 (x 0.859) | 20 (0.050) | 1 | positive regulation of transcription from RNA polymerase II promoter | Bro |
| 1391 | GO:0009966 | P | 4, 5, | 3 | 5.997 (x 0.500) | 103 (0.029) | 1 | regulation of signal transduction | EG:52C10.2 PGRP-SA rho |
| 1392 | GO:0006635 | P | 8, 9, | 1 | 1.281 (x 0.781) | 22 (0.045) | 1 | fatty acid beta-oxidation | CG8778 |
| 1393 | GO:0008643 | P | 5, 6, | 4 | 4.716 (x 0.848) | 81 (0.049) | 1 | carbohydrate transport | CG1213 CG15408 CG3036 CG4288 |
| 1394 | GO:0001584 | F | 6, | 11 | 12.402 (x 0.887) | 213 (0.052) | 1 | rhodopsin-like receptor activity | CG4187 Gr61a Gr98c Or46a Or59a Or85a Or94a Or98b Rh7 Takr99D TyrR |
| 1395 | GO:0035295 | P | 3, | 2 | 4.483 (x 0.446) | 77 (0.026) | 1 | tube development | apt btl |
| 1396 | GO:0019395 | P | 7, 8, | 1 | 1.339 (x 0.747) | 23 (0.043) | 1 | fatty acid oxidation | CG8778 |
| 1397 | GO:0019200 | F | 6, | 1 | 1.921 (x 0.520) | 33 (0.030) | 1 | carbohydrate kinase activity | CG2964 |
| 1398 | GO:0006959 | P | 5, 6, | 4 | 4.541 (x 0.881) | 78 (0.051) | 1 | humoral immune response | Def PGRP-SA Tehao upd3 |
| 1399 | GO:0042277 | F | 3, | 2 | 3.435 (x 0.582) | 59 (0.034) | 1 | peptide binding | CG4187 Takr99D |
| 1400 | GO:0003697 | F | 6, | 1 | 1.164 (x 0.859) | 20 (0.050) | 1 | single-stranded DNA binding | Ssb-c31a |
| 1401 | GO:0046148 | P | 6, | 2 | 2.271 (x 0.881) | 39 (0.051) | 1 | pigment biosynthesis | CG1885 CG5037 |
| 1402 | GO:0048627 | P | 5, 6, 8, 9, | 1 | 1.281 (x 0.781) | 22 (0.045) | 1 | myoblast development | CG6803 |
| 1403 | GO:0007309 | P | 5, 7, 8, 10, | 2 | 4.425 (x 0.452) | 76 (0.026) | 1 | oocyte axis determination | Tm1 pip |
| 1404 | GO:0043632 | P | 7, | 2 | 4.483 (x 0.446) | 77 (0.026) | 1 | modification-dependent macromolecule catabolism | CG32479 Prosbeta5 |
| 1405 | GO:0019752 | P | 6, | 21 | 22.823 (x 0.920) | 392 (0.054) | 1 | carboxylic acid metabolism | Aats-his CG10092 CG10184 CG11251 CG1140 CG17821 CG4095 CG5122 CG5535 CG6432 CG6461 CG6921 CG8498 CG8778 CG9413 CG9804 Dhfr Gs2 Las gatA mtacp1 |
| 1406 | GO:0006401 | P | 7, | 1 | 1.339 (x 0.747) | 23 (0.043) | 1 | RNA catabolism | pum |
| 1407 | GO:0048522 | P | 4, | 8 | 11.004 (x 0.727) | 189 (0.042) | 1 | positive regulation of cellular process | Bro Eip93F InR mirr pum rho rpr skpA |
| 1408 | GO:0043565 | F | 5, | 1 | 1.921 (x 0.520) | 33 (0.030) | 1 | sequence-specific DNA binding | skpA |
| 1409 | GO:0016331 | P | 5, | 3 | 4.774 (x 0.628) | 82 (0.037) | 1 | morphogenesis of embryonic epithelium | Abl InR hep |
| 1410 | GO:0007254 | P | 7, 8, | 1 | 2.736 (x 0.365) | 47 (0.021) | 1 | JNK cascade | hep |
| 1411 | GO:0005184 | F | 5, 6, | 1 | 1.980 (x 0.505) | 34 (0.029) | 1 | neuropeptide hormone activity | CG15361 |
| 1412 | GO:0006790 | P | 5, | 2 | 3.435 (x 0.582) | 59 (0.034) | 1 | sulfur metabolism | Act57B CG8993 |
| 1413 | GO:0007186 | P | 6, | 16 | 17.758 (x 0.901) | 305 (0.052) | 1 | G-protein coupled receptor protein signaling pathway | Ac78C Arf84F Arr2 CG11318 CG15361 CG4187 Dgkepsilon EG:52C10.2 GABA-B-R2 Or46a Or59a Or85a Or94a Rh7 Takr99D TyrR |
| 1414 | GO:0000902 | P | 4, 5, | 17 | 19.679 (x 0.864) | 338 (0.050) | 1 | cellular morphogenesis | Abl CG14825 CG2069 Con Cpn Dab Doa InR Mp20 SIP1 Tig btl chp hep mirr veli vvl |
| 1415 | GO:0043039 | P | 8, 9, | 3 | 3.493 (x 0.859) | 60 (0.050) | 1 | tRNA aminoacylation | Aats-his CG10092 gatA |
| 1416 | GO:0006637 | P | 7, 8, | 1 | 1.164 (x 0.859) | 20 (0.050) | 1 | acyl-CoA metabolism | CG8498 |
| 1417 | GO:0009792 | P | 4, | 7 | 11.353 (x 0.617) | 195 (0.036) | 1 | embryonic development (sensu Metazoa) | Abl Hmgs InR hep mirr pum rpr |
| 1418 | GO:0009408 | P | 4, 5, | 2 | 2.736 (x 0.731) | 47 (0.043) | 1 | response to heat | CG5001 Hsp22 |
| 1419 | GO:0019941 | P | 8, 9, 10, | 2 | 4.483 (x 0.446) | 77 (0.026) | 1 | modification-dependent protein catabolism | CG32479 Prosbeta5 |
| 1420 | GO:0007446 | P | 4, 5, | 1 | 1.281 (x 0.781) | 22 (0.045) | 1 | imaginal disc growth | InR |
| 1421 | GO:0015203 | F | 4, | 2 | 2.271 (x 0.881) | 39 (0.051) | 1 | polyamine transporter activity | CG5535 CG9413 |
| 1422 | GO:0009064 | P | 7, 8, | 1 | 1.106 (x 0.904) | 19 (0.053) | 1 | glutamine family amino acid metabolism | Gs2 |
| 1423 | GO:0000151 | C | 3, 4, 5, 6, | 5 | 7.336 (x 0.682) | 126 (0.040) | 1 | ubiquitin ligase complex | BG:DS02740.5 CG11360 CG5382 Iap2 skpA |
| 1424 | GO:0045165 | P | 4, | 8 | 11.004 (x 0.727) | 189 (0.042) | 1 | cell fate commitment | Dab insc mira pum rho salm trol vvl |
| 1425 | GO:0003774 | F | 2, | 4 | 5.065 (x 0.790) | 87 (0.046) | 1 | motor activity | Act57B Dhc98D Mlc2 Myo28B1 |
| 1426 | GO:0007424 | P | 4, | 5 | 6.346 (x 0.788) | 109 (0.046) | 1 | tracheal system development (sensu Insecta) | apt btl rho salm vvl |
| 1427 | GO:0007619 | P | 5, 6, | 1 | 1.921 (x 0.520) | 33 (0.030) | 1 | courtship behavior | tko |
| 1428 | GO:0031098 | P | 6, | 1 | 2.736 (x 0.365) | 47 (0.021) | 1 | stress-activated protein kinase signaling pathway | hep |
| 1429 | GO:0043549 | P | 5, | 1 | 1.339 (x 0.747) | 23 (0.043) | 1 | regulation of kinase activity | Dgkepsilon |
| 1430 | GO:0006082 | P | 5, | 21 | 22.823 (x 0.920) | 392 (0.054) | 1 | organic acid metabolism | Aats-his CG10092 CG10184 CG11251 CG1140 CG17821 CG4095 CG5122 CG5535 CG6432 CG6461 CG6921 CG8498 CG8778 CG9413 CG9804 Dhfr Gs2 Las gatA mtacp1 |
| 1431 | GO:0050909 | P | 5, 7, | 2 | 3.493 (x 0.573) | 60 (0.033) | 1 | sensory perception of taste | Gr61a Gr98c |
| 1432 | GO:0008361 | P | 5, 6, | 1 | 1.980 (x 0.505) | 34 (0.029) | 1 | regulation of cell size | InR |
| 1433 | GO:0043285 | P | 6, | 4 | 7.394 (x 0.541) | 127 (0.031) | 1 | biopolymer catabolism | CG32479 Chit Prosbeta5 pum |
| 1434 | GO:0008406 | P | 5, | 1 | 1.456 (x 0.687) | 25 (0.040) | 1 | gonad development | CG7194 |
| 1435 | GO:0051049 | P | 5, 6, | 2 | 3.435 (x 0.582) | 59 (0.034) | 1 | regulation of transport | Arf84F Rab3 |
| 1436 | GO:0009880 | P | 4, | 6 | 7.511 (x 0.799) | 129 (0.047) | 1 | embryonic pattern specification | Doa gt ksr pum salm tsl |
| 1437 | GO:0048592 | P | 5, 6, | 6 | 8.675 (x 0.692) | 149 (0.040) | 1 | eye morphogenesis | Cpn Dab Doa chp mirr salm |
| 1438 | GO:0015031 | P | 5, 6, | 28 | 30.101 (x 0.930) | 517 (0.054) | 1 | protein transport | AnnX Arf84F Arr2 CG10950 CG17266 CG2185 CG32677 CG33066 CG3529 CG4071 CG4187 CG4673 CG5559 CG8004 CG8219 CG9326 CLIP-190 Myo28B1 Nxt1 PNUTS Rab3 SytIV Syx16 Syx8 Tim17b2 Tim9a cyp33 ran-like |
| 1439 | GO:0000785 | C | 5, 6, 7, 8, 9, 10, | 2 | 4.483 (x 0.446) | 77 (0.026) | 1 | chromatin | CG31611 HP1c |
| 1440 | GO:0043119 | P | 4, | 7 | 9.840 (x 0.711) | 169 (0.041) | 1 | positive regulation of physiological process | Bro Eip93F InR mirr pum rpr skpA |
| 1441 | GO:0007292 | P | 5, | 14 | 18.049 (x 0.776) | 310 (0.045) | 1 | female gamete generation | CG5162 Cp36 Doa Femcoat InR Tbh Tm1 Vm34Ca hep l(3)01239 pip pum rho shu |
| 1442 | GO:0016811 | F | 5, | 2 | 2.736 (x 0.731) | 47 (0.043) | 1 | hydrolase activity, acting on carbon-nitrogen (but not peptide) bonds, in linear amides | PGRP-SA gatA |
| 1443 | GO:0005643 | C | 3, 5, 6, 7, 8, 9, 10, 11, 12, 13, | 2 | 2.329 (x 0.859) | 40 (0.050) | 1 | nuclear pore | CG4673 CG8219 |
| 1444 | GO:0006418 | P | 8, 9, 10, | 3 | 3.493 (x 0.859) | 60 (0.050) | 1 | tRNA aminoacylation for protein translation | Aats-his CG10092 gatA |
| 1445 | GO:0008037 | P | 3, | 1 | 1.281 (x 0.781) | 22 (0.045) | 1 | cell recognition | Con |
| 1446 | GO:0009889 | P | 5, | 4 | 4.600 (x 0.870) | 79 (0.051) | 1 | regulation of biosynthesis | CG40068 apt pum rpr |
| 1447 | GO:0005604 | C | 3, 4, 5, | 1 | 1.164 (x 0.859) | 20 (0.050) | 1 | basement membrane | trol |
| 1448 | GO:0009057 | P | 5, | 10 | 11.062 (x 0.904) | 190 (0.053) | 1 | macromolecule catabolism | CG2964 CG30499 CG32479 CG5103 Chit ImpL3 PGRP-SA Pglym78 Prosbeta5 pum |
| 1449 | GO:0009060 | P | 8, | 1 | 2.736 (x 0.365) | 47 (0.021) | 1 | aerobic respiration | CG4095 |
| 1450 | GO:0001752 | P | 7, 8, 9, 10, | 1 | 1.921 (x 0.520) | 33 (0.030) | 1 | eye photoreceptor fate commitment (sensu Endopterygota) | Dab |
| 1451 | GO:0044272 | P | 6, | 1 | 1.106 (x 0.904) | 19 (0.053) | 1 | sulfur compound biosynthesis | Act57B |
| 1452 | GO:0048488 | P | 7, 8, | 1 | 1.980 (x 0.505) | 34 (0.029) | 1 | synaptic vesicle endocytosis | Arf84F |
| 1453 | GO:0051338 | P | 4, | 1 | 1.339 (x 0.747) | 23 (0.043) | 1 | regulation of transferase activity | Dgkepsilon |
| 1454 | GO:0000228 | C | 5, 6, 7, 8, 9, 10, | 2 | 2.678 (x 0.747) | 46 (0.043) | 1 | nuclear chromosome | CG15220 Orc6 |
| 1455 | GO:0004970 | F | 6, | 1 | 1.456 (x 0.687) | 25 (0.040) | 1 | ionotropic glutamate receptor activity | CG14076 |
| 1456 | GO:0007423 | P | 4, | 10 | 13.391 (x 0.747) | 230 (0.043) | 1 | sensory organ development | Bro Cpn Dab Doa Gp150 chp insc mirr salm toy |
| 1457 | GO:0016758 | F | 5, | 6 | 7.511 (x 0.799) | 129 (0.047) | 1 | transferase activity, transferring hexosyl groups | Act57B CG10166 CG18869 CG30438 Ugt86De Ugt86Di |
| 1458 | GO:0004872 | F | 3, | 30 | 32.197 (x 0.932) | 553 (0.054) | 1 | receptor activity | CG10738 CG11318 CG14076 CG1504 CG18249 CG2789 CG3212 CG4187 CG8916 EG:30B8.6 GABA-B-R2 Gr61a Gr98c Hr38 Hr4 InR Or46a Or59a Or85a Or94a Or98b PGRP-SA Rh7 Takr99D Tehao Toll-7 TyrR btl chp unc-13 |
| 1459 | GO:0045893 | P | 9, | 2 | 2.736 (x 0.731) | 47 (0.043) | 1 | positive regulation of transcription, DNA-dependent | Bro Eip93F |
| 1460 | GO:0046530 | P | 4, | 5 | 5.706 (x 0.876) | 98 (0.051) | 1 | photoreceptor cell differentiation | Cpn Dab Doa chp salm |
| 1461 | GO:0006099 | P | 8, 9, | 1 | 2.736 (x 0.365) | 47 (0.021) | 1 | tricarboxylic acid cycle | CG4095 |
| 1462 | GO:0008038 | P | 4, | 1 | 1.281 (x 0.781) | 22 (0.045) | 1 | neuron recognition | Con |
| 1463 | GO:0046930 | C | 6, 7, 8, | 2 | 2.329 (x 0.859) | 40 (0.050) | 1 | pore complex | CG4673 CG8219 |
| 1464 | GO:0005249 | F | 6, 7, 8, | 1 | 1.921 (x 0.520) | 33 (0.030) | 1 | voltage-gated potassium channel activity | CG1756 |
| 1465 | GO:0016284 | F | 7, | 1 | 1.164 (x 0.859) | 20 (0.050) | 1 | alanine aminopeptidase activity | sda |
| 1466 | GO:0031326 | P | 6, | 4 | 4.600 (x 0.870) | 79 (0.051) | 1 | regulation of cellular biosynthesis | CG40068 apt pum rpr |
| 1467 | GO:0043068 | P | 6, 7, | 2 | 4.367 (x 0.458) | 75 (0.027) | 1 | positive regulation of programmed cell death | Eip93F rpr |
| 1468 | GO:0016458 | P | 6, | 1 | 2.562 (x 0.390) | 44 (0.023) | 1 | gene silencing | Orc6 |
| 1469 | GO:0007456 | P | 6, | 9 | 10.073 (x 0.894) | 173 (0.052) | 1 | eye development (sensu Endopterygota) | Bro Cpn Dab Doa Gp150 chp mirr salm toy |
| 1470 | GO:0031300 | C | 4, 5, 6, 7, 8, 9, | 1 | 1.106 (x 0.904) | 19 (0.053) | 1 | intrinsic to organelle membrane | Sras |
| 1471 | GO:0009967 | P | 5, 6, | 1 | 1.339 (x 0.747) | 23 (0.043) | 1 | positive regulation of signal transduction | rho |
| 1472 | GO:0007265 | P | 7, | 1 | 1.456 (x 0.687) | 25 (0.040) | 1 | Ras protein signal transduction | ksr |
| 1473 | GO:0015837 | P | 5, 6, | 2 | 2.678 (x 0.747) | 46 (0.043) | 1 | amine transport | CG5535 CG9413 |
| 1474 | GO:0007552 | P | 4, | 10 | 14.730 (x 0.679) | 253 (0.040) | 1 | metamorphosis | Cpn Dab Doa Hr4 Kr-h1 chp hep mirr rho salm |
| 1475 | GO:0008652 | P | 7, 8, | 2 | 4.192 (x 0.477) | 72 (0.028) | 1 | amino acid biosynthesis | Dhfr Gs2 |
| 1476 | GO:0046356 | P | 8, | 1 | 2.736 (x 0.365) | 47 (0.021) | 1 | acetyl-CoA catabolism | CG4095 |
| 1477 | GO:0016407 | F | 7, | 2 | 2.736 (x 0.731) | 47 (0.043) | 1 | acetyltransferase activity | CG5122 Rpb4 |
| 1478 | GO:0006511 | P | 9, 10, 11, | 2 | 4.367 (x 0.458) | 75 (0.027) | 1 | ubiquitin-dependent protein catabolism | CG32479 Prosbeta5 |
| 1479 | GO:0042303 | P | 4, | 1 | 1.921 (x 0.520) | 33 (0.030) | 1 | molting cycle | Sgs4 |
| 1480 | GO:0004003 | F | 5, 11, | 1 | 1.223 (x 0.818) | 21 (0.048) | 1 | ATP-dependent DNA helicase activity | hay |
| 1481 | GO:0004553 | F | 5, | 5 | 5.706 (x 0.876) | 98 (0.051) | 1 | hydrolase activity, hydrolyzing O-glycosyl compounds | Amyrel CG11909 CG14935 CG15012 Chit |
| 1482 | GO:0045927 | P | 4, | 1 | 1.281 (x 0.781) | 22 (0.045) | 1 | positive regulation of growth | InR |
| 1483 | GO:0006260 | P | 7, | 4 | 7.045 (x 0.568) | 121 (0.033) | 1 | DNA replication | CG15220 CG18013 Orc6 skpA |
| 1484 | GO:0004540 | F | 6, | 1 | 2.562 (x 0.390) | 44 (0.023) | 1 | ribonuclease activity | CG3931 |
| 1485 | GO:0015103 | F | 5, | 2 | 2.329 (x 0.859) | 40 (0.050) | 1 | inorganic anion transporter activity | CG3036 CG4288 |
| 1486 | GO:0016337 | P | 4, | 4 | 7.220 (x 0.554) | 124 (0.032) | 1 | cell-cell adhesion | Cad99C Con chp trol |
| 1487 | GO:0016903 | F | 4, | 1 | 1.397 (x 0.716) | 24 (0.042) | 1 | oxidoreductase activity, acting on the aldehyde or oxo group of donors | CG9629 |
| 1488 | GO:0016028 | C | 5, 6, 7, | 1 | 1.164 (x 0.859) | 20 (0.050) | 1 | rhabdomere | Arr2 |
| 1489 | GO:0007447 | P | 4, 5, | 1 | 2.504 (x 0.399) | 43 (0.023) | 1 | imaginal disc pattern formation | rho |
| 1490 | GO:0005231 | F | 7, 8, | 1 | 2.620 (x 0.382) | 45 (0.022) | 1 | excitatory extracellular ligand-gated ion channel activity | CG14076 |
| 1491 | GO:0003712 | F | 3, 5, | 2 | 4.309 (x 0.464) | 74 (0.027) | 1 | transcription cofactor activity | Bro Ssb-c31a |
| 1492 | GO:0045445 | P | 5, 7, 8, | 1 | 1.339 (x 0.747) | 23 (0.043) | 1 | myoblast differentiation | CG6803 |
| 1493 | GO:0043566 | F | 5, | 1 | 1.456 (x 0.687) | 25 (0.040) | 1 | structure-specific DNA binding | Ssb-c31a |
| 1494 | GO:0006865 | P | 6, 7, 8, | 2 | 2.678 (x 0.747) | 46 (0.043) | 1 | amino acid transport | CG5535 CG9413 |
| 1495 | GO:0005657 | C | 5, 6, 7, 8, 9, 10, | 1 | 1.106 (x 0.904) | 19 (0.053) | 1 | replication fork | CG15220 |
| 1496 | GO:0016616 | F | 5, | 4 | 4.891 (x 0.818) | 84 (0.048) | 1 | oxidoreductase activity, acting on the CH-OH group of donors, NAD or NADP as acceptor | CG10638 CG10962 CG3215 ImpL3 |
| 1497 | GO:0004871 | F | 2, | 51 | 61.309 (x 0.832) | 1053 (0.048) | 1 | signal transducer activity | Arf84F BG:DS00180.7 CG10738 CG11318 CG14076 CG1504 CG15361 CG18249 CG2789 CG31146 CG3153 CG3212 CG3529 CG4187 CG6124 CG8916 Chit Dab Doa EG:30B8.6 EG:52C10.2 EG:80H7.10 GABA-B-R2 Gr61a Gr98c Hr38 Hr4 Idgf1 Idgf2 InR Or46a Or59a Or85a Or94a Or98b PGRP-SA Rh7 Takr99D Tehao Toll-7 Tsp42El TyrR btl chp hep ksr rho sra tsl unc-13 upd3 |
| 1498 | GO:0006512 | P | 8, | 9 | 13.624 (x 0.661) | 234 (0.038) | 1 | ubiquitin cycle | BG:DS02740.5 CG11360 CG32479 CG40045 CG5382 CG9602 Iap2 Prosbeta5 rpr |
| 1499 | GO:0042165 | F | 3, | 3 | 3.668 (x 0.818) | 63 (0.048) | 1 | neurotransmitter binding | CG2789 CG4187 Takr99D |
| 1500 | GO:0009993 | P | 7, | 11 | 16.128 (x 0.682) | 277 (0.040) | 1 | oogenesis (sensu Insecta) | Cp36 Doa Femcoat Tm1 Vm34Ca hep l(3)01239 pip pum rho shu |
| 1501 | GO:0045333 | P | 7, | 1 | 2.736 (x 0.365) | 47 (0.021) | 1 | cellular respiration | CG4095 |
| 1502 | GO:0051674 | P | 4, | 10 | 14.963 (x 0.668) | 257 (0.039) | 1 | localization of cell | Abl Dhc98D InR Tig btl hep pum robl salm vvl |
| 1503 | GO:0006817 | P | 8, 9, | 2 | 2.504 (x 0.799) | 43 (0.047) | 1 | phosphate transport | CG3036 CG4288 |
| 1504 | GO:0018988 | P | 5, | 1 | 1.921 (x 0.520) | 33 (0.030) | 1 | molting cycle (sensu Protostomia and Nematoda) | Sgs4 |
| 1505 | GO:0045934 | P | 7, | 4 | 7.045 (x 0.568) | 121 (0.033) | 1 | negative regulation of nucleobase, nucleoside, nucleotide and nucleic acid metabolism | Orc6 gt pum salm |
| 1506 | GO:0001653 | F | 4, | 2 | 2.736 (x 0.731) | 47 (0.043) | 1 | peptide receptor activity | CG4187 Takr99D |
| 1507 | GO:0005102 | F | 3, 4, | 14 | 16.244 (x 0.862) | 279 (0.050) | 1 | receptor binding | BG:DS00180.7 CG15361 CG31146 CG3153 CG3529 CG6124 Chit Dab Idgf1 Idgf2 rho sra tsl upd3 |
| 1508 | GO:0045892 | P | 9, | 3 | 5.648 (x 0.531) | 97 (0.031) | 1 | negative regulation of transcription, DNA-dependent | Orc6 gt pum |
| 1509 | GO:0007399 | P | 4, | 24 | 27.248 (x 0.881) | 468 (0.051) | 1 | nervous system development | Abl BG:DS02740.9 CG12361 CG31146 Con Dab Doa Gs2 InR Tig Tm1 Tsp42El apt btl insc mira mirr pum rho robl rpr toy trol vvl |
| 1510 | GO:0046698 | P | 5, | 10 | 14.614 (x 0.684) | 251 (0.040) | 1 | metamorphosis (sensu Insecta) | Cpn Dab Doa Hr4 Kr-h1 chp hep mirr rho salm |
| 1511 | GO:0007346 | P | 6, 7, | 1 | 1.223 (x 0.818) | 21 (0.048) | 1 | regulation of progression through mitotic cell cycle | skpA |
| 1512 | GO:0048628 | P | 6, 7, 9, 10, | 1 | 1.281 (x 0.781) | 22 (0.045) | 1 | myoblast maturation | CG6803 |
| 1513 | GO:0006814 | P | 8, 9, | 1 | 2.504 (x 0.399) | 43 (0.023) | 1 | sodium ion transport | CG4805 |
| 1514 | GO:0019898 | C | 4, 5, 6, | 1 | 2.620 (x 0.382) | 45 (0.022) | 1 | extrinsic to membrane | Abl |
| 1515 | GO:0009953 | P | 4, | 2 | 4.309 (x 0.464) | 74 (0.027) | 1 | dorsal/ventral pattern formation | Tehao pip |
| 1516 | GO:0007494 | P | 5, | 1 | 1.397 (x 0.716) | 24 (0.042) | 1 | midgut development | sisA |
| 1517 | GO:0007507 | P | 5, | 2 | 3.552 (x 0.563) | 61 (0.033) | 1 | heart development | Act57B apt |
| 1518 | GO:0051276 | P | 6, | 6 | 10.014 (x 0.599) | 172 (0.035) | 1 | chromosome organization and biogenesis | CG31611 Doa EG:BACR7A4.18 HP1c Orc6 skpA |
| 1519 | GO:0008360 | P | 5, 6, | 3 | 4.949 (x 0.606) | 85 (0.035) | 1 | regulation of cell shape | Abl Mp20 SIP1 |
| 1520 | GO:0008632 | P | 7, | 1 | 1.164 (x 0.859) | 20 (0.050) | 1 | apoptotic program | rpr |
| 1521 | GO:0048513 | P | 3, | 31 | 39.009 (x 0.795) | 670 (0.046) | 1 | organ development | Act57B Bro CG10861 CG12361 CG6803 CG7194 CG9650 Cpn Dab Doa Eip93F Gp150 Idgf1 Idgf2 InR Mp20 apt btl chp dmrt93B gt hep insc mirr oho23B rho robl rpr salm sisA toy |
| 1522 | GO:0005262 | F | 6, 7, | 1 | 1.456 (x 0.687) | 25 (0.040) | 1 | calcium channel activity | na |
| 1523 | GO:0016319 | P | 5, 7, | 1 | 1.339 (x 0.747) | 23 (0.043) | 1 | mushroom body development | robl |
| 1524 | GO:0015290 | F | 4, | 11 | 12.634 (x 0.871) | 217 (0.051) | 1 | electrochemical potential-driven transporter activity | CG10804 CG1213 CG3036 CG4288 CG5535 CG6574 CG6723 CG7084 CG7333 CG8271 CG9413 |
| 1525 | GO:0006928 | P | 4, 5, | 10 | 14.963 (x 0.668) | 257 (0.039) | 1 | cell motility | Abl Dhc98D InR Tig btl hep pum robl salm vvl |
| 1526 | GO:0030534 | P | 4, | 2 | 2.678 (x 0.747) | 46 (0.043) | 1 | adult behavior | Tbh na |
| 1527 | GO:0003702 | F | 3, | 14 | 15.487 (x 0.904) | 266 (0.053) | 1 | RNA polymerase II transcription factor activity | BEST:LD29214 CG15398 Rpb4 Taf10b Taf11 TfIIEalpha Tfb2 Trap36 apt gt hay salm toy vvl |
| 1528 | GO:0044271 | P | 5, 6, | 4 | 4.891 (x 0.818) | 84 (0.048) | 1 | nitrogen compound biosynthesis | Dhfr Gs2 SamDC Tbh |
| 1529 | GO:0006414 | P | 8, 9, | 1 | 1.106 (x 0.904) | 19 (0.053) | 1 | translational elongation | RpP1 |
| 1530 | GO:0030594 | F | 4, | 3 | 3.668 (x 0.818) | 63 (0.048) | 1 | neurotransmitter receptor activity | CG2789 CG4187 Takr99D |
| 1531 | GO:0006139 | P | 5, | 90 | 103.113 (x 0.873) | 1771 (0.051) | 1 | nucleobase, nucleoside, nucleotide and nucleic acid metabolism | Aats-his Ac78C Ahcy13 Bro CG10092 CG10418 CG10466 CG10669 CG10738 CG11360 CG12175 CG12361 CG1249 CG12605 CG13277 CG15220 CG15398 CG17224 CG17266 CG17385 CG17768 CG18013 CG18522 CG18619 CG2021 CG2277 CG30499 CG31184 CG31477 CG31611 CG32105 CG32549 CG32626 CG3931 CG4279 CG4827 CG5103 CG5382 CG5537 CG6272 CG6610 CG7211 CG7339 CG8360 CG9326 CG9650 CG9862 CycG DebB Dhfr Doa EG:BACR7A4.18 EG:BACR7A4.8 Eip93F HP1c Hr38 Hr4 Kr-h1 Nipped-B Orc6 RpII18 Rpb10 Rpb4 SmB Ssb-c31a Taf10b Taf11 TfIIEalpha Tfb2 Trap36 Vha36 agt apt bbx dmrt93B e(y)2 gatA gt hay hep l(2)06225 mirr nmdyn-D6 pum salm skpA sun toy trol vvl |
| 1532 | GO:0019098 | P | 3, 4, | 2 | 3.377 (x 0.592) | 58 (0.034) | 1 | reproductive behavior | sra tko |
| 1533 | GO:0015370 | F | 6, 8, | 3 | 3.610 (x 0.831) | 62 (0.048) | 1 | solute:sodium symporter activity | CG10804 CG4288 CG6723 |
| 1534 | GO:0008354 | P | 5, 6, 7, | 1 | 1.921 (x 0.520) | 33 (0.030) | 1 | germ cell migration | pum |
| 1535 | GO:0030036 | P | 8, | 3 | 5.648 (x 0.531) | 97 (0.031) | 1 | actin cytoskeleton organization and biogenesis | Abl CG6803 hep |
| 1536 | GO:0017157 | P | 6, 7, 8, | 2 | 2.504 (x 0.799) | 43 (0.047) | 1 | regulation of exocytosis | Arf84F Rab3 |
| 1537 | GO:0051649 | P | 5, 6, | 34 | 36.972 (x 0.920) | 635 (0.054) | 1 | establishment of cellular localization | AnnX Arf84F Arr2 CG10320 CG10950 CG17266 CG2185 CG32677 CG33066 CG3529 CG4071 CG4187 CG4673 CG5559 CG8004 CG8219 CG9326 CLIP-190 Dhc98D Myo28B1 Nxt1 PNUTS Rab3 SytIV Syx16 Syx8 Tim17b2 Tim9a cyp33 dynactin-subunit-p25 insc porin ran-like robl |
| 1538 | GO:0044453 | C | 4, 5, 6, 7, 8, 9, 10, 11, 12, | 2 | 2.736 (x 0.731) | 47 (0.043) | 1 | nuclear membrane part | CG4673 CG8219 |
| 1539 | GO:0007530 | P | 3, | 1 | 2.504 (x 0.399) | 43 (0.023) | 1 | sex determination | sisA |
| 1540 | GO:0016614 | F | 4, | 6 | 6.870 (x 0.873) | 118 (0.051) | 1 | oxidoreductase activity, acting on CH-OH group of donors | CG10638 CG10962 CG3215 CG7322 EG:BACR7A4.14 ImpL3 |
| 1541 | GO:0006960 | P | 7, 8, | 1 | 2.620 (x 0.382) | 45 (0.022) | 1 | antimicrobial humoral response (sensu Protostomia) | Def |
| 1542 | GO:0018193 | P | 8, | 1 | 2.038 (x 0.491) | 35 (0.029) | 1 | peptidyl-amino acid modification | CG18749 |
| 1543 | GO:0044421 | C | 2, 3, | 3 | 4.949 (x 0.606) | 85 (0.035) | 1 | extracellular region part | Tig Timp trol |
| 1544 | GO:0008154 | P | 6, 9, | 1 | 1.223 (x 0.818) | 21 (0.048) | 1 | actin polymerization and/or depolymerization | Abl |
| 1545 | GO:0040003 | P | 8, | 1 | 1.281 (x 0.781) | 22 (0.045) | 1 | cuticle biosynthesis (sensu Insecta) | Doa |
| 1546 | GO:0005253 | F | 5, 6, | 1 | 1.397 (x 0.716) | 24 (0.042) | 1 | anion channel activity | porin |
| 1547 | GO:0007455 | P | 6, 7, | 6 | 8.501 (x 0.706) | 146 (0.041) | 1 | eye-antennal disc morphogenesis | Cpn Dab Doa chp mirr salm |
| 1548 | GO:0005700 | C | 6, 7, 8, 9, | 2 | 2.620 (x 0.763) | 45 (0.044) | 1 | polytene chromosome | Eip93F Rpb4 |
| 1549 | GO:0008049 | P | 6, 7, | 1 | 1.164 (x 0.859) | 20 (0.050) | 1 | male courtship behavior | tko |
| 1550 | GO:0045137 | P | 4, | 1 | 1.456 (x 0.687) | 25 (0.040) | 1 | development of primary sexual characteristics | CG7194 |
| 1551 | GO:0006304 | P | 7, | 1 | 1.339 (x 0.747) | 23 (0.043) | 1 | DNA modification | trol |
| 1552 | GO:0015291 | F | 5, | 11 | 12.634 (x 0.871) | 217 (0.051) | 1 | porter activity | CG10804 CG1213 CG3036 CG4288 CG5535 CG6574 CG6723 CG7084 CG7333 CG8271 CG9413 |
| 1553 | GO:0005200 | F | 3, | 13 | 17.001 (x 0.765) | 292 (0.045) | 1 | structural constituent of cytoskeleton | Act57B Arp11 BG:DS02740.9 CG13889 CG31551 CG8918 CLIP-190 Dhc98D Mp20 Myo28B1 mira robl zormin |
| 1554 | GO:0051704 | P | 2, | 2 | 3.377 (x 0.592) | 58 (0.034) | 1 | interaction between organisms | sra tko |
| 1555 | GO:0009309 | P | 6, 7, | 4 | 4.891 (x 0.818) | 84 (0.048) | 1 | amine biosynthesis | Dhfr Gs2 SamDC Tbh |
| 1556 | GO:0042706 | P | 6, 7, 8, | 1 | 1.921 (x 0.520) | 33 (0.030) | 1 | eye photoreceptor cell fate commitment | Dab |
| 1557 | GO:0030029 | P | 7, | 3 | 5.648 (x 0.531) | 97 (0.031) | 1 | actin filament-based process | Abl CG6803 hep |
| 1558 | GO:0016790 | F | 5, | 1 | 1.863 (x 0.537) | 32 (0.031) | 1 | thiolester hydrolase activity | CG32479 |
| 1559 | GO:0050662 | F | 4, | 1 | 1.572 (x 0.636) | 27 (0.037) | 1 | coenzyme binding | CG8498 |
| 1560 | GO:0008372 | C | 2, | 44 | 47.394 (x 0.928) | 814 (0.054) | 1 | cellular component unknown | CG10674 CG11722 CG12022 CG13691 CG14701 CG14825 CG16817 CG2069 CG30105 CG30126 CG30154 CG30334 CG30343 CG30476 CG31601 CG31715 CG31957 CG32023 CG32160 CG32175 CG32202 CG32207 CG32442 CG32448 CG32582 CG32625 CG32637 CG32692 CG32710 CG32856 CG33322 CG3887 CG7194 CG7949 CG9381 EG:63B12.12 JhI-26 Karl NP15.6 Rep2 SIP1 SelG fau retinin |
| 1561 | GO:0015294 | F | 5, 7, | 3 | 3.668 (x 0.818) | 63 (0.048) | 1 | solute:cation symporter activity | CG10804 CG4288 CG6723 |
| 1562 | GO:0009620 | P | 5, | 1 | 1.106 (x 0.904) | 19 (0.053) | 1 | response to fungus | Tehao |
| 1563 | GO:0005635 | C | 4, 5, 6, 7, 8, 9, 10, | 3 | 3.610 (x 0.831) | 62 (0.048) | 1 | nuclear envelope | CG4673 CG8219 Nxt1 |
| 1564 | GO:0007315 | P | 7, 9, 10, 12, | 1 | 2.504 (x 0.399) | 43 (0.023) | 1 | pole plasm assembly | Tm1 |
| 1565 | GO:0048112 | P | 7, 9, 10, 12, | 1 | 2.620 (x 0.382) | 45 (0.022) | 1 | oocyte anterior/posterior axis determination (sensu Insecta) | Tm1 |
| 1566 | GO:0015171 | F | 4, 5, | 2 | 2.736 (x 0.731) | 47 (0.043) | 1 | amino acid transporter activity | CG5535 CG9413 |
| 1567 | GO:0001736 | P | 5, 6, | 2 | 2.504 (x 0.799) | 43 (0.047) | 1 | establishment of planar polarity | hep mirr |
| 1568 | GO:0009583 | P | 5, 6, | 1 | 2.038 (x 0.491) | 35 (0.029) | 1 | detection of light stimulus | Arr2 |
| 1569 | GO:0008610 | P | 5, 6, 7, | 3 | 4.949 (x 0.606) | 85 (0.035) | 1 | lipid biosynthesis | CG10268 fu12 mtacp1 |
| 1570 | GO:0019748 | P | 4, | 3 | 3.959 (x 0.758) | 68 (0.044) | 1 | secondary metabolism | Bc CG1885 CG5037 |
| 1571 | GO:0044238 | P | 4, | 261 | 266.079 (x 0.981) | 4570 (0.057) | 1 | primary metabolism | Aats-his Abl Ac78C Act57B Ahcy13 Amyrel AnnX Arf84F BG:DS02740.5 BcDNA:GH08420 BcDNA:GH08902 Bro CG10092 CG10104 CG10166 CG10184 CG10237 CG10268 CG10418 CG10466 CG10669 CG10738 CG11251 CG11313 CG11360 CG1140 CG11597 CG11909 CG1213 CG12133 CG12175 CG12361 CG1249 CG12605 CG12775 CG1299 CG1304 CG13277 CG13318 CG14894 CG14935 CG15220 CG15398 CG15408 CG17224 CG17266 CG17385 CG17768 CG17821 CG18013 CG18223 CG18522 CG18530 CG18619 CG18749 CG18767 CG1883 CG1885 CG18869 CG2021 CG2056 CG2277 CG2789 CG2964 CG2998 CG30283 CG3036 CG30438 CG30499 CG31184 CG31272 CG31477 CG31611 CG31704 CG32105 CG3215 CG32479 CG32549 CG32626 CG32627 CG33002 CG33128 CG33177 CG3355 CG3843 CG3931 CG40045 CG40068 CG4046 CG4095 CG4279 CG4288 CG4386 CG4408 CG4592 CG4827 CG4866 CG5001 CG5103 CG5122 CG5162 CG5177 CG5338 CG5382 CG5535 CG5537 CG6214 CG6272 CG6296 CG6432 CG6461 CG6574 CG6610 CG6723 CG6763 CG6764 CG6803 CG6921 CG6947 CG7014 CG7211 CG7298 CG7322 CG7339 CG7770 CG8360 CG8415 CG8498 CG8550 CG8756 CG8778 CG8857 CG8918 CG9267 CG9326 CG9372 CG9413 CG9602 CG9650 CG9804 CG9862 Chit CycG Cyp12e1 Cyp28d2 Cyp311a1 Cyp49a1 Dab DebB Dgkepsilon Dhc98D Dhfr Doa EG:9D2.4 EG:BACR7A4.18 EG:BACR7A4.8 Eip93F Gasp Gs2 HP1c Hmgs Hr38 Hr4 Hsp22 Iap2 ImpL3 InR Kr-h1 Las Lip1 Nipped-B Obp58b Orc6 PGRP-SA PNUTS Peritrophin-A Pglym78 Pld Pof Prosbeta5 REG RN-tre RpII18 RpL11 RpL17A RpL27A RpL38 RpL46 RpL8 RpL9 RpP1 RpS17 RpS18 RpS4 RpS9 Rpb10 Rpb4 SamDC Ser7 SmB Spn6 Sras Ssb-c31a Taf10b Taf11 Takl2 Tbh TfIIEalpha Tfb2 Timp Trap36 Ugt86De Ugt86Di Vha36 agt apt bbx betaggt-II btl cyp33 dmrt93B e(y)2 fu12 gatA gt hay hep jdp ksr l(2)06225 l(3)01239 mRpL11 mRpL14 mRpL2 mRpL21 mRpL22 mRpL22-24 mRpL33 mRpS14 mRpS21 mRpS24 mRpS26 mRpS32 mirr mtacp1 na nmdyn-D6 oho23B pip pum rpr salm sda shu skpA sop sun tko toy trol vvl |
| 1572 | GO:0015036 | F | 4, | 1 | 1.397 (x 0.716) | 24 (0.042) | 1 | disulfide oxidoreductase activity | CG8993 |
| 1573 | GO:0045941 | P | 8, | 3 | 3.726 (x 0.805) | 64 (0.047) | 1 | positive regulation of transcription | Bro Eip93F mirr |
| 1574 | GO:0007592 | P | 7, | 1 | 1.281 (x 0.781) | 22 (0.045) | 1 | cuticle biosynthesis (sensu Protostomia and Nematoda) | Doa |
| 1575 | GO:0051242 | P | 5, | 7 | 9.723 (x 0.720) | 167 (0.042) | 1 | positive regulation of cellular physiological process | Bro Eip93F InR mirr pum rpr skpA |
| 1576 | GO:0010033 | P | 5, | 1 | 1.223 (x 0.818) | 21 (0.048) | 1 | response to organic substance | Tbh |
| 1577 | GO:0005839 | C | 3, 4, 5, 6, 7, | 1 | 1.456 (x 0.687) | 25 (0.040) | 1 | proteasome core complex (sensu Eukaryota) | Prosbeta5 |
| 1578 | GO:0007623 | P | 4, | 1 | 2.096 (x 0.477) | 36 (0.028) | 1 | circadian rhythm | na |
| 1579 | GO:0016876 | F | 5, | 2 | 3.377 (x 0.592) | 58 (0.034) | 1 | ligase activity, forming aminoacyl-tRNA and related compounds | Aats-his CG10092 |
| 1580 | GO:0009072 | P | 6, 7, 8, | 1 | 1.164 (x 0.859) | 20 (0.050) | 1 | aromatic amino acid family metabolism | CG10184 |
| 1581 | GO:0000139 | C | 4, 5, 6, 7, 8, 9, 10, | 1 | 1.339 (x 0.747) | 23 (0.043) | 1 | Golgi membrane | Syx16 |
| 1582 | GO:0016706 | F | 5, | 1 | 1.921 (x 0.520) | 33 (0.030) | 1 | oxidoreductase activity, acting on paired donors, with incorporation or reduction of molecular oxygen, 2-oxoglutarate as one donor, and incorporation of one atom each of oxygen into both donors | CG18749 |
| 1583 | GO:0016301 | F | 5, | 19 | 23.347 (x 0.814) | 401 (0.047) | 1 | kinase activity | Abl CG10268 CG10738 CG14721 CG2056 CG2846 CG2964 CG5537 CG6214 CG9326 CG9790 Dgkepsilon Doa InR Takl2 btl hep ksr nmdyn-D6 |
| 1584 | GO:0019208 | F | 3, | 1 | 1.863 (x 0.537) | 32 (0.031) | 1 | phosphatase regulator activity | PNUTS |
| 1585 | GO:0007163 | P | 5, 6, | 2 | 4.134 (x 0.484) | 71 (0.028) | 1 | establishment and/or maintenance of cell polarity | mirr veli |
| 1586 | GO:0046907 | P | 5, 6, 7, | 33 | 35.865 (x 0.920) | 616 (0.054) | 1 | intracellular transport | AnnX Arf84F Arr2 CG10320 CG10950 CG17266 CG2185 CG32677 CG33066 CG3529 CG4071 CG4187 CG4673 CG5559 CG8004 CG8219 CG9326 CLIP-190 Dhc98D Myo28B1 Nxt1 PNUTS Rab3 SytIV Syx16 Syx8 Tim17b2 Tim9a cyp33 dynactin-subunit-p25 porin ran-like robl |
| 1587 | GO:0042127 | P | 5, | 1 | 1.572 (x 0.636) | 27 (0.037) | 1 | regulation of cell proliferation | InR |
| 1588 | GO:0006508 | P | 7, | 36 | 44.133 (x 0.816) | 758 (0.047) | 1 | proteolysis | BcDNA:GH08420 CG10104 CG10466 CG11313 CG12133 CG1299 CG1304 CG13318 CG18223 CG1885 CG2056 CG30283 CG31704 CG32479 CG32627 CG33128 CG3355 CG4386 CG4408 CG6461 CG6763 CG8550 CG9372 Dab EG:9D2.4 Obp58b Prosbeta5 REG RN-tre Ser7 Spn6 Sras Timp rpr sda skpA |
| 1589 | GO:0007411 | P | 6, 7, 9, 10, 12, | 4 | 4.774 (x 0.838) | 82 (0.049) | 1 | axon guidance | Abl InR Tig vvl |
| 1590 | GO:0019204 | F | 7, | 1 | 2.038 (x 0.491) | 35 (0.029) | 1 | nucleotide phosphatase activity | CG2277 |
| 1591 | GO:0031301 | C | 5, 6, 7, 8, 9, 10, | 1 | 1.106 (x 0.904) | 19 (0.053) | 1 | integral to organelle membrane | Sras |
| 1592 | GO:0042981 | P | 6, 7, | 4 | 6.346 (x 0.630) | 109 (0.037) | 1 | regulation of apoptosis | CG7188 Eip93F Iap2 rpr |
| 1593 | GO:0051641 | P | 4, 5, | 34 | 37.030 (x 0.918) | 636 (0.053) | 1 | cellular localization | AnnX Arf84F Arr2 CG10320 CG10950 CG17266 CG2185 CG32677 CG33066 CG3529 CG4071 CG4187 CG4673 CG5559 CG8004 CG8219 CG9326 CLIP-190 Dhc98D Myo28B1 Nxt1 PNUTS Rab3 SytIV Syx16 Syx8 Tim17b2 Tim9a cyp33 dynactin-subunit-p25 insc porin ran-like robl |
| 1594 | GO:0031965 | C | 5, 6, 7, 8, 9, 10, 11, | 2 | 2.736 (x 0.731) | 47 (0.043) | 1 | nuclear membrane | CG4673 CG8219 |
| 1595 | GO:0006810 | P | 4, 5, | 75 | 85.995 (x 0.872) | 1477 (0.051) | 1 | transport | AnnX Arf84F Arr2 Bc CG10237 CG10320 CG10804 CG10950 CG11897 CG11898 CG1213 CG14691 CG15408 CG17266 CG1756 CG2185 CG2789 CG3036 CG31272 CG31477 CG32677 CG33066 CG3397 CG3529 CG4071 CG4187 CG4288 CG4673 CG4805 CG5535 CG5559 CG6214 CG6574 CG6723 CG7084 CG7211 CG7333 CG7777 CG8004 CG8193 CG8219 CG8271 CG8498 CG8916 CG8925 CG9326 CG9413 CLIP-190 Dhc98D EG:9D2.4 Eip93F Myo28B1 Nxt1 Obp58b PNUTS Rab3 SytIV Syx16 Syx8 Tim17b2 Tim9a Tsf1 Vha36 bbx cyp33 dynactin-subunit-p25 glob1 l(2)03659 l(2)06225 na porin ran-like robl sun unc-13 |
| 1596 | GO:0044454 | C | 5, 6, 7, 8, 9, 10, 11, | 2 | 2.504 (x 0.799) | 43 (0.047) | 1 | nuclear chromosome part | CG15220 Orc6 |
| 1597 | GO:0043067 | P | 5, 6, | 4 | 6.929 (x 0.577) | 119 (0.034) | 1 | regulation of programmed cell death | CG7188 Eip93F Iap2 rpr |
| 1598 | GO:0030261 | P | 7, | 1 | 1.397 (x 0.716) | 24 (0.042) | 1 | chromosome condensation | skpA |
| 1599 | GO:0043037 | P | 7, 8, | 8 | 10.829 (x 0.739) | 186 (0.043) | 1 | translation | Aats-his CG10092 CG40068 RpP1 RpS18 apt gatA pum |
| 1600 | GO:0030097 | P | 5, | 3 | 3.726 (x 0.805) | 64 (0.047) | 1 | hemopoiesis | CG12361 CG9650 hep |
| 1601 | GO:0008076 | C | 3, 5, 6, 7, 8, 9, | 1 | 1.281 (x 0.781) | 22 (0.045) | 1 | voltage-gated potassium channel complex | CG3397 |
| 1602 | GO:0043235 | C | 3, | 1 | 2.096 (x 0.477) | 36 (0.028) | 1 | receptor complex | InR |
| 1603 | GO:0006405 | P | 7, 8, 9, 10, | 1 | 1.223 (x 0.818) | 21 (0.048) | 1 | RNA export from nucleus | Nxt1 |
| 1604 | GO:0009892 | P | 5, | 6 | 9.607 (x 0.625) | 165 (0.036) | 1 | negative regulation of metabolism | Orc6 apt gt pum rpr salm |
| 1605 | GO:0004812 | F | 6, | 2 | 3.377 (x 0.592) | 58 (0.034) | 1 | aminoacyl-tRNA ligase activity | Aats-his CG10092 |
| 1606 | GO:0016796 | F | 7, | 1 | 1.456 (x 0.687) | 25 (0.040) | 1 | exonuclease activity, active with either ribo- or deoxyribonucleic acids and producing 5'-phosphomonoesters | CG3931 |
| 1607 | GO:0035214 | P | 5, | 7 | 9.316 (x 0.751) | 160 (0.044) | 1 | eye-antennal disc development | Cpn Dab Doa chp mirr salm toy |
| 1608 | GO:0005774 | C | 5, 6, 7, 8, 9, 10, | 1 | 1.863 (x 0.537) | 32 (0.031) | 1 | vacuolar membrane | Vha36 |
| 1609 | GO:0004245 | F | 7, | 1 | 1.339 (x 0.747) | 23 (0.043) | 1 | neprilysin activity | CG8550 |
| 1610 | GO:0005326 | F | 3, | 1 | 1.164 (x 0.859) | 20 (0.050) | 1 | neurotransmitter transporter activity | CG10804 |
| 1611 | GO:0005507 | F | 6, | 1 | 1.572 (x 0.636) | 27 (0.037) | 1 | copper ion binding | Tbh |
| 1612 | GO:0043066 | P | 7, 8, | 2 | 2.445 (x 0.818) | 42 (0.048) | 1 | negative regulation of apoptosis | CG7188 Iap2 |
| 1613 | GO:0051656 | P | 5, | 1 | 1.747 (x 0.573) | 30 (0.033) | 1 | establishment of organelle localization | insc |
| 1614 | GO:0040029 | P | 3, | 2 | 4.076 (x 0.491) | 70 (0.029) | 1 | regulation of gene expression, epigenetic | Orc6 trol |
| 1615 | GO:0005216 | F | 4, 5, | 6 | 9.549 (x 0.628) | 164 (0.037) | 1 | ion channel activity | CG14076 CG1756 CG4805 CG8916 na porin |
| 1616 | GO:0006519 | P | 5, | 14 | 17.059 (x 0.821) | 293 (0.048) | 1 | amino acid and derivative metabolism | Aats-his CG10092 CG10184 CG11251 CG18749 CG5122 CG5535 CG6461 CG9413 Dhfr Gs2 SamDC Tbh gatA |
| 1617 | GO:0051119 | F | 4, | 2 | 2.736 (x 0.731) | 47 (0.043) | 1 | sugar transporter activity | CG1213 CG15408 |
| 1618 | GO:0030707 | P | 8, | 5 | 7.220 (x 0.693) | 124 (0.040) | 1 | ovarian follicle cell development (sensu Insecta) | Cp36 Femcoat Vm34Ca hep rho |
| 1619 | GO:0046915 | F | 5, | 1 | 1.106 (x 0.904) | 19 (0.053) | 1 | transition metal ion transporter activity | Tsf1 |
| 1620 | GO:0007164 | P | 4, | 2 | 2.504 (x 0.799) | 43 (0.047) | 1 | establishment of tissue polarity | hep mirr |
| 1621 | GO:0007154 | P | 3, | 77 | 88.324 (x 0.872) | 1517 (0.051) | 1 | cell communication | Abl Ac78C Arf84F Arr2 BG:DS00180.7 CG10738 CG11318 CG14691 CG1504 CG15361 CG17262 CG1756 CG1796 CG18249 CG2185 CG31146 CG31272 CG3529 CG4187 CG5559 CG5819 CG6124 CG7646 CG8916 CG9326 Cad99C Chit Con Dab Dgkepsilon EG:52C10.2 GABA-B-R2 Gp150 Gs2 Hr38 Hr4 Idgf1 Idgf2 InR Or46a Or59a Or85a Or94a PGRP-SA Pld Rab3 Rh7 SytIV Syx16 Syx8 Takr99D Tbh Tehao Toll-7 TpnC41C Tsp42El TyrR apt btl chp gt hep inx7 ksr mirr pip pum ran-like regucalcin rho sra trol tsl unc-13 upd3 veli zpg |
| 1622 | GO:0015698 | P | 7, 8, | 2 | 3.144 (x 0.636) | 54 (0.037) | 1 | inorganic anion transport | CG3036 CG4288 |
| 1623 | GO:0019897 | C | 5, 6, 7, | 1 | 2.096 (x 0.477) | 36 (0.028) | 1 | extrinsic to plasma membrane | Abl |
| 1624 | GO:0007391 | P | 6, | 3 | 4.425 (x 0.678) | 76 (0.039) | 1 | dorsal closure | Abl InR hep |
| 1625 | GO:0016323 | C | 5, 6, 7, | 1 | 1.397 (x 0.716) | 24 (0.042) | 1 | basolateral plasma membrane | Abl |
| 1626 | GO:0005886 | C | 4, 5, | 26 | 31.091 (x 0.836) | 534 (0.049) | 1 | plasma membrane | Abl Arr2 BG:DS01219.1 CG10738 CG10804 CG12918 CG3212 CG3397 Cad99C Con Gp150 InR PGRP-SA SytIV Syx16 Syx8 Takr99D Tehao Toll-7 TyrR btl chp inx7 rho veli zpg |
| 1627 | GO:0008237 | F | 5, | 7 | 10.771 (x 0.650) | 185 (0.038) | 1 | metallopeptidase activity | CG10466 CG32627 CG4408 CG6763 CG8550 Sras sda |
| 1628 | GO:0006399 | P | 7, | 4 | 5.124 (x 0.781) | 88 (0.045) | 1 | tRNA metabolism | Aats-his CG10092 EG:BACR7A4.8 gatA |
| 1629 | GO:0016875 | F | 4, | 2 | 3.377 (x 0.592) | 58 (0.034) | 1 | ligase activity, forming carbon-oxygen bonds | Aats-his CG10092 |
| 1630 | GO:0044420 | C | 2, 3, | 1 | 1.281 (x 0.781) | 22 (0.045) | 1 | extracellular matrix part | trol |
| 1631 | GO:0009056 | P | 4, | 17 | 19.039 (x 0.893) | 327 (0.052) | 1 | catabolism | CG10184 CG11251 CG1140 CG17224 CG2964 CG30499 CG32479 CG4095 CG4827 CG5103 Chit Gs2 ImpL3 PGRP-SA Pglym78 Prosbeta5 pum |
| 1632 | GO:0004888 | F | 4, | 21 | 24.745 (x 0.849) | 425 (0.049) | 1 | transmembrane receptor activity | CG11318 CG14076 CG3212 CG4187 CG8916 EG:30B8.6 GABA-B-R2 Gr61a Gr98c InR Or46a Or59a Or85a Or94a Or98b Rh7 Takr99D Tehao Toll-7 TyrR btl |
| 1633 | GO:0050808 | P | 5, | 1 | 1.863 (x 0.537) | 32 (0.031) | 1 | synapse organization and biogenesis | Gs2 |
| 1634 | GO:0035265 | P | 3, | 1 | 1.456 (x 0.687) | 25 (0.040) | 1 | organ growth | InR |
| 1635 | GO:0007469 | P | 6, | 1 | 1.223 (x 0.818) | 21 (0.048) | 1 | antennal development | salm |
| 1636 | GO:0046467 | P | 6, 7, 8, | 1 | 1.805 (x 0.554) | 31 (0.032) | 1 | membrane lipid biosynthesis | fu12 |
| 1637 | GO:0000267 | C | 3, 4, | 3 | 5.589 (x 0.537) | 96 (0.031) | 1 | cell fraction | Arr2 Cyp28d2 Cyp311a1 |
| 1638 | GO:0051082 | F | 4, | 2 | 3.610 (x 0.554) | 62 (0.032) | 1 | unfolded protein binding | CG5001 jdp |
| 1639 | GO:0031507 | P | 11, | 1 | 1.572 (x 0.636) | 27 (0.037) | 1 | heterochromatin formation | Orc6 |
| 1640 | GO:0004532 | F | 7, | 1 | 1.339 (x 0.747) | 23 (0.043) | 1 | exoribonuclease activity | CG3931 |
| 1641 | GO:0012502 | P | 7, 8, | 2 | 4.076 (x 0.491) | 70 (0.029) | 1 | induction of programmed cell death | Eip93F rpr |
| 1642 | GO:0009952 | P | 4, | 6 | 7.569 (x 0.793) | 130 (0.046) | 1 | anterior/posterior pattern formation | Tm1 gt ksr pum regucalcin tsl |
| 1643 | GO:0007591 | P | 6, | 1 | 1.747 (x 0.573) | 30 (0.033) | 1 | molting cycle (sensu Insecta) | Sgs4 |
| 1644 | GO:0008227 | F | 7, | 1 | 1.164 (x 0.859) | 20 (0.050) | 1 | amine receptor activity | TyrR |
| 1645 | GO:0005275 | F | 3, | 2 | 3.028 (x 0.661) | 52 (0.038) | 1 | amine transporter activity | CG5535 CG9413 |
| 1646 | GO:0043069 | P | 6, 7, | 2 | 2.445 (x 0.818) | 42 (0.048) | 1 | negative regulation of programmed cell death | CG7188 Iap2 |
| 1647 | GO:0004222 | F | 6, | 3 | 4.134 (x 0.726) | 71 (0.042) | 1 | metalloendopeptidase activity | CG6763 CG8550 Sras |
| 1648 | GO:0008528 | F | 5, 7, | 2 | 2.736 (x 0.731) | 47 (0.043) | 1 | peptide receptor activity, G-protein coupled | CG4187 Takr99D |
| 1649 | GO:0043190 | C | 3, 4, | 1 | 2.445 (x 0.409) | 42 (0.024) | 1 | ATP-binding cassette (ABC) transporter complex | l(2)03659 |
| 1650 | GO:0007166 | P | 5, | 32 | 38.369 (x 0.834) | 659 (0.049) | 1 | cell surface receptor linked signal transduction | Ac78C Arf84F Arr2 CG11318 CG1504 CG15361 CG18249 CG4187 Dab Dgkepsilon EG:52C10.2 GABA-B-R2 Gp150 InR Or46a Or59a Or85a Or94a PGRP-SA Rh7 Takr99D Tehao Toll-7 TyrR btl chp gt ksr mirr pip rho tsl |
| 1651 | GO:0048193 | P | 6, 7, 8, | 1 | 2.096 (x 0.477) | 36 (0.028) | 1 | Golgi vesicle transport | CG3529 |
| 1652 | GO:0015849 | P | 5, 6, | 2 | 3.144 (x 0.636) | 54 (0.037) | 1 | organic acid transport | CG5535 CG9413 |
| 1653 | GO:0004221 | F | 6, 8, | 1 | 1.514 (x 0.661) | 26 (0.038) | 1 | ubiquitin thiolesterase activity | CG32479 |
| 1654 | GO:0004180 | F | 6, | 2 | 2.504 (x 0.799) | 43 (0.047) | 1 | carboxypeptidase activity | CG32627 CG4408 |
| 1655 | GO:0006644 | P | 7, 8, | 3 | 4.425 (x 0.678) | 76 (0.039) | 1 | phospholipid metabolism | CG5162 Pld fu12 |
| 1656 | GO:0031545 | F | 7, | 1 | 1.106 (x 0.904) | 19 (0.053) | 1 | peptidyl-proline 4-dioxygenase activity | CG18749 |
| 1657 | GO:0008509 | F | 4, | 4 | 5.473 (x 0.731) | 94 (0.043) | 1 | anion transporter activity | CG3036 CG4288 CG6723 porin |
| 1658 | GO:0006869 | P | 5, 6, | 2 | 3.377 (x 0.592) | 58 (0.034) | 1 | lipid transport | CG2789 CG8498 |
| 1659 | GO:0008407 | P | 4, 5, | 1 | 1.397 (x 0.716) | 24 (0.042) | 1 | bristle morphogenesis | Doa |
| 1660 | GO:0000070 | P | 6, 8, | 1 | 1.863 (x 0.537) | 32 (0.031) | 1 | mitotic sister chromatid segregation | Nipped-B |
| 1661 | GO:0030003 | P | 6, | 1 | 1.281 (x 0.781) | 22 (0.045) | 1 | cation homeostasis | Tsf1 |
| 1662 | GO:0046620 | P | 4, | 1 | 1.456 (x 0.687) | 25 (0.040) | 1 | regulation of organ size | InR |
| 1663 | GO:0006917 | P | 8, 9, | 2 | 3.610 (x 0.554) | 62 (0.032) | 1 | induction of apoptosis | Eip93F rpr |
| 1664 | GO:0006333 | P | 9, | 4 | 5.822 (x 0.687) | 100 (0.040) | 1 | chromatin assembly or disassembly | CG31611 EG:BACR7A4.18 HP1c Orc6 |
| 1665 | GO:0007224 | P | 6, | 1 | 1.805 (x 0.554) | 31 (0.032) | 1 | smoothened signaling pathway | mirr |
| 1666 | GO:0009110 | P | 6, | 1 | 1.223 (x 0.818) | 21 (0.048) | 1 | vitamin biosynthesis | CG2846 |
| 1667 | GO:0042692 | P | 4, | 1 | 1.572 (x 0.636) | 27 (0.037) | 1 | muscle cell differentiation | CG6803 |
| 1668 | GO:0042386 | P | 4, 6, | 1 | 1.747 (x 0.573) | 30 (0.033) | 1 | hemocyte differentiation (sensu Arthropoda) | hep |
| 1669 | GO:0016874 | F | 3, | 15 | 18.457 (x 0.813) | 317 (0.047) | 1 | ligase activity | Aats-his BG:DS02740.5 CG10092 CG11360 CG18155 CG40045 CG5382 CG6432 CG8918 CG9602 CG9804 Gs2 Iap2 Las gatA |
| 1670 | GO:0005941 | C | 3, | 2 | 3.028 (x 0.661) | 52 (0.038) | 1 | unlocalized protein complex | CG18749 CG3215 |
| 1671 | GO:0006955 | P | 4, 5, | 5 | 7.103 (x 0.704) | 122 (0.041) | 1 | immune response | Def PGRP-SA Tehao Toll-7 upd3 |
| 1672 | GO:0007167 | P | 6, | 8 | 10.014 (x 0.799) | 172 (0.047) | 1 | enzyme linked receptor protein signaling pathway | Dab Gp150 InR btl gt ksr rho tsl |
| 1673 | GO:0048812 | P | 7, 8, 10, | 5 | 7.045 (x 0.710) | 121 (0.041) | 1 | neurite morphogenesis | Abl Con InR Tig vvl |
| 1674 | GO:0003704 | F | 4, | 3 | 4.600 (x 0.652) | 79 (0.038) | 1 | specific RNA polymerase II transcription factor activity | gt salm toy |
| 1675 | GO:0030005 | P | 7, | 1 | 1.164 (x 0.859) | 20 (0.050) | 1 | di-, tri-valent inorganic cation homeostasis | Tsf1 |
| 1676 | GO:0048637 | P | 6, | 1 | 2.445 (x 0.409) | 42 (0.024) | 1 | skeletal muscle development | CG6803 |
| 1677 | GO:0008092 | F | 4, | 11 | 13.857 (x 0.794) | 238 (0.046) | 1 | cytoskeletal protein binding | AnnX Arp11 BG:DS02740.9 CG6891 CG8397 CLIP-190 Mp20 Myo28B1 Tm1 insc mira |
| 1678 | GO:0000502 | C | 3, 4, 5, 6, | 2 | 3.144 (x 0.636) | 54 (0.037) | 1 | proteasome complex (sensu Eukaryota) | Prosbeta5 REG |
| 1679 | GO:0006950 | P | 3, | 16 | 19.446 (x 0.823) | 334 (0.048) | 1 | response to stress | Bc CG13889 CG5001 CycG Def GstE1 Hsp22 PGRP-SA Tehao Tfb2 Toll-7 agt hay hep rpr upd3 |
| 1680 | GO:0008235 | F | 6, | 3 | 4.425 (x 0.678) | 76 (0.039) | 1 | metalloexopeptidase activity | CG32627 CG4408 sda |
| 1681 | GO:0006813 | P | 8, 9, | 2 | 3.377 (x 0.592) | 58 (0.034) | 1 | potassium ion transport | CG1756 CG3397 |
| 1682 | GO:0051128 | P | 5, | 1 | 1.514 (x 0.661) | 26 (0.038) | 1 | regulation of cell organization and biogenesis | Abl |
| 1683 | GO:0015268 | F | 4, | 8 | 10.597 (x 0.755) | 182 (0.044) | 1 | alpha-type channel activity | CG14076 CG1756 CG4805 CG8916 inx7 na porin zpg |
| 1684 | GO:0044437 | C | 4, 5, 6, 7, 8, 9, | 1 | 1.863 (x 0.537) | 32 (0.031) | 1 | vacuolar part | Vha36 |
| 1685 | GO:0015082 | F | 5, | 1 | 1.397 (x 0.716) | 24 (0.042) | 1 | di-, tri-valent inorganic cation transporter activity | Tsf1 |
| 1686 | GO:0031543 | F | 6, | 1 | 1.106 (x 0.904) | 19 (0.053) | 1 | peptidyl-proline dioxygenase activity | CG18749 |
| 1687 | GO:0007617 | P | 4, 5, | 2 | 3.202 (x 0.625) | 55 (0.036) | 1 | mating behavior | sra tko |
| 1688 | GO:0006820 | P | 6, 7, | 4 | 5.822 (x 0.687) | 100 (0.040) | 1 | anion transport | CG3036 CG4288 CG8916 porin |
| 1689 | GO:0016410 | F | 7, | 1 | 1.805 (x 0.554) | 31 (0.032) | 1 | N-acyltransferase activity | Rpb4 |
| 1690 | GO:0006875 | P | 7, | 1 | 1.281 (x 0.781) | 22 (0.045) | 1 | metal ion homeostasis | Tsf1 |
| 1691 | GO:0048748 | P | 6, 7, | 6 | 8.209 (x 0.731) | 141 (0.043) | 1 | eye morphogenesis (sensu Endopterygota) | Cpn Dab Doa chp mirr salm |
| 1692 | GO:0016477 | P | 5, 6, | 8 | 10.189 (x 0.785) | 175 (0.046) | 1 | cell migration | Abl InR Tig btl hep pum salm vvl |
| 1693 | GO:0012506 | C | 4, 5, 6, 7, 8, 9, | 1 | 1.747 (x 0.573) | 30 (0.033) | 1 | vesicle membrane | CG31272 |
| 1694 | GO:0050801 | P | 4, | 1 | 1.572 (x 0.636) | 27 (0.037) | 1 | ion homeostasis | Tsf1 |
| 1695 | GO:0016339 | P | 5, | 1 | 1.223 (x 0.818) | 21 (0.048) | 1 | calcium-dependent cell-cell adhesion | Cad99C |
| 1696 | GO:0016471 | C | 3, 4, 5, 6, 7, 8, 9, 10, 11, | 1 | 1.688 (x 0.592) | 29 (0.034) | 1 | hydrogen-translocating V-type ATPase complex | Vha36 |
| 1697 | GO:0044432 | C | 4, 5, 6, 7, 8, 9, | 2 | 3.028 (x 0.661) | 52 (0.038) | 1 | endoplasmic reticulum part | CG11909 Sras |
| 1698 | GO:0045595 | P | 4, | 1 | 2.445 (x 0.409) | 42 (0.024) | 1 | regulation of cell differentiation | hep |
| 1699 | GO:0048667 | P | 6, 7, 9, | 5 | 7.045 (x 0.710) | 121 (0.041) | 1 | neuron morphogenesis during differentiation | Abl Con InR Tig vvl |
| 1700 | GO:0005694 | C | 5, 6, 7, 8, | 8 | 10.713 (x 0.747) | 184 (0.043) | 1 | chromosome | CG15220 CG31611 Eip93F HP1c Orc6 Pof Rpb4 pip |
| 1701 | GO:0004620 | F | 7, | 2 | 2.387 (x 0.838) | 41 (0.049) | 1 | phospholipase activity | CG6296 Pld |
| 1702 | GO:0004179 | F | 7, 8, | 1 | 1.164 (x 0.859) | 20 (0.050) | 1 | membrane alanyl aminopeptidase activity | sda |
| 1703 | GO:0008233 | F | 4, | 33 | 37.903 (x 0.871) | 651 (0.051) | 1 | peptidase activity | BG:DS01068.5 BcDNA:GH08420 CG10104 CG10466 CG11313 CG12133 CG1299 CG1304 CG13318 CG18223 CG1885 CG2056 CG30283 CG32479 CG32627 CG33128 CG3355 CG4386 CG4408 CG6357 CG6461 CG6763 CG8550 CG9372 Dab EG:9D2.4 Obp58b Prosbeta5 RN-tre Ser7 Sras rho sda |
| 1704 | GO:0007028 | P | 5, | 2 | 3.144 (x 0.636) | 54 (0.037) | 1 | cytoplasm organization and biogenesis | CG32409 CG6764 |
| 1705 | GO:0004527 | F | 6, | 1 | 2.387 (x 0.419) | 41 (0.024) | 1 | exonuclease activity | CG3931 |
| 1706 | GO:0051301 | P | 4, | 6 | 8.093 (x 0.741) | 139 (0.043) | 1 | cell division | Act57B insc mira pum rho trol |
| 1707 | GO:0006643 | P | 6, 7, | 3 | 5.065 (x 0.592) | 87 (0.034) | 1 | membrane lipid metabolism | CG5162 Pld fu12 |
| 1708 | GO:0015267 | F | 3, | 8 | 10.597 (x 0.755) | 182 (0.044) | 1 | channel or pore class transporter activity | CG14076 CG1756 CG4805 CG8916 inx7 na porin zpg |
| 1709 | GO:0008654 | P | 7, 8, 9, | 1 | 1.514 (x 0.661) | 26 (0.038) | 1 | phospholipid biosynthesis | fu12 |
| 1710 | GO:0046873 | F | 4, | 1 | 1.863 (x 0.537) | 32 (0.031) | 1 | metal ion transporter activity | Tsf1 |
| 1711 | GO:0051705 | P | 3, | 2 | 3.202 (x 0.625) | 55 (0.036) | 1 | behavioral interaction between organisms | sra tko |
| 1712 | GO:0016053 | P | 6, | 1 | 1.805 (x 0.554) | 31 (0.032) | 1 | organic acid biosynthesis | mtacp1 |
| 1713 | GO:0006461 | P | 6, | 4 | 6.812 (x 0.587) | 117 (0.034) | 1 | protein complex assembly | CG14894 CG31611 CG6803 EG:BACR7A4.18 |
| 1714 | GO:0048749 | P | 7, | 6 | 7.744 (x 0.775) | 133 (0.045) | 1 | compound eye development (sensu Endopterygota) | Cpn Dab Doa chp mirr salm |
| 1715 | GO:0008194 | F | 5, | 3 | 5.357 (x 0.560) | 92 (0.033) | 1 | UDP-glycosyltransferase activity | Act57B Ugt86De Ugt86Di |
| 1716 | GO:0048511 | P | 3, | 1 | 2.154 (x 0.464) | 37 (0.027) | 1 | rhythmic process | na |
| 1717 | GO:0016049 | P | 3, 4, 6, 7, | 1 | 1.747 (x 0.573) | 30 (0.033) | 1 | cell growth | InR |
| 1718 | GO:0015672 | P | 7, 8, | 8 | 10.189 (x 0.785) | 175 (0.046) | 1 | monovalent inorganic cation transport | CG1756 CG31477 CG3397 CG4805 CG7211 Vha36 l(2)06225 sun |
| 1719 | GO:0006342 | P | 5, 7, 10, 12, | 1 | 1.572 (x 0.636) | 27 (0.037) | 1 | chromatin silencing | Orc6 |
| 1720 | GO:0006281 | P | 5, 7, | 4 | 6.405 (x 0.625) | 110 (0.036) | 1 | DNA repair | CycG Tfb2 agt hay |
| 1721 | GO:0048747 | P | 5, | 1 | 2.445 (x 0.409) | 42 (0.024) | 1 | muscle fiber development | CG6803 |
| 1722 | GO:0042157 | P | 7, | 1 | 1.688 (x 0.592) | 29 (0.034) | 1 | lipoprotein metabolism | betaggt-II |
| 1723 | GO:0001738 | P | 5, | 2 | 3.028 (x 0.661) | 52 (0.038) | 1 | morphogenesis of a polarized epithelium | hep mirr |
| 1724 | GO:0019783 | F | 6, | 1 | 1.630 (x 0.613) | 28 (0.036) | 1 | small conjugating protein-specific protease activity | CG32479 |
| 1725 | GO:0046552 | P | 5, | 1 | 2.329 (x 0.429) | 40 (0.025) | 1 | photoreceptor cell fate commitment | Dab |
| 1726 | GO:0016567 | P | 9, | 5 | 6.579 (x 0.760) | 113 (0.044) | 1 | protein ubiquitination | BG:DS02740.5 CG11360 CG5382 Iap2 rpr |
| 1727 | GO:0048542 | P | 5, | 1 | 1.223 (x 0.818) | 21 (0.048) | 1 | lymph gland development (sensu Arthropoda) | oho23B |
| 1728 | GO:0007409 | P | 8, 9, 11, | 5 | 7.045 (x 0.710) | 121 (0.041) | 1 | axonogenesis | Abl Con InR Tig vvl |
| 1729 | GO:0016779 | F | 5, | 4 | 5.298 (x 0.755) | 91 (0.044) | 1 | nucleotidyltransferase activity | CG7339 RpII18 Rpb10 Rpb4 |
| 1730 | GO:0042175 | C | 4, 5, 6, | 1 | 2.387 (x 0.419) | 41 (0.024) | 1 | nuclear envelope-endoplasmic reticulum network | Sras |
| 1731 | GO:0005624 | C | 4, 5, | 3 | 5.415 (x 0.554) | 93 (0.032) | 1 | membrane fraction | Arr2 Cyp28d2 Cyp311a1 |
| 1732 | GO:0009266 | P | 4, | 2 | 3.144 (x 0.636) | 54 (0.037) | 1 | response to temperature stimulus | CG5001 Hsp22 |
| 1733 | GO:0016757 | F | 4, | 8 | 9.257 (x 0.864) | 159 (0.050) | 1 | transferase activity, transferring glycosyl groups | Act57B CG10166 CG17224 CG18869 CG30438 CG5537 Ugt86De Ugt86Di |
| 1734 | GO:0043038 | P | 7, 8, | 3 | 3.552 (x 0.845) | 61 (0.049) | 1 | amino acid activation | Aats-his CG10092 gatA |
| 1735 | GO:0030286 | C | 4, 6, 7, 8, 9, 10, 11, | 2 | 2.387 (x 0.838) | 41 (0.049) | 1 | dynein complex | Dhc98D robl |
| 1736 | GO:0019888 | F | 4, | 1 | 1.863 (x 0.537) | 32 (0.031) | 1 | protein phosphatase regulator activity | PNUTS |
| 1737 | GO:0005272 | F | 6, 7, | 1 | 1.514 (x 0.661) | 26 (0.038) | 1 | sodium channel activity | CG4805 |
| 1738 | GO:0046394 | P | 7, | 1 | 1.805 (x 0.554) | 31 (0.032) | 1 | carboxylic acid biosynthesis | mtacp1 |
| 1739 | GO:0019094 | P | 6, 10, 12, 13, 15, | 1 | 2.154 (x 0.464) | 37 (0.027) | 1 | pole plasm mRNA localization | Tm1 |
| 1740 | GO:0005875 | C | 3, 5, 6, 7, 8, 9, 10, | 4 | 6.754 (x 0.592) | 116 (0.034) | 1 | microtubule associated complex | CLIP-190 Dhc98D dynactin-subunit-p25 robl |
| 1741 | GO:0001745 | P | 7, 8, | 6 | 7.744 (x 0.775) | 133 (0.045) | 1 | compound eye morphogenesis (sensu Endopterygota) | Cpn Dab Doa chp mirr salm |
| 1742 | GO:0030162 | P | 6, 7, 8, | 1 | 1.747 (x 0.573) | 30 (0.033) | 1 | regulation of proteolysis | rpr |
| 1743 | GO:0048741 | P | 6, 7, | 1 | 2.445 (x 0.409) | 42 (0.024) | 1 | skeletal muscle fiber development | CG6803 |
| 1744 | GO:0044433 | C | 4, 5, 6, 7, 8, 9, | 1 | 1.572 (x 0.636) | 27 (0.037) | 1 | cytoplasmic vesicle part | CG31272 |
| 1745 | GO:0043414 | P | 7, | 1 | 2.329 (x 0.429) | 40 (0.025) | 1 | biopolymer methylation | trol |
| 1746 | GO:0006497 | P | 8, 9, | 1 | 1.688 (x 0.592) | 29 (0.034) | 1 | protein amino acid lipidation | betaggt-II |
| 1747 | GO:0016051 | P | 6, 7, | 2 | 2.853 (x 0.701) | 49 (0.041) | 1 | carbohydrate biosynthesis | Act57B CG5177 |
| 1748 | GO:0008408 | F | 7, | 1 | 1.630 (x 0.613) | 28 (0.036) | 1 | 3'-5' exonuclease activity | CG3931 |
| 1749 | GO:0019787 | F | 6, | 5 | 8.093 (x 0.618) | 139 (0.036) | 1 | small conjugating protein ligase activity | BG:DS02740.5 CG11360 CG5382 CG9602 Iap2 |
| 1750 | GO:0048113 | P | 8, 10, 11, 13, | 1 | 2.387 (x 0.419) | 41 (0.024) | 1 | pole plasm assembly (sensu Insecta) | Tm1 |
| 1751 | GO:0016579 | P | 9, | 1 | 1.223 (x 0.818) | 21 (0.048) | 1 | protein deubiquitination | CG32479 |
| 1752 | GO:0007350 | P | 4, 5, | 5 | 5.939 (x 0.842) | 102 (0.049) | 1 | blastoderm segmentation | Doa gt ksr pum tsl |
| 1753 | GO:0046942 | P | 6, 7, | 2 | 3.144 (x 0.636) | 54 (0.037) | 1 | carboxylic acid transport | CG5535 CG9413 |
| 1754 | GO:0005230 | F | 6, 7, | 2 | 2.911 (x 0.687) | 50 (0.040) | 1 | extracellular ligand-gated ion channel activity | CG14076 CG8916 |
| 1755 | GO:0000819 | P | 5, | 1 | 1.863 (x 0.537) | 32 (0.031) | 1 | sister chromatid segregation | Nipped-B |
| 1756 | GO:0009798 | P | 4, | 8 | 9.257 (x 0.864) | 159 (0.050) | 1 | axis specification | Tehao Tm1 gt ksr pip pum regucalcin tsl |
| 1757 | GO:0044431 | C | 4, 5, 6, 7, 8, 9, | 2 | 3.784 (x 0.528) | 65 (0.031) | 1 | Golgi apparatus part | CG3529 Syx16 |
| 1758 | GO:0007310 | P | 6, 8, 9, 11, | 1 | 1.514 (x 0.661) | 26 (0.038) | 1 | oocyte dorsal/ventral axis determination | pip |
| 1759 | GO:0006261 | P | 8, | 3 | 3.552 (x 0.845) | 61 (0.049) | 1 | DNA-dependent DNA replication | CG18013 Orc6 skpA |
| 1760 | GO:0007127 | P | 8, | 1 | 1.805 (x 0.554) | 31 (0.032) | 1 | meiosis I | sra |
| 1761 | GO:0016327 | C | 5, 6, 7, | 1 | 2.154 (x 0.464) | 37 (0.027) | 1 | apicolateral plasma membrane | veli |
| 1762 | GO:0007264 | P | 6, | 4 | 6.754 (x 0.592) | 116 (0.034) | 1 | small GTPase mediated signal transduction | Arf84F Rab3 ksr ran-like |
| 1763 | GO:0005874 | C | 5, 6, 7, 8, 9, 10, | 1 | 2.445 (x 0.409) | 42 (0.024) | 1 | microtubule | CLIP-190 |
| 1764 | GO:0048518 | P | 3, | 9 | 12.693 (x 0.709) | 218 (0.041) | 1 | positive regulation of biological process | Bro Dgkepsilon Eip93F InR mirr pum rho rpr skpA |
| 1765 | GO:0043065 | P | 7, 8, | 2 | 3.726 (x 0.537) | 64 (0.031) | 1 | positive regulation of apoptosis | Eip93F rpr |
| 1766 | GO:0048589 | P | 3, | 1 | 2.329 (x 0.429) | 40 (0.025) | 1 | developmental growth | InR |
| 1767 | GO:0051246 | P | 5, 6, | 6 | 7.744 (x 0.775) | 133 (0.045) | 1 | regulation of protein metabolism | Abl CG40068 PNUTS apt pum rpr |
| 1768 | GO:0016455 | F | 5, | 1 | 1.572 (x 0.636) | 27 (0.037) | 1 | RNA polymerase II transcription mediator activity | Trap36 |
| 1769 | GO:0050875 | P | 3, | 354 | 361.914 (x 0.978) | 6216 (0.057) | 1 | cellular physiological process | Aats-his Abl Ac78C Act57B Ahcy13 AnnX Arf84F Arp11 Arr2 BG:DS02740.5 Bc BcDNA:GH08420 BcDNA:GH08902 Bro CAH2 CG10092 CG10104 CG10166 CG10184 CG10237 CG10268 CG10320 CG10418 CG10466 CG10638 CG10669 CG10738 CG10804 CG10861 CG10950 CG11015 CG11251 CG11313 CG11360 CG1140 CG11455 CG11597 CG11897 CG11898 CG11909 CG1213 CG12133 CG12175 CG12361 CG12400 CG1249 CG12605 CG12775 CG1299 CG1304 CG13277 CG13318 CG13889 CG14482 CG14508 CG14691 CG14721 CG14825 CG14894 CG15220 CG15398 CG15408 CG17224 CG17266 CG17280 CG17385 CG1756 CG17768 CG17821 CG18011 CG18013 CG18223 CG18522 CG18619 CG18749 CG18767 CG1883 CG1885 CG18869 CG2021 CG2056 CG2069 CG2185 CG2277 CG2750 CG2789 CG2846 CG2964 CG2998 CG30022 CG30283 CG3036 CG30438 CG30499 CG31184 CG31272 CG31477 CG31551 CG31611 CG31704 CG32105 CG3215 CG32174 CG32230 CG32409 CG32479 CG32549 CG32626 CG32627 CG32677 CG33002 CG33066 CG33128 CG33177 CG3355 CG3397 CG3529 CG3843 CG3931 CG40045 CG40068 CG4046 CG4071 CG4095 CG4169 CG4187 CG4279 CG4288 CG4386 CG4408 CG4511 CG4673 CG4769 CG4805 CG4827 CG4866 CG5001 CG5037 CG5103 CG5122 CG5162 CG5177 CG5338 CG5382 CG5535 CG5537 CG5548 CG5559 CG6214 CG6272 CG6432 CG6461 CG6574 CG6610 CG6723 CG6763 CG6764 CG6803 CG6921 CG6947 CG7014 CG7084 CG7181 CG7188 CG7211 CG7298 CG7333 CG7339 CG7770 CG7777 CG7834 CG8004 CG8193 CG8219 CG8271 CG8360 CG8415 CG8498 CG8550 CG8756 CG8778 CG8857 CG8916 CG8918 CG8925 CG8993 CG9267 CG9326 CG9372 CG9413 CG9602 CG9650 CG9790 CG9804 CG9862 CLIP-190 Chit CoVa Con Cpn CycG Cyp12e1 Cyp28d2 Cyp311a1 Cyp49a1 Dab DebB Dgkepsilon Dhc98D Dhfr Doa EG:152A3.7 EG:9D2.4 EG:BACR7A4.18 EG:BACR7A4.8 Eip93F Gasp Gs2 GstE1 GstE5 GstE6 GstE7 HP1c Hmgs Hr38 Hr4 Hsp22 Iap2 ImpL3 InR Kr-h1 Las Mp20 Myo28B1 Nipped-B Nxt1 Obp58b Or59a Orc6 PGRP-SA PNUTS Pdsw Peritrophin-A Pglym78 Pld Pof Prosbeta5 Prx6005 REG RN-tre Rab3 RpII18 RpL11 RpL17A RpL27A RpL38 RpL46 RpL8 RpL9 RpP1 RpS17 RpS18 RpS4 RpS9 Rpb10 Rpb4 SIP1 SamDC SelG Ser7 SmB Sod Spn6 Sras Ssb-c31a SytIV Syx16 Syx8 Taf10b Taf11 Takl2 Tbh TfIIEalpha Tfb2 Tig Tim17b2 Tim9a Timp Trap36 Tsf1 Ugt86De Ugt86Di Vha36 agt apt bbx betaggt-II btl chp cyp33 dmrt93B dynactin-subunit-p25 e(y)2 fu12 gatA glob1 gt hay hep insc jdp ksr l(2)03659 l(2)06225 l(3)01239 l(3)02640 mRpL11 mRpL14 mRpL2 mRpL21 mRpL22 mRpL22-24 mRpL33 mRpS14 mRpS21 mRpS24 mRpS26 mRpS32 mira mirr mtacp1 na nmdyn-D6 oho23B pip porin pum ran-like rho robl rpr salm sda shu skpA sop sra sun tko toy trol unc-13 veli vvl |
| 1770 | GO:0005795 | C | 5, 6, 7, 8, 9, 10, | 1 | 1.688 (x 0.592) | 29 (0.034) | 1 | Golgi stack | CG3529 |
| 1771 | GO:0016791 | F | 6, | 7 | 10.597 (x 0.661) | 182 (0.038) | 1 | phosphoric monoester hydrolase activity | CG11597 CG2277 CG32549 CG4827 CG5177 CG9267 Pld |
| 1772 | GO:0004842 | F | 7, | 5 | 8.093 (x 0.618) | 139 (0.036) | 1 | ubiquitin-protein ligase activity | BG:DS02740.5 CG11360 CG5382 CG9602 Iap2 |
| 1773 | GO:0046943 | F | 4, | 3 | 4.192 (x 0.716) | 72 (0.042) | 1 | carboxylic acid transporter activity | CG5535 CG8271 CG9413 |
| 1774 | GO:0008080 | F | 8, | 1 | 1.630 (x 0.613) | 28 (0.036) | 1 | N-acetyltransferase activity | Rpb4 |
| 1775 | GO:0045184 | P | 5, | 28 | 30.276 (x 0.925) | 520 (0.054) | 1 | establishment of protein localization | AnnX Arf84F Arr2 CG10950 CG17266 CG2185 CG32677 CG33066 CG3529 CG4071 CG4187 CG4673 CG5559 CG8004 CG8219 CG9326 CLIP-190 Myo28B1 Nxt1 PNUTS Rab3 SytIV Syx16 Syx8 Tim17b2 Tim9a cyp33 ran-like |
| 1776 | GO:0008298 | P | 5, | 2 | 2.853 (x 0.701) | 49 (0.041) | 1 | intracellular mRNA localization | Tm1 mira |
| 1777 | GO:0031324 | P | 6, | 6 | 9.025 (x 0.665) | 155 (0.039) | 1 | negative regulation of cellular metabolism | Orc6 apt gt pum rpr salm |
| 1778 | GO:0003676 | F | 3, | 95 | 101.483 (x 0.936) | 1743 (0.055) | 1 | nucleic acid binding | Aats-his BEST:LD29214 BcDNA:GH11110 CG10092 CG10466 CG10669 CG11360 CG12361 CG1249 CG12605 CG12775 CG15220 CG15398 CG17385 CG17768 CG18011 CG18619 CG1883 CG2998 CG31184 CG31611 CG31922 CG31957 CG32105 CG3843 CG3931 CG40068 CG4046 CG4866 CG5338 CG6272 CG6764 CG7014 CG7339 CG7911 CG8152 CG8415 CG8506 CG8778 CG8857 CG9650 CG9862 DebB EG:BACR7A4.8 Eip93F Hr38 Hr4 Kr-h1 Orc6 PNUTS Pof RpII18 RpL11 RpL17A RpL27A RpL38 RpL46 RpL8 RpL9 RpP1 RpS17 RpS18 RpS4 RpS9 Rpb10 Rpb4 SmB Ssb-c31a Taf10b Taf11 Tfb2 agt apt bbx cyp33 dmrt93B e(y)2 gt hay l(2)k10201 mRpL11 mRpL2 mRpL21 mRpS14 mirr oho23B pum salm sisA skpA sop tko toy trol vvl |
| 1779 | GO:0048468 | P | 4, | 15 | 19.796 (x 0.758) | 340 (0.044) | 1 | cell development | Abl CG6803 Con Cpn Dab Doa InR Tig Tm1 chp pip pum robl vvl zpg |
| 1780 | GO:0003700 | F | 3, 5, | 17 | 22.649 (x 0.751) | 389 (0.044) | 1 | transcription factor activity | CG12361 CG15398 CG32105 Eip93F Hr38 Hr4 Kr-h1 Taf10b Taf11 Tfb2 bbx dmrt93B e(y)2 mirr sisA toy vvl |
| 1781 | GO:0051640 | P | 4, | 1 | 1.863 (x 0.537) | 32 (0.031) | 1 | organelle localization | insc |
| 1782 | GO:0035239 | P | 4, | 2 | 3.784 (x 0.528) | 65 (0.031) | 1 | tube morphogenesis | apt btl |
| 1783 | GO:0016860 | F | 4, | 1 | 1.514 (x 0.661) | 26 (0.038) | 1 | intramolecular oxidoreductase activity | CG4592 |
| 1784 | GO:0008094 | F | 10, | 1 | 2.212 (x 0.452) | 38 (0.026) | 1 | DNA-dependent ATPase activity | hay |
| 1785 | GO:0043062 | P | 3, | 1 | 2.445 (x 0.409) | 42 (0.024) | 1 | extracellular structure organization and biogenesis | Gs2 |
| 1786 | GO:0016481 | P | 8, | 4 | 6.463 (x 0.619) | 111 (0.036) | 1 | negative regulation of transcription | Orc6 gt pum salm |
| 1787 | GO:0009888 | P | 3, | 18 | 23.173 (x 0.777) | 398 (0.045) | 1 | tissue development | BG:DS00180.7 BG:DS02740.9 CG12361 CG1942 CG31146 CG5397 CG9650 Dab Doa Hr38 Tsp42El btl dmrt93B mirr rho sisA toy vvl |
| 1788 | GO:0005789 | C | 4, 5, 6, 7, 8, 9, 10, | 1 | 2.329 (x 0.429) | 40 (0.025) | 1 | endoplasmic reticulum membrane | Sras |
| 1789 | GO:0044274 | P | 5, | 1 | 1.572 (x 0.636) | 27 (0.037) | 1 | organismal biosynthesis | Doa |
| 1790 | GO:0007416 | P | 5, 6, | 1 | 1.688 (x 0.592) | 29 (0.034) | 1 | synaptogenesis | Gs2 |
| 1791 | GO:0006445 | P | 7, 8, 9, | 3 | 4.192 (x 0.716) | 72 (0.042) | 1 | regulation of translation | CG40068 apt pum |
| 1792 | GO:0001501 | P | 4, | 1 | 1.630 (x 0.613) | 28 (0.036) | 1 | skeletal development | dmrt93B |
| 1793 | GO:0019199 | F | 5, 7, | 2 | 2.853 (x 0.701) | 49 (0.041) | 1 | transmembrane receptor protein kinase activity | InR btl |
| 1794 | GO:0030198 | P | 4, | 1 | 1.863 (x 0.537) | 32 (0.031) | 1 | extracellular matrix organization and biogenesis | Gs2 |
| 1795 | GO:0006338 | P | 10, | 1 | 2.445 (x 0.409) | 42 (0.024) | 1 | chromatin remodeling | Orc6 |
| 1796 | GO:0007316 | P | 5, 9, 11, 12, 14, | 1 | 2.212 (x 0.452) | 38 (0.026) | 1 | pole plasm RNA localization | Tm1 |
| 1797 | GO:0044459 | C | 4, 5, 6, | 14 | 18.806 (x 0.744) | 323 (0.043) | 1 | plasma membrane part | Abl Arr2 CG10804 CG3212 CG3397 Cad99C InR PGRP-SA Takr99D TyrR inx7 rho veli zpg |
| 1798 | GO:0008287 | C | 3, 4, | 1 | 1.514 (x 0.661) | 26 (0.038) | 1 | protein serine/threonine phosphatase complex | CG11597 |
| 1799 | GO:0004177 | F | 6, | 1 | 2.329 (x 0.429) | 40 (0.025) | 1 | aminopeptidase activity | sda |
| 1800 | GO:0006520 | P | 6, 7, | 11 | 15.196 (x 0.724) | 261 (0.042) | 1 | amino acid metabolism | Aats-his CG10092 CG10184 CG11251 CG5122 CG5535 CG6461 CG9413 Dhfr Gs2 gatA |
| 1801 | GO:0007173 | P | 8, | 1 | 2.271 (x 0.440) | 39 (0.026) | 1 | epidermal growth factor receptor signaling pathway | rho |
| 1802 | GO:0007349 | P | 3, 4, | 1 | 1.688 (x 0.592) | 29 (0.034) | 1 | cellularization | CLIP-190 |
| 1803 | GO:0051168 | P | 7, 8, 9, | 1 | 1.572 (x 0.636) | 27 (0.037) | 1 | nuclear export | Nxt1 |
| 1804 | GO:0016881 | F | 5, | 6 | 9.199 (x 0.652) | 158 (0.038) | 1 | acid-amino acid ligase activity | BG:DS02740.5 CG11360 CG5382 CG8918 CG9602 Iap2 |
| 1805 | GO:0004843 | F | 7, | 1 | 1.630 (x 0.613) | 28 (0.036) | 1 | ubiquitin-specific protease activity | CG32479 |
| 1806 | GO:0007420 | P | 4, 6, | 2 | 2.853 (x 0.701) | 49 (0.041) | 1 | brain development | rho robl |
| 1807 | GO:0042623 | F | 9, | 14 | 19.214 (x 0.729) | 330 (0.042) | 1 | ATPase activity, coupled | CG11897 CG11898 CG31477 CG6214 CG7211 Dhc98D Mlc2 Myo28B1 Vha36 hay l(2)03659 l(2)06225 robl sun |
| 1808 | GO:0012505 | C | 4, 5, | 6 | 8.384 (x 0.716) | 144 (0.042) | 1 | endomembrane system | CG31272 CG4673 CG8219 Nxt1 Sras Syx16 |
| 1809 | GO:0007602 | P | 6, 7, | 1 | 1.863 (x 0.537) | 32 (0.031) | 1 | phototransduction | Arr2 |
| 1810 | GO:0007281 | P | 5, | 4 | 6.521 (x 0.613) | 112 (0.036) | 1 | germ cell development | Tm1 pip pum zpg |
| 1811 | GO:0007389 | P | 3, | 12 | 14.905 (x 0.805) | 256 (0.047) | 1 | pattern specification | Doa Tehao Tm1 gt hep ksr pip pum regucalcin rho salm tsl |
| 1812 | GO:0005351 | F | 5, 6, | 1 | 2.329 (x 0.429) | 40 (0.025) | 1 | sugar porter activity | CG1213 |
| 1813 | GO:0006812 | P | 6, 7, | 17 | 22.299 (x 0.762) | 383 (0.044) | 1 | cation transport | CG1756 CG3036 CG31477 CG3397 CG4288 CG4805 CG6723 CG7084 CG7211 CG7333 CG8271 CG8925 Tsf1 Vha36 l(2)06225 na sun |
| 1814 | GO:0008344 | P | 5, | 1 | 1.514 (x 0.661) | 26 (0.038) | 1 | adult locomotory behavior | na |
| 1815 | GO:0004722 | F | 8, | 1 | 2.271 (x 0.440) | 39 (0.026) | 1 | protein serine/threonine phosphatase activity | CG11597 |
| 1816 | GO:0006633 | P | 6, 7, 8, | 1 | 1.688 (x 0.592) | 29 (0.034) | 1 | fatty acid biosynthesis | mtacp1 |
| 1817 | GO:0048469 | P | 5, | 1 | 1.572 (x 0.636) | 27 (0.037) | 1 | cell maturation | CG6803 |
| 1818 | GO:0004091 | F | 6, | 1 | 1.630 (x 0.613) | 28 (0.036) | 1 | carboxylesterase activity | alpha-Est8 |
| 1819 | GO:0045451 | P | 7, 11, 13, 14, 16, | 1 | 1.863 (x 0.537) | 32 (0.031) | 1 | pole plasm oskar mRNA localization | Tm1 |
| 1820 | GO:0016772 | F | 4, | 23 | 29.170 (x 0.788) | 501 (0.046) | 1 | transferase activity, transferring phosphorus-containing groups | Abl CG10268 CG10738 CG14721 CG2056 CG2846 CG2964 CG5537 CG6214 CG7339 CG9326 CG9790 Dgkepsilon Doa InR RpII18 Rpb10 Rpb4 Takl2 btl hep ksr nmdyn-D6 |
| 1821 | GO:0007444 | P | 4, | 13 | 17.583 (x 0.739) | 302 (0.043) | 1 | imaginal disc development | Cpn Dab Doa Idgf1 Idgf2 InR btl chp hep mirr rho salm toy |
| 1822 | GO:0009950 | P | 5, | 2 | 3.319 (x 0.603) | 57 (0.035) | 1 | dorsal/ventral axis specification | Tehao pip |
| 1823 | GO:0007459 | P | 6, | 1 | 2.329 (x 0.429) | 40 (0.025) | 1 | photoreceptor fate commitment (sensu Endopterygota) | Dab |
| 1824 | GO:0035218 | P | 5, | 1 | 2.271 (x 0.440) | 39 (0.026) | 1 | leg disc development | rho |
| 1825 | GO:0048731 | P | 3, | 28 | 34.584 (x 0.810) | 594 (0.047) | 1 | system development | Abl BG:DS02740.9 CG10861 CG12361 CG31146 Con Dab Doa Eip93F Gs2 InR Tig Tm1 Tsp42El apt btl gt insc mira mirr pum rho robl rpr salm toy trol vvl |
| 1826 | GO:0007379 | P | 4, 5, | 1 | 1.688 (x 0.592) | 29 (0.034) | 1 | segment specification | salm |
| 1827 | GO:0042335 | P | 6, | 1 | 1.572 (x 0.636) | 27 (0.037) | 1 | cuticle biosynthesis | Doa |
| 1828 | GO:0005819 | C | 5, 6, 7, 8, 9, 10, | 1 | 1.630 (x 0.613) | 28 (0.036) | 1 | spindle | CLIP-190 |
| 1829 | GO:0015276 | F | 5, 6, | 2 | 3.901 (x 0.513) | 67 (0.030) | 1 | ligand-gated ion channel activity | CG14076 CG8916 |
| 1830 | GO:0043296 | C | 6, 7, 8, 9, | 1 | 1.863 (x 0.537) | 32 (0.031) | 1 | apical junction complex | veli |
| 1831 | GO:0015674 | P | 7, 8, | 1 | 2.329 (x 0.429) | 40 (0.025) | 1 | di-, tri-valent inorganic cation transport | Tsf1 |
| 1832 | GO:0008527 | F | 7, | 2 | 3.086 (x 0.648) | 53 (0.038) | 1 | taste receptor activity | Gr61a Gr98c |
| 1833 | GO:0031226 | C | 5, 6, 7, | 9 | 11.528 (x 0.781) | 198 (0.045) | 1 | intrinsic to plasma membrane | CG10804 CG3212 CG3397 Cad99C InR PGRP-SA Takr99D TyrR rho |
| 1834 | GO:0042158 | P | 7, 8, | 1 | 1.688 (x 0.592) | 29 (0.034) | 1 | lipoprotein biosynthesis | betaggt-II |
| 1835 | GO:0019207 | F | 3, | 2 | 3.901 (x 0.513) | 67 (0.030) | 1 | kinase regulator activity | CG9790 CycG |
| 1836 | GO:0005234 | F | 8, 9, | 1 | 1.572 (x 0.636) | 27 (0.037) | 1 | glutamate-gated ion channel activity | CG14076 |
| 1837 | GO:0000119 | C | 3, 4, 6, 7, 8, 9, 10, 11, 12, 13, 14, | 1 | 1.630 (x 0.613) | 28 (0.036) | 1 | mediator complex | Trap36 |
| 1838 | GO:0005887 | C | 6, 7, 8, | 9 | 11.412 (x 0.789) | 196 (0.046) | 1 | integral to plasma membrane | CG10804 CG3212 CG3397 Cad99C InR PGRP-SA Takr99D TyrR rho |
| 1839 | GO:0005509 | F | 5, | 10 | 12.693 (x 0.788) | 218 (0.046) | 1 | calcium ion binding | AnnX CG10126 CG2185 CG6426 CG7646 Cad99C Cpn Mlc2 Mp20 TpnC41C |
| 1840 | GO:0006974 | P | 4, | 5 | 6.870 (x 0.728) | 118 (0.042) | 1 | response to DNA damage stimulus | CycG Tfb2 agt hay rpr |
| 1841 | GO:0045814 | P | 4, | 1 | 1.572 (x 0.636) | 27 (0.037) | 1 | negative regulation of gene expression, epigenetic | Orc6 |
| 1842 | GO:0005342 | F | 3, | 3 | 4.309 (x 0.696) | 74 (0.041) | 1 | organic acid transporter activity | CG5535 CG8271 CG9413 |
| 1843 | GO:0006897 | P | 6, 7, | 5 | 6.754 (x 0.740) | 116 (0.043) | 1 | endocytosis | AnnX Arf84F Arr2 Eip93F Rab3 |

  

---

Regulated Genes that don't have GO terms
  

312 BEST:CK00246 BG:DS04641.8 BG:DS05899.3 BG:DS07295.5 BcDNA:GH05536 BcDNA:LD37196 CG10035 CG10075 CG10165 CG10195 CG10208 CG10233 CG10337 CG10365 CG10428 CG10581 CG10732 CG10912 CG10916 CG10969 CG11137 CG11279 CG11345 CG11350 CG11370 CG1143 CG11523 CG1157 CG11630 CG11755 CG11788 CG11825 CG11852 CG11975 CG12379 CG12402 CG12481 CG12519 CG12716 CG12848 CG12868 CG12873 CG1288 CG12936 CG12960 CG13004 CG13014 CG13018 CG13031 CG13044 CG13067 CG13126 CG13157 CG13200 CG13220 CG13226 CG13235 CG13339 CG13365 CG13407 CG13434 CG13482 CG13533 CG13568 CG13589 CG13603 CG13623 CG13636 CG13663 CG13785 CG13843 CG13914 CG13966 CG13993 CG14095 CG14104 CG14121 CG14125 CG14149 CG14187 CG14190 CG14210 CG14270 CG14321 CG14391 CG14394 CG14479 CG14483 CG14572 CG14615 CG14693 CG14731 CG14774 CG14843 CG14847 CG14898 CG14903 CG14966 CG1503 CG15031 CG15032 CG15168 CG15353 CG15362 CG15366 CG1537 CG15395 CG15525 CG15536 CG15593 CG1572 CG15784 CG15863 CG15888 CG15908 CG15922 CG16790 CG16820 CG17059 CG17327 CG17376 CG17490 CG17570 CG17625 CG17681 CG17734 CG17996 CG18294 CG18358 CG18428 CG1850 CG18538 CG18600 CG18635 CG18643 CG18678 CG18748 CG18765 CG2010 CG2124 CG2909 CG30053 CG30196 CG30219 CG30271 CG30373 CG30393 CG30412 CG31076 CG31330 CG31391 CG31436 CG31663 CG31781 CG31806 CG31873 CG31955 CG3199 CG32069 CG32206 CG32407 CG32500 CG32584 CG32633 CG3271 CG32729 CG32779 CG3280 CG32850 CG32986 CG33051 CG33120 CG33156 CG3448 CG3500 CG3566 CG3624 CG3984 CG40115 CG40127 CG40164 CG40169 CG40177 CG40192 CG40216 CG40228 CG40260 CG40270 CG40295 CG40329 CG40420 CG4186 CG4623 CG4786 CG4911 CG5013 CG5043 CG5056 CG5074 CG5156 CG5172 CG5174 CG5207 CG5237 CG5325 CG5360 CG5664 CG5866 CG5910 CG5922 CG5937 CG5961 CG6171 CG6353 CG6497 CG6691 CG7006 CG7011 CG7168 CG7201 CG7206 CG7231 CG7341 CG7484 CG7552 CG7603 CG7630 CG7637 CG7671 CG7759 CG8008 CG8204 CG8369 CG8386 CG8538 CG8620 CG8927 CG9034 CG9084 CG9130 CG9154 CG9231 CG9328 CG9336 CG9338 CG9350 CG9422 CG9617 CG9667 CG9766 CG9922 CG9948 CG9996 EG:131F2.3 EG:171E4.4 EG:34F3.10 EG:63B12.11 Frq His2B:CG17949 Max-element l(2)44Db l(3)87Df
