# Supplementary material for: Functional dissection of the ash2 and ash1 transcriptomes provides insights into the transcriptional basis of wing phenotypes and reveals conserved protein interactions
Source: Genome Biol. 2007 Apr 28;8(4):R67. doi: 10.1186/gb-2007-8-4-r67 (PMC1896016; doi:10.1186/gb-2007-8-4-r67)
Supplement: Additional data file 11 — GO annotations of the genes downregulated over 2.0-fold in ash2112411 [file gb-2007-8-4-r67-S11.html]

  

---

  

|  |  |
| --- | --- |
| Go Statistics | Reg File: **ash2112411\_D2.0x.txt.fbgns** (174 genes -- 35 skipped)  Ref File: **ref.fbgns** (13577 genes -- 4663 skipped)  Database: **go\_200507-termdb.rdf-xml** |

---

  

Fields Description

| Pos | Go Term | Ontology | Levels | Observed | Expected | Possibles | p-value(Adj) | Go term description | Genes with the GO term |
| --- | --- | --- | --- | --- | --- | --- | --- | --- | --- |
| 1 | GO:0009653 | P | 3, | 33 | 10.011 (x 3.296) | 642 (0.051) | 7.51e-07 | morphogenesis | Abi Arf79F Cdk4 Dll Dr Eip63E ImpE2 ImpE3 Lam Moe Mtl NetA Sb Scm ap ash2 dac dve edl fax fra fz gft gol grn klu ninA pbl rin scrib sqd th tkv |
| 2 | GO:0007275 | P | 2, | 51 | 23.156 (x 2.202) | 1485 (0.034) | 5.14e-06 | development | Abi Act5C Akap200 Arf79F Cdk4 CkIIalpha CycA Dll Dr Eip63E Hrb27C Hsp26 Hsp27 ImpE2 ImpE3 Lam Moe Mtl NetA Sb Scm Trxr-1 ap ash2 betaTub56D bib dac dve edl esn fax fra fz gft gol grn klu msi ninA pbl polo rin scrib serpin-27A smi35A sqd stai th tkv tok wbl |
| 3 | GO:0007552 | P | 4, | 16 | 3.945 (x 4.056) | 253 (0.063) | 0.000592 | metamorphosis | Dr Eip63E ImpE2 ImpE3 Moe Sb ap ash2 dac dve fz gft klu rin scrib tkv |
| 4 | GO:0046698 | P | 5, | 16 | 3.914 (x 4.088) | 251 (0.064) | 0.000711 | metamorphosis (sensu Insecta) | Dr Eip63E ImpE2 ImpE3 Moe Sb ap ash2 dac dve fz gft klu rin scrib tkv |
| 5 | GO:0007560 | P | 5, 6, | 15 | 3.742 (x 4.008) | 240 (0.062) | 0.000839 | imaginal disc morphogenesis | Dr ImpE2 ImpE3 Moe Sb ap ash2 dac dve fz gft klu rin scrib tkv |
| 6 | GO:0009887 | P | 4, | 18 | 5.239 (x 3.436) | 336 (0.054) | 0.00093 | organ morphogenesis | Dll Dr ImpE2 ImpE3 Moe Sb ap ash2 dac dve fz gft grn klu pbl rin scrib tkv |
| 7 | GO:0007444 | P | 4, | 17 | 4.709 (x 3.610) | 302 (0.056) | 0.00109 | imaginal disc development | Dll Dr ImpE2 ImpE3 Moe Sb ap ash2 dac dve fz gft klu rin scrib th tkv |
| 8 | GO:0050791 | P | 3, | 40 | 20.880 (x 1.916) | 1339 (0.030) | 0.00348 | regulation of physiological process | Aac11 Abi B52 Brf CG10990 CG15141 CG15835 CREG Caf1 Cdk4 CkIalpha CycA D19A Dll Dr Dsp1 Eb1 Eip63E Hrb27C Nek2 Scm ap ash2 dve eIF-4B eIF3-S9 edl gft gol grn klu msi rin scrib serpin-27A smi35A sqd th tkv yps |
| 9 | GO:0002009 | P | 4, | 10 | 2.074 (x 4.822) | 133 (0.075) | 0.00348 | morphogenesis of an epithelium | Eip63E Moe Mtl Sb Scm fz rin scrib sqd tkv |
| 10 | GO:0002165 | P | 4, | 17 | 5.380 (x 3.160) | 345 (0.049) | 0.00348 | larval or pupal development (sensu Insecta) | Akap200 Dr Eip63E ImpE2 ImpE3 Moe Sb ap ash2 dac dve fz gft klu rin scrib tkv |
| 11 | GO:0035107 | P | 4, | 10 | 2.074 (x 4.822) | 133 (0.075) | 0.00373 | appendage morphogenesis | Dll Dr ap ash2 dac dve fz gft th tkv |
| 12 | GO:0009791 | P | 3, | 17 | 5.551 (x 3.062) | 356 (0.048) | 0.00389 | post-embryonic development | Akap200 Dr Eip63E ImpE2 ImpE3 Moe Sb ap ash2 dac dve fz gft klu rin scrib tkv |
| 13 | GO:0048737 | P | 4, | 10 | 2.027 (x 4.933) | 130 (0.077) | 0.0039 | appendage development (sensu Endopterygota) | Dll Dr ap ash2 dac dve fz gft th tkv |
| 14 | GO:0048736 | P | 3, | 10 | 2.074 (x 4.822) | 133 (0.075) | 0.00401 | appendage development | Dll Dr ap ash2 dac dve fz gft th tkv |
| 15 | GO:0035114 | P | 5, | 10 | 2.027 (x 4.933) | 130 (0.077) | 0.00429 | appendage morphogenesis (sensu Endopterygota) | Dll Dr ap ash2 dac dve fz gft th tkv |
| 16 | GO:0005622 | C | 3, 4, | 74 | 51.380 (x 1.440) | 3295 (0.022) | 0.00482 | intracellular | Aats-glupro Act42A Act5C ApepP B52 BcDNA:LD41548 Brf CBP CG10423 CG10657 CG13895 CG17838 CG2118 CG31617 CG3605 CG3823 CG9057 CG9894 CREG Caf1 CkIIalpha CkIalpha CycA D19A Dll DnaJ-1 Dr Dsp1 Eb1 Fur1 Gapdh2 Hrb27C Idh ImpE2 Lam Mcm5 Mcm7 Moe NUCB1 Nek2 Pep Scm Snap Top2 Trxr-1 Uch ap ash2 betaTub56D d dac dpa dve eIF-4B eIF3-S9 edl esn fz gft gol grn klu l(2)dtl lig pbl polo rin scrib smi35A sqd stai th wbl yps |
| 17 | GO:0050789 | P | 2, | 42 | 23.468 (x 1.790) | 1505 (0.028) | 0.0049 | regulation of biological process | Aac11 Abi Akap200 B52 Brf CG10990 CG15141 CG15835 CREG Caf1 Cdk4 CkIalpha CycA D19A Dll Dr Dsp1 Eb1 Eip63E Hrb27C Nek2 Scm ap ash2 dve eIF-4B eIF3-S9 edl gft gol grn klu msi pbl rin scrib serpin-27A smi35A sqd th tkv yps |
| 18 | GO:0016335 | P | 5, 6, 7, | 3 | 0.094 (x 32.065) | 6 (0.500) | 0.00512 | morphogenesis of larval imaginal disc epithelium | Moe Sb scrib |
| 19 | GO:0050794 | P | 3, | 39 | 21.472 (x 1.816) | 1377 (0.028) | 0.00645 | regulation of cellular process | Aac11 Abi Akap200 B52 Brf CG10990 CG15141 CG15835 CREG Caf1 Cdk4 CkIalpha CycA D19A Dll Dr Dsp1 Eb1 Eip63E Nek2 Scm ap ash2 dve eIF-4B eIF3-S9 edl gft gol grn klu msi rin scrib smi35A sqd th tkv yps |
| 20 | GO:0046578 | P | 6, 7, 8, | 3 | 0.109 (x 27.484) | 7 (0.429) | 0.00797 | regulation of Ras protein signal transduction | Akap200 edl klu |
| 21 | GO:0051244 | P | 4, | 37 | 20.225 (x 1.829) | 1297 (0.029) | 0.0081 | regulation of cellular physiological process | Aac11 Abi B52 Brf CG10990 CG15141 CG15835 CREG Caf1 Cdk4 CycA D19A Dll Dr Dsp1 Eb1 Eip63E Nek2 Scm ap ash2 dve eIF-4B eIF3-S9 edl gft gol grn klu msi rin scrib smi35A sqd th tkv yps |
| 22 | GO:0044424 | C | 3, 4, 5, | 71 | 49.743 (x 1.427) | 3190 (0.022) | 0.00832 | intracellular part | Aats-glupro Act42A Act5C ApepP B52 BcDNA:LD41548 Brf CBP CG10423 CG13895 CG17838 CG2118 CG31617 CG3605 CG9057 CG9894 CREG Caf1 CkIIalpha CkIalpha CycA D19A Dll DnaJ-1 Dr Dsp1 Eb1 Fur1 Gapdh2 Hrb27C Idh ImpE2 Lam Mcm5 Mcm7 Moe NUCB1 Nek2 Pep Scm Snap Top2 Trxr-1 ap ash2 betaTub56D d dac dpa dve eIF-4B eIF3-S9 edl esn fz gft gol grn klu l(2)dtl lig pbl polo rin scrib smi35A sqd stai th wbl yps |
| 23 | GO:0007389 | P | 3, | 13 | 3.992 (x 3.257) | 256 (0.051) | 0.00967 | pattern specification | Cdk4 Dll Dr Hrb27C Moe ap dac edl serpin-27A sqd tkv tok wbl |
| 24 | GO:0051056 | P | 5, 6, 7, | 3 | 0.125 (x 24.049) | 8 (0.375) | 0.0105 | regulation of small GTPase mediated signal transduction | Akap200 edl klu |
| 25 | GO:0048468 | P | 4, | 15 | 5.302 (x 2.829) | 340 (0.044) | 0.0122 | cell development | Act5C Hrb27C Moe NetA ap dac edl fax fra ninA pbl sqd th tkv wbl |
| 26 | GO:0000902 | P | 4, 5, | 15 | 5.271 (x 2.846) | 338 (0.044) | 0.0124 | cellular morphogenesis | Abi Arf79F Cdk4 Moe NetA ap dac edl fax fra fz ninA pbl rin scrib |
| 27 | GO:0048513 | P | 3, | 23 | 10.448 (x 2.201) | 670 (0.034) | 0.0126 | organ development | Akap200 CkIIalpha Dll Dr ImpE2 ImpE3 Moe Sb ap ash2 betaTub56D dac dve edl fz gft grn klu pbl rin scrib th tkv |
| 28 | GO:0006073 | P | 7, 8, | 3 | 0.140 (x 21.376) | 9 (0.333) | 0.0129 | glucan metabolism | CG33138 CG6904 GlyP |
| 29 | GO:0005977 | P | 8, 9, | 3 | 0.140 (x 21.376) | 9 (0.333) | 0.0134 | glycogen metabolism | CG33138 CG6904 GlyP |
| 30 | GO:0030154 | P | 3, | 19 | 7.953 (x 2.389) | 510 (0.037) | 0.0145 | cell differentiation | Act5C Dr Hrb27C Moe NetA ap dac edl fax fra fz msi ninA pbl rin sqd th tkv wbl |
| 31 | GO:0006767 | P | 6, | 4 | 0.359 (x 11.153) | 23 (0.174) | 0.0165 | water-soluble vitamin metabolism | BEST:LD22483 CG31472 CG8036 ESTS:39C10S |
| 32 | GO:0019842 | F | 3, | 4 | 0.374 (x 10.688) | 24 (0.167) | 0.0184 | vitamin binding | CG10657 CG13848 CG2118 CG3823 |
| 33 | GO:0016043 | P | 4, | 37 | 21.503 (x 1.721) | 1379 (0.027) | 0.0185 | cell organization and biogenesis | Abi Act42A Act5C Arf79F CG2852 CG31617 CG9057 Caf1 Cdk4 Dsp1 Eb1 Lam Mcm5 Moe Mtl NetA Sb Snap ap ash2 betaTub56D d dac dpa edl esn fax fra fz ninA pbl polo rin scrib sqd stai wbl |
| 34 | GO:0015980 | P | 6, | 8 | 1.840 (x 4.348) | 118 (0.068) | 0.0187 | energy derivation by oxidation of organic compounds | BEST:LD22483 CG1544 CG33138 CG6904 CG8036 Gapdh2 GlyP Idh |
| 35 | GO:0007447 | P | 4, 5, | 5 | 0.671 (x 7.457) | 43 (0.116) | 0.0188 | imaginal disc pattern formation | Dll Dr ap dac tkv |
| 36 | GO:0051225 | P | 7, 10, | 3 | 0.172 (x 17.490) | 11 (0.273) | 0.0194 | spindle assembly | Eb1 pbl polo |
| 37 | GO:0007265 | P | 7, | 4 | 0.390 (x 10.261) | 25 (0.160) | 0.0198 | Ras protein signal transduction | Akap200 edl klu rin |
| 38 | GO:0050875 | P | 3, | 114 | 96.929 (x 1.176) | 6216 (0.018) | 0.0219 | cellular physiological process | Aac11 Aats-glupro Abi Act42A Act5C Akap200 ApepP Arf79F B52 BEST:LD22483 BcDNA:GH02901 BcDNA:LD22910 BcDNA:LD41548 Brf CG10423 CG10657 CG10960 CG10990 CG14439 CG15141 CG1544 CG15835 CG17419 CG2118 CG2852 CG31169 CG31472 CG31617 CG33138 CG3590 CG3605 CG3823 CG4670 CG5873 CG6680 CG6767 CG6854 CG6904 CG7461 CG8036 CG9027 CG9057 CREG Caf1 Cdk4 CkIIalpha CkIalpha CycA D19A Dll DnaJ-1 Dr Dsp1 ESTS:39C10S Eb1 Eip63E Fur1 Gapdh2 GlyP Hrb27C Hsp26 Hsp27 Idh Lam Mcm5 Mcm7 Moe Mtl Nek2 NetA Sb Scm Snap Top2 Trxr-1 Uch ap ash2 betaTub56D bib d dac deltaTry dpa dve eIF-4B eIF3-S9 edl endos esn fax fra fz gft gol grn klu l(2)dtl msi ninA pbl polo rin rpk scrib serpin-27A smi35A sqd stai th tkv tok wbl yps |
| 39 | GO:0002168 | P | 5, | 4 | 0.421 (x 9.501) | 27 (0.148) | 0.0248 | larval development (sensu Insecta) | Eip63E Moe Sb scrib |
| 40 | GO:0035214 | P | 5, | 9 | 2.495 (x 3.607) | 160 (0.056) | 0.0278 | eye-antennal disc development | Dll Moe dac fz gft klu rin th tkv |
| 41 | GO:0009953 | P | 4, | 6 | 1.154 (x 5.200) | 74 (0.081) | 0.0306 | dorsal/ventral pattern formation | Dr ap serpin-27A sqd tok wbl |
| 42 | GO:0007051 | P | 9, | 4 | 0.452 (x 8.845) | 29 (0.138) | 0.0311 | spindle organization and biogenesis | Eb1 dpa pbl polo |
| 43 | GO:0035223 | P | 5, 6, | 3 | 0.218 (x 13.742) | 14 (0.214) | 0.0319 | leg disc pattern formation | Dll ap dac |
| 44 | GO:0007472 | P | 6, 7, | 7 | 1.637 (x 4.275) | 105 (0.067) | 0.0323 | wing disc morphogenesis | Dr ap ash2 dve fz gft tkv |
| 45 | GO:0003688 | F | 6, | 3 | 0.218 (x 13.742) | 14 (0.214) | 0.0326 | DNA replication origin binding | Mcm5 Mcm7 dpa |
| 46 | GO:0007561 | P | 6, 7, | 2 | 0.062 (x 32.065) | 4 (0.500) | 0.0326 | imaginal disc eversion | ImpE2 ImpE3 |
| 47 | GO:0000904 | P | 5, 6, | 9 | 2.635 (x 3.415) | 169 (0.053) | 0.0327 | cellular morphogenesis during differentiation | Moe NetA ap dac edl fax fra ninA pbl |
| 48 | GO:0007476 | P | 6, 7, 8, | 7 | 1.606 (x 4.358) | 103 (0.068) | 0.0328 | wing morphogenesis | Dr ap ash2 dve fz gft tkv |
| 49 | GO:0008615 | P | 9, | 2 | 0.062 (x 32.065) | 4 (0.500) | 0.0332 | pyridoxine biosynthesis | CG31472 ESTS:39C10S |
| 50 | GO:0007479 | P | 6, 7, | 3 | 0.218 (x 13.742) | 14 (0.214) | 0.0333 | leg disc proximal/distal pattern formation | Dll ap dac |
| 51 | GO:0016336 | P | 6, 7, 8, | 2 | 0.062 (x 32.065) | 4 (0.500) | 0.0338 | establishment and/or maintenance of polarity of larval imaginal disc epithelium | Moe scrib |
| 52 | GO:0006112 | P | 7, | 3 | 0.218 (x 13.742) | 14 (0.214) | 0.034 | energy reserve metabolism | CG33138 CG6904 GlyP |
| 53 | GO:0008614 | P | 8, | 2 | 0.062 (x 32.065) | 4 (0.500) | 0.0344 | pyridoxine metabolism | CG31472 ESTS:39C10S |
| 54 | GO:0006270 | P | 9, | 3 | 0.218 (x 13.742) | 14 (0.214) | 0.0348 | DNA replication initiation | Mcm5 Mcm7 dpa |
| 55 | GO:0042816 | P | 7, | 2 | 0.062 (x 32.065) | 4 (0.500) | 0.0351 | vitamin B6 metabolism | CG31472 ESTS:39C10S |
| 56 | GO:0005634 | C | 5, 6, 7, 8, | 38 | 23.764 (x 1.599) | 1524 (0.025) | 0.0354 | nucleus | B52 Brf CG13895 CG17838 CG31617 CG3605 CG9894 CREG Caf1 CkIIalpha CkIalpha CycA D19A Dll Dr Dsp1 Hrb27C Lam Mcm5 Mcm7 Pep Scm Top2 ap ash2 dac dpa dve edl esn gft gol grn klu pbl rin sqd yps |
| 57 | GO:0043565 | F | 5, | 4 | 0.515 (x 7.773) | 33 (0.121) | 0.0355 | sequence-specific DNA binding | Mcm5 Mcm7 dpa dve |
| 58 | GO:0042819 | P | 8, | 2 | 0.062 (x 32.065) | 4 (0.500) | 0.0358 | vitamin B6 biosynthesis | CG31472 ESTS:39C10S |
| 59 | GO:0007010 | P | 6, | 16 | 7.017 (x 2.280) | 450 (0.036) | 0.0358 | cytoskeleton organization and biogenesis | Abi Act42A Act5C CG9057 Eb1 Lam Moe Mtl Sb betaTub56D dpa esn fra pbl polo stai |
| 60 | GO:0016477 | P | 5, 6, | 9 | 2.729 (x 3.298) | 175 (0.051) | 0.036 | cell migration | NetA ap fra ninA pbl scrib sqd stai th |
| 61 | GO:0006996 | P | 5, | 22 | 11.258 (x 1.954) | 722 (0.030) | 0.0363 | organelle organization and biogenesis | Abi Act42A Act5C CG31617 CG9057 Caf1 Cdk4 Dsp1 Eb1 Lam Mcm5 Moe Mtl Sb ash2 betaTub56D dpa esn fra pbl polo stai |
| 62 | GO:0000910 | P | 5, | 6 | 1.310 (x 4.581) | 84 (0.071) | 0.0395 | cytokinesis | Act42A Act5C Caf1 Snap pbl polo |
| 63 | GO:0000003 | P | 2, | 17 | 7.812 (x 2.176) | 501 (0.034) | 0.0396 | reproduction | Act5C Caf1 Hrb27C Moe Scm bib endos esn lig polo scrib sqd stai th tkv wbl yps |
| 64 | GO:0019953 | P | 3, | 16 | 7.111 (x 2.250) | 456 (0.035) | 0.0397 | sexual reproduction | Act5C Caf1 Hrb27C Moe Scm bib endos esn polo scrib sqd stai th tkv wbl yps |
| 65 | GO:0051674 | P | 4, | 11 | 4.008 (x 2.745) | 257 (0.043) | 0.0426 | localization of cell | Abi NetA ap betaTub56D fra ninA pbl scrib sqd stai th |
| 66 | GO:0006766 | P | 5, | 4 | 0.561 (x 7.125) | 36 (0.111) | 0.0428 | vitamin metabolism | BEST:LD22483 CG31472 CG8036 ESTS:39C10S |
| 67 | GO:0006461 | P | 6, | 7 | 1.824 (x 3.837) | 117 (0.060) | 0.0428 | protein complex assembly | B52 CG31617 Caf1 Eb1 pbl polo scrib |
| 68 | GO:0030332 | F | 4, | 2 | 0.078 (x 25.652) | 5 (0.400) | 0.043 | cyclin binding | Cdk4 Eip63E |
| 69 | GO:0006928 | P | 4, 5, | 11 | 4.008 (x 2.745) | 257 (0.043) | 0.0433 | cell motility | Abi NetA ap betaTub56D fra ninA pbl scrib sqd stai th |
| 70 | GO:0035220 | P | 5, | 7 | 1.809 (x 3.870) | 116 (0.060) | 0.0433 | wing disc development | Dr ap ash2 dve fz gft tkv |
| 71 | GO:0009954 | P | 4, | 3 | 0.281 (x 10.688) | 18 (0.167) | 0.0437 | proximal/distal pattern formation | Dll ap dac |
| 72 | GO:0005654 | C | 5, 6, 7, 8, 9, 10, 11, | 10 | 3.493 (x 2.863) | 224 (0.045) | 0.0438 | nucleoplasm | B52 Brf Caf1 Hrb27C Mcm5 Mcm7 Top2 ap dac sqd |
| 73 | GO:0044428 | C | 4, 5, 6, 7, 8, 9, | 17 | 8.031 (x 2.117) | 515 (0.033) | 0.044 | nuclear part | B52 Brf CG17838 CG3605 Caf1 CycA Hrb27C Lam Mcm5 Mcm7 Pep Scm Top2 ap dac gft sqd |
| 74 | GO:0045197 | P | 7, 8, | 3 | 0.281 (x 10.688) | 18 (0.167) | 0.0443 | establishment and/or maintenance of epithelial cell polarity | Moe fz scrib |
| 75 | GO:0007449 | P | 5, 6, | 3 | 0.281 (x 10.688) | 18 (0.167) | 0.0449 | proximal/distal pattern formation, imaginal disc | Dll ap dac |
| 76 | GO:0043170 | P | 4, | 65 | 48.589 (x 1.338) | 3116 (0.021) | 0.045 | macromolecule metabolism | Aats-glupro ApepP Arf79F B52 BEST:LD22483 BcDNA:LD22910 BcDNA:LD41548 CG10423 CG10657 CG10960 CG10990 CG15141 CG1544 CG2852 CG31169 CG31617 CG33138 CG3605 CG4670 CG6680 CG6904 CG8036 Caf1 Cdk4 CkIIalpha CkIalpha D19A DnaJ-1 Dsp1 Eb1 Eip63E Fur1 GNBP3 Gapdh2 GlyP Hrb27C Hsp26 Hsp27 Idh Mcm5 Mcm7 Nek2 Sb Top2 Uch ash2 betaTub56D deltaTry dpa eIF-4B eIF3-S9 fra gft gol msi pbl polo rin scrib smi35A sqd th tkv tok wbl |
| 77 | GO:0040011 | P | 3, | 11 | 4.070 (x 2.703) | 261 (0.042) | 0.0454 | locomotion | Abi NetA ap betaTub56D fra ninA pbl scrib sqd stai th |
| 78 | GO:0035088 | P | 6, 7, | 3 | 0.296 (x 10.126) | 19 (0.158) | 0.0493 | establishment and/or maintenance of apical/basal cell polarity | Moe fz scrib |
| 79 | GO:0003697 | F | 6, | 3 | 0.312 (x 9.619) | 20 (0.150) | 0.0559 | single-stranded DNA binding | Dsp1 Hrb27C Pep |
| 80 | GO:0046580 | P | 7, 8, 9, | 2 | 0.094 (x 21.376) | 6 (0.333) | 0.0565 | negative regulation of Ras protein signal transduction | Akap200 klu |
| 81 | GO:0009888 | P | 3, | 14 | 6.206 (x 2.256) | 398 (0.035) | 0.0571 | tissue development | Dll Dr NetA ap bib dac fra fz gol grn msi ninA pbl smi35A |
| 82 | GO:0007398 | P | 4, | 10 | 3.696 (x 2.706) | 237 (0.042) | 0.0609 | ectoderm development | Dll Dr NetA ap bib dac fra fz msi smi35A |
| 83 | GO:0007276 | P | 4, | 15 | 6.970 (x 2.152) | 447 (0.034) | 0.0619 | gametogenesis | Act5C Caf1 Hrb27C Moe Scm bib endos esn scrib sqd stai th tkv wbl yps |
| 84 | GO:0019222 | P | 4, | 27 | 15.968 (x 1.691) | 1024 (0.026) | 0.0619 | regulation of metabolism | B52 Brf CG15141 CG15835 CREG Caf1 CkIalpha D19A Dll Dr Dsp1 Scm ap ash2 dve eIF-4B eIF3-S9 edl gft gol grn klu msi rin serpin-27A sqd yps |
| 85 | GO:0007469 | P | 6, | 3 | 0.327 (x 9.161) | 21 (0.143) | 0.0622 | antennal development | Dll dac th |
| 86 | GO:0043118 | P | 4, | 10 | 3.758 (x 2.661) | 241 (0.041) | 0.0653 | negative regulation of physiological process | Aac11 CREG Caf1 CycA edl msi scrib serpin-27A sqd th |
| 87 | GO:0006267 | P | 9, | 2 | 0.109 (x 18.323) | 7 (0.286) | 0.0665 | pre-replicative complex formation and maintenance | Mcm5 Mcm7 |
| 88 | GO:0048731 | P | 3, | 18 | 9.263 (x 1.943) | 594 (0.030) | 0.0671 | system development | Akap200 Cdk4 CycA Dll Dr Lam NetA ap bib dac fax fra msi ninA pbl smi35A stai tkv |
| 89 | GO:0051058 | P | 6, 7, 8, | 2 | 0.109 (x 18.323) | 7 (0.286) | 0.0673 | negative regulation of small GTPase mediated signal transduction | Akap200 klu |
| 90 | GO:0048749 | P | 7, | 7 | 2.074 (x 3.375) | 133 (0.053) | 0.0678 | compound eye development (sensu Endopterygota) | Moe dac fz gft klu rin tkv |
| 91 | GO:0016607 | C | 7, 8, 9, 10, 11, 12, 13, 14, | 2 | 0.109 (x 18.323) | 7 (0.286) | 0.068 | nuclear speck | B52 sqd |
| 92 | GO:0001745 | P | 7, 8, | 7 | 2.074 (x 3.375) | 133 (0.053) | 0.0685 | compound eye morphogenesis (sensu Endopterygota) | Moe dac fz gft klu rin tkv |
| 93 | GO:0030530 | C | 4, 5, 6, 7, 8, 9, 10, | 2 | 0.109 (x 18.323) | 7 (0.286) | 0.0688 | heterogeneous nuclear ribonucleoprotein complex | CG17838 Pep |
| 94 | GO:0016333 | P | 5, | 3 | 0.359 (x 8.365) | 23 (0.130) | 0.0693 | morphogenesis of follicular epithelium | Scm scrib sqd |
| 95 | GO:0051246 | P | 5, 6, | 7 | 2.074 (x 3.375) | 133 (0.053) | 0.0693 | regulation of protein metabolism | CkIalpha eIF-4B eIF3-S9 gft msi rin sqd |
| 96 | GO:0002164 | P | 4, | 4 | 0.702 (x 5.700) | 45 (0.089) | 0.0695 | larval development | Eip63E Moe Sb scrib |
| 97 | GO:0006445 | P | 7, 8, 9, | 5 | 1.123 (x 4.453) | 72 (0.069) | 0.0696 | regulation of translation | eIF-4B eIF3-S9 msi rin sqd |
| 98 | GO:0048477 | P | 6, | 11 | 4.507 (x 2.441) | 289 (0.038) | 0.0712 | oogenesis | Caf1 Hrb27C Moe Scm bib endos sqd th tkv wbl yps |
| 99 | GO:0007456 | P | 6, | 8 | 2.698 (x 2.966) | 173 (0.046) | 0.0725 | eye development (sensu Endopterygota) | CkIIalpha Moe dac fz gft klu rin tkv |
| 100 | GO:0048748 | P | 6, 7, | 7 | 2.199 (x 3.184) | 141 (0.050) | 0.0759 | eye morphogenesis (sensu Endopterygota) | Moe dac fz gft klu rin tkv |
| 101 | GO:0004702 | F | 4, 8, | 8 | 2.760 (x 2.899) | 177 (0.045) | 0.0761 | receptor signaling protein serine/threonine kinase activity | Cdk4 CkIIalpha CkIalpha Eip63E Nek2 polo smi35A tkv |
| 102 | GO:0051301 | P | 4, | 7 | 2.167 (x 3.230) | 139 (0.050) | 0.0766 | cell division | Act42A Act5C Caf1 Snap pbl polo tkv |
| 103 | GO:0043226 | C | 2, | 56 | 41.853 (x 1.338) | 2684 (0.021) | 0.0768 | organelle | Act42A Act5C B52 Brf CBP CG10423 CG13895 CG17838 CG2118 CG31617 CG3605 CG9894 CREG Caf1 CkIIalpha CkIalpha CycA D19A Dll Dr Dsp1 Eb1 Fur1 Hrb27C Idh Lam Mcm5 Mcm7 Moe NUCB1 Nek2 Pep Scm Snap Top2 Trxr-1 ap ash2 betaTub56D d dac dpa dve edl esn gft gol grn klu pbl polo rin sqd stai wbl yps |
| 104 | GO:0043229 | C | 3, 4, 5, 6, | 56 | 41.853 (x 1.338) | 2684 (0.021) | 0.0775 | intracellular organelle | Act42A Act5C B52 Brf CBP CG10423 CG13895 CG17838 CG2118 CG31617 CG3605 CG9894 CREG Caf1 CkIIalpha CkIalpha CycA D19A Dll Dr Dsp1 Eb1 Fur1 Hrb27C Idh Lam Mcm5 Mcm7 Moe NUCB1 Nek2 Pep Scm Snap Top2 Trxr-1 ap ash2 betaTub56D d dac dpa dve edl esn gft gol grn klu pbl polo rin sqd stai wbl yps |
| 105 | GO:0043566 | F | 5, | 3 | 0.390 (x 7.696) | 25 (0.120) | 0.0776 | structure-specific DNA binding | Dsp1 Hrb27C Pep |
| 106 | GO:0051226 | P | 7, 8, 11, | 2 | 0.125 (x 16.032) | 8 (0.250) | 0.0778 | meiotic spindle assembly | pbl polo |
| 107 | GO:0006417 | P | 6, 7, 8, | 5 | 1.170 (x 4.275) | 75 (0.067) | 0.0779 | regulation of protein biosynthesis | eIF-4B eIF3-S9 msi rin sqd |
| 108 | GO:0007399 | P | 4, | 15 | 7.298 (x 2.055) | 468 (0.032) | 0.0783 | nervous system development | CycA Dll Dr NetA ap bib dac fax fra msi ninA pbl smi35A stai tkv |
| 109 | GO:0016604 | C | 6, 7, 8, 9, 10, 11, 12, 13, | 2 | 0.125 (x 16.032) | 8 (0.250) | 0.0785 | nuclear body | B52 sqd |
| 110 | GO:0046879 | P | 5, 6, | 2 | 0.125 (x 16.032) | 8 (0.250) | 0.0793 | hormone secretion | CG15835 stai |
| 111 | GO:0009987 | P | 2, | 117 | 104.570 (x 1.119) | 6706 (0.017) | 0.0817 | cellular process | Aac11 Aats-glupro Abi Act42A Act5C Akap200 ApepP Arf79F B52 BEST:LD22483 BcDNA:GH02901 BcDNA:LD22910 BcDNA:LD41548 Brf CG10423 CG10657 CG10960 CG10990 CG14439 CG15141 CG1544 CG15835 CG17064 CG17419 CG2118 CG2852 CG30440 CG31169 CG31472 CG31617 CG33138 CG3590 CG3605 CG3823 CG4670 CG5873 CG6680 CG6767 CG6854 CG6904 CG6954 CG7461 CG8036 CG9027 CG9057 CREG Caf1 Cdk4 CkIIalpha CkIalpha CycA D19A Dll DnaJ-1 Dr Dsp1 ESTS:39C10S Eb1 Eip63E Fur1 Gapdh2 GlyP Hrb27C Hsp26 Hsp27 Idh Lam Mcm5 Mcm7 Moe Mtl Nek2 NetA Sb Scm Snap Top2 Trxr-1 Uch ap ash2 betaTub56D bib d dac deltaTry dpa dve eIF-4B eIF3-S9 edl endos esn fax fra fz gft gol grn klu l(2)dtl msi ninA pbl polo rin rpk scrib serpin-27A smi35A sqd stai th tkv tok wbl yps |
| 112 | GO:0001654 | P | 5, | 8 | 2.822 (x 2.834) | 181 (0.044) | 0.0829 | eye development | CkIIalpha Moe dac fz gft klu rin tkv |
| 113 | GO:0044238 | P | 4, | 86 | 71.262 (x 1.207) | 4570 (0.019) | 0.0829 | primary metabolism | Aats-glupro ApepP Arf79F B52 BEST:LD22483 BcDNA:GH02901 BcDNA:LD22910 BcDNA:LD41548 Brf CG10423 CG10657 CG10960 CG10990 CG15141 CG1544 CG15835 CG2118 CG2852 CG31169 CG31617 CG33138 CG3590 CG3605 CG4670 CG6680 CG6767 CG6854 CG6904 CG7461 CG8036 CG9057 CREG Caf1 Cdk4 CkIIalpha CkIalpha D19A Dll DnaJ-1 Dr Dsp1 ESTS:39C10S Eb1 Eip63E Fur1 GNBP3 Gapdh2 GlyP Hrb27C Hsp26 Hsp27 Idh Mcm5 Mcm7 Nek2 Sb Scm Top2 Uch ap ash2 betaTub56D dac deltaTry dpa dve eIF-4B eIF3-S9 edl fra gft gol grn klu msi pbl polo rin scrib smi35A sqd th tkv tok wbl yps |
| 114 | GO:0005703 | C | 5, 6, 7, 8, 9, 10, | 2 | 0.140 (x 14.251) | 9 (0.222) | 0.0845 | polytene chromosome puff | Pep sqd |
| 115 | GO:0005656 | C | 3, 6, 7, 8, 9, 10, 11, 12, 13, | 2 | 0.140 (x 14.251) | 9 (0.222) | 0.0852 | pre-replicative complex | Mcm5 Mcm7 |
| 116 | GO:0007455 | P | 6, 7, | 7 | 2.277 (x 3.075) | 146 (0.048) | 0.0858 | eye-antennal disc morphogenesis | Moe dac fz gft klu rin tkv |
| 117 | GO:0009889 | P | 5, | 5 | 1.232 (x 4.059) | 79 (0.063) | 0.0858 | regulation of biosynthesis | eIF-4B eIF3-S9 msi rin sqd |
| 118 | GO:0035003 | C | 7, 8, 9, 10, | 2 | 0.140 (x 14.251) | 9 (0.222) | 0.0859 | subapical complex | Moe scrib |
| 119 | GO:0044427 | C | 4, 5, 6, 7, 8, 9, | 7 | 2.308 (x 3.033) | 148 (0.047) | 0.0863 | chromosomal part | CG13895 CG31617 Caf1 Dsp1 Pep polo sqd |
| 120 | GO:0031326 | P | 6, | 5 | 1.232 (x 4.059) | 79 (0.063) | 0.0866 | regulation of cellular biosynthesis | eIF-4B eIF3-S9 msi rin sqd |
| 121 | GO:0042364 | P | 7, | 2 | 0.140 (x 14.251) | 9 (0.222) | 0.0866 | water-soluble vitamin biosynthesis | CG31472 ESTS:39C10S |
| 122 | GO:0008431 | F | 4, | 2 | 0.140 (x 14.251) | 9 (0.222) | 0.0873 | vitamin E binding | CG13848 CG3823 |
| 123 | GO:0048519 | P | 3, | 11 | 4.756 (x 2.313) | 305 (0.036) | 0.0875 | negative regulation of biological process | Aac11 CREG Caf1 CkIalpha CycA edl msi scrib serpin-27A sqd th |
| 124 | GO:0005694 | C | 5, 6, 7, 8, | 8 | 2.869 (x 2.788) | 184 (0.043) | 0.0881 | chromosome | CG13895 CG31617 Caf1 Dsp1 Pep Top2 polo sqd |
| 125 | GO:0048592 | P | 5, 6, | 7 | 2.323 (x 3.013) | 149 (0.047) | 0.0886 | eye morphogenesis | Moe dac fz gft klu rin tkv |
| 126 | GO:0008152 | P | 3, | 93 | 78.856 (x 1.179) | 5057 (0.018) | 0.0887 | metabolism | Aats-glupro Abi ApepP Arf79F B52 BEST:LD22483 BcDNA:GH02901 BcDNA:LD22910 BcDNA:LD41548 Brf CG10423 CG10657 CG10960 CG10990 CG15141 CG1544 CG15835 CG2118 CG2852 CG31169 CG31472 CG31617 CG33138 CG3590 CG3605 CG4670 CG5873 CG6680 CG6767 CG6854 CG6904 CG7461 CG7675 CG8036 CG9027 CG9057 CREG Caf1 Cdk4 CkIIalpha CkIalpha D19A Dll DnaJ-1 Dr Dsp1 ESTS:39C10S Eb1 Eip63E Fur1 GNBP3 Gapdh2 GlyP Hrb27C Hsp26 Hsp27 Idh Mcm5 Mcm7 Nek2 Sb Scm Top2 Trxr-1 Uch ap ash2 betaTub56D dac deltaTry dpa dve eIF-4B eIF3-S9 edl fra gft gol grn klu msi pbl polo rin scrib serpin-27A smi35A sqd th tkv tok wbl yps |
| 127 | GO:0042048 | P | 5, 6, | 4 | 0.826 (x 4.840) | 53 (0.075) | 0.0908 | olfactory behavior | CG8588 scrib smi21F smi35A |
| 128 | GO:0000278 | P | 5, | 10 | 4.179 (x 2.393) | 268 (0.037) | 0.0908 | mitotic cell cycle | Cdk4 CycA Eb1 Eip63E Lam Nek2 Top2 betaTub56D dpa polo |
| 129 | GO:0035110 | P | 6, | 3 | 0.437 (x 6.871) | 28 (0.107) | 0.091 | leg morphogenesis | Dll ap dac |
| 130 | GO:0007292 | P | 5, | 11 | 4.834 (x 2.276) | 310 (0.035) | 0.0913 | female gamete generation | Caf1 Hrb27C Moe Scm bib endos sqd th tkv wbl yps |
| 131 | GO:0003682 | F | 3, | 5 | 1.294 (x 3.863) | 83 (0.060) | 0.0924 | chromatin binding | Caf1 Dsp1 Mcm5 Mcm7 Scm |
| 132 | GO:0007635 | P | 4, 5, | 4 | 0.842 (x 4.750) | 54 (0.074) | 0.0933 | chemosensory behavior | CG8588 scrib smi21F smi35A |
| 133 | GO:0003729 | F | 5, | 11 | 4.881 (x 2.254) | 313 (0.035) | 0.0933 | mRNA binding | Aats-glupro B52 CG17838 Hrb27C Top2 eIF-4B eIF3-S9 msi rin sqd yps |
| 134 | GO:0007423 | P | 4, | 9 | 3.586 (x 2.509) | 230 (0.039) | 0.0936 | sensory organ development | CkIIalpha Moe dac edl fz gft klu rin tkv |
| 135 | GO:0009057 | P | 5, | 8 | 2.963 (x 2.700) | 190 (0.042) | 0.0938 | macromolecule catabolism | BEST:LD22483 BcDNA:LD22910 CG8036 CkIalpha Gapdh2 Uch sqd th |
| 136 | GO:0035108 | P | 5, | 3 | 0.452 (x 6.634) | 29 (0.103) | 0.0952 | limb morphogenesis | Dll ap dac |
| 137 | GO:0051243 | P | 5, | 9 | 3.618 (x 2.488) | 232 (0.039) | 0.0965 | negative regulation of cellular physiological process | Aac11 CREG Caf1 CycA edl msi scrib sqd th |
| 138 | GO:0003844 | F | 6, | 1 | 0.016 (x 64.129) | 1 (1.000) | 0.0982 | 1,4-alpha-glucan branching enzyme activity | CG33138 |
| 139 | GO:0031887 | P | 8, 9, 10, | 1 | 0.016 (x 64.129) | 1 (1.000) | 0.0987 | lipid particle transport along microtubule | CG9057 |
| 140 | GO:0043067 | P | 5, 6, | 6 | 1.856 (x 3.233) | 119 (0.050) | 0.0989 | regulation of programmed cell death | Aac11 CG10990 gft klu smi35A th |
| 141 | GO:0031981 | C | 4, 5, 6, 7, 8, 9, 10, | 10 | 4.288 (x 2.332) | 275 (0.036) | 0.0992 | nuclear lumen | B52 Brf Caf1 Hrb27C Mcm5 Mcm7 Top2 ap dac sqd |
| 142 | GO:0015249 | F | 5, | 1 | 0.016 (x 64.129) | 1 (1.000) | 0.0992 | nonselective channel activity | bib |
| 143 | GO:0035035 | F | 5, | 1 | 0.016 (x 64.129) | 1 (1.000) | 0.0997 | histone acetyltransferase binding | Caf1 |
| 144 | GO:0007181 | P | 9, | 1 | 0.016 (x 64.129) | 1 (1.000) | 0.1 | transforming growth factor beta receptor complex assembly | tkv |
| 145 | GO:0003677 | F | 4, | 21 | 12.818 (x 1.638) | 822 (0.026) | 0.1 | DNA binding | CG13895 CG15141 CG31169 CG31617 CG6854 Dll Dr Dsp1 Hrb27C Mcm5 Mcm7 NUCB1 Pep Top2 ap ash2 dpa dve edl grn yps |
| 146 | GO:0003883 | F | 5, | 1 | 0.016 (x 64.129) | 1 (1.000) | 0.101 | CTP synthase activity | CG6854 |
| 147 | GO:0006260 | P | 7, | 6 | 1.887 (x 3.180) | 121 (0.050) | 0.101 | DNA replication | Caf1 Dsp1 Mcm5 Mcm7 Top2 dpa |
| 148 | GO:0007481 | P | 6, 7, | 1 | 0.016 (x 64.129) | 1 (1.000) | 0.101 | haltere disc morphogenesis | ap |
| 149 | GO:0051656 | P | 5, | 3 | 0.468 (x 6.413) | 30 (0.100) | 0.102 | establishment of organelle localization | Eb1 Lam polo |
| 150 | GO:0048812 | P | 7, 8, 10, | 6 | 1.887 (x 3.180) | 121 (0.050) | 0.102 | neurite morphogenesis | NetA ap fax fra ninA pbl |
| 151 | GO:0004450 | F | 7, | 1 | 0.016 (x 64.129) | 1 (1.000) | 0.102 | isocitrate dehydrogenase (NADP+) activity | Idh |
| 152 | GO:0007058 | P | 9, 10, 13, | 1 | 0.016 (x 64.129) | 1 (1.000) | 0.102 | female meiosis II spindle assembly (sensu Metazoa) | polo |
| 153 | GO:0048667 | P | 6, 7, 9, | 6 | 1.887 (x 3.180) | 121 (0.050) | 0.102 | neuron morphogenesis during differentiation | NetA ap fax fra ninA pbl |
| 154 | GO:0043119 | P | 4, | 7 | 2.635 (x 2.656) | 169 (0.041) | 0.102 | positive regulation of physiological process | CG10990 ash2 gft klu scrib smi35A tkv |
| 155 | GO:0009993 | P | 7, | 10 | 4.319 (x 2.315) | 277 (0.036) | 0.103 | oogenesis (sensu Insecta) | Caf1 Hrb27C Moe Scm bib endos sqd th tkv yps |
| 156 | GO:0016265 | P | 3, | 9 | 3.914 (x 2.299) | 251 (0.036) | 0.103 | death | Aac11 Akap200 CG10990 CG6680 ap gft klu smi35A th |
| 157 | GO:0015038 | F | 6, | 1 | 0.016 (x 64.129) | 1 (1.000) | 0.103 | glutathione disulfide oxidoreductase activity | Trxr-1 |
| 158 | GO:0007409 | P | 8, 9, 11, | 6 | 1.887 (x 3.180) | 121 (0.050) | 0.103 | axonogenesis | NetA ap fax fra ninA pbl |
| 159 | GO:0009950 | P | 5, | 4 | 0.889 (x 4.500) | 57 (0.070) | 0.103 | dorsal/ventral axis specification | serpin-27A sqd tok wbl |
| 160 | GO:0045841 | P | 8, 9, 10, | 1 | 0.016 (x 64.129) | 1 (1.000) | 0.103 | negative regulation of mitotic metaphase/anaphase transition | CycA |
| 161 | GO:0000226 | P | 8, | 5 | 1.357 (x 3.686) | 87 (0.057) | 0.103 | microtubule cytoskeleton organization and biogenesis | Eb1 betaTub56D dpa pbl polo |
| 162 | GO:0016667 | F | 4, | 2 | 0.172 (x 11.660) | 11 (0.182) | 0.104 | oxidoreductase activity, acting on sulfur group of donors | CG4670 Trxr-1 |
| 163 | GO:0006513 | P | 10, | 1 | 0.016 (x 64.129) | 1 (1.000) | 0.104 | protein monoubiquitination | th |
| 164 | GO:0004362 | F | 3, 5, 6, 7, | 1 | 0.016 (x 64.129) | 1 (1.000) | 0.104 | glutathione-disulfide reductase activity | Trxr-1 |
| 165 | GO:0004680 | F | 8, | 2 | 0.172 (x 11.660) | 11 (0.182) | 0.104 | casein kinase activity | CkIIalpha CkIalpha |
| 166 | GO:0048523 | P | 4, | 10 | 4.366 (x 2.290) | 280 (0.036) | 0.105 | negative regulation of cellular process | Aac11 CREG Caf1 CkIalpha CycA edl msi scrib sqd th |
| 167 | GO:0044430 | C | 4, 5, 6, 7, 8, 9, | 9 | 3.742 (x 2.405) | 240 (0.037) | 0.105 | cytoskeletal part | Act42A Act5C Eb1 Lam Nek2 betaTub56D d polo stai |
| 168 | GO:0006335 | P | 8, 12, | 1 | 0.016 (x 64.129) | 1 (1.000) | 0.105 | DNA replication-dependent nucleosome assembly | Caf1 |
| 169 | GO:0051726 | P | 5, | 8 | 3.103 (x 2.578) | 199 (0.040) | 0.105 | regulation of cell cycle | Abi Cdk4 CycA Eb1 Eip63E Nek2 gft scrib |
| 170 | GO:0031348 | P | 6, | 1 | 0.016 (x 64.129) | 1 (1.000) | 0.105 | negative regulation of defense response | serpin-27A |
| 171 | GO:0005057 | F | 3, | 9 | 3.742 (x 2.405) | 240 (0.037) | 0.106 | receptor signaling protein activity | CG17419 Cdk4 CkIIalpha CkIalpha Eip63E Nek2 polo smi35A tkv |
| 172 | GO:0030707 | P | 8, | 6 | 1.934 (x 3.103) | 124 (0.048) | 0.106 | ovarian follicle cell development (sensu Insecta) | Caf1 Scm bib sqd th tkv |
| 173 | GO:0000074 | P | 6, | 8 | 3.103 (x 2.578) | 199 (0.040) | 0.106 | regulation of progression through cell cycle | Abi Cdk4 CycA Eb1 Eip63E Nek2 gft scrib |
| 174 | GO:0004018 | F | 6, | 1 | 0.016 (x 64.129) | 1 (1.000) | 0.106 | adenylosuccinate lyase activity | CG3590 |
| 175 | GO:0044237 | P | 4, | 85 | 72.182 (x 1.178) | 4629 (0.018) | 0.106 | cellular metabolism | Aats-glupro ApepP Arf79F B52 BEST:LD22483 BcDNA:GH02901 BcDNA:LD22910 BcDNA:LD41548 Brf CG10423 CG10657 CG10990 CG15141 CG1544 CG15835 CG2118 CG2852 CG31169 CG31472 CG31617 CG33138 CG3590 CG3605 CG4670 CG5873 CG6680 CG6767 CG6854 CG6904 CG7461 CG8036 CG9027 CREG Caf1 Cdk4 CkIIalpha CkIalpha D19A Dll DnaJ-1 Dr Dsp1 ESTS:39C10S Eip63E Fur1 Gapdh2 GlyP Hrb27C Hsp26 Hsp27 Idh Mcm5 Mcm7 Nek2 Sb Scm Top2 Trxr-1 Uch ap ash2 betaTub56D dac deltaTry dpa dve eIF-4B eIF3-S9 edl fra gft gol grn klu msi polo rin serpin-27A smi35A sqd th tkv tok wbl yps |
| 176 | GO:0003723 | F | 4, | 12 | 5.754 (x 2.086) | 369 (0.033) | 0.106 | RNA binding | Aats-glupro B52 CG17838 Hrb27C Pep Top2 eIF-4B eIF3-S9 msi rin sqd yps |
| 177 | GO:0050431 | F | 5, | 1 | 0.016 (x 64.129) | 1 (1.000) | 0.107 | transforming growth factor beta binding | tkv |
| 178 | GO:0051640 | P | 4, | 3 | 0.499 (x 6.012) | 32 (0.094) | 0.107 | organelle localization | Eb1 Lam polo |
| 179 | GO:0017098 | F | 4, 5, | 1 | 0.016 (x 64.129) | 1 (1.000) | 0.107 | sulfonylurea receptor binding | endos |
| 180 | GO:0008105 | P | 5, | 3 | 0.499 (x 6.012) | 32 (0.094) | 0.108 | asymmetric protein localization | Moe fz scrib |
| 181 | GO:0006097 | P | 7, 8, | 1 | 0.016 (x 64.129) | 1 (1.000) | 0.108 | glyoxylate cycle | Idh |
| 182 | GO:0031323 | P | 5, | 24 | 15.375 (x 1.561) | 986 (0.024) | 0.108 | regulation of cellular metabolism | B52 Brf CG15141 CG15835 CREG Caf1 D19A Dll Dr Dsp1 Scm ap ash2 dve eIF-4B eIF3-S9 edl gol grn klu msi rin sqd yps |
| 183 | GO:0019538 | P | 5, | 46 | 34.040 (x 1.351) | 2183 (0.021) | 0.108 | protein metabolism | Aats-glupro ApepP Arf79F B52 BcDNA:LD22910 BcDNA:LD41548 CG10423 CG10657 CG10990 CG15141 CG2852 CG31617 CG4670 CG6680 Caf1 Cdk4 CkIIalpha CkIalpha D19A DnaJ-1 Eb1 Eip63E Fur1 Hsp26 Hsp27 Nek2 Sb Uch betaTub56D deltaTry eIF-4B eIF3-S9 fra gft gol msi pbl polo rin scrib smi35A sqd th tkv tok wbl |
| 184 | GO:0016978 | F | 6, | 1 | 0.016 (x 64.129) | 1 (1.000) | 0.108 | lipoate-protein ligase B activity | CG6767 |
| 185 | GO:0004674 | F | 7, | 8 | 3.306 (x 2.420) | 212 (0.038) | 0.108 | protein serine/threonine kinase activity | Cdk4 CkIIalpha CkIalpha Eip63E Nek2 polo smi35A tkv |
| 186 | GO:0004648 | F | 6, | 1 | 0.016 (x 64.129) | 1 (1.000) | 0.109 | phosphoserine transaminase activity | ESTS:39C10S |
| 187 | GO:0044265 | P | 6, | 7 | 2.682 (x 2.610) | 172 (0.041) | 0.109 | cellular macromolecule catabolism | BEST:LD22483 BcDNA:LD22910 CG8036 Gapdh2 Uch sqd th |
| 188 | GO:0004290 | F | 8, | 1 | 0.016 (x 64.129) | 1 (1.000) | 0.109 | kexin activity | Fur1 |
| 189 | GO:0005626 | C | 4, 5, | 1 | 0.016 (x 64.129) | 1 (1.000) | 0.11 | insoluble fraction | Top2 |
| 190 | GO:0008354 | P | 5, 6, 7, | 3 | 0.515 (x 5.830) | 33 (0.091) | 0.111 | germ cell migration | scrib stai th |
| 191 | GO:0046487 | P | 6, 7, | 1 | 0.016 (x 64.129) | 1 (1.000) | 0.111 | glyoxylate metabolism | Idh |
| 192 | GO:0046530 | P | 4, | 5 | 1.528 (x 3.272) | 98 (0.051) | 0.111 | photoreceptor cell differentiation | Moe dac edl fz rin |
| 193 | GO:0015281 | F | 6, 7, | 1 | 0.016 (x 64.129) | 1 (1.000) | 0.111 | nonselective cation channel activity | bib |
| 194 | GO:0006740 | P | 10, 11, | 2 | 0.187 (x 10.688) | 12 (0.167) | 0.111 | NADPH regeneration | BEST:LD22483 CG8036 |
| 195 | GO:0008184 | F | 7, | 1 | 0.016 (x 64.129) | 1 (1.000) | 0.112 | glycogen phosphorylase activity | GlyP |
| 196 | GO:0031519 | C | 3, 5, 6, 7, 8, 9, 10, | 2 | 0.187 (x 10.688) | 12 (0.167) | 0.112 | PcG protein complex | Caf1 Scm |
| 197 | GO:0004373 | F | 7, 8, | 1 | 0.016 (x 64.129) | 1 (1.000) | 0.113 | glycogen (starch) synthase activity | CG6904 |
| 198 | GO:0006739 | P | 9, 10, | 2 | 0.187 (x 10.688) | 12 (0.167) | 0.113 | NADP metabolism | BEST:LD22483 CG8036 |
| 199 | GO:0043228 | C | 3, | 19 | 11.087 (x 1.714) | 711 (0.027) | 0.113 | non-membrane-bound organelle | Act42A Act5C CG10423 CG13895 CG31617 Caf1 CycA Dsp1 Eb1 Lam Moe Nek2 Pep Top2 betaTub56D d polo sqd stai |
| 200 | GO:0008418 | F | 7, | 1 | 0.016 (x 64.129) | 1 (1.000) | 0.113 | protein N-terminal asparagine amidohydrolase activity | SP2637 |
| 201 | GO:0006098 | P | 8, 10, 11, 12, | 2 | 0.187 (x 10.688) | 12 (0.167) | 0.113 | pentose-phosphate shunt | BEST:LD22483 CG8036 |
| 202 | GO:0043232 | C | 4, 5, 6, 7, | 19 | 11.087 (x 1.714) | 711 (0.027) | 0.114 | intracellular non-membrane-bound organelle | Act42A Act5C CG10423 CG13895 CG31617 Caf1 CycA Dsp1 Eb1 Lam Moe Nek2 Pep Top2 betaTub56D d polo sqd stai |
| 203 | GO:0007049 | P | 4, | 14 | 7.345 (x 1.906) | 471 (0.030) | 0.114 | cell cycle | Abi Cdk4 CycA Eb1 Eip63E Lam Nek2 Top2 betaTub56D dpa gft pbl polo scrib |
| 204 | GO:0006261 | P | 8, | 4 | 0.951 (x 4.205) | 61 (0.066) | 0.114 | DNA-dependent DNA replication | Dsp1 Mcm5 Mcm7 dpa |
| 205 | GO:0015037 | F | 5, | 1 | 0.016 (x 64.129) | 1 (1.000) | 0.114 | peptide disulfide oxidoreductase activity | Trxr-1 |
| 206 | GO:0000212 | P | 6, 10, | 2 | 0.187 (x 10.688) | 12 (0.167) | 0.114 | meiotic spindle organization and biogenesis | pbl polo |
| 207 | GO:0005856 | C | 5, 6, 7, 8, | 10 | 4.522 (x 2.211) | 290 (0.034) | 0.114 | cytoskeleton | Act42A Act5C Eb1 Lam Moe Nek2 betaTub56D d polo stai |
| 208 | GO:0035009 | P | 6, 7, 9, | 1 | 0.016 (x 64.129) | 1 (1.000) | 0.115 | negative regulation of melanization defense response | serpin-27A |
| 209 | GO:0017099 | F | 6, | 1 | 0.016 (x 64.129) | 1 (1.000) | 0.115 | very-long-chain-acyl-CoA dehydrogenase activity | CG7461 |
| 210 | GO:0035011 | P | 6, 7, 8, | 1 | 0.016 (x 64.129) | 1 (1.000) | 0.116 | melanotic encapsulation of foreign target | serpin-27A |
| 211 | GO:0007147 | P | 9, | 1 | 0.016 (x 64.129) | 1 (1.000) | 0.116 | female meiosis II | polo |
| 212 | GO:0006769 | P | 8, 9, | 2 | 0.218 (x 9.161) | 14 (0.143) | 0.117 | nicotinamide metabolism | BEST:LD22483 CG8036 |
| 213 | GO:0018987 | P | 4, | 1 | 0.016 (x 64.129) | 1 (1.000) | 0.117 | osmoregulation | endos |
| 214 | GO:0006997 | P | 6, | 2 | 0.218 (x 9.161) | 14 (0.143) | 0.117 | nuclear organization and biogenesis | Lam polo |
| 215 | GO:0005488 | F | 2, | 77 | 64.463 (x 1.194) | 4134 (0.019) | 0.118 | binding | Aats-glupro Abi Akap200 Arf79F B52 BcDNA:LD41548 Brf CBP CG10423 CG10657 CG10990 CG13848 CG13895 CG15141 CG15835 CG17419 CG17838 CG2118 CG31169 CG31617 CG3823 CG6854 CG9027 CG9057 CG9598 CREG Caf1 Cdk4 CkIIalpha CkIalpha D19A Dll DnaJ-1 Dr Dsp1 Eb1 Eip63E GNBP3 Hrb27C Lam Mcm5 Mcm7 Moe Mtl NUCB1 Nek2 Pep Scm Top2 Trxr-1 ap ash2 betaTub56D bib d dpa dve eIF-4B eIF3-S9 edl endos esn fz gol grn klu msi polo rin scrib smi35A sqd stai th tkv tok yps |
| 216 | GO:0048518 | P | 3, | 8 | 3.399 (x 2.353) | 218 (0.037) | 0.122 | positive regulation of biological process | Abi CG10990 ash2 gft klu scrib smi35A tkv |
| 217 | GO:0009790 | P | 3, | 11 | 5.473 (x 2.010) | 351 (0.031) | 0.124 | embryonic development | Cdk4 CycA Eip63E Mtl edl gol pbl polo scrib tkv wbl |
| 218 | GO:0008094 | F | 10, | 3 | 0.593 (x 5.063) | 38 (0.079) | 0.125 | DNA-dependent ATPase activity | Mcm5 Mcm7 dpa |
| 219 | GO:0035215 | P | 5, | 2 | 0.234 (x 8.551) | 15 (0.133) | 0.128 | genital disc development | Dll dac |
| 220 | GO:0048699 | P | 6, | 7 | 2.807 (x 2.494) | 180 (0.039) | 0.128 | generation of neurons | Dr NetA ap fax fra ninA pbl |
| 221 | GO:0004693 | F | 8, | 2 | 0.234 (x 8.551) | 15 (0.133) | 0.128 | cyclin-dependent protein kinase activity | Cdk4 Eip63E |
| 222 | GO:0035218 | P | 5, | 3 | 0.608 (x 4.933) | 39 (0.077) | 0.129 | leg disc development | Dll ap dac |
| 223 | GO:0007052 | P | 6, 10, | 2 | 0.234 (x 8.551) | 15 (0.133) | 0.129 | mitotic spindle organization and biogenesis | Eb1 dpa |
| 224 | GO:0007450 | P | 5, 6, | 2 | 0.234 (x 8.551) | 15 (0.133) | 0.13 | dorsal/ventral pattern formation, imaginal disc | Dr ap |
| 225 | GO:0019362 | P | 7, 8, | 2 | 0.234 (x 8.551) | 15 (0.133) | 0.13 | pyridine nucleotide metabolism | BEST:LD22483 CG8036 |
| 226 | GO:0006092 | P | 7, | 5 | 1.622 (x 3.083) | 104 (0.048) | 0.131 | main pathways of carbohydrate metabolism | BEST:LD22483 CG1544 CG8036 Gapdh2 Idh |
| 227 | GO:0048666 | P | 5, 8, | 6 | 2.214 (x 2.710) | 142 (0.042) | 0.132 | neuron development | NetA ap fax fra ninA pbl |
| 228 | GO:0031175 | P | 6, 9, | 6 | 2.214 (x 2.710) | 142 (0.042) | 0.133 | neurite development | NetA ap fax fra ninA pbl |
| 229 | GO:0007163 | P | 5, 6, | 4 | 1.107 (x 3.613) | 71 (0.056) | 0.138 | establishment and/or maintenance of cell polarity | Moe fz rin scrib |
| 230 | GO:0005829 | C | 5, 6, 7, 8, | 7 | 2.869 (x 2.440) | 184 (0.038) | 0.139 | cytosol | ApepP CG10423 CkIIalpha eIF-4B eIF3-S9 rin smi35A |
| 231 | GO:0007097 | P | 7, 8, | 2 | 0.249 (x 8.016) | 16 (0.125) | 0.14 | nuclear migration | Lam polo |
| 232 | GO:0007222 | P | 7, | 2 | 0.281 (x 7.125) | 18 (0.111) | 0.142 | frizzled signaling pathway | CkIIalpha fz |
| 233 | GO:0009056 | P | 4, | 10 | 5.099 (x 1.961) | 327 (0.031) | 0.142 | catabolism | BEST:LD22483 BcDNA:LD22910 CG1544 CG8036 CkIalpha Gapdh2 Idh Uch sqd th |
| 234 | GO:0004591 | F | 6, | 1 | 0.031 (x 32.065) | 2 (0.500) | 0.142 | oxoglutarate dehydrogenase (succinyl-transferring) activity | CG1544 |
| 235 | GO:0007582 | P | 2, | 116 | 106.534 (x 1.089) | 6832 (0.017) | 0.142 | physiological process | Aac11 Aats-glupro Abi Act42A Act5C Akap200 ApepP Arf79F B52 BEST:LD22483 BcDNA:GH02901 BcDNA:LD22910 BcDNA:LD41548 Brf CG10423 CG10657 CG10960 CG10990 CG14439 CG15141 CG1544 CG15835 CG17419 CG2118 CG2852 CG31169 CG31472 CG31617 CG33138 CG3590 CG3605 CG3823 CG4670 CG5873 CG6680 CG6767 CG6854 CG6904 CG7461 CG7675 CG8036 CG9027 CG9057 CREG Caf1 Cdk4 CkIIalpha CkIalpha CycA D19A Dll DnaJ-1 Dr Dsp1 ESTS:39C10S Eb1 Eip63E Fur1 GNBP3 Gapdh2 GlyP Hrb27C Hsp26 Hsp27 Idh Lam Mcm5 Mcm7 Moe Mtl Nek2 NetA Sb Scm Snap Top2 Trxr-1 Uch ap ash2 betaTub56D bib d dac deltaTry dpa dve eIF-4B eIF3-S9 edl endos esn fax fra fz gft gol grn klu l(2)dtl msi ninA pbl polo rin rpk scrib serpin-27A smi35A sqd stai th tkv tok wbl yps |
| 236 | GO:0048113 | P | 8, 10, 11, 13, | 3 | 0.639 (x 4.692) | 41 (0.073) | 0.142 | pole plasm assembly (sensu Insecta) | Hrb27C Moe sqd |
| 237 | GO:0051647 | P | 5, 6, | 2 | 0.281 (x 7.125) | 18 (0.111) | 0.142 | nucleus localization | Lam polo |
| 238 | GO:0045313 | P | 9, 10, 11, 12, | 1 | 0.031 (x 32.065) | 2 (0.500) | 0.143 | rhabdomere membrane biogenesis | Moe |
| 239 | GO:0022008 | P | 5, | 7 | 3.010 (x 2.326) | 193 (0.036) | 0.143 | neurogenesis | Dr NetA ap fax fra ninA pbl |
| 240 | GO:0005884 | C | 5, 6, 7, 8, 9, 10, | 2 | 0.281 (x 7.125) | 18 (0.111) | 0.143 | actin filament | Act42A Act5C |
| 241 | GO:0044260 | P | 5, | 44 | 33.557 (x 1.311) | 2152 (0.020) | 0.143 | cellular macromolecule metabolism | Aats-glupro ApepP Arf79F BcDNA:LD22910 BcDNA:LD41548 CG10423 CG10657 CG10990 CG15141 CG2852 CG33138 CG4670 CG6680 CG6904 Caf1 Cdk4 CkIIalpha CkIalpha D19A DnaJ-1 Eip63E Fur1 GlyP Hsp26 Hsp27 Nek2 Sb Uch betaTub56D deltaTry eIF-4B eIF3-S9 fra gft gol msi polo rin smi35A sqd th tkv tok wbl |
| 242 | GO:0045111 | C | 6, 7, 8, 9, | 1 | 0.031 (x 32.065) | 2 (0.500) | 0.143 | intermediate filament cytoskeleton | Lam |
| 243 | GO:0005882 | C | 5, 6, 7, 8, 9, 10, | 1 | 0.031 (x 32.065) | 2 (0.500) | 0.144 | intermediate filament | Lam |
| 244 | GO:0004645 | F | 6, | 1 | 0.031 (x 32.065) | 2 (0.500) | 0.144 | phosphorylase activity | GlyP |
| 245 | GO:0031099 | P | 4, | 1 | 0.031 (x 32.065) | 2 (0.500) | 0.145 | regeneration | ninA |
| 246 | GO:0009792 | P | 4, | 7 | 3.041 (x 2.302) | 195 (0.036) | 0.145 | embryonic development (sensu Metazoa) | Eip63E Mtl gol pbl scrib tkv wbl |
| 247 | GO:0004802 | F | 5, | 1 | 0.031 (x 32.065) | 2 (0.500) | 0.145 | transketolase activity | CG8036 |
| 248 | GO:0005700 | C | 6, 7, 8, 9, | 3 | 0.702 (x 4.275) | 45 (0.067) | 0.145 | polytene chromosome | Caf1 Pep sqd |
| 249 | GO:0006433 | P | 9, 10, 11, | 1 | 0.031 (x 32.065) | 2 (0.500) | 0.146 | prolyl-tRNA aminoacylation | Aats-glupro |
| 250 | GO:0019898 | C | 4, 5, 6, | 3 | 0.702 (x 4.275) | 45 (0.067) | 0.146 | extrinsic to membrane | ImpE2 ImpE3 Snap |
| 251 | GO:0042246 | P | 4, 5, 6, | 1 | 0.031 (x 32.065) | 2 (0.500) | 0.146 | tissue regeneration | ninA |
| 252 | GO:0048112 | P | 7, 9, 10, 12, | 3 | 0.702 (x 4.275) | 45 (0.067) | 0.146 | oocyte anterior/posterior axis determination (sensu Insecta) | Hrb27C Moe sqd |
| 253 | GO:0005811 | C | 5, 6, 7, 8, | 1 | 0.031 (x 32.065) | 2 (0.500) | 0.147 | lipid particle | CG9057 |
| 254 | GO:0035216 | P | 5, | 1 | 0.031 (x 32.065) | 2 (0.500) | 0.147 | haltere disc development | ap |
| 255 | GO:0000126 | C | 4, 7, 8, 9, 10, 11, 12, 13, 14, | 1 | 0.031 (x 32.065) | 2 (0.500) | 0.148 | transcription factor TFIIIB complex | Brf |
| 256 | GO:0007017 | P | 7, | 7 | 3.056 (x 2.290) | 196 (0.036) | 0.148 | microtubule-based process | CG9057 Eb1 betaTub56D dpa pbl polo stai |
| 257 | GO:0007289 | P | 6, 9, | 1 | 0.031 (x 32.065) | 2 (0.500) | 0.148 | spermatid nuclear differentiation | th |
| 258 | GO:0043231 | C | 4, 5, 6, 7, | 46 | 35.974 (x 1.279) | 2307 (0.020) | 0.149 | intracellular membrane-bound organelle | B52 Brf CBP CG13895 CG17838 CG2118 CG31617 CG3605 CG9894 CREG Caf1 CkIIalpha CkIalpha CycA D19A Dll Dr Dsp1 Fur1 Hrb27C Idh Lam Mcm5 Mcm7 NUCB1 Pep Scm Snap Top2 Trxr-1 ap ash2 dac dpa dve edl esn gft gol grn klu pbl rin sqd wbl yps |
| 259 | GO:0035320 | P | 7, 8, 9, 10, 11, 12, | 1 | 0.031 (x 32.065) | 2 (0.500) | 0.149 | wing hair site selection | fz |
| 260 | GO:0035044 | P | 6, 9, | 1 | 0.031 (x 32.065) | 2 (0.500) | 0.149 | sperm aster formation | polo |
| 261 | GO:0051240 | P | 5, | 1 | 0.031 (x 32.065) | 2 (0.500) | 0.15 | positive regulation of organismal physiological process | scrib |
| 262 | GO:0007308 | P | 6, 7, 9, | 4 | 1.232 (x 3.247) | 79 (0.051) | 0.15 | oocyte construction | Hrb27C Moe sqd wbl |
| 263 | GO:0007264 | P | 6, | 5 | 1.809 (x 2.764) | 116 (0.043) | 0.15 | small GTPase mediated signal transduction | Akap200 Mtl edl klu rin |
| 264 | GO:0043227 | C | 3, | 46 | 36.005 (x 1.278) | 2309 (0.020) | 0.15 | membrane-bound organelle | B52 Brf CBP CG13895 CG17838 CG2118 CG31617 CG3605 CG9894 CREG Caf1 CkIIalpha CkIalpha CycA D19A Dll Dr Dsp1 Fur1 Hrb27C Idh Lam Mcm5 Mcm7 NUCB1 Pep Scm Snap Top2 Trxr-1 ap ash2 dac dpa dve edl esn gft gol grn klu pbl rin sqd wbl yps |
| 265 | GO:0006081 | P | 5, | 1 | 0.031 (x 32.065) | 2 (0.500) | 0.151 | aldehyde metabolism | Idh |
| 266 | GO:0008340 | P | 4, | 3 | 0.717 (x 4.182) | 46 (0.065) | 0.151 | determination of adult life span | Hsp26 Hsp27 Trxr-1 |
| 267 | GO:0005638 | C | 5, 6, 7, 8, 9, 10, 11, 12, 13, 14, | 1 | 0.031 (x 32.065) | 2 (0.500) | 0.151 | lamin filament | Lam |
| 268 | GO:0007568 | P | 3, | 3 | 0.717 (x 4.182) | 46 (0.065) | 0.151 | aging | Hsp26 Hsp27 Trxr-1 |
| 269 | GO:0042981 | P | 6, 7, | 5 | 1.700 (x 2.942) | 109 (0.046) | 0.151 | regulation of apoptosis | Aac11 CG10990 gft smi35A th |
| 270 | GO:0004485 | F | 6, | 1 | 0.031 (x 32.065) | 2 (0.500) | 0.152 | methylcrotonoyl-CoA carboxylase activity | CG2118 |
| 271 | GO:0030730 | P | 5, 7, | 1 | 0.031 (x 32.065) | 2 (0.500) | 0.152 | sequestering of triacylglycerol | CG9057 |
| 272 | GO:0016478 | P | 8, 9, 10, | 2 | 0.265 (x 7.545) | 17 (0.118) | 0.153 | negative regulation of translation | msi sqd |
| 273 | GO:0005623 | C | 2, | 83 | 71.979 (x 1.153) | 4616 (0.018) | 0.153 | cell | Aats-glupro Act42A Act5C ApepP B52 BcDNA:LD41548 Brf CBP CG10423 CG10657 CG10960 CG13895 CG14439 CG17838 CG2118 CG31617 CG3605 CG3823 CG9057 CG9894 CREG Caf1 CkIIalpha CkIalpha CycA D19A Dll DnaJ-1 Dr Dsp1 Eb1 Fur1 Gapdh2 Hrb27C Idh ImpE2 ImpE3 Lam Mcm5 Mcm7 Moe NUCB1 Nek2 Pep Sb Scm Snap Top2 Trxr-1 Uch ap ash2 betaTub56D bib d dac dpa dve eIF-4B eIF3-S9 edl esn fra fz gft gol grn klu l(2)dtl lig ninA pbl polo rin rpk scrib smi35A sqd stai th tkv wbl yps |
| 274 | GO:0007483 | P | 6, 7, | 1 | 0.031 (x 32.065) | 2 (0.500) | 0.153 | genital disc morphogenesis | dac |
| 275 | GO:0016334 | P | 6, | 2 | 0.265 (x 7.545) | 17 (0.118) | 0.153 | establishment and/or maintenance of polarity of follicular epithelium | scrib sqd |
| 276 | GO:0044464 | C | 2, 3, | 83 | 71.979 (x 1.153) | 4616 (0.018) | 0.153 | cell part | Aats-glupro Act42A Act5C ApepP B52 BcDNA:LD41548 Brf CBP CG10423 CG10657 CG10960 CG13895 CG14439 CG17838 CG2118 CG31617 CG3605 CG3823 CG9057 CG9894 CREG Caf1 CkIIalpha CkIalpha CycA D19A Dll DnaJ-1 Dr Dsp1 Eb1 Fur1 Gapdh2 Hrb27C Idh ImpE2 ImpE3 Lam Mcm5 Mcm7 Moe NUCB1 Nek2 Pep Sb Scm Snap Top2 Trxr-1 Uch ap ash2 betaTub56D bib d dac dpa dve eIF-4B eIF3-S9 edl esn fra fz gft gol grn klu l(2)dtl lig ninA pbl polo rin rpk scrib smi35A sqd stai th tkv wbl yps |
| 277 | GO:0004791 | F | 3, 5, 6, | 1 | 0.031 (x 32.065) | 2 (0.500) | 0.154 | thioredoxin-disulfide reductase activity | Trxr-1 |
| 278 | GO:0040023 | P | 6, 7, | 2 | 0.265 (x 7.545) | 17 (0.118) | 0.154 | establishment of nucleus localization | Lam polo |
| 279 | GO:0008442 | F | 6, | 1 | 0.031 (x 32.065) | 2 (0.500) | 0.154 | 3-hydroxyisobutyrate dehydrogenase activity | BEST:LD22483 |
| 280 | GO:0048599 | P | 5, 6, 8, | 4 | 1.247 (x 3.206) | 80 (0.050) | 0.154 | oocyte development | Hrb27C Moe sqd wbl |
| 281 | GO:0004749 | F | 6, 7, | 1 | 0.031 (x 32.065) | 2 (0.500) | 0.155 | ribose phosphate diphosphokinase activity | CG6767 |
| 282 | GO:0006125 | P | 7, | 1 | 0.031 (x 32.065) | 2 (0.500) | 0.155 | thioredoxin pathway | Trxr-1 |
| 283 | GO:0048729 | P | 4, | 3 | 0.671 (x 4.474) | 43 (0.070) | 0.156 | tissue morphogenesis | fz gol pbl |
| 284 | GO:0030182 | P | 4, 7, | 6 | 2.323 (x 2.582) | 149 (0.040) | 0.156 | neuron differentiation | NetA ap fax fra ninA pbl |
| 285 | GO:0004827 | F | 7, | 1 | 0.031 (x 32.065) | 2 (0.500) | 0.156 | proline-tRNA ligase activity | Aats-glupro |
| 286 | GO:0009408 | P | 4, 5, | 3 | 0.733 (x 4.093) | 47 (0.064) | 0.156 | response to heat | DnaJ-1 Hsp26 Hsp27 |
| 287 | GO:0007315 | P | 7, 9, 10, 12, | 3 | 0.671 (x 4.474) | 43 (0.070) | 0.156 | pole plasm assembly | Hrb27C Moe sqd |
| 288 | GO:0031347 | P | 5, | 1 | 0.031 (x 32.065) | 2 (0.500) | 0.157 | regulation of defense response | serpin-27A |
| 289 | GO:0043068 | P | 6, 7, | 4 | 1.170 (x 3.420) | 75 (0.053) | 0.157 | positive regulation of programmed cell death | CG10990 gft klu smi35A |
| 290 | GO:0000022 | P | 7, 11, | 1 | 0.031 (x 32.065) | 2 (0.500) | 0.157 | mitotic spindle elongation | Eb1 |
| 291 | GO:0016654 | F | 5, | 1 | 0.031 (x 32.065) | 2 (0.500) | 0.158 | oxidoreductase activity, acting on NADH or NADPH, disulfide as acceptor | Trxr-1 |
| 292 | GO:0035007 | P | 5, 6, 8, | 1 | 0.031 (x 32.065) | 2 (0.500) | 0.158 | regulation of melanization defense response | serpin-27A |
| 293 | GO:0042787 | P | 10, 11, 12, | 1 | 0.031 (x 32.065) | 2 (0.500) | 0.159 | protein ubiquitination during ubiquitin-dependent protein catabolism | th |
| 294 | GO:0007084 | P | 6, 8, 9, | 1 | 0.031 (x 32.065) | 2 (0.500) | 0.16 | mitotic nuclear envelope reassembly | Lam |
| 295 | GO:0044446 | C | 3, 4, 5, 6, 7, | 31 | 22.205 (x 1.396) | 1424 (0.022) | 0.16 | intracellular organelle part | Act42A Act5C B52 Brf CG10423 CG13895 CG17838 CG2118 CG31617 CG3605 Caf1 CycA Dsp1 Eb1 Fur1 Hrb27C Lam Mcm5 Mcm7 Nek2 Pep Scm Top2 ap betaTub56D d dac gft polo sqd stai |
| 296 | GO:0005042 | F | 6, | 1 | 0.031 (x 32.065) | 2 (0.500) | 0.16 | netrin receptor activity | fra |
| 297 | GO:0007309 | P | 5, 7, 8, 10, | 4 | 1.185 (x 3.375) | 76 (0.053) | 0.161 | oocyte axis determination | Hrb27C Moe sqd wbl |
| 298 | GO:0007281 | P | 5, | 5 | 1.746 (x 2.863) | 112 (0.045) | 0.161 | germ cell development | Hrb27C Moe sqd tkv wbl |
| 299 | GO:0044422 | C | 2, 3, | 31 | 22.205 (x 1.396) | 1424 (0.022) | 0.161 | organelle part | Act42A Act5C B52 Brf CG10423 CG13895 CG17838 CG2118 CG31617 CG3605 Caf1 CycA Dsp1 Eb1 Fur1 Hrb27C Lam Mcm5 Mcm7 Nek2 Pep Scm Top2 ap betaTub56D d dac gft polo sqd stai |
| 300 | GO:0035222 | P | 5, 6, | 2 | 0.312 (x 6.413) | 20 (0.100) | 0.161 | wing disc pattern formation | ap tkv |
| 301 | GO:0035211 | P | 5, | 1 | 0.031 (x 32.065) | 2 (0.500) | 0.161 | spermathecum morphogenesis | dac |
| 302 | GO:0009798 | P | 4, | 6 | 2.479 (x 2.420) | 159 (0.038) | 0.161 | axis specification | Hrb27C Moe serpin-27A sqd tok wbl |
| 303 | GO:0006733 | P | 7, | 2 | 0.312 (x 6.413) | 20 (0.100) | 0.161 | oxidoreduction coenzyme metabolism | BEST:LD22483 CG8036 |
| 304 | GO:0045823 | P | 6, | 1 | 0.031 (x 32.065) | 2 (0.500) | 0.162 | positive regulation of heart contraction | scrib |
| 305 | GO:0016318 | P | 7, 8, 9, 10, | 2 | 0.312 (x 6.413) | 20 (0.100) | 0.162 | ommatidial rotation | fz rin |
| 306 | GO:0007411 | P | 6, 7, 9, 10, 12, | 4 | 1.279 (x 3.128) | 82 (0.049) | 0.163 | axon guidance | NetA ap fra ninA |
| 307 | GO:0008135 | F | 3, 4, | 4 | 1.279 (x 3.128) | 82 (0.049) | 0.163 | translation factor activity, nucleic acid binding | CG10990 eIF-4B eIF3-S9 msi |
| 308 | GO:0044451 | C | 5, 6, 7, 8, 9, 10, 11, 12, | 7 | 3.165 (x 2.211) | 203 (0.034) | 0.164 | nucleoplasm part | B52 Brf Caf1 Mcm5 Mcm7 Top2 sqd |
| 309 | GO:0012501 | P | 5, | 8 | 3.867 (x 2.069) | 248 (0.032) | 0.166 | programmed cell death | Aac11 Akap200 CG10990 CG6680 gft klu smi35A th |
| 310 | GO:0005652 | C | 5, 6, 7, 8, 9, 10, 11, | 1 | 0.047 (x 21.376) | 3 (0.333) | 0.167 | nuclear lamina | Lam |
| 311 | GO:0051242 | P | 5, | 6 | 2.604 (x 2.304) | 167 (0.036) | 0.167 | positive regulation of cellular physiological process | CG10990 ash2 gft klu smi35A tkv |
| 312 | GO:0035062 | C | 8, 9, 10, 11, 12, 13, 14, 15, | 1 | 0.047 (x 21.376) | 3 (0.333) | 0.167 | omega speckle | sqd |
| 313 | GO:0051227 | P | 7, 8, 11, | 1 | 0.047 (x 21.376) | 3 (0.333) | 0.168 | mitotic spindle assembly | Eb1 |
| 314 | GO:0009994 | P | 4, 7, | 4 | 1.357 (x 2.948) | 87 (0.046) | 0.168 | oocyte differentiation | Hrb27C Moe sqd wbl |
| 315 | GO:0035006 | P | 5, 7, | 1 | 0.047 (x 21.376) | 3 (0.333) | 0.168 | melanization defense response | serpin-27A |
| 316 | GO:0003680 | F | 6, | 1 | 0.047 (x 21.376) | 3 (0.333) | 0.169 | AT DNA binding | dve |
| 317 | GO:0001738 | P | 5, | 3 | 0.811 (x 3.700) | 52 (0.058) | 0.169 | morphogenesis of a polarized epithelium | fz rin scrib |
| 318 | GO:0007313 | P | 7, 9, 10, 12, | 1 | 0.047 (x 21.376) | 3 (0.333) | 0.169 | maternal determination of dorsal/ventral axis, oocyte, soma encoded | wbl |
| 319 | GO:0042461 | P | 5, 6, 7, | 3 | 0.811 (x 3.700) | 52 (0.058) | 0.169 | photoreceptor cell development | Moe dac edl |
| 320 | GO:0004276 | F | 7, | 1 | 0.047 (x 21.376) | 3 (0.333) | 0.17 | furin activity | Fur1 |
| 321 | GO:0048098 | P | 7, | 1 | 0.047 (x 21.376) | 3 (0.333) | 0.17 | antennal joint development | dac |
| 322 | GO:0009374 | F | 4, | 1 | 0.047 (x 21.376) | 3 (0.333) | 0.171 | biotin binding | CG2118 |
| 323 | GO:0006424 | P | 9, 10, 11, | 1 | 0.047 (x 21.376) | 3 (0.333) | 0.171 | glutamyl-tRNA aminoacylation | Aats-glupro |
| 324 | GO:0017148 | P | 7, 8, 9, | 2 | 0.327 (x 6.108) | 21 (0.095) | 0.172 | negative regulation of protein biosynthesis | msi sqd |
| 325 | GO:0042826 | F | 5, | 1 | 0.047 (x 21.376) | 3 (0.333) | 0.172 | histone deacetylase binding | Caf1 |
| 326 | GO:0035010 | P | 5, 6, | 1 | 0.047 (x 21.376) | 3 (0.333) | 0.172 | encapsulation of foreign target | serpin-27A |
| 327 | GO:0009110 | P | 6, | 2 | 0.327 (x 6.108) | 21 (0.095) | 0.172 | vitamin biosynthesis | CG31472 ESTS:39C10S |
| 328 | GO:0043285 | P | 6, | 5 | 1.980 (x 2.525) | 127 (0.039) | 0.172 | biopolymer catabolism | BcDNA:LD22910 CkIalpha Uch sqd th |
| 329 | GO:0008219 | P | 4, | 8 | 3.898 (x 2.052) | 250 (0.032) | 0.172 | cell death | Aac11 Akap200 CG10990 CG6680 gft klu smi35A th |
| 330 | GO:0003709 | F | 3, | 1 | 0.047 (x 21.376) | 3 (0.333) | 0.173 | RNA polymerase III transcription factor activity | Brf |
| 331 | GO:0016579 | P | 9, | 2 | 0.327 (x 6.108) | 21 (0.095) | 0.173 | protein deubiquitination | BcDNA:LD22910 Uch |
| 332 | GO:0000900 | F | 4, 5, | 1 | 0.047 (x 21.376) | 3 (0.333) | 0.173 | translation repressor activity, nucleic acid binding | msi |
| 333 | GO:0015280 | F | 6, 7, 8, | 1 | 0.047 (x 21.376) | 3 (0.333) | 0.174 | amiloride-sensitive sodium channel activity | rpk |
| 334 | GO:0006139 | P | 5, | 36 | 27.616 (x 1.304) | 1771 (0.020) | 0.174 | nucleobase, nucleoside, nucleotide and nucleic acid metabolism | Aats-glupro B52 BEST:LD22483 Brf CG15141 CG15835 CG31617 CG3590 CG3605 CG6767 CG6854 CG8036 CREG Caf1 CkIalpha D19A Dll Dr Dsp1 Hrb27C Mcm5 Mcm7 Scm Top2 ap ash2 dac dpa dve edl gol grn klu msi sqd yps |
| 335 | GO:0004367 | F | 6, | 1 | 0.047 (x 21.376) | 3 (0.333) | 0.174 | glycerol-3-phosphate dehydrogenase (NAD+) activity | CG31169 |
| 336 | GO:0008451 | F | 7, | 1 | 0.047 (x 21.376) | 3 (0.333) | 0.175 | X-Pro aminopeptidase activity | ApepP |
| 337 | GO:0007548 | P | 3, | 3 | 0.780 (x 3.848) | 50 (0.060) | 0.175 | sex differentiation | Dll dac scrib |
| 338 | GO:0016979 | F | 5, | 1 | 0.047 (x 21.376) | 3 (0.333) | 0.175 | lipoate-protein ligase activity | CG6767 |
| 339 | GO:0048111 | P | 6, 8, 9, 11, | 3 | 0.826 (x 3.630) | 53 (0.057) | 0.175 | oocyte axis determination (sensu Insecta) | Hrb27C Moe sqd |
| 340 | GO:0004733 | F | 6, | 1 | 0.047 (x 21.376) | 3 (0.333) | 0.176 | pyridoxamine-phosphate oxidase activity | CG31472 |
| 341 | GO:0044248 | P | 5, | 9 | 4.772 (x 1.886) | 306 (0.029) | 0.176 | cellular catabolism | BEST:LD22483 BcDNA:LD22910 CG1544 CG8036 Gapdh2 Idh Uch sqd th |
| 342 | GO:0005737 | C | 4, 5, 6, | 31 | 23.156 (x 1.339) | 1485 (0.021) | 0.176 | cytoplasm | Aats-glupro ApepP BcDNA:LD41548 CBP CG10423 CG2118 CG9057 CkIIalpha CkIalpha CycA DnaJ-1 Fur1 Gapdh2 Idh ImpE2 Moe NUCB1 Nek2 Snap Trxr-1 eIF-4B eIF3-S9 fz l(2)dtl lig polo rin scrib smi35A sqd wbl |
| 343 | GO:0019915 | P | 6, | 1 | 0.047 (x 21.376) | 3 (0.333) | 0.176 | sequestering of lipid | CG9057 |
| 344 | GO:0035046 | P | 6, 8, 9, | 1 | 0.047 (x 21.376) | 3 (0.333) | 0.177 | pronuclear migration | polo |
| 345 | GO:0045839 | P | 7, 8, 9, | 1 | 0.047 (x 21.376) | 3 (0.333) | 0.177 | negative regulation of mitosis | CycA |
| 346 | GO:0045182 | F | 2, | 4 | 1.325 (x 3.018) | 85 (0.047) | 0.177 | translation regulator activity | CG10990 eIF-4B eIF3-S9 msi |
| 347 | GO:0016668 | F | 5, | 1 | 0.047 (x 21.376) | 3 (0.333) | 0.178 | oxidoreductase activity, acting on sulfur group of donors, NAD or NADP as acceptor | Trxr-1 |
| 348 | GO:0009250 | P | 8, 9, | 1 | 0.047 (x 21.376) | 3 (0.333) | 0.178 | glucan biosynthesis | CG6904 |
| 349 | GO:0005025 | F | 7, 8, 10, 11, | 1 | 0.047 (x 21.376) | 3 (0.333) | 0.179 | transforming growth factor beta receptor activity, type I | tkv |
| 350 | GO:0009892 | P | 5, | 6 | 2.573 (x 2.332) | 165 (0.036) | 0.179 | negative regulation of metabolism | CREG Caf1 edl msi serpin-27A sqd |
| 351 | GO:0016744 | F | 4, | 1 | 0.047 (x 21.376) | 3 (0.333) | 0.179 | transferase activity, transferring aldehyde or ketonic groups | CG8036 |
| 352 | GO:0046164 | P | 6, | 3 | 0.795 (x 3.772) | 51 (0.059) | 0.18 | alcohol catabolism | BEST:LD22483 CG8036 Gapdh2 |
| 353 | GO:0005978 | P | 9, 10, | 1 | 0.047 (x 21.376) | 3 (0.333) | 0.18 | glycogen biosynthesis | CG6904 |
| 354 | GO:0046365 | P | 7, 8, | 3 | 0.795 (x 3.772) | 51 (0.059) | 0.18 | monosaccharide catabolism | BEST:LD22483 CG8036 Gapdh2 |
| 355 | GO:0051231 | P | 10, | 1 | 0.047 (x 21.376) | 3 (0.333) | 0.18 | spindle elongation | Eb1 |
| 356 | GO:0006007 | P | 9, 10, | 3 | 0.795 (x 3.772) | 51 (0.059) | 0.181 | glucose catabolism | BEST:LD22483 CG8036 Gapdh2 |
| 357 | GO:0010092 | P | 5, | 1 | 0.047 (x 21.376) | 3 (0.333) | 0.181 | specification of organ identity | Dll |
| 358 | GO:0031327 | P | 7, | 2 | 0.343 (x 5.830) | 22 (0.091) | 0.181 | negative regulation of cellular biosynthesis | msi sqd |
| 359 | GO:0030163 | P | 6, 7, | 4 | 1.403 (x 2.850) | 90 (0.044) | 0.181 | protein catabolism | BcDNA:LD22910 CkIalpha Uch th |
| 360 | GO:0003678 | F | 4, | 3 | 0.795 (x 3.772) | 51 (0.059) | 0.181 | DNA helicase activity | Mcm5 Mcm7 dpa |
| 361 | GO:0017101 | C | 3, 4, 5, 6, | 1 | 0.047 (x 21.376) | 3 (0.333) | 0.182 | aminoacyl-tRNA synthetase multienzyme complex | Aats-glupro |
| 362 | GO:0009890 | P | 6, | 2 | 0.343 (x 5.830) | 22 (0.091) | 0.182 | negative regulation of biosynthesis | msi sqd |
| 363 | GO:0048110 | P | 7, 8, 10, | 3 | 0.842 (x 3.563) | 54 (0.056) | 0.182 | oocyte construction (sensu Insecta) | Hrb27C Moe sqd |
| 364 | GO:0019320 | P | 8, 9, | 3 | 0.795 (x 3.772) | 51 (0.059) | 0.182 | hexose catabolism | BEST:LD22483 CG8036 Gapdh2 |
| 365 | GO:0009266 | P | 4, | 3 | 0.842 (x 3.563) | 54 (0.056) | 0.182 | response to temperature stimulus | DnaJ-1 Hsp26 Hsp27 |
| 366 | GO:0004682 | F | 9, | 1 | 0.047 (x 21.376) | 3 (0.333) | 0.182 | protein kinase CK2 activity | CkIIalpha |
| 367 | GO:0016842 | F | 5, | 1 | 0.047 (x 21.376) | 3 (0.333) | 0.183 | amidine-lyase activity | CG3590 |
| 368 | GO:0016903 | F | 4, | 2 | 0.374 (x 5.344) | 24 (0.083) | 0.186 | oxidoreductase activity, acting on the aldehyde or oxo group of donors | CG1544 Gapdh2 |
| 369 | GO:0006334 | P | 7, 11, | 2 | 0.374 (x 5.344) | 24 (0.083) | 0.186 | nucleosome assembly | CG31617 Caf1 |
| 370 | GO:0044267 | P | 6, | 41 | 32.419 (x 1.265) | 2079 (0.020) | 0.187 | cellular protein metabolism | Aats-glupro ApepP Arf79F BcDNA:LD22910 BcDNA:LD41548 CG10423 CG10657 CG10990 CG15141 CG2852 CG4670 CG6680 Caf1 Cdk4 CkIIalpha CkIalpha D19A DnaJ-1 Eip63E Fur1 Hsp26 Hsp27 Nek2 Sb Uch betaTub56D deltaTry eIF-4B eIF3-S9 fra gft gol msi polo rin smi35A sqd th tkv tok wbl |
| 371 | GO:0016055 | P | 6, | 3 | 0.858 (x 3.498) | 55 (0.055) | 0.187 | Wnt receptor signaling pathway | CkIIalpha CkIalpha fz |
| 372 | GO:0016584 | P | 11, | 1 | 0.062 (x 16.032) | 4 (0.250) | 0.193 | nucleosome spacing | Caf1 |
| 373 | GO:0004221 | F | 6, 8, | 2 | 0.405 (x 4.933) | 26 (0.077) | 0.193 | ubiquitin thiolesterase activity | BcDNA:LD22910 Uch |
| 374 | GO:0004818 | F | 7, | 1 | 0.062 (x 16.032) | 4 (0.250) | 0.193 | glutamate-tRNA ligase activity | Aats-glupro |
| 375 | GO:0048332 | P | 5, | 2 | 0.405 (x 4.933) | 26 (0.077) | 0.194 | mesoderm morphogenesis | gol pbl |
| 376 | GO:0005625 | C | 4, 5, | 1 | 0.062 (x 16.032) | 4 (0.250) | 0.194 | soluble fraction | Top2 |
| 377 | GO:0001707 | P | 6, 7, | 2 | 0.405 (x 4.933) | 26 (0.077) | 0.194 | mesoderm formation | gol pbl |
| 378 | GO:0004616 | F | 6, | 1 | 0.062 (x 16.032) | 4 (0.250) | 0.194 | phosphogluconate dehydrogenase (decarboxylating) activity | BEST:LD22483 |
| 379 | GO:0007056 | P | 8, 9, 12, | 1 | 0.062 (x 16.032) | 4 (0.250) | 0.195 | female meiotic spindle assembly (sensu Metazoa) | polo |
| 380 | GO:0003918 | F | 5, 6, | 1 | 0.062 (x 16.032) | 4 (0.250) | 0.195 | DNA topoisomerase (ATP-hydrolyzing) activity | Top2 |
| 381 | GO:0007314 | P | 6, 8, 9, 11, | 3 | 0.873 (x 3.436) | 56 (0.054) | 0.195 | oocyte anterior/posterior axis determination | Hrb27C Moe sqd |
| 382 | GO:0031228 | C | 5, 6, 7, 8, 9, 10, 11, | 1 | 0.062 (x 16.032) | 4 (0.250) | 0.196 | intrinsic to Golgi membrane | Fur1 |
| 383 | GO:0016528 | C | 5, 6, 7, | 1 | 0.062 (x 16.032) | 4 (0.250) | 0.196 | sarcoplasm | CBP |
| 384 | GO:0042813 | F | 5, | 1 | 0.062 (x 16.032) | 4 (0.250) | 0.197 | Wnt receptor activity | fz |
| 385 | GO:0006551 | P | 8, 9, | 1 | 0.062 (x 16.032) | 4 (0.250) | 0.197 | leucine metabolism | CG2118 |
| 386 | GO:0016585 | C | 3, 6, 7, 8, 9, 10, 11, 12, 13, | 2 | 0.390 (x 5.130) | 25 (0.080) | 0.198 | chromatin remodeling complex | Caf1 Top2 |
| 387 | GO:0016589 | C | 5, 8, 9, 10, 11, 12, 13, 14, 15, | 1 | 0.062 (x 16.032) | 4 (0.250) | 0.198 | NURF complex | Caf1 |
| 388 | GO:0007067 | P | 7, | 7 | 3.524 (x 1.986) | 226 (0.031) | 0.198 | mitosis | Cdk4 CycA Eip63E Nek2 Top2 betaTub56D polo |
| 389 | GO:0016573 | P | 9, 10, 12, | 1 | 0.062 (x 16.032) | 4 (0.250) | 0.198 | histone acetylation | Caf1 |
| 390 | GO:0016421 | F | 5, | 1 | 0.062 (x 16.032) | 4 (0.250) | 0.199 | CoA carboxylase activity | CG2118 |
| 391 | GO:0030173 | C | 6, 7, 8, 9, 10, 11, 12, | 1 | 0.062 (x 16.032) | 4 (0.250) | 0.199 | integral to Golgi membrane | Fur1 |
| 392 | GO:0031010 | C | 4, 7, 8, 9, 10, 11, 12, 13, 14, | 1 | 0.062 (x 16.032) | 4 (0.250) | 0.2 | ISWI complex | Caf1 |
| 393 | GO:0016328 | C | 5, 6, 7, | 1 | 0.062 (x 16.032) | 4 (0.250) | 0.2 | lateral plasma membrane | scrib |
| 394 | GO:0007111 | P | 6, 7, | 1 | 0.062 (x 16.032) | 4 (0.250) | 0.201 | cytokinesis after meiosis II | pbl |
| 395 | GO:0016879 | F | 4, | 6 | 2.838 (x 2.114) | 182 (0.033) | 0.201 | ligase activity, forming carbon-nitrogen bonds | CG15141 CG6767 CG6854 gft gol th |
| 396 | GO:0000087 | P | 6, | 7 | 3.540 (x 1.978) | 227 (0.031) | 0.201 | M phase of mitotic cell cycle | Cdk4 CycA Eip63E Nek2 Top2 betaTub56D polo |
| 397 | GO:0005515 | F | 3, | 26 | 19.242 (x 1.351) | 1234 (0.021) | 0.201 | protein binding | Aats-glupro Abi Akap200 B52 Brf CG15835 CG9057 CG9598 CREG Caf1 Cdk4 DnaJ-1 Dsp1 Eb1 Eip63E Lam Moe betaTub56D d edl endos fz rin scrib stai tkv |
| 398 | GO:0008078 | P | 6, 7, 8, 9, | 1 | 0.062 (x 16.032) | 4 (0.250) | 0.201 | mesodermal cell migration | pbl |
| 399 | GO:0030036 | P | 8, | 4 | 1.513 (x 2.645) | 97 (0.041) | 0.201 | actin cytoskeleton organization and biogenesis | Abi Moe Sb pbl |
| 400 | GO:0019219 | P | 6, | 20 | 13.847 (x 1.444) | 888 (0.023) | 0.202 | regulation of nucleobase, nucleoside, nucleotide and nucleic acid metabolism | B52 Brf CG15141 CG15835 CREG Caf1 D19A Dll Dr Dsp1 Scm ap ash2 dve edl gol grn klu sqd yps |
| 401 | GO:0030029 | P | 7, | 4 | 1.513 (x 2.645) | 97 (0.041) | 0.202 | actin filament-based process | Abi Moe Sb pbl |
| 402 | GO:0016529 | C | 6, 7, 8, 9, | 1 | 0.062 (x 16.032) | 4 (0.250) | 0.202 | sarcoplasmic reticulum | CBP |
| 403 | GO:0035263 | P | 4, 6, | 1 | 0.062 (x 16.032) | 4 (0.250) | 0.202 | genital disc sexually dimorphic development | dac |
| 404 | GO:0044262 | P | 6, | 8 | 4.288 (x 1.866) | 275 (0.029) | 0.203 | cellular carbohydrate metabolism | BEST:LD22483 CG1544 CG33138 CG6904 CG8036 Gapdh2 GlyP Idh |
| 405 | GO:0000775 | C | 5, 6, 7, 8, 9, 10, | 2 | 0.421 (x 4.750) | 27 (0.074) | 0.203 | chromosome, pericentric region | CG13895 polo |
| 406 | GO:0030371 | F | 3, | 1 | 0.062 (x 16.032) | 4 (0.250) | 0.203 | translation repressor activity | msi |
| 407 | GO:0007060 | P | 6, 9, | 1 | 0.062 (x 16.032) | 4 (0.250) | 0.203 | male meiosis chromosome segregation | polo |
| 408 | GO:0006915 | P | 6, | 6 | 2.776 (x 2.162) | 178 (0.034) | 0.204 | apoptosis | Aac11 CG10990 CG6680 gft smi35A th |
| 409 | GO:0016874 | F | 3, | 9 | 4.943 (x 1.821) | 317 (0.028) | 0.204 | ligase activity | Aats-glupro BcDNA:GH02901 CG15141 CG2118 CG6767 CG6854 gft gol th |
| 410 | GO:0051018 | F | 7, | 1 | 0.062 (x 16.032) | 4 (0.250) | 0.204 | protein kinase A binding | Akap200 |
| 411 | GO:0046670 | P | 7, 8, 9, | 1 | 0.062 (x 16.032) | 4 (0.250) | 0.204 | positive regulation of retinal programmed cell death | klu |
| 412 | GO:0000301 | P | 7, 8, 9, 10, | 1 | 0.062 (x 16.032) | 4 (0.250) | 0.205 | retrograde transport, vesicle recycling within Golgi | wbl |
| 413 | GO:0043283 | P | 5, | 34 | 26.259 (x 1.295) | 1684 (0.020) | 0.205 | biopolymer metabolism | Aats-glupro Arf79F B52 BcDNA:LD22910 CG15141 CG31617 CG33138 CG3605 CG4670 CG6904 Caf1 Cdk4 CkIIalpha CkIalpha Dsp1 Eip63E GNBP3 GlyP Hrb27C Mcm5 Mcm7 Nek2 Top2 Uch ash2 dpa gol msi polo smi35A sqd th tkv wbl |
| 414 | GO:0006006 | P | 8, 9, | 3 | 0.936 (x 3.206) | 60 (0.050) | 0.205 | glucose metabolism | BEST:LD22483 CG8036 Gapdh2 |
| 415 | GO:0019955 | F | 4, | 1 | 0.062 (x 16.032) | 4 (0.250) | 0.206 | cytokine binding | tkv |
| 416 | GO:0031468 | P | 7, 8, | 1 | 0.062 (x 16.032) | 4 (0.250) | 0.206 | nuclear envelope reassembly | Lam |
| 417 | GO:0046672 | P | 8, 9, 10, 11, | 1 | 0.062 (x 16.032) | 4 (0.250) | 0.207 | positive regulation of retinal cell programmed cell death (sensu Endopterygota) | klu |
| 418 | GO:0000042 | P | 8, 9, 10, 11, | 1 | 0.062 (x 16.032) | 4 (0.250) | 0.207 | protein targeting to Golgi | wbl |
| 419 | GO:0001704 | P | 5, 6, | 2 | 0.437 (x 4.581) | 28 (0.071) | 0.214 | formation of primary germ layer | gol pbl |
| 420 | GO:0019783 | F | 6, | 2 | 0.437 (x 4.581) | 28 (0.071) | 0.215 | small conjugating protein-specific protease activity | BcDNA:LD22910 Uch |
| 421 | GO:0017124 | F | 5, | 1 | 0.078 (x 12.826) | 5 (0.200) | 0.215 | SH3 domain binding | rin |
| 422 | GO:0043037 | P | 7, 8, | 6 | 2.900 (x 2.069) | 186 (0.032) | 0.215 | translation | Aats-glupro eIF-4B eIF3-S9 msi rin sqd |
| 423 | GO:0004843 | F | 7, | 2 | 0.437 (x 4.581) | 28 (0.071) | 0.215 | ubiquitin-specific protease activity | BcDNA:LD22910 Uch |
| 424 | GO:0007110 | P | 6, 7, | 1 | 0.078 (x 12.826) | 5 (0.200) | 0.215 | cytokinesis after meiosis I | pbl |
| 425 | GO:0016972 | F | 6, | 1 | 0.078 (x 12.826) | 5 (0.200) | 0.216 | thiol oxidase activity | CG4670 |
| 426 | GO:0006359 | P | 9, | 1 | 0.078 (x 12.826) | 5 (0.200) | 0.216 | regulation of transcription from RNA polymerase III promoter | Brf |
| 427 | GO:0006998 | P | 6, 7, | 1 | 0.078 (x 12.826) | 5 (0.200) | 0.217 | nuclear membrane organization and biogenesis | Lam |
| 428 | GO:0007451 | P | 6, 7, | 1 | 0.078 (x 12.826) | 5 (0.200) | 0.217 | dorsal/ventral lineage restriction, imaginal disc | ap |
| 429 | GO:0042325 | P | 8, | 1 | 0.078 (x 12.826) | 5 (0.200) | 0.218 | regulation of phosphorylation | edl |
| 430 | GO:0007344 | P | 6, 8, | 1 | 0.078 (x 12.826) | 5 (0.200) | 0.218 | pronuclear fusion | polo |
| 431 | GO:0006468 | P | 8, | 8 | 4.444 (x 1.800) | 285 (0.028) | 0.218 | protein amino acid phosphorylation | Cdk4 CkIIalpha CkIalpha Eip63E Nek2 polo smi35A tkv |
| 432 | GO:0009966 | P | 4, 5, | 4 | 1.606 (x 2.490) | 103 (0.039) | 0.218 | regulation of signal transduction | Akap200 CkIalpha edl klu |
| 433 | GO:0008368 | F | 4, | 1 | 0.078 (x 12.826) | 5 (0.200) | 0.219 | Gram-negative bacterial binding | GNBP3 |
| 434 | GO:0045887 | P | 5, 6, 8, 9, 10, 11, | 1 | 0.078 (x 12.826) | 5 (0.200) | 0.219 | positive regulation of synaptic growth at neuromuscular junction | tkv |
| 435 | GO:0006917 | P | 8, 9, | 3 | 0.967 (x 3.103) | 62 (0.048) | 0.219 | induction of apoptosis | CG10990 gft smi35A |
| 436 | GO:0043065 | P | 7, 8, | 3 | 0.998 (x 3.006) | 64 (0.047) | 0.22 | positive regulation of apoptosis | CG10990 gft smi35A |
| 437 | GO:0030071 | P | 8, 9, | 1 | 0.078 (x 12.826) | 5 (0.200) | 0.22 | regulation of mitotic metaphase/anaphase transition | CycA |
| 438 | GO:0007610 | P | 3, | 6 | 2.978 (x 2.015) | 191 (0.031) | 0.22 | behavior | CG8588 CkIIalpha lig scrib smi21F smi35A |
| 439 | GO:0046976 | F | 9, 10, | 1 | 0.078 (x 12.826) | 5 (0.200) | 0.22 | histone lysine N-methyltransferase activity (H3-K27 specific) | Caf1 |
| 440 | GO:0016778 | F | 5, | 1 | 0.078 (x 12.826) | 5 (0.200) | 0.221 | diphosphotransferase activity | CG6767 |
| 441 | GO:0000940 | C | 5, 6, 7, 8, 9, 10, 11, 12, 13, | 1 | 0.078 (x 12.826) | 5 (0.200) | 0.221 | outer kinetochore of condensed chromosome | polo |
| 442 | GO:0008063 | P | 6, | 2 | 0.468 (x 4.275) | 30 (0.067) | 0.221 | Toll signaling pathway | serpin-27A wbl |
| 443 | GO:0031974 | C | 2, | 11 | 6.721 (x 1.637) | 431 (0.026) | 0.221 | membrane-enclosed lumen | B52 Brf CG2118 Caf1 Hrb27C Mcm5 Mcm7 Top2 ap dac sqd |
| 444 | GO:0016971 | F | 7, | 1 | 0.078 (x 12.826) | 5 (0.200) | 0.222 | flavin-linked sulfhydryl oxidase activity | CG4670 |
| 445 | GO:0008283 | P | 4, | 8 | 4.397 (x 1.819) | 282 (0.028) | 0.222 | cell proliferation | Cdk4 CkIIalpha D19A dac eIF-4B klu scrib smi35A |
| 446 | GO:0048646 | P | 4, | 2 | 0.468 (x 4.275) | 30 (0.067) | 0.222 | anatomical structure formation | gol pbl |
| 447 | GO:0043233 | C | 3, 4, | 11 | 6.721 (x 1.637) | 431 (0.026) | 0.222 | organelle lumen | B52 Brf CG2118 Caf1 Hrb27C Mcm5 Mcm7 Top2 ap dac sqd |
| 448 | GO:0017026 | F | 7, | 1 | 0.078 (x 12.826) | 5 (0.200) | 0.222 | procollagen C-endopeptidase activity | tok |
| 449 | GO:0000279 | P | 5, | 8 | 4.475 (x 1.788) | 287 (0.028) | 0.222 | M phase | Cdk4 CycA Eip63E Nek2 Top2 betaTub56D pbl polo |
| 450 | GO:0030162 | P | 6, 7, 8, | 2 | 0.468 (x 4.275) | 30 (0.067) | 0.222 | regulation of proteolysis | CkIalpha gft |
| 451 | GO:0006732 | P | 6, | 7 | 3.727 (x 1.878) | 239 (0.029) | 0.223 | coenzyme metabolism | BEST:LD22483 CG10657 CG1544 CG31472 CG7461 CG8036 Idh |
| 452 | GO:0005024 | F | 6, 7, 9, 10, | 1 | 0.078 (x 12.826) | 5 (0.200) | 0.223 | transforming growth factor beta receptor activity | tkv |
| 453 | GO:0007091 | P | 8, | 1 | 0.078 (x 12.826) | 5 (0.200) | 0.223 | mitotic metaphase/anaphase transition | CycA |
| 454 | GO:0008623 | C | 4, 7, 8, 9, 10, 11, 12, 13, 14, | 1 | 0.078 (x 12.826) | 5 (0.200) | 0.224 | chromatin accessibility complex | Top2 |
| 455 | GO:0035098 | C | 4, 6, 7, 8, 9, 10, 11, 12, 13, 14, | 1 | 0.078 (x 12.826) | 5 (0.200) | 0.224 | ESC/E(Z) complex | Caf1 |
| 456 | GO:0016885 | F | 4, | 1 | 0.078 (x 12.826) | 5 (0.200) | 0.225 | ligase activity, forming carbon-carbon bonds | CG2118 |
| 457 | GO:0048522 | P | 4, | 6 | 2.947 (x 2.036) | 189 (0.032) | 0.225 | positive regulation of cellular process | CG10990 ash2 gft klu smi35A tkv |
| 458 | GO:0008367 | F | 3, | 1 | 0.078 (x 12.826) | 5 (0.200) | 0.225 | bacterial binding | GNBP3 |
| 459 | GO:0045165 | P | 4, | 6 | 2.947 (x 2.036) | 189 (0.032) | 0.226 | cell fate commitment | Dr ap fz msi rin tkv |
| 460 | GO:0005637 | C | 5, 6, 7, 8, 9, 10, 11, 12, 13, | 1 | 0.078 (x 12.826) | 5 (0.200) | 0.226 | nuclear inner membrane | Lam |
| 461 | GO:0000741 | P | 7, | 1 | 0.078 (x 12.826) | 5 (0.200) | 0.226 | karyogamy | polo |
| 462 | GO:0015926 | F | 6, | 2 | 0.483 (x 4.137) | 31 (0.065) | 0.232 | glucosidase activity | CG4670 GNBP3 |
| 463 | GO:0048598 | P | 4, | 4 | 1.653 (x 2.420) | 106 (0.038) | 0.232 | embryonic morphogenesis | Mtl pbl scrib tkv |
| 464 | GO:0007318 | P | 6, 9, 11, 12, 14, | 1 | 0.094 (x 10.688) | 6 (0.167) | 0.234 | pole plasm protein localization | Moe |
| 465 | GO:0000779 | C | 6, 7, 8, 9, 10, 11, | 1 | 0.094 (x 10.688) | 6 (0.167) | 0.234 | condensed chromosome, pericentric region | polo |
| 466 | GO:0016670 | F | 5, | 1 | 0.094 (x 10.688) | 6 (0.167) | 0.235 | oxidoreductase activity, acting on sulfur group of donors, oxygen as acceptor | CG4670 |
| 467 | GO:0030708 | P | 7, 8, 9, | 1 | 0.094 (x 10.688) | 6 (0.167) | 0.235 | female germ-line cyst encapsulation (sensu Insecta) | Scm |
| 468 | GO:0045198 | P | 6, 7, 8, 9, | 1 | 0.094 (x 10.688) | 6 (0.167) | 0.236 | establishment of epithelial cell polarity | fz |
| 469 | GO:0048190 | P | 6, 7, | 1 | 0.094 (x 10.688) | 6 (0.167) | 0.236 | wing disc dorsal/ventral pattern formation | ap |
| 470 | GO:0009794 | P | 5, 7, 8, | 1 | 0.094 (x 10.688) | 6 (0.167) | 0.237 | regulation of progression through embryonic mitotic cell cycle | CycA |
| 471 | GO:0000777 | C | 6, 7, 8, 9, 10, 11, 12, | 1 | 0.094 (x 10.688) | 6 (0.167) | 0.237 | condensed chromosome kinetochore | polo |
| 472 | GO:0008329 | F | 4, | 1 | 0.094 (x 10.688) | 6 (0.167) | 0.238 | pattern recognition receptor activity | GNBP3 |
| 473 | GO:0030859 | P | 5, 6, | 1 | 0.094 (x 10.688) | 6 (0.167) | 0.238 | polarized epithelial cell differentiation | fz |
| 474 | GO:0005956 | C | 3, 4, 5, 6, | 1 | 0.094 (x 10.688) | 6 (0.167) | 0.238 | protein kinase CK2 complex | CkIIalpha |
| 475 | GO:0015276 | F | 5, 6, | 3 | 1.045 (x 2.871) | 67 (0.045) | 0.239 | ligand-gated ion channel activity | CG8533 bib rpk |
| 476 | GO:0007487 | P | 6, | 1 | 0.094 (x 10.688) | 6 (0.167) | 0.239 | analia development (sensu Endopterygota) | Dll |
| 477 | GO:0044275 | P | 7, | 3 | 1.045 (x 2.871) | 67 (0.045) | 0.239 | cellular carbohydrate catabolism | BEST:LD22483 CG8036 Gapdh2 |
| 478 | GO:0005483 | F | 4, | 1 | 0.094 (x 10.688) | 6 (0.167) | 0.239 | soluble NSF attachment protein activity | Snap |
| 479 | GO:0042067 | P | 6, 7, 8, 9, | 2 | 0.515 (x 3.887) | 33 (0.061) | 0.239 | establishment of ommatidial polarity (sensu Endopterygota) | fz rin |
| 480 | GO:0016052 | P | 6, | 3 | 1.045 (x 2.871) | 67 (0.045) | 0.24 | carbohydrate catabolism | BEST:LD22483 CG8036 Gapdh2 |
| 481 | GO:0007297 | P | 6, 7, 9, | 2 | 0.515 (x 3.887) | 33 (0.061) | 0.24 | follicle cell migration (sensu Insecta) | sqd th |
| 482 | GO:0000307 | C | 3, 4, 5, 6, | 1 | 0.094 (x 10.688) | 6 (0.167) | 0.24 | cyclin-dependent protein kinase holoenzyme complex | CycA |
| 483 | GO:0001709 | P | 5, | 4 | 1.715 (x 2.332) | 110 (0.036) | 0.24 | cell fate determination | Dr fz msi tkv |
| 484 | GO:0005813 | C | 5, 6, 7, 8, 9, 10, | 2 | 0.515 (x 3.887) | 33 (0.061) | 0.24 | centrosome | Nek2 polo |
| 485 | GO:0016840 | F | 4, | 1 | 0.094 (x 10.688) | 6 (0.167) | 0.24 | carbon-nitrogen lyase activity | CG3590 |
| 486 | GO:0009611 | P | 4, | 2 | 0.515 (x 3.887) | 33 (0.061) | 0.241 | response to wounding | ninA serpin-27A |
| 487 | GO:0019908 | C | 4, 5, 6, 7, 8, 9, 10, | 1 | 0.094 (x 10.688) | 6 (0.167) | 0.241 | nuclear cyclin-dependent protein kinase holoenzyme complex | CycA |
| 488 | GO:0016918 | F | 4, 5, | 1 | 0.094 (x 10.688) | 6 (0.167) | 0.241 | retinal binding | CG10657 |
| 489 | GO:0016790 | F | 5, | 2 | 0.499 (x 4.008) | 32 (0.062) | 0.242 | thiolester hydrolase activity | BcDNA:LD22910 Uch |
| 490 | GO:0030496 | C | 3, 4, | 1 | 0.094 (x 10.688) | 6 (0.167) | 0.242 | midbody | Nek2 |
| 491 | GO:0007154 | P | 3, | 30 | 23.655 (x 1.268) | 1517 (0.020) | 0.242 | cell communication | Abi Akap200 Arf79F CG15835 CG17064 CG30440 CG6954 Cdk4 CkIIalpha CkIalpha Mtl Nek2 NetA Snap bib edl endos fax fra fz klu msi pbl polo rin scrib serpin-27A stai tkv wbl |
| 492 | GO:0045449 | P | 7, | 18 | 12.958 (x 1.389) | 831 (0.022) | 0.242 | regulation of transcription | Brf CG15141 CG15835 CREG Caf1 D19A Dll Dr Dsp1 Scm ap ash2 dve edl gol grn klu yps |
| 493 | GO:0006206 | P | 7, | 2 | 0.499 (x 4.008) | 32 (0.062) | 0.242 | pyrimidine base metabolism | CG6767 CG6854 |
| 494 | GO:0031324 | P | 6, | 5 | 2.417 (x 2.069) | 155 (0.032) | 0.242 | negative regulation of cellular metabolism | CREG Caf1 edl msi sqd |
| 495 | GO:0007517 | P | 4, | 4 | 1.684 (x 2.375) | 108 (0.037) | 0.242 | muscle development | Dr ap betaTub56D tkv |
| 496 | GO:0042026 | P | 8, | 1 | 0.094 (x 10.688) | 6 (0.167) | 0.242 | protein refolding | Hsp27 |
| 497 | GO:0043296 | C | 6, 7, 8, 9, | 2 | 0.499 (x 4.008) | 32 (0.062) | 0.243 | apical junction complex | Moe scrib |
| 498 | GO:0003676 | F | 3, | 34 | 27.179 (x 1.251) | 1743 (0.020) | 0.243 | nucleic acid binding | Aats-glupro B52 CG10423 CG10990 CG13895 CG15141 CG17838 CG31169 CG31617 CG6854 D19A Dll Dr Dsp1 Hrb27C Mcm5 Mcm7 NUCB1 Pep Top2 ap ash2 dpa dve eIF-4B eIF3-S9 edl esn grn klu msi rin sqd yps |
| 499 | GO:0015250 | F | 4, 5, | 1 | 0.094 (x 10.688) | 6 (0.167) | 0.243 | water channel activity | bib |
| 500 | GO:0045170 | C | 5, 6, 7, 8, | 1 | 0.094 (x 10.688) | 6 (0.167) | 0.244 | spectrosome | CycA |
| 501 | GO:0004867 | F | 6, | 3 | 1.092 (x 2.748) | 70 (0.043) | 0.244 | serine-type endopeptidase inhibitor activity | CG6680 Spn43Aa serpin-27A |
| 502 | GO:0008046 | F | 5, | 1 | 0.094 (x 10.688) | 6 (0.167) | 0.244 | axon guidance receptor activity | fra |
| 503 | GO:0012502 | P | 7, 8, | 3 | 1.092 (x 2.748) | 70 (0.043) | 0.244 | induction of programmed cell death | CG10990 gft smi35A |
| 504 | GO:0005678 | C | 4, 7, 8, 9, 10, 11, 12, 13, 14, | 1 | 0.094 (x 10.688) | 6 (0.167) | 0.245 | chromatin assembly complex | Caf1 |
| 505 | GO:0004672 | F | 6, | 8 | 4.600 (x 1.739) | 295 (0.027) | 0.245 | protein kinase activity | Cdk4 CkIIalpha CkIalpha Eip63E Nek2 polo smi35A tkv |
| 506 | GO:0006350 | P | 6, | 20 | 14.736 (x 1.357) | 945 (0.021) | 0.245 | transcription | Brf CG15141 CG15835 CREG Caf1 D19A Dll Dr Dsp1 Scm Top2 ap ash2 dac dve edl gol grn klu yps |
| 507 | GO:0045815 | P | 4, | 1 | 0.109 (x 9.161) | 7 (0.143) | 0.245 | positive regulation of gene expression, epigenetic | ash2 |
| 508 | GO:0004448 | F | 6, | 1 | 0.109 (x 9.161) | 7 (0.143) | 0.246 | isocitrate dehydrogenase activity | Idh |
| 509 | GO:0048806 | P | 4, | 1 | 0.109 (x 9.161) | 7 (0.143) | 0.246 | genitalia development | Dll |
| 510 | GO:0035102 | C | 4, 6, 7, 8, 9, 10, 11, | 1 | 0.109 (x 9.161) | 7 (0.143) | 0.247 | PRC1 complex | Scm |
| 511 | GO:0006265 | P | 7, | 1 | 0.109 (x 9.161) | 7 (0.143) | 0.247 | DNA topological change | Top2 |
| 512 | GO:0001751 | P | 6, 7, 8, 9, | 3 | 1.138 (x 2.635) | 73 (0.041) | 0.247 | eye photoreceptor cell differentiation (sensu Endopterygota) | Moe dac fz |
| 513 | GO:0048138 | P | 5, 6, | 1 | 0.109 (x 9.161) | 7 (0.143) | 0.248 | germ-line cyst encapsulation | Scm |
| 514 | GO:0051179 | P | 3, | 34 | 27.616 (x 1.231) | 1771 (0.019) | 0.248 | localization | Abi Akap200 Arf79F CG10657 CG10960 CG14439 CG15835 CG17419 CG2852 CG3823 CG9057 Eb1 Hrb27C Lam Moe NetA Snap ap betaTub56D bib d fra fz l(2)dtl ninA pbl polo rin rpk scrib sqd stai th wbl |
| 515 | GO:0017147 | F | 4, | 1 | 0.109 (x 9.161) | 7 (0.143) | 0.248 | Wnt-protein binding | fz |
| 516 | GO:0016624 | F | 5, | 1 | 0.109 (x 9.161) | 7 (0.143) | 0.248 | oxidoreductase activity, acting on the aldehyde or oxo group of donors, disulfide as acceptor | CG1544 |
| 517 | GO:0001763 | P | 4, | 2 | 0.530 (x 3.772) | 34 (0.059) | 0.248 | morphogenesis of a branching structure | Lam tkv |
| 518 | GO:0000922 | C | 5, 6, 7, 8, 9, 10, 11, | 1 | 0.109 (x 9.161) | 7 (0.143) | 0.249 | spindle pole | polo |
| 519 | GO:0009331 | C | 4, | 1 | 0.109 (x 9.161) | 7 (0.143) | 0.249 | glycerol-3-phosphate dehydrogenase complex | CG31169 |
| 520 | GO:0007484 | P | 5, 6, | 1 | 0.109 (x 9.161) | 7 (0.143) | 0.25 | genitalia development (sensu Endopterygota) | Dll |
| 521 | GO:0035097 | C | 3, 6, 7, 8, 9, 10, 11, 12, 13, | 1 | 0.109 (x 9.161) | 7 (0.143) | 0.25 | histone methyltransferase complex | Caf1 |
| 522 | GO:0016199 | P | 6, 8, 9, 11, 12, 14, | 1 | 0.109 (x 9.161) | 7 (0.143) | 0.251 | axon midline choice point recognition | fra |
| 523 | GO:0007286 | P | 5, 8, | 2 | 0.561 (x 3.563) | 36 (0.056) | 0.251 | spermatid development | Act5C th |
| 524 | GO:0004785 | F | 6, | 1 | 0.109 (x 9.161) | 7 (0.143) | 0.251 | copper, zinc superoxide dismutase activity | CG9027 |
| 525 | GO:0048515 | P | 4, 7, | 2 | 0.561 (x 3.563) | 36 (0.056) | 0.251 | spermatid differentiation | Act5C th |
| 526 | GO:0015629 | C | 6, 7, 8, 9, | 3 | 1.154 (x 2.600) | 74 (0.041) | 0.252 | actin cytoskeleton | Act42A Act5C d |
| 527 | GO:0048096 | P | 5, 10, 11, | 1 | 0.109 (x 9.161) | 7 (0.143) | 0.252 | chromatin-mediated maintenance of transcription | ash2 |
| 528 | GO:0007088 | P | 7, 8, | 2 | 0.561 (x 3.563) | 36 (0.056) | 0.252 | regulation of mitosis | CycA Nek2 |
| 529 | GO:0016209 | F | 2, | 2 | 0.561 (x 3.563) | 36 (0.056) | 0.252 | antioxidant activity | CG5873 Trxr-1 |
| 530 | GO:0016641 | F | 5, | 1 | 0.109 (x 9.161) | 7 (0.143) | 0.252 | oxidoreductase activity, acting on the CH-NH2 group of donors, oxygen as acceptor | CG31472 |
| 531 | GO:0048193 | P | 6, 7, 8, | 2 | 0.561 (x 3.563) | 36 (0.056) | 0.253 | Golgi vesicle transport | Snap wbl |
| 532 | GO:0030865 | P | 7, | 1 | 0.109 (x 9.161) | 7 (0.143) | 0.253 | cortical cytoskeleton organization and biogenesis | Abi |
| 533 | GO:0004467 | F | 6, | 1 | 0.109 (x 9.161) | 7 (0.143) | 0.253 | long-chain-fatty-acid-CoA ligase activity | BcDNA:GH02901 |
| 534 | GO:0035089 | P | 7, 8, | 1 | 0.109 (x 9.161) | 7 (0.143) | 0.254 | establishment of apical/basal cell polarity | fz |
| 535 | GO:0005524 | F | 6, | 15 | 10.479 (x 1.431) | 672 (0.022) | 0.254 | ATP binding | Aats-glupro CG2118 Cdk4 CkIIalpha CkIalpha Eip63E Mcm5 Mcm7 Nek2 Top2 d dpa polo smi35A tkv |
| 536 | GO:0051186 | P | 5, | 7 | 3.945 (x 1.774) | 253 (0.028) | 0.254 | cofactor metabolism | BEST:LD22483 CG10657 CG1544 CG31472 CG7461 CG8036 Idh |
| 537 | GO:0019838 | F | 4, | 1 | 0.109 (x 9.161) | 7 (0.143) | 0.254 | growth factor binding | tkv |
| 538 | GO:0007155 | P | 3, | 8 | 4.725 (x 1.693) | 303 (0.026) | 0.254 | cell adhesion | Arf79F CG17419 NetA fra fz ninA pbl scrib |
| 539 | GO:0030866 | P | 8, 9, | 1 | 0.109 (x 9.161) | 7 (0.143) | 0.255 | cortical actin cytoskeleton organization and biogenesis | Abi |
| 540 | GO:0048139 | P | 6, 7, | 1 | 0.109 (x 9.161) | 7 (0.143) | 0.255 | female germ-line cyst encapsulation | Scm |
| 541 | GO:0017076 | F | 4, | 18 | 13.239 (x 1.360) | 849 (0.021) | 0.255 | purine nucleotide binding | Aats-glupro Arf79F CG2118 Cdk4 CkIIalpha CkIalpha Eip63E Mcm5 Mcm7 Mtl Nek2 Top2 betaTub56D d dpa polo smi35A tkv |
| 542 | GO:0030145 | F | 6, | 1 | 0.109 (x 9.161) | 7 (0.143) | 0.256 | manganese ion binding | BcDNA:LD41548 |
| 543 | GO:0042766 | P | 10, | 1 | 0.109 (x 9.161) | 7 (0.143) | 0.256 | nucleosome mobilization | Caf1 |
| 544 | GO:0007620 | P | 6, 7, | 1 | 0.109 (x 9.161) | 7 (0.143) | 0.257 | copulation | lig |
| 545 | GO:0046974 | F | 9, 10, | 1 | 0.109 (x 9.161) | 7 (0.143) | 0.257 | histone lysine N-methyltransferase activity (H3-K9 specific) | Caf1 |
| 546 | GO:0046669 | P | 7, 8, 9, 10, | 1 | 0.109 (x 9.161) | 7 (0.143) | 0.258 | regulation of retinal cell programmed cell death (sensu Endopterygota) | klu |
| 547 | GO:0005501 | F | 4, | 1 | 0.109 (x 9.161) | 7 (0.143) | 0.258 | retinoid binding | CG10657 |
| 548 | GO:0009117 | P | 6, | 5 | 2.479 (x 2.017) | 159 (0.031) | 0.258 | nucleotide metabolism | BEST:LD22483 CG3590 CG6767 CG6854 CG8036 |
| 549 | GO:0006511 | P | 9, 10, 11, | 3 | 1.170 (x 2.565) | 75 (0.040) | 0.258 | ubiquitin-dependent protein catabolism | BcDNA:LD22910 Uch th |
| 550 | GO:0006268 | P | 9, | 1 | 0.109 (x 9.161) | 7 (0.143) | 0.259 | DNA unwinding during replication | Dsp1 |
| 551 | GO:0044264 | P | 6, 7, | 3 | 1.201 (x 2.499) | 77 (0.039) | 0.259 | cellular polysaccharide metabolism | CG33138 CG6904 GlyP |
| 552 | GO:0009081 | P | 7, 8, | 1 | 0.109 (x 9.161) | 7 (0.143) | 0.259 | branched chain family amino acid metabolism | CG2118 |
| 553 | GO:0016614 | F | 4, | 4 | 1.840 (x 2.174) | 118 (0.034) | 0.259 | oxidoreductase activity, acting on CH-OH group of donors | BEST:LD22483 CG31169 CG7675 Idh |
| 554 | GO:0043632 | P | 7, | 3 | 1.201 (x 2.499) | 77 (0.039) | 0.259 | modification-dependent macromolecule catabolism | BcDNA:LD22910 Uch th |
| 555 | GO:0019840 | F | 3, | 1 | 0.109 (x 9.161) | 7 (0.143) | 0.26 | isoprenoid binding | CG10657 |
| 556 | GO:0019094 | P | 6, 10, 12, 13, 15, | 2 | 0.577 (x 3.466) | 37 (0.054) | 0.26 | pole plasm mRNA localization | Hrb27C sqd |
| 557 | GO:0019941 | P | 8, 9, 10, | 3 | 1.201 (x 2.499) | 77 (0.039) | 0.26 | modification-dependent protein catabolism | BcDNA:LD22910 Uch th |
| 558 | GO:0015645 | F | 5, | 1 | 0.109 (x 9.161) | 7 (0.143) | 0.26 | fatty-acid ligase activity | BcDNA:GH02901 |
| 559 | GO:0000184 | P | 9, | 1 | 0.125 (x 8.016) | 8 (0.125) | 0.26 | mRNA catabolism, nonsense-mediated decay | sqd |
| 560 | GO:0016327 | C | 5, 6, 7, | 2 | 0.577 (x 3.466) | 37 (0.054) | 0.26 | apicolateral plasma membrane | Moe scrib |
| 561 | GO:0000785 | C | 5, 6, 7, 8, 9, 10, | 3 | 1.201 (x 2.499) | 77 (0.039) | 0.26 | chromatin | CG31617 Dsp1 sqd |
| 562 | GO:0045705 | P | 8, | 1 | 0.125 (x 8.016) | 8 (0.125) | 0.261 | negative regulation of salivary gland determination | tkv |
| 563 | GO:0048284 | P | 6, | 1 | 0.109 (x 9.161) | 7 (0.143) | 0.261 | organelle fusion | polo |
| 564 | GO:0005089 | F | 5, | 1 | 0.125 (x 8.016) | 8 (0.125) | 0.261 | Rho guanyl-nucleotide exchange factor activity | pbl |
| 565 | GO:0007584 | P | 5, 6, | 1 | 0.109 (x 9.161) | 7 (0.143) | 0.261 | response to nutrient | endos |
| 566 | GO:0006582 | P | 6, | 1 | 0.125 (x 8.016) | 8 (0.125) | 0.261 | melanin metabolism | serpin-27A |
| 567 | GO:0042393 | F | 4, | 1 | 0.109 (x 9.161) | 7 (0.143) | 0.262 | histone binding | Caf1 |
| 568 | GO:0045879 | P | 6, 7, 8, | 1 | 0.125 (x 8.016) | 8 (0.125) | 0.262 | negative regulation of smoothened signaling pathway | CkIalpha |
| 569 | GO:0006376 | P | 8, 11, 13, | 1 | 0.125 (x 8.016) | 8 (0.125) | 0.262 | mRNA splice site selection | B52 |
| 570 | GO:0000271 | P | 7, 8, | 1 | 0.125 (x 8.016) | 8 (0.125) | 0.263 | polysaccharide biosynthesis | CG6904 |
| 571 | GO:0043284 | P | 6, 7, | 1 | 0.125 (x 8.016) | 8 (0.125) | 0.263 | biopolymer biosynthesis | CG6904 |
| 572 | GO:0001754 | P | 5, 6, 7, | 3 | 1.185 (x 2.531) | 76 (0.039) | 0.263 | eye photoreceptor cell differentiation | Moe dac fz |
| 573 | GO:0006801 | P | 6, | 1 | 0.125 (x 8.016) | 8 (0.125) | 0.264 | superoxide metabolism | CG9027 |
| 574 | GO:0007391 | P | 6, | 3 | 1.185 (x 2.531) | 76 (0.039) | 0.264 | dorsal closure | Mtl scrib tkv |
| 575 | GO:0005372 | F | 3, | 1 | 0.125 (x 8.016) | 8 (0.125) | 0.264 | water transporter activity | bib |
| 576 | GO:0008235 | F | 6, | 3 | 1.185 (x 2.531) | 76 (0.039) | 0.264 | metalloexopeptidase activity | ApepP BcDNA:LD41548 fra |
| 577 | GO:0004067 | F | 6, | 1 | 0.125 (x 8.016) | 8 (0.125) | 0.265 | asparaginase activity | SP2637 |
| 578 | GO:0016721 | F | 4, | 1 | 0.125 (x 8.016) | 8 (0.125) | 0.265 | oxidoreductase activity, acting on superoxide radicals as acceptor | CG9027 |
| 579 | GO:0007242 | P | 5, | 12 | 8.171 (x 1.469) | 524 (0.023) | 0.265 | intracellular signaling cascade | Akap200 CG30440 Cdk4 CkIIalpha CkIalpha Mtl Nek2 edl klu polo rin stai |
| 580 | GO:0004365 | F | 7, | 1 | 0.125 (x 8.016) | 8 (0.125) | 0.266 | glyceraldehyde-3-phosphate dehydrogenase (phosphorylating) activity | Gapdh2 |
| 581 | GO:0009112 | P | 6, | 3 | 1.216 (x 2.467) | 78 (0.038) | 0.266 | nucleobase metabolism | CG3590 CG6767 CG6854 |
| 582 | GO:0030528 | F | 2, | 17 | 12.553 (x 1.354) | 805 (0.021) | 0.266 | transcription regulator activity | Brf CG15835 CG6854 CREG D19A Dll Dr Dsp1 Scm ap ash2 dac dve gol grn klu yps |
| 583 | GO:0045704 | P | 7, | 1 | 0.125 (x 8.016) | 8 (0.125) | 0.266 | regulation of salivary gland determination | tkv |
| 584 | GO:0004681 | F | 9, | 1 | 0.125 (x 8.016) | 8 (0.125) | 0.266 | casein kinase I activity | CkIalpha |
| 585 | GO:0000086 | P | 7, | 1 | 0.125 (x 8.016) | 8 (0.125) | 0.267 | G2/M transition of mitotic cell cycle | CycA |
| 586 | GO:0030554 | F | 5, | 15 | 10.744 (x 1.396) | 689 (0.022) | 0.267 | adenyl nucleotide binding | Aats-glupro CG2118 Cdk4 CkIIalpha CkIalpha Eip63E Mcm5 Mcm7 Nek2 Top2 d dpa polo smi35A tkv |
| 587 | GO:0004784 | F | 5, | 1 | 0.125 (x 8.016) | 8 (0.125) | 0.267 | superoxide dismutase activity | CG9027 |
| 588 | GO:0008943 | F | 6, | 1 | 0.125 (x 8.016) | 8 (0.125) | 0.268 | glyceraldehyde-3-phosphate dehydrogenase activity | Gapdh2 |
| 589 | GO:0005815 | C | 5, 6, 7, 8, | 2 | 0.608 (x 3.289) | 39 (0.051) | 0.268 | microtubule organizing center | Nek2 polo |
| 590 | GO:0007316 | P | 5, 9, 11, 12, 14, | 2 | 0.593 (x 3.375) | 38 (0.053) | 0.268 | pole plasm RNA localization | Hrb27C sqd |
| 591 | GO:0006916 | P | 8, 9, | 2 | 0.593 (x 3.375) | 38 (0.053) | 0.269 | anti-apoptosis | Aac11 th |
| 592 | GO:0003704 | F | 4, | 3 | 1.232 (x 2.435) | 79 (0.038) | 0.272 | specific RNA polymerase II transcription factor activity | Dll Scm ap |
| 593 | GO:0006259 | P | 6, | 9 | 5.785 (x 1.556) | 371 (0.024) | 0.272 | DNA metabolism | CG31617 Caf1 CkIalpha Dsp1 Mcm5 Mcm7 Top2 ash2 dpa |
| 594 | GO:0006402 | P | 8, | 1 | 0.140 (x 7.125) | 9 (0.111) | 0.274 | mRNA catabolism | sqd |
| 595 | GO:0003995 | F | 5, | 1 | 0.140 (x 7.125) | 9 (0.111) | 0.275 | acyl-CoA dehydrogenase activity | CG7461 |
| 596 | GO:0046845 | P | 5, 6, | 1 | 0.140 (x 7.125) | 9 (0.111) | 0.275 | branched duct epithelial cell fate determination (sensu Insecta) | tkv |
| 597 | GO:0046552 | P | 5, | 2 | 0.624 (x 3.206) | 40 (0.050) | 0.276 | photoreceptor cell fate commitment | fz rin |
| 598 | GO:0007509 | P | 7, 8, | 1 | 0.140 (x 7.125) | 9 (0.111) | 0.276 | mesoderm migration | pbl |
| 599 | GO:0019318 | P | 7, 8, | 3 | 1.247 (x 2.405) | 80 (0.037) | 0.276 | hexose metabolism | BEST:LD22483 CG8036 Gapdh2 |
| 600 | GO:0048589 | P | 3, | 2 | 0.624 (x 3.206) | 40 (0.050) | 0.276 | developmental growth | ninA tkv |
| 601 | GO:0000776 | C | 5, 6, 7, 8, 9, 10, 11, | 1 | 0.140 (x 7.125) | 9 (0.111) | 0.276 | kinetochore | polo |
| 602 | GO:0004177 | F | 6, | 2 | 0.624 (x 3.206) | 40 (0.050) | 0.277 | aminopeptidase activity | ApepP BcDNA:LD41548 |
| 603 | GO:0003730 | F | 6, | 1 | 0.140 (x 7.125) | 9 (0.111) | 0.277 | mRNA 3'-UTR binding | sqd |
| 604 | GO:0005351 | F | 5, 6, | 2 | 0.624 (x 3.206) | 40 (0.050) | 0.277 | sugar porter activity | CG10960 l(2)dtl |
| 605 | GO:0008069 | P | 6, 7, 9, | 1 | 0.140 (x 7.125) | 9 (0.111) | 0.277 | dorsal/ventral axis determination, follicular epithelium (sensu Insecta) | sqd |
| 606 | GO:0007459 | P | 6, | 2 | 0.624 (x 3.206) | 40 (0.050) | 0.277 | photoreceptor fate commitment (sensu Endopterygota) | fz rin |
| 607 | GO:0007618 | P | 5, 6, | 1 | 0.140 (x 7.125) | 9 (0.111) | 0.278 | mating | lig |
| 608 | GO:0007059 | P | 4, | 4 | 1.934 (x 2.069) | 124 (0.032) | 0.278 | chromosome segregation | CycA Eb1 betaTub56D polo |
| 609 | GO:0007464 | P | 7, 8, 9, 10, 11, | 1 | 0.140 (x 7.125) | 9 (0.111) | 0.278 | R3/R4 cell fate commitment | fz |
| 610 | GO:0048056 | P | 7, 8, 9, 10, | 1 | 0.140 (x 7.125) | 9 (0.111) | 0.278 | R3/R4 cell differentiation (sensu Endopterygota) | fz |
| 611 | GO:0035161 | P | 5, 6, | 1 | 0.140 (x 7.125) | 9 (0.111) | 0.279 | imaginal disc lineage restriction | ap |
| 612 | GO:0007430 | P | 5, 6, | 1 | 0.140 (x 7.125) | 9 (0.111) | 0.279 | terminal branching of trachea, cytoplasmic projection extension (sensu Insecta) | Lam |
| 613 | GO:0035251 | F | 6, 7, | 1 | 0.140 (x 7.125) | 9 (0.111) | 0.28 | UDP-glucosyltransferase activity | CG6904 |
| 614 | GO:0051276 | P | 6, | 5 | 2.682 (x 1.864) | 172 (0.029) | 0.28 | chromosome organization and biogenesis | CG31617 Caf1 Dsp1 Mcm5 ash2 |
| 615 | GO:0006072 | P | 8, | 1 | 0.140 (x 7.125) | 9 (0.111) | 0.28 | glycerol-3-phosphate metabolism | CG31169 |
| 616 | GO:0006221 | P | 7, 8, | 1 | 0.140 (x 7.125) | 9 (0.111) | 0.281 | pyrimidine nucleotide biosynthesis | CG6854 |
| 617 | GO:0016331 | P | 5, | 3 | 1.279 (x 2.346) | 82 (0.037) | 0.281 | morphogenesis of embryonic epithelium | Mtl scrib tkv |
| 618 | GO:0004289 | F | 7, | 1 | 0.140 (x 7.125) | 9 (0.111) | 0.281 | subtilase activity | Fur1 |
| 619 | GO:0005975 | P | 5, | 11 | 7.563 (x 1.454) | 485 (0.023) | 0.281 | carbohydrate metabolism | BEST:LD22483 CG10960 CG1544 CG31169 CG33138 CG6904 CG8036 GNBP3 Gapdh2 GlyP Idh |
| 620 | GO:0051603 | P | 8, 9, | 3 | 1.279 (x 2.346) | 82 (0.037) | 0.281 | proteolysis during cellular protein catabolism | BcDNA:LD22910 Uch th |
| 621 | GO:0040001 | P | 6, 7, 8, 10, | 1 | 0.140 (x 7.125) | 9 (0.111) | 0.282 | establishment of mitotic spindle localization | Eb1 |
| 622 | GO:0044257 | P | 7, 8, | 3 | 1.279 (x 2.346) | 82 (0.037) | 0.282 | cellular protein catabolism | BcDNA:LD22910 Uch th |
| 623 | GO:0043066 | P | 7, 8, | 2 | 0.655 (x 3.054) | 42 (0.048) | 0.285 | negative regulation of apoptosis | Aac11 th |
| 624 | GO:0000166 | F | 3, | 18 | 13.691 (x 1.315) | 878 (0.021) | 0.285 | nucleotide binding | Aats-glupro Arf79F CG2118 Cdk4 CkIIalpha CkIalpha Eip63E Mcm5 Mcm7 Mtl Nek2 Top2 betaTub56D d dpa polo smi35A tkv |
| 625 | GO:0043069 | P | 6, 7, | 2 | 0.655 (x 3.054) | 42 (0.048) | 0.285 | negative regulation of programmed cell death | Aac11 th |
| 626 | GO:0005874 | C | 5, 6, 7, 8, 9, 10, | 2 | 0.655 (x 3.054) | 42 (0.048) | 0.286 | microtubule | Eb1 betaTub56D |
| 627 | GO:0006338 | P | 10, | 2 | 0.655 (x 3.054) | 42 (0.048) | 0.286 | chromatin remodeling | Caf1 ash2 |
| 628 | GO:0001737 | P | 6, 7, 8, 9, 10, 11, | 1 | 0.156 (x 6.413) | 10 (0.100) | 0.288 | establishment of wing hair orientation | fz |
| 629 | GO:0051017 | P | 10, | 1 | 0.156 (x 6.413) | 10 (0.100) | 0.289 | actin filament bundle formation | Sb |
| 630 | GO:0006564 | P | 9, 10, | 1 | 0.156 (x 6.413) | 10 (0.100) | 0.289 | L-serine biosynthesis | ESTS:39C10S |
| 631 | GO:0004178 | F | 7, | 1 | 0.156 (x 6.413) | 10 (0.100) | 0.289 | leucyl aminopeptidase activity | BcDNA:LD41548 |
| 632 | GO:0008360 | P | 5, 6, | 3 | 1.325 (x 2.263) | 85 (0.035) | 0.29 | regulation of cell shape | Abi Arf79F pbl |
| 633 | GO:0006071 | P | 7, | 1 | 0.156 (x 6.413) | 10 (0.100) | 0.29 | glycerol metabolism | CG31169 |
| 634 | GO:0004866 | F | 5, | 3 | 1.325 (x 2.263) | 85 (0.035) | 0.29 | endopeptidase inhibitor activity | CG6680 Spn43Aa serpin-27A |
| 635 | GO:0008016 | P | 5, | 1 | 0.156 (x 6.413) | 10 (0.100) | 0.29 | regulation of heart contraction | scrib |
| 636 | GO:0019751 | P | 6, | 1 | 0.156 (x 6.413) | 10 (0.100) | 0.291 | polyol metabolism | CG31169 |
| 637 | GO:0001700 | P | 5, | 4 | 2.012 (x 1.989) | 129 (0.031) | 0.291 | embryonic development (sensu Insecta) | Eip63E Mtl scrib tkv |
| 638 | GO:0019901 | F | 6, | 1 | 0.156 (x 6.413) | 10 (0.100) | 0.291 | protein kinase binding | Akap200 |
| 639 | GO:0006457 | P | 7, | 4 | 2.043 (x 1.958) | 131 (0.031) | 0.292 | protein folding | CG2852 DnaJ-1 Hsp26 Hsp27 |
| 640 | GO:0030713 | P | 9, | 1 | 0.156 (x 6.413) | 10 (0.100) | 0.292 | stalk formation (sensu Insecta) | Scm |
| 641 | GO:0051653 | P | 5, 6, | 1 | 0.156 (x 6.413) | 10 (0.100) | 0.292 | spindle localization | Eb1 |
| 642 | GO:0016616 | F | 5, | 3 | 1.310 (x 2.290) | 84 (0.036) | 0.292 | oxidoreductase activity, acting on the CH-OH group of donors, NAD or NADP as acceptor | BEST:LD22483 CG31169 Idh |
| 643 | GO:0030010 | P | 6, 7, | 1 | 0.156 (x 6.413) | 10 (0.100) | 0.293 | establishment of cell polarity | fz |
| 644 | GO:0008015 | P | 4, | 1 | 0.156 (x 6.413) | 10 (0.100) | 0.293 | circulation | scrib |
| 645 | GO:0051293 | P | 6, 7, 9, | 1 | 0.156 (x 6.413) | 10 (0.100) | 0.294 | establishment of spindle localization | Eb1 |
| 646 | GO:0001736 | P | 5, 6, | 2 | 0.671 (x 2.983) | 43 (0.047) | 0.294 | establishment of planar polarity | fz rin |
| 647 | GO:0016458 | P | 6, | 2 | 0.686 (x 2.915) | 44 (0.045) | 0.294 | gene silencing | Caf1 Scm |
| 648 | GO:0006220 | P | 7, | 1 | 0.156 (x 6.413) | 10 (0.100) | 0.294 | pyrimidine nucleotide metabolism | CG6854 |
| 649 | GO:0007164 | P | 4, | 2 | 0.671 (x 2.983) | 43 (0.047) | 0.294 | establishment of tissue polarity | fz rin |
| 650 | GO:0006950 | P | 3, | 8 | 5.208 (x 1.536) | 334 (0.024) | 0.294 | response to stress | Caf1 CkIalpha DnaJ-1 Hsp26 Hsp27 ninA scrib serpin-27A |
| 651 | GO:0005911 | C | 6, 7, 8, | 2 | 0.686 (x 2.915) | 44 (0.045) | 0.294 | intercellular junction | Moe scrib |
| 652 | GO:0016332 | P | 6, | 1 | 0.156 (x 6.413) | 10 (0.100) | 0.294 | establishment and/or maintenance of polarity of embryonic epithelium | scrib |
| 653 | GO:0009952 | P | 4, | 4 | 2.027 (x 1.973) | 130 (0.031) | 0.294 | anterior/posterior pattern formation | Hrb27C Moe sqd tkv |
| 654 | GO:0051248 | P | 6, 7, | 2 | 0.671 (x 2.983) | 43 (0.047) | 0.295 | negative regulation of protein metabolism | msi sqd |
| 655 | GO:0030414 | F | 4, | 3 | 1.341 (x 2.237) | 86 (0.035) | 0.295 | protease inhibitor activity | CG6680 Spn43Aa serpin-27A |
| 656 | GO:0042051 | P | 7, 8, 9, 10, | 2 | 0.686 (x 2.915) | 44 (0.045) | 0.295 | eye photoreceptor development (sensu Endopterygota) | Moe dac |
| 657 | GO:0003916 | F | 4, 5, | 1 | 0.156 (x 6.413) | 10 (0.100) | 0.295 | DNA topoisomerase activity | Top2 |
| 658 | GO:0007467 | P | 5, | 3 | 1.357 (x 2.211) | 87 (0.034) | 0.301 | photoreceptor cell differentiation (sensu Endopterygota) | Moe dac fz |
| 659 | GO:0003774 | F | 2, | 3 | 1.357 (x 2.211) | 87 (0.034) | 0.301 | motor activity | Act42A Act5C d |
| 660 | GO:0042462 | P | 6, 7, 8, | 2 | 0.702 (x 2.850) | 45 (0.044) | 0.302 | eye photoreceptor cell development | Moe dac |
| 661 | GO:0019904 | F | 4, | 1 | 0.172 (x 5.830) | 11 (0.091) | 0.304 | protein domain specific binding | rin |
| 662 | GO:0042060 | P | 5, | 1 | 0.172 (x 5.830) | 11 (0.091) | 0.305 | wound healing | ninA |
| 663 | GO:0030855 | P | 4, 5, | 1 | 0.172 (x 5.830) | 11 (0.091) | 0.305 | epithelial cell differentiation | fz |
| 664 | GO:0006383 | P | 8, | 1 | 0.172 (x 5.830) | 11 (0.091) | 0.306 | transcription from RNA polymerase III promoter | Brf |
| 665 | GO:0045186 | P | 8, 9, | 1 | 0.172 (x 5.830) | 11 (0.091) | 0.306 | zonula adherens assembly | scrib |
| 666 | GO:0006563 | P | 8, 9, | 1 | 0.172 (x 5.830) | 11 (0.091) | 0.306 | L-serine metabolism | ESTS:39C10S |
| 667 | GO:0031461 | C | 4, 5, 6, 7, | 1 | 0.172 (x 5.830) | 11 (0.091) | 0.307 | cullin-RING ubiquitin ligase complex | gft |
| 668 | GO:0030509 | P | 8, | 1 | 0.172 (x 5.830) | 11 (0.091) | 0.307 | BMP signaling pathway | tkv |
| 669 | GO:0019005 | C | 5, 6, 7, 8, | 1 | 0.172 (x 5.830) | 11 (0.091) | 0.308 | SCF ubiquitin ligase complex | gft |
| 670 | GO:0007267 | P | 4, | 10 | 6.986 (x 1.431) | 448 (0.022) | 0.308 | cell-cell signaling | Arf79F CG15835 CG17064 NetA Snap fax fz msi scrib stai |
| 671 | GO:0046668 | P | 6, 7, 8, | 1 | 0.172 (x 5.830) | 11 (0.091) | 0.308 | regulation of retinal programmed cell death | klu |
| 672 | GO:0008134 | F | 4, | 3 | 1.388 (x 2.162) | 89 (0.034) | 0.309 | transcription factor binding | Brf CG15835 Dsp1 |
| 673 | GO:0030718 | P | 4, | 1 | 0.172 (x 5.830) | 11 (0.091) | 0.309 | germ-line stem cell maintenance | tkv |
| 674 | GO:0008237 | F | 5, | 5 | 2.885 (x 1.733) | 185 (0.027) | 0.31 | metallopeptidase activity | ApepP BcDNA:LD41548 D19A fra tok |
| 675 | GO:0008104 | P | 4, | 12 | 8.826 (x 1.360) | 566 (0.021) | 0.31 | protein localization | Akap200 Arf79F CG2852 Moe Snap betaTub56D d fz polo rin scrib wbl |
| 676 | GO:0046527 | F | 6, | 1 | 0.187 (x 5.344) | 12 (0.083) | 0.31 | glucosyltransferase activity | CG6904 |
| 677 | GO:0046785 | P | 8, 10, | 1 | 0.187 (x 5.344) | 12 (0.083) | 0.311 | microtubule polymerization | betaTub56D |
| 678 | GO:0000915 | P | 7, 11, | 1 | 0.187 (x 5.344) | 12 (0.083) | 0.311 | cytokinesis, contractile ring formation | pbl |
| 679 | GO:0051321 | P | 5, | 3 | 1.435 (x 2.091) | 92 (0.033) | 0.311 | meiotic cell cycle | Top2 pbl polo |
| 680 | GO:0046667 | P | 7, 8, 9, | 1 | 0.187 (x 5.344) | 12 (0.083) | 0.312 | retinal cell programmed cell death (sensu Endopterygota) | klu |
| 681 | GO:0009060 | P | 8, | 2 | 0.733 (x 2.729) | 47 (0.043) | 0.312 | aerobic respiration | CG1544 Idh |
| 682 | GO:0000912 | P | 6, 10, | 1 | 0.187 (x 5.344) | 12 (0.083) | 0.312 | cytokinesis, formation of actomyosin apparatus | pbl |
| 683 | GO:0006355 | P | 8, | 16 | 12.272 (x 1.304) | 787 (0.020) | 0.312 | regulation of transcription, DNA-dependent | Brf CG15141 CG15835 Caf1 D19A Dll Dr Dsp1 ap ash2 dve edl gol grn klu yps |
| 684 | GO:0016301 | F | 5, | 9 | 6.253 (x 1.439) | 401 (0.022) | 0.312 | kinase activity | CG6767 Cdk4 CkIIalpha CkIalpha Eip63E Nek2 polo smi35A tkv |
| 685 | GO:0006099 | P | 8, 9, | 2 | 0.733 (x 2.729) | 47 (0.043) | 0.312 | tricarboxylic acid cycle | CG1544 Idh |
| 686 | GO:0006091 | P | 5, | 11 | 7.875 (x 1.397) | 505 (0.022) | 0.313 | generation of precursor metabolites and energy | BEST:LD22483 CG1544 CG33138 CG4670 CG6904 CG7461 CG8036 Gapdh2 GlyP Idh Trxr-1 |
| 687 | GO:0009991 | P | 4, | 1 | 0.187 (x 5.344) | 12 (0.083) | 0.313 | response to extracellular stimulus | endos |
| 688 | GO:0030030 | P | 5, 6, | 2 | 0.733 (x 2.729) | 47 (0.043) | 0.313 | cell projection organization and biogenesis | Abi fz |
| 689 | GO:0031523 | C | 3, 5, 6, 7, 8, 9, 10, | 1 | 0.187 (x 5.344) | 12 (0.083) | 0.313 | Myb complex | Caf1 |
| 690 | GO:0040007 | P | 2, | 3 | 1.403 (x 2.138) | 90 (0.033) | 0.313 | growth | Cdk4 ninA tkv |
| 691 | GO:0015630 | C | 6, 7, 8, 9, | 5 | 2.900 (x 1.724) | 186 (0.027) | 0.313 | microtubule cytoskeleton | Eb1 Nek2 betaTub56D polo stai |
| 692 | GO:0046356 | P | 8, | 2 | 0.733 (x 2.729) | 47 (0.043) | 0.313 | acetyl-CoA catabolism | CG1544 Idh |
| 693 | GO:0048100 | P | 6, 7, | 1 | 0.187 (x 5.344) | 12 (0.083) | 0.313 | wing disc anterior/posterior pattern formation | tkv |
| 694 | GO:0005794 | C | 5, 6, 7, 8, | 3 | 1.403 (x 2.138) | 90 (0.033) | 0.314 | Golgi apparatus | Fur1 NUCB1 Snap |
| 695 | GO:0008092 | F | 4, | 6 | 3.711 (x 1.617) | 238 (0.025) | 0.314 | cytoskeletal protein binding | Aats-glupro Eb1 Moe betaTub56D d stai |
| 696 | GO:0051119 | F | 4, | 2 | 0.733 (x 2.729) | 47 (0.043) | 0.314 | sugar transporter activity | CG10960 l(2)dtl |
| 697 | GO:0045786 | P | 6, 7, | 1 | 0.187 (x 5.344) | 12 (0.083) | 0.314 | negative regulation of progression through cell cycle | CycA |
| 698 | GO:0045333 | P | 7, | 2 | 0.733 (x 2.729) | 47 (0.043) | 0.314 | cellular respiration | CG1544 Idh |
| 699 | GO:0016082 | P | 8, 9, 10, | 1 | 0.187 (x 5.344) | 12 (0.083) | 0.314 | synaptic vesicle priming | Snap |
| 700 | GO:0016638 | F | 4, | 1 | 0.187 (x 5.344) | 12 (0.083) | 0.315 | oxidoreductase activity, acting on the CH-NH2 group of donors | CG31472 |
| 701 | GO:0006323 | P | 7, | 4 | 2.152 (x 1.859) | 138 (0.029) | 0.315 | DNA packaging | CG31617 Caf1 Dsp1 ash2 |
| 702 | GO:0051327 | P | 6, | 3 | 1.419 (x 2.114) | 91 (0.033) | 0.315 | M phase of meiotic cell cycle | Top2 pbl polo |
| 703 | GO:0008589 | P | 5, 6, 7, | 1 | 0.187 (x 5.344) | 12 (0.083) | 0.315 | regulation of smoothened signaling pathway | CkIalpha |
| 704 | GO:0000381 | P | 10, 11, 13, | 2 | 0.764 (x 2.618) | 49 (0.041) | 0.315 | regulation of alternative nuclear mRNA splicing, via spliceosome | B52 sqd |
| 705 | GO:0006325 | P | 8, | 4 | 2.152 (x 1.859) | 138 (0.029) | 0.315 | establishment and/or maintenance of chromatin architecture | CG31617 Caf1 Dsp1 ash2 |
| 706 | GO:0015631 | F | 5, | 3 | 1.419 (x 2.114) | 91 (0.033) | 0.316 | tubulin binding | Eb1 betaTub56D stai |
| 707 | GO:0016198 | P | 5, 7, 8, 10, 11, 13, | 1 | 0.187 (x 5.344) | 12 (0.083) | 0.316 | axon choice point recognition | fra |
| 708 | GO:0008298 | P | 5, | 2 | 0.764 (x 2.618) | 49 (0.041) | 0.316 | intracellular mRNA localization | Hrb27C sqd |
| 709 | GO:0006952 | P | 4, | 11 | 8.015 (x 1.372) | 514 (0.021) | 0.316 | defense response | CG2852 CG5873 CG9027 DnaJ-1 GNBP3 Hsp26 Hsp27 Sb fra scrib serpin-27A |
| 710 | GO:0006366 | P | 8, | 14 | 10.635 (x 1.316) | 682 (0.021) | 0.316 | transcription from RNA polymerase II promoter | Brf CG15835 Caf1 D19A Dll Dr Dsp1 Top2 ap ash2 gol grn klu yps |
| 711 | GO:0031667 | P | 5, | 1 | 0.187 (x 5.344) | 12 (0.083) | 0.316 | response to nutrient levels | endos |
| 712 | GO:0005912 | C | 6, 7, 8, | 2 | 0.764 (x 2.618) | 49 (0.041) | 0.316 | adherens junction | Moe scrib |
| 713 | GO:0008045 | P | 7, 8, 10, 11, 13, | 1 | 0.187 (x 5.344) | 12 (0.083) | 0.317 | motor axon guidance | fra |
| 714 | GO:0008594 | P | 6, 7, 8, | 2 | 0.764 (x 2.618) | 49 (0.041) | 0.317 | photoreceptor cell morphogenesis (sensu Endopterygota) | Moe dac |
| 715 | GO:0031032 | P | 9, | 1 | 0.187 (x 5.344) | 12 (0.083) | 0.317 | actomyosin structure organization and biogenesis | pbl |
| 716 | GO:0000380 | P | 10, 12, | 2 | 0.764 (x 2.618) | 49 (0.041) | 0.317 | alternative nuclear mRNA splicing, via spliceosome | B52 sqd |
| 717 | GO:0045298 | C | 3, 5, 6, 7, 8, 9, 10, 11, | 1 | 0.187 (x 5.344) | 12 (0.083) | 0.318 | tubulin | betaTub56D |
| 718 | GO:0007420 | P | 4, 6, | 2 | 0.764 (x 2.618) | 49 (0.041) | 0.318 | brain development | Dr dac |
| 719 | GO:0009109 | P | 7, | 2 | 0.748 (x 2.672) | 48 (0.042) | 0.318 | coenzyme catabolism | CG1544 Idh |
| 720 | GO:0051187 | P | 6, | 2 | 0.764 (x 2.618) | 49 (0.041) | 0.318 | cofactor catabolism | CG1544 Idh |
| 721 | GO:0006084 | P | 7, | 2 | 0.748 (x 2.672) | 48 (0.042) | 0.318 | acetyl-CoA metabolism | CG1544 Idh |
| 722 | GO:0005200 | F | 3, | 7 | 4.553 (x 1.537) | 292 (0.024) | 0.318 | structural constituent of cytoskeleton | Act42A Act5C Lam Moe betaTub56D d esn |
| 723 | GO:0019787 | F | 6, | 4 | 2.167 (x 1.845) | 139 (0.029) | 0.318 | small conjugating protein ligase activity | CG15141 gft gol th |
| 724 | GO:0004842 | F | 7, | 4 | 2.167 (x 1.845) | 139 (0.029) | 0.319 | ubiquitin-protein ligase activity | CG15141 gft gol th |
| 725 | GO:0045448 | P | 4, 6, | 1 | 0.203 (x 4.933) | 13 (0.077) | 0.321 | mitotic cell cycle, embryonic | CycA |
| 726 | GO:0016571 | P | 9, 10, 12, | 1 | 0.203 (x 4.933) | 13 (0.077) | 0.322 | histone methylation | Caf1 |
| 727 | GO:0035286 | P | 5, 6, 7, | 1 | 0.203 (x 4.933) | 13 (0.077) | 0.322 | leg segmentation | ap |
| 728 | GO:0045169 | C | 5, 6, 7, 8, | 1 | 0.203 (x 4.933) | 13 (0.077) | 0.322 | fusome | CycA |
| 729 | GO:0006891 | P | 6, 7, 8, 9, | 1 | 0.203 (x 4.933) | 13 (0.077) | 0.323 | intra-Golgi vesicle-mediated transport | wbl |
| 730 | GO:0019220 | P | 7, | 1 | 0.203 (x 4.933) | 13 (0.077) | 0.323 | regulation of phosphate metabolism | edl |
| 731 | GO:0016278 | F | 7, | 1 | 0.203 (x 4.933) | 13 (0.077) | 0.324 | lysine N-methyltransferase activity | Caf1 |
| 732 | GO:0019900 | F | 5, | 1 | 0.203 (x 4.933) | 13 (0.077) | 0.324 | kinase binding | Akap200 |
| 733 | GO:0016279 | F | 7, 8, | 1 | 0.203 (x 4.933) | 13 (0.077) | 0.325 | protein-lysine N-methyltransferase activity | Caf1 |
| 734 | GO:0051174 | P | 6, | 1 | 0.203 (x 4.933) | 13 (0.077) | 0.325 | regulation of phosphorus metabolism | edl |
| 735 | GO:0007338 | P | 5, | 1 | 0.203 (x 4.933) | 13 (0.077) | 0.325 | fertilization (sensu Metazoa) | polo |
| 736 | GO:0008582 | P | 4, 5, 7, 8, 9, 10, | 1 | 0.203 (x 4.933) | 13 (0.077) | 0.326 | regulation of synaptic growth at neuromuscular junction | tkv |
| 737 | GO:0035285 | P | 4, 5, | 1 | 0.203 (x 4.933) | 13 (0.077) | 0.326 | appendage segmentation | ap |
| 738 | GO:0008533 | F | 7, | 1 | 0.203 (x 4.933) | 13 (0.077) | 0.327 | astacin activity | tok |
| 739 | GO:0031497 | P | 10, | 2 | 0.795 (x 2.515) | 51 (0.039) | 0.327 | chromatin assembly | CG31617 Caf1 |
| 740 | GO:0006473 | P | 9, | 1 | 0.203 (x 4.933) | 13 (0.077) | 0.327 | protein amino acid acetylation | Caf1 |
| 741 | GO:0018024 | F | 8, 9, | 1 | 0.203 (x 4.933) | 13 (0.077) | 0.328 | histone-lysine N-methyltransferase activity | Caf1 |
| 742 | GO:0051649 | P | 5, 6, | 13 | 9.902 (x 1.313) | 635 (0.020) | 0.33 | establishment of cellular localization | Arf79F CG2852 CG9057 Eb1 Lam Snap betaTub56D d polo rin scrib sqd wbl |
| 743 | GO:0009607 | P | 3, | 11 | 8.140 (x 1.351) | 522 (0.021) | 0.33 | response to biotic stimulus | CG2852 CG5873 CG9027 DnaJ-1 GNBP3 Hsp26 Hsp27 Sb fra scrib serpin-27A |
| 744 | GO:0051641 | P | 4, 5, | 13 | 9.917 (x 1.311) | 636 (0.020) | 0.331 | cellular localization | Arf79F CG2852 CG9057 Eb1 Lam Snap betaTub56D d polo rin scrib sqd wbl |
| 745 | GO:0050803 | P | 7, | 1 | 0.218 (x 4.581) | 14 (0.071) | 0.333 | regulation of synapse structure and function | scrib |
| 746 | GO:0004556 | F | 7, | 1 | 0.218 (x 4.581) | 14 (0.071) | 0.334 | alpha-amylase activity | CG33138 |
| 747 | GO:0016773 | F | 5, | 8 | 5.567 (x 1.437) | 357 (0.022) | 0.334 | phosphotransferase activity, alcohol group as acceptor | Cdk4 CkIIalpha CkIalpha Eip63E Nek2 polo smi35A tkv |
| 748 | GO:0016197 | P | 6, 7, 8, | 1 | 0.218 (x 4.581) | 14 (0.071) | 0.334 | endosome transport | Arf79F |
| 749 | GO:0005686 | C | 5, 6, 7, 8, 9, 10, 11, 12, | 1 | 0.218 (x 4.581) | 14 (0.071) | 0.335 | snRNP U2 | CG3605 |
| 750 | GO:0019899 | F | 4, | 2 | 0.811 (x 2.467) | 52 (0.038) | 0.335 | enzyme binding | Akap200 Caf1 |
| 751 | GO:0000245 | P | 7, 10, 12, | 1 | 0.218 (x 4.581) | 14 (0.071) | 0.335 | spliceosome assembly | B52 |
| 752 | GO:0016160 | F | 6, | 1 | 0.218 (x 4.581) | 14 (0.071) | 0.335 | amylase activity | CG33138 |
| 753 | GO:0016203 | P | 5, | 1 | 0.218 (x 4.581) | 14 (0.071) | 0.336 | muscle attachment | betaTub56D |
| 754 | GO:0042052 | P | 8, 9, 10, 11, | 1 | 0.218 (x 4.581) | 14 (0.071) | 0.336 | rhabdomere development | Moe |
| 755 | GO:0050684 | P | 8, 9, | 2 | 0.826 (x 2.420) | 53 (0.038) | 0.336 | regulation of mRNA processing | B52 sqd |
| 756 | GO:0009070 | P | 8, 9, | 1 | 0.218 (x 4.581) | 14 (0.071) | 0.337 | serine family amino acid biosynthesis | ESTS:39C10S |
| 757 | GO:0048024 | P | 9, 10, 12, | 2 | 0.826 (x 2.420) | 53 (0.038) | 0.337 | regulation of nuclear mRNA splicing, via spliceosome | B52 sqd |
| 758 | GO:0009566 | P | 4, | 1 | 0.218 (x 4.581) | 14 (0.071) | 0.337 | fertilization | polo |
| 759 | GO:0035317 | P | 7, 8, 9, 10, | 1 | 0.218 (x 4.581) | 14 (0.071) | 0.338 | wing hair organization and biogenesis | fz |
| 760 | GO:0004553 | F | 5, | 3 | 1.528 (x 1.963) | 98 (0.031) | 0.338 | hydrolase activity, hydrolyzing O-glycosyl compounds | CG33138 CG4670 GNBP3 |
| 761 | GO:0006397 | P | 8, | 5 | 3.088 (x 1.619) | 198 (0.025) | 0.338 | mRNA processing | B52 CG3605 Hrb27C msi sqd |
| 762 | GO:0007448 | P | 5, 6, | 1 | 0.218 (x 4.581) | 14 (0.071) | 0.338 | anterior/posterior pattern formation, imaginal disc | tkv |
| 763 | GO:0051124 | P | 4, 6, 7, 8, 9, | 1 | 0.218 (x 4.581) | 14 (0.071) | 0.339 | synaptic growth at neuromuscular junction | tkv |
| 764 | GO:0006508 | P | 7, | 15 | 11.820 (x 1.269) | 758 (0.020) | 0.339 | proteolysis | ApepP BcDNA:LD22910 BcDNA:LD41548 CG6680 CkIalpha D19A Fur1 Sb Uch deltaTry fra gft gol th tok |
| 765 | GO:0016360 | P | 6, | 1 | 0.218 (x 4.581) | 14 (0.071) | 0.339 | sensory organ precursor cell fate determination | fz |
| 766 | GO:0007165 | P | 4, | 24 | 20.131 (x 1.192) | 1291 (0.019) | 0.342 | signal transduction | Abi Akap200 Arf79F CG15835 CG30440 CG6954 Cdk4 CkIIalpha CkIalpha Mtl Nek2 NetA bib edl fra fz klu pbl polo rin serpin-27A stai tkv wbl |
| 767 | GO:0006333 | P | 9, | 3 | 1.559 (x 1.924) | 100 (0.030) | 0.342 | chromatin assembly or disassembly | CG31617 Caf1 Dsp1 |
| 768 | GO:0006464 | P | 7, | 17 | 13.675 (x 1.243) | 877 (0.019) | 0.342 | protein modification | Arf79F BcDNA:LD22910 CG15141 CG4670 Caf1 Cdk4 CkIIalpha CkIalpha Eip63E Nek2 Uch gol polo smi35A th tkv wbl |
| 769 | GO:0005996 | P | 6, 7, | 3 | 1.559 (x 1.924) | 100 (0.030) | 0.343 | monosaccharide metabolism | BEST:LD22483 CG8036 Gapdh2 |
| 770 | GO:0051252 | P | 7, | 2 | 0.842 (x 2.375) | 54 (0.037) | 0.343 | regulation of RNA metabolism | B52 sqd |
| 771 | GO:0005355 | F | 7, | 1 | 0.234 (x 4.275) | 15 (0.067) | 0.346 | glucose transporter activity | CG10960 |
| 772 | GO:0007307 | P | 9, 10, 11, | 1 | 0.234 (x 4.275) | 15 (0.067) | 0.347 | chorion gene amplification | Caf1 |
| 773 | GO:0000786 | C | 3, 5, 6, 7, 8, 9, 10, 11, | 1 | 0.234 (x 4.275) | 15 (0.067) | 0.347 | nucleosome | CG31617 |
| 774 | GO:0000793 | C | 6, 7, 8, 9, | 1 | 0.234 (x 4.275) | 15 (0.067) | 0.348 | condensed chromosome | polo |
| 775 | GO:0006144 | P | 7, | 2 | 0.858 (x 2.332) | 55 (0.036) | 0.348 | purine base metabolism | CG3590 CG6767 |
| 776 | GO:0019827 | P | 3, | 1 | 0.234 (x 4.275) | 15 (0.067) | 0.348 | stem cell maintenance | tkv |
| 777 | GO:0005852 | C | 3, 4, 5, 6, 7, 8, 9, | 1 | 0.234 (x 4.275) | 15 (0.067) | 0.348 | eukaryotic translation initiation factor 3 complex | eIF3-S9 |
| 778 | GO:0008285 | P | 6, | 1 | 0.234 (x 4.275) | 15 (0.067) | 0.349 | negative regulation of cell proliferation | scrib |
| 779 | GO:0007432 | P | 6, | 1 | 0.234 (x 4.275) | 15 (0.067) | 0.349 | salivary gland determination | tkv |
| 780 | GO:0006963 | P | 7, 8, 9, | 1 | 0.234 (x 4.275) | 15 (0.067) | 0.35 | positive regulation of antibacterial peptide biosynthesis | scrib |
| 781 | GO:0006888 | P | 6, 7, 8, 9, | 1 | 0.234 (x 4.275) | 15 (0.067) | 0.35 | ER to Golgi vesicle-mediated transport | Snap |
| 782 | GO:0006631 | P | 6, 7, | 3 | 1.606 (x 1.868) | 103 (0.029) | 0.356 | fatty acid metabolism | BcDNA:GH02901 CG2118 CG7461 |
| 783 | GO:0003743 | F | 4, 5, | 2 | 0.873 (x 2.290) | 56 (0.036) | 0.356 | translation initiation factor activity | eIF-4B eIF3-S9 |
| 784 | GO:0043543 | P | 8, | 1 | 0.249 (x 4.008) | 16 (0.062) | 0.36 | protein amino acid acylation | Caf1 |
| 785 | GO:0016564 | F | 3, | 2 | 0.889 (x 2.250) | 57 (0.035) | 0.36 | transcriptional repressor activity | CREG Dsp1 |
| 786 | GO:0006277 | P | 8, | 1 | 0.249 (x 4.008) | 16 (0.062) | 0.36 | DNA amplification | Caf1 |
| 787 | GO:0046666 | P | 6, 7, | 1 | 0.249 (x 4.008) | 16 (0.062) | 0.361 | retinal cell programmed cell death | klu |
| 788 | GO:0042054 | F | 7, | 1 | 0.249 (x 4.008) | 16 (0.062) | 0.361 | histone methyltransferase activity | Caf1 |
| 789 | GO:0016071 | P | 7, | 5 | 3.243 (x 1.542) | 208 (0.024) | 0.361 | mRNA metabolism | B52 CG3605 Hrb27C msi sqd |
| 790 | GO:0016620 | F | 5, | 1 | 0.249 (x 4.008) | 16 (0.062) | 0.361 | oxidoreductase activity, acting on the aldehyde or oxo group of donors, NAD or NADP as acceptor | Gapdh2 |
| 791 | GO:0009628 | P | 3, | 8 | 5.801 (x 1.379) | 372 (0.022) | 0.362 | response to abiotic stimulus | CG8588 DnaJ-1 Hsp26 Hsp27 endos scrib smi21F smi35A |
| 792 | GO:0009605 | P | 3, | 3 | 1.637 (x 1.832) | 105 (0.029) | 0.362 | response to external stimulus | endos ninA serpin-27A |
| 793 | GO:0016538 | F | 5, | 1 | 0.249 (x 4.008) | 16 (0.062) | 0.362 | cyclin-dependent protein kinase regulator activity | CycA |
| 794 | GO:0051347 | P | 5, | 1 | 0.249 (x 4.008) | 16 (0.062) | 0.362 | positive regulation of transferase activity | Abi |
| 795 | GO:0045860 | P | 6, 7, | 1 | 0.249 (x 4.008) | 16 (0.062) | 0.363 | positive regulation of protein kinase activity | Abi |
| 796 | GO:0007291 | P | 6, 9, | 1 | 0.249 (x 4.008) | 16 (0.062) | 0.363 | sperm individualization | Act5C |
| 797 | GO:0000152 | C | 4, 5, 6, 7, 8, 9, 10, | 1 | 0.249 (x 4.008) | 16 (0.062) | 0.364 | nuclear ubiquitin ligase complex | gft |
| 798 | GO:0005918 | C | 7, 8, 9, 10, | 1 | 0.249 (x 4.008) | 16 (0.062) | 0.364 | septate junction | scrib |
| 799 | GO:0042058 | P | 5, 6, 9, | 1 | 0.249 (x 4.008) | 16 (0.062) | 0.365 | regulation of epidermal growth factor receptor signaling pathway | edl |
| 800 | GO:0030529 | C | 3, 4, 5, 6, | 7 | 4.959 (x 1.412) | 318 (0.022) | 0.366 | ribonucleoprotein complex | B52 CG10423 CG17838 CG3605 Hrb27C Pep sqd |
| 801 | GO:0005085 | F | 4, | 2 | 0.904 (x 2.211) | 58 (0.034) | 0.366 | guanyl-nucleotide exchange factor activity | CG30440 pbl |
| 802 | GO:0007369 | P | 5, | 2 | 0.904 (x 2.211) | 58 (0.034) | 0.367 | gastrulation | gol pbl |
| 803 | GO:0007001 | P | 7, | 4 | 2.448 (x 1.634) | 157 (0.025) | 0.367 | chromosome organization and biogenesis (sensu Eukaryota) | CG31617 Caf1 Dsp1 ash2 |
| 804 | GO:0009913 | P | 4, 6, 7, | 1 | 0.265 (x 3.772) | 17 (0.059) | 0.368 | epidermal cell differentiation | fz |
| 805 | GO:0003702 | F | 3, | 6 | 4.148 (x 1.447) | 266 (0.023) | 0.368 | RNA polymerase II transcription factor activity | Brf Dll Scm ap dac grn |
| 806 | GO:0051028 | P | 7, 8, 9, | 1 | 0.265 (x 3.772) | 17 (0.059) | 0.368 | mRNA transport | sqd |
| 807 | GO:0006351 | P | 7, | 17 | 13.972 (x 1.217) | 896 (0.019) | 0.369 | transcription, DNA-dependent | Brf CG15141 CG15835 Caf1 D19A Dll Dr Dsp1 Top2 ap ash2 dve edl gol grn klu yps |
| 808 | GO:0003746 | F | 4, 5, | 1 | 0.265 (x 3.772) | 17 (0.059) | 0.369 | translation elongation factor activity | CG10990 |
| 809 | GO:0007259 | P | 7, | 1 | 0.265 (x 3.772) | 17 (0.059) | 0.369 | JAK-STAT cascade | Cdk4 |
| 810 | GO:0050770 | P | 5, 8, 9, 10, 12, | 1 | 0.265 (x 3.772) | 17 (0.059) | 0.37 | regulation of axonogenesis | pbl |
| 811 | GO:0006406 | P | 8, 9, 10, 11, | 1 | 0.265 (x 3.772) | 17 (0.059) | 0.37 | mRNA export from nucleus | sqd |
| 812 | GO:0035315 | P | 5, 7, 8, | 1 | 0.265 (x 3.772) | 17 (0.059) | 0.37 | hair cell differentiation | fz |
| 813 | GO:0008052 | P | 5, | 1 | 0.265 (x 3.772) | 17 (0.059) | 0.371 | sensory organ determination | fz |
| 814 | GO:0048730 | P | 5, 6, | 1 | 0.265 (x 3.772) | 17 (0.059) | 0.371 | epidermis morphogenesis | fz |
| 815 | GO:0016881 | F | 5, | 4 | 2.464 (x 1.624) | 158 (0.025) | 0.372 | acid-amino acid ligase activity | CG15141 gft gol th |
| 816 | GO:0006471 | P | 8, | 1 | 0.265 (x 3.772) | 17 (0.059) | 0.372 | protein amino acid ADP-ribosylation | Arf79F |
| 817 | GO:0016798 | F | 4, | 3 | 1.668 (x 1.798) | 107 (0.028) | 0.372 | hydrolase activity, acting on glycosyl bonds | CG33138 CG4670 GNBP3 |
| 818 | GO:0007280 | P | 6, 7, 8, | 1 | 0.265 (x 3.772) | 17 (0.059) | 0.372 | pole cell migration | scrib |
| 819 | GO:0035316 | P | 6, 7, 8, 9, | 1 | 0.265 (x 3.772) | 17 (0.059) | 0.373 | trichome organization and biogenesis (sensu Insecta) | fz |
| 820 | GO:0003714 | F | 4, 6, | 1 | 0.265 (x 3.772) | 17 (0.059) | 0.373 | transcription corepressor activity | Dsp1 |
| 821 | GO:0006413 | P | 8, 9, | 2 | 0.920 (x 2.174) | 59 (0.034) | 0.373 | translational initiation | eIF-4B eIF3-S9 |
| 822 | GO:0016282 | C | 3, 5, 6, 7, 8, | 2 | 0.936 (x 2.138) | 60 (0.033) | 0.376 | eukaryotic 43S preinitiation complex | CG10423 eIF3-S9 |
| 823 | GO:0008238 | F | 5, | 3 | 1.700 (x 1.765) | 109 (0.028) | 0.377 | exopeptidase activity | ApepP BcDNA:LD41548 fra |
| 824 | GO:0007424 | P | 4, | 3 | 1.700 (x 1.765) | 109 (0.028) | 0.377 | tracheal system development (sensu Insecta) | Cdk4 Lam tkv |
| 825 | GO:0006066 | P | 5, | 4 | 2.511 (x 1.593) | 161 (0.025) | 0.378 | alcohol metabolism | BEST:LD22483 CG31169 CG8036 Gapdh2 |
| 826 | GO:0051235 | P | 4, | 1 | 0.281 (x 3.563) | 18 (0.056) | 0.379 | maintenance of localization | CG9057 |
| 827 | GO:0007005 | P | 6, | 1 | 0.281 (x 3.563) | 18 (0.056) | 0.379 | mitochondrion organization and biogenesis | Cdk4 |
| 828 | GO:0015149 | F | 6, | 1 | 0.281 (x 3.563) | 18 (0.056) | 0.38 | hexose transporter activity | CG10960 |
| 829 | GO:0009069 | P | 7, 8, | 1 | 0.281 (x 3.563) | 18 (0.056) | 0.38 | serine family amino acid metabolism | ESTS:39C10S |
| 830 | GO:0009116 | P | 6, | 1 | 0.281 (x 3.563) | 18 (0.056) | 0.38 | nucleoside metabolism | CG6767 |
| 831 | GO:0042659 | P | 5, 6, | 1 | 0.281 (x 3.563) | 18 (0.056) | 0.381 | regulation of cell fate specification | Dr |
| 832 | GO:0005976 | P | 6, | 4 | 2.526 (x 1.583) | 162 (0.025) | 0.381 | polysaccharide metabolism | CG33138 CG6904 GNBP3 GlyP |
| 833 | GO:0019209 | F | 4, | 1 | 0.281 (x 3.563) | 18 (0.056) | 0.381 | kinase activator activity | CycA |
| 834 | GO:0007507 | P | 5, | 2 | 0.951 (x 2.103) | 61 (0.033) | 0.381 | heart development | fz tkv |
| 835 | GO:0016568 | P | 9, | 2 | 0.951 (x 2.103) | 61 (0.033) | 0.382 | chromatin modification | Caf1 ash2 |
| 836 | GO:0009165 | P | 6, 7, | 3 | 1.715 (x 1.749) | 110 (0.027) | 0.382 | nucleotide biosynthesis | CG3590 CG6767 CG6854 |
| 837 | GO:0040008 | P | 3, | 2 | 0.951 (x 2.103) | 61 (0.033) | 0.382 | regulation of growth | Cdk4 tkv |
| 838 | GO:0016481 | P | 8, | 3 | 1.731 (x 1.733) | 111 (0.027) | 0.383 | negative regulation of transcription | CREG Caf1 edl |
| 839 | GO:0048565 | P | 4, | 2 | 0.967 (x 2.069) | 62 (0.032) | 0.386 | gut development | dve pbl |
| 840 | GO:0004386 | F | 3, | 3 | 1.746 (x 1.718) | 112 (0.027) | 0.389 | helicase activity | Mcm5 Mcm7 dpa |
| 841 | GO:0031300 | C | 4, 5, 6, 7, 8, 9, | 1 | 0.296 (x 3.375) | 19 (0.053) | 0.392 | intrinsic to organelle membrane | Fur1 |
| 842 | GO:0009620 | P | 5, | 1 | 0.296 (x 3.375) | 19 (0.053) | 0.392 | response to fungus | GNBP3 |
| 843 | GO:0031301 | C | 5, 6, 7, 8, 9, 10, | 1 | 0.296 (x 3.375) | 19 (0.053) | 0.392 | integral to organelle membrane | Fur1 |
| 844 | GO:0007140 | P | 8, | 1 | 0.296 (x 3.375) | 19 (0.053) | 0.393 | male meiosis | polo |
| 845 | GO:0016769 | F | 4, | 1 | 0.296 (x 3.375) | 19 (0.053) | 0.393 | transferase activity, transferring nitrogenous groups | ESTS:39C10S |
| 846 | GO:0031109 | P | 9, | 1 | 0.296 (x 3.375) | 19 (0.053) | 0.394 | microtubule polymerization or depolymerization | betaTub56D |
| 847 | GO:0008483 | F | 5, | 1 | 0.296 (x 3.375) | 19 (0.053) | 0.394 | transaminase activity | ESTS:39C10S |
| 848 | GO:0043412 | P | 6, | 17 | 14.299 (x 1.189) | 917 (0.019) | 0.394 | biopolymer modification | Arf79F BcDNA:LD22910 CG15141 CG4670 Caf1 Cdk4 CkIIalpha CkIalpha Eip63E Nek2 Uch gol polo smi35A th tkv wbl |
| 849 | GO:0005783 | C | 5, 6, 7, 8, | 3 | 1.762 (x 1.703) | 113 (0.027) | 0.395 | endoplasmic reticulum | CBP Snap wbl |
| 850 | GO:0051234 | P | 4, | 30 | 26.634 (x 1.126) | 1708 (0.018) | 0.397 | establishment of localization | Abi Arf79F CG10657 CG10960 CG14439 CG15835 CG17419 CG2852 CG3823 CG9057 Eb1 Lam NetA Snap ap betaTub56D bib d fra l(2)dtl ninA pbl polo rin rpk scrib sqd stai th wbl |
| 851 | GO:0001558 | P | 4, 5, 7, 8, | 1 | 0.312 (x 3.206) | 20 (0.050) | 0.401 | regulation of cell growth | Cdk4 |
| 852 | GO:0006637 | P | 7, 8, | 1 | 0.312 (x 3.206) | 20 (0.050) | 0.402 | acyl-CoA metabolism | CG7461 |
| 853 | GO:0007528 | P | 7, 8, | 1 | 0.312 (x 3.206) | 20 (0.050) | 0.402 | neuromuscular junction development | tkv |
| 854 | GO:0009156 | P | 8, 9, | 1 | 0.312 (x 3.206) | 20 (0.050) | 0.402 | ribonucleoside monophosphate biosynthesis | CG6767 |
| 855 | GO:0016491 | F | 3, | 12 | 9.730 (x 1.233) | 624 (0.019) | 0.403 | oxidoreductase activity | BEST:LD22483 CG1544 CG31169 CG31472 CG4670 CG5873 CG7461 CG7675 CG9027 Gapdh2 Idh Trxr-1 |
| 856 | GO:0043297 | P | 8, | 1 | 0.312 (x 3.206) | 20 (0.050) | 0.403 | apical junction assembly | scrib |
| 857 | GO:0009161 | P | 8, | 1 | 0.312 (x 3.206) | 20 (0.050) | 0.403 | ribonucleoside monophosphate metabolism | CG6767 |
| 858 | GO:0045475 | P | 5, 6, | 1 | 0.312 (x 3.206) | 20 (0.050) | 0.404 | locomotor rhythm | CkIIalpha |
| 859 | GO:0008276 | F | 6, | 1 | 0.312 (x 3.206) | 20 (0.050) | 0.404 | protein methyltransferase activity | Caf1 |
| 860 | GO:0015145 | F | 5, | 1 | 0.312 (x 3.206) | 20 (0.050) | 0.405 | monosaccharide transporter activity | CG10960 |
| 861 | GO:0004675 | F | 5, 6, 8, 9, | 1 | 0.312 (x 3.206) | 20 (0.050) | 0.405 | transmembrane receptor protein serine/threonine kinase activity | tkv |
| 862 | GO:0007317 | P | 4, 8, 12, 14, 15, 17, | 1 | 0.312 (x 3.206) | 20 (0.050) | 0.406 | regulation of pole plasm oskar mRNA localization | Hrb27C |
| 863 | GO:0035239 | P | 4, | 2 | 1.014 (x 1.973) | 65 (0.031) | 0.406 | tube morphogenesis | Lam pbl |
| 864 | GO:0016877 | F | 4, | 1 | 0.312 (x 3.206) | 20 (0.050) | 0.406 | ligase activity, forming carbon-sulfur bonds | BcDNA:GH02901 |
| 865 | GO:0007605 | P | 5, 7, | 1 | 0.312 (x 3.206) | 20 (0.050) | 0.407 | sensory perception of sound | d |
| 866 | GO:0051169 | P | 6, 7, 8, | 2 | 1.029 (x 1.943) | 66 (0.030) | 0.408 | nuclear transport | rin sqd |
| 867 | GO:0030054 | C | 5, 6, 7, | 2 | 1.029 (x 1.943) | 66 (0.030) | 0.408 | cell junction | Moe scrib |
| 868 | GO:0050896 | P | 2, | 21 | 18.229 (x 1.152) | 1169 (0.018) | 0.408 | response to stimulus | CG2852 CG5873 CG8588 CG9027 Caf1 CkIIalpha CkIalpha DnaJ-1 GNBP3 Hsp26 Hsp27 Sb d endos fra lig ninA scrib serpin-27A smi21F smi35A |
| 869 | GO:0007346 | P | 6, 7, | 1 | 0.327 (x 3.054) | 21 (0.048) | 0.411 | regulation of progression through mitotic cell cycle | CycA |
| 870 | GO:0009123 | P | 7, | 1 | 0.327 (x 3.054) | 21 (0.048) | 0.412 | nucleoside monophosphate metabolism | CG6767 |
| 871 | GO:0051329 | P | 6, | 1 | 0.327 (x 3.054) | 21 (0.048) | 0.412 | interphase of mitotic cell cycle | CycA |
| 872 | GO:0006405 | P | 7, 8, 9, 10, | 1 | 0.327 (x 3.054) | 21 (0.048) | 0.413 | RNA export from nucleus | sqd |
| 873 | GO:0003755 | F | 5, | 1 | 0.327 (x 3.054) | 21 (0.048) | 0.413 | peptidyl-prolyl cis-trans isomerase activity | CG2852 |
| 874 | GO:0007043 | P | 7, | 1 | 0.327 (x 3.054) | 21 (0.048) | 0.414 | intercellular junction assembly | scrib |
| 875 | GO:0009124 | P | 7, 8, | 1 | 0.327 (x 3.054) | 21 (0.048) | 0.414 | nucleoside monophosphate biosynthesis | CG6767 |
| 876 | GO:0009948 | P | 5, | 3 | 1.856 (x 1.617) | 119 (0.025) | 0.414 | anterior/posterior axis specification | Hrb27C Moe sqd |
| 877 | GO:0051325 | P | 5, | 1 | 0.327 (x 3.054) | 21 (0.048) | 0.415 | interphase | CycA |
| 878 | GO:0051258 | P | 7, | 1 | 0.327 (x 3.054) | 21 (0.048) | 0.415 | protein polymerization | betaTub56D |
| 879 | GO:0006800 | P | 5, | 2 | 1.045 (x 1.914) | 67 (0.030) | 0.415 | oxygen and reactive oxygen species metabolism | CG5873 CG9027 |
| 880 | GO:0050954 | P | 4, 6, | 1 | 0.327 (x 3.054) | 21 (0.048) | 0.416 | sensory perception of mechanical stimulus | d |
| 881 | GO:0008544 | P | 5, | 1 | 0.343 (x 2.915) | 22 (0.045) | 0.419 | epidermis development | fz |
| 882 | GO:0048558 | P | 6, 7, | 1 | 0.343 (x 2.915) | 22 (0.045) | 0.419 | embryonic gut morphogenesis | pbl |
| 883 | GO:0048619 | P | 8, 9, | 1 | 0.343 (x 2.915) | 22 (0.045) | 0.42 | embryonic hindgut morphogenesis | pbl |
| 884 | GO:0043085 | P | 4, | 1 | 0.343 (x 2.915) | 22 (0.045) | 0.42 | positive regulation of enzyme activity | Abi |
| 885 | GO:0007443 | P | 5, 9, 10, | 1 | 0.343 (x 2.915) | 22 (0.045) | 0.421 | Malpighian tubule morphogenesis | pbl |
| 886 | GO:0042078 | P | 6, | 1 | 0.343 (x 2.915) | 22 (0.045) | 0.421 | germ-line stem cell division | tkv |
| 887 | GO:0009401 | P | 6, 7, | 1 | 0.343 (x 2.915) | 22 (0.045) | 0.422 | phosphoenolpyruvate-dependent sugar phosphotransferase system | l(2)dtl |
| 888 | GO:0003727 | F | 5, | 1 | 0.343 (x 2.915) | 22 (0.045) | 0.422 | single-stranded RNA binding | Pep |
| 889 | GO:0048557 | P | 6, | 1 | 0.343 (x 2.915) | 22 (0.045) | 0.422 | embryonic digestive tract morphogenesis | pbl |
| 890 | GO:0008037 | P | 3, | 1 | 0.343 (x 2.915) | 22 (0.045) | 0.423 | cell recognition | fra |
| 891 | GO:0008038 | P | 4, | 1 | 0.343 (x 2.915) | 22 (0.045) | 0.423 | neuron recognition | fra |
| 892 | GO:0045927 | P | 4, | 1 | 0.343 (x 2.915) | 22 (0.045) | 0.424 | positive regulation of growth | tkv |
| 893 | GO:0048611 | P | 6, | 1 | 0.343 (x 2.915) | 22 (0.045) | 0.424 | embryonic ectodermal gut development | pbl |
| 894 | GO:0048566 | P | 5, | 1 | 0.343 (x 2.915) | 22 (0.045) | 0.425 | embryonic gut development | pbl |
| 895 | GO:0048613 | P | 7, 8, | 1 | 0.343 (x 2.915) | 22 (0.045) | 0.425 | embryonic ectodermal gut morphogenesis | pbl |
| 896 | GO:0046903 | P | 5, | 5 | 3.602 (x 1.388) | 231 (0.022) | 0.425 | secretion | Arf79F CG15835 Snap stai wbl |
| 897 | GO:0045934 | P | 7, | 3 | 1.887 (x 1.590) | 121 (0.025) | 0.426 | negative regulation of nucleobase, nucleoside, nucleotide and nucleic acid metabolism | CREG Caf1 edl |
| 898 | GO:0016310 | P | 7, | 9 | 7.189 (x 1.252) | 461 (0.020) | 0.426 | phosphorylation | Cdk4 CkIIalpha CkIalpha Eip63E Nek2 edl polo smi35A tkv |
| 899 | GO:0040029 | P | 3, | 2 | 1.092 (x 1.832) | 70 (0.029) | 0.426 | regulation of gene expression, epigenetic | Caf1 ash2 |
| 900 | GO:0009299 | P | 8, | 1 | 0.359 (x 2.788) | 23 (0.043) | 0.428 | mRNA transcription | Top2 |
| 901 | GO:0006913 | P | 6, 7, 8, | 2 | 1.107 (x 1.806) | 71 (0.028) | 0.428 | nucleocytoplasmic transport | rin sqd |
| 902 | GO:0016859 | F | 4, | 1 | 0.359 (x 2.788) | 23 (0.043) | 0.428 | cis-trans isomerase activity | CG2852 |
| 903 | GO:0045859 | P | 6, | 1 | 0.359 (x 2.788) | 23 (0.043) | 0.429 | regulation of protein kinase activity | Abi |
| 904 | GO:0008213 | P | 8, | 1 | 0.359 (x 2.788) | 23 (0.043) | 0.429 | protein amino acid alkylation | Caf1 |
| 905 | GO:0006479 | P | 8, 9, | 1 | 0.359 (x 2.788) | 23 (0.043) | 0.43 | protein amino acid methylation | Caf1 |
| 906 | GO:0006401 | P | 7, | 1 | 0.359 (x 2.788) | 23 (0.043) | 0.43 | RNA catabolism | sqd |
| 907 | GO:0006512 | P | 8, | 5 | 3.649 (x 1.370) | 234 (0.021) | 0.43 | ubiquitin cycle | BcDNA:LD22910 CG15141 Uch gol th |
| 908 | GO:0045216 | P | 6, | 1 | 0.359 (x 2.788) | 23 (0.043) | 0.431 | intercellular junction assembly and maintenance | scrib |
| 909 | GO:0043549 | P | 5, | 1 | 0.359 (x 2.788) | 23 (0.043) | 0.431 | regulation of kinase activity | Abi |
| 910 | GO:0051338 | P | 4, | 1 | 0.359 (x 2.788) | 23 (0.043) | 0.432 | regulation of transferase activity | Abi |
| 911 | GO:0016319 | P | 5, 7, | 1 | 0.359 (x 2.788) | 23 (0.043) | 0.432 | mushroom body development | dac |
| 912 | GO:0035109 | P | 6, | 1 | 0.359 (x 2.788) | 23 (0.043) | 0.433 | limb morphogenesis (sensu Endopterygota) | Dll |
| 913 | GO:0000139 | C | 4, 5, 6, 7, 8, 9, 10, | 1 | 0.359 (x 2.788) | 23 (0.043) | 0.433 | Golgi membrane | Fur1 |
| 914 | GO:0016271 | P | 4, | 2 | 1.123 (x 1.781) | 72 (0.028) | 0.435 | tissue death | Akap200 ap |
| 915 | GO:0031202 | F | 5, | 1 | 0.374 (x 2.672) | 24 (0.042) | 0.435 | RNA splicing factor activity, transesterification mechanism | B52 |
| 916 | GO:0007559 | P | 5, | 2 | 1.123 (x 1.781) | 72 (0.028) | 0.436 | histolysis | Akap200 ap |
| 917 | GO:0007494 | P | 5, | 1 | 0.374 (x 2.672) | 24 (0.042) | 0.436 | midgut development | dve |
| 918 | GO:0015036 | F | 4, | 1 | 0.374 (x 2.672) | 24 (0.042) | 0.436 | disulfide oxidoreductase activity | Trxr-1 |
| 919 | GO:0050658 | P | 6, 7, 8, | 1 | 0.374 (x 2.672) | 24 (0.042) | 0.437 | RNA transport | sqd |
| 920 | GO:0030261 | P | 7, | 1 | 0.374 (x 2.672) | 24 (0.042) | 0.437 | chromosome condensation | Mcm5 |
| 921 | GO:0016323 | C | 5, 6, 7, | 1 | 0.374 (x 2.672) | 24 (0.042) | 0.437 | basolateral plasma membrane | scrib |
| 922 | GO:0016570 | P | 8, 11, | 1 | 0.374 (x 2.672) | 24 (0.042) | 0.438 | histone modification | Caf1 |
| 923 | GO:0048512 | P | 5, | 1 | 0.374 (x 2.672) | 24 (0.042) | 0.438 | circadian behavior | CkIIalpha |
| 924 | GO:0044249 | P | 5, | 15 | 12.943 (x 1.159) | 830 (0.018) | 0.439 | cellular biosynthesis | Aats-glupro CG10423 CG10990 CG2118 CG31472 CG3590 CG6767 CG6854 CG6904 ESTS:39C10S eIF-4B eIF3-S9 msi rin sqd |
| 925 | GO:0051236 | P | 5, | 1 | 0.374 (x 2.672) | 24 (0.042) | 0.439 | establishment of RNA localization | sqd |
| 926 | GO:0004857 | F | 3, | 3 | 1.980 (x 1.515) | 127 (0.024) | 0.439 | enzyme inhibitor activity | CG6680 Spn43Aa serpin-27A |
| 927 | GO:0005913 | C | 7, 8, 9, | 1 | 0.374 (x 2.672) | 24 (0.042) | 0.439 | cell-cell adherens junction | scrib |
| 928 | GO:0000151 | C | 3, 4, 5, 6, | 3 | 1.965 (x 1.527) | 126 (0.024) | 0.439 | ubiquitin ligase complex | gft gol th |
| 929 | GO:0016569 | P | 10, | 1 | 0.374 (x 2.672) | 24 (0.042) | 0.44 | covalent chromatin modification | Caf1 |
| 930 | GO:0050657 | P | 6, 7, | 1 | 0.374 (x 2.672) | 24 (0.042) | 0.44 | nucleic acid transport | sqd |
| 931 | GO:0003712 | F | 3, 5, | 2 | 1.154 (x 1.733) | 74 (0.027) | 0.443 | transcription cofactor activity | CG15835 Dsp1 |
| 932 | GO:0030234 | F | 2, | 7 | 5.567 (x 1.257) | 357 (0.020) | 0.445 | enzyme regulator activity | Abi CG30440 CG6680 CycA Spn43Aa pbl serpin-27A |
| 933 | GO:0008406 | P | 5, | 1 | 0.390 (x 2.565) | 25 (0.040) | 0.446 | gonad development | scrib |
| 934 | GO:0016459 | C | 3, 5, 6, 7, 8, 9, 10, | 1 | 0.390 (x 2.565) | 25 (0.040) | 0.446 | myosin | d |
| 935 | GO:0045137 | P | 4, | 1 | 0.390 (x 2.565) | 25 (0.040) | 0.447 | development of primary sexual characteristics | scrib |
| 936 | GO:0016758 | F | 5, | 3 | 2.012 (x 1.491) | 129 (0.023) | 0.447 | transferase activity, transferring hexosyl groups | CG33138 CG6904 GlyP |
| 937 | GO:0048562 | P | 5, | 1 | 0.390 (x 2.565) | 25 (0.040) | 0.447 | embryonic organ morphogenesis | pbl |
| 938 | GO:0051239 | P | 4, | 1 | 0.390 (x 2.565) | 25 (0.040) | 0.448 | regulation of organismal physiological process | scrib |
| 939 | GO:0050767 | P | 4, 7, | 1 | 0.390 (x 2.565) | 25 (0.040) | 0.448 | regulation of neurogenesis | pbl |
| 940 | GO:0007166 | P | 5, | 12 | 10.276 (x 1.168) | 659 (0.018) | 0.451 | cell surface receptor linked signal transduction | Arf79F CG30440 CkIIalpha CkIalpha Mtl bib edl fz pbl serpin-27A tkv wbl |
| 941 | GO:0003924 | F | 8, | 3 | 2.027 (x 1.480) | 130 (0.023) | 0.452 | GTPase activity | Arf79F Mtl betaTub56D |
| 942 | GO:0030695 | F | 3, | 3 | 2.027 (x 1.480) | 130 (0.023) | 0.452 | GTPase regulator activity | Abi CG30440 pbl |
| 943 | GO:0008017 | F | 6, | 2 | 1.185 (x 1.688) | 76 (0.026) | 0.452 | microtubule binding | Eb1 stai |
| 944 | GO:0007422 | P | 5, | 2 | 1.185 (x 1.688) | 76 (0.026) | 0.453 | peripheral nervous system development | CycA pbl |
| 945 | GO:0042592 | P | 3, | 2 | 1.185 (x 1.688) | 76 (0.026) | 0.453 | homeostasis | bib endos |
| 946 | GO:0007622 | P | 4, | 1 | 0.405 (x 2.467) | 26 (0.038) | 0.455 | rhythmic behavior | CkIIalpha |
| 947 | GO:0051707 | P | 4, | 3 | 2.043 (x 1.469) | 131 (0.023) | 0.455 | response to other organism | GNBP3 scrib serpin-27A |
| 948 | GO:0005272 | F | 6, 7, | 1 | 0.405 (x 2.467) | 26 (0.038) | 0.455 | sodium channel activity | rpk |
| 949 | GO:0007310 | P | 6, 8, 9, 11, | 1 | 0.405 (x 2.467) | 26 (0.038) | 0.456 | oocyte dorsal/ventral axis determination | wbl |
| 950 | GO:0035295 | P | 3, | 2 | 1.201 (x 1.666) | 77 (0.026) | 0.458 | tube development | Lam pbl |
| 951 | GO:0042127 | P | 5, | 1 | 0.421 (x 2.375) | 27 (0.037) | 0.464 | regulation of cell proliferation | scrib |
| 952 | GO:0031507 | P | 11, | 1 | 0.421 (x 2.375) | 27 (0.037) | 0.464 | heterochromatin formation | Caf1 |
| 953 | GO:0007419 | P | 4, 6, | 1 | 0.421 (x 2.375) | 27 (0.037) | 0.465 | ventral cord development | Dr |
| 954 | GO:0006342 | P | 5, 7, 10, 12, | 1 | 0.421 (x 2.375) | 27 (0.037) | 0.465 | chromatin silencing | Caf1 |
| 955 | GO:0007298 | P | 7, 8, 10, | 1 | 0.421 (x 2.375) | 27 (0.037) | 0.466 | border follicle cell migration (sensu Insecta) | th |
| 956 | GO:0006357 | P | 9, | 10 | 8.530 (x 1.172) | 547 (0.018) | 0.466 | regulation of transcription from RNA polymerase II promoter | CG15835 Caf1 D19A Dll Dr Dsp1 ap grn klu yps |
| 957 | GO:0051168 | P | 7, 8, 9, | 1 | 0.421 (x 2.375) | 27 (0.037) | 0.466 | nuclear export | sqd |
| 958 | GO:0005234 | F | 8, 9, | 1 | 0.421 (x 2.375) | 27 (0.037) | 0.467 | glutamate-gated ion channel activity | CG8533 |
| 959 | GO:0044444 | C | 4, 5, 6, 7, | 20 | 18.042 (x 1.109) | 1157 (0.017) | 0.467 | cytoplasmic part | ApepP CBP CG10423 CG2118 CG9057 CkIIalpha CycA Fur1 Idh NUCB1 Nek2 Snap Trxr-1 eIF-4B eIF3-S9 fz polo rin smi35A wbl |
| 960 | GO:0045814 | P | 4, | 1 | 0.421 (x 2.375) | 27 (0.037) | 0.467 | negative regulation of gene expression, epigenetic | Caf1 |
| 961 | GO:0050793 | P | 3, | 2 | 1.232 (x 1.624) | 79 (0.025) | 0.468 | regulation of development | Dr pbl |
| 962 | GO:0048489 | P | 6, 7, | 2 | 1.247 (x 1.603) | 80 (0.025) | 0.475 | synaptic vesicle transport | Arf79F Snap |
| 963 | GO:0005819 | C | 5, 6, 7, 8, 9, 10, | 1 | 0.437 (x 2.290) | 28 (0.036) | 0.476 | spindle | polo |
| 964 | GO:0015144 | F | 3, | 2 | 1.263 (x 1.583) | 81 (0.025) | 0.481 | carbohydrate transporter activity | CG10960 l(2)dtl |
| 965 | GO:0008643 | P | 5, 6, | 2 | 1.263 (x 1.583) | 81 (0.025) | 0.481 | carbohydrate transport | CG10960 l(2)dtl |
| 966 | GO:0007156 | P | 5, | 1 | 0.452 (x 2.211) | 29 (0.034) | 0.483 | homophilic cell adhesion | fz |
| 967 | GO:0014016 | P | 4, 7, | 1 | 0.452 (x 2.211) | 29 (0.034) | 0.484 | neuroblast differentiation | Dr |
| 968 | GO:0007179 | P | 8, | 1 | 0.452 (x 2.211) | 29 (0.034) | 0.484 | transforming growth factor beta receptor signaling pathway | tkv |
| 969 | GO:0007400 | P | 6, 9, | 1 | 0.452 (x 2.211) | 29 (0.034) | 0.485 | neuroblast fate determination | Dr |
| 970 | GO:0005478 | F | 3, | 1 | 0.452 (x 2.211) | 29 (0.034) | 0.485 | intracellular transporter activity | Snap |
| 971 | GO:0005795 | C | 5, 6, 7, 8, 9, 10, | 1 | 0.452 (x 2.211) | 29 (0.034) | 0.486 | Golgi stack | Fur1 |
| 972 | GO:0007416 | P | 5, 6, | 1 | 0.452 (x 2.211) | 29 (0.034) | 0.486 | synaptogenesis | tkv |
| 973 | GO:0014017 | P | 5, 8, | 1 | 0.452 (x 2.211) | 29 (0.034) | 0.487 | neuroblast fate commitment | Dr |
| 974 | GO:0006633 | P | 6, 7, 8, | 1 | 0.452 (x 2.211) | 29 (0.034) | 0.487 | fatty acid biosynthesis | CG2118 |
| 975 | GO:0006396 | P | 7, | 5 | 3.976 (x 1.257) | 255 (0.020) | 0.487 | RNA processing | B52 CG3605 Hrb27C msi sqd |
| 976 | GO:0007498 | P | 4, | 4 | 3.088 (x 1.296) | 198 (0.020) | 0.491 | mesoderm development | bib gol grn pbl |
| 977 | GO:0046483 | P | 5, | 3 | 2.183 (x 1.374) | 140 (0.021) | 0.491 | heterocycle metabolism | CG3590 CG6767 CG6854 |
| 978 | GO:0016485 | P | 8, | 1 | 0.468 (x 2.138) | 30 (0.033) | 0.492 | protein processing | wbl |
| 979 | GO:0031589 | P | 4, | 1 | 0.468 (x 2.138) | 30 (0.033) | 0.492 | cell-substrate adhesion | NetA |
| 980 | GO:0007160 | P | 5, | 1 | 0.468 (x 2.138) | 30 (0.033) | 0.493 | cell-matrix adhesion | NetA |
| 981 | GO:0016627 | F | 4, | 1 | 0.468 (x 2.138) | 30 (0.033) | 0.493 | oxidoreductase activity, acting on the CH-CH group of donors | CG7461 |
| 982 | GO:0019201 | F | 7, | 1 | 0.468 (x 2.138) | 30 (0.033) | 0.494 | nucleotide kinase activity | CG6767 |
| 983 | GO:0016049 | P | 3, 4, 6, 7, | 1 | 0.468 (x 2.138) | 30 (0.033) | 0.494 | cell growth | Cdk4 |
| 984 | GO:0004871 | F | 2, | 18 | 16.420 (x 1.096) | 1053 (0.017) | 0.494 | signal transducer activity | Abi CG17419 Cdk4 CkIIalpha CkIalpha Eb1 Eip63E GNBP3 Nek2 endos fra fz pbl polo rin scrib smi35A tkv |
| 985 | GO:0016772 | F | 4, | 9 | 7.812 (x 1.152) | 501 (0.018) | 0.496 | transferase activity, transferring phosphorus-containing groups | CG6767 Cdk4 CkIIalpha CkIalpha Eip63E Nek2 polo smi35A tkv |
| 986 | GO:0005667 | C | 3, 6, 7, 8, 9, 10, 11, 12, 13, | 2 | 1.325 (x 1.509) | 85 (0.024) | 0.5 | transcription factor complex | Brf Caf1 |
| 987 | GO:0048754 | P | 5, | 1 | 0.483 (x 2.069) | 31 (0.032) | 0.501 | branching morphogenesis of a tube | Lam |
| 988 | GO:0007224 | P | 6, | 1 | 0.483 (x 2.069) | 31 (0.032) | 0.502 | smoothened signaling pathway | CkIalpha |
| 989 | GO:0016053 | P | 6, | 1 | 0.483 (x 2.069) | 31 (0.032) | 0.502 | organic acid biosynthesis | CG2118 |
| 990 | GO:0046394 | P | 7, | 1 | 0.483 (x 2.069) | 31 (0.032) | 0.503 | carboxylic acid biosynthesis | CG2118 |
| 991 | GO:0030031 | P | 6, 7, | 1 | 0.499 (x 2.004) | 32 (0.031) | 0.506 | cell projection biogenesis | Abi |
| 992 | GO:0004181 | F | 7, | 1 | 0.499 (x 2.004) | 32 (0.031) | 0.507 | metallocarboxypeptidase activity | fra |
| 993 | GO:0050808 | P | 5, | 1 | 0.499 (x 2.004) | 32 (0.031) | 0.507 | synapse organization and biogenesis | tkv |
| 994 | GO:0042221 | P | 4, | 5 | 4.101 (x 1.219) | 263 (0.019) | 0.507 | response to chemical stimulus | CG8588 endos scrib smi21F smi35A |
| 995 | GO:0000070 | P | 6, 8, | 1 | 0.499 (x 2.004) | 32 (0.031) | 0.508 | mitotic sister chromatid segregation | CycA |
| 996 | GO:0019205 | F | 6, | 1 | 0.499 (x 2.004) | 32 (0.031) | 0.508 | nucleobase, nucleoside, nucleotide kinase activity | CG6767 |
| 997 | GO:0016684 | F | 4, | 1 | 0.499 (x 2.004) | 32 (0.031) | 0.509 | oxidoreductase activity, acting on peroxide as acceptor | CG5873 |
| 998 | GO:0000819 | P | 5, | 1 | 0.499 (x 2.004) | 32 (0.031) | 0.509 | sister chromatid segregation | CycA |
| 999 | GO:0030198 | P | 4, | 1 | 0.499 (x 2.004) | 32 (0.031) | 0.51 | extracellular matrix organization and biogenesis | tkv |
| 1000 | GO:0045451 | P | 7, 11, 13, 14, 16, | 1 | 0.499 (x 2.004) | 32 (0.031) | 0.51 | pole plasm oskar mRNA localization | Hrb27C |
| 1001 | GO:0016044 | P | 5, | 1 | 0.499 (x 2.004) | 32 (0.031) | 0.511 | membrane organization and biogenesis | Lam |
| 1002 | GO:0006725 | P | 5, | 3 | 2.261 (x 1.327) | 145 (0.021) | 0.511 | aromatic compound metabolism | CG3590 CG6767 CG6854 |
| 1003 | GO:0004182 | F | 8, | 1 | 0.499 (x 2.004) | 32 (0.031) | 0.511 | carboxypeptidase A activity | fra |
| 1004 | GO:0007126 | P | 7, | 2 | 1.357 (x 1.474) | 87 (0.023) | 0.511 | meiosis | Top2 polo |
| 1005 | GO:0004601 | F | 3, 5, | 1 | 0.499 (x 2.004) | 32 (0.031) | 0.512 | peroxidase activity | CG5873 |
| 1006 | GO:0019200 | F | 6, | 1 | 0.515 (x 1.943) | 33 (0.030) | 0.517 | carbohydrate kinase activity | CG6767 |
| 1007 | GO:0001752 | P | 7, 8, 9, 10, | 1 | 0.515 (x 1.943) | 33 (0.030) | 0.517 | eye photoreceptor fate commitment (sensu Endopterygota) | fz |
| 1008 | GO:0042706 | P | 6, 7, 8, | 1 | 0.515 (x 1.943) | 33 (0.030) | 0.518 | eye photoreceptor cell fate commitment | fz |
| 1009 | GO:0000398 | P | 9, 11, | 3 | 2.323 (x 1.291) | 149 (0.020) | 0.523 | nuclear mRNA splicing, via spliceosome | B52 CG3605 sqd |
| 1010 | GO:0000377 | P | 10, | 3 | 2.323 (x 1.291) | 149 (0.020) | 0.524 | RNA splicing, via transesterification reactions with bulged adenosine as nucleophile | B52 CG3605 sqd |
| 1011 | GO:0000375 | P | 9, | 3 | 2.323 (x 1.291) | 149 (0.020) | 0.524 | RNA splicing, via transesterification reactions | B52 CG3605 sqd |
| 1012 | GO:0008170 | F | 6, | 1 | 0.530 (x 1.886) | 34 (0.029) | 0.525 | N-methyltransferase activity | Caf1 |
| 1013 | GO:0043234 | C | 2, | 26 | 24.622 (x 1.056) | 1579 (0.016) | 0.525 | protein complex | Aats-glupro B52 Brf CG10423 CG17838 CG31169 CG31617 CG3605 Caf1 CkIIalpha CycA Eb1 Hrb27C Mcm5 Mcm7 Pep Scm Top2 betaTub56D d eIF3-S9 gft gol sqd stai th |
| 1014 | GO:0008361 | P | 5, 6, | 1 | 0.530 (x 1.886) | 34 (0.029) | 0.525 | regulation of cell size | Cdk4 |
| 1015 | GO:0009260 | P | 7, 8, | 2 | 1.419 (x 1.409) | 91 (0.022) | 0.526 | ribonucleotide biosynthesis | CG3590 CG6767 |
| 1016 | GO:0048488 | P | 7, 8, | 1 | 0.530 (x 1.886) | 34 (0.029) | 0.526 | synaptic vesicle endocytosis | Arf79F |
| 1017 | GO:0016853 | F | 3, | 2 | 1.419 (x 1.409) | 91 (0.022) | 0.526 | isomerase activity | CG2852 Top2 |
| 1018 | GO:0005083 | F | 4, | 2 | 1.419 (x 1.409) | 91 (0.022) | 0.527 | small GTPase regulator activity | Abi pbl |
| 1019 | GO:0009259 | P | 7, | 2 | 1.435 (x 1.394) | 92 (0.022) | 0.532 | ribonucleotide metabolism | CG3590 CG6767 |
| 1020 | GO:0005681 | C | 4, 5, 6, 7, 8, 9, 10, | 2 | 1.435 (x 1.394) | 92 (0.022) | 0.532 | spliceosome complex | B52 CG3605 |
| 1021 | GO:0019887 | F | 4, | 1 | 0.546 (x 1.832) | 35 (0.029) | 0.532 | protein kinase regulator activity | CycA |
| 1022 | GO:0016358 | P | 7, 10, | 1 | 0.546 (x 1.832) | 35 (0.029) | 0.533 | dendrite development | fra |
| 1023 | GO:0048568 | P | 4, | 1 | 0.546 (x 1.832) | 35 (0.029) | 0.533 | embryonic organ development | pbl |
| 1024 | GO:0009059 | P | 5, 6, | 9 | 8.140 (x 1.106) | 522 (0.017) | 0.536 | macromolecule biosynthesis | Aats-glupro CG10423 CG10990 CG6904 eIF-4B eIF3-S9 msi rin sqd |
| 1025 | GO:0008234 | F | 5, | 2 | 1.450 (x 1.379) | 93 (0.022) | 0.536 | cysteine-type peptidase activity | BcDNA:LD22910 Uch |
| 1026 | GO:0006403 | P | 4, | 2 | 1.450 (x 1.379) | 93 (0.022) | 0.536 | RNA localization | Hrb27C sqd |
| 1027 | GO:0009058 | P | 4, | 15 | 14.003 (x 1.071) | 898 (0.017) | 0.538 | biosynthesis | Aats-glupro CG10423 CG10990 CG2118 CG31472 CG3590 CG6767 CG6854 CG6904 ESTS:39C10S eIF-4B eIF3-S9 msi rin sqd |
| 1028 | GO:0007442 | P | 7, 8, | 1 | 0.561 (x 1.781) | 36 (0.028) | 0.54 | hindgut morphogenesis | pbl |
| 1029 | GO:0007623 | P | 4, | 1 | 0.561 (x 1.781) | 36 (0.028) | 0.541 | circadian rhythm | CkIIalpha |
| 1030 | GO:0008233 | F | 4, | 11 | 10.151 (x 1.084) | 651 (0.017) | 0.544 | peptidase activity | ApepP BcDNA:LD22910 BcDNA:LD41548 D19A Fur1 Sb Uch deltaTry fra gol tok |
| 1031 | GO:0008380 | P | 8, | 3 | 2.417 (x 1.241) | 155 (0.019) | 0.545 | RNA splicing | B52 CG3605 sqd |
| 1032 | GO:0045132 | P | 5, 8, | 1 | 0.577 (x 1.733) | 37 (0.027) | 0.548 | meiotic chromosome segregation | polo |
| 1033 | GO:0015931 | P | 5, 6, | 1 | 0.577 (x 1.733) | 37 (0.027) | 0.548 | nucleobase, nucleoside, nucleotide and nucleic acid transport | sqd |
| 1034 | GO:0016740 | F | 3, | 16 | 15.079 (x 1.061) | 967 (0.017) | 0.548 | transferase activity | Abi CG33138 CG6767 CG6904 CG8036 Caf1 Cdk4 CkIIalpha CkIalpha ESTS:39C10S Eip63E GlyP Nek2 polo smi35A tkv |
| 1035 | GO:0017145 | P | 5, | 1 | 0.577 (x 1.733) | 37 (0.027) | 0.549 | stem cell division | tkv |
| 1036 | GO:0048511 | P | 3, | 1 | 0.577 (x 1.733) | 37 (0.027) | 0.549 | rhythmic process | CkIIalpha |
| 1037 | GO:0019731 | P | 6, 7, 8, | 1 | 0.593 (x 1.688) | 38 (0.026) | 0.558 | antibacterial humoral response | scrib |
| 1038 | GO:0016757 | F | 4, | 3 | 2.479 (x 1.210) | 159 (0.019) | 0.561 | transferase activity, transferring glycosyl groups | CG33138 CG6904 GlyP |
| 1039 | GO:0005886 | C | 4, 5, | 9 | 8.327 (x 1.081) | 534 (0.017) | 0.562 | plasma membrane | Fur1 ImpE3 Moe Sb bib fra fz scrib tkv |
| 1040 | GO:0007143 | P | 8, | 1 | 0.608 (x 1.644) | 39 (0.026) | 0.564 | female meiosis | polo |
| 1041 | GO:0007173 | P | 8, | 1 | 0.608 (x 1.644) | 39 (0.026) | 0.565 | epidermal growth factor receptor signaling pathway | edl |
| 1042 | GO:0008150 | P | 1, | 127 | 126.026 (x 1.008) | 8082 (0.016) | 0.565 | biological\_process | Aac11 Aats-glupro Abi Act42A Act5C Akap200 ApepP Arf79F B52 BEST:LD22483 BcDNA:GH02901 BcDNA:LD22910 BcDNA:LD41548 Brf CG10423 CG10657 CG10960 CG10990 CG14439 CG15141 CG1544 CG15835 CG17064 CG17419 CG2118 CG2852 CG30440 CG31125 CG31169 CG31472 CG31617 CG31658 CG32663 CG33138 CG3590 CG3605 CG3823 CG4670 CG5873 CG6680 CG6767 CG6854 CG6904 CG6954 CG7461 CG7675 CG8036 CG8588 CG9027 CG9057 CREG Caf1 Cdk4 CkIIalpha CkIalpha CycA D19A Dll DnaJ-1 Dr Dsp1 ESTS:39C10S Eb1 Eip63E Fur1 GNBP3 Gapdh2 GlyP Hrb27C Hsp26 Hsp27 Idh ImpE2 ImpE3 Lam Mcm5 Mcm7 Moe Mtl Nek2 NetA Sb Scm Snap Top2 Trxr-1 Uch ap ash2 betaTub56D bib d dac deltaTry dpa dve eIF-4B eIF3-S9 edl endos esn fax fra fz gft gol grn klu l(2)dtl lig msi ninA pbl polo rin rpk scrib serpin-27A smi21F smi35A sqd stai th tkv tok wbl yps |
| 1043 | GO:0005843 | C | 4, 5, 6, 7, 8, 9, 10, 11, | 1 | 0.608 (x 1.644) | 39 (0.026) | 0.565 | cytosolic small ribosomal subunit (sensu Eukaryota) | CG10423 |
| 1044 | GO:0005525 | F | 6, | 3 | 2.511 (x 1.195) | 161 (0.019) | 0.566 | GTP binding | Arf79F Mtl betaTub56D |
| 1045 | GO:0006096 | P | 8, 10, 11, | 1 | 0.608 (x 1.644) | 39 (0.026) | 0.566 | glycolysis | Gapdh2 |
| 1046 | GO:0016283 | C | 3, 5, 6, 7, 8, | 1 | 0.608 (x 1.644) | 39 (0.026) | 0.567 | eukaryotic 48S initiation complex | CG10423 |
| 1047 | GO:0008355 | P | 6, 7, | 1 | 0.608 (x 1.644) | 39 (0.026) | 0.567 | olfactory learning | CG8588 |
| 1048 | GO:0009613 | P | 4, 5, | 2 | 1.559 (x 1.283) | 100 (0.020) | 0.569 | response to pest, pathogen or parasite | scrib serpin-27A |
| 1049 | GO:0048547 | P | 5, 6, | 1 | 0.624 (x 1.603) | 40 (0.025) | 0.569 | gut morphogenesis | pbl |
| 1050 | GO:0019001 | F | 5, | 3 | 2.526 (x 1.188) | 162 (0.019) | 0.57 | guanyl nucleotide binding | Arf79F Mtl betaTub56D |
| 1051 | GO:0048546 | P | 5, | 1 | 0.624 (x 1.603) | 40 (0.025) | 0.57 | digestive tract morphogenesis | pbl |
| 1052 | GO:0043414 | P | 7, | 1 | 0.624 (x 1.603) | 40 (0.025) | 0.57 | biopolymer methylation | Caf1 |
| 1053 | GO:0048567 | P | 6, 7, | 1 | 0.624 (x 1.603) | 40 (0.025) | 0.571 | ectodermal gut morphogenesis | pbl |
| 1054 | GO:0016251 | F | 4, | 2 | 1.575 (x 1.270) | 101 (0.020) | 0.571 | general RNA polymerase II transcription factor activity | Brf grn |
| 1055 | GO:0007439 | P | 5, | 1 | 0.624 (x 1.603) | 40 (0.025) | 0.572 | ectodermal gut development | pbl |
| 1056 | GO:0008010 | F | 5, | 1 | 0.624 (x 1.603) | 40 (0.025) | 0.572 | structural constituent of larval cuticle (sensu Insecta) | CG32029 |
| 1057 | GO:0005216 | F | 4, 5, | 3 | 2.557 (x 1.173) | 164 (0.018) | 0.575 | ion channel activity | CG8533 bib rpk |
| 1058 | GO:0005684 | C | 5, 6, 7, 8, 9, 10, 11, | 1 | 0.639 (x 1.564) | 41 (0.024) | 0.577 | major (U2-dependent) spliceosome | CG3605 |
| 1059 | GO:0007219 | P | 6, | 1 | 0.639 (x 1.564) | 41 (0.024) | 0.578 | Notch signaling pathway | bib |
| 1060 | GO:0007018 | P | 7, 8, 9, | 2 | 1.606 (x 1.245) | 103 (0.019) | 0.582 | microtubule-based movement | CG9057 betaTub56D |
| 1061 | GO:0048637 | P | 6, | 1 | 0.655 (x 1.527) | 42 (0.024) | 0.583 | skeletal muscle development | tkv |
| 1062 | GO:0030705 | P | 6, 7, 8, | 2 | 1.622 (x 1.233) | 104 (0.019) | 0.584 | cytoskeleton-dependent intracellular transport | CG9057 betaTub56D |
| 1063 | GO:0045595 | P | 4, | 1 | 0.655 (x 1.527) | 42 (0.024) | 0.584 | regulation of cell differentiation | Dr |
| 1064 | GO:0048747 | P | 5, | 1 | 0.655 (x 1.527) | 42 (0.024) | 0.585 | muscle fiber development | tkv |
| 1065 | GO:0007306 | P | 9, 10, | 1 | 0.655 (x 1.527) | 42 (0.024) | 0.585 | insect chorion formation | Caf1 |
| 1066 | GO:0048741 | P | 6, 7, | 1 | 0.655 (x 1.527) | 42 (0.024) | 0.586 | skeletal muscle fiber development | tkv |
| 1067 | GO:0043062 | P | 3, | 1 | 0.655 (x 1.527) | 42 (0.024) | 0.586 | extracellular structure organization and biogenesis | tkv |
| 1068 | GO:0006605 | P | 7, 8, 9, | 4 | 3.602 (x 1.110) | 231 (0.017) | 0.587 | protein targeting | CG2852 rin scrib wbl |
| 1069 | GO:0007269 | P | 6, 7, 8, | 2 | 1.637 (x 1.222) | 105 (0.019) | 0.588 | neurotransmitter secretion | Arf79F Snap |
| 1070 | GO:0016651 | F | 4, | 1 | 0.671 (x 1.491) | 43 (0.023) | 0.589 | oxidoreductase activity, acting on NADH or NADPH | Trxr-1 |
| 1071 | GO:0045055 | P | 6, 7, | 2 | 1.637 (x 1.222) | 105 (0.019) | 0.589 | regulated secretory pathway | Arf79F Snap |
| 1072 | GO:0006814 | P | 8, 9, | 1 | 0.671 (x 1.491) | 43 (0.023) | 0.589 | sodium ion transport | rpk |
| 1073 | GO:0004180 | F | 6, | 1 | 0.671 (x 1.491) | 43 (0.023) | 0.59 | carboxypeptidase activity | fra |
| 1074 | GO:0007612 | P | 5, | 1 | 0.671 (x 1.491) | 43 (0.023) | 0.59 | learning | CG8588 |
| 1075 | GO:0046907 | P | 5, 6, 7, | 10 | 9.606 (x 1.041) | 616 (0.016) | 0.592 | intracellular transport | Arf79F CG2852 CG9057 Snap betaTub56D d rin scrib sqd wbl |
| 1076 | GO:0050790 | P | 3, | 1 | 0.686 (x 1.457) | 44 (0.023) | 0.597 | regulation of catalytic activity | Abi |
| 1077 | GO:0019226 | P | 5, | 6 | 5.660 (x 1.060) | 363 (0.017) | 0.598 | transmission of nerve impulse | Arf79F NetA Snap fax msi scrib |
| 1078 | GO:0005231 | F | 7, 8, | 1 | 0.702 (x 1.425) | 45 (0.022) | 0.604 | excitatory extracellular ligand-gated ion channel activity | CG8533 |
| 1079 | GO:0006412 | P | 6, 7, | 8 | 7.703 (x 1.039) | 494 (0.016) | 0.604 | protein biosynthesis | Aats-glupro CG10423 CG10990 eIF-4B eIF3-S9 msi rin sqd |
| 1080 | GO:0007431 | P | 5, | 2 | 1.700 (x 1.177) | 109 (0.018) | 0.604 | salivary gland development | Akap200 tkv |
| 1081 | GO:0005578 | C | 3, 4, | 1 | 0.702 (x 1.425) | 45 (0.022) | 0.604 | extracellular matrix (sensu Metazoa) | NetA |
| 1082 | GO:0035272 | P | 4, | 2 | 1.700 (x 1.177) | 109 (0.018) | 0.605 | exocrine system development | Akap200 tkv |
| 1083 | GO:0031012 | C | 2, | 1 | 0.702 (x 1.425) | 45 (0.022) | 0.605 | extracellular matrix | NetA |
| 1084 | GO:0005938 | C | 5, 6, 7, 8, | 1 | 0.702 (x 1.425) | 45 (0.022) | 0.605 | cell cortex | fz |
| 1085 | GO:0016070 | P | 6, | 6 | 5.723 (x 1.048) | 367 (0.016) | 0.607 | RNA metabolism | Aats-glupro B52 CG3605 Hrb27C msi sqd |
| 1086 | GO:0006281 | P | 5, 7, | 2 | 1.715 (x 1.166) | 110 (0.018) | 0.609 | DNA repair | Caf1 CkIalpha |
| 1087 | GO:0016079 | P | 7, 8, 9, | 1 | 0.717 (x 1.394) | 46 (0.022) | 0.609 | synaptic vesicle exocytosis | Snap |
| 1088 | GO:0042440 | P | 5, | 1 | 0.717 (x 1.394) | 46 (0.022) | 0.609 | pigment metabolism | serpin-27A |
| 1089 | GO:0007283 | P | 6, | 2 | 1.731 (x 1.155) | 111 (0.018) | 0.612 | spermatogenesis | Act5C th |
| 1090 | GO:0048232 | P | 5, | 2 | 1.731 (x 1.155) | 111 (0.018) | 0.613 | male gamete generation | Act5C th |
| 1091 | GO:0007293 | P | 8, | 1 | 0.733 (x 1.364) | 47 (0.021) | 0.613 | egg chamber formation (sensu Insecta) | Scm |
| 1092 | GO:0016811 | F | 5, | 1 | 0.733 (x 1.364) | 47 (0.021) | 0.614 | hydrolase activity, acting on carbon-nitrogen (but not peptide) bonds, in linear amides | SP2637 |
| 1093 | GO:0006606 | P | 7, 8, 9, 10, | 1 | 0.733 (x 1.364) | 47 (0.021) | 0.615 | protein import into nucleus | rin |
| 1094 | GO:0045893 | P | 9, | 1 | 0.733 (x 1.364) | 47 (0.021) | 0.615 | positive regulation of transcription, DNA-dependent | ash2 |
| 1095 | GO:0044453 | C | 4, 5, 6, 7, 8, 9, 10, 11, 12, | 1 | 0.733 (x 1.364) | 47 (0.021) | 0.616 | nuclear membrane part | Lam |
| 1096 | GO:0031965 | C | 5, 6, 7, 8, 9, 10, 11, | 1 | 0.733 (x 1.364) | 47 (0.021) | 0.616 | nuclear membrane | Lam |
| 1097 | GO:0016567 | P | 9, | 2 | 1.762 (x 1.135) | 113 (0.018) | 0.62 | protein ubiquitination | gol th |
| 1098 | GO:0051170 | P | 7, 8, 9, | 1 | 0.748 (x 1.336) | 48 (0.021) | 0.621 | nuclear import | rin |
| 1099 | GO:0016051 | P | 6, 7, | 1 | 0.764 (x 1.309) | 49 (0.020) | 0.627 | carbohydrate biosynthesis | CG6904 |
| 1100 | GO:0005261 | F | 5, 6, | 2 | 1.793 (x 1.115) | 115 (0.017) | 0.627 | cation channel activity | bib rpk |
| 1101 | GO:0006886 | P | 6, 7, 8, | 8 | 7.890 (x 1.014) | 506 (0.016) | 0.627 | intracellular protein transport | Arf79F CG2852 Snap betaTub56D d rin scrib wbl |
| 1102 | GO:0009968 | P | 5, 6, | 1 | 0.764 (x 1.309) | 49 (0.020) | 0.627 | negative regulation of signal transduction | CkIalpha |
| 1103 | GO:0007519 | P | 5, | 1 | 0.764 (x 1.309) | 49 (0.020) | 0.628 | striated muscle development | tkv |
| 1104 | GO:0019199 | F | 5, 7, | 1 | 0.764 (x 1.309) | 49 (0.020) | 0.628 | transmembrane receptor protein kinase activity | tkv |
| 1105 | GO:0005875 | C | 3, 5, 6, 7, 8, 9, 10, | 2 | 1.809 (x 1.106) | 116 (0.017) | 0.631 | microtubule associated complex | Eb1 stai |
| 1106 | GO:0015268 | F | 4, | 3 | 2.838 (x 1.057) | 182 (0.016) | 0.631 | alpha-type channel activity | CG8533 bib rpk |
| 1107 | GO:0015267 | F | 3, | 3 | 2.838 (x 1.057) | 182 (0.016) | 0.632 | channel or pore class transporter activity | CG8533 bib rpk |
| 1108 | GO:0005230 | F | 6, 7, | 1 | 0.780 (x 1.283) | 50 (0.020) | 0.632 | extracellular ligand-gated ion channel activity | CG8533 |
| 1109 | GO:0001505 | P | 7, | 2 | 1.824 (x 1.096) | 117 (0.017) | 0.635 | regulation of neurotransmitter levels | Arf79F Snap |
| 1110 | GO:0005575 | C | 1, | 90 | 90.161 (x 0.998) | 5782 (0.016) | 0.637 | cellular\_component | Aats-glupro Act42A Act5C ApepP B52 BcDNA:LD41548 Brf CBP CG10423 CG10657 CG10960 CG13895 CG14439 CG17838 CG2118 CG31125 CG31169 CG31617 CG31658 CG32663 CG3605 CG3823 CG8588 CG9057 CG9894 CREG Caf1 CkIIalpha CkIalpha CycA D19A Dll DnaJ-1 Dr Dsp1 Eb1 Fur1 Gapdh2 Hrb27C Idh ImpE2 ImpE3 Lam Mcm5 Mcm7 Moe NUCB1 Nek2 NetA Pep Sb Scm Snap Top2 Trxr-1 Uch ap ash2 betaTub56D bib d dac deltaTry dpa dve eIF-4B eIF3-S9 edl esn fra fz gft gol grn klu l(2)dtl lig ninA pbl polo rin rpk scrib smi35A sqd stai th tkv wbl yps |
| 1111 | GO:0006974 | P | 4, | 2 | 1.840 (x 1.087) | 118 (0.017) | 0.638 | response to DNA damage stimulus | Caf1 CkIalpha |
| 1112 | GO:0007178 | P | 7, | 1 | 0.795 (x 1.257) | 51 (0.020) | 0.639 | transmembrane receptor protein serine/threonine kinase signaling pathway | tkv |
| 1113 | GO:0000122 | P | 10, | 1 | 0.811 (x 1.233) | 52 (0.019) | 0.645 | negative regulation of transcription from RNA polymerase II promoter | Caf1 |
| 1114 | GO:0005941 | C | 3, | 1 | 0.811 (x 1.233) | 52 (0.019) | 0.646 | unlocalized protein complex | CG31169 |
| 1115 | GO:0015031 | P | 5, 6, | 8 | 8.062 (x 0.992) | 517 (0.015) | 0.647 | protein transport | Arf79F CG2852 Snap betaTub56D d rin scrib wbl |
| 1116 | GO:0008356 | P | 5, | 1 | 0.826 (x 1.210) | 53 (0.019) | 0.65 | asymmetric cell division | tkv |
| 1117 | GO:0001708 | P | 5, | 1 | 0.826 (x 1.210) | 53 (0.019) | 0.651 | cell fate specification | Dr |
| 1118 | GO:0005179 | F | 4, 5, | 1 | 0.826 (x 1.210) | 53 (0.019) | 0.651 | hormone activity | scrib |
| 1119 | GO:0045184 | P | 5, | 8 | 8.109 (x 0.987) | 520 (0.015) | 0.652 | establishment of protein localization | Arf79F CG2852 Snap betaTub56D d rin scrib wbl |
| 1120 | GO:0009055 | F | 4, | 1 | 0.826 (x 1.210) | 53 (0.019) | 0.652 | electron carrier activity | Trxr-1 |
| 1121 | GO:0007417 | P | 5, | 2 | 1.902 (x 1.051) | 122 (0.016) | 0.653 | central nervous system development | Dr dac |
| 1122 | GO:0003700 | F | 3, 5, | 6 | 6.066 (x 0.989) | 389 (0.015) | 0.653 | transcription factor activity | CG6854 Dll Dr ap dve grn |
| 1123 | GO:0006955 | P | 4, 5, | 2 | 1.902 (x 1.051) | 122 (0.016) | 0.654 | immune response | scrib serpin-27A |
| 1124 | GO:0007028 | P | 5, | 1 | 0.842 (x 1.188) | 54 (0.019) | 0.655 | cytoplasm organization and biogenesis | scrib |
| 1125 | GO:0017111 | F | 7, | 8 | 8.155 (x 0.981) | 523 (0.015) | 0.656 | nucleoside-triphosphatase activity | Arf79F Mcm5 Mcm7 Mtl Top2 betaTub56D d dpa |
| 1126 | GO:0003779 | F | 5, | 2 | 1.918 (x 1.043) | 123 (0.016) | 0.656 | actin binding | Aats-glupro Moe |
| 1127 | GO:0007304 | P | 8, 9, | 1 | 0.858 (x 1.166) | 55 (0.018) | 0.657 | eggshell formation (sensu Insecta) | Caf1 |
| 1128 | GO:0007617 | P | 4, 5, | 1 | 0.858 (x 1.166) | 55 (0.018) | 0.658 | mating behavior | lig |
| 1129 | GO:0019752 | P | 6, | 6 | 6.113 (x 0.982) | 392 (0.015) | 0.658 | carboxylic acid metabolism | Aats-glupro BcDNA:GH02901 CG2118 CG7461 ESTS:39C10S Idh |
| 1130 | GO:0017038 | P | 6, 7, | 1 | 0.858 (x 1.166) | 55 (0.018) | 0.658 | protein import | rin |
| 1131 | GO:0006082 | P | 5, | 6 | 6.113 (x 0.982) | 392 (0.015) | 0.659 | organic acid metabolism | Aats-glupro BcDNA:GH02901 CG2118 CG7461 ESTS:39C10S Idh |
| 1132 | GO:0030703 | P | 7, | 1 | 0.858 (x 1.166) | 55 (0.018) | 0.659 | eggshell formation | Caf1 |
| 1133 | GO:0051705 | P | 3, | 1 | 0.858 (x 1.166) | 55 (0.018) | 0.659 | behavioral interaction between organisms | lig |
| 1134 | GO:0016337 | P | 4, | 2 | 1.934 (x 1.034) | 124 (0.016) | 0.66 | cell-cell adhesion | NetA fz |
| 1135 | GO:0006887 | P | 6, 7, | 2 | 1.949 (x 1.026) | 125 (0.016) | 0.661 | exocytosis | Snap wbl |
| 1136 | GO:0035282 | P | 3, | 2 | 1.965 (x 1.018) | 126 (0.016) | 0.666 | segmentation | Cdk4 ap |
| 1137 | GO:0016462 | F | 6, | 8 | 8.280 (x 0.966) | 531 (0.015) | 0.669 | pyrophosphatase activity | Arf79F Mcm5 Mcm7 Mtl Top2 betaTub56D d dpa |
| 1138 | GO:0007243 | P | 6, | 2 | 1.980 (x 1.010) | 127 (0.016) | 0.669 | protein kinase cascade | CG30440 Cdk4 |
| 1139 | GO:0019098 | P | 3, 4, | 1 | 0.904 (x 1.106) | 58 (0.017) | 0.673 | reproductive behavior | lig |
| 1140 | GO:0008270 | F | 6, | 9 | 9.387 (x 0.959) | 602 (0.015) | 0.673 | zinc ion binding | CG17419 D19A Pep ap esn gol klu th tok |
| 1141 | GO:0046872 | F | 4, | 14 | 14.533 (x 0.963) | 932 (0.015) | 0.674 | metal ion binding | BcDNA:LD41548 CBP CG17419 CG9027 D19A NUCB1 Pep Trxr-1 ap esn gol klu th tok |
| 1142 | GO:0009880 | P | 4, | 2 | 2.012 (x 0.994) | 129 (0.016) | 0.674 | embryonic pattern specification | Cdk4 edl |
| 1143 | GO:0051704 | P | 2, | 1 | 0.904 (x 1.106) | 58 (0.017) | 0.674 | interaction between organisms | lig |
| 1144 | GO:0043167 | F | 3, | 14 | 14.533 (x 0.963) | 932 (0.015) | 0.674 | ion binding | BcDNA:LD41548 CBP CG17419 CG9027 D19A NUCB1 Pep Trxr-1 ap esn gol klu th tok |
| 1145 | GO:0016876 | F | 5, | 1 | 0.904 (x 1.106) | 58 (0.017) | 0.674 | ligase activity, forming aminoacyl-tRNA and related compounds | Aats-glupro |
| 1146 | GO:0003824 | F | 2, | 58 | 58.959 (x 0.984) | 3781 (0.015) | 0.675 | catalytic activity | Aats-glupro Abi ApepP Arf79F BEST:LD22483 BcDNA:GH02901 BcDNA:LD22910 BcDNA:LD41548 CG15141 CG1544 CG2118 CG2852 CG31169 CG31472 CG33138 CG3590 CG4670 CG5873 CG6767 CG6854 CG6904 CG7461 CG7675 CG8036 CG9027 Caf1 Cdk4 CkIIalpha CkIalpha D19A ESTS:39C10S Eip63E Fur1 GNBP3 Gapdh2 GlyP Idh Mcm5 Mcm7 Mtl Nek2 SP2637 Sb Top2 Trxr-1 Uch betaTub56D d deltaTry dpa fra gft gol polo smi35A th tkv tok |
| 1147 | GO:0004812 | F | 6, | 1 | 0.904 (x 1.106) | 58 (0.017) | 0.675 | aminoacyl-tRNA ligase activity | Aats-glupro |
| 1148 | GO:0016875 | F | 4, | 1 | 0.904 (x 1.106) | 58 (0.017) | 0.676 | ligase activity, forming carbon-oxygen bonds | Aats-glupro |
| 1149 | GO:0006796 | P | 6, | 9 | 9.418 (x 0.956) | 604 (0.015) | 0.676 | phosphate metabolism | Cdk4 CkIIalpha CkIalpha Eip63E Nek2 edl polo smi35A tkv |
| 1150 | GO:0006793 | P | 5, | 9 | 9.418 (x 0.956) | 604 (0.015) | 0.676 | phosphorus metabolism | Cdk4 CkIIalpha CkIalpha Eip63E Nek2 edl polo smi35A tkv |
| 1151 | GO:0008757 | F | 6, | 1 | 0.920 (x 1.087) | 59 (0.017) | 0.677 | S-adenosylmethionine-dependent methyltransferase activity | Caf1 |
| 1152 | GO:0006790 | P | 5, | 1 | 0.920 (x 1.087) | 59 (0.017) | 0.677 | sulfur metabolism | Trxr-1 |
| 1153 | GO:0051189 | P | 5, 7, | 1 | 0.936 (x 1.069) | 60 (0.017) | 0.679 | prosthetic group metabolism | CG10657 |
| 1154 | GO:0048732 | P | 4, | 2 | 2.043 (x 0.979) | 131 (0.015) | 0.679 | gland development | Akap200 tkv |
| 1155 | GO:0007015 | P | 9, | 1 | 0.936 (x 1.069) | 60 (0.017) | 0.679 | actin filament organization | Sb |
| 1156 | GO:0043039 | P | 8, 9, | 1 | 0.936 (x 1.069) | 60 (0.017) | 0.68 | tRNA aminoacylation | Aats-glupro |
| 1157 | GO:0007611 | P | 4, | 1 | 0.936 (x 1.069) | 60 (0.017) | 0.681 | learning and/or memory | CG8588 |
| 1158 | GO:0006418 | P | 8, 9, 10, | 1 | 0.936 (x 1.069) | 60 (0.017) | 0.681 | tRNA aminoacylation for protein translation | Aats-glupro |
| 1159 | GO:0046914 | F | 5, | 10 | 10.541 (x 0.949) | 676 (0.015) | 0.683 | transition metal ion binding | BcDNA:LD41548 CG17419 D19A Pep ap esn gol klu th tok |
| 1160 | GO:0009719 | P | 3, | 2 | 2.074 (x 0.964) | 133 (0.015) | 0.684 | response to endogenous stimulus | Caf1 CkIalpha |
| 1161 | GO:0043038 | P | 7, 8, | 1 | 0.951 (x 1.051) | 61 (0.016) | 0.684 | amino acid activation | Aats-glupro |
| 1162 | GO:0051082 | F | 4, | 1 | 0.967 (x 1.034) | 62 (0.016) | 0.689 | unfolded protein binding | DnaJ-1 |
| 1163 | GO:0005635 | C | 4, 5, 6, 7, 8, 9, 10, | 1 | 0.967 (x 1.034) | 62 (0.016) | 0.689 | nuclear envelope | Lam |
| 1164 | GO:0030532 | C | 4, 5, 6, 7, 8, 9, 10, | 1 | 0.967 (x 1.034) | 62 (0.016) | 0.69 | small nuclear ribonucleoprotein complex | CG3605 |
| 1165 | GO:0016817 | F | 4, | 8 | 8.545 (x 0.936) | 548 (0.015) | 0.692 | hydrolase activity, acting on acid anhydrides | Arf79F Mcm5 Mcm7 Mtl Top2 betaTub56D d dpa |
| 1166 | GO:0016818 | F | 5, | 8 | 8.545 (x 0.936) | 548 (0.015) | 0.693 | hydrolase activity, acting on acid anhydrides, in phosphorus-containing anhydrides | Arf79F Mcm5 Mcm7 Mtl Top2 betaTub56D d dpa |
| 1167 | GO:0019730 | P | 6, 7, | 1 | 0.982 (x 1.018) | 63 (0.016) | 0.693 | antimicrobial humoral response | scrib |
| 1168 | GO:0045941 | P | 8, | 1 | 0.998 (x 1.002) | 64 (0.016) | 0.699 | positive regulation of transcription | ash2 |
| 1169 | GO:0030097 | P | 5, | 1 | 0.998 (x 1.002) | 64 (0.016) | 0.699 | hemopoiesis | grn |
| 1170 | GO:0005102 | F | 3, 4, | 4 | 4.351 (x 0.919) | 279 (0.014) | 0.7 | receptor binding | Eb1 endos rin scrib |
| 1171 | GO:0045935 | P | 7, | 1 | 1.014 (x 0.987) | 65 (0.015) | 0.703 | positive regulation of nucleobase, nucleoside, nucleotide and nucleic acid metabolism | ash2 |
| 1172 | GO:0044431 | C | 4, 5, 6, 7, 8, 9, | 1 | 1.014 (x 0.987) | 65 (0.015) | 0.704 | Golgi apparatus part | Fur1 |
| 1173 | GO:0019725 | P | 4, | 1 | 1.029 (x 0.972) | 66 (0.015) | 0.709 | cell homeostasis | bib |
| 1174 | GO:0019207 | F | 3, | 1 | 1.045 (x 0.957) | 67 (0.015) | 0.714 | kinase regulator activity | CycA |
| 1175 | GO:0045045 | P | 5, 6, | 3 | 3.353 (x 0.895) | 215 (0.014) | 0.718 | secretory pathway | Arf79F Snap wbl |
| 1176 | GO:0019748 | P | 4, | 1 | 1.060 (x 0.943) | 68 (0.015) | 0.719 | secondary metabolism | serpin-27A |
| 1177 | GO:0012505 | C | 4, 5, | 2 | 2.245 (x 0.891) | 144 (0.014) | 0.722 | endomembrane system | Fur1 Lam |
| 1178 | GO:0015935 | C | 3, 4, 5, 6, 7, 8, 9, | 1 | 1.076 (x 0.929) | 69 (0.014) | 0.723 | small ribosomal subunit | CG10423 |
| 1179 | GO:0042742 | P | 5, 6, | 1 | 1.076 (x 0.929) | 69 (0.014) | 0.724 | defense response to bacterium | scrib |
| 1180 | GO:0005509 | F | 5, | 3 | 3.399 (x 0.883) | 218 (0.014) | 0.725 | calcium ion binding | CBP NUCB1 tok |
| 1181 | GO:0007626 | P | 4, | 1 | 1.092 (x 0.916) | 70 (0.014) | 0.727 | locomotory behavior | CkIIalpha |
| 1182 | GO:0048102 | P | 6, | 1 | 1.107 (x 0.903) | 71 (0.014) | 0.731 | autophagic cell death | Akap200 |
| 1183 | GO:0035070 | P | 6, | 1 | 1.107 (x 0.903) | 71 (0.014) | 0.731 | salivary gland histolysis | Akap200 |
| 1184 | GO:0035071 | P | 7, | 1 | 1.107 (x 0.903) | 71 (0.014) | 0.732 | salivary gland cell autophagic cell death | Akap200 |
| 1185 | GO:0004222 | F | 6, | 1 | 1.107 (x 0.903) | 71 (0.014) | 0.733 | metalloendopeptidase activity | tok |
| 1186 | GO:0008652 | P | 7, 8, | 1 | 1.123 (x 0.891) | 72 (0.014) | 0.736 | amino acid biosynthesis | ESTS:39C10S |
| 1187 | GO:0016192 | P | 5, 6, | 4 | 4.616 (x 0.867) | 296 (0.014) | 0.74 | vesicle-mediated transport | Arf79F Snap d wbl |
| 1188 | GO:0007268 | P | 6, | 3 | 3.524 (x 0.851) | 226 (0.013) | 0.742 | synaptic transmission | Arf79F Snap scrib |
| 1189 | GO:0009617 | P | 5, | 1 | 1.154 (x 0.867) | 74 (0.014) | 0.742 | response to bacterium | scrib |
| 1190 | GO:0031325 | P | 6, | 1 | 1.154 (x 0.867) | 74 (0.014) | 0.743 | positive regulation of cellular metabolism | ash2 |
| 1191 | GO:0009893 | P | 5, | 1 | 1.154 (x 0.867) | 74 (0.014) | 0.743 | positive regulation of metabolism | ash2 |
| 1192 | GO:0007601 | P | 5, 7, | 1 | 1.154 (x 0.867) | 74 (0.014) | 0.744 | visual perception | d |
| 1193 | GO:0050953 | P | 4, 6, | 1 | 1.154 (x 0.867) | 74 (0.014) | 0.745 | sensory perception of light stimulus | d |
| 1194 | GO:0016887 | F | 8, | 5 | 5.785 (x 0.864) | 371 (0.013) | 0.745 | ATPase activity | Mcm5 Mcm7 Top2 d dpa |
| 1195 | GO:0048534 | P | 4, | 1 | 1.154 (x 0.867) | 74 (0.014) | 0.745 | hemopoietic or lymphoid organ development | grn |
| 1196 | GO:0008565 | F | 3, | 1 | 1.170 (x 0.855) | 75 (0.013) | 0.745 | protein transporter activity | rin |
| 1197 | GO:0007186 | P | 6, | 4 | 4.756 (x 0.841) | 305 (0.013) | 0.758 | G-protein coupled receptor protein signaling pathway | CG30440 Mtl fz pbl |
| 1198 | GO:0006959 | P | 5, 6, | 1 | 1.216 (x 0.822) | 78 (0.013) | 0.759 | humoral immune response | scrib |
| 1199 | GO:0005386 | F | 3, | 6 | 7.064 (x 0.849) | 453 (0.013) | 0.768 | carrier activity | CG10657 CG10960 CG13848 CG3823 bib l(2)dtl |
| 1200 | GO:0005214 | F | 4, | 1 | 1.263 (x 0.792) | 81 (0.012) | 0.772 | structural constituent of cuticle (sensu Insecta) | CG32029 |
| 1201 | GO:0004197 | F | 6, | 1 | 1.263 (x 0.792) | 81 (0.012) | 0.773 | cysteine-type endopeptidase activity | BcDNA:LD22910 |
| 1202 | GO:0005198 | F | 2, | 10 | 11.508 (x 0.869) | 738 (0.014) | 0.775 | structural molecule activity | Act42A Act5C CG10423 CG32029 Lam Moe NetA betaTub56D d esn |
| 1203 | GO:0006367 | P | 9, | 1 | 1.310 (x 0.763) | 84 (0.012) | 0.783 | transcription initiation from RNA polymerase II promoter | Brf |
| 1204 | GO:0044271 | P | 5, 6, | 1 | 1.310 (x 0.763) | 84 (0.012) | 0.783 | nitrogen compound biosynthesis | ESTS:39C10S |
| 1205 | GO:0016810 | F | 4, | 1 | 1.325 (x 0.754) | 85 (0.012) | 0.784 | hydrolase activity, acting on carbon-nitrogen (but not peptide) bonds | SP2637 |
| 1206 | GO:0009309 | P | 6, 7, | 1 | 1.310 (x 0.763) | 84 (0.012) | 0.784 | amine biosynthesis | ESTS:39C10S |
| 1207 | GO:0016741 | F | 4, | 1 | 1.325 (x 0.754) | 85 (0.012) | 0.785 | transferase activity, transferring one-carbon groups | Caf1 |
| 1208 | GO:0008168 | F | 5, | 1 | 1.310 (x 0.763) | 84 (0.012) | 0.785 | methyltransferase activity | Caf1 |
| 1209 | GO:0043169 | F | 4, | 12 | 13.831 (x 0.868) | 887 (0.014) | 0.785 | cation binding | BcDNA:LD41548 CBP CG17419 D19A NUCB1 Pep ap esn gol klu th tok |
| 1210 | GO:0044421 | C | 2, 3, | 1 | 1.325 (x 0.754) | 85 (0.012) | 0.785 | extracellular region part | NetA |
| 1211 | GO:0008610 | P | 5, 6, 7, | 1 | 1.325 (x 0.754) | 85 (0.012) | 0.786 | lipid biosynthesis | CG2118 |
| 1212 | GO:0004713 | F | 7, | 1 | 1.325 (x 0.754) | 85 (0.012) | 0.786 | protein-tyrosine kinase activity | Eip63E |
| 1213 | GO:0006352 | P | 8, | 1 | 1.341 (x 0.746) | 86 (0.012) | 0.787 | transcription initiation | Brf |
| 1214 | GO:0044459 | C | 4, 5, 6, | 4 | 5.037 (x 0.794) | 323 (0.012) | 0.791 | plasma membrane part | Moe bib fz scrib |
| 1215 | GO:0006399 | P | 7, | 1 | 1.372 (x 0.729) | 88 (0.011) | 0.794 | tRNA metabolism | Aats-glupro |
| 1216 | GO:0007167 | P | 6, | 2 | 2.682 (x 0.746) | 172 (0.012) | 0.796 | enzyme linked receptor protein signaling pathway | edl tkv |
| 1217 | GO:0009152 | P | 8, 9, | 1 | 1.388 (x 0.721) | 89 (0.011) | 0.797 | purine ribonucleotide biosynthesis | CG3590 |
| 1218 | GO:0005830 | C | 5, 6, 7, 8, 9, 10, | 1 | 1.403 (x 0.713) | 90 (0.011) | 0.8 | cytosolic ribosome (sensu Eukaryota) | CG10423 |
| 1219 | GO:0009150 | P | 8, | 1 | 1.403 (x 0.713) | 90 (0.011) | 0.8 | purine ribonucleotide metabolism | CG3590 |
| 1220 | GO:0006164 | P | 7, 8, | 1 | 1.419 (x 0.705) | 91 (0.011) | 0.802 | purine nucleotide biosynthesis | CG3590 |
| 1221 | GO:0042623 | F | 9, | 4 | 5.146 (x 0.777) | 330 (0.012) | 0.803 | ATPase activity, coupled | Mcm5 Mcm7 d dpa |
| 1222 | GO:0008194 | F | 5, | 1 | 1.435 (x 0.697) | 92 (0.011) | 0.805 | UDP-glycosyltransferase activity | CG6904 |
| 1223 | GO:0006163 | P | 7, | 1 | 1.435 (x 0.697) | 92 (0.011) | 0.806 | purine nucleotide metabolism | CG3590 |
| 1224 | GO:0004252 | F | 6, | 3 | 4.008 (x 0.749) | 257 (0.012) | 0.808 | serine-type endopeptidase activity | Fur1 Sb deltaTry |
| 1225 | GO:0008047 | F | 3, | 1 | 1.481 (x 0.675) | 95 (0.011) | 0.815 | enzyme activator activity | CycA |
| 1226 | GO:0006520 | P | 6, 7, | 3 | 4.070 (x 0.737) | 261 (0.011) | 0.816 | amino acid metabolism | Aats-glupro CG2118 ESTS:39C10S |
| 1227 | GO:0000267 | C | 3, 4, | 1 | 1.497 (x 0.668) | 96 (0.010) | 0.818 | cell fraction | Top2 |
| 1228 | GO:0045892 | P | 9, | 1 | 1.513 (x 0.661) | 97 (0.010) | 0.82 | negative regulation of transcription, DNA-dependent | Caf1 |
| 1229 | GO:0042302 | F | 3, | 1 | 1.544 (x 0.648) | 99 (0.010) | 0.827 | structural constituent of cuticle | CG32029 |
| 1230 | GO:0007350 | P | 4, 5, | 1 | 1.591 (x 0.629) | 102 (0.010) | 0.836 | blastoderm segmentation | Cdk4 |
| 1231 | GO:0000165 | P | 7, | 1 | 1.606 (x 0.623) | 103 (0.010) | 0.839 | MAPKKK cascade | CG30440 |
| 1232 | GO:0005887 | C | 6, 7, 8, | 2 | 3.056 (x 0.654) | 196 (0.010) | 0.85 | integral to plasma membrane | bib fz |
| 1233 | GO:0031226 | C | 5, 6, 7, | 2 | 3.088 (x 0.648) | 198 (0.010) | 0.854 | intrinsic to plasma membrane | bib fz |
| 1234 | GO:0008236 | F | 5, | 3 | 4.569 (x 0.657) | 293 (0.010) | 0.873 | serine-type peptidase activity | Fur1 Sb deltaTry |
| 1235 | GO:0006519 | P | 5, | 3 | 4.569 (x 0.657) | 293 (0.010) | 0.874 | amino acid and derivative metabolism | Aats-glupro CG2118 ESTS:39C10S |
| 1236 | GO:0006897 | P | 6, 7, | 1 | 1.809 (x 0.553) | 116 (0.009) | 0.874 | endocytosis | Arf79F |
| 1237 | GO:0050874 | P | 3, | 11 | 14.034 (x 0.784) | 900 (0.012) | 0.875 | organismal physiological process | Arf79F Hsp26 Hsp27 NetA Snap Trxr-1 d fax msi scrib serpin-27A |
| 1238 | GO:0004263 | F | 7, | 2 | 3.243 (x 0.617) | 208 (0.010) | 0.875 | chymotrypsin activity | Sb deltaTry |
| 1239 | GO:0044445 | C | 5, 6, 7, 8, 9, | 1 | 1.840 (x 0.543) | 118 (0.008) | 0.877 | cytosolic part | CG10423 |
| 1240 | GO:0050877 | P | 4, | 7 | 9.606 (x 0.729) | 616 (0.011) | 0.885 | neurophysiological process | Arf79F NetA Snap d fax msi scrib |
| 1241 | GO:0015290 | F | 4, | 2 | 3.384 (x 0.591) | 217 (0.009) | 0.886 | electrochemical potential-driven transporter activity | CG10960 l(2)dtl |
| 1242 | GO:0015291 | F | 5, | 2 | 3.384 (x 0.591) | 217 (0.009) | 0.887 | porter activity | CG10960 l(2)dtl |
| 1243 | GO:0005576 | C | 2, | 4 | 6.019 (x 0.665) | 386 (0.010) | 0.887 | extracellular region | ImpE2 NetA deltaTry scrib |
| 1244 | GO:0007169 | P | 7, | 1 | 1.934 (x 0.517) | 124 (0.008) | 0.888 | transmembrane receptor protein tyrosine kinase signaling pathway | edl |
| 1245 | GO:0004175 | F | 5, | 5 | 7.345 (x 0.681) | 471 (0.011) | 0.893 | endopeptidase activity | BcDNA:LD22910 Fur1 Sb deltaTry tok |
| 1246 | GO:0016787 | F | 3, | 23 | 28.006 (x 0.821) | 1796 (0.013) | 0.909 | hydrolase activity | ApepP Arf79F BcDNA:LD22910 BcDNA:LD41548 CG33138 CG4670 D19A Fur1 GNBP3 Mcm5 Mcm7 Mtl SP2637 Sb Top2 Uch betaTub56D d deltaTry dpa fra gol tok |
| 1247 | GO:0004295 | F | 7, | 2 | 3.664 (x 0.546) | 235 (0.009) | 0.913 | trypsin activity | Sb deltaTry |
| 1248 | GO:0006629 | P | 5, | 5 | 7.641 (x 0.654) | 490 (0.010) | 0.913 | lipid metabolism | BcDNA:GH02901 CG2118 CG31169 CG7461 CG9057 |
| 1249 | GO:0006118 | P | 6, | 3 | 5.193 (x 0.578) | 333 (0.009) | 0.923 | electron transport | CG4670 CG7461 Trxr-1 |
| 1250 | GO:0031980 | C | 4, 5, 6, 7, 8, 9, 10, | 1 | 2.261 (x 0.442) | 145 (0.007) | 0.923 | mitochondrial lumen | CG2118 |
| 1251 | GO:0006810 | P | 4, 5, | 18 | 23.032 (x 0.782) | 1477 (0.012) | 0.924 | transport | Arf79F CG10657 CG10960 CG14439 CG17419 CG2852 CG3823 CG9057 Snap betaTub56D bib d l(2)dtl rin rpk scrib sqd wbl |
| 1252 | GO:0005759 | C | 5, 6, 7, 8, 9, 10, 11, | 1 | 2.261 (x 0.442) | 145 (0.007) | 0.924 | mitochondrial matrix | CG2118 |
| 1253 | GO:0030001 | P | 7, 8, | 1 | 2.292 (x 0.436) | 147 (0.007) | 0.924 | metal ion transport | rpk |
| 1254 | GO:0005215 | F | 2, | 11 | 15.094 (x 0.729) | 968 (0.011) | 0.924 | transporter activity | CG10657 CG10960 CG13848 CG14439 CG3823 CG8533 Snap bib l(2)dtl rin rpk |
| 1255 | GO:0044255 | P | 5, 6, | 3 | 5.224 (x 0.574) | 335 (0.009) | 0.924 | cellular lipid metabolism | BcDNA:GH02901 CG2118 CG7461 |
| 1256 | GO:0004888 | F | 4, | 4 | 6.627 (x 0.604) | 425 (0.009) | 0.925 | transmembrane receptor activity | fra fz pbl tkv |
| 1257 | GO:0004930 | F | 5, | 2 | 4.008 (x 0.499) | 257 (0.008) | 0.934 | G-protein coupled receptor activity | fz pbl |
| 1258 | GO:0016829 | F | 3, | 1 | 2.448 (x 0.408) | 157 (0.006) | 0.937 | lyase activity | CG3590 |
| 1259 | GO:0004872 | F | 3, | 5 | 8.623 (x 0.580) | 553 (0.009) | 0.955 | receptor activity | GNBP3 fra fz pbl tkv |
| 1260 | GO:0009308 | P | 5, | 3 | 5.863 (x 0.512) | 376 (0.008) | 0.955 | amine metabolism | Aats-glupro CG2118 ESTS:39C10S |
| 1261 | GO:0015672 | P | 7, 8, | 1 | 2.729 (x 0.366) | 175 (0.006) | 0.955 | monovalent inorganic cation transport | rpk |
| 1262 | GO:0003674 | F | 1, | 125 | 129.207 (x 0.967) | 8286 (0.015) | 0.955 | molecular\_function | Aats-glupro Abi Act42A Act5C Akap200 ApepP Arf79F B52 BEST:LD22483 BcDNA:GH02901 BcDNA:LD22910 BcDNA:LD41548 Brf CBP CG10423 CG10657 CG10960 CG10990 CG13848 CG13895 CG14439 CG15141 CG1544 CG15835 CG17419 CG17838 CG2118 CG2852 CG30440 CG31125 CG31169 CG31472 CG31617 CG31658 CG32029 CG32663 CG33138 CG3590 CG3823 CG4670 CG5873 CG6680 CG6767 CG6854 CG6904 CG7461 CG7675 CG8036 CG8533 CG8588 CG9027 CG9057 CG9598 CREG Caf1 Cdk4 CkIIalpha CkIalpha CycA D19A Dll DnaJ-1 Dr Dsp1 ESTS:39C10S Eb1 Eip63E Fur1 GNBP3 Gapdh2 GlyP Hrb27C Idh Lam Mcm5 Mcm7 Moe Mtl NUCB1 Nek2 NetA Pep SP2637 Sb Scm Snap Spn43Aa Top2 Trxr-1 Uch ap ash2 betaTub56D bib d dac deltaTry dpa dve eIF-4B eIF3-S9 edl endos esn fra fz gft gol grn klu l(2)dtl msi pbl polo rin rpk scrib serpin-27A smi35A sqd stai th tkv tok yps |
| 1263 | GO:0019866 | C | 4, 5, 6, 7, 8, 9, | 1 | 2.713 (x 0.369) | 174 (0.006) | 0.955 | organelle inner membrane | Lam |
| 1264 | GO:0006807 | P | 4, | 3 | 6.097 (x 0.492) | 391 (0.008) | 0.963 | nitrogen compound metabolism | Aats-glupro CG2118 ESTS:39C10S |
| 1265 | GO:0003735 | F | 3, | 1 | 2.932 (x 0.341) | 188 (0.005) | 0.965 | structural constituent of ribosome | CG10423 |
| 1266 | GO:0005840 | C | 4, 5, 6, 7, 8, | 1 | 2.947 (x 0.339) | 189 (0.005) | 0.965 | ribosome | CG10423 |
| 1267 | GO:0044425 | C | 3, 4, 5, | 13 | 18.962 (x 0.686) | 1216 (0.011) | 0.966 | membrane part | CG10960 CG14439 Fur1 ImpE2 ImpE3 Lam Moe Snap bib fz ninA pbl scrib |
| 1268 | GO:0001584 | F | 6, | 1 | 3.321 (x 0.301) | 213 (0.005) | 0.979 | rhodopsin-like receptor activity | pbl |
| 1269 | GO:0005739 | C | 5, 6, 7, 8, | 3 | 7.204 (x 0.416) | 462 (0.006) | 0.99 | mitochondrion | CG2118 Idh Trxr-1 |
| 1270 | GO:0031090 | C | 4, 5, 6, 7, 8, | 2 | 5.629 (x 0.355) | 361 (0.006) | 0.99 | organelle membrane | Fur1 Lam |
| 1271 | GO:0007600 | P | 3, 5, | 1 | 4.023 (x 0.249) | 258 (0.004) | 0.994 | sensory perception | d |
| 1272 | GO:0015075 | F | 3, | 3 | 7.563 (x 0.397) | 485 (0.006) | 0.994 | ion transporter activity | CG8533 bib rpk |
| 1273 | GO:0031975 | C | 2, | 1 | 4.163 (x 0.240) | 267 (0.004) | 0.994 | envelope | Lam |
| 1274 | GO:0031967 | C | 3, 4, 5, 6, 7, 8, | 1 | 4.163 (x 0.240) | 267 (0.004) | 0.995 | organelle envelope | Lam |
| 1275 | GO:0008324 | F | 4, | 2 | 6.222 (x 0.321) | 399 (0.005) | 0.995 | cation transporter activity | bib rpk |
| 1276 | GO:0008372 | C | 2, | 4 | 12.693 (x 0.315) | 814 (0.005) | 0.999 | cellular component unknown | CG31125 CG31658 CG32663 CG8588 |
| 1277 | GO:0005554 | F | 2, | 4 | 11.726 (x 0.341) | 752 (0.005) | 1 | molecular function unknown | CG31125 CG31658 CG32663 CG8588 |
| 1278 | GO:0006811 | P | 5, 6, | 2 | 7.204 (x 0.278) | 462 (0.004) | 1 | ion transport | CG17419 rpk |
| 1279 | GO:0016788 | F | 4, | 2 | 7.095 (x 0.282) | 455 (0.004) | 1 | hydrolase activity, acting on ester bonds | BcDNA:LD22910 Uch |
| 1280 | GO:0000004 | P | 2, | 3 | 10.931 (x 0.274) | 701 (0.004) | 1 | biological process unknown | CG31125 CG31658 CG32663 |
| 1281 | GO:0044429 | C | 4, 5, 6, 7, 8, 9, | 1 | 5.380 (x 0.186) | 345 (0.003) | 1 | mitochondrial part | CG2118 |
| 1282 | GO:0031224 | C | 4, 5, 6, | 7 | 14.767 (x 0.474) | 947 (0.007) | 1 | intrinsic to membrane | CG10960 CG14439 Fur1 bib fz ninA pbl |
| 1283 | GO:0006812 | P | 6, 7, | 1 | 5.972 (x 0.167) | 383 (0.003) | 1 | cation transport | rpk |
| 1284 | GO:0016020 | C | 3, 4, | 17 | 29.097 (x 0.584) | 1866 (0.009) | 1 | membrane | CG10960 CG14439 Fur1 ImpE2 ImpE3 Lam Moe Sb Snap bib fra fz ninA pbl rpk scrib tkv |
| 1285 | GO:0016021 | C | 5, 6, 7, | 7 | 14.720 (x 0.476) | 944 (0.007) | 1 | integral to membrane | CG10960 CG14439 Fur1 bib fz ninA pbl |

  

---

Regulated Genes that don't have GO terms
  

BG:DS07721.3 BcDNA:LD24702 CG10555 CG11120 CG11138 CG12643 CG13252 CG13676 CG13679 CG15905 CG16786 CG17032 CG17153 CG17255 CG18349 CG1962 CG2083 CG2469 CG32373 CG33936 CG3570 CG40354 CG4098 CG4751 CG4877 CG5175 CG5514 CG5521 CG6169 CG6234 CG7802 CG9416 CG9628 CG9883 miple
