# Supplementary material for: Functional dissection of the ash2 and ash1 transcriptomes provides insights into the transcriptional basis of wing phenotypes and reveals conserved protein interactions
Source: Genome Biol. 2007 Apr 28;8(4):R67. doi: 10.1186/gb-2007-8-4-r67 (PMC1896016; doi:10.1186/gb-2007-8-4-r67)
Supplement: Additional data file 12 — GO annotations of the genes upregulated over 2.0-fold in ash2112411 [file gb-2007-8-4-r67-S12.html]

  

---

  

|  |  |
| --- | --- |
| Go Statistics | Reg File: **ash2112411\_U2.0x.txt.fbgns** (272 genes -- 94 skipped)  Ref File: **ref.fbgns** (13577 genes -- 4663 skipped)  Database: **go\_200507-termdb.rdf-xml** |

---

  

Fields Description

| Pos | Go Term | Ontology | Levels | Observed | Expected | Possibles | p-value(Adj) | Go term description | Genes with the GO term |
| --- | --- | --- | --- | --- | --- | --- | --- | --- | --- |
| 1 | GO:0005840 | C | 4, 5, 6, 7, 8, | 21 | 3.774 (x 5.564) | 189 (0.111) | 7.21e-08 | ribosome | CG33002 CG4046 CG4866 CG5338 CG7014 CG8415 RpL11 RpL17A RpL38 RpL46 RpS17 RpS18 mRpL21 mRpL22 mRpL54 mRpS14 mRpS21 mRpS24 na oho23B tko |
| 2 | GO:0003735 | F | 3, | 21 | 3.754 (x 5.594) | 188 (0.112) | 1.3e-07 | structural constituent of ribosome | CG33002 CG4046 CG4866 CG5338 CG7014 CG8415 RpL11 RpL17A RpL38 RpL46 RpS17 RpS18 mRpL21 mRpL22 mRpL54 mRpS14 mRpS21 mRpS24 na oho23B tko |
| 3 | GO:0030529 | C | 3, 4, 5, 6, | 25 | 6.350 (x 3.937) | 318 (0.079) | 1.27e-06 | ribonucleoprotein complex | CG13277 CG31922 CG31950 CG33002 CG4046 CG4866 CG5338 CG6610 CG7014 CG8415 RpL11 RpL17A RpL38 RpL46 RpS17 RpS18 mRpL21 mRpL22 mRpL54 mRpS14 mRpS21 mRpS24 na oho23B tko |
| 4 | GO:0005198 | F | 2, | 37 | 14.737 (x 2.511) | 738 (0.050) | 2.46e-05 | structural molecule activity | Act57B BG:DS02740.9 CG2555 CG31876 CG33002 CG4046 CG4673 CG4866 CG5162 CG5338 CG7014 CG7941 CG8415 CG8511 CG8515 CLIP-190 Edg91 Femcoat Lcp65Ae RpL11 RpL17A RpL38 RpL46 RpS17 RpS18 Vm34Ca mRpL21 mRpL22 mRpL54 mRpS14 mRpS21 mRpS24 mira na oho23B robl tko |
| 5 | GO:0015935 | C | 3, 4, 5, 6, 7, 8, 9, | 11 | 1.378 (x 7.984) | 69 (0.159) | 2.61e-05 | small ribosomal subunit | CG4046 CG5338 CG7014 CG8415 RpS17 RpS18 mRpS14 mRpS21 mRpS24 oho23B tko |
| 6 | GO:0044445 | C | 5, 6, 7, 8, 9, | 13 | 2.356 (x 5.517) | 118 (0.110) | 0.000103 | cytosolic part | CG4046 CG5338 CG7014 CG7770 CG8415 RpL11 RpL17A RpL38 RpL46 RpS17 RpS18 l(3)01239 oho23B |
| 7 | GO:0005830 | C | 5, 6, 7, 8, 9, 10, | 11 | 1.797 (x 6.121) | 90 (0.122) | 0.000237 | cytosolic ribosome (sensu Eukaryota) | CG4046 CG5338 CG7014 CG8415 RpL11 RpL17A RpL38 RpL46 RpS17 RpS18 oho23B |
| 8 | GO:0005843 | C | 4, 5, 6, 7, 8, 9, 10, 11, | 7 | 0.779 (x 8.988) | 39 (0.179) | 0.00119 | cytosolic small ribosomal subunit (sensu Eukaryota) | CG4046 CG5338 CG7014 CG8415 RpS17 RpS18 oho23B |
| 9 | GO:0016283 | C | 3, 5, 6, 7, 8, | 7 | 0.779 (x 8.988) | 39 (0.179) | 0.00134 | eukaryotic 48S initiation complex | CG4046 CG5338 CG7014 CG8415 RpS17 RpS18 oho23B |
| 10 | GO:0005761 | C | 5, 6, 7, 8, 9, 10, 11, 12, | 9 | 1.478 (x 6.091) | 74 (0.122) | 0.00144 | mitochondrial ribosome | CG33002 CG4866 mRpL21 mRpL22 mRpL54 mRpS14 mRpS21 mRpS24 tko |
| 11 | GO:0044429 | C | 4, 5, 6, 7, 8, 9, | 20 | 6.889 (x 2.903) | 345 (0.058) | 0.0015 | mitochondrial part | CG11015 CG12400 CG14482 CG31477 CG32174 CG32230 CG33002 CG4866 CG5037 CG5548 EG:152A3.7 Pdsw Tim9a mRpL21 mRpL22 mRpL54 mRpS14 mRpS21 mRpS24 tko |
| 12 | GO:0000313 | C | 5, 6, 7, 8, 9, | 9 | 1.478 (x 6.091) | 74 (0.122) | 0.00158 | organellar ribosome | CG33002 CG4866 mRpL21 mRpL22 mRpL54 mRpS14 mRpS21 mRpS24 tko |
| 13 | GO:0044444 | C | 4, 5, 6, 7, | 42 | 23.104 (x 1.818) | 1157 (0.036) | 0.00537 | cytoplasmic part | CG11015 CG12400 CG14482 CG31477 CG32174 CG32230 CG33002 CG4046 CG4866 CG5037 CG5189 CG5338 CG5548 CG7014 CG7770 CG8415 CLIP-190 EG:152A3.7 Mlc2 Pdsw Rab3 RpL11 RpL17A RpL38 RpL46 RpS17 RpS18 Tim9a Tm1 Vha36 l(3)01239 mRpL21 mRpL22 mRpL54 mRpS14 mRpS21 mRpS24 mira na oho23B tko unc-13 |
| 14 | GO:0005829 | C | 5, 6, 7, 8, | 13 | 3.674 (x 3.538) | 184 (0.071) | 0.00579 | cytosol | CG4046 CG5338 CG7014 CG7770 CG8415 RpL11 RpL17A RpL38 RpL46 RpS17 RpS18 l(3)01239 oho23B |
| 15 | GO:0043228 | C | 3, | 29 | 14.198 (x 2.043) | 711 (0.041) | 0.0106 | non-membrane-bound organelle | Act57B CG13277 CG31950 CG33002 CG4046 CG4866 CG5338 CG6610 CG7014 CG8415 CLIP-190 Mlc2 RpL11 RpL17A RpL38 RpL46 RpS17 RpS18 Tm1 mRpL21 mRpL22 mRpL54 mRpS14 mRpS21 mRpS24 na oho23B robl tko |
| 16 | GO:0044446 | C | 3, 4, 5, 6, 7, | 47 | 28.435 (x 1.653) | 1424 (0.033) | 0.0109 | intracellular organelle part | Act57B CG11015 CG12400 CG13277 CG14482 CG15398 CG31477 CG31922 CG31950 CG32174 CG32230 CG33002 CG4046 CG4673 CG4866 CG5037 CG5338 CG5548 CG6610 CG7014 CG8415 CLIP-190 EG:152A3.7 Mlc2 Pdsw RpII18 RpL11 RpL17A RpL38 RpL46 RpS17 RpS18 Rpb10 Ssb-c31a Tim9a Tm1 Trap36 Vha36 mRpL21 mRpL22 mRpL54 mRpS14 mRpS21 mRpS24 oho23B robl tko |
| 17 | GO:0016282 | C | 3, 5, 6, 7, 8, | 7 | 1.198 (x 5.843) | 60 (0.117) | 0.0111 | eukaryotic 43S preinitiation complex | CG4046 CG5338 CG7014 CG8415 RpS17 RpS18 oho23B |
| 18 | GO:0044249 | P | 5, | 32 | 16.574 (x 1.931) | 830 (0.039) | 0.0111 | cellular biosynthesis | Ac78C Act57B CG10092 CG31477 CG32174 CG33002 CG4046 CG4866 CG5037 CG5338 CG7014 CG8415 CG9804 Dhfr Gs2 RpL11 RpL17A RpL38 RpL46 RpS17 RpS18 Tbh Vha36 mRpL21 mRpL22 mRpS14 mRpS21 mRpS24 na oho23B pum tko |
| 19 | GO:0043232 | C | 4, 5, 6, 7, | 29 | 14.198 (x 2.043) | 711 (0.041) | 0.0113 | intracellular non-membrane-bound organelle | Act57B CG13277 CG31950 CG33002 CG4046 CG4866 CG5338 CG6610 CG7014 CG8415 CLIP-190 Mlc2 RpL11 RpL17A RpL38 RpL46 RpS17 RpS18 Tm1 mRpL21 mRpL22 mRpL54 mRpS14 mRpS21 mRpS24 na oho23B robl tko |
| 20 | GO:0044422 | C | 2, 3, | 47 | 28.435 (x 1.653) | 1424 (0.033) | 0.0114 | organelle part | Act57B CG11015 CG12400 CG13277 CG14482 CG15398 CG31477 CG31922 CG31950 CG32174 CG32230 CG33002 CG4046 CG4673 CG4866 CG5037 CG5338 CG5548 CG6610 CG7014 CG8415 CLIP-190 EG:152A3.7 Mlc2 Pdsw RpII18 RpL11 RpL17A RpL38 RpL46 RpS17 RpS18 Rpb10 Ssb-c31a Tim9a Tm1 Trap36 Vha36 mRpL21 mRpL22 mRpL54 mRpS14 mRpS21 mRpS24 oho23B robl tko |
| 21 | GO:0042773 | P | 7, 9, | 7 | 1.238 (x 5.654) | 62 (0.113) | 0.0116 | ATP synthesis coupled electron transport | CG11015 CG12400 CG14482 CG32230 CG5548 EG:152A3.7 Pdsw |
| 22 | GO:0042775 | P | 8, 10, | 7 | 1.218 (x 5.747) | 61 (0.115) | 0.0116 | ATP synthesis coupled electron transport (sensu Eukaryota) | CG11015 CG12400 CG14482 CG32230 CG5548 EG:152A3.7 Pdsw |
| 23 | GO:0009059 | P | 5, 6, | 23 | 10.424 (x 2.207) | 522 (0.044) | 0.0127 | macromolecule biosynthesis | Act57B CG10092 CG33002 CG4046 CG4866 CG5338 CG7014 CG8415 RpL11 RpL17A RpL38 RpL46 RpS17 RpS18 mRpL21 mRpL22 mRpS14 mRpS21 mRpS24 na oho23B pum tko |
| 24 | GO:0006412 | P | 6, 7, | 22 | 9.864 (x 2.230) | 494 (0.045) | 0.0138 | protein biosynthesis | CG10092 CG33002 CG4046 CG4866 CG5338 CG7014 CG8415 RpL11 RpL17A RpL38 RpL46 RpS17 RpS18 mRpL21 mRpL22 mRpS14 mRpS21 mRpS24 na oho23B pum tko |
| 25 | GO:0005737 | C | 4, 5, 6, | 48 | 29.653 (x 1.619) | 1485 (0.032) | 0.0138 | cytoplasm | Abl CG11015 CG12400 CG14482 CG31477 CG32174 CG32230 CG33002 CG4046 CG4866 CG5037 CG5189 CG5338 CG5548 CG7014 CG7770 CG8415 CLIP-190 Cpn EG:152A3.7 Femcoat Gs2 Mlc2 Pdsw Rab3 RpL11 RpL17A RpL38 RpL46 RpS17 RpS18 Sod Tim9a Tm1 Vha36 l(3)01239 mRpL21 mRpL22 mRpL54 mRpS14 mRpS21 mRpS24 mira na oho23B pum tko unc-13 |
| 26 | GO:0044455 | C | 4, 5, 6, 7, 8, 9, 10, 11, 12, | 9 | 2.197 (x 4.097) | 110 (0.082) | 0.0138 | mitochondrial membrane part | CG11015 CG12400 CG14482 CG31477 CG32230 CG5548 EG:152A3.7 Pdsw Tim9a |
| 27 | GO:0009058 | P | 4, | 33 | 17.932 (x 1.840) | 898 (0.037) | 0.0149 | biosynthesis | Ac78C Act57B CG10092 CG31477 CG32174 CG33002 CG4046 CG4866 CG5037 CG5338 CG7014 CG8415 CG9804 Dhfr Gs2 RpL11 RpL17A RpL38 RpL46 RpS17 RpS18 Tbh Vha36 fu12 mRpL21 mRpL22 mRpS14 mRpS21 mRpS24 na oho23B pum tko |
| 28 | GO:0005746 | C | 5, 6, 7, 8, 9, 10, 11, 12, 13, | 7 | 1.378 (x 5.080) | 69 (0.101) | 0.0162 | mitochondrial electron transport chain | CG11015 CG12400 CG14482 CG32230 CG5548 EG:152A3.7 Pdsw |
| 29 | GO:0006120 | P | 9, 11, | 5 | 0.699 (x 7.154) | 35 (0.143) | 0.0219 | mitochondrial electron transport, NADH to ubiquinone | CG12400 CG32230 CG5548 EG:152A3.7 Pdsw |
| 30 | GO:0015934 | C | 3, 4, 5, 6, 7, 8, 9, | 8 | 1.917 (x 4.173) | 96 (0.083) | 0.0227 | large ribosomal subunit | CG33002 RpL11 RpL17A RpL38 RpL46 mRpL21 mRpL22 mRpL54 |
| 31 | GO:0003954 | F | 5, | 5 | 0.719 (x 6.955) | 36 (0.139) | 0.0227 | NADH dehydrogenase activity | CG12400 CG32230 CG5548 EG:152A3.7 Pdsw |
| 32 | GO:0004364 | F | 5, | 5 | 0.719 (x 6.955) | 36 (0.139) | 0.0234 | glutathione transferase activity | CG5224 GstD9 GstE5 GstE6 GstE7 |
| 33 | GO:0005747 | C | 4, 5, 6, 7, 8, 9, 10, 11, 12, 13, 14, | 5 | 0.739 (x 6.767) | 37 (0.135) | 0.0243 | respiratory chain complex I (sensu Eukaryota) | CG12400 CG32230 CG5548 EG:152A3.7 Pdsw |
| 34 | GO:0045271 | C | 3, 4, 5, 6, | 5 | 0.739 (x 6.767) | 37 (0.135) | 0.025 | respiratory chain complex I | CG12400 CG32230 CG5548 EG:152A3.7 Pdsw |
| 35 | GO:0005739 | C | 5, 6, 7, 8, | 20 | 9.225 (x 2.168) | 462 (0.043) | 0.0266 | mitochondrion | CG11015 CG12400 CG14482 CG31477 CG32174 CG32230 CG33002 CG4866 CG5037 CG5548 EG:152A3.7 Pdsw Tim9a mRpL21 mRpL22 mRpL54 mRpS14 mRpS21 mRpS24 tko |
| 36 | GO:0009636 | P | 5, | 9 | 2.576 (x 3.494) | 129 (0.070) | 0.0319 | response to toxin | CG11898 CG30438 CG31146 CG5224 GstD9 GstE5 GstE6 GstE7 Ugt86Di |
| 37 | GO:0005214 | F | 4, | 7 | 1.617 (x 4.328) | 81 (0.086) | 0.0323 | structural constituent of cuticle (sensu Insecta) | CG2555 CG31876 CG7941 CG8511 CG8515 Edg91 Lcp65Ae |
| 38 | GO:0016765 | F | 4, | 6 | 1.198 (x 5.008) | 60 (0.100) | 0.0333 | transferase activity, transferring alkyl or aryl (other than methyl) groups | CG5037 CG5224 GstD9 GstE5 GstE6 GstE7 |
| 39 | GO:0016651 | F | 4, | 5 | 0.859 (x 5.823) | 43 (0.116) | 0.0425 | oxidoreductase activity, acting on NADH or NADPH | CG12400 CG32230 CG5548 EG:152A3.7 Pdsw |
| 40 | GO:0050136 | F | 6, | 4 | 0.559 (x 7.154) | 28 (0.143) | 0.051 | NADH dehydrogenase (quinone) activity | CG12400 CG5548 EG:152A3.7 Pdsw |
| 41 | GO:0016655 | F | 5, | 4 | 0.559 (x 7.154) | 28 (0.143) | 0.0521 | oxidoreductase activity, acting on NADH or NADPH, quinone or similar compound as acceptor | CG12400 CG5548 EG:152A3.7 Pdsw |
| 42 | GO:0043234 | C | 2, | 47 | 31.530 (x 1.491) | 1579 (0.030) | 0.0523 | protein complex | CG11015 CG12400 CG13277 CG14482 CG15398 CG31477 CG31922 CG31950 CG32230 CG33002 CG4046 CG4673 CG4866 CG5338 CG5548 CG6610 CG7014 CG7770 CG8415 CLIP-190 EG:152A3.7 Mlc2 Pdsw RpII18 RpL11 RpL17A RpL38 RpL46 RpS17 RpS18 Rpb10 Ssb-c31a Tim9a Trap36 Vha36 l(3)01239 mRpL21 mRpL22 mRpL54 mRpS14 mRpS21 mRpS24 na oho23B robl skpA tko |
| 43 | GO:0005743 | C | 5, 6, 7, 8, 9, 10, 11, 12, | 10 | 3.375 (x 2.963) | 169 (0.059) | 0.0524 | mitochondrial inner membrane | CG11015 CG12400 CG14482 CG31477 CG32174 CG32230 CG5548 EG:152A3.7 Pdsw Tim9a |
| 44 | GO:0008137 | F | 6, 7, | 4 | 0.559 (x 7.154) | 28 (0.143) | 0.0533 | NADH dehydrogenase (ubiquinone) activity | CG12400 CG5548 EG:152A3.7 Pdsw |
| 45 | GO:0006119 | P | 6, 8, | 9 | 2.816 (x 3.197) | 141 (0.064) | 0.0534 | oxidative phosphorylation | CG11015 CG12400 CG14482 CG31477 CG32230 CG5548 EG:152A3.7 Pdsw Vha36 |
| 46 | GO:0006800 | P | 5, | 6 | 1.338 (x 4.485) | 67 (0.090) | 0.0535 | oxygen and reactive oxygen species metabolism | CG18522 GstE5 GstE6 GstE7 Prx6005 Sod |
| 47 | GO:0031980 | C | 4, 5, 6, 7, 8, 9, 10, | 9 | 2.895 (x 3.108) | 145 (0.062) | 0.0539 | mitochondrial lumen | CG33002 CG4866 mRpL21 mRpL22 mRpL54 mRpS14 mRpS21 mRpS24 tko |
| 48 | GO:0019866 | C | 4, 5, 6, 7, 8, 9, | 10 | 3.475 (x 2.878) | 174 (0.057) | 0.0543 | organelle inner membrane | CG11015 CG12400 CG14482 CG31477 CG32174 CG32230 CG5548 EG:152A3.7 Pdsw Tim9a |
| 49 | GO:0005759 | C | 5, 6, 7, 8, 9, 10, 11, | 9 | 2.895 (x 3.108) | 145 (0.062) | 0.0551 | mitochondrial matrix | CG33002 CG4866 mRpL21 mRpL22 mRpL54 mRpS14 mRpS21 mRpS24 tko |
| 50 | GO:0005740 | C | 4, 5, 6, 7, 8, 9, 10, | 11 | 4.094 (x 2.687) | 205 (0.054) | 0.0563 | mitochondrial envelope | CG11015 CG12400 CG14482 CG31477 CG32174 CG32230 CG5037 CG5548 EG:152A3.7 Pdsw Tim9a |
| 51 | GO:0000314 | C | 4, 5, 6, 7, 8, 9, 10, | 4 | 0.599 (x 6.677) | 30 (0.133) | 0.0572 | organellar small ribosomal subunit | mRpS14 mRpS21 mRpS24 tko |
| 52 | GO:0005763 | C | 5, 6, 7, 8, 9, 10, 11, 12, 13, | 4 | 0.599 (x 6.677) | 30 (0.133) | 0.0583 | mitochondrial small ribosomal subunit | mRpS14 mRpS21 mRpS24 tko |
| 53 | GO:0006952 | P | 4, | 20 | 10.264 (x 1.949) | 514 (0.039) | 0.0626 | defense response | CG11898 CG18249 CG18522 CG30438 CG31146 CG5224 CG5397 CG6426 CG8193 GstD9 GstE5 GstE6 GstE7 Prx6005 Sod Tehao Tsf1 Ugt86Di hig upd3 |
| 54 | GO:0042302 | F | 3, | 7 | 1.977 (x 3.541) | 99 (0.071) | 0.0703 | structural constituent of cuticle | CG2555 CG31876 CG7941 CG8511 CG8515 Edg91 Lcp65Ae |
| 55 | GO:0009607 | P | 3, | 20 | 10.424 (x 1.919) | 522 (0.038) | 0.0719 | response to biotic stimulus | CG11898 CG18249 CG18522 CG30438 CG31146 CG5224 CG5397 CG6426 CG8193 GstD9 GstE5 GstE6 GstE7 Prx6005 Sod Tehao Tsf1 Ugt86Di hig upd3 |
| 56 | GO:0009055 | F | 4, | 5 | 1.058 (x 4.724) | 53 (0.094) | 0.0751 | electron carrier activity | CG12400 CG32230 CG5548 EG:152A3.7 Pdsw |
| 57 | GO:0031966 | C | 5, 6, 7, 8, 9, 10, 11, | 10 | 3.734 (x 2.678) | 187 (0.053) | 0.0783 | mitochondrial membrane | CG11015 CG12400 CG14482 CG31477 CG32174 CG32230 CG5548 EG:152A3.7 Pdsw Tim9a |
| 58 | GO:0031974 | C | 2, | 17 | 8.606 (x 1.975) | 431 (0.039) | 0.0999 | membrane-enclosed lumen | CG13277 CG15398 CG31950 CG33002 CG4866 CG6610 RpII18 Rpb10 Ssb-c31a Trap36 mRpL21 mRpL22 mRpL54 mRpS14 mRpS21 mRpS24 tko |
| 59 | GO:0043233 | C | 3, 4, | 17 | 8.606 (x 1.975) | 431 (0.039) | 0.102 | organelle lumen | CG13277 CG15398 CG31950 CG33002 CG4866 CG6610 RpII18 Rpb10 Ssb-c31a Trap36 mRpL21 mRpL22 mRpL54 mRpS14 mRpS21 mRpS24 tko |
| 60 | GO:0031975 | C | 2, | 12 | 5.332 (x 2.251) | 267 (0.045) | 0.121 | envelope | CG11015 CG12400 CG14482 CG31477 CG32174 CG32230 CG4673 CG5037 CG5548 EG:152A3.7 Pdsw Tim9a |
| 61 | GO:0008374 | F | 7, | 3 | 0.399 (x 7.512) | 20 (0.150) | 0.121 | O-acyltransferase activity | CG5122 CG5397 fu12 |
| 62 | GO:0031967 | C | 3, 4, 5, 6, 7, 8, | 12 | 5.332 (x 2.251) | 267 (0.045) | 0.123 | organelle envelope | CG11015 CG12400 CG14482 CG31477 CG32174 CG32230 CG4673 CG5037 CG5548 EG:152A3.7 Pdsw Tim9a |
| 63 | GO:0005732 | C | 4, 5, 6, 7, 8, 9, 10, 11, 12, 13, | 3 | 0.399 (x 7.512) | 20 (0.150) | 0.123 | small nucleolar ribonucleoprotein complex | CG13277 CG31950 CG6610 |
| 64 | GO:0008011 | F | 5, | 2 | 0.140 (x 14.308) | 7 (0.286) | 0.127 | structural constituent of pupal cuticle (sensu Insecta) | CG31876 Edg91 |
| 65 | GO:0016272 | C | 3, 6, 7, 8, 9, 10, | 2 | 0.140 (x 14.308) | 7 (0.286) | 0.129 | prefoldin complex | CG7770 l(3)01239 |
| 66 | GO:0005762 | C | 5, 6, 7, 8, 9, 10, 11, 12, 13, | 4 | 0.899 (x 4.451) | 45 (0.089) | 0.192 | mitochondrial large ribosomal subunit | CG33002 mRpL21 mRpL22 mRpL54 |
| 67 | GO:0000315 | C | 4, 5, 6, 7, 8, 9, 10, | 4 | 0.899 (x 4.451) | 45 (0.089) | 0.194 | organellar large ribosomal subunit | CG33002 mRpL21 mRpL22 mRpL54 |
| 68 | GO:0005688 | C | 5, 6, 7, 8, 9, 10, 11, 12, | 2 | 0.180 (x 11.129) | 9 (0.222) | 0.203 | snRNP U6 | CG13277 CG6610 |
| 69 | GO:0043487 | P | 8, | 1 | 0.020 (x 50.079) | 1 (1.000) | 0.223 | regulation of RNA stability | pum |
| 70 | GO:0044452 | C | 5, 6, 7, 8, 9, 10, 11, 12, | 3 | 0.519 (x 5.778) | 26 (0.115) | 0.223 | nucleolar part | CG13277 CG31950 CG6610 |
| 71 | GO:0006307 | P | 6, 8, | 1 | 0.020 (x 50.079) | 1 (1.000) | 0.225 | DNA dealkylation | agt |
| 72 | GO:0008495 | F | 7, | 1 | 0.020 (x 50.079) | 1 (1.000) | 0.228 | protoheme IX farnesyltransferase activity | CG5037 |
| 73 | GO:0048033 | P | 7, 8, | 1 | 0.020 (x 50.079) | 1 (1.000) | 0.23 | heme o metabolism | CG5037 |
| 74 | GO:0007497 | P | 6, | 1 | 0.020 (x 50.079) | 1 (1.000) | 0.233 | posterior midgut development | sisA |
| 75 | GO:0006542 | P | 9, 10, | 1 | 0.020 (x 50.079) | 1 (1.000) | 0.235 | glutamine biosynthesis | Gs2 |
| 76 | GO:0006589 | P | 7, 8, 9, 10, | 1 | 0.020 (x 50.079) | 1 (1.000) | 0.238 | octopamine biosynthesis | Tbh |
| 77 | GO:0004836 | F | 4, | 1 | 0.020 (x 50.079) | 1 (1.000) | 0.241 | tyramine-beta hydroxylase activity | Tbh |
| 78 | GO:0042221 | P | 4, | 11 | 5.252 (x 2.095) | 263 (0.042) | 0.242 | response to chemical stimulus | CG11898 CG30438 CG31146 CG5224 GstD9 GstE5 GstE6 GstE7 Obp56a Tbh Ugt86Di |
| 79 | GO:0051087 | F | 4, | 2 | 0.200 (x 10.016) | 10 (0.200) | 0.243 | chaperone binding | CG7770 l(3)01239 |
| 80 | GO:0045931 | P | 7, 8, | 1 | 0.020 (x 50.079) | 1 (1.000) | 0.243 | positive regulation of progression through mitotic cell cycle | skpA |
| 81 | GO:0006545 | P | 9, 10, | 1 | 0.020 (x 50.079) | 1 (1.000) | 0.246 | glycine biosynthesis | Dhfr |
| 82 | GO:0043488 | P | 8, 9, | 1 | 0.020 (x 50.079) | 1 (1.000) | 0.249 | regulation of mRNA stability | pum |
| 83 | GO:0000288 | P | 9, 10, | 1 | 0.020 (x 50.079) | 1 (1.000) | 0.252 | mRNA catabolism, deadenylylation-dependent decay | pum |
| 84 | GO:0015949 | P | 6, | 1 | 0.020 (x 50.079) | 1 (1.000) | 0.255 | nucleobase, nucleoside and nucleotide interconversion | Dhfr |
| 85 | GO:0007496 | P | 6, | 1 | 0.020 (x 50.079) | 1 (1.000) | 0.258 | anterior midgut development | sisA |
| 86 | GO:0004146 | F | 6, | 1 | 0.020 (x 50.079) | 1 (1.000) | 0.261 | dihydrofolate reductase activity | Dhfr |
| 87 | GO:0003908 | F | 7, | 1 | 0.020 (x 50.079) | 1 (1.000) | 0.265 | methylated-DNA-[protein]-cysteine S-methyltransferase activity | agt |
| 88 | GO:0015955 | P | 8, | 1 | 0.020 (x 50.079) | 1 (1.000) | 0.268 | pyrimidine deoxyribonucleotide interconversion | Dhfr |
| 89 | GO:0048034 | P | 8, 9, | 1 | 0.020 (x 50.079) | 1 (1.000) | 0.271 | heme o biosynthesis | CG5037 |
| 90 | GO:0005842 | C | 4, 5, 6, 7, 8, 9, 10, 11, | 4 | 1.018 (x 3.928) | 51 (0.078) | 0.273 | cytosolic large ribosomal subunit (sensu Eukaryota) | RpL11 RpL17A RpL38 RpL46 |
| 91 | GO:0006458 | P | 8, | 2 | 0.220 (x 9.105) | 11 (0.182) | 0.274 | 'de novo' protein folding | CG7770 l(3)01239 |
| 92 | GO:0005927 | C | 8, 9, 10, | 1 | 0.020 (x 50.079) | 1 (1.000) | 0.275 | muscle tendon junction | Abl |
| 93 | GO:0044449 | C | 5, 6, 7, 8, 9, | 2 | 0.220 (x 9.105) | 11 (0.182) | 0.278 | contractile fiber part | Mlc2 Tm1 |
| 94 | GO:0015953 | P | 7, | 1 | 0.020 (x 50.079) | 1 (1.000) | 0.279 | pyrimidine nucleotide interconversion | Dhfr |
| 95 | GO:0005665 | C | 4, 5, 6, 7, 8, 9, 10, 11, 12, 13, 14, | 2 | 0.220 (x 9.105) | 11 (0.182) | 0.281 | DNA-directed RNA polymerase II, core complex | RpII18 Rpb10 |
| 96 | GO:0043292 | C | 5, 6, 7, 8, | 2 | 0.260 (x 7.704) | 13 (0.154) | 0.296 | contractile fiber | Mlc2 Tm1 |
| 97 | GO:0016358 | P | 7, 10, | 3 | 0.699 (x 4.292) | 35 (0.086) | 0.351 | dendrite development | Tm1 pum robl |
| 98 | GO:0007492 | P | 4, | 2 | 0.319 (x 6.260) | 16 (0.125) | 0.378 | endoderm development | sisA toy |
| 99 | GO:0030532 | C | 4, 5, 6, 7, 8, 9, 10, | 4 | 1.238 (x 3.231) | 62 (0.065) | 0.378 | small nuclear ribonucleoprotein complex | CG13277 CG31922 CG31950 CG6610 |
| 100 | GO:0019430 | P | 7, | 1 | 0.040 (x 25.039) | 2 (0.500) | 0.381 | removal of superoxide radicals | Sod |
| 101 | GO:0004802 | F | 5, | 1 | 0.040 (x 25.039) | 2 (0.500) | 0.385 | transketolase activity | CG5103 |
| 102 | GO:0045787 | P | 6, 7, | 1 | 0.040 (x 25.039) | 2 (0.500) | 0.388 | positive regulation of progression through cell cycle | skpA |
| 103 | GO:0004311 | F | 6, | 1 | 0.040 (x 25.039) | 2 (0.500) | 0.392 | farnesyltranstransferase activity | CG5037 |
| 104 | GO:0046333 | P | 6, 7, 8, 9, | 1 | 0.040 (x 25.039) | 2 (0.500) | 0.395 | octopamine metabolism | Tbh |
| 105 | GO:0006081 | P | 5, | 1 | 0.040 (x 25.039) | 2 (0.500) | 0.399 | aldehyde metabolism | CG10638 |
| 106 | GO:0009107 | P | 8, 9, | 1 | 0.040 (x 25.039) | 2 (0.500) | 0.403 | lipoate biosynthesis | CG9804 |
| 107 | GO:0005122 | F | 4, 5, | 1 | 0.040 (x 25.039) | 2 (0.500) | 0.407 | torso binding | tsl |
| 108 | GO:0005862 | C | 5, 6, 7, 8, 9, 10, 11, 12, | 1 | 0.040 (x 25.039) | 2 (0.500) | 0.411 | muscle thin filament tropomyosin | Tm1 |
| 109 | GO:0019740 | P | 5, | 1 | 0.040 (x 25.039) | 2 (0.500) | 0.415 | nitrogen utilization | CG9836 |
| 110 | GO:0009106 | P | 7, 8, | 1 | 0.040 (x 25.039) | 2 (0.500) | 0.419 | lipoate metabolism | CG9804 |
| 111 | GO:0009105 | P | 7, 8, | 1 | 0.040 (x 25.039) | 2 (0.500) | 0.423 | lipoic acid biosynthesis | CG9804 |
| 112 | GO:0008010 | F | 5, | 3 | 0.799 (x 3.756) | 40 (0.075) | 0.427 | structural constituent of larval cuticle (sensu Insecta) | CG2555 CG7941 Lcp65Ae |
| 113 | GO:0051188 | P | 6, | 5 | 2.097 (x 2.385) | 105 (0.048) | 0.442 | cofactor biosynthesis | CG31477 CG32174 CG5037 CG9804 Vha36 |
| 114 | GO:0006733 | P | 7, | 2 | 0.399 (x 5.008) | 20 (0.100) | 0.445 | oxidoreduction coenzyme metabolism | CG32174 CG5103 |
| 115 | GO:0004092 | F | 9, | 1 | 0.060 (x 16.693) | 3 (0.333) | 0.445 | carnitine O-acetyltransferase activity | CG5122 |
| 116 | GO:0000146 | F | 3, | 1 | 0.060 (x 16.693) | 3 (0.333) | 0.448 | microfilament motor activity | Mlc2 |
| 117 | GO:0045213 | P | 7, 8, | 1 | 0.060 (x 16.693) | 3 (0.333) | 0.451 | neurotransmitter receptor metabolism | Gs2 |
| 118 | GO:0046483 | P | 5, | 6 | 2.796 (x 2.146) | 140 (0.043) | 0.454 | heterocycle metabolism | CG18522 CG2277 CG5037 CG9804 Dhfr Vha36 |
| 119 | GO:0000900 | F | 4, 5, | 1 | 0.060 (x 16.693) | 3 (0.333) | 0.454 | translation repressor activity, nucleic acid binding | pum |
| 120 | GO:0017056 | F | 3, | 1 | 0.060 (x 16.693) | 3 (0.333) | 0.458 | structural constituent of nuclear pore | CG4673 |
| 121 | GO:0043112 | P | 7, | 1 | 0.060 (x 16.693) | 3 (0.333) | 0.461 | receptor metabolism | Gs2 |
| 122 | GO:0004356 | F | 7, | 1 | 0.060 (x 16.693) | 3 (0.333) | 0.465 | glutamate-ammonia ligase activity | Gs2 |
| 123 | GO:0004814 | F | 7, | 1 | 0.060 (x 16.693) | 3 (0.333) | 0.468 | arginine-tRNA ligase activity | CG10092 |
| 124 | GO:0006732 | P | 6, | 9 | 4.772 (x 1.886) | 239 (0.038) | 0.469 | coenzyme metabolism | CG10237 CG31477 CG32174 CG5037 CG5103 CG6574 CG9804 Dhfr Vha36 |
| 125 | GO:0005542 | F | 4, | 1 | 0.060 (x 16.693) | 3 (0.333) | 0.472 | folic acid binding | CG6574 |
| 126 | GO:0000273 | P | 6, 7, | 1 | 0.060 (x 16.693) | 3 (0.333) | 0.475 | lipoic acid metabolism | CG9804 |
| 127 | GO:0016716 | F | 5, | 1 | 0.060 (x 16.693) | 3 (0.333) | 0.479 | oxidoreductase activity, acting on paired donors, with incorporation or reduction of molecular oxygen, another compound as one donor, and incorporation of one atom of oxygen | CG8193 |
| 128 | GO:0006420 | P | 9, 10, 11, | 1 | 0.060 (x 16.693) | 3 (0.333) | 0.483 | arginyl-tRNA aminoacylation | CG10092 |
| 129 | GO:0016979 | F | 5, | 1 | 0.060 (x 16.693) | 3 (0.333) | 0.486 | lipoate-protein ligase activity | CG9804 |
| 130 | GO:0016211 | F | 6, | 1 | 0.060 (x 16.693) | 3 (0.333) | 0.49 | ammonia ligase activity | Gs2 |
| 131 | GO:0051186 | P | 5, | 9 | 5.052 (x 1.781) | 253 (0.036) | 0.491 | cofactor metabolism | CG10237 CG31477 CG32174 CG5037 CG5103 CG6574 CG9804 Dhfr Vha36 |
| 132 | GO:0005859 | C | 5, 6, 7, 8, 9, 10, 11, 12, | 1 | 0.060 (x 16.693) | 3 (0.333) | 0.494 | muscle myosin | Mlc2 |
| 133 | GO:0005516 | F | 4, | 4 | 1.418 (x 2.821) | 71 (0.056) | 0.495 | calmodulin binding | CG7646 Mlc2 TpnC41C unc-13 |
| 134 | GO:0008172 | F | 6, | 1 | 0.060 (x 16.693) | 3 (0.333) | 0.498 | S-methyltransferase activity | agt |
| 135 | GO:0006744 | P | 8, 9, | 1 | 0.060 (x 16.693) | 3 (0.333) | 0.502 | ubiquinone biosynthesis | CG32174 |
| 136 | GO:0009628 | P | 3, | 12 | 7.428 (x 1.615) | 372 (0.032) | 0.503 | response to abiotic stimulus | CG11898 CG30438 CG31146 CG5224 GstD9 GstE5 GstE6 GstE7 Obp56a Tbh Ugt86Di tko |
| 137 | GO:0008518 | F | 6, | 1 | 0.060 (x 16.693) | 3 (0.333) | 0.506 | reduced folate carrier activity | CG6574 |
| 138 | GO:0051298 | P | 6, 8, 9, | 1 | 0.060 (x 16.693) | 3 (0.333) | 0.51 | centrosome duplication | skpA |
| 139 | GO:0009112 | P | 6, | 4 | 1.558 (x 2.568) | 78 (0.051) | 0.51 | nucleobase metabolism | CG18522 CG2277 Dhfr Vha36 |
| 140 | GO:0016744 | F | 4, | 1 | 0.060 (x 16.693) | 3 (0.333) | 0.514 | transferase activity, transferring aldehyde or ketonic groups | CG5103 |
| 141 | GO:0006541 | P | 8, 9, | 1 | 0.060 (x 16.693) | 3 (0.333) | 0.519 | glutamine metabolism | Gs2 |
| 142 | GO:0016880 | F | 5, | 1 | 0.060 (x 16.693) | 3 (0.333) | 0.523 | acid-ammonia (or amide) ligase activity | Gs2 |
| 143 | GO:0050910 | P | 6, 7, 8, | 1 | 0.080 (x 12.520) | 4 (0.250) | 0.523 | detection of mechanical stimulus during sensory perception of sound | tko |
| 144 | GO:0042375 | P | 6, | 1 | 0.080 (x 12.520) | 4 (0.250) | 0.527 | quinone cofactor metabolism | CG32174 |
| 145 | GO:0009399 | P | 5, | 1 | 0.060 (x 16.693) | 3 (0.333) | 0.528 | nitrogen fixation | Gs2 |
| 146 | GO:0031090 | C | 4, 5, 6, 7, 8, | 12 | 7.209 (x 1.665) | 361 (0.033) | 0.53 | organelle membrane | CG11015 CG12400 CG14482 CG31477 CG32174 CG32230 CG4673 CG5548 EG:152A3.7 Pdsw Tim9a Vha36 |
| 147 | GO:0007064 | P | 6, 7, 9, | 1 | 0.080 (x 12.520) | 4 (0.250) | 0.53 | mitotic sister chromatid cohesion | Nipped-B |
| 148 | GO:0004503 | F | 6, | 1 | 0.060 (x 16.693) | 3 (0.333) | 0.532 | monophenol monooxygenase activity | CG8193 |
| 149 | GO:0045426 | P | 7, 8, | 1 | 0.080 (x 12.520) | 4 (0.250) | 0.534 | quinone cofactor biosynthesis | CG32174 |
| 150 | GO:0004772 | F | 8, | 1 | 0.060 (x 16.693) | 3 (0.333) | 0.537 | sterol O-acyltransferase activity | CG5397 |
| 151 | GO:0008415 | F | 6, | 5 | 2.296 (x 2.177) | 115 (0.043) | 0.537 | acyltransferase activity | CG5037 CG5122 CG5397 CG6921 fu12 |
| 152 | GO:0030728 | P | 6, | 1 | 0.080 (x 12.520) | 4 (0.250) | 0.537 | ovulation | Tbh |
| 153 | GO:0016460 | C | 4, 6, 7, 8, 9, 10, 11, | 1 | 0.080 (x 12.520) | 4 (0.250) | 0.541 | myosin II | Mlc2 |
| 154 | GO:0042043 | F | 4, | 1 | 0.080 (x 12.520) | 4 (0.250) | 0.544 | neurexin binding | CG31146 |
| 155 | GO:0030880 | C | 3, 4, 5, 6, | 2 | 0.479 (x 4.173) | 24 (0.083) | 0.544 | RNA polymerase complex | RpII18 Rpb10 |
| 156 | GO:0007268 | P | 6, | 8 | 4.513 (x 1.773) | 226 (0.035) | 0.546 | synaptic transmission | Arf84F CG31146 GABA-B-R2 Gs2 Rab3 Tbh pum unc-13 |
| 157 | GO:0019842 | F | 3, | 2 | 0.479 (x 4.173) | 24 (0.083) | 0.548 | vitamin binding | CG10237 CG6574 |
| 158 | GO:0006538 | P | 9, 10, | 1 | 0.080 (x 12.520) | 4 (0.250) | 0.548 | glutamate catabolism | Gs2 |
| 159 | GO:0016591 | C | 3, 6, 7, 8, 9, 10, 11, 12, 13, | 4 | 1.657 (x 2.413) | 83 (0.048) | 0.55 | DNA-directed RNA polymerase II, holoenzyme | CG15398 RpII18 Rpb10 Trap36 |
| 160 | GO:0030371 | F | 3, | 1 | 0.080 (x 12.520) | 4 (0.250) | 0.552 | translation repressor activity | pum |
| 161 | GO:0008316 | F | 3, | 1 | 0.080 (x 12.520) | 4 (0.250) | 0.555 | structural constituent of vitelline membrane (sensu Insecta) | Vm34Ca |
| 162 | GO:0006826 | P | 9, 10, | 1 | 0.080 (x 12.520) | 4 (0.250) | 0.559 | iron ion transport | Tsf1 |
| 163 | GO:0006144 | P | 7, | 3 | 1.098 (x 2.732) | 55 (0.055) | 0.57 | purine base metabolism | CG18522 CG2277 Vha36 |
| 164 | GO:0050982 | P | 5, 6, | 1 | 0.100 (x 10.016) | 5 (0.200) | 0.571 | detection of mechanical stimulus | tko |
| 165 | GO:0016563 | F | 3, | 3 | 1.098 (x 2.732) | 55 (0.055) | 0.573 | transcriptional activator activity | Nipped-B Ssb-c31a mirr |
| 166 | GO:0007613 | P | 5, | 2 | 0.499 (x 4.006) | 25 (0.080) | 0.573 | memory | Tbh pum |
| 167 | GO:0007540 | P | 6, | 1 | 0.100 (x 10.016) | 5 (0.200) | 0.575 | sex determination, establishment of X:A ratio | sisA |
| 168 | GO:0042721 | C | 3, 5, 6, 7, 8, 9, 10, 11, 12, 13, | 1 | 0.100 (x 10.016) | 5 (0.200) | 0.578 | mitochondrial inner membrane protein insertion complex | Tim9a |
| 169 | GO:0009008 | F | 6, | 1 | 0.100 (x 10.016) | 5 (0.200) | 0.581 | DNA-methyltransferase activity | agt |
| 170 | GO:0005865 | C | 5, 6, 7, 8, 9, 10, 11, | 1 | 0.100 (x 10.016) | 5 (0.200) | 0.585 | striated muscle thin filament | Tm1 |
| 171 | GO:0050974 | P | 5, 6, 7, | 1 | 0.100 (x 10.016) | 5 (0.200) | 0.588 | detection of mechanical stimulus during sensory perception | tko |
| 172 | GO:0045180 | C | 4, 5, 6, 7, 8, 9, 10, | 1 | 0.100 (x 10.016) | 5 (0.200) | 0.591 | basal cortex | mira |
| 173 | GO:0016413 | F | 8, | 1 | 0.100 (x 10.016) | 5 (0.200) | 0.595 | O-acetyltransferase activity | CG5122 |
| 174 | GO:0045454 | P | 5, | 1 | 0.100 (x 10.016) | 5 (0.200) | 0.598 | cell redox homeostasis | Prx6005 |
| 175 | GO:0005730 | C | 5, 6, 7, 8, 9, 10, 11, | 3 | 1.078 (x 2.782) | 54 (0.056) | 0.6 | nucleolus | CG13277 CG31950 CG6610 |
| 176 | GO:0005381 | F | 6, | 1 | 0.100 (x 10.016) | 5 (0.200) | 0.602 | iron ion transporter activity | Tsf1 |
| 177 | GO:0007217 | P | 7, | 1 | 0.100 (x 10.016) | 5 (0.200) | 0.605 | tachykinin signaling pathway | Takr99D |
| 178 | GO:0016747 | F | 5, | 5 | 2.416 (x 2.069) | 121 (0.041) | 0.607 | transferase activity, transferring groups other than amino-acyl groups | CG5037 CG5122 CG5397 CG6921 fu12 |
| 179 | GO:0007638 | P | 4, 5, | 1 | 0.100 (x 10.016) | 5 (0.200) | 0.609 | mechanosensory behavior | tko |
| 180 | GO:0006743 | P | 8, | 1 | 0.100 (x 10.016) | 5 (0.200) | 0.613 | ubiquinone metabolism | CG32174 |
| 181 | GO:0015355 | F | 6, | 1 | 0.120 (x 8.346) | 6 (0.167) | 0.613 | monocarboxylate porter activity | CG8271 |
| 182 | GO:0045946 | P | 8, 9, 10, | 1 | 0.140 (x 7.154) | 7 (0.143) | 0.615 | positive regulation of translation | pum |
| 183 | GO:0016406 | F | 8, | 1 | 0.120 (x 8.346) | 6 (0.167) | 0.617 | carnitine O-acyltransferase activity | CG5122 |
| 184 | GO:0046916 | P | 8, | 1 | 0.140 (x 7.154) | 7 (0.143) | 0.618 | transition metal ion homeostasis | Tsf1 |
| 185 | GO:0004192 | F | 7, | 1 | 0.120 (x 8.346) | 6 (0.167) | 0.62 | cathepsin D activity | CG10104 |
| 186 | GO:0006879 | P | 8, 9, | 1 | 0.140 (x 7.154) | 7 (0.143) | 0.621 | iron ion homeostasis | Tsf1 |
| 187 | GO:0003841 | F | 9, | 1 | 0.120 (x 8.346) | 6 (0.167) | 0.623 | 1-acylglycerol-3-phosphate O-acyltransferase activity | fu12 |
| 188 | GO:0050654 | P | 6, 8, | 1 | 0.140 (x 7.154) | 7 (0.143) | 0.623 | chondroitin sulfate proteoglycan metabolism | Act57B |
| 189 | GO:0042023 | P | 9, | 1 | 0.140 (x 7.154) | 7 (0.143) | 0.626 | DNA endoreduplication | skpA |
| 190 | GO:0045317 | P | 7, 8, 9, 10, | 1 | 0.120 (x 8.346) | 6 (0.167) | 0.626 | equator specification | mirr |
| 191 | GO:0008199 | F | 7, | 1 | 0.140 (x 7.154) | 7 (0.143) | 0.629 | ferric iron binding | Tsf1 |
| 192 | GO:0004500 | F | 6, | 1 | 0.120 (x 8.346) | 6 (0.167) | 0.629 | dopamine beta-monooxygenase activity | Tbh |
| 193 | GO:0004785 | F | 6, | 1 | 0.140 (x 7.154) | 7 (0.143) | 0.632 | copper, zinc superoxide dismutase activity | Sod |
| 194 | GO:0007007 | P | 7, 8, | 1 | 0.120 (x 8.346) | 6 (0.167) | 0.633 | inner mitochondrial membrane organization and biogenesis | Tim9a |
| 195 | GO:0009108 | P | 7, | 4 | 1.877 (x 2.131) | 94 (0.043) | 0.634 | coenzyme biosynthesis | CG31477 CG32174 CG9804 Vha36 |
| 196 | GO:0016411 | F | 8, | 1 | 0.140 (x 7.154) | 7 (0.143) | 0.635 | acylglycerol O-acyltransferase activity | fu12 |
| 197 | GO:0004032 | F | 7, | 1 | 0.120 (x 8.346) | 6 (0.167) | 0.636 | aldehyde reductase activity | CG10638 |
| 198 | GO:0006189 | P | 11, 12, | 1 | 0.140 (x 7.154) | 7 (0.143) | 0.638 | 'de novo' IMP biosynthesis | Dhfr |
| 199 | GO:0003899 | F | 6, | 2 | 0.599 (x 3.339) | 30 (0.067) | 0.639 | DNA-directed RNA polymerase activity | RpII18 Rpb10 |
| 200 | GO:0045039 | P | 7, 8, 9, 10, 11, | 1 | 0.120 (x 8.346) | 6 (0.167) | 0.639 | protein import into mitochondrial inner membrane | Tim9a |
| 201 | GO:0004965 | F | 6, 7, | 1 | 0.140 (x 7.154) | 7 (0.143) | 0.64 | GABA-B receptor activity | GABA-B-R2 |
| 202 | GO:0016918 | F | 4, 5, | 1 | 0.120 (x 8.346) | 6 (0.167) | 0.643 | retinal binding | CG10237 |
| 203 | GO:0019226 | P | 5, | 11 | 7.249 (x 1.518) | 363 (0.030) | 0.643 | transmission of nerve impulse | Arf84F CG18249 CG31146 GABA-B-R2 Gs2 Rab3 Takr99D Tbh Tsp42El pum unc-13 |
| 204 | GO:0030206 | P | 8, 9, 10, | 1 | 0.140 (x 7.154) | 7 (0.143) | 0.643 | chondroitin sulfate biosynthesis | Act57B |
| 205 | GO:0016746 | F | 4, | 5 | 2.536 (x 1.972) | 127 (0.039) | 0.645 | transferase activity, transferring acyl groups | CG5037 CG5122 CG5397 CG6921 fu12 |
| 206 | GO:0045178 | C | 3, 4, | 1 | 0.120 (x 8.346) | 6 (0.167) | 0.646 | basal part of cell | mira |
| 207 | GO:0006760 | P | 6, 7, 8, | 1 | 0.140 (x 7.154) | 7 (0.143) | 0.646 | folic acid and derivative metabolism | Dhfr |
| 208 | GO:0046040 | P | 10, | 1 | 0.140 (x 7.154) | 7 (0.143) | 0.649 | IMP metabolism | Dhfr |
| 209 | GO:0006544 | P | 8, 9, | 1 | 0.120 (x 8.346) | 6 (0.167) | 0.65 | glycine metabolism | Dhfr |
| 210 | GO:0007267 | P | 4, | 13 | 8.946 (x 1.453) | 448 (0.029) | 0.651 | cell-cell signaling | Arf84F CG18249 CG31146 GABA-B-R2 Gs2 Or46a Or59a Rab3 Takr99D Tbh Tsp42El pum unc-13 |
| 211 | GO:0030204 | P | 7, 8, 9, | 1 | 0.140 (x 7.154) | 7 (0.143) | 0.652 | chondroitin sulfate metabolism | Act57B |
| 212 | GO:0004995 | F | 6, 7, 9, | 1 | 0.120 (x 8.346) | 6 (0.167) | 0.653 | tachykinin receptor activity | Takr99D |
| 213 | GO:0005554 | F | 2, | 20 | 15.016 (x 1.332) | 752 (0.027) | 0.654 | molecular function unknown | CG13691 CG14825 CG16817 CG30154 CG30343 CG31601 CG31715 CG32023 CG32174 CG32175 CG32207 CG32448 CG32582 CG32625 CG32856 EG:63B12.12 JhI-26 NP15.6 Rep2 SIP1 |
| 214 | GO:0009065 | P | 8, 9, | 1 | 0.140 (x 7.154) | 7 (0.143) | 0.656 | glutamine family amino acid catabolism | Gs2 |
| 215 | GO:0007305 | P | 9, 10, | 1 | 0.140 (x 7.154) | 7 (0.143) | 0.659 | vitelline membrane formation (sensu Insecta) | Vm34Ca |
| 216 | GO:0005501 | F | 4, | 1 | 0.140 (x 7.154) | 7 (0.143) | 0.662 | retinoid binding | CG10237 |
| 217 | GO:0007402 | P | 5, 6, | 1 | 0.140 (x 7.154) | 7 (0.143) | 0.665 | ganglion mother cell fate determination | mira |
| 218 | GO:0042136 | P | 6, 9, | 1 | 0.160 (x 6.260) | 8 (0.125) | 0.667 | neurotransmitter biosynthesis | Tbh |
| 219 | GO:0031328 | P | 7, | 1 | 0.140 (x 7.154) | 7 (0.143) | 0.668 | positive regulation of cellular biosynthesis | pum |
| 220 | GO:0004602 | F | 4, 6, | 1 | 0.160 (x 6.260) | 8 (0.125) | 0.67 | glutathione peroxidase activity | Prx6005 |
| 221 | GO:0015078 | F | 6, | 4 | 2.057 (x 1.945) | 103 (0.039) | 0.67 | hydrogen ion transporter activity | CG11015 CG14482 CG31477 Vha36 |
| 222 | GO:0045727 | P | 7, 8, 9, | 1 | 0.140 (x 7.154) | 7 (0.143) | 0.671 | positive regulation of protein biosynthesis | pum |
| 223 | GO:0006967 | P | 7, 8, 9, | 1 | 0.160 (x 6.260) | 8 (0.125) | 0.672 | positive regulation of antifungal peptide biosynthesis | Tehao |
| 224 | GO:0009891 | P | 6, | 1 | 0.140 (x 7.154) | 7 (0.143) | 0.675 | positive regulation of biosynthesis | pum |
| 225 | GO:0006801 | P | 6, | 1 | 0.160 (x 6.260) | 8 (0.125) | 0.675 | superoxide metabolism | Sod |
| 226 | GO:0045167 | P | 5, 6, | 1 | 0.180 (x 5.564) | 9 (0.111) | 0.677 | asymmetric protein localization during cell fate commitment | mira |
| 227 | GO:0019840 | F | 3, | 1 | 0.140 (x 7.154) | 7 (0.143) | 0.678 | isoprenoid binding | CG10237 |
| 228 | GO:0008559 | F | 4, 5, 7, 12, | 1 | 0.160 (x 6.260) | 8 (0.125) | 0.678 | xenobiotic-transporting ATPase activity | CG11898 |
| 229 | GO:0006402 | P | 8, | 1 | 0.180 (x 5.564) | 9 (0.111) | 0.68 | mRNA catabolism | pum |
| 230 | GO:0007006 | P | 6, 7, | 1 | 0.140 (x 7.154) | 7 (0.143) | 0.681 | mitochondrial membrane organization and biogenesis | Tim9a |
| 231 | GO:0005344 | F | 3, | 1 | 0.160 (x 6.260) | 8 (0.125) | 0.681 | oxygen transporter activity | CG8193 |
| 232 | GO:0016715 | F | 5, | 1 | 0.180 (x 5.564) | 9 (0.111) | 0.682 | oxidoreductase activity, acting on paired donors, with incorporation or reduction of molecular oxygen, reduced ascorbate as one donor, and incorporation of one atom of oxygen | Tbh |
| 233 | GO:0017145 | P | 5, | 2 | 0.739 (x 2.707) | 37 (0.054) | 0.683 | stem cell division | mira pum |
| 234 | GO:0016721 | F | 4, | 1 | 0.160 (x 6.260) | 8 (0.125) | 0.684 | oxidoreductase activity, acting on superoxide radicals as acceptor | Sod |
| 235 | GO:0015020 | F | 6, | 2 | 0.679 (x 2.946) | 34 (0.059) | 0.684 | glucuronosyltransferase activity | Act57B Ugt86Di |
| 236 | GO:0004033 | F | 6, | 1 | 0.140 (x 7.154) | 7 (0.143) | 0.684 | aldo-keto reductase activity | CG10638 |
| 237 | GO:0003730 | F | 6, | 1 | 0.180 (x 5.564) | 9 (0.111) | 0.685 | mRNA 3'-UTR binding | pum |
| 238 | GO:0006118 | P | 6, | 10 | 6.650 (x 1.504) | 333 (0.030) | 0.687 | electron transport | CG11015 CG12400 CG14482 CG18522 CG32230 CG4511 CG5548 EG:152A3.7 Or59a Pdsw |
| 239 | GO:0042910 | F | 3, | 1 | 0.160 (x 6.260) | 8 (0.125) | 0.687 | xenobiotic transporter activity | CG11898 |
| 240 | GO:0030017 | C | 6, 7, 8, 9, 10, | 1 | 0.180 (x 5.564) | 9 (0.111) | 0.688 | sarcomere | Tm1 |
| 241 | GO:0050650 | P | 7, 8, 9, | 1 | 0.140 (x 7.154) | 7 (0.143) | 0.688 | chondroitin sulfate proteoglycan biosynthesis | Act57B |
| 242 | GO:0004784 | F | 5, | 1 | 0.160 (x 6.260) | 8 (0.125) | 0.69 | superoxide dismutase activity | Sod |
| 243 | GO:0015012 | P | 7, 8, 9, | 1 | 0.180 (x 5.564) | 9 (0.111) | 0.69 | heparan sulfate proteoglycan biosynthesis | Act57B |
| 244 | GO:0030704 | P | 8, | 1 | 0.140 (x 7.154) | 7 (0.143) | 0.691 | vitelline membrane formation | Vm34Ca |
| 245 | GO:0045261 | C | 3, 4, 5, 6, 7, | 1 | 0.180 (x 5.564) | 9 (0.111) | 0.693 | proton-transporting ATP synthase complex, catalytic core F(1) | CG31477 |
| 246 | GO:0006188 | P | 10, 11, | 1 | 0.140 (x 7.154) | 7 (0.143) | 0.695 | IMP biosynthesis | Dhfr |
| 247 | GO:0051247 | P | 6, 7, | 1 | 0.180 (x 5.564) | 9 (0.111) | 0.696 | positive regulation of protein metabolism | pum |
| 248 | GO:0008439 | F | 4, 7, | 1 | 0.180 (x 5.564) | 9 (0.111) | 0.699 | monophenol monooxygenase activator activity | CG11313 |
| 249 | GO:0006752 | P | 7, | 3 | 1.438 (x 2.087) | 72 (0.042) | 0.7 | group transfer coenzyme metabolism | CG31477 Dhfr Vha36 |
| 250 | GO:0015077 | F | 5, | 4 | 2.097 (x 1.908) | 105 (0.038) | 0.701 | monovalent inorganic cation transporter activity | CG11015 CG14482 CG31477 Vha36 |
| 251 | GO:0050896 | P | 2, | 28 | 23.343 (x 1.199) | 1169 (0.024) | 0.701 | response to stimulus | CG11898 CG18249 CG18522 CG30438 CG31146 CG5224 CG5397 CG6426 CG8193 GstD9 GstE5 GstE6 GstE7 Obp56a Or46a Or59a Prx6005 Sod Tbh Tehao Tsf1 Ugt86Di agt hig na pum tko upd3 |
| 252 | GO:0000275 | C | 4, 5, 6, 7, 8, 9, 10, 11, 12, 13, 14, | 1 | 0.180 (x 5.564) | 9 (0.111) | 0.701 | proton-transporting ATP synthase complex, catalytic core F(1) (sensu Eukaryota) | CG31477 |
| 253 | GO:0007608 | P | 5, 7, | 3 | 1.438 (x 2.087) | 72 (0.042) | 0.703 | sensory perception of smell | Obp56a Or46a Or59a |
| 254 | GO:0050906 | P | 4, 5, 6, | 1 | 0.180 (x 5.564) | 9 (0.111) | 0.704 | detection of stimulus during sensory perception | tko |
| 255 | GO:0006023 | P | 7, 8, | 1 | 0.200 (x 5.008) | 10 (0.100) | 0.707 | aminoglycan biosynthesis | Act57B |
| 256 | GO:0030201 | P | 6, 8, | 1 | 0.180 (x 5.564) | 9 (0.111) | 0.707 | heparan sulfate proteoglycan metabolism | Act57B |
| 257 | GO:0015629 | C | 6, 7, 8, 9, | 3 | 1.478 (x 2.030) | 74 (0.041) | 0.708 | actin cytoskeleton | Act57B Mlc2 Tm1 |
| 258 | GO:0031981 | C | 4, 5, 6, 7, 8, 9, 10, | 8 | 5.491 (x 1.457) | 275 (0.029) | 0.709 | nuclear lumen | CG13277 CG15398 CG31950 CG6610 RpII18 Rpb10 Ssb-c31a Trap36 |
| 259 | GO:0009165 | P | 6, 7, | 4 | 2.197 (x 1.821) | 110 (0.036) | 0.709 | nucleotide biosynthesis | Ac78C CG31477 Dhfr Vha36 |
| 260 | GO:0009612 | P | 4, | 1 | 0.200 (x 5.008) | 10 (0.100) | 0.71 | response to mechanical stimulus | tko |
| 261 | GO:0030016 | C | 6, 7, 8, 9, | 1 | 0.180 (x 5.564) | 9 (0.111) | 0.71 | myofibril | Tm1 |
| 262 | GO:0000004 | P | 2, | 18 | 13.998 (x 1.286) | 701 (0.026) | 0.711 | biological process unknown | CG13691 CG16817 CG30154 CG30343 CG31601 CG31715 CG31922 CG32023 CG32175 CG32207 CG32448 CG32582 CG32625 CG32856 EG:63B12.12 JhI-26 NP15.6 Rep2 |
| 263 | GO:0016209 | F | 2, | 2 | 0.719 (x 2.782) | 36 (0.056) | 0.712 | antioxidant activity | Prx6005 Sod |
| 264 | GO:0006783 | P | 7, 8, | 1 | 0.200 (x 5.008) | 10 (0.100) | 0.712 | heme biosynthesis | CG5037 |
| 265 | GO:0005213 | F | 3, | 1 | 0.180 (x 5.564) | 9 (0.111) | 0.713 | structural constituent of chorion (sensu Insecta) | Femcoat |
| 266 | GO:0048149 | P | 5, 7, | 1 | 0.200 (x 5.008) | 10 (0.100) | 0.715 | behavioral response to ethanol | Tbh |
| 267 | GO:0008121 | F | 6, 7, | 1 | 0.180 (x 5.564) | 9 (0.111) | 0.716 | ubiquinol-cytochrome-c reductase activity | CG14482 |
| 268 | GO:0006024 | P | 8, 9, | 1 | 0.200 (x 5.008) | 10 (0.100) | 0.717 | glycosaminoglycan biosynthesis | Act57B |
| 269 | GO:0006536 | P | 8, 9, | 1 | 0.180 (x 5.564) | 9 (0.111) | 0.719 | glutamate metabolism | Gs2 |
| 270 | GO:0006725 | P | 5, | 5 | 2.895 (x 1.727) | 145 (0.034) | 0.719 | aromatic compound metabolism | CG18522 CG2277 Dhfr Tbh Vha36 |
| 271 | GO:0006022 | P | 6, 7, | 1 | 0.200 (x 5.008) | 10 (0.100) | 0.72 | aminoglycan metabolism | Act57B |
| 272 | GO:0016681 | F | 5, | 1 | 0.180 (x 5.564) | 9 (0.111) | 0.721 | oxidoreductase activity, acting on diphenols and related substances as donors, cytochrome as acceptor | CG14482 |
| 273 | GO:0016491 | F | 3, | 16 | 12.460 (x 1.284) | 624 (0.026) | 0.722 | oxidoreductase activity | CG10638 CG10962 CG11015 CG12400 CG14482 CG18522 CG32230 CG5548 CG8193 Dhfr EG:152A3.7 Or59a Pdsw Prx6005 Sod Tbh |
| 274 | GO:0022008 | P | 5, | 6 | 3.854 (x 1.557) | 193 (0.031) | 0.722 | neurogenesis | Abl BG:DS02740.9 Tm1 mira pum robl |
| 275 | GO:0005858 | C | 5, 6, 7, 8, 9, 10, 11, 12, | 1 | 0.220 (x 4.553) | 11 (0.091) | 0.723 | axonemal dynein complex | robl |
| 276 | GO:0030203 | P | 7, 8, | 1 | 0.200 (x 5.008) | 10 (0.100) | 0.723 | glycosaminoglycan metabolism | Act57B |
| 277 | GO:0008431 | F | 4, | 1 | 0.180 (x 5.564) | 9 (0.111) | 0.724 | vitamin E binding | CG10237 |
| 278 | GO:0005750 | C | 4, 5, 6, 7, 8, 9, 10, 11, 12, 13, 14, | 1 | 0.220 (x 4.553) | 11 (0.091) | 0.725 | respiratory chain complex III (sensu Eukaryota) | CG14482 |
| 279 | GO:0009190 | P | 7, 8, | 1 | 0.200 (x 5.008) | 10 (0.100) | 0.726 | cyclic nucleotide biosynthesis | Ac78C |
| 280 | GO:0045285 | C | 3, 4, 5, 6, 7, | 1 | 0.220 (x 4.553) | 11 (0.091) | 0.728 | ubiquinol-cytochrome-c reductase complex | CG14482 |
| 281 | GO:0005930 | C | 4, 5, 6, 7, 8, | 1 | 0.220 (x 4.553) | 11 (0.091) | 0.73 | axoneme | robl |
| 282 | GO:0006122 | P | 9, 11, | 1 | 0.240 (x 4.173) | 12 (0.083) | 0.731 | mitochondrial electron transport, ubiquinol to cytochrome c | CG14482 |
| 283 | GO:0044447 | C | 4, 5, 6, 7, 8, 9, | 1 | 0.220 (x 4.553) | 11 (0.091) | 0.733 | axoneme part | robl |
| 284 | GO:0019237 | F | 6, | 1 | 0.240 (x 4.173) | 12 (0.083) | 0.733 | centromeric DNA binding | skpA |
| 285 | GO:0045275 | C | 3, 4, 5, 6, | 1 | 0.220 (x 4.553) | 11 (0.091) | 0.735 | respiratory chain complex III | CG14482 |
| 286 | GO:0015239 | F | 4, | 1 | 0.240 (x 4.173) | 12 (0.083) | 0.736 | multidrug transporter activity | CG11898 |
| 287 | GO:0006936 | P | 4, | 3 | 1.717 (x 1.747) | 86 (0.035) | 0.737 | muscle contraction | Mlc2 Tm1 TpnC41C |
| 288 | GO:0000041 | P | 8, 9, | 1 | 0.220 (x 4.553) | 11 (0.091) | 0.738 | transition metal ion transport | Tsf1 |
| 289 | GO:0008067 | F | 6, | 1 | 0.240 (x 4.173) | 12 (0.083) | 0.738 | metabotropic glutamate, GABA-B-like receptor activity | GABA-B-R2 |
| 290 | GO:0004556 | F | 7, | 1 | 0.280 (x 3.577) | 14 (0.071) | 0.739 | alpha-amylase activity | CG14935 |
| 291 | GO:0003779 | F | 5, | 4 | 2.456 (x 1.629) | 123 (0.033) | 0.74 | actin binding | BG:DS02740.9 CLIP-190 Tm1 mira |
| 292 | GO:0009084 | P | 8, 9, | 1 | 0.240 (x 4.173) | 12 (0.083) | 0.74 | glutamine family amino acid biosynthesis | Gs2 |
| 293 | GO:0031461 | C | 4, 5, 6, 7, | 1 | 0.220 (x 4.553) | 11 (0.091) | 0.74 | cullin-RING ubiquitin ligase complex | skpA |
| 294 | GO:0050877 | P | 4, | 15 | 12.301 (x 1.219) | 616 (0.024) | 0.741 | neurophysiological process | Arf84F CG18249 CG31146 GABA-B-R2 Gs2 Obp56a Or46a Or59a Rab3 Takr99D Tbh Tsp42El pum tko unc-13 |
| 295 | GO:0016197 | P | 6, 7, 8, | 1 | 0.280 (x 3.577) | 14 (0.071) | 0.741 | endosome transport | Arf84F |
| 296 | GO:0004558 | F | 7, | 1 | 0.260 (x 3.852) | 13 (0.077) | 0.741 | alpha-glucosidase activity | CG14935 |
| 297 | GO:0006123 | P | 9, 11, | 1 | 0.240 (x 4.173) | 12 (0.083) | 0.743 | mitochondrial electron transport, cytochrome c to oxygen | CG11015 |
| 298 | GO:0042168 | P | 6, 7, | 1 | 0.220 (x 4.553) | 11 (0.091) | 0.743 | heme metabolism | CG5037 |
| 299 | GO:0006769 | P | 8, 9, | 1 | 0.280 (x 3.577) | 14 (0.071) | 0.743 | nicotinamide metabolism | CG5103 |
| 300 | GO:0045169 | C | 5, 6, 7, 8, | 1 | 0.260 (x 3.852) | 13 (0.077) | 0.744 | fusome | CLIP-190 |
| 301 | GO:0001505 | P | 7, | 4 | 2.336 (x 1.712) | 117 (0.034) | 0.744 | regulation of neurotransmitter levels | Arf84F Rab3 Tbh unc-13 |
| 302 | GO:0006740 | P | 10, 11, | 1 | 0.240 (x 4.173) | 12 (0.083) | 0.745 | NADPH regeneration | CG5103 |
| 303 | GO:0019732 | P | 6, 7, 8, | 1 | 0.280 (x 3.577) | 14 (0.071) | 0.746 | antifungal humoral response | Tehao |
| 304 | GO:0019005 | C | 5, 6, 7, 8, | 1 | 0.220 (x 4.553) | 11 (0.091) | 0.746 | SCF ubiquitin ligase complex | skpA |
| 305 | GO:0007098 | P | 5, 8, | 1 | 0.260 (x 3.852) | 13 (0.077) | 0.746 | centrosome cycle | skpA |
| 306 | GO:0030534 | P | 4, | 2 | 0.919 (x 2.177) | 46 (0.043) | 0.747 | adult behavior | Tbh na |
| 307 | GO:0004263 | F | 7, | 6 | 4.153 (x 1.445) | 208 (0.029) | 0.747 | chymotrypsin activity | CG11313 CG1304 CG18223 CG2056 CG4386 CG9372 |
| 308 | GO:0044271 | P | 5, 6, | 3 | 1.677 (x 1.789) | 84 (0.036) | 0.747 | nitrogen compound biosynthesis | Dhfr Gs2 Tbh |
| 309 | GO:0045786 | P | 6, 7, | 1 | 0.240 (x 4.173) | 12 (0.083) | 0.748 | negative regulation of progression through cell cycle | pum |
| 310 | GO:0030166 | P | 7, 8, | 1 | 0.280 (x 3.577) | 14 (0.071) | 0.748 | proteoglycan biosynthesis | Act57B |
| 311 | GO:0043226 | C | 2, | 59 | 53.596 (x 1.101) | 2684 (0.022) | 0.748 | organelle | Act57B BEST:LD29214 CG11015 CG12400 CG13277 CG14482 CG15398 CG31477 CG31922 CG31950 CG32174 CG32230 CG32409 CG33002 CG4046 CG4673 CG4866 CG5037 CG5189 CG5338 CG5548 CG6610 CG7014 CG7911 CG8415 CG9650 CLIP-190 EG:152A3.7 Mlc2 Nipped-B Pdsw Rab3 RpII18 RpL11 RpL17A RpL38 RpL46 RpS17 RpS18 Rpb10 Ssb-c31a Tim9a Tm1 Trap36 Vha36 mRpL21 mRpL22 mRpL54 mRpS14 mRpS21 mRpS24 mirr na oho23B robl sisA tko toy unc-13 |
| 312 | GO:0016679 | F | 4, | 1 | 0.260 (x 3.852) | 13 (0.077) | 0.748 | oxidoreductase activity, acting on diphenols and related substances as donors | CG14482 |
| 313 | GO:0006779 | P | 7, | 1 | 0.220 (x 4.553) | 11 (0.091) | 0.748 | porphyrin biosynthesis | CG5037 |
| 314 | GO:0006091 | P | 5, | 13 | 10.084 (x 1.289) | 505 (0.026) | 0.749 | generation of precursor metabolites and energy | CG11015 CG12400 CG14482 CG18522 CG31477 CG32230 CG4511 CG5103 CG5548 EG:152A3.7 Or59a Pdsw Vha36 |
| 315 | GO:0009309 | P | 6, 7, | 3 | 1.677 (x 1.789) | 84 (0.036) | 0.75 | amine biosynthesis | Dhfr Gs2 Tbh |
| 316 | GO:0016160 | F | 6, | 1 | 0.280 (x 3.577) | 14 (0.071) | 0.75 | amylase activity | CG14935 |
| 317 | GO:0042384 | P | 7, 8, | 1 | 0.240 (x 4.173) | 12 (0.083) | 0.75 | cilium biogenesis | CG14825 |
| 318 | GO:0043229 | C | 3, 4, 5, 6, | 59 | 53.596 (x 1.101) | 2684 (0.022) | 0.75 | intracellular organelle | Act57B BEST:LD29214 CG11015 CG12400 CG13277 CG14482 CG15398 CG31477 CG31922 CG31950 CG32174 CG32230 CG32409 CG33002 CG4046 CG4673 CG4866 CG5037 CG5189 CG5338 CG5548 CG6610 CG7014 CG7911 CG8415 CG9650 CLIP-190 EG:152A3.7 Mlc2 Nipped-B Pdsw Rab3 RpII18 RpL11 RpL17A RpL38 RpL46 RpS17 RpS18 Rpb10 Ssb-c31a Tim9a Tm1 Trap36 Vha36 mRpL21 mRpL22 mRpL54 mRpS14 mRpS21 mRpS24 mirr na oho23B robl sisA tko toy unc-13 |
| 319 | GO:0005549 | F | 3, | 4 | 2.296 (x 1.742) | 115 (0.035) | 0.75 | odorant binding | Obp56a Obp58b Or46a Or59a |
| 320 | GO:0006778 | P | 6, | 1 | 0.260 (x 3.852) | 13 (0.077) | 0.75 | porphyrin metabolism | CG5037 |
| 321 | GO:0005245 | F | 6, 7, 8, | 1 | 0.220 (x 4.553) | 11 (0.091) | 0.751 | voltage-gated calcium channel activity | na |
| 322 | GO:0005684 | C | 5, 6, 7, 8, 9, 10, 11, | 2 | 0.819 (x 2.443) | 41 (0.049) | 0.752 | major (U2-dependent) spliceosome | CG13277 CG6610 |
| 323 | GO:0042052 | P | 8, 9, 10, 11, | 1 | 0.280 (x 3.577) | 14 (0.071) | 0.752 | rhabdomere development | Cpn |
| 324 | GO:0016082 | P | 8, 9, 10, | 1 | 0.240 (x 4.173) | 12 (0.083) | 0.752 | synaptic vesicle priming | unc-13 |
| 325 | GO:0005744 | C | 3, 5, 6, 7, 8, 9, 10, 11, 12, 13, | 1 | 0.260 (x 3.852) | 13 (0.077) | 0.753 | mitochondrial inner membrane presequence translocase complex | Tim9a |
| 326 | GO:0005876 | C | 6, 7, 8, 9, 10, 11, | 1 | 0.220 (x 4.553) | 11 (0.091) | 0.754 | spindle microtubule | CLIP-190 |
| 327 | GO:0007062 | P | 5, | 1 | 0.280 (x 3.577) | 14 (0.071) | 0.754 | sister chromatid cohesion | Nipped-B |
| 328 | GO:0009117 | P | 6, | 5 | 3.175 (x 1.575) | 159 (0.031) | 0.754 | nucleotide metabolism | Ac78C CG31477 CG5103 Dhfr Vha36 |
| 329 | GO:0006739 | P | 9, 10, | 1 | 0.240 (x 4.173) | 12 (0.083) | 0.755 | NADP metabolism | CG5103 |
| 330 | GO:0008064 | P | 6, 7, 10, | 1 | 0.260 (x 3.852) | 13 (0.077) | 0.755 | regulation of actin polymerization and/or depolymerization | Abl |
| 331 | GO:0017157 | P | 6, 7, 8, | 2 | 0.859 (x 2.329) | 43 (0.047) | 0.756 | regulation of exocytosis | Arf84F Rab3 |
| 332 | GO:0044424 | C | 3, 4, 5, | 69 | 63.700 (x 1.083) | 3190 (0.022) | 0.756 | intracellular part | Abl Act57B BEST:LD29214 CG11015 CG12400 CG13277 CG14482 CG15398 CG31477 CG31922 CG31950 CG32174 CG32230 CG32409 CG33002 CG4046 CG4673 CG4866 CG5037 CG5189 CG5338 CG5548 CG6610 CG7014 CG7770 CG7911 CG8415 CG9650 CLIP-190 Cpn EG:152A3.7 Femcoat Gs2 Mlc2 Nipped-B Pdsw Rab3 RpII18 RpL11 RpL17A RpL38 RpL46 RpS17 RpS18 Rpb10 Sod Ssb-c31a Tim9a Tm1 Trap36 Vha36 l(3)01239 mRpL21 mRpL22 mRpL54 mRpS14 mRpS21 mRpS24 mira mirr na oho23B pum robl sisA skpA tko toy unc-13 |
| 333 | GO:0005924 | C | 7, 8, 9, | 1 | 0.280 (x 3.577) | 14 (0.071) | 0.756 | cell-substrate adherens junction | Abl |
| 334 | GO:0004252 | F | 6, | 7 | 5.132 (x 1.364) | 257 (0.027) | 0.756 | serine-type endopeptidase activity | BG:DS01068.5 CG11313 CG1304 CG18223 CG2056 CG4386 CG9372 |
| 335 | GO:0005355 | F | 7, | 1 | 0.300 (x 3.339) | 15 (0.067) | 0.757 | glucose transporter activity | CG1213 |
| 336 | GO:0050874 | P | 3, | 21 | 17.972 (x 1.169) | 900 (0.023) | 0.757 | organismal physiological process | Arf84F CG18249 CG31146 GABA-B-R2 Gs2 Mlc2 Obp56a Or46a Or59a Rab3 Sod Takr99D Tbh Tehao Tm1 TpnC41C Tsp42El pum tko unc-13 upd3 |
| 337 | GO:0008595 | P | 6, 7, | 2 | 0.998 (x 2.003) | 50 (0.040) | 0.757 | determination of anterior/posterior axis, embryo | pum tsl |
| 338 | GO:0016646 | F | 5, | 1 | 0.260 (x 3.852) | 13 (0.077) | 0.757 | oxidoreductase activity, acting on the CH-NH group of donors, NAD or NADP as acceptor | Dhfr |
| 339 | GO:0006098 | P | 8, 10, 11, 12, | 1 | 0.240 (x 4.173) | 12 (0.083) | 0.757 | pentose-phosphate shunt | CG5103 |
| 340 | GO:0009152 | P | 8, 9, | 3 | 1.777 (x 1.688) | 89 (0.034) | 0.758 | purine ribonucleotide biosynthesis | CG31477 Dhfr Vha36 |
| 341 | GO:0008298 | P | 5, | 2 | 0.978 (x 2.044) | 49 (0.041) | 0.759 | intracellular mRNA localization | Tm1 mira |
| 342 | GO:0045034 | P | 6, 7, 10, | 1 | 0.280 (x 3.577) | 14 (0.071) | 0.759 | neuroblast division | mira |
| 343 | GO:0005125 | F | 4, 5, | 1 | 0.300 (x 3.339) | 15 (0.067) | 0.759 | cytokine activity | upd3 |
| 344 | GO:0007351 | P | 5, 6, | 2 | 0.998 (x 2.003) | 50 (0.040) | 0.759 | regional subdivision | pum tsl |
| 345 | GO:0007616 | P | 6, | 1 | 0.260 (x 3.852) | 13 (0.077) | 0.76 | long-term memory | pum |
| 346 | GO:0019236 | P | 5, | 1 | 0.240 (x 4.173) | 12 (0.083) | 0.76 | response to pheromone | Obp56a |
| 347 | GO:0008553 | F | 6, 7, 9, 14, | 2 | 0.978 (x 2.044) | 49 (0.041) | 0.761 | hydrogen-exporting ATPase activity, phosphorylative mechanism | CG31477 Vha36 |
| 348 | GO:0009070 | P | 8, 9, | 1 | 0.280 (x 3.577) | 14 (0.071) | 0.761 | serine family amino acid biosynthesis | Dhfr |
| 349 | GO:0030055 | C | 6, 7, 8, | 1 | 0.300 (x 3.339) | 15 (0.067) | 0.761 | cell-matrix junction | Abl |
| 350 | GO:0030832 | P | 9, | 1 | 0.260 (x 3.852) | 13 (0.077) | 0.762 | regulation of actin filament length | Abl |
| 351 | GO:0045471 | P | 6, | 1 | 0.240 (x 4.173) | 12 (0.083) | 0.763 | response to ethanol | Tbh |
| 352 | GO:0007538 | P | 4, | 1 | 0.300 (x 3.339) | 15 (0.067) | 0.763 | primary sex determination | sisA |
| 353 | GO:0005751 | C | 4, 5, 6, 7, 8, 9, 10, 11, 12, 13, 14, | 1 | 0.280 (x 3.577) | 14 (0.071) | 0.763 | respiratory chain complex IV (sensu Eukaryota) | CG11015 |
| 354 | GO:0016645 | F | 4, | 1 | 0.319 (x 3.130) | 16 (0.062) | 0.764 | oxidoreductase activity, acting on the CH-NH group of donors | Dhfr |
| 355 | GO:0007539 | P | 5, | 1 | 0.260 (x 3.852) | 13 (0.077) | 0.765 | primary sex determination, soma | sisA |
| 356 | GO:0005938 | C | 5, 6, 7, 8, | 2 | 0.899 (x 2.226) | 45 (0.044) | 0.765 | cell cortex | CLIP-190 mira |
| 357 | GO:0006029 | P | 7, | 1 | 0.300 (x 3.339) | 15 (0.067) | 0.765 | proteoglycan metabolism | Act57B |
| 358 | GO:0018958 | P | 6, | 1 | 0.280 (x 3.577) | 14 (0.071) | 0.765 | phenol metabolism | Tbh |
| 359 | GO:0016675 | F | 4, | 1 | 0.319 (x 3.130) | 16 (0.062) | 0.766 | oxidoreductase activity, acting on heme group of donors | CG11015 |
| 360 | GO:0051297 | P | 7, | 1 | 0.260 (x 3.852) | 13 (0.077) | 0.767 | centrosome organization and biogenesis | skpA |
| 361 | GO:0009150 | P | 8, | 3 | 1.797 (x 1.669) | 90 (0.033) | 0.767 | purine ribonucleotide metabolism | CG31477 Dhfr Vha36 |
| 362 | GO:0009259 | P | 7, | 3 | 1.837 (x 1.633) | 92 (0.033) | 0.767 | ribonucleotide metabolism | CG31477 Dhfr Vha36 |
| 363 | GO:0019362 | P | 7, 8, | 1 | 0.300 (x 3.339) | 15 (0.067) | 0.767 | pyridine nucleotide metabolism | CG5103 |
| 364 | GO:0042401 | P | 7, 8, | 1 | 0.280 (x 3.577) | 14 (0.071) | 0.767 | biogenic amine biosynthesis | Tbh |
| 365 | GO:0004659 | F | 5, | 1 | 0.319 (x 3.130) | 16 (0.062) | 0.768 | prenyltransferase activity | CG5037 |
| 366 | GO:0006163 | P | 7, | 3 | 1.837 (x 1.633) | 92 (0.033) | 0.769 | purine nucleotide metabolism | CG31477 Dhfr Vha36 |
| 367 | GO:0004715 | F | 8, | 1 | 0.300 (x 3.339) | 15 (0.067) | 0.769 | non-membrane spanning protein tyrosine kinase activity | Abl |
| 368 | GO:0042133 | P | 5, 8, | 1 | 0.260 (x 3.852) | 13 (0.077) | 0.769 | neurotransmitter metabolism | Tbh |
| 369 | GO:0008372 | C | 2, | 19 | 16.254 (x 1.169) | 814 (0.023) | 0.77 | cellular component unknown | CG13691 CG14825 CG16817 CG30154 CG30343 CG31601 CG31715 CG32023 CG32175 CG32207 CG32448 CG32582 CG32625 CG32856 EG:63B12.12 JhI-26 NP15.6 Rep2 SIP1 |
| 370 | GO:0044463 | C | 3, 4, 5, | 1 | 0.280 (x 3.577) | 14 (0.071) | 0.77 | cell projection part | robl |
| 371 | GO:0004016 | F | 4, 5, | 1 | 0.319 (x 3.130) | 16 (0.062) | 0.77 | adenylate cyclase activity | Ac78C |
| 372 | GO:0008021 | C | 8, 9, 10, 11, 12, | 2 | 1.018 (x 1.964) | 51 (0.039) | 0.77 | synaptic vesicle | Rab3 unc-13 |
| 373 | GO:0005509 | F | 5, | 6 | 4.353 (x 1.378) | 218 (0.028) | 0.771 | calcium ion binding | CG10126 CG6426 CG7646 Cpn Mlc2 TpnC41C |
| 374 | GO:0006584 | P | 7, 8, | 1 | 0.260 (x 3.852) | 13 (0.077) | 0.772 | catecholamine metabolism | Tbh |
| 375 | GO:0016676 | F | 5, | 1 | 0.319 (x 3.130) | 16 (0.062) | 0.772 | oxidoreductase activity, acting on heme group of donors, oxygen as acceptor | CG11015 |
| 376 | GO:0031023 | P | 6, | 1 | 0.280 (x 3.577) | 14 (0.071) | 0.772 | microtubule organizing center organization and biogenesis | skpA |
| 377 | GO:0005622 | C | 3, 4, | 71 | 65.796 (x 1.079) | 3295 (0.022) | 0.772 | intracellular | Abl Act57B BEST:LD29214 BG:DS02740.9 CG10237 CG11015 CG12400 CG13277 CG14482 CG15398 CG31477 CG31922 CG31950 CG32174 CG32230 CG32409 CG33002 CG4046 CG4673 CG4866 CG5037 CG5189 CG5338 CG5548 CG6610 CG7014 CG7770 CG7911 CG8415 CG9650 CLIP-190 Cpn EG:152A3.7 Femcoat Gs2 Mlc2 Nipped-B Pdsw Rab3 RpII18 RpL11 RpL17A RpL38 RpL46 RpS17 RpS18 Rpb10 Sod Ssb-c31a Tim9a Tm1 Trap36 Vha36 l(3)01239 mRpL21 mRpL22 mRpL54 mRpS14 mRpS21 mRpS24 mira mirr na oho23B pum robl sisA skpA tko toy unc-13 |
| 378 | GO:0009260 | P | 7, 8, | 3 | 1.817 (x 1.651) | 91 (0.033) | 0.772 | ribonucleotide biosynthesis | CG31477 Dhfr Vha36 |
| 379 | GO:0042398 | P | 6, 7, | 1 | 0.319 (x 3.130) | 16 (0.062) | 0.774 | amino acid derivative biosynthesis | Tbh |
| 380 | GO:0004194 | F | 7, | 1 | 0.260 (x 3.852) | 13 (0.077) | 0.774 | pepsin A activity | CG10104 |
| 381 | GO:0006164 | P | 7, 8, | 3 | 1.817 (x 1.651) | 91 (0.033) | 0.774 | purine nucleotide biosynthesis | CG31477 Dhfr Vha36 |
| 382 | GO:0045277 | C | 3, 4, 5, 6, | 1 | 0.280 (x 3.577) | 14 (0.071) | 0.774 | respiratory chain complex IV | CG11015 |
| 383 | GO:0003674 | F | 1, | 168 | 165.460 (x 1.015) | 8286 (0.020) | 0.774 | molecular\_function | Abl Ac78C Act57B Arf84F BEST:LD29214 BG:DS01068.5 BG:DS02740.9 BcDNA:GH11110 CG10092 CG10104 CG10126 CG10237 CG10466 CG10638 CG10950 CG10962 CG11015 CG11313 CG11898 CG1213 CG12400 CG1304 CG1342 CG13691 CG14482 CG14825 CG14935 CG15361 CG15398 CG15820 CG16817 CG18223 CG18249 CG18522 CG18530 CG1939 CG2056 CG2277 CG2555 CG30154 CG30343 CG30438 CG31146 CG31477 CG31601 CG31704 CG31715 CG31876 CG31922 CG32023 CG32174 CG32175 CG32207 CG32230 CG32448 CG32582 CG32625 CG32856 CG33002 CG33096 CG4046 CG4115 CG4288 CG4386 CG4511 CG4673 CG4866 CG5037 CG5103 CG5122 CG5162 CG5224 CG5338 CG5397 CG5548 CG6426 CG6574 CG6921 CG7014 CG7646 CG7770 CG7911 CG7941 CG8193 CG8271 CG8415 CG8511 CG8515 CG8520 CG9372 CG9650 CG9804 CLIP-190 Cpn Dhfr EG:152A3.7 EG:63B12.12 EG:BACH7M4.1 Edg91 Femcoat GABA-B-R2 Gs2 GstD9 GstE5 GstE6 GstE7 JhI-26 Lcp65Ae Mlc2 MtnA NP15.6 Nipped-B Obp56a Obp58b Or46a Or59a Pdsw Prx6005 RN-tre Rab3 Rep2 RpII18 RpL11 RpL17A RpL38 RpL46 RpS17 RpS18 Rpb10 SIP1 Sod Ssb-c31a Takr99D Tbh Tehao Tim9a Tm1 TpnC41C Trap36 Tsf1 Tsp42El Ugt86Di Vha36 Vm34Ca agt alpha-Est8 fu12 l(3)01239 mRpL21 mRpL22 mRpL54 mRpS14 mRpS21 mRpS24 mira mirr na oho23B pum ran-like robl sisA skpA tko toy tsl unc-13 upd3 |
| 384 | GO:0015002 | F | 4, | 1 | 0.319 (x 3.130) | 16 (0.062) | 0.776 | heme-copper terminal oxidase activity | CG11015 |
| 385 | GO:0000221 | C | 4, 5, 6, 7, 8, 9, 10, 11, 12, | 1 | 0.280 (x 3.577) | 14 (0.071) | 0.777 | hydrogen-transporting ATPase V1 domain | Vha36 |
| 386 | GO:0006403 | P | 4, | 3 | 1.857 (x 1.615) | 93 (0.032) | 0.778 | RNA localization | Tm1 mira ran-like |
| 387 | GO:0004129 | F | 5, 6, 7, | 1 | 0.319 (x 3.130) | 16 (0.062) | 0.778 | cytochrome-c oxidase activity | CG11015 |
| 388 | GO:0048699 | P | 6, | 5 | 3.594 (x 1.391) | 180 (0.028) | 0.78 | generation of neurons | Abl Tm1 mira pum robl |
| 389 | GO:0008356 | P | 5, | 2 | 1.058 (x 1.890) | 53 (0.038) | 0.781 | asymmetric cell division | mira pum |
| 390 | GO:0018993 | P | 4, | 1 | 0.339 (x 2.946) | 17 (0.059) | 0.782 | somatic sex determination | sisA |
| 391 | GO:0007259 | P | 7, | 1 | 0.339 (x 2.946) | 17 (0.059) | 0.784 | JAK-STAT cascade | upd3 |
| 392 | GO:0016478 | P | 8, 9, 10, | 1 | 0.339 (x 2.946) | 17 (0.059) | 0.786 | negative regulation of translation | pum |
| 393 | GO:0006471 | P | 8, | 1 | 0.339 (x 2.946) | 17 (0.059) | 0.788 | protein amino acid ADP-ribosylation | Arf84F |
| 394 | GO:0007280 | P | 6, 7, 8, | 1 | 0.339 (x 2.946) | 17 (0.059) | 0.79 | pole cell migration | pum |
| 395 | GO:0008028 | F | 5, | 1 | 0.339 (x 2.946) | 17 (0.059) | 0.792 | monocarboxylic acid transporter activity | CG8271 |
| 396 | GO:0009127 | P | 8, 9, | 1 | 0.359 (x 2.782) | 18 (0.056) | 0.792 | purine nucleoside monophosphate biosynthesis | Dhfr |
| 397 | GO:0007005 | P | 6, | 1 | 0.359 (x 2.782) | 18 (0.056) | 0.794 | mitochondrion organization and biogenesis | Tim9a |
| 398 | GO:0050832 | P | 5, 6, | 1 | 0.359 (x 2.782) | 18 (0.056) | 0.796 | defense response to fungus | Tehao |
| 399 | GO:0015149 | F | 6, | 1 | 0.359 (x 2.782) | 18 (0.056) | 0.798 | hexose transporter activity | CG1213 |
| 400 | GO:0030136 | C | 7, 8, 9, 10, 11, | 2 | 1.118 (x 1.789) | 56 (0.036) | 0.799 | clathrin-coated vesicle | Rab3 unc-13 |
| 401 | GO:0009167 | P | 9, | 1 | 0.359 (x 2.782) | 18 (0.056) | 0.799 | purine ribonucleoside monophosphate metabolism | Dhfr |
| 402 | GO:0007304 | P | 8, 9, | 2 | 1.098 (x 1.821) | 55 (0.036) | 0.801 | eggshell formation (sensu Insecta) | Femcoat Vm34Ca |
| 403 | GO:0009069 | P | 7, 8, | 1 | 0.359 (x 2.782) | 18 (0.056) | 0.801 | serine family amino acid metabolism | Dhfr |
| 404 | GO:0017038 | P | 6, 7, | 2 | 1.098 (x 1.821) | 55 (0.036) | 0.803 | protein import | CG10950 Tim9a |
| 405 | GO:0009168 | P | 9, 10, | 1 | 0.359 (x 2.782) | 18 (0.056) | 0.803 | purine ribonucleoside monophosphate biosynthesis | Dhfr |
| 406 | GO:0030703 | P | 7, | 2 | 1.098 (x 1.821) | 55 (0.036) | 0.805 | eggshell formation | Femcoat Vm34Ca |
| 407 | GO:0009126 | P | 8, | 1 | 0.359 (x 2.782) | 18 (0.056) | 0.805 | purine nucleoside monophosphate metabolism | Dhfr |
| 408 | GO:0009064 | P | 7, 8, | 1 | 0.379 (x 2.636) | 19 (0.053) | 0.806 | glutamine family amino acid metabolism | Gs2 |
| 409 | GO:0005316 | F | 7, 8, 9, | 1 | 0.359 (x 2.782) | 18 (0.056) | 0.807 | high affinity inorganic phosphate:sodium symporter activity | CG4288 |
| 410 | GO:0044272 | P | 6, | 1 | 0.379 (x 2.636) | 19 (0.053) | 0.808 | sulfur compound biosynthesis | Act57B |
| 411 | GO:0005884 | C | 5, 6, 7, 8, 9, 10, | 1 | 0.359 (x 2.782) | 18 (0.056) | 0.809 | actin filament | Act57B |
| 412 | GO:0009620 | P | 5, | 1 | 0.379 (x 2.636) | 19 (0.053) | 0.81 | response to fungus | Tehao |
| 413 | GO:0048666 | P | 5, 8, | 4 | 2.836 (x 1.411) | 142 (0.028) | 0.811 | neuron development | Abl Tm1 pum robl |
| 414 | GO:0004190 | F | 6, | 1 | 0.379 (x 2.636) | 19 (0.053) | 0.812 | aspartic-type endopeptidase activity | CG10104 |
| 415 | GO:0007399 | P | 4, | 11 | 9.345 (x 1.177) | 468 (0.024) | 0.812 | nervous system development | Abl BG:DS02740.9 CG31146 Gs2 Tm1 Tsp42El mira mirr pum robl toy |
| 416 | GO:0031175 | P | 6, 9, | 4 | 2.836 (x 1.411) | 142 (0.028) | 0.813 | neurite development | Abl Tm1 pum robl |
| 417 | GO:0003697 | F | 6, | 1 | 0.399 (x 2.504) | 20 (0.050) | 0.813 | single-stranded DNA binding | Ssb-c31a |
| 418 | GO:0016917 | F | 5, | 1 | 0.379 (x 2.636) | 19 (0.053) | 0.814 | GABA receptor activity | GABA-B-R2 |
| 419 | GO:0007610 | P | 3, | 5 | 3.814 (x 1.311) | 191 (0.026) | 0.814 | behavior | Obp56a Tbh na pum tko |
| 420 | GO:0016310 | P | 7, | 11 | 9.206 (x 1.195) | 461 (0.024) | 0.814 | phosphorylation | Abl CG11015 CG12400 CG14482 CG2056 CG31477 CG32230 CG5548 EG:152A3.7 Pdsw Vha36 |
| 421 | GO:0005753 | C | 4, 5, 6, 7, 8, 9, 10, 11, 12, 13, | 1 | 0.399 (x 2.504) | 20 (0.050) | 0.815 | proton-transporting ATP synthase complex (sensu Eukaryota) | CG31477 |
| 422 | GO:0046915 | F | 5, | 1 | 0.379 (x 2.636) | 19 (0.053) | 0.816 | transition metal ion transporter activity | Tsf1 |
| 423 | GO:0007362 | P | 8, 9, | 1 | 0.419 (x 2.385) | 21 (0.048) | 0.816 | terminal region determination | tsl |
| 424 | GO:0007398 | P | 4, | 6 | 4.733 (x 1.268) | 237 (0.025) | 0.817 | ectoderm development | BG:DS02740.9 CG31146 CG5397 Tsp42El mirr toy |
| 425 | GO:0051189 | P | 5, 7, | 2 | 1.198 (x 1.669) | 60 (0.033) | 0.817 | prosthetic group metabolism | CG10237 CG6574 |
| 426 | GO:0008320 | F | 4, | 1 | 0.399 (x 2.504) | 20 (0.050) | 0.817 | protein carrier activity | CG10950 |
| 427 | GO:0030182 | P | 4, 7, | 4 | 2.975 (x 1.344) | 149 (0.027) | 0.818 | neuron differentiation | Abl Tm1 pum robl |
| 428 | GO:0007346 | P | 6, 7, | 1 | 0.419 (x 2.385) | 21 (0.048) | 0.818 | regulation of progression through mitotic cell cycle | skpA |
| 429 | GO:0015238 | F | 3, | 1 | 0.379 (x 2.636) | 19 (0.053) | 0.818 | drug transporter activity | CG11898 |
| 430 | GO:0007611 | P | 4, | 2 | 1.198 (x 1.669) | 60 (0.033) | 0.819 | learning and/or memory | Tbh pum |
| 431 | GO:0009156 | P | 8, 9, | 1 | 0.399 (x 2.504) | 20 (0.050) | 0.819 | ribonucleoside monophosphate biosynthesis | Dhfr |
| 432 | GO:0017148 | P | 7, 8, 9, | 1 | 0.419 (x 2.385) | 21 (0.048) | 0.82 | negative regulation of protein biosynthesis | pum |
| 433 | GO:0007269 | P | 6, 7, 8, | 3 | 2.097 (x 1.431) | 105 (0.029) | 0.82 | neurotransmitter secretion | Arf84F Rab3 unc-13 |
| 434 | GO:0008049 | P | 6, 7, | 1 | 0.399 (x 2.504) | 20 (0.050) | 0.821 | male courtship behavior | tko |
| 435 | GO:0008154 | P | 6, 9, | 1 | 0.419 (x 2.385) | 21 (0.048) | 0.821 | actin polymerization and/or depolymerization | Abl |
| 436 | GO:0045055 | P | 6, 7, | 3 | 2.097 (x 1.431) | 105 (0.029) | 0.822 | regulated secretory pathway | Arf84F Rab3 unc-13 |
| 437 | GO:0009161 | P | 8, | 1 | 0.399 (x 2.504) | 20 (0.050) | 0.823 | ribonucleoside monophosphate metabolism | Dhfr |
| 438 | GO:0009123 | P | 7, | 1 | 0.419 (x 2.385) | 21 (0.048) | 0.823 | nucleoside monophosphate metabolism | Dhfr |
| 439 | GO:0046961 | F | 6, 7, 9, 14, | 2 | 1.258 (x 1.590) | 63 (0.032) | 0.825 | hydrogen-transporting ATPase activity, rotational mechanism | CG31477 Vha36 |
| 440 | GO:0015145 | F | 5, | 1 | 0.399 (x 2.504) | 20 (0.050) | 0.825 | monosaccharide transporter activity | CG1213 |
| 441 | GO:0010033 | P | 5, | 1 | 0.419 (x 2.385) | 21 (0.048) | 0.825 | response to organic substance | Tbh |
| 442 | GO:0046933 | F | 7, | 2 | 1.258 (x 1.590) | 63 (0.032) | 0.827 | hydrogen-transporting ATP synthase activity, rotational mechanism | CG31477 Vha36 |
| 443 | GO:0030005 | P | 7, | 1 | 0.399 (x 2.504) | 20 (0.050) | 0.827 | di-, tri-valent inorganic cation homeostasis | Tsf1 |
| 444 | GO:0005436 | F | 6, 7, 8, | 1 | 0.419 (x 2.385) | 21 (0.048) | 0.827 | sodium:phosphate symporter activity | CG4288 |
| 445 | GO:0019730 | P | 6, 7, | 2 | 1.258 (x 1.590) | 63 (0.032) | 0.828 | antimicrobial humoral response | Tehao upd3 |
| 446 | GO:0031327 | P | 7, | 1 | 0.439 (x 2.276) | 22 (0.045) | 0.828 | negative regulation of cellular biosynthesis | pum |
| 447 | GO:0045255 | C | 4, 7, 8, 9, | 1 | 0.399 (x 2.504) | 20 (0.050) | 0.829 | hydrogen-translocating F-type ATPase complex | CG31477 |
| 448 | GO:0009124 | P | 7, 8, | 1 | 0.419 (x 2.385) | 21 (0.048) | 0.829 | nucleoside monophosphate biosynthesis | Dhfr |
| 449 | GO:0000578 | P | 5, | 2 | 1.178 (x 1.698) | 59 (0.034) | 0.83 | embryonic axis specification | pum tsl |
| 450 | GO:0004295 | F | 7, | 6 | 4.693 (x 1.279) | 235 (0.026) | 0.83 | trypsin activity | CG11313 CG1304 CG18223 CG2056 CG4386 CG9372 |
| 451 | GO:0006873 | P | 5, | 1 | 0.439 (x 2.276) | 22 (0.045) | 0.83 | cell ion homeostasis | Tsf1 |
| 452 | GO:0007605 | P | 5, 7, | 1 | 0.399 (x 2.504) | 20 (0.050) | 0.831 | sensory perception of sound | tko |
| 453 | GO:0048542 | P | 5, | 1 | 0.419 (x 2.385) | 21 (0.048) | 0.831 | lymph gland development (sensu Arthropoda) | oho23B |
| 454 | GO:0051049 | P | 5, 6, | 2 | 1.178 (x 1.698) | 59 (0.034) | 0.832 | regulation of transport | Arf84F Rab3 |
| 455 | GO:0042078 | P | 6, | 1 | 0.439 (x 2.276) | 22 (0.045) | 0.832 | germ-line stem cell division | pum |
| 456 | GO:0006754 | P | 7, 8, 9, 10, 11, | 2 | 1.278 (x 1.565) | 64 (0.031) | 0.832 | ATP biosynthesis | CG31477 Vha36 |
| 457 | GO:0045259 | C | 3, 4, 5, 6, | 1 | 0.399 (x 2.504) | 20 (0.050) | 0.833 | proton-transporting ATP synthase complex | CG31477 |
| 458 | GO:0050954 | P | 4, 6, | 1 | 0.419 (x 2.385) | 21 (0.048) | 0.833 | sensory perception of mechanical stimulus | tko |
| 459 | GO:0009299 | P | 8, | 1 | 0.459 (x 2.177) | 23 (0.043) | 0.833 | mRNA transcription | RpII18 |
| 460 | GO:0004984 | F | 7, | 2 | 1.218 (x 1.642) | 61 (0.033) | 0.833 | olfactory receptor activity | Or46a Or59a |
| 461 | GO:0009890 | P | 6, | 1 | 0.439 (x 2.276) | 22 (0.045) | 0.834 | negative regulation of biosynthesis | pum |
| 462 | GO:0015986 | P | 7, 8, 9, 10, 11, 12, | 2 | 1.278 (x 1.565) | 64 (0.031) | 0.834 | ATP synthesis coupled proton transport | CG31477 Vha36 |
| 463 | GO:0008258 | P | 6, | 1 | 0.459 (x 2.177) | 23 (0.043) | 0.834 | head involution | pum |
| 464 | GO:0003713 | F | 4, 6, | 1 | 0.439 (x 2.276) | 22 (0.045) | 0.836 | transcription coactivator activity | Ssb-c31a |
| 465 | GO:0008236 | F | 5, | 7 | 5.851 (x 1.196) | 293 (0.024) | 0.836 | serine-type peptidase activity | BG:DS01068.5 CG11313 CG1304 CG18223 CG2056 CG4386 CG9372 |
| 466 | GO:0015985 | P | 7, 8, 9, 10, | 2 | 1.278 (x 1.565) | 64 (0.031) | 0.836 | energy coupled proton transport, down electrochemical gradient | CG31477 Vha36 |
| 467 | GO:0046034 | P | 6, 10, | 2 | 1.298 (x 1.541) | 65 (0.031) | 0.836 | ATP metabolism | CG31477 Vha36 |
| 468 | GO:0006767 | P | 6, | 1 | 0.459 (x 2.177) | 23 (0.043) | 0.836 | water-soluble vitamin metabolism | CG5103 |
| 469 | GO:0030003 | P | 6, | 1 | 0.439 (x 2.276) | 22 (0.045) | 0.838 | cation homeostasis | Tsf1 |
| 470 | GO:0019722 | P | 7, | 2 | 1.278 (x 1.565) | 64 (0.031) | 0.838 | calcium-mediated signaling | CG7646 TpnC41C |
| 471 | GO:0006401 | P | 7, | 1 | 0.459 (x 2.177) | 23 (0.043) | 0.838 | RNA catabolism | pum |
| 472 | GO:0006875 | P | 7, | 1 | 0.439 (x 2.276) | 22 (0.045) | 0.839 | metal ion homeostasis | Tsf1 |
| 473 | GO:0006753 | P | 8, | 2 | 1.278 (x 1.565) | 64 (0.031) | 0.839 | nucleoside phosphate metabolism | CG31477 Vha36 |
| 474 | GO:0016319 | P | 5, 7, | 1 | 0.459 (x 2.177) | 23 (0.043) | 0.84 | mushroom body development | robl |
| 475 | GO:0015450 | F | 4, 6, | 1 | 0.479 (x 2.087) | 24 (0.042) | 0.841 | protein translocase activity | Tim9a |
| 476 | GO:0044425 | C | 3, 4, 5, | 26 | 24.282 (x 1.071) | 1216 (0.021) | 0.841 | membrane part | Abl Ac78C CG11015 CG11898 CG1213 CG12400 CG14482 CG31477 CG32230 CG4288 CG4673 CG5037 CG5548 CG6921 CG7188 CG8271 EG:152A3.7 GABA-B-R2 Or46a Or59a Pdsw Takr99D Tehao Tim9a Tsp42El Vha36 |
| 477 | GO:0007494 | P | 5, | 1 | 0.479 (x 2.087) | 24 (0.042) | 0.843 | midgut development | sisA |
| 478 | GO:0042063 | P | 6, | 1 | 0.479 (x 2.087) | 24 (0.042) | 0.845 | gliogenesis | BG:DS02740.9 |
| 479 | GO:0030261 | P | 7, | 1 | 0.479 (x 2.087) | 24 (0.042) | 0.847 | chromosome condensation | skpA |
| 480 | GO:0016323 | C | 5, 6, 7, | 1 | 0.479 (x 2.087) | 24 (0.042) | 0.848 | basolateral plasma membrane | Abl |
| 481 | GO:0044451 | C | 5, 6, 7, 8, 9, 10, 11, 12, | 5 | 4.054 (x 1.233) | 203 (0.025) | 0.848 | nucleoplasm part | CG15398 RpII18 Rpb10 Ssb-c31a Trap36 |
| 482 | GO:0016705 | F | 4, | 2 | 1.318 (x 1.518) | 66 (0.030) | 0.849 | oxidoreductase activity, acting on paired donors, with incorporation or reduction of molecular oxygen | CG8193 Tbh |
| 483 | GO:0007264 | P | 6, | 3 | 2.316 (x 1.295) | 116 (0.026) | 0.85 | small GTPase mediated signal transduction | Arf84F Rab3 ran-like |
| 484 | GO:0015082 | F | 5, | 1 | 0.479 (x 2.087) | 24 (0.042) | 0.85 | di-, tri-valent inorganic cation transporter activity | Tsf1 |
| 485 | GO:0019725 | P | 4, | 2 | 1.318 (x 1.518) | 66 (0.030) | 0.85 | cell homeostasis | Prx6005 Tsf1 |
| 486 | GO:0043566 | F | 5, | 1 | 0.499 (x 2.003) | 25 (0.040) | 0.851 | structure-specific DNA binding | Ssb-c31a |
| 487 | GO:0004867 | F | 6, | 2 | 1.398 (x 1.431) | 70 (0.029) | 0.851 | serine-type endopeptidase inhibitor activity | CG1342 CG31704 |
| 488 | GO:0019992 | F | 4, | 1 | 0.519 (x 1.926) | 26 (0.038) | 0.851 | diacylglycerol binding | unc-13 |
| 489 | GO:0009205 | P | 9, | 2 | 1.378 (x 1.452) | 69 (0.029) | 0.851 | purine ribonucleoside triphosphate metabolism | CG31477 Vha36 |
| 490 | GO:0046982 | F | 5, | 1 | 0.479 (x 2.087) | 24 (0.042) | 0.852 | protein heterodimerization activity | sisA |
| 491 | GO:0016459 | C | 3, 5, 6, 7, 8, 9, 10, | 1 | 0.499 (x 2.003) | 25 (0.040) | 0.852 | myosin | Mlc2 |
| 492 | GO:0051128 | P | 5, | 1 | 0.519 (x 1.926) | 26 (0.038) | 0.852 | regulation of cell organization and biogenesis | Abl |
| 493 | GO:0009142 | P | 7, 8, | 2 | 1.378 (x 1.452) | 69 (0.029) | 0.853 | nucleoside triphosphate biosynthesis | CG31477 Vha36 |
| 494 | GO:0009206 | P | 9, 10, | 2 | 1.358 (x 1.473) | 68 (0.029) | 0.853 | purine ribonucleoside triphosphate biosynthesis | CG31477 Vha36 |
| 495 | GO:0004383 | F | 4, 5, | 1 | 0.519 (x 1.926) | 26 (0.038) | 0.854 | guanylate cyclase activity | Ac78C |
| 496 | GO:0005262 | F | 6, 7, | 1 | 0.499 (x 2.003) | 25 (0.040) | 0.854 | calcium channel activity | na |
| 497 | GO:0009144 | P | 8, | 2 | 1.378 (x 1.452) | 69 (0.029) | 0.855 | purine nucleoside triphosphate metabolism | CG31477 Vha36 |
| 498 | GO:0009145 | P | 8, 9, | 2 | 1.358 (x 1.473) | 68 (0.029) | 0.855 | purine nucleoside triphosphate biosynthesis | CG31477 Vha36 |
| 499 | GO:0008654 | P | 7, 8, 9, | 1 | 0.519 (x 1.926) | 26 (0.038) | 0.856 | phospholipid biosynthesis | fu12 |
| 500 | GO:0009888 | P | 3, | 9 | 7.947 (x 1.132) | 398 (0.023) | 0.856 | tissue development | BG:DS02740.9 CG1942 CG31146 CG5397 CG9650 Tsp42El mirr sisA toy |
| 501 | GO:0019829 | F | 5, 6, 8, 13, | 2 | 1.378 (x 1.452) | 69 (0.029) | 0.856 | cation-transporting ATPase activity | CG31477 Vha36 |
| 502 | GO:0009798 | P | 4, | 4 | 3.175 (x 1.260) | 159 (0.025) | 0.856 | axis specification | Tehao Tm1 pum tsl |
| 503 | GO:0016469 | C | 3, 6, 7, 8, | 2 | 1.358 (x 1.473) | 68 (0.029) | 0.857 | proton-transporting two-sector ATPase complex | CG31477 Vha36 |
| 504 | GO:0045177 | C | 3, 4, | 1 | 0.519 (x 1.926) | 26 (0.038) | 0.858 | apical part of cell | mira |
| 505 | GO:0009199 | P | 8, | 2 | 1.378 (x 1.452) | 69 (0.029) | 0.858 | ribonucleoside triphosphate metabolism | CG31477 Vha36 |
| 506 | GO:0009201 | P | 8, 9, | 2 | 1.358 (x 1.473) | 68 (0.029) | 0.859 | ribonucleoside triphosphate biosynthesis | CG31477 Vha36 |
| 507 | GO:0015114 | F | 6, | 1 | 0.519 (x 1.926) | 26 (0.038) | 0.859 | phosphate transporter activity | CG4288 |
| 508 | GO:0042742 | P | 5, 6, | 2 | 1.378 (x 1.452) | 69 (0.029) | 0.86 | defense response to bacterium | CG6426 Tehao |
| 509 | GO:0030135 | C | 6, 7, 8, 9, 10, | 2 | 1.358 (x 1.473) | 68 (0.029) | 0.86 | coated vesicle | Rab3 unc-13 |
| 510 | GO:0008344 | P | 5, | 1 | 0.519 (x 1.926) | 26 (0.038) | 0.861 | adult locomotory behavior | na |
| 511 | GO:0009141 | P | 7, | 2 | 1.418 (x 1.411) | 71 (0.028) | 0.862 | nucleoside triphosphate metabolism | CG31477 Vha36 |
| 512 | GO:0005507 | F | 6, | 1 | 0.539 (x 1.855) | 27 (0.037) | 0.862 | copper ion binding | Tbh |
| 513 | GO:0007186 | P | 6, | 7 | 6.090 (x 1.149) | 305 (0.023) | 0.863 | G-protein coupled receptor protein signaling pathway | Ac78C Arf84F CG15361 GABA-B-R2 Or46a Or59a Takr99D |
| 514 | GO:0050801 | P | 4, | 1 | 0.539 (x 1.855) | 27 (0.037) | 0.864 | ion homeostasis | Tsf1 |
| 515 | GO:0008652 | P | 7, 8, | 2 | 1.438 (x 1.391) | 72 (0.028) | 0.864 | amino acid biosynthesis | Dhfr Gs2 |
| 516 | GO:0009948 | P | 5, | 3 | 2.376 (x 1.262) | 119 (0.025) | 0.864 | anterior/posterior axis specification | Tm1 pum tsl |
| 517 | GO:0007292 | P | 5, | 7 | 6.190 (x 1.131) | 310 (0.023) | 0.865 | female gamete generation | CG5162 Femcoat Tbh Tm1 Vm34Ca l(3)01239 pum |
| 518 | GO:0044267 | P | 6, | 43 | 41.515 (x 1.036) | 2079 (0.021) | 0.865 | cellular protein metabolism | Abl Act57B Arf84F CG10092 CG10104 CG10237 CG10466 CG11313 CG1304 CG18223 CG2056 CG31704 CG33002 CG4046 CG4386 CG4866 CG5338 CG6574 CG7014 CG7770 CG8415 CG9372 CG9804 Gs2 Obp58b RN-tre RpL11 RpL17A RpL38 RpL46 RpS17 RpS18 l(3)01239 mRpL21 mRpL22 mRpS14 mRpS21 mRpS24 na oho23B pum skpA tko |
| 519 | GO:0005386 | F | 3, | 10 | 9.046 (x 1.105) | 453 (0.022) | 0.865 | carrier activity | CG10237 CG10950 CG1213 CG31477 CG4288 CG6574 CG8271 Tim9a Tsf1 Vha36 |
| 520 | GO:0016455 | F | 5, | 1 | 0.539 (x 1.855) | 27 (0.037) | 0.865 | RNA polymerase II transcription mediator activity | Trap36 |
| 521 | GO:0005669 | C | 4, 7, 8, 9, 10, 11, 12, 13, 14, | 1 | 0.559 (x 1.789) | 28 (0.036) | 0.866 | transcription factor TFIID complex | CG15398 |
| 522 | GO:0015662 | F | 5, 6, 8, 13, | 2 | 1.438 (x 1.391) | 72 (0.028) | 0.866 | ATPase activity, coupled to transmembrane movement of ions, phosphorylative mechanism | CG31477 Vha36 |
| 523 | GO:0008565 | F | 3, | 2 | 1.498 (x 1.335) | 75 (0.027) | 0.866 | protein transporter activity | CG10950 Tim9a |
| 524 | GO:0007354 | P | 7, 8, | 1 | 0.539 (x 1.855) | 27 (0.037) | 0.867 | zygotic determination of anterior/posterior axis, embryo | tsl |
| 525 | GO:0014016 | P | 4, 7, | 1 | 0.579 (x 1.727) | 29 (0.034) | 0.867 | neuroblast differentiation | mira |
| 526 | GO:0004091 | F | 6, | 1 | 0.559 (x 1.789) | 28 (0.036) | 0.867 | carboxylesterase activity | alpha-Est8 |
| 527 | GO:0009617 | P | 5, | 2 | 1.478 (x 1.353) | 74 (0.027) | 0.867 | response to bacterium | CG6426 Tehao |
| 528 | GO:0007400 | P | 6, 9, | 1 | 0.579 (x 1.727) | 29 (0.034) | 0.869 | neuroblast fate determination | mira |
| 529 | GO:0005819 | C | 5, 6, 7, 8, 9, 10, | 1 | 0.559 (x 1.789) | 28 (0.036) | 0.869 | spindle | CLIP-190 |
| 530 | GO:0031325 | P | 6, | 2 | 1.478 (x 1.353) | 74 (0.027) | 0.869 | positive regulation of cellular metabolism | mirr pum |
| 531 | GO:0003729 | F | 5, | 7 | 6.250 (x 1.120) | 313 (0.022) | 0.87 | mRNA binding | BcDNA:GH11110 CG10092 CG10466 CG4866 RpS18 mRpL21 pum |
| 532 | GO:0015399 | F | 4, | 3 | 2.396 (x 1.252) | 120 (0.025) | 0.87 | primary active transporter activity | CG31477 Tim9a Vha36 |
| 533 | GO:0016471 | C | 3, 4, 5, 6, 7, 8, 9, 10, 11, | 1 | 0.579 (x 1.727) | 29 (0.034) | 0.87 | hydrogen-translocating V-type ATPase complex | Vha36 |
| 534 | GO:0000119 | C | 3, 4, 6, 7, 8, 9, 10, 11, 12, 13, 14, | 1 | 0.559 (x 1.789) | 28 (0.036) | 0.871 | mediator complex | Trap36 |
| 535 | GO:0009893 | P | 5, | 2 | 1.478 (x 1.353) | 74 (0.027) | 0.871 | positive regulation of metabolism | mirr pum |
| 536 | GO:0015405 | F | 5, | 3 | 2.396 (x 1.252) | 120 (0.025) | 0.872 | P-P-bond-hydrolysis-driven transporter activity | CG31477 Tim9a Vha36 |
| 537 | GO:0007416 | P | 5, 6, | 1 | 0.579 (x 1.727) | 29 (0.034) | 0.872 | synaptogenesis | Gs2 |
| 538 | GO:0048534 | P | 4, | 2 | 1.478 (x 1.353) | 74 (0.027) | 0.872 | hemopoietic or lymphoid organ development | CG9650 oho23B |
| 539 | GO:0016789 | F | 5, | 3 | 2.396 (x 1.252) | 120 (0.025) | 0.874 | carboxylic ester hydrolase activity | CG18530 CG5162 alpha-Est8 |
| 540 | GO:0014017 | P | 5, 8, | 1 | 0.579 (x 1.727) | 29 (0.034) | 0.874 | neuroblast fate commitment | mira |
| 541 | GO:0007349 | P | 3, 4, | 1 | 0.579 (x 1.727) | 29 (0.034) | 0.875 | cellularization | CLIP-190 |
| 542 | GO:0006644 | P | 7, 8, | 2 | 1.518 (x 1.318) | 76 (0.026) | 0.876 | phospholipid metabolism | CG5162 fu12 |
| 543 | GO:0009975 | F | 3, | 1 | 0.579 (x 1.727) | 29 (0.034) | 0.877 | cyclase activity | Ac78C |
| 544 | GO:0042592 | P | 3, | 2 | 1.518 (x 1.318) | 76 (0.026) | 0.878 | homeostasis | Prx6005 Tsf1 |
| 545 | GO:0007274 | P | 7, | 1 | 0.579 (x 1.727) | 29 (0.034) | 0.878 | neuromuscular synaptic transmission | CG31146 |
| 546 | GO:0008063 | P | 6, | 1 | 0.599 (x 1.669) | 30 (0.033) | 0.879 | Toll signaling pathway | Tehao |
| 547 | GO:0007218 | P | 7, | 1 | 0.659 (x 1.518) | 33 (0.030) | 0.88 | neuropeptide signaling pathway | CG15361 |
| 548 | GO:0006576 | P | 6, 7, | 1 | 0.599 (x 1.669) | 30 (0.033) | 0.881 | biogenic amine metabolism | Tbh |
| 549 | GO:0001700 | P | 5, | 3 | 2.576 (x 1.165) | 129 (0.023) | 0.881 | embryonic development (sensu Insecta) | Abl mirr pum |
| 550 | GO:0051707 | P | 4, | 3 | 2.616 (x 1.147) | 131 (0.023) | 0.882 | response to other organism | CG6426 Tehao upd3 |
| 551 | GO:0042067 | P | 6, 7, 8, 9, | 1 | 0.659 (x 1.518) | 33 (0.030) | 0.882 | establishment of ommatidial polarity (sensu Endopterygota) | mirr |
| 552 | GO:0030031 | P | 6, 7, | 1 | 0.639 (x 1.565) | 32 (0.031) | 0.882 | cell projection biogenesis | CG14825 |
| 553 | GO:0016849 | F | 4, | 1 | 0.599 (x 1.669) | 30 (0.033) | 0.882 | phosphorus-oxygen lyase activity | Ac78C |
| 554 | GO:0048489 | P | 6, 7, | 2 | 1.597 (x 1.252) | 80 (0.025) | 0.883 | synaptic vesicle transport | Arf84F unc-13 |
| 555 | GO:0016758 | F | 5, | 3 | 2.576 (x 1.165) | 129 (0.023) | 0.883 | transferase activity, transferring hexosyl groups | Act57B CG30438 Ugt86Di |
| 556 | GO:0006887 | P | 6, 7, | 3 | 2.496 (x 1.202) | 125 (0.024) | 0.883 | exocytosis | Arf84F Rab3 unc-13 |
| 557 | GO:0043565 | F | 5, | 1 | 0.659 (x 1.518) | 33 (0.030) | 0.883 | sequence-specific DNA binding | skpA |
| 558 | GO:0005774 | C | 5, 6, 7, 8, 9, 10, | 1 | 0.639 (x 1.565) | 32 (0.031) | 0.884 | vacuolar membrane | Vha36 |
| 559 | GO:0007619 | P | 5, 6, | 1 | 0.659 (x 1.518) | 33 (0.030) | 0.885 | courtship behavior | tko |
| 560 | GO:0050808 | P | 5, | 1 | 0.639 (x 1.565) | 32 (0.031) | 0.886 | synapse organization and biogenesis | Gs2 |
| 561 | GO:0046467 | P | 6, 7, 8, | 1 | 0.619 (x 1.615) | 31 (0.032) | 0.886 | membrane lipid biosynthesis | fu12 |
| 562 | GO:0008293 | P | 8, | 1 | 0.659 (x 1.518) | 33 (0.030) | 0.886 | torso signaling pathway | tsl |
| 563 | GO:0006605 | P | 7, 8, 9, | 5 | 4.613 (x 1.084) | 231 (0.022) | 0.887 | protein targeting | CG10950 CG4673 CLIP-190 Tim9a ran-like |
| 564 | GO:0008643 | P | 5, 6, | 2 | 1.617 (x 1.237) | 81 (0.025) | 0.887 | carbohydrate transport | CG1213 CG4288 |
| 565 | GO:0006626 | P | 8, 9, 10, | 1 | 0.679 (x 1.473) | 34 (0.029) | 0.887 | protein targeting to mitochondrion | Tim9a |
| 566 | GO:0000070 | P | 6, 8, | 1 | 0.639 (x 1.565) | 32 (0.031) | 0.887 | mitotic sister chromatid segregation | Nipped-B |
| 567 | GO:0015926 | F | 6, | 1 | 0.619 (x 1.615) | 31 (0.032) | 0.887 | glucosidase activity | CG14935 |
| 568 | GO:0003924 | F | 8, | 3 | 2.596 (x 1.156) | 130 (0.023) | 0.888 | GTPase activity | Arf84F Rab3 ran-like |
| 569 | GO:0019221 | P | 6, | 1 | 0.659 (x 1.518) | 33 (0.030) | 0.888 | cytokine and chemokine mediated signaling pathway | Tehao |
| 570 | GO:0009187 | P | 7, | 1 | 0.679 (x 1.473) | 34 (0.029) | 0.888 | cyclic nucleotide metabolism | Ac78C |
| 571 | GO:0044437 | C | 4, 5, 6, 7, 8, 9, | 1 | 0.639 (x 1.565) | 32 (0.031) | 0.889 | vacuolar part | Vha36 |
| 572 | GO:0007224 | P | 6, | 1 | 0.619 (x 1.615) | 31 (0.032) | 0.889 | smoothened signaling pathway | mirr |
| 573 | GO:0009952 | P | 4, | 3 | 2.596 (x 1.156) | 130 (0.023) | 0.889 | anterior/posterior pattern formation | Tm1 pum tsl |
| 574 | GO:0044448 | C | 5, 6, 7, 8, 9, | 1 | 0.659 (x 1.518) | 33 (0.030) | 0.889 | cell cortex part | mira |
| 575 | GO:0005184 | F | 5, 6, | 1 | 0.679 (x 1.473) | 34 (0.029) | 0.89 | neuropeptide hormone activity | CG15361 |
| 576 | GO:0046873 | F | 4, | 1 | 0.639 (x 1.565) | 32 (0.031) | 0.89 | metal ion transporter activity | Tsf1 |
| 577 | GO:0042995 | C | 3, 4, | 1 | 0.619 (x 1.615) | 31 (0.032) | 0.891 | cell projection | robl |
| 578 | GO:0006959 | P | 5, 6, | 2 | 1.558 (x 1.284) | 78 (0.026) | 0.891 | humoral immune response | Tehao upd3 |
| 579 | GO:0005654 | C | 5, 6, 7, 8, 9, 10, 11, | 5 | 4.473 (x 1.118) | 224 (0.022) | 0.891 | nucleoplasm | CG15398 RpII18 Rpb10 Ssb-c31a Trap36 |
| 580 | GO:0008354 | P | 5, 6, 7, | 1 | 0.659 (x 1.518) | 33 (0.030) | 0.891 | germ cell migration | pum |
| 581 | GO:0048488 | P | 7, 8, | 1 | 0.679 (x 1.473) | 34 (0.029) | 0.891 | synaptic vesicle endocytosis | Arf84F |
| 582 | GO:0042254 | P | 6, | 1 | 0.639 (x 1.565) | 32 (0.031) | 0.892 | ribosome biogenesis and assembly | CG32409 |
| 583 | GO:0016023 | C | 5, 6, 7, 8, 9, | 2 | 1.657 (x 1.207) | 83 (0.024) | 0.892 | cytoplasmic membrane-bound vesicle | Rab3 unc-13 |
| 584 | GO:0015992 | P | 6, 7, 8, 9, | 2 | 1.558 (x 1.284) | 78 (0.026) | 0.892 | proton transport | CG31477 Vha36 |
| 585 | GO:0016684 | F | 4, | 1 | 0.639 (x 1.565) | 32 (0.031) | 0.893 | oxidoreductase activity, acting on peroxide as acceptor | Prx6005 |
| 586 | GO:0031410 | C | 4, 5, 6, 7, 8, | 2 | 1.657 (x 1.207) | 83 (0.024) | 0.893 | cytoplasmic vesicle | Rab3 unc-13 |
| 587 | GO:0006818 | P | 5, 6, | 2 | 1.558 (x 1.284) | 78 (0.026) | 0.894 | hydrogen transport | CG31477 Vha36 |
| 588 | GO:0006367 | P | 9, | 2 | 1.677 (x 1.192) | 84 (0.024) | 0.894 | transcription initiation from RNA polymerase II promoter | CG15398 Trap36 |
| 589 | GO:0000819 | P | 5, | 1 | 0.639 (x 1.565) | 32 (0.031) | 0.895 | sister chromatid segregation | Nipped-B |
| 590 | GO:0016616 | F | 5, | 2 | 1.677 (x 1.192) | 84 (0.024) | 0.896 | oxidoreductase activity, acting on the CH-OH group of donors, NAD or NADP as acceptor | CG10638 CG10962 |
| 591 | GO:0030198 | P | 4, | 1 | 0.639 (x 1.565) | 32 (0.031) | 0.897 | extracellular matrix organization and biogenesis | Gs2 |
| 592 | GO:0045451 | P | 7, 11, 13, 14, 16, | 1 | 0.639 (x 1.565) | 32 (0.031) | 0.898 | pole plasm oskar mRNA localization | Tm1 |
| 593 | GO:0008360 | P | 5, 6, | 2 | 1.697 (x 1.178) | 85 (0.024) | 0.899 | regulation of cell shape | Abl SIP1 |
| 594 | GO:0006206 | P | 7, | 1 | 0.639 (x 1.565) | 32 (0.031) | 0.9 | pyrimidine base metabolism | Dhfr |
| 595 | GO:0019204 | F | 7, | 1 | 0.699 (x 1.431) | 35 (0.029) | 0.9 | nucleotide phosphatase activity | CG2277 |
| 596 | GO:0005667 | C | 3, 6, 7, 8, 9, 10, 11, 12, 13, | 2 | 1.697 (x 1.178) | 85 (0.024) | 0.901 | transcription factor complex | CG15398 Ssb-c31a |
| 597 | GO:0016044 | P | 5, | 1 | 0.639 (x 1.565) | 32 (0.031) | 0.901 | membrane organization and biogenesis | Tim9a |
| 598 | GO:0004866 | F | 5, | 2 | 1.697 (x 1.178) | 85 (0.024) | 0.902 | endopeptidase inhibitor activity | CG1342 CG31704 |
| 599 | GO:0007623 | P | 4, | 1 | 0.719 (x 1.391) | 36 (0.028) | 0.903 | circadian rhythm | na |
| 600 | GO:0008105 | P | 5, | 1 | 0.639 (x 1.565) | 32 (0.031) | 0.903 | asymmetric protein localization | mira |
| 601 | GO:0019897 | C | 5, 6, 7, | 1 | 0.719 (x 1.391) | 36 (0.028) | 0.904 | extrinsic to plasma membrane | Abl |
| 602 | GO:0031982 | C | 3, | 2 | 1.717 (x 1.165) | 86 (0.023) | 0.904 | vesicle | Rab3 unc-13 |
| 603 | GO:0004601 | F | 3, 5, | 1 | 0.639 (x 1.565) | 32 (0.031) | 0.905 | peroxidase activity | Prx6005 |
| 604 | GO:0006766 | P | 5, | 1 | 0.719 (x 1.391) | 36 (0.028) | 0.906 | vitamin metabolism | CG5103 |
| 605 | GO:0030414 | F | 4, | 2 | 1.717 (x 1.165) | 86 (0.023) | 0.906 | protease inhibitor activity | CG1342 CG31704 |
| 606 | GO:0051301 | P | 4, | 3 | 2.776 (x 1.081) | 139 (0.022) | 0.907 | cell division | Act57B mira pum |
| 607 | GO:0006352 | P | 8, | 2 | 1.717 (x 1.165) | 86 (0.023) | 0.908 | transcription initiation | CG15398 Trap36 |
| 608 | GO:0003774 | F | 2, | 2 | 1.737 (x 1.151) | 87 (0.023) | 0.908 | motor activity | Act57B Mlc2 |
| 609 | GO:0016298 | F | 6, | 2 | 1.757 (x 1.138) | 88 (0.023) | 0.908 | lipase activity | CG18530 CG5162 |
| 610 | GO:0031988 | C | 4, | 2 | 1.717 (x 1.165) | 86 (0.023) | 0.909 | membrane-bound vesicle | Rab3 unc-13 |
| 611 | GO:0006643 | P | 6, 7, | 2 | 1.737 (x 1.151) | 87 (0.023) | 0.909 | membrane lipid metabolism | CG5162 fu12 |
| 612 | GO:0019932 | P | 6, | 2 | 1.757 (x 1.138) | 88 (0.023) | 0.91 | second-messenger-mediated signaling | CG7646 TpnC41C |
| 613 | GO:0048511 | P | 3, | 1 | 0.739 (x 1.353) | 37 (0.027) | 0.911 | rhythmic process | na |
| 614 | GO:0044260 | P | 5, | 43 | 42.972 (x 1.001) | 2152 (0.020) | 0.911 | cellular macromolecule metabolism | Abl Act57B Arf84F CG10092 CG10104 CG10237 CG10466 CG11313 CG1304 CG18223 CG2056 CG31704 CG33002 CG4046 CG4386 CG4866 CG5338 CG6574 CG7014 CG7770 CG8415 CG9372 CG9804 Gs2 Obp58b RN-tre RpL11 RpL17A RpL38 RpL46 RpS17 RpS18 l(3)01239 mRpL21 mRpL22 mRpS14 mRpS21 mRpS24 na oho23B pum skpA tko |
| 615 | GO:0004806 | F | 7, | 1 | 0.739 (x 1.353) | 37 (0.027) | 0.912 | triacylglycerol lipase activity | CG18530 |
| 616 | GO:0019094 | P | 6, 10, 12, 13, 15, | 1 | 0.739 (x 1.353) | 37 (0.027) | 0.914 | pole plasm mRNA localization | Tm1 |
| 617 | GO:0044430 | C | 4, 5, 6, 7, 8, 9, | 5 | 4.792 (x 1.043) | 240 (0.021) | 0.914 | cytoskeletal part | Act57B CLIP-190 Mlc2 Tm1 robl |
| 618 | GO:0007316 | P | 5, 9, 11, 12, 14, | 1 | 0.759 (x 1.318) | 38 (0.026) | 0.917 | pole plasm RNA localization | Tm1 |
| 619 | GO:0006916 | P | 8, 9, | 1 | 0.759 (x 1.318) | 38 (0.026) | 0.918 | anti-apoptosis | CG7188 |
| 620 | GO:0008066 | F | 5, | 1 | 0.759 (x 1.318) | 38 (0.026) | 0.92 | glutamate receptor activity | GABA-B-R2 |
| 621 | GO:0005794 | C | 5, 6, 7, 8, | 2 | 1.797 (x 1.113) | 90 (0.022) | 0.922 | Golgi apparatus | CG5189 CLIP-190 |
| 622 | GO:0016779 | F | 5, | 2 | 1.817 (x 1.101) | 91 (0.022) | 0.927 | nucleotidyltransferase activity | RpII18 Rpb10 |
| 623 | GO:0015296 | F | 5, 7, | 1 | 0.779 (x 1.284) | 39 (0.026) | 0.928 | anion:cation symporter activity | CG4288 |
| 624 | GO:0005643 | C | 3, 5, 6, 7, 8, 9, 10, 11, 12, 13, | 1 | 0.799 (x 1.252) | 40 (0.025) | 0.929 | nuclear pore | CG4673 |
| 625 | GO:0046148 | P | 6, | 1 | 0.779 (x 1.284) | 39 (0.026) | 0.929 | pigment biosynthesis | CG5037 |
| 626 | GO:0046930 | C | 6, 7, 8, | 1 | 0.799 (x 1.252) | 40 (0.025) | 0.93 | pore complex | CG4673 |
| 627 | GO:0008194 | F | 5, | 2 | 1.837 (x 1.089) | 92 (0.022) | 0.931 | UDP-glycosyltransferase activity | Act57B Ugt86Di |
| 628 | GO:0005351 | F | 5, 6, | 1 | 0.799 (x 1.252) | 40 (0.025) | 0.932 | sugar porter activity | CG1213 |
| 629 | GO:0008234 | F | 5, | 2 | 1.857 (x 1.077) | 93 (0.022) | 0.932 | cysteine-type peptidase activity | Obp58b RN-tre |
| 630 | GO:0006796 | P | 6, | 12 | 12.061 (x 0.995) | 604 (0.020) | 0.933 | phosphate metabolism | Abl CG11015 CG12400 CG14482 CG2056 CG31477 CG32230 CG4288 CG5548 EG:152A3.7 Pdsw Vha36 |
| 631 | GO:0005681 | C | 4, 5, 6, 7, 8, 9, 10, | 2 | 1.837 (x 1.089) | 92 (0.022) | 0.933 | spliceosome complex | CG13277 CG6610 |
| 632 | GO:0015674 | P | 7, 8, | 1 | 0.799 (x 1.252) | 40 (0.025) | 0.933 | di-, tri-valent inorganic cation transport | Tsf1 |
| 633 | GO:0006793 | P | 5, | 12 | 12.061 (x 0.995) | 604 (0.020) | 0.934 | phosphorus metabolism | Abl CG11015 CG12400 CG14482 CG2056 CG31477 CG32230 CG4288 CG5548 EG:152A3.7 Pdsw Vha36 |
| 634 | GO:0015103 | F | 5, | 1 | 0.799 (x 1.252) | 40 (0.025) | 0.934 | inorganic anion transporter activity | CG4288 |
| 635 | GO:0043066 | P | 7, 8, | 1 | 0.839 (x 1.192) | 42 (0.024) | 0.939 | negative regulation of apoptosis | CG7188 |
| 636 | GO:0030286 | C | 4, 6, 7, 8, 9, 10, 11, | 1 | 0.819 (x 1.221) | 41 (0.024) | 0.939 | dynein complex | robl |
| 637 | GO:0051726 | P | 5, | 4 | 3.974 (x 1.007) | 199 (0.020) | 0.939 | regulation of cell cycle | Abl CG2750 pum skpA |
| 638 | GO:0016740 | F | 3, | 19 | 19.310 (x 0.984) | 967 (0.020) | 0.94 | transferase activity | Abl Act57B CG2056 CG30438 CG5037 CG5103 CG5122 CG5224 CG5397 CG6921 GstD9 GstE5 GstE6 GstE7 RpII18 Rpb10 Ugt86Di agt fu12 |
| 639 | GO:0043069 | P | 6, 7, | 1 | 0.839 (x 1.192) | 42 (0.024) | 0.94 | negative regulation of programmed cell death | CG7188 |
| 640 | GO:0048113 | P | 8, 10, 11, 13, | 1 | 0.819 (x 1.221) | 41 (0.024) | 0.941 | pole plasm assembly (sensu Insecta) | Tm1 |
| 641 | GO:0000074 | P | 6, | 4 | 3.974 (x 1.007) | 199 (0.020) | 0.941 | regulation of progression through cell cycle | Abl CG2750 pum skpA |
| 642 | GO:0007306 | P | 9, 10, | 1 | 0.839 (x 1.192) | 42 (0.024) | 0.942 | insect chorion formation | Femcoat |
| 643 | GO:0006817 | P | 8, 9, | 1 | 0.859 (x 1.165) | 43 (0.023) | 0.943 | phosphate transport | CG4288 |
| 644 | GO:0042625 | F | 4, 5, 7, 12, | 2 | 1.897 (x 1.054) | 95 (0.021) | 0.943 | ATPase activity, coupled to transmembrane movement of ions | CG31477 Vha36 |
| 645 | GO:0005874 | C | 5, 6, 7, 8, 9, 10, | 1 | 0.839 (x 1.192) | 42 (0.024) | 0.943 | microtubule | CLIP-190 |
| 646 | GO:0042923 | F | 4, | 1 | 0.879 (x 1.138) | 44 (0.023) | 0.943 | neuropeptide binding | Takr99D |
| 647 | GO:0019538 | P | 5, | 43 | 43.591 (x 0.986) | 2183 (0.020) | 0.944 | protein metabolism | Abl Act57B Arf84F CG10092 CG10104 CG10237 CG10466 CG11313 CG1304 CG18223 CG2056 CG31704 CG33002 CG4046 CG4386 CG4866 CG5338 CG6574 CG7014 CG7770 CG8415 CG9372 CG9804 Gs2 Obp58b RN-tre RpL11 RpL17A RpL38 RpL46 RpS17 RpS18 l(3)01239 mRpL21 mRpL22 mRpS14 mRpS21 mRpS24 na oho23B pum skpA tko |
| 648 | GO:0001736 | P | 5, 6, | 1 | 0.859 (x 1.165) | 43 (0.023) | 0.944 | establishment of planar polarity | mirr |
| 649 | GO:0043492 | F | 3, 10, | 3 | 3.015 (x 0.995) | 151 (0.020) | 0.944 | ATPase activity, coupled to movement of substances | CG11898 CG31477 Vha36 |
| 650 | GO:0043062 | P | 3, | 1 | 0.839 (x 1.192) | 42 (0.024) | 0.945 | extracellular structure organization and biogenesis | Gs2 |
| 651 | GO:0008188 | F | 5, 6, 8, | 1 | 0.879 (x 1.138) | 44 (0.023) | 0.945 | neuropeptide receptor activity | Takr99D |
| 652 | GO:0044428 | C | 4, 5, 6, 7, 8, 9, | 10 | 10.284 (x 0.972) | 515 (0.019) | 0.945 | nuclear part | CG13277 CG15398 CG31922 CG31950 CG4673 CG6610 RpII18 Rpb10 Ssb-c31a Trap36 |
| 653 | GO:0005506 | F | 6, | 1 | 0.859 (x 1.165) | 43 (0.023) | 0.946 | iron ion binding | Tsf1 |
| 654 | GO:0042626 | F | 4, 6, 11, | 3 | 3.015 (x 0.995) | 151 (0.020) | 0.946 | ATPase activity, coupled to transmembrane movement of substances | CG11898 CG31477 Vha36 |
| 655 | GO:0009582 | P | 4, 5, | 1 | 0.879 (x 1.138) | 44 (0.023) | 0.946 | detection of abiotic stimulus | tko |
| 656 | GO:0007164 | P | 4, | 1 | 0.859 (x 1.165) | 43 (0.023) | 0.947 | establishment of tissue polarity | mirr |
| 657 | GO:0005911 | C | 6, 7, 8, | 1 | 0.879 (x 1.138) | 44 (0.023) | 0.948 | intercellular junction | Abl |
| 658 | GO:0006606 | P | 7, 8, 9, 10, | 1 | 0.939 (x 1.066) | 47 (0.021) | 0.948 | protein import into nucleus | CG10950 |
| 659 | GO:0007530 | P | 3, | 1 | 0.859 (x 1.165) | 43 (0.023) | 0.949 | sex determination | sisA |
| 660 | GO:0042051 | P | 7, 8, 9, 10, | 1 | 0.879 (x 1.138) | 44 (0.023) | 0.949 | eye photoreceptor development (sensu Endopterygota) | Cpn |
| 661 | GO:0016407 | F | 7, | 1 | 0.939 (x 1.066) | 47 (0.021) | 0.949 | acetyltransferase activity | CG5122 |
| 662 | GO:0042462 | P | 6, 7, 8, | 1 | 0.899 (x 1.113) | 45 (0.022) | 0.949 | eye photoreceptor cell development | Cpn |
| 663 | GO:0051248 | P | 6, 7, | 1 | 0.859 (x 1.165) | 43 (0.023) | 0.95 | negative regulation of protein metabolism | pum |
| 664 | GO:0016820 | F | 5, | 3 | 3.035 (x 0.988) | 152 (0.020) | 0.95 | hydrolase activity, acting on acid anhydrides, catalyzing transmembrane movement of substances | CG11898 CG31477 Vha36 |
| 665 | GO:0001653 | F | 4, | 1 | 0.939 (x 1.066) | 47 (0.021) | 0.951 | peptide receptor activity | Takr99D |
| 666 | GO:0009581 | P | 4, 5, | 1 | 0.899 (x 1.113) | 45 (0.022) | 0.951 | detection of external stimulus | tko |
| 667 | GO:0008340 | P | 4, | 1 | 0.919 (x 1.089) | 46 (0.022) | 0.951 | determination of adult life span | Sod |
| 668 | GO:0007315 | P | 7, 9, 10, 12, | 1 | 0.859 (x 1.165) | 43 (0.023) | 0.952 | pole plasm assembly | Tm1 |
| 669 | GO:0030030 | P | 5, 6, | 1 | 0.939 (x 1.066) | 47 (0.021) | 0.952 | cell projection organization and biogenesis | CG14825 |
| 670 | GO:0007270 | P | 7, | 1 | 0.919 (x 1.089) | 46 (0.022) | 0.952 | nerve-nerve synaptic transmission | GABA-B-R2 |
| 671 | GO:0019898 | C | 4, 5, 6, | 1 | 0.899 (x 1.113) | 45 (0.022) | 0.952 | extrinsic to membrane | Abl |
| 672 | GO:0016071 | P | 7, | 4 | 4.153 (x 0.963) | 208 (0.019) | 0.953 | mRNA metabolism | CG10466 CG13277 CG6610 pum |
| 673 | GO:0005529 | F | 4, | 1 | 0.939 (x 1.066) | 47 (0.021) | 0.953 | sugar binding | CG4115 |
| 674 | GO:0007350 | P | 4, 5, | 2 | 2.037 (x 0.982) | 102 (0.020) | 0.954 | blastoderm segmentation | pum tsl |
| 675 | GO:0007568 | P | 3, | 1 | 0.919 (x 1.089) | 46 (0.022) | 0.954 | aging | Sod |
| 676 | GO:0048112 | P | 7, 9, 10, 12, | 1 | 0.899 (x 1.113) | 45 (0.022) | 0.954 | oocyte anterior/posterior axis determination (sensu Insecta) | Tm1 |
| 677 | GO:0004175 | F | 5, | 9 | 9.405 (x 0.957) | 471 (0.019) | 0.954 | endopeptidase activity | BG:DS01068.5 CG10104 CG11313 CG1304 CG18223 CG2056 CG4386 CG9372 Obp58b |
| 678 | GO:0003723 | F | 4, | 7 | 7.368 (x 0.950) | 369 (0.019) | 0.954 | RNA binding | BcDNA:GH11110 CG10092 CG10466 CG4866 RpS18 mRpL21 pum |
| 679 | GO:0044453 | C | 4, 5, 6, 7, 8, 9, 10, 11, 12, | 1 | 0.939 (x 1.066) | 47 (0.021) | 0.955 | nuclear membrane part | CG4673 |
| 680 | GO:0016051 | P | 6, 7, | 1 | 0.978 (x 1.022) | 49 (0.020) | 0.955 | carbohydrate biosynthesis | Act57B |
| 681 | GO:0009613 | P | 4, 5, | 2 | 1.997 (x 1.002) | 100 (0.020) | 0.955 | response to pest, pathogen or parasite | Tehao upd3 |
| 682 | GO:0016251 | F | 4, | 2 | 2.017 (x 0.992) | 101 (0.020) | 0.955 | general RNA polymerase II transcription factor activity | BEST:LD29214 Trap36 |
| 683 | GO:0016079 | P | 7, 8, 9, | 1 | 0.919 (x 1.089) | 46 (0.022) | 0.955 | synaptic vesicle exocytosis | unc-13 |
| 684 | GO:0016757 | F | 4, | 3 | 3.175 (x 0.945) | 159 (0.019) | 0.956 | transferase activity, transferring glycosyl groups | Act57B CG30438 Ugt86Di |
| 685 | GO:0031965 | C | 5, 6, 7, 8, 9, 10, 11, | 1 | 0.939 (x 1.066) | 47 (0.021) | 0.956 | nuclear membrane | CG4673 |
| 686 | GO:0046983 | F | 4, | 1 | 0.978 (x 1.022) | 49 (0.020) | 0.956 | protein dimerization activity | sisA |
| 687 | GO:0042440 | P | 5, | 1 | 0.919 (x 1.089) | 46 (0.022) | 0.957 | pigment metabolism | CG5037 |
| 688 | GO:0051170 | P | 7, 8, 9, | 1 | 0.958 (x 1.043) | 48 (0.021) | 0.957 | nuclear import | CG10950 |
| 689 | GO:0051119 | F | 4, | 1 | 0.939 (x 1.066) | 47 (0.021) | 0.958 | sugar transporter activity | CG1213 |
| 690 | GO:0006575 | P | 6, | 1 | 0.978 (x 1.022) | 49 (0.020) | 0.958 | amino acid derivative metabolism | Tbh |
| 691 | GO:0019001 | F | 5, | 3 | 3.235 (x 0.927) | 162 (0.019) | 0.959 | guanyl nucleotide binding | Arf84F Rab3 ran-like |
| 692 | GO:0015290 | F | 4, | 4 | 4.333 (x 0.923) | 217 (0.018) | 0.959 | electrochemical potential-driven transporter activity | CG1213 CG4288 CG6574 CG8271 |
| 693 | GO:0005912 | C | 6, 7, 8, | 1 | 0.978 (x 1.022) | 49 (0.020) | 0.959 | adherens junction | Abl |
| 694 | GO:0008528 | F | 5, 7, | 1 | 0.939 (x 1.066) | 47 (0.021) | 0.959 | peptide receptor activity, G-protein coupled | Takr99D |
| 695 | GO:0035214 | P | 5, | 3 | 3.195 (x 0.939) | 160 (0.019) | 0.96 | eye-antennal disc development | Cpn mirr toy |
| 696 | GO:0015291 | F | 5, | 4 | 4.333 (x 0.923) | 217 (0.018) | 0.96 | porter activity | CG1213 CG4288 CG6574 CG8271 |
| 697 | GO:0051606 | P | 3, 4, | 1 | 0.978 (x 1.022) | 49 (0.020) | 0.96 | detection of stimulus | tko |
| 698 | GO:0008594 | P | 6, 7, 8, | 1 | 0.978 (x 1.022) | 49 (0.020) | 0.962 | photoreceptor cell morphogenesis (sensu Endopterygota) | Cpn |
| 699 | GO:0009063 | P | 7, 8, | 1 | 0.978 (x 1.022) | 49 (0.020) | 0.963 | amino acid catabolism | Gs2 |
| 700 | GO:0007420 | P | 4, 6, | 1 | 0.978 (x 1.022) | 49 (0.020) | 0.965 | brain development | robl |
| 701 | GO:0005525 | F | 6, | 3 | 3.215 (x 0.933) | 161 (0.019) | 0.965 | GTP binding | Arf84F Rab3 ran-like |
| 702 | GO:0044270 | P | 5, 6, | 1 | 1.038 (x 0.963) | 52 (0.019) | 0.966 | nitrogen compound catabolism | Gs2 |
| 703 | GO:0004497 | F | 4, | 2 | 2.177 (x 0.919) | 109 (0.018) | 0.966 | monooxygenase activity | CG8193 Tbh |
| 704 | GO:0046164 | P | 6, | 1 | 1.018 (x 0.982) | 51 (0.020) | 0.966 | alcohol catabolism | CG5103 |
| 705 | GO:0009993 | P | 7, | 5 | 5.531 (x 0.904) | 277 (0.018) | 0.967 | oogenesis (sensu Insecta) | Femcoat Tm1 Vm34Ca l(3)01239 pum |
| 706 | GO:0001738 | P | 5, | 1 | 1.038 (x 0.963) | 52 (0.019) | 0.967 | morphogenesis of a polarized epithelium | mirr |
| 707 | GO:0051242 | P | 5, | 3 | 3.335 (x 0.900) | 167 (0.018) | 0.968 | positive regulation of cellular physiological process | mirr pum skpA |
| 708 | GO:0005244 | F | 5, 6, | 1 | 1.018 (x 0.982) | 51 (0.020) | 0.968 | voltage-gated ion channel activity | na |
| 709 | GO:0048731 | P | 3, | 11 | 11.861 (x 0.927) | 594 (0.019) | 0.969 | system development | Abl BG:DS02740.9 CG31146 Gs2 Tm1 Tsp42El mira mirr pum robl toy |
| 710 | GO:0009310 | P | 6, 7, | 1 | 1.038 (x 0.963) | 52 (0.019) | 0.969 | amine catabolism | Gs2 |
| 711 | GO:0046365 | P | 7, 8, | 1 | 1.018 (x 0.982) | 51 (0.020) | 0.969 | monosaccharide catabolism | CG5103 |
| 712 | GO:0015698 | P | 7, 8, | 1 | 1.078 (x 0.927) | 54 (0.019) | 0.969 | inorganic anion transport | CG4288 |
| 713 | GO:0001709 | P | 5, | 2 | 2.197 (x 0.911) | 110 (0.018) | 0.969 | cell fate determination | mira pum |
| 714 | GO:0042048 | P | 5, 6, | 1 | 1.058 (x 0.945) | 53 (0.019) | 0.97 | olfactory behavior | Obp56a |
| 715 | GO:0007606 | P | 4, 6, | 3 | 3.315 (x 0.905) | 166 (0.018) | 0.97 | sensory perception of chemical stimulus | Obp56a Or46a Or59a |
| 716 | GO:0042461 | P | 5, 6, 7, | 1 | 1.038 (x 0.963) | 52 (0.019) | 0.97 | photoreceptor cell development | Cpn |
| 717 | GO:0007281 | P | 5, | 2 | 2.236 (x 0.894) | 112 (0.018) | 0.97 | germ cell development | Tm1 pum |
| 718 | GO:0051252 | P | 7, | 1 | 1.078 (x 0.927) | 54 (0.019) | 0.97 | regulation of RNA metabolism | pum |
| 719 | GO:0006007 | P | 9, 10, | 1 | 1.018 (x 0.982) | 51 (0.020) | 0.97 | glucose catabolism | CG5103 |
| 720 | GO:0005179 | F | 4, 5, | 1 | 1.058 (x 0.945) | 53 (0.019) | 0.971 | hormone activity | CG15361 |
| 721 | GO:0043119 | P | 4, | 3 | 3.375 (x 0.889) | 169 (0.018) | 0.971 | positive regulation of physiological process | mirr pum skpA |
| 722 | GO:0007028 | P | 5, | 1 | 1.078 (x 0.927) | 54 (0.019) | 0.972 | cytoplasm organization and biogenesis | CG32409 |
| 723 | GO:0019320 | P | 8, 9, | 1 | 1.018 (x 0.982) | 51 (0.020) | 0.972 | hexose catabolism | CG5103 |
| 724 | GO:0043231 | C | 4, 5, 6, 7, | 44 | 46.068 (x 0.955) | 2307 (0.019) | 0.972 | intracellular membrane-bound organelle | BEST:LD29214 CG11015 CG12400 CG13277 CG14482 CG15398 CG31477 CG31922 CG31950 CG32174 CG32230 CG32409 CG33002 CG4673 CG4866 CG5037 CG5189 CG5548 CG6610 CG7911 CG9650 CLIP-190 EG:152A3.7 Nipped-B Pdsw Rab3 RpII18 Rpb10 Ssb-c31a Tim9a Trap36 Vha36 mRpL21 mRpL22 mRpL54 mRpS14 mRpS21 mRpS24 mirr robl sisA tko toy unc-13 |
| 725 | GO:0007617 | P | 4, 5, | 1 | 1.098 (x 0.911) | 55 (0.018) | 0.972 | mating behavior | tko |
| 726 | GO:0048111 | P | 6, 8, 9, 11, | 1 | 1.058 (x 0.945) | 53 (0.019) | 0.972 | oocyte axis determination (sensu Insecta) | Tm1 |
| 727 | GO:0006807 | P | 4, | 7 | 7.808 (x 0.897) | 391 (0.018) | 0.973 | nitrogen compound metabolism | Act57B CG10092 CG5122 CG9836 Dhfr Gs2 Tbh |
| 728 | GO:0048110 | P | 7, 8, 10, | 1 | 1.078 (x 0.927) | 54 (0.019) | 0.973 | oocyte construction (sensu Insecta) | Tm1 |
| 729 | GO:0051705 | P | 3, | 1 | 1.098 (x 0.911) | 55 (0.018) | 0.974 | behavioral interaction between organisms | tko |
| 730 | GO:0043227 | C | 3, | 44 | 46.107 (x 0.954) | 2309 (0.019) | 0.974 | membrane-bound organelle | BEST:LD29214 CG11015 CG12400 CG13277 CG14482 CG15398 CG31477 CG31922 CG31950 CG32174 CG32230 CG32409 CG33002 CG4673 CG4866 CG5037 CG5189 CG5548 CG6610 CG7911 CG9650 CLIP-190 EG:152A3.7 Nipped-B Pdsw Rab3 RpII18 Rpb10 Ssb-c31a Tim9a Trap36 Vha36 mRpL21 mRpL22 mRpL54 mRpS14 mRpS21 mRpS24 mirr robl sisA tko toy unc-13 |
| 731 | GO:0005773 | C | 5, 6, 7, 8, | 1 | 1.078 (x 0.927) | 54 (0.019) | 0.974 | vacuole | Vha36 |
| 732 | GO:0007635 | P | 4, 5, | 1 | 1.078 (x 0.927) | 54 (0.019) | 0.976 | chemosensory behavior | Obp56a |
| 733 | GO:0007314 | P | 6, 8, 9, 11, | 1 | 1.118 (x 0.894) | 56 (0.018) | 0.976 | oocyte anterior/posterior axis determination | Tm1 |
| 734 | GO:0005875 | C | 3, 5, 6, 7, 8, 9, 10, | 2 | 2.316 (x 0.863) | 116 (0.017) | 0.978 | microtubule associated complex | CLIP-190 robl |
| 735 | GO:0007456 | P | 6, | 3 | 3.455 (x 0.868) | 173 (0.017) | 0.978 | eye development (sensu Endopterygota) | Cpn mirr toy |
| 736 | GO:0006897 | P | 6, 7, | 2 | 2.316 (x 0.863) | 116 (0.017) | 0.979 | endocytosis | Arf84F Rab3 |
| 737 | GO:0019098 | P | 3, 4, | 1 | 1.158 (x 0.863) | 58 (0.017) | 0.983 | reproductive behavior | tko |
| 738 | GO:0051704 | P | 2, | 1 | 1.158 (x 0.863) | 58 (0.017) | 0.984 | interaction between organisms | tko |
| 739 | GO:0009950 | P | 5, | 1 | 1.138 (x 0.879) | 57 (0.018) | 0.984 | dorsal/ventral axis specification | Tehao |
| 740 | GO:0005856 | C | 5, 6, 7, 8, | 5 | 5.791 (x 0.863) | 290 (0.017) | 0.984 | cytoskeleton | Act57B CLIP-190 Mlc2 Tm1 robl |
| 741 | GO:0048565 | P | 4, | 1 | 1.238 (x 0.808) | 62 (0.016) | 0.985 | gut development | sisA |
| 742 | GO:0016876 | F | 5, | 1 | 1.158 (x 0.863) | 58 (0.017) | 0.985 | ligase activity, forming aminoacyl-tRNA and related compounds | CG10092 |
| 743 | GO:0031224 | C | 4, 5, 6, | 17 | 18.910 (x 0.899) | 947 (0.018) | 0.986 | intrinsic to membrane | Ac78C CG11898 CG1213 CG31477 CG4288 CG4673 CG5037 CG6921 CG7188 CG8271 GABA-B-R2 Or46a Or59a Takr99D Tehao Tsp42El Vha36 |
| 744 | GO:0016192 | P | 5, 6, | 5 | 5.911 (x 0.846) | 296 (0.017) | 0.986 | vesicle-mediated transport | Arf84F CLIP-190 Rab3 ran-like unc-13 |
| 745 | GO:0007507 | P | 5, | 1 | 1.218 (x 0.821) | 61 (0.016) | 0.986 | heart development | Act57B |
| 746 | GO:0042277 | F | 3, | 1 | 1.178 (x 0.849) | 59 (0.017) | 0.986 | peptide binding | Takr99D |
| 747 | GO:0001654 | P | 5, | 3 | 3.614 (x 0.830) | 181 (0.017) | 0.987 | eye development | Cpn mirr toy |
| 748 | GO:0007059 | P | 4, | 2 | 2.476 (x 0.808) | 124 (0.016) | 0.987 | chromosome segregation | Nipped-B skpA |
| 749 | GO:0016614 | F | 4, | 2 | 2.356 (x 0.849) | 118 (0.017) | 0.987 | oxidoreductase activity, acting on CH-OH group of donors | CG10638 CG10962 |
| 750 | GO:0015370 | F | 6, 8, | 1 | 1.238 (x 0.808) | 62 (0.016) | 0.987 | solute:sodium symporter activity | CG4288 |
| 751 | GO:0004812 | F | 6, | 1 | 1.158 (x 0.863) | 58 (0.017) | 0.987 | aminoacyl-tRNA ligase activity | CG10092 |
| 752 | GO:0016021 | C | 5, 6, 7, | 17 | 18.850 (x 0.902) | 944 (0.018) | 0.987 | integral to membrane | Ac78C CG11898 CG1213 CG31477 CG4288 CG4673 CG5037 CG6921 CG7188 CG8271 GABA-B-R2 Or46a Or59a Takr99D Tehao Tsp42El Vha36 |
| 753 | GO:0008092 | F | 4, | 4 | 4.753 (x 0.842) | 238 (0.017) | 0.987 | cytoskeletal protein binding | BG:DS02740.9 CLIP-190 Tm1 mira |
| 754 | GO:0005200 | F | 3, | 5 | 5.831 (x 0.858) | 292 (0.017) | 0.987 | structural constituent of cytoskeleton | Act57B BG:DS02740.9 CLIP-190 mira robl |
| 755 | GO:0043038 | P | 7, 8, | 1 | 1.218 (x 0.821) | 61 (0.016) | 0.988 | amino acid activation | CG10092 |
| 756 | GO:0006790 | P | 5, | 1 | 1.178 (x 0.849) | 59 (0.017) | 0.988 | sulfur metabolism | Act57B |
| 757 | GO:0035282 | P | 3, | 2 | 2.516 (x 0.795) | 126 (0.016) | 0.988 | segmentation | pum tsl |
| 758 | GO:0007417 | P | 5, | 2 | 2.436 (x 0.821) | 122 (0.016) | 0.988 | central nervous system development | Abl robl |
| 759 | GO:0030707 | P | 8, | 2 | 2.476 (x 0.808) | 124 (0.016) | 0.988 | ovarian follicle cell development (sensu Insecta) | Femcoat Vm34Ca |
| 760 | GO:0005635 | C | 4, 5, 6, 7, 8, 9, 10, | 1 | 1.238 (x 0.808) | 62 (0.016) | 0.988 | nuclear envelope | CG4673 |
| 761 | GO:0007242 | P | 5, | 9 | 10.464 (x 0.860) | 524 (0.017) | 0.988 | intracellular signaling cascade | Abl Ac78C Arf84F CG7646 Rab3 TpnC41C ran-like unc-13 upd3 |
| 762 | GO:0016875 | F | 4, | 1 | 1.158 (x 0.863) | 58 (0.017) | 0.988 | ligase activity, forming carbon-oxygen bonds | CG10092 |
| 763 | GO:0042165 | F | 3, | 1 | 1.258 (x 0.795) | 63 (0.016) | 0.988 | neurotransmitter binding | Takr99D |
| 764 | GO:0045941 | P | 8, | 1 | 1.278 (x 0.782) | 64 (0.016) | 0.988 | positive regulation of transcription | mirr |
| 765 | GO:0006006 | P | 8, 9, | 1 | 1.198 (x 0.835) | 60 (0.017) | 0.988 | glucose metabolism | CG5103 |
| 766 | GO:0043037 | P | 7, 8, | 3 | 3.714 (x 0.808) | 186 (0.016) | 0.989 | translation | CG10092 RpS18 pum |
| 767 | GO:0006261 | P | 8, | 1 | 1.218 (x 0.821) | 61 (0.016) | 0.989 | DNA-dependent DNA replication | skpA |
| 768 | GO:0006413 | P | 8, 9, | 1 | 1.178 (x 0.849) | 59 (0.017) | 0.989 | translational initiation | RpS18 |
| 769 | GO:0006955 | P | 4, 5, | 2 | 2.436 (x 0.821) | 122 (0.016) | 0.989 | immune response | Tehao upd3 |
| 770 | GO:0008324 | F | 4, | 7 | 7.967 (x 0.879) | 399 (0.018) | 0.989 | cation transporter activity | CG11015 CG14482 CG31477 CG4288 Tsf1 Vha36 na |
| 771 | GO:0030594 | F | 4, | 1 | 1.258 (x 0.795) | 63 (0.016) | 0.989 | neurotransmitter receptor activity | Takr99D |
| 772 | GO:0004857 | F | 3, | 2 | 2.536 (x 0.789) | 127 (0.016) | 0.989 | enzyme inhibitor activity | CG1342 CG31704 |
| 773 | GO:0030097 | P | 5, | 1 | 1.278 (x 0.782) | 64 (0.016) | 0.989 | hemopoiesis | CG9650 |
| 774 | GO:0048477 | P | 6, | 5 | 5.771 (x 0.866) | 289 (0.017) | 0.99 | oogenesis | Femcoat Tm1 Vm34Ca l(3)01239 pum |
| 775 | GO:0043039 | P | 8, 9, | 1 | 1.198 (x 0.835) | 60 (0.017) | 0.99 | tRNA aminoacylation | CG10092 |
| 776 | GO:0006858 | P | 5, 6, | 2 | 2.456 (x 0.814) | 123 (0.016) | 0.99 | extracellular transport | CG11898 CG4288 |
| 777 | GO:0006519 | P | 5, | 5 | 5.851 (x 0.855) | 293 (0.017) | 0.99 | amino acid and derivative metabolism | CG10092 CG5122 Dhfr Gs2 Tbh |
| 778 | GO:0017111 | F | 7, | 9 | 10.444 (x 0.862) | 523 (0.017) | 0.99 | nucleoside-triphosphatase activity | Arf84F CG11898 CG31477 CG8520 Mlc2 Rab3 Vha36 ran-like robl |
| 779 | GO:0015294 | F | 5, 7, | 1 | 1.258 (x 0.795) | 63 (0.016) | 0.991 | solute:cation symporter activity | CG4288 |
| 780 | GO:0006418 | P | 8, 9, 10, | 1 | 1.198 (x 0.835) | 60 (0.017) | 0.991 | tRNA aminoacylation for protein translation | CG10092 |
| 781 | GO:0009880 | P | 4, | 2 | 2.576 (x 0.776) | 129 (0.016) | 0.991 | embryonic pattern specification | pum tsl |
| 782 | GO:0048522 | P | 4, | 3 | 3.774 (x 0.795) | 189 (0.016) | 0.992 | positive regulation of cellular process | mirr pum skpA |
| 783 | GO:0003676 | F | 3, | 32 | 34.805 (x 0.919) | 1743 (0.018) | 0.993 | nucleic acid binding | BEST:LD29214 BcDNA:GH11110 CG10092 CG10466 CG15398 CG31922 CG4046 CG4866 CG5338 CG7014 CG7911 CG8415 CG9650 RpII18 RpL11 RpL17A RpL38 RpL46 RpS17 RpS18 Rpb10 Ssb-c31a agt mRpL21 mRpS14 mirr oho23B pum sisA skpA tko toy |
| 784 | GO:0051169 | P | 6, 7, 8, | 1 | 1.318 (x 0.759) | 66 (0.015) | 0.993 | nuclear transport | CG10950 |
| 785 | GO:0045935 | P | 7, | 1 | 1.298 (x 0.770) | 65 (0.015) | 0.993 | positive regulation of nucleobase, nucleoside, nucleotide and nucleic acid metabolism | mirr |
| 786 | GO:0008289 | F | 3, | 1 | 1.318 (x 0.759) | 66 (0.015) | 0.994 | lipid binding | unc-13 |
| 787 | GO:0044275 | P | 7, | 1 | 1.338 (x 0.747) | 67 (0.015) | 0.995 | cellular carbohydrate catabolism | CG5103 |
| 788 | GO:0030054 | C | 5, 6, 7, | 1 | 1.318 (x 0.759) | 66 (0.015) | 0.995 | cell junction | Abl |
| 789 | GO:0016462 | F | 6, | 9 | 10.603 (x 0.849) | 531 (0.017) | 0.996 | pyrophosphatase activity | Arf84F CG11898 CG31477 CG8520 Mlc2 Rab3 Vha36 ran-like robl |
| 790 | GO:0016052 | P | 6, | 1 | 1.338 (x 0.747) | 67 (0.015) | 0.996 | carbohydrate catabolism | CG5103 |
| 791 | GO:0006457 | P | 7, | 2 | 2.616 (x 0.765) | 131 (0.015) | 0.997 | protein folding | CG7770 l(3)01239 |
| 792 | GO:0048749 | P | 7, | 2 | 2.656 (x 0.753) | 133 (0.015) | 0.997 | compound eye development (sensu Endopterygota) | Cpn mirr |
| 793 | GO:0005515 | F | 3, | 22 | 24.641 (x 0.893) | 1234 (0.018) | 0.998 | protein binding | Abl BG:DS02740.9 CG10950 CG15361 CG31146 CG7646 CG7770 CLIP-190 EG:BACH7M4.1 Mlc2 RpL11 Ssb-c31a Tm1 TpnC41C l(3)01239 mira mirr pum sisA tsl unc-13 upd3 |
| 794 | GO:0002009 | P | 4, | 2 | 2.656 (x 0.753) | 133 (0.015) | 0.998 | morphogenesis of an epithelium | Abl mirr |
| 795 | GO:0009792 | P | 4, | 3 | 3.894 (x 0.770) | 195 (0.015) | 0.999 | embryonic development (sensu Metazoa) | Abl mirr pum |
| 796 | GO:0007600 | P | 3, 5, | 4 | 5.152 (x 0.776) | 258 (0.016) | 0.999 | sensory perception | Obp56a Or46a Or59a tko |
| 797 | GO:0001745 | P | 7, 8, | 2 | 2.656 (x 0.753) | 133 (0.015) | 0.999 | compound eye morphogenesis (sensu Endopterygota) | Cpn mirr |
| 798 | GO:0045182 | F | 2, | 1 | 1.697 (x 0.589) | 85 (0.012) | 1 | translation regulator activity | pum |
| 799 | GO:0043412 | P | 6, | 4 | 18.311 (x 0.218) | 917 (0.004) | 1 | biopolymer modification | Abl Arf84F CG2056 CG9804 |
| 800 | GO:0007163 | P | 5, 6, | 1 | 1.418 (x 0.705) | 71 (0.014) | 1 | establishment and/or maintenance of cell polarity | mirr |
| 801 | GO:0019748 | P | 4, | 1 | 1.358 (x 0.736) | 68 (0.015) | 1 | secondary metabolism | CG5037 |
| 802 | GO:0051246 | P | 5, 6, | 2 | 2.656 (x 0.753) | 133 (0.015) | 1 | regulation of protein metabolism | Abl pum |
| 803 | GO:0015144 | F | 3, | 1 | 1.617 (x 0.618) | 81 (0.012) | 1 | carbohydrate transporter activity | CG1213 |
| 804 | GO:0016741 | F | 4, | 1 | 1.697 (x 0.589) | 85 (0.012) | 1 | transferase activity, transferring one-carbon groups | agt |
| 805 | GO:0000902 | P | 4, 5, | 5 | 6.749 (x 0.741) | 338 (0.015) | 1 | cellular morphogenesis | Abl CG14825 Cpn SIP1 mirr |
| 806 | GO:0044237 | P | 4, | 87 | 92.435 (x 0.941) | 4629 (0.019) | 1 | cellular metabolism | Abl Ac78C Act57B Arf84F CG10092 CG10104 CG10237 CG10466 CG10638 CG11015 CG11313 CG12400 CG1304 CG13277 CG14482 CG15398 CG18223 CG18522 CG2056 CG2277 CG30438 CG31477 CG31704 CG32174 CG32230 CG33002 CG4046 CG4288 CG4386 CG4511 CG4866 CG5037 CG5103 CG5122 CG5162 CG5338 CG5548 CG6574 CG6610 CG6921 CG7014 CG7770 CG8415 CG9372 CG9650 CG9804 Dhfr EG:152A3.7 Gs2 GstE5 GstE6 GstE7 Nipped-B Obp58b Or59a Pdsw Prx6005 RN-tre RpII18 RpL11 RpL17A RpL38 RpL46 RpS17 RpS18 Rpb10 Sod Ssb-c31a Tbh Trap36 Ugt86Di Vha36 agt fu12 l(3)01239 mRpL21 mRpL22 mRpS14 mRpS21 mRpS24 mirr na oho23B pum skpA tko toy |
| 807 | GO:0016331 | P | 5, | 1 | 1.637 (x 0.611) | 82 (0.012) | 1 | morphogenesis of embryonic epithelium | Abl |
| 808 | GO:0043283 | P | 5, | 14 | 33.627 (x 0.416) | 1684 (0.008) | 1 | biopolymer metabolism | Abl Arf84F CG10092 CG10466 CG13277 CG2056 CG30438 CG6610 CG9804 Dhfr Ugt86Di agt pum skpA |
| 809 | GO:0000910 | P | 5, | 1 | 1.677 (x 0.596) | 84 (0.012) | 1 | cytokinesis | Act57B |
| 810 | GO:0005215 | F | 2, | 16 | 19.330 (x 0.828) | 968 (0.017) | 1 | transporter activity | CG10237 CG10950 CG11015 CG11898 CG1213 CG14482 CG14935 CG31477 CG4288 CG6574 CG8193 CG8271 Tim9a Tsf1 Vha36 na |
| 811 | GO:0006397 | P | 8, | 3 | 3.954 (x 0.759) | 198 (0.015) | 1 | mRNA processing | CG10466 CG13277 CG6610 |
| 812 | GO:0019953 | P | 3, | 7 | 9.106 (x 0.769) | 456 (0.015) | 1 | sexual reproduction | CG5162 Femcoat Tbh Tm1 Vm34Ca l(3)01239 pum |
| 813 | GO:0005102 | F | 3, 4, | 4 | 5.571 (x 0.718) | 279 (0.014) | 1 | receptor binding | CG15361 CG31146 tsl upd3 |
| 814 | GO:0006913 | P | 6, 7, 8, | 1 | 1.418 (x 0.705) | 71 (0.014) | 1 | nucleocytoplasmic transport | CG10950 |
| 815 | GO:0005623 | C | 2, | 87 | 92.175 (x 0.944) | 4616 (0.019) | 1 | cell | Abl Ac78C Act57B BEST:LD29214 BG:DS02740.9 CG10237 CG11015 CG11898 CG1213 CG12400 CG13277 CG14482 CG15398 CG31477 CG31922 CG31950 CG32174 CG32230 CG32409 CG33002 CG4046 CG4288 CG4673 CG4866 CG5037 CG5189 CG5338 CG5548 CG6574 CG6610 CG6921 CG7014 CG7188 CG7770 CG7911 CG8271 CG8415 CG9650 CLIP-190 Cpn EG:152A3.7 Femcoat GABA-B-R2 Gs2 Mlc2 Nipped-B Or46a Or59a Pdsw Rab3 RpII18 RpL11 RpL17A RpL38 RpL46 RpS17 RpS18 Rpb10 Sod Ssb-c31a Takr99D Tehao Tim9a Tm1 Trap36 Tsp42El Vha36 fu12 hig l(3)01239 mRpL21 mRpL22 mRpL54 mRpS14 mRpS21 mRpS24 mira mirr na oho23B pum robl sisA skpA tko toy unc-13 |
| 816 | GO:0048518 | P | 3, | 3 | 4.353 (x 0.689) | 218 (0.014) | 1 | positive regulation of biological process | mirr pum skpA |
| 817 | GO:0006464 | P | 7, | 4 | 17.512 (x 0.228) | 877 (0.005) | 1 | protein modification | Abl Arf84F CG2056 CG9804 |
| 818 | GO:0004197 | F | 6, | 1 | 1.617 (x 0.618) | 81 (0.012) | 1 | cysteine-type endopeptidase activity | Obp58b |
| 819 | GO:0008610 | P | 5, 6, 7, | 1 | 1.697 (x 0.589) | 85 (0.012) | 1 | lipid biosynthesis | fu12 |
| 820 | GO:0045045 | P | 5, 6, | 3 | 4.293 (x 0.699) | 215 (0.014) | 1 | secretory pathway | Arf84F Rab3 unc-13 |
| 821 | GO:0008017 | F | 6, | 1 | 1.518 (x 0.659) | 76 (0.013) | 1 | microtubule binding | CLIP-190 |
| 822 | GO:0048748 | P | 6, 7, | 2 | 2.816 (x 0.710) | 141 (0.014) | 1 | eye morphogenesis (sensu Endopterygota) | Cpn mirr |
| 823 | GO:0007411 | P | 6, 7, 9, 10, 12, | 1 | 1.637 (x 0.611) | 82 (0.012) | 1 | axon guidance | Abl |
| 824 | GO:0008168 | F | 5, | 1 | 1.677 (x 0.596) | 84 (0.012) | 1 | methyltransferase activity | agt |
| 825 | GO:0048468 | P | 4, | 5 | 6.789 (x 0.736) | 340 (0.015) | 1 | cell development | Abl Cpn Tm1 pum robl |
| 826 | GO:0007498 | P | 4, | 3 | 3.954 (x 0.759) | 198 (0.015) | 1 | mesoderm development | CG1942 CG9650 toy |
| 827 | GO:0009308 | P | 5, | 6 | 7.508 (x 0.799) | 376 (0.016) | 1 | amine metabolism | Act57B CG10092 CG5122 Dhfr Gs2 Tbh |
| 828 | GO:0007626 | P | 4, | 1 | 1.398 (x 0.715) | 70 (0.014) | 1 | locomotory behavior | na |
| 829 | GO:0008380 | P | 8, | 2 | 3.095 (x 0.646) | 155 (0.013) | 1 | RNA splicing | CG13277 CG6610 |
| 830 | GO:0003712 | F | 3, 5, | 1 | 1.478 (x 0.677) | 74 (0.014) | 1 | transcription cofactor activity | Ssb-c31a |
| 831 | GO:0001584 | F | 6, | 3 | 4.253 (x 0.705) | 213 (0.014) | 1 | rhodopsin-like receptor activity | Or46a Or59a Takr99D |
| 832 | GO:0050789 | P | 2, | 13 | 30.053 (x 0.433) | 1505 (0.009) | 1 | regulation of biological process | Abl Arf84F CG15398 CG2750 CG7188 CG9650 Nipped-B Rab3 Ssb-c31a mirr pum skpA toy |
| 833 | GO:0008233 | F | 4, | 11 | 13.000 (x 0.846) | 651 (0.017) | 1 | peptidase activity | BG:DS01068.5 CG10104 CG10466 CG11313 CG1304 CG18223 CG2056 CG4386 CG9372 Obp58b RN-tre |
| 834 | GO:0044464 | C | 2, 3, | 87 | 92.175 (x 0.944) | 4616 (0.019) | 1 | cell part | Abl Ac78C Act57B BEST:LD29214 BG:DS02740.9 CG10237 CG11015 CG11898 CG1213 CG12400 CG13277 CG14482 CG15398 CG31477 CG31922 CG31950 CG32174 CG32230 CG32409 CG33002 CG4046 CG4288 CG4673 CG4866 CG5037 CG5189 CG5338 CG5548 CG6574 CG6610 CG6921 CG7014 CG7188 CG7770 CG7911 CG8271 CG8415 CG9650 CLIP-190 Cpn EG:152A3.7 Femcoat GABA-B-R2 Gs2 Mlc2 Nipped-B Or46a Or59a Pdsw Rab3 RpII18 RpL11 RpL17A RpL38 RpL46 RpS17 RpS18 Rpb10 Sod Ssb-c31a Takr99D Tehao Tim9a Tm1 Trap36 Tsp42El Vha36 fu12 hig l(3)01239 mRpL21 mRpL22 mRpL54 mRpS14 mRpS21 mRpS24 mira mirr na oho23B pum robl sisA skpA tko toy unc-13 |
| 835 | GO:0046943 | F | 4, | 1 | 1.438 (x 0.696) | 72 (0.014) | 1 | carboxylic acid transporter activity | CG8271 |
| 836 | GO:0004713 | F | 7, | 1 | 1.697 (x 0.589) | 85 (0.012) | 1 | protein-tyrosine kinase activity | Abl |
| 837 | GO:0001754 | P | 5, 6, 7, | 1 | 1.518 (x 0.659) | 76 (0.013) | 1 | eye photoreceptor cell differentiation | Cpn |
| 838 | GO:0004930 | F | 5, | 4 | 5.132 (x 0.779) | 257 (0.016) | 1 | G-protein coupled receptor activity | GABA-B-R2 Or46a Or59a Takr99D |
| 839 | GO:0008135 | F | 3, 4, | 1 | 1.637 (x 0.611) | 82 (0.012) | 1 | translation factor activity, nucleic acid binding | pum |
| 840 | GO:0000398 | P | 9, 11, | 2 | 2.975 (x 0.672) | 149 (0.013) | 1 | nuclear mRNA splicing, via spliceosome | CG13277 CG6610 |
| 841 | GO:0007389 | P | 3, | 4 | 5.112 (x 0.782) | 256 (0.016) | 1 | pattern specification | Tehao Tm1 pum tsl |
| 842 | GO:0007166 | P | 5, | 11 | 13.159 (x 0.836) | 659 (0.017) | 1 | cell surface receptor linked signal transduction | Ac78C Arf84F CG15361 CG18249 GABA-B-R2 Or46a Or59a Takr99D Tehao mirr tsl |
| 843 | GO:0031323 | P | 5, | 7 | 19.689 (x 0.356) | 986 (0.007) | 1 | regulation of cellular metabolism | CG15398 CG9650 Nipped-B Ssb-c31a mirr pum toy |
| 844 | GO:0044255 | P | 5, 6, | 5 | 6.689 (x 0.747) | 335 (0.015) | 1 | cellular lipid metabolism | CG30438 CG5162 CG6921 Ugt86Di fu12 |
| 845 | GO:0005342 | F | 3, | 1 | 1.478 (x 0.677) | 74 (0.014) | 1 | organic acid transporter activity | CG8271 |
| 846 | GO:0008202 | P | 6, 7, | 2 | 2.716 (x 0.736) | 136 (0.015) | 1 | steroid metabolism | CG30438 Ugt86Di |
| 847 | GO:0015293 | F | 6, | 1 | 1.458 (x 0.686) | 73 (0.014) | 1 | symporter activity | CG4288 |
| 848 | GO:0016887 | F | 8, | 6 | 7.408 (x 0.810) | 371 (0.016) | 1 | ATPase activity | CG11898 CG31477 CG8520 Mlc2 Vha36 robl |
| 849 | GO:0016817 | F | 4, | 9 | 10.943 (x 0.822) | 548 (0.016) | 1 | hydrolase activity, acting on acid anhydrides | Arf84F CG11898 CG31477 CG8520 Mlc2 Rab3 Vha36 ran-like robl |
| 850 | GO:0006445 | P | 7, 8, 9, | 1 | 1.438 (x 0.696) | 72 (0.014) | 1 | regulation of translation | pum |
| 851 | GO:0007391 | P | 6, | 1 | 1.518 (x 0.659) | 76 (0.013) | 1 | dorsal closure | Abl |
| 852 | GO:0019222 | P | 4, | 8 | 20.448 (x 0.391) | 1024 (0.008) | 1 | regulation of metabolism | Abl CG15398 CG9650 Nipped-B Ssb-c31a mirr pum toy |
| 853 | GO:0048592 | P | 5, 6, | 2 | 2.975 (x 0.672) | 149 (0.013) | 1 | eye morphogenesis | Cpn mirr |
| 854 | GO:0006520 | P | 6, 7, | 4 | 5.212 (x 0.767) | 261 (0.015) | 1 | amino acid metabolism | CG10092 CG5122 Dhfr Gs2 |
| 855 | GO:0007467 | P | 5, | 1 | 1.737 (x 0.576) | 87 (0.011) | 1 | photoreceptor cell differentiation (sensu Endopterygota) | Cpn |
| 856 | GO:0009953 | P | 4, | 1 | 1.478 (x 0.677) | 74 (0.014) | 1 | dorsal/ventral pattern formation | Tehao |
| 857 | GO:0001751 | P | 6, 7, 8, 9, | 1 | 1.458 (x 0.686) | 73 (0.014) | 1 | eye photoreceptor cell differentiation (sensu Endopterygota) | Cpn |
| 858 | GO:0050794 | P | 3, | 13 | 27.497 (x 0.473) | 1377 (0.009) | 1 | regulation of cellular process | Abl Arf84F CG15398 CG2750 CG7188 CG9650 Nipped-B Rab3 Ssb-c31a mirr pum skpA toy |
| 859 | GO:0016818 | F | 5, | 9 | 10.943 (x 0.822) | 548 (0.016) | 1 | hydrolase activity, acting on acid anhydrides, in phosphorus-containing anhydrides | Arf84F CG11898 CG31477 CG8520 Mlc2 Rab3 Vha36 ran-like robl |
| 860 | GO:0007422 | P | 5, | 1 | 1.518 (x 0.659) | 76 (0.013) | 1 | peripheral nervous system development | mirr |
| 861 | GO:0005576 | C | 2, | 6 | 7.708 (x 0.778) | 386 (0.016) | 1 | extracellular region | CG15361 Obp56a Tsf1 hig tsl upd3 |
| 862 | GO:0000377 | P | 10, | 2 | 2.975 (x 0.672) | 149 (0.013) | 1 | RNA splicing, via transesterification reactions with bulged adenosine as nucleophile | CG13277 CG6610 |
| 863 | GO:0009994 | P | 4, 7, | 1 | 1.737 (x 0.576) | 87 (0.011) | 1 | oocyte differentiation | Tm1 |
| 864 | GO:0006417 | P | 6, 7, 8, | 1 | 1.498 (x 0.668) | 75 (0.013) | 1 | regulation of protein biosynthesis | pum |
| 865 | GO:0000003 | P | 2, | 8 | 10.004 (x 0.800) | 501 (0.016) | 1 | reproduction | CG5162 Femcoat Tbh Tm1 Vm34Ca l(3)01239 pum tko |
| 866 | GO:0003702 | F | 3, | 4 | 5.312 (x 0.753) | 266 (0.015) | 1 | RNA polymerase II transcription factor activity | BEST:LD29214 CG15398 Trap36 toy |
| 867 | GO:0008270 | F | 6, | 3 | 12.021 (x 0.250) | 602 (0.005) | 1 | zinc ion binding | CG10466 CG31922 CG9650 |
| 868 | GO:0007309 | P | 5, 7, 8, 10, | 1 | 1.518 (x 0.659) | 76 (0.013) | 1 | oocyte axis determination | Tm1 |
| 869 | GO:0000375 | P | 9, | 2 | 2.975 (x 0.672) | 149 (0.013) | 1 | RNA splicing, via transesterification reactions | CG13277 CG6610 |
| 870 | GO:0050791 | P | 3, | 13 | 26.738 (x 0.486) | 1339 (0.010) | 1 | regulation of physiological process | Abl Arf84F CG15398 CG2750 CG7188 CG9650 Nipped-B Rab3 Ssb-c31a mirr pum skpA toy |
| 871 | GO:0009889 | P | 5, | 1 | 1.578 (x 0.634) | 79 (0.013) | 1 | regulation of biosynthesis | pum |
| 872 | GO:0005575 | C | 1, | 111 | 115.458 (x 0.961) | 5782 (0.019) | 1 | cellular\_component | Abl Ac78C Act57B BEST:LD29214 BG:DS02740.9 CG10237 CG11015 CG11898 CG1213 CG12400 CG13277 CG13691 CG14482 CG14825 CG15361 CG15398 CG16817 CG30154 CG30343 CG31477 CG31601 CG31715 CG31922 CG31950 CG32023 CG32174 CG32175 CG32207 CG32230 CG32409 CG32448 CG32582 CG32625 CG32856 CG33002 CG4046 CG4288 CG4673 CG4866 CG5037 CG5189 CG5338 CG5548 CG6574 CG6610 CG6921 CG7014 CG7188 CG7770 CG7911 CG8271 CG8415 CG9650 CLIP-190 Cpn EG:152A3.7 EG:63B12.12 Femcoat GABA-B-R2 Gs2 JhI-26 Mlc2 NP15.6 Nipped-B Obp56a Or46a Or59a Pdsw Rab3 Rep2 RpII18 RpL11 RpL17A RpL38 RpL46 RpS17 RpS18 Rpb10 SIP1 Sod Ssb-c31a Takr99D Tehao Tim9a Tm1 Trap36 Tsf1 Tsp42El Vha36 fu12 hig l(3)01239 mRpL21 mRpL22 mRpL54 mRpS14 mRpS21 mRpS24 mira mirr na oho23B pum robl sisA skpA tko toy tsl unc-13 upd3 |
| 873 | GO:0006812 | P | 6, 7, | 6 | 7.648 (x 0.785) | 383 (0.016) | 1 | cation transport | CG31477 CG4288 CG8271 Tsf1 Vha36 na |
| 874 | GO:0048599 | P | 5, 6, 8, | 1 | 1.597 (x 0.626) | 80 (0.013) | 1 | oocyte development | Tm1 |
| 875 | GO:0019219 | P | 6, | 7 | 17.732 (x 0.395) | 888 (0.008) | 1 | regulation of nucleobase, nucleoside, nucleotide and nucleic acid metabolism | CG15398 CG9650 Nipped-B Ssb-c31a mirr pum toy |
| 876 | GO:0006399 | P | 7, | 1 | 1.757 (x 0.569) | 88 (0.011) | 1 | tRNA metabolism | CG10092 |
| 877 | GO:0042623 | F | 9, | 5 | 6.590 (x 0.759) | 330 (0.015) | 1 | ATPase activity, coupled | CG11898 CG31477 Mlc2 Vha36 robl |
| 878 | GO:0007455 | P | 6, 7, | 2 | 2.915 (x 0.686) | 146 (0.014) | 1 | eye-antennal disc morphogenesis | Cpn mirr |
| 879 | GO:0007308 | P | 6, 7, 9, | 1 | 1.578 (x 0.634) | 79 (0.013) | 1 | oocyte construction | Tm1 |
| 880 | GO:0051244 | P | 4, | 13 | 25.899 (x 0.502) | 1297 (0.010) | 1 | regulation of cellular physiological process | Abl Arf84F CG15398 CG2750 CG7188 CG9650 Nipped-B Rab3 Ssb-c31a mirr pum skpA toy |
| 881 | GO:0000087 | P | 6, | 3 | 4.533 (x 0.662) | 227 (0.013) | 1 | M phase of mitotic cell cycle | Nipped-B pum skpA |
| 882 | GO:0019318 | P | 7, 8, | 1 | 1.597 (x 0.626) | 80 (0.013) | 1 | hexose metabolism | CG5103 |
| 883 | GO:0019752 | P | 6, | 6 | 7.828 (x 0.767) | 392 (0.015) | 1 | carboxylic acid metabolism | CG10092 CG5122 CG6921 CG9804 Dhfr Gs2 |
| 884 | GO:0005976 | P | 6, | 2 | 3.235 (x 0.618) | 162 (0.012) | 1 | polysaccharide metabolism | CG30438 Ugt86Di |
| 885 | GO:0005886 | C | 4, 5, | 3 | 10.663 (x 0.281) | 534 (0.006) | 1 | plasma membrane | Abl Takr99D Tehao |
| 886 | GO:0007276 | P | 4, | 7 | 8.926 (x 0.784) | 447 (0.016) | 1 | gametogenesis | CG5162 Femcoat Tbh Tm1 Vm34Ca l(3)01239 pum |
| 887 | GO:0007067 | P | 7, | 3 | 4.513 (x 0.665) | 226 (0.013) | 1 | mitosis | Nipped-B pum skpA |
| 888 | GO:0006066 | P | 5, | 2 | 3.215 (x 0.622) | 161 (0.012) | 1 | alcohol metabolism | CG5103 Tbh |
| 889 | GO:0045449 | P | 7, | 7 | 16.594 (x 0.422) | 831 (0.008) | 1 | regulation of transcription | CG15398 CG9650 Nipped-B Ssb-c31a mirr pum toy |
| 890 | GO:0003704 | F | 4, | 1 | 1.578 (x 0.634) | 79 (0.013) | 1 | specific RNA polymerase II transcription factor activity | toy |
| 891 | GO:0008134 | F | 4, | 1 | 1.777 (x 0.563) | 89 (0.011) | 1 | transcription factor binding | Ssb-c31a |
| 892 | GO:0006508 | P | 7, | 12 | 15.136 (x 0.793) | 758 (0.016) | 1 | proteolysis | CG10104 CG10466 CG11313 CG1304 CG18223 CG2056 CG31704 CG4386 CG9372 Obp58b RN-tre skpA |
| 893 | GO:0006082 | P | 5, | 6 | 7.828 (x 0.767) | 392 (0.015) | 1 | organic acid metabolism | CG10092 CG5122 CG6921 CG9804 Dhfr Gs2 |
| 894 | GO:0046914 | F | 5, | 5 | 13.499 (x 0.370) | 676 (0.007) | 1 | transition metal ion binding | CG10466 CG31922 CG9650 Tbh Tsf1 |
| 895 | GO:0031326 | P | 6, | 1 | 1.578 (x 0.634) | 79 (0.013) | 1 | regulation of cellular biosynthesis | pum |
| 896 | GO:0044238 | P | 4, | 73 | 91.256 (x 0.800) | 4570 (0.016) | 1 | primary metabolism | Abl Ac78C Act57B Arf84F CG10092 CG10104 CG10237 CG10466 CG11313 CG1213 CG1304 CG13277 CG14935 CG15398 CG18223 CG18522 CG18530 CG2056 CG2277 CG30438 CG31477 CG31704 CG33002 CG4046 CG4288 CG4386 CG4866 CG5103 CG5122 CG5162 CG5338 CG6574 CG6610 CG6921 CG7014 CG7770 CG8415 CG9372 CG9650 CG9804 Dhfr Gs2 Nipped-B Obp58b RN-tre RpII18 RpL11 RpL17A RpL38 RpL46 RpS17 RpS18 Rpb10 Ssb-c31a Tbh Trap36 Ugt86Di Vha36 agt fu12 l(3)01239 mRpL21 mRpL22 mRpS14 mRpS21 mRpS24 mirr na oho23B pum skpA tko toy |
| 897 | GO:0015631 | F | 5, | 1 | 1.817 (x 0.550) | 91 (0.011) | 1 | tubulin binding | CLIP-190 |
| 898 | GO:0007423 | P | 4, | 3 | 4.593 (x 0.653) | 230 (0.013) | 1 | sensory organ development | Cpn mirr toy |
| 899 | GO:0050875 | P | 3, | 107 | 124.125 (x 0.862) | 6216 (0.017) | 1 | cellular physiological process | Abl Ac78C Act57B Arf84F CG10092 CG10104 CG10237 CG10466 CG10638 CG10950 CG11015 CG11313 CG11898 CG1213 CG12400 CG1304 CG13277 CG14482 CG14825 CG15398 CG18223 CG18522 CG2056 CG2277 CG2750 CG30438 CG31477 CG31704 CG32174 CG32230 CG32409 CG33002 CG4046 CG4288 CG4386 CG4511 CG4673 CG4866 CG5037 CG5103 CG5122 CG5162 CG5338 CG5548 CG6574 CG6610 CG6921 CG7014 CG7188 CG7770 CG8193 CG8271 CG8415 CG9372 CG9650 CG9804 CLIP-190 Cpn Dhfr EG:152A3.7 Gs2 GstE5 GstE6 GstE7 Nipped-B Obp58b Or59a Pdsw Prx6005 RN-tre Rab3 RpII18 RpL11 RpL17A RpL38 RpL46 RpS17 RpS18 Rpb10 SIP1 Sod Ssb-c31a Tbh Tim9a Trap36 Tsf1 Ugt86Di Vha36 agt fu12 l(3)01239 mRpL21 mRpL22 mRpS14 mRpS21 mRpS24 mira mirr na oho23B pum ran-like robl skpA tko toy unc-13 |
| 900 | GO:0016301 | F | 5, | 2 | 8.007 (x 0.250) | 401 (0.005) | 1 | kinase activity | Abl CG2056 |
| 901 | GO:0046903 | P | 5, | 3 | 4.613 (x 0.650) | 231 (0.013) | 1 | secretion | Arf84F Rab3 unc-13 |
| 902 | GO:0005634 | C | 5, 6, 7, 8, | 18 | 30.432 (x 0.591) | 1524 (0.012) | 1 | nucleus | BEST:LD29214 CG13277 CG15398 CG31922 CG31950 CG32409 CG4673 CG6610 CG7911 CG9650 Nipped-B RpII18 Rpb10 Ssb-c31a Trap36 mirr sisA toy |
| 903 | GO:0006355 | P | 8, | 7 | 15.715 (x 0.445) | 787 (0.009) | 1 | regulation of transcription, DNA-dependent | CG15398 CG9650 Nipped-B Ssb-c31a mirr pum toy |
| 904 | GO:0006357 | P | 9, | 4 | 10.923 (x 0.366) | 547 (0.007) | 1 | regulation of transcription from RNA polymerase II promoter | CG9650 Ssb-c31a mirr toy |
| 905 | GO:0030554 | F | 5, | 6 | 13.758 (x 0.436) | 689 (0.009) | 1 | adenyl nucleotide binding | Abl CG10092 CG11898 CG1939 CG2056 CG4511 |
| 906 | GO:0006139 | P | 5, | 23 | 35.364 (x 0.650) | 1771 (0.013) | 1 | nucleobase, nucleoside, nucleotide and nucleic acid metabolism | Ac78C CG10092 CG10466 CG13277 CG15398 CG18522 CG2277 CG31477 CG5103 CG6610 CG9650 Dhfr Nipped-B RpII18 Rpb10 Ssb-c31a Trap36 Vha36 agt mirr pum skpA toy |
| 907 | GO:0003677 | F | 4, | 8 | 16.414 (x 0.487) | 822 (0.010) | 1 | DNA binding | BEST:LD29214 CG15398 RpII18 Ssb-c31a mirr sisA skpA toy |
| 908 | GO:0016773 | F | 5, | 2 | 7.129 (x 0.281) | 357 (0.006) | 1 | phosphotransferase activity, alcohol group as acceptor | Abl CG2056 |
| 909 | GO:0009791 | P | 3, | 2 | 7.109 (x 0.281) | 356 (0.006) | 1 | post-embryonic development | Cpn mirr |
| 910 | GO:0016265 | P | 3, | 1 | 5.012 (x 0.200) | 251 (0.004) | 1 | death | CG7188 |
| 911 | GO:0008219 | P | 4, | 1 | 4.992 (x 0.200) | 250 (0.004) | 1 | cell death | CG7188 |
| 912 | GO:0008509 | F | 4, | 1 | 1.877 (x 0.533) | 94 (0.011) | 1 | anion transporter activity | CG4288 |
| 913 | GO:0016070 | P | 6, | 5 | 7.328 (x 0.682) | 367 (0.014) | 1 | RNA metabolism | CG10092 CG10466 CG13277 CG6610 pum |
| 914 | GO:0005524 | F | 6, | 6 | 13.419 (x 0.447) | 672 (0.009) | 1 | ATP binding | Abl CG10092 CG11898 CG1939 CG2056 CG4511 |
| 915 | GO:0012501 | P | 5, | 1 | 4.952 (x 0.202) | 248 (0.004) | 1 | programmed cell death | CG7188 |
| 916 | GO:0046530 | P | 4, | 1 | 1.957 (x 0.511) | 98 (0.010) | 1 | photoreceptor cell differentiation | Cpn |
| 917 | GO:0007155 | P | 3, | 4 | 6.050 (x 0.661) | 303 (0.013) | 1 | cell adhesion | CG18249 RN-tre Tehao hig |
| 918 | GO:0000166 | F | 3, | 9 | 17.532 (x 0.513) | 878 (0.010) | 1 | nucleotide binding | Abl Arf84F CG10092 CG11898 CG1939 CG2056 CG4511 Rab3 ran-like |
| 919 | GO:0004553 | F | 5, | 1 | 1.957 (x 0.511) | 98 (0.010) | 1 | hydrolase activity, hydrolyzing O-glycosyl compounds | CG14935 |
| 920 | GO:0016477 | P | 5, 6, | 2 | 3.495 (x 0.572) | 175 (0.011) | 1 | cell migration | Abl pum |
| 921 | GO:0008047 | F | 3, | 1 | 1.897 (x 0.527) | 95 (0.011) | 1 | enzyme activator activity | CG11313 |
| 922 | GO:0045892 | P | 9, | 1 | 1.937 (x 0.516) | 97 (0.010) | 1 | negative regulation of transcription, DNA-dependent | pum |
| 923 | GO:0006350 | P | 6, | 10 | 18.870 (x 0.530) | 945 (0.011) | 1 | transcription | CG15398 CG9650 Nipped-B RpII18 Rpb10 Ssb-c31a Trap36 mirr pum toy |
| 924 | GO:0044265 | P | 6, | 2 | 3.435 (x 0.582) | 172 (0.012) | 1 | cellular macromolecule catabolism | CG5103 pum |
| 925 | GO:0000904 | P | 5, 6, | 2 | 3.375 (x 0.593) | 169 (0.012) | 1 | cellular morphogenesis during differentiation | Abl Cpn |
| 926 | GO:0002165 | P | 4, | 2 | 6.889 (x 0.290) | 345 (0.006) | 1 | larval or pupal development (sensu Insecta) | Cpn mirr |
| 927 | GO:0015672 | P | 7, 8, | 2 | 3.495 (x 0.572) | 175 (0.011) | 1 | monovalent inorganic cation transport | CG31477 Vha36 |
| 928 | GO:0030036 | P | 8, | 1 | 1.937 (x 0.516) | 97 (0.010) | 1 | actin cytoskeleton organization and biogenesis | Abl |
| 929 | GO:0005057 | F | 3, | 1 | 4.792 (x 0.209) | 240 (0.004) | 1 | receptor signaling protein activity | Tsp42El |
| 930 | GO:0015075 | F | 3, | 7 | 9.685 (x 0.723) | 485 (0.014) | 1 | ion transporter activity | CG11015 CG14482 CG31477 CG4288 Tsf1 Vha36 na |
| 931 | GO:0006820 | P | 6, 7, | 1 | 1.997 (x 0.501) | 100 (0.010) | 1 | anion transport | CG4288 |
| 932 | GO:0030029 | P | 7, | 1 | 1.937 (x 0.516) | 97 (0.010) | 1 | actin filament-based process | Abl |
| 933 | GO:0016043 | P | 4, | 17 | 27.537 (x 0.617) | 1379 (0.012) | 1 | cell organization and biogenesis | Abl Act57B Arf84F CG10950 CG14825 CG32409 CG4673 CLIP-190 Cpn Rab3 SIP1 Tim9a mira mirr ran-like robl skpA |
| 934 | GO:0005975 | P | 5, | 7 | 9.685 (x 0.723) | 485 (0.014) | 1 | carbohydrate metabolism | Act57B CG1213 CG14935 CG30438 CG4288 CG5103 Ugt86Di |
| 935 | GO:0005996 | P | 6, 7, | 1 | 1.997 (x 0.501) | 100 (0.010) | 1 | monosaccharide metabolism | CG5103 |
| 936 | GO:0007582 | P | 2, | 123 | 136.425 (x 0.902) | 6832 (0.018) | 1 | physiological process | Abl Ac78C Act57B Arf84F CG10092 CG10104 CG10237 CG10466 CG10638 CG10950 CG10962 CG11015 CG11313 CG11898 CG1213 CG12400 CG1304 CG13277 CG14482 CG14825 CG14935 CG15398 CG18223 CG18249 CG18522 CG18530 CG2056 CG2277 CG2750 CG30438 CG31146 CG31477 CG31704 CG32174 CG32230 CG32409 CG33002 CG4046 CG4288 CG4386 CG4511 CG4673 CG4866 CG5037 CG5103 CG5122 CG5162 CG5338 CG5548 CG6574 CG6610 CG6921 CG7014 CG7188 CG7770 CG8193 CG8271 CG8415 CG9372 CG9650 CG9804 CG9836 CLIP-190 Cpn Dhfr EG:152A3.7 GABA-B-R2 Gs2 GstE5 GstE6 GstE7 Mlc2 Nipped-B Obp56a Obp58b Or46a Or59a Pdsw Prx6005 RN-tre Rab3 RpII18 RpL11 RpL17A RpL38 RpL46 RpS17 RpS18 Rpb10 SIP1 Sod Ssb-c31a Takr99D Tbh Tehao Tim9a Tm1 TpnC41C Trap36 Tsf1 Tsp42El Ugt86Di Vha36 agt fu12 l(3)01239 mRpL21 mRpL22 mRpS14 mRpS21 mRpS24 mira mirr na oho23B pum ran-like robl skpA tko toy unc-13 upd3 |
| 937 | GO:0030528 | F | 2, | 9 | 16.075 (x 0.560) | 805 (0.011) | 1 | transcription regulator activity | BEST:LD29214 CG15398 CG9650 Nipped-B Ssb-c31a Trap36 mirr sisA toy |
| 938 | GO:0017076 | F | 4, | 9 | 16.953 (x 0.531) | 849 (0.011) | 1 | purine nucleotide binding | Abl Arf84F CG10092 CG11898 CG1939 CG2056 CG4511 Rab3 ran-like |
| 939 | GO:0016772 | F | 4, | 4 | 10.004 (x 0.400) | 501 (0.008) | 1 | transferase activity, transferring phosphorus-containing groups | Abl CG2056 RpII18 Rpb10 |
| 940 | GO:0051179 | P | 3, | 25 | 35.364 (x 0.707) | 1771 (0.014) | 1 | localization | Abl Arf84F CG10237 CG10950 CG11898 CG1213 CG31477 CG4288 CG4673 CG6574 CG8193 CG8271 CLIP-190 Obp58b Rab3 Tim9a Tm1 Tsf1 Vha36 mira na pum ran-like robl unc-13 |
| 941 | GO:0009653 | P | 3, | 6 | 12.820 (x 0.468) | 642 (0.009) | 1 | morphogenesis | Abl Act57B CG14825 Cpn SIP1 mirr |
| 942 | GO:0048519 | P | 3, | 2 | 6.090 (x 0.328) | 305 (0.007) | 1 | negative regulation of biological process | CG7188 pum |
| 943 | GO:0042578 | F | 5, | 1 | 3.994 (x 0.250) | 200 (0.005) | 1 | phosphoric ester hydrolase activity | CG2277 |
| 944 | GO:0051234 | P | 4, | 23 | 34.106 (x 0.674) | 1708 (0.013) | 1 | establishment of localization | Abl Arf84F CG10237 CG10950 CG11898 CG1213 CG31477 CG4288 CG4673 CG6574 CG8193 CG8271 CLIP-190 Obp58b Rab3 Tim9a Tsf1 Vha36 na pum ran-like robl unc-13 |
| 945 | GO:0030246 | F | 3, | 1 | 2.256 (x 0.443) | 113 (0.009) | 1 | carbohydrate binding | CG4115 |
| 946 | GO:0015031 | P | 5, 6, | 7 | 10.324 (x 0.678) | 517 (0.014) | 1 | protein transport | Arf84F CG10950 CG4673 CLIP-190 Rab3 Tim9a ran-like |
| 947 | GO:0007049 | P | 4, | 6 | 9.405 (x 0.638) | 471 (0.013) | 1 | cell cycle | Abl CG2750 Nipped-B pum ran-like skpA |
| 948 | GO:0007476 | P | 6, 7, 8, | 1 | 2.057 (x 0.486) | 103 (0.010) | 1 | wing morphogenesis | mirr |
| 949 | GO:0044459 | C | 4, 5, 6, | 2 | 6.450 (x 0.310) | 323 (0.006) | 1 | plasma membrane part | Abl Takr99D |
| 950 | GO:0004672 | F | 6, | 2 | 5.891 (x 0.340) | 295 (0.007) | 1 | protein kinase activity | Abl CG2056 |
| 951 | GO:0006260 | P | 7, | 1 | 2.416 (x 0.414) | 121 (0.008) | 1 | DNA replication | skpA |
| 952 | GO:0006351 | P | 7, | 10 | 17.892 (x 0.559) | 896 (0.011) | 1 | transcription, DNA-dependent | CG15398 CG9650 Nipped-B RpII18 Rpb10 Ssb-c31a Trap36 mirr pum toy |
| 953 | GO:0016020 | C | 3, 4, | 31 | 37.261 (x 0.832) | 1866 (0.017) | 1 | membrane | Abl Ac78C CG11015 CG11898 CG1213 CG12400 CG14482 CG31477 CG32174 CG32230 CG4288 CG4673 CG5037 CG5548 CG6574 CG6921 CG7188 CG8271 EG:152A3.7 GABA-B-R2 Or46a Or59a Pdsw Takr99D Tehao Tim9a Tsp42El Vha36 fu12 hig na |
| 954 | GO:0009987 | P | 2, | 121 | 133.909 (x 0.904) | 6706 (0.018) | 1 | cellular process | Abl Ac78C Act57B Arf84F CG10092 CG10104 CG10237 CG10466 CG10638 CG10950 CG11015 CG11313 CG11898 CG1213 CG12400 CG1304 CG13277 CG14482 CG14825 CG15361 CG15398 CG18223 CG18249 CG18522 CG2056 CG2277 CG2750 CG30438 CG31146 CG31477 CG31704 CG32174 CG32230 CG32409 CG33002 CG4046 CG4288 CG4386 CG4511 CG4673 CG4866 CG5037 CG5103 CG5122 CG5162 CG5338 CG5548 CG6574 CG6610 CG6921 CG7014 CG7188 CG7646 CG7770 CG8193 CG8271 CG8415 CG9372 CG9650 CG9804 CLIP-190 Cpn Dhfr EG:152A3.7 GABA-B-R2 Gs2 GstE5 GstE6 GstE7 Nipped-B Obp58b Or46a Or59a Pdsw Prx6005 RN-tre Rab3 RpII18 RpL11 RpL17A RpL38 RpL46 RpS17 RpS18 Rpb10 SIP1 Sod Ssb-c31a Takr99D Tbh Tehao Tim9a Tm1 TpnC41C Trap36 Tsf1 Tsp42El Ugt86Di Vha36 agt fu12 hig l(3)01239 mRpL21 mRpL22 mRpS14 mRpS21 mRpS24 mira mirr na oho23B pum ran-like robl skpA tko toy tsl unc-13 upd3 |
| 955 | GO:0004871 | F | 2, | 13 | 21.027 (x 0.618) | 1053 (0.012) | 1 | signal transducer activity | Arf84F CG15361 CG18249 CG31146 GABA-B-R2 Or46a Or59a Takr99D Tehao Tsp42El tsl unc-13 upd3 |
| 956 | GO:0030705 | P | 6, 7, 8, | 1 | 2.077 (x 0.482) | 104 (0.010) | 1 | cytoskeleton-dependent intracellular transport | robl |
| 957 | GO:0006974 | P | 4, | 1 | 2.356 (x 0.424) | 118 (0.008) | 1 | response to DNA damage stimulus | agt |
| 958 | GO:0005261 | F | 5, 6, | 1 | 2.296 (x 0.435) | 115 (0.009) | 1 | cation channel activity | na |
| 959 | GO:0008152 | P | 3, | 93 | 100.981 (x 0.921) | 5057 (0.018) | 1 | metabolism | Abl Ac78C Act57B Arf84F CG10092 CG10104 CG10237 CG10466 CG10638 CG10962 CG11015 CG11313 CG1213 CG12400 CG1304 CG13277 CG14482 CG14935 CG15398 CG18223 CG18522 CG18530 CG2056 CG2277 CG30438 CG31477 CG31704 CG32174 CG32230 CG33002 CG4046 CG4288 CG4386 CG4511 CG4866 CG5037 CG5103 CG5122 CG5162 CG5338 CG5548 CG6574 CG6610 CG6921 CG7014 CG7770 CG8193 CG8415 CG9372 CG9650 CG9804 CG9836 Dhfr EG:152A3.7 Gs2 GstE5 GstE6 GstE7 Nipped-B Obp58b Or59a Pdsw Prx6005 RN-tre RpII18 RpL11 RpL17A RpL38 RpL46 RpS17 RpS18 Rpb10 Sod Ssb-c31a Tbh Trap36 Ugt86Di Vha36 agt fu12 l(3)01239 mRpL21 mRpL22 mRpS14 mRpS21 mRpS24 mirr na oho23B pum skpA tko toy |
| 960 | GO:0006281 | P | 5, 7, | 1 | 2.197 (x 0.455) | 110 (0.009) | 1 | DNA repair | agt |
| 961 | GO:0016798 | F | 4, | 1 | 2.137 (x 0.468) | 107 (0.009) | 1 | hydrolase activity, acting on glycosyl bonds | CG14935 |
| 962 | GO:0007018 | P | 7, 8, 9, | 1 | 2.057 (x 0.486) | 103 (0.010) | 1 | microtubule-based movement | robl |
| 963 | GO:0045184 | P | 5, | 7 | 10.384 (x 0.674) | 520 (0.013) | 1 | establishment of protein localization | Arf84F CG10950 CG4673 CLIP-190 Rab3 Tim9a ran-like |
| 964 | GO:0009605 | P | 3, | 1 | 2.097 (x 0.477) | 105 (0.010) | 1 | response to external stimulus | tko |
| 965 | GO:0051674 | P | 4, | 3 | 5.132 (x 0.585) | 257 (0.012) | 1 | localization of cell | Abl pum robl |
| 966 | GO:0048812 | P | 7, 8, 10, | 1 | 2.416 (x 0.414) | 121 (0.008) | 1 | neurite morphogenesis | Abl |
| 967 | GO:0006811 | P | 5, 6, | 6 | 9.225 (x 0.650) | 462 (0.013) | 1 | ion transport | CG31477 CG4288 CG8271 Tsf1 Vha36 na |
| 968 | GO:0007017 | P | 7, | 2 | 3.914 (x 0.511) | 196 (0.010) | 1 | microtubule-based process | CLIP-190 robl |
| 969 | GO:0031226 | C | 5, 6, 7, | 1 | 3.954 (x 0.253) | 198 (0.005) | 1 | intrinsic to plasma membrane | Takr99D |
| 970 | GO:0040011 | P | 3, | 3 | 5.212 (x 0.576) | 261 (0.011) | 1 | locomotion | Abl pum robl |
| 971 | GO:0000278 | P | 5, | 3 | 5.352 (x 0.561) | 268 (0.011) | 1 | mitotic cell cycle | Nipped-B pum skpA |
| 972 | GO:0009057 | P | 5, | 2 | 3.794 (x 0.527) | 190 (0.011) | 1 | macromolecule catabolism | CG5103 pum |
| 973 | GO:0005887 | C | 6, 7, 8, | 1 | 3.914 (x 0.256) | 196 (0.005) | 1 | integral to plasma membrane | Takr99D |
| 974 | GO:0043067 | P | 5, 6, | 1 | 2.376 (x 0.421) | 119 (0.008) | 1 | regulation of programmed cell death | CG7188 |
| 975 | GO:0048598 | P | 4, | 1 | 2.117 (x 0.472) | 106 (0.009) | 1 | embryonic morphogenesis | Abl |
| 976 | GO:0006092 | P | 7, | 1 | 2.077 (x 0.482) | 104 (0.010) | 1 | main pathways of carbohydrate metabolism | CG5103 |
| 977 | GO:0015980 | P | 6, | 1 | 2.356 (x 0.424) | 118 (0.008) | 1 | energy derivation by oxidation of organic compounds | CG5103 |
| 978 | GO:0045165 | P | 4, | 2 | 3.774 (x 0.530) | 189 (0.011) | 1 | cell fate commitment | mira pum |
| 979 | GO:0016879 | F | 4, | 2 | 3.634 (x 0.550) | 182 (0.011) | 1 | ligase activity, forming carbon-nitrogen bonds | CG9804 Gs2 |
| 980 | GO:0042981 | P | 6, 7, | 1 | 2.177 (x 0.459) | 109 (0.009) | 1 | regulation of apoptosis | CG7188 |
| 981 | GO:0035220 | P | 5, | 1 | 2.316 (x 0.432) | 116 (0.009) | 1 | wing disc development | mirr |
| 982 | GO:0006996 | P | 5, | 8 | 14.417 (x 0.555) | 722 (0.011) | 1 | organelle organization and biogenesis | Abl Act57B CG32409 CLIP-190 Tim9a mira robl skpA |
| 983 | GO:0006259 | P | 6, | 3 | 7.408 (x 0.405) | 371 (0.008) | 1 | DNA metabolism | Dhfr agt skpA |
| 984 | GO:0006886 | P | 6, 7, 8, | 7 | 10.104 (x 0.693) | 506 (0.014) | 1 | intracellular protein transport | Arf84F CG10950 CG4673 CLIP-190 Rab3 Tim9a ran-like |
| 985 | GO:0006396 | P | 7, | 3 | 5.092 (x 0.589) | 255 (0.012) | 1 | RNA processing | CG10466 CG13277 CG6610 |
| 986 | GO:0006631 | P | 6, 7, | 1 | 2.057 (x 0.486) | 103 (0.010) | 1 | fatty acid metabolism | CG6921 |
| 987 | GO:0007472 | P | 6, 7, | 1 | 2.097 (x 0.477) | 105 (0.010) | 1 | wing disc morphogenesis | mirr |
| 988 | GO:0006928 | P | 4, 5, | 3 | 5.132 (x 0.585) | 257 (0.012) | 1 | cell motility | Abl pum robl |
| 989 | GO:0048667 | P | 6, 7, 9, | 1 | 2.416 (x 0.414) | 121 (0.008) | 1 | neuron morphogenesis during differentiation | Abl |
| 990 | GO:0016481 | P | 8, | 1 | 2.217 (x 0.451) | 111 (0.009) | 1 | negative regulation of transcription | pum |
| 991 | GO:0015630 | C | 6, 7, 8, 9, | 2 | 3.714 (x 0.538) | 186 (0.011) | 1 | microtubule cytoskeleton | CLIP-190 robl |
| 992 | GO:0008283 | P | 4, | 2 | 5.631 (x 0.355) | 282 (0.007) | 1 | cell proliferation | CG2750 toy |
| 993 | GO:0007275 | P | 2, | 24 | 29.653 (x 0.809) | 1485 (0.016) | 1 | development | Abl Act57B BG:DS02740.9 CG14825 CG1942 CG31146 CG5397 CG9650 CLIP-190 Cpn Gs2 SIP1 Sod Tehao Tm1 Tsp42El mira mirr oho23B pum robl sisA toy tsl |
| 994 | GO:0007517 | P | 4, | 1 | 2.157 (x 0.464) | 108 (0.009) | 1 | muscle development | toy |
| 995 | GO:0035107 | P | 4, | 1 | 2.656 (x 0.377) | 133 (0.008) | 1 | appendage morphogenesis | mirr |
| 996 | GO:0006468 | P | 8, | 2 | 5.691 (x 0.351) | 285 (0.007) | 1 | protein amino acid phosphorylation | Abl CG2056 |
| 997 | GO:0048523 | P | 4, | 2 | 5.591 (x 0.358) | 280 (0.007) | 1 | negative regulation of cellular process | CG7188 pum |
| 998 | GO:0008237 | F | 5, | 1 | 3.694 (x 0.271) | 185 (0.005) | 1 | metallopeptidase activity | CG10466 |
| 999 | GO:0008104 | P | 4, | 8 | 11.302 (x 0.708) | 566 (0.014) | 1 | protein localization | Arf84F CG10950 CG4673 CLIP-190 Rab3 Tim9a mira ran-like |
| 1000 | GO:0030234 | F | 2, | 3 | 7.129 (x 0.421) | 357 (0.008) | 1 | enzyme regulator activity | CG11313 CG1342 CG31704 |
| 1001 | GO:0045934 | P | 7, | 1 | 2.416 (x 0.414) | 121 (0.008) | 1 | negative regulation of nucleobase, nucleoside, nucleotide and nucleic acid metabolism | pum |
| 1002 | GO:0044262 | P | 6, | 2 | 5.491 (x 0.364) | 275 (0.007) | 1 | cellular carbohydrate metabolism | Act57B CG5103 |
| 1003 | GO:0009719 | P | 3, | 1 | 2.656 (x 0.377) | 133 (0.008) | 1 | response to endogenous stimulus | agt |
| 1004 | GO:0007169 | P | 7, | 1 | 2.476 (x 0.404) | 124 (0.008) | 1 | transmembrane receptor protein tyrosine kinase signaling pathway | tsl |
| 1005 | GO:0015268 | F | 4, | 1 | 3.634 (x 0.275) | 182 (0.005) | 1 | alpha-type channel activity | na |
| 1006 | GO:0007409 | P | 8, 9, 11, | 1 | 2.416 (x 0.414) | 121 (0.008) | 1 | axonogenesis | Abl |
| 1007 | GO:0046907 | P | 5, 6, 7, | 8 | 12.301 (x 0.650) | 616 (0.013) | 1 | intracellular transport | Arf84F CG10950 CG4673 CLIP-190 Rab3 Tim9a ran-like robl |
| 1008 | GO:0048736 | P | 3, | 1 | 2.656 (x 0.377) | 133 (0.008) | 1 | appendage development | mirr |
| 1009 | GO:0006366 | P | 8, | 8 | 13.619 (x 0.587) | 682 (0.012) | 1 | transcription from RNA polymerase II promoter | CG15398 CG9650 RpII18 Rpb10 Ssb-c31a Trap36 mirr toy |
| 1010 | GO:0015267 | F | 3, | 1 | 3.634 (x 0.275) | 182 (0.005) | 1 | channel or pore class transporter activity | na |
| 1011 | GO:0009790 | P | 3, | 4 | 7.009 (x 0.571) | 351 (0.011) | 1 | embryonic development | Abl mirr pum tsl |
| 1012 | GO:0006629 | P | 5, | 6 | 9.785 (x 0.613) | 490 (0.012) | 1 | lipid metabolism | CG18530 CG30438 CG5162 CG6921 Ugt86Di fu12 |
| 1013 | GO:0006810 | P | 4, 5, | 21 | 29.494 (x 0.712) | 1477 (0.014) | 1 | transport | Arf84F CG10237 CG10950 CG11898 CG1213 CG31477 CG4288 CG4673 CG6574 CG8193 CG8271 CLIP-190 Obp58b Rab3 Tim9a Tsf1 Vha36 na ran-like robl unc-13 |
| 1014 | GO:0051276 | P | 6, | 1 | 3.435 (x 0.291) | 172 (0.006) | 1 | chromosome organization and biogenesis | skpA |
| 1015 | GO:0043285 | P | 6, | 1 | 2.536 (x 0.394) | 127 (0.008) | 1 | biopolymer catabolism | pum |
| 1016 | GO:0007165 | P | 4, | 18 | 25.779 (x 0.698) | 1291 (0.014) | 1 | signal transduction | Abl Ac78C Arf84F CG15361 CG18249 CG7646 GABA-B-R2 Or46a Or59a Rab3 Takr99D Tehao TpnC41C mirr ran-like tsl unc-13 upd3 |
| 1017 | GO:0000151 | C | 3, 4, 5, 6, | 1 | 2.516 (x 0.397) | 126 (0.008) | 1 | ubiquitin ligase complex | skpA |
| 1018 | GO:0006915 | P | 6, | 1 | 3.554 (x 0.281) | 178 (0.006) | 1 | apoptosis | CG7188 |
| 1019 | GO:0016791 | F | 6, | 1 | 3.634 (x 0.275) | 182 (0.005) | 1 | phosphoric monoester hydrolase activity | CG2277 |
| 1020 | GO:0043169 | F | 4, | 11 | 17.712 (x 0.621) | 887 (0.012) | 1 | cation binding | CG10126 CG10466 CG31922 CG6426 CG7646 CG9650 Cpn Mlc2 Tbh TpnC41C Tsf1 |
| 1021 | GO:0009887 | P | 4, | 3 | 6.709 (x 0.447) | 336 (0.009) | 1 | organ morphogenesis | Act57B Cpn mirr |
| 1022 | GO:0004888 | F | 4, | 5 | 8.487 (x 0.589) | 425 (0.012) | 1 | transmembrane receptor activity | GABA-B-R2 Or46a Or59a Takr99D Tehao |
| 1023 | GO:0007167 | P | 6, | 1 | 3.435 (x 0.291) | 172 (0.006) | 1 | enzyme linked receptor protein signaling pathway | tsl |
| 1024 | GO:0007243 | P | 6, | 1 | 2.536 (x 0.394) | 127 (0.008) | 1 | protein kinase cascade | upd3 |
| 1025 | GO:0048732 | P | 4, | 1 | 2.616 (x 0.382) | 131 (0.008) | 1 | gland development | oho23B |
| 1026 | GO:0008150 | P | 1, | 154 | 161.386 (x 0.954) | 8082 (0.019) | 1 | biological\_process | Abl Ac78C Act57B Arf84F BG:DS02740.9 CG10092 CG10104 CG10237 CG10466 CG10638 CG10950 CG10962 CG11015 CG11313 CG11898 CG1213 CG12400 CG1304 CG13277 CG13691 CG14482 CG14825 CG14935 CG15361 CG15398 CG16817 CG18223 CG18249 CG18522 CG18530 CG1942 CG2056 CG2277 CG2750 CG30154 CG30343 CG30438 CG31146 CG31477 CG31601 CG31704 CG31715 CG31922 CG32023 CG32174 CG32175 CG32207 CG32230 CG32409 CG32448 CG32582 CG32625 CG32856 CG33002 CG4046 CG4288 CG4386 CG4511 CG4673 CG4866 CG5037 CG5103 CG5122 CG5162 CG5224 CG5338 CG5397 CG5548 CG6426 CG6574 CG6610 CG6921 CG7014 CG7188 CG7646 CG7770 CG8193 CG8271 CG8415 CG9372 CG9650 CG9804 CG9836 CLIP-190 Cpn Dhfr EG:152A3.7 EG:63B12.12 Femcoat GABA-B-R2 Gs2 GstD9 GstE5 GstE6 GstE7 JhI-26 Mlc2 NP15.6 Nipped-B Obp56a Obp58b Or46a Or59a Pdsw Prx6005 RN-tre Rab3 Rep2 RpII18 RpL11 RpL17A RpL38 RpL46 RpS17 RpS18 Rpb10 SIP1 Sod Ssb-c31a Takr99D Tbh Tehao Tim9a Tm1 TpnC41C Trap36 Tsf1 Tsp42El Ugt86Di Vha36 Vm34Ca agt fu12 hig l(3)01239 mRpL21 mRpL22 mRpS14 mRpS21 mRpS24 mira mirr na oho23B pum ran-like robl sisA skpA tko toy tsl unc-13 upd3 |
| 1027 | GO:0004872 | F | 3, | 7 | 11.043 (x 0.634) | 553 (0.013) | 1 | receptor activity | CG18249 GABA-B-R2 Or46a Or59a Takr99D Tehao unc-13 |
| 1028 | GO:0006950 | P | 3, | 3 | 6.670 (x 0.450) | 334 (0.009) | 1 | response to stress | Tehao agt upd3 |
| 1029 | GO:0005488 | F | 2, | 71 | 82.550 (x 0.860) | 4134 (0.017) | 1 | binding | Abl Arf84F BEST:LD29214 BG:DS02740.9 BcDNA:GH11110 CG10092 CG10126 CG10237 CG10466 CG10950 CG11898 CG15361 CG15398 CG1939 CG2056 CG31146 CG31922 CG4046 CG4115 CG4511 CG4866 CG5338 CG6426 CG6574 CG7014 CG7646 CG7770 CG7911 CG8415 CG9650 CLIP-190 Cpn EG:BACH7M4.1 Mlc2 MtnA Obp56a Obp58b Or46a Or59a Rab3 RpII18 RpL11 RpL17A RpL38 RpL46 RpS17 RpS18 Rpb10 Sod Ssb-c31a Takr99D Tbh Tm1 TpnC41C Tsf1 agt l(3)01239 mRpL21 mRpS14 mira mirr oho23B pum ran-like sisA skpA tko toy tsl unc-13 upd3 |
| 1030 | GO:0048737 | P | 4, | 1 | 2.596 (x 0.385) | 130 (0.008) | 1 | appendage development (sensu Endopterygota) | mirr |
| 1031 | GO:0044248 | P | 5, | 3 | 6.110 (x 0.491) | 306 (0.010) | 1 | cellular catabolism | CG5103 Gs2 pum |
| 1032 | GO:0043118 | P | 4, | 2 | 4.812 (x 0.416) | 241 (0.008) | 1 | negative regulation of physiological process | CG7188 pum |
| 1033 | GO:0012505 | C | 4, 5, | 1 | 2.875 (x 0.348) | 144 (0.007) | 1 | endomembrane system | CG4673 |
| 1034 | GO:0007010 | P | 6, | 5 | 8.986 (x 0.556) | 450 (0.011) | 1 | cytoskeleton organization and biogenesis | Abl Act57B CLIP-190 mira robl |
| 1035 | GO:0031324 | P | 6, | 1 | 3.095 (x 0.323) | 155 (0.006) | 1 | negative regulation of cellular metabolism | pum |
| 1036 | GO:0000279 | P | 5, | 3 | 5.731 (x 0.523) | 287 (0.010) | 1 | M phase | Nipped-B pum skpA |
| 1037 | GO:0016874 | F | 3, | 3 | 6.330 (x 0.474) | 317 (0.009) | 1 | ligase activity | CG10092 CG9804 Gs2 |
| 1038 | GO:0009892 | P | 5, | 1 | 3.295 (x 0.304) | 165 (0.006) | 1 | negative regulation of metabolism | pum |
| 1039 | GO:0046872 | F | 4, | 13 | 18.611 (x 0.699) | 932 (0.014) | 1 | metal ion binding | CG10126 CG10466 CG31922 CG6426 CG7646 CG9650 Cpn Mlc2 MtnA Sod Tbh TpnC41C Tsf1 |
| 1040 | GO:0030001 | P | 7, 8, | 1 | 2.935 (x 0.341) | 147 (0.007) | 1 | metal ion transport | Tsf1 |
| 1041 | GO:0051243 | P | 5, | 2 | 4.633 (x 0.432) | 232 (0.009) | 1 | negative regulation of cellular physiological process | CG7188 pum |
| 1042 | GO:0016829 | F | 3, | 1 | 3.135 (x 0.319) | 157 (0.006) | 1 | lyase activity | Ac78C |
| 1043 | GO:0035114 | P | 5, | 1 | 2.596 (x 0.385) | 130 (0.008) | 1 | appendage morphogenesis (sensu Endopterygota) | mirr |
| 1044 | GO:0030154 | P | 3, | 6 | 10.184 (x 0.589) | 510 (0.012) | 1 | cell differentiation | Abl Cpn Tm1 mira pum robl |
| 1045 | GO:0016787 | F | 3, | 27 | 35.864 (x 0.753) | 1796 (0.015) | 1 | hydrolase activity | Arf84F BG:DS01068.5 CG10104 CG10466 CG11313 CG11898 CG1304 CG14935 CG15820 CG18223 CG18530 CG2056 CG2277 CG31477 CG4386 CG5162 CG5397 CG8520 CG9372 Mlc2 Obp58b RN-tre Rab3 Vha36 alpha-Est8 ran-like robl |
| 1046 | GO:0007560 | P | 5, 6, | 2 | 4.792 (x 0.417) | 240 (0.008) | 1 | imaginal disc morphogenesis | Cpn mirr |
| 1047 | GO:0043167 | F | 3, | 13 | 18.611 (x 0.699) | 932 (0.014) | 1 | ion binding | CG10126 CG10466 CG31922 CG6426 CG7646 CG9650 Cpn Mlc2 MtnA Sod Tbh TpnC41C Tsf1 |
| 1048 | GO:0007154 | P | 3, | 23 | 30.292 (x 0.759) | 1517 (0.015) | 1 | cell communication | Abl Ac78C Arf84F CG15361 CG18249 CG31146 CG7646 GABA-B-R2 Gs2 Or46a Or59a Rab3 Takr99D Tbh Tehao TpnC41C Tsp42El mirr pum ran-like tsl unc-13 upd3 |
| 1049 | GO:0005216 | F | 4, 5, | 1 | 3.275 (x 0.305) | 164 (0.006) | 1 | ion channel activity | na |
| 1050 | GO:0043170 | P | 4, | 53 | 62.222 (x 0.852) | 3116 (0.017) | 1 | macromolecule metabolism | Abl Act57B Arf84F CG10092 CG10104 CG10237 CG10466 CG11313 CG1213 CG1304 CG13277 CG14935 CG18223 CG2056 CG30438 CG31704 CG33002 CG4046 CG4288 CG4386 CG4866 CG5103 CG5338 CG6574 CG6610 CG7014 CG7770 CG8415 CG9372 CG9804 Dhfr Gs2 Obp58b RN-tre RpL11 RpL17A RpL38 RpL46 RpS17 RpS18 Ugt86Di agt l(3)01239 mRpL21 mRpL22 mRpS14 mRpS21 mRpS24 na oho23B pum skpA tko |
| 1051 | GO:0051641 | P | 4, 5, | 8 | 12.700 (x 0.630) | 636 (0.013) | 1 | cellular localization | Arf84F CG10950 CG4673 CLIP-190 Rab3 Tim9a ran-like robl |
| 1052 | GO:0046698 | P | 5, | 2 | 5.012 (x 0.399) | 251 (0.008) | 1 | metamorphosis (sensu Insecta) | Cpn mirr |
| 1053 | GO:0051649 | P | 5, 6, | 8 | 12.680 (x 0.631) | 635 (0.013) | 1 | establishment of cellular localization | Arf84F CG10950 CG4673 CLIP-190 Rab3 Tim9a ran-like robl |
| 1054 | GO:0003700 | F | 3, 5, | 4 | 7.768 (x 0.515) | 389 (0.010) | 1 | transcription factor activity | CG15398 mirr sisA toy |
| 1055 | GO:0016788 | F | 4, | 5 | 9.086 (x 0.550) | 455 (0.011) | 1 | hydrolase activity, acting on ester bonds | CG18530 CG2277 CG5162 CG5397 alpha-Est8 |
| 1056 | GO:0007552 | P | 4, | 2 | 5.052 (x 0.396) | 253 (0.008) | 1 | metamorphosis | Cpn mirr |
| 1057 | GO:0007444 | P | 4, | 3 | 6.031 (x 0.497) | 302 (0.010) | 1 | imaginal disc development | Cpn mirr toy |
| 1058 | GO:0003824 | F | 2, | 65 | 75.501 (x 0.861) | 3781 (0.017) | 1 | catalytic activity | Abl Ac78C Act57B Arf84F BG:DS01068.5 CG10092 CG10104 CG10466 CG10638 CG10962 CG11015 CG11313 CG11898 CG12400 CG1304 CG14482 CG14935 CG15820 CG18223 CG18522 CG18530 CG2056 CG2277 CG30438 CG31477 CG32230 CG33096 CG4386 CG5037 CG5103 CG5122 CG5162 CG5224 CG5397 CG5548 CG6921 CG8193 CG8520 CG9372 CG9804 Dhfr EG:152A3.7 Gs2 GstD9 GstE5 GstE6 GstE7 Mlc2 Obp58b Or59a Pdsw Prx6005 RN-tre Rab3 RpII18 Rpb10 Sod Tbh Ugt86Di Vha36 agt alpha-Est8 fu12 ran-like robl |
| 1059 | GO:0048513 | P | 3, | 8 | 13.379 (x 0.598) | 670 (0.012) | 1 | organ development | Act57B CG9650 Cpn mirr oho23B robl sisA toy |
| 1060 | GO:0009056 | P | 4, | 3 | 6.530 (x 0.459) | 327 (0.009) | 1 | catabolism | CG5103 Gs2 pum |

  

---

Regulated Genes that don't have GO terms
  

312 BG:DS07295.5 BcDNA:GH05536 CG10337 CG10581 CG11137 CG11279 CG11345 CG1143 CG11852 CG12481 CG12868 CG1288 CG13014 CG13018 CG13044 CG13067 CG13157 CG13434 CG13589 CG13623 CG13636 CG13663 CG13843 CG14104 CG14210 CG14270 CG14321 CG14394 CG14479 CG14483 CG14572 CG14731 CG14774 CG14898 CG14903 CG15032 CG15353 CG15525 CG15784 CG15863 CG15888 CG17681 CG17996 CG18294 CG18643 CG2909 CG30053 CG30196 CG30219 CG30412 CG31330 CG31436 CG32069 CG32500 CG32633 CG33120 CG33156 CG3566 CG40115 CG40169 CG40177 CG40216 CG40228 CG40260 CG40295 CG40329 CG40420 CG4186 CG5156 CG5174 CG5360 CG5866 CG5961 CG6353 CG7168 CG7201 CG7630 CG7637 CG7671 CG8369 CG8386 CG8538 CG9034 CG9231 CG9328 CG9336 CG9350 CG9617 CG9667 CG9922 EG:171E4.4 EG:34F3.10 Max-element
