# Supplementary material for: Functional dissection of the ash2 and ash1 transcriptomes provides insights into the transcriptional basis of wing phenotypes and reveals conserved protein interactions
Source: Genome Biol. 2007 Apr 28;8(4):R67. doi: 10.1186/gb-2007-8-4-r67 (PMC1896016; doi:10.1186/gb-2007-8-4-r67)
Supplement: Additional data file 13 — GO annotations of the genes downregulated over 1.5-fold in ash122 [file gb-2007-8-4-r67-S13.html]

  

---

  

|  |  |
| --- | --- |
| Go Statistics | Reg File: **ash1\_D1.5x.txt.fbgns** (115 genes -- 22 skipped)  Ref File: **ref.fbgns** (13577 genes -- 4663 skipped)  Database: **go\_200507-termdb.rdf-xml** |

---

  

Fields Description

| Pos | Go Term | Ontology | Levels | Observed | Expected | Possibles | p-value(Adj) | Go term description | Genes with the GO term |
| --- | --- | --- | --- | --- | --- | --- | --- | --- | --- |
| 1 | GO:0009653 | P | 3, | 22 | 6.698 (x 3.285) | 642 (0.034) | 0.000208 | morphogenesis | Abi CG6982 Dll Dr Fs(2)Ket ImpE2 ImpE3 Lam POSH Pi3K21B Pk61C ap bowl edl ena eyg fax hth klu kn plexA sqd |
| 2 | GO:0007275 | P | 2, | 36 | 15.493 (x 2.324) | 1485 (0.024) | 0.00024 | development | Abi Akap200 CG6982 Dll Dr Fs(2)Ket Hrb27C Hsp26 Hsp27 ImpE2 ImpE3 Lam POSH Pi3K21B Pk61C ana ap bowl edl ena eyg fax hth hts kis klu kn msi ogre pdm2 plexA smi35A spdo sqd stai toy |
| 3 | GO:0009408 | P | 4, 5, | 6 | 0.490 (x 12.236) | 47 (0.128) | 0.00183 | response to heat | CG7182 Hsp23 Hsp26 Hsp27 Hsp67Ba Hsp70Bc |
| 4 | GO:0006457 | P | 7, | 9 | 1.367 (x 6.585) | 131 (0.069) | 0.00241 | protein folding | CG11267 CG7182 Fkbp13 Hop Hsp23 Hsp26 Hsp27 Hsp67Ba Hsp70Bc |
| 5 | GO:0009266 | P | 4, | 6 | 0.563 (x 10.650) | 54 (0.111) | 0.00332 | response to temperature stimulus | CG7182 Hsp23 Hsp26 Hsp27 Hsp67Ba Hsp70Bc |
| 6 | GO:0007399 | P | 4, | 16 | 4.883 (x 3.277) | 468 (0.034) | 0.00339 | nervous system development | Dll Dr ana ap ena eyg fax hth msi ogre pdm2 plexA smi35A spdo stai toy |
| 7 | GO:0046578 | P | 6, 7, 8, | 3 | 0.073 (x 41.078) | 7 (0.429) | 0.0041 | regulation of Ras protein signal transduction | Akap200 edl klu |
| 8 | GO:0048731 | P | 3, | 18 | 6.197 (x 2.905) | 594 (0.030) | 0.00425 | system development | Akap200 Dll Dr Lam ana ap ena eyg fax hth msi ogre pdm2 plexA smi35A spdo stai toy |
| 9 | GO:0007389 | P | 3, | 11 | 2.671 (x 4.119) | 256 (0.043) | 0.00536 | pattern specification | Dll Dr Hrb27C POSH ap bowl edl hth kis kn sqd |
| 10 | GO:0007444 | P | 4, | 12 | 3.151 (x 3.809) | 302 (0.040) | 0.00575 | imaginal disc development | Dll Dr ImpE2 ImpE3 POSH ap bowl eyg hth klu kn toy |
| 11 | GO:0051056 | P | 5, 6, 7, | 3 | 0.083 (x 35.944) | 8 (0.375) | 0.00578 | regulation of small GTPase mediated signal transduction | Akap200 edl klu |
| 12 | GO:0007560 | P | 5, 6, | 10 | 2.504 (x 3.994) | 240 (0.042) | 0.0138 | imaginal disc morphogenesis | Dr ImpE2 ImpE3 POSH ap bowl eyg hth klu kn |
| 13 | GO:0007552 | P | 4, | 10 | 2.640 (x 3.789) | 253 (0.040) | 0.0181 | metamorphosis | Dr ImpE2 ImpE3 POSH ap bowl eyg hth klu kn |
| 14 | GO:0046698 | P | 5, | 10 | 2.619 (x 3.819) | 251 (0.040) | 0.0183 | metamorphosis (sensu Insecta) | Dr ImpE2 ImpE3 POSH ap bowl eyg hth klu kn |
| 15 | GO:0010092 | P | 5, | 2 | 0.031 (x 63.900) | 3 (0.667) | 0.0188 | specification of organ identity | Dll hth |
| 16 | GO:0007479 | P | 6, 7, | 3 | 0.146 (x 20.539) | 14 (0.214) | 0.019 | leg disc proximal/distal pattern formation | Dll ap hth |
| 17 | GO:0035223 | P | 5, 6, | 3 | 0.146 (x 20.539) | 14 (0.214) | 0.0202 | leg disc pattern formation | Dll ap hth |
| 18 | GO:0009628 | P | 3, | 12 | 3.881 (x 3.092) | 372 (0.032) | 0.0224 | response to abiotic stimulus | CG30437 CG7182 CG8588 Hsp23 Hsp26 Hsp27 Hsp67Ba Hsp70Bc Obp99a Ugt86Di ogre smi35A |
| 19 | GO:0007449 | P | 5, 6, | 3 | 0.188 (x 15.975) | 18 (0.167) | 0.027 | proximal/distal pattern formation, imaginal disc | Dll ap hth |
| 20 | GO:0009954 | P | 4, | 3 | 0.188 (x 15.975) | 18 (0.167) | 0.0281 | proximal/distal pattern formation | Dll ap hth |
| 21 | GO:0009887 | P | 4, | 11 | 3.505 (x 3.138) | 336 (0.033) | 0.0284 | organ morphogenesis | Dll Dr ImpE2 ImpE3 POSH ap bowl eyg hth klu kn |
| 22 | GO:0000902 | P | 4, 5, | 11 | 3.526 (x 3.119) | 338 (0.033) | 0.0285 | cellular morphogenesis | Abi CG6982 Fs(2)Ket POSH Pi3K21B Pk61C ap edl ena fax plexA |
| 23 | GO:0002165 | P | 4, | 11 | 3.599 (x 3.056) | 345 (0.032) | 0.0287 | larval or pupal development (sensu Insecta) | Akap200 Dr ImpE2 ImpE3 POSH ap bowl eyg hth klu kn |
| 24 | GO:0007398 | P | 4, | 9 | 2.473 (x 3.640) | 237 (0.038) | 0.0288 | ectoderm development | Dll Dr ap eyg msi pdm2 plexA smi35A toy |
| 25 | GO:0035218 | P | 5, | 4 | 0.407 (x 9.831) | 39 (0.103) | 0.0289 | leg disc development | Dll ap bowl hth |
| 26 | GO:0005622 | C | 3, 4, | 50 | 34.377 (x 1.454) | 3295 (0.015) | 0.0294 | intracellular | B52 BcDNA:LD41548 CBP CG11267 CG11500 CG12054 CG17735 CG17952 CG30059 CG31363 CG31611 CG3823 CG4759 CG5028 CG6654 CG8089 CG9057 Dll Dr Fs(2)Ket Hrb27C ImpE2 Lam POSH Pi3K21B Pk61C Prx5037 Sox102F Su(z)2 Tbp-1 Ufd1-like ap aru bip1 bowl edl eyg garz hth hts kis klu kn lmg pdm2 smi35A spdo sqd stai toy |
| 27 | GO:0007561 | P | 6, 7, | 2 | 0.042 (x 47.925) | 4 (0.500) | 0.0295 | imaginal disc eversion | ImpE2 ImpE3 |
| 28 | GO:0007447 | P | 4, 5, | 4 | 0.449 (x 8.916) | 43 (0.093) | 0.0315 | imaginal disc pattern formation | Dll Dr ap hth |
| 29 | GO:0050789 | P | 2, | 28 | 15.702 (x 1.783) | 1505 (0.019) | 0.0318 | regulation of biological process | Abi Akap200 B52 CG6654 Dll Dr Hrb27C Pi3K21B Pk61C Sox102F Su(z)2 ana ap bowl charybde edl ena eyg hth kis klu kn msi pdm2 scylla smi35A sqd toy |
| 30 | GO:0005637 | C | 5, 6, 7, 8, 9, 10, 11, 12, 13, | 2 | 0.052 (x 38.340) | 5 (0.400) | 0.0319 | nuclear inner membrane | CG17952 Lam |
| 31 | GO:0009791 | P | 3, | 11 | 3.714 (x 2.962) | 356 (0.031) | 0.0323 | post-embryonic development | Akap200 Dr ImpE2 ImpE3 POSH ap bowl eyg hth klu kn |
| 32 | GO:0044424 | C | 3, 4, 5, | 48 | 33.281 (x 1.442) | 3190 (0.015) | 0.0333 | intracellular part | B52 BcDNA:LD41548 CBP CG11267 CG11500 CG12054 CG17952 CG30059 CG31363 CG31611 CG4759 CG5028 CG6654 CG8089 CG9057 Dll Dr Fs(2)Ket Hrb27C ImpE2 Lam POSH Pi3K21B Pk61C Prx5037 Sox102F Su(z)2 Tbp-1 Ufd1-like ap aru bip1 bowl edl eyg garz hth hts kis klu kn lmg pdm2 smi35A spdo sqd stai toy |
| 33 | GO:0003704 | F | 4, | 5 | 0.824 (x 6.066) | 79 (0.063) | 0.0362 | specific RNA polymerase II transcription factor activity | Dll ap kn pdm2 toy |
| 34 | GO:0046580 | P | 7, 8, 9, | 2 | 0.063 (x 31.950) | 6 (0.333) | 0.0406 | negative regulation of Ras protein signal transduction | Akap200 klu |
| 35 | GO:0051058 | P | 6, 7, 8, | 2 | 0.073 (x 27.386) | 7 (0.286) | 0.0505 | negative regulation of small GTPase mediated signal transduction | Akap200 klu |
| 36 | GO:0016607 | C | 7, 8, 9, 10, 11, 12, 13, 14, | 2 | 0.073 (x 27.386) | 7 (0.286) | 0.0519 | nuclear speck | B52 sqd |
| 37 | GO:0007265 | P | 7, | 3 | 0.261 (x 11.502) | 25 (0.120) | 0.0522 | Ras protein signal transduction | Akap200 edl klu |
| 38 | GO:0030154 | P | 3, | 13 | 5.321 (x 2.443) | 510 (0.025) | 0.0523 | cell differentiation | Dr Hrb27C POSH ap edl ena fax hth hts kn msi plexA sqd |
| 39 | GO:0050791 | P | 3, | 25 | 13.970 (x 1.790) | 1339 (0.019) | 0.0526 | regulation of physiological process | Abi B52 CG6654 Dll Dr Hrb27C Pi3K21B Pk61C Sox102F Su(z)2 ana ap bowl edl ena eyg hth kis klu kn msi pdm2 smi35A sqd toy |
| 40 | GO:0035110 | P | 6, | 3 | 0.292 (x 10.270) | 28 (0.107) | 0.0623 | leg morphogenesis | Dll ap bowl |
| 41 | GO:0030036 | P | 8, | 5 | 1.012 (x 4.941) | 97 (0.052) | 0.0628 | actin cytoskeleton organization and biogenesis | Abi Fs(2)Ket Pk61C ena hts |
| 42 | GO:0050794 | P | 3, | 25 | 14.366 (x 1.740) | 1377 (0.018) | 0.0631 | regulation of cellular process | Abi Akap200 B52 CG6654 Dll Dr Pi3K21B Pk61C Sox102F Su(z)2 ana ap bowl edl ena eyg hth kis klu kn msi pdm2 smi35A sqd toy |
| 43 | GO:0016604 | C | 6, 7, 8, 9, 10, 11, 12, 13, | 2 | 0.083 (x 23.962) | 8 (0.250) | 0.0635 | nuclear body | B52 sqd |
| 44 | GO:0051244 | P | 4, | 24 | 13.532 (x 1.774) | 1297 (0.019) | 0.0637 | regulation of cellular physiological process | Abi B52 CG6654 Dll Dr Pi3K21B Pk61C Sox102F Su(z)2 ana ap bowl edl ena eyg hth kis klu kn msi pdm2 smi35A sqd toy |
| 45 | GO:0030029 | P | 7, | 5 | 1.012 (x 4.941) | 97 (0.052) | 0.0641 | actin filament-based process | Abi Fs(2)Ket Pk61C ena hts |
| 46 | GO:0035108 | P | 5, | 3 | 0.303 (x 9.915) | 29 (0.103) | 0.0644 | limb morphogenesis | Dll ap bowl |
| 47 | GO:0007379 | P | 4, 5, | 3 | 0.303 (x 9.915) | 29 (0.103) | 0.0659 | segment specification | bowl kis kn |
| 48 | GO:0040008 | P | 3, | 4 | 0.636 (x 6.285) | 61 (0.066) | 0.0673 | regulation of growth | Pi3K21B Pk61C charybde scylla |
| 49 | GO:0006357 | P | 9, | 13 | 5.707 (x 2.278) | 547 (0.024) | 0.0762 | regulation of transcription from RNA polymerase II promoter | CG6654 Dll Dr Sox102F Su(z)2 ap bowl eyg hth kis klu pdm2 toy |
| 50 | GO:0005634 | C | 5, 6, 7, 8, | 26 | 15.900 (x 1.635) | 1524 (0.017) | 0.103 | nucleus | B52 CG12054 CG17952 CG31611 CG6654 CG8089 Dll Dr Fs(2)Ket Hrb27C Lam Sox102F Su(z)2 ap bip1 bowl edl eyg hth kis klu kn lmg pdm2 sqd toy |
| 51 | GO:0035214 | P | 5, | 6 | 1.669 (x 3.594) | 160 (0.037) | 0.111 | eye-antennal disc development | Dll POSH eyg hth klu toy |
| 52 | GO:0019094 | P | 6, 10, 12, 13, 15, | 3 | 0.386 (x 7.772) | 37 (0.081) | 0.112 | pole plasm mRNA localization | Hrb27C POSH sqd |
| 53 | GO:0016066 |  | 6, 7, | 1 | 0.010 (x 95.849) | 1 (1.000) | 0.112 |  | kn |
| 54 | GO:0031887 | P | 8, 9, 10, | 1 | 0.010 (x 95.849) | 1 (1.000) | 0.113 | lipid particle transport along microtubule | CG9057 |
| 55 | GO:0006366 | P | 8, | 14 | 7.115 (x 1.968) | 682 (0.021) | 0.113 | transcription from RNA polymerase II promoter | CG6654 Dll Dr Sox102F Su(z)2 ap bowl eyg hth kis klu kn pdm2 toy |
| 56 | GO:0005521 | F | 4, | 1 | 0.010 (x 95.849) | 1 (1.000) | 0.115 | lamin binding | CG17952 |
| 57 | GO:0006952 | P | 4, | 12 | 5.363 (x 2.238) | 514 (0.023) | 0.115 | defense response | CG30437 CG5873 CG7182 Hop Hsp23 Hsp26 Hsp27 Hsp67Ba Hsp70Bc Prx5037 Ugt86Di kn |
| 58 | GO:0035114 | P | 5, | 5 | 1.356 (x 3.687) | 130 (0.038) | 0.115 | appendage morphogenesis (sensu Endopterygota) | Dll Dr ap bowl kn |
| 59 | GO:0007316 | P | 5, 9, 11, 12, 14, | 3 | 0.396 (x 7.567) | 38 (0.079) | 0.116 | pole plasm RNA localization | Hrb27C POSH sqd |
| 60 | GO:0044453 | C | 4, 5, 6, 7, 8, 9, 10, 11, 12, | 3 | 0.490 (x 6.118) | 47 (0.064) | 0.116 | nuclear membrane part | CG17952 Fs(2)Ket Lam |
| 61 | GO:0003883 | F | 5, | 1 | 0.010 (x 95.849) | 1 (1.000) | 0.116 | CTP synthase activity | CG6854 |
| 62 | GO:0035286 | P | 5, 6, 7, | 2 | 0.136 (x 14.746) | 13 (0.154) | 0.116 | leg segmentation | ap bowl |
| 63 | GO:0048737 | P | 4, | 5 | 1.356 (x 3.687) | 130 (0.038) | 0.117 | appendage development (sensu Endopterygota) | Dll Dr ap bowl kn |
| 64 | GO:0048699 | P | 6, | 6 | 1.878 (x 3.195) | 180 (0.033) | 0.117 | generation of neurons | Dr ana ap ena fax plexA |
| 65 | GO:0035107 | P | 4, | 5 | 1.388 (x 3.603) | 133 (0.038) | 0.117 | appendage morphogenesis | Dll Dr ap bowl kn |
| 66 | GO:0031965 | C | 5, 6, 7, 8, 9, 10, 11, | 3 | 0.490 (x 6.118) | 47 (0.064) | 0.117 | nuclear membrane | CG17952 Fs(2)Ket Lam |
| 67 | GO:0007481 | P | 6, 7, | 1 | 0.010 (x 95.849) | 1 (1.000) | 0.117 | haltere disc morphogenesis | ap |
| 68 | GO:0048112 | P | 7, 9, 10, 12, | 3 | 0.469 (x 6.390) | 45 (0.067) | 0.117 | oocyte anterior/posterior axis determination (sensu Insecta) | Hrb27C POSH sqd |
| 69 | GO:0008340 | P | 4, | 3 | 0.480 (x 6.251) | 46 (0.065) | 0.118 | determination of adult life span | Hsp26 Hsp27 POSH |
| 70 | GO:0031323 | P | 5, | 18 | 10.287 (x 1.750) | 986 (0.018) | 0.118 | regulation of cellular metabolism | B52 CG6654 Dll Dr Sox102F Su(z)2 ap bowl edl eyg hth kis klu kn msi pdm2 sqd toy |
| 71 | GO:0004866 | F | 5, | 4 | 0.887 (x 4.511) | 85 (0.047) | 0.118 | endopeptidase inhibitor activity | CG7722 CG8066 Spn43Aa plexA |
| 72 | GO:0035285 | P | 4, 5, | 2 | 0.136 (x 14.746) | 13 (0.154) | 0.118 | appendage segmentation | ap bowl |
| 73 | GO:0048736 | P | 3, | 5 | 1.388 (x 3.603) | 133 (0.038) | 0.118 | appendage development | Dll Dr ap bowl kn |
| 74 | GO:0009994 | P | 4, 7, | 4 | 0.908 (x 4.407) | 87 (0.046) | 0.118 | oocyte differentiation | Hrb27C POSH hts sqd |
| 75 | GO:0016478 | P | 8, 9, 10, | 2 | 0.177 (x 11.276) | 17 (0.118) | 0.119 | negative regulation of translation | msi sqd |
| 76 | GO:0006461 | P | 6, | 5 | 1.221 (x 4.096) | 117 (0.043) | 0.119 | protein complex assembly | B52 CG31611 Hop Hsp70Bc hts |
| 77 | GO:0008283 | P | 4, | 8 | 2.942 (x 2.719) | 282 (0.028) | 0.119 | cell proliferation | CG6654 Pi3K21B ana bowl eyg klu smi35A toy |
| 78 | GO:0030414 | F | 4, | 4 | 0.897 (x 4.458) | 86 (0.047) | 0.119 | protease inhibitor activity | CG7722 CG8066 Spn43Aa plexA |
| 79 | GO:0035291 | P | 6, 7, 8, | 1 | 0.010 (x 95.849) | 1 (1.000) | 0.119 | specification of segmental identity, intercalary segment | kn |
| 80 | GO:0007568 | P | 3, | 3 | 0.480 (x 6.251) | 46 (0.065) | 0.119 | aging | Hsp26 Hsp27 POSH |
| 81 | GO:0048513 | P | 3, | 14 | 6.990 (x 2.003) | 670 (0.021) | 0.119 | organ development | Akap200 Dll Dr ImpE2 ImpE3 POSH ap bowl edl eyg hth klu kn toy |
| 82 | GO:0007169 | P | 7, | 5 | 1.294 (x 3.865) | 124 (0.040) | 0.12 | transmembrane receptor protein tyrosine kinase signaling pathway | Pi3K21B Pk61C aru edl plexA |
| 83 | GO:0019900 | F | 5, | 2 | 0.136 (x 14.746) | 13 (0.154) | 0.12 | kinase binding | Akap200 Pi3K21B |
| 84 | GO:0008286 | P | 8, | 2 | 0.146 (x 13.693) | 14 (0.143) | 0.12 | insulin receptor signaling pathway | Pi3K21B Pk61C |
| 85 | GO:0035014 | F | 4, | 1 | 0.010 (x 95.849) | 1 (1.000) | 0.121 | phosphoinositide 3-kinase regulator activity | Pi3K21B |
| 86 | GO:0019222 | P | 4, | 19 | 10.683 (x 1.778) | 1024 (0.019) | 0.121 | regulation of metabolism | B52 CG6654 Dll Dr Sox102F Su(z)2 ap bowl edl ena eyg hth kis klu kn msi pdm2 sqd toy |
| 87 | GO:0000785 | C | 5, 6, 7, 8, 9, 10, | 4 | 0.803 (x 4.979) | 77 (0.052) | 0.121 | chromatin | CG31611 Su(z)2 kis sqd |
| 88 | GO:0048468 | P | 4, | 9 | 3.547 (x 2.537) | 340 (0.026) | 0.121 | cell development | Hrb27C POSH ap edl ena fax hts plexA sqd |
| 89 | GO:0019219 | P | 6, | 17 | 9.265 (x 1.835) | 888 (0.019) | 0.121 | regulation of nucleobase, nucleoside, nucleotide and nucleic acid metabolism | B52 CG6654 Dll Dr Sox102F Su(z)2 ap bowl edl eyg hth kis klu kn pdm2 sqd toy |
| 90 | GO:0000904 | P | 5, 6, | 6 | 1.763 (x 3.403) | 169 (0.036) | 0.122 | cellular morphogenesis during differentiation | POSH ap edl ena fax plexA |
| 91 | GO:0045793 | P | 6, 7, | 2 | 0.146 (x 13.693) | 14 (0.143) | 0.122 | positive regulation of cell size | Pi3K21B Pk61C |
| 92 | GO:0009607 | P | 3, | 12 | 5.446 (x 2.203) | 522 (0.023) | 0.122 | response to biotic stimulus | CG30437 CG5873 CG7182 Hop Hsp23 Hsp26 Hsp27 Hsp67Ba Hsp70Bc Prx5037 Ugt86Di kn |
| 93 | GO:0042087 | P | 7, 8, | 1 | 0.010 (x 95.849) | 1 (1.000) | 0.122 | cell-mediated immune response | kn |
| 94 | GO:0048113 | P | 8, 10, 11, 13, | 3 | 0.428 (x 7.013) | 41 (0.073) | 0.123 | pole plasm assembly (sensu Insecta) | Hrb27C POSH sqd |
| 95 | GO:0008411 | F | 6, | 1 | 0.010 (x 95.849) | 1 (1.000) | 0.124 | 4-hydroxybutyrate CoA-transferase activity | CG7920 |
| 96 | GO:0007315 | P | 7, 9, 10, 12, | 3 | 0.449 (x 6.687) | 43 (0.070) | 0.124 | pole plasm assembly | Hrb27C POSH sqd |
| 97 | GO:0007406 | P | 6, 7, 8, 9, | 1 | 0.010 (x 95.849) | 1 (1.000) | 0.125 | negative regulation of neuroblast proliferation | ana |
| 98 | GO:0040007 | P | 2, | 4 | 0.939 (x 4.260) | 90 (0.044) | 0.126 | growth | Pi3K21B Pk61C charybde scylla |
| 99 | GO:0007450 | P | 5, 6, | 2 | 0.156 (x 12.780) | 15 (0.133) | 0.126 | dorsal/ventral pattern formation, imaginal disc | Dr ap |
| 100 | GO:0008298 | P | 5, | 3 | 0.511 (x 5.868) | 49 (0.061) | 0.127 | intracellular mRNA localization | Hrb27C POSH sqd |
| 101 | GO:0005488 | F | 2, | 54 | 43.130 (x 1.252) | 4134 (0.013) | 0.132 | binding | Abi Akap200 B52 BcDNA:LD41548 CBP CG11267 CG12054 CG17735 CG17952 CG30437 CG31611 CG3823 CG4759 CG6654 CG6854 CG7182 CG8089 CG9057 Dll Dr Fkbp13 Fs(2)Ket Hop Hrb27C Hsp23 Hsp70Bc Lam Obp99a POSH Pi3K21B Pk61C RhoGAP71E Sox102F Su(z)2 Tbp-1 ap bip1 bowl edl ena eyg hth hts kis klu kn lmg msi pdm2 smi35A spdo sqd stai toy |
| 102 | GO:0022008 | P | 5, | 6 | 2.014 (x 2.980) | 193 (0.031) | 0.132 | neurogenesis | Dr ana ap ena fax plexA |
| 103 | GO:0006355 | P | 8, | 15 | 8.211 (x 1.827) | 787 (0.019) | 0.134 | regulation of transcription, DNA-dependent | CG6654 Dll Dr Sox102F Su(z)2 ap bowl edl eyg hth kis klu kn pdm2 toy |
| 104 | GO:0035030 | C | 4, 7, 8, 9, 10, 11, | 1 | 0.021 (x 47.925) | 2 (0.500) | 0.142 | phosphoinositide 3-kinase complex, class IA | Pi3K21B |
| 105 | GO:0017154 | F | 5, | 1 | 0.021 (x 47.925) | 2 (0.500) | 0.144 | semaphorin receptor activity | plexA |
| 106 | GO:0043226 | C | 2, | 38 | 28.002 (x 1.357) | 2684 (0.014) | 0.144 | organelle | B52 CBP CG11267 CG11500 CG12054 CG17952 CG30059 CG31363 CG31611 CG4759 CG5028 CG6654 CG8089 Dll Dr Fs(2)Ket Hrb27C Lam Prx5037 Sox102F Su(z)2 ap aru bip1 bowl edl eyg garz hth hts kis klu kn lmg pdm2 sqd stai toy |
| 107 | GO:0030159 | F | 4, | 1 | 0.021 (x 47.925) | 2 (0.500) | 0.145 | receptor signaling complex scaffold activity | POSH |
| 108 | GO:0042048 | P | 5, 6, | 3 | 0.553 (x 5.425) | 53 (0.057) | 0.145 | olfactory behavior | CG8588 Obp99a smi35A |
| 109 | GO:0043229 | C | 3, 4, 5, 6, | 38 | 28.002 (x 1.357) | 2684 (0.014) | 0.145 | intracellular organelle | B52 CBP CG11267 CG11500 CG12054 CG17952 CG30059 CG31363 CG31611 CG4759 CG5028 CG6654 CG8089 Dll Dr Fs(2)Ket Hrb27C Lam Prx5037 Sox102F Su(z)2 ap aru bip1 bowl edl eyg garz hth hts kis klu kn lmg pdm2 sqd stai toy |
| 110 | GO:0031327 | P | 7, | 2 | 0.230 (x 8.714) | 22 (0.091) | 0.146 | negative regulation of cellular biosynthesis | msi sqd |
| 111 | GO:0006607 | P | 8, 9, 10, 11, | 1 | 0.021 (x 47.925) | 2 (0.500) | 0.146 | NLS-bearing substrate import into nucleus | Fs(2)Ket |
| 112 | GO:0048111 | P | 6, 8, 9, 11, | 3 | 0.553 (x 5.425) | 53 (0.057) | 0.146 | oocyte axis determination (sensu Insecta) | Hrb27C POSH sqd |
| 113 | GO:0045927 | P | 4, | 2 | 0.230 (x 8.714) | 22 (0.091) | 0.147 | positive regulation of growth | Pi3K21B Pk61C |
| 114 | GO:0045111 | C | 6, 7, 8, 9, | 1 | 0.021 (x 47.925) | 2 (0.500) | 0.147 | intermediate filament cytoskeleton | Lam |
| 115 | GO:0007635 | P | 4, 5, | 3 | 0.563 (x 5.325) | 54 (0.056) | 0.147 | chemosensory behavior | CG8588 Obp99a smi35A |
| 116 | GO:0045926 | P | 4, | 2 | 0.209 (x 9.585) | 20 (0.100) | 0.147 | negative regulation of growth | charybde scylla |
| 117 | GO:0009890 | P | 6, | 2 | 0.230 (x 8.714) | 22 (0.091) | 0.148 | negative regulation of biosynthesis | msi sqd |
| 118 | GO:0005882 | C | 5, 6, 7, 8, 9, 10, | 1 | 0.021 (x 47.925) | 2 (0.500) | 0.148 | intermediate filament | Lam |
| 119 | GO:0048110 | P | 7, 8, 10, | 3 | 0.563 (x 5.325) | 54 (0.056) | 0.148 | oocyte construction (sensu Insecta) | Hrb27C POSH sqd |
| 120 | GO:0007010 | P | 6, | 10 | 4.695 (x 2.130) | 450 (0.022) | 0.149 | cytoskeleton organization and biogenesis | Abi CG8177 CG9057 Fs(2)Ket Lam POSH Pk61C ena hts stai |
| 121 | GO:0003677 | F | 4, | 15 | 8.576 (x 1.749) | 822 (0.018) | 0.149 | DNA binding | CG31611 CG6854 Dll Dr Hrb27C Sox102F Su(z)2 ap edl eyg hth kis kn pdm2 toy |
| 122 | GO:0005811 | C | 5, 6, 7, 8, | 1 | 0.021 (x 47.925) | 2 (0.500) | 0.149 | lipid particle | CG9057 |
| 123 | GO:0009888 | P | 3, | 9 | 4.152 (x 2.167) | 398 (0.023) | 0.15 | tissue development | Dll Dr ap eyg msi pdm2 plexA smi35A toy |
| 124 | GO:0035216 | P | 5, | 1 | 0.021 (x 47.925) | 2 (0.500) | 0.151 | haltere disc development | ap |
| 125 | GO:0006950 | P | 3, | 8 | 3.485 (x 2.296) | 334 (0.024) | 0.151 | response to stress | CG7182 Hop Hsp23 Hsp26 Hsp27 Hsp67Ba Hsp70Bc kn |
| 126 | GO:0005452 | F | 7, 8, 9, | 1 | 0.021 (x 47.925) | 2 (0.500) | 0.152 | inorganic anion exchanger activity | CG8177 |
| 127 | GO:0035203 | P | 6, 7, 8, 9, 10, | 1 | 0.021 (x 47.925) | 2 (0.500) | 0.153 | regulation of lamellocyte differentiation | kn |
| 128 | GO:0035109 | P | 6, | 2 | 0.240 (x 8.335) | 23 (0.087) | 0.154 | limb morphogenesis (sensu Endopterygota) | Dll bowl |
| 129 | GO:0007084 | P | 6, 8, 9, | 1 | 0.021 (x 47.925) | 2 (0.500) | 0.154 | mitotic nuclear envelope reassembly | Lam |
| 130 | GO:0017148 | P | 7, 8, 9, | 2 | 0.219 (x 9.129) | 21 (0.095) | 0.154 | negative regulation of protein biosynthesis | msi sqd |
| 131 | GO:0003700 | F | 3, 5, | 9 | 4.058 (x 2.218) | 389 (0.023) | 0.156 | transcription factor activity | CG6854 Dll Dr Sox102F ap eyg hth pdm2 toy |
| 132 | GO:0005638 | C | 5, 6, 7, 8, 9, 10, 11, 12, 13, 14, | 1 | 0.021 (x 47.925) | 2 (0.500) | 0.156 | lamin filament | Lam |
| 133 | GO:0008410 | F | 5, | 1 | 0.021 (x 47.925) | 2 (0.500) | 0.157 | CoA-transferase activity | CG7920 |
| 134 | GO:0007015 | P | 9, | 3 | 0.626 (x 4.792) | 60 (0.050) | 0.157 | actin filament organization | Fs(2)Ket ena hts |
| 135 | GO:0007314 | P | 6, 8, 9, 11, | 3 | 0.584 (x 5.135) | 56 (0.054) | 0.158 | oocyte anterior/posterior axis determination | Hrb27C POSH sqd |
| 136 | GO:0045449 | P | 7, | 15 | 8.670 (x 1.730) | 831 (0.018) | 0.158 | regulation of transcription | CG6654 Dll Dr Sox102F Su(z)2 ap bowl edl eyg hth kis klu kn pdm2 toy |
| 137 | GO:0030730 | P | 5, 7, | 1 | 0.021 (x 47.925) | 2 (0.500) | 0.158 | sequestering of triacylglycerol | CG9057 |
| 138 | GO:0043231 | C | 4, 5, 6, 7, | 33 | 24.069 (x 1.371) | 2307 (0.014) | 0.159 | intracellular membrane-bound organelle | B52 CBP CG11267 CG11500 CG12054 CG17952 CG30059 CG31611 CG5028 CG6654 CG8089 Dll Dr Fs(2)Ket Hrb27C Lam Prx5037 Sox102F Su(z)2 ap bip1 bowl edl eyg garz hth kis klu kn lmg pdm2 sqd toy |
| 139 | GO:0043227 | C | 3, | 33 | 24.090 (x 1.370) | 2309 (0.014) | 0.16 | membrane-bound organelle | B52 CBP CG11267 CG11500 CG12054 CG17952 CG30059 CG31611 CG5028 CG6654 CG8089 Dll Dr Fs(2)Ket Hrb27C Lam Prx5037 Sox102F Su(z)2 ap bip1 bowl edl eyg garz hth kis klu kn lmg pdm2 sqd toy |
| 140 | GO:0005635 | C | 4, 5, 6, 7, 8, 9, 10, | 3 | 0.647 (x 4.638) | 62 (0.048) | 0.167 | nuclear envelope | CG17952 Fs(2)Ket Lam |
| 141 | GO:0035265 | P | 3, | 2 | 0.261 (x 7.668) | 25 (0.080) | 0.171 | organ growth | Pi3K21B Pk61C |
| 142 | GO:0046620 | P | 4, | 2 | 0.261 (x 7.668) | 25 (0.080) | 0.172 | regulation of organ size | Pi3K21B Pk61C |
| 143 | GO:0005652 | C | 5, 6, 7, 8, 9, 10, 11, | 1 | 0.031 (x 31.950) | 3 (0.333) | 0.174 | nuclear lamina | Lam |
| 144 | GO:0035062 | C | 8, 9, 10, 11, 12, 13, 14, 15, | 1 | 0.031 (x 31.950) | 3 (0.333) | 0.175 | omega speckle | sqd |
| 145 | GO:0015380 | F | 6, 7, 8, | 1 | 0.031 (x 31.950) | 3 (0.333) | 0.177 | anion exchanger activity | CG8177 |
| 146 | GO:0035183 | C | 3, 4, 5, 6, | 1 | 0.031 (x 31.950) | 3 (0.333) | 0.178 | ring canal inner rim | hts |
| 147 | GO:0042127 | P | 5, | 2 | 0.282 (x 7.100) | 27 (0.074) | 0.178 | regulation of cell proliferation | Pi3K21B ana |
| 148 | GO:0019915 | P | 6, | 1 | 0.031 (x 31.950) | 3 (0.333) | 0.179 | sequestering of lipid | CG9057 |
| 149 | GO:0000900 | F | 4, 5, | 1 | 0.031 (x 31.950) | 3 (0.333) | 0.18 | translation repressor activity, nucleic acid binding | msi |
| 150 | GO:0007281 | P | 5, | 4 | 1.168 (x 3.423) | 112 (0.036) | 0.18 | germ cell development | Hrb27C POSH hts sqd |
| 151 | GO:0030234 | F | 2, | 8 | 3.725 (x 2.148) | 357 (0.022) | 0.18 | enzyme regulator activity | Abi CG30440 CG7722 CG8066 Pi3K21B Spn43Aa garz plexA |
| 152 | GO:0007423 | P | 4, | 6 | 2.400 (x 2.500) | 230 (0.026) | 0.181 | sensory organ development | POSH edl eyg hth klu toy |
| 153 | GO:0016922 | F | 4, 5, | 1 | 0.031 (x 31.950) | 3 (0.333) | 0.181 | ligand-dependent nuclear receptor binding | CG17735 |
| 154 | GO:0005923 | C | 7, 8, 9, 10, | 1 | 0.031 (x 31.950) | 3 (0.333) | 0.183 | tight junction | CG6982 |
| 155 | GO:0015108 | F | 6, | 1 | 0.031 (x 31.950) | 3 (0.333) | 0.184 | chloride transporter activity | CG8177 |
| 156 | GO:0007167 | P | 6, | 5 | 1.794 (x 2.786) | 172 (0.029) | 0.185 | enzyme linked receptor protein signaling pathway | Pi3K21B Pk61C aru edl plexA |
| 157 | GO:0008449 | F | 6, | 1 | 0.031 (x 31.950) | 3 (0.333) | 0.185 | N-acetylglucosamine-6-sulfatase activity | CG30059 |
| 158 | GO:0008302 | P | 7, 10, | 1 | 0.031 (x 31.950) | 3 (0.333) | 0.186 | ring canal formation, actin assembly | hts |
| 159 | GO:0007456 | P | 6, | 5 | 1.805 (x 2.770) | 173 (0.029) | 0.188 | eye development (sensu Endopterygota) | POSH eyg hth klu toy |
| 160 | GO:0015106 | F | 6, | 1 | 0.031 (x 31.950) | 3 (0.333) | 0.188 | bicarbonate transporter activity | CG8177 |
| 161 | GO:0008471 | F | 6, | 1 | 0.031 (x 31.950) | 3 (0.333) | 0.189 | laccase activity | CG30437 |
| 162 | GO:0044428 | C | 4, 5, 6, 7, 8, 9, | 10 | 5.373 (x 1.861) | 515 (0.019) | 0.193 | nuclear part | B52 CG17952 Fs(2)Ket Hrb27C Lam Su(z)2 ap eyg lmg sqd |
| 163 | GO:0017064 | F | 6, | 1 | 0.042 (x 23.962) | 4 (0.250) | 0.193 | fatty acid amide hydrolase activity | CG7900 |
| 164 | GO:0016879 | F | 4, | 5 | 1.899 (x 2.633) | 182 (0.027) | 0.193 | ligase activity, forming carbon-nitrogen bonds | CG17735 CG6854 POSH Su(z)2 lmg |
| 165 | GO:0009953 | P | 4, | 3 | 0.772 (x 3.886) | 74 (0.041) | 0.194 | dorsal/ventral pattern formation | Dr ap sqd |
| 166 | GO:0009003 | F | 5, | 1 | 0.042 (x 23.962) | 4 (0.250) | 0.194 | signal peptidase activity | CG11500 |
| 167 | GO:0016477 | P | 5, 6, | 5 | 1.826 (x 2.739) | 175 (0.029) | 0.194 | cell migration | ap ena plexA sqd stai |
| 168 | GO:0048135 | P | 6, 7, | 1 | 0.042 (x 23.962) | 4 (0.250) | 0.195 | female germ-line cyst formation | hts |
| 169 | GO:0035282 | P | 3, | 4 | 1.315 (x 3.043) | 126 (0.032) | 0.195 | segmentation | ap bowl kis kn |
| 170 | GO:0006351 | P | 7, | 15 | 9.348 (x 1.605) | 896 (0.017) | 0.196 | transcription, DNA-dependent | CG6654 Dll Dr Sox102F Su(z)2 ap bowl edl eyg hth kis klu kn pdm2 toy |
| 171 | GO:0016529 | C | 6, 7, 8, 9, | 1 | 0.042 (x 23.962) | 4 (0.250) | 0.196 | sarcoplasmic reticulum | CBP |
| 172 | GO:0004867 | F | 6, | 3 | 0.730 (x 4.108) | 70 (0.043) | 0.196 | serine-type endopeptidase inhibitor activity | CG7722 Spn43Aa plexA |
| 173 | GO:0016684 | F | 4, | 2 | 0.334 (x 5.991) | 32 (0.062) | 0.196 | oxidoreductase activity, acting on peroxide as acceptor | CG5873 Prx5037 |
| 174 | GO:0006465 | P | 6, 9, | 1 | 0.042 (x 23.962) | 4 (0.250) | 0.197 | signal peptide processing | CG11500 |
| 175 | GO:0045451 | P | 7, 11, 13, 14, 16, | 2 | 0.334 (x 5.991) | 32 (0.062) | 0.197 | pole plasm oskar mRNA localization | Hrb27C POSH |
| 176 | GO:0048667 | P | 6, 7, 9, | 4 | 1.262 (x 3.169) | 121 (0.033) | 0.198 | neuron morphogenesis during differentiation | ap ena fax plexA |
| 177 | GO:0030371 | F | 3, | 1 | 0.042 (x 23.962) | 4 (0.250) | 0.198 | translation repressor activity | msi |
| 178 | GO:0004601 | F | 3, 5, | 2 | 0.334 (x 5.991) | 32 (0.062) | 0.198 | peroxidase activity | CG5873 Prx5037 |
| 179 | GO:0004857 | F | 3, | 4 | 1.325 (x 3.019) | 127 (0.031) | 0.199 | enzyme inhibitor activity | CG7722 CG8066 Spn43Aa plexA |
| 180 | GO:0048812 | P | 7, 8, 10, | 4 | 1.262 (x 3.169) | 121 (0.033) | 0.199 | neurite morphogenesis | ap ena fax plexA |
| 181 | GO:0051018 | F | 7, | 1 | 0.042 (x 23.962) | 4 (0.250) | 0.199 | protein kinase A binding | Akap200 |
| 182 | GO:0007309 | P | 5, 7, 8, 10, | 3 | 0.793 (x 3.784) | 76 (0.039) | 0.2 | oocyte axis determination | Hrb27C POSH sqd |
| 183 | GO:0046670 | P | 7, 8, 9, | 1 | 0.042 (x 23.962) | 4 (0.250) | 0.2 | positive regulation of retinal programmed cell death | klu |
| 184 | GO:0007409 | P | 8, 9, 11, | 4 | 1.262 (x 3.169) | 121 (0.033) | 0.201 | axonogenesis | ap ena fax plexA |
| 185 | GO:0016528 | C | 5, 6, 7, | 1 | 0.042 (x 23.962) | 4 (0.250) | 0.202 | sarcoplasm | CBP |
| 186 | GO:0009880 | P | 4, | 4 | 1.346 (x 2.972) | 129 (0.031) | 0.202 | embryonic pattern specification | bowl edl kis kn |
| 187 | GO:0005787 | C | 3, 4, 5, 6, 7, 8, 9, 10, 11, | 1 | 0.042 (x 23.962) | 4 (0.250) | 0.203 | signal peptidase complex | CG11500 |
| 188 | GO:0016682 | F | 5, | 1 | 0.042 (x 23.962) | 4 (0.250) | 0.204 | oxidoreductase activity, acting on diphenols and related substances as donors, oxygen as acceptor | CG30437 |
| 189 | GO:0030528 | F | 2, | 14 | 8.399 (x 1.667) | 805 (0.017) | 0.204 | transcription regulator activity | CG6654 CG6854 Dll Dr Sox102F Su(z)2 ap bowl eyg hth klu kn pdm2 toy |
| 190 | GO:0030723 | P | 7, 9, | 1 | 0.042 (x 23.962) | 4 (0.250) | 0.205 | ovarian fusome organization and biogenesis | hts |
| 191 | GO:0031468 | P | 7, 8, | 1 | 0.042 (x 23.962) | 4 (0.250) | 0.206 | nuclear envelope reassembly | Lam |
| 192 | GO:0046672 | P | 8, 9, 10, 11, | 1 | 0.042 (x 23.962) | 4 (0.250) | 0.207 | positive regulation of retinal cell programmed cell death (sensu Endopterygota) | klu |
| 193 | GO:0007405 | P | 5, 7, | 1 | 0.052 (x 19.170) | 5 (0.200) | 0.208 | neuroblast proliferation | ana |
| 194 | GO:0048519 | P | 3, | 7 | 3.182 (x 2.200) | 305 (0.023) | 0.208 | negative regulation of biological process | Pk61C ana charybde edl msi scylla sqd |
| 195 | GO:0006928 | P | 4, 5, | 6 | 2.681 (x 2.238) | 257 (0.023) | 0.208 | cell motility | Abi ap ena plexA sqd stai |
| 196 | GO:0001654 | P | 5, | 5 | 1.888 (x 2.648) | 181 (0.028) | 0.208 | eye development | POSH eyg hth klu toy |
| 197 | GO:0030721 | P | 6, | 1 | 0.042 (x 23.962) | 4 (0.250) | 0.209 | spectrosome organization and biogenesis | hts |
| 198 | GO:0050768 | P | 5, 8, | 1 | 0.052 (x 19.170) | 5 (0.200) | 0.209 | negative regulation of neurogenesis | ana |
| 199 | GO:0045165 | P | 4, | 5 | 1.972 (x 2.536) | 189 (0.026) | 0.209 | cell fate commitment | Dr ap hth hts msi |
| 200 | GO:0044464 | C | 2, 3, | 57 | 48.159 (x 1.184) | 4616 (0.012) | 0.209 | cell part | B52 BEST:CK02137 BcDNA:LD41548 CBP CG10960 CG11267 CG11500 CG12054 CG17735 CG17952 CG30059 CG31363 CG31611 CG3823 CG4759 CG5028 CG6654 CG6982 CG8089 CG8177 CG9057 Dll Dr Fs(2)Ket Hrb27C ImpE2 ImpE3 Lam POSH Pi3K21B Pk61C Prx5037 Sox102F Su(z)2 Tbp-1 Ufd1-like ap aru bip1 bowl edl eyg garz hth hts kis klu kn lmg ogre pdm2 plexA smi35A spdo sqd stai toy |
| 201 | GO:0051674 | P | 4, | 6 | 2.681 (x 2.238) | 257 (0.023) | 0.209 | localization of cell | Abi ap ena plexA sqd stai |
| 202 | GO:0045454 | P | 5, | 1 | 0.052 (x 19.170) | 5 (0.200) | 0.21 | cell redox homeostasis | Prx5037 |
| 203 | GO:0008379 | F | 4, 6, | 1 | 0.042 (x 23.962) | 4 (0.250) | 0.21 | thioredoxin peroxidase activity | Prx5037 |
| 204 | GO:0048732 | P | 4, | 4 | 1.367 (x 2.927) | 131 (0.031) | 0.21 | gland development | Akap200 eyg hth kn |
| 205 | GO:0005623 | C | 2, | 57 | 48.159 (x 1.184) | 4616 (0.012) | 0.21 | cell | B52 BEST:CK02137 BcDNA:LD41548 CBP CG10960 CG11267 CG11500 CG12054 CG17735 CG17952 CG30059 CG31363 CG31611 CG3823 CG4759 CG5028 CG6654 CG6982 CG8089 CG8177 CG9057 Dll Dr Fs(2)Ket Hrb27C ImpE2 ImpE3 Lam POSH Pi3K21B Pk61C Prx5037 Sox102F Su(z)2 Tbp-1 Ufd1-like ap aru bip1 bowl edl eyg garz hth hts kis klu kn lmg ogre pdm2 plexA smi35A spdo sqd stai toy |
| 206 | GO:0016303 | F | 8, | 1 | 0.052 (x 19.170) | 5 (0.200) | 0.211 | phosphatidylinositol 3-kinase activity | Pi3K21B |
| 207 | GO:0006998 | P | 6, 7, | 1 | 0.052 (x 19.170) | 5 (0.200) | 0.212 | nuclear membrane organization and biogenesis | Lam |
| 208 | GO:0007451 | P | 6, 7, | 1 | 0.052 (x 19.170) | 5 (0.200) | 0.213 | dorsal/ventral lineage restriction, imaginal disc | ap |
| 209 | GO:0008361 | P | 5, 6, | 2 | 0.355 (x 5.638) | 34 (0.059) | 0.213 | regulation of cell size | Pi3K21B Pk61C |
| 210 | GO:0050793 | P | 3, | 3 | 0.824 (x 3.640) | 79 (0.038) | 0.213 | regulation of development | Dr ana kn |
| 211 | GO:0000287 | F | 5, | 1 | 0.052 (x 19.170) | 5 (0.200) | 0.214 | magnesium ion binding | RhoGAP71E |
| 212 | GO:0007308 | P | 6, 7, 9, | 3 | 0.824 (x 3.640) | 79 (0.038) | 0.214 | oocyte construction | Hrb27C POSH sqd |
| 213 | GO:0048749 | P | 7, | 4 | 1.388 (x 2.883) | 133 (0.030) | 0.214 | compound eye development (sensu Endopterygota) | POSH eyg hth klu |
| 214 | GO:0042325 | P | 8, | 1 | 0.052 (x 19.170) | 5 (0.200) | 0.215 | regulation of phosphorylation | edl |
| 215 | GO:0007411 | P | 6, 7, 9, 10, 12, | 3 | 0.856 (x 3.507) | 82 (0.037) | 0.215 | axon guidance | ap ena plexA |
| 216 | GO:0001745 | P | 7, 8, | 4 | 1.388 (x 2.883) | 133 (0.030) | 0.215 | compound eye morphogenesis (sensu Endopterygota) | POSH eyg hth klu |
| 217 | GO:0046854 | P | 8, 9, 10, 11, | 1 | 0.052 (x 19.170) | 5 (0.200) | 0.216 | phosphoinositide phosphorylation | Pi3K21B |
| 218 | GO:0016209 | F | 2, | 2 | 0.376 (x 5.325) | 36 (0.056) | 0.216 | antioxidant activity | CG5873 Prx5037 |
| 219 | GO:0048599 | P | 5, 6, 8, | 3 | 0.835 (x 3.594) | 80 (0.037) | 0.217 | oocyte development | Hrb27C POSH sqd |
| 220 | GO:0005942 | C | 3, 6, 7, 8, 9, 10, | 1 | 0.052 (x 19.170) | 5 (0.200) | 0.217 | phosphoinositide 3-kinase complex | Pi3K21B |
| 221 | GO:0019538 | P | 5, | 30 | 22.775 (x 1.317) | 2183 (0.014) | 0.217 | protein metabolism | B52 BcDNA:LD41548 CG11267 CG11500 CG17735 CG31611 CG4759 CG7182 CG7722 Fkbp13 Hop Hsp23 Hsp26 Hsp27 Hsp67Ba Hsp70Bc POSH Pi3K21B Pk61C Su(z)2 Tbp-1 Ufd1-like aru ena hts lmg msi plexA smi35A sqd |
| 222 | GO:0040011 | P | 3, | 6 | 2.723 (x 2.203) | 261 (0.023) | 0.217 | locomotion | Abi ap ena plexA sqd stai |
| 223 | GO:0005527 | F | 4, | 1 | 0.063 (x 15.975) | 6 (0.167) | 0.223 | macrolide binding | Fkbp13 |
| 224 | GO:0019787 | F | 6, | 4 | 1.450 (x 2.758) | 139 (0.029) | 0.224 | small conjugating protein ligase activity | CG17735 POSH Su(z)2 lmg |
| 225 | GO:0048190 | P | 6, 7, | 1 | 0.063 (x 15.975) | 6 (0.167) | 0.224 | wing disc dorsal/ventral pattern formation | ap |
| 226 | GO:0004842 | F | 7, | 4 | 1.450 (x 2.758) | 139 (0.029) | 0.225 | ubiquitin-protein ligase activity | CG17735 POSH Su(z)2 lmg |
| 227 | GO:0005528 | F | 5, | 1 | 0.063 (x 15.975) | 6 (0.167) | 0.225 | FK506 binding | Fkbp13 |
| 228 | GO:0042026 | P | 8, | 1 | 0.063 (x 15.975) | 6 (0.167) | 0.226 | protein refolding | Hsp27 |
| 229 | GO:0007173 | P | 8, | 2 | 0.407 (x 4.915) | 39 (0.051) | 0.227 | epidermal growth factor receptor signaling pathway | aru edl |
| 230 | GO:0004449 | F | 7, | 1 | 0.063 (x 15.975) | 6 (0.167) | 0.227 | isocitrate dehydrogenase (NAD+) activity | CG5028 |
| 231 | GO:0005725 | C | 8, 9, 10, 11, 12, 13, 14, | 1 | 0.063 (x 15.975) | 6 (0.167) | 0.228 | intercalary heterochromatin | Su(z)2 |
| 232 | GO:0045170 | C | 5, 6, 7, 8, | 1 | 0.063 (x 15.975) | 6 (0.167) | 0.229 | spectrosome | hts |
| 233 | GO:0003702 | F | 3, | 6 | 2.775 (x 2.162) | 266 (0.023) | 0.23 | RNA polymerase II transcription factor activity | Dll ap bowl kn pdm2 toy |
| 234 | GO:0008360 | P | 5, 6, | 3 | 0.887 (x 3.383) | 85 (0.035) | 0.23 | regulation of cell shape | Abi Fs(2)Ket ena |
| 235 | GO:0008046 | F | 5, | 1 | 0.063 (x 15.975) | 6 (0.167) | 0.23 | axon guidance receptor activity | plexA |
| 236 | GO:0012505 | C | 4, 5, | 4 | 1.502 (x 2.662) | 144 (0.028) | 0.23 | endomembrane system | CG11500 CG17952 Fs(2)Ket Lam |
| 237 | GO:0048748 | P | 6, 7, | 4 | 1.471 (x 2.719) | 141 (0.028) | 0.231 | eye morphogenesis (sensu Endopterygota) | POSH eyg hth klu |
| 238 | GO:0004040 | F | 6, | 1 | 0.063 (x 15.975) | 6 (0.167) | 0.231 | amidase activity | CG7900 |
| 239 | GO:0006350 | P | 6, | 15 | 9.859 (x 1.521) | 945 (0.016) | 0.231 | transcription | CG6654 Dll Dr Sox102F Su(z)2 ap bowl edl eyg hth kis klu kn pdm2 toy |
| 240 | GO:0007487 | P | 6, | 1 | 0.063 (x 15.975) | 6 (0.167) | 0.232 | analia development (sensu Endopterygota) | Dll |
| 241 | GO:0046834 | P | 7, 8, | 1 | 0.063 (x 15.975) | 6 (0.167) | 0.233 | lipid phosphorylation | Pi3K21B |
| 242 | GO:0048666 | P | 5, 8, | 4 | 1.481 (x 2.700) | 142 (0.028) | 0.233 | neuron development | ap ena fax plexA |
| 243 | GO:0031175 | P | 6, 9, | 4 | 1.481 (x 2.700) | 142 (0.028) | 0.234 | neurite development | ap ena fax plexA |
| 244 | GO:0007455 | P | 6, 7, | 4 | 1.523 (x 2.626) | 146 (0.027) | 0.238 | eye-antennal disc morphogenesis | POSH eyg hth klu |
| 245 | GO:0045595 | P | 4, | 2 | 0.438 (x 4.564) | 42 (0.048) | 0.238 | regulation of cell differentiation | Dr kn |
| 246 | GO:0004448 | F | 6, | 1 | 0.073 (x 13.693) | 7 (0.143) | 0.239 | isocitrate dehydrogenase activity | CG5028 |
| 247 | GO:0006996 | P | 5, | 12 | 7.533 (x 1.593) | 722 (0.017) | 0.239 | organelle organization and biogenesis | Abi CG31611 CG8177 CG9057 Fs(2)Ket Lam POSH Pk61C ena hts kis stai |
| 248 | GO:0030145 | F | 6, | 1 | 0.073 (x 13.693) | 7 (0.143) | 0.24 | manganese ion binding | BcDNA:LD41548 |
| 249 | GO:0048806 | P | 4, | 1 | 0.073 (x 13.693) | 7 (0.143) | 0.241 | genitalia development | Dll |
| 250 | GO:0005385 | F | 6, | 1 | 0.073 (x 13.693) | 7 (0.143) | 0.242 | zinc ion transporter activity | BEST:CK02137 |
| 251 | GO:0016348 | P | 7, 8, 9, | 1 | 0.073 (x 13.693) | 7 (0.143) | 0.243 | leg joint morphogenesis (sensu Endopterygota) | bowl |
| 252 | GO:0006403 | P | 4, | 3 | 0.970 (x 3.092) | 93 (0.032) | 0.244 | RNA localization | Hrb27C POSH sqd |
| 253 | GO:0046669 | P | 7, 8, 9, 10, | 1 | 0.073 (x 13.693) | 7 (0.143) | 0.244 | regulation of retinal cell programmed cell death (sensu Endopterygota) | klu |
| 254 | GO:0030258 | P | 6, 7, | 1 | 0.073 (x 13.693) | 7 (0.143) | 0.245 | lipid modification | Pi3K21B |
| 255 | GO:0051248 | P | 6, 7, | 2 | 0.449 (x 4.458) | 43 (0.047) | 0.246 | negative regulation of protein metabolism | msi sqd |
| 256 | GO:0007484 | P | 5, 6, | 1 | 0.073 (x 13.693) | 7 (0.143) | 0.246 | genitalia development (sensu Endopterygota) | Dll |
| 257 | GO:0044427 | C | 4, 5, 6, 7, 8, 9, | 4 | 1.544 (x 2.591) | 148 (0.027) | 0.246 | chromosomal part | CG31611 Su(z)2 kis sqd |
| 258 | GO:0009993 | P | 7, | 6 | 2.890 (x 2.076) | 277 (0.022) | 0.247 | oogenesis (sensu Insecta) | Fs(2)Ket Hrb27C POSH Pk61C hts sqd |
| 259 | GO:0035171 | P | 6, 7, 8, 9, | 1 | 0.073 (x 13.693) | 7 (0.143) | 0.247 | lamellocyte differentiation | kn |
| 260 | GO:0016043 | P | 4, | 20 | 14.387 (x 1.390) | 1379 (0.015) | 0.247 | cell organization and biogenesis | Abi CG31611 CG6982 CG8177 CG9057 Fs(2)Ket Lam POSH Pi3K21B Pk61C ap edl ena fax garz hts kis plexA sqd stai |
| 261 | GO:0035288 | P | 6, 7, | 1 | 0.073 (x 13.693) | 7 (0.143) | 0.248 | anterior head segmentation | kn |
| 262 | GO:0048592 | P | 5, 6, | 4 | 1.555 (x 2.573) | 149 (0.027) | 0.248 | eye morphogenesis | POSH eyg hth klu |
| 263 | GO:0030865 | P | 7, | 1 | 0.073 (x 13.693) | 7 (0.143) | 0.249 | cortical cytoskeleton organization and biogenesis | Abi |
| 264 | GO:0030182 | P | 4, 7, | 4 | 1.555 (x 2.573) | 149 (0.027) | 0.249 | neuron differentiation | ap ena fax plexA |
| 265 | GO:0030866 | P | 8, 9, | 1 | 0.073 (x 13.693) | 7 (0.143) | 0.25 | cortical actin cytoskeleton organization and biogenesis | Abi |
| 266 | GO:0005911 | C | 6, 7, 8, | 2 | 0.459 (x 4.357) | 44 (0.045) | 0.254 | intercellular junction | CG6982 ogre |
| 267 | GO:0048518 | P | 3, | 5 | 2.274 (x 2.198) | 218 (0.023) | 0.254 | positive regulation of biological process | Abi Pi3K21B Pk61C klu smi35A |
| 268 | GO:0015301 | F | 5, 7, | 1 | 0.083 (x 11.981) | 8 (0.125) | 0.254 | anion:anion antiporter activity | CG8177 |
| 269 | GO:0048477 | P | 6, | 6 | 3.015 (x 1.990) | 289 (0.021) | 0.255 | oogenesis | Fs(2)Ket Hrb27C POSH Pk61C hts sqd |
| 270 | GO:0000184 | P | 9, | 1 | 0.083 (x 11.981) | 8 (0.125) | 0.255 | mRNA catabolism, nonsense-mediated decay | sqd |
| 271 | GO:0006518 | P | 5, | 1 | 0.083 (x 11.981) | 8 (0.125) | 0.256 | peptide metabolism | CG11500 |
| 272 | GO:0005243 | F | 5, | 1 | 0.083 (x 11.981) | 8 (0.125) | 0.257 | gap-junction forming channel activity | ogre |
| 273 | GO:0006376 | P | 8, 11, 13, | 1 | 0.083 (x 11.981) | 8 (0.125) | 0.258 | mRNA splice site selection | B52 |
| 274 | GO:0046530 | P | 4, | 3 | 1.022 (x 2.934) | 98 (0.031) | 0.259 | photoreceptor cell differentiation | POSH edl hth |
| 275 | GO:0016881 | F | 5, | 4 | 1.648 (x 2.427) | 158 (0.025) | 0.259 | acid-amino acid ligase activity | CG17735 POSH Su(z)2 lmg |
| 276 | GO:0035004 | F | 7, | 1 | 0.083 (x 11.981) | 8 (0.125) | 0.259 | phosphoinositide 3-kinase activity | Pi3K21B |
| 277 | GO:0005720 | C | 7, 8, 9, 10, 11, 12, 13, | 1 | 0.083 (x 11.981) | 8 (0.125) | 0.26 | nuclear heterochromatin | Su(z)2 |
| 278 | GO:0005703 | C | 5, 6, 7, 8, 9, 10, | 1 | 0.094 (x 10.650) | 9 (0.111) | 0.26 | polytene chromosome puff | sqd |
| 279 | GO:0005921 | C | 7, 8, 9, | 1 | 0.083 (x 11.981) | 8 (0.125) | 0.261 | gap junction | ogre |
| 280 | GO:0035161 | P | 5, 6, | 1 | 0.094 (x 10.650) | 9 (0.111) | 0.261 | imaginal disc lineage restriction | ap |
| 281 | GO:0019898 | C | 4, 5, 6, | 2 | 0.469 (x 4.260) | 45 (0.044) | 0.262 | extrinsic to membrane | ImpE2 ImpE3 |
| 282 | GO:0005680 | C | 5, 6, 7, 8, 9, 10, 11, | 1 | 0.083 (x 11.981) | 8 (0.125) | 0.262 | anaphase-promoting complex | lmg |
| 283 | GO:0007430 | P | 5, 6, | 1 | 0.094 (x 10.650) | 9 (0.111) | 0.262 | terminal branching of trachea, cytoplasmic projection extension (sensu Insecta) | Lam |
| 284 | GO:0005654 | C | 5, 6, 7, 8, 9, 10, 11, | 5 | 2.337 (x 2.139) | 224 (0.022) | 0.262 | nucleoplasm | B52 Hrb27C ap eyg sqd |
| 285 | GO:0003676 | F | 3, | 24 | 18.185 (x 1.320) | 1743 (0.014) | 0.263 | nucleic acid binding | B52 CG12054 CG31611 CG4759 CG6654 CG6854 CG8089 Dll Dr Hrb27C Sox102F Su(z)2 ap bowl edl eyg hth kis klu kn msi pdm2 sqd toy |
| 286 | GO:0046914 | F | 5, | 11 | 7.053 (x 1.560) | 676 (0.016) | 0.263 | transition metal ion binding | BcDNA:LD41548 CG12054 CG30437 CG6654 CG8089 POSH Su(z)2 ap bowl klu lmg |
| 287 | GO:0046879 | P | 5, 6, | 1 | 0.083 (x 11.981) | 8 (0.125) | 0.263 | hormone secretion | stai |
| 288 | GO:0007476 | P | 6, 7, 8, | 3 | 1.075 (x 2.792) | 103 (0.029) | 0.263 | wing morphogenesis | Dr ap kn |
| 289 | GO:0006402 | P | 8, | 1 | 0.094 (x 10.650) | 9 (0.111) | 0.263 | mRNA catabolism | sqd |
| 290 | GO:0009966 | P | 4, 5, | 3 | 1.075 (x 2.792) | 103 (0.029) | 0.264 | regulation of signal transduction | Akap200 edl klu |
| 291 | GO:0003730 | F | 6, | 1 | 0.094 (x 10.650) | 9 (0.111) | 0.264 | mRNA 3'-UTR binding | sqd |
| 292 | GO:0005515 | F | 3, | 18 | 12.874 (x 1.398) | 1234 (0.015) | 0.264 | protein binding | Abi Akap200 B52 CG11267 CG17735 CG17952 CG9057 Fs(2)Ket Hop Hsp23 Lam Pi3K21B bip1 edl ena hts spdo stai |
| 293 | GO:0000381 | P | 10, 11, 13, | 2 | 0.511 (x 3.912) | 49 (0.041) | 0.264 | regulation of alternative nuclear mRNA splicing, via spliceosome | B52 sqd |
| 294 | GO:0007380 | P | 5, 6, 7, | 1 | 0.094 (x 10.650) | 9 (0.111) | 0.265 | specification of segmental identity, head | kn |
| 295 | GO:0000380 | P | 10, 12, | 2 | 0.511 (x 3.912) | 49 (0.041) | 0.265 | alternative nuclear mRNA splicing, via spliceosome | B52 sqd |
| 296 | GO:0045478 | P | 6, | 1 | 0.094 (x 10.650) | 9 (0.111) | 0.265 | fusome organization and biogenesis | hts |
| 297 | GO:0007420 | P | 4, 6, | 2 | 0.511 (x 3.912) | 49 (0.041) | 0.266 | brain development | Dr hth |
| 298 | GO:0008069 | P | 6, 7, 9, | 1 | 0.094 (x 10.650) | 9 (0.111) | 0.266 | dorsal/ventral axis determination, follicular epithelium (sensu Insecta) | sqd |
| 299 | GO:0006073 | P | 7, 8, | 1 | 0.094 (x 10.650) | 9 (0.111) | 0.267 | glucan metabolism | Pk61C |
| 300 | GO:0051243 | P | 5, | 5 | 2.420 (x 2.066) | 232 (0.022) | 0.267 | negative regulation of cellular physiological process | Pk61C ana edl msi sqd |
| 301 | GO:0007632 | P | 4, 6, | 1 | 0.094 (x 10.650) | 9 (0.111) | 0.268 | visual behavior | ogre |
| 302 | GO:0050896 | P | 2, | 17 | 12.196 (x 1.394) | 1169 (0.015) | 0.268 | response to stimulus | CG17121 CG30437 CG5873 CG7182 CG8588 Hop Hsp23 Hsp26 Hsp27 Hsp67Ba Hsp70Bc Obp99a Prx5037 Ugt86Di kn ogre smi35A |
| 303 | GO:0008484 | F | 5, | 1 | 0.094 (x 10.650) | 9 (0.111) | 0.269 | sulfuric ester hydrolase activity | CG30059 |
| 304 | GO:0007548 | P | 3, | 2 | 0.522 (x 3.834) | 50 (0.040) | 0.269 | sex differentiation | Dll Pk61C |
| 305 | GO:0007282 | P | 6, | 1 | 0.094 (x 10.650) | 9 (0.111) | 0.27 | cystoblast division | hts |
| 306 | GO:0007472 | P | 6, 7, | 3 | 1.095 (x 2.739) | 105 (0.029) | 0.27 | wing disc morphogenesis | Dr ap kn |
| 307 | GO:0006221 | P | 7, 8, | 1 | 0.094 (x 10.650) | 9 (0.111) | 0.271 | pyrimidine nucleotide biosynthesis | CG6854 |
| 308 | GO:0006512 | P | 8, | 5 | 2.441 (x 2.048) | 234 (0.021) | 0.271 | ubiquitin cycle | CG17735 POSH Su(z)2 Ufd1-like lmg |
| 309 | GO:0005112 | F | 4, 5, | 1 | 0.094 (x 10.650) | 9 (0.111) | 0.272 | Notch binding | spdo |
| 310 | GO:0030725 | P | 5, | 1 | 0.104 (x 9.585) | 10 (0.100) | 0.272 | ring canal formation | hts |
| 311 | GO:0000090 | P | 7, 8, | 1 | 0.094 (x 10.650) | 9 (0.111) | 0.273 | mitotic anaphase | lmg |
| 312 | GO:0035168 | P | 5, 6, 7, 8, | 1 | 0.104 (x 9.585) | 10 (0.100) | 0.273 | lymph gland hemocyte differentiation (sensu Arthropoda) | kn |
| 313 | GO:0008431 | F | 4, | 1 | 0.094 (x 10.650) | 9 (0.111) | 0.274 | vitamin E binding | CG3823 |
| 314 | GO:0019901 | F | 6, | 1 | 0.104 (x 9.585) | 10 (0.100) | 0.274 | protein kinase binding | Akap200 |
| 315 | GO:0005977 | P | 8, 9, | 1 | 0.094 (x 10.650) | 9 (0.111) | 0.275 | glycogen metabolism | Pk61C |
| 316 | GO:0006084 | P | 7, | 2 | 0.501 (x 3.994) | 48 (0.042) | 0.275 | acetyl-CoA metabolism | CG5028 CG7920 |
| 317 | GO:0006220 | P | 7, | 1 | 0.104 (x 9.585) | 10 (0.100) | 0.275 | pyrimidine nucleotide metabolism | CG6854 |
| 318 | GO:0004869 | F | 6, | 1 | 0.094 (x 10.650) | 9 (0.111) | 0.276 | cysteine protease inhibitor activity | CG8066 |
| 319 | GO:0004178 | F | 7, | 1 | 0.104 (x 9.585) | 10 (0.100) | 0.276 | leucyl aminopeptidase activity | BcDNA:LD41548 |
| 320 | GO:0007301 | P | 6, 9, | 1 | 0.094 (x 10.650) | 9 (0.111) | 0.277 | ovarian ring canal formation | hts |
| 321 | GO:0043063 | P | 4, | 1 | 0.104 (x 9.585) | 10 (0.100) | 0.277 | intercellular bridge organization and biogenesis | hts |
| 322 | GO:0007517 | P | 4, | 3 | 1.127 (x 2.662) | 108 (0.028) | 0.277 | muscle development | Dr ap toy |
| 323 | GO:0019899 | F | 4, | 2 | 0.543 (x 3.687) | 52 (0.038) | 0.278 | enzyme binding | Akap200 Pi3K21B |
| 324 | GO:0046872 | F | 4, | 14 | 9.724 (x 1.440) | 932 (0.015) | 0.278 | metal ion binding | BcDNA:LD41548 CBP CG12054 CG30437 CG6654 CG8089 Fkbp13 POSH RhoGAP71E Su(z)2 ap bowl klu lmg |
| 325 | GO:0048569 | P | 4, | 1 | 0.115 (x 8.714) | 11 (0.091) | 0.278 | post-embryonic organ development | kn |
| 326 | GO:0042461 | P | 5, 6, 7, | 2 | 0.543 (x 3.687) | 52 (0.038) | 0.279 | photoreceptor cell development | POSH edl |
| 327 | GO:0043167 | F | 3, | 14 | 9.724 (x 1.440) | 932 (0.015) | 0.279 | ion binding | BcDNA:LD41548 CBP CG12054 CG30437 CG6654 CG8089 Fkbp13 POSH RhoGAP71E Su(z)2 ap bowl klu lmg |
| 328 | GO:0050684 | P | 8, 9, | 2 | 0.553 (x 3.617) | 53 (0.038) | 0.279 | regulation of mRNA processing | B52 sqd |
| 329 | GO:0006458 | P | 8, | 1 | 0.115 (x 8.714) | 11 (0.091) | 0.279 | 'de novo' protein folding | CG11267 |
| 330 | GO:0008356 | P | 5, | 2 | 0.553 (x 3.617) | 53 (0.038) | 0.28 | asymmetric cell division | hts spdo |
| 331 | GO:0046668 | P | 6, 7, 8, | 1 | 0.115 (x 8.714) | 11 (0.091) | 0.28 | regulation of retinal programmed cell death | klu |
| 332 | GO:0035272 | P | 4, | 3 | 1.137 (x 2.638) | 109 (0.028) | 0.281 | exocrine system development | Akap200 eyg hth |
| 333 | GO:0048024 | P | 9, 10, 12, | 2 | 0.553 (x 3.617) | 53 (0.038) | 0.281 | regulation of nuclear mRNA splicing, via spliceosome | B52 sqd |
| 334 | GO:0001727 | F | 6, | 1 | 0.115 (x 8.714) | 11 (0.091) | 0.281 | lipid kinase activity | Pi3K21B |
| 335 | GO:0007292 | P | 5, | 6 | 3.234 (x 1.855) | 310 (0.019) | 0.281 | female gamete generation | Fs(2)Ket Hrb27C POSH Pk61C hts sqd |
| 336 | GO:0007431 | P | 5, | 3 | 1.137 (x 2.638) | 109 (0.028) | 0.281 | salivary gland development | Akap200 eyg hth |
| 337 | GO:0035111 | P | 6, 7, 8, | 1 | 0.115 (x 8.714) | 11 (0.091) | 0.282 | leg joint morphogenesis | bowl |
| 338 | GO:0043118 | P | 4, | 5 | 2.514 (x 1.989) | 241 (0.021) | 0.282 | negative regulation of physiological process | Pk61C ana edl msi sqd |
| 339 | GO:0001709 | P | 5, | 3 | 1.148 (x 2.614) | 110 (0.027) | 0.282 | cell fate determination | Dr hts msi |
| 340 | GO:0008540 | C | 3, 4, 5, 6, 7, 8, | 1 | 0.115 (x 8.714) | 11 (0.091) | 0.282 | proteasome regulatory particle, base subcomplex (sensu Eukaryota) | Tbp-1 |
| 341 | GO:0035167 | P | 5, 6, 7, | 1 | 0.115 (x 8.714) | 11 (0.091) | 0.283 | lymph gland hemopoiesis (sensu Arthropoda) | kn |
| 342 | GO:0035166 | P | 4, 5, 6, | 1 | 0.115 (x 8.714) | 11 (0.091) | 0.284 | post-embryonic hemopoiesis | kn |
| 343 | GO:0000502 | C | 3, 4, 5, 6, | 2 | 0.563 (x 3.550) | 54 (0.037) | 0.284 | proteasome complex (sensu Eukaryota) | Tbp-1 Ufd1-like |
| 344 | GO:0051252 | P | 7, | 2 | 0.563 (x 3.550) | 54 (0.037) | 0.285 | regulation of RNA metabolism | B52 sqd |
| 345 | GO:0016567 | P | 9, | 3 | 1.179 (x 2.545) | 113 (0.027) | 0.29 | protein ubiquitination | POSH Su(z)2 lmg |
| 346 | GO:0051322 | P | 6, | 1 | 0.125 (x 7.987) | 12 (0.083) | 0.296 | anaphase | lmg |
| 347 | GO:0046667 | P | 7, 8, 9, | 1 | 0.125 (x 7.987) | 12 (0.083) | 0.297 | retinal cell programmed cell death (sensu Endopterygota) | klu |
| 348 | GO:0030307 | P | 5, 6, 7, 8, 9, | 1 | 0.125 (x 7.987) | 12 (0.083) | 0.298 | positive regulation of cell growth | Pk61C |
| 349 | GO:0019236 | P | 5, | 1 | 0.125 (x 7.987) | 12 (0.083) | 0.299 | response to pheromone | Obp99a |
| 350 | GO:0008045 | P | 7, 8, 10, 11, 13, | 1 | 0.125 (x 7.987) | 12 (0.083) | 0.299 | motor axon guidance | plexA |
| 351 | GO:0007264 | P | 6, | 3 | 1.210 (x 2.479) | 116 (0.026) | 0.3 | small GTPase mediated signal transduction | Akap200 edl klu |
| 352 | GO:0006968 | P | 5, 6, | 1 | 0.125 (x 7.987) | 12 (0.083) | 0.3 | cellular defense response | kn |
| 353 | GO:0016265 | P | 3, | 5 | 2.619 (x 1.909) | 251 (0.020) | 0.301 | death | Akap200 Pk61C ap klu smi35A |
| 354 | GO:0035220 | P | 5, | 3 | 1.210 (x 2.479) | 116 (0.026) | 0.301 | wing disc development | Dr ap kn |
| 355 | GO:0005085 | F | 4, | 2 | 0.605 (x 3.305) | 58 (0.034) | 0.303 | guanyl-nucleotide exchange factor activity | CG30440 garz |
| 356 | GO:0043067 | P | 5, 6, | 3 | 1.242 (x 2.416) | 119 (0.025) | 0.304 | regulation of programmed cell death | Pk61C klu smi35A |
| 357 | GO:0009948 | P | 5, | 3 | 1.242 (x 2.416) | 119 (0.025) | 0.305 | anterior/posterior axis specification | Hrb27C POSH sqd |
| 358 | GO:0030832 | P | 9, | 1 | 0.136 (x 7.373) | 13 (0.077) | 0.306 | regulation of actin filament length | ena |
| 359 | GO:0048134 | P | 6, | 1 | 0.136 (x 7.373) | 13 (0.077) | 0.307 | germ-line cyst formation | hts |
| 360 | GO:0045169 | C | 5, 6, 7, 8, | 1 | 0.136 (x 7.373) | 13 (0.077) | 0.308 | fusome | hts |
| 361 | GO:0016679 | F | 4, | 1 | 0.136 (x 7.373) | 13 (0.077) | 0.308 | oxidoreductase activity, acting on diphenols and related substances as donors | CG30437 |
| 362 | GO:0006891 | P | 6, 7, 8, 9, | 1 | 0.136 (x 7.373) | 13 (0.077) | 0.309 | intra-Golgi vesicle-mediated transport | garz |
| 363 | GO:0005694 | C | 5, 6, 7, 8, | 4 | 1.920 (x 2.084) | 184 (0.022) | 0.31 | chromosome | CG31611 Su(z)2 kis sqd |
| 364 | GO:0019220 | P | 7, | 1 | 0.136 (x 7.373) | 13 (0.077) | 0.31 | regulation of phosphate metabolism | edl |
| 365 | GO:0008103 | P | 6, 8, 9, 10, 11, | 1 | 0.136 (x 7.373) | 13 (0.077) | 0.311 | oocyte microtubule cytoskeleton polarization | POSH |
| 366 | GO:0043169 | F | 4, | 13 | 9.254 (x 1.405) | 887 (0.015) | 0.311 | cation binding | BcDNA:LD41548 CBP CG12054 CG30437 CG6654 CG8089 Fkbp13 POSH Su(z)2 ap bowl klu lmg |
| 367 | GO:0000792 | C | 6, 7, 8, 9, 10, 11, | 1 | 0.136 (x 7.373) | 13 (0.077) | 0.312 | heterochromatin | Su(z)2 |
| 368 | GO:0051174 | P | 6, | 1 | 0.136 (x 7.373) | 13 (0.077) | 0.313 | regulation of phosphorus metabolism | edl |
| 369 | GO:0008064 | P | 6, 7, 10, | 1 | 0.136 (x 7.373) | 13 (0.077) | 0.314 | regulation of actin polymerization and/or depolymerization | ena |
| 370 | GO:0007474 | P | 7, 8, 9, | 1 | 0.146 (x 6.846) | 14 (0.071) | 0.32 | wing vein specification | kn |
| 371 | GO:0003779 | F | 5, | 3 | 1.283 (x 2.338) | 123 (0.024) | 0.32 | actin binding | Hsp23 ena hts |
| 372 | GO:0006112 | P | 7, | 1 | 0.146 (x 6.846) | 14 (0.071) | 0.321 | energy reserve metabolism | Pk61C |
| 373 | GO:0006997 | P | 6, | 1 | 0.146 (x 6.846) | 14 (0.071) | 0.322 | nuclear organization and biogenesis | Lam |
| 374 | GO:0000245 | P | 7, 10, 12, | 1 | 0.146 (x 6.846) | 14 (0.071) | 0.323 | spliceosome assembly | B52 |
| 375 | GO:0007610 | P | 3, | 4 | 1.993 (x 2.007) | 191 (0.021) | 0.323 | behavior | CG8588 Obp99a ogre smi35A |
| 376 | GO:0042052 | P | 8, 9, 10, 11, | 1 | 0.146 (x 6.846) | 14 (0.071) | 0.323 | rhabdomere development | POSH |
| 377 | GO:0051082 | F | 4, | 2 | 0.647 (x 3.092) | 62 (0.032) | 0.324 | unfolded protein binding | CG11267 Hop |
| 378 | GO:0042221 | P | 4, | 5 | 2.744 (x 1.822) | 263 (0.019) | 0.325 | response to chemical stimulus | CG30437 CG8588 Obp99a Ugt86Di smi35A |
| 379 | GO:0000786 | C | 3, 5, 6, 7, 8, 9, 10, 11, | 1 | 0.156 (x 6.390) | 15 (0.067) | 0.33 | nucleosome | CG31611 |
| 380 | GO:0005355 | F | 7, | 1 | 0.156 (x 6.390) | 15 (0.067) | 0.331 | glucose transporter activity | CG10960 |
| 381 | GO:0035215 | P | 5, | 1 | 0.156 (x 6.390) | 15 (0.067) | 0.332 | genital disc development | Dll |
| 382 | GO:0007435 | P | 6, | 1 | 0.156 (x 6.390) | 15 (0.067) | 0.333 | salivary gland morphogenesis | eyg |
| 383 | GO:0035289 | P | 6, 7, | 1 | 0.156 (x 6.390) | 15 (0.067) | 0.334 | posterior head segmentation | kn |
| 384 | GO:0000151 | C | 3, 4, 5, 6, | 3 | 1.315 (x 2.282) | 126 (0.024) | 0.334 | ubiquitin ligase complex | POSH Su(z)2 lmg |
| 385 | GO:0008285 | P | 6, | 1 | 0.156 (x 6.390) | 15 (0.067) | 0.335 | negative regulation of cell proliferation | ana |
| 386 | GO:0007432 | P | 6, | 1 | 0.156 (x 6.390) | 15 (0.067) | 0.336 | salivary gland determination | hth |
| 387 | GO:0006888 | P | 6, 7, 8, 9, | 1 | 0.156 (x 6.390) | 15 (0.067) | 0.337 | ER to Golgi vesicle-mediated transport | garz |
| 388 | GO:0007492 | P | 4, | 1 | 0.167 (x 5.991) | 16 (0.062) | 0.337 | endoderm development | toy |
| 389 | GO:0045171 | C | 3, 4, | 1 | 0.167 (x 5.991) | 16 (0.062) | 0.338 | intercellular bridge | hts |
| 390 | GO:0045172 | C | 4, 5, | 1 | 0.167 (x 5.991) | 16 (0.062) | 0.339 | ring canal (sensu Insecta) | hts |
| 391 | GO:0051347 | P | 5, | 1 | 0.167 (x 5.991) | 16 (0.062) | 0.339 | positive regulation of transferase activity | Abi |
| 392 | GO:0051169 | P | 6, 7, 8, | 2 | 0.689 (x 2.905) | 66 (0.030) | 0.34 | nuclear transport | Fs(2)Ket sqd |
| 393 | GO:0046666 | P | 6, 7, | 1 | 0.167 (x 5.991) | 16 (0.062) | 0.34 | retinal cell programmed cell death | klu |
| 394 | GO:0019725 | P | 4, | 2 | 0.689 (x 2.905) | 66 (0.030) | 0.34 | cell homeostasis | CG8177 Prx5037 |
| 395 | GO:0045860 | P | 6, 7, | 1 | 0.167 (x 5.991) | 16 (0.062) | 0.341 | positive regulation of protein kinase activity | Abi |
| 396 | GO:0006397 | P | 8, | 4 | 2.066 (x 1.936) | 198 (0.020) | 0.341 | mRNA processing | B52 Hrb27C msi sqd |
| 397 | GO:0030054 | C | 5, 6, 7, | 2 | 0.689 (x 2.905) | 66 (0.030) | 0.341 | cell junction | CG6982 ogre |
| 398 | GO:0007097 | P | 7, 8, | 1 | 0.167 (x 5.991) | 16 (0.062) | 0.342 | nuclear migration | Lam |
| 399 | GO:0000152 | C | 4, 5, 6, 7, 8, 9, 10, | 1 | 0.167 (x 5.991) | 16 (0.062) | 0.343 | nuclear ubiquitin ligase complex | lmg |
| 400 | GO:0042058 | P | 5, 6, 9, | 1 | 0.167 (x 5.991) | 16 (0.062) | 0.344 | regulation of epidermal growth factor receptor signaling pathway | edl |
| 401 | GO:0009952 | P | 4, | 3 | 1.356 (x 2.212) | 130 (0.023) | 0.344 | anterior/posterior pattern formation | Hrb27C POSH sqd |
| 402 | GO:0006800 | P | 5, | 2 | 0.699 (x 2.861) | 67 (0.030) | 0.344 | oxygen and reactive oxygen species metabolism | CG5873 Prx5037 |
| 403 | GO:0030695 | F | 3, | 3 | 1.356 (x 2.212) | 130 (0.023) | 0.345 | GTPase regulator activity | Abi CG30440 garz |
| 404 | GO:0031981 | C | 4, 5, 6, 7, 8, 9, 10, | 5 | 2.869 (x 1.743) | 275 (0.018) | 0.346 | nuclear lumen | B52 Hrb27C ap eyg sqd |
| 405 | GO:0051028 | P | 7, 8, 9, | 1 | 0.177 (x 5.638) | 17 (0.059) | 0.346 | mRNA transport | sqd |
| 406 | GO:0008152 | P | 3, | 58 | 52.760 (x 1.099) | 5057 (0.011) | 0.346 | metabolism | Abi B52 BcDNA:LD41548 CG10960 CG11267 CG11500 CG17121 CG17735 CG30059 CG31611 CG4759 CG5028 CG5873 CG6654 CG6854 CG7182 CG7722 CG7900 CG7920 CG9057 Dll Dr Fkbp13 Hop Hrb27C Hsp23 Hsp26 Hsp27 Hsp67Ba Hsp70Bc POSH Pi3K21B Pk61C Prx5037 RhoGAP71E Sox102F Su(z)2 Tbp-1 Ufd1-like Ugt86Di ap aru bowl edl ena eyg hth hts kis klu kn lmg msi pdm2 plexA smi35A sqd toy |
| 407 | GO:0000059 | P | 8, 9, 10, 11, | 1 | 0.177 (x 5.638) | 17 (0.059) | 0.347 | protein import into nucleus, docking | Fs(2)Ket |
| 408 | GO:0006406 | P | 8, 9, 10, 11, | 1 | 0.177 (x 5.638) | 17 (0.059) | 0.347 | mRNA export from nucleus | sqd |
| 409 | GO:0004428 | F | 6, | 1 | 0.177 (x 5.638) | 17 (0.059) | 0.348 | inositol or phosphatidylinositol kinase activity | Pi3K21B |
| 410 | GO:0044260 | P | 5, | 27 | 22.452 (x 1.203) | 2152 (0.013) | 0.349 | cellular macromolecule metabolism | BcDNA:LD41548 CG11267 CG11500 CG17735 CG4759 CG7182 CG7722 Fkbp13 Hop Hsp23 Hsp26 Hsp27 Hsp67Ba Hsp70Bc POSH Pi3K21B Pk61C Su(z)2 Tbp-1 Ufd1-like aru ena lmg msi plexA smi35A sqd |
| 411 | GO:0031974 | C | 2, | 7 | 4.497 (x 1.557) | 431 (0.016) | 0.349 | membrane-enclosed lumen | B52 CG11267 CG5028 Hrb27C ap eyg sqd |
| 412 | GO:0016334 | P | 6, | 1 | 0.177 (x 5.638) | 17 (0.059) | 0.349 | establishment and/or maintenance of polarity of follicular epithelium | sqd |
| 413 | GO:0051246 | P | 5, 6, | 3 | 1.388 (x 2.162) | 133 (0.023) | 0.349 | regulation of protein metabolism | ena msi sqd |
| 414 | GO:0043233 | C | 3, 4, | 7 | 4.497 (x 1.557) | 431 (0.016) | 0.35 | organelle lumen | B52 CG11267 CG5028 Hrb27C ap eyg sqd |
| 415 | GO:0040023 | P | 6, 7, | 1 | 0.177 (x 5.638) | 17 (0.059) | 0.35 | establishment of nucleus localization | Lam |
| 416 | GO:0044267 | P | 6, | 26 | 21.690 (x 1.199) | 2079 (0.013) | 0.353 | cellular protein metabolism | BcDNA:LD41548 CG11267 CG11500 CG17735 CG4759 CG7182 CG7722 Fkbp13 Hop Hsp23 Hsp26 Hsp27 Hsp67Ba Hsp70Bc POSH Pi3K21B Pk61C Su(z)2 Tbp-1 Ufd1-like aru lmg msi plexA smi35A sqd |
| 417 | GO:0006445 | P | 7, 8, 9, | 2 | 0.751 (x 2.662) | 72 (0.028) | 0.354 | regulation of translation | msi sqd |
| 418 | GO:0048523 | P | 4, | 5 | 2.921 (x 1.712) | 280 (0.018) | 0.354 | negative regulation of cellular process | Pk61C ana edl msi sqd |
| 419 | GO:0016271 | P | 4, | 2 | 0.751 (x 2.662) | 72 (0.028) | 0.355 | tissue death | Akap200 ap |
| 420 | GO:0045610 | P | 5, 7, | 1 | 0.188 (x 5.325) | 18 (0.056) | 0.355 | regulation of hemocyte differentiation | kn |
| 421 | GO:0007559 | P | 5, | 2 | 0.751 (x 2.662) | 72 (0.028) | 0.356 | histolysis | Akap200 ap |
| 422 | GO:0051235 | P | 4, | 1 | 0.188 (x 5.325) | 18 (0.056) | 0.356 | maintenance of localization | CG9057 |
| 423 | GO:0006913 | P | 6, 7, 8, | 2 | 0.741 (x 2.700) | 71 (0.028) | 0.356 | nucleocytoplasmic transport | Fs(2)Ket sqd |
| 424 | GO:0042659 | P | 5, 6, | 1 | 0.188 (x 5.325) | 18 (0.056) | 0.357 | regulation of cell fate specification | Dr |
| 425 | GO:0008270 | F | 6, | 9 | 6.281 (x 1.433) | 602 (0.015) | 0.357 | zinc ion binding | CG12054 CG6654 CG8089 POSH Su(z)2 ap bowl klu lmg |
| 426 | GO:0035287 | P | 5, 6, | 1 | 0.188 (x 5.325) | 18 (0.056) | 0.357 | head segmentation | kn |
| 427 | GO:0030384 | P | 9, 10, | 1 | 0.188 (x 5.325) | 18 (0.056) | 0.358 | phosphoinositide metabolism | Pi3K21B |
| 428 | GO:0001751 | P | 6, 7, 8, 9, | 2 | 0.762 (x 2.626) | 73 (0.027) | 0.359 | eye photoreceptor cell differentiation (sensu Endopterygota) | POSH hth |
| 429 | GO:0007294 | P | 6, 7, 9, 10, | 1 | 0.188 (x 5.325) | 18 (0.056) | 0.359 | oocyte fate determination (sensu Insecta) | hts |
| 430 | GO:0051647 | P | 5, 6, | 1 | 0.188 (x 5.325) | 18 (0.056) | 0.36 | nucleus localization | Lam |
| 431 | GO:0015149 | F | 6, | 1 | 0.188 (x 5.325) | 18 (0.056) | 0.361 | hexose transporter activity | CG10960 |
| 432 | GO:0016071 | P | 7, | 4 | 2.170 (x 1.843) | 208 (0.019) | 0.361 | mRNA metabolism | B52 Hrb27C msi sqd |
| 433 | GO:0030706 | P | 5, 8, 9, | 1 | 0.198 (x 5.045) | 19 (0.053) | 0.365 | oocyte differentiation (sensu Insecta) | hts |
| 434 | GO:0046915 | F | 5, | 1 | 0.198 (x 5.045) | 19 (0.053) | 0.366 | transition metal ion transporter activity | BEST:CK02137 |
| 435 | GO:0006650 | P | 8, 9, | 1 | 0.198 (x 5.045) | 19 (0.053) | 0.367 | glycerophospholipid metabolism | Pi3K21B |
| 436 | GO:0001558 | P | 4, 5, 7, 8, | 1 | 0.209 (x 4.792) | 20 (0.050) | 0.37 | regulation of cell growth | Pk61C |
| 437 | GO:0043068 | P | 6, 7, | 2 | 0.782 (x 2.556) | 75 (0.027) | 0.37 | positive regulation of programmed cell death | klu smi35A |
| 438 | GO:0007276 | P | 4, | 7 | 4.664 (x 1.501) | 447 (0.016) | 0.37 | gametogenesis | Fs(2)Ket Hrb27C POSH Pk61C hts sqd stai |
| 439 | GO:0035222 | P | 5, 6, | 1 | 0.209 (x 4.792) | 20 (0.050) | 0.37 | wing disc pattern formation | ap |
| 440 | GO:0006417 | P | 6, 7, 8, | 2 | 0.782 (x 2.556) | 75 (0.027) | 0.371 | regulation of protein biosynthesis | msi sqd |
| 441 | GO:0030716 | P | 6, | 1 | 0.209 (x 4.792) | 20 (0.050) | 0.371 | oocyte fate determination | hts |
| 442 | GO:0003697 | F | 6, | 1 | 0.209 (x 4.792) | 20 (0.050) | 0.372 | single-stranded DNA binding | Hrb27C |
| 443 | GO:0015145 | F | 5, | 1 | 0.209 (x 4.792) | 20 (0.050) | 0.373 | monosaccharide transporter activity | CG10960 |
| 444 | GO:0008320 | F | 4, | 1 | 0.209 (x 4.792) | 20 (0.050) | 0.374 | protein carrier activity | Fs(2)Ket |
| 445 | GO:0007422 | P | 5, | 2 | 0.793 (x 2.522) | 76 (0.026) | 0.374 | peripheral nervous system development | hth spdo |
| 446 | GO:0008144 | F | 3, | 1 | 0.209 (x 4.792) | 20 (0.050) | 0.375 | drug binding | Fkbp13 |
| 447 | GO:0042592 | P | 3, | 2 | 0.793 (x 2.522) | 76 (0.026) | 0.375 | homeostasis | CG8177 Prx5037 |
| 448 | GO:0016782 | F | 4, | 1 | 0.209 (x 4.792) | 20 (0.050) | 0.375 | transferase activity, transferring sulfur-containing groups | CG7920 |
| 449 | GO:0001754 | P | 5, 6, 7, | 2 | 0.793 (x 2.522) | 76 (0.026) | 0.376 | eye photoreceptor cell differentiation | POSH hth |
| 450 | GO:0007317 | P | 4, 8, 12, 14, 15, 17, | 1 | 0.209 (x 4.792) | 20 (0.050) | 0.376 | regulation of pole plasm oskar mRNA localization | Hrb27C |
| 451 | GO:0016325 | P | 7, 8, 9, 10, | 1 | 0.219 (x 4.564) | 21 (0.048) | 0.377 | oocyte microtubule cytoskeleton organization | POSH |
| 452 | GO:0006405 | P | 7, 8, 9, 10, | 1 | 0.219 (x 4.564) | 21 (0.048) | 0.378 | RNA export from nucleus | sqd |
| 453 | GO:0009889 | P | 5, | 2 | 0.824 (x 2.427) | 79 (0.025) | 0.378 | regulation of biosynthesis | msi sqd |
| 454 | GO:0007469 | P | 6, | 1 | 0.219 (x 4.564) | 21 (0.048) | 0.379 | antennal development | Dll |
| 455 | GO:0031326 | P | 6, | 2 | 0.824 (x 2.427) | 79 (0.025) | 0.379 | regulation of cellular biosynthesis | msi sqd |
| 456 | GO:0003755 | F | 5, | 1 | 0.219 (x 4.564) | 21 (0.048) | 0.379 | peptidyl-prolyl cis-trans isomerase activity | Fkbp13 |
| 457 | GO:0030952 | P | 7, | 1 | 0.219 (x 4.564) | 21 (0.048) | 0.38 | establishment and/or maintenance of cytoskeleton polarity | POSH |
| 458 | GO:0030951 | P | 8, 9, | 1 | 0.219 (x 4.564) | 21 (0.048) | 0.381 | establishment and/or maintenance of microtubule cytoskeleton polarity | POSH |
| 459 | GO:0008154 | P | 6, 9, | 1 | 0.219 (x 4.564) | 21 (0.048) | 0.382 | actin polymerization and/or depolymerization | ena |
| 460 | GO:0048542 | P | 5, | 1 | 0.219 (x 4.564) | 21 (0.048) | 0.383 | lymph gland development (sensu Arthropoda) | kn |
| 461 | GO:0007480 | P | 7, 8, | 1 | 0.219 (x 4.564) | 21 (0.048) | 0.384 | leg morphogenesis (sensu Endopterygota) | bowl |
| 462 | GO:0019953 | P | 3, | 7 | 4.757 (x 1.471) | 456 (0.015) | 0.384 | sexual reproduction | Fs(2)Ket Hrb27C POSH Pk61C hts sqd stai |
| 463 | GO:0005737 | C | 4, 5, 6, | 19 | 15.493 (x 1.226) | 1485 (0.013) | 0.384 | cytoplasm | BcDNA:LD41548 CBP CG11267 CG11500 CG30059 CG4759 CG5028 CG9057 Fs(2)Ket ImpE2 Pi3K21B Pk61C Prx5037 aru garz hts smi35A spdo sqd |
| 464 | GO:0043085 | P | 4, | 1 | 0.230 (x 4.357) | 22 (0.045) | 0.385 | positive regulation of enzyme activity | Abi |
| 465 | GO:0005798 | C | 5, 6, 7, 8, 9, 10, | 1 | 0.230 (x 4.357) | 22 (0.045) | 0.386 | Golgi-associated vesicle | garz |
| 466 | GO:0008076 | C | 3, 5, 6, 7, 8, 9, | 1 | 0.230 (x 4.357) | 22 (0.045) | 0.387 | voltage-gated potassium channel complex | Pk61C |
| 467 | GO:0043228 | C | 3, | 10 | 7.418 (x 1.348) | 711 (0.014) | 0.388 | non-membrane-bound organelle | CG31363 CG31611 CG4759 Lam Su(z)2 aru hts kis sqd stai |
| 468 | GO:0000790 | C | 6, 7, 8, 9, 10, 11, 12, | 1 | 0.230 (x 4.357) | 22 (0.045) | 0.388 | nuclear chromatin | Su(z)2 |
| 469 | GO:0043232 | C | 4, 5, 6, 7, | 10 | 7.418 (x 1.348) | 711 (0.014) | 0.388 | intracellular non-membrane-bound organelle | CG31363 CG31611 CG4759 Lam Su(z)2 aru hts kis sqd stai |
| 470 | GO:0050875 | P | 3, | 69 | 64.852 (x 1.064) | 6216 (0.011) | 0.388 | cellular physiological process | Abi Akap200 B52 BEST:CK02137 BcDNA:LD41548 CG10960 CG11267 CG11500 CG17735 CG30059 CG31611 CG3823 CG4759 CG5028 CG5873 CG6654 CG6854 CG6982 CG7182 CG7722 CG7920 CG8177 CG9057 Dll Dr Fkbp13 Fs(2)Ket Hop Hrb27C Hsp23 Hsp26 Hsp27 Hsp67Ba Hsp70Bc Lam Obp99a POSH Pi3K21B Pk61C Prx5037 Sox102F Su(z)2 Tbp-1 Ufd1-like Ugt86Di ana ap aru bowl edl ena eyg fax garz hth hts kis klu kn lmg msi ogre pdm2 plexA smi35A spdo sqd stai toy |
| 471 | GO:0016859 | F | 4, | 1 | 0.240 (x 4.167) | 23 (0.043) | 0.394 | cis-trans isomerase activity | Fkbp13 |
| 472 | GO:0043549 | P | 5, | 1 | 0.240 (x 4.167) | 23 (0.043) | 0.395 | regulation of kinase activity | Abi |
| 473 | GO:0051338 | P | 4, | 1 | 0.240 (x 4.167) | 23 (0.043) | 0.396 | regulation of transferase activity | Abi |
| 474 | GO:0045859 | P | 6, | 1 | 0.240 (x 4.167) | 23 (0.043) | 0.397 | regulation of protein kinase activity | Abi |
| 475 | GO:0005838 | C | 3, 4, 5, 6, 7, | 1 | 0.240 (x 4.167) | 23 (0.043) | 0.397 | proteasome regulatory particle (sensu Eukaryota) | Tbp-1 |
| 476 | GO:0016333 | P | 5, | 1 | 0.240 (x 4.167) | 23 (0.043) | 0.398 | morphogenesis of follicular epithelium | sqd |
| 477 | GO:0007478 | P | 6, 7, | 1 | 0.240 (x 4.167) | 23 (0.043) | 0.399 | leg disc morphogenesis | bowl |
| 478 | GO:0006401 | P | 7, | 1 | 0.240 (x 4.167) | 23 (0.043) | 0.4 | RNA catabolism | sqd |
| 479 | GO:0031202 | F | 5, | 1 | 0.250 (x 3.994) | 24 (0.042) | 0.4 | RNA splicing factor activity, transesterification mechanism | B52 |
| 480 | GO:0016616 | F | 5, | 2 | 0.876 (x 2.282) | 84 (0.024) | 0.4 | oxidoreductase activity, acting on the CH-OH group of donors, NAD or NADP as acceptor | CG17121 CG5028 |
| 481 | GO:0006334 | P | 7, 11, | 1 | 0.250 (x 3.994) | 24 (0.042) | 0.401 | nucleosome assembly | CG31611 |
| 482 | GO:0051236 | P | 5, | 1 | 0.250 (x 3.994) | 24 (0.042) | 0.402 | establishment of RNA localization | sqd |
| 483 | GO:0031324 | P | 6, | 3 | 1.617 (x 1.855) | 155 (0.019) | 0.402 | negative regulation of cellular metabolism | edl msi sqd |
| 484 | GO:0015082 | F | 5, | 1 | 0.250 (x 3.994) | 24 (0.042) | 0.402 | di-, tri-valent inorganic cation transporter activity | BEST:CK02137 |
| 485 | GO:0019842 | F | 3, | 1 | 0.250 (x 3.994) | 24 (0.042) | 0.403 | vitamin binding | CG3823 |
| 486 | GO:0044446 | C | 3, 4, 5, 6, 7, | 18 | 14.857 (x 1.212) | 1424 (0.013) | 0.404 | intracellular organelle part | B52 CG11267 CG11500 CG17952 CG31611 CG4759 CG5028 Fs(2)Ket Hrb27C Lam Su(z)2 ap eyg garz kis lmg sqd stai |
| 487 | GO:0050658 | P | 6, 7, 8, | 1 | 0.250 (x 3.994) | 24 (0.042) | 0.404 | RNA transport | sqd |
| 488 | GO:0044422 | C | 2, 3, | 18 | 14.857 (x 1.212) | 1424 (0.013) | 0.405 | organelle part | B52 CG11267 CG11500 CG17952 CG31611 CG4759 CG5028 Fs(2)Ket Hrb27C Lam Su(z)2 ap eyg garz kis lmg sqd stai |
| 489 | GO:0050657 | P | 6, 7, | 1 | 0.250 (x 3.994) | 24 (0.042) | 0.405 | nucleic acid transport | sqd |
| 490 | GO:0000323 | C | 6, 7, 8, 9, | 1 | 0.261 (x 3.834) | 25 (0.040) | 0.41 | lytic vacuole | CG30059 |
| 491 | GO:0043566 | F | 5, | 1 | 0.261 (x 3.834) | 25 (0.040) | 0.41 | structure-specific DNA binding | Hrb27C |
| 492 | GO:0050767 | P | 4, 7, | 1 | 0.261 (x 3.834) | 25 (0.040) | 0.411 | regulation of neurogenesis | ana |
| 493 | GO:0007467 | P | 5, | 2 | 0.908 (x 2.203) | 87 (0.023) | 0.412 | photoreceptor cell differentiation (sensu Endopterygota) | POSH hth |
| 494 | GO:0005764 | C | 7, 8, 9, 10, | 1 | 0.261 (x 3.834) | 25 (0.040) | 0.412 | lysosome | CG30059 |
| 495 | GO:0009798 | P | 4, | 3 | 1.659 (x 1.808) | 159 (0.019) | 0.412 | axis specification | Hrb27C POSH sqd |
| 496 | GO:0016874 | F | 3, | 5 | 3.307 (x 1.512) | 317 (0.016) | 0.417 | ligase activity | CG17735 CG6854 POSH Su(z)2 lmg |
| 497 | GO:0008092 | F | 4, | 4 | 2.483 (x 1.611) | 238 (0.017) | 0.418 | cytoskeletal protein binding | Hsp23 ena hts stai |
| 498 | GO:0051093 | P | 4, | 1 | 0.271 (x 3.687) | 26 (0.038) | 0.419 | negative regulation of development | ana |
| 499 | GO:0030529 | C | 3, 4, 5, 6, | 5 | 3.318 (x 1.507) | 318 (0.016) | 0.419 | ribonucleoprotein complex | B52 CG4759 Hrb27C aru sqd |
| 500 | GO:0051128 | P | 5, | 1 | 0.271 (x 3.687) | 26 (0.038) | 0.42 | regulation of cell organization and biogenesis | ena |
| 501 | GO:0005976 | P | 6, | 3 | 1.690 (x 1.775) | 162 (0.019) | 0.42 | polysaccharide metabolism | CG30059 Pk61C Ugt86Di |
| 502 | GO:0009892 | P | 5, | 3 | 1.721 (x 1.743) | 165 (0.018) | 0.429 | negative regulation of metabolism | edl msi sqd |
| 503 | GO:0005507 | F | 6, | 1 | 0.282 (x 3.550) | 27 (0.037) | 0.429 | copper ion binding | CG30437 |
| 504 | GO:0005575 | C | 1, | 64 | 60.324 (x 1.061) | 5782 (0.011) | 0.43 | cellular\_component | B52 BEST:CK02137 BcDNA:LD41548 CBP CG10960 CG11267 CG11500 CG12054 CG17735 CG17952 CG30059 CG31363 CG31611 CG32267 CG3823 CG4759 CG5028 CG6654 CG6982 CG8089 CG8177 CG8588 CG9057 Dll Dr Fs(2)Ket Hrb27C ImpE2 ImpE3 ImpL2 Lam MESR6 Obp99a POSH Pi3K21B Pk61C Prx5037 Sox102F Su(z)2 Tbp-1 Ufd1-like Xe7 ana ap aru bip1 bowl edl eyg garz hth hts kis klu kn lmg ogre pdm2 plexA smi35A spdo sqd stai toy |
| 505 | GO:0007419 | P | 4, 6, | 1 | 0.282 (x 3.550) | 27 (0.037) | 0.43 | ventral cord development | Dr |
| 506 | GO:0051168 | P | 7, 8, 9, | 1 | 0.282 (x 3.550) | 27 (0.037) | 0.431 | nuclear export | sqd |
| 507 | GO:0051242 | P | 5, | 3 | 1.742 (x 1.722) | 167 (0.018) | 0.438 | positive regulation of cellular physiological process | Pk61C klu smi35A |
| 508 | GO:0007300 | P | 8, | 1 | 0.292 (x 3.423) | 28 (0.036) | 0.44 | nurse cell to oocyte transport (sensu Insecta) | hts |
| 509 | GO:0043170 | P | 4, | 36 | 32.509 (x 1.107) | 3116 (0.012) | 0.44 | macromolecule metabolism | B52 BcDNA:LD41548 CG10960 CG11267 CG11500 CG17735 CG30059 CG31611 CG4759 CG5028 CG7182 CG7722 Fkbp13 Hop Hrb27C Hsp23 Hsp26 Hsp27 Hsp67Ba Hsp70Bc POSH Pi3K21B Pk61C Su(z)2 Tbp-1 Ufd1-like Ugt86Di aru ena hts kis lmg msi plexA smi35A sqd |
| 510 | GO:0043119 | P | 4, | 3 | 1.763 (x 1.701) | 169 (0.018) | 0.445 | positive regulation of physiological process | Pk61C klu smi35A |
| 511 | GO:0012501 | P | 5, | 4 | 2.587 (x 1.546) | 248 (0.016) | 0.446 | programmed cell death | Akap200 Pk61C klu smi35A |
| 512 | GO:0014017 | P | 5, 8, | 1 | 0.303 (x 3.305) | 29 (0.034) | 0.448 | neuroblast fate commitment | Dr |
| 513 | GO:0014016 | P | 4, 7, | 1 | 0.303 (x 3.305) | 29 (0.034) | 0.449 | neuroblast differentiation | Dr |
| 514 | GO:0008219 | P | 4, | 4 | 2.608 (x 1.534) | 250 (0.016) | 0.449 | cell death | Akap200 Pk61C klu smi35A |
| 515 | GO:0007400 | P | 6, 9, | 1 | 0.303 (x 3.305) | 29 (0.034) | 0.449 | neuroblast fate determination | Dr |
| 516 | GO:0004714 | F | 6, 8, | 1 | 0.303 (x 3.305) | 29 (0.034) | 0.45 | transmembrane receptor protein tyrosine kinase activity | plexA |
| 517 | GO:0000003 | P | 2, | 7 | 5.227 (x 1.339) | 501 (0.014) | 0.454 | reproduction | Fs(2)Ket Hrb27C POSH Pk61C hts sqd stai |
| 518 | GO:0042386 | P | 4, 6, | 1 | 0.313 (x 3.195) | 30 (0.033) | 0.456 | hemocyte differentiation (sensu Arthropoda) | kn |
| 519 | GO:0051656 | P | 5, | 1 | 0.313 (x 3.195) | 30 (0.033) | 0.456 | establishment of organelle localization | Lam |
| 520 | GO:0016485 | P | 8, | 1 | 0.313 (x 3.195) | 30 (0.033) | 0.457 | protein processing | CG11500 |
| 521 | GO:0016049 | P | 3, 4, 6, 7, | 1 | 0.313 (x 3.195) | 30 (0.033) | 0.458 | cell growth | Pk61C |
| 522 | GO:0006396 | P | 7, | 4 | 2.660 (x 1.504) | 255 (0.016) | 0.464 | RNA processing | B52 Hrb27C msi sqd |
| 523 | GO:0044238 | P | 4, | 51 | 47.679 (x 1.070) | 4570 (0.011) | 0.466 | primary metabolism | B52 BcDNA:LD41548 CG10960 CG11267 CG11500 CG17735 CG30059 CG31611 CG4759 CG5028 CG6654 CG6854 CG7182 CG7722 CG9057 Dll Dr Fkbp13 Hop Hrb27C Hsp23 Hsp26 Hsp27 Hsp67Ba Hsp70Bc POSH Pi3K21B Pk61C Sox102F Su(z)2 Tbp-1 Ufd1-like Ugt86Di ap aru bowl edl ena eyg hth hts kis klu kn lmg msi pdm2 plexA smi35A sqd toy |
| 524 | GO:0015297 | F | 6, | 1 | 0.323 (x 3.092) | 31 (0.032) | 0.466 | antiporter activity | CG8177 |
| 525 | GO:0048754 | P | 5, | 1 | 0.323 (x 3.092) | 31 (0.032) | 0.467 | branching morphogenesis of a tube | Lam |
| 526 | GO:0006333 | P | 9, | 2 | 1.043 (x 1.917) | 100 (0.020) | 0.468 | chromatin assembly or disassembly | CG31611 kis |
| 527 | GO:0030031 | P | 6, 7, | 1 | 0.334 (x 2.995) | 32 (0.031) | 0.47 | cell projection biogenesis | Abi |
| 528 | GO:0051640 | P | 4, | 1 | 0.334 (x 2.995) | 32 (0.031) | 0.471 | organelle localization | Lam |
| 529 | GO:0007602 | P | 6, 7, | 1 | 0.334 (x 2.995) | 32 (0.031) | 0.472 | phototransduction | ogre |
| 530 | GO:0006206 | P | 7, | 1 | 0.334 (x 2.995) | 32 (0.031) | 0.473 | pyrimidine base metabolism | CG6854 |
| 531 | GO:0007350 | P | 4, 5, | 2 | 1.064 (x 1.879) | 102 (0.020) | 0.474 | blastoderm segmentation | kis kn |
| 532 | GO:0046873 | F | 4, | 1 | 0.334 (x 2.995) | 32 (0.031) | 0.474 | metal ion transporter activity | BEST:CK02137 |
| 533 | GO:0016044 | P | 5, | 1 | 0.334 (x 2.995) | 32 (0.031) | 0.475 | membrane organization and biogenesis | Lam |
| 534 | GO:0009611 | P | 4, | 1 | 0.344 (x 2.905) | 33 (0.030) | 0.476 | response to wounding | kn |
| 535 | GO:0043296 | C | 6, 7, 8, 9, | 1 | 0.334 (x 2.995) | 32 (0.031) | 0.476 | apical junction complex | CG6982 |
| 536 | GO:0007297 | P | 6, 7, 9, | 1 | 0.344 (x 2.905) | 33 (0.030) | 0.476 | follicle cell migration (sensu Insecta) | sqd |
| 537 | GO:0001752 | P | 7, 8, 9, 10, | 1 | 0.344 (x 2.905) | 33 (0.030) | 0.477 | eye photoreceptor fate commitment (sensu Endopterygota) | hth |
| 538 | GO:0008354 | P | 5, 6, 7, | 1 | 0.344 (x 2.905) | 33 (0.030) | 0.478 | germ cell migration | stai |
| 539 | GO:0042706 | P | 6, 7, 8, | 1 | 0.344 (x 2.905) | 33 (0.030) | 0.479 | eye photoreceptor cell fate commitment | hth |
| 540 | GO:0006139 | P | 5, | 21 | 18.477 (x 1.137) | 1771 (0.012) | 0.48 | nucleobase, nucleoside, nucleotide and nucleic acid metabolism | B52 CG31611 CG6654 CG6854 Dll Dr Hrb27C Sox102F Su(z)2 ap bowl edl eyg hth kis klu kn msi pdm2 sqd toy |
| 541 | GO:0005249 | F | 6, 7, 8, | 1 | 0.344 (x 2.905) | 33 (0.030) | 0.48 | voltage-gated potassium channel activity | Pk61C |
| 542 | GO:0015020 | F | 6, | 1 | 0.355 (x 2.819) | 34 (0.029) | 0.485 | glucuronosyltransferase activity | Ugt86Di |
| 543 | GO:0005829 | C | 5, 6, 7, 8, | 3 | 1.920 (x 1.563) | 184 (0.016) | 0.485 | cytosol | CG4759 Pi3K21B smi35A |
| 544 | GO:0009605 | P | 3, | 2 | 1.095 (x 1.826) | 105 (0.019) | 0.485 | response to external stimulus | kn ogre |
| 545 | GO:0001763 | P | 4, | 1 | 0.355 (x 2.819) | 34 (0.029) | 0.486 | morphogenesis of a branching structure | Lam |
| 546 | GO:0009790 | P | 3, | 5 | 3.662 (x 1.365) | 351 (0.014) | 0.488 | embryonic development | bowl edl ena kis kn |
| 547 | GO:0007242 | P | 5, | 7 | 5.467 (x 1.280) | 524 (0.013) | 0.491 | intracellular signaling cascade | Akap200 CG30440 Pi3K21B Pk61C edl klu stai |
| 548 | GO:0009583 | P | 5, 6, | 1 | 0.365 (x 2.739) | 35 (0.029) | 0.492 | detection of light stimulus | ogre |
| 549 | GO:0016358 | P | 7, 10, | 1 | 0.365 (x 2.739) | 35 (0.029) | 0.493 | dendrite development | ena |
| 550 | GO:0048522 | P | 4, | 3 | 1.972 (x 1.521) | 189 (0.016) | 0.501 | positive regulation of cellular process | Pk61C klu smi35A |
| 551 | GO:0042981 | P | 6, 7, | 2 | 1.137 (x 1.759) | 109 (0.018) | 0.501 | regulation of apoptosis | Pk61C smi35A |
| 552 | GO:0007442 | P | 7, 8, | 1 | 0.376 (x 2.662) | 36 (0.028) | 0.502 | hindgut morphogenesis | bowl |
| 553 | GO:0048193 | P | 6, 7, 8, | 1 | 0.376 (x 2.662) | 36 (0.028) | 0.503 | Golgi vesicle transport | garz |
| 554 | GO:0015931 | P | 5, 6, | 1 | 0.386 (x 2.591) | 37 (0.027) | 0.51 | nucleobase, nucleoside, nucleotide and nucleic acid transport | sqd |
| 555 | GO:0044237 | P | 4, | 51 | 48.294 (x 1.056) | 4629 (0.011) | 0.51 | cellular metabolism | B52 BcDNA:LD41548 CG11267 CG11500 CG17735 CG30059 CG31611 CG4759 CG5028 CG5873 CG6654 CG6854 CG7182 CG7722 CG7920 Dll Dr Fkbp13 Hop Hrb27C Hsp23 Hsp26 Hsp27 Hsp67Ba Hsp70Bc POSH Pi3K21B Pk61C Prx5037 Sox102F Su(z)2 Tbp-1 Ufd1-like Ugt86Di ap aru bowl edl ena eyg hth kis klu kn lmg msi pdm2 plexA smi35A sqd toy |
| 556 | GO:0016327 | C | 5, 6, 7, | 1 | 0.386 (x 2.591) | 37 (0.027) | 0.511 | apicolateral plasma membrane | CG6982 |
| 557 | GO:0006916 | P | 8, 9, | 1 | 0.396 (x 2.522) | 38 (0.026) | 0.519 | anti-apoptosis | Pk61C |
| 558 | GO:0005783 | C | 5, 6, 7, 8, | 2 | 1.179 (x 1.696) | 113 (0.018) | 0.52 | endoplasmic reticulum | CBP CG11500 |
| 559 | GO:0048547 | P | 5, 6, | 1 | 0.417 (x 2.396) | 40 (0.025) | 0.527 | gut morphogenesis | bowl |
| 560 | GO:0007017 | P | 7, | 3 | 2.045 (x 1.467) | 196 (0.015) | 0.527 | microtubule-based process | CG9057 POSH stai |
| 561 | GO:0008355 | P | 6, 7, | 1 | 0.407 (x 2.458) | 39 (0.026) | 0.527 | olfactory learning | CG8588 |
| 562 | GO:0048546 | P | 5, | 1 | 0.417 (x 2.396) | 40 (0.025) | 0.528 | digestive tract morphogenesis | bowl |
| 563 | GO:0048567 | P | 6, 7, | 1 | 0.417 (x 2.396) | 40 (0.025) | 0.529 | ectodermal gut morphogenesis | bowl |
| 564 | GO:0007439 | P | 5, | 1 | 0.417 (x 2.396) | 40 (0.025) | 0.53 | ectodermal gut development | bowl |
| 565 | GO:0046552 | P | 5, | 1 | 0.417 (x 2.396) | 40 (0.025) | 0.531 | photoreceptor cell fate commitment | hth |
| 566 | GO:0006468 | P | 8, | 4 | 2.973 (x 1.345) | 285 (0.014) | 0.531 | protein amino acid phosphorylation | Pi3K21B Pk61C plexA smi35A |
| 567 | GO:0005643 | C | 3, 5, 6, 7, 8, 9, 10, 11, 12, 13, | 1 | 0.417 (x 2.396) | 40 (0.025) | 0.532 | nuclear pore | Fs(2)Ket |
| 568 | GO:0007219 | P | 6, | 1 | 0.428 (x 2.338) | 41 (0.024) | 0.532 | Notch signaling pathway | spdo |
| 569 | GO:0005789 | C | 4, 5, 6, 7, 8, 9, 10, | 1 | 0.417 (x 2.396) | 40 (0.025) | 0.532 | endoplasmic reticulum membrane | CG11500 |
| 570 | GO:0042175 | C | 4, 5, 6, | 1 | 0.428 (x 2.338) | 41 (0.024) | 0.533 | nuclear envelope-endoplasmic reticulum network | CG11500 |
| 571 | GO:0016614 | F | 4, | 2 | 1.231 (x 1.625) | 118 (0.017) | 0.533 | oxidoreductase activity, acting on CH-OH group of donors | CG17121 CG5028 |
| 572 | GO:0004177 | F | 6, | 1 | 0.417 (x 2.396) | 40 (0.025) | 0.533 | aminopeptidase activity | BcDNA:LD41548 |
| 573 | GO:0044445 | C | 5, 6, 7, 8, 9, | 2 | 1.231 (x 1.625) | 118 (0.017) | 0.534 | cytosolic part | CG4759 Pi3K21B |
| 574 | GO:0015103 | F | 5, | 1 | 0.417 (x 2.396) | 40 (0.025) | 0.534 | inorganic anion transporter activity | CG8177 |
| 575 | GO:0015980 | P | 6, | 2 | 1.231 (x 1.625) | 118 (0.017) | 0.535 | energy derivation by oxidation of organic compounds | CG5028 Pk61C |
| 576 | GO:0046930 | C | 6, 7, 8, | 1 | 0.417 (x 2.396) | 40 (0.025) | 0.535 | pore complex | Fs(2)Ket |
| 577 | GO:0005351 | F | 5, 6, | 1 | 0.417 (x 2.396) | 40 (0.025) | 0.536 | sugar porter activity | CG10960 |
| 578 | GO:0007459 | P | 6, | 1 | 0.417 (x 2.396) | 40 (0.025) | 0.537 | photoreceptor fate commitment (sensu Endopterygota) | hth |
| 579 | GO:0043066 | P | 7, 8, | 1 | 0.438 (x 2.282) | 42 (0.024) | 0.538 | negative regulation of apoptosis | Pk61C |
| 580 | GO:0044451 | C | 5, 6, 7, 8, 9, 10, 11, 12, | 3 | 2.118 (x 1.416) | 203 (0.015) | 0.539 | nucleoplasm part | B52 eyg sqd |
| 581 | GO:0043062 | P | 3, | 1 | 0.438 (x 2.282) | 42 (0.024) | 0.539 | extracellular structure organization and biogenesis | hts |
| 582 | GO:0043069 | P | 6, 7, | 1 | 0.438 (x 2.282) | 42 (0.024) | 0.54 | negative regulation of programmed cell death | Pk61C |
| 583 | GO:0007612 | P | 5, | 1 | 0.449 (x 2.229) | 43 (0.023) | 0.546 | learning | CG8588 |
| 584 | GO:0007417 | P | 5, | 2 | 1.273 (x 1.571) | 122 (0.016) | 0.546 | central nervous system development | Dr hth |
| 585 | GO:0044454 | C | 5, 6, 7, 8, 9, 10, 11, | 1 | 0.449 (x 2.229) | 43 (0.023) | 0.547 | nuclear chromosome part | Su(z)2 |
| 586 | GO:0005200 | F | 3, | 4 | 3.046 (x 1.313) | 292 (0.014) | 0.547 | structural constituent of cytoskeleton | Lam POSH ena hts |
| 587 | GO:0009582 | P | 4, 5, | 1 | 0.459 (x 2.178) | 44 (0.023) | 0.552 | detection of abiotic stimulus | ogre |
| 588 | GO:0042051 | P | 7, 8, 9, 10, | 1 | 0.459 (x 2.178) | 44 (0.023) | 0.553 | eye photoreceptor development (sensu Endopterygota) | POSH |
| 589 | GO:0030707 | P | 8, | 2 | 1.294 (x 1.546) | 124 (0.016) | 0.554 | ovarian follicle cell development (sensu Insecta) | Fs(2)Ket sqd |
| 590 | GO:0050790 | P | 3, | 1 | 0.459 (x 2.178) | 44 (0.023) | 0.554 | regulation of catalytic activity | Abi |
| 591 | GO:0002164 | P | 4, | 1 | 0.469 (x 2.130) | 45 (0.022) | 0.556 | larval development | kn |
| 592 | GO:0005700 | C | 6, 7, 8, 9, | 1 | 0.469 (x 2.130) | 45 (0.022) | 0.557 | polytene chromosome | sqd |
| 593 | GO:0042462 | P | 6, 7, 8, | 1 | 0.469 (x 2.130) | 45 (0.022) | 0.558 | eye photoreceptor cell development | POSH |
| 594 | GO:0009581 | P | 4, 5, | 1 | 0.469 (x 2.130) | 45 (0.022) | 0.559 | detection of external stimulus | ogre |
| 595 | GO:0005576 | C | 2, | 5 | 4.027 (x 1.242) | 386 (0.013) | 0.559 | extracellular region | ImpE2 ImpL2 Obp99a ana hts |
| 596 | GO:0000228 | C | 5, 6, 7, 8, 9, 10, | 1 | 0.480 (x 2.084) | 46 (0.022) | 0.564 | nuclear chromosome | Su(z)2 |
| 597 | GO:0009636 | P | 5, | 2 | 1.346 (x 1.486) | 129 (0.016) | 0.564 | response to toxin | CG30437 Ugt86Di |
| 598 | GO:0043285 | P | 6, | 2 | 1.325 (x 1.509) | 127 (0.016) | 0.565 | biopolymer catabolism | Ufd1-like sqd |
| 599 | GO:0006606 | P | 7, 8, 9, 10, | 1 | 0.490 (x 2.039) | 47 (0.021) | 0.565 | protein import into nucleus | Fs(2)Ket |
| 600 | GO:0001700 | P | 5, | 2 | 1.346 (x 1.486) | 129 (0.016) | 0.565 | embryonic development (sensu Insecta) | ena kn |
| 601 | GO:0006099 | P | 8, 9, | 1 | 0.490 (x 2.039) | 47 (0.021) | 0.566 | tricarboxylic acid cycle | CG5028 |
| 602 | GO:0007293 | P | 8, | 1 | 0.490 (x 2.039) | 47 (0.021) | 0.567 | egg chamber formation (sensu Insecta) | hts |
| 603 | GO:0016811 | F | 5, | 1 | 0.490 (x 2.039) | 47 (0.021) | 0.568 | hydrolase activity, acting on carbon-nitrogen (but not peptide) bonds, in linear amides | CG7900 |
| 604 | GO:0030030 | P | 5, 6, | 1 | 0.490 (x 2.039) | 47 (0.021) | 0.569 | cell projection organization and biogenesis | Abi |
| 605 | GO:0009416 | P | 5, | 1 | 0.501 (x 1.997) | 48 (0.021) | 0.57 | response to light stimulus | ogre |
| 606 | GO:0046356 | P | 8, | 1 | 0.490 (x 2.039) | 47 (0.021) | 0.57 | acetyl-CoA catabolism | CG5028 |
| 607 | GO:0051170 | P | 7, 8, 9, | 1 | 0.501 (x 1.997) | 48 (0.021) | 0.57 | nuclear import | Fs(2)Ket |
| 608 | GO:0009060 | P | 8, | 1 | 0.490 (x 2.039) | 47 (0.021) | 0.571 | aerobic respiration | CG5028 |
| 609 | GO:0009109 | P | 7, | 1 | 0.501 (x 1.997) | 48 (0.021) | 0.571 | coenzyme catabolism | CG5028 |
| 610 | GO:0051119 | F | 4, | 1 | 0.490 (x 2.039) | 47 (0.021) | 0.572 | sugar transporter activity | CG10960 |
| 611 | GO:0045333 | P | 7, | 1 | 0.490 (x 2.039) | 47 (0.021) | 0.573 | cellular respiration | CG5028 |
| 612 | GO:0005524 | F | 6, | 8 | 7.011 (x 1.141) | 672 (0.012) | 0.574 | ATP binding | CG11267 CG7182 Hsp70Bc Pk61C Su(z)2 Tbp-1 kis smi35A |
| 613 | GO:0019199 | F | 5, 7, | 1 | 0.511 (x 1.956) | 49 (0.020) | 0.575 | transmembrane receptor protein kinase activity | plexA |
| 614 | GO:0051187 | P | 6, | 1 | 0.511 (x 1.956) | 49 (0.020) | 0.576 | cofactor catabolism | CG5028 |
| 615 | GO:0051606 | P | 3, 4, | 1 | 0.511 (x 1.956) | 49 (0.020) | 0.577 | detection of stimulus | ogre |
| 616 | GO:0002009 | P | 4, | 2 | 1.388 (x 1.441) | 133 (0.015) | 0.577 | morphogenesis of an epithelium | ena sqd |
| 617 | GO:0008594 | P | 6, 7, 8, | 1 | 0.511 (x 1.956) | 49 (0.020) | 0.578 | photoreceptor cell morphogenesis (sensu Endopterygota) | POSH |
| 618 | GO:0005244 | F | 5, 6, | 1 | 0.532 (x 1.879) | 51 (0.020) | 0.587 | voltage-gated ion channel activity | Pk61C |
| 619 | GO:0003729 | F | 5, | 4 | 3.266 (x 1.225) | 313 (0.013) | 0.587 | mRNA binding | B52 Hrb27C msi sqd |
| 620 | GO:0005842 | C | 4, 5, 6, 7, 8, 9, 10, 11, | 1 | 0.532 (x 1.879) | 51 (0.020) | 0.588 | cytosolic large ribosomal subunit (sensu Eukaryota) | CG4759 |
| 621 | GO:0031497 | P | 10, | 1 | 0.532 (x 1.879) | 51 (0.020) | 0.589 | chromatin assembly | CG31611 |
| 622 | GO:0005267 | F | 6, 7, | 1 | 0.543 (x 1.843) | 52 (0.019) | 0.594 | potassium channel activity | Pk61C |
| 623 | GO:0044432 | C | 4, 5, 6, 7, 8, 9, | 1 | 0.543 (x 1.843) | 52 (0.019) | 0.595 | endoplasmic reticulum part | CG11500 |
| 624 | GO:0006323 | P | 7, | 2 | 1.440 (x 1.389) | 138 (0.014) | 0.595 | DNA packaging | CG31611 kis |
| 625 | GO:0006325 | P | 8, | 2 | 1.440 (x 1.389) | 138 (0.014) | 0.596 | establishment and/or maintenance of chromatin architecture | CG31611 kis |
| 626 | GO:0009314 | P | 4, | 1 | 0.553 (x 1.808) | 53 (0.019) | 0.597 | response to radiation | ogre |
| 627 | GO:0001708 | P | 5, | 1 | 0.553 (x 1.808) | 53 (0.019) | 0.598 | cell fate specification | Dr |
| 628 | GO:0051301 | P | 4, | 2 | 1.450 (x 1.379) | 139 (0.014) | 0.599 | cell division | hts spdo |
| 629 | GO:0030554 | F | 5, | 8 | 7.188 (x 1.113) | 689 (0.012) | 0.601 | adenyl nucleotide binding | CG11267 CG7182 Hsp70Bc Pk61C Su(z)2 Tbp-1 kis smi35A |
| 630 | GO:0005773 | C | 5, 6, 7, 8, | 1 | 0.563 (x 1.775) | 54 (0.019) | 0.602 | vacuole | CG30059 |
| 631 | GO:0044444 | C | 4, 5, 6, 7, | 13 | 12.071 (x 1.077) | 1157 (0.011) | 0.603 | cytoplasmic part | CBP CG11267 CG11500 CG30059 CG4759 CG5028 CG9057 Pi3K21B Prx5037 aru garz hts smi35A |
| 632 | GO:0006464 | P | 7, | 10 | 9.150 (x 1.093) | 877 (0.011) | 0.603 | protein modification | CG11500 CG17735 POSH Pi3K21B Pk61C Su(z)2 Ufd1-like lmg plexA smi35A |
| 633 | GO:0007304 | P | 8, 9, | 1 | 0.574 (x 1.743) | 55 (0.018) | 0.607 | eggshell formation (sensu Insecta) | Fs(2)Ket |
| 634 | GO:0017038 | P | 6, 7, | 1 | 0.574 (x 1.743) | 55 (0.018) | 0.608 | protein import | Fs(2)Ket |
| 635 | GO:0030703 | P | 7, | 1 | 0.574 (x 1.743) | 55 (0.018) | 0.609 | eggshell formation | Fs(2)Ket |
| 636 | GO:0005759 | C | 5, 6, 7, 8, 9, 10, 11, | 2 | 1.513 (x 1.322) | 145 (0.014) | 0.618 | mitochondrial matrix | CG11267 CG5028 |
| 637 | GO:0031980 | C | 4, 5, 6, 7, 8, 9, 10, | 2 | 1.513 (x 1.322) | 145 (0.014) | 0.619 | mitochondrial lumen | CG11267 CG5028 |
| 638 | GO:0009950 | P | 5, | 1 | 0.595 (x 1.682) | 57 (0.018) | 0.621 | dorsal/ventral axis specification | sqd |
| 639 | GO:0006813 | P | 8, 9, | 1 | 0.605 (x 1.653) | 58 (0.017) | 0.627 | potassium ion transport | Pk61C |
| 640 | GO:0009987 | P | 2, | 71 | 69.964 (x 1.015) | 6706 (0.011) | 0.627 | cellular process | Abi Akap200 B52 BEST:CK02137 BcDNA:LD41548 CG10960 CG11267 CG11500 CG17735 CG30059 CG30440 CG31611 CG3823 CG4759 CG5028 CG5873 CG6654 CG6854 CG6982 CG7182 CG7722 CG7920 CG8177 CG9057 Dll Dr Fkbp13 Fs(2)Ket Hop Hrb27C Hsp23 Hsp26 Hsp27 Hsp67Ba Hsp70Bc ImpL2 Lam Obp99a POSH Pi3K21B Pk61C Prx5037 Sox102F Su(z)2 Tbp-1 Ufd1-like Ugt86Di ana ap aru bowl edl ena eyg fax garz hth hts kis klu kn lmg msi ogre pdm2 plexA smi35A spdo sqd stai toy |
| 641 | GO:0005057 | F | 3, | 3 | 2.504 (x 1.198) | 240 (0.013) | 0.63 | receptor signaling protein activity | POSH Pk61C smi35A |
| 642 | GO:0000377 | P | 10, | 2 | 1.555 (x 1.287) | 149 (0.013) | 0.63 | RNA splicing, via transesterification reactions with bulged adenosine as nucleophile | B52 sqd |
| 643 | GO:0000398 | P | 9, 11, | 2 | 1.555 (x 1.287) | 149 (0.013) | 0.631 | nuclear mRNA splicing, via spliceosome | B52 sqd |
| 644 | GO:0000375 | P | 9, | 2 | 1.555 (x 1.287) | 149 (0.013) | 0.632 | RNA splicing, via transesterification reactions | B52 sqd |
| 645 | GO:0006790 | P | 5, | 1 | 0.616 (x 1.625) | 59 (0.017) | 0.633 | sulfur metabolism | CG30059 |
| 646 | GO:0007611 | P | 4, | 1 | 0.626 (x 1.597) | 60 (0.017) | 0.636 | learning and/or memory | CG8588 |
| 647 | GO:0048565 | P | 4, | 1 | 0.647 (x 1.546) | 62 (0.016) | 0.649 | gut development | bowl |
| 648 | GO:0006917 | P | 8, 9, | 1 | 0.647 (x 1.546) | 62 (0.016) | 0.65 | induction of apoptosis | smi35A |
| 649 | GO:0008380 | P | 8, | 2 | 1.617 (x 1.237) | 155 (0.013) | 0.654 | RNA splicing | B52 sqd |
| 650 | GO:0043234 | C | 2, | 17 | 16.474 (x 1.032) | 1579 (0.011) | 0.656 | protein complex | B52 CG11500 CG31611 CG4759 Fs(2)Ket Hrb27C POSH Pi3K21B Pk61C Su(z)2 Tbp-1 Ufd1-like aru eyg lmg sqd stai |
| 651 | GO:0051179 | P | 3, | 19 | 18.477 (x 1.028) | 1771 (0.011) | 0.656 | localization | Abi Akap200 BEST:CK02137 CG10960 CG3823 CG8177 CG9057 Fs(2)Ket Hrb27C Lam Obp99a POSH Pk61C ap ena garz plexA sqd stai |
| 652 | GO:0007582 | P | 2, | 72 | 71.278 (x 1.010) | 6832 (0.011) | 0.657 | physiological process | Abi Akap200 B52 BEST:CK02137 BcDNA:LD41548 CG10960 CG11267 CG11500 CG17121 CG17735 CG30059 CG31611 CG3823 CG4759 CG5028 CG5873 CG6654 CG6854 CG6982 CG7182 CG7722 CG7900 CG7920 CG8177 CG9057 Dll Dr Fkbp13 Fs(2)Ket Hop Hrb27C Hsp23 Hsp26 Hsp27 Hsp67Ba Hsp70Bc Lam Obp99a POSH Pi3K21B Pk61C Prx5037 RhoGAP71E Sox102F Su(z)2 Tbp-1 Ufd1-like Ugt86Di ana ap aru bowl edl ena eyg fax garz hth hts kis klu kn lmg msi ogre pdm2 plexA smi35A spdo sqd stai toy |
| 653 | GO:0043065 | P | 7, 8, | 1 | 0.668 (x 1.498) | 64 (0.016) | 0.657 | positive regulation of apoptosis | smi35A |
| 654 | GO:0043412 | P | 6, | 10 | 9.567 (x 1.045) | 917 (0.011) | 0.657 | biopolymer modification | CG11500 CG17735 POSH Pi3K21B Pk61C Su(z)2 Ufd1-like lmg plexA smi35A |
| 655 | GO:0030097 | P | 5, | 1 | 0.668 (x 1.498) | 64 (0.016) | 0.658 | hemopoiesis | kn |
| 656 | GO:0007001 | P | 7, | 2 | 1.638 (x 1.221) | 157 (0.013) | 0.659 | chromosome organization and biogenesis (sensu Eukaryota) | CG31611 kis |
| 657 | GO:0008150 | P | 1, | 85 | 84.320 (x 1.008) | 8082 (0.011) | 0.659 | biological\_process | Abi Akap200 B52 BEST:CK02137 BcDNA:LD41548 CG10960 CG11267 CG11500 CG17121 CG17735 CG30059 CG30437 CG30440 CG31363 CG31611 CG32267 CG3823 CG4759 CG5028 CG5873 CG6654 CG6854 CG6982 CG7182 CG7722 CG7900 CG7920 CG8177 CG8588 CG9057 Dll Dr Fkbp13 Fs(2)Ket Hop Hrb27C Hsp23 Hsp26 Hsp27 Hsp67Ba Hsp70Bc ImpE2 ImpE3 ImpL2 Lam MESR6 Obp99a POSH Pi3K21B Pk61C Prx5037 RhoGAP71E Sox102F Su(z)2 Tbp-1 Ufd1-like Ugt86Di Xe7 ana ap aru bip1 bowl charybde edl ena eyg fax garz hth hts kis klu kn lmg msi ogre pdm2 plexA scylla smi35A spdo sqd stai toy |
| 658 | GO:0044431 | C | 4, 5, 6, 7, 8, 9, | 1 | 0.678 (x 1.475) | 65 (0.015) | 0.66 | Golgi apparatus part | garz |
| 659 | GO:0043283 | P | 5, | 18 | 17.569 (x 1.025) | 1684 (0.011) | 0.66 | biopolymer metabolism | B52 CG11500 CG17735 CG30059 CG31611 Hrb27C POSH Pi3K21B Pk61C Su(z)2 Ufd1-like Ugt86Di kis lmg msi plexA smi35A sqd |
| 660 | GO:0035239 | P | 4, | 1 | 0.678 (x 1.475) | 65 (0.015) | 0.661 | tube morphogenesis | Lam |
| 661 | GO:0019207 | F | 3, | 1 | 0.699 (x 1.431) | 67 (0.015) | 0.672 | kinase regulator activity | Pi3K21B |
| 662 | GO:0005198 | F | 2, | 8 | 7.700 (x 1.039) | 738 (0.011) | 0.675 | structural molecule activity | CG4759 CG6982 CG8634 Lam POSH aru ena hts |
| 663 | GO:0016773 | F | 5, | 4 | 3.725 (x 1.074) | 357 (0.011) | 0.682 | phosphotransferase activity, alcohol group as acceptor | Pi3K21B Pk61C plexA smi35A |
| 664 | GO:0048102 | P | 6, | 1 | 0.741 (x 1.350) | 71 (0.014) | 0.689 | autophagic cell death | Akap200 |
| 665 | GO:0012502 | P | 7, 8, | 1 | 0.730 (x 1.369) | 70 (0.014) | 0.69 | induction of programmed cell death | smi35A |
| 666 | GO:0007163 | P | 5, 6, | 1 | 0.741 (x 1.350) | 71 (0.014) | 0.69 | establishment and/or maintenance of cell polarity | CG6982 |
| 667 | GO:0006040 | P | 6, 7, | 1 | 0.751 (x 1.331) | 72 (0.014) | 0.69 | amino sugar metabolism | CG30059 |
| 668 | GO:0035071 | P | 7, | 1 | 0.741 (x 1.350) | 71 (0.014) | 0.691 | salivary gland cell autophagic cell death | Akap200 |
| 669 | GO:0008652 | P | 7, 8, | 1 | 0.751 (x 1.331) | 72 (0.014) | 0.691 | amino acid biosynthesis | CG5028 |
| 670 | GO:0031090 | C | 4, 5, 6, 7, 8, | 4 | 3.766 (x 1.062) | 361 (0.011) | 0.691 | organelle membrane | CG11500 CG17952 Fs(2)Ket Lam |
| 671 | GO:0031975 | C | 2, | 3 | 2.786 (x 1.077) | 267 (0.011) | 0.692 | envelope | CG17952 Fs(2)Ket Lam |
| 672 | GO:0035070 | P | 6, | 1 | 0.741 (x 1.350) | 71 (0.014) | 0.692 | salivary gland histolysis | Akap200 |
| 673 | GO:0031967 | C | 3, 4, 5, 6, 7, 8, | 3 | 2.786 (x 1.077) | 267 (0.011) | 0.693 | organelle envelope | CG17952 Fs(2)Ket Lam |
| 674 | GO:0016310 | P | 7, | 5 | 4.810 (x 1.040) | 461 (0.011) | 0.693 | phosphorylation | Pi3K21B Pk61C edl plexA smi35A |
| 675 | GO:0006044 | P | 8, 9, | 1 | 0.741 (x 1.350) | 71 (0.014) | 0.693 | N-acetylglucosamine metabolism | CG30059 |
| 676 | GO:0006041 | P | 7, 8, | 1 | 0.741 (x 1.350) | 71 (0.014) | 0.694 | glucosamine metabolism | CG30059 |
| 677 | GO:0007601 | P | 5, 7, | 1 | 0.772 (x 1.295) | 74 (0.014) | 0.695 | visual perception | CG17121 |
| 678 | GO:0050953 | P | 4, 6, | 1 | 0.772 (x 1.295) | 74 (0.014) | 0.696 | sensory perception of light stimulus | CG17121 |
| 679 | GO:0007166 | P | 5, | 7 | 6.875 (x 1.018) | 659 (0.011) | 0.696 | cell surface receptor linked signal transduction | CG30440 Pi3K21B Pk61C aru edl plexA spdo |
| 680 | GO:0051276 | P | 6, | 2 | 1.794 (x 1.115) | 172 (0.012) | 0.696 | chromosome organization and biogenesis | CG31611 kis |
| 681 | GO:0016070 | P | 6, | 4 | 3.829 (x 1.045) | 367 (0.011) | 0.696 | RNA metabolism | B52 Hrb27C msi sqd |
| 682 | GO:0048534 | P | 4, | 1 | 0.772 (x 1.295) | 74 (0.014) | 0.697 | hemopoietic or lymphoid organ development | kn |
| 683 | GO:0044265 | P | 6, | 2 | 1.794 (x 1.115) | 172 (0.012) | 0.697 | cellular macromolecule catabolism | Ufd1-like sqd |
| 684 | GO:0003723 | F | 4, | 4 | 3.850 (x 1.039) | 369 (0.011) | 0.698 | RNA binding | B52 Hrb27C msi sqd |
| 685 | GO:0008565 | F | 3, | 1 | 0.782 (x 1.278) | 75 (0.013) | 0.698 | protein transporter activity | Fs(2)Ket |
| 686 | GO:0006511 | P | 9, 10, 11, | 1 | 0.782 (x 1.278) | 75 (0.013) | 0.699 | ubiquitin-dependent protein catabolism | Ufd1-like |
| 687 | GO:0019866 | C | 4, 5, 6, 7, 8, 9, | 2 | 1.815 (x 1.102) | 174 (0.011) | 0.699 | organelle inner membrane | CG17952 Lam |
| 688 | GO:0044262 | P | 6, | 3 | 2.869 (x 1.046) | 275 (0.011) | 0.699 | cellular carbohydrate metabolism | CG30059 CG5028 Pk61C |
| 689 | GO:0044264 | P | 6, 7, | 1 | 0.803 (x 1.245) | 77 (0.013) | 0.7 | cellular polysaccharide metabolism | Pk61C |
| 690 | GO:0008017 | F | 6, | 1 | 0.793 (x 1.261) | 76 (0.013) | 0.7 | microtubule binding | stai |
| 691 | GO:0019941 | P | 8, 9, 10, | 1 | 0.803 (x 1.245) | 77 (0.013) | 0.701 | modification-dependent protein catabolism | Ufd1-like |
| 692 | GO:0006644 | P | 7, 8, | 1 | 0.793 (x 1.261) | 76 (0.013) | 0.701 | phospholipid metabolism | Pi3K21B |
| 693 | GO:0006915 | P | 6, | 2 | 1.857 (x 1.077) | 178 (0.011) | 0.702 | apoptosis | Pk61C smi35A |
| 694 | GO:0035295 | P | 3, | 1 | 0.803 (x 1.245) | 77 (0.013) | 0.702 | tube development | Lam |
| 695 | GO:0008235 | F | 6, | 1 | 0.793 (x 1.261) | 76 (0.013) | 0.702 | metalloexopeptidase activity | BcDNA:LD41548 |
| 696 | GO:0004702 | F | 4, 8, | 2 | 1.847 (x 1.083) | 177 (0.011) | 0.703 | receptor signaling protein serine/threonine kinase activity | Pk61C smi35A |
| 697 | GO:0043632 | P | 7, | 1 | 0.803 (x 1.245) | 77 (0.013) | 0.703 | modification-dependent macromolecule catabolism | Ufd1-like |
| 698 | GO:0007391 | P | 6, | 1 | 0.793 (x 1.261) | 76 (0.013) | 0.703 | dorsal closure | ena |
| 699 | GO:0009112 | P | 6, | 1 | 0.814 (x 1.229) | 78 (0.013) | 0.704 | nucleobase metabolism | CG6854 |
| 700 | GO:0015268 | F | 4, | 2 | 1.899 (x 1.053) | 182 (0.011) | 0.714 | alpha-type channel activity | Pk61C ogre |
| 701 | GO:0016810 | F | 4, | 1 | 0.887 (x 1.128) | 85 (0.012) | 0.715 | hydrolase activity, acting on carbon-nitrogen (but not peptide) bonds | CG7900 |
| 702 | GO:0015267 | F | 3, | 2 | 1.899 (x 1.053) | 182 (0.011) | 0.715 | channel or pore class transporter activity | Pk61C ogre |
| 703 | GO:0008026 | F | 4, 10, | 1 | 0.866 (x 1.155) | 83 (0.012) | 0.715 | ATP-dependent helicase activity | kis |
| 704 | GO:0045182 | F | 2, | 1 | 0.887 (x 1.128) | 85 (0.012) | 0.716 | translation regulator activity | msi |
| 705 | GO:0003735 | F | 3, | 2 | 1.961 (x 1.020) | 188 (0.011) | 0.716 | structural constituent of ribosome | CG4759 aru |
| 706 | GO:0009057 | P | 5, | 2 | 1.982 (x 1.009) | 190 (0.011) | 0.716 | macromolecule catabolism | Ufd1-like sqd |
| 707 | GO:0031982 | C | 3, | 1 | 0.897 (x 1.115) | 86 (0.012) | 0.716 | vesicle | garz |
| 708 | GO:0005792 | C | 6, 7, | 1 | 0.866 (x 1.155) | 83 (0.012) | 0.716 | microsome | CG11500 |
| 709 | GO:0005856 | C | 5, 6, 7, 8, | 3 | 3.026 (x 0.992) | 290 (0.010) | 0.716 | cytoskeleton | CG31363 Lam stai |
| 710 | GO:0044421 | C | 2, 3, | 1 | 0.887 (x 1.128) | 85 (0.012) | 0.717 | extracellular region part | hts |
| 711 | GO:0006352 | P | 8, | 1 | 0.897 (x 1.115) | 86 (0.012) | 0.717 | transcription initiation | kn |
| 712 | GO:0016023 | C | 5, 6, 7, 8, 9, | 1 | 0.866 (x 1.155) | 83 (0.012) | 0.717 | cytoplasmic membrane-bound vesicle | garz |
| 713 | GO:0008643 | P | 5, 6, | 1 | 0.845 (x 1.183) | 81 (0.012) | 0.717 | carbohydrate transport | CG10960 |
| 714 | GO:0006367 | P | 9, | 1 | 0.876 (x 1.141) | 84 (0.012) | 0.717 | transcription initiation from RNA polymerase II promoter | kn |
| 715 | GO:0008135 | F | 3, 4, | 1 | 0.856 (x 1.169) | 82 (0.012) | 0.717 | translation factor activity, nucleic acid binding | msi |
| 716 | GO:0005667 | C | 3, 6, 7, 8, 9, 10, 11, 12, 13, | 1 | 0.887 (x 1.128) | 85 (0.012) | 0.718 | transcription factor complex | eyg |
| 717 | GO:0031988 | C | 4, | 1 | 0.897 (x 1.115) | 86 (0.012) | 0.718 | membrane-bound vesicle | garz |
| 718 | GO:0003682 | F | 3, | 1 | 0.866 (x 1.155) | 83 (0.012) | 0.718 | chromatin binding | kis |
| 719 | GO:0015144 | F | 3, | 1 | 0.845 (x 1.183) | 81 (0.012) | 0.718 | carbohydrate transporter activity | CG10960 |
| 720 | GO:0000226 | P | 8, | 1 | 0.908 (x 1.102) | 87 (0.011) | 0.718 | microtubule cytoskeleton organization and biogenesis | POSH |
| 721 | GO:0005840 | C | 4, 5, 6, 7, 8, | 2 | 1.972 (x 1.014) | 189 (0.011) | 0.718 | ribosome | CG4759 aru |
| 722 | GO:0044271 | P | 5, 6, | 1 | 0.876 (x 1.141) | 84 (0.012) | 0.718 | nitrogen compound biosynthesis | CG5028 |
| 723 | GO:0016331 | P | 5, | 1 | 0.856 (x 1.169) | 82 (0.012) | 0.718 | morphogenesis of embryonic epithelium | ena |
| 724 | GO:0005975 | P | 5, | 5 | 5.060 (x 0.988) | 485 (0.010) | 0.718 | carbohydrate metabolism | CG10960 CG30059 CG5028 Pk61C Ugt86Di |
| 725 | GO:0004713 | F | 7, | 1 | 0.887 (x 1.128) | 85 (0.012) | 0.719 | protein-tyrosine kinase activity | plexA |
| 726 | GO:0004672 | F | 6, | 3 | 3.078 (x 0.975) | 295 (0.010) | 0.719 | protein kinase activity | Pk61C plexA smi35A |
| 727 | GO:0042598 | C | 5, 6, | 1 | 0.866 (x 1.155) | 83 (0.012) | 0.719 | vesicular fraction | CG11500 |
| 728 | GO:0006643 | P | 6, 7, | 1 | 0.908 (x 1.102) | 87 (0.011) | 0.719 | membrane lipid metabolism | Pi3K21B |
| 729 | GO:0009309 | P | 6, 7, | 1 | 0.876 (x 1.141) | 84 (0.012) | 0.719 | amine biosynthesis | CG5028 |
| 730 | GO:0043037 | P | 7, 8, | 2 | 1.941 (x 1.031) | 186 (0.011) | 0.719 | translation | msi sqd |
| 731 | GO:0051603 | P | 8, 9, | 1 | 0.856 (x 1.169) | 82 (0.012) | 0.719 | proteolysis during cellular protein catabolism | Ufd1-like |
| 732 | GO:0031410 | C | 4, 5, 6, 7, 8, | 1 | 0.866 (x 1.155) | 83 (0.012) | 0.72 | cytoplasmic vesicle | garz |
| 733 | GO:0015630 | C | 6, 7, 8, 9, | 2 | 1.941 (x 1.031) | 186 (0.011) | 0.72 | microtubule cytoskeleton | CG31363 stai |
| 734 | GO:0044257 | P | 7, 8, | 1 | 0.856 (x 1.169) | 82 (0.012) | 0.72 | cellular protein catabolism | Ufd1-like |
| 735 | GO:0016301 | F | 5, | 4 | 4.184 (x 0.956) | 401 (0.010) | 0.725 | kinase activity | Pi3K21B Pk61C plexA smi35A |
| 736 | GO:0009792 | P | 4, | 2 | 2.034 (x 0.983) | 195 (0.010) | 0.726 | embryonic development (sensu Metazoa) | ena kn |
| 737 | GO:0005830 | C | 5, 6, 7, 8, 9, 10, | 1 | 0.939 (x 1.065) | 90 (0.011) | 0.728 | cytosolic ribosome (sensu Eukaryota) | CG4759 |
| 738 | GO:0030163 | P | 6, 7, | 1 | 0.939 (x 1.065) | 90 (0.011) | 0.729 | protein catabolism | Ufd1-like |
| 739 | GO:0016853 | F | 3, | 1 | 0.949 (x 1.053) | 91 (0.011) | 0.73 | isomerase activity | Fkbp13 |
| 740 | GO:0005794 | C | 5, 6, 7, 8, | 1 | 0.939 (x 1.065) | 90 (0.011) | 0.73 | Golgi apparatus | garz |
| 741 | GO:0005083 | F | 4, | 1 | 0.949 (x 1.053) | 91 (0.011) | 0.731 | small GTPase regulator activity | Abi |
| 742 | GO:0015631 | F | 5, | 1 | 0.949 (x 1.053) | 91 (0.011) | 0.732 | tubulin binding | stai |
| 743 | GO:0008194 | F | 5, | 1 | 0.960 (x 1.042) | 92 (0.011) | 0.733 | UDP-glycosyltransferase activity | Ugt86Di |
| 744 | GO:0005681 | C | 4, 5, 6, 7, 8, 9, 10, | 1 | 0.960 (x 1.042) | 92 (0.011) | 0.734 | spliceosome complex | B52 |
| 745 | GO:0005624 | C | 4, 5, | 1 | 0.970 (x 1.031) | 93 (0.011) | 0.735 | membrane fraction | CG11500 |
| 746 | GO:0044248 | P | 5, | 3 | 3.193 (x 0.940) | 306 (0.010) | 0.736 | cellular catabolism | CG5028 Ufd1-like sqd |
| 747 | GO:0008509 | F | 4, | 1 | 0.981 (x 1.020) | 94 (0.011) | 0.739 | anion transporter activity | CG8177 |
| 748 | GO:0015934 | C | 3, 4, 5, 6, 7, 8, 9, | 1 | 1.002 (x 0.998) | 96 (0.010) | 0.746 | large ribosomal subunit | CG4759 |
| 749 | GO:0000267 | C | 3, 4, | 1 | 1.002 (x 0.998) | 96 (0.010) | 0.747 | cell fraction | CG11500 |
| 750 | GO:0016491 | F | 3, | 6 | 6.510 (x 0.922) | 624 (0.010) | 0.751 | oxidoreductase activity | CG17121 CG30427 CG30437 CG5028 CG5873 Prx5037 |
| 751 | GO:0042302 | F | 3, | 1 | 1.033 (x 0.968) | 99 (0.010) | 0.758 | structural constituent of cuticle | CG8634 |
| 752 | GO:0009613 | P | 4, 5, | 1 | 1.043 (x 0.958) | 100 (0.010) | 0.76 | response to pest, pathogen or parasite | kn |
| 753 | GO:0004674 | F | 7, | 2 | 2.212 (x 0.904) | 212 (0.009) | 0.761 | protein serine/threonine kinase activity | Pk61C smi35A |
| 754 | GO:0006820 | P | 6, 7, | 1 | 1.043 (x 0.958) | 100 (0.010) | 0.761 | anion transport | CG8177 |
| 755 | GO:0000165 | P | 7, | 1 | 1.075 (x 0.931) | 103 (0.010) | 0.768 | MAPKKK cascade | CG30440 |
| 756 | GO:0030705 | P | 6, 7, 8, | 1 | 1.085 (x 0.922) | 104 (0.010) | 0.768 | cytoskeleton-dependent intracellular transport | CG9057 |
| 757 | GO:0044459 | C | 4, 5, 6, | 3 | 3.370 (x 0.890) | 323 (0.009) | 0.768 | plasma membrane part | CG6982 Pk61C ogre |
| 758 | GO:0007018 | P | 7, 8, 9, | 1 | 1.075 (x 0.931) | 103 (0.010) | 0.769 | microtubule-based movement | CG9057 |
| 759 | GO:0006092 | P | 7, | 1 | 1.085 (x 0.922) | 104 (0.010) | 0.769 | main pathways of carbohydrate metabolism | CG5028 |
| 760 | GO:0009056 | P | 4, | 3 | 3.412 (x 0.879) | 327 (0.009) | 0.769 | catabolism | CG5028 Ufd1-like sqd |
| 761 | GO:0005886 | C | 4, 5, | 5 | 5.571 (x 0.897) | 534 (0.009) | 0.769 | plasma membrane | CG6982 ImpE3 Pk61C ogre spdo |
| 762 | GO:0005509 | F | 5, | 2 | 2.274 (x 0.879) | 218 (0.009) | 0.769 | calcium ion binding | CBP Fkbp13 |
| 763 | GO:0015291 | F | 5, | 2 | 2.264 (x 0.883) | 217 (0.009) | 0.769 | porter activity | CG10960 CG8177 |
| 764 | GO:0015290 | F | 4, | 2 | 2.264 (x 0.883) | 217 (0.009) | 0.77 | electrochemical potential-driven transporter activity | CG10960 CG8177 |
| 765 | GO:0017076 | F | 4, | 8 | 8.858 (x 0.903) | 849 (0.009) | 0.771 | purine nucleotide binding | CG11267 CG7182 Hsp70Bc Pk61C Su(z)2 Tbp-1 kis smi35A |
| 766 | GO:0048598 | P | 4, | 1 | 1.106 (x 0.904) | 106 (0.009) | 0.772 | embryonic morphogenesis | ena |
| 767 | GO:0008238 | F | 5, | 1 | 1.137 (x 0.879) | 109 (0.009) | 0.781 | exopeptidase activity | BcDNA:LD41548 |
| 768 | GO:0007424 | P | 4, | 1 | 1.137 (x 0.879) | 109 (0.009) | 0.782 | tracheal system development (sensu Insecta) | Lam |
| 769 | GO:0009165 | P | 6, 7, | 1 | 1.148 (x 0.871) | 110 (0.009) | 0.784 | nucleotide biosynthesis | CG6854 |
| 770 | GO:0048232 | P | 5, | 1 | 1.158 (x 0.864) | 111 (0.009) | 0.785 | male gamete generation | Pk61C |
| 771 | GO:0007283 | P | 6, | 1 | 1.158 (x 0.864) | 111 (0.009) | 0.786 | spermatogenesis | Pk61C |
| 772 | GO:0016481 | P | 8, | 1 | 1.158 (x 0.864) | 111 (0.009) | 0.787 | negative regulation of transcription | edl |
| 773 | GO:0004386 | F | 3, | 1 | 1.168 (x 0.856) | 112 (0.009) | 0.788 | helicase activity | kis |
| 774 | GO:0046903 | P | 5, | 2 | 2.410 (x 0.830) | 231 (0.009) | 0.793 | secretion | garz stai |
| 775 | GO:0005549 | F | 3, | 1 | 1.200 (x 0.833) | 115 (0.009) | 0.794 | odorant binding | Obp99a |
| 776 | GO:0005261 | F | 5, 6, | 1 | 1.200 (x 0.833) | 115 (0.009) | 0.795 | cation channel activity | Pk61C |
| 777 | GO:0005386 | F | 3, | 4 | 4.726 (x 0.846) | 453 (0.009) | 0.796 | carrier activity | CG10960 CG3823 CG8177 Fs(2)Ket |
| 778 | GO:0005875 | C | 3, 5, 6, 7, 8, 9, 10, | 1 | 1.210 (x 0.826) | 116 (0.009) | 0.797 | microtubule associated complex | stai |
| 779 | GO:0000166 | F | 3, | 8 | 9.160 (x 0.873) | 878 (0.009) | 0.799 | nucleotide binding | CG11267 CG7182 Hsp70Bc Pk61C Su(z)2 Tbp-1 kis smi35A |
| 780 | GO:0007165 | P | 4, | 12 | 13.469 (x 0.891) | 1291 (0.009) | 0.802 | signal transduction | Abi Akap200 CG30440 Pi3K21B Pk61C aru edl klu ogre plexA spdo stai |
| 781 | GO:0006732 | P | 6, | 2 | 2.493 (x 0.802) | 239 (0.008) | 0.806 | coenzyme metabolism | CG5028 CG7920 |
| 782 | GO:0044430 | C | 4, 5, 6, 7, 8, 9, | 2 | 2.504 (x 0.799) | 240 (0.008) | 0.808 | cytoskeletal part | Lam stai |
| 783 | GO:0045934 | P | 7, | 1 | 1.262 (x 0.792) | 121 (0.008) | 0.809 | negative regulation of nucleobase, nucleoside, nucleotide and nucleic acid metabolism | edl |
| 784 | GO:0051234 | P | 4, | 16 | 17.820 (x 0.898) | 1708 (0.009) | 0.811 | establishment of localization | Abi BEST:CK02137 CG10960 CG3823 CG8177 CG9057 Fs(2)Ket Lam Obp99a Pk61C ap ena garz plexA sqd stai |
| 785 | GO:0006955 | P | 4, 5, | 1 | 1.273 (x 0.786) | 122 (0.008) | 0.811 | immune response | kn |
| 786 | GO:0006887 | P | 6, 7, | 1 | 1.304 (x 0.767) | 125 (0.008) | 0.819 | exocytosis | garz |
| 787 | GO:0007154 | P | 3, | 14 | 15.827 (x 0.885) | 1517 (0.009) | 0.819 | cell communication | Abi Akap200 CG30440 Pi3K21B Pk61C aru edl fax klu msi ogre plexA spdo stai |
| 788 | GO:0050874 | P | 3, | 8 | 9.390 (x 0.852) | 900 (0.009) | 0.82 | organismal physiological process | CG17121 Hsp26 Hsp27 Obp99a POSH fax kn msi |
| 789 | GO:0007243 | P | 6, | 1 | 1.325 (x 0.755) | 127 (0.008) | 0.822 | protein kinase cascade | CG30440 |
| 790 | GO:0044425 | C | 3, 4, 5, | 11 | 12.687 (x 0.867) | 1216 (0.009) | 0.822 | membrane part | CG10960 CG11500 CG17952 CG6982 Fs(2)Ket ImpE2 ImpE3 Lam Pk61C ogre spdo |
| 791 | GO:0016758 | F | 5, | 1 | 1.346 (x 0.743) | 129 (0.008) | 0.826 | transferase activity, transferring hexosyl groups | Ugt86Di |
| 792 | GO:0051186 | P | 5, | 2 | 2.640 (x 0.758) | 253 (0.008) | 0.827 | cofactor metabolism | CG5028 CG7920 |
| 793 | GO:0016887 | F | 8, | 3 | 3.871 (x 0.775) | 371 (0.008) | 0.829 | ATPase activity | CG11267 Tbp-1 kis |
| 794 | GO:0051707 | P | 4, | 1 | 1.367 (x 0.732) | 131 (0.008) | 0.83 | response to other organism | kn |
| 795 | GO:0007600 | P | 3, 5, | 2 | 2.692 (x 0.743) | 258 (0.008) | 0.835 | sensory perception | CG17121 Obp99a |
| 796 | GO:0008202 | P | 6, 7, | 1 | 1.419 (x 0.705) | 136 (0.007) | 0.841 | steroid metabolism | Ugt86Di |
| 797 | GO:0006796 | P | 6, | 5 | 6.302 (x 0.793) | 604 (0.008) | 0.841 | phosphate metabolism | Pi3K21B Pk61C edl plexA smi35A |
| 798 | GO:0006412 | P | 6, 7, | 4 | 5.154 (x 0.776) | 494 (0.008) | 0.841 | protein biosynthesis | CG4759 aru msi sqd |
| 799 | GO:0006793 | P | 5, | 5 | 6.302 (x 0.793) | 604 (0.008) | 0.842 | phosphorus metabolism | Pi3K21B Pk61C edl plexA smi35A |
| 800 | GO:0046483 | P | 5, | 1 | 1.461 (x 0.685) | 140 (0.007) | 0.848 | heterocycle metabolism | CG6854 |
| 801 | GO:0016772 | F | 4, | 4 | 5.227 (x 0.765) | 501 (0.008) | 0.849 | transferase activity, transferring phosphorus-containing groups | Pi3K21B Pk61C plexA smi35A |
| 802 | GO:0000278 | P | 5, | 2 | 2.796 (x 0.715) | 268 (0.007) | 0.849 | mitotic cell cycle | Lam lmg |
| 803 | GO:0006807 | P | 4, | 3 | 4.079 (x 0.735) | 391 (0.008) | 0.854 | nitrogen compound metabolism | CG30059 CG5028 CG7900 |
| 804 | GO:0006725 | P | 5, | 1 | 1.513 (x 0.661) | 145 (0.007) | 0.856 | aromatic compound metabolism | CG6854 |
| 805 | GO:0030001 | P | 7, 8, | 1 | 1.534 (x 0.652) | 147 (0.007) | 0.86 | metal ion transport | Pk61C |
| 806 | GO:0005102 | F | 3, 4, | 2 | 2.911 (x 0.687) | 279 (0.007) | 0.864 | receptor binding | CG17735 spdo |
| 807 | GO:0051641 | P | 4, 5, | 5 | 6.635 (x 0.754) | 636 (0.008) | 0.871 | cellular localization | CG9057 Fs(2)Ket Lam garz sqd |
| 808 | GO:0009059 | P | 5, 6, | 4 | 5.446 (x 0.734) | 522 (0.008) | 0.872 | macromolecule biosynthesis | CG4759 aru msi sqd |
| 809 | GO:0051649 | P | 5, 6, | 5 | 6.625 (x 0.755) | 635 (0.008) | 0.872 | establishment of cellular localization | CG9057 Fs(2)Ket Lam garz sqd |
| 810 | GO:0009117 | P | 6, | 1 | 1.659 (x 0.603) | 159 (0.006) | 0.88 | nucleotide metabolism | CG6854 |
| 811 | GO:0016462 | F | 6, | 4 | 5.540 (x 0.722) | 531 (0.008) | 0.881 | pyrophosphatase activity | CG11267 RhoGAP71E Tbp-1 kis |
| 812 | GO:0016757 | F | 4, | 1 | 1.659 (x 0.603) | 159 (0.006) | 0.881 | transferase activity, transferring glycosyl groups | Ugt86Di |
| 813 | GO:0005216 | F | 4, 5, | 1 | 1.711 (x 0.584) | 164 (0.006) | 0.89 | ion channel activity | Pk61C |
| 814 | GO:0007606 | P | 4, 6, | 1 | 1.732 (x 0.577) | 166 (0.006) | 0.893 | sensory perception of chemical stimulus | Obp99a |
| 815 | GO:0016818 | F | 5, | 4 | 5.717 (x 0.700) | 548 (0.007) | 0.895 | hydrolase activity, acting on acid anhydrides, in phosphorus-containing anhydrides | CG11267 RhoGAP71E Tbp-1 kis |
| 816 | GO:0016817 | F | 4, | 4 | 5.717 (x 0.700) | 548 (0.007) | 0.896 | hydrolase activity, acting on acid anhydrides | CG11267 RhoGAP71E Tbp-1 kis |
| 817 | GO:0015672 | P | 7, 8, | 1 | 1.826 (x 0.548) | 175 (0.006) | 0.906 | monovalent inorganic cation transport | Pk61C |
| 818 | GO:0008104 | P | 4, | 4 | 5.905 (x 0.677) | 566 (0.007) | 0.913 | protein localization | Akap200 Fs(2)Ket POSH garz |
| 819 | GO:0007267 | P | 4, | 3 | 4.674 (x 0.642) | 448 (0.007) | 0.915 | cell-cell signaling | fax msi stai |
| 820 | GO:0008237 | F | 5, | 1 | 1.930 (x 0.518) | 185 (0.005) | 0.92 | metallopeptidase activity | BcDNA:LD41548 |
| 821 | GO:0042623 | F | 9, | 2 | 3.443 (x 0.581) | 330 (0.006) | 0.924 | ATPase activity, coupled | CG11267 kis |
| 822 | GO:0005739 | C | 5, 6, 7, 8, | 3 | 4.820 (x 0.622) | 462 (0.006) | 0.925 | mitochondrion | CG11267 CG5028 Prx5037 |
| 823 | GO:0000004 | P | 2, | 5 | 7.314 (x 0.684) | 701 (0.007) | 0.925 | biological process unknown | CG31363 CG32267 MESR6 Xe7 bip1 |
| 824 | GO:0006811 | P | 5, 6, | 3 | 4.820 (x 0.622) | 462 (0.006) | 0.926 | ion transport | BEST:CK02137 CG8177 Pk61C |
| 825 | GO:0044255 | P | 5, 6, | 2 | 3.495 (x 0.572) | 335 (0.006) | 0.926 | cellular lipid metabolism | Pi3K21B Ugt86Di |
| 826 | GO:0051726 | P | 5, | 1 | 2.076 (x 0.482) | 199 (0.005) | 0.926 | regulation of cell cycle | Abi |
| 827 | GO:0044429 | C | 4, 5, 6, 7, 8, 9, | 2 | 3.599 (x 0.556) | 345 (0.006) | 0.927 | mitochondrial part | CG11267 CG5028 |
| 828 | GO:0031226 | C | 5, 6, 7, | 1 | 2.066 (x 0.484) | 198 (0.005) | 0.927 | intrinsic to plasma membrane | Pk61C |
| 829 | GO:0000074 | P | 6, | 1 | 2.076 (x 0.482) | 199 (0.005) | 0.927 | regulation of progression through cell cycle | Abi |
| 830 | GO:0007049 | P | 4, | 3 | 4.914 (x 0.611) | 471 (0.006) | 0.928 | cell cycle | Abi Lam lmg |
| 831 | GO:0004871 | F | 2, | 8 | 10.986 (x 0.728) | 1053 (0.008) | 0.928 | signal transducer activity | Abi CG17735 CG17952 POSH Pk61C plexA smi35A spdo |
| 832 | GO:0007498 | P | 4, | 1 | 2.066 (x 0.484) | 198 (0.005) | 0.928 | mesoderm development | toy |
| 833 | GO:0044249 | P | 5, | 6 | 8.659 (x 0.693) | 830 (0.007) | 0.929 | cellular biosynthesis | CG4759 CG5028 CG6854 aru msi sqd |
| 834 | GO:0005887 | C | 6, 7, 8, | 1 | 2.045 (x 0.489) | 196 (0.005) | 0.929 | integral to plasma membrane | Pk61C |
| 835 | GO:0046907 | P | 5, 6, 7, | 4 | 6.427 (x 0.622) | 616 (0.006) | 0.933 | intracellular transport | CG9057 Fs(2)Ket garz sqd |
| 836 | GO:0015075 | F | 3, | 3 | 5.060 (x 0.593) | 485 (0.006) | 0.934 | ion transporter activity | BEST:CK02137 CG8177 Pk61C |
| 837 | GO:0050877 | P | 4, | 4 | 6.427 (x 0.622) | 616 (0.006) | 0.934 | neurophysiological process | CG17121 Obp99a fax msi |
| 838 | GO:0005215 | F | 2, | 7 | 10.099 (x 0.693) | 968 (0.007) | 0.935 | transporter activity | BEST:CK02137 CG10960 CG3823 CG8177 Fs(2)Ket Pk61C ogre |
| 839 | GO:0006629 | P | 5, | 3 | 5.112 (x 0.587) | 490 (0.006) | 0.935 | lipid metabolism | CG9057 Pi3K21B Ugt86Di |
| 840 | GO:0016740 | F | 3, | 7 | 10.089 (x 0.694) | 967 (0.007) | 0.935 | transferase activity | Abi CG7920 Pi3K21B Pk61C Ugt86Di plexA smi35A |
| 841 | GO:0045045 | P | 5, 6, | 1 | 2.243 (x 0.446) | 215 (0.005) | 0.936 | secretory pathway | garz |
| 842 | GO:0019226 | P | 5, | 2 | 3.787 (x 0.528) | 363 (0.006) | 0.937 | transmission of nerve impulse | fax msi |
| 843 | GO:0005554 | F | 2, | 5 | 7.846 (x 0.637) | 752 (0.007) | 0.94 | molecular function unknown | CG31363 CG32267 CG8588 MESR6 Xe7 |
| 844 | GO:0006259 | P | 6, | 2 | 3.871 (x 0.517) | 371 (0.005) | 0.941 | DNA metabolism | CG31611 kis |
| 845 | GO:0000087 | P | 6, | 1 | 2.368 (x 0.422) | 227 (0.004) | 0.944 | M phase of mitotic cell cycle | lmg |
| 846 | GO:0007067 | P | 7, | 1 | 2.358 (x 0.424) | 226 (0.004) | 0.944 | mitosis | lmg |
| 847 | GO:0009308 | P | 5, | 2 | 3.923 (x 0.510) | 376 (0.005) | 0.944 | amine metabolism | CG30059 CG5028 |
| 848 | GO:0006605 | P | 7, 8, 9, | 1 | 2.410 (x 0.415) | 231 (0.004) | 0.945 | protein targeting | Fs(2)Ket |
| 849 | GO:0006812 | P | 6, 7, | 2 | 3.996 (x 0.501) | 383 (0.005) | 0.946 | cation transport | BEST:CK02137 Pk61C |
| 850 | GO:0009058 | P | 4, | 6 | 9.369 (x 0.640) | 898 (0.007) | 0.947 | biosynthesis | CG4759 CG5028 CG6854 aru msi sqd |
| 851 | GO:0017111 | F | 7, | 3 | 5.456 (x 0.550) | 523 (0.006) | 0.947 | nucleoside-triphosphatase activity | CG11267 Tbp-1 kis |
| 852 | GO:0008324 | F | 4, | 2 | 4.163 (x 0.480) | 399 (0.005) | 0.954 | cation transporter activity | BEST:CK02137 Pk61C |
| 853 | GO:0016021 | C | 5, 6, 7, | 6 | 9.849 (x 0.609) | 944 (0.006) | 0.965 | integral to membrane | CG10960 CG11500 Fs(2)Ket Pk61C ogre spdo |
| 854 | GO:0031224 | C | 4, 5, 6, | 6 | 9.880 (x 0.607) | 947 (0.006) | 0.965 | intrinsic to membrane | CG10960 CG11500 Fs(2)Ket Pk61C ogre spdo |
| 855 | GO:0006520 | P | 6, 7, | 1 | 2.723 (x 0.367) | 261 (0.004) | 0.965 | amino acid metabolism | CG5028 |
| 856 | GO:0016020 | C | 3, 4, | 14 | 19.468 (x 0.719) | 1866 (0.008) | 0.966 | membrane | BEST:CK02137 CG10960 CG11500 CG17952 CG6982 CG8177 Fs(2)Ket ImpE2 ImpE3 Lam Pk61C ogre plexA spdo |
| 857 | GO:0000279 | P | 5, | 1 | 2.994 (x 0.334) | 287 (0.003) | 0.976 | M phase | lmg |
| 858 | GO:0006810 | P | 4, 5, | 10 | 15.410 (x 0.649) | 1477 (0.007) | 0.978 | transport | BEST:CK02137 CG10960 CG3823 CG8177 CG9057 Fs(2)Ket Obp99a Pk61C garz sqd |
| 859 | GO:0006519 | P | 5, | 1 | 3.057 (x 0.327) | 293 (0.003) | 0.978 | amino acid and derivative metabolism | CG5028 |
| 860 | GO:0006508 | P | 7, | 4 | 7.908 (x 0.506) | 758 (0.005) | 0.979 | proteolysis | BcDNA:LD41548 CG7722 Tbp-1 Ufd1-like |
| 861 | GO:0016192 | P | 5, 6, | 1 | 3.088 (x 0.324) | 296 (0.003) | 0.979 | vesicle-mediated transport | garz |
| 862 | GO:0007186 | P | 6, | 1 | 3.182 (x 0.314) | 305 (0.003) | 0.979 | G-protein coupled receptor protein signaling pathway | CG30440 |
| 863 | GO:0007155 | P | 3, | 1 | 3.161 (x 0.316) | 303 (0.003) | 0.98 | cell adhesion | ImpL2 |
| 864 | GO:0006886 | P | 6, 7, 8, | 2 | 5.279 (x 0.379) | 506 (0.004) | 0.984 | intracellular protein transport | Fs(2)Ket garz |
| 865 | GO:0008372 | C | 2, | 4 | 8.492 (x 0.471) | 814 (0.005) | 0.984 | cellular component unknown | CG32267 CG8588 MESR6 Xe7 |
| 866 | GO:0006091 | P | 5, | 2 | 5.269 (x 0.380) | 505 (0.004) | 0.985 | generation of precursor metabolites and energy | CG5028 Pk61C |
| 867 | GO:0003674 | F | 1, | 82 | 86.448 (x 0.949) | 8286 (0.010) | 0.985 | molecular\_function | Abi Akap200 B52 BEST:CK02137 BcDNA:LD41548 CBP CG10960 CG11267 CG11500 CG12054 CG17121 CG17735 CG17952 CG30059 CG30427 CG30437 CG30440 CG31363 CG31611 CG32267 CG3823 CG4759 CG5028 CG5873 CG6654 CG6854 CG6982 CG7182 CG7722 CG7900 CG7920 CG8066 CG8089 CG8177 CG8588 CG8634 CG9057 Dll Dr Fkbp13 Fs(2)Ket Hop Hrb27C Hsp23 Hsp70Bc Lam MESR6 Obp99a POSH Pi3K21B Pk61C Prx5037 RhoGAP71E Sox102F Spn43Aa Su(z)2 Tbp-1 Ugt86Di Xe7 ap aru bip1 bowl edl ena eyg garz hth hts kis klu kn lmg msi ogre pdm2 plexA smi35A spdo sqd stai toy |
| 868 | GO:0045184 | P | 5, | 2 | 5.425 (x 0.369) | 520 (0.004) | 0.985 | establishment of protein localization | Fs(2)Ket garz |
| 869 | GO:0015031 | P | 5, 6, | 2 | 5.394 (x 0.371) | 517 (0.004) | 0.986 | protein transport | Fs(2)Ket garz |
| 870 | GO:0008233 | F | 4, | 3 | 6.792 (x 0.442) | 651 (0.005) | 0.986 | peptidase activity | BcDNA:LD41548 CG11500 Tbp-1 |
| 871 | GO:0004872 | F | 3, | 2 | 5.769 (x 0.347) | 553 (0.004) | 0.99 | receptor activity | CG17952 plexA |
| 872 | GO:0019752 | P | 6, | 1 | 4.090 (x 0.245) | 392 (0.003) | 0.991 | carboxylic acid metabolism | CG5028 |
| 873 | GO:0006082 | P | 5, | 1 | 4.090 (x 0.245) | 392 (0.003) | 0.992 | organic acid metabolism | CG5028 |
| 874 | GO:0004888 | F | 4, | 1 | 4.434 (x 0.226) | 425 (0.002) | 0.994 | transmembrane receptor activity | plexA |
| 875 | GO:0016788 | F | 4, | 1 | 4.747 (x 0.211) | 455 (0.002) | 0.996 | hydrolase activity, acting on ester bonds | CG30059 |
| 876 | GO:0004175 | F | 5, | 1 | 4.914 (x 0.204) | 471 (0.002) | 0.996 | endopeptidase activity | Tbp-1 |
| 877 | GO:0003824 | F | 2, | 27 | 39.447 (x 0.684) | 3781 (0.007) | 0.998 | catalytic activity | Abi BcDNA:LD41548 CG11267 CG11500 CG17121 CG17735 CG30059 CG30427 CG30437 CG5028 CG5873 CG6854 CG7900 CG7920 Fkbp13 POSH Pi3K21B Pk61C Prx5037 RhoGAP71E Su(z)2 Tbp-1 Ugt86Di kis lmg plexA smi35A |
| 878 | GO:0016787 | F | 3, | 8 | 18.738 (x 0.427) | 1796 (0.004) | 0.999 | hydrolase activity | BcDNA:LD41548 CG11267 CG11500 CG30059 CG7900 RhoGAP71E Tbp-1 kis |

  

---

Regulated Genes that don't have GO terms
  

BG:DS07721.3 BcDNA:GH11415 CG15905 CG18349 CG18731 CG2083 CG2469 CG32373 CG40294 CG4686 CG5281 CG6234 CG6900 CG6959 CG7694 CG7802 CG8031 CG8828 CG9186 CG9416 EG:152A3.3 Mkrn1
