# Supplementary material for: Functional dissection of the ash2 and ash1 transcriptomes provides insights into the transcriptional basis of wing phenotypes and reveals conserved protein interactions
Source: Genome Biol. 2007 Apr 28;8(4):R67. doi: 10.1186/gb-2007-8-4-r67 (PMC1896016; doi:10.1186/gb-2007-8-4-r67)
Supplement: Additional data file 14 — GO annotations of the genes upregulated over 1.5-fold in ash122 [file gb-2007-8-4-r67-S14.html]

  

---

  

|  |  |
| --- | --- |
| Go Statistics | Reg File: **ash1\_U1.5x.txt.fbgns** (194 genes -- 52 skipped)  Ref File: **ref.fbgns** (13577 genes -- 4663 skipped)  Database: **go\_200507-termdb.rdf-xml** |

---

  

Fields Description

| Pos | Go Term | Ontology | Levels | Observed | Expected | Possibles | p-value(Adj) | Go term description | Genes with the GO term |
| --- | --- | --- | --- | --- | --- | --- | --- | --- | --- |
| 1 | GO:0005830 | C | 5, 6, 7, 8, 9, 10, | 15 | 1.434 (x 10.462) | 90 (0.167) | 7.95e-09 | cytosolic ribosome (sensu Eukaryota) | CG11522 CG6764 CG7283 CG9282 RpL11 RpL27A RpL36 RpL38 RpL8 RpP1 RpS12 RpS18 RpS3 RpS9 sop |
| 2 | GO:0005840 | C | 4, 5, 6, 7, 8, | 19 | 3.011 (x 6.311) | 189 (0.101) | 3.79e-08 | ribosome | CG11522 CG33002 CG4866 CG6764 CG7283 CG9282 RpL11 RpL27A RpL36 RpL38 RpL8 RpP1 RpS12 RpS18 RpS3 RpS9 mRpL21 mRpS21 sop |
| 3 | GO:0003735 | F | 3, | 19 | 2.995 (x 6.344) | 188 (0.101) | 5.18e-08 | structural constituent of ribosome | CG11522 CG33002 CG4866 CG6764 CG7283 CG9282 RpL11 RpL27A RpL36 RpL38 RpL8 RpP1 RpS12 RpS18 RpS3 RpS9 mRpL21 mRpS21 sop |
| 4 | GO:0044445 | C | 5, 6, 7, 8, 9, | 15 | 1.880 (x 7.980) | 118 (0.127) | 1.08e-07 | cytosolic part | CG11522 CG6764 CG7283 CG9282 RpL11 RpL27A RpL36 RpL38 RpL8 RpP1 RpS12 RpS18 RpS3 RpS9 sop |
| 5 | GO:0030529 | C | 3, 4, 5, 6, | 23 | 5.066 (x 4.540) | 318 (0.072) | 1.72e-07 | ribonucleoprotein complex | CG11522 CG13277 CG17838 CG33002 CG3436 CG4866 CG6610 CG6764 CG7283 CG9282 RpL11 RpL27A RpL36 RpL38 RpL8 RpP1 RpS12 RpS18 RpS3 RpS9 mRpL21 mRpS21 sop |
| 6 | GO:0005198 | F | 2, | 35 | 11.756 (x 2.977) | 738 (0.047) | 3.95e-07 | structural molecule activity | Act57B BG:DS02740.9 CG11522 CG15006 CG2555 CG31876 CG33002 CG4673 CG4866 CG6764 CG7283 CG7846 CG7941 CG9282 Edg91 Gasp Lcp2 Lcp3 Mhc Mp20 RpL11 RpL27A RpL36 RpL38 RpL8 RpP1 RpS12 RpS18 RpS3 RpS9 fat-spondin mRpL21 mRpS21 robl sop |
| 7 | GO:0005842 | C | 4, 5, 6, 7, 8, 9, 10, 11, | 10 | 0.812 (x 12.309) | 51 (0.196) | 7.53e-07 | cytosolic large ribosomal subunit (sensu Eukaryota) | CG11522 CG6764 CG7283 CG9282 RpL11 RpL27A RpL36 RpL38 RpL8 RpP1 |
| 8 | GO:0015934 | C | 3, 4, 5, 6, 7, 8, 9, | 12 | 1.529 (x 7.847) | 96 (0.125) | 3.92e-06 | large ribosomal subunit | CG11522 CG33002 CG6764 CG7283 CG9282 RpL11 RpL27A RpL36 RpL38 RpL8 RpP1 mRpL21 |
| 9 | GO:0043228 | C | 3, | 31 | 11.326 (x 2.737) | 711 (0.044) | 1.64e-05 | non-membrane-bound organelle | Act57B CG11522 CG13277 CG31617 CG33002 CG4866 CG6610 CG6764 CG7283 CG7846 CG9282 Mhc Mlc1 Mlc2 Msp-300 RpL11 RpL27A RpL36 RpL38 RpL8 RpP1 RpS12 RpS18 RpS3 RpS9 Tm1 corto mRpL21 mRpS21 robl sop |
| 10 | GO:0005829 | C | 5, 6, 7, 8, | 15 | 2.931 (x 5.117) | 184 (0.082) | 1.76e-05 | cytosol | CG11522 CG6764 CG7283 CG9282 RpL11 RpL27A RpL36 RpL38 RpL8 RpP1 RpS12 RpS18 RpS3 RpS9 sop |
| 11 | GO:0043232 | C | 4, 5, 6, 7, | 31 | 11.326 (x 2.737) | 711 (0.044) | 1.83e-05 | intracellular non-membrane-bound organelle | Act57B CG11522 CG13277 CG31617 CG33002 CG4866 CG6610 CG6764 CG7283 CG7846 CG9282 Mhc Mlc1 Mlc2 Msp-300 RpL11 RpL27A RpL36 RpL38 RpL8 RpP1 RpS12 RpS18 RpS3 RpS9 Tm1 corto mRpL21 mRpS21 robl sop |
| 12 | GO:0044249 | P | 5, | 33 | 13.222 (x 2.496) | 830 (0.040) | 4.31e-05 | cellular biosynthesis | Act57B CG10932 CG11522 CG33002 CG4866 CG5037 CG5177 CG6188 CG6764 CG7283 CG7846 CG7860 CG9282 CG9804 Dhfr Gs2 RpL11 RpL27A RpL36 RpL38 RpL8 RpP1 RpS12 RpS18 RpS3 RpS9 Tbh Thor Vha36 desat1 mRpL21 mRpS21 sop |
| 13 | GO:0043292 | C | 5, 6, 7, 8, | 5 | 0.207 (x 24.144) | 13 (0.385) | 8.04e-05 | contractile fiber | Mhc Mlc1 Mlc2 Mp20 Tm1 |
| 14 | GO:0044446 | C | 3, 4, 5, 6, 7, | 45 | 22.684 (x 1.984) | 1424 (0.032) | 0.00013 | intracellular organelle part | Acf1 Act57B CG11522 CG12400 CG13277 CG15398 CG17838 CG2789 CG31617 CG33002 CG3436 CG4673 CG4866 CG5037 CG6610 CG6764 CG7283 CG7846 CG9282 EG:152A3.7 Mhc Mlc1 Mlc2 RpII18 RpL11 RpL27A RpL36 RpL38 RpL8 RpP1 RpS12 RpS18 RpS3 RpS9 Ssb-c31a Tfb2 Tm1 Trap36 Vha36 corto mRpL21 mRpS21 robl sop wal |
| 15 | GO:0044422 | C | 2, 3, | 45 | 22.684 (x 1.984) | 1424 (0.032) | 0.00014 | organelle part | Acf1 Act57B CG11522 CG12400 CG13277 CG15398 CG17838 CG2789 CG31617 CG33002 CG3436 CG4673 CG4866 CG5037 CG6610 CG6764 CG7283 CG7846 CG9282 EG:152A3.7 Mhc Mlc1 Mlc2 RpII18 RpL11 RpL27A RpL36 RpL38 RpL8 RpP1 RpS12 RpS18 RpS3 RpS9 Ssb-c31a Tfb2 Tm1 Trap36 Vha36 corto mRpL21 mRpS21 robl sop wal |
| 16 | GO:0009058 | P | 4, | 33 | 14.305 (x 2.307) | 898 (0.037) | 0.000189 | biosynthesis | Act57B CG10932 CG11522 CG33002 CG4866 CG5037 CG5177 CG6188 CG6764 CG7283 CG7846 CG7860 CG9282 CG9804 Dhfr Gs2 RpL11 RpL27A RpL36 RpL38 RpL8 RpP1 RpS12 RpS18 RpS3 RpS9 Tbh Thor Vha36 desat1 mRpL21 mRpS21 sop |
| 17 | GO:0000146 | F | 3, | 3 | 0.048 (x 62.775) | 3 (1.000) | 0.000207 | microfilament motor activity | Mhc Mlc1 Mlc2 |
| 18 | GO:0005859 | C | 5, 6, 7, 8, 9, 10, 11, 12, | 3 | 0.048 (x 62.775) | 3 (1.000) | 0.000219 | muscle myosin | Mhc Mlc1 Mlc2 |
| 19 | GO:0009059 | P | 5, 6, | 23 | 8.315 (x 2.766) | 522 (0.044) | 0.000367 | macromolecule biosynthesis | Act57B CG11522 CG33002 CG4866 CG5177 CG6764 CG7283 CG7860 CG9282 RpL11 RpL27A RpL36 RpL38 RpL8 RpP1 RpS12 RpS18 RpS3 RpS9 Thor mRpL21 mRpS21 sop |
| 20 | GO:0016460 | C | 4, 6, 7, 8, 9, 10, 11, | 3 | 0.064 (x 47.081) | 4 (0.750) | 0.000736 | myosin II | Mhc Mlc1 Mlc2 |
| 21 | GO:0044449 | C | 5, 6, 7, 8, 9, | 4 | 0.175 (x 22.827) | 11 (0.364) | 0.000837 | contractile fiber part | Mhc Mlc1 Mlc2 Tm1 |
| 22 | GO:0006412 | P | 6, 7, | 21 | 7.869 (x 2.669) | 494 (0.043) | 0.00142 | protein biosynthesis | CG11522 CG33002 CG4866 CG6764 CG7283 CG7860 CG9282 RpL11 RpL27A RpL36 RpL38 RpL8 RpP1 RpS12 RpS18 RpS3 RpS9 Thor mRpL21 mRpS21 sop |
| 23 | GO:0044444 | C | 4, 5, 6, 7, | 36 | 18.431 (x 1.953) | 1157 (0.031) | 0.00181 | cytoplasmic part | BG:DS00004.12 CG10932 CG11079 CG11522 CG12400 CG2789 CG33002 CG4866 CG5037 CG5189 CG6764 CG7283 CG9282 EG:152A3.7 Mhc Mlc1 Mlc2 Mp20 RpL11 RpL27A RpL36 RpL38 RpL8 RpP1 RpS12 RpS18 RpS3 RpS9 Tm1 Vha36 corto desat1 mRpL21 mRpS21 sop wal |
| 24 | GO:0043234 | C | 2, | 43 | 25.153 (x 1.710) | 1579 (0.027) | 0.00617 | protein complex | Acf1 CG11522 CG12400 CG13277 CG15398 CG17838 CG31617 CG33002 CG3436 CG4673 CG4866 CG6610 CG6764 CG7283 CG7846 CG9282 EG:152A3.7 Iap2 Lsp1beta Mhc Mlc1 Mlc2 Msp-300 RpII18 RpL11 RpL27A RpL36 RpL38 RpL8 RpP1 RpS12 RpS18 RpS3 RpS9 Ssb-c31a Tfb2 Trap36 Vha36 mRpL21 mRpS21 robl sop wal |
| 25 | GO:0015629 | C | 6, 7, 8, 9, | 7 | 1.179 (x 5.938) | 74 (0.095) | 0.00624 | actin cytoskeleton | Act57B CG7846 Mhc Mlc1 Mlc2 Msp-300 Tm1 |
| 26 | GO:0005214 | F | 4, | 7 | 1.290 (x 5.425) | 81 (0.086) | 0.0106 | structural constituent of cuticle (sensu Insecta) | CG15006 CG2555 CG31876 CG7941 Edg91 Lcp2 Lcp3 |
| 27 | GO:0016283 | C | 3, 5, 6, 7, 8, | 5 | 0.621 (x 8.048) | 39 (0.128) | 0.012 | eukaryotic 48S initiation complex | RpS12 RpS18 RpS3 RpS9 sop |
| 28 | GO:0005737 | C | 4, 5, 6, | 40 | 23.656 (x 1.691) | 1485 (0.027) | 0.012 | cytoplasm | BG:DS00004.12 CG10932 CG11079 CG11522 CG12400 CG2789 CG33002 CG4866 CG5037 CG5189 CG6764 CG7283 CG9282 EG:152A3.7 Gs2 Mhc Mlc1 Mlc2 Mlp60A Mlp84B Mp20 Pcmt RpL11 RpL27A RpL36 RpL38 RpL8 RpP1 RpS12 RpS18 RpS3 RpS9 Tm1 Vha36 corto desat1 mRpL21 mRpS21 sop wal |
| 29 | GO:0005843 | C | 4, 5, 6, 7, 8, 9, 10, 11, | 5 | 0.621 (x 8.048) | 39 (0.128) | 0.0124 | cytosolic small ribosomal subunit (sensu Eukaryota) | RpS12 RpS18 RpS3 RpS9 sop |
| 30 | GO:0008010 | F | 5, | 5 | 0.637 (x 7.847) | 40 (0.125) | 0.0126 | structural constituent of larval cuticle (sensu Insecta) | CG15006 CG2555 CG7941 Lcp2 Lcp3 |
| 31 | GO:0015935 | C | 3, 4, 5, 6, 7, 8, 9, | 6 | 1.099 (x 5.459) | 69 (0.087) | 0.0235 | small ribosomal subunit | RpS12 RpS18 RpS3 RpS9 mRpS21 sop |
| 32 | GO:0042302 | F | 3, | 7 | 1.577 (x 4.439) | 99 (0.071) | 0.029 | structural constituent of cuticle | CG15006 CG2555 CG31876 CG7941 Edg91 Lcp2 Lcp3 |
| 33 | GO:0016282 | C | 3, 5, 6, 7, 8, | 5 | 0.956 (x 5.231) | 60 (0.083) | 0.0737 | eukaryotic 43S preinitiation complex | RpS12 RpS18 RpS3 RpS9 sop |
| 34 | GO:0008011 | F | 5, | 2 | 0.112 (x 17.936) | 7 (0.286) | 0.139 | structural constituent of pupal cuticle (sensu Insecta) | CG31876 Edg91 |
| 35 | GO:0045735 | F | 2, | 2 | 0.127 (x 15.694) | 8 (0.250) | 0.164 | nutrient reservoir activity | Fbp2 Lsp1beta |
| 36 | GO:0005344 | F | 3, | 2 | 0.127 (x 15.694) | 8 (0.250) | 0.169 | oxygen transporter activity | CG8193 Lsp1beta |
| 37 | GO:0016459 | C | 3, 5, 6, 7, 8, 9, 10, | 3 | 0.398 (x 7.533) | 25 (0.120) | 0.17 | myosin | Mhc Mlc1 Mlc2 |
| 38 | GO:0043226 | C | 2, | 57 | 42.756 (x 1.333) | 2684 (0.021) | 0.171 | organelle | Acf1 Act57B BEST:LD29214 BG:DS00004.12 CG10932 CG11079 CG11522 CG12400 CG13277 CG15398 CG17838 CG18619 CG2789 CG31617 CG33002 CG3436 CG4673 CG4866 CG5037 CG5189 CG6610 CG6764 CG7283 CG7846 CG7911 CG9282 EG:152A3.7 Hr4 Mhc Mlc1 Mlc2 Mlp60A Mlp84B Msp-300 RpII18 RpL11 RpL27A RpL36 RpL38 RpL8 RpP1 RpS12 RpS18 RpS3 RpS9 Ssb-c31a Tfb2 Tm1 Trap36 Vha36 corto desat1 mRpL21 mRpS21 robl sop wal |
| 39 | GO:0043229 | C | 3, 4, 5, 6, | 57 | 42.756 (x 1.333) | 2684 (0.021) | 0.176 | intracellular organelle | Acf1 Act57B BEST:LD29214 BG:DS00004.12 CG10932 CG11079 CG11522 CG12400 CG13277 CG15398 CG17838 CG18619 CG2789 CG31617 CG33002 CG3436 CG4673 CG4866 CG5037 CG5189 CG6610 CG6764 CG7283 CG7846 CG7911 CG9282 EG:152A3.7 Hr4 Mhc Mlc1 Mlc2 Mlp60A Mlp84B Msp-300 RpII18 RpL11 RpL27A RpL36 RpL38 RpL8 RpP1 RpS12 RpS18 RpS3 RpS9 Ssb-c31a Tfb2 Tm1 Trap36 Vha36 corto desat1 mRpL21 mRpS21 robl sop wal |
| 40 | GO:0030016 | C | 6, 7, 8, 9, | 2 | 0.143 (x 13.950) | 9 (0.222) | 0.189 | myofibril | Mhc Tm1 |
| 41 | GO:0030017 | C | 6, 7, 8, 9, 10, | 2 | 0.143 (x 13.950) | 9 (0.222) | 0.193 | sarcomere | Mhc Tm1 |
| 42 | GO:0005688 | C | 5, 6, 7, 8, 9, 10, 11, 12, | 2 | 0.143 (x 13.950) | 9 (0.222) | 0.198 | snRNP U6 | CG13277 CG6610 |
| 43 | GO:0045978 | P | 8, | 1 | 0.016 (x 62.775) | 1 (1.000) | 0.203 | negative regulation of nucleoside metabolism | CG11079 |
| 44 | GO:0004836 | F | 4, | 1 | 0.016 (x 62.775) | 1 (1.000) | 0.205 | tyramine-beta hydroxylase activity | Tbh |
| 45 | GO:0045947 | P | 9, 10, 11, | 1 | 0.016 (x 62.775) | 1 (1.000) | 0.208 | negative regulation of translational initiation | Thor |
| 46 | GO:0030091 | P | 7, | 1 | 0.016 (x 62.775) | 1 (1.000) | 0.211 | protein repair | Pcmt |
| 47 | GO:0008190 | F | 5, | 1 | 0.016 (x 62.775) | 1 (1.000) | 0.214 | eukaryotic initiation factor 4E binding | Thor |
| 48 | GO:0006545 | P | 9, 10, | 1 | 0.016 (x 62.775) | 1 (1.000) | 0.217 | glycine biosynthesis | Dhfr |
| 49 | GO:0008415 | F | 6, | 6 | 1.832 (x 3.275) | 115 (0.052) | 0.219 | acyltransferase activity | CG10932 CG1969 CG5037 CG5122 CG5397 CG6921 |
| 50 | GO:0006307 | P | 6, 8, | 1 | 0.016 (x 62.775) | 1 (1.000) | 0.22 | DNA dealkylation | agt |
| 51 | GO:0008503 | F | 5, | 1 | 0.016 (x 62.775) | 1 (1.000) | 0.224 | benzodiazepine receptor activity | CG2789 |
| 52 | GO:0009118 | P | 7, | 1 | 0.016 (x 62.775) | 1 (1.000) | 0.227 | regulation of nucleoside metabolism | CG11079 |
| 53 | GO:0004750 | F | 6, | 1 | 0.016 (x 62.775) | 1 (1.000) | 0.231 | ribulose-phosphate 3-epimerase activity | CG30499 |
| 54 | GO:0004045 | F | 6, | 1 | 0.016 (x 62.775) | 1 (1.000) | 0.234 | aminoacyl-tRNA hydrolase activity | BG:DS00004.12 |
| 55 | GO:0004719 | F | 7, | 1 | 0.016 (x 62.775) | 1 (1.000) | 0.238 | protein-L-isoaspartate (D-aspartate) O-methyltransferase activity | Pcmt |
| 56 | GO:0031974 | C | 2, | 13 | 6.866 (x 1.893) | 431 (0.030) | 0.239 | membrane-enclosed lumen | Acf1 CG13277 CG15398 CG33002 CG4866 CG6610 RpII18 Ssb-c31a Tfb2 Trap36 mRpL21 mRpS21 wal |
| 57 | GO:0004364 | F | 5, | 3 | 0.573 (x 5.231) | 36 (0.083) | 0.242 | glutathione transferase activity | CG5224 GstD9 GstE1 |
| 58 | GO:0015949 | P | 6, | 1 | 0.016 (x 62.775) | 1 (1.000) | 0.242 | nucleobase, nucleoside and nucleotide interconversion | Dhfr |
| 59 | GO:0043233 | C | 3, 4, | 13 | 6.866 (x 1.893) | 431 (0.030) | 0.242 | organelle lumen | Acf1 CG13277 CG15398 CG33002 CG4866 CG6610 RpII18 Ssb-c31a Tfb2 Trap36 mRpL21 mRpS21 wal |
| 60 | GO:0051186 | P | 5, | 9 | 4.030 (x 2.233) | 253 (0.036) | 0.243 | cofactor metabolism | CG10237 CG2789 CG30499 CG5037 CG6574 CG7846 CG9804 Dhfr Vha36 |
| 61 | GO:0008495 | F | 7, | 1 | 0.016 (x 62.775) | 1 (1.000) | 0.246 | protoheme IX farnesyltransferase activity | CG5037 |
| 62 | GO:0004146 | F | 6, | 1 | 0.016 (x 62.775) | 1 (1.000) | 0.25 | dihydrofolate reductase activity | Dhfr |
| 63 | GO:0006936 | P | 4, | 5 | 1.370 (x 3.650) | 86 (0.058) | 0.254 | muscle contraction | Mhc Mlc1 Mlc2 Mp20 Tm1 |
| 64 | GO:0017174 | F | 7, | 1 | 0.016 (x 62.775) | 1 (1.000) | 0.254 | glycine N-methyltransferase activity | CG6188 |
| 65 | GO:0004343 | F | 9, | 1 | 0.016 (x 62.775) | 1 (1.000) | 0.258 | glucosamine 6-phosphate N-acetyltransferase activity | CG1969 |
| 66 | GO:0003908 | F | 7, | 1 | 0.016 (x 62.775) | 1 (1.000) | 0.263 | methylated-DNA-[protein]-cysteine S-methyltransferase activity | agt |
| 67 | GO:0016747 | F | 5, | 6 | 1.928 (x 3.113) | 121 (0.050) | 0.265 | transferase activity, transferring groups other than amino-acyl groups | CG10932 CG1969 CG5037 CG5122 CG5397 CG6921 |
| 68 | GO:0048033 | P | 7, 8, | 1 | 0.016 (x 62.775) | 1 (1.000) | 0.268 | heme o metabolism | CG5037 |
| 69 | GO:0006542 | P | 9, 10, | 1 | 0.016 (x 62.775) | 1 (1.000) | 0.273 | glutamine biosynthesis | Gs2 |
| 70 | GO:0015955 | P | 8, | 1 | 0.016 (x 62.775) | 1 (1.000) | 0.278 | pyrimidine deoxyribonucleotide interconversion | Dhfr |
| 71 | GO:0031369 | F | 4, | 1 | 0.016 (x 62.775) | 1 (1.000) | 0.283 | translation initiation factor binding | Thor |
| 72 | GO:0006732 | P | 6, | 9 | 3.807 (x 2.364) | 239 (0.038) | 0.287 | coenzyme metabolism | CG10237 CG2789 CG30499 CG5037 CG6574 CG7846 CG9804 Dhfr Vha36 |
| 73 | GO:0048034 | P | 8, 9, | 1 | 0.016 (x 62.775) | 1 (1.000) | 0.288 | heme o biosynthesis | CG5037 |
| 74 | GO:0006941 | P | 5, | 1 | 0.016 (x 62.775) | 1 (1.000) | 0.294 | striated muscle contraction | Mhc |
| 75 | GO:0015953 | P | 7, | 1 | 0.016 (x 62.775) | 1 (1.000) | 0.3 | pyrimidine nucleotide interconversion | Dhfr |
| 76 | GO:0016765 | F | 4, | 4 | 0.956 (x 4.185) | 60 (0.067) | 0.303 | transferase activity, transferring alkyl or aryl (other than methyl) groups | CG5037 CG5224 GstD9 GstE1 |
| 77 | GO:0006589 | P | 7, 8, 9, 10, | 1 | 0.016 (x 62.775) | 1 (1.000) | 0.306 | octopamine biosynthesis | Tbh |
| 78 | GO:0016746 | F | 4, | 6 | 2.023 (x 2.966) | 127 (0.047) | 0.309 | transferase activity, transferring acyl groups | CG10932 CG1969 CG5037 CG5122 CG5397 CG6921 |
| 79 | GO:0017133 | C | 4, 5, 6, 7, 8, 9, 10, 11, 12, | 1 | 0.032 (x 31.387) | 2 (0.500) | 0.31 | electron transfer flavoprotein complex (sensu Eukaryota) | wal |
| 80 | GO:0016590 | C | 5, 8, 9, 10, 11, 12, 13, 14, 15, | 1 | 0.032 (x 31.387) | 2 (0.500) | 0.313 | ACF complex | Acf1 |
| 81 | GO:0030272 | F | 6, | 1 | 0.032 (x 31.387) | 2 (0.500) | 0.316 | 5-formyltetrahydrofolate cyclo-ligase activity | CG11079 |
| 82 | GO:0005884 | C | 5, 6, 7, 8, 9, 10, | 2 | 0.287 (x 6.975) | 18 (0.111) | 0.317 | actin filament | Act57B CG7846 |
| 83 | GO:0005122 | F | 4, 5, | 1 | 0.032 (x 31.387) | 2 (0.500) | 0.32 | torso binding | tsl |
| 84 | GO:0003985 | F | 9, | 1 | 0.032 (x 31.387) | 2 (0.500) | 0.323 | acetyl-CoA C-acetyltransferase activity | CG10932 |
| 85 | GO:0005684 | C | 5, 6, 7, 8, 9, 10, 11, | 3 | 0.653 (x 4.593) | 41 (0.073) | 0.323 | major (U2-dependent) spliceosome | CG13277 CG3436 CG6610 |
| 86 | GO:0005542 | F | 4, | 1 | 0.048 (x 20.925) | 3 (0.333) | 0.325 | folic acid binding | CG6574 |
| 87 | GO:0004311 | F | 6, | 1 | 0.032 (x 31.387) | 2 (0.500) | 0.327 | farnesyltranstransferase activity | CG5037 |
| 88 | GO:0008171 | F | 6, | 1 | 0.048 (x 20.925) | 3 (0.333) | 0.328 | O-methyltransferase activity | Pcmt |
| 89 | GO:0000273 | P | 6, 7, | 1 | 0.048 (x 20.925) | 3 (0.333) | 0.33 | lipoic acid metabolism | CG9804 |
| 90 | GO:0005862 | C | 5, 6, 7, 8, 9, 10, 11, 12, | 1 | 0.032 (x 31.387) | 2 (0.500) | 0.33 | muscle thin filament tropomyosin | Tm1 |
| 91 | GO:0030414 | F | 4, | 4 | 1.370 (x 2.920) | 86 (0.047) | 0.33 | protease inhibitor activity | CG1342 CG16712 Cys fat-spondin |
| 92 | GO:0046351 | P | 7, 8, | 1 | 0.048 (x 20.925) | 3 (0.333) | 0.333 | disaccharide biosynthesis | CG5177 |
| 93 | GO:0030522 | P | 6, | 1 | 0.032 (x 31.387) | 2 (0.500) | 0.334 | intracellular receptor-mediated signaling pathway | Hr4 |
| 94 | GO:0016716 | F | 5, | 1 | 0.048 (x 20.925) | 3 (0.333) | 0.335 | oxidoreductase activity, acting on paired donors, with incorporation or reduction of molecular oxygen, another compound as one donor, and incorporation of one atom of oxygen | CG8193 |
| 95 | GO:0004092 | F | 9, | 1 | 0.048 (x 20.925) | 3 (0.333) | 0.338 | carnitine O-acetyltransferase activity | CG5122 |
| 96 | GO:0030518 | P | 7, | 1 | 0.032 (x 31.387) | 2 (0.500) | 0.338 | steroid hormone receptor signaling pathway | Hr4 |
| 97 | GO:0003774 | F | 2, | 4 | 1.386 (x 2.886) | 87 (0.046) | 0.34 | motor activity | Act57B Mhc Mlc1 Mlc2 |
| 98 | GO:0016979 | F | 5, | 1 | 0.048 (x 20.925) | 3 (0.333) | 0.34 | lipoate-protein ligase activity | CG9804 |
| 99 | GO:0008534 | F | 6, 8, | 1 | 0.032 (x 31.387) | 2 (0.500) | 0.342 | purine-specific oxidized base lesion DNA N-glycosylase activity | RpS3 |
| 100 | GO:0016211 | F | 6, | 1 | 0.048 (x 20.925) | 3 (0.333) | 0.343 | ammonia ligase activity | Gs2 |
| 101 | GO:0008172 | F | 6, | 1 | 0.048 (x 20.925) | 3 (0.333) | 0.346 | S-methyltransferase activity | agt |
| 102 | GO:0046333 | P | 6, 7, 8, 9, | 1 | 0.032 (x 31.387) | 2 (0.500) | 0.346 | octopamine metabolism | Tbh |
| 103 | GO:0045213 | P | 7, 8, | 1 | 0.048 (x 20.925) | 3 (0.333) | 0.349 | neurotransmitter receptor metabolism | Gs2 |
| 104 | GO:0005761 | C | 5, 6, 7, 8, 9, 10, 11, 12, | 4 | 1.179 (x 3.393) | 74 (0.054) | 0.35 | mitochondrial ribosome | CG33002 CG4866 mRpL21 mRpS21 |
| 105 | GO:0009055 | F | 4, | 3 | 0.844 (x 3.553) | 53 (0.057) | 0.35 | electron carrier activity | CG12400 EG:152A3.7 wal |
| 106 | GO:0009106 | P | 7, 8, | 1 | 0.032 (x 31.387) | 2 (0.500) | 0.35 | lipoate metabolism | CG9804 |
| 107 | GO:0008518 | F | 6, | 1 | 0.048 (x 20.925) | 3 (0.333) | 0.351 | reduced folate carrier activity | CG6574 |
| 108 | GO:0004857 | F | 3, | 5 | 2.023 (x 2.471) | 127 (0.039) | 0.352 | enzyme inhibitor activity | CG11079 CG1342 CG16712 Cys fat-spondin |
| 109 | GO:0009105 | P | 7, 8, | 1 | 0.032 (x 31.387) | 2 (0.500) | 0.354 | lipoic acid biosynthesis | CG9804 |
| 110 | GO:0017056 | F | 3, | 1 | 0.048 (x 20.925) | 3 (0.333) | 0.354 | structural constituent of nuclear pore | CG4673 |
| 111 | GO:0000313 | C | 5, 6, 7, 8, 9, | 4 | 1.179 (x 3.393) | 74 (0.054) | 0.354 | organellar ribosome | CG33002 CG4866 mRpL21 mRpS21 |
| 112 | GO:0005863 | C | 6, 7, 8, 9, 10, 11, 12, | 1 | 0.048 (x 20.925) | 3 (0.333) | 0.357 | striated muscle thick filament | Mhc |
| 113 | GO:0045251 | C | 3, 5, 6, 7, 8, | 1 | 0.032 (x 31.387) | 2 (0.500) | 0.358 | electron transfer flavoprotein complex | wal |
| 114 | GO:0004805 | F | 8, | 1 | 0.048 (x 20.925) | 3 (0.333) | 0.36 | trehalose-phosphatase activity | CG5177 |
| 115 | GO:0009107 | P | 8, 9, | 1 | 0.032 (x 31.387) | 2 (0.500) | 0.363 | lipoate biosynthesis | CG9804 |
| 116 | GO:0016453 | F | 8, | 1 | 0.048 (x 20.925) | 3 (0.333) | 0.363 | C-acetyltransferase activity | CG10932 |
| 117 | GO:0044430 | C | 4, 5, 6, 7, 8, 9, | 8 | 3.823 (x 2.092) | 240 (0.033) | 0.363 | cytoskeletal part | Act57B CG7846 Mhc Mlc1 Mlc2 Tm1 corto robl |
| 118 | GO:0006334 | P | 7, 11, | 2 | 0.382 (x 5.231) | 24 (0.083) | 0.364 | nucleosome assembly | Acf1 CG31617 |
| 119 | GO:0006541 | P | 8, 9, | 1 | 0.048 (x 20.925) | 3 (0.333) | 0.366 | glutamine metabolism | Gs2 |
| 120 | GO:0009636 | P | 5, | 5 | 2.055 (x 2.433) | 129 (0.039) | 0.367 | response to toxin | CG30438 CG31146 CG5224 GstD9 GstE1 |
| 121 | GO:0016407 | F | 7, | 3 | 0.749 (x 4.007) | 47 (0.064) | 0.367 | acetyltransferase activity | CG10932 CG1969 CG5122 |
| 122 | GO:0019842 | F | 3, | 2 | 0.382 (x 5.231) | 24 (0.083) | 0.367 | vitamin binding | CG10237 CG6574 |
| 123 | GO:0016880 | F | 5, | 1 | 0.048 (x 20.925) | 3 (0.333) | 0.369 | acid-ammonia (or amide) ligase activity | Gs2 |
| 124 | GO:0008374 | F | 7, | 2 | 0.319 (x 6.277) | 20 (0.100) | 0.37 | O-acyltransferase activity | CG5122 CG5397 |
| 125 | GO:0043112 | P | 7, | 1 | 0.048 (x 20.925) | 3 (0.333) | 0.372 | receptor metabolism | Gs2 |
| 126 | GO:0016584 | P | 11, | 1 | 0.064 (x 15.694) | 4 (0.250) | 0.373 | nucleosome spacing | Acf1 |
| 127 | GO:0005732 | C | 4, 5, 6, 7, 8, 9, 10, 11, 12, 13, | 2 | 0.319 (x 6.277) | 20 (0.100) | 0.374 | small nucleolar ribonucleoprotein complex | CG13277 CG6610 |
| 128 | GO:0009399 | P | 5, | 1 | 0.048 (x 20.925) | 3 (0.333) | 0.375 | nitrogen fixation | Gs2 |
| 129 | GO:0006538 | P | 9, 10, | 1 | 0.064 (x 15.694) | 4 (0.250) | 0.375 | glutamate catabolism | Gs2 |
| 130 | GO:0005622 | C | 3, 4, | 63 | 52.489 (x 1.200) | 3295 (0.019) | 0.376 | intracellular | Acf1 Act57B BEST:LD29214 BG:DS00004.12 BG:DS02740.9 CG10237 CG10932 CG11079 CG11522 CG12400 CG13277 CG15398 CG17838 CG18619 CG2789 CG31617 CG33002 CG3436 CG4673 CG4866 CG5037 CG5189 CG6610 CG6764 CG7283 CG7846 CG7911 CG9282 EG:152A3.7 Gs2 Hr4 Iap2 Mhc Mlc1 Mlc2 Mlp60A Mlp84B Mp20 Msp-300 Pcmt RpII18 RpL11 RpL27A RpL36 RpL38 RpL8 RpP1 RpS12 RpS18 RpS3 RpS9 Ssb-c31a Tfb2 Tm1 Trap36 Vha36 corto desat1 mRpL21 mRpS21 robl sop wal |
| 131 | GO:0006032 | P | 8, 9, 10, 11, | 1 | 0.064 (x 15.694) | 4 (0.250) | 0.378 | chitin catabolism | Chit |
| 132 | GO:0004503 | F | 6, | 1 | 0.048 (x 20.925) | 3 (0.333) | 0.378 | monophenol monooxygenase activity | CG8193 |
| 133 | GO:0006036 | P | 9, 10, 11, 12, | 1 | 0.064 (x 15.694) | 4 (0.250) | 0.38 | cuticle chitin catabolism | Chit |
| 134 | GO:0044452 | C | 5, 6, 7, 8, 9, 10, 11, 12, | 2 | 0.414 (x 4.829) | 26 (0.077) | 0.38 | nucleolar part | CG13277 CG6610 |
| 135 | GO:0005739 | C | 5, 6, 7, 8, | 12 | 7.360 (x 1.631) | 462 (0.026) | 0.382 | mitochondrion | BG:DS00004.12 CG10932 CG11079 CG12400 CG2789 CG33002 CG4866 CG5037 EG:152A3.7 mRpL21 mRpS21 wal |
| 136 | GO:0008511 | F | 7, 9, | 1 | 0.048 (x 20.925) | 3 (0.333) | 0.382 | sodium:potassium:chloride symporter activity | CG31547 |
| 137 | GO:0000272 | P | 7, | 1 | 0.064 (x 15.694) | 4 (0.250) | 0.383 | polysaccharide catabolism | Chit |
| 138 | GO:0005856 | C | 5, 6, 7, 8, | 9 | 4.620 (x 1.948) | 290 (0.031) | 0.383 | cytoskeleton | Act57B CG7846 Mhc Mlc1 Mlc2 Msp-300 Tm1 corto robl |
| 139 | GO:0000702 | F | 7, | 1 | 0.048 (x 20.925) | 3 (0.333) | 0.385 | oxidized base lesion DNA N-glycosylase activity | RpS3 |
| 140 | GO:0030728 | P | 6, | 1 | 0.064 (x 15.694) | 4 (0.250) | 0.385 | ovulation | Tbh |
| 141 | GO:0016591 | C | 3, 6, 7, 8, 9, 10, 11, 12, 13, | 4 | 1.322 (x 3.025) | 83 (0.048) | 0.387 | DNA-directed RNA polymerase II, holoenzyme | CG15398 RpII18 Tfb2 Trap36 |
| 142 | GO:0007519 | P | 5, | 3 | 0.781 (x 3.843) | 49 (0.061) | 0.388 | striated muscle development | Mhc Mlp60A Mlp84B |
| 143 | GO:0046348 | P | 7, 8, | 1 | 0.064 (x 15.694) | 4 (0.250) | 0.388 | amino sugar catabolism | Chit |
| 144 | GO:0031672 | C | 6, 7, 8, 9, 10, 11, | 1 | 0.048 (x 20.925) | 3 (0.333) | 0.388 | A band | Mhc |
| 145 | GO:0006046 | P | 9, 10, | 1 | 0.064 (x 15.694) | 4 (0.250) | 0.39 | N-acetylglucosamine catabolism | Chit |
| 146 | GO:0004356 | F | 7, | 1 | 0.048 (x 20.925) | 3 (0.333) | 0.392 | glutamate-ammonia ligase activity | Gs2 |
| 147 | GO:0044424 | C | 3, 4, 5, | 61 | 50.817 (x 1.200) | 3190 (0.019) | 0.392 | intracellular part | Acf1 Act57B BEST:LD29214 BG:DS00004.12 CG10932 CG11079 CG11522 CG12400 CG13277 CG15398 CG17838 CG18619 CG2789 CG31617 CG33002 CG3436 CG4673 CG4866 CG5037 CG5189 CG6610 CG6764 CG7283 CG7846 CG7911 CG9282 EG:152A3.7 Gs2 Hr4 Iap2 Mhc Mlc1 Mlc2 Mlp60A Mlp84B Mp20 Msp-300 Pcmt RpII18 RpL11 RpL27A RpL36 RpL38 RpL8 RpP1 RpS12 RpS18 RpS3 RpS9 Ssb-c31a Tfb2 Tm1 Trap36 Vha36 corto desat1 mRpL21 mRpS21 robl sop wal |
| 148 | GO:0006043 | P | 8, 9, | 1 | 0.064 (x 15.694) | 4 (0.250) | 0.393 | glucosamine catabolism | Chit |
| 149 | GO:0044271 | P | 5, 6, | 4 | 1.338 (x 2.989) | 84 (0.048) | 0.394 | nitrogen compound biosynthesis | CG6188 Dhfr Gs2 Tbh |
| 150 | GO:0016882 | F | 5, | 1 | 0.048 (x 20.925) | 3 (0.333) | 0.395 | cyclo-ligase activity | CG11079 |
| 151 | GO:0004563 | F | 7, | 1 | 0.064 (x 15.694) | 4 (0.250) | 0.396 | beta-N-acetylhexosaminidase activity | CG15012 |
| 152 | GO:0009309 | P | 6, 7, | 4 | 1.338 (x 2.989) | 84 (0.048) | 0.398 | amine biosynthesis | CG6188 Dhfr Gs2 Tbh |
| 153 | GO:0006034 | P | 8, 9, 10, 11, | 1 | 0.064 (x 15.694) | 4 (0.250) | 0.398 | cuticle chitin metabolism | Chit |
| 154 | GO:0005992 | P | 8, 9, | 1 | 0.048 (x 20.925) | 3 (0.333) | 0.399 | trehalose biosynthesis | CG5177 |
| 155 | GO:0004866 | F | 5, | 4 | 1.354 (x 2.954) | 85 (0.047) | 0.401 | endopeptidase inhibitor activity | CG1342 CG16712 Cys fat-spondin |
| 156 | GO:0042043 | F | 4, | 1 | 0.064 (x 15.694) | 4 (0.250) | 0.401 | neurexin binding | CG31146 |
| 157 | GO:0004772 | F | 8, | 1 | 0.048 (x 20.925) | 3 (0.333) | 0.402 | sterol O-acyltransferase activity | CG5397 |
| 158 | GO:0006826 | P | 9, 10, | 1 | 0.064 (x 15.694) | 4 (0.250) | 0.404 | iron ion transport | Tsf1 |
| 159 | GO:0006633 | P | 6, 7, 8, | 2 | 0.462 (x 4.329) | 29 (0.069) | 0.404 | fatty acid biosynthesis | CG10932 desat1 |
| 160 | GO:0051766 | F | 7, | 1 | 0.080 (x 12.555) | 5 (0.200) | 0.406 | inositol trisphosphate kinase activity | CG18854 |
| 161 | GO:0044247 | P | 7, 8, | 1 | 0.064 (x 15.694) | 4 (0.250) | 0.407 | cellular polysaccharide catabolism | Chit |
| 162 | GO:0005991 | P | 8, | 1 | 0.080 (x 12.555) | 5 (0.200) | 0.408 | trehalose metabolism | CG5177 |
| 163 | GO:0009008 | F | 6, | 1 | 0.080 (x 12.555) | 5 (0.200) | 0.41 | DNA-methyltransferase activity | agt |
| 164 | GO:0019104 | F | 6, | 1 | 0.080 (x 12.555) | 5 (0.200) | 0.413 | DNA N-glycosylase activity | RpS3 |
| 165 | GO:0051189 | P | 5, 7, | 3 | 0.956 (x 3.139) | 60 (0.050) | 0.413 | prosthetic group metabolism | CG10237 CG2789 CG6574 |
| 166 | GO:0005865 | C | 5, 6, 7, 8, 9, 10, 11, | 1 | 0.080 (x 12.555) | 5 (0.200) | 0.415 | striated muscle thin filament | Tm1 |
| 167 | GO:0016413 | F | 8, | 1 | 0.080 (x 12.555) | 5 (0.200) | 0.417 | O-acetyltransferase activity | CG5122 |
| 168 | GO:0016655 | F | 5, | 2 | 0.446 (x 4.484) | 28 (0.071) | 0.418 | oxidoreductase activity, acting on NADH or NADPH, quinone or similar compound as acceptor | CG12400 EG:152A3.7 |
| 169 | GO:0003906 | F | 5, | 1 | 0.080 (x 12.555) | 5 (0.200) | 0.42 | DNA-(apurinic or apyrimidinic site) lyase activity | RpS3 |
| 170 | GO:0008137 | F | 6, 7, | 2 | 0.446 (x 4.484) | 28 (0.071) | 0.42 | NADH dehydrogenase (ubiquinone) activity | CG12400 EG:152A3.7 |
| 171 | GO:0005381 | F | 6, | 1 | 0.080 (x 12.555) | 5 (0.200) | 0.422 | iron ion transporter activity | Tsf1 |
| 172 | GO:0005759 | C | 5, 6, 7, 8, 9, 10, 11, | 5 | 2.310 (x 2.165) | 145 (0.034) | 0.422 | mitochondrial matrix | CG33002 CG4866 mRpL21 mRpS21 wal |
| 173 | GO:0050136 | F | 6, | 2 | 0.446 (x 4.484) | 28 (0.071) | 0.423 | NADH dehydrogenase (quinone) activity | CG12400 EG:152A3.7 |
| 174 | GO:0031981 | C | 4, 5, 6, 7, 8, 9, 10, | 8 | 4.381 (x 1.826) | 275 (0.029) | 0.423 | nuclear lumen | Acf1 CG13277 CG15398 CG6610 RpII18 Ssb-c31a Tfb2 Trap36 |
| 175 | GO:0006119 | P | 6, 8, | 5 | 2.246 (x 2.226) | 141 (0.035) | 0.424 | oxidative phosphorylation | CG12400 CG7846 EG:152A3.7 Vha36 wal |
| 176 | GO:0031980 | C | 4, 5, 6, 7, 8, 9, 10, | 5 | 2.310 (x 2.165) | 145 (0.034) | 0.425 | mitochondrial lumen | CG33002 CG4866 mRpL21 mRpS21 wal |
| 177 | GO:0045792 | P | 6, 7, | 1 | 0.080 (x 12.555) | 5 (0.200) | 0.425 | negative regulation of cell size | Thor |
| 178 | GO:0008623 | C | 4, 7, 8, 9, 10, 11, 12, 13, 14, | 1 | 0.080 (x 12.555) | 5 (0.200) | 0.427 | chromatin accessibility complex | Acf1 |
| 179 | GO:0008440 | F | 8, | 1 | 0.080 (x 12.555) | 5 (0.200) | 0.43 | inositol trisphosphate 3-kinase activity | CG18854 |
| 180 | GO:0030532 | C | 4, 5, 6, 7, 8, 9, 10, | 3 | 0.988 (x 3.037) | 62 (0.048) | 0.43 | small nuclear ribonucleoprotein complex | CG13277 CG3436 CG6610 |
| 181 | GO:0015377 | F | 6, 8, | 1 | 0.080 (x 12.555) | 5 (0.200) | 0.432 | cation:chloride symporter activity | CG31547 |
| 182 | GO:0005984 | P | 7, | 1 | 0.096 (x 10.462) | 6 (0.167) | 0.434 | disaccharide metabolism | CG5177 |
| 183 | GO:0006516 | P | 8, 9, 10, | 1 | 0.080 (x 12.555) | 5 (0.200) | 0.435 | glycoprotein catabolism | CG7860 |
| 184 | GO:0005527 | F | 4, | 1 | 0.096 (x 10.462) | 6 (0.167) | 0.437 | macrolide binding | CG14715 |
| 185 | GO:0019203 | F | 7, | 1 | 0.096 (x 10.462) | 6 (0.167) | 0.439 | carbohydrate phosphatase activity | CG5177 |
| 186 | GO:0007517 | P | 4, | 4 | 1.720 (x 2.325) | 108 (0.037) | 0.44 | muscle development | Mhc Mlp60A Mlp84B Mp20 |
| 187 | GO:0005616 | C | 3, 4, 5, | 1 | 0.096 (x 10.462) | 6 (0.167) | 0.441 | larval serum protein complex | Lsp1beta |
| 188 | GO:0016053 | P | 6, | 2 | 0.494 (x 4.050) | 31 (0.065) | 0.442 | organic acid biosynthesis | CG10932 desat1 |
| 189 | GO:0030239 | P | 7, 8, 10, 11, | 1 | 0.096 (x 10.462) | 6 (0.167) | 0.443 | myofibril assembly | Mhc |
| 190 | GO:0006752 | P | 7, | 3 | 1.147 (x 2.616) | 72 (0.042) | 0.444 | group transfer coenzyme metabolism | CG7846 Dhfr Vha36 |
| 191 | GO:0005576 | C | 2, | 10 | 6.149 (x 1.626) | 386 (0.026) | 0.444 | extracellular region | CG11051 CG15361 Chit Gasp Lsp1beta Obp99b Tsf1 fat-spondin hig tsl |
| 192 | GO:0046394 | P | 7, | 2 | 0.494 (x 4.050) | 31 (0.065) | 0.444 | carboxylic acid biosynthesis | CG10932 desat1 |
| 193 | GO:0016705 | F | 4, | 3 | 1.051 (x 2.853) | 66 (0.045) | 0.444 | oxidoreductase activity, acting on paired donors, with incorporation or reduction of molecular oxygen | CG8193 Tbh desat1 |
| 194 | GO:0051188 | P | 6, | 4 | 1.673 (x 2.391) | 105 (0.038) | 0.444 | cofactor biosynthesis | CG5037 CG7846 CG9804 Vha36 |
| 195 | GO:0005528 | F | 5, | 1 | 0.096 (x 10.462) | 6 (0.167) | 0.446 | FK506 binding | CG14715 |
| 196 | GO:0008652 | P | 7, 8, | 3 | 1.147 (x 2.616) | 72 (0.042) | 0.446 | amino acid biosynthesis | CG6188 Dhfr Gs2 |
| 197 | GO:0016358 | P | 7, 10, | 2 | 0.558 (x 3.587) | 35 (0.057) | 0.446 | dendrite development | Tm1 robl |
| 198 | GO:0016406 | F | 8, | 1 | 0.096 (x 10.462) | 6 (0.167) | 0.448 | carnitine O-acyltransferase activity | CG5122 |
| 199 | GO:0006120 | P | 9, 11, | 2 | 0.558 (x 3.587) | 35 (0.057) | 0.448 | mitochondrial electron transport, NADH to ubiquinone | CG12400 EG:152A3.7 |
| 200 | GO:0042766 | P | 10, | 1 | 0.112 (x 8.968) | 7 (0.143) | 0.449 | nucleosome mobilization | Acf1 |
| 201 | GO:0016918 | F | 4, 5, | 1 | 0.096 (x 10.462) | 6 (0.167) | 0.45 | retinal binding | CG10237 |
| 202 | GO:0016202 | P | 6, | 1 | 0.112 (x 8.968) | 7 (0.143) | 0.451 | regulation of striated muscle development | Mlp60A |
| 203 | GO:0007218 | P | 7, | 2 | 0.526 (x 3.805) | 33 (0.061) | 0.452 | neuropeptide signaling pathway | CG11051 CG15361 |
| 204 | GO:0004192 | F | 7, | 1 | 0.096 (x 10.462) | 6 (0.167) | 0.453 | cathepsin D activity | CG10104 |
| 205 | GO:0005501 | F | 4, | 1 | 0.112 (x 8.968) | 7 (0.143) | 0.453 | retinoid binding | CG10237 |
| 206 | GO:0046916 | P | 8, | 1 | 0.112 (x 8.968) | 7 (0.143) | 0.455 | transition metal ion homeostasis | Tsf1 |
| 207 | GO:0004500 | F | 6, | 1 | 0.096 (x 10.462) | 6 (0.167) | 0.455 | dopamine beta-monooxygenase activity | Tbh |
| 208 | GO:0030530 | C | 4, 5, 6, 7, 8, 9, 10, | 1 | 0.112 (x 8.968) | 7 (0.143) | 0.457 | heterogeneous nuclear ribonucleoprotein complex | CG17838 |
| 209 | GO:0006544 | P | 8, 9, | 1 | 0.096 (x 10.462) | 6 (0.167) | 0.457 | glycine metabolism | Dhfr |
| 210 | GO:0006879 | P | 8, 9, | 1 | 0.112 (x 8.968) | 7 (0.143) | 0.459 | iron ion homeostasis | Tsf1 |
| 211 | GO:0005678 | C | 4, 7, 8, 9, 10, 11, 12, 13, 14, | 1 | 0.096 (x 10.462) | 6 (0.167) | 0.46 | chromatin assembly complex | Acf1 |
| 212 | GO:0050654 | P | 6, 8, | 1 | 0.112 (x 8.968) | 7 (0.143) | 0.461 | chondroitin sulfate proteoglycan metabolism | Act57B |
| 213 | GO:0003954 | F | 5, | 2 | 0.573 (x 3.487) | 36 (0.056) | 0.462 | NADH dehydrogenase activity | CG12400 EG:152A3.7 |
| 214 | GO:0044429 | C | 4, 5, 6, 7, 8, 9, | 9 | 5.496 (x 1.638) | 345 (0.026) | 0.463 | mitochondrial part | CG12400 CG2789 CG33002 CG4866 CG5037 EG:152A3.7 mRpL21 mRpS21 wal |
| 215 | GO:0008199 | F | 7, | 1 | 0.112 (x 8.968) | 7 (0.143) | 0.463 | ferric iron binding | Tsf1 |
| 216 | GO:0019840 | F | 3, | 1 | 0.112 (x 8.968) | 7 (0.143) | 0.465 | isoprenoid binding | CG10237 |
| 217 | GO:0004867 | F | 6, | 3 | 1.115 (x 2.690) | 70 (0.043) | 0.466 | serine-type endopeptidase inhibitor activity | CG1342 CG16712 fat-spondin |
| 218 | GO:0006189 | P | 11, 12, | 1 | 0.112 (x 8.968) | 7 (0.143) | 0.468 | 'de novo' IMP biosynthesis | Dhfr |
| 219 | GO:0005184 | F | 5, 6, | 2 | 0.542 (x 3.693) | 34 (0.059) | 0.469 | neuropeptide hormone activity | CG11051 CG15361 |
| 220 | GO:0030206 | P | 8, 9, 10, | 1 | 0.112 (x 8.968) | 7 (0.143) | 0.47 | chondroitin sulfate biosynthesis | Act57B |
| 221 | GO:0006760 | P | 6, 7, 8, | 1 | 0.112 (x 8.968) | 7 (0.143) | 0.472 | folic acid and derivative metabolism | Dhfr |
| 222 | GO:0015929 | F | 6, | 1 | 0.112 (x 8.968) | 7 (0.143) | 0.474 | hexosaminidase activity | CG15012 |
| 223 | GO:0050650 | P | 7, 8, 9, | 1 | 0.112 (x 8.968) | 7 (0.143) | 0.477 | chondroitin sulfate proteoglycan biosynthesis | Act57B |
| 224 | GO:0046040 | P | 10, | 1 | 0.112 (x 8.968) | 7 (0.143) | 0.479 | IMP metabolism | Dhfr |
| 225 | GO:0005747 | C | 4, 5, 6, 7, 8, 9, 10, 11, 12, 13, 14, | 2 | 0.589 (x 3.393) | 37 (0.054) | 0.479 | respiratory chain complex I (sensu Eukaryota) | CG12400 EG:152A3.7 |
| 226 | GO:0045271 | C | 3, 4, 5, 6, | 2 | 0.589 (x 3.393) | 37 (0.054) | 0.481 | respiratory chain complex I | CG12400 EG:152A3.7 |
| 227 | GO:0030204 | P | 7, 8, 9, | 1 | 0.112 (x 8.968) | 7 (0.143) | 0.481 | chondroitin sulfate metabolism | Act57B |
| 228 | GO:0006821 | P | 8, 9, | 1 | 0.127 (x 7.847) | 8 (0.125) | 0.483 | chloride transport | CG31547 |
| 229 | GO:0044451 | C | 5, 6, 7, 8, 9, 10, 11, 12, | 6 | 3.234 (x 1.855) | 203 (0.030) | 0.483 | nucleoplasm part | Acf1 CG15398 RpII18 Ssb-c31a Tfb2 Trap36 |
| 230 | GO:0009065 | P | 8, 9, | 1 | 0.112 (x 8.968) | 7 (0.143) | 0.483 | glutamine family amino acid catabolism | Gs2 |
| 231 | GO:0042136 | P | 6, 9, | 1 | 0.127 (x 7.847) | 8 (0.125) | 0.485 | neurotransmitter biosynthesis | Tbh |
| 232 | GO:0006188 | P | 10, 11, | 1 | 0.112 (x 8.968) | 7 (0.143) | 0.486 | IMP biosynthesis | Dhfr |
| 233 | GO:0016215 | F | 5, | 1 | 0.127 (x 7.847) | 8 (0.125) | 0.487 | CoA desaturase activity | desat1 |
| 234 | GO:0006916 | P | 8, 9, | 2 | 0.605 (x 3.304) | 38 (0.053) | 0.488 | anti-apoptosis | CG7188 Iap2 |
| 235 | GO:0004768 | F | 6, | 1 | 0.127 (x 7.847) | 8 (0.125) | 0.489 | stearoyl-CoA 9-desaturase activity | desat1 |
| 236 | GO:0004067 | F | 6, | 1 | 0.127 (x 7.847) | 8 (0.125) | 0.491 | asparaginase activity | CG7860 |
| 237 | GO:0042221 | P | 4, | 7 | 4.190 (x 1.671) | 263 (0.027) | 0.503 | response to chemical stimulus | CG30438 CG31146 CG5224 GstD9 GstE1 Obp99b Tbh |
| 238 | GO:0005682 | C | 5, 6, 7, 8, 9, 10, 11, 12, | 1 | 0.143 (x 6.975) | 9 (0.111) | 0.513 | snRNP U5 | CG3436 |
| 239 | GO:0016715 | F | 5, | 1 | 0.143 (x 6.975) | 9 (0.111) | 0.515 | oxidoreductase activity, acting on paired donors, with incorporation or reduction of molecular oxygen, reduced ascorbate as one donor, and incorporation of one atom of oxygen | Tbh |
| 240 | GO:0030201 | P | 6, 8, | 1 | 0.143 (x 6.975) | 9 (0.111) | 0.517 | heparan sulfate proteoglycan metabolism | Act57B |
| 241 | GO:0006536 | P | 8, 9, | 1 | 0.143 (x 6.975) | 9 (0.111) | 0.519 | glutamate metabolism | Gs2 |
| 242 | GO:0016408 | F | 7, | 1 | 0.143 (x 6.975) | 9 (0.111) | 0.521 | C-acyltransferase activity | CG10932 |
| 243 | GO:0015012 | P | 7, 8, 9, | 1 | 0.143 (x 6.975) | 9 (0.111) | 0.523 | heparan sulfate proteoglycan biosynthesis | Act57B |
| 244 | GO:0003779 | F | 5, | 4 | 1.959 (x 2.041) | 123 (0.033) | 0.525 | actin binding | BG:DS02740.9 Mp20 Msp-300 Tm1 |
| 245 | GO:0005675 | C | 4, 7, 8, 9, 10, 11, 12, 13, 14, | 1 | 0.143 (x 6.975) | 9 (0.111) | 0.526 | transcription factor TFIIH complex | Tfb2 |
| 246 | GO:0008431 | F | 4, | 1 | 0.143 (x 6.975) | 9 (0.111) | 0.528 | vitamin E binding | CG10237 |
| 247 | GO:0006367 | P | 9, | 3 | 1.338 (x 2.242) | 84 (0.036) | 0.53 | transcription initiation from RNA polymerase II promoter | CG15398 Tfb2 Trap36 |
| 248 | GO:0004869 | F | 6, | 1 | 0.143 (x 6.975) | 9 (0.111) | 0.53 | cysteine protease inhibitor activity | Cys |
| 249 | GO:0000910 | P | 5, | 3 | 1.338 (x 2.242) | 84 (0.036) | 0.532 | cytokinesis | Act57B Mhc Msp-300 |
| 250 | GO:0044260 | P | 5, | 40 | 34.281 (x 1.167) | 2152 (0.019) | 0.533 | cellular macromolecule metabolism | Act57B CG10104 CG10237 CG10862 CG11522 CG1304 CG13991 CG14715 CG2789 CG33002 CG4386 CG4408 CG4866 CG6574 CG6764 CG7283 CG7860 CG9267 CG9282 CG9804 Chit Gasp Gs2 Iap2 Mlc1 Pcmt RpL11 RpL27A RpL36 RpL38 RpL8 RpP1 RpS12 RpS18 RpS3 RpS9 Thor mRpL21 mRpS21 sop |
| 251 | GO:0008168 | F | 5, | 3 | 1.338 (x 2.242) | 84 (0.036) | 0.534 | methyltransferase activity | CG6188 Pcmt agt |
| 252 | GO:0016651 | F | 4, | 2 | 0.685 (x 2.920) | 43 (0.047) | 0.535 | oxidoreductase activity, acting on NADH or NADPH | CG12400 EG:152A3.7 |
| 253 | GO:0005506 | F | 6, | 2 | 0.685 (x 2.920) | 43 (0.047) | 0.537 | iron ion binding | Tsf1 desat1 |
| 254 | GO:0016741 | F | 4, | 3 | 1.354 (x 2.216) | 85 (0.035) | 0.537 | transferase activity, transferring one-carbon groups | CG6188 Pcmt agt |
| 255 | GO:0006023 | P | 7, 8, | 1 | 0.159 (x 6.277) | 10 (0.100) | 0.537 | aminoglycan biosynthesis | Act57B |
| 256 | GO:0006814 | P | 8, 9, | 2 | 0.685 (x 2.920) | 43 (0.047) | 0.539 | sodium ion transport | CG31547 CG4805 |
| 257 | GO:0005667 | C | 3, 6, 7, 8, 9, 10, 11, 12, 13, | 3 | 1.354 (x 2.216) | 85 (0.035) | 0.539 | transcription factor complex | CG15398 Ssb-c31a Tfb2 |
| 258 | GO:0006024 | P | 8, 9, | 1 | 0.159 (x 6.277) | 10 (0.100) | 0.539 | glycosaminoglycan biosynthesis | Act57B |
| 259 | GO:0006783 | P | 7, 8, | 1 | 0.159 (x 6.277) | 10 (0.100) | 0.541 | heme biosynthesis | CG5037 |
| 260 | GO:0043066 | P | 7, 8, | 2 | 0.669 (x 2.989) | 42 (0.048) | 0.542 | negative regulation of apoptosis | CG7188 Iap2 |
| 261 | GO:0005858 | C | 5, 6, 7, 8, 9, 10, 11, 12, | 1 | 0.175 (x 5.707) | 11 (0.091) | 0.543 | axonemal dynein complex | robl |
| 262 | GO:0006022 | P | 6, 7, | 1 | 0.159 (x 6.277) | 10 (0.100) | 0.543 | aminoglycan metabolism | Act57B |
| 263 | GO:0016799 | F | 5, | 2 | 0.669 (x 2.989) | 42 (0.048) | 0.544 | hydrolase activity, hydrolyzing N-glycosyl compounds | Chit RpS3 |
| 264 | GO:0008307 | F | 3, | 1 | 0.175 (x 5.707) | 11 (0.091) | 0.544 | structural constituent of muscle | Mhc |
| 265 | GO:0009308 | P | 5, | 9 | 5.990 (x 1.503) | 376 (0.024) | 0.545 | amine metabolism | Act57B CG5122 CG6188 CG7860 Chit Dhfr Gasp Gs2 Tbh |
| 266 | GO:0008305 | C | 4, 5, 6, 7, 8, 9, | 1 | 0.159 (x 6.277) | 10 (0.100) | 0.546 | integrin complex | Msp-300 |
| 267 | GO:0043069 | P | 6, 7, | 2 | 0.669 (x 2.989) | 42 (0.048) | 0.546 | negative regulation of programmed cell death | CG7188 Iap2 |
| 268 | GO:0005930 | C | 4, 5, 6, 7, 8, | 1 | 0.175 (x 5.707) | 11 (0.091) | 0.546 | axoneme | robl |
| 269 | GO:0030203 | P | 7, 8, | 1 | 0.159 (x 6.277) | 10 (0.100) | 0.548 | glycosaminoglycan metabolism | Act57B |
| 270 | GO:0006352 | P | 8, | 3 | 1.370 (x 2.190) | 86 (0.035) | 0.548 | transcription initiation | CG15398 Tfb2 Trap36 |
| 271 | GO:0006779 | P | 7, | 1 | 0.175 (x 5.707) | 11 (0.091) | 0.548 | porphyrin biosynthesis | CG5037 |
| 272 | GO:0016857 | F | 5, | 1 | 0.159 (x 6.277) | 10 (0.100) | 0.55 | racemase and epimerase activity, acting on carbohydrates and derivatives | CG30499 |
| 273 | GO:0006446 | P | 8, 9, 10, | 1 | 0.175 (x 5.707) | 11 (0.091) | 0.55 | regulation of translational initiation | Thor |
| 274 | GO:0005654 | C | 5, 6, 7, 8, 9, 10, 11, | 6 | 3.568 (x 1.681) | 224 (0.027) | 0.551 | nucleoplasm | Acf1 CG15398 RpII18 Ssb-c31a Tfb2 Trap36 |
| 275 | GO:0048149 | P | 5, 7, | 1 | 0.159 (x 6.277) | 10 (0.100) | 0.552 | behavioral response to ethanol | Tbh |
| 276 | GO:0005665 | C | 4, 5, 6, 7, 8, 9, 10, 11, 12, 13, 14, | 1 | 0.175 (x 5.707) | 11 (0.091) | 0.552 | DNA-directed RNA polymerase II, core complex | RpII18 |
| 277 | GO:0044447 | C | 4, 5, 6, 7, 8, 9, | 1 | 0.175 (x 5.707) | 11 (0.091) | 0.554 | axoneme part | robl |
| 278 | GO:0005762 | C | 5, 6, 7, 8, 9, 10, 11, 12, 13, | 2 | 0.717 (x 2.790) | 45 (0.044) | 0.556 | mitochondrial large ribosomal subunit | CG33002 mRpL21 |
| 279 | GO:0000041 | P | 8, 9, | 1 | 0.175 (x 5.707) | 11 (0.091) | 0.556 | transition metal ion transport | Tsf1 |
| 280 | GO:0000315 | C | 4, 5, 6, 7, 8, 9, 10, | 2 | 0.717 (x 2.790) | 45 (0.044) | 0.558 | organellar large ribosomal subunit | CG33002 mRpL21 |
| 281 | GO:0042168 | P | 6, 7, | 1 | 0.175 (x 5.707) | 11 (0.091) | 0.558 | heme metabolism | CG5037 |
| 282 | GO:0009260 | P | 7, 8, | 3 | 1.450 (x 2.069) | 91 (0.033) | 0.562 | ribonucleotide biosynthesis | CG7846 Dhfr Vha36 |
| 283 | GO:0019538 | P | 5, | 40 | 34.775 (x 1.150) | 2183 (0.018) | 0.562 | protein metabolism | Acf1 Act57B CG10104 CG10237 CG10862 CG11522 CG1304 CG13991 CG14715 CG2789 CG31617 CG33002 CG4386 CG4408 CG4866 CG6574 CG6764 CG7283 CG7860 CG9267 CG9282 CG9804 Gs2 Iap2 Mlc1 Pcmt RpL11 RpL27A RpL36 RpL38 RpL8 RpP1 RpS12 RpS18 RpS3 RpS9 Thor mRpL21 mRpS21 sop |
| 284 | GO:0006740 | P | 10, 11, | 1 | 0.191 (x 5.231) | 12 (0.083) | 0.563 | NADPH regeneration | CG30499 |
| 285 | GO:0009152 | P | 8, 9, | 3 | 1.418 (x 2.116) | 89 (0.034) | 0.564 | purine ribonucleotide biosynthesis | CG7846 Dhfr Vha36 |
| 286 | GO:0006164 | P | 7, 8, | 3 | 1.450 (x 2.069) | 91 (0.033) | 0.564 | purine nucleotide biosynthesis | CG7846 Dhfr Vha36 |
| 287 | GO:0019237 | F | 6, | 1 | 0.191 (x 5.231) | 12 (0.083) | 0.565 | centromeric DNA binding | Msp-300 |
| 288 | GO:0006739 | P | 9, 10, | 1 | 0.191 (x 5.231) | 12 (0.083) | 0.567 | NADP metabolism | CG30499 |
| 289 | GO:0009259 | P | 7, | 3 | 1.466 (x 2.047) | 92 (0.033) | 0.569 | ribonucleotide metabolism | CG7846 Dhfr Vha36 |
| 290 | GO:0019752 | P | 6, | 9 | 6.245 (x 1.441) | 392 (0.023) | 0.569 | carboxylic acid metabolism | CG10932 CG5122 CG6188 CG6921 CG7860 CG9804 Dhfr Gs2 desat1 |
| 291 | GO:0006098 | P | 8, 10, 11, 12, | 1 | 0.191 (x 5.231) | 12 (0.083) | 0.569 | pentose-phosphate shunt | CG30499 |
| 292 | GO:0006807 | P | 4, | 9 | 6.229 (x 1.445) | 391 (0.023) | 0.569 | nitrogen compound metabolism | Act57B CG5122 CG6188 CG7860 Chit Dhfr Gasp Gs2 Tbh |
| 293 | GO:0006163 | P | 7, | 3 | 1.466 (x 2.047) | 92 (0.033) | 0.571 | purine nucleotide metabolism | CG7846 Dhfr Vha36 |
| 294 | GO:0019236 | P | 5, | 1 | 0.191 (x 5.231) | 12 (0.083) | 0.571 | response to pheromone | Obp99b |
| 295 | GO:0006082 | P | 5, | 9 | 6.245 (x 1.441) | 392 (0.023) | 0.571 | organic acid metabolism | CG10932 CG5122 CG6188 CG6921 CG7860 CG9804 Dhfr Gs2 desat1 |
| 296 | GO:0046483 | P | 5, | 4 | 2.230 (x 1.794) | 140 (0.029) | 0.572 | heterocycle metabolism | CG5037 CG9804 Dhfr Vha36 |
| 297 | GO:0044267 | P | 6, | 38 | 33.118 (x 1.147) | 2079 (0.018) | 0.573 | cellular protein metabolism | Act57B CG10104 CG10237 CG10862 CG11522 CG1304 CG13991 CG14715 CG2789 CG33002 CG4386 CG4408 CG4866 CG6574 CG6764 CG7283 CG7860 CG9267 CG9282 CG9804 Gs2 Iap2 Mlc1 Pcmt RpL11 RpL27A RpL36 RpL38 RpL8 RpP1 RpS12 RpS18 RpS3 RpS9 Thor mRpL21 mRpS21 sop |
| 298 | GO:0009150 | P | 8, | 3 | 1.434 (x 2.092) | 90 (0.033) | 0.573 | purine ribonucleotide metabolism | CG7846 Dhfr Vha36 |
| 299 | GO:0005681 | C | 4, 5, 6, 7, 8, 9, 10, | 3 | 1.466 (x 2.047) | 92 (0.033) | 0.573 | spliceosome complex | CG13277 CG3436 CG6610 |
| 300 | GO:0009888 | P | 3, | 9 | 6.340 (x 1.420) | 398 (0.023) | 0.573 | tissue development | BG:DS02740.9 CG31146 CG5397 Mlc1 Mlp60A Mlp84B Msp-300 fat-spondin mfas |
| 301 | GO:0009084 | P | 8, 9, | 1 | 0.191 (x 5.231) | 12 (0.083) | 0.573 | glutamine family amino acid biosynthesis | Gs2 |
| 302 | GO:0009108 | P | 7, | 3 | 1.497 (x 2.003) | 94 (0.032) | 0.573 | coenzyme biosynthesis | CG7846 CG9804 Vha36 |
| 303 | GO:0009063 | P | 7, 8, | 2 | 0.781 (x 2.562) | 49 (0.041) | 0.573 | amino acid catabolism | CG7860 Gs2 |
| 304 | GO:0016646 | F | 5, | 1 | 0.207 (x 4.829) | 13 (0.077) | 0.574 | oxidoreductase activity, acting on the CH-NH group of donors, NAD or NADP as acceptor | Dhfr |
| 305 | GO:0045471 | P | 6, | 1 | 0.191 (x 5.231) | 12 (0.083) | 0.575 | response to ethanol | Tbh |
| 306 | GO:0016051 | P | 6, 7, | 2 | 0.781 (x 2.562) | 49 (0.041) | 0.575 | carbohydrate biosynthesis | Act57B CG5177 |
| 307 | GO:0006778 | P | 6, | 1 | 0.207 (x 4.829) | 13 (0.077) | 0.576 | porphyrin metabolism | CG5037 |
| 308 | GO:0005689 | C | 5, 6, 7, 8, 9, 10, 11, | 1 | 0.207 (x 4.829) | 13 (0.077) | 0.578 | minor (U12-dependent) spliceosome complex | CG3436 |
| 309 | GO:0042133 | P | 5, 8, | 1 | 0.207 (x 4.829) | 13 (0.077) | 0.58 | neurotransmitter metabolism | Tbh |
| 310 | GO:0009070 | P | 8, 9, | 1 | 0.223 (x 4.484) | 14 (0.071) | 0.58 | serine family amino acid biosynthesis | Dhfr |
| 311 | GO:0006112 | P | 7, | 1 | 0.223 (x 4.484) | 14 (0.071) | 0.582 | energy reserve metabolism | CG5177 |
| 312 | GO:0006584 | P | 7, 8, | 1 | 0.207 (x 4.829) | 13 (0.077) | 0.582 | catecholamine metabolism | Tbh |
| 313 | GO:0004623 | F | 8, | 1 | 0.223 (x 4.484) | 14 (0.071) | 0.583 | phospholipase A2 activity | CG10133 |
| 314 | GO:0004194 | F | 7, | 1 | 0.207 (x 4.829) | 13 (0.077) | 0.584 | pepsin A activity | CG10104 |
| 315 | GO:0018958 | P | 6, | 1 | 0.223 (x 4.484) | 14 (0.071) | 0.585 | phenol metabolism | Tbh |
| 316 | GO:0042401 | P | 7, 8, | 1 | 0.223 (x 4.484) | 14 (0.071) | 0.587 | biogenic amine biosynthesis | Tbh |
| 317 | GO:0031497 | P | 10, | 2 | 0.812 (x 2.462) | 51 (0.039) | 0.588 | chromatin assembly | Acf1 CG31617 |
| 318 | GO:0006769 | P | 8, 9, | 1 | 0.223 (x 4.484) | 14 (0.071) | 0.589 | nicotinamide metabolism | CG30499 |
| 319 | GO:0006952 | P | 4, | 11 | 8.188 (x 1.343) | 514 (0.021) | 0.589 | defense response | CG30438 CG31146 CG5224 CG5397 CG6426 CG8193 GstD9 GstE1 Thor Tsf1 hig |
| 320 | GO:0044463 | C | 3, 4, 5, | 1 | 0.223 (x 4.484) | 14 (0.071) | 0.591 | cell projection part | robl |
| 321 | GO:0044428 | C | 4, 5, 6, 7, 8, 9, | 11 | 8.204 (x 1.341) | 515 (0.021) | 0.592 | nuclear part | Acf1 CG13277 CG15398 CG17838 CG3436 CG4673 CG6610 RpII18 Ssb-c31a Tfb2 Trap36 |
| 322 | GO:0030166 | P | 7, 8, | 1 | 0.223 (x 4.484) | 14 (0.071) | 0.592 | proteoglycan biosynthesis | Act57B |
| 323 | GO:0005179 | F | 4, 5, | 2 | 0.844 (x 2.369) | 53 (0.038) | 0.593 | hormone activity | CG11051 CG15361 |
| 324 | GO:0016854 | F | 4, | 1 | 0.223 (x 4.484) | 14 (0.071) | 0.594 | racemase and epimerase activity | CG30499 |
| 325 | GO:0000221 | C | 4, 5, 6, 7, 8, 9, 10, 11, 12, | 1 | 0.223 (x 4.484) | 14 (0.071) | 0.596 | hydrogen-transporting ATPase V1 domain | Vha36 |
| 326 | GO:0044270 | P | 5, 6, | 2 | 0.828 (x 2.414) | 52 (0.038) | 0.598 | nitrogen compound catabolism | CG7860 Gs2 |
| 327 | GO:0004568 | F | 6, | 1 | 0.223 (x 4.484) | 14 (0.071) | 0.598 | chitinase activity | Chit |
| 328 | GO:0009607 | P | 3, | 11 | 8.315 (x 1.323) | 522 (0.021) | 0.599 | response to biotic stimulus | CG30438 CG31146 CG5224 CG5397 CG6426 CG8193 GstD9 GstE1 Thor Tsf1 hig |
| 329 | GO:0009310 | P | 6, 7, | 2 | 0.828 (x 2.414) | 52 (0.038) | 0.6 | amine catabolism | CG7860 Gs2 |
| 330 | GO:0000786 | C | 3, 5, 6, 7, 8, 9, 10, 11, | 1 | 0.239 (x 4.185) | 15 (0.067) | 0.602 | nucleosome | CG31617 |
| 331 | GO:0006029 | P | 7, | 1 | 0.239 (x 4.185) | 15 (0.067) | 0.604 | proteoglycan metabolism | Act57B |
| 332 | GO:0006090 | P | 7, | 1 | 0.239 (x 4.185) | 15 (0.067) | 0.605 | pyruvate metabolism | CG10932 |
| 333 | GO:0005730 | C | 5, 6, 7, 8, 9, 10, 11, | 2 | 0.860 (x 2.325) | 54 (0.037) | 0.606 | nucleolus | CG13277 CG6610 |
| 334 | GO:0004022 | F | 6, | 1 | 0.239 (x 4.185) | 15 (0.067) | 0.607 | alcohol dehydrogenase activity | Fbp2 |
| 335 | GO:0019362 | P | 7, 8, | 1 | 0.239 (x 4.185) | 15 (0.067) | 0.609 | pyridine nucleotide metabolism | CG30499 |
| 336 | GO:0016251 | F | 4, | 3 | 1.609 (x 1.865) | 101 (0.030) | 0.609 | general RNA polymerase II transcription factor activity | BEST:LD29214 Tfb2 Trap36 |
| 337 | GO:0003725 | F | 5, | 1 | 0.255 (x 3.923) | 16 (0.062) | 0.624 | double-stranded RNA binding | Msp-300 |
| 338 | GO:0016645 | F | 4, | 1 | 0.255 (x 3.923) | 16 (0.062) | 0.626 | oxidoreductase activity, acting on the CH-NH group of donors | Dhfr |
| 339 | GO:0042398 | P | 6, 7, | 1 | 0.255 (x 3.923) | 16 (0.062) | 0.628 | amino acid derivative biosynthesis | Tbh |
| 340 | GO:0004659 | F | 5, | 1 | 0.255 (x 3.923) | 16 (0.062) | 0.629 | prenyltransferase activity | CG5037 |
| 341 | GO:0006631 | P | 6, 7, | 3 | 1.641 (x 1.828) | 103 (0.029) | 0.631 | fatty acid metabolism | CG10932 CG6921 desat1 |
| 342 | GO:0004089 | F | 6, | 1 | 0.255 (x 3.923) | 16 (0.062) | 0.631 | carbonate dehydratase activity | CAH2 |
| 343 | GO:0006979 | P | 4, 5, 6, | 1 | 0.271 (x 3.693) | 17 (0.059) | 0.652 | response to oxidative stress | GstE1 |
| 344 | GO:0004428 | F | 6, | 1 | 0.271 (x 3.693) | 17 (0.059) | 0.654 | inositol or phosphatidylinositol kinase activity | CG18854 |
| 345 | GO:0008757 | F | 6, | 2 | 0.940 (x 2.128) | 59 (0.034) | 0.656 | S-adenosylmethionine-dependent methyltransferase activity | CG6188 Pcmt |
| 346 | GO:0016478 | P | 8, 9, 10, | 1 | 0.271 (x 3.693) | 17 (0.059) | 0.656 | negative regulation of translation | Thor |
| 347 | GO:0016798 | F | 4, | 3 | 1.705 (x 1.760) | 107 (0.028) | 0.657 | hydrolase activity, acting on glycosyl bonds | CG15012 Chit RpS3 |
| 348 | GO:0006413 | P | 8, 9, | 2 | 0.940 (x 2.128) | 59 (0.034) | 0.658 | translational initiation | RpS18 Thor |
| 349 | GO:0009168 | P | 9, 10, | 1 | 0.287 (x 3.487) | 18 (0.056) | 0.664 | purine ribonucleoside monophosphate biosynthesis | Dhfr |
| 350 | GO:0006368 | P | 9, | 1 | 0.287 (x 3.487) | 18 (0.056) | 0.666 | RNA elongation from RNA polymerase II promoter | Tfb2 |
| 351 | GO:0009116 | P | 6, | 1 | 0.287 (x 3.487) | 18 (0.056) | 0.668 | nucleoside metabolism | CG11079 |
| 352 | GO:0009117 | P | 6, | 4 | 2.533 (x 1.579) | 159 (0.025) | 0.668 | nucleotide metabolism | CG30499 CG7846 Dhfr Vha36 |
| 353 | GO:0009142 | P | 7, 8, | 2 | 1.099 (x 1.820) | 69 (0.029) | 0.668 | nucleoside triphosphate biosynthesis | CG7846 Vha36 |
| 354 | GO:0042775 | P | 8, 10, | 2 | 0.972 (x 2.058) | 61 (0.033) | 0.669 | ATP synthesis coupled electron transport (sensu Eukaryota) | CG12400 EG:152A3.7 |
| 355 | GO:0009126 | P | 8, | 1 | 0.287 (x 3.487) | 18 (0.056) | 0.67 | purine nucleoside monophosphate metabolism | Dhfr |
| 356 | GO:0005746 | C | 5, 6, 7, 8, 9, 10, 11, 12, 13, | 2 | 1.099 (x 1.820) | 69 (0.029) | 0.67 | mitochondrial electron transport chain | CG12400 EG:152A3.7 |
| 357 | GO:0009165 | P | 6, 7, | 3 | 1.752 (x 1.712) | 110 (0.027) | 0.67 | nucleotide biosynthesis | CG7846 Dhfr Vha36 |
| 358 | GO:0009141 | P | 7, | 2 | 1.131 (x 1.768) | 71 (0.028) | 0.671 | nucleoside triphosphate metabolism | CG7846 Vha36 |
| 359 | GO:0009205 | P | 9, | 2 | 1.099 (x 1.820) | 69 (0.029) | 0.671 | purine ribonucleoside triphosphate metabolism | CG7846 Vha36 |
| 360 | GO:0009127 | P | 8, 9, | 1 | 0.287 (x 3.487) | 18 (0.056) | 0.672 | purine nucleoside monophosphate biosynthesis | Dhfr |
| 361 | GO:0009299 | P | 8, | 1 | 0.366 (x 2.729) | 23 (0.043) | 0.672 | mRNA transcription | RpII18 |
| 362 | GO:0006281 | P | 5, 7, | 3 | 1.752 (x 1.712) | 110 (0.027) | 0.672 | DNA repair | RpS3 Tfb2 agt |
| 363 | GO:0048102 | P | 6, | 2 | 1.131 (x 1.768) | 71 (0.028) | 0.672 | autophagic cell death | CG7860 Obp99b |
| 364 | GO:0048557 | P | 6, | 1 | 0.350 (x 2.853) | 22 (0.045) | 0.672 | embryonic digestive tract morphogenesis | wal |
| 365 | GO:0043170 | P | 4, | 53 | 49.638 (x 1.068) | 3116 (0.017) | 0.672 | macromolecule metabolism | Acf1 Act57B BG:DS00004.12 CG10104 CG10237 CG10862 CG11522 CG11594 CG1304 CG13277 CG13991 CG14715 CG2789 CG30438 CG30499 CG31617 CG33002 CG3436 CG4386 CG4408 CG4866 CG5177 CG6574 CG6610 CG6764 CG7283 CG7860 CG9267 CG9282 CG9804 Chit Dhfr Gasp Gs2 Iap2 Mlc1 Pcmt RpL11 RpL27A RpL36 RpL38 RpL8 RpP1 RpS12 RpS18 RpS3 RpS9 Tfb2 Thor agt mRpL21 mRpS21 sop |
| 366 | GO:0015672 | P | 7, 8, | 4 | 2.788 (x 1.435) | 175 (0.023) | 0.672 | monovalent inorganic cation transport | CG31547 CG4805 CG7846 Vha36 |
| 367 | GO:0009144 | P | 8, | 2 | 1.099 (x 1.820) | 69 (0.029) | 0.673 | purine nucleoside triphosphate metabolism | CG7846 Vha36 |
| 368 | GO:0008258 | P | 6, | 1 | 0.366 (x 2.729) | 23 (0.043) | 0.673 | head involution | wal |
| 369 | GO:0009167 | P | 9, | 1 | 0.287 (x 3.487) | 18 (0.056) | 0.674 | purine ribonucleoside monophosphate metabolism | Dhfr |
| 370 | GO:0035071 | P | 7, | 2 | 1.131 (x 1.768) | 71 (0.028) | 0.674 | salivary gland cell autophagic cell death | CG7860 Obp99b |
| 371 | GO:0001558 | P | 4, 5, 7, 8, | 1 | 0.319 (x 3.139) | 20 (0.050) | 0.674 | regulation of cell growth | Thor |
| 372 | GO:0048627 | P | 5, 6, 8, 9, | 1 | 0.350 (x 2.853) | 22 (0.045) | 0.674 | myoblast development | Mhc |
| 373 | GO:0015082 | F | 5, | 1 | 0.382 (x 2.616) | 24 (0.042) | 0.674 | di-, tri-valent inorganic cation transporter activity | Tsf1 |
| 374 | GO:0019829 | F | 5, 6, 8, 13, | 2 | 1.099 (x 1.820) | 69 (0.029) | 0.675 | cation-transporting ATPase activity | CG7846 Vha36 |
| 375 | GO:0009064 | P | 7, 8, | 1 | 0.303 (x 3.304) | 19 (0.053) | 0.675 | glutamine family amino acid metabolism | Gs2 |
| 376 | GO:0016859 | F | 4, | 1 | 0.366 (x 2.729) | 23 (0.043) | 0.675 | cis-trans isomerase activity | CG14715 |
| 377 | GO:0035070 | P | 6, | 2 | 1.131 (x 1.768) | 71 (0.028) | 0.675 | salivary gland histolysis | CG7860 Obp99b |
| 378 | GO:0006030 | P | 7, 8, 9, 10, | 2 | 1.004 (x 1.993) | 63 (0.032) | 0.675 | chitin metabolism | Chit Gasp |
| 379 | GO:0009069 | P | 7, 8, | 1 | 0.287 (x 3.487) | 18 (0.056) | 0.675 | serine family amino acid metabolism | Dhfr |
| 380 | GO:0005200 | F | 3, | 6 | 4.652 (x 1.290) | 292 (0.021) | 0.675 | structural constituent of cytoskeleton | Act57B BG:DS02740.9 CG7846 Mhc Mp20 robl |
| 381 | GO:0042063 | P | 6, | 1 | 0.382 (x 2.616) | 24 (0.042) | 0.675 | gliogenesis | BG:DS02740.9 |
| 382 | GO:0048558 | P | 6, 7, | 1 | 0.350 (x 2.853) | 22 (0.045) | 0.676 | embryonic gut morphogenesis | wal |
| 383 | GO:0009161 | P | 8, | 1 | 0.319 (x 3.139) | 20 (0.050) | 0.676 | ribonucleoside monophosphate metabolism | Dhfr |
| 384 | GO:0009199 | P | 8, | 2 | 1.099 (x 1.820) | 69 (0.029) | 0.676 | ribonucleoside triphosphate metabolism | CG7846 Vha36 |
| 385 | GO:0006961 | P | 7, 8, 9, | 1 | 0.366 (x 2.729) | 23 (0.043) | 0.676 | antibacterial humoral response (sensu Protostomia) | Thor |
| 386 | GO:0046915 | F | 5, | 1 | 0.303 (x 3.304) | 19 (0.053) | 0.677 | transition metal ion transporter activity | Tsf1 |
| 387 | GO:0042773 | P | 7, 9, | 2 | 0.988 (x 2.025) | 62 (0.032) | 0.677 | ATP synthesis coupled electron transport | CG12400 EG:152A3.7 |
| 388 | GO:0006044 | P | 8, 9, | 2 | 1.131 (x 1.768) | 71 (0.028) | 0.677 | N-acetylglucosamine metabolism | Chit Gasp |
| 389 | GO:0016490 | F | 3, | 1 | 0.382 (x 2.616) | 24 (0.042) | 0.677 | structural constituent of peritrophic membrane (sensu Insecta) | Gasp |
| 390 | GO:0048619 | P | 8, 9, | 1 | 0.350 (x 2.853) | 22 (0.045) | 0.677 | embryonic hindgut morphogenesis | wal |
| 391 | GO:0046961 | F | 6, 7, 9, 14, | 2 | 1.004 (x 1.993) | 63 (0.032) | 0.677 | hydrogen-transporting ATPase activity, rotational mechanism | CG7846 Vha36 |
| 392 | GO:0006733 | P | 7, | 1 | 0.319 (x 3.139) | 20 (0.050) | 0.677 | oxidoreduction coenzyme metabolism | CG30499 |
| 393 | GO:0042742 | P | 5, 6, | 2 | 1.099 (x 1.820) | 69 (0.029) | 0.678 | defense response to bacterium | CG6426 Thor |
| 394 | GO:0046034 | P | 6, 10, | 2 | 1.035 (x 1.932) | 65 (0.031) | 0.678 | ATP metabolism | CG7846 Vha36 |
| 395 | GO:0045445 | P | 5, 7, 8, | 1 | 0.366 (x 2.729) | 23 (0.043) | 0.678 | myoblast differentiation | Mhc |
| 396 | GO:0006041 | P | 7, 8, | 2 | 1.131 (x 1.768) | 71 (0.028) | 0.678 | glucosamine metabolism | Chit Gasp |
| 397 | GO:0044272 | P | 6, | 1 | 0.303 (x 3.304) | 19 (0.053) | 0.679 | sulfur compound biosynthesis | Act57B |
| 398 | GO:0004879 | F | 4, | 1 | 0.382 (x 2.616) | 24 (0.042) | 0.679 | ligand-dependent nuclear receptor activity | Hr4 |
| 399 | GO:0016271 | P | 4, | 2 | 1.147 (x 1.744) | 72 (0.028) | 0.679 | tissue death | CG7860 Obp99b |
| 400 | GO:0016835 | F | 4, | 2 | 0.988 (x 2.025) | 62 (0.032) | 0.679 | carbon-oxygen lyase activity | CAH2 RpS3 |
| 401 | GO:0005615 | C | 3, 4, | 1 | 0.350 (x 2.853) | 22 (0.045) | 0.679 | extracellular space | Lsp1beta |
| 402 | GO:0046933 | F | 7, | 2 | 1.004 (x 1.993) | 63 (0.032) | 0.679 | hydrogen-transporting ATP synthase activity, rotational mechanism | CG7846 Vha36 |
| 403 | GO:0003697 | F | 6, | 1 | 0.319 (x 3.139) | 20 (0.050) | 0.679 | single-stranded DNA binding | Ssb-c31a |
| 404 | GO:0016319 | P | 5, 7, | 1 | 0.366 (x 2.729) | 23 (0.043) | 0.68 | mushroom body development | robl |
| 405 | GO:0006519 | P | 5, | 6 | 4.667 (x 1.285) | 293 (0.020) | 0.68 | amino acid and derivative metabolism | CG5122 CG6188 CG7860 Dhfr Gs2 Tbh |
| 406 | GO:0030880 | C | 3, 4, 5, 6, | 1 | 0.382 (x 2.616) | 24 (0.042) | 0.68 | RNA polymerase complex | RpII18 |
| 407 | GO:0007559 | P | 5, | 2 | 1.147 (x 1.744) | 72 (0.028) | 0.68 | histolysis | CG7860 Obp99b |
| 408 | GO:0006414 | P | 8, 9, | 1 | 0.303 (x 3.304) | 19 (0.053) | 0.68 | translational elongation | RpP1 |
| 409 | GO:0031327 | P | 7, | 1 | 0.350 (x 2.853) | 22 (0.045) | 0.68 | negative regulation of cellular biosynthesis | Thor |
| 410 | GO:0008276 | F | 6, | 1 | 0.319 (x 3.139) | 20 (0.050) | 0.681 | protein methyltransferase activity | Pcmt |
| 411 | GO:0006754 | P | 7, 8, 9, 10, 11, | 2 | 1.020 (x 1.962) | 64 (0.031) | 0.681 | ATP biosynthesis | CG7846 Vha36 |
| 412 | GO:0006767 | P | 6, | 1 | 0.366 (x 2.729) | 23 (0.043) | 0.681 | water-soluble vitamin metabolism | CG30499 |
| 413 | GO:0016585 | C | 3, 6, 7, 8, 9, 10, 11, 12, 13, | 1 | 0.398 (x 2.511) | 25 (0.040) | 0.682 | chromatin remodeling complex | Acf1 |
| 414 | GO:0006040 | P | 6, 7, | 2 | 1.147 (x 1.744) | 72 (0.028) | 0.682 | amino sugar metabolism | Chit Gasp |
| 415 | GO:0006873 | P | 5, | 1 | 0.350 (x 2.853) | 22 (0.045) | 0.682 | cell ion homeostasis | Tsf1 |
| 416 | GO:0003707 | F | 5, | 1 | 0.303 (x 3.304) | 19 (0.053) | 0.682 | steroid hormone receptor activity | Hr4 |
| 417 | GO:0007398 | P | 4, | 5 | 3.775 (x 1.324) | 237 (0.021) | 0.682 | ectoderm development | BG:DS02740.9 CG31146 CG5397 fat-spondin mfas |
| 418 | GO:0008144 | F | 3, | 1 | 0.319 (x 3.139) | 20 (0.050) | 0.683 | drug binding | CG14715 |
| 419 | GO:0015986 | P | 7, 8, 9, 10, 11, 12, | 2 | 1.020 (x 1.962) | 64 (0.031) | 0.683 | ATP synthesis coupled proton transport | CG7846 Vha36 |
| 420 | GO:0048562 | P | 5, | 1 | 0.398 (x 2.511) | 25 (0.040) | 0.683 | embryonic organ morphogenesis | wal |
| 421 | GO:0007443 | P | 5, 9, 10, | 1 | 0.350 (x 2.853) | 22 (0.045) | 0.684 | Malpighian tubule morphogenesis | wal |
| 422 | GO:0004190 | F | 6, | 1 | 0.303 (x 3.304) | 19 (0.053) | 0.684 | aspartic-type endopeptidase activity | CG10104 |
| 423 | GO:0009156 | P | 8, 9, | 1 | 0.319 (x 3.139) | 20 (0.050) | 0.684 | ribonucleoside monophosphate biosynthesis | Dhfr |
| 424 | GO:0007613 | P | 5, | 1 | 0.398 (x 2.511) | 25 (0.040) | 0.685 | memory | Tbh |
| 425 | GO:0015985 | P | 7, 8, 9, 10, | 2 | 1.020 (x 1.962) | 64 (0.031) | 0.685 | energy coupled proton transport, down electrochemical gradient | CG7846 Vha36 |
| 426 | GO:0009890 | P | 6, | 1 | 0.350 (x 2.853) | 22 (0.045) | 0.685 | negative regulation of biosynthesis | Thor |
| 427 | GO:0043566 | F | 5, | 1 | 0.398 (x 2.511) | 25 (0.040) | 0.686 | structure-specific DNA binding | Ssb-c31a |
| 428 | GO:0030005 | P | 7, | 1 | 0.319 (x 3.139) | 20 (0.050) | 0.686 | di-, tri-valent inorganic cation homeostasis | Tsf1 |
| 429 | GO:0009617 | P | 5, | 2 | 1.179 (x 1.697) | 74 (0.027) | 0.686 | response to bacterium | CG6426 Thor |
| 430 | GO:0016879 | F | 4, | 4 | 2.899 (x 1.380) | 182 (0.022) | 0.686 | ligase activity, forming carbon-nitrogen bonds | CG11079 CG9804 Gs2 Iap2 |
| 431 | GO:0019722 | P | 7, | 2 | 1.020 (x 1.962) | 64 (0.031) | 0.687 | calcium-mediated signaling | CG14715 regucalcin |
| 432 | GO:0048628 | P | 6, 7, 9, 10, | 1 | 0.350 (x 2.853) | 22 (0.045) | 0.687 | myoblast maturation | Mhc |
| 433 | GO:0008092 | F | 4, | 5 | 3.791 (x 1.319) | 238 (0.021) | 0.688 | cytoskeletal protein binding | BG:DS02740.9 Mhc Mp20 Msp-300 Tm1 |
| 434 | GO:0007362 | P | 8, 9, | 1 | 0.335 (x 2.989) | 21 (0.048) | 0.688 | terminal region determination | tsl |
| 435 | GO:0006730 | P | 5, | 1 | 0.319 (x 3.139) | 20 (0.050) | 0.688 | one-carbon compound metabolism | CAH2 |
| 436 | GO:0006753 | P | 8, | 2 | 1.020 (x 1.962) | 64 (0.031) | 0.689 | nucleoside phosphate metabolism | CG7846 Vha36 |
| 437 | GO:0048611 | P | 6, | 1 | 0.350 (x 2.853) | 22 (0.045) | 0.689 | embryonic ectodermal gut development | wal |
| 438 | GO:0010033 | P | 5, | 1 | 0.335 (x 2.989) | 21 (0.048) | 0.69 | response to organic substance | Tbh |
| 439 | GO:0048566 | P | 5, | 1 | 0.350 (x 2.853) | 22 (0.045) | 0.69 | embryonic gut development | wal |
| 440 | GO:0016052 | P | 6, | 2 | 1.067 (x 1.874) | 67 (0.030) | 0.691 | carbohydrate catabolism | CG30499 Chit |
| 441 | GO:0017148 | P | 7, 8, 9, | 1 | 0.335 (x 2.989) | 21 (0.048) | 0.691 | negative regulation of protein biosynthesis | Thor |
| 442 | GO:0006974 | P | 4, | 3 | 1.880 (x 1.596) | 118 (0.025) | 0.692 | response to DNA damage stimulus | RpS3 Tfb2 agt |
| 443 | GO:0003713 | F | 4, 6, | 1 | 0.350 (x 2.853) | 22 (0.045) | 0.692 | transcription coactivator activity | Ssb-c31a |
| 444 | GO:0044275 | P | 7, | 2 | 1.067 (x 1.874) | 67 (0.030) | 0.693 | cellular carbohydrate catabolism | CG30499 Chit |
| 445 | GO:0016339 | P | 5, | 1 | 0.335 (x 2.989) | 21 (0.048) | 0.693 | calcium-dependent cell-cell adhesion | Cad99C |
| 446 | GO:0006354 | P | 8, | 1 | 0.350 (x 2.853) | 22 (0.045) | 0.694 | RNA elongation | Tfb2 |
| 447 | GO:0009206 | P | 9, 10, | 2 | 1.083 (x 1.846) | 68 (0.029) | 0.694 | purine ribonucleoside triphosphate biosynthesis | CG7846 Vha36 |
| 448 | GO:0003755 | F | 5, | 1 | 0.335 (x 2.989) | 21 (0.048) | 0.695 | peptidyl-prolyl cis-trans isomerase activity | CG14715 |
| 449 | GO:0048613 | P | 7, 8, | 1 | 0.350 (x 2.853) | 22 (0.045) | 0.696 | embryonic ectodermal gut morphogenesis | wal |
| 450 | GO:0009145 | P | 8, 9, | 2 | 1.083 (x 1.846) | 68 (0.029) | 0.696 | purine nucleoside triphosphate biosynthesis | CG7846 Vha36 |
| 451 | GO:0009124 | P | 7, 8, | 1 | 0.335 (x 2.989) | 21 (0.048) | 0.697 | nucleoside monophosphate biosynthesis | Dhfr |
| 452 | GO:0030003 | P | 6, | 1 | 0.350 (x 2.853) | 22 (0.045) | 0.697 | cation homeostasis | Tsf1 |
| 453 | GO:0016469 | C | 3, 6, 7, 8, | 2 | 1.083 (x 1.846) | 68 (0.029) | 0.698 | proton-transporting two-sector ATPase complex | CG7846 Vha36 |
| 454 | GO:0009123 | P | 7, | 1 | 0.335 (x 2.989) | 21 (0.048) | 0.698 | nucleoside monophosphate metabolism | Dhfr |
| 455 | GO:0006875 | P | 7, | 1 | 0.350 (x 2.853) | 22 (0.045) | 0.699 | metal ion homeostasis | Tsf1 |
| 456 | GO:0009201 | P | 8, 9, | 2 | 1.083 (x 1.846) | 68 (0.029) | 0.7 | ribonucleoside triphosphate biosynthesis | CG7846 Vha36 |
| 457 | GO:0009948 | P | 5, | 3 | 1.896 (x 1.583) | 119 (0.025) | 0.7 | anterior/posterior axis specification | Tm1 regucalcin tsl |
| 458 | GO:0005272 | F | 6, 7, | 1 | 0.414 (x 2.414) | 26 (0.038) | 0.701 | sodium channel activity | CG4805 |
| 459 | GO:0009952 | P | 4, | 3 | 2.071 (x 1.449) | 130 (0.023) | 0.701 | anterior/posterior pattern formation | Tm1 regucalcin tsl |
| 460 | GO:0004520 | F | 7, | 1 | 0.414 (x 2.414) | 26 (0.038) | 0.702 | endodeoxyribonuclease activity | RpS3 |
| 461 | GO:0009112 | P | 6, | 2 | 1.243 (x 1.610) | 78 (0.026) | 0.708 | nucleobase metabolism | Dhfr Vha36 |
| 462 | GO:0016455 | F | 5, | 1 | 0.430 (x 2.325) | 27 (0.037) | 0.71 | RNA polymerase II transcription mediator activity | Trap36 |
| 463 | GO:0006818 | P | 5, 6, | 2 | 1.243 (x 1.610) | 78 (0.026) | 0.71 | hydrogen transport | CG7846 Vha36 |
| 464 | GO:0044264 | P | 6, 7, | 2 | 1.227 (x 1.631) | 77 (0.026) | 0.71 | cellular polysaccharide metabolism | Chit Gasp |
| 465 | GO:0009719 | P | 3, | 3 | 2.119 (x 1.416) | 133 (0.023) | 0.711 | response to endogenous stimulus | RpS3 Tfb2 agt |
| 466 | GO:0005507 | F | 6, | 1 | 0.430 (x 2.325) | 27 (0.037) | 0.711 | copper ion binding | Tbh |
| 467 | GO:0015992 | P | 6, 7, 8, 9, | 2 | 1.243 (x 1.610) | 78 (0.026) | 0.711 | proton transport | CG7846 Vha36 |
| 468 | GO:0048469 | P | 5, | 1 | 0.430 (x 2.325) | 27 (0.037) | 0.713 | cell maturation | Mhc |
| 469 | GO:0042692 | P | 4, | 1 | 0.430 (x 2.325) | 27 (0.037) | 0.714 | muscle cell differentiation | Mhc |
| 470 | GO:0007354 | P | 7, 8, | 1 | 0.430 (x 2.325) | 27 (0.037) | 0.716 | zygotic determination of anterior/posterior axis, embryo | tsl |
| 471 | GO:0050801 | P | 4, | 1 | 0.430 (x 2.325) | 27 (0.037) | 0.717 | ion homeostasis | Tsf1 |
| 472 | GO:0005669 | C | 4, 7, 8, 9, 10, 11, 12, 13, 14, | 1 | 0.446 (x 2.242) | 28 (0.036) | 0.72 | transcription factor TFIID complex | CG15398 |
| 473 | GO:0008080 | F | 8, | 1 | 0.446 (x 2.242) | 28 (0.036) | 0.721 | N-acetyltransferase activity | CG1969 |
| 474 | GO:0000119 | C | 3, 4, 6, 7, 8, 9, 10, 11, 12, 13, 14, | 1 | 0.446 (x 2.242) | 28 (0.036) | 0.723 | mediator complex | Trap36 |
| 475 | GO:0003723 | F | 4, | 7 | 5.878 (x 1.191) | 369 (0.019) | 0.732 | RNA binding | CG17838 CG4866 Msp-300 RpL8 RpS18 RpS9 mRpL21 |
| 476 | GO:0007416 | P | 5, 6, | 1 | 0.462 (x 2.165) | 29 (0.034) | 0.732 | synaptogenesis | Gs2 |
| 477 | GO:0022008 | P | 5, | 4 | 3.074 (x 1.301) | 193 (0.021) | 0.732 | neurogenesis | BG:DS02740.9 Tm1 mfas robl |
| 478 | GO:0007156 | P | 5, | 1 | 0.462 (x 2.165) | 29 (0.034) | 0.734 | homophilic cell adhesion | Cad99C |
| 479 | GO:0007274 | P | 7, | 1 | 0.462 (x 2.165) | 29 (0.034) | 0.735 | neuromuscular synaptic transmission | CG31146 |
| 480 | GO:0016471 | C | 3, 4, 5, 6, 7, 8, 9, 10, 11, | 1 | 0.462 (x 2.165) | 29 (0.034) | 0.737 | hydrogen-translocating V-type ATPase complex | Vha36 |
| 481 | GO:0006576 | P | 6, 7, | 1 | 0.478 (x 2.092) | 30 (0.033) | 0.738 | biogenic amine metabolism | Tbh |
| 482 | GO:0003899 | F | 6, | 1 | 0.478 (x 2.092) | 30 (0.033) | 0.74 | DNA-directed RNA polymerase activity | RpII18 |
| 483 | GO:0016049 | P | 3, 4, 6, 7, | 1 | 0.478 (x 2.092) | 30 (0.033) | 0.741 | cell growth | Thor |
| 484 | GO:0000314 | C | 4, 5, 6, 7, 8, 9, 10, | 1 | 0.478 (x 2.092) | 30 (0.033) | 0.743 | organellar small ribosomal subunit | mRpS21 |
| 485 | GO:0051301 | P | 4, | 3 | 2.214 (x 1.355) | 139 (0.022) | 0.744 | cell division | Act57B Mhc Msp-300 |
| 486 | GO:0005763 | C | 5, 6, 7, 8, 9, 10, 11, 12, 13, | 1 | 0.478 (x 2.092) | 30 (0.033) | 0.744 | mitochondrial small ribosomal subunit | mRpS21 |
| 487 | GO:0003729 | F | 5, | 6 | 4.986 (x 1.203) | 313 (0.019) | 0.745 | mRNA binding | CG17838 CG4866 RpL8 RpS18 RpS9 mRpL21 |
| 488 | GO:0009628 | P | 3, | 7 | 5.926 (x 1.181) | 372 (0.019) | 0.746 | response to abiotic stimulus | CG30438 CG31146 CG5224 GstD9 GstE1 Obp99b Tbh |
| 489 | GO:0007498 | P | 4, | 4 | 3.154 (x 1.268) | 198 (0.020) | 0.746 | mesoderm development | Mlc1 Mlp60A Mlp84B Msp-300 |
| 490 | GO:0006520 | P | 6, 7, | 5 | 4.158 (x 1.203) | 261 (0.019) | 0.749 | amino acid metabolism | CG5122 CG6188 CG7860 Dhfr Gs2 |
| 491 | GO:0042254 | P | 6, | 1 | 0.510 (x 1.962) | 32 (0.031) | 0.75 | ribosome biogenesis and assembly | CG6764 |
| 492 | GO:0048666 | P | 5, 8, | 3 | 2.262 (x 1.326) | 142 (0.021) | 0.751 | neuron development | Tm1 mfas robl |
| 493 | GO:0004181 | F | 7, | 1 | 0.510 (x 1.962) | 32 (0.031) | 0.751 | metallocarboxypeptidase activity | CG4408 |
| 494 | GO:0008360 | P | 5, 6, | 2 | 1.354 (x 1.477) | 85 (0.024) | 0.752 | regulation of cell shape | Mp20 SIP1 |
| 495 | GO:0031175 | P | 6, 9, | 3 | 2.262 (x 1.326) | 142 (0.021) | 0.752 | neurite development | Tm1 mfas robl |
| 496 | GO:0042995 | C | 3, 4, | 1 | 0.494 (x 2.025) | 31 (0.032) | 0.753 | cell projection | robl |
| 497 | GO:0005774 | C | 5, 6, 7, 8, 9, 10, | 1 | 0.510 (x 1.962) | 32 (0.031) | 0.753 | vacuolar membrane | Vha36 |
| 498 | GO:0008610 | P | 5, 6, 7, | 2 | 1.354 (x 1.477) | 85 (0.024) | 0.753 | lipid biosynthesis | CG10932 desat1 |
| 499 | GO:0016410 | F | 7, | 1 | 0.494 (x 2.025) | 31 (0.032) | 0.754 | N-acyltransferase activity | CG1969 |
| 500 | GO:0050808 | P | 5, | 1 | 0.510 (x 1.962) | 32 (0.031) | 0.754 | synapse organization and biogenesis | Gs2 |
| 501 | GO:0008083 | F | 4, 5, | 1 | 0.526 (x 1.902) | 33 (0.030) | 0.756 | growth factor activity | Chit |
| 502 | GO:0030198 | P | 4, | 1 | 0.510 (x 1.962) | 32 (0.031) | 0.756 | extracellular matrix organization and biogenesis | Gs2 |
| 503 | GO:0005740 | C | 4, 5, 6, 7, 8, 9, 10, | 4 | 3.266 (x 1.225) | 205 (0.020) | 0.756 | mitochondrial envelope | CG12400 CG2789 CG5037 EG:152A3.7 |
| 504 | GO:0006725 | P | 5, | 3 | 2.310 (x 1.299) | 145 (0.021) | 0.757 | aromatic compound metabolism | Dhfr Tbh Vha36 |
| 505 | GO:0008293 | P | 8, | 1 | 0.526 (x 1.902) | 33 (0.030) | 0.757 | torso signaling pathway | tsl |
| 506 | GO:0045451 | P | 7, 11, 13, 14, 16, | 1 | 0.510 (x 1.962) | 32 (0.031) | 0.757 | pole plasm oskar mRNA localization | Tm1 |
| 507 | GO:0043565 | F | 5, | 1 | 0.526 (x 1.902) | 33 (0.030) | 0.759 | sequence-specific DNA binding | Msp-300 |
| 508 | GO:0040007 | P | 2, | 2 | 1.434 (x 1.395) | 90 (0.022) | 0.759 | growth | Hr4 Thor |
| 509 | GO:0006206 | P | 7, | 1 | 0.510 (x 1.962) | 32 (0.031) | 0.759 | pyrimidine base metabolism | Dhfr |
| 510 | GO:0000377 | P | 10, | 3 | 2.374 (x 1.264) | 149 (0.020) | 0.759 | RNA splicing, via transesterification reactions with bulged adenosine as nucleophile | CG13277 CG3436 CG6610 |
| 511 | GO:0004840 | F | 4, | 1 | 0.526 (x 1.902) | 33 (0.030) | 0.76 | ubiquitin conjugating enzyme activity | CG10862 |
| 512 | GO:0031975 | C | 2, | 5 | 4.253 (x 1.176) | 267 (0.019) | 0.76 | envelope | CG12400 CG2789 CG4673 CG5037 EG:152A3.7 |
| 513 | GO:0000398 | P | 9, 11, | 3 | 2.374 (x 1.264) | 149 (0.020) | 0.76 | nuclear mRNA splicing, via spliceosome | CG13277 CG3436 CG6610 |
| 514 | GO:0044437 | C | 4, 5, 6, 7, 8, 9, | 1 | 0.510 (x 1.962) | 32 (0.031) | 0.76 | vacuolar part | Vha36 |
| 515 | GO:0019932 | P | 6, | 2 | 1.402 (x 1.427) | 88 (0.023) | 0.761 | second-messenger-mediated signaling | CG14715 regucalcin |
| 516 | GO:0030001 | P | 7, 8, | 3 | 2.342 (x 1.281) | 147 (0.020) | 0.761 | metal ion transport | CG31547 CG4805 Tsf1 |
| 517 | GO:0016853 | F | 3, | 2 | 1.450 (x 1.380) | 91 (0.022) | 0.761 | isomerase activity | CG14715 CG30499 |
| 518 | GO:0031967 | C | 3, 4, 5, 6, 7, 8, | 5 | 4.253 (x 1.176) | 267 (0.019) | 0.761 | organelle envelope | CG12400 CG2789 CG4673 CG5037 EG:152A3.7 |
| 519 | GO:0005813 | C | 5, 6, 7, 8, 9, 10, | 1 | 0.526 (x 1.902) | 33 (0.030) | 0.762 | centrosome | corto |
| 520 | GO:0000375 | P | 9, | 3 | 2.374 (x 1.264) | 149 (0.020) | 0.762 | RNA splicing, via transesterification reactions | CG13277 CG3436 CG6610 |
| 521 | GO:0046873 | F | 4, | 1 | 0.510 (x 1.962) | 32 (0.031) | 0.762 | metal ion transporter activity | Tsf1 |
| 522 | GO:0008361 | P | 5, 6, | 1 | 0.542 (x 1.846) | 34 (0.029) | 0.762 | regulation of cell size | Thor |
| 523 | GO:0048568 | P | 4, | 1 | 0.558 (x 1.794) | 35 (0.029) | 0.763 | embryonic organ development | wal |
| 524 | GO:0003702 | F | 3, | 5 | 4.237 (x 1.180) | 266 (0.019) | 0.763 | RNA polymerase II transcription factor activity | BEST:LD29214 CG15398 Tfb2 Trap36 corto |
| 525 | GO:0030182 | P | 4, 7, | 3 | 2.374 (x 1.264) | 149 (0.020) | 0.763 | neuron differentiation | Tm1 mfas robl |
| 526 | GO:0004182 | F | 8, | 1 | 0.510 (x 1.962) | 32 (0.031) | 0.763 | carboxypeptidase A activity | CG4408 |
| 527 | GO:0042623 | F | 9, | 6 | 5.257 (x 1.141) | 330 (0.018) | 0.764 | ATPase activity, coupled | CG7846 Mhc Mlc1 Mlc2 Vha36 robl |
| 528 | GO:0015020 | F | 6, | 1 | 0.542 (x 1.846) | 34 (0.029) | 0.764 | glucuronosyltransferase activity | Act57B |
| 529 | GO:0003676 | F | 3, | 29 | 27.766 (x 1.044) | 1743 (0.017) | 0.764 | nucleic acid binding | Acf1 BEST:LD29214 CG11522 CG15398 CG17838 CG18619 CG31617 CG4866 CG6764 CG7283 CG7911 CG9282 Hr4 Msp-300 RpII18 RpL11 RpL27A RpL36 RpL38 RpL8 RpP1 RpS18 RpS3 RpS9 Ssb-c31a Tfb2 agt mRpL21 sop |
| 530 | GO:0008170 | F | 6, | 1 | 0.542 (x 1.846) | 34 (0.029) | 0.765 | N-methyltransferase activity | CG6188 |
| 531 | GO:0008639 | F | 3, | 1 | 0.542 (x 1.846) | 34 (0.029) | 0.767 | small protein conjugating enzyme activity | CG10862 |
| 532 | GO:0007442 | P | 7, 8, | 1 | 0.573 (x 1.744) | 36 (0.028) | 0.773 | hindgut morphogenesis | wal |
| 533 | GO:0043235 | C | 3, | 1 | 0.573 (x 1.744) | 36 (0.028) | 0.775 | receptor complex | Msp-300 |
| 534 | GO:0006403 | P | 4, | 2 | 1.481 (x 1.350) | 93 (0.022) | 0.775 | RNA localization | Tm1 ran-like |
| 535 | GO:0006766 | P | 5, | 1 | 0.573 (x 1.744) | 36 (0.028) | 0.776 | vitamin metabolism | CG30499 |
| 536 | GO:0042625 | F | 4, 5, 7, 12, | 2 | 1.513 (x 1.322) | 95 (0.021) | 0.784 | ATPase activity, coupled to transmembrane movement of ions | CG7846 Vha36 |
| 537 | GO:0044262 | P | 6, | 5 | 4.381 (x 1.141) | 275 (0.018) | 0.784 | cellular carbohydrate metabolism | Act57B CG30499 CG5177 Chit Gasp |
| 538 | GO:0008380 | P | 8, | 3 | 2.469 (x 1.215) | 155 (0.019) | 0.785 | RNA splicing | CG13277 CG3436 CG6610 |
| 539 | GO:0004536 | F | 6, | 1 | 0.589 (x 1.697) | 37 (0.027) | 0.785 | deoxyribonuclease activity | RpS3 |
| 540 | GO:0003674 | F | 1, | 133 | 131.996 (x 1.008) | 8286 (0.016) | 0.786 | molecular\_function | Acf1 Act57B BEST:LD29214 BG:DS00004.12 BG:DS01068.5 BG:DS02740.9 CAH2 CG10104 CG10133 CG10237 CG10862 CG10932 CG11051 CG11079 CG11522 CG12400 CG1304 CG1342 CG13991 CG14715 CG14946 CG15006 CG15012 CG15361 CG15398 CG16712 CG17838 CG18619 CG18854 CG1969 CG2555 CG2789 CG30438 CG30499 CG31146 CG31538 CG31547 CG31617 CG31715 CG31775 CG31876 CG32175 CG32207 CG32212 CG32249 CG3262 CG33002 CG33096 CG33493 CG4386 CG4408 CG4511 CG4673 CG4805 CG4866 CG5037 CG5122 CG5177 CG5224 CG5397 CG6188 CG6426 CG6574 CG6764 CG6921 CG7283 CG7846 CG7860 CG7911 CG7941 CG8193 CG9267 CG9282 CG9804 Cad99C Chit Cys Dhfr EG:152A3.7 EG:63B12.12 EG:80H7.10 Edg91 Fbp2 Gasp Gs2 GstD9 GstE1 Hr4 Iap2 Lcp2 Lcp3 Lsp1beta Mhc Mlc1 Mlc2 Mlp60A Mlp84B Mp20 Msp-300 Obp99b Pcmt RpII18 RpL11 RpL27A RpL36 RpL38 RpL8 RpP1 RpS12 RpS18 RpS3 RpS9 SIP1 Ssb-c31a Tbh Tfb2 Thor Tm1 Trap36 Tsf1 Vha36 agt corto desat1 fat-spondin fau mRpL21 mRpS21 ran-like robl sop tsl wal |
| 541 | GO:0019094 | P | 6, 10, 12, 13, 15, | 1 | 0.589 (x 1.697) | 37 (0.027) | 0.786 | pole plasm mRNA localization | Tm1 |
| 542 | GO:0007316 | P | 5, 9, 11, 12, 14, | 1 | 0.605 (x 1.652) | 38 (0.026) | 0.793 | pole plasm RNA localization | Tm1 |
| 543 | GO:0005509 | F | 5, | 4 | 3.473 (x 1.152) | 218 (0.018) | 0.793 | calcium ion binding | CG6426 Cad99C Mlc2 Mp20 |
| 544 | GO:0019731 | P | 6, 7, 8, | 1 | 0.605 (x 1.652) | 38 (0.026) | 0.794 | antibacterial humoral response | Thor |
| 545 | GO:0005102 | F | 3, 4, | 5 | 4.444 (x 1.125) | 279 (0.018) | 0.795 | receptor binding | CG11051 CG15361 CG31146 Chit tsl |
| 546 | GO:0009798 | P | 4, | 3 | 2.533 (x 1.184) | 159 (0.019) | 0.796 | axis specification | Tm1 regucalcin tsl |
| 547 | GO:0015296 | F | 5, 7, | 1 | 0.621 (x 1.610) | 39 (0.026) | 0.796 | anion:cation symporter activity | CG31547 |
| 548 | GO:0005279 | F | 5, 6, | 1 | 0.621 (x 1.610) | 39 (0.026) | 0.798 | amino acid-polyamine transporter activity | CG31547 |
| 549 | GO:0016740 | F | 3, | 16 | 15.404 (x 1.039) | 967 (0.017) | 0.798 | transferase activity | Act57B CG10932 CG18854 CG1969 CG30438 CG5037 CG5122 CG5224 CG5397 CG6188 CG6921 GstD9 GstE1 Pcmt RpII18 agt |
| 550 | GO:0006333 | P | 9, | 2 | 1.593 (x 1.255) | 100 (0.020) | 0.799 | chromatin assembly or disassembly | Acf1 CG31617 |
| 551 | GO:0046148 | P | 6, | 1 | 0.621 (x 1.610) | 39 (0.026) | 0.799 | pigment biosynthesis | CG5037 |
| 552 | GO:0015674 | P | 7, 8, | 1 | 0.637 (x 1.569) | 40 (0.025) | 0.799 | di-, tri-valent inorganic cation transport | Tsf1 |
| 553 | GO:0015203 | F | 4, | 1 | 0.621 (x 1.610) | 39 (0.026) | 0.8 | polyamine transporter activity | CG31547 |
| 554 | GO:0048547 | P | 5, 6, | 1 | 0.637 (x 1.569) | 40 (0.025) | 0.8 | gut morphogenesis | wal |
| 555 | GO:0004553 | F | 5, | 2 | 1.561 (x 1.281) | 98 (0.020) | 0.801 | hydrolase activity, hydrolyzing O-glycosyl compounds | CG15012 Chit |
| 556 | GO:0005815 | C | 5, 6, 7, 8, | 1 | 0.621 (x 1.610) | 39 (0.026) | 0.802 | microtubule organizing center | corto |
| 557 | GO:0048546 | P | 5, | 1 | 0.637 (x 1.569) | 40 (0.025) | 0.802 | digestive tract morphogenesis | wal |
| 558 | GO:0005976 | P | 6, | 3 | 2.581 (x 1.162) | 162 (0.019) | 0.802 | polysaccharide metabolism | CG30438 Chit Gasp |
| 559 | GO:0048567 | P | 6, 7, | 1 | 0.637 (x 1.569) | 40 (0.025) | 0.803 | ectodermal gut morphogenesis | wal |
| 560 | GO:0030286 | C | 4, 6, 7, 8, 9, 10, 11, | 1 | 0.653 (x 1.531) | 41 (0.024) | 0.805 | dynein complex | robl |
| 561 | GO:0007439 | P | 5, | 1 | 0.637 (x 1.569) | 40 (0.025) | 0.805 | ectodermal gut development | wal |
| 562 | GO:0004620 | F | 7, | 1 | 0.653 (x 1.531) | 41 (0.024) | 0.806 | phospholipase activity | CG10133 |
| 563 | GO:0005643 | C | 3, 5, 6, 7, 8, 9, 10, 11, 12, 13, | 1 | 0.637 (x 1.569) | 40 (0.025) | 0.806 | nuclear pore | CG4673 |
| 564 | GO:0048113 | P | 8, 10, 11, 13, | 1 | 0.653 (x 1.531) | 41 (0.024) | 0.807 | pole plasm assembly (sensu Insecta) | Tm1 |
| 565 | GO:0046930 | C | 6, 7, 8, | 1 | 0.637 (x 1.569) | 40 (0.025) | 0.808 | pore complex | CG4673 |
| 566 | GO:0048637 | P | 6, | 1 | 0.669 (x 1.495) | 42 (0.024) | 0.811 | skeletal muscle development | Mhc |
| 567 | GO:0048741 | P | 6, 7, | 1 | 0.669 (x 1.495) | 42 (0.024) | 0.813 | skeletal muscle fiber development | Mhc |
| 568 | GO:0043062 | P | 3, | 1 | 0.669 (x 1.495) | 42 (0.024) | 0.814 | extracellular structure organization and biogenesis | Gs2 |
| 569 | GO:0048747 | P | 5, | 1 | 0.669 (x 1.495) | 42 (0.024) | 0.815 | muscle fiber development | Mhc |
| 570 | GO:0015078 | F | 6, | 2 | 1.641 (x 1.219) | 103 (0.019) | 0.816 | hydrogen ion transporter activity | CG7846 Vha36 |
| 571 | GO:0004180 | F | 6, | 1 | 0.685 (x 1.460) | 43 (0.023) | 0.82 | carboxypeptidase activity | CG4408 |
| 572 | GO:0015077 | F | 5, | 2 | 1.673 (x 1.196) | 105 (0.019) | 0.822 | monovalent inorganic cation transporter activity | CG7846 Vha36 |
| 573 | GO:0051248 | P | 6, 7, | 1 | 0.685 (x 1.460) | 43 (0.023) | 0.822 | negative regulation of protein metabolism | Thor |
| 574 | GO:0007315 | P | 7, 9, 10, 12, | 1 | 0.685 (x 1.460) | 43 (0.023) | 0.823 | pole plasm assembly | Tm1 |
| 575 | GO:0007275 | P | 2, | 24 | 23.656 (x 1.015) | 1485 (0.016) | 0.825 | development | Act57B BG:DS02740.9 CG31146 CG5397 CG7860 EG:80H7.10 Gs2 Hr4 Mhc Mlc1 Mlp60A Mlp84B Mp20 Msp-300 Obp99b SIP1 Thor Tm1 fat-spondin mfas regucalcin robl tsl wal |
| 576 | GO:0051243 | P | 5, | 4 | 3.696 (x 1.082) | 232 (0.017) | 0.83 | negative regulation of cellular physiological process | CG11079 CG7188 Iap2 Thor |
| 577 | GO:0042440 | P | 5, | 1 | 0.733 (x 1.365) | 46 (0.022) | 0.837 | pigment metabolism | CG5037 |
| 578 | GO:0006865 | P | 6, 7, 8, | 1 | 0.733 (x 1.365) | 46 (0.022) | 0.839 | amino acid transport | CG31547 |
| 579 | GO:0006960 | P | 7, 8, | 1 | 0.717 (x 1.395) | 45 (0.022) | 0.839 | antimicrobial humoral response (sensu Protostomia) | Thor |
| 580 | GO:0044237 | P | 4, | 74 | 73.740 (x 1.004) | 4629 (0.016) | 0.84 | cellular metabolism | Acf1 Act57B BG:DS00004.12 CAH2 CG10104 CG10237 CG10862 CG10932 CG11079 CG11522 CG12400 CG1304 CG13277 CG13991 CG14715 CG15398 CG18619 CG2789 CG30438 CG30499 CG31617 CG33002 CG3436 CG4386 CG4408 CG4511 CG4866 CG5037 CG5122 CG5177 CG6188 CG6574 CG6610 CG6764 CG6921 CG7283 CG7846 CG7860 CG9267 CG9282 CG9804 Chit Dhfr EG:152A3.7 Gasp Gs2 GstE1 Hr4 Iap2 Mlc1 Pcmt RpII18 RpL11 RpL27A RpL36 RpL38 RpL8 RpP1 RpS12 RpS18 RpS3 RpS9 Ssb-c31a Tbh Tfb2 Thor Trap36 Vha36 agt desat1 mRpL21 mRpS21 sop wal |
| 581 | GO:0004497 | F | 4, | 2 | 1.736 (x 1.152) | 109 (0.018) | 0.84 | monooxygenase activity | CG8193 Tbh |
| 582 | GO:0030534 | P | 4, | 1 | 0.733 (x 1.365) | 46 (0.022) | 0.84 | adult behavior | Tbh |
| 583 | GO:0044455 | C | 4, 5, 6, 7, 8, 9, 10, 11, 12, | 2 | 1.752 (x 1.141) | 110 (0.018) | 0.84 | mitochondrial membrane part | CG12400 EG:152A3.7 |
| 584 | GO:0048112 | P | 7, 9, 10, 12, | 1 | 0.717 (x 1.395) | 45 (0.022) | 0.841 | oocyte anterior/posterior axis determination (sensu Insecta) | Tm1 |
| 585 | GO:0035272 | P | 4, | 2 | 1.736 (x 1.152) | 109 (0.018) | 0.841 | exocrine system development | CG7860 Obp99b |
| 586 | GO:0015837 | P | 5, 6, | 1 | 0.733 (x 1.365) | 46 (0.022) | 0.842 | amine transport | CG31547 |
| 587 | GO:0016811 | F | 5, | 1 | 0.749 (x 1.336) | 47 (0.021) | 0.842 | hydrolase activity, acting on carbon-nitrogen (but not peptide) bonds, in linear amides | CG7860 |
| 588 | GO:0044265 | P | 6, | 3 | 2.740 (x 1.095) | 172 (0.017) | 0.842 | cellular macromolecule catabolism | CG30499 CG7860 Chit |
| 589 | GO:0042981 | P | 6, 7, | 2 | 1.736 (x 1.152) | 109 (0.018) | 0.843 | regulation of apoptosis | CG7188 Iap2 |
| 590 | GO:0007155 | P | 3, | 5 | 4.827 (x 1.036) | 303 (0.017) | 0.843 | cell adhesion | Cad99C Mp20 fat-spondin hig mfas |
| 591 | GO:0044453 | C | 4, 5, 6, 7, 8, 9, 10, 11, 12, | 1 | 0.749 (x 1.336) | 47 (0.021) | 0.844 | nuclear membrane part | CG4673 |
| 592 | GO:0007431 | P | 5, | 2 | 1.736 (x 1.152) | 109 (0.018) | 0.844 | salivary gland development | CG7860 Obp99b |
| 593 | GO:0015171 | F | 4, 5, | 1 | 0.749 (x 1.336) | 47 (0.021) | 0.845 | amino acid transporter activity | CG31547 |
| 594 | GO:0031965 | C | 5, 6, 7, 8, 9, 10, 11, | 1 | 0.749 (x 1.336) | 47 (0.021) | 0.847 | nuclear membrane | CG4673 |
| 595 | GO:0005575 | C | 1, | 92 | 92.107 (x 0.999) | 5782 (0.016) | 0.851 | cellular\_component | Acf1 Act57B BEST:LD29214 BG:DS00004.12 BG:DS02740.9 CG10237 CG10932 CG11051 CG11079 CG11522 CG12400 CG13277 CG13991 CG15361 CG15398 CG17838 CG18619 CG2789 CG31538 CG31547 CG31617 CG31715 CG31775 CG32175 CG32207 CG32212 CG33002 CG33493 CG3436 CG4673 CG4805 CG4866 CG5037 CG5189 CG6574 CG6610 CG6764 CG6921 CG7188 CG7283 CG7846 CG7911 CG9282 Cad99C Chit EG:152A3.7 EG:63B12.12 EG:80H7.10 Gasp Gs2 Hr4 Iap2 Lsp1beta Mhc Mlc1 Mlc2 Mlp60A Mlp84B Mp20 Msp-300 Obp99b Pcmt RpII18 RpL11 RpL27A RpL36 RpL38 RpL8 RpP1 RpS12 RpS18 RpS3 RpS9 SIP1 Ssb-c31a Tfb2 Tm1 Trap36 Tsf1 Vha36 corto desat1 fat-spondin fau hig mRpL21 mRpS21 mfas robl sop tsl wal |
| 596 | GO:0043118 | P | 4, | 4 | 3.839 (x 1.042) | 241 (0.017) | 0.851 | negative regulation of physiological process | CG11079 CG7188 Iap2 Thor |
| 597 | GO:0004519 | F | 6, | 1 | 0.781 (x 1.281) | 49 (0.020) | 0.852 | endonuclease activity | RpS3 |
| 598 | GO:0008298 | P | 5, | 1 | 0.781 (x 1.281) | 49 (0.020) | 0.853 | intracellular mRNA localization | Tm1 |
| 599 | GO:0046983 | F | 4, | 1 | 0.781 (x 1.281) | 49 (0.020) | 0.854 | protein dimerization activity | CG18619 |
| 600 | GO:0048699 | P | 6, | 3 | 2.867 (x 1.046) | 180 (0.017) | 0.856 | generation of neurons | Tm1 mfas robl |
| 601 | GO:0006575 | P | 6, | 1 | 0.781 (x 1.281) | 49 (0.020) | 0.856 | amino acid derivative metabolism | Tbh |
| 602 | GO:0008595 | P | 6, 7, | 1 | 0.796 (x 1.255) | 50 (0.020) | 0.857 | determination of anterior/posterior axis, embryo | tsl |
| 603 | GO:0007420 | P | 4, 6, | 1 | 0.781 (x 1.281) | 49 (0.020) | 0.857 | brain development | robl |
| 604 | GO:0006259 | P | 6, | 6 | 5.910 (x 1.015) | 371 (0.016) | 0.858 | DNA metabolism | Acf1 CG31617 Dhfr RpS3 Tfb2 agt |
| 605 | GO:0007351 | P | 5, 6, | 1 | 0.796 (x 1.255) | 50 (0.020) | 0.859 | regional subdivision | tsl |
| 606 | GO:0008553 | F | 6, 7, 9, 14, | 1 | 0.781 (x 1.281) | 49 (0.020) | 0.859 | hydrogen-exporting ATPase activity, phosphorylative mechanism | Vha36 |
| 607 | GO:0016065 | P | 6, 7, | 1 | 0.844 (x 1.184) | 53 (0.019) | 0.859 | humoral defense mechanism (sensu Protostomia) | Thor |
| 608 | GO:0016887 | F | 8, | 6 | 5.910 (x 1.015) | 371 (0.016) | 0.859 | ATPase activity | CG7846 Mhc Mlc1 Mlc2 Vha36 robl |
| 609 | GO:0007635 | P | 4, 5, | 1 | 0.860 (x 1.162) | 54 (0.019) | 0.86 | chemosensory behavior | Obp99b |
| 610 | GO:0012501 | P | 5, | 4 | 3.951 (x 1.012) | 248 (0.016) | 0.86 | programmed cell death | CG7188 CG7860 Iap2 Obp99b |
| 611 | GO:0046164 | P | 6, | 1 | 0.812 (x 1.231) | 51 (0.020) | 0.86 | alcohol catabolism | CG30499 |
| 612 | GO:0042048 | P | 5, 6, | 1 | 0.844 (x 1.184) | 53 (0.019) | 0.86 | olfactory behavior | Obp99b |
| 613 | GO:0015698 | P | 7, 8, | 1 | 0.860 (x 1.162) | 54 (0.019) | 0.861 | inorganic anion transport | CG31547 |
| 614 | GO:0046365 | P | 7, 8, | 1 | 0.812 (x 1.231) | 51 (0.020) | 0.861 | monosaccharide catabolism | CG30499 |
| 615 | GO:0048111 | P | 6, 8, 9, 11, | 1 | 0.844 (x 1.184) | 53 (0.019) | 0.862 | oocyte axis determination (sensu Insecta) | Tm1 |
| 616 | GO:0015399 | F | 4, | 2 | 1.912 (x 1.046) | 120 (0.017) | 0.862 | primary active transporter activity | CG7846 Vha36 |
| 617 | GO:0015849 | P | 5, 6, | 1 | 0.860 (x 1.162) | 54 (0.019) | 0.862 | organic acid transport | CG31547 |
| 618 | GO:0006007 | P | 9, 10, | 1 | 0.812 (x 1.231) | 51 (0.020) | 0.863 | glucose catabolism | CG30499 |
| 619 | GO:0015980 | P | 6, | 2 | 1.880 (x 1.064) | 118 (0.017) | 0.863 | energy derivation by oxidation of organic compounds | CG30499 CG5177 |
| 620 | GO:0015405 | F | 5, | 2 | 1.912 (x 1.046) | 120 (0.017) | 0.863 | P-P-bond-hydrolysis-driven transporter activity | CG7846 Vha36 |
| 621 | GO:0007028 | P | 5, | 1 | 0.860 (x 1.162) | 54 (0.019) | 0.864 | cytoplasm organization and biogenesis | CG6764 |
| 622 | GO:0019320 | P | 8, 9, | 1 | 0.812 (x 1.231) | 51 (0.020) | 0.864 | hexose catabolism | CG30499 |
| 623 | GO:0006461 | P | 6, | 2 | 1.864 (x 1.073) | 117 (0.017) | 0.865 | protein complex assembly | Acf1 CG31617 |
| 624 | GO:0016265 | P | 3, | 4 | 3.998 (x 1.000) | 251 (0.016) | 0.865 | death | CG7188 CG7860 Iap2 Obp99b |
| 625 | GO:0006091 | P | 5, | 8 | 8.045 (x 0.994) | 505 (0.016) | 0.865 | generation of precursor metabolites and energy | CG12400 CG30499 CG4511 CG5177 CG7846 EG:152A3.7 Vha36 wal |
| 626 | GO:0016789 | F | 5, | 2 | 1.912 (x 1.046) | 120 (0.017) | 0.865 | carboxylic ester hydrolase activity | BG:DS00004.12 CG10133 |
| 627 | GO:0044464 | C | 2, 3, | 73 | 73.533 (x 0.993) | 4616 (0.016) | 0.865 | cell part | Acf1 Act57B BEST:LD29214 BG:DS00004.12 BG:DS02740.9 CG10237 CG10932 CG11079 CG11522 CG12400 CG13277 CG13991 CG15398 CG17838 CG18619 CG2789 CG31547 CG31617 CG33002 CG3436 CG4673 CG4805 CG4866 CG5037 CG5189 CG6574 CG6610 CG6764 CG6921 CG7188 CG7283 CG7846 CG7911 CG9282 Cad99C EG:152A3.7 EG:80H7.10 Gs2 Hr4 Iap2 Mhc Mlc1 Mlc2 Mlp60A Mlp84B Mp20 Msp-300 Pcmt RpII18 RpL11 RpL27A RpL36 RpL38 RpL8 RpP1 RpS12 RpS18 RpS3 RpS9 Ssb-c31a Tfb2 Tm1 Trap36 Vha36 corto desat1 hig mRpL21 mRpS21 mfas robl sop wal |
| 628 | GO:0046942 | P | 6, 7, | 1 | 0.860 (x 1.162) | 54 (0.019) | 0.865 | carboxylic acid transport | CG31547 |
| 629 | GO:0008219 | P | 4, | 4 | 3.982 (x 1.004) | 250 (0.016) | 0.865 | cell death | CG7188 CG7860 Iap2 Obp99b |
| 630 | GO:0006144 | P | 7, | 1 | 0.876 (x 1.141) | 55 (0.018) | 0.865 | purine base metabolism | Vha36 |
| 631 | GO:0016874 | F | 3, | 5 | 5.050 (x 0.990) | 317 (0.016) | 0.865 | ligase activity | CG10862 CG11079 CG9804 Gs2 Iap2 |
| 632 | GO:0043067 | P | 5, 6, | 2 | 1.896 (x 1.055) | 119 (0.017) | 0.866 | regulation of programmed cell death | CG7188 Iap2 |
| 633 | GO:0005623 | C | 2, | 73 | 73.533 (x 0.993) | 4616 (0.016) | 0.866 | cell | Acf1 Act57B BEST:LD29214 BG:DS00004.12 BG:DS02740.9 CG10237 CG10932 CG11079 CG11522 CG12400 CG13277 CG13991 CG15398 CG17838 CG18619 CG2789 CG31547 CG31617 CG33002 CG3436 CG4673 CG4805 CG4866 CG5037 CG5189 CG6574 CG6610 CG6764 CG6921 CG7188 CG7283 CG7846 CG7911 CG9282 Cad99C EG:152A3.7 EG:80H7.10 Gs2 Hr4 Iap2 Mhc Mlc1 Mlc2 Mlp60A Mlp84B Mp20 Msp-300 Pcmt RpII18 RpL11 RpL27A RpL36 RpL38 RpL8 RpP1 RpS12 RpS18 RpS3 RpS9 Ssb-c31a Tfb2 Tm1 Trap36 Vha36 corto desat1 hig mRpL21 mRpS21 mfas robl sop wal |
| 634 | GO:0043037 | P | 7, 8, | 3 | 2.963 (x 1.012) | 186 (0.016) | 0.866 | translation | RpP1 RpS18 Thor |
| 635 | GO:0048110 | P | 7, 8, 10, | 1 | 0.860 (x 1.162) | 54 (0.019) | 0.866 | oocyte construction (sensu Insecta) | Tm1 |
| 636 | GO:0005275 | F | 3, | 1 | 0.828 (x 1.207) | 52 (0.019) | 0.866 | amine transporter activity | CG31547 |
| 637 | GO:0016563 | F | 3, | 1 | 0.876 (x 1.141) | 55 (0.018) | 0.867 | transcriptional activator activity | Ssb-c31a |
| 638 | GO:0009057 | P | 5, | 3 | 3.027 (x 0.991) | 190 (0.016) | 0.867 | macromolecule catabolism | CG30499 CG7860 Chit |
| 639 | GO:0043231 | C | 4, 5, 6, 7, | 36 | 36.751 (x 0.980) | 2307 (0.016) | 0.867 | intracellular membrane-bound organelle | Acf1 BEST:LD29214 BG:DS00004.12 CG10932 CG11079 CG12400 CG13277 CG15398 CG17838 CG18619 CG2789 CG31617 CG33002 CG3436 CG4673 CG4866 CG5037 CG5189 CG6610 CG7911 EG:152A3.7 Hr4 Mlp60A Mlp84B RpII18 RpS3 Ssb-c31a Tfb2 Trap36 Vha36 corto desat1 mRpL21 mRpS21 robl wal |
| 640 | GO:0005773 | C | 5, 6, 7, 8, | 1 | 0.860 (x 1.162) | 54 (0.019) | 0.868 | vacuole | Vha36 |
| 641 | GO:0016337 | P | 4, | 2 | 1.975 (x 1.012) | 124 (0.016) | 0.868 | cell-cell adhesion | Cad99C mfas |
| 642 | GO:0043227 | C | 3, | 36 | 36.782 (x 0.979) | 2309 (0.016) | 0.868 | membrane-bound organelle | Acf1 BEST:LD29214 BG:DS00004.12 CG10932 CG11079 CG12400 CG13277 CG15398 CG17838 CG18619 CG2789 CG31617 CG33002 CG3436 CG4673 CG4866 CG5037 CG5189 CG6610 CG7911 EG:152A3.7 Hr4 Mlp60A Mlp84B RpII18 RpS3 Ssb-c31a Tfb2 Trap36 Vha36 corto desat1 mRpL21 mRpS21 robl wal |
| 643 | GO:0016836 | F | 5, | 1 | 0.892 (x 1.121) | 56 (0.018) | 0.868 | hydro-lyase activity | CAH2 |
| 644 | GO:0007314 | P | 6, 8, 9, 11, | 1 | 0.892 (x 1.121) | 56 (0.018) | 0.87 | oocyte anterior/posterior axis determination | Tm1 |
| 645 | GO:0043285 | P | 6, | 2 | 2.023 (x 0.989) | 127 (0.016) | 0.882 | biopolymer catabolism | CG7860 Chit |
| 646 | GO:0006869 | P | 5, 6, | 1 | 0.924 (x 1.082) | 58 (0.017) | 0.884 | lipid transport | CG2789 |
| 647 | GO:0006790 | P | 5, | 1 | 0.940 (x 1.064) | 59 (0.017) | 0.888 | sulfur metabolism | Act57B |
| 648 | GO:0048731 | P | 3, | 9 | 9.462 (x 0.951) | 594 (0.015) | 0.889 | system development | BG:DS02740.9 CG31146 CG7860 Gs2 Obp99b Tm1 mfas robl wal |
| 649 | GO:0006397 | P | 8, | 3 | 3.154 (x 0.951) | 198 (0.015) | 0.889 | mRNA processing | CG13277 CG3436 CG6610 |
| 650 | GO:0000578 | P | 5, | 1 | 0.940 (x 1.064) | 59 (0.017) | 0.889 | embryonic axis specification | tsl |
| 651 | GO:0016758 | F | 5, | 2 | 2.055 (x 0.973) | 129 (0.016) | 0.89 | transferase activity, transferring hexosyl groups | Act57B CG30438 |
| 652 | GO:0048732 | P | 4, | 2 | 2.087 (x 0.958) | 131 (0.015) | 0.891 | gland development | CG7860 Obp99b |
| 653 | GO:0006950 | P | 3, | 5 | 5.321 (x 0.940) | 334 (0.015) | 0.892 | response to stress | GstE1 RpS3 Tfb2 Thor agt |
| 654 | GO:0051707 | P | 4, | 2 | 2.087 (x 0.958) | 131 (0.015) | 0.893 | response to other organism | CG6426 Thor |
| 655 | GO:0007611 | P | 4, | 1 | 0.956 (x 1.046) | 60 (0.017) | 0.893 | learning and/or memory | Tbh |
| 656 | GO:0006006 | P | 8, 9, | 1 | 0.956 (x 1.046) | 60 (0.017) | 0.894 | glucose metabolism | CG30499 |
| 657 | GO:0040008 | P | 3, | 1 | 0.972 (x 1.029) | 61 (0.016) | 0.895 | regulation of growth | Thor |
| 658 | GO:0007507 | P | 5, | 1 | 0.972 (x 1.029) | 61 (0.016) | 0.896 | heart development | Act57B |
| 659 | GO:0005635 | C | 4, 5, 6, 7, 8, 9, 10, | 1 | 0.988 (x 1.012) | 62 (0.016) | 0.901 | nuclear envelope | CG4673 |
| 660 | GO:0048565 | P | 4, | 1 | 0.988 (x 1.012) | 62 (0.016) | 0.902 | gut development | wal |
| 661 | GO:0042165 | F | 3, | 1 | 1.004 (x 0.996) | 63 (0.016) | 0.904 | neurotransmitter binding | CG2789 |
| 662 | GO:0008061 | F | 5, | 1 | 1.004 (x 0.996) | 63 (0.016) | 0.905 | chitin binding | Gasp |
| 663 | GO:0019730 | P | 6, 7, | 1 | 1.004 (x 0.996) | 63 (0.016) | 0.906 | antimicrobial humoral response | Thor |
| 664 | GO:0030594 | F | 4, | 1 | 1.004 (x 0.996) | 63 (0.016) | 0.908 | neurotransmitter receptor activity | CG2789 |
| 665 | GO:0006323 | P | 7, | 2 | 2.198 (x 0.910) | 138 (0.014) | 0.915 | DNA packaging | Acf1 CG31617 |
| 666 | GO:0006325 | P | 8, | 2 | 2.198 (x 0.910) | 138 (0.014) | 0.917 | establishment and/or maintenance of chromatin architecture | Acf1 CG31617 |
| 667 | GO:0035239 | P | 4, | 1 | 1.035 (x 0.966) | 65 (0.015) | 0.917 | tube morphogenesis | wal |
| 668 | GO:0016071 | P | 7, | 3 | 3.313 (x 0.905) | 208 (0.014) | 0.918 | mRNA metabolism | CG13277 CG3436 CG6610 |
| 669 | GO:0019725 | P | 4, | 1 | 1.051 (x 0.951) | 66 (0.015) | 0.921 | cell homeostasis | Tsf1 |
| 670 | GO:0048523 | P | 4, | 4 | 4.460 (x 0.897) | 280 (0.014) | 0.923 | negative regulation of cellular process | CG11079 CG7188 Iap2 Thor |
| 671 | GO:0030247 | F | 4, | 1 | 1.067 (x 0.937) | 67 (0.015) | 0.923 | polysaccharide binding | Gasp |
| 672 | GO:0006800 | P | 5, | 1 | 1.067 (x 0.937) | 67 (0.015) | 0.925 | oxygen and reactive oxygen species metabolism | GstE1 |
| 673 | GO:0005975 | P | 5, | 7 | 7.726 (x 0.906) | 485 (0.014) | 0.926 | carbohydrate metabolism | Act57B CG11594 CG30438 CG30499 CG5177 Chit Gasp |
| 674 | GO:0019748 | P | 4, | 1 | 1.083 (x 0.923) | 68 (0.015) | 0.93 | secondary metabolism | CG5037 |
| 675 | GO:0016491 | F | 3, | 9 | 9.940 (x 0.905) | 624 (0.014) | 0.934 | oxidoreductase activity | CG12400 CG14946 CG8193 Dhfr EG:152A3.7 Fbp2 Tbh desat1 wal |
| 676 | GO:0030234 | F | 2, | 5 | 5.687 (x 0.879) | 357 (0.014) | 0.944 | enzyme regulator activity | CG11079 CG1342 CG16712 Cys fat-spondin |
| 677 | GO:0005516 | F | 4, | 1 | 1.131 (x 0.884) | 71 (0.014) | 0.947 | calmodulin binding | Mlc2 |
| 678 | GO:0006445 | P | 7, 8, 9, | 1 | 1.147 (x 0.872) | 72 (0.014) | 0.95 | regulation of translation | Thor |
| 679 | GO:0043492 | F | 3, 10, | 2 | 2.405 (x 0.831) | 151 (0.013) | 0.952 | ATPase activity, coupled to movement of substances | CG7846 Vha36 |
| 680 | GO:0015662 | F | 5, 6, 8, 13, | 1 | 1.147 (x 0.872) | 72 (0.014) | 0.952 | ATPase activity, coupled to transmembrane movement of ions, phosphorylative mechanism | Vha36 |
| 681 | GO:0006417 | P | 6, 7, 8, | 1 | 1.195 (x 0.837) | 75 (0.013) | 0.953 | regulation of protein biosynthesis | Thor |
| 682 | GO:0042626 | F | 4, 6, 11, | 2 | 2.405 (x 0.831) | 151 (0.013) | 0.953 | ATPase activity, coupled to transmembrane movement of substances | CG7846 Vha36 |
| 683 | GO:0003712 | F | 3, 5, | 1 | 1.179 (x 0.848) | 74 (0.014) | 0.953 | transcription cofactor activity | Ssb-c31a |
| 684 | GO:0046943 | F | 4, | 1 | 1.147 (x 0.872) | 72 (0.014) | 0.953 | carboxylic acid transporter activity | CG31547 |
| 685 | GO:0008152 | P | 3, | 78 | 80.558 (x 0.968) | 5057 (0.015) | 0.953 | metabolism | Acf1 Act57B BG:DS00004.12 CAH2 CG10104 CG10237 CG10862 CG10932 CG11079 CG11522 CG11594 CG12400 CG1304 CG13277 CG13991 CG14715 CG14946 CG15398 CG18619 CG2789 CG30438 CG30499 CG31617 CG33002 CG3436 CG4386 CG4408 CG4511 CG4866 CG5037 CG5122 CG5177 CG6188 CG6574 CG6610 CG6764 CG6921 CG7283 CG7846 CG7860 CG8193 CG9267 CG9282 CG9804 Chit Dhfr EG:152A3.7 Fbp2 Gasp Gs2 GstE1 Hr4 Iap2 Mlc1 Pcmt RpII18 RpL11 RpL27A RpL36 RpL38 RpL8 RpP1 RpS12 RpS18 RpS3 RpS9 Ssb-c31a Tbh Tfb2 Thor Trap36 Vha36 agt desat1 mRpL21 mRpS21 sop wal |
| 686 | GO:0007268 | P | 6, | 3 | 3.600 (x 0.833) | 226 (0.013) | 0.953 | synaptic transmission | CG31146 Gs2 Tbh |
| 687 | GO:0016820 | F | 5, | 2 | 2.421 (x 0.826) | 152 (0.013) | 0.954 | hydrolase activity, acting on acid anhydrides, catalyzing transmembrane movement of substances | CG7846 Vha36 |
| 688 | GO:0008235 | F | 6, | 1 | 1.211 (x 0.826) | 76 (0.013) | 0.954 | metalloexopeptidase activity | CG4408 |
| 689 | GO:0005342 | F | 3, | 1 | 1.179 (x 0.848) | 74 (0.014) | 0.954 | organic acid transporter activity | CG31547 |
| 690 | GO:0015293 | F | 6, | 1 | 1.163 (x 0.860) | 73 (0.014) | 0.955 | symporter activity | CG31547 |
| 691 | GO:0042592 | P | 3, | 1 | 1.211 (x 0.826) | 76 (0.013) | 0.955 | homeostasis | Tsf1 |
| 692 | GO:0007601 | P | 5, 7, | 1 | 1.179 (x 0.848) | 74 (0.014) | 0.956 | visual perception | CG14946 |
| 693 | GO:0008236 | F | 5, | 4 | 4.667 (x 0.857) | 293 (0.014) | 0.956 | serine-type peptidase activity | BG:DS01068.5 CG1304 CG13991 CG4386 |
| 694 | GO:0050896 | P | 2, | 17 | 18.622 (x 0.913) | 1169 (0.015) | 0.956 | response to stimulus | CG14946 CG30438 CG31146 CG5224 CG5397 CG6426 CG8193 GstD9 GstE1 Obp99b RpS3 Tbh Tfb2 Thor Tsf1 agt hig |
| 695 | GO:0005515 | F | 3, | 18 | 19.658 (x 0.916) | 1234 (0.015) | 0.956 | protein binding | BG:DS02740.9 CG11051 CG15361 CG18619 CG31146 Chit Mhc Mlc2 Mlp60A Mlp84B Mp20 Msp-300 RpL11 Ssb-c31a Thor Tm1 corto tsl |
| 696 | GO:0031324 | P | 6, | 2 | 2.469 (x 0.810) | 155 (0.013) | 0.956 | negative regulation of cellular metabolism | CG11079 Thor |
| 697 | GO:0007309 | P | 5, 7, 8, 10, | 1 | 1.211 (x 0.826) | 76 (0.013) | 0.956 | oocyte axis determination | Tm1 |
| 698 | GO:0050953 | P | 4, 6, | 1 | 1.179 (x 0.848) | 74 (0.014) | 0.957 | sensory perception of light stimulus | CG14946 |
| 699 | GO:0035295 | P | 3, | 1 | 1.227 (x 0.815) | 77 (0.013) | 0.957 | tube development | wal |
| 700 | GO:0019318 | P | 7, 8, | 1 | 1.274 (x 0.785) | 80 (0.013) | 0.958 | hexose metabolism | CG30499 |
| 701 | GO:0000785 | C | 5, 6, 7, 8, 9, 10, | 1 | 1.227 (x 0.815) | 77 (0.013) | 0.959 | chromatin | CG31617 |
| 702 | GO:0001871 | F | 3, | 1 | 1.274 (x 0.785) | 80 (0.013) | 0.959 | pattern binding | Gasp |
| 703 | GO:0043413 | P | 7, | 1 | 1.290 (x 0.775) | 81 (0.012) | 0.96 | biopolymer glycosylation | CG7860 |
| 704 | GO:0009889 | P | 5, | 1 | 1.258 (x 0.795) | 79 (0.013) | 0.96 | regulation of biosynthesis | Thor |
| 705 | GO:0048519 | P | 3, | 4 | 4.859 (x 0.823) | 305 (0.013) | 0.96 | negative regulation of biological process | CG11079 CG7188 Iap2 Thor |
| 706 | GO:0009101 | P | 7, 8, | 1 | 1.322 (x 0.756) | 83 (0.012) | 0.96 | glycoprotein biosynthesis | CG7860 |
| 707 | GO:0016788 | F | 4, | 6 | 7.248 (x 0.828) | 455 (0.013) | 0.96 | hydrolase activity, acting on ester bonds | BG:DS00004.12 CG10133 CG5177 CG5397 CG9267 RpS3 |
| 708 | GO:0048599 | P | 5, 6, 8, | 1 | 1.274 (x 0.785) | 80 (0.013) | 0.961 | oocyte development | Tm1 |
| 709 | GO:0006066 | P | 5, | 2 | 2.565 (x 0.780) | 161 (0.012) | 0.961 | alcohol metabolism | CG30499 Tbh |
| 710 | GO:0006959 | P | 5, 6, | 1 | 1.243 (x 0.805) | 78 (0.013) | 0.961 | humoral immune response | Thor |
| 711 | GO:0006812 | P | 6, 7, | 5 | 6.101 (x 0.820) | 383 (0.013) | 0.961 | cation transport | CG31547 CG4805 CG7846 Tsf1 Vha36 |
| 712 | GO:0017111 | F | 7, | 7 | 8.331 (x 0.840) | 523 (0.013) | 0.961 | nucleoside-triphosphatase activity | CG7846 Mhc Mlc1 Mlc2 Vha36 ran-like robl |
| 713 | GO:0004197 | F | 6, | 1 | 1.290 (x 0.775) | 81 (0.012) | 0.961 | cysteine-type endopeptidase activity | Mlc1 |
| 714 | GO:0007001 | P | 7, | 2 | 2.501 (x 0.800) | 157 (0.013) | 0.961 | chromosome organization and biogenesis (sensu Eukaryota) | Acf1 CG31617 |
| 715 | GO:0051603 | P | 8, 9, | 1 | 1.306 (x 0.766) | 82 (0.012) | 0.961 | proteolysis during cellular protein catabolism | CG7860 |
| 716 | GO:0031326 | P | 6, | 1 | 1.258 (x 0.795) | 79 (0.013) | 0.961 | regulation of cellular biosynthesis | Thor |
| 717 | GO:0016757 | F | 4, | 2 | 2.533 (x 0.790) | 159 (0.013) | 0.962 | transferase activity, transferring glycosyl groups | Act57B CG30438 |
| 718 | GO:0003682 | F | 3, | 1 | 1.322 (x 0.756) | 83 (0.012) | 0.962 | chromatin binding | Acf1 |
| 719 | GO:0005386 | F | 3, | 6 | 7.216 (x 0.831) | 453 (0.013) | 0.962 | carrier activity | CG10237 CG31547 CG6574 CG7846 Tsf1 Vha36 |
| 720 | GO:0044248 | P | 5, | 4 | 4.875 (x 0.821) | 306 (0.013) | 0.962 | cellular catabolism | CG30499 CG7860 Chit Gs2 |
| 721 | GO:0006486 | P | 8, 9, | 1 | 1.290 (x 0.775) | 81 (0.012) | 0.962 | protein amino acid glycosylation | CG7860 |
| 722 | GO:0016829 | F | 3, | 2 | 2.501 (x 0.800) | 157 (0.013) | 0.963 | lyase activity | CAH2 RpS3 |
| 723 | GO:0044257 | P | 7, 8, | 1 | 1.306 (x 0.766) | 82 (0.012) | 0.963 | cellular protein catabolism | CG7860 |
| 724 | GO:0007308 | P | 6, 7, 9, | 1 | 1.258 (x 0.795) | 79 (0.013) | 0.963 | oocyte construction | Tm1 |
| 725 | GO:0016616 | F | 5, | 1 | 1.338 (x 0.747) | 84 (0.012) | 0.963 | oxidoreductase activity, acting on the CH-OH group of donors, NAD or NADP as acceptor | Fbp2 |
| 726 | GO:0009892 | P | 5, | 2 | 2.628 (x 0.761) | 165 (0.012) | 0.964 | negative regulation of metabolism | CG11079 Thor |
| 727 | GO:0009100 | P | 7, | 1 | 1.370 (x 0.730) | 86 (0.012) | 0.965 | glycoprotein metabolism | CG7860 |
| 728 | GO:0016810 | F | 4, | 1 | 1.354 (x 0.739) | 85 (0.012) | 0.965 | hydrolase activity, acting on carbon-nitrogen (but not peptide) bonds | CG7860 |
| 729 | GO:0016310 | P | 7, | 6 | 7.344 (x 0.817) | 461 (0.013) | 0.966 | phosphorylation | CG12400 CG7846 CG9267 EG:152A3.7 Vha36 wal |
| 730 | GO:0044421 | C | 2, 3, | 1 | 1.354 (x 0.739) | 85 (0.012) | 0.966 | extracellular region part | Lsp1beta |
| 731 | GO:0048513 | P | 3, | 9 | 10.673 (x 0.843) | 670 (0.013) | 0.967 | organ development | Act57B CG7860 Mhc Mlp60A Mlp84B Mp20 Obp99b robl wal |
| 732 | GO:0016462 | F | 6, | 7 | 8.459 (x 0.828) | 531 (0.013) | 0.968 | pyrophosphatase activity | CG7846 Mhc Mlc1 Mlc2 Vha36 ran-like robl |
| 733 | GO:0009056 | P | 4, | 4 | 5.209 (x 0.768) | 327 (0.012) | 0.968 | catabolism | CG30499 CG7860 Chit Gs2 |
| 734 | GO:0005743 | C | 5, 6, 7, 8, 9, 10, 11, 12, | 2 | 2.692 (x 0.743) | 169 (0.012) | 0.968 | mitochondrial inner membrane | CG12400 EG:152A3.7 |
| 735 | GO:0016779 | F | 5, | 1 | 1.450 (x 0.690) | 91 (0.011) | 0.968 | nucleotidyltransferase activity | RpII18 |
| 736 | GO:0009994 | P | 4, 7, | 1 | 1.386 (x 0.722) | 87 (0.011) | 0.968 | oocyte differentiation | Tm1 |
| 737 | GO:0019866 | C | 4, 5, 6, 7, 8, 9, | 2 | 2.772 (x 0.722) | 174 (0.011) | 0.969 | organelle inner membrane | CG12400 EG:152A3.7 |
| 738 | GO:0006399 | P | 7, | 1 | 1.402 (x 0.713) | 88 (0.011) | 0.97 | tRNA metabolism | BG:DS00004.12 |
| 739 | GO:0044238 | P | 4, | 69 | 72.800 (x 0.948) | 4570 (0.015) | 0.97 | primary metabolism | Acf1 Act57B BG:DS00004.12 CG10104 CG10237 CG10862 CG10932 CG11079 CG11522 CG11594 CG1304 CG13277 CG13991 CG14715 CG15398 CG18619 CG2789 CG30438 CG30499 CG31617 CG33002 CG3436 CG4386 CG4408 CG4866 CG5122 CG5177 CG6188 CG6574 CG6610 CG6764 CG6921 CG7283 CG7846 CG7860 CG9267 CG9282 CG9804 Chit Dhfr Fbp2 Gasp Gs2 Hr4 Iap2 Mlc1 Pcmt RpII18 RpL11 RpL27A RpL36 RpL38 RpL8 RpP1 RpS12 RpS18 RpS3 RpS9 Ssb-c31a Tbh Tfb2 Thor Trap36 Vha36 agt desat1 mRpL21 mRpS21 sop |
| 740 | GO:0004518 | F | 5, | 1 | 1.434 (x 0.697) | 90 (0.011) | 0.97 | nuclease activity | RpS3 |
| 741 | GO:0008194 | F | 5, | 1 | 1.466 (x 0.682) | 92 (0.011) | 0.97 | UDP-glycosyltransferase activity | Act57B |
| 742 | GO:0008324 | F | 4, | 5 | 6.356 (x 0.787) | 399 (0.013) | 0.97 | cation transporter activity | CG31547 CG4805 CG7846 Tsf1 Vha36 |
| 743 | GO:0008509 | F | 4, | 1 | 1.497 (x 0.668) | 94 (0.011) | 0.971 | anion transporter activity | CG31547 |
| 744 | GO:0005554 | F | 2, | 10 | 11.979 (x 0.835) | 752 (0.013) | 0.971 | molecular function unknown | CG31538 CG31715 CG31775 CG32175 CG32207 CG32212 CG33493 EG:63B12.12 SIP1 fau |
| 745 | GO:0006118 | P | 6, | 4 | 5.305 (x 0.754) | 333 (0.012) | 0.971 | electron transport | CG12400 CG4511 EG:152A3.7 wal |
| 746 | GO:0004252 | F | 6, | 3 | 4.094 (x 0.733) | 257 (0.012) | 0.971 | serine-type endopeptidase activity | BG:DS01068.5 CG1304 CG4386 |
| 747 | GO:0016298 | F | 6, | 1 | 1.402 (x 0.713) | 88 (0.011) | 0.971 | lipase activity | CG10133 |
| 748 | GO:0016818 | F | 5, | 7 | 8.730 (x 0.802) | 548 (0.013) | 0.971 | hydrolase activity, acting on acid anhydrides, in phosphorus-containing anhydrides | CG7846 Mhc Mlc1 Mlc2 Vha36 ran-like robl |
| 749 | GO:0006915 | P | 6, | 2 | 2.836 (x 0.705) | 178 (0.011) | 0.971 | apoptosis | CG7188 Iap2 |
| 750 | GO:0051276 | P | 6, | 2 | 2.740 (x 0.730) | 172 (0.012) | 0.971 | chromosome organization and biogenesis | Acf1 CG31617 |
| 751 | GO:0030163 | P | 6, 7, | 1 | 1.434 (x 0.697) | 90 (0.011) | 0.971 | protein catabolism | CG7860 |
| 752 | GO:0007399 | P | 4, | 6 | 7.455 (x 0.805) | 468 (0.013) | 0.972 | nervous system development | BG:DS02740.9 CG31146 Gs2 Tm1 mfas robl |
| 753 | GO:0007389 | P | 3, | 3 | 4.078 (x 0.736) | 256 (0.012) | 0.972 | pattern specification | Tm1 regucalcin tsl |
| 754 | GO:0008134 | F | 4, | 1 | 1.418 (x 0.705) | 89 (0.011) | 0.972 | transcription factor binding | Ssb-c31a |
| 755 | GO:0016817 | F | 4, | 7 | 8.730 (x 0.802) | 548 (0.013) | 0.973 | hydrolase activity, acting on acid anhydrides | CG7846 Mhc Mlc1 Mlc2 Vha36 ran-like robl |
| 756 | GO:0005794 | C | 5, 6, 7, 8, | 1 | 1.434 (x 0.697) | 90 (0.011) | 0.973 | Golgi apparatus | CG5189 |
| 757 | GO:0006396 | P | 7, | 3 | 4.062 (x 0.739) | 255 (0.012) | 0.973 | RNA processing | CG13277 CG3436 CG6610 |
| 758 | GO:0004721 | F | 7, | 1 | 1.418 (x 0.705) | 89 (0.011) | 0.973 | phosphoprotein phosphatase activity | CG9267 |
| 759 | GO:0008234 | F | 5, | 1 | 1.481 (x 0.675) | 93 (0.011) | 0.974 | cysteine-type peptidase activity | Mlc1 |
| 760 | GO:0044255 | P | 5, 6, | 4 | 5.337 (x 0.750) | 335 (0.012) | 0.974 | cellular lipid metabolism | CG10932 CG30438 CG6921 desat1 |
| 761 | GO:0016791 | F | 6, | 2 | 2.899 (x 0.690) | 182 (0.011) | 0.978 | phosphoric monoester hydrolase activity | CG5177 CG9267 |
| 762 | GO:0000902 | P | 4, 5, | 4 | 5.384 (x 0.743) | 338 (0.012) | 0.979 | cellular morphogenesis | Mp20 SIP1 Thor mfas |
| 763 | GO:0009613 | P | 4, 5, | 1 | 1.593 (x 0.628) | 100 (0.010) | 0.98 | response to pest, pathogen or parasite | Thor |
| 764 | GO:0048468 | P | 4, | 4 | 5.416 (x 0.739) | 340 (0.012) | 0.981 | cell development | Mhc Tm1 mfas robl |
| 765 | GO:0000004 | P | 2, | 9 | 11.167 (x 0.806) | 701 (0.013) | 0.981 | biological process unknown | CG31538 CG31715 CG31775 CG32175 CG32207 CG32212 CG33493 EG:63B12.12 fau |
| 766 | GO:0006820 | P | 6, 7, | 1 | 1.593 (x 0.628) | 100 (0.010) | 0.982 | anion transport | CG31547 |
| 767 | GO:0031966 | C | 5, 6, 7, 8, 9, 10, 11, | 2 | 2.979 (x 0.671) | 187 (0.011) | 0.982 | mitochondrial membrane | CG12400 EG:152A3.7 |
| 768 | GO:0005996 | P | 6, 7, | 1 | 1.593 (x 0.628) | 100 (0.010) | 0.983 | monosaccharide metabolism | CG30499 |
| 769 | GO:0015630 | C | 6, 7, 8, 9, | 2 | 2.963 (x 0.675) | 186 (0.011) | 0.984 | microtubule cytoskeleton | corto robl |
| 770 | GO:0006629 | P | 5, | 6 | 7.806 (x 0.769) | 490 (0.012) | 0.984 | lipid metabolism | CG10932 CG2789 CG30438 CG6921 Fbp2 desat1 |
| 771 | GO:0007350 | P | 4, 5, | 1 | 1.625 (x 0.615) | 102 (0.010) | 0.985 | blastoderm segmentation | tsl |
| 772 | GO:0030705 | P | 6, 7, 8, | 1 | 1.657 (x 0.604) | 104 (0.010) | 0.988 | cytoskeleton-dependent intracellular transport | robl |
| 773 | GO:0007018 | P | 7, 8, 9, | 1 | 1.641 (x 0.609) | 103 (0.010) | 0.988 | microtubule-based movement | robl |
| 774 | GO:0006092 | P | 7, | 1 | 1.657 (x 0.604) | 104 (0.010) | 0.989 | main pathways of carbohydrate metabolism | CG30499 |
| 775 | GO:0007610 | P | 3, | 2 | 3.043 (x 0.657) | 191 (0.010) | 0.989 | behavior | Obp99b Tbh |
| 776 | GO:0048598 | P | 4, | 1 | 1.689 (x 0.592) | 106 (0.009) | 0.994 | embryonic morphogenesis | wal |
| 777 | GO:0005887 | C | 6, 7, 8, | 2 | 3.122 (x 0.641) | 196 (0.010) | 0.998 | integral to plasma membrane | Cad99C Msp-300 |
| 778 | GO:0031226 | C | 5, 6, 7, | 2 | 3.154 (x 0.634) | 198 (0.010) | 0.999 | intrinsic to plasma membrane | Cad99C Msp-300 |
| 779 | GO:0050789 | P | 2, | 11 | 23.975 (x 0.459) | 1505 (0.007) | 1 | regulation of biological process | Acf1 CG11079 CG14715 CG15398 CG18619 CG7188 Hr4 Iap2 Ssb-c31a Tfb2 Thor |
| 780 | GO:0051234 | P | 4, | 14 | 27.208 (x 0.515) | 1708 (0.008) | 1 | establishment of localization | CG10237 CG2789 CG31547 CG4673 CG4805 CG6574 CG7846 CG8193 Lsp1beta Obp99b Tsf1 Vha36 ran-like robl |
| 781 | GO:0008238 | F | 5, | 1 | 1.736 (x 0.576) | 109 (0.009) | 1 | exopeptidase activity | CG4408 |
| 782 | GO:0008104 | P | 4, | 2 | 9.016 (x 0.222) | 566 (0.004) | 1 | protein localization | CG4673 ran-like |
| 783 | GO:0007424 | P | 4, | 1 | 1.736 (x 0.576) | 109 (0.009) | 1 | tracheal system development (sensu Insecta) | wal |
| 784 | GO:0051179 | P | 3, | 15 | 28.212 (x 0.532) | 1771 (0.008) | 1 | localization | CG10237 CG2789 CG31547 CG4673 CG4805 CG6574 CG7846 CG8193 Lsp1beta Obp99b Tm1 Tsf1 Vha36 ran-like robl |
| 785 | GO:0031090 | C | 4, 5, 6, 7, 8, | 4 | 5.751 (x 0.696) | 361 (0.011) | 1 | organelle membrane | CG12400 CG4673 EG:152A3.7 Vha36 |
| 786 | GO:0006811 | P | 5, 6, | 5 | 7.360 (x 0.679) | 462 (0.011) | 1 | ion transport | CG31547 CG4805 CG7846 Tsf1 Vha36 |
| 787 | GO:0007059 | P | 4, | 1 | 1.975 (x 0.506) | 124 (0.008) | 1 | chromosome segregation | Msp-300 |
| 788 | GO:0050874 | P | 3, | 11 | 14.337 (x 0.767) | 900 (0.012) | 1 | organismal physiological process | CG14946 CG31146 Gs2 Mhc Mlc1 Mlc2 Mp20 Obp99b Tbh Thor Tm1 |
| 789 | GO:0042578 | F | 5, | 2 | 3.186 (x 0.628) | 200 (0.010) | 1 | phosphoric ester hydrolase activity | CG5177 CG9267 |
| 790 | GO:0030246 | F | 3, | 1 | 1.800 (x 0.556) | 113 (0.009) | 1 | carbohydrate binding | Gasp |
| 791 | GO:0004872 | F | 3, | 2 | 8.809 (x 0.227) | 553 (0.004) | 1 | receptor activity | CG2789 Hr4 |
| 792 | GO:0001505 | P | 7, | 1 | 1.864 (x 0.537) | 117 (0.009) | 1 | regulation of neurotransmitter levels | Tbh |
| 793 | GO:0006955 | P | 4, 5, | 1 | 1.943 (x 0.515) | 122 (0.008) | 1 | immune response | Thor |
| 794 | GO:0007169 | P | 7, | 1 | 1.975 (x 0.506) | 124 (0.008) | 1 | transmembrane receptor protein tyrosine kinase signaling pathway | tsl |
| 795 | GO:0000151 | C | 3, 4, 5, 6, | 1 | 2.007 (x 0.498) | 126 (0.008) | 1 | ubiquitin ligase complex | Iap2 |
| 796 | GO:0016614 | F | 4, | 1 | 1.880 (x 0.532) | 118 (0.008) | 1 | oxidoreductase activity, acting on CH-OH group of donors | Fbp2 |
| 797 | GO:0000166 | F | 3, | 5 | 13.987 (x 0.357) | 878 (0.006) | 1 | nucleotide binding | CG3262 CG4511 CG7846 Mhc ran-like |
| 798 | GO:0003824 | F | 2, | 55 | 60.231 (x 0.913) | 3781 (0.015) | 1 | catalytic activity | Act57B BG:DS00004.12 BG:DS01068.5 CAH2 CG10104 CG10133 CG10862 CG10932 CG11079 CG12400 CG1304 CG13991 CG14715 CG14946 CG15012 CG18854 CG1969 CG30438 CG30499 CG33096 CG4386 CG4408 CG5037 CG5122 CG5177 CG5224 CG5397 CG6188 CG6921 CG7846 CG7860 CG8193 CG9267 CG9804 Chit Dhfr EG:152A3.7 Fbp2 Gs2 GstD9 GstE1 Iap2 Mhc Mlc1 Mlc2 Pcmt RpII18 RpS3 Tbh Vha36 agt desat1 ran-like robl wal |
| 799 | GO:0004263 | F | 7, | 2 | 3.313 (x 0.604) | 208 (0.010) | 1 | chymotrypsin activity | CG1304 CG4386 |
| 800 | GO:0005783 | C | 5, 6, 7, 8, | 1 | 1.800 (x 0.556) | 113 (0.009) | 1 | endoplasmic reticulum | desat1 |
| 801 | GO:0007010 | P | 6, | 5 | 7.168 (x 0.697) | 450 (0.011) | 1 | cytoskeleton organization and biogenesis | Act57B CG7846 Msp-300 fat-spondin robl |
| 802 | GO:0007264 | P | 6, | 1 | 1.848 (x 0.541) | 116 (0.009) | 1 | small GTPase mediated signal transduction | ran-like |
| 803 | GO:0015291 | F | 5, | 2 | 3.457 (x 0.579) | 217 (0.009) | 1 | porter activity | CG31547 CG6574 |
| 804 | GO:0005488 | F | 2, | 60 | 65.855 (x 0.911) | 4134 (0.015) | 1 | binding | Acf1 BEST:LD29214 BG:DS02740.9 CAH2 CG10237 CG11051 CG11522 CG14715 CG15361 CG15398 CG17838 CG18619 CG2789 CG31146 CG31617 CG3262 CG4511 CG4866 CG6426 CG6574 CG6764 CG7283 CG7846 CG7911 CG9282 Cad99C Chit Gasp Hr4 Iap2 Mhc Mlc2 Mlp60A Mlp84B Mp20 Msp-300 Obp99b RpII18 RpL11 RpL27A RpL36 RpL38 RpL8 RpP1 RpS18 RpS3 RpS9 Ssb-c31a Tbh Tfb2 Thor Tm1 Tsf1 agt corto desat1 mRpL21 ran-like sop tsl |
| 805 | GO:0007281 | P | 5, | 1 | 1.784 (x 0.560) | 112 (0.009) | 1 | germ cell development | Tm1 |
| 806 | GO:0007166 | P | 5, | 3 | 10.498 (x 0.286) | 659 (0.005) | 1 | cell surface receptor linked signal transduction | CG11051 CG15361 tsl |
| 807 | GO:0007417 | P | 5, | 1 | 1.943 (x 0.515) | 122 (0.008) | 1 | central nervous system development | robl |
| 808 | GO:0035282 | P | 3, | 1 | 2.007 (x 0.498) | 126 (0.008) | 1 | segmentation | tsl |
| 809 | GO:0005549 | F | 3, | 1 | 1.832 (x 0.546) | 115 (0.009) | 1 | odorant binding | Obp99b |
| 810 | GO:0043169 | F | 4, | 11 | 14.130 (x 0.778) | 887 (0.012) | 1 | cation binding | CAH2 CG6426 Cad99C Iap2 Mlc2 Mlp60A Mlp84B Mp20 Tbh Tsf1 desat1 |
| 811 | GO:0016567 | P | 9, | 1 | 1.800 (x 0.556) | 113 (0.009) | 1 | protein ubiquitination | Iap2 |
| 812 | GO:0005875 | C | 3, 5, 6, 7, 8, 9, 10, | 1 | 1.848 (x 0.541) | 116 (0.009) | 1 | microtubule associated complex | robl |
| 813 | GO:0015290 | F | 4, | 2 | 3.457 (x 0.579) | 217 (0.009) | 1 | electrochemical potential-driven transporter activity | CG31547 CG6574 |
| 814 | GO:0016070 | P | 6, | 4 | 5.846 (x 0.684) | 367 (0.011) | 1 | RNA metabolism | BG:DS00004.12 CG13277 CG3436 CG6610 |
| 815 | GO:0005634 | C | 5, 6, 7, 8, | 20 | 24.277 (x 0.824) | 1524 (0.013) | 1 | nucleus | Acf1 BEST:LD29214 CG13277 CG15398 CG17838 CG18619 CG31617 CG3436 CG4673 CG6610 CG7911 Hr4 Mlp60A Mlp84B RpII18 RpS3 Ssb-c31a Tfb2 Trap36 corto |
| 816 | GO:0016301 | F | 5, | 1 | 6.388 (x 0.157) | 401 (0.002) | 1 | kinase activity | CG18854 |
| 817 | GO:0048667 | P | 6, 7, 9, | 1 | 1.928 (x 0.519) | 121 (0.008) | 1 | neuron morphogenesis during differentiation | mfas |
| 818 | GO:0005261 | F | 5, 6, | 1 | 1.832 (x 0.546) | 115 (0.009) | 1 | cation channel activity | CG4805 |
| 819 | GO:0017076 | F | 4, | 5 | 13.525 (x 0.370) | 849 (0.006) | 1 | purine nucleotide binding | CG3262 CG4511 CG7846 Mhc ran-like |
| 820 | GO:0003677 | F | 4, | 10 | 13.094 (x 0.764) | 822 (0.012) | 1 | DNA binding | Acf1 BEST:LD29214 CG15398 CG18619 CG31617 Hr4 Msp-300 RpII18 Ssb-c31a Tfb2 |
| 821 | GO:0008372 | C | 2, | 10 | 12.967 (x 0.771) | 814 (0.012) | 1 | cellular component unknown | CG31538 CG31715 CG31775 CG32175 CG32207 CG32212 CG33493 EG:63B12.12 SIP1 fau |
| 822 | GO:0045934 | P | 7, | 1 | 1.928 (x 0.519) | 121 (0.008) | 1 | negative regulation of nucleobase, nucleoside, nucleotide and nucleic acid metabolism | CG11079 |
| 823 | GO:0045184 | P | 5, | 2 | 8.284 (x 0.241) | 520 (0.004) | 1 | establishment of protein localization | CG4673 ran-like |
| 824 | GO:0006457 | P | 7, | 1 | 2.087 (x 0.479) | 131 (0.008) | 1 | protein folding | CG14715 |
| 825 | GO:0003924 | F | 8, | 1 | 2.071 (x 0.483) | 130 (0.008) | 1 | GTPase activity | ran-like |
| 826 | GO:0048812 | P | 7, 8, 10, | 1 | 1.928 (x 0.519) | 121 (0.008) | 1 | neurite morphogenesis | mfas |
| 827 | GO:0000087 | P | 6, | 2 | 3.616 (x 0.553) | 227 (0.009) | 1 | M phase of mitotic cell cycle | Mhc Msp-300 |
| 828 | GO:0009880 | P | 4, | 1 | 2.055 (x 0.487) | 129 (0.008) | 1 | embryonic pattern specification | tsl |
| 829 | GO:0004175 | F | 5, | 5 | 7.503 (x 0.666) | 471 (0.011) | 1 | endopeptidase activity | BG:DS01068.5 CG10104 CG1304 CG4386 Mlc1 |
| 830 | GO:0051641 | P | 4, 5, | 3 | 10.131 (x 0.296) | 636 (0.005) | 1 | cellular localization | CG4673 ran-like robl |
| 831 | GO:0002009 | P | 4, | 1 | 2.119 (x 0.472) | 133 (0.008) | 1 | morphogenesis of an epithelium | wal |
| 832 | GO:0019787 | F | 6, | 1 | 2.214 (x 0.452) | 139 (0.007) | 1 | small conjugating protein ligase activity | Iap2 |
| 833 | GO:0007067 | P | 7, | 2 | 3.600 (x 0.556) | 226 (0.009) | 1 | mitosis | Mhc Msp-300 |
| 834 | GO:0051649 | P | 5, 6, | 3 | 10.116 (x 0.297) | 635 (0.005) | 1 | establishment of cellular localization | CG4673 ran-like robl |
| 835 | GO:0007409 | P | 8, 9, 11, | 1 | 1.928 (x 0.519) | 121 (0.008) | 1 | axonogenesis | mfas |
| 836 | GO:0001700 | P | 5, | 1 | 2.055 (x 0.487) | 129 (0.008) | 1 | embryonic development (sensu Insecta) | wal |
| 837 | GO:0051246 | P | 5, 6, | 1 | 2.119 (x 0.472) | 133 (0.008) | 1 | regulation of protein metabolism | Thor |
| 838 | GO:0004842 | F | 7, | 1 | 2.214 (x 0.452) | 139 (0.007) | 1 | ubiquitin-protein ligase activity | Iap2 |
| 839 | GO:0008202 | P | 6, 7, | 1 | 2.166 (x 0.462) | 136 (0.007) | 1 | steroid metabolism | CG30438 |
| 840 | GO:0050794 | P | 3, | 11 | 21.936 (x 0.501) | 1377 (0.008) | 1 | regulation of cellular process | Acf1 CG11079 CG14715 CG15398 CG18619 CG7188 Hr4 Iap2 Ssb-c31a Tfb2 Thor |
| 841 | GO:0004295 | F | 7, | 2 | 3.744 (x 0.534) | 235 (0.009) | 1 | trypsin activity | CG1304 CG4386 |
| 842 | GO:0016021 | C | 5, 6, 7, | 11 | 15.038 (x 0.731) | 944 (0.012) | 1 | integral to membrane | CG2789 CG31547 CG4673 CG5037 CG6921 CG7188 CG7846 Cad99C EG:80H7.10 Msp-300 Vha36 |
| 843 | GO:0009653 | P | 3, | 7 | 10.227 (x 0.684) | 642 (0.011) | 1 | morphogenesis | Act57B Hr4 Mp20 SIP1 Thor mfas wal |
| 844 | GO:0015075 | F | 3, | 5 | 7.726 (x 0.647) | 485 (0.010) | 1 | ion transporter activity | CG31547 CG4805 CG7846 Tsf1 Vha36 |
| 845 | GO:0046872 | F | 4, | 11 | 14.847 (x 0.741) | 932 (0.012) | 1 | metal ion binding | CAH2 CG6426 Cad99C Iap2 Mlc2 Mlp60A Mlp84B Mp20 Tbh Tsf1 desat1 |
| 846 | GO:0006605 | P | 7, 8, 9, | 2 | 3.680 (x 0.544) | 231 (0.009) | 1 | protein targeting | CG4673 ran-like |
| 847 | GO:0006512 | P | 8, | 2 | 3.728 (x 0.537) | 234 (0.009) | 1 | ubiquitin cycle | CG10862 Iap2 |
| 848 | GO:0015031 | P | 5, 6, | 2 | 8.236 (x 0.243) | 517 (0.004) | 1 | protein transport | CG4673 ran-like |
| 849 | GO:0006996 | P | 5, | 8 | 11.501 (x 0.696) | 722 (0.011) | 1 | organelle organization and biogenesis | Acf1 Act57B CG31617 CG6764 CG7846 Msp-300 fat-spondin robl |
| 850 | GO:0031224 | C | 4, 5, 6, | 11 | 15.086 (x 0.729) | 947 (0.012) | 1 | intrinsic to membrane | CG2789 CG31547 CG4673 CG5037 CG6921 CG7188 CG7846 Cad99C EG:80H7.10 Msp-300 Vha36 |
| 851 | GO:0012505 | C | 4, 5, | 1 | 2.294 (x 0.436) | 144 (0.007) | 1 | endomembrane system | CG4673 |
| 852 | GO:0043167 | F | 3, | 11 | 14.847 (x 0.741) | 932 (0.012) | 1 | ion binding | CAH2 CG6426 Cad99C Iap2 Mlc2 Mlp60A Mlp84B Mp20 Tbh Tsf1 desat1 |
| 853 | GO:0008233 | F | 4, | 7 | 10.370 (x 0.675) | 651 (0.011) | 1 | peptidase activity | BG:DS01068.5 CG10104 CG1304 CG13991 CG4386 CG4408 Mlc1 |
| 854 | GO:0007165 | P | 4, | 10 | 20.566 (x 0.486) | 1291 (0.008) | 1 | signal transduction | CG11051 CG14715 CG15361 Cad99C Chit Hr4 mfas ran-like regucalcin tsl |
| 855 | GO:0006886 | P | 6, 7, 8, | 2 | 8.061 (x 0.248) | 506 (0.004) | 1 | intracellular protein transport | CG4673 ran-like |
| 856 | GO:0043412 | P | 6, | 6 | 14.608 (x 0.411) | 917 (0.007) | 1 | biopolymer modification | CG10862 CG7860 CG9267 CG9804 Iap2 Pcmt |
| 857 | GO:0046907 | P | 5, 6, 7, | 3 | 9.813 (x 0.306) | 616 (0.005) | 1 | intracellular transport | CG4673 ran-like robl |
| 858 | GO:0000003 | P | 2, | 2 | 7.981 (x 0.251) | 501 (0.004) | 1 | reproduction | Tbh Tm1 |
| 859 | GO:0016772 | F | 4, | 2 | 7.981 (x 0.251) | 501 (0.004) | 1 | transferase activity, transferring phosphorus-containing groups | CG18854 RpII18 |
| 860 | GO:0044427 | C | 4, 5, 6, 7, 8, 9, | 1 | 2.358 (x 0.424) | 148 (0.007) | 1 | chromosomal part | CG31617 |
| 861 | GO:0007154 | P | 3, | 13 | 24.166 (x 0.538) | 1517 (0.009) | 1 | cell communication | CG11051 CG14715 CG15361 CG31146 Cad99C Chit Gs2 Hr4 Tbh mfas ran-like regucalcin tsl |
| 862 | GO:0050791 | P | 3, | 11 | 21.330 (x 0.516) | 1339 (0.008) | 1 | regulation of physiological process | Acf1 CG11079 CG14715 CG15398 CG18619 CG7188 Hr4 Iap2 Ssb-c31a Tfb2 Thor |
| 863 | GO:0016773 | F | 5, | 1 | 5.687 (x 0.176) | 357 (0.003) | 1 | phosphotransferase activity, alcohol group as acceptor | CG18854 |
| 864 | GO:0008150 | P | 1, | 119 | 128.746 (x 0.924) | 8082 (0.015) | 1 | biological\_process | Acf1 Act57B BG:DS00004.12 BG:DS02740.9 CAH2 CG10104 CG10237 CG10862 CG10932 CG11051 CG11079 CG11522 CG11594 CG12400 CG1304 CG13277 CG13991 CG14715 CG14946 CG15361 CG15398 CG18619 CG2789 CG30438 CG30499 CG31146 CG31538 CG31547 CG31617 CG31715 CG31775 CG32175 CG32207 CG32212 CG33002 CG33493 CG3436 CG4386 CG4408 CG4511 CG4673 CG4805 CG4866 CG5037 CG5122 CG5177 CG5224 CG5397 CG6188 CG6426 CG6574 CG6610 CG6764 CG6921 CG7188 CG7283 CG7846 CG7860 CG8193 CG9267 CG9282 CG9804 Cad99C Chit Dhfr EG:152A3.7 EG:63B12.12 EG:80H7.10 Fbp2 Gasp Gs2 GstD9 GstE1 Hr4 Iap2 Lsp1beta Mhc Mlc1 Mlc2 Mlp60A Mlp84B Mp20 Msp-300 Obp99b Pcmt RpII18 RpL11 RpL27A RpL36 RpL38 RpL8 RpP1 RpS12 RpS18 RpS3 RpS9 SIP1 Ssb-c31a Tbh Tfb2 Thor Tm1 Trap36 Tsf1 Vha36 agt desat1 fat-spondin fau hig mRpL21 mRpS21 mfas ran-like regucalcin robl sop tsl wal |
| 865 | GO:0030554 | F | 5, | 4 | 10.976 (x 0.364) | 689 (0.006) | 1 | adenyl nucleotide binding | CG3262 CG4511 CG7846 Mhc |
| 866 | GO:0050875 | P | 3, | 92 | 99.021 (x 0.929) | 6216 (0.015) | 1 | cellular physiological process | Acf1 Act57B BG:DS00004.12 CAH2 CG10104 CG10237 CG10862 CG10932 CG11079 CG11522 CG12400 CG1304 CG13277 CG13991 CG14715 CG15398 CG18619 CG2789 CG30438 CG30499 CG31547 CG31617 CG33002 CG3436 CG4386 CG4408 CG4511 CG4673 CG4805 CG4866 CG5037 CG5122 CG5177 CG6188 CG6574 CG6610 CG6764 CG6921 CG7188 CG7283 CG7846 CG7860 CG8193 CG9267 CG9282 CG9804 Chit Dhfr EG:152A3.7 Gasp Gs2 GstE1 Hr4 Iap2 Lsp1beta Mhc Mlc1 Mlp60A Mlp84B Mp20 Msp-300 Obp99b Pcmt RpII18 RpL11 RpL27A RpL36 RpL38 RpL8 RpP1 RpS12 RpS18 RpS3 RpS9 SIP1 Ssb-c31a Tbh Tfb2 Thor Trap36 Tsf1 Vha36 agt desat1 fat-spondin mRpL21 mRpS21 mfas ran-like robl sop wal |
| 867 | GO:0019001 | F | 5, | 1 | 2.581 (x 0.387) | 162 (0.006) | 1 | guanyl nucleotide binding | ran-like |
| 868 | GO:0006464 | P | 7, | 6 | 13.971 (x 0.429) | 877 (0.007) | 1 | protein modification | CG10862 CG7860 CG9267 CG9804 Iap2 Pcmt |
| 869 | GO:0019226 | P | 5, | 3 | 5.783 (x 0.519) | 363 (0.008) | 1 | transmission of nerve impulse | CG31146 Gs2 Tbh |
| 870 | GO:0007606 | P | 4, 6, | 1 | 2.644 (x 0.378) | 166 (0.006) | 1 | sensory perception of chemical stimulus | Obp99b |
| 871 | GO:0051244 | P | 4, | 11 | 20.661 (x 0.532) | 1297 (0.008) | 1 | regulation of cellular physiological process | Acf1 CG11079 CG14715 CG15398 CG18619 CG7188 Hr4 Iap2 Ssb-c31a Tfb2 Thor |
| 872 | GO:0002165 | P | 4, | 3 | 5.496 (x 0.546) | 345 (0.009) | 1 | larval or pupal development (sensu Insecta) | CG7860 Hr4 Obp99b |
| 873 | GO:0009791 | P | 3, | 3 | 5.671 (x 0.529) | 356 (0.008) | 1 | post-embryonic development | CG7860 Hr4 Obp99b |
| 874 | GO:0007267 | P | 4, | 4 | 7.137 (x 0.560) | 448 (0.009) | 1 | cell-cell signaling | CG31146 Chit Gs2 Tbh |
| 875 | GO:0030154 | P | 3, | 5 | 8.124 (x 0.615) | 510 (0.010) | 1 | cell differentiation | Mhc Mlp84B Tm1 mfas robl |
| 876 | GO:0000278 | P | 5, | 2 | 4.269 (x 0.468) | 268 (0.007) | 1 | mitotic cell cycle | Mhc Msp-300 |
| 877 | GO:0005525 | F | 6, | 1 | 2.565 (x 0.390) | 161 (0.006) | 1 | GTP binding | ran-like |
| 878 | GO:0005216 | F | 4, 5, | 1 | 2.613 (x 0.383) | 164 (0.006) | 1 | ion channel activity | CG4805 |
| 879 | GO:0000904 | P | 5, 6, | 1 | 2.692 (x 0.371) | 169 (0.006) | 1 | cellular morphogenesis during differentiation | mfas |
| 880 | GO:0005524 | F | 6, | 4 | 10.705 (x 0.374) | 672 (0.006) | 1 | ATP binding | CG3262 CG4511 CG7846 Mhc |
| 881 | GO:0016881 | F | 5, | 1 | 2.517 (x 0.397) | 158 (0.006) | 1 | acid-amino acid ligase activity | Iap2 |
| 882 | GO:0006796 | P | 6, | 6 | 9.622 (x 0.624) | 604 (0.010) | 1 | phosphate metabolism | CG12400 CG7846 CG9267 EG:152A3.7 Vha36 wal |
| 883 | GO:0016787 | F | 3, | 22 | 28.610 (x 0.769) | 1796 (0.012) | 1 | hydrolase activity | BG:DS00004.12 BG:DS01068.5 CG10104 CG10133 CG1304 CG13991 CG15012 CG4386 CG4408 CG5177 CG5397 CG7846 CG7860 CG9267 Chit Mhc Mlc1 Mlc2 RpS3 Vha36 ran-like robl |
| 884 | GO:0007167 | P | 6, | 1 | 2.740 (x 0.365) | 172 (0.006) | 1 | enzyme linked receptor protein signaling pathway | tsl |
| 885 | GO:0019953 | P | 3, | 2 | 7.264 (x 0.275) | 456 (0.004) | 1 | sexual reproduction | Tbh Tm1 |
| 886 | GO:0009987 | P | 2, | 100 | 106.827 (x 0.936) | 6706 (0.015) | 1 | cellular process | Acf1 Act57B BG:DS00004.12 CAH2 CG10104 CG10237 CG10862 CG10932 CG11051 CG11079 CG11522 CG12400 CG1304 CG13277 CG13991 CG14715 CG15361 CG15398 CG18619 CG2789 CG30438 CG30499 CG31146 CG31547 CG31617 CG33002 CG3436 CG4386 CG4408 CG4511 CG4673 CG4805 CG4866 CG5037 CG5122 CG5177 CG6188 CG6574 CG6610 CG6764 CG6921 CG7188 CG7283 CG7846 CG7860 CG8193 CG9267 CG9282 CG9804 Cad99C Chit Dhfr EG:152A3.7 Gasp Gs2 GstE1 Hr4 Iap2 Lsp1beta Mhc Mlc1 Mlp60A Mlp84B Mp20 Msp-300 Obp99b Pcmt RpII18 RpL11 RpL27A RpL36 RpL38 RpL8 RpP1 RpS12 RpS18 RpS3 RpS9 SIP1 Ssb-c31a Tbh Tfb2 Thor Tm1 Trap36 Tsf1 Vha36 agt desat1 fat-spondin hig mRpL21 mRpS21 mfas ran-like regucalcin robl sop tsl wal |
| 887 | GO:0046914 | F | 5, | 7 | 10.769 (x 0.650) | 676 (0.010) | 1 | transition metal ion binding | CAH2 Iap2 Mlp60A Mlp84B Tbh Tsf1 desat1 |
| 888 | GO:0007276 | P | 4, | 2 | 7.121 (x 0.281) | 447 (0.004) | 1 | gametogenesis | Tbh Tm1 |
| 889 | GO:0006793 | P | 5, | 6 | 9.622 (x 0.624) | 604 (0.010) | 1 | phosphorus metabolism | CG12400 CG7846 CG9267 EG:152A3.7 Vha36 wal |
| 890 | GO:0007600 | P | 3, 5, | 2 | 4.110 (x 0.487) | 258 (0.008) | 1 | sensory perception | CG14946 Obp99b |
| 891 | GO:0006366 | P | 8, | 7 | 10.864 (x 0.644) | 682 (0.010) | 1 | transcription from RNA polymerase II promoter | Acf1 CG15398 Hr4 RpII18 Ssb-c31a Tfb2 Trap36 |
| 892 | GO:0019222 | P | 4, | 8 | 16.312 (x 0.490) | 1024 (0.008) | 1 | regulation of metabolism | Acf1 CG11079 CG15398 CG18619 Hr4 Ssb-c31a Tfb2 Thor |
| 893 | GO:0006357 | P | 9, | 3 | 8.714 (x 0.344) | 547 (0.005) | 1 | regulation of transcription from RNA polymerase II promoter | Acf1 Hr4 Ssb-c31a |
| 894 | GO:0045449 | P | 7, | 6 | 13.238 (x 0.453) | 831 (0.007) | 1 | regulation of transcription | Acf1 CG15398 CG18619 Hr4 Ssb-c31a Tfb2 |
| 895 | GO:0003700 | F | 3, 5, | 3 | 6.197 (x 0.484) | 389 (0.008) | 1 | transcription factor activity | CG15398 Hr4 Tfb2 |
| 896 | GO:0016020 | C | 3, 4, | 19 | 29.725 (x 0.639) | 1866 (0.010) | 1 | membrane | CG12400 CG13991 CG2789 CG31547 CG4673 CG4805 CG5037 CG6574 CG6921 CG7188 CG7846 Cad99C EG:152A3.7 EG:80H7.10 Msp-300 Vha36 desat1 hig mfas |
| 897 | GO:0008283 | P | 4, | 2 | 4.492 (x 0.445) | 282 (0.007) | 1 | cell proliferation | Mlp60A Mlp84B |
| 898 | GO:0008237 | F | 5, | 1 | 2.947 (x 0.339) | 185 (0.005) | 1 | metallopeptidase activity | CG4408 |
| 899 | GO:0005886 | C | 4, 5, | 3 | 8.507 (x 0.353) | 534 (0.006) | 1 | plasma membrane | Cad99C Msp-300 mfas |
| 900 | GO:0007292 | P | 5, | 2 | 4.938 (x 0.405) | 310 (0.006) | 1 | female gamete generation | Tbh Tm1 |
| 901 | GO:0015268 | F | 4, | 1 | 2.899 (x 0.345) | 182 (0.005) | 1 | alpha-type channel activity | CG4805 |
| 902 | GO:0030528 | F | 2, | 8 | 12.824 (x 0.624) | 805 (0.010) | 1 | transcription regulator activity | Acf1 BEST:LD29214 CG15398 Hr4 Ssb-c31a Tfb2 Trap36 corto |
| 903 | GO:0005215 | F | 2, | 10 | 15.420 (x 0.648) | 968 (0.010) | 1 | transporter activity | CG10237 CG2789 CG31547 CG4805 CG6574 CG7846 CG8193 Lsp1beta Tsf1 Vha36 |
| 904 | GO:0006810 | P | 4, 5, | 14 | 23.529 (x 0.595) | 1477 (0.009) | 1 | transport | CG10237 CG2789 CG31547 CG4673 CG4805 CG6574 CG7846 CG8193 Lsp1beta Obp99b Tsf1 Vha36 ran-like robl |
| 905 | GO:0005694 | C | 5, 6, 7, 8, | 1 | 2.931 (x 0.341) | 184 (0.005) | 1 | chromosome | CG31617 |
| 906 | GO:0044425 | C | 3, 4, 5, | 13 | 19.371 (x 0.671) | 1216 (0.011) | 1 | membrane part | CG12400 CG2789 CG31547 CG4673 CG5037 CG6921 CG7188 CG7846 Cad99C EG:152A3.7 EG:80H7.10 Msp-300 Vha36 |
| 907 | GO:0015267 | F | 3, | 1 | 2.899 (x 0.345) | 182 (0.005) | 1 | channel or pore class transporter activity | CG4805 |
| 908 | GO:0007049 | P | 4, | 4 | 7.503 (x 0.533) | 471 (0.008) | 1 | cell cycle | CG14715 Mhc Msp-300 ran-like |
| 909 | GO:0006508 | P | 7, | 7 | 12.075 (x 0.580) | 758 (0.009) | 1 | proteolysis | CG10104 CG1304 CG13991 CG4386 CG4408 CG7860 Mlc1 |
| 910 | GO:0016192 | P | 5, 6, | 1 | 4.715 (x 0.212) | 296 (0.003) | 1 | vesicle-mediated transport | ran-like |
| 911 | GO:0051726 | P | 5, | 1 | 3.170 (x 0.315) | 199 (0.005) | 1 | regulation of cell cycle | CG14715 |
| 912 | GO:0000279 | P | 5, | 2 | 4.572 (x 0.437) | 287 (0.007) | 1 | M phase | Mhc Msp-300 |
| 913 | GO:0007017 | P | 7, | 1 | 3.122 (x 0.320) | 196 (0.005) | 1 | microtubule-based process | robl |
| 914 | GO:0031323 | P | 5, | 8 | 15.707 (x 0.509) | 986 (0.008) | 1 | regulation of cellular metabolism | Acf1 CG11079 CG15398 CG18619 Hr4 Ssb-c31a Tfb2 Thor |
| 915 | GO:0007186 | P | 6, | 2 | 4.859 (x 0.412) | 305 (0.007) | 1 | G-protein coupled receptor protein signaling pathway | CG11051 CG15361 |
| 916 | GO:0048477 | P | 6, | 1 | 4.604 (x 0.217) | 289 (0.003) | 1 | oogenesis | Tm1 |
| 917 | GO:0000074 | P | 6, | 1 | 3.170 (x 0.315) | 199 (0.005) | 1 | regulation of progression through cell cycle | CG14715 |
| 918 | GO:0019219 | P | 6, | 7 | 14.146 (x 0.495) | 888 (0.008) | 1 | regulation of nucleobase, nucleoside, nucleotide and nucleic acid metabolism | Acf1 CG11079 CG15398 CG18619 Hr4 Ssb-c31a Tfb2 |
| 919 | GO:0009792 | P | 4, | 1 | 3.106 (x 0.322) | 195 (0.005) | 1 | embryonic development (sensu Metazoa) | wal |
| 920 | GO:0043283 | P | 5, | 19 | 26.826 (x 0.708) | 1684 (0.011) | 1 | biopolymer metabolism | Acf1 BG:DS00004.12 CG10862 CG13277 CG30438 CG31617 CG3436 CG6610 CG7860 CG9267 CG9804 Chit Dhfr Gasp Iap2 Pcmt RpS3 Tfb2 agt |
| 921 | GO:0009887 | P | 4, | 2 | 5.352 (x 0.374) | 336 (0.006) | 1 | organ morphogenesis | Act57B wal |
| 922 | GO:0006468 | P | 8, | 1 | 4.540 (x 0.220) | 285 (0.004) | 1 | protein amino acid phosphorylation | CG9267 |
| 923 | GO:0044459 | C | 4, 5, 6, | 2 | 5.145 (x 0.389) | 323 (0.006) | 1 | plasma membrane part | Cad99C Msp-300 |
| 924 | GO:0050877 | P | 4, | 5 | 9.813 (x 0.510) | 616 (0.008) | 1 | neurophysiological process | CG14946 CG31146 Gs2 Obp99b Tbh |
| 925 | GO:0004871 | F | 2, | 9 | 16.774 (x 0.537) | 1053 (0.009) | 1 | signal transducer activity | CG11051 CG15361 CG2789 CG31146 CG32249 Chit EG:80H7.10 Hr4 tsl |
| 926 | GO:0009993 | P | 7, | 1 | 4.413 (x 0.227) | 277 (0.004) | 1 | oogenesis (sensu Insecta) | Tm1 |
| 927 | GO:0006139 | P | 5, | 20 | 28.212 (x 0.709) | 1771 (0.011) | 1 | nucleobase, nucleoside, nucleotide and nucleic acid metabolism | Acf1 BG:DS00004.12 CG11079 CG13277 CG15398 CG18619 CG30499 CG31617 CG3436 CG6610 CG7846 Dhfr Hr4 RpII18 RpS3 Ssb-c31a Tfb2 Trap36 Vha36 agt |
| 928 | GO:0007242 | P | 5, | 4 | 8.347 (x 0.479) | 524 (0.008) | 1 | intracellular signaling cascade | CG14715 Hr4 ran-like regucalcin |
| 929 | GO:0006355 | P | 8, | 6 | 12.537 (x 0.479) | 787 (0.008) | 1 | regulation of transcription, DNA-dependent | Acf1 CG15398 CG18619 Hr4 Ssb-c31a Tfb2 |
| 930 | GO:0006350 | P | 6, | 8 | 15.054 (x 0.531) | 945 (0.008) | 1 | transcription | Acf1 CG15398 CG18619 Hr4 RpII18 Ssb-c31a Tfb2 Trap36 |
| 931 | GO:0007582 | P | 2, | 98 | 108.834 (x 0.900) | 6832 (0.014) | 1 | physiological process | Acf1 Act57B BG:DS00004.12 CAH2 CG10104 CG10237 CG10862 CG10932 CG11079 CG11522 CG11594 CG12400 CG1304 CG13277 CG13991 CG14715 CG14946 CG15398 CG18619 CG2789 CG30438 CG30499 CG31146 CG31547 CG31617 CG33002 CG3436 CG4386 CG4408 CG4511 CG4673 CG4805 CG4866 CG5037 CG5122 CG5177 CG6188 CG6574 CG6610 CG6764 CG6921 CG7188 CG7283 CG7846 CG7860 CG8193 CG9267 CG9282 CG9804 Chit Dhfr EG:152A3.7 Fbp2 Gasp Gs2 GstE1 Hr4 Iap2 Lsp1beta Mhc Mlc1 Mlc2 Mlp60A Mlp84B Mp20 Msp-300 Obp99b Pcmt RpII18 RpL11 RpL27A RpL36 RpL38 RpL8 RpP1 RpS12 RpS18 RpS3 RpS9 SIP1 Ssb-c31a Tbh Tfb2 Thor Tm1 Trap36 Tsf1 Vha36 agt desat1 fat-spondin mRpL21 mRpS21 mfas ran-like robl sop wal |
| 932 | GO:0008270 | F | 6, | 4 | 9.590 (x 0.417) | 602 (0.007) | 1 | zinc ion binding | CAH2 Iap2 Mlp60A Mlp84B |
| 933 | GO:0040011 | P | 3, | 1 | 4.158 (x 0.241) | 261 (0.004) | 1 | locomotion | robl |
| 934 | GO:0006928 | P | 4, 5, | 1 | 4.094 (x 0.244) | 257 (0.004) | 1 | cell motility | robl |
| 935 | GO:0005057 | F | 3, | 1 | 3.823 (x 0.262) | 240 (0.004) | 1 | receptor signaling protein activity | EG:80H7.10 |
| 936 | GO:0016043 | P | 4, | 14 | 21.967 (x 0.637) | 1379 (0.010) | 1 | cell organization and biogenesis | Acf1 Act57B CG31617 CG4673 CG6764 CG7846 Mp20 Msp-300 SIP1 Thor fat-spondin mfas ran-like robl |
| 937 | GO:0006351 | P | 7, | 8 | 14.273 (x 0.560) | 896 (0.009) | 1 | transcription, DNA-dependent | Acf1 CG15398 CG18619 Hr4 RpII18 Ssb-c31a Tfb2 Trap36 |
| 938 | GO:0009790 | P | 3, | 2 | 5.591 (x 0.358) | 351 (0.006) | 1 | embryonic development | tsl wal |
| 939 | GO:0051674 | P | 4, | 1 | 4.094 (x 0.244) | 257 (0.004) | 1 | localization of cell | robl |
| 940 | GO:0007552 | P | 4, | 1 | 4.030 (x 0.248) | 253 (0.004) | 1 | metamorphosis | Hr4 |
| 941 | GO:0046698 | P | 5, | 1 | 3.998 (x 0.250) | 251 (0.004) | 1 | metamorphosis (sensu Insecta) | Hr4 |

  

---

Regulated Genes that don't have GO terms
  

BG:DS00810.3 BG:DS01368.1 CG10038 CG10200 CG10581 CG12310 CG13053 CG13067 CG14104 CG14270 CG1572 CG15784 CG15888 CG17478 CG17681 CG18294 CG18410 CG2233 CG2444 CG2641 CG30412 CG30423 CG31955 CG32500 CG32633 CG32850 CG33156 CG3566 CG40178 CG40228 CG40260 CG40329 CG40386 CG40420 CG4989 CG5167 CG5174 CG5360 CG5773 CG5866 CG5961 CG6845 CG7603 CG7671 CG8369 CG8818 CG9231 CG9336 CG9338 CG9617 CG9766 EG:171E4.4
