# Supplementary material for: Functional dissection of the ash2 and ash1 transcriptomes provides insights into the transcriptional basis of wing phenotypes and reveals conserved protein interactions
Source: Genome Biol. 2007 Apr 28;8(4):R67. doi: 10.1186/gb-2007-8-4-r67 (PMC1896016; doi:10.1186/gb-2007-8-4-r67)
Supplement: Additional data file 15 — GO annotations of the genes downregulated over 2.0-fold in ash122 [file gb-2007-8-4-r67-S15.html]

  

---

  

|  |  |
| --- | --- |
| Go Statistics | Reg File: **ash1\_D2.0x.txt.fbgns** (22 genes -- 3 skipped)  Ref File: **ref.fbgns** (13577 genes -- 4663 skipped)  Database: **go\_200507-termdb.rdf-xml** |

---

  

Fields Description

| Pos | Go Term | Ontology | Levels | Observed | Expected | Possibles | p-value(Adj) | Go term description | Genes with the GO term |
| --- | --- | --- | --- | --- | --- | --- | --- | --- | --- |
| 1 | GO:0007635 | P | 4, 5, | 3 | 0.115 (x 26.064) | 54 (0.056) | 0.00991 | chemosensory behavior | CG8588 Obp99a smi35A |
| 2 | GO:0009408 | P | 4, 5, | 3 | 0.100 (x 29.946) | 47 (0.064) | 0.0114 | response to heat | Hsp23 Hsp26 Hsp67Ba |
| 3 | GO:0009266 | P | 4, | 3 | 0.115 (x 26.064) | 54 (0.056) | 0.0116 | response to temperature stimulus | Hsp23 Hsp26 Hsp67Ba |
| 4 | GO:0042048 | P | 5, 6, | 3 | 0.113 (x 26.556) | 53 (0.057) | 0.0131 | olfactory behavior | CG8588 Obp99a smi35A |
| 5 | GO:0016604 | C | 6, 7, 8, 9, 10, 11, 12, 13, | 2 | 0.017 (x 117.289) | 8 (0.250) | 0.0146 | nuclear body | B52 sqd |
| 6 | GO:0016607 | C | 7, 8, 9, 10, 11, 12, 13, 14, | 2 | 0.015 (x 134.045) | 7 (0.286) | 0.0164 | nuclear speck | B52 sqd |
| 7 | GO:0009628 | P | 3, | 6 | 0.793 (x 7.567) | 372 (0.016) | 0.0316 | response to abiotic stimulus | CG8588 Hsp23 Hsp26 Hsp67Ba Obp99a smi35A |
| 8 | GO:0045926 | P | 4, | 2 | 0.043 (x 46.916) | 20 (0.100) | 0.0365 | negative regulation of growth | charybde scylla |
| 9 | GO:0005521 | F | 4, | 1 | 0.002 (x 469.158) | 1 (1.000) | 0.0778 | lamin binding | CG17952 |
| 10 | GO:0006457 | P | 7, | 3 | 0.279 (x 10.744) | 131 (0.023) | 0.084 | protein folding | Hsp23 Hsp26 Hsp67Ba |
| 11 | GO:0008411 | F | 6, | 1 | 0.002 (x 469.158) | 1 (1.000) | 0.0864 | 4-hydroxybutyrate CoA-transferase activity | CG7920 |
| 12 | GO:0000381 | P | 10, 11, 13, | 2 | 0.104 (x 19.149) | 49 (0.041) | 0.116 | regulation of alternative nuclear mRNA splicing, via spliceosome | B52 sqd |
| 13 | GO:0051252 | P | 7, | 2 | 0.115 (x 17.376) | 54 (0.037) | 0.117 | regulation of RNA metabolism | B52 sqd |
| 14 | GO:0048024 | P | 9, 10, 12, | 2 | 0.113 (x 17.704) | 53 (0.038) | 0.119 | regulation of nuclear mRNA splicing, via spliceosome | B52 sqd |
| 15 | GO:0035062 | C | 8, 9, 10, 11, 12, 13, 14, 15, | 1 | 0.006 (x 156.386) | 3 (0.333) | 0.123 | omega speckle | sqd |
| 16 | GO:0000380 | P | 10, 12, | 2 | 0.104 (x 19.149) | 49 (0.041) | 0.124 | alternative nuclear mRNA splicing, via spliceosome | B52 sqd |
| 17 | GO:0050684 | P | 8, 9, | 2 | 0.113 (x 17.704) | 53 (0.038) | 0.127 | regulation of mRNA processing | B52 sqd |
| 18 | GO:0040008 | P | 3, | 2 | 0.130 (x 15.382) | 61 (0.033) | 0.127 | regulation of growth | charybde scylla |
| 19 | GO:0006084 | P | 7, | 2 | 0.102 (x 19.548) | 48 (0.042) | 0.129 | acetyl-CoA metabolism | CG5028 CG7920 |
| 20 | GO:0008410 | F | 5, | 1 | 0.004 (x 234.579) | 2 (0.500) | 0.13 | CoA-transferase activity | CG7920 |
| 21 | GO:0007610 | P | 3, | 3 | 0.407 (x 7.369) | 191 (0.016) | 0.133 | behavior | CG8588 Obp99a smi35A |
| 22 | GO:0007561 | P | 6, 7, | 1 | 0.009 (x 117.289) | 4 (0.250) | 0.141 | imaginal disc eversion | ImpE2 |
| 23 | GO:0005637 | C | 5, 6, 7, 8, 9, 10, 11, 12, 13, | 1 | 0.011 (x 93.832) | 5 (0.200) | 0.168 | nuclear inner membrane | CG17952 |
| 24 | GO:0000785 | C | 5, 6, 7, 8, 9, 10, | 2 | 0.164 (x 12.186) | 77 (0.026) | 0.174 | chromatin | kis sqd |
| 25 | GO:0004449 | F | 7, | 1 | 0.013 (x 78.193) | 6 (0.167) | 0.186 | isocitrate dehydrogenase (NAD+) activity | CG5028 |
| 26 | GO:0005703 | C | 5, 6, 7, 8, 9, 10, | 1 | 0.019 (x 52.129) | 9 (0.111) | 0.198 | polytene chromosome puff | sqd |
| 27 | GO:0006402 | P | 8, | 1 | 0.019 (x 52.129) | 9 (0.111) | 0.204 | mRNA catabolism | sqd |
| 28 | GO:0004448 | F | 6, | 1 | 0.015 (x 67.023) | 7 (0.143) | 0.208 | isocitrate dehydrogenase activity | CG5028 |
| 29 | GO:0040007 | P | 2, | 2 | 0.192 (x 10.426) | 90 (0.022) | 0.208 | growth | charybde scylla |
| 30 | GO:0008431 | F | 4, | 1 | 0.019 (x 52.129) | 9 (0.111) | 0.21 | vitamin E binding | CG3823 |
| 31 | GO:0042221 | P | 4, | 3 | 0.561 (x 5.352) | 263 (0.011) | 0.211 | response to chemical stimulus | CG8588 Obp99a smi35A |
| 32 | GO:0000184 | P | 9, | 1 | 0.017 (x 58.645) | 8 (0.125) | 0.213 | mRNA catabolism, nonsense-mediated decay | sqd |
| 33 | GO:0003730 | F | 6, | 1 | 0.019 (x 52.129) | 9 (0.111) | 0.217 | mRNA 3'-UTR binding | sqd |
| 34 | GO:0006376 | P | 8, 11, 13, | 1 | 0.017 (x 58.645) | 8 (0.125) | 0.221 | mRNA splice site selection | B52 |
| 35 | GO:0044446 | C | 3, 4, 5, 6, 7, | 7 | 3.035 (x 2.306) | 1424 (0.005) | 0.222 | intracellular organelle part | B52 CG17952 CG4759 CG5028 garz kis sqd |
| 36 | GO:0008069 | P | 6, 7, 9, | 1 | 0.019 (x 52.129) | 9 (0.111) | 0.224 | dorsal/ventral axis determination, follicular epithelium (sensu Insecta) | sqd |
| 37 | GO:0044422 | C | 2, 3, | 7 | 3.035 (x 2.306) | 1424 (0.005) | 0.228 | organelle part | B52 CG17952 CG4759 CG5028 garz kis sqd |
| 38 | GO:0048519 | P | 3, | 3 | 0.650 (x 4.615) | 305 (0.010) | 0.24 | negative regulation of biological process | charybde scylla sqd |
| 39 | GO:0019236 | P | 5, | 1 | 0.026 (x 39.096) | 12 (0.083) | 0.243 | response to pheromone | Obp99a |
| 40 | GO:0050896 | P | 2, | 6 | 2.492 (x 2.408) | 1169 (0.005) | 0.249 | response to stimulus | CG8588 Hsp23 Hsp26 Hsp67Ba Obp99a smi35A |
| 41 | GO:0006891 | P | 6, 7, 8, 9, | 1 | 0.028 (x 36.089) | 13 (0.077) | 0.25 | intra-Golgi vesicle-mediated transport | garz |
| 42 | GO:0050789 | P | 2, | 7 | 3.208 (x 2.182) | 1505 (0.005) | 0.253 | regulation of biological process | B52 charybde kis scylla smi35A sqd toy |
| 43 | GO:0030529 | C | 3, 4, 5, 6, | 3 | 0.678 (x 4.426) | 318 (0.009) | 0.254 | ribonucleoprotein complex | B52 CG4759 sqd |
| 44 | GO:0051028 | P | 7, 8, 9, | 1 | 0.036 (x 27.598) | 17 (0.059) | 0.255 | mRNA transport | sqd |
| 45 | GO:0006888 | P | 6, 7, 8, 9, | 1 | 0.032 (x 31.277) | 15 (0.067) | 0.256 | ER to Golgi vesicle-mediated transport | garz |
| 46 | GO:0000245 | P | 7, 10, 12, | 1 | 0.030 (x 33.511) | 14 (0.071) | 0.256 | spliceosome assembly | B52 |
| 47 | GO:0006950 | P | 3, | 3 | 0.712 (x 4.214) | 334 (0.009) | 0.257 | response to stress | Hsp23 Hsp26 Hsp67Ba |
| 48 | GO:0016478 | P | 8, 9, 10, | 1 | 0.036 (x 27.598) | 17 (0.059) | 0.26 | negative regulation of translation | sqd |
| 49 | GO:0007492 | P | 4, | 1 | 0.034 (x 29.322) | 16 (0.062) | 0.261 | endoderm development | toy |
| 50 | GO:0000377 | P | 10, | 2 | 0.318 (x 6.297) | 149 (0.013) | 0.262 | RNA splicing, via transesterification reactions with bulged adenosine as nucleophile | B52 sqd |
| 51 | GO:0050658 | P | 6, 7, 8, | 1 | 0.051 (x 19.548) | 24 (0.042) | 0.264 | RNA transport | sqd |
| 52 | GO:0016334 | P | 6, | 1 | 0.036 (x 27.598) | 17 (0.059) | 0.266 | establishment and/or maintenance of polarity of follicular epithelium | sqd |
| 53 | GO:0000398 | P | 9, 11, | 2 | 0.318 (x 6.297) | 149 (0.013) | 0.266 | nuclear mRNA splicing, via spliceosome | B52 sqd |
| 54 | GO:0005622 | C | 3, 4, | 11 | 7.023 (x 1.566) | 3295 (0.003) | 0.268 | intracellular | B52 CG17952 CG3823 CG4759 CG5028 ImpE2 garz kis smi35A sqd toy |
| 55 | GO:0031202 | F | 5, | 1 | 0.051 (x 19.548) | 24 (0.042) | 0.268 | RNA splicing factor activity, transesterification mechanism | B52 |
| 56 | GO:0009890 | P | 6, | 1 | 0.047 (x 21.325) | 22 (0.045) | 0.27 | negative regulation of biosynthesis | sqd |
| 57 | GO:0008380 | P | 8, | 2 | 0.330 (x 6.054) | 155 (0.013) | 0.271 | RNA splicing | B52 sqd |
| 58 | GO:0006406 | P | 8, 9, 10, 11, | 1 | 0.036 (x 27.598) | 17 (0.059) | 0.271 | mRNA export from nucleus | sqd |
| 59 | GO:0017148 | P | 7, 8, 9, | 1 | 0.045 (x 22.341) | 21 (0.048) | 0.271 | negative regulation of protein biosynthesis | sqd |
| 60 | GO:0000375 | P | 9, | 2 | 0.318 (x 6.297) | 149 (0.013) | 0.271 | RNA splicing, via transesterification reactions | B52 sqd |
| 61 | GO:0051236 | P | 5, | 1 | 0.051 (x 19.548) | 24 (0.042) | 0.272 | establishment of RNA localization | sqd |
| 62 | GO:0016782 | F | 4, | 1 | 0.043 (x 23.458) | 20 (0.050) | 0.273 | transferase activity, transferring sulfur-containing groups | CG7920 |
| 63 | GO:0044427 | C | 4, 5, 6, 7, 8, 9, | 2 | 0.315 (x 6.340) | 148 (0.014) | 0.273 | chromosomal part | kis sqd |
| 64 | GO:0016333 | P | 5, | 1 | 0.049 (x 20.398) | 23 (0.043) | 0.273 | morphogenesis of follicular epithelium | sqd |
| 65 | GO:0031327 | P | 7, | 1 | 0.047 (x 21.325) | 22 (0.045) | 0.275 | negative regulation of cellular biosynthesis | sqd |
| 66 | GO:0006405 | P | 7, 8, 9, 10, | 1 | 0.045 (x 22.341) | 21 (0.048) | 0.276 | RNA export from nucleus | sqd |
| 67 | GO:0050657 | P | 6, 7, | 1 | 0.051 (x 19.548) | 24 (0.042) | 0.276 | nucleic acid transport | sqd |
| 68 | GO:0006401 | P | 7, | 1 | 0.049 (x 20.398) | 23 (0.043) | 0.278 | RNA catabolism | sqd |
| 69 | GO:0005798 | C | 5, 6, 7, 8, 9, 10, | 1 | 0.047 (x 21.325) | 22 (0.045) | 0.279 | Golgi-associated vesicle | garz |
| 70 | GO:0019842 | F | 3, | 1 | 0.051 (x 19.548) | 24 (0.042) | 0.281 | vitamin binding | CG3823 |
| 71 | GO:0005829 | C | 5, 6, 7, 8, | 2 | 0.392 (x 5.100) | 184 (0.011) | 0.288 | cytosol | CG4759 smi35A |
| 72 | GO:0051168 | P | 7, 8, 9, | 1 | 0.058 (x 17.376) | 27 (0.037) | 0.288 | nuclear export | sqd |
| 73 | GO:0005694 | C | 5, 6, 7, 8, | 2 | 0.392 (x 5.100) | 184 (0.011) | 0.292 | chromosome | kis sqd |
| 74 | GO:0043233 | C | 3, 4, | 3 | 0.919 (x 3.266) | 431 (0.007) | 0.294 | organelle lumen | B52 CG5028 sqd |
| 75 | GO:0007379 | P | 4, 5, | 1 | 0.062 (x 16.178) | 29 (0.034) | 0.296 | segment specification | kis |
| 76 | GO:0031974 | C | 2, | 3 | 0.919 (x 3.266) | 431 (0.007) | 0.298 | membrane-enclosed lumen | B52 CG5028 sqd |
| 77 | GO:0006397 | P | 8, | 2 | 0.422 (x 4.739) | 198 (0.010) | 0.311 | mRNA processing | B52 sqd |
| 78 | GO:0044451 | C | 5, 6, 7, 8, 9, 10, 11, 12, | 2 | 0.433 (x 4.622) | 203 (0.010) | 0.316 | nucleoplasm part | B52 sqd |
| 79 | GO:0007297 | P | 6, 7, 9, | 1 | 0.070 (x 14.217) | 33 (0.030) | 0.319 | follicle cell migration (sensu Insecta) | sqd |
| 80 | GO:0051186 | P | 5, | 2 | 0.539 (x 3.709) | 253 (0.008) | 0.323 | cofactor metabolism | CG5028 CG7920 |
| 81 | GO:0007389 | P | 3, | 2 | 0.546 (x 3.665) | 256 (0.008) | 0.324 | pattern specification | kis sqd |
| 82 | GO:0006396 | P | 7, | 2 | 0.544 (x 3.680) | 255 (0.008) | 0.325 | RNA processing | B52 sqd |
| 83 | GO:0044424 | C | 3, 4, 5, | 10 | 6.799 (x 1.471) | 3190 (0.003) | 0.325 | intracellular part | B52 CG17952 CG4759 CG5028 ImpE2 garz kis smi35A sqd toy |
| 84 | GO:0005842 | C | 4, 5, 6, 7, 8, 9, 10, 11, | 1 | 0.109 (x 9.199) | 51 (0.020) | 0.325 | cytosolic large ribosomal subunit (sensu Eukaryota) | CG4759 |
| 85 | GO:0009607 | P | 3, | 3 | 1.113 (x 2.696) | 522 (0.006) | 0.326 | response to biotic stimulus | Hsp23 Hsp26 Hsp67Ba |
| 86 | GO:0016071 | P | 7, | 2 | 0.443 (x 4.511) | 208 (0.010) | 0.326 | mRNA metabolism | B52 sqd |
| 87 | GO:0006099 | P | 8, 9, | 1 | 0.100 (x 9.982) | 47 (0.021) | 0.326 | tricarboxylic acid cycle | CG5028 |
| 88 | GO:0009109 | P | 7, | 1 | 0.102 (x 9.774) | 48 (0.021) | 0.327 | coenzyme catabolism | CG5028 |
| 89 | GO:0008298 | P | 5, | 1 | 0.104 (x 9.575) | 49 (0.020) | 0.327 | intracellular mRNA localization | sqd |
| 90 | GO:0044453 | C | 4, 5, 6, 7, 8, 9, 10, 11, 12, | 1 | 0.100 (x 9.982) | 47 (0.021) | 0.329 | nuclear membrane part | CG17952 |
| 91 | GO:0051187 | P | 6, | 1 | 0.104 (x 9.575) | 49 (0.020) | 0.33 | cofactor catabolism | CG5028 |
| 92 | GO:0046356 | P | 8, | 1 | 0.100 (x 9.982) | 47 (0.021) | 0.333 | acetyl-CoA catabolism | CG5028 |
| 93 | GO:0048193 | P | 6, 7, 8, | 1 | 0.077 (x 13.032) | 36 (0.028) | 0.334 | Golgi vesicle transport | garz |
| 94 | GO:0015931 | P | 5, 6, | 1 | 0.079 (x 12.680) | 37 (0.027) | 0.334 | nucleobase, nucleoside, nucleotide and nucleic acid transport | sqd |
| 95 | GO:0048111 | P | 6, 8, 9, 11, | 1 | 0.113 (x 8.852) | 53 (0.019) | 0.335 | oocyte axis determination (sensu Insecta) | sqd |
| 96 | GO:0009060 | P | 8, | 1 | 0.100 (x 9.982) | 47 (0.021) | 0.336 | aerobic respiration | CG5028 |
| 97 | GO:0048110 | P | 7, 8, 10, | 1 | 0.115 (x 8.688) | 54 (0.019) | 0.338 | oocyte construction (sensu Insecta) | sqd |
| 98 | GO:0019094 | P | 6, 10, 12, 13, 15, | 1 | 0.079 (x 12.680) | 37 (0.027) | 0.339 | pole plasm mRNA localization | sqd |
| 99 | GO:0008340 | P | 4, | 1 | 0.098 (x 10.199) | 46 (0.022) | 0.339 | determination of adult life span | Hsp26 |
| 100 | GO:0031965 | C | 5, 6, 7, 8, 9, 10, 11, | 1 | 0.100 (x 9.982) | 47 (0.021) | 0.339 | nuclear membrane | CG17952 |
| 101 | GO:0007316 | P | 5, 9, 11, 12, 14, | 1 | 0.081 (x 12.346) | 38 (0.026) | 0.339 | pole plasm RNA localization | sqd |
| 102 | GO:0005737 | C | 4, 5, 6, | 6 | 3.165 (x 1.896) | 1485 (0.004) | 0.34 | cytoplasm | CG4759 CG5028 ImpE2 garz smi35A sqd |
| 103 | GO:0007568 | P | 3, | 1 | 0.098 (x 10.199) | 46 (0.022) | 0.342 | aging | Hsp26 |
| 104 | GO:0045333 | P | 7, | 1 | 0.100 (x 9.982) | 47 (0.021) | 0.342 | cellular respiration | CG5028 |
| 105 | GO:0008355 | P | 6, 7, | 1 | 0.083 (x 12.030) | 39 (0.026) | 0.344 | olfactory learning | CG8588 |
| 106 | GO:0031981 | C | 4, 5, 6, 7, 8, 9, 10, | 2 | 0.586 (x 3.412) | 275 (0.007) | 0.344 | nuclear lumen | B52 sqd |
| 107 | GO:0044428 | C | 4, 5, 6, 7, 8, 9, | 3 | 1.098 (x 2.733) | 515 (0.006) | 0.344 | nuclear part | B52 CG17952 sqd |
| 108 | GO:0048113 | P | 8, 10, 11, 13, | 1 | 0.087 (x 11.443) | 41 (0.024) | 0.344 | pole plasm assembly (sensu Insecta) | sqd |
| 109 | GO:0007275 | P | 2, | 6 | 3.165 (x 1.896) | 1485 (0.004) | 0.344 | development | Hsp26 ImpE2 kis smi35A sqd toy |
| 110 | GO:0005654 | C | 5, 6, 7, 8, 9, 10, 11, | 2 | 0.477 (x 4.189) | 224 (0.009) | 0.345 | nucleoplasm | B52 sqd |
| 111 | GO:0005700 | C | 6, 7, 8, 9, | 1 | 0.096 (x 10.426) | 45 (0.022) | 0.345 | polytene chromosome | sqd |
| 112 | GO:0019219 | P | 6, | 4 | 1.893 (x 2.113) | 888 (0.005) | 0.345 | regulation of nucleobase, nucleoside, nucleotide and nucleic acid metabolism | B52 kis sqd toy |
| 113 | GO:0006952 | P | 4, | 3 | 1.096 (x 2.738) | 514 (0.006) | 0.346 | defense response | Hsp23 Hsp26 Hsp67Ba |
| 114 | GO:0007314 | P | 6, 8, 9, 11, | 1 | 0.119 (x 8.378) | 56 (0.018) | 0.346 | oocyte anterior/posterior axis determination | sqd |
| 115 | GO:0009950 | P | 5, | 1 | 0.121 (x 8.231) | 57 (0.018) | 0.346 | dorsal/ventral axis specification | sqd |
| 116 | GO:0005085 | F | 4, | 1 | 0.124 (x 8.089) | 58 (0.017) | 0.346 | guanyl-nucleotide exchange factor activity | garz |
| 117 | GO:0007612 | P | 5, | 1 | 0.092 (x 10.911) | 43 (0.023) | 0.349 | learning | CG8588 |
| 118 | GO:0048112 | P | 7, 9, 10, 12, | 1 | 0.096 (x 10.426) | 45 (0.022) | 0.349 | oocyte anterior/posterior axis determination (sensu Insecta) | sqd |
| 119 | GO:0007398 | P | 4, | 2 | 0.505 (x 3.959) | 237 (0.008) | 0.351 | ectoderm development | smi35A toy |
| 120 | GO:0007611 | P | 4, | 1 | 0.128 (x 7.819) | 60 (0.017) | 0.352 | learning and/or memory | CG8588 |
| 121 | GO:0051248 | P | 6, 7, | 1 | 0.092 (x 10.911) | 43 (0.023) | 0.352 | negative regulation of protein metabolism | sqd |
| 122 | GO:0019898 | C | 4, 5, 6, | 1 | 0.096 (x 10.426) | 45 (0.022) | 0.353 | extrinsic to membrane | ImpE2 |
| 123 | GO:0006732 | P | 6, | 2 | 0.509 (x 3.926) | 239 (0.008) | 0.353 | coenzyme metabolism | CG5028 CG7920 |
| 124 | GO:0008283 | P | 4, | 2 | 0.601 (x 3.327) | 282 (0.007) | 0.353 | cell proliferation | smi35A toy |
| 125 | GO:0007315 | P | 7, 9, 10, 12, | 1 | 0.092 (x 10.911) | 43 (0.023) | 0.356 | pole plasm assembly | sqd |
| 126 | GO:0005635 | C | 4, 5, 6, 7, 8, 9, 10, | 1 | 0.132 (x 7.567) | 62 (0.016) | 0.357 | nuclear envelope | CG17952 |
| 127 | GO:0006917 | P | 8, 9, | 1 | 0.132 (x 7.567) | 62 (0.016) | 0.36 | induction of apoptosis | smi35A |
| 128 | GO:0043065 | P | 7, 8, | 1 | 0.136 (x 7.331) | 64 (0.016) | 0.365 | positive regulation of apoptosis | smi35A |
| 129 | GO:0051169 | P | 6, 7, 8, | 1 | 0.141 (x 7.108) | 66 (0.015) | 0.367 | nuclear transport | sqd |
| 130 | GO:0051244 | P | 4, | 5 | 2.765 (x 1.809) | 1297 (0.004) | 0.368 | regulation of cellular physiological process | B52 kis smi35A sqd toy |
| 131 | GO:0044431 | C | 4, 5, 6, 7, 8, 9, | 1 | 0.139 (x 7.218) | 65 (0.015) | 0.368 | Golgi apparatus part | garz |
| 132 | GO:0007444 | P | 4, | 2 | 0.644 (x 3.107) | 302 (0.007) | 0.371 | imaginal disc development | ImpE2 toy |
| 133 | GO:0044248 | P | 5, | 2 | 0.652 (x 3.066) | 306 (0.007) | 0.376 | cellular catabolism | CG5028 sqd |
| 134 | GO:0006445 | P | 7, 8, 9, | 1 | 0.153 (x 6.516) | 72 (0.014) | 0.378 | regulation of translation | sqd |
| 135 | GO:0044271 | P | 5, 6, | 1 | 0.179 (x 5.585) | 84 (0.012) | 0.378 | nitrogen compound biosynthesis | CG5028 |
| 136 | GO:0012502 | P | 7, 8, | 1 | 0.149 (x 6.702) | 70 (0.014) | 0.379 | induction of programmed cell death | smi35A |
| 137 | GO:0031988 | C | 4, | 1 | 0.183 (x 5.455) | 86 (0.012) | 0.379 | membrane-bound vesicle | garz |
| 138 | GO:0019222 | P | 4, | 4 | 2.183 (x 1.833) | 1024 (0.004) | 0.38 | regulation of metabolism | B52 kis sqd toy |
| 139 | GO:0050791 | P | 3, | 5 | 2.854 (x 1.752) | 1339 (0.004) | 0.38 | regulation of physiological process | B52 kis smi35A sqd toy |
| 140 | GO:0009309 | P | 6, 7, | 1 | 0.179 (x 5.585) | 84 (0.012) | 0.381 | amine biosynthesis | CG5028 |
| 141 | GO:0008652 | P | 7, 8, | 1 | 0.153 (x 6.516) | 72 (0.014) | 0.381 | amino acid biosynthesis | CG5028 |
| 142 | GO:0009994 | P | 4, 7, | 1 | 0.185 (x 5.393) | 87 (0.011) | 0.381 | oocyte differentiation | sqd |
| 143 | GO:0016023 | C | 5, 6, 7, 8, 9, | 1 | 0.177 (x 5.653) | 83 (0.012) | 0.381 | cytoplasmic membrane-bound vesicle | garz |
| 144 | GO:0006913 | P | 6, 7, 8, | 1 | 0.151 (x 6.608) | 71 (0.014) | 0.381 | nucleocytoplasmic transport | sqd |
| 145 | GO:0009889 | P | 5, | 1 | 0.168 (x 5.939) | 79 (0.013) | 0.382 | regulation of biosynthesis | sqd |
| 146 | GO:0043068 | P | 6, 7, | 1 | 0.160 (x 6.255) | 75 (0.013) | 0.382 | positive regulation of programmed cell death | smi35A |
| 147 | GO:0003729 | F | 5, | 2 | 0.667 (x 2.998) | 313 (0.006) | 0.382 | mRNA binding | B52 sqd |
| 148 | GO:0031982 | C | 3, | 1 | 0.183 (x 5.455) | 86 (0.012) | 0.382 | vesicle | garz |
| 149 | GO:0050794 | P | 3, | 5 | 2.935 (x 1.704) | 1377 (0.004) | 0.382 | regulation of cellular process | B52 kis smi35A sqd toy |
| 150 | GO:0009953 | P | 4, | 1 | 0.158 (x 6.340) | 74 (0.014) | 0.382 | dorsal/ventral pattern formation | sqd |
| 151 | GO:0031323 | P | 5, | 4 | 2.102 (x 1.903) | 986 (0.004) | 0.383 | regulation of cellular metabolism | B52 kis sqd toy |
| 152 | GO:0016616 | F | 5, | 1 | 0.179 (x 5.585) | 84 (0.012) | 0.383 | oxidoreductase activity, acting on the CH-OH group of donors, NAD or NADP as acceptor | CG5028 |
| 153 | GO:0048599 | P | 5, 6, 8, | 1 | 0.171 (x 5.864) | 80 (0.013) | 0.383 | oocyte development | sqd |
| 154 | GO:0007309 | P | 5, 7, 8, 10, | 1 | 0.162 (x 6.173) | 76 (0.013) | 0.384 | oocyte axis determination | sqd |
| 155 | GO:0008026 | F | 4, 10, | 1 | 0.177 (x 5.653) | 83 (0.012) | 0.384 | ATP-dependent helicase activity | kis |
| 156 | GO:0009056 | P | 4, | 2 | 0.697 (x 2.869) | 327 (0.006) | 0.384 | catabolism | CG5028 sqd |
| 157 | GO:0031326 | P | 6, | 1 | 0.168 (x 5.939) | 79 (0.013) | 0.384 | regulation of cellular biosynthesis | sqd |
| 158 | GO:0006417 | P | 6, 7, 8, | 1 | 0.160 (x 6.255) | 75 (0.013) | 0.384 | regulation of protein biosynthesis | sqd |
| 159 | GO:0003682 | F | 3, | 1 | 0.177 (x 5.653) | 83 (0.012) | 0.386 | chromatin binding | kis |
| 160 | GO:0019538 | P | 5, | 7 | 4.653 (x 1.504) | 2183 (0.003) | 0.387 | protein metabolism | B52 CG4759 Hsp23 Hsp26 Hsp67Ba smi35A sqd |
| 161 | GO:0007308 | P | 6, 7, 9, | 1 | 0.168 (x 5.939) | 79 (0.013) | 0.387 | oocyte construction | sqd |
| 162 | GO:0005830 | C | 5, 6, 7, 8, 9, 10, | 1 | 0.192 (x 5.213) | 90 (0.011) | 0.388 | cytosolic ribosome (sensu Eukaryota) | CG4759 |
| 163 | GO:0031410 | C | 4, 5, 6, 7, 8, | 1 | 0.177 (x 5.653) | 83 (0.012) | 0.389 | cytoplasmic vesicle | garz |
| 164 | GO:0003704 | F | 4, | 1 | 0.168 (x 5.939) | 79 (0.013) | 0.389 | specific RNA polymerase II transcription factor activity | toy |
| 165 | GO:0005794 | C | 5, 6, 7, 8, | 1 | 0.192 (x 5.213) | 90 (0.011) | 0.391 | Golgi apparatus | garz |
| 166 | GO:0043226 | C | 2, | 8 | 5.721 (x 1.398) | 2684 (0.003) | 0.392 | organelle | B52 CG17952 CG4759 CG5028 garz kis sqd toy |
| 167 | GO:0015934 | C | 3, 4, 5, 6, 7, 8, 9, | 1 | 0.205 (x 4.887) | 96 (0.010) | 0.393 | large ribosomal subunit | CG4759 |
| 168 | GO:0005681 | C | 4, 5, 6, 7, 8, 9, 10, | 1 | 0.196 (x 5.100) | 92 (0.011) | 0.394 | spliceosome complex | B52 |
| 169 | GO:0043229 | C | 3, 4, 5, 6, | 8 | 5.721 (x 1.398) | 2684 (0.003) | 0.394 | intracellular organelle | B52 CG17952 CG4759 CG5028 garz kis sqd toy |
| 170 | GO:0006403 | P | 4, | 1 | 0.198 (x 5.045) | 93 (0.011) | 0.395 | RNA localization | sqd |
| 171 | GO:0043228 | C | 3, | 3 | 1.515 (x 1.980) | 711 (0.004) | 0.395 | non-membrane-bound organelle | CG4759 kis sqd |
| 172 | GO:0003723 | F | 4, | 2 | 0.787 (x 2.543) | 369 (0.005) | 0.396 | RNA binding | B52 sqd |
| 173 | GO:0043232 | C | 4, 5, 6, 7, | 3 | 1.515 (x 1.980) | 711 (0.004) | 0.398 | intracellular non-membrane-bound organelle | CG4759 kis sqd |
| 174 | GO:0016070 | P | 6, | 2 | 0.782 (x 2.557) | 367 (0.005) | 0.398 | RNA metabolism | B52 sqd |
| 175 | GO:0043170 | P | 4, | 9 | 6.642 (x 1.355) | 3116 (0.003) | 0.398 | macromolecule metabolism | B52 CG4759 CG5028 Hsp23 Hsp26 Hsp67Ba kis smi35A sqd |
| 176 | GO:0006333 | P | 9, | 1 | 0.213 (x 4.692) | 100 (0.010) | 0.4 | chromatin assembly or disassembly | kis |
| 177 | GO:0043227 | C | 3, | 7 | 4.922 (x 1.422) | 2309 (0.003) | 0.404 | membrane-bound organelle | B52 CG17952 CG5028 garz kis sqd toy |
| 178 | GO:0007350 | P | 4, 5, | 1 | 0.217 (x 4.600) | 102 (0.010) | 0.405 | blastoderm segmentation | kis |
| 179 | GO:0005576 | C | 2, | 2 | 0.823 (x 2.431) | 386 (0.005) | 0.406 | extracellular region | ImpE2 Obp99a |
| 180 | GO:0006092 | P | 7, | 1 | 0.222 (x 4.511) | 104 (0.010) | 0.406 | main pathways of carbohydrate metabolism | CG5028 |
| 181 | GO:0043231 | C | 4, 5, 6, 7, | 7 | 4.917 (x 1.424) | 2307 (0.003) | 0.407 | intracellular membrane-bound organelle | B52 CG17952 CG5028 garz kis sqd toy |
| 182 | GO:0009888 | P | 3, | 2 | 0.848 (x 2.358) | 398 (0.005) | 0.413 | tissue development | smi35A toy |
| 183 | GO:0042981 | P | 6, 7, | 1 | 0.232 (x 4.304) | 109 (0.009) | 0.414 | regulation of apoptosis | smi35A |
| 184 | GO:0007517 | P | 4, | 1 | 0.230 (x 4.344) | 108 (0.009) | 0.415 | muscle development | toy |
| 185 | GO:0005634 | C | 5, 6, 7, 8, | 5 | 3.248 (x 1.539) | 1524 (0.003) | 0.418 | nucleus | B52 CG17952 kis sqd toy |
| 186 | GO:0004386 | F | 3, | 1 | 0.239 (x 4.189) | 112 (0.009) | 0.419 | helicase activity | kis |
| 187 | GO:0007281 | P | 5, | 1 | 0.239 (x 4.189) | 112 (0.009) | 0.422 | germ cell development | sqd |
| 188 | GO:0044444 | C | 4, 5, 6, 7, | 4 | 2.466 (x 1.622) | 1157 (0.003) | 0.424 | cytoplasmic part | CG4759 CG5028 garz smi35A |
| 189 | GO:0043067 | P | 5, 6, | 1 | 0.254 (x 3.943) | 119 (0.008) | 0.424 | regulation of programmed cell death | smi35A |
| 190 | GO:0005549 | F | 3, | 1 | 0.245 (x 4.080) | 115 (0.009) | 0.425 | odorant binding | Obp99a |
| 191 | GO:0016614 | F | 4, | 1 | 0.252 (x 3.976) | 118 (0.008) | 0.426 | oxidoreductase activity, acting on CH-OH group of donors | CG5028 |
| 192 | GO:0009948 | P | 5, | 1 | 0.254 (x 3.943) | 119 (0.008) | 0.427 | anterior/posterior axis specification | sqd |
| 193 | GO:0044445 | C | 5, 6, 7, 8, 9, | 1 | 0.252 (x 3.976) | 118 (0.008) | 0.428 | cytosolic part | CG4759 |
| 194 | GO:0006461 | P | 6, | 1 | 0.249 (x 4.010) | 117 (0.009) | 0.429 | protein complex assembly | B52 |
| 195 | GO:0015980 | P | 6, | 1 | 0.252 (x 3.976) | 118 (0.008) | 0.43 | energy derivation by oxidation of organic compounds | CG5028 |
| 196 | GO:0003779 | F | 5, | 1 | 0.262 (x 3.814) | 123 (0.008) | 0.432 | actin binding | Hsp23 |
| 197 | GO:0030707 | P | 8, | 1 | 0.264 (x 3.784) | 124 (0.008) | 0.433 | ovarian follicle cell development (sensu Insecta) | sqd |
| 198 | GO:0006887 | P | 6, 7, | 1 | 0.266 (x 3.753) | 125 (0.008) | 0.434 | exocytosis | garz |
| 199 | GO:0035282 | P | 3, | 1 | 0.269 (x 3.723) | 126 (0.008) | 0.435 | segmentation | kis |
| 200 | GO:0043285 | P | 6, | 1 | 0.271 (x 3.694) | 127 (0.008) | 0.436 | biopolymer catabolism | sqd |
| 201 | GO:0009952 | P | 4, | 1 | 0.277 (x 3.609) | 130 (0.008) | 0.438 | anterior/posterior pattern formation | sqd |
| 202 | GO:0009880 | P | 4, | 1 | 0.275 (x 3.637) | 129 (0.008) | 0.44 | embryonic pattern specification | kis |
| 203 | GO:0030695 | F | 3, | 1 | 0.277 (x 3.609) | 130 (0.008) | 0.44 | GTPase regulator activity | garz |
| 204 | GO:0002009 | P | 4, | 1 | 0.283 (x 3.528) | 133 (0.008) | 0.443 | morphogenesis of an epithelium | sqd |
| 205 | GO:0051246 | P | 5, 6, | 1 | 0.283 (x 3.528) | 133 (0.008) | 0.445 | regulation of protein metabolism | sqd |
| 206 | GO:0044249 | P | 5, | 3 | 1.769 (x 1.696) | 830 (0.004) | 0.452 | cellular biosynthesis | CG4759 CG5028 sqd |
| 207 | GO:0006325 | P | 8, | 1 | 0.294 (x 3.400) | 138 (0.007) | 0.453 | establishment and/or maintenance of chromatin architecture | kis |
| 208 | GO:0006323 | P | 7, | 1 | 0.294 (x 3.400) | 138 (0.007) | 0.455 | DNA packaging | kis |
| 209 | GO:0007399 | P | 4, | 2 | 0.998 (x 2.005) | 468 (0.004) | 0.46 | nervous system development | smi35A toy |
| 210 | GO:0005759 | C | 5, 6, 7, 8, 9, 10, 11, | 1 | 0.309 (x 3.236) | 145 (0.007) | 0.461 | mitochondrial matrix | CG5028 |
| 211 | GO:0012505 | C | 4, 5, | 1 | 0.307 (x 3.258) | 144 (0.007) | 0.463 | endomembrane system | CG17952 |
| 212 | GO:0044267 | P | 6, | 6 | 4.431 (x 1.354) | 2079 (0.003) | 0.463 | cellular protein metabolism | CG4759 Hsp23 Hsp26 Hsp67Ba smi35A sqd |
| 213 | GO:0031980 | C | 4, 5, 6, 7, 8, 9, 10, | 1 | 0.309 (x 3.236) | 145 (0.007) | 0.464 | mitochondrial lumen | CG5028 |
| 214 | GO:0006412 | P | 6, 7, | 2 | 1.053 (x 1.899) | 494 (0.004) | 0.482 | protein biosynthesis | CG4759 sqd |
| 215 | GO:0031324 | P | 6, | 1 | 0.330 (x 3.027) | 155 (0.006) | 0.484 | negative regulation of cellular metabolism | sqd |
| 216 | GO:0007001 | P | 7, | 1 | 0.335 (x 2.988) | 157 (0.006) | 0.485 | chromosome organization and biogenesis (sensu Eukaryota) | kis |
| 217 | GO:0009798 | P | 4, | 1 | 0.339 (x 2.951) | 159 (0.006) | 0.488 | axis specification | sqd |
| 218 | GO:0035214 | P | 5, | 1 | 0.341 (x 2.932) | 160 (0.006) | 0.488 | eye-antennal disc development | toy |
| 219 | GO:0007606 | P | 4, 6, | 1 | 0.354 (x 2.826) | 166 (0.006) | 0.494 | sensory perception of chemical stimulus | Obp99a |
| 220 | GO:0051242 | P | 5, | 1 | 0.356 (x 2.809) | 167 (0.006) | 0.494 | positive regulation of cellular physiological process | smi35A |
| 221 | GO:0044260 | P | 5, | 6 | 4.587 (x 1.308) | 2152 (0.003) | 0.496 | cellular macromolecule metabolism | CG4759 Hsp23 Hsp26 Hsp67Ba smi35A sqd |
| 222 | GO:0051276 | P | 6, | 1 | 0.367 (x 2.728) | 172 (0.006) | 0.496 | chromosome organization and biogenesis | kis |
| 223 | GO:0007456 | P | 6, | 1 | 0.369 (x 2.712) | 173 (0.006) | 0.496 | eye development (sensu Endopterygota) | toy |
| 224 | GO:0019866 | C | 4, 5, 6, 7, 8, 9, | 1 | 0.371 (x 2.696) | 174 (0.006) | 0.496 | organelle inner membrane | CG17952 |
| 225 | GO:0009892 | P | 5, | 1 | 0.352 (x 2.843) | 165 (0.006) | 0.496 | negative regulation of metabolism | sqd |
| 226 | GO:0016477 | P | 5, 6, | 1 | 0.373 (x 2.681) | 175 (0.006) | 0.496 | cell migration | sqd |
| 227 | GO:0043119 | P | 4, | 1 | 0.360 (x 2.776) | 169 (0.006) | 0.497 | positive regulation of physiological process | smi35A |
| 228 | GO:0009058 | P | 4, | 3 | 1.914 (x 1.567) | 898 (0.003) | 0.497 | biosynthesis | CG4759 CG5028 sqd |
| 229 | GO:0009059 | P | 5, 6, | 2 | 1.113 (x 1.798) | 522 (0.004) | 0.498 | macromolecule biosynthesis | CG4759 sqd |
| 230 | GO:0044265 | P | 6, | 1 | 0.367 (x 2.728) | 172 (0.006) | 0.498 | cellular macromolecule catabolism | sqd |
| 231 | GO:0003676 | F | 3, | 5 | 3.715 (x 1.346) | 1743 (0.003) | 0.498 | nucleic acid binding | B52 CG4759 kis sqd toy |
| 232 | GO:0004702 | F | 4, 8, | 1 | 0.377 (x 2.651) | 177 (0.006) | 0.499 | receptor signaling protein serine/threonine kinase activity | smi35A |
| 233 | GO:0006915 | P | 6, | 1 | 0.379 (x 2.636) | 178 (0.006) | 0.499 | apoptosis | smi35A |
| 234 | GO:0001654 | P | 5, | 1 | 0.386 (x 2.592) | 181 (0.006) | 0.504 | eye development | toy |
| 235 | GO:0006357 | P | 9, | 2 | 1.166 (x 1.715) | 547 (0.004) | 0.508 | regulation of transcription from RNA polymerase II promoter | kis toy |
| 236 | GO:0043037 | P | 7, 8, | 1 | 0.396 (x 2.522) | 186 (0.005) | 0.511 | translation | sqd |
| 237 | GO:0005840 | C | 4, 5, 6, 7, 8, | 1 | 0.403 (x 2.482) | 189 (0.005) | 0.511 | ribosome | CG4759 |
| 238 | GO:0009057 | P | 5, | 1 | 0.405 (x 2.469) | 190 (0.005) | 0.511 | macromolecule catabolism | sqd |
| 239 | GO:0003735 | F | 3, | 1 | 0.401 (x 2.496) | 188 (0.005) | 0.513 | structural constituent of ribosome | CG4759 |
| 240 | GO:0048522 | P | 4, | 1 | 0.403 (x 2.482) | 189 (0.005) | 0.513 | positive regulation of cellular process | smi35A |
| 241 | GO:0007498 | P | 4, | 1 | 0.422 (x 2.369) | 198 (0.005) | 0.527 | mesoderm development | toy |
| 242 | GO:0048731 | P | 3, | 2 | 1.266 (x 1.580) | 594 (0.003) | 0.55 | system development | smi35A toy |
| 243 | GO:0004674 | F | 7, | 1 | 0.452 (x 2.213) | 212 (0.005) | 0.552 | protein serine/threonine kinase activity | smi35A |
| 244 | GO:0045045 | P | 5, 6, | 1 | 0.458 (x 2.182) | 215 (0.005) | 0.556 | secretory pathway | garz |
| 245 | GO:0048518 | P | 3, | 1 | 0.465 (x 2.152) | 218 (0.005) | 0.557 | positive regulation of biological process | smi35A |
| 246 | GO:0005488 | F | 2, | 10 | 8.812 (x 1.135) | 4134 (0.002) | 0.557 | binding | B52 CG17952 CG3823 CG4759 Hsp23 Obp99a kis smi35A sqd toy |
| 247 | GO:0005623 | C | 2, | 11 | 9.839 (x 1.118) | 4616 (0.002) | 0.56 | cell | B52 CG17952 CG3823 CG4759 CG5028 ImpE2 garz kis smi35A sqd toy |
| 248 | GO:0044464 | C | 2, 3, | 11 | 9.839 (x 1.118) | 4616 (0.002) | 0.562 | cell part | B52 CG17952 CG3823 CG4759 CG5028 ImpE2 garz kis smi35A sqd toy |
| 249 | GO:0046907 | P | 5, 6, 7, | 2 | 1.313 (x 1.523) | 616 (0.003) | 0.564 | intracellular transport | garz sqd |
| 250 | GO:0006810 | P | 4, 5, | 4 | 3.148 (x 1.271) | 1477 (0.003) | 0.565 | transport | CG3823 Obp99a garz sqd |
| 251 | GO:0044237 | P | 4, | 11 | 9.867 (x 1.115) | 4629 (0.002) | 0.565 | cellular metabolism | B52 CG4759 CG5028 CG7920 Hsp23 Hsp26 Hsp67Ba kis smi35A sqd toy |
| 252 | GO:0051641 | P | 4, 5, | 2 | 1.356 (x 1.475) | 636 (0.003) | 0.567 | cellular localization | garz sqd |
| 253 | GO:0051243 | P | 5, | 1 | 0.495 (x 2.022) | 232 (0.004) | 0.567 | negative regulation of cellular physiological process | sqd |
| 254 | GO:0046903 | P | 5, | 1 | 0.492 (x 2.031) | 231 (0.004) | 0.567 | secretion | garz |
| 255 | GO:0007423 | P | 4, | 1 | 0.490 (x 2.040) | 230 (0.004) | 0.567 | sensory organ development | toy |
| 256 | GO:0051649 | P | 5, 6, | 2 | 1.353 (x 1.478) | 635 (0.003) | 0.568 | establishment of cellular localization | garz sqd |
| 257 | GO:0043118 | P | 4, | 1 | 0.514 (x 1.947) | 241 (0.004) | 0.568 | negative regulation of physiological process | sqd |
| 258 | GO:0007560 | P | 5, 6, | 1 | 0.512 (x 1.955) | 240 (0.004) | 0.568 | imaginal disc morphogenesis | ImpE2 |
| 259 | GO:0008092 | F | 4, | 1 | 0.507 (x 1.971) | 238 (0.004) | 0.569 | cytoskeletal protein binding | Hsp23 |
| 260 | GO:0005057 | F | 3, | 1 | 0.512 (x 1.955) | 240 (0.004) | 0.571 | receptor signaling protein activity | smi35A |
| 261 | GO:0009653 | P | 3, | 2 | 1.368 (x 1.462) | 642 (0.003) | 0.571 | morphogenesis | ImpE2 sqd |
| 262 | GO:0007600 | P | 3, 5, | 1 | 0.550 (x 1.818) | 258 (0.004) | 0.576 | sensory perception | Obp99a |
| 263 | GO:0006366 | P | 8, | 2 | 1.454 (x 1.376) | 682 (0.003) | 0.577 | transcription from RNA polymerase II promoter | kis toy |
| 264 | GO:0031967 | C | 3, 4, 5, 6, 7, 8, | 1 | 0.569 (x 1.757) | 267 (0.004) | 0.577 | organelle envelope | CG17952 |
| 265 | GO:0051674 | P | 4, | 1 | 0.548 (x 1.826) | 257 (0.004) | 0.577 | localization of cell | sqd |
| 266 | GO:0006520 | P | 6, 7, | 1 | 0.556 (x 1.798) | 261 (0.004) | 0.577 | amino acid metabolism | CG5028 |
| 267 | GO:0016265 | P | 3, | 1 | 0.535 (x 1.869) | 251 (0.004) | 0.577 | death | smi35A |
| 268 | GO:0012501 | P | 5, | 1 | 0.529 (x 1.892) | 248 (0.004) | 0.579 | programmed cell death | smi35A |
| 269 | GO:0007552 | P | 4, | 1 | 0.539 (x 1.854) | 253 (0.004) | 0.579 | metamorphosis | ImpE2 |
| 270 | GO:0031975 | C | 2, | 1 | 0.569 (x 1.757) | 267 (0.004) | 0.579 | envelope | CG17952 |
| 271 | GO:0006928 | P | 4, 5, | 1 | 0.548 (x 1.826) | 257 (0.004) | 0.579 | cell motility | sqd |
| 272 | GO:0005524 | F | 6, | 2 | 1.432 (x 1.396) | 672 (0.003) | 0.579 | ATP binding | kis smi35A |
| 273 | GO:0048513 | P | 3, | 2 | 1.428 (x 1.400) | 670 (0.003) | 0.579 | organ development | ImpE2 toy |
| 274 | GO:0030554 | F | 5, | 2 | 1.469 (x 1.362) | 689 (0.003) | 0.579 | adenyl nucleotide binding | kis smi35A |
| 275 | GO:0040011 | P | 3, | 1 | 0.556 (x 1.798) | 261 (0.004) | 0.579 | locomotion | sqd |
| 276 | GO:0046698 | P | 5, | 1 | 0.535 (x 1.869) | 251 (0.004) | 0.579 | metamorphosis (sensu Insecta) | ImpE2 |
| 277 | GO:0008219 | P | 4, | 1 | 0.533 (x 1.877) | 250 (0.004) | 0.58 | cell death | smi35A |
| 278 | GO:0003702 | F | 3, | 1 | 0.567 (x 1.764) | 266 (0.004) | 0.581 | RNA polymerase II transcription factor activity | toy |
| 279 | GO:0044262 | P | 6, | 1 | 0.586 (x 1.706) | 275 (0.004) | 0.587 | cellular carbohydrate metabolism | CG5028 |
| 280 | GO:0009993 | P | 7, | 1 | 0.590 (x 1.694) | 277 (0.004) | 0.588 | oogenesis (sensu Insecta) | sqd |
| 281 | GO:0048523 | P | 4, | 1 | 0.597 (x 1.676) | 280 (0.004) | 0.591 | negative regulation of cellular process | sqd |
| 282 | GO:0008150 | P | 1, | 18 | 17.227 (x 1.045) | 8082 (0.002) | 0.594 | biological\_process | B52 CG3823 CG4759 CG5028 CG7920 CG8588 Hsp23 Hsp26 Hsp67Ba ImpE2 Obp99a charybde garz kis scylla smi35A sqd toy |
| 283 | GO:0006468 | P | 8, | 1 | 0.607 (x 1.646) | 285 (0.004) | 0.595 | protein amino acid phosphorylation | smi35A |
| 284 | GO:0050875 | P | 3, | 14 | 13.249 (x 1.057) | 6216 (0.002) | 0.596 | cellular physiological process | B52 CG3823 CG4759 CG5028 CG7920 Hsp23 Hsp26 Hsp67Ba Obp99a garz kis smi35A sqd toy |
| 285 | GO:0048477 | P | 6, | 1 | 0.616 (x 1.623) | 289 (0.003) | 0.596 | oogenesis | sqd |
| 286 | GO:0006519 | P | 5, | 1 | 0.625 (x 1.601) | 293 (0.003) | 0.6 | amino acid and derivative metabolism | CG5028 |
| 287 | GO:0016192 | P | 5, 6, | 1 | 0.631 (x 1.585) | 296 (0.003) | 0.601 | vesicle-mediated transport | garz |
| 288 | GO:0004672 | F | 6, | 1 | 0.629 (x 1.590) | 295 (0.003) | 0.601 | protein kinase activity | smi35A |
| 289 | GO:0005575 | C | 1, | 13 | 12.324 (x 1.055) | 5782 (0.002) | 0.601 | cellular\_component | B52 CG17952 CG3823 CG4759 CG5028 CG8588 ImpE2 Obp99a garz kis smi35A sqd toy |
| 290 | GO:0007292 | P | 5, | 1 | 0.661 (x 1.513) | 310 (0.003) | 0.617 | female gamete generation | sqd |
| 291 | GO:0043283 | P | 5, | 4 | 3.589 (x 1.114) | 1684 (0.002) | 0.622 | biopolymer metabolism | B52 kis smi35A sqd |
| 292 | GO:0005515 | F | 3, | 3 | 2.630 (x 1.141) | 1234 (0.002) | 0.626 | protein binding | B52 CG17952 Hsp23 |
| 293 | GO:0051234 | P | 4, | 4 | 3.641 (x 1.099) | 1708 (0.002) | 0.633 | establishment of localization | CG3823 Obp99a garz sqd |
| 294 | GO:0006355 | P | 8, | 2 | 1.677 (x 1.192) | 787 (0.003) | 0.633 | regulation of transcription, DNA-dependent | kis toy |
| 295 | GO:0042623 | F | 9, | 1 | 0.703 (x 1.422) | 330 (0.003) | 0.634 | ATPase activity, coupled | kis |
| 296 | GO:0009887 | P | 4, | 1 | 0.716 (x 1.396) | 336 (0.003) | 0.639 | organ morphogenesis | ImpE2 |
| 297 | GO:0048468 | P | 4, | 1 | 0.725 (x 1.380) | 340 (0.003) | 0.642 | cell development | sqd |
| 298 | GO:0016773 | F | 5, | 1 | 0.761 (x 1.314) | 357 (0.003) | 0.642 | phosphotransferase activity, alcohol group as acceptor | smi35A |
| 299 | GO:0044238 | P | 4, | 10 | 9.741 (x 1.027) | 4570 (0.002) | 0.643 | primary metabolism | B52 CG4759 CG5028 Hsp23 Hsp26 Hsp67Ba kis smi35A sqd toy |
| 300 | GO:0002165 | P | 4, | 1 | 0.735 (x 1.360) | 345 (0.003) | 0.645 | larval or pupal development (sensu Insecta) | ImpE2 |
| 301 | GO:0030234 | F | 2, | 1 | 0.761 (x 1.314) | 357 (0.003) | 0.645 | enzyme regulator activity | garz |
| 302 | GO:0031090 | C | 4, 5, 6, 7, 8, | 1 | 0.769 (x 1.300) | 361 (0.003) | 0.645 | organelle membrane | CG17952 |
| 303 | GO:0045449 | P | 7, | 2 | 1.771 (x 1.129) | 831 (0.002) | 0.646 | regulation of transcription | kis toy |
| 304 | GO:0044429 | C | 4, 5, 6, 7, 8, 9, | 1 | 0.735 (x 1.360) | 345 (0.003) | 0.647 | mitochondrial part | CG5028 |
| 305 | GO:0008152 | P | 3, | 11 | 10.779 (x 1.021) | 5057 (0.002) | 0.648 | metabolism | B52 CG4759 CG5028 CG7920 Hsp23 Hsp26 Hsp67Ba kis smi35A sqd toy |
| 306 | GO:0009791 | P | 3, | 1 | 0.759 (x 1.318) | 356 (0.003) | 0.648 | post-embryonic development | ImpE2 |
| 307 | GO:0009790 | P | 3, | 1 | 0.748 (x 1.337) | 351 (0.003) | 0.648 | embryonic development | kis |
| 308 | GO:0006259 | P | 6, | 1 | 0.791 (x 1.265) | 371 (0.003) | 0.649 | DNA metabolism | kis |
| 309 | GO:0006139 | P | 5, | 4 | 3.775 (x 1.060) | 1771 (0.002) | 0.65 | nucleobase, nucleoside, nucleotide and nucleic acid metabolism | B52 kis sqd toy |
| 310 | GO:0003677 | F | 4, | 2 | 1.752 (x 1.142) | 822 (0.002) | 0.65 | DNA binding | kis toy |
| 311 | GO:0017076 | F | 4, | 2 | 1.810 (x 1.105) | 849 (0.002) | 0.65 | purine nucleotide binding | kis smi35A |
| 312 | GO:0009308 | P | 5, | 1 | 0.801 (x 1.248) | 376 (0.003) | 0.65 | amine metabolism | CG5028 |
| 313 | GO:0016887 | F | 8, | 1 | 0.791 (x 1.265) | 371 (0.003) | 0.651 | ATPase activity | kis |
| 314 | GO:0051179 | P | 3, | 4 | 3.775 (x 1.060) | 1771 (0.002) | 0.652 | localization | CG3823 Obp99a garz sqd |
| 315 | GO:0019752 | P | 6, | 1 | 0.836 (x 1.197) | 392 (0.003) | 0.658 | carboxylic acid metabolism | CG5028 |
| 316 | GO:0006082 | P | 5, | 1 | 0.836 (x 1.197) | 392 (0.003) | 0.66 | organic acid metabolism | CG5028 |
| 317 | GO:0003700 | F | 3, 5, | 1 | 0.829 (x 1.206) | 389 (0.003) | 0.661 | transcription factor activity | toy |
| 318 | GO:0006807 | P | 4, | 1 | 0.833 (x 1.200) | 391 (0.003) | 0.661 | nitrogen compound metabolism | CG5028 |
| 319 | GO:0016301 | F | 5, | 1 | 0.855 (x 1.170) | 401 (0.002) | 0.661 | kinase activity | smi35A |
| 320 | GO:0050874 | P | 3, | 2 | 1.918 (x 1.043) | 900 (0.002) | 0.662 | organismal physiological process | Hsp26 Obp99a |
| 321 | GO:0000166 | F | 3, | 2 | 1.871 (x 1.069) | 878 (0.002) | 0.662 | nucleotide binding | kis smi35A |
| 322 | GO:0006351 | P | 7, | 2 | 1.910 (x 1.047) | 896 (0.002) | 0.663 | transcription, DNA-dependent | kis toy |
| 323 | GO:0016043 | P | 4, | 3 | 2.939 (x 1.021) | 1379 (0.002) | 0.663 | cell organization and biogenesis | garz kis sqd |
| 324 | GO:0006350 | P | 6, | 2 | 2.014 (x 0.993) | 945 (0.002) | 0.691 | transcription | kis toy |
| 325 | GO:0007276 | P | 4, | 1 | 0.953 (x 1.050) | 447 (0.002) | 0.701 | gametogenesis | sqd |
| 326 | GO:0016740 | F | 3, | 2 | 2.061 (x 0.970) | 967 (0.002) | 0.702 | transferase activity | CG7920 smi35A |
| 327 | GO:0005386 | F | 3, | 1 | 0.966 (x 1.036) | 453 (0.002) | 0.702 | carrier activity | CG3823 |
| 328 | GO:0019953 | P | 3, | 1 | 0.972 (x 1.029) | 456 (0.002) | 0.703 | sexual reproduction | sqd |
| 329 | GO:0005739 | C | 5, 6, 7, 8, | 1 | 0.985 (x 1.015) | 462 (0.002) | 0.704 | mitochondrion | CG5028 |
| 330 | GO:0016310 | P | 7, | 1 | 0.983 (x 1.018) | 461 (0.002) | 0.705 | phosphorylation | smi35A |
| 331 | GO:0005975 | P | 5, | 1 | 1.034 (x 0.967) | 485 (0.002) | 0.722 | carbohydrate metabolism | CG5028 |
| 332 | GO:0045184 | P | 5, | 1 | 1.108 (x 0.902) | 520 (0.002) | 0.729 | establishment of protein localization | garz |
| 333 | GO:0017111 | F | 7, | 1 | 1.115 (x 0.897) | 523 (0.002) | 0.729 | nucleoside-triphosphatase activity | kis |
| 334 | GO:0043234 | C | 2, | 3 | 3.366 (x 0.891) | 1579 (0.002) | 0.73 | protein complex | B52 CG4759 sqd |
| 335 | GO:0009987 | P | 2, | 14 | 14.294 (x 0.979) | 6706 (0.002) | 0.731 | cellular process | B52 CG3823 CG4759 CG5028 CG7920 Hsp23 Hsp26 Hsp67Ba Obp99a garz kis smi35A sqd toy |
| 336 | GO:0006886 | P | 6, 7, 8, | 1 | 1.079 (x 0.927) | 506 (0.002) | 0.731 | intracellular protein transport | garz |
| 337 | GO:0004871 | F | 2, | 2 | 2.244 (x 0.891) | 1053 (0.002) | 0.731 | signal transducer activity | CG17952 smi35A |
| 338 | GO:0015031 | P | 5, 6, | 1 | 1.102 (x 0.907) | 517 (0.002) | 0.731 | protein transport | garz |
| 339 | GO:0000003 | P | 2, | 1 | 1.068 (x 0.936) | 501 (0.002) | 0.731 | reproduction | sqd |
| 340 | GO:0030154 | P | 3, | 1 | 1.087 (x 0.920) | 510 (0.002) | 0.732 | cell differentiation | sqd |
| 341 | GO:0006091 | P | 5, | 1 | 1.076 (x 0.929) | 505 (0.002) | 0.732 | generation of precursor metabolites and energy | CG5028 |
| 342 | GO:0016462 | F | 6, | 1 | 1.132 (x 0.884) | 531 (0.002) | 0.733 | pyrophosphatase activity | kis |
| 343 | GO:0016772 | F | 4, | 1 | 1.068 (x 0.936) | 501 (0.002) | 0.734 | transferase activity, transferring phosphorus-containing groups | smi35A |
| 344 | GO:0016818 | F | 5, | 1 | 1.168 (x 0.856) | 548 (0.002) | 0.741 | hydrolase activity, acting on acid anhydrides, in phosphorus-containing anhydrides | kis |
| 345 | GO:0004872 | F | 3, | 1 | 1.179 (x 0.848) | 553 (0.002) | 0.743 | receptor activity | CG17952 |
| 346 | GO:0016817 | F | 4, | 1 | 1.168 (x 0.856) | 548 (0.002) | 0.744 | hydrolase activity, acting on acid anhydrides | kis |
| 347 | GO:0008104 | P | 4, | 1 | 1.206 (x 0.829) | 566 (0.002) | 0.75 | protein localization | garz |
| 348 | GO:0007582 | P | 2, | 14 | 14.562 (x 0.961) | 6832 (0.002) | 0.764 | physiological process | B52 CG3823 CG4759 CG5028 CG7920 Hsp23 Hsp26 Hsp67Ba Obp99a garz kis smi35A sqd toy |
| 349 | GO:0006796 | P | 6, | 1 | 1.287 (x 0.777) | 604 (0.002) | 0.768 | phosphate metabolism | smi35A |
| 350 | GO:0006793 | P | 5, | 1 | 1.287 (x 0.777) | 604 (0.002) | 0.77 | phosphorus metabolism | smi35A |
| 351 | GO:0050877 | P | 4, | 1 | 1.313 (x 0.762) | 616 (0.002) | 0.774 | neurophysiological process | Obp99a |
| 352 | GO:0016491 | F | 3, | 1 | 1.330 (x 0.752) | 624 (0.002) | 0.776 | oxidoreductase activity | CG5028 |
| 353 | GO:0044425 | C | 3, 4, 5, | 2 | 2.592 (x 0.772) | 1216 (0.002) | 0.779 | membrane part | CG17952 ImpE2 |
| 354 | GO:0006996 | P | 5, | 1 | 1.539 (x 0.650) | 722 (0.001) | 0.824 | organelle organization and biogenesis | kis |
| 355 | GO:0005198 | F | 2, | 1 | 1.573 (x 0.636) | 738 (0.001) | 0.829 | structural molecule activity | CG4759 |
| 356 | GO:0005554 | F | 2, | 1 | 1.603 (x 0.624) | 752 (0.001) | 0.833 | molecular function unknown | CG8588 |
| 357 | GO:0030528 | F | 2, | 1 | 1.716 (x 0.583) | 805 (0.001) | 0.853 | transcription regulator activity | toy |
| 358 | GO:0008372 | C | 2, | 1 | 1.735 (x 0.576) | 814 (0.001) | 0.855 | cellular component unknown | CG8588 |
| 359 | GO:0006464 | P | 7, | 1 | 1.869 (x 0.535) | 877 (0.001) | 0.875 | protein modification | smi35A |
| 360 | GO:0043412 | P | 6, | 1 | 1.955 (x 0.512) | 917 (0.001) | 0.885 | biopolymer modification | smi35A |
| 361 | GO:0005215 | F | 2, | 1 | 2.063 (x 0.485) | 968 (0.001) | 0.898 | transporter activity | CG3823 |
| 362 | GO:0016020 | C | 3, 4, | 2 | 3.977 (x 0.503) | 1866 (0.001) | 0.938 | membrane | CG17952 ImpE2 |
| 363 | GO:0003824 | F | 2, | 4 | 8.059 (x 0.496) | 3781 (0.001) | 0.989 | catalytic activity | CG5028 CG7920 kis smi35A |
| 364 | GO:0016787 | F | 3, | 1 | 3.828 (x 0.261) | 1796 (0.001) | 0.992 | hydrolase activity | kis |
| 365 | GO:0003674 | F | 1, | 14 | 17.661 (x 0.793) | 8286 (0.002) | 0.999 | molecular\_function | B52 CG17952 CG3823 CG4759 CG5028 CG7920 CG8588 Hsp23 Obp99a garz kis smi35A sqd toy |

  

---

Regulated Genes that don't have GO terms
  

CG2469 CG32373 CG9186
