# Supplementary material for: Functional dissection of the ash2 and ash1 transcriptomes provides insights into the transcriptional basis of wing phenotypes and reveals conserved protein interactions
Source: Genome Biol. 2007 Apr 28;8(4):R67. doi: 10.1186/gb-2007-8-4-r67 (PMC1896016; doi:10.1186/gb-2007-8-4-r67)
Supplement: Additional data file 16 — GO annotations of the genes upregulated over 2.0-fold in ash122 [file gb-2007-8-4-r67-S16.html]

  

---

  

|  |  |
| --- | --- |
| Go Statistics | Reg File: **ash1\_U2.0x.txt.fbgns** (59 genes -- 18 skipped)  Ref File: **ref.fbgns** (13577 genes -- 4663 skipped)  Database: **go\_200507-termdb.rdf-xml** |

---

  

Fields Description

| Pos | Go Term | Ontology | Levels | Observed | Expected | Possibles | p-value(Adj) | Go term description | Genes with the GO term |
| --- | --- | --- | --- | --- | --- | --- | --- | --- | --- |
| 1 | GO:0043292 | C | 5, 6, 7, 8, | 4 | 0.060 (x 66.897) | 13 (0.308) | 0.000143 | contractile fiber | Mhc Mlc1 Mp20 Tm1 |
| 2 | GO:0044449 | C | 5, 6, 7, 8, 9, | 3 | 0.051 (x 59.295) | 11 (0.273) | 0.00389 | contractile fiber part | Mhc Mlc1 Tm1 |
| 3 | GO:0000146 | F | 3, | 2 | 0.014 (x 144.943) | 3 (0.667) | 0.00826 | microfilament motor activity | Mhc Mlc1 |
| 4 | GO:0005859 | C | 5, 6, 7, 8, 9, 10, 11, 12, | 2 | 0.014 (x 144.943) | 3 (0.667) | 0.011 | muscle myosin | Mhc Mlc1 |
| 5 | GO:0016460 | C | 4, 6, 7, 8, 9, 10, 11, | 2 | 0.018 (x 108.707) | 4 (0.500) | 0.0132 | myosin II | Mhc Mlc1 |
| 6 | GO:0043228 | C | 3, | 11 | 3.270 (x 3.364) | 711 (0.015) | 0.0202 | non-membrane-bound organelle | Act57B CG11522 CG31617 Mhc Mlc1 RpL38 RpL8 Tm1 corto mRpS21 robl |
| 7 | GO:0015629 | C | 6, 7, 8, 9, | 4 | 0.340 (x 11.752) | 74 (0.054) | 0.0235 | actin cytoskeleton | Act57B Mhc Mlc1 Tm1 |
| 8 | GO:0043232 | C | 4, 5, 6, 7, | 11 | 3.270 (x 3.364) | 711 (0.015) | 0.0235 | intracellular non-membrane-bound organelle | Act57B CG11522 CG31617 Mhc Mlc1 RpL38 RpL8 Tm1 corto mRpS21 robl |
| 9 | GO:0044430 | C | 4, 5, 6, 7, 8, 9, | 6 | 1.104 (x 5.435) | 240 (0.025) | 0.0279 | cytoskeletal part | Act57B Mhc Mlc1 Tm1 corto robl |
| 10 | GO:0030016 | C | 6, 7, 8, 9, | 2 | 0.041 (x 48.314) | 9 (0.222) | 0.03 | myofibril | Mhc Tm1 |
| 11 | GO:0006936 | P | 4, | 4 | 0.396 (x 10.112) | 86 (0.047) | 0.0303 | muscle contraction | Mhc Mlc1 Mp20 Tm1 |
| 12 | GO:0045735 | F | 2, | 2 | 0.037 (x 54.354) | 8 (0.250) | 0.0304 | nutrient reservoir activity | Fbp2 Lsp1beta |
| 13 | GO:0030017 | C | 6, 7, 8, 9, 10, | 2 | 0.041 (x 48.314) | 9 (0.222) | 0.0325 | sarcomere | Mhc Tm1 |
| 14 | GO:0005344 | F | 3, | 2 | 0.037 (x 54.354) | 8 (0.250) | 0.0338 | oxygen transporter activity | CG8193 Lsp1beta |
| 15 | GO:0044446 | C | 3, 4, 5, 6, 7, | 15 | 6.550 (x 2.290) | 1424 (0.011) | 0.0358 | intracellular organelle part | Acf1 Act57B CG11522 CG12400 CG15398 CG17838 CG31617 Mhc Mlc1 RpL38 RpL8 Tm1 corto mRpS21 robl |
| 16 | GO:0044422 | C | 2, 3, | 15 | 6.550 (x 2.290) | 1424 (0.011) | 0.0382 | organelle part | Acf1 Act57B CG11522 CG12400 CG15398 CG17838 CG31617 Mhc Mlc1 RpL38 RpL8 Tm1 corto mRpS21 robl |
| 17 | GO:0005842 | C | 4, 5, 6, 7, 8, 9, 10, 11, | 3 | 0.235 (x 12.789) | 51 (0.059) | 0.0508 | cytosolic large ribosomal subunit (sensu Eukaryota) | CG11522 RpL38 RpL8 |
| 18 | GO:0005856 | C | 5, 6, 7, 8, | 6 | 1.334 (x 4.498) | 290 (0.021) | 0.0576 | cytoskeleton | Act57B Mhc Mlc1 Tm1 corto robl |
| 19 | GO:0045947 | P | 9, 10, 11, | 1 | 0.005 (x 217.415) | 1 (1.000) | 0.103 | negative regulation of translational initiation | Thor |
| 20 | GO:0008190 | F | 5, | 1 | 0.005 (x 217.415) | 1 (1.000) | 0.107 | eukaryotic initiation factor 4E binding | Thor |
| 21 | GO:0004343 | F | 9, | 1 | 0.005 (x 217.415) | 1 (1.000) | 0.112 | glucosamine 6-phosphate N-acetyltransferase activity | CG1969 |
| 22 | GO:0005198 | F | 2, | 9 | 3.394 (x 2.651) | 738 (0.012) | 0.112 | structural molecule activity | Act57B CG11522 Lcp3 Mhc Mp20 RpL38 RpL8 mRpS21 robl |
| 23 | GO:0006334 | P | 7, 11, | 2 | 0.110 (x 18.118) | 24 (0.083) | 0.114 | nucleosome assembly | Acf1 CG31617 |
| 24 | GO:0016459 | C | 3, 5, 6, 7, 8, 9, 10, | 2 | 0.115 (x 17.393) | 25 (0.080) | 0.115 | myosin | Mhc Mlc1 |
| 25 | GO:0006542 | P | 9, 10, | 1 | 0.005 (x 217.415) | 1 (1.000) | 0.117 | glutamine biosynthesis | Gs2 |
| 26 | GO:0031369 | F | 4, | 1 | 0.005 (x 217.415) | 1 (1.000) | 0.123 | translation initiation factor binding | Thor |
| 27 | GO:0006941 | P | 5, | 1 | 0.005 (x 217.415) | 1 (1.000) | 0.13 | striated muscle contraction | Mhc |
| 28 | GO:0046351 | P | 7, 8, | 1 | 0.014 (x 72.472) | 3 (0.333) | 0.139 | disaccharide biosynthesis | CG5177 |
| 29 | GO:0003774 | F | 2, | 3 | 0.400 (x 7.497) | 87 (0.034) | 0.14 | motor activity | Act57B Mhc Mlc1 |
| 30 | GO:0016716 | F | 5, | 1 | 0.014 (x 72.472) | 3 (0.333) | 0.141 | oxidoreductase activity, acting on paired donors, with incorporation or reduction of molecular oxygen, another compound as one donor, and incorporation of one atom of oxygen | CG8193 |
| 31 | GO:0016211 | F | 6, | 1 | 0.014 (x 72.472) | 3 (0.333) | 0.144 | ammonia ligase activity | Gs2 |
| 32 | GO:0030529 | C | 3, 4, 5, 6, | 5 | 1.463 (x 3.418) | 318 (0.016) | 0.145 | ribonucleoprotein complex | CG11522 CG17838 RpL38 RpL8 mRpS21 |
| 33 | GO:0016584 | P | 11, | 1 | 0.018 (x 54.354) | 4 (0.250) | 0.146 | nucleosome spacing | Acf1 |
| 34 | GO:0045213 | P | 7, 8, | 1 | 0.014 (x 72.472) | 3 (0.333) | 0.147 | neurotransmitter receptor metabolism | Gs2 |
| 35 | GO:0006538 | P | 9, 10, | 1 | 0.018 (x 54.354) | 4 (0.250) | 0.148 | glutamate catabolism | Gs2 |
| 36 | GO:0005830 | C | 5, 6, 7, 8, 9, 10, | 3 | 0.414 (x 7.247) | 90 (0.033) | 0.148 | cytosolic ribosome (sensu Eukaryota) | CG11522 RpL38 RpL8 |
| 37 | GO:0005863 | C | 6, 7, 8, 9, 10, 11, 12, | 1 | 0.014 (x 72.472) | 3 (0.333) | 0.15 | striated muscle thick filament | Mhc |
| 38 | GO:0006032 | P | 8, 9, 10, 11, | 1 | 0.018 (x 54.354) | 4 (0.250) | 0.15 | chitin catabolism | Chit |
| 39 | GO:0006036 | P | 9, 10, 11, 12, | 1 | 0.018 (x 54.354) | 4 (0.250) | 0.153 | cuticle chitin catabolism | Chit |
| 40 | GO:0004805 | F | 8, | 1 | 0.014 (x 72.472) | 3 (0.333) | 0.153 | trehalose-phosphatase activity | CG5177 |
| 41 | GO:0016590 | C | 5, 8, 9, 10, 11, 12, 13, 14, 15, | 1 | 0.009 (x 108.707) | 2 (0.500) | 0.153 | ACF complex | Acf1 |
| 42 | GO:0000272 | P | 7, | 1 | 0.018 (x 54.354) | 4 (0.250) | 0.155 | polysaccharide catabolism | Chit |
| 43 | GO:0015934 | C | 3, 4, 5, 6, 7, 8, 9, | 3 | 0.442 (x 6.794) | 96 (0.031) | 0.156 | large ribosomal subunit | CG11522 RpL38 RpL8 |
| 44 | GO:0006541 | P | 8, 9, | 1 | 0.014 (x 72.472) | 3 (0.333) | 0.156 | glutamine metabolism | Gs2 |
| 45 | GO:0046348 | P | 7, 8, | 1 | 0.018 (x 54.354) | 4 (0.250) | 0.158 | amino sugar catabolism | Chit |
| 46 | GO:0005862 | C | 5, 6, 7, 8, 9, 10, 11, 12, | 1 | 0.009 (x 108.707) | 2 (0.500) | 0.158 | muscle thin filament tropomyosin | Tm1 |
| 47 | GO:0016880 | F | 5, | 1 | 0.014 (x 72.472) | 3 (0.333) | 0.16 | acid-ammonia (or amide) ligase activity | Gs2 |
| 48 | GO:0009059 | P | 5, 6, | 7 | 2.401 (x 2.916) | 522 (0.013) | 0.16 | macromolecule biosynthesis | Act57B CG11522 CG5177 RpL38 RpL8 Thor mRpS21 |
| 49 | GO:0006046 | P | 9, 10, | 1 | 0.018 (x 54.354) | 4 (0.250) | 0.16 | N-acetylglucosamine catabolism | Chit |
| 50 | GO:0031497 | P | 10, | 2 | 0.235 (x 8.526) | 51 (0.039) | 0.161 | chromatin assembly | Acf1 CG31617 |
| 51 | GO:0005991 | P | 8, | 1 | 0.023 (x 43.483) | 5 (0.200) | 0.163 | trehalose metabolism | CG5177 |
| 52 | GO:0044445 | C | 5, 6, 7, 8, 9, | 3 | 0.543 (x 5.527) | 118 (0.025) | 0.163 | cytosolic part | CG11522 RpL38 RpL8 |
| 53 | GO:0006043 | P | 8, 9, | 1 | 0.018 (x 54.354) | 4 (0.250) | 0.163 | glucosamine catabolism | Chit |
| 54 | GO:0043112 | P | 7, | 1 | 0.014 (x 72.472) | 3 (0.333) | 0.163 | receptor metabolism | Gs2 |
| 55 | GO:0016051 | P | 6, 7, | 2 | 0.225 (x 8.874) | 49 (0.041) | 0.164 | carbohydrate biosynthesis | Act57B CG5177 |
| 56 | GO:0005840 | C | 4, 5, 6, 7, 8, | 4 | 0.869 (x 4.601) | 189 (0.021) | 0.164 | ribosome | CG11522 RpL38 RpL8 mRpS21 |
| 57 | GO:0005865 | C | 5, 6, 7, 8, 9, 10, 11, | 1 | 0.023 (x 43.483) | 5 (0.200) | 0.165 | striated muscle thin filament | Tm1 |
| 58 | GO:0004563 | F | 7, | 1 | 0.018 (x 54.354) | 4 (0.250) | 0.166 | beta-N-acetylhexosaminidase activity | CG15012 |
| 59 | GO:0043234 | C | 2, | 13 | 7.263 (x 1.790) | 1579 (0.008) | 0.166 | protein complex | Acf1 CG11522 CG12400 CG15398 CG17838 CG31617 Lsp1beta Mhc Mlc1 RpL38 RpL8 mRpS21 robl |
| 60 | GO:0016358 | P | 7, 10, | 2 | 0.161 (x 12.424) | 35 (0.057) | 0.166 | dendrite development | Tm1 robl |
| 61 | GO:0003735 | F | 3, | 4 | 0.865 (x 4.626) | 188 (0.021) | 0.166 | structural constituent of ribosome | CG11522 RpL38 RpL8 mRpS21 |
| 62 | GO:0009399 | P | 5, | 1 | 0.014 (x 72.472) | 3 (0.333) | 0.167 | nitrogen fixation | Gs2 |
| 63 | GO:0005381 | F | 6, | 1 | 0.023 (x 43.483) | 5 (0.200) | 0.167 | iron ion transporter activity | Tsf1 |
| 64 | GO:0006034 | P | 8, 9, 10, 11, | 1 | 0.018 (x 54.354) | 4 (0.250) | 0.169 | cuticle chitin metabolism | Chit |
| 65 | GO:0045792 | P | 6, 7, | 1 | 0.023 (x 43.483) | 5 (0.200) | 0.169 | negative regulation of cell size | Thor |
| 66 | GO:0004364 | F | 5, | 2 | 0.166 (x 12.079) | 36 (0.056) | 0.17 | glutathione transferase activity | GstD9 GstE1 |
| 67 | GO:0004503 | F | 6, | 1 | 0.014 (x 72.472) | 3 (0.333) | 0.171 | monophenol monooxygenase activity | CG8193 |
| 68 | GO:0006826 | P | 9, 10, | 1 | 0.018 (x 54.354) | 4 (0.250) | 0.172 | iron ion transport | Tsf1 |
| 69 | GO:0008623 | C | 4, 7, 8, 9, 10, 11, 12, 13, 14, | 1 | 0.023 (x 43.483) | 5 (0.200) | 0.172 | chromatin accessibility complex | Acf1 |
| 70 | GO:0015377 | F | 6, 8, | 1 | 0.023 (x 43.483) | 5 (0.200) | 0.174 | cation:chloride symporter activity | CG31547 |
| 71 | GO:0044247 | P | 7, 8, | 1 | 0.018 (x 54.354) | 4 (0.250) | 0.175 | cellular polysaccharide catabolism | Chit |
| 72 | GO:0008511 | F | 7, 9, | 1 | 0.014 (x 72.472) | 3 (0.333) | 0.175 | sodium:potassium:chloride symporter activity | CG31547 |
| 73 | GO:0042766 | P | 10, | 1 | 0.032 (x 31.059) | 7 (0.143) | 0.177 | nucleosome mobilization | Acf1 |
| 74 | GO:0046916 | P | 8, | 1 | 0.032 (x 31.059) | 7 (0.143) | 0.179 | transition metal ion homeostasis | Tsf1 |
| 75 | GO:0031672 | C | 6, 7, 8, 9, 10, 11, | 1 | 0.014 (x 72.472) | 3 (0.333) | 0.179 | A band | Mhc |
| 76 | GO:0005984 | P | 7, | 1 | 0.028 (x 36.236) | 6 (0.167) | 0.18 | disaccharide metabolism | CG5177 |
| 77 | GO:0030530 | C | 4, 5, 6, 7, 8, 9, 10, | 1 | 0.032 (x 31.059) | 7 (0.143) | 0.181 | heterogeneous nuclear ribonucleoprotein complex | CG17838 |
| 78 | GO:0044249 | P | 5, | 8 | 3.818 (x 2.096) | 830 (0.010) | 0.181 | cellular biosynthesis | Act57B CG11522 CG5177 Gs2 RpL38 RpL8 Thor mRpS21 |
| 79 | GO:0044444 | C | 4, 5, 6, 7, | 10 | 5.322 (x 1.879) | 1157 (0.009) | 0.182 | cytoplasmic part | CG11522 CG12400 Mhc Mlc1 Mp20 RpL38 RpL8 Tm1 corto mRpS21 |
| 80 | GO:0019203 | F | 7, | 1 | 0.028 (x 36.236) | 6 (0.167) | 0.182 | carbohydrate phosphatase activity | CG5177 |
| 81 | GO:0006879 | P | 8, 9, | 1 | 0.032 (x 31.059) | 7 (0.143) | 0.183 | iron ion homeostasis | Tsf1 |
| 82 | GO:0004356 | F | 7, | 1 | 0.014 (x 72.472) | 3 (0.333) | 0.184 | glutamate-ammonia ligase activity | Gs2 |
| 83 | GO:0050654 | P | 6, 8, | 1 | 0.032 (x 31.059) | 7 (0.143) | 0.185 | chondroitin sulfate proteoglycan metabolism | Act57B |
| 84 | GO:0005616 | C | 3, 4, 5, | 1 | 0.028 (x 36.236) | 6 (0.167) | 0.185 | larval serum protein complex | Lsp1beta |
| 85 | GO:0008199 | F | 7, | 1 | 0.032 (x 31.059) | 7 (0.143) | 0.187 | ferric iron binding | Tsf1 |
| 86 | GO:0030239 | P | 7, 8, 10, 11, | 1 | 0.028 (x 36.236) | 6 (0.167) | 0.187 | myofibril assembly | Mhc |
| 87 | GO:0006952 | P | 4, | 6 | 2.364 (x 2.538) | 514 (0.012) | 0.188 | defense response | CG5397 CG8193 GstD9 GstE1 Thor Tsf1 |
| 88 | GO:0005992 | P | 8, 9, | 1 | 0.014 (x 72.472) | 3 (0.333) | 0.188 | trehalose biosynthesis | CG5177 |
| 89 | GO:0030206 | P | 8, 9, 10, | 1 | 0.032 (x 31.059) | 7 (0.143) | 0.189 | chondroitin sulfate biosynthesis | Act57B |
| 90 | GO:0005678 | C | 4, 7, 8, 9, 10, 11, 12, 13, 14, | 1 | 0.028 (x 36.236) | 6 (0.167) | 0.19 | chromatin assembly complex | Acf1 |
| 91 | GO:0015929 | F | 6, | 1 | 0.032 (x 31.059) | 7 (0.143) | 0.191 | hexosaminidase activity | CG15012 |
| 92 | GO:0050650 | P | 7, 8, 9, | 1 | 0.032 (x 31.059) | 7 (0.143) | 0.193 | chondroitin sulfate proteoglycan biosynthesis | Act57B |
| 93 | GO:0004772 | F | 8, | 1 | 0.014 (x 72.472) | 3 (0.333) | 0.193 | sterol O-acyltransferase activity | CG5397 |
| 94 | GO:0005576 | C | 2, | 5 | 1.775 (x 2.816) | 386 (0.013) | 0.194 | extracellular region | CG11051 Chit Lsp1beta Obp99b Tsf1 |
| 95 | GO:0030204 | P | 7, 8, 9, | 1 | 0.032 (x 31.059) | 7 (0.143) | 0.195 | chondroitin sulfate metabolism | Act57B |
| 96 | GO:0006821 | P | 8, 9, | 1 | 0.037 (x 27.177) | 8 (0.125) | 0.196 | chloride transport | CG31547 |
| 97 | GO:0016765 | F | 4, | 2 | 0.276 (x 7.247) | 60 (0.033) | 0.197 | transferase activity, transferring alkyl or aryl (other than methyl) groups | GstD9 GstE1 |
| 98 | GO:0009065 | P | 8, 9, | 1 | 0.032 (x 31.059) | 7 (0.143) | 0.198 | glutamine family amino acid catabolism | Gs2 |
| 99 | GO:0009607 | P | 3, | 6 | 2.401 (x 2.499) | 522 (0.011) | 0.198 | response to biotic stimulus | CG5397 CG8193 GstD9 GstE1 Thor Tsf1 |
| 100 | GO:0030201 | P | 6, 8, | 1 | 0.041 (x 24.157) | 9 (0.111) | 0.213 | heparan sulfate proteoglycan metabolism | Act57B |
| 101 | GO:0006536 | P | 8, 9, | 1 | 0.041 (x 24.157) | 9 (0.111) | 0.215 | glutamate metabolism | Gs2 |
| 102 | GO:0015012 | P | 7, 8, 9, | 1 | 0.041 (x 24.157) | 9 (0.111) | 0.218 | heparan sulfate proteoglycan biosynthesis | Act57B |
| 103 | GO:0006023 | P | 7, 8, | 1 | 0.046 (x 21.741) | 10 (0.100) | 0.225 | aminoglycan biosynthesis | Act57B |
| 104 | GO:0006024 | P | 8, 9, | 1 | 0.046 (x 21.741) | 10 (0.100) | 0.228 | glycosaminoglycan biosynthesis | Act57B |
| 105 | GO:0005200 | F | 3, | 4 | 1.343 (x 2.978) | 292 (0.014) | 0.23 | structural constituent of cytoskeleton | Act57B Mhc Mp20 robl |
| 106 | GO:0006022 | P | 6, 7, | 1 | 0.046 (x 21.741) | 10 (0.100) | 0.23 | aminoglycan metabolism | Act57B |
| 107 | GO:0030203 | P | 7, 8, | 1 | 0.046 (x 21.741) | 10 (0.100) | 0.232 | glycosaminoglycan metabolism | Act57B |
| 108 | GO:0005858 | C | 5, 6, 7, 8, 9, 10, 11, 12, | 1 | 0.051 (x 19.765) | 11 (0.091) | 0.232 | axonemal dynein complex | robl |
| 109 | GO:0008307 | F | 3, | 1 | 0.051 (x 19.765) | 11 (0.091) | 0.234 | structural constituent of muscle | Mhc |
| 110 | GO:0005930 | C | 4, 5, 6, 7, 8, | 1 | 0.051 (x 19.765) | 11 (0.091) | 0.236 | axoneme | robl |
| 111 | GO:0006446 | P | 8, 9, 10, | 1 | 0.051 (x 19.765) | 11 (0.091) | 0.238 | regulation of translational initiation | Thor |
| 112 | GO:0044447 | C | 4, 5, 6, 7, 8, 9, | 1 | 0.051 (x 19.765) | 11 (0.091) | 0.241 | axoneme part | robl |
| 113 | GO:0005829 | C | 5, 6, 7, 8, | 3 | 0.846 (x 3.545) | 184 (0.016) | 0.242 | cytosol | CG11522 RpL38 RpL8 |
| 114 | GO:0000041 | P | 8, 9, | 1 | 0.051 (x 19.765) | 11 (0.091) | 0.243 | transition metal ion transport | Tsf1 |
| 115 | GO:0009058 | P | 4, | 8 | 4.130 (x 1.937) | 898 (0.009) | 0.243 | biosynthesis | Act57B CG11522 CG5177 Gs2 RpL38 RpL8 Thor mRpS21 |
| 116 | GO:0019236 | P | 5, | 1 | 0.055 (x 18.118) | 12 (0.083) | 0.246 | response to pheromone | Obp99b |
| 117 | GO:0009084 | P | 8, 9, | 1 | 0.055 (x 18.118) | 12 (0.083) | 0.248 | glutamine family amino acid biosynthesis | Gs2 |
| 118 | GO:0000910 | P | 5, | 2 | 0.386 (x 5.177) | 84 (0.024) | 0.257 | cytokinesis | Act57B Mhc |
| 119 | GO:0006112 | P | 7, | 1 | 0.064 (x 15.530) | 14 (0.071) | 0.274 | energy reserve metabolism | CG5177 |
| 120 | GO:0044463 | C | 3, 4, 5, | 1 | 0.064 (x 15.530) | 14 (0.071) | 0.277 | cell projection part | robl |
| 121 | GO:0030166 | P | 7, 8, | 1 | 0.064 (x 15.530) | 14 (0.071) | 0.279 | proteoglycan biosynthesis | Act57B |
| 122 | GO:0004568 | F | 6, | 1 | 0.064 (x 15.530) | 14 (0.071) | 0.281 | chitinase activity | Chit |
| 123 | GO:0007275 | P | 2, | 11 | 6.830 (x 1.610) | 1485 (0.007) | 0.285 | development | Act57B CG5397 Gs2 Mhc Mlc1 Mp20 Obp99b Thor Tm1 regucalcin robl |
| 124 | GO:0000786 | C | 3, 5, 6, 7, 8, 9, 10, 11, | 1 | 0.069 (x 14.494) | 15 (0.067) | 0.286 | nucleosome | CG31617 |
| 125 | GO:0005737 | C | 4, 5, 6, | 11 | 6.830 (x 1.610) | 1485 (0.007) | 0.287 | cytoplasm | CG11522 CG12400 Gs2 Mhc Mlc1 Mp20 RpL38 RpL8 Tm1 corto mRpS21 |
| 126 | GO:0006029 | P | 7, | 1 | 0.069 (x 14.494) | 15 (0.067) | 0.288 | proteoglycan metabolism | Act57B |
| 127 | GO:0004022 | F | 6, | 1 | 0.069 (x 14.494) | 15 (0.067) | 0.291 | alcohol dehydrogenase activity | Fbp2 |
| 128 | GO:0006979 | P | 4, 5, 6, | 1 | 0.078 (x 12.789) | 17 (0.059) | 0.308 | response to oxidative stress | GstE1 |
| 129 | GO:0004553 | F | 5, | 2 | 0.451 (x 4.437) | 98 (0.020) | 0.309 | hydrolase activity, hydrolyzing O-glycosyl compounds | CG15012 Chit |
| 130 | GO:0016478 | P | 8, 9, 10, | 1 | 0.078 (x 12.789) | 17 (0.059) | 0.31 | negative regulation of translation | Thor |
| 131 | GO:0006412 | P | 6, 7, | 5 | 2.272 (x 2.201) | 494 (0.010) | 0.311 | protein biosynthesis | CG11522 RpL38 RpL8 Thor mRpS21 |
| 132 | GO:0006333 | P | 9, | 2 | 0.460 (x 4.348) | 100 (0.020) | 0.312 | chromatin assembly or disassembly | Acf1 CG31617 |
| 133 | GO:0005884 | C | 5, 6, 7, 8, 9, 10, | 1 | 0.083 (x 12.079) | 18 (0.056) | 0.321 | actin filament | Act57B |
| 134 | GO:0009064 | P | 7, 8, | 1 | 0.087 (x 11.443) | 19 (0.053) | 0.33 | glutamine family amino acid metabolism | Gs2 |
| 135 | GO:0046915 | F | 5, | 1 | 0.087 (x 11.443) | 19 (0.053) | 0.333 | transition metal ion transporter activity | Tsf1 |
| 136 | GO:0001558 | P | 4, 5, 7, 8, | 1 | 0.092 (x 10.871) | 20 (0.050) | 0.335 | regulation of cell growth | Thor |
| 137 | GO:0044272 | P | 6, | 1 | 0.087 (x 11.443) | 19 (0.053) | 0.335 | sulfur compound biosynthesis | Act57B |
| 138 | GO:0008374 | F | 7, | 1 | 0.092 (x 10.871) | 20 (0.050) | 0.337 | O-acyltransferase activity | CG5397 |
| 139 | GO:0016798 | F | 4, | 2 | 0.492 (x 4.064) | 107 (0.019) | 0.338 | hydrolase activity, acting on glycosyl bonds | CG15012 Chit |
| 140 | GO:0030005 | P | 7, | 1 | 0.092 (x 10.871) | 20 (0.050) | 0.339 | di-, tri-valent inorganic cation homeostasis | Tsf1 |
| 141 | GO:0007517 | P | 4, | 2 | 0.497 (x 4.026) | 108 (0.019) | 0.341 | muscle development | Mhc Mp20 |
| 142 | GO:0048627 | P | 5, 6, 8, 9, | 1 | 0.101 (x 9.882) | 22 (0.045) | 0.342 | myoblast development | Mhc |
| 143 | GO:0008415 | F | 6, | 2 | 0.529 (x 3.781) | 115 (0.017) | 0.344 | acyltransferase activity | CG1969 CG5397 |
| 144 | GO:0005615 | C | 3, 4, | 1 | 0.101 (x 9.882) | 22 (0.045) | 0.344 | extracellular space | Lsp1beta |
| 145 | GO:0006961 | P | 7, 8, 9, | 1 | 0.106 (x 9.453) | 23 (0.043) | 0.345 | antibacterial humoral response (sensu Protostomia) | Thor |
| 146 | GO:0031327 | P | 7, | 1 | 0.101 (x 9.882) | 22 (0.045) | 0.347 | negative regulation of cellular biosynthesis | Thor |
| 147 | GO:0045445 | P | 5, 7, 8, | 1 | 0.106 (x 9.453) | 23 (0.043) | 0.348 | myoblast differentiation | Mhc |
| 148 | GO:0017148 | P | 7, 8, 9, | 1 | 0.097 (x 10.353) | 21 (0.048) | 0.348 | negative regulation of protein biosynthesis | Thor |
| 149 | GO:0006873 | P | 5, | 1 | 0.101 (x 9.882) | 22 (0.045) | 0.349 | cell ion homeostasis | Tsf1 |
| 150 | GO:0016319 | P | 5, 7, | 1 | 0.106 (x 9.453) | 23 (0.043) | 0.35 | mushroom body development | robl |
| 151 | GO:0009890 | P | 6, | 1 | 0.101 (x 9.882) | 22 (0.045) | 0.351 | negative regulation of biosynthesis | Thor |
| 152 | GO:0006461 | P | 6, | 2 | 0.538 (x 3.716) | 117 (0.017) | 0.352 | protein complex assembly | Acf1 CG31617 |
| 153 | GO:0009948 | P | 5, | 2 | 0.547 (x 3.654) | 119 (0.017) | 0.353 | anterior/posterior axis specification | Tm1 regucalcin |
| 154 | GO:0048628 | P | 6, 7, 9, 10, | 1 | 0.101 (x 9.882) | 22 (0.045) | 0.354 | myoblast maturation | Mhc |
| 155 | GO:0015082 | F | 5, | 1 | 0.110 (x 9.059) | 24 (0.042) | 0.355 | di-, tri-valent inorganic cation transporter activity | Tsf1 |
| 156 | GO:0030003 | P | 6, | 1 | 0.101 (x 9.882) | 22 (0.045) | 0.356 | cation homeostasis | Tsf1 |
| 157 | GO:0008092 | F | 4, | 3 | 1.095 (x 2.741) | 238 (0.013) | 0.358 | cytoskeletal protein binding | Mhc Mp20 Tm1 |
| 158 | GO:0016747 | F | 5, | 2 | 0.557 (x 3.594) | 121 (0.017) | 0.358 | transferase activity, transferring groups other than amino-acyl groups | CG1969 CG5397 |
| 159 | GO:0006875 | P | 7, | 1 | 0.101 (x 9.882) | 22 (0.045) | 0.359 | metal ion homeostasis | Tsf1 |
| 160 | GO:0003779 | F | 5, | 2 | 0.566 (x 3.535) | 123 (0.016) | 0.364 | actin binding | Mp20 Tm1 |
| 161 | GO:0016585 | C | 3, 6, 7, 8, 9, 10, 11, 12, 13, | 1 | 0.115 (x 8.697) | 25 (0.040) | 0.364 | chromatin remodeling complex | Acf1 |
| 162 | GO:0016655 | F | 5, | 1 | 0.129 (x 7.765) | 28 (0.036) | 0.373 | oxidoreductase activity, acting on NADH or NADPH, quinone or similar compound as acceptor | CG12400 |
| 163 | GO:0005669 | C | 4, 7, 8, 9, 10, 11, 12, 13, 14, | 1 | 0.129 (x 7.765) | 28 (0.036) | 0.375 | transcription factor TFIID complex | CG15398 |
| 164 | GO:0050874 | P | 3, | 7 | 4.140 (x 1.691) | 900 (0.008) | 0.376 | organismal physiological process | Gs2 Mhc Mlc1 Mp20 Obp99b Thor Tm1 |
| 165 | GO:0008080 | F | 8, | 1 | 0.129 (x 7.765) | 28 (0.036) | 0.377 | N-acetyltransferase activity | CG1969 |
| 166 | GO:0048469 | P | 5, | 1 | 0.124 (x 8.052) | 27 (0.037) | 0.378 | cell maturation | Mhc |
| 167 | GO:0016746 | F | 4, | 2 | 0.584 (x 3.424) | 127 (0.016) | 0.379 | transferase activity, transferring acyl groups | CG1969 CG5397 |
| 168 | GO:0009636 | P | 5, | 2 | 0.593 (x 3.371) | 129 (0.016) | 0.379 | response to toxin | GstD9 GstE1 |
| 169 | GO:0008137 | F | 6, 7, | 1 | 0.129 (x 7.765) | 28 (0.036) | 0.379 | NADH dehydrogenase (ubiquinone) activity | CG12400 |
| 170 | GO:0009952 | P | 4, | 2 | 0.598 (x 3.345) | 130 (0.015) | 0.38 | anterior/posterior pattern formation | Tm1 regucalcin |
| 171 | GO:0042692 | P | 4, | 1 | 0.124 (x 8.052) | 27 (0.037) | 0.38 | muscle cell differentiation | Mhc |
| 172 | GO:0042221 | P | 4, | 3 | 1.210 (x 2.480) | 263 (0.011) | 0.381 | response to chemical stimulus | GstD9 GstE1 Obp99b |
| 173 | GO:0050136 | F | 6, | 1 | 0.129 (x 7.765) | 28 (0.036) | 0.382 | NADH dehydrogenase (quinone) activity | CG12400 |
| 174 | GO:0050801 | P | 4, | 1 | 0.124 (x 8.052) | 27 (0.037) | 0.382 | ion homeostasis | Tsf1 |
| 175 | GO:0007416 | P | 5, 6, | 1 | 0.133 (x 7.497) | 29 (0.034) | 0.383 | synaptogenesis | Gs2 |
| 176 | GO:0016049 | P | 3, 4, 6, 7, | 1 | 0.138 (x 7.247) | 30 (0.033) | 0.389 | cell growth | Thor |
| 177 | GO:0051301 | P | 4, | 2 | 0.639 (x 3.128) | 139 (0.014) | 0.389 | cell division | Act57B Mhc |
| 178 | GO:0048666 | P | 5, 8, | 2 | 0.653 (x 3.062) | 142 (0.014) | 0.39 | neuron development | Tm1 robl |
| 179 | GO:0042995 | C | 3, 4, | 1 | 0.143 (x 7.013) | 31 (0.032) | 0.39 | cell projection | robl |
| 180 | GO:0006323 | P | 7, | 2 | 0.635 (x 3.151) | 138 (0.014) | 0.391 | DNA packaging | Acf1 CG31617 |
| 181 | GO:0050808 | P | 5, | 1 | 0.147 (x 6.794) | 32 (0.031) | 0.391 | synapse organization and biogenesis | Gs2 |
| 182 | GO:0000314 | C | 4, 5, 6, 7, 8, 9, 10, | 1 | 0.138 (x 7.247) | 30 (0.033) | 0.391 | organellar small ribosomal subunit | mRpS21 |
| 183 | GO:0008083 | F | 4, 5, | 1 | 0.152 (x 6.588) | 33 (0.030) | 0.392 | growth factor activity | Chit |
| 184 | GO:0031175 | P | 6, 9, | 2 | 0.653 (x 3.062) | 142 (0.014) | 0.392 | neurite development | Tm1 robl |
| 185 | GO:0016410 | F | 7, | 1 | 0.143 (x 7.013) | 31 (0.032) | 0.392 | N-acyltransferase activity | CG1969 |
| 186 | GO:0006325 | P | 8, | 2 | 0.635 (x 3.151) | 138 (0.014) | 0.393 | establishment and/or maintenance of chromatin architecture | Acf1 CG31617 |
| 187 | GO:0030198 | P | 4, | 1 | 0.147 (x 6.794) | 32 (0.031) | 0.393 | extracellular matrix organization and biogenesis | Gs2 |
| 188 | GO:0005763 | C | 5, 6, 7, 8, 9, 10, 11, 12, 13, | 1 | 0.138 (x 7.247) | 30 (0.033) | 0.393 | mitochondrial small ribosomal subunit | mRpS21 |
| 189 | GO:0007218 | P | 7, | 1 | 0.152 (x 6.588) | 33 (0.030) | 0.394 | neuropeptide signaling pathway | CG11051 |
| 190 | GO:0044262 | P | 6, | 3 | 1.265 (x 2.372) | 275 (0.011) | 0.394 | cellular carbohydrate metabolism | Act57B CG5177 Chit |
| 191 | GO:0045451 | P | 7, 11, 13, 14, 16, | 1 | 0.147 (x 6.794) | 32 (0.031) | 0.395 | pole plasm oskar mRNA localization | Tm1 |
| 192 | GO:0005813 | C | 5, 6, 7, 8, 9, 10, | 1 | 0.152 (x 6.588) | 33 (0.030) | 0.396 | centrosome | corto |
| 193 | GO:0008361 | P | 5, 6, | 1 | 0.156 (x 6.395) | 34 (0.029) | 0.397 | regulation of cell size | Thor |
| 194 | GO:0046873 | F | 4, | 1 | 0.147 (x 6.794) | 32 (0.031) | 0.397 | metal ion transporter activity | Tsf1 |
| 195 | GO:0030001 | P | 7, 8, | 2 | 0.676 (x 2.958) | 147 (0.014) | 0.397 | metal ion transport | CG31547 Tsf1 |
| 196 | GO:0015020 | F | 6, | 1 | 0.156 (x 6.395) | 34 (0.029) | 0.399 | glucuronosyltransferase activity | Act57B |
| 197 | GO:0005184 | F | 5, 6, | 1 | 0.156 (x 6.395) | 34 (0.029) | 0.401 | neuropeptide hormone activity | CG11051 |
| 198 | GO:0030182 | P | 4, 7, | 2 | 0.685 (x 2.918) | 149 (0.013) | 0.402 | neuron differentiation | Tm1 robl |
| 199 | GO:0006120 | P | 9, 11, | 1 | 0.161 (x 6.212) | 35 (0.029) | 0.403 | mitochondrial electron transport, NADH to ubiquinone | CG12400 |
| 200 | GO:0003954 | F | 5, | 1 | 0.166 (x 6.039) | 36 (0.028) | 0.41 | NADH dehydrogenase activity | CG12400 |
| 201 | GO:0005747 | C | 4, 5, 6, 7, 8, 9, 10, 11, 12, 13, 14, | 1 | 0.170 (x 5.876) | 37 (0.027) | 0.414 | respiratory chain complex I (sensu Eukaryota) | CG12400 |
| 202 | GO:0045271 | C | 3, 4, 5, 6, | 1 | 0.170 (x 5.876) | 37 (0.027) | 0.416 | respiratory chain complex I | CG12400 |
| 203 | GO:0019094 | P | 6, 10, 12, 13, 15, | 1 | 0.170 (x 5.876) | 37 (0.027) | 0.418 | pole plasm mRNA localization | Tm1 |
| 204 | GO:0009798 | P | 4, | 2 | 0.731 (x 2.735) | 159 (0.013) | 0.42 | axis specification | Tm1 regucalcin |
| 205 | GO:0015296 | F | 5, 7, | 1 | 0.179 (x 5.575) | 39 (0.026) | 0.42 | anion:cation symporter activity | CG31547 |
| 206 | GO:0007316 | P | 5, 9, 11, 12, 14, | 1 | 0.175 (x 5.721) | 38 (0.026) | 0.42 | pole plasm RNA localization | Tm1 |
| 207 | GO:0007001 | P | 7, | 2 | 0.722 (x 2.770) | 157 (0.013) | 0.422 | chromosome organization and biogenesis (sensu Eukaryota) | Acf1 CG31617 |
| 208 | GO:0005279 | F | 5, 6, | 1 | 0.179 (x 5.575) | 39 (0.026) | 0.422 | amino acid-polyamine transporter activity | CG31547 |
| 209 | GO:0019731 | P | 6, 7, 8, | 1 | 0.175 (x 5.721) | 38 (0.026) | 0.422 | antibacterial humoral response | Thor |
| 210 | GO:0015674 | P | 7, 8, | 1 | 0.184 (x 5.435) | 40 (0.025) | 0.424 | di-, tri-valent inorganic cation transport | Tsf1 |
| 211 | GO:0015203 | F | 4, | 1 | 0.179 (x 5.575) | 39 (0.026) | 0.424 | polyamine transporter activity | CG31547 |
| 212 | GO:0008010 | F | 5, | 1 | 0.184 (x 5.435) | 40 (0.025) | 0.426 | structural constituent of larval cuticle (sensu Insecta) | Lcp3 |
| 213 | GO:0005815 | C | 5, 6, 7, 8, | 1 | 0.179 (x 5.575) | 39 (0.026) | 0.426 | microtubule organizing center | corto |
| 214 | GO:0016651 | F | 4, | 1 | 0.198 (x 5.056) | 43 (0.023) | 0.428 | oxidoreductase activity, acting on NADH or NADPH | CG12400 |
| 215 | GO:0048637 | P | 6, | 1 | 0.193 (x 5.177) | 42 (0.024) | 0.429 | skeletal muscle development | Mhc |
| 216 | GO:0030286 | C | 4, 6, 7, 8, 9, 10, 11, | 1 | 0.189 (x 5.303) | 41 (0.024) | 0.429 | dynein complex | robl |
| 217 | GO:0051248 | P | 6, 7, | 1 | 0.198 (x 5.056) | 43 (0.023) | 0.43 | negative regulation of protein metabolism | Thor |
| 218 | GO:0048741 | P | 6, 7, | 1 | 0.193 (x 5.177) | 42 (0.024) | 0.431 | skeletal muscle fiber development | Mhc |
| 219 | GO:0048113 | P | 8, 10, 11, 13, | 1 | 0.189 (x 5.303) | 41 (0.024) | 0.431 | pole plasm assembly (sensu Insecta) | Tm1 |
| 220 | GO:0005506 | F | 6, | 1 | 0.198 (x 5.056) | 43 (0.023) | 0.432 | iron ion binding | Tsf1 |
| 221 | GO:0043062 | P | 3, | 1 | 0.193 (x 5.177) | 42 (0.024) | 0.433 | extracellular structure organization and biogenesis | Gs2 |
| 222 | GO:0006814 | P | 8, 9, | 1 | 0.198 (x 5.056) | 43 (0.023) | 0.434 | sodium ion transport | CG31547 |
| 223 | GO:0016799 | F | 5, | 1 | 0.193 (x 5.177) | 42 (0.024) | 0.435 | hydrolase activity, hydrolyzing N-glycosyl compounds | Chit |
| 224 | GO:0007315 | P | 7, 9, 10, 12, | 1 | 0.198 (x 5.056) | 43 (0.023) | 0.436 | pole plasm assembly | Tm1 |
| 225 | GO:0048747 | P | 5, | 1 | 0.193 (x 5.177) | 42 (0.024) | 0.437 | muscle fiber development | Mhc |
| 226 | GO:0006960 | P | 7, 8, | 1 | 0.207 (x 4.831) | 45 (0.022) | 0.441 | antimicrobial humoral response (sensu Protostomia) | Thor |
| 227 | GO:0048112 | P | 7, 9, 10, 12, | 1 | 0.207 (x 4.831) | 45 (0.022) | 0.443 | oocyte anterior/posterior axis determination (sensu Insecta) | Tm1 |
| 228 | GO:0051276 | P | 6, | 2 | 0.791 (x 2.528) | 172 (0.012) | 0.443 | chromosome organization and biogenesis | Acf1 CG31617 |
| 229 | GO:0048513 | P | 3, | 5 | 3.082 (x 1.622) | 670 (0.007) | 0.444 | organ development | Act57B Mhc Mp20 Obp99b robl |
| 230 | GO:0042623 | F | 9, | 3 | 1.518 (x 1.976) | 330 (0.009) | 0.445 | ATPase activity, coupled | Mhc Mlc1 robl |
| 231 | GO:0006865 | P | 6, 7, 8, | 1 | 0.212 (x 4.726) | 46 (0.022) | 0.446 | amino acid transport | CG31547 |
| 232 | GO:0016407 | F | 7, | 1 | 0.216 (x 4.626) | 47 (0.021) | 0.446 | acetyltransferase activity | CG1969 |
| 233 | GO:0015837 | P | 5, 6, | 1 | 0.212 (x 4.726) | 46 (0.022) | 0.447 | amine transport | CG31547 |
| 234 | GO:0015171 | F | 4, 5, | 1 | 0.216 (x 4.626) | 47 (0.021) | 0.448 | amino acid transporter activity | CG31547 |
| 235 | GO:0009063 | P | 7, 8, | 1 | 0.225 (x 4.437) | 49 (0.020) | 0.454 | amino acid catabolism | Gs2 |
| 236 | GO:0048468 | P | 4, | 3 | 1.564 (x 1.918) | 340 (0.009) | 0.455 | cell development | Mhc Tm1 robl |
| 237 | GO:0008298 | P | 5, | 1 | 0.225 (x 4.437) | 49 (0.020) | 0.456 | intracellular mRNA localization | Tm1 |
| 238 | GO:0048699 | P | 6, | 2 | 0.828 (x 2.416) | 180 (0.011) | 0.456 | generation of neurons | Tm1 robl |
| 239 | GO:0003674 | F | 1, | 40 | 38.112 (x 1.050) | 8286 (0.005) | 0.456 | molecular\_function | Acf1 Act57B CG11051 CG11522 CG12400 CG15012 CG15398 CG17838 CG1969 CG31547 CG31617 CG31775 CG32207 CG32212 CG32249 CG4386 CG4511 CG5177 CG5397 CG8193 Chit Fbp2 Gs2 GstD9 GstE1 Lcp3 Lsp1beta Mhc Mlc1 Mp20 Obp99b RpL38 RpL8 Thor Tm1 Tsf1 corto fau mRpS21 robl |
| 240 | GO:0007420 | P | 4, 6, | 1 | 0.225 (x 4.437) | 49 (0.020) | 0.458 | brain development | robl |
| 241 | GO:0007519 | P | 5, | 1 | 0.225 (x 4.437) | 49 (0.020) | 0.459 | striated muscle development | Mhc |
| 242 | GO:0007635 | P | 4, 5, | 1 | 0.248 (x 4.026) | 54 (0.019) | 0.464 | chemosensory behavior | Obp99b |
| 243 | GO:0022008 | P | 5, | 2 | 0.888 (x 2.253) | 193 (0.010) | 0.465 | neurogenesis | Tm1 robl |
| 244 | GO:0016065 | P | 6, 7, | 1 | 0.244 (x 4.102) | 53 (0.019) | 0.465 | humoral defense mechanism (sensu Protostomia) | Thor |
| 245 | GO:0015698 | P | 7, 8, | 1 | 0.248 (x 4.026) | 54 (0.019) | 0.465 | inorganic anion transport | CG31547 |
| 246 | GO:0015630 | C | 6, 7, 8, 9, | 2 | 0.856 (x 2.338) | 186 (0.011) | 0.466 | microtubule cytoskeleton | corto robl |
| 247 | GO:0044270 | P | 5, 6, | 1 | 0.239 (x 4.181) | 52 (0.019) | 0.467 | nitrogen compound catabolism | Gs2 |
| 248 | GO:0042048 | P | 5, 6, | 1 | 0.244 (x 4.102) | 53 (0.019) | 0.467 | olfactory behavior | Obp99b |
| 249 | GO:0015849 | P | 5, 6, | 1 | 0.248 (x 4.026) | 54 (0.019) | 0.467 | organic acid transport | CG31547 |
| 250 | GO:0005275 | F | 3, | 1 | 0.239 (x 4.181) | 52 (0.019) | 0.469 | amine transporter activity | CG31547 |
| 251 | GO:0005179 | F | 4, 5, | 1 | 0.244 (x 4.102) | 53 (0.019) | 0.469 | hormone activity | CG11051 |
| 252 | GO:0046942 | P | 6, 7, | 1 | 0.248 (x 4.026) | 54 (0.019) | 0.469 | carboxylic acid transport | CG31547 |
| 253 | GO:0009310 | P | 6, 7, | 1 | 0.239 (x 4.181) | 52 (0.019) | 0.47 | amine catabolism | Gs2 |
| 254 | GO:0048111 | P | 6, 8, 9, 11, | 1 | 0.244 (x 4.102) | 53 (0.019) | 0.471 | oocyte axis determination (sensu Insecta) | Tm1 |
| 255 | GO:0048110 | P | 7, 8, 10, | 1 | 0.248 (x 4.026) | 54 (0.019) | 0.471 | oocyte construction (sensu Insecta) | Tm1 |
| 256 | GO:0007314 | P | 6, 8, 9, 11, | 1 | 0.258 (x 3.882) | 56 (0.018) | 0.471 | oocyte anterior/posterior axis determination | Tm1 |
| 257 | GO:0009055 | F | 4, | 1 | 0.244 (x 4.102) | 53 (0.019) | 0.473 | electron carrier activity | CG12400 |
| 258 | GO:0043226 | C | 2, | 15 | 12.345 (x 1.215) | 2684 (0.006) | 0.473 | organelle | Acf1 Act57B CG11522 CG12400 CG15398 CG17838 CG31617 Mhc Mlc1 RpL38 RpL8 Tm1 corto mRpS21 robl |
| 259 | GO:0043229 | C | 3, 4, 5, 6, | 15 | 12.345 (x 1.215) | 2684 (0.006) | 0.475 | intracellular organelle | Acf1 Act57B CG11522 CG12400 CG15398 CG17838 CG31617 Mhc Mlc1 RpL38 RpL8 Tm1 corto mRpS21 robl |
| 260 | GO:0044451 | C | 5, 6, 7, 8, 9, 10, 11, 12, | 2 | 0.934 (x 2.142) | 203 (0.010) | 0.489 | nucleoplasm part | Acf1 CG15398 |
| 261 | GO:0006790 | P | 5, | 1 | 0.271 (x 3.685) | 59 (0.017) | 0.49 | sulfur metabolism | Act57B |
| 262 | GO:0006413 | P | 8, 9, | 1 | 0.271 (x 3.685) | 59 (0.017) | 0.491 | translational initiation | Thor |
| 263 | GO:0040008 | P | 3, | 1 | 0.281 (x 3.564) | 61 (0.016) | 0.493 | regulation of growth | Thor |
| 264 | GO:0016887 | F | 8, | 3 | 1.706 (x 1.758) | 371 (0.008) | 0.493 | ATPase activity | Mhc Mlc1 robl |
| 265 | GO:0009628 | P | 3, | 3 | 1.711 (x 1.753) | 372 (0.008) | 0.494 | response to abiotic stimulus | GstD9 GstE1 Obp99b |
| 266 | GO:0007507 | P | 5, | 1 | 0.281 (x 3.564) | 61 (0.016) | 0.494 | heart development | Act57B |
| 267 | GO:0042773 | P | 7, 9, | 1 | 0.285 (x 3.507) | 62 (0.016) | 0.496 | ATP synthesis coupled electron transport | CG12400 |
| 268 | GO:0042775 | P | 8, 10, | 1 | 0.281 (x 3.564) | 61 (0.016) | 0.496 | ATP synthesis coupled electron transport (sensu Eukaryota) | CG12400 |
| 269 | GO:0009308 | P | 5, | 3 | 1.729 (x 1.735) | 376 (0.008) | 0.497 | amine metabolism | Act57B Chit Gs2 |
| 270 | GO:0006030 | P | 7, 8, 9, 10, | 1 | 0.290 (x 3.451) | 63 (0.016) | 0.499 | chitin metabolism | Chit |
| 271 | GO:0019730 | P | 6, 7, | 1 | 0.290 (x 3.451) | 63 (0.016) | 0.501 | antimicrobial humoral response | Thor |
| 272 | GO:0019722 | P | 7, | 1 | 0.294 (x 3.397) | 64 (0.016) | 0.504 | calcium-mediated signaling | regucalcin |
| 273 | GO:0016705 | F | 4, | 1 | 0.304 (x 3.294) | 66 (0.015) | 0.514 | oxidoreductase activity, acting on paired donors, with incorporation or reduction of molecular oxygen | CG8193 |
| 274 | GO:0006807 | P | 4, | 3 | 1.798 (x 1.668) | 391 (0.008) | 0.515 | nitrogen compound metabolism | Act57B Chit Gs2 |
| 275 | GO:0016052 | P | 6, | 1 | 0.308 (x 3.245) | 67 (0.015) | 0.515 | carbohydrate catabolism | Chit |
| 276 | GO:0019725 | P | 4, | 1 | 0.304 (x 3.294) | 66 (0.015) | 0.516 | cell homeostasis | Tsf1 |
| 277 | GO:0006445 | P | 7, 8, 9, | 1 | 0.331 (x 3.020) | 72 (0.014) | 0.516 | regulation of translation | Thor |
| 278 | GO:0048731 | P | 3, | 4 | 2.732 (x 1.464) | 594 (0.007) | 0.517 | system development | Gs2 Obp99b Tm1 robl |
| 279 | GO:0044275 | P | 7, | 1 | 0.308 (x 3.245) | 67 (0.015) | 0.517 | cellular carbohydrate catabolism | Chit |
| 280 | GO:0005761 | C | 5, 6, 7, 8, 9, 10, 11, 12, | 1 | 0.340 (x 2.938) | 74 (0.014) | 0.517 | mitochondrial ribosome | mRpS21 |
| 281 | GO:0016271 | P | 4, | 1 | 0.331 (x 3.020) | 72 (0.014) | 0.518 | tissue death | Obp99b |
| 282 | GO:0005746 | C | 5, 6, 7, 8, 9, 10, 11, 12, 13, | 1 | 0.317 (x 3.151) | 69 (0.014) | 0.519 | mitochondrial electron transport chain | CG12400 |
| 283 | GO:0006800 | P | 5, | 1 | 0.308 (x 3.245) | 67 (0.015) | 0.519 | oxygen and reactive oxygen species metabolism | GstE1 |
| 284 | GO:0050896 | P | 2, | 7 | 5.377 (x 1.302) | 1169 (0.006) | 0.519 | response to stimulus | CG5397 CG8193 GstD9 GstE1 Obp99b Thor Tsf1 |
| 285 | GO:0009617 | P | 5, | 1 | 0.340 (x 2.938) | 74 (0.014) | 0.519 | response to bacterium | Thor |
| 286 | GO:0007559 | P | 5, | 1 | 0.331 (x 3.020) | 72 (0.014) | 0.519 | histolysis | Obp99b |
| 287 | GO:0006417 | P | 6, 7, 8, | 1 | 0.345 (x 2.899) | 75 (0.013) | 0.52 | regulation of protein biosynthesis | Thor |
| 288 | GO:0015293 | F | 6, | 1 | 0.336 (x 2.978) | 73 (0.014) | 0.52 | symporter activity | CG31547 |
| 289 | GO:0048102 | P | 6, | 1 | 0.327 (x 3.062) | 71 (0.014) | 0.52 | autophagic cell death | Obp99b |
| 290 | GO:0015935 | C | 3, 4, 5, 6, 7, 8, 9, | 1 | 0.317 (x 3.151) | 69 (0.014) | 0.52 | small ribosomal subunit | mRpS21 |
| 291 | GO:0005342 | F | 3, | 1 | 0.340 (x 2.938) | 74 (0.014) | 0.521 | organic acid transporter activity | CG31547 |
| 292 | GO:0005654 | C | 5, 6, 7, 8, 9, 10, 11, | 2 | 1.030 (x 1.941) | 224 (0.009) | 0.521 | nucleoplasm | Acf1 CG15398 |
| 293 | GO:0006040 | P | 6, 7, | 1 | 0.331 (x 3.020) | 72 (0.014) | 0.521 | amino sugar metabolism | Chit |
| 294 | GO:0042592 | P | 3, | 1 | 0.350 (x 2.861) | 76 (0.013) | 0.522 | homeostasis | Tsf1 |
| 295 | GO:0044424 | C | 3, 4, 5, | 17 | 14.672 (x 1.159) | 3190 (0.005) | 0.522 | intracellular part | Acf1 Act57B CG11522 CG12400 CG15398 CG17838 CG31617 Gs2 Mhc Mlc1 Mp20 RpL38 RpL8 Tm1 corto mRpS21 robl |
| 296 | GO:0035071 | P | 7, | 1 | 0.327 (x 3.062) | 71 (0.014) | 0.522 | salivary gland cell autophagic cell death | Obp99b |
| 297 | GO:0042742 | P | 5, 6, | 1 | 0.317 (x 3.151) | 69 (0.014) | 0.522 | defense response to bacterium | Thor |
| 298 | GO:0000313 | C | 5, 6, 7, 8, 9, | 1 | 0.340 (x 2.938) | 74 (0.014) | 0.522 | organellar ribosome | mRpS21 |
| 299 | GO:0008652 | P | 7, 8, | 1 | 0.331 (x 3.020) | 72 (0.014) | 0.523 | amino acid biosynthesis | Gs2 |
| 300 | GO:0007309 | P | 5, 7, 8, 10, | 1 | 0.350 (x 2.861) | 76 (0.013) | 0.524 | oocyte axis determination | Tm1 |
| 301 | GO:0035070 | P | 6, | 1 | 0.327 (x 3.062) | 71 (0.014) | 0.524 | salivary gland histolysis | Obp99b |
| 302 | GO:0044264 | P | 6, 7, | 1 | 0.354 (x 2.824) | 77 (0.013) | 0.524 | cellular polysaccharide metabolism | Chit |
| 303 | GO:0046943 | F | 4, | 1 | 0.331 (x 3.020) | 72 (0.014) | 0.525 | carboxylic acid transporter activity | CG31547 |
| 304 | GO:0006044 | P | 8, 9, | 1 | 0.327 (x 3.062) | 71 (0.014) | 0.526 | N-acetylglucosamine metabolism | Chit |
| 305 | GO:0000785 | C | 5, 6, 7, 8, 9, 10, | 1 | 0.354 (x 2.824) | 77 (0.013) | 0.526 | chromatin | CG31617 |
| 306 | GO:0006041 | P | 7, 8, | 1 | 0.327 (x 3.062) | 71 (0.014) | 0.528 | glucosamine metabolism | Chit |
| 307 | GO:0006959 | P | 5, 6, | 1 | 0.359 (x 2.787) | 78 (0.013) | 0.528 | humoral immune response | Thor |
| 308 | GO:0009889 | P | 5, | 1 | 0.363 (x 2.752) | 79 (0.013) | 0.529 | regulation of biosynthesis | Thor |
| 309 | GO:0031326 | P | 6, | 1 | 0.363 (x 2.752) | 79 (0.013) | 0.53 | regulation of cellular biosynthesis | Thor |
| 310 | GO:0007308 | P | 6, 7, 9, | 1 | 0.363 (x 2.752) | 79 (0.013) | 0.532 | oocyte construction | Tm1 |
| 311 | GO:0048599 | P | 5, 6, 8, | 1 | 0.368 (x 2.718) | 80 (0.013) | 0.533 | oocyte development | Tm1 |
| 312 | GO:0005214 | F | 4, | 1 | 0.373 (x 2.684) | 81 (0.012) | 0.535 | structural constituent of cuticle (sensu Insecta) | Lcp3 |
| 313 | GO:0004197 | F | 6, | 1 | 0.373 (x 2.684) | 81 (0.012) | 0.536 | cysteine-type endopeptidase activity | Mlc1 |
| 314 | GO:0006367 | P | 9, | 1 | 0.386 (x 2.588) | 84 (0.012) | 0.537 | transcription initiation from RNA polymerase II promoter | CG15398 |
| 315 | GO:0008360 | P | 5, 6, | 1 | 0.391 (x 2.558) | 85 (0.012) | 0.537 | regulation of cell shape | Mp20 |
| 316 | GO:0016591 | C | 3, 6, 7, 8, 9, 10, 11, 12, 13, | 1 | 0.382 (x 2.619) | 83 (0.012) | 0.539 | DNA-directed RNA polymerase II, holoenzyme | CG15398 |
| 317 | GO:0044271 | P | 5, 6, | 1 | 0.386 (x 2.588) | 84 (0.012) | 0.539 | nitrogen compound biosynthesis | Gs2 |
| 318 | GO:0044421 | C | 2, 3, | 1 | 0.391 (x 2.558) | 85 (0.012) | 0.539 | extracellular region part | Lsp1beta |
| 319 | GO:0005622 | C | 3, 4, | 17 | 15.155 (x 1.122) | 3295 (0.005) | 0.539 | intracellular | Acf1 Act57B CG11522 CG12400 CG15398 CG17838 CG31617 Gs2 Mhc Mlc1 Mp20 RpL38 RpL8 Tm1 corto mRpS21 robl |
| 320 | GO:0006352 | P | 8, | 1 | 0.396 (x 2.528) | 86 (0.012) | 0.539 | transcription initiation | CG15398 |
| 321 | GO:0003682 | F | 3, | 1 | 0.382 (x 2.619) | 83 (0.012) | 0.54 | chromatin binding | Acf1 |
| 322 | GO:0007389 | P | 3, | 2 | 1.177 (x 1.699) | 256 (0.008) | 0.54 | pattern specification | Tm1 regucalcin |
| 323 | GO:0009309 | P | 6, 7, | 1 | 0.386 (x 2.588) | 84 (0.012) | 0.541 | amine biosynthesis | Gs2 |
| 324 | GO:0031974 | C | 2, | 3 | 1.982 (x 1.513) | 431 (0.007) | 0.541 | membrane-enclosed lumen | Acf1 CG15398 mRpS21 |
| 325 | GO:0005667 | C | 3, 6, 7, 8, 9, 10, 11, 12, 13, | 1 | 0.391 (x 2.558) | 85 (0.012) | 0.541 | transcription factor complex | CG15398 |
| 326 | GO:0009994 | P | 4, 7, | 1 | 0.400 (x 2.499) | 87 (0.011) | 0.541 | oocyte differentiation | Tm1 |
| 327 | GO:0016616 | F | 5, | 1 | 0.386 (x 2.588) | 84 (0.012) | 0.542 | oxidoreductase activity, acting on the CH-OH group of donors, NAD or NADP as acceptor | Fbp2 |
| 328 | GO:0043233 | C | 3, 4, | 3 | 1.982 (x 1.513) | 431 (0.007) | 0.542 | organelle lumen | Acf1 CG15398 mRpS21 |
| 329 | GO:0019932 | P | 6, | 1 | 0.405 (x 2.471) | 88 (0.011) | 0.544 | second-messenger-mediated signaling | regucalcin |
| 330 | GO:0005515 | F | 3, | 7 | 5.676 (x 1.233) | 1234 (0.006) | 0.547 | protein binding | CG11051 Chit Mhc Mp20 Thor Tm1 corto |
| 331 | GO:0040007 | P | 2, | 1 | 0.414 (x 2.416) | 90 (0.011) | 0.551 | growth | Thor |
| 332 | GO:0003702 | F | 3, | 2 | 1.223 (x 1.635) | 266 (0.008) | 0.558 | RNA polymerase II transcription factor activity | CG15398 corto |
| 333 | GO:0008234 | F | 5, | 1 | 0.428 (x 2.338) | 93 (0.011) | 0.559 | cysteine-type peptidase activity | Mlc1 |
| 334 | GO:0008194 | F | 5, | 1 | 0.423 (x 2.363) | 92 (0.011) | 0.559 | UDP-glycosyltransferase activity | Act57B |
| 335 | GO:0006403 | P | 4, | 1 | 0.428 (x 2.338) | 93 (0.011) | 0.561 | RNA localization | Tm1 |
| 336 | GO:0008509 | F | 4, | 1 | 0.432 (x 2.313) | 94 (0.011) | 0.562 | anion transporter activity | CG31547 |
| 337 | GO:0031981 | C | 4, 5, 6, 7, 8, 9, 10, | 2 | 1.265 (x 1.581) | 275 (0.007) | 0.575 | nuclear lumen | Acf1 CG15398 |
| 338 | GO:0007399 | P | 4, | 3 | 2.153 (x 1.394) | 468 (0.006) | 0.579 | nervous system development | Gs2 Tm1 robl |
| 339 | GO:0009613 | P | 4, 5, | 1 | 0.460 (x 2.174) | 100 (0.010) | 0.58 | response to pest, pathogen or parasite | Thor |
| 340 | GO:0005102 | F | 3, 4, | 2 | 1.283 (x 1.559) | 279 (0.007) | 0.581 | receptor binding | CG11051 Chit |
| 341 | GO:0042302 | F | 3, | 1 | 0.455 (x 2.196) | 99 (0.010) | 0.581 | structural constituent of cuticle | Lcp3 |
| 342 | GO:0006820 | P | 6, 7, | 1 | 0.460 (x 2.174) | 100 (0.010) | 0.582 | anion transport | CG31547 |
| 343 | GO:0007018 | P | 7, 8, 9, | 1 | 0.474 (x 2.111) | 103 (0.010) | 0.592 | microtubule-based movement | robl |
| 344 | GO:0030705 | P | 6, 7, 8, | 1 | 0.478 (x 2.091) | 104 (0.010) | 0.595 | cytoskeleton-dependent intracellular transport | robl |
| 345 | GO:0005975 | P | 5, | 3 | 2.231 (x 1.345) | 485 (0.006) | 0.601 | carbohydrate metabolism | Act57B CG5177 Chit |
| 346 | GO:0004497 | F | 4, | 1 | 0.501 (x 1.995) | 109 (0.009) | 0.61 | monooxygenase activity | CG8193 |
| 347 | GO:0035272 | P | 4, | 1 | 0.501 (x 1.995) | 109 (0.009) | 0.612 | exocrine system development | Obp99b |
| 348 | GO:0044455 | C | 4, 5, 6, 7, 8, 9, 10, 11, 12, | 1 | 0.506 (x 1.976) | 110 (0.009) | 0.613 | mitochondrial membrane part | CG12400 |
| 349 | GO:0007431 | P | 5, | 1 | 0.501 (x 1.995) | 109 (0.009) | 0.614 | salivary gland development | Obp99b |
| 350 | GO:0007281 | P | 5, | 1 | 0.515 (x 1.941) | 112 (0.009) | 0.618 | germ cell development | Tm1 |
| 351 | GO:0000004 | P | 2, | 4 | 3.224 (x 1.241) | 701 (0.006) | 0.619 | biological process unknown | CG31775 CG32207 CG32212 fau |
| 352 | GO:0005549 | F | 3, | 1 | 0.529 (x 1.891) | 115 (0.009) | 0.625 | odorant binding | Obp99b |
| 353 | GO:0044248 | P | 5, | 2 | 1.407 (x 1.421) | 306 (0.007) | 0.627 | cellular catabolism | Chit Gs2 |
| 354 | GO:0006091 | P | 5, | 3 | 2.323 (x 1.292) | 505 (0.006) | 0.627 | generation of precursor metabolites and energy | CG12400 CG4511 CG5177 |
| 355 | GO:0005875 | C | 3, 5, 6, 7, 8, 9, 10, | 1 | 0.534 (x 1.874) | 116 (0.009) | 0.627 | microtubule associated complex | robl |
| 356 | GO:0019538 | P | 5, | 11 | 10.041 (x 1.096) | 2183 (0.005) | 0.629 | protein metabolism | Acf1 Act57B CG11522 CG31617 CG4386 Gs2 Mlc1 RpL38 RpL8 Thor mRpS21 |
| 357 | GO:0030154 | P | 3, | 3 | 2.346 (x 1.279) | 510 (0.006) | 0.63 | cell differentiation | Mhc Tm1 robl |
| 358 | GO:0044428 | C | 4, 5, 6, 7, 8, 9, | 3 | 2.369 (x 1.266) | 515 (0.006) | 0.63 | nuclear part | Acf1 CG15398 CG17838 |
| 359 | GO:0016614 | F | 4, | 1 | 0.543 (x 1.842) | 118 (0.008) | 0.63 | oxidoreductase activity, acting on CH-OH group of donors | Fbp2 |
| 360 | GO:0003729 | F | 5, | 2 | 1.440 (x 1.389) | 313 (0.006) | 0.631 | mRNA binding | CG17838 RpL8 |
| 361 | GO:0006996 | P | 5, | 4 | 3.321 (x 1.205) | 722 (0.006) | 0.632 | organelle organization and biogenesis | Acf1 Act57B CG31617 robl |
| 362 | GO:0015980 | P | 6, | 1 | 0.543 (x 1.842) | 118 (0.008) | 0.632 | energy derivation by oxidation of organic compounds | CG5177 |
| 363 | GO:0006955 | P | 4, 5, | 1 | 0.561 (x 1.782) | 122 (0.008) | 0.635 | immune response | Thor |
| 364 | GO:0007417 | P | 5, | 1 | 0.561 (x 1.782) | 122 (0.008) | 0.637 | central nervous system development | robl |
| 365 | GO:0017111 | F | 7, | 3 | 2.406 (x 1.247) | 523 (0.006) | 0.638 | nucleoside-triphosphatase activity | Mhc Mlc1 robl |
| 366 | GO:0043285 | P | 6, | 1 | 0.584 (x 1.712) | 127 (0.008) | 0.649 | biopolymer catabolism | Chit |
| 367 | GO:0009056 | P | 4, | 2 | 1.504 (x 1.330) | 327 (0.006) | 0.65 | catabolism | Chit Gs2 |
| 368 | GO:0016462 | F | 6, | 3 | 2.442 (x 1.228) | 531 (0.006) | 0.651 | pyrophosphatase activity | Mhc Mlc1 robl |
| 369 | GO:0016758 | F | 5, | 1 | 0.593 (x 1.685) | 129 (0.008) | 0.653 | transferase activity, transferring hexosyl groups | Act57B |
| 370 | GO:0006118 | P | 6, | 2 | 1.532 (x 1.306) | 333 (0.006) | 0.656 | electron transport | CG12400 CG4511 |
| 371 | GO:0005554 | F | 2, | 4 | 3.459 (x 1.156) | 752 (0.005) | 0.657 | molecular function unknown | CG31775 CG32207 CG32212 fau |
| 372 | GO:0006950 | P | 3, | 2 | 1.536 (x 1.302) | 334 (0.006) | 0.657 | response to stress | GstE1 Thor |
| 373 | GO:0048732 | P | 4, | 1 | 0.603 (x 1.660) | 131 (0.008) | 0.657 | gland development | Obp99b |
| 374 | GO:0051246 | P | 5, 6, | 1 | 0.612 (x 1.635) | 133 (0.008) | 0.657 | regulation of protein metabolism | Thor |
| 375 | GO:0016818 | F | 5, | 3 | 2.521 (x 1.190) | 548 (0.005) | 0.658 | hydrolase activity, acting on acid anhydrides, in phosphorus-containing anhydrides | Mhc Mlc1 robl |
| 376 | GO:0016740 | F | 3, | 5 | 4.448 (x 1.124) | 967 (0.005) | 0.659 | transferase activity | Act57B CG1969 CG5397 GstD9 GstE1 |
| 377 | GO:0000902 | P | 4, 5, | 2 | 1.555 (x 1.286) | 338 (0.006) | 0.659 | cellular morphogenesis | Mp20 Thor |
| 378 | GO:0051707 | P | 4, | 1 | 0.603 (x 1.660) | 131 (0.008) | 0.659 | response to other organism | Thor |
| 379 | GO:0016817 | F | 4, | 3 | 2.521 (x 1.190) | 548 (0.005) | 0.659 | hydrolase activity, acting on acid anhydrides | Mhc Mlc1 robl |
| 380 | GO:0044429 | C | 4, 5, 6, 7, 8, 9, | 2 | 1.587 (x 1.260) | 345 (0.006) | 0.669 | mitochondrial part | CG12400 mRpS21 |
| 381 | GO:0006119 | P | 6, 8, | 1 | 0.649 (x 1.542) | 141 (0.007) | 0.675 | oxidative phosphorylation | CG12400 |
| 382 | GO:0005759 | C | 5, 6, 7, 8, 9, 10, 11, | 1 | 0.667 (x 1.499) | 145 (0.007) | 0.685 | mitochondrial matrix | mRpS21 |
| 383 | GO:0031980 | C | 4, 5, 6, 7, 8, 9, 10, | 1 | 0.667 (x 1.499) | 145 (0.007) | 0.687 | mitochondrial lumen | mRpS21 |
| 384 | GO:0044427 | C | 4, 5, 6, 7, 8, 9, | 1 | 0.681 (x 1.469) | 148 (0.007) | 0.693 | chromosomal part | CG31617 |
| 385 | GO:0003723 | F | 4, | 2 | 1.697 (x 1.178) | 369 (0.005) | 0.71 | RNA binding | CG17838 RpL8 |
| 386 | GO:0006259 | P | 6, | 2 | 1.706 (x 1.172) | 371 (0.005) | 0.71 | DNA metabolism | Acf1 CG31617 |
| 387 | GO:0031324 | P | 6, | 1 | 0.713 (x 1.403) | 155 (0.006) | 0.712 | negative regulation of cellular metabolism | Thor |
| 388 | GO:0005575 | C | 1, | 27 | 26.594 (x 1.015) | 5782 (0.005) | 0.715 | cellular\_component | Acf1 Act57B CG11051 CG11522 CG12400 CG15398 CG17838 CG31547 CG31617 CG31775 CG32207 CG32212 Chit Gs2 Lsp1beta Mhc Mlc1 Mp20 Obp99b RpL38 RpL8 Tm1 Tsf1 corto fau mRpS21 robl |
| 389 | GO:0008372 | C | 2, | 4 | 3.744 (x 1.068) | 814 (0.005) | 0.717 | cellular component unknown | CG31775 CG32207 CG32212 fau |
| 390 | GO:0016757 | F | 4, | 1 | 0.731 (x 1.367) | 159 (0.006) | 0.719 | transferase activity, transferring glycosyl groups | Act57B |
| 391 | GO:0005976 | P | 6, | 1 | 0.745 (x 1.342) | 162 (0.006) | 0.724 | polysaccharide metabolism | Chit |
| 392 | GO:0006812 | P | 6, 7, | 2 | 1.762 (x 1.135) | 383 (0.005) | 0.725 | cation transport | CG31547 Tsf1 |
| 393 | GO:0009892 | P | 5, | 1 | 0.759 (x 1.318) | 165 (0.006) | 0.73 | negative regulation of metabolism | Thor |
| 394 | GO:0007606 | P | 4, 6, | 1 | 0.764 (x 1.310) | 166 (0.006) | 0.731 | sensory perception of chemical stimulus | Obp99b |
| 395 | GO:0044260 | P | 5, | 10 | 9.898 (x 1.010) | 2152 (0.005) | 0.737 | cellular macromolecule metabolism | Act57B CG11522 CG4386 Chit Gs2 Mlc1 RpL38 RpL8 Thor mRpS21 |
| 396 | GO:0005743 | C | 5, 6, 7, 8, 9, 10, 11, 12, | 1 | 0.777 (x 1.286) | 169 (0.006) | 0.738 | mitochondrial inner membrane | CG12400 |
| 397 | GO:0019866 | C | 4, 5, 6, 7, 8, 9, | 1 | 0.800 (x 1.250) | 174 (0.006) | 0.741 | organelle inner membrane | CG12400 |
| 398 | GO:0015672 | P | 7, 8, | 1 | 0.805 (x 1.242) | 175 (0.006) | 0.742 | monovalent inorganic cation transport | CG31547 |
| 399 | GO:0016491 | F | 3, | 3 | 2.870 (x 1.045) | 624 (0.005) | 0.742 | oxidoreductase activity | CG12400 CG8193 Fbp2 |
| 400 | GO:0008324 | F | 4, | 2 | 1.835 (x 1.090) | 399 (0.005) | 0.742 | cation transporter activity | CG31547 Tsf1 |
| 401 | GO:0009888 | P | 3, | 2 | 1.831 (x 1.093) | 398 (0.005) | 0.742 | tissue development | CG5397 Mlc1 |
| 402 | GO:0044265 | P | 6, | 1 | 0.791 (x 1.264) | 172 (0.006) | 0.742 | cellular macromolecule catabolism | Chit |
| 403 | GO:0016791 | F | 6, | 1 | 0.837 (x 1.195) | 182 (0.005) | 0.757 | phosphoric monoester hydrolase activity | CG5177 |
| 404 | GO:0005694 | C | 5, 6, 7, 8, | 1 | 0.846 (x 1.182) | 184 (0.005) | 0.759 | chromosome | CG31617 |
| 405 | GO:0016879 | F | 4, | 1 | 0.837 (x 1.195) | 182 (0.005) | 0.759 | ligase activity, forming carbon-nitrogen bonds | Gs2 |
| 406 | GO:0009653 | P | 3, | 3 | 2.953 (x 1.016) | 642 (0.005) | 0.759 | morphogenesis | Act57B Mp20 Thor |
| 407 | GO:0043037 | P | 7, 8, | 1 | 0.856 (x 1.169) | 186 (0.005) | 0.762 | translation | Thor |
| 408 | GO:0031966 | C | 5, 6, 7, 8, 9, 10, 11, | 1 | 0.860 (x 1.163) | 187 (0.005) | 0.763 | mitochondrial membrane | CG12400 |
| 409 | GO:0009057 | P | 5, | 1 | 0.874 (x 1.144) | 190 (0.005) | 0.768 | macromolecule catabolism | Chit |
| 410 | GO:0007610 | P | 3, | 1 | 0.879 (x 1.138) | 191 (0.005) | 0.769 | behavior | Obp99b |
| 411 | GO:0007017 | P | 7, | 1 | 0.902 (x 1.109) | 196 (0.005) | 0.78 | microtubule-based process | robl |
| 412 | GO:0007498 | P | 4, | 1 | 0.911 (x 1.098) | 198 (0.005) | 0.781 | mesoderm development | Mlc1 |
| 413 | GO:0016787 | F | 3, | 8 | 8.261 (x 0.968) | 1796 (0.004) | 0.783 | hydrolase activity | CG15012 CG4386 CG5177 CG5397 Chit Mhc Mlc1 robl |
| 414 | GO:0042578 | F | 5, | 1 | 0.920 (x 1.087) | 200 (0.005) | 0.784 | phosphoric ester hydrolase activity | CG5177 |
| 415 | GO:0005740 | C | 4, 5, 6, 7, 8, 9, 10, | 1 | 0.943 (x 1.061) | 205 (0.005) | 0.794 | mitochondrial envelope | CG12400 |
| 416 | GO:0007267 | P | 4, | 2 | 2.061 (x 0.971) | 448 (0.004) | 0.795 | cell-cell signaling | Chit Gs2 |
| 417 | GO:0004263 | F | 7, | 1 | 0.957 (x 1.045) | 208 (0.005) | 0.795 | chymotrypsin activity | CG4386 |
| 418 | GO:0007010 | P | 6, | 2 | 2.070 (x 0.966) | 450 (0.004) | 0.796 | cytoskeleton organization and biogenesis | Act57B robl |
| 419 | GO:0016788 | F | 4, | 2 | 2.093 (x 0.956) | 455 (0.004) | 0.796 | hydrolase activity, acting on ester bonds | CG5177 CG5397 |
| 420 | GO:0016043 | P | 4, | 6 | 6.343 (x 0.946) | 1379 (0.004) | 0.797 | cell organization and biogenesis | Acf1 Act57B CG31617 Mp20 Thor robl |
| 421 | GO:0005386 | F | 3, | 2 | 2.084 (x 0.960) | 453 (0.004) | 0.797 | carrier activity | CG31547 Tsf1 |
| 422 | GO:0015291 | F | 5, | 1 | 0.998 (x 1.002) | 217 (0.005) | 0.802 | porter activity | CG31547 |
| 423 | GO:0005509 | F | 5, | 1 | 1.003 (x 0.997) | 218 (0.005) | 0.802 | calcium ion binding | Mp20 |
| 424 | GO:0005739 | C | 5, 6, 7, 8, | 2 | 2.125 (x 0.941) | 462 (0.004) | 0.803 | mitochondrion | CG12400 mRpS21 |
| 425 | GO:0015290 | F | 4, | 1 | 0.998 (x 1.002) | 217 (0.005) | 0.803 | electrochemical potential-driven transporter activity | CG31547 |
| 426 | GO:0044267 | P | 6, | 9 | 9.562 (x 0.941) | 2079 (0.004) | 0.805 | cellular protein metabolism | Act57B CG11522 CG4386 Gs2 Mlc1 RpL38 RpL8 Thor mRpS21 |
| 427 | GO:0006811 | P | 5, 6, | 2 | 2.125 (x 0.941) | 462 (0.004) | 0.805 | ion transport | CG31547 Tsf1 |
| 428 | GO:0004175 | F | 5, | 2 | 2.166 (x 0.923) | 471 (0.004) | 0.807 | endopeptidase activity | CG4386 Mlc1 |
| 429 | GO:0007268 | P | 6, | 1 | 1.039 (x 0.962) | 226 (0.004) | 0.811 | synaptic transmission | Gs2 |
| 430 | GO:0000087 | P | 6, | 1 | 1.044 (x 0.958) | 227 (0.004) | 0.811 | M phase of mitotic cell cycle | Mhc |
| 431 | GO:0007067 | P | 7, | 1 | 1.039 (x 0.962) | 226 (0.004) | 0.813 | mitosis | Mhc |
| 432 | GO:0015075 | F | 3, | 2 | 2.231 (x 0.897) | 485 (0.004) | 0.818 | ion transporter activity | CG31547 Tsf1 |
| 433 | GO:0005215 | F | 2, | 4 | 4.452 (x 0.898) | 968 (0.004) | 0.819 | transporter activity | CG31547 CG8193 Lsp1beta Tsf1 |
| 434 | GO:0051243 | P | 5, | 1 | 1.067 (x 0.937) | 232 (0.004) | 0.819 | negative regulation of cellular physiological process | Thor |
| 435 | GO:0004295 | F | 7, | 1 | 1.081 (x 0.925) | 235 (0.004) | 0.82 | trypsin activity | CG4386 |
| 436 | GO:0007398 | P | 4, | 1 | 1.090 (x 0.917) | 237 (0.004) | 0.822 | ectoderm development | CG5397 |
| 437 | GO:0043118 | P | 4, | 1 | 1.108 (x 0.902) | 241 (0.004) | 0.827 | negative regulation of physiological process | Thor |
| 438 | GO:0012501 | P | 5, | 1 | 1.141 (x 0.877) | 248 (0.004) | 0.838 | programmed cell death | Obp99b |
| 439 | GO:0016265 | P | 3, | 1 | 1.154 (x 0.866) | 251 (0.004) | 0.84 | death | Obp99b |
| 440 | GO:0008219 | P | 4, | 1 | 1.150 (x 0.870) | 250 (0.004) | 0.84 | cell death | Obp99b |
| 441 | GO:0007600 | P | 3, 5, | 1 | 1.187 (x 0.843) | 258 (0.004) | 0.843 | sensory perception | Obp99b |
| 442 | GO:0006928 | P | 4, 5, | 1 | 1.182 (x 0.846) | 257 (0.004) | 0.843 | cell motility | robl |
| 443 | GO:0006810 | P | 4, 5, | 6 | 6.793 (x 0.883) | 1477 (0.004) | 0.843 | transport | CG31547 CG8193 Lsp1beta Obp99b Tsf1 robl |
| 444 | GO:0006520 | P | 6, 7, | 1 | 1.200 (x 0.833) | 261 (0.004) | 0.844 | amino acid metabolism | Gs2 |
| 445 | GO:0004252 | F | 6, | 1 | 1.182 (x 0.846) | 257 (0.004) | 0.845 | serine-type endopeptidase activity | CG4386 |
| 446 | GO:0040011 | P | 3, | 1 | 1.200 (x 0.833) | 261 (0.004) | 0.846 | locomotion | robl |
| 447 | GO:0051674 | P | 4, | 1 | 1.182 (x 0.846) | 257 (0.004) | 0.847 | localization of cell | robl |
| 448 | GO:0003676 | F | 3, | 7 | 8.017 (x 0.873) | 1743 (0.004) | 0.85 | nucleic acid binding | Acf1 CG11522 CG15398 CG17838 CG31617 RpL38 RpL8 |
| 449 | GO:0000278 | P | 5, | 1 | 1.233 (x 0.811) | 268 (0.004) | 0.85 | mitotic cell cycle | Mhc |
| 450 | GO:0031975 | C | 2, | 1 | 1.228 (x 0.814) | 267 (0.004) | 0.85 | envelope | CG12400 |
| 451 | GO:0031967 | C | 3, 4, 5, 6, 7, 8, | 1 | 1.228 (x 0.814) | 267 (0.004) | 0.852 | organelle envelope | CG12400 |
| 452 | GO:0003824 | F | 2, | 16 | 17.391 (x 0.920) | 3781 (0.004) | 0.854 | catalytic activity | Act57B CG12400 CG15012 CG1969 CG4386 CG5177 CG5397 CG8193 Chit Fbp2 Gs2 GstD9 GstE1 Mhc Mlc1 robl |
| 453 | GO:0043170 | P | 4, | 13 | 14.332 (x 0.907) | 3116 (0.004) | 0.855 | macromolecule metabolism | Acf1 Act57B CG11522 CG31617 CG4386 CG5177 Chit Gs2 Mlc1 RpL38 RpL8 Thor mRpS21 |
| 454 | GO:0009993 | P | 7, | 1 | 1.274 (x 0.785) | 277 (0.004) | 0.856 | oogenesis (sensu Insecta) | Tm1 |
| 455 | GO:0048523 | P | 4, | 1 | 1.288 (x 0.776) | 280 (0.004) | 0.857 | negative regulation of cellular process | Thor |
| 456 | GO:0030528 | F | 2, | 3 | 3.703 (x 0.810) | 805 (0.004) | 0.857 | transcription regulator activity | Acf1 CG15398 corto |
| 457 | GO:0051179 | P | 3, | 7 | 8.146 (x 0.859) | 1771 (0.004) | 0.858 | localization | CG31547 CG8193 Lsp1beta Obp99b Tm1 Tsf1 robl |
| 458 | GO:0003677 | F | 4, | 3 | 3.781 (x 0.793) | 822 (0.004) | 0.864 | DNA binding | Acf1 CG15398 CG31617 |
| 459 | GO:0000279 | P | 5, | 1 | 1.320 (x 0.758) | 287 (0.003) | 0.864 | M phase | Mhc |
| 460 | GO:0048477 | P | 6, | 1 | 1.329 (x 0.752) | 289 (0.003) | 0.865 | oogenesis | Tm1 |
| 461 | GO:0006519 | P | 5, | 1 | 1.348 (x 0.742) | 293 (0.003) | 0.865 | amino acid and derivative metabolism | Gs2 |
| 462 | GO:0008236 | F | 5, | 1 | 1.348 (x 0.742) | 293 (0.003) | 0.867 | serine-type peptidase activity | CG4386 |
| 463 | GO:0048519 | P | 3, | 1 | 1.403 (x 0.713) | 305 (0.003) | 0.875 | negative regulation of biological process | Thor |
| 464 | GO:0007155 | P | 3, | 1 | 1.394 (x 0.718) | 303 (0.003) | 0.877 | cell adhesion | Mp20 |
| 465 | GO:0007186 | P | 6, | 1 | 1.403 (x 0.713) | 305 (0.003) | 0.877 | G-protein coupled receptor protein signaling pathway | CG11051 |
| 466 | GO:0007292 | P | 5, | 1 | 1.426 (x 0.701) | 310 (0.003) | 0.88 | female gamete generation | Tm1 |
| 467 | GO:0016874 | F | 3, | 1 | 1.458 (x 0.686) | 317 (0.003) | 0.887 | ligase activity | Gs2 |
| 468 | GO:0050877 | P | 4, | 2 | 2.833 (x 0.706) | 616 (0.003) | 0.897 | neurophysiological process | Gs2 Obp99b |
| 469 | GO:0005488 | F | 2, | 17 | 19.014 (x 0.894) | 4134 (0.004) | 0.897 | binding | Acf1 CG11051 CG11522 CG15398 CG17838 CG31617 CG4511 Chit Mhc Mp20 Obp99b RpL38 RpL8 Thor Tm1 Tsf1 corto |
| 470 | GO:0009887 | P | 4, | 1 | 1.545 (x 0.647) | 336 (0.003) | 0.904 | organ morphogenesis | Act57B |
| 471 | GO:0002165 | P | 4, | 1 | 1.587 (x 0.630) | 345 (0.003) | 0.912 | larval or pupal development (sensu Insecta) | Obp99b |
| 472 | GO:0009791 | P | 3, | 1 | 1.637 (x 0.611) | 356 (0.003) | 0.919 | post-embryonic development | Obp99b |
| 473 | GO:0008233 | F | 4, | 2 | 2.994 (x 0.668) | 651 (0.003) | 0.92 | peptidase activity | CG4386 Mlc1 |
| 474 | GO:0031090 | C | 4, 5, 6, 7, 8, | 1 | 1.660 (x 0.602) | 361 (0.003) | 0.922 | organelle membrane | CG12400 |
| 475 | GO:0019226 | P | 5, | 1 | 1.670 (x 0.599) | 363 (0.003) | 0.922 | transmission of nerve impulse | Gs2 |
| 476 | GO:0005524 | F | 6, | 2 | 3.091 (x 0.647) | 672 (0.003) | 0.926 | ATP binding | CG4511 Mhc |
| 477 | GO:0051234 | P | 4, | 6 | 7.856 (x 0.764) | 1708 (0.004) | 0.927 | establishment of localization | CG31547 CG8193 Lsp1beta Obp99b Tsf1 robl |
| 478 | GO:0006366 | P | 8, | 2 | 3.137 (x 0.638) | 682 (0.003) | 0.932 | transcription from RNA polymerase II promoter | Acf1 CG15398 |
| 479 | GO:0030554 | F | 5, | 2 | 3.169 (x 0.631) | 689 (0.003) | 0.935 | adenyl nucleotide binding | CG4511 Mhc |
| 480 | GO:0019752 | P | 6, | 1 | 1.803 (x 0.555) | 392 (0.003) | 0.935 | carboxylic acid metabolism | Gs2 |
| 481 | GO:0003700 | F | 3, 5, | 1 | 1.789 (x 0.559) | 389 (0.003) | 0.936 | transcription factor activity | CG15398 |
| 482 | GO:0006082 | P | 5, | 1 | 1.803 (x 0.555) | 392 (0.003) | 0.937 | organic acid metabolism | Gs2 |
| 483 | GO:0031323 | P | 5, | 3 | 4.535 (x 0.662) | 986 (0.003) | 0.938 | regulation of cellular metabolism | Acf1 CG15398 Thor |
| 484 | GO:0005634 | C | 5, 6, 7, 8, | 5 | 7.010 (x 0.713) | 1524 (0.003) | 0.943 | nucleus | Acf1 CG15398 CG17838 CG31617 corto |
| 485 | GO:0019222 | P | 4, | 3 | 4.710 (x 0.637) | 1024 (0.003) | 0.955 | regulation of metabolism | Acf1 CG15398 Thor |
| 486 | GO:0043227 | C | 3, | 8 | 10.620 (x 0.753) | 2309 (0.003) | 0.955 | membrane-bound organelle | Acf1 CG12400 CG15398 CG17838 CG31617 corto mRpS21 robl |
| 487 | GO:0007276 | P | 4, | 1 | 2.056 (x 0.486) | 447 (0.002) | 0.956 | gametogenesis | Tm1 |
| 488 | GO:0043231 | C | 4, 5, 6, 7, | 8 | 10.611 (x 0.754) | 2307 (0.003) | 0.956 | intracellular membrane-bound organelle | Acf1 CG12400 CG15398 CG17838 CG31617 corto mRpS21 robl |
| 489 | GO:0044464 | C | 2, 3, | 18 | 21.231 (x 0.848) | 4616 (0.004) | 0.958 | cell part | Acf1 Act57B CG11522 CG12400 CG15398 CG17838 CG31547 CG31617 Gs2 Mhc Mlc1 Mp20 RpL38 RpL8 Tm1 corto mRpS21 robl |
| 490 | GO:0006355 | P | 8, | 2 | 3.620 (x 0.553) | 787 (0.003) | 0.958 | regulation of transcription, DNA-dependent | Acf1 CG15398 |
| 491 | GO:0009987 | P | 2, | 28 | 30.844 (x 0.908) | 6706 (0.004) | 0.959 | cellular process | Acf1 Act57B CG11051 CG11522 CG12400 CG15398 CG31547 CG31617 CG4386 CG4511 CG5177 CG8193 Chit Gs2 GstE1 Lsp1beta Mhc Mlc1 Mp20 Obp99b RpL38 RpL8 Thor Tm1 Tsf1 mRpS21 regucalcin robl |
| 492 | GO:0016310 | P | 7, | 1 | 2.120 (x 0.472) | 461 (0.002) | 0.959 | phosphorylation | CG12400 |
| 493 | GO:0006508 | P | 7, | 2 | 3.486 (x 0.574) | 758 (0.003) | 0.959 | proteolysis | CG4386 Mlc1 |
| 494 | GO:0005623 | C | 2, | 18 | 21.231 (x 0.848) | 4616 (0.004) | 0.96 | cell | Acf1 Act57B CG11522 CG12400 CG15398 CG17838 CG31547 CG31617 Gs2 Mhc Mlc1 Mp20 RpL38 RpL8 Tm1 corto mRpS21 robl |
| 495 | GO:0019953 | P | 3, | 1 | 2.097 (x 0.477) | 456 (0.002) | 0.96 | sexual reproduction | Tm1 |
| 496 | GO:0004871 | F | 2, | 3 | 4.843 (x 0.619) | 1053 (0.003) | 0.961 | signal transducer activity | CG11051 CG32249 Chit |
| 497 | GO:0007049 | P | 4, | 1 | 2.166 (x 0.462) | 471 (0.002) | 0.961 | cell cycle | Mhc |
| 498 | GO:0006629 | P | 5, | 1 | 2.254 (x 0.444) | 490 (0.002) | 0.969 | lipid metabolism | Fbp2 |
| 499 | GO:0000003 | P | 2, | 1 | 2.304 (x 0.434) | 501 (0.002) | 0.971 | reproduction | Tm1 |
| 500 | GO:0045449 | P | 7, | 2 | 3.822 (x 0.523) | 831 (0.002) | 0.972 | regulation of transcription | Acf1 CG15398 |
| 501 | GO:0008150 | P | 1, | 35 | 37.173 (x 0.942) | 8082 (0.004) | 0.974 | biological\_process | Acf1 Act57B CG11051 CG11522 CG12400 CG15398 CG31547 CG31617 CG31775 CG32207 CG32212 CG4386 CG4511 CG5177 CG5397 CG8193 Chit Fbp2 Gs2 GstD9 GstE1 Lsp1beta Mhc Mlc1 Mp20 Obp99b RpL38 RpL8 Thor Tm1 Tsf1 fau mRpS21 regucalcin robl |
| 502 | GO:0017076 | F | 4, | 2 | 3.905 (x 0.512) | 849 (0.002) | 0.975 | purine nucleotide binding | CG4511 Mhc |
| 503 | GO:0006357 | P | 9, | 1 | 2.516 (x 0.397) | 547 (0.002) | 0.975 | regulation of transcription from RNA polymerase II promoter | Acf1 |
| 504 | GO:0006351 | P | 7, | 2 | 4.121 (x 0.485) | 896 (0.002) | 0.975 | transcription, DNA-dependent | Acf1 CG15398 |
| 505 | GO:0007242 | P | 5, | 1 | 2.410 (x 0.415) | 524 (0.002) | 0.975 | intracellular signaling cascade | regucalcin |
| 506 | GO:0051641 | P | 4, 5, | 1 | 2.925 (x 0.342) | 636 (0.002) | 0.976 | cellular localization | robl |
| 507 | GO:0050875 | P | 3, | 25 | 28.591 (x 0.874) | 6216 (0.004) | 0.976 | cellular physiological process | Acf1 Act57B CG11522 CG12400 CG15398 CG31547 CG31617 CG4386 CG4511 CG5177 CG8193 Chit Gs2 GstE1 Lsp1beta Mhc Mlc1 Mp20 Obp99b RpL38 RpL8 Thor Tsf1 mRpS21 robl |
| 508 | GO:0046872 | F | 4, | 2 | 4.287 (x 0.467) | 932 (0.002) | 0.976 | metal ion binding | Mp20 Tsf1 |
| 509 | GO:0019219 | P | 6, | 2 | 4.084 (x 0.490) | 888 (0.002) | 0.977 | regulation of nucleobase, nucleoside, nucleotide and nucleic acid metabolism | Acf1 CG15398 |
| 510 | GO:0006796 | P | 6, | 1 | 2.778 (x 0.360) | 604 (0.002) | 0.977 | phosphate metabolism | CG12400 |
| 511 | GO:0000166 | F | 3, | 2 | 4.038 (x 0.495) | 878 (0.002) | 0.977 | nucleotide binding | CG4511 Mhc |
| 512 | GO:0044237 | P | 4, | 17 | 21.291 (x 0.798) | 4629 (0.004) | 0.977 | cellular metabolism | Acf1 Act57B CG11522 CG12400 CG15398 CG31617 CG4386 CG4511 CG5177 Chit Gs2 GstE1 Mlc1 RpL38 RpL8 Thor mRpS21 |
| 513 | GO:0051244 | P | 4, | 3 | 5.966 (x 0.503) | 1297 (0.002) | 0.977 | regulation of cellular physiological process | Acf1 CG15398 Thor |
| 514 | GO:0007582 | P | 2, | 27 | 31.424 (x 0.859) | 6832 (0.004) | 0.978 | physiological process | Acf1 Act57B CG11522 CG12400 CG15398 CG31547 CG31617 CG4386 CG4511 CG5177 CG8193 Chit Fbp2 Gs2 GstE1 Lsp1beta Mhc Mlc1 Mp20 Obp99b RpL38 RpL8 Thor Tm1 Tsf1 mRpS21 robl |
| 515 | GO:0051649 | P | 5, 6, | 1 | 2.921 (x 0.342) | 635 (0.002) | 0.978 | establishment of cellular localization | robl |
| 516 | GO:0050791 | P | 3, | 3 | 6.159 (x 0.487) | 1339 (0.002) | 0.978 | regulation of physiological process | Acf1 CG15398 Thor |
| 517 | GO:0007154 | P | 3, | 4 | 6.977 (x 0.573) | 1517 (0.003) | 0.978 | cell communication | CG11051 Chit Gs2 regucalcin |
| 518 | GO:0006350 | P | 6, | 2 | 4.347 (x 0.460) | 945 (0.002) | 0.978 | transcription | Acf1 CG15398 |
| 519 | GO:0050794 | P | 3, | 3 | 6.334 (x 0.474) | 1377 (0.002) | 0.978 | regulation of cellular process | Acf1 CG15398 Thor |
| 520 | GO:0043167 | F | 3, | 2 | 4.287 (x 0.467) | 932 (0.002) | 0.978 | ion binding | Mp20 Tsf1 |
| 521 | GO:0007165 | P | 4, | 3 | 5.938 (x 0.505) | 1291 (0.002) | 0.978 | signal transduction | CG11051 Chit regucalcin |
| 522 | GO:0043169 | F | 4, | 2 | 4.080 (x 0.490) | 887 (0.002) | 0.978 | cation binding | Mp20 Tsf1 |
| 523 | GO:0046907 | P | 5, 6, 7, | 1 | 2.833 (x 0.353) | 616 (0.002) | 0.978 | intracellular transport | robl |
| 524 | GO:0008152 | P | 3, | 19 | 23.260 (x 0.817) | 5057 (0.004) | 0.979 | metabolism | Acf1 Act57B CG11522 CG12400 CG15398 CG31617 CG4386 CG4511 CG5177 CG8193 Chit Fbp2 Gs2 GstE1 Mlc1 RpL38 RpL8 Thor mRpS21 |
| 525 | GO:0006793 | P | 5, | 1 | 2.778 (x 0.360) | 604 (0.002) | 0.979 | phosphorus metabolism | CG12400 |
| 526 | GO:0046914 | F | 5, | 1 | 3.109 (x 0.322) | 676 (0.001) | 0.979 | transition metal ion binding | Tsf1 |
| 527 | GO:0007166 | P | 5, | 1 | 3.031 (x 0.330) | 659 (0.002) | 0.979 | cell surface receptor linked signal transduction | CG11051 |
| 528 | GO:0044238 | P | 4, | 15 | 21.020 (x 0.714) | 4570 (0.003) | 0.991 | primary metabolism | Acf1 Act57B CG11522 CG15398 CG31617 CG4386 CG5177 Chit Fbp2 Gs2 Mlc1 RpL38 RpL8 Thor mRpS21 |
| 529 | GO:0044425 | C | 3, 4, 5, | 2 | 5.593 (x 0.358) | 1216 (0.002) | 0.991 | membrane part | CG12400 CG31547 |
| 530 | GO:0050789 | P | 2, | 3 | 6.922 (x 0.433) | 1505 (0.002) | 0.991 | regulation of biological process | Acf1 CG15398 Thor |
| 531 | GO:0031224 | C | 4, 5, 6, | 1 | 4.356 (x 0.230) | 947 (0.001) | 0.994 | intrinsic to membrane | CG31547 |
| 532 | GO:0006139 | P | 5, | 3 | 8.146 (x 0.368) | 1771 (0.002) | 0.995 | nucleobase, nucleoside, nucleotide and nucleic acid metabolism | Acf1 CG15398 CG31617 |
| 533 | GO:0016021 | C | 5, 6, 7, | 1 | 4.342 (x 0.230) | 944 (0.001) | 0.996 | integral to membrane | CG31547 |
| 534 | GO:0043283 | P | 5, | 3 | 7.746 (x 0.387) | 1684 (0.002) | 0.997 | biopolymer metabolism | Acf1 CG31617 Chit |
| 535 | GO:0016020 | C | 3, 4, | 2 | 8.583 (x 0.233) | 1866 (0.001) | 0.999 | membrane | CG12400 CG31547 |

  

---

Regulated Genes that don't have GO terms
  

BG:DS00810.3 CG10038 CG13053 CG13067 CG17478 CG17681 CG18294 CG2233 CG30412 CG40178 CG40228 CG5174 CG5773 CG5961 CG8369 CG9231 CG9336 CG9766
