# Supplementary material for: Functional dissection of the ash2 and ash1 transcriptomes provides insights into the transcriptional basis of wing phenotypes and reveals conserved protein interactions
Source: Genome Biol. 2007 Apr 28;8(4):R67. doi: 10.1186/gb-2007-8-4-r67 (PMC1896016; doi:10.1186/gb-2007-8-4-r67)
Supplement: Additional data file 17 — GO annotations of wing disc genes identified by Klebes et al. [37] [file gb-2007-8-4-r67-S17.html]

  

---

  

|  |  |
| --- | --- |
| Go Statistics | Reg File: **Klebes\_Wing.txt.fbgns** (67 genes -- 12 skipped)  Ref File: **ref.fbgns** (13577 genes -- 4663 skipped)  Database: **go\_200507-termdb.rdf-xml** |

---

  

Fields Description

| Pos | Go Term | Ontology | Levels | Observed | Expected | Possibles | p-value(Adj) | Go term description | Genes with the GO term |
| --- | --- | --- | --- | --- | --- | --- | --- | --- | --- |
| 1 | GO:0009888 | P | 3, | 17 | 2.456 (x 6.923) | 398 (0.043) | 7.96e-08 | tissue development | CG5397 Doc1 Doc2 Doc3 Dr NetA Sox15 ap dnt drl ds eya pdm2 rost toe tup ush |
| 2 | GO:0007398 | P | 4, | 12 | 1.462 (x 8.206) | 237 (0.051) | 5.27e-06 | ectoderm development | CG5397 Dr NetA Sox15 ap dnt drl ds pdm2 toe tup ush |
| 3 | GO:0007275 | P | 2, | 26 | 9.163 (x 2.838) | 1485 (0.018) | 2.77e-05 | development | CG3132 CG5397 CG9358 Doc1 Doc2 Doc3 Dr NetA Sox15 ap b dnt drl ds eya if kn nvy pdm2 regucalcin rost rpr toe tup ush vg |
| 4 | GO:0003700 | F | 3, 5, | 13 | 2.400 (x 5.416) | 389 (0.033) | 8.14e-05 | transcription factor activity | CG11835 CG4914 Doc1 Doc2 Doc3 Dr Sox15 ap nvy pdm2 toe tup ush |
| 5 | GO:0007411 | P | 6, 7, 9, 10, 12, | 7 | 0.506 (x 13.835) | 82 (0.085) | 8.56e-05 | axon guidance | NetA ap dnt drl if nvy tup |
| 6 | GO:0000904 | P | 5, 6, | 9 | 1.043 (x 8.631) | 169 (0.053) | 8.98e-05 | cellular morphogenesis during differentiation | NetA ap dnt drl ds eya if nvy tup |
| 7 | GO:0009653 | P | 3, | 16 | 3.961 (x 4.039) | 642 (0.025) | 9.13e-05 | morphogenesis | CG9358 Doc1 Dr NetA ap dnt drl ds eya if kn nvy rpr tup ush vg |
| 8 | GO:0048667 | P | 6, 7, 9, | 7 | 0.747 (x 9.376) | 121 (0.058) | 0.000591 | neuron morphogenesis during differentiation | NetA ap dnt drl if nvy tup |
| 9 | GO:0030154 | P | 3, | 13 | 3.147 (x 4.131) | 510 (0.025) | 0.000606 | cell differentiation | Dr NetA ap dnt drl ds eya if kn nvy rost tup ush |
| 10 | GO:0016477 | P | 5, 6, | 8 | 1.080 (x 7.409) | 175 (0.046) | 0.000608 | cell migration | NetA ap dnt drl eya if nvy tup |
| 11 | GO:0048812 | P | 7, 8, 10, | 7 | 0.747 (x 9.376) | 121 (0.058) | 0.000656 | neurite morphogenesis | NetA ap dnt drl if nvy tup |
| 12 | GO:0048699 | P | 6, | 8 | 1.111 (x 7.203) | 180 (0.044) | 0.00069 | generation of neurons | Dr NetA ap dnt drl if nvy tup |
| 13 | GO:0007409 | P | 8, 9, 11, | 7 | 0.747 (x 9.376) | 121 (0.058) | 0.000738 | axonogenesis | NetA ap dnt drl if nvy tup |
| 14 | GO:0048666 | P | 5, 8, | 7 | 0.876 (x 7.990) | 142 (0.049) | 0.00099 | neuron development | NetA ap dnt drl if nvy tup |
| 15 | GO:0022008 | P | 5, | 8 | 1.191 (x 6.718) | 193 (0.041) | 0.000993 | neurogenesis | Dr NetA ap dnt drl if nvy tup |
| 16 | GO:0031175 | P | 6, 9, | 7 | 0.876 (x 7.990) | 142 (0.049) | 0.00105 | neurite development | NetA ap dnt drl if nvy tup |
| 17 | GO:0007399 | P | 4, | 12 | 2.888 (x 4.156) | 468 (0.026) | 0.00106 | nervous system development | Dr NetA Sox15 ap dnt drl if nvy pdm2 rpr toe tup |
| 18 | GO:0030182 | P | 4, 7, | 7 | 0.919 (x 7.614) | 149 (0.047) | 0.00128 | neuron differentiation | NetA ap dnt drl if nvy tup |
| 19 | GO:0007472 | P | 6, 7, | 6 | 0.648 (x 9.261) | 105 (0.057) | 0.00128 | wing disc morphogenesis | Dr ap ds if kn vg |
| 20 | GO:0048513 | P | 3, | 14 | 4.134 (x 3.387) | 670 (0.021) | 0.00128 | organ development | CG3132 Doc1 Dr ap dnt drl ds eya if kn rost rpr ush vg |
| 21 | GO:0002165 | P | 4, | 10 | 2.129 (x 4.698) | 345 (0.029) | 0.0013 | larval or pupal development (sensu Insecta) | CG3132 CG9358 Dr ap ds eya if kn rpr vg |
| 22 | GO:0007476 | P | 6, 7, 8, | 6 | 0.636 (x 9.441) | 103 (0.058) | 0.00132 | wing morphogenesis | Dr ap ds if kn vg |
| 23 | GO:0048468 | P | 4, | 10 | 2.098 (x 4.767) | 340 (0.029) | 0.00133 | cell development | NetA ap dnt drl ds eya if nvy rost tup |
| 24 | GO:0007517 | P | 4, | 6 | 0.666 (x 9.004) | 108 (0.056) | 0.00138 | muscle development | Dr ap dnt drl if rost |
| 25 | GO:0048731 | P | 3, | 13 | 3.665 (x 3.547) | 594 (0.022) | 0.0014 | system development | CG3132 Dr NetA Sox15 ap dnt drl if nvy pdm2 rpr toe tup |
| 26 | GO:0009791 | P | 3, | 10 | 2.197 (x 4.553) | 356 (0.028) | 0.00144 | post-embryonic development | CG3132 CG9358 Dr ap ds eya if kn rpr vg |
| 27 | GO:0035220 | P | 5, | 6 | 0.716 (x 8.383) | 116 (0.052) | 0.00191 | wing disc development | Dr ap ds if kn vg |
| 28 | GO:0016203 | P | 5, | 3 | 0.086 (x 34.730) | 14 (0.214) | 0.00193 | muscle attachment | dnt drl if |
| 29 | GO:0003677 | F | 4, | 15 | 5.072 (x 2.958) | 822 (0.018) | 0.00222 | DNA binding | CG11835 CG4914 Doc1 Doc2 Doc3 Dr GV1 Sox15 ap kn nvy pdm2 toe tup ush |
| 30 | GO:0045595 | P | 4, | 4 | 0.259 (x 15.435) | 42 (0.095) | 0.00285 | regulation of cell differentiation | Dr eya kn ush |
| 31 | GO:0006928 | P | 4, 5, | 8 | 1.586 (x 5.045) | 257 (0.031) | 0.00289 | cell motility | NetA ap dnt drl eya if nvy tup |
| 32 | GO:0035114 | P | 5, | 6 | 0.802 (x 7.480) | 130 (0.046) | 0.00293 | appendage morphogenesis (sensu Endopterygota) | Dr ap ds if kn vg |
| 33 | GO:0007389 | P | 3, | 8 | 1.580 (x 5.065) | 256 (0.031) | 0.00297 | pattern specification | CG9358 Dr ap ds kn regucalcin tup ush |
| 34 | GO:0051674 | P | 4, | 8 | 1.586 (x 5.045) | 257 (0.031) | 0.00297 | localization of cell | NetA ap dnt drl eya if nvy tup |
| 35 | GO:0042659 | P | 5, 6, | 3 | 0.111 (x 27.012) | 18 (0.167) | 0.00297 | regulation of cell fate specification | Dr eya ush |
| 36 | GO:0007552 | P | 4, | 8 | 1.561 (x 5.125) | 253 (0.032) | 0.00298 | metamorphosis | CG9358 Dr ap ds eya if kn vg |
| 37 | GO:0048737 | P | 4, | 6 | 0.802 (x 7.480) | 130 (0.046) | 0.00303 | appendage development (sensu Endopterygota) | Dr ap ds if kn vg |
| 38 | GO:0035107 | P | 4, | 6 | 0.821 (x 7.312) | 133 (0.045) | 0.00305 | appendage morphogenesis | Dr ap ds if kn vg |
| 39 | GO:0040011 | P | 3, | 8 | 1.610 (x 4.968) | 261 (0.031) | 0.00306 | locomotion | NetA ap dnt drl eya if nvy tup |
| 40 | GO:0046698 | P | 5, | 8 | 1.549 (x 5.166) | 251 (0.032) | 0.00309 | metamorphosis (sensu Insecta) | CG9358 Dr ap ds eya if kn vg |
| 41 | GO:0048736 | P | 3, | 6 | 0.821 (x 7.312) | 133 (0.045) | 0.00314 | appendage development | Dr ap ds if kn vg |
| 42 | GO:0009887 | P | 4, | 9 | 2.073 (x 4.341) | 336 (0.027) | 0.00316 | organ morphogenesis | Doc1 Dr ap ds eya if kn ush vg |
| 43 | GO:0000902 | P | 4, 5, | 9 | 2.085 (x 4.316) | 338 (0.027) | 0.00322 | cellular morphogenesis | NetA ap dnt drl ds eya if nvy tup |
| 44 | GO:0030528 | F | 2, | 14 | 4.967 (x 2.819) | 805 (0.017) | 0.00432 | transcription regulator activity | CG11835 CG4914 Doc1 Doc2 Doc3 Dr Sox15 ap kn nvy pdm2 toe tup ush |
| 45 | GO:0007362 | P | 8, 9, | 3 | 0.130 (x 23.153) | 21 (0.143) | 0.00435 | terminal region determination | CG9358 tup ush |
| 46 | GO:0046665 | P | 6, | 2 | 0.031 (x 64.829) | 5 (0.400) | 0.00562 | amnioserosa maintenance | tup ush |
| 47 | GO:0045449 | P | 7, | 14 | 5.127 (x 2.730) | 831 (0.017) | 0.00575 | regulation of transcription | CG11835 CG4914 Doc1 Doc2 Doc3 Dr Sox15 ap kn nvy pdm2 toe tup ush |
| 48 | GO:0006350 | P | 6, | 15 | 5.831 (x 2.573) | 945 (0.016) | 0.00618 | transcription | CG11835 CG4914 Doc1 Doc2 Doc3 Dr Sox15 ap eya kn nvy pdm2 toe tup ush |
| 49 | GO:0006357 | P | 9, | 11 | 3.375 (x 3.259) | 547 (0.020) | 0.00618 | regulation of transcription from RNA polymerase II promoter | CG11835 Doc1 Doc2 Doc3 Dr Sox15 ap nvy pdm2 toe tup |
| 50 | GO:0007354 | P | 7, 8, | 3 | 0.167 (x 18.008) | 27 (0.111) | 0.0082 | zygotic determination of anterior/posterior axis, embryo | CG9358 tup ush |
| 51 | GO:0007560 | P | 5, 6, | 7 | 1.481 (x 4.727) | 240 (0.029) | 0.00869 | imaginal disc morphogenesis | Dr ap ds eya if kn vg |
| 52 | GO:0031323 | P | 5, | 15 | 6.084 (x 2.466) | 986 (0.015) | 0.00916 | regulation of cellular metabolism | CG11835 CG4914 Doc1 Doc2 Doc3 Dr Sox15 ap kn nvy pdm2 rpr toe tup ush |
| 53 | GO:0019219 | P | 6, | 14 | 5.479 (x 2.555) | 888 (0.016) | 0.00981 | regulation of nucleobase, nucleoside, nucleotide and nucleic acid metabolism | CG11835 CG4914 Doc1 Doc2 Doc3 Dr Sox15 ap kn nvy pdm2 toe tup ush |
| 54 | GO:0006366 | P | 8, | 12 | 4.208 (x 2.852) | 682 (0.018) | 0.0099 | transcription from RNA polymerase II promoter | CG11835 Doc1 Doc2 Doc3 Dr Sox15 ap kn nvy pdm2 toe tup |
| 55 | GO:0006355 | P | 8, | 13 | 4.856 (x 2.677) | 787 (0.017) | 0.01 | regulation of transcription, DNA-dependent | CG11835 CG4914 Doc1 Doc2 Doc3 Dr Sox15 ap kn nvy pdm2 toe tup |
| 56 | GO:0007169 | P | 7, | 5 | 0.765 (x 6.535) | 124 (0.040) | 0.012 | transmembrane receptor protein tyrosine kinase signaling pathway | CG9358 dnt drl tup ush |
| 57 | GO:0019222 | P | 4, | 15 | 6.318 (x 2.374) | 1024 (0.015) | 0.0124 | regulation of metabolism | CG11835 CG4914 Doc1 Doc2 Doc3 Dr Sox15 ap kn nvy pdm2 rpr toe tup ush |
| 58 | GO:0035282 | P | 3, | 5 | 0.777 (x 6.431) | 126 (0.040) | 0.0124 | segmentation | CG9358 ap kn tup ush |
| 59 | GO:0050794 | P | 3, | 18 | 8.496 (x 2.119) | 1377 (0.013) | 0.0124 | regulation of cellular process | CG11835 CG30069 CG4914 Doc1 Doc2 Doc3 Dr Mmp2 Sox15 ap eya kn nvy pdm2 rpr toe tup ush |
| 60 | GO:0008293 | P | 8, | 3 | 0.204 (x 14.734) | 33 (0.091) | 0.0126 | torso signaling pathway | CG9358 tup ush |
| 61 | GO:0001700 | P | 5, | 5 | 0.796 (x 6.282) | 129 (0.039) | 0.0131 | embryonic development (sensu Insecta) | Doc1 kn rpr tup ush |
| 62 | GO:0009792 | P | 4, | 6 | 1.203 (x 4.987) | 195 (0.031) | 0.0136 | embryonic development (sensu Metazoa) | CG9358 Doc1 kn rpr tup ush |
| 63 | GO:0003704 | F | 4, | 4 | 0.487 (x 8.206) | 79 (0.051) | 0.0151 | specific RNA polymerase II transcription factor activity | ap kn pdm2 tup |
| 64 | GO:0050793 | P | 3, | 4 | 0.487 (x 8.206) | 79 (0.051) | 0.0154 | regulation of development | Dr eya kn ush |
| 65 | GO:0051244 | P | 4, | 17 | 8.003 (x 2.124) | 1297 (0.013) | 0.0162 | regulation of cellular physiological process | CG11835 CG30069 CG4914 Doc1 Doc2 Doc3 Dr Mmp2 Sox15 ap kn nvy pdm2 rpr toe tup ush |
| 66 | GO:0030855 | P | 4, 5, | 2 | 0.068 (x 29.468) | 11 (0.182) | 0.0207 | epithelial cell differentiation | ds ush |
| 67 | GO:0007390 | P | 6, | 2 | 0.068 (x 29.468) | 11 (0.182) | 0.021 | germ-band shortening | tup ush |
| 68 | GO:0050791 | P | 3, | 17 | 8.262 (x 2.058) | 1339 (0.013) | 0.022 | regulation of physiological process | CG11835 CG30069 CG4914 Doc1 Doc2 Doc3 Dr Mmp2 Sox15 ap kn nvy pdm2 rpr toe tup ush |
| 69 | GO:0007447 | P | 4, 5, | 3 | 0.265 (x 11.307) | 43 (0.070) | 0.0234 | imaginal disc pattern formation | Dr ap ds |
| 70 | GO:0007444 | P | 4, | 7 | 1.863 (x 3.757) | 302 (0.023) | 0.024 | imaginal disc development | Dr ap ds eya if kn vg |
| 71 | GO:0006351 | P | 7, | 13 | 5.528 (x 2.352) | 896 (0.015) | 0.0255 | transcription, DNA-dependent | CG11835 CG4914 Doc1 Doc2 Doc3 Dr Sox15 ap kn nvy pdm2 toe tup |
| 72 | GO:0009996 | P | 6, 7, | 2 | 0.080 (x 24.934) | 13 (0.154) | 0.0271 | negative regulation of cell fate specification | eya ush |
| 73 | GO:0050789 | P | 2, | 18 | 9.286 (x 1.938) | 1505 (0.012) | 0.0287 | regulation of biological process | CG11835 CG30069 CG4914 Doc1 Doc2 Doc3 Dr Mmp2 Sox15 ap eya kn nvy pdm2 rpr toe tup ush |
| 74 | GO:0008595 | P | 6, 7, | 3 | 0.309 (x 9.724) | 50 (0.060) | 0.0327 | determination of anterior/posterior axis, embryo | CG9358 tup ush |
| 75 | GO:0007351 | P | 5, 6, | 3 | 0.309 (x 9.724) | 50 (0.060) | 0.0331 | regional subdivision | CG9358 tup ush |
| 76 | GO:0007350 | P | 4, 5, | 4 | 0.629 (x 6.356) | 102 (0.039) | 0.0333 | blastoderm segmentation | CG9358 kn tup ush |
| 77 | GO:0007450 | P | 5, 6, | 2 | 0.093 (x 21.610) | 15 (0.133) | 0.0339 | dorsal/ventral pattern formation, imaginal disc | Dr ap |
| 78 | GO:0007167 | P | 6, | 5 | 1.061 (x 4.711) | 172 (0.029) | 0.0361 | enzyme linked receptor protein signaling pathway | CG9358 dnt drl tup ush |
| 79 | GO:0001708 | P | 5, | 3 | 0.327 (x 9.174) | 53 (0.057) | 0.0371 | cell fate specification | Dr eya ush |
| 80 | GO:0016066 |  | 6, 7, | 1 | 0.006 (x 162.073) | 1 (1.000) | 0.0432 |  | kn |
| 81 | GO:0006208 | P | 7, 8, | 1 | 0.006 (x 162.073) | 1 (1.000) | 0.0436 | pyrimidine base catabolism | b |
| 82 | GO:0030104 | P | 4, 5, | 1 | 0.006 (x 162.073) | 1 (1.000) | 0.0441 | water homeostasis | Drip |
| 83 | GO:0019483 | P | 9, 10, | 1 | 0.006 (x 162.073) | 1 (1.000) | 0.0445 | beta-alanine biosynthesis | b |
| 84 | GO:0007481 | P | 6, 7, | 1 | 0.006 (x 162.073) | 1 (1.000) | 0.045 | haltere disc morphogenesis | ap |
| 85 | GO:0004249 | F | 7, | 1 | 0.006 (x 162.073) | 1 (1.000) | 0.0455 | stromelysin 3 activity | Mmp2 |
| 86 | GO:0035222 | P | 5, 6, | 2 | 0.123 (x 16.207) | 20 (0.100) | 0.0458 | wing disc pattern formation | ap ds |
| 87 | GO:0035291 | P | 6, 7, 8, | 1 | 0.006 (x 162.073) | 1 (1.000) | 0.0459 | specification of segmental identity, intercalary segment | kn |
| 88 | GO:0006212 | P | 8, 9, | 1 | 0.006 (x 162.073) | 1 (1.000) | 0.0464 | uracil catabolism | b |
| 89 | GO:0019860 | P | 8, | 1 | 0.006 (x 162.073) | 1 (1.000) | 0.0469 | uracil metabolism | b |
| 90 | GO:0045610 | P | 5, 7, | 2 | 0.111 (x 18.008) | 18 (0.111) | 0.047 | regulation of hemocyte differentiation | kn ush |
| 91 | GO:0042087 | P | 7, 8, | 1 | 0.006 (x 162.073) | 1 (1.000) | 0.0475 | cell-mediated immune response | kn |
| 92 | GO:0009790 | P | 3, | 7 | 2.166 (x 3.232) | 351 (0.020) | 0.0476 | embryonic development | CG9358 Doc1 eya kn rpr tup ush |
| 93 | GO:0019795 | P | 8, 9, | 1 | 0.006 (x 162.073) | 1 (1.000) | 0.048 | nonprotein amino acid biosynthesis | b |
| 94 | GO:0000578 | P | 5, | 3 | 0.364 (x 8.241) | 59 (0.051) | 0.0483 | embryonic axis specification | CG9358 tup ush |
| 95 | GO:0046113 | P | 6, 7, | 1 | 0.006 (x 162.073) | 1 (1.000) | 0.0485 | nucleobase catabolism | b |
| 96 | GO:0004068 | F | 6, | 1 | 0.006 (x 162.073) | 1 (1.000) | 0.0491 | aspartate 1-decarboxylase activity | b |
| 97 | GO:0019482 | P | 8, 9, | 1 | 0.006 (x 162.073) | 1 (1.000) | 0.0496 | beta-alanine metabolism | b |
| 98 | GO:0050839 | F | 4, | 2 | 0.117 (x 17.060) | 19 (0.105) | 0.0498 | cell adhesion molecule binding | ds if |
| 99 | GO:0007498 | P | 4, | 5 | 1.222 (x 4.093) | 198 (0.025) | 0.0499 | mesoderm development | Doc1 Doc2 Doc3 eya rost |
| 100 | GO:0009948 | P | 5, | 4 | 0.734 (x 5.448) | 119 (0.034) | 0.0502 | anterior/posterior axis specification | CG9358 regucalcin tup ush |
| 101 | GO:0017114 | F | 6, | 1 | 0.006 (x 162.073) | 1 (1.000) | 0.0502 | wide-spectrum protease inhibitor activity | TepII |
| 102 | GO:0045596 | P | 5, | 2 | 0.117 (x 17.060) | 19 (0.105) | 0.0504 | negative regulation of cell differentiation | eya ush |
| 103 | GO:0048627 | P | 5, 6, 8, 9, | 2 | 0.136 (x 14.734) | 22 (0.091) | 0.0537 | myoblast development | if rost |
| 104 | GO:0009880 | P | 4, | 4 | 0.796 (x 5.026) | 129 (0.031) | 0.0539 | embryonic pattern specification | CG9358 kn tup ush |
| 105 | GO:0048628 | P | 6, 7, 9, 10, | 2 | 0.136 (x 14.734) | 22 (0.091) | 0.0542 | myoblast maturation | if rost |
| 106 | GO:0005634 | C | 5, 6, 7, 8, | 17 | 9.403 (x 1.808) | 1524 (0.011) | 0.0547 | nucleus | CG11835 CG4914 Doc1 Doc2 Doc3 Dr GV1 Sox15 ap eya kn nvy pdm2 toe tup ush vg |
| 107 | GO:0009952 | P | 4, | 4 | 0.802 (x 4.987) | 130 (0.031) | 0.0548 | anterior/posterior pattern formation | CG9358 regucalcin tup ush |
| 108 | GO:0048732 | P | 4, | 4 | 0.808 (x 4.949) | 131 (0.031) | 0.0552 | gland development | CG3132 if kn rpr |
| 109 | GO:0008258 | P | 6, | 2 | 0.142 (x 14.093) | 23 (0.087) | 0.0554 | head involution | tup ush |
| 110 | GO:0045445 | P | 5, 7, 8, | 2 | 0.142 (x 14.093) | 23 (0.087) | 0.0559 | myoblast differentiation | if rost |
| 111 | GO:0004867 | F | 6, | 3 | 0.432 (x 6.946) | 70 (0.043) | 0.0573 | serine-type endopeptidase inhibitor activity | CG5392 CG6680 TepII |
| 112 | GO:0008587 | P | 7, 8, 9, | 2 | 0.148 (x 13.506) | 24 (0.083) | 0.0591 | wing margin morphogenesis | if vg |
| 113 | GO:0016271 | P | 4, | 3 | 0.444 (x 6.753) | 72 (0.042) | 0.0603 | tissue death | CG3132 ap rpr |
| 114 | GO:0007559 | P | 5, | 3 | 0.444 (x 6.753) | 72 (0.042) | 0.0608 | histolysis | CG3132 ap rpr |
| 115 | GO:0008627 | P | 10, 11, | 1 | 0.012 (x 81.036) | 2 (0.500) | 0.0668 | induction of apoptosis by ionic changes | rpr |
| 116 | GO:0051093 | P | 4, | 2 | 0.160 (x 12.467) | 26 (0.077) | 0.0673 | negative regulation of development | eya ush |
| 117 | GO:0008442 | F | 6, | 1 | 0.012 (x 81.036) | 2 (0.500) | 0.0673 | 3-hydroxyisobutyrate dehydrogenase activity | CG15093 |
| 118 | GO:0001743 | P | 5, | 1 | 0.012 (x 81.036) | 2 (0.500) | 0.0678 | optic placode formation | eya |
| 119 | GO:0035096 | P | 6, 7, | 1 | 0.012 (x 81.036) | 2 (0.500) | 0.0683 | larval midgut cell programmed cell death | rpr |
| 120 | GO:0004091 | F | 6, | 2 | 0.173 (x 11.577) | 28 (0.071) | 0.0687 | carboxylesterase activity | CG4382 alpha-Est5 |
| 121 | GO:0009341 | C | 4, | 1 | 0.012 (x 81.036) | 2 (0.500) | 0.0689 | beta-galactosidase complex | CG3132 |
| 122 | GO:0042690 | P | 7, 9, | 1 | 0.012 (x 81.036) | 2 (0.500) | 0.0695 | negative regulation of crystal cell differentiation | ush |
| 123 | GO:0035216 | P | 5, | 1 | 0.012 (x 81.036) | 2 (0.500) | 0.07 | haltere disc development | ap |
| 124 | GO:0004565 | F | 7, | 1 | 0.012 (x 81.036) | 2 (0.500) | 0.0706 | beta-galactosidase activity | CG3132 |
| 125 | GO:0048469 | P | 5, | 2 | 0.167 (x 12.005) | 27 (0.074) | 0.0711 | cell maturation | if rost |
| 126 | GO:0001744 | P | 6, 8, 9, | 1 | 0.012 (x 81.036) | 2 (0.500) | 0.0712 | optic placode formation (sensu Endopterygota) | eya |
| 127 | GO:0042692 | P | 4, | 2 | 0.167 (x 12.005) | 27 (0.074) | 0.0717 | muscle cell differentiation | if rost |
| 128 | GO:0035203 | P | 6, 7, 8, 9, 10, | 1 | 0.012 (x 81.036) | 2 (0.500) | 0.0718 | regulation of lamellocyte differentiation | kn |
| 129 | GO:0006833 | P | 6, 7, | 1 | 0.012 (x 81.036) | 2 (0.500) | 0.0724 | water transport | Drip |
| 130 | GO:0004714 | F | 6, 8, | 2 | 0.179 (x 11.177) | 29 (0.069) | 0.0729 | transmembrane receptor protein tyrosine kinase activity | dnt drl |
| 131 | GO:0004351 | F | 6, | 1 | 0.012 (x 81.036) | 2 (0.500) | 0.073 | glutamate decarboxylase activity | b |
| 132 | GO:0007160 | P | 5, | 2 | 0.185 (x 10.805) | 30 (0.067) | 0.0761 | cell-matrix adhesion | NetA if |
| 133 | GO:0042386 | P | 4, 6, | 2 | 0.185 (x 10.805) | 30 (0.067) | 0.0766 | hemocyte differentiation (sensu Arthropoda) | kn ush |
| 134 | GO:0031589 | P | 4, | 2 | 0.185 (x 10.805) | 30 (0.067) | 0.0772 | cell-substrate adhesion | NetA if |
| 135 | GO:0004866 | F | 5, | 3 | 0.524 (x 5.720) | 85 (0.035) | 0.0797 | endopeptidase inhibitor activity | CG5392 CG6680 TepII |
| 136 | GO:0005886 | C | 4, 5, | 8 | 3.295 (x 2.428) | 534 (0.015) | 0.0812 | plasma membrane | CG1698 CG9358 Mmp2 dnt drl ds if rost |
| 137 | GO:0030414 | F | 4, | 3 | 0.531 (x 5.654) | 86 (0.035) | 0.0816 | protease inhibitor activity | CG5392 CG6680 TepII |
| 138 | GO:0009798 | P | 4, | 4 | 0.981 (x 4.077) | 159 (0.025) | 0.0833 | axis specification | CG9358 regucalcin tup ush |
| 139 | GO:0016831 | F | 5, | 2 | 0.210 (x 9.534) | 34 (0.059) | 0.084 | carboxy-lyase activity | amd b |
| 140 | GO:0019794 | P | 7, 8, | 1 | 0.019 (x 54.024) | 3 (0.333) | 0.0842 | nonprotein amino acid metabolism | b |
| 141 | GO:0018149 | P | 8, | 1 | 0.019 (x 54.024) | 3 (0.333) | 0.0847 | peptide cross-linking | ds |
| 142 | GO:0016265 | P | 3, | 5 | 1.549 (x 3.229) | 251 (0.020) | 0.0851 | death | CG3132 CG6680 Mmp2 ap rpr |
| 143 | GO:0048049 | P | 7, 8, | 1 | 0.019 (x 54.024) | 3 (0.333) | 0.0853 | embryonic eye morphogenesis (sensu Endopterygota) | eya |
| 144 | GO:0015280 | F | 6, 7, 8, | 1 | 0.019 (x 54.024) | 3 (0.333) | 0.0859 | amiloride-sensitive sodium channel activity | rpk |
| 145 | GO:0048048 | P | 6, 7, | 1 | 0.019 (x 54.024) | 3 (0.333) | 0.0864 | embryonic eye morphogenesis | eya |
| 146 | GO:0035027 | P | 5, 6, 7, | 1 | 0.019 (x 54.024) | 3 (0.333) | 0.087 | leading edge cell fate commitment | ush |
| 147 | GO:0007393 | P | 7, 8, 9, 10, | 1 | 0.019 (x 54.024) | 3 (0.333) | 0.0876 | dorsal closure, leading edge cell fate determination | ush |
| 148 | GO:0035069 | P | 6, | 1 | 0.019 (x 54.024) | 3 (0.333) | 0.0882 | larval midgut histolysis | rpr |
| 149 | GO:0045611 | P | 6, 8, | 1 | 0.019 (x 54.024) | 3 (0.333) | 0.0888 | negative regulation of hemocyte differentiation | ush |
| 150 | GO:0006573 | P | 8, 9, | 1 | 0.019 (x 54.024) | 3 (0.333) | 0.0894 | valine metabolism | CG15093 |
| 151 | GO:0035028 | P | 6, 7, 8, | 1 | 0.019 (x 54.024) | 3 (0.333) | 0.0901 | leading edge cell fate determination | ush |
| 152 | GO:0035029 | P | 6, 7, 8, 9, | 1 | 0.019 (x 54.024) | 3 (0.333) | 0.0907 | dorsal closure, leading edge cell fate commitment | ush |
| 153 | GO:0015925 | F | 6, | 1 | 0.019 (x 54.024) | 3 (0.333) | 0.0913 | galactosidase activity | CG3132 |
| 154 | GO:0008624 | P | 9, 10, | 1 | 0.019 (x 54.024) | 3 (0.333) | 0.092 | induction of apoptosis by extracellular signals | rpr |
| 155 | GO:0004772 | F | 8, | 1 | 0.019 (x 54.024) | 3 (0.333) | 0.0927 | sterol O-acyltransferase activity | CG5397 |
| 156 | GO:0006520 | P | 6, 7, | 5 | 1.610 (x 3.105) | 261 (0.019) | 0.0983 | amino acid metabolism | CG15093 CG1698 Sox15 amd b |
| 157 | GO:0042302 | F | 3, | 3 | 0.611 (x 4.911) | 99 (0.030) | 0.102 | structural constituent of cuticle | CG7160 CG8502 Edg91 |
| 158 | GO:0003702 | F | 3, | 5 | 1.641 (x 3.046) | 266 (0.019) | 0.104 | RNA polymerase II transcription factor activity | CG11835 ap kn pdm2 tup |
| 159 | GO:0004616 | F | 6, | 1 | 0.025 (x 40.518) | 4 (0.250) | 0.105 | phosphogluconate dehydrogenase (decarboxylating) activity | CG15093 |
| 160 | GO:0042689 | P | 6, 8, | 1 | 0.025 (x 40.518) | 4 (0.250) | 0.106 | regulation of crystal cell differentiation | ush |
| 161 | GO:0046546 | P | 5, | 1 | 0.025 (x 40.518) | 4 (0.250) | 0.106 | development of primary male sexual characteristics | eya |
| 162 | GO:0008584 | P | 6, | 1 | 0.025 (x 40.518) | 4 (0.250) | 0.107 | male gonad development | eya |
| 163 | GO:0004058 | F | 6, | 1 | 0.025 (x 40.518) | 4 (0.250) | 0.108 | aromatic-L-amino-acid decarboxylase activity | amd |
| 164 | GO:0009308 | P | 5, | 6 | 2.320 (x 2.586) | 376 (0.016) | 0.115 | amine metabolism | CG15093 CG1698 CG9307 Sox15 amd b |
| 165 | GO:0048637 | P | 6, | 2 | 0.259 (x 7.718) | 42 (0.048) | 0.115 | skeletal muscle development | if rost |
| 166 | GO:0048741 | P | 6, 7, | 2 | 0.259 (x 7.718) | 42 (0.048) | 0.116 | skeletal muscle fiber development | if rost |
| 167 | GO:0048747 | P | 5, | 2 | 0.259 (x 7.718) | 42 (0.048) | 0.117 | muscle fiber development | if rost |
| 168 | GO:0048598 | P | 4, | 3 | 0.654 (x 4.587) | 106 (0.028) | 0.117 | embryonic morphogenesis | eya tup ush |
| 169 | GO:0045165 | P | 4, | 4 | 1.166 (x 3.430) | 189 (0.021) | 0.119 | cell fate commitment | Dr ap eya ush |
| 170 | GO:0035272 | P | 4, | 3 | 0.673 (x 4.461) | 109 (0.028) | 0.121 | exocrine system development | CG3132 if rpr |
| 171 | GO:0007451 | P | 6, 7, | 1 | 0.031 (x 32.415) | 5 (0.200) | 0.121 | dorsal/ventral lineage restriction, imaginal disc | ap |
| 172 | GO:0007431 | P | 5, | 3 | 0.673 (x 4.461) | 109 (0.028) | 0.121 | salivary gland development | CG3132 if rpr |
| 173 | GO:0035026 | P | 5, 6, | 1 | 0.031 (x 32.415) | 5 (0.200) | 0.122 | leading edge cell differentiation | ush |
| 174 | GO:0016830 | F | 4, | 2 | 0.278 (x 7.203) | 45 (0.044) | 0.122 | carbon-carbon lyase activity | amd b |
| 175 | GO:0007638 | P | 4, 5, | 1 | 0.031 (x 32.415) | 5 (0.200) | 0.123 | mechanosensory behavior | sda |
| 176 | GO:0031012 | C | 2, | 2 | 0.278 (x 7.203) | 45 (0.044) | 0.123 | extracellular matrix | Mmp2 NetA |
| 177 | GO:0017032 | F | 7, 8, 9, | 1 | 0.031 (x 32.415) | 5 (0.200) | 0.123 | potassium:amino acid symporter activity | CG1698 |
| 178 | GO:0005578 | C | 3, 4, | 2 | 0.278 (x 7.203) | 45 (0.044) | 0.124 | extracellular matrix (sensu Metazoa) | Mmp2 NetA |
| 179 | GO:0046663 | P | 6, 7, 8, | 1 | 0.031 (x 32.415) | 5 (0.200) | 0.124 | dorsal closure, leading edge cell differentiation | ush |
| 180 | GO:0006807 | P | 4, | 6 | 2.412 (x 2.487) | 391 (0.015) | 0.126 | nitrogen compound metabolism | CG15093 CG1698 CG9307 Sox15 amd b |
| 181 | GO:0006519 | P | 5, | 5 | 1.808 (x 2.766) | 293 (0.017) | 0.131 | amino acid and derivative metabolism | CG15093 CG1698 Sox15 amd b |
| 182 | GO:0006139 | P | 5, | 17 | 10.927 (x 1.556) | 1771 (0.010) | 0.132 | nucleobase, nucleoside, nucleotide and nucleic acid metabolism | CG11835 CG15093 CG4914 Doc1 Doc2 Doc3 Dr Sox15 ap b eya kn nvy pdm2 toe tup ush |
| 183 | GO:0019199 | F | 5, 7, | 2 | 0.302 (x 6.615) | 49 (0.041) | 0.132 | transmembrane receptor protein kinase activity | dnt drl |
| 184 | GO:0007519 | P | 5, | 2 | 0.302 (x 6.615) | 49 (0.041) | 0.133 | striated muscle development | if rost |
| 185 | GO:0045198 | P | 6, 7, 8, 9, | 1 | 0.037 (x 27.012) | 6 (0.167) | 0.133 | establishment of epithelial cell polarity | ds |
| 186 | GO:0042688 | P | 5, 7, | 1 | 0.037 (x 27.012) | 6 (0.167) | 0.134 | crystal cell differentiation | ush |
| 187 | GO:0030239 | P | 7, 8, 10, 11, | 1 | 0.037 (x 27.012) | 6 (0.167) | 0.134 | myofibril assembly | if |
| 188 | GO:0016789 | F | 5, | 3 | 0.740 (x 4.052) | 120 (0.025) | 0.135 | carboxylic ester hydrolase activity | CG4382 CG5966 alpha-Est5 |
| 189 | GO:0048190 | P | 6, 7, | 1 | 0.037 (x 27.012) | 6 (0.167) | 0.135 | wing disc dorsal/ventral pattern formation | ap |
| 190 | GO:0004034 | F | 6, | 1 | 0.037 (x 27.012) | 6 (0.167) | 0.136 | aldose 1-epimerase activity | BG:DS00797.2 |
| 191 | GO:0045317 | P | 7, 8, 9, 10, | 1 | 0.037 (x 27.012) | 6 (0.167) | 0.136 | equator specification | ds |
| 192 | GO:0042044 | P | 5, 6, | 1 | 0.037 (x 27.012) | 6 (0.167) | 0.137 | fluid transport | Drip |
| 193 | GO:0015250 | F | 4, 5, | 1 | 0.037 (x 27.012) | 6 (0.167) | 0.138 | water channel activity | Drip |
| 194 | GO:0030859 | P | 5, 6, | 1 | 0.037 (x 27.012) | 6 (0.167) | 0.139 | polarized epithelial cell differentiation | ds |
| 195 | GO:0042417 | P | 6, 8, 9, | 1 | 0.037 (x 27.012) | 6 (0.167) | 0.139 | dopamine metabolism | amd |
| 196 | GO:0035193 | P | 5, 6, | 1 | 0.043 (x 23.153) | 7 (0.143) | 0.145 | central nervous system remodeling (sensu Insecta) | rpr |
| 197 | GO:0008011 | F | 5, | 1 | 0.043 (x 23.153) | 7 (0.143) | 0.146 | structural constituent of pupal cuticle (sensu Insecta) | Edg91 |
| 198 | GO:0016337 | P | 4, | 3 | 0.765 (x 3.921) | 124 (0.024) | 0.146 | cell-cell adhesion | NetA ds if |
| 199 | GO:0017147 | F | 4, | 1 | 0.043 (x 23.153) | 7 (0.143) | 0.146 | Wnt-protein binding | drl |
| 200 | GO:0035171 | P | 6, 7, 8, 9, | 1 | 0.043 (x 23.153) | 7 (0.143) | 0.147 | lamellocyte differentiation | kn |
| 201 | GO:0004857 | F | 3, | 3 | 0.784 (x 3.828) | 127 (0.024) | 0.147 | enzyme inhibitor activity | CG5392 CG6680 TepII |
| 202 | GO:0035288 | P | 6, 7, | 1 | 0.043 (x 23.153) | 7 (0.143) | 0.148 | anterior head segmentation | kn |
| 203 | GO:0016199 | P | 6, 8, 9, 11, 12, 14, | 1 | 0.043 (x 23.153) | 7 (0.143) | 0.148 | axon midline choice point recognition | drl |
| 204 | GO:0009081 | P | 7, 8, | 1 | 0.043 (x 23.153) | 7 (0.143) | 0.149 | branched chain family amino acid metabolism | CG15093 |
| 205 | GO:0035089 | P | 7, 8, | 1 | 0.043 (x 23.153) | 7 (0.143) | 0.15 | establishment of apical/basal cell polarity | ds |
| 206 | GO:0046661 | P | 4, | 1 | 0.043 (x 23.153) | 7 (0.143) | 0.151 | male sex differentiation | eya |
| 207 | GO:0051707 | P | 4, | 3 | 0.808 (x 3.712) | 131 (0.023) | 0.158 | response to other organism | CG9358 TepII kn |
| 208 | GO:0009886 | P | 4, | 1 | 0.049 (x 20.259) | 8 (0.125) | 0.162 | post-embryonic morphogenesis | rpr |
| 209 | GO:0002009 | P | 4, | 3 | 0.821 (x 3.656) | 133 (0.023) | 0.162 | morphogenesis of an epithelium | ds tup ush |
| 210 | GO:0005372 | F | 3, | 1 | 0.049 (x 20.259) | 8 (0.125) | 0.163 | water transporter activity | Drip |
| 211 | GO:0007507 | P | 5, | 2 | 0.376 (x 5.314) | 61 (0.033) | 0.173 | heart development | Doc1 ush |
| 212 | GO:0035161 | P | 5, 6, | 1 | 0.056 (x 18.008) | 9 (0.111) | 0.174 | imaginal disc lineage restriction | ap |
| 213 | GO:0050878 | P | 4, | 1 | 0.056 (x 18.008) | 9 (0.111) | 0.175 | regulation of body fluids | Drip |
| 214 | GO:0007380 | P | 5, 6, 7, | 1 | 0.056 (x 18.008) | 9 (0.111) | 0.176 | specification of segmental identity, head | kn |
| 215 | GO:0007632 | P | 4, 6, | 1 | 0.056 (x 18.008) | 9 (0.111) | 0.176 | visual behavior | b |
| 216 | GO:0043231 | C | 4, 5, 6, 7, | 20 | 14.234 (x 1.405) | 2307 (0.009) | 0.177 | intracellular membrane-bound organelle | CG11835 CG15093 CG3132 CG4914 Doc1 Doc2 Doc3 Dr GV1 Sox15 ap eya kn nvy pdm2 rpr toe tup ush vg |
| 217 | GO:0043227 | C | 3, | 20 | 14.247 (x 1.404) | 2309 (0.009) | 0.177 | membrane-bound organelle | CG11835 CG15093 CG3132 CG4914 Doc1 Doc2 Doc3 Dr GV1 Sox15 ap eya kn nvy pdm2 rpr toe tup ush vg |
| 218 | GO:0035072 | P | 7, 8, 9, 10, 11, | 1 | 0.056 (x 18.008) | 9 (0.111) | 0.177 | ecdysone-mediated induction of salivary gland cell autophagic cell death | rpr |
| 219 | GO:0045185 | P | 5, | 1 | 0.056 (x 18.008) | 9 (0.111) | 0.178 | maintenance of protein localization | if |
| 220 | GO:0008431 | F | 4, | 1 | 0.056 (x 18.008) | 9 (0.111) | 0.179 | vitamin E binding | CG2663 |
| 221 | GO:0006012 | P | 8, 9, | 1 | 0.056 (x 18.008) | 9 (0.111) | 0.18 | galactose metabolism | BG:DS00797.2 |
| 222 | GO:0009612 | P | 4, | 1 | 0.062 (x 16.207) | 10 (0.100) | 0.183 | response to mechanical stimulus | sda |
| 223 | GO:0035168 | P | 5, 6, 7, 8, | 1 | 0.062 (x 16.207) | 10 (0.100) | 0.184 | lymph gland hemocyte differentiation (sensu Arthropoda) | kn |
| 224 | GO:0001746 | P | 7, 8, | 1 | 0.062 (x 16.207) | 10 (0.100) | 0.184 | Bolwig's organ morphogenesis | eya |
| 225 | GO:0001737 | P | 6, 7, 8, 9, 10, 11, | 1 | 0.062 (x 16.207) | 10 (0.100) | 0.185 | establishment of wing hair orientation | ds |
| 226 | GO:0008305 | C | 4, 5, 6, 7, 8, 9, | 1 | 0.062 (x 16.207) | 10 (0.100) | 0.186 | integrin complex | if |
| 227 | GO:0030097 | P | 5, | 2 | 0.395 (x 5.065) | 64 (0.031) | 0.186 | hemopoiesis | kn ush |
| 228 | GO:0016857 | F | 5, | 1 | 0.062 (x 16.207) | 10 (0.100) | 0.187 | racemase and epimerase activity, acting on carbohydrates and derivatives | BG:DS00797.2 |
| 229 | GO:0030010 | P | 6, 7, | 1 | 0.062 (x 16.207) | 10 (0.100) | 0.188 | establishment of cell polarity | ds |
| 230 | GO:0035078 | P | 6, 7, 8, 9, 10, | 1 | 0.062 (x 16.207) | 10 (0.100) | 0.188 | induction of programmed cell death by ecdysone | rpr |
| 231 | GO:0007157 | P | 5, | 1 | 0.068 (x 14.734) | 11 (0.091) | 0.196 | heterophilic cell adhesion | if |
| 232 | GO:0012501 | P | 5, | 4 | 1.530 (x 2.614) | 248 (0.016) | 0.196 | programmed cell death | CG3132 CG6680 Mmp2 rpr |
| 233 | GO:0048569 | P | 4, | 1 | 0.068 (x 14.734) | 11 (0.091) | 0.197 | post-embryonic organ development | kn |
| 234 | GO:0035081 | P | 8, 9, | 1 | 0.068 (x 14.734) | 11 (0.091) | 0.198 | induction of programmed cell death by hormones | rpr |
| 235 | GO:0035167 | P | 5, 6, 7, | 1 | 0.068 (x 14.734) | 11 (0.091) | 0.199 | lymph gland hemopoiesis (sensu Arthropoda) | kn |
| 236 | GO:0035166 | P | 4, 5, 6, | 1 | 0.068 (x 14.734) | 11 (0.091) | 0.2 | post-embryonic hemopoiesis | kn |
| 237 | GO:0008219 | P | 4, | 4 | 1.543 (x 2.593) | 250 (0.016) | 0.2 | cell death | CG3132 CG6680 Mmp2 rpr |
| 238 | GO:0006740 | P | 10, 11, | 1 | 0.074 (x 13.506) | 12 (0.083) | 0.202 | NADPH regeneration | CG15093 |
| 239 | GO:0016198 | P | 5, 7, 8, 10, 11, 13, | 1 | 0.074 (x 13.506) | 12 (0.083) | 0.203 | axon choice point recognition | drl |
| 240 | GO:0016829 | F | 3, | 3 | 0.969 (x 3.097) | 157 (0.019) | 0.204 | lyase activity | CAH1 amd b |
| 241 | GO:0006739 | P | 9, 10, | 1 | 0.074 (x 13.506) | 12 (0.083) | 0.204 | NADP metabolism | CG15093 |
| 242 | GO:0007426 | P | 5, | 1 | 0.074 (x 13.506) | 12 (0.083) | 0.205 | tracheal outgrowth (sensu Insecta) | if |
| 243 | GO:0006098 | P | 8, 10, 11, 12, | 1 | 0.074 (x 13.506) | 12 (0.083) | 0.205 | pentose-phosphate shunt | CG15093 |
| 244 | GO:0008045 | P | 7, 8, 10, 11, 13, | 1 | 0.074 (x 13.506) | 12 (0.083) | 0.206 | motor axon guidance | tup |
| 245 | GO:0048102 | P | 6, | 2 | 0.438 (x 4.565) | 71 (0.028) | 0.207 | autophagic cell death | CG3132 rpr |
| 246 | GO:0007392 | P | 7, | 1 | 0.074 (x 13.506) | 12 (0.083) | 0.207 | initiation of dorsal closure | ush |
| 247 | GO:0035286 | P | 5, 6, 7, | 1 | 0.080 (x 12.467) | 13 (0.077) | 0.207 | leg segmentation | ap |
| 248 | GO:0035071 | P | 7, | 2 | 0.438 (x 4.565) | 71 (0.028) | 0.208 | salivary gland cell autophagic cell death | CG3132 rpr |
| 249 | GO:0006968 | P | 5, 6, | 1 | 0.074 (x 13.506) | 12 (0.083) | 0.208 | cellular defense response | kn |
| 250 | GO:0005416 | F | 6, 7, 8, | 1 | 0.080 (x 12.467) | 13 (0.077) | 0.208 | cation:amino acid symporter activity | CG1698 |
| 251 | GO:0035070 | P | 6, | 2 | 0.438 (x 4.565) | 71 (0.028) | 0.209 | salivary gland histolysis | CG3132 rpr |
| 252 | GO:0043292 | C | 5, 6, 7, 8, | 1 | 0.080 (x 12.467) | 13 (0.077) | 0.209 | contractile fiber | if |
| 253 | GO:0035285 | P | 4, 5, | 1 | 0.080 (x 12.467) | 13 (0.077) | 0.21 | appendage segmentation | ap |
| 254 | GO:0009617 | P | 5, | 2 | 0.457 (x 4.380) | 74 (0.027) | 0.21 | response to bacterium | CG9358 TepII |
| 255 | GO:0042133 | P | 5, 8, | 1 | 0.080 (x 12.467) | 13 (0.077) | 0.211 | neurotransmitter metabolism | amd |
| 256 | GO:0009953 | P | 4, | 2 | 0.457 (x 4.380) | 74 (0.027) | 0.211 | dorsal/ventral pattern formation | Dr ap |
| 257 | GO:0006066 | P | 5, | 3 | 0.993 (x 3.020) | 161 (0.019) | 0.211 | alcohol metabolism | BG:DS00797.2 CG15093 amd |
| 258 | GO:0006584 | P | 7, 8, | 1 | 0.080 (x 12.467) | 13 (0.077) | 0.212 | catecholamine metabolism | amd |
| 259 | GO:0007601 | P | 5, 7, | 2 | 0.457 (x 4.380) | 74 (0.027) | 0.212 | visual perception | CG11835 eya |
| 260 | GO:0050953 | P | 4, 6, | 2 | 0.457 (x 4.380) | 74 (0.027) | 0.212 | sensory perception of light stimulus | CG11835 eya |
| 261 | GO:0007391 | P | 6, | 2 | 0.469 (x 4.265) | 76 (0.026) | 0.213 | dorsal closure | tup ush |
| 262 | GO:0048534 | P | 4, | 2 | 0.457 (x 4.380) | 74 (0.027) | 0.213 | hemopoietic or lymphoid organ development | kn ush |
| 263 | GO:0007474 | P | 7, 8, 9, | 1 | 0.086 (x 11.577) | 14 (0.071) | 0.215 | wing vein specification | kn |
| 264 | GO:0007479 | P | 6, 7, | 1 | 0.086 (x 11.577) | 14 (0.071) | 0.215 | leg disc proximal/distal pattern formation | ap |
| 265 | GO:0035317 | P | 7, 8, 9, 10, | 1 | 0.086 (x 11.577) | 14 (0.071) | 0.216 | wing hair organization and biogenesis | ds |
| 266 | GO:0018958 | P | 6, | 1 | 0.086 (x 11.577) | 14 (0.071) | 0.217 | phenol metabolism | amd |
| 267 | GO:0006769 | P | 8, 9, | 1 | 0.086 (x 11.577) | 14 (0.071) | 0.218 | nicotinamide metabolism | CG15093 |
| 268 | GO:0035223 | P | 5, 6, | 1 | 0.086 (x 11.577) | 14 (0.071) | 0.219 | leg disc pattern formation | ap |
| 269 | GO:0007475 | P | 8, 9, 10, | 1 | 0.086 (x 11.577) | 14 (0.071) | 0.219 | apposition of dorsal and ventral wing surfaces | if |
| 270 | GO:0016854 | F | 4, | 1 | 0.086 (x 11.577) | 14 (0.071) | 0.22 | racemase and epimerase activity | BG:DS00797.2 |
| 271 | GO:0004568 | F | 6, | 1 | 0.086 (x 11.577) | 14 (0.071) | 0.221 | chitinase activity | CG9307 |
| 272 | GO:0019318 | P | 7, 8, | 2 | 0.494 (x 4.052) | 80 (0.025) | 0.224 | hexose metabolism | BG:DS00797.2 CG15093 |
| 273 | GO:0005214 | F | 4, | 2 | 0.500 (x 4.002) | 81 (0.025) | 0.225 | structural constituent of cuticle (sensu Insecta) | CG8502 Edg91 |
| 274 | GO:0035075 | P | 5, 6, 7, | 1 | 0.093 (x 10.805) | 15 (0.067) | 0.225 | response to ecdysone | rpr |
| 275 | GO:0048545 | P | 5, 6, | 1 | 0.093 (x 10.805) | 15 (0.067) | 0.226 | response to steroid hormone stimulus | rpr |
| 276 | GO:0035289 | P | 6, 7, | 1 | 0.093 (x 10.805) | 15 (0.067) | 0.227 | posterior head segmentation | kn |
| 277 | GO:0019362 | P | 7, 8, | 1 | 0.093 (x 10.805) | 15 (0.067) | 0.228 | pyridine nucleotide metabolism | CG15093 |
| 278 | GO:0016331 | P | 5, | 2 | 0.506 (x 3.953) | 82 (0.024) | 0.228 | morphogenesis of embryonic epithelium | tup ush |
| 279 | GO:0007456 | P | 6, | 3 | 1.067 (x 2.811) | 173 (0.017) | 0.228 | eye development (sensu Endopterygota) | ds eya ush |
| 280 | GO:0006367 | P | 9, | 2 | 0.518 (x 3.859) | 84 (0.024) | 0.231 | transcription initiation from RNA polymerase II promoter | CG11835 kn |
| 281 | GO:0016616 | F | 5, | 2 | 0.518 (x 3.859) | 84 (0.024) | 0.232 | oxidoreductase activity, acting on the CH-OH group of donors, NAD or NADP as acceptor | CG10962 CG15093 |
| 282 | GO:0007520 | P | 7, 8, 10, 11, | 1 | 0.099 (x 10.130) | 16 (0.062) | 0.232 | myoblast fusion | rost |
| 283 | GO:0019752 | P | 6, | 5 | 2.419 (x 2.067) | 392 (0.013) | 0.232 | carboxylic acid metabolism | CG15093 CG1698 Sox15 amd b |
| 284 | GO:0048523 | P | 4, | 4 | 1.728 (x 2.315) | 280 (0.014) | 0.232 | negative regulation of cellular process | Mmp2 eya rpr ush |
| 285 | GO:0004089 | F | 6, | 1 | 0.099 (x 10.130) | 16 (0.062) | 0.233 | carbonate dehydratase activity | CAH1 |
| 286 | GO:0006082 | P | 5, | 5 | 2.419 (x 2.067) | 392 (0.013) | 0.233 | organic acid metabolism | CG15093 CG1698 Sox15 amd b |
| 287 | GO:0009725 | P | 4, 5, | 1 | 0.099 (x 10.130) | 16 (0.062) | 0.233 | response to hormone stimulus | rpr |
| 288 | GO:0006915 | P | 6, | 3 | 1.098 (x 2.732) | 178 (0.017) | 0.234 | apoptosis | CG6680 Mmp2 rpr |
| 289 | GO:0044421 | C | 2, 3, | 2 | 0.524 (x 3.813) | 85 (0.024) | 0.234 | extracellular region part | Mmp2 NetA |
| 290 | GO:0004713 | F | 7, | 2 | 0.524 (x 3.813) | 85 (0.024) | 0.235 | protein-tyrosine kinase activity | dnt drl |
| 291 | GO:0009913 | P | 4, 6, 7, | 1 | 0.105 (x 9.534) | 17 (0.059) | 0.235 | epidermal cell differentiation | ds |
| 292 | GO:0001654 | P | 5, | 3 | 1.117 (x 2.686) | 181 (0.017) | 0.235 | eye development | ds eya ush |
| 293 | GO:0007280 | P | 6, 7, 8, | 1 | 0.105 (x 9.534) | 17 (0.059) | 0.236 | pole cell migration | eya |
| 294 | GO:0050874 | P | 3, | 9 | 5.553 (x 1.621) | 900 (0.010) | 0.236 | organismal physiological process | CG11835 CG9358 Drip NetA TepII amd b eya kn |
| 295 | GO:0006352 | P | 8, | 2 | 0.531 (x 3.769) | 86 (0.023) | 0.236 | transcription initiation | CG11835 kn |
| 296 | GO:0035315 | P | 5, 7, 8, | 1 | 0.105 (x 9.534) | 17 (0.059) | 0.236 | hair cell differentiation | ds |
| 297 | GO:0035316 | P | 6, 7, 8, 9, | 1 | 0.105 (x 9.534) | 17 (0.059) | 0.237 | trichome organization and biogenesis (sensu Insecta) | ds |
| 298 | GO:0005328 | F | 4, 7, 9, | 1 | 0.105 (x 9.534) | 17 (0.059) | 0.238 | neurotransmitter:sodium symporter activity | CG1698 |
| 299 | GO:0048730 | P | 5, 6, | 1 | 0.105 (x 9.534) | 17 (0.059) | 0.239 | epidermis morphogenesis | ds |
| 300 | GO:0003676 | F | 3, | 15 | 10.754 (x 1.395) | 1743 (0.009) | 0.242 | nucleic acid binding | CG11835 CG4914 Doc1 Doc2 Doc3 Dr GV1 Sox15 ap kn nvy pdm2 toe tup ush |
| 301 | GO:0051235 | P | 4, | 1 | 0.111 (x 9.004) | 18 (0.056) | 0.242 | maintenance of localization | if |
| 302 | GO:0045197 | P | 7, 8, | 1 | 0.111 (x 9.004) | 18 (0.056) | 0.243 | establishment and/or maintenance of epithelial cell polarity | ds |
| 303 | GO:0035287 | P | 5, 6, | 1 | 0.111 (x 9.004) | 18 (0.056) | 0.244 | head segmentation | kn |
| 304 | GO:0007449 | P | 5, 6, | 1 | 0.111 (x 9.004) | 18 (0.056) | 0.245 | proximal/distal pattern formation, imaginal disc | ap |
| 305 | GO:0009954 | P | 4, | 1 | 0.111 (x 9.004) | 18 (0.056) | 0.245 | proximal/distal pattern formation | ap |
| 306 | GO:0035088 | P | 6, 7, | 1 | 0.117 (x 8.530) | 19 (0.053) | 0.254 | establishment and/or maintenance of apical/basal cell polarity | ds |
| 307 | GO:0007610 | P | 3, | 3 | 1.178 (x 2.546) | 191 (0.016) | 0.259 | behavior | b drl sda |
| 308 | GO:0006733 | P | 7, | 1 | 0.123 (x 8.104) | 20 (0.050) | 0.259 | oxidoreduction coenzyme metabolism | CG15093 |
| 309 | GO:0008374 | F | 7, | 1 | 0.123 (x 8.104) | 20 (0.050) | 0.26 | O-acyltransferase activity | CG5397 |
| 310 | GO:0016284 | F | 7, | 1 | 0.123 (x 8.104) | 20 (0.050) | 0.261 | alanine aminopeptidase activity | sda |
| 311 | GO:0005326 | F | 3, | 1 | 0.123 (x 8.104) | 20 (0.050) | 0.261 | neurotransmitter transporter activity | CG1698 |
| 312 | GO:0048519 | P | 3, | 4 | 1.882 (x 2.126) | 305 (0.013) | 0.262 | negative regulation of biological process | Mmp2 eya rpr ush |
| 313 | GO:0016318 | P | 7, 8, 9, 10, | 1 | 0.123 (x 8.104) | 20 (0.050) | 0.262 | ommatidial rotation | ds |
| 314 | GO:0008632 | P | 7, | 1 | 0.123 (x 8.104) | 20 (0.050) | 0.263 | apoptotic program | rpr |
| 315 | GO:0004179 | F | 7, 8, | 1 | 0.123 (x 8.104) | 20 (0.050) | 0.264 | membrane alanyl aminopeptidase activity | sda |
| 316 | GO:0006730 | P | 5, | 1 | 0.123 (x 8.104) | 20 (0.050) | 0.265 | one-carbon compound metabolism | CAH1 |
| 317 | GO:0005887 | C | 6, 7, 8, | 3 | 1.209 (x 2.481) | 196 (0.015) | 0.265 | integral to plasma membrane | CG1698 ds if |
| 318 | GO:0008544 | P | 5, | 1 | 0.136 (x 7.367) | 22 (0.045) | 0.265 | epidermis development | ds |
| 319 | GO:0031226 | C | 5, 6, 7, | 3 | 1.222 (x 2.456) | 198 (0.015) | 0.266 | intrinsic to plasma membrane | CG1698 ds if |
| 320 | GO:0016021 | C | 5, 6, 7, | 9 | 5.825 (x 1.545) | 944 (0.010) | 0.266 | integral to membrane | CG11835 CG1698 Drip Tsp42Ei dnt drl ds if rost |
| 321 | GO:0008037 | P | 3, | 1 | 0.136 (x 7.367) | 22 (0.045) | 0.266 | cell recognition | drl |
| 322 | GO:0004553 | F | 5, | 2 | 0.605 (x 3.308) | 98 (0.020) | 0.267 | hydrolase activity, hydrolyzing O-glycosyl compounds | CG3132 CG9307 |
| 323 | GO:0017148 | P | 7, 8, 9, | 1 | 0.130 (x 7.718) | 21 (0.048) | 0.267 | negative regulation of protein biosynthesis | rpr |
| 324 | GO:0031327 | P | 7, | 1 | 0.136 (x 7.367) | 22 (0.045) | 0.267 | negative regulation of cellular biosynthesis | rpr |
| 325 | GO:0004872 | F | 3, | 6 | 3.412 (x 1.758) | 553 (0.011) | 0.267 | receptor activity | CG11835 dnt drl ds if ush |
| 326 | GO:0031224 | C | 4, 5, 6, | 9 | 5.843 (x 1.540) | 947 (0.010) | 0.268 | intrinsic to membrane | CG11835 CG1698 Drip Tsp42Ei dnt drl ds if rost |
| 327 | GO:0016339 | P | 5, | 1 | 0.130 (x 7.718) | 21 (0.048) | 0.268 | calcium-dependent cell-cell adhesion | ds |
| 328 | GO:0006836 | P | 5, 6, | 1 | 0.136 (x 7.367) | 22 (0.045) | 0.268 | neurotransmitter transport | CG1698 |
| 329 | GO:0048542 | P | 5, | 1 | 0.130 (x 7.718) | 21 (0.048) | 0.269 | lymph gland development (sensu Arthropoda) | kn |
| 330 | GO:0008038 | P | 4, | 1 | 0.136 (x 7.367) | 22 (0.045) | 0.269 | neuron recognition | drl |
| 331 | GO:0009890 | P | 6, | 1 | 0.136 (x 7.367) | 22 (0.045) | 0.27 | negative regulation of biosynthesis | rpr |
| 332 | GO:0040003 | P | 8, | 1 | 0.136 (x 7.367) | 22 (0.045) | 0.27 | cuticle biosynthesis (sensu Insecta) | amd |
| 333 | GO:0009613 | P | 4, 5, | 2 | 0.617 (x 3.241) | 100 (0.020) | 0.271 | response to pest, pathogen or parasite | TepII kn |
| 334 | GO:0007592 | P | 7, | 1 | 0.136 (x 7.367) | 22 (0.045) | 0.271 | cuticle biosynthesis (sensu Protostomia and Nematoda) | amd |
| 335 | GO:0005996 | P | 6, 7, | 2 | 0.617 (x 3.241) | 100 (0.020) | 0.271 | monosaccharide metabolism | BG:DS00797.2 CG15093 |
| 336 | GO:0007427 | P | 5, 6, 7, | 1 | 0.136 (x 7.367) | 22 (0.045) | 0.272 | tracheal epithelial cell migration (sensu Insecta) | if |
| 337 | GO:0006961 | P | 7, 8, 9, | 1 | 0.142 (x 7.047) | 23 (0.043) | 0.275 | antibacterial humoral response (sensu Protostomia) | TepII |
| 338 | GO:0006767 | P | 6, | 1 | 0.142 (x 7.047) | 23 (0.043) | 0.276 | water-soluble vitamin metabolism | CG15093 |
| 339 | GO:0007494 | P | 5, | 1 | 0.148 (x 6.753) | 24 (0.042) | 0.282 | midgut development | if |
| 340 | GO:0009605 | P | 3, | 2 | 0.648 (x 3.087) | 105 (0.019) | 0.282 | response to external stimulus | kn sda |
| 341 | GO:0019842 | F | 3, | 1 | 0.148 (x 6.753) | 24 (0.042) | 0.283 | vitamin binding | CG2663 |
| 342 | GO:0044459 | C | 4, 5, 6, | 4 | 1.993 (x 2.007) | 323 (0.012) | 0.283 | plasma membrane part | CG1698 CG9358 ds if |
| 343 | GO:0004879 | F | 4, | 1 | 0.148 (x 6.753) | 24 (0.042) | 0.284 | ligand-dependent nuclear receptor activity | ush |
| 344 | GO:0000323 | C | 6, 7, 8, 9, | 1 | 0.154 (x 6.483) | 25 (0.040) | 0.285 | lytic vacuole | CG3132 |
| 345 | GO:0045137 | P | 4, | 1 | 0.154 (x 6.483) | 25 (0.040) | 0.286 | development of primary sexual characteristics | eya |
| 346 | GO:0007267 | P | 4, | 5 | 2.764 (x 1.809) | 448 (0.011) | 0.287 | cell-cell signaling | NetA amd b dnt drl |
| 347 | GO:0016798 | F | 4, | 2 | 0.660 (x 3.029) | 107 (0.019) | 0.287 | hydrolase activity, acting on glycosyl bonds | CG3132 CG9307 |
| 348 | GO:0048562 | P | 5, | 1 | 0.154 (x 6.483) | 25 (0.040) | 0.287 | embryonic organ morphogenesis | eya |
| 349 | GO:0008406 | P | 5, | 1 | 0.154 (x 6.483) | 25 (0.040) | 0.288 | gonad development | eya |
| 350 | GO:0042981 | P | 6, 7, | 2 | 0.673 (x 2.974) | 109 (0.018) | 0.288 | regulation of apoptosis | Mmp2 rpr |
| 351 | GO:0007613 | P | 5, | 1 | 0.154 (x 6.483) | 25 (0.040) | 0.289 | memory | drl |
| 352 | GO:0007265 | P | 7, | 1 | 0.154 (x 6.483) | 25 (0.040) | 0.29 | Ras protein signal transduction | CG9358 |
| 353 | GO:0005764 | C | 7, 8, 9, 10, | 1 | 0.154 (x 6.483) | 25 (0.040) | 0.29 | lysosome | CG3132 |
| 354 | GO:0001709 | P | 5, | 2 | 0.679 (x 2.947) | 110 (0.018) | 0.291 | cell fate determination | Dr ush |
| 355 | GO:0048232 | P | 5, | 2 | 0.685 (x 2.920) | 111 (0.018) | 0.292 | male gamete generation | CG9358 eya |
| 356 | GO:0016788 | F | 4, | 5 | 2.807 (x 1.781) | 455 (0.011) | 0.292 | hydrolase activity, acting on ester bonds | CG4382 CG5397 CG5966 alpha-Est5 eya |
| 357 | GO:0005272 | F | 6, 7, | 1 | 0.160 (x 6.234) | 26 (0.038) | 0.292 | sodium channel activity | rpk |
| 358 | GO:0007283 | P | 6, | 2 | 0.685 (x 2.920) | 111 (0.018) | 0.292 | spermatogenesis | CG9358 eya |
| 359 | GO:0019992 | F | 4, | 1 | 0.160 (x 6.234) | 26 (0.038) | 0.293 | diacylglycerol binding | CG9358 |
| 360 | GO:0044274 | P | 5, | 1 | 0.167 (x 6.003) | 27 (0.037) | 0.298 | organismal biosynthesis | amd |
| 361 | GO:0007419 | P | 4, 6, | 1 | 0.167 (x 6.003) | 27 (0.037) | 0.299 | ventral cord development | Dr |
| 362 | GO:0042335 | P | 6, | 1 | 0.167 (x 6.003) | 27 (0.037) | 0.3 | cuticle biosynthesis | amd |
| 363 | GO:0035110 | P | 6, | 1 | 0.173 (x 5.788) | 28 (0.036) | 0.307 | leg morphogenesis | ap |
| 364 | GO:0008270 | F | 6, | 6 | 3.714 (x 1.615) | 602 (0.010) | 0.311 | zinc ion binding | CAH1 Mmp2 ap sda tup ush |
| 365 | GO:0016614 | F | 4, | 2 | 0.728 (x 2.747) | 118 (0.017) | 0.312 | oxidoreductase activity, acting on CH-OH group of donors | CG10962 CG15093 |
| 366 | GO:0007156 | P | 5, | 1 | 0.179 (x 5.589) | 29 (0.034) | 0.312 | homophilic cell adhesion | ds |
| 367 | GO:0014017 | P | 5, 8, | 1 | 0.179 (x 5.589) | 29 (0.034) | 0.313 | neuroblast fate commitment | Dr |
| 368 | GO:0014016 | P | 4, 7, | 1 | 0.179 (x 5.589) | 29 (0.034) | 0.314 | neuroblast differentiation | Dr |
| 369 | GO:0043067 | P | 5, 6, | 2 | 0.734 (x 2.724) | 119 (0.017) | 0.314 | regulation of programmed cell death | Mmp2 rpr |
| 370 | GO:0035108 | P | 5, | 1 | 0.179 (x 5.589) | 29 (0.034) | 0.315 | limb morphogenesis | ap |
| 371 | GO:0048646 | P | 4, | 1 | 0.185 (x 5.402) | 30 (0.033) | 0.315 | anatomical structure formation | eya |
| 372 | GO:0007400 | P | 6, 9, | 1 | 0.179 (x 5.589) | 29 (0.034) | 0.316 | neuroblast fate determination | Dr |
| 373 | GO:0004725 | F | 8, | 1 | 0.185 (x 5.402) | 30 (0.033) | 0.316 | protein tyrosine phosphatase activity | eya |
| 374 | GO:0007379 | P | 4, 5, | 1 | 0.179 (x 5.589) | 29 (0.034) | 0.316 | segment specification | kn |
| 375 | GO:0007423 | P | 4, | 3 | 1.419 (x 2.114) | 230 (0.013) | 0.317 | sensory organ development | ds eya ush |
| 376 | GO:0006576 | P | 6, 7, | 1 | 0.185 (x 5.402) | 30 (0.033) | 0.317 | biogenic amine metabolism | amd |
| 377 | GO:0030162 | P | 6, 7, 8, | 1 | 0.185 (x 5.402) | 30 (0.033) | 0.318 | regulation of proteolysis | rpr |
| 378 | GO:0006955 | P | 4, 5, | 2 | 0.753 (x 2.657) | 122 (0.016) | 0.32 | immune response | TepII kn |
| 379 | GO:0007417 | P | 5, | 2 | 0.753 (x 2.657) | 122 (0.016) | 0.321 | central nervous system development | Dr rpr |
| 380 | GO:0050877 | P | 4, | 6 | 3.801 (x 1.579) | 616 (0.010) | 0.326 | neurophysiological process | CG11835 CG9358 NetA amd b eya |
| 381 | GO:0050896 | P | 2, | 10 | 7.213 (x 1.386) | 1169 (0.009) | 0.326 | response to stimulus | CG11835 CG5397 CG9358 TepII b drl eya kn rpr sda |
| 382 | GO:0006206 | P | 7, | 1 | 0.197 (x 5.065) | 32 (0.031) | 0.33 | pyrimidine base metabolism | b |
| 383 | GO:0009611 | P | 4, | 1 | 0.204 (x 4.911) | 33 (0.030) | 0.336 | response to wounding | kn |
| 384 | GO:0044238 | P | 4, | 32 | 28.197 (x 1.135) | 4570 (0.007) | 0.336 | primary metabolism | BG:DS00797.2 CG11835 CG15093 CG1698 CG2663 CG3132 CG4914 CG5966 CG6680 CG9307 CG9358 Doc1 Doc2 Doc3 Dr Mmp2 Sox15 amd ap b dnt drl ds eya kn nvy pdm2 rpr sda toe tup ush |
| 385 | GO:0042067 | P | 6, 7, 8, 9, | 1 | 0.204 (x 4.911) | 33 (0.030) | 0.336 | establishment of ommatidial polarity (sensu Endopterygota) | ds |
| 386 | GO:0008152 | P | 3, | 35 | 31.202 (x 1.122) | 5057 (0.007) | 0.337 | metabolism | BG:DS00797.2 CAH1 CG10962 CG11835 CG15093 CG1698 CG2663 CG30069 CG3132 CG4914 CG5966 CG6680 CG9307 CG9358 Doc1 Doc2 Doc3 Dr Mmp2 Sox15 amd ap b dnt drl ds eya kn nvy pdm2 rpr sda toe tup ush |
| 387 | GO:0008354 | P | 5, 6, 7, | 1 | 0.204 (x 4.911) | 33 (0.030) | 0.337 | germ cell migration | eya |
| 388 | GO:0043226 | C | 2, | 20 | 16.560 (x 1.208) | 2684 (0.007) | 0.345 | organelle | CG11835 CG15093 CG3132 CG4914 Doc1 Doc2 Doc3 Dr GV1 Sox15 ap eya kn nvy pdm2 rpr toe tup ush vg |
| 389 | GO:0043229 | C | 3, 4, 5, 6, | 20 | 16.560 (x 1.208) | 2684 (0.007) | 0.346 | intracellular organelle | CG11835 CG15093 CG3132 CG4914 Doc1 Doc2 Doc3 Dr GV1 Sox15 ap eya kn nvy pdm2 rpr toe tup ush vg |
| 390 | GO:0048568 | P | 4, | 1 | 0.216 (x 4.631) | 35 (0.029) | 0.35 | embryonic organ development | eya |
| 391 | GO:0009628 | P | 3, | 4 | 2.295 (x 1.743) | 372 (0.011) | 0.351 | response to abiotic stimulus | b drl rpr sda |
| 392 | GO:0048749 | P | 7, | 2 | 0.821 (x 2.437) | 133 (0.015) | 0.353 | compound eye development (sensu Endopterygota) | ds eya |
| 393 | GO:0001745 | P | 7, 8, | 2 | 0.821 (x 2.437) | 133 (0.015) | 0.353 | compound eye morphogenesis (sensu Endopterygota) | ds eya |
| 394 | GO:0043235 | C | 3, | 1 | 0.222 (x 4.502) | 36 (0.028) | 0.354 | receptor complex | if |
| 395 | GO:0019897 | C | 5, 6, 7, | 1 | 0.222 (x 4.502) | 36 (0.028) | 0.355 | extrinsic to plasma membrane | CG9358 |
| 396 | GO:0006766 | P | 5, | 1 | 0.222 (x 4.502) | 36 (0.028) | 0.355 | vitamin metabolism | CG15093 |
| 397 | GO:0004806 | F | 7, | 1 | 0.228 (x 4.380) | 37 (0.027) | 0.362 | triacylglycerol lipase activity | CG5966 |
| 398 | GO:0044464 | C | 2, 3, | 32 | 28.481 (x 1.124) | 4616 (0.007) | 0.363 | cell part | CG11835 CG15093 CG1698 CG2663 CG3132 CG4914 CG9358 Doc1 Doc2 Doc3 Dr Drip GV1 Mmp2 Sox15 Tsp42Ei ap dnt drl ds eya if kn nvy pdm2 rost rpk rpr toe tup ush vg |
| 399 | GO:0044425 | C | 3, 4, 5, | 10 | 7.503 (x 1.333) | 1216 (0.008) | 0.364 | membrane part | CG11835 CG1698 CG9358 Drip Tsp42Ei dnt drl ds if rost |
| 400 | GO:0005623 | C | 2, | 32 | 28.481 (x 1.124) | 4616 (0.007) | 0.364 | cell | CG11835 CG15093 CG1698 CG2663 CG3132 CG4914 CG9358 Doc1 Doc2 Doc3 Dr Drip GV1 Mmp2 Sox15 Tsp42Ei ap dnt drl ds eya if kn nvy pdm2 rost rpk rpr toe tup ush vg |
| 401 | GO:0048066 | P | 3, | 1 | 0.234 (x 4.265) | 38 (0.026) | 0.365 | pigmentation during development | b |
| 402 | GO:0019731 | P | 6, 7, 8, | 1 | 0.234 (x 4.265) | 38 (0.026) | 0.366 | antibacterial humoral response | TepII |
| 403 | GO:0006916 | P | 8, 9, | 1 | 0.234 (x 4.265) | 38 (0.026) | 0.367 | anti-apoptosis | Mmp2 |
| 404 | GO:0043473 | P | 2, | 1 | 0.234 (x 4.265) | 38 (0.026) | 0.367 | pigmentation | b |
| 405 | GO:0007600 | P | 3, 5, | 3 | 1.592 (x 1.885) | 258 (0.012) | 0.367 | sensory perception | CG11835 CG9358 eya |
| 406 | GO:0008355 | P | 6, 7, | 1 | 0.241 (x 4.156) | 39 (0.026) | 0.368 | olfactory learning | drl |
| 407 | GO:0035218 | P | 5, | 1 | 0.241 (x 4.156) | 39 (0.026) | 0.369 | leg disc development | ap |
| 408 | GO:0048748 | P | 6, 7, | 2 | 0.870 (x 2.299) | 141 (0.014) | 0.369 | eye morphogenesis (sensu Endopterygota) | ds eya |
| 409 | GO:0005279 | F | 5, 6, | 1 | 0.241 (x 4.156) | 39 (0.026) | 0.37 | amino acid-polyamine transporter activity | CG1698 |
| 410 | GO:0015203 | F | 4, | 1 | 0.241 (x 4.156) | 39 (0.026) | 0.37 | polyamine transporter activity | CG1698 |
| 411 | GO:0008010 | F | 5, | 1 | 0.247 (x 4.052) | 40 (0.025) | 0.372 | structural constituent of larval cuticle (sensu Insecta) | CG8502 |
| 412 | GO:0007166 | P | 5, | 6 | 4.066 (x 1.476) | 659 (0.009) | 0.373 | cell surface receptor linked signal transduction | CG11835 CG9358 dnt drl tup ush |
| 413 | GO:0004177 | F | 6, | 1 | 0.247 (x 4.052) | 40 (0.025) | 0.373 | aminopeptidase activity | sda |
| 414 | GO:0006725 | P | 5, | 2 | 0.895 (x 2.235) | 145 (0.014) | 0.381 | aromatic compound metabolism | amd b |
| 415 | GO:0043066 | P | 7, 8, | 1 | 0.259 (x 3.859) | 42 (0.024) | 0.383 | negative regulation of apoptosis | Mmp2 |
| 416 | GO:0007455 | P | 6, 7, | 2 | 0.901 (x 2.220) | 146 (0.014) | 0.384 | eye-antennal disc morphogenesis | ds eya |
| 417 | GO:0005874 | C | 5, 6, 7, 8, 9, 10, | 1 | 0.259 (x 3.859) | 42 (0.024) | 0.384 | microtubule | vg |
| 418 | GO:0048592 | P | 5, 6, | 2 | 0.919 (x 2.175) | 149 (0.013) | 0.385 | eye morphogenesis | ds eya |
| 419 | GO:0016799 | F | 5, | 1 | 0.259 (x 3.859) | 42 (0.024) | 0.385 | hydrolase activity, hydrolyzing N-glycosyl compounds | CG9307 |
| 420 | GO:0007164 | P | 4, | 1 | 0.265 (x 3.769) | 43 (0.023) | 0.386 | establishment of tissue polarity | ds |
| 421 | GO:0043069 | P | 6, 7, | 1 | 0.259 (x 3.859) | 42 (0.024) | 0.386 | negative regulation of programmed cell death | Mmp2 |
| 422 | GO:0048729 | P | 4, | 1 | 0.265 (x 3.769) | 43 (0.023) | 0.387 | tissue morphogenesis | ds |
| 423 | GO:0046914 | F | 5, | 6 | 4.171 (x 1.439) | 676 (0.009) | 0.387 | transition metal ion binding | CAH1 Mmp2 ap sda tup ush |
| 424 | GO:0007612 | P | 5, | 1 | 0.265 (x 3.769) | 43 (0.023) | 0.387 | learning | drl |
| 425 | GO:0001736 | P | 5, 6, | 1 | 0.265 (x 3.769) | 43 (0.023) | 0.388 | establishment of planar polarity | ds |
| 426 | GO:0051248 | P | 6, 7, | 1 | 0.265 (x 3.769) | 43 (0.023) | 0.389 | negative regulation of protein metabolism | rpr |
| 427 | GO:0006814 | P | 8, 9, | 1 | 0.265 (x 3.769) | 43 (0.023) | 0.39 | sodium ion transport | rpk |
| 428 | GO:0042051 | P | 7, 8, 9, 10, | 1 | 0.271 (x 3.683) | 44 (0.023) | 0.391 | eye photoreceptor development (sensu Endopterygota) | eya |
| 429 | GO:0044262 | P | 6, | 3 | 1.697 (x 1.768) | 275 (0.011) | 0.392 | cellular carbohydrate metabolism | BG:DS00797.2 CG15093 CG9307 |
| 430 | GO:0051234 | P | 4, | 13 | 10.538 (x 1.234) | 1708 (0.008) | 0.394 | establishment of localization | CG1698 CG2663 Drip NetA ap dnt drl eya if nvy rpk tup vg |
| 431 | GO:0006960 | P | 7, 8, | 1 | 0.278 (x 3.602) | 45 (0.022) | 0.394 | antimicrobial humoral response (sensu Protostomia) | TepII |
| 432 | GO:0002164 | P | 4, | 1 | 0.278 (x 3.602) | 45 (0.022) | 0.395 | larval development | kn |
| 433 | GO:0042462 | P | 6, 7, 8, | 1 | 0.278 (x 3.602) | 45 (0.022) | 0.396 | eye photoreceptor cell development | eya |
| 434 | GO:0019898 | C | 4, 5, 6, | 1 | 0.278 (x 3.602) | 45 (0.022) | 0.397 | extrinsic to membrane | CG9358 |
| 435 | GO:0042440 | P | 5, | 1 | 0.284 (x 3.523) | 46 (0.022) | 0.398 | pigment metabolism | ush |
| 436 | GO:0006865 | P | 6, 7, 8, | 1 | 0.284 (x 3.523) | 46 (0.022) | 0.399 | amino acid transport | CG1698 |
| 437 | GO:0015837 | P | 5, 6, | 1 | 0.284 (x 3.523) | 46 (0.022) | 0.4 | amine transport | CG1698 |
| 438 | GO:0030030 | P | 5, 6, | 1 | 0.290 (x 3.448) | 47 (0.021) | 0.402 | cell projection organization and biogenesis | ds |
| 439 | GO:0008283 | P | 4, | 3 | 1.740 (x 1.724) | 282 (0.011) | 0.403 | cell proliferation | CG30069 ds toe |
| 440 | GO:0015171 | F | 4, 5, | 1 | 0.290 (x 3.448) | 47 (0.021) | 0.403 | amino acid transporter activity | CG1698 |
| 441 | GO:0006468 | P | 8, | 3 | 1.758 (x 1.706) | 285 (0.011) | 0.408 | protein amino acid phosphorylation | CG9358 dnt drl |
| 442 | GO:0009416 | P | 5, | 1 | 0.296 (x 3.377) | 48 (0.021) | 0.408 | response to light stimulus | b |
| 443 | GO:0035214 | P | 5, | 2 | 0.987 (x 2.026) | 160 (0.013) | 0.41 | eye-antennal disc development | ds eya |
| 444 | GO:0006575 | P | 6, | 1 | 0.302 (x 3.308) | 49 (0.020) | 0.412 | amino acid derivative metabolism | amd |
| 445 | GO:0007420 | P | 4, 6, | 1 | 0.302 (x 3.308) | 49 (0.020) | 0.412 | brain development | Dr |
| 446 | GO:0008594 | P | 6, 7, 8, | 1 | 0.302 (x 3.308) | 49 (0.020) | 0.413 | photoreceptor cell morphogenesis (sensu Endopterygota) | eya |
| 447 | GO:0007548 | P | 3, | 1 | 0.309 (x 3.241) | 50 (0.020) | 0.418 | sex differentiation | eya |
| 448 | GO:0046164 | P | 6, | 1 | 0.315 (x 3.178) | 51 (0.020) | 0.42 | alcohol catabolism | CG15093 |
| 449 | GO:0005622 | C | 3, 4, | 23 | 20.330 (x 1.131) | 3295 (0.007) | 0.421 | intracellular | CG11835 CG15093 CG2663 CG3132 CG4914 CG9358 Doc1 Doc2 Doc3 Dr GV1 Sox15 ap eya if kn nvy pdm2 rpr toe tup ush vg |
| 450 | GO:0046365 | P | 7, 8, | 1 | 0.315 (x 3.178) | 51 (0.020) | 0.421 | monosaccharide catabolism | CG15093 |
| 451 | GO:0006007 | P | 9, 10, | 1 | 0.315 (x 3.178) | 51 (0.020) | 0.422 | glucose catabolism | CG15093 |
| 452 | GO:0005275 | F | 3, | 1 | 0.321 (x 3.117) | 52 (0.019) | 0.423 | amine transporter activity | CG1698 |
| 453 | GO:0019320 | P | 8, 9, | 1 | 0.315 (x 3.178) | 51 (0.020) | 0.423 | hexose catabolism | CG15093 |
| 454 | GO:0004672 | F | 6, | 3 | 1.820 (x 1.648) | 295 (0.010) | 0.423 | protein kinase activity | CG9358 dnt drl |
| 455 | GO:0005941 | C | 3, | 1 | 0.321 (x 3.117) | 52 (0.019) | 0.423 | unlocalized protein complex | CG3132 |
| 456 | GO:0001738 | P | 5, | 1 | 0.321 (x 3.117) | 52 (0.019) | 0.424 | morphogenesis of a polarized epithelium | ds |
| 457 | GO:0042461 | P | 5, 6, 7, | 1 | 0.321 (x 3.117) | 52 (0.019) | 0.425 | photoreceptor cell development | eya |
| 458 | GO:0016065 | P | 6, 7, | 1 | 0.327 (x 3.058) | 53 (0.019) | 0.427 | humoral defense mechanism (sensu Protostomia) | TepII |
| 459 | GO:0042048 | P | 5, 6, | 1 | 0.327 (x 3.058) | 53 (0.019) | 0.428 | olfactory behavior | drl |
| 460 | GO:0009314 | P | 4, | 1 | 0.327 (x 3.058) | 53 (0.019) | 0.428 | response to radiation | b |
| 461 | GO:0007635 | P | 4, 5, | 1 | 0.333 (x 3.001) | 54 (0.019) | 0.43 | chemosensory behavior | drl |
| 462 | GO:0015849 | P | 5, 6, | 1 | 0.333 (x 3.001) | 54 (0.019) | 0.431 | organic acid transport | CG1698 |
| 463 | GO:0046942 | P | 6, 7, | 1 | 0.333 (x 3.001) | 54 (0.019) | 0.432 | carboxylic acid transport | CG1698 |
| 464 | GO:0007155 | P | 3, | 3 | 1.870 (x 1.605) | 303 (0.010) | 0.432 | cell adhesion | NetA ds if |
| 465 | GO:0005773 | C | 5, 6, 7, 8, | 1 | 0.333 (x 3.001) | 54 (0.019) | 0.432 | vacuole | CG3132 |
| 466 | GO:0016055 | P | 6, | 1 | 0.339 (x 2.947) | 55 (0.018) | 0.434 | Wnt receptor signaling pathway | drl |
| 467 | GO:0051179 | P | 3, | 13 | 10.927 (x 1.190) | 1771 (0.007) | 0.434 | localization | CG1698 CG2663 Drip NetA ap dnt drl eya if nvy rpk tup vg |
| 468 | GO:0016836 | F | 5, | 1 | 0.346 (x 2.894) | 56 (0.018) | 0.439 | hydro-lyase activity | CAH1 |
| 469 | GO:0005198 | F | 2, | 6 | 4.554 (x 1.318) | 738 (0.008) | 0.446 | structural molecule activity | CG7160 CG8502 Edg91 Mmp2 NetA vg |
| 470 | GO:0004812 | F | 6, | 1 | 0.358 (x 2.794) | 58 (0.017) | 0.447 | aminoacyl-tRNA ligase activity | Sox15 |
| 471 | GO:0007369 | P | 5, | 1 | 0.358 (x 2.794) | 58 (0.017) | 0.448 | gastrulation | CG9358 |
| 472 | GO:0044424 | C | 3, 4, 5, | 22 | 19.683 (x 1.118) | 3190 (0.007) | 0.448 | intracellular part | CG11835 CG15093 CG3132 CG4914 CG9358 Doc1 Doc2 Doc3 Dr GV1 Sox15 ap eya if kn nvy pdm2 rpr toe tup ush vg |
| 473 | GO:0043169 | F | 4, | 7 | 5.473 (x 1.279) | 887 (0.008) | 0.448 | cation binding | CAH1 Mmp2 ap ds sda tup ush |
| 474 | GO:0005386 | F | 3, | 4 | 2.795 (x 1.431) | 453 (0.009) | 0.448 | carrier activity | CG1698 CG2663 CG9358 Drip |
| 475 | GO:0044237 | P | 4, | 31 | 28.561 (x 1.085) | 4629 (0.007) | 0.448 | cellular metabolism | BG:DS00797.2 CAH1 CG11835 CG15093 CG1698 CG2663 CG4914 CG6680 CG9307 CG9358 Doc1 Doc2 Doc3 Dr Mmp2 Sox15 amd ap b dnt drl ds eya kn nvy pdm2 rpr sda toe tup ush |
| 476 | GO:0016875 | F | 4, | 1 | 0.358 (x 2.794) | 58 (0.017) | 0.448 | ligase activity, forming carbon-oxygen bonds | Sox15 |
| 477 | GO:0043039 | P | 8, 9, | 1 | 0.370 (x 2.701) | 60 (0.017) | 0.449 | tRNA aminoacylation | Sox15 |
| 478 | GO:0006790 | P | 5, | 1 | 0.364 (x 2.747) | 59 (0.017) | 0.449 | sulfur metabolism | b |
| 479 | GO:0016876 | F | 5, | 1 | 0.358 (x 2.794) | 58 (0.017) | 0.449 | ligase activity, forming aminoacyl-tRNA and related compounds | Sox15 |
| 480 | GO:0007611 | P | 4, | 1 | 0.370 (x 2.701) | 60 (0.017) | 0.45 | learning and/or memory | drl |
| 481 | GO:0051189 | P | 5, 7, | 1 | 0.370 (x 2.701) | 60 (0.017) | 0.451 | prosthetic group metabolism | CG2663 |
| 482 | GO:0006006 | P | 8, 9, | 1 | 0.370 (x 2.701) | 60 (0.017) | 0.452 | glucose metabolism | CG15093 |
| 483 | GO:0015268 | F | 4, | 2 | 1.123 (x 1.781) | 182 (0.011) | 0.452 | alpha-type channel activity | Drip rpk |
| 484 | GO:0006418 | P | 8, 9, 10, | 1 | 0.370 (x 2.701) | 60 (0.017) | 0.453 | tRNA aminoacylation for protein translation | Sox15 |
| 485 | GO:0015267 | F | 3, | 2 | 1.123 (x 1.781) | 182 (0.011) | 0.453 | channel or pore class transporter activity | Drip rpk |
| 486 | GO:0043038 | P | 7, 8, | 1 | 0.376 (x 2.657) | 61 (0.016) | 0.454 | amino acid activation | Sox15 |
| 487 | GO:0015370 | F | 6, 8, | 1 | 0.383 (x 2.614) | 62 (0.016) | 0.455 | solute:sodium symporter activity | CG1698 |
| 488 | GO:0008237 | F | 5, | 2 | 1.141 (x 1.752) | 185 (0.011) | 0.455 | metallopeptidase activity | Mmp2 sda |
| 489 | GO:0005635 | C | 4, 5, 6, 7, 8, 9, 10, | 1 | 0.383 (x 2.614) | 62 (0.016) | 0.456 | nuclear envelope | GV1 |
| 490 | GO:0048565 | P | 4, | 1 | 0.383 (x 2.614) | 62 (0.016) | 0.457 | gut development | if |
| 491 | GO:0006030 | P | 7, 8, 9, 10, | 1 | 0.389 (x 2.573) | 63 (0.016) | 0.457 | chitin metabolism | CG9307 |
| 492 | GO:0006917 | P | 8, 9, | 1 | 0.383 (x 2.614) | 62 (0.016) | 0.457 | induction of apoptosis | rpr |
| 493 | GO:0008061 | F | 5, | 1 | 0.389 (x 2.573) | 63 (0.016) | 0.458 | chitin binding | CG9307 |
| 494 | GO:0016835 | F | 4, | 1 | 0.383 (x 2.614) | 62 (0.016) | 0.458 | carbon-oxygen lyase activity | CAH1 |
| 495 | GO:0019730 | P | 6, 7, | 1 | 0.389 (x 2.573) | 63 (0.016) | 0.459 | antimicrobial humoral response | TepII |
| 496 | GO:0015294 | F | 5, 7, | 1 | 0.389 (x 2.573) | 63 (0.016) | 0.46 | solute:cation symporter activity | CG1698 |
| 497 | GO:0043065 | P | 7, 8, | 1 | 0.395 (x 2.532) | 64 (0.016) | 0.461 | positive regulation of apoptosis | rpr |
| 498 | GO:0007154 | P | 3, | 11 | 9.360 (x 1.175) | 1517 (0.007) | 0.462 | cell communication | CG11835 CG9358 NetA amd b dnt drl ds regucalcin tup ush |
| 499 | GO:0019722 | P | 7, | 1 | 0.395 (x 2.532) | 64 (0.016) | 0.462 | calcium-mediated signaling | regucalcin |
| 500 | GO:0019725 | P | 4, | 1 | 0.407 (x 2.456) | 66 (0.015) | 0.47 | cell homeostasis | Drip |
| 501 | GO:0008289 | F | 3, | 1 | 0.407 (x 2.456) | 66 (0.015) | 0.471 | lipid binding | CG9358 |
| 502 | GO:0016052 | P | 6, | 1 | 0.413 (x 2.419) | 67 (0.015) | 0.471 | carbohydrate catabolism | CG15093 |
| 503 | GO:0015276 | F | 5, 6, | 1 | 0.413 (x 2.419) | 67 (0.015) | 0.472 | ligand-gated ion channel activity | rpk |
| 504 | GO:0016043 | P | 4, | 10 | 8.509 (x 1.175) | 1379 (0.007) | 0.472 | cell organization and biogenesis | NetA ap dnt drl ds eya if nvy tup vg |
| 505 | GO:0044275 | P | 7, | 1 | 0.413 (x 2.419) | 67 (0.015) | 0.473 | cellular carbohydrate catabolism | CG15093 |
| 506 | GO:0030247 | F | 4, | 1 | 0.413 (x 2.419) | 67 (0.015) | 0.474 | polysaccharide binding | CG9307 |
| 507 | GO:0006950 | P | 3, | 3 | 2.061 (x 1.456) | 334 (0.009) | 0.474 | response to stress | TepII kn rpr |
| 508 | GO:0019748 | P | 4, | 1 | 0.420 (x 2.383) | 68 (0.015) | 0.475 | secondary metabolism | ush |
| 509 | GO:0042742 | P | 5, 6, | 1 | 0.426 (x 2.349) | 69 (0.014) | 0.479 | defense response to bacterium | TepII |
| 510 | GO:0046872 | F | 4, | 7 | 5.751 (x 1.217) | 932 (0.008) | 0.48 | metal ion binding | CAH1 Mmp2 ap ds sda tup ush |
| 511 | GO:0043167 | F | 3, | 7 | 5.751 (x 1.217) | 932 (0.008) | 0.481 | ion binding | CAH1 Mmp2 ap ds sda tup ush |
| 512 | GO:0012502 | P | 7, 8, | 1 | 0.432 (x 2.315) | 70 (0.014) | 0.481 | induction of programmed cell death | rpr |
| 513 | GO:0005975 | P | 5, | 4 | 2.992 (x 1.337) | 485 (0.008) | 0.482 | carbohydrate metabolism | BG:DS00797.2 CG15093 CG3132 CG9307 |
| 514 | GO:0007163 | P | 5, 6, | 1 | 0.438 (x 2.283) | 71 (0.014) | 0.483 | establishment and/or maintenance of cell polarity | ds |
| 515 | GO:0004222 | F | 6, | 1 | 0.438 (x 2.283) | 71 (0.014) | 0.484 | metalloendopeptidase activity | Mmp2 |
| 516 | GO:0006044 | P | 8, 9, | 1 | 0.438 (x 2.283) | 71 (0.014) | 0.485 | N-acetylglucosamine metabolism | CG9307 |
| 517 | GO:0016020 | C | 3, 4, | 13 | 11.513 (x 1.129) | 1866 (0.007) | 0.485 | membrane | CG11835 CG1698 CG9358 Drip GV1 Mmp2 Tsp42Ei dnt drl ds if rost rpk |
| 518 | GO:0006040 | P | 6, 7, | 1 | 0.444 (x 2.251) | 72 (0.014) | 0.486 | amino sugar metabolism | CG9307 |
| 519 | GO:0006041 | P | 7, 8, | 1 | 0.438 (x 2.283) | 71 (0.014) | 0.486 | glucosamine metabolism | CG9307 |
| 520 | GO:0008652 | P | 7, 8, | 1 | 0.444 (x 2.251) | 72 (0.014) | 0.487 | amino acid biosynthesis | b |
| 521 | GO:0046943 | F | 4, | 1 | 0.444 (x 2.251) | 72 (0.014) | 0.487 | carboxylic acid transporter activity | CG1698 |
| 522 | GO:0001751 | P | 6, 7, 8, 9, | 1 | 0.450 (x 2.220) | 73 (0.014) | 0.488 | eye photoreceptor cell differentiation (sensu Endopterygota) | eya |
| 523 | GO:0015293 | F | 6, | 1 | 0.450 (x 2.220) | 73 (0.014) | 0.489 | symporter activity | CG1698 |
| 524 | GO:0005342 | F | 3, | 1 | 0.457 (x 2.190) | 74 (0.014) | 0.492 | organic acid transporter activity | CG1698 |
| 525 | GO:0043068 | P | 6, 7, | 1 | 0.463 (x 2.161) | 75 (0.013) | 0.496 | positive regulation of programmed cell death | rpr |
| 526 | GO:0006417 | P | 6, 7, 8, | 1 | 0.463 (x 2.161) | 75 (0.013) | 0.497 | regulation of protein biosynthesis | rpr |
| 527 | GO:0008235 | F | 6, | 1 | 0.469 (x 2.133) | 76 (0.013) | 0.498 | metalloexopeptidase activity | sda |
| 528 | GO:0042592 | P | 3, | 1 | 0.469 (x 2.133) | 76 (0.013) | 0.499 | homeostasis | Drip |
| 529 | GO:0016773 | F | 5, | 3 | 2.203 (x 1.362) | 357 (0.008) | 0.5 | phosphotransferase activity, alcohol group as acceptor | CG9358 dnt drl |
| 530 | GO:0001754 | P | 5, 6, 7, | 1 | 0.469 (x 2.133) | 76 (0.013) | 0.5 | eye photoreceptor cell differentiation | eya |
| 531 | GO:0044264 | P | 6, 7, | 1 | 0.475 (x 2.105) | 77 (0.013) | 0.5 | cellular polysaccharide metabolism | CG9307 |
| 532 | GO:0030234 | F | 2, | 3 | 2.203 (x 1.362) | 357 (0.008) | 0.501 | enzyme regulator activity | CG5392 CG6680 TepII |
| 533 | GO:0009112 | P | 6, | 1 | 0.481 (x 2.078) | 78 (0.013) | 0.504 | nucleobase metabolism | b |
| 534 | GO:0006959 | P | 5, 6, | 1 | 0.481 (x 2.078) | 78 (0.013) | 0.505 | humoral immune response | TepII |
| 535 | GO:0009889 | P | 5, | 1 | 0.487 (x 2.052) | 79 (0.013) | 0.507 | regulation of biosynthesis | rpr |
| 536 | GO:0019226 | P | 5, | 3 | 2.240 (x 1.339) | 363 (0.008) | 0.508 | transmission of nerve impulse | NetA amd b |
| 537 | GO:0031326 | P | 6, | 1 | 0.487 (x 2.052) | 79 (0.013) | 0.508 | regulation of cellular biosynthesis | rpr |
| 538 | GO:0001871 | F | 3, | 1 | 0.494 (x 2.026) | 80 (0.013) | 0.51 | pattern binding | CG9307 |
| 539 | GO:0007165 | P | 4, | 9 | 7.966 (x 1.130) | 1291 (0.007) | 0.522 | signal transduction | CG11835 CG9358 NetA dnt drl ds regucalcin tup ush |
| 540 | GO:0005654 | C | 5, 6, 7, 8, 9, 10, 11, | 2 | 1.382 (x 1.447) | 224 (0.009) | 0.523 | nucleoplasm | ap toe |
| 541 | GO:0009607 | P | 3, | 4 | 3.221 (x 1.242) | 522 (0.008) | 0.523 | response to biotic stimulus | CG5397 CG9358 TepII kn |
| 542 | GO:0044271 | P | 5, 6, | 1 | 0.518 (x 1.929) | 84 (0.012) | 0.525 | nitrogen compound biosynthesis | b |
| 543 | GO:0008360 | P | 5, 6, | 1 | 0.524 (x 1.907) | 85 (0.012) | 0.525 | regulation of cell shape | if |
| 544 | GO:0009309 | P | 6, 7, | 1 | 0.518 (x 1.929) | 84 (0.012) | 0.525 | amine biosynthesis | b |
| 545 | GO:0007268 | P | 6, | 2 | 1.394 (x 1.434) | 226 (0.009) | 0.526 | synaptic transmission | amd b |
| 546 | GO:0006470 | P | 8, | 1 | 0.524 (x 1.907) | 85 (0.012) | 0.526 | protein amino acid dephosphorylation | eya |
| 547 | GO:0005667 | C | 3, 6, 7, 8, 9, 10, 11, 12, 13, | 1 | 0.524 (x 1.907) | 85 (0.012) | 0.527 | transcription factor complex | toe |
| 548 | GO:0005575 | C | 1, | 37 | 35.675 (x 1.037) | 5782 (0.006) | 0.527 | cellular\_component | CG11835 CG15064 CG15093 CG1698 CG2663 CG3132 CG4914 CG9307 CG9358 Doc1 Doc2 Doc3 Dr Drip GV1 Mmp2 NetA Sox15 Tsp42Ei ap dnt drl ds eya if kn mab-2 nvy pdm2 rost rpk rpr toe tup ush vg yellow-e2 |
| 549 | GO:0007467 | P | 5, | 1 | 0.537 (x 1.863) | 87 (0.011) | 0.533 | photoreceptor cell differentiation (sensu Endopterygota) | eya |
| 550 | GO:0019932 | P | 6, | 1 | 0.543 (x 1.842) | 88 (0.011) | 0.534 | second-messenger-mediated signaling | regucalcin |
| 551 | GO:0006399 | P | 7, | 1 | 0.543 (x 1.842) | 88 (0.011) | 0.535 | tRNA metabolism | Sox15 |
| 552 | GO:0016298 | F | 6, | 1 | 0.543 (x 1.842) | 88 (0.011) | 0.535 | lipase activity | CG5966 |
| 553 | GO:0008134 | F | 4, | 1 | 0.549 (x 1.821) | 89 (0.011) | 0.536 | transcription factor binding | ush |
| 554 | GO:0051243 | P | 5, | 2 | 1.431 (x 1.397) | 232 (0.009) | 0.536 | negative regulation of cellular physiological process | Mmp2 rpr |
| 555 | GO:0004721 | F | 7, | 1 | 0.549 (x 1.821) | 89 (0.011) | 0.537 | phosphoprotein phosphatase activity | eya |
| 556 | GO:0005576 | C | 2, | 3 | 2.382 (x 1.260) | 386 (0.008) | 0.539 | extracellular region | CG9307 Mmp2 NetA |
| 557 | GO:0016853 | F | 3, | 1 | 0.561 (x 1.781) | 91 (0.011) | 0.543 | isomerase activity | BG:DS00797.2 |
| 558 | GO:0006732 | P | 6, | 2 | 1.475 (x 1.356) | 239 (0.008) | 0.547 | coenzyme metabolism | CG15093 CG2663 |
| 559 | GO:0043118 | P | 4, | 2 | 1.487 (x 1.345) | 241 (0.008) | 0.552 | negative regulation of physiological process | Mmp2 rpr |
| 560 | GO:0016301 | F | 5, | 3 | 2.474 (x 1.213) | 401 (0.007) | 0.566 | kinase activity | CG9358 dnt drl |
| 561 | GO:0046530 | P | 4, | 1 | 0.605 (x 1.654) | 98 (0.010) | 0.57 | photoreceptor cell differentiation | eya |
| 562 | GO:0006464 | P | 7, | 6 | 5.411 (x 1.109) | 877 (0.007) | 0.572 | protein modification | CG9358 dnt drl ds eya rpr |
| 563 | GO:0051186 | P | 5, | 2 | 1.561 (x 1.281) | 253 (0.008) | 0.579 | cofactor metabolism | CG15093 CG2663 |
| 564 | GO:0016251 | F | 4, | 1 | 0.623 (x 1.605) | 101 (0.010) | 0.579 | general RNA polymerase II transcription factor activity | CG11835 |
| 565 | GO:0007018 | P | 7, 8, 9, | 1 | 0.636 (x 1.574) | 103 (0.010) | 0.586 | microtubule-based movement | vg |
| 566 | GO:0030705 | P | 6, 7, 8, | 1 | 0.642 (x 1.558) | 104 (0.010) | 0.588 | cytoskeleton-dependent intracellular transport | vg |
| 567 | GO:0006092 | P | 7, | 1 | 0.642 (x 1.558) | 104 (0.010) | 0.589 | main pathways of carbohydrate metabolism | CG15093 |
| 568 | GO:0042221 | P | 4, | 2 | 1.623 (x 1.232) | 263 (0.008) | 0.599 | response to chemical stimulus | drl rpr |
| 569 | GO:0016311 | P | 7, | 1 | 0.666 (x 1.501) | 108 (0.009) | 0.602 | dephosphorylation | eya |
| 570 | GO:0008238 | F | 5, | 1 | 0.673 (x 1.487) | 109 (0.009) | 0.603 | exopeptidase activity | sda |
| 571 | GO:0004888 | F | 4, | 3 | 2.622 (x 1.144) | 425 (0.007) | 0.603 | transmembrane receptor activity | CG11835 dnt drl |
| 572 | GO:0007424 | P | 4, | 1 | 0.673 (x 1.487) | 109 (0.009) | 0.604 | tracheal system development (sensu Insecta) | if |
| 573 | GO:0030246 | F | 3, | 1 | 0.697 (x 1.434) | 113 (0.009) | 0.615 | carbohydrate binding | CG9307 |
| 574 | GO:0043412 | P | 6, | 6 | 5.658 (x 1.060) | 917 (0.007) | 0.616 | biopolymer modification | CG9358 dnt drl ds eya rpr |
| 575 | GO:0006508 | P | 7, | 5 | 4.677 (x 1.069) | 758 (0.007) | 0.616 | proteolysis | CG4914 CG6680 Mmp2 rpr sda |
| 576 | GO:0016567 | P | 9, | 1 | 0.697 (x 1.434) | 113 (0.009) | 0.616 | protein ubiquitination | rpr |
| 577 | GO:0005261 | F | 5, 6, | 1 | 0.710 (x 1.409) | 115 (0.009) | 0.618 | cation channel activity | rpk |
| 578 | GO:0031981 | C | 4, 5, 6, 7, 8, 9, 10, | 2 | 1.697 (x 1.179) | 275 (0.007) | 0.618 | nuclear lumen | ap toe |
| 579 | GO:0008415 | F | 6, | 1 | 0.710 (x 1.409) | 115 (0.009) | 0.619 | acyltransferase activity | CG5397 |
| 580 | GO:0007264 | P | 6, | 1 | 0.716 (x 1.397) | 116 (0.009) | 0.62 | small GTPase mediated signal transduction | CG9358 |
| 581 | GO:0009993 | P | 7, | 2 | 1.709 (x 1.170) | 277 (0.007) | 0.62 | oogenesis (sensu Insecta) | CG9358 Mmp2 |
| 582 | GO:0001505 | P | 7, | 1 | 0.722 (x 1.385) | 117 (0.009) | 0.62 | regulation of neurotransmitter levels | amd |
| 583 | GO:0006796 | P | 6, | 4 | 3.727 (x 1.073) | 604 (0.007) | 0.621 | phosphate metabolism | CG9358 dnt drl eya |
| 584 | GO:0006974 | P | 4, | 1 | 0.728 (x 1.373) | 118 (0.008) | 0.622 | response to DNA damage stimulus | rpr |
| 585 | GO:0006793 | P | 5, | 4 | 3.727 (x 1.073) | 604 (0.007) | 0.622 | phosphorus metabolism | CG9358 dnt drl eya |
| 586 | GO:0015980 | P | 6, | 1 | 0.728 (x 1.373) | 118 (0.008) | 0.623 | energy derivation by oxidation of organic compounds | CG15093 |
| 587 | GO:0007276 | P | 4, | 3 | 2.758 (x 1.088) | 447 (0.007) | 0.627 | gametogenesis | CG9358 Mmp2 eya |
| 588 | GO:0016747 | F | 5, | 1 | 0.747 (x 1.339) | 121 (0.008) | 0.63 | transferase activity, transferring groups other than amino-acyl groups | CG5397 |
| 589 | GO:0006858 | P | 5, 6, | 1 | 0.759 (x 1.318) | 123 (0.008) | 0.636 | extracellular transport | CG1698 |
| 590 | GO:0048477 | P | 6, | 2 | 1.783 (x 1.122) | 289 (0.007) | 0.637 | oogenesis | CG9358 Mmp2 |
| 591 | GO:0030707 | P | 8, | 1 | 0.765 (x 1.307) | 124 (0.008) | 0.638 | ovarian follicle cell development (sensu Insecta) | CG9358 |
| 592 | GO:0019953 | P | 3, | 3 | 2.814 (x 1.066) | 456 (0.007) | 0.638 | sexual reproduction | CG9358 Mmp2 eya |
| 593 | GO:0016310 | P | 7, | 3 | 2.844 (x 1.055) | 461 (0.007) | 0.645 | phosphorylation | CG9358 dnt drl |
| 594 | GO:0016746 | F | 4, | 1 | 0.784 (x 1.276) | 127 (0.008) | 0.646 | transferase activity, transferring acyl groups | CG5397 |
| 595 | GO:0009719 | P | 3, | 1 | 0.821 (x 1.219) | 133 (0.008) | 0.662 | response to endogenous stimulus | rpr |
| 596 | GO:0051246 | P | 5, 6, | 1 | 0.821 (x 1.219) | 133 (0.008) | 0.663 | regulation of protein metabolism | rpr |
| 597 | GO:0044248 | P | 5, | 2 | 1.888 (x 1.059) | 306 (0.007) | 0.666 | cellular catabolism | CG15093 b |
| 598 | GO:0007292 | P | 5, | 2 | 1.913 (x 1.046) | 310 (0.006) | 0.673 | female gamete generation | CG9358 Mmp2 |
| 599 | GO:0046483 | P | 5, | 1 | 0.864 (x 1.158) | 140 (0.007) | 0.681 | heterocycle metabolism | b |
| 600 | GO:0044260 | P | 5, | 13 | 13.278 (x 0.979) | 2152 (0.006) | 0.685 | cellular macromolecule metabolism | CG2663 CG4914 CG6680 CG9307 CG9358 Mmp2 Sox15 dnt drl ds eya rpr sda |
| 601 | GO:0012505 | C | 4, 5, | 1 | 0.888 (x 1.126) | 144 (0.007) | 0.69 | endomembrane system | GV1 |
| 602 | GO:0030001 | P | 7, 8, | 1 | 0.907 (x 1.103) | 147 (0.007) | 0.698 | metal ion transport | rpk |
| 603 | GO:0005488 | F | 2, | 25 | 25.507 (x 0.980) | 4134 (0.006) | 0.699 | binding | CAH1 CG11835 CG2663 CG4914 CG9307 CG9358 Doc1 Doc2 Doc3 Dr GV1 Mmp2 Sox15 ap dnt drl ds if kn nvy pdm2 sda toe tup ush |
| 604 | GO:0009056 | P | 4, | 2 | 2.018 (x 0.991) | 327 (0.006) | 0.7 | catabolism | CG15093 b |
| 605 | GO:0050875 | P | 3, | 38 | 38.353 (x 0.991) | 6216 (0.006) | 0.7 | cellular physiological process | BG:DS00797.2 CAH1 CG11835 CG15093 CG1698 CG2663 CG30069 CG3132 CG4914 CG6680 CG9307 CG9358 Doc1 Doc2 Doc3 Dr Drip Mmp2 NetA Sox15 amd ap b dnt drl ds eya if kn nvy pdm2 rpk rpr sda toe tup ush vg |
| 606 | GO:0000003 | P | 2, | 3 | 3.091 (x 0.970) | 501 (0.006) | 0.701 | reproduction | CG9358 Mmp2 eya |
| 607 | GO:0016772 | F | 4, | 3 | 3.091 (x 0.970) | 501 (0.006) | 0.702 | transferase activity, transferring phosphorus-containing groups | CG9358 dnt drl |
| 608 | GO:0031324 | P | 6, | 1 | 0.956 (x 1.046) | 155 (0.006) | 0.714 | negative regulation of cellular metabolism | rpr |
| 609 | GO:0006952 | P | 4, | 3 | 3.171 (x 0.946) | 514 (0.006) | 0.715 | defense response | CG5397 TepII kn |
| 610 | GO:0044428 | C | 4, 5, 6, 7, 8, 9, | 3 | 3.178 (x 0.944) | 515 (0.006) | 0.716 | nuclear part | GV1 ap toe |
| 611 | GO:0009117 | P | 6, | 1 | 0.981 (x 1.019) | 159 (0.006) | 0.721 | nucleotide metabolism | CG15093 |
| 612 | GO:0005976 | P | 6, | 1 | 1.000 (x 1.000) | 162 (0.006) | 0.728 | polysaccharide metabolism | CG9307 |
| 613 | GO:0000004 | P | 2, | 4 | 4.325 (x 0.925) | 701 (0.006) | 0.728 | biological process unknown | CG15064 GV1 mab-2 yellow-e2 |
| 614 | GO:0005216 | F | 4, 5, | 1 | 1.012 (x 0.988) | 164 (0.006) | 0.731 | ion channel activity | rpk |
| 615 | GO:0009892 | P | 5, | 1 | 1.018 (x 0.982) | 165 (0.006) | 0.732 | negative regulation of metabolism | rpr |
| 616 | GO:0004871 | F | 2, | 6 | 6.497 (x 0.923) | 1053 (0.006) | 0.733 | signal transducer activity | CG11835 dnt drl ds if ush |
| 617 | GO:0051242 | P | 5, | 1 | 1.030 (x 0.970) | 167 (0.006) | 0.735 | positive regulation of cellular physiological process | rpr |
| 618 | GO:0043119 | P | 4, | 1 | 1.043 (x 0.959) | 169 (0.006) | 0.739 | positive regulation of physiological process | rpr |
| 619 | GO:0003674 | F | 1, | 51 | 51.125 (x 0.998) | 8286 (0.006) | 0.74 | molecular\_function | BG:DS00797.2 CAH1 CG10962 CG11835 CG15064 CG15093 CG1698 CG2663 CG30069 CG3132 CG4382 CG4914 CG5392 CG5397 CG5966 CG6680 CG7160 CG8502 CG9307 CG9358 Doc1 Doc2 Doc3 Dr Drip Edg91 GV1 Mmp2 NetA Sox15 TepII alpha-Est5 amd ap b dnt drl ds eya if kn mab-2 nvy pdm2 rpk sda toe tup ush vg yellow-e2 |
| 620 | GO:0044267 | P | 6, | 12 | 12.828 (x 0.935) | 2079 (0.006) | 0.74 | cellular protein metabolism | CG2663 CG4914 CG6680 CG9358 Mmp2 Sox15 dnt drl ds eya rpr sda |
| 621 | GO:0044265 | P | 6, | 1 | 1.061 (x 0.942) | 172 (0.006) | 0.742 | cellular macromolecule catabolism | CG15093 |
| 622 | GO:0015672 | P | 7, 8, | 1 | 1.080 (x 0.926) | 175 (0.006) | 0.748 | monovalent inorganic cation transport | rpk |
| 623 | GO:0016791 | F | 6, | 1 | 1.123 (x 0.891) | 182 (0.005) | 0.764 | phosphoric monoester hydrolase activity | eya |
| 624 | GO:0043037 | P | 7, 8, | 1 | 1.148 (x 0.871) | 186 (0.005) | 0.77 | translation | Sox15 |
| 625 | GO:0015630 | C | 6, 7, 8, 9, | 1 | 1.148 (x 0.871) | 186 (0.005) | 0.771 | microtubule cytoskeleton | vg |
| 626 | GO:0006812 | P | 6, 7, | 2 | 2.363 (x 0.846) | 383 (0.005) | 0.773 | cation transport | CG1698 rpk |
| 627 | GO:0009057 | P | 5, | 1 | 1.172 (x 0.853) | 190 (0.005) | 0.774 | macromolecule catabolism | CG15093 |
| 628 | GO:0016787 | F | 3, | 10 | 11.081 (x 0.902) | 1796 (0.006) | 0.774 | hydrolase activity | CG3132 CG4382 CG4914 CG5397 CG5966 CG9307 Mmp2 alpha-Est5 eya sda |
| 629 | GO:0048522 | P | 4, | 1 | 1.166 (x 0.858) | 189 (0.005) | 0.774 | positive regulation of cellular process | rpr |
| 630 | GO:0007017 | P | 7, | 1 | 1.209 (x 0.827) | 196 (0.005) | 0.785 | microtubule-based process | vg |
| 631 | GO:0007582 | P | 2, | 41 | 42.154 (x 0.973) | 6832 (0.006) | 0.786 | physiological process | BG:DS00797.2 CAH1 CG10962 CG11835 CG15093 CG1698 CG2663 CG30069 CG3132 CG4914 CG5966 CG6680 CG9307 CG9358 Doc1 Doc2 Doc3 Dr Drip Mmp2 NetA Sox15 TepII amd ap b dnt drl ds eya if kn nvy pdm2 rpk rpr sda toe tup ush vg |
| 632 | GO:0008324 | F | 4, | 2 | 2.462 (x 0.812) | 399 (0.005) | 0.787 | cation transporter activity | CG1698 rpk |
| 633 | GO:0042578 | F | 5, | 1 | 1.234 (x 0.810) | 200 (0.005) | 0.787 | phosphoric ester hydrolase activity | eya |
| 634 | GO:0051726 | P | 5, | 1 | 1.228 (x 0.814) | 199 (0.005) | 0.788 | regulation of cell cycle | CG30069 |
| 635 | GO:0000074 | P | 6, | 1 | 1.228 (x 0.814) | 199 (0.005) | 0.789 | regulation of progression through cell cycle | CG30069 |
| 636 | GO:0044451 | C | 5, 6, 7, 8, 9, 10, 11, 12, | 1 | 1.253 (x 0.798) | 203 (0.005) | 0.792 | nucleoplasm part | toe |
| 637 | GO:0004263 | F | 7, | 1 | 1.283 (x 0.779) | 208 (0.005) | 0.796 | chymotrypsin activity | CG4914 |
| 638 | GO:0005215 | F | 2, | 5 | 5.973 (x 0.837) | 968 (0.005) | 0.797 | transporter activity | CG1698 CG2663 CG9358 Drip rpk |
| 639 | GO:0009987 | P | 2, | 40 | 41.376 (x 0.967) | 6706 (0.006) | 0.798 | cellular process | BG:DS00797.2 CAH1 CG11835 CG15093 CG1698 CG2663 CG30069 CG3132 CG4914 CG6680 CG9307 CG9358 Doc1 Doc2 Doc3 Dr Drip Mmp2 NetA Sox15 amd ap b dnt drl ds eya if kn nvy pdm2 regucalcin rost rpk rpr sda toe tup ush vg |
| 640 | GO:0019538 | P | 5, | 12 | 13.469 (x 0.891) | 2183 (0.005) | 0.798 | protein metabolism | CG2663 CG4914 CG6680 CG9358 Mmp2 Sox15 dnt drl ds eya rpr sda |
| 641 | GO:0004674 | F | 7, | 1 | 1.308 (x 0.764) | 212 (0.005) | 0.803 | protein serine/threonine kinase activity | CG9358 |
| 642 | GO:0001584 | F | 6, | 1 | 1.314 (x 0.761) | 213 (0.005) | 0.803 | rhodopsin-like receptor activity | CG11835 |
| 643 | GO:0048518 | P | 3, | 1 | 1.345 (x 0.743) | 218 (0.005) | 0.807 | positive regulation of biological process | rpr |
| 644 | GO:0015291 | F | 5, | 1 | 1.339 (x 0.747) | 217 (0.005) | 0.808 | porter activity | CG1698 |
| 645 | GO:0005509 | F | 5, | 1 | 1.345 (x 0.743) | 218 (0.005) | 0.808 | calcium ion binding | ds |
| 646 | GO:0015290 | F | 4, | 1 | 1.339 (x 0.747) | 217 (0.005) | 0.809 | electrochemical potential-driven transporter activity | CG1698 |
| 647 | GO:0031974 | C | 2, | 2 | 2.659 (x 0.752) | 431 (0.005) | 0.812 | membrane-enclosed lumen | ap toe |
| 648 | GO:0016491 | F | 3, | 3 | 3.850 (x 0.779) | 624 (0.005) | 0.812 | oxidoreductase activity | CG10962 CG15093 CG30069 |
| 649 | GO:0043233 | C | 3, 4, | 2 | 2.659 (x 0.752) | 431 (0.005) | 0.813 | organelle lumen | ap toe |
| 650 | GO:0006512 | P | 8, | 1 | 1.444 (x 0.693) | 234 (0.004) | 0.829 | ubiquitin cycle | rpr |
| 651 | GO:0004295 | F | 7, | 1 | 1.450 (x 0.690) | 235 (0.004) | 0.829 | trypsin activity | CG4914 |
| 652 | GO:0008233 | F | 4, | 3 | 4.017 (x 0.747) | 651 (0.005) | 0.833 | peptidase activity | CG4914 Mmp2 sda |
| 653 | GO:0044430 | C | 4, 5, 6, 7, 8, 9, | 1 | 1.481 (x 0.675) | 240 (0.004) | 0.834 | cytoskeletal part | vg |
| 654 | GO:0003824 | F | 2, | 21 | 23.329 (x 0.900) | 3781 (0.006) | 0.834 | catalytic activity | BG:DS00797.2 CAH1 CG10962 CG15093 CG30069 CG3132 CG4382 CG4914 CG5397 CG5966 CG9307 CG9358 Mmp2 Sox15 alpha-Est5 amd b dnt drl eya sda |
| 655 | GO:0005739 | C | 5, 6, 7, 8, | 2 | 2.851 (x 0.702) | 462 (0.004) | 0.839 | mitochondrion | CG15093 rpr |
| 656 | GO:0006811 | P | 5, 6, | 2 | 2.851 (x 0.702) | 462 (0.004) | 0.84 | ion transport | CG1698 rpk |
| 657 | GO:0004175 | F | 5, | 2 | 2.906 (x 0.688) | 471 (0.004) | 0.846 | endopeptidase activity | CG4914 Mmp2 |
| 658 | GO:0005524 | F | 6, | 3 | 4.146 (x 0.724) | 672 (0.004) | 0.847 | ATP binding | Sox15 dnt drl |
| 659 | GO:0004252 | F | 6, | 1 | 1.586 (x 0.631) | 257 (0.004) | 0.849 | serine-type endopeptidase activity | CG4914 |
| 660 | GO:0004930 | F | 5, | 1 | 1.586 (x 0.631) | 257 (0.004) | 0.851 | G-protein coupled receptor activity | CG11835 |
| 661 | GO:0015075 | F | 3, | 2 | 2.992 (x 0.668) | 485 (0.004) | 0.855 | ion transporter activity | CG1698 rpk |
| 662 | GO:0030554 | F | 5, | 3 | 4.251 (x 0.706) | 689 (0.004) | 0.857 | adenyl nucleotide binding | Sox15 dnt drl |
| 663 | GO:0031975 | C | 2, | 1 | 1.647 (x 0.607) | 267 (0.004) | 0.857 | envelope | GV1 |
| 664 | GO:0031967 | C | 3, 4, 5, 6, 7, 8, | 1 | 1.647 (x 0.607) | 267 (0.004) | 0.859 | organelle envelope | GV1 |
| 665 | GO:0006412 | P | 6, 7, | 2 | 3.048 (x 0.656) | 494 (0.004) | 0.86 | protein biosynthesis | Sox15 rpr |
| 666 | GO:0009058 | P | 4, | 4 | 5.541 (x 0.722) | 898 (0.004) | 0.86 | biosynthesis | Sox15 amd b rpr |
| 667 | GO:0007242 | P | 5, | 2 | 3.233 (x 0.619) | 524 (0.004) | 0.879 | intracellular signaling cascade | CG9358 regucalcin |
| 668 | GO:0008236 | F | 5, | 1 | 1.808 (x 0.553) | 293 (0.003) | 0.879 | serine-type peptidase activity | CG4914 |
| 669 | GO:0005856 | C | 5, 6, 7, 8, | 1 | 1.789 (x 0.559) | 290 (0.003) | 0.88 | cytoskeleton | vg |
| 670 | GO:0043283 | P | 5, | 8 | 10.390 (x 0.770) | 1684 (0.005) | 0.88 | biopolymer metabolism | CG9307 CG9358 Sox15 dnt drl ds eya rpr |
| 671 | GO:0009059 | P | 5, 6, | 2 | 3.221 (x 0.621) | 522 (0.004) | 0.881 | macromolecule biosynthesis | Sox15 rpr |
| 672 | GO:0043170 | P | 4, | 16 | 19.226 (x 0.832) | 3116 (0.005) | 0.888 | macromolecule metabolism | BG:DS00797.2 CG15093 CG2663 CG3132 CG4914 CG6680 CG9307 CG9358 Mmp2 Sox15 dnt drl ds eya rpr sda |
| 673 | GO:0005554 | F | 2, | 3 | 4.640 (x 0.647) | 752 (0.004) | 0.889 | molecular function unknown | CG15064 mab-2 yellow-e2 |
| 674 | GO:0007186 | P | 6, | 1 | 1.882 (x 0.531) | 305 (0.003) | 0.889 | G-protein coupled receptor protein signaling pathway | CG11835 |
| 675 | GO:0016740 | F | 3, | 4 | 5.966 (x 0.670) | 967 (0.004) | 0.894 | transferase activity | CG5397 CG9358 dnt drl |
| 676 | GO:0016874 | F | 3, | 1 | 1.956 (x 0.511) | 317 (0.003) | 0.894 | ligase activity | Sox15 |
| 677 | GO:0008150 | P | 1, | 48 | 49.867 (x 0.963) | 8082 (0.006) | 0.894 | biological\_process | BG:DS00797.2 CAH1 CG10962 CG11835 CG15064 CG15093 CG1698 CG2663 CG30069 CG3132 CG4914 CG5397 CG5966 CG6680 CG9307 CG9358 Doc1 Doc2 Doc3 Dr Drip GV1 Mmp2 NetA Sox15 TepII amd ap b dnt drl ds eya if kn mab-2 nvy pdm2 regucalcin rost rpk rpr sda toe tup ush vg yellow-e2 |
| 678 | GO:0008372 | C | 2, | 3 | 5.022 (x 0.597) | 814 (0.004) | 0.919 | cellular component unknown | CG15064 mab-2 yellow-e2 |
| 679 | GO:0044249 | P | 5, | 3 | 5.121 (x 0.586) | 830 (0.004) | 0.925 | cellular biosynthesis | Sox15 b rpr |
| 680 | GO:0016070 | P | 6, | 1 | 2.264 (x 0.442) | 367 (0.003) | 0.928 | RNA metabolism | Sox15 |
| 681 | GO:0017076 | F | 4, | 3 | 5.238 (x 0.573) | 849 (0.004) | 0.931 | purine nucleotide binding | Sox15 dnt drl |
| 682 | GO:0000166 | F | 3, | 3 | 5.417 (x 0.554) | 878 (0.003) | 0.942 | nucleotide binding | Sox15 dnt drl |
| 683 | GO:0044444 | C | 4, 5, 6, 7, | 4 | 7.139 (x 0.560) | 1157 (0.003) | 0.962 | cytoplasmic part | CG15093 CG3132 if rpr |
| 684 | GO:0007010 | P | 6, | 1 | 2.777 (x 0.360) | 450 (0.002) | 0.965 | cytoskeleton organization and biogenesis | vg |
| 685 | GO:0007049 | P | 4, | 1 | 2.906 (x 0.344) | 471 (0.002) | 0.971 | cell cycle | CG30069 |
| 686 | GO:0005515 | F | 3, | 4 | 7.614 (x 0.525) | 1234 (0.003) | 0.975 | protein binding | drl ds if ush |
| 687 | GO:0006629 | P | 5, | 1 | 3.023 (x 0.331) | 490 (0.002) | 0.975 | lipid metabolism | CG5966 |
| 688 | GO:0006091 | P | 5, | 1 | 3.116 (x 0.321) | 505 (0.002) | 0.977 | generation of precursor metabolites and energy | CG15093 |
| 689 | GO:0005737 | C | 4, 5, 6, | 5 | 9.163 (x 0.546) | 1485 (0.003) | 0.978 | cytoplasm | CG15093 CG3132 CG9358 if rpr |
| 690 | GO:0006810 | P | 4, 5, | 5 | 9.113 (x 0.549) | 1477 (0.003) | 0.978 | transport | CG1698 CG2663 Drip rpk vg |
| 691 | GO:0008104 | P | 4, | 1 | 3.492 (x 0.286) | 566 (0.002) | 0.986 | protein localization | if |
| 692 | GO:0044446 | C | 3, 4, 5, 6, 7, | 4 | 8.786 (x 0.455) | 1424 (0.003) | 0.989 | intracellular organelle part | GV1 ap toe vg |
| 693 | GO:0044422 | C | 2, 3, | 4 | 8.786 (x 0.455) | 1424 (0.003) | 0.99 | organelle part | GV1 ap toe vg |
| 694 | GO:0051641 | P | 4, 5, | 1 | 3.924 (x 0.255) | 636 (0.002) | 0.992 | cellular localization | vg |
| 695 | GO:0006996 | P | 5, | 1 | 4.455 (x 0.224) | 722 (0.001) | 0.992 | organelle organization and biogenesis | vg |
| 696 | GO:0046907 | P | 5, 6, 7, | 1 | 3.801 (x 0.263) | 616 (0.002) | 0.992 | intracellular transport | vg |
| 697 | GO:0043228 | C | 3, | 1 | 4.387 (x 0.228) | 711 (0.001) | 0.993 | non-membrane-bound organelle | vg |
| 698 | GO:0051649 | P | 5, 6, | 1 | 3.918 (x 0.255) | 635 (0.002) | 0.993 | establishment of cellular localization | vg |
| 699 | GO:0043232 | C | 4, 5, 6, 7, | 1 | 4.387 (x 0.228) | 711 (0.001) | 0.994 | intracellular non-membrane-bound organelle | vg |
| 700 | GO:0043234 | C | 2, | 3 | 9.743 (x 0.308) | 1579 (0.002) | 0.998 | protein complex | CG3132 if toe |

  

---

Regulated Genes that don't have GO terms
  

BcDNA:GH11415 CG10570 CG11905 CG16885 CG16959 CG17032 CG6044 CG7924 CG8501 CG9266 CG9427 CG9812
