# Supplementary material for: Functional dissection of the ash2 and ash1 transcriptomes provides insights into the transcriptional basis of wing phenotypes and reveals conserved protein interactions
Source: Genome Biol. 2007 Apr 28;8(4):R67. doi: 10.1186/gb-2007-8-4-r67 (PMC1896016; doi:10.1186/gb-2007-8-4-r67)
Supplement: Additional data file 18 — Genes misregulated in ash2 and ash1 mutants preferentially expressed in the wing disc and in the prospective wing-hinge or body wall region [file gb-2007-8-4-r67-S18.pdf]

Wing Disc	
ash2	***ash1***
ap	ap
CG15093	
CG17032	
CG4382	
CG4766	CG4766
CG4914	
CG5392	
CG6044	
CG6680	
CG7160	
CG8502	
CG9266	
CG9307	
CG9427	
Doc1	
Doc2	
Doc3	
kn	kn
msh, Dr	msh, Dr
NetA	
pdm2	pdm2
rost	
rpk	
toe	
vg	
CAH1	
CG10570	
CG10962	
CG5397	
Edg91	
GV1	
Phk-3	
regucalcin	
rpr	
sda	

Wing-Hinge	
ash2	***ash1***
ana	ana
BcDNA:SD04019	
CG14301	
CG16868	
CG32029	
CG6234	CG6234
CG7160	
CG9057	CG9057
Cyp310a1	
Dll	Dll
Doc1	
Doc2	
dve	
ed	
en	
hh	
inv	
kn	kn
NetA	
opa	
pdm2	pdm2
Poxn	
vg	
wgn	
Gasp	Gasp
pip	
Ugt86Di	Ugt86Di
vvl	

Body Wall	
ash2	***ash1***
Ance	
CG13023	
CG13053	CG13053
CG15786	
CG31640	
CG3770	
CG6199	
CG7860	CG7860
Fas2	
ImpL2	ImpL2
Obp99a	Obp99a
ss	
toe	
	eyg
	Sox102F
Act57B	Act57B
apt	
BcDNA:GH07269	
CG10126	
CG11370	
CG12481	
CG12505	
CG13044	
CG15353	
CG1572	CG1572
CG16820	
CG3244	
CG3624	
CG4386	CG4386
CG5397	CG5397
CG6921	CG6921
CG9192	
CG9338	CG9338
CG9358	
eiger	
Gs2	Gs2
Obp56a	
regucalcin	regucalcin
Traf1	
trol	
wun2	
	CG10200
