# Supplementary material for: Functional dissection of the ash2 and ash1 transcriptomes provides insights into the transcriptional basis of wing phenotypes and reveals conserved protein interactions
Source: Genome Biol. 2007 Apr 28;8(4):R67. doi: 10.1186/gb-2007-8-4-r67 (PMC1896016; doi:10.1186/gb-2007-8-4-r67)
Supplement: Additional data file 19 — GO annotations of the genes upregulated in Sin3A deficient cells according to Pile et al. [49] [file gb-2007-8-4-r67-S19.html]

  

---

  

|  |  |
| --- | --- |
| Go Statistics | Reg File: **Sin3\_Up.txt.fbgns** (360 genes -- 75 skipped)  Ref File: **ref.fbgns** (13577 genes -- 4663 skipped)  Database: **go\_200507-termdb.rdf-xml** |

---

  

Fields Description

| Pos | Go Term | Ontology | Levels | Observed | Expected | Possibles | p-value(Adj) | Go term description | Genes with the GO term |
| --- | --- | --- | --- | --- | --- | --- | --- | --- | --- |
| 1 | GO:0005739 | C | 5, 6, 7, 8, | 60 | 14.771 (x 4.062) | 462 (0.130) | 2.05e-18 | mitochondrion | Aats-ala-m Alas CG1140 CG12013 CG12264 CG12534 CG12954 CG1319 CG1673 CG2789 CG31075 CG33066 CG3476 CG3499 CG3731 CG4225 CG4589 CG4769 CG4963 CG5261 CG6512 CG6608 CG7598 CG7842 CG8728 CG9065 CG9240 Cyt-c-p EG:100G10.7 EfTuM Idh JhI-1 Shawn Thiolase Tim10 Trxr-1 aralar1 att-ORFA bcn92 ferrochelatase mRpL11 mRpL13 mRpL19 mRpL21 mRpL22 mRpL22-24 mRpL28 mRpL3 mRpL48 mRpL55 mRpS18a mRpS18b mRpS2 mRpS22 mRpS25 mRpS32 mRpS33 mRpS9 milton yip2 |
| 2 | GO:0005737 | C | 4, 5, 6, | 112 | 47.479 (x 2.359) | 1485 (0.075) | 9.88e-18 | cytoplasm | AP-2sigma Aats-ala-m Aats-asp Aats-his Adk1 Alas Aprt BcDNA:GH07485 CG10268 CG10306 CG1140 CG11876 CG12013 CG12264 CG12534 CG12954 CG1319 CG13887 CG14040 CG1673 CG2263 CG2789 CG2846 CG31075 CG32549 CG33066 CG3476 CG3499 CG3731 CG3931 CG4225 CG4589 CG4769 CG4963 CG5261 CG6512 CG6608 CG6718 CG7598 CG7809 CG7842 CG8412 CG8636 CG8728 CG9065 CG9240 CG9298 Cat Cyt-c-p Dip-B EG:100G10.7 EfTuM Eno Gapdh1 Gapdh2 Gclm Gfat2 Idh Inos Jafrac1 JhI-1 Jra Nsf2 PEK Pcd Pfk PyK Rep Shawn Sply Sras Thiolase Tim10 Transaldolase Trxr-1 Updo Vha36 Vha44 aralar1 att-ORFA bcn92 bor bsf cnn eIF-2alpha eIF2B-alpha eIF2B-delta eIF2B-gamma fbl ferrochelatase gammaSnap mRpL11 mRpL13 mRpL19 mRpL21 mRpL22 mRpL22-24 mRpL28 mRpL3 mRpL48 mRpL55 mRpS18a mRpS18b mRpS2 mRpS22 mRpS25 mRpS32 mRpS33 mRpS9 milton snapin yip2 |
| 3 | GO:0044444 | C | 4, 5, 6, 7, | 93 | 36.992 (x 2.514) | 1157 (0.080) | 8.5e-16 | cytoplasmic part | AP-2sigma Aats-ala-m Alas BcDNA:GH07485 CG10306 CG1140 CG11876 CG12013 CG12264 CG12534 CG12954 CG1319 CG13887 CG14040 CG1673 CG2789 CG31075 CG32549 CG33066 CG3476 CG3499 CG3731 CG3931 CG4225 CG4589 CG4769 CG4963 CG5261 CG6512 CG6608 CG6718 CG7598 CG7809 CG7842 CG8412 CG8636 CG8728 CG9065 CG9240 CG9298 Cat Cyt-c-p Dip-B EG:100G10.7 EfTuM Eno Gclm Idh Jafrac1 JhI-1 PEK Pcd Pfk PyK Rep Shawn Sply Sras Thiolase Tim10 Trxr-1 Vha36 Vha44 aralar1 att-ORFA bcn92 cnn eIF-2alpha eIF2B-alpha eIF2B-delta eIF2B-gamma ferrochelatase mRpL11 mRpL13 mRpL19 mRpL21 mRpL22 mRpL22-24 mRpL28 mRpL3 mRpL48 mRpL55 mRpS18a mRpS18b mRpS2 mRpS22 mRpS25 mRpS32 mRpS33 mRpS9 milton snapin yip2 |
| 4 | GO:0044429 | C | 4, 5, 6, 7, 8, 9, | 45 | 11.030 (x 4.080) | 345 (0.130) | 9.62e-14 | mitochondrial part | Alas CG1140 CG12954 CG1319 CG2789 CG31075 CG33066 CG3476 CG3499 CG3731 CG4225 CG4769 CG4963 CG5261 CG6608 CG7598 CG8728 CG9065 CG9240 Cyt-c-p Shawn Thiolase Tim10 aralar1 att-ORFA bcn92 ferrochelatase mRpL11 mRpL13 mRpL19 mRpL21 mRpL22 mRpL22-24 mRpL28 mRpL3 mRpL48 mRpL55 mRpS18a mRpS18b mRpS2 mRpS22 mRpS25 mRpS32 mRpS33 mRpS9 |
| 5 | GO:0031980 | C | 4, 5, 6, 7, 8, 9, 10, | 28 | 4.636 (x 6.040) | 145 (0.193) | 1.99e-12 | mitochondrial lumen | Alas CG1140 CG12954 CG1319 CG31075 CG3731 CG5261 CG8728 Cyt-c-p Thiolase mRpL11 mRpL13 mRpL19 mRpL21 mRpL22 mRpL22-24 mRpL28 mRpL3 mRpL48 mRpL55 mRpS18a mRpS18b mRpS2 mRpS22 mRpS25 mRpS32 mRpS33 mRpS9 |
| 6 | GO:0005759 | C | 5, 6, 7, 8, 9, 10, 11, | 28 | 4.636 (x 6.040) | 145 (0.193) | 2.39e-12 | mitochondrial matrix | Alas CG1140 CG12954 CG1319 CG31075 CG3731 CG5261 CG8728 Cyt-c-p Thiolase mRpL11 mRpL13 mRpL19 mRpL21 mRpL22 mRpL22-24 mRpL28 mRpL3 mRpL48 mRpL55 mRpS18a mRpS18b mRpS2 mRpS22 mRpS25 mRpS32 mRpS33 mRpS9 |
| 7 | GO:0009058 | P | 4, | 70 | 28.711 (x 2.438) | 898 (0.078) | 1.12e-10 | biosynthesis | Aats-ala-m Aats-asp Aats-cys Aats-gly Aats-his Aats-thr Aats-trp Aats-tyr Aats-val Alas BcDNA:LD32788 CG10268 CG10306 CG10802 CG12264 CG12954 CG13645 CG1673 CG2246 CG2263 CG2846 CG32380 CG3522 CG4225 CG4447 CG6016 CG7842 CG8412 CG8636 Dgp-1 EfTuM Gclm Gfat2 Inos Mocs1 PEK Paps Pcd Rep Thor Tpi Updo Vha36 Vha44 betaggt-II eIF-2alpha eIF2B-alpha eIF2B-delta eIF2B-gamma fbl ferrochelatase l(2)k01209 l(3)02640 mRpL11 mRpL13 mRpL19 mRpL21 mRpL22 mRpL22-24 mRpL28 mRpL3 mRpL48 mRpS18a mRpS18b mRpS2 mRpS22 mRpS25 mRpS32 mRpS33 mRpS9 |
| 8 | GO:0005761 | C | 5, 6, 7, 8, 9, 10, 11, 12, | 19 | 2.366 (x 8.031) | 74 (0.257) | 1.46e-10 | mitochondrial ribosome | CG12954 mRpL11 mRpL13 mRpL19 mRpL21 mRpL22 mRpL22-24 mRpL28 mRpL3 mRpL48 mRpL55 mRpS18a mRpS18b mRpS2 mRpS22 mRpS25 mRpS32 mRpS33 mRpS9 |
| 9 | GO:0000313 | C | 5, 6, 7, 8, 9, | 19 | 2.366 (x 8.031) | 74 (0.257) | 1.64e-10 | organellar ribosome | CG12954 mRpL11 mRpL13 mRpL19 mRpL21 mRpL22 mRpL22-24 mRpL28 mRpL3 mRpL48 mRpL55 mRpS18a mRpS18b mRpS2 mRpS22 mRpS25 mRpS32 mRpS33 mRpS9 |
| 10 | GO:0003824 | F | 2, | 179 | 120.887 (x 1.481) | 3781 (0.047) | 2.16e-10 | catalytic activity | Aats-ala-m Aats-asp Aats-cys Aats-gly Aats-his Aats-thr Aats-trp Aats-tyr Aats-val Adk1 Ahcy13 Alas Aprt Ate1 BG:BACR48E02.4 BcDNA:GH07485 BcDNA:LD32788 CG10268 CG10333 CG10414 CG10638 CG10639 CG10721 CG10802 CG11309 CG1140 CG11876 CG11883 CG11897 CG11967 CG11989 CG12013 CG12163 CG12171 CG12177 CG12264 CG12534 CG1319 CG13645 CG1598 CG1635 CG1673 CG17266 CG17294 CG17760 CG18522 CG18547 CG1882 CG1969 CG2064 CG2107 CG2246 CG2263 CG2846 CG31063 CG31075 CG31549 CG31694 CG31793 CG32549 CG32687 CG33138 CG3397 CG3499 CG3561 CG3608 CG3609 CG3731 CG3931 CG4225 CG4407 CG4769 CG4802 CG4860 CG5044 CG5224 CG5261 CG5380 CG5567 CG5604 CG6016 CG6428 CG6512 CG6523 CG6668 CG6718 CG6744 CG6746 CG6805 CG6984 CG7627 CG7632 CG7789 CG7842 CG8128 CG8360 CG8412 CG8417 CG8446 CG8479 CG8728 CG8778 CG8839 CG9232 CG9240 CG9249 CG9882 Cat Cyp6a20 Cyp6d5 Cyt-b5 Cyt-c-p Dak1 Dgp-1 Dip-B EG:100G10.7 EG:65F1.1 Eno Gapdh1 Gapdh2 Gclm Gfat2 GstE1 GstE6 GstE7 GstE9 Hmgs Ide Idh Inos Jafrac1 JhI-1 Men Mocs1 NTPase Nedd4 Nsf2 PEK Paps Past1 Pcd Pfk Pgm Pi4KIIalpha Prx6005 PyK Rep Sply Sras Thiolase Tpi Transaldolase Trxr-1 Ugt86Da Updo Vha36 Vha44 alpha-Est3 bcn92 betaTub60D betaggt-II bor bsf cathD cl cnn eIF2B-gamma fbl ferrochelatase ire-1 kraken l(2)05070 l(2)k01209 l(3)02640 mRpL28 mus205 poe ref(2)P yip2 |
| 11 | GO:0044249 | P | 5, | 65 | 26.537 (x 2.449) | 830 (0.078) | 4.95e-10 | cellular biosynthesis | Aats-ala-m Aats-asp Aats-cys Aats-gly Aats-his Aats-thr Aats-trp Aats-tyr Aats-val Alas BcDNA:LD32788 CG10306 CG10802 CG12264 CG12954 CG13645 CG1673 CG2246 CG2263 CG2846 CG4225 CG4447 CG7842 CG8412 CG8636 Dgp-1 EfTuM Gclm Gfat2 Inos Mocs1 PEK Paps Pcd Rep Thor Tpi Updo Vha36 Vha44 betaggt-II eIF-2alpha eIF2B-alpha eIF2B-delta eIF2B-gamma fbl ferrochelatase l(3)02640 mRpL11 mRpL13 mRpL19 mRpL21 mRpL22 mRpL22-24 mRpL28 mRpL3 mRpL48 mRpS18a mRpS18b mRpS2 mRpS22 mRpS25 mRpS32 mRpS33 mRpS9 |
| 12 | GO:0009059 | P | 5, 6, | 48 | 16.689 (x 2.876) | 522 (0.092) | 2.08e-09 | macromolecule biosynthesis | Aats-ala-m Aats-asp Aats-cys Aats-gly Aats-his Aats-thr Aats-trp Aats-tyr Aats-val CG10306 CG10802 CG12954 CG2263 CG4225 CG4447 CG8412 CG8636 Dgp-1 EfTuM Gfat2 Inos PEK Pcd Rep Thor Tpi betaggt-II eIF-2alpha eIF2B-alpha eIF2B-delta eIF2B-gamma mRpL11 mRpL13 mRpL19 mRpL21 mRpL22 mRpL22-24 mRpL28 mRpL3 mRpL48 mRpS18a mRpS18b mRpS2 mRpS22 mRpS25 mRpS32 mRpS33 mRpS9 |
| 13 | GO:0006412 | P | 6, 7, | 45 | 15.794 (x 2.849) | 494 (0.091) | 1.24e-08 | protein biosynthesis | Aats-ala-m Aats-asp Aats-cys Aats-gly Aats-his Aats-thr Aats-trp Aats-tyr Aats-val CG10306 CG10802 CG12954 CG2263 CG4225 CG4447 CG8412 CG8636 Dgp-1 EfTuM PEK Pcd Rep Thor betaggt-II eIF-2alpha eIF2B-alpha eIF2B-delta eIF2B-gamma mRpL11 mRpL13 mRpL19 mRpL21 mRpL22 mRpL22-24 mRpL28 mRpL3 mRpL48 mRpS18a mRpS18b mRpS2 mRpS22 mRpS25 mRpS32 mRpS33 mRpS9 |
| 14 | GO:0005762 | C | 5, 6, 7, 8, 9, 10, 11, 12, 13, | 12 | 1.439 (x 8.341) | 45 (0.267) | 9.39e-07 | mitochondrial large ribosomal subunit | CG12954 mRpL11 mRpL13 mRpL19 mRpL21 mRpL22 mRpL22-24 mRpL28 mRpL3 mRpL48 mRpL55 mRpS32 |
| 15 | GO:0000315 | C | 4, 5, 6, 7, 8, 9, 10, | 12 | 1.439 (x 8.341) | 45 (0.267) | 1.01e-06 | organellar large ribosomal subunit | CG12954 mRpL11 mRpL13 mRpL19 mRpL21 mRpL22 mRpL22-24 mRpL28 mRpL3 mRpL48 mRpL55 mRpS32 |
| 16 | GO:0008152 | P | 3, | 207 | 161.683 (x 1.280) | 5057 (0.041) | 1.1e-06 | metabolism | Aats-ala-m Aats-asp Aats-cys Aats-gly Aats-his Aats-thr Aats-trp Aats-tyr Aats-val Adk1 Ahcy13 Alas Aprt Ate1 BG:BACR48E02.4 BcDNA:GH07485 BcDNA:LD32788 CG10042 CG10268 CG10306 CG10333 CG10340 CG10414 CG10638 CG10639 CG10721 CG10802 CG1140 CG11710 CG11876 CG11883 CG11897 CG11967 CG11989 CG12013 CG12163 CG12171 CG12177 CG12264 CG12534 CG12954 CG1319 CG13645 CG15094 CG1635 CG1673 CG17187 CG17266 CG17294 CG17760 CG18522 CG1882 CG2064 CG2107 CG2246 CG2263 CG2789 CG2846 CG31075 CG31549 CG32380 CG32549 CG32687 CG33138 CG3476 CG3499 CG3522 CG3561 CG3608 CG3609 CG3731 CG3931 CG4225 CG4407 CG4447 CG4769 CG4802 CG4860 CG5044 CG5261 CG5380 CG5567 CG5604 CG6000 CG6016 CG6272 CG6428 CG6512 CG6523 CG6718 CG6746 CG6805 CG6984 CG7417 CG7598 CG7789 CG7842 CG8128 CG8360 CG8412 CG8417 CG8446 CG8636 CG8728 CG8778 CG8839 CG9065 CG9215 CG9232 CG9240 CG9249 CG9836 CG9882 Cat Cyp6a20 Cyp6d5 Cyt-b5 Cyt-c-p Dak1 Dgp-1 Dip-B EG:100G10.7 EfTuM Eno Gapdh1 Gapdh2 Gclm Gfat2 Gp93 GstE1 GstE6 GstE7 Hmgs Ide Idh Inos Jafrac1 JhI-1 Jra MTF-1 Men Mocs1 NTPase Nedd4 PEK Paps Pcd Pfk Pgm Pi4KIIalpha Prx6005 PyK Rep RhoGEF2 SCAP Sply Sras TMS1 TfIIEbeta Thiolase Thor Tpi Transaldolase Trxr-1 Ugt86Da Updo Vha36 Vha44 aralar1 bcn92 betaTub60D betaggt-II cathD cl eIF-2alpha eIF2B-alpha eIF2B-delta eIF2B-gamma fbl ferrochelatase ire-1 kraken l(2)05070 l(2)k01209 l(3)02640 mRpL11 mRpL13 mRpL19 mRpL21 mRpL22 mRpL22-24 mRpL28 mRpL3 mRpL48 mRpS18a mRpS18b mRpS2 mRpS22 mRpS25 mRpS32 mRpS33 mRpS9 mio mus205 poe ref(2)P yip2 |
| 17 | GO:0019752 | P | 6, | 35 | 12.533 (x 2.793) | 392 (0.089) | 2.19e-06 | carboxylic acid metabolism | Aats-ala-m Aats-asp Aats-cys Aats-gly Aats-his Aats-thr Aats-trp Aats-tyr Aats-val Alas BcDNA:GH07485 CG10802 CG1140 CG11876 CG12264 CG1673 CG2107 CG2263 CG31075 CG4225 CG4802 CG4860 CG5044 CG5261 CG6428 CG6984 CG7842 CG8778 Idh Men Paps Sply Thiolase Tpi yip2 |
| 18 | GO:0006082 | P | 5, | 35 | 12.533 (x 2.793) | 392 (0.089) | 2.32e-06 | organic acid metabolism | Aats-ala-m Aats-asp Aats-cys Aats-gly Aats-his Aats-thr Aats-trp Aats-tyr Aats-val Alas BcDNA:GH07485 CG10802 CG1140 CG11876 CG12264 CG1673 CG2107 CG2263 CG31075 CG4225 CG4802 CG4860 CG5044 CG5261 CG6428 CG6984 CG7842 CG8778 Idh Men Paps Sply Thiolase Tpi yip2 |
| 19 | GO:0044237 | P | 4, | 192 | 147.999 (x 1.297) | 4629 (0.041) | 4.19e-06 | cellular metabolism | Aats-ala-m Aats-asp Aats-cys Aats-gly Aats-his Aats-thr Aats-trp Aats-tyr Aats-val Adk1 Ahcy13 Alas Aprt Ate1 BG:BACR48E02.4 BcDNA:GH07485 BcDNA:LD32788 CG10042 CG10268 CG10306 CG10333 CG10414 CG10638 CG10639 CG10721 CG10802 CG1140 CG11710 CG11876 CG11883 CG11989 CG12013 CG12163 CG12177 CG12264 CG12534 CG12954 CG1319 CG13645 CG15094 CG1673 CG17187 CG17266 CG17760 CG18522 CG1882 CG2107 CG2246 CG2263 CG2789 CG2846 CG31075 CG32380 CG32549 CG32687 CG33138 CG3499 CG3522 CG3561 CG3608 CG3609 CG3731 CG3931 CG4225 CG4447 CG4769 CG4802 CG4860 CG5044 CG5261 CG5380 CG5604 CG6000 CG6016 CG6272 CG6428 CG6512 CG6523 CG6718 CG6746 CG6805 CG6984 CG7417 CG7598 CG7789 CG7842 CG8128 CG8360 CG8412 CG8417 CG8446 CG8636 CG8728 CG8778 CG9215 CG9232 CG9240 CG9249 CG9882 Cat Cyp6a20 Cyp6d5 Cyt-b5 Cyt-c-p Dak1 Dgp-1 Dip-B EG:100G10.7 EfTuM Eno Gapdh1 Gapdh2 Gclm Gfat2 Gp93 GstE1 GstE6 GstE7 Hmgs Ide Idh Inos Jafrac1 JhI-1 Jra MTF-1 Men Mocs1 NTPase Nedd4 PEK Paps Pcd Pfk Pgm Pi4KIIalpha Prx6005 PyK Rep RhoGEF2 SCAP Sply Sras TMS1 TfIIEbeta Thiolase Thor Tpi Transaldolase Trxr-1 Ugt86Da Updo Vha36 Vha44 bcn92 betaTub60D betaggt-II cathD cl eIF-2alpha eIF2B-alpha eIF2B-delta eIF2B-gamma fbl ferrochelatase ire-1 kraken l(2)05070 l(2)k01209 l(3)02640 mRpL11 mRpL13 mRpL19 mRpL21 mRpL22 mRpL22-24 mRpL28 mRpL3 mRpL48 mRpS18a mRpS18b mRpS2 mRpS22 mRpS25 mRpS32 mRpS33 mRpS9 mio mus205 poe ref(2)P yip2 |
| 20 | GO:0016876 | F | 5, | 12 | 1.854 (x 6.471) | 58 (0.207) | 1.37e-05 | ligase activity, forming aminoacyl-tRNA and related compounds | Aats-ala-m Aats-asp Aats-cys Aats-gly Aats-his Aats-thr Aats-trp Aats-tyr Aats-val CG10802 CG2263 CG4225 |
| 21 | GO:0000314 | C | 4, 5, 6, 7, 8, 9, 10, | 9 | 0.959 (x 9.383) | 30 (0.300) | 1.41e-05 | organellar small ribosomal subunit | mRpL3 mRpS18a mRpS18b mRpS2 mRpS22 mRpS25 mRpS32 mRpS33 mRpS9 |
| 22 | GO:0004812 | F | 6, | 12 | 1.854 (x 6.471) | 58 (0.207) | 1.43e-05 | aminoacyl-tRNA ligase activity | Aats-ala-m Aats-asp Aats-cys Aats-gly Aats-his Aats-thr Aats-trp Aats-tyr Aats-val CG10802 CG2263 CG4225 |
| 23 | GO:0005763 | C | 5, 6, 7, 8, 9, 10, 11, 12, 13, | 9 | 0.959 (x 9.383) | 30 (0.300) | 1.47e-05 | mitochondrial small ribosomal subunit | mRpL3 mRpS18a mRpS18b mRpS2 mRpS22 mRpS25 mRpS32 mRpS33 mRpS9 |
| 24 | GO:0016875 | F | 4, | 12 | 1.854 (x 6.471) | 58 (0.207) | 1.51e-05 | ligase activity, forming carbon-oxygen bonds | Aats-ala-m Aats-asp Aats-cys Aats-gly Aats-his Aats-thr Aats-trp Aats-tyr Aats-val CG10802 CG2263 CG4225 |
| 25 | GO:0031974 | C | 2, | 35 | 13.780 (x 2.540) | 431 (0.081) | 1.59e-05 | membrane-enclosed lumen | Alas CG1140 CG12954 CG1319 CG31075 CG3731 CG5134 CG5261 CG5380 CG8728 CG9065 Cyt-c-p MTF-1 PEK TfIIEbeta Thiolase mRpL11 mRpL13 mRpL19 mRpL21 mRpL22 mRpL22-24 mRpL28 mRpL3 mRpL48 mRpL55 mRpS18a mRpS18b mRpS2 mRpS22 mRpS25 mRpS32 mRpS33 mRpS9 mus205 |
| 26 | GO:0043039 | P | 8, 9, | 12 | 1.918 (x 6.255) | 60 (0.200) | 1.59e-05 | tRNA aminoacylation | Aats-ala-m Aats-asp Aats-cys Aats-gly Aats-his Aats-thr Aats-trp Aats-tyr Aats-val CG10802 CG2263 CG4225 |
| 27 | GO:0006418 | P | 8, 9, 10, | 12 | 1.918 (x 6.255) | 60 (0.200) | 1.65e-05 | tRNA aminoacylation for protein translation | Aats-ala-m Aats-asp Aats-cys Aats-gly Aats-his Aats-thr Aats-trp Aats-tyr Aats-val CG10802 CG2263 CG4225 |
| 28 | GO:0043233 | C | 3, 4, | 35 | 13.780 (x 2.540) | 431 (0.081) | 1.65e-05 | organelle lumen | Alas CG1140 CG12954 CG1319 CG31075 CG3731 CG5134 CG5261 CG5380 CG8728 CG9065 Cyt-c-p MTF-1 PEK TfIIEbeta Thiolase mRpL11 mRpL13 mRpL19 mRpL21 mRpL22 mRpL22-24 mRpL28 mRpL3 mRpL48 mRpL55 mRpS18a mRpS18b mRpS2 mRpS22 mRpS25 mRpS32 mRpS33 mRpS9 mus205 |
| 29 | GO:0043038 | P | 7, 8, | 12 | 1.950 (x 6.153) | 61 (0.197) | 1.86e-05 | amino acid activation | Aats-ala-m Aats-asp Aats-cys Aats-gly Aats-his Aats-thr Aats-trp Aats-tyr Aats-val CG10802 CG2263 CG4225 |
| 30 | GO:0006399 | P | 7, | 14 | 2.814 (x 4.976) | 88 (0.159) | 3.01e-05 | tRNA metabolism | Aats-ala-m Aats-asp Aats-cys Aats-gly Aats-his Aats-thr Aats-trp Aats-tyr Aats-val CG10802 CG2263 CG4225 CG5380 JhI-1 |
| 31 | GO:0050875 | P | 3, | 234 | 198.739 (x 1.177) | 6216 (0.038) | 4.21e-05 | cellular physiological process | AP-2sigma Aats-ala-m Aats-asp Aats-cys Aats-gly Aats-his Aats-thr Aats-trp Aats-tyr Aats-val Adk1 Ahcy13 Alas Aprt Ate1 BG:BACR48E02.4 BcDNA:GH07485 BcDNA:LD32788 CG10042 CG10068 CG10268 CG10306 CG10333 CG10414 CG10535 CG10638 CG10639 CG10721 CG10802 CG1140 CG11710 CG11876 CG11880 CG11883 CG11897 CG11989 CG12013 CG12163 CG12177 CG12264 CG12534 CG12876 CG12954 CG1319 CG13645 CG13887 CG14040 CG15094 CG1598 CG1673 CG17184 CG17187 CG17266 CG17760 CG18522 CG1882 CG2107 CG2246 CG2263 CG2789 CG2846 CG31075 CG31793 CG32380 CG32549 CG32687 CG33066 CG33138 CG33193 CG3397 CG3476 CG3499 CG3522 CG3561 CG3608 CG3609 CG3731 CG3931 CG4225 CG4447 CG4769 CG4802 CG4860 CG4963 CG5044 CG5261 CG5380 CG5604 CG6000 CG6016 CG6272 CG6428 CG6512 CG6523 CG6608 CG6718 CG6746 CG6805 CG6984 CG7417 CG7598 CG7627 CG7789 CG7816 CG7842 CG8128 CG8360 CG8412 CG8417 CG8446 CG8479 CG8602 CG8636 CG8728 CG8778 CG9065 CG9139 CG9215 CG9232 CG9240 CG9249 CG9298 CG9882 Cat Cyp6a20 Cyp6d5 Cyt-b5 Cyt-c-p Dak1 Dgp-1 Dip-B EG:100G10.7 EfTuM Eno Fer1HCH Fer2LCH Gapdh1 Gapdh2 Gclm Gfat2 Gp93 GstE1 GstE6 GstE7 Hmgs Ide Idh Inos Jafrac1 JhI-1 Jra MTF-1 Men Mocs1 NTPase Nedd4 Nsf2 PEK Paps Past1 Pcd Pfk Pgm Pi4KIIalpha Prx6005 PyK Rep RhoGEF2 SCAP SH3PX1 Shawn Sply Sras Syx13 TMS1 TepIV TfIIEbeta Thiolase Thor Tim10 Tpi Transaldolase Trxr-1 Ugt86Da Unc-76 Updo Vha36 Vha44 aralar1 att-ORFA bcn92 betaTub60D betaggt-II cathD cl cnn dynactin-subunit-p25 eIF-2alpha eIF2B-alpha eIF2B-delta eIF2B-gamma fbl ferrochelatase foi gammaSnap ire-1 kraken l(2)05070 l(2)k01209 l(3)02640 mRpL11 mRpL13 mRpL19 mRpL21 mRpL22 mRpL22-24 mRpL28 mRpL3 mRpL48 mRpS18a mRpS18b mRpS2 mRpS22 mRpS25 mRpS32 mRpS33 mRpS9 milton mio mus205 poe ref(2)P snapin yip2 |
| 32 | GO:0051186 | P | 5, | 24 | 8.089 (x 2.967) | 253 (0.095) | 7.34e-05 | cofactor metabolism | Adk1 Alas CG11876 CG12264 CG13645 CG2789 CG3608 CG4860 CG5261 CG8446 CG9249 Gclm Idh Men Mocs1 Pcd Tpi Transaldolase Updo Vha36 Vha44 fbl ferrochelatase l(3)02640 |
| 33 | GO:0043037 | P | 7, 8, | 20 | 5.947 (x 3.363) | 186 (0.108) | 7.88e-05 | translation | Aats-ala-m Aats-asp Aats-cys Aats-gly Aats-his Aats-thr Aats-trp Aats-tyr Aats-val CG10306 CG10802 CG2263 CG4225 CG8636 EfTuM Thor eIF-2alpha eIF2B-alpha eIF2B-delta eIF2B-gamma |
| 34 | GO:0003735 | F | 3, | 20 | 6.011 (x 3.327) | 188 (0.106) | 9.04e-05 | structural constituent of ribosome | CG12954 Pcd mRpL11 mRpL13 mRpL19 mRpL21 mRpL22 mRpL22-24 mRpL28 mRpL3 mRpL48 mRpL55 mRpS18a mRpS18b mRpS2 mRpS22 mRpS25 mRpS32 mRpS33 mRpS9 |
| 35 | GO:0005840 | C | 4, 5, 6, 7, 8, | 20 | 6.043 (x 3.310) | 189 (0.106) | 9.54e-05 | ribosome | CG12954 Pcd mRpL11 mRpL13 mRpL19 mRpL21 mRpL22 mRpL22-24 mRpL28 mRpL3 mRpL48 mRpL55 mRpS18a mRpS18b mRpS2 mRpS22 mRpS25 mRpS32 mRpS33 mRpS9 |
| 36 | GO:0006091 | P | 5, | 36 | 16.146 (x 2.230) | 505 (0.071) | 0.00017 | generation of precursor metabolites and energy | CG10639 CG10721 CG11876 CG12534 CG18522 CG33138 CG3609 CG3731 CG4769 CG4860 CG5261 CG6523 CG7598 CG8728 Cat Cyp6a20 Cyp6d5 Cyt-b5 Cyt-c-p Eno Gapdh1 Gapdh2 Idh Men PEK Pfk PyK TMS1 Tpi Transaldolase Trxr-1 Vha36 Vha44 bcn92 cl ire-1 |
| 37 | GO:0009112 | P | 6, | 12 | 2.494 (x 4.812) | 78 (0.154) | 0.000224 | nucleobase metabolism | Adk1 Ahcy13 Aprt CG12177 CG18522 CG2246 CG32549 CG4802 CG8360 Dak1 Vha36 l(2)k01209 |
| 38 | GO:0046483 | P | 5, | 16 | 4.476 (x 3.575) | 140 (0.114) | 0.000344 | heterocycle metabolism | Adk1 Ahcy13 Alas Aprt CG12177 CG18522 CG2246 CG32549 CG4802 CG8360 Dak1 Updo Vha36 ferrochelatase l(2)k01209 l(3)02640 |
| 39 | GO:0016740 | F | 3, | 55 | 30.917 (x 1.779) | 967 (0.057) | 0.000454 | transferase activity | Adk1 Alas Aprt Ate1 BG:BACR48E02.4 CG10268 CG10414 CG1140 CG11989 CG12264 CG13645 CG1673 CG1969 CG2107 CG2246 CG2846 CG32687 CG33138 CG3608 CG4407 CG4802 CG5224 CG5261 CG5380 CG6016 CG7842 CG8412 CG8446 CG9232 CG9249 CG9882 Dak1 Gfat2 GstE1 GstE6 GstE7 GstE9 Hmgs PEK Paps Pfk Pi4KIIalpha PyK Rep Thiolase Transaldolase Ugt86Da betaggt-II eIF2B-gamma fbl ire-1 l(2)k01209 l(3)02640 mus205 yip2 |
| 40 | GO:0006725 | P | 5, | 16 | 4.636 (x 3.451) | 145 (0.110) | 0.000512 | aromatic compound metabolism | Adk1 Ahcy13 Aprt CG12177 CG18522 CG1882 CG2246 CG32549 CG4802 CG8360 Dak1 Mocs1 Pcd Vha36 kraken l(2)k01209 |
| 41 | GO:0005996 | P | 6, 7, | 13 | 3.197 (x 4.066) | 100 (0.130) | 0.000553 | monosaccharide metabolism | CG5261 CG8417 CG9232 Eno Gapdh1 Gapdh2 Gfat2 Inos Pfk Pgm PyK Tpi Transaldolase |
| 42 | GO:0007582 | P | 2, | 246 | 218.434 (x 1.126) | 6832 (0.036) | 0.000811 | physiological process | AP-2sigma Aats-ala-m Aats-asp Aats-cys Aats-gly Aats-his Aats-thr Aats-trp Aats-tyr Aats-val Adk1 Ahcy13 Alas Aprt Ate1 BG:BACR48E02.4 BcDNA:GH07485 BcDNA:LD32788 CG10042 CG10068 CG10268 CG10306 CG10333 CG10340 CG10414 CG10535 CG10638 CG10639 CG10721 CG10802 CG1140 CG11710 CG11876 CG11880 CG11883 CG11897 CG11967 CG11989 CG12013 CG12163 CG12171 CG12177 CG12264 CG12534 CG12876 CG12954 CG1319 CG13645 CG13887 CG14040 CG15094 CG1598 CG1635 CG1673 CG17184 CG17187 CG17266 CG17294 CG17760 CG18522 CG1882 CG2064 CG2107 CG2246 CG2263 CG2789 CG2846 CG31075 CG31549 CG31793 CG32380 CG32549 CG32687 CG33066 CG33138 CG33193 CG3397 CG3476 CG3499 CG3522 CG3561 CG3608 CG3609 CG3731 CG3931 CG4225 CG4407 CG4447 CG4769 CG4802 CG4860 CG4963 CG5044 CG5261 CG5380 CG5567 CG5604 CG6000 CG6016 CG6272 CG6428 CG6512 CG6523 CG6608 CG6668 CG6718 CG6746 CG6805 CG6984 CG7417 CG7598 CG7627 CG7789 CG7816 CG7842 CG8128 CG8360 CG8412 CG8417 CG8446 CG8479 CG8602 CG8636 CG8728 CG8778 CG8839 CG9065 CG9139 CG9215 CG9232 CG9240 CG9249 CG9298 CG9836 CG9882 Cat Cyp6a20 Cyp6d5 Cyt-b5 Cyt-c-p Dak1 Dgp-1 Dip-B EG:100G10.7 EfTuM Eno Fer1HCH Fer2LCH Gapdh1 Gapdh2 Gclm Gfat2 Gp93 GstE1 GstE6 GstE7 Hmgs Ide Idh Inos Jafrac1 JhI-1 Jra MTF-1 Men Mocs1 NTPase Nedd4 Nsf2 PEK Paps Past1 Pcd Pfk Pgm Pi4KIIalpha Prx6005 PyK Rep RhoGEF2 SCAP SH3PX1 Shawn Sply Sras Syx13 TMS1 TepIV TfIIEbeta Thiolase Thor Tim10 Tpi Transaldolase Trxr-1 Ugt86Da Unc-76 Updo Vha36 Vha44 aralar1 att-ORFA bcn92 betaTub60D betaggt-II cathD cl cnn dynactin-subunit-p25 eIF-2alpha eIF2B-alpha eIF2B-delta eIF2B-gamma fbl ferrochelatase foi gammaSnap ire-1 kraken l(2)05070 l(2)k01209 l(3)02640 mRpL11 mRpL13 mRpL19 mRpL21 mRpL22 mRpL22-24 mRpL28 mRpL3 mRpL48 mRpS18a mRpS18b mRpS2 mRpS22 mRpS25 mRpS32 mRpS33 mRpS9 milton mio mus205 poe ref(2)P snapin yip2 |
| 43 | GO:0005740 | C | 4, 5, 6, 7, 8, 9, 10, | 19 | 6.554 (x 2.899) | 205 (0.093) | 0.000943 | mitochondrial envelope | CG2789 CG33066 CG3476 CG3499 CG3731 CG4225 CG4769 CG4963 CG6608 CG7598 CG9065 CG9240 Cyt-c-p Shawn Tim10 aralar1 att-ORFA bcn92 ferrochelatase |
| 44 | GO:0005851 | C | 3, 5, 6, 7, 8, | 4 | 0.224 (x 17.873) | 7 (0.571) | 0.00105 | eukaryotic translation initiation factor 2B complex | eIF-2alpha eIF2B-alpha eIF2B-delta eIF2B-gamma |
| 45 | GO:0016491 | F | 3, | 39 | 19.951 (x 1.955) | 624 (0.062) | 0.00113 | oxidoreductase activity | BcDNA:GH07485 CG10638 CG10639 CG10721 CG11876 CG11897 CG12013 CG12171 CG12534 CG1319 CG18522 CG18547 CG2064 CG31075 CG31549 CG3397 CG3609 CG3731 CG4769 CG4860 CG5044 CG6523 CG8778 Cat Cyp6a20 Cyp6d5 Cyt-b5 Cyt-c-p Gapdh1 Gapdh2 Idh Jafrac1 Men Prx6005 Thiolase Trxr-1 bcn92 bsf cl |
| 46 | GO:0006629 | P | 5, | 33 | 15.666 (x 2.106) | 490 (0.067) | 0.00113 | lipid metabolism | BcDNA:GH07485 CG10268 CG10639 CG1140 CG12013 CG12171 CG1635 CG2789 CG31549 CG32380 CG3476 CG3522 CG4860 CG5044 CG6016 CG6718 CG6805 CG6984 CG7789 CG7842 CG8778 Cyp6a20 Cyp6d5 Cyt-b5 Hmgs Inos Pi4KIIalpha Sply Thiolase Ugt86Da Vha44 aralar1 yip2 |
| 47 | GO:0006635 | P | 8, 9, | 6 | 0.703 (x 8.530) | 22 (0.273) | 0.00145 | fatty acid beta-oxidation | BcDNA:GH07485 CG5044 CG6984 CG8778 Thiolase yip2 |
| 48 | GO:0015934 | C | 3, 4, 5, 6, 7, 8, 9, | 12 | 3.069 (x 3.910) | 96 (0.125) | 0.00149 | large ribosomal subunit | CG12954 mRpL11 mRpL13 mRpL19 mRpL21 mRpL22 mRpL22-24 mRpL28 mRpL3 mRpL48 mRpL55 mRpS32 |
| 49 | GO:0006144 | P | 7, | 9 | 1.758 (x 5.118) | 55 (0.164) | 0.00152 | purine base metabolism | Adk1 Ahcy13 Aprt CG12177 CG18522 CG2246 CG32549 CG4802 Vha36 |
| 50 | GO:0019395 | P | 7, 8, | 6 | 0.735 (x 8.159) | 23 (0.261) | 0.0018 | fatty acid oxidation | BcDNA:GH07485 CG5044 CG6984 CG8778 Thiolase yip2 |
| 51 | GO:0044255 | P | 5, 6, | 25 | 10.711 (x 2.334) | 335 (0.075) | 0.00183 | cellular lipid metabolism | BcDNA:GH07485 CG10268 CG1140 CG12013 CG32380 CG3522 CG4860 CG5044 CG6016 CG6718 CG6984 CG7789 CG7842 CG8778 Cyp6a20 Cyp6d5 Cyt-b5 Hmgs Inos Pi4KIIalpha Sply Thiolase Ugt86Da Vha44 yip2 |
| 52 | GO:0006520 | P | 6, 7, | 21 | 8.345 (x 2.517) | 261 (0.080) | 0.00242 | amino acid metabolism | Aats-ala-m Aats-asp Aats-cys Aats-gly Aats-his Aats-thr Aats-trp Aats-tyr Aats-val Alas CG10802 CG12264 CG1673 CG2107 CG2263 CG31075 CG4225 CG4802 CG6428 Paps Sply |
| 53 | GO:0005743 | C | 5, 6, 7, 8, 9, 10, 11, 12, | 16 | 5.403 (x 2.961) | 169 (0.095) | 0.00255 | mitochondrial inner membrane | CG33066 CG3476 CG3499 CG3731 CG4225 CG4769 CG4963 CG6608 CG7598 Cyt-c-p Shawn Tim10 aralar1 att-ORFA bcn92 ferrochelatase |
| 54 | GO:0019866 | C | 4, 5, 6, 7, 8, 9, | 16 | 5.563 (x 2.876) | 174 (0.092) | 0.00353 | organelle inner membrane | CG33066 CG3476 CG3499 CG3731 CG4225 CG4769 CG4963 CG6608 CG7598 Cyt-c-p Shawn Tim10 aralar1 att-ORFA bcn92 ferrochelatase |
| 55 | GO:0044446 | C | 3, 4, 5, 6, 7, | 69 | 45.528 (x 1.516) | 1424 (0.048) | 0.00415 | intracellular organelle part | AP-2sigma Alas CG10333 CG1140 CG12954 CG1319 CG14040 CG17266 CG2789 CG31075 CG33066 CG3476 CG3499 CG3731 CG3931 CG4225 CG4769 CG4963 CG5134 CG5261 CG5380 CG6608 CG7598 CG7809 CG8728 CG9065 CG9240 CG9298 CG9424 Cyt-c-p MTF-1 PEK Rep Shawn Sply Sras TfIIEbeta Thiolase Tim10 Unc-76 Vha36 Vha44 aralar1 att-ORFA bcn92 betaTub60D cnn dynactin-subunit-p25 fbl ferrochelatase mRpL11 mRpL13 mRpL19 mRpL21 mRpL22 mRpL22-24 mRpL28 mRpL3 mRpL48 mRpL55 mRpS18a mRpS18b mRpS2 mRpS22 mRpS25 mRpS32 mRpS33 mRpS9 mus205 |
| 56 | GO:0044422 | C | 2, 3, | 69 | 45.528 (x 1.516) | 1424 (0.048) | 0.00422 | organelle part | AP-2sigma Alas CG10333 CG1140 CG12954 CG1319 CG14040 CG17266 CG2789 CG31075 CG33066 CG3476 CG3499 CG3731 CG3931 CG4225 CG4769 CG4963 CG5134 CG5261 CG5380 CG6608 CG7598 CG7809 CG8728 CG9065 CG9240 CG9298 CG9424 Cyt-c-p MTF-1 PEK Rep Shawn Sply Sras TfIIEbeta Thiolase Tim10 Unc-76 Vha36 Vha44 aralar1 att-ORFA bcn92 betaTub60D cnn dynactin-subunit-p25 fbl ferrochelatase mRpL11 mRpL13 mRpL19 mRpL21 mRpL22 mRpL22-24 mRpL28 mRpL3 mRpL48 mRpL55 mRpS18a mRpS18b mRpS2 mRpS22 mRpS25 mRpS32 mRpS33 mRpS9 mus205 |
| 57 | GO:0006066 | P | 5, | 15 | 5.148 (x 2.914) | 161 (0.093) | 0.00436 | alcohol metabolism | CG10268 CG5261 CG8417 CG9232 Eno Gapdh1 Gapdh2 Gfat2 Hmgs Inos Pfk Pgm PyK Tpi Transaldolase |
| 58 | GO:0046164 | P | 6, | 8 | 1.631 (x 4.906) | 51 (0.157) | 0.00441 | alcohol catabolism | CG5261 Eno Gapdh1 Gapdh2 Pfk PyK Tpi Transaldolase |
| 59 | GO:0006096 | P | 8, 10, 11, | 7 | 1.247 (x 5.614) | 39 (0.179) | 0.00447 | glycolysis | CG5261 Eno Gapdh1 Gapdh2 Pfk PyK Tpi |
| 60 | GO:0046365 | P | 7, 8, | 8 | 1.631 (x 4.906) | 51 (0.157) | 0.00449 | monosaccharide catabolism | CG5261 Eno Gapdh1 Gapdh2 Pfk PyK Tpi Transaldolase |
| 61 | GO:0016746 | F | 4, | 13 | 4.060 (x 3.202) | 127 (0.102) | 0.00452 | transferase activity, transferring acyl groups | Alas Ate1 CG10414 CG11989 CG1969 CG2107 CG5261 CG7842 Hmgs Rep Thiolase betaggt-II yip2 |
| 62 | GO:0044424 | C | 3, 4, 5, | 131 | 101.991 (x 1.284) | 3190 (0.041) | 0.00454 | intracellular part | AP-2sigma Aats-ala-m Aats-asp Aats-his Adk1 Alas Aprt BcDNA:GH07485 CG10042 CG10268 CG10306 CG10333 CG1140 CG11876 CG12013 CG12264 CG12534 CG12954 CG1319 CG13887 CG14040 CG1673 CG17266 CG2263 CG2789 CG2846 CG31075 CG32549 CG33066 CG3476 CG3499 CG3731 CG3931 CG4225 CG4589 CG4769 CG4882 CG4963 CG5134 CG5261 CG5380 CG5604 CG6272 CG6512 CG6608 CG6718 CG7417 CG7598 CG7809 CG7842 CG8412 CG8636 CG8728 CG9065 CG9215 CG9240 CG9298 CG9424 Cat Cyt-c-p Dip-B EG:100G10.7 EfTuM Eno Gapdh1 Gapdh2 Gclm Gfat2 Idh Inos Jafrac1 JhI-1 Jra MTF-1 Nsf2 PEK Pcd Pfk PyK Rep Shawn Sply Sras TfIIEbeta Thiolase Tim10 Transaldolase Trxr-1 Unc-76 Updo Vha36 Vha44 aralar1 att-ORFA bcn92 betaTub60D bor bsf cnn dynactin-subunit-p25 eIF-2alpha eIF2B-alpha eIF2B-delta eIF2B-gamma fbl ferrochelatase gammaSnap l(2)05070 mRpL11 mRpL13 mRpL19 mRpL21 mRpL22 mRpL22-24 mRpL28 mRpL3 mRpL48 mRpL55 mRpS18a mRpS18b mRpS2 mRpS22 mRpS25 mRpS32 mRpS33 mRpS9 milton mus205 ref(2)P snapin yip2 |
| 63 | GO:0006007 | P | 9, 10, | 8 | 1.631 (x 4.906) | 51 (0.157) | 0.00456 | glucose catabolism | CG5261 Eno Gapdh1 Gapdh2 Pfk PyK Tpi Transaldolase |
| 64 | GO:0019318 | P | 7, 8, | 10 | 2.558 (x 3.910) | 80 (0.125) | 0.00464 | hexose metabolism | CG5261 CG9232 Eno Gapdh1 Gapdh2 Inos Pfk PyK Tpi Transaldolase |
| 65 | GO:0019320 | P | 8, 9, | 8 | 1.631 (x 4.906) | 51 (0.157) | 0.00464 | hexose catabolism | CG5261 Eno Gapdh1 Gapdh2 Pfk PyK Tpi Transaldolase |
| 66 | GO:0006779 | P | 7, | 4 | 0.352 (x 11.374) | 11 (0.364) | 0.00595 | porphyrin biosynthesis | Alas Updo ferrochelatase l(3)02640 |
| 67 | GO:0031966 | C | 5, 6, 7, 8, 9, 10, 11, | 16 | 5.979 (x 2.676) | 187 (0.086) | 0.00652 | mitochondrial membrane | CG33066 CG3476 CG3499 CG3731 CG4225 CG4769 CG4963 CG6608 CG7598 Cyt-c-p Shawn Tim10 aralar1 att-ORFA bcn92 ferrochelatase |
| 68 | GO:0015935 | C | 3, 4, 5, 6, 7, 8, 9, | 9 | 2.206 (x 4.080) | 69 (0.130) | 0.00661 | small ribosomal subunit | mRpL3 mRpS18a mRpS18b mRpS2 mRpS22 mRpS25 mRpS32 mRpS33 mRpS9 |
| 69 | GO:0031975 | C | 2, | 20 | 8.537 (x 2.343) | 267 (0.075) | 0.00687 | envelope | CG2789 CG33066 CG3476 CG3499 CG3731 CG4225 CG4769 CG4963 CG6608 CG7598 CG9065 CG9240 CG9424 Cyt-c-p Shawn Tim10 aralar1 att-ORFA bcn92 ferrochelatase |
| 70 | GO:0031967 | C | 3, 4, 5, 6, 7, 8, | 20 | 8.537 (x 2.343) | 267 (0.075) | 0.00697 | organelle envelope | CG2789 CG33066 CG3476 CG3499 CG3731 CG4225 CG4769 CG4963 CG6608 CG7598 CG9065 CG9240 CG9424 Cyt-c-p Shawn Tim10 aralar1 att-ORFA bcn92 ferrochelatase |
| 71 | GO:0015980 | P | 6, | 12 | 3.773 (x 3.181) | 118 (0.102) | 0.00734 | energy derivation by oxidation of organic compounds | CG11876 CG33138 CG5261 Eno Gapdh1 Gapdh2 Idh Men Pfk PyK Tpi Transaldolase |
| 72 | GO:0005622 | C | 3, 4, | 133 | 105.348 (x 1.262) | 3295 (0.040) | 0.00795 | intracellular | AP-2sigma Aats-ala-m Aats-asp Aats-his Adk1 Alas Aprt BcDNA:GH07485 CG10042 CG10268 CG10306 CG10333 CG1140 CG11876 CG12013 CG12264 CG12534 CG12954 CG1319 CG13887 CG14040 CG1673 CG17266 CG2263 CG2789 CG2846 CG31075 CG32549 CG33066 CG3476 CG3499 CG3731 CG3931 CG4225 CG4589 CG4769 CG4882 CG4963 CG5134 CG5261 CG5380 CG5604 CG6272 CG6512 CG6608 CG6718 CG6744 CG7417 CG7598 CG7809 CG7842 CG8412 CG8636 CG8728 CG9065 CG9215 CG9240 CG9298 CG9424 Cat Cyt-c-p Dip-B EG:100G10.7 EfTuM Eno Gapdh1 Gapdh2 Gclm Gfat2 Idh Inos Jafrac1 JhI-1 Jra MTF-1 Nedd4 Nsf2 PEK Pcd Pfk PyK Rep Shawn Sply Sras TfIIEbeta Thiolase Tim10 Transaldolase Trxr-1 Unc-76 Updo Vha36 Vha44 aralar1 att-ORFA bcn92 betaTub60D bor bsf cnn dynactin-subunit-p25 eIF-2alpha eIF2B-alpha eIF2B-delta eIF2B-gamma fbl ferrochelatase gammaSnap l(2)05070 mRpL11 mRpL13 mRpL19 mRpL21 mRpL22 mRpL22-24 mRpL28 mRpL3 mRpL48 mRpL55 mRpS18a mRpS18b mRpS2 mRpS22 mRpS25 mRpS32 mRpS33 mRpS9 milton mus205 ref(2)P snapin yip2 |
| 73 | GO:0006519 | P | 5, | 21 | 9.368 (x 2.242) | 293 (0.072) | 0.00851 | amino acid and derivative metabolism | Aats-ala-m Aats-asp Aats-cys Aats-gly Aats-his Aats-thr Aats-trp Aats-tyr Aats-val Alas CG10802 CG12264 CG1673 CG2107 CG2263 CG31075 CG4225 CG4802 CG6428 Paps Sply |
| 74 | GO:0006206 | P | 7, | 6 | 1.023 (x 5.864) | 32 (0.188) | 0.00855 | pyrimidine base metabolism | Adk1 CG12177 CG2246 CG8360 Dak1 l(2)k01209 |
| 75 | GO:0006092 | P | 7, | 11 | 3.325 (x 3.308) | 104 (0.106) | 0.00866 | main pathways of carbohydrate metabolism | CG11876 CG5261 Eno Gapdh1 Gapdh2 Idh Men Pfk PyK Tpi Transaldolase |
| 76 | GO:0000166 | F | 3, | 46 | 28.072 (x 1.639) | 878 (0.052) | 0.00903 | nucleotide binding | Aats-ala-m Aats-asp Aats-cys Aats-gly Aats-his Aats-thr Aats-trp Aats-tyr Aats-val Adk1 BcDNA:LD32788 CG10333 CG10802 CG11897 CG1598 CG17760 CG17904 CG2263 CG31793 CG3499 CG3561 CG3608 CG4225 CG4858 CG6512 CG6668 CG7627 CG8479 Dak1 Dgp-1 EG:100G10.7 EfTuM Gp93 Nsf2 PEK Paps Past1 Vha44 betaTub60D bor eIF-2alpha eIF2B-alpha eIF2B-delta ire-1 l(2)k01209 mus205 |
| 77 | GO:0030529 | C | 3, 4, 5, 6, | 22 | 10.167 (x 2.164) | 318 (0.069) | 0.00961 | ribonucleoprotein complex | CG10333 CG12954 CG17266 Pcd mRpL11 mRpL13 mRpL19 mRpL21 mRpL22 mRpL22-24 mRpL28 mRpL3 mRpL48 mRpL55 mRpS18a mRpS18b mRpS2 mRpS22 mRpS25 mRpS32 mRpS33 mRpS9 |
| 78 | GO:0006778 | P | 6, | 4 | 0.416 (x 9.624) | 13 (0.308) | 0.0104 | porphyrin metabolism | Alas Updo ferrochelatase l(3)02640 |
| 79 | GO:0006006 | P | 8, 9, | 8 | 1.918 (x 4.170) | 60 (0.133) | 0.0104 | glucose metabolism | CG5261 Eno Gapdh1 Gapdh2 Pfk PyK Tpi Transaldolase |
| 80 | GO:0016765 | F | 4, | 8 | 1.918 (x 4.170) | 60 (0.133) | 0.0105 | transferase activity, transferring alkyl or aryl (other than methyl) groups | CG5224 GstE1 GstE6 GstE7 GstE9 Rep betaggt-II l(3)02640 |
| 81 | GO:0006732 | P | 6, | 18 | 7.641 (x 2.356) | 239 (0.075) | 0.011 | coenzyme metabolism | CG11876 CG13645 CG2789 CG3608 CG4860 CG5261 CG8446 CG9249 Gclm Idh Men Mocs1 Pcd Tpi Transaldolase Vha36 Vha44 fbl |
| 82 | GO:0009987 | P | 2, | 237 | 214.405 (x 1.105) | 6706 (0.035) | 0.0119 | cellular process | AP-2sigma Aats-ala-m Aats-asp Aats-cys Aats-gly Aats-his Aats-thr Aats-trp Aats-tyr Aats-val Adk1 Ahcy13 Alas Aprt Ate1 BG:BACR48E02.4 BcDNA:GH07485 BcDNA:LD32788 CG10042 CG10068 CG10268 CG10306 CG10333 CG10414 CG10535 CG10638 CG10639 CG10721 CG10802 CG1140 CG11710 CG11876 CG11880 CG11883 CG11897 CG11989 CG12013 CG12163 CG12177 CG12264 CG12534 CG12876 CG12954 CG1319 CG13645 CG13887 CG14040 CG15094 CG1598 CG1673 CG17184 CG17187 CG17266 CG17760 CG18522 CG1882 CG2107 CG2246 CG2263 CG2789 CG2846 CG31075 CG31793 CG32380 CG32549 CG32687 CG33066 CG33138 CG33193 CG3397 CG3476 CG3499 CG3522 CG3561 CG3608 CG3609 CG3731 CG3931 CG4225 CG4447 CG4769 CG4802 CG4860 CG4963 CG5044 CG5261 CG5380 CG5604 CG6000 CG6016 CG6272 CG6428 CG6512 CG6523 CG6608 CG6718 CG6746 CG6805 CG6984 CG7417 CG7598 CG7627 CG7789 CG7816 CG7842 CG8128 CG8360 CG8412 CG8417 CG8446 CG8479 CG8602 CG8636 CG8728 CG8778 CG9065 CG9139 CG9215 CG9232 CG9240 CG9249 CG9298 CG9882 Cat Cyp6a20 Cyp6d5 Cyt-b5 Cyt-c-p Dak1 Dgp-1 Dip-B EG:100G10.7 EfTuM Eno Fer1HCH Fer2LCH Gapdh1 Gapdh2 Gclm Gfat2 Gp93 GstE1 GstE6 GstE7 Hmgs Ide Idh Inos Jafrac1 JhI-1 Jra MTF-1 Men Mocs1 NTPase Nedd4 Nsf2 PEK Paps Past1 Pcd Pfk Pgm Pi4KIIalpha Prx6005 PyK Rep RhoGEF2 SCAP SH3PX1 Shawn Sply Sras Syx13 TMS1 TepIV TfIIEbeta Thiolase Thor Tim10 Tpi Transaldolase Trxr-1 Tsp86D Ugt86Da Unc-76 Updo Vha36 Vha44 aralar1 att-ORFA bcn92 betaTub60D betaggt-II cactin cathD cl cnn dynactin-subunit-p25 eIF-2alpha eIF2B-alpha eIF2B-delta eIF2B-gamma fbl ferrochelatase foi gammaSnap ire-1 kraken l(2)05070 l(2)k01209 l(3)02640 mRpL11 mRpL13 mRpL19 mRpL21 mRpL22 mRpL22-24 mRpL28 mRpL3 mRpL48 mRpS18a mRpS18b mRpS2 mRpS22 mRpS25 mRpS32 mRpS33 mRpS9 milton mio mus205 poe ref(2)P snapin yip2 ytr |
| 83 | GO:0009636 | P | 5, | 12 | 4.124 (x 2.910) | 129 (0.093) | 0.0139 | response to toxin | CG11897 CG12013 CG1598 CG31793 CG5224 CG7627 GstE1 GstE6 GstE7 GstE9 Ugt86Da kraken |
| 84 | GO:0017076 | F | 4, | 44 | 27.144 (x 1.621) | 849 (0.052) | 0.0139 | purine nucleotide binding | Aats-ala-m Aats-asp Aats-cys Aats-gly Aats-his Aats-thr Aats-trp Aats-tyr Aats-val Adk1 BcDNA:LD32788 CG10333 CG10802 CG11897 CG1598 CG17760 CG2263 CG31793 CG3499 CG3561 CG3608 CG4225 CG4858 CG6512 CG6668 CG7627 CG8479 Dak1 Dgp-1 EG:100G10.7 EfTuM Gp93 Nsf2 PEK Paps Past1 Vha44 betaTub60D bor eIF-2alpha eIF2B-alpha eIF2B-delta ire-1 l(2)k01209 |
| 85 | GO:0006081 | P | 5, | 2 | 0.064 (x 31.277) | 2 (1.000) | 0.0163 | aldehyde metabolism | CG10638 Idh |
| 86 | GO:0019740 | P | 5, | 2 | 0.064 (x 31.277) | 2 (1.000) | 0.0165 | nitrogen utilization | CG12264 CG9836 |
| 87 | GO:0004240 | F | 7, | 2 | 0.064 (x 31.277) | 2 (1.000) | 0.0166 | mitochondrial processing peptidase activity | CG3731 CG8728 |
| 88 | GO:0006090 | P | 7, | 4 | 0.480 (x 8.341) | 15 (0.267) | 0.0167 | pyruvate metabolism | CG11876 CG31075 CG5261 Tpi |
| 89 | GO:0044238 | P | 4, | 172 | 146.113 (x 1.177) | 4570 (0.038) | 0.0168 | primary metabolism | Aats-ala-m Aats-asp Aats-cys Aats-gly Aats-his Aats-thr Aats-trp Aats-tyr Aats-val Adk1 Ahcy13 Alas Aprt Ate1 BG:BACR48E02.4 BcDNA:GH07485 BcDNA:LD32788 CG10042 CG10268 CG10306 CG10333 CG10340 CG10414 CG10639 CG10802 CG1140 CG11710 CG11876 CG11883 CG11989 CG12013 CG12163 CG12171 CG12177 CG12264 CG12954 CG1319 CG13645 CG15094 CG1635 CG1673 CG17187 CG17266 CG17760 CG18522 CG2107 CG2246 CG2263 CG2789 CG31075 CG31549 CG32380 CG32549 CG32687 CG33138 CG3476 CG3499 CG3522 CG3561 CG3608 CG3731 CG3931 CG4225 CG4447 CG4802 CG4860 CG5044 CG5261 CG5380 CG5604 CG6000 CG6016 CG6272 CG6428 CG6512 CG6718 CG6746 CG6805 CG6984 CG7417 CG7598 CG7789 CG7842 CG8128 CG8360 CG8412 CG8417 CG8446 CG8636 CG8728 CG8778 CG9065 CG9215 CG9232 CG9240 CG9882 Cyp6a20 Cyp6d5 Cyt-b5 Dak1 Dgp-1 Dip-B EG:100G10.7 EfTuM Eno Gapdh1 Gapdh2 Gfat2 Gp93 Hmgs Ide Idh Inos JhI-1 Jra MTF-1 Men NTPase Nedd4 PEK Paps Pcd Pfk Pgm Pi4KIIalpha PyK Rep RhoGEF2 SCAP Sply Sras TfIIEbeta Thiolase Thor Tpi Transaldolase Ugt86Da Vha36 Vha44 aralar1 betaTub60D betaggt-II cathD eIF-2alpha eIF2B-alpha eIF2B-delta eIF2B-gamma ire-1 l(2)05070 l(2)k01209 mRpL11 mRpL13 mRpL19 mRpL21 mRpL22 mRpL22-24 mRpL28 mRpL3 mRpL48 mRpS18a mRpS18b mRpS2 mRpS22 mRpS25 mRpS32 mRpS33 mRpS9 mio mus205 poe ref(2)P yip2 |
| 90 | GO:0008415 | F | 6, | 11 | 3.677 (x 2.992) | 115 (0.096) | 0.0169 | acyltransferase activity | Alas CG10414 CG11989 CG1969 CG2107 CG5261 CG7842 Rep Thiolase betaggt-II yip2 |
| 91 | GO:0044275 | P | 7, | 8 | 2.142 (x 3.735) | 67 (0.119) | 0.0185 | cellular carbohydrate catabolism | CG5261 Eno Gapdh1 Gapdh2 Pfk PyK Tpi Transaldolase |
| 92 | GO:0016052 | P | 6, | 8 | 2.142 (x 3.735) | 67 (0.119) | 0.0187 | carbohydrate catabolism | CG5261 Eno Gapdh1 Gapdh2 Pfk PyK Tpi Transaldolase |
| 93 | GO:0031090 | C | 4, 5, 6, 7, 8, | 23 | 11.542 (x 1.993) | 361 (0.064) | 0.0189 | organelle membrane | AP-2sigma CG14040 CG33066 CG3476 CG3499 CG3731 CG4225 CG4769 CG4963 CG6608 CG7598 CG7809 Cyt-c-p Shawn Sply Sras Tim10 Vha36 Vha44 aralar1 att-ORFA bcn92 ferrochelatase |
| 94 | GO:0006800 | P | 5, | 8 | 2.142 (x 3.735) | 67 (0.119) | 0.0189 | oxygen and reactive oxygen species metabolism | CG12013 CG18522 Cat GstE1 GstE6 GstE7 Jafrac1 Prx6005 |
| 95 | GO:0016829 | F | 3, | 13 | 5.020 (x 2.590) | 157 (0.083) | 0.0224 | lyase activity | CG11967 CG12264 CG5044 CG6984 CG8778 Eno Hmgs Inos Men Pcd Sply Updo ferrochelatase |
| 96 | GO:0006807 | P | 4, | 24 | 12.501 (x 1.920) | 391 (0.061) | 0.0232 | nitrogen compound metabolism | Aats-ala-m Aats-asp Aats-cys Aats-gly Aats-his Aats-thr Aats-trp Aats-tyr Aats-val Alas CG10802 CG11897 CG12264 CG1673 CG2107 CG2263 CG31075 CG4225 CG4802 CG6428 CG8839 CG9836 Paps Sply |
| 97 | GO:0008252 | F | 7, | 3 | 0.256 (x 11.729) | 8 (0.375) | 0.0233 | nucleotidase activity | CG11883 CG32549 CG7789 |
| 98 | GO:0016747 | F | 5, | 11 | 3.869 (x 2.843) | 121 (0.091) | 0.0235 | transferase activity, transferring groups other than amino-acyl groups | Alas CG10414 CG11989 CG1969 CG2107 CG5261 CG7842 Rep Thiolase betaggt-II yip2 |
| 99 | GO:0051188 | P | 6, | 10 | 3.357 (x 2.979) | 105 (0.095) | 0.0265 | cofactor biosynthesis | Alas CG13645 Gclm Mocs1 Updo Vha36 Vha44 fbl ferrochelatase l(3)02640 |
| 100 | GO:0003743 | F | 4, 5, | 7 | 1.790 (x 3.910) | 56 (0.125) | 0.0267 | translation initiation factor activity | CG10306 CG8636 PEK eIF-2alpha eIF2B-alpha eIF2B-delta eIF2B-gamma |
| 101 | GO:0006118 | P | 6, | 21 | 10.647 (x 1.972) | 333 (0.063) | 0.0312 | electron transport | CG10639 CG10721 CG12534 CG18522 CG3609 CG3731 CG4769 CG4860 CG6523 CG8728 Cat Cyp6a20 Cyp6d5 Cyt-b5 Cyt-c-p PEK TMS1 Trxr-1 bcn92 cl ire-1 |
| 102 | GO:0004869 | F | 6, | 3 | 0.288 (x 10.426) | 9 (0.333) | 0.0321 | cysteine protease inhibitor activity | CG10460 CG12163 Cys |
| 103 | GO:0019201 | F | 7, | 5 | 0.959 (x 5.213) | 30 (0.167) | 0.0321 | nucleotide kinase activity | Adk1 CG2246 Dak1 Paps l(2)k01209 |
| 104 | GO:0006413 | P | 8, 9, | 7 | 1.886 (x 3.711) | 59 (0.119) | 0.0348 | translational initiation | CG10306 CG8636 Thor eIF-2alpha eIF2B-alpha eIF2B-delta eIF2B-gamma |
| 105 | GO:0016769 | F | 4, | 4 | 0.607 (x 6.585) | 19 (0.211) | 0.0355 | transferase activity, transferring nitrogenous groups | Alas CG12264 CG1673 Gfat2 |
| 106 | GO:0008483 | F | 5, | 4 | 0.607 (x 6.585) | 19 (0.211) | 0.0359 | transaminase activity | Alas CG12264 CG1673 Gfat2 |
| 107 | GO:0004849 | F | 8, | 2 | 0.096 (x 20.851) | 3 (0.667) | 0.0368 | uridine kinase activity | Adk1 l(2)k01209 |
| 108 | GO:0004300 | F | 6, | 2 | 0.096 (x 20.851) | 3 (0.667) | 0.0371 | enoyl-CoA hydratase activity | CG6984 CG8778 |
| 109 | GO:0006419 | P | 9, 10, 11, | 2 | 0.096 (x 20.851) | 3 (0.667) | 0.0374 | alanyl-tRNA aminoacylation | Aats-ala-m CG10802 |
| 110 | GO:0008043 | C | 4, | 2 | 0.096 (x 20.851) | 3 (0.667) | 0.0378 | ferritin complex | Fer1HCH Fer2LCH |
| 111 | GO:0004813 | F | 7, | 2 | 0.096 (x 20.851) | 3 (0.667) | 0.0381 | alanine-tRNA ligase activity | Aats-ala-m CG10802 |
| 112 | GO:0003988 | F | 8, | 2 | 0.096 (x 20.851) | 3 (0.667) | 0.0385 | acetyl-CoA C-acyltransferase activity | Thiolase yip2 |
| 113 | GO:0008198 | F | 7, | 2 | 0.096 (x 20.851) | 3 (0.667) | 0.0388 | ferrous iron binding | Fer1HCH Fer2LCH |
| 114 | GO:0019205 | F | 6, | 5 | 1.023 (x 4.887) | 32 (0.156) | 0.039 | nucleobase, nucleoside, nucleotide kinase activity | Adk1 CG2246 Dak1 Paps l(2)k01209 |
| 115 | GO:0006733 | P | 7, | 4 | 0.639 (x 6.255) | 20 (0.200) | 0.0396 | oxidoreduction coenzyme metabolism | CG13645 CG9249 Tpi Transaldolase |
| 116 | GO:0006783 | P | 7, 8, | 3 | 0.320 (x 9.383) | 10 (0.300) | 0.0397 | heme biosynthesis | Alas Updo ferrochelatase |
| 117 | GO:0042168 | P | 6, 7, | 3 | 0.352 (x 8.530) | 11 (0.273) | 0.0523 | heme metabolism | Alas Updo ferrochelatase |
| 118 | GO:0008135 | F | 3, 4, | 8 | 2.622 (x 3.051) | 82 (0.098) | 0.0535 | translation factor activity, nucleic acid binding | CG10306 CG8636 EfTuM PEK eIF-2alpha eIF2B-alpha eIF2B-delta eIF2B-gamma |
| 119 | GO:0016209 | F | 2, | 5 | 1.151 (x 4.344) | 36 (0.139) | 0.0624 | antioxidant activity | CG12013 Cat Jafrac1 Prx6005 Trxr-1 |
| 120 | GO:0004364 | F | 5, | 5 | 1.151 (x 4.344) | 36 (0.139) | 0.063 | glutathione transferase activity | CG5224 GstE1 GstE6 GstE7 GstE9 |
| 121 | GO:0006767 | P | 6, | 4 | 0.735 (x 5.440) | 23 (0.174) | 0.0638 | water-soluble vitamin metabolism | CG13645 CG2846 Tpi Transaldolase |
| 122 | GO:0045182 | F | 2, | 8 | 2.718 (x 2.944) | 85 (0.094) | 0.0638 | translation regulator activity | CG10306 CG8636 EfTuM PEK eIF-2alpha eIF2B-alpha eIF2B-delta eIF2B-gamma |
| 123 | GO:0006631 | P | 6, 7, | 9 | 3.293 (x 2.733) | 103 (0.087) | 0.0641 | fatty acid metabolism | BcDNA:GH07485 CG1140 CG4860 CG5044 CG6984 CG7842 CG8778 Thiolase yip2 |
| 124 | GO:0043231 | C | 4, 5, 6, 7, | 93 | 73.760 (x 1.261) | 2307 (0.040) | 0.0642 | intracellular membrane-bound organelle | AP-2sigma Aats-ala-m Alas BcDNA:GH07485 CG10042 CG10306 CG10333 CG1140 CG12013 CG12264 CG12534 CG12954 CG1319 CG13887 CG14040 CG1673 CG17266 CG2789 CG31075 CG33066 CG3476 CG3499 CG3731 CG3931 CG4225 CG4589 CG4769 CG4882 CG4963 CG5134 CG5261 CG5380 CG6272 CG6512 CG6608 CG7417 CG7598 CG7809 CG7842 CG8412 CG8728 CG9065 CG9215 CG9240 CG9298 CG9424 Cat Cyt-c-p Dip-B EG:100G10.7 EfTuM Idh JhI-1 Jra MTF-1 PEK Rep Shawn Sply Sras TfIIEbeta Thiolase Tim10 Trxr-1 Vha36 Vha44 aralar1 att-ORFA bcn92 ferrochelatase mRpL11 mRpL13 mRpL19 mRpL21 mRpL22 mRpL22-24 mRpL28 mRpL3 mRpL48 mRpL55 mRpS18a mRpS18b mRpS2 mRpS22 mRpS25 mRpS32 mRpS33 mRpS9 milton mus205 ref(2)P snapin yip2 |
| 125 | GO:0016082 | P | 8, 9, 10, | 3 | 0.384 (x 7.819) | 12 (0.250) | 0.0643 | synaptic vesicle priming | Nsf2 gammaSnap snapin |
| 126 | GO:0045254 | C | 3, 5, 6, 7, 8, | 2 | 0.128 (x 15.639) | 4 (0.500) | 0.0646 | pyruvate dehydrogenase complex | CG11876 CG5261 |
| 127 | GO:0043227 | C | 3, | 93 | 73.824 (x 1.260) | 2309 (0.040) | 0.0649 | membrane-bound organelle | AP-2sigma Aats-ala-m Alas BcDNA:GH07485 CG10042 CG10306 CG10333 CG1140 CG12013 CG12264 CG12534 CG12954 CG1319 CG13887 CG14040 CG1673 CG17266 CG2789 CG31075 CG33066 CG3476 CG3499 CG3731 CG3931 CG4225 CG4589 CG4769 CG4882 CG4963 CG5134 CG5261 CG5380 CG6272 CG6512 CG6608 CG7417 CG7598 CG7809 CG7842 CG8412 CG8728 CG9065 CG9215 CG9240 CG9298 CG9424 Cat Cyt-c-p Dip-B EG:100G10.7 EfTuM Idh JhI-1 Jra MTF-1 PEK Rep Shawn Sply Sras TfIIEbeta Thiolase Tim10 Trxr-1 Vha36 Vha44 aralar1 att-ORFA bcn92 ferrochelatase mRpL11 mRpL13 mRpL19 mRpL21 mRpL22 mRpL22-24 mRpL28 mRpL3 mRpL48 mRpL55 mRpS18a mRpS18b mRpS2 mRpS22 mRpS25 mRpS32 mRpS33 mRpS9 milton mus205 ref(2)P snapin yip2 |
| 128 | GO:0016903 | F | 4, | 4 | 0.767 (x 5.213) | 24 (0.167) | 0.0706 | oxidoreductase activity, acting on the aldehyde or oxo group of donors | CG11876 CG31075 Gapdh1 Gapdh2 |
| 129 | GO:0015036 | F | 4, | 4 | 0.767 (x 5.213) | 24 (0.167) | 0.0712 | disulfide oxidoreductase activity | CG10721 CG6523 Trxr-1 cl |
| 130 | GO:0044262 | P | 6, | 17 | 8.792 (x 1.933) | 275 (0.062) | 0.0753 | cellular carbohydrate metabolism | CG11876 CG33138 CG5261 CG8417 CG9232 Eno Gapdh1 Gapdh2 Gfat2 Idh Inos Men Pfk Pgm PyK Tpi Transaldolase |
| 131 | GO:0004222 | F | 6, | 7 | 2.270 (x 3.084) | 71 (0.099) | 0.0779 | metalloendopeptidase activity | CG3499 CG3731 CG6512 CG8728 EG:100G10.7 Ide Sras |
| 132 | GO:0005524 | F | 6, | 33 | 21.485 (x 1.536) | 672 (0.049) | 0.09 | ATP binding | Aats-ala-m Aats-asp Aats-cys Aats-gly Aats-his Aats-thr Aats-trp Aats-tyr Aats-val Adk1 CG10333 CG10802 CG11897 CG1598 CG2263 CG31793 CG3499 CG3561 CG3608 CG4225 CG4858 CG6512 CG7627 Dak1 EG:100G10.7 Gp93 Nsf2 PEK Paps Vha44 bor ire-1 l(2)k01209 |
| 133 | GO:0016836 | F | 5, | 6 | 1.790 (x 3.351) | 56 (0.107) | 0.0905 | hydro-lyase activity | CG11967 CG5044 CG6984 CG8778 Eno Pcd |
| 134 | GO:0009308 | P | 5, | 21 | 12.022 (x 1.747) | 376 (0.056) | 0.0928 | amine metabolism | Aats-ala-m Aats-asp Aats-cys Aats-gly Aats-his Aats-thr Aats-trp Aats-tyr Aats-val Alas CG10802 CG12264 CG1673 CG2107 CG2263 CG31075 CG4225 CG4802 CG6428 Paps Sply |
| 135 | GO:0016417 | F | 7, | 2 | 0.160 (x 12.511) | 5 (0.400) | 0.0928 | S-acyltransferase activity | CG5261 CG7842 |
| 136 | GO:0009607 | P | 3, | 27 | 16.689 (x 1.618) | 522 (0.052) | 0.093 | response to biotic stimulus | BG:BACR48E02.4 CG10535 CG11897 CG12013 CG13887 CG1598 CG17266 CG18522 CG31793 CG32687 CG5224 CG6523 CG6668 CG7627 Cat Gp93 GstE1 GstE6 GstE7 GstE9 Jafrac1 PEK Prx6005 TepIV Thor Ugt86Da cactin |
| 137 | GO:0006769 | P | 8, 9, | 3 | 0.448 (x 6.702) | 14 (0.214) | 0.0932 | nicotinamide metabolism | CG13645 Tpi Transaldolase |
| 138 | GO:0042721 | C | 3, 5, 6, 7, 8, 9, 10, 11, 12, 13, | 2 | 0.160 (x 12.511) | 5 (0.400) | 0.0935 | mitochondrial inner membrane protein insertion complex | CG33066 Tim10 |
| 139 | GO:0044248 | P | 5, | 18 | 9.783 (x 1.840) | 306 (0.059) | 0.0938 | cellular catabolism | Ate1 CG1140 CG11876 CG11883 CG31075 CG5261 CG6428 Eno Gapdh1 Gapdh2 Idh Men Pfk PyK Sply Tpi Transaldolase l(2)05070 |
| 140 | GO:0045454 | P | 5, | 2 | 0.160 (x 12.511) | 5 (0.400) | 0.0941 | cell redox homeostasis | Jafrac1 Prx6005 |
| 141 | GO:0051181 | P | 5, 6, | 2 | 0.160 (x 12.511) | 5 (0.400) | 0.0948 | cofactor transport | CG3476 CG4225 |
| 142 | GO:0008253 | F | 8, | 2 | 0.160 (x 12.511) | 5 (0.400) | 0.0955 | 5'-nucleotidase activity | CG11883 CG32549 |
| 143 | GO:0000049 | F | 5, | 2 | 0.160 (x 12.511) | 5 (0.400) | 0.0962 | tRNA binding | Aats-tyr eIF-2alpha |
| 144 | GO:0006644 | P | 7, 8, | 7 | 2.430 (x 2.881) | 76 (0.092) | 0.102 | phospholipid metabolism | CG32380 CG6016 CG6718 CG7789 Inos Pi4KIIalpha Sply |
| 145 | GO:0019362 | P | 7, 8, | 3 | 0.480 (x 6.255) | 15 (0.200) | 0.106 | pyridine nucleotide metabolism | CG13645 Tpi Transaldolase |
| 146 | GO:0008408 | F | 7, | 4 | 0.895 (x 4.468) | 28 (0.143) | 0.109 | 3'-5' exonuclease activity | CG3931 CG6744 mRpL28 mus205 |
| 147 | GO:0030554 | F | 5, | 33 | 22.029 (x 1.498) | 689 (0.048) | 0.116 | adenyl nucleotide binding | Aats-ala-m Aats-asp Aats-cys Aats-gly Aats-his Aats-thr Aats-trp Aats-tyr Aats-val Adk1 CG10333 CG10802 CG11897 CG1598 CG2263 CG31793 CG3499 CG3561 CG3608 CG4225 CG4858 CG6512 CG7627 Dak1 EG:100G10.7 Gp93 Nsf2 PEK Paps Vha44 bor ire-1 l(2)k01209 |
| 148 | GO:0016874 | F | 3, | 18 | 10.135 (x 1.776) | 317 (0.057) | 0.122 | ligase activity | Aats-ala-m Aats-asp Aats-cys Aats-gly Aats-his Aats-thr Aats-trp Aats-tyr Aats-val BcDNA:LD32788 CG10802 CG2263 CG4225 CG5604 CG8446 Gclm Nedd4 poe |
| 149 | GO:0016620 | F | 5, | 3 | 0.512 (x 5.864) | 16 (0.188) | 0.124 | oxidoreductase activity, acting on the aldehyde or oxo group of donors, NAD or NADP as acceptor | CG31075 Gapdh1 Gapdh2 |
| 150 | GO:0005525 | F | 6, | 11 | 5.148 (x 2.137) | 161 (0.068) | 0.126 | GTP binding | BcDNA:LD32788 CG17760 CG6668 CG8479 Dgp-1 EfTuM Past1 betaTub60D eIF-2alpha eIF2B-alpha eIF2B-delta |
| 151 | GO:0004661 | F | 7, | 2 | 0.192 (x 10.426) | 6 (0.333) | 0.127 | protein geranylgeranyltransferase activity | Rep betaggt-II |
| 152 | GO:0016861 | F | 5, | 2 | 0.192 (x 10.426) | 6 (0.333) | 0.127 | intramolecular oxidoreductase activity, interconverting aldoses and ketoses | CG8417 Tpi |
| 153 | GO:0007007 | P | 7, 8, | 2 | 0.192 (x 10.426) | 6 (0.333) | 0.128 | inner mitochondrial membrane organization and biogenesis | CG33066 Tim10 |
| 154 | GO:0016835 | F | 4, | 6 | 1.982 (x 3.027) | 62 (0.097) | 0.129 | carbon-oxygen lyase activity | CG11967 CG5044 CG6984 CG8778 Eno Pcd |
| 155 | GO:0045039 | P | 7, 8, 9, 10, 11, | 2 | 0.192 (x 10.426) | 6 (0.333) | 0.129 | protein import into mitochondrial inner membrane | CG33066 Tim10 |
| 156 | GO:0016485 | P | 8, | 4 | 0.959 (x 4.170) | 30 (0.133) | 0.13 | protein processing | CG3731 CG8728 CG9240 SCAP |
| 157 | GO:0019001 | F | 5, | 11 | 5.179 (x 2.124) | 162 (0.068) | 0.13 | guanyl nucleotide binding | BcDNA:LD32788 CG17760 CG6668 CG8479 Dgp-1 EfTuM Past1 betaTub60D eIF-2alpha eIF2B-alpha eIF2B-delta |
| 158 | GO:0006979 | P | 4, 5, 6, | 3 | 0.544 (x 5.520) | 17 (0.176) | 0.139 | response to oxidative stress | CG12013 Cat GstE1 |
| 159 | GO:0016410 | F | 7, | 4 | 0.991 (x 4.036) | 31 (0.129) | 0.142 | N-acyltransferase activity | Alas CG10414 CG11989 CG1969 |
| 160 | GO:0016598 | P | 8, 10, 11, 12, | 1 | 0.032 (x 31.277) | 1 (1.000) | 0.146 | protein arginylation | Ate1 |
| 161 | GO:0003844 | F | 6, | 1 | 0.032 (x 31.277) | 1 (1.000) | 0.147 | 1,4-alpha-glucan branching enzyme activity | CG33138 |
| 162 | GO:0004631 | F | 6, | 1 | 0.032 (x 31.277) | 1 (1.000) | 0.147 | phosphomevalonate kinase activity | CG10268 |
| 163 | GO:0046950 | P | 5, | 1 | 0.032 (x 31.277) | 1 (1.000) | 0.148 | ketone body metabolism | CG1140 |
| 164 | GO:0016732 | F | 5, | 1 | 0.032 (x 31.277) | 1 (1.000) | 0.148 | oxidoreductase activity, acting on iron-sulfur proteins as donors, dinitrogen as acceptor | CG11897 |
| 165 | GO:0003894 | F | 7, | 1 | 0.032 (x 31.277) | 1 (1.000) | 0.149 | zeta DNA polymerase activity | mus205 |
| 166 | GO:0008503 | F | 5, | 1 | 0.032 (x 31.277) | 1 (1.000) | 0.149 | benzodiazepine receptor activity | CG2789 |
| 167 | GO:0003999 | F | 6, | 1 | 0.032 (x 31.277) | 1 (1.000) | 0.15 | adenine phosphoribosyltransferase activity | Aprt |
| 168 | GO:0006791 | P | 6, | 1 | 0.032 (x 31.277) | 1 (1.000) | 0.15 | sulfur utilization | Paps |
| 169 | GO:0019058 | P | 3, | 1 | 0.032 (x 31.277) | 1 (1.000) | 0.151 | viral infectious cycle | ref(2)P |
| 170 | GO:0009056 | P | 4, | 18 | 10.455 (x 1.722) | 327 (0.055) | 0.151 | catabolism | Ate1 CG1140 CG11876 CG11883 CG31075 CG5261 CG6428 Eno Gapdh1 Gapdh2 Idh Men Pfk PyK Sply Tpi Transaldolase l(2)05070 |
| 171 | GO:0046952 | P | 6, | 1 | 0.032 (x 31.277) | 1 (1.000) | 0.151 | ketone body catabolism | CG1140 |
| 172 | GO:0000009 | F | 7, | 1 | 0.032 (x 31.277) | 1 (1.000) | 0.152 | alpha-1,6-mannosyltransferase activity | CG8412 |
| 173 | GO:0016032 | P | 2, | 1 | 0.032 (x 31.277) | 1 (1.000) | 0.152 | viral life cycle | ref(2)P |
| 174 | GO:0015937 | P | 8, 9, | 1 | 0.032 (x 31.277) | 1 (1.000) | 0.153 | coenzyme A biosynthesis | fbl |
| 175 | GO:0008108 | F | 6, | 1 | 0.032 (x 31.277) | 1 (1.000) | 0.153 | UDP-glucose:hexose-1-phosphate uridylyltransferase activity | CG9232 |
| 176 | GO:0044267 | P | 6, | 82 | 66.470 (x 1.234) | 2079 (0.039) | 0.153 | cellular protein metabolism | Aats-ala-m Aats-asp Aats-cys Aats-gly Aats-his Aats-thr Aats-trp Aats-tyr Aats-val Ate1 BG:BACR48E02.4 CG10306 CG10802 CG11883 CG11989 CG12163 CG12954 CG1319 CG13645 CG17187 CG17266 CG17760 CG2263 CG2789 CG32687 CG3499 CG3608 CG3731 CG4225 CG4447 CG5604 CG6000 CG6512 CG6746 CG8412 CG8446 CG8636 CG8728 CG9240 CG9882 Dgp-1 Dip-B EG:100G10.7 EfTuM Gp93 Ide Nedd4 PEK Pcd Rep SCAP Sras Thor betaTub60D betaggt-II cathD eIF-2alpha eIF2B-alpha eIF2B-delta eIF2B-gamma ire-1 l(2)05070 mRpL11 mRpL13 mRpL19 mRpL21 mRpL22 mRpL22-24 mRpL28 mRpL3 mRpL48 mRpS18a mRpS18b mRpS2 mRpS22 mRpS25 mRpS32 mRpS33 mRpS9 mio poe ref(2)P |
| 177 | GO:0004476 | F | 6, | 1 | 0.032 (x 31.277) | 1 (1.000) | 0.154 | mannose-6-phosphate isomerase activity | CG8417 |
| 178 | GO:0004614 | F | 6, | 1 | 0.032 (x 31.277) | 1 (1.000) | 0.154 | phosphoglucomutase activity | Pgm |
| 179 | GO:0046916 | P | 8, | 2 | 0.224 (x 8.936) | 7 (0.286) | 0.154 | transition metal ion homeostasis | Fer1HCH Fer2LCH |
| 180 | GO:0004821 | F | 7, | 1 | 0.032 (x 31.277) | 1 (1.000) | 0.155 | histidine-tRNA ligase activity | Aats-his |
| 181 | GO:0016684 | F | 4, | 4 | 1.023 (x 3.910) | 32 (0.125) | 0.155 | oxidoreductase activity, acting on peroxide as acceptor | CG12013 Cat Jafrac1 Prx6005 |
| 182 | GO:0006086 | P | 8, 9, | 1 | 0.032 (x 31.277) | 1 (1.000) | 0.155 | acetyl-CoA biosynthesis from pyruvate | CG5261 |
| 183 | GO:0006879 | P | 8, 9, | 2 | 0.224 (x 8.936) | 7 (0.286) | 0.155 | iron ion homeostasis | Fer1HCH Fer2LCH |
| 184 | GO:0003860 | F | 7, | 1 | 0.032 (x 31.277) | 1 (1.000) | 0.156 | 3-hydroxyisobutyryl-CoA hydrolase activity | CG5044 |
| 185 | GO:0004601 | F | 3, 5, | 4 | 1.023 (x 3.910) | 32 (0.125) | 0.156 | peroxidase activity | CG12013 Cat Jafrac1 Prx6005 |
| 186 | GO:0019894 | F | 5, | 2 | 0.224 (x 8.936) | 7 (0.286) | 0.156 | kinesin binding | Unc-76 milton |
| 187 | GO:0009082 | P | 8, 9, | 1 | 0.032 (x 31.277) | 1 (1.000) | 0.156 | branched chain family amino acid biosynthesis | CG1673 |
| 188 | GO:0009116 | P | 6, | 3 | 0.575 (x 5.213) | 18 (0.167) | 0.157 | nucleoside metabolism | Aprt CG2246 CG8360 |
| 189 | GO:0000302 | P | 5, 6, 7, | 1 | 0.032 (x 31.277) | 1 (1.000) | 0.157 | response to reactive oxygen species | Cat |
| 190 | GO:0008199 | F | 7, | 2 | 0.224 (x 8.936) | 7 (0.286) | 0.157 | ferric iron binding | Fer1HCH Fer2LCH |
| 191 | GO:0004307 | F | 7, | 1 | 0.032 (x 31.277) | 1 (1.000) | 0.157 | ethanolaminephosphotransferase activity | CG6016 |
| 192 | GO:0008610 | P | 5, 6, 7, | 7 | 2.718 (x 2.576) | 85 (0.082) | 0.158 | lipid biosynthesis | CG10268 CG32380 CG3522 CG6016 CG7842 Inos Vha44 |
| 193 | GO:0006021 | P | 9, 10, | 1 | 0.032 (x 31.277) | 1 (1.000) | 0.158 | myo-inositol biosynthesis | Inos |
| 194 | GO:0007006 | P | 6, 7, | 2 | 0.224 (x 8.936) | 7 (0.286) | 0.158 | mitochondrial membrane organization and biogenesis | CG33066 Tim10 |
| 195 | GO:0006983 | P | 4, 7, | 1 | 0.032 (x 31.277) | 1 (1.000) | 0.159 | ER overload response | PEK |
| 196 | GO:0019206 | F | 7, | 2 | 0.224 (x 8.936) | 7 (0.286) | 0.159 | nucleoside kinase activity | Adk1 l(2)k01209 |
| 197 | GO:0016163 | F | 6, | 1 | 0.032 (x 31.277) | 1 (1.000) | 0.159 | nitrogenase activity | CG11897 |
| 198 | GO:0006984 | P | 6, | 1 | 0.032 (x 31.277) | 1 (1.000) | 0.16 | ER-nuclear signaling pathway | PEK |
| 199 | GO:0008088 | P | 8, 9, 10, | 2 | 0.224 (x 8.936) | 7 (0.286) | 0.16 | axon cargo transport | Unc-76 milton |
| 200 | GO:0000103 | P | 7, | 1 | 0.032 (x 31.277) | 1 (1.000) | 0.16 | sulfate assimilation | Paps |
| 201 | GO:0006522 | P | 8, 9, | 1 | 0.032 (x 31.277) | 1 (1.000) | 0.161 | alanine metabolism | CG12264 |
| 202 | GO:0009119 | P | 7, | 2 | 0.224 (x 8.936) | 7 (0.286) | 0.161 | ribonucleoside metabolism | Aprt CG8360 |
| 203 | GO:0009078 | P | 7, 8, | 1 | 0.032 (x 31.277) | 1 (1.000) | 0.161 | pyruvate family amino acid metabolism | CG12264 |
| 204 | GO:0004450 | F | 7, | 1 | 0.032 (x 31.277) | 1 (1.000) | 0.162 | isocitrate dehydrogenase (NADP+) activity | Idh |
| 205 | GO:0004418 | F | 5, | 1 | 0.032 (x 31.277) | 1 (1.000) | 0.163 | hydroxymethylbilane synthase activity | l(3)02640 |
| 206 | GO:0006686 | P | 8, 9, 10, | 1 | 0.032 (x 31.277) | 1 (1.000) | 0.163 | sphingomyelin biosynthesis | CG32380 |
| 207 | GO:0015485 | F | 4, | 1 | 0.032 (x 31.277) | 1 (1.000) | 0.164 | cholesterol binding | CG3522 |
| 208 | GO:0006426 | P | 9, 10, 11, | 1 | 0.032 (x 31.277) | 1 (1.000) | 0.164 | glycyl-tRNA aminoacylation | Aats-gly |
| 209 | GO:0016872 | F | 4, | 1 | 0.032 (x 31.277) | 1 (1.000) | 0.165 | intramolecular lyase activity | Inos |
| 210 | GO:0016749 | F | 8, | 1 | 0.032 (x 31.277) | 1 (1.000) | 0.166 | N-succinyltransferase activity | Alas |
| 211 | GO:0004781 | F | 7, | 1 | 0.032 (x 31.277) | 1 (1.000) | 0.166 | sulfate adenylyltransferase (ATP) activity | Paps |
| 212 | GO:0008490 | F | 6, 7, | 1 | 0.032 (x 31.277) | 1 (1.000) | 0.167 | arsenite porter activity | CG1598 |
| 213 | GO:0006986 | P | 4, 6, | 1 | 0.032 (x 31.277) | 1 (1.000) | 0.168 | response to unfolded protein | PEK |
| 214 | GO:0006643 | P | 6, 7, | 7 | 2.782 (x 2.517) | 87 (0.080) | 0.168 | membrane lipid metabolism | CG32380 CG6016 CG6718 CG7789 Inos Pi4KIIalpha Sply |
| 215 | GO:0004820 | F | 7, | 1 | 0.032 (x 31.277) | 1 (1.000) | 0.168 | glycine-tRNA ligase activity | Aats-gly |
| 216 | GO:0042542 | P | 6, 7, 8, | 1 | 0.032 (x 31.277) | 1 (1.000) | 0.169 | response to hydrogen peroxide | Cat |
| 217 | GO:0019538 | P | 5, | 85 | 69.795 (x 1.218) | 2183 (0.039) | 0.169 | protein metabolism | Aats-ala-m Aats-asp Aats-cys Aats-gly Aats-his Aats-thr Aats-trp Aats-tyr Aats-val Ate1 BG:BACR48E02.4 CG10306 CG10340 CG10802 CG11883 CG11989 CG12163 CG12954 CG1319 CG13645 CG17187 CG17266 CG17760 CG2263 CG2789 CG32687 CG3499 CG3608 CG3731 CG4225 CG4447 CG5604 CG6000 CG6512 CG6746 CG7598 CG8412 CG8446 CG8636 CG8728 CG9065 CG9240 CG9882 Dgp-1 Dip-B EG:100G10.7 EfTuM Gp93 Ide Nedd4 PEK Pcd Rep SCAP Sras Thor betaTub60D betaggt-II cathD eIF-2alpha eIF2B-alpha eIF2B-delta eIF2B-gamma ire-1 l(2)05070 mRpL11 mRpL13 mRpL19 mRpL21 mRpL22 mRpL22-24 mRpL28 mRpL3 mRpL48 mRpS18a mRpS18b mRpS2 mRpS22 mRpS25 mRpS32 mRpS33 mRpS9 mio poe ref(2)P |
| 218 | GO:0015038 | F | 6, | 1 | 0.032 (x 31.277) | 1 (1.000) | 0.17 | glutathione disulfide oxidoreductase activity | Trxr-1 |
| 219 | GO:0042221 | P | 4, | 15 | 8.409 (x 1.784) | 263 (0.057) | 0.17 | response to chemical stimulus | CG11897 CG12013 CG1598 CG31793 CG5224 CG7627 Cat Gclm GstE1 GstE6 GstE7 GstE9 PEK Ugt86Da kraken |
| 220 | GO:0019896 | P | 9, 10, 11, | 1 | 0.032 (x 31.277) | 1 (1.000) | 0.17 | axon transport of mitochondrion | milton |
| 221 | GO:0017118 | F | 4, | 1 | 0.032 (x 31.277) | 1 (1.000) | 0.171 | lipoyltransferase activity | CG8446 |
| 222 | GO:0004362 | F | 3, 5, 6, 7, | 1 | 0.032 (x 31.277) | 1 (1.000) | 0.171 | glutathione-disulfide reductase activity | Trxr-1 |
| 223 | GO:0004634 | F | 6, | 1 | 0.032 (x 31.277) | 1 (1.000) | 0.172 | phosphopyruvate hydratase activity | Eno |
| 224 | GO:0018348 | P | 11, 12, | 1 | 0.032 (x 31.277) | 1 (1.000) | 0.173 | protein amino acid geranylgeranylation | Rep |
| 225 | GO:0051351 | P | 5, | 1 | 0.032 (x 31.277) | 1 (1.000) | 0.173 | positive regulation of ligase activity | Gclm |
| 226 | GO:0006097 | P | 7, 8, | 1 | 0.032 (x 31.277) | 1 (1.000) | 0.174 | glyoxylate cycle | Idh |
| 227 | GO:0004020 | F | 6, | 1 | 0.032 (x 31.277) | 1 (1.000) | 0.175 | adenylylsulfate kinase activity | Paps |
| 228 | GO:0046165 | P | 6, | 2 | 0.320 (x 6.255) | 10 (0.200) | 0.175 | alcohol biosynthesis | Inos Tpi |
| 229 | GO:0004594 | F | 6, | 1 | 0.032 (x 31.277) | 1 (1.000) | 0.176 | pantothenate kinase activity | fbl |
| 230 | GO:0046364 | P | 7, 8, | 2 | 0.320 (x 6.255) | 10 (0.200) | 0.176 | monosaccharide biosynthesis | Inos Tpi |
| 231 | GO:0019319 | P | 8, 9, | 2 | 0.320 (x 6.255) | 10 (0.200) | 0.176 | hexose biosynthesis | Inos Tpi |
| 232 | GO:0008319 | F | 7, | 1 | 0.032 (x 31.277) | 1 (1.000) | 0.176 | prenyl protein specific endopeptidase activity | Sras |
| 233 | GO:0009079 | P | 8, 9, | 1 | 0.032 (x 31.277) | 1 (1.000) | 0.177 | pyruvate family amino acid biosynthesis | CG12264 |
| 234 | GO:0035229 | P | 6, | 1 | 0.032 (x 31.277) | 1 (1.000) | 0.178 | positive regulation of glutamate-cysteine ligase activity | Gclm |
| 235 | GO:0045947 | P | 9, 10, 11, | 1 | 0.032 (x 31.277) | 1 (1.000) | 0.178 | negative regulation of translational initiation | Thor |
| 236 | GO:0008190 | F | 5, | 1 | 0.032 (x 31.277) | 1 (1.000) | 0.179 | eukaryotic initiation factor 4E binding | Thor |
| 237 | GO:0004231 | F | 7, | 1 | 0.032 (x 31.277) | 1 (1.000) | 0.18 | insulysin activity | Ide |
| 238 | GO:0003870 | F | 9, | 1 | 0.032 (x 31.277) | 1 (1.000) | 0.181 | 5-aminolevulinate synthase activity | Alas |
| 239 | GO:0004019 | F | 5, | 1 | 0.032 (x 31.277) | 1 (1.000) | 0.181 | adenylosuccinate synthase activity | BcDNA:LD32788 |
| 240 | GO:0004779 | F | 6, | 1 | 0.032 (x 31.277) | 1 (1.000) | 0.182 | sulfate adenylyltransferase activity | Paps |
| 241 | GO:0008531 | F | 6, | 1 | 0.032 (x 31.277) | 1 (1.000) | 0.183 | riboflavin kinase activity | CG2846 |
| 242 | GO:0006982 | P | 5, 6, 7, | 1 | 0.032 (x 31.277) | 1 (1.000) | 0.184 | response to lipid hydroperoxide | CG12013 |
| 243 | GO:0006952 | P | 4, | 25 | 16.434 (x 1.521) | 514 (0.049) | 0.184 | defense response | BG:BACR48E02.4 CG10535 CG11897 CG12013 CG13887 CG1598 CG17266 CG18522 CG31793 CG32687 CG5224 CG6668 CG7627 Cat Gp93 GstE1 GstE6 GstE7 GstE9 Jafrac1 Prx6005 TepIV Thor Ugt86Da cactin |
| 244 | GO:0051340 | P | 4, | 1 | 0.032 (x 31.277) | 1 (1.000) | 0.184 | regulation of ligase activity | Gclm |
| 245 | GO:0004527 | F | 6, | 4 | 1.311 (x 3.051) | 41 (0.098) | 0.185 | exonuclease activity | CG3931 CG6744 mRpL28 mus205 |
| 246 | GO:0004801 | F | 5, | 1 | 0.032 (x 31.277) | 1 (1.000) | 0.185 | transaldolase activity | Transaldolase |
| 247 | GO:0015936 | P | 8, | 1 | 0.032 (x 31.277) | 1 (1.000) | 0.186 | coenzyme A metabolism | fbl |
| 248 | GO:0018344 | P | 10, 11, | 1 | 0.032 (x 31.277) | 1 (1.000) | 0.187 | protein geranylgeranylation | Rep |
| 249 | GO:0046487 | P | 6, 7, | 1 | 0.032 (x 31.277) | 1 (1.000) | 0.187 | glyoxylate metabolism | Idh |
| 250 | GO:0008117 | F | 6, | 1 | 0.032 (x 31.277) | 1 (1.000) | 0.188 | sphinganine-1-phosphate aldolase activity | Sply |
| 251 | GO:0017091 | F | 5, | 1 | 0.032 (x 31.277) | 1 (1.000) | 0.189 | AU-specific RNA binding | CG8778 |
| 252 | GO:0004602 | F | 4, 6, | 2 | 0.256 (x 7.819) | 8 (0.250) | 0.19 | glutathione peroxidase activity | CG12013 Prx6005 |
| 253 | GO:0006427 | P | 9, 10, 11, | 1 | 0.032 (x 31.277) | 1 (1.000) | 0.19 | histidyl-tRNA aminoacylation | Aats-his |
| 254 | GO:0008559 | F | 4, 5, 7, 12, | 2 | 0.256 (x 7.819) | 8 (0.250) | 0.191 | xenobiotic-transporting ATPase activity | CG11897 CG7627 |
| 255 | GO:0004742 | F | 9, | 1 | 0.032 (x 31.277) | 1 (1.000) | 0.191 | dihydrolipoyllysine-residue acetyltransferase activity | CG5261 |
| 256 | GO:0005941 | C | 3, | 5 | 1.663 (x 3.007) | 52 (0.096) | 0.191 | unlocalized protein complex | CG2263 Fer1HCH Fer2LCH Mocs1 Rep |
| 257 | GO:0019895 | F | 6, | 1 | 0.032 (x 31.277) | 1 (1.000) | 0.191 | kinesin-associated mitochondrial adaptor activity | milton |
| 258 | GO:0004365 | F | 7, | 2 | 0.256 (x 7.819) | 8 (0.250) | 0.192 | glyceraldehyde-3-phosphate dehydrogenase (phosphorylating) activity | Gapdh1 Gapdh2 |
| 259 | GO:0019204 | F | 7, | 4 | 1.119 (x 3.575) | 35 (0.114) | 0.192 | nucleotide phosphatase activity | CG11883 CG1598 CG8128 NTPase |
| 260 | GO:0004807 | F | 6, | 1 | 0.032 (x 31.277) | 1 (1.000) | 0.192 | triose-phosphate isomerase activity | Tpi |
| 261 | GO:0042910 | F | 3, | 2 | 0.256 (x 7.819) | 8 (0.250) | 0.193 | xenobiotic transporter activity | CG11897 CG7627 |
| 262 | GO:0017087 | C | 3, 5, 6, 7, 8, 9, 10, 11, 12, | 1 | 0.032 (x 31.277) | 1 (1.000) | 0.193 | mitochondrial processing peptidase complex | CG3731 |
| 263 | GO:0008943 | F | 6, | 2 | 0.256 (x 7.819) | 8 (0.250) | 0.194 | glyceraldehyde-3-phosphate dehydrogenase activity | Gapdh1 Gapdh2 |
| 264 | GO:0006523 | P | 9, 10, | 1 | 0.032 (x 31.277) | 1 (1.000) | 0.194 | alanine biosynthesis | CG12264 |
| 265 | GO:0006695 | P | 8, 9, 10, | 1 | 0.032 (x 31.277) | 1 (1.000) | 0.195 | cholesterol biosynthesis | CG10268 |
| 266 | GO:0016779 | F | 5, | 7 | 2.909 (x 2.406) | 91 (0.077) | 0.196 | nucleotidyltransferase activity | CG13645 CG4407 CG5380 CG9232 Paps eIF2B-gamma mus205 |
| 267 | GO:0043123 | P | 6, 7, 9, | 1 | 0.032 (x 31.277) | 1 (1.000) | 0.196 | positive regulation of I-kappaB kinase/NF-kappaB cascade | CG7417 |
| 268 | GO:0016070 | P | 6, | 19 | 11.734 (x 1.619) | 367 (0.052) | 0.196 | RNA metabolism | Aats-ala-m Aats-asp Aats-cys Aats-gly Aats-his Aats-thr Aats-trp Aats-tyr Aats-val CG10333 CG10802 CG17266 CG2263 CG3931 CG4225 CG5380 CG8128 JhI-1 mRpL28 |
| 269 | GO:0015037 | F | 5, | 1 | 0.032 (x 31.277) | 1 (1.000) | 0.197 | peptide disulfide oxidoreductase activity | Trxr-1 |
| 270 | GO:0043234 | C | 2, | 62 | 50.484 (x 1.228) | 1579 (0.039) | 0.197 | protein complex | CG10306 CG10333 CG11876 CG12954 CG17266 CG17760 CG2263 CG31793 CG33066 CG3397 CG3731 CG3931 CG4225 CG4769 CG5134 CG5261 CG5380 CG5604 CG7598 CG7627 CG8636 Eno Fer1HCH Fer2LCH Gclm Mocs1 Pcd Pfk Pgm Rep TfIIEbeta Thiolase Tim10 Vha36 Vha44 betaTub60D cnn dynactin-subunit-p25 eIF-2alpha eIF2B-alpha eIF2B-delta eIF2B-gamma l(2)05070 mRpL11 mRpL13 mRpL19 mRpL21 mRpL22 mRpL22-24 mRpL28 mRpL3 mRpL48 mRpL55 mRpS18a mRpS18b mRpS2 mRpS22 mRpS25 mRpS32 mRpS33 mRpS9 mus205 |
| 271 | GO:0005945 | C | 3, 6, 7, 8, 9, 10, | 1 | 0.032 (x 31.277) | 1 (1.000) | 0.197 | 6-phosphofructokinase complex | Pfk |
| 272 | GO:0006488 | P | 10, 11, | 1 | 0.032 (x 31.277) | 1 (1.000) | 0.198 | dolichol-linked oligosaccharide biosynthesis | CG8412 |
| 273 | GO:0004127 | F | 8, | 1 | 0.032 (x 31.277) | 1 (1.000) | 0.199 | cytidylate kinase activity | Dak1 |
| 274 | GO:0004343 | F | 9, | 1 | 0.032 (x 31.277) | 1 (1.000) | 0.2 | glucosamine 6-phosphate N-acetyltransferase activity | CG1969 |
| 275 | GO:0006766 | P | 5, | 4 | 1.151 (x 3.475) | 36 (0.111) | 0.2 | vitamin metabolism | CG13645 CG2846 Tpi Transaldolase |
| 276 | GO:0003872 | F | 7, 8, | 1 | 0.032 (x 31.277) | 1 (1.000) | 0.201 | 6-phosphofructokinase activity | Pfk |
| 277 | GO:0005084 | F | 5, | 1 | 0.032 (x 31.277) | 1 (1.000) | 0.202 | Rab escort protein activity | Rep |
| 278 | GO:0030176 | C | 6, 7, 8, 9, 10, 11, 12, | 2 | 0.352 (x 5.687) | 11 (0.182) | 0.202 | integral to endoplasmic reticulum membrane | Sply Sras |
| 279 | GO:0004084 | F | 6, | 1 | 0.032 (x 31.277) | 1 (1.000) | 0.203 | branched-chain-amino-acid transaminase activity | CG1673 |
| 280 | GO:0030508 | F | 5, | 2 | 0.352 (x 5.687) | 11 (0.182) | 0.203 | thiol-disulfide exchange intermediate activity | CG6523 cl |
| 281 | GO:0005750 | C | 4, 5, 6, 7, 8, 9, 10, 11, 12, 13, 14, | 2 | 0.352 (x 5.687) | 11 (0.182) | 0.204 | respiratory chain complex III (sensu Eukaryota) | CG3731 CG4769 |
| 282 | GO:0004395 | F | 7, | 1 | 0.032 (x 31.277) | 1 (1.000) | 0.204 | hexaprenyldihydroxybenzoate methyltransferase activity | CG9249 |
| 283 | GO:0045285 | C | 3, 4, 5, 6, 7, | 2 | 0.352 (x 5.687) | 11 (0.182) | 0.204 | ubiquinol-cytochrome-c reductase complex | CG3731 CG4769 |
| 284 | GO:0000015 | C | 3, 6, 7, 8, 9, 10, | 1 | 0.032 (x 31.277) | 1 (1.000) | 0.205 | phosphopyruvate hydratase complex | Eno |
| 285 | GO:0016667 | F | 4, | 2 | 0.352 (x 5.687) | 11 (0.182) | 0.205 | oxidoreductase activity, acting on sulfur group of donors | CG12534 Trxr-1 |
| 286 | GO:0031227 | C | 5, 6, 7, 8, 9, 10, 11, | 2 | 0.352 (x 5.687) | 11 (0.182) | 0.206 | intrinsic to endoplasmic reticulum membrane | Sply Sras |
| 287 | GO:0004057 | F | 6, | 1 | 0.032 (x 31.277) | 1 (1.000) | 0.206 | arginyltransferase activity | Ate1 |
| 288 | GO:0045275 | C | 3, 4, 5, 6, | 2 | 0.352 (x 5.687) | 11 (0.182) | 0.206 | respiratory chain complex III | CG3731 CG4769 |
| 289 | GO:0004584 | F | 7, | 1 | 0.032 (x 31.277) | 1 (1.000) | 0.207 | dolichyl-phosphate-mannose-glycolipid alpha-mannosyltransferase activity | CG8412 |
| 290 | GO:0000041 | P | 8, 9, | 2 | 0.352 (x 5.687) | 11 (0.182) | 0.207 | transition metal ion transport | CG9065 Fer1HCH |
| 291 | GO:0016866 | F | 4, | 2 | 0.352 (x 5.687) | 11 (0.182) | 0.208 | intramolecular transferase activity | Pgm Tpi |
| 292 | GO:0016776 | F | 5, | 3 | 0.671 (x 4.468) | 21 (0.143) | 0.208 | phosphotransferase activity, phosphate group as acceptor | Adk1 CG10268 Dak1 |
| 293 | GO:0035227 | P | 5, | 1 | 0.032 (x 31.277) | 1 (1.000) | 0.208 | regulation of glutamate-cysteine ligase activity | Gclm |
| 294 | GO:0009041 | F | 6, 8, | 1 | 0.032 (x 31.277) | 1 (1.000) | 0.208 | uridylate kinase activity | Dak1 |
| 295 | GO:0006891 | P | 6, 7, 8, 9, | 2 | 0.416 (x 4.812) | 13 (0.154) | 0.209 | intra-Golgi vesicle-mediated transport | Nsf2 SCAP |
| 296 | GO:0005744 | C | 3, 5, 6, 7, 8, 9, 10, 11, 12, 13, | 2 | 0.416 (x 4.812) | 13 (0.154) | 0.209 | mitochondrial inner membrane presequence translocase complex | CG33066 Tim10 |
| 297 | GO:0004382 | F | 8, | 1 | 0.032 (x 31.277) | 1 (1.000) | 0.209 | guanosine-diphosphatase activity | NTPase |
| 298 | GO:0042558 | P | 6, | 2 | 0.416 (x 4.812) | 13 (0.154) | 0.21 | pteridine and derivative metabolism | Mocs1 Pcd |
| 299 | GO:0009052 | P | 9, 11, 12, 13, | 1 | 0.064 (x 15.639) | 2 (0.500) | 0.21 | pentose-phosphate shunt, non-oxidative branch | Transaldolase |
| 300 | GO:0006782 | P | 8, 9, | 1 | 0.032 (x 31.277) | 1 (1.000) | 0.21 | protoporphyrinogen IX biosynthesis | ferrochelatase |
| 301 | GO:0016226 | P | 7, | 1 | 0.064 (x 15.639) | 2 (0.500) | 0.211 | iron-sulfur cluster assembly | CG12264 |
| 302 | GO:0015227 | F | 5, | 1 | 0.064 (x 15.639) | 2 (0.500) | 0.211 | acyl carnitine transporter activity | CG3476 |
| 303 | GO:0044260 | P | 5, | 83 | 68.804 (x 1.206) | 2152 (0.039) | 0.211 | cellular macromolecule metabolism | Aats-ala-m Aats-asp Aats-cys Aats-gly Aats-his Aats-thr Aats-trp Aats-tyr Aats-val Ate1 BG:BACR48E02.4 CG10306 CG10802 CG11883 CG11989 CG12163 CG12954 CG1319 CG13645 CG17187 CG17266 CG17760 CG2263 CG2789 CG32687 CG33138 CG3499 CG3608 CG3731 CG4225 CG4447 CG5604 CG6000 CG6512 CG6746 CG8412 CG8446 CG8636 CG8728 CG9240 CG9882 Dgp-1 Dip-B EG:100G10.7 EfTuM Gp93 Ide Nedd4 PEK Pcd Rep SCAP Sras Thor betaTub60D betaggt-II cathD eIF-2alpha eIF2B-alpha eIF2B-delta eIF2B-gamma ire-1 l(2)05070 mRpL11 mRpL13 mRpL19 mRpL21 mRpL22 mRpL22-24 mRpL28 mRpL3 mRpL48 mRpS18a mRpS18b mRpS2 mRpS22 mRpS25 mRpS32 mRpS33 mRpS9 mio poe ref(2)P |
| 304 | GO:0015446 | F | 6, 7, 8, 9, 14, | 1 | 0.032 (x 31.277) | 1 (1.000) | 0.211 | arsenite-transporting ATPase activity | CG1598 |
| 305 | GO:0004829 | F | 7, | 1 | 0.064 (x 15.639) | 2 (0.500) | 0.212 | threonine-tRNA ligase activity | Aats-thr |
| 306 | GO:0043226 | C | 2, | 99 | 85.813 (x 1.154) | 2684 (0.037) | 0.212 | organelle | AP-2sigma Aats-ala-m Alas BcDNA:GH07485 CG10042 CG10306 CG10333 CG1140 CG12013 CG12264 CG12534 CG12954 CG1319 CG13887 CG14040 CG1673 CG17266 CG2789 CG31075 CG33066 CG3476 CG3499 CG3731 CG3931 CG4225 CG4589 CG4769 CG4882 CG4963 CG5134 CG5261 CG5380 CG6272 CG6512 CG6608 CG7417 CG7598 CG7809 CG7842 CG8412 CG8728 CG9065 CG9215 CG9240 CG9298 CG9424 Cat Cyt-c-p Dip-B EG:100G10.7 EfTuM Idh JhI-1 Jra MTF-1 PEK Pcd Rep Shawn Sply Sras TfIIEbeta Thiolase Tim10 Trxr-1 Unc-76 Vha36 Vha44 aralar1 att-ORFA bcn92 betaTub60D cnn dynactin-subunit-p25 fbl ferrochelatase mRpL11 mRpL13 mRpL19 mRpL21 mRpL22 mRpL22-24 mRpL28 mRpL3 mRpL48 mRpL55 mRpS18a mRpS18b mRpS2 mRpS22 mRpS25 mRpS32 mRpS33 mRpS9 milton mus205 ref(2)P snapin yip2 |
| 307 | GO:0016416 | F | 8, | 1 | 0.064 (x 15.639) | 2 (0.500) | 0.212 | O-palmitoyltransferase activity | CG2107 |
| 308 | GO:0008654 | P | 7, 8, 9, | 3 | 0.831 (x 3.609) | 26 (0.115) | 0.212 | phospholipid biosynthesis | CG32380 CG6016 Inos |
| 309 | GO:0044265 | P | 6, | 10 | 5.499 (x 1.818) | 172 (0.058) | 0.212 | cellular macromolecule catabolism | Ate1 CG5261 Eno Gapdh1 Gapdh2 Pfk PyK Tpi Transaldolase l(2)05070 |
| 310 | GO:0031369 | F | 4, | 1 | 0.032 (x 31.277) | 1 (1.000) | 0.212 | translation initiation factor binding | Thor |
| 311 | GO:0043229 | C | 3, 4, 5, 6, | 99 | 85.813 (x 1.154) | 2684 (0.037) | 0.213 | intracellular organelle | AP-2sigma Aats-ala-m Alas BcDNA:GH07485 CG10042 CG10306 CG10333 CG1140 CG12013 CG12264 CG12534 CG12954 CG1319 CG13887 CG14040 CG1673 CG17266 CG2789 CG31075 CG33066 CG3476 CG3499 CG3731 CG3931 CG4225 CG4589 CG4769 CG4882 CG4963 CG5134 CG5261 CG5380 CG6272 CG6512 CG6608 CG7417 CG7598 CG7809 CG7842 CG8412 CG8728 CG9065 CG9215 CG9240 CG9298 CG9424 Cat Cyt-c-p Dip-B EG:100G10.7 EfTuM Idh JhI-1 Jra MTF-1 PEK Pcd Rep Shawn Sply Sras TfIIEbeta Thiolase Tim10 Trxr-1 Unc-76 Vha36 Vha44 aralar1 att-ORFA bcn92 betaTub60D cnn dynactin-subunit-p25 fbl ferrochelatase mRpL11 mRpL13 mRpL19 mRpL21 mRpL22 mRpL22-24 mRpL28 mRpL3 mRpL48 mRpL55 mRpS18a mRpS18b mRpS2 mRpS22 mRpS25 mRpS32 mRpS33 mRpS9 milton mus205 ref(2)P snapin yip2 |
| 312 | GO:0046087 | P | 9, | 1 | 0.064 (x 15.639) | 2 (0.500) | 0.213 | cytidine metabolism | CG8360 |
| 313 | GO:0003723 | F | 4, | 18 | 11.798 (x 1.526) | 369 (0.049) | 0.213 | RNA binding | Aats-ala-m Aats-asp Aats-cys Aats-his Aats-thr Aats-tyr Aats-val CG10802 CG3931 CG8636 CG8778 Pcd bsf eIF-2alpha mRpL21 mRpL28 mRpL55 ytr |
| 314 | GO:0031163 | P | 6, | 1 | 0.064 (x 15.639) | 2 (0.500) | 0.213 | metallo-sulfur cluster assembly | CG12264 |
| 315 | GO:0017127 | F | 5, | 1 | 0.032 (x 31.277) | 1 (1.000) | 0.214 | cholesterol transporter activity | CG3522 |
| 316 | GO:0048471 | C | 5, 6, 7, 8, | 1 | 0.064 (x 15.639) | 2 (0.500) | 0.214 | perinuclear region | Rep |
| 317 | GO:0015879 | P | 6, 7, | 1 | 0.064 (x 15.639) | 2 (0.500) | 0.214 | carnitine transport | CG3476 |
| 318 | GO:0035226 | F | 5, | 1 | 0.032 (x 31.277) | 1 (1.000) | 0.215 | glutamate-cysteine ligase catalytic subunit binding | Gclm |
| 319 | GO:0006627 | P | 9, 10, 11, | 1 | 0.064 (x 15.639) | 2 (0.500) | 0.215 | mitochondrial protein processing | CG9240 |
| 320 | GO:0015886 | P | 6, 7, | 1 | 0.064 (x 15.639) | 2 (0.500) | 0.215 | heme transport | CG4225 |
| 321 | GO:0004325 | F | 4, | 1 | 0.032 (x 31.277) | 1 (1.000) | 0.216 | ferrochelatase activity | ferrochelatase |
| 322 | GO:0007557 | P | 6, 7, 8, 9, 10, 11, 12, 13, | 1 | 0.064 (x 15.639) | 2 (0.500) | 0.216 | regulation of juvenile hormone biosynthesis | Vha44 |
| 323 | GO:0017171 | F | 4, | 1 | 0.064 (x 15.639) | 2 (0.500) | 0.216 | serine hydrolase activity | kraken |
| 324 | GO:0005967 | C | 4, 5, 6, 7, 8, 9, 10, 11, 12, | 1 | 0.032 (x 31.277) | 1 (1.000) | 0.217 | pyruvate dehydrogenase complex (sensu Eukaryota) | CG5261 |
| 325 | GO:0004832 | F | 7, | 1 | 0.064 (x 15.639) | 2 (0.500) | 0.217 | valine-tRNA ligase activity | Aats-val |
| 326 | GO:0016772 | F | 4, | 23 | 16.018 (x 1.436) | 501 (0.046) | 0.217 | transferase activity, transferring phosphorus-containing groups | Adk1 BG:BACR48E02.4 CG10268 CG13645 CG2246 CG2846 CG32687 CG3608 CG4407 CG5380 CG6016 CG9232 Dak1 PEK Paps Pfk Pi4KIIalpha PyK eIF2B-gamma fbl ire-1 l(2)k01209 mus205 |
| 327 | GO:0005673 | C | 4, 7, 8, 9, 10, 11, 12, 13, 14, | 1 | 0.064 (x 15.639) | 2 (0.500) | 0.217 | transcription factor TFIIE complex | TfIIEbeta |
| 328 | GO:0004512 | F | 5, | 1 | 0.032 (x 31.277) | 1 (1.000) | 0.218 | inositol-3-phosphate synthase activity | Inos |
| 329 | GO:0004314 | F | 8, 9, | 1 | 0.064 (x 15.639) | 2 (0.500) | 0.218 | [acyl-carrier protein] S-malonyltransferase activity | CG7842 |
| 330 | GO:0004830 | F | 7, | 1 | 0.064 (x 15.639) | 2 (0.500) | 0.219 | tryptophan-tRNA ligase activity | Aats-trp |
| 331 | GO:0004853 | F | 6, | 1 | 0.032 (x 31.277) | 1 (1.000) | 0.219 | uroporphyrinogen decarboxylase activity | Updo |
| 332 | GO:0004096 | F | 4, 6, | 1 | 0.064 (x 15.639) | 2 (0.500) | 0.219 | catalase activity | Cat |
| 333 | GO:0009109 | P | 7, | 4 | 1.535 (x 2.606) | 48 (0.083) | 0.219 | coenzyme catabolism | CG11876 CG5261 Idh Men |
| 334 | GO:0004360 | F | 6, | 1 | 0.064 (x 15.639) | 2 (0.500) | 0.22 | glutamine-fructose-6-phosphate transaminase (isomerizing) activity | Gfat2 |
| 335 | GO:0006084 | P | 7, | 4 | 1.535 (x 2.606) | 48 (0.083) | 0.22 | acetyl-CoA metabolism | CG11876 CG5261 Idh Men |
| 336 | GO:0016791 | F | 6, | 10 | 5.819 (x 1.719) | 182 (0.055) | 0.22 | phosphoric monoester hydrolase activity | CG11883 CG1598 CG17294 CG32549 CG5567 CG6746 CG6805 CG7789 CG8128 NTPase |
| 337 | GO:0008260 | F | 6, | 1 | 0.032 (x 31.277) | 1 (1.000) | 0.22 | 3-oxoacid CoA-transferase activity | CG1140 |
| 338 | GO:0047499 | F | 9, | 1 | 0.064 (x 15.639) | 2 (0.500) | 0.22 | calcium-independent phospholipase A2 activity | CG6718 |
| 339 | GO:0004596 | F | 9, | 1 | 0.064 (x 15.639) | 2 (0.500) | 0.221 | peptide alpha-N-acetyltransferase activity | CG11989 |
| 340 | GO:0004421 | F | 6, | 1 | 0.032 (x 31.277) | 1 (1.000) | 0.221 | hydroxymethylglutaryl-CoA synthase activity | Hmgs |
| 341 | GO:0046885 | P | 7, | 1 | 0.064 (x 15.639) | 2 (0.500) | 0.221 | regulation of hormone biosynthesis | Vha44 |
| 342 | GO:0019008 | C | 4, | 1 | 0.064 (x 15.639) | 2 (0.500) | 0.222 | molybdopterin synthase complex | Mocs1 |
| 343 | GO:0008124 | F | 6, | 1 | 0.032 (x 31.277) | 1 (1.000) | 0.222 | 4-alpha-hydroxytetrahydrobiopterin dehydratase activity | Pcd |
| 344 | GO:0006436 | P | 9, 10, 11, | 1 | 0.064 (x 15.639) | 2 (0.500) | 0.222 | tryptophanyl-tRNA aminoacylation | Aats-trp |
| 345 | GO:0043170 | P | 4, | 112 | 99.625 (x 1.124) | 3116 (0.036) | 0.223 | macromolecule metabolism | Aats-ala-m Aats-asp Aats-cys Aats-gly Aats-his Aats-thr Aats-trp Aats-tyr Aats-val Ate1 BG:BACR48E02.4 CG10306 CG10333 CG10340 CG10802 CG11876 CG11883 CG11989 CG12163 CG12954 CG1319 CG13645 CG15094 CG17187 CG17266 CG17760 CG2263 CG2789 CG32687 CG33138 CG3499 CG3608 CG3731 CG3931 CG4225 CG4447 CG5261 CG5380 CG5604 CG6000 CG6512 CG6746 CG7598 CG8128 CG8412 CG8417 CG8446 CG8636 CG8728 CG9065 CG9232 CG9240 CG9882 Dgp-1 Dip-B EG:100G10.7 EfTuM Eno Gapdh1 Gapdh2 Gfat2 Gp93 Ide Idh Inos JhI-1 Men Nedd4 PEK Pcd Pfk Pgm Pi4KIIalpha PyK Rep RhoGEF2 SCAP Sras Thor Tpi Transaldolase Ugt86Da betaTub60D betaggt-II cathD eIF-2alpha eIF2B-alpha eIF2B-delta eIF2B-gamma ire-1 l(2)05070 mRpL11 mRpL13 mRpL19 mRpL21 mRpL22 mRpL22-24 mRpL28 mRpL3 mRpL48 mRpS18a mRpS18b mRpS2 mRpS22 mRpS25 mRpS32 mRpS33 mRpS9 mio mus205 poe ref(2)P |
| 346 | GO:0006437 | P | 9, 10, 11, | 1 | 0.064 (x 15.639) | 2 (0.500) | 0.223 | tyrosyl-tRNA aminoacylation | Aats-tyr |
| 347 | GO:0046501 | P | 7, | 1 | 0.032 (x 31.277) | 1 (1.000) | 0.223 | protoporphyrinogen IX metabolism | ferrochelatase |
| 348 | GO:0017004 | P | 7, | 1 | 0.064 (x 15.639) | 2 (0.500) | 0.224 | cytochrome complex assembly | CG7598 |
| 349 | GO:0009231 | P | 8, 9, | 1 | 0.064 (x 15.639) | 2 (0.500) | 0.224 | riboflavin biosynthesis | CG2846 |
| 350 | GO:0015248 | F | 4, | 1 | 0.032 (x 31.277) | 1 (1.000) | 0.224 | sterol transporter activity | CG3522 |
| 351 | GO:0045153 | F | 5, | 1 | 0.064 (x 15.639) | 2 (0.500) | 0.225 | electron transporter, transferring electrons within CoQH2-cytochrome c reductase complex activity | CG4769 |
| 352 | GO:0009117 | P | 6, | 9 | 5.084 (x 1.770) | 159 (0.057) | 0.225 | nucleotide metabolism | Adk1 BcDNA:LD32788 CG11883 CG13645 CG2246 Tpi Transaldolase Vha36 Vha44 |
| 353 | GO:0042727 | P | 8, | 1 | 0.064 (x 15.639) | 2 (0.500) | 0.225 | riboflavin and derivative biosynthesis | CG2846 |
| 354 | GO:0051789 | P | 5, | 1 | 0.032 (x 31.277) | 1 (1.000) | 0.225 | response to protein stimulus | PEK |
| 355 | GO:0008410 | F | 5, | 1 | 0.064 (x 15.639) | 2 (0.500) | 0.226 | CoA-transferase activity | CG1140 |
| 356 | GO:0006873 | P | 5, | 3 | 0.703 (x 4.265) | 22 (0.136) | 0.226 | cell ion homeostasis | Fer1HCH Fer2LCH MTF-1 |
| 357 | GO:0003674 | F | 1, | 272 | 264.921 (x 1.027) | 8286 (0.033) | 0.226 | molecular\_function | Aats-ala-m Aats-asp Aats-cys Aats-gly Aats-his Aats-thr Aats-trp Aats-tyr Aats-val Adk1 Ahcy13 Alas Aprt Ate1 BG:BACR48E02.4 BcDNA:GH07485 BcDNA:LD32788 CG10042 CG10268 CG10306 CG10333 CG10414 CG10460 CG10535 CG10638 CG10639 CG10721 CG10802 CG11309 CG1140 CG11710 CG11722 CG11876 CG11880 CG11883 CG11897 CG11967 CG11989 CG12013 CG12163 CG12171 CG12177 CG12264 CG12534 CG12788 CG12876 CG12954 CG1319 CG13645 CG13887 CG14040 CG15094 CG1598 CG1635 CG1673 CG17184 CG17187 CG17266 CG17294 CG17760 CG17904 CG18522 CG18547 CG1882 CG1969 CG2064 CG2107 CG2246 CG2263 CG2789 CG2846 CG30152 CG31063 CG31075 CG31126 CG31549 CG31694 CG31793 CG32380 CG32549 CG32675 CG32687 CG33066 CG33138 CG33193 CG3397 CG3476 CG3499 CG3522 CG3561 CG3608 CG3609 CG3731 CG3781 CG3931 CG4225 CG4407 CG4589 CG4769 CG4802 CG4858 CG4860 CG4882 CG4963 CG5044 CG5224 CG5261 CG5380 CG5469 CG5567 CG5604 CG6000 CG6016 CG6272 CG6428 CG6512 CG6523 CG6608 CG6668 CG6718 CG6734 CG6744 CG6746 CG6805 CG6984 CG7291 CG7417 CG7627 CG7632 CG7789 CG7809 CG7816 CG7842 CG8128 CG8360 CG8412 CG8417 CG8446 CG8479 CG8602 CG8636 CG8728 CG8778 CG8839 CG9065 CG9139 CG9215 CG9232 CG9240 CG9249 CG9298 CG9882 Cat Cyp6a20 Cyp6d5 Cys Cyt-b5 Cyt-c-p Dak1 Dgp-1 Dip-B EG:100G10.7 EG:65F1.1 EfTuM Eno Fer1HCH Fer2LCH Gapdh1 Gapdh2 Gclm Gfat2 Gp93 GstE1 GstE6 GstE7 GstE9 Hmgs Ide Idh Inos Jafrac1 JhI-1 JhI-26 Jra MTF-1 Men Mocs1 NTPase Nedd4 Nsf2 PEK Paps Past1 Pcd Pfk Pgm Pi4KIIalpha Prx6005 PyK Rep RhoGAP16F RhoGEF2 Shawn Sply Sras Syx13 TMS1 TepIV TfIIEbeta Thiolase Thor Tim10 Tpi Transaldolase Trxr-1 Tsp86D Ugt86Da Unc-76 Updo Vha36 Vha44 alpha-Est3 aralar1 att-ORFA bcn92 betaTub60D betaggt-II blp bor bsf c11.1 cathD cl cnn eIF-2alpha eIF2B-alpha eIF2B-delta eIF2B-gamma fbl ferrochelatase foi gammaSnap ire-1 kraken l(2)05070 l(2)k01209 l(3)02640 mRpL11 mRpL13 mRpL19 mRpL21 mRpL22 mRpL22-24 mRpL28 mRpL3 mRpL48 mRpL55 mRpS18a mRpS18b mRpS2 mRpS22 mRpS25 mRpS32 mRpS33 mRpS9 milton mio mus205 poe ref(2)P yip2 ytr |
| 358 | GO:0006122 | P | 9, 11, | 2 | 0.384 (x 5.213) | 12 (0.167) | 0.226 | mitochondrial electron transport, ubiquinol to cytochrome c | CG3731 CG4769 |
| 359 | GO:0004053 | F | 6, | 1 | 0.064 (x 15.639) | 2 (0.500) | 0.226 | arginase activity | EG:65F1.1 |
| 360 | GO:0016035 | C | 4, 5, 6, 7, 8, 9, 10, 11, 12, 13, | 1 | 0.032 (x 31.277) | 1 (1.000) | 0.227 | zeta DNA polymerase complex | mus205 |
| 361 | GO:0006694 | P | 6, 7, 8, | 2 | 0.384 (x 5.213) | 12 (0.167) | 0.227 | steroid biosynthesis | CG10268 CG3522 |
| 362 | GO:0004123 | F | 5, | 1 | 0.064 (x 15.639) | 2 (0.500) | 0.227 | cystathionine gamma-lyase activity | CG12264 |
| 363 | GO:0030003 | P | 6, | 3 | 0.703 (x 4.265) | 22 (0.136) | 0.227 | cation homeostasis | Fer1HCH Fer2LCH MTF-1 |
| 364 | GO:0015105 | F | 6, | 1 | 0.064 (x 15.639) | 2 (0.500) | 0.228 | arsenite transporter activity | CG1598 |
| 365 | GO:0042559 | P | 7, | 2 | 0.384 (x 5.213) | 12 (0.167) | 0.228 | pteridine and derivative biosynthesis | Mocs1 Pcd |
| 366 | GO:0007556 | P | 5, 7, 8, 9, 10, 11, 12, | 1 | 0.064 (x 15.639) | 2 (0.500) | 0.228 | regulation of juvenile hormone metabolism | Vha44 |
| 367 | GO:0008202 | P | 6, 7, | 8 | 4.348 (x 1.840) | 136 (0.059) | 0.228 | steroid metabolism | CG10268 CG12013 CG3522 Cyp6a20 Cyp6d5 Cyt-b5 Hmgs Ugt86Da |
| 368 | GO:0015239 | F | 4, | 2 | 0.384 (x 5.213) | 12 (0.167) | 0.228 | multidrug transporter activity | CG11897 CG7627 |
| 369 | GO:0006875 | P | 7, | 3 | 0.703 (x 4.265) | 22 (0.136) | 0.229 | metal ion homeostasis | Fer1HCH Fer2LCH MTF-1 |
| 370 | GO:0016408 | F | 7, | 2 | 0.288 (x 6.950) | 9 (0.222) | 0.229 | C-acyltransferase activity | Thiolase yip2 |
| 371 | GO:0019674 | P | 9, 10, | 1 | 0.064 (x 15.639) | 2 (0.500) | 0.229 | NAD metabolism | CG13645 |
| 372 | GO:0051184 | F | 3, | 2 | 0.384 (x 5.213) | 12 (0.167) | 0.229 | cofactor transporter activity | CG3476 CG4225 |
| 373 | GO:0004791 | F | 3, 5, 6, | 1 | 0.064 (x 15.639) | 2 (0.500) | 0.229 | thioredoxin-disulfide reductase activity | Trxr-1 |
| 374 | GO:0050801 | P | 4, | 3 | 0.863 (x 3.475) | 27 (0.111) | 0.23 | ion homeostasis | Fer1HCH Fer2LCH MTF-1 |
| 375 | GO:0006740 | P | 10, 11, | 2 | 0.384 (x 5.213) | 12 (0.167) | 0.23 | NADPH regeneration | Tpi Transaldolase |
| 376 | GO:0003869 | F | 7, | 2 | 0.288 (x 6.950) | 9 (0.222) | 0.23 | 4-nitrophenylphosphatase activity | CG17294 CG5567 |
| 377 | GO:0009435 | P | 8, 9, 10, 11, | 1 | 0.064 (x 15.639) | 2 (0.500) | 0.23 | NAD biosynthesis | CG13645 |
| 378 | GO:0005829 | C | 5, 6, 7, 8, | 10 | 5.883 (x 1.700) | 184 (0.054) | 0.23 | cytosol | CG32549 CG6718 Eno Jafrac1 Pfk PyK eIF-2alpha eIF2B-alpha eIF2B-delta eIF2B-gamma |
| 379 | GO:0051187 | P | 6, | 4 | 1.567 (x 2.553) | 49 (0.082) | 0.23 | cofactor catabolism | CG11876 CG5261 Idh Men |
| 380 | GO:0006739 | P | 9, 10, | 2 | 0.384 (x 5.213) | 12 (0.167) | 0.23 | NADP metabolism | Tpi Transaldolase |
| 381 | GO:0004749 | F | 6, 7, | 1 | 0.064 (x 15.639) | 2 (0.500) | 0.231 | ribose phosphate diphosphokinase activity | CG2246 |
| 382 | GO:0042364 | P | 7, | 2 | 0.288 (x 6.950) | 9 (0.222) | 0.231 | water-soluble vitamin biosynthesis | CG13645 CG2846 |
| 383 | GO:0006098 | P | 8, 10, 11, 12, | 2 | 0.384 (x 5.213) | 12 (0.167) | 0.231 | pentose-phosphate shunt | Tpi Transaldolase |
| 384 | GO:0006125 | P | 7, | 1 | 0.064 (x 15.639) | 2 (0.500) | 0.231 | thioredoxin pathway | Trxr-1 |
| 385 | GO:0017061 | F | 6, | 1 | 0.064 (x 15.639) | 2 (0.500) | 0.232 | S-methyl-5-thioadenosine phosphorylase activity | CG4802 |
| 386 | GO:0008318 | F | 6, | 2 | 0.384 (x 5.213) | 12 (0.167) | 0.232 | protein prenyltransferase activity | Rep betaggt-II |
| 387 | GO:0016420 | F | 7, | 1 | 0.064 (x 15.639) | 2 (0.500) | 0.232 | malonyltransferase activity | CG7842 |
| 388 | GO:0019438 | P | 6, | 2 | 0.448 (x 4.468) | 14 (0.143) | 0.233 | aromatic compound biosynthesis | Mocs1 Pcd |
| 389 | GO:0000175 | F | 8, 9, | 2 | 0.448 (x 4.468) | 14 (0.143) | 0.233 | 3'-5'-exoribonuclease activity | CG3931 mRpL28 |
| 390 | GO:0004817 | F | 7, | 1 | 0.064 (x 15.639) | 2 (0.500) | 0.233 | cysteine-tRNA ligase activity | Aats-cys |
| 391 | GO:0000221 | C | 4, 5, 6, 7, 8, 9, 10, 11, 12, | 2 | 0.448 (x 4.468) | 14 (0.143) | 0.234 | hydrogen-transporting ATPase V1 domain | Vha36 Vha44 |
| 392 | GO:0004662 | F | 8, | 1 | 0.064 (x 15.639) | 2 (0.500) | 0.234 | CAAX-protein geranylgeranyltransferase activity | betaggt-II |
| 393 | GO:0030695 | F | 3, | 8 | 4.156 (x 1.925) | 130 (0.062) | 0.234 | GTPase regulator activity | BG:BACR48E02.4 CG17184 CG9139 Rep RhoGAP16F RhoGEF2 eIF2B-delta eIF2B-gamma |
| 394 | GO:0006085 | P | 8, | 1 | 0.064 (x 15.639) | 2 (0.500) | 0.234 | acetyl-CoA biosynthesis | CG5261 |
| 395 | GO:0016419 | F | 8, | 1 | 0.064 (x 15.639) | 2 (0.500) | 0.235 | S-malonyltransferase activity | CG7842 |
| 396 | GO:0006844 | P | 6, 7, | 1 | 0.064 (x 15.639) | 2 (0.500) | 0.236 | acyl carnitine transport | CG3476 |
| 397 | GO:0016509 | F | 6, | 1 | 0.064 (x 15.639) | 2 (0.500) | 0.236 | long-chain-3-hydroxyacyl-CoA dehydrogenase activity | Thiolase |
| 398 | GO:0017110 | F | 7, | 1 | 0.064 (x 15.639) | 2 (0.500) | 0.237 | nucleoside-diphosphatase activity | NTPase |
| 399 | GO:0043122 | P | 5, 6, 8, | 1 | 0.064 (x 15.639) | 2 (0.500) | 0.238 | regulation of I-kappaB kinase/NF-kappaB cascade | CG7417 |
| 400 | GO:0006771 | P | 8, | 1 | 0.064 (x 15.639) | 2 (0.500) | 0.238 | riboflavin metabolism | CG2846 |
| 401 | GO:0004686 | F | 9, | 1 | 0.064 (x 15.639) | 2 (0.500) | 0.239 | eukaryotic elongation factor-2 kinase activity | PEK |
| 402 | GO:0008441 | F | 8, | 1 | 0.064 (x 15.639) | 2 (0.500) | 0.239 | 3'(2'),5'-bisphosphate nucleotidase activity | CG7789 |
| 403 | GO:0003919 | F | 6, | 1 | 0.064 (x 15.639) | 2 (0.500) | 0.24 | FMN adenylyltransferase activity | CG4407 |
| 404 | GO:0045155 | F | 5, | 1 | 0.064 (x 15.639) | 2 (0.500) | 0.241 | electron transporter, transferring electrons from CoQH2-cytochrome c reductase complex and cytochrome c oxidase complex activity | Cyt-c-p |
| 405 | GO:0016079 | P | 7, 8, 9, | 4 | 1.471 (x 2.720) | 46 (0.087) | 0.241 | synaptic vesicle exocytosis | Nsf2 Syx13 gammaSnap snapin |
| 406 | GO:0001677 | P | 9, 10, | 1 | 0.064 (x 15.639) | 2 (0.500) | 0.241 | formation of translation initiation ternary complex | eIF-2alpha |
| 407 | GO:0008080 | F | 8, | 3 | 0.895 (x 3.351) | 28 (0.107) | 0.242 | N-acetyltransferase activity | CG10414 CG11989 CG1969 |
| 408 | GO:0004831 | F | 7, | 1 | 0.064 (x 15.639) | 2 (0.500) | 0.242 | tyrosine-tRNA ligase activity | Aats-tyr |
| 409 | GO:0046467 | P | 6, 7, 8, | 3 | 0.991 (x 3.027) | 31 (0.097) | 0.243 | membrane lipid biosynthesis | CG32380 CG6016 Inos |
| 410 | GO:0016748 | F | 7, | 1 | 0.064 (x 15.639) | 2 (0.500) | 0.243 | succinyltransferase activity | Alas |
| 411 | GO:0019725 | P | 4, | 5 | 2.110 (x 2.369) | 66 (0.076) | 0.243 | cell homeostasis | Fer1HCH Fer2LCH Jafrac1 MTF-1 Prx6005 |
| 412 | GO:0006438 | P | 9, 10, 11, | 1 | 0.064 (x 15.639) | 2 (0.500) | 0.243 | valyl-tRNA aminoacylation | Aats-val |
| 413 | GO:0030523 | F | 8, | 1 | 0.064 (x 15.639) | 2 (0.500) | 0.244 | dihydrolipoamide S-acyltransferase activity | CG5261 |
| 414 | GO:0016507 | C | 3, 5, 6, 7, 8, 9, 10, 11, 12, | 1 | 0.064 (x 15.639) | 2 (0.500) | 0.245 | fatty acid beta-oxidation multienzyme complex | Thiolase |
| 415 | GO:0016654 | F | 5, | 1 | 0.064 (x 15.639) | 2 (0.500) | 0.246 | oxidoreductase activity, acting on NADH or NADPH, disulfide as acceptor | Trxr-1 |
| 416 | GO:0016230 | F | 4, | 1 | 0.064 (x 15.639) | 2 (0.500) | 0.246 | sphingomyelin phosphodiesterase activator activity | CG6734 |
| 417 | GO:0003729 | F | 5, | 15 | 10.007 (x 1.499) | 313 (0.048) | 0.247 | mRNA binding | Aats-ala-m Aats-asp Aats-cys Aats-his Aats-thr Aats-val CG10802 CG3931 CG8636 Pcd bsf mRpL21 mRpL28 mRpL55 ytr |
| 418 | GO:0046131 | P | 8, | 1 | 0.064 (x 15.639) | 2 (0.500) | 0.247 | pyrimidine ribonucleoside metabolism | CG8360 |
| 419 | GO:0019747 | P | 6, 7, 8, | 1 | 0.064 (x 15.639) | 2 (0.500) | 0.248 | regulation of isoprenoid metabolism | Vha44 |
| 420 | GO:0042726 | P | 7, | 1 | 0.064 (x 15.639) | 2 (0.500) | 0.248 | riboflavin and derivative metabolism | CG2846 |
| 421 | GO:0016418 | F | 8, | 1 | 0.064 (x 15.639) | 2 (0.500) | 0.249 | S-acetyltransferase activity | CG5261 |
| 422 | GO:0006423 | P | 9, 10, 11, | 1 | 0.064 (x 15.639) | 2 (0.500) | 0.25 | cysteinyl-tRNA aminoacylation | Aats-cys |
| 423 | GO:0006435 | P | 9, 10, 11, | 1 | 0.064 (x 15.639) | 2 (0.500) | 0.25 | threonyl-tRNA aminoacylation | Aats-thr |
| 424 | GO:0009328 | C | 4, | 1 | 0.064 (x 15.639) | 2 (0.500) | 0.251 | phenylalanine-tRNA ligase complex | CG2263 |
| 425 | GO:0005801 | C | 5, 6, 7, 8, 9, 10, | 1 | 0.064 (x 15.639) | 2 (0.500) | 0.252 | Golgi cis-face | CG9298 |
| 426 | GO:0009060 | P | 8, | 4 | 1.503 (x 2.662) | 47 (0.085) | 0.252 | aerobic respiration | CG11876 CG5261 Idh Men |
| 427 | GO:0005476 | F | 8, | 1 | 0.064 (x 15.639) | 2 (0.500) | 0.253 | carnitine:acyl carnitine antiporter activity | CG3476 |
| 428 | GO:0006099 | P | 8, 9, | 4 | 1.503 (x 2.662) | 47 (0.085) | 0.253 | tricarboxylic acid cycle | CG11876 CG5261 Idh Men |
| 429 | GO:0004095 | F | 9, | 1 | 0.064 (x 15.639) | 2 (0.500) | 0.253 | carnitine O-palmitoyltransferase activity | CG2107 |
| 430 | GO:0016407 | F | 7, | 4 | 1.503 (x 2.662) | 47 (0.085) | 0.253 | acetyltransferase activity | CG10414 CG11989 CG1969 CG5261 |
| 431 | GO:0046356 | P | 8, | 4 | 1.503 (x 2.662) | 47 (0.085) | 0.254 | acetyl-CoA catabolism | CG11876 CG5261 Idh Men |
| 432 | GO:0045333 | P | 7, | 4 | 1.503 (x 2.662) | 47 (0.085) | 0.255 | cellular respiration | CG11876 CG5261 Idh Men |
| 433 | GO:0009108 | P | 7, | 6 | 3.005 (x 1.996) | 94 (0.064) | 0.257 | coenzyme biosynthesis | CG13645 Gclm Mocs1 Vha36 Vha44 fbl |
| 434 | GO:0006665 | P | 7, 8, | 2 | 0.480 (x 4.170) | 15 (0.133) | 0.258 | sphingolipid metabolism | CG32380 Sply |
| 435 | GO:0005852 | C | 3, 4, 5, 6, 7, 8, 9, | 2 | 0.480 (x 4.170) | 15 (0.133) | 0.259 | eukaryotic translation initiation factor 3 complex | CG10306 CG8636 |
| 436 | GO:0006888 | P | 6, 7, 8, 9, | 2 | 0.480 (x 4.170) | 15 (0.133) | 0.259 | ER to Golgi vesicle-mediated transport | CG9298 Nsf2 |
| 437 | GO:0046873 | F | 4, | 3 | 1.023 (x 2.932) | 32 (0.094) | 0.259 | metal ion transporter activity | CG7816 CG9065 foi |
| 438 | GO:0016044 | P | 5, | 3 | 1.023 (x 2.932) | 32 (0.094) | 0.26 | membrane organization and biogenesis | CG33066 Tim10 fbl |
| 439 | GO:0004357 | F | 6, | 1 | 0.096 (x 10.426) | 3 (0.333) | 0.262 | glutamate-cysteine ligase activity | Gclm |
| 440 | GO:0008443 | F | 6, 7, | 1 | 0.096 (x 10.426) | 3 (0.333) | 0.262 | phosphofructokinase activity | Pfk |
| 441 | GO:0006626 | P | 8, 9, 10, | 3 | 1.087 (x 2.760) | 34 (0.088) | 0.263 | protein targeting to mitochondrion | CG33066 CG9240 Tim10 |
| 442 | GO:0016802 | F | 5, | 1 | 0.096 (x 10.426) | 3 (0.333) | 0.263 | trialkylsulfonium hydrolase activity | Ahcy13 |
| 443 | GO:0016831 | F | 5, | 3 | 1.087 (x 2.760) | 34 (0.088) | 0.263 | carboxy-lyase activity | Men Sply Updo |
| 444 | GO:0035246 | P | 10, 11, | 1 | 0.096 (x 10.426) | 3 (0.333) | 0.263 | peptidyl-arginine N-methylation | CG9882 |
| 445 | GO:0004029 | F | 6, | 1 | 0.096 (x 10.426) | 3 (0.333) | 0.264 | aldehyde dehydrogenase (NAD) activity | CG31075 |
| 446 | GO:0009057 | P | 5, | 10 | 6.075 (x 1.646) | 190 (0.053) | 0.264 | macromolecule catabolism | Ate1 CG5261 Eno Gapdh1 Gapdh2 Pfk PyK Tpi Transaldolase l(2)05070 |
| 447 | GO:0009241 | P | 7, 8, 9, | 1 | 0.096 (x 10.426) | 3 (0.333) | 0.264 | polyisoprenoid biosynthesis | Vha44 |
| 448 | GO:0046246 | P | 7, | 1 | 0.096 (x 10.426) | 3 (0.333) | 0.265 | terpene biosynthesis | Vha44 |
| 449 | GO:0016114 | P | 6, 8, 9, 10, | 1 | 0.096 (x 10.426) | 3 (0.333) | 0.265 | terpenoid biosynthesis | Vha44 |
| 450 | GO:0018195 | P | 9, | 1 | 0.096 (x 10.426) | 3 (0.333) | 0.266 | peptidyl-arginine modification | CG9882 |
| 451 | GO:0006432 | P | 9, 10, 11, | 1 | 0.096 (x 10.426) | 3 (0.333) | 0.267 | phenylalanyl-tRNA aminoacylation | CG2263 |
| 452 | GO:0035247 | P | 11, 12, | 1 | 0.096 (x 10.426) | 3 (0.333) | 0.267 | peptidyl-arginine omega-N-methylation | CG9882 |
| 453 | GO:0042592 | P | 3, | 5 | 2.430 (x 2.058) | 76 (0.066) | 0.267 | homeostasis | Fer1HCH Fer2LCH Jafrac1 MTF-1 Prx6005 |
| 454 | GO:0051762 | P | 8, | 1 | 0.096 (x 10.426) | 3 (0.333) | 0.268 | sesquiterpene biosynthesis | Vha44 |
| 455 | GO:0016530 | F | 5, | 1 | 0.096 (x 10.426) | 3 (0.333) | 0.268 | metallochaperone activity | CG9065 |
| 456 | GO:0050908 | P | 6, 7, 8, | 1 | 0.096 (x 10.426) | 3 (0.333) | 0.269 | detection of light stimulus during visual perception | Pgm |
| 457 | GO:0019363 | P | 7, 8, 9, | 1 | 0.096 (x 10.426) | 3 (0.333) | 0.269 | pyridine nucleotide biosynthesis | CG13645 |
| 458 | GO:0019919 | P | 12, 13, | 1 | 0.096 (x 10.426) | 3 (0.333) | 0.27 | peptidyl-arginine methylation, to asymmetrical-dimethyl arginine | CG9882 |
| 459 | GO:0006166 | P | 7, 9, | 1 | 0.096 (x 10.426) | 3 (0.333) | 0.27 | purine ribonucleoside salvage | Aprt |
| 460 | GO:0000281 | P | 6, 7, | 1 | 0.096 (x 10.426) | 3 (0.333) | 0.271 | cytokinesis after mitosis | Syx13 |
| 461 | GO:0016289 | F | 6, | 1 | 0.096 (x 10.426) | 3 (0.333) | 0.272 | CoA hydrolase activity | CG5044 |
| 462 | GO:0016401 | F | 7, | 1 | 0.096 (x 10.426) | 3 (0.333) | 0.272 | palmitoyl-CoA oxidase activity | BcDNA:GH07485 |
| 463 | GO:0043174 | P | 6, | 1 | 0.096 (x 10.426) | 3 (0.333) | 0.273 | nucleoside salvage | Aprt |
| 464 | GO:0046890 | P | 6, 7, 8, | 1 | 0.096 (x 10.426) | 3 (0.333) | 0.273 | regulation of lipid biosynthesis | Vha44 |
| 465 | GO:0016106 | P | 7, 9, 10, 11, | 1 | 0.096 (x 10.426) | 3 (0.333) | 0.274 | sesquiterpenoid biosynthesis | Vha44 |
| 466 | GO:0043450 | P | 6, | 1 | 0.096 (x 10.426) | 3 (0.333) | 0.275 | alkene biosynthesis | Vha44 |
| 467 | GO:0043094 | P | 5, | 1 | 0.096 (x 10.426) | 3 (0.333) | 0.275 | metabolic compound salvage | Aprt |
| 468 | GO:0006718 | P | 7, 8, 10, 11, 12, | 1 | 0.096 (x 10.426) | 3 (0.333) | 0.276 | juvenile hormone biosynthesis | Vha44 |
| 469 | GO:0019200 | F | 6, | 3 | 1.055 (x 2.843) | 33 (0.091) | 0.276 | carbohydrate kinase activity | CG2246 Pfk PyK |
| 470 | GO:0009628 | P | 3, | 17 | 11.894 (x 1.429) | 372 (0.046) | 0.276 | response to abiotic stimulus | CG11897 CG12013 CG1598 CG31793 CG5224 CG7627 Cat Gclm GstE1 GstE6 GstE7 GstE9 PEK Pgm Ugt86Da betaTub60D kraken |
| 471 | GO:0017109 | C | 3, 5, 6, 7, 8, | 1 | 0.096 (x 10.426) | 3 (0.333) | 0.276 | glutamate-cysteine ligase complex | Gclm |
| 472 | GO:0043101 | P | 6, | 1 | 0.096 (x 10.426) | 3 (0.333) | 0.277 | purine salvage | Aprt |
| 473 | GO:0016064 | P | 6, 7, | 1 | 0.096 (x 10.426) | 3 (0.333) | 0.277 | immunoglobulin mediated immune response | CG13887 |
| 474 | GO:0050962 | P | 5, 6, 7, | 1 | 0.096 (x 10.426) | 3 (0.333) | 0.278 | detection of light stimulus during sensory perception | Pgm |
| 475 | GO:0009055 | F | 4, | 4 | 1.695 (x 2.361) | 53 (0.075) | 0.278 | electron carrier activity | CG1319 CG4769 Cyt-c-p Trxr-1 |
| 476 | GO:0009894 | P | 5, | 1 | 0.096 (x 10.426) | 3 (0.333) | 0.279 | regulation of catabolism | Ate1 |
| 477 | GO:0004739 | F | 6, | 1 | 0.096 (x 10.426) | 3 (0.333) | 0.279 | pyruvate dehydrogenase (acetyl-transferring) activity | CG11876 |
| 478 | GO:0004013 | F | 6, | 1 | 0.096 (x 10.426) | 3 (0.333) | 0.28 | adenosylhomocysteinase activity | Ahcy13 |
| 479 | GO:0016531 | F | 6, 7, | 1 | 0.096 (x 10.426) | 3 (0.333) | 0.28 | copper chaperone activity | CG9065 |
| 480 | GO:0016668 | F | 5, | 1 | 0.096 (x 10.426) | 3 (0.333) | 0.281 | oxidoreductase activity, acting on sulfur group of donors, NAD or NADP as acceptor | Trxr-1 |
| 481 | GO:0004126 | F | 4, 6, | 1 | 0.096 (x 10.426) | 3 (0.333) | 0.282 | cytidine deaminase activity | CG8360 |
| 482 | GO:0016744 | F | 4, | 1 | 0.096 (x 10.426) | 3 (0.333) | 0.282 | transferase activity, transferring aldehyde or ketonic groups | Transaldolase |
| 483 | GO:0006839 | P | 6, 7, 8, | 2 | 0.544 (x 3.680) | 17 (0.118) | 0.283 | mitochondrial transport | CG3476 aralar1 |
| 484 | GO:0016922 | F | 4, 5, | 1 | 0.096 (x 10.426) | 3 (0.333) | 0.283 | ligand-dependent nuclear receptor binding | CG11710 |
| 485 | GO:0018216 | P | 9, 10, | 1 | 0.096 (x 10.426) | 3 (0.333) | 0.284 | peptidyl-arginine methylation | CG9882 |
| 486 | GO:0019724 | P | 7, 8, | 1 | 0.096 (x 10.426) | 3 (0.333) | 0.284 | B cell mediated immunity | CG13887 |
| 487 | GO:0042176 | P | 6, 7, 8, | 1 | 0.096 (x 10.426) | 3 (0.333) | 0.285 | regulation of protein catabolism | Ate1 |
| 488 | GO:0009399 | P | 5, | 1 | 0.096 (x 10.426) | 3 (0.333) | 0.285 | nitrogen fixation | CG11897 |
| 489 | GO:0004659 | F | 5, | 2 | 0.512 (x 3.910) | 16 (0.125) | 0.286 | prenyltransferase activity | Rep betaggt-II |
| 490 | GO:0004826 | F | 7, | 1 | 0.096 (x 10.426) | 3 (0.333) | 0.286 | phenylalanine-tRNA ligase activity | CG2263 |
| 491 | GO:0035103 | P | 9, | 1 | 0.096 (x 10.426) | 3 (0.333) | 0.287 | sterol regulatory element binding-protein cleavage | SCAP |
| 492 | GO:0005818 | C | 5, 6, 7, 8, 9, 10, 11, | 1 | 0.096 (x 10.426) | 3 (0.333) | 0.287 | aster | Rep |
| 493 | GO:0016788 | F | 4, | 20 | 14.547 (x 1.375) | 455 (0.044) | 0.287 | hydrolase activity, acting on ester bonds | CG11883 CG1598 CG1635 CG17294 CG32549 CG3931 CG5044 CG5567 CG6428 CG6718 CG6744 CG6746 CG6805 CG7789 CG8128 JhI-1 NTPase alpha-Est3 mRpL28 mus205 |
| 494 | GO:0016813 | F | 5, | 1 | 0.096 (x 10.426) | 3 (0.333) | 0.288 | hydrolase activity, acting on carbon-nitrogen (but not peptide) bonds, in linear amidines | EG:65F1.1 |
| 495 | GO:0016730 | F | 4, | 1 | 0.096 (x 10.426) | 3 (0.333) | 0.289 | oxidoreductase activity, acting on iron-sulfur proteins as donors | CG11897 |
| 496 | GO:0004430 | F | 8, | 1 | 0.096 (x 10.426) | 3 (0.333) | 0.289 | 1-phosphatidylinositol 4-kinase activity | Pi4KIIalpha |
| 497 | GO:0004738 | F | 5, | 1 | 0.096 (x 10.426) | 3 (0.333) | 0.29 | pyruvate dehydrogenase activity | CG11876 |
| 498 | GO:0048193 | P | 6, 7, 8, | 3 | 1.151 (x 2.606) | 36 (0.083) | 0.298 | Golgi vesicle transport | CG9298 Nsf2 SCAP |
| 499 | GO:0030234 | F | 2, | 16 | 11.414 (x 1.402) | 357 (0.045) | 0.3 | enzyme regulator activity | BG:BACR48E02.4 CG10460 CG10535 CG12163 CG15094 CG17184 CG6734 CG9139 Cys Gclm Rep RhoGAP16F RhoGEF2 TepIV eIF2B-delta eIF2B-gamma |
| 500 | GO:0009889 | P | 5, | 5 | 2.526 (x 1.980) | 79 (0.063) | 0.3 | regulation of biosynthesis | EfTuM PEK Thor Vha44 eIF2B-delta |
| 501 | GO:0042578 | F | 5, | 10 | 6.394 (x 1.564) | 200 (0.050) | 0.3 | phosphoric ester hydrolase activity | CG11883 CG1598 CG17294 CG32549 CG5567 CG6746 CG6805 CG7789 CG8128 NTPase |
| 502 | GO:0031326 | P | 6, | 5 | 2.526 (x 1.980) | 79 (0.063) | 0.3 | regulation of cellular biosynthesis | EfTuM PEK Thor Vha44 eIF2B-delta |
| 503 | GO:0007005 | P | 6, | 2 | 0.575 (x 3.475) | 18 (0.111) | 0.307 | mitochondrion organization and biogenesis | CG33066 Tim10 |
| 504 | GO:0016896 | F | 8, | 2 | 0.575 (x 3.475) | 18 (0.111) | 0.308 | exoribonuclease activity, producing 5'-phosphomonoesters | CG3931 mRpL28 |
| 505 | GO:0004437 | F | 7, | 2 | 0.607 (x 3.292) | 19 (0.105) | 0.308 | inositol or phosphatidylinositol phosphatase activity | CG6805 CG7789 |
| 506 | GO:0031300 | C | 4, 5, 6, 7, 8, 9, | 2 | 0.607 (x 3.292) | 19 (0.105) | 0.309 | intrinsic to organelle membrane | Sply Sras |
| 507 | GO:0031301 | C | 5, 6, 7, 8, 9, 10, | 2 | 0.607 (x 3.292) | 19 (0.105) | 0.309 | integral to organelle membrane | Sply Sras |
| 508 | GO:0048489 | P | 6, 7, | 5 | 2.558 (x 1.955) | 80 (0.062) | 0.309 | synaptic vesicle transport | AP-2sigma Nsf2 Syx13 gammaSnap snapin |
| 509 | GO:0006510 | P | 8, | 2 | 0.607 (x 3.292) | 19 (0.105) | 0.31 | ATP-dependent proteolysis | CG6512 EG:100G10.7 |
| 510 | GO:0046915 | F | 5, | 2 | 0.607 (x 3.292) | 19 (0.105) | 0.31 | transition metal ion transporter activity | CG9065 foi |
| 511 | GO:0016763 | F | 5, | 2 | 0.607 (x 3.292) | 19 (0.105) | 0.311 | transferase activity, transferring pentosyl groups | Aprt CG4802 |
| 512 | GO:0015238 | F | 3, | 2 | 0.607 (x 3.292) | 19 (0.105) | 0.312 | drug transporter activity | CG11897 CG7627 |
| 513 | GO:0004470 | F | 6, | 1 | 0.128 (x 7.819) | 4 (0.250) | 0.312 | malic enzyme activity | Men |
| 514 | GO:0005085 | F | 4, | 4 | 1.854 (x 2.157) | 58 (0.069) | 0.312 | guanyl-nucleotide exchange factor activity | CG9139 RhoGEF2 eIF2B-delta eIF2B-gamma |
| 515 | GO:0015232 | F | 4, | 1 | 0.128 (x 7.819) | 4 (0.250) | 0.312 | heme transporter activity | CG4225 |
| 516 | GO:0006869 | P | 5, 6, | 4 | 1.854 (x 2.157) | 58 (0.069) | 0.312 | lipid transport | CG2789 CG3476 CG3522 aralar1 |
| 517 | GO:0004818 | F | 7, | 1 | 0.128 (x 7.819) | 4 (0.250) | 0.313 | glutamate-tRNA ligase activity | Aats-val |
| 518 | GO:0030149 | P | 8, 9, | 1 | 0.128 (x 7.819) | 4 (0.250) | 0.314 | sphingolipid catabolism | Sply |
| 519 | GO:0005758 | C | 5, 6, 7, 8, 9, 10, 11, | 1 | 0.128 (x 7.819) | 4 (0.250) | 0.314 | mitochondrial intermembrane space | CG9065 |
| 520 | GO:0051189 | P | 5, 7, | 4 | 1.918 (x 2.085) | 60 (0.067) | 0.314 | prosthetic group metabolism | CG13645 CG2789 CG3608 Pcd |
| 521 | GO:0046146 | P | 7, | 1 | 0.128 (x 7.819) | 4 (0.250) | 0.315 | tetrahydrobiopterin metabolism | Pcd |
| 522 | GO:0019216 | P | 5, 6, | 1 | 0.128 (x 7.819) | 4 (0.250) | 0.315 | regulation of lipid metabolism | Vha44 |
| 523 | GO:0030148 | P | 7, 8, 9, | 1 | 0.128 (x 7.819) | 4 (0.250) | 0.316 | sphingolipid biosynthesis | CG32380 |
| 524 | GO:0004473 | F | 6, 7, | 1 | 0.128 (x 7.819) | 4 (0.250) | 0.316 | malate dehydrogenase (oxaloacetate-decarboxylating) (NADP+) activity | Men |
| 525 | GO:0005968 | C | 4, | 1 | 0.128 (x 7.819) | 4 (0.250) | 0.317 | Rab-protein geranylgeranyltransferase complex | Rep |
| 526 | GO:0008379 | F | 4, 6, | 1 | 0.128 (x 7.819) | 4 (0.250) | 0.318 | thioredoxin peroxidase activity | Jafrac1 |
| 527 | GO:0046466 | P | 7, 8, | 1 | 0.128 (x 7.819) | 4 (0.250) | 0.318 | membrane lipid catabolism | Sply |
| 528 | GO:0006729 | P | 8, | 1 | 0.128 (x 7.819) | 4 (0.250) | 0.319 | tetrahydrobiopterin biosynthesis | Pcd |
| 529 | GO:0017064 | F | 6, | 1 | 0.128 (x 7.819) | 4 (0.250) | 0.319 | fatty acid amide hydrolase activity | CG8839 |
| 530 | GO:0046906 | F | 3, | 1 | 0.128 (x 7.819) | 4 (0.250) | 0.32 | tetrapyrrole binding | Cat |
| 531 | GO:0046912 | F | 5, | 1 | 0.128 (x 7.819) | 4 (0.250) | 0.321 | transferase activity, transferring acyl groups, acyl groups converted into alkyl on transfer | Hmgs |
| 532 | GO:0046148 | P | 6, | 3 | 1.247 (x 2.406) | 39 (0.077) | 0.321 | pigment biosynthesis | Alas Updo ferrochelatase |
| 533 | GO:0007375 | P | 8, | 1 | 0.128 (x 7.819) | 4 (0.250) | 0.321 | anterior midgut invagination | RhoGEF2 |
| 534 | GO:0016409 | F | 7, | 1 | 0.128 (x 7.819) | 4 (0.250) | 0.322 | palmitoyltransferase activity | CG2107 |
| 535 | GO:0004622 | F | 8, | 1 | 0.128 (x 7.819) | 4 (0.250) | 0.323 | lysophospholipase activity | CG6428 |
| 536 | GO:0042575 | C | 3, 4, 5, 6, | 1 | 0.128 (x 7.819) | 4 (0.250) | 0.323 | DNA polymerase complex | mus205 |
| 537 | GO:0040020 | P | 8, | 1 | 0.128 (x 7.819) | 4 (0.250) | 0.324 | regulation of meiosis | mio |
| 538 | GO:0006213 | P | 7, | 1 | 0.128 (x 7.819) | 4 (0.250) | 0.324 | pyrimidine nucleoside metabolism | CG8360 |
| 539 | GO:0019985 | P | 6, 8, | 1 | 0.128 (x 7.819) | 4 (0.250) | 0.325 | bypass DNA synthesis | mus205 |
| 540 | GO:0007269 | P | 6, 7, 8, | 6 | 3.357 (x 1.787) | 105 (0.057) | 0.326 | neurotransmitter secretion | AP-2sigma Nsf2 Rep Syx13 gammaSnap snapin |
| 541 | GO:0000301 | P | 7, 8, 9, 10, | 1 | 0.128 (x 7.819) | 4 (0.250) | 0.326 | retrograde transport, vesicle recycling within Golgi | SCAP |
| 542 | GO:0006790 | P | 5, | 4 | 1.886 (x 2.120) | 59 (0.068) | 0.326 | sulfur metabolism | CG6523 Gclm Paps Trxr-1 |
| 543 | GO:0045055 | P | 6, 7, | 6 | 3.357 (x 1.787) | 105 (0.057) | 0.326 | regulated secretory pathway | AP-2sigma Nsf2 Rep Syx13 gammaSnap snapin |
| 544 | GO:0020037 | F | 4, | 1 | 0.128 (x 7.819) | 4 (0.250) | 0.326 | heme binding | Cat |
| 545 | GO:0007586 | P | 4, | 1 | 0.128 (x 7.819) | 4 (0.250) | 0.327 | digestion | kraken |
| 546 | GO:0046844 | P | 10, 11, | 1 | 0.128 (x 7.819) | 4 (0.250) | 0.328 | micropyle formation | Jra |
| 547 | GO:0046330 | P | 6, 7, 9, 10, | 1 | 0.128 (x 7.819) | 4 (0.250) | 0.328 | positive regulation of JNK cascade | CG7417 |
| 548 | GO:0006749 | P | 6, 7, | 1 | 0.128 (x 7.819) | 4 (0.250) | 0.329 | glutathione metabolism | Gclm |
| 549 | GO:0006826 | P | 9, 10, | 1 | 0.128 (x 7.819) | 4 (0.250) | 0.329 | iron ion transport | Fer1HCH |
| 550 | GO:0004663 | F | 8, | 1 | 0.128 (x 7.819) | 4 (0.250) | 0.33 | Rab-protein geranylgeranyltransferase activity | Rep |
| 551 | GO:0000042 | P | 8, 9, 10, 11, | 1 | 0.128 (x 7.819) | 4 (0.250) | 0.331 | protein targeting to Golgi | SCAP |
| 552 | GO:0006750 | P | 7, 8, | 1 | 0.128 (x 7.819) | 4 (0.250) | 0.331 | glutathione biosynthesis | Gclm |
| 553 | GO:0004675 | F | 5, 6, 8, 9, | 2 | 0.639 (x 3.128) | 20 (0.100) | 0.333 | transmembrane receptor protein serine/threonine kinase activity | BG:BACR48E02.4 CG32687 |
| 554 | GO:0030005 | P | 7, | 2 | 0.639 (x 3.128) | 20 (0.100) | 0.334 | di-, tri-valent inorganic cation homeostasis | Fer1HCH Fer2LCH |
| 555 | GO:0004866 | F | 5, | 5 | 2.718 (x 1.840) | 85 (0.059) | 0.339 | endopeptidase inhibitor activity | CG10460 CG12163 CG15094 Cys TepIV |
| 556 | GO:0051082 | F | 4, | 4 | 1.982 (x 2.018) | 62 (0.065) | 0.34 | unfolded protein binding | CG17187 CG6000 Gp93 mio |
| 557 | GO:0030414 | F | 4, | 5 | 2.750 (x 1.818) | 86 (0.058) | 0.35 | protease inhibitor activity | CG10460 CG12163 CG15094 Cys TepIV |
| 558 | GO:0017148 | P | 7, 8, 9, | 2 | 0.671 (x 2.979) | 21 (0.095) | 0.358 | negative regulation of protein biosynthesis | PEK Thor |
| 559 | GO:0009110 | P | 6, | 2 | 0.671 (x 2.979) | 21 (0.095) | 0.358 | vitamin biosynthesis | CG13645 CG2846 |
| 560 | GO:0051180 | P | 5, 6, | 1 | 0.160 (x 6.255) | 5 (0.200) | 0.36 | vitamin transport | CG3476 |
| 561 | GO:0007110 | P | 6, 7, | 1 | 0.160 (x 6.255) | 5 (0.200) | 0.36 | cytokinesis after meiosis I | Syx13 |
| 562 | GO:0004017 | F | 6, 8, | 1 | 0.160 (x 6.255) | 5 (0.200) | 0.361 | adenylate kinase activity | Adk1 |
| 563 | GO:0016972 | F | 6, | 1 | 0.160 (x 6.255) | 5 (0.200) | 0.362 | thiol oxidase activity | CG12534 |
| 564 | GO:0007374 | P | 8, | 1 | 0.160 (x 6.255) | 5 (0.200) | 0.362 | posterior midgut invagination | RhoGEF2 |
| 565 | GO:0046854 | P | 8, 9, 10, 11, | 1 | 0.160 (x 6.255) | 5 (0.200) | 0.363 | phosphoinositide phosphorylation | Pi4KIIalpha |
| 566 | GO:0006388 | P | 9, 10, | 1 | 0.160 (x 6.255) | 5 (0.200) | 0.364 | tRNA splicing | JhI-1 |
| 567 | GO:0015976 | P | 4, | 1 | 0.160 (x 6.255) | 5 (0.200) | 0.364 | carbon utilization | CG11967 |
| 568 | GO:0000394 | P | 9, | 1 | 0.160 (x 6.255) | 5 (0.200) | 0.365 | RNA splicing, via endonucleolytic cleavage and ligation | JhI-1 |
| 569 | GO:0016778 | F | 5, | 1 | 0.160 (x 6.255) | 5 (0.200) | 0.366 | diphosphotransferase activity | CG2246 |
| 570 | GO:0005459 | F | 6, | 1 | 0.160 (x 6.255) | 5 (0.200) | 0.366 | UDP-galactose transporter activity | CG14040 |
| 571 | GO:0016971 | F | 7, | 1 | 0.160 (x 6.255) | 5 (0.200) | 0.367 | flavin-linked sulfhydryl oxidase activity | CG12534 |
| 572 | GO:0005783 | C | 5, 6, 7, 8, | 6 | 3.613 (x 1.661) | 113 (0.053) | 0.367 | endoplasmic reticulum | CG13887 CG6512 CG8412 PEK Sply Sras |
| 573 | GO:0045792 | P | 6, 7, | 1 | 0.160 (x 6.255) | 5 (0.200) | 0.367 | negative regulation of cell size | Thor |
| 574 | GO:0006684 | P | 8, 9, | 1 | 0.160 (x 6.255) | 5 (0.200) | 0.368 | sphingomyelin metabolism | CG32380 |
| 575 | GO:0016832 | F | 5, | 1 | 0.160 (x 6.255) | 5 (0.200) | 0.369 | aldehyde-lyase activity | Sply |
| 576 | GO:0046128 | P | 8, | 1 | 0.160 (x 6.255) | 5 (0.200) | 0.369 | purine ribonucleoside metabolism | Aprt |
| 577 | GO:0005850 | C | 3, 4, 5, 6, 7, 8, 9, | 1 | 0.160 (x 6.255) | 5 (0.200) | 0.37 | eukaryotic translation initiation factor 2 complex | eIF-2alpha |
| 578 | GO:0031327 | P | 7, | 2 | 0.703 (x 2.843) | 22 (0.091) | 0.37 | negative regulation of cellular biosynthesis | PEK Thor |
| 579 | GO:0006743 | P | 8, | 1 | 0.160 (x 6.255) | 5 (0.200) | 0.371 | ubiquinone metabolism | CG9249 |
| 580 | GO:0009890 | P | 6, | 2 | 0.703 (x 2.843) | 22 (0.091) | 0.371 | negative regulation of biosynthesis | PEK Thor |
| 581 | GO:0042278 | P | 7, | 1 | 0.160 (x 6.255) | 5 (0.200) | 0.371 | purine nucleoside metabolism | Aprt |
| 582 | GO:0043190 | C | 3, 4, | 3 | 1.343 (x 2.234) | 42 (0.071) | 0.372 | ATP-binding cassette (ABC) transporter complex | CG31793 CG4225 CG7627 |
| 583 | GO:0005506 | F | 6, | 3 | 1.375 (x 2.182) | 43 (0.070) | 0.375 | iron ion binding | Fer1HCH Fer2LCH Mocs1 |
| 584 | GO:0016817 | F | 4, | 22 | 17.521 (x 1.256) | 548 (0.040) | 0.377 | hydrolase activity, acting on acid anhydrides | CG10333 CG11897 CG1598 CG17760 CG31063 CG31793 CG3561 CG4225 CG6512 CG6668 CG7627 CG8479 Dgp-1 EG:100G10.7 NTPase Nsf2 Past1 Vha36 Vha44 betaTub60D bor cnn |
| 585 | GO:0016818 | F | 5, | 22 | 17.521 (x 1.256) | 548 (0.040) | 0.378 | hydrolase activity, acting on acid anhydrides, in phosphorus-containing anhydrides | CG10333 CG11897 CG1598 CG17760 CG31063 CG31793 CG3561 CG4225 CG6512 CG6668 CG7627 CG8479 Dgp-1 EG:100G10.7 NTPase Nsf2 Past1 Vha36 Vha44 betaTub60D bor cnn |
| 586 | GO:0004518 | F | 5, | 5 | 2.877 (x 1.738) | 90 (0.056) | 0.381 | nuclease activity | CG3931 CG6744 JhI-1 mRpL28 mus205 |
| 587 | GO:0019239 | F | 3, | 2 | 0.735 (x 2.720) | 23 (0.087) | 0.39 | deaminase activity | CG8360 l(3)02640 |
| 588 | GO:0006961 | P | 7, 8, 9, | 2 | 0.735 (x 2.720) | 23 (0.087) | 0.391 | antibacterial humoral response (sensu Protostomia) | TepIV Thor |
| 589 | GO:0004540 | F | 6, | 3 | 1.407 (x 2.133) | 44 (0.068) | 0.391 | ribonuclease activity | CG3931 JhI-1 mRpL28 |
| 590 | GO:0006119 | P | 6, 8, | 7 | 4.508 (x 1.553) | 141 (0.050) | 0.391 | oxidative phosphorylation | CG12534 CG3731 CG4769 CG7598 Cyt-c-p Vha36 Vha44 |
| 591 | GO:0016853 | F | 3, | 5 | 2.909 (x 1.719) | 91 (0.055) | 0.391 | isomerase activity | CG17266 CG8417 Inos Pgm Tpi |
| 592 | GO:0000139 | C | 4, 5, 6, 7, 8, 9, 10, | 2 | 0.735 (x 2.720) | 23 (0.087) | 0.391 | Golgi membrane | CG14040 CG7809 |
| 593 | GO:0004532 | F | 7, | 2 | 0.735 (x 2.720) | 23 (0.087) | 0.392 | exoribonuclease activity | CG3931 mRpL28 |
| 594 | GO:0015450 | F | 4, 6, | 2 | 0.767 (x 2.606) | 24 (0.083) | 0.396 | protein translocase activity | CG33066 Tim10 |
| 595 | GO:0000280 | P | 4, | 1 | 0.192 (x 5.213) | 6 (0.167) | 0.396 | nuclear division | cnn |
| 596 | GO:0015082 | F | 5, | 2 | 0.767 (x 2.606) | 24 (0.083) | 0.396 | di-, tri-valent inorganic cation transporter activity | CG9065 foi |
| 597 | GO:0019720 | P | 8, | 1 | 0.192 (x 5.213) | 6 (0.167) | 0.396 | Mo-molybdopterin cofactor metabolism | Mocs1 |
| 598 | GO:0046982 | F | 5, | 2 | 0.767 (x 2.606) | 24 (0.083) | 0.397 | protein heterodimerization activity | CG6272 Jra |
| 599 | GO:0016670 | F | 5, | 1 | 0.192 (x 5.213) | 6 (0.167) | 0.397 | oxidoreductase activity, acting on sulfur group of donors, oxygen as acceptor | CG12534 |
| 600 | GO:0004176 | F | 5, | 1 | 0.192 (x 5.213) | 6 (0.167) | 0.398 | ATP-dependent peptidase activity | CG3499 |
| 601 | GO:0006124 | P | 7, | 1 | 0.192 (x 5.213) | 6 (0.167) | 0.398 | ferredoxin metabolism | CG1319 |
| 602 | GO:0016406 | F | 8, | 1 | 0.192 (x 5.213) | 6 (0.167) | 0.399 | carnitine O-acyltransferase activity | CG2107 |
| 603 | GO:0031970 | C | 4, 5, 6, 7, 8, 9, | 1 | 0.192 (x 5.213) | 6 (0.167) | 0.4 | organelle envelope lumen | CG9065 |
| 604 | GO:0003997 | F | 6, | 1 | 0.192 (x 5.213) | 6 (0.167) | 0.4 | acyl-CoA oxidase activity | BcDNA:GH07485 |
| 605 | GO:0043545 | P | 7, | 1 | 0.192 (x 5.213) | 6 (0.167) | 0.401 | molybdopterin cofactor metabolism | Mocs1 |
| 606 | GO:0001505 | P | 7, | 6 | 3.741 (x 1.604) | 117 (0.051) | 0.401 | regulation of neurotransmitter levels | AP-2sigma Nsf2 Rep Syx13 gammaSnap snapin |
| 607 | GO:0004192 | F | 7, | 1 | 0.192 (x 5.213) | 6 (0.167) | 0.401 | cathepsin D activity | cathD |
| 608 | GO:0006907 | P | 7, 8, | 1 | 0.192 (x 5.213) | 6 (0.167) | 0.402 | pinocytosis | AP-2sigma |
| 609 | GO:0019748 | P | 4, | 4 | 2.174 (x 1.840) | 68 (0.059) | 0.402 | secondary metabolism | Alas Updo Vha44 ferrochelatase |
| 610 | GO:0042440 | P | 5, | 3 | 1.471 (x 2.040) | 46 (0.065) | 0.402 | pigment metabolism | Alas Updo ferrochelatase |
| 611 | GO:0006825 | P | 9, 10, | 1 | 0.192 (x 5.213) | 6 (0.167) | 0.403 | copper ion transport | CG9065 |
| 612 | GO:0046834 | P | 7, 8, | 1 | 0.192 (x 5.213) | 6 (0.167) | 0.403 | lipid phosphorylation | Pi4KIIalpha |
| 613 | GO:0016830 | F | 4, | 3 | 1.439 (x 2.085) | 45 (0.067) | 0.404 | carbon-carbon lyase activity | Men Sply Updo |
| 614 | GO:0004032 | F | 7, | 1 | 0.192 (x 5.213) | 6 (0.167) | 0.404 | aldehyde reductase activity | CG10638 |
| 615 | GO:0006777 | P | 8, 9, | 1 | 0.192 (x 5.213) | 6 (0.167) | 0.405 | Mo-molybdopterin cofactor biosynthesis | Mocs1 |
| 616 | GO:0006094 | P | 8, 9, 10, | 1 | 0.192 (x 5.213) | 6 (0.167) | 0.405 | gluconeogenesis | Tpi |
| 617 | GO:0005483 | F | 4, | 1 | 0.192 (x 5.213) | 6 (0.167) | 0.406 | soluble NSF attachment protein activity | gammaSnap |
| 618 | GO:0004743 | F | 6, | 1 | 0.192 (x 5.213) | 6 (0.167) | 0.407 | pyruvate kinase activity | PyK |
| 619 | GO:0016126 | P | 7, 8, 9, | 1 | 0.192 (x 5.213) | 6 (0.167) | 0.407 | sterol biosynthesis | CG10268 |
| 620 | GO:0006422 | P | 9, 10, 11, | 1 | 0.192 (x 5.213) | 6 (0.167) | 0.408 | aspartyl-tRNA aminoacylation | Aats-asp |
| 621 | GO:0015226 | F | 4, | 1 | 0.192 (x 5.213) | 6 (0.167) | 0.409 | carnitine transporter activity | CG3476 |
| 622 | GO:0004815 | F | 7, | 1 | 0.192 (x 5.213) | 6 (0.167) | 0.409 | aspartate-tRNA ligase activity | Aats-asp |
| 623 | GO:0016846 | F | 4, | 1 | 0.192 (x 5.213) | 6 (0.167) | 0.41 | carbon-sulfur lyase activity | CG12264 |
| 624 | GO:0017169 | F | 6, | 1 | 0.192 (x 5.213) | 6 (0.167) | 0.411 | CDP-alcohol phosphatidyltransferase activity | CG6016 |
| 625 | GO:0005788 | C | 4, 5, 6, 7, 8, 9, 10, | 1 | 0.192 (x 5.213) | 6 (0.167) | 0.412 | endoplasmic reticulum lumen | PEK |
| 626 | GO:0000242 | C | 5, 6, 7, 8, 9, 10, 11, | 1 | 0.192 (x 5.213) | 6 (0.167) | 0.412 | pericentriolar material | cnn |
| 627 | GO:0016796 | F | 7, | 2 | 0.799 (x 2.502) | 25 (0.080) | 0.42 | exonuclease activity, active with either ribo- or deoxyribonucleic acids and producing 5'-phosphomonoesters | CG3931 mRpL28 |
| 628 | GO:0004448 | F | 6, | 1 | 0.224 (x 4.468) | 7 (0.143) | 0.435 | isocitrate dehydrogenase activity | Idh |
| 629 | GO:0016755 | F | 5, | 1 | 0.224 (x 4.468) | 7 (0.143) | 0.436 | transferase activity, transferring amino-acyl groups | Ate1 |
| 630 | GO:0016624 | F | 5, | 1 | 0.224 (x 4.468) | 7 (0.143) | 0.436 | oxidoreductase activity, acting on the aldehyde or oxo group of donors, disulfide as acceptor | CG11876 |
| 631 | GO:0000922 | C | 5, 6, 7, 8, 9, 10, 11, | 1 | 0.224 (x 4.468) | 7 (0.143) | 0.437 | spindle pole | Rep |
| 632 | GO:0006714 | P | 6, 8, 9, 10, | 1 | 0.224 (x 4.468) | 7 (0.143) | 0.438 | sesquiterpenoid metabolism | Vha44 |
| 633 | GO:0006020 | P | 8, 9, | 1 | 0.224 (x 4.468) | 7 (0.143) | 0.438 | myo-inositol metabolism | Inos |
| 634 | GO:0030145 | F | 6, | 1 | 0.224 (x 4.468) | 7 (0.143) | 0.439 | manganese ion binding | Dip-B |
| 635 | GO:0016051 | P | 6, 7, | 3 | 1.567 (x 1.915) | 49 (0.061) | 0.439 | carbohydrate biosynthesis | Gfat2 Inos Tpi |
| 636 | GO:0005385 | F | 6, | 1 | 0.224 (x 4.468) | 7 (0.143) | 0.44 | zinc ion transporter activity | foi |
| 637 | GO:0016801 | F | 4, | 1 | 0.224 (x 4.468) | 7 (0.143) | 0.44 | hydrolase activity, acting on ether bonds | Ahcy13 |
| 638 | GO:0030258 | P | 6, 7, | 1 | 0.224 (x 4.468) | 7 (0.143) | 0.441 | lipid modification | Pi4KIIalpha |
| 639 | GO:0004312 | F | 7, | 1 | 0.224 (x 4.468) | 7 (0.143) | 0.442 | fatty-acid synthase activity | CG7842 |
| 640 | GO:0009584 | P | 6, 7, | 1 | 0.224 (x 4.468) | 7 (0.143) | 0.442 | detection of visible light | Pgm |
| 641 | GO:0009081 | P | 7, 8, | 1 | 0.224 (x 4.468) | 7 (0.143) | 0.443 | branched chain family amino acid metabolism | CG1673 |
| 642 | GO:0006858 | P | 5, 6, | 6 | 3.933 (x 1.526) | 123 (0.049) | 0.444 | extracellular transport | CG11897 CG15094 CG31793 CG4225 CG7627 CG8602 |
| 643 | GO:0006716 | P | 6, 7, 9, 10, 11, | 1 | 0.224 (x 4.468) | 7 (0.143) | 0.444 | juvenile hormone metabolism | Vha44 |
| 644 | GO:0008203 | P | 7, 8, 9, | 2 | 0.831 (x 2.406) | 26 (0.077) | 0.444 | cholesterol metabolism | CG10268 Hmgs |
| 645 | GO:0043492 | F | 3, 10, | 7 | 4.828 (x 1.450) | 151 (0.046) | 0.444 | ATPase activity, coupled to movement of substances | CG11897 CG1598 CG31793 CG4225 CG7627 Vha36 Vha44 |
| 646 | GO:0051183 | F | 3, | 1 | 0.224 (x 4.468) | 7 (0.143) | 0.444 | vitamin transporter activity | CG3476 |
| 647 | GO:0005975 | P | 5, | 19 | 15.507 (x 1.225) | 485 (0.039) | 0.445 | carbohydrate metabolism | CG11876 CG15094 CG33138 CG5261 CG8417 CG9232 Eno Gapdh1 Gapdh2 Gfat2 Idh Inos Men Pfk Pgm PyK Tpi Transaldolase Ugt86Da |
| 648 | GO:0016860 | F | 4, | 2 | 0.831 (x 2.406) | 26 (0.077) | 0.445 | intramolecular oxidoreductase activity | CG8417 Tpi |
| 649 | GO:0042626 | F | 4, 6, 11, | 7 | 4.828 (x 1.450) | 151 (0.046) | 0.445 | ATPase activity, coupled to transmembrane movement of substances | CG11897 CG1598 CG31793 CG4225 CG7627 Vha36 Vha44 |
| 650 | GO:0046030 | F | 8, | 1 | 0.224 (x 4.468) | 7 (0.143) | 0.445 | inositol trisphosphate phosphatase activity | CG6805 |
| 651 | GO:0051761 | P | 7, | 1 | 0.224 (x 4.468) | 7 (0.143) | 0.446 | sesquiterpene metabolism | Vha44 |
| 652 | GO:0005375 | F | 6, | 1 | 0.224 (x 4.468) | 7 (0.143) | 0.447 | copper ion transporter activity | CG9065 |
| 653 | GO:0004033 | F | 6, | 1 | 0.224 (x 4.468) | 7 (0.143) | 0.447 | aldo-keto reductase activity | CG10638 |
| 654 | GO:0015165 | F | 5, | 1 | 0.224 (x 4.468) | 7 (0.143) | 0.448 | pyrimidine nucleotide sugar transporter activity | CG14040 |
| 655 | GO:0016820 | F | 5, | 7 | 4.860 (x 1.440) | 152 (0.046) | 0.452 | hydrolase activity, acting on acid anhydrides, catalyzing transmembrane movement of substances | CG11897 CG1598 CG31793 CG4225 CG7627 Vha36 Vha44 |
| 656 | GO:0016301 | F | 5, | 16 | 12.821 (x 1.248) | 401 (0.040) | 0.452 | kinase activity | Adk1 BG:BACR48E02.4 CG10268 CG2246 CG2846 CG32687 CG3608 Dak1 PEK Paps Pfk Pi4KIIalpha PyK fbl ire-1 l(2)k01209 |
| 657 | GO:0006417 | P | 6, 7, 8, | 4 | 2.398 (x 1.668) | 75 (0.053) | 0.461 | regulation of protein biosynthesis | EfTuM PEK Thor eIF2B-delta |
| 658 | GO:0016476 | P | 6, 7, | 1 | 0.256 (x 3.910) | 8 (0.125) | 0.468 | shape changes of embryonic cells | RhoGEF2 |
| 659 | GO:0005089 | F | 5, | 1 | 0.256 (x 3.910) | 8 (0.125) | 0.469 | Rho guanyl-nucleotide exchange factor activity | RhoGEF2 |
| 660 | GO:0000177 | C | 4, 5, 6, 7, 8, | 1 | 0.256 (x 3.910) | 8 (0.125) | 0.47 | cytoplasmic exosome (RNase complex) | CG3931 |
| 661 | GO:0008021 | C | 8, 9, 10, 11, 12, | 3 | 1.631 (x 1.840) | 51 (0.059) | 0.47 | synaptic vesicle | AP-2sigma Rep snapin |
| 662 | GO:0043449 | P | 5, | 1 | 0.256 (x 3.910) | 8 (0.125) | 0.471 | alkene metabolism | Vha44 |
| 663 | GO:0008535 | P | 7, | 1 | 0.256 (x 3.910) | 8 (0.125) | 0.471 | cytochrome c oxidase complex assembly | CG9065 |
| 664 | GO:0035004 | F | 7, | 1 | 0.256 (x 3.910) | 8 (0.125) | 0.472 | phosphoinositide 3-kinase activity | Pi4KIIalpha |
| 665 | GO:0044432 | C | 4, 5, 6, 7, 8, 9, | 3 | 1.663 (x 1.804) | 52 (0.058) | 0.472 | endoplasmic reticulum part | PEK Sply Sras |
| 666 | GO:0005666 | C | 4, 5, 6, 7, 8, 9, 10, 11, 12, 13, | 1 | 0.256 (x 3.910) | 8 (0.125) | 0.473 | DNA-directed RNA polymerase III complex | CG5380 |
| 667 | GO:0042214 | P | 6, | 1 | 0.256 (x 3.910) | 8 (0.125) | 0.473 | terpene metabolism | Vha44 |
| 668 | GO:0004683 | F | 8, | 1 | 0.256 (x 3.910) | 8 (0.125) | 0.474 | calmodulin regulated protein kinase activity | PEK |
| 669 | GO:0005819 | C | 5, 6, 7, 8, 9, 10, | 2 | 0.895 (x 2.234) | 28 (0.071) | 0.474 | spindle | Rep fbl |
| 670 | GO:0016868 | F | 5, | 1 | 0.256 (x 3.910) | 8 (0.125) | 0.475 | intramolecular transferase activity, phosphotransferases | Pgm |
| 671 | GO:0017111 | F | 7, | 20 | 16.721 (x 1.196) | 523 (0.038) | 0.475 | nucleoside-triphosphatase activity | CG10333 CG11897 CG1598 CG17760 CG31793 CG3561 CG4225 CG6512 CG6668 CG7627 CG8479 EG:100G10.7 NTPase Nsf2 Past1 Vha36 Vha44 betaTub60D bor cnn |
| 672 | GO:0016350 | P | 9, | 1 | 0.256 (x 3.910) | 8 (0.125) | 0.475 | maintenance of oocyte identity (sensu Insecta) | mio |
| 673 | GO:0004067 | F | 6, | 1 | 0.256 (x 3.910) | 8 (0.125) | 0.476 | asparaginase activity | CG6428 |
| 674 | GO:0045735 | F | 2, | 1 | 0.256 (x 3.910) | 8 (0.125) | 0.477 | nutrient reservoir activity | CG6718 |
| 675 | GO:0007506 | P | 5, 6, | 1 | 0.256 (x 3.910) | 8 (0.125) | 0.478 | gonadal mesoderm development | foi |
| 676 | GO:0005338 | F | 4, | 1 | 0.256 (x 3.910) | 8 (0.125) | 0.478 | nucleotide-sugar transporter activity | CG14040 |
| 677 | GO:0007370 | P | 8, | 1 | 0.256 (x 3.910) | 8 (0.125) | 0.479 | ventral furrow formation | RhoGEF2 |
| 678 | GO:0016780 | F | 5, | 1 | 0.256 (x 3.910) | 8 (0.125) | 0.48 | phosphotransferase activity, for other substituted phosphate groups | CG6016 |
| 679 | GO:0016471 | C | 3, 4, 5, 6, 7, 8, 9, 10, 11, | 2 | 0.927 (x 2.157) | 29 (0.069) | 0.48 | hydrogen-translocating V-type ATPase complex | Vha36 Vha44 |
| 680 | GO:0042157 | P | 7, | 2 | 0.927 (x 2.157) | 29 (0.069) | 0.48 | lipoprotein metabolism | Rep betaggt-II |
| 681 | GO:0016096 | P | 7, 8, | 1 | 0.256 (x 3.910) | 8 (0.125) | 0.48 | polyisoprenoid metabolism | Vha44 |
| 682 | GO:0005478 | F | 3, | 2 | 0.927 (x 2.157) | 29 (0.069) | 0.481 | intracellular transporter activity | Syx13 gammaSnap |
| 683 | GO:0007377 | P | 8, | 1 | 0.256 (x 3.910) | 8 (0.125) | 0.481 | germ-band extension | RhoGEF2 |
| 684 | GO:0006497 | P | 8, 9, | 2 | 0.927 (x 2.157) | 29 (0.069) | 0.482 | protein amino acid lipidation | Rep betaggt-II |
| 685 | GO:0006721 | P | 5, 7, 8, 9, | 1 | 0.256 (x 3.910) | 8 (0.125) | 0.482 | terpenoid metabolism | Vha44 |
| 686 | GO:0016192 | P | 5, 6, | 12 | 9.464 (x 1.268) | 296 (0.041) | 0.482 | vesicle-mediated transport | AP-2sigma CG8479 CG9139 CG9298 Nsf2 Past1 Rep RhoGEF2 SCAP Syx13 gammaSnap snapin |
| 687 | GO:0042158 | P | 7, 8, | 2 | 0.927 (x 2.157) | 29 (0.069) | 0.483 | lipoprotein biosynthesis | Rep betaggt-II |
| 688 | GO:0008237 | F | 5, | 8 | 5.915 (x 1.353) | 185 (0.043) | 0.485 | metallopeptidase activity | CG3499 CG3731 CG6512 CG8728 Dip-B EG:100G10.7 Ide Sras |
| 689 | GO:0016462 | F | 6, | 20 | 16.977 (x 1.178) | 531 (0.038) | 0.492 | pyrophosphatase activity | CG10333 CG11897 CG1598 CG17760 CG31793 CG3561 CG4225 CG6512 CG6668 CG7627 CG8479 EG:100G10.7 NTPase Nsf2 Past1 Vha36 Vha44 betaTub60D bor cnn |
| 690 | GO:0005682 | C | 5, 6, 7, 8, 9, 10, 11, 12, | 1 | 0.288 (x 3.475) | 9 (0.111) | 0.492 | snRNP U5 | CG10333 |
| 691 | GO:0009796 | P | 4, 5, | 1 | 0.288 (x 3.475) | 9 (0.111) | 0.493 | cellularization (sensu Metazoa) | RhoGEF2 |
| 692 | GO:0003995 | F | 5, | 1 | 0.288 (x 3.475) | 9 (0.111) | 0.493 | acyl-CoA dehydrogenase activity | CG4860 |
| 693 | GO:0016274 | F | 8, | 1 | 0.288 (x 3.475) | 9 (0.111) | 0.494 | protein-arginine N-methyltransferase activity | CG9882 |
| 694 | GO:0003730 | F | 6, | 1 | 0.288 (x 3.475) | 9 (0.111) | 0.495 | mRNA 3'-UTR binding | bsf |
| 695 | GO:0008079 | F | 4, 5, | 1 | 0.288 (x 3.475) | 9 (0.111) | 0.496 | translation termination factor activity | EfTuM |
| 696 | GO:0006073 | P | 7, 8, | 1 | 0.288 (x 3.475) | 9 (0.111) | 0.496 | glucan metabolism | CG33138 |
| 697 | GO:0018346 | P | 10, 11, | 1 | 0.288 (x 3.475) | 9 (0.111) | 0.497 | protein amino acid prenylation | Rep |
| 698 | GO:0006108 | P | 8, 9, | 1 | 0.288 (x 3.475) | 9 (0.111) | 0.498 | malate metabolism | Men |
| 699 | GO:0046907 | P | 5, 6, 7, | 23 | 19.695 (x 1.168) | 616 (0.037) | 0.498 | intracellular transport | AP-2sigma CG10535 CG13887 CG17266 CG33066 CG3476 CG8479 CG9139 CG9240 CG9298 Nsf2 Rep RhoGEF2 SCAP SH3PX1 Syx13 Tim10 Unc-76 aralar1 betaTub60D dynactin-subunit-p25 gammaSnap milton |
| 700 | GO:0007464 | P | 7, 8, 9, 10, 11, | 1 | 0.288 (x 3.475) | 9 (0.111) | 0.498 | R3/R4 cell fate commitment | Jra |
| 701 | GO:0016615 | F | 5, | 1 | 0.288 (x 3.475) | 9 (0.111) | 0.499 | malate dehydrogenase activity | Men |
| 702 | GO:0048056 | P | 7, 8, 9, 10, | 1 | 0.288 (x 3.475) | 9 (0.111) | 0.5 | R3/R4 cell differentiation (sensu Endopterygota) | Jra |
| 703 | GO:0005773 | C | 5, 6, 7, 8, | 3 | 1.726 (x 1.738) | 54 (0.056) | 0.5 | vacuole | Dip-B Vha36 Vha44 |
| 704 | GO:0016627 | F | 4, | 2 | 0.959 (x 2.085) | 30 (0.067) | 0.5 | oxidoreductase activity, acting on the CH-CH group of donors | BcDNA:GH07485 CG4860 |
| 705 | GO:0005869 | C | 4, 6, 7, 8, 9, 10, 11, | 1 | 0.288 (x 3.475) | 9 (0.111) | 0.5 | dynactin complex | dynactin-subunit-p25 |
| 706 | GO:0003747 | F | 5, 6, | 1 | 0.288 (x 3.475) | 9 (0.111) | 0.501 | translation release factor activity | EfTuM |
| 707 | GO:0005977 | P | 8, 9, | 1 | 0.288 (x 3.475) | 9 (0.111) | 0.502 | glycogen metabolism | CG33138 |
| 708 | GO:0006012 | P | 8, 9, | 1 | 0.288 (x 3.475) | 9 (0.111) | 0.503 | galactose metabolism | CG9232 |
| 709 | GO:0050906 | P | 4, 5, 6, | 1 | 0.288 (x 3.475) | 9 (0.111) | 0.503 | detection of stimulus during sensory perception | Pgm |
| 710 | GO:0018342 | P | 9, 10, | 1 | 0.288 (x 3.475) | 9 (0.111) | 0.504 | protein prenylation | Rep |
| 711 | GO:0016273 | F | 7, | 1 | 0.288 (x 3.475) | 9 (0.111) | 0.505 | arginine N-methyltransferase activity | CG9882 |
| 712 | GO:0045570 | P | 5, 6, | 1 | 0.288 (x 3.475) | 9 (0.111) | 0.506 | regulation of imaginal disc growth | CG33193 |
| 713 | GO:0008299 | P | 6, 7, 8, | 1 | 0.288 (x 3.475) | 9 (0.111) | 0.506 | isoprenoid biosynthesis | Vha44 |
| 714 | GO:0008121 | F | 6, 7, | 1 | 0.288 (x 3.475) | 9 (0.111) | 0.507 | ubiquinol-cytochrome-c reductase activity | CG3731 |
| 715 | GO:0016027 | C | 3, 6, 7, 8, | 1 | 0.288 (x 3.475) | 9 (0.111) | 0.508 | inaD signaling complex | Pgm |
| 716 | GO:0016681 | F | 5, | 1 | 0.288 (x 3.475) | 9 (0.111) | 0.508 | oxidoreductase activity, acting on diphenols and related substances as donors, cytochrome as acceptor | CG3731 |
| 717 | GO:0043228 | C | 3, | 26 | 22.732 (x 1.144) | 711 (0.037) | 0.509 | non-membrane-bound organelle | CG12954 Pcd Rep Unc-76 betaTub60D cnn dynactin-subunit-p25 fbl mRpL11 mRpL13 mRpL19 mRpL21 mRpL22 mRpL22-24 mRpL28 mRpL3 mRpL48 mRpL55 mRpS18a mRpS18b mRpS2 mRpS22 mRpS25 mRpS32 mRpS33 mRpS9 |
| 718 | GO:0009166 | P | 6, 7, | 1 | 0.288 (x 3.475) | 9 (0.111) | 0.509 | nucleotide catabolism | CG11883 |
| 719 | GO:0043232 | C | 4, 5, 6, 7, | 26 | 22.732 (x 1.144) | 711 (0.037) | 0.509 | intracellular non-membrane-bound organelle | CG12954 Pcd Rep Unc-76 betaTub60D cnn dynactin-subunit-p25 fbl mRpL11 mRpL13 mRpL19 mRpL21 mRpL22 mRpL22-24 mRpL28 mRpL3 mRpL48 mRpL55 mRpS18a mRpS18b mRpS2 mRpS22 mRpS25 mRpS32 mRpS33 mRpS9 |
| 720 | GO:0030136 | C | 7, 8, 9, 10, 11, | 3 | 1.790 (x 1.676) | 56 (0.054) | 0.512 | clathrin-coated vesicle | AP-2sigma Rep snapin |
| 721 | GO:0008150 | P | 1, | 262 | 258.399 (x 1.014) | 8082 (0.032) | 0.512 | biological\_process | AP-2sigma Aats-ala-m Aats-asp Aats-cys Aats-gly Aats-his Aats-thr Aats-trp Aats-tyr Aats-val Adk1 Ahcy13 Alas Aprt Ate1 BG:BACR48E02.4 BcDNA:GH07485 BcDNA:LD32788 CG10042 CG10068 CG10268 CG10306 CG10333 CG10340 CG10414 CG10460 CG10535 CG10638 CG10639 CG10721 CG10802 CG1140 CG11710 CG11722 CG11876 CG11880 CG11883 CG11897 CG11967 CG11989 CG12013 CG12163 CG12171 CG12177 CG12264 CG12534 CG12788 CG12876 CG12954 CG1319 CG13645 CG13887 CG14040 CG15094 CG1598 CG1635 CG1673 CG17184 CG17187 CG17266 CG17294 CG17760 CG18522 CG1882 CG2064 CG2107 CG2246 CG2263 CG2789 CG2846 CG30152 CG31075 CG31126 CG31549 CG31694 CG31793 CG32380 CG32549 CG32675 CG32687 CG33066 CG33138 CG33193 CG3397 CG3476 CG3499 CG3522 CG3561 CG3608 CG3609 CG3731 CG3781 CG3931 CG4225 CG4407 CG4447 CG4769 CG4802 CG4860 CG4963 CG5044 CG5224 CG5261 CG5380 CG5567 CG5604 CG6000 CG6016 CG6272 CG6428 CG6512 CG6523 CG6608 CG6668 CG6718 CG6746 CG6805 CG6984 CG7417 CG7598 CG7627 CG7789 CG7816 CG7842 CG8128 CG8360 CG8412 CG8417 CG8446 CG8479 CG8602 CG8636 CG8728 CG8778 CG8839 CG9065 CG9139 CG9215 CG9232 CG9240 CG9249 CG9298 CG9836 CG9882 Cat Cyp6a20 Cyp6d5 Cyt-b5 Cyt-c-p Dak1 Dgp-1 Dip-B EG:100G10.7 EfTuM Eno Fer1HCH Fer2LCH Gapdh1 Gapdh2 Gclm Gfat2 Gp93 GstE1 GstE6 GstE7 GstE9 Hmgs Ide Idh Inos Jafrac1 JhI-1 JhI-26 Jra MTF-1 Men Mocs1 NTPase Nedd4 Nsf2 PEK Paps Past1 Pcd Pfk Pgm Pi4KIIalpha Prx6005 PyK Rep RhoGEF2 SCAP SH3PX1 Shawn Sply Sras Syx13 TMS1 TepIV TfIIEbeta Thiolase Thor Tim10 Tpi Transaldolase Trxr-1 Tsp86D Ugt86Da Unc-76 Updo Vha36 Vha44 aralar1 att-ORFA bcn92 betaTub60D betaggt-II blp c11.1 cactin cathD cl cnn dynactin-subunit-p25 eIF-2alpha eIF2B-alpha eIF2B-delta eIF2B-gamma fbl ferrochelatase foi gammaSnap ire-1 kraken l(2)05070 l(2)k01209 l(3)02640 mRpL11 mRpL13 mRpL19 mRpL21 mRpL22 mRpL22-24 mRpL28 mRpL3 mRpL48 mRpS18a mRpS18b mRpS2 mRpS22 mRpS25 mRpS32 mRpS33 mRpS9 milton mio mus205 poe ref(2)P snapin yip2 ytr |
| 722 | GO:0016787 | F | 3, | 62 | 57.422 (x 1.080) | 1796 (0.035) | 0.514 | hydrolase activity | Ahcy13 CG10333 CG11309 CG11883 CG11897 CG12163 CG12177 CG1598 CG1635 CG17294 CG17760 CG1882 CG31063 CG31694 CG31793 CG32549 CG33138 CG3499 CG3561 CG3731 CG3931 CG4225 CG5044 CG5567 CG6428 CG6512 CG6668 CG6718 CG6744 CG6746 CG6805 CG7627 CG7632 CG7789 CG8128 CG8360 CG8479 CG8728 CG8839 CG9240 Dgp-1 Dip-B EG:100G10.7 EG:65F1.1 Ide JhI-1 NTPase Nsf2 Past1 Sras Vha36 Vha44 alpha-Est3 betaTub60D bor cathD cnn kraken l(2)05070 mRpL28 mus205 ref(2)P |
| 723 | GO:0005774 | C | 5, 6, 7, 8, 9, 10, | 2 | 1.023 (x 1.955) | 32 (0.062) | 0.523 | vacuolar membrane | Vha36 Vha44 |
| 724 | GO:0044437 | C | 4, 5, 6, 7, 8, 9, | 2 | 1.023 (x 1.955) | 32 (0.062) | 0.524 | vacuolar part | Vha36 Vha44 |
| 725 | GO:0016891 | F | 8, | 1 | 0.320 (x 3.128) | 10 (0.100) | 0.526 | endoribonuclease activity, producing 5'-phosphomonoesters | JhI-1 |
| 726 | GO:0046621 | P | 5, | 1 | 0.320 (x 3.128) | 10 (0.100) | 0.527 | negative regulation of organ size | CG33193 |
| 727 | GO:0009165 | P | 6, 7, | 5 | 3.517 (x 1.422) | 110 (0.045) | 0.527 | nucleotide biosynthesis | BcDNA:LD32788 CG13645 CG2246 Vha36 Vha44 |
| 728 | GO:0004178 | F | 7, | 1 | 0.320 (x 3.128) | 10 (0.100) | 0.527 | leucyl aminopeptidase activity | Dip-B |
| 729 | GO:0044455 | C | 4, 5, 6, 7, 8, 9, 10, 11, 12, | 5 | 3.517 (x 1.422) | 110 (0.045) | 0.528 | mitochondrial membrane part | CG33066 CG3731 CG4769 CG7598 Tim10 |
| 730 | GO:0000030 | F | 6, | 1 | 0.320 (x 3.128) | 10 (0.100) | 0.528 | mannosyltransferase activity | CG8412 |
| 731 | GO:0005623 | C | 2, | 153 | 147.584 (x 1.037) | 4616 (0.033) | 0.528 | cell | AP-2sigma Aats-ala-m Aats-asp Aats-his Adk1 Alas Aprt BcDNA:GH07485 CG10042 CG10268 CG10306 CG10333 CG1140 CG11876 CG11883 CG11897 CG12013 CG12264 CG12534 CG12954 CG1319 CG13887 CG14040 CG15094 CG1598 CG1673 CG17266 CG17760 CG2263 CG2789 CG2846 CG31075 CG31793 CG32380 CG32549 CG33066 CG3397 CG3476 CG3499 CG3731 CG3931 CG4225 CG4589 CG4769 CG4882 CG4963 CG5134 CG5261 CG5380 CG5604 CG6272 CG6512 CG6608 CG6718 CG6744 CG7417 CG7598 CG7627 CG7809 CG7816 CG7842 CG8412 CG8602 CG8636 CG8728 CG9065 CG9215 CG9240 CG9298 CG9424 Cat Cyp6a20 Cyp6d5 Cyt-b5 Cyt-c-p Dip-B EG:100G10.7 EfTuM Eno Gapdh1 Gapdh2 Gclm Gfat2 Ide Idh Inos Jafrac1 JhI-1 Jra MTF-1 Nedd4 Nsf2 PEK Pcd Pfk Pgm PyK Rep Shawn Sply Sras Syx13 TMS1 TfIIEbeta Thiolase Tim10 Transaldolase Trxr-1 Tsp86D Unc-76 Updo Vha36 Vha44 aralar1 att-ORFA bcn92 betaTub60D bor bsf cnn dynactin-subunit-p25 eIF-2alpha eIF2B-alpha eIF2B-delta eIF2B-gamma fbl ferrochelatase foi gammaSnap l(2)05070 mRpL11 mRpL13 mRpL19 mRpL21 mRpL22 mRpL22-24 mRpL28 mRpL3 mRpL48 mRpL55 mRpS18a mRpS18b mRpS2 mRpS22 mRpS25 mRpS32 mRpS33 mRpS9 milton mus205 ref(2)P snapin yip2 |
| 732 | GO:0044242 | P | 6, 7, | 1 | 0.320 (x 3.128) | 10 (0.100) | 0.529 | cellular lipid catabolism | Sply |
| 733 | GO:0044464 | C | 2, 3, | 153 | 147.584 (x 1.037) | 4616 (0.033) | 0.529 | cell part | AP-2sigma Aats-ala-m Aats-asp Aats-his Adk1 Alas Aprt BcDNA:GH07485 CG10042 CG10268 CG10306 CG10333 CG1140 CG11876 CG11883 CG11897 CG12013 CG12264 CG12534 CG12954 CG1319 CG13887 CG14040 CG15094 CG1598 CG1673 CG17266 CG17760 CG2263 CG2789 CG2846 CG31075 CG31793 CG32380 CG32549 CG33066 CG3397 CG3476 CG3499 CG3731 CG3931 CG4225 CG4589 CG4769 CG4882 CG4963 CG5134 CG5261 CG5380 CG5604 CG6272 CG6512 CG6608 CG6718 CG6744 CG7417 CG7598 CG7627 CG7809 CG7816 CG7842 CG8412 CG8602 CG8636 CG8728 CG9065 CG9215 CG9240 CG9298 CG9424 Cat Cyp6a20 Cyp6d5 Cyt-b5 Cyt-c-p Dip-B EG:100G10.7 EfTuM Eno Gapdh1 Gapdh2 Gclm Gfat2 Ide Idh Inos Jafrac1 JhI-1 Jra MTF-1 Nedd4 Nsf2 PEK Pcd Pfk Pgm PyK Rep Shawn Sply Sras Syx13 TMS1 TfIIEbeta Thiolase Tim10 Transaldolase Trxr-1 Tsp86D Unc-76 Updo Vha36 Vha44 aralar1 att-ORFA bcn92 betaTub60D bor bsf cnn dynactin-subunit-p25 eIF-2alpha eIF2B-alpha eIF2B-delta eIF2B-gamma fbl ferrochelatase foi gammaSnap l(2)05070 mRpL11 mRpL13 mRpL19 mRpL21 mRpL22 mRpL22-24 mRpL28 mRpL3 mRpL48 mRpL55 mRpS18a mRpS18b mRpS2 mRpS22 mRpS25 mRpS32 mRpS33 mRpS9 milton mus205 ref(2)P snapin yip2 |
| 734 | GO:0016616 | F | 5, | 4 | 2.686 (x 1.489) | 84 (0.048) | 0.532 | oxidoreductase activity, acting on the CH-OH group of donors, NAD or NADP as acceptor | CG10638 Idh Men Thiolase |
| 735 | GO:0016125 | P | 6, 7, 8, | 2 | 1.055 (x 1.896) | 33 (0.061) | 0.538 | sterol metabolism | CG10268 Hmgs |
| 736 | GO:0016810 | F | 4, | 4 | 2.718 (x 1.472) | 85 (0.047) | 0.544 | hydrolase activity, acting on carbon-nitrogen (but not peptide) bonds | CG6428 CG8360 CG8839 EG:65F1.1 |
| 737 | GO:0051649 | P | 5, 6, | 23 | 20.302 (x 1.133) | 635 (0.036) | 0.557 | establishment of cellular localization | AP-2sigma CG10535 CG13887 CG17266 CG33066 CG3476 CG8479 CG9139 CG9240 CG9298 Nsf2 Rep RhoGEF2 SCAP SH3PX1 Syx13 Tim10 Unc-76 aralar1 betaTub60D dynactin-subunit-p25 gammaSnap milton |
| 738 | GO:0016282 | C | 3, 5, 6, 7, 8, | 3 | 1.918 (x 1.564) | 60 (0.050) | 0.558 | eukaryotic 43S preinitiation complex | CG10306 CG8636 eIF-2alpha |
| 739 | GO:0042579 | C | 5, 6, 7, 8, | 2 | 1.087 (x 1.840) | 34 (0.059) | 0.558 | microbody | BcDNA:GH07485 Cat |
| 740 | GO:0001727 | F | 6, | 1 | 0.352 (x 2.843) | 11 (0.091) | 0.558 | lipid kinase activity | Pi4KIIalpha |
| 741 | GO:0005777 | C | 6, 7, 8, 9, | 2 | 1.087 (x 1.840) | 34 (0.059) | 0.559 | peroxisome | BcDNA:GH07485 Cat |
| 742 | GO:0006446 | P | 8, 9, 10, | 1 | 0.352 (x 2.843) | 11 (0.091) | 0.559 | regulation of translational initiation | Thor |
| 743 | GO:0051641 | P | 4, 5, | 23 | 20.334 (x 1.131) | 636 (0.036) | 0.56 | cellular localization | AP-2sigma CG10535 CG13887 CG17266 CG33066 CG3476 CG8479 CG9139 CG9240 CG9298 Nsf2 Rep RhoGEF2 SCAP SH3PX1 Syx13 Tim10 Unc-76 aralar1 betaTub60D dynactin-subunit-p25 gammaSnap milton |
| 744 | GO:0006383 | P | 8, | 1 | 0.352 (x 2.843) | 11 (0.091) | 0.56 | transcription from RNA polymerase III promoter | CG5380 |
| 745 | GO:0016634 | F | 5, | 1 | 0.352 (x 2.843) | 11 (0.091) | 0.561 | oxidoreductase activity, acting on the CH-CH group of donors, oxygen as acceptor | BcDNA:GH07485 |
| 746 | GO:0007126 | P | 7, | 4 | 2.782 (x 1.438) | 87 (0.046) | 0.561 | meiosis | Syx13 cnn fbl mio |
| 747 | GO:0035147 | P | 5, 7, | 1 | 0.352 (x 2.843) | 11 (0.091) | 0.561 | tracheal branch fusion | foi |
| 748 | GO:0035146 | P | 6, | 1 | 0.352 (x 2.843) | 11 (0.091) | 0.562 | tube fusion | foi |
| 749 | GO:0005544 | F | 5, | 1 | 0.352 (x 2.843) | 11 (0.091) | 0.563 | calcium-dependent phospholipid binding | CG7632 |
| 750 | GO:0007249 | P | 7, | 1 | 0.352 (x 2.843) | 11 (0.091) | 0.564 | I-kappaB kinase/NF-kappaB cascade | CG7417 |
| 751 | GO:0012505 | C | 4, 5, | 6 | 4.604 (x 1.303) | 144 (0.042) | 0.578 | endomembrane system | AP-2sigma CG14040 CG7809 CG9424 Sply Sras |
| 752 | GO:0006897 | P | 6, 7, | 5 | 3.709 (x 1.348) | 116 (0.043) | 0.579 | endocytosis | AP-2sigma CG8479 CG9139 Past1 RhoGEF2 |
| 753 | GO:0046785 | P | 8, 10, | 1 | 0.384 (x 2.606) | 12 (0.083) | 0.59 | microtubule polymerization | betaTub60D |
| 754 | GO:0042446 | P | 6, | 1 | 0.384 (x 2.606) | 12 (0.083) | 0.591 | hormone biosynthesis | Vha44 |
| 755 | GO:0019897 | C | 5, 6, 7, | 2 | 1.151 (x 1.738) | 36 (0.056) | 0.591 | extrinsic to plasma membrane | CG17760 Pgm |
| 756 | GO:0005496 | F | 3, | 1 | 0.384 (x 2.606) | 12 (0.083) | 0.592 | steroid binding | CG3522 |
| 757 | GO:0000176 | C | 4, 5, 6, 7, 8, 9, 10, | 1 | 0.384 (x 2.606) | 12 (0.083) | 0.593 | nuclear exosome (RNase complex) | CG3931 |
| 758 | GO:0043087 | P | 5, | 1 | 0.384 (x 2.606) | 12 (0.083) | 0.594 | regulation of GTPase activity | Rep |
| 759 | GO:0005486 | F | 5, | 1 | 0.384 (x 2.606) | 12 (0.083) | 0.594 | t-SNARE activity | Syx13 |
| 760 | GO:0016614 | F | 4, | 5 | 3.773 (x 1.325) | 118 (0.042) | 0.595 | oxidoreductase activity, acting on CH-OH group of donors | CG10638 CG12171 Idh Men Thiolase |
| 761 | GO:0045298 | C | 3, 5, 6, 7, 8, 9, 10, 11, | 1 | 0.384 (x 2.606) | 12 (0.083) | 0.595 | tubulin | betaTub60D |
| 762 | GO:0030001 | P | 7, 8, | 6 | 4.700 (x 1.277) | 147 (0.041) | 0.602 | metal ion transport | CG15094 CG3397 CG7816 CG9065 Fer1HCH foi |
| 763 | GO:0051327 | P | 6, | 4 | 2.909 (x 1.375) | 91 (0.044) | 0.604 | M phase of meiotic cell cycle | Syx13 cnn fbl mio |
| 764 | GO:0005083 | F | 4, | 4 | 2.909 (x 1.375) | 91 (0.044) | 0.604 | small GTPase regulator activity | BG:BACR48E02.4 CG17184 Rep RhoGEF2 |
| 765 | GO:0044431 | C | 4, 5, 6, 7, 8, 9, | 3 | 2.078 (x 1.444) | 65 (0.046) | 0.612 | Golgi apparatus part | CG14040 CG7809 CG9298 |
| 766 | GO:0046034 | P | 6, 10, | 3 | 2.078 (x 1.444) | 65 (0.046) | 0.613 | ATP metabolism | Adk1 Vha36 Vha44 |
| 767 | GO:0000178 | C | 3, 4, 5, 6, | 1 | 0.416 (x 2.406) | 13 (0.077) | 0.613 | exosome (RNase complex) | CG3931 |
| 768 | GO:0051321 | P | 5, | 4 | 2.941 (x 1.360) | 92 (0.043) | 0.614 | meiotic cell cycle | Syx13 cnn fbl mio |
| 769 | GO:0015399 | F | 4, | 5 | 3.837 (x 1.303) | 120 (0.042) | 0.614 | primary active transporter activity | CG1598 CG33066 Tim10 Vha36 Vha44 |
| 770 | GO:0007098 | P | 5, 8, | 1 | 0.416 (x 2.406) | 13 (0.077) | 0.614 | centrosome cycle | cnn |
| 771 | GO:0006163 | P | 7, | 4 | 2.941 (x 1.360) | 92 (0.043) | 0.614 | purine nucleotide metabolism | Adk1 BcDNA:LD32788 Vha36 Vha44 |
| 772 | GO:0015405 | F | 5, | 5 | 3.837 (x 1.303) | 120 (0.042) | 0.615 | P-P-bond-hydrolysis-driven transporter activity | CG1598 CG33066 Tim10 Vha36 Vha44 |
| 773 | GO:0016679 | F | 4, | 1 | 0.416 (x 2.406) | 13 (0.077) | 0.615 | oxidoreductase activity, acting on diphenols and related substances as donors | CG3731 |
| 774 | GO:0005689 | C | 5, 6, 7, 8, 9, 10, 11, | 1 | 0.416 (x 2.406) | 13 (0.077) | 0.615 | minor (U12-dependent) spliceosome complex | CG10333 |
| 775 | GO:0016183 | P | 8, 9, 10, | 1 | 0.416 (x 2.406) | 13 (0.077) | 0.616 | synaptic vesicle coating | AP-2sigma |
| 776 | GO:0006901 | P | 7, 8, | 1 | 0.416 (x 2.406) | 13 (0.077) | 0.617 | vesicle coating | AP-2sigma |
| 777 | GO:0016893 | F | 7, | 1 | 0.416 (x 2.406) | 13 (0.077) | 0.618 | endonuclease activity, active with either ribo- or deoxyribonucleic acids and producing 5'-phosphomonoesters | JhI-1 |
| 778 | GO:0007616 | P | 6, | 1 | 0.416 (x 2.406) | 13 (0.077) | 0.619 | long-term memory | CG10460 |
| 779 | GO:0046328 | P | 5, 6, 8, 9, | 1 | 0.416 (x 2.406) | 13 (0.077) | 0.619 | regulation of JNK cascade | CG7417 |
| 780 | GO:0051297 | P | 7, | 1 | 0.416 (x 2.406) | 13 (0.077) | 0.62 | centrosome organization and biogenesis | cnn |
| 781 | GO:0006473 | P | 9, | 1 | 0.416 (x 2.406) | 13 (0.077) | 0.621 | protein amino acid acetylation | CG11989 |
| 782 | GO:0019731 | P | 6, 7, 8, | 2 | 1.215 (x 1.646) | 38 (0.053) | 0.622 | antibacterial humoral response | TepIV Thor |
| 783 | GO:0004194 | F | 7, | 1 | 0.416 (x 2.406) | 13 (0.077) | 0.622 | pepsin A activity | cathD |
| 784 | GO:0006886 | P | 6, 7, 8, | 18 | 16.178 (x 1.113) | 506 (0.036) | 0.627 | intracellular protein transport | AP-2sigma CG10535 CG13887 CG17266 CG33066 CG8479 CG9139 CG9240 CG9298 Nsf2 Rep RhoGEF2 SCAP SH3PX1 Syx13 Tim10 betaTub60D gammaSnap |
| 785 | GO:0007143 | P | 8, | 2 | 1.247 (x 1.604) | 39 (0.051) | 0.63 | female meiosis | Syx13 cnn |
| 786 | GO:0004004 | F | 5, 11, | 2 | 1.279 (x 1.564) | 40 (0.050) | 0.636 | ATP-dependent RNA helicase activity | CG10333 CG3561 |
| 787 | GO:0043414 | P | 7, | 2 | 1.279 (x 1.564) | 40 (0.050) | 0.636 | biopolymer methylation | CG9882 RhoGEF2 |
| 788 | GO:0005789 | C | 4, 5, 6, 7, 8, 9, 10, | 2 | 1.279 (x 1.564) | 40 (0.050) | 0.637 | endoplasmic reticulum membrane | Sply Sras |
| 789 | GO:0005351 | F | 5, 6, | 2 | 1.279 (x 1.564) | 40 (0.050) | 0.638 | sugar porter activity | CG14040 TepIV |
| 790 | GO:0030125 | C | 6, 7, 8, 9, 10, 11, 12, 13, | 1 | 0.448 (x 2.234) | 14 (0.071) | 0.638 | clathrin vesicle coat | AP-2sigma |
| 791 | GO:0015674 | P | 7, 8, | 2 | 1.279 (x 1.564) | 40 (0.050) | 0.639 | di-, tri-valent inorganic cation transport | CG9065 Fer1HCH |
| 792 | GO:0004556 | F | 7, | 1 | 0.448 (x 2.234) | 14 (0.071) | 0.639 | alpha-amylase activity | CG33138 |
| 793 | GO:0008186 | F | 10, | 2 | 1.279 (x 1.564) | 40 (0.050) | 0.64 | RNA-dependent ATPase activity | CG10333 CG3561 |
| 794 | GO:0004623 | F | 8, | 1 | 0.448 (x 2.234) | 14 (0.071) | 0.64 | phospholipase A2 activity | CG6718 |
| 795 | GO:0015103 | F | 5, | 2 | 1.279 (x 1.564) | 40 (0.050) | 0.64 | inorganic anion transporter activity | CG15094 CG1598 |
| 796 | GO:0008033 | P | 8, | 1 | 0.448 (x 2.234) | 14 (0.071) | 0.641 | tRNA processing | JhI-1 |
| 797 | GO:0030135 | C | 6, 7, 8, 9, 10, | 3 | 2.174 (x 1.380) | 68 (0.044) | 0.641 | coated vesicle | AP-2sigma Rep snapin |
| 798 | GO:0016160 | F | 6, | 1 | 0.448 (x 2.234) | 14 (0.071) | 0.641 | amylase activity | CG33138 |
| 799 | GO:0006720 | P | 6, 7, | 1 | 0.448 (x 2.234) | 14 (0.071) | 0.642 | isoprenoid metabolism | Vha44 |
| 800 | GO:0006487 | P | 9, 10, | 1 | 0.448 (x 2.234) | 14 (0.071) | 0.643 | protein amino acid N-linked glycosylation | CG8412 |
| 801 | GO:0006112 | P | 7, | 1 | 0.448 (x 2.234) | 14 (0.071) | 0.644 | energy reserve metabolism | CG33138 |
| 802 | GO:0051336 | P | 4, | 1 | 0.448 (x 2.234) | 14 (0.071) | 0.645 | regulation of hydrolase activity | Rep |
| 803 | GO:0030665 | C | 7, 8, 9, 10, 11, 12, | 1 | 0.448 (x 2.234) | 14 (0.071) | 0.645 | clathrin coated vesicle membrane | AP-2sigma |
| 804 | GO:0031023 | P | 6, | 1 | 0.448 (x 2.234) | 14 (0.071) | 0.646 | microtubule organizing center organization and biogenesis | cnn |
| 805 | GO:0009205 | P | 9, | 3 | 2.206 (x 1.360) | 69 (0.043) | 0.652 | purine ribonucleoside triphosphate metabolism | Adk1 Vha36 Vha44 |
| 806 | GO:0005746 | C | 5, 6, 7, 8, 9, 10, 11, 12, 13, | 3 | 2.206 (x 1.360) | 69 (0.043) | 0.652 | mitochondrial electron transport chain | CG3731 CG4769 CG7598 |
| 807 | GO:0004620 | F | 7, | 2 | 1.311 (x 1.526) | 41 (0.049) | 0.653 | phospholipase activity | CG6428 CG6718 |
| 808 | GO:0009144 | P | 8, | 3 | 2.206 (x 1.360) | 69 (0.043) | 0.653 | purine nucleoside triphosphate metabolism | Adk1 Vha36 Vha44 |
| 809 | GO:0042175 | C | 4, 5, 6, | 2 | 1.311 (x 1.526) | 41 (0.049) | 0.654 | nuclear envelope-endoplasmic reticulum network | Sply Sras |
| 810 | GO:0009199 | P | 8, | 3 | 2.206 (x 1.360) | 69 (0.043) | 0.654 | ribonucleoside triphosphate metabolism | Adk1 Vha36 Vha44 |
| 811 | GO:0045045 | P | 5, 6, | 8 | 6.874 (x 1.164) | 215 (0.037) | 0.654 | secretory pathway | AP-2sigma CG9298 Nsf2 Rep SCAP Syx13 gammaSnap snapin |
| 812 | GO:0004857 | F | 3, | 5 | 4.060 (x 1.231) | 127 (0.039) | 0.655 | enzyme inhibitor activity | CG10460 CG12163 CG15094 Cys TepIV |
| 813 | GO:0016185 | P | 7, 8, 9, | 1 | 0.480 (x 2.085) | 15 (0.067) | 0.656 | synaptic vesicle budding | AP-2sigma |
| 814 | GO:0007052 | P | 6, 10, | 1 | 0.480 (x 2.085) | 15 (0.067) | 0.657 | mitotic spindle organization and biogenesis | cnn |
| 815 | GO:0043648 | P | 7, | 1 | 0.480 (x 2.085) | 15 (0.067) | 0.658 | dicarboxylic acid metabolism | Men |
| 816 | GO:0008285 | P | 6, | 1 | 0.480 (x 2.085) | 15 (0.067) | 0.659 | negative regulation of cell proliferation | CG33193 |
| 817 | GO:0016806 | F | 6, | 1 | 0.480 (x 2.085) | 15 (0.067) | 0.66 | dipeptidyl-peptidase and tripeptidyl-peptidase activity | Dip-B |
| 818 | GO:0015031 | P | 5, 6, | 18 | 16.530 (x 1.089) | 517 (0.035) | 0.66 | protein transport | AP-2sigma CG10535 CG13887 CG17266 CG33066 CG8479 CG9139 CG9240 CG9298 Nsf2 Rep RhoGEF2 SCAP SH3PX1 Syx13 Tim10 betaTub60D gammaSnap |
| 819 | GO:0005874 | C | 5, 6, 7, 8, 9, 10, | 2 | 1.343 (x 1.489) | 42 (0.048) | 0.662 | microtubule | Unc-76 betaTub60D |
| 820 | GO:0009141 | P | 7, | 3 | 2.270 (x 1.322) | 71 (0.042) | 0.673 | nucleoside triphosphate metabolism | Adk1 Vha36 Vha44 |
| 821 | GO:0045184 | P | 5, | 18 | 16.626 (x 1.083) | 520 (0.035) | 0.674 | establishment of protein localization | AP-2sigma CG10535 CG13887 CG17266 CG33066 CG8479 CG9139 CG9240 CG9298 Nsf2 Rep RhoGEF2 SCAP SH3PX1 Syx13 Tim10 betaTub60D gammaSnap |
| 822 | GO:0016757 | F | 4, | 6 | 5.084 (x 1.180) | 159 (0.038) | 0.674 | transferase activity, transferring glycosyl groups | Aprt CG33138 CG4802 CG8412 Ugt86Da l(2)k01209 |
| 823 | GO:0006752 | P | 7, | 3 | 2.302 (x 1.303) | 72 (0.042) | 0.675 | group transfer coenzyme metabolism | Vha36 Vha44 fbl |
| 824 | GO:0006445 | P | 7, 8, 9, | 3 | 2.302 (x 1.303) | 72 (0.042) | 0.675 | regulation of translation | EfTuM Thor eIF2B-delta |
| 825 | GO:0008652 | P | 7, 8, | 3 | 2.302 (x 1.303) | 72 (0.042) | 0.676 | amino acid biosynthesis | CG12264 CG1673 Paps |
| 826 | GO:0043543 | P | 8, | 1 | 0.512 (x 1.955) | 16 (0.062) | 0.676 | protein amino acid acylation | CG11989 |
| 827 | GO:0044450 | C | 5, 6, 7, 8, 9, | 1 | 0.512 (x 1.955) | 16 (0.062) | 0.677 | microtubule organizing center part | cnn |
| 828 | GO:0016651 | F | 4, | 2 | 1.375 (x 1.455) | 43 (0.047) | 0.677 | oxidoreductase activity, acting on NADH or NADPH | Trxr-1 bcn92 |
| 829 | GO:0003924 | F | 8, | 5 | 4.156 (x 1.203) | 130 (0.038) | 0.677 | GTPase activity | CG17760 CG6668 CG8479 Past1 betaTub60D |
| 830 | GO:0046666 | P | 6, 7, | 1 | 0.512 (x 1.955) | 16 (0.062) | 0.678 | retinal cell programmed cell death | CG33193 |
| 831 | GO:0051248 | P | 6, 7, | 2 | 1.375 (x 1.455) | 43 (0.047) | 0.678 | negative regulation of protein metabolism | PEK Thor |
| 832 | GO:0006457 | P | 7, | 5 | 4.188 (x 1.194) | 131 (0.038) | 0.678 | protein folding | CG17187 CG17266 CG6000 Gp93 mio |
| 833 | GO:0004089 | F | 6, | 1 | 0.512 (x 1.955) | 16 (0.062) | 0.679 | carbonate dehydratase activity | CG11967 |
| 834 | GO:0016310 | P | 7, | 16 | 14.739 (x 1.086) | 461 (0.035) | 0.679 | phosphorylation | BG:BACR48E02.4 CG12534 CG32687 CG3608 CG3731 CG4769 CG4802 CG6746 CG7598 Cyt-c-p PEK Pi4KIIalpha PyK Vha36 Vha44 ire-1 |
| 835 | GO:0030537 | P | 4, | 1 | 0.512 (x 1.955) | 16 (0.062) | 0.68 | larval behavior | betaTub60D |
| 836 | GO:0030118 | C | 5, 6, 7, 8, 9, | 1 | 0.512 (x 1.955) | 16 (0.062) | 0.681 | clathrin coat | AP-2sigma |
| 837 | GO:0007291 | P | 6, 9, | 1 | 0.512 (x 1.955) | 16 (0.062) | 0.681 | sperm individualization | poe |
| 838 | GO:0050790 | P | 3, | 2 | 1.407 (x 1.422) | 44 (0.045) | 0.685 | regulation of catalytic activity | Gclm Rep |
| 839 | GO:0046843 | P | 10, 11, | 1 | 0.544 (x 1.840) | 17 (0.059) | 0.693 | dorsal appendage formation | Jra |
| 840 | GO:0003746 | F | 4, 5, | 1 | 0.544 (x 1.840) | 17 (0.059) | 0.694 | translation elongation factor activity | EfTuM |
| 841 | GO:0007018 | P | 7, 8, 9, | 4 | 3.293 (x 1.215) | 103 (0.039) | 0.695 | microtubule-based movement | Unc-76 betaTub60D dynactin-subunit-p25 milton |
| 842 | GO:0050770 | P | 5, 8, 9, 10, 12, | 1 | 0.544 (x 1.840) | 17 (0.059) | 0.695 | regulation of axonogenesis | RhoGEF2 |
| 843 | GO:0006909 | P | 7, 8, | 1 | 0.544 (x 1.840) | 17 (0.059) | 0.696 | phagocytosis | RhoGEF2 |
| 844 | GO:0030705 | P | 6, 7, 8, | 4 | 3.325 (x 1.203) | 104 (0.038) | 0.696 | cytoskeleton-dependent intracellular transport | Unc-76 betaTub60D dynactin-subunit-p25 milton |
| 845 | GO:0016478 | P | 8, 9, 10, | 1 | 0.544 (x 1.840) | 17 (0.059) | 0.697 | negative regulation of translation | Thor |
| 846 | GO:0051246 | P | 5, 6, | 5 | 4.252 (x 1.176) | 133 (0.038) | 0.697 | regulation of protein metabolism | Ate1 EfTuM PEK Thor eIF2B-delta |
| 847 | GO:0006471 | P | 8, | 1 | 0.544 (x 1.840) | 17 (0.059) | 0.697 | protein amino acid ADP-ribosylation | CG17760 |
| 848 | GO:0007280 | P | 6, 7, 8, | 1 | 0.544 (x 1.840) | 17 (0.059) | 0.698 | pole cell migration | foi |
| 849 | GO:0004428 | F | 6, | 1 | 0.544 (x 1.840) | 17 (0.059) | 0.699 | inositol or phosphatidylinositol kinase activity | Pi4KIIalpha |
| 850 | GO:0006900 | P | 6, 7, | 1 | 0.544 (x 1.840) | 17 (0.059) | 0.7 | vesicle budding | AP-2sigma |
| 851 | GO:0019898 | C | 4, 5, 6, | 2 | 1.439 (x 1.390) | 45 (0.044) | 0.7 | extrinsic to membrane | CG17760 Pgm |
| 852 | GO:0006960 | P | 7, 8, | 2 | 1.439 (x 1.390) | 45 (0.044) | 0.701 | antimicrobial humoral response (sensu Protostomia) | TepIV Thor |
| 853 | GO:0008340 | P | 4, | 2 | 1.471 (x 1.360) | 46 (0.043) | 0.708 | determination of adult life span | Cat Trxr-1 |
| 854 | GO:0007568 | P | 3, | 2 | 1.471 (x 1.360) | 46 (0.043) | 0.709 | aging | Cat Trxr-1 |
| 855 | GO:0030384 | P | 9, 10, | 1 | 0.575 (x 1.738) | 18 (0.056) | 0.716 | phosphoinositide metabolism | Pi4KIIalpha |
| 856 | GO:0006305 | P | 8, | 1 | 0.575 (x 1.738) | 18 (0.056) | 0.717 | DNA alkylation | RhoGEF2 |
| 857 | GO:0016811 | F | 5, | 2 | 1.503 (x 1.331) | 47 (0.043) | 0.717 | hydrolase activity, acting on carbon-nitrogen (but not peptide) bonds, in linear amides | CG6428 CG8839 |
| 858 | GO:0019209 | F | 4, | 1 | 0.575 (x 1.738) | 18 (0.056) | 0.717 | kinase activator activity | CG10535 |
| 859 | GO:0007254 | P | 7, 8, | 2 | 1.503 (x 1.331) | 47 (0.043) | 0.718 | JNK cascade | CG7417 Jra |
| 860 | GO:0006306 | P | 4, 8, 9, | 1 | 0.575 (x 1.738) | 18 (0.056) | 0.718 | DNA methylation | RhoGEF2 |
| 861 | GO:0031098 | P | 6, | 2 | 1.503 (x 1.331) | 47 (0.043) | 0.719 | stress-activated protein kinase signaling pathway | CG7417 Jra |
| 862 | GO:0005316 | F | 7, 8, 9, | 1 | 0.575 (x 1.738) | 18 (0.056) | 0.719 | high affinity inorganic phosphate:sodium symporter activity | CG15094 |
| 863 | GO:0051119 | F | 4, | 2 | 1.503 (x 1.331) | 47 (0.043) | 0.72 | sugar transporter activity | CG14040 TepIV |
| 864 | GO:0007465 | P | 8, 9, 10, 11, | 1 | 0.575 (x 1.738) | 18 (0.056) | 0.72 | R7 cell fate commitment | Jra |
| 865 | GO:0006959 | P | 5, 6, | 3 | 2.494 (x 1.203) | 78 (0.038) | 0.733 | humoral immune response | CG13887 TepIV Thor |
| 866 | GO:0044272 | P | 6, | 1 | 0.607 (x 1.646) | 19 (0.053) | 0.733 | sulfur compound biosynthesis | Gclm |
| 867 | GO:0009416 | P | 5, | 2 | 1.535 (x 1.303) | 48 (0.042) | 0.734 | response to light stimulus | Pgm betaTub60D |
| 868 | GO:0006414 | P | 8, 9, | 1 | 0.607 (x 1.646) | 19 (0.053) | 0.734 | translational elongation | EfTuM |
| 869 | GO:0004190 | F | 6, | 1 | 0.607 (x 1.646) | 19 (0.053) | 0.735 | aspartic-type endopeptidase activity | cathD |
| 870 | GO:0007140 | P | 8, | 1 | 0.607 (x 1.646) | 19 (0.053) | 0.736 | male meiosis | Syx13 |
| 871 | GO:0031109 | P | 9, | 1 | 0.607 (x 1.646) | 19 (0.053) | 0.737 | microtubule polymerization or depolymerization | betaTub60D |
| 872 | GO:0046903 | P | 5, | 8 | 7.386 (x 1.083) | 231 (0.035) | 0.737 | secretion | AP-2sigma CG9298 Nsf2 Rep SCAP Syx13 gammaSnap snapin |
| 873 | GO:0006650 | P | 8, 9, | 1 | 0.607 (x 1.646) | 19 (0.053) | 0.738 | glycerophospholipid metabolism | Pi4KIIalpha |
| 874 | GO:0046983 | F | 4, | 2 | 1.567 (x 1.277) | 49 (0.041) | 0.741 | protein dimerization activity | CG6272 Jra |
| 875 | GO:0008553 | F | 6, 7, 9, 14, | 2 | 1.567 (x 1.277) | 49 (0.041) | 0.742 | hydrogen-exporting ATPase activity, phosphorylative mechanism | Vha36 Vha44 |
| 876 | GO:0009063 | P | 7, 8, | 2 | 1.567 (x 1.277) | 49 (0.041) | 0.742 | amino acid catabolism | CG31075 CG6428 |
| 877 | GO:0019199 | F | 5, 7, | 2 | 1.567 (x 1.277) | 49 (0.041) | 0.743 | transmembrane receptor protein kinase activity | BG:BACR48E02.4 CG32687 |
| 878 | GO:0001558 | P | 4, 5, 7, 8, | 1 | 0.639 (x 1.564) | 20 (0.050) | 0.748 | regulation of cell growth | Thor |
| 879 | GO:0045926 | P | 4, | 1 | 0.639 (x 1.564) | 20 (0.050) | 0.749 | negative regulation of growth | CG33193 |
| 880 | GO:0030716 | P | 6, | 1 | 0.639 (x 1.564) | 20 (0.050) | 0.75 | oocyte fate determination | mio |
| 881 | GO:0006637 | P | 7, 8, | 1 | 0.639 (x 1.564) | 20 (0.050) | 0.751 | acyl-CoA metabolism | CG4860 |
| 882 | GO:0016028 | C | 5, 6, 7, | 1 | 0.639 (x 1.564) | 20 (0.050) | 0.751 | rhabdomere | Pgm |
| 883 | GO:0006810 | P | 4, 5, | 48 | 47.223 (x 1.016) | 1477 (0.032) | 0.752 | transport | AP-2sigma CG10535 CG11880 CG11897 CG13887 CG14040 CG15094 CG1598 CG17266 CG2789 CG31793 CG33066 CG3397 CG3476 CG3522 CG4225 CG4963 CG6608 CG7627 CG7816 CG8479 CG8602 CG9065 CG9139 CG9240 CG9298 Fer1HCH Nsf2 Past1 Rep RhoGEF2 SCAP SH3PX1 Shawn Syx13 TepIV Tim10 Unc-76 Vha36 Vha44 aralar1 att-ORFA betaTub60D dynactin-subunit-p25 foi gammaSnap milton snapin |
| 884 | GO:0004197 | F | 6, | 3 | 2.590 (x 1.158) | 81 (0.037) | 0.752 | cysteine-type endopeptidase activity | CG12163 Dip-B ref(2)P |
| 885 | GO:0016814 | F | 5, | 1 | 0.639 (x 1.564) | 20 (0.050) | 0.752 | hydrolase activity, acting on carbon-nitrogen (but not peptide) bonds, in cyclic amidines | CG8360 |
| 886 | GO:0005386 | F | 3, | 15 | 14.483 (x 1.036) | 453 (0.033) | 0.753 | carrier activity | CG14040 CG15094 CG1598 CG33066 CG3476 CG3522 CG4963 CG6608 Shawn TepIV Tim10 Vha36 Vha44 aralar1 att-ORFA |
| 887 | GO:0008643 | P | 5, 6, | 3 | 2.590 (x 1.158) | 81 (0.037) | 0.753 | carbohydrate transport | CG14040 CG15094 TepIV |
| 888 | GO:0008374 | F | 7, | 1 | 0.639 (x 1.564) | 20 (0.050) | 0.753 | O-acyltransferase activity | CG2107 |
| 889 | GO:0016782 | F | 4, | 1 | 0.639 (x 1.564) | 20 (0.050) | 0.754 | transferase activity, transferring sulfur-containing groups | CG1140 |
| 890 | GO:0005484 | F | 4, | 1 | 0.639 (x 1.564) | 20 (0.050) | 0.755 | SNAP receptor activity | Syx13 |
| 891 | GO:0006730 | P | 5, | 1 | 0.639 (x 1.564) | 20 (0.050) | 0.756 | one-carbon compound metabolism | Ahcy13 |
| 892 | GO:0007178 | P | 7, | 2 | 1.631 (x 1.227) | 51 (0.039) | 0.761 | transmembrane receptor protein serine/threonine kinase signaling pathway | BG:BACR48E02.4 CG32687 |
| 893 | GO:0015300 | F | 7, | 1 | 0.671 (x 1.489) | 21 (0.048) | 0.762 | solute:solute antiporter activity | CG3476 |
| 894 | GO:0005834 | C | 3, 6, 7, 8, | 1 | 0.671 (x 1.489) | 21 (0.048) | 0.763 | heterotrimeric G-protein complex | CG17760 |
| 895 | GO:0044270 | P | 5, 6, | 2 | 1.663 (x 1.203) | 52 (0.038) | 0.763 | nitrogen compound catabolism | CG31075 CG6428 |
| 896 | GO:0016081 | P | 8, 9, 10, | 1 | 0.671 (x 1.489) | 21 (0.048) | 0.764 | synaptic vesicle docking during exocytosis | Syx13 |
| 897 | GO:0005792 | C | 6, 7, | 3 | 2.654 (x 1.131) | 83 (0.036) | 0.764 | microsome | Cyp6a20 Cyp6d5 Cyt-b5 |
| 898 | GO:0009310 | P | 6, 7, | 2 | 1.663 (x 1.203) | 52 (0.038) | 0.764 | amine catabolism | CG31075 CG6428 |
| 899 | GO:0004521 | F | 7, | 1 | 0.671 (x 1.489) | 21 (0.048) | 0.765 | endoribonuclease activity | JhI-1 |
| 900 | GO:0016023 | C | 5, 6, 7, 8, 9, | 3 | 2.654 (x 1.131) | 83 (0.036) | 0.765 | cytoplasmic membrane-bound vesicle | AP-2sigma Rep snapin |
| 901 | GO:0016339 | P | 5, | 1 | 0.671 (x 1.489) | 21 (0.048) | 0.766 | calcium-dependent cell-cell adhesion | Cat |
| 902 | GO:0042598 | C | 5, 6, | 3 | 2.654 (x 1.131) | 83 (0.036) | 0.766 | vesicular fraction | Cyp6a20 Cyp6d5 Cyt-b5 |
| 903 | GO:0003755 | F | 5, | 1 | 0.671 (x 1.489) | 21 (0.048) | 0.766 | peptidyl-prolyl cis-trans isomerase activity | CG17266 |
| 904 | GO:0031410 | C | 4, 5, 6, 7, 8, | 3 | 2.654 (x 1.131) | 83 (0.036) | 0.767 | cytoplasmic vesicle | AP-2sigma Rep snapin |
| 905 | GO:0005436 | F | 6, 7, 8, | 1 | 0.671 (x 1.489) | 21 (0.048) | 0.767 | sodium:phosphate symporter activity | CG15094 |
| 906 | GO:0006100 | P | 8, | 1 | 0.671 (x 1.489) | 21 (0.048) | 0.768 | tricarboxylic acid cycle intermediate metabolism | Men |
| 907 | GO:0016042 | P | 5, 6, | 1 | 0.671 (x 1.489) | 21 (0.048) | 0.769 | lipid catabolism | Sply |
| 908 | GO:0051258 | P | 7, | 1 | 0.671 (x 1.489) | 21 (0.048) | 0.77 | protein polymerization | betaTub60D |
| 909 | GO:0044271 | P | 5, 6, | 3 | 2.686 (x 1.117) | 84 (0.036) | 0.772 | nitrogen compound biosynthesis | CG12264 CG1673 Paps |
| 910 | GO:0043085 | P | 4, | 1 | 0.703 (x 1.422) | 22 (0.045) | 0.773 | positive regulation of enzyme activity | Gclm |
| 911 | GO:0009309 | P | 6, 7, | 3 | 2.686 (x 1.117) | 84 (0.036) | 0.773 | amine biosynthesis | CG12264 CG1673 Paps |
| 912 | GO:0008081 | F | 6, | 1 | 0.703 (x 1.422) | 22 (0.045) | 0.773 | phosphoric diester hydrolase activity | CG11883 |
| 913 | GO:0000910 | P | 5, | 3 | 2.686 (x 1.117) | 84 (0.036) | 0.774 | cytokinesis | CG10068 Syx13 fbl |
| 914 | GO:0009401 | P | 6, 7, | 1 | 0.703 (x 1.422) | 22 (0.045) | 0.774 | phosphoenolpyruvate-dependent sugar phosphotransferase system | TepIV |
| 915 | GO:0016065 | P | 6, 7, | 2 | 1.695 (x 1.180) | 53 (0.038) | 0.775 | humoral defense mechanism (sensu Protostomia) | TepIV Thor |
| 916 | GO:0005319 | F | 3, | 1 | 0.703 (x 1.422) | 22 (0.045) | 0.775 | lipid transporter activity | CG3522 |
| 917 | GO:0009314 | P | 4, | 2 | 1.695 (x 1.180) | 53 (0.038) | 0.776 | response to radiation | Pgm betaTub60D |
| 918 | GO:0007446 | P | 4, 5, | 1 | 0.703 (x 1.422) | 22 (0.045) | 0.776 | imaginal disc growth | CG33193 |
| 919 | GO:0008076 | C | 3, 5, 6, 7, 8, 9, | 1 | 0.703 (x 1.422) | 22 (0.045) | 0.777 | voltage-gated potassium channel complex | CG3397 |
| 920 | GO:0006461 | P | 6, | 4 | 3.741 (x 1.069) | 117 (0.034) | 0.782 | protein complex assembly | CG10340 CG7598 CG9065 Gp93 |
| 921 | GO:0000502 | C | 3, 4, 5, 6, | 2 | 1.726 (x 1.158) | 54 (0.037) | 0.783 | proteasome complex (sensu Eukaryota) | CG5604 l(2)05070 |
| 922 | GO:0031982 | C | 3, | 3 | 2.750 (x 1.091) | 86 (0.035) | 0.786 | vesicle | AP-2sigma Rep snapin |
| 923 | GO:0016859 | F | 4, | 1 | 0.735 (x 1.360) | 23 (0.043) | 0.786 | cis-trans isomerase activity | CG17266 |
| 924 | GO:0031988 | C | 4, | 3 | 2.750 (x 1.091) | 86 (0.035) | 0.787 | membrane-bound vesicle | AP-2sigma Rep snapin |
| 925 | GO:0005838 | C | 3, 4, 5, 6, 7, | 1 | 0.735 (x 1.360) | 23 (0.043) | 0.787 | proteasome regulatory particle (sensu Eukaryota) | CG5604 |
| 926 | GO:0008213 | P | 8, | 1 | 0.735 (x 1.360) | 23 (0.043) | 0.788 | protein amino acid alkylation | CG9882 |
| 927 | GO:0006479 | P | 8, 9, | 1 | 0.735 (x 1.360) | 23 (0.043) | 0.789 | protein amino acid methylation | CG9882 |
| 928 | GO:0017038 | P | 6, 7, | 2 | 1.758 (x 1.137) | 55 (0.036) | 0.789 | protein import | CG33066 Tim10 |
| 929 | GO:0042445 | P | 5, | 1 | 0.735 (x 1.360) | 23 (0.043) | 0.789 | hormone metabolism | Vha44 |
| 930 | GO:0009967 | P | 5, 6, | 1 | 0.735 (x 1.360) | 23 (0.043) | 0.79 | positive regulation of signal transduction | CG7417 |
| 931 | GO:0016887 | F | 8, | 12 | 11.862 (x 1.012) | 371 (0.032) | 0.791 | ATPase activity | CG10333 CG11897 CG1598 CG31793 CG3561 CG4225 CG6512 CG7627 EG:100G10.7 Vha36 Vha44 cnn |
| 932 | GO:0006304 | P | 7, | 1 | 0.735 (x 1.360) | 23 (0.043) | 0.791 | DNA modification | RhoGEF2 |
| 933 | GO:0007494 | P | 5, | 1 | 0.767 (x 1.303) | 24 (0.042) | 0.802 | midgut development | cnn |
| 934 | GO:0010004 | P | 7, | 1 | 0.767 (x 1.303) | 24 (0.042) | 0.803 | gastrulation (sensu Insecta) | RhoGEF2 |
| 935 | GO:0030880 | C | 3, 4, 5, 6, | 1 | 0.767 (x 1.303) | 24 (0.042) | 0.803 | RNA polymerase complex | CG5380 |
| 936 | GO:0030659 | C | 5, 6, 7, 8, 9, 10, | 1 | 0.767 (x 1.303) | 24 (0.042) | 0.804 | cytoplasmic vesicle membrane | AP-2sigma |
| 937 | GO:0001703 | P | 6, | 1 | 0.767 (x 1.303) | 24 (0.042) | 0.805 | gastrulation (sensu Protostomia) | RhoGEF2 |
| 938 | GO:0008104 | P | 4, | 18 | 18.096 (x 0.995) | 566 (0.032) | 0.806 | protein localization | AP-2sigma CG10535 CG13887 CG17266 CG33066 CG8479 CG9139 CG9240 CG9298 Nsf2 Rep RhoGEF2 SCAP SH3PX1 Syx13 Tim10 betaTub60D gammaSnap |
| 939 | GO:0030662 | C | 6, 7, 8, 9, 10, 11, | 1 | 0.767 (x 1.303) | 24 (0.042) | 0.806 | coated vesicle membrane | AP-2sigma |
| 940 | GO:0030120 | C | 5, 6, 7, 8, 9, 10, 11, 12, | 1 | 0.767 (x 1.303) | 24 (0.042) | 0.807 | vesicle coat | AP-2sigma |
| 941 | GO:0000323 | C | 6, 7, 8, 9, | 1 | 0.799 (x 1.251) | 25 (0.040) | 0.809 | lytic vacuole | Dip-B |
| 942 | GO:0008406 | P | 5, | 1 | 0.799 (x 1.251) | 25 (0.040) | 0.81 | gonad development | foi |
| 943 | GO:0016321 | P | 6, 9, | 1 | 0.799 (x 1.251) | 25 (0.040) | 0.811 | female meiosis chromosome segregation | cnn |
| 944 | GO:0016459 | C | 3, 5, 6, 7, 8, 9, 10, | 1 | 0.799 (x 1.251) | 25 (0.040) | 0.811 | myosin | cnn |
| 945 | GO:0015630 | C | 6, 7, 8, 9, | 6 | 5.947 (x 1.009) | 186 (0.032) | 0.812 | microtubule cytoskeleton | Rep Unc-76 betaTub60D cnn dynactin-subunit-p25 fbl |
| 946 | GO:0005764 | C | 7, 8, 9, 10, | 1 | 0.799 (x 1.251) | 25 (0.040) | 0.812 | lysosome | Dip-B |
| 947 | GO:0006955 | P | 4, 5, | 4 | 3.901 (x 1.025) | 122 (0.033) | 0.812 | immune response | CG13887 CG6668 TepIV Thor |
| 948 | GO:0009150 | P | 8, | 3 | 2.877 (x 1.043) | 90 (0.033) | 0.813 | purine ribonucleotide metabolism | Adk1 Vha36 Vha44 |
| 949 | GO:0045137 | P | 4, | 1 | 0.799 (x 1.251) | 25 (0.040) | 0.813 | development of primary sexual characteristics | foi |
| 950 | GO:0006164 | P | 7, 8, | 3 | 2.909 (x 1.031) | 91 (0.033) | 0.814 | purine nucleotide biosynthesis | BcDNA:LD32788 Vha36 Vha44 |
| 951 | GO:0040007 | P | 2, | 3 | 2.877 (x 1.043) | 90 (0.033) | 0.814 | growth | Aats-asp CG33193 Thor |
| 952 | GO:0005839 | C | 3, 4, 5, 6, 7, | 1 | 0.799 (x 1.251) | 25 (0.040) | 0.814 | proteasome core complex (sensu Eukaryota) | l(2)05070 |
| 953 | GO:0005794 | C | 5, 6, 7, 8, | 3 | 2.877 (x 1.043) | 90 (0.033) | 0.815 | Golgi apparatus | CG14040 CG7809 CG9298 |
| 954 | GO:0007613 | P | 5, | 1 | 0.799 (x 1.251) | 25 (0.040) | 0.815 | memory | CG10460 |
| 955 | GO:0035265 | P | 3, | 1 | 0.799 (x 1.251) | 25 (0.040) | 0.816 | organ growth | CG33193 |
| 956 | GO:0050767 | P | 4, 7, | 1 | 0.799 (x 1.251) | 25 (0.040) | 0.817 | regulation of neurogenesis | RhoGEF2 |
| 957 | GO:0046620 | P | 4, | 1 | 0.799 (x 1.251) | 25 (0.040) | 0.817 | regulation of organ size | CG33193 |
| 958 | GO:0019992 | F | 4, | 1 | 0.831 (x 1.203) | 26 (0.038) | 0.821 | diacylglycerol binding | RhoGEF2 |
| 959 | GO:0008757 | F | 6, | 2 | 1.886 (x 1.060) | 59 (0.034) | 0.822 | S-adenosylmethionine-dependent methyltransferase activity | CG9249 CG9882 |
| 960 | GO:0030117 | C | 4, 5, 6, 7, 8, | 1 | 0.831 (x 1.203) | 26 (0.038) | 0.822 | membrane coat | AP-2sigma |
| 961 | GO:0000279 | P | 5, | 9 | 9.176 (x 0.981) | 287 (0.031) | 0.822 | M phase | CG3499 CG6512 EG:100G10.7 Nsf2 Syx13 betaTub60D cnn fbl mio |
| 962 | GO:0009259 | P | 7, | 3 | 2.941 (x 1.020) | 92 (0.033) | 0.822 | ribonucleotide metabolism | Adk1 Vha36 Vha44 |
| 963 | GO:0003724 | F | 4, | 2 | 1.886 (x 1.060) | 59 (0.034) | 0.823 | RNA helicase activity | CG10333 CG3561 |
| 964 | GO:0003887 | F | 6, | 1 | 0.831 (x 1.203) | 26 (0.038) | 0.823 | DNA-directed DNA polymerase activity | mus205 |
| 965 | GO:0048475 | C | 4, 5, | 1 | 0.831 (x 1.203) | 26 (0.038) | 0.823 | coated membrane | AP-2sigma |
| 966 | GO:0015114 | F | 6, | 1 | 0.831 (x 1.203) | 26 (0.038) | 0.824 | phosphate transporter activity | CG15094 |
| 967 | GO:0006887 | P | 6, 7, | 4 | 3.997 (x 1.001) | 125 (0.032) | 0.824 | exocytosis | Nsf2 Syx13 gammaSnap snapin |
| 968 | GO:0008234 | F | 5, | 3 | 2.973 (x 1.009) | 93 (0.032) | 0.825 | cysteine-type peptidase activity | CG12163 Dip-B ref(2)P |
| 969 | GO:0007611 | P | 4, | 2 | 1.918 (x 1.043) | 60 (0.033) | 0.826 | learning and/or memory | CG10460 Gclm |
| 970 | GO:0005624 | C | 4, 5, | 3 | 2.973 (x 1.009) | 93 (0.032) | 0.826 | membrane fraction | Cyp6a20 Cyp6d5 Cyt-b5 |
| 971 | GO:0040008 | P | 3, | 2 | 1.950 (x 1.025) | 61 (0.033) | 0.832 | regulation of growth | CG33193 Thor |
| 972 | GO:0005543 | F | 4, | 1 | 0.863 (x 1.158) | 27 (0.037) | 0.832 | phospholipid binding | CG7632 |
| 973 | GO:0042775 | P | 8, 10, | 2 | 1.950 (x 1.025) | 61 (0.033) | 0.832 | ATP synthesis coupled electron transport (sensu Eukaryota) | CG3731 CG4769 |
| 974 | GO:0042127 | P | 5, | 1 | 0.863 (x 1.158) | 27 (0.037) | 0.833 | regulation of cell proliferation | CG33193 |
| 975 | GO:0005507 | F | 6, | 1 | 0.863 (x 1.158) | 27 (0.037) | 0.834 | copper ion binding | CG9065 |
| 976 | GO:0044433 | C | 4, 5, 6, 7, 8, 9, | 1 | 0.863 (x 1.158) | 27 (0.037) | 0.835 | cytoplasmic vesicle part | AP-2sigma |
| 977 | GO:0007067 | P | 7, | 7 | 7.226 (x 0.969) | 226 (0.031) | 0.835 | mitosis | CG3499 CG6512 EG:100G10.7 Nsf2 Syx13 betaTub60D fbl |
| 978 | GO:0005198 | F | 2, | 23 | 23.595 (x 0.975) | 738 (0.031) | 0.835 | structural molecule activity | CG12954 CG8479 Pcd Unc-76 betaTub60D mRpL11 mRpL13 mRpL19 mRpL21 mRpL22 mRpL22-24 mRpL28 mRpL3 mRpL48 mRpL55 mRpS18a mRpS18b mRpS2 mRpS22 mRpS25 mRpS32 mRpS33 mRpS9 |
| 979 | GO:0042625 | F | 4, 5, 7, 12, | 3 | 3.037 (x 0.988) | 95 (0.032) | 0.835 | ATPase activity, coupled to transmembrane movement of ions | CG1598 Vha36 Vha44 |
| 980 | GO:0002168 | P | 5, | 1 | 0.863 (x 1.158) | 27 (0.037) | 0.835 | larval development (sensu Insecta) | blp |
| 981 | GO:0008047 | F | 3, | 3 | 3.037 (x 0.988) | 95 (0.032) | 0.836 | enzyme activator activity | CG10535 CG6734 RhoGAP16F |
| 982 | GO:0042773 | P | 7, 9, | 2 | 1.982 (x 1.009) | 62 (0.032) | 0.837 | ATP synthesis coupled electron transport | CG3731 CG4769 |
| 983 | GO:0006917 | P | 8, 9, | 2 | 1.982 (x 1.009) | 62 (0.032) | 0.838 | induction of apoptosis | CG12876 CG33193 |
| 984 | GO:0045466 | P | 7, 8, 9, 10, | 1 | 0.895 (x 1.117) | 28 (0.036) | 0.839 | R7 cell differentiation | Jra |
| 985 | GO:0000087 | P | 6, | 7 | 7.258 (x 0.964) | 227 (0.031) | 0.839 | M phase of mitotic cell cycle | CG3499 CG6512 EG:100G10.7 Nsf2 Syx13 betaTub60D fbl |
| 986 | GO:0030532 | C | 4, 5, 6, 7, 8, 9, 10, | 2 | 1.982 (x 1.009) | 62 (0.032) | 0.839 | small nuclear ribonucleoprotein complex | CG10333 CG17266 |
| 987 | GO:0004091 | F | 6, | 1 | 0.895 (x 1.117) | 28 (0.036) | 0.84 | carboxylesterase activity | alpha-Est3 |
| 988 | GO:0016773 | F | 5, | 11 | 11.414 (x 0.964) | 357 (0.031) | 0.84 | phosphotransferase activity, alcohol group as acceptor | BG:BACR48E02.4 CG2846 CG32687 CG3608 PEK Paps Pfk Pi4KIIalpha PyK fbl ire-1 |
| 989 | GO:0000267 | C | 3, 4, | 3 | 3.069 (x 0.977) | 96 (0.031) | 0.84 | cell fraction | Cyp6a20 Cyp6d5 Cyt-b5 |
| 990 | GO:0000119 | C | 3, 4, 6, 7, 8, 9, 10, 11, 12, 13, 14, | 1 | 0.895 (x 1.117) | 28 (0.036) | 0.841 | mediator complex | CG5134 |
| 991 | GO:0019730 | P | 6, 7, | 2 | 2.014 (x 0.993) | 63 (0.032) | 0.845 | antimicrobial humoral response | TepIV Thor |
| 992 | GO:0043065 | P | 7, 8, | 2 | 2.046 (x 0.977) | 64 (0.031) | 0.847 | positive regulation of apoptosis | CG12876 CG33193 |
| 993 | GO:0006754 | P | 7, 8, 9, 10, 11, | 2 | 2.046 (x 0.977) | 64 (0.031) | 0.848 | ATP biosynthesis | Vha36 Vha44 |
| 994 | GO:0015986 | P | 7, 8, 9, 10, 11, 12, | 2 | 2.046 (x 0.977) | 64 (0.031) | 0.849 | ATP synthesis coupled proton transport | Vha36 Vha44 |
| 995 | GO:0030097 | P | 5, | 2 | 2.046 (x 0.977) | 64 (0.031) | 0.85 | hemopoiesis | CG17760 ytr |
| 996 | GO:0015985 | P | 7, 8, 9, 10, | 2 | 2.046 (x 0.977) | 64 (0.031) | 0.851 | energy coupled proton transport, down electrochemical gradient | Vha36 Vha44 |
| 997 | GO:0042623 | F | 9, | 10 | 10.551 (x 0.948) | 330 (0.030) | 0.851 | ATPase activity, coupled | CG10333 CG11897 CG1598 CG31793 CG3561 CG4225 CG7627 Vha36 Vha44 cnn |
| 998 | GO:0006753 | P | 8, | 2 | 2.046 (x 0.977) | 64 (0.031) | 0.852 | nucleoside phosphate metabolism | Vha36 Vha44 |
| 999 | GO:0006904 | P | 7, 8, | 1 | 0.927 (x 1.079) | 29 (0.034) | 0.852 | vesicle docking during exocytosis | Syx13 |
| 1000 | GO:0006605 | P | 7, 8, 9, | 7 | 7.386 (x 0.948) | 231 (0.030) | 0.852 | protein targeting | CG10535 CG17266 CG33066 CG9240 SCAP Syx13 Tim10 |
| 1001 | GO:0007051 | P | 9, | 1 | 0.927 (x 1.079) | 29 (0.034) | 0.853 | spindle organization and biogenesis | cnn |
| 1002 | GO:0007349 | P | 3, 4, | 1 | 0.927 (x 1.079) | 29 (0.034) | 0.854 | cellularization | RhoGEF2 |
| 1003 | GO:0006633 | P | 6, 7, 8, | 1 | 0.927 (x 1.079) | 29 (0.034) | 0.854 | fatty acid biosynthesis | CG7842 |
| 1004 | GO:0048278 | P | 6, 7, | 1 | 0.927 (x 1.079) | 29 (0.034) | 0.855 | vesicle docking | Syx13 |
| 1005 | GO:0042386 | P | 4, 6, | 1 | 0.959 (x 1.043) | 30 (0.033) | 0.858 | hemocyte differentiation (sensu Arthropoda) | ytr |
| 1006 | GO:0016849 | F | 4, | 1 | 0.959 (x 1.043) | 30 (0.033) | 0.859 | phosphorus-oxygen lyase activity | Inos |
| 1007 | GO:0009613 | P | 4, 5, | 3 | 3.197 (x 0.938) | 100 (0.030) | 0.859 | response to pest, pathogen or parasite | CG13887 TepIV Thor |
| 1008 | GO:0012506 | C | 4, 5, 6, 7, 8, 9, | 1 | 0.959 (x 1.043) | 30 (0.033) | 0.86 | vesicle membrane | AP-2sigma |
| 1009 | GO:0003899 | F | 6, | 1 | 0.959 (x 1.043) | 30 (0.033) | 0.861 | DNA-directed RNA polymerase activity | CG5380 |
| 1010 | GO:0016049 | P | 3, 4, 6, 7, | 1 | 0.959 (x 1.043) | 30 (0.033) | 0.862 | cell growth | Thor |
| 1011 | GO:0008289 | F | 3, | 2 | 2.110 (x 0.948) | 66 (0.030) | 0.864 | lipid binding | CG7632 RhoGEF2 |
| 1012 | GO:0000278 | P | 5, | 8 | 8.569 (x 0.934) | 268 (0.030) | 0.864 | mitotic cell cycle | CG3499 CG6512 EG:100G10.7 Nsf2 Syx13 betaTub60D cnn fbl |
| 1013 | GO:0048754 | P | 5, | 1 | 0.991 (x 1.009) | 31 (0.032) | 0.869 | branching morphogenesis of a tube | foi |
| 1014 | GO:0015297 | F | 6, | 1 | 0.991 (x 1.009) | 31 (0.032) | 0.87 | antiporter activity | CG3476 |
| 1015 | GO:0019207 | F | 3, | 2 | 2.142 (x 0.934) | 67 (0.030) | 0.87 | kinase regulator activity | BG:BACR48E02.4 CG10535 |
| 1016 | GO:0016053 | P | 6, | 1 | 0.991 (x 1.009) | 31 (0.032) | 0.87 | organic acid biosynthesis | CG7842 |
| 1017 | GO:0046394 | P | 7, | 1 | 0.991 (x 1.009) | 31 (0.032) | 0.871 | carboxylic acid biosynthesis | CG7842 |
| 1018 | GO:0015078 | F | 6, | 3 | 3.293 (x 0.911) | 103 (0.029) | 0.876 | hydrogen ion transporter activity | CG3731 Vha36 Vha44 |
| 1019 | GO:0000165 | P | 7, | 3 | 3.293 (x 0.911) | 103 (0.029) | 0.877 | MAPKKK cascade | BG:BACR48E02.4 CG7417 Jra |
| 1020 | GO:0016790 | F | 5, | 1 | 1.023 (x 0.977) | 32 (0.031) | 0.877 | thiolester hydrolase activity | CG5044 |
| 1021 | GO:0009206 | P | 9, 10, | 2 | 2.174 (x 0.920) | 68 (0.029) | 0.877 | purine ribonucleoside triphosphate biosynthesis | Vha36 Vha44 |
| 1022 | GO:0008105 | P | 5, | 1 | 1.023 (x 0.977) | 32 (0.031) | 0.878 | asymmetric protein localization | CG10535 |
| 1023 | GO:0009145 | P | 8, 9, | 2 | 2.174 (x 0.920) | 68 (0.029) | 0.878 | purine nucleoside triphosphate biosynthesis | Vha36 Vha44 |
| 1024 | GO:0016469 | C | 3, 6, 7, 8, | 2 | 2.174 (x 0.920) | 68 (0.029) | 0.879 | proton-transporting two-sector ATPase complex | Vha36 Vha44 |
| 1025 | GO:0009201 | P | 8, 9, | 2 | 2.174 (x 0.920) | 68 (0.029) | 0.88 | ribonucleoside triphosphate biosynthesis | Vha36 Vha44 |
| 1026 | GO:0042067 | P | 6, 7, 8, 9, | 1 | 1.055 (x 0.948) | 33 (0.030) | 0.882 | establishment of ommatidial polarity (sensu Endopterygota) | Jra |
| 1027 | GO:0009142 | P | 7, 8, | 2 | 2.206 (x 0.907) | 69 (0.029) | 0.882 | nucleoside triphosphate biosynthesis | Vha36 Vha44 |
| 1028 | GO:0001752 | P | 7, 8, 9, 10, | 1 | 1.055 (x 0.948) | 33 (0.030) | 0.883 | eye photoreceptor fate commitment (sensu Endopterygota) | Jra |
| 1029 | GO:0019829 | F | 5, 6, 8, 13, | 2 | 2.206 (x 0.907) | 69 (0.029) | 0.883 | cation-transporting ATPase activity | CG1598 Vha36 |
| 1030 | GO:0005813 | C | 5, 6, 7, 8, 9, 10, | 1 | 1.055 (x 0.948) | 33 (0.030) | 0.884 | centrosome | cnn |
| 1031 | GO:0042742 | P | 5, 6, | 2 | 2.206 (x 0.907) | 69 (0.029) | 0.884 | defense response to bacterium | TepIV Thor |
| 1032 | GO:0012502 | P | 7, 8, | 2 | 2.238 (x 0.894) | 70 (0.029) | 0.884 | induction of programmed cell death | CG12876 CG33193 |
| 1033 | GO:0019221 | P | 6, | 1 | 1.055 (x 0.948) | 33 (0.030) | 0.885 | cytokine and chemokine mediated signaling pathway | CG10535 |
| 1034 | GO:0008354 | P | 5, 6, 7, | 1 | 1.055 (x 0.948) | 33 (0.030) | 0.885 | germ cell migration | foi |
| 1035 | GO:0042706 | P | 6, 7, 8, | 1 | 1.055 (x 0.948) | 33 (0.030) | 0.886 | eye photoreceptor cell fate commitment | Jra |
| 1036 | GO:0006796 | P | 6, | 18 | 19.311 (x 0.932) | 604 (0.030) | 0.886 | phosphate metabolism | BG:BACR48E02.4 CG12534 CG15094 CG32687 CG3608 CG3731 CG4769 CG4802 CG6746 CG6805 CG7598 Cyt-c-p PEK Pi4KIIalpha PyK Vha36 Vha44 ire-1 |
| 1037 | GO:0006793 | P | 5, | 18 | 19.311 (x 0.932) | 604 (0.030) | 0.887 | phosphorus metabolism | BG:BACR48E02.4 CG12534 CG15094 CG32687 CG3608 CG3731 CG4769 CG4802 CG6746 CG6805 CG7598 Cyt-c-p PEK Pi4KIIalpha PyK Vha36 Vha44 ire-1 |
| 1038 | GO:0015077 | F | 5, | 3 | 3.357 (x 0.894) | 105 (0.029) | 0.888 | monovalent inorganic cation transporter activity | CG3731 Vha36 Vha44 |
| 1039 | GO:0008170 | F | 6, | 1 | 1.087 (x 0.920) | 34 (0.029) | 0.888 | N-methyltransferase activity | CG9882 |
| 1040 | GO:0008361 | P | 5, 6, | 1 | 1.087 (x 0.920) | 34 (0.029) | 0.889 | regulation of cell size | Thor |
| 1041 | GO:0015020 | F | 6, | 1 | 1.087 (x 0.920) | 34 (0.029) | 0.89 | glucuronosyltransferase activity | Ugt86Da |
| 1042 | GO:0048488 | P | 7, 8, | 1 | 1.087 (x 0.920) | 34 (0.029) | 0.891 | synaptic vesicle endocytosis | AP-2sigma |
| 1043 | GO:0048102 | P | 6, | 2 | 2.270 (x 0.881) | 71 (0.028) | 0.891 | autophagic cell death | CG12163 cathD |
| 1044 | GO:0001763 | P | 4, | 1 | 1.087 (x 0.920) | 34 (0.029) | 0.891 | morphogenesis of a branching structure | foi |
| 1045 | GO:0035070 | P | 6, | 2 | 2.270 (x 0.881) | 71 (0.028) | 0.892 | salivary gland histolysis | CG12163 cathD |
| 1046 | GO:0005516 | F | 4, | 2 | 2.270 (x 0.881) | 71 (0.028) | 0.892 | calmodulin binding | Past1 poe |
| 1047 | GO:0016271 | P | 4, | 2 | 2.302 (x 0.869) | 72 (0.028) | 0.893 | tissue death | CG12163 cathD |
| 1048 | GO:0035071 | P | 7, | 2 | 2.270 (x 0.881) | 71 (0.028) | 0.893 | salivary gland cell autophagic cell death | CG12163 cathD |
| 1049 | GO:0007559 | P | 5, | 2 | 2.302 (x 0.869) | 72 (0.028) | 0.894 | histolysis | CG12163 cathD |
| 1050 | GO:0015662 | F | 5, 6, 8, 13, | 2 | 2.302 (x 0.869) | 72 (0.028) | 0.895 | ATPase activity, coupled to transmembrane movement of ions, phosphorylative mechanism | Vha36 Vha44 |
| 1051 | GO:0005215 | F | 2, | 29 | 30.949 (x 0.937) | 968 (0.030) | 0.895 | transporter activity | CG11880 CG11897 CG14040 CG15094 CG1598 CG2789 CG31793 CG33066 CG3476 CG3522 CG3608 CG3731 CG4225 CG4963 CG6608 CG7627 CG7816 CG8602 CG9065 Shawn Syx13 TepIV Tim10 Vha36 Vha44 aralar1 att-ORFA foi gammaSnap |
| 1052 | GO:0018193 | P | 8, | 1 | 1.119 (x 0.894) | 35 (0.029) | 0.897 | peptidyl-amino acid modification | CG9882 |
| 1053 | GO:0009583 | P | 5, 6, | 1 | 1.119 (x 0.894) | 35 (0.029) | 0.898 | detection of light stimulus | Pgm |
| 1054 | GO:0007286 | P | 5, 8, | 1 | 1.151 (x 0.869) | 36 (0.028) | 0.905 | spermatid development | poe |
| 1055 | GO:0048515 | P | 4, 7, | 1 | 1.151 (x 0.869) | 36 (0.028) | 0.906 | spermatid differentiation | poe |
| 1056 | GO:0006898 | P | 7, 8, | 1 | 1.151 (x 0.869) | 36 (0.028) | 0.907 | receptor mediated endocytosis | AP-2sigma |
| 1057 | GO:0009617 | P | 5, | 2 | 2.366 (x 0.845) | 74 (0.027) | 0.908 | response to bacterium | TepIV Thor |
| 1058 | GO:0015629 | C | 6, 7, 8, 9, | 2 | 2.366 (x 0.845) | 74 (0.027) | 0.909 | actin cytoskeleton | cnn dynactin-subunit-p25 |
| 1059 | GO:0048534 | P | 4, | 2 | 2.366 (x 0.845) | 74 (0.027) | 0.91 | hemopoietic or lymphoid organ development | CG17760 ytr |
| 1060 | GO:0043068 | P | 6, 7, | 2 | 2.398 (x 0.834) | 75 (0.027) | 0.911 | positive regulation of programmed cell death | CG12876 CG33193 |
| 1061 | GO:0006511 | P | 9, 10, 11, | 2 | 2.398 (x 0.834) | 75 (0.027) | 0.912 | ubiquitin-dependent protein catabolism | Ate1 l(2)05070 |
| 1062 | GO:0005747 | C | 4, 5, 6, 7, 8, 9, 10, 11, 12, 13, 14, | 1 | 1.183 (x 0.845) | 37 (0.027) | 0.912 | respiratory chain complex I (sensu Eukaryota) | CG7598 |
| 1063 | GO:0008565 | F | 3, | 2 | 2.398 (x 0.834) | 75 (0.027) | 0.913 | protein transporter activity | CG33066 Tim10 |
| 1064 | GO:0016879 | F | 4, | 5 | 5.819 (x 0.859) | 182 (0.027) | 0.913 | ligase activity, forming carbon-nitrogen bonds | BcDNA:LD32788 CG5604 Gclm Nedd4 poe |
| 1065 | GO:0045132 | P | 5, 8, | 1 | 1.183 (x 0.845) | 37 (0.027) | 0.913 | meiotic chromosome segregation | cnn |
| 1066 | GO:0045271 | C | 3, 4, 5, 6, | 1 | 1.183 (x 0.845) | 37 (0.027) | 0.914 | respiratory chain complex I | CG7598 |
| 1067 | GO:0043632 | P | 7, | 2 | 2.462 (x 0.812) | 77 (0.026) | 0.925 | modification-dependent macromolecule catabolism | Ate1 l(2)05070 |
| 1068 | GO:0019941 | P | 8, 9, 10, | 2 | 2.462 (x 0.812) | 77 (0.026) | 0.926 | modification-dependent protein catabolism | Ate1 l(2)05070 |
| 1069 | GO:0015296 | F | 5, 7, | 1 | 1.247 (x 0.802) | 39 (0.026) | 0.931 | anion:cation symporter activity | CG15094 |
| 1070 | GO:0015992 | P | 6, 7, 8, 9, | 2 | 2.494 (x 0.802) | 78 (0.026) | 0.932 | proton transport | Vha36 Vha44 |
| 1071 | GO:0005815 | C | 5, 6, 7, 8, | 1 | 1.247 (x 0.802) | 39 (0.026) | 0.932 | microtubule organizing center | cnn |
| 1072 | GO:0006818 | P | 5, 6, | 2 | 2.494 (x 0.802) | 78 (0.026) | 0.933 | hydrogen transport | Vha36 Vha44 |
| 1073 | GO:0008355 | P | 6, 7, | 1 | 1.247 (x 0.802) | 39 (0.026) | 0.933 | olfactory learning | Gclm |
| 1074 | GO:0003704 | F | 4, | 2 | 2.526 (x 0.792) | 79 (0.025) | 0.937 | specific RNA polymerase II transcription factor activity | Jra MTF-1 |
| 1075 | GO:0046552 | P | 5, | 1 | 1.279 (x 0.782) | 40 (0.025) | 0.938 | photoreceptor cell fate commitment | Jra |
| 1076 | GO:0048589 | P | 3, | 1 | 1.279 (x 0.782) | 40 (0.025) | 0.939 | developmental growth | CG33193 |
| 1077 | GO:0004177 | F | 6, | 1 | 1.279 (x 0.782) | 40 (0.025) | 0.94 | aminopeptidase activity | Dip-B |
| 1078 | GO:0007459 | P | 6, | 1 | 1.279 (x 0.782) | 40 (0.025) | 0.941 | photoreceptor fate commitment (sensu Endopterygota) | Jra |
| 1079 | GO:0007268 | P | 6, | 6 | 7.226 (x 0.830) | 226 (0.027) | 0.946 | synaptic transmission | AP-2sigma Nsf2 Rep Syx13 gammaSnap snapin |
| 1080 | GO:0015144 | F | 3, | 2 | 2.590 (x 0.772) | 81 (0.025) | 0.947 | carbohydrate transporter activity | CG14040 TepIV |
| 1081 | GO:0005684 | C | 5, 6, 7, 8, 9, 10, 11, | 1 | 1.311 (x 0.763) | 41 (0.024) | 0.948 | major (U2-dependent) spliceosome | CG10333 |
| 1082 | GO:0006817 | P | 8, 9, | 1 | 1.375 (x 0.727) | 43 (0.023) | 0.951 | phosphate transport | CG15094 |
| 1083 | GO:0016591 | C | 3, 6, 7, 8, 9, 10, 11, 12, 13, | 2 | 2.654 (x 0.754) | 83 (0.024) | 0.951 | DNA-directed RNA polymerase II, holoenzyme | CG5134 TfIIEbeta |
| 1084 | GO:0001736 | P | 5, 6, | 1 | 1.375 (x 0.727) | 43 (0.023) | 0.952 | establishment of planar polarity | Jra |
| 1085 | GO:0008168 | F | 5, | 2 | 2.686 (x 0.745) | 84 (0.024) | 0.952 | methyltransferase activity | CG9249 CG9882 |
| 1086 | GO:0016789 | F | 5, | 3 | 3.837 (x 0.782) | 120 (0.025) | 0.952 | carboxylic ester hydrolase activity | CG6428 CG6718 alpha-Est3 |
| 1087 | GO:0008026 | F | 4, 10, | 2 | 2.654 (x 0.754) | 83 (0.024) | 0.952 | ATP-dependent helicase activity | CG10333 CG3561 |
| 1088 | GO:0007411 | P | 6, 7, 9, 10, 12, | 2 | 2.622 (x 0.763) | 82 (0.024) | 0.952 | axon guidance | Nedd4 betaTub60D |
| 1089 | GO:0006814 | P | 8, 9, | 1 | 1.375 (x 0.727) | 43 (0.023) | 0.952 | sodium ion transport | CG15094 |
| 1090 | GO:0016799 | F | 5, | 1 | 1.343 (x 0.745) | 42 (0.024) | 0.952 | hydrolase activity, hydrolyzing N-glycosyl compounds | CG31694 |
| 1091 | GO:0007017 | P | 7, | 5 | 6.267 (x 0.798) | 196 (0.026) | 0.953 | microtubule-based process | Unc-76 betaTub60D cnn dynactin-subunit-p25 milton |
| 1092 | GO:0016881 | F | 5, | 4 | 5.052 (x 0.792) | 158 (0.025) | 0.953 | acid-amino acid ligase activity | CG5604 Gclm Nedd4 poe |
| 1093 | GO:0051603 | P | 8, 9, | 2 | 2.622 (x 0.763) | 82 (0.024) | 0.953 | proteolysis during cellular protein catabolism | Ate1 l(2)05070 |
| 1094 | GO:0048812 | P | 7, 8, 10, | 3 | 3.869 (x 0.775) | 121 (0.025) | 0.953 | neurite morphogenesis | Nedd4 RhoGEF2 betaTub60D |
| 1095 | GO:0007164 | P | 4, | 1 | 1.375 (x 0.727) | 43 (0.023) | 0.953 | establishment of tissue polarity | Jra |
| 1096 | GO:0007306 | P | 9, 10, | 1 | 1.343 (x 0.745) | 42 (0.024) | 0.953 | insect chorion formation | Jra |
| 1097 | GO:0050896 | P | 2, | 34 | 37.375 (x 0.910) | 1169 (0.029) | 0.954 | response to stimulus | BG:BACR48E02.4 CG10460 CG10535 CG11897 CG12013 CG13887 CG1598 CG17266 CG17760 CG18522 CG31793 CG32687 CG5224 CG6523 CG6668 CG7627 Cat Gclm Gp93 GstE1 GstE6 GstE7 GstE9 Jafrac1 PEK Pgm Prx6005 TepIV Thor Ugt86Da betaTub60D cactin kraken mus205 |
| 1098 | GO:0044257 | P | 7, 8, | 2 | 2.622 (x 0.763) | 82 (0.024) | 0.954 | cellular protein catabolism | Ate1 l(2)05070 |
| 1099 | GO:0048667 | P | 6, 7, 9, | 3 | 3.869 (x 0.775) | 121 (0.025) | 0.954 | neuron morphogenesis during differentiation | Nedd4 RhoGEF2 betaTub60D |
| 1100 | GO:0006950 | P | 3, | 9 | 10.679 (x 0.843) | 334 (0.027) | 0.954 | response to stress | CG12013 CG13887 Cat Gp93 GstE1 PEK TepIV Thor mus205 |
| 1101 | GO:0007612 | P | 5, | 1 | 1.375 (x 0.727) | 43 (0.023) | 0.954 | learning | Gclm |
| 1102 | GO:0007409 | P | 8, 9, 11, | 3 | 3.869 (x 0.775) | 121 (0.025) | 0.955 | axonogenesis | Nedd4 RhoGEF2 betaTub60D |
| 1103 | GO:0016072 | P | 7, | 1 | 1.375 (x 0.727) | 43 (0.023) | 0.955 | rRNA metabolism | CG5380 |
| 1104 | GO:0009582 | P | 4, 5, | 1 | 1.407 (x 0.711) | 44 (0.023) | 0.957 | detection of abiotic stimulus | Pgm |
| 1105 | GO:0016741 | F | 4, | 2 | 2.718 (x 0.736) | 85 (0.024) | 0.957 | transferase activity, transferring one-carbon groups | CG9249 CG9882 |
| 1106 | GO:0007059 | P | 4, | 3 | 3.965 (x 0.757) | 124 (0.024) | 0.959 | chromosome segregation | betaTub60D cnn fbl |
| 1107 | GO:0005096 | F | 4, | 1 | 1.439 (x 0.695) | 45 (0.022) | 0.963 | GTPase activator activity | RhoGAP16F |
| 1108 | GO:0009581 | P | 4, 5, | 1 | 1.439 (x 0.695) | 45 (0.022) | 0.964 | detection of external stimulus | Pgm |
| 1109 | GO:0002164 | P | 4, | 1 | 1.439 (x 0.695) | 45 (0.022) | 0.965 | larval development | blp |
| 1110 | GO:0000226 | P | 8, | 2 | 2.782 (x 0.719) | 87 (0.023) | 0.966 | microtubule cytoskeleton organization and biogenesis | betaTub60D cnn |
| 1111 | GO:0016298 | F | 6, | 2 | 2.814 (x 0.711) | 88 (0.023) | 0.971 | lipase activity | CG6428 CG6718 |
| 1112 | GO:0006139 | P | 5, | 52 | 56.623 (x 0.918) | 1771 (0.029) | 0.972 | nucleobase, nucleoside, nucleotide and nucleic acid metabolism | Aats-ala-m Aats-asp Aats-cys Aats-gly Aats-his Aats-thr Aats-trp Aats-tyr Aats-val Adk1 Ahcy13 Aprt BcDNA:LD32788 CG10042 CG10333 CG10414 CG10802 CG11710 CG11876 CG11883 CG12177 CG13645 CG17266 CG18522 CG2246 CG2263 CG32549 CG3561 CG3931 CG4225 CG4802 CG5380 CG6272 CG7417 CG8128 CG8360 CG9215 Dak1 JhI-1 Jra MTF-1 NTPase Pcd RhoGEF2 TfIIEbeta Tpi Transaldolase Vha36 Vha44 l(2)k01209 mRpL28 mus205 |
| 1113 | GO:0007243 | P | 6, | 3 | 4.060 (x 0.739) | 127 (0.024) | 0.972 | protein kinase cascade | BG:BACR48E02.4 CG7417 Jra |
| 1114 | GO:0007293 | P | 8, | 1 | 1.503 (x 0.665) | 47 (0.021) | 0.974 | egg chamber formation (sensu Insecta) | mio |
| 1115 | GO:0051234 | P | 4, | 50 | 54.608 (x 0.916) | 1708 (0.029) | 0.974 | establishment of localization | AP-2sigma CG10535 CG11880 CG11897 CG13887 CG14040 CG15094 CG1598 CG17184 CG17266 CG2789 CG31793 CG33066 CG3397 CG3476 CG3522 CG4225 CG4963 CG6608 CG7627 CG7816 CG8479 CG8602 CG9065 CG9139 CG9240 CG9298 Fer1HCH Nedd4 Nsf2 Past1 Rep RhoGEF2 SCAP SH3PX1 Shawn Syx13 TepIV Tim10 Unc-76 Vha36 Vha44 aralar1 att-ORFA betaTub60D dynactin-subunit-p25 foi gammaSnap milton snapin |
| 1116 | GO:0001700 | P | 5, | 3 | 4.124 (x 0.727) | 129 (0.023) | 0.974 | embryonic development (sensu Insecta) | Hmgs Jra blp |
| 1117 | GO:0008134 | F | 4, | 2 | 2.846 (x 0.703) | 89 (0.022) | 0.975 | transcription factor binding | CG11710 Jra |
| 1118 | GO:0005529 | F | 4, | 1 | 1.503 (x 0.665) | 47 (0.021) | 0.975 | sugar binding | Gfat2 |
| 1119 | GO:0044430 | C | 4, 5, 6, 7, 8, 9, | 6 | 7.673 (x 0.782) | 240 (0.025) | 0.975 | cytoskeletal part | Rep Unc-76 betaTub60D cnn dynactin-subunit-p25 fbl |
| 1120 | GO:0016758 | F | 5, | 3 | 4.124 (x 0.727) | 129 (0.023) | 0.975 | transferase activity, transferring hexosyl groups | CG33138 CG8412 Ugt86Da |
| 1121 | GO:0006812 | P | 6, 7, | 10 | 12.245 (x 0.817) | 383 (0.026) | 0.975 | cation transport | CG15094 CG3397 CG6608 CG7816 CG8602 CG9065 Fer1HCH Vha36 Vha44 foi |
| 1122 | GO:0009152 | P | 8, 9, | 2 | 2.846 (x 0.703) | 89 (0.022) | 0.975 | purine ribonucleotide biosynthesis | Vha36 Vha44 |
| 1123 | GO:0030163 | P | 6, 7, | 2 | 2.877 (x 0.695) | 90 (0.022) | 0.976 | protein catabolism | Ate1 l(2)05070 |
| 1124 | GO:0009260 | P | 7, 8, | 2 | 2.909 (x 0.687) | 91 (0.022) | 0.98 | ribonucleotide biosynthesis | Vha36 Vha44 |
| 1125 | GO:0015631 | F | 5, | 2 | 2.909 (x 0.687) | 91 (0.022) | 0.98 | tubulin binding | betaTub60D cnn |
| 1126 | GO:0051707 | P | 4, | 3 | 4.188 (x 0.716) | 131 (0.023) | 0.981 | response to other organism | CG13887 TepIV Thor |
| 1127 | GO:0004519 | F | 6, | 1 | 1.567 (x 0.638) | 49 (0.020) | 0.982 | endonuclease activity | JhI-1 |
| 1128 | GO:0005681 | C | 4, 5, 6, 7, 8, 9, 10, | 2 | 2.941 (x 0.680) | 92 (0.022) | 0.982 | spliceosome complex | CG10333 CG17266 |
| 1129 | GO:0051606 | P | 3, 4, | 1 | 1.567 (x 0.638) | 49 (0.020) | 0.983 | detection of stimulus | Pgm |
| 1130 | GO:0007548 | P | 3, | 1 | 1.599 (x 0.626) | 50 (0.020) | 0.988 | sex differentiation | foi |
| 1131 | GO:0008509 | F | 4, | 2 | 3.005 (x 0.665) | 94 (0.021) | 0.992 | anion transporter activity | CG15094 CG1598 |
| 1132 | GO:0012501 | P | 5, | 6 | 7.929 (x 0.757) | 248 (0.024) | 0.995 | programmed cell death | CG12163 CG12876 CG13887 CG33193 RhoGEF2 cathD |
| 1133 | GO:0008219 | P | 4, | 6 | 7.993 (x 0.751) | 250 (0.024) | 0.998 | cell death | CG12163 CG12876 CG13887 CG33193 RhoGEF2 cathD |
| 1134 | GO:0019899 | F | 4, | 1 | 1.663 (x 0.601) | 52 (0.019) | 0.999 | enzyme binding | Gclm |
| 1135 | GO:0001738 | P | 5, | 1 | 1.663 (x 0.601) | 52 (0.019) | 1 | morphogenesis of a polarized epithelium | Jra |
| 1136 | GO:0005634 | C | 5, 6, 7, 8, | 19 | 48.726 (x 0.390) | 1524 (0.012) | 1 | nucleus | CG10042 CG10306 CG10333 CG17266 CG3931 CG4882 CG5134 CG5380 CG6272 CG7417 CG9215 CG9424 JhI-1 Jra MTF-1 TfIIEbeta mRpL55 mus205 ref(2)P |
| 1137 | GO:0015672 | P | 7, 8, | 4 | 5.595 (x 0.715) | 175 (0.023) | 1 | monovalent inorganic cation transport | CG15094 CG3397 Vha36 Vha44 |
| 1138 | GO:0050794 | P | 3, | 17 | 44.026 (x 0.386) | 1377 (0.012) | 1 | regulation of cellular process | CG10042 CG10414 CG11710 CG11876 CG12876 CG33193 CG6272 CG6523 CG7417 CG9215 EfTuM Jra MTF-1 PEK Thor Vha44 eIF2B-delta |
| 1139 | GO:0016265 | P | 3, | 6 | 8.025 (x 0.748) | 251 (0.024) | 1 | death | CG12163 CG12876 CG13887 CG33193 RhoGEF2 cathD |
| 1140 | GO:0051244 | P | 4, | 16 | 41.468 (x 0.386) | 1297 (0.012) | 1 | regulation of cellular physiological process | CG10042 CG10414 CG11710 CG11876 CG12876 CG33193 CG6272 CG7417 CG9215 EfTuM Jra MTF-1 PEK Thor Vha44 eIF2B-delta |
| 1141 | GO:0050791 | P | 3, | 17 | 42.811 (x 0.397) | 1339 (0.013) | 1 | regulation of physiological process | Ate1 CG10042 CG10414 CG11710 CG11876 CG12876 CG33193 CG6272 CG7417 CG9215 EfTuM Jra MTF-1 PEK Thor Vha44 eIF2B-delta |
| 1142 | GO:0007165 | P | 4, | 16 | 41.276 (x 0.388) | 1291 (0.012) | 1 | signal transduction | BG:BACR48E02.4 CG10535 CG12876 CG17760 CG32687 CG33193 CG6523 CG6805 CG7417 Ide Jra PEK RhoGEF2 SH3PX1 Tsp86D cactin |
| 1143 | GO:0042048 | P | 5, 6, | 1 | 1.695 (x 0.590) | 53 (0.019) | 1 | olfactory behavior | Gclm |
| 1144 | GO:0050789 | P | 2, | 21 | 48.118 (x 0.436) | 1505 (0.014) | 1 | regulation of biological process | Ate1 CG10042 CG10414 CG11710 CG11876 CG12876 CG33193 CG6272 CG6523 CG7417 CG9215 EfTuM Gclm Jra MTF-1 PEK Rep RhoGEF2 Thor Vha44 eIF2B-delta |
| 1145 | GO:0015290 | F | 4, | 5 | 6.938 (x 0.721) | 217 (0.023) | 1 | electrochemical potential-driven transporter activity | CG14040 CG15094 CG1598 CG3476 TepIV |
| 1146 | GO:0006396 | P | 7, | 6 | 8.153 (x 0.736) | 255 (0.024) | 1 | RNA processing | CG10333 CG17266 CG3931 CG8128 JhI-1 mRpL28 |
| 1147 | GO:0007275 | P | 2, | 21 | 47.479 (x 0.442) | 1485 (0.014) | 1 | development | CG12163 CG17760 CG31694 CG33193 Cat Hmgs Jra Nedd4 RhoGEF2 Thor Trxr-1 Vha44 betaTub60D blp cactin cathD cnn foi mio poe ytr |
| 1148 | GO:0015698 | P | 7, 8, | 1 | 1.726 (x 0.579) | 54 (0.019) | 1 | inorganic anion transport | CG15094 |
| 1149 | GO:0015291 | F | 5, | 5 | 6.938 (x 0.721) | 217 (0.023) | 1 | porter activity | CG14040 CG15094 CG1598 CG3476 TepIV |
| 1150 | GO:0004175 | F | 5, | 12 | 15.059 (x 0.797) | 471 (0.025) | 1 | endopeptidase activity | CG12163 CG3499 CG3731 CG6512 CG8728 Dip-B EG:100G10.7 Ide Sras cathD l(2)05070 ref(2)P |
| 1151 | GO:0005509 | F | 5, | 5 | 6.970 (x 0.717) | 218 (0.023) | 1 | calcium ion binding | CG11309 CG4589 CG7632 Past1 aralar1 |
| 1152 | GO:0007304 | P | 8, 9, | 1 | 1.758 (x 0.569) | 55 (0.018) | 1 | eggshell formation (sensu Insecta) | Jra |
| 1153 | GO:0019219 | P | 6, | 9 | 28.391 (x 0.317) | 888 (0.010) | 1 | regulation of nucleobase, nucleoside, nucleotide and nucleic acid metabolism | CG10042 CG10414 CG11710 CG11876 CG6272 CG7417 CG9215 Jra MTF-1 |
| 1154 | GO:0007635 | P | 4, 5, | 1 | 1.726 (x 0.579) | 54 (0.019) | 1 | chemosensory behavior | Gclm |
| 1155 | GO:0006820 | P | 6, 7, | 2 | 3.197 (x 0.626) | 100 (0.020) | 1 | anion transport | CG15094 CG1598 |
| 1156 | GO:0019787 | F | 6, | 3 | 4.444 (x 0.675) | 139 (0.022) | 1 | small conjugating protein ligase activity | CG5604 Nedd4 poe |
| 1157 | GO:0005576 | C | 2, | 1 | 12.341 (x 0.081) | 386 (0.003) | 1 | extracellular region | CG7291 |
| 1158 | GO:0030703 | P | 7, | 1 | 1.758 (x 0.569) | 55 (0.018) | 1 | eggshell formation | Jra |
| 1159 | GO:0005730 | C | 5, 6, 7, 8, 9, 10, 11, | 1 | 1.726 (x 0.579) | 54 (0.019) | 1 | nucleolus | mRpL55 |
| 1160 | GO:0007154 | P | 3, | 23 | 48.502 (x 0.474) | 1517 (0.015) | 1 | cell communication | AP-2sigma BG:BACR48E02.4 CG10535 CG12876 CG17760 CG32687 CG33193 CG6523 CG6805 CG7417 Ide Jra Nsf2 PEK Rep RhoGEF2 SH3PX1 Syx13 Tsp86D Unc-76 cactin gammaSnap snapin |
| 1161 | GO:0051301 | P | 4, | 3 | 4.444 (x 0.675) | 139 (0.022) | 1 | cell division | CG10068 Syx13 fbl |
| 1162 | GO:0007166 | P | 5, | 5 | 21.070 (x 0.237) | 659 (0.008) | 1 | cell surface receptor linked signal transduction | BG:BACR48E02.4 CG10535 CG17760 CG32687 Ide |
| 1163 | GO:0048666 | P | 5, 8, | 3 | 4.540 (x 0.661) | 142 (0.021) | 1 | neuron development | Nedd4 RhoGEF2 betaTub60D |
| 1164 | GO:0004842 | F | 7, | 3 | 4.444 (x 0.675) | 139 (0.022) | 1 | ubiquitin-protein ligase activity | CG5604 Nedd4 poe |
| 1165 | GO:0006915 | P | 6, | 4 | 5.691 (x 0.703) | 178 (0.022) | 1 | apoptosis | CG12876 CG13887 CG33193 RhoGEF2 |
| 1166 | GO:0004871 | F | 2, | 13 | 33.667 (x 0.386) | 1053 (0.012) | 1 | signal transducer activity | BG:BACR48E02.4 CG11710 CG12876 CG13887 CG17760 CG2789 CG31694 CG32687 CG7291 Ide PEK TMS1 Tsp86D |
| 1167 | GO:0031175 | P | 6, 9, | 3 | 4.540 (x 0.661) | 142 (0.021) | 1 | neurite development | Nedd4 RhoGEF2 betaTub60D |
| 1168 | GO:0006366 | P | 8, | 6 | 21.805 (x 0.275) | 682 (0.009) | 1 | transcription from RNA polymerase II promoter | CG11710 CG9215 Jra MTF-1 Pcd TfIIEbeta |
| 1169 | GO:0006355 | P | 8, | 8 | 25.162 (x 0.318) | 787 (0.010) | 1 | regulation of transcription, DNA-dependent | CG10042 CG10414 CG11710 CG11876 CG6272 CG9215 Jra MTF-1 |
| 1170 | GO:0045449 | P | 7, | 9 | 26.569 (x 0.339) | 831 (0.011) | 1 | regulation of transcription | CG10042 CG10414 CG11710 CG11876 CG6272 CG7417 CG9215 Jra MTF-1 |
| 1171 | GO:0004872 | F | 3, | 4 | 17.681 (x 0.226) | 553 (0.007) | 1 | receptor activity | BG:BACR48E02.4 CG2789 CG32687 Ide |
| 1172 | GO:0006357 | P | 9, | 4 | 17.489 (x 0.229) | 547 (0.007) | 1 | regulation of transcription from RNA polymerase II promoter | CG11710 CG9215 Jra MTF-1 |
| 1173 | GO:0006350 | P | 6, | 12 | 30.214 (x 0.397) | 945 (0.013) | 1 | transcription | CG10042 CG10414 CG11710 CG11876 CG5380 CG6272 CG7417 CG9215 Jra MTF-1 Pcd TfIIEbeta |
| 1174 | GO:0006351 | P | 7, | 11 | 28.647 (x 0.384) | 896 (0.012) | 1 | transcription, DNA-dependent | CG10042 CG10414 CG11710 CG11876 CG5380 CG6272 CG9215 Jra MTF-1 Pcd TfIIEbeta |
| 1175 | GO:0006259 | P | 6, | 2 | 11.862 (x 0.169) | 371 (0.005) | 1 | DNA metabolism | RhoGEF2 mus205 |
| 1176 | GO:0009950 | P | 5, | 1 | 1.822 (x 0.549) | 57 (0.018) | 1 | dorsal/ventral axis specification | cactin |
| 1177 | GO:0031323 | P | 5, | 14 | 31.525 (x 0.444) | 986 (0.014) | 1 | regulation of cellular metabolism | CG10042 CG10414 CG11710 CG11876 CG6272 CG7417 CG9215 EfTuM Jra MTF-1 PEK Thor Vha44 eIF2B-delta |
| 1178 | GO:0005554 | F | 2, | 9 | 24.043 (x 0.374) | 752 (0.012) | 1 | molecular function unknown | CG11722 CG12788 CG30152 CG32380 CG32675 CG3781 JhI-26 blp c11.1 |
| 1179 | GO:0019222 | P | 4, | 15 | 32.740 (x 0.458) | 1024 (0.015) | 1 | regulation of metabolism | Ate1 CG10042 CG10414 CG11710 CG11876 CG6272 CG7417 CG9215 EfTuM Jra MTF-1 PEK Thor Vha44 eIF2B-delta |
| 1180 | GO:0009966 | P | 4, 5, | 2 | 3.293 (x 0.607) | 103 (0.019) | 1 | regulation of signal transduction | CG6523 CG7417 |
| 1181 | GO:0007369 | P | 5, | 1 | 1.854 (x 0.539) | 58 (0.017) | 1 | gastrulation | RhoGEF2 |
| 1182 | GO:0000004 | P | 2, | 8 | 22.412 (x 0.357) | 701 (0.011) | 1 | biological process unknown | CG11722 CG12788 CG30152 CG31126 CG32675 CG3781 JhI-26 c11.1 |
| 1183 | GO:0004888 | F | 4, | 3 | 13.588 (x 0.221) | 425 (0.007) | 1 | transmembrane receptor activity | BG:BACR48E02.4 CG32687 Ide |
| 1184 | GO:0006813 | P | 8, 9, | 1 | 1.854 (x 0.539) | 58 (0.017) | 1 | potassium ion transport | CG3397 |
| 1185 | GO:0009653 | P | 3, | 7 | 20.526 (x 0.341) | 642 (0.011) | 1 | morphogenesis | CG33193 Jra Nedd4 RhoGEF2 Thor betaTub60D foi |
| 1186 | GO:0005654 | C | 5, 6, 7, 8, 9, 10, 11, | 5 | 7.162 (x 0.698) | 224 (0.022) | 1 | nucleoplasm | CG5134 CG5380 MTF-1 TfIIEbeta mus205 |
| 1187 | GO:0030528 | F | 2, | 11 | 25.738 (x 0.427) | 805 (0.014) | 1 | transcription regulator activity | CG11710 CG11876 CG31126 CG4882 CG7417 CG9215 Jra MTF-1 TfIIEbeta mRpL55 ref(2)P |
| 1188 | GO:0009888 | P | 3, | 3 | 12.725 (x 0.236) | 398 (0.008) | 1 | tissue development | CG17760 CG31694 foi |
| 1189 | GO:0004930 | F | 5, | 1 | 8.217 (x 0.122) | 257 (0.004) | 1 | G-protein coupled receptor activity | Ide |
| 1190 | GO:0007389 | P | 3, | 1 | 8.185 (x 0.122) | 256 (0.004) | 1 | pattern specification | cactin |
| 1191 | GO:0007552 | P | 4, | 1 | 8.089 (x 0.124) | 253 (0.004) | 1 | metamorphosis | Jra |
| 1192 | GO:0046698 | P | 5, | 1 | 8.025 (x 0.125) | 251 (0.004) | 1 | metamorphosis (sensu Insecta) | Jra |
| 1193 | GO:0000003 | P | 2, | 5 | 16.018 (x 0.312) | 501 (0.010) | 1 | reproduction | Jra fbl foi mio poe |
| 1194 | GO:0007560 | P | 5, 6, | 1 | 7.673 (x 0.130) | 240 (0.004) | 1 | imaginal disc morphogenesis | Jra |
| 1195 | GO:0007398 | P | 4, | 1 | 7.577 (x 0.132) | 237 (0.004) | 1 | ectoderm development | CG31694 |
| 1196 | GO:0007292 | P | 5, | 2 | 9.911 (x 0.202) | 310 (0.006) | 1 | female gamete generation | Jra mio |
| 1197 | GO:0006996 | P | 5, | 10 | 23.084 (x 0.433) | 722 (0.014) | 1 | organelle organization and biogenesis | CG17184 CG33066 CG6805 RhoGEF2 Tim10 Unc-76 betaTub60D cnn dynactin-subunit-p25 milton |
| 1198 | GO:0051179 | P | 3, | 50 | 56.623 (x 0.883) | 1771 (0.028) | 1 | localization | AP-2sigma CG10535 CG11880 CG11897 CG13887 CG14040 CG15094 CG1598 CG17184 CG17266 CG2789 CG31793 CG33066 CG3397 CG3476 CG3522 CG4225 CG4963 CG6608 CG7627 CG7816 CG8479 CG8602 CG9065 CG9139 CG9240 CG9298 Fer1HCH Nedd4 Nsf2 Past1 Rep RhoGEF2 SCAP SH3PX1 Shawn Syx13 TepIV Tim10 Unc-76 Vha36 Vha44 aralar1 att-ORFA betaTub60D dynactin-subunit-p25 foi gammaSnap milton snapin |
| 1199 | GO:0007015 | P | 9, | 1 | 1.918 (x 0.521) | 60 (0.017) | 1 | actin filament organization | RhoGEF2 |
| 1200 | GO:0030182 | P | 4, 7, | 3 | 4.764 (x 0.630) | 149 (0.020) | 1 | neuron differentiation | Nedd4 RhoGEF2 betaTub60D |
| 1201 | GO:0007186 | P | 6, | 2 | 9.752 (x 0.205) | 305 (0.007) | 1 | G-protein coupled receptor protein signaling pathway | CG17760 Ide |
| 1202 | GO:0016798 | F | 4, | 2 | 3.421 (x 0.585) | 107 (0.019) | 1 | hydrolase activity, acting on glycosyl bonds | CG31694 CG33138 |
| 1203 | GO:0050909 | P | 5, 7, | 1 | 1.918 (x 0.521) | 60 (0.017) | 1 | sensory perception of taste | CG17760 |
| 1204 | GO:0048513 | P | 3, | 9 | 21.421 (x 0.420) | 670 (0.013) | 1 | organ development | CG12163 CG17760 CG33193 Jra betaTub60D cathD cnn foi ytr |
| 1205 | GO:0042165 | F | 3, | 1 | 2.014 (x 0.496) | 63 (0.016) | 1 | neurotransmitter binding | CG2789 |
| 1206 | GO:0007507 | P | 5, | 1 | 1.950 (x 0.513) | 61 (0.016) | 1 | heart development | betaTub60D |
| 1207 | GO:0004497 | F | 4, | 2 | 3.485 (x 0.574) | 109 (0.018) | 1 | monooxygenase activity | Cyp6a20 Cyp6d5 |
| 1208 | GO:0007444 | P | 4, | 2 | 9.656 (x 0.207) | 302 (0.007) | 1 | imaginal disc development | CG33193 Jra |
| 1209 | GO:0004386 | F | 3, | 2 | 3.581 (x 0.559) | 112 (0.018) | 1 | helicase activity | CG10333 CG3561 |
| 1210 | GO:0019722 | P | 7, | 1 | 2.046 (x 0.489) | 64 (0.016) | 1 | calcium-mediated signaling | CG6805 |
| 1211 | GO:0031981 | C | 4, 5, 6, 7, 8, 9, 10, | 6 | 8.792 (x 0.682) | 275 (0.022) | 1 | nuclear lumen | CG5134 CG5380 MTF-1 TfIIEbeta mRpL55 mus205 |
| 1212 | GO:0006811 | P | 5, 6, | 11 | 14.771 (x 0.745) | 462 (0.024) | 1 | ion transport | CG15094 CG1598 CG3397 CG6608 CG7816 CG8602 CG9065 Fer1HCH Vha36 Vha44 foi |
| 1213 | GO:0009792 | P | 4, | 4 | 6.235 (x 0.642) | 195 (0.021) | 1 | embryonic development (sensu Metazoa) | Hmgs Jra RhoGEF2 blp |
| 1214 | GO:0035239 | P | 4, | 1 | 2.078 (x 0.481) | 65 (0.015) | 1 | tube morphogenesis | foi |
| 1215 | GO:0046961 | F | 6, 7, 9, 14, | 1 | 2.014 (x 0.496) | 63 (0.016) | 1 | hydrogen-transporting ATPase activity, rotational mechanism | Vha36 |
| 1216 | GO:0008380 | P | 8, | 3 | 4.956 (x 0.605) | 155 (0.019) | 1 | RNA splicing | CG10333 CG17266 JhI-1 |
| 1217 | GO:0007399 | P | 4, | 5 | 14.963 (x 0.334) | 468 (0.011) | 1 | nervous system development | CG31694 Nedd4 RhoGEF2 betaTub60D cnn |
| 1218 | GO:0007283 | P | 6, | 2 | 3.549 (x 0.564) | 111 (0.018) | 1 | spermatogenesis | fbl poe |
| 1219 | GO:0042981 | P | 6, 7, | 2 | 3.485 (x 0.574) | 109 (0.018) | 1 | regulation of apoptosis | CG12876 CG33193 |
| 1220 | GO:0043412 | P | 6, | 24 | 29.318 (x 0.819) | 917 (0.026) | 1 | biopolymer modification | Ate1 BG:BACR48E02.4 CG11989 CG17760 CG32687 CG3608 CG3731 CG5604 CG6746 CG8412 CG8446 CG8728 CG9240 CG9882 Nedd4 PEK Pi4KIIalpha Rep RhoGEF2 SCAP betaggt-II ire-1 l(2)05070 poe |
| 1221 | GO:0008236 | F | 5, | 2 | 9.368 (x 0.213) | 293 (0.007) | 1 | serine-type peptidase activity | CG11883 CG9240 |
| 1222 | GO:0046933 | F | 7, | 1 | 2.014 (x 0.496) | 63 (0.016) | 1 | hydrogen-transporting ATP synthase activity, rotational mechanism | Vha36 |
| 1223 | GO:0006512 | P | 8, | 5 | 7.481 (x 0.668) | 234 (0.021) | 1 | ubiquitin cycle | Ate1 CG5604 Nedd4 l(2)05070 poe |
| 1224 | GO:0048232 | P | 5, | 2 | 3.549 (x 0.564) | 111 (0.018) | 1 | male gamete generation | fbl poe |
| 1225 | GO:0007431 | P | 5, | 2 | 3.485 (x 0.574) | 109 (0.018) | 1 | salivary gland development | CG12163 cathD |
| 1226 | GO:0006397 | P | 8, | 4 | 6.330 (x 0.632) | 198 (0.020) | 1 | mRNA processing | CG10333 CG17266 CG3931 CG8128 |
| 1227 | GO:0005200 | F | 3, | 2 | 9.336 (x 0.214) | 292 (0.007) | 1 | structural constituent of cytoskeleton | CG8479 betaTub60D |
| 1228 | GO:0030594 | F | 4, | 1 | 2.014 (x 0.496) | 63 (0.016) | 1 | neurotransmitter receptor activity | CG2789 |
| 1229 | GO:0048565 | P | 4, | 1 | 1.982 (x 0.504) | 62 (0.016) | 1 | gut development | cnn |
| 1230 | GO:0035272 | P | 4, | 2 | 3.485 (x 0.574) | 109 (0.018) | 1 | exocrine system development | CG12163 cathD |
| 1231 | GO:0048477 | P | 6, | 2 | 9.240 (x 0.216) | 289 (0.007) | 1 | oogenesis | Jra mio |
| 1232 | GO:0015294 | F | 5, 7, | 1 | 2.014 (x 0.496) | 63 (0.016) | 1 | solute:cation symporter activity | CG15094 |
| 1233 | GO:0015370 | F | 6, 8, | 1 | 1.982 (x 0.504) | 62 (0.016) | 1 | solute:sodium symporter activity | CG15094 |
| 1234 | GO:0019953 | P | 3, | 5 | 14.579 (x 0.343) | 456 (0.011) | 1 | sexual reproduction | Jra fbl foi mio poe |
| 1235 | GO:0003677 | F | 4, | 13 | 26.281 (x 0.495) | 822 (0.016) | 1 | DNA binding | CG10042 CG10414 CG11876 CG4882 CG5380 CG6272 CG7417 CG9139 Jra MTF-1 RhoGEF2 mRpL55 mus205 |
| 1236 | GO:0005635 | C | 4, 5, 6, 7, 8, 9, 10, | 1 | 1.982 (x 0.504) | 62 (0.016) | 1 | nuclear envelope | CG9424 |
| 1237 | GO:0001584 | F | 6, | 1 | 6.810 (x 0.147) | 213 (0.005) | 1 | rhodopsin-like receptor activity | Ide |
| 1238 | GO:0048731 | P | 3, | 8 | 18.991 (x 0.421) | 594 (0.013) | 1 | system development | CG12163 CG31694 Nedd4 RhoGEF2 betaTub60D cathD cnn foi |
| 1239 | GO:0007276 | P | 4, | 5 | 14.292 (x 0.350) | 447 (0.011) | 1 | gametogenesis | Jra fbl foi mio poe |
| 1240 | GO:0005057 | F | 3, | 5 | 7.673 (x 0.652) | 240 (0.021) | 1 | receptor signaling protein activity | BG:BACR48E02.4 CG12876 CG32687 PEK TMS1 |
| 1241 | GO:0009993 | P | 7, | 2 | 8.856 (x 0.226) | 277 (0.007) | 1 | oogenesis (sensu Insecta) | Jra mio |
| 1242 | GO:0051726 | P | 5, | 1 | 6.362 (x 0.157) | 199 (0.005) | 1 | regulation of cell cycle | CG33193 |
| 1243 | GO:0000074 | P | 6, | 1 | 6.362 (x 0.157) | 199 (0.005) | 1 | regulation of progression through cell cycle | CG33193 |
| 1244 | GO:0005875 | C | 3, 5, 6, 7, 8, 9, 10, | 2 | 3.709 (x 0.539) | 116 (0.017) | 1 | microtubule associated complex | cnn dynactin-subunit-p25 |
| 1245 | GO:0031226 | C | 5, 6, 7, | 1 | 6.330 (x 0.158) | 198 (0.005) | 1 | intrinsic to plasma membrane | CG3397 |
| 1246 | GO:0005887 | C | 6, 7, 8, | 1 | 6.267 (x 0.160) | 196 (0.005) | 1 | integral to plasma membrane | CG3397 |
| 1247 | GO:0044459 | C | 4, 5, 6, | 3 | 10.327 (x 0.291) | 323 (0.009) | 1 | plasma membrane part | CG17760 CG3397 Pgm |
| 1248 | GO:0050877 | P | 4, | 9 | 19.695 (x 0.457) | 616 (0.015) | 1 | neurophysiological process | AP-2sigma CG17760 Nsf2 Pgm Rep Syx13 Unc-76 gammaSnap snapin |
| 1249 | GO:0007242 | P | 5, | 7 | 16.753 (x 0.418) | 524 (0.013) | 1 | intracellular signaling cascade | BG:BACR48E02.4 CG6805 CG7417 Jra PEK RhoGEF2 SH3PX1 |
| 1250 | GO:0007600 | P | 3, 5, | 2 | 8.249 (x 0.242) | 258 (0.008) | 1 | sensory perception | CG17760 Pgm |
| 1251 | GO:0007049 | P | 4, | 11 | 15.059 (x 0.730) | 471 (0.023) | 1 | cell cycle | CG33193 CG3499 CG6512 EG:100G10.7 Nsf2 RhoGEF2 Syx13 betaTub60D cnn fbl mio |
| 1252 | GO:0006468 | P | 8, | 6 | 9.112 (x 0.658) | 285 (0.021) | 1 | protein amino acid phosphorylation | BG:BACR48E02.4 CG32687 CG3608 CG6746 PEK ire-1 |
| 1253 | GO:0044451 | C | 5, 6, 7, 8, 9, 10, 11, 12, | 4 | 6.490 (x 0.616) | 203 (0.020) | 1 | nucleoplasm part | CG5134 CG5380 TfIIEbeta mus205 |
| 1254 | GO:0044445 | C | 5, 6, 7, 8, 9, | 2 | 3.773 (x 0.530) | 118 (0.017) | 1 | cytosolic part | Eno Pfk |
| 1255 | GO:0050874 | P | 3, | 16 | 28.775 (x 0.556) | 900 (0.018) | 1 | organismal physiological process | AP-2sigma CG13887 CG17760 CG6668 Cat Nsf2 Pgm Rep Syx13 TepIV Thor Trxr-1 Unc-76 gammaSnap kraken snapin |
| 1256 | GO:0043067 | P | 5, 6, | 2 | 3.805 (x 0.526) | 119 (0.017) | 1 | regulation of programmed cell death | CG12876 CG33193 |
| 1257 | GO:0008270 | F | 6, | 9 | 19.247 (x 0.468) | 602 (0.015) | 1 | zinc ion binding | CG10042 CG11967 CG8360 CG8417 CG9139 CG9215 Ide MTF-1 ref(2)P |
| 1258 | GO:0040029 | P | 3, | 1 | 2.238 (x 0.447) | 70 (0.014) | 1 | regulation of gene expression, epigenetic | RhoGEF2 |
| 1259 | GO:0030154 | P | 3, | 7 | 16.306 (x 0.429) | 510 (0.014) | 1 | cell differentiation | Jra Nedd4 RhoGEF2 betaTub60D mio poe ytr |
| 1260 | GO:0004867 | F | 6, | 1 | 2.238 (x 0.447) | 70 (0.014) | 1 | serine-type endopeptidase inhibitor activity | CG15094 |
| 1261 | GO:0007163 | P | 5, 6, | 1 | 2.270 (x 0.441) | 71 (0.014) | 1 | establishment and/or maintenance of cell polarity | Jra |
| 1262 | GO:0043283 | P | 5, | 46 | 53.841 (x 0.854) | 1684 (0.027) | 1 | biopolymer metabolism | Aats-ala-m Aats-asp Aats-cys Aats-gly Aats-his Aats-thr Aats-trp Aats-tyr Aats-val Ate1 BG:BACR48E02.4 CG10333 CG10802 CG11989 CG17266 CG17760 CG2263 CG32687 CG33138 CG3608 CG3731 CG3931 CG4225 CG5380 CG5604 CG6746 CG8128 CG8412 CG8446 CG8728 CG9240 CG9882 JhI-1 Nedd4 PEK Pi4KIIalpha Rep RhoGEF2 SCAP Ugt86Da betaggt-II ire-1 l(2)05070 mRpL28 mus205 poe |
| 1263 | GO:0008372 | C | 2, | 14 | 26.025 (x 0.538) | 814 (0.017) | 1 | cellular component unknown | CG11722 CG12788 CG30152 CG31126 CG32675 CG3781 CG4802 CG9232 CG9882 Dgp-1 JhI-26 blp c11.1 kraken |
| 1264 | GO:0048519 | P | 3, | 3 | 9.752 (x 0.308) | 305 (0.010) | 1 | negative regulation of biological process | CG33193 PEK Thor |
| 1265 | GO:0008017 | F | 6, | 1 | 2.430 (x 0.412) | 76 (0.013) | 1 | microtubule binding | cnn |
| 1266 | GO:0000904 | P | 5, 6, | 3 | 5.403 (x 0.555) | 169 (0.018) | 1 | cellular morphogenesis during differentiation | Nedd4 RhoGEF2 betaTub60D |
| 1267 | GO:0004674 | F | 7, | 4 | 6.778 (x 0.590) | 212 (0.019) | 1 | protein serine/threonine kinase activity | BG:BACR48E02.4 CG32687 PEK ire-1 |
| 1268 | GO:0007155 | P | 3, | 3 | 9.688 (x 0.310) | 303 (0.010) | 1 | cell adhesion | Cat RhoGEF2 Tsp86D |
| 1269 | GO:0044264 | P | 6, 7, | 1 | 2.462 (x 0.406) | 77 (0.013) | 1 | cellular polysaccharide metabolism | CG33138 |
| 1270 | GO:0016337 | P | 4, | 2 | 3.965 (x 0.504) | 124 (0.016) | 1 | cell-cell adhesion | Cat Tsp86D |
| 1271 | GO:0043285 | P | 6, | 2 | 4.060 (x 0.493) | 127 (0.016) | 1 | biopolymer catabolism | Ate1 l(2)05070 |
| 1272 | GO:0009790 | P | 3, | 4 | 11.222 (x 0.356) | 351 (0.011) | 1 | embryonic development | Hmgs Jra RhoGEF2 blp |
| 1273 | GO:0001754 | P | 5, 6, 7, | 1 | 2.430 (x 0.412) | 76 (0.013) | 1 | eye photoreceptor cell differentiation | Jra |
| 1274 | GO:0016071 | P | 7, | 4 | 6.650 (x 0.601) | 208 (0.019) | 1 | mRNA metabolism | CG10333 CG17266 CG3931 CG8128 |
| 1275 | GO:0003712 | F | 3, 5, | 1 | 2.366 (x 0.423) | 74 (0.014) | 1 | transcription cofactor activity | CG11710 |
| 1276 | GO:0035295 | P | 3, | 1 | 2.462 (x 0.406) | 77 (0.013) | 1 | tube development | foi |
| 1277 | GO:0007456 | P | 6, | 1 | 5.531 (x 0.181) | 173 (0.006) | 1 | eye development (sensu Endopterygota) | Jra |
| 1278 | GO:0007391 | P | 6, | 1 | 2.430 (x 0.412) | 76 (0.013) | 1 | dorsal closure | Jra |
| 1279 | GO:0005886 | C | 4, 5, | 8 | 17.073 (x 0.469) | 534 (0.015) | 1 | plasma membrane | CG17760 CG3397 Cat Pgm Syx13 TMS1 fbl foi |
| 1280 | GO:0005488 | F | 2, | 121 | 132.173 (x 0.915) | 4134 (0.029) | 1 | binding | Aats-ala-m Aats-asp Aats-cys Aats-gly Aats-his Aats-thr Aats-trp Aats-tyr Aats-val Adk1 BcDNA:LD32788 CG10042 CG10306 CG10333 CG10414 CG10802 CG11309 CG11710 CG11876 CG11897 CG11967 CG13887 CG1598 CG17187 CG17760 CG17904 CG2263 CG2789 CG31063 CG31694 CG31793 CG33193 CG3476 CG3499 CG3522 CG3561 CG3608 CG3931 CG4225 CG4589 CG4858 CG4882 CG4963 CG5261 CG5380 CG5469 CG6000 CG6272 CG6512 CG6608 CG6668 CG6744 CG7291 CG7417 CG7627 CG7632 CG7809 CG8360 CG8417 CG8479 CG8636 CG8778 CG9065 CG9139 CG9215 CG9298 Cat Dak1 Dgp-1 Dip-B EG:100G10.7 EfTuM Fer1HCH Fer2LCH Gclm Gfat2 Gp93 Ide Jra MTF-1 Mocs1 Nsf2 PEK Paps Past1 Pcd Pgm RhoGEF2 Shawn Thor Trxr-1 Tsp86D Unc-76 Vha44 aralar1 att-ORFA betaTub60D bor bsf cnn eIF-2alpha eIF2B-alpha eIF2B-delta eIF2B-gamma ire-1 l(2)k01209 mRpL11 mRpL13 mRpL21 mRpL28 mRpL3 mRpL55 mRpS2 mRpS25 mRpS9 milton mio mus205 poe ref(2)P ytr |
| 1281 | GO:0007606 | P | 4, 6, | 1 | 5.307 (x 0.188) | 166 (0.006) | 1 | sensory perception of chemical stimulus | CG17760 |
| 1282 | GO:0016020 | C | 3, 4, | 51 | 59.660 (x 0.855) | 1866 (0.027) | 1 | membrane | AP-2sigma CG11883 CG11897 CG13887 CG14040 CG15094 CG1598 CG17760 CG2789 CG31793 CG32380 CG33066 CG3397 CG3476 CG3499 CG3731 CG4225 CG4769 CG4963 CG6512 CG6608 CG7598 CG7627 CG7809 CG7816 CG8602 CG9424 Cat Cyp6a20 Cyp6d5 Cyt-b5 Cyt-c-p EG:100G10.7 Ide Pgm Rep Shawn Sply Sras Syx13 TMS1 Tim10 Tsp86D Vha36 Vha44 aralar1 att-ORFA bcn92 fbl ferrochelatase foi |
| 1283 | GO:0007601 | P | 5, 7, | 1 | 2.366 (x 0.423) | 74 (0.014) | 1 | visual perception | Pgm |
| 1284 | GO:0015293 | F | 6, | 1 | 2.334 (x 0.428) | 73 (0.014) | 1 | symporter activity | CG15094 |
| 1285 | GO:0016477 | P | 5, 6, | 3 | 5.595 (x 0.536) | 175 (0.017) | 1 | cell migration | Nedd4 betaTub60D foi |
| 1286 | GO:0008235 | F | 6, | 1 | 2.430 (x 0.412) | 76 (0.013) | 1 | metalloexopeptidase activity | Dip-B |
| 1287 | GO:0048468 | P | 4, | 4 | 10.871 (x 0.368) | 340 (0.012) | 1 | cell development | Nedd4 RhoGEF2 betaTub60D poe |
| 1288 | GO:0048518 | P | 3, | 4 | 6.970 (x 0.574) | 218 (0.018) | 1 | positive regulation of biological process | CG12876 CG33193 CG7417 Gclm |
| 1289 | GO:0050793 | P | 3, | 1 | 2.526 (x 0.396) | 79 (0.013) | 1 | regulation of development | RhoGEF2 |
| 1290 | GO:0050953 | P | 4, 6, | 1 | 2.366 (x 0.423) | 74 (0.014) | 1 | sensory perception of light stimulus | Pgm |
| 1291 | GO:0001751 | P | 6, 7, 8, 9, | 1 | 2.334 (x 0.428) | 73 (0.014) | 1 | eye photoreceptor cell differentiation (sensu Endopterygota) | Jra |
| 1292 | GO:0007422 | P | 5, | 1 | 2.430 (x 0.412) | 76 (0.013) | 1 | peripheral nervous system development | cnn |
| 1293 | GO:0005856 | C | 5, 6, 7, 8, | 6 | 9.272 (x 0.647) | 290 (0.021) | 1 | cytoskeleton | Rep Unc-76 betaTub60D cnn dynactin-subunit-p25 fbl |
| 1294 | GO:0007423 | P | 4, | 2 | 7.354 (x 0.272) | 230 (0.009) | 1 | sensory organ development | CG33193 Jra |
| 1295 | GO:0004702 | F | 4, 8, | 3 | 5.659 (x 0.530) | 177 (0.017) | 1 | receptor signaling protein serine/threonine kinase activity | BG:BACR48E02.4 CG32687 PEK |
| 1296 | GO:0009953 | P | 4, | 1 | 2.366 (x 0.423) | 74 (0.014) | 1 | dorsal/ventral pattern formation | cactin |
| 1297 | GO:0009887 | P | 4, | 4 | 10.743 (x 0.372) | 336 (0.012) | 1 | organ morphogenesis | CG33193 Jra betaTub60D foi |
| 1298 | GO:0048732 | P | 4, | 2 | 4.188 (x 0.478) | 131 (0.015) | 1 | gland development | CG12163 cathD |
| 1299 | GO:0035214 | P | 5, | 1 | 5.116 (x 0.195) | 160 (0.006) | 1 | eye-antennal disc development | Jra |
| 1300 | GO:0006464 | P | 7, | 22 | 28.040 (x 0.785) | 877 (0.025) | 1 | protein modification | Ate1 BG:BACR48E02.4 CG11989 CG17760 CG32687 CG3608 CG3731 CG5604 CG6746 CG8412 CG8446 CG8728 CG9240 CG9882 Nedd4 PEK Rep SCAP betaggt-II ire-1 l(2)05070 poe |
| 1301 | GO:0005515 | F | 3, | 26 | 39.454 (x 0.659) | 1234 (0.021) | 1 | protein binding | CG11710 CG13887 CG17187 CG31694 CG33193 CG5261 CG5469 CG6000 CG6272 CG7291 CG7809 CG9298 Gclm Gp93 Jra Past1 Pgm RhoGEF2 Thor Tsp86D Unc-76 betaTub60D cnn milton mio poe |
| 1302 | GO:0043413 | P | 7, | 1 | 2.590 (x 0.386) | 81 (0.012) | 1 | biopolymer glycosylation | CG8412 |
| 1303 | GO:0003702 | F | 3, | 3 | 8.505 (x 0.353) | 266 (0.011) | 1 | RNA polymerase II transcription factor activity | Jra MTF-1 TfIIEbeta |
| 1304 | GO:0007267 | P | 4, | 7 | 14.324 (x 0.489) | 448 (0.016) | 1 | cell-cell signaling | AP-2sigma Nsf2 Rep Syx13 Unc-76 gammaSnap snapin |
| 1305 | GO:0048523 | P | 4, | 3 | 8.952 (x 0.335) | 280 (0.011) | 1 | negative regulation of cellular process | CG33193 PEK Thor |
| 1306 | GO:0007455 | P | 6, 7, | 1 | 4.668 (x 0.214) | 146 (0.007) | 1 | eye-antennal disc morphogenesis | Jra |
| 1307 | GO:0006486 | P | 8, 9, | 1 | 2.590 (x 0.386) | 81 (0.012) | 1 | protein amino acid glycosylation | CG8412 |
| 1308 | GO:0048699 | P | 6, | 3 | 5.755 (x 0.521) | 180 (0.017) | 1 | generation of neurons | Nedd4 RhoGEF2 betaTub60D |
| 1309 | GO:0009791 | P | 3, | 5 | 11.382 (x 0.439) | 356 (0.014) | 1 | post-embryonic development | CG12163 Jra Vha44 blp cathD |
| 1310 | GO:0002165 | P | 4, | 5 | 11.030 (x 0.453) | 345 (0.014) | 1 | larval or pupal development (sensu Insecta) | CG12163 Jra Vha44 blp cathD |
| 1311 | GO:0009798 | P | 4, | 1 | 5.084 (x 0.197) | 159 (0.006) | 1 | axis specification | cactin |
| 1312 | GO:0007498 | P | 4, | 2 | 6.330 (x 0.316) | 198 (0.010) | 1 | mesoderm development | CG17760 foi |
| 1313 | GO:0015075 | F | 3, | 8 | 15.507 (x 0.516) | 485 (0.016) | 1 | ion transporter activity | CG15094 CG1598 CG3731 CG7816 CG9065 Vha36 Vha44 foi |
| 1314 | GO:0048748 | P | 6, 7, | 1 | 4.508 (x 0.222) | 141 (0.007) | 1 | eye morphogenesis (sensu Endopterygota) | Jra |
| 1315 | GO:0016331 | P | 5, | 1 | 2.622 (x 0.381) | 82 (0.012) | 1 | morphogenesis of embryonic epithelium | Jra |
| 1316 | GO:0003700 | F | 3, 5, | 6 | 12.437 (x 0.482) | 389 (0.015) | 1 | transcription factor activity | CG11876 CG4882 CG7417 Jra MTF-1 mRpL55 |
| 1317 | GO:0016043 | P | 4, | 30 | 44.090 (x 0.680) | 1379 (0.022) | 1 | cell organization and biogenesis | AP-2sigma CG10535 CG13887 CG17184 CG17266 CG33066 CG3476 CG6805 CG8479 CG9139 CG9240 CG9298 Jra Nedd4 Nsf2 Rep RhoGEF2 SCAP SH3PX1 Syx13 Thor Tim10 Unc-76 aralar1 betaTub60D cnn dynactin-subunit-p25 fbl gammaSnap milton |
| 1318 | GO:0048749 | P | 7, | 1 | 4.252 (x 0.235) | 133 (0.008) | 1 | compound eye development (sensu Endopterygota) | Jra |
| 1319 | GO:0009101 | P | 7, 8, | 1 | 2.654 (x 0.377) | 83 (0.012) | 1 | glycoprotein biosynthesis | CG8412 |
| 1320 | GO:0002009 | P | 4, | 1 | 4.252 (x 0.235) | 133 (0.008) | 1 | morphogenesis of an epithelium | Jra |
| 1321 | GO:0006367 | P | 9, | 1 | 2.686 (x 0.372) | 84 (0.012) | 1 | transcription initiation from RNA polymerase II promoter | TfIIEbeta |
| 1322 | GO:0009719 | P | 3, | 1 | 4.252 (x 0.235) | 133 (0.008) | 1 | response to endogenous stimulus | mus205 |
| 1323 | GO:0008360 | P | 5, 6, | 1 | 2.718 (x 0.368) | 85 (0.012) | 1 | regulation of cell shape | RhoGEF2 |
| 1324 | GO:0008283 | P | 4, | 4 | 9.016 (x 0.444) | 282 (0.014) | 1 | cell proliferation | CG33193 CG8128 CG9215 MTF-1 |
| 1325 | GO:0031224 | C | 4, 5, 6, | 20 | 30.278 (x 0.661) | 947 (0.021) | 1 | intrinsic to membrane | CG11897 CG13887 CG14040 CG15094 CG2789 CG31793 CG32380 CG3397 CG4225 CG7627 CG7816 CG8602 Ide Shawn Sply Sras Tsp86D Vha36 Vha44 aralar1 |
| 1326 | GO:0001745 | P | 7, 8, | 1 | 4.252 (x 0.235) | 133 (0.008) | 1 | compound eye morphogenesis (sensu Endopterygota) | Jra |
| 1327 | GO:0016021 | C | 5, 6, 7, | 20 | 30.182 (x 0.663) | 944 (0.021) | 1 | integral to membrane | CG11897 CG13887 CG14040 CG15094 CG2789 CG31793 CG32380 CG3397 CG4225 CG7627 CG7816 CG8602 Ide Shawn Sply Sras Tsp86D Vha36 Vha44 aralar1 |
| 1328 | GO:0030707 | P | 8, | 1 | 3.965 (x 0.252) | 124 (0.008) | 1 | ovarian follicle cell development (sensu Insecta) | Jra |
| 1329 | GO:0030246 | F | 3, | 1 | 3.613 (x 0.277) | 113 (0.009) | 1 | carbohydrate binding | Gfat2 |
| 1330 | GO:0007167 | P | 6, | 2 | 5.499 (x 0.364) | 172 (0.012) | 1 | enzyme linked receptor protein signaling pathway | BG:BACR48E02.4 CG32687 |
| 1331 | GO:0006352 | P | 8, | 1 | 2.750 (x 0.364) | 86 (0.012) | 1 | transcription initiation | TfIIEbeta |
| 1332 | GO:0007417 | P | 5, | 1 | 3.901 (x 0.256) | 122 (0.008) | 1 | central nervous system development | cnn |
| 1333 | GO:0000902 | P | 4, 5, | 5 | 10.807 (x 0.463) | 338 (0.015) | 1 | cellular morphogenesis | Jra Nedd4 RhoGEF2 Thor betaTub60D |
| 1334 | GO:0005667 | C | 3, 6, 7, 8, 9, 10, 11, 12, 13, | 1 | 2.718 (x 0.368) | 85 (0.012) | 1 | transcription factor complex | TfIIEbeta |
| 1335 | GO:0016567 | P | 9, | 1 | 3.613 (x 0.277) | 113 (0.009) | 1 | protein ubiquitination | Nedd4 |
| 1336 | GO:0045165 | P | 4, | 2 | 6.043 (x 0.331) | 189 (0.011) | 1 | cell fate commitment | Jra mio |
| 1337 | GO:0001654 | P | 5, | 2 | 5.787 (x 0.346) | 181 (0.011) | 1 | eye development | CG33193 Jra |
| 1338 | GO:0008324 | F | 4, | 7 | 12.757 (x 0.549) | 399 (0.018) | 1 | cation transporter activity | CG15094 CG1598 CG3731 CG9065 Vha36 Vha44 foi |
| 1339 | GO:0043119 | P | 4, | 2 | 5.403 (x 0.370) | 169 (0.012) | 1 | positive regulation of physiological process | CG12876 CG33193 |
| 1340 | GO:0009100 | P | 7, | 1 | 2.750 (x 0.364) | 86 (0.012) | 1 | glycoprotein metabolism | CG8412 |
| 1341 | GO:0051243 | P | 5, | 3 | 7.418 (x 0.404) | 232 (0.013) | 1 | negative regulation of cellular physiological process | CG33193 PEK Thor |
| 1342 | GO:0001709 | P | 5, | 1 | 3.517 (x 0.284) | 110 (0.009) | 1 | cell fate determination | mio |
| 1343 | GO:0043118 | P | 4, | 3 | 7.705 (x 0.389) | 241 (0.012) | 1 | negative regulation of physiological process | CG33193 PEK Thor |
| 1344 | GO:0046914 | F | 5, | 14 | 21.613 (x 0.648) | 676 (0.021) | 1 | transition metal ion binding | CG10042 CG11967 CG8360 CG8417 CG9065 CG9139 CG9215 Dip-B Fer1HCH Fer2LCH Ide MTF-1 Mocs1 ref(2)P |
| 1345 | GO:0007467 | P | 5, | 1 | 2.782 (x 0.360) | 87 (0.011) | 1 | photoreceptor cell differentiation (sensu Endopterygota) | Jra |
| 1346 | GO:0006974 | P | 4, | 1 | 3.773 (x 0.265) | 118 (0.008) | 1 | response to DNA damage stimulus | mus205 |
| 1347 | GO:0006260 | P | 7, | 1 | 3.869 (x 0.258) | 121 (0.008) | 1 | DNA replication | mus205 |
| 1348 | GO:0006281 | P | 5, 7, | 1 | 3.517 (x 0.284) | 110 (0.009) | 1 | DNA repair | mus205 |
| 1349 | GO:0007010 | P | 6, | 8 | 14.387 (x 0.556) | 450 (0.018) | 1 | cytoskeleton organization and biogenesis | CG17184 CG6805 RhoGEF2 Unc-76 betaTub60D cnn dynactin-subunit-p25 milton |
| 1350 | GO:0051242 | P | 5, | 2 | 5.339 (x 0.375) | 167 (0.012) | 1 | positive regulation of cellular physiological process | CG12876 CG33193 |
| 1351 | GO:0019932 | P | 6, | 1 | 2.814 (x 0.355) | 88 (0.011) | 1 | second-messenger-mediated signaling | CG6805 |
| 1352 | GO:0008238 | F | 5, | 1 | 3.485 (x 0.287) | 109 (0.009) | 1 | exopeptidase activity | Dip-B |
| 1353 | GO:0043169 | F | 4, | 19 | 28.359 (x 0.670) | 887 (0.021) | 1 | cation binding | CG10042 CG11309 CG11967 CG4589 CG7632 CG8360 CG8417 CG9065 CG9139 CG9215 Dip-B Fer1HCH Fer2LCH Ide MTF-1 Mocs1 Past1 aralar1 ref(2)P |
| 1354 | GO:0048522 | P | 4, | 3 | 6.043 (x 0.496) | 189 (0.016) | 1 | positive regulation of cellular process | CG12876 CG33193 CG7417 |
| 1355 | GO:0004721 | F | 7, | 1 | 2.846 (x 0.351) | 89 (0.011) | 1 | phosphoprotein phosphatase activity | CG6746 |
| 1356 | GO:0007424 | P | 4, | 1 | 3.485 (x 0.287) | 109 (0.009) | 1 | tracheal system development (sensu Insecta) | foi |
| 1357 | GO:0008194 | F | 5, | 1 | 2.941 (x 0.340) | 92 (0.011) | 1 | UDP-glycosyltransferase activity | Ugt86Da |
| 1358 | GO:0008092 | F | 4, | 4 | 7.609 (x 0.526) | 238 (0.017) | 1 | cytoskeletal protein binding | Unc-76 betaTub60D cnn milton |
| 1359 | GO:0044428 | C | 4, 5, 6, 7, 8, 9, | 10 | 16.466 (x 0.607) | 515 (0.019) | 1 | nuclear part | CG10333 CG17266 CG3931 CG5134 CG5380 CG9424 MTF-1 TfIIEbeta mRpL55 mus205 |
| 1360 | GO:0005102 | F | 3, 4, | 5 | 8.920 (x 0.561) | 279 (0.018) | 1 | receptor binding | CG11710 CG13887 CG31694 CG7291 Tsp86D |
| 1361 | GO:0007610 | P | 3, | 3 | 6.107 (x 0.491) | 191 (0.016) | 1 | behavior | CG10460 Gclm betaTub60D |
| 1362 | GO:0022008 | P | 5, | 3 | 6.171 (x 0.486) | 193 (0.016) | 1 | neurogenesis | Nedd4 RhoGEF2 betaTub60D |
| 1363 | GO:0046872 | F | 4, | 21 | 29.798 (x 0.705) | 932 (0.023) | 1 | metal ion binding | CG10042 CG11309 CG11967 CG31063 CG4589 CG7632 CG8360 CG8417 CG9065 CG9139 CG9215 Dip-B Fer1HCH Fer2LCH Ide MTF-1 Mocs1 Past1 Trxr-1 aralar1 ref(2)P |
| 1364 | GO:0000398 | P | 9, 11, | 2 | 4.764 (x 0.420) | 149 (0.013) | 1 | nuclear mRNA splicing, via spliceosome | CG10333 CG17266 |
| 1365 | GO:0019226 | P | 5, | 7 | 11.606 (x 0.603) | 363 (0.019) | 1 | transmission of nerve impulse | AP-2sigma Nsf2 Rep Syx13 Unc-76 gammaSnap snapin |
| 1366 | GO:0046530 | P | 4, | 1 | 3.133 (x 0.319) | 98 (0.010) | 1 | photoreceptor cell differentiation | Jra |
| 1367 | GO:0043167 | F | 3, | 21 | 29.798 (x 0.705) | 932 (0.023) | 1 | ion binding | CG10042 CG11309 CG11967 CG31063 CG4589 CG7632 CG8360 CG8417 CG9065 CG9139 CG9215 Dip-B Fer1HCH Fer2LCH Ide MTF-1 Mocs1 Past1 Trxr-1 aralar1 ref(2)P |
| 1368 | GO:0048592 | P | 5, 6, | 2 | 4.764 (x 0.420) | 149 (0.013) | 1 | eye morphogenesis | CG33193 Jra |
| 1369 | GO:0031324 | P | 6, | 2 | 4.956 (x 0.404) | 155 (0.013) | 1 | negative regulation of cellular metabolism | PEK Thor |
| 1370 | GO:0009892 | P | 5, | 2 | 5.275 (x 0.379) | 165 (0.012) | 1 | negative regulation of metabolism | PEK Thor |
| 1371 | GO:0004553 | F | 5, | 1 | 3.133 (x 0.319) | 98 (0.010) | 1 | hydrolase activity, hydrolyzing O-glycosyl compounds | CG33138 |
| 1372 | GO:0016251 | F | 4, | 1 | 3.229 (x 0.310) | 101 (0.010) | 1 | general RNA polymerase II transcription factor activity | TfIIEbeta |
| 1373 | GO:0003676 | F | 3, | 45 | 55.728 (x 0.808) | 1743 (0.026) | 1 | nucleic acid binding | Aats-ala-m Aats-asp Aats-cys Aats-his Aats-thr Aats-tyr Aats-val CG10042 CG10306 CG10414 CG10802 CG11876 CG3561 CG3931 CG4882 CG5380 CG6272 CG6744 CG7417 CG8636 CG8778 CG9139 CG9215 EfTuM Jra MTF-1 PEK Pcd RhoGEF2 bsf eIF-2alpha eIF2B-alpha eIF2B-delta eIF2B-gamma mRpL11 mRpL13 mRpL21 mRpL28 mRpL3 mRpL55 mRpS2 mRpS25 mRpS9 mus205 ytr |
| 1374 | GO:0005575 | C | 1, | 171 | 184.863 (x 0.925) | 5782 (0.030) | 1 | cellular\_component | AP-2sigma Aats-ala-m Aats-asp Aats-his Adk1 Alas Aprt BcDNA:GH07485 CG10042 CG10268 CG10306 CG10333 CG1140 CG11722 CG11876 CG11883 CG11897 CG12013 CG12264 CG12534 CG12788 CG12954 CG1319 CG13887 CG14040 CG15094 CG1598 CG1673 CG17266 CG17760 CG2263 CG2789 CG2846 CG30152 CG31075 CG31126 CG31793 CG32380 CG32549 CG32675 CG33066 CG3397 CG3476 CG3499 CG3731 CG3781 CG3931 CG4225 CG4589 CG4769 CG4802 CG4882 CG4963 CG5134 CG5261 CG5380 CG5604 CG6272 CG6512 CG6608 CG6718 CG6744 CG7291 CG7417 CG7598 CG7627 CG7809 CG7816 CG7842 CG8412 CG8602 CG8636 CG8728 CG9065 CG9215 CG9232 CG9240 CG9298 CG9424 CG9882 Cat Cyp6a20 Cyp6d5 Cyt-b5 Cyt-c-p Dgp-1 Dip-B EG:100G10.7 EfTuM Eno Fer1HCH Fer2LCH Gapdh1 Gapdh2 Gclm Gfat2 Ide Idh Inos Jafrac1 JhI-1 JhI-26 Jra MTF-1 Mocs1 Nedd4 Nsf2 PEK Pcd Pfk Pgm PyK Rep Shawn Sply Sras Syx13 TMS1 TfIIEbeta Thiolase Tim10 Transaldolase Trxr-1 Tsp86D Unc-76 Updo Vha36 Vha44 aralar1 att-ORFA bcn92 betaTub60D blp bor bsf c11.1 cnn dynactin-subunit-p25 eIF-2alpha eIF2B-alpha eIF2B-delta eIF2B-gamma fbl ferrochelatase foi gammaSnap kraken l(2)05070 mRpL11 mRpL13 mRpL19 mRpL21 mRpL22 mRpL22-24 mRpL28 mRpL3 mRpL48 mRpL55 mRpS18a mRpS18b mRpS2 mRpS22 mRpS25 mRpS32 mRpS33 mRpS9 milton mus205 ref(2)P snapin yip2 |
| 1375 | GO:0004672 | F | 6, | 5 | 9.432 (x 0.530) | 295 (0.017) | 1 | protein kinase activity | BG:BACR48E02.4 CG32687 CG3608 PEK ire-1 |
| 1376 | GO:0008233 | F | 4, | 14 | 20.814 (x 0.673) | 651 (0.022) | 1 | peptidase activity | CG11883 CG12163 CG3499 CG3731 CG6512 CG8728 CG9240 Dip-B EG:100G10.7 Ide Sras cathD l(2)05070 ref(2)P |
| 1377 | GO:0006508 | P | 7, | 17 | 24.235 (x 0.701) | 758 (0.022) | 1 | proteolysis | Ate1 CG11883 CG12163 CG3499 CG3731 CG5604 CG6512 CG8728 CG9240 Dip-B EG:100G10.7 Ide Nedd4 Sras cathD l(2)05070 ref(2)P |
| 1378 | GO:0000377 | P | 10, | 2 | 4.764 (x 0.420) | 149 (0.013) | 1 | RNA splicing, via transesterification reactions with bulged adenosine as nucleophile | CG10333 CG17266 |
| 1379 | GO:0016311 | P | 7, | 1 | 3.453 (x 0.290) | 108 (0.009) | 1 | dephosphorylation | CG6805 |
| 1380 | GO:0040011 | P | 3, | 4 | 8.345 (x 0.479) | 261 (0.015) | 1 | locomotion | CG17184 Nedd4 betaTub60D foi |
| 1381 | GO:0030036 | P | 8, | 1 | 3.101 (x 0.322) | 97 (0.010) | 1 | actin cytoskeleton organization and biogenesis | RhoGEF2 |
| 1382 | GO:0048598 | P | 4, | 1 | 3.389 (x 0.295) | 106 (0.009) | 1 | embryonic morphogenesis | Jra |
| 1383 | GO:0044425 | C | 3, 4, 5, | 29 | 38.878 (x 0.746) | 1216 (0.024) | 1 | membrane part | AP-2sigma CG11897 CG13887 CG14040 CG15094 CG17760 CG2789 CG31793 CG32380 CG33066 CG3397 CG3731 CG4225 CG4769 CG7598 CG7627 CG7809 CG7816 CG8602 Ide Pgm Shawn Sply Sras Tim10 Tsp86D Vha36 Vha44 aralar1 |
| 1384 | GO:0005976 | P | 6, | 2 | 5.179 (x 0.386) | 162 (0.012) | 1 | polysaccharide metabolism | CG33138 Ugt86Da |
| 1385 | GO:0000375 | P | 9, | 2 | 4.764 (x 0.420) | 149 (0.013) | 1 | RNA splicing, via transesterification reactions | CG10333 CG17266 |
| 1386 | GO:0009605 | P | 3, | 1 | 3.357 (x 0.298) | 105 (0.010) | 1 | response to external stimulus | Pgm |
| 1387 | GO:0030029 | P | 7, | 1 | 3.101 (x 0.322) | 97 (0.010) | 1 | actin filament-based process | RhoGEF2 |
| 1388 | GO:0051674 | P | 4, | 4 | 8.217 (x 0.487) | 257 (0.016) | 1 | localization of cell | CG17184 Nedd4 betaTub60D foi |
| 1389 | GO:0006928 | P | 4, 5, | 4 | 8.217 (x 0.487) | 257 (0.016) | 1 | cell motility | CG17184 Nedd4 betaTub60D foi |

  

---

Regulated Genes that don't have GO terms
  

CG10195 CG10343 CG10383 CG11347 CG11699 CG11885 CG12118 CG12125 CG12379 CG12505 CG12797 CG13533 CG13623 CG13941 CG14084 CG14270 CG14483 CG14696 CG14883 CG14907 CG14985 CG15027 CG15083 CG15099 CG1553 CG15784 CG15863 CG15908 CG16787 CG17327 CG17680 CG17726 CG17734 CG18445 CG18596 CG1983 CG2076 CG2794 CG2909 CG30344 CG30493 CG31098 CG3271 CG33137 CG3448 CG4611 CG4679 CG5010 CG5118 CG5862 CG5989 CG6196 CG6353 CG6443 CG6568 CG7394 CG7506 CG7611 CG7739 CG7841 CG7950 CG8297 CG8607 CG8678 CG9034 CG9067 CG9186 CG9288 CG9646 CG9773 CG9987 Chmp1 l(1)G0136 l(2)k14505 rho-7
