# Supplementary material for: Functional dissection of the ash2 and ash1 transcriptomes provides insights into the transcriptional basis of wing phenotypes and reveals conserved protein interactions
Source: Genome Biol. 2007 Apr 28;8(4):R67. doi: 10.1186/gb-2007-8-4-r67 (PMC1896016; doi:10.1186/gb-2007-8-4-r67)
Supplement: Additional data file 20 — Genes misregulated in Sin3A deficient cells that are also misexpressed in ash2 and/or ash1 mutants [file gb-2007-8-4-r67-S20.pdf]

Sin3A Up	
ash2	***ash1***
Ahcy13	
Ate1	
BcDNA:LD32788	
bcn92	
betaggt-II	
CG10195	
CG10268	
CG10306	
CG10460	
CG10638	
CG1140	
CG11722	
CG11897	
CG12264	
CG12379	
CG12505	
CG12876	
CG12954	
CG13533	
CG13623	
CG13645	
CG13941	
CG14270	CG14270
CG14483	
CG14696	
CG14907	
CG15784	CG15784
CG15863	
CG15908	
CG17266	
CG17327	
CG17530	
CG17531	
CG17734	
CG17904	
CG18522	
CG18547	
CG18596	
CG1882	
CG2064	
CG2246	
CG2789	CG2789
CG2846	
CG2909	
CG30152	
CG30493	
CG31063	
CG31793	
CG32549	
CG3271	
CG33066	
CG3397	
CG3448	
CG3931	
CG4769	
CG4858	
CG5044	
CG5224	CG5224
CG5989	
CG6272	
CG6335	
CG6353	
CG6512	
CG7506	
CG7627	
CG7842	
CG8198	
CG8360	
CG8778	
CG9034	
CG9646	
CG9836	
Chmp1	
Cys	Cys
Dgp-1	
dynactin-subunit-p25	
GstE1	GstE1
Hmgs	
Ide	
Jafrac1	
Jhl-1	
Jhl-26	
l(3)02640	
Mocs1	
mRpL11	
mRpL21	mRpL21
mRpL22	
mRpL22-24	
mRpS18a	
mRpS32	
mRpS33	
mus205	
Prx6005	
Rep	
Sras	
TepIV	
Thor	Thor
Tim10	
TMS1	
Ugt86Da	
Vha36	Vha36
bsf	
Cat	
CG17184	
CG1969	CG1969
CG33138	
CG7611	
CG7739	
CG9186	CG9186
Eno	
Gapdh1	
Gapdh2	
Idh	
NTPase	
Thiolase	
Tpi	
Trxr-1	
Vha44	

Sin3A Down	
ash2	***ash1***
ApepP	
CG11120	
CG1544	
CG1998	
CG33214	
CG9471	
HmgZ	
Mgstl	
Pepck	
Taf6L	
Ts	
CG10912	
Karl	
Mlc2	Mlc2
