# Supplementary material for: Functional dissection of the ash2 and ash1 transcriptomes provides insights into the transcriptional basis of wing phenotypes and reveals conserved protein interactions
Source: Genome Biol. 2007 Apr 28;8(4):R67. doi: 10.1186/gb-2007-8-4-r67 (PMC1896016; doi:10.1186/gb-2007-8-4-r67)
Supplement: Additional data file 21 — FDR adjusted p values for the GO terms displayed in Figure 2a [file gb-2007-8-4-r67-S21.pdf]

GO TERM	Downregulated Sets			Upregulated Sets		
	ash2^{I1}	*ash2*^{112411}	*ash1*^{22}	*ash2*^{I1}	*ash2*^{112411}	*ash1*^{22}
**Development**	2.51E-16	1.57E-21	2.40E-04	8.18E-01	1.00E+00	8.25E-01
**Organ development**	3.21E-10	1.45E-11	1.19E-01	5.63E-01	1.00E+00	9.67E-01
**Wing disc development**	7.98E-04	2.44E-05	3.01E-01	9.04E-01	1.00E+00	none
**Dorsal/ventral pattern formation, imaginal disc**	1.99E-01	2.79E-01	1.26E-01	6.93E-01	none	none
**Anterior/posterior pattern formation, imaginal disc**	4.93E-02	1.09E-01	none	none	none	none
**Death**	2.06E-04	5.37E-03	3.01E-01	3.64E-01	1.00E+00	8.65E-01
**Regulation of growth**	1.49E-02	1.23E-02	6.73E-02	8.30E-01	1.00E+00	8.95E-01
**Mitochondrion**	1.00E+00	1.00E+00	9.25E-01	4.16E-04	2.45E-05	3.82E-01
**Mitochondrial ribosome**	none	none	none	1.38E-02	1.99E-05	3.50E-01

(orange)	FDR adjusted P value < 0.01
(yellow)	FDR adjusted P value < 0.05
