# Supplementary figures and images for: Functional dissection of the ash2 and ash1 transcriptomes provides insights into the transcriptional basis of wing phenotypes and reveals conserved protein interactions
Source: Genome Biol. 2007 Apr 28;8(4):R67. doi: 10.1186/gb-2007-8-4-r67 (PMC1896016; doi:10.1186/gb-2007-8-4-r67)

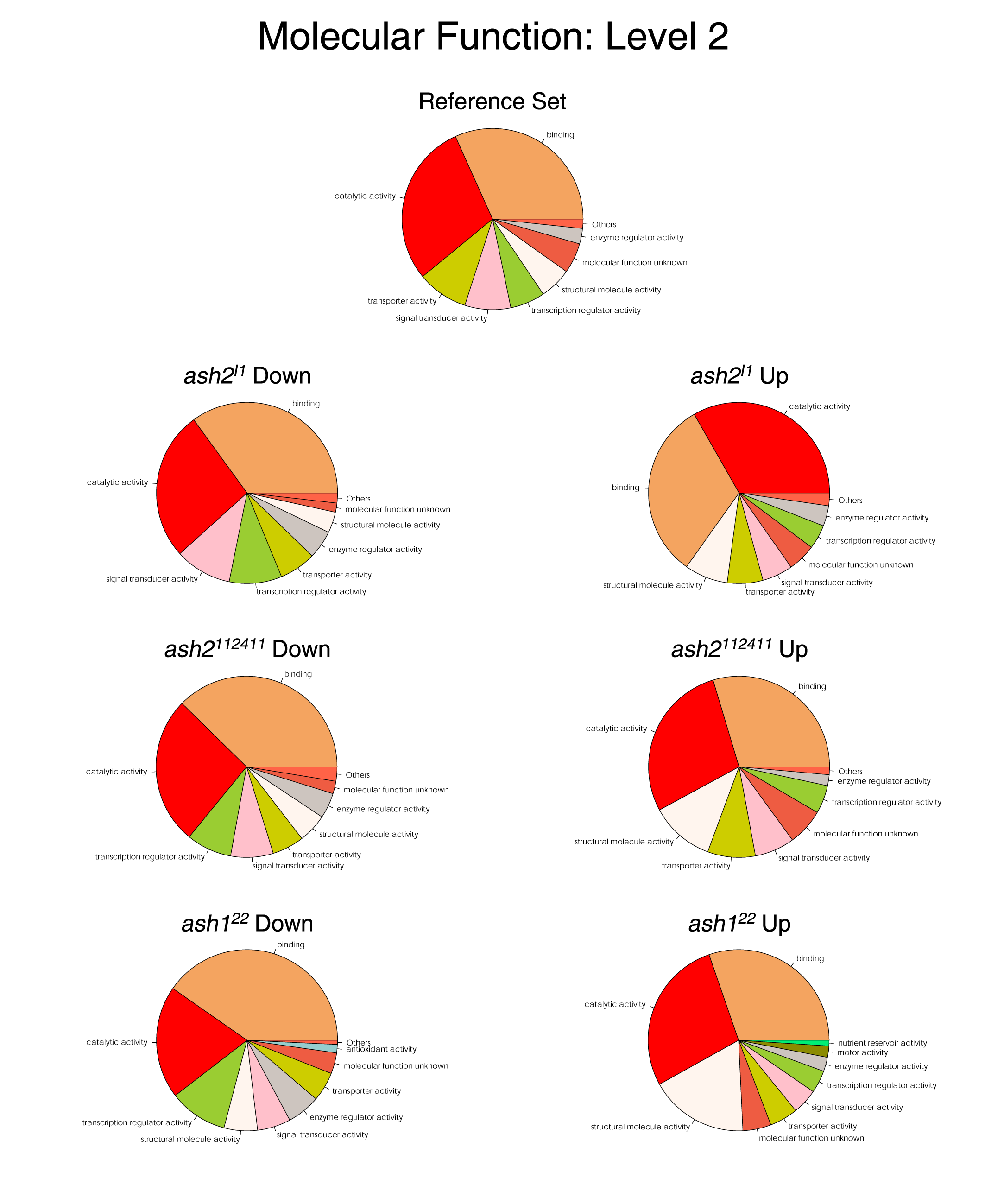

Supplement: Additional data file 22 — Distribution of regulated genes in Molecular function GO classes [file gb-2007-8-4-r67-S22.tiff]

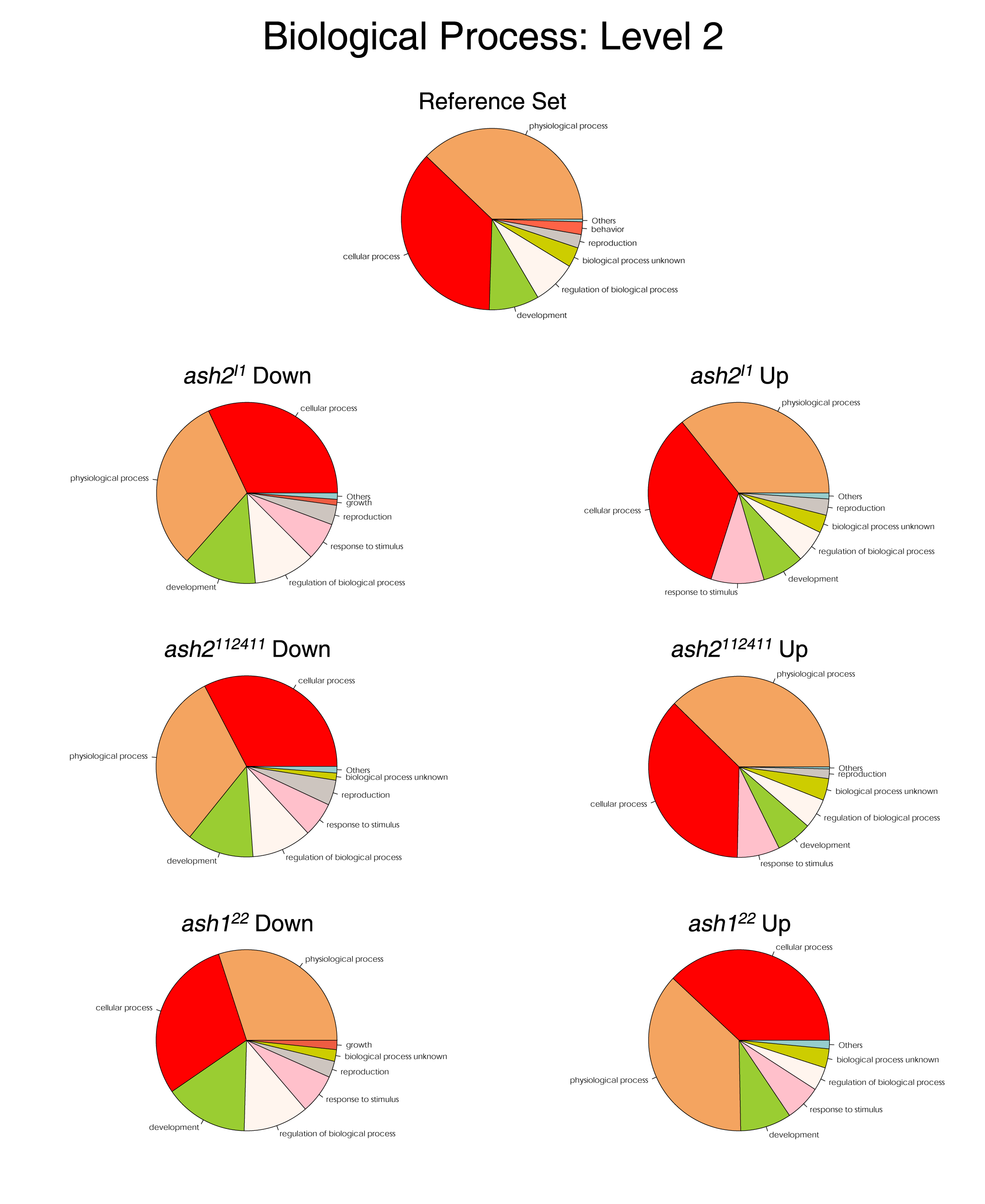

Supplement: Additional data file 23 — Distribution of regulated genes in Biological process GO classes [file gb-2007-8-4-r67-S23.tiff]

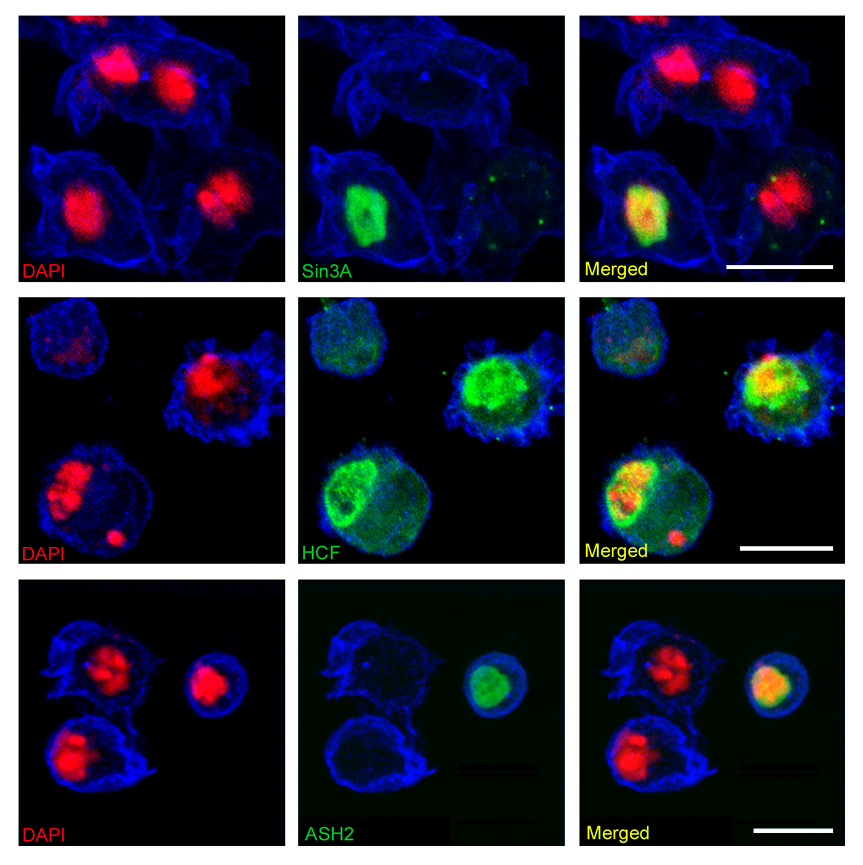

Supplement: Additional data file 24 — ASH2, HCF and Sin3A are found in the nucleus [file gb-2007-8-4-r67-S24.tiff]
